# Comprehensive Analysis of GDF10 Methylation Site-Associated Genes as Prognostic Markers for Endometrial Cancer

**DOI:** 10.1155/2022/7117083

**Published:** 2022-10-10

**Authors:** Jingyi Fan, Huaijun Zhou

**Affiliations:** Department of Gynecology, Nanjing Drum Tower Hospital Clinical College of Nanjing Medical University, Nanjing 210008, China

## Abstract

Growth differentiation factor-10 (GDF10) with its methylation trait has recently been found to play a crucial regulatory and communication role in cancers. This investigation aims to identify GDF10 methylation site-associated genes that are closely associated with endometrial cancer (EC) patients' survival based on normal and UCEC samples from the UCSC Xena database. Our study revealed for the first time that EC exhibited significantly higher levels of GDF10 promoter methylation in comparison with normal tissues. Multiple differentiated methylation sites, which have prognostic value due to their apparent survival differences, were found in the GDF10 promoter region. We performed weighted gene coexpression network analysis (WGCNA) on EC tissues and paraneoplastic tissues while using these differentially methylated sites as phenotypes for selecting the most correlated key modules and their internal genes. To obtain a gene set, the key module genes and differentially expressed genes (DEGs) of EC were intersected. The least absolute shrinkage and selection operator (LASSO) regression along with multivariate Cox regression were performed from the gene set and we screened out the key genes B4GALNT3, DNAJC22, and GREB1. Finally, a prognostic model was validated for effectiveness based on these genes. Additionally, Kaplan-Meier analysis and time-dependent receiver operating characteristics (ROC) were applied to assess and verify the model, and they showed good prognosis prediction. Moreover, the differences in risk scores were statistically significant with age, tumor stage, and grade. They may be related to the immune infiltration of tumors as well. In conclusion, based on the methylation-related genes associated with GDF10, we developed a prognosis model for EC patients. It might provide a fresh view for further research and treatment of EC.

## 1. Introduction

In the gynecological field, endometrial cancer (EC) is among the most frequent cancers [1]. There is a continuous increase in EC incidence and mortality. According to a recent study conducted by the American Cancer Society (ACS), 65,950 new cases of EC will be diagnosed in 2022, and 12,550 will die from the disease [2]. Over 95% of EC patients with an early-stage diagnosis will live for more than five years, suggesting that EC patients in early stage typically have a good prognosis [3]. However, the mortality rate will be significantly higher once recurrence occurs, and less than 30% of people survive over five years [4]. Therefore, finding reliable biomarkers for diagnosis is extraordinarily critical. Even so, the selection of prognostic markers for EC remains limited. Studies have shown that the combined diagnosis of HE4, CA125, CA724, and CA19-9 levels in the serum has a high diagnostic value for early-stage EC, and HE4 is a sensitive target to predict the recurrence risk and overall survival [5, 6]. Except for CA125 and HE4, which have demonstrated some accuracy in clinical diagnosis, other potential biomarkers are still in the initial stage of development. In that case, a new perspective is urgently needed on molecular therapeutic targets.

Growth and differentiation factor-10 (GDF10) is one of the members of transforming growth factor-*β* (TGF-*β*) superfamily. GDF10 is vital in cell proliferation and differentiation, and it inhibits several types of cancer by acting as a tumor suppressor too. For instance, by upregulating Smad7, the epithelial-mesenchymal transition (EMT) of triple-negative breast cancer is restrained by GDF10 [7]. In nasopharyngeal carcinoma, GDF10 is regulated downward due to its promoter's aberrant methylation, which can be reversed when treated with 5-Aza-2′-deoxycytidine. NF-*κ*B and Smad2 are reduced in the nucleus when GDF10 is overexpressed [8]. Likewise, the role of GDF10 in epigenetics should not be underestimated. GDF10 and BMP6 have aberrant promoter methylation in malignant pleural mesothelioma [9]. The histone H3K9-specific methyltransferase Suv39h1 is recruited to the GDF10 proximal promoter in lung cancer cells by Runx2 [10]. It remains unclear, however, whether the methylation of GDF10 promoter can affect EC progression.

Among the major epigenetic changes in DNA, methylation of DNA is crucial to the occurrence and development of cancer [11]. Transcriptional silencing occurs during the early stage of cancer when CpG islands (CGIs) in tumor suppressor genes (TSGs) are hypermethylated, while having repeat-rich regions hypomethylated causes genomic instability [12, 13]. In the last few decades, a series of genes including BHLHE22, CDO1, CELF4, SHP1, and TMEFF2, which undergo aberrant methylation have been potentially assessed for the diagnosis of EC [14–16]. These results suggest that DNA methylation-related molecules have great possibility to be served as candidate prognostic biomarkers for EC. However, up to now, far too little attention has been paid to mine methylation-related molecules from characteristic DNA methylation sites in EC.

In this study, we found several methylation sites of GDF10, which were notably associated with EC prognosis. As phenotypes, these sites were chosen to further screen for key methylation site-associated genes. All in all, our findings of GDF10 methylation site-associated genes may probably provide some reference value for EC therapy.

## 2. Materials and Methods

### 2.1. Data Source

Methylation data of 46 normal and 432 UCEC samples, of which 428 UCEC samples have survival information, and transcriptome data of 35 normal and 548 UCEC samples, of which a total of 425 UCCE samples with survival data were all retrieved from the UCSC Xena database (https://xenabrowser.net/datapages/).

### 2.2. Screening and Evaluation of Differential Methylation Sites of GDF10

Rank-sum test was used to analyze different methylation of GDF10 between UCEC and normal samples. Univariate Cox regression analysis was applied to detect methylation sites with *P*value < 0.05; Kaplan-Meier (K-M) survival analysis was further applied to evaluate the prognostic value of methylation sites in GDF10 using the survival software package in the R platform [17].

### 2.3. Coexpression Network Construction for Identifying GDF10 Methylation-Related Genes (GMRGs)

With the WGCNA R package, we constructed a gene coexpression network [18]. We first checked the association between 35 normal samples and 548 UCEC samples by performing cluster analysis and removing the outlier samples. After selecting the appropriate soft threshold, we ensured that the gene interaction conformed maximally to the scale-free distribution. For the gene dendrogram, hierarchical clustering was performed using a dynamic tree-cutting algorithm with a module size of thirty at least. To obtain the ultimate network, some modules were merged based on the dissimilarity of their eigengenes. Finally, by searching for the association between each module and the methylation level of prognosis-related methylation sites, the module that correlates best with the methylation level of prognosis-related methylation sites was defined as GDF10 methylation-related module, and genes in this module were regarded as GDF10 methylation-related genes (GMRGs).

### 2.4. Detection of Differentially Expressed Genes Associated with GDF10 Methylation

By selectively using the Limma R package (|log2 fold change (*FC*)| ≥ 1 and *P* < 0.05) [19], 35 normal samples and 548 UCEC samples with transcriptome data were compared for differentially expressed genes (DEGs). Then they were plotted in the form of volcano plots and heat map using the ggplot2 R package [20]. Moreover, the genes in the result of overlapping DEGs and GMRGs were defined as differentially expressed GDF10 methylation-related genes (DEGMRGs). Venn showed the DEGMRGs plotted by the TBtools software [21].

### 2.5. Analyzing, Evaluating, and Validating the Prognostic Model

A TCGA database containing complete survival information and transcriptome data for 425 UCEC samples was used to evaluate a risk model. Firstly, the samples were randomly divided into a training and verification set according to the ratio of 3 : 1. Next, to identify prognostic genes, multivariate Cox regression was conducted on the training set [22]. Prognostic models were constructed using genes derived from the multivariate Cox regression. Based on the expression of prognostic genes, the multivariate Cox analysis calculated the regression coefficient (coef) and risk score for each UCEC patient. Risk scores are calculated using the following equation:

(1) $$\begin{array}{c} \text{risk score}={\text{coef}}_{{\text{gene}}_{1}}\times {\text{expression}}_{{\text{gene}}_{1}}+{\text{coef}}_{{\text{gene}}_{2}}\times {\text{expression}}_{{\text{gene}}_{2}}+\cdots +{\text{coef}}_{{\text{gene}}_{n}}\times {\text{expression}}_{{\text{gene}}_{n}}. \end{array}$$

Moreover, by using the median risk values separately for the training and verification sets, a group of high-risk and a group of low-risk was divided. The overall survival rates between the two groups were compared from K-M survival analysis using the survival R package (the log-rank *P* < 0.05). We plotted time-dependent receiver operating characteristic (ROC) curves using survival ROC R package to evaluate risk model's effectiveness [23].

### 2.6. Risk Score-Based Independent Prognosis

The clinical characteristics of UCEC (age, tumor stage, and grade) were combined with risk scores in the Cox regression analysis. Results of *P* value less than 0.05 as the standard from the univariate Cox regression analysis would be carried out in conjunction with multivariate Cox regression analyses. Then, we considered variables with *P* < 0.05 as independent prognostic factors from the multivariate Cox regression analysis. Clinical characteristics were also examined in relation to risk scores. In addition, to explore the diagnostic capability of risk score level in different levels of clinical characteristics, the K-M survival analysis was performed to compare the differences in different subgroups of clinical characteristics which included the following variables: age (>65 years old or ≤65 years old); grade (G1, G3, and G3); stage (Stage I, Stage II, Stage III, and Stage IV).

### 2.7. Functional Enrichment Identified

Gene Set Enrichment Analysis (GSEA) was demonstrated based on the training set of genes from high-risk and low-risk groups [24]. Using the clusterProfiler R package, Gene Ontology (GO) and Kyoto Encyclopedia of Genes and Genomes (KEGG) terms enriched between these two groups were obtained [25], and the significance of difference was determined at *P* < 0.05. Additionally, Reactome pathway analysis was conducted by clusterProfiler package for pathway analysis.

### 2.8. Analysis of the Immune Cell Patterns in the Microenvironment

On the basis of normalized gene expression profiles of UCEC samples from the training set, CIBERSORT was used for us analyzing immune cell fraction [26]. We selected samples that had a threshold *P* value of less than 0.05 for the analysis. With the help of online analytical platform CIBERSORT, our comparison consisted of 22 immunological cell subtypes (LM22) sorted from a reference set (https://cibersort.stanford.edu/). Additionally, the differential expression of immune checkpoint molecules was plotted using the ggplot2 R package [20]. The correlations among 22 immune cell types and model genes were analyzed by gsva package.

### 2.9. Statistical Analysis

For all analyses, we used R software. Besides, a log-rank test was used to test whether OS variables differed significantly among groups. Measurement of prognosis accuracy was based on area under the ROC curve (AUC). In all cases, the *P* value less than 0.05 was viewed as statistically significant.

## 3. Results

### 3.1. Screening and Evaluation of Differential Methylation Sites of GDF10

**Figure 1:**
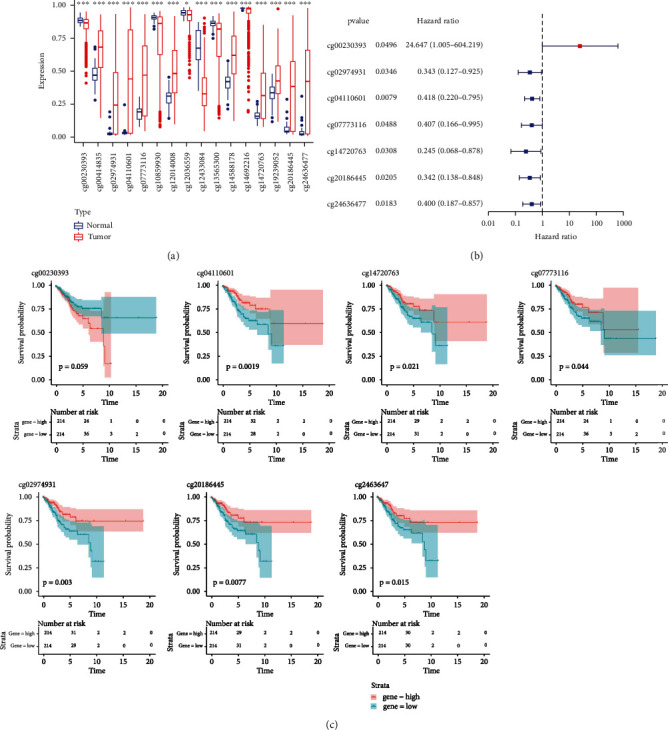
Analysing differential methylation sites of GDF10. (a) Different expression of DNA methylation between UCEC samples and normal samples in 16 GDF10 methylation sites. (b) Forest plot of the univariate Cox regression analysis. The left side represents genes and the corresponding *P* values and HR values. The red square on the right side indicates HR value greater than 1, and the blue squares on the left indicate HR value less than 1. The lines on either side of the squares are 95% confidence intervals for the HR values. (c) K-M curves show overall survival analyses of UCEC patients with different methylation levels between 7 methylation sites.

Through the rank-sum test, 16 methylation sites of GDF10 were markedly disordered between UCEC and normal samples (Figure 1(a)). Using univariate Cox regression as well as Kaplan-Meier survival analysis, six methylation sites of GDF10, including cg02974931 (*P* = 0.003), cg04110601 (*P* = 0.0019), cg07773116 (*P* = 0.044), cg14720763 (*P* = 0.021), cg20186445 (*P* = 0.0077), and cg24636477 (*P* = 0.015) were all found to have strong correlation with UCEC patients' prognosis (Figures 1(b) and 1(c)). Notably, high methylation levels at these six sites were associated with a better prognosis in UCEC patients (Figure 1(c)).

### 3.2. Identification of GMRGs Based on WGCNA

**Figure 2:**
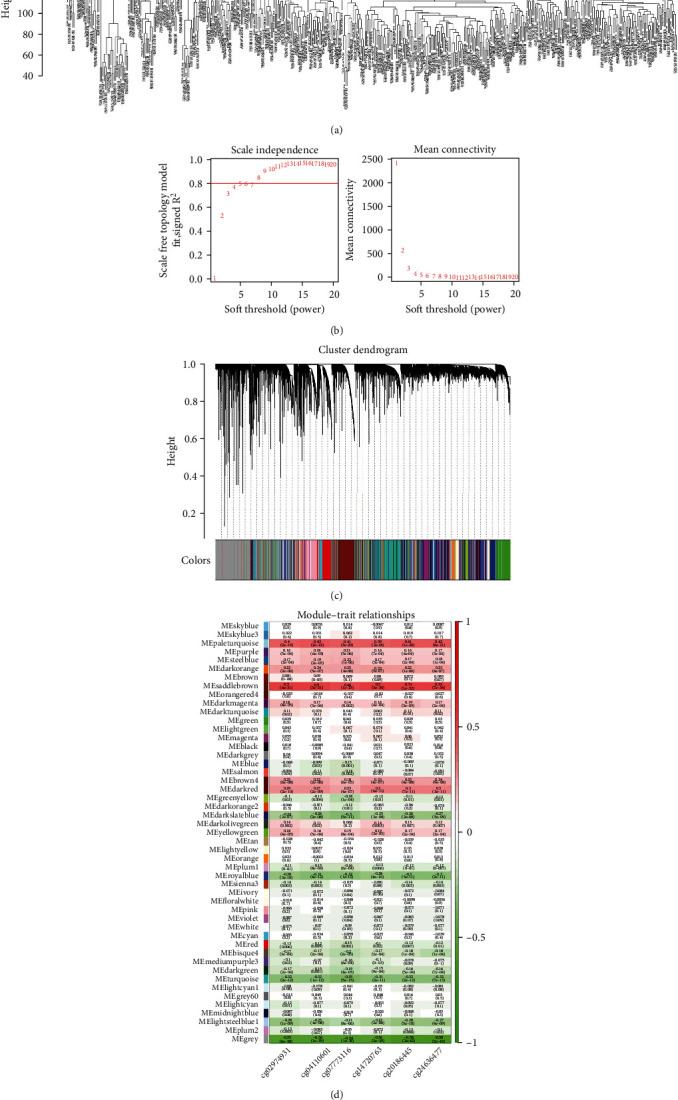
WGCNA-based identification of GMRGs. (a) Sample clustering of the dataset. The branches in the figure represent the samples and the vertical coordinate represents the height of the hierarchical clustering. The overall clustering of the combined data samples can be seen in the graph. (b) The distribution of scale-free soft threshold. The horizontal axis represents the weight parameter power value in both two graphs. The vertical axis of the left graph shows the scale-free fit index, i.e., signed R2 and the higher R2, the more the network will approximate the scale-free distribution. The vertical axis of the right graph represents the mean value of all gene adjacency functions in the corresponding gene module. (c) Module clustering tree diagram. Genes are classified into various modules by hierarchical clustering, with different colors representing different modules. The gray is for genes that cannot be classified in any module by default. (d) Heat map of correlations between modules and methylation sites. The vertical coordinate is for different modules, and the horizontal coordinate is for different sites. Each square indicates the correlation coefficient and significant *P* value for one module and one site. Red is for positive correlation, and green is for negative correlation. As the correlation level increases, the color becomes darker.

The cluster of all samples showed that there was no outlier sample (Figure 2(a)). Soft threshold analysis revealed that the soft threshold was set to 8 (Figure 2(b)). Moreover, in a hierarchical clustering and dynamic tree clipping analysis, 48 modules were identified (Figure 2(c) and Table S1), and the saddlebrown module was most positively correlated with the six prognostic methylation sites of GDF10 (Figure 2(d) and Table S2). Therefore, this module was selected as GDF10 methylation-related module, and 139 genes in this module were defined as GMRGs.

### 3.3. Identification of DEGMRGs

**Figure 3:**
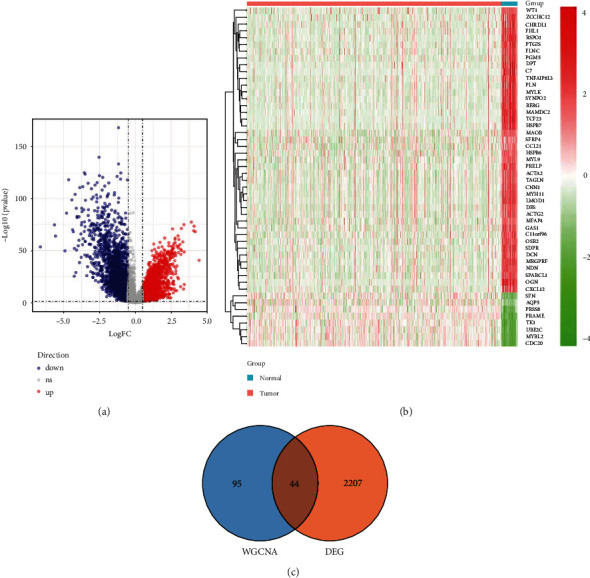
The DEGMRGs are identified by overlapping DEGs and genes from the selected module. (a) Volcano plot of DEGs between UCEC and normal samples. Each dot represents a differential gene. Red dots represent 991 upregulated genes, blue dots represent 1260 downregulated genes, and gray dots represent genes with no significance in UCEC samples compared with normal samples. (b) Heat map of top 50 upregulated and downregulated differential genes. Each square indicates each gene, and its color indicates the expression level of that gene. Red is for high expression, and green is for low expression. As the expression level increases, the color becomes darker. The first row indicates the sample grouping, with blue indicating normal samples and red indicating tumor samples. Each row shows the expression of each gene in different samples, and each column shows the expression levels of all differential genes in each sample. The left side of the tree shows the results of the clustering analysis of different genes from different samples. (c) Venn diagram by overlapping DEGs and module genes.

Differential expression analysis was conducted using expression profiles of 548 UCEC samples and 35 normal samples. Our analysis of UCEC and normal samples using the limma R package identified 2251 DEGs in total. Among them, genes were upregulated in 991 cases (Table S3) and downregulated in 1260 cases (Figures 3(a) and 3(b) and Table S4). Subsequently, by overlapping (Figure 3(c)), 44 DEGMRGs (Table S5) were identified.

### 3.4. Prognostic Model Construction, Evaluation, and Validation

**Figure 4:**
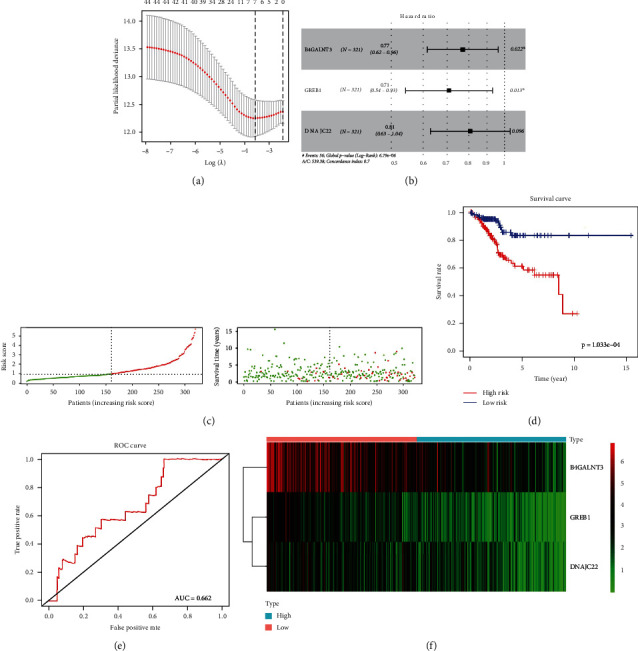
A prognostic model is built and gets verified. (a) LASSO regression analysis. The horizontal coordinate is log (lambda) and the vertical coordinate represents the cross-validation error, which we want to minimize in the actual analysis. The dashed position on the left is the location with the lowest cross-validation error, and the corresponding horizontal coordinate log (lambda) is determined from here (lambda.min). The top side shows the number of characteristic genes. According to the optimal log (lambda) value, the corresponding genes can be found. (b) The forest plot of multivariate Cox analysis. The squares indicate HR values and the lines are 95% confidence intervals for the HR values. (c) Risk curve and scatter plot for high- and low-risk groups in the training set. The horizontal coordinate in both two graphs shows the samples of patients ranked according to their risk scores. The vertical coordinates are the risk score and survival time, respectively. The dashed line shows the median risk score and its corresponding number of patients. Red is for the high-risk group, and green is for the low-risk group. (d) Survival curves for the high- and low-risk groups in the training set. (e) ROC curve in the training set. The larger the AUC is, the higher the prediction accuracy will be. (f) Heat map of the model genes' expression for high- and low-risk groups in the training set.

**Figure 5:**
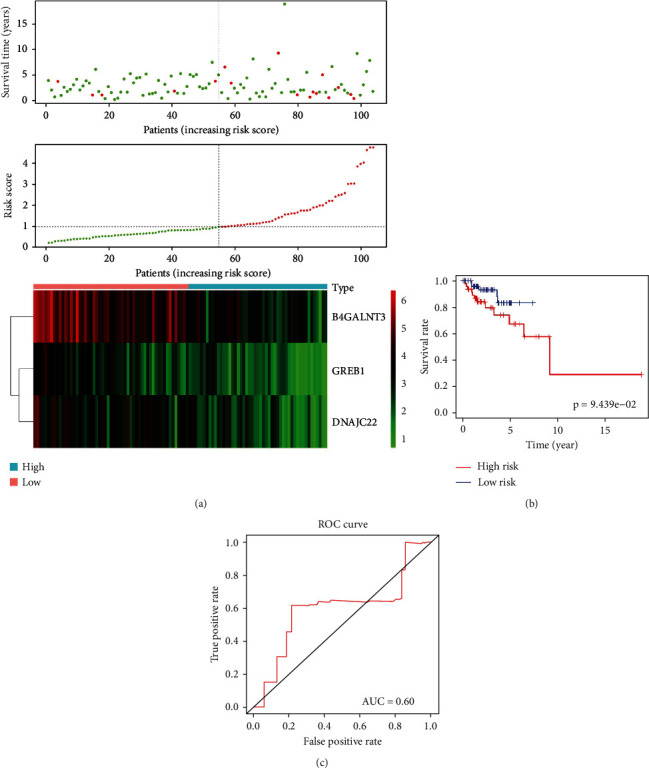
TCGA internal validation set for risk model validation. (a) Risk curve, scatter plot, and heat map of model genes' expression for high- and low-risk groups of UCEC patients in the TCGA internal validation set. (b) Survival curves for the high- and low-risk groups in the TCGA internal validation set. (c) ROC curve in the TCGA internal validation set.

In the training set, LASSO regression together with multivariate Cox regression were used to better identify DEGMRGs associated with UCEC survival. Based on the lambda set to 0.02358713, seven variables associated with survival were screened, including B4GALNT3, GREB1, NCMAP, PGR, CLDN6, MAL, and DNAJC22 (Figure 4(a)). Furthermore, B4GALNT3 (HR = 0.77028, *P* = 0.02169), GREB1 (HR = 0.70639, *P* = 0.01286), and DNAJC22 (HR = 0.80822, *P* = 0.09576) (Figure 4(b)) within the Cox model were identified as the optimal prognostic DEGMRGs by sophisticated calculations of multivariate Cox analysis with stepwise regression. For each patient in the training set, the risk score was separately calculated according to the formula below: risk score = (−0.26^∗^ expression of B4GALNT3) + (−0.35^∗^ expression of GREB1) + (−0.21^∗^ expression of DNAJC22). By analyzing median risk scores of UCEC samples in the training set, a classification of samples based on their risk levels was conducted (Table S6). In Figure 4(c), as compared to patients at high-risk, low-risk UCEC patients had relatively longer OS. K-M survival analysis confirmed better survival rates in low-risk group (*P* = 1.033*e* − 04; Figure 4(d)). Further, for better OS prediction, ROC curve was used with an AUC of 0.6622 (Figure 4(e)). Moreover, the heat map showed that B4GALNT3, GREB1, and DNAJC22 were relatively highly expressed from the group of low-risk (Figure 4(f)). In TCGA internal verification set, patients with UCEC were also categorized into high- and low-risk groups by using the formula above. The survival rate of low-risk patients was significantly higher than that of high-risk patients (Figure 5(a)). The risk score system wasable to differentiate the outcome status of UCEC, with a low-risk score implying a better likelihood of survival (*P* = 9.439*e* − 02; Figure 5(b)). Meanwhile, the AUC showed as 0.60 (Figure 5(c)).

### 3.5. Risk Score Independent Prognostic Analysis

We assessed the predictive ability of risk model combined with age, tumor stage, and grade using the training set. To determine if UCEC patients' outcome can be predicted based on the risk score corresponding to their clinical characteristics (age, tumor stage, and grade), Cox regression was performed (Fig. S1a, b). Age, tumor stage, grade, and risk score significantly impacted prognosis in UCEC patients (*P* < 0.05) according to the univariate Cox regression analysis. To be specific, risk scores significantly differed among patients categorized by age (*P* = 0.0024), stage I and stage II (*P* = 0.027), stage I and stage III (*P* = 0.00084), stage I and stage IV (*P* = 0.00017), stage II and stage IV (*P* = 0.028), grade 1 and grade 3 (*P* = 2.5*e* − 12), grade 2 and grade 3 (*P* = 4.3*e* − 08). However, the risk scores between patients classified by stage II and stage III, stage III and stage IV, or grade1 and grade 2 were differentially insignificant (Fig. S2 a, b, c Table S7). Moreover, we performed a K-M survival analysis to verify the prognostic value of three signatures in different risk groups of UCEC patients. The results suggested that the patients with high-risk scores had significant worse OS in age > 65 years old (*P* = 0.033), age ≤ 65 years old (*P* = 0.0013), G3 (*P* = 0.012), and Stage III (*P* = 0.014) subgroups than patients with low risk scores (Fig. S3).

### 3.6. Functional Enrichment Analysis

**Figure 6:**
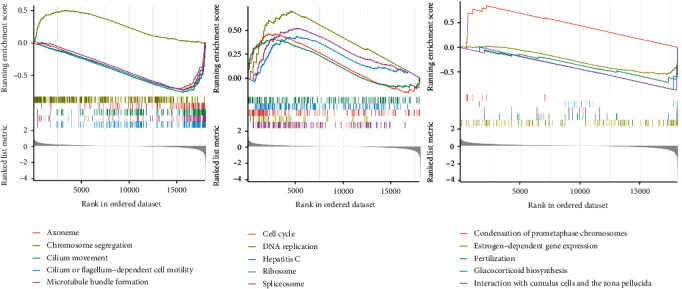
GSEA functional enrichment analysis for high- and low-risk groups. (a and b) The left graph is the plot of top 5 terms in GO enrichment for high- and low-risk groups, and the right graph is the plot of top 5 terms in KEGG enrichment. Each short vertical line represents a gene. (c) Reactome pathway analysis was conducted to delineate the metabolic pathways.

In the training set, we conducted GSEA to examine biological functions related to risk scores. The clusterProfiler R package was used to identify GO and KEGG terms. GSEA analysis showed 5 significant GO terms and 5 significant KEGG pathways associated with risk score, GO terms included “axoneme,” “chromosome segregation,” “cilium movement,” “cilium or flagellum-dependent cell motility,” and “microtubule bundle formation” in the high-risk group were highly enriched (Figure 6(a)). Besides, the KEGG terms revealed that high-risk genes mainly participated in pathways like “cell cycle,” “DNA replication,” “Hepatitis C,” “Ribosome,” and “Spliceosome” (Figure 6(b)). Subsequently, Reactome pathway analysis was conducted to delineate the metabolic pathways. The top 5 significantly enriched Reactome pathways listed as “Condensation of Prometaphase Chromosomes,” “Estrogen-dependent gene expression,” “Fertilization,” “Glucocorticoid biosynthesis,” “Interaction With Cumulus Cells,” and “The Zona Pellucida” were visualized in Figure 6(c).

### 3.7. Associations between Risk Model and Immune Characteristics

**Figure 7:**
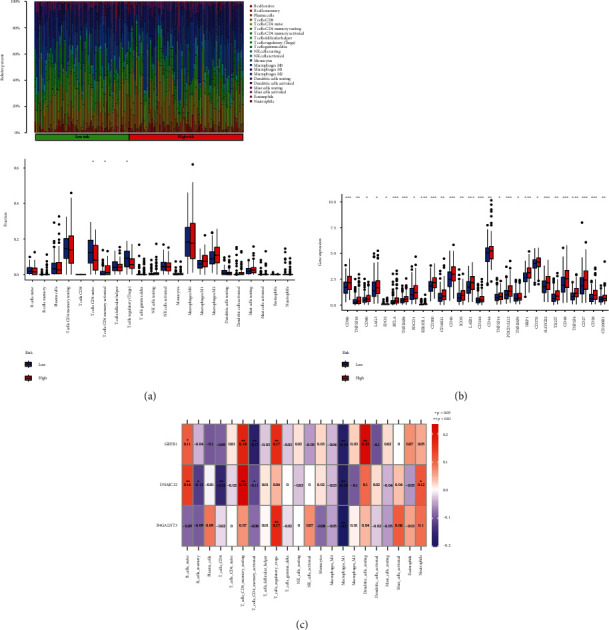
Risk model and immune characteristics. (a) CIBERSORT immune cell scale bar graph and CIBERSORT immune cell box line graph for high- and low-risk groups. (b) Box line graph of immune checkpoint genes for high- and low-risk groups. (c) Heat map of the correlations among three model genes and 22 immune cell types.

During our investigation of the relationship between immune cells and risk scores, 22 different types of immune cells were analyzed in each UCEC training sample based on the CIBERSORT algorithm. The CIBERSORT analysis was conducted on 73 UCEC patients at high-risk and 61 UCEC patients at low-risk with *P* < 0.05 used to screen. The two risk-differentiated groups displayed dysregulation of CD4 memory-activated T cells, CD4 memory-resting T cells, and regulatory T cells (Tregs) (Figure 7(a)). The immune checkpoint molecules expressed by these two groups were also different (Figure 7(b)). Three model genes (B4GALAT3, DNAJC22, and GREB1) were all found to be significantly negative correlated to macrophages M0 (Figure 7(c)).

### 3.8. UCEC Transcriptome Validation of Model Genes

**Figure 8:**
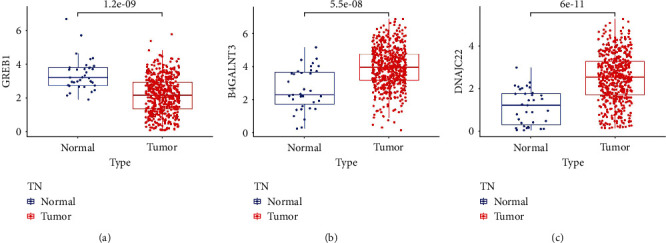
Evaluation of model genes' expression in UCEC transcriptome. (a–c) Differential expression analyses of GREB1, B4GALNT3, and DNAJC22 between UCEC and normal samples in TCGA database.

Finally, we examined TCGA database for model genes' expression. Comparing UCEC samples with normal samples, the expression level of GREB1 was significantly decreased (*P* = 1.2*e* − 09; Figure 8(a)), whereas B4GALNT3 (*P* = 5.5*e* − 08; Figure 8(b)) and DNAJC22 (*P* = 6*e* − 11; Figure 8(c)) were significantly overexpressed in UCEC (*P* < 0.05).

## 4. Discussion

In this study, we propose the possibility of GDF10 methylation site-associated genes as prognostic markers for EC.

GDF10 was initially found to enhance nervous system's development and effectively relieve neuropathic pain [27, 28]. With the deepening of genetic research, gene silencing caused by aberrant methylation of GDF10 has been confirmed in several studies. Since it is closely associated with bone morphogenetic protein-3 (BMP3), GDF10 is also known as BMP3B [29]. In particular, BMP3 has been found to have promoter hypermethylated and therefore inactive in several cancers, especially including colorectal cancer (CRC). CRC can be detected by BMP3, which suppresses colon tumorigenesis through TAK1/JNK and ActRIIB/SMAD2-dependent pathways [30]. In addition, through repressing methylation of BMP3 promoter, the role for 1,25-dihydroxyvitamin D3 in the progression of gastric cancer has been identified [31].

Our study found hypermethylation in the CGIs of GDF10, which is consistent with previous findings of elevated methylation of GDF10 promoter in NSCLC, NPC, and MPM. That is, GDF10 may function via the DNA methylation pathway in EC as well. Although extensive research has been carried out on GDF10 being aberrantly methylated in various cancers, no single study reported this gene's specific methylation sites in much detail. It is common for tumor suppressor genes to have high level methylation at CpG sites in their promoters [32]. The high methylation of CpG sites can alter chromatin conformation and close tumor suppressor genes' expression, thus leading to the loss of apoptosis, defective DNA repair, dysregulation of cell differentiation, dysfunction of cell adhesion, and ultimately tumorigenesis [33]. This evidence suggests that tumor suppressor genes are inactivated, and cancer usually develops as a result of promoter hypermethylation. Therefore, identifying the differentially methylated sites becomes the primary basis for methylation studies. To assess the prognostic value of methylation sites, we screened six GDF10 differentially methylated sites that were strongly associated with EC prognosis and found these sites had never been reported before. Here is the first time that the specific methylation sites of GDF10 been able to draw on some systematic research into cancer.

Therefore, we hope to identify methylation site-associated genes with independent prognostic value through the deep mining of methylation sites. GREB1 is a target gene for ER regulation, and it relates to estrogen level in patients with breast cancer [34–36]. GREB1 can limit the growth of hormone-sensitive breast cancer cells by modulating the PI3K/Akt signaling pathway [37]. It is also a target gene of AR and has responsive effects to androgen in prostate cancer cells [38]. According to recent reports, GREB1 can be responsive to progesterone in human endometrial cells too [39]. In a word, GREB1 is a pan-hormone-reactive gene. As a critical decidual molecular modulator, GREB1 may help to ameliorate poor implantation attributed to inadequate endometrial decidua [40]. In addition, chemotherapeutic resistance in EC may be predicted by GREB1 deletion [41]. As we know, estrogen-dependent type of EC is in the predominant status, and the methylation modification of GREB1 may act on EC via estrogen-related signaling, bridging the gap between epigenetic and hormonal pathways. Regarding B4GALNT3, it has been shown that B4GALNT3 may be targeted by m6A methylation in EC patients, the expression of which is elevated in epithelial ovarian cancer [42, 43]. By the way, there is still a lack of studies on DNAJC22, and we would like to explore it further in the future.

Building a prognostic model facilitated our further evaluation of prognosis. In the current study, we found significant differences in risk score regarding age, tumor stage, and grade through risk modeling. Interestingly, the differences between stages II and III, III and IV, and grades G1 and G2 were not statistically significant, suggesting that the risk model may better evaluate early, low-grade EC. In addition, the results of GSEA suggested that the prognostic model may be connected to DNA replication and cell cycle mechanisms. It is well known that DNA replication commonly happens in the S segment of cell cycle. Once the regulatory mechanism of cell cycle out of order, normal cells will show uncontrolled growth and thus have more potential to transform into tumor cells [44, 45]. This further implies the predictive value of the prognostic model we constructed in the early formation of EC. From immune microenvironment analyses, the findings suggested that GDF10 methylation site-related genes may closely associated with CD4+ T cells. By directly inhibiting cell cycle of tumor cells, which is a novel antitumor immune mechanism, the antitumor effects of CD4+ T cells have been reported recently [46]. Surface markers of Tregs in secretion include TGF-*β* and IL-10 [47]. Tregs are important for tumor immune escape because they can inhibit effector T cells' (Teffs') activity in the body. Meanwhile, Tregs and their productive cytokines in the tumor microenvironment can prevent the activation and chemotaxis of Teffs and promote tumor growth. Tregs are significantly increased in the serum of EC patients, and the number of Tregs may become a new factor for evaluating EC prognosis [48, 49].

It is important to note that all these results need further caution as well. On the one hand, we used the TCGA database only, so the data source is relatively homogeneous. On the other hand, considerably more work need to be done to verify the outcome with experimental studies. In addition, different from the role of miRNA or IncRNA, DNA methylation affects the occurrence and development of key tumors by downregulating the protein expression of genes. The impact of protein can be seen from the ROC survival curve, and this is what we will work on in the future. We will put more effort on studying the value of protein deletion regarding to DNA methylation. Anyway, the current results imply that the use of methylation site-related genes as therapeutic targets for cancer is still of high value; and the variability of epigenetic markers may be an immensely valuable prognostic tool for further in-depth information on cancer. Taken together, this study demonstrates the potential value of GDF10 methylation site-associated genes as EC prognostic markers.

## 5. Conclusions

This study was set out to investigate GDF10 methylation site-associated genes related to EC survival. The clinical prognostic model based on GDF10 methylation site-associated genes B4GALNT3, DNAJC22, and GREB1 revealed the prognostic value of GDF10 methylation sites. The findings of this study will provide us with a deeper understanding of methylation site-associated genes as prognostic markers in EC.

## Data Availability

The data used to support the findings of this study are included within the article.

## Supplementary Materials

Supplementary Materials Supplementary materials are shown as Supplemental Figures S1-3 and Supplemental Tables S1-7. Supplemental Figure S1: Cox regression independent prognostic analyses. Supplemental Figure S2: Analysis of expression correlations between the risk model and clinicopathological factors. Supplemental Figure S3: Kaplan-Meier survival analysis of patients with different clinicopathologic features between high- and low-risk groups. (a–i) The different OS of UCEC patients with different ages, tumor stages, and grades between high-risk and low-risk groups according to GDF10 methylation. The abscissa shows survival months, and the vertical axis shows overall survival probability. Supplemental Table S1: 48 modules were identified. Supplemental Table S2: The saddlebrown module positively correlated with the methylation sites the most. Supplemental Table S3: 991 genes were upregulated in DEGs. Supplemental Table S4: 1260 genes were downregulated in DEGs. Supplemental Table S5: 44 DEGMRGs were identified. Supplemental Table S6: All UCEC samples in the training set were divided into the high- and low-risk groups. Supplemental Table S7: Risk scores were significantly different between patients classified by age, tumor stage, and grade.
