# Supplementary material for: Comprehensive Analysis of GDF10 Methylation Site-Associated Genes as Prognostic Markers for Endometrial Cancer
Source: J Oncol. 2022 Oct 10;2022:7117083. doi: 10.1155/2022/7117083 (PMC9576415; doi:10.1155/2022/7117083)
Supplement: Supplementary Materials — are shown as Supplemental Figures S1-3 and Supplemental Tables S1-7. Supplemental Figure S1: Cox regression independent prognostic analyses. Supplemental Figure S2: Analysis of expression correlations between the risk model and clinicopathological factors. Supplemental Figure S3: Kaplan-Meier survival analysis of patients with different clinicopathologic features between high- and low-risk groups. (a–i) The different OS of UCEC patients with different ages, tumor stages, and grades between high-risk and low-risk groups according to GDF10 methylation. The abscissa shows survival months, and the vertical axis shows overall survival probability. Supplemental Table S1: 48 modules were identified. Supplemental Table S2: The saddlebrown module positively correlated with the methylation sites the most. Supplemental Table S3: 991 genes were upregulated in DEGs. Supplemental Table S4: 1260 genes were downregulated in DEGs. Supplemental Table S5: 44 DEGMRGs were identified. Supplemental Table S6: All UCEC samples in the training set were divided into the high- and low-risk groups. Supplemental Table S7: Risk scores were significantly different between patients classified by age, tumor stage, and grade. [file 7117083.f1.zip › Supplemental Figure S1.docx]

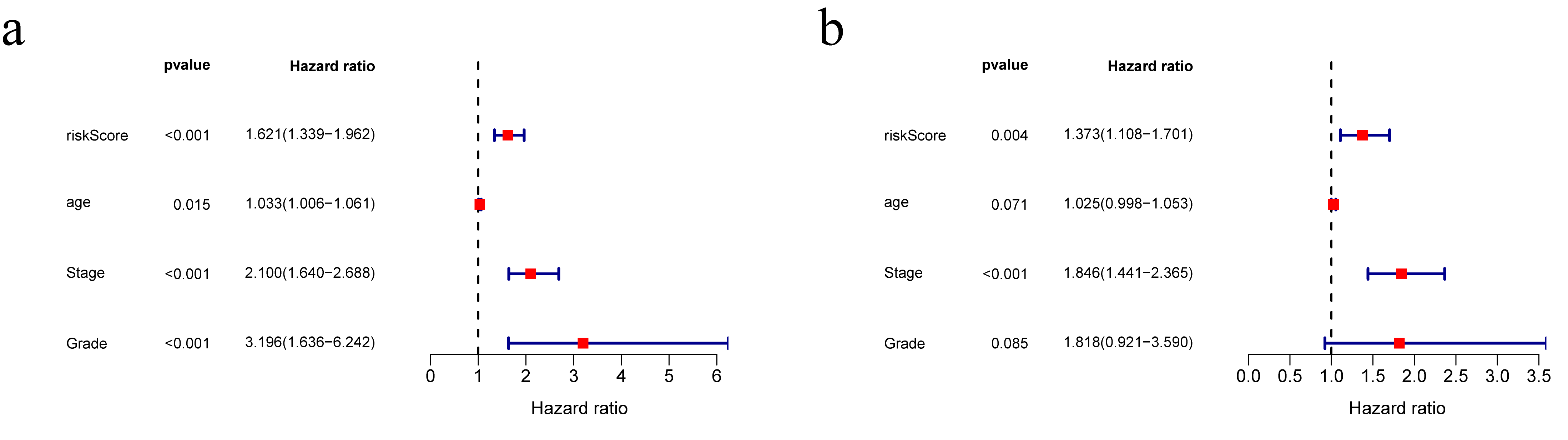


Supplemental Figure S1: Cox regression independent prognostic analyses. (a) Univariate Cox independent prognostic analysis (left) based on TCGA-UCEC. (b) Multivariate Cox independent prognostic analysis (right) based on TCGA-UCEC. Genes and corresponding P values and HR values are represented on the left of each graph, and the line segments on either side of the square are 95% confidence intervals of HR values.
