# Supplementary material for: Comprehensive Analysis of GDF10 Methylation Site-Associated Genes as Prognostic Markers for Endometrial Cancer
Source: J Oncol. 2022 Oct 10;2022:7117083. doi: 10.1155/2022/7117083 (PMC9576415; doi:10.1155/2022/7117083)
Supplement: Supplementary Materials — are shown as Supplemental Figures S1-3 and Supplemental Tables S1-7. Supplemental Figure S1: Cox regression independent prognostic analyses. Supplemental Figure S2: Analysis of expression correlations between the risk model and clinicopathological factors. Supplemental Figure S3: Kaplan-Meier survival analysis of patients with different clinicopathologic features between high- and low-risk groups. (a–i) The different OS of UCEC patients with different ages, tumor stages, and grades between high-risk and low-risk groups according to GDF10 methylation. The abscissa shows survival months, and the vertical axis shows overall survival probability. Supplemental Table S1: 48 modules were identified. Supplemental Table S2: The saddlebrown module positively correlated with the methylation sites the most. Supplemental Table S3: 991 genes were upregulated in DEGs. Supplemental Table S4: 1260 genes were downregulated in DEGs. Supplemental Table S5: 44 DEGMRGs were identified. Supplemental Table S6: All UCEC samples in the training set were divided into the high- and low-risk groups. Supplemental Table S7: Risk scores were significantly different between patients classified by age, tumor stage, and grade. [file 7117083.f1.zip › Supplemental Table S2.pdf]

probes	moduleCc	p.GS.cg0297	p.GS.cg04110	p.GS.cg07773	p.GS.cg14720	p.GS.cg20186
AACS	saddlebro	1.20E-08	3.17E-09	5.45E-11	6.39E-12	9.19E-10
AC11449	saddlebro	1.67E-09	1.48E-10	1.47E-09	6.52E-10	2.15E-10
ACAA2	saddlebro	0.00013385	5.68E-05	5.49E-07	3.39E-05	5.36E-05
ACSL5	saddlebro	0.00047823	0.000852152	0.003396996	0.000164423	0.000188923
ADAM28	saddlebro	1.65E-11	1.70E-09	3.54E-08	1.00E-10	1.32E-11
ANO1	saddlebro	2.65E-06	4.80E-06	2.31E-05	5.60E-07	2.98E-06
AQP5	saddlebro	2.52E-07	2.59E-08	2.67E-06	1.00E-05	1.46E-07
ARF1	saddlebro	5.06E-13	2.02E-14	3.33E-14	1.38E-13	3.28E-13
ARFIP2	saddlebro	4.98E-19	1.13E-19	3.78E-14	2.12E-17	2.96E-19
ARSD	saddlebro	1.46E-19	3.30E-19	1.41E-14	6.24E-16	1.66E-18
ATP2C2	saddlebro	1.14E-22	3.58E-22	4.36E-21	5.95E-23	2.48E-24
B4GALN	saddlebro	2.05E-15	4.79E-16	1.03E-14	1.21E-15	4.60E-16
B4GALT	saddlebro	3.49E-13	2.18E-13	3.62E-14	1.21E-14	5.63E-14
BACE2	saddlebro	0.00727027	0.005334748	0.029598518	0.007689129	0.005224749
BCAS4	saddlebro	1.04E-09	1.30E-08	1.98E-06	1.61E-08	3.96E-10
C1orf64	saddlebro	3.00E-07	8.07E-09	2.63E-06	5.83E-07	7.58E-08
C4BPA	saddlebro	1.35E-08	1.27E-08	1.57E-07	2.66E-11	1.16E-08
C5AR2	saddlebro	1.35E-06	4.25E-07	1.23E-06	1.71E-06	3.69E-07
C9orf152	saddlebro	1.39E-23	1.84E-24	4.25E-20	2.48E-25	7.14E-25
CA8	saddlebro	8.57E-11	9.65E-09	6.45E-06	4.63E-09	2.15E-10
CAPN6	saddlebro	3.42E-08	2.45E-09	2.08E-05	6.87E-08	4.96E-08
CCDC12	saddlebro	0.00817695	0.025939194	0.146400424	0.009890312	0.00256002
CEACAM	saddlebro	1.14E-16	1.95E-16	3.15E-17	8.22E-18	3.09E-18
CES3	saddlebro	1.47E-18	2.27E-17	7.04E-14	7.85E-18	7.64E-19
CHDH	saddlebro	4.00E-14	8.53E-13	4.29E-15	2.94E-16	3.87E-14
CISH	saddlebro	9.58E-12	9.15E-12	7.51E-13	1.88E-13	3.14E-12
CLDN6	saddlebro	3.63E-14	5.23E-15	8.03E-15	4.17E-16	4.83E-16
CPVL	saddlebro	2.61E-26	4.07E-26	1.88E-20	5.68E-23	9.48E-28
CREB3L1	saddlebro	3.60E-12	8.69E-13	8.14E-09	1.14E-11	3.28E-13
CREB3L4	saddlebro	1.10E-19	5.15E-20	2.91E-16	6.01E-19	3.25E-18
CRYL1	saddlebro	5.13E-14	1.46E-14	1.73E-12	1.51E-14	3.34E-15
CTNNBII	saddlebro	1.83E-17	5.60E-17	1.21E-18	3.44E-16	1.61E-18
CYB561	saddlebro	2.00E-12	2.14E-13	4.02E-13	2.14E-12	2.77E-12
CYB5R1	saddlebro	1.35E-06	2.47E-06	6.81E-05	9.70E-06	1.63E-06
CYP2J2	saddlebro	0.0010991	0.001722326	0.000488614	0.000315861	0.000933692
CYP4X1	saddlebro	1.57E-09	2.16E-08	7.00E-07	3.44E-08	2.26E-09
DACT2	saddlebro	0.00040858	0.002310851	0.045289983	0.00825904	0.002185472
DEPTOR	saddlebro	6.17E-08	5.85E-08	7.09E-09	3.46E-09	8.18E-08
DNAJC22	saddlebro	8.20E-11	1.55E-11	3.91E-09	1.66E-11	2.47E-10
DOCK5	saddlebro	9.99E-08	2.84E-07	4.24E-06	6.23E-09	3.74E-08
ENTPD3	saddlebro	3.80E-13	4.50E-12	6.02E-08	4.34E-10	1.45E-13
EPHB2	saddlebro	2.57E-16	1.56E-16	7.64E-13	6.79E-17	9.11E-18
EPHX1	saddlebro	1.48E-18	6.73E-19	8.13E-17	2.06E-20	5.84E-19
ERGIC1	saddlebro	6.47E-15	5.06E-16	3.52E-15	1.20E-16	1.54E-15

ERMN	saddlebro	1.30E-11	3.48E-10	7.04E-09	2.16E-10	7.84E-12
ESR1	saddlebro	1.84E-07	1.05E-07	5.58E-05	1.31E-07	7.91E-08
FAM174I	saddlebro	3.28E-08	1.86E-08	8.52E-06	1.35E-06	8.40E-09
FAM189/	saddlebro	0.00069879	0.001950755	0.028063929	0.023238273	0.001177517
FAM46C	saddlebro	7.09E-14	1.05E-14	9.65E-12	2.52E-13	3.08E-16
FBLN1	saddlebro	0.03263034	0.031434013	0.309803044	0.093835951	0.020330734
FCHO1	saddlebro	4.47E-09	4.52E-08	0.000102654	5.62E-06	1.05E-09
FOLH1	saddlebro	7.09E-07	3.96E-07	0.000230197	5.65E-06	5.56E-07
FOXA2	saddlebro	4.15E-20	2.42E-19	6.18E-19	1.13E-19	2.18E-21
GALNT1(	saddlebro	1.44E-24	3.15E-25	1.75E-19	4.95E-23	2.60E-27
GALNT1	saddlebro	1.86E-09	2.69E-10	1.82E-08	1.81E-10	2.78E-08
GCNT1	saddlebro	6.24E-10	1.03E-08	1.54E-06	6.96E-08	5.91E-10
GCNT2	saddlebro	5.31E-12	1.25E-12	3.56E-10	1.19E-11	3.31E-12
GCNT3	saddlebro	2.01E-08	2.20E-10	5.78E-10	3.14E-12	1.49E-09
GDF5	saddlebro	0.00245687	0.001660165	0.020090303	0.005065583	0.002248345
GLIS2	saddlebro	3.90E-21	1.78E-19	1.48E-17	9.81E-20	4.00E-22
GLYATL	saddlebro	1.63E-18	1.79E-18	1.41E-17	3.33E-17	7.71E-20
GNB5	saddlebro	0.00078817	0.001445746	0.031706866	0.005064505	0.000639156
GREB1	saddlebro	0.00333977	0.027395452	0.209237526	0.0289628	0.007526102
GRIN1	saddlebro	3.28E-19	2.15E-19	4.27E-19	7.63E-18	1.44E-19
HADH	saddlebro	2.93E-12	1.09E-12	1.01E-11	5.58E-13	3.69E-13
HGD	saddlebro	9.70E-17	1.13E-17	6.83E-14	6.77E-18	2.40E-17
HID1	saddlebro	1.24E-07	1.98E-07	3.81E-07	1.09E-06	2.46E-08
HIF3A	saddlebro	4.65E-29	2.53E-30	9.16E-30	1.03E-30	1.28E-32
HMGA2	saddlebro	3.08E-05	1.32E-05	3.12E-07	5.33E-07	3.99E-06
HOXA4	saddlebro	5.28E-09	1.23E-09	1.06E-08	2.24E-09	6.34E-10
HPX	saddlebro	3.13E-05	1.69E-05	1.33E-05	1.58E-05	5.57E-05
IGF2BP2	saddlebro	1.29E-15	4.85E-16	6.20E-16	2.40E-16	1.06E-16
IHH	saddlebro	1.61E-09	2.36E-08	7.62E-06	1.40E-07	1.64E-09
IL20RA	saddlebro	1.51E-13	8.42E-14	1.67E-09	3.01E-12	2.31E-14
IQCJ-SCI	saddlebro	2.62E-12	2.86E-13	8.14E-12	7.24E-12	1.57E-12
ITM2C	saddlebro	1.63E-11	8.52E-13	2.17E-14	2.85E-14	9.81E-14
KDM4B	saddlebro	3.12E-30	4.08E-29	8.26E-21	5.61E-27	5.60E-32
KIAA132	saddlebro	3.19E-20	3.13E-21	2.64E-17	1.25E-20	2.00E-21
L1CAM	saddlebro	2.77E-18	2.47E-19	8.10E-17	3.80E-19	2.02E-20
LRP5L	saddlebro	2.89E-06	1.04E-05	0.000190056	8.05E-06	5.39E-06
LRRC26	saddlebro	5.49E-20	1.20E-20	1.84E-19	4.76E-19	6.12E-21
LRRC3	saddlebro	1.02E-09	3.14E-11	9.46E-08	6.99E-10	1.52E-11
MAL	saddlebro	0.00010431	0.000269861	0.001141542	0.00033423	4.15E-05
MANSC1	saddlebro	7.32E-17	4.11E-16	4.23E-12	1.97E-15	2.75E-17
MAP2K6	saddlebro	2.10E-11	1.46E-11	2.56E-06	4.16E-09	1.52E-11
MCF2L	saddlebro	3.50E-11	4.28E-10	3.66E-07	5.08E-09	2.50E-11
MLPH	saddlebro	1.85E-14	7.61E-16	1.30E-16	1.09E-16	7.92E-16
MMP26	saddlebro	0.00738811	0.080893071	0.176442965	0.103967914	0.023482272
MUC5AC	saddlebro	0.00251807	5.41E-05	0.001889998	0.00111948	0.002169339

MUC5B	saddlebro	2.82E-13	2.07E-14	2.33E-13	3.54E-16	3.06E-13
NAAA	saddlebro	4.42E-05	2.51E-05	0.000197582	2.15E-05	1.23E-05
NCMAP	saddlebro	5.59E-09	9.88E-09	1.07E-08	1.70E-09	6.58E-09
PGR	saddlebro	1.91E-05	0.000269747	0.010795956	0.000110497	2.17E-05
PIGR	saddlebro	1.37E-13	2.98E-15	1.38E-14	1.61E-17	1.52E-14
PIK3R3	saddlebro	1.17E-22	1.94E-24	7.17E-19	1.47E-21	1.64E-24
PLLP	saddlebro	5.11E-06	1.02E-06	5.71E-05	6.37E-05	3.64E-06
POC1B-G	saddlebro	1.74E-09	5.48E-09	8.79E-07	4.95E-09	3.78E-10
PORCN	saddlebro	0.00163427	0.003966484	0.002815702	0.002469534	0.001070004
PPAP2C	saddlebro	5.29E-22	1.04E-23	1.37E-21	1.27E-22	3.35E-23
RBBP7	saddlebro	3.08E-11	8.50E-11	9.26E-13	3.41E-11	4.61E-12
RNF183	saddlebro	7.93E-11	1.16E-11	6.67E-10	3.18E-10	3.00E-11
RSPO4	saddlebro	2.01E-22	7.41E-24	6.52E-21	6.98E-23	1.49E-25
SCGB2A	saddlebro	2.46E-14	4.47E-15	5.34E-11	9.00E-12	7.25E-15
SELENBI	saddlebro	2.07E-05	1.91E-05	9.47E-05	6.08E-06	2.21E-05
SERINC2	saddlebro	4.92E-14	5.28E-16	1.15E-14	3.17E-13	1.64E-14
SH2D4A	saddlebro	7.05E-15	4.65E-14	2.31E-13	1.42E-16	6.25E-15
SLC25A3	saddlebro	2.27E-10	1.16E-09	4.19E-07	2.11E-08	1.24E-10
SLC43A1	saddlebro	7.68E-09	6.64E-08	8.51E-06	7.04E-08	2.53E-08
SLC44A3	saddlebro	1.55E-06	8.72E-06	0.000124974	8.65E-06	1.27E-06
SLC47A1	saddlebro	1.96E-11	2.02E-09	4.48E-08	1.77E-09	2.80E-11
SLC5A1	saddlebro	1.43E-14	9.37E-14	1.05E-11	3.66E-13	1.22E-15
SLC6A12	saddlebro	4.80E-16	1.07E-15	2.03E-15	3.85E-15	1.10E-17
SLCO3A1	saddlebro	3.54E-18	1.15E-17	1.53E-13	1.74E-14	1.17E-18
SPDEF	saddlebro	1.20E-24	6.85E-27	2.92E-23	1.70E-25	2.37E-26
SPINK4	saddlebro	1.86E-07	4.47E-08	1.26E-08	2.76E-07	5.39E-08
SRD5A3	saddlebro	4.07E-17	3.43E-18	1.02E-14	1.52E-16	7.47E-19
ST6GALN	saddlebro	6.80E-06	9.13E-08	4.47E-06	2.99E-07	1.08E-06
STEAP1	saddlebro	1.00E-11	1.08E-10	3.62E-12	5.29E-13	9.07E-13
STX19	saddlebro	5.69E-08	3.85E-08	4.46E-06	1.40E-06	2.17E-08
STXBP6	saddlebro	5.72E-10	2.42E-10	5.43E-05	6.24E-08	7.52E-10
TBC1D3C	saddlebro	2.73E-13	2.17E-12	7.60E-11	2.05E-15	2.05E-14
TFF3	saddlebro	1.33E-22	2.27E-25	4.13E-21	4.84E-23	4.72E-24
TMC5	saddlebro	5.47E-11	6.13E-12	7.70E-12	7.95E-15	3.42E-12
TMEM21	saddlebro	7.15E-18	1.31E-16	8.19E-17	3.10E-17	5.98E-18
TMEM21	saddlebro	5.64E-11	1.51E-11	1.37E-09	6.76E-12	1.86E-11
TMEM62	saddlebro	2.23E-22	1.00E-23	3.30E-21	4.58E-25	1.54E-23
TMEM63	saddlebro	1.51E-11	8.02E-12	2.74E-10	7.45E-12	5.05E-12
TMPRSS	saddlebro	2.78E-09	3.27E-08	4.27E-08	1.55E-08	8.89E-11
TNNT1	saddlebro	9.52E-09	2.10E-08	1.94E-06	2.66E-07	1.43E-09
TRIM3	saddlebro	1.79E-11	4.02E-12	5.73E-10	1.66E-12	5.34E-12
TRPM4	saddlebro	5.15E-20	3.67E-21	1.50E-18	1.78E-19	4.63E-21
TSKU	saddlebro	2.28E-10	3.60E-11	1.44E-08	1.23E-11	1.60E-11
TSPAN8	saddlebro	3.26E-07	2.76E-07	7.35E-06	4.63E-07	4.78E-07
TUBA3D	saddlebro	0.78841172	0.862347395	0.472416707	0.632739907	0.970578046

TUBB4A	saddlebro	8.54E-10	1.08E-10	6.11E-12	2.99E-12	1.74E-11
VIM	saddlebro	0.00318946	0.001281465	0.116437433	0.039486128	0.001867284
VMAC	saddlebro	1.38E-21	5.27E-23	2.47E-19	2.92E-22	4.68E-22
ZG16B	saddlebro	4.91E-10	2.12E-10	4.10E-10	6.02E-11	9.98E-11
ZNF516	saddlebro	0.34848677	0.645478837	0.460479263	0.920830812	0.414197364

p.GS.cg24636	p.MMskyblue	p.MMskyblue	p.MMpaleturq	p.MMpurple	p.MMsteelblu
9.72E-11	0.001119714	2.42E-07	1.23E-10	0.249131844	2.67E-05
3.21E-11	5.53E-14	0.686684291	0.040644089	0.002763564	0.433933613
1.80E-05	0.709206926	0.731532269	0.000890067	0.239777489	0.277636309
8.02E-05	0.013351055	7.30E-07	0.124211611	0.095231561	0.017791163
1.20E-11	0.202511093	7.01E-07	0.066532626	0.001015391	0.00129236
4.96E-07	0.119684063	5.73E-07	0.098136524	6.54E-06	0.00088364
8.56E-08	0.35812775	4.76E-06	6.27E-11	2.74E-08	2.24E-10
9.45E-14	1.51E-05	2.80E-09	3.15E-17	0.021240512	5.46E-09
7.48E-20	0.000100766	9.26E-05	2.48E-31	6.36E-12	8.62E-14
3.70E-19	0.692219273	0.185933123	3.67E-08	0.968980725	0.855722311
1.72E-24	0.858049865	0.039300505	1.50E-21	1.77E-05	2.46E-06
2.90E-16	0.560582521	0.000632966	1.10E-22	1.54E-08	1.62E-09
8.07E-16	0.169325915	0.525397921	0.002778913	0.608784504	0.196680539
0.004333209	0.281639433	0.014423279	0.354154122	0.061691997	0.072889654
2.42E-10	0.894296838	2.09E-06	0.234711798	0.003772408	0.002944841
1.05E-08	0.212831064	0.114029694	7.45E-06	0.282479897	0.270833754
5.35E-09	0.119838871	5.31E-06	0.080372551	0.029085462	0.011044107
5.26E-08	0.191177157	0.009733944	6.98E-06	0.642956821	0.636809039
7.76E-26	0.355421339	0.001387679	8.73E-10	0.848518102	0.944172307
5.31E-10	0.532086219	0.025602174	0.512920286	0.09950434	0.006734838
2.55E-08	0.234748649	2.75E-05	0.019700763	0.019412134	0.1486459
0.001939253	0.448328653	0.091527552	0.641288906	0.062148648	0.047555255
4.23E-18	9.21E-06	0.000640621	0.002130906	0.239440053	0.295837447
2.14E-19	0.003402582	0.409033361	2.03E-07	0.000125696	0.000279228
4.65E-15	0.338066598	0.050949815	9.44E-09	0.550063226	0.052091033
1.26E-13	0.099427764	0.64465532	6.52E-06	0.971450971	0.126884238
5.46E-16	0.412655379	1.37E-05	9.59E-11	0.830019027	0.386208199
7.41E-27	0.747470059	0.909371964	1.95E-12	0.064243175	0.001990605
1.72E-13	0.10728125	6.82E-07	7.33E-06	0.329270421	0.357289772
1.69E-19	0.024531511	0.031529919	2.95E-11	1.62E-06	1.07E-05
3.78E-16	0.587306057	0.003028294	1.34E-11	0.181238067	0.803711127
1.07E-17	0.580299036	2.73E-06	4.75E-24	7.91E-08	4.75E-11
6.28E-13	0.647943845	2.91E-05	6.06E-18	1.54E-08	3.59E-09
7.72E-07	0.123663289	0.174050958	2.58E-05	0.936769566	0.863638536
0.000774664	0.044047009	0.423206154	0.093137896	0.052039598	0.23663723
2.38E-09	8.95E-06	0.18187838	0.637901607	0.049364634	0.135643426
0.002098234	0.022643211	0.422828037	0.000109339	0.363173004	0.013143266
7.67E-09	0.28707146	0.206290439	0.019121446	0.506486701	0.265363608
7.02E-11	0.345096487	0.821325513	0.000493854	0.1129439	0.294436517
1.91E-08	0.223445873	1.58E-09	0.112579266	0.000760914	0.001382394
4.67E-13	0.238627948	0.018318484	0.000808578	0.29257715	0.227153529
9.12E-18	3.23E-05	2.22E-12	2.70E-07	0.891130268	0.041770856
7.54E-20	0.646138673	0.060212738	1.38E-13	1.92E-05	0.000181184
5.43E-16	2.14E-05	0.304599025	2.82E-05	0.066452528	0.49191315

2.87E-12	0.949309865	0.479791715	0.000271383	0.234134849	0.378911747
2.57E-08	0.792055048	1.65E-09	0.9549871	0.000104643	9.61E-06
2.65E-09	0.065828276	0.023158324	1.79E-13	5.11E-08	9.34E-08
0.001275835	0.239466399	1.64E-07	0.080935915	0.00045251	0.000330824
7.71E-16	0.012662061	0.00011825	0.000148784	0.024731737	0.026815159
0.010057836	0.038152591	0.47201444	3.48E-07	0.519852287	0.016594728
1.44E-09	0.224909895	7.00E-16	0.080657396	5.46E-05	1.36E-06
1.12E-07	0.049034056	0.066623629	0.004799638	0.032464994	0.38664317
1.30E-21	0.017291956	0.607745671	1.51E-15	0.000420647	0.000292355
2.37E-26	0.711695799	0.061764647	1.98E-11	0.706970888	0.394414278
2.79E-08	0.277108606	0.498113087	0.001861002	0.069000707	0.267486363
2.35E-10	0.28975146	0.000962812	0.161212581	0.069207593	0.098844009
4.18E-13	0.033997783	0.759640806	0.008251392	0.022855831	0.218784833
2.76E-10	0.000149251	0.003153249	0.000500296	0.262771899	0.333316819
0.001188792	0.097841353	0.010316155	2.30E-05	4.90E-06	1.55E-05
5.65E-22	0.000710102	0.322040306	7.18E-11	7.44E-08	0.005336863
7.75E-20	0.320081951	0.177387122	1.95E-06	0.056064723	0.155408807
0.000576445	0.741042049	1.22E-07	0.007102864	2.02E-05	0.00032234
0.007442375	0.863789106	3.76E-10	0.270080007	3.19E-15	4.45E-12
3.25E-19	0.759167491	0.002975031	1.58E-15	9.73E-05	5.81E-06
1.52E-13	0.000209209	0.366711397	1.01E-09	0.171809812	0.188522474
2.54E-19	0.013512499	0.56289789	1.06E-10	0.013126291	0.020166445
4.07E-08	0.143240039	2.84E-09	4.37E-14	3.89E-07	3.58E-11
3.42E-33	0.104826494	0.269379357	2.06E-19	0.000596557	0.00230126
1.93E-06	0.251719709	0.383722847	5.73E-10	0.121505278	0.090608457
2.08E-10	0.041569947	0.232780613	1.82E-06	0.956561063	0.392379015
2.43E-05	0.690179429	0.436432824	0.000107857	0.023307747	0.104658561
7.92E-18	0.080126195	0.180910975	5.70E-10	0.119076961	0.108112781
1.14E-09	0.001069912	0.00050123	4.39E-07	0.947810485	0.642518549
6.69E-14	0.12852852	5.51E-07	4.26E-07	0.236872886	0.097206129
4.92E-13	8.90E-08	0.416152744	6.45E-12	1.31E-09	4.52E-06
5.66E-14	0.05737106	6.69E-05	0.004026851	0.781231182	0.283302365
1.15E-31	0.870714237	0.934208519	8.60E-27	0.670811649	0.021330307
4.12E-22	0.832619197	0.152447362	3.30E-13	0.179559988	0.058698978
2.34E-20	0.00166411	8.55E-05	2.61E-15	0.267449555	0.543311471
3.76E-06	0.111823336	0.63139311	6.32E-06	0.647264355	0.02546191
6.50E-21	0.941444008	0.031996756	2.67E-19	2.85E-06	1.83E-07
6.17E-12	0.749553443	0.004573557	1.19E-05	0.11245136	0.536360445
9.02E-05	0.251723285	3.70E-09	0.145073759	0.000191096	0.000373246
8.63E-18	0.085592708	0.003332925	0.000522559	0.122219069	0.080196948
8.16E-13	0.057732553	0.232944672	0.002726583	0.278831524	0.550762355
4.95E-11	4.86E-06	0.948356725	7.43E-05	0.008054023	0.744810005
6.10E-17	0.320314924	0.296470495	5.69E-12	0.00428663	0.010445254
0.028286152	0.04233405	7.03E-05	0.916332729	0.071295709	0.023854272
0.000929261	1.31E-05	0.961894527	0.001610499	0.44235388	0.41263004

3.65E-14	0.00040233	0.357900664	1.12E-07	0.46006544	0.362943419
7.93E-06	0.761121961	0.024867112	0.07460095	0.411224645	0.112419885
1.31E-09	0.007709002	0.858794011	1.10E-05	0.361247786	0.073260066
2.30E-05	0.198923997	1.81E-17	0.76370008	1.75E-10	3.89E-11
3.05E-16	0.082209538	0.014405073	2.39E-05	0.54115748	0.606952909
1.14E-24	0.002015935	0.97844427	6.85E-08	0.020706293	0.056199061
1.92E-06	0.257387333	0.000187341	1.68E-12	2.67E-08	9.66E-09
3.13E-10	0.025578969	0.056211391	0.166590624	0.022293736	0.094483015
0.000984999	0.046440864	0.0585187	0.022843428	0.392008499	0.01337377
9.74E-25	0.783074227	0.001925743	1.96E-34	2.30E-06	2.59E-09
8.35E-13	0.145973285	0.59480085	2.80E-05	0.000648892	0.003170238
2.20E-12	0.693482825	0.16802676	2.79E-09	0.000101421	0.000666386
8.83E-26	0.056785861	1.82E-06	2.61E-13	0.249644034	0.308585534
4.21E-15	0.406972323	0.155400778	2.15E-05	0.117898324	0.359465039
2.31E-05	0.319353265	0.04753821	0.001831641	0.0122769	0.012933012
1.08E-15	0.039642326	2.00E-09	1.53E-23	7.54E-09	3.71E-16
1.61E-15	0.430522204	0.872361217	8.73E-05	0.023592191	0.170325247
5.47E-11	0.028740774	0.75636661	1.41E-13	0.001722438	0.000169122
1.04E-08	0.003692554	0.413609509	3.83E-10	0.048653419	0.027148219
1.06E-06	0.214521151	0.051000264	0.069040682	0.976385407	0.810191405
7.83E-12	0.009834033	0.000196691	4.53E-05	0.420214283	0.78415032
1.68E-14	0.027105774	0.015132688	1.33E-05	0.335649576	0.420460453
1.19E-16	0.004864477	0.00027977	6.24E-09	0.246617772	0.481182444
6.08E-19	0.000295804	0.068276939	4.62E-12	0.000906543	0.029504803
7.34E-28	0.225797609	0.982410544	1.43E-20	4.15E-05	3.11E-05
9.77E-09	0.000233071	0.598489053	4.35E-05	0.000106881	0.00411026
3.73E-19	0.41847934	0.807539217	7.41E-10	0.00024447	0.002730817
2.49E-07	0.001079259	0.673648678	0.001437631	0.374638981	0.291518368
1.70E-12	0.555710559	0.000692985	0.015721863	0.850385762	0.381451381
3.57E-09	0.527641502	3.67E-06	0.455804788	0.09878955	0.227754313
1.96E-10	0.349476951	0.106996147	0.004698667	0.002722065	0.024092529
8.68E-15	0.121026551	0.000186693	0.061127798	0.476226885	0.098468782
3.92E-25	0.212747251	0.823907635	7.50E-25	1.17E-05	6.21E-06
6.67E-13	0.000953527	0.001142055	0.001735927	0.286411413	0.275581479
8.67E-18	0.706756565	0.069543264	1.03E-12	3.25E-05	5.66E-05
1.99E-11	0.135569971	0.380934667	6.44E-09	0.008940506	0.015533431
1.94E-25	0.64812663	0.190761126	1.65E-11	3.83E-06	0.012347048
2.23E-12	2.11E-08	0.025018927	4.15E-06	0.568806345	0.034686747
1.90E-10	0.113137394	0.000881903	9.40E-07	0.000268934	6.00E-05
2.98E-09	0.279550647	1.24E-15	0.055880138	9.13E-09	1.88E-06
3.68E-12	6.79E-05	0.238903167	3.90E-09	0.091893066	0.425779805
3.75E-21	0.069991938	0.007444253	1.33E-24	3.16E-06	5.60E-10
2.03E-12	0.003384665	0.273211697	7.54E-09	0.213639791	0.248109046
7.27E-08	0.012510061	1.17E-07	0.009414043	0.644512852	0.102137949
0.850410315	0.000368155	0.149283049	0.042189353	0.636992544	0.943532075

2.07E-11	0.246258453	5.75E-05	2.42E-07	0.456652993	0.488063433
0.00130855	2.86E-08	1.11E-06	2.92E-05	0.220148472	0.104948233
1.10E-22	0.478711895	0.403215818	3.69E-18	0.005900373	0.007590463
1.83E-10	0.586624491	0.135367347	2.77E-06	0.439817858	0.366873626
0.411876689	0.659542101	2.04E-09	0.579004952	3.90E-12	1.62E-09

p.MMdarkora	p.MMbrown	p.MMsaddl	p.MMorangen	p.MMdarl	p.MMdarkturc	p.MMgree
5.62E-18	0.000552894	1.49E-28	0.326665113	0.04749	0.00508868	0.06766
6.36E-13	3.91E-20	1.37E-57	0.558956945	0.66906	0.707495443	1.51E-05
6.05E-05	4.76E-07	5.30E-35	0.280811487	0.79382	0.99908585	9.98E-12
0.007064401	2.62E-05	8.79E-37	0.09739826	0.01711	1.18E-17	2.97E-12
8.48E-11	4.79E-09	6.49E-53	0.082462129	0.0001	3.05E-15	4.76E-06
0.002055263	6.60E-22	3.14E-68	0.857179745	0.07388	0.16532012	9.31E-17
2.30E-25	2.89E-06	5.81E-33	0.3812909	0.00596	0.038556527	0.86883
6.75E-21	0.017654567	1.27E-30	0.559715234	0.11845	0.085981442	0.22897
2.54E-16	3.83E-16	2.96E-57	0.90211961	0.6135	0.273801537	0.9115
6.75E-27	4.11E-14	5.17E-49	0.193523203	7.48E-05	0.002342717	1.25E-05
2.00E-19	4.04E-21	3.44E-79	0.620692737	0.01733	0.029174522	0.12692
5.22E-22	1.04E-10	2.39E-65	0.723909996	0.03546	0.933529963	0.05688
1.46E-21	1.13E-10	4.12E-61	0.132429487	0.32683	0.116133782	0.03552
5.10E-11	0.000375187	1.39E-32	0.023692438	2.27E-05	4.70E-20	0.001
0.045706828	0.000251252	4.00E-38	0.17308905	0.19267	1.14E-12	0.02782
0.046181656	4.98E-09	6.83E-53	0.912252912	0.30718	1.17E-05	1.20E-09
1.35E-10	1.95E-12	4.08E-41	0.409352663	0.04889	0.018887341	3.60E-11
0.004158352	5.39E-15	1.03E-55	0.028956408	0.54835	1.76E-05	5.26E-10
5.68E-23	1.16E-21	8.99E-131	0.23140715	0.00286	6.11E-05	0.00015
2.04E-11	2.24E-11	1.76E-39	0.197889203	0.0001	2.79E-08	0.02901
0.054323653	5.50E-08	7.45E-28	0.767645719	0.77376	0.462401378	1.99E-07
0.006685379	3.61E-07	1.08E-32	0.052702626	0.03269	2.35E-11	0.00087
3.82E-11	0.002374069	4.62E-61	0.081075904	6.83E-09	8.50E-06	0.00141
1.36E-11	8.93E-07	3.20E-37	0.889035955	0.11387	0.000648188	0.0845
4.19E-07	1.61E-06	5.11E-45	0.710556988	0.30562	0.104702372	0.17376
4.30E-11	2.35E-05	2.59E-41	0.896638978	0.02044	0.245717776	3.88E-05
0.073308846	6.16E-05	1.35E-41	0.739139924	0.4808	0.002148871	3.82E-14
1.92E-07	9.09E-06	1.46E-60	0.175700961	5.70E-06	9.02E-05	0.00143
0.007526204	2.35E-05	3.88E-61	0.248745183	0.01105	4.24E-09	1.24E-19
1.03E-21	2.14E-19	1.47E-80	0.668224826	0.53288	0.202838185	0.09292
0.004004354	1.08E-11	2.42E-39	0.26225367	0.55856	0.442555433	2.31E-12
2.07E-19	0.000197177	7.02E-35	0.125675359	5.86E-10	7.93E-05	0.34818
1.01E-67	4.35E-35	5.86E-62	0.956427731	0.08031	0.486946179	0.97131
0.007586684	4.93E-08	4.29E-36	0.098999647	0.00023	2.68E-21	4.46E-10
1.37E-15	8.48E-12	2.28E-36	0.710452374	0.12259	0.158879535	0.50908
1.27E-12	6.95E-13	8.80E-38	0.160192659	0.00053	1.15E-06	0.91544
2.10E-13	3.40E-12	5.46E-30	0.121385531	0.48139	5.08E-05	0.00115
1.37E-16	1.36E-09	1.26E-35	0.305856212	9.48E-05	1.48E-05	0.35284
1.29E-23	4.70E-12	3.95E-36	0.61354461	0.6382	0.490367633	0.52016
0.591354972	8.36E-12	1.41E-42	0.426546244	0.88823	8.96E-07	9.35E-11
2.75E-12	2.61E-12	1.53E-85	0.035934246	4.09E-05	3.61E-15	1.36E-05
0.025434929	1.22E-05	1.28E-51	0.361373377	0.77163	0.000138826	4.60E-16
4.97E-11	0.000627319	7.06E-32	0.326880009	0.80192	0.29465401	0.02827
1.83E-25	0.000863606	8.95E-45	0.807506231	0.71766	0.445914445	0.56971

0.000135349	2.28E-12	2.29E-61	0.296473871	0.22439	0.000107773	0.83873
0.006300225	1.47E-19	3.08E-69	0.716729337	0.01876	0.475087912	1.34E-10
2.14E-24	8.04E-17	4.15E-46	0.925795914	0.26671	0.986136506	0.11952
0.939076104	2.83E-28	4.57E-47	0.781583994	0.40905	0.001900076	1.79E-27
0.002879773	3.37E-08	1.57E-44	0.173465167	0.01421	5.36E-10	1.29E-05
0.126910403	0.032716246	1.19E-21	0.931366063	0.35073	4.40E-05	4.19E-07
0.000336715	3.26E-09	2.00E-36	0.372622277	0.05681	2.17E-10	9.67E-13
1.67E-10	2.50E-13	2.66E-44	0.342940213	0.44125	0.14802426	0.89138
2.11E-05	2.83E-10	6.28E-81	0.062578901	0.23211	1.74E-16	0.00857
3.35E-05	0.001926297	3.00E-65	0.026553347	9.58E-06	1.89E-10	0.08809
2.78E-27	1.10E-07	1.14E-21	0.396726941	0.00601	0.03568292	0.4335
6.84E-08	3.86E-08	1.58E-46	0.208513922	0.16093	6.16E-10	0.47244
1.53E-15	5.71E-17	1.20E-44	0.515918354	0.32456	0.068882792	0.06593
7.53E-21	6.41E-13	2.35E-32	0.395591419	0.01602	0.447392163	7.34E-06
2.12E-05	4.76E-07	1.41E-28	0.822999132	0.2212	0.239910506	0.30577
0.001377992	8.82E-09	6.56E-44	0.550341905	0.12605	4.88E-05	0.30724
8.05E-11	3.76E-11	7.85E-86	0.186733303	0.02908	0.009732923	0.01995
0.024723378	5.22E-24	3.93E-45	0.908175029	0.03552	0.02472349	3.45E-24
0.015581271	3.11E-14	6.90E-30	0.94922694	0.13752	0.000440212	8.16E-15
2.47E-08	7.55E-07	1.98E-44	0.891309936	0.34047	0.221831813	0.07773
0.418207136	2.43E-10	5.73E-33	0.507095086	0.38159	0.806143333	0.96385
8.90E-27	6.20E-21	8.51E-62	0.608290263	0.90234	0.296510162	0.03418
6.61E-56	1.24E-09	1.31E-48	0.613605496	0.21875	0.307465384	0.13072
0.000801988	0.003616099	3.85E-44	0.388038642	0.00254	0.000301619	0.08655
0.684373803	8.12E-05	2.04E-24	0.77819535	0.64346	0.770915005	1.26E-17
0.058891542	0.000924624	1.33E-37	0.15175661	0.02286	2.27E-06	0.00351
0.223869951	8.03E-06	1.40E-19	0.938580543	0.38625	0.13736607	5.83E-07
0.806244456	2.00E-07	7.09E-54	0.392497097	0.71855	0.001329135	1.09E-08
0.071438402	1.13E-19	8.60E-84	0.459057355	0.9262	4.18E-11	3.24E-15
0.000399299	1.12E-24	4.78E-80	0.047348668	6.45E-06	4.55E-11	7.00E-18
2.95E-06	1.63E-09	6.83E-40	0.503648828	0.31451	0.002872908	0.00639
0.885279053	0.001153327	1.28E-34	0.164543895	0.17002	0.009028854	5.59E-07
1.48E-06	4.60E-13	1.01E-67	0.170837571	0.01378	0.000105815	5.30E-06
9.77E-11	1.84E-25	2.27E-185	0.278998369	0.47202	2.80E-05	1.87E-07
0.201534001	1.08E-09	1.43E-61	0.485753104	0.05583	3.72E-06	7.90E-09
6.71E-06	2.75E-09	6.87E-32	0.40615139	0.19735	0.007603802	7.38E-07
2.73E-10	1.22E-07	1.80E-69	0.956911995	0.17843	0.021541981	1.01E-05
1.70E-15	5.05E-14	1.84E-53	0.225020384	0.00295	1.08E-05	0.00026
1.59E-07	2.11E-07	1.80E-25	0.994152292	0.25824	2.95E-08	1.68E-11
2.81E-20	2.13E-11	1.52E-73	0.670630372	0.02647	0.281156877	0.1398
1.02E-14	4.26E-08	2.32E-39	0.232808338	0.13447	0.000138135	0.00179
3.06E-09	1.93E-07	4.83E-43	0.754758332	0.56746	0.004220724	0.01103
8.73E-13	7.00E-19	1.71E-108	0.721543989	0.64497	0.135478336	1.06E-12
0.343627699	0.001320041	1.82E-18	0.462170114	0.81281	5.54E-06	4.14E-06
5.08E-11	0.000154942	6.50E-07	0.283528669	0.07666	0.000748045	0.00019

8.93E-19	3.22E-06	4.39E-37	0.919758287	0.00802	0.706233447	4.76E-10
0.873342692	1.22E-09	8.00E-32	0.596661703	0.01879	0.565024768	9.07E-05
9.31E-25	4.93E-05	1.49E-32	0.020671262	6.32E-07	3.12E-07	0.50849
0.104174877	1.43E-25	4.17E-61	0.61779514	0.04787	0.000871133	2.66E-18
2.39E-17	1.14E-13	2.51E-84	0.59140745	0.28495	0.745004582	5.83E-10
1.05E-07	0.001416901	4.28E-38	0.447991077	0.00322	0.662723631	0.0014
2.63E-24	2.24E-10	5.92E-27	0.7033767	0.10866	0.037257863	0.00696
0.008982936	1.11E-07	2.13E-29	0.909914017	0.28112	0.98431545	0.64944
2.10E-09	2.80E-08	3.97E-28	0.680316096	0.70363	0.89964969	0.0176
2.18E-36	5.98E-16	1.72E-111	0.280846951	0.00241	6.22E-06	0.001
0.000103164	5.21E-06	2.24E-43	0.91737484	0.7053	0.836920962	0.00012
3.70E-21	6.78E-20	9.79E-64	0.880080631	0.63475	0.121218245	0.17113
0.701988947	1.60E-05	1.97E-40	0.409486335	0.50272	0.783616417	1.53E-09
1.59E-08	0.000172014	2.07E-34	0.762816051	0.00023	0.031141416	2.42E-07
1.17E-13	1.49E-06	1.48E-29	0.899004104	0.21622	0.046583481	0.0005
2.97E-60	4.16E-14	1.94E-80	0.739488074	0.04021	0.353588539	0.00769
3.62E-29	1.86E-08	2.21E-38	0.373434328	6.51E-06	0.002136916	0.24321
0.000323097	1.16E-19	2.01E-66	0.021424885	0.35386	2.53E-14	3.42E-11
0.684531482	6.33E-20	1.21E-53	0.667559333	0.00356	0.899633471	2.63E-12
2.98E-23	6.57E-09	6.65E-33	0.694265683	0.36321	0.006741614	0.97391
0.747377168	1.52E-08	3.02E-71	0.078908602	0.88241	9.12E-14	1.10E-09
1.36E-14	8.09E-12	2.12E-60	0.043425146	4.85E-10	1.59E-10	9.84E-10
0.004717359	0.000213522	3.11E-40	0.559842376	0.02492	6.86E-10	0.08416
0.808670746	0.002313838	5.89E-39	0.267936548	0.52247	0.029437072	0.01474
1.74E-20	2.93E-23	1.69E-179	0.807264857	0.23758	0.105590504	1.52E-06
0.005549853	3.45E-06	5.69E-35	0.52037734	0.82065	0.022911817	4.17E-06
3.40E-20	8.89E-11	6.44E-55	0.419953347	0.00104	0.803112693	0.09547
4.85E-32	4.25E-21	1.92E-50	0.974717353	0.83106	0.014170303	0.01037
8.57E-06	0.008016033	1.05E-32	0.111854209	1.34E-05	5.60E-07	0.77289
3.35E-14	8.72E-13	1.04E-34	0.497457378	0.03642	0.029110641	0.17911
1.09E-15	3.17E-05	1.11E-31	0.903858512	0.67996	0.108888834	1.63E-07
1.84E-10	2.94E-10	9.29E-59	0.525287643	0.29641	0.002343651	0.17816
6.13E-11	5.91E-19	4.15E-109	0.751999116	0.18649	0.014061156	8.24E-08
3.22E-30	1.39E-16	1.34E-43	0.101968624	1.80E-08	0.000965297	0.00117
1.26E-06	0.015644408	9.11E-40	0.912419018	0.51017	0.267456052	0.07862
8.98E-11	0.176442761	1.53E-36	0.97575645	0.00024	3.73E-08	0.00067
9.17E-19	0.000168119	3.94E-41	0.400747935	0.00295	0.005633153	0.41515
2.06E-32	4.60E-18	1.25E-45	0.80801101	0.37875	0.525325373	0.00043
1.42E-22	0.000607539	5.60E-49	0.037094929	1.35E-08	1.24E-07	0.51546
0.00084015	3.95E-07	2.65E-38	0.187508876	0.02489	1.49E-08	1.04E-14
0.492240462	6.71E-08	2.60E-40	0.508664826	0.2825	0.918207025	7.96E-12
4.35E-33	5.78E-14	6.45E-85	0.069154597	0.00344	0.270973731	4.99E-05
4.27E-05	0.000331106	2.75E-42	0.140351431	0.00494	4.16E-06	3.26E-05
0.080375956	7.50E-06	2.32E-34	0.395967387	0.27917	5.20E-09	1.46E-08
0.040404934	1.40E-07	5.02E-16	0.937323307	0.25717	2.19E-12	2.17E-13

0.07051846	4.48E-06	5.34E-39	0.526938259	0.00898	0.000210081	2.74E-14
9.40E-07	5.44E-08	3.15E-19	0.545892308	0.19123	3.08E-06	3.05E-14
7.72E-11	2.47E-31	1.65E-105	0.353316149	0.82495	0.012248246	8.33E-09
1.41E-10	3.14E-06	4.40E-38	0.716612306	0.00019	0.001171945	7.85E-11
0.007685463	1.47E-17	2.06E-25	0.378732955	0.12167	4.32E-07	5.56E-17

p.MMlightgre	p.MMmag	p.MMblack	p.MMdarl	p.MMblue	p.MMsalmon	p.MMbrown4
9.01E-10	1.96E-15	1.50E-06	0.0004	0.026154353	0.108237758	0.022468842
6.58E-13	0.02082	0.007603649	0.06816	0.084708058	1.79E-07	0.143954299
3.45E-17	3.70E-06	3.18E-06	4.34E-12	0.003674305	0.00899237	0.71011258
3.18E-06	0.09158	0.451681627	0.01925	0.110451385	0.402298566	0.003731266
0.000109136	0.0855	0.002009081	0.28252	0.000342887	0.037191496	8.78E-10
1.37E-07	0.11099	0.013623725	0.2452	0.009336706	0.024970939	0.000793581
0.244537197	0.11934	0.003123285	0.00028	6.51E-07	3.02E-05	0.601370982
0.536547768	1.40E-08	0.144840814	0.58623	1.93E-07	0.008395126	0.976466953
5.31E-06	0.00647	0.783106603	0.384	0.000157511	2.40E-06	0.000753852
0.273124993	0.00319	0.010062909	0.3214	0.395373483	0.753810693	0.000321623
0.859723325	0.24985	0.822141337	0.30627	0.067735321	0.069232543	0.000365932
0.071007287	0.03304	0.000667106	3.61E-05	1.29E-05	1.14E-05	0.968490499
0.022646138	0.98875	0.715488097	0.30932	0.573263465	0.439167126	0.022606233
0.295570256	0.18578	0.027595398	0.58709	0.261385746	0.493680197	0.002779592
0.000921086	0.17515	0.007762033	0.06207	0.002039176	0.174193219	1.08E-07
0.000131321	0.19104	0.061671781	0.00036	0.185324046	0.022710094	0.178866361
0.00026275	0.51449	0.187269092	0.33152	0.071601883	0.32311108	0.060465247
6.15E-05	0.06263	0.059108958	0.00038	0.212484223	0.062501163	0.043015299
0.584372874	0.00338	0.00181026	0.0569	0.081980392	0.591687315	5.72E-08
0.022288777	0.8749	0.018812339	0.11783	0.000676432	0.193948057	1.19E-06
0.000273864	0.12471	0.06118792	0.44478	0.136390256	0.317668605	0.182033214
7.27E-05	0.75781	0.338819283	0.38769	0.086525607	0.190383872	0.001264268
0.002420535	0.8436	0.199021097	0.68508	0.321298091	0.429677857	2.61E-06
0.018831035	0.09655	0.017686553	0.02297	0.102513489	0.001403644	0.264710003
0.936266209	0.00015	0.018126388	0.20984	0.750209922	0.343572131	0.002525003
0.055155991	0.08011	0.636095571	0.05139	0.045266685	0.576856965	0.019216184
2.60E-05	0.31882	0.205677486	0.00563	0.944758088	0.151116716	3.68E-08
7.91E-06	0.67332	0.30259234	0.01315	0.039506496	0.077966844	0.003393268
2.40E-15	0.19655	0.149549979	4.30E-06	0.726612235	0.097415161	0.62712588
0.351940889	0.95171	0.176953515	0.13255	0.045194681	0.000115627	0.304121426
0.917342171	0.0003	0.028680806	0.86028	0.558407601	0.60829176	0.000469108
0.002677426	0.05778	0.588544362	0.75911	1.76E-06	1.05E-05	0.013256542
0.003673622	0.05756	0.784490365	0.48313	0.004338557	0.000259211	0.122395404
0.890566391	0.68541	0.952986487	0.15066	0.302000763	0.049579838	1.90E-05
0.001367006	0.08743	0.384823901	0.15567	0.982544834	0.345274799	0.540163547
0.943871879	0.00014	3.64E-07	0.00022	0.001090367	0.002240891	1.84E-09
0.000575011	0.90946	0.406355262	0.02004	0.139177318	0.024813965	0.150556065
0.62255746	0.07748	0.022728613	0.01693	0.087221071	0.477821827	0.000479159
0.842015339	0.18889	0.040202548	0.02842	0.121109447	0.68304275	0.010015961
5.34E-16	0.31676	0.469366091	0.04708	0.000158922	0.014697001	3.26E-08
0.006357105	0.25045	0.027650595	0.9586	0.053904405	0.518215735	2.22E-08
7.62E-10	0.00027	0.013049588	0.00069	0.144547435	0.017383228	2.78E-07
0.250278388	0.9556	0.207138006	0.13615	0.001946761	8.72E-06	0.005764671
0.000131537	9.98E-18	1.26E-11	1.28E-06	0.676374395	0.019516375	0.022718795

0.066674815	0.19371	0.825768058	0.87894	0.319826462	0.831827412	1.21E-08
3.39E-07	0.61202	0.002469865	0.51697	9.04E-09	0.001362915	8.61E-08
0.234105355	0.08349	0.010210238	0.00539	0.001056817	4.17E-05	0.439481776
1.15E-11	0.53253	0.877025494	0.00259	0.029485257	0.919291848	0.000459192
0.182841295	0.00119	8.50E-05	0.04164	0.000683783	0.002419814	4.73E-10
0.006660621	0.21997	0.00752747	8.88E-07	0.000357385	0.000768307	0.350169826
4.01E-08	0.41541	0.098612408	0.55427	3.22E-06	0.067579248	7.40E-11
0.425528032	0.3078	0.767993623	0.43931	0.084527381	0.2610029	0.005893433
0.057159094	0.89494	0.289127864	0.17587	0.191156083	0.002171624	4.55E-07
0.326254938	0.01031	0.003226465	0.04963	0.357085866	0.551733058	3.20E-13
0.120368644	0.05176	0.025401619	0.00175	0.219568768	0.848975552	0.029420768
0.610443956	0.07241	4.28E-05	0.00014	1.13E-05	0.005869348	1.50E-14
0.351482114	0.19357	0.323530164	0.363	0.012796697	0.28184273	1.90E-07
0.143744388	0.05664	0.039347849	0.80596	0.539233839	0.166951909	0.128128447
0.248494996	0.00109	0.000279726	7.38E-05	0.002031109	0.000350403	0.32730983
0.054573235	4.13E-09	2.94E-06	0.00038	0.012133994	0.000174448	0.018984167
0.0015271	0.20883	0.829257605	0.21773	0.783024238	0.321690285	4.46E-06
3.08E-10	0.44793	0.214716773	0.01322	0.002576036	0.105998927	1.84E-05
0.002874639	7.80E-07	3.52E-10	0.00496	1.16E-10	6.99E-08	7.47E-09
0.034891522	0.93909	0.093514102	0.02952	0.005444393	0.021467316	0.052944193
1.69E-06	6.88E-05	0.000353203	4.37E-06	0.012812223	0.938438285	3.50E-17
0.016291558	0.51144	0.548520499	0.1085	0.336440731	0.180140079	0.013945567
0.266078161	0.51549	0.082239054	0.00126	1.78E-07	0.000574952	0.026113171
0.697505807	0.11062	0.07452504	0.1134	0.008604029	0.001688032	2.91E-07
9.69E-06	0.01808	4.66E-05	7.54E-10	0.00014584	4.47E-05	0.361853668
0.022381677	0.9713	0.752414684	0.173	0.584225221	0.685076293	4.46E-05
1.42E-14	7.97E-05	1.50E-05	1.85E-08	0.09734682	0.015246513	0.075404692
0.001500086	0.187	0.043049793	0.00088	0.048090099	0.028630354	5.14E-06
1.28E-08	0.14435	0.100687577	0.00018	0.781671694	0.023542476	0.032377812
0.000135784	0.81271	0.552086969	0.14699	0.170437006	0.390411458	4.28E-06
0.001487025	2.90E-07	8.56E-08	2.54E-06	0.00011476	5.75E-08	0.106746724
1.54E-11	5.85E-06	0.003346247	8.87E-05	0.721132807	0.38394177	0.007436932
0.02811096	1.57E-05	0.013200209	0.78498	0.902957844	0.613177127	4.50E-06
0.000364678	0.81096	0.990979523	0.04866	0.740116414	0.274328436	1.84E-06
0.00968574	0.43136	0.417283005	0.12376	0.949771996	0.035024714	1.14E-09
6.52E-19	0.34837	0.021309854	4.94E-08	0.063741078	0.617266975	0.011771467
2.85E-05	0.10865	0.00046559	9.08E-07	1.89E-05	0.00013747	0.913345787
8.22E-05	0.04485	0.248269818	0.06575	0.980941752	0.068628576	0.063683353
2.16E-06	0.4814	0.198724758	0.48503	0.001394612	0.164429745	0.000155607
0.047404316	4.31E-06	5.64E-09	5.06E-07	3.03E-05	0.120940015	7.31E-12
0.054289455	0.10681	0.000381785	5.53E-05	0.00315962	0.090250587	5.92E-08
0.007179397	5.60E-09	3.89E-07	0.03507	0.003952818	0.000123761	6.57E-07
6.61E-08	0.00344	0.000528933	8.26E-08	0.001381882	0.000846702	0.087686965
7.99E-05	0.23551	0.913882656	0.24005	0.020480905	0.971753627	0.010914681
0.086100392	0.34811	0.985295329	0.0193	0.001175492	0.973395771	0.00938678

0.005172797	0.10299	0.94165232	0.00535	0.002044471	0.605510745	0.234528542
0.180331538	0.83663	0.325753235	0.88601	0.029329344	0.318751147	0.000170925
0.685803111	0.49453	0.438800175	0.75997	0.195850436	0.572480326	0.260738418
3.27E-06	0.06121	1.64E-05	0.13206	6.48E-13	4.13E-05	1.00E-09
1.02E-08	0.01332	0.04742139	1.22E-05	0.157107769	0.110146026	0.079691974
0.032993924	0.02251	0.001006725	0.00013	0.021873274	0.132657706	6.38E-17
0.004088929	0.00389	2.36E-05	1.10E-07	2.34E-06	1.87E-06	0.024605628
0.732164832	0.00063	5.16E-06	0.00089	0.000116673	5.34E-07	2.40E-07
0.006236438	0.62966	0.217793535	0.00113	0.005296506	0.212602532	0.64972214
0.447587853	0.57168	0.131856818	0.00485	6.99E-06	5.38E-05	0.004878045
0.000592072	0.09877	0.05803402	0.00023	0.221024242	0.308767914	2.14E-09
0.006317836	0.02581	0.023246017	0.00372	0.015334088	0.01404864	0.039032793
0.133447256	0.21939	0.177050672	0.95282	0.130948448	0.988767913	3.14E-09
4.56E-05	0.00999	0.017319096	0.0002	0.064112017	0.025841641	0.204351574
0.388952055	0.57033	0.34204301	0.07238	0.022218626	0.001373368	0.131820936
0.001251349	0.45793	0.000405047	6.96E-09	4.87E-13	2.76E-07	0.184731955
0.16095581	0.08305	0.02947839	0.00364	0.264638461	0.794185385	2.33E-19
3.80E-12	0.00334	6.17E-06	1.32E-10	0.000574659	4.78E-06	0.668611761
9.83E-05	0.03413	0.001724645	1.97E-06	0.02684347	0.001460529	0.219089626
0.939330427	0.03568	0.001612083	0.01142	0.002668743	0.13025711	0.039486814
2.62E-07	0.21736	0.440411263	0.00951	0.673322848	0.206683665	3.11E-05
0.00050994	0.25565	0.211737651	0.23793	0.926759215	0.7926725	0.030127204
0.33933735	0.76801	0.80229173	0.67208	0.244013082	0.204671511	1.50E-15
0.012395969	0.00041	0.002172215	0.00444	0.266543214	0.000372608	1.73E-07
0.001509623	0.17343	0.025714648	0.00021	0.003463447	0.000137678	0.001730114
3.44E-07	1.16E-10	4.06E-10	5.97E-12	2.60E-05	1.54E-06	0.591377997
0.536262731	0.11474	0.136433048	0.09183	0.021825312	0.007972299	0.037444203
0.38110486	0.27645	0.187221071	0.88146	0.962134392	0.944978198	0.597143631
0.037386081	0.36099	0.016910809	0.00495	0.030578825	0.686681832	2.83E-13
1.41E-05	2.87E-06	0.177699473	0.2663	0.00522675	0.569332756	0.001195116
1.39E-05	0.06147	0.002049544	1.87E-06	0.014601023	0.847381446	1.68E-08
0.051508324	0.27149	0.005137345	0.03375	0.000142467	0.030060097	2.89E-08
0.008044335	0.13946	0.004953802	2.29E-05	5.57E-05	2.99E-05	0.170726763
0.133713747	0.30468	0.057206576	0.68403	0.382922282	0.146302387	0.001349658
0.026846604	0.24927	0.019371625	0.00672	0.00528348	0.018302632	0.164581279
0.112233739	0.13074	0.033045053	0.00816	0.002085063	0.000330846	0.995772699
0.832648727	0.06	0.373595352	0.65148	0.641569324	0.044441941	1.01E-05
2.74E-08	0.36211	0.955796658	0.00102	0.154147882	0.189988583	0.130692137
0.027898044	0.0324	0.017978246	0.00057	0.000310459	0.004983774	0.35046079
4.83E-10	0.05292	0.001971814	0.83866	3.89E-05	0.001237783	2.79E-06
7.83E-05	8.56E-06	0.049242638	0.09715	0.635678595	0.262040426	0.468710606
1.73E-05	0.39464	0.004773072	1.18E-06	1.56E-05	0.000349976	0.531769417
0.994772905	1.62E-05	0.005724028	0.71539	0.26635834	0.967199294	0.003851007
0.000183536	0.05226	0.379414763	0.04102	0.29077005	0.26269956	0.009927215
2.96E-06	0.01309	0.003038811	2.55E-07	0.124799289	0.036850789	0.968254435

6.85E-05	0.19453	0.273497394	0.00196	0.65132142	0.290622512	0.000717278
0.000225537	0.01736	0.082313069	0.00063	0.741876448	0.008520462	9.87E-06
3.34E-06	0.26131	0.101320944	0.0009	0.313024641	0.074345988	0.046723488
0.000762652	0.39941	0.098658226	0.00041	0.031150326	0.041875954	0.84561899
9.92E-05	0.0008	2.19E-06	0.18153	1.77E-09	8.57E-06	1.33E-11

p.MMdarkred	p.MMgreenye	p.MMdarl	p.MMdarkslat	p.MMdarl	p.MMyellowg	p.MMtan
1.92E-05	3.87E-06	0.05325	7.59E-07	0.38431	0.195845144	5.57E-07
3.93E-10	0.000829943	0.05553	0.697087885	0.00015	0.173774344	1.15E-20
0.007484447	0.495101541	0.00847	7.48E-06	0.27471	0.007286458	1.12E-07
7.34E-14	0.082844751	0.54783	0.007393797	0.44114	0.064827576	0.03231
4.03E-23	0.000421987	0.07313	0.128132988	3.10E-10	0.010193755	0.00138
2.75E-16	0.326244279	0.01267	0.346499501	4.76E-05	0.934010458	2.09E-11
0.133265505	0.01121949	0.15399	7.17E-06	0.04825	0.5408052	0.04745
0.003356229	7.59E-09	0.00033	2.74E-10	4.56E-05	3.14E-07	2.94E-13
1.78E-09	0.006666909	0.37871	2.98E-07	0.86095	0.006411892	0.85158
6.87E-21	0.677678061	0.3834	0.004052989	5.78E-06	0.003787095	0.00093
9.01E-12	0.709782149	0.75226	2.84E-06	0.10531	0.046171567	0.02734
3.31E-05	0.025685136	0.03014	2.39E-11	0.18348	0.869228782	0.04147
2.57E-17	0.675288103	0.13978	1.78E-05	3.11E-05	0.00050711	0.00712
6.31E-11	0.61375459	0.91696	0.000951916	0.00535	0.242040922	0.02475
1.32E-16	0.001012574	0.19484	0.005297183	4.76E-06	0.02965686	0.05832
3.09E-07	0.727998906	0.5062	0.004716851	0.89145	0.224294494	0.00054
8.51E-20	0.432809858	0.84073	0.052483176	2.37E-09	0.002434565	0.00024
2.98E-08	0.403010801	0.11195	3.41E-05	0.19718	0.817203442	0.0001
6.19E-38	0.691663042	0.13882	0.001333425	1.48E-05	0.013513035	0.00746
5.17E-19	0.000286113	0.08411	0.704059043	6.22E-14	2.00E-08	0.97871
8.78E-10	0.84497151	0.11071	0.959248673	0.02745	0.350651075	1.43E-05
2.87E-07	0.017663392	0.2283	0.352998714	0.00019	0.160051481	0.00391
2.11E-21	0.278397193	0.21978	5.71E-07	0.00055	0.219843666	0.00232
3.25E-07	0.152472496	0.52645	0.057251218	0.00754	0.000768382	0.7219
1.36E-10	0.968048116	0.89845	0.000681846	0.00051	0.007306495	0.00049
1.03E-06	0.010424147	0.00941	1.10E-08	0.17918	0.891485591	1.81E-08
2.92E-11	0.058834839	0.57335	7.70E-07	0.97966	0.050946207	6.69E-05
1.19E-06	0.355096142	0.12241	3.61E-08	0.46688	0.735515348	1.68E-06
6.16E-12	0.605391728	0.18782	0.001673479	0.01321	0.345452108	6.88E-10
1.37E-10	0.206781341	0.33799	0.010361757	2.34E-10	1.40E-16	0.38066
1.64E-18	0.002085133	0.20086	6.35E-06	0.72035	0.154830689	2.32E-05
0.000530543	2.75E-07	0.00108	2.27E-16	0.87472	0.024933387	0.22952
2.41E-16	0.070927586	0.1071	7.08E-09	0.23132	0.003014011	0.89466
1.02E-07	0.000219718	0.05275	7.76E-08	9.25E-05	4.36E-05	0.01894
2.30E-09	0.021282527	0.84135	0.170093591	0.00243	0.019105505	0.55837
2.08E-17	0.047136909	0.0041	0.352070126	4.48E-10	3.59E-05	0.05315
0.003703769	0.185182947	0.90664	0.18471387	0.8434	0.145598422	0.00015
2.80E-19	0.103115076	0.04434	0.222032014	0.00303	0.011360539	0.73634
7.91E-19	0.010917965	0.1952	0.097685795	0.00015	0.004154726	0.91147
5.51E-18	4.95E-06	0.07597	0.979773873	0.00107	0.07548832	5.84E-06
3.34E-20	0.104732628	0.03186	0.022231296	1.64E-10	9.41E-06	0.01553
1.20E-16	0.151676417	0.59088	0.001501585	0.014	0.453323342	0.21498
1.54E-07	1.58E-05	0.00095	5.97E-13	0.00128	2.57E-08	0.8391
1.23E-18	0.000525173	0.55142	0.000803305	7.46E-05	0.009937588	2.11E-07

2.84E-13	0.169313017	0.28121	0.007473976	0.00233	0.014831256	0.99562
5.25E-29	4.30E-06	7.02E-08	0.339928251	7.23E-07	0.358687881	0.01394
1.11E-06	0.256137318	0.55528	5.16E-05	0.3468	0.820342326	0.38205
1.01E-11	0.052750337	0.00755	0.511410887	0.01898	0.149977695	9.35E-08
1.22E-23	0.908101553	0.08044	0.200656556	6.50E-08	0.010399517	0.00112
0.423772087	0.002570655	0.09529	1.61E-05	0.00013	2.45E-05	4.58E-06
2.73E-16	0.005571681	0.00079	0.846362197	0.00114	0.458482544	0.04529
3.32E-18	0.00010413	0.00161	0.751515379	6.41E-07	7.30E-08	0.02775
1.16E-12	0.867099119	0.94297	0.000153737	0.22219	0.199635891	0.12622
2.37E-19	0.029624974	0.53515	0.000296615	0.00223	0.031474599	0.08196
8.55E-17	0.149534706	0.04319	0.306174625	0.00022	0.000135201	0.50886
3.71E-24	0.000226203	0.00088	0.916366249	5.55E-09	8.52E-05	0.71858
6.42E-24	0.002932019	0.25308	0.286425859	2.27E-09	8.50E-09	0.01706
3.08E-20	0.088410896	0.06287	0.000745237	0.01069	0.961126368	6.17E-05
0.119490384	0.573223139	0.88727	0.015796529	0.77736	0.033429599	0.87025
5.79E-07	0.29953728	0.16408	2.49E-06	0.03904	1.66E-05	0.12558
5.19E-19	0.503907022	0.38748	0.000653826	9.58E-06	0.00043839	0.17546
2.58E-11	0.080215639	0.04924	0.340370214	0.11057	0.003416078	1.43E-10
3.48E-14	0.002153802	8.45E-07	0.118135881	1.95E-05	0.316542033	8.18E-08
3.10E-05	0.346529932	0.30436	8.62E-05	0.09956	0.074491534	0.00202
7.57E-19	0.638342793	0.00975	0.017692657	0.03467	0.002218458	0.0763
4.03E-20	0.820699136	0.00529	1.13E-07	0.1131	0.520180459	0.03955
0.006880354	0.00755389	0.0239	1.06E-08	0.60341	0.56739537	1.24E-05
4.56E-11	1.75E-05	5.11E-08	1.73E-26	0.95276	0.336034495	0.25535
0.006801869	3.72E-06	0.00027	5.10E-14	0.00402	0.00114902	1.30E-07
3.39E-06	0.029138608	0.22897	0.000136619	0.24011	0.952621971	0.00016
0.019972808	0.012885586	0.11912	0.841292658	0.23016	0.94270847	0.00047
4.51E-06	0.006371629	0.34228	1.43E-07	0.36814	0.485856828	5.31E-05
1.48E-11	0.322707135	0.6987	0.020783221	0.9585	0.014873029	4.34E-06
4.72E-22	0.675174959	0.85733	0.000652737	0.06154	0.394315848	0.00019
0.000129734	0.61087741	0.85635	3.78E-06	0.33419	0.368105555	0.91488
3.34E-10	0.620565628	0.43351	0.000225658	0.2183	0.607770109	0.04461
6.34E-12	0.636522592	0.06271	0.001995464	0.01636	0.921914422	1.32E-10
4.13E-21	0.933289073	0.2729	0.000344646	0.06092	0.962068888	1.13E-05
4.67E-13	0.176737683	0.76234	8.86E-07	0.88112	0.399416134	0.01717
0.081853	8.65E-05	0.44794	0.779210992	0.58076	0.005304302	3.51E-15
6.16E-05	0.014887333	0.03914	2.99E-07	0.54197	0.590214467	3.23E-06
1.02E-18	0.124513092	0.18407	5.14E-06	0.01346	0.117225407	0.35673
3.55E-08	0.03771196	0.00973	0.772330639	0.09554	0.0431043	8.12E-05
3.65E-49	0.486337543	0.06168	0.004767051	2.15E-14	8.89E-10	0.8387
1.22E-19	0.001916254	0.02455	0.315952456	7.79E-09	2.93E-09	0.01742
1.54E-11	0.001537432	7.94E-07	0.865609807	8.68E-07	0.255047929	8.04E-09
1.07E-09	0.080677272	0.2517	4.30E-08	0.47855	0.90038887	4.07E-07
1.71E-06	0.001316276	0.01237	0.483748356	0.00311	0.290452474	0.214
0.600649075	1.53E-08	0.00224	0.000230126	0.03308	0.000341008	9.07E-11

9.03E-06	6.40E-07	0.0053	1.01E-06	0.99256	0.045483965	2.67E-16
1.31E-13	0.564928065	0.3384	0.170875159	7.59E-05	0.004450551	0.27895
2.96E-12	0.007643137	0.00011	8.92E-09	0.88176	0.449269954	0.0258
3.87E-31	1.59E-05	3.81E-08	0.158067309	1.72E-06	0.745583116	0.00086
1.24E-16	0.197638447	0.00111	8.96E-10	0.00021	0.038907513	1.68E-06
1.38E-19	0.315154416	0.00102	0.03941563	2.30E-07	6.90E-08	0.10123
0.017700393	0.162469547	0.09446	3.62E-05	0.07481	0.580715069	0.0733
5.35E-13	0.03426567	0.00102	0.90667069	0.00099	0.444777255	0.03348
0.08477827	0.101524581	0.18459	0.000138941	0.29032	0.304864769	2.31E-06
9.25E-10	0.000161024	0.014	4.29E-14	0.8313	0.352119557	2.67E-05
8.53E-18	0.179309529	0.04401	0.119709424	0.00328	1.14E-06	0.00074
1.07E-07	0.436025137	0.09191	7.47E-07	0.07244	0.021166944	0.0932
4.07E-19	0.043264803	0.60713	6.09E-08	0.80588	0.042022285	0.00302
3.10E-08	0.018615122	0.04238	2.11E-05	0.73207	0.466728541	0.00364
0.001246995	0.353787607	0.43704	0.000220147	0.0004	2.32E-06	0.02217
0.001513585	0.000153322	0.00011	1.81E-16	0.10199	0.313669754	8.65E-10
2.12E-23	0.354216309	0.73992	0.000191771	1.90E-05	1.43E-05	0.13181
0.000112492	0.842946164	0.03014	1.10E-05	0.14117	0.007480735	1.87E-07
0.000291875	0.179140525	0.58882	0.006997802	0.50115	0.198467674	6.77E-05
1.11E-23	0.000419398	0.21947	0.895822342	6.95E-06	0.003836515	0.76845
6.01E-11	0.474648048	0.85603	0.002626577	0.04539	0.946977739	7.45E-05
1.33E-16	0.083674236	0.2238	1.88E-05	0.00044	0.378803208	2.91E-07
3.96E-09	0.732840156	0.06153	0.077197195	0.07012	0.122733181	0.98858
1.89E-08	0.889866596	0.85022	7.63E-06	0.81608	0.507036161	0.61025
4.28E-16	0.066032042	0.17189	7.76E-09	0.35472	0.431478431	0.00042
0.041821302	0.053792979	0.0009	1.58E-05	0.41029	0.94323877	0.06758
2.67E-14	0.005573873	0.02173	1.98E-09	0.0078	0.000412427	0.36033
9.34E-18	0.771310803	0.73189	0.005147455	0.15756	0.794233646	0.02115
1.66E-21	0.691943537	0.99076	0.000543024	0.00071	0.00110651	0.03846
3.30E-19	1.15E-08	0.00052	0.640422457	0.00011	0.006399908	0.00134
1.85E-14	0.000689504	1.92E-05	0.645634223	1.86E-10	4.01E-18	4.45E-07
2.32E-31	9.29E-05	0.11657	0.217695149	7.69E-13	5.65E-07	0.87036
2.38E-10	9.65E-05	0.05826	7.00E-09	0.56431	0.665758021	2.15E-05
2.21E-23	0.385262758	0.01424	2.04E-06	0.00031	0.236539404	0.00267
0.00019374	0.108390288	0.16936	4.77E-05	0.50372	0.29476077	0.018
4.39E-05	0.004241511	0.00339	1.75E-07	0.89425	0.670696745	0.02388
8.53E-28	0.354181178	0.19606	5.35E-06	9.32E-05	1.48E-06	0.00096
1.37E-06	0.354839567	0.07679	0.001018122	5.01E-05	0.18208177	9.39E-14
0.001697895	0.19372065	0.07318	9.27E-08	0.56643	0.228532184	0.02671
1.52E-14	0.084692398	0.01308	0.643014687	0.00527	0.022731026	6.63E-10
5.33E-05	0.172222249	0.33638	0.077715186	0.12849	0.093518316	1.84E-21
1.03E-08	0.065506316	0.09954	2.60E-10	0.55673	0.200774368	2.87E-08
2.86E-09	6.74E-06	0.00831	2.35E-08	0.55211	0.021452511	7.12E-09
2.68E-12	0.550040405	0.82917	0.243132508	0.00571	0.29047254	0.12455
0.787391666	0.876694678	0.53112	0.058066538	0.1131	0.004943794	3.08E-05

1.01E-10	0.011312758	0.02449	8.55E-10	0.21982	0.000365399	1.53E-05
0.0008317	0.529964699	0.89921	0.021579608	0.01893	3.84E-05	0.1096
2.84E-15	0.877230905	0.39216	0.001172167	0.01393	0.251958284	2.81E-05
1.20E-07	0.005315949	0.02326	1.60E-05	0.53569	0.454858436	8.28E-06
8.33E-11	0.000428064	2.84E-08	0.037214625	5.63E-05	0.393630684	1.44E-06

p.MMlightyel	p.MMorange	p.MMplum1	p.MMroyalblu	p.MMsien	p.MMivory	p.MMflor
0.745500748	3.82E-06	0.027552234	0.000123701	0.22928	0.054618932	0.73582
0.004129508	1.32E-08	0.001974062	2.09E-06	0.44325	0.744863166	1.89E-06
1.09E-08	9.21E-05	6.30E-05	4.28E-16	0.00978	0.003248847	0.97729
0.586815035	0.040299261	0.006401235	7.23E-09	0.00114	3.38E-05	0.00292
0.166505943	0.08763082	0.111821475	0.000195588	0.01052	0.000237547	0.26018
0.266148691	0.314825471	0.000532265	2.71E-09	0.03443	0.003038575	0.1535
0.000272467	0.00042717	0.001287671	1.59E-07	0.00414	0.267560833	0.15425
1.85E-05	3.05E-10	1.04E-06	2.20E-13	0.34234	0.010906435	0.26757
0.646883597	0.643070477	7.52E-06	2.75E-12	3.68E-05	0.012358882	0.03344
0.685767004	0.489432264	0.003258271	1.20E-07	0.0008	1.00E-06	0.08343
0.193287501	0.233799592	0.003458758	9.53E-13	0.52541	0.001410166	0.3066
0.001103139	0.009010599	0.000114473	2.15E-16	0.0049	0.003236087	0.91863
0.920341911	0.592762939	0.049634385	6.33E-07	0.03715	0.041122636	0.8812
0.474843267	0.624415873	0.221263967	0.002732068	0.00283	1.14E-05	0.03108
0.054600047	0.02484766	0.711055513	0.001056527	0.21674	0.000937997	0.43567
0.006475066	0.331379308	8.94E-05	2.47E-14	0.06004	0.058057939	0.26394
0.865589054	0.723016093	0.002369334	2.59E-07	0.08022	0.010819945	0.60774
0.006942052	0.109783439	1.01E-05	7.22E-15	0.73272	0.117873353	0.94255
0.083021583	0.087990147	0.004351232	7.40E-10	0.00215	0.000199125	0.69411
0.002846025	0.002461617	0.003898583	0.350227642	0.0124	0.097871323	0.55315
0.447063859	0.887503332	0.000744646	3.78E-06	0.01832	0.822847711	0.04309
0.813164194	0.585645173	0.538160908	0.003953147	0.0119	0.256006942	0.87496
0.520331589	0.721745767	0.293012566	2.57E-08	0.85093	0.002204824	0.95813
0.187924232	0.651061143	0.022306796	1.21E-07	0.16914	0.312826672	0.10563
0.942289872	0.203132837	0.37945075	5.92E-07	0.76447	0.004721847	0.1069
0.004098536	0.00076891	1.72E-06	4.34E-13	1.09E-05	0.084205498	0.32699
0.036784545	0.910824656	2.19E-05	5.19E-21	0.50274	0.00183355	0.00591
0.002040905	0.01874583	5.12E-06	8.35E-17	0.00021	0.015513143	0.56663
0.000250652	0.138764668	8.87E-12	2.69E-21	0.01268	0.001644953	0.0019
0.174038287	0.926868744	0.001435424	1.16E-08	0.07691	0.211853651	0.47081
0.908972146	0.565737159	0.0013615	2.25E-14	7.68E-07	3.39E-08	0.0005
0.122981338	0.062242512	0.002245293	4.50E-11	0.30325	0.000759249	0.10048
0.78855246	0.899987723	0.164263425	4.29E-07	2.24E-05	0.011560695	0.44836
0.322818575	0.902109109	0.007100188	4.94E-11	0.16557	0.001138989	0.00268
0.744674666	0.679639205	0.940181702	0.023593206	0.2223	0.04566079	0.80106
0.002095416	0.361581729	0.317593643	0.802405244	0.09555	0.095951141	0.83737
0.003373783	0.095144708	2.08E-05	1.07E-06	0.00187	2.46E-05	0.05813
0.003331363	0.024186681	1.19E-05	0.158463194	0.02009	0.003712077	0.08676
0.008967194	0.013565043	0.990285224	0.083901827	0.00333	0.006142577	0.9973
0.554289855	0.507588143	0.022080248	5.49E-07	0.1876	0.028540935	0.52556
0.305338236	0.40527897	0.827690437	1.56E-05	0.00304	0.001004828	0.14229
0.556666674	0.001455443	0.000174267	3.14E-16	0.00302	0.000223261	0.00627
0.418950837	0.922691673	0.091257995	3.83E-10	0.00084	9.38E-05	0.03736
0.104016159	0.145619128	0.056939869	0.000669208	9.27E-05	0.015809242	0.02609

0.138022924	0.286705248	0.153149967	0.00069801	0.04361	0.069213772	0.54511
0.015345439	0.000236568	0.973644273	0.003427361	9.21E-05	0.004448664	0.29761
0.034246177	0.536778258	0.000333825	2.09E-09	0.00605	0.099715256	0.59002
0.046666228	0.726572022	0.001415443	2.27E-10	0.02766	0.029348946	0.00685
0.035501747	0.189831649	0.667058487	0.000127121	0.37027	0.074166959	0.85852
4.03E-07	0.000397283	2.99E-05	1.24E-14	0.00092	0.009787747	0.00107
0.104142835	0.002060049	0.675650897	4.61E-05	0.00659	0.441741317	0.24575
0.007007367	0.000137084	0.63892685	0.074809054	0.01578	0.059498151	0.87684
0.33706199	0.420208393	8.47E-05	1.42E-14	0.0097	0.008095122	0.03569
0.030654372	0.16843133	0.114117605	9.91E-08	0.00155	0.190718823	0.0436
0.004506849	0.010244766	0.229836347	0.208907394	0.02953	0.001019791	0.83399
1.38E-06	0.000627207	0.006568262	0.755709375	0.18328	0.123931153	0.17172
9.49E-05	0.007666811	0.001891319	0.847234655	0.00878	0.643851305	6.39E-07
0.900574261	0.536945491	0.00076478	1.60E-07	0.05171	0.018357042	0.16127
0.017149552	0.130641963	0.155209556	4.29E-05	0.03515	0.467551873	0.4493
0.275740266	0.95678726	0.079991062	1.89E-11	0.45367	0.046849349	0.00354
0.748311398	0.545952808	0.311126255	3.32E-08	0.02168	0.02624151	0.82588
0.054118486	0.813965812	0.000976128	6.89E-11	0.01793	0.317719174	0.18265
0.062643341	0.074010826	0.417910704	0.042891041	0.01086	0.011517319	0.00439
0.008441631	0.019048783	0.004132966	1.61E-12	0.69028	0.399797741	0.86537
1.03E-05	2.43E-07	0.328456803	0.002383291	0.75229	0.061725637	0.00827
0.612703036	0.611950151	0.037604873	5.62E-10	0.00071	0.005672916	0.00608
2.52E-06	1.89E-07	4.11E-05	7.86E-12	2.60E-06	7.59E-05	0.70617
0.453368183	0.711265231	0.000141734	4.30E-20	0.08475	0.002212218	0.98921
7.32E-09	0.001690934	3.68E-10	1.65E-27	0.78724	0.000475427	0.00215
0.214339964	0.040445011	0.028404377	1.22E-09	0.00073	0.001414971	0.24881
9.70E-06	0.028143995	5.75E-06	2.95E-09	0.51928	0.85081804	0.73391
0.001143743	0.070841581	2.73E-06	6.92E-22	0.93902	0.005730926	0.26142
0.003481096	0.822626962	1.16E-07	9.09E-19	0.00494	0.003901496	0.01523
0.504059381	0.167806299	7.62E-05	6.40E-16	0.00811	0.000221137	0.02296
0.003552679	0.960309557	4.40E-06	6.45E-14	0.02982	0.000141729	0.96011
0.172618401	0.765816904	0.009461762	2.91E-11	0.21361	0.039598559	0.96831
0.091832849	0.056278007	1.12E-06	8.36E-16	0.00082	0.019249077	0.06923
0.11677378	0.416384737	0.000127126	2.07E-18	0.00379	0.000991444	0.4245
0.334897974	0.12486099	0.000252611	2.46E-19	0.14545	0.026808608	0.00172
2.44E-10	8.12E-08	2.56E-10	1.82E-12	0.09657	0.077835994	0.09509
5.83E-07	0.000307025	3.65E-09	5.34E-23	0.06621	0.017241058	0.23407
0.879264723	0.197607968	0.008967738	6.19E-08	0.00909	9.21E-07	0.03732
0.723007589	0.270588368	0.012303928	1.50E-06	0.54317	0.213915331	0.00028
1.79E-08	3.72E-06	0.031982367	0.096940902	7.68E-05	5.97E-06	0.03285
7.24E-07	0.00054649	0.002771903	0.683533911	0.0048	0.497797644	0.00084
0.776163921	0.221556217	0.584570818	0.002170208	0.1706	0.023020786	0.56809
1.75E-06	0.002974716	1.08E-12	3.08E-27	0.00074	2.82E-05	0.67476
0.879588251	0.049631625	0.286433278	0.002473971	0.14719	0.250698989	0.24394
2.97E-05	1.07E-08	9.25E-09	4.46E-08	0.20985	0.142262679	0.48527

2.84E-06	6.97E-08	2.64E-13	6.20E-19	0.0769	0.002277963	0.48715
0.201786055	0.192841553	0.243708789	0.001523766	0.063	0.992537817	0.82012
0.95042798	0.264836354	0.1172481	9.65E-07	0.54126	0.00557725	0.39043
0.003516947	1.79E-05	0.824885969	0.001239853	0.00334	0.080920962	0.04359
0.002629761	0.023279747	3.53E-06	1.53E-18	0.00324	0.001043602	0.9897
5.77E-06	0.006904517	0.102669567	0.294482302	0.03869	0.2863062	0.07113
3.92E-05	0.007567037	0.000939605	1.83E-10	9.14E-05	0.063437171	0.13575
0.005336349	0.646123785	0.433049973	0.246598969	0.07066	0.185920574	1.29E-06
0.000142577	1.26E-06	0.000692293	9.60E-07	0.24324	0.260057352	0.3563
0.000383563	0.001501897	1.07E-06	6.89E-25	0.04493	6.62E-06	0.21268
1.13E-05	4.39E-05	0.048018474	0.284080987	0.16124	0.017805824	0.51984
0.024384809	0.06131889	0.008560525	2.29E-10	0.05745	0.001632875	0.03858
0.542677479	0.054392194	0.003162042	1.14E-14	1.55E-06	0.04161306	0.09908
0.021695986	0.261461833	0.000450546	5.92E-13	0.08719	0.020902444	0.98552
0.010207526	0.270051139	0.000246142	1.13E-06	3.24E-05	0.003931033	0.00107
5.35E-11	1.40E-12	1.84E-11	1.21E-25	0.00011	0.001020988	0.91692
0.001089227	0.003870945	0.513512887	0.020955477	0.01157	1.38E-06	0.47932
3.50E-09	0.003814107	2.26E-12	6.71E-26	0.65545	0.005191311	0.12772
3.76E-05	0.073286136	2.18E-06	2.46E-19	0.08502	0.117900811	0.47029
0.000647686	0.005478346	0.616075497	0.395906236	0.00285	3.55E-05	0.43607
0.089128336	0.952419455	3.79E-05	2.78E-14	0.00553	0.002474303	0.00869
0.177118909	0.152753846	0.000924755	3.29E-12	0.18823	0.008875844	0.27152
0.260700841	0.010147809	0.202225586	1.30E-06	0.70529	0.957789848	0.01625
0.73906715	0.11656051	0.071852987	7.46E-11	1.16E-05	0.695827093	0.15985
0.002759778	0.138609204	1.27E-08	1.18E-24	0.00026	0.000175749	0.5825
5.36E-06	0.042080391	1.47E-05	1.68E-15	0.26312	0.230444668	0.63398
0.497572009	0.551552111	0.033103539	1.85E-10	0.07393	0.01674138	0.46052
0.85617711	0.640393721	0.044561659	0.000196689	0.02908	0.000521013	0.26259
2.13E-05	0.000131978	0.507940336	0.031341151	0.00524	0.016966646	0.19834
0.00628817	6.61E-06	0.301755607	0.407831021	0.0168	0.00899543	2.29E-05
1.16E-09	5.49E-07	3.73E-07	0.020653306	0.00786	0.287116632	0.08158
0.00051727	0.005488311	0.848867559	0.075559439	0.00438	0.038717568	0.11328
5.96E-05	0.017124219	4.61E-12	1.81E-29	0.08956	0.002162938	0.47183
0.429122367	0.790147941	0.010262565	5.79E-07	0.03315	0.00037789	0.03581
0.010375652	0.040019107	0.001583767	1.30E-11	0.34088	0.388416425	0.51132
0.01119657	0.268945106	1.07E-05	1.33E-13	0.04699	0.008484354	0.08344
0.002705666	3.07E-05	0.827037879	1.55E-05	0.00324	9.18E-05	0.0734
2.13E-05	9.93E-10	0.000443933	2.32E-09	0.02399	0.004921582	0.00031
0.006169743	0.000201894	0.245305971	4.06E-08	0.90928	0.000370695	0.28612
0.977537307	0.707014053	0.000104199	1.60E-07	0.28238	0.521759529	0.03128
1.42E-05	4.13E-06	8.73E-14	6.46E-16	0.07628	0.620332224	0.01725
7.32E-07	1.11E-05	1.54E-09	2.32E-25	0.00493	0.000582551	0.68786
0.174312866	0.018407192	1.07E-06	5.49E-13	0.00011	0.006868345	0.0016
0.704976289	0.056963372	0.000791925	1.71E-07	0.08823	0.094505236	0.02234
3.89E-06	0.019068178	3.71E-06	3.75E-10	0.33091	0.145698201	0.04272

0.021656381	0.501415668	2.76E-06	9.53E-19	0.00648	0.000495854	0.51324
0.178178581	0.056586059	0.01618987	2.91E-10	2.32E-05	0.128457498	0.00052
0.008385986	0.250269364	2.31E-06	2.09E-18	0.04401	0.012017184	0.75322
0.001434474	0.049207499	5.78E-07	1.90E-15	0.42636	0.013465455	0.04103
0.189315058	0.05081159	0.737537122	0.026609584	0.06469	0.063756644	0.01094

p.MMpink	p.MMviol	p.MMwhite	p.MMcyan	p.MMred	p.MMbisque4	p.MMmedium
0.659220318	0.12413	0.175656775	0.178128643	0.02719141	9.38E-05	0.021393205
1.64E-06	6.12E-07	0.00012273	1.25E-05	2.80E-10	0.01674898	0.476685831
0.272745138	0.2277	0.181003933	0.454832703	0.857081464	0.014379331	0.003976296
0.130822802	0.51658	0.823478066	0.269046446	0.387533298	0.072266852	0.002294731
0.586511608	0.94112	0.981308257	0.65520249	0.269769386	0.000794342	0.000295869
0.921508269	0.6178	0.576324642	0.969556859	0.945741585	0.006517025	0.013893768
0.638580249	0.99549	0.67464947	0.565618132	0.006347116	1.89E-06	0.028685072
0.626155014	0.00315	0.081386679	0.00822611	1.67E-12	0.000137874	0.176625208
0.097914659	0.00016	0.020430338	4.96E-05	4.96E-10	7.08E-05	4.93E-06
0.612652081	0.43635	0.676347977	0.344971065	0.016431287	0.066402253	0.312735746
0.902044521	0.69041	0.926111781	0.524287162	0.174995165	8.76E-05	0.000302049
0.334519848	0.22793	0.594965921	0.09502869	0.008128533	2.00E-10	0.012334984
0.815155772	0.87186	0.854013752	0.40139584	0.258301737	3.39E-05	0.003340647
0.103435809	0.26071	0.206785567	0.341962793	0.189392542	3.92E-07	0.045682769
0.334510183	0.41861	0.476882131	0.442772402	0.335843098	0.017039746	0.003935432
0.524375805	0.46131	0.677227355	0.25742262	0.115796986	0.045811656	0.601594754
0.738574814	0.57663	0.616928528	0.74758933	0.902048803	0.080676316	0.672302385
0.460629578	0.31615	0.278134111	0.720338986	0.72471494	0.077724663	0.662178217
0.143448356	0.27285	0.292724286	0.26546544	0.09817409	2.33E-05	0.600497593
0.659886506	0.36406	0.722563138	0.281776066	0.092444526	0.000486899	0.932513022
0.910789077	0.90377	0.665926048	0.626904419	0.159559213	0.204974778	2.48E-07
0.130302194	0.055	0.136621408	0.154863803	0.236455287	0.22297049	0.679133935
0.479136509	0.67766	0.979325949	0.728646018	0.47052704	7.87E-05	0.345022554
0.930934975	0.47406	0.97923397	0.492746673	0.268062936	0.002997542	0.002776368
0.655913693	0.44114	0.649979884	0.367344633	0.19247646	0.019651443	0.019215695
0.779862116	0.4735	0.858430102	0.290870638	0.009670074	0.007608483	0.715003965
0.056523858	0.22963	0.160066904	0.261821429	0.033447113	0.844646957	0.360373164
0.691546058	0.23715	0.856418248	0.083048889	0.116607445	0.003316302	0.398022936
0.953376966	0.28293	0.981236025	0.16100011	0.035472763	0.066540002	8.52E-11
0.781556051	0.18086	0.82437514	0.083909749	0.005440609	0.000747564	1.00E-05
0.00231726	0.0019	0.002797856	0.013630679	0.000103421	0.120578979	0.520074256
0.296833155	0.03508	0.084109555	0.074524959	7.97E-07	0.000123515	0.40829228
0.02184392	0.00437	0.045717763	0.00226074	1.37E-06	9.80E-13	7.38E-09
0.012451989	0.05508	0.04412166	0.100311966	0.000481923	0.318074345	0.714279316
0.867182932	0.42662	0.477343453	0.470051261	0.742157492	9.52E-05	0.005919201
0.638991679	0.38931	0.952921054	0.290909376	0.983681924	0.001091969	0.415898592
0.864537173	0.20566	0.943376663	0.034413315	0.044216993	9.44E-06	0.237665905
0.381765786	0.68022	0.498101272	0.911110182	0.366355993	0.003934479	1.26E-06
0.885004024	0.86588	0.960489394	0.624611112	0.694296793	3.64E-06	0.28274594
0.390479646	0.07019	0.115357443	0.182671058	0.001385536	0.719727929	0.001253427
0.428295664	0.86587	0.858094746	0.665234694	0.457021146	0.00039341	0.095719453
0.029931096	0.19016	0.294433281	0.228992203	0.258033685	0.109143171	0.027809127
0.128809174	0.05462	0.165934189	0.076304155	3.48E-08	0.245405514	8.56E-06
0.237546747	0.02119	0.021541574	0.111492617	0.000400051	0.000104781	0.359412943

0.931689147	0.32004	0.713399861	0.336023215	0.974100059	0.005681373	0.561673995
0.39705849	0.45454	0.972368439	0.516512727	0.130860516	0.008043606	0.342459109
0.676052532	0.79023	0.69299105	0.377312798	0.020149123	0.000417829	0.001467122
0.598514719	0.35706	0.976328694	0.053076105	0.135759717	0.058769411	0.10561571
0.994683955	0.33706	0.83396465	0.321423688	0.530613553	0.123915412	0.393441165
0.114802786	0.0405	0.156023017	0.016298125	0.000339126	0.19774177	2.31E-05
0.014866909	0.74868	0.379039849	0.932173152	0.633538642	0.689147604	1.83E-07
0.360091147	0.65557	0.72359177	0.431784732	0.616731605	0.000192245	0.658369636
0.444936006	0.30776	0.597002891	0.176298475	0.012370669	9.31E-05	0.652228714
0.087577146	0.62051	0.623079938	0.49062978	0.043043341	0.265977137	0.019726298
0.235012098	0.57944	0.702581869	0.40775933	0.425800692	1.87E-06	0.007169732
0.378230599	0.64657	0.903797966	0.887696695	0.33325685	1.06E-05	0.026498563
0.410889435	0.02794	0.077609696	0.093189165	0.027920649	2.41E-06	5.91E-06
0.678116978	0.75939	0.94179426	0.581386469	0.845801654	0.001511387	0.437573073
0.644955856	0.53929	0.389852347	0.813285215	0.136129892	0.010778042	0.000541996
0.354099083	0.54041	0.910968004	0.519707246	0.605025393	5.74E-05	8.50E-07
0.893829034	0.80193	0.994345773	0.553238186	0.193715038	0.000684603	0.143433911
0.477491412	0.2607	0.246100587	0.844232445	0.815929815	0.00172071	0.003839608
0.687011785	0.89693	0.843466583	0.82550463	0.355705455	1.78E-06	1.06E-06
0.015719486	0.5983	0.054258368	0.562497536	0.248960013	0.016399432	0.001281879
0.053184647	0.16396	0.098566964	0.334607565	0.001707683	0.793078565	0.063061731
0.612392389	0.86815	0.807922101	0.82501832	0.676117964	1.25E-07	0.045823148
0.846836371	0.04602	0.500021982	0.009365568	0.000166155	7.91E-17	0.001903437
0.002424373	0.02411	0.010521063	0.100375657	0.003701815	8.65E-09	0.636163749
0.782643882	0.31765	0.490220108	0.268407965	2.58E-06	0.982717263	0.112172992
0.731757269	0.75146	0.996922644	0.935101999	0.637874398	0.018603775	0.353091595
0.001101938	0.09846	0.009407905	0.43953551	0.49146502	0.347337459	0.008222289
0.822128083	0.76567	0.678654757	0.983636446	0.046157101	0.673608225	0.744145099
0.282924852	0.23704	0.733748224	0.067047187	0.053049093	0.007065453	0.004521334
0.334696786	0.37693	0.60455516	0.326636555	0.128732411	0.300582035	0.045489756
0.79388956	0.81273	0.97213095	0.703294624	0.058309871	0.141115638	7.99E-10
0.614050069	0.13013	0.27025103	0.113500324	0.42150076	0.42820601	0.227893156
0.31496891	0.46236	0.52796376	0.086410647	0.019865788	0.025059419	0.343478806
0.448542428	0.43718	0.751699296	0.198923226	0.052627017	7.41E-05	0.631694047
0.005175328	0.05312	0.065973736	0.072242146	0.004078206	0.539907491	0.081102221
0.00874612	0.68457	0.193635135	0.803227275	0.023749813	0.00478125	0.271405398
0.928085407	0.2558	0.736036802	0.161834591	0.02026096	0.002825847	0.021798308
0.260584564	0.68773	0.270705688	0.851398183	0.62115448	8.02E-11	0.189318588
0.438688914	0.66392	0.74850907	0.566207362	0.931896936	0.117081328	8.83E-07
0.013418859	0.04428	0.055463162	0.043165784	0.001078325	0.000457942	0.648154146
0.223770134	0.38849	0.883640559	0.397842213	0.197306509	6.28E-06	0.949318435
0.615032128	0.82326	0.781320595	0.482743106	0.505729229	0.000434888	0.022448091
0.958577961	0.47671	0.988618465	0.156040865	0.066223653	0.002550379	0.022542613
0.99699763	0.90265	0.656036638	0.534668983	0.905148061	0.335647692	0.004649745
0.364493645	0.37918	0.293149004	0.597877392	0.252258796	0.095386909	0.797358689

0.819072308	0.15516	0.199567254	0.334731282	0.070320931	0.008341795	0.87197569
0.315697118	0.83011	0.652810645	0.551975858	0.944158338	0.126968869	0.520422394
0.319966739	0.46932	0.620779491	0.260705202	0.37775442	1.72E-06	0.208036358
0.396377759	0.66575	0.986878574	0.743428436	0.169277988	0.860255473	4.78E-05
0.701726051	0.48744	0.590574829	0.601753413	0.313373699	0.000104248	0.004204115
0.685562747	0.52743	0.957637653	0.447728528	0.86392068	1.69E-06	0.185138244
0.28003127	0.95047	0.419540579	0.534278707	0.005580614	7.75E-08	1.69E-05
0.63367516	0.62369	0.926147816	0.502801394	0.000219216	0.050075618	0.48610059
0.245268271	0.33365	0.92496191	0.146284793	0.256965734	0.002992685	0.034353254
0.626763194	0.16403	0.495078725	0.097174765	0.00011135	1.87E-10	0.168419114
0.045724231	0.15419	0.092257202	0.349010894	0.018215552	0.202562788	0.000865642
0.743096331	0.60496	0.71413551	0.893293818	0.652356264	6.01E-06	8.42E-06
0.010634222	0.22451	0.221851107	0.206100321	0.043420352	0.388060254	0.007510871
0.604159066	0.77816	0.954497621	0.758300931	0.170862038	0.022162222	0.772054949
0.93203707	0.06652	0.374113269	0.042474395	2.11E-05	0.13189074	1.59E-05
0.878320768	0.09803	0.67846526	0.023541849	2.83E-06	3.06E-18	0.004740124
0.044763649	0.07298	0.14882519	0.049412802	0.219158436	0.320631403	0.00011798
0.195084877	0.98183	0.08321283	0.094863169	0.033323404	0.002713818	0.335152864
0.975149202	0.43253	0.697620477	0.061847941	0.037141925	0.67404384	0.697119521
0.05419065	0.27156	0.179478981	0.562600563	0.520158352	2.01E-06	0.904762779
0.696073386	0.65306	0.667168695	0.169042322	0.095251032	0.137680965	0.00101211
0.960550749	0.56338	0.805088505	0.52724627	0.163342697	0.006720893	0.418737225
0.027889642	0.29007	0.306246429	0.32164418	0.107063445	0.92500539	0.009878668
0.072038135	0.244	0.435826214	0.207442295	0.007347971	0.063442434	0.138585448
0.40423917	0.29283	0.644617471	0.159499843	0.001986125	6.83E-07	0.051027873
0.021725911	0.30903	0.085782518	0.97814729	0.652950019	0.04188416	9.82E-06
0.256326244	0.24665	0.212995446	0.552975616	0.045589316	9.96E-08	0.016968489
0.549605039	0.9049	0.67889374	0.823565945	0.162154491	9.01E-06	0.003493784
0.007187152	0.10362	0.069532724	0.166338002	0.143805527	0.014090998	0.188699957
0.640550102	0.02315	0.087408808	0.082931703	0.00311391	7.35E-05	0.251755987
0.884809918	0.51609	0.78119204	0.293590166	0.999238187	5.22E-05	0.000853933
0.594889522	0.61894	0.952881409	0.616080988	0.04779439	0.000134168	0.18307423
0.669918238	0.29892	0.716064184	0.148934298	0.001111489	0.000810056	0.134402733
0.298252291	0.96901	0.646941542	0.993762043	0.477209438	3.12E-05	0.096836525
0.298930078	0.41394	0.156959895	0.920895369	0.420643429	0.048526513	0.010473444
0.476303236	0.90051	0.686815291	0.64545341	0.025977568	0.002378985	0.339289059
0.08413336	0.82664	0.652270365	0.843523765	0.957746991	3.65E-07	0.099218149
0.143481333	0.36109	0.419667781	0.465488592	0.074139574	3.46E-05	0.000754345
0.712603249	0.8172	0.869288178	0.580946105	0.113969177	1.76E-06	0.012690718
0.970099485	0.89297	0.79098931	0.884076979	0.790162856	0.211399191	8.27E-09
0.87437764	0.26099	0.794331606	0.143663533	0.053836947	0.919337919	0.008345179
0.723542468	0.18752	0.816298827	0.016437413	0.001533047	2.64E-12	0.017439665
0.037789297	0.00964	0.025093801	0.017922863	6.72E-05	0.083242185	7.97E-06
0.972049185	0.74029	0.888066335	0.610010181	0.978594118	0.280140076	0.412634251
0.930632707	0.75341	0.90592962	0.480945428	0.853714788	0.7387459	0.472745108

0.086704893	0.25219	0.163213692	0.273032129	0.356183602	0.034226398	0.096407564
0.056636233	0.29884	0.62451815	0.059231004	0.01689709	0.044048674	9.60E-13
0.425622163	0.62034	0.285518692	0.750359546	0.636527708	0.033427085	0.202984093
0.62703833	0.21302	0.58599635	0.140594459	0.040634343	0.007531301	0.845030579
0.717788465	0.87886	0.930324509	0.915720537	0.173296947	0.100114927	5.20E-10

p.MMdarkgre	p.MMturq	p.MMlightcya	p.MMgrey60	p.MMlightcya	p.MMmidnigl	p.MMligh
9.76E-06	1.04E-12	0.04173492	0.499455356	0.301869305	0.838244401	0.08092
0.119907103	2.29E-05	0.678587836	0.902519547	0.748704552	0.177543792	2.45E-05
0.414722807	0.00044	0.141189279	0.116693164	0.380160088	0.959856557	0.00694
0.000273153	0.29526	0.624176611	8.10E-05	0.001152242	0.021814555	0.88281
0.167959258	0.12174	0.106710812	0.550888645	0.531310504	0.318586584	0.00353
2.43E-05	0.04382	0.039037502	0.567921263	0.844219992	0.239537551	4.39E-05
0.001177929	3.06E-14	0.012645854	0.545373894	0.001292366	0.922960789	0.10987
0.032939084	4.32E-11	0.155029408	0.399750985	0.010679385	0.912822852	0.00813
6.69E-08	1.49E-14	0.000984462	0.403522593	2.15E-05	0.228784772	5.99E-05
0.007005254	1.69E-06	0.089777446	0.135547636	0.00318237	0.009107486	0.0005
0.000371211	1.24E-11	0.170314363	0.47901984	0.001863305	0.068761106	5.70E-09
0.001764114	2.19E-14	0.000600865	0.80501717	0.000759899	0.369873014	4.71E-06
0.000740743	5.40E-14	0.346640508	0.953124822	0.902847945	0.509496611	0.11021
0.595554456	0.0075	0.000973138	0.041438886	0.283732884	0.166969433	0.62549
0.682331046	0.02035	0.36312991	0.052784262	0.052114272	5.89E-05	9.96E-07
0.247800135	0.13148	0.371370033	0.082029102	0.549387543	0.794099656	1.91E-07
0.032538319	0.15872	0.528878335	0.357487668	0.079902019	0.168407632	0.35243
0.102998092	0.02163	0.56832119	0.01989515	0.073009598	0.015626832	0.00035
0.178523338	1.49E-06	0.066123427	0.825856525	0.168565869	0.06188216	1.32E-06
0.030029562	4.57E-05	0.078800033	3.33E-06	0.000711332	4.30E-06	1.85E-05
1.33E-05	0.02283	0.130659778	0.978732412	0.499372328	0.144333871	0.00042
0.967921492	0.06337	0.640470783	0.192726002	0.580842366	0.184178849	0.00221
0.287503775	0.00024	0.703847978	0.02136657	0.037636069	0.310735367	0.32753
0.005154489	4.80E-07	0.362085979	0.878036573	0.007649526	0.159698948	0.00041
0.359408677	0.00084	0.739751127	0.747600816	0.1966922	0.097182204	1.30E-05
0.91812466	7.33E-05	0.83399529	0.075338464	0.377771127	0.009807642	0.30632
0.000479578	0.14338	0.570144135	0.004359993	0.432111262	0.993550925	3.17E-08
0.001130079	7.38E-08	4.49E-05	2.34E-05	1.43E-14	1.94E-11	6.22E-21
1.94E-07	0.12917	0.391023255	0.392385239	0.105228353	0.04926955	1.75E-11
4.00E-05	2.74E-12	0.017629125	0.006370785	1.33E-07	0.000641842	2.89E-05
0.000223534	0.31355	0.438178877	0.09259042	0.471195115	0.687945608	4.99E-09
4.97E-06	1.59E-14	1.38E-06	0.016729486	3.28E-07	0.010039795	0.00011
1.40E-12	4.26E-30	0.000536571	0.376243158	0.00106745	0.663992064	0.26278
0.404312585	0.14267	0.432162436	0.044022939	0.243465384	0.544982017	0.00344
0.023081894	2.63E-07	0.730955637	0.960678641	0.557289746	0.490320294	0.1194
0.002956584	3.87E-05	0.010140858	0.000702659	0.044429541	0.003147358	0.25348
0.683985218	0.00855	0.052677555	0.043533138	3.24E-05	0.01874104	0.00039
0.000603173	1.21E-08	0.018233831	0.488466333	0.220716334	0.085640134	0.27388
6.07E-05	2.51E-10	0.585533819	0.213685642	0.04101477	0.048849286	0.02106
0.001131412	0.3561	0.566738767	0.005554894	0.012030997	0.754441965	0.0001
0.489987967	0.0002	0.018975902	0.000520541	0.000928271	4.76E-05	2.40E-05
3.81E-06	0.04968	0.544928998	0.236545749	0.476620701	0.015240923	1.37E-18
0.00931254	2.45E-07	0.178744032	0.000580978	7.43E-07	0.005857244	0.00176
0.013115824	1.51E-07	0.13335706	0.037242446	0.009452514	0.007116909	0.01479

0.032399984	1.36E-06	0.197551497	0.437765168	0.226702671	0.076013947	0.00736
0.000651323	0.03164	0.017288614	0.050602066	0.325747566	0.000471891	0.00113
0.000169042	4.89E-12	0.001857378	0.315411152	0.000326916	0.415709083	0.00059
0.000344941	0.00256	0.034012292	0.144211021	0.139952183	0.062689271	1.11E-05
0.063002601	0.33212	0.055254133	2.95E-08	6.83E-06	0.091771875	0.01205
0.00680235	0.35798	0.009882891	0.235998808	0.005772792	0.34511948	2.16E-06
7.86E-06	0.00018	0.201659825	0.000167846	0.004855072	3.27E-09	1.92E-14
0.143497027	2.43E-06	0.065214654	0.763810837	0.869483618	0.594445137	0.05805
0.147628109	2.46E-05	0.718049016	0.639652619	0.001884449	0.01980728	9.72E-14
0.029548101	5.48E-05	0.09466516	0.152722041	0.048331787	0.011293627	3.02E-07
0.000587859	6.16E-08	0.49320619	0.824028108	0.228995756	0.447098329	0.90648
0.091903117	4.23E-05	0.128967148	0.080445172	0.574691172	0.022154762	0.00588
7.45E-05	2.42E-14	0.347636274	0.265798708	0.770776152	0.241795452	0.4338
0.494681272	1.31E-05	0.750948183	0.039081505	0.037774198	0.055605601	0.02266
0.008703679	3.68E-07	0.064296325	0.478737171	0.20227062	0.243192732	0.60952
0.000278224	3.66E-14	0.293483823	0.097288876	0.673842061	0.434149135	3.42E-06
0.056053422	1.79E-08	0.053650221	0.304694295	0.03273703	0.023610449	0.00041
9.51E-09	2.96E-07	0.448561579	0.74978608	0.902539943	0.35853532	1.27E-06
1.37E-06	8.02E-10	0.126491484	0.004949133	0.181004289	8.13E-06	0.00016
0.003777553	1.96E-08	0.831161563	0.919899178	0.000431929	0.028839606	7.89E-09
0.71264547	0.93845	0.000989276	0.026049141	0.018824141	0.000907539	2.77E-05
0.003060057	7.42E-17	0.854018434	0.024237647	0.396616336	0.070114175	0.01102
0.00080075	8.26E-18	0.002795289	0.631379633	0.000124788	0.759285427	0.06031
0.264856733	5.86E-10	0.001443173	0.000170253	0.16309651	0.053525841	3.83E-08
0.000120137	0.40778	0.309422436	0.000400635	0.098963634	0.000146629	0.0031
0.611302137	0.00269	0.432123364	0.898851136	0.22019854	0.135130592	3.79E-05
0.639296777	0.05876	1.72E-21	0.030399282	0.982264488	0.854256646	6.14E-05
0.185540094	0.24474	0.379175251	0.705302718	0.367706537	0.97804813	6.28E-11
0.00128535	0.77196	0.835356185	0.373219083	0.183190194	0.278488387	1.32E-11
0.011241535	0.51192	0.06910529	0.222052701	0.017091561	0.000329103	1.68E-11
8.39E-09	3.66E-15	0.585642554	0.571263531	0.045410583	0.5064169	3.00E-05
0.701639873	0.07766	0.930637535	0.015338277	0.092311655	0.57502603	5.06E-07
0.11622653	0.001	0.01030063	0.212438061	1.45E-05	0.000223644	5.25E-19
0.846562431	0.00011	0.132442119	0.958731474	0.040189914	0.099760741	5.00E-10
0.009148242	0.79032	0.354162513	0.307922815	0.753304313	0.449620984	1.27E-10
0.610033269	0.00436	0.594957149	0.482610651	0.013576206	0.185121778	8.58E-07
0.10630065	1.25E-07	0.337851599	0.771309657	0.000152644	0.020578524	6.71E-12
0.384317182	2.18E-08	0.115828372	0.18546804	0.92258288	0.96088149	0.00158
1.87E-06	2.82E-06	0.72788082	0.808977119	0.941554476	0.01399022	3.14E-07
0.82317703	0.0005	0.001163574	0.009380085	0.063761072	0.000543548	0.14394
4.69E-06	7.59E-14	0.005780489	0.00582961	0.066313448	0.004350561	0.74892
0.666489597	0.13011	0.027812236	0.012984322	0.000112258	1.05E-06	4.40E-08
0.321629156	2.64E-06	0.073730886	0.346534719	0.206900118	0.556440694	2.25E-06
0.011461446	0.04556	0.507506525	0.166237899	0.094271838	0.001771712	3.97E-05
0.773115183	0.00617	0.279639657	0.187659105	0.58321531	0.006593335	0.77447

0.519480497	0.00046	0.364475307	0.03250064	0.524713178	0.011655182	0.03571
0.004610593	0.74074	0.029026219	0.004817803	0.001679472	0.321912537	0.13313
0.024453775	7.87E-12	0.147446818	0.028874651	0.326337864	0.161630336	0.36606
3.33E-11	4.49E-09	0.094962044	0.383540959	0.720522942	0.001647446	1.04E-06
0.722667183	5.25E-07	0.444017776	0.004645388	0.089571374	0.009170788	0.9418
0.032844048	1.30E-08	0.025748403	0.286692645	0.284441342	0.053991813	0.08793
0.002477797	1.98E-12	0.016561057	0.928766913	0.002370361	0.850809489	0.00323
0.179307715	0.02344	0.562389633	0.741751411	0.790488532	0.047983827	0.00077
0.187849801	1.67E-05	0.129408323	0.319445336	0.095658942	0.78718796	0.14387
0.001186414	9.31E-16	0.000220947	0.491613436	5.20E-05	0.210589038	9.70E-09
8.28E-05	5.66E-08	0.008945879	0.019623102	0.00324022	0.000130338	0.00366
5.41E-07	5.68E-19	0.018172296	0.293412453	0.007347896	0.46895279	0.1152
0.000289709	0.51162	0.314142048	0.052710065	0.176584435	0.924808052	3.28E-11
0.959291318	0.00037	0.582065378	0.116212322	0.871071809	0.730044583	0.00098
0.04010997	0.00195	0.019060086	0.000526335	6.30E-07	0.010067441	0.24165
9.14E-07	2.42E-29	0.011553702	0.879540943	8.02E-05	0.79656084	6.66E-05
9.51E-10	2.29E-16	0.009372411	0.080187539	0.099613216	0.262205269	0.32838
0.057304887	0.01929	0.775626509	0.025356619	0.455178402	0.080387193	3.15E-07
0.00289683	0.89995	0.402698959	0.116196856	0.439694604	0.729852452	1.14E-09
0.032309942	2.95E-06	0.059189994	0.720074826	0.39544798	0.019077358	0.51772
0.011121833	0.98204	0.607560336	0.266007743	0.048176367	0.045422801	3.42E-10
0.891562049	0.00066	0.135150401	0.633277785	0.258366624	0.196957142	1.28E-05
0.289314278	0.74698	0.04074744	0.01455487	0.000300295	2.45E-07	1.43E-11
0.066754608	0.0001	0.216643563	0.204261612	0.000917944	0.001572079	2.20E-10
0.118121499	2.22E-09	0.015596612	0.602865995	0.000738556	0.125082455	1.53E-08
0.117186809	1.83E-06	0.619021609	0.704610031	0.114797872	0.880719356	1.89E-05
0.066505473	7.11E-14	0.189699125	0.194546953	0.436286948	0.025398816	0.07252
0.211182954	3.53E-08	0.058627638	0.969969732	0.879038733	0.459379439	0.35599
0.343528524	0.00104	0.123025873	0.478631216	0.025160883	0.299113155	0.60197
0.016251658	4.11E-07	0.033157182	0.65739944	0.893601368	0.333274462	0.76526
2.41E-08	6.81E-13	0.000751428	1.08E-05	4.98E-05	1.22E-05	0.88027
0.148526008	5.00E-06	0.528561853	0.423013563	0.89754923	0.022037598	0.02055
0.643092461	9.43E-07	0.154020064	0.563588447	0.013471188	0.561266962	3.04E-12
0.034773905	2.00E-10	0.313144259	0.344405211	0.038815401	0.020771342	0.35287
0.032341357	4.52E-07	0.948040496	0.747610319	0.003512509	0.034064157	1.15E-09
0.464766227	9.10E-05	0.070378288	0.076528716	0.000123058	0.012981646	1.91E-05
0.00212467	3.65E-14	0.594531142	0.003235753	0.093385411	0.22242781	0.38015
0.001067447	5.80E-14	0.424876449	0.2404253	0.753842802	0.527826842	0.07443
7.98E-07	4.83E-20	0.071291464	0.035174029	0.000171695	0.053718868	0.00863
3.25E-09	1.58E-05	0.928994294	0.61584241	0.351846947	0.049229698	5.03E-10
7.07E-06	0.01995	0.639316418	0.284070195	0.142497871	0.14292624	6.59E-09
0.033873626	4.25E-14	0.079228475	0.119414446	0.018185109	0.612863062	3.76E-06
0.118825908	0.113	0.060777787	0.739594659	0.423461741	0.930698162	0.00115
0.253344772	0.78264	0.608931342	0.973682851	0.679794818	0.188344383	0.00011
0.006817402	0.03238	0.543305024	0.867836972	0.385221275	0.93753574	0.00149

0.002339684	0.87388	0.510630852	0.005025606	0.145544184	0.374449157	6.14E-07
1.45E-08	1.02E-06	0.228312264	0.682966658	0.9023433	0.896768546	2.78E-09
0.742184416	0.00033	0.530938905	0.233883371	0.153748006	0.530731259	8.56E-11
0.366935999	0.00547	0.770699648	0.097705259	0.838325197	0.341529638	0.00011
1.83E-09	8.12E-13	0.089730076	0.005682522	0.213186	0.000277661	6.48E-07

p.MMplum2	p.MMgrey
0.403242374	3.29E-13
0.193593842	6.80E-16
0.002973652	3.28E-18
1.78E-11	1.62E-24
0.557656911	7.71E-24
0.82184764	5.99E-24
0.621390893	1.57E-07
0.045988682	2.26E-13
0.613547308	8.06E-26
0.92575632	1.27E-19
0.086031625	3.82E-31
0.961847673	7.96E-30
0.002317518	1.15E-29
0.000171516	5.92E-16
0.192195175	8.10E-38
0.769852519	1.13E-22
0.000493961	2.06E-21
0.013401248	2.18E-27
0.60457092	1.10E-51
0.002774523	7.45E-13
0.260924492	5.64E-13
0.991555566	2.93E-11
0.000983193	8.22E-44
0.923716725	3.28E-12
0.815509936	1.04E-22
1.18E-06	3.06E-22
0.367023048	4.58E-56
8.16E-05	1.48E-40
0.23315686	4.70E-29
0.35265561	5.82E-16
0.874811027	4.41E-38
0.514930384	4.34E-22
0.01693103	2.23E-21
0.011654382	5.65E-23
0.261370612	7.57E-11
0.167468587	1.39E-11
0.469045594	2.69E-06
0.607881807	1.29E-18
0.382045467	2.26E-11
0.787993292	4.25E-27
0.538734089	4.83E-30
0.002173218	7.80E-58
0.502139539	4.65E-20
0.716681216	1.37E-19

0.78437303	6.08E-31
0.974609001	2.10E-31
0.677074997	4.87E-13
0.450024496	2.46E-19
0.511693145	5.10E-28
0.990812141	2.86E-12
9.68E-07	1.77E-40
0.978096529	4.38E-15
0.017122167	2.65E-36
0.052616291	5.47E-42
0.928528691	8.22E-07
0.424286519	4.35E-25
0.774564821	6.44E-23
0.076633699	8.75E-23
0.006101036	6.26E-08
0.552583811	9.04E-47
0.895214006	3.58E-42
0.383276823	1.56E-24
0.343176918	2.41E-14
0.06901095	1.29E-17
0.139943713	2.61E-30
0.019314069	2.52E-37
0.063174711	2.97E-12
0.22196772	2.27E-91
4.12E-06	1.79E-29
0.967166877	7.66E-31
0.049292996	2.21E-06
0.6873756	7.43E-38
0.995955491	3.10E-35
0.167498854	2.58E-48
0.428077524	1.91E-24
0.640953787	1.47E-45
0.000257737	9.56E-37
0.686362383	1.12E-62
0.739923056	8.45E-69
0.337642278	1.27E-08
0.044572423	2.49E-27
0.122669178	1.56E-37
0.312775545	2.78E-21
0.86578285	4.60E-39
0.997284578	4.37E-19
0.001602811	9.53E-11
0.1623276	2.37E-44
0.453302983	5.39E-09
0.013094271	0.00017

0.00232692	2.41E-19
0.308207663	5.55E-20
0.000780172	8.82E-25
0.573902451	1.00E-34
1.51E-12	2.06E-41
0.058594066	1.03E-26
0.080903904	1.60E-09
0.013068331	7.51E-17
0.024730415	9.35E-08
0.67777227	3.40E-44
0.01148658	2.84E-25
0.004432804	1.45E-25
0.757854111	2.71E-76
0.468405955	4.86E-22
0.204152238	0.00011
0.040410936	3.07E-24
0.644993063	1.01E-26
0.006265829	2.06E-25
0.388525105	7.23E-26
0.359080639	2.32E-14
0.954307079	5.68E-39
0.618910873	1.39E-28
1.58E-05	1.23E-37
0.011234348	1.13E-44
0.630151259	2.88E-55
0.331137119	4.18E-17
0.027933618	3.52E-36
0.000675374	2.21E-18
0.047328039	1.05E-34
0.971882356	5.13E-17
0.12351996	4.87E-08
0.297310006	9.43E-31
0.259586342	4.24E-46
1.34E-09	1.88E-32
0.001622697	4.18E-18
0.459781426	2.56E-19
0.01585496	4.70E-43
0.01302258	4.05E-16
0.845396739	1.36E-17
0.034686436	1.41E-40
0.073710293	3.16E-17
0.074618942	3.24E-35
0.267269781	3.94E-28
0.450372055	1.71E-19
0.075950761	1.74E-05

0.263207333	3.55E-58
0.152993757	3.40E-18
0.290401636	4.15E-39
0.370810337	1.81E-20
0.418183334	1.49E-09
