# Supplementary material for: Comprehensive Analysis of GDF10 Methylation Site-Associated Genes as Prognostic Markers for Endometrial Cancer
Source: J Oncol. 2022 Oct 10;2022:7117083. doi: 10.1155/2022/7117083 (PMC9576415; doi:10.1155/2022/7117083)
Supplement: Supplementary Materials — are shown as Supplemental Figures S1-3 and Supplemental Tables S1-7. Supplemental Figure S1: Cox regression independent prognostic analyses. Supplemental Figure S2: Analysis of expression correlations between the risk model and clinicopathological factors. Supplemental Figure S3: Kaplan-Meier survival analysis of patients with different clinicopathologic features between high- and low-risk groups. (a–i) The different OS of UCEC patients with different ages, tumor stages, and grades between high-risk and low-risk groups according to GDF10 methylation. The abscissa shows survival months, and the vertical axis shows overall survival probability. Supplemental Table S1: 48 modules were identified. Supplemental Table S2: The saddlebrown module positively correlated with the methylation sites the most. Supplemental Table S3: 991 genes were upregulated in DEGs. Supplemental Table S4: 1260 genes were downregulated in DEGs. Supplemental Table S5: 44 DEGMRGs were identified. Supplemental Table S6: All UCEC samples in the training set were divided into the high- and low-risk groups. Supplemental Table S7: Risk scores were significantly different between patients classified by age, tumor stage, and grade. [file 7117083.f1.zip › Supplemental Table S3.pdf]

sym	logFC	AveExpr	P.Value	adj.P.Val	direction
SFN	4.44651	6.24188	2.27E-41	7.39E-40	up
PRAME	4.18655	5.61342	8.52E-69	1.06E-66	up
MYBL2	4.10161	4.7251	3.03E-74	4.77E-72	up
UBE2C	4.06901	5.41418	9.49E-70	1.24E-67	up
CDC20	3.91155	5.05006	3.51E-78	7.35E-76	up
AQP5	3.41182	5.03457	3.88E-18	3.59E-17	up
PRSS8	3.40318	6.14769	1.96E-49	9.81E-48	up
TK1	3.38706	5.33633	1.06E-75	1.87E-73	up
PI3	3.36068	4.10456	8.23E-14	5.48E-13	up
TPX2	3.31175	4.23489	4.95E-59	3.98E-57	up
ASF1B	3.20888	4.18118	5.83E-72	8.45E-70	up
KRT8	3.20528	7.66641	8.97E-51	4.81E-49	up
SDC1	3.17532	5.307	4.89E-47	2.08E-45	up
EPCAM	3.16581	7.45711	5.92E-52	3.34E-50	up
WFDC2	3.15698	9.73168	4.79E-16	3.82E-15	up
BIRC5	3.10852	3.71717	1.53E-56	1.09E-54	up
AURKB	3.03605	3.65929	1.93E-61	1.73E-59	up
SPP1	3.00643	6.04265	1.27E-23	1.69E-22	up
TOP2A	3.00191	3.95452	8.74E-49	4.14E-47	up
ST14	2.98827	6.29176	7.77E-55	5.07E-53	up
PTTG1	2.98583	4.19463	2.91E-53	1.74E-51	up
ZWINT	2.97727	4.32032	6.93E-65	7.19E-63	up
CLDN3	2.94916	7.67434	6.05E-25	8.69E-24	up
LAD1	2.899	5.27901	7.31E-36	1.86E-34	up
KIFC1	2.89238	3.86948	3.54E-55	2.34E-53	up
SLPI	2.88075	9.27341	2.69E-12	1.58E-11	up
TMPRSS4	2.82778	3.33229	3.18E-31	6.53E-30	up
CLDN7	2.81913	5.72585	7.45E-46	2.98E-44	up
SERINC2	2.81784	6.43638	1.48E-28	2.62E-27	up
CLDN4	2.79929	6.10603	3.35E-34	7.82E-33	up
SPINT1	2.79187	6.18886	6.47E-48	2.93E-46	up
CCNB1	2.78003	4.78748	2.48E-58	1.93E-56	up
CKS2	2.77704	6.0979	1.29E-62	1.23E-60	up
CCNB2	2.7712	3.58735	1.61E-61	1.45E-59	up
S100A9	2.77024	5.44912	6.06E-12	3.45E-11	up
KRT18	2.74907	7.74876	5.93E-41	1.90E-39	up
AP1M2	2.74183	5.87663	4.38E-49	2.12E-47	up
RRM2	2.72241	3.26474	3.34E-46	1.36E-44	up
DLX5	2.71569	5.31716	6.32E-14	4.23E-13	up
S100A2	2.69264	3.58741	3.98E-18	3.67E-17	up
UBE2T	2.68859	4.37632	2.32E-57	1.75E-55	up
CDCA8	2.67642	3.47098	4.28E-53	2.53E-51	up
CD24	2.67434	7.91332	1.11E-35	2.80E-34	up
GDF15	2.65876	4.78714	2.74E-21	3.17E-20	up

TACSTD2	2.65566	6.66791	3.88E-21	4.45E-20	up
RAB25	2.64885	5.95797	5.81E-28	1.00E-26	up
SAPCD2	2.6398	3.33606	3.30E-55	2.19E-53	up
LCN2	2.63873	6.05471	6.23E-10	2.97E-09	up
FOXM1	2.63045	3.16385	3.68E-44	1.34E-42	up
ISG15	2.61797	7.02444	8.08E-19	7.80E-18	up
NUSAP1	2.61449	3.83598	5.23E-48	2.39E-46	up
SCD	2.60548	5.33216	7.39E-38	2.09E-36	up
TROAP	2.60145	2.9989	2.96E-52	1.69E-50	up
HN1	2.59963	5.27592	1.50E-71	2.16E-69	up
CTSV	2.59757	3.23973	1.37E-19	1.40E-18	up
KIF20A	2.58032	3.15681	1.18E-51	6.59E-50	up
SMPDL3B	2.57825	4.35456	8.84E-48	3.97E-46	up
CEP55	2.57602	3.08091	2.49E-48	1.16E-46	up
HOXB9	2.57465	3.41064	5.23E-14	3.53E-13	up
SPC24	2.57173	3.27388	8.57E-47	3.59E-45	up
CCNE1	2.57119	3.27565	6.65E-30	1.27E-28	up
MKI67	2.52911	3.00672	5.51E-51	3.03E-49	up
KIF2C	2.52598	3.23755	1.28E-44	4.86E-43	up
PLK1	2.51949	3.1279	5.94E-50	3.05E-48	up
TACC3	2.51908	3.77283	1.23E-54	7.93E-53	up
KRT7	2.50585	5.54465	3.53E-13	2.22E-12	up
SLC7A5	2.50388	4.48682	8.22E-29	1.48E-27	up
C4orf48	2.50376	4.68866	1.38E-24	1.94E-23	up
SBK1	2.50242	3.6832	3.90E-37	1.06E-35	up
TFF3	2.4701	5.90644	3.03E-05	8.66E-05	up
MT1G	2.46656	3.60727	2.62E-14	1.81E-13	up
GYLTL1B	2.46301	4.61635	9.86E-32	2.06E-30	up
PBK	2.45553	2.87509	2.94E-37	8.01E-36	up
MELK	2.4544	2.80882	5.11E-48	2.34E-46	up
KIAA010	2.43822	3.00454	1.67E-43	5.90E-42	up
CDCA5	2.43759	3.14984	5.96E-43	2.07E-41	up
ELF3	2.42539	5.09571	2.48E-33	5.56E-32	up
LMNB1	2.41909	4.43666	5.45E-44	1.97E-42	up
KPNA2	2.41722	5.35669	8.41E-47	3.53E-45	up
CDT1	2.41575	3.24278	2.16E-48	1.01E-46	up
NEK2	2.41039	2.84192	1.74E-47	7.66E-46	up
CENPM	2.40605	3.66744	1.92E-47	8.45E-46	up
HIST1H2	2.40255	2.83301	1.53E-18	1.45E-17	up
PVRL4	2.40224	3.44677	5.26E-34	1.21E-32	up
TMEM13	2.39833	4.77149	2.03E-45	7.96E-44	up
PIGR	2.38231	5.64638	3.20E-05	9.14E-05	up
MMP9	2.38162	3.44293	2.78E-15	2.08E-14	up
SAA1	2.37753	3.60535	1.08E-08	4.57E-08	up
LEFTY1	2.3762	2.62928	1.33E-08	5.53E-08	up

CENPF	2.37076	2.95734	3.45E-41	1.11E-39	up
POC1A	2.34082	3.22818	3.29E-58	2.55E-56	up
FAM83D	2.34073	2.82744	6.52E-35	1.58E-33	up
FAM83H	2.34024	4.44326	2.85E-55	1.91E-53	up
ESPN	2.339	3.71524	7.46E-21	8.41E-20	up
HMGA1	2.33789	6.69344	1.56E-28	2.77E-27	up
AOC1	2.33594	2.65896	4.72E-09	2.07E-08	up
PKP3	2.33391	4.43627	5.91E-36	1.51E-34	up
HJURP	2.33261	2.67064	3.14E-49	1.54E-47	up
CBLC	2.33162	4.1439	1.48E-30	2.93E-29	up
CDC45	2.29906	2.63212	5.43E-50	2.80E-48	up
HES6	2.29753	4.51175	1.07E-21	1.27E-20	up
RDH10	2.29278	4.22018	6.06E-20	6.36E-19	up
HGD	2.28909	3.46507	6.51E-15	4.72E-14	up
CDKN2A	2.28828	2.74697	2.31E-14	1.60E-13	up
CDH1	2.28535	5.48256	2.25E-31	4.66E-30	up
VTCN1	2.27902	4.43969	4.73E-12	2.73E-11	up
NCAPH	2.2765	2.6596	2.23E-47	9.75E-46	up
CDK1	2.27133	3.49103	5.71E-37	1.53E-35	up
DLGAP5	2.26654	2.5753	1.91E-38	5.53E-37	up
CXCL17	2.26375	3.87388	3.08E-07	1.11E-06	up
ALDH3B	2.25503	3.49199	3.13E-16	2.53E-15	up
SCGB2A	2.25063	7.67599	2.31E-05	6.72E-05	up
MISP	2.24751	3.90199	3.13E-18	2.90E-17	up
PRR15L	2.24736	4.17111	2.07E-22	2.57E-21	up
PYCR1	2.24443	4.63084	1.84E-34	4.38E-33	up
JSRP1	2.24	2.5494	3.82E-16	3.07E-15	up
NUP210	2.23791	3.94329	1.47E-48	6.93E-47	up
PSAT1	2.22591	4.92556	3.05E-20	3.28E-19	up
SPAG5	2.22485	2.98421	2.85E-49	1.41E-47	up
UCP2	2.22377	6.66866	3.81E-30	7.35E-29	up
PAFAH1I	2.22232	5.85477	1.55E-48	7.30E-47	up
SPINT2	2.22097	6.82134	6.50E-40	2.00E-38	up
MUC1	2.22036	6.42018	8.01E-15	5.76E-14	up
E2F1	2.21731	3.33361	3.51E-32	7.50E-31	up
TFAP2A	2.20639	2.27879	7.27E-27	1.18E-25	up
RECQL4	2.20507	3.39657	3.82E-42	1.29E-40	up
PAX8	2.19436	5.41355	7.66E-18	6.91E-17	up
ESRP1	2.19203	4.19351	5.19E-37	1.40E-35	up
AURKA	2.18485	2.91283	6.28E-39	1.85E-37	up
PKMYT1	2.18199	2.42506	2.26E-47	9.88E-46	up
PODXL2	2.17858	5.10057	1.99E-28	3.50E-27	up
PHLDA2	2.17397	3.61326	1.03E-18	9.83E-18	up
CENPA	2.17352	2.4477	9.37E-41	2.96E-39	up
CCNA2	2.17248	3.2021	4.68E-37	1.27E-35	up

IQGAP3	2.17136	2.48596	1.26E-43	4.49E-42	up
TM7SF2	2.16667	4.56062	1.68E-32	3.62E-31	up
RHPN1	2.16483	4.0825	1.10E-39	3.36E-38	up
NMU	2.15059	2.42741	2.44E-19	2.45E-18	up
KRT17	2.14522	4.11654	1.11E-08	4.67E-08	up
TJP3	2.14318	4.2042	5.32E-25	7.66E-24	up
S100A11	2.14054	9.91647	7.79E-34	1.77E-32	up
KIF1A	2.13656	2.29999	8.94E-13	5.46E-12	up
BUB1	2.12847	2.47517	1.18E-40	3.71E-39	up
PRR36	2.12269	3.42943	2.17E-29	4.01E-28	up
RAC3	2.11082	3.47621	4.64E-29	8.46E-28	up
DBNDD1	2.10411	3.46582	3.32E-41	1.08E-39	up
GLYATL	2.09704	2.35636	1.19E-08	5.01E-08	up
KIF11	2.09623	2.91726	1.87E-37	5.14E-36	up
KIF4A	2.09261	2.58918	2.27E-43	8.03E-42	up
VAV3	2.09004	3.01529	5.04E-24	6.86E-23	up
BUB1B	2.07931	2.51711	6.94E-38	1.97E-36	up
BSPRY	2.07881	3.59619	7.61E-36	1.94E-34	up
DSP	2.07783	4.68869	6.77E-32	1.43E-30	up
ABRACL	2.07005	5.4119	6.31E-41	2.01E-39	up
PITX1	2.06996	2.23746	3.90E-16	3.13E-15	up
KRT5	2.06952	2.80873	2.41E-07	8.79E-07	up
HMGB3	2.06446	4.63668	8.96E-51	4.81E-49	up
MAL2	2.06241	5.02376	3.02E-24	4.17E-23	up
B3GNT3	2.06172	2.72271	1.06E-11	5.90E-11	up
MEX3A	2.06051	3.79364	1.59E-28	2.80E-27	up
RHOV	2.05706	2.97051	6.99E-20	7.30E-19	up
TRAF4	2.05489	4.74078	9.69E-63	9.32E-61	up
TLCD1	2.05405	3.55863	4.04E-41	1.29E-39	up
KDF1	2.0442	3.98484	8.01E-46	3.20E-44	up
CCNO	2.04354	3.6033	5.01E-15	3.67E-14	up
FGF18	2.03856	3.47912	1.07E-14	7.61E-14	up
GGH	2.03613	3.4269	1.60E-25	2.39E-24	up
LSR	2.02602	6.38734	4.17E-43	1.46E-41	up
KIF18B	2.02559	2.25898	1.21E-40	3.78E-39	up
CRB3	2.0168	3.94758	1.07E-45	4.24E-44	up
CENPU	2.00987	2.75376	2.34E-38	6.73E-37	up
C16orf59	2.00944	2.54067	1.27E-51	7.11E-50	up
GTSE1	2.00384	2.33407	5.08E-42	1.70E-40	up
UPK1B	2.00281	2.85793	9.75E-07	3.33E-06	up
CDC6	2.00152	2.4588	4.23E-44	1.54E-42	up
CAPN13	1.99817	2.34814	1.00E-11	5.60E-11	up
CDKN3	1.99791	2.68812	2.13E-35	5.32E-34	up
ALPPL2	1.99644	2.50402	2.54E-06	8.28E-06	up
IDH2	1.98923	6.43829	2.13E-37	5.86E-36	up

SKA3	1.98271	2.33813	2.62E-48	1.22E-46	up
VAX2	1.98119	2.53176	7.51E-21	8.46E-20	up
SPDEF	1.97731	4.83815	3.45E-06	1.11E-05	up
HK2	1.97683	3.60214	8.26E-34	1.88E-32	up
STC2	1.9707	2.9522	4.55E-21	5.20E-20	up
HOXB8	1.96235	4.11837	3.74E-09	1.66E-08	up
MYH14	1.96105	4.20294	5.59E-19	5.47E-18	up
TMPRSS	1.95895	2.28772	6.19E-20	6.48E-19	up
LLGL2	1.95579	4.40118	8.56E-39	2.51E-37	up
TMEM12	1.95528	4.14989	1.64E-30	3.24E-29	up
SMIM22	1.95456	4.50653	1.20E-10	6.09E-10	up
ESRP2	1.95426	3.19697	9.47E-47	3.96E-45	up
GRHL2	1.95287	3.55135	6.45E-33	1.42E-31	up
C1orf210	1.94966	3.48884	2.10E-35	5.25E-34	up
MAL	1.94966	3.24838	2.96E-06	9.57E-06	up
CRACR2	1.94237	3.7099	2.19E-20	2.38E-19	up
KRTCAP	1.94049	4.8144	3.93E-21	4.50E-20	up
SCNN1A	1.93775	5.1023	1.02E-18	9.73E-18	up
IDO1	1.93711	3.49847	2.04E-11	1.12E-10	up
CA9	1.93665	2.63992	7.26E-11	3.78E-10	up
ECT2	1.93632	3.1366	1.00E-26	1.61E-25	up
H2AFX	1.93509	5.98255	2.12E-41	6.92E-40	up
MSX1	1.93018	6.32895	3.31E-05	9.44E-05	up
HIST1H2	1.92991	3.17949	1.99E-17	1.74E-16	up
SEL1L3	1.92418	4.05363	1.96E-33	4.40E-32	up
FXYD3	1.92384	4.07522	1.13E-10	5.76E-10	up
UHRF1	1.9229	2.2322	1.67E-34	3.98E-33	up
MT1F	1.89177	3.78102	1.39E-19	1.42E-18	up
IFI6	1.88971	7.90971	1.84E-08	7.55E-08	up
ABHD11	1.88934	5.25933	9.98E-39	2.92E-37	up
S100A8	1.88729	3.06138	3.65E-07	1.31E-06	up
CYBA	1.88619	6.15299	5.92E-25	8.50E-24	up
CDCP1	1.88277	3.5847	8.23E-31	1.66E-29	up
FEN1	1.87685	4.32167	2.17E-39	6.51E-38	up
VAMP8	1.87679	6.76009	5.19E-40	1.60E-38	up
IL4I1	1.87252	3.85051	5.53E-12	3.17E-11	up
FAM110	1.87104	3.41368	2.38E-54	1.53E-52	up
TYMS	1.86776	3.54116	8.77E-27	1.42E-25	up
ANLN	1.8649	2.36334	7.34E-30	1.40E-28	up
DLX6	1.86374	3.16941	3.86E-15	2.86E-14	up
KRT23	1.86105	3.6973	2.91E-10	1.43E-09	up
OCIAD2	1.85572	5.34588	2.81E-34	6.60E-33	up
PRC1	1.84957	3.01831	1.27E-34	3.05E-33	up
NUF2	1.84953	2.19307	1.02E-29	1.93E-28	up
COL26A1	1.84463	2.4135	1.66E-09	7.61E-09	up

HIST1H3	1.84298	2.42226	5.76E-24	7.80E-23	up
HIST1H1	1.84253	5.74594	2.72E-11	1.47E-10	up
TUBA1C	1.83673	4.38468	2.55E-34	6.02E-33	up
IGSF9	1.83227	2.98662	1.70E-19	1.73E-18	up
C1orf186	1.83175	4.48637	3.24E-08	1.30E-07	up
CRABP1	1.82809	2.02177	2.40E-06	7.85E-06	up
EZH2	1.82714	2.69756	1.43E-45	5.64E-44	up
SKA1	1.81787	2.09057	4.04E-41	1.29E-39	up
ALPL	1.8132	4.74742	6.29E-09	2.72E-08	up
TTK	1.81297	2.12285	8.89E-31	1.78E-29	up
SDF2L1	1.81187	5.71469	2.09E-26	3.30E-25	up
SULT2B1	1.81151	2.14641	9.13E-14	6.06E-13	up
SPC25	1.803	2.14895	1.35E-35	3.41E-34	up
CCDC167	1.80122	5.48365	3.47E-49	1.69E-47	up
FOXJ1	1.79972	5.11473	5.12E-08	2.01E-07	up
EIF4EBP1	1.79968	5.89804	4.32E-25	6.26E-24	up
CDCA3	1.79851	2.23295	1.46E-40	4.55E-39	up
NCAPG	1.79619	2.06777	3.35E-34	7.82E-33	up
FAM64A	1.79475	2.18339	2.39E-29	4.39E-28	up
SERPINA	1.7833	4.96132	2.16E-06	7.10E-06	up
STXBP2	1.78305	3.69094	1.10E-44	4.22E-43	up
TNFRSF1	1.78172	2.61731	2.39E-12	1.41E-11	up
TNNC1	1.78105	2.97708	2.48E-09	1.12E-08	up
GALE	1.78082	4.08968	6.31E-40	1.94E-38	up
TMEM8A	1.77916	5.10484	8.43E-42	2.79E-40	up
PGD	1.77487	6.15204	4.30E-42	1.44E-40	up
MND1	1.77026	2.20367	4.03E-31	8.20E-30	up
TSTA3	1.76848	5.2675	2.27E-46	9.30E-45	up
FOLR1	1.76831	4.70481	6.98E-06	2.16E-05	up
ANPEP	1.76066	2.86314	7.49E-08	2.89E-07	up
SPTBN2	1.75967	3.16606	2.54E-25	3.73E-24	up
BIK	1.75632	3.35043	2.22E-18	2.08E-17	up
HIST1H3	1.75582	2.22285	1.00E-20	1.12E-19	up
HAPLN3	1.75514	4.09247	3.16E-15	2.36E-14	up
MFSD3	1.75346	4.22317	4.59E-33	1.02E-31	up
NQO1	1.74388	4.30775	1.44E-08	5.96E-08	up
RNF183	1.74326	2.3989	2.24E-14	1.56E-13	up
GINS1	1.74222	2.58973	9.32E-33	2.03E-31	up
HIST1H2	1.73686	4.5729	1.32E-19	1.36E-18	up
RIPK4	1.73128	3.40511	1.24E-24	1.74E-23	up
SLC16A3	1.73122	3.33929	3.76E-21	4.32E-20	up
TTYH3	1.72841	4.83045	4.38E-30	8.42E-29	up
PAQR4	1.72821	2.85791	7.43E-29	1.34E-27	up
ADM	1.72671	3.76203	1.34E-13	8.77E-13	up
DPP3	1.72642	4.20163	1.28E-58	1.01E-56	up

PNP	1.72602	4.03873	2.97E-34	6.97E-33	up
MESP1	1.72359	1.93783	8.46E-13	5.18E-12	up
MCM2	1.72295	4.1415	1.11E-24	1.56E-23	up
TSPAN1	1.72022	5.57009	5.37E-08	2.10E-07	up
MCM4	1.72003	3.92351	2.28E-28	3.99E-27	up
RAP1GAI	1.71521	3.41315	8.30E-16	6.48E-15	up
CLDN6	1.71413	1.78963	8.42E-05	0.00023	up
SLC39A4	1.71223	3.66702	1.70E-22	2.12E-21	up
CAMSAP	1.7092	3.31543	8.19E-38	2.30E-36	up
RAD54L	1.70231	1.99838	5.86E-34	1.34E-32	up
PROM2	1.69962	3.37622	2.37E-15	1.79E-14	up
RNASEH	1.6996	4.65	4.03E-32	8.60E-31	up
RBM47	1.69771	3.57345	1.29E-27	2.19E-26	up
SLC2A1	1.69732	5.27448	3.02E-17	2.61E-16	up
NME1	1.69391	4.64877	3.90E-41	1.25E-39	up
GRIN2D	1.69307	1.79386	2.19E-13	1.40E-12	up
BAIAP2L	1.68928	3.55704	1.43E-47	6.35E-46	up
SLC52A2	1.68663	5.00536	1.55E-46	6.43E-45	up
KRT6A	1.68658	1.82003	2.02E-06	6.66E-06	up
ESPL1	1.6862	1.87474	2.03E-33	4.55E-32	up
ORC6	1.68454	2.10449	1.59E-41	5.21E-40	up
RACGAP	1.68219	3.08636	4.86E-30	9.34E-29	up
LPAR2	1.68109	3.62532	5.79E-36	1.48E-34	up
GOLT1A	1.6808	2.55438	3.05E-13	1.93E-12	up
FAM132E	1.67758	1.81345	3.07E-13	1.94E-12	up
MAP2K6	1.6751	2.6703	2.46E-18	2.31E-17	up
CBX2	1.6679	2.63083	1.67E-19	1.70E-18	up
CP	1.6654	3.75884	6.92E-07	2.41E-06	up
NR2F6	1.66193	5.72584	2.59E-30	5.05E-29	up
CDC25C	1.65633	1.81917	4.79E-39	1.41E-37	up
ERBB3	1.65552	3.95757	1.03E-27	1.75E-26	up
ENO1	1.6535	8.65396	9.86E-35	2.37E-33	up
GAPDH	1.65344	10.1886	1.02E-39	3.10E-38	up
TRIP13	1.6505	2.83759	2.43E-28	4.25E-27	up
PRSS22	1.64541	3.03472	4.69E-14	3.18E-13	up
ARHGEF	1.64414	2.773	1.77E-36	4.64E-35	up
SLC29A2	1.64409	3.52139	1.09E-25	1.65E-24	up
RPS6KA1	1.6428	3.97872	1.58E-45	6.20E-44	up
F2RL1	1.63723	3.30486	8.35E-19	8.04E-18	up
SLC34A2	1.63134	5.38583	5.36E-07	1.89E-06	up
SYNE4	1.63106	3.1921	6.15E-27	1.00E-25	up
S100P	1.63052	2.02774	2.50E-07	9.10E-07	up
EFNA4	1.62619	4.43598	3.99E-31	8.13E-30	up
CCNA1	1.62528	3.21066	1.06E-06	3.62E-06	up
RCC2	1.62505	6.19721	1.20E-53	7.31E-52	up

LGR5	1.62293	3.52273	4.18E-06	1.33E-05	up
MMP12	1.62258	1.85895	4.46E-11	2.37E-10	up
F11R	1.61571	5.13588	1.47E-37	4.06E-36	up
RAD51	1.61361	2.09475	2.49E-35	6.16E-34	up
CCL20	1.61256	2.32542	2.09E-07	7.69E-07	up
GPT2	1.61147	3.91531	4.09E-32	8.70E-31	up
CNFN	1.6108	3.24606	4.70E-11	2.49E-10	up
FASN	1.6095	4.83783	1.37E-21	1.61E-20	up
PRR7	1.60925	3.0661	1.32E-22	1.66E-21	up
TRPM2	1.60804	2.30753	9.88E-33	2.15E-31	up
TMEM23	1.60782	3.08566	2.25E-17	1.96E-16	up
EVPL	1.60709	3.20279	2.62E-19	2.62E-18	up
CAPS	1.6064	6.09571	1.50E-05	4.49E-05	up
NXPH4	1.59752	1.72795	6.36E-09	2.75E-08	up
RBBP8NI	1.59738	2.47147	1.91E-25	2.83E-24	up
OIP5	1.59676	2.03036	1.96E-29	3.63E-28	up
CDCA2	1.59322	1.75585	6.36E-30	1.22E-28	up
NCMAP	1.5913	2.3296	6.11E-14	4.10E-13	up
TNFAIP2	1.5894	5.46866	8.32E-11	4.29E-10	up
DDX39A	1.58439	4.93529	4.12E-38	1.17E-36	up
RHPN2	1.57758	3.72472	3.35E-24	4.61E-23	up
SYTL1	1.57375	3.36477	6.08E-18	5.53E-17	up
CCDC64I	1.5718	2.87531	1.12E-21	1.33E-20	up
TMSB10	1.57155	11.6514	1.10E-26	1.76E-25	up
PPP1R14I	1.57126	5.79228	6.99E-29	1.26E-27	up
ANKRD2	1.56607	1.95542	2.21E-14	1.54E-13	up
ASRGL1	1.5653	5.01149	4.15E-08	1.64E-07	up
PKM	1.56515	7.50892	2.27E-36	5.94E-35	up
TPI1	1.56447	8.0317	1.28E-37	3.53E-36	up
ADM2	1.56348	1.77628	5.97E-20	6.28E-19	up
PRR11	1.56304	2.21635	4.47E-28	7.76E-27	up
GGT6	1.56064	2.7906	2.39E-11	1.30E-10	up
TFCP2L1	1.56025	2.0329	5.94E-16	4.69E-15	up
MEST	1.55999	4.37666	4.28E-18	3.94E-17	up
LAMB3	1.55096	3.78504	2.46E-08	9.99E-08	up
ECE2	1.55	2.00321	2.42E-44	8.97E-43	up
CGN	1.54974	3.18532	4.08E-20	4.35E-19	up
CA2	1.54941	2.1956	3.34E-11	1.79E-10	up
HILPDA	1.54923	3.91917	1.29E-16	1.07E-15	up
EXO1	1.54795	1.74094	1.57E-31	3.27E-30	up
APOC1	1.54448	4.12406	5.25E-09	2.29E-08	up
PRRG2	1.54438	3.10416	1.10E-33	2.49E-32	up
ANO9	1.54374	2.63523	1.92E-27	3.22E-26	up
APOBEC	1.54266	2.25095	9.13E-17	7.71E-16	up
DTL	1.54165	2.14801	1.09E-29	2.05E-28	up

COL9A2	1.54108	3.89253	3.39E-10	1.65E-09	up
MTFP1	1.54065	2.37398	7.89E-33	1.73E-31	up
RAB11FI	1.53896	2.61955	2.95E-25	4.32E-24	up
CSTB	1.53725	6.70783	5.37E-27	8.82E-26	up
DEPDC1I	1.53642	1.74716	1.82E-26	2.89E-25	up
SLC25A3	1.53633	6.18898	1.33E-47	5.94E-46	up
KRT16	1.53571	1.80301	1.68E-06	5.60E-06	up
SOX17	1.53566	6.3361	3.87E-08	1.54E-07	up
PLS1	1.53553	2.46679	8.80E-21	9.88E-20	up
CXCL14	1.53501	4.61488	0.00087	0.00203	up
NACC1	1.53382	5.12358	3.81E-43	1.34E-41	up
RMI2	1.53344	2.82765	9.46E-27	1.53E-25	up
PDZK1IP	1.53261	4.16783	3.89E-05	0.00011	up
C10orf99	1.53259	1.61672	9.14E-07	3.14E-06	up
HIST1H4	1.52887	2.38969	3.99E-12	2.31E-11	up
SGPP2	1.52715	3.21491	1.29E-13	8.41E-13	up
ZDHHC1	1.52551	4.62603	4.73E-34	1.10E-32	up
GINS2	1.5254	2.38398	1.41E-23	1.87E-22	up
RASSF7	1.52	4.96701	2.29E-21	2.66E-20	up
FOXA1	1.51896	1.89132	8.00E-08	3.08E-07	up
LTF	1.51838	3.6726	0.00596	0.012	up
CITED4	1.51747	6.10595	1.40E-09	6.46E-09	up
MYO10	1.51726	3.17167	6.51E-33	1.43E-31	up
MMP11	1.51508	4.92867	1.95E-09	8.88E-09	up
TIMELES	1.51465	3.26444	2.02E-28	3.55E-27	up
HIST1H2	1.51388	2.34785	1.38E-12	8.33E-12	up
REC8	1.5133	4.31434	1.00E-08	4.24E-08	up
WNT7A	1.51168	2.29474	2.58E-05	7.46E-05	up
CDC25A	1.50717	1.95814	4.86E-31	9.86E-30	up
GGT1	1.507	2.53106	4.83E-13	3.01E-12	up
GALNT6	1.50416	2.79869	2.75E-17	2.38E-16	up
SPAG4	1.50392	2.82734	3.66E-17	3.16E-16	up
RAD51AI	1.50348	2.20439	9.36E-23	1.20E-21	up
ARHGAP	1.49737	1.99413	1.21E-28	2.16E-27	up
PPAP2C	1.49728	4.30501	4.87E-10	2.35E-09	up
CHGA	1.49727	2.63528	0.00148	0.00332	up
CHMP4C	1.49414	3.38321	4.32E-25	6.26E-24	up
MYCN	1.49379	2.09262	6.84E-09	2.95E-08	up
UBE2S	1.4934	3.45169	5.66E-19	5.54E-18	up
SFTA2	1.4916	2.10586	4.35E-06	1.38E-05	up
EFNA3	1.49063	2.4508	5.49E-19	5.38E-18	up
ORC1	1.49022	1.83044	1.26E-30	2.50E-29	up
KRT19	1.48982	7.55349	2.72E-08	1.10E-07	up
IRF7	1.48736	4.14512	3.98E-16	3.19E-15	up
CXCL10	1.48696	3.44117	7.00E-07	2.43E-06	up

PROM1	1.48684	3.18697	3.54E-07	1.27E-06	up
LDHA	1.48561	6.63238	2.17E-24	3.01E-23	up
EYA2	1.48422	5.21376	1.79E-05	5.27E-05	up
PSRC1	1.48275	2.78654	2.48E-24	3.45E-23	up
HMMR	1.47905	1.81306	3.06E-24	4.21E-23	up
PACSIN1	1.47682	2.11765	2.96E-16	2.40E-15	up
DNMT3B	1.4738	2.11558	7.10E-24	9.55E-23	up
CCNF	1.47362	2.23957	1.66E-30	3.27E-29	up
LAMTOR	1.47252	5.65304	5.81E-33	1.29E-31	up
SHCBP1	1.47182	1.7801	1.46E-34	3.50E-33	up
PRR15	1.47079	4.52347	3.63E-11	1.94E-10	up
AUNIP	1.46986	1.75106	1.14E-29	2.14E-28	up
TMC4	1.46967	5.00999	7.27E-15	5.26E-14	up
KLF5	1.46795	4.5286	5.20E-14	3.51E-13	up
GMIP	1.46794	3.19913	2.72E-49	1.35E-47	up
ASCL2	1.46726	2.53534	1.42E-09	6.55E-09	up
C2orf54	1.45982	1.87213	3.89E-12	2.26E-11	up
TSTD1	1.45796	5.12226	4.18E-18	3.85E-17	up
ABCG1	1.45717	3.17913	4.00E-19	3.96E-18	up
STXBP6	1.45716	3.20562	9.45E-13	5.76E-12	up
MIF	1.45487	5.76181	2.90E-14	2.00E-13	up
TYMP	1.45308	4.02943	4.23E-12	2.44E-11	up
HOXB5	1.45291	4.03983	2.55E-06	8.32E-06	up
EZR	1.45209	6.99768	1.55E-22	1.94E-21	up
MTFR2	1.45015	1.6461	6.36E-32	1.35E-30	up
MAPK13	1.44967	3.09036	6.17E-26	9.49E-25	up
KIF23	1.44933	2.11779	3.82E-27	6.33E-26	up
S100A14	1.44848	4.01487	1.26E-05	3.78E-05	up
MARCKS	1.44778	8.28174	1.30E-25	1.95E-24	up
SAMD10	1.44735	3.08907	2.20E-37	6.03E-36	up
DKK4	1.44602	1.79659	0.00864	0.01695	up
C6orf132	1.44597	2.64064	3.37E-23	4.40E-22	up
PGP	1.44507	3.50628	8.98E-33	1.96E-31	up
LAPTM4	1.44348	7.19403	9.33E-14	6.19E-13	up
CDK2AP	1.44053	6.1302	1.32E-24	1.86E-23	up
IKBKE	1.43935	2.76155	1.54E-35	3.87E-34	up
NDC80	1.43817	2.44395	3.99E-19	3.95E-18	up
HIST1H2	1.43408	2.0259	1.51E-12	9.07E-12	up
PDIA4	1.43331	7.14238	2.77E-31	5.69E-30	up
TMPRSS	1.43249	2.40347	2.82E-13	1.79E-12	up
MCM10	1.42983	1.52863	8.24E-27	1.33E-25	up
PPIF	1.42811	4.91336	5.13E-28	8.88E-27	up
KIF15	1.42744	1.78626	1.49E-25	2.22E-24	up
IFI27	1.4263	6.14701	6.34E-05	0.00017	up
MREG	1.42603	2.90296	3.84E-29	7.01E-28	up

AP2S1	1.42461	6.16378	5.98E-36	1.53E-34	up
MDK	1.42299	8.3894	5.51E-12	3.16E-11	up
ATP5J2	1.42154	5.8705	1.17E-36	3.12E-35	up
SCX	1.42099	2.79718	1.27E-08	5.30E-08	up
FUT2	1.42026	2.68606	7.13E-12	4.04E-11	up
RGL3	1.41787	3.6246	3.01E-15	2.25E-14	up
CXADR	1.41573	3.55095	6.20E-19	6.03E-18	up
SLC4A11	1.415	2.29508	8.96E-12	5.03E-11	up
MAD2L1	1.41494	2.34274	8.24E-21	9.26E-20	up
HOXB13	1.41445	1.36038	6.66E-11	3.47E-10	up
FANCI	1.41368	2.46612	8.99E-31	1.80E-29	up
CXCL1	1.41355	2.66971	5.53E-05	0.00015	up
REEP4	1.41231	4.27577	2.63E-32	5.64E-31	up
HIST3H2.	1.41023	4.23728	1.34E-12	8.08E-12	up
COMTD1	1.40754	3.52525	9.42E-18	8.45E-17	up
EXOSC4	1.4063	5.02198	4.56E-25	6.59E-24	up
CCL18	1.40275	1.7789	2.30E-07	8.41E-07	up
HIST1H2.	1.40275	1.57668	5.14E-15	3.76E-14	up
ST6GALN	1.40117	3.37979	6.47E-07	2.26E-06	up
GIPC1	1.40081	5.60816	5.45E-34	1.25E-32	up
TXNDC1	1.39979	3.84642	1.38E-40	4.30E-39	up
HOXB6	1.3971	4.28826	7.33E-06	2.27E-05	up
PTPN6	1.39674	3.76487	2.68E-34	6.31E-33	up
DEFB1	1.39672	4.43137	0.0011	0.00251	up
NEK6	1.39501	3.76562	3.51E-27	5.82E-26	up
DSG2	1.39442	4.17867	1.26E-16	1.05E-15	up
OVOL2	1.3941	2.88724	1.69E-21	1.98E-20	up
AGR2	1.39049	4.36272	0.0003	0.00075	up
CENPW	1.38997	4.67357	1.99E-13	1.28E-12	up
CDC42BI	1.38971	2.60209	2.15E-30	4.21E-29	up
KCNH3	1.38962	2.48684	5.81E-08	2.27E-07	up
LAMC2	1.38943	4.39495	2.62E-09	1.19E-08	up
ENC1	1.3894	3.64973	3.31E-13	2.09E-12	up
FAM195A	1.38802	3.28533	3.72E-24	5.10E-23	up
PPP1CA	1.38744	6.379	4.37E-46	1.77E-44	up
IDH1	1.38713	4.8358	2.24E-16	1.83E-15	up
SLC2A6	1.38546	2.24549	2.88E-22	3.54E-21	up
MMP15	1.38508	4.35658	5.92E-22	7.15E-21	up
RIPPLY3	1.38083	1.52841	1.27E-10	6.40E-10	up
B4GALT1	1.37551	6.36778	1.20E-10	6.11E-10	up
GJB2	1.3748	2.86916	7.77E-07	2.69E-06	up
BOP1	1.37424	4.76233	9.99E-26	1.52E-24	up
SAT1	1.37353	7.88023	9.95E-12	5.57E-11	up
DNAJC22	1.37266	2.40934	6.46E-12	3.67E-11	up
SCNN1B	1.37039	2.19162	7.01E-06	2.17E-05	up

ROMO1	1.37005	7.20142	3.43E-20	3.67E-19	up
GPI	1.36831	5.79397	6.17E-32	1.31E-30	up
HIST2H2.	1.36808	2.64409	3.04E-14	2.09E-13	up
ETV4	1.36779	2.94136	1.25E-07	4.71E-07	up
SCGB1D2	1.36405	4.85955	0.00603	0.01213	up
HIST1H4	1.36383	1.87644	3.84E-12	2.23E-11	up
DHCR24	1.36244	5.57354	1.09E-11	6.09E-11	up
GPR56	1.36138	4.53619	1.76E-15	1.34E-14	up
ASPM	1.35988	1.50528	1.42E-23	1.89E-22	up
TONSL	1.35513	2.51136	6.62E-26	1.01E-24	up
NRTN	1.35493	2.64671	9.04E-08	3.46E-07	up
ARRDC1	1.35477	4.57767	2.42E-30	4.72E-29	up
P4HB	1.35458	7.67199	8.79E-35	2.12E-33	up
FUT3	1.35417	1.65487	1.29E-09	5.98E-09	up
B4GALN	1.3535	3.8677	4.87E-10	2.34E-09	up
RTKN	1.35336	3.86055	5.20E-44	1.88E-42	up
TSPAN33	1.35289	3.32422	1.61E-17	1.42E-16	up
ESM1	1.35242	2.15783	3.53E-12	2.06E-11	up
GLDC	1.35151	1.68935	1.61E-07	5.99E-07	up
FLAD1	1.35136	4.1724	5.75E-52	3.26E-50	up
ATP6V0E	1.35034	5.60727	1.55E-44	5.83E-43	up
FBXL6	1.35012	3.52032	3.21E-31	6.58E-30	up
LSM4	1.34975	5.54306	1.08E-30	2.16E-29	up
C1orf53	1.34951	2.73786	3.13E-20	3.37E-19	up
C10orf35	1.34835	3.44436	2.37E-29	4.36E-28	up
APBA2	1.3481	2.3776	3.84E-15	2.85E-14	up
E2F2	1.34798	1.51947	1.44E-28	2.55E-27	up
MAP7	1.34767	3.01299	4.29E-27	7.08E-26	up
CHAF1A	1.34716	3.0788	1.66E-31	3.45E-30	up
SMIM24	1.34646	1.98055	1.21E-06	4.08E-06	up
CHST6	1.3461	2.08167	2.04E-09	9.29E-09	up
SST	1.346	2.1292	0.00345	0.00724	up
SMARCA	1.34555	4.86937	2.26E-30	4.42E-29	up
EPHA1	1.3429	2.88939	3.83E-27	6.34E-26	up
SLC9A3R	1.34221	5.47874	2.81E-12	1.65E-11	up
HID1	1.34172	4.54258	1.56E-16	1.29E-15	up
CALR	1.34118	9.1315	2.68E-36	6.98E-35	up
SLC50A1	1.34115	4.96851	2.48E-31	5.11E-30	up
EPS8L2	1.33863	4.39955	1.15E-20	1.29E-19	up
EBP	1.338	4.57149	1.39E-15	1.07E-14	up
TUBG1	1.33764	4.71418	3.14E-26	4.91E-25	up
MSI1	1.3367	3.39424	5.47E-10	2.62E-09	up
AMH	1.33421	1.51299	4.78E-10	2.30E-09	up
FAM111E	1.33378	1.64691	5.11E-20	5.41E-19	up
CHTF18	1.33374	3.06626	2.09E-22	2.60E-21	up

MST1R	1.33346	2.08854	2.67E-13	1.70E-12	up
SGOL1	1.33158	1.45326	1.56E-28	2.77E-27	up
PROSER2	1.32532	2.92516	5.87E-24	7.93E-23	up
TMEM63	1.32448	4.11322	2.45E-36	6.38E-35	up
WWC1	1.32233	3.07314	2.11E-30	4.14E-29	up
PPP1R14	1.32105	2.69636	1.14E-07	4.33E-07	up
PFKFB4	1.32087	2.13018	2.80E-22	3.45E-21	up
PABPC1L	1.31995	2.98869	7.24E-15	5.25E-14	up
EPS8L1	1.31886	3.86512	2.63E-11	1.42E-10	up
EHF	1.31732	3.51765	1.16E-07	4.41E-07	up
PCNA	1.31666	6.17725	2.59E-21	3.01E-20	up
MYO5B	1.31635	2.5163	2.98E-20	3.21E-19	up
ATP6V0E	1.3147	4.21844	9.96E-13	6.05E-12	up
KNSTRN	1.31467	2.81798	3.07E-27	5.11E-26	up
RCOR2	1.31405	2.82967	9.47E-10	4.44E-09	up
TMC5	1.31326	2.36632	4.57E-08	1.80E-07	up
GRB7	1.31261	3.59685	5.91E-09	2.56E-08	up
FZD5	1.31236	2.49885	4.61E-16	3.68E-15	up
NBEAL2	1.31236	3.53476	1.67E-23	2.22E-22	up
IL32	1.31179	4.26957	9.48E-08	3.62E-07	up
TNNT1	1.31147	2.26646	0.00052	0.00126	up
ALPP	1.31146	1.96317	0.00019	0.00049	up
TFRC	1.31135	4.31853	8.28E-12	4.66E-11	up
F12	1.31104	1.61264	3.34E-13	2.11E-12	up
MUC5B	1.31051	2.14609	0.00105	0.00241	up
PLEKHS1	1.30818	2.36813	7.55E-06	2.33E-05	up
C15orf48	1.3078	2.31129	1.27E-09	5.92E-09	up
CENPN	1.30581	1.99575	6.25E-29	1.13E-27	up
TIMM8B	1.30416	5.41933	5.02E-33	1.12E-31	up
GMDS	1.30226	3.4398	4.65E-19	4.59E-18	up
STIL	1.30022	1.70935	6.53E-33	1.44E-31	up
KIAA132	1.29996	4.17995	0.00425	0.00878	up
CNKSR1	1.29995	3.37744	1.76E-25	2.62E-24	up
C17orf53	1.29873	1.97523	1.46E-29	2.73E-28	up
NHSL1	1.29788	2.02714	1.20E-21	1.42E-20	up
GGCT	1.29745	5.11497	3.22E-21	3.70E-20	up
HIST1H2	1.29568	1.89881	7.68E-12	4.33E-11	up
DEPDC1	1.29548	1.43854	4.45E-24	6.08E-23	up
AGRN	1.29373	5.45891	5.67E-17	4.85E-16	up
STAP2	1.29314	4.32206	6.41E-12	3.64E-11	up
KIF22	1.28922	4.18927	5.87E-27	9.60E-26	up
SIX1	1.28904	1.37162	1.04E-09	4.86E-09	up
MARVEL	1.28897	2.78867	7.77E-26	1.19E-24	up
FAM136A	1.28884	4.63958	5.96E-42	1.98E-40	up
TBC1D7	1.28536	2.09353	5.17E-57	3.86E-55	up

CYP4X1	1.28492	2.71403	1.20E-07	4.55E-07	up
PVRL1	1.28402	2.93086	5.62E-19	5.50E-18	up
CASP6	1.28397	4.1469	2.68E-30	5.22E-29	up
DTYMK	1.28387	4.15287	1.45E-29	2.71E-28	up
DTNB	1.28372	2.6949	6.55E-34	1.50E-32	up
EPN3	1.28369	2.68755	1.49E-13	9.67E-13	up
STMN1	1.28114	5.52048	1.59E-13	1.04E-12	up
NKD2	1.28085	1.85716	7.12E-09	3.06E-08	up
GAD1	1.28084	1.47022	1.15E-07	4.35E-07	up
CXCL5	1.27883	1.58873	1.13E-05	3.41E-05	up
CST1	1.27849	2.37189	0.00838	0.01647	up
C1orf116	1.27734	1.82564	1.17E-11	6.53E-11	up
ITGB6	1.27672	2.16855	8.19E-09	3.50E-08	up
SAA2	1.27595	1.6881	6.47E-06	2.02E-05	up
LYPLA2	1.27399	5.7164	7.04E-35	1.70E-33	up
TUBB4B	1.27374	8.18311	7.80E-17	6.62E-16	up
ARHGEF	1.27298	3.64384	5.89E-18	5.36E-17	up
TKT	1.27207	5.51545	9.07E-20	9.42E-19	up
PPP4C	1.2694	5.9346	1.03E-37	2.89E-36	up
MMP1	1.26866	1.58543	1.19E-07	4.49E-07	up
EGLN3	1.26863	3.18624	3.38E-10	1.65E-09	up
TALDO1	1.26735	6.3166	1.34E-22	1.69E-21	up
MRPL12	1.26545	5.10302	1.41E-15	1.08E-14	up
PCSK9	1.26527	1.46762	1.51E-09	6.93E-09	up
CTHRC1	1.26486	3.19093	1.28E-07	4.84E-07	up
MAPK15	1.26396	3.15711	3.82E-07	1.36E-06	up
ALG3	1.26244	4.4268	8.29E-29	1.49E-27	up
RCC1	1.26233	4.41248	9.98E-30	1.88E-28	up
FRAT2	1.26162	3.68963	1.90E-25	2.82E-24	up
HMBS	1.26089	3.21452	1.90E-36	4.98E-35	up
IRF6	1.26007	4.07973	6.03E-15	4.38E-14	up
TRAIP	1.25797	2.20742	8.74E-28	1.50E-26	up
RNFT2	1.25594	1.56404	4.80E-33	1.07E-31	up
GFRA3	1.2552	1.79309	9.23E-05	0.00025	up
AGPAT2	1.25492	6.11418	8.00E-15	5.76E-14	up
OVOL1	1.25431	1.51832	4.87E-15	3.57E-14	up
LTB	1.25429	2.90382	1.22E-06	4.13E-06	up
DUS1L	1.25358	4.63188	2.65E-31	5.47E-30	up
PIF1	1.25132	1.57088	2.83E-24	3.91E-23	up
CDK16	1.25019	5.00827	2.81E-27	4.67E-26	up
MT1H	1.24975	1.39959	3.68E-07	1.32E-06	up
HSPE1	1.2489	5.58927	1.67E-22	2.09E-21	up
PCK2	1.24826	3.28468	8.92E-20	9.27E-19	up
MECOM	1.24713	4.13259	3.03E-13	1.92E-12	up
COX5A	1.24551	6.21394	6.72E-33	1.48E-31	up

PLK4	1.24354	1.95225	1.66E-23	2.19E-22	up
SSR4	1.24317	6.24396	9.74E-19	9.33E-18	up
C2CD4A	1.24185	1.80406	1.90E-06	6.30E-06	up
NUDT8	1.24131	3.04971	3.62E-15	2.69E-14	up
TMEM13	1.2402	3.33887	8.88E-13	5.43E-12	up
TRPM4	1.23861	3.87061	7.02E-11	3.65E-10	up
TP53I3	1.23808	4.38329	5.93E-19	5.79E-18	up
COPE	1.23744	6.34347	1.39E-26	2.22E-25	up
PRAC2	1.23657	1.17422	4.70E-08	1.85E-07	up
METTL7I	1.23566	2.1462	4.51E-07	1.60E-06	up
DHCR7	1.23555	4.1708	4.76E-22	5.80E-21	up
CCDC64	1.23515	2.12075	1.14E-19	1.17E-18	up
ANAPC1	1.23186	4.94807	7.87E-21	8.85E-20	up
ALDH18A	1.23183	5.47158	1.41E-27	2.39E-26	up
SOX9	1.23167	4.11561	2.95E-07	1.07E-06	up
RNF208	1.23143	4.14997	2.15E-12	1.27E-11	up
HIP1R	1.22963	3.54599	9.34E-30	1.77E-28	up
DSC2	1.22939	2.09459	3.89E-10	1.89E-09	up
ATP13A2	1.22852	4.21737	4.41E-24	6.03E-23	up
SREBF1	1.22851	4.48472	1.98E-17	1.73E-16	up
TMEM16	1.22733	4.02426	1.02E-13	6.77E-13	up
SHMT2	1.22665	4.78179	3.94E-23	5.11E-22	up
WDR34	1.22617	5.76722	2.75E-17	2.37E-16	up
UQCRQ	1.22379	6.63542	5.57E-26	8.59E-25	up
TMC6	1.22203	3.08167	3.25E-19	3.24E-18	up
PSD4	1.22156	2.7089	1.13E-27	1.92E-26	up
SAC3D1	1.22101	3.75577	1.01E-19	1.05E-18	up
ATHL1	1.21991	4.37351	3.05E-06	9.85E-06	up
RAB26	1.2197	1.44498	9.23E-15	6.61E-14	up
CKAP2	1.21887	2.83026	2.36E-17	2.06E-16	up
FADS2	1.21717	4.23849	2.13E-09	9.68E-09	up
KIAA152	1.21709	5.07892	7.79E-22	9.36E-21	up
UNC93B1	1.21699	4.17937	1.09E-21	1.30E-20	up
DBI	1.21686	5.18661	1.43E-25	2.14E-24	up
CLIC3	1.21543	2.82755	5.73E-07	2.01E-06	up
SYK	1.21512	3.60956	2.22E-15	1.68E-14	up
NDUFS8	1.2149	5.13034	1.40E-21	1.65E-20	up
TNFRSF1	1.21344	4.63156	1.65E-10	8.22E-10	up
LAMA1	1.21317	1.54889	5.79E-09	2.52E-08	up
CKAP2L	1.21312	1.38451	2.86E-23	3.75E-22	up
LRRC45	1.21299	3.655	3.75E-21	4.30E-20	up
MARVEL	1.21291	2.18308	6.79E-29	1.23E-27	up
KIF18A	1.21267	1.47756	9.99E-25	1.42E-23	up
PLEKHB	1.21231	3.32126	1.44E-08	5.99E-08	up
TMPRSS	1.21173	2.59367	2.49E-07	9.09E-07	up

SLC29A4	1.2094	2.50931	5.94E-10	2.83E-09	up
GPR160	1.20936	2.66294	6.71E-10	3.19E-09	up
THEMIS2	1.20919	2.66668	9.55E-13	5.82E-12	up
CEACAM	1.20856	1.26591	9.42E-05	0.00025	up
FANCD2	1.20801	1.90102	2.20E-26	3.47E-25	up
TMEM20	1.20735	2.60063	1.53E-30	3.03E-29	up
TMEM18	1.20726	2.21019	9.80E-14	6.49E-13	up
C12orf75	1.20725	4.35336	2.94E-07	1.06E-06	up
OLFM4	1.20665	1.89822	0.00352	0.00736	up
NUAK2	1.20606	3.21448	7.66E-14	5.11E-13	up
CHI3L1	1.2054	3.67546	7.04E-05	0.00019	up
NUDT5	1.20312	3.81505	5.64E-46	2.26E-44	up
MTHFD2	1.20116	3.69768	1.93E-15	1.46E-14	up
TPD52	1.20099	3.25905	8.26E-17	6.99E-16	up
ECEL1	1.2009	2.60935	2.82E-05	8.10E-05	up
CIT	1.20001	1.6303	2.43E-32	5.23E-31	up
HMGCR	1.19982	3.91487	3.27E-11	1.75E-10	up
ZIC1	1.19731	1.14866	1.77E-07	6.58E-07	up
C14orf80	1.19679	2.29371	9.62E-25	1.37E-23	up
SLC39A1	1.19626	3.01238	2.38E-25	3.51E-24	up
HOOK1	1.19568	2.514	1.00E-17	8.98E-17	up
AGMAT	1.19315	1.79773	1.69E-20	1.85E-19	up
DIAPH3	1.19033	1.33585	2.37E-22	2.93E-21	up
NLRP2	1.18954	2.03933	0.00018	0.00045	up
SCNN1G	1.18884	2.41907	3.00E-05	8.58E-05	up
IGSF8	1.18814	5.74885	3.23E-13	2.04E-12	up
NUDT14	1.18811	4.30434	9.26E-18	8.31E-17	up
E2F8	1.18768	1.44571	8.34E-22	9.98E-21	up
LRRC26	1.18725	1.39311	6.70E-06	2.08E-05	up
SLC37A1	1.18393	2.80872	8.76E-22	1.05E-20	up
CMTM7	1.18232	4.34492	2.39E-11	1.30E-10	up
PLEK2	1.18041	2.39982	2.64E-13	1.68E-12	up
ACY3	1.18032	2.40723	1.11E-06	3.78E-06	up
SRD5A3	1.17939	3.4399	9.57E-11	4.92E-10	up
ITPR3	1.17863	3.45412	3.57E-10	1.74E-09	up
H2AFZ	1.17815	6.69986	2.38E-19	2.40E-18	up
CREB3L4	1.17798	4.37635	8.77E-10	4.13E-09	up
SLC5A6	1.17684	3.3867	2.19E-24	3.04E-23	up
PSMG3	1.17527	4.89456	5.64E-24	7.65E-23	up
TNNI3	1.17465	1.65353	1.10E-06	3.75E-06	up
GPRC5A	1.17418	3.26932	2.27E-06	7.44E-06	up
PHGDH	1.17356	4.50769	1.14E-09	5.31E-09	up
RANGAP	1.17337	4.92708	1.49E-29	2.77E-28	up
HOMER2	1.17322	2.78409	3.70E-09	1.65E-08	up
INA	1.17263	1.19891	4.15E-09	1.84E-08	up

PCCB	1.17159	3.07682	4.92E-28	8.52E-27	up
SCGB2A:	1.17067	1.41933	0.00071	0.00168	up
KAZALD	1.16993	3.0063	3.68E-08	1.46E-07	up
BCL2L12	1.1691	3.63613	4.24E-24	5.81E-23	up
VDR	1.16859	2.05038	1.55E-16	1.28E-15	up
NCCRP1	1.16839	1.38803	7.98E-07	2.75E-06	up
RAB20	1.16838	4.24818	8.16E-18	7.34E-17	up
UBALD2	1.16817	5.34899	4.68E-21	5.34E-20	up
GSTP1	1.16762	8.84315	1.40E-17	1.24E-16	up
PSMB3	1.16676	7.10195	1.32E-21	1.56E-20	up
PRKCD	1.16665	4.52605	3.93E-18	3.63E-17	up
YIF1B	1.1643	4.19158	1.19E-27	2.03E-26	up
NOXA1	1.16256	4.52254	2.87E-09	1.29E-08	up
MANF	1.16212	5.54177	1.27E-19	1.31E-18	up
LRG1	1.16162	3.47891	1.69E-05	4.99E-05	up
TOMM40	1.16073	4.67209	1.55E-26	2.46E-25	up
TGFA	1.16064	2.20862	4.45E-09	1.96E-08	up
BRI3BP	1.15974	2.57349	4.37E-22	5.33E-21	up
PAQR6	1.15839	1.87438	8.78E-14	5.84E-13	up
YDJC	1.15746	4.61937	1.43E-22	1.80E-21	up
AIFM1	1.15712	4.98507	1.04E-17	9.28E-17	up
HOXB7	1.15711	4.47218	4.66E-06	1.48E-05	up
SNRPF	1.15689	4.8031	9.54E-27	1.54E-25	up
THEM6	1.15671	4.21917	5.13E-16	4.07E-15	up
TMEM10	1.1557	2.89938	1.83E-21	2.14E-20	up
TSPAN13	1.15447	5.13714	1.87E-10	9.31E-10	up
SLC25A1	1.15333	2.04092	1.11E-50	5.93E-49	up
PYCRL	1.15276	3.51763	1.62E-21	1.90E-20	up
PLEKHG	1.15197	1.72421	6.29E-18	5.72E-17	up
SLC19A1	1.15137	2.34082	2.22E-33	4.99E-32	up
IL1RN	1.15115	1.74617	1.23E-09	5.71E-09	up
CHCHD1	1.1493	5.0439	1.02E-11	5.70E-11	up
FAAH	1.14913	3.78922	5.45E-14	3.67E-13	up
PSME2	1.14906	5.68666	1.62E-16	1.34E-15	up
RAB17	1.14878	2.84277	1.41E-15	1.08E-14	up
GDA	1.14736	1.96299	8.63E-06	2.64E-05	up
TMEM97	1.1435	3.93373	1.52E-15	1.16E-14	up
NFE2L3	1.14159	2.77892	8.32E-10	3.93E-09	up
USP18	1.14067	2.2934	5.15E-12	2.96E-11	up
CEACAM	1.13845	1.51903	5.93E-05	0.00016	up
DNMT1	1.13843	3.66265	1.70E-18	1.61E-17	up
SEMA4A	1.1381	2.60371	1.04E-15	8.04E-15	up
AKR1B1(	1.13661	1.26492	0.00013	0.00034	up
CTB-55O	1.13601	2.45758	5.54E-12	3.17E-11	up
HKDC1	1.13533	2.26062	8.40E-07	2.90E-06	up

GRB14	1.13159	1.61177	3.07E-07	1.11E-06	up
EME1	1.13152	1.38089	3.44E-26	5.35E-25	up
KRT80	1.13144	2.1177	1.10E-08	4.62E-08	up
OPLAH	1.13082	2.77916	2.18E-12	1.29E-11	up
FBN2	1.13051	1.78701	2.48E-05	7.18E-05	up
QPCTL	1.13023	3.69637	1.97E-22	2.45E-21	up
ATAD3A	1.13008	3.94665	3.93E-26	6.11E-25	up
CKS1B	1.12996	3.36033	6.09E-12	3.46E-11	up
HDGF	1.12961	6.82135	7.68E-23	9.85E-22	up
EMC9	1.12937	4.03028	4.40E-21	5.03E-20	up
CXCL9	1.1279	2.32935	0.00033	0.00081	up
SLC12A8	1.12776	1.99939	4.91E-24	6.69E-23	up
EDN2	1.12741	2.03979	8.69E-07	2.99E-06	up
CELSR1	1.12661	3.48463	8.28E-12	4.66E-11	up
ISG20	1.12617	2.21165	2.29E-15	1.73E-14	up
MCCC2	1.12528	4.30755	3.22E-17	2.78E-16	up
STON2	1.12387	2.48394	4.70E-12	2.71E-11	up
ABCA7	1.12314	2.14643	3.47E-14	2.37E-13	up
SIX4	1.12219	2.16049	4.64E-20	4.93E-19	up
COX5B	1.12215	6.92061	1.04E-19	1.08E-18	up
CDK5	1.12159	3.1602	4.45E-34	1.03E-32	up
CYB561D	1.12156	3.63748	1.98E-23	2.62E-22	up
FGFBP1	1.1212	1.74033	2.75E-05	7.90E-05	up
STRA13	1.12051	4.95278	1.23E-17	1.09E-16	up
PPP1R13l	1.12009	3.21352	2.21E-16	1.80E-15	up
DARS2	1.11894	3.05404	1.51E-20	1.66E-19	up
ERO1L	1.11893	3.82271	2.11E-13	1.36E-12	up
IGFBP2	1.1166	6.00563	0.00013	0.00034	up
HIST2H2	1.11593	3.64262	1.10E-07	4.16E-07	up
GPX1	1.11539	7.68138	1.73E-15	1.32E-14	up
C1orf106	1.11449	1.74284	9.49E-10	4.45E-09	up
LGALS7E	1.11423	1.18128	1.05E-07	3.99E-07	up
GPX2	1.11364	1.82064	0.00316	0.00668	up
PIK3R3	1.11355	3.71706	8.98E-12	5.04E-11	up
GSR	1.11216	5.33502	7.37E-13	4.53E-12	up
ULBP3	1.11108	1.63886	6.64E-15	4.82E-14	up
BATF	1.11091	2.22057	8.53E-09	3.64E-08	up
E2F3	1.11055	2.73547	1.03E-25	1.56E-24	up
LRFN4	1.10993	3.42014	2.67E-14	1.85E-13	up
CYB561	1.1077	4.81958	2.39E-17	2.08E-16	up
CAPG	1.10729	5.45055	1.17E-10	5.95E-10	up
BLM	1.10583	1.42728	2.03E-29	3.75E-28	up
SLC26A6	1.10469	2.39319	1.74E-20	1.91E-19	up
TRIB3	1.10446	2.43066	2.97E-08	1.19E-07	up
LAGE3	1.10393	4.77258	8.12E-17	6.87E-16	up

GTF3C6	1.10372	5.89585	2.49E-19	2.50E-18	up
ORMDL2	1.10288	3.7885	3.19E-30	6.18E-29	up
NEU1	1.10223	4.57025	4.31E-24	5.89E-23	up
OAS3	1.10176	3.06234	4.71E-08	1.85E-07	up
C9orf16	1.10093	5.91531	4.91E-14	3.33E-13	up
MIEN1	1.10011	4.79471	3.72E-13	2.34E-12	up
GCNT2	1.09997	2.11582	5.97E-13	3.69E-12	up
LMNB2	1.09811	4.46047	7.08E-18	6.41E-17	up
KIF14	1.0966	1.2066	5.45E-24	7.41E-23	up
ARL4C	1.09401	3.89981	2.25E-07	8.24E-07	up
MYO6	1.09311	3.69354	8.63E-14	5.74E-13	up
ABHD12	1.09254	4.99173	9.01E-31	1.80E-29	up
NDUFA3	1.09235	5.10506	1.33E-15	1.02E-14	up
LRRC59	1.09168	5.15819	6.19E-25	8.88E-24	up
ABHD17(	1.09164	3.24445	3.92E-18	3.63E-17	up
RASAL1	1.09122	1.99148	4.08E-08	1.62E-07	up
LIMD2	1.09098	3.13997	2.72E-11	1.47E-10	up
OTX1	1.0893	1.05768	1.85E-20	2.01E-19	up
MRPL14	1.08901	6.04198	7.71E-26	1.18E-24	up
GRN	1.08796	7.36437	2.75E-14	1.90E-13	up
RNF223	1.08499	1.40215	4.13E-11	2.19E-10	up
MXD3	1.08453	2.06714	1.91E-19	1.94E-18	up
BAK1	1.0834	3.63944	8.30E-20	8.65E-19	up
RPN2	1.0832	7.32563	2.02E-21	2.35E-20	up
C19orf48	1.08302	5.01838	1.52E-16	1.26E-15	up
ILDR1	1.08269	2.5933	1.49E-14	1.05E-13	up
GSG2	1.08232	1.23787	6.95E-23	8.93E-22	up
POLR2L	1.08189	6.7117	4.79E-15	3.52E-14	up
RFXANK	1.08163	5.23997	1.55E-16	1.29E-15	up
ABCC3	1.08147	2.23804	2.18E-07	8.00E-07	up
PLAUR	1.08127	3.40771	4.70E-10	2.27E-09	up
HGH1	1.08037	4.2368	5.18E-25	7.47E-24	up
POLR2H	1.07852	4.82653	3.96E-30	7.63E-29	up
UQCR10	1.07815	6.79823	2.70E-24	3.74E-23	up
HN1L	1.07729	5.37351	8.69E-23	1.11E-21	up
S100A7	1.07647	1.15392	0.00086	0.00201	up
IGSF3	1.07571	2.63351	4.20E-13	2.63E-12	up
CASC5	1.07501	1.20955	4.19E-23	5.42E-22	up
DECR2	1.0743	3.24449	1.50E-15	1.15E-14	up
DCXR	1.07339	5.2328	6.03E-12	3.43E-11	up
HSH2D	1.07294	2.29324	4.92E-11	2.60E-10	up
PFDN2	1.07266	6.71366	1.93E-19	1.95E-18	up
KANK4	1.07102	1.17065	1.96E-07	7.25E-07	up
ABCC1	1.06994	3.97925	1.11E-25	1.68E-24	up
CENPO	1.06944	1.86976	6.44E-27	1.05E-25	up

C1QL4	1.06929	1.16549	1.40E-06	4.70E-06	up
EFNA1	1.06913	5.25612	2.24E-10	1.11E-09	up
ARPC5L	1.06911	4.09851	1.14E-35	2.86E-34	up
PITPNM1	1.06874	3.71734	1.20E-11	6.66E-11	up
CXCL8	1.06843	2.21203	2.29E-05	6.66E-05	up
INCENP	1.06837	2.89689	5.27E-16	4.18E-15	up
FAAH2	1.06805	2.74707	1.16E-15	8.93E-15	up
TMEM63	1.06714	3.42195	7.95E-15	5.72E-14	up
ENTPD7	1.06606	2.20714	2.10E-18	1.98E-17	up
ARTN	1.066	1.61317	1.03E-17	9.21E-17	up
NARF	1.0655	3.4744	6.53E-33	1.44E-31	up
ZNF296	1.06441	1.76901	1.30E-19	1.34E-18	up
ECHS1	1.06426	7.09457	3.61E-21	4.15E-20	up
LMAN2	1.06386	6.91519	9.12E-22	1.09E-20	up
CENPH	1.0629	2.70972	3.28E-18	3.04E-17	up
CDC20B	1.0625	1.33057	0.00013	0.00034	up
FA2H	1.06222	1.59562	7.19E-12	4.07E-11	up
FAM174I	1.06217	3.79178	8.99E-09	3.83E-08	up
TIMM10	1.06192	5.2969	1.34E-18	1.28E-17	up
MRPS12	1.06185	4.4642	4.05E-20	4.32E-19	up
OASL	1.06179	1.68791	6.86E-08	2.66E-07	up
CHEK1	1.061	1.91456	8.58E-20	8.93E-19	up
ERCC6L	1.0608	1.22238	3.42E-25	4.98E-24	up
FANCA	1.05758	1.3897	3.00E-26	4.68E-25	up
GLB1	1.0565	4.76118	4.10E-16	3.28E-15	up
APOE	1.05554	6.21013	0.00015	0.0004	up
E2F7	1.05324	1.18954	1.42E-17	1.26E-16	up
KIFC2	1.05318	2.42322	2.95E-11	1.59E-10	up
PLEKHJ1	1.05292	3.95987	3.28E-19	3.27E-18	up
DNA2	1.05259	1.51032	1.76E-20	1.92E-19	up
PPP1R1B	1.05165	3.44597	0.00138	0.0031	up
PDE7A	1.05121	2.617	5.25E-20	5.55E-19	up
ALDOA	1.05119	7.81947	1.84E-17	1.62E-16	up
TRABD	1.05076	4.50904	5.40E-18	4.94E-17	up
ENTPD6	1.05047	4.25015	2.72E-27	4.53E-26	up
CDCA7	1.04996	3.09341	2.56E-08	1.04E-07	up
GCNT1	1.04992	2.92286	5.42E-07	1.91E-06	up
NAT8L	1.04877	2.12216	1.58E-06	5.27E-06	up
HS6ST1	1.04855	4.508	2.44E-12	1.43E-11	up
MOGS	1.04768	5.0982	2.19E-39	6.57E-38	up
PDIA2	1.04664	1.21503	7.63E-06	2.35E-05	up
GPX4	1.04652	7.37429	3.30E-14	2.26E-13	up
HIST1H2.	1.04604	4.58339	2.90E-07	1.05E-06	up
FBXW9	1.04561	3.66067	2.22E-12	1.31E-11	up
AP1S1	1.0456	5.24655	7.80E-27	1.26E-25	up

PNKD	1.04534	4.62936	2.70E-18	2.52E-17	up
ZNF750	1.04506	1.21754	4.33E-09	1.91E-08	up
C14orf2	1.04459	4.79743	1.17E-21	1.38E-20	up
GALNT1	1.04433	1.70646	1.46E-05	4.35E-05	up
CDCA4	1.04324	3.64494	1.42E-20	1.57E-19	up
PGAM5	1.04307	4.20675	4.49E-24	6.12E-23	up
CIB1	1.04036	6.85514	1.39E-12	8.34E-12	up
SLC27A4	1.04023	4.16228	9.34E-24	1.25E-22	up
SYT17	1.03911	1.71431	3.03E-16	2.45E-15	up
MRPS15	1.03846	4.85707	8.12E-19	7.83E-18	up
SLC25A2	1.03775	3.55804	1.35E-24	1.90E-23	up
MCM7	1.03749	5.3095	5.25E-15	3.83E-14	up
LAMP3	1.03746	1.56868	6.21E-09	2.69E-08	up
GINS4	1.03687	1.4691	1.27E-16	1.06E-15	up
C12orf45	1.0367	3.67821	1.33E-18	1.27E-17	up
SDSL	1.03667	3.30939	6.61E-14	4.42E-13	up
CENPE	1.03637	1.30286	7.79E-20	8.13E-19	up
SDS	1.03531	1.41347	4.23E-16	3.39E-15	up
SH3BGRI	1.03522	6.86795	1.08E-17	9.65E-17	up
TICRR	1.0344	1.27523	1.85E-25	2.76E-24	up
FAM71E1	1.03256	2.31429	6.29E-12	3.58E-11	up
OGDHL	1.03229	2.42173	5.19E-08	2.04E-07	up
MYEOV	1.03213	1.12729	6.61E-08	2.57E-07	up
SOX4	1.03182	5.73838	1.18E-10	5.99E-10	up
DDX49	1.03157	4.98424	1.20E-25	1.81E-24	up
HIST1H2	1.03156	1.07702	7.23E-15	5.24E-14	up
GCAT	1.0309	3.78053	2.29E-11	1.25E-10	up
STRBP	1.03031	2.8809	1.71E-23	2.27E-22	up
CST6	1.02957	2.09915	6.57E-05	0.00018	up
COX6B1	1.02733	7.97959	4.83E-18	4.43E-17	up
SPRR1B	1.02726	1.01422	0.00081	0.00189	up
WDR62	1.02703	1.48012	5.11E-22	6.21E-21	up
CLDN9	1.02647	1.57375	6.09E-05	0.00017	up
PADI2	1.0262	1.96175	1.79E-06	5.95E-06	up
ARVCF	1.02613	3.33888	1.35E-16	1.13E-15	up
PYY	1.02594	1.8762	0.00034	0.00084	up
SLC52A3	1.0259	2.59085	1.74E-08	7.17E-08	up
SAMD1	1.02585	5.3805	2.03E-20	2.21E-19	up
ATG4D	1.0255	3.57127	2.53E-18	2.36E-17	up
RUNX3	1.02546	2.14946	5.05E-08	1.98E-07	up
FOXA2	1.02538	3.18509	0.0013	0.00294	up
ATP6V1E	1.025	2.49988	0.00017	0.00044	up
STX19	1.02499	1.98732	6.80E-08	2.64E-07	up
NDUFS6	1.02308	5.81714	3.30E-16	2.66E-15	up
VDAC1	1.02195	5.85638	1.05E-24	1.48E-23	up

MFAP2	1.02116	4.71707	0.00011	0.00029	up
SMPD2	1.01991	3.61798	7.92E-18	7.14E-17	up
DDIT4	1.01908	5.53267	9.37E-06	2.86E-05	up
UQCC3	1.0189	4.08805	4.87E-16	3.88E-15	up
ATAD2	1.01834	2.41024	6.93E-11	3.61E-10	up
CCDC137	1.01832	3.91848	7.03E-23	9.03E-22	up
HNRNPA	1.018	6.62043	4.10E-28	7.14E-27	up
SEMA4B	1.01771	4.42136	7.76E-11	4.02E-10	up
NT5DC2	1.01658	4.41196	3.47E-10	1.69E-09	up
ADAMDI	1.01602	1.18405	8.03E-09	3.44E-08	up
IER5L	1.01481	4.21918	4.68E-08	1.84E-07	up
FOLH1	1.01395	2.14388	4.35E-08	1.72E-07	up
NOTUM	1.01363	2.2931	0.0213	0.03863	up
BEX5	1.01292	3.57003	0.00696	0.01387	up
TXNL4A	1.01229	3.8562	1.45E-28	2.57E-27	up
WHSC1	1.01215	2.69494	2.82E-21	3.26E-20	up
MROH6	1.01184	2.14543	1.98E-07	7.31E-07	up
SEPHS2	1.01157	5.2488	7.54E-19	7.30E-18	up
TUFT1	1.0115	3.09135	5.46E-17	4.67E-16	up
TFF1	1.01063	1.25584	0.0005	0.00121	up
LGALS9	1.01046	4.12647	1.55E-07	5.80E-07	up
RUVBL2	1.0104	5.6958	3.42E-16	2.75E-15	up
LRRTM1	1.01013	2.03609	0.00057	0.00137	up
TTC39A	1.00941	1.61692	5.24E-13	3.25E-12	up
IMPDH1	1.00934	4.33702	9.43E-17	7.96E-16	up
IVNS1AE	1.00883	4.72539	1.11E-13	7.30E-13	up
C1orf233	1.00851	2.63204	1.30E-06	4.37E-06	up
NIPSNAF	1.0085	5.01517	3.70E-17	3.18E-16	up
APOL4	1.00768	2.9974	3.35E-06	1.08E-05	up
NUDT1	1.00652	3.40741	1.07E-14	7.60E-14	up
ENTPD3	1.00609	2.40151	1.36E-05	4.07E-05	up
IGLL5	1.00566	2.25438	0.00364	0.00761	up
PERP	1.005	5.69584	1.81E-08	7.43E-08	up
RPL22L1	1.00453	4.34854	2.59E-07	9.43E-07	up
XRCC2	1.00444	1.36536	2.86E-21	3.30E-20	up
ILF2	1.00425	6.78195	5.31E-20	5.61E-19	up
FABP5	1.00384	3.09582	4.71E-06	1.49E-05	up
CDC7	1.00304	1.88225	1.03E-12	6.25E-12	up
SEC23B	1.00237	4.26891	4.95E-14	3.35E-13	up
FGFR4	1.00198	2.15989	3.33E-06	1.07E-05	up
CENPK	1.00138	1.40646	1.96E-19	1.98E-18	up
TMED3	1.00117	3.15667	3.97E-19	3.93E-18	up
DENND2	1.00106	2.69407	1.21E-18	1.15E-17	up
ZBTB7B	1.00076	4.16982	2.47E-17	2.14E-16	up
SERF2	1.00049	6.61793	8.13E-15	5.85E-14	up

CYP2D6	1.00048	1.43356	1.82E-10	9.04E-10	up
MPDU1	1.00034	4.45026	7.55E-22	9.07E-21	up
