# Supplementary material for: Comprehensive Analysis of GDF10 Methylation Site-Associated Genes as Prognostic Markers for Endometrial Cancer
Source: J Oncol. 2022 Oct 10;2022:7117083. doi: 10.1155/2022/7117083 (PMC9576415; doi:10.1155/2022/7117083)
Supplement: Supplementary Materials — are shown as Supplemental Figures S1-3 and Supplemental Tables S1-7. Supplemental Figure S1: Cox regression independent prognostic analyses. Supplemental Figure S2: Analysis of expression correlations between the risk model and clinicopathological factors. Supplemental Figure S3: Kaplan-Meier survival analysis of patients with different clinicopathologic features between high- and low-risk groups. (a–i) The different OS of UCEC patients with different ages, tumor stages, and grades between high-risk and low-risk groups according to GDF10 methylation. The abscissa shows survival months, and the vertical axis shows overall survival probability. Supplemental Table S1: 48 modules were identified. Supplemental Table S2: The saddlebrown module positively correlated with the methylation sites the most. Supplemental Table S3: 991 genes were upregulated in DEGs. Supplemental Table S4: 1260 genes were downregulated in DEGs. Supplemental Table S5: 44 DEGMRGs were identified. Supplemental Table S6: All UCEC samples in the training set were divided into the high- and low-risk groups. Supplemental Table S7: Risk scores were significantly different between patients classified by age, tumor stage, and grade. [file 7117083.f1.zip › Supplemental Table S4.pdf]

sym	logFC	AveExpr	P.Value	adj.P.Val	direction
DES	-6.6295224	2.99897898	3.08E-54	1.93E-52	down
MYH11	-5.6381323	2.19126495	1.66E-75	2.87E-73	down
CNN1	-5.564228	2.86773734	1.45E-64	1.47E-62	down
ACTG2	-4.9111959	2.75064467	1.75E-50	9.28E-49	down
LMOD1	-4.7988264	2.0529793	1.09E-86	3.46E-84	down
OGN	-4.6541784	0.82412764	8.36E-119	1.50E-115	down
DPT	-4.4273048	1.04616496	2.31E-99	1.34E-96	down
SPARCL1	-4.3801293	4.68879831	8.27E-71	1.15E-68	down
ZCCHC12	-4.3315396	1.3905076	8.85E-69	1.10E-66	down
SFRP4	-4.2635941	3.45527023	4.95E-26	7.65E-25	down
ACTA2	-4.2539146	5.33010091	8.72E-53	5.03E-51	down
C7	-4.2013227	0.67167319	8.62E-105	7.08E-102	down
CCL21	-4.1488455	2.81217019	1.56E-29	2.90E-28	down
PTGIS	-4.1249081	1.19534336	2.23E-91	9.35E-89	down
TAGLN	-4.1038195	4.83964412	3.21E-51	1.78E-49	down
PLN	-4.0699044	1.07436937	1.48E-83	3.93E-81	down
FHL1	-4.0597478	1.96585374	1.53E-82	3.81E-80	down
WT1	-3.9853029	1.48050797	2.65E-55	1.78E-53	down
DCN	-3.9376128	2.81980055	1.43E-60	1.23E-58	down
MRGPRF	-3.9312323	2.0237471	2.81E-83	7.28E-81	down
MFAP4	-3.9032507	3.29841314	4.54E-39	1.34E-37	down
RERG	-3.8813596	1.11998935	2.70E-111	2.80E-108	down
PRELP	-3.7976749	1.78262941	1.15E-66	1.31E-64	down
HSPB6	-3.7799606	2.92788051	2.94E-44	1.08E-42	down
CHRDL1	-3.7595358	0.99625146	2.16E-75	3.70E-73	down
TNFAIP8L3	-3.7475926	1.16463452	1.90E-91	8.16E-89	down
PGM5	-3.74144	1.3818591	1.07E-77	2.23E-75	down
MYLK	-3.7291174	1.97299388	3.80E-88	1.34E-85	down
GAS1	-3.6914579	1.81877843	2.21E-56	1.56E-54	down
RSPO1	-3.6748218	0.76349595	4.12E-112	4.51E-109	down
OSR2	-3.6477188	2.60387937	4.69E-62	4.34E-60	down
SYNPO2	-3.6070345	1.4091196	5.20E-90	2.09E-87	down
TCF23	-3.5637414	0.54171729	1.78E-125	8.75E-122	down
FLNC	-3.5615037	1.40253283	4.71E-69	5.96E-67	down
SDPR	-3.5472019	1.52083247	2.05E-78	4.35E-76	down
MAOB	-3.5290887	2.90559312	1.51E-46	6.29E-45	down
NDN	-3.508885	2.52271783	9.63E-74	1.48E-71	down
C11orf96	-3.5056694	2.79318614	2.06E-59	1.68E-57	down
MAMDC2	-3.4986909	0.67807543	3.47E-124	1.14E-120	down
CXCL12	-3.4859844	2.05629466	1.70E-59	1.40E-57	down
HSPB7	-3.4805103	0.96039391	4.67E-99	2.63E-96	down
MYL9	-3.4010709	5.65700371	4.15E-45	1.60E-43	down
APOD	-3.3834288	2.49254132	1.30E-26	2.08E-25	down
LTBP4	-3.3385096	3.61523994	1.80E-46	7.45E-45	down

RAMP1	-3.327669	4.21312456	4.00E-35	9.76E-34	down
ADH1B	-3.2899824	0.40906106	1.84E-115	2.41E-112	down
HAND2	-3.2849401	1.13983516	1.86E-84	5.30E-82	down
CLEC3B	-3.2721337	1.43177653	1.88E-70	2.54E-68	down
PPP1R12B	-3.2687364	1.46510484	5.09E-90	2.09E-87	down
ITM2A	-3.2134543	1.88390417	9.00E-57	6.50E-55	down
PDLIM3	-3.2056811	0.95023847	1.07E-79	2.38E-77	down
ALDH1A2	-3.1992857	1.33277741	5.90E-65	6.18E-63	down
DUSP1	-3.1885865	5.33559062	1.46E-44	5.52E-43	down
PODN	-3.1729396	1.60359429	6.81E-57	4.97E-55	down
AOC3	-3.1612321	1.52571527	2.91E-70	3.86E-68	down
CAV1	-3.150177	3.14749385	2.71E-67	3.22E-65	down
EFEMP1	-3.1471242	2.18197667	9.37E-45	3.59E-43	down
TCEAL7	-3.1309623	1.32768267	2.34E-74	3.75E-72	down
SMOC2	-3.1164206	3.23651681	8.54E-43	2.94E-41	down
FOSB	-3.1053914	1.96817506	2.07E-30	4.07E-29	down
CHRDL2	-3.1036578	0.74308077	1.41E-56	1.01E-54	down
SLC24A3	-3.0628385	1.49898272	8.27E-76	1.47E-73	down
ASPN	-3.0593242	1.71383474	3.88E-39	1.15E-37	down
BCHE	-3.0556513	0.4948339	3.31E-110	3.26E-107	down
FBXO32	-3.0485058	2.26841115	1.13E-64	1.17E-62	down
MSRB3	-3.0478774	1.57471179	5.84E-69	7.33E-67	down
TNXB	-3.0430978	0.6972988	2.97E-100	1.77E-97	down
TMEM200B	-3.0257382	1.13966647	5.65E-77	1.08E-74	down
KLF2	-3.0152808	2.67756192	7.55E-62	6.91E-60	down
TSPAN2	-2.9805697	1.07612013	4.18E-75	7.04E-73	down
RGS2	-2.9700351	2.90434208	1.58E-42	5.43E-41	down
IGFBP5	-2.9621733	5.35165366	6.19E-25	8.88E-24	down
ACKR1	-2.9582672	2.43302022	2.13E-29	3.92E-28	down
IGFBP6	-2.9572595	3.13615534	3.37E-37	9.17E-36	down
LEFTY2	-2.9400814	1.53651138	5.53E-25	7.96E-24	down
SFRP1	-2.9367421	2.71211605	8.49E-29	1.52E-27	down
PEG3	-2.9331489	0.54795528	2.70E-102	1.77E-99	down
CACNA1H	-2.9274647	1.23477833	4.58E-76	8.44E-74	down
CSDC2	-2.9178732	1.17981568	1.17E-67	1.41E-65	down
TGFB1I1	-2.9084515	2.41461156	1.55E-62	1.47E-60	down
PTRF	-2.9010001	4.97024752	4.64E-61	4.10E-59	down
RASL12	-2.8969988	1.93329892	6.71E-71	9.52E-69	down
TNS1	-2.8967802	2.60661712	4.08E-72	6.14E-70	down
CLMP	-2.8864916	1.28063351	3.02E-76	5.62E-74	down
KCTD12	-2.8841599	3.04901773	2.16E-38	6.23E-37	down
EMILIN1	-2.8736271	3.54430738	1.58E-38	4.57E-37	down
RPRM	-2.8662685	1.05750398	1.67E-47	7.40E-46	down
CYP1B1	-2.8409282	1.55161525	5.71E-54	3.55E-52	down
JPH2	-2.8377872	0.73343893	4.42E-89	1.71E-86	down

GAS6	-2.8368926	3.49578967	9.73E-38	2.72E-36	down
LRRN4CL	-2.8192005	1.37411802	1.54E-69	1.98E-67	down
RSPO3	-2.8186574	1.25937423	4.41E-35	1.07E-33	down
CYBRD1	-2.8141492	3.07530919	8.66E-54	5.32E-52	down
JPH4	-2.8087617	0.57232511	1.09E-113	1.34E-110	down
CLIP3	-2.7940549	2.57228575	5.41E-50	2.79E-48	down
MRVI1	-2.7938075	1.57673362	3.24E-70	4.26E-68	down
TGFBR3	-2.785189	1.53084817	8.68E-66	9.40E-64	down
CCDC80	-2.7827459	2.3263868	1.29E-44	4.89E-43	down
KCNMB1	-2.7806767	0.92777709	4.84E-75	8.08E-73	down
FOS	-2.7772754	5.43413719	5.14E-22	6.25E-21	down
EGR1	-2.7720811	5.51297544	1.52E-28	2.69E-27	down
FXYD6	-2.7373589	1.98420234	1.82E-39	5.49E-38	down
SORBS1	-2.7199045	1.36680891	1.03E-74	1.68E-72	down
CALD1	-2.7150732	3.70499788	3.44E-53	2.05E-51	down
CBX7	-2.7101799	1.55277127	2.90E-104	2.28E-101	down
ANGPTL1	-2.7073598	0.9610632	4.29E-47	1.84E-45	down
ZCCHC24	-2.6967652	1.95839499	4.80E-72	7.13E-70	down
SLC22A3	-2.693832	0.54135556	1.67E-97	9.16E-95	down
TPM2	-2.6834544	5.21579427	6.04E-33	1.34E-31	down
TACC1	-2.6708297	2.00625597	5.24E-72	7.65E-70	down
BNC2	-2.6627301	0.61487698	4.30E-116	6.52E-113	down
MASP1	-2.6586628	0.50248179	5.16E-101	3.18E-98	down
NEXN	-2.6524362	1.0283952	4.45E-77	8.60E-75	down
TCEAL2	-2.648994	1.26513619	5.45E-27	8.94E-26	down
PLSCR4	-2.64591	1.18710973	1.90E-88	6.94E-86	down
SRPX	-2.6424259	1.39414237	2.72E-46	1.11E-44	down
CYR61	-2.6408244	5.36847744	1.19E-36	3.16E-35	down
DACT3	-2.6295802	0.95973875	8.44E-85	2.45E-82	down
GATA6	-2.6218261	1.01953432	7.95E-71	1.11E-68	down
PMP22	-2.6212363	3.11972628	2.51E-44	9.26E-43	down
CSRP1	-2.6184542	4.66659783	3.31E-82	8.06E-80	down
KIAA1462	-2.6012334	1.37328209	9.13E-92	4.09E-89	down
OLFML3	-2.6004225	3.68396474	5.32E-29	9.63E-28	down
PTGER3	-2.5991448	0.48348548	3.08E-72	4.67E-70	down
EDNRA	-2.5867362	1.8382873	4.51E-56	3.13E-54	down
KANK2	-2.5861363	3.77086207	5.41E-59	4.33E-57	down
NCAM1	-2.58592	0.93097343	4.25E-49	2.07E-47	down
F10	-2.5791794	1.12541557	2.44E-59	1.97E-57	down
CES1	-2.5773726	1.46295357	1.64E-20	1.79E-19	down
SERPINF1	-2.5553349	4.29170032	4.00E-28	6.96E-27	down
KIAA1644	-2.5541063	0.6984314	4.81E-72	7.13E-70	down
ANTXR2	-2.5418796	1.76946565	4.28E-65	4.52E-63	down
PDK4	-2.5397378	2.16861674	2.12E-38	6.13E-37	down
GPR124	-2.5330814	1.95486234	3.16E-51	1.75E-49	down

GSTM5	-2.5329738	0.47413027	1.67E-140	1.65E-136	down
FILIP1L	-2.5282292	2.18013976	2.32E-39	6.94E-38	down
MAGEH1	-2.5221001	2.63668708	1.31E-36	3.45E-35	down
ABCG2	-2.5151977	0.99075804	1.36E-69	1.76E-67	down
GPRASP1	-2.5013367	0.83718727	1.00E-122	2.82E-119	down
PRUNE2	-2.4980227	0.80700182	5.68E-75	9.41E-73	down
TSHZ3	-2.4941947	1.12326801	4.55E-82	1.07E-79	down
EHD2	-2.4935838	3.5718294	5.24E-47	2.22E-45	down
GYPC	-2.4771871	2.07322613	1.05E-54	6.82E-53	down
SERTM1	-2.4768319	1.05996185	3.74E-49	1.82E-47	down
PSD	-2.4685624	1.54890592	1.69E-60	1.44E-58	down
JAM2	-2.4680435	1.30961098	6.21E-57	4.55E-55	down
HSPB8	-2.4679019	2.63280873	5.95E-27	9.72E-26	down
CCND2	-2.4607255	2.46258484	4.12E-31	8.37E-30	down
JAM3	-2.4455901	1.73191932	3.58E-66	3.93E-64	down
PRNP	-2.4445226	3.89181561	3.55E-31	7.25E-30	down
KCNMA1	-2.4440765	1.10393999	6.11E-61	5.36E-59	down
ARMCX1	-2.4417722	2.30178504	6.50E-32	1.38E-30	down
MEIS3	-2.4401269	1.24900854	1.59E-66	1.77E-64	down
ECM2	-2.4395643	0.85747152	2.08E-84	5.77E-82	down
AQP1	-2.4337694	3.78619859	2.17E-35	5.39E-34	down
FGF7	-2.4277015	0.48259104	1.76E-102	1.19E-99	down
ABI3BP	-2.4221141	0.86966923	7.89E-71	1.11E-68	down
LIFR	-2.4210241	1.40552612	9.95E-67	1.14E-64	down
LONRF2	-2.4196224	1.19386576	5.82E-76	1.05E-73	down
FOXL2	-2.4185071	1.1774701	6.25E-51	3.41E-49	down
C16orf45	-2.4159574	1.7802202	1.03E-56	7.37E-55	down
ARHGEF25	-2.4100313	1.99797414	2.71E-55	1.81E-53	down
TSPYL5	-2.4071732	1.31420126	1.53E-32	3.32E-31	down
EMCN	-2.39839	1.05060771	8.83E-71	1.22E-68	down
PLIN4	-2.3938771	1.0931566	1.06E-52	6.07E-51	down
LDB2	-2.3935144	1.67524799	3.69E-60	3.12E-58	down
PLAC9	-2.3880756	1.08569621	4.27E-61	3.79E-59	down
NR2F1	-2.3832451	2.27893306	5.19E-34	1.20E-32	down
DPYSL3	-2.3817555	3.27748405	3.38E-22	4.14E-21	down
FBXL7	-2.3807565	1.72379956	2.00E-45	7.83E-44	down
DDR2	-2.3807299	1.28419284	9.96E-64	9.81E-62	down
ADAM33	-2.364488	1.23443187	5.39E-35	1.31E-33	down
GEM	-2.3630053	2.37318767	1.38E-32	3.00E-31	down
NR4A1	-2.3625254	2.87068608	2.09E-26	3.30E-25	down
TPM1	-2.3558163	3.46425981	4.41E-53	2.59E-51	down
NPR1	-2.3515781	2.08589598	7.98E-21	8.97E-20	down
MGP	-2.3452731	4.89664339	2.99E-21	3.45E-20	down
TRH	-2.3426622	0.76069288	3.42E-15	2.54E-14	down
CPED1	-2.3347168	0.83399498	6.68E-83	1.69E-80	down

TIMP2	-2.3324469	4.89298254	5.21E-34	1.20E-32	down
OLFML2A	-2.3306483	1.86102558	4.94E-49	2.39E-47	down
COX7A1	-2.3298429	2.32606602	2.72E-48	1.25E-46	down
HLF	-2.3296843	0.63432796	3.01E-88	1.08E-85	down
AKT3	-2.3269404	1.0479013	9.32E-84	2.55E-81	down
CNRIP1	-2.3265661	1.25979304	5.21E-87	1.71E-84	down
FLNA	-2.3209839	6.2514306	3.15E-31	6.46E-30	down
PDE2A	-2.3154777	0.61049614	6.04E-109	5.67E-106	down
GNG11	-2.3085443	2.95307297	2.09E-35	5.21E-34	down
TXNIP	-2.3028168	6.34831045	2.01E-32	4.32E-31	down
CTSK	-2.298425	4.05924454	5.26E-29	9.54E-28	down
TUB	-2.2983613	0.98673918	2.61E-64	2.62E-62	down
TWIST2	-2.296487	1.81904644	2.69E-25	3.95E-24	down
TMEM132C	-2.2878465	0.43402553	2.17E-82	5.35E-80	down
LIMS2	-2.2857658	2.13089634	7.82E-47	3.29E-45	down
FGF2	-2.2837076	0.71740346	1.46E-92	6.86E-90	down
TSPAN18	-2.2807391	1.39215101	2.45E-49	1.22E-47	down
ZEB1	-2.2803369	1.39242246	7.37E-57	5.34E-55	down
ENPP2	-2.2795737	2.55020017	1.54E-27	2.59E-26	down
ALDH1A1	-2.2777821	4.35655009	2.29E-13	1.47E-12	down
COL14A1	-2.277162	2.09666659	5.41E-26	8.36E-25	down
MYOCD	-2.2756494	0.33154958	4.54E-121	9.93E-118	down
THBS1	-2.2711787	3.49835696	2.23E-23	2.94E-22	down
AHNAK	-2.2677337	4.30731755	1.63E-42	5.59E-41	down
PID1	-2.2663492	0.70830592	2.77E-77	5.47E-75	down
FXYD1	-2.2533837	0.51732635	2.42E-87	8.22E-85	down
SNCG	-2.2511454	2.35836901	7.36E-16	5.77E-15	down
IL33	-2.2466656	2.26663604	1.36E-22	1.72E-21	down
ATP1B2	-2.2425415	1.20841571	3.91E-47	1.68E-45	down
TGM2	-2.239027	4.32552498	2.22E-26	3.49E-25	down
IGFBP4	-2.2379178	7.00574577	6.76E-21	7.64E-20	down
SSPN	-2.2358811	1.32586282	2.24E-65	2.39E-63	down
SVIL	-2.2250014	2.75561915	1.61E-44	6.03E-43	down
pk	-2.2233248	2.36746783	3.42E-62	3.21E-60	down
MAF	-2.2219521	1.72745746	3.49E-52	1.99E-50	down
LHFP	-2.2084811	3.05530454	1.68E-40	5.25E-39	down
TNS2	-2.205488	2.79509147	1.09E-61	9.88E-60	down
NXPH3	-2.2002552	0.67095464	1.52E-77	3.09E-75	down
RERGL	-2.1949998	0.43931371	1.24E-85	3.77E-83	down
CDO1	-2.1880286	0.74154787	5.55E-44	2.00E-42	down
CPE	-2.1879675	3.20929022	1.16E-25	1.75E-24	down
OLFML1	-2.1843591	1.37739696	1.84E-54	1.19E-52	down
CYS1	-2.1834737	1.4047769	8.00E-18	7.21E-17	down
PHYHIP	-2.1833034	0.72527651	1.47E-91	6.42E-89	down
KLF9	-2.183003	2.28383265	1.85E-55	1.25E-53	down

TCEAL1	-2.1784861	3.1730996	7.52E-60	6.28E-58	down
PDGFRA	-2.1777523	1.58180562	7.24E-36	1.84E-34	down
DPP6	-2.1676992	0.5711129	6.96E-59	5.53E-57	down
DIRAS1	-2.1675571	0.73116109	3.52E-51	1.94E-49	down
FAXDC2	-2.1673242	1.48719348	7.57E-62	6.91E-60	down
CCDC69	-2.1666353	2.19148692	1.55E-46	6.43E-45	down
CALHM2	-2.1621426	2.39788102	5.32E-50	2.75E-48	down
SOCS2	-2.1614203	1.25704184	3.97E-65	4.20E-63	down
APBB1	-2.1607822	2.1433313	7.68E-42	2.55E-40	down
RHOJ	-2.1520526	1.64378968	3.21E-63	3.15E-61	down
DIXDC1	-2.1489209	1.28616194	5.80E-92	2.66E-89	down
SMIM10	-2.1469579	1.5197043	1.90E-58	1.49E-56	down
KCNE4	-2.1439572	0.98425201	2.92E-54	1.84E-52	down
TMEM47	-2.1432414	2.65771211	1.57E-22	1.97E-21	down
RBM24	-2.1361993	1.10475321	1.87E-40	5.82E-39	down
PTGFR	-2.1350833	0.29843713	9.39E-113	1.09E-109	down
PPP1R3C	-2.1334393	0.99939246	1.20E-60	1.04E-58	down
OMD	-2.1303188	0.34403137	4.62E-96	2.40E-93	down
POSTN	-2.1298196	2.06488761	3.10E-18	2.88E-17	down
ISLR	-2.1288339	5.06930026	8.22E-18	7.39E-17	down
LAMA4	-2.1253832	2.03456633	8.96E-47	3.75E-45	down
NR2F2	-2.1242567	3.4521149	3.28E-42	1.11E-40	down
CYYR1	-2.119715	1.91019946	3.57E-41	1.15E-39	down
MYCT1	-2.1194423	1.09499059	3.83E-74	5.99E-72	down
GPRASP2	-2.1144968	1.37817365	1.71E-77	3.43E-75	down
COL4A6	-2.1122795	0.48506078	5.56E-89	2.11E-86	down
PI16	-2.1109373	0.40986073	7.01E-53	4.06E-51	down
MEDAG	-2.1091284	0.82338292	5.28E-64	5.25E-62	down
FBLN5	-2.1086527	3.33417884	1.80E-24	2.51E-23	down
CTGF	-2.100056	5.71213427	1.03E-20	1.15E-19	down
SYTL4	-2.0961637	1.28201286	1.49E-74	2.41E-72	down
PROS1	-2.0940607	1.60113321	7.30E-47	3.08E-45	down
SYNM	-2.0860597	0.81912536	1.33E-80	3.01E-78	down
GNG7	-2.0789847	0.92775045	1.04E-60	8.97E-59	down
CXorf36	-2.0777098	1.39236643	1.46E-57	1.11E-55	down
GSN	-2.0764619	5.20348746	9.58E-38	2.68E-36	down
CFL2	-2.0702591	1.95652187	1.26E-70	1.73E-68	down
NAP1L3	-2.0700519	0.89057993	2.15E-60	1.82E-58	down
FRMD6	-2.0687577	1.38839989	2.69E-48	1.24E-46	down
RASSF8	-2.065474	1.23095061	4.02E-56	2.81E-54	down
CRISP3	-2.0649302	0.97728808	8.40E-13	5.15E-12	down
ZFP36	-2.0644901	5.7681503	2.52E-18	2.36E-17	down
TBX3	-2.0568025	1.79684292	1.25E-33	2.83E-32	down
LEPR	-2.0555878	0.91825376	1.26E-66	1.42E-64	down
CORO2B	-2.0549638	0.89756726	8.34E-51	4.50E-49	down

SGCA	-2.053479	0.56984356	3.54E-58	2.74E-56	down
AVPR1A	-2.0491656	0.78962058	1.49E-47	6.63E-46	down
CITED2	-2.04727	3.87080443	1.30E-26	2.08E-25	down
GIMAP7	-2.046698	2.2002822	3.06E-30	5.95E-29	down
SLC7A2	-2.0448365	1.60660983	1.89E-15	1.44E-14	down
PGR	-2.0349665	2.7691466	6.05E-11	3.17E-10	down
FBXL22	-2.0319934	0.72987846	1.27E-93	6.26E-91	down
MMRN1	-2.0274612	1.14366438	2.87E-39	8.53E-38	down
REM1	-2.0269674	0.97642913	1.22E-62	1.16E-60	down
STON1	-2.0238966	0.9970254	9.93E-59	7.86E-57	down
SCARA3	-2.0237983	4.18256907	2.28E-19	2.30E-18	down
MUM1L1	-2.0218739	0.63994147	3.30E-53	1.97E-51	down
AKAP12	-2.0214971	2.16034208	4.14E-20	4.41E-19	down
CASQ2	-2.0214791	0.46306927	1.35E-74	2.20E-72	down
FAM198B	-2.0182102	2.26980499	7.35E-22	8.84E-21	down
JAZF1	-2.0168742	2.05405545	5.35E-56	3.70E-54	down
MATN2	-2.0158176	2.95539538	5.19E-19	5.10E-18	down
PAMR1	-2.0097075	2.6467489	3.29E-18	3.05E-17	down
SSC5D	-2.0084261	1.3997605	2.63E-34	6.20E-33	down
RBP7	-2.0069508	1.83158169	8.31E-22	9.95E-21	down
ATOH8	-2.0057679	1.03620368	4.82E-42	1.61E-40	down
WISP2	-2.0000923	0.49946659	2.75E-54	1.74E-52	down
SGCE	-1.9996155	2.54701115	1.28E-25	1.93E-24	down
PALLD	-1.9976184	3.95918773	2.12E-24	2.95E-23	down
IGF2	-1.9973323	4.24967567	3.04E-06	9.82E-06	down
C8orf88	-1.9941257	0.57193796	1.30E-64	1.32E-62	down
ITPR1	-1.9935074	1.06322021	5.17E-67	6.00E-65	down
SMTN	-1.9934903	2.88946701	7.83E-48	3.54E-46	down
FMOD	-1.9932222	4.38993583	1.15E-13	7.55E-13	down
MFAP5	-1.992665	1.70365562	4.75E-14	3.22E-13	down
GLT8D2	-1.9820207	1.36340929	1.37E-53	8.26E-52	down
SEPP1	-1.9798244	2.24040674	1.87E-25	2.78E-24	down
VSTM4	-1.9756036	0.87506104	7.24E-66	7.88E-64	down
ADAMTS1	-1.9679366	3.56732462	2.33E-19	2.35E-18	down
TMOD1	-1.9668009	2.0560798	1.30E-19	1.34E-18	down
C1S	-1.965601	4.05554847	6.74E-21	7.62E-20	down
ZFPM2	-1.9645086	0.4168103	1.53E-102	1.08E-99	down
TPSB2	-1.9621772	1.26505382	2.77E-22	3.41E-21	down
PI15	-1.959728	0.55121166	6.91E-53	4.02E-51	down
PTPRN2	-1.9573448	0.87607961	8.08E-64	8.00E-62	down
KLF4	-1.9562468	2.37928062	3.08E-31	6.34E-30	down
PTN	-1.9554898	3.16212632	1.00E-10	5.14E-10	down
CA11	-1.9546613	3.03531353	2.70E-26	4.23E-25	down
CRISPLD2	-1.9545467	1.83209168	5.75E-42	1.91E-40	down
MXRA8	-1.952271	3.8136883	8.10E-19	7.81E-18	down

NBL1	-1.9516096	3.61724595	2.49E-21	2.89E-20	down
COL6A3	-1.949558	3.14629215	2.68E-20	2.90E-19	down
SNAI2	-1.9487946	1.85276207	1.61E-33	3.63E-32	down
DENND2A	-1.9487038	1.70204122	3.09E-44	1.14E-42	down
PKD2	-1.9474052	2.62288307	7.42E-51	4.03E-49	down
LRRC4B	-1.945903	1.23687176	8.46E-31	1.70E-29	down
ABCA8	-1.9393771	0.43126857	9.97E-104	7.28E-101	down
LGI4	-1.9372536	0.57239064	5.47E-93	2.63E-90	down
HSD17B6	-1.9361625	0.68019776	1.14E-68	1.40E-66	down
EPDR1	-1.935637	2.75459736	1.09E-19	1.13E-18	down
INMT	-1.9355609	0.98628803	2.38E-47	1.04E-45	down
C3orf70	-1.9353967	0.52354928	5.55E-83	1.42E-80	down
SELP	-1.9347982	0.94355943	5.04E-47	2.15E-45	down
WBSCR17	-1.9318617	0.91476035	6.91E-30	1.32E-28	down
AOX1	-1.9309461	0.46160553	2.87E-77	5.61E-75	down
SH3BP5	-1.9308204	1.3052968	1.16E-52	6.67E-51	down
TMEM246	-1.9303877	0.9597822	3.37E-31	6.89E-30	down
OLFM1	-1.9295641	0.78154406	2.52E-35	6.22E-34	down
EVA1C	-1.9235677	1.69491817	2.44E-38	6.99E-37	down
HSPA12B	-1.9226763	1.40711529	2.47E-55	1.67E-53	down
GIMAP6	-1.9097359	1.61581634	8.21E-44	2.94E-42	down
PLAT	-1.9064684	2.73821508	7.61E-21	8.56E-20	down
MMP28	-1.9037655	0.61714167	2.57E-74	4.09E-72	down
CDH13	-1.9016225	0.81876648	1.18E-57	9.00E-56	down
AEBP1	-1.9012261	4.87008287	1.70E-17	1.50E-16	down
VSIG2	-1.9009693	1.3078696	4.31E-14	2.93E-13	down
PALM	-1.8988374	2.69456379	4.85E-15	3.55E-14	down
MYADM	-1.8942542	4.99049574	1.25E-33	2.82E-32	down
CCBE1	-1.8921242	0.39120926	4.13E-69	5.25E-67	down
GPIHBP1	-1.8916826	0.41737424	1.54E-85	4.54E-83	down
CLIC2	-1.8903859	1.55554788	5.31E-53	3.10E-51	down
PPM1K	-1.8896472	1.23102267	8.81E-67	1.02E-64	down
PTGDS	-1.8886616	4.67107138	1.35E-08	5.63E-08	down
TMEM200A	-1.8871453	1.31273181	5.73E-38	1.63E-36	down
C1QTNF7	-1.887078	0.59374549	6.06E-79	1.33E-76	down
AFAP1L2	-1.8870274	2.33455996	1.17E-26	1.87E-25	down
ADAMTS5	-1.8850031	1.004032	4.15E-43	1.45E-41	down
ABCC9	-1.8793361	0.47099643	2.26E-105	1.93E-102	down
CRYAB	-1.8773761	3.12568196	6.30E-12	3.58E-11	down
NR3C1	-1.8756466	1.24980396	1.82E-48	8.52E-47	down
SOX15	-1.8750207	0.92831592	1.20E-59	9.92E-58	down
KCNJ8	-1.8743255	1.37690736	1.23E-53	7.47E-52	down
SLC2A4	-1.8727057	0.53986244	1.98E-84	5.57E-82	down
ST5	-1.8670594	2.37906224	2.15E-51	1.20E-49	down
LAYN	-1.8667895	1.42658241	5.96E-57	4.40E-55	down

MEF2C	-1.8665956	1.03070105	1.34E-83	3.62E-81	down
NPR2	-1.8633053	1.81218387	7.95E-51	4.31E-49	down
HIC1	-1.8624252	1.17668116	8.44E-54	5.22E-52	down
MDFIC	-1.8599823	1.60836376	1.55E-44	5.83E-43	down
RFTN1	-1.8596172	2.31447405	1.63E-39	4.92E-38	down
RARRES2	-1.8586891	4.6195285	4.40E-15	3.24E-14	down
PAGE4	-1.8571272	0.54062875	8.91E-34	2.02E-32	down
CCL14	-1.8534637	0.21422844	1.01E-115	1.42E-112	down
PDZD4	-1.8516563	0.70956726	6.58E-43	2.28E-41	down
UST	-1.8452582	0.95600839	7.84E-43	2.71E-41	down
ATF3	-1.843315	3.15016397	3.42E-16	2.76E-15	down
AXL	-1.8372545	2.53061482	2.58E-35	6.36E-34	down
NDP	-1.8371838	1.91972853	2.99E-16	2.42E-15	down
TEF	-1.8363691	2.22458764	6.45E-61	5.63E-59	down
SYDE1	-1.8360372	2.01919785	2.87E-41	9.30E-40	down
SNCA	-1.8341733	0.7812363	5.58E-56	3.83E-54	down
KDR	-1.8314903	1.89417873	9.40E-40	2.87E-38	down
PPP1R14A	-1.8284476	2.80611358	1.32E-15	1.01E-14	down
SUSD3	-1.8280516	2.41238257	1.82E-19	1.84E-18	down
COLEC12	-1.8270055	0.96116966	4.81E-35	1.17E-33	down
MMRN2	-1.8258808	2.14607201	2.73E-41	8.87E-40	down
LPP	-1.8193964	2.25250962	2.57E-47	1.12E-45	down
PRKG1	-1.818561	0.56008617	1.54E-85	4.54E-83	down
THBS2	-1.8178876	2.4098475	1.84E-16	1.51E-15	down
HTR2B	-1.8146563	1.0773635	3.53E-30	6.84E-29	down
HOXD8	-1.8138519	1.70624823	2.23E-23	2.94E-22	down
GUCY1A2	-1.8111015	0.64585273	1.39E-64	1.41E-62	down
PDE8B	-1.8101408	0.98687854	2.11E-70	2.81E-68	down
PBX1	-1.8099954	2.57277269	4.15E-34	9.67E-33	down
ASB2	-1.8057715	1.09456556	2.66E-50	1.39E-48	down
ARHGAP6	-1.8054996	0.45285964	3.72E-95	1.88E-92	down
SETD7	-1.8017011	1.63738279	2.07E-44	7.68E-43	down
MITF	-1.8013848	1.88436784	4.13E-36	1.07E-34	down
PROCR	-1.8000306	2.74035895	2.42E-29	4.44E-28	down
CTSF	-1.7986986	4.18726557	4.23E-20	4.50E-19	down
C2orf40	-1.7932551	1.30349475	1.68E-15	1.28E-14	down
DPYD	-1.7928499	1.068126	2.14E-47	9.41E-46	down
CDC42EP3	-1.7897093	2.19699373	1.41E-44	5.32E-43	down
CLIC4	-1.7896229	4.46584217	5.99E-26	9.22E-25	down
SPEG	-1.7866885	0.8173964	1.82E-46	7.49E-45	down
LIX1L	-1.7863513	2.75752183	7.62E-40	2.33E-38	down
TRPC1	-1.7835871	0.73393159	6.70E-68	8.11E-66	down
FOXL2NB	-1.7811045	0.62963719	4.82E-61	4.24E-59	down
PDGFRL	-1.774968	1.96393765	8.70E-23	1.11E-21	down
GIMAP8	-1.7720694	1.24341421	1.06E-50	5.64E-49	down

EFS	-1.7677798	1.94022122	2.28E-17	1.99E-16	down
SOD3	-1.7651565	4.25597545	4.95E-12	2.85E-11	down
PRKCDBP	-1.7643667	3.04482632	6.96E-21	7.86E-20	down
FAM110D	-1.7618464	1.0769482	4.63E-55	3.04E-53	down
CLU	-1.7610189	6.68017963	3.79E-07	1.35E-06	down
FERMT2	-1.7573931	2.36489512	2.29E-45	8.96E-44	down
RGS5	-1.7571081	3.21443674	2.73E-19	2.73E-18	down
DPYSL2	-1.7564646	2.68654274	1.11E-24	1.56E-23	down
GSPT2	-1.7537247	0.97881866	2.25E-29	4.15E-28	down
EBF4	-1.752393	2.28576071	4.26E-20	4.53E-19	down
PARM1	-1.7496274	2.27640176	4.59E-16	3.68E-15	down
S1PR1	-1.7458947	2.08945337	3.16E-32	6.75E-31	down
LUM	-1.7452796	5.16891117	4.97E-11	2.62E-10	down
PRRX1	-1.7448892	2.43581599	6.55E-13	4.04E-12	down
PRKAR2B	-1.7447022	1.30764373	1.25E-29	2.34E-28	down
COPZ2	-1.7434369	1.72027628	7.39E-38	2.09E-36	down
FAM107A	-1.738628	2.50021815	2.11E-09	9.60E-09	down
MXRA7	-1.7385648	2.78683863	1.14E-38	3.32E-37	down
PCDH18	-1.7369116	1.65765953	4.82E-28	8.36E-27	down
SULF2	-1.7367818	3.92667466	2.05E-14	1.43E-13	down
CAV2	-1.7340863	1.60718152	2.47E-41	8.03E-40	down
PDGFRB	-1.7333186	3.48969502	2.31E-22	2.85E-21	down
SELM	-1.7313302	4.07445096	8.16E-19	7.87E-18	down
ADAMTSL5	-1.729235	0.65505985	6.49E-49	3.11E-47	down
CLDN5	-1.7290174	2.39586944	6.07E-18	5.52E-17	down
TACR2	-1.7246611	0.75193568	6.27E-54	3.89E-52	down
PER1	-1.7222241	2.31372864	3.68E-38	1.05E-36	down
TEK	-1.7216429	1.30367695	2.21E-38	6.36E-37	down
STAT5B	-1.7207856	3.25070575	2.31E-67	2.75E-65	down
IRS1	-1.7198443	2.17076262	7.05E-24	9.49E-23	down
CD34	-1.7174915	2.43059779	2.97E-37	8.08E-36	down
CDC42EP5	-1.7168743	2.40074801	1.17E-20	1.30E-19	down
METTL7A	-1.7167691	3.59143381	1.56E-23	2.08E-22	down
THBD	-1.7125241	1.74222924	9.24E-29	1.65E-27	down
FAM69A	-1.7122174	1.33718125	3.30E-46	1.35E-44	down
CPQ	-1.7106391	2.86368108	1.55E-38	4.49E-37	down
DMD	-1.7069522	0.75824268	5.59E-59	4.46E-57	down
FAM65B	-1.705853	0.7796157	2.95E-64	2.95E-62	down
SYNE1	-1.702237	0.64036119	1.66E-75	2.87E-73	down
COL15A1	-1.700759	2.80084379	3.44E-19	3.42E-18	down
CBX6	-1.7004723	2.89192774	3.11E-25	4.54E-24	down
SNCAIP	-1.6998602	1.20193561	1.67E-35	4.17E-34	down
AR	-1.6992439	2.07900533	3.33E-19	3.31E-18	down
CILP	-1.6950307	0.80338966	4.65E-27	7.66E-26	down
PIANP	-1.6949432	1.07233627	1.05E-24	1.48E-23	down

ITGA11	-1.6940528	1.14887942	1.30E-26	2.07E-25	down
SLC16A2	-1.6921332	1.7704245	1.34E-28	2.39E-27	down
TBL1X	-1.6908878	2.85774829	2.52E-37	6.91E-36	down
C1QTNF1	-1.6898522	1.59125167	7.24E-37	1.93E-35	down
BCL2	-1.6895426	1.82901781	9.73E-27	1.57E-25	down
TGFBR2	-1.6886625	3.51913538	1.26E-25	1.91E-24	down
GNA14	-1.6885786	1.16703352	5.08E-43	1.77E-41	down
SHC2	-1.6884681	1.95816283	1.02E-16	8.59E-16	down
EPHB6	-1.6884369	1.48970865	2.29E-21	2.66E-20	down
CPA3	-1.6807891	0.8318001	6.56E-24	8.85E-23	down
APOLD1	-1.6792014	1.2100372	3.68E-40	1.14E-38	down
PDZRN3	-1.6790188	2.57275705	2.78E-17	2.40E-16	down
DCHS1	-1.6787884	1.7038544	2.36E-27	3.95E-26	down
PRICKLE2	-1.675755	1.51177579	2.98E-43	1.05E-41	down
PTH1R	-1.6752802	1.10919033	9.89E-22	1.18E-20	down
TSPAN7	-1.6743587	2.6077891	1.44E-11	7.96E-11	down
RASSF2	-1.6742314	2.00773019	1.07E-30	2.14E-29	down
ANKRD29	-1.6722323	0.66398754	5.04E-72	7.42E-70	down
TCEAL4	-1.6677415	5.16404762	5.27E-46	2.12E-44	down
NES	-1.6671237	3.28906863	1.50E-20	1.66E-19	down
FBN1	-1.6666102	1.52845795	3.15E-28	5.50E-27	down
GJC1	-1.665366	1.07152035	2.44E-35	6.07E-34	down
RECK	-1.6647296	1.01854613	6.45E-65	6.73E-63	down
CRIM1	-1.6625115	2.7124232	8.99E-32	1.89E-30	down
PAEP	-1.662488	0.91877114	2.25E-10	1.11E-09	down
C10orf54	-1.6608797	2.57617331	3.23E-29	5.91E-28	down
FHL5	-1.655835	0.36865381	1.22E-89	4.80E-87	down
STXBP1	-1.6555467	2.01574531	7.74E-30	1.47E-28	down
DNAJB4	-1.6537745	1.91627315	6.36E-38	1.80E-36	down
COL16A1	-1.6537719	2.00327542	9.65E-27	1.56E-25	down
EDIL3	-1.6496448	1.3183557	1.78E-27	3.00E-26	down
SERINC1	-1.6493396	5.06613823	2.98E-35	7.32E-34	down
RAB23	-1.6485612	1.42356419	1.17E-38	3.42E-37	down
SH3BGRL	-1.6483391	5.17072321	5.02E-30	9.63E-29	down
COL6A2	-1.6468725	6.06098019	2.28E-14	1.59E-13	down
REEP1	-1.6435076	0.98293888	1.28E-38	3.72E-37	down
ZNF521	-1.6417807	1.01922023	2.42E-26	3.79E-25	down
ELTD1	-1.6415352	1.9423566	2.49E-32	5.34E-31	down
BOC	-1.6399825	1.80621018	1.11E-19	1.14E-18	down
CLEC14A	-1.6397149	2.88221554	5.52E-27	9.04E-26	down
SLIT3	-1.6392362	1.40689401	9.48E-28	1.62E-26	down
SNED1	-1.6355412	1.09273119	1.64E-42	5.61E-41	down
ITGA7	-1.6353226	1.56864083	4.09E-27	6.77E-26	down
FKBP7	-1.6337746	2.03605023	1.67E-38	4.83E-37	down
FBXO17	-1.6335021	1.28804031	6.82E-18	6.19E-17	down

TCEAL3	-1.6283003	3.44366179	3.31E-40	1.03E-38	down
ADAMTSL4	-1.6277388	0.9764949	5.10E-44	1.85E-42	down
APOBEC3C	-1.6275981	3.40325936	9.30E-18	8.34E-17	down
ABCB1	-1.6252969	0.98529021	3.48E-36	8.99E-35	down
TUBB6	-1.6251849	3.29138587	3.35E-19	3.33E-18	down
HNMT	-1.6246068	2.4256536	1.13E-25	1.71E-24	down
STMN3	-1.6226852	3.07425871	5.16E-14	3.49E-13	down
ITGA8	-1.6223832	0.58260915	4.75E-74	7.38E-72	down
GLI3	-1.6196677	0.92792904	7.72E-38	2.17E-36	down
LAMA2	-1.6188874	0.96038823	1.05E-33	2.38E-32	down
RGMA	-1.6183441	1.35327576	9.16E-28	1.57E-26	down
PCP4	-1.6151467	1.79019348	2.75E-08	1.11E-07	down
TPSAB1	-1.6137071	1.33484931	3.51E-16	2.83E-15	down
CALCRL	-1.6123878	1.57571215	3.61E-23	4.69E-22	down
FZD4	-1.6076204	1.7590348	2.13E-42	7.23E-41	down
EBF1	-1.6060754	1.09859131	1.50E-35	3.76E-34	down
TMEM119	-1.605305	1.76756317	5.64E-20	5.95E-19	down
FIBIN	-1.6049698	1.15881046	1.62E-17	1.42E-16	down
MEIS2	-1.6038223	1.28314044	1.03E-24	1.46E-23	down
SOX18	-1.6033314	2.45148877	1.44E-22	1.82E-21	down
LRP1	-1.6028779	3.33154548	6.60E-26	1.01E-24	down
THRA	-1.5969175	3.0250133	1.44E-31	2.99E-30	down
EFHD1	-1.596254	2.88433771	1.11E-13	7.28E-13	down
CD248	-1.5947018	3.81537469	5.66E-18	5.16E-17	down
ENPP1	-1.5939646	1.11336586	2.75E-34	6.45E-33	down
GJA4	-1.5916477	2.92376753	3.93E-14	2.68E-13	down
ST6GALNA	-1.5869046	3.07287283	8.18E-45	3.14E-43	down
RAI2	-1.5858985	3.88745182	1.39E-11	7.68E-11	down
RAB3IL1	-1.5857302	2.90003772	6.89E-30	1.31E-28	down
ENPEP	-1.5802352	0.78900605	5.79E-49	2.79E-47	down
VAMP2	-1.5779945	3.98861258	6.05E-34	1.38E-32	down
TBX2	-1.5776747	1.80494388	3.26E-22	4.00E-21	down
NID1	-1.5771621	3.17575045	7.93E-17	6.72E-16	down
NUDT10	-1.5705426	0.7846621	1.94E-36	5.07E-35	down
ROBO4	-1.5701234	1.56647137	5.84E-45	2.25E-43	down
LMCD1	-1.5700445	1.61900234	1.78E-42	6.08E-41	down
EDNRB	-1.568897	0.85181636	1.77E-39	5.32E-38	down
LYVE1	-1.5643623	0.55111384	2.20E-75	3.74E-73	down
ZBTB38	-1.5622286	2.0379953	1.82E-42	6.21E-41	down
RBPMS	-1.5622189	3.05586567	6.97E-22	8.40E-21	down
HEPH	-1.5621279	1.62198627	7.05E-23	9.05E-22	down
OSBPL1A	-1.5619804	1.96559616	4.06E-44	1.48E-42	down
ANO6	-1.5616056	2.62049286	7.58E-38	2.13E-36	down
HTRA3	-1.5584277	3.01066732	1.57E-14	1.11E-13	down
KANK3	-1.5567355	0.95916802	8.19E-55	5.33E-53	down

PER3	-1.5564864	1.39446468	3.05E-36	7.91E-35	down
PBX3	-1.5564282	1.14805309	3.60E-31	7.34E-30	down
NDRG2	-1.5562965	3.26622066	1.34E-15	1.03E-14	down
LGI2	-1.5562527	0.72168989	2.47E-35	6.12E-34	down
EMP3	-1.5551497	3.95501622	1.12E-21	1.33E-20	down
RCAN2	-1.555065	1.61027985	5.68E-23	7.32E-22	down
SLC25A25	-1.554192	2.09415285	2.23E-35	5.55E-34	down
RHOB	-1.5520953	6.41678903	1.27E-19	1.31E-18	down
CTSO	-1.5516855	3.18845117	9.90E-27	1.59E-25	down
ARHGEF15	-1.5510022	1.37224206	3.66E-40	1.13E-38	down
KCND3	-1.5491057	0.65798863	1.25E-45	4.96E-44	down
PPAP2B	-1.5477715	3.57387748	9.36E-15	6.70E-14	down
SYNPO	-1.544344	2.43105724	1.51E-27	2.54E-26	down
ANXA6	-1.5437151	4.23379583	9.59E-23	1.22E-21	down
GFRA1	-1.5436495	0.55021555	7.37E-24	9.91E-23	down
LRRC32	-1.5431621	2.53363092	5.83E-24	7.89E-23	down
NOSTRIN	-1.5419035	1.12358504	1.63E-44	6.09E-43	down
PYGM	-1.5415005	0.60023028	1.05E-42	3.60E-41	down
HSD17B11	-1.5360036	3.46109002	3.64E-20	3.89E-19	down
FAM65C	-1.5343076	0.80481579	3.35E-49	1.64E-47	down
PRIMA1	-1.5322326	1.28211442	7.61E-19	7.37E-18	down
FYCO1	-1.5319294	2.46324913	3.12E-50	1.62E-48	down
HSPB2	-1.5315685	0.57183786	2.11E-59	1.72E-57	down
RAB31	-1.5296596	2.83140957	2.38E-26	3.74E-25	down
HEYL	-1.528771	1.99549928	2.46E-25	3.62E-24	down
ATP8B2	-1.527995	2.35730479	1.67E-27	2.81E-26	down
NGF	-1.5275068	0.80720674	1.35E-39	4.08E-38	down
TIE1	-1.5273694	2.06505158	4.17E-29	7.60E-28	down
NRXN2	-1.5262371	0.94256148	9.05E-36	2.30E-34	down
CCDC3	-1.5256181	2.683443	2.51E-14	1.74E-13	down
A2M	-1.5243063	5.71533359	8.74E-20	9.09E-19	down
IL6ST	-1.5241545	3.40166195	1.42E-26	2.25E-25	down
WNT4	-1.5236248	1.09222895	5.79E-24	7.84E-23	down
FZD7	-1.5226173	2.18577582	1.80E-11	9.87E-11	down
TIPARP	-1.5212737	2.10973916	3.06E-39	9.10E-38	down
NAP1L2	-1.520496	0.68849811	1.84E-44	6.85E-43	down
PDE5A	-1.5189222	1.21863827	1.24E-37	3.43E-36	down
DIO3	-1.5177799	0.4881836	1.20E-44	4.57E-43	down
ILK	-1.5173562	1.49576913	1.20E-40	3.77E-39	down
FOXO1	-1.5160617	2.8928372	1.60E-28	2.81E-27	down
GIMAP4	-1.5157497	2.75739059	1.22E-19	1.26E-18	down
ANTXR1	-1.5138609	3.55489712	2.43E-18	2.27E-17	down
CLEC2B	-1.5135731	1.62235857	5.95E-31	1.20E-29	down
SPG20	-1.5127794	2.6884246	3.25E-25	4.74E-24	down
ACOX2	-1.5113715	1.21778548	1.63E-30	3.22E-29	down

DZIP1	-1.5111884	1.39034574	4.70E-29	8.56E-28	down
TBC1D2B	-1.5110861	1.67997009	1.99E-57	1.51E-55	down
SERPING1	-1.5106641	6.20032639	1.15E-12	6.96E-12	down
CELF2	-1.5080955	1.50709207	1.16E-23	1.55E-22	down
JUN	-1.5040863	6.05803815	7.47E-18	6.75E-17	down
LOXL4	-1.5026705	1.43579126	2.73E-18	2.54E-17	down
BTG2	-1.5019807	5.58884374	1.56E-14	1.10E-13	down
PPAPDC3	-1.5014228	0.6668126	2.15E-59	1.75E-57	down
KLF6	-1.5009513	3.49169831	5.05E-21	5.76E-20	down
TIMP3	-1.5002809	0.51171058	2.48E-50	1.30E-48	down
NUDT11	-1.4992257	0.66184069	7.14E-28	1.23E-26	down
GAB2	-1.4991672	2.20445495	1.17E-27	1.98E-26	down
RAMP2	-1.4947962	3.94544294	1.18E-18	1.12E-17	down
CDON	-1.4947281	0.90593738	1.78E-44	6.66E-43	down
GLIPR1	-1.4934725	1.34493572	2.28E-39	6.83E-38	down
NLGN3	-1.4927385	0.71194121	3.22E-61	2.87E-59	down
KNDC1	-1.4916355	0.47724403	3.96E-36	1.02E-34	down
MBNL2	-1.4905957	2.80615889	1.37E-30	2.71E-29	down
PRDM8	-1.4888632	0.38650524	1.63E-80	3.65E-78	down
VGLL3	-1.4887836	0.36328422	3.28E-67	3.87E-65	down
LYPLAL1	-1.4876461	1.15180622	9.29E-21	1.04E-19	down
MARVELD1	-1.4874895	3.67633637	1.51E-22	1.89E-21	down
CA3	-1.485692	0.61270636	2.13E-44	7.89E-43	down
TNFSF12	-1.4853242	3.41865486	4.10E-23	5.31E-22	down
P3H3	-1.4842899	2.62106415	6.99E-14	4.67E-13	down
DSTN	-1.4807772	6.13561673	7.27E-46	2.91E-44	down
VAMP5	-1.4804111	4.26534832	4.51E-18	4.15E-17	down
MT-ND6	-1.4786348	10.36742	5.88E-09	2.56E-08	down
PEAR1	-1.4779926	0.8602809	1.25E-55	8.53E-54	down
C1R	-1.4764162	4.69094042	2.82E-16	2.28E-15	down
HSPB3	-1.474098	0.29822488	2.34E-57	1.76E-55	down
KCNB1	-1.4716561	0.2196596	3.16E-104	2.39E-101	down
RUSC2	-1.4712808	2.35389335	9.00E-42	2.97E-40	down
FGD5	-1.4711168	1.58289456	4.89E-27	8.04E-26	down
MYOM1	-1.4704089	0.47708678	1.22E-64	1.25E-62	down
RRAD	-1.4697542	2.60126873	1.11E-06	3.77E-06	down
NEURL1B	-1.4692456	2.38070451	1.13E-29	2.13E-28	down
RBPMS2	-1.4680368	2.51323325	4.70E-21	5.37E-20	down
SMARCA2	-1.4679554	3.18199054	1.43E-21	1.68E-20	down
HAAO	-1.467864	1.33854296	6.69E-32	1.41E-30	down
FLT4	-1.4676842	1.15005062	1.82E-49	9.15E-48	down
IGIP	-1.4662453	1.68597445	8.69E-48	3.91E-46	down
SLC16A9	-1.4660597	1.92548645	2.89E-15	2.16E-14	down
ZBTB4	-1.4648831	3.8072341	3.00E-49	1.48E-47	down
GPM6A	-1.4648584	0.29075914	5.89E-51	3.23E-49	down

TLE4	-1.4644736	1.09344414	1.88E-31	3.89E-30	down
NLRP1	-1.4639875	1.29342003	5.96E-33	1.32E-31	down
IGFBP7	-1.4631246	7.1501542	6.20E-16	4.89E-15	down
TCEAL6	-1.4574296	0.1560737	2.08E-106	1.86E-103	down
DAB2	-1.4571361	1.99614075	1.04E-25	1.58E-24	down
MRC2	-1.4559444	3.49071014	4.37E-15	3.22E-14	down
TMEM55A	-1.4553133	1.33770385	2.78E-36	7.23E-35	down
GREM2	-1.4528419	1.30674656	5.61E-08	2.20E-07	down
ADRA2C	-1.4521783	3.29636673	5.28E-09	2.30E-08	down
CCL23	-1.4521084	0.37323752	5.31E-57	3.95E-55	down
ADAMTSL1	-1.4510517	0.79338554	5.03E-30	9.64E-29	down
STOM	-1.4503373	4.75047231	3.77E-23	4.89E-22	down
FAM129A	-1.4495984	1.70525923	6.80E-16	5.34E-15	down
TMEM204	-1.4492004	3.00954559	2.33E-20	2.53E-19	down
APOL3	-1.447262	1.68835227	2.55E-21	2.95E-20	down
CD200	-1.4463813	2.9544145	1.38E-12	8.30E-12	down
C9orf3	-1.4463601	1.80133558	2.63E-43	9.27E-42	down
PDE7B	-1.4452573	0.78411349	1.38E-49	6.94E-48	down
ID4	-1.4445997	4.51802093	2.68E-11	1.45E-10	down
SNAP25	-1.4428344	0.61413753	1.22E-30	2.42E-29	down
TMEM240	-1.4416595	0.90002808	6.54E-53	3.81E-51	down
TRPC4	-1.4410417	0.4451041	6.79E-58	5.21E-56	down
GBP2	-1.4405153	3.0391963	6.03E-16	4.76E-15	down
CRMP1	-1.4398668	1.93768318	2.31E-22	2.85E-21	down
NDNF	-1.4389312	0.7795685	1.34E-25	2.01E-24	down
CLDN1	-1.4377885	3.52424051	9.04E-07	3.10E-06	down
KLHL13	-1.4318167	1.47544213	6.83E-19	6.64E-18	down
ELK3	-1.4286767	3.01070592	3.49E-23	4.55E-22	down
MAP6	-1.428183	1.23082406	1.55E-20	1.70E-19	down
NECAB1	-1.4274178	0.30873324	2.30E-87	7.94E-85	down
EZH1	-1.4254294	2.40998278	4.45E-62	4.13E-60	down
PJA2	-1.4231554	3.79671553	1.57E-28	2.77E-27	down
GPR133	-1.4190384	0.31713099	1.95E-78	4.18E-76	down
ATP1A2	-1.4181607	0.85111698	2.60E-12	1.53E-11	down
C1orf21	-1.4156845	1.64591628	1.48E-31	3.08E-30	down
NT5E	-1.4152492	3.19801472	5.46E-07	1.92E-06	down
CD93	-1.4148516	2.58619902	7.94E-22	9.52E-21	down
TUSC1	-1.4133982	2.47781289	5.83E-16	4.61E-15	down
COL6A1	-1.4133221	5.86580506	4.85E-12	2.79E-11	down
KIF26A	-1.4115115	0.63293013	4.12E-33	9.20E-32	down
CACNA2D1	-1.4113337	0.45840035	5.40E-56	3.72E-54	down
RAB8B	-1.4105822	1.7701754	2.22E-36	5.79E-35	down
DLC1	-1.4102443	1.47504387	2.56E-34	6.03E-33	down
RRAS	-1.4098665	5.05773525	4.38E-20	4.65E-19	down
FBXL3	-1.4097417	2.28208693	1.08E-38	3.17E-37	down

FABP3	-1.4074886	3.02630261	6.02E-08	2.35E-07	down
SLITRK3	-1.4072887	0.11314487	7.66E-89	2.85E-86	down
GATA5	-1.4070994	0.6503043	6.51E-16	5.13E-15	down
SLC22A17	-1.406313	2.4300017	4.29E-13	2.68E-12	down
UBL3	-1.4058342	3.2709598	1.10E-29	2.08E-28	down
DKK3	-1.405623	2.68738693	7.48E-15	5.41E-14	down
NFASC	-1.4033382	0.53189716	7.55E-50	3.84E-48	down
IRS2	-1.4027305	1.43738211	9.03E-15	6.47E-14	down
GABBR1	-1.4019019	1.49115683	1.86E-18	1.76E-17	down
GFPT2	-1.4013539	1.13607153	1.26E-22	1.60E-21	down
CHRD	-1.4011026	1.40223302	3.48E-17	3.00E-16	down
HPSE2	-1.4010323	0.33154834	3.63E-44	1.33E-42	down
LRCH2	-1.4007528	0.56498692	3.67E-31	7.49E-30	down
FILIP1	-1.3952181	0.98188825	1.65E-30	3.26E-29	down
CACNA1C	-1.3939771	0.58059895	1.19E-49	6.02E-48	down
GPR161	-1.3939378	1.40194164	8.06E-28	1.38E-26	down
RASA3	-1.3909193	1.94425258	1.40E-25	2.10E-24	down
SLC45A1	-1.39072	0.83556139	4.66E-58	3.59E-56	down
PPAP2A	-1.3902148	3.6364199	1.89E-20	2.06E-19	down
TM4SF18	-1.3888776	0.98246975	9.28E-43	3.19E-41	down
MAN1C1	-1.38886	1.05917816	1.33E-38	3.88E-37	down
RGAG4	-1.3886937	1.66438717	2.46E-27	4.11E-26	down
IFFO1	-1.3877705	1.15396641	4.34E-47	1.86E-45	down
GRASP	-1.3859941	2.12092215	1.42E-23	1.89E-22	down
FOXO4	-1.3850574	2.66456105	3.94E-42	1.32E-40	down
BCL6B	-1.3833851	1.27035151	2.51E-42	8.52E-41	down
RASD2	-1.3833796	1.16508318	1.32E-29	2.48E-28	down
GIMAP1	-1.3829854	0.92893591	1.32E-44	5.02E-43	down
MAPK10	-1.3826566	0.86151414	1.13E-32	2.45E-31	down
CDKN1B	-1.3819911	3.80649451	2.45E-14	1.70E-13	down
TMEM88	-1.3795376	2.17107782	9.25E-24	1.24E-22	down
BEX4	-1.3786917	4.57787133	3.39E-08	1.36E-07	down
FREM1	-1.378667	0.84348596	9.10E-30	1.72E-28	down
DST	-1.377378	1.70174437	8.45E-15	6.07E-14	down
SLC23A2	-1.3763302	2.03180567	9.26E-35	2.23E-33	down
SLMAP	-1.3747789	2.02821161	3.33E-39	9.88E-38	down
SAMD4A	-1.3718321	0.9464203	9.48E-39	2.78E-37	down
ZNF25	-1.37177	1.61196188	8.90E-51	4.80E-49	down
PKNOX2	-1.3715842	0.50500606	8.76E-36	2.22E-34	down
ARHGAP23	-1.3715395	2.1046237	1.74E-21	2.04E-20	down
TRIM4	-1.3714105	2.25490243	1.31E-21	1.55E-20	down
SH3PXD2A	-1.3708216	2.60162674	1.46E-30	2.90E-29	down
RNF150	-1.3707644	1.65733387	1.45E-17	1.28E-16	down
TCEAL5	-1.3680584	0.80663919	9.72E-15	6.94E-14	down
N4BP2L1	-1.3678156	1.12215286	3.48E-45	1.35E-43	down

RASL11A	-1.3674614	1.97000188	8.26E-19	7.96E-18	down
ABCA9	-1.3659233	0.34940087	1.80E-70	2.44E-68	down
ITPKB	-1.3653879	3.21199331	8.90E-26	1.36E-24	down
TSPYL2	-1.3648697	2.93704985	2.01E-29	3.71E-28	down
PLEKHH2	-1.3624957	0.83955752	9.35E-31	1.87E-29	down
CAB39L	-1.360079	1.52661793	2.49E-48	1.16E-46	down
AFAP1L1	-1.3587521	1.21984539	6.11E-48	2.78E-46	down
FAM162B	-1.3587377	0.9180696	7.25E-35	1.75E-33	down
ATP2B4	-1.3579581	4.47687169	1.46E-18	1.39E-17	down
EID1	-1.3572332	5.68102136	1.46E-33	3.29E-32	down
EGFL7	-1.355111	3.6224564	4.07E-11	2.16E-10	down
CGNL1	-1.3544192	2.22598555	1.20E-17	1.07E-16	down
CD99L2	-1.3536574	2.95068421	4.48E-36	1.15E-34	down
PRKCA	-1.3532936	1.47047097	2.66E-27	4.43E-26	down
SHE	-1.352911	0.5752646	1.58E-65	1.70E-63	down
FGL2	-1.3524351	1.78531622	8.49E-14	5.65E-13	down
MCAM	-1.3492051	3.36463239	3.29E-19	3.28E-18	down
TMEM245	-1.3489395	2.70132298	3.07E-38	8.79E-37	down
HOXD9	-1.3488062	2.97897419	4.92E-14	3.33E-13	down
PRX	-1.3468948	0.87288664	6.47E-49	3.10E-47	down
CALCOCO1	-1.3433686	2.80444197	1.95E-39	5.86E-38	down
CDH11	-1.3431705	1.78022462	4.28E-17	3.68E-16	down
FYN	-1.3419418	2.48160415	1.17E-19	1.20E-18	down
KCNH2	-1.3416575	1.28933914	2.79E-13	1.78E-12	down
SCN7A	-1.340451	0.21552085	2.42E-54	1.55E-52	down
CAP2	-1.3396374	2.30505715	6.93E-25	9.90E-24	down
HEG1	-1.3362071	2.14467408	4.77E-26	7.39E-25	down
ADCY9	-1.3350499	1.17431595	4.40E-24	6.02E-23	down
FNBP1	-1.3333112	3.19064984	8.23E-20	8.59E-19	down
GNG12	-1.3310736	3.83152382	5.95E-19	5.80E-18	down
SCN4B	-1.3280072	0.46165104	3.40E-51	1.87E-49	down
KCTD15	-1.3276572	2.05458839	8.72E-16	6.79E-15	down
PCDHB5	-1.3274325	0.65272757	3.87E-25	5.62E-24	down
OVGP1	-1.3271551	2.55913794	0.00255831	0.00548981	down
BAG2	-1.3267669	1.91105519	3.98E-20	4.25E-19	down
ESAM	-1.324631	2.98204009	4.21E-24	5.78E-23	down
BHMT2	-1.3236604	0.83280408	7.86E-17	6.67E-16	down
STK26	-1.3225527	2.36925407	2.45E-12	1.44E-11	down
SH3D19	-1.3212109	2.23848498	2.69E-29	4.92E-28	down
GADD45G	-1.3211411	3.47634408	1.65E-10	8.24E-10	down
HOXD10	-1.3204603	1.86170843	5.15E-14	3.48E-13	down
RBMS3	-1.3192512	0.71474346	2.69E-45	1.04E-43	down
MYC	-1.3188351	3.94898185	5.01E-10	2.41E-09	down
RNF144A	-1.3169872	2.31005153	5.14E-25	7.42E-24	down
TMEM43	-1.3149146	4.45843173	6.58E-42	2.18E-40	down

MEOX1	-1.3147047	0.98835415	1.76E-11	9.65E-11	down
RBFOX2	-1.3144133	3.05913543	1.20E-37	3.32E-36	down
WIPF3	-1.3143897	1.11574165	4.76E-29	8.66E-28	down
MAOA	-1.3128708	2.19177529	1.77E-14	1.24E-13	down
SLC7A8	-1.3119224	2.7884081	1.33E-08	5.53E-08	down
SLCO2A1	-1.3108574	2.61704408	5.24E-09	2.29E-08	down
NAP1L5	-1.3107633	1.50304249	1.92E-34	4.57E-33	down
ZMYM6NB	-1.309668	1.72750865	2.60E-23	3.42E-22	down
GPR116	-1.3094349	1.52628083	8.00E-30	1.52E-28	down
PCDHB15	-1.3091072	0.61105781	3.70E-53	2.20E-51	down
GJC2	-1.3088329	1.2991777	2.94E-16	2.38E-15	down
EHBP1	-1.3085293	2.18469007	2.61E-45	1.02E-43	down
SOX7	-1.3074321	0.89059915	1.80E-34	4.30E-33	down
FOXN3	-1.3071441	1.97621917	4.76E-39	1.41E-37	down
TFPI	-1.3063422	1.03847615	1.63E-17	1.44E-16	down
ZEB2	-1.3044063	0.68760065	2.83E-54	1.79E-52	down
LYNX1	-1.3034026	1.6360142	1.36E-09	6.28E-09	down
COL3A1	-1.3030362	6.25380687	5.55E-07	1.95E-06	down
NR4A3	-1.3025922	0.61413225	9.51E-25	1.35E-23	down
SETBP1	-1.3008736	1.06666469	2.56E-36	6.67E-35	down
FNDC5	-1.3007459	0.60959853	3.01E-35	7.38E-34	down
HSD17B14	-1.3001046	2.91756298	2.15E-10	1.06E-09	down
RUNX1T1	-1.2992171	0.38019647	3.64E-73	5.56E-71	down
GRK5	-1.2968882	1.00982214	1.27E-43	4.51E-42	down
CCDC8	-1.2964945	3.26238659	1.41E-07	5.29E-07	down
CDIP1	-1.2963228	2.61561056	5.85E-22	7.07E-21	down
IFI16	-1.2940656	3.49858937	9.64E-11	4.95E-10	down
ECM1	-1.2928651	3.3110033	6.28E-08	2.44E-07	down
MYZAP	-1.2919449	0.54043058	1.68E-69	2.15E-67	down
COL5A1	-1.2915515	3.25993055	7.42E-11	3.85E-10	down
NGFR	-1.291005	0.64511366	5.33E-22	6.47E-21	down
GLIPR2	-1.2904769	3.36804457	3.92E-13	2.46E-12	down
OTUD1	-1.2902078	2.29300861	1.15E-22	1.46E-21	down
NRIP2	-1.2897935	1.42243201	6.90E-41	2.20E-39	down
FAM110B	-1.2894536	1.23053185	1.43E-14	1.01E-13	down
PDGFD	-1.2887252	1.88078486	7.45E-15	5.39E-14	down
RAB9B	-1.2877967	0.49580948	3.66E-63	3.55E-61	down
ARHGAP31	-1.287101	1.15947839	4.67E-42	1.56E-40	down
TMEM35	-1.2869826	1.13193468	1.02E-20	1.14E-19	down
ANK2	-1.2853845	0.81431268	6.14E-27	1.00E-25	down
CCDC184	-1.2850684	0.86763923	1.80E-34	4.30E-33	down
INSR	-1.2847674	2.65071915	7.01E-25	1.00E-23	down
EVC	-1.2841239	1.53732694	4.57E-27	7.54E-26	down
S1PR3	-1.2830023	1.45899244	2.60E-27	4.34E-26	down
LDOC1	-1.2817486	4.41856599	4.10E-05	0.00011552	down

C10orf10	-1.2802653	3.78844578	3.35E-11	1.79E-10	down
NPTXR	-1.2778336	2.24010381	6.20E-09	2.69E-08	down
PDLIM7	-1.2776949	4.42232684	2.21E-16	1.80E-15	down
SEPT10	-1.2773747	2.90005863	1.34E-21	1.58E-20	down
WASF3	-1.2772433	1.41310287	2.78E-21	3.22E-20	down
ARHGAP20	-1.2772028	0.22874411	5.31E-122	1.31E-118	down
PTPN21	-1.2762411	1.72173714	5.12E-34	1.18E-32	down
CNTNAP1	-1.2754083	1.43151268	1.79E-26	2.84E-25	down
POLR2M	-1.2752062	1.97127369	8.85E-41	2.81E-39	down
CYP2U1	-1.2744032	1.16752523	5.40E-48	2.46E-46	down
RGN	-1.2742266	0.64993733	1.41E-25	2.12E-24	down
TCF4	-1.2739529	1.65103097	2.53E-25	3.71E-24	down
NPY1R	-1.2732769	0.52241102	1.56E-24	2.18E-23	down
EPB41L3	-1.2729927	1.02142626	3.80E-38	1.08E-36	down
ERG	-1.2728186	1.24778659	3.21E-27	5.33E-26	down
CYTL1	-1.272174	1.15877517	1.30E-08	5.44E-08	down
ANGPTL2	-1.2720378	3.15584724	4.45E-15	3.28E-14	down
OSR1	-1.2716149	0.57667568	6.54E-25	9.35E-24	down
RAB11FIP2	-1.2703455	2.05446987	2.39E-17	2.08E-16	down
MPDZ	-1.2695848	1.38206751	6.79E-35	1.64E-33	down
INPP5A	-1.2687589	2.77994868	1.35E-37	3.71E-36	down
SSBP2	-1.2673755	1.39219295	5.02E-34	1.16E-32	down
SLC51B	-1.2667717	0.79423208	1.21E-19	1.25E-18	down
PTGER2	-1.2658823	1.31579708	5.85E-19	5.71E-18	down
GNAZ	-1.2641961	1.51539224	6.10E-21	6.92E-20	down
PPP1R16B	-1.2641358	0.85948151	3.44E-35	8.41E-34	down
ZNF502	-1.2638628	0.90991176	1.45E-24	2.04E-23	down
MGARP	-1.2618021	0.46622277	1.15E-51	6.48E-50	down
CCDC89	-1.2614656	0.81558147	4.14E-24	5.68E-23	down
PALMD	-1.2608885	1.89109262	1.08E-13	7.13E-13	down
CRY2	-1.2606917	2.70431325	1.55E-35	3.88E-34	down
LPAR6	-1.2595205	2.07597405	1.01E-18	9.69E-18	down
ADCY5	-1.2582679	1.390814	1.28E-16	1.07E-15	down
CFD	-1.2580885	2.55087402	3.93E-08	1.56E-07	down
ACACB	-1.2571617	0.93111472	8.02E-49	3.82E-47	down
MAP3K3	-1.2564963	2.32511913	1.05E-43	3.76E-42	down
PPP2R2C	-1.2563707	1.55983662	1.30E-09	6.04E-09	down
VCL	-1.2549628	3.99576883	4.20E-20	4.47E-19	down
CD22	-1.2541533	0.73208477	1.27E-16	1.06E-15	down
ARHGEF6	-1.2522884	1.22339197	6.49E-35	1.57E-33	down
FEZ1	-1.2522133	0.82378952	2.18E-41	7.11E-40	down
FLI1	-1.2519916	1.07519303	2.59E-37	7.09E-36	down
SBDS	-1.2508435	4.45370086	1.83E-25	2.73E-24	down
GREB1	-1.2494901	2.2126687	4.55E-11	2.41E-10	down
GABARAPL	-1.2489335	3.06542501	1.69E-16	1.39E-15	down

MAN1A1	-1.2481366	2.5377596	3.89E-09	1.72E-08	down
NR3C2	-1.2476299	0.84900226	2.13E-34	5.04E-33	down
NRP1	-1.2466958	2.66026083	2.67E-14	1.85E-13	down
PLXDC2	-1.2464656	1.76804845	1.86E-16	1.53E-15	down
ABLIM3	-1.2458214	0.52086536	3.19E-47	1.37E-45	down
EGR2	-1.2455161	1.35474556	2.02E-20	2.19E-19	down
FAM198A	-1.2452796	0.97301397	1.27E-22	1.60E-21	down
CRTAP	-1.2441212	4.59625398	1.34E-37	3.71E-36	down
PHLDB2	-1.2430951	0.93830112	2.14E-22	2.66E-21	down
GLI2	-1.2428143	0.49348064	6.66E-50	3.41E-48	down
CCM2L	-1.2420714	1.17801089	6.80E-28	1.17E-26	down
MAMSTR	-1.2407865	1.34104047	6.25E-18	5.68E-17	down
PCDHGC3	-1.2391266	1.46410712	2.10E-18	1.98E-17	down
EPHX2	-1.2389386	2.13668546	6.66E-16	5.24E-15	down
SLC7A3	-1.2367436	0.63788588	2.64E-23	3.47E-22	down
NBEA	-1.2358203	0.56544885	1.42E-41	4.68E-40	down
HIPK3	-1.2356378	3.1912853	2.12E-23	2.80E-22	down
GDF7	-1.2355472	0.56319999	6.23E-28	1.07E-26	down
CREBRF	-1.234711	1.2100543	2.53E-50	1.32E-48	down
P3H2	-1.2341835	1.68493848	5.77E-13	3.57E-12	down
MEOX2	-1.2338286	0.27502753	4.21E-28	7.33E-27	down
AMOTL2	-1.2334413	3.40518327	4.96E-16	3.94E-15	down
AMIGO2	-1.2326345	2.48311581	6.64E-08	2.58E-07	down
RPS6KA2	-1.2325495	2.65762222	1.07E-25	1.63E-24	down
PDPN	-1.2316117	2.00067008	4.38E-10	2.12E-09	down
ZYX	-1.2313373	6.18117629	1.04E-26	1.67E-25	down
NCS1	-1.230148	3.06715178	6.46E-17	5.51E-16	down
GLI1	-1.2297253	0.74184724	4.98E-24	6.79E-23	down
SNX21	-1.2262576	2.12171134	1.22E-39	3.72E-38	down
PCDHB4	-1.2249332	0.65225886	1.48E-45	5.84E-44	down
NR4A2	-1.2245488	1.99922256	4.47E-13	2.79E-12	down
STARD13	-1.2234647	1.42382112	7.57E-37	2.02E-35	down
GSTM2	-1.2231359	1.20782348	5.20E-20	5.51E-19	down
FAM49A	-1.2229425	1.0471246	5.80E-36	1.48E-34	down
MPRIP	-1.2218538	2.98299403	2.23E-43	7.90E-42	down
KLHDC1	-1.2198075	0.63239041	3.74E-82	8.88E-80	down
MOB3B	-1.2187356	1.8103937	2.15E-24	2.99E-23	down
CSGALNAC	-1.2180972	1.19904383	1.41E-25	2.12E-24	down
SLC9A9	-1.2171766	0.79124158	2.76E-47	1.20E-45	down
EFEMP2	-1.2122774	3.61634135	2.93E-09	1.32E-08	down
STX2	-1.2117453	1.88211373	3.29E-22	4.03E-21	down
NACAD	-1.2109602	1.12570494	1.08E-19	1.12E-18	down
ABO	-1.210638	0.91396746	4.70E-12	2.71E-11	down
NTN4	-1.2102516	2.55301982	2.80E-11	1.51E-10	down
CHPT1	-1.2097867	1.64301027	2.08E-24	2.90E-23	down

MAMLD1	-1.2096167	1.07699126	3.28E-23	4.28E-22	down
PTPRB	-1.2093643	1.03626988	7.02E-33	1.54E-31	down
EPAS1	-1.2093328	3.85429821	1.82E-13	1.18E-12	down
ZNF516	-1.2088245	3.03543514	5.67E-10	2.71E-09	down
AKNA	-1.2063289	2.37384385	5.61E-24	7.61E-23	down
SPON1	-1.205033	3.61959797	0.0021688	0.00470934	down
RCBTB2	-1.2050113	2.32234285	5.24E-28	9.06E-27	down
KATNAL1	-1.2046889	0.97464855	2.20E-53	1.32E-51	down
ADARB1	-1.2042649	1.27334562	5.06E-43	1.76E-41	down
PARVA	-1.2042144	3.51952768	3.27E-35	8.00E-34	down
LCN6	-1.2038863	0.17197622	4.09E-56	2.85E-54	down
NYNRIN	-1.2035336	2.54663972	5.48E-11	2.88E-10	down
AASS	-1.2034447	0.8728184	2.77E-35	6.81E-34	down
TGFB3	-1.20292	2.00360621	2.29E-18	2.15E-17	down
NR1D2	-1.2020725	2.73646173	8.91E-24	1.19E-22	down
NLGN2	-1.2015059	2.9469885	3.89E-15	2.88E-14	down
TAGAP	-1.2013805	0.8731492	1.95E-29	3.61E-28	down
PLCL1	-1.2009632	0.30783885	2.92E-86	8.99E-84	down
STARD8	-1.1994707	0.91306701	3.53E-42	1.19E-40	down
FAM50B	-1.1989675	2.29319635	3.01E-11	1.62E-10	down
NAALADL1	-1.1989438	0.87751128	1.57E-34	3.75E-33	down
KLF11	-1.1970174	2.14272709	9.48E-23	1.21E-21	down
EGR3	-1.1968864	1.41051448	1.40E-11	7.71E-11	down
NFATC4	-1.1967977	2.57902653	1.04E-17	9.29E-17	down
SH2D3C	-1.1959673	1.92787273	1.47E-22	1.84E-21	down
GAS7	-1.1959078	1.94739582	4.26E-15	3.14E-14	down
HIGD1B	-1.1958964	0.96390768	3.08E-33	6.91E-32	down
LRCH1	-1.1958843	1.76491737	7.00E-37	1.87E-35	down
TMEM98	-1.1954838	3.75346107	5.34E-11	2.82E-10	down
PKIG	-1.1953655	4.50904062	2.89E-24	3.99E-23	down
PTPN13	-1.1947521	1.52943871	1.35E-13	8.80E-13	down
CSRNP1	-1.1945096	3.95919076	4.76E-17	4.08E-16	down
HSPA1A	-1.1938554	3.9838754	1.75E-06	5.81E-06	down
CADM3	-1.1938495	0.51711536	2.05E-16	1.68E-15	down
PRLR	-1.193499	1.03802496	1.35E-16	1.12E-15	down
TBC1D1	-1.1932251	3.34739896	9.18E-28	1.57E-26	down
MEF2A	-1.1919088	2.48169726	1.10E-27	1.87E-26	down
ZNF835	-1.1910956	0.36469609	5.95E-57	4.40E-55	down
CNTN4	-1.1903625	0.50815768	1.19E-40	3.75E-39	down
SERP2	-1.1900853	1.11112373	9.78E-32	2.05E-30	down
F8	-1.1897897	0.78259441	1.71E-56	1.21E-54	down
JUNB	-1.18948	6.52544933	2.51E-10	1.24E-09	down
MCC	-1.1881372	1.5038584	8.15E-14	5.43E-13	down
FOXJ2	-1.1875593	2.22118322	7.60E-38	2.14E-36	down
NRP2	-1.1856508	2.2597758	5.29E-11	2.79E-10	down

LINGO2	-1.1853305	0.31372359	1.55E-35	3.88E-34	down
ZBTB16	-1.1852013	0.16693792	5.56E-97	2.96E-94	down
HOXA11	-1.1838365	2.66508149	3.07E-06	9.89E-06	down
TWSG1	-1.1829896	3.66490719	1.90E-14	1.33E-13	down
WSCD2	-1.1810255	0.27019147	8.60E-44	3.08E-42	down
NFIX	-1.1794349	4.27745873	1.34E-07	5.05E-07	down
IFI27L2	-1.1787777	2.87940737	2.49E-10	1.22E-09	down
FAM8A1	-1.1782748	3.07045043	1.00E-25	1.53E-24	down
IL11RA	-1.1774451	1.50648644	2.53E-23	3.33E-22	down
ZNF662	-1.1768221	0.71128305	4.93E-25	7.13E-24	down
SLC16A11	-1.1759833	0.59504379	9.24E-22	1.10E-20	down
LTBP2	-1.1745494	2.78210521	1.34E-11	7.43E-11	down
ALDH1B1	-1.1745075	3.7828351	6.85E-20	7.16E-19	down
KLF15	-1.1742522	1.48588968	9.53E-18	8.53E-17	down
CTF1	-1.1736967	2.89447709	1.05E-13	6.95E-13	down
PPP1R12A	-1.1731122	2.18630986	7.37E-30	1.40E-28	down
ASPA	-1.1727671	0.12486013	6.95E-169	1.37E-164	down
TSC22D3	-1.1726979	3.59839002	1.86E-14	1.30E-13	down
SELE	-1.1713926	0.40685983	1.31E-26	2.08E-25	down
TLN1	-1.1694259	5.05307153	1.87E-27	3.15E-26	down
KLHL4	-1.1685591	0.13223145	4.95E-134	3.25E-130	down
FIGF	-1.1681476	0.31167862	3.16E-49	1.55E-47	down
RASIP1	-1.1672202	2.23641435	2.25E-12	1.33E-11	down
SVEP1	-1.1669834	0.81544534	5.52E-27	9.04E-26	down
TMEM64	-1.1667681	1.64720908	3.36E-19	3.33E-18	down
CLIP4	-1.1661124	1.36526129	1.33E-21	1.56E-20	down
ST3GAL5	-1.1652263	1.55683655	1.25E-20	1.39E-19	down
IQSEC3	-1.1649131	0.47053163	8.27E-32	1.74E-30	down
PIK3IP1	-1.1638051	3.09593906	1.84E-21	2.16E-20	down
CIRBP	-1.1634459	5.2650667	1.99E-22	2.47E-21	down
FAP	-1.1627375	0.8683213	5.76E-22	6.97E-21	down
C11orf95	-1.1626896	2.57432075	2.31E-25	3.41E-24	down
EPM2AIP1	-1.1623906	2.02132931	2.05E-11	1.12E-10	down
DAAM2	-1.1614964	1.49274627	1.59E-17	1.40E-16	down
GNAO1	-1.1611791	0.41445305	2.87E-50	1.49E-48	down
HVCN1	-1.1610905	1.027622	3.57E-41	1.15E-39	down
BCL2L2	-1.1585795	2.8606082	9.73E-28	1.66E-26	down
RAP2C	-1.1562602	2.71465865	1.88E-21	2.19E-20	down
CPXM2	-1.1562113	1.83105896	2.53E-06	8.25E-06	down
NACC2	-1.1556815	2.35292644	3.17E-23	4.14E-22	down
THSD1	-1.1554827	0.97681278	3.90E-38	1.11E-36	down
MICU3	-1.155028	0.60286416	4.60E-34	1.07E-32	down
CACNB2	-1.1545797	0.32830121	3.46E-101	2.20E-98	down
TRANK1	-1.1545463	1.60610435	2.94E-26	4.60E-25	down
B4GAT1	-1.1529274	3.64697631	2.19E-25	3.23E-24	down

VEGFC	-1.1522348	2.22685256	1.71E-14	1.20E-13	down
PHF1	-1.1518074	3.92557057	6.78E-35	1.64E-33	down
POU6F1	-1.1517043	1.2504527	4.48E-47	1.91E-45	down
CTSG	-1.1513338	0.24108367	4.61E-46	1.86E-44	down
MMP19	-1.1508438	1.16723079	2.75E-23	3.60E-22	down
ZFP36L1	-1.1507484	6.38303157	6.31E-15	4.58E-14	down
TSPAN5	-1.1490566	1.63150461	7.04E-15	5.10E-14	down
ACTN1	-1.14905	4.42507628	4.23E-23	5.46E-22	down
ZBTB47	-1.1480822	2.59888463	4.40E-25	6.37E-24	down
MTURN	-1.1477649	2.66956438	8.46E-16	6.60E-15	down
PFKM	-1.1466084	2.62951515	9.45E-16	7.33E-15	down
SLC8A2	-1.1464251	0.29096769	8.73E-53	5.03E-51	down
MBNL1	-1.145739	3.19517672	6.37E-28	1.10E-26	down
RASGRP2	-1.1446169	0.75674609	1.52E-39	4.59E-38	down
RAB12	-1.1440466	3.3413507	3.69E-23	4.79E-22	down
MAP7D3	-1.1437628	1.07317021	1.28E-32	2.77E-31	down
OSBPL5	-1.1435706	2.1900491	7.73E-27	1.25E-25	down
RGL1	-1.1432701	2.45270683	1.24E-15	9.51E-15	down
CLEC1A	-1.1432063	0.74143763	1.33E-49	6.70E-48	down
ZNF486	-1.1430764	0.89813804	1.04E-10	5.31E-10	down
SCARA5	-1.1430453	0.17939377	2.24E-47	9.78E-46	down
DCAF12L2	-1.1427129	0.34245059	4.94E-37	1.34E-35	down
ADHFE1	-1.1421029	0.73321675	1.51E-30	2.99E-29	down
POPDC2	-1.1411297	0.92349501	9.83E-46	3.91E-44	down
PRKD1	-1.1395861	1.2349815	2.28E-26	3.59E-25	down
ZBTB46	-1.1394877	1.61498861	1.55E-22	1.94E-21	down
HOXD11	-1.139431	0.79126335	1.76E-12	1.05E-11	down
PNRC1	-1.1394227	4.71809176	1.04E-25	1.58E-24	down
PRICKLE1	-1.1391105	0.92772091	1.34E-28	2.39E-27	down
PPP1R12C	-1.1388966	3.36807263	1.20E-37	3.34E-36	down
CD97	-1.1381493	3.95562952	2.47E-12	1.45E-11	down
ST13	-1.1379213	5.40652936	5.48E-22	6.65E-21	down
CD302	-1.137686	0.59305992	1.13E-49	5.73E-48	down
FAM180A	-1.1376333	0.3001253	3.06E-55	2.04E-53	down
FRY	-1.1375121	0.72114731	2.75E-50	1.43E-48	down
RNF38	-1.1355497	2.70909726	1.16E-26	1.86E-25	down
DFNA5	-1.1355275	0.96689437	7.79E-33	1.71E-31	down
PINLYP	-1.1346414	0.66463624	2.62E-45	1.02E-43	down
NPNT	-1.1337952	1.92988268	2.41E-08	9.78E-08	down
ESD	-1.1324058	4.20755966	3.30E-19	3.28E-18	down
EMILIN3	-1.1323281	0.6888939	1.29E-18	1.23E-17	down
AVPR2	-1.1313779	0.59302371	2.21E-17	1.93E-16	down
KIRREL	-1.1297665	2.49495673	8.71E-12	4.89E-11	down
SORBS3	-1.1293894	3.97112374	6.79E-11	3.54E-10	down
ADCY4	-1.1290905	1.17421583	6.80E-23	8.74E-22	down

C1QTNF2	-1.1289126	0.46330859	6.07E-57	4.46E-55	down
ZNF853	-1.1287724	2.34767776	2.73E-15	2.05E-14	down
PKD1	-1.1271504	2.23399654	2.12E-28	3.72E-27	down
PPM1F	-1.1265659	1.85064675	1.26E-44	4.82E-43	down
GAB1	-1.1260802	1.43884213	3.47E-31	7.09E-30	down
DMPK	-1.1242507	2.89960894	1.15E-20	1.28E-19	down
CPXM1	-1.1241174	4.26637704	6.74E-06	2.09E-05	down
PECAM1	-1.1233956	3.33951142	4.34E-15	3.20E-14	down
TMOD2	-1.1226578	0.46610343	1.89E-65	2.02E-63	down
KDELC2	-1.1224018	2.93335849	1.70E-15	1.30E-14	down
KLF7	-1.1219509	1.34853732	1.15E-29	2.16E-28	down
SOBP	-1.121545	0.92183738	2.28E-19	2.30E-18	down
LRRC2	-1.1213493	0.46640836	5.21E-44	1.88E-42	down
RIC3	-1.1213059	0.23337092	1.34E-77	2.76E-75	down
ITIH5	-1.1198947	1.39805033	2.06E-09	9.39E-09	down
ADCY3	-1.1190593	2.11033017	7.64E-19	7.40E-18	down
GHR	-1.1184295	0.31138602	2.43E-83	6.40E-81	down
ITGA9	-1.117665	1.71215846	2.60E-11	1.41E-10	down
FAM63B	-1.1167575	1.50809952	3.65E-28	6.37E-27	down
SLC8A1	-1.1165832	0.6915585	1.84E-25	2.74E-24	down
GALNT15	-1.1156405	0.33663871	1.09E-41	3.59E-40	down
C2orf74	-1.1150551	0.52949569	3.55E-35	8.67E-34	down
GARNL3	-1.1148023	0.60188935	1.95E-70	2.62E-68	down
AHNAK2	-1.1147602	1.45897835	2.94E-09	1.32E-08	down
RBMS1	-1.1145764	2.3908531	2.04E-20	2.22E-19	down
PLXDC1	-1.114016	1.10682898	1.25E-31	2.61E-30	down
HOXA5	-1.1137614	2.31839846	8.86E-05	0.00023874	down
SATB1	-1.1120627	1.01636823	1.62E-18	1.54E-17	down
LATS2	-1.1116913	2.02799278	7.73E-25	1.10E-23	down
LYSMD2	-1.1116368	1.73508141	1.58E-19	1.61E-18	down
ITGB1BP2	-1.1115811	0.57363573	5.34E-46	2.15E-44	down
C3orf18	-1.1102934	1.55669151	9.95E-26	1.52E-24	down
PDLIM4	-1.1100948	3.36362271	4.48E-09	1.97E-08	down
ZNF302	-1.1097985	2.05867145	2.64E-16	2.15E-15	down
MYOC	-1.1095532	0.85267751	2.28E-12	1.35E-11	down
MAGEE1	-1.1094126	1.04150104	1.53E-24	2.14E-23	down
DNAJB5	-1.1086336	1.6318718	6.11E-21	6.93E-20	down
RAB11FIP5	-1.1076136	2.42192581	7.55E-24	1.01E-22	down
BEX1	-1.1073472	2.61339538	0.00077744	0.00181877	down
POMGNT2	-1.1064624	3.70563215	2.55E-17	2.21E-16	down
PCDHB7	-1.106123	0.66020381	4.69E-29	8.55E-28	down
HDAC5	-1.1042092	3.22092088	1.19E-26	1.91E-25	down
CLEC11A	-1.1016841	3.50725659	1.83E-09	8.37E-09	down
PYGO1	-1.1011678	0.90059176	7.89E-22	9.47E-21	down
ACSS3	-1.0996825	0.9243588	4.18E-18	3.86E-17	down

ARRDC4	-1.0966547	2.45848626	2.80E-13	1.78E-12	down
TMEM252	-1.0964821	0.86469074	3.80E-09	1.69E-08	down
MACF1	-1.0964441	2.23810743	9.85E-27	1.59E-25	down
LEPROT	-1.0959892	3.90569809	1.43E-27	2.41E-26	down
ZC2HC1A	-1.0959335	1.81696242	1.46E-19	1.49E-18	down
SCARF1	-1.0944204	1.31276336	1.17E-37	3.25E-36	down
NR1D1	-1.0922374	3.09492451	1.91E-18	1.80E-17	down
BTG1	-1.0919982	4.45657787	2.19E-22	2.71E-21	down
ROR2	-1.0905377	2.50349882	5.18E-09	2.26E-08	down
FCER1A	-1.0887711	0.77904498	3.87E-16	3.11E-15	down
APH1B	-1.0883438	1.4615989	3.25E-27	5.38E-26	down
NEURL1	-1.0872023	0.52744285	2.35E-17	2.04E-16	down
BST1	-1.0866186	0.87358859	3.08E-24	4.24E-23	down
HBB	-1.0856406	3.26668812	0.00027804	0.00069693	down
WISP1	-1.0855058	0.90530099	2.19E-22	2.71E-21	down
OAF	-1.0847286	3.90661269	1.71E-15	1.30E-14	down
RBM43	-1.084509	1.57573952	4.34E-26	6.74E-25	down
CBLN1	-1.0844147	0.42297368	2.92E-22	3.58E-21	down
RFX2	-1.0842797	1.46926116	2.94E-13	1.87E-12	down
PLA2R1	-1.0834526	0.96035855	4.17E-30	8.03E-29	down
SYT1	-1.082606	0.61012205	3.06E-15	2.29E-14	down
SMPDL3A	-1.0816556	1.52517213	1.04E-16	8.73E-16	down
TOM1L2	-1.0810529	2.54870371	1.58E-28	2.79E-27	down
TEAD1	-1.0809865	2.6724961	1.02E-16	8.60E-16	down
ENOX1	-1.0809403	0.58736002	6.30E-33	1.39E-31	down
LRRK2	-1.0787273	0.34912646	5.18E-48	2.37E-46	down
RBP5	-1.078669	2.0866048	1.16E-14	8.22E-14	down
SPON2	-1.0783892	3.09297595	9.13E-09	3.88E-08	down
LCA5	-1.0782699	0.99638915	2.32E-23	3.06E-22	down
WWTR1	-1.0781319	3.13942226	2.73E-15	2.05E-14	down
ELN	-1.07803	2.60406097	9.47E-06	2.89E-05	down
SAP30L	-1.0779611	2.39791994	2.52E-50	1.32E-48	down
MAP1A	-1.0760287	1.53427685	1.32E-13	8.65E-13	down
MXI1	-1.0759951	3.2265051	8.82E-20	9.16E-19	down
CREB3L2	-1.0756475	3.17369121	1.39E-20	1.54E-19	down
ARHGEF17	-1.0755179	2.8825482	2.99E-20	3.22E-19	down
VCAM1	-1.0747794	1.18867834	1.38E-13	9.01E-13	down
P2RY1	-1.0745135	0.81973645	4.18E-33	9.32E-32	down
PDCD4	-1.0736709	4.16752362	1.71E-16	1.41E-15	down
TTYH2	-1.0734364	0.67535253	9.81E-36	2.48E-34	down
ADAMTS10	-1.073073	1.1252602	5.71E-19	5.59E-18	down
ANKRD33B	-1.0720757	0.57663407	8.05E-22	9.64E-21	down
MAP1B	-1.0705687	2.00024702	1.16E-08	4.88E-08	down
PRR16	-1.0692065	0.87169263	4.67E-15	3.43E-14	down
FOSL2	-1.0682708	4.32054212	6.90E-14	4.61E-13	down

HBEGF	-1.0681519	1.77961006	5.55E-20	5.86E-19	down
STEAP4	-1.0675525	0.54022962	9.85E-24	1.32E-22	down
NXPE3	-1.0674727	0.93390634	3.89E-42	1.31E-40	down
NIPAL2	-1.0662011	1.74859705	4.81E-22	5.86E-21	down
SNTB2	-1.0660532	1.94156013	4.65E-26	7.21E-25	down
TCF21	-1.0648741	0.23556639	1.48E-39	4.49E-38	down
KLF10	-1.0644114	3.59679975	6.07E-15	4.41E-14	down
MMP2	-1.0637451	5.49382355	6.28E-05	0.00017252	down
PXDC1	-1.0626997	3.0307142	5.96E-10	2.85E-09	down
PRKACB	-1.0626835	2.15608447	2.43E-18	2.27E-17	down
CRISP2	-1.0626827	0.22007223	5.12E-21	5.83E-20	down
SRF	-1.0604708	4.13006274	2.70E-31	5.55E-30	down
SYNDIG1	-1.0599807	0.98073651	2.32E-09	1.05E-08	down
USP51	-1.0594013	0.97480506	1.84E-28	3.24E-27	down
NMNAT3	-1.0589061	1.42895613	2.43E-17	2.11E-16	down
SLC27A6	-1.0582692	1.05892584	9.52E-11	4.89E-10	down
CSPG4	-1.0582508	1.67613087	1.64E-14	1.16E-13	down
FAM89A	-1.057122	1.59483319	5.49E-18	5.01E-17	down
KLHDC8B	-1.0568209	3.55082007	2.57E-17	2.23E-16	down
ARHGEF26	-1.0566773	0.95022603	1.87E-32	4.02E-31	down
KIAA1377	-1.0565915	0.76808734	2.28E-22	2.82E-21	down
DLL1	-1.0565889	1.67338456	1.06E-14	7.55E-14	down
HRCT1	-1.0562162	0.91870718	2.03E-15	1.54E-14	down
FAM43A	-1.0561741	2.22906399	1.95E-11	1.07E-10	down
ARMCX4	-1.0561372	0.56270072	1.13E-46	4.70E-45	down
MEF2D	-1.0561138	3.24854639	4.34E-28	7.54E-27	down
FEM1B	-1.05594	2.86515528	3.92E-30	7.56E-29	down
TSPYL4	-1.0558898	3.02568732	3.51E-19	3.48E-18	down
CCL19	-1.0554876	1.5507866	8.99E-06	2.75E-05	down
TPPP	-1.0553007	1.0307977	6.72E-12	3.81E-11	down
MED13L	-1.0549445	1.97458343	5.78E-24	7.83E-23	down
YAP1	-1.0543293	4.12762748	3.27E-15	2.43E-14	down
EIF3L	-1.0541673	4.95697204	6.02E-10	2.87E-09	down
CCDC50	-1.0534711	2.82887724	1.74E-27	2.92E-26	down
EIF4B	-1.0523456	5.51652876	1.44E-20	1.59E-19	down
LIN7A	-1.0511285	0.54215133	3.49E-25	5.08E-24	down
KIAA1755	-1.0504544	0.60091724	1.43E-36	3.75E-35	down
CALCOCO2	-1.0492188	2.88074435	1.73E-32	3.73E-31	down
PDLIM5	-1.0488661	3.21868617	6.22E-14	4.17E-13	down
CREBL2	-1.0483666	3.14032461	5.15E-25	7.43E-24	down
SALL2	-1.0479901	2.18279467	1.64E-09	7.54E-09	down
CMYA5	-1.0478011	0.48169115	7.72E-40	2.36E-38	down
H6PD	-1.0473953	3.50778126	5.37E-24	7.30E-23	down
HTRA1	-1.0471073	5.709765	2.96E-09	1.33E-08	down
GATA2	-1.0455136	3.22545192	1.22E-05	3.66E-05	down

CNTFR	-1.0451828	1.07369905	2.61E-06	8.49E-06	down
RNF11	-1.0443195	4.37920565	1.68E-18	1.60E-17	down
ENG	-1.044105	5.01597493	1.78E-14	1.25E-13	down
BTBD19	-1.0439808	1.01411812	1.57E-24	2.20E-23	down
GNAL	-1.0430758	0.60823025	2.71E-43	9.55E-42	down
TTYH1	-1.0416557	0.4802098	9.45E-16	7.33E-15	down
KCNK3	-1.0413483	0.5527293	3.49E-18	3.23E-17	down
SMAD9	-1.0394097	1.64039883	5.00E-10	2.40E-09	down
PRRT2	-1.0388858	0.65318718	7.57E-32	1.60E-30	down
JADE2	-1.0386541	1.85855665	1.69E-18	1.60E-17	down
SYT11	-1.0383165	2.40778509	6.34E-09	2.74E-08	down
RUNDC3B	-1.0381454	0.59910607	1.32E-27	2.23E-26	down
AGPAT9	-1.0381386	0.80624937	1.60E-16	1.32E-15	down
PDE1A	-1.0380357	0.68457357	3.30E-30	6.39E-29	down
C14orf28	-1.0376202	0.96213815	2.64E-54	1.68E-52	down
NEDD4	-1.0372569	0.66584141	1.95E-44	7.27E-43	down
RPL22	-1.0368732	5.56072548	5.61E-15	4.09E-14	down
ETS1	-1.0365067	2.79504988	1.67E-13	1.08E-12	down
TACR1	-1.0363603	0.64843295	7.23E-15	5.24E-14	down
TMEM158	-1.0356643	2.58131562	1.55E-06	5.16E-06	down
ROBO3	-1.035527	0.68128054	4.95E-20	5.25E-19	down
GULP1	-1.0342215	1.15969591	1.63E-17	1.44E-16	down
FAM117A	-1.0341039	2.56000453	1.00E-24	1.42E-23	down
FAM13C	-1.0335891	0.46017805	5.19E-47	2.21E-45	down
FLRT2	-1.0319522	0.33808536	1.69E-58	1.33E-56	down
NPTN	-1.0314363	4.45597699	3.49E-20	3.74E-19	down
NEK9	-1.0308012	2.74084144	1.20E-23	1.60E-22	down
GUCY1B3	-1.0303355	2.07904742	1.98E-12	1.18E-11	down
IL17RD	-1.0302698	2.18472989	3.51E-08	1.40E-07	down
WFIKKN2	-1.0276058	0.1955731	2.09E-27	3.51E-26	down
PABPC5	-1.0275271	0.20776686	1.98E-119	3.90E-116	down
DUSP26	-1.0270176	0.4378834	4.22E-22	5.15E-21	down
CEP68	-1.0259019	2.15228785	6.07E-31	1.23E-29	down
WFS1	-1.0257047	4.7400795	6.24E-09	2.70E-08	down
GSTM1	-1.0251602	1.67605043	0.00061001	0.00145259	down
PLS3	-1.0250903	3.82982788	8.74E-10	4.11E-09	down
HERC5	-1.0225685	1.58518193	1.38E-08	5.75E-08	down
TM4SF1	-1.0225653	4.51302385	6.44E-07	2.25E-06	down
HOXD4	-1.022342	0.50598834	2.47E-19	2.48E-18	down
DNAJC18	-1.0220949	1.24536799	3.07E-35	7.52E-34	down
ZDBF2	-1.0213213	0.40090695	2.30E-26	3.62E-25	down
EPS8	-1.0209852	2.70296656	1.27E-11	7.02E-11	down
FAM19A5	-1.0208617	1.53352169	4.85E-07	1.72E-06	down
GRIK5	-1.0203406	1.09379726	1.32E-07	4.96E-07	down
C1QTNF4	-1.0190529	0.57434295	4.90E-26	7.58E-25	down

ADCYAP1R	-1.0189437	1.6392078	5.22E-05	0.0001452	down
MTMR11	-1.0182379	1.69665957	3.28E-20	3.52E-19	down
CLSTN2	-1.0181026	0.80044401	1.17E-12	7.04E-12	down
VIM	-1.0179497	7.83791475	2.20E-05	6.42E-05	down
P2RY14	-1.0167284	0.62577061	2.69E-18	2.51E-17	down
CD109	-1.0166562	1.36031755	7.33E-09	3.15E-08	down
FGF10	-1.0161312	0.13678345	2.77E-77	5.47E-75	down
KCNN3	-1.015693	0.6677842	9.30E-27	1.50E-25	down
KITLG	-1.0151415	1.58457467	5.16E-09	2.26E-08	down
PENK	-1.0148774	0.14897088	1.13E-21	1.34E-20	down
PPP1R15A	-1.0145063	4.76633343	4.27E-14	2.90E-13	down
FSTL3	-1.0142942	2.39263531	1.71E-09	7.82E-09	down
LDB3	-1.0127745	0.4392939	7.38E-29	1.33E-27	down
SYNC	-1.0127158	0.35133672	1.96E-59	1.61E-57	down
EDA2R	-1.0125656	0.6671133	4.22E-27	6.98E-26	down
ITM2B	-1.0122728	5.71764529	2.71E-14	1.87E-13	down
ARID5B	-1.0122186	3.15058689	1.59E-14	1.12E-13	down
ARHGEF10	-1.0119215	1.54008006	1.09E-22	1.39E-21	down
AXIN2	-1.0118255	2.45278505	2.51E-06	8.18E-06	down
VWA7	-1.0104648	1.26386679	4.16E-11	2.21E-10	down
CAMK2A	-1.0093442	0.13183803	2.86E-125	1.13E-121	down
RBFOX3	-1.0088629	0.24912729	7.26E-38	2.05E-36	down
ADAMTSL3	-1.0081016	0.54787725	6.14E-20	6.44E-19	down
SERTAD4	-1.0076278	1.40733337	3.60E-10	1.75E-09	down
MPV17L	-1.0074254	0.80625525	1.33E-13	8.69E-13	down
SPRY1	-1.0068231	3.75046214	7.23E-09	3.11E-08	down
FSTL1	-1.0066224	4.57983629	4.62E-09	2.03E-08	down
SLC9B2	-1.0064178	0.93449785	9.23E-37	2.45E-35	down
SMAD3	-1.0062925	2.46057378	2.17E-19	2.19E-18	down
AKAP13	-1.005735	2.40274332	1.15E-25	1.74E-24	down
RPS3A	-1.0050962	7.13667625	7.22E-12	4.08E-11	down
EMP1	-1.0045953	2.99120616	1.88E-06	6.23E-06	down
ZFP36L2	-1.0045197	5.95951149	4.01E-12	2.32E-11	down
NUAK1	-1.004145	1.61482127	7.30E-15	5.29E-14	down
ATRNL1	-1.0039015	0.50969274	2.96E-19	2.96E-18	down
TUBA1A	-1.0037371	6.00110884	9.03E-07	3.10E-06	down
SESN1	-1.0036461	2.24665611	2.67E-20	2.88E-19	down
TCF7L2	-1.0030235	2.69067598	3.14E-13	1.99E-12	down
SDC2	-1.0029821	3.96862307	7.69E-07	2.66E-06	down
JAK1	-1.0028413	4.04281746	1.79E-13	1.16E-12	down
EVC2	-1.0026123	1.01119817	2.10E-23	2.78E-22	down
FBXO21	-1.002596	3.43683711	4.35E-18	4.00E-17	down
RBL2	-1.0022838	2.6697075	9.47E-17	7.99E-16	down
EFCC1	-1.0009608	0.9875934	1.39E-22	1.75E-21	down
KAT2B	-1.0008303	1.71539945	5.75E-18	5.24E-17	down

AGFG2	-1.0000005	2.30006896	1.15E-22	1.46E-21	down
