# Supplementary material for: Comprehensive Analysis of GDF10 Methylation Site-Associated Genes as Prognostic Markers for Endometrial Cancer
Source: J Oncol. 2022 Oct 10;2022:7117083. doi: 10.1155/2022/7117083 (PMC9576415; doi:10.1155/2022/7117083)
Supplement: Supplementary Materials — are shown as Supplemental Figures S1-3 and Supplemental Tables S1-7. Supplemental Figure S1: Cox regression independent prognostic analyses. Supplemental Figure S2: Analysis of expression correlations between the risk model and clinicopathological factors. Supplemental Figure S3: Kaplan-Meier survival analysis of patients with different clinicopathologic features between high- and low-risk groups. (a–i) The different OS of UCEC patients with different ages, tumor stages, and grades between high-risk and low-risk groups according to GDF10 methylation. The abscissa shows survival months, and the vertical axis shows overall survival probability. Supplemental Table S1: 48 modules were identified. Supplemental Table S2: The saddlebrown module positively correlated with the methylation sites the most. Supplemental Table S3: 991 genes were upregulated in DEGs. Supplemental Table S4: 1260 genes were downregulated in DEGs. Supplemental Table S5: 44 DEGMRGs were identified. Supplemental Table S6: All UCEC samples in the training set were divided into the high- and low-risk groups. Supplemental Table S7: Risk scores were significantly different between patients classified by age, tumor stage, and grade. [file 7117083.f1.zip › Supplemental Table S5.pdf]

SPDEF
TMPRSS13
TMEM63A
B4GALNT3
GREB1
NCMAP
MUC5B
HID1
PGR
MAP2K6
PPAP2C
AQP5
STXBP6
TRPM4
HGD
CLDN6
GLYATL2
PIGR
TMC5
LRRC26
MAL
SRD5A3
ST6GALNAC1
KIAA1324
ENTPD3
PIK3R3
STX19
FOXA2
FAM174B
VIM
CREB3L4
GCNT1
GCNT2
CYB561
RNF183
SERINC2
ZNF516
SCGB2A2
CYP4X1
TNNT1
TFF3
FOLH1
DNAJC22
B4GALT1
