# Supplementary material for: Comprehensive Analysis of GDF10 Methylation Site-Associated Genes as Prognostic Markers for Endometrial Cancer
Source: J Oncol. 2022 Oct 10;2022:7117083. doi: 10.1155/2022/7117083 (PMC9576415; doi:10.1155/2022/7117083)
Supplement: Supplementary Materials — are shown as Supplemental Figures S1-3 and Supplemental Tables S1-7. Supplemental Figure S1: Cox regression independent prognostic analyses. Supplemental Figure S2: Analysis of expression correlations between the risk model and clinicopathological factors. Supplemental Figure S3: Kaplan-Meier survival analysis of patients with different clinicopathologic features between high- and low-risk groups. (a–i) The different OS of UCEC patients with different ages, tumor stages, and grades between high-risk and low-risk groups according to GDF10 methylation. The abscissa shows survival months, and the vertical axis shows overall survival probability. Supplemental Table S1: 48 modules were identified. Supplemental Table S2: The saddlebrown module positively correlated with the methylation sites the most. Supplemental Table S3: 991 genes were upregulated in DEGs. Supplemental Table S4: 1260 genes were downregulated in DEGs. Supplemental Table S5: 44 DEGMRGs were identified. Supplemental Table S6: All UCEC samples in the training set were divided into the high- and low-risk groups. Supplemental Table S7: Risk scores were significantly different between patients classified by age, tumor stage, and grade. [file 7117083.f1.zip › Supplemental Table S6 (1).pdf]

id	OS.time	OS	B4GALNT3	GREB1	DNAJC22	riskScore	risk
TCGA-2E-A9G8	3.4219	0.0000	2.9385	1.2434	1.9208	1.9441	high
TCGA-A5-A0G1	8.9068	1.0000	3.7154	0.0442	1.7385	2.5036	high
TCGA-A5-A0GG	10.3068	0.0000	4.2929	1.3301	2.8657	1.0833	high
TCGA-A5-A0GQ	6.1562	0.0000	3.9136	1.9380	3.0361	0.9337	high
TCGA-A5-A0GR	3.0685	0.0000	2.1124	0.1341	1.2308	4.1079	high
TCGA-A5-A1OF	2.6658	0.0000	2.4107	2.5015	3.0975	1.1216	high
TCGA-A5-A1OG	2.6658	1.0000	2.9465	1.3197	2.5947	1.6368	high
TCGA-A5-A1OH	3.0274	0.0000	1.3753	1.7183	4.6888	1.3749	high
TCGA-A5-A1OJ	2.7562	0.0000	2.4232	2.9101	2.3367	1.1405	high
TCGA-A5-A2K2	9.8493	0.0000	2.6524	0.9440	0.6477	3.0483	high
TCGA-A5-A2K3	5.5123	0.0000	2.3560	0.1764	0.1501	4.7814	high
TCGA-A5-A3LP	1.2466	0.0000	2.3022	0.2642	2.0347	3.1487	high
TCGA-A5-A7WJ	0.9342	0.0000	4.9634	0.3066	4.0745	1.0034	high
TCGA-AJ-A23N	1.2027	1.0000	2.7210	1.7325	3.0137	1.3756	high
TCGA-AJ-A2QM	2.4493	0.0000	3.0424	2.6985	1.6811	1.2008	high
TCGA-AJ-A3BD	2.8466	0.0000	4.5166	1.0128	3.3888	1.0208	high
TCGA-AJ-A3EJ	0.1370	1.0000	2.4315	1.9940	1.0337	2.0650	high
TCGA-AJ-A3EL	1.8740	0.0000	2.9269	3.4572	1.6768	0.9515	high
TCGA-AJ-A3I9	1.4219	1.0000	5.6362	1.3472	1.6212	0.9885	high
TCGA-AJ-A3IA	1.7342	0.0000	2.6214	0.6658	1.3975	2.8858	high
TCGA-AJ-A3NC	3.0822	0.0000	3.5597	1.8951	2.6434	1.1301	high
TCGA-AJ-A3NF	1.7671	0.0000	2.3516	2.5410	1.8642	1.4609	high
TCGA-AJ-A3NG	1.3014	0.0000	3.2204	0.9517	1.4164	2.2256	high
TCGA-AJ-A3QS	1.4055	0.0000	2.5298	2.0718	1.4425	1.7957	high
TCGA-AJ-A3TW	1.4055	0.0000	5.0833	0.0468	2.0675	1.6319	high
TCGA-AP-A0LF	7.7945	0.0000	5.3919	0.4167	0.2656	1.9431	high
TCGA-AP-A0LS	7.9562	0.0000	4.0678	1.0466	2.0907	1.4953	high
TCGA-AP-A1DK	7.5808	0.0000	4.5664	0.6688	3.2205	1.1770	high
TCGA-AP-A1E3	2.3205	0.0000	5.0773	2.7314	0.2221	0.9523	high
TCGA-AP-A1E4	4.2603	0.0000	3.4294	1.5490	1.6581	1.6265	high
TCGA-AP-A3K1	1.8247	1.0000	3.1743	1.4278	0.9232	2.1204	high
TCGA-AX-A06D	1.0219	1.0000	3.6623	0.3452	2.2286	2.0598	high
TCGA-AX-A0IS	5.0904	1.0000	3.0632	2.5054	2.4916	1.0747	high
TCGA-AX-A0IZ	3.6521	0.0000	3.9570	1.4131	3.0652	1.1012	high
TCGA-AX-A0J0	3.3425	0.0000	2.1684	1.4532	2.6103	1.9080	high
TCGA-AX-A1C7	2.8877	0.0000	2.6882	1.6709	2.2250	1.6766	high
TCGA-AX-A1C8	2.1260	0.0000	1.5296	0.4081	2.1630	3.5655	high
TCGA-AX-A1CA	2.6685	0.0000	3.1759	0.7850	1.2917	2.4502	high
TCGA-AX-A1CC	2.1589	0.0000	2.5261	3.0591	2.2035	1.0846	high
TCGA-AX-A1CE	2.8411	0.0000	3.5990	1.9909	1.7847	1.2990	high
TCGA-AX-A1CJ	6.1178	0.0000	4.1661	2.3110	1.6161	1.0390	high
TCGA-AX-A1CN	3.1315	0.0000	4.2401	0.6990	0.5530	2.2380	high
TCGA-AX-A1CP	3.0329	0.0000	3.1750	1.3411	3.7912	1.1864	high

TCGA-AX-A1CR	1.9205	0.0000	2.0583	2.7439	2.5505	1.2699	high
TCGA-AX-A2H4	2.5096	1.0000	3.3678	0.0492	0.1949	3.8013	high
TCGA-AX-A2H5	5.9370	0.0000	3.2881	0.7244	3.7374	1.4437	high
TCGA-AX-A2H7	2.1151	1.0000	2.9578	1.6926	0.4396	2.2683	high
TCGA-AX-A2HC	2.8603	0.0000	0.4466	3.9012	1.1100	1.7577	high
TCGA-AX-A2HF	2.3233	0.0000	3.1917	1.2029	0.8381	2.3241	high
TCGA-AX-A2HG	3.1863	0.0000	3.9936	1.1787	2.8562	1.2371	high
TCGA-AX-A2HH	1.4795	0.0000	4.5485	2.2818	1.3728	1.0004	high
TCGA-AX-A2IN	2.5945	1.0000	1.2198	0.9056	0.4444	4.6887	high
TCGA-AX-A3FS	0.0192	0.0000	2.6805	0.0983	0.7007	4.0147	high
TCGA-AX-A3FT	0.0164	0.0000	3.2293	0.9933	2.5796	1.7084	high
TCGA-AX-A3FZ	0.0164	0.0000	0.2744	1.1286	2.1311	3.8778	high
TCGA-AX-A3G1	1.7096	1.0000	3.1907	2.6043	0.6906	1.4739	high
TCGA-AX-A3G3	0.1151	0.0000	1.6906	0.4534	0.7401	4.5562	high
TCGA-AX-A3G6	0.0219	0.0000	2.7190	1.0348	0.7313	2.8516	high
TCGA-AX-A3G7	0.0247	0.0000	2.6294	2.3199	2.4661	1.2908	high
TCGA-AX-A3G8	0.0986	0.0000	5.9197	0.3324	0.5945	1.6255	high
TCGA-AX-A3G9	1.5288	0.0000	4.5285	0.8273	3.0000	1.1791	high
TCGA-AX-A3GI	0.0329	0.0000	2.5644	2.3871	0.1072	2.1193	high
TCGA-B5-A0K8	2.3205	0.0000	4.0973	2.5941	1.3770	1.0087	high
TCGA-B5-A0KB	2.2575	0.0000	3.7911	1.4032	1.7938	1.5125	high
TCGA-B5-A11E	4.9863	0.0000	3.9141	1.4207	2.0995	1.3642	high
TCGA-B5-A11I	6.3096	0.0000	1.3682	2.9789	0.9435	1.9729	high
TCGA-B5-A11L	6.1370	1.0000	3.8395	0.7204	1.3003	2.1034	high
TCGA-B5-A11P	2.2603	0.0000	3.7223	2.6984	1.6032	1.0224	high
TCGA-B5-A1MS	8.5260	1.0000	2.8290	1.2124	2.5798	1.7575	high
TCGA-B5-A1MU	4.2411	1.0000	2.0891	2.2467	1.7193	1.7873	high
TCGA-B5-A1MY	0.9890	1.0000	3.2338	1.5485	2.4893	1.4343	high
TCGA-B5-A1N2	2.6137	1.0000	2.6309	1.7769	0.8650	2.1912	high
TCGA-B5-A5OD	2.8438	0.0000	5.1559	1.3661	1.2909	1.1943	high
TCGA-B5-A5OE	2.9123	0.0000	2.0407	0.4206	0.2340	4.6847	high
TCGA-BG-A0YV	1.0466	0.0000	2.7919	1.3124	2.9413	1.5870	high
TCGA-BG-A221	0.1507	0.0000	2.9215	1.1744	2.5037	1.7668	high
TCGA-BG-A2L7	1.6767	0.0000	3.0835	2.6437	0.8899	1.4330	high
TCGA-BG-A3EW	0.9425	0.0000	3.4448	1.3837	3.3357	1.2004	high
TCGA-BG-A3PP	1.8658	0.0000	2.0654	0.9645	4.4383	1.5740	high
TCGA-BK-A139	2.5836	1.0000	3.4179	2.5513	1.4248	1.2101	high
TCGA-BK-A26L	2.0110	0.0000	2.9354	2.7554	2.2643	1.0692	high
TCGA-BS-A0T9	3.9123	1.0000	3.9404	1.7987	2.1303	1.1803	high
TCGA-BS-A0U5	8.0411	0.0000	3.3490	2.1756	1.6825	1.3290	high
TCGA-BS-A0V8	6.9699	0.0000	3.2444	1.8034	3.7457	1.0018	high
TCGA-BS-A0VI	7.2493	0.0000	3.6662	0.8187	3.2278	1.4109	high
TCGA-BS-A0WQ	5.5397	0.0000	3.2788	2.3010	2.9684	0.9855	high
TCGA-D1-A0ZS	0.4055	0.0000	1.3075	1.0425	0.9067	3.9601	high

TCGA-D1-A0ZV	1.9945	0.0000	3.4814	1.6508	2.0868	1.4137	high
TCGA-D1-A0ZZ	1.8795	0.0000	2.4867	1.4395	4.2467	1.2453	high
TCGA-D1-A15V	3.2904	0.0000	4.2183	2.1117	0.4315	1.4136	high
TCGA-D1-A15X	2.4904	0.0000	2.9444	0.0999	1.8700	2.9199	high
TCGA-D1-A16G	2.2849	1.0000	3.1380	0.3849	3.8929	1.6344	high
TCGA-D1-A16J	2.4932	0.0000	3.3760	0.3679	2.8634	1.9237	high
TCGA-D1-A16Q	0.8877	0.0000	3.3316	2.2346	1.8597	1.2595	high
TCGA-D1-A16S	1.5562	0.0000	2.2799	1.6154	3.2325	1.5344	high
TCGA-D1-A16Y	1.4575	0.0000	3.7128	1.9158	1.9050	1.2616	high
TCGA-D1-A17C	0.0822	0.0000	3.9281	2.8179	1.5066	0.9488	high
TCGA-D1-A1NU	1.1014	0.0000	3.2433	0.7645	1.4755	2.3316	high
TCGA-D1-A1NW	0.0603	0.0000	2.8864	1.0918	2.6999	1.7599	high
TCGA-D1-A1NX	1.5205	0.0000	3.3839	1.2000	2.5889	1.5241	high
TCGA-D1-A1O0	0.1726	0.0000	4.0039	2.0162	1.8922	1.1323	high
TCGA-D1-A1O8	0.3288	1.0000	2.9680	0.3040	0.2882	3.7859	high
TCGA-D1-A2G0	4.0658	0.0000	3.7200	0.9001	0.3511	2.4953	high
TCGA-D1-A2G6	0.7068	1.0000	2.7975	1.5545	4.4122	1.0651	high
TCGA-D1-A3DG	0.3562	1.0000	3.4850	1.0402	1.5534	1.9563	high
TCGA-D1-A3JQ	1.4411	0.0000	3.1828	1.7286	3.6863	1.0582	high
TCGA-DF-A2KN	0.1836	0.0000	4.3944	1.3299	1.8899	1.2987	high
TCGA-DF-A2KU	8.4027	0.0000	4.5003	0.5017	0.1306	2.4502	high
TCGA-DF-A2KZ	2.2630	0.0000	3.9007	1.1609	2.2553	1.4494	high
TCGA-DF-A2L0	2.1589	1.0000	6.7200	0.5285	1.0176	1.1260	high
TCGA-DI-A1NN	2.9479	0.0000	3.0413	1.6408	1.3396	1.8657	high
TCGA-DI-A1NO	0.7808	1.0000	1.2837	0.9299	1.9826	3.2954	high
TCGA-DI-A2QU	2.5918	1.0000	2.1164	2.7320	0.3101	2.0237	high
TCGA-E6-A1LZ	6.5918	0.0000	2.0623	0.3713	1.6743	3.4873	high
TCGA-E6-A1M0	4.9123	0.0000	3.0255	2.5590	1.2547	1.3864	high
TCGA-E6-A2P8	0.1973	1.0000	4.7961	1.1453	0.1729	1.7973	high
TCGA-E6-A8L9	2.7753	0.0000	3.1209	1.4349	1.5799	1.8650	high
TCGA-EO-A1Y5	0.9096	0.0000	2.7266	0.6163	2.3583	2.3279	high
TCGA-EO-A1Y8	2.4685	0.0000	3.5597	1.3010	1.5202	1.7647	high
TCGA-EO-A22T	5.5753	0.0000	4.5879	0.8051	1.7363	1.5311	high
TCGA-EO-A2CH	1.6164	1.0000	2.8360	1.2748	3.5457	1.3975	high
TCGA-EO-A3AV	2.9288	0.0000	3.6907	0.2461	3.9570	1.4646	high
TCGA-EO-A3AZ	2.2630	0.0000	3.4696	0.9024	2.2181	1.7887	high
TCGA-EO-A3KU	1.8548	0.0000	2.5944	1.4849	2.5423	1.7132	high
TCGA-EO-A3KW	1.3616	0.0000	3.6239	1.5029	2.5029	1.3123	high
TCGA-EY-A1G8	1.2493	1.0000	3.3891	1.6924	3.7683	0.9978	high
TCGA-EY-A1GL	2.7781	0.0000	5.3353	1.2417	1.0441	1.2542	high
TCGA-EY-A1GM	2.7260	1.0000	2.9651	0.6416	1.1803	2.7863	high
TCGA-EY-A1GS	1.8356	1.0000	0.5926	0.2506	1.7406	5.2623	high
TCGA-EY-A1GX	3.0877	1.0000	3.7424	0.6137	3.3857	1.4362	high
TCGA-EY-A210	1.0493	0.0000	4.1251	1.1017	0.7858	1.9080	high

TCGA-EY-A212	0.8493	1.0000	2.5943	2.1135	2.0798	1.5195	high
TCGA-EY-A215	1.5726	0.0000	3.0842	1.8178	3.0604	1.2026	high
TCGA-EY-A2ON	1.6712	1.0000	3.5008	0.8714	0.5826	2.5404	high
TCGA-EY-A3L3	2.2795	0.0000	0.8850	2.4690	1.0788	2.5962	high
TCGA-EY-A4KR	0.9479	1.0000	2.7317	0.5418	0.3540	3.6557	high
TCGA-EY-A54A	1.2247	0.0000	2.6989	1.7253	3.1143	1.3577	high
TCGA-EY-A72D	0.4904	0.0000	4.0461	0.7926	1.4340	1.8891	high
TCGA-FI-A2CY	5.6986	0.0000	1.9814	2.4069	1.8381	1.6952	high
TCGA-FI-A2D5	1.1534	1.0000	0.9801	0.3542	0.7475	5.6679	high
TCGA-FI-A2D6	2.1014	1.0000	1.5735	2.0802	0.1413	3.0316	high
TCGA-FI-A2EU	8.0575	0.0000	1.6004	1.3873	2.3424	2.3972	high
TCGA-FI-A2EW	4.9178	0.0000	2.9522	0.5620	0.8295	3.0971	high
TCGA-FI-A2EX	0.7781	0.0000	2.9673	0.8514	0.4632	3.0160	high
TCGA-FI-A2EY	2.4110	1.0000	2.9564	1.1862	1.0012	2.4008	high
TCGA-FI-A2F8	2.5562	0.0000	3.2142	2.3656	1.6329	1.3023	high
TCGA-FI-A2F9	4.9397	0.0000	0.7153	1.6438	1.1849	3.5346	high
TCGA-FI-A3PV	3.4219	1.0000	3.8328	1.6003	1.3015	1.5515	high
TCGA-H5-A2HR	0.1452	0.0000	2.1299	0.6435	0.7895	3.7632	high
TCGA-JU-AAVI	1.4795	1.0000	2.2542	0.5511	0.4357	4.0563	high
TCGA-K6-A3WQ	1.1699	1.0000	1.9774	1.0608	3.1381	2.0543	high
TCGA-KP-A3W0	1.1068	0.0000	3.6271	1.4429	1.9538	1.5049	high
TCGA-KP-A3W1	0.7890	0.0000	2.3859	0.0609	0.1147	4.9761	high
TCGA-KP-A3W3	2.1753	0.0000	2.4677	1.9749	1.4931	1.8673	high
TCGA-PG-A5BC	1.9370	0.0000	2.4372	2.1041	2.4766	1.4596	high
TCGA-PG-A6IB	1.7753	0.0000	1.6025	0.3910	0.2230	5.3190	high
TCGA-PG-A7D5	3.2247	0.0000	1.8983	1.4268	1.9492	2.3787	high
TCGA-PG-A915	0.6822	0.0000	3.0337	2.1166	1.8420	1.4237	high
TCGA-QS-A8F1	0.3699	1.0000	2.8719	0.4545	1.3824	2.9184	high
TCGA-SL-A6J9	0.0055	0.0000	1.0636	1.4099	0.6001	3.9650	high
TCGA-4E-A92E	0.0356	0.0000	4.7534	2.7324	3.6252	0.5019	low
TCGA-5B-A90C	3.2932	0.0000	4.6235	1.9441	3.5009	0.7013	low
TCGA-5S-A9Q8	0.8575	0.0000	4.3695	2.9520	2.0497	0.7189	low
TCGA-A5-A0VO	2.3973	1.0000	3.8784	2.4659	3.0295	0.7855	low
TCGA-A5-A1OK	2.5123	0.0000	3.4506	4.4593	3.3641	0.4090	low
TCGA-A5-A2K5	2.0603	0.0000	5.2433	0.6583	3.9396	0.8494	low
TCGA-A5-A2K7	2.9123	0.0000	3.1603	2.5009	4.0881	0.7471	low
TCGA-A5-A3LO	2.1260	0.0000	4.9209	2.9884	2.5418	0.5536	low
TCGA-AJ-A23O	0.8548	1.0000	3.5968	2.3785	3.9333	0.7189	low
TCGA-AJ-A2QK	1.7616	0.0000	5.2066	3.2001	2.2435	0.5087	low
TCGA-AJ-A2QL	1.6493	0.0000	4.5590	4.1561	1.8170	0.4731	low
TCGA-AJ-A2QO	2.8630	1.0000	5.5489	1.1751	2.6584	0.8609	low
TCGA-AJ-A3BG	1.5068	0.0000	5.4794	2.1403	4.3447	0.4377	low
TCGA-AJ-A3BI	1.5753	0.0000	5.8558	2.1702	1.7660	0.6800	low
TCGA-AJ-A3BK	2.8247	0.0000	5.8570	1.8412	2.6147	0.6361	low

TCGA-AJ-A3NE	1.7205	0.0000	4.6895	2.9325	2.8274	0.5642	low
TCGA-AJ-A3NH	0.0027	1.0000	4.3202	2.5700	2.5768	0.7434	low
TCGA-AJ-A3OK	1.3288	0.0000	6.6641	2.0711	2.3430	0.5040	low
TCGA-AJ-A3OL	1.2301	1.0000	3.3682	1.7525	4.0321	0.9289	low
TCGA-AJ-A5DV	1.8603	0.0000	3.2536	3.1206	2.9007	0.7569	low
TCGA-AJ-A5DW	1.0685	0.0000	3.9376	3.2464	1.7840	0.7687	low
TCGA-AJ-A8CV	2.9260	0.0000	4.0256	2.8479	3.6795	0.5763	low
TCGA-AJ-A8CW	0.0110	0.0000	5.7258	3.1173	3.1664	0.3756	low
TCGA-AP-A05O	1.0521	0.0000	4.3788	2.2047	2.4361	0.8565	low
TCGA-AP-A1DP	2.9616	1.0000	4.5694	3.4566	3.8117	0.3935	low
TCGA-AP-A1DQ	3.8986	1.0000	3.3403	2.3658	3.8370	0.7881	low
TCGA-AP-A1E0	5.0192	0.0000	4.2278	3.6923	0.6663	0.7743	low
TCGA-AP-A1E1	3.8219	0.0000	5.2394	2.5756	3.6686	0.4626	low
TCGA-AP-A5FX	3.0110	1.0000	5.9821	2.4777	1.6009	0.6124	low
TCGA-AW-A1PO	0.0466	0.0000	6.3004	0.4781	3.6085	0.7364	low
TCGA-AX-A06H	2.2822	0.0000	4.0733	1.9346	3.0843	0.8875	low
TCGA-AX-A1C4	1.1068	0.0000	4.0674	2.6786	4.9763	0.4588	low
TCGA-AX-A1C5	2.7425	0.0000	3.1899	4.0189	1.5000	0.7589	low
TCGA-AX-A1CF	6.4027	0.0000	6.2085	1.4391	2.6616	0.6608	low
TCGA-AX-A1CI	7.1233	0.0000	4.5939	2.5229	3.1621	0.6211	low
TCGA-AX-A1CK	6.6877	0.0000	3.9274	2.9584	2.8631	0.6771	low
TCGA-AX-A2H8	5.0986	0.0000	3.7164	2.5111	3.9367	0.6650	low
TCGA-AX-A2HA	6.7808	0.0000	5.2144	2.1072	2.6885	0.6751	low
TCGA-AX-A2HD	3.8740	0.0000	4.4302	3.6884	4.0769	0.3558	low
TCGA-AX-A3G4	0.0247	0.0000	4.3931	2.4488	1.8047	0.8967	low
TCGA-AX-A3GB	1.2055	0.0000	4.4849	2.8052	2.1206	0.7231	low
TCGA-B5-A11F	15.4822	0.0000	4.0295	4.5075	1.5685	0.5069	low
TCGA-B5-A11G	9.4466	0.0000	6.2343	3.5176	2.7690	0.3115	low
TCGA-B5-A11J	4.5370	0.0000	5.6135	2.4275	2.8987	0.5204	low
TCGA-B5-A11N	5.1397	0.0000	3.9920	2.4107	2.6177	0.8485	low
TCGA-B5-A11O	7.1479	0.0000	3.8795	2.0147	3.2894	0.8691	low
TCGA-B5-A11Q	2.6740	0.0000	5.6176	2.8922	3.5301	0.3867	low
TCGA-B5-A11R	6.4356	0.0000	3.7879	2.6686	3.1583	0.7293	low
TCGA-B5-A11S	6.1068	0.0000	4.8458	3.4647	2.6616	0.4664	low
TCGA-B5-A11V	1.6301	0.0000	4.7643	3.7392	3.3197	0.3764	low
TCGA-B5-A11W	1.6247	0.0000	5.8602	2.2011	3.2320	0.4918	low
TCGA-B5-A11Z	1.4466	0.0000	3.1268	3.9204	3.0834	0.5699	low
TCGA-B5-A121	1.3808	0.0000	3.8100	2.6376	3.2324	0.7215	low
TCGA-B5-A1MW	1.2247	0.0000	5.5568	2.0249	1.6950	0.7850	low
TCGA-B5-A3F9	11.3836	0.0000	4.3451	3.4836	2.3141	0.5686	low
TCGA-B5-A3FB	7.7918	0.0000	5.1539	3.5023	2.5247	0.4373	low
TCGA-B5-A3S1	2.4712	0.0000	3.4730	1.8861	3.8277	0.9012	low
TCGA-B5-A5OC	3.4219	0.0000	3.7498	2.5649	3.6886	0.6821	low
TCGA-BG-A0MA	0.8932	1.0000	3.1259	3.4379	3.0194	0.6833	low

TCGA-BG-A0MH	5.2877	0.0000	4.0222	2.0773	2.7052	0.9278	low
TCGA-BG-A0VV	4.2548	0.0000	5.6204	2.7684	2.7389	0.4774	low
TCGA-BG-A0VW	4.3342	0.0000	4.7261	1.7469	3.3525	0.7546	low
TCGA-BG-A0VX	2.8575	0.0000	3.5865	3.5274	2.0001	0.7297	low
TCGA-BG-A0VZ	4.3863	0.0000	6.1106	3.6766	2.3510	0.3327	low
TCGA-BG-A0W1	0.3342	0.0000	6.9092	3.4636	2.6297	0.2741	low
TCGA-BG-A0W2	4.7151	0.0000	3.5352	3.2521	1.8250	0.8448	low
TCGA-BG-A0YU	1.2356	0.0000	5.0920	4.2302	3.2302	0.2970	low
TCGA-BG-A187	1.2274	0.0000	5.2423	3.8455	2.2803	0.3995	low
TCGA-BG-A18A	1.1671	0.0000	4.0953	3.5096	2.7798	0.5446	low
TCGA-BG-A18B	1.2877	0.0000	6.5294	4.3346	4.3360	0.1555	low
TCGA-BG-A18C	1.2877	0.0000	4.9619	3.9810	1.8237	0.4520	low
TCGA-BG-A220	1.1890	0.0000	4.2610	3.2794	4.6954	0.3758	low
TCGA-BG-A222	0.7973	0.0000	2.0846	3.6285	4.4837	0.6145	low
TCGA-BG-A2AD	1.6685	0.0000	6.3900	1.7255	2.4828	0.5926	low
TCGA-BG-A2AE	1.9753	0.0000	4.1139	3.0925	4.4030	0.4435	low
TCGA-BK-A0CA	2.9151	0.0000	3.9079	3.2473	4.1842	0.4646	low
TCGA-BK-A13B	2.6466	0.0000	3.8052	2.5757	3.0715	0.7638	low
TCGA-BK-A13C	2.0822	0.0000	4.4989	3.5543	2.2650	0.5385	low
TCGA-BK-A4ZD	1.3863	0.0000	5.0182	4.3503	3.6788	0.2639	low
TCGA-BK-A56F	1.0986	0.0000	5.3438	3.2567	3.6102	0.3597	low
TCGA-BK-A6W4	0.8219	0.0000	4.4360	3.5657	2.9377	0.4725	low
TCGA-BS-A0UA	9.5753	0.0000	6.5187	2.3350	4.6091	0.2948	low
TCGA-BS-A0UL	6.7973	0.0000	4.5803	2.6110	2.5592	0.6873	low
TCGA-BS-A0V6	7.4658	0.0000	4.3082	2.8083	3.9136	0.5164	low
TCGA-BS-A0V7	5.2986	0.0000	5.1906	2.1532	1.9012	0.7906	low
TCGA-D1-A0ZO	1.6493	0.0000	5.1275	3.4973	3.3960	0.3664	low
TCGA-D1-A0ZP	2.4959	0.0000	3.4858	3.2602	1.9466	0.8315	low
TCGA-D1-A0ZR	0.0466	0.0000	5.7357	3.3566	3.1075	0.3491	low
TCGA-D1-A0ZU	2.3890	0.0000	5.1011	3.7456	3.8134	0.3097	low
TCGA-D1-A101	0.1452	0.0000	4.0935	2.4437	2.7194	0.7994	low
TCGA-D1-A102	0.3699	0.0000	3.7091	3.5492	2.5364	0.6257	low
TCGA-D1-A103	1.8329	0.0000	3.8154	2.9579	2.9912	0.6785	low
TCGA-D1-A15W	2.5068	0.0000	4.8579	2.2048	3.2095	0.6410	low
TCGA-D1-A15Z	0.1589	1.0000	3.8604	2.9087	2.3766	0.7775	low
TCGA-D1-A162	3.1370	0.0000	5.7853	3.4034	3.2533	0.3287	low
TCGA-D1-A165	0.0548	0.0000	5.7623	4.0314	2.9608	0.2829	low
TCGA-D1-A167	1.3096	0.0000	4.6973	3.8088	3.6727	0.3468	low
TCGA-D1-A169	3.1096	0.0000	5.8513	4.1775	2.2714	0.3043	low
TCGA-D1-A16E	3.0658	0.0000	4.6523	1.5964	3.5855	0.7714	low
TCGA-D1-A16N	2.5890	0.0000	5.4786	4.8412	0.7396	0.3689	low
TCGA-D1-A16O	2.8027	0.0000	6.0459	3.5367	4.7308	0.2140	low
TCGA-D1-A16R	0.0575	0.0000	3.5787	2.9513	3.0007	0.7219	low

TCGA-D1-A16V	0.3288	1.0000	5.0653	3.1422	0.3887	0.7993	low
TCGA-D1-A176	2.5123	0.0000	5.9157	2.8278	2.5880	0.4471	low
TCGA-D1-A177	1.5014	0.0000	4.7291	0.2947	5.1721	0.8479	low
TCGA-D1-A17A	0.1315	0.0000	3.9857	4.3222	1.5728	0.5463	low
TCGA-D1-A17B	0.5836	0.0000	5.7000	3.8502	1.9689	0.3782	low
TCGA-D1-A17F	0.1068	0.0000	4.3419	2.0351	3.6416	0.7096	low
TCGA-D1-A17H	2.3342	0.0000	4.4758	2.3840	3.7985	0.5870	low
TCGA-D1-A17L	2.1973	0.0000	5.5704	2.6873	3.2944	0.4420	low
TCGA-D1-A17Q	0.1479	0.0000	6.5559	4.5547	3.2357	0.1808	low
TCGA-D1-A17S	2.0247	0.0000	5.1680	2.5389	2.0377	0.6755	low
TCGA-D1-A17T	0.1945	0.0000	4.4269	2.1207	3.1331	0.7508	low
TCGA-D1-A1NS	0.1178	0.0000	4.6101	2.7832	3.9657	0.4761	low
TCGA-D1-A1NY	1.4055	0.0000	3.8749	2.7828	4.2753	0.5401	low
TCGA-D1-A1O5	1.2548	0.0000	6.1123	3.9650	1.6899	0.3463	low
TCGA-D1-A1O7	0.0877	0.0000	4.5359	2.1013	3.7276	0.6473	low
TCGA-D1-A2G5	3.9863	0.0000	5.1491	2.9398	3.9967	0.3892	low
TCGA-D1-A3DH	1.5699	0.0000	4.8805	2.4611	2.6413	0.6579	low
TCGA-DF-A2KR	5.1096	0.0000	5.7360	2.4260	3.0894	0.4842	low
TCGA-DF-A2KV	7.4438	0.0000	5.2169	1.5075	4.8939	0.5197	low
TCGA-DI-A1BY	3.0521	0.0000	4.4040	1.5807	3.2981	0.8797	low
TCGA-E6-A1LX	1.9479	0.0000	3.7813	3.1064	3.6672	0.5630	low
TCGA-EC-A1NJ	1.3370	0.0000	5.0531	2.4427	2.7235	0.6220	low
TCGA-EC-A24G	1.4000	0.0000	6.1620	3.2214	4.1814	0.2605	low
TCGA-EO-A1Y7	4.0959	0.0000	6.9160	2.2611	1.4070	0.5392	low
TCGA-EO-A22U	3.8548	0.0000	3.1409	3.7161	1.7482	0.8100	low
TCGA-EO-A22X	4.8000	0.0000	4.3065	2.6233	3.9655	0.5449	low
TCGA-EO-A22Y	4.2932	0.0000	4.5072	2.0713	3.1551	0.7444	low
TCGA-EO-A2CG	4.2849	0.0000	3.3897	2.4317	3.0086	0.9070	low
TCGA-EO-A3AS	2.8658	0.0000	4.0783	1.8176	3.8196	0.7894	low
TCGA-EO-A3AU	4.2274	0.0000	4.8854	1.5069	3.8689	0.7049	low
TCGA-EO-A3B0	3.8685	0.0000	4.9931	2.6943	1.7767	0.7082	low
TCGA-EO-A3KX	3.2055	0.0000	5.0726	2.5989	3.9974	0.4469	low
TCGA-EO-A3L0	4.2575	0.0000	5.1769	2.8808	2.4713	0.5457	low
TCGA-EY-A1GC	4.5123	0.0000	5.2994	1.9220	1.3648	0.9335	low
TCGA-EY-A1GD	4.4904	0.0000	3.1746	5.7990	2.1258	0.3592	low
TCGA-EY-A1GF	2.2630	0.0000	4.2019	3.0010	4.3763	0.4499	low
TCGA-EY-A1GH	1.9452	0.0000	3.6867	4.2855	4.7935	0.3014	low
TCGA-EY-A1GI	1.9452	0.0000	4.1480	2.7828	2.3860	0.7520	low
TCGA-EY-A1GT	1.3425	0.0000	4.6400	2.2848	3.4847	0.6224	low
TCGA-EY-A1GU	2.7315	1.0000	4.3446	2.1947	3.0791	0.7562	low
TCGA-EY-A214	1.2000	0.0000	5.8172	3.0753	1.5311	0.5271	low
TCGA-EY-A2OP	2.5863	0.0000	5.5039	2.8041	1.6940	0.6072	low
TCGA-EY-A2OQ	2.4247	0.0000	4.7343	3.0231	2.8961	0.5325	low
TCGA-EY-A3QX	2.7096	1.0000	4.8149	0.9059	3.9848	0.8633	low

TCGA-EY-A547	1.4932	0.0000	5.9009	1.8011	2.7018	0.6260	low
TCGA-EY-A548	1.1781	0.0000	6.0254	3.1210	2.9374	0.3643	low
TCGA-EY-A549	1.6575	0.0000	4.4131	3.9643	3.3282	0.3808	low
TCGA-EY-A5W2	1.4137	0.0000	5.4910	1.9276	4.6145	0.4437	low
TCGA-FI-A2D4	0.6658	1.0000	5.1167	3.1529	0.9443	0.6980	low
TCGA-FI-A2F4	6.1096	0.0000	5.8916	2.1473	3.5671	0.4627	low
TCGA-PG-A916	1.4301	0.0000	6.1973	3.2273	3.2214	0.3159	low
TCGA-PG-A917	0.5808	0.0000	4.7247	2.8492	1.6522	0.7391	low
TCGA-QF-A5YS	1.8877	0.0000	4.4822	1.5992	2.9976	0.9130	low
TCGA-QF-A5YT	3.3288	0.0000	4.8599	2.3863	2.1609	0.7520	low
TCGA-QS-A5YQ	2.0795	0.0000	4.9317	2.3395	2.2204	0.7407	low
TCGA-QS-A5YR	1.9699	0.0000	5.2436	2.0903	3.0931	0.6183	low
TCGA-SJ-A6ZI	1.7288	0.0000	4.5633	2.7575	3.8847	0.4948	low
TCGA-SJ-A6ZJ	1.5233	0.0000	4.3367	2.5236	2.2325	0.8094	low
TCGA-SL-A6JA	0.0192	0.0000	5.6598	1.7253	3.4071	0.5890	low
