# Supplementary material for: Comprehensive Analysis of GDF10 Methylation Site-Associated Genes as Prognostic Markers for Endometrial Cancer
Source: J Oncol. 2022 Oct 10;2022:7117083. doi: 10.1155/2022/7117083 (PMC9576415; doi:10.1155/2022/7117083)
Supplement: Supplementary Materials — are shown as Supplemental Figures S1-3 and Supplemental Tables S1-7. Supplemental Figure S1: Cox regression independent prognostic analyses. Supplemental Figure S2: Analysis of expression correlations between the risk model and clinicopathological factors. Supplemental Figure S3: Kaplan-Meier survival analysis of patients with different clinicopathologic features between high- and low-risk groups. (a–i) The different OS of UCEC patients with different ages, tumor stages, and grades between high-risk and low-risk groups according to GDF10 methylation. The abscissa shows survival months, and the vertical axis shows overall survival probability. Supplemental Table S1: 48 modules were identified. Supplemental Table S2: The saddlebrown module positively correlated with the methylation sites the most. Supplemental Table S3: 991 genes were upregulated in DEGs. Supplemental Table S4: 1260 genes were downregulated in DEGs. Supplemental Table S5: 44 DEGMRGs were identified. Supplemental Table S6: All UCEC samples in the training set were divided into the high- and low-risk groups. Supplemental Table S7: Risk scores were significantly different between patients classified by age, tumor stage, and grade. [file Supplemental Table S1.pdf › Supplemental Table S1.pdf]

| probes | moduleCol( | GS.cg02974931 | p.GS.cg02974931 | GS.cg04110601 | p.GS.cg04110601 |
|---|---|---|---|---|---|
| ACVR1C | bisque4 | -0.123305307 | 0.007637327 | -0.117436074 | 0.011091346 |
| ADRB3 | bisque4 | -0.160004233 | 0.000518831 | -0.164111384 | 0.00036924 |
| AMHR2 | bisque4 | -0.129158428 | 0.005183963 | -0.128238138 | 0.005515251 |
| ANGPTL: | bisque4 | -0.08996741 | 0.052022997 | -0.091843254 | 0.047297706 |
| ASIP | bisque4 | -0.275507906 | 1.40E-09 | -0.27693963 | 1.14E-09 |
| C21orf62 | bisque4 | -0.13098366 | 0.004579423 | -0.130048549 | 0.004880698 |
| C2orf71 | bisque4 | -0.126055441 | 0.006378359 | -0.138451856 | 0.002713973 |
| CDK15 | bisque4 | -0.137826499 | 0.002838286 | -0.136539732 | 0.003110522 |
| CRYGD | bisque4 | -0.103562646 | 0.025219051 | -0.102170224 | 0.027259587 |
| CYP11A1 | bisque4 | -0.260122833 | 1.17E-08 | -0.261170052 | 1.01E-08 |
| DCAF8L2 | bisque4 | -0.097207432 | 0.035727416 | -0.087733898 | 0.058156151 |
| DPF3 | bisque4 | -0.17910641 | 9.96E-05 | -0.163707192 | 0.000381945 |
| DUSP26 | bisque4 | -0.222317118 | 1.22E-06 | -0.22715569 | 7.02E-07 |
| EML5 | bisque4 | -0.071310521 | 0.123837721 | -0.105149289 | 0.023055808 |
| ENPP6 | bisque4 | -0.134705431 | 0.003539701 | -0.135578102 | 0.003329235 |
| FAM153E | bisque4 | -0.129897344 | 0.004931059 | -0.145432802 | 0.001626144 |
| FRMPD3 | bisque4 | -0.105361724 | 0.022778731 | -0.098623255 | 0.033109597 |
| FSHR | bisque4 | -0.085119016 | 0.066085331 | -0.068502778 | 0.139373781 |
| GATA4 | bisque4 | -0.097227695 | 0.035688744 | -0.115727299 | 0.012328497 |
| GPR88 | bisque4 | -0.066078947 | 0.153952131 | -0.042648373 | 0.357788296 |
| HPCAL4 | bisque4 | -0.194678247 | 2.27E-05 | -0.198707661 | 1.52E-05 |
| INHA | bisque4 | -0.10643882 | 0.021417834 | -0.116314697 | 0.011890123 |
| LRRTM3 | bisque4 | -0.028381081 | 0.540670142 | -0.028800165 | 0.534706432 |
| MCF2 | bisque4 | -0.043981885 | 0.342941966 | -0.057178173 | 0.21745322 |
| NR5A1 | bisque4 | 0.008775091 | 0.84999347 | -0.001902266 | 0.967297337 |
| NRK | bisque4 | -0.191980084 | 2.96E-05 | -0.193328415 | 2.59E-05 |
| NT5C1A | bisque4 | -0.085582081 | 0.064619785 | -0.083596002 | 0.071099147 |
| NXPH2 | bisque4 | -0.20679784 | 6.61E-06 | -0.205983603 | 7.20E-06 |
| PCSK2 | bisque4 | -0.13444679 | 0.003604357 | -0.136530608 | 0.003112534 |
| PLA2G1E | bisque4 | -0.075526049 | 0.103083204 | -0.063310085 | 0.171987173 |
| PLA2G5 | bisque4 | -0.118472558 | 0.010395682 | -0.117793652 | 0.010846825 |
| PLCXD3 | bisque4 | -0.200384134 | 1.28E-05 | -0.213992047 | 3.07E-06 |
| PON3 | bisque4 | -0.19687709 | 1.83E-05 | -0.196226 | 1.95E-05 |
| PROK1 | bisque4 | -0.10195057 | 0.027594114 | -0.079478863 | 0.08622343 |
| RGAG1 | bisque4 | -0.008661632 | 0.851910498 | -0.012705855 | 0.784199089 |
| SCUBE1 | bisque4 | -0.036463998 | 0.431783514 | -0.026299534 | 0.570773513 |
| SGCZ | bisque4 | -0.13137697 | 0.004457801 | -0.126567003 | 0.00616586 |
| SIGLEC1 | bisque4 | -0.033828359 | 0.465826354 | -0.032693492 | 0.480930146 |
| STAR | bisque4 | -0.02553216 | 0.582068571 | -0.036665816 | 0.42923738 |
| STXBP5L | bisque4 | -0.121979135 | 0.008320316 | -0.145164867 | 0.001659119 |
| VIT | bisque4 | -0.109589032 | 0.017834736 | -0.097071186 | 0.035988369 |
| WFIKKN: | bisque4 | -0.186180238 | 5.16E-05 | -0.195867304 | 2.02E-05 |
| ABCC4 | black | -0.230914837 | 4.53E-07 | -0.234439173 | 2.99E-07 |
| ABHD16/ | black | -0.10442609 | 0.024021051 | -0.138253571 | 0.002752842 |

| | | | | | |
|---|---|---|---|---|---|
| ABHD2 | black | 0.164874735 | 0.000346313 | 0.136418234 | 0.003137416 |
| ABL1 | black | 0.129641367 | 0.005017383 | 0.103499128 | 0.025309181 |
| ABTB2 | black | -0.025110126 | 0.588324578 | -0.031954155 | 0.490911089 |
| AC025048 | black | 0.023458156 | 0.613105147 | 0.010828311 | 0.81546517 |
| AC131263 | black | 0.009075545 | 0.844921209 | -0.017822422 | 0.700871615 |
| ACAD8 | black | 0.211930988 | 3.83E-06 | 0.180071175 | 9.12E-05 |
| ACBD7 | black | -0.039032478 | 0.40003475 | -0.048178814 | 0.298820479 |
| ACER2 | black | 0.131095826 | 0.004544436 | 0.096011444 | 0.03807393 |
| ACO1 | black | 0.100602889 | 0.029724829 | 0.100277958 | 0.03025916 |
| ACOX1 | black | 0.248534537 | 5.27E-08 | 0.247271195 | 6.18E-08 |
| ACTR8 | black | 0.126385527 | 0.006240505 | 0.122318902 | 0.008140332 |
| ACVR1 | black | 0.094034736 | 0.042238314 | 0.080793977 | 0.081133684 |
| ADAM17 | black | -0.135168124 | 0.003426654 | -0.162812708 | 0.000411523 |
| ADAM9 | black | 0.250116759 | 4.30E-08 | 0.233951166 | 3.17E-07 |
| ADAR | black | -0.017160563 | 0.711474663 | -0.023463815 | 0.613019475 |
| ADAT1 | black | 0.341555657 | 3.18E-14 | 0.349596107 | 7.17E-15 |
| ADCY6 | black | 0.140176277 | 0.002396437 | 0.112598397 | 0.014912489 |
| ADNP | black | -0.173775988 | 0.000160639 | -0.199360546 | 1.42E-05 |
| AFAP1 | black | -0.043472474 | 0.348566404 | -0.052885935 | 0.254032166 |
| AGER | black | -0.084018756 | 0.069677218 | -0.09046497 | 0.050732814 |
| AGFG1 | black | -0.119532312 | 0.009724685 | -0.126089695 | 0.006363927 |
| AHCYL1 | black | 0.205438243 | 7.62E-06 | 0.221547383 | 1.33E-06 |
| AHCYL2 | black | 0.03799863 | 0.412642321 | 0.005095434 | 0.912553028 |
| AIFM3 | black | -0.007749916 | 0.867345833 | -0.018353755 | 0.692401342 |
| AJUBA | black | -0.208915205 | 5.29E-06 | -0.221494791 | 1.34E-06 |
| AKAP12 | black | -0.166774591 | 0.000294885 | -0.159690628 | 0.000532307 |
| ALKBH5 | black | 0.190494663 | 3.42E-05 | 0.203328973 | 9.49E-06 |
| AMER1 | black | 0.007217216 | 0.87638821 | 0.005479083 | 0.90599828 |
| AMMECR | black | -0.121460011 | 0.008602167 | -0.162040157 | 0.000438772 |
| AMOTL1 | black | 0.002467722 | 0.957584435 | -0.003611574 | 0.937957033 |
| ANGEL1 | black | -0.167138655 | 0.000285883 | -0.211807617 | 3.89E-06 |
| ANKRD1 | black | 0.096480959 | 0.037137617 | 0.067064454 | 0.147891156 |
| ANKS1A | black | -0.110529135 | 0.016872291 | -0.147738924 | 0.001366214 |
| ANKS3 | black | -0.016373484 | 0.724156374 | -0.006544325 | 0.887833166 |
| ANO6 | black | -0.054171604 | 0.242653922 | -0.08135632 | 0.079032827 |
| APAF1 | black | 0.081392823 | 0.078897994 | 0.072007025 | 0.120200538 |
| APLP2 | black | 0.116189216 | 0.011982595 | 0.082950107 | 0.073317321 |
| APOPT1 | black | 0.054713705 | 0.237964998 | 0.062826134 | 0.17529494 |
| AREL1 | black | -0.032038911 | 0.489761289 | -0.0537193 | 0.246615403 |
| ARHGAP | black | 0.075181591 | 0.104668327 | 0.079908015 | 0.084535006 |
| ARID1B | black | 0.003188952 | 0.945205078 | -0.016923529 | 0.715285671 |
| ARIH2 | black | -0.006637864 | 0.886240742 | -0.032160713 | 0.488111466 |
| ARL2BP | black | 0.03028235 | 0.513883255 | 0.012234118 | 0.792024296 |
| ARNT | black | -0.090141239 | 0.05156918 | -0.11981414 | 0.009552846 |
| ARNT2 | black | 0.012150133 | 0.793419667 | -0.020525232 | 0.658191153 |

| | | | | | |
|---|---|---|---|---|---|
| ARPC4-T | black | 0.000374674 | 0.993557093 | -0.019223597 | 0.678617493 |
| ARSB | black | 0.107080931 | 0.020640576 | 0.093343744 | 0.04378187 |
| ASAP1 | black | -0.223260824 | 1.10E-06 | -0.255508739 | 2.14E-08 |
| ASAP2 | black | -0.070922044 | 0.12590325 | -0.072491568 | 0.117719783 |
| ASCC3 | black | 0.122951184 | 0.00781462 | 0.1241053 | 0.007249973 |
| ASH1L | black | -0.015210457 | 0.743033542 | -0.046389941 | 0.317142565 |
| ATG16L1 | black | 0.046250445 | 0.318601557 | 0.036599553 | 0.430072389 |
| ATP10D | black | -0.16040942 | 0.00050189 | -0.163238988 | 0.000397171 |
| ATRN | black | -0.08037128 | 0.082742397 | -0.116172118 | 0.011995244 |
| ATXN7L: | black | -0.145498049 | 0.001618206 | -0.169570041 | 0.000232037 |
| B3GNT9 | black | 0.028241597 | 0.542662369 | 0.045638689 | 0.325051529 |
| B4GALT: | black | -0.05929599 | 0.200865797 | -0.082538732 | 0.074759275 |
| BACE1 | black | -0.116020398 | 0.012108005 | -0.128813934 | 0.005305816 |
| BAG5 | black | -0.012944165 | 0.78025418 | -0.012175689 | 0.792995004 |
| BAG6 | black | -0.085335049 | 0.06539825 | -0.09126118 | 0.048723863 |
| BAHD1 | black | 0.094569882 | 0.041074653 | 0.066106432 | 0.153780584 |
| BCL2L11 | black | 0.074677391 | 0.107023261 | 0.034389075 | 0.458461936 |
| BCL2L2 | black | -0.131045379 | 0.004560142 | -0.142392391 | 0.002038186 |
| BCL9 | black | 0.098700331 | 0.03297191 | 0.047951976 | 0.301104003 |
| BCORL1 | black | -0.210247699 | 4.59E-06 | -0.230704922 | 4.65E-07 |
| BGLAP | black | 0.027064078 | 0.559624639 | 0.022799793 | 0.623107393 |
| BICD2 | black | 0.006593587 | 0.886994465 | -0.010434169 | 0.82206785 |
| BLOC1S1 | black | 0.081655321 | 0.077933879 | 0.096070216 | 0.037955642 |
| BPTF | black | -0.077275205 | 0.095325501 | -0.111767479 | 0.015674048 |
| BRD1 | black | 0.164673108 | 0.000352236 | 0.127970127 | 0.005615248 |
| BRD4 | black | -0.094589222 | 0.041033112 | -0.089606862 | 0.052974905 |
| BTD | black | 0.139724667 | 0.002476145 | 0.13880583 | 0.002645826 |
| BTRC | black | 0.089764802 | 0.052556146 | 0.05717591 | 0.217471456 |
| C12orf49 | black | 0.052116553 | 0.261015608 | 0.050858369 | 0.27271872 |
| C19orf43 | black | 0.030733932 | 0.507623621 | 0.049359911 | 0.287117196 |
| C19orf71 | black | 0.139471113 | 0.002521948 | 0.115382024 | 0.012592782 |
| C1orf123 | black | -0.179664854 | 9.46E-05 | -0.18866956 | 4.08E-05 |
| C1orf233 | black | 0.014427377 | 0.755831878 | 0.032170611 | 0.487977521 |
| C1orf86 | black | -0.003931472 | 0.932474088 | -0.007703824 | 0.868127554 |
| C2CD3 | black | 0.044473754 | 0.337566383 | 0.028637194 | 0.537021635 |
| C3orf62 | black | 0.092684842 | 0.045297698 | 0.078029765 | 0.092126175 |
| C6orf89 | black | -0.004248474 | 0.927043929 | -0.01838588 | 0.691890428 |
| C7orf61 | black | -0.262579755 | 8.38E-09 | -0.255277998 | 2.21E-08 |
| C8G | black | -0.099856157 | 0.03096504 | -0.070316904 | 0.12917393 |
| C9orf16 | black | 0.197448326 | 1.72E-05 | 0.218541351 | 1.86E-06 |
| CACHD1 | black | -0.17303341 | 0.000171521 | -0.201826422 | 1.11E-05 |
| CAMKK2 | black | 0.061272926 | 0.186230503 | 0.042620185 | 0.358106403 |
| CASK | black | -0.037996467 | 0.412668939 | -0.064184514 | 0.166128921 |
| CASP2 | black | 0.21013013 | 4.65E-06 | 0.203487561 | 9.33E-06 |
| CBLL1 | black | -0.081042108 | 0.080201188 | -0.084450836 | 0.068248056 |

| | | | | | |
|---|---|---|---|---|---|
| CCDC101 | black | -0.090610973 | 0.050359333 | -0.0723354 | 0.11851492 |
| CCDC117 | black | 0.076238908 | 0.099863244 | 0.04637053 | 0.317345322 |
| CCDC12 | black | 0.027548966 | 0.552608818 | 0.04593692 | 0.321896662 |
| CCDC130 | black | 0.030092181 | 0.516531214 | 0.022370162 | 0.629672349 |
| CCDC159 | black | 0.275542495 | 1.39E-09 | 0.270420107 | 2.86E-09 |
| CCDC28I | black | -0.161897728 | 0.000443975 | -0.148069163 | 0.001332291 |
| CCDC61 | black | 0.010548163 | 0.820156903 | 0.032676157 | 0.481162892 |
| CCDC93 | black | -0.162772814 | 0.000412891 | -0.187773631 | 4.44E-05 |
| CCER2 | black | -0.087395597 | 0.059135361 | -0.100642998 | 0.029659436 |
| CCNI | black | 0.033573391 | 0.469196634 | 0.024878149 | 0.591776422 |
| CCNK | black | -0.042146278 | 0.363481191 | -0.061536537 | 0.184339932 |
| CCS | black | -0.014309714 | 0.757760803 | -0.017405791 | 0.707539472 |
| CDK10 | black | 0.218115822 | 1.95E-06 | 0.229829554 | 5.15E-07 |
| CDK12 | black | 0.034120253 | 0.461984486 | 0.008724867 | 0.850841963 |
| CDK5R1 | black | 0.123736536 | 0.007426289 | 0.083636776 | 0.070960979 |
| CERK | black | -0.045431758 | 0.327252325 | -0.093941209 | 0.042444513 |
| CERS2 | black | 0.001904353 | 0.967261479 | -0.02827491 | 0.542186234 |
| CHADL | black | 0.22772229 | 6.58E-07 | 0.242119028 | 1.18E-07 |
| CHMP1B | black | -0.074990695 | 0.105555061 | -0.084115929 | 0.069353688 |
| CHMP4A | black | -0.055984229 | 0.227226427 | -0.047865809 | 0.301974457 |
| CIPC | black | 0.026272266 | 0.571173079 | 0.011270355 | 0.80807556 |
| CIRH1A | black | 0.102419625 | 0.026883989 | 0.109774481 | 0.017641171 |
| CITED2 | black | -0.111901988 | 0.0155485 | -0.114970083 | 0.012914626 |
| CLASRP | black | -0.0941738 | 0.041933281 | -0.114350586 | 0.013412289 |
| CMTM4 | black | 0.137009887 | 0.003008416 | 0.139398689 | 0.002535173 |
| CNN3 | black | -0.07342073 | 0.113074885 | -0.067712388 | 0.144006443 |
| CNNM4 | black | -0.083919509 | 0.070008927 | -0.104056374 | 0.024527861 |
| CNOT1 | black | 0.135182926 | 0.003423092 | 0.127402701 | 0.005832357 |
| CNPY2 | black | 0.091095558 | 0.049136177 | 0.081872326 | 0.077144091 |
| CORO1C | black | 0.049707881 | 0.283728853 | 0.052126256 | 0.260926727 |
| CORO7 | black | 0.150714406 | 0.001087256 | 0.201966757 | 1.09E-05 |
| CPSF7 | black | -0.108593858 | 0.018905473 | -0.13326476 | 0.003913685 |
| CRADD | black | 0.132220005 | 0.004206887 | 0.115306616 | 0.012651162 |
| CREB3L2 | black | 0.040819701 | 0.378792131 | 0.041408594 | 0.371947083 |
| CRISPLD | black | 0.046409907 | 0.316934107 | 0.017681832 | 0.703119118 |
| CRTC3 | black | -0.015200643 | 0.743193508 | -0.042972438 | 0.354143824 |
| CRYBB3 | black | -0.054641801 | 0.238583235 | -0.051513204 | 0.266583794 |
| CSDE1 | black | -0.149057633 | 0.001235326 | -0.180600045 | 8.69E-05 |
| CSNK1A | black | 0.134512769 | 0.003587762 | 0.118162731 | 0.01059946 |
| CSNK1G | black | 0.182036108 | 7.61E-05 | 0.183531844 | 6.62E-05 |
| CSNK2A | black | -0.071797465 | 0.121285991 | -0.099833379 | 0.031003557 |
| CTB-102I | black | 0.050080099 | 0.280134374 | 0.048556931 | 0.295039733 |
| CTBP2 | black | 0.125866294 | 0.006458578 | 0.107460624 | 0.020192598 |
| CTNNB1 | black | 0.034213291 | 0.460763646 | 0.013406677 | 0.772614067 |
| CTNND1 | black | 0.023914004 | 0.606221301 | -0.000929092 | 0.984024184 |

| | | | | | |
|---|---|---|---|---|---|
| CYTH3 | black | -0.229016713 | 5.66E-07 | -0.21695174 | 2.22E-06 |
| DAGLA | black | -0.213525385 | 3.23E-06 | -0.210055852 | 4.69E-06 |
| DAZAP2 | black | 0.111656854 | 0.015777969 | 0.116791929 | 0.011544168 |
| DCP1A | black | -0.052268006 | 0.259630564 | -0.076823208 | 0.097284047 |
| DCTN1 | black | -0.103211698 | 0.025720517 | -0.112923018 | 0.014623909 |
| DDX42 | black | -0.05701485 | 0.218772049 | -0.085600795 | 0.064561123 |
| DENND5. | black | 0.100746323 | 0.029491546 | 0.079513491 | 0.086086195 |
| DEPDC5 | black | 0.021965723 | 0.635879124 | -0.00497457 | 0.914619266 |
| DGKD | black | -0.028862912 | 0.533816381 | -0.063340328 | 0.171782022 |
| DHX35 | black | -0.21845956 | 1.88E-06 | -0.243794243 | 9.55E-08 |
| DIAPH1 | black | 0.068712527 | 0.138163808 | 0.081807238 | 0.077380293 |
| DIDO1 | black | -0.200015596 | 1.33E-05 | -0.218552329 | 1.86E-06 |
| DIP2B | black | 0.113382167 | 0.014224122 | 0.093748214 | 0.042872694 |
| DMAP1 | black | -0.18652742 | 5.00E-05 | -0.18572724 | 5.39E-05 |
| DOCK1 | black | 0.047244789 | 0.308297281 | 0.012244065 | 0.791859081 |
| DOCK7 | black | 0.013292295 | 0.774501498 | -0.024681611 | 0.594708153 |
| DOCK9 | black | -0.009584272 | 0.836347602 | -0.021830912 | 0.637953725 |
| DOPEY2 | black | 0.200558356 | 1.26E-05 | 0.186375852 | 5.07E-05 |
| DPAGT1 | black | 0.181108874 | 8.29E-05 | 0.1671723 | 0.000285064 |
| DUSP16 | black | 0.034269654 | 0.460024931 | 0.025519907 | 0.582249767 |
| DUSP7 | black | -0.059855072 | 0.196644587 | -0.070837008 | 0.12635893 |
| DVL2 | black | -0.00377048 | 0.935233049 | -0.036032287 | 0.437259192 |
| DYM | black | 0.048452443 | 0.296081287 | 0.028732723 | 0.535663913 |
| DYNC1I2 | black | -0.029694715 | 0.522088323 | -0.059458451 | 0.199632437 |
| DYNC1L | black | 0.047392031 | 0.306790326 | 0.032545916 | 0.482913555 |
| EDEM1 | black | 0.13775191 | 0.002853454 | 0.139765446 | 0.002468849 |
| EEF2K | black | 0.035294055 | 0.446714386 | 0.044868568 | 0.333290778 |
| EGFR | black | 0.024223501 | 0.601567274 | 0.000686584 | 0.988193774 |
| EHF | black | 0.167658015 | 0.000273484 | 0.168381304 | 0.000257053 |
| EHMT1 | black | 0.224958408 | 9.04E-07 | 0.232989947 | 3.55E-07 |
| EIF2S3L | black | 0.05103112 | 0.271091004 | 0.043353349 | 0.349890058 |
| ELF1 | black | 0.078884274 | 0.0886075 | 0.065981675 | 0.154560428 |
| ELK1 | black | -0.134124937 | 0.003686309 | -0.127297563 | 0.005873404 |
| ELMO2 | black | -0.184032266 | 6.32E-05 | -0.203453191 | 9.37E-06 |
| EMG1 | black | -0.101171772 | 0.028808799 | -0.089638221 | 0.052891541 |
| EMP1 | black | -0.12485468 | 0.006903166 | -0.122891174 | 0.007845025 |
| ENDOD1 | black | 0.052777629 | 0.255007315 | 0.037581101 | 0.417799818 |
| ENOX2 | black | -0.139875742 | 0.002449215 | -0.147001743 | 0.001444824 |
| ENTPD7 | black | 0.265878356 | 5.36E-09 | 0.239936748 | 1.54E-07 |
| EPHA4 | black | -0.116714457 | 0.011599717 | -0.123068452 | 0.007755506 |
| EPN2 | black | -0.043767888 | 0.345297635 | -0.064502314 | 0.164037292 |
| ERAP1 | black | -0.052935518 | 0.253586612 | -0.068729949 | 0.13806367 |
| ERC1 | black | -0.026797249 | 0.563503661 | -0.044102477 | 0.34161901 |
| ERCC3 | black | -0.119153408 | 0.009960039 | -0.147762888 | 0.001363726 |
| ERCC5 | black | 0.033153497 | 0.474776164 | 0.02201982 | 0.635047428 |

| | | | | | |
|---|---|---|---|---|---|
| ERCC6-P | black | -0.057153494 | 0.217652139 | -0.075997348 | 0.100945282 |
| ERMP1 | black | 0.023062925 | 0.619101276 | 0.025243844 | 0.586339072 |
| ESYT2 | black | -0.108879397 | 0.018592665 | -0.110118683 | 0.017286763 |
| ETV2 | black | 0.06126142 | 0.186313351 | 0.050025862 | 0.280656206 |
| ETV3 | black | -0.009857563 | 0.831749737 | -0.048631732 | 0.294295605 |
| EVC | black | -0.205051007 | 7.94E-06 | -0.234239174 | 3.06E-07 |
| EXOC2 | black | 0.127966605 | 0.005616573 | 0.121877544 | 0.008374817 |
| EXOC4 | black | -0.017851101 | 0.700413482 | -0.023518307 | 0.612194822 |
| EXOC6B | black | -0.152794618 | 0.000924525 | -0.177246362 | 0.000117833 |
| EXOSC1 | black | 0.182926054 | 7.01E-05 | 0.175712572 | 0.000135227 |
| EYA3 | black | 0.016779213 | 0.717609437 | 0.004842144 | 0.916883823 |
| FAM168A | black | 0.04769298 | 0.303725388 | 0.020607259 | 0.656912195 |
| FAM193A | black | 0.149049128 | 0.001236132 | 0.137213584 | 0.002965132 |
| FAM193B | black | -0.05622313 | 0.225246306 | -0.060270242 | 0.193552103 |
| FAM222B | black | 0.173338213 | 0.000166973 | 0.160505578 | 0.000497946 |
| FAN1 | black | -0.01098406 | 0.812859629 | -0.057847171 | 0.212110605 |
| FARP1 | black | 0.086818999 | 0.060835738 | 0.059036855 | 0.202844537 |
| FARP2 | black | 0.117063942 | 0.011350991 | 0.107771956 | 0.019831609 |
| FAT1 | black | 0.172472542 | 0.000180194 | 0.182457281 | 7.32E-05 |
| FBF1 | black | 0.115334718 | 0.012629379 | 0.118383457 | 0.010453926 |
| FBXL20 | black | 0.02722821 | 0.557244995 | 0.021926654 | 0.636480068 |
| FBXO11 | black | -0.1221572 | 0.008225552 | -0.141712463 | 0.002142517 |
| FBXO21 | black | -0.1764072 | 0.00012707 | -0.196103422 | 1.97E-05 |
| FBXO7 | black | -0.033088679 | 0.475640687 | -0.022706834 | 0.62452534 |
| FBXW11 | black | -0.097556153 | 0.035066828 | -0.117930978 | 0.010754195 |
| FKBP15 | black | 0.202749127 | 1.01E-05 | 0.198725074 | 1.52E-05 |
| FKBP2 | black | 0.03126803 | 0.500272016 | 0.025996014 | 0.57522861 |
| FKBP9 | black | -0.069476434 | 0.133825186 | -0.101537878 | 0.028232187 |
| FLCN | black | 0.062619507 | 0.176721551 | 0.048205413 | 0.298553464 |
| FLNB | black | 0.084299583 | 0.068745588 | 0.088103886 | 0.057100639 |
| FOCAD | black | 0.1711578 | 0.000202164 | 0.153642631 | 0.00086489 |
| FOXJ3 | black | -0.02030591 | 0.661615698 | -0.038103251 | 0.411355914 |
| FRAS1 | black | 0.170435864 | 0.00021527 | 0.145704485 | 0.001593323 |
| FRG1B | black | -0.236690315 | 2.28E-07 | -0.241981727 | 1.20E-07 |
| FRRS1 | black | 0.182419068 | 7.34E-05 | 0.157742373 | 0.000623574 |
| FZD1 | black | 0.186469272 | 5.03E-05 | 0.172787518 | 0.000175274 |
| FZD5 | black | 0.016734587 | 0.718328539 | 0.036275791 | 0.434165783 |
| GALK2 | black | 0.175189505 | 0.00014169 | 0.167930787 | 0.000267176 |
| GALNT4 | black | 0.262293071 | 8.71E-09 | 0.217585319 | 2.07E-06 |
| GATAD2 | black | 0.026060515 | 0.574280482 | -0.011150784 | 0.810072771 |
| GGCX | black | -0.183842811 | 6.43E-05 | -0.195487152 | 2.10E-05 |
| GID4 | black | 0.07480294 | 0.106432991 | 0.053989581 | 0.244242764 |
| GIGYF2 | black | -0.068467143 | 0.139580158 | -0.112295807 | 0.015185974 |
| GLCE | black | 0.07008766 | 0.13043 | 0.028376334 | 0.540737879 |
| GLTSCR1 | black | -0.027611012 | 0.551714179 | -0.053584148 | 0.247807837 |

| | | | | | |
|---|---|---|---|---|---|
| GLUD2 | black | -0.092395188 | 0.045977879 | -0.107311359 | 0.020367689 |
| GMEB1 | black | 0.158695573 | 0.000577246 | 0.145955013 | 0.001563597 |
| GNB1 | black | 0.178057745 | 0.000109505 | 0.174345743 | 0.000152732 |
| GNE | black | 0.083812833 | 0.070366904 | 0.088689476 | 0.055462545 |
| GNS | black | 0.042716933 | 0.357015308 | 0.014314361 | 0.757684581 |
| GOLGA2 | black | 0.113636968 | 0.014006436 | 0.098177154 | 0.033916178 |
| GPATCH | black | -0.011542283 | 0.803538182 | -0.04582258 | 0.323103852 |
| GPHN | black | 0.260433789 | 1.12E-08 | 0.238468375 | 1.84E-07 |
| GRB10 | black | 0.043754878 | 0.345441186 | 0.054997201 | 0.235538448 |
| GSTO1 | black | 0.107104981 | 0.020611947 | 0.109956051 | 0.017453434 |
| GTF2I | black | -0.004551224 | 0.921861066 | -0.037869588 | 0.414232281 |
| GTPBP1 | black | 0.006565565 | 0.887471533 | 0.010869343 | 0.814778539 |
| HAGHL | black | -0.007645271 | 0.869120815 | 0.014333251 | 0.757374826 |
| HBP1 | black | -0.12403876 | 0.007281508 | -0.132368518 | 0.004164031 |
| HCFC1R1 | black | -0.01619086 | 0.727109858 | 0.003723709 | 0.936034731 |
| HDAC10 | black | 0.222482764 | 1.20E-06 | 0.237775602 | 2.00E-07 |
| HDAC4 | black | -0.093053909 | 0.044443304 | -0.125363362 | 0.006676294 |
| HDHD2 | black | 0.018677506 | 0.687258892 | -0.021996827 | 0.635400865 |
| HEATR1 | black | -0.024607794 | 0.595810991 | -0.060972432 | 0.18840297 |
| HECTD1 | black | 0.001594785 | 0.972581113 | -0.017070828 | 0.712916578 |
| HIF1AN | black | -0.019075282 | 0.680960346 | -0.025716945 | 0.579339214 |
| HIP1 | black | 0.051887412 | 0.263120787 | 0.037074238 | 0.424111408 |
| HIPK1 | black | 0.156485096 | 0.00068996 | 0.140927345 | 0.002269046 |
| HIST1H4. | black | -0.034686633 | 0.454580402 | -0.018649077 | 0.687709875 |
| HMG20A | black | 0.009615709 | 0.835818429 | -0.034312465 | 0.45946427 |
| HSD11B1 | black | 0.054872117 | 0.236606922 | 0.053088133 | 0.252218585 |
| HSD3B7 | black | -0.107001239 | 0.02073569 | -0.102247734 | 0.027142376 |
| HSPG2 | black | -0.072941723 | 0.115451145 | -0.077421513 | 0.094698319 |
| HYOU1 | black | 0.136755898 | 0.003063191 | 0.112450892 | 0.01504526 |
| ICMT | black | 0.212872751 | 3.46E-06 | 0.205196213 | 7.82E-06 |
| IGSF3 | black | 0.005012412 | 0.913972277 | -0.04115369 | 0.374900552 |
| INAFM1 | black | -0.001943053 | 0.966596567 | 0.019628017 | 0.672244879 |
| INO80B | black | -0.060665935 | 0.190637979 | -0.054389825 | 0.240758682 |
| INPP5B | black | -0.118828088 | 0.010166131 | -0.131225692 | 0.004504231 |
| IPO9 | black | 0.118207752 | 0.010569631 | 0.086045705 | 0.063179358 |
| IPP | black | 0.161968446 | 0.000441385 | 0.14787017 | 0.001352638 |
| IQGAP1 | black | 0.057871629 | 0.211917085 | 0.043124471 | 0.352442129 |
| ISY1 | black | -0.219069177 | 1.76E-06 | -0.200037849 | 1.33E-05 |
| ITGA2 | black | 0.121834962 | 0.008397755 | 0.103798512 | 0.024886784 |
| ITGA6 | black | 0.202554349 | 1.03E-05 | 0.164319069 | 0.000362866 |
| ITGB1 | black | 0.065004799 | 0.160770596 | 0.029203447 | 0.528998993 |
| ITPR3 | black | 0.093233694 | 0.044032033 | 0.097705823 | 0.03478651 |
| ITPRIPL2 | black | -0.079496117 | 0.086155026 | -0.076695883 | 0.097841511 |
| JAG1 | black | 0.078197469 | 0.091426923 | 0.080217609 | 0.083333592 |
| JAK1 | black | -0.27107202 | 2.62E-09 | -0.274840871 | 1.54E-09 |

| | | | | | |
|---|---|---|---|---|---|
| JARID2 | black | -0.031868263 | 0.492077777 | -0.058239148 | 0.209024534 |
| JOSD1 | black | 0.054364125 | 0.24098134 | 0.033264662 | 0.47329549 |
| JUND | black | -0.128593191 | 0.005385246 | -0.136919781 | 0.003027745 |
| KANK1 | black | -0.028902597 | 0.533253842 | -0.070940245 | 0.125805882 |
| KANSL1 | black | 0.047171574 | 0.309048424 | 0.017584457 | 0.704677282 |
| KAT6B | black | -0.091438013 | 0.048286841 | -0.123045751 | 0.007766918 |
| KAT7 | black | -0.061397227 | 0.185337269 | -0.08357876 | 0.071157641 |
| KCTD18 | black | -0.01115562 | 0.809991965 | -0.046034384 | 0.320869963 |
| KCTD20 | black | 0.032146926 | 0.488298061 | 0.020521183 | 0.658254313 |
| KDM2A | black | -0.051154749 | 0.269930214 | -0.068784428 | 0.137750891 |
| KDM2B | black | 0.05770901 | 0.213206134 | 0.056437666 | 0.223478627 |
| KDM4A | black | 0.00035591 | 0.993879748 | -0.024091962 | 0.603543321 |
| KDM6A | black | -0.005026601 | 0.913729688 | -0.022321013 | 0.630425246 |
| KDM6B | black | 0.239095023 | 1.70E-07 | 0.230173342 | 4.95E-07 |
| KIAA010 | black | 0.002033783 | 0.965037782 | -0.006626603 | 0.886432423 |
| KIAA092 | black | -0.053773767 | 0.246135972 | -0.042159681 | 0.363328494 |
| KIAA114 | black | 0.084388343 | 0.068453261 | 0.075163353 | 0.104752791 |
| KIAA121 | black | -0.065290255 | 0.158936781 | -0.092498391 | 0.045734554 |
| KIAA121 | black | 0.035616644 | 0.442568516 | 0.022110467 | 0.633654836 |
| KIAA146 | black | 0.097455498 | 0.035256425 | 0.070214682 | 0.129732861 |
| KIAA154 | black | 0.043002702 | 0.35380466 | 0.016312763 | 0.725137941 |
| KIAA167 | black | 0.066153165 | 0.153489232 | 0.070266757 | 0.129447892 |
| KIAA195 | black | 0.088497999 | 0.055993821 | 0.070069897 | 0.130527717 |
| KIDINS2 | black | -0.127487532 | 0.005799427 | -0.1590928 | 0.0005589 |
| KIF13A | black | -0.176294078 | 0.000128366 | -0.196755671 | 1.85E-05 |
| KIF13B | black | 0.362114813 | 6.46E-16 | 0.355845169 | 2.19E-15 |
| KIF1B | black | -0.178370947 | 0.000106441 | -0.203078008 | 9.74E-06 |
| KIF3C | black | -0.282286335 | 5.28E-10 | -0.307137234 | 1.17E-11 |
| KLF10 | black | -0.039276098 | 0.397097808 | -0.072730494 | 0.116511373 |
| KLF11 | black | -0.196889607 | 1.82E-05 | -0.225719482 | 8.29E-07 |
| KLHL18 | black | 0.233480069 | 3.35E-07 | 0.237415608 | 2.09E-07 |
| KLHL20 | black | -0.011698482 | 0.800934815 | -0.035503077 | 0.44402556 |
| KMT2E | black | -0.027232763 | 0.557179058 | -0.045689715 | 0.324510335 |
| KRTCAP | black | 0.113042384 | 0.014519038 | 0.090652481 | 0.050253576 |
| LAMC1 | black | -0.115686467 | 0.012359494 | -0.132258898 | 0.004195625 |
| LANCL1 | black | -0.060543141 | 0.19153884 | -0.087660429 | 0.058367659 |
| LARGE | black | 0.098756119 | 0.032872556 | 0.080431526 | 0.082511551 |
| LASP1 | black | -0.065878991 | 0.155204533 | -0.080611669 | 0.081824387 |
| LDLR | black | 0.182461073 | 7.32E-05 | 0.169386652 | 0.000235741 |
| LENG8 | black | -0.111136711 | 0.016274728 | -0.12479321 | 0.006931044 |
| LIMD1 | black | 0.2527237 | 3.08E-08 | 0.243433309 | 9.99E-08 |
| LIN7B | black | 0.162584771 | 0.000419395 | 0.173259828 | 0.000168132 |
| LMO7 | black | -0.037202894 | 0.422504113 | -0.033439597 | 0.470970546 |
| LMTK2 | black | -0.031444367 | 0.49785725 | -0.032817049 | 0.479272946 |
| LNX2 | black | 0.171403444 | 0.000197877 | 0.166459235 | 0.000302896 |

| | | | | | |
|---|---|---|---|---|---|
| LPIN2 | black | -0.238466775 | 1.84E-07 | -0.237431182 | 2.08E-07 |
| LRBA | black | 0.288098154 | 2.24E-10 | 0.278083395 | 9.70E-10 |
| LRIG3 | black | -0.020759611 | 0.6545394 | -0.010368038 | 0.823176931 |
| LRP10 | black | 0.190828928 | 3.31E-05 | 0.193805713 | 2.48E-05 |
| LRRC16A | black | 0.070646266 | 0.127385718 | 0.080998552 | 0.080364244 |
| LRRC8A | black | 0.147980184 | 0.001341354 | 0.152644128 | 0.000935497 |
| LRRC8E | black | 0.260922677 | 1.05E-08 | 0.237317954 | 2.11E-07 |
| LTBP1 | black | 0.028233778 | 0.542774159 | 0.021567522 | 0.642015175 |
| LUC7L | black | 0.06115551 | 0.187077175 | 0.054356769 | 0.241045103 |
| LUZP1 | black | -0.064352195 | 0.16502284 | -0.078265031 | 0.091146424 |
| LYSMD1 | black | -0.040216758 | 0.385879983 | -0.070198341 | 0.129822385 |
| MAFK | black | -0.020091434 | 0.6649714 | -0.038162477 | 0.41062874 |
| MAGIX | black | 0.093365026 | 0.04373363 | 0.117736581 | 0.010885529 |
| MAML2 | black | -0.092748906 | 0.045148406 | -0.110170094 | 0.017234365 |
| MAML3 | black | 0.09882367 | 0.032752594 | 0.075773345 | 0.101956984 |
| MAP1LC3 | black | 0.004907292 | 0.915769673 | 0.0305818 | 0.509727937 |
| MAP2 | black | 0.03010794 | 0.516311525 | 0.017792879 | 0.701343687 |
| MAP2K5 | black | 0.009959932 | 0.830028942 | -0.00924505 | 0.842062443 |
| MAP4 | black | -0.032259642 | 0.486773658 | -0.035329054 | 0.446263531 |
| MAP4K4 | black | 0.07457802 | 0.107492289 | 0.076183523 | 0.100110517 |
| MAPK1 | black | 0.119556311 | 0.009709946 | 0.098100736 | 0.034056013 |
| 8-Mar | black | -0.173109031 | 0.000170382 | -0.193937267 | 2.44E-05 |
| MARK2 | black | 0.07393227 | 0.110579832 | 0.062564517 | 0.177102661 |
| MAU2 | black | -0.092034279 | 0.046837361 | -0.107494569 | 0.020152963 |
| MBLAC1 | black | -0.011016486 | 0.812317424 | 0.009776275 | 0.833116741 |
| MBOAT1 | black | 0.072046234 | 0.119998287 | 0.055389644 | 0.232208256 |
| MBP | black | -0.016721585 | 0.718538085 | 0.017698185 | 0.702857564 |
| MED1 | black | 0.017805277 | 0.701145562 | -0.019734132 | 0.670576636 |
| MED14 | black | 0.100699156 | 0.029568085 | 0.085764109 | 0.064051059 |
| METTL24 | black | -0.192289911 | 2.87E-05 | -0.1884002 | 4.18E-05 |
| METTL3 | black | -0.004011138 | 0.931109119 | -0.017792593 | 0.701348251 |
| MFAP3 | black | 0.015775621 | 0.733840274 | -0.010929688 | 0.813768987 |
| MFN2 | black | -0.056263619 | 0.22491193 | -0.065635092 | 0.156742583 |
| MFSD2B | black | -0.199777441 | 1.36E-05 | -0.183149898 | 6.86E-05 |
| MFSD9 | black | 0.139675848 | 0.002484904 | 0.138150658 | 0.002773215 |
| MGMT | black | 0.07362584 | 0.112069194 | 0.097713497 | 0.034772188 |
| MIIP | black | -0.036128933 | 0.436029917 | -0.033515168 | 0.46996814 |
| MINOS1 | black | -0.007040209 | 0.879396452 | 0.006215688 | 0.893431467 |
| MMS19 | black | 0.071072477 | 0.125100248 | 0.042440789 | 0.360135123 |
| MOAP1 | black | -0.040923649 | 0.3775783 | -0.028078346 | 0.544998688 |
| MRPS21 | black | -0.067564282 | 0.14488747 | -0.081000511 | 0.080356905 |
| MSRB2 | black | 0.169260174 | 0.000238328 | 0.193525075 | 2.54E-05 |
| MTERF4 | black | -0.123854216 | 0.007369611 | -0.138350796 | 0.002733721 |
| MTF1 | black | -0.084021428 | 0.069668304 | -0.090427482 | 0.05082908 |
| MTG1 | black | 0.010809582 | 0.815778637 | 0.015812344 | 0.733244227 |

| | | | | | |
|---|---|---|---|---|---|
| MTMR1 | black | 0.006477004 | 0.888979527 | -0.000948441 | 0.983691538 |
| MTMR12 | black | -0.136595253 | 0.003098302 | -0.14544913 | 0.001624154 |
| MTOR | black | 0.165363847 | 0.000332328 | 0.122406671 | 0.008094407 |
| MYC | black | -0.194523138 | 2.31E-05 | -0.240770178 | 1.39E-07 |
| MYH10 | black | -0.078315862 | 0.09093584 | -0.096164572 | 0.037766386 |
| MYH9 | black | 0.028622042 | 0.53723713 | 0.0378057 | 0.415020792 |
| MYL5 | black | 0.093704365 | 0.042970484 | 0.098303103 | 0.033686775 |
| MYO1E | black | 0.186612114 | 4.96E-05 | 0.202146234 | 1.07E-05 |
| MYOF | black | 0.136677984 | 0.003080175 | 0.123256167 | 0.007661712 |
| NATD1 | black | 0.019676665 | 0.671479884 | 0.027884529 | 0.547778889 |
| NAV2 | black | 0.173323418 | 0.000167191 | 0.142676224 | 0.001996025 |
| NBAS | black | 0.13894305 | 0.00261983 | 0.134075239 | 0.003699113 |
| NBPF3 | black | 0.036301988 | 0.433833738 | 0.010612596 | 0.819077249 |
| NCOA1 | black | -0.11953477 | 0.009723175 | -0.146192823 | 0.001535852 |
| NCOA4 | black | 0.041226452 | 0.374056015 | -0.002602263 | 0.955274278 |
| NCOA6 | black | -0.013445 | 0.771981987 | -0.039977244 | 0.388717844 |
| NCOR1 | black | 0.190187449 | 3.52E-05 | 0.175493205 | 0.000137903 |
| NDEL1 | black | 0.158027341 | 0.000609374 | 0.142914172 | 0.001961296 |
| NDUFA7 | black | 0.114214966 | 0.013523465 | 0.125482087 | 0.006624314 |
| NDUFB1 | black | 0.141561842 | 0.002166279 | 0.164933397 | 0.000344607 |
| NDUFB8 | black | 0.137094793 | 0.002990304 | 0.147535917 | 0.00138746 |
| NDUFV2 | black | -0.020689392 | 0.655632602 | -0.013372766 | 0.773173501 |
| NEDD9 | black | -0.08991207 | 0.052168172 | -0.112612739 | 0.014899634 |
| NENF | black | 0.186663613 | 4.93E-05 | 0.195910319 | 2.01E-05 |
| NEO1 | black | -0.282494912 | 5.12E-10 | -0.287716467 | 2.37E-10 |
| NFATC3 | black | 0.121786785 | 0.008423775 | 0.128369194 | 0.005466939 |
| NFKB1 | black | 0.056591133 | 0.222220209 | 0.049828885 | 0.282556925 |
| NFRKB | black | 0.054159181 | 0.242762129 | 0.038825514 | 0.402539985 |
| NFX1 | black | 0.057681282 | 0.213426492 | 0.05542548 | 0.231905827 |
| NHEJ1 | black | 0.039225618 | 0.397705299 | -0.003074393 | 0.947170598 |
| NIPAL3 | black | 0.007617558 | 0.869590995 | 0.016358304 | 0.724401714 |
| NME3 | black | 0.181952126 | 7.67E-05 | 0.224049835 | 1.00E-06 |
| NOL6 | black | 0.001343088 | 0.97690718 | 0.009996771 | 0.829409878 |
| NOL9 | black | -0.008687051 | 0.851480939 | -0.021969923 | 0.635814537 |
| NOTCH2 | black | -0.161597839 | 0.000455119 | -0.176961816 | 0.000120892 |
| NOTCH3 | black | -0.06818105 | 0.141245511 | -0.075488279 | 0.103256083 |
| NPEPL1 | black | -0.016537207 | 0.721512057 | -0.006542468 | 0.887864781 |
| NPTN | black | -0.004448241 | 0.923623691 | -0.020339075 | 0.6610974 |
| NSD1 | black | -0.026074452 | 0.574075716 | -0.045528278 | 0.326224602 |
| NT5C2 | black | 0.086343647 | 0.062267727 | 0.054171886 | 0.242651465 |
| NUMB | black | 0.143572978 | 0.001868008 | 0.147528583 | 0.001388233 |
| NUP153 | black | -0.150389456 | 0.00111494 | -0.168451081 | 0.000255517 |
| NUP160 | black | -0.024756675 | 0.593587651 | -0.056680738 | 0.2214878 |
| NUP188 | black | 0.385713284 | 5.15E-18 | 0.381130648 | 1.36E-17 |
| NUP214 | black | -0.042378223 | 0.360844347 | -0.058206619 | 0.209279399 |

| | | | | | |
|---|---|---|---|---|---|
| NUP98 | black | 0.076601393 | 0.098256852 | 0.056702046 | 0.221313884 |
| NUPR1 | black | 0.000562235 | 0.990331919 | 0.021716689 | 0.639713715 |
| OCEL1 | black | -0.015947049 | 0.731059165 | -0.001908834 | 0.967184494 |
| OCLN | black | 0.181894168 | 7.71E-05 | 0.181398318 | 8.07E-05 |
| OGFR | black | -0.199491891 | 1.40E-05 | -0.165802136 | 0.000320245 |
| ORAI3 | black | -0.070726995 | 0.126950357 | -0.03783719 | 0.414632026 |
| OSBPL10 | black | -0.091190009 | 0.048900686 | -0.083901746 | 0.070068432 |
| P2RY11 | black | 0.267288603 | 4.42E-09 | 0.272434333 | 2.16E-09 |
| PABPN1 | black | -0.068286575 | 0.140629486 | -0.084547426 | 0.067931879 |
| PARD3 | black | -0.120176975 | 0.009335592 | -0.134745866 | 0.003529689 |
| PARP4 | black | 0.108674002 | 0.018817215 | 0.08938192 | 0.053576105 |
| PATL1 | black | 0.043165831 | 0.351980083 | 0.036160819 | 0.435624779 |
| PCDH7 | black | 0.184517522 | 6.04E-05 | 0.169166501 | 0.000240262 |
| PCDHAC | black | 0.042053925 | 0.364534435 | 0.046520855 | 0.315777327 |
| PCDHGB | black | -0.065423007 | 0.15808935 | -0.083069179 | 0.072904205 |
| PCDHGC | black | -0.067155214 | 0.147342244 | -0.067823987 | 0.143345288 |
| PCGF3 | black | 0.147668819 | 0.001373517 | 0.140013501 | 0.002424893 |
| PCP2 | black | 0.114936245 | 0.012941383 | 0.100679774 | 0.029599586 |
| PDK2 | black | -0.158024848 | 0.000609497 | -0.165595003 | 0.000325903 |
| PDP2 | black | 0.173300337 | 0.000167532 | 0.177604948 | 0.000114082 |
| PERM1 | black | -0.069007704 | 0.136474709 | -0.05403744 | 0.243824303 |
| PET100 | black | 0.099878179 | 0.030927841 | 0.108969428 | 0.018494977 |
| PEX26 | black | 0.090415739 | 0.050859268 | 0.074737171 | 0.106741885 |
| PEX5 | black | -0.087715247 | 0.058209784 | -0.131998637 | 0.004271506 |
| PFAS | black | 0.170296076 | 0.000217898 | 0.138894623 | 0.002628978 |
| PFKM | black | -0.306203847 | 1.36E-11 | -0.330185534 | 2.43E-13 |
| PGS1 | black | -0.233302975 | 3.42E-07 | -0.256841824 | 1.80E-08 |
| PHACTR4 | black | 0.010297479 | 0.824360655 | -0.006398043 | 0.890324388 |
| PHC1 | black | -0.06901901 | 0.136410329 | -0.106196983 | 0.02171709 |
| PHF12 | black | 0.004728504 | 0.91882767 | -0.009522388 | 0.837389538 |
| PHF2 | black | 0.096517979 | 0.03706463 | 0.072818932 | 0.116066551 |
| PHF8 | black | 0.131278967 | 0.00448783 | 0.114154926 | 0.013572942 |
| PHKA1 | black | 0.088166516 | 0.056923548 | 0.074694721 | 0.10694163 |
| PHKG2 | black | 0.025133789 | 0.587973 | 0.047533929 | 0.305342663 |
| PHLDB1 | black | -0.188233945 | 4.25E-05 | -0.198324419 | 1.58E-05 |
| PHRF1 | black | 0.12517631 | 0.00675893 | 0.116353281 | 0.011861816 |
| PI4KB | black | -0.00731613 | 0.874707931 | -0.021759734 | 0.639050228 |
| PIAS3 | black | 0.245259627 | 7.96E-08 | 0.207179559 | 6.35E-06 |
| PIGG | black | 0.155560831 | 0.000742876 | 0.138999702 | 0.002609166 |
| PIGO | black | 0.133350403 | 0.00389049 | 0.122755114 | 0.007914355 |
| PIK3C2B | black | -0.103820956 | 0.024855364 | -0.09949622 | 0.031578486 |
| PIK3R2 | black | 0.016885248 | 0.715901819 | 0.04396596 | 0.343116908 |
| PIK3R4 | black | -0.165425925 | 0.000330591 | -0.191446228 | 3.12E-05 |
| PIP4K2C | black | 0.017852054 | 0.700398256 | 0.017477332 | 0.706392905 |
| PIP5K1B | black | 0.08129019 | 0.07927757 | 0.073529552 | 0.112540434 |

| | | | | | |
|---|---|---|---|---|---|
| PITPNA | black | 0.234055457 | 3.13E-07 | 0.226445385 | 7.62E-07 |
| PITPNM3 | black | 0.080709503 | 0.08145314 | 0.070186449 | 0.129887564 |
| PKP2 | black | 0.079243964 | 0.087159036 | 0.075124032 | 0.104935075 |
| PLAGL2 | black | -0.179565836 | 9.55E-05 | -0.21225127 | 3.70E-06 |
| PLCG1 | black | 0.077306369 | 0.095191632 | 0.057425163 | 0.215469637 |
| PLEKHA' | black | 0.048811049 | 0.292516865 | 0.033806004 | 0.466121313 |
| PLEKHB2 | black | 0.075407176 | 0.10362807 | 0.085454363 | 0.065021301 |
| PLK2 | black | 0.044508008 | 0.337194049 | 0.020380209 | 0.660454778 |
| PNMA1 | black | -0.263879973 | 7.03E-09 | -0.270897752 | 2.68E-09 |
| POFUT1 | black | -0.198325432 | 1.58E-05 | -0.223541564 | 1.06E-06 |
| POLD4 | black | 0.035252454 | 0.447250636 | 0.040479559 | 0.382780772 |
| POLH | black | 0.036943902 | 0.425743323 | 0.029198283 | 0.529071879 |
| PPAP2B | black | -0.126906548 | 0.006028346 | -0.133841123 | 0.003759972 |
| PPFIA1 | black | -0.016157286 | 0.727653265 | -0.046922764 | 0.31161006 |
| PPRC1 | black | 0.05959809 | 0.198576753 | 0.01402761 | 0.762391592 |
| PRDM10 | black | 0.093805573 | 0.042745058 | 0.067650817 | 0.144372207 |
| PREP | black | 0.206320173 | 6.95E-06 | 0.184234821 | 6.20E-05 |
| PROSER1 | black | 0.130004557 | 0.004895302 | 0.122448899 | 0.008072393 |
| PROSER2 | black | -0.037729034 | 0.415968177 | -0.051977989 | 0.262287244 |
| PRPF40B | black | -0.090713053 | 0.050099577 | -0.141815415 | 0.002126412 |
| PRR14L | black | 0.117444533 | 0.011085506 | 0.093111065 | 0.044312206 |
| PRR22 | black | 0.242412093 | 1.13E-07 | 0.236909498 | 2.22E-07 |
| PRR5 | black | 0.026112879 | 0.573511314 | 0.054191136 | 0.242483863 |
| PRRC2A | black | -0.101729513 | 0.027934337 | -0.117881424 | 0.010787539 |
| PRUNE | black | -0.058177681 | 0.209506316 | -0.094775336 | 0.04063515 |
| PTPLAD1 | black | 0.100344951 | 0.030148325 | 0.082456354 | 0.075050783 |
| PTPN1 | black | -0.065777648 | 0.155842216 | -0.062926767 | 0.174603259 |
| PTPRA | black | -0.144552788 | 0.001736761 | -0.164347898 | 0.000361989 |
| PTPRJ | black | 0.079616212 | 0.085680125 | 0.075840053 | 0.10165486 |
| PTPRK | black | -0.075962786 | 0.101100858 | -0.054015807 | 0.244013392 |
| PUM1 | black | 0.013742935 | 0.767073224 | 0.001971102 | 0.966114659 |
| RAB11FI | black | 0.205392511 | 7.66E-06 | 0.20437355 | 8.52E-06 |
| RAB4B | black | 0.068206036 | 0.141099462 | 0.09705895 | 0.036011883 |
| RAF1 | black | 0.005191208 | 0.910916122 | -0.019716478 | 0.670854064 |
| RANGRF | black | 0.271318748 | 2.53E-09 | 0.247074984 | 6.33E-08 |
| RAP2B | black | -0.062724024 | 0.175998865 | -0.05374502 | 0.24638893 |
| RASA1 | black | 0.009907482 | 0.830910505 | -0.027825206 | 0.548631229 |
| RBL2 | black | 0.02737223 | 0.55516101 | -0.005544515 | 0.904880992 |
| RBSN | black | -0.120667789 | 0.009048704 | -0.147362367 | 0.001405864 |
| RC3H2 | black | -0.008324673 | 0.857608958 | -0.011063 | 0.811539809 |
| RCN3 | black | -0.158218689 | 0.000600009 | -0.137991402 | 0.002805012 |
| RCOR1 | black | -0.001707308 | 0.970647347 | -0.0257593 | 0.578714449 |
| REPS1 | black | 0.005585137 | 0.904187441 | 0.008560964 | 0.853612144 |
| RERE | black | -0.189806151 | 3.65E-05 | -0.211619342 | 3.96E-06 |
| RHBDD1 | black | -0.112149445 | 0.01531983 | -0.122255666 | 0.008173565 |

| | | | | | |
|---|---|---|---|---|---|
| RILP | black | 0.01264111 | 0.785271812 | 0.026569112 | 0.566830403 |
| RIN2 | black | -0.247206895 | 6.23E-08 | -0.251625702 | 3.55E-08 |
| RIPK1 | black | -0.034854105 | 0.452403925 | -0.03103752 | 0.503437961 |
| RNASEH | black | -0.118080614 | 0.01065406 | -0.102783896 | 0.026343404 |
| RNASET: | black | -0.076877634 | 0.097046527 | -0.070420658 | 0.128608535 |
| RNF111 | black | 0.062400841 | 0.178240644 | 0.046224971 | 0.318868461 |
| RNF214 | black | 0.049143301 | 0.289240128 | 0.003132413 | 0.946175085 |
| RNF38 | black | -0.138450067 | 0.002714322 | -0.152455936 | 0.000949386 |
| RNMT | black | -0.149135247 | 0.001227995 | -0.18531619 | 5.60E-05 |
| ROBO1 | black | 0.086081202 | 0.063070173 | 0.085423345 | 0.065119124 |
| RP11-514 | black | 0.125491069 | 0.006620396 | 0.146131264 | 0.00154299 |
| RP11-544 | black | 0.161058756 | 0.000475809 | 0.140477804 | 0.00234453 |
| RP11-729 | black | -0.000840413 | 0.985548856 | -0.026490188 | 0.567983475 |
| RPRD2 | black | 0.005855287 | 0.899576929 | -0.014870627 | 0.74857912 |
| RREB1 | black | 0.283671267 | 4.31E-10 | 0.273536001 | 1.85E-09 |
| S100A1 | black | -0.181206341 | 8.21E-05 | -0.184214522 | 6.21E-05 |
| S100A13 | black | -0.115983139 | 0.01213584 | -0.099395139 | 0.031752619 |
| SALL1 | black | 0.299827851 | 3.72E-11 | 0.275287931 | 1.45E-09 |
| SALL2 | black | -0.259047109 | 1.34E-08 | -0.27326866 | 1.92E-09 |
| SAP25 | black | -0.147836365 | 0.001356123 | -0.145321314 | 0.001639791 |
| SARM1 | black | -0.099525619 | 0.031527994 | -0.107497943 | 0.020149028 |
| SARS2 | black | -0.132437463 | 0.004144271 | -0.144897672 | 0.001692612 |
| SBNO1 | black | -0.002565001 | 0.955914054 | -0.01020312 | 0.825944286 |
| SCAF4 | black | -0.080881807 | 0.080802613 | -0.10533223 | 0.022817027 |
| SCFD2 | black | 0.204534387 | 8.37E-06 | 0.201056371 | 1.20E-05 |
| SCT | black | -0.033018954 | 0.47657162 | -0.045225667 | 0.329453745 |
| SEC16A | black | 0.41002939 | 2.31E-20 | 0.418943997 | 2.85E-21 |
| SEC23B | black | 0.250072004 | 4.33E-08 | 0.251257624 | 3.72E-08 |
| SEC24B | black | 0.092531535 | 0.04565664 | 0.074606068 | 0.107359739 |
| SEC24D | black | 0.16224522 | 0.00043138 | 0.166433773 | 0.000303552 |
| SELK | black | 0.060446157 | 0.192252561 | 0.079858258 | 0.084729386 |
| SERGEF | black | 0.194913714 | 2.22E-05 | 0.183250299 | 6.80E-05 |
| SETD2 | black | 0.021503606 | 0.643002368 | -0.004257039 | 0.926897255 |
| SETD5 | black | 0.031158587 | 0.501773846 | -0.003006877 | 0.948329133 |
| SETDB1 | black | -0.034113222 | 0.462076813 | -0.069680107 | 0.132686355 |
| SETSIP | black | 0.091291491 | 0.048648719 | 0.073737067 | 0.111526778 |
| SF3B3 | black | 0.120372325 | 0.009220452 | 0.136517285 | 0.003115475 |
| SFXN4 | black | 0.063635247 | 0.169791006 | 0.043102155 | 0.352691585 |
| SGPL1 | black | 0.118985423 | 0.010065991 | 0.116932907 | 0.011443689 |
| SH3BP4 | black | 0.241427844 | 1.28E-07 | 0.237799697 | 1.99E-07 |
| SH3PXD: | black | 0.109947737 | 0.017461991 | 0.089556564 | 0.053108845 |
| SH3RF1 | black | 0.138521955 | 0.002700352 | 0.138663516 | 0.002673035 |
| SHROOM | black | 0.033210664 | 0.474014407 | 0.049180268 | 0.288877082 |
| SIN3A | black | -0.020748223 | 0.654716642 | -0.044415432 | 0.338200939 |
| SIN3B | black | -0.213231786 | 3.33E-06 | -0.232110066 | 3.94E-07 |

| | | | | | |
|---|---|---|---|---|---|
| SIPA1L3 | black | 0.142946602 | 0.001956606 | 0.144534748 | 0.001739099 |
| SKI | black | -0.152983872 | 0.000910896 | -0.175440931 | 0.000138548 |
| SLC12A2 | black | 0.337769599 | 6.32E-14 | 0.319701199 | 1.47E-12 |
| SLC12A6 | black | 0.088527246 | 0.0559124 | 0.066067143 | 0.154025853 |
| SLC24A1 | black | 0.102422014 | 0.026880412 | 0.082087952 | 0.076365776 |
| SLC25A1 | black | 0.183751119 | 6.49E-05 | 0.153766491 | 0.000856484 |
| SLC25A2 | black | 0.120300651 | 0.009262549 | 0.125672323 | 0.00654178 |
| SLC25A5 | black | 0.06702844 | 0.148109401 | 0.060482077 | 0.191987993 |
| SLC2A12 | black | -0.046011846 | 0.321107187 | -0.05647601 | 0.22316373 |
| SLC35C2 | black | -0.136660838 | 0.003083924 | -0.137639676 | 0.002876416 |
| SLC35E1 | black | -0.039742385 | 0.391512802 | -0.044073533 | 0.341936238 |
| SLC35E2 | black | 0.03374594 | 0.466914336 | -0.003203829 | 0.944949859 |
| SLC36A1 | black | 0.048805043 | 0.292576325 | 0.02343339 | 0.613480116 |
| SLC39A1 | black | 0.09546337 | 0.039192144 | 0.065544843 | 0.157314614 |
| SLC39A1 | black | -0.073722253 | 0.111598902 | -0.095116908 | 0.039913259 |
| SLC39A6 | black | 0.17578986 | 0.000134295 | 0.147247399 | 0.001418178 |
| SLC41A1 | black | 0.002699578 | 0.953603551 | -0.013785621 | 0.766370686 |
| SLC7A1 | black | 0.167092804 | 0.000287003 | 0.159512906 | 0.000540087 |
| SLC7A6C | black | 0.045542539 | 0.326072924 | 0.037536239 | 0.41835622 |
| SMAD1 | black | -0.061632424 | 0.183655769 | -0.081251268 | 0.079421907 |
| SMAD7 | black | -0.178073646 | 0.000109347 | -0.190293574 | 3.49E-05 |
| SMARCC | black | 0.134348293 | 0.00362926 | 0.099552231 | 0.031482349 |
| SMCR8 | black | 0.067585882 | 0.144758722 | 0.052886228 | 0.254029531 |
| SMDT1 | black | 0.28958892 | 1.79E-10 | 0.315012043 | 3.23E-12 |
| SMIM22 | black | 0.143642021 | 0.00185847 | 0.175066838 | 0.000143248 |
| SMURF1 | black | -0.139026063 | 0.002604217 | -0.151746665 | 0.001003462 |
| SNN | black | -0.138234162 | 0.002756674 | -0.131677263 | 0.004366915 |
| SNRNP35 | black | 0.085728095 | 0.064163252 | 0.093702642 | 0.042974331 |
| SNX1 | black | 0.038035922 | 0.412183514 | 0.025550356 | 0.58179954 |
| SNX18 | black | 0.018400043 | 0.691665219 | 0.008259981 | 0.858703845 |
| SNX19 | black | 0.050681963 | 0.274387735 | 0.030489055 | 0.511013044 |
| SNX22 | black | 0.154960165 | 0.000779253 | 0.167078381 | 0.000287355 |
| SNX27 | black | 0.079638482 | 0.085592291 | 0.057264867 | 0.216755494 |
| SNX29 | black | -0.016581965 | 0.720789736 | -0.037400849 | 0.420038016 |
| SNX30 | black | 0.088627566 | 0.055633863 | 0.042367703 | 0.360963683 |
| SON | black | -0.032282217 | 0.486468653 | -0.050603132 | 0.275135815 |
| SORL1 | black | 0.081884043 | 0.077101634 | 0.06082354 | 0.189486287 |
| SOWAHB | black | 0.042806019 | 0.356012459 | 0.026581195 | 0.566653967 |
| SPG11 | black | 0.291986463 | 1.25E-10 | 0.271343047 | 2.52E-09 |
| SPG7 | black | 0.240186857 | 1.49E-07 | 0.261852103 | 9.24E-09 |
| SPIDR | black | -0.0194951 | 0.67433676 | -0.062607407 | 0.176805354 |
| SPRED2 | black | -0.054637973 | 0.238616187 | -0.085247748 | 0.065675195 |
| SPRN | black | 0.024121907 | 0.603093216 | 0.035185749 | 0.448111252 |
| SPRY1 | black | 0.074196898 | 0.109306219 | 0.052723412 | 0.255496435 |
| SPRY2 | black | 0.236317187 | 2.38E-07 | 0.211651065 | 3.95E-06 |

| | | | | | |
|---|---|---|---|---|---|
| SPSB2 | black | 0.023634539 | 0.61043747 | 0.041225759 | 0.374064054 |
| SPTBN1 | black | 0.065220155 | 0.159385648 | 0.051211899 | 0.269394758 |
| SRCAP | black | 0.034166029 | 0.461383581 | 0.027395413 | 0.554825904 |
| SREBF2 | black | 0.290278681 | 1.61E-10 | 0.252113581 | 3.33E-08 |
| SRPR | black | 0.172192536 | 0.000184677 | 0.16926213 | 0.000238288 |
| SSH1 | black | -0.128163956 | 0.005542768 | -0.148066178 | 0.001332594 |
| ST20 | black | -0.003747349 | 0.935629511 | -0.001969728 | 0.966138255 |
| STAG1 | black | -0.065808182 | 0.155649879 | -0.089013729 | 0.054572419 |
| STAT3 | black | 0.101470629 | 0.028337355 | 0.116612055 | 0.011673503 |
| STIM1 | black | 0.244629137 | 8.61E-08 | 0.254129123 | 2.57E-08 |
| STK35 | black | -0.046930046 | 0.311534887 | -0.054397631 | 0.240691083 |
| STK40 | black | -0.018258371 | 0.693919123 | -0.003123343 | 0.946330697 |
| STRADA | black | -0.070712456 | 0.127028678 | -0.087752763 | 0.058101946 |
| STT3A | black | 0.265509948 | 5.63E-09 | 0.240684421 | 1.40E-07 |
| SUFU | black | 0.136408549 | 0.003139569 | 0.081247962 | 0.079434175 |
| SUMF2 | black | 0.183708856 | 6.51E-05 | 0.163262135 | 0.000396405 |
| SUPT6H | black | 0.000165892 | 0.997147297 | 0.005728993 | 0.901731917 |
| SUSD1 | black | 0.038957836 | 0.400937189 | 0.025542934 | 0.581909276 |
| SYNRG | black | 0.086769557 | 0.060983403 | 0.068202399 | 0.141120717 |
| SYT11 | black | -0.362938621 | 5.50E-16 | -0.356494152 | 1.93E-15 |
| TACC2 | black | 0.121761687 | 0.008437358 | 0.114896569 | 0.012972819 |
| TAF1 | black | -0.085287313 | 0.065549564 | -0.123492022 | 0.007545302 |
| TAF8 | black | -0.191071858 | 3.23E-05 | -0.185420184 | 5.55E-05 |
| TANGO6 | black | 0.2286744 | 5.89E-07 | 0.211837623 | 3.87E-06 |
| TAX1BP3 | black | -0.062645557 | 0.176541218 | -0.039843332 | 0.390309984 |
| TAZ | black | 0.031400164 | 0.498461982 | 0.040586526 | 0.381523673 |
| TBC1D14 | black | -0.012404316 | 0.789198589 | -0.042449146 | 0.36004046 |
| TBC1D5 | black | 0.063176117 | 0.172898141 | 0.037667334 | 0.416731544 |
| TCF15 | black | -0.215283216 | 2.67E-06 | -0.241863215 | 1.21E-07 |
| TCF20 | black | 0.083233911 | 0.072335809 | 0.045535752 | 0.326145099 |
| TCHP | black | 0.044591601 | 0.33628651 | 0.009751392 | 0.833535296 |
| TET3 | black | 0.069798148 | 0.132029758 | 0.055439044 | 0.231791432 |
| TEX2 | black | -0.161629353 | 0.000453936 | -0.177597541 | 0.000114158 |
| TEX261 | black | 0.043006625 | 0.353760716 | 0.050895313 | 0.272370062 |
| TFE3 | black | -0.144367501 | 0.001760913 | -0.136505846 | 0.003118002 |
| TGFB2 | black | -0.054574541 | 0.23916257 | -0.05135446 | 0.268062246 |
| TGFBRAP1 | black | -0.029326196 | 0.52726794 | -0.050972283 | 0.271644631 |
| TGOLN2 | black | 0.000473009 | 0.99186617 | -0.02753055 | 0.552874482 |
| TIMM23B | black | 0.002812972 | 0.951657034 | -0.032481548 | 0.483780038 |
| TIMP1 | black | 0.0003253 | 0.994406113 | 0.04114838 | 0.374962227 |
| TIRAP | black | 0.334099683 | 1.22E-13 | 0.308907257 | 8.78E-12 |
| TJP1 | black | -0.072924938 | 0.11553512 | -0.096014424 | 0.038067925 |
| TJP2 | black | 0.192000082 | 2.95E-05 | 0.193030646 | 2.67E-05 |
| TM9SF4 | black | -0.174932314 | 0.000144974 | -0.19769863 | 1.68E-05 |
| TMCO3 | black | 0.040864954 | 0.378263403 | 0.042522879 | 0.359205914 |

| | | | | | |
|---|---|---|---|---|---|
| TMED4 | black | -0.101430032 | 0.028401004 | -0.112160732 | 0.015309471 |
| TMEFF1 | black | -0.171371118 | 0.000198437 | -0.19593087 | 2.01E-05 |
| TMEM13 | black | 0.15069486 | 0.001088903 | 0.132432714 | 0.004145629 |
| TMEM18 | black | -0.182593407 | 7.23E-05 | -0.193969576 | 2.44E-05 |
| TMEM18 | black | -0.171799457 | 0.000191146 | -0.192770523 | 2.74E-05 |
| TMEM21 | black | 0.146290456 | 0.001524592 | 0.186205901 | 5.15E-05 |
| TMEM24 | black | 0.14648475 | 0.001502411 | 0.11864344 | 0.010284783 |
| TMEM24 | black | -0.112191052 | 0.015281673 | -0.095100547 | 0.039947588 |
| TMEM86 | black | -0.045356277 | 0.328057482 | -0.032753265 | 0.480128055 |
| TMEM91 | black | 0.03295432 | 0.477435461 | 0.027429277 | 0.554336587 |
| TMSB10 | black | 0.153183007 | 0.000896756 | 0.187640973 | 4.50E-05 |
| TNFAIP1 | black | 0.0267448 | 0.564267656 | 0.017966068 | 0.698577964 |
| TNFRSF1 | black | 0.262272198 | 8.74E-09 | 0.249222 | 4.82E-08 |
| TNNI3 | black | -0.161121076 | 0.000473373 | -0.1559929 | 0.000717688 |
| TOB2 | black | 0.215370312 | 2.64E-06 | 0.180689876 | 8.61E-05 |
| TP53BP1 | black | 0.091163404 | 0.048966924 | 0.056173524 | 0.225656452 |
| TPP1 | black | 0.081207862 | 0.07958312 | 0.083556758 | 0.071232339 |
| TPR | black | 0.083694047 | 0.07076728 | 0.047589912 | 0.304772762 |
| TRAK1 | black | 0.004649495 | 0.920179434 | -0.009905821 | 0.830938432 |
| TRAPPC1 | black | -0.072510291 | 0.117624738 | -0.086124867 | 0.062936076 |
| TRAPPC2 | black | -0.033539187 | 0.469649777 | -0.014088175 | 0.761396682 |
| TRIM35 | black | 0.138682796 | 0.002669334 | 0.130309697 | 0.004794812 |
| TRIM68 | black | 0.266315499 | 5.05E-09 | 0.244614217 | 8.63E-08 |
| TRIP12 | black | 0.011263911 | 0.808183163 | -0.018581368 | 0.688784443 |
| TRRAP | black | -0.043187265 | 0.351740778 | -0.050903554 | 0.272292331 |
| TSC22D1 | black | -0.037563312 | 0.418020397 | -0.067480709 | 0.145386429 |
| TSHZ1 | black | 0.163930517 | 0.000374876 | 0.152731744 | 0.000929094 |
| TSPYL1 | black | 0.00290265 | 0.950117826 | -0.01305174 | 0.778475258 |
| TSTD2 | black | 0.025632993 | 0.580578485 | 0.0020959 | 0.963970644 |
| TTC5 | black | -0.00415135 | 0.928707284 | -0.016599212 | 0.720511457 |
| TTC7B | black | -0.216759865 | 2.27E-06 | -0.248211249 | 5.49E-08 |
| TUBGCP: | black | 0.282396105 | 5.19E-10 | 0.25282939 | 3.04E-08 |
| TULP3 | black | 0.11143039 | 0.015992594 | 0.091919806 | 0.047112772 |
| TXLNA | black | 0.036770615 | 0.42791867 | 0.048046778 | 0.300148246 |
| U2AF1L4 | black | -0.148957446 | 0.00124485 | -0.156009175 | 0.000716755 |
| UBAP2 | black | 0.082308686 | 0.075575648 | 0.088995358 | 0.054622529 |
| UBE4B | black | -0.123596432 | 0.007494275 | -0.178521499 | 0.000104997 |
| UBQLN2 | black | -0.078663592 | 0.08950571 | -0.099614804 | 0.031375245 |
| UBR4 | black | -0.074519548 | 0.107769029 | -0.08687064 | 0.060681823 |
| UCN | black | 0.26430616 | 6.64E-09 | 0.292420969 | 1.17E-10 |
| UGGT1 | black | 0.15218522 | 0.0009697 | 0.137381273 | 0.002929925 |
| UHRF1BI | black | 0.011031722 | 0.812062697 | 0.008220026 | 0.859380209 |
| ULBP3 | black | 0.193476605 | 2.56E-05 | 0.183254061 | 6.80E-05 |
| UNC13B | black | 0.312180411 | 5.15E-12 | 0.293535273 | 9.84E-11 |
| UNC5B | black | 0.144551997 | 0.001736863 | 0.14157276 | 0.002164549 |

| | | | | | |
|---|---|---|---|---|---|
| UNKL | black | -0.139641939 | 0.002491005 | -0.141760607 | 0.002134972 |
| UPF1 | black | 0.113898934 | 0.01378569 | 0.111302773 | 0.016114664 |
| URB2 | black | 0.112057817 | 0.015404155 | 0.093827249 | 0.042696908 |
| USMG5 | black | 0.107999183 | 0.019571696 | 0.117861897 | 0.010800704 |
| USP10 | black | 0.147071 | 0.001437266 | 0.139117661 | 0.002587088 |
| USP30 | black | -0.085411694 | 0.065155902 | -0.108542365 | 0.018962369 |
| USP36 | black | -0.146451368 | 0.001506201 | -0.185783929 | 5.36E-05 |
| USP54 | black | 0.285627957 | 3.23E-10 | 0.257976567 | 1.55E-08 |
| USP6NL | black | 0.151314371 | 0.001037801 | 0.117323052 | 0.011169643 |
| USP9X | black | -0.058209013 | 0.209260636 | -0.033086451 | 0.475670422 |
| UTP14C | black | -0.003976639 | 0.931700197 | -0.028100913 | 0.544675424 |
| VANGL1 | black | 0.047609749 | 0.304570994 | 0.040449082 | 0.383139407 |
| VCL | black | -0.116012285 | 0.012114061 | -0.116912697 | 0.011458046 |
| VEZF1 | black | 0.092452664 | 0.045842234 | 0.071369249 | 0.123527776 |
| VGLL4 | black | 0.006688844 | 0.885373042 | -0.0155442 | 0.73760018 |
| VHL | black | 0.14884838 | 0.001255294 | 0.13808944 | 0.002785398 |
| VPS11 | black | 0.023860337 | 0.607029954 | 0.007859154 | 0.865493652 |
| WDR37 | black | -0.107836806 | 0.019757125 | -0.139912175 | 0.002442761 |
| WDR45B | black | 0.145407019 | 0.001629291 | 0.140112318 | 0.002407582 |
| WDR82 | black | 0.27241782 | 2.17E-09 | 0.254577376 | 2.42E-08 |
| WDR83 | black | -0.024161265 | 0.602501849 | -0.020434562 | 0.659606043 |
| WIPF2 | black | 0.195518702 | 2.09E-05 | 0.179411672 | 9.68E-05 |
| WLS | black | -0.036007628 | 0.437573158 | -0.044372213 | 0.338671665 |
| WNK1 | black | -0.055561134 | 0.230763534 | -0.081990127 | 0.076718088 |
| YLPM1 | black | -0.013751409 | 0.766933733 | -0.050764802 | 0.27360311 |
| ZBED3 | black | 0.13031115 | 0.004794338 | 0.136441817 | 0.003132179 |
| ZBED4 | black | 0.29067574 | 1.52E-10 | 0.266798105 | 4.72E-09 |
| ZBTB40 | black | 0.071126952 | 0.124810444 | 0.054808932 | 0.23714796 |
| ZBTB48 | black | -0.017688731 | 0.703008768 | -0.035076749 | 0.449519563 |
| ZBTB49 | black | -0.101078256 | 0.028957695 | -0.133957768 | 0.003729537 |
| ZBTB9 | black | -0.014443857 | 0.755561826 | 0.009140775 | 0.843820843 |
| ZC3H14 | black | 0.09930703 | 0.031905072 | 0.057629862 | 0.213835568 |
| ZCCHC2 | black | -0.056250324 | 0.225021689 | -0.064334591 | 0.1651387 |
| ZDHHC3 | black | -0.048316097 | 0.297444087 | -0.03870308 | 0.404026407 |
| ZDHHC5 | black | 0.15046707 | 0.001108269 | 0.137610328 | 0.002882448 |
| ZDHHC7 | black | 0.170996844 | 0.000205019 | 0.180148023 | 9.05E-05 |
| ZFAT | black | -0.213207425 | 3.34E-06 | -0.217161138 | 2.17E-06 |
| ZFP36L1 | black | 0.069968602 | 0.131086054 | 0.037244585 | 0.421984029 |
| ZFR | black | -0.091352934 | 0.048496692 | -0.123029191 | 0.007775252 |
| ZFYVE1 | black | 0.018907145 | 0.68362002 | 0.033663039 | 0.468010107 |
| ZFYVE9 | black | -0.118877532 | 0.010134566 | -0.161874924 | 0.000444814 |
| ZGLP1 | black | 0.046010848 | 0.3211177 | 0.040876421 | 0.378129498 |
| ZHX1-C8 | black | -0.210613028 | 4.42E-06 | -0.211757201 | 3.91E-06 |
| ZHX2 | black | -0.184408762 | 6.10E-05 | -0.213544837 | 3.22E-06 |
| ZMIZ1 | black | -0.148561962 | 0.001283107 | -0.167076654 | 0.000287398 |

| | | | | | |
|---|---|---|---|---|---|
| ZMYM3 | black | -0.148846541 | 0.001255471 | -0.162668106 | 0.000416501 |
| ZNF142 | black | 0.047011375 | 0.310696166 | 0.033602523 | 0.468810883 |
| ZNF143 | black | 0.122874173 | 0.007853658 | 0.100122677 | 0.030517401 |
| ZNF28 | black | 0.130576535 | 0.004708465 | 0.127216416 | 0.005905262 |
| ZNF317 | black | 0.02473033 | 0.593980803 | 0.013493759 | 0.771178002 |
| ZNF318 | black | -0.133643987 | 0.003811921 | -0.172625417 | 0.00017779 |
| ZNF398 | black | 0.183951691 | 6.37E-05 | 0.166545928 | 0.000300674 |
| ZNF425 | black | -0.067139328 | 0.147438212 | -0.105919508 | 0.022064907 |
| ZNF468 | black | 0.186510054 | 5.01E-05 | 0.182454464 | 7.32E-05 |
| ZNF511 | black | 0.210476411 | 4.48E-06 | 0.217488791 | 2.09E-06 |
| ZNF512B | black | -0.15777737 | 0.000621813 | -0.158921686 | 0.000566735 |
| ZNF532 | black | -0.167996488 | 0.000265676 | -0.192369332 | 2.85E-05 |
| ZNF608 | black | -0.167132562 | 0.000286032 | -0.161241892 | 0.000468684 |
| ZNF609 | black | 0.098422358 | 0.033470784 | 0.090470054 | 0.050719769 |
| ZNF618 | black | -0.119750156 | 0.009591621 | -0.144946936 | 0.001686391 |
| ZNF619 | black | -0.027737487 | 0.549892764 | -0.051732413 | 0.264551409 |
| ZNF687 | black | 0.02805029 | 0.5454007 | 0.006426977 | 0.889831546 |
| ZNF688 | black | 0.084956105 | 0.066607372 | 0.114469404 | 0.01331555 |
| ZNF689 | black | -0.083338219 | 0.071977776 | -0.094484164 | 0.041259203 |
| ZNF692 | black | 0.118010698 | 0.010700744 | 0.09473289 | 0.040725623 |
| ZNF746 | black | 0.151113837 | 0.001054095 | 0.132366554 | 0.004164595 |
| ZNF770 | black | -0.03573033 | 0.441112675 | -0.061237802 | 0.186483484 |
| ZNF775 | black | 0.035949116 | 0.438318657 | 0.027197587 | 0.557688609 |
| ZNF786 | black | 0.070293966 | 0.129299188 | 0.048871342 | 0.291920405 |
| ZNF79 | black | 0.107913401 | 0.019669467 | 0.091242422 | 0.048770415 |
| ZNF792 | black | -0.03346824 | 0.470590474 | -0.066560516 | 0.150967329 |
| ZNF821 | black | 0.043082056 | 0.35291635 | 0.043876455 | 0.344101246 |
| ZNF860 | black | 0.021399389 | 0.644613369 | 0.032838706 | 0.478982792 |
| ZRANB1 | black | -0.118652987 | 0.010278618 | -0.159484348 | 0.000541347 |
| ZRSR2 | black | 0.22648801 | 7.59E-07 | 0.227007933 | 7.14E-07 |
| ZSCAN2[illegible] | black | 0.104293372 | 0.024201931 | 0.087745109 | 0.058123933 |
| ZSCAN2[illegible] | black | -0.190625066 | 3.38E-05 | -0.211028811 | 4.22E-06 |
| ZXDC | black | -0.228637008 | 5.92E-07 | -0.253990714 | 2.61E-08 |
| AAED1 | blue | -0.095779348 | 0.03854412 | -0.082580116 | 0.074613179 |
| AASDH | blue | -0.038752539 | 0.403425553 | -0.072391877 | 0.11822688 |
| ABCA5 | blue | 0.101598083 | 0.028138321 | 0.089062858 | 0.054438591 |
| ABI2 | blue | -0.176332655 | 0.000127923 | -0.201391231 | 1.16E-05 |
| AC02406[illegible] | blue | -0.070514156 | 0.128100668 | -0.073819883 | 0.111124257 |
| AC08735[illegible] | blue | -0.223349082 | 1.09E-06 | -0.250701418 | 3.99E-08 |
| ACADSB | blue | 0.034474509 | 0.457345577 | 0.019281856 | 0.677698061 |
| ACAP2 | blue | -0.182835826 | 7.07E-05 | -0.200477023 | 1.27E-05 |
| ACER3 | blue | 0.041449539 | 0.371474012 | 0.028813475 | 0.534517571 |
| ACOT8 | blue | -0.113091598 | 0.014475992 | -0.070753628 | 0.126806982 |
| ACP2 | blue | 0.132619426 | 0.004092525 | 0.136455604 | 0.003129121 |
| ACSL1 | blue | -0.017935771 | 0.699061502 | -0.078054222 | 0.092023932 |

| | | | | | |
|---|---|---|---|---|---|
| ACSL4 | blue | 0.067002967 | 0.148263915 | 0.037666255 | 0.416744897 |
| ACTL10 | blue | -0.046573042 | 0.315234161 | -0.026888742 | 0.562172127 |
| ACVR2A | blue | -0.14366852 | 0.001854821 | -0.15846254 | 0.000588267 |
| ACVR2B | blue | -0.248239336 | 5.47E-08 | -0.268958241 | 3.51E-09 |
| ADAMTS | blue | -0.172242604 | 0.000183868 | -0.189120822 | 3.90E-05 |
| ADCK1 | blue | 0.124387912 | 0.007117393 | 0.138476499 | 0.002709178 |
| ADD3 | blue | 0.092966737 | 0.044643876 | 0.06738268 | 0.145973359 |
| ADRBK2 | blue | -0.093429768 | 0.043587156 | -0.124525979 | 0.007053415 |
| ADRM1 | blue | -0.071689921 | 0.121845998 | -0.050851928 | 0.272779539 |
| AFF1 | blue | 0.074878135 | 0.106080696 | 0.03802137 | 0.412362517 |
| AFF4 | blue | 0.051904949 | 0.262959259 | 0.021444041 | 0.643922928 |
| AGAP3 | blue | -0.057544479 | 0.214516073 | -0.056288882 | 0.224703485 |
| AGK | blue | 0.069225349 | 0.135239485 | 0.036384436 | 0.432789663 |
| AGO4 | blue | -0.123930624 | 0.007333018 | -0.152729914 | 0.000929228 |
| AGPAT2 | blue | 0.149993269 | 0.001149571 | 0.152187917 | 0.000969496 |
| AGTPBP | blue | 0.037481368 | 0.419037343 | 0.002902652 | 0.950117787 |
| AGTRAP | blue | 0.041795002 | 0.36749745 | 0.055805633 | 0.228714777 |
| AHR | blue | -0.04173554 | 0.368180016 | -0.007839533 | 0.865826283 |
| AIP | blue | -0.132180258 | 0.004218424 | -0.116117928 | 0.012035412 |
| AKAP10 | blue | 0.010868072 | 0.814799805 | -0.013649732 | 0.768607834 |
| AKAP11 | blue | -0.057024604 | 0.218693124 | -0.077915784 | 0.092603866 |
| AKAP5 | blue | -0.061795544 | 0.182496211 | -0.057288999 | 0.21656156 |
| AKAP9 | blue | -0.0411143 | 0.375358231 | -0.052416781 | 0.25827495 |
| AL36518 | blue | -0.169456074 | 0.000234332 | -0.178844199 | 0.000101963 |
| ALG10B | blue | -0.08936786 | 0.053613873 | -0.104949146 | 0.023319516 |
| ALG11 | blue | -0.006227606 | 0.893228339 | -0.019852388 | 0.66871943 |
| ALG9 | blue | 0.125768049 | 0.0065006 | 0.073965779 | 0.110417919 |
| ALKBH8 | blue | -0.042180164 | 0.363095214 | -0.072335409 | 0.118514872 |
| ALMS1 | blue | -0.050697412 | 0.274241293 | -0.06966035 | 0.132796497 |
| ALS2 | blue | -0.030682323 | 0.508336975 | -0.047704361 | 0.303609878 |
| AMDHD | blue | 0.0059795 | 0.89745819 | 0.046670411 | 0.314222383 |
| AMN1 | blue | -0.164438407 | 0.00035925 | -0.162323473 | 0.00042859 |
| ANK3 | blue | 0.17389685 | 0.00015893 | 0.147088628 | 0.001435348 |
| ANKIB1 | blue | -0.035087481 | 0.449380786 | -0.057923003 | 0.211511024 |
| ANKMY | blue | 0.005477603 | 0.906023562 | 0.006856716 | 0.882516771 |
| ANKRD1 | blue | -0.074765761 | 0.106607522 | -0.095231572 | 0.039673368 |
| ANKRD1 | blue | -0.244801421 | 8.43E-08 | -0.230935489 | 4.52E-07 |
| ANKRD1 | blue | 0.01050059 | 0.820954275 | -0.029220519 | 0.52875806 |
| ANKRD2 | blue | -0.117075673 | 0.011342724 | -0.132298564 | 0.004184168 |
| ANKRD4 | blue | -0.111366874 | 0.016053248 | -0.135828926 | 0.003270881 |
| ANKRD5 | blue | 0.051388493 | 0.267744814 | 0.054663635 | 0.238395386 |
| ANKS1B | blue | -0.219116829 | 1.75E-06 | -0.228317432 | 6.14E-07 |
| AP4E1 | blue | 0.114462747 | 0.013320953 | 0.109089896 | 0.018364963 |
| AP5Z1 | blue | 0.067409105 | 0.145814971 | 0.109344498 | 0.018092808 |
| APBB2 | blue | -0.052951002 | 0.253447574 | -0.092397311 | 0.045972862 |

| | | | | | |
|---|---|---|---|---|---|
| APC | blue | -0.005204082 | 0.910696121 | -0.030952435 | 0.504609223 |
| APH1B | blue | -0.140719582 | 0.002303652 | -0.148848871 | 0.001255247 |
| APOOL | blue | -0.019110825 | 0.680398618 | -0.045004879 | 0.331822728 |
| APPBP2 | blue | -0.06871644 | 0.138141313 | -0.100639446 | 0.029665223 |
| APPL1 | blue | 0.019398198 | 0.675863392 | -0.006486238 | 0.888822272 |
| APPL2 | blue | 0.027356751 | 0.555384812 | -0.007332085 | 0.874436947 |
| AQR | blue | 0.186293341 | 5.11E-05 | 0.152860298 | 0.000919774 |
| ARAP2 | blue | -0.037554706 | 0.418127128 | -0.038930696 | 0.401265613 |
| ARFGAP | blue | -0.123527911 | 0.007527728 | -0.097647835 | 0.034894888 |
| ARFRP1 | blue | -0.225300963 | 8.69E-07 | -0.194023312 | 2.42E-05 |
| ARHGAP | blue | 0.049760868 | 0.283215266 | 0.017339638 | 0.708600272 |
| ARHGAP | blue | -0.074579345 | 0.10748602 | -0.111564468 | 0.015865219 |
| ARHGAP | blue | -0.279812133 | 7.56E-10 | -0.300855095 | 3.17E-11 |
| ARHGAP | blue | -0.068080856 | 0.141832327 | -0.064299778 | 0.165368004 |
| ARHGAP | blue | -0.071594468 | 0.12234473 | -0.106980161 | 0.020760909 |
| ARHGEF | blue | 0.055216504 | 0.233673351 | 0.032663632 | 0.481331103 |
| ARHGEF | blue | -0.020438138 | 0.659550205 | -0.033689508 | 0.467660086 |
| ARHGEF | blue | 0.079267744 | 0.08706395 | 0.06801913 | 0.142194775 |
| ARID2 | blue | 0.031852166 | 0.49229658 | -0.000852459 | 0.985341752 |
| ARID4A | blue | -0.069625196 | 0.132992649 | -0.096575262 | 0.036951934 |
| ARID5B | blue | -0.178073701 | 0.000109347 | -0.193767418 | 2.49E-05 |
| ARIH1 | blue | 0.015492633 | 0.738438852 | -0.008963116 | 0.846818497 |
| ARL13B | blue | -0.127143692 | 0.005933944 | -0.147289342 | 0.001413674 |
| ARL2 | blue | -0.193608772 | 2.52E-05 | -0.193694353 | 2.50E-05 |
| ARL8B | blue | -0.026109579 | 0.573559765 | -0.045261852 | 0.329066532 |
| ARMC5 | blue | -0.066887339 | 0.148966829 | -0.045746065 | 0.323913335 |
| ARMCX3 | blue | 0.08798301 | 0.057443716 | 0.081080645 | 0.080057142 |
| ARNTL | blue | 0.002702017 | 0.953561686 | -0.005346074 | 0.908270077 |
| ARPC1B | blue | 0.014445258 | 0.755538884 | 0.054494909 | 0.239849748 |
| ARPIN | blue | 0.122666061 | 0.007960028 | 0.120705749 | 0.009026845 |
| ARPP19 | blue | 0.12127863 | 0.008702633 | 0.107096195 | 0.020622402 |
| ARRDC1 | blue | 0.258040041 | 1.54E-08 | 0.294349985 | 8.69E-11 |
| ARRDC3 | blue | 0.052983994 | 0.253151519 | 0.047808876 | 0.30255051 |
| ASB7 | blue | -0.135044009 | 0.003456652 | -0.154557796 | 0.000804536 |
| ASB8 | blue | -0.107857346 | 0.019733586 | -0.120367977 | 0.009223 |
| ATAD2B | blue | 0.005045279 | 0.913410384 | -0.014840984 | 0.749063478 |
| ATE1 | blue | 0.048929916 | 0.291341729 | 0.014899532 | 0.748106921 |
| ATF2 | blue | -0.036143384 | 0.435846278 | -0.062657391 | 0.176459342 |
| ATF7 | blue | 0.192251153 | 2.88E-05 | 0.171055508 | 0.000203974 |
| ATF7IP | blue | -0.103634893 | 0.025116871 | -0.114450011 | 0.013331297 |
| ATG2B | blue | -0.099900248 | 0.0308906 | -0.126896017 | 0.006032569 |
| ATG4B | blue | 0.00934661 | 0.840350591 | 0.018933836 | 0.683197547 |
| ATL3 | blue | 0.046592661 | 0.315030131 | 0.042657338 | 0.357687158 |
| ATM | blue | -0.128967665 | 0.005251124 | -0.157000814 | 0.000661975 |
| ATP11B | blue | -0.22646169 | 7.61E-07 | -0.213404663 | 3.27E-06 |

| | | | | | |
|---|---|---|---|---|---|
| ATP11C | blue | -0.148222117 | 0.001316843 | -0.155616551 | 0.000739582 |
| ATP2B1 | blue | -0.053707759 | 0.246717064 | -0.070680406 | 0.127201467 |
| ATP2B4 | blue | -0.013680391 | 0.768102927 | -0.03386109 | 0.465394672 |
| ATP7A | blue | -0.012930248 | 0.780484405 | -0.01947791 | 0.674607477 |
| ATP8A1 | blue | 0.059262398 | 0.201121511 | 0.03660673 | 0.429981905 |
| ATR | blue | -0.071883564 | 0.120839105 | -0.087823973 | 0.057897707 |
| ATRX | blue | 0.003069672 | 0.947251588 | -0.019802203 | 0.669507346 |
| ATXN1 | blue | -0.032076949 | 0.48924574 | -0.031875755 | 0.491975947 |
| ATXN7 | blue | 0.083745723 | 0.070592874 | 0.059119501 | 0.202211922 |
| B3GNT5 | blue | -0.053405736 | 0.249388105 | -0.078221247 | 0.091328125 |
| B4GALT( | blue | -0.289618848 | 1.78E-10 | -0.322078468 | 9.86E-13 |
| B4GALT' | blue | 0.082888102 | 0.073533199 | 0.111407133 | 0.016014779 |
| BACH1 | blue | -0.18192619 | 7.69E-05 | -0.197214128 | 1.77E-05 |
| BAZ2B | blue | -0.0350461 | 0.449915997 | -0.064116404 | 0.166579783 |
| BBIP1 | blue | 0.024055095 | 0.604097681 | 0.016018089 | 0.729907714 |
| BBS10 | blue | -0.279762093 | 7.61E-10 | -0.303714344 | 2.02E-11 |
| BBS9 | blue | -0.062282394 | 0.179067544 | -0.091620709 | 0.047838783 |
| BBX | blue | 0.010413151 | 0.822420309 | -0.009817888 | 0.832416876 |
| BCAP29 | blue | 0.069863203 | 0.131668969 | 0.061967336 | 0.18128086 |
| BCAP31 | blue | -0.029061688 | 0.531001701 | -0.00898999 | 0.846364903 |
| BDP1 | blue | -0.040553685 | 0.381909369 | -0.069426176 | 0.134107364 |
| BEND7 | blue | -0.509802663 | 2.94E-32 | -0.523690075 | 3.01E-34 |
| BHLHB9 | blue | -0.239156646 | 1.69E-07 | -0.256223995 | 1.95E-08 |
| BICD1 | blue | -0.146719846 | 0.001475968 | -0.16804058 | 0.000264675 |
| BIVM | blue | -0.119899138 | 0.009501553 | -0.148301943 | 0.001308846 |
| BLOC1S( | blue | 0.059761221 | 0.197348621 | 0.039994288 | 0.388515475 |
| BLVRB | blue | 0.067020551 | 0.148157239 | 0.103254748 | 0.025658544 |
| BMPR1A | blue | 0.086560949 | 0.061609695 | 0.038938552 | 0.40117054 |
| BMPR2 | blue | -0.098167503 | 0.033933812 | -0.116654242 | 0.011643055 |
| BNIP2 | blue | -0.078378602 | 0.090676459 | -0.10388467 | 0.024766358 |
| BOLA2B | blue | 0.046838013 | 0.312485795 | 0.086386099 | 0.062138721 |
| BRAF | blue | -0.082303104 | 0.075595545 | -0.115145495 | 0.012776703 |
| BRI3 | blue | -0.063827301 | 0.168503738 | -0.013499738 | 0.771079434 |
| BRWD3 | blue | 0.026321837 | 0.570446786 | -0.002020884 | 0.965259382 |
| BTAF1 | blue | 0.030887436 | 0.505504942 | -0.007548027 | 0.870770851 |
| BTBD3 | blue | -0.005726938 | 0.901766989 | -0.005145103 | 0.911704079 |
| BTBD7 | blue | 0.004748215 | 0.918490467 | -0.031711856 | 0.494206064 |
| BTC | blue | -0.110924324 | 0.016481474 | -0.134942 | 0.003481486 |
| BTG2 | blue | 0.145109861 | 0.001665964 | 0.098095771 | 0.034065116 |
| C11orf24 | blue | 0.163986171 | 0.000373133 | 0.179617503 | 9.50E-05 |
| C11orf30 | blue | -0.026559264 | 0.56697422 | -0.059151575 | 0.2019668 |
| C11orf54 | blue | 0.025495002 | 0.58261814 | -0.001093836 | 0.981191892 |
| C12orf57 | blue | 0.118987896 | 0.010064424 | 0.145876658 | 0.001572839 |
| C14orf37 | blue | -0.169743555 | 0.000228582 | -0.186670223 | 4.93E-05 |
| C15orf41 | blue | -0.068383861 | 0.140063383 | -0.130320867 | 0.004791169 |

| | | | | | |
|---|---|---|---|---|---|
| C17orf67 | blue | -0.062128846 | 0.18014372 | -0.050608914 | 0.275080902 |
| C17orf80 | blue | -0.151808708 | 0.000998621 | -0.178537059 | 0.000104849 |
| C17orf85 | blue | 0.167370965 | 0.000280274 | 0.126624818 | 0.006142248 |
| C18orf25 | blue | -0.106997878 | 0.020739709 | -0.115423249 | 0.012560966 |
| C20orf194 | blue | -0.162662643 | 0.00041669 | -0.179606141 | 9.51E-05 |
| C21orf91 | blue | -0.154679256 | 0.000796825 | -0.170447445 | 0.000215054 |
| C2orf42 | blue | -0.220020189 | 1.58E-06 | -0.241593022 | 1.25E-07 |
| C3orf58 | blue | -0.112523561 | 0.014979721 | -0.093463014 | 0.0435121 |
| C4orf29 | blue | 0.126477423 | 0.006202605 | 0.142269697 | 0.002056662 |
| C4orf48 | blue | 0.102410589 | 0.026897518 | 0.127357168 | 0.005850102 |
| C5orf24 | blue | -0.010626991 | 0.818836093 | -0.039188376 | 0.398153834 |
| C6orf1 | blue | 0.112324627 | 0.015159737 | 0.138986685 | 0.002611613 |
| C6orf120 | blue | -0.079427673 | 0.086426628 | -0.100458195 | 0.029961762 |
| C7orf60 | blue | -0.087243155 | 0.059581041 | -0.099775852 | 0.031101014 |
| C8orf37 | blue | 0.026103848 | 0.573643936 | 0.006500583 | 0.888577993 |
| C9orf72 | blue | 0.003225862 | 0.944571874 | -0.010382121 | 0.822940711 |
| C9orf89 | blue | 0.011895399 | 0.797655966 | 0.04858786 | 0.2947319 |
| CA13 | blue | 0.022285432 | 0.630970524 | -0.01011168 | 0.827479595 |
| CACFD1 | blue | 0.189982096 | 3.59E-05 | 0.203021328 | 9.79E-06 |
| CALCOC | blue | -0.093364024 | 0.043735902 | -0.11277604 | 0.014753956 |
| CAMK2D | blue | 0.161630772 | 0.000453883 | 0.137605214 | 0.002883501 |
| CAP2 | blue | -0.126043576 | 0.006383364 | -0.152958057 | 0.000912744 |
| CAPN2 | blue | 0.181081979 | 8.31E-05 | 0.191379105 | 3.14E-05 |
| CAPN7 | blue | -0.046153273 | 0.31962046 | -0.076629004 | 0.09813534 |
| CAPNS1 | blue | 0.177633591 | 0.000113787 | 0.210093403 | 4.67E-06 |
| CARF | blue | -0.100533142 | 0.029838837 | -0.121047582 | 0.008832121 |
| CASD1 | blue | 0.059380381 | 0.200224435 | 0.033644549 | 0.468254693 |
| CASP8AP | blue | -0.064953143 | 0.161104138 | -0.091872283 | 0.047227506 |
| CBLB | blue | 0.104218266 | 0.024304812 | 0.098822568 | 0.032754549 |
| CCDC121 | blue | -0.033155401 | 0.474750789 | -0.06144462 | 0.18499753 |
| CCDC122 | blue | -0.014338838 | 0.757283211 | -0.019105022 | 0.680490314 |
| CCDC124 | blue | 0.018641018 | 0.687837753 | 0.03460209 | 0.45568135 |
| CCDC132 | blue | -0.036001571 | 0.437650289 | -0.066300032 | 0.152576326 |
| CCDC169 | blue | -0.066233819 | 0.152987385 | -0.07315475 | 0.114389575 |
| CCDC171 | blue | 0.014029268 | 0.762364365 | -0.008343778 | 0.857285657 |
| CCDC186 | blue | 0.071504617 | 0.122815643 | 0.054433371 | 0.240381734 |
| CCDC50 | blue | -0.271318828 | 2.53E-09 | -0.283072117 | 4.70E-10 |
| CCDC68 | blue | -0.09009666 | 0.051685247 | -0.094369546 | 0.041507068 |
| CCDC71L | blue | -0.142601487 | 0.002007048 | -0.133971901 | 0.003725865 |
| CCDC82 | blue | -0.096035431 | 0.038025615 | -0.124361225 | 0.007129819 |
| CCDC85B | blue | -0.121519722 | 0.00856932 | -0.127166756 | 0.005924834 |
| CCDC88A | blue | -0.202671251 | 1.02E-05 | -0.208073331 | 5.78E-06 |
| CCDC91 | blue | -0.132301891 | 0.004183208 | -0.15156067 | 0.001018106 |
| CCM2 | blue | -0.010601398 | 0.819264869 | 0.030969405 | 0.504375499 |
| CCNG2 | blue | -0.03241437 | 0.484685251 | -0.050168831 | 0.279282074 |

| | | | | | |
|---|---|---|---|---|---|
| CCNT1 | blue | -0.117905751 | 0.010771159 | -0.15397385 | 0.000842579 |
| CCP110 | blue | 0.061854925 | 0.182075437 | 0.039792736 | 0.390912579 |
| CCSER2 | blue | 0.231374661 | 4.29E-07 | 0.217072502 | 2.19E-06 |
| CD109 | blue | -0.074900697 | 0.10597517 | -0.080819103 | 0.081038862 |
| CD151 | blue | 0.143260697 | 0.001911709 | 0.160804726 | 0.000485858 |
| CDC14A | blue | 0.089054258 | 0.054461999 | 0.067145154 | 0.147403012 |
| CDC14B | blue | -0.087190139 | 0.059736689 | -0.110634245 | 0.01676756 |
| CDK13 | blue | -0.128208695 | 0.005526158 | -0.150802408 | 0.001079868 |
| CDK14 | blue | -0.080494458 | 0.082270967 | -0.096500684 | 0.037098714 |
| CDK17 | blue | -0.184420552 | 6.09E-05 | -0.209707356 | 4.86E-06 |
| CDK19 | blue | -0.111025537 | 0.016382664 | -0.132731326 | 0.004060994 |
| CDK2AP1 | blue | 0.162976373 | 0.000405957 | 0.181327081 | 8.12E-05 |
| CDS2 | blue | -0.180575311 | 8.71E-05 | -0.187014266 | 4.77E-05 |
| CENPB | blue | 0.015896301 | 0.731882094 | 0.019714381 | 0.67088703 |
| CENPC | blue | -0.08832544 | 0.056476219 | -0.120107163 | 0.009377049 |
| CEP120 | blue | 0.022680364 | 0.624929358 | -0.003485432 | 0.940119873 |
| CEP128 | blue | -0.05237039 | 0.258697133 | -0.065596192 | 0.15698895 |
| CEP135 | blue | -0.103717405 | 0.025000612 | -0.128935273 | 0.005262605 |
| CEP152 | blue | 0.083339498 | 0.071973395 | 0.061690656 | 0.183241196 |
| CEP192 | blue | -0.096380034 | 0.037337218 | -0.125537977 | 0.00659997 |
| CEP295 | blue | -0.029338831 | 0.527089919 | -0.059073605 | 0.202563057 |
| CEP44 | blue | -0.141754756 | 0.002135887 | -0.158782925 | 0.000573164 |
| CEP68 | blue | -0.171134038 | 0.000202583 | -0.207418231 | 6.20E-06 |
| CEP85L | blue | -0.183356024 | 6.73E-05 | -0.202753615 | 1.01E-05 |
| CEP97 | blue | -0.01050716 | 0.820844142 | -0.023822101 | 0.607606381 |
| CEPT1 | blue | -0.044071498 | 0.34195855 | -0.071632577 | 0.122145426 |
| CFAP97 | blue | 0.095370393 | 0.039384571 | 0.057172107 | 0.217502104 |
| CFLAR | blue | -0.079076931 | 0.087829289 | -0.065898769 | 0.155080314 |
| CGGBP1 | blue | -0.040504925 | 0.382482438 | -0.069858092 | 0.13169729 |
| CHD1 | blue | 0.015440557 | 0.739286116 | -0.017144744 | 0.711728778 |
| CHD2 | blue | 0.023353681 | 0.614687675 | -0.011969241 | 0.796427325 |
| CHD6 | blue | -0.088440266 | 0.056154835 | -0.120824556 | 0.008958739 |
| CHD9 | blue | 0.047428217 | 0.306420716 | 0.027662624 | 0.550970545 |
| CHIC1 | blue | -0.10166865 | 0.028028639 | -0.109647349 | 0.017773668 |
| CHID1 | blue | 0.144700714 | 0.001717697 | 0.144459187 | 0.001748923 |
| CHM | blue | -0.188129362 | 4.29E-05 | -0.215286691 | 2.66E-06 |
| CHMP4B | blue | -0.14871433 | 0.001268241 | -0.130249703 | 0.004814421 |
| CHORDC | blue | -0.046811422 | 0.312760896 | -0.072493384 | 0.117710561 |
| CHPF | blue | 0.212622395 | 3.56E-06 | 0.210881859 | 4.29E-06 |
| CHSY1 | blue | -0.058561579 | 0.206510422 | -0.092141196 | 0.046581351 |
| CHURC1 | blue | 0.035961495 | 0.438160877 | 0.016151187 | 0.727751996 |
| CIB1 | blue | 0.365345864 | 3.41E-16 | 0.408999469 | 2.94E-20 |
| CLASP2 | blue | 0.04707911 | 0.309998762 | 0.011865641 | 0.798151226 |
| CLCN5 | blue | -0.086252426 | 0.062545683 | -0.121528422 | 0.008564544 |
| CLCN6 | blue | -0.322730084 | 8.82E-13 | -0.352498292 | 4.14E-15 |

| | | | | | |
|---|---|---|---|---|---|
| CLDN12 | blue | 0.083453832 | 0.071582632 | 0.076514226 | 0.09864125 |
| CLDND1 | blue | -0.136984266 | 0.0030139 | -0.168672367 | 0.000250704 |
| CLIC4 | blue | -0.150279379 | 0.001124464 | -0.170537147 | 0.000213384 |
| CLIP1 | blue | 0.068883338 | 0.137184424 | 0.056117179 | 0.226122955 |
| CLIP4 | blue | -0.356868587 | 1.79E-15 | -0.385830181 | 5.02E-18 |
| CLOCK | blue | -0.023742548 | 0.60880645 | -0.033619326 | 0.468588464 |
| CLTB | blue | 0.147715193 | 0.001368682 | 0.197247442 | 1.76E-05 |
| CMTR2 | blue | 0.052089179 | 0.261266494 | 0.040552616 | 0.381921926 |
| CNFN | blue | 0.160842631 | 0.000484346 | 0.180793867 | 8.53E-05 |
| CNKSR3 | blue | 0.052213687 | 0.260126726 | 0.041397792 | 0.372071952 |
| CNOT2 | blue | -0.074161247 | 0.109477127 | -0.098325177 | 0.033646707 |
| CNOT4 | blue | -0.112796642 | 0.014735666 | -0.129510536 | 0.005062027 |
| CNOT6L | blue | 0.033764007 | 0.466675725 | 0.006508365 | 0.888445478 |
| CNTLN | blue | -0.042316226 | 0.361547985 | -0.055062683 | 0.234980449 |
| CNTRL | blue | 0.061786974 | 0.182556997 | 0.044061521 | 0.342067949 |
| COG5 | blue | -0.035624264 | 0.442470846 | -0.062128406 | 0.180146811 |
| COL4A3I | blue | 0.180703918 | 8.60E-05 | 0.185054645 | 5.74E-05 |
| COL4A5 | blue | 0.100018122 | 0.030692344 | 0.05225448 | 0.259754055 |
| COMT | blue | 0.301906629 | 2.69E-11 | 0.305853399 | 1.43E-11 |
| COPE | blue | 0.230291006 | 4.88E-07 | 0.266144712 | 5.17E-09 |
| CORO1B | blue | 0.248198428 | 5.49E-08 | 0.271617983 | 2.42E-09 |
| CPEB2 | blue | -0.028971318 | 0.53228042 | -0.043918225 | 0.343641655 |
| CPEB3 | blue | 0.120293995 | 0.009266467 | 0.124280317 | 0.00716761 |
| CPEB4 | blue | 0.098245083 | 0.033792288 | 0.080215 | 0.083343659 |
| CPNE8 | blue | -0.333322146 | 1.40E-13 | -0.330867609 | 2.16E-13 |
| CPSF3L | blue | 0.0679136 | 0.142816072 | 0.072135097 | 0.119540904 |
| CPTP | blue | 0.065356268 | 0.158514954 | 0.090076204 | 0.05173858 |
| CRAMP1 | blue | -0.098321601 | 0.033653195 | -0.127734661 | 0.005704445 |
| CRBN | blue | -0.2126343 | 3.55E-06 | -0.235910276 | 2.50E-07 |
| CREB1 | blue | -0.176945063 | 0.000121074 | -0.202173651 | 1.07E-05 |
| CREB5 | blue | 0.10807746 | 0.019482849 | 0.103399523 | 0.025451077 |
| CREBL2 | blue | -0.068615376 | 0.138723236 | -0.067126379 | 0.147516467 |
| CREBZF | blue | -0.107313618 | 0.020365029 | -0.140172853 | 0.002397033 |
| CRIP2 | blue | 0.121224467 | 0.008732835 | 0.153672186 | 0.000862877 |
| CRK | blue | 0.090288336 | 0.051187737 | 0.094200235 | 0.041875507 |
| CRLF3 | blue | 0.058782227 | 0.204802605 | 0.044111375 | 0.341521517 |
| CROT | blue | -0.001847294 | 0.968241891 | -0.030749575 | 0.5074075 |
| CSGALN | blue | -0.068973495 | 0.136669647 | -0.101290893 | 0.028620085 |
| CSNK1G | blue | -0.022971313 | 0.620494775 | -0.065314823 | 0.158779691 |
| CSRNP2 | blue | 0.008311788 | 0.857827013 | -0.02711229 | 0.558925139 |
| CSRNP3 | blue | -0.11358222 | 0.01405296 | -0.134915944 | 0.003487855 |
| CTBS | blue | -0.100872325 | 0.029287911 | -0.100886884 | 0.029264459 |
| CTD-310 | blue | -0.129681195 | 0.005003862 | -0.132678214 | 0.004075932 |
| CTDSPL2 | blue | 0.026122804 | 0.573365571 | -0.009475234 | 0.838183646 |
| CTTNBP | blue | -0.154145688 | 0.000831215 | -0.174768552 | 0.000147102 |

| | | | | | |
|---|---|---|---|---|---|
| CUL4B | blue | -0.134761638 | 0.00352579 | -0.144014217 | 0.001807818 |
| CUTA | blue | -0.00691055 | 0.881601129 | 0.011158062 | 0.809951168 |
| CWF19L2 | blue | -0.174224342 | 0.000154385 | -0.207573664 | 6.10E-06 |
| CXXC1 | blue | 0.135737205 | 0.003292111 | 0.135942506 | 0.003244763 |
| CYB561D | blue | 0.303174622 | 2.20E-11 | 0.338145774 | 5.91E-14 |
| CYBA | blue | 0.151374703 | 0.001032944 | 0.195614761 | 2.07E-05 |
| CYP20A1 | blue | 0.111527316 | 0.015900424 | 0.109468695 | 0.017961331 |
| CYP39A1 | blue | 0.044676544 | 0.335365915 | 0.029779472 | 0.520900747 |
| DAAM1 | blue | 0.044553485 | 0.336700121 | 0.025571653 | 0.581484747 |
| DACH1 | blue | -0.032955754 | 0.477416286 | -0.037702233 | 0.416299663 |
| DCAF10 | blue | -0.015272567 | 0.742021404 | -0.034317925 | 0.459392795 |
| DCAF16 | blue | -0.062178659 | 0.179794074 | -0.079862142 | 0.084714202 |
| DCAF17 | blue | -0.108058627 | 0.019504194 | -0.137337602 | 0.002939057 |
| DCAF5 | blue | 0.028850017 | 0.533999234 | 0.021003537 | 0.650747639 |
| DCBLD2 | blue | -0.008510387 | 0.854467335 | -0.007961986 | 0.863750768 |
| DCP2 | blue | 0.017210135 | 0.710678552 | -0.004923091 | 0.915499506 |
| DCTN4 | blue | 0.110674527 | 0.016727574 | 0.078408007 | 0.090555097 |
| DCUN1D | blue | 0.005921496 | 0.898447495 | -0.009026895 | 0.845742103 |
| DCUN1D | blue | 0.063761761 | 0.16894221 | 0.022847249 | 0.622384072 |
| DDAH2 | blue | -0.139885038 | 0.002447567 | -0.154444425 | 0.000811795 |
| DDHD1 | blue | 0.012089395 | 0.794429206 | -8.80E-05 | 0.998486746 |
| DDI2 | blue | 0.064504217 | 0.164024833 | 0.039051361 | 0.399806644 |
| DDRGK1 | blue | 0.078746645 | 0.089166817 | 0.104993741 | 0.023260533 |
| DDX41 | blue | 0.07810917 | 0.091794559 | 0.111360979 | 0.016058887 |
| DDX49 | blue | 0.063639225 | 0.169764271 | 0.092700481 | 0.045261216 |
| DDX5 | blue | 0.060668814 | 0.190616897 | 0.033504656 | 0.470107506 |
| DDX6 | blue | 0.039179891 | 0.39825607 | 0.003130368 | 0.946210176 |
| DEAF1 | blue | 0.162049948 | 0.000438416 | 0.151146488 | 0.001051426 |
| DECR2 | blue | 0.383928901 | 7.53E-18 | 0.394802236 | 7.19E-19 |
| DENND2 | blue | -0.139495809 | 0.002517453 | -0.156569245 | 0.00068532 |
| DENND4 | blue | -0.00724046 | 0.875993302 | -0.020208001 | 0.663146748 |
| DENND4 | blue | -0.045270957 | 0.32896914 | -0.056719598 | 0.2211707 |
| DENND5 | blue | -0.277369771 | 1.07E-09 | -0.283982711 | 4.12E-10 |
| DENND6 | blue | -0.03416017 | 0.461460476 | -0.053543012 | 0.248171572 |
| DGKE | blue | 0.040191661 | 0.386176749 | 0.052718003 | 0.255545271 |
| DGKZ | blue | 0.019209895 | 0.678833807 | 0.03711209 | 0.423638152 |
| DHFRL1 | blue | 0.055035168 | 0.235214795 | 0.037377217 | 0.420331983 |
| DHPS | blue | 0.018389977 | 0.691825275 | 0.029806174 | 0.520526893 |
| DHRS1 | blue | 0.023191455 | 0.617148521 | 0.037680653 | 0.416566693 |
| DHRS4 | blue | 0.075768777 | 0.101977696 | 0.095110329 | 0.03992706 |
| DHX40 | blue | -0.007455057 | 0.872348888 | -0.043413652 | 0.349219603 |
| DIAPH2 | blue | -0.097316714 | 0.035519273 | -0.117680369 | 0.010923769 |
| DICER1 | blue | -0.016549671 | 0.721310873 | -0.038760814 | 0.403325072 |
| DIS3L | blue | 0.135605029 | 0.003322926 | 0.115209322 | 0.01272684 |
| DMXL1 | blue | -0.004148527 | 0.928755642 | -0.03439531 | 0.458380408 |

| | | | | | |
|---|---|---|---|---|---|
| DNAJB14 | blue | 0.003583307 | 0.938441655 | -0.009268531 | 0.841666602 |
| DNAJB2 | blue | 0.056475138 | 0.223170892 | 0.073430687 | 0.113025902 |
| DNAJB4 | blue | -0.183810195 | 6.45E-05 | -0.191462973 | 3.11E-05 |
| DNAJB6 | blue | -0.208002244 | 5.83E-06 | -0.226892596 | 7.24E-07 |
| DNAJC13 | blue | -0.163880276 | 0.000376456 | -0.19810116 | 1.62E-05 |
| DNAJC16 | blue | 0.042839473 | 0.355636324 | 0.025729168 | 0.57915888 |
| DNAJC24 | blue | -0.019159955 | 0.679622435 | -0.041234974 | 0.373957181 |
| DNAJC27 | blue | -0.176306229 | 0.000128226 | -0.207760202 | 5.98E-06 |
| DNAJC4 | blue | 0.231314205 | 4.33E-07 | 0.236950991 | 2.21E-07 |
| DNASE1L | blue | 0.131586435 | 0.004394226 | 0.16189221 | 0.000444178 |
| DOCK11 | blue | -0.122586214 | 0.00800118 | -0.125547836 | 0.006595684 |
| DOPEY1 | blue | -0.041114521 | 0.375355657 | -0.059321618 | 0.200670869 |
| DPH6 | blue | -0.028658979 | 0.536711856 | -0.017997783 | 0.698071929 |
| DPM3 | blue | 0.287797473 | 2.34E-10 | 0.299329797 | 4.02E-11 |
| DPP7 | blue | 0.216133624 | 2.43E-06 | 0.217801831 | 2.02E-06 |
| DPP8 | blue | 0.025958358 | 0.575782466 | 0.004563779 | 0.921646189 |
| DST | blue | -0.116998513 | 0.011397194 | -0.117390978 | 0.011122528 |
| DSTYK | blue | -0.161401993 | 0.000462537 | -0.186590497 | 4.97E-05 |
| DTWD1 | blue | 0.096926778 | 0.03626672 | 0.070153375 | 0.130068972 |
| DTWD2 | blue | 0.123863704 | 0.007365058 | 0.110094248 | 0.017311716 |
| DTX2 | blue | 0.179792948 | 9.35E-05 | 0.219303916 | 1.71E-06 |
| DVL1 | blue | 0.004760831 | 0.918274664 | 0.003447085 | 0.940777463 |
| DYRK1A | blue | -0.085404441 | 0.065178804 | -0.102497141 | 0.026768161 |
| ECSIT | blue | -0.057111082 | 0.217994288 | -0.051932336 | 0.262707142 |
| EDRF1 | blue | 0.178834888 | 0.00010205 | 0.14104391 | 0.002249839 |
| EEA1 | blue | -0.023016646 | 0.619805048 | -0.04178004 | 0.367669124 |
| EEFSEC | blue | -0.078703261 | 0.089343712 | -0.073755025 | 0.111439396 |
| EFCAB14 | blue | -0.003377347 | 0.94197347 | -0.034678839 | 0.454681826 |
| EFCAB7 | blue | -0.060286189 | 0.193434036 | -0.080984652 | 0.080416335 |
| EFR3A | blue | -0.125856753 | 0.006462648 | -0.15728245 | 0.000647139 |
| EHBP1 | blue | -0.216957454 | 2.22E-06 | -0.249795465 | 4.48E-08 |
| EID1 | blue | -0.003935912 | 0.932398009 | -0.024500367 | 0.597417598 |
| EIF2AK4 | blue | 0.140830751 | 0.002285076 | 0.12372918 | 0.007429845 |
| EIF2B4 | blue | 0.132936661 | 0.004003706 | 0.142750578 | 0.001985113 |
| EIF3G | blue | -0.044157924 | 0.34101182 | -0.028948673 | 0.532601073 |
| EIF3K | blue | -0.157142001 | 0.000654499 | -0.14146826 | 0.002181164 |
| EIF4A1 | blue | 0.005962862 | 0.897741955 | -0.037501324 | 0.418789554 |
| EIF4A2 | blue | -0.234766026 | 2.87E-07 | -0.269753912 | 3.14E-09 |
| EIF4E3 | blue | -0.174351884 | 0.000152648 | -0.187044791 | 4.76E-05 |
| EIF4G3 | blue | -0.043574765 | 0.347432335 | -0.072827663 | 0.116022708 |
| EIF6 | blue | -0.070429264 | 0.128561719 | -0.057313153 | 0.216367581 |
| ELF2 | blue | -0.033656784 | 0.468092839 | -0.057101663 | 0.218070322 |
| ELK4 | blue | -0.06341777 | 0.171257524 | -0.074483265 | 0.107941034 |
| ELOF1 | blue | -0.069313503 | 0.134741632 | -0.047081981 | 0.309969232 |
| ELP2 | blue | -0.016930706 | 0.715170174 | -0.051040202 | 0.271005612 |

| | | | | | |
|---|---|---|---|---|---|
| EMC10 | blue | 0.18334789 | 6.74E-05 | 0.165896673 | 0.000317693 |
| EML4 | blue | -0.073942002 | 0.110532787 | -0.077244386 | 0.095458033 |
| ENTPD5 | blue | 0.204269606 | 8.61E-06 | 0.175540513 | 0.000137322 |
| EOGT | blue | 0.106655627 | 0.021152602 | 0.093378584 | 0.043702923 |
| EPB41L2 | blue | -0.050046136 | 0.280461072 | -0.052053259 | 0.261595949 |
| EPB41L5 | blue | -0.067659572 | 0.144320154 | -0.108533417 | 0.018972272 |
| EPC1 | blue | -0.042440436 | 0.360139127 | -0.066750793 | 0.149800164 |
| EPM2AIP | blue | -0.540263414 | 9.60E-37 | -0.539680075 | 1.18E-36 |
| EPN1 | blue | -0.02470862 | 0.594304873 | 0.010783173 | 0.816220677 |
| EPS15 | blue | -0.145032886 | 0.001675586 | -0.153354956 | 0.00088471 |
| EPS8 | blue | 0.1615638 | 0.000456401 | 0.144533077 | 0.001739316 |
| ERCC2 | blue | 0.079787646 | 0.085005861 | 0.073528305 | 0.112546546 |
| ERCC4 | blue | -0.093471761 | 0.043492373 | -0.103619305 | 0.025138888 |
| ERCC6 | blue | 0.012367535 | 0.789809018 | 0.012789552 | 0.782812957 |
| ERCC6L2 | blue | -0.002051031 | 0.964741469 | -0.024686093 | 0.594641228 |
| ERGIC3 | blue | 0.019933504 | 0.667446685 | 0.026640019 | 0.565795422 |
| ERMAP | blue | -0.071757528 | 0.121493721 | -0.114879876 | 0.012986065 |
| ERN1 | blue | 0.131522221 | 0.004413628 | 0.108679769 | 0.018810879 |
| ERRFI1 | blue | 0.107569611 | 0.020065582 | 0.084256018 | 0.068889439 |
| ERV3-1 | blue | 0.064706477 | 0.162704053 | 0.051273956 | 0.268814161 |
| ETAA1 | blue | 0.049308941 | 0.287615793 | 0.033982522 | 0.463795081 |
| ETFB | blue | 0.132553619 | 0.004111171 | 0.158896058 | 0.000567917 |
| EVI5 | blue | -0.027641318 | 0.551277467 | -0.03794124 | 0.413348991 |
| EXOC1 | blue | -0.040951862 | 0.377249257 | -0.061711352 | 0.183094019 |
| EXPH5 | blue | 0.254704947 | 2.38E-08 | 0.252116187 | 3.33E-08 |
| EXTL2 | blue | -0.037463028 | 0.419265144 | -0.071577988 | 0.122430997 |
| FAM102E | blue | 0.0918705 | 0.047231816 | 0.060417439 | 0.192464267 |
| FAM120E | blue | 0.035890569 | 0.439065337 | 0.016495292 | 0.722188724 |
| FAM120C | blue | -0.12518689 | 0.006754232 | -0.117060534 | 0.011353393 |
| FAM122A | blue | 0.027789639 | 0.549142567 | 0.022552893 | 0.62687653 |
| FAM122E | blue | -0.145560411 | 0.001610651 | -0.157628152 | 0.000629351 |
| FAM122C | blue | -0.074514623 | 0.107792362 | -0.080306901 | 0.082989662 |
| FAM126A | blue | -0.033253168 | 0.473448474 | -0.050739055 | 0.273846819 |
| FAM135A | blue | -0.160350766 | 0.00050431 | -0.177906186 | 0.000111017 |
| FAM13A | blue | 0.159726665 | 0.000530742 | 0.143304317 | 0.001905549 |
| FAM13B | blue | -0.059211888 | 0.201506455 | -0.084301445 | 0.068739444 |
| FAM160E | blue | 0.033914408 | 0.464691952 | 0.017950379 | 0.698828347 |
| FAM161A | blue | -0.142335325 | 0.002046761 | -0.174562092 | 0.000149826 |
| FAM171E | blue | -0.004493336 | 0.922851814 | -0.011226152 | 0.808813748 |
| FAM172A | blue | -0.041945557 | 0.365772738 | -0.07142493 | 0.123234462 |
| FAM175A | blue | -0.132058291 | 0.004254005 | -0.168725765 | 0.000249556 |
| FAM178A | blue | -0.002997079 | 0.948497265 | -0.042181782 | 0.363076793 |
| FAM184A | blue | -0.319612095 | 1.50E-12 | -0.313461306 | 4.17E-12 |
| FAM188A | blue | -0.035002203 | 0.450484144 | -0.065389837 | 0.158300776 |
| FAM19A2 | blue | -0.048911286 | 0.291525703 | -0.040037801 | 0.38799915 |

| | | | | | |
|---|---|---|---|---|---|
| FAM207A | blue | -0.0170502 | 0.713248172 | -0.002309829 | 0.960296015 |
| FAM208A | blue | -0.06013611 | 0.194547294 | -0.090500599 | 0.05064146 |
| FAM208B | blue | -0.068214388 | 0.141050671 | -0.097770465 | 0.034666031 |
| FAM214A | blue | 0.187991462 | 4.35E-05 | 0.189452937 | 3.78E-05 |
| FAM217B | blue | -0.161510633 | 0.000458409 | -0.183501197 | 6.64E-05 |
| FAM227B | blue | 0.019628544 | 0.672236601 | 0.006955849 | 0.880830778 |
| FAM3A | blue | -0.076847332 | 0.09717871 | -0.075301121 | 0.104116104 |
| FAM58A | blue | -0.06660088 | 0.150719159 | -0.026844444 | 0.56281662 |
| FAM63B | blue | -0.148173258 | 0.001321759 | -0.155121174 | 0.000769344 |
| FAM73A | blue | -0.183449464 | 6.67E-05 | -0.193625346 | 2.52E-05 |
| FAM76A | blue | -0.00806909 | 0.861936172 | -0.023569053 | 0.611427309 |
| FAM8A1 | blue | -0.04682211 | 0.312650301 | -0.061737422 | 0.182908758 |
| FAS | blue | 0.11424686 | 0.013497247 | 0.100717277 | 0.029538659 |
| FBL | blue | -0.145826128 | 0.001578825 | -0.142390741 | 0.002038434 |
| FBXL15 | blue | 0.174984206 | 0.000144306 | 0.200968054 | 1.21E-05 |
| FBXL17 | blue | 0.021997152 | 0.635395867 | -0.0141857 | 0.759795466 |
| FBXL3 | blue | -0.182026539 | 7.62E-05 | -0.199549353 | 1.40E-05 |
| FBXL4 | blue | -0.010192202 | 0.826127557 | -0.018386848 | 0.691875036 |
| FBXL5 | blue | 0.146735007 | 0.001474277 | 0.143632674 | 0.001859758 |
| FBXO3 | blue | 0.008443349 | 0.85560112 | -0.011242574 | 0.80853947 |
| FBXO30 | blue | -0.109185941 | 0.01826188 | -0.126774695 | 0.006081415 |
| FBXO38 | blue | 0.029851172 | 0.519897188 | -0.000952199 | 0.983626913 |
| FBXO42 | blue | 0.030864217 | 0.505825121 | 0.009283441 | 0.841415256 |
| FBXO44 | blue | -0.157829339 | 0.000619208 | -0.165816906 | 0.000319845 |
| FBXO48 | blue | -0.140568403 | 0.002329135 | -0.139174797 | 0.002576455 |
| FBXO8 | blue | -0.056573632 | 0.222363456 | -0.063125297 | 0.173244651 |
| FBXO9 | blue | 0.009286505 | 0.841363615 | -0.002968745 | 0.948983508 |
| FBXW5 | blue | 0.261893736 | 9.19E-09 | 0.29619177 | 6.55E-11 |
| FCHO2 | blue | 0.051596459 | 0.265810649 | 0.043605353 | 0.347093672 |
| FCHSD2 | blue | -0.106908125 | 0.020847304 | -0.101705734 | 0.027971149 |
| FEM1B | blue | -0.113150653 | 0.014424487 | -0.130056299 | 0.00487813 |
| FEM1C | blue | 0.097625348 | 0.034936994 | 0.075295752 | 0.10414086 |
| FER | blue | -0.064463522 | 0.164291545 | -0.098923919 | 0.032575256 |
| FEZ2 | blue | -0.224300075 | 9.75E-07 | -0.222319428 | 1.22E-06 |
| FGD4 | blue | 0.008192947 | 0.859838655 | -0.000297776 | 0.994879417 |
| FGD6 | blue | 0.086249149 | 0.062555686 | 0.089610793 | 0.052964449 |
| FIS1 | blue | -0.060487414 | 0.191948704 | -0.026273323 | 0.571157592 |
| FIZ1 | blue | -0.001745188 | 0.969996412 | -0.000836662 | 0.985613344 |
| FKBP14 | blue | 0.144748733 | 0.00171155 | 0.135794586 | 0.003278815 |
| FKBP8 | blue | 0.018524147 | 0.689693059 | 0.04487202 | 0.333253553 |
| FKTN | blue | 0.106540718 | 0.021292819 | 0.074452651 | 0.108086333 |
| FLOT1 | blue | -0.203825862 | 9.01E-06 | -0.210714901 | 4.37E-06 |
| FLOT2 | blue | 0.111240683 | 0.016174349 | 0.128095539 | 0.005568255 |
| FMNL2 | blue | -0.105249282 | 0.022925027 | -0.114369695 | 0.01339669 |
| FNDC3A | blue | 0.10200015 | 0.027518299 | 0.070779236 | 0.126669247 |

| | | | | | |
|---|---|---|---|---|---|
| FNDC3B | blue | 0.046744076 | 0.313458337 | 0.026306956 | 0.570664768 |
| FNIP1 | blue | -0.026093456 | 0.57379656 | -0.03924523 | 0.397469215 |
| FNIP2 | blue | 0.129755675 | 0.004978667 | 0.112724144 | 0.014800115 |
| FOXN2 | blue | -0.094779665 | 0.040625932 | -0.123555969 | 0.007514013 |
| FOXO3 | blue | -0.042405397 | 0.360536206 | -0.06981449 | 0.131939057 |
| FPGS | blue | 0.117150291 | 0.011290268 | 0.154456311 | 0.000811031 |
| FRMD4B | blue | 0.056169749 | 0.225687683 | 0.017997133 | 0.698082298 |
| FRS2 | blue | -0.044981916 | 0.33206975 | -0.070724276 | 0.126965001 |
| FRYL | blue | 0.112807962 | 0.014725625 | 0.100836727 | 0.029345319 |
| FUT11 | blue | 0.142602924 | 0.002006836 | 0.156068714 | 0.000713351 |
| FZD6 | blue | 0.062867193 | 0.175012487 | 0.031180908 | 0.501467356 |
| G6PC3 | blue | 0.279433613 | 7.98E-10 | 0.280711128 | 6.64E-10 |
| GAB1 | blue | -0.056500738 | 0.222960828 | -0.087689575 | 0.058283675 |
| GABPB2 | blue | 0.097114038 | 0.035906119 | 0.089443167 | 0.053411851 |
| GALNT1 | blue | 0.08471361 | 0.067390702 | 0.045219229 | 0.329522661 |
| GANC | blue | 0.20622121 | 7.03E-06 | 0.211131794 | 4.18E-06 |
| GAPVD1 | blue | 0.115617181 | 0.012412248 | 0.112359635 | 0.015127921 |
| GAREM | blue | 0.048388459 | 0.296720303 | 0.037093115 | 0.423875354 |
| GATAD1 | blue | 0.088564244 | 0.055809541 | 0.059216744 | 0.201469422 |
| GCC2 | blue | -0.071834424 | 0.121094006 | -0.090656614 | 0.050243055 |
| GCLC | blue | -0.063734516 | 0.169124732 | -0.098434012 | 0.03344974 |
| GDAP1 | blue | -0.248708395 | 5.15E-08 | -0.271948004 | 2.31E-09 |
| GDAP2 | blue | -0.053481321 | 0.248717759 | -0.053326707 | 0.250090338 |
| GK5 | blue | 0.106823525 | 0.020949165 | 0.091184887 | 0.048913432 |
| GLCCI1 | blue | -0.026750241 | 0.564188363 | -0.038537606 | 0.406040568 |
| GLS | blue | -0.207076697 | 6.42E-06 | -0.217934176 | 1.99E-06 |
| GNA13 | blue | -0.159968581 | 0.000520347 | -0.185295762 | 5.61E-05 |
| GNAI1 | blue | -0.130979315 | 0.004580783 | -0.139692935 | 0.002481835 |
| GNAQ | blue | 0.072921597 | 0.115551838 | 0.069394417 | 0.13428591 |
| GNB1L | blue | -0.06189234 | 0.181810686 | -0.041438775 | 0.37159834 |
| GNG12 | blue | 0.020435227 | 0.659595649 | -0.014190261 | 0.7597206 |
| GNPDA2 | blue | -0.027745397 | 0.54977896 | -0.043555877 | 0.347641568 |
| GNPTAB | blue | 0.162255534 | 0.000431011 | 0.169639469 | 0.000230648 |
| GOLGA1 | blue | 0.292869254 | 1.09E-10 | 0.285810523 | 3.14E-10 |
| GOLGA8 | blue | -0.144568451 | 0.001734733 | -0.154164641 | 0.00082997 |
| GOLGA8 | blue | -0.086679383 | 0.06125348 | -0.091087466 | 0.049156394 |
| GOLGB1 | blue | -0.003010489 | 0.948267141 | -0.023695422 | 0.60951785 |
| GOLIM4 | blue | 0.062791401 | 0.17553415 | 0.046786235 | 0.313021612 |
| GOSR1 | blue | 0.084926026 | 0.066704128 | 0.071644499 | 0.122083123 |
| GPATCH | blue | 0.13357485 | 0.003830293 | 0.115694867 | 0.012353111 |
| GPC6 | blue | -0.016236906 | 0.72636479 | -0.060435973 | 0.192327616 |
| GPD1L | blue | 0.134503118 | 0.003590185 | 0.116316844 | 0.011888546 |
| GPM6B | blue | -0.00057685 | 0.99008062 | 0.006172249 | 0.89417185 |
| GPR125 | blue | -0.122196958 | 0.008204526 | -0.169567331 | 0.000232091 |
| GPR126 | blue | -0.037401596 | 0.420028728 | -0.029176682 | 0.529376826 |

| | | | | | |
|---|---|---|---|---|---|
| GPX1 | blue | 0.038301818 | 0.408920893 | 0.091366542 | 0.048463076 |
| GPX8 | blue | -0.170130722 | 0.000221045 | -0.144812634 | 0.001703401 |
| GRIP1 | blue | 0.063953612 | 0.167661115 | 0.038065566 | 0.411819013 |
| GRN | blue | 0.062508424 | 0.177492048 | 0.076827492 | 0.097265334 |
| GSDMD | blue | 0.016545199 | 0.721383061 | 0.042584731 | 0.358506773 |
| GSTP1 | blue | -0.032974732 | 0.47716256 | -0.002891812 | 0.950303829 |
| GTF3C3 | blue | -0.081905879 | 0.07702256 | -0.106610062 | 0.021208105 |
| GTF3C5 | blue | 0.197019719 | 1.80E-05 | 0.191092855 | 3.23E-05 |
| GTPBP6 | blue | 0.06445578 | 0.164342318 | 0.072112758 | 0.119655754 |
| GUK1 | blue | 0.15611151 | 0.000710914 | 0.208298244 | 5.65E-06 |
| GULP1 | blue | 0.052867808 | 0.254195195 | 0.047930618 | 0.301319608 |
| GVQW1 | blue | -0.099033255 | 0.032382778 | -0.110660583 | 0.016741407 |
| GXYLT2 | blue | -0.062071517 | 0.180546751 | -0.076004399 | 0.100913569 |
| GZF1 | blue | -0.141729385 | 0.002139862 | -0.159040061 | 0.000561304 |
| HACE1 | blue | -0.096919689 | 0.036280431 | -0.137150792 | 0.002978414 |
| HCFC2 | blue | -0.078727502 | 0.089244839 | -0.09221536 | 0.04640446 |
| HDAC9 | blue | 0.04101798 | 0.376478838 | 0.034189425 | 0.461076643 |
| HDX | blue | -0.394124691 | 8.35E-19 | -0.414521915 | 8.11E-21 |
| HEATR5A | blue | 0.045349816 | 0.328126462 | 0.025870216 | 0.577079868 |
| HEATR5B | blue | 0.125115179 | 0.006786135 | 0.104162554 | 0.024381373 |
| HEATR6 | blue | -0.097319494 | 0.035513991 | -0.132776329 | 0.004048375 |
| HECA | blue | -0.126648727 | 0.006132507 | -0.124891844 | 0.00688636 |
| HECTD2 | blue | -0.134231095 | 0.003659093 | -0.155232342 | 0.00076257 |
| HELZ | blue | -0.031912337 | 0.49147892 | -0.051596701 | 0.2658084 |
| HERC3 | blue | 0.077303751 | 0.09520287 | 0.079640918 | 0.085582687 |
| HERC4 | blue | 0.038606235 | 0.405204486 | 0.006258546 | 0.892701071 |
| HERPUD | blue | -0.13149565 | 0.004421679 | -0.152166053 | 0.000971154 |
| HFM1 | blue | -0.289069886 | 1.93E-10 | -0.312165016 | 5.16E-12 |
| HIATL2 | blue | 0.116816475 | 0.011526618 | 0.121632973 | 0.008507329 |
| HIPK3 | blue | -0.091430806 | 0.048304588 | -0.093114156 | 0.044305127 |
| HM13 | blue | -0.089859985 | 0.052305114 | -0.076083212 | 0.100559606 |
| HOOK3 | blue | -0.01471764 | 0.751079937 | -0.045542288 | 0.326075597 |
| HP1BP3 | blue | -0.089278128 | 0.05385542 | -0.130606178 | 0.00469896 |
| HPS5 | blue | -0.004524167 | 0.922324131 | -0.017209124 | 0.710694793 |
| HS6ST2 | blue | -0.034915333 | 0.451609685 | -0.040060346 | 0.387731779 |
| HSD17B8 | blue | -0.029839236 | 0.52006419 | -0.016699726 | 0.71889045 |
| HSPB1 | blue | 0.062310919 | 0.178868146 | 0.10238529 | 0.026935432 |
| HYAL2 | blue | 0.087327389 | 0.059334433 | 0.083679985 | 0.070814801 |
| IBTK | blue | 0.105261777 | 0.02290873 | 0.091919748 | 0.047112911 |
| ICE2 | blue | 0.10831425 | 0.019216219 | 0.087120026 | 0.059943045 |
| ICK | blue | 0.144168205 | 0.001787234 | 0.12075388 | 0.008999199 |
| IDS | blue | -0.150688834 | 0.001089411 | -0.167113163 | 0.000286505 |
| IFNAR1 | blue | -0.056721514 | 0.221155072 | -0.05263657 | 0.256281234 |
| IFNGR1 | blue | -0.109002089 | 0.018459649 | -0.112906135 | 0.014638797 |
| IKZF2 | blue | 0.037455663 | 0.419356646 | 0.035388371 | 0.445499981 |

| | | | | | |
|---|---|---|---|---|---|
| IKZF4 | blue | -0.214059125 | 3.05E-06 | -0.216547698 | 2.32E-06 |
| IKZF5 | blue | -0.04133748 | 0.3727696 | -0.072738604 | 0.116470521 |
| IL1R1 | blue | 0.003612545 | 0.937940386 | 0.013337152 | 0.773761147 |
| IL1RAP | blue | -0.123436718 | 0.007572455 | -0.118681965 | 0.010259926 |
| IL6ST | blue | 0.040661537 | 0.380643645 | 0.011774243 | 0.799672909 |
| IMPDH1 | blue | 0.18970667 | 3.69E-05 | 0.223981843 | 1.01E-06 |
| INADL | blue | 0.127711143 | 0.005713423 | 0.092501225 | 0.045727889 |
| ING3 | blue | -0.099503779 | 0.031565497 | -0.122908874 | 0.007836046 |
| INO80D | blue | -0.029228248 | 0.528649002 | -0.0461472 | 0.319684202 |
| INPP4B | blue | -0.07241941 | 0.118086658 | -0.052340446 | 0.258969889 |
| INPP5F | blue | -0.010495658 | 0.821036947 | -0.023149119 | 0.617791432 |
| INTS6 | blue | -0.02084285 | 0.653244465 | -0.031778434 | 0.493299514 |
| INVS | blue | 0.099825027 | 0.031017689 | 0.06708126 | 0.147789401 |
| IRAK1BP | blue | -0.046958073 | 0.311245682 | -0.058602295 | 0.206194505 |
| IRAK4 | blue | 0.164211264 | 0.000366162 | 0.155346316 | 0.000755683 |
| ITCH | blue | -0.11108557 | 0.016324302 | -0.133127255 | 0.003951187 |
| ITFG3 | blue | 0.160328369 | 0.000505237 | 0.196250417 | 1.94E-05 |
| ITGAV | blue | -0.109504913 | 0.017923148 | -0.122101914 | 0.008254871 |
| ITGB8 | blue | -0.121982782 | 0.008318365 | -0.127883433 | 0.005647942 |
| ITPR2 | blue | 0.024283546 | 0.600666214 | 0.00105714 | 0.981822747 |
| ITSN1 | blue | -0.143284829 | 0.001908299 | -0.18018908 | 9.02E-05 |
| ITSN2 | blue | -0.012464384 | 0.78820197 | -0.017615786 | 0.704175837 |
| JAK2 | blue | -0.042936651 | 0.354545133 | -0.032186559 | 0.487761761 |
| JMJD1C | blue | -0.043543213 | 0.34778189 | -0.078449721 | 0.090383159 |
| JMJD7-PI | blue | 0.151942095 | 0.000988285 | 0.130867771 | 0.004615826 |
| JMJD8 | blue | 0.118229298 | 0.010555382 | 0.145340207 | 0.001637471 |
| JMY | blue | -0.20688386 | 6.55E-06 | -0.236349629 | 2.38E-07 |
| JOSD2 | blue | 0.075040689 | 0.105322261 | 0.098074796 | 0.034103593 |
| JRKL | blue | -0.013663853 | 0.768375267 | -0.05197975 | 0.262271053 |
| KANSL1l | blue | -0.166348961 | 0.000305745 | -0.188128001 | 4.29E-05 |
| KANSL3 | blue | -0.323759926 | 7.40E-13 | -0.370119045 | 1.31E-16 |
| KAT2B | blue | 0.01405536 | 0.761935694 | 0.00331986 | 0.942959472 |
| KBTBD2 | blue | -0.149111885 | 0.001230197 | -0.176511089 | 0.000125891 |
| KBTBD3 | blue | 0.015545384 | 0.737580929 | -0.001516016 | 0.973934881 |
| KBTBD6 | blue | 0.053411566 | 0.249336355 | 0.05855026 | 0.206598308 |
| KBTBD7 | blue | 0.136759956 | 0.003062309 | 0.1248462 | 0.006907006 |
| KCNIP4 | blue | -0.192624871 | 2.78E-05 | -0.212121258 | 3.76E-06 |
| KCNT2 | blue | -0.348807286 | 8.31E-15 | -0.336711952 | 7.64E-14 |
| KCTD6 | blue | -0.068047547 | 0.142027829 | -0.06788393 | 0.142991125 |
| KDELC2 | blue | 0.150429862 | 0.001111462 | 0.124629123 | 0.007005956 |
| KDM3A | blue | -0.078782597 | 0.089020435 | -0.10424858 | 0.024263243 |
| KDM4C | blue | 0.117996241 | 0.01071042 | 0.106141985 | 0.02178565 |
| KDM7A | blue | 0.321289349 | 1.13E-12 | 0.302696804 | 2.37E-11 |
| KIAA023: | blue | 0.056872615 | 0.219925241 | 0.033137891 | 0.474984243 |
| KIAA039 | blue | 0.175583711 | 0.000136793 | 0.169041638 | 0.000242861 |

| | | | | | |
|---|---|---|---|---|---|
| KIAA043( | blue | -0.105604138 | 0.022466082 | -0.11096737 | 0.016439386 |
| KIAA058( | blue | 0.191790344 | 3.02E-05 | 0.164326804 | 0.000362631 |
| KIAA075: | blue | 0.062125297 | 0.180168651 | 0.031762479 | 0.493516684 |
| KIAA089. | blue | 0.037106674 | 0.42370585 | 0.020672864 | 0.655890013 |
| KIAA102· | blue | 0.007391835 | 0.873422293 | -0.002477863 | 0.957410292 |
| KIAA103: | blue | -0.037456332 | 0.419348342 | -0.060027937 | 0.195352608 |
| KIAA110' | blue | -0.033586875 | 0.469018059 | -0.068582688 | 0.13891185 |
| KIAA132· | blue | -0.190787452 | 3.32E-05 | -0.219319519 | 1.71E-06 |
| KIAA132: | blue | -0.174983831 | 0.00014431 | -0.207341469 | 6.25E-06 |
| KIAA146· | blue | 0.137498245 | 0.002905592 | 0.118583578 | 0.010323512 |
| KIAA155 | blue | -0.164297583 | 0.000363521 | -0.163158395 | 0.000399848 |
| KIAA201: | blue | 0.139121181 | 0.002586432 | 0.159449404 | 0.000542892 |
| KIAA202( | blue | -0.031564451 | 0.496216347 | -0.046638529 | 0.314553442 |
| KIF16B | blue | -0.004281905 | 0.926471464 | -0.019827877 | 0.669104219 |
| KIF21A | blue | 0.009519346 | 0.837440759 | -0.010478474 | 0.821325027 |
| KIF3A | blue | 0.066194313 | 0.153233046 | 0.042959782 | 0.354285707 |
| KITLG | blue | -0.045264888 | 0.329034058 | -0.040628526 | 0.38103078 |
| KLF12 | blue | -0.26084253 | 1.06E-08 | -0.271120456 | 2.60E-09 |
| KLF3 | blue | 0.043777314 | 0.345193658 | 0.02534727 | 0.584805467 |
| KLF6 | blue | -0.0886621 | 0.055538247 | -0.123403952 | 0.007588584 |
| KLF8 | blue | -0.22031132 | 1.53E-06 | -0.208569291 | 5.49E-06 |
| KLHDC1( | blue | 0.031095004 | 0.50264746 | 0.010769393 | 0.816451358 |
| KLHDC3 | blue | -0.121390849 | 0.008640353 | -0.096502055 | 0.037096011 |
| KLHL15 | blue | -0.083582506 | 0.071144929 | -0.103251915 | 0.025662618 |
| KLHL28 | blue | 0.022464306 | 0.62823126 | -0.001446383 | 0.975131687 |
| KLHL42 | blue | -0.066361505 | 0.15219544 | -0.109559454 | 0.01786578 |
| KLHL5 | blue | -0.330954875 | 2.12E-13 | -0.321338351 | 1.12E-12 |
| KLHL9 | blue | -0.023472747 | 0.612884266 | -0.032214761 | 0.487380336 |
| KMT2C | blue | 0.08910223 | 0.05433154 | 0.063659477 | 0.169628199 |
| KPNA5 | blue | -0.130861289 | 0.00461787 | -0.154371906 | 0.00081647 |
| KRBOX4 | blue | -0.296459205 | 6.28E-11 | -0.301222603 | 2.99E-11 |
| KRIT1 | blue | -0.115010307 | 0.012882883 | -0.149055003 | 0.001235575 |
| L3MBTL: | blue | -0.064287598 | 0.165448286 | -0.084441985 | 0.068277086 |
| L3MBTL· | blue | -0.089132001 | 0.054250713 | -0.148466983 | 0.001292454 |
| LACE1 | blue | -0.029245811 | 0.528401237 | -0.03177373 | 0.49336353 |
| LAPTM4. | blue | -0.179700357 | 9.43E-05 | -0.189396472 | 3.80E-05 |
| LARP4 | blue | -0.007844097 | 0.865748913 | -0.021263247 | 0.646720383 |
| LATS1 | blue | 0.046687955 | 0.314040312 | 0.023342093 | 0.614863303 |
| LCMT1 | blue | -0.013639657 | 0.768773772 | 0.003403635 | 0.941522615 |
| LCOR | blue | -0.056229325 | 0.22519512 | -0.070410973 | 0.128661229 |
| LDLRAD | blue | -0.090770755 | 0.049953242 | -0.152764693 | 0.000926697 |
| LEMD3 | blue | 0.032259088 | 0.486781148 | -0.007671121 | 0.868682282 |
| LEPROT | blue | -0.114040072 | 0.013668034 | -0.132922354 | 0.004007674 |
| LHFPL2 | blue | -0.041275711 | 0.37348494 | -0.055680748 | 0.229759619 |
| LIG4 | blue | 0.065424643 | 0.158078928 | 0.046351527 | 0.317543906 |

| | | | | | |
|---|---|---|---|---|---|
| LIMA1 | blue | 0.14266559 | 0.00199759 | 0.119716285 | 0.009612203 |
| LIMCH1 | blue | -0.070320487 | 0.129154374 | -0.082698775 | 0.074195571 |
| LIMS1 | blue | 0.020659596 | 0.656096689 | 0.006699385 | 0.885193655 |
| LINS | blue | 0.018884265 | 0.683982243 | 0.012205589 | 0.79249822 |
| LMAN2 | blue | 0.143217226 | 0.001917866 | 0.152720976 | 0.000929879 |
| LMBRD2 | blue | 0.028348257 | 0.541138639 | 0.016361172 | 0.724355373 |
| LNPEP | blue | -0.091372086 | 0.048449386 | -0.08332816 | 0.07201224 |
| LONP2 | blue | 0.183388182 | 6.71E-05 | 0.197688998 | 1.68E-05 |
| LPIN1 | blue | -0.003134644 | 0.946136802 | -0.038217571 | 0.409952971 |
| LRCH1 | blue | -0.07101065 | 0.125429789 | -0.082098599 | 0.07632751 |
| LRCH3 | blue | -0.224571197 | 9.45E-07 | -0.231894416 | 4.04E-07 |
| LRIG2 | blue | -0.197401097 | 1.73E-05 | -0.210025134 | 4.70E-06 |
| LRP12 | blue | -0.120728418 | 0.009013815 | -0.158140391 | 0.000603825 |
| LRP6 | blue | -0.097044622 | 0.036039434 | -0.120356899 | 0.009229498 |
| LRRC37E | blue | -0.100186346 | 0.030411288 | -0.128231727 | 0.005517625 |
| LRRFIP1 | blue | 0.091338978 | 0.048531188 | 0.102291286 | 0.027076708 |
| LRRFIP2 | blue | 0.059830303 | 0.196830218 | 0.040672669 | 0.380513143 |
| LSM11 | blue | -0.070468276 | 0.128349687 | -0.087637047 | 0.058435103 |
| LTN1 | blue | -0.143873767 | 0.001826781 | -0.180175219 | 9.03E-05 |
| LYRM7 | blue | 0.086893088 | 0.060615014 | 0.065655752 | 0.156611857 |
| LYSMD3 | blue | 0.081865755 | 0.077167911 | 0.074696213 | 0.106934607 |
| LYST | blue | 0.105903609 | 0.022084983 | 0.100179796 | 0.030422191 |
| MACROI | blue | 0.206013137 | 7.18E-06 | 0.207166149 | 6.36E-06 |
| MAD1L1 | blue | 0.02772439 | 0.550081252 | 0.052313363 | 0.259216758 |
| MAGI3 | blue | 0.040340782 | 0.384415469 | 0.013441657 | 0.772037115 |
| MALT1 | blue | -0.092806243 | 0.045015142 | -0.102890402 | 0.026187128 |
| MAN1A2 | blue | 0.124065509 | 0.007268816 | 0.093933479 | 0.042461595 |
| MAN1B1 | blue | 0.217067891 | 2.19E-06 | 0.218655213 | 1.84E-06 |
| MAN2A1 | blue | 0.192352934 | 2.85E-05 | 0.155996678 | 0.000717472 |
| MANEA | blue | -0.030146883 | 0.515768806 | -0.041684967 | 0.368761162 |
| MAP1S | blue | -0.020664353 | 0.656022592 | -0.004465444 | 0.92332923 |
| MAP2K4 | blue | 0.238338157 | 1.87E-07 | 0.211907221 | 3.84E-06 |
| MAP3K1 | blue | -0.035886258 | 0.439120351 | -0.049741724 | 0.283400748 |
| MAP3K2 | blue | -0.067830847 | 0.143304725 | -0.077675568 | 0.093617092 |
| MAP3K4 | blue | 0.001601451 | 0.972466535 | -0.005639929 | 0.903252059 |
| MAP3K5 | blue | -0.091221276 | 0.048822938 | -0.084347861 | 0.068586459 |
| MAP3K7 | blue | -0.108578892 | 0.018921994 | -0.113280395 | 0.014311898 |
| MAP4K3 | blue | -0.117994097 | 0.010711855 | -0.15908858 | 0.000559092 |
| MAP4K5 | blue | -0.064395424 | 0.164738581 | -0.098457758 | 0.033406896 |
| MAP9 | blue | -0.008126421 | 0.860965156 | -0.017318031 | 0.708946866 |
| MAPK14 | blue | -0.198812497 | 1.50E-05 | -0.230049716 | 5.02E-07 |
| MAPK8 | blue | 0.129755235 | 0.004978816 | 0.088693818 | 0.055450547 |
| MBLAC2 | blue | -0.022824705 | 0.622727637 | -0.042652617 | 0.357740418 |
| MBNL1 | blue | -0.104049774 | 0.024536992 | -0.114348696 | 0.013413833 |
| MBNL2 | blue | -0.292051457 | 1.23E-10 | -0.304100224 | 1.90E-11 |

| | | | | | |
|---|---|---|---|---|---|
| MBNL3 | blue | -0.076313162 | 0.09953249 | -0.068880657 | 0.137199753 |
| MBTPS2 | blue | -0.093914536 | 0.042503476 | -0.119762123 | 0.009584358 |
| MCC | blue | 0.106469925 | 0.021379606 | 0.070907884 | 0.125979035 |
| MCM9 | blue | 0.056548356 | 0.222570464 | 0.034441882 | 0.457771736 |
| MDK | blue | 0.034623632 | 0.455400672 | 0.050685215 | 0.274356902 |
| MDN1 | blue | -0.161329638 | 0.000465306 | -0.194720344 | 2.26E-05 |
| MECOM | blue | -0.040381913 | 0.383930533 | -0.050403694 | 0.277034612 |
| MED13 | blue | -0.059096147 | 0.202390545 | -0.076786457 | 0.097444691 |
| MED13L | blue | -0.171120829 | 0.000202816 | -0.187745 | 4.45E-05 |
| MED23 | blue | 0.060125109 | 0.194629082 | 0.0428461 | 0.355561844 |
| MED28 | blue | -0.095644921 | 0.038818691 | -0.119727646 | 0.009605295 |
| MERTK | blue | 0.08023425 | 0.083269411 | 0.047753272 | 0.303113806 |
| METTL1: | blue | 0.128122281 | 0.00555828 | 0.129493153 | 0.005067986 |
| METTL2 | blue | 0.039991906 | 0.388543761 | 0.008616649 | 0.852670786 |
| METTL2: | blue | 0.008859472 | 0.848568327 | -0.004762418 | 0.918247502 |
| MEX3C | blue | -0.041940011 | 0.365836184 | -0.072476172 | 0.117797988 |
| MFSD10 | blue | 0.158736019 | 0.000575352 | 0.167009256 | 0.000289053 |
| MFSD5 | blue | 0.078485489 | 0.090235931 | 0.096463735 | 0.037171617 |
| MFSD8 | blue | -0.085010457 | 0.066432829 | -0.100667435 | 0.029619656 |
| MGA | blue | -0.045177192 | 0.329972928 | -0.0774009 | 0.094786482 |
| MGEA5 | blue | 0.060052969 | 0.195166035 | 0.027367196 | 0.55523379 |
| MIB1 | blue | -0.078843155 | 0.08877431 | -0.107936721 | 0.019642845 |
| MID1 | blue | 0.034687986 | 0.454562796 | 0.016237377 | 0.726357177 |
| MIER1 | blue | -0.036818771 | 0.427313504 | -0.048487647 | 0.295730094 |
| MIER3 | blue | 0.003431088 | 0.941051806 | -0.018646847 | 0.687745259 |
| MIF | blue | 0.189832516 | 3.64E-05 | 0.176465757 | 0.000126404 |
| MKLN1 | blue | -0.101800687 | 0.027824403 | -0.114503676 | 0.01328776 |
| MLH3 | blue | -0.086113038 | 0.062972381 | -0.121869561 | 0.008379113 |
| MLLT10 | blue | -0.11413333 | 0.013590778 | -0.152643496 | 0.000935543 |
| MLLT4 | blue | 0.139257361 | 0.002561161 | 0.100849723 | 0.029324348 |
| MOB1B | blue | -0.045148455 | 0.330280961 | -0.078317046 | 0.09093094 |
| MOB2 | blue | 0.02137004 | 0.645067354 | 0.036547186 | 0.430732958 |
| MOB3B | blue | -0.247024433 | 6.37E-08 | -0.255508576 | 2.14E-08 |
| MON1A | blue | 0.225324257 | 8.67E-07 | 0.254812585 | 2.35E-08 |
| MON2 | blue | -0.056250383 | 0.225021202 | -0.084795556 | 0.067125153 |
| MORC3 | blue | -0.109487383 | 0.01794162 | -0.132791587 | 0.004044105 |
| MOSPD1 | blue | -0.144467186 | 0.001747881 | -0.166428335 | 0.000303692 |
| MOSPD2 | blue | 0.120814127 | 0.008964699 | 0.110352221 | 0.017049859 |
| MPDZ | blue | -0.097979274 | 0.034279285 | -0.110046719 | 0.017360341 |
| MPHOSP | blue | 0.055018203 | 0.235359377 | 0.041410379 | 0.371926453 |
| MPP7 | blue | -0.06535457 | 0.158525797 | -0.083194146 | 0.072472685 |
| MPST | blue | 0.246110192 | 7.15E-08 | 0.259050582 | 1.34E-08 |
| MRPL2 | blue | -0.183348158 | 6.74E-05 | -0.158210967 | 0.000600384 |
| MRPL43 | blue | 0.261302242 | 9.95E-09 | 0.266551253 | 4.89E-09 |
| MRPL55 | blue | 0.172207806 | 0.00018443 | 0.199557476 | 1.39E-05 |

| | | | | | |
|---|---|---|---|---|---|
| MRPS2 | blue | 0.10985849 | 0.01755409 | 0.117014216 | 0.01138609 |
| MSANTD | blue | -0.05415079 | 0.242835234 | -0.066176099 | 0.153346404 |
| MSANTD | blue | -0.198541206 | 1.55E-05 | -0.216578239 | 2.31E-06 |
| MSI2 | blue | 0.14100586 | 0.002256092 | 0.116994778 | 0.011399836 |
| MSL2 | blue | -0.177579065 | 0.000114349 | -0.202517155 | 1.03E-05 |
| MSRB1 | blue | 0.022306833 | 0.630642529 | 0.051254712 | 0.268994113 |
| MT1X | blue | 0.095215242 | 0.039707458 | 0.088256105 | 0.056671019 |
| MT2A | blue | 0.107723632 | 0.019887269 | 0.121771479 | 0.008432056 |
| MTCP1 | blue | -0.077688989 | 0.093560252 | -0.068804467 | 0.137635983 |
| MTM1 | blue | -0.181997814 | 7.64E-05 | -0.19454376 | 2.30E-05 |
| MTMR10 | blue | -0.090807963 | 0.049859069 | -0.133355963 | 0.003888989 |
| MTMR3 | blue | -0.109536067 | 0.017890359 | -0.124356565 | 0.00713199 |
| MTMR6 | blue | 0.156113494 | 0.000710801 | 0.128062788 | 0.005580493 |
| MTR | blue | -0.035761529 | 0.440713623 | -0.060655356 | 0.190715467 |
| MTRR | blue | -0.2411595 | 1.32E-07 | -0.26728508 | 4.42E-09 |
| MTURN | blue | -0.057071267 | 0.21831584 | -0.073962121 | 0.110435582 |
| MTUS1 | blue | 0.270511615 | 2.83E-09 | 0.239420029 | 1.64E-07 |
| MTX3 | blue | -0.126953034 | 0.006009735 | -0.153894392 | 0.000847882 |
| MXD1 | blue | 0.02788695 | 0.547744113 | 0.006953893 | 0.880864039 |
| MXI1 | blue | -0.099535317 | 0.031511354 | -0.126783269 | 0.006077952 |
| MYEF2 | blue | 0.04533402 | 0.328295151 | 0.010370778 | 0.823130977 |
| MYLIP | blue | -0.096690754 | 0.036725602 | -0.099973544 | 0.030767193 |
| MYO6 | blue | 0.096998468 | 0.036128306 | 0.086260691 | 0.062520455 |
| MYO9A | blue | -0.113130587 | 0.014441969 | -0.135372565 | 0.003377756 |
| MYRIP | blue | 0.001020747 | 0.982448427 | 0.005369966 | 0.907861951 |
| N4BP2 | blue | 0.039090095 | 0.399338985 | 0.015805451 | 0.733356094 |
| N6AMT1 | blue | -0.017578519 | 0.704772346 | -0.031673474 | 0.494729091 |
| NAA60 | blue | 0.227849145 | 6.48E-07 | 0.268529738 | 3.72E-09 |
| NAALAD | blue | 0.019879298 | 0.668297096 | 0.011969787 | 0.796418249 |
| NAB1 | blue | -0.230894851 | 4.54E-07 | -0.246118036 | 7.14E-08 |
| NAGLU | blue | 0.185211281 | 5.66E-05 | 0.193768698 | 2.48E-05 |
| NAGPA | blue | 0.022949995 | 0.620819235 | 0.029034025 | 0.531392966 |
| NAPEPLI | blue | 0.096871689 | 0.036373386 | 0.094197668 | 0.041881114 |
| NAPG | blue | -0.051783453 | 0.264079732 | -0.086330078 | 0.062309008 |
| NBPF1 | blue | -0.05662225 | 0.221965668 | -0.07166839 | 0.121958358 |
| NBPF10 | blue | -0.040865627 | 0.378255543 | -0.056826671 | 0.220298666 |
| NBPF11 | blue | -0.021895981 | 0.636952025 | -0.059389917 | 0.20015206 |
| NBPF12 | blue | -0.059463185 | 0.199596583 | -0.08726308 | 0.059522629 |
| NBPF14 | blue | -0.124019065 | 0.007290865 | -0.145191845 | 0.001655771 |
| NBPF15 | blue | 0.264285453 | 6.66E-09 | 0.226888159 | 7.24E-07 |
| NBPF19 | blue | -0.052738632 | 0.255359062 | -0.076816587 | 0.097312972 |
| NBPF8 | blue | 0.021816521 | 0.638175358 | -0.003280306 | 0.943637931 |
| NBR1 | blue | 0.114043176 | 0.013665457 | 0.099298999 | 0.031918999 |
| NCKAP1 | blue | 0.006884861 | 0.882038046 | -0.01440099 | 0.756264332 |
| NCKAP5 | blue | 0.013729128 | 0.767300491 | -0.001204912 | 0.979282388 |

| | | | | | |
|---|---|---|---|---|---|
| NCOA3 | blue | -0.039891762 | 0.389733725 | -0.049935582 | 0.281526286 |
| NCOA7 | blue | -0.139536607 | 0.002510044 | -0.153115696 | 0.000901513 |
| NDUFAF | blue | 0.117713654 | 0.010901111 | 0.141493517 | 0.002177137 |
| NEK1 | blue | 0.04881514 | 0.292476366 | 0.014077295 | 0.761575389 |
| NEK7 | blue | -0.015704611 | 0.734993312 | -0.020355474 | 0.660841168 |
| NEK9 | blue | -0.072456189 | 0.117899554 | -0.091013148 | 0.049342418 |
| NEMF | blue | -0.033931252 | 0.46447008 | -0.059502121 | 0.199301854 |
| NEU3 | blue | -0.052832779 | 0.25451044 | -0.090999707 | 0.049376124 |
| NF1 | blue | 0.001282554 | 0.977947723 | -0.026708899 | 0.564790882 |
| NFE2L2 | blue | -0.044300757 | 0.339450861 | -0.057857528 | 0.21202864 |
| NFIA | blue | 0.246932122 | 6.45E-08 | 0.253483142 | 2.79E-08 |
| NFKB2 | blue | 0.00595121 | 0.897940683 | 0.008573574 | 0.853398964 |
| NFKBIB | blue | 0.084722324 | 0.067362425 | 0.11541806 | 0.012564967 |
| NFXL1 | blue | 0.072335602 | 0.118513885 | 0.03882896 | 0.402498189 |
| NHLRC2 | blue | 0.056315438 | 0.224484514 | 0.037011986 | 0.424890397 |
| NHLRC3 | blue | 0.000752778 | 0.987055624 | -0.026948488 | 0.561303439 |
| NHS | blue | 0.07566835 | 0.102433948 | 0.06010766 | 0.194758863 |
| NIN | blue | 0.065442949 | 0.157962346 | 0.041705858 | 0.368521034 |
| NIPAL1 | blue | 0.12868294 | 0.005352823 | 0.098063503 | 0.034124323 |
| NIPBL | blue | -0.197962944 | 1.64E-05 | -0.229086732 | 5.61E-07 |
| NME4 | blue | 0.258667703 | 1.41E-08 | 0.259871292 | 1.20E-08 |
| NPAT | blue | -0.041961473 | 0.365590704 | -0.071318242 | 0.123796936 |
| NPM3 | blue | 0.126006132 | 0.006399184 | 0.116694158 | 0.01161431 |
| NPRL3 | blue | 0.197773934 | 1.67E-05 | 0.210636035 | 4.41E-06 |
| NR1D2 | blue | -0.199488568 | 1.40E-05 | -0.220921912 | 1.43E-06 |
| NR2C2 | blue | -0.054518904 | 0.239642543 | -0.088074392 | 0.057184194 |
| NRDE2 | blue | -0.05678621 | 0.220627905 | -0.07789855 | 0.092676265 |
| NRIP1 | blue | 0.132272962 | 0.00419156 | 0.104932134 | 0.023342051 |
| NSDHL | blue | 0.03714887 | 0.423178593 | 0.046361836 | 0.317436166 |
| NSMAF | blue | -0.087842675 | 0.057844166 | -0.117793719 | 0.01084678 |
| NSMCE1 | blue | 0.087656195 | 0.058379865 | 0.115094298 | 0.012816823 |
| NSUN3 | blue | 0.00176701 | 0.969621431 | -0.007320234 | 0.874638228 |
| NUBPL | blue | -0.086615852 | 0.06144435 | -0.122096512 | 0.008257741 |
| NUDT14 | blue | 0.019020631 | 0.681824415 | 0.042164966 | 0.363268292 |
| NUDT22 | blue | 0.228534557 | 5.99E-07 | 0.249939658 | 4.40E-08 |
| NUDT8 | blue | 0.220963613 | 1.42E-06 | 0.257487185 | 1.65E-08 |
| NUFIP2 | blue | -0.012596621 | 0.786009166 | -0.032744935 | 0.480239787 |
| OAT | blue | -0.05449113 | 0.239882395 | -0.10616669 | 0.02175483 |
| OCRL | blue | -0.026048795 | 0.574452709 | -0.073131192 | 0.114506591 |
| OGFOD3 | blue | 0.071564586 | 0.122501185 | 0.062798147 | 0.175487666 |
| OGFRL1 | blue | -0.065443432 | 0.157959271 | -0.082164311 | 0.07609169 |
| OPHN1 | blue | 0.02627035 | 0.571201164 | -0.030647841 | 0.508813891 |
| OR2A7 | blue | 0.009245492 | 0.842054999 | -0.017873897 | 0.700049389 |
| OSBPL8 | blue | -0.055585416 | 0.230559486 | -0.069333143 | 0.134630908 |
| OSBPL9 | blue | -0.198619334 | 1.53E-05 | -0.20964596 | 4.90E-06 |

| | | | | | |
|---|---|---|---|---|---|
| OST4 | blue | 0.049066242 | 0.289997894 | 0.077751267 | 0.093296842 |
| OSTM1 | blue | 0.027216946 | 0.557408152 | 0.021754024 | 0.63913821 |
| OTUD1 | blue | -0.337571688 | 6.55E-14 | -0.353789808 | 3.24E-15 |
| OTUD3 | blue | -0.044511936 | 0.337151372 | -0.076436965 | 0.098982965 |
| OTUD4 | blue | -0.096630253 | 0.036844019 | -0.116982232 | 0.011408717 |
| OXR1 | blue | -0.011861735 | 0.798216246 | -0.059894553 | 0.196348962 |
| P4HB | blue | 0.280489442 | 6.85E-10 | 0.269692871 | 3.17E-09 |
| PABPC4I | blue | -0.223439359 | 1.07E-06 | -0.230443805 | 4.79E-07 |
| PAIP2B | blue | -0.236347758 | 2.38E-07 | -0.256435356 | 1.90E-08 |
| PAPD4 | blue | 0.081888619 | 0.077085057 | 0.057983578 | 0.21103296 |
| PAPOLG | blue | -0.177962057 | 0.000110457 | -0.192639972 | 2.78E-05 |
| PAQR3 | blue | 0.02992181 | 0.518909483 | 0.002316185 | 0.960186847 |
| PARD6G | blue | -0.065245781 | 0.159221443 | -0.073671542 | 0.111846072 |
| PARP10 | blue | -0.005442258 | 0.906627183 | 0.00853235 | 0.854095954 |
| PARP11 | blue | -0.222306989 | 1.22E-06 | -0.227886811 | 6.45E-07 |
| PARP8 | blue | -0.018123133 | 0.696073187 | -0.014303751 | 0.757858586 |
| PAWR | blue | -0.084744342 | 0.067291013 | -0.11880411 | 0.01018147 |
| PBX1 | blue | -0.125462117 | 0.006633031 | -0.143708806 | 0.001849287 |
| PCBD2 | blue | -0.00455618 | 0.921776243 | -0.006800584 | 0.883471671 |
| PCBP4 | blue | 0.075023585 | 0.105401861 | 0.088873985 | 0.054954571 |
| PCGF5 | blue | -0.019274841 | 0.677808744 | -0.019600582 | 0.67267646 |
| PCNXL4 | blue | 0.013903976 | 0.764423769 | -0.01326605 | 0.774934763 |
| PCYOX1 | blue | -0.044335463 | 0.339072265 | -0.072863747 | 0.115841651 |
| PDCD4 | blue | 0.000787283 | 0.986462349 | -0.009169865 | 0.843330219 |
| PDCD6IP | blue | 0.082663142 | 0.074320778 | 0.066343787 | 0.152305148 |
| PDE3A | blue | -0.022128696 | 0.633374942 | -0.012755778 | 0.783372213 |
| PDE4D | blue | 0.07515749 | 0.104779955 | 0.055885359 | 0.228049522 |
| PDE8A | blue | 0.012160457 | 0.793248102 | 0.004900132 | 0.915892105 |
| PDGFC | blue | -0.077292752 | 0.095250105 | -0.082146311 | 0.076156228 |
| PDLIM5 | blue | 0.128570455 | 0.005393487 | 0.145802725 | 0.001581605 |
| PDP1 | blue | 0.091234283 | 0.048790626 | 0.066349428 | 0.152270215 |
| PDPK1 | blue | -0.006270398 | 0.892499101 | -0.008890369 | 0.848046607 |
| PDPR | blue | -0.060191236 | 0.194137839 | -0.073857634 | 0.11094115 |
| PDS5B | blue | -0.156102461 | 0.000711428 | -0.184554276 | 6.02E-05 |
| PDXK | blue | -0.034214373 | 0.460749449 | -0.008470185 | 0.855147215 |
| PELI1 | blue | -0.050783706 | 0.27342427 | -0.060692959 | 0.190440142 |
| PELI2 | blue | -0.376965728 | 3.24E-17 | -0.382446664 | 1.03E-17 |
| PEX1 | blue | 0.141002233 | 0.002256689 | 0.103716612 | 0.025001726 |
| PEX12 | blue | -0.015982982 | 0.730476669 | -0.028411063 | 0.540242388 |
| PEX14 | blue | 0.011033398 | 0.812034665 | 0.017947055 | 0.698881395 |
| PEX16 | blue | 0.12183145 | 0.008399649 | 0.14662041 | 0.0014871 |
| PFKL | blue | 0.204432097 | 8.46E-06 | 0.229235453 | 5.52E-07 |
| PFN1 | blue | 0.249945115 | 4.40E-08 | 0.289661126 | 1.77E-10 |
| PGAP1 | blue | -0.068683675 | 0.138329763 | -0.100904179 | 0.029236622 |
| PGBD4 | blue | -0.009008658 | 0.846049853 | -0.004018682 | 0.930979885 |

| | | | | | |
|---|---|---|---|---|---|
| PGM2L1 | blue | 0.047510137 | 0.305585084 | 0.055451402 | 0.231687234 |
| PHACTR: | blue | -0.156039311 | 0.00071503 | -0.142778344 | 0.001981052 |
| PHF20L1 | blue | -0.100344584 | 0.030148931 | -0.127244404 | 0.005894256 |
| PHF21A | blue | -0.119755588 | 0.009588323 | -0.153544363 | 0.000871614 |
| PHF3 | blue | -0.173169767 | 0.000169472 | -0.203133843 | 9.68E-06 |
| PHIP | blue | -0.11067362 | 0.016728474 | -0.118866935 | 0.010141324 |
| PHKB | blue | 0.369955146 | 1.36E-16 | 0.372562279 | 7.99E-17 |
| PIAS1 | blue | -0.106302738 | 0.021585782 | -0.114925831 | 0.012949628 |
| PICALM | blue | -0.046830843 | 0.312559958 | -0.059976966 | 0.195732912 |
| PIGQ | blue | 0.216118222 | 2.43E-06 | 0.236597297 | 2.31E-07 |
| PIK3C2A | blue | 0.030073564 | 0.51679083 | -9.79E-05 | 0.998316804 |
| PIK3C3 | blue | -0.157574109 | 0.000632101 | -0.18498592 | 5.78E-05 |
| PIKFYVE | blue | 0.004131422 | 0.929048617 | -0.023359405 | 0.614600916 |
| PIN1 | blue | 0.063580109 | 0.170161929 | 0.085812906 | 0.063899299 |
| PJA2 | blue | 0.032682944 | 0.481071762 | 0.012637844 | 0.785325947 |
| PKN2 | blue | -0.094959849 | 0.040243834 | -0.128704732 | 0.005344977 |
| PLA2G4E | blue | 0.050602376 | 0.275143 | 0.017523556 | 0.705652437 |
| PLAGL1 | blue | -0.331090477 | 2.07E-13 | -0.348798082 | 8.33E-15 |
| PLCB1 | blue | 0.096220286 | 0.037655012 | 0.069532276 | 0.133512203 |
| PLCB4 | blue | 0.206082316 | 7.13E-06 | 0.175315478 | 0.000140108 |
| PLCE1 | blue | 0.207568243 | 6.10E-06 | 0.204323891 | 8.56E-06 |
| PLCL2 | blue | -0.059722353 | 0.197640738 | -0.083737386 | 0.070620988 |
| PLD1 | blue | -0.199328575 | 1.43E-05 | -0.19469938 | 2.27E-05 |
| PLEKHA | blue | 0.150896915 | 0.001071986 | 0.111026568 | 0.01638166 |
| PLEKHA: | blue | -0.147705068 | 0.001369737 | -0.154780855 | 0.000790428 |
| PLEKHA: | blue | 0.186002358 | 5.25E-05 | 0.1541408 | 0.000831536 |
| PLEKHF1 | blue | -0.309917754 | 7.45E-12 | -0.260719954 | 1.08E-08 |
| PLEKHG | blue | 0.021214872 | 0.647469752 | 0.028163095 | 0.543785201 |
| PLOD3 | blue | 0.026240081 | 0.57164487 | 0.0285438 | 0.538350664 |
| PM20D2 | blue | -0.241105022 | 1.33E-07 | -0.266673013 | 4.81E-09 |
| PNRC1 | blue | -0.248053614 | 5.60E-08 | -0.246321022 | 6.96E-08 |
| POGLUT | blue | -0.206388768 | 6.90E-06 | -0.263462946 | 7.44E-09 |
| POLK | blue | 0.109534756 | 0.017891738 | 0.086429979 | 0.062005608 |
| POLR2M | blue | -0.067239797 | 0.146832088 | -0.090394127 | 0.050914863 |
| POU2F1 | blue | -0.074238229 | 0.10910835 | -0.097375364 | 0.035407991 |
| PPDPF | blue | 0.067594882 | 0.144705103 | 0.095192095 | 0.03975582 |
| PPFIBP1 | blue | -0.23539486 | 2.66E-07 | -0.26684475 | 4.69E-09 |
| PPIP5K2 | blue | 0.029414022 | 0.526031159 | -0.001057111 | 0.981823246 |
| PPM1A | blue | -0.036162193 | 0.435607325 | -0.059845182 | 0.196718686 |
| PPM1D | blue | -0.048258449 | 0.298021554 | -0.064259855 | 0.165631257 |
| PPM1L | blue | 0.105272921 | 0.022894203 | 0.097231152 | 0.03568215 |
| PPP1R12 | blue | -0.113939907 | 0.013751442 | -0.138066054 | 0.002790066 |
| PPP1R21 | blue | -0.200065834 | 1.32E-05 | -0.223618868 | 1.05E-06 |
| PPP1R3B | blue | 0.187922088 | 4.38E-05 | 0.159176115 | 0.000555121 |
| PPP1R3D | blue | 0.054955981 | 0.235890173 | 0.040921669 | 0.377601399 |

| | | | | | |
|---|---|---|---|---|---|
| PPP1R9A | blue | 0.056985108 | 0.219012827 | 0.029481826 | 0.525077337 |
| PPP2R1A | blue | 0.187230457 | 4.68E-05 | 0.204465782 | 8.43E-06 |
| PPP2R3A | blue | -0.289361276 | 1.85E-10 | -0.311708739 | 5.56E-12 |
| PPP2R3B | blue | -0.009808589 | 0.832573264 | -0.006458945 | 0.889287081 |
| PPP3CA | blue | -0.009840198 | 0.83204172 | -0.03069414 | 0.508173594 |
| PPP3CB | blue | -0.012806503 | 0.782532308 | -0.038444624 | 0.407174972 |
| PPP4R1 | blue | -0.110914926 | 0.016490675 | -0.126664622 | 0.006126039 |
| PPP6R3 | blue | 0.044547284 | 0.336767447 | 0.011747812 | 0.8001131 |
| PQLC3 | blue | -0.089769442 | 0.052543885 | -0.057912331 | 0.211595332 |
| PRDM2 | blue | -0.119820323 | 0.009549107 | -0.157387919 | 0.000641663 |
| PREPL | blue | -0.061553927 | 0.184215716 | -0.074326629 | 0.108686083 |
| PREX2 | blue | 0.048482499 | 0.295781433 | 0.033332108 | 0.472398374 |
| PRKAA1 | blue | -0.086473124 | 0.061874952 | -0.105774991 | 0.022247967 |
| PRKAA2 | blue | -0.152196648 | 0.000968835 | -0.161799564 | 0.000447595 |
| PRKAB2 | blue | 0.01102624 | 0.81215435 | -0.028332417 | 0.541364785 |
| PRKACB | blue | -0.070872308 | 0.126169611 | -0.089337335 | 0.05369594 |
| PRKAR1A | blue | -0.034337733 | 0.459133535 | -0.058615726 | 0.206090369 |
| PRKCSH | blue | -0.079711077 | 0.085306485 | -0.086879113 | 0.060656599 |
| PRKD2 | blue | -0.008189304 | 0.859900338 | 0.03910575 | 0.399150064 |
| PRKD3 | blue | -0.15554312 | 0.000743926 | -0.172871714 | 0.00017398 |
| PRKRIR | blue | -0.156312896 | 0.000699546 | -0.191197596 | 3.19E-05 |
| PRMT1 | blue | 0.023533672 | 0.611962381 | 0.032702385 | 0.480810764 |
| PRMT9 | blue | 0.016131938 | 0.728063639 | -0.006800681 | 0.88347002 |
| PRPF4B | blue | -0.069852528 | 0.131728121 | -0.099518751 | 0.031539785 |
| PRPF6 | blue | -0.215798843 | 2.52E-06 | -0.199730604 | 1.37E-05 |
| PRRG1 | blue | -0.289509169 | 1.81E-10 | -0.311240446 | 6.00E-12 |
| PRTG | blue | -0.103753285 | 0.024950201 | -0.124459287 | 0.007084254 |
| PSD3 | blue | -0.067687764 | 0.144152636 | -0.101486022 | 0.028313252 |
| PTAR1 | blue | -0.027673478 | 0.550814223 | -0.047415754 | 0.30654798 |
| PTCD1 | blue | 0.093293779 | 0.0438953 | 0.097631149 | 0.034926127 |
| PTDSS2 | blue | 0.01136417 | 0.806509438 | 0.008710438 | 0.851085753 |
| PTEN | blue | 0.004767628 | 0.918158394 | 0.008377183 | 0.856720439 |
| PTPDC1 | blue | -0.000189576 | 0.996740021 | -0.040196072 | 0.386124589 |
| PTPN12 | blue | 0.089650392 | 0.052859215 | 0.074626203 | 0.107264664 |
| PTPN13 | blue | -0.257777369 | 1.59E-08 | -0.266791127 | 4.73E-09 |
| PTPN4 | blue | 0.062009605 | 0.180982748 | 0.034685523 | 0.454594842 |
| PTPRG | blue | 0.010243798 | 0.825261503 | -0.003382858 | 0.94187895 |
| PUM2 | blue | -0.046663749 | 0.314291542 | -0.0778421 | 0.092913728 |
| PURA | blue | -0.05868671 | 0.205540644 | -0.052500244 | 0.257516597 |
| PUS1 | blue | 0.078978412 | 0.088226552 | 0.084772073 | 0.067201162 |
| PUS7L | blue | 0.013515149 | 0.770825386 | -0.013855254 | 0.76522505 |
| PUSL1 | blue | 0.086161577 | 0.062823519 | 0.11461333 | 0.013199191 |
| PVRL2 | blue | -0.080477288 | 0.08233655 | -0.06884869 | 0.137382655 |
| PWWP2A | blue | -0.007371125 | 0.873773959 | -0.040636203 | 0.380940729 |
| PWWP2B | blue | 0.115112493 | 0.012802552 | 0.125721275 | 0.006520693 |

| | | | | | |
|---|---|---|---|---|---|
| PXK | blue | -0.132948767 | 0.004000352 | -0.15745469 | 0.000638219 |
| PYGO1 | blue | -0.177022627 | 0.000120232 | -0.198032093 | 1.63E-05 |
| QARS | blue | 0.182456264 | 7.32E-05 | 0.176119998 | 0.000130385 |
| QKI | blue | -0.239974556 | 1.53E-07 | -0.24692087 | 6.46E-08 |
| QSER1 | blue | 0.001783111 | 0.969344751 | -0.01487423 | 0.748520251 |
| QTRT1 | blue | -0.043510993 | 0.348139081 | -0.048033894 | 0.300278019 |
| R3HDM2 | blue | -0.100443289 | 0.029986263 | -0.146382195 | 0.001514082 |
| RAB11FI | blue | -0.000332238 | 0.994286817 | -0.057361937 | 0.215976165 |
| RAB2B | blue | -0.099006844 | 0.032429183 | -0.112602289 | 0.014908999 |
| RAB30 | blue | 0.019327497 | 0.676978086 | -0.006479144 | 0.888943077 |
| RAB33B | blue | -0.015903298 | 0.731768619 | -0.034665914 | 0.454850069 |
| RAB3GA | blue | -0.152584124 | 0.000939905 | -0.174572878 | 0.000149682 |
| RAB8B | blue | -0.125864249 | 0.00645945 | -0.140747217 | 0.002299022 |
| RABAC1 | blue | -0.124522575 | 0.007054986 | -0.103790213 | 0.024898411 |
| RABEP1 | blue | 0.097565755 | 0.035048786 | 0.070732847 | 0.126918844 |
| RABGAP | blue | 0.018932705 | 0.683215445 | -0.015915919 | 0.731563942 |
| RABGGT | blue | 0.172699625 | 0.000176634 | 0.182948745 | 6.99E-05 |
| RAD50 | blue | -0.014500223 | 0.754638433 | -0.036667745 | 0.429213078 |
| RAD51B | blue | 0.086738913 | 0.061075073 | 0.050561483 | 0.275531616 |
| RAD9B | blue | -0.256016873 | 2.01E-08 | -0.264603246 | 6.37E-09 |
| RAG1 | blue | -0.098006999 | 0.034228211 | -0.119188708 | 0.009937901 |
| RAI14 | blue | -0.216801731 | 2.26E-06 | -0.221327759 | 1.36E-06 |
| RALGAP | blue | 0.04507691 | 0.331048657 | 0.009160901 | 0.843481405 |
| RALGPS2 | blue | -0.1055791 | 0.022498201 | -0.112148582 | 0.015320622 |
| RALY | blue | -0.040846588 | 0.378477928 | -0.021204702 | 0.647627324 |
| RANBP2 | blue | 0.091105915 | 0.049110307 | 0.044791615 | 0.334121384 |
| RANBP6 | blue | 0.033790795 | 0.466322049 | 0.028527971 | 0.538576073 |
| RAP1B | blue | -0.085272076 | 0.065597924 | -0.088858793 | 0.054996249 |
| RAP2C | blue | -0.155130069 | 0.0007688 | -0.16075125 | 0.000487999 |
| RAPGEF2 | blue | 0.079534089 | 0.086004642 | 0.045991236 | 0.321324223 |
| RAPGEF5 | blue | -0.206272068 | 6.99E-06 | -0.209523244 | 4.96E-06 |
| RAPGEF6 | blue | -0.072738978 | 0.116468638 | -0.081645362 | 0.077970283 |
| RARB | blue | -0.141058194 | 0.002247496 | -0.12737944 | 0.005841416 |
| RASSF3 | blue | -0.018007168 | 0.697922204 | -0.040304539 | 0.384843094 |
| RASSF9 | blue | -0.231892514 | 4.04E-07 | -0.213752998 | 3.15E-06 |
| RAVER2 | blue | 0.146885785 | 0.001457561 | 0.125258261 | 0.006722614 |
| RBAK | blue | -0.019418662 | 0.675540887 | -0.027530905 | 0.552869361 |
| RBCK1 | blue | -0.104994619 | 0.023259373 | -0.072220162 | 0.119104343 |
| RBM10 | blue | -0.017179972 | 0.711162932 | -0.008344155 | 0.857279285 |
| RBM12B | blue | -0.140894826 | 0.002274431 | -0.17326044 | 0.000168123 |
| RBM26 | blue | -0.018931198 | 0.683239289 | -0.045550637 | 0.325986826 |
| RBM41 | blue | -0.040849948 | 0.378438679 | -0.055291518 | 0.233037793 |
| RBM42 | blue | -0.173080868 | 0.000170805 | -0.152647855 | 0.000935223 |
| RBM43 | blue | -0.109568273 | 0.01785652 | -0.123395225 | 0.007592885 |
| RBM48 | blue | -0.167600374 | 0.000274835 | -0.189375063 | 3.81E-05 |

| | | | | | |
|---|---|---|---|---|---|
| RBMS1 | blue | -0.388695113 | 2.72E-18 | -0.385211873 | 5.73E-18 |
| RBMXL1 | blue | -0.110392672 | 0.017009116 | -0.127243512 | 0.005894607 |
| RBPJ | blue | 0.101536926 | 0.028233674 | 0.094374822 | 0.041495631 |
| RCBTB1 | blue | 0.14198441 | 0.002100216 | 0.123689893 | 0.007448861 |
| RCHY1 | blue | 0.054217942 | 0.242250607 | 0.036352417 | 0.43319497 |
| REV1 | blue | -0.027468841 | 0.553765171 | -0.065012095 | 0.160723528 |
| REV3L | blue | 0.013175919 | 0.776423201 | -0.031975417 | 0.490622513 |
| RFX7 | blue | 0.109276534 | 0.018165111 | 0.095462699 | 0.039193529 |
| RFXANK | blue | -0.068744916 | 0.137977684 | -0.052366618 | 0.258731483 |
| RHBDD2 | blue | 0.091362945 | 0.048471962 | 0.141993887 | 0.002098756 |
| RHOBTB | blue | 0.004669828 | 0.919831524 | 0.000420194 | 0.992774343 |
| RHOBTB | blue | -0.170859642 | 0.000207483 | -0.207030113 | 6.45E-06 |
| RHOG | blue | 0.10725137 | 0.020438428 | 0.149344033 | 0.001208471 |
| RHOQ | blue | -0.342284419 | 2.78E-14 | -0.355956443 | 2.14E-15 |
| RHOU | blue | 0.000720372 | 0.987612815 | -0.013987457 | 0.763051416 |
| RIC1 | blue | -0.098848023 | 0.03270944 | -0.108699625 | 0.018789074 |
| RIC8B | blue | -0.083798712 | 0.070414404 | -0.110362653 | 0.017039344 |
| RICTOR | blue | -0.299373311 | 4.00E-11 | -0.312109605 | 5.21E-12 |
| RIF1 | blue | -0.201000403 | 1.20E-05 | -0.23108761 | 4.44E-07 |
| RINT1 | blue | 0.042857864 | 0.355429647 | 0.010023862 | 0.828954712 |
| RLF | blue | -0.115161847 | 0.012763912 | -0.145272619 | 0.001645785 |
| RLIM | blue | -0.062453754 | 0.177872172 | -0.069423114 | 0.134124567 |
| RMDN2 | blue | -0.259835952 | 1.21E-08 | -0.273545402 | 1.85E-09 |
| RMND5A | blue | -0.045820257 | 0.323128416 | -0.05996378 | 0.195831384 |
| RNASEL | blue | 0.057655304 | 0.213633089 | 0.067983802 | 0.142402533 |
| RND3 | blue | -0.196962995 | 1.81E-05 | -0.212068025 | 3.78E-06 |
| RNF103 | blue | 0.016884183 | 0.715918949 | 0.009961259 | 0.830006637 |
| RNF11 | blue | -0.095657661 | 0.038792599 | -0.110924192 | 0.016481602 |
| RNF125 | blue | -0.026385399 | 0.569516149 | -0.01566548 | 0.735628957 |
| RNF13 | blue | -0.061011374 | 0.188120391 | -0.086850636 | 0.060741406 |
| RNF141 | blue | 0.157210328 | 0.000650909 | 0.136780224 | 0.003057906 |
| RNF146 | blue | -0.192499776 | 2.81E-05 | -0.214253567 | 2.98E-06 |
| RNF150 | blue | -0.321690624 | 1.05E-12 | -0.314500353 | 3.51E-12 |
| RNF170 | blue | -0.030114889 | 0.516214653 | -0.05664489 | 0.221780602 |
| RNF180 | blue | -0.026433117 | 0.568817975 | -0.027672907 | 0.550822438 |
| RNF187 | blue | 0.113131075 | 0.014441544 | 0.133232614 | 0.003922423 |
| RNF19A | blue | -0.19011437 | 3.55E-05 | -0.206775196 | 6.63E-06 |
| RNF208 | blue | 0.194148201 | 2.39E-05 | 0.216200345 | 2.41E-06 |
| RNF217 | blue | -0.304334585 | 1.83E-11 | -0.304050379 | 1.91E-11 |
| RNPEPL1 | blue | 0.239472554 | 1.63E-07 | 0.280814262 | 6.54E-10 |
| ROCK1 | blue | -0.083345162 | 0.071953995 | -0.120553061 | 0.009115052 |
| ROCK2 | blue | -0.087584927 | 0.058585681 | -0.114900624 | 0.012969602 |
| ROR1 | blue | -0.027417308 | 0.554509501 | -0.037049677 | 0.424418648 |
| RORA | blue | -0.062612289 | 0.176771536 | -0.084677704 | 0.067507329 |
| RP11-111 | blue | -0.087711598 | 0.058220284 | -0.103766171 | 0.024932118 |

| | | | | | |
|---|---|---|---|---|---|
| RP2 | blue | -0.001018715 | 0.982483361 | -0.000979271 | 0.98316148 |
| RPAP2 | blue | -0.00191352 | 0.967103972 | -0.02407896 | 0.603738804 |
| RPL8 | blue | 0.004414149 | 0.92420729 | -0.009875522 | 0.831447793 |
| RPP14 | blue | -0.080426667 | 0.082530151 | -0.093180878 | 0.044152521 |
| RPRD1B | blue | -0.2484331 | 5.33E-08 | -0.289456327 | 1.82E-10 |
| RPS21 | blue | -0.13203578 | 0.004260601 | -0.122346187 | 0.00812603 |
| RPS6KA3 | blue | -0.129652256 | 0.005013683 | -0.134794869 | 0.003517589 |
| RPS6KA4 | blue | 0.039103027 | 0.399182918 | 0.060072676 | 0.195019249 |
| RPS6KA5 | blue | 0.142016573 | 0.002095264 | 0.113508937 | 0.014115448 |
| RRAGB | blue | -0.247031906 | 6.37E-08 | -0.277303019 | 1.08E-09 |
| RRP9 | blue | 0.057920182 | 0.211533306 | 0.059981312 | 0.195700468 |
| RSBN1 | blue | -0.091504171 | 0.048124186 | -0.114310771 | 0.013444845 |
| RSBN1L | blue | -0.019312943 | 0.677207646 | -0.032766767 | 0.479946975 |
| RSF1 | blue | -0.135065073 | 0.003451545 | -0.175748943 | 0.000134788 |
| RSPRY1 | blue | 0.280082427 | 7.27E-10 | 0.280752906 | 6.60E-10 |
| RTN4 | blue | -0.046141205 | 0.319747142 | -0.05333284 | 0.25003579 |
| RTTN | blue | 0.106165316 | 0.021756543 | 0.088096864 | 0.057120522 |
| RUFY2 | blue | 0.028122252 | 0.544369853 | -0.008069916 | 0.861922176 |
| RUFY3 | blue | -0.283295187 | 4.55E-10 | -0.298367071 | 4.67E-11 |
| RYBP | blue | 0.065880409 | 0.155195621 | 0.021778804 | 0.638756371 |
| RYK | blue | -0.168788935 | 0.000248203 | -0.211349375 | 4.08E-06 |
| S100PBP | blue | -0.118609159 | 0.010306946 | -0.14350109 | 0.001877986 |
| SACM1L | blue | 0.037902399 | 0.41382767 | 0.009202476 | 0.842780293 |
| SACS | blue | -0.08323712 | 0.072324774 | -0.112010489 | 0.01544787 |
| SAMD8 | blue | -0.071930841 | 0.120594263 | -0.095745503 | 0.038613093 |
| SART1 | blue | -0.130016711 | 0.004891264 | -0.124389539 | 0.007116636 |
| SASH1 | blue | -0.179675809 | 9.45E-05 | -0.193122786 | 2.65E-05 |
| SBF2 | blue | 0.075977932 | 0.101032661 | 0.039812768 | 0.390673927 |
| SCAF11 | blue | -0.060541892 | 0.191548022 | -0.088500528 | 0.055986777 |
| SCAF8 | blue | -0.115302274 | 0.012654532 | -0.153093565 | 0.000903082 |
| SCAI | blue | -0.11930457 | 0.009865546 | -0.145820354 | 0.001579511 |
| SCAMP1 | blue | 0.08487167 | 0.066879271 | 0.056684984 | 0.221453135 |
| SCAPER | blue | -0.031771366 | 0.49339571 | -0.061468457 | 0.184826828 |
| SCO2 | blue | 0.202837027 | 9.98E-06 | 0.228210093 | 6.22E-07 |
| SCRN3 | blue | 0.083120293 | 0.072727453 | 0.077304621 | 0.095199135 |
| SDC2 | blue | -0.059943909 | 0.19597985 | -0.08538005 | 0.06525587 |
| SDSL | blue | 0.1751882 | 0.000141707 | 0.205577451 | 7.51E-06 |
| SEC23A | blue | -0.00580545 | 0.900427234 | -0.018701889 | 0.686872174 |
| SEC24A | blue | 0.141692777 | 0.002145609 | 0.132305545 | 0.004182154 |
| SEC31A | blue | -0.028849612 | 0.534004971 | -0.036608981 | 0.429953525 |
| SECISBP2 | blue | 0.05413785 | 0.242948007 | 0.017101836 | 0.712418211 |
| SECISBP2 | blue | 0.10031039 | 0.03020546 | 0.072029489 | 0.120084628 |
| SEL1L | blue | -0.052424705 | 0.258202883 | -0.070727974 | 0.126945084 |
| SEMA3C | blue | 0.036798976 | 0.427562195 | 0.024022028 | 0.604595078 |
| SENP8 | blue | 0.006239772 | 0.893021015 | 0.002804675 | 0.95179944 |

| | | | | | |
|---|---|---|---|---|---|
| SEPP1 | blue | -0.247222097 | 6.22E-08 | -0.280692584 | 6.65E-10 |
| SEPT10 | blue | -0.054789527 | 0.23731429 | -0.079446547 | 0.086351664 |
| SEPT11 | blue | -0.061334414 | 0.185788249 | -0.061114525 | 0.187373377 |
| SEPT2 | blue | 0.011282671 | 0.807869918 | -0.010045524 | 0.828590786 |
| SERAC1 | blue | 0.16029758 | 0.000506514 | 0.148924603 | 0.001247986 |
| SERINC1 | blue | -0.170076055 | 0.000222095 | -0.180726975 | 8.58E-05 |
| SERINC3 | blue | -0.096862161 | 0.036391861 | -0.100722114 | 0.029530809 |
| SERTAD | blue | -0.012475068 | 0.788024738 | 0.006774139 | 0.883921589 |
| SERTAD | blue | -0.078873743 | 0.088650196 | -0.113120789 | 0.014450513 |
| SESN1 | blue | 0.179024968 | 0.000100301 | 0.158019464 | 0.000609763 |
| SESN3 | blue | 0.18547749 | 5.52E-05 | 0.140765276 | 0.002296 |
| SESTD1 | blue | 0.144215635 | 0.001780938 | 0.125996502 | 0.006403259 |
| SETDB2 | blue | -0.034700797 | 0.454396101 | -0.05190203 | 0.262986144 |
| SETMAR | blue | 0.07982987 | 0.08484045 | 0.047819934 | 0.302438566 |
| SETX | blue | 0.033172958 | 0.47451677 | 0.018105083 | 0.696360871 |
| SF3B1 | blue | -0.066570131 | 0.150908185 | -0.11020488 | 0.017198991 |
| SFT2D2 | blue | -0.045116405 | 0.330624723 | -0.036007274 | 0.437577659 |
| SGK494 | blue | -0.08127635 | 0.07932887 | -0.092121823 | 0.046627654 |
| SGMS1 | blue | 0.034620019 | 0.455447748 | 0.019660402 | 0.67173558 |
| SGMS2 | blue | 0.025379701 | 0.584324965 | 0.051830949 | 0.263641321 |
| SGPP1 | blue | 0.011431007 | 0.805394135 | 0.001470757 | 0.974712763 |
| SGTB | blue | -0.143424418 | 0.001888682 | -0.156552366 | 0.000686249 |
| SH2B2 | blue | -0.082700056 | 0.074191072 | -0.082385089 | 0.075303713 |
| SH3BGRI | blue | -0.05093777 | 0.271969748 | -0.061380075 | 0.185460337 |
| SH3D19 | blue | -0.107386165 | 0.020279775 | -0.114591209 | 0.013217017 |
| SHB | blue | 0.211665142 | 3.95E-06 | 0.209259371 | 5.10E-06 |
| SHISA5 | blue | 0.057447841 | 0.215288163 | 0.089711829 | 0.052696288 |
| SHKBP1 | blue | -0.1109308 | 0.016475136 | -0.094749148 | 0.040690948 |
| SHOC2 | blue | 0.105783247 | 0.022237473 | 0.08369351 | 0.070769093 |
| SIGIRR | blue | 0.144027206 | 0.001806073 | 0.170073959 | 0.000222135 |
| SIMC1 | blue | -0.325336542 | 5.65E-13 | -0.364575498 | 3.98E-16 |
| SIPA1 | blue | 0.056283909 | 0.224744509 | 0.088344028 | 0.056424093 |
| SIRT1 | blue | -0.041079768 | 0.375759743 | -0.078601745 | 0.089758743 |
| SKIL | blue | -0.175297964 | 0.000140327 | -0.204335585 | 8.55E-06 |
| SLAIN2 | blue | 0.093072088 | 0.044401572 | 0.086732605 | 0.061093958 |
| SLC10A7 | blue | 0.132605817 | 0.004096374 | 0.126621314 | 0.006143676 |
| SLC15A4 | blue | 0.047684467 | 0.30381181 | 0.03426718 | 0.46005734 |
| SLC16A4 | blue | -0.157652746 | 0.000628103 | -0.176079642 | 0.000130857 |
| SLC19A2 | blue | 0.043892217 | 0.343927778 | 0.014322856 | 0.757545276 |
| SLC25A1 | blue | 0.278986064 | 8.52E-10 | 0.28620724 | 2.96E-10 |
| SLC25A1 | blue | 0.170056608 | 0.00022247 | 0.206941092 | 6.51E-06 |
| SLC25A1 | blue | -0.237871758 | 1.98E-07 | -0.248124711 | 5.55E-08 |
| SLC25A1 | blue | 0.050458017 | 0.276516542 | 0.056998048 | 0.218908045 |
| SLC25A3 | blue | 0.066345071 | 0.152297197 | 0.047252498 | 0.308218263 |
| SLC25A4 | blue | -0.134337951 | 0.003631883 | -0.126624218 | 0.006142493 |

| | | | | | |
|---|---|---|---|---|---|
| SLC25A6 | blue | 0.132716911 | 0.004065043 | 0.132654321 | 0.004082668 |
| SLC27A5 | blue | 0.071767115 | 0.121443829 | 0.05844309 | 0.207431769 |
| SLC2A13 | blue | -0.203723244 | 9.11E-06 | -0.211740106 | 3.91E-06 |
| SLC2A4R | blue | 0.013792937 | 0.766250289 | 0.029292693 | 0.527740132 |
| SLC30A4 | blue | -0.150697689 | 0.001088664 | -0.147785834 | 0.001361348 |
| SLC30A7 | blue | 0.07351505 | 0.112611542 | 0.061134889 | 0.187226166 |
| SLC35B4 | blue | -0.143121036 | 0.001931555 | -0.175002093 | 0.000144076 |
| SLC35D1 | blue | 0.013170562 | 0.776511687 | 0.003425401 | 0.941149327 |
| SLC35F5 | blue | -0.175483744 | 0.000138019 | -0.200769358 | 1.23E-05 |
| SLC36A4 | blue | -0.243978311 | 9.34E-08 | -0.243766904 | 9.59E-08 |
| SLC37A3 | blue | 0.142424312 | 0.002033404 | 0.117322699 | 0.011169888 |
| SLC38A2 | blue | -0.252935112 | 3.00E-08 | -0.262171271 | 8.86E-09 |
| SLC38A9 | blue | -0.244620785 | 8.62E-08 | -0.251558661 | 3.58E-08 |
| SLC39A1 | blue | -0.104116955 | 0.024444189 | -0.140440738 | 0.002350855 |
| SLC4A7 | blue | 0.094507246 | 0.041209439 | 0.085862911 | 0.063744088 |
| SLC5A3 | blue | -0.13673114 | 0.003068579 | -0.145853229 | 0.001575612 |
| SLC9A6 | blue | -0.010223351 | 0.825604691 | -0.012445591 | 0.788513731 |
| SLC9B1 | blue | -0.041756199 | 0.36794279 | -0.060534792 | 0.191600204 |
| SLFN12 | blue | -0.048335583 | 0.297249072 | -0.057022403 | 0.218710932 |
| SLK | blue | 0.091446 | 0.04826718 | 0.071263222 | 0.124087793 |
| SLMAP | blue | -0.066048829 | 0.154140281 | -0.080686566 | 0.081540054 |
| SLTM | blue | 0.053625252 | 0.24744475 | 0.027847641 | 0.548308806 |
| SMAD2 | blue | -0.016056169 | 0.729290725 | -0.034326834 | 0.459276175 |
| SMAD4 | blue | -0.058526512 | 0.206782785 | -0.086223624 | 0.062633655 |
| SMAD5 | blue | 0.032032322 | 0.489850628 | 0.005016652 | 0.913899778 |
| SMARCA | blue | -0.057531234 | 0.214621774 | -0.040782354 | 0.379228822 |
| SMARCA | blue | 0.05689515 | 0.219742249 | 0.021826301 | 0.638024736 |
| SMC5 | blue | 0.006153732 | 0.89448747 | -0.024508334 | 0.597298383 |
| SMCHD1 | blue | -0.166002151 | 0.000314868 | -0.201008204 | 1.20E-05 |
| SMG1 | blue | -0.141401289 | 0.002191873 | -0.159612806 | 0.000535701 |
| SMIM17 | blue | -0.109493461 | 0.017935213 | -0.105912414 | 0.022073862 |
| SMURF2 | blue | -0.006854093 | 0.882561392 | -0.024046212 | 0.604231275 |
| SNRK | blue | -0.109523653 | 0.017903419 | -0.117660533 | 0.010937291 |
| SNTB2 | blue | -0.180946116 | 8.41E-05 | -0.163378544 | 0.000392575 |
| SNX13 | blue | -0.054602793 | 0.238919106 | -0.065648526 | 0.156657569 |
| SNX16 | blue | -0.123306351 | 0.007636809 | -0.180269188 | 8.95E-05 |
| SNX8 | blue | 0.005204565 | 0.910687864 | 0.036533934 | 0.430900218 |
| SNX9 | blue | -0.075602949 | 0.102731942 | -0.087844828 | 0.057838004 |
| SOCS5 | blue | -0.19461517 | 2.29E-05 | -0.219778471 | 1.62E-06 |
| SOCS6 | blue | 0.115155979 | 0.012768501 | 0.103664648 | 0.025074892 |
| SOS1 | blue | -0.209229661 | 5.12E-06 | -0.22934088 | 5.45E-07 |
| SOS2 | blue | -0.117806012 | 0.010838459 | -0.1369629 | 0.003018481 |
| SOWAH( | blue | -0.21293115 | 3.44E-06 | -0.239691934 | 1.58E-07 |
| SOX5 | blue | -0.092252894 | 0.04631515 | -0.099707495 | 0.03121716 |
| SP1 | blue | 0.047724899 | 0.303401515 | 0.017302505 | 0.709195963 |

| | | | | | |
|---|---|---|---|---|---|
| SP3 | blue | -0.158633146 | 0.000580179 | -0.189465031 | 3.78E-05 |
| SP4 | blue | -0.116624029 | 0.011664854 | -0.135160379 | 0.003428519 |
| SPAG9 | blue | -0.004351128 | 0.9252862 | -0.022050723 | 0.634572524 |
| SPATA13 | blue | -0.0095871 | 0.836300002 | -0.033374747 | 0.471831691 |
| SPATA5 | blue | -0.029276778 | 0.527964501 | -0.038041197 | 0.41211864 |
| SPG20 | blue | -0.175219997 | 0.000141306 | -0.182167425 | 7.52E-05 |
| SPICE1 | blue | -0.095381895 | 0.039360724 | -0.11986669 | 0.009521105 |
| SPIN1 | blue | 0.015743405 | 0.73436332 | -0.01741025 | 0.70746798 |
| SPIN3 | blue | -0.122482264 | 0.008055038 | -0.148494078 | 0.001289781 |
| SPOPL | blue | -0.201533783 | 1.14E-05 | -0.202940693 | 9.87E-06 |
| SPRED1 | blue | 0.037382191 | 0.420270102 | 0.024319952 | 0.600120196 |
| SPTLC3 | blue | -0.187981121 | 4.35E-05 | -0.159039537 | 0.000561328 |
| SRGAP2( | blue | -0.211559602 | 3.99E-06 | -0.252895929 | 3.01E-08 |
| SRSF8 | blue | 0.066191418 | 0.15325106 | 0.05579097 | 0.228837281 |
| SS18 | blue | -0.015790706 | 0.733595406 | -0.032059426 | 0.489483208 |
| SS18L1 | blue | -0.060854407 | 0.189261331 | -0.084211906 | 0.069035348 |
| SSFA2 | blue | 0.143985656 | 0.00181166 | 0.142526643 | 0.002018144 |
| SSH2 | blue | 0.043444518 | 0.348876747 | 0.034864725 | 0.452266116 |
| STAG2 | blue | -0.031058528 | 0.503148984 | -0.051626442 | 0.265532587 |
| STAP2 | blue | 0.348313172 | 9.12E-15 | 0.37385059 | 6.14E-17 |
| STARD1( | blue | 0.221922673 | 1.28E-06 | 0.239219195 | 1.68E-07 |
| STIM2 | blue | 0.230479973 | 4.77E-07 | 0.215743315 | 2.54E-06 |
| STK26 | blue | -0.218957952 | 1.78E-06 | -0.205402352 | 7.65E-06 |
| STK4 | blue | -0.175993371 | 0.000131872 | -0.186003319 | 5.25E-05 |
| STOX2 | blue | -0.177635376 | 0.000113768 | -0.207281145 | 6.29E-06 |
| STRN | blue | 0.01750064 | 0.706019501 | -0.01306112 | 0.778320197 |
| STRN3 | blue | -0.030059587 | 0.516985768 | -0.042096182 | 0.36405228 |
| STX17 | blue | 0.100672295 | 0.02961175 | 0.073223509 | 0.114048567 |
| STX4 | blue | -0.126245649 | 0.006298592 | -0.088765522 | 0.055252713 |
| STX5 | blue | 0.053053783 | 0.252526053 | 0.067086663 | 0.147756695 |
| STX7 | blue | -0.137058827 | 0.002997964 | -0.158929601 | 0.00056637 |
| STXBP4 | blue | -0.001606416 | 0.972381216 | -0.015754986 | 0.734175277 |
| STXBP5 | blue | 0.123312976 | 0.007633527 | 0.094208676 | 0.041857074 |
| SUGP1 | blue | -0.042933689 | 0.354578366 | -0.042395714 | 0.360645983 |
| SULT1C4 | blue | -0.165143925 | 0.00033855 | -0.198358789 | 1.57E-05 |
| SUN1 | blue | -0.018876813 | 0.684100249 | -0.032422121 | 0.484580765 |
| SUV420H | blue | -0.023245258 | 0.616331889 | -0.047637271 | 0.304291205 |
| SWAP70 | blue | 0.25070858 | 3.99E-08 | 0.21984924 | 1.61E-06 |
| SWI5 | blue | -0.049368838 | 0.287029931 | -0.044785897 | 0.334183165 |
| SYMPK | blue | 0.048281156 | 0.297794009 | 0.046792141 | 0.31296046 |
| SYNJ1 | blue | -0.019244076 | 0.678294245 | -0.027665252 | 0.550932689 |
| SYNJ2BP | blue | 0.026123394 | 0.573356912 | 0.031301422 | 0.49981427 |
| SYTL2 | blue | -0.068529456 | 0.139219439 | -0.081178432 | 0.079692577 |
| TAB2 | blue | -0.223639894 | 1.05E-06 | -0.231958655 | 4.01E-07 |
| TAB3 | blue | -0.134597609 | 0.003566526 | -0.149994867 | 0.001149429 |

| | | | | | |
|---|---|---|---|---|---|
| TAF4 | blue | -0.168417561 | 0.000256254 | -0.183059832 | 6.92E-05 |
| TAF9B | blue | -0.021765807 | 0.638956641 | -0.04312656 | 0.352418776 |
| TAOK1 | blue | 0.019453852 | 0.674986431 | -0.006903281 | 0.881724755 |
| TAOK3 | blue | -0.113180474 | 0.01439854 | -0.123803484 | 0.007393997 |
| TAPT1 | blue | 0.176850762 | 0.000122106 | 0.165241369 | 0.00033578 |
| TARSL2 | blue | -0.058605825 | 0.206167137 | -0.101123726 | 0.028885217 |
| TAS2R5 | blue | -0.175123366 | 0.000142528 | -0.186291387 | 5.11E-05 |
| TASP1 | blue | -0.171777869 | 0.000191507 | -0.184541026 | 6.03E-05 |
| TBC1D12 | blue | 0.106674367 | 0.02112981 | 0.082920242 | 0.073421234 |
| TBC1D15 | blue | -0.210573975 | 4.43E-06 | -0.228815935 | 5.79E-07 |
| TBC1D19 | blue | -0.057533785 | 0.214601419 | -0.074515716 | 0.107787186 |
| TBC1D23 | blue | -0.130034976 | 0.0048852 | -0.154943502 | 0.000780285 |
| TBC1D4 | blue | -0.003734747 | 0.935845516 | -0.039557664 | 0.393719615 |
| TBC1D8E | blue | -0.082815756 | 0.073785732 | -0.104940966 | 0.02333035 |
| TBCB | blue | -0.107227494 | 0.020466641 | -0.104316466 | 0.024170373 |
| TBCEL | blue | -0.135963167 | 0.003240033 | -0.150326501 | 0.001120378 |
| TBCK | blue | 0.013797143 | 0.766181086 | -0.02843866 | 0.539848816 |
| TCAF1 | blue | 0.049566753 | 0.285099812 | 0.013093971 | 0.777777216 |
| TCEANC | blue | 0.00715166 | 0.877502133 | 0.011297835 | 0.807616728 |
| TCEANC | blue | 0.062464523 | 0.177797244 | 0.052502467 | 0.257496415 |
| TCF12 | blue | 0.158621896 | 0.000580709 | 0.117979859 | 0.010721393 |
| TDRD3 | blue | 0.145899912 | 0.001570091 | 0.128732352 | 0.005335048 |
| TDRD7 | blue | -0.056598941 | 0.222156314 | -0.035544596 | 0.443492562 |
| TEAD1 | blue | -0.174067937 | 0.00015654 | -0.198261687 | 1.59E-05 |
| TECR | blue | 0.120860989 | 0.008937945 | 0.127058414 | 0.005967737 |
| TET2 | blue | 0.187037456 | 4.76E-05 | 0.150316157 | 0.001121273 |
| TGFBR1 | blue | -0.070391192 | 0.128768912 | -0.090668484 | 0.050212851 |
| THAP2 | blue | -0.164035483 | 0.000371595 | -0.190842139 | 3.31E-05 |
| THAP4 | blue | 0.041738935 | 0.368141028 | 0.040114044 | 0.387095429 |
| THAP6 | blue | 0.077217652 | 0.095573121 | 0.076372516 | 0.09926873 |
| THAP8 | blue | -0.210040983 | 4.69E-06 | -0.211428211 | 4.05E-06 |
| THSD7A | blue | -0.113348337 | 0.014253247 | -0.110931581 | 0.016474371 |
| THUMPD | blue | 0.055634831 | 0.23014463 | 0.042556626 | 0.358824349 |
| TIGD7 | blue | -0.380472804 | 1.56E-17 | -0.400850232 | 1.88E-19 |
| TIPARP | blue | -0.147015437 | 0.001443327 | -0.176086132 | 0.000130781 |
| TMCC1 | blue | -0.001959418 | 0.966315399 | -0.028295409 | 0.541893346 |
| TMCC3 | blue | -0.313319504 | 4.27E-12 | -0.313641876 | 4.05E-12 |
| TMED8 | blue | 0.014463167 | 0.755245446 | -0.015971592 | 0.730661289 |
| TMED9 | blue | 0.103200182 | 0.025737117 | 0.108650354 | 0.01884322 |
| TMEM10 | blue | -0.089541959 | 0.053147791 | -0.093324493 | 0.043825544 |
| TMEM12 | blue | 0.003402554 | 0.941541155 | 0.015872165 | 0.732273602 |
| TMEM13 | blue | -0.042893866 | 0.355025295 | -0.031873344 | 0.49200871 |
| TMEM16 | blue | 0.129034658 | 0.005227449 | 0.107281464 | 0.020402914 |
| TMEM16 | blue | -0.008220476 | 0.859372576 | -0.031272827 | 0.500206245 |
| TMEM16 | blue | 0.044842373 | 0.333573374 | 0.008612162 | 0.852746633 |

| | | | | | |
|---|---|---|---|---|---|
| TMEM17 | blue | -0.292561242 | 1.14E-10 | -0.328050078 | 3.53E-13 |
| TMEM18 | blue | 0.145712777 | 0.00159233 | 0.134386347 | 0.00361962 |
| TMEM18 | blue | 0.163977511 | 0.000373404 | 0.171216641 | 0.000201129 |
| TMEM19 | blue | 0.06751139 | 0.145203098 | 0.069299078 | 0.134822998 |
| TMEM20 | blue | 0.100602717 | 0.02972511 | 0.105693423 | 0.022351868 |
| TMEM22 | blue | 0.180601225 | 8.68E-05 | 0.217926398 | 1.99E-06 |
| TMEM24 | blue | -0.199274675 | 1.44E-05 | -0.229179241 | 5.55E-07 |
| TMEM30 | blue | 0.061272339 | 0.186234733 | 0.058127431 | 0.209900768 |
| TMEM39 | blue | -0.120736522 | 0.009009161 | -0.148860761 | 0.001254104 |
| TMEM45 | blue | -0.224322076 | 9.72E-07 | -0.234058406 | 3.13E-07 |
| TMEM53 | blue | 0.140743385 | 0.002299663 | 0.145783182 | 0.00158393 |
| TMEM54 | blue | 0.031671233 | 0.494759646 | 0.060786273 | 0.189758156 |
| TMEM56 | blue | 0.182056802 | 7.59E-05 | 0.154529911 | 0.000806316 |
| TMEM56 | blue | -0.124840248 | 0.006909702 | -0.156789807 | 0.000673295 |
| TMEM64 | blue | -0.176399695 | 0.000127156 | -0.208579419 | 5.48E-06 |
| TMEM9 | blue | 0.313063388 | 4.45E-12 | 0.316471214 | 2.53E-12 |
| TMF1 | blue | 0.108323691 | 0.019205655 | 0.091667608 | 0.047724328 |
| TMTC1 | blue | -0.260894965 | 1.05E-08 | -0.264664876 | 6.32E-09 |
| TMTC2 | blue | 0.168001798 | 0.000265556 | 0.142474601 | 0.002025891 |
| TMX3 | blue | 0.074220891 | 0.109191318 | 0.040537983 | 0.382093855 |
| TNIP2 | blue | 0.15537973 | 0.000753675 | 0.159919823 | 0.000522427 |
| TNKS2 | blue | 0.048905718 | 0.291580696 | 0.024489147 | 0.597585513 |
| TNRC6A | blue | -0.106037255 | 0.021916724 | -0.113618539 | 0.014022081 |
| TOP1MT | blue | -0.112651443 | 0.014864993 | -0.165428911 | 0.000330508 |
| TOPORS | blue | -0.057972024 | 0.211124086 | -0.088257084 | 0.056668264 |
| TOR2A | blue | 0.266228926 | 5.11E-09 | 0.27153961 | 2.45E-09 |
| TP53INP1 | blue | 0.04356434 | 0.347547805 | 0.030717438 | 0.507851545 |
| TPP2 | blue | -0.015092513 | 0.744956743 | -0.030438221 | 0.511718128 |
| TPRA1 | blue | -0.157768061 | 0.000622281 | -0.150198758 | 0.001131487 |
| TPST1 | blue | -0.436652122 | 3.66E-23 | -0.427619211 | 3.48E-22 |
| TRABD | blue | 0.314567198 | 3.48E-12 | 0.329285704 | 2.85E-13 |
| TRAF2 | blue | 0.09833748 | 0.03362439 | 0.100793531 | 0.02941511 |
| TRAF6 | blue | -0.012640813 | 0.785276734 | -0.044604346 | 0.336148277 |
| TRAK2 | blue | -0.151393708 | 0.001031419 | -0.164179891 | 0.000367126 |
| TRAPPC1 | blue | 0.077276345 | 0.0953206 | 0.053478067 | 0.248746594 |
| TRAPPC8 | blue | 0.010577364 | 0.819667566 | -0.02464998 | 0.595180611 |
| TRDMT1 | blue | 0.001934704 | 0.966740011 | -0.00612209 | 0.895026873 |
| TRIM13 | blue | 0.173036561 | 0.000171473 | 0.151922404 | 0.000989805 |
| TRIM23 | blue | -0.073370315 | 0.113323165 | -0.095044083 | 0.040066254 |
| TRIM28 | blue | 0.000247404 | 0.9957456 | 0.000806957 | 0.986124085 |
| TRIM33 | blue | -0.028158919 | 0.543844974 | -0.077303908 | 0.095202197 |
| TRIM44 | blue | -0.18064497 | 8.65E-05 | -0.201632629 | 1.13E-05 |
| TRIM5 | blue | 0.045537665 | 0.326124763 | 0.050155175 | 0.279413125 |
| TRIM6 | blue | -0.047561285 | 0.305064096 | -0.038641412 | 0.404776338 |
| TRIM66 | blue | 0.099897437 | 0.030895341 | 0.087221265 | 0.059645266 |

| | | | | | |
|---|---|---|---|---|---|
| TRIP11 | blue | -0.000606989 | 0.989562375 | -0.01993573 | 0.667411773 |
| TRIQK | blue | 0.009507761 | 0.837635844 | -0.034710364 | 0.454271634 |
| TRMT1 | blue | -0.184413486 | 6.10E-05 | -0.197039073 | 1.80E-05 |
| TRMT11 | blue | -0.138875032 | 0.002632687 | -0.174174993 | 0.000155062 |
| TRMT61/ | blue | -0.180198717 | 9.01E-05 | -0.158368163 | 0.000592786 |
| TRPM7 | blue | 0.109334348 | 0.01810359 | 0.08178735 | 0.077452584 |
| TRPS1 | blue | 0.24688299 | 6.49E-08 | 0.211940974 | 3.83E-06 |
| TRPT1 | blue | 0.241681568 | 1.24E-07 | 0.262925278 | 8.00E-09 |
| TSC1 | blue | -0.005192935 | 0.910886611 | -0.035201632 | 0.447906254 |
| TSC22D2 | blue | -0.289985052 | 1.69E-10 | -0.31061157 | 6.65E-12 |
| TSPO | blue | 0.293485211 | 9.91E-11 | 0.302034573 | 2.63E-11 |
| TSPYL4 | blue | -0.224764283 | 9.24E-07 | -0.241965656 | 1.20E-07 |
| TSSC4 | blue | -0.005926468 | 0.898362689 | 0.014186987 | 0.75977434 |
| TST | blue | 0.136775305 | 0.003058974 | 0.151000185 | 0.001063434 |
| TTBK2 | blue | -0.053299381 | 0.250333465 | -0.07615263 | 0.10024865 |
| TTC17 | blue | -0.177219743 | 0.000118116 | -0.224605745 | 9.41E-07 |
| TTC21B | blue | -0.097636446 | 0.034916207 | -0.121035905 | 0.008838711 |
| TTC3 | blue | 0.066296559 | 0.152597864 | 0.031700768 | 0.494357131 |
| TTC33 | blue | -0.082548467 | 0.074724887 | -0.120768051 | 0.008991073 |
| TTC37 | blue | -0.040386189 | 0.383880142 | -0.074036764 | 0.110075533 |
| TTC39B | blue | -0.00322624 | 0.944565381 | 0.013446576 | 0.771956001 |
| TUBGCP: | blue | -0.057514039 | 0.21475906 | -0.083135218 | 0.072675906 |
| TWF2 | blue | 0.153446713 | 0.000878343 | 0.16923051 | 0.000238939 |
| TWSG1 | blue | -0.23138736 | 4.29E-07 | -0.261761255 | 9.36E-09 |
| TXNRD2 | blue | 0.20425775 | 8.62E-06 | 0.212437235 | 3.63E-06 |
| UACA | blue | -0.049461518 | 0.286125006 | -0.070125491 | 0.130222069 |
| UBA6 | blue | 0.068846797 | 0.137393493 | 0.038166741 | 0.410576406 |
| UBALD1 | blue | 0.000364705 | 0.993728521 | 0.026459933 | 0.568425787 |
| UBALD2 | blue | -0.063496496 | 0.170725564 | -0.035527343 | 0.443714001 |
| UBE2F-S( | blue | 0.042270055 | 0.362072552 | 0.040973418 | 0.376997978 |
| UBE3A | blue | 0.0444302 | 0.338040196 | 0.018299706 | 0.693261229 |
| UBE4A | blue | 0.010673363 | 0.818059338 | -0.01468929 | 0.751543638 |
| UBL3 | blue | -0.025496466 | 0.582596486 | -0.049403702 | 0.286689294 |
| UBR1 | blue | 0.166528771 | 0.000301112 | 0.146833481 | 0.00146334 |
| UBR2 | blue | -0.164117989 | 0.000369036 | -0.176423557 | 0.000126884 |
| UBR3 | blue | 0.035168498 | 0.448333969 | 0.00526925 | 0.90958258 |
| UBTD1 | blue | -0.02246336 | 0.628245738 | 6.94E-06 | 0.999880601 |
| UEVLD | blue | 0.179573108 | 9.54E-05 | 0.159041064 | 0.000561258 |
| UFSP1 | blue | -0.093965191 | 0.04239156 | -0.077617472 | 0.093863468 |
| UGGT2 | blue | -0.085097663 | 0.066153562 | -0.117579152 | 0.010992926 |
| UHRF1BI | blue | 0.135205051 | 0.003417775 | 0.117721214 | 0.010895971 |
| UHRF2 | blue | -0.092873866 | 0.044858398 | -0.128505868 | 0.005416961 |
| ULK2 | blue | 0.029700447 | 0.522007958 | -0.004779097 | 0.917962203 |
| UQCRC1 | blue | 0.192363218 | 2.85E-05 | 0.223403385 | 1.08E-06 |
| UROD | blue | -0.081031096 | 0.080242385 | -0.056392883 | 0.223846799 |

| | | | | | |
|---|---|---|---|---|---|
| USF2 | blue | -0.187964741 | 4.36E-05 | -0.190543736 | 3.40E-05 |
| USP12 | blue | -0.111943633 | 0.015509808 | -0.142487338 | 0.002023993 |
| USP13 | blue | -0.073189693 | 0.114216177 | -0.08863883 | 0.055602661 |
| USP15 | blue | -0.027963206 | 0.546649461 | -0.041869097 | 0.366648013 |
| USP25 | blue | -0.118707494 | 0.010243485 | -0.142382162 | 0.002039721 |
| USP3 | blue | 0.131550926 | 0.004404946 | 0.100373649 | 0.030100954 |
| USP33 | blue | -0.121244015 | 0.008721924 | -0.141032415 | 0.002251727 |
| USP4 | blue | -0.183903562 | 6.40E-05 | -0.167596254 | 0.000274932 |
| USP46 | blue | -0.006430206 | 0.889776548 | -0.041445423 | 0.371521551 |
| USP47 | blue | 0.044911313 | 0.332829974 | 0.018915956 | 0.683480538 |
| USP49 | blue | -0.157910777 | 0.000615146 | -0.173860671 | 0.000159439 |
| USP53 | blue | 0.074769027 | 0.106592182 | 0.033205038 | 0.474089346 |
| USP8 | blue | 0.094652204 | 0.040898072 | 0.072468766 | 0.117835623 |
| UTP20 | blue | 0.063737132 | 0.1691072 | 0.053361595 | 0.249780161 |
| UTRN | blue | -0.033313436 | 0.472646639 | -0.049807226 | 0.282766455 |
| UVRAG | blue | -0.364476807 | 4.06E-16 | -0.389897748 | 2.10E-18 |
| UVSSA | blue | 0.152331837 | 0.000958649 | 0.147183127 | 0.001425106 |
| VAMP3 | blue | -0.187359487 | 4.62E-05 | -0.199629762 | 1.38E-05 |
| VAX2 | blue | 0.058092695 | 0.210173759 | 0.071385388 | 0.123442703 |
| VCPIP1 | blue | -0.111800012 | 0.015643602 | -0.123348848 | 0.007615778 |
| VEGFB | blue | -0.081150554 | 0.079796374 | -0.061303985 | 0.186007015 |
| VMP1 | blue | -0.097787564 | 0.034634224 | -0.113983746 | 0.013714882 |
| VPS13A | blue | 0.013369 | 0.773235628 | 0.003405114 | 0.941497258 |
| VPS13C | blue | 0.094911825 | 0.040345375 | 0.075267546 | 0.104270985 |
| VPS13D | blue | -0.016685786 | 0.719115195 | -0.028942473 | 0.532688893 |
| VPS16 | blue | -0.014948091 | 0.747313858 | -0.017634271 | 0.703880029 |
| VPS36 | blue | 0.080017036 | 0.084110355 | 0.055017246 | 0.235367534 |
| VPS41 | blue | -0.056653983 | 0.221706303 | -0.085134364 | 0.066036323 |
| VPS54 | blue | 0.021086347 | 0.649462404 | -0.008635874 | 0.852345846 |
| VPS8 | blue | -0.185082863 | 5.73E-05 | -0.213596028 | 3.20E-06 |
| VWA8 | blue | 0.188487902 | 4.15E-05 | 0.154438017 | 0.000812207 |
| VWDE | blue | -0.055783218 | 0.228902059 | -0.060338578 | 0.193046519 |
| WASF3 | blue | -0.07492984 | 0.105838989 | -0.107505629 | 0.020140064 |
| WASL | blue | 0.019452858 | 0.67500209 | 0.018595783 | 0.68855562 |
| WDFY1 | blue | -0.075402829 | 0.103648037 | -0.083366876 | 0.071879667 |
| WDFY2 | blue | 0.120971049 | 0.00887539 | 0.118448613 | 0.010411306 |
| WDFY3 | blue | 0.059719482 | 0.197662324 | 0.028463379 | 0.539496397 |
| WDR11 | blue | 0.051675851 | 0.265074805 | 0.01412685 | 0.760761577 |
| WDR20 | blue | -0.05544392 | 0.231750314 | -0.073333276 | 0.11350584 |
| WDR35 | blue | -0.158646634 | 0.000579544 | -0.186452801 | 5.03E-05 |
| WDR36 | blue | 0.016167138 | 0.7274938 | -0.015504918 | 0.738239021 |
| WDR44 | blue | -0.042879569 | 0.355185843 | -0.074361008 | 0.10852221 |
| WDR47 | blue | -0.051277935 | 0.268776966 | -0.067877983 | 0.143026232 |
| WDR48 | blue | -0.074749173 | 0.106685461 | -0.106626283 | 0.021188332 |
| WDR73 | blue | -0.000459291 | 0.992102061 | -0.025172409 | 0.587399381 |

| | | | | | |
|---|---|---|---|---|---|
| WDR83O | blue | -0.02018975 | 0.663432303 | -0.000280924 | 0.9951692 |
| WHAMM | blue | 0.143026763 | 0.001945057 | 0.149027262 | 0.001238206 |
| WRN | blue | 0.020796087 | 0.653971818 | -0.02446807 | 0.597901003 |
| WWC2 | blue | -0.289375637 | 1.85E-10 | -0.297877055 | 5.04E-11 |
| WWP1 | blue | 0.03488657 | 0.451982694 | -0.022730309 | 0.624167138 |
| XIAP | blue | 0.107908189 | 0.019675422 | 0.099924512 | 0.030849701 |
| XPO4 | blue | -0.018041347 | 0.697377046 | -0.040285789 | 0.385064433 |
| XRN1 | blue | -0.149327545 | 0.001210002 | -0.160358465 | 0.000503992 |
| YAP1 | blue | -0.077149446 | 0.095867236 | -0.113288208 | 0.014305142 |
| YPEL2 | blue | -0.225258937 | 8.73E-07 | -0.237105739 | 2.17E-07 |
| YPEL5 | blue | -0.196652046 | 1.87E-05 | -0.218037434 | 1.97E-06 |
| YTHDC1 | blue | -0.095976759 | 0.038143886 | -0.139600051 | 0.00249856 |
| YTHDC2 | blue | 0.233444695 | 3.36E-07 | 0.183272998 | 6.78E-05 |
| YTHDF3 | blue | -0.122326609 | 0.00813629 | -0.156369456 | 0.000696384 |
| YY2 | blue | 0.022347808 | 0.630014727 | 0.013130683 | 0.777170553 |
| ZBTB1 | blue | -0.020178548 | 0.663607606 | -0.056625544 | 0.221938737 |
| ZBTB11 | blue | -0.113302308 | 0.014292958 | -0.153160411 | 0.00089835 |
| ZBTB14 | blue | -0.106914438 | 0.02083972 | -0.141160104 | 0.002230841 |
| ZBTB17 | blue | 0.112985901 | 0.014568579 | 0.135697062 | 0.003301442 |
| ZBTB21 | blue | -0.007653597 | 0.868979561 | -0.04014433 | 0.386736805 |
| ZBTB26 | blue | -0.108781288 | 0.01869963 | -0.122050046 | 0.008282462 |
| ZBTB34 | blue | -0.111211185 | 0.016202773 | -0.145836676 | 0.001577574 |
| ZBTB43 | blue | -0.015851638 | 0.732606608 | -0.036133606 | 0.435970529 |
| ZBTB44 | blue | -0.128649008 | 0.005365061 | -0.161428651 | 0.000461521 |
| ZBTB45 | blue | 0.210646544 | 4.40E-06 | 0.21191459 | 3.84E-06 |
| ZBTB8A | blue | -0.009292025 | 0.841270572 | -0.000261888 | 0.995496544 |
| ZC2HC1A | blue | -0.10088855 | 0.029261777 | -0.150720563 | 0.001086737 |
| ZC3H12C | blue | -0.243785734 | 9.56E-08 | -0.26040402 | 1.12E-08 |
| ZC3H13 | blue | -0.031330451 | 0.499416517 | -0.073513768 | 0.11261783 |
| ZC3H6 | blue | -0.10340724 | 0.025440059 | -0.110196595 | 0.01720741 |
| ZC3H7A | blue | -0.004503442 | 0.922678835 | -0.017718758 | 0.702528559 |
| ZC3HAV | blue | 0.038547678 | 0.405917808 | 0.025256498 | 0.586151334 |
| ZCCHC4 | blue | 0.068089027 | 0.141784404 | 0.044918197 | 0.332755803 |
| ZCCHC6 | blue | 0.149895043 | 0.001158309 | 0.131676305 | 0.004367202 |
| ZCWPW2 | blue | 0.053583883 | 0.247810178 | 0.038605438 | 0.405214199 |
| ZDHHC1: | blue | 0.289459484 | 1.82E-10 | 0.319223589 | 1.60E-12 |
| ZDHHC2 | blue | 0.109148518 | 0.018301986 | 0.106917972 | 0.020835477 |
| ZDHHC2 | blue | -0.072419345 | 0.118086991 | -0.09117909 | 0.048927862 |
| ZDHHC2 | blue | 0.223536422 | 1.06E-06 | 0.257425166 | 1.67E-08 |
| ZFAND2B | blue | 0.041295791 | 0.373252308 | 0.047148394 | 0.309286485 |
| ZFAND4 | blue | -0.033887126 | 0.465051449 | -0.046679455 | 0.31412851 |
| ZFAND5 | blue | 0.038625767 | 0.404966724 | 0.009727856 | 0.833931232 |
| ZFP1 | blue | 0.031038319 | 0.503426961 | 0.015896641 | 0.731876592 |
| ZFP2 | blue | 0.031916189 | 0.491426602 | -0.002056781 | 0.964642683 |
| ZFP3 | blue | -0.416942062 | 4.58E-21 | -0.408959379 | 2.96E-20 |

| | | | | | |
|---|---|---|---|---|---|
| ZFP36L2 | blue | -0.032467454 | 0.483969884 | -0.047629945 | 0.30436567 |
| ZFP37 | blue | 0.074734909 | 0.106752522 | 0.035438296 | 0.444857908 |
| ZFP90 | blue | 0.094800631 | 0.040581315 | 0.079089313 | 0.087779459 |
| ZFX | blue | -0.1027433 | 0.026403183 | -0.123166907 | 0.007706185 |
| ZFYVE16 | blue | 0.051448531 | 0.267185452 | 0.012986559 | 0.779552993 |
| ZHX1 | blue | -0.141045359 | 0.002249601 | -0.160215884 | 0.000509917 |
| ZKSCAN | blue | 0.028432714 | 0.539933602 | 0.003433663 | 0.941007643 |
| ZKSCAN | blue | -0.027216322 | 0.557417189 | -0.046337974 | 0.317685575 |
| ZKSCAN | blue | -0.352086503 | 4.48E-15 | -0.362653943 | 5.81E-16 |
| ZKSCAN | blue | -0.22257808 | 1.18E-06 | -0.243349342 | 1.01E-07 |
| ZMAT3 | blue | 0.050858336 | 0.272719027 | 0.053251562 | 0.25075933 |
| ZMYM1 | blue | -0.130432386 | 0.004754935 | -0.158243947 | 0.000598782 |
| ZMYM2 | blue | 0.034765844 | 0.453550238 | -0.002657927 | 0.9543186 |
| ZMYM4 | blue | -0.081407745 | 0.078842929 | -0.113900539 | 0.013784347 |
| ZNF106 | blue | 0.085482598 | 0.064932362 | 0.062877387 | 0.17494241 |
| ZNF107 | blue | -0.038833315 | 0.402445388 | -0.047224438 | 0.30850595 |
| ZNF117 | blue | 0.036179888 | 0.435382604 | 0.02410608 | 0.603331091 |
| ZNF12 | blue | -0.156071012 | 0.00071322 | -0.161217704 | 0.000469619 |
| ZNF121 | blue | 0.092246851 | 0.046329518 | 0.086381422 | 0.062152923 |
| ZNF131 | blue | -0.086230685 | 0.062612079 | -0.117222906 | 0.011239427 |
| ZNF136 | blue | -0.138460787 | 0.002712234 | -0.151308372 | 0.001038285 |
| ZNF148 | blue | -0.143500418 | 0.001878079 | -0.16072547 | 0.000489034 |
| ZNF160 | blue | 0.01383572 | 0.765546383 | -0.004951347 | 0.915016342 |
| ZNF169 | blue | -0.056430189 | 0.223540064 | -0.070613373 | 0.127563439 |
| ZNF175 | blue | -0.184278017 | 6.18E-05 | -0.19397345 | 2.44E-05 |
| ZNF182 | blue | -0.140480838 | 0.002344013 | -0.158126364 | 0.000604511 |
| ZNF189 | blue | 0.032134871 | 0.488461247 | -0.007915059 | 0.864546041 |
| ZNF197 | blue | -0.020635235 | 0.656476229 | -0.052129098 | 0.260900694 |
| ZNF2 | blue | -0.0858591 | 0.063755908 | -0.112144696 | 0.01532419 |
| ZNF200 | blue | 0.147652367 | 0.001375236 | 0.127795652 | 0.005681219 |
| ZNF205 | blue | 0.133233521 | 0.003922176 | 0.141374639 | 0.002196148 |
| ZNF214 | blue | -0.261273245 | 9.99E-09 | -0.280221296 | 7.12E-10 |
| ZNF217 | blue | -0.084125433 | 0.069322112 | -0.097514004 | 0.035146115 |
| ZNF23 | blue | -0.014352334 | 0.75706193 | 0.000106503 | 0.998168556 |
| ZNF24 | blue | -0.024045952 | 0.604235183 | -0.057293857 | 0.216522538 |
| ZNF248 | blue | -0.303338903 | 2.14E-11 | -0.336753988 | 7.59E-14 |
| ZNF267 | blue | -0.107234358 | 0.020458527 | -0.10125737 | 0.028673086 |
| ZNF274 | blue | -0.213338446 | 3.29E-06 | -0.223836985 | 1.03E-06 |
| ZNF280C | blue | -0.01294162 | 0.780296275 | -0.03736123 | 0.420530914 |
| ZNF280D | blue | 0.022915542 | 0.621343775 | -0.004048677 | 0.930466024 |
| ZNF286A | blue | 0.000786477 | 0.986476205 | -0.036732233 | 0.428401359 |
| ZNF286B | blue | -0.095807133 | 0.038487574 | -0.134648771 | 0.003553774 |
| ZNF287 | blue | 0.050843496 | 0.272859169 | 0.018715537 | 0.68665575 |
| ZNF292 | blue | -0.013222206 | 0.775658698 | -0.035227135 | 0.447577185 |
| ZNF320 | blue | 0.023531595 | 0.61199381 | 0.020632377 | 0.656520771 |

| | | | | | |
|---|---|---|---|---|---|
| ZNF333 | blue | -0.194154737 | 2.39E-05 | -0.221462962 | 1.34E-06 |
| ZNF337 | blue | -0.102822582 | 0.026286547 | -0.133587761 | 0.003826856 |
| ZNF33A | blue | -0.03048289 | 0.511098524 | -0.065245297 | 0.159224544 |
| ZNF33B | blue | 0.026727496 | 0.564519816 | 8.95E-05 | 0.998460277 |
| ZNF354C | blue | -0.363882897 | 4.56E-16 | -0.380895819 | 1.43E-17 |
| ZNF37A | blue | -0.132742634 | 0.00405782 | -0.161926111 | 0.000442934 |
| ZNF397 | blue | -0.038410973 | 0.407585987 | -0.065166095 | 0.159732456 |
| ZNF408 | blue | 0.001090593 | 0.981247641 | 0.003617843 | 0.937849542 |
| ZNF41 | blue | -0.04893324 | 0.291308913 | -0.071970762 | 0.12038782 |
| ZNF426 | blue | 0.028988662 | 0.532034879 | 0.020028741 | 0.665953574 |
| ZNF436 | blue | -0.000584912 | 0.989941988 | -0.026411657 | 0.569131905 |
| ZNF441 | blue | -0.203850452 | 8.99E-06 | -0.211794157 | 3.89E-06 |
| ZNF442 | blue | -0.05427456 | 0.241758456 | -0.051009712 | 0.271292353 |
| ZNF445 | blue | -0.012295927 | 0.790997799 | -0.042080966 | 0.364225851 |
| ZNF451 | blue | -0.133457883 | 0.003861557 | -0.136901156 | 0.003031755 |
| ZNF484 | blue | -0.002515256 | 0.956768208 | -0.016303505 | 0.725287626 |
| ZNF491 | blue | 0.177472099 | 0.000115458 | 0.171397799 | 0.000197975 |
| ZNF510 | blue | 0.07935907 | 0.086699553 | 0.041104307 | 0.375474394 |
| ZNF518A | blue | 0.070093587 | 0.130397408 | 0.053025949 | 0.252775376 |
| ZNF518B | blue | -0.076273844 | 0.099707517 | -0.102069411 | 0.027412688 |
| ZNF519 | blue | -0.232583822 | 3.72E-07 | -0.258702295 | 1.41E-08 |
| ZNF525 | blue | 0.056473193 | 0.223186852 | 0.044071469 | 0.341958871 |
| ZNF555 | blue | 0.05961875 | 0.198420904 | 0.032396628 | 0.484924478 |
| ZNF558 | blue | -0.04678508 | 0.313033571 | -0.079521682 | 0.086053755 |
| ZNF561 | blue | 0.120501057 | 0.009145269 | 0.096915565 | 0.036288409 |
| ZNF562 | blue | 0.165113206 | 0.000339427 | 0.156808276 | 0.000672297 |
| ZNF579 | blue | -0.141615154 | 0.002157841 | -0.123523091 | 0.007530086 |
| ZNF580 | blue | 0.09378021 | 0.042801456 | 0.116211174 | 0.011966368 |
| ZNF594 | blue | -0.064530702 | 0.163851425 | -0.087026468 | 0.060219324 |
| ZNF595 | blue | -0.13508273 | 0.003447268 | -0.153426909 | 0.000879714 |
| ZNF597 | blue | 0.167331649 | 0.000281216 | 0.165870818 | 0.000318389 |
| ZNF621 | blue | 0.102874542 | 0.026210347 | 0.078175565 | 0.09151801 |
| ZNF624 | blue | -0.013640804 | 0.768754887 | -0.021887018 | 0.637089958 |
| ZNF627 | blue | -0.120524203 | 0.009131809 | -0.138015211 | 0.002800237 |
| ZNF628 | blue | -0.030308658 | 0.513517481 | -0.017241256 | 0.710178921 |
| ZNF630 | blue | -0.040490893 | 0.38264746 | -0.045086517 | 0.330945503 |
| ZNF641 | blue | 0.00109656 | 0.981145061 | -0.029547056 | 0.524160561 |
| ZNF644 | blue | -0.207281979 | 6.29E-06 | -0.231500415 | 4.23E-07 |
| ZNF652 | blue | 0.179276782 | 9.80E-05 | 0.186926437 | 4.81E-05 |
| ZNF653 | blue | 0.086659495 | 0.061313179 | 0.094659754 | 0.04088191 |
| ZNF654 | blue | 0.057918986 | 0.211542753 | 0.051053964 | 0.270876252 |
| ZNF655 | blue | -0.101601798 | 0.028132537 | -0.119550584 | 0.009713461 |
| ZNF658 | blue | -0.03525602 | 0.447204654 | -0.086898725 | 0.060598248 |
| ZNF660 | blue | -0.371694064 | 9.53E-17 | -0.401683964 | 1.56E-19 |
| ZNF674 | blue | -0.167854764 | 0.00026892 | -0.161917204 | 0.00044326 |

| | | | | | |
|---|---|---|---|---|---|
| ZNF697 | blue | -0.042845584 | 0.355567647 | -0.078756252 | 0.089127683 |
| ZNF701 | blue | 0.03667515 | 0.429119835 | 0.039544998 | 0.393871203 |
| ZNF704 | blue | 0.082430562 | 0.075142243 | 0.039098288 | 0.399240104 |
| ZNF711 | blue | -0.218735138 | 1.82E-06 | -0.236638018 | 2.29E-07 |
| ZNF713 | blue | 0.049982331 | 0.281075518 | 0.046127079 | 0.319895477 |
| ZNF717 | blue | -0.420998028 | 1.74E-21 | -0.417792361 | 3.74E-21 |
| ZNF718 | blue | -0.275496864 | 1.40E-09 | -0.272148027 | 2.25E-09 |
| ZNF720 | blue | -0.047469337 | 0.306001075 | -0.049250131 | 0.288191803 |
| ZNF730 | blue | -0.174631845 | 0.0001489 | -0.167263735 | 0.00028285 |
| ZNF737 | blue | 0.002400117 | 0.958745396 | 0.003741694 | 0.935726448 |
| ZNF75D | blue | 0.015746358 | 0.734315368 | 0.000487768 | 0.991612383 |
| ZNF761 | blue | 0.066618591 | 0.150610366 | 0.052244943 | 0.259841147 |
| ZNF772 | blue | -0.333588245 | 1.33E-13 | -0.332266223 | 1.69E-13 |
| ZNF774 | blue | -0.101947854 | 0.027598273 | -0.128641293 | 0.005367847 |
| ZNF791 | blue | -0.104173467 | 0.024366359 | -0.116249205 | 0.011938307 |
| ZNF800 | blue | 0.02271296 | 0.624431861 | -0.003625039 | 0.937726177 |
| ZNF816 | blue | 0.251084048 | 3.80E-08 | 0.25297179 | 2.98E-08 |
| ZNF839 | blue | 0.04021719 | 0.38587488 | 0.01653427 | 0.721559462 |
| ZNF841 | blue | 0.05849896 | 0.206996965 | 0.054364468 | 0.240978371 |
| ZNF844 | blue | -0.041067231 | 0.37590558 | -0.048633692 | 0.29427613 |
| ZNF846 | blue | -0.130125272 | 0.004855323 | -0.165192459 | 0.000337167 |
| ZNF865 | blue | -0.019093162 | 0.680677733 | 0.000710006 | 0.987791046 |
| ZNF91 | blue | -0.034978659 | 0.450789046 | -0.03812729 | 0.411060674 |
| ZP3 | blue | -0.041645668 | 0.369213159 | -0.017900034 | 0.699632024 |
| ZSCAN12 | blue | -0.394747471 | 7.28E-19 | -0.418584911 | 3.10E-21 |
| ZSCAN21 | blue | -0.014587277 | 0.753212978 | -0.053755619 | 0.246295647 |
| ZSCAN26 | blue | -0.273818364 | 1.78E-09 | -0.301485715 | 2.87E-11 |
| ZSCAN29 | blue | 0.146511561 | 0.001499373 | 0.116011951 | 0.012114311 |
| ZSCAN32 | blue | 0.035058491 | 0.449755697 | 0.00356396 | 0.938773363 |
| ZSWIM6 | blue | 0.035819363 | 0.439974452 | 0.028055497 | 0.545326076 |
| ZXDA | blue | -0.222496773 | 1.20E-06 | -0.241081594 | 1.34E-07 |
| ZXDB | blue | -0.108356684 | 0.019168775 | -0.139265414 | 0.002559673 |
| ZYG11B | blue | -0.206858234 | 6.57E-06 | -0.227660706 | 6.63E-07 |
| ZZZ3 | blue | -0.134453523 | 0.00360266 | -0.176181928 | 0.000129663 |
| ABAT | brown | 0.067898754 | 0.142903645 | 0.0807202 | 0.081412628 |
| ABCB11 | brown | -0.060076673 | 0.194989486 | -0.04812815 | 0.2993295 |
| ABCC11 | brown | -0.066297292 | 0.152593318 | -0.080250137 | 0.083208171 |
| ABCC6 | brown | -0.095221614 | 0.039694152 | -0.086460345 | 0.061913628 |
| ABCD1 | brown | -0.216144547 | 2.43E-06 | -0.204711535 | 8.22E-06 |
| ABHD6 | brown | 0.194301311 | 2.36E-05 | 0.15921113 | 0.00055354 |
| ABLIM2 | brown | -0.106879911 | 0.020881227 | -0.081766217 | 0.077529459 |
| AC002398 | brown | 0.132449811 | 0.004140741 | 0.10668528 | 0.021116547 |
| AC006547 | brown | 0.061290734 | 0.186102342 | 0.076492821 | 0.09873583 |
| AC006994 | brown | -0.050276978 | 0.27824566 | -0.053679243 | 0.246968404 |
| AC010547 | brown | -0.100330933 | 0.030171487 | -0.106956801 | 0.020788892 |

| | | | | | |
|---|---|---|---|---|---|
| AC09238‹ | brown | -0.061668504 | 0.183398826 | -0.037447469 | 0.419458467 |
| AC10453: | brown | 0.167402079 | 0.00027953 | 0.185370988 | 5.57E-05 |
| AC10480! | brown | 0.089184586 | 0.054108185 | 0.101017209 | 0.029055252 |
| AC10893! | brown | -0.030882728 | 0.505569857 | -0.039598173 | 0.393235024 |
| AC24510( | brown | -0.021429075 | 0.644154311 | -0.014018928 | 0.76253425 |
| ACAD10 | brown | 0.132486786 | 0.004130186 | 0.116628914 | 0.011661326 |
| ACADL | brown | 0.047722554 | 0.3034253 | 0.014247248 | 0.758785448 |
| ACCS | brown | -0.080915431 | 0.08067616 | -0.081562226 | 0.078274703 |
| ACOT7 | brown | 0.032468017 | 0.483962295 | 0.037960127 | 0.41311635 |
| ACSBG1 | brown | 0.00776398 | 0.867107332 | 0.000771634 | 0.986731423 |
| ACSM1 | brown | 0.079081832 | 0.087809565 | 0.07275444 | 0.116390796 |
| ACSS1 | brown | -0.096701565 | 0.036704476 | -0.072696525 | 0.116682582 |
| ACYP2 | brown | -0.131582287 | 0.004395477 | -0.125512832 | 0.006610912 |
| ADAMTS | brown | -0.037928333 | 0.413508023 | -0.037708054 | 0.416227654 |
| ADAMTS | brown | 0.114146126 | 0.013580207 | 0.091605221 | 0.047876632 |
| ADCYAP | brown | -0.005447841 | 0.906531835 | 0.002370719 | 0.959250266 |
| ADGB | brown | 0.030183124 | 0.515264022 | 0.042856154 | 0.355448863 |
| ADH1C | brown | 0.065470725 | 0.157785575 | 0.08144497 | 0.0787057 |
| ADH6 | brown | 0.147668758 | 0.001373524 | 0.156505545 | 0.00068883 |
| ADH7 | brown | 0.11105915 | 0.016349964 | 0.097128813 | 0.035877798 |
| ADPRHL | brown | 0.09254201 | 0.04563204 | 0.100690536 | 0.029582092 |
| AGBL2 | brown | -0.001633734 | 0.971911728 | 0.008076394 | 0.861812451 |
| AGPAT4 | brown | -0.145293331 | 0.001643233 | -0.142199963 | 0.002067231 |
| AGPAT6 | brown | -0.068453712 | 0.139658002 | -0.096273364 | 0.037549169 |
| AGR2 | brown | 0.302186429 | 2.57E-11 | 0.340387653 | 3.93E-14 |
| AGR3 | brown | 0.065406157 | 0.158196724 | 0.079954501 | 0.084353724 |
| AK7 | brown | 0.126007227 | 0.006398721 | 0.122490966 | 0.008050517 |
| AK8 | brown | 0.132395446 | 0.004156303 | 0.140040118 | 0.002420219 |
| AK9 | brown | 0.109394983 | 0.018039263 | 0.115795757 | 0.012276683 |
| AKAP14 | brown | 0.081199743 | 0.079613305 | 0.097218484 | 0.035706318 |
| AKNA | brown | 0.017766303 | 0.70176844 | 0.040540703 | 0.38206189 |
| AKR1C3 | brown | 0.081836586 | 0.077273719 | 0.079489922 | 0.086179583 |
| AKR1C4 | brown | 0.002015409 | 0.965353437 | 0.019299063 | 0.677426594 |
| ALDH3A | brown | 0.163015888 | 0.000404623 | 0.197907918 | 1.65E-05 |
| ALDH3B | brown | -0.189064639 | 3.92E-05 | -0.161010462 | 0.000477704 |
| ALDOC | brown | 0.058977707 | 0.203298166 | 0.031725377 | 0.494021889 |
| ALG14 | brown | 0.15286801 | 0.000919218 | 0.156005153 | 0.000716986 |
| ALG1L | brown | 0.050803707 | 0.273235143 | 0.066689834 | 0.15017334 |
| ALOX15 | brown | 0.129100235 | 0.005204368 | 0.132578801 | 0.004104027 |
| ALPK1 | brown | 0.086629512 | 0.061403271 | 0.089495697 | 0.053271308 |
| ALPP | brown | 0.109192146 | 0.018255238 | 0.126809287 | 0.006067452 |
| ALS2CR1 | brown | -0.117522058 | 0.011032107 | -0.113704705 | 0.013949061 |
| AMPD3 | brown | 0.052185762 | 0.260382056 | 0.062363323 | 0.178502264 |
| AMT | brown | -0.008469546 | 0.855158022 | -0.017282671 | 0.709514223 |
| ANAPC4 | brown | 0.144585229 | 0.001732564 | 0.12800351 | 0.005602704 |

| | | | | | |
|---|---|---|---|---|---|
| ANKDD1 | brown | 0.029840054 | 0.520052742 | 0.042512353 | 0.359324976 |
| ANKFN1 | brown | 0.26049975 | 1.11E-08 | 0.253755734 | 2.69E-08 |
| ANKK1 | brown | 0.087978565 | 0.057456366 | 0.092789662 | 0.045053648 |
| ANKMY1 | brown | 0.114710062 | 0.013121492 | 0.119432453 | 0.009786228 |
| ANKRD3 | brown | -0.152815794 | 0.000922991 | -0.155357966 | 0.000754982 |
| ANKRD3 | brown | 0.209226995 | 5.12E-06 | 0.214520554 | 2.90E-06 |
| ANKRD3 | brown | 0.299084269 | 4.18E-11 | 0.314658394 | 3.42E-12 |
| ANKRD4 | brown | 0.102692911 | 0.026477543 | 0.094528741 | 0.041163143 |
| ANKRD4 | brown | -0.04780709 | 0.302568587 | -0.049567926 | 0.285088405 |
| ANKRD5 | brown | -0.204709726 | 8.22E-06 | -0.198103472 | 1.61E-05 |
| ANKRD5 | brown | 0.218711515 | 1.83E-06 | 0.245265031 | 7.95E-08 |
| ANKRD6 | brown | -0.147016866 | 0.001443171 | -0.110866673 | 0.016537988 |
| ANKRD6 | brown | 0.075250845 | 0.104348095 | 0.08467519 | 0.0675155 |
| ANKS4B | brown | 0.003106288 | 0.946623332 | 0.017962359 | 0.698637156 |
| ANKUB1 | brown | 0.021015622 | 0.650560002 | 0.035629398 | 0.442405052 |
| ANXA13 | brown | -0.167746611 | 0.00027142 | -0.168013134 | 0.000265298 |
| ANXA8 | brown | 0.046097077 | 0.320210668 | 0.07148328 | 0.122927677 |
| ANXA8L | brown | -0.031026613 | 0.503588016 | -0.006435527 | 0.889685914 |
| APLN | brown | 0.195674033 | 2.06E-05 | 0.150008438 | 0.001148227 |
| APOBEC | brown | 0.116825618 | 0.011520087 | 0.126401236 | 0.006234012 |
| APOO | brown | 0.261997883 | 9.07E-09 | 0.269091776 | 3.44E-09 |
| AQP10 | brown | -0.045539721 | 0.326102891 | -0.036845505 | 0.426977745 |
| AQP3 | brown | 0.19967043 | 1.38E-05 | 0.216148406 | 2.43E-06 |
| AQP7 | brown | -0.066620143 | 0.150600833 | -0.045717302 | 0.324217974 |
| ARHGAP | brown | 0.102606054 | 0.026606144 | 0.117566262 | 0.011001761 |
| ARHGAP | brown | 0.217341896 | 2.13E-06 | 0.22927844 | 5.49E-07 |
| ARHGAP | brown | -0.090433463 | 0.050813711 | -0.146510807 | 0.001499459 |
| ARHGEF | brown | -0.217980557 | 1.98E-06 | -0.214213142 | 3.00E-06 |
| ARHGEF | brown | 0.231209421 | 4.38E-07 | 0.239704372 | 1.58E-07 |
| ARL3 | brown | 0.112120497 | 0.015346426 | 0.092169992 | 0.046512602 |
| ARL6 | brown | -0.012338516 | 0.790290708 | -0.025997242 | 0.575210552 |
| ARMC2 | brown | 0.018021801 | 0.697688784 | 0.024112908 | 0.603228456 |
| ARMC3 | brown | 0.146215881 | 0.001533186 | 0.157249468 | 0.00064886 |
| ARMC4 | brown | 0.034768916 | 0.453510308 | 0.031341588 | 0.499263961 |
| ART3 | brown | 0.159539445 | 0.000538918 | 0.146498855 | 0.001500812 |
| ASB12 | brown | 0.072085814 | 0.119794397 | 0.068656641 | 0.138485405 |
| ASRGL1 | brown | 0.24721205 | 6.23E-08 | 0.243792379 | 9.56E-08 |
| ASTN2 | brown | 0.023704495 | 0.609380866 | 0.009289985 | 0.841304957 |
| ATF7IP2 | brown | -0.117385399 | 0.011126391 | -0.120218034 | 0.009311286 |
| ATG9B | brown | -0.038553961 | 0.405841234 | -0.026775522 | 0.563820082 |
| ATP2B3 | brown | -0.149093668 | 0.001231917 | -0.150129403 | 0.001137561 |
| ATP4B | brown | -0.020779659 | 0.654227425 | -0.012308508 | 0.79078891 |
| ATP5S | brown | -0.020670792 | 0.655922286 | -0.022621047 | 0.625835129 |
| ATP6AP1 | brown | 0.093984969 | 0.042347931 | 0.074466422 | 0.108020955 |
| ATXN7L | brown | 0.075315709 | 0.104048868 | 0.072233804 | 0.119034448 |

| | | | | | |
|---|---|---|---|---|---|
| AVPI1 | brown | -0.007787923 | 0.866701327 | -0.024073456 | 0.603821558 |
| AXDND1 | brown | 0.080537619 | 0.082106292 | 0.087389517 | 0.059153084 |
| AZU1 | brown | 0.151743334 | 0.001003723 | 0.167722441 | 0.000271982 |
| B3GALT5 | brown | 0.139955721 | 0.002435067 | 0.159676461 | 0.000532923 |
| B3GNTL1 | brown | 0.016211026 | 0.726783518 | 0.00023998 | 0.995873262 |
| B9D1 | brown | 0.113857222 | 0.013820632 | 0.122941905 | 0.007819314 |
| B9D2 | brown | 0.166471301 | 0.000302586 | 0.182215562 | 7.48E-05 |
| BAIAP3 | brown | 0.095573516 | 0.038965211 | 0.094339434 | 0.041572395 |
| BARX1 | brown | 8.24E-05 | 0.998583452 | 0.006821505 | 0.88311575 |
| BBOX1 | brown | 0.055649087 | 0.230025047 | 0.063543742 | 0.170406904 |
| BBS12 | brown | -0.044453442 | 0.337787301 | -0.039797833 | 0.39085185 |
| BBS4 | brown | 0.233707155 | 3.26E-07 | 0.211226001 | 4.14E-06 |
| BBS5 | brown | 0.033247022 | 0.473530277 | 0.038443688 | 0.407186401 |
| BCAS1 | brown | -0.042485938 | 0.359623879 | -0.029656856 | 0.52261924 |
| BCAS3 | brown | -0.054726768 | 0.237852799 | -0.058120001 | 0.209959139 |
| BCO2 | brown | -0.126035469 | 0.006386787 | -0.117722815 | 0.010894883 |
| BEST4 | brown | 0.03427843 | 0.459909967 | 0.041269976 | 0.373551409 |
| BLCAP | brown | -0.376119286 | 3.85E-17 | -0.350123062 | 6.49E-15 |
| BMP15 | brown | 0.047632346 | 0.304341263 | 0.07212159 | 0.119610336 |
| BMP2 | brown | 0.02453636 | 0.596879088 | 0.016113343 | 0.728364719 |
| BMPR1B | brown | 0.197143847 | 1.78E-05 | 0.207294359 | 6.28E-06 |
| BTBD9 | brown | -0.077831756 | 0.092957293 | -0.064352984 | 0.165017644 |
| BTG4 | brown | -0.005550365 | 0.904781111 | -0.007093515 | 0.878490332 |
| C10orf10 | brown | 0.048864162 | 0.291991391 | 0.051545121 | 0.266287219 |
| C10orf53 | brown | -0.123398032 | 0.007591501 | -0.121264706 | 0.008710388 |
| C10orf67 | brown | -0.097852199 | 0.034514208 | -0.093731662 | 0.042909585 |
| C11orf16 | brown | 0.121003252 | 0.008857161 | 0.139538852 | 0.002509637 |
| C11orf49 | brown | 0.198644041 | 1.53E-05 | 0.194070842 | 2.41E-05 |
| C11orf52 | brown | 0.062775426 | 0.175644247 | 0.07476141 | 0.106627962 |
| C11orf63 | brown | 0.152698686 | 0.000931505 | 0.138095179 | 0.002784254 |
| C11orf70 | brown | -0.023432149 | 0.613498913 | -0.021517269 | 0.642791288 |
| C11orf74 | brown | 0.008576423 | 0.853350787 | -0.002684678 | 0.953859346 |
| C11orf88 | brown | -0.056973112 | 0.219109998 | -0.061094513 | 0.187518132 |
| C11orf91 | brown | -0.061617492 | 0.183762187 | -0.04483107 | 0.333695357 |
| C12orf60 | brown | -0.078437506 | 0.09043348 | -0.062867636 | 0.175009439 |
| C12orf74 | brown | 0.151208591 | 0.001046367 | 0.144691608 | 0.001718865 |
| C12orf76 | brown | -0.073851335 | 0.110971686 | -0.072459826 | 0.11788106 |
| C14orf10 | brown | -0.180068668 | 9.12E-05 | -0.171233364 | 0.000200836 |
| C14orf14 | brown | 0.101732744 | 0.027929338 | 0.129933194 | 0.004919076 |
| C14orf15 | brown | 0.042138526 | 0.363569526 | 0.060594134 | 0.191164358 |
| C14orf79 | brown | 0.002220221 | 0.961835109 | 0.004416678 | 0.924164003 |
| C14orf93 | brown | -7.78E-05 | 0.998661583 | -0.010502738 | 0.820918263 |
| C15orf26 | brown | 0.119330956 | 0.009849133 | 0.122923084 | 0.007828844 |
| C15orf65 | brown | 0.280189376 | 7.16E-10 | 0.295978563 | 6.76E-11 |
| C16orf46 | brown | 0.173860014 | 0.000159449 | 0.164522809 | 0.000356713 |

| | | | | | |
|---|---|---|---|---|---|
| C16orf71 | brown | 0.122230688 | 0.008186724 | 0.14732945 | 0.00140938 |
| C16orf93 | brown | 0.13743792 | 0.002918118 | 0.148049408 | 0.001334298 |
| C17orf50 | brown | 0.053508888 | 0.248473592 | 0.075499211 | 0.103206023 |
| C17orf97 | brown | 0.173887604 | 0.00015906 | 0.183201048 | 6.83E-05 |
| C18orf65 | brown | 0.260866088 | 1.06E-08 | 0.257395495 | 1.67E-08 |
| C19orf18 | brown | 0.125948539 | 0.006423586 | 0.125341216 | 0.00668603 |
| C19orf26 | brown | 0.288200419 | 2.20E-10 | 0.279312498 | 8.12E-10 |
| C19orf44 | brown | -0.145099551 | 0.00166725 | -0.161045848 | 0.000476315 |
| C1orf101 | brown | -0.01199789 | 0.795950787 | -0.015483256 | 0.738591386 |
| C1orf110 | brown | 0.075216394 | 0.104507302 | 0.088341602 | 0.056430893 |
| C1orf137 | brown | 0.139209963 | 0.002569931 | 0.13506112 | 0.003452503 |
| C1orf158 | brown | 0.098455553 | 0.033410873 | 0.111477893 | 0.015947364 |
| C1orf168 | brown | 0.039145706 | 0.398668117 | 0.036591998 | 0.430167658 |
| C1orf186 | brown | -0.024689312 | 0.594593162 | -0.004679162 | 0.919671838 |
| C1orf189 | brown | 0.11607489 | 0.012067398 | 0.119511327 | 0.009737589 |
| C1orf194 | brown | 0.139086759 | 0.002592855 | 0.14916031 | 0.001225636 |
| C1orf228 | brown | 0.11030212 | 0.017100442 | 0.121194884 | 0.00874937 |
| C1orf87 | brown | -0.044263018 | 0.339862853 | -0.036656665 | 0.429352631 |
| C1QL1 | brown | -0.099573168 | 0.031446476 | -0.100335324 | 0.03016423 |
| C1QTNF8 | brown | 0.107921385 | 0.019660349 | 0.113626145 | 0.014015623 |
| C20orf19 | brown | -0.114104582 | 0.013614552 | -0.109022865 | 0.018437207 |
| C20orf85 | brown | 0.056626664 | 0.221929575 | 0.069381762 | 0.134357104 |
| C20orf96 | brown | -0.022335222 | 0.630207545 | -0.028265335 | 0.542323062 |
| C21orf58 | brown | 0.095993982 | 0.038109134 | 0.097248695 | 0.035648703 |
| C21orf59 | brown | 0.102646187 | 0.026546656 | 0.099016261 | 0.03241263 |
| C22orf15 | brown | 0.104249701 | 0.024261707 | 0.107708782 | 0.019904402 |
| C22orf23 | brown | -0.079729966 | 0.085232243 | -0.081011105 | 0.080317222 |
| C22orf42 | brown | -0.024967475 | 0.590446144 | -0.039865652 | 0.390044344 |
| C2CD2L | brown | 0.233445797 | 3.36E-07 | 0.256445394 | 1.90E-08 |
| C2orf50 | brown | 0.06111471 | 0.187372042 | 0.086642679 | 0.061363692 |
| C2orf70 | brown | -0.037787752 | 0.415242468 | -0.02461627 | 0.595684306 |
| C2orf73 | brown | 0.019370443 | 0.67630091 | 0.031082617 | 0.502817751 |
| C2orf81 | brown | 0.100725733 | 0.029524937 | 0.124967806 | 0.006852123 |
| C2orf88 | brown | 0.097558816 | 0.035061823 | 0.116880224 | 0.011481147 |
| C4orf22 | brown | 0.034144233 | 0.461669648 | 0.045204784 | 0.329677342 |
| C4orf33 | brown | 0.183664155 | 6.54E-05 | 0.210659021 | 4.39E-06 |
| C4orf47 | brown | 0.123020501 | 0.007779629 | 0.129645324 | 0.005016038 |
| C5orf49 | brown | 0.034631219 | 0.455301849 | 0.055337105 | 0.232652145 |
| C5orf52 | brown | -0.004360508 | 0.925125614 | 0.01466674 | 0.751912544 |
| C6 | brown | -0.043053865 | 0.35323177 | -0.035040516 | 0.449988251 |
| C6orf118 | brown | 0.096190235 | 0.037715051 | 0.102949938 | 0.026100118 |
| C6orf165 | brown | 0.024129088 | 0.602985299 | 0.040539553 | 0.382075405 |
| C6orf201 | brown | 0.000605714 | 0.989584308 | 0.011738221 | 0.800272845 |
| C6orf223 | brown | 0.07772047 | 0.093427028 | 0.050210074 | 0.278886517 |
| C6orf229 | brown | -0.028671212 | 0.536537947 | -0.025023628 | 0.5896106 |

| | | | | | |
|---|---|---|---|---|---|
| C6orf52 | brown | 0.297100366 | 5.69E-11 | 0.318578982 | 1.78E-12 |
| C6orf57 | brown | 0.008951871 | 0.847008315 | 0.031338794 | 0.499302236 |
| C7orf57 | brown | 0.07862722 | 0.08965445 | 0.088166941 | 0.056922348 |
| C8B | brown | 0.031179476 | 0.501487023 | 0.027766497 | 0.549475407 |
| C8orf34 | brown | 0.137423413 | 0.002921137 | 0.134399305 | 0.003616343 |
| C8orf48 | brown | -0.181686228 | 7.86E-05 | -0.194087921 | 2.41E-05 |
| C9orf116 | brown | 0.25185467 | 3.44E-08 | 0.272342915 | 2.19E-09 |
| C9orf117 | brown | 0.119663342 | 0.009644453 | 0.123510644 | 0.007536178 |
| C9orf135 | brown | -0.07813776 | 0.091675395 | -0.064557232 | 0.163677862 |
| C9orf171 | brown | 0.087456831 | 0.058957115 | 0.101879763 | 0.027702701 |
| C9orf24 | brown | 0.109047807 | 0.018410296 | 0.122547888 | 0.008020999 |
| C9orf43 | brown | 0.031779975 | 0.493278545 | 0.019539619 | 0.673635824 |
| C9orf50 | brown | -0.037703981 | 0.416278041 | -0.035392747 | 0.445443676 |
| C9orf9 | brown | 0.188670003 | 4.08E-05 | 0.187886916 | 4.39E-05 |
| CABYR | brown | 0.061682272 | 0.183300846 | 0.074131995 | 0.109617512 |
| CACNA2 | brown | -0.258154132 | 1.51E-08 | -0.288273885 | 2.18E-10 |
| CACNG6 | brown | -0.266504687 | 4.92E-09 | -0.280285653 | 7.06E-10 |
| CALM1 | brown | 0.15296537 | 0.00091222 | 0.178533423 | 0.000104883 |
| CALML4 | brown | 0.324098956 | 6.98E-13 | 0.324121416 | 6.96E-13 |
| CALN1 | brown | 0.062423816 | 0.178080581 | 0.066847512 | 0.149209524 |
| CAPN9 | brown | 0.069650164 | 0.13285331 | 0.076595877 | 0.098281142 |
| CAPS | brown | 0.305981683 | 1.41E-11 | 0.313687009 | 4.02E-12 |
| CAPS2 | brown | -0.041653359 | 0.369124678 | -0.044130481 | 0.341312252 |
| CAPSL | brown | 0.108862583 | 0.018610959 | 0.122620783 | 0.00798334 |
| CARKD | brown | 0.042937938 | 0.354530701 | 0.034844799 | 0.452524711 |
| CASC1 | brown | 0.096708428 | 0.03669107 | 0.109459085 | 0.017971474 |
| CASP16 | brown | 0.024469727 | 0.597876198 | 0.02312464 | 0.618163312 |
| CATIP | brown | 0.089559958 | 0.053099799 | 0.105718493 | 0.022319889 |
| CATSPEI | brown | 0.031319246 | 0.499570032 | 0.053414325 | 0.24931187 |
| CBY1 | brown | 0.259191927 | 1.32E-08 | 0.259160939 | 1.32E-08 |
| CC2D2A | brown | 0.025516905 | 0.582294161 | 0.03021892 | 0.514765681 |
| CCDC103 | brown | 0.000892484 | 0.984653594 | 0.001022961 | 0.98241035 |
| CCDC108 | brown | 0.067870651 | 0.143069523 | 0.071777739 | 0.121388559 |
| CCDC113 | brown | 0.162045594 | 0.000438575 | 0.178885028 | 0.000101586 |
| CCDC114 | brown | 0.106641112 | 0.021170269 | 0.115617473 | 0.012412026 |
| CCDC125 | brown | 0.390634649 | 1.79E-18 | 0.405209313 | 7.00E-20 |
| CCDC13 | brown | 0.058873064 | 0.204102507 | 0.070033884 | 0.130726011 |
| CCDC138 | brown | 0.094009543 | 0.042293774 | 0.09141523 | 0.048342963 |
| CCDC146 | brown | 0.130572202 | 0.004709856 | 0.11964159 | 0.009657731 |
| CCDC148 | brown | 0.074976986 | 0.10561897 | 0.058572976 | 0.206421955 |
| CCDC151 | brown | -0.037532898 | 0.418397674 | -0.029737798 | 0.521484487 |
| CCDC153 | brown | -0.023520074 | 0.612168089 | -0.011631289 | 0.802054461 |
| CCDC157 | brown | 0.043024663 | 0.353558691 | 0.046425531 | 0.316771038 |
| CCDC160 | brown | 0.284402394 | 3.87E-10 | 0.292322012 | 1.18E-10 |
| CCDC17 | brown | 0.036499072 | 0.431340401 | 0.046148512 | 0.319670431 |

| | | | | | |
|---|---|---|---|---|---|
| CCDC17( | brown | 0.109108389 | 0.018345076 | 0.117915209 | 0.010764796 |
| CCDC173 | brown | 0.080515132 | 0.082192055 | 0.087595379 | 0.058555459 |
| CCDC176 | brown | 0.088382371 | 0.056316688 | 0.093640611 | 0.043112999 |
| CCDC18( | brown | -0.098013111 | 0.03421696 | -0.094370083 | 0.041505904 |
| CCDC24 | brown | 0.013522908 | 0.770697493 | 0.026721629 | 0.56460532 |
| CCDC28/ | brown | 0.074270686 | 0.10895316 | 0.088690517 | 0.055459669 |
| CCDC30 | brown | -0.057380994 | 0.215823401 | -0.054883267 | 0.236511542 |
| CCDC33 | brown | 0.011985857 | 0.796150927 | 0.025208739 | 0.586860027 |
| CCDC37 | brown | 0.081867519 | 0.077161519 | 0.095789901 | 0.038522635 |
| CCDC39 | brown | -0.112709212 | 0.01481342 | -0.109316644 | 0.01812241 |
| CCDC40 | brown | 0.14257231 | 0.002011367 | 0.151888498 | 0.000992426 |
| CCDC42I | brown | 0.120227767 | 0.009305533 | 0.135726486 | 0.003294601 |
| CCDC57 | brown | 0.134905287 | 0.003490463 | 0.131228804 | 0.004503271 |
| CCDC60 | brown | 0.068593479 | 0.138849565 | 0.092123622 | 0.046623353 |
| CCDC65 | brown | 0.029564215 | 0.523919538 | 0.033119823 | 0.475225194 |
| CCDC67 | brown | -0.30077487 | 3.21E-11 | -0.296002984 | 6.74E-11 |
| CCDC74/ | brown | -0.003905017 | 0.932927395 | -0.006798715 | 0.883503459 |
| CCDC74I | brown | -0.055906869 | 0.227870269 | -0.064495152 | 0.164084216 |
| CCDC78 | brown | 0.090178092 | 0.051473394 | 0.11209379 | 0.015371001 |
| CCDC81 | brown | -0.216476344 | 2.34E-06 | -0.209865521 | 4.78E-06 |
| CCDC87 | brown | 2.06E-05 | 0.999645282 | 0.001907607 | 0.967205575 |
| CCDC88( | brown | 0.24279337 | 1.08E-07 | 0.23665103 | 2.29E-07 |
| CCDC89 | brown | -0.226885363 | 7.25E-07 | -0.220963253 | 1.42E-06 |
| CCDC96 | brown | 0.143841424 | 0.001831174 | 0.166834243 | 0.000293392 |
| CCL15 | brown | -0.057727597 | 0.213058519 | -0.042017422 | 0.364951263 |
| CCL15-C | brown | -0.07937871 | 0.086621347 | -0.077285916 | 0.095279471 |
| CCL20 | brown | 0.211092669 | 4.20E-06 | 0.227309052 | 6.90E-07 |
| CCNA1 | brown | 0.16749828 | 0.000277243 | 0.18521241 | 5.66E-05 |
| CCNO | brown | 0.246987157 | 6.40E-08 | 0.277761643 | 1.02E-09 |
| CCT6B | brown | 0.162965388 | 0.000406328 | 0.146933936 | 0.00145226 |
| CD164L2 | brown | 0.080884495 | 0.080792498 | 0.091535849 | 0.048046466 |
| CDC20B | brown | 0.18950712 | 3.76E-05 | 0.196823203 | 1.84E-05 |
| CDC25B | brown | -0.215208275 | 2.69E-06 | -0.226607794 | 7.48E-07 |
| CDHR2 | brown | -0.003811197 | 0.934535197 | 0.011059808 | 0.811593171 |
| CDHR3 | brown | 0.084185762 | 0.069121945 | 0.09655229 | 0.036997093 |
| CDHR4 | brown | 0.137094913 | 0.002990279 | 0.148631078 | 0.001276344 |
| CDK20 | brown | 0.163378768 | 0.000392567 | 0.155567223 | 0.000742497 |
| CDKL1 | brown | -0.079160175 | 0.087494739 | -0.075300834 | 0.10411743 |
| CDKN1A | brown | 0.417207433 | 4.30E-21 | 0.440846302 | 1.26E-23 |
| CDNF | brown | -0.038776771 | 0.403131362 | -0.032965292 | 0.477288757 |
| CDRT1 | brown | 0.118462738 | 0.010402087 | 0.136821428 | 0.003048973 |
| CDS1 | brown | 0.180242802 | 8.97E-05 | 0.176359854 | 0.000127611 |
| CEACAM | brown | -0.025274648 | 0.585882108 | -0.010107908 | 0.827542942 |
| CEL | brown | 0.155751389 | 0.000731668 | 0.182647181 | 7.19E-05 |
| CELF3 | brown | -0.029349512 | 0.526939462 | -0.026321519 | 0.57045145 |

| | | | | | |
|---|---|---|---|---|---|
| CEP164 | brown | -0.180159002 | 9.04E-05 | -0.22440496 | 9.63E-07 |
| CEP19 | brown | -0.132662801 | 0.004080276 | -0.132017588 | 0.004265939 |
| CEP41 | brown | 0.033100369 | 0.475484708 | 0.033838844 | 0.46568805 |
| CEP83 | brown | 0.092845529 | 0.044924025 | 0.083069839 | 0.07290192 |
| CERCAM | brown | -0.117019393 | 0.011382431 | -0.131341472 | 0.004468657 |
| CERKL | brown | 0.238692267 | 1.79E-07 | 0.263787786 | 7.12E-09 |
| CES4A | brown | -0.134537286 | 0.003581614 | -0.142681462 | 0.001995254 |
| CETN2 | brown | -0.030406928 | 0.51215242 | -0.024308453 | 0.60029263 |
| CFAP221 | brown | 0.039424772 | 0.395311864 | 0.051807138 | 0.263861047 |
| CFAP36 | brown | 0.081974611 | 0.076774087 | 0.066893138 | 0.148931515 |
| CFAP43 | brown | 0.089228932 | 0.053988233 | 0.094907539 | 0.040354449 |
| CFAP45 | brown | 0.146953176 | 0.001450147 | 0.158756152 | 0.000574412 |
| CFAP46 | brown | 0.005082305 | 0.912777439 | 0.012530232 | 0.787109845 |
| CFAP52 | brown | -0.049105065 | 0.28961596 | -0.037192239 | 0.422637093 |
| CFAP53 | brown | 0.05814446 | 0.209767036 | 0.06423417 | 0.165800793 |
| CFAP54 | brown | -0.073729283 | 0.111564671 | -0.06560008 | 0.156964314 |
| CFAP57 | brown | 0.065606541 | 0.156923378 | 0.080236326 | 0.083261404 |
| CFAP58 | brown | -0.01014883 | 0.826855742 | -0.006983839 | 0.880354832 |
| CFAP61 | brown | 0.03532395 | 0.446329264 | 0.052748735 | 0.255267901 |
| CFAP69 | brown | 0.007499834 | 0.871588792 | 0.006590708 | 0.887043471 |
| CFAP70 | brown | -0.003552345 | 0.938972517 | 0.005459393 | 0.906334544 |
| CFAP74 | brown | 0.081553512 | 0.078306668 | 0.089437503 | 0.053427023 |
| CFAP99 | brown | 0.104555495 | 0.023845814 | 0.124110964 | 0.007247294 |
| CFI | brown | -0.173724033 | 0.000161378 | -0.180837354 | 8.50E-05 |
| CFP | brown | 0.021769235 | 0.638903816 | 0.046563524 | 0.315333181 |
| CH507-39 | brown | 0.101336303 | 0.028548427 | 0.09981661 | 0.031031937 |
| CHGA | brown | 0.109482358 | 0.017946919 | 0.145099924 | 0.001667203 |
| CHIA | brown | 0.042749132 | 0.356652635 | 0.053256177 | 0.250718212 |
| CHN2 | brown | -0.076129996 | 0.100349957 | -0.074350602 | 0.108571789 |
| CHRM4 | brown | 0.145297411 | 0.001642731 | 0.146368571 | 0.001515639 |
| CHST4 | brown | 0.077233521 | 0.095504792 | 0.117238841 | 0.011228297 |
| CHST5 | brown | 0.016765475 | 0.717830777 | 0.033677745 | 0.467815614 |
| CHST6 | brown | 0.319021455 | 1.65E-12 | 0.366681002 | 2.62E-16 |
| CHST9 | brown | -0.096373281 | 0.037350605 | -0.096506148 | 0.037087942 |
| CLEC19A | brown | -0.076850753 | 0.097163781 | -0.072331972 | 0.118532416 |
| CLGN | brown | 0.058921947 | 0.203726483 | 0.050975476 | 0.271614573 |
| CLHC1 | brown | 0.085336439 | 0.065393848 | 0.075107627 | 0.105011196 |
| CLIC6 | brown | -0.079282576 | 0.087004687 | -0.069370023 | 0.134423175 |
| CLMN | brown | 0.101801828 | 0.027822645 | 0.088627974 | 0.055632732 |
| CLUAP1 | brown | 0.000749931 | 0.98710458 | -0.002085349 | 0.964151908 |
| CNGA4 | brown | 0.101233358 | 0.028711101 | 0.107984672 | 0.019588205 |
| CNIH2 | brown | 0.101546518 | 0.028218701 | 0.102568405 | 0.026662054 |
| CNTD1 | brown | 0.062021436 | 0.18089937 | 0.089904254 | 0.052188701 |
| CNTF | brown | -0.025906743 | 0.576542045 | -0.023322946 | 0.615153563 |
| CNTN3 | brown | -0.08190715 | 0.077017957 | -0.101825247 | 0.027786555 |

| | | | | | |
|---|---|---|---|---|---|
| CNTN5 | brown | -0.095829499 | 0.038442107 | -0.085297182 | 0.065518258 |
| CNTNAP | brown | -0.030879376 | 0.50561607 | -0.044853383 | 0.333454573 |
| CNTNAP | brown | -0.015193323 | 0.743312819 | -0.006158663 | 0.894403424 |
| COBL | brown | 0.132865903 | 0.004023364 | 0.155273975 | 0.000760048 |
| COG7 | brown | 0.245206905 | 8.01E-08 | 0.272165736 | 2.24E-09 |
| COL21A1 | brown | 0.02956689 | 0.523881967 | 0.044332377 | 0.339105926 |
| COL28A1 | brown | 0.096022361 | 0.038051934 | 0.081409368 | 0.078836944 |
| COLCA2 | brown | -0.008944603 | 0.847131002 | -0.025702854 | 0.579547134 |
| COLQ | brown | -0.166460664 | 0.000302859 | -0.182947428 | 6.99E-05 |
| COQ10A | brown | 0.007494413 | 0.871680816 | -0.008245395 | 0.858950736 |
| COQ4 | brown | 0.216190308 | 2.41E-06 | 0.24720285 | 6.23E-08 |
| COX19 | brown | 0.012949218 | 0.780170602 | 0.018474436 | 0.690482783 |
| CPM | brown | 0.09766811 | 0.034856961 | 0.095533844 | 0.039046818 |
| CPNE2 | brown | 0.243088369 | 1.04E-07 | 0.254429056 | 2.47E-08 |
| CPXCR1 | brown | 0.073542599 | 0.112476492 | 0.075433601 | 0.103506751 |
| CR1L | brown | -0.073914669 | 0.110664955 | -0.045431357 | 0.327256601 |
| CRISP2 | brown | -0.142735501 | 0.001987321 | -0.14270278 | 0.001992122 |
| CRISP3 | brown | -0.052402061 | 0.258408857 | -0.045176275 | 0.329982755 |
| CRLF1 | brown | -0.016553241 | 0.721253257 | 0.007801323 | 0.866474107 |
| CROCC | brown | -0.086481194 | 0.06185054 | -0.097159919 | 0.035818234 |
| CSPP1 | brown | -0.016695825 | 0.718953337 | -0.03489782 | 0.451836773 |
| CT62 | brown | -0.077222342 | 0.095552921 | -0.064391593 | 0.164763758 |
| CTD-250 | brown | -0.021320537 | 0.645833391 | -0.007212203 | 0.87647339 |
| CTNNA2 | brown | 0.020171899 | 0.663711646 | 0.030939859 | 0.504782461 |
| CTXN1 | brown | 0.062405268 | 0.178209795 | 0.071692454 | 0.121832786 |
| CUBN | brown | 0.010315631 | 0.824056103 | 0.014868756 | 0.748609692 |
| CUEDC1 | brown | 0.274353725 | 1.65E-09 | 0.294382814 | 8.64E-11 |
| CWH43 | brown | 0.104183334 | 0.024352792 | 0.130543378 | 0.004719117 |
| CXCL1 | brown | -0.069487249 | 0.133764527 | -0.048023028 | 0.300387494 |
| CXCL17 | brown | 0.142595418 | 0.002007946 | 0.166136706 | 0.000311299 |
| CXCL2 | brown | 0.11974794 | 0.009592966 | 0.118277223 | 0.010523748 |
| CXCL3 | brown | 0.245312884 | 7.90E-08 | 0.258452648 | 1.45E-08 |
| CXCL5 | brown | 0.132980284 | 0.00399163 | 0.147577183 | 0.001383117 |
| CXCR2 | brown | 0.080969223 | 0.080474192 | 0.110619 | 0.016782715 |
| CXorf22 | brown | 0.00172442 | 0.970353298 | 0.027261441 | 0.556763808 |
| CXorf58 | brown | -0.011256739 | 0.808302923 | 0.014765137 | 0.750303243 |
| CXXC4 | brown | 0.068897013 | 0.137106248 | 0.04350214 | 0.34823727 |
| CYP2C8 | brown | -0.177514917 | 0.000115013 | -0.18539198 | 5.56E-05 |
| CYP4B1 | brown | -0.026767817 | 0.56393231 | -0.03324043 | 0.473618035 |
| CYSTM1 | brown | 0.205930219 | 7.24E-06 | 0.247167259 | 6.26E-08 |
| CYTH1 | brown | 0.12270989 | 0.00793752 | 0.127001316 | 0.00599046 |
| DAW1 | brown | -0.132938744 | 0.004003129 | -0.132381981 | 0.004160166 |
| DCAKD | brown | 0.341545185 | 3.19E-14 | 0.330956806 | 2.12E-13 |
| DCDC1 | brown | 0.019956463 | 0.667086615 | 0.020493507 | 0.658686085 |
| DCDC2 | brown | -0.12891785 | 0.005268791 | -0.119797754 | 0.009562763 |

| | | | | | |
|---|---|---|---|---|---|
| DCDC2B | brown | -0.029660761 | 0.522564456 | -0.027243808 | 0.557019115 |
| DDO | brown | -0.078534987 | 0.090032514 | -0.049700892 | 0.283796636 |
| DENND6 | brown | 0.220749949 | 1.46E-06 | 0.236199801 | 2.42E-07 |
| DFNB31 | brown | 0.18979558 | 3.66E-05 | 0.168816038 | 0.000247625 |
| DHRS3 | brown | 0.07802286 | 0.092155057 | 0.120431426 | 0.009185867 |
| DHRS7B | brown | 0.382045841 | 1.12E-17 | 0.39494704 | 6.97E-19 |
| DIO1 | brown | -0.109100076 | 0.018354013 | -0.089767988 | 0.052547726 |
| DIS3L2 | brown | 0.100721734 | 0.029531425 | 0.109371741 | 0.018063897 |
| DLEC1 | brown | 0.055822668 | 0.228572515 | 0.056367338 | 0.224057 |
| DMBT1 | brown | 0.097830371 | 0.034554698 | 0.089675766 | 0.052791874 |
| DNAAF1 | brown | 0.142939399 | 0.001957647 | 0.151355549 | 0.001034484 |
| DNAAF3 | brown | 0.113546179 | 0.014083662 | 0.119957216 | 0.009466646 |
| DNAH10 | brown | 0.034605536 | 0.455636443 | 0.038103427 | 0.411353759 |
| DNAH11 | brown | 0.123383079 | 0.007598875 | 0.132435642 | 0.004144792 |
| DNAH12 | brown | 0.066240361 | 0.152946733 | 0.075317401 | 0.10404107 |
| DNAH2 | brown | -0.077376952 | 0.09488899 | -0.072752517 | 0.116400477 |
| DNAH3 | brown | 0.089085403 | 0.054377273 | 0.097138051 | 0.0358601 |
| DNAH5 | brown | 0.039568675 | 0.393587857 | 0.048009738 | 0.300521435 |
| DNAH6 | brown | 0.02765058 | 0.551144039 | 0.032016471 | 0.49006557 |
| DNAH7 | brown | -0.030440061 | 0.511692596 | -0.023147377 | 0.617817902 |
| DNAH9 | brown | 0.07543015 | 0.103522588 | 0.081967322 | 0.076800406 |
| DNAI1 | brown | 0.075308544 | 0.104081889 | 0.091881155 | 0.047206069 |
| DNAI2 | brown | 0.009445666 | 0.838681661 | 0.016262843 | 0.72594523 |
| DNAJA4 | brown | 0.100839408 | 0.029340992 | 0.10546461 | 0.022645578 |
| DNAJB13 | brown | 0.122787925 | 0.007897586 | 0.138928518 | 0.002622572 |
| DNAJC1( | brown | 0.118155475 | 0.010604274 | 0.091823843 | 0.047344693 |
| DNAL1 | brown | 0.075417942 | 0.103578632 | 0.068975648 | 0.136657374 |
| DNAL4 | brown | 0.165663079 | 0.000324034 | 0.175222001 | 0.00014128 |
| DNALI1 | brown | -0.076310993 | 0.099542137 | -0.057337897 | 0.216168987 |
| DOC2A | brown | -0.029578882 | 0.523713572 | -0.024054055 | 0.604113315 |
| DPCD | brown | 0.274040538 | 1.72E-09 | 0.288284761 | 2.17E-10 |
| DPP10 | brown | -0.084316011 | 0.068691406 | -0.093254546 | 0.043984541 |
| DRC1 | brown | 0.043029727 | 0.353501983 | 0.055411435 | 0.232024323 |
| DRC7 | brown | 0.122343634 | 0.008127368 | 0.138259298 | 0.002751712 |
| DRICH1 | brown | 0.045156241 | 0.330197482 | 0.028258842 | 0.542415866 |
| DTHD1 | brown | 0.036056213 | 0.436954689 | 0.049748435 | 0.283335724 |
| DUOX1 | brown | 0.093669628 | 0.043048086 | 0.10435377 | 0.02411947 |
| DUOX2 | brown | 0.095477445 | 0.039163082 | 0.11075472 | 0.016648219 |
| DUOXA1 | brown | 0.082470098 | 0.075002083 | 0.085625594 | 0.064483456 |
| DUOXA2 | brown | 0.013956157 | 0.763565871 | 0.059970682 | 0.195779837 |
| DUSP14 | brown | 0.25760628 | 1.63E-08 | 0.239419019 | 1.64E-07 |
| DUSP18 | brown | 0.012963129 | 0.779940494 | 0.02426491 | 0.600945821 |
| DUSP19 | brown | -0.052746431 | 0.255288688 | -0.055508471 | 0.231206512 |
| DUSP5 | brown | -0.065552108 | 0.157268508 | -0.06306253 | 0.17367333 |
| DYDC1 | brown | 0.048220174 | 0.29840536 | 0.07233604 | 0.118511652 |

| | | | | | |
|---|---|---|---|---|---|
| DYDC2 | brown | -0.032534974 | 0.483060788 | -0.019257845 | 0.678076941 |
| DYNC2H | brown | -0.137919275 | 0.002819521 | -0.142095107 | 0.002083217 |
| DYNC2L | brown | -0.023798767 | 0.607958271 | -0.03246088 | 0.484058445 |
| DYNLL1 | brown | 0.103435526 | 0.025399708 | 0.135387891 | 0.003374116 |
| DYNLRB | brown | 0.075769674 | 0.10197363 | 0.087351221 | 0.059264814 |
| DYNLT1 | brown | 0.340627202 | 3.77E-14 | 0.379105977 | 2.07E-17 |
| DYRK3 | brown | 0.043418821 | 0.349162173 | 0.013137742 | 0.777053914 |
| DYX1C1 | brown | 0.137505089 | 0.002904174 | 0.142353133 | 0.002044081 |
| DZANK1 | brown | -0.068534703 | 0.139189096 | -0.084855832 | 0.066930373 |
| DZIP1L | brown | -0.058228634 | 0.209106887 | -0.053055283 | 0.252512616 |
| DZIP3 | brown | 0.078540326 | 0.090010593 | 0.062787965 | 0.175557823 |
| ECEL1 | brown | 0.150078929 | 0.001142 | 0.168319711 | 0.000258415 |
| ECHDC2 | brown | -0.029280357 | 0.527914046 | -0.044452668 | 0.337795721 |
| ECI2 | brown | 0.062260355 | 0.179221721 | 0.072260275 | 0.118898912 |
| ECT2L | brown | -0.04095198 | 0.377247883 | -0.027295311 | 0.556273565 |
| EEPD1 | brown | 0.196056297 | 1.98E-05 | 0.18331797 | 6.76E-05 |
| EFCAB1 | brown | 0.00055013 | 0.990540059 | 0.003393566 | 0.941695301 |
| EFCAB1( | brown | 0.077410024 | 0.094747451 | 0.085093707 | 0.066166213 |
| EFCAB11 | brown | 0.227550177 | 6.71E-07 | 0.228765271 | 5.83E-07 |
| EFCAB12 | brown | 0.068223058 | 0.141000032 | 0.079209644 | 0.087296416 |
| EFCAB2 | brown | 0.011729119 | 0.800424451 | 0.012103179 | 0.794200082 |
| EFCAB6 | brown | 0.006037579 | 0.896467759 | 0.005539215 | 0.904971491 |
| EFHB | brown | 0.019097007 | 0.680616976 | 0.029718082 | 0.521760778 |
| EFHC1 | brown | 0.00606836 | 0.895942912 | 0.014485194 | 0.7548846 |
| EFHC2 | brown | -0.107597438 | 0.020033264 | -0.096382045 | 0.037333232 |
| EGLN3 | brown | 0.197421954 | 1.73E-05 | 0.21994972 | 1.59E-06 |
| EIF4EBP | brown | -0.239738807 | 1.57E-07 | -0.231100495 | 4.44E-07 |
| ELL3 | brown | 0.244206628 | 9.08E-08 | 0.251058811 | 3.82E-08 |
| ELMOD1 | brown | -0.155960464 | 0.000719551 | -0.137981855 | 0.002806928 |
| ELP3 | brown | 0.354631889 | 2.76E-15 | 0.331675443 | 1.87E-13 |
| ENKD1 | brown | 0.192569472 | 2.79E-05 | 0.213801953 | 3.13E-06 |
| ENKUR | brown | 0.098191994 | 0.03388908 | 0.106664655 | 0.021141619 |
| ENO4 | brown | 0.091154001 | 0.048990351 | 0.098852456 | 0.032701588 |
| ENPP5 | brown | 0.116261003 | 0.011929614 | 0.098397939 | 0.033514916 |
| EPPIN | brown | 0.077434786 | 0.094641584 | 0.08468642 | 0.067479002 |
| EPX | brown | -0.114323155 | 0.013434711 | -0.11616926 | 0.01199736 |
| ERICH2 | brown | 0.158400599 | 0.000591229 | 0.160729986 | 0.000488852 |
| ERICH3 | brown | 0.161558349 | 0.000456606 | 0.148863178 | 0.001253872 |
| ERICH5 | brown | -0.17059268 | 0.000212357 | -0.149908343 | 0.001157122 |
| ERMARD | brown | 0.09631407 | 0.037468167 | 0.105131984 | 0.023078507 |
| ERP27 | brown | -0.329588428 | 2.70E-13 | -0.321099919 | 1.16E-12 |
| ETV4 | brown | 0.135477547 | 0.003352894 | 0.118915947 | 0.010110102 |
| ETV5 | brown | 0.049318914 | 0.287518197 | 0.033845438 | 0.465601079 |
| EYA2 | brown | 0.238277255 | 1.88E-07 | 0.24979563 | 4.48E-08 |
| F7 | brown | -0.094146223 | 0.041993622 | -0.082147543 | 0.076151807 |

| | | | | | |
|---|---|---|---|---|---|
| FABP6 | brown | -0.14579073 | 0.001583031 | -0.143220503 | 0.001917401 |
| FAIM | brown | -0.081471372 | 0.078608488 | -0.082773244 | 0.073934457 |
| FAM101/ | brown | -0.107465772 | 0.020186582 | -0.097713847 | 0.034771536 |
| FAM102/ | brown | 0.020348785 | 0.660945676 | 0.034887153 | 0.451975133 |
| FAM104E | brown | -0.021969944 | 0.635814215 | -0.020366118 | 0.660674881 |
| FAM110C | brown | 0.070731913 | 0.126923872 | 0.086880451 | 0.060652617 |
| FAM149/ | brown | 0.261361866 | 9.87E-09 | 0.260828609 | 1.06E-08 |
| FAM150E | brown | 0.086372839 | 0.062178991 | 0.07629952 | 0.099593192 |
| FAM151E | brown | 0.228195939 | 6.23E-07 | 0.220451987 | 1.51E-06 |
| FAM154/ | brown | -0.018262242 | 0.693857507 | -0.0149487 | 0.747303909 |
| FAM154E | brown | 0.080141608 | 0.083627237 | 0.084608513 | 0.067732537 |
| FAM160/ | brown | 0.140696181 | 0.00230758 | 0.12622746 | 0.006306181 |
| FAM161E | brown | -0.052768378 | 0.255090728 | -0.080848637 | 0.080927515 |
| FAM163/ | brown | -0.017070576 | 0.71292062 | -0.014379477 | 0.756616941 |
| FAM166E | brown | 0.107975493 | 0.019598655 | 0.128934404 | 0.005262914 |
| FAM170/ | brown | 0.084152348 | 0.069232753 | 0.121509886 | 0.008574723 |
| FAM174/ | brown | 0.206951863 | 6.51E-06 | 0.228950707 | 5.70E-07 |
| FAM179/ | brown | 0.019141259 | 0.679917771 | 0.030266685 | 0.514101114 |
| FAM179E | brown | -0.320175574 | 1.36E-12 | -0.338836836 | 5.21E-14 |
| FAM181/ | brown | -0.023925889 | 0.606042298 | -0.006107269 | 0.895279538 |
| FAM181E | brown | 0.020922538 | 0.652005745 | 0.015631893 | 0.736174688 |
| FAM182E | brown | -0.049898158 | 0.281887489 | -0.052726018 | 0.255472906 |
| FAM183/ | brown | 0.087029818 | 0.060209412 | 0.100717469 | 0.029538348 |
| FAM188E | brown | -0.048580405 | 0.294806073 | -0.027972606 | 0.546514603 |
| FAM206/ | brown | 0.120736348 | 0.00900926 | 0.110205638 | 0.01719822 |
| FAM216E | brown | 0.119224589 | 0.009915444 | 0.127759911 | 0.005694819 |
| FAM221/ | brown | -0.024542299 | 0.596790252 | -0.018301779 | 0.693228249 |
| FAM222/ | brown | 0.028520484 | 0.538682713 | 0.011375892 | 0.806313794 |
| FAM227/ | brown | 0.028755533 | 0.535339983 | 0.034060975 | 0.462763258 |
| FAM229E | brown | -0.025204385 | 0.586924658 | -0.01465855 | 0.752046556 |
| FAM27C | brown | -0.026649545 | 0.565656433 | -0.022909831 | 0.621430727 |
| FAM47E | brown | 0.090990086 | 0.049400263 | 0.10414769 | 0.024401833 |
| FAM81A | brown | 0.174035725 | 0.000156987 | 0.169047057 | 0.000242748 |
| FAM81B | brown | 0.069972975 | 0.131061914 | 0.084514301 | 0.068040172 |
| FAM83C | brown | -0.002330808 | 0.959935703 | 0.002476213 | 0.957438633 |
| FAM86B1 | brown | 0.121142322 | 0.008778819 | 0.114935149 | 0.012942251 |
| FAM86B2 | brown | 0.033765806 | 0.466651963 | 0.01764154 | 0.703763716 |
| FAM92B | brown | 0.116218473 | 0.011960978 | 0.130034657 | 0.004885306 |
| FANCF | brown | 0.097788455 | 0.034632567 | 0.060927219 | 0.188731456 |
| FANK1 | brown | 0.14613267 | 0.001542827 | 0.15592722 | 0.000721466 |
| FBXL13 | brown | -0.037029866 | 0.42466657 | -0.029413041 | 0.526044962 |
| FBXL2 | brown | -0.187706025 | 4.47E-05 | -0.180039347 | 9.14E-05 |
| FBXO15 | brown | 0.004397883 | 0.924485747 | 0.000751872 | 0.98707121 |
| FBXO16 | brown | 0.346010789 | 1.40E-14 | 0.339136092 | 4.94E-14 |
| FBXO36 | brown | 0.158620016 | 0.000580798 | 0.155478263 | 0.000747781 |

| | | | | | |
|---|---|---|---|---|---|
| FBXW10 | brown | 0.016298474 | 0.725368985 | 0.012593463 | 0.786061516 |
| FBXW9 | brown | 0.114536264 | 0.013261384 | 0.125362254 | 0.00667678 |
| FCGBP | brown | 0.083130663 | 0.072691635 | 0.097054186 | 0.036021041 |
| FER1L6 | brown | 0.008722389 | 0.850883834 | 0.021788682 | 0.638604188 |
| FFAR4 | brown | -0.037821723 | 0.414822954 | -0.01641957 | 0.723411708 |
| FGF14 | brown | 0.043776078 | 0.345207295 | 0.032540645 | 0.482984477 |
| FGFR1OI | brown | -0.047445517 | 0.306244124 | -0.052962712 | 0.253342464 |
| FGGY | brown | 0.11011096 | 0.017294646 | 0.093347576 | 0.043773181 |
| FHAD1 | brown | 0.041528283 | 0.370565278 | 0.056373838 | 0.2240035 |
| FHIT | brown | 0.099733552 | 0.031172843 | 0.086226886 | 0.062623686 |
| FHOD3 | brown | -0.166325468 | 0.000306355 | -0.167690017 | 0.000272737 |
| FKBP6 | brown | -0.062559971 | 0.177134197 | -0.032465568 | 0.483995292 |
| FMN1 | brown | 0.06209891 | 0.180354088 | 0.038452989 | 0.407072834 |
| FMO2 | brown | -0.06787608 | 0.143037469 | -0.066807477 | 0.149453793 |
| FMO4 | brown | -0.012361455 | 0.789909923 | -0.004672105 | 0.919792575 |
| FMO6P | brown | 0.047067026 | 0.310123106 | 0.055072184 | 0.234899562 |
| FOSL1 | brown | 0.097787724 | 0.034633925 | 0.084190051 | 0.069107734 |
| FOXA1 | brown | 0.181448744 | 8.03E-05 | 0.215866101 | 2.50E-06 |
| FOXB1 | brown | 0.070380348 | 0.128827972 | 0.072431262 | 0.118026337 |
| FOXB2 | brown | -0.021025068 | 0.650413375 | -0.015303854 | 0.741511724 |
| FOXD4L3 | brown | 0.112286796 | 0.015194185 | 0.108916211 | 0.018552665 |
| FOXJ1 | brown | 0.130604032 | 0.004699647 | 0.146887726 | 0.001457347 |
| FOXN4 | brown | 0.109390282 | 0.018044244 | 0.11562376 | 0.012407231 |
| FRAT1 | brown | 0.062781065 | 0.175605379 | 0.056784455 | 0.220642188 |
| FRMPD2 | brown | 0.153627473 | 0.000865924 | 0.155794017 | 0.000729182 |
| FSD1L | brown | 0.117532063 | 0.011025232 | 0.106080613 | 0.021862378 |
| FSIP1 | brown | -0.114748473 | 0.01309075 | -0.119943795 | 0.009474702 |
| FST | brown | -0.078760988 | 0.089108396 | -0.07604469 | 0.100732491 |
| FUCA1 | brown | 0.244499539 | 8.75E-08 | 0.255815725 | 2.06E-08 |
| FUZ | brown | -0.261497515 | 9.70E-09 | -0.256301598 | 1.93E-08 |
| GABRP | brown | 0.022603608 | 0.626101532 | 0.029265549 | 0.528122852 |
| GALC | brown | -0.191050984 | 3.24E-05 | -0.204666884 | 8.26E-06 |
| GALNS | brown | 0.149593653 | 0.001185503 | 0.158376836 | 0.000592369 |
| GALR2 | brown | 0.028009963 | 0.545978807 | 0.046129135 | 0.319873888 |
| GAS2L2 | brown | 0.140774931 | 0.002294386 | 0.153529757 | 0.000872617 |
| GAS8 | brown | 0.275854559 | 1.33E-09 | 0.283543239 | 4.39E-10 |
| GATS | brown | -0.213766004 | 3.14E-06 | -0.226173444 | 7.86E-07 |
| GEMIN8 | brown | 0.248906932 | 5.02E-08 | 0.255144532 | 2.25E-08 |
| GFOD2 | brown | 0.251038418 | 3.83E-08 | 0.269036444 | 3.47E-09 |
| GIPR | brown | 0.016699515 | 0.718893858 | 0.039182623 | 0.398223154 |
| GJB7 | brown | -0.101833884 | 0.027773256 | -0.095658748 | 0.038790374 |
| GK | brown | 0.018876843 | 0.684099775 | 0.028960838 | 0.532428802 |
| GLB1L | brown | 0.030682867 | 0.508329463 | 0.017472343 | 0.706472834 |
| GLB1L2 | brown | 0.097007491 | 0.036110918 | 0.084390961 | 0.068444654 |
| GLRA2 | brown | -0.089739281 | 0.052623623 | -0.103252529 | 0.025661735 |

| | | | | | |
|---|---|---|---|---|---|
| GLRB | brown | 0.139466911 | 0.002522714 | 0.134443149 | 0.003605275 |
| GLT8D1 | brown | 0.11656007 | 0.01171112 | 0.110698272 | 0.016704043 |
| GLYATL | brown | 0.215321706 | 2.65E-06 | 0.196881002 | 1.83E-05 |
| GLYCTK | brown | 0.176410643 | 0.000127031 | 0.179237123 | 9.84E-05 |
| GMNC | brown | 0.039973617 | 0.388760916 | 0.04824922 | 0.298114063 |
| GMPR | brown | 0.116910021 | 0.011459948 | 0.116653182 | 0.011643819 |
| GNL1 | brown | -0.234964248 | 2.81E-07 | -0.235905904 | 2.51E-07 |
| GNMT | brown | -0.001925563 | 0.966897058 | 0.030211692 | 0.514866288 |
| GOLGA8 | brown | -0.036919036 | 0.426055075 | -0.035680384 | 0.441751934 |
| GOLGA8 | brown | -0.051222246 | 0.269297897 | -0.056528787 | 0.222730826 |
| GOLGA8 | brown | -0.035217139 | 0.447706151 | -0.045767482 | 0.323686632 |
| GOLGA8 | brown | -0.004619247 | 0.920696996 | -0.002240371 | 0.961489004 |
| GOLGA8 | brown | -0.095522108 | 0.039070986 | -0.115368006 | 0.012603617 |
| GOLM1 | brown | 0.305983137 | 1.41E-11 | 0.297966008 | 4.97E-11 |
| GORASP | brown | 0.269682866 | 3.17E-09 | 0.271328343 | 2.52E-09 |
| GP2 | brown | 0.070400139 | 0.128720194 | 0.076755465 | 0.097580327 |
| GP6 | brown | -0.08361763 | 0.071025832 | -0.079280347 | 0.087013591 |
| GPC1 | brown | 0.038449008 | 0.407121437 | 0.022678335 | 0.624960337 |
| GPR123 | brown | -0.151666101 | 0.001009781 | -0.155397398 | 0.000752615 |
| GPR135 | brown | -0.172339292 | 0.000182315 | -0.168213032 | 0.000260791 |
| GPR162 | brown | -0.1382316 | 0.00275718 | -0.13784832 | 0.002833862 |
| GPR3 | brown | 0.016363874 | 0.724311696 | -0.012757439 | 0.783344709 |
| GPR97 | brown | -0.011269469 | 0.808090358 | 0.011821064 | 0.798893301 |
| GRAMD1 | brown | -0.058379794 | 0.207925161 | -0.088625565 | 0.055639408 |
| GRAMD2 | brown | -0.05628856 | 0.224706141 | -0.039626269 | 0.392899137 |
| GRIA2 | brown | -0.009454032 | 0.838540749 | 0.001738476 | 0.970111755 |
| GRIK4 | brown | -0.034875694 | 0.452123785 | -0.058217418 | 0.209194764 |
| GRIN3B | brown | 0.026089606 | 0.573853105 | 0.035915847 | 0.43874287 |
| GRIP2 | brown | -0.111159999 | 0.016252197 | -0.097860774 | 0.034498312 |
| GSAP | brown | 0.119194783 | 0.009934096 | 0.11208802 | 0.015376314 |
| GSTA1 | brown | -0.079394047 | 0.086560317 | -0.068824273 | 0.137522479 |
| GSTA3 | brown | 0.002411404 | 0.958551567 | 0.008588493 | 0.853146751 |
| GTF2IRD | brown | -0.104314863 | 0.024172562 | -0.113242228 | 0.014344938 |
| GUCY2F | brown | -0.016129622 | 0.728101123 | -0.00714772 | 0.877569084 |
| H1FOO | brown | 0.035242943 | 0.447373289 | 0.045452638 | 0.32702982 |
| HABP2 | brown | -0.013124271 | 0.777276498 | -0.008916777 | 0.847600746 |
| HCN4 | brown | -0.150435794 | 0.001110952 | -0.168678473 | 0.000250573 |
| HEATR9 | brown | -0.096211583 | 0.037672392 | -0.086531506 | 0.061698517 |
| HES5 | brown | 0.231242885 | 4.36E-07 | 0.217777857 | 2.03E-06 |
| HEY2 | brown | -0.07703788 | 0.09634989 | -0.084479994 | 0.068152482 |
| HHAT | brown | 0.136163881 | 0.003194402 | 0.091027166 | 0.049307284 |
| HHATL | brown | -0.093849083 | 0.042648453 | -0.088140486 | 0.056997094 |
| HHLA2 | brown | -0.10274702 | 0.026397699 | -0.095428106 | 0.039265033 |
| HIVEP3 | brown | 0.073851059 | 0.110973023 | 0.049466452 | 0.28607688 |
| HMGXB3 | brown | 0.23250831 | 3.76E-07 | 0.197861127 | 1.65E-05 |

| | | | | | |
|---|---|---|---|---|---|
| HNF1A | brown | 0.215263604 | 2.67E-06 | 0.227246857 | 6.95E-07 |
| HOMER2 | brown | 0.181240628 | 8.19E-05 | 0.196638545 | 1.87E-05 |
| HOXB1 | brown | -0.041109148 | 0.375418111 | 0.001983981 | 0.965893375 |
| HOXB2 | brown | -0.16007566 | 0.000515807 | -0.120969793 | 0.008876102 |
| HOXB3 | brown | -0.108188724 | 0.019357165 | -0.092317739 | 0.046161194 |
| HOXB4 | brown | -0.15055623 | 0.001100651 | -0.133300459 | 0.003904001 |
| HOXB5 | brown | -0.026943678 | 0.561373345 | 0.007280273 | 0.875316984 |
| HOXB6 | brown | -0.03149847 | 0.4971176 | 0.007037824 | 0.879437012 |
| HRASLS2 | brown | -0.134696078 | 0.003542021 | -0.090388465 | 0.050929437 |
| HS3ST6 | brown | -0.165380246 | 0.000331868 | -0.15499495 | 0.000777102 |
| HSBP1 | brown | 0.318982943 | 1.66E-12 | 0.358425316 | 1.33E-15 |
| HSD17B3 | brown | -0.025141401 | 0.587859915 | -0.018476519 | 0.690449676 |
| HSPA1L | brown | -0.119117858 | 0.009982378 | -0.113517095 | 0.01410848 |
| HYDIN | brown | -0.069933251 | 0.131281345 | -0.060174404 | 0.194262792 |
| ICA1L | brown | -0.095697815 | 0.038710458 | -0.090115533 | 0.051636082 |
| IDNK | brown | 0.330747313 | 2.20E-13 | 0.341171393 | 3.41E-14 |
| IFT122 | brown | -0.003723871 | 0.936031938 | -0.001507721 | 0.97407745 |
| IFT140 | brown | 0.098496422 | 0.033337238 | 0.099942826 | 0.03081886 |
| IFT172 | brown | -0.026813993 | 0.563259864 | -0.02890613 | 0.533203763 |
| IFT22 | brown | 0.048754361 | 0.293078404 | 0.060803613 | 0.18963162 |
| IFT27 | brown | 0.065029646 | 0.160610344 | 0.06088999 | 0.189002248 |
| IFT43 | brown | 0.08885815 | 0.054998015 | 0.111652847 | 0.015781744 |
| IFT46 | brown | 0.037272473 | 0.421636347 | 0.023541501 | 0.611843961 |
| IFT57 | brown | -0.184831345 | 5.86E-05 | -0.19434148 | 2.35E-05 |
| IFT74 | brown | -0.048894161 | 0.29169488 | -0.058050618 | 0.210504775 |
| IFT88 | brown | 0.078767896 | 0.089080269 | 0.069487445 | 0.133763431 |
| IGSF5 | brown | 0.135198977 | 0.003419234 | 0.12222309 | 0.008190731 |
| IK | brown | 0.004247769 | 0.927055995 | -0.001329492 | 0.977140881 |
| IL12A | brown | -0.139733312 | 0.002474596 | -0.144862006 | 0.00169713 |
| IL18R1 | brown | 0.154015578 | 0.000839806 | 0.147326764 | 0.001409667 |
| IL1R2 | brown | 0.155017097 | 0.000775736 | 0.172444719 | 0.000180635 |
| IL2 | brown | 0.068829023 | 0.137495273 | 0.071544263 | 0.12260768 |
| IL4 | brown | 0.036552224 | 0.430669384 | 0.061508016 | 0.184543797 |
| IL5RA | brown | 0.057310865 | 0.21638595 | 0.070149393 | 0.130090825 |
| IL7 | brown | -0.033214736 | 0.473960176 | -0.018438534 | 0.691053329 |
| INPP5E | brown | 0.080525181 | 0.08215372 | 0.070371362 | 0.128876927 |
| INSL5 | brown | -0.015146134 | 0.744082196 | 0.021126002 | 0.648847327 |
| INTU | brown | 0.056863796 | 0.219996888 | 0.041641612 | 0.369259828 |
| IPCEF1 | brown | -0.0216958 | 0.640035789 | -0.001870059 | 0.96785073 |
| IQCC | brown | -0.01279152 | 0.782780373 | -0.030300916 | 0.513625116 |
| IQCD | brown | 0.083257609 | 0.072254339 | 0.0989984 | 0.032444032 |
| IQCE | brown | 0.117913155 | 0.010766177 | 0.126638597 | 0.006136632 |
| IQCG | brown | 0.045886112 | 0.322432728 | 0.056983917 | 0.219022468 |
| IQCH | brown | 0.101660097 | 0.028041914 | 0.087204604 | 0.059694187 |
| IQCK | brown | 0.040642607 | 0.380865612 | 0.021271913 | 0.646586185 |

| | | | | | |
|---|---|---|---|---|---|
| IQGAP2 | brown | 0.163392656 | 0.000392113 | 0.166007124 | 0.000314736 |
| IQUB | brown | 0.122098506 | 0.008256681 | 0.132947691 | 0.00400065 |
| IRX3 | brown | 0.030470089 | 0.511276049 | 0.058707803 | 0.205377497 |
| ITIH2 | brown | 0.002271188 | 0.960959692 | 0.008263181 | 0.858649672 |
| ITIH4 | brown | -0.042192403 | 0.36295587 | -0.047015925 | 0.310649286 |
| KATNAL | brown | -0.066746407 | 0.149826994 | -0.079902748 | 0.084555564 |
| KATNB1 | brown | 0.247587523 | 5.94E-08 | 0.266534204 | 4.90E-09 |
| KCNC3 | brown | -0.116181344 | 0.011988417 | -0.115011457 | 0.012881977 |
| KCNE1 | brown | 0.078366092 | 0.09072813 | 0.093019754 | 0.044521799 |
| KCNH3 | brown | 0.15108614 | 0.001056364 | 0.149375486 | 0.001205554 |
| KCNH6 | brown | -0.017893796 | 0.699731626 | -0.017572268 | 0.704872423 |
| KCNMB2 | brown | -0.192231144 | 2.89E-05 | -0.198835837 | 1.50E-05 |
| KCNN3 | brown | -0.120318901 | 0.009251814 | -0.118291393 | 0.010514411 |
| KCNRG | brown | 0.170702022 | 0.000210348 | 0.182411257 | 7.35E-05 |
| KHK | brown | -0.16081839 | 0.000485313 | -0.174165421 | 0.000155194 |
| KIAA022 | brown | 0.037895035 | 0.413918449 | 0.029298014 | 0.527665123 |
| KIAA031 | brown | -0.070822515 | 0.126436721 | -0.051498635 | 0.26671925 |
| KIAA055 | brown | 0.05763763 | 0.213773727 | 0.065530821 | 0.157403634 |
| KIAA082 | brown | 0.008073515 | 0.861861221 | 0.004750074 | 0.918458665 |
| KIAA121 | brown | -0.051849493 | 0.26347029 | -0.028297733 | 0.541860149 |
| KIAA125 | brown | 0.08300646 | 0.073121569 | 0.090720292 | 0.050081199 |
| KIAA137 | brown | -0.062378194 | 0.178398529 | -0.067249258 | 0.146775113 |
| KIAA140 | brown | 0.03200984 | 0.490155514 | 0.001161369 | 0.980030914 |
| KIAA145 | brown | -0.015913893 | 0.731596803 | -0.040392621 | 0.383804349 |
| KIAA184 | brown | 0.125429458 | 0.00664731 | 0.127455647 | 0.005811785 |
| KIAA187 | brown | -0.027337473 | 0.555663608 | -0.027117332 | 0.558852006 |
| KIAA201 | brown | -0.096816202 | 0.036481093 | -0.092663882 | 0.045346633 |
| KIF17 | brown | -0.160033612 | 0.000517585 | -0.150659033 | 0.001091928 |
| KIF19 | brown | 0.025610702 | 0.580907746 | 0.04246246 | 0.359889673 |
| KIF24 | brown | 0.11913957 | 0.00996873 | 0.128022515 | 0.005595575 |
| KIF27 | brown | 0.142821048 | 0.001974821 | 0.125228777 | 0.006735659 |
| KIF6 | brown | 0.003178193 | 0.945389664 | -0.009127682 | 0.844041682 |
| KIF9 | brown | 0.185044663 | 5.75E-05 | 0.212654172 | 3.55E-06 |
| KLB | brown | -0.010996786 | 0.812646827 | 0.013607441 | 0.769304474 |
| KLHDC9 | brown | 0.219507605 | 1.67E-06 | 0.249736402 | 4.52E-08 |
| KLHL3 | brown | 0.042733402 | 0.35682978 | 0.011931862 | 0.797049203 |
| KLHL32 | brown | -0.166936961 | 0.000290838 | -0.155891879 | 0.000723506 |
| KLHL41 | brown | -0.017320585 | 0.708905894 | -0.006870695 | 0.88227899 |
| KLHL6 | brown | 0.003185562 | 0.945263233 | 0.035864414 | 0.439399146 |
| KLK11 | brown | 0.104584165 | 0.023807138 | 0.105562803 | 0.022519128 |
| KLLN | brown | 0.194323867 | 2.35E-05 | 0.186253733 | 5.13E-05 |
| KNCN | brown | 0.060150496 | 0.194440382 | 0.068567744 | 0.138998148 |
| KNDC1 | brown | -0.138660926 | 0.002673532 | -0.137132831 | 0.002982223 |
| KPNA7 | brown | 0.036097716 | 0.436426755 | 0.059776026 | 0.197237437 |
| LAMB4 | brown | 0.128309201 | 0.005489007 | 0.134964946 | 0.003475886 |

| | | | | | |
|---|---|---|---|---|---|
| LBHD1 | brown | 0.088480782 | 0.056041799 | 0.077680026 | 0.093598209 |
| LCA5 | brown | -0.099765238 | 0.031119024 | -0.111536431 | 0.01589178 |
| LCA5L | brown | 0.094237322 | 0.041794567 | 0.087299521 | 0.059415926 |
| LDLRAD | brown | 0.082367435 | 0.075366475 | 0.103717212 | 0.025000882 |
| LEKR1 | brown | -0.178107186 | 0.000109016 | -0.166663452 | 0.000297686 |
| LIPT2 | brown | -0.08633698 | 0.062288007 | -0.071014346 | 0.125410071 |
| LKAAEA | brown | -0.056074788 | 0.226474381 | -0.042855479 | 0.355456445 |
| LMLN | brown | 0.096281127 | 0.037533709 | 0.091771093 | 0.047472587 |
| LMNTD1 | brown | 0.032071478 | 0.489319882 | 0.033988392 | 0.463717837 |
| LMX1A | brown | 0.093802605 | 0.042751655 | 0.096747408 | 0.036615007 |
| LPA | brown | -0.072363857 | 0.118369715 | -0.056252208 | 0.225006133 |
| LPAR3 | brown | -0.164759821 | 0.000349677 | -0.156517996 | 0.000688143 |
| LPPR3 | brown | 0.089412719 | 0.053493456 | 0.103635599 | 0.025115875 |
| LRGUK | brown | 0.10755127 | 0.020086908 | 0.121516163 | 0.008571275 |
| LRIG1 | brown | 0.088818458 | 0.055107034 | 0.096235515 | 0.037624618 |
| LRP11 | brown | 0.173439822 | 0.000165482 | 0.167296225 | 0.000282067 |
| LRP2BP | brown | 0.096179628 | 0.037736261 | 0.09546162 | 0.039195756 |
| LRRC10E | brown | 0.051852969 | 0.263438237 | 0.056874973 | 0.219906084 |
| LRRC17 | brown | 0.088821562 | 0.055098503 | 0.075491074 | 0.103243279 |
| LRRC18 | brown | 0.091093594 | 0.049141082 | 0.108114072 | 0.019441414 |
| LRRC23 | brown | 0.076400629 | 0.099143998 | 0.095969569 | 0.038158401 |
| LRRC27 | brown | 0.078316675 | 0.090932476 | 0.073066773 | 0.114827058 |
| LRRC34 | brown | -0.232094303 | 3.95E-07 | -0.22494378 | 9.06E-07 |
| LRRC36 | brown | 0.152894725 | 0.000917293 | 0.147129616 | 0.001430897 |
| LRRC3B | brown | 0.034385368 | 0.458510404 | 0.059889161 | 0.196389311 |
| LRRC43 | brown | 0.105200198 | 0.022989144 | 0.121224589 | 0.008732767 |
| LRRC46 | brown | 0.162223552 | 0.000432156 | 0.173920243 | 0.000158601 |
| LRRC48 | brown | 0.119982661 | 0.009451388 | 0.127543114 | 0.005777941 |
| LRRC49 | brown | 0.014254841 | 0.75866087 | -0.013181179 | 0.776336308 |
| LRRC56 | brown | 0.206953284 | 6.51E-06 | 0.210132209 | 4.65E-06 |
| LRRC6 | brown | 0.080670366 | 0.081601485 | 0.087717822 | 0.058202378 |
| LRRC63 | brown | -0.035149901 | 0.448574146 | -0.029241865 | 0.528456899 |
| LRRC71 | brown | 0.125894904 | 0.006446386 | 0.134617991 | 0.003561441 |
| LRRC73 | brown | 0.059461065 | 0.199612642 | 0.083547085 | 0.0712652 |
| LRRC74E | brown | 0.084995986 | 0.066479263 | 0.088212585 | 0.056793573 |
| LRRIQ1 | brown | -0.077279569 | 0.095306744 | -0.074210607 | 0.109240559 |
| LRRIQ3 | brown | -0.021985903 | 0.635568812 | -0.020404912 | 0.660068973 |
| LRTOMT | brown | 0.203940679 | 8.91E-06 | 0.209021468 | 5.23E-06 |
| LRWD1 | brown | 0.16816939 | 0.000261769 | 0.179517909 | 9.59E-05 |
| LTF | brown | 0.104737991 | 0.02360056 | 0.130534317 | 0.004722032 |
| LY6G5C | brown | 0.271504987 | 2.46E-09 | 0.300215972 | 3.50E-11 |
| LY75 | brown | -0.005778694 | 0.900883767 | 0.031640331 | 0.495180967 |
| LZTFL1 | brown | -0.005970975 | 0.897603572 | -0.027754507 | 0.549647881 |
| MAATS1 | brown | -0.212425323 | 3.64E-06 | -0.222619563 | 1.18E-06 |
| MAGI1 | brown | 0.242926655 | 1.06E-07 | 0.218979777 | 1.77E-06 |

| | | | | | |
|---|---|---|---|---|---|
| MAK | brown | -0.000507351 | 0.991275654 | 0.017596567 | 0.704483435 |
| MAP1A | brown | -0.023967455 | 0.605416394 | -0.017263979 | 0.709814195 |
| MAP3K1! | brown | 0.091939608 | 0.047065034 | 0.100920697 | 0.029210056 |
| MAP6 | brown | -0.116083688 | 0.012060854 | -0.107994775 | 0.01957671 |
| MAPK12 | brown | 0.088199378 | 0.05683081 | 0.088418153 | 0.056216608 |
| MAPK15 | brown | -0.000782419 | 0.986545988 | -0.010452194 | 0.821765619 |
| MAPK8IF | brown | -0.035469862 | 0.444452211 | -0.061762814 | 0.18272844 |
| MAPRE3 | brown | 0.040747334 | 0.379638587 | 0.043547414 | 0.347735344 |
| 10-Mar | brown | -0.089367961 | 0.053613601 | -0.084429084 | 0.068319423 |
| 4-Mar | brown | -0.04553826 | 0.326118436 | -0.056780342 | 0.22067568 |
| MARK4 | brown | -0.158464991 | 0.00058815 | -0.170825806 | 0.000208095 |
| MAST2 | brown | -0.005960489 | 0.897782417 | -0.032533217 | 0.483084434 |
| MB | brown | 0.178612898 | 0.000104129 | 0.184500733 | 6.05E-05 |
| MBD3L1 | brown | -0.08369729 | 0.070756325 | -0.063007307 | 0.174051144 |
| MBOAT4 | brown | 0.055878677 | 0.228105225 | 0.035374485 | 0.445678651 |
| MCIDAS | brown | 0.166178569 | 0.000310196 | 0.179212179 | 9.86E-05 |
| MDH1B | brown | -0.004947018 | 0.915090367 | -0.018465162 | 0.690630145 |
| MDM1 | brown | 0.018769831 | 0.685795033 | 0.012829484 | 0.782151876 |
| MDM2 | brown | 0.35791118 | 1.47E-15 | 0.36968689 | 1.43E-16 |
| MED25 | brown | -0.011950318 | 0.796742139 | -0.014226241 | 0.759130131 |
| MEIG1 | brown | -0.026730974 | 0.564469123 | -0.019508568 | 0.674124681 |
| MEIS1 | brown | -0.23978006 | 1.57E-07 | -0.214152876 | 3.02E-06 |
| METTL2( | brown | 0.053902243 | 0.245007694 | 0.047977074 | 0.300850777 |
| MFAP3L | brown | 0.216206675 | 2.41E-06 | 0.221713983 | 1.31E-06 |
| MGC507: | brown | 0.030974364 | 0.504307211 | 0.034551058 | 0.456346629 |
| MGST2 | brown | 0.2564813 | 1.89E-08 | 0.268538983 | 3.72E-09 |
| MID1IP1 | brown | 0.105613766 | 0.022453741 | 0.104945924 | 0.023323783 |
| MIPEP | brown | 0.230485229 | 4.77E-07 | 0.221517622 | 1.34E-06 |
| MKS1 | brown | 0.025510466 | 0.582389398 | 0.022455971 | 0.628358793 |
| MKX | brown | -0.02882328 | 0.534378461 | -0.04354874 | 0.347720642 |
| MMP1 | brown | 0.072389387 | 0.11823957 | 0.071021603 | 0.125371362 |
| MNS1 | brown | -0.031878571 | 0.491937679 | -0.033044835 | 0.476225956 |
| MOBP | brown | -0.189076408 | 3.92E-05 | -0.169269751 | 0.000238132 |
| MOK | brown | 0.024493424 | 0.597521498 | 0.035567919 | 0.443193305 |
| MORN1 | brown | -0.089942975 | 0.052087055 | -0.081105398 | 0.079964733 |
| MORN2 | brown | 0.092163494 | 0.046528108 | 0.109504932 | 0.017923128 |
| MORN3 | brown | 0.110234279 | 0.017169144 | 0.117511614 | 0.011039287 |
| MORN4 | brown | 0.149810534 | 0.001165876 | 0.127501707 | 0.005793941 |
| MORN5 | brown | 0.040015019 | 0.388269423 | 0.050949373 | 0.271860417 |
| MPPED2 | brown | 0.143909142 | 0.001821988 | 0.144023165 | 0.001806616 |
| MRAP2 | brown | 0.256274275 | 1.94E-08 | 0.266273491 | 5.08E-09 |
| MROH9 | brown | 0.060597875 | 0.191136906 | 0.067923555 | 0.142757375 |
| MS4A8 | brown | -0.002028733 | 0.96512454 | 0.010753946 | 0.816709954 |
| MSH5-SA | brown | -0.122116688 | 0.008247027 | -0.125789794 | 0.006491278 |
| MSMB | brown | 0.137199238 | 0.002968161 | 0.140108649 | 0.002408223 |

| | | | | | |
|---|---|---|---|---|---|
| MSX1 | brown | 0.333979615 | 1.25E-13 | 0.341292801 | 3.34E-14 |
| MTHFSD | brown | 0.190051946 | 3.57E-05 | 0.190127328 | 3.54E-05 |
| MTMR8 | brown | 0.00634061 | 0.891302788 | 0.008289914 | 0.858197199 |
| MUC13 | brown | 0.182221023 | 7.48E-05 | 0.196906356 | 1.82E-05 |
| MUC4 | brown | -0.174669012 | 0.000148409 | -0.141614443 | 0.002157953 |
| MYB | brown | 0.221444039 | 1.35E-06 | 0.213513773 | 3.23E-06 |
| MYCBP | brown | 0.210499261 | 4.47E-06 | 0.230130332 | 4.97E-07 |
| MYCBPA | brown | 0.10141733 | 0.028420945 | 0.102293166 | 0.027073876 |
| MYLK3 | brown | 0.029533872 | 0.5243458 | 0.029387156 | 0.526409336 |
| N4BP3 | brown | -0.220470591 | 1.50E-06 | -0.225381809 | 8.61E-07 |
| N6AMT2 | brown | 0.093862084 | 0.042619624 | 0.086991665 | 0.060322364 |
| NCBP2L | brown | -0.071336918 | 0.12369833 | -0.059165136 | 0.201863227 |
| NCKAP5l | brown | -0.100248791 | 0.030307523 | -0.09247819 | 0.045782099 |
| NDRG1 | brown | -0.073262373 | 0.113856179 | -0.068494015 | 0.139424509 |
| NEIL1 | brown | 0.264793699 | 6.21E-09 | 0.260300487 | 1.14E-08 |
| NEK10 | brown | 0.101145404 | 0.028850716 | 0.107075841 | 0.02064664 |
| NEK11 | brown | 0.021479001 | 0.643382567 | 0.017809574 | 0.701076911 |
| NEK5 | brown | 0.077247599 | 0.09544421 | 0.09100056 | 0.049373984 |
| NGB | brown | -0.21547911 | 2.61E-06 | -0.21603486 | 2.46E-06 |
| NHLRC4 | brown | 0.099859259 | 0.030959798 | 0.107024948 | 0.020707353 |
| NICN1 | brown | 0.083382569 | 0.071825984 | 0.091182979 | 0.04891818 |
| NMBR | brown | -0.080589605 | 0.081908301 | -0.070195516 | 0.129837867 |
| NME5 | brown | 0.156820748 | 0.000671624 | 0.166422655 | 0.000303838 |
| NME7 | brown | 0.002993886 | 0.948552061 | -0.013351851 | 0.773518589 |
| NME9 | brown | 0.033089015 | 0.475636206 | 0.042092805 | 0.364090793 |
| NOX5 | brown | 0.018234465 | 0.694299718 | 0.029614612 | 0.523211971 |
| NOXRED | brown | -0.041609157 | 0.369633384 | -0.032071903 | 0.489314116 |
| NPAS3 | brown | 0.089850253 | 0.052330734 | 0.092136723 | 0.04659204 |
| NPHP1 | brown | 0.01258756 | 0.786159362 | 0.012238121 | 0.791957808 |
| NPHP4 | brown | 0.06200866 | 0.180989406 | 0.044191328 | 0.340646349 |
| NQO1 | brown | 0.349509922 | 7.29E-15 | 0.384311856 | 6.94E-18 |
| NR2E3 | brown | -0.084274934 | 0.068826948 | -0.067825553 | 0.14333603 |
| NRF1 | brown | -0.088430671 | 0.056181633 | -0.088600567 | 0.055708713 |
| NRG4 | brown | -0.18707615 | 4.74E-05 | -0.173119307 | 0.000170228 |
| NRM | brown | -0.041160876 | 0.374817089 | -0.036182688 | 0.435347049 |
| NRTN | brown | 0.210485795 | 4.48E-06 | 0.220474827 | 1.50E-06 |
| NSUN7 | brown | -0.050632952 | 0.274852672 | -0.060617191 | 0.190995212 |
| NTN1 | brown | 0.093578158 | 0.043252992 | 0.080813612 | 0.081059575 |
| NUCB2 | brown | 0.199507303 | 1.40E-05 | 0.192071046 | 2.93E-05 |
| NUP62Cl | brown | 0.135497941 | 0.003348083 | 0.140474585 | 0.002345079 |
| NWD1 | brown | 0.185892433 | 5.31E-05 | 0.195898077 | 2.01E-05 |
| NXF3 | brown | 0.050832992 | 0.272958388 | 0.073253875 | 0.113898222 |
| NXNL2 | brown | 0.051226268 | 0.269260248 | 0.072368602 | 0.118345517 |
| ODF2 | brown | 0.286005545 | 3.05E-10 | 0.278691055 | 8.88E-10 |
| ODF2L | brown | 0.092554368 | 0.045603029 | 0.070850077 | 0.126288815 |

| | | | | | |
|---|---|---|---|---|---|
| ODF3B | brown | 0.076155048 | 0.100237833 | 0.115298646 | 0.012657347 |
| ODF3L1 | brown | -0.07522153 | 0.104483556 | -0.064127995 | 0.166502987 |
| OLFM4 | brown | 0.116098898 | 0.012049546 | 0.12385698 | 0.007368284 |
| OMG | brown | 0.158805768 | 0.000572101 | 0.175815289 | 0.00013399 |
| OR2C1 | brown | -0.028591315 | 0.537674296 | -0.003690145 | 0.936610059 |
| OR6B3 | brown | 0.107138755 | 0.0205718 | 0.105899023 | 0.022090775 |
| OSCP1 | brown | 0.132029656 | 0.004262397 | 0.147798175 | 0.00136007 |
| OTUB2 | brown | 0.138400492 | 0.002723994 | 0.108651767 | 0.018841666 |
| OVGP1 | brown | 0.034879917 | 0.452068996 | 0.040942369 | 0.377359951 |
| OXTR | brown | -0.231579645 | 4.19E-07 | -0.246240059 | 7.04E-08 |
| P2RX2 | brown | -0.172663551 | 0.000177195 | -0.168000652 | 0.000265582 |
| P4HTM | brown | 0.205808235 | 7.34E-06 | 0.209799039 | 4.82E-06 |
| PACRG | brown | -0.024306024 | 0.600329063 | -0.033764651 | 0.466667218 |
| PAK7 | brown | 0.063282439 | 0.172174866 | 0.035424179 | 0.445039404 |
| PALMD | brown | 0.126171148 | 0.006329728 | 0.128958865 | 0.005254241 |
| PAM | brown | 0.105123096 | 0.023090172 | 0.090234981 | 0.051325821 |
| PAPSS1 | brown | 0.198121569 | 1.61E-05 | 0.174899184 | 0.000145402 |
| PBLD | brown | 0.204475654 | 8.43E-06 | 0.199055531 | 1.47E-05 |
| PCDHB1 | brown | -0.252757469 | 3.07E-08 | -0.250772409 | 3.96E-08 |
| PCSK4 | brown | 0.183206142 | 6.83E-05 | 0.190730463 | 3.34E-05 |
| PCYOX1l | brown | 0.072849216 | 0.115914534 | 0.055967496 | 0.227365587 |
| PCYT1B | brown | 0.066929924 | 0.148707654 | 0.075430742 | 0.103519871 |
| PDLIM1 | brown | 0.24861739 | 5.21E-08 | 0.269164883 | 3.41E-09 |
| PEX11A | brown | 0.154728812 | 0.000793699 | 0.131250675 | 0.004496533 |
| PEX6 | brown | -0.039015009 | 0.400245845 | -0.028859505 | 0.533864684 |
| PF4V1 | brown | 0.101931214 | 0.027623761 | 0.123002611 | 0.007788646 |
| PFN4 | brown | -0.047977816 | 0.300843297 | -0.066264445 | 0.15279715 |
| PHF19 | brown | -0.132981921 | 0.003991178 | -0.133964513 | 0.003727784 |
| PHF7 | brown | 0.291225066 | 1.40E-10 | 0.308877798 | 8.82E-12 |
| PHTF1 | brown | 0.031494387 | 0.497173395 | 0.028602186 | 0.537519615 |
| PHYHIPL | brown | 0.122694357 | 0.00794549 | 0.127776152 | 0.005688635 |
| PIFO | brown | 0.076082399 | 0.10056325 | 0.09412715 | 0.042035399 |
| PIH1D2 | brown | 0.080030742 | 0.084057088 | 0.08764949 | 0.058399203 |
| PIH1D3 | brown | 0.06587511 | 0.155228916 | 0.077639955 | 0.093768061 |
| PIK3R1 | brown | 0.167990436 | 0.000265814 | 0.1674934 | 0.000277359 |
| PIP4K2A | brown | 0.196550114 | 1.89E-05 | 0.175946032 | 0.000132432 |
| PITPNM1 | brown | 0.061289437 | 0.186111674 | 0.083978669 | 0.069811046 |
| PKHD1L1 | brown | 0.02072875 | 0.655019771 | 0.018091394 | 0.696579076 |
| PKP4 | brown | 0.152886205 | 0.000917906 | 0.119953553 | 0.009468844 |
| PLA2G10 | brown | 0.224682586 | 9.33E-07 | 0.265044228 | 6.00E-09 |
| PLAC8 | brown | 0.003630782 | 0.937627721 | 0.043500548 | 0.34825493 |
| PLCB3 | brown | 0.129158573 | 0.005183912 | 0.119649625 | 0.009652825 |
| PLCH1 | brown | 0.191173489 | 3.20E-05 | 0.191189005 | 3.20E-05 |
| PLEKHB | brown | 0.102862515 | 0.026227968 | 0.100413224 | 0.030035731 |
| PLEKHG | brown | 0.17713424 | 0.00011903 | 0.175130635 | 0.000142436 |

| | | | | | |
|---|---|---|---|---|---|
| PLEKHH | brown | 0.213825661 | 3.12E-06 | 0.172568965 | 0.000178675 |
| PLEKHS1 | brown | 0.314375519 | 3.59E-12 | 0.33432434 | 1.17E-13 |
| PLG | brown | 0.028061128 | 0.545245383 | 0.028011716 | 0.545953669 |
| PLIN2 | brown | -0.216471033 | 2.34E-06 | -0.221753478 | 1.30E-06 |
| PLXNB1 | brown | -0.009401754 | 0.839421423 | -0.030766782 | 0.507169827 |
| PNLDC1 | brown | -0.023201569 | 0.616994976 | -0.002523805 | 0.956621409 |
| PODXL | brown | 0.012382108 | 0.789567142 | 0.02217406 | 0.632678639 |
| POFUT2 | brown | -0.072030231 | 0.120080801 | -0.087595927 | 0.058553873 |
| PPAPDC1 | brown | 0.330444568 | 2.32E-13 | 0.336725404 | 7.62E-14 |
| PPIL6 | brown | 0.048463309 | 0.295972856 | 0.064630565 | 0.163198822 |
| PPM1E | brown | -0.048445956 | 0.296146028 | -0.042998622 | 0.353850381 |
| PPOX | brown | 0.09915702 | 0.032166072 | 0.099107992 | 0.032251769 |
| PPP1R13I | brown | -0.018457839 | 0.690746513 | -0.008274707 | 0.858454591 |
| PPP1R14( | brown | -0.077372558 | 0.094907806 | -0.085199299 | 0.065829306 |
| PPP1R32 | brown | 0.108750513 | 0.018733294 | 0.120397801 | 0.00920553 |
| PPP1R36 | brown | 0.081255413 | 0.079406525 | 0.093481031 | 0.043471471 |
| PPP1R42 | brown | 0.02193706 | 0.636319982 | 0.029086955 | 0.530644464 |
| PRM2 | brown | 0.03613744 | 0.435921807 | 0.037793892 | 0.415166626 |
| PRMT8 | brown | -0.078889086 | 0.088587995 | -0.088456997 | 0.056108135 |
| PROC | brown | -0.000216587 | 0.996275542 | 0.025092575 | 0.588585415 |
| PRR18 | brown | -0.083163961 | 0.072576724 | -0.069904108 | 0.131442505 |
| PRR29 | brown | 0.015361054 | 0.740580215 | 0.029991722 | 0.517932884 |
| PRR32 | brown | 0.043245614 | 0.351089877 | 0.031671502 | 0.494755977 |
| PRRX2 | brown | -0.153673289 | 0.000862802 | -0.143835971 | 0.001831915 |
| PRSS12 | brown | -0.194632128 | 2.28E-05 | -0.165645576 | 0.000324513 |
| PSENEN | brown | -0.000176552 | 0.996963976 | 0.010310631 | 0.824139977 |
| PTGR2 | brown | -0.009042683 | 0.845475683 | -0.0033471 | 0.942492238 |
| PTPLAD2 | brown | -0.068064115 | 0.14193056 | -0.030366581 | 0.512712651 |
| PTPRQ | brown | -0.070786439 | 0.126630521 | -0.077315111 | 0.095154105 |
| PTPRT | brown | -0.256118276 | 1.98E-08 | -0.258854381 | 1.38E-08 |
| PYY | brown | 0.149991317 | 0.001149744 | 0.141485843 | 0.00217836 |
| PZP | brown | 0.098847927 | 0.032709609 | 0.092516168 | 0.045692752 |
| QRICH2 | brown | 0.169703812 | 0.000229369 | 0.140921811 | 0.002269962 |
| RAB36 | brown | 0.073185016 | 0.114239374 | 0.074484619 | 0.107934614 |
| RAB44 | brown | -0.066451345 | 0.151640096 | -0.060833896 | 0.189410794 |
| RABL2A | brown | -0.101950835 | 0.027593709 | -0.105564508 | 0.022516938 |
| RABL2B | brown | 0.147025936 | 0.00144218 | 0.140276039 | 0.002379149 |
| RAET1E | brown | 0.067451826 | 0.145559172 | 0.091256319 | 0.048735924 |
| RALGPS1 | brown | 0.107097272 | 0.02062112 | 0.090835808 | 0.049788693 |
| RASGEF1 | brown | -0.203031314 | 9.78E-06 | -0.218927322 | 1.78E-06 |
| RBKS | brown | 0.063991188 | 0.167411058 | 0.069340508 | 0.134589402 |
| RCAN3 | brown | 0.166766755 | 0.000295082 | 0.163401482 | 0.000391824 |
| RDH12 | brown | 0.026211798 | 0.572059629 | 0.032903353 | 0.478117253 |
| REM2 | brown | 0.092049484 | 0.046800883 | 0.076733477 | 0.097676647 |
| RFX2 | brown | 0.034746668 | 0.453799506 | 0.041103808 | 0.375480195 |

| | | | | | |
|---|---|---|---|---|---|
| RFX3 | brown | 0.115393888 | 0.012583619 | 0.108666263 | 0.018825723 |
| RGS12 | brown | 0.105244362 | 0.022931447 | 0.08132563 | 0.079146334 |
| RGS22 | brown | -0.180462219 | 8.80E-05 | -0.181360192 | 8.10E-05 |
| RGS6 | brown | -0.040492816 | 0.382624839 | -0.049290318 | 0.287798118 |
| RGS7BP | brown | -0.061617262 | 0.18376383 | -0.081948019 | 0.076870145 |
| RHEBL1 | brown | -0.10340047 | 0.025449724 | -0.097094438 | 0.03594372 |
| RIBC1 | brown | 0.176893156 | 0.000121641 | 0.188807904 | 4.02E-05 |
| RIBC2 | brown | 0.119185506 | 0.009939908 | 0.125470509 | 0.006629367 |
| RIIAD1 | brown | 0.092623871 | 0.045440166 | 0.101368866 | 0.028497135 |
| RILPL2 | brown | 0.141985773 | 0.002100006 | 0.163471121 | 0.000389553 |
| RNF122 | brown | 0.059006097 | 0.203080337 | 0.059465166 | 0.199581582 |
| RNF135 | brown | 0.131795576 | 0.004331568 | 0.151493682 | 0.001023428 |
| RNF32 | brown | -0.194911418 | 2.22E-05 | -0.184674329 | 5.95E-05 |
| ROBO2 | brown | 0.139509036 | 0.002515049 | 0.157188974 | 0.000652029 |
| ROPN1B | brown | 0.049762163 | 0.283202722 | 0.057689706 | 0.213359532 |
| ROPN1L | brown | 0.127566574 | 0.005768894 | 0.142509126 | 0.002020748 |
| RP1 | brown | -0.058741585 | 0.205116401 | -0.057493356 | 0.21492427 |
| RP11-190 | brown | 0.093403664 | 0.043646166 | 0.087195485 | 0.059720978 |
| RP11-307 | brown | -0.08349452 | 0.071443987 | -0.076051918 | 0.100700035 |
| RP11-444 | brown | -0.135912707 | 0.003251597 | -0.139518453 | 0.002513338 |
| RP11-505 | brown | 0.006329118 | 0.891498587 | 0.041918384 | 0.366083658 |
| RP11-569 | brown | 0.184555511 | 6.02E-05 | 0.190711451 | 3.35E-05 |
| RP11-697 | brown | 0.023745778 | 0.60875771 | 0.014628117 | 0.752544529 |
| RP11-723 | brown | -0.012176935 | 0.792974288 | -0.008685623 | 0.851505069 |
| RP11-766 | brown | 0.138919427 | 0.002624289 | 0.117213601 | 0.011245931 |
| RP11-77K | brown | -0.041704879 | 0.368532285 | -0.025996074 | 0.575227723 |
| RP5-877J | brown | -0.105492717 | 0.02260932 | -0.131443146 | 0.004437627 |
| RP5-966M | brown | -0.010386758 | 0.822862949 | -0.020634792 | 0.656483128 |
| RPGR | brown | -0.054416294 | 0.240529511 | -0.055464735 | 0.231574866 |
| RPGRIP1 | brown | 0.126423246 | 0.006224924 | 0.121393992 | 0.008638614 |
| RRAD | brown | -0.063506687 | 0.170656789 | -0.029166742 | 0.52951717 |
| RSPH1 | brown | 0.141137965 | 0.002234449 | 0.1589423 | 0.000565786 |
| RSPH10B | brown | -0.000873003 | 0.984988535 | 0.011162695 | 0.809873759 |
| RSPH10B | brown | -0.005391814 | 0.907488749 | 0.00297206 | 0.948926617 |
| RSPH14 | brown | 0.048924864 | 0.291391607 | 0.056706702 | 0.221275899 |
| RSPH3 | brown | 0.09709298 | 0.035946518 | 0.100932068 | 0.029191781 |
| RSPH4A | brown | 0.102939706 | 0.026115054 | 0.115076473 | 0.012830818 |
| RSPH9 | brown | -0.079496432 | 0.086153778 | -0.073903082 | 0.110721022 |
| RTN1 | brown | -0.021636923 | 0.64094397 | -0.021898777 | 0.63690899 |
| RUFY1 | brown | 0.125051667 | 0.006814503 | 0.095393901 | 0.039335844 |
| RUNDC1 | brown | 0.263956529 | 6.96E-09 | 0.276473404 | 1.22E-09 |
| SAMD15 | brown | 0.048903215 | 0.291605425 | 0.046865809 | 0.312198399 |
| SAT1 | brown | 0.375219075 | 4.64E-17 | 0.384261248 | 7.01E-18 |
| SCAMP4 | brown | 0.398375416 | 3.26E-19 | 0.416540095 | 5.04E-21 |
| SCGB1A1 | brown | 0.105691629 | 0.022354158 | 0.129886355 | 0.004934737 |

| | | | | | |
|---|---|---|---|---|---|
| SCGB1D2 | brown | 0.169898943 | 0.000225528 | 0.180523321 | 8.75E-05 |
| SCGB1D4 | brown | 0.069236969 | 0.135173778 | 0.080343328 | 0.082849682 |
| SCGB2A1 | brown | 0.293411417 | 1.00E-10 | 0.294626402 | 8.33E-11 |
| SCGB3A1 | brown | 0.217381402 | 2.12E-06 | 0.234773866 | 2.87E-07 |
| SCN1A | brown | -0.067413105 | 0.145791002 | -0.073262939 | 0.113853378 |
| SCNN1B | brown | 0.169302536 | 0.000237459 | 0.195155433 | 2.17E-05 |
| SCNN1G | brown | 0.067351374 | 0.14616118 | 0.074603408 | 0.107372303 |
| SDC3 | brown | -0.293796323 | 9.45E-11 | -0.298678226 | 4.45E-11 |
| SDR42E2 | brown | 0.091247295 | 0.048758317 | 0.101633684 | 0.028082941 |
| SEC14L3 | brown | 0.042100819 | 0.363999398 | 0.047884372 | 0.301786794 |
| SEC14L5 | brown | 0.042680399 | 0.357427082 | 0.04946566 | 0.286084609 |
| SEMA3D | brown | 0.039237786 | 0.39755881 | 0.053419778 | 0.249263478 |
| SEMA3E | brown | -0.028359659 | 0.54097587 | 0.003385945 | 0.941826 |
| SERPINA | brown | 0.07024588 | 0.129562078 | 0.068960681 | 0.136742723 |
| SERPINA | brown | 0.204332606 | 8.55E-06 | 0.207641545 | 6.05E-06 |
| SERPINA | brown | 0.092306762 | 0.046187225 | 0.103230674 | 0.025693184 |
| SERPINE | brown | 0.004821591 | 0.917235346 | 0.019070615 | 0.681034109 |
| SERPINH | brown | -0.142011727 | 0.002096009 | -0.122349956 | 0.008124056 |
| SERPINI2 | brown | -0.11397876 | 0.013719036 | -0.103155869 | 0.025801078 |
| SFI1 | brown | 0.149623499 | 0.001182784 | 0.140971721 | 0.002261716 |
| SGK3 | brown | 0.024676439 | 0.594785398 | 0.008000455 | 0.863098928 |
| SH2D5 | brown | -0.100428798 | 0.030010097 | -0.120204068 | 0.009319547 |
| SH3BGR | brown | 0.101169845 | 0.02881186 | 0.094940219 | 0.040285314 |
| SH3BP1 | brown | -0.082477422 | 0.074976143 | -0.069881834 | 0.131565784 |
| SH3GL3 | brown | -0.069538869 | 0.133475285 | -0.066053419 | 0.154111597 |
| SH3RF2 | brown | 0.275704748 | 1.36E-09 | 0.297261438 | 5.55E-11 |
| SH3RF3 | brown | -0.095899521 | 0.038300058 | -0.094154561 | 0.04197537 |
| SH3YL1 | brown | 0.209756709 | 4.84E-06 | 0.177273956 | 0.00011754 |
| SHANK2 | brown | 0.095535604 | 0.039043194 | 0.087446505 | 0.058987141 |
| SHISA8 | brown | 0.235652788 | 2.58E-07 | 0.237523049 | 2.06E-07 |
| SIAH3 | brown | -0.100294252 | 0.030232169 | -0.08867086 | 0.055514013 |
| SIDT1 | brown | 0.193056448 | 2.66E-05 | 0.208764928 | 5.38E-06 |
| SIPA1L1 | brown | 0.240863464 | 1.37E-07 | 0.232537368 | 3.74E-07 |
| SLAIN1 | brown | -0.002515502 | 0.956763978 | -0.008749799 | 0.850420729 |
| SLC15A2 | brown | 0.204234772 | 8.64E-06 | 0.206994504 | 6.48E-06 |
| SLC17A8 | brown | 0.048339756 | 0.29720732 | 0.043939678 | 0.343405761 |
| SLC22A2 | brown | 0.165041647 | 0.00034148 | 0.149056359 | 0.001235447 |
| SLC22A4 | brown | 0.047766504 | 0.302979702 | 0.04676163 | 0.313276443 |
| SLC22A5 | brown | 0.189466822 | 3.78E-05 | 0.161701097 | 0.000451253 |
| SLC23A1 | brown | 0.054648649 | 0.238524309 | 0.077123591 | 0.09597892 |
| SLC25A1 | brown | 0.048256512 | 0.298040961 | 0.05977903 | 0.197214886 |
| SLC27A2 | brown | 0.27012013 | 2.99E-09 | 0.261785221 | 9.33E-09 |
| SLC27A6 | brown | -0.121065216 | 0.008822178 | -0.120710705 | 0.009023995 |
| SLC28A3 | brown | 0.107234814 | 0.020457988 | 0.113136017 | 0.014437237 |
| SLC32A1 | brown | 0.048530063 | 0.295307323 | 0.057426389 | 0.215459825 |

| | | | | | |
|---|---|---|---|---|---|
| SLC35F3 | brown | 0.032039254 | 0.489756642 | 0.054086128 | 0.243399115 |
| SLC38A3 | brown | -0.171262913 | 0.000200319 | -0.17866316 | 0.000103655 |
| SLC3A2 | brown | -0.001284997 | 0.977905714 | -0.031493802 | 0.497181397 |
| SLC40A1 | brown | 0.134901091 | 0.00349149 | 0.142136753 | 0.002076855 |
| SLC44A4 | brown | -0.002852623 | 0.950976444 | 0.028494623 | 0.539051126 |
| SLC46A1 | brown | -0.123556248 | 0.007513877 | -0.121730927 | 0.008454033 |
| SLC46A2 | brown | 0.114219424 | 0.013519798 | 0.098346878 | 0.033607354 |
| SLC47A2 | brown | 0.040152243 | 0.38664314 | 0.046610044 | 0.314849417 |
| SLC48A1 | brown | -0.160790569 | 0.000486424 | -0.184075234 | 6.29E-05 |
| SLC4A4 | brown | -0.011231295 | 0.808727851 | -0.003898037 | 0.933047011 |
| SLC4A8 | brown | 0.007570593 | 0.870387897 | 0.025775042 | 0.578482329 |
| SLC51A | brown | -0.049214793 | 0.288538299 | -0.040949747 | 0.377273913 |
| SLC52A1 | brown | 0.052345361 | 0.258925102 | 0.072272657 | 0.118835555 |
| SLC6A16 | brown | 0.06175211 | 0.182804434 | 0.07027536 | 0.129400861 |
| SLC9A3R | brown | 0.179919515 | 9.24E-05 | 0.203760845 | 9.07E-06 |
| SLC9C2 | brown | -0.113904602 | 0.013780948 | -0.111568615 | 0.015861293 |
| SLCO4A1 | brown | 0.021262206 | 0.6467365 | 0.031219354 | 0.500939681 |
| SLITRK5 | brown | -0.018360575 | 0.692292854 | -0.018204532 | 0.694776368 |
| SMIM5 | brown | -0.01500766 | 0.74634135 | -0.009843793 | 0.83198127 |
| SMIM6 | brown | 0.156934644 | 0.000665506 | 0.170494187 | 0.000214182 |
| SMKR1 | brown | -0.126363652 | 0.006249557 | -0.112757537 | 0.014770399 |
| SMOC1 | brown | -0.088367142 | 0.056359324 | -0.063281025 | 0.172184471 |
| SMOX | brown | 0.031453158 | 0.497737021 | 0.059466449 | 0.199571863 |
| SMPD2 | brown | 0.148381973 | 0.001300874 | 0.172313089 | 0.000182734 |
| SMPD3 | brown | 0.298733555 | 4.41E-11 | 0.281564079 | 5.86E-10 |
| SNAPC4 | brown | 0.063869913 | 0.168219119 | 0.054422675 | 0.240474281 |
| SNTN | brown | 0.043642347 | 0.346684362 | 0.057204033 | 0.217244933 |
| SNX31 | brown | 0.009554782 | 0.836844088 | -0.002336214 | 0.959842861 |
| SORBS2 | brown | 0.324761138 | 6.23E-13 | 0.316634155 | 2.47E-12 |
| SOWAH/ | brown | -0.148149226 | 0.001324184 | -0.151800103 | 0.000999291 |
| SOX17 | brown | 0.203139938 | 9.67E-06 | 0.201465627 | 1.15E-05 |
| SOX3 | brown | -0.133719756 | 0.003791878 | -0.140505008 | 0.002339898 |
| SOX30 | brown | -0.226903672 | 7.23E-07 | -0.222504485 | 1.19E-06 |
| SPA17 | brown | 0.269265913 | 3.36E-09 | 0.27090876 | 2.68E-09 |
| SPAG1 | brown | 0.007784596 | 0.866757746 | 0.010612983 | 0.819070776 |
| SPAG16 | brown | -0.338026074 | 6.03E-14 | -0.342750318 | 2.56E-14 |
| SPAG17 | brown | -0.12229454 | 0.008153121 | -0.124134236 | 0.007236297 |
| SPAG6 | brown | -0.245246054 | 7.97E-08 | -0.257774905 | 1.59E-08 |
| SPAG8 | brown | 0.052353298 | 0.258852794 | 0.064111587 | 0.166611701 |
| SPATA17 | brown | 0.123390523 | 0.007595203 | 0.127458096 | 0.005810835 |
| SPATA18 | brown | 0.291348566 | 1.37E-10 | 0.302907793 | 2.29E-11 |
| SPATA24 | brown | -0.078113632 | 0.091775953 | -0.067733011 | 0.14388409 |
| SPATA33 | brown | 0.265280855 | 5.81E-09 | 0.2862849 | 2.93E-10 |
| SPATA4 | brown | -0.078783046 | 0.089018611 | -0.069712547 | 0.132505656 |
| SPATA6 | brown | 0.157031077 | 0.000660365 | 0.134250368 | 0.003654172 |

| | | | | | |
|---|---|---|---|---|---|
| SPATA6L | brown | -0.015614966 | 0.73644978 | 0.002669724 | 0.954116074 |
| SPATA7 | brown | -0.117717106 | 0.010898764 | -0.137587632 | 0.002887121 |
| SPATS1 | brown | 0.074583205 | 0.107467776 | 0.078736985 | 0.089206179 |
| SPECC1 | brown | 0.123713977 | 0.007437199 | 0.141036201 | 0.002251105 |
| SPEF1 | brown | 0.071921144 | 0.120644455 | 0.08841652 | 0.056221174 |
| SPEF2 | brown | -0.041509907 | 0.370777215 | -0.049661964 | 0.284174418 |
| SPIRE2 | brown | -0.026651674 | 0.565625386 | -0.038528787 | 0.406148086 |
| SPNS3 | brown | -0.049203463 | 0.288649444 | -0.0286862 | 0.536324918 |
| SPPL2B | brown | 0.332291071 | 1.68E-13 | 0.334257397 | 1.19E-13 |
| SPRY3 | brown | 0.251837585 | 3.45E-08 | 0.235727016 | 2.56E-07 |
| SRCIN1 | brown | -0.039715211 | 0.391836976 | -0.054287583 | 0.241645353 |
| SRD5A2 | brown | 0.05675197 | 0.220906797 | 0.066850699 | 0.149190093 |
| SRGAP3 | brown | -0.003993313 | 0.931414506 | -0.007194088 | 0.876781173 |
| SSUH2 | brown | -0.177600574 | 0.000114127 | -0.175546006 | 0.000137254 |
| ST3GAL6 | brown | -0.17213216 | 0.000185657 | -0.14645029 | 0.001506323 |
| ST6GALN | brown | 0.145311228 | 0.001641031 | 0.140916618 | 0.002270821 |
| STAG3 | brown | -0.166879361 | 0.000292268 | -0.166813776 | 0.000293904 |
| STK33 | brown | -0.080931247 | 0.080616733 | -0.081791377 | 0.077437943 |
| STK36 | brown | 0.052327292 | 0.259089769 | 0.031865778 | 0.492111548 |
| STMND1 | brown | 0.094818313 | 0.040543717 | 0.101211019 | 0.028746506 |
| STOML3 | brown | 0.058069479 | 0.210356352 | 0.076111503 | 0.100432786 |
| STOX1 | brown | 0.062047941 | 0.180712687 | 0.04061496 | 0.381189939 |
| STPG1 | brown | 0.242851199 | 1.07E-07 | 0.248462184 | 5.31E-08 |
| STX18 | brown | 0.286859825 | 2.69E-10 | 0.286265916 | 2.94E-10 |
| SUGCT | brown | -0.283766141 | 4.25E-10 | -0.297898749 | 5.03E-11 |
| SULT1A1 | brown | -0.011492587 | 0.804366914 | 0.023600845 | 0.610946672 |
| SUOX | brown | 0.147759114 | 0.001364118 | 0.137850001 | 0.002833522 |
| SVOPL | brown | -0.217498445 | 2.09E-06 | -0.211913474 | 3.84E-06 |
| SYBU | brown | -0.090739328 | 0.050032899 | -0.061232262 | 0.186523408 |
| SYS1 | brown | -0.224046347 | 1.00E-06 | -0.217247599 | 2.15E-06 |
| SYT5 | brown | -0.047452781 | 0.306169995 | -0.05322596 | 0.25098754 |
| SYT8 | brown | 0.174602203 | 0.000149293 | 0.194896278 | 2.22E-05 |
| SYTL3 | brown | 0.209438158 | 5.00E-06 | 0.221719958 | 1.31E-06 |
| TAAR1 | brown | -0.105981242 | 0.021987109 | -0.108826474 | 0.018650299 |
| TACR1 | brown | -0.078534159 | 0.090035911 | -0.087922727 | 0.057615453 |
| TAS2R1 | brown | 0.003458099 | 0.940588584 | -0.003596186 | 0.938220851 |
| TBC1D8 | brown | 0.226109266 | 7.92E-07 | 0.216856351 | 2.24E-06 |
| TCF7 | brown | 0.120662041 | 0.009052017 | 0.102919999 | 0.026143842 |
| TCP10L | brown | -0.193661572 | 2.51E-05 | -0.184929513 | 5.81E-05 |
| TCTE1 | brown | 0.08100969 | 0.080322521 | 0.090419679 | 0.050849138 |
| TCTEX1D | brown | -0.194845523 | 2.23E-05 | -0.19174904 | 3.03E-05 |
| TCTEX1D | brown | 0.098324223 | 0.033648436 | 0.105319332 | 0.022833792 |
| TCTEX1D | brown | 0.100701465 | 0.029564335 | 0.119198757 | 0.009931607 |
| TCTN1 | brown | 0.089800751 | 0.052461217 | 0.097763655 | 0.034678707 |
| TCTN2 | brown | 0.079703425 | 0.085336573 | 0.070928952 | 0.125866287 |

| | | | | | |
|---|---|---|---|---|---|
| TDRP | brown | -0.264495458 | 6.47E-09 | -0.287472484 | 2.46E-10 |
| TEKT1 | brown | 0.016543158 | 0.721415996 | 0.025939997 | 0.576052609 |
| TEKT2 | brown | 0.070366952 | 0.12890096 | 0.08311343 | 0.072751163 |
| TEKT3 | brown | 0.044904495 | 0.33290345 | 0.046912921 | 0.311711681 |
| TEKT4 | brown | 0.141413642 | 0.002189894 | 0.159953558 | 0.000520987 |
| TEX26 | brown | -0.068789011 | 0.137724607 | -0.060703528 | 0.190362806 |
| TEX9 | brown | 0.055478468 | 0.231459156 | 0.056923638 | 0.219511067 |
| TFF1 | brown | 0.248601904 | 5.22E-08 | 0.303900081 | 1.96E-11 |
| TGM3 | brown | 0.24238396 | 1.14E-07 | 0.23142946 | 4.27E-07 |
| THSD4 | brown | -0.212053718 | 3.78E-06 | -0.203802081 | 9.03E-06 |
| TIGD4 | brown | 0.063199033 | 0.172742056 | 0.059447508 | 0.199715343 |
| TMC1 | brown | 0.099026765 | 0.032394176 | 0.121112435 | 0.008795603 |
| TMC3 | brown | -0.064214887 | 0.165928162 | -0.070929758 | 0.125861972 |
| TMCO5A | brown | 0.027219989 | 0.557364071 | 0.046864419 | 0.31221277 |
| TMED6 | brown | 0.199023508 | 1.47E-05 | 0.210294315 | 4.57E-06 |
| TMEM10 | brown | 0.117693084 | 0.010915109 | 0.107560593 | 0.020076066 |
| TMEM10 | brown | 0.269761798 | 3.14E-09 | 0.280709655 | 6.64E-10 |
| TMEM11 | brown | 0.024183533 | 0.602167389 | 0.05390494 | 0.244984048 |
| TMEM11 | brown | 0.036391357 | 0.432702089 | 0.036798398 | 0.427569465 |
| TMEM12 | brown | 0.028228095 | 0.54285541 | -0.001700873 | 0.970757942 |
| TMEM13 | brown | 0.026097463 | 0.573737701 | 0.054935914 | 0.236061539 |
| TMEM15 | brown | 0.224649993 | 9.36E-07 | 0.219913863 | 1.60E-06 |
| TMEM17 | brown | -0.078305361 | 0.090979313 | -0.077843745 | 0.092906798 |
| TMEM17 | brown | -0.117538407 | 0.011020874 | -0.112367273 | 0.015120987 |
| TMEM17 | brown | -0.011673457 | 0.801351761 | -0.02513712 | 0.587923509 |
| TMEM19 | brown | -0.033728751 | 0.467141425 | -0.030518567 | 0.510603934 |
| TMEM20 | brown | -0.097760747 | 0.034684121 | -0.104173131 | 0.024366821 |
| TMEM21 | brown | -0.051989485 | 0.26218158 | -0.037062418 | 0.424259251 |
| TMEM23 | brown | 0.145692734 | 0.00159473 | 0.153014879 | 0.000908681 |
| TMEM23 | brown | 0.031245947 | 0.500574867 | 0.033938592 | 0.464373412 |
| TMEM23 | brown | -0.076712965 | 0.097766571 | -0.070530257 | 0.128013366 |
| TMEM25 | brown | 0.34237764 | 2.74E-14 | 0.310618042 | 6.65E-12 |
| TMEM25 | brown | -0.100439139 | 0.029993086 | -0.109805445 | 0.017609031 |
| TMEM31 | brown | -0.105431669 | 0.022688137 | -0.114942818 | 0.012936182 |
| TMEM40 | brown | -0.000194113 | 0.996662002 | 0.036856696 | 0.426837247 |
| TMEM45 | brown | 0.114710787 | 0.013120911 | 0.125727987 | 0.006517806 |
| TMEM67 | brown | -0.05177834 | 0.264126959 | -0.086007682 | 0.063296489 |
| TMPRSS: | brown | 0.304833787 | 1.69E-11 | 0.299893615 | 3.68E-11 |
| TMPRSS: | brown | -0.074955102 | 0.105721054 | -0.055951748 | 0.227496604 |
| TMPRSS' | brown | -0.101420824 | 0.028415458 | -0.092370583 | 0.04603605 |
| TNFAIP8 | brown | 0.047515285 | 0.305532614 | 0.066865779 | 0.149098171 |
| TNNC2 | brown | -0.067400358 | 0.145867385 | -0.060956532 | 0.188518439 |
| TNNI2 | brown | 0.04365239 | 0.346573297 | 0.075304795 | 0.104099168 |
| TP53AIP1 | brown | 0.127187173 | 0.00591678 | 0.142307947 | 0.002050886 |
| TP73 | brown | 0.339715653 | 4.45E-14 | 0.347760658 | 1.01E-14 |

| | | | | | |
|---|---|---|---|---|---|
| TPCN2 | brown | -0.151578525 | 0.001016692 | -0.187382889 | 4.61E-05 |
| TPPP | brown | -0.107169439 | 0.020535386 | -0.093411188 | 0.04362915 |
| TPPP3 | brown | 0.093273019 | 0.043942503 | 0.118183504 | 0.010585687 |
| TPRXL | brown | -0.036359529 | 0.433104928 | -0.016945992 | 0.714924212 |
| TRAF3IP | brown | 0.064827585 | 0.161917044 | 0.062738992 | 0.175895545 |
| TREH | brown | 0.206238473 | 7.01E-06 | 0.205369784 | 7.68E-06 |
| TRIB3 | brown | -0.109424585 | 0.018007931 | -0.09116014 | 0.048975053 |
| TRIM10 | brown | 0.007445588 | 0.872509648 | 0.009682682 | 0.834691302 |
| TRIM15 | brown | 0.056743128 | 0.220978853 | 0.056276689 | 0.224804076 |
| TRIM31 | brown | 0.069277432 | 0.13494517 | 0.092678173 | 0.045313262 |
| TRIM41 | brown | 0.061170746 | 0.186967151 | 0.044489055 | 0.337400034 |
| TRIM55 | brown | 0.021607218 | 0.641402381 | 0.041540281 | 0.370426936 |
| TRIM73 | brown | 0.022558885 | 0.626784949 | 0.022131162 | 0.633337078 |
| TRIM74 | brown | -0.056000651 | 0.227089919 | -0.055169059 | 0.234075967 |
| TSGA10 | brown | -0.010432175 | 0.822101296 | -0.020579969 | 0.657337593 |
| TSNAXIP | brown | 0.111081143 | 0.016328599 | 0.125986512 | 0.006407488 |
| TSPAN1 | brown | 0.139189462 | 0.002573733 | 0.172914392 | 0.000173328 |
| TSPAN19 | brown | -0.103471162 | 0.025348952 | -0.102369458 | 0.026959181 |
| TSPAN3 | brown | 0.021806729 | 0.638326179 | 0.023829883 | 0.607489044 |
| TTC12 | brown | 0.225148182 | 8.85E-07 | 0.21038147 | 4.53E-06 |
| TTC16 | brown | 0.029856402 | 0.519824034 | 0.042530114 | 0.359124089 |
| TTC21A | brown | 0.110798478 | 0.016605059 | 0.120476962 | 0.009159299 |
| TTC23L | brown | 0.01800317 | 0.697985987 | 0.01578719 | 0.73365249 |
| TTC25 | brown | 0.099081588 | 0.032298 | 0.107411168 | 0.020250465 |
| TTC26 | brown | 0.088710681 | 0.055403969 | 0.079700586 | 0.085347739 |
| TTC29 | brown | 0.063098484 | 0.173427677 | 0.07401458 | 0.110182441 |
| TTC30A | brown | -0.280584342 | 6.76E-10 | -0.271405768 | 2.50E-09 |
| TTC30B | brown | -0.142333516 | 0.002047033 | -0.125526729 | 0.006604862 |
| TTC34 | brown | -0.169960358 | 0.000224332 | -0.163833655 | 0.000377927 |
| TTC39C | brown | -0.055650431 | 0.230013778 | -0.044540531 | 0.336840771 |
| TTC6 | brown | 0.065498881 | 0.15760654 | 0.077710096 | 0.09347091 |
| TTC8 | brown | 0.114822929 | 0.013031342 | 0.086370567 | 0.062185893 |
| TTLL1 | brown | 0.258310575 | 1.48E-08 | 0.252393612 | 3.21E-08 |
| TTLL10 | brown | 0.088979786 | 0.054665038 | 0.104954515 | 0.023312408 |
| TTLL5 | brown | 0.03820466 | 0.410111277 | 0.017822245 | 0.70087445 |
| TTLL6 | brown | -0.10084506 | 0.029331872 | -0.093121879 | 0.044287439 |
| TTLL7 | brown | 0.064681586 | 0.162866163 | 0.03703902 | 0.424552013 |
| TTLL9 | brown | -0.020577117 | 0.657382052 | -0.013193435 | 0.776133874 |
| TUBA4A | brown | 0.065159932 | 0.159772029 | 0.066307644 | 0.152529124 |
| UBE3D | brown | -0.136748114 | 0.003064884 | -0.146188189 | 0.001536388 |
| UBXN10 | brown | 0.17159095 | 0.000194663 | 0.173808013 | 0.000160184 |
| UBXN11 | brown | 0.072145153 | 0.119489231 | 0.089978958 | 0.051992746 |
| UCN3 | brown | -0.014762309 | 0.75034947 | 0.006939916 | 0.881101718 |
| UFC1 | brown | 0.243178852 | 1.03E-07 | 0.229099378 | 5.61E-07 |
| UGT2B7 | brown | -0.156508854 | 0.000688647 | -0.127995837 | 0.005605585 |

| | | | | | |
|---|---|---|---|---|---|
| ULBP2 | brown | 0.217656028 | 2.05E-06 | 0.227461584 | 6.78E-07 |
| ULK4 | brown | -0.112497994 | 0.015002751 | -0.106423392 | 0.021436818 |
| UMODL1 | brown | -0.039486041 | 0.39457729 | -0.04170334 | 0.368549972 |
| UNC79 | brown | -0.106728242 | 0.021064407 | -0.118840797 | 0.01015801 |
| UPK1B | brown | 0.160869888 | 0.000483262 | 0.178163462 | 0.000108461 |
| UROC1 | brown | -0.067664531 | 0.144290678 | -0.046357236 | 0.317484236 |
| USH1C | brown | 0.038693664 | 0.404140861 | 0.026958666 | 0.56115552 |
| USH1G | brown | 0.009515067 | 0.837512814 | -0.014498025 | 0.754674434 |
| USP2 | brown | -0.127089211 | 0.005955514 | -0.118371824 | 0.010461552 |
| USP40 | brown | 0.095601267 | 0.038908211 | 0.055705426 | 0.229552887 |
| VEPH1 | brown | -0.090060588 | 0.051779324 | -0.081031866 | 0.080239505 |
| VGF | brown | -0.05229285 | 0.259403846 | -0.040646917 | 0.380815066 |
| VNN3 | brown | 0.001208595 | 0.979219069 | 0.006734573 | 0.884594835 |
| VRK3 | brown | 0.040191277 | 0.386181297 | 0.062431008 | 0.178030496 |
| VSIG10L | brown | -0.111263649 | 0.016152249 | -0.128853574 | 0.005291665 |
| VWA3A | brown | 0.093344385 | 0.043780417 | 0.089785897 | 0.052500424 |
| VWA3B | brown | 0.062307651 | 0.178890986 | 0.077913262 | 0.092614455 |
| VWA5B1 | brown | -0.093415362 | 0.043619714 | -0.100873752 | 0.029285611 |
| VWA5B2 | brown | 0.174219928 | 0.000154446 | 0.181381691 | 8.08E-05 |
| VWA7 | brown | -0.17250457 | 0.000179688 | -0.170709724 | 0.000210207 |
| WBSCR2 | brown | 0.058145849 | 0.209756131 | 0.082149516 | 0.076144734 |
| WBSCR2 | brown | 0.002138461 | 0.963239515 | 0.024441835 | 0.598293798 |
| WDR19 | brown | -0.085798508 | 0.063944045 | -0.100008751 | 0.030708066 |
| WDR31 | brown | 0.307390823 | 1.12E-11 | 0.289160188 | 1.91E-10 |
| WDR38 | brown | 0.096141365 | 0.037812858 | 0.108892682 | 0.018578222 |
| WDR49 | brown | 0.033266247 | 0.47327439 | 0.047930736 | 0.301318414 |
| WDR54 | brown | 0.07518514 | 0.1046519 | 0.093829731 | 0.042691397 |
| WDR5B | brown | -0.104390254 | 0.024069776 | -0.108282504 | 0.019251781 |
| WDR60 | brown | 0.001952403 | 0.966435914 | -0.015669335 | 0.735566332 |
| WDR63 | brown | 0.052594087 | 0.256665766 | 0.058875757 | 0.204081782 |
| WDR66 | brown | 0.113770371 | 0.013893638 | 0.121653835 | 0.008495953 |
| WDR77 | brown | 0.045986739 | 0.321371591 | 0.049084579 | 0.289817453 |
| WDR78 | brown | 0.071017341 | 0.125394094 | 0.074853316 | 0.106196873 |
| WDR90 | brown | 0.215163671 | 2.70E-06 | 0.224974502 | 9.02E-07 |
| WDR92 | brown | -0.118654101 | 0.010277899 | -0.111233042 | 0.016181707 |
| WDR93 | brown | 0.065183535 | 0.15962051 | 0.080940227 | 0.080583008 |
| WFDC3 | brown | -6.46E-05 | 0.998889269 | 0.025315838 | 0.585271344 |
| WFDC6 | brown | 0.129548261 | 0.005049117 | 0.131707143 | 0.004357963 |
| WNT9A | brown | -0.120483628 | 0.009155416 | -0.104255178 | 0.024254203 |
| WRB | brown | 0.118335052 | 0.010485689 | 0.104799019 | 0.023519033 |
| XG | brown | 0.264848892 | 6.17E-09 | 0.264566195 | 6.41E-09 |
| XPNPEP3 | brown | 0.203479382 | 9.34E-06 | 0.215436063 | 2.62E-06 |
| ZBBX | brown | 0.023497721 | 0.612506309 | 0.033904308 | 0.464825031 |
| ZBTB18 | brown | 0.226561683 | 7.52E-07 | 0.219907549 | 1.60E-06 |
| ZC2HC1C | brown | 0.022053335 | 0.634532393 | 0.043498625 | 0.348276252 |

| | | | | | |
|---|---|---|---|---|---|
| ZCWPW1 | brown | -0.248516777 | 5.28E-08 | -0.256160267 | 1.97E-08 |
| ZDHHC1 | brown | 0.266785013 | 4.73E-09 | 0.28404228 | 4.08E-10 |
| ZDHHC1 | brown | -0.114013486 | 0.013690129 | -0.110123126 | 0.017282229 |
| ZDHHC1 | brown | -0.243616544 | 9.77E-08 | -0.242072762 | 1.18E-07 |
| ZDHHC1 | brown | 0.142561338 | 0.002012993 | 0.127717368 | 0.005711045 |
| ZFHX2 | brown | 0.096270456 | 0.03755496 | 0.100582494 | 0.029758128 |
| ZFYVE27 | brown | 0.081496955 | 0.078514384 | 0.055609904 | 0.230353839 |
| ZFYVE28 | brown | -0.077419608 | 0.094706464 | -0.072607543 | 0.117131998 |
| ZMYND1 | brown | 0.155050559 | 0.000773675 | 0.173761307 | 0.000160847 |
| ZMYND1 | brown | 0.101276899 | 0.0286422 | 0.099238356 | 0.03202433 |
| ZNF132 | brown | -0.290285012 | 1.61E-10 | -0.293394413 | 1.01E-10 |
| ZNF19 | brown | 0.064267606 | 0.165580121 | 0.079721315 | 0.08526624 |
| ZNF20 | brown | 0.022582337 | 0.626426538 | 0.030684883 | 0.508301585 |
| ZNF3 | brown | -0.00310053 | 0.946722117 | -0.014737774 | 0.750750668 |
| ZNF391 | brown | 0.054259814 | 0.241886565 | 0.045237944 | 0.329322335 |
| ZNF396 | brown | 0.038489871 | 0.406622717 | 0.032414467 | 0.484683951 |
| ZNF440 | brown | 0.046775322 | 0.313134617 | 0.049390772 | 0.286815599 |
| ZNF446 | brown | 0.149158752 | 0.001225782 | 0.142164659 | 0.002072601 |
| ZNF473 | brown | -0.070293332 | 0.129302652 | -0.066484828 | 0.151433515 |
| ZNF474 | brown | 0.109073904 | 0.018382176 | 0.120093662 | 0.009385085 |
| ZNF487 | brown | 0.087381013 | 0.059177879 | 0.101718006 | 0.027952145 |
| ZNF497 | brown | 0.16753178 | 0.000276451 | 0.161827498 | 0.000446562 |
| ZNF584 | brown | 0.021627363 | 0.641091481 | 0.011603903 | 0.802510912 |
| ZNF599 | brown | -0.204987932 | 7.99E-06 | -0.215771031 | 2.53E-06 |
| ZNF671 | brown | 0.071727944 | 0.121647774 | 0.081426588 | 0.078773442 |
| ZNF69 | brown | 0.164319058 | 0.000362866 | 0.165511019 | 0.000328224 |
| ZNF812 | brown | 0.164301513 | 0.000363401 | 0.171579688 | 0.000194855 |
| ZSCAN4 | brown | -0.035417806 | 0.445121362 | -0.022444166 | 0.628539435 |
| ZSCAN5 | brown | -0.099519411 | 0.031538651 | -0.101825353 | 0.027786393 |
| AGPAT5 | brown4 | 0.160373159 | 0.000503385 | 0.113871706 | 0.01380849 |
| ASAH1 | brown4 | 0.196643661 | 1.87E-05 | 0.177054802 | 0.000119884 |
| ASH2L | brown4 | 0.080704376 | 0.08147256 | 0.056201531 | 0.225424823 |
| ATP6V1E | brown4 | 0.30173682 | 2.76E-11 | 0.285280787 | 3.40E-10 |
| BAG4 | brown4 | 0.035837228 | 0.439746266 | 0.015687148 | 0.735276953 |
| BIN3 | brown4 | 0.135722399 | 0.00329555 | 0.119415953 | 0.00979643 |
| BNIP3L | brown4 | 0.243498897 | 9.91E-08 | 0.216243805 | 2.40E-06 |
| CCAR2 | brown4 | 0.2555343 | 2.14E-08 | 0.24663417 | 6.70E-08 |
| CCDC25 | brown4 | 0.225431001 | 8.56E-07 | 0.200335117 | 1.29E-05 |
| CHMP7 | brown4 | 0.29045647 | 1.57E-10 | 0.285913873 | 3.09E-10 |
| CLN8 | brown4 | 0.254122057 | 2.57E-08 | 0.258926612 | 1.37E-08 |
| CNOT7 | brown4 | 0.123754055 | 0.007417827 | 0.097864979 | 0.03449052 |
| DCTN6 | brown4 | 0.179735757 | 9.40E-05 | 0.155622686 | 0.000739221 |
| DDHD2 | brown4 | 0.081399998 | 0.078871516 | 0.050973963 | 0.271628819 |
| ENTPD4 | brown4 | 0.279774342 | 7.60E-10 | 0.246821135 | 6.54E-08 |
| ERICH1 | brown4 | 0.072027052 | 0.120097199 | 0.053121198 | 0.251922873 |

| | | | | | |
|---|---|---|---|---|---|
| ERLIN2 | brown4 | 0.227768522 | 6.54E-07 | 0.205236498 | 7.79E-06 |
| FBXO25 | brown4 | 0.190823671 | 3.31E-05 | 0.195334356 | 2.13E-05 |
| FDFT1 | brown4 | 0.382185116 | 1.09E-17 | 0.361230133 | 7.69E-16 |
| FUT10 | brown4 | 0.25408035 | 2.58E-08 | 0.217988169 | 1.98E-06 |
| GTF2E2 | brown4 | 0.263994213 | 6.92E-09 | 0.262673647 | 8.28E-09 |
| HMBOX1 | brown4 | 0.069125302 | 0.13580622 | 0.045987754 | 0.321360901 |
| INTS10 | brown4 | 0.322509938 | 9.16E-13 | 0.302795226 | 2.33E-11 |
| INTS9 | brown4 | 0.339682167 | 4.47E-14 | 0.321784638 | 1.04E-12 |
| KCTD9 | brown4 | 0.143454591 | 0.001884466 | 0.121063836 | 0.008822956 |
| LEPROTI | brown4 | 0.211796288 | 3.89E-06 | 0.206384753 | 6.91E-06 |
| LONRF1 | brown4 | 0.016848792 | 0.716488751 | -0.021200565 | 0.647691442 |
| LSM1 | brown4 | 0.146629927 | 0.001486031 | 0.133289241 | 0.003907042 |
| MAK16 | brown4 | 0.103714142 | 0.0250052 | 0.08255361 | 0.074706725 |
| MCPH1 | brown4 | 0.253685331 | 2.72E-08 | 0.218379036 | 1.90E-06 |
| MTMR9 | brown4 | -0.024544881 | 0.596751631 | -0.057426584 | 0.215458264 |
| NEIL2 | brown4 | 0.228414429 | 6.07E-07 | 0.217907178 | 2.00E-06 |
| PCM1 | brown4 | 0.177250825 | 0.000117786 | 0.15184155 | 0.000996067 |
| PLEKHA: | brown4 | 0.040839065 | 0.378565832 | 0.019730803 | 0.670628956 |
| POLR3D | brown4 | 0.111768023 | 0.015673539 | 0.10058453 | 0.029754802 |
| PPP2CB | brown4 | 0.095231678 | 0.039673147 | 0.080627348 | 0.0817648 |
| PPP2R2A | brown4 | 0.203098272 | 9.72E-06 | 0.161939402 | 0.000442447 |
| PPP3CC | brown4 | 0.072760824 | 0.116358669 | 0.068146628 | 0.141446905 |
| PROSC | brown4 | 0.084764173 | 0.067226747 | 0.06804614 | 0.142036091 |
| SARAF | brown4 | 0.136853524 | 0.003042031 | 0.125461069 | 0.006633489 |
| TM2D2 | brown4 | 0.117355475 | 0.011147131 | 0.10928323 | 0.018157977 |
| TNFRSF1 | brown4 | 0.413097246 | 1.13E-20 | 0.386757438 | 4.12E-18 |
| TNKS | brown4 | 0.15428096 | 0.000822369 | 0.126943571 | 0.00601352 |
| TTI2 | brown4 | 0.302384816 | 2.49E-11 | 0.288067883 | 2.25E-10 |
| UBXN8 | brown4 | 0.051530642 | 0.26642173 | 0.025369958 | 0.58446931 |
| VPS37A | brown4 | 0.187166896 | 4.70E-05 | 0.167049606 | 0.000288061 |
| WHSC1L | brown4 | 0.048031567 | 0.30030146 | 0.017970395 | 0.698508912 |
| XPO7 | brown4 | 0.21385622 | 3.11E-06 | 0.187046545 | 4.76E-05 |
| ZNF395 | brown4 | 0.156929898 | 0.00066576 | 0.143315426 | 0.001903983 |
| AC01115: | cyan | -0.015986352 | 0.73042205 | 0.001793595 | 0.969164602 |
| AC01326( | cyan | -0.062658171 | 0.176453949 | -0.046862433 | 0.3122333 |
| AC01675: | cyan | -0.012156669 | 0.793311054 | 0.001129742 | 0.980574614 |
| AC10982( | cyan | -0.062807539 | 0.175422978 | -0.034391977 | 0.458423986 |
| ADAM18 | cyan | -0.102156366 | 0.027280589 | -0.090065176 | 0.051767352 |
| ADAM2 | cyan | 0.019721716 | 0.670771746 | 0.029252079 | 0.528312817 |
| ADAM29 | cyan | -0.116544194 | 0.011722629 | -0.10194956 | 0.027595661 |
| AKAP4 | cyan | -0.060671802 | 0.190595014 | -0.040703758 | 0.380148848 |
| AL13347: | cyan | -0.03644545 | 0.432017957 | -0.017102606 | 0.712405823 |
| AL13584: | cyan | -0.035925804 | 0.438615883 | -0.015976404 | 0.730583298 |
| ALDOB | cyan | 0.008884506 | 0.848145609 | 0.00910874 | 0.844361217 |
| AMY1C | cyan | -0.053606748 | 0.247608162 | -0.043058468 | 0.353180262 |

| | | | | | |
|---|---|---|---|---|---|
| ANKRD3 | cyan | -0.043095477 | 0.352766252 | -0.023991341 | 0.605056858 |
| ANKRD6 | cyan | -0.012152081 | 0.793387289 | 0.020909611 | 0.652206631 |
| AP00524 | cyan | -0.047712719 | 0.303525074 | -0.030958522 | 0.504525376 |
| AQPEP | cyan | -0.101762778 | 0.027882911 | -0.099764764 | 0.031119829 |
| ASAH2 | cyan | 0.03954382 | 0.393885309 | 0.038924877 | 0.401336061 |
| ASIC5 | cyan | -0.0490657 | 0.290003229 | -0.040335231 | 0.38448095 |
| ATP1B4 | cyan | -0.04943167 | 0.286416232 | -0.042774922 | 0.356362319 |
| BMP10 | cyan | -0.041149272 | 0.374951859 | -0.026906777 | 0.56190983 |
| BTNL3 | cyan | -0.066626855 | 0.150559621 | -0.047132001 | 0.309454914 |
| C11orf44 | cyan | -0.041671019 | 0.368921545 | -0.017853945 | 0.70036805 |
| C12orf42 | cyan | -0.150405629 | 0.001113546 | -0.131154576 | 0.004526208 |
| C4orf17 | cyan | 0.011345405 | 0.806822635 | 0.026671314 | 0.565338909 |
| C7orf34 | cyan | -0.127737836 | 0.005703234 | -0.133651912 | 0.00380982 |
| C7orf62 | cyan | -0.068913397 | 0.137012628 | -0.055770752 | 0.229006268 |
| C7orf72 | cyan | -0.02062144 | 0.656691186 | -0.006396621 | 0.890348607 |
| CCDC10 | cyan | -0.155521781 | 0.000745192 | -0.144765703 | 0.001709383 |
| CCDC17 | cyan | -0.052463945 | 0.257846227 | -0.033557312 | 0.469409631 |
| CCER1 | cyan | -0.038780823 | 0.403082177 | -0.029240099 | 0.528481809 |
| CLDN25 | cyan | 0.013272874 | 0.774822101 | 0.010345763 | 0.823550578 |
| CLEC12B | cyan | -0.117001576 | 0.011395027 | -0.111571874 | 0.015858208 |
| CLEC4C | cyan | -0.054557328 | 0.23931099 | -0.006789731 | 0.883656319 |
| CLRN1 | cyan | -0.059017672 | 0.202991573 | -0.020506584 | 0.65848205 |
| CNGB3 | cyan | -0.235556253 | 2.61E-07 | -0.2321145 | 3.94E-07 |
| CSN1S1 | cyan | -0.038706628 | 0.403983289 | -0.028906436 | 0.533199436 |
| CTAGE6 | cyan | 0.038769443 | 0.403220311 | 0.031228421 | 0.500815279 |
| CTAGE9 | cyan | 0.017386124 | 0.707854785 | 0.031749054 | 0.493699457 |
| CTC-554I | cyan | -0.021020155 | 0.65048963 | -0.022353556 | 0.629926687 |
| CTXN2 | cyan | -0.178320185 | 0.000106932 | -0.17672225 | 0.000123525 |
| CYLC1 | cyan | -0.043044474 | 0.353336883 | -0.024955626 | 0.59062252 |
| DEFB112 | cyan | -0.040994241 | 0.376755342 | -0.019484932 | 0.674496875 |
| DEFB114 | cyan | -0.013659001 | 0.768455181 | 0.016796327 | 0.71733373 |
| DEFB115 | cyan | -0.038562406 | 0.405738324 | -0.017466807 | 0.70656154 |
| DEFB127 | cyan | -0.008148599 | 0.86058957 | 0.011308123 | 0.807444982 |
| DEFB129 | cyan | -0.01963951 | 0.672064123 | -0.028084474 | 0.544910896 |
| DEFB131 | cyan | -0.016806561 | 0.717168877 | -0.007507706 | 0.871455175 |
| DYTN | cyan | -0.142858093 | 0.001969431 | -0.134157059 | 0.003678055 |
| EBLN1 | cyan | -0.097322386 | 0.035508497 | -0.114191529 | 0.01354276 |
| EVX2 | cyan | -0.059169585 | 0.201829257 | -0.057582411 | 0.214213558 |
| EXD1 | cyan | -0.040493022 | 0.382622419 | -0.020431236 | 0.659657957 |
| F13B | cyan | -0.050605867 | 0.275109837 | -0.04339855 | 0.349387437 |
| F9 | cyan | -0.042879481 | 0.355186829 | -0.032099387 | 0.488941757 |
| FAM156A | cyan | -0.075645453 | 0.102538195 | -0.076243185 | 0.099844169 |
| FAM25G | cyan | 0.094114571 | 0.04206297 | 0.069467781 | 0.133873739 |
| FAM71A | cyan | -0.040866744 | 0.378242503 | -0.039413701 | 0.395444692 |
| FAM71B | cyan | -0.049956486 | 0.281324663 | -0.03069693 | 0.508135023 |

| | | | | | |
|---|---|---|---|---|---|
| FSHB | cyan | -0.055987538 | 0.227198924 | -0.038571408 | 0.405628641 |
| GABRA4 | cyan | -0.069509783 | 0.133638206 | -0.05088679 | 0.272450468 |
| GALNTL( | cyan | -0.10113477 | 0.028867636 | -0.079357818 | 0.08670454 |
| GC | cyan | -0.034051918 | 0.462882316 | -0.025490173 | 0.582689581 |
| GJD3 | cyan | 0.018045505 | 0.697310733 | 0.015051759 | 0.745621653 |
| GKN2 | cyan | -0.066057394 | 0.154086757 | -0.050689465 | 0.274316617 |
| GLRA1 | cyan | -0.052985539 | 0.253137656 | -0.035629853 | 0.442399219 |
| GLRA3 | cyan | -0.138579555 | 0.002689206 | -0.124987925 | 0.006843081 |
| GOLGA6 | cyan | -0.094444217 | 0.041345448 | -0.082150645 | 0.076140684 |
| GPR179 | cyan | -0.148698844 | 0.001269745 | -0.142596732 | 0.002007752 |
| GPRC6A | cyan | -0.116887897 | 0.011475684 | -0.105634719 | 0.022426906 |
| GPX6 | cyan | -0.104625894 | 0.023750945 | -0.106426638 | 0.021432823 |
| GRXCR2 | cyan | 0.002263748 | 0.961087476 | 0.014049927 | 0.762024953 |
| GSTA5 | cyan | -0.012963892 | 0.77992788 | -0.001658323 | 0.971489153 |
| HHLA1 | cyan | -0.045285305 | 0.328815717 | -0.030126291 | 0.516055747 |
| HIST1H2. | cyan | -0.005130915 | 0.911946561 | -0.019515389 | 0.674017283 |
| HIST1H2 | cyan | 0.024192615 | 0.602031 | 0.037665949 | 0.416748692 |
| IFNA10 | cyan | -0.01707484 | 0.712852089 | 0.009621238 | 0.835725357 |
| IFNA14 | cyan | -0.024532359 | 0.596938944 | 0.00213933 | 0.963224591 |
| IFNA16 | cyan | -0.032276851 | 0.486541145 | -0.012269724 | 0.791432929 |
| IFNA17 | cyan | -0.037074282 | 0.424110857 | -0.041429808 | 0.37170194 |
| IFNA4 | cyan | -0.03481771 | 0.452876425 | -0.025073207 | 0.588873318 |
| IFNA5 | cyan | -0.056969902 | 0.219135999 | -0.062659599 | 0.176444068 |
| IFNA7 | cyan | -0.057581298 | 0.214222431 | -0.044579856 | 0.336413923 |
| IL9 | cyan | 0.044576144 | 0.336454196 | 0.049808502 | 0.28275411 |
| IQCJ | cyan | -0.087601508 | 0.058537742 | -0.080882288 | 0.080800803 |
| KCNA10 | cyan | -0.018928807 | 0.683277139 | -0.014197182 | 0.75960701 |
| KCNH7 | cyan | -0.041134032 | 0.37512891 | -0.025225997 | 0.58660389 |
| KRBOX1 | cyan | -0.245533902 | 7.69E-08 | -0.242445822 | 1.13E-07 |
| KRT12 | cyan | -0.070939166 | 0.125811649 | -0.055924135 | 0.227726456 |
| KRTAP13 | cyan | -0.058672276 | 0.205652343 | -0.044208624 | 0.340457218 |
| KRTAP13 | cyan | -0.086184333 | 0.062753832 | -0.087966704 | 0.057490128 |
| KRTAP15 | cyan | -0.038932594 | 0.401242645 | -0.018770825 | 0.68577927 |
| KRTAP19 | cyan | -0.076761709 | 0.097552988 | -0.066045942 | 0.154158327 |
| KRTAP19 | cyan | -0.04544306 | 0.327131873 | -0.014177784 | 0.759925398 |
| KRTAP19 | cyan | -0.041661081 | 0.36903585 | -0.029842848 | 0.52001365 |
| KRTAP21 | cyan | -0.013364869 | 0.773303789 | -0.005324694 | 0.908635318 |
| KRTAP21 | cyan | -0.084426535 | 0.068327792 | -0.089035118 | 0.054514123 |
| KRTAP25 | cyan | -0.038064905 | 0.411827142 | -0.025958452 | 0.575781089 |
| LACRT | cyan | 0.05560957 | 0.230356639 | 0.055882395 | 0.228074229 |
| LCE1B | cyan | 0.023073048 | 0.618947388 | 0.03515585 | 0.448497304 |
| LCE3B | cyan | -0.058604762 | 0.206175374 | -0.04919704 | 0.288712467 |
| LCE6A | cyan | 0.017162291 | 0.711446911 | 0.037612617 | 0.417409212 |
| LGALS16 | cyan | -0.030264675 | 0.514129072 | 0.042725337 | 0.356920618 |
| LRIT1 | cyan | -0.025024419 | 0.589598821 | 0.019878129 | 0.668315447 |

| | | | | | |
|---|---|---|---|---|---|
| LRRC72 | cyan | -0.001051503 | 0.981919655 | 0.018344106 | 0.692554819 |
| LRRC74A | cyan | -0.049511033 | 0.285642322 | -0.017151085 | 0.71162692 |
| LUZP4 | cyan | -0.101281071 | 0.028635606 | -0.087176652 | 0.059776336 |
| MBD3L4 | cyan | -0.060120403 | 0.194664077 | -0.042750521 | 0.356636992 |
| MC2R | cyan | -0.006383837 | 0.890566385 | 0.01622744 | 0.72651794 |
| MEPE | cyan | -0.08267399 | 0.074282641 | -0.063680465 | 0.169487267 |
| MGAT4C | cyan | -0.069802793 | 0.132003973 | -0.059411794 | 0.199986081 |
| MTNR1B | cyan | 0.023348631 | 0.614764212 | 0.032421904 | 0.484583684 |
| MTRNR2 | cyan | 0.047107063 | 0.309711265 | 0.069519435 | 0.133584126 |
| NEUROD | cyan | -0.016279184 | 0.725680936 | 0.006962804 | 0.880712515 |
| NMS | cyan | 0.008443877 | 0.855592188 | 0.02140774 | 0.644484225 |
| NPY2R | cyan | 0.021220579 | 0.647381314 | -0.001446166 | 0.975135429 |
| OR10A7 | cyan | -0.04163001 | 0.36939334 | -0.027051782 | 0.559803098 |
| OR10AG | cyan | -0.040022259 | 0.388183524 | -0.020196337 | 0.66332924 |
| OR10D3 | cyan | -0.023989134 | 0.605090078 | 0.003343791 | 0.942549008 |
| OR10G6 | cyan | -0.019469104 | 0.674746177 | -0.010769502 | 0.816449522 |
| OR10G8 | cyan | -0.039179898 | 0.398255982 | -0.016770485 | 0.717750068 |
| OR10H4 | cyan | -0.023657484 | 0.61009083 | -0.007759728 | 0.867179444 |
| OR10J1 | cyan | -0.001099776 | 0.981089775 | 0.015405106 | 0.739863071 |
| OR10T2 | cyan | -0.039414192 | 0.395438803 | -0.039104715 | 0.399162547 |
| OR10Z1 | cyan | 0.047098709 | 0.309797165 | 0.052898549 | 0.253918765 |
| OR11H2 | cyan | -0.039113557 | 0.39905586 | -0.025428775 | 0.583598228 |
| OR13C3 | cyan | -0.076142504 | 0.100293963 | -0.050434258 | 0.276743044 |
| OR13C5 | cyan | 0.019811145 | 0.669366933 | -0.0065357 | 0.887980019 |
| OR14A16 | cyan | -0.002833534 | 0.951304086 | 0.011064687 | 0.811511609 |
| OR14C36 | cyan | -0.064612222 | 0.16331855 | -0.04668732 | 0.3140469 |
| OR1C1 | cyan | -0.107458047 | 0.020195609 | -0.095148031 | 0.039848024 |
| OR1J1 | cyan | -0.064438158 | 0.164457944 | -0.048658098 | 0.294033617 |
| OR1J2 | cyan | -0.028432983 | 0.53992977 | -0.011978326 | 0.796276208 |
| OR1J4 | cyan | -0.049856449 | 0.282290431 | -0.062528238 | 0.177354431 |
| OR1L1 | cyan | -0.063859677 | 0.168287458 | -0.060528927 | 0.191643326 |
| OR1L3 | cyan | -0.073077183 | 0.114775222 | -0.055810831 | 0.228671362 |
| OR1S2 | cyan | -0.066121243 | 0.1536882 | -0.061838938 | 0.182188651 |
| OR2A14 | cyan | -0.042317276 | 0.361536061 | -0.031011141 | 0.503800929 |
| OR2A25 | cyan | -0.094734918 | 0.040721296 | -0.090218212 | 0.051369283 |
| OR2A4 | cyan | 0.03961303 | 0.393057379 | 0.049377603 | 0.286944273 |
| OR2AJ1 | cyan | -0.042932766 | 0.354588719 | -0.019789806 | 0.669702031 |
| OR2AK2 | cyan | -0.03281507 | 0.479299465 | -0.030556418 | 0.510079473 |
| OR2AP1 | cyan | -0.019572513 | 0.673118109 | -0.009556427 | 0.836816397 |
| OR2C3 | cyan | -0.110452751 | 0.016948758 | -0.088337068 | 0.056443605 |
| OR2J3 | cyan | -0.064313201 | 0.165279562 | -0.057791602 | 0.212550745 |
| OR2L3 | cyan | -0.09310779 | 0.04431971 | -0.077946459 | 0.092475114 |
| OR2L5 | cyan | -0.072007869 | 0.12019618 | -0.068459042 | 0.139627105 |
| OR2M2 | cyan | -0.056865791 | 0.219980681 | -0.041492437 | 0.370978779 |
| OR2T1 | cyan | -0.032546752 | 0.482902309 | -0.007755867 | 0.867244915 |

| | | | | | |
|---|---|---|---|---|---|
| OR2T5 | cyan | -0.03898085 | 0.400658809 | -0.01223622 | 0.791989384 |
| OR2V2 | cyan | -0.049046994 | 0.290187384 | -0.055361998 | 0.232441755 |
| OR2W1 | cyan | -0.067378124 | 0.146000683 | -0.060210119 | 0.193997728 |
| OR4A15 | cyan | -0.051147986 | 0.269993621 | -0.030035062 | 0.517327933 |
| OR4A16 | cyan | -0.052142405 | 0.260778831 | -0.032182635 | 0.487814842 |
| OR4A47 | cyan | -0.027498969 | 0.553330218 | -0.003352665 | 0.942396796 |
| OR4A5 | cyan | -0.021984045 | 0.635597392 | 0.006489038 | 0.888774586 |
| OR4C13 | cyan | -0.021244596 | 0.64700926 | -0.013554756 | 0.770172589 |
| OR4C3 | cyan | -0.003669833 | 0.936958263 | 0.013982374 | 0.763134945 |
| OR4D6 | cyan | -0.043105081 | 0.352658871 | -0.026417681 | 0.569043769 |
| OR4E2 | cyan | -0.031362008 | 0.498984313 | -0.021512792 | 0.642860446 |
| OR4F15 | cyan | -0.050262548 | 0.278383795 | -0.030110595 | 0.516274515 |
| OR4F17 | cyan | -0.027745878 | 0.549772035 | -0.015420684 | 0.73960952 |
| OR4F21 | cyan | -0.008169843 | 0.86022984 | 0.001771494 | 0.969544379 |
| OR4F5 | cyan | -0.043279305 | 0.350714384 | -0.044751784 | 0.334551838 |
| OR4F6 | cyan | -0.066289677 | 0.152640557 | -0.048017978 | 0.300438386 |
| OR4K1 | cyan | -0.038839026 | 0.402376135 | -0.009214581 | 0.842576169 |
| OR4N5 | cyan | -0.061236874 | 0.186490168 | -0.040769017 | 0.379384849 |
| OR4P4 | cyan | -0.017423979 | 0.70724791 | 0.000684793 | 0.988224576 |
| OR4S1 | cyan | -0.026589726 | 0.566529419 | -0.013250223 | 0.775196072 |
| OR51A4 | cyan | -0.05522187 | 0.233627846 | -0.032346592 | 0.485599476 |
| OR51B5 | cyan | -0.068723482 | 0.138100831 | -0.04963069 | 0.284478156 |
| OR51G2 | cyan | 0.048996716 | 0.290682729 | 0.055739683 | 0.229266127 |
| OR51T1 | cyan | -0.008123407 | 0.861016189 | 0.006089139 | 0.895588631 |
| OR52E2 | cyan | -0.084796251 | 0.067122904 | -0.070654046 | 0.127343712 |
| OR52J3 | cyan | 0.043730489 | 0.345710378 | 0.054720138 | 0.237909735 |
| OR52K2 | cyan | -0.054392021 | 0.240739662 | -0.038547553 | 0.405919331 |
| OR52L1 | cyan | -0.087269967 | 0.059502452 | -0.072553338 | 0.117406437 |
| OR52N4 | cyan | -0.12030564 | 0.009259614 | -0.112519357 | 0.014983505 |
| OR56A4 | cyan | -0.107009195 | 0.020726176 | -0.087462034 | 0.05894199 |
| OR5A2 | cyan | -0.047774754 | 0.302896099 | -0.034285247 | 0.45982067 |
| OR5AC2 | cyan | 0.048808098 | 0.292546081 | 0.043652869 | 0.346568009 |
| OR5AK2 | cyan | -0.044694036 | 0.335176543 | -0.040978714 | 0.376936256 |
| OR5B17 | cyan | -0.035395232 | 0.445411707 | -0.013938865 | 0.76385013 |
| OR5B2 | cyan | -0.059710736 | 0.197728103 | -0.042819466 | 0.355861241 |
| OR5B3 | cyan | -0.059088289 | 0.20245067 | -0.036010587 | 0.437535473 |
| OR5D14 | cyan | -0.04943274 | 0.286405788 | -0.029231776 | 0.528599235 |
| OR5H1 | cyan | -0.035273266 | 0.446982324 | -0.023911416 | 0.606260289 |
| OR5H14 | cyan | -0.036810315 | 0.427419731 | -0.019744659 | 0.670411241 |
| OR5H15 | cyan | -0.037261459 | 0.421773638 | -0.013597659 | 0.769465637 |
| OR5I1 | cyan | -0.058453935 | 0.207347313 | -0.051251344 | 0.269025614 |
| OR5K1 | cyan | -0.019786995 | 0.669746179 | -0.007100386 | 0.878373552 |
| OR5K4 | cyan | 0.045717604 | 0.324214775 | 0.042975747 | 0.354106725 |
| OR5M1 | cyan | -0.018661484 | 0.687513042 | -0.001332429 | 0.977090394 |
| OR5M10 | cyan | -0.072695699 | 0.116686747 | -0.021455406 | 0.643747241 |

| | | | | | |
|---|---|---|---|---|---|
| OR5M3 | cyan | -0.034920927 | 0.451537157 | -0.019609818 | 0.672531157 |
| OR5P2 | cyan | -0.062532043 | 0.177328012 | -0.031252184 | 0.500489319 |
| OR5W2 | cyan | 0.003816977 | 0.934436138 | 0.014460166 | 0.755294614 |
| OR6C1 | cyan | -0.027773523 | 0.549374346 | -0.012373068 | 0.789717176 |
| OR6C3 | cyan | -0.006551809 | 0.887705741 | -8.43E-05 | 0.998550581 |
| OR6C4 | cyan | 0.024781793 | 0.593212919 | 0.046965259 | 0.311171566 |
| OR6C65 | cyan | -0.05666646 | 0.221604385 | -0.04179467 | 0.367501256 |
| OR6C74 | cyan | 0.004179628 | 0.928222956 | 0.02323018 | 0.616560699 |
| OR6F1 | cyan | 0.05585112 | 0.22833505 | 0.056238618 | 0.225118357 |
| OR6K3 | cyan | 0.078524929 | 0.090073819 | 0.075817256 | 0.10175803 |
| OR6P1 | cyan | -0.011283014 | 0.807864184 | 0.011485322 | 0.804488096 |
| OR8A1 | cyan | -0.066781662 | 0.149611463 | -0.056511388 | 0.22287348 |
| OR8B2 | cyan | -0.035852707 | 0.439548602 | -0.009692024 | 0.834534097 |
| OR8B8 | cyan | -0.040831376 | 0.378655683 | -0.011846639 | 0.798467534 |
| OR8G1 | cyan | -0.032221014 | 0.487295788 | -0.007905886 | 0.864701517 |
| OR8G5 | cyan | -0.094457161 | 0.041317486 | -0.070811741 | 0.126494578 |
| OR8H3 | cyan | -0.036806147 | 0.4274721 | -0.020037039 | 0.665823536 |
| OR8I2 | cyan | -0.045970698 | 0.321540597 | -0.051032701 | 0.271076133 |
| OR8J1 | cyan | -0.053429722 | 0.249175239 | -0.031182405 | 0.501446808 |
| OR8U1 | cyan | -0.025314443 | 0.585292029 | 0.002118526 | 0.963581977 |
| OR9G1 | cyan | -0.059858113 | 0.1966218 | -0.045140322 | 0.330368174 |
| OR9I1 | cyan | -0.017176147 | 0.711224352 | -0.008846074 | 0.84879457 |
| OR9K2 | cyan | -0.038138044 | 0.410928631 | -0.025369394 | 0.584477668 |
| OR9Q2 | cyan | -0.016706561 | 0.71878027 | -0.001629958 | 0.971976627 |
| OTOL1 | cyan | -0.067425265 | 0.145718167 | -0.065286688 | 0.158959598 |
| PDHA2 | cyan | 0.007948181 | 0.863984713 | 0.032479358 | 0.483809545 |
| PLSCR5 | cyan | -0.046253622 | 0.318568273 | -0.037750051 | 0.415708333 |
| PM20D1 | cyan | -0.148155333 | 0.001323567 | -0.131906957 | 0.00429853 |
| POTEB3 | cyan | -0.04053636 | 0.382112926 | -0.016284241 | 0.725599151 |
| POTED | cyan | 0.040960234 | 0.377151653 | 0.059192884 | 0.201651422 |
| PPIAL4D | cyan | -0.013803569 | 0.766075336 | -0.003206301 | 0.944907451 |
| PPP1R3A | cyan | -0.036134403 | 0.435960405 | -0.023503941 | 0.61241218 |
| PSKH2 | cyan | -0.026771429 | 0.563879696 | 0.000543596 | 0.990652409 |
| PYDC2 | cyan | 0.003706777 | 0.936324962 | 0.014486098 | 0.754869802 |
| RBP3 | cyan | -0.074092705 | 0.109806299 | -0.053740876 | 0.246425404 |
| RNASE12 | cyan | 0.010541707 | 0.820265111 | 0.026199334 | 0.572242447 |
| RNASE8 | cyan | -0.089221415 | 0.05400855 | -0.076607221 | 0.098231194 |
| RNASE9 | cyan | -0.029170718 | 0.529461034 | -0.02086972 | 0.652826684 |
| RP11-148 | cyan | -0.150412384 | 0.001112965 | -0.126999963 | 0.005991 |
| RP11-166 | cyan | -0.052656199 | 0.256103703 | -0.00440173 | 0.924419885 |
| RP11-457 | cyan | -0.00236472 | 0.959353282 | 0.001999531 | 0.965626235 |
| RP11-500 | cyan | 0.050721951 | 0.274008787 | 0.060228705 | 0.193859891 |
| SCP2D1 | cyan | -0.050951553 | 0.271839877 | -0.032540123 | 0.482991499 |
| SI | cyan | -0.046264258 | 0.318456883 | -0.044770524 | 0.334349269 |
| SLC17A6 | cyan | -0.057970626 | 0.211135109 | -0.044265268 | 0.339838277 |

| | | | | | |
|---|---|---|---|---|---|
| SLC6A5 | cyan | -0.058714228 | 0.205327822 | -0.058843691 | 0.204328701 |
| SLC7A13 | cyan | 0.014543982 | 0.753921799 | 0.04182781 | 0.367121181 |
| SPACA5I | cyan | -0.025455857 | 0.583197361 | -0.010414398 | 0.82239939 |
| SPANXN | cyan | 0.036064769 | 0.436845821 | 0.044637923 | 0.335784282 |
| SPATA31 | cyan | 0.002103677 | 0.963837056 | 0.006331708 | 0.891454457 |
| SSX4 | cyan | -0.03579116 | 0.440334829 | -0.034238416 | 0.460434259 |
| SYT10 | cyan | -0.004528264 | 0.922254009 | 0.007841111 | 0.865799526 |
| TAAR2 | cyan | -0.017595712 | 0.704497132 | -0.009816209 | 0.832445124 |
| TAS2R16 | cyan | -0.013963725 | 0.763441477 | -0.015540754 | 0.73765622 |
| TECRL | cyan | 0.019180298 | 0.679301153 | 0.027538764 | 0.552755983 |
| TEX33 | cyan | -0.028578564 | 0.537855761 | -0.016702072 | 0.718852632 |
| TMEM17 | cyan | -0.064160975 | 0.166284634 | -0.058150632 | 0.209718579 |
| TMEM78 | cyan | 0.047959326 | 0.301029832 | 0.061878536 | 0.181908331 |
| TNP1 | cyan | -0.06810125 | 0.141712736 | -0.070000446 | 0.130910333 |
| TRIM72 | cyan | -0.110295329 | 0.017107308 | -0.109766455 | 0.01764951 |
| TSPY4 | cyan | -0.043279305 | 0.350714384 | -0.044751784 | 0.334551838 |
| UBTFL1 | cyan | -0.053332686 | 0.25003716 | -0.032492985 | 0.483626021 |
| UGT2A1 | cyan | -0.077137832 | 0.095917391 | -0.057023692 | 0.218700499 |
| UGT2B28 | cyan | 0.002881552 | 0.950479921 | 0.025024311 | 0.589600436 |
| UNC5D | cyan | -0.112531741 | 0.014972358 | -0.088664792 | 0.0555308 |
| VN1R2 | cyan | -0.069250063 | 0.135099769 | -0.06186399 | 0.182011268 |
| VN1R4 | cyan | -0.036827181 | 0.427207859 | -0.024658785 | 0.595049079 |
| XIRP2 | cyan | -0.075960556 | 0.101110904 | -0.06143458 | 0.185069464 |
| ZNF479 | cyan | -0.062156657 | 0.179948448 | -0.055987631 | 0.227198149 |
| ZNF705B | cyan | 0.035158757 | 0.448459758 | 0.047290838 | 0.307825466 |
| ZNF705D | cyan | 0.013453824 | 0.771836478 | 0.043848286 | 0.344411418 |
| ZNF705G | cyan | -0.049596046 | 0.284814886 | -0.027197646 | 0.55768776 |
| A2M | darkgreen | -0.145699478 | 0.001593922 | -0.106626558 | 0.021187997 |
| ABCB1 | darkgreen | -0.2077847 | 5.96E-06 | -0.196441345 | 1.91E-05 |
| ABCG2 | darkgreen | -0.247009179 | 6.39E-08 | -0.244833325 | 8.39E-08 |
| AC00933 | darkgreen | -0.132506017 | 0.004124707 | -0.164373548 | 0.000361211 |
| ACKR1 | darkgreen | -0.110030182 | 0.017377289 | -0.081094305 | 0.080006134 |
| ACVRL1 | darkgreen | -0.123374136 | 0.007603287 | -0.075022545 | 0.105406706 |
| ADAMTS | darkgreen | 0.066287939 | 0.152651337 | 0.070023711 | 0.130782066 |
| ADCY4 | darkgreen | -0.12062691 | 0.009072295 | -0.09142758 | 0.048312534 |
| ADPRH | darkgreen | -0.343269575 | 2.32E-14 | -0.305116681 | 1.61E-11 |
| ADRB2 | darkgreen | -0.038936888 | 0.401190668 | -0.029721986 | 0.521706054 |
| APLNR | darkgreen | 0.05160658 | 0.265716762 | 0.064279395 | 0.165502369 |
| AQP1 | darkgreen | -0.145880291 | 0.001572409 | -0.115930088 | 0.012175569 |
| ARHGAP | darkgreen | -0.163149481 | 0.000400146 | -0.148184686 | 0.001320608 |
| ARHGEF | darkgreen | -0.128196333 | 0.005530743 | -0.116978892 | 0.011411082 |
| ARHGEF | darkgreen | -0.142136272 | 0.002076928 | -0.114642373 | 0.013175821 |
| ARID5A | darkgreen | -0.224227174 | 9.83E-07 | -0.20873479 | 5.39E-06 |
| ARL15 | darkgreen | 0.178493007 | 0.000105269 | 0.175439402 | 0.000138567 |
| BCL6B | darkgreen | -0.173704196 | 0.000161662 | -0.166380929 | 0.000304917 |

| | | | | | |
|---|---|---|---|---|---|
| BDKRB2 | darkgreen | -0.164048396 | 0.000371194 | -0.14782497 | 0.0013573 |
| BMP8A | darkgreen | -0.052453959 | 0.257936955 | -0.047043548 | 0.310364784 |
| C10orf10 | darkgreen | -0.198846285 | 1.50E-05 | -0.170616602 | 0.000211916 |
| C10orf128 | darkgreen | 0.03236398 | 0.485364845 | 0.041412791 | 0.371898572 |
| C10orf54 | darkgreen | -0.23899297 | 1.72E-07 | -0.191272414 | 3.17E-05 |
| CALCRL | darkgreen | -0.07879888 | 0.088954202 | -0.060216195 | 0.193952657 |
| CAPN11 | darkgreen | -0.091817033 | 0.047361188 | -0.09884206 | 0.032720001 |
| CBFA2T3 | darkgreen | -0.161371354 | 0.000463708 | -0.148048806 | 0.001334359 |
| CCDC69 | darkgreen | -0.184824183 | 5.87E-05 | -0.150639685 | 0.001093565 |
| CCM2L | darkgreen | -0.319216771 | 1.60E-12 | -0.316063454 | 2.71E-12 |
| CD34 | darkgreen | -0.162574692 | 0.000419746 | -0.148655852 | 0.001273928 |
| CD93 | darkgreen | -0.066538379 | 0.151103565 | -0.054204965 | 0.242363505 |
| CD97 | darkgreen | -0.171720408 | 0.000192472 | -0.131373774 | 0.004458777 |
| CDH5 | darkgreen | 0.001385609 | 0.976176298 | 0.009111991 | 0.844306364 |
| CLDN15 | darkgreen | -0.113971191 | 0.013725344 | -0.100296563 | 0.030228343 |
| CLDN5 | darkgreen | -0.184516119 | 6.04E-05 | -0.150660646 | 0.001091792 |
| CLEC14A | darkgreen | -0.130089991 | 0.004866977 | -0.099843893 | 0.030985773 |
| CLEC1A | darkgreen | -0.186797723 | 4.87E-05 | -0.177671129 | 0.000113402 |
| CLEC2B | darkgreen | -0.218901206 | 1.79E-06 | -0.182096675 | 7.57E-05 |
| CLEC3B | darkgreen | -0.170302351 | 0.000217779 | -0.16083918 | 0.000484484 |
| CLEC9A | darkgreen | -0.186357867 | 5.08E-05 | -0.169312175 | 0.000237261 |
| CLIC2 | darkgreen | -0.209202682 | 5.13E-06 | -0.183689791 | 6.53E-05 |
| COL5A3 | darkgreen | -0.078331916 | 0.090869413 | -0.047563949 | 0.305036975 |
| CSPG4 | darkgreen | 0.016233248 | 0.726423982 | 0.012902967 | 0.780935766 |
| CXorf36 | darkgreen | -0.207394835 | 6.21E-06 | -0.194578656 | 2.29E-05 |
| CYGB | darkgreen | 0.101756052 | 0.027893302 | 0.117048999 | 0.011361529 |
| CYYR1 | darkgreen | -0.328470876 | 3.28E-13 | -0.313841308 | 3.92E-12 |
| DHH | darkgreen | -0.036195985 | 0.435178229 | -0.004692785 | 0.919438757 |
| DLL4 | darkgreen | -0.022053723 | 0.634526424 | -0.023489509 | 0.612630578 |
| DYSF | darkgreen | -0.107755814 | 0.019850187 | -0.091091424 | 0.049146505 |
| ECSCR | darkgreen | -0.122904587 | 0.00783822 | -0.099641 | 0.031330498 |
| EDN1 | darkgreen | -0.144489036 | 0.001745037 | -0.148820451 | 0.001257981 |
| EGFL7 | darkgreen | -0.16806004 | 0.000264234 | -0.160076115 | 0.000515788 |
| ELFN1 | darkgreen | -0.083193524 | 0.072474827 | -0.065216731 | 0.159407598 |
| ELMO1 | darkgreen | -0.108237484 | 0.019302308 | -0.086993599 | 0.060316635 |
| ELTD1 | darkgreen | -0.122396539 | 0.008099697 | -0.110578335 | 0.016823197 |
| EMCN | darkgreen | -0.206402452 | 6.89E-06 | -0.19198754 | 2.96E-05 |
| ENG | darkgreen | -0.160503981 | 0.000498011 | -0.114653102 | 0.013167196 |
| ENPP2 | darkgreen | -0.278830055 | 8.71E-10 | -0.270914364 | 2.67E-09 |
| ENTPD1 | darkgreen | 0.019109521 | 0.680419216 | 0.012414676 | 0.789026685 |
| EPAS1 | darkgreen | 0.084639504 | 0.06763159 | 0.092984667 | 0.044602558 |
| ERG | darkgreen | -0.003653566 | 0.937237123 | -0.013559433 | 0.770095499 |
| ESAM | darkgreen | -0.080194581 | 0.083422478 | -0.055720473 | 0.229426896 |
| ETS1 | darkgreen | -0.059640036 | 0.198260432 | -0.047500313 | 0.305685213 |
| EXOC3L1 | darkgreen | 0.086678393 | 0.06125645 | 0.118385501 | 0.010452587 |

| | | | | | |
|---|---|---|---|---|---|
| EXOC3L2 | darkgreen | -0.058858125 | 0.204217528 | -0.029325545 | 0.527277115 |
| FAM110D | darkgreen | -0.161149127 | 0.00047228 | -0.148132789 | 0.001325844 |
| FAM124B | darkgreen | -0.131520746 | 0.004414075 | -0.141983532 | 0.002100351 |
| FAM167B | darkgreen | -0.051850138 | 0.263464335 | -0.034796439 | 0.453152689 |
| FAM212A | darkgreen | -0.154757709 | 0.000791881 | -0.124702477 | 0.006972378 |
| FGD5 | darkgreen | -0.21771771 | 2.04E-06 | -0.210858346 | 4.30E-06 |
| FLI1 | darkgreen | -0.194852747 | 2.23E-05 | -0.168687089 | 0.000250387 |
| FLT1 | darkgreen | 0.015075109 | 0.74524066 | 0.012278569 | 0.791286033 |
| FLT4 | darkgreen | -0.186676007 | 4.93E-05 | -0.176492596 | 0.0001261 |
| FOXF1 | darkgreen | 0.023446819 | 0.613276776 | 0.030231239 | 0.514594248 |
| FOXS1 | darkgreen | 0.040515378 | 0.382359545 | 0.075825126 | 0.101722404 |
| GIMAP1 | darkgreen | -0.169596477 | 0.000231507 | -0.140112641 | 0.002407525 |
| GIMAP2 | darkgreen | -0.043880714 | 0.344054375 | -0.023022745 | 0.619712275 |
| GIMAP4 | darkgreen | -0.121695097 | 0.008473493 | -0.086980108 | 0.060356614 |
| GIMAP5 | darkgreen | -0.160220694 | 0.000509716 | -0.160175628 | 0.000511601 |
| GIMAP6 | darkgreen | -0.15012425 | 0.001138013 | -0.124716666 | 0.006965899 |
| GIMAP7 | darkgreen | -0.125641906 | 0.006554915 | -0.099294576 | 0.031926671 |
| GIMAP8 | darkgreen | -0.182416943 | 7.35E-05 | -0.16259279 | 0.000419115 |
| GIPC3 | darkgreen | -0.117300982 | 0.011184988 | -0.091241308 | 0.048773181 |
| GJA4 | darkgreen | -0.113264383 | 0.014325751 | -0.102046317 | 0.027447863 |
| GJA5 | darkgreen | -0.058067568 | 0.210371384 | -0.051323596 | 0.268350352 |
| GNAI2 | darkgreen | 0.075974333 | 0.101048864 | 0.109673797 | 0.017746033 |
| GPR116 | darkgreen | -0.125495049 | 0.006618661 | -0.111293534 | 0.016123533 |
| GPR4 | darkgreen | 0.067195022 | 0.147101978 | 0.094341391 | 0.041568147 |
| GPR85 | darkgreen | -0.242847409 | 1.07E-07 | -0.217349928 | 2.12E-06 |
| GRAP | darkgreen | -0.054056508 | 0.243657729 | -0.057884182 | 0.211817818 |
| GUCY1A | darkgreen | 0.12132359 | 0.008677632 | 0.112722146 | 0.014801894 |
| GYPC | darkgreen | -0.221387739 | 1.36E-06 | -0.18297045 | 6.98E-05 |
| HEG1 | darkgreen | -0.209914359 | 4.76E-06 | -0.184598729 | 5.99E-05 |
| HEYL | darkgreen | -0.075808214 | 0.101798972 | -0.068804364 | 0.137636573 |
| HHEX | darkgreen | -0.337878055 | 6.20E-14 | -0.319499454 | 1.53E-12 |
| HIGD1B | darkgreen | -0.098019025 | 0.034206077 | -0.085329892 | 0.065414584 |
| HOXD1 | darkgreen | -0.120106954 | 0.009377173 | -0.139487493 | 0.002518966 |
| HOXD10 | darkgreen | -0.130321457 | 0.004790977 | -0.134003687 | 0.003717618 |
| HOXD11 | darkgreen | -0.097963384 | 0.034308587 | -0.112783325 | 0.014747486 |
| HOXD3 | darkgreen | -0.146042329 | 0.001553357 | -0.163788792 | 0.000379348 |
| HOXD4 | darkgreen | -0.15060659 | 0.00109637 | -0.179913211 | 9.25E-05 |
| HOXD8 | darkgreen | -0.138819757 | 0.002643177 | -0.153073261 | 0.000904524 |
| HOXD9 | darkgreen | -0.125786916 | 0.006492511 | -0.133093066 | 0.003960562 |
| HPCAL1 | darkgreen | -0.137212686 | 0.002965321 | -0.103672358 | 0.025064025 |
| HVCN1 | darkgreen | -0.264940577 | 6.09E-09 | -0.243827065 | 9.51E-08 |
| HYAL1 | darkgreen | -0.146590072 | 0.001490511 | -0.141020936 | 0.002253613 |
| ICAM2 | darkgreen | -0.23934433 | 1.65E-07 | -0.211236952 | 4.13E-06 |
| IFFO1 | darkgreen | -0.208160796 | 5.73E-06 | -0.167510547 | 0.000276953 |
| IGFBP7 | darkgreen | -0.107960009 | 0.019616293 | -0.077432339 | 0.094652041 |

| | | | | | |
|---|---|---|---|---|---|
| IL33 | darkgreen | 0.002013964 | 0.965378272 | 0.002600416 | 0.955305999 |
| IL34 | darkgreen | -0.16918802 | 0.000239816 | -0.144339733 | 0.001764559 |
| IL3RA | darkgreen | -0.041587975 | 0.369877321 | -0.02040012 | 0.660143808 |
| INSR | darkgreen | -0.202581562 | 1.02E-05 | -0.202763347 | 1.01E-05 |
| IRAK3 | darkgreen | -0.203720331 | 9.11E-06 | -0.196193165 | 1.95E-05 |
| ITGA1 | darkgreen | 0.010773993 | 0.816374338 | 0.006462925 | 0.889219292 |
| ITGA5 | darkgreen | -0.090486789 | 0.050676854 | -0.070355815 | 0.128961662 |
| KANK3 | darkgreen | -0.157808287 | 0.000620262 | -0.134466353 | 0.003599429 |
| KDR | darkgreen | -0.11663735 | 0.011655238 | -0.114128742 | 0.01359457 |
| KL | darkgreen | -0.227459622 | 6.78E-07 | -0.236408031 | 2.36E-07 |
| LHX6 | darkgreen | -0.247823761 | 5.76E-08 | -0.238450002 | 1.84E-07 |
| LYL1 | darkgreen | -0.2047734 | 8.17E-06 | -0.157611578 | 0.000630193 |
| MAPK11 | darkgreen | 0.122254156 | 0.008174359 | 0.131468855 | 0.004429811 |
| MCAM | darkgreen | -0.171957317 | 0.000188523 | -0.188637732 | 4.09E-05 |
| MFNG | darkgreen | 0.008942263 | 0.847170509 | 0.042163914 | 0.363280274 |
| MMRN1 | darkgreen | -0.201457792 | 1.15E-05 | -0.180276517 | 8.95E-05 |
| MMRN2 | darkgreen | -0.165429427 | 0.000330493 | -0.146872396 | 0.001459039 |
| MYCT1 | darkgreen | -0.208725775 | 5.40E-06 | -0.197834841 | 1.66E-05 |
| NOS3 | darkgreen | -0.032612552 | 0.482017422 | -0.002361487 | 0.959408821 |
| NOTCH4 | darkgreen | -0.081194264 | 0.079633679 | -0.065402818 | 0.158218006 |
| NOVA2 | darkgreen | -0.277137434 | 1.11E-09 | -0.270584797 | 2.80E-09 |
| NRP1 | darkgreen | -0.093775403 | 0.042812152 | -0.050431815 | 0.276766341 |
| OR51E1 | darkgreen | 0.222108764 | 1.25E-06 | 0.246797358 | 6.56E-08 |
| OR51E2 | darkgreen | 0.101540855 | 0.02822754 | 0.093442793 | 0.043557739 |
| P2RX7 | darkgreen | -0.186184487 | 5.16E-05 | -0.168216938 | 0.000260704 |
| PCDH12 | darkgreen | -0.088349167 | 0.056409687 | -0.077411983 | 0.094739071 |
| PCDH17 | darkgreen | -0.111400872 | 0.016020757 | -0.088337157 | 0.056443358 |
| PECAM1 | darkgreen | -0.067363782 | 0.146086721 | -0.033688544 | 0.467672833 |
| PLA2G4C | darkgreen | -0.255555517 | 2.13E-08 | -0.271992118 | 2.30E-09 |
| PLVAP | darkgreen | 0.099005327 | 0.03243185 | 0.123635174 | 0.00747542 |
| PLXND1 | darkgreen | -0.1833714 | 6.72E-05 | -0.174385321 | 0.000152196 |
| PPAP2A | darkgreen | -0.051730901 | 0.264565395 | -0.035760828 | 0.440722585 |
| PPM1M | darkgreen | 0.097886109 | 0.034451384 | 0.119163381 | 0.00995378 |
| PPP1R16I | darkgreen | -0.15492217 | 0.000781609 | -0.143124195 | 0.001931104 |
| PRKCH | darkgreen | 0.119051299 | 0.010024323 | 0.137623803 | 0.002879677 |
| PTGIR | darkgreen | -0.134641454 | 0.003555596 | -0.07193096 | 0.120593649 |
| PTPRB | darkgreen | -0.092993994 | 0.044581079 | -0.088851346 | 0.055016689 |
| RAMP2 | darkgreen | -0.159687249 | 0.000532454 | -0.127497783 | 0.005795459 |
| RAMP3 | darkgreen | 0.047978415 | 0.30083725 | 0.090308957 | 0.051134453 |
| RASGRP | darkgreen | -0.195695269 | 2.05E-05 | -0.165293698 | 0.000334301 |
| RASIP1 | darkgreen | -0.155740753 | 0.00073229 | -0.142247854 | 0.002059967 |
| RBP5 | darkgreen | -0.1715411 | 0.000195513 | -0.147133426 | 0.001430484 |
| RFTN1 | darkgreen | -0.273238833 | 1.93E-09 | -0.241272215 | 1.31E-07 |
| RGCC | darkgreen | -0.068494699 | 0.139420551 | -0.057701861 | 0.213262936 |
| RGS5 | darkgreen | -0.018383305 | 0.691931376 | -0.020168335 | 0.663767429 |

| | | | | | |
|---|---|---|---|---|---|
| RHOJ | darkgreen | -0.238707071 | 1.79E-07 | -0.219443808 | 1.68E-06 |
| ROBO4 | darkgreen | -0.110844201 | 0.016560063 | -0.098529101 | 0.033278457 |
| S1PR1 | darkgreen | -0.04765647 | 0.304096135 | -0.050446477 | 0.276626538 |
| SCARF1 | darkgreen | -0.124709107 | 0.00696935 | -0.113944973 | 0.013747213 |
| SELP | darkgreen | -0.118289764 | 0.010515484 | -0.090879245 | 0.049679073 |
| SEMA3G | darkgreen | 0.048077028 | 0.29984371 | 0.036343438 | 0.433308664 |
| SEMA6B | darkgreen | -0.072220528 | 0.119102466 | -0.05655812 | 0.222490486 |
| SERPINE | darkgreen | 0.017311646 | 0.709049309 | 0.023340215 | 0.614891782 |
| SFMBT2 | darkgreen | -0.132027815 | 0.004262937 | -0.127830436 | 0.005668012 |
| SH2B3 | darkgreen | -0.172668918 | 0.000177112 | -0.147951833 | 0.001344254 |
| SH2D3C | darkgreen | -0.111270498 | 0.016145664 | -0.072694023 | 0.116695201 |
| SHE | darkgreen | -0.225186294 | 8.81E-07 | -0.214668351 | 2.85E-06 |
| SLC16A1 | darkgreen | -0.172485604 | 0.000179988 | -0.162385373 | 0.000426395 |
| SLC2A1 | darkgreen | 0.038184938 | 0.410353159 | 0.046739935 | 0.313501247 |
| SLC9A9 | darkgreen | -0.210849333 | 4.31E-06 | -0.19863567 | 1.53E-05 |
| SOX18 | darkgreen | -0.257089555 | 1.74E-08 | -0.230576237 | 4.72E-07 |
| STARD8 | darkgreen | -0.195559241 | 2.08E-05 | -0.203267332 | 9.55E-06 |
| STOM | darkgreen | -0.135748232 | 0.003289552 | -0.11023177 | 0.017171689 |
| SULT1B1 | darkgreen | -0.021887342 | 0.637084972 | -0.01548915 | 0.738495513 |
| TAGAP | darkgreen | -0.165305532 | 0.000333967 | -0.134819346 | 0.003511559 |
| TBXA2R | darkgreen | -0.104837788 | 0.023467368 | -0.077504185 | 0.094345389 |
| TEK | darkgreen | -0.073592702 | 0.112231202 | -0.066134559 | 0.153605177 |
| TGFB1 | darkgreen | -0.147808292 | 0.001359023 | -0.09981188 | 0.031039947 |
| TGFBR2 | darkgreen | -0.145409186 | 0.001629026 | -0.145529382 | 0.001614406 |
| THBD | darkgreen | -0.093688004 | 0.043007018 | -0.098571009 | 0.033203207 |
| THSD1 | darkgreen | -0.027232263 | 0.557186302 | -0.043779534 | 0.345169175 |
| TIE1 | darkgreen | -0.239331426 | 1.66E-07 | -0.21137329 | 4.07E-06 |
| TLR4 | darkgreen | 0.141237763 | 0.002218225 | 0.11221669 | 0.015258202 |
| TM4SF18 | darkgreen | -0.123870745 | 0.007361681 | -0.125194946 | 0.006750656 |
| TMEM20 | darkgreen | -0.09697179 | 0.036179761 | -0.070668159 | 0.127267537 |
| TMEM25 | darkgreen | -0.170870596 | 0.000207286 | -0.137884855 | 0.00282647 |
| TP53INP2 | darkgreen | -0.021358935 | 0.645239171 | 0.009533265 | 0.837206372 |
| USHBP1 | darkgreen | -0.160245478 | 0.000508682 | -0.156145286 | 0.000708995 |
| VEGFC | darkgreen | -0.086065836 | 0.063117418 | -0.080645263 | 0.081696755 |
| VWF | darkgreen | -0.000790426 | 0.986408315 | 0.026790532 | 0.563601471 |
| WFDC1 | darkgreen | -0.002707938 | 0.953460041 | 0.022930128 | 0.621121679 |
| ZNF366 | darkgreen | -0.120231964 | 0.009303052 | -0.112399081 | 0.015092142 |
| ABCB7 | darkgrey | -0.041201086 | 0.3743503 | -0.043750324 | 0.345491441 |
| ABHD17I | darkgrey | 0.152237116 | 0.000965775 | 0.137115865 | 0.002985825 |
| ADNP2 | darkgrey | 0.182857431 | 7.05E-05 | 0.156720346 | 0.000677061 |
| ANAPC1 | darkgrey | -0.128001443 | 0.00560348 | -0.161959035 | 0.000441729 |
| BAZ1B | darkgrey | 0.004385308 | 0.92470103 | -0.017619626 | 0.704114376 |
| BCL7A | darkgrey | 0.104801673 | 0.023515493 | 0.073008436 | 0.115117871 |
| BCOR | darkgrey | 0.094485455 | 0.04125642 | 0.063838301 | 0.168430233 |
| BPHL | darkgrey | 0.038654852 | 0.404612835 | 0.024303894 | 0.600361015 |

| | | | | | |
|---|---|---|---|---|---|
| BRD8 | darkgrey | -0.172194542 | 0.000184644 | -0.200027566 | 1.33E-05 |
| C1orf109 | darkgrey | -0.07726413 | 0.095373111 | -0.107407883 | 0.020254314 |
| CAMSAP | darkgrey | 0.034200653 | 0.460929371 | 0.007148988 | 0.877547538 |
| CAND1 | darkgrey | -0.028453046 | 0.539643709 | -0.05794986 | 0.211298966 |
| CAPRIN1 | darkgrey | 0.104659201 | 0.023706176 | 0.074848406 | 0.106219867 |
| CCDC97 | darkgrey | 0.085518351 | 0.064819881 | 0.08205929 | 0.076468866 |
| CDC23 | darkgrey | -0.06542849 | 0.158054426 | -0.090729197 | 0.050058599 |
| CDC40 | darkgrey | -0.052323825 | 0.259121372 | -0.074597915 | 0.107398254 |
| CDK5RA | darkgrey | 0.069224431 | 0.135244676 | 0.038640364 | 0.404789093 |
| CHAMP1 | darkgrey | 0.071520386 | 0.122732891 | 0.050076233 | 0.280171556 |
| CHD4 | darkgrey | -0.201950353 | 1.09E-05 | -0.212989478 | 3.42E-06 |
| CHERP | darkgrey | -0.138612877 | 0.002682778 | -0.165238314 | 0.000335866 |
| CLTC | darkgrey | 0.125885378 | 0.006450443 | 0.111134546 | 0.016276825 |
| CPSF6 | darkgrey | -0.039001098 | 0.400413989 | -0.057794146 | 0.212530576 |
| CS | darkgrey | 0.145799042 | 0.001582043 | 0.10374267 | 0.024965106 |
| CTCF | darkgrey | 0.112585809 | 0.014923779 | 0.109815732 | 0.017598364 |
| CUL3 | darkgrey | -0.021413468 | 0.644395648 | -0.045407913 | 0.327506546 |
| DBR1 | darkgrey | -0.013859372 | 0.765157319 | -0.017442618 | 0.706949166 |
| DCAF7 | darkgrey | 0.048395491 | 0.296650028 | 0.030107439 | 0.5163185 |
| DDX31 | darkgrey | 0.054514357 | 0.239681795 | 0.022016934 | 0.635091777 |
| DHX15 | darkgrey | 0.07599126 | 0.100972675 | 0.046844828 | 0.31241531 |
| DHX33 | darkgrey | 0.032178755 | 0.487867336 | -0.004381173 | 0.924771814 |
| DHX9 | darkgrey | 0.037586355 | 0.417734694 | 0.003156215 | 0.945766727 |
| DIABLO | darkgrey | -0.030937597 | 0.504813621 | -0.05630506 | 0.224570069 |
| DNAJC11 | darkgrey | -0.025978097 | 0.575492104 | -0.060532321 | 0.19161837 |
| DNAJC14 | darkgrey | -0.06076285 | 0.189929171 | -0.094222197 | 0.04182756 |
| DSCR3 | darkgrey | 0.020304228 | 0.661641985 | 0.004234183 | 0.927288664 |
| EIF3A | darkgrey | 0.068528618 | 0.139224283 | 0.027955347 | 0.546762222 |
| EPB41 | darkgrey | -0.074931994 | 0.105828929 | -0.095319352 | 0.039490546 |
| EPS15L1 | darkgrey | -0.087883046 | 0.05772873 | -0.103732863 | 0.024978883 |
| FAM104A | darkgrey | 0.179647482 | 9.48E-05 | 0.165653776 | 0.000324288 |
| FAM53C | darkgrey | -0.029388519 | 0.526390141 | -0.066489162 | 0.151406788 |
| FBXW2 | darkgrey | 0.022517042 | 0.62742464 | 0.011159871 | 0.809920934 |
| FCF1 | darkgrey | -0.106146283 | 0.021780285 | -0.110820645 | 0.016583231 |
| FIP1L1 | darkgrey | 0.003018992 | 0.948121228 | -0.043774342 | 0.34522644 |
| FUBP1 | darkgrey | -0.063145021 | 0.173110104 | -0.087874103 | 0.057754283 |
| FUBP3 | darkgrey | -0.008180129 | 0.86005568 | -0.035220869 | 0.447658021 |
| GEMIN4 | darkgrey | 0.074014035 | 0.110185067 | 0.059310812 | 0.200753044 |
| GEMIN5 | darkgrey | -0.07941617 | 0.086472342 | -0.109048304 | 0.018409761 |
| GLE1 | darkgrey | 0.306513703 | 1.29E-11 | 0.300541317 | 3.33E-11 |
| GLOD4 | darkgrey | 0.171352003 | 0.000198768 | 0.158934812 | 0.000566131 |
| GNAI3 | darkgrey | -0.054299454 | 0.241542286 | -0.072690984 | 0.116710527 |
| GPR107 | darkgrey | 0.008327038 | 0.857568938 | 0.005232712 | 0.910206885 |
| GRB2 | darkgrey | -0.050673091 | 0.274471859 | -0.054089011 | 0.243373962 |
| GSTCD | darkgrey | 0.133142754 | 0.003946944 | 0.107582452 | 0.020050663 |

| | | | | | |
|---|---|---|---|---|---|
| GTF2H2C | darkgrey | 0.147389118 | 0.001403013 | 0.131850689 | 0.004315192 |
| GTF3C4 | darkgrey | 0.031083781 | 0.502801749 | 0.005071082 | 0.912969281 |
| HMGN4 | darkgrey | -0.154149468 | 0.000830967 | -0.171910409 | 0.000189299 |
| HMGXB4 | darkgrey | 0.060553732 | 0.191461019 | 0.024876696 | 0.591798069 |
| HNRNPA | darkgrey | 0.007564617 | 0.870489309 | -0.029546309 | 0.524171055 |
| HNRNPF | darkgrey | 0.279814104 | 7.56E-10 | 0.258714808 | 1.41E-08 |
| HNRNPK | darkgrey | 0.123234708 | 0.007672383 | 0.09382579 | 0.042700147 |
| HNRNPU | darkgrey | -0.074917613 | 0.105896106 | -0.09329001 | 0.043903867 |
| IARS | darkgrey | 0.129055904 | 0.005219961 | 0.107428022 | 0.020230728 |
| IDH3A | darkgrey | 0.068219326 | 0.141021826 | 0.043686715 | 0.346193869 |
| IKBKAP | darkgrey | 0.109569696 | 0.017855025 | 0.061759428 | 0.182752479 |
| INIP | darkgrey | 0.163700188 | 0.000382169 | 0.136140587 | 0.003199667 |
| INTS2 | darkgrey | 0.038276884 | 0.409226196 | 0.015270305 | 0.742058252 |
| KDM1B | darkgrey | -0.023276551 | 0.615857139 | -0.04352715 | 0.347959943 |
| KDM3B | darkgrey | 0.085298821 | 0.065513061 | 0.052525303 | 0.257289205 |
| KDM4D | darkgrey | -0.155550384 | 0.000743495 | -0.170324607 | 0.000217359 |
| KHSRP | darkgrey | 0.218053362 | 1.97E-06 | 0.184785472 | 5.89E-05 |
| KIAA019 | darkgrey | -0.104834257 | 0.02347207 | -0.141138465 | 0.002234368 |
| KIAA036 | darkgrey | 0.167097981 | 0.000286876 | 0.135594357 | 0.003325425 |
| KIF2A | darkgrey | 0.193407617 | 2.57E-05 | 0.162629417 | 0.000417842 |
| KPNA6 | darkgrey | -0.039936385 | 0.38920322 | -0.04480322 | 0.333996045 |
| LARP1 | darkgrey | 0.06953947 | 0.133471922 | 0.050872917 | 0.272581388 |
| LSM14B | darkgrey | 0.00560788 | 0.903799169 | -0.017773934 | 0.701646459 |
| MAML1 | darkgrey | 0.023014408 | 0.619839096 | -0.002952614 | 0.949260332 |
| MAPKAP | darkgrey | -0.093491995 | 0.043446762 | -0.088714679 | 0.055392929 |
| MARS2 | darkgrey | 0.029198392 | 0.529070345 | 0.016804364 | 0.717204271 |
| MAT2A | darkgrey | -0.07685398 | 0.097149697 | -0.100270355 | 0.030271761 |
| MCMBP | darkgrey | 0.059527877 | 0.199107064 | 0.02555515 | 0.581728672 |
| MIEF1 | darkgrey | -0.033598265 | 0.468867256 | -0.047612833 | 0.304539641 |
| MTMR4 | darkgrey | 0.005925102 | 0.898385993 | -0.014287952 | 0.758117725 |
| NAA35 | darkgrey | 0.158498625 | 0.000586548 | 0.133945497 | 0.003732729 |
| NCBP1 | darkgrey | 0.159547954 | 0.000538544 | 0.136353907 | 0.00315174 |
| NDE1 | darkgrey | 0.164657947 | 0.000352685 | 0.162641845 | 0.000417411 |
| NDUFS1 | darkgrey | 0.101026022 | 0.029041151 | 0.095946072 | 0.038205869 |
| NEK4 | darkgrey | 0.122256848 | 0.008172942 | 0.100843576 | 0.029334266 |
| NFYA | darkgrey | -0.010533432 | 0.8204038 | -0.035569603 | 0.443171703 |
| NIPA2 | darkgrey | 0.11770996 | 0.010903624 | 0.094035203 | 0.042237286 |
| NOM1 | darkgrey | 0.076896005 | 0.09696646 | 0.059323585 | 0.200655918 |
| NONO | darkgrey | -0.019178293 | 0.679332818 | -0.046411586 | 0.316916576 |
| NUP205 | darkgrey | 0.024678594 | 0.594753218 | -0.01098454 | 0.812851595 |
| PALB2 | darkgrey | -0.049992331 | 0.280979155 | -0.075064012 | 0.105213795 |
| PARG | darkgrey | 0.046949756 | 0.31133149 | 0.026328552 | 0.570348443 |
| PAXIP1 | darkgrey | 0.155347442 | 0.000755615 | 0.118721725 | 0.010234329 |
| PDCL | darkgrey | 0.152063494 | 0.000978965 | 0.13868164 | 0.002669556 |
| PMS2 | darkgrey | -0.060876732 | 0.189098753 | -0.080944911 | 0.080565424 |

| | | | | | |
|---|---|---|---|---|---|
| POLR1B | darkgrey | -0.129433057 | 0.005088634 | -0.15773034 | 0.00062418 |
| POLR2B | darkgrey | 0.00942692 | 0.83899746 | -0.015048281 | 0.745678406 |
| POLR3B | darkgrey | -0.049692995 | 0.283873243 | -0.074860361 | 0.106163885 |
| PPP1R8 | darkgrey | 0.043589707 | 0.347266882 | 0.022278406 | 0.631078226 |
| PPP6C | darkgrey | 0.196519643 | 1.89E-05 | 0.182002532 | 7.63E-05 |
| PRKDC | darkgrey | -0.103141026 | 0.025822532 | -0.139414854 | 0.002532215 |
| PSMC1 | darkgrey | -0.068043297 | 0.142052783 | -0.079556349 | 0.08591658 |
| PSMD5 | darkgrey | 0.140969854 | 0.002262024 | 0.117972975 | 0.010726007 |
| RAB14 | darkgrey | 0.150284693 | 0.001124002 | 0.134091433 | 0.003694936 |
| RAD23B | darkgrey | 0.194153121 | 2.39E-05 | 0.181410137 | 8.06E-05 |
| RALBP1 | darkgrey | -0.070534688 | 0.12798935 | -0.096794005 | 0.036524256 |
| RBBP4 | darkgrey | 0.028948369 | 0.532605376 | -0.002142305 | 0.963173489 |
| RBM12 | darkgrey | -0.026359644 | 0.569893158 | -0.047127729 | 0.309498821 |
| RBM18 | darkgrey | 0.109758944 | 0.017657317 | 0.09485568 | 0.040464362 |
| RFC1 | darkgrey | 0.009350892 | 0.840278434 | -0.012850761 | 0.781799691 |
| RNF20 | darkgrey | 0.054380544 | 0.24083907 | 0.029210459 | 0.528900024 |
| RNF4 | darkgrey | 0.116952527 | 0.011429766 | 0.10570149 | 0.022341574 |
| RNF41 | darkgrey | -0.047724648 | 0.303404061 | -0.074662712 | 0.107092446 |
| RPA1 | darkgrey | 0.135478174 | 0.003352746 | 0.103468062 | 0.025353363 |
| SART3 | darkgrey | 0.000789199 | 0.986429419 | -0.030419379 | 0.511979591 |
| SDAD1 | darkgrey | -0.065228529 | 0.159331976 | -0.096077796 | 0.037940408 |
| SDR39U1 | darkgrey | -0.013890658 | 0.764642769 | -0.056593549 | 0.222200439 |
| SLC31A1 | darkgrey | 0.296964597 | 5.81E-11 | 0.293168342 | 1.04E-10 |
| SMC1A | darkgrey | 0.101161755 | 0.028824718 | 0.081124493 | 0.079893505 |
| SMC3 | darkgrey | -0.011158985 | 0.809935746 | -0.050693788 | 0.274275638 |
| SMG8 | darkgrey | -0.015221991 | 0.742845548 | -0.043601262 | 0.347138954 |
| SPTLC1 | darkgrey | -0.020342186 | 0.661048788 | -0.051387583 | 0.267753301 |
| STAMBP | darkgrey | 0.009672223 | 0.834867298 | 0.011979018 | 0.796264699 |
| STK24 | darkgrey | 0.006839639 | 0.882807253 | -0.013895972 | 0.764555387 |
| TFDP1 | darkgrey | 0.140925812 | 0.0022693 | 0.123619456 | 0.007483065 |
| TGS1 | darkgrey | -0.156104803 | 0.000711295 | -0.19631361 | 1.93E-05 |
| THRAP3 | darkgrey | 0.033199943 | 0.47415721 | 0.002726328 | 0.953144343 |
| TMEM16 | darkgrey | -0.004590502 | 0.921188882 | -0.02190369 | 0.636833398 |
| TMEM20 | darkgrey | 0.037734121 | 0.415905269 | 0.001730592 | 0.97024724 |
| TNPO3 | darkgrey | 0.051169793 | 0.269789188 | 0.035022149 | 0.45022594 |
| TOR1A | darkgrey | 0.144226138 | 0.001779546 | 0.133708234 | 0.003794919 |
| TOR1B | darkgrey | 0.129491405 | 0.005068585 | 0.120695099 | 0.009032973 |
| TRIM32 | darkgrey | 0.1138448 | 0.013831054 | 0.103634815 | 0.025116982 |
| TRIM37 | darkgrey | 0.090488546 | 0.05067235 | 0.054561722 | 0.239273098 |
| TRMT61I | darkgrey | -0.094245279 | 0.041777219 | -0.124113256 | 0.007246211 |
| TSR1 | darkgrey | 0.149778179 | 0.001168785 | 0.114282648 | 0.013467882 |
| UBE3C | darkgrey | 0.113259694 | 0.01432981 | 0.094303503 | 0.041650458 |
| UBFD1 | darkgrey | -0.010672349 | 0.818076324 | -0.009427302 | 0.838991015 |
| UBQLN1 | darkgrey | 0.123783925 | 0.007403419 | 0.110407938 | 0.016993761 |
| UBXN2B | darkgrey | -0.087158251 | 0.059830467 | -0.122718692 | 0.007933007 |

| | | | | | |
|---|---|---|---|---|---|
| USP32 | darkgrey | -0.158046573 | 0.000608427 | -0.184924653 | 5.81E-05 |
| USP42 | darkgrey | -0.07140088 | 0.123361086 | -0.063747438 | 0.169038141 |
| WBP11 | darkgrey | -0.144613792 | 0.001728876 | -0.154074352 | 0.000835915 |
| WIZ | darkgrey | -0.126274856 | 0.006286423 | -0.144452128 | 0.001749844 |
| XRN2 | darkgrey | -0.125854813 | 0.006463476 | -0.156491183 | 0.000689624 |
| YWHAG | darkgrey | 0.078906491 | 0.088517475 | 0.065789593 | 0.155766952 |
| YWHAQ | darkgrey | -0.027119031 | 0.558827366 | -0.047587224 | 0.304800114 |
| ZBTB5 | darkgrey | -0.008319707 | 0.857692983 | -0.024507261 | 0.597314435 |
| ZBTB6 | darkgrey | 0.02082489 | 0.653523785 | 0.005889558 | 0.898992299 |
| ZC3H18 | darkgrey | 0.162214088 | 0.000432495 | 0.15571671 | 0.000733696 |
| ZNF207 | darkgrey | 0.09711805 | 0.035898428 | 0.087935777 | 0.05757824 |
| A2ML1 | darkmagent | 0.159256924 | 0.000551479 | 0.1532148 | 0.000894517 |
| C15orf62 | darkmagent | 0.176248594 | 0.000128891 | 0.178316093 | 0.000106972 |
| C1orf204 | darkmagent | -0.02251776 | 0.627413652 | -0.03613965 | 0.43589372 |
| C1orf68 | darkmagent | 0.01837868 | 0.692004925 | 0.012418529 | 0.788962744 |
| C6orf15 | darkmagent | 0.181276241 | 8.16E-05 | 0.166327112 | 0.000306312 |
| CALML5 | darkmagent | 0.049097673 | 0.289688653 | 0.044519335 | 0.337070983 |
| CCL24 | darkmagent | 0.218936797 | 1.78E-06 | 0.217502943 | 2.09E-06 |
| CCR3 | darkmagent | 0.035438492 | 0.444855384 | -0.010659251 | 0.818295702 |
| CDSN | darkmagent | 0.081352025 | 0.079048703 | 0.111660833 | 0.01577422 |
| CRCT1 | darkmagent | 0.069170758 | 0.135548496 | 0.066340537 | 0.152325278 |
| CYP4F22 | darkmagent | 0.04194673 | 0.365759325 | 0.055653239 | 0.229990231 |
| DSG1 | darkmagent | 0.151946526 | 0.000987944 | 0.127785747 | 0.005684985 |
| DSG3 | darkmagent | 0.171564967 | 0.000195105 | 0.140587435 | 0.002325913 |
| DSG4 | darkmagent | 0.118084 | 0.010651803 | 0.101857841 | 0.027736395 |
| ENDOU | darkmagent | 0.022955259 | 0.620739111 | 0.010650278 | 0.818446001 |
| FABP4 | darkmagent | 0.146972142 | 0.001448066 | 0.113210972 | 0.014372046 |
| FABP9 | darkmagent | 0.095678767 | 0.038749404 | 0.099361886 | 0.031810083 |
| FAM25A | darkmagent | 0.199216303 | 1.44E-05 | 0.166883504 | 0.000292165 |
| FGFBP1 | darkmagent | 0.250983921 | 3.85E-08 | 0.2658173 | 5.40E-09 |
| GJB2 | darkmagent | 0.237781819 | 2.00E-07 | 0.251110599 | 3.79E-08 |
| GJB6 | darkmagent | 0.098277491 | 0.033733317 | 0.075590473 | 0.102788864 |
| GPR87 | darkmagent | 0.073604365 | 0.11217416 | 0.069072898 | 0.136103799 |
| GRHL3 | darkmagent | 0.058223508 | 0.209147049 | 0.050281115 | 0.278206061 |
| IL1RN | darkmagent | -0.013912176 | 0.764288941 | 0.01984945 | 0.668765559 |
| IL36RN | darkmagent | -0.00802519 | 0.862679858 | 0.000962349 | 0.983452416 |
| IVL | darkmagent | 0.185671778 | 5.42E-05 | 0.167816723 | 0.000269797 |
| KPRP | darkmagent | 0.075223174 | 0.104475954 | 0.070343482 | 0.129028911 |
| KRT1 | darkmagent | 0.079939141 | 0.084413589 | 0.071745815 | 0.121554694 |
| KRT13 | darkmagent | 0.071645804 | 0.12207631 | 0.082624515 | 0.074456695 |
| KRT14 | darkmagent | 0.058455701 | 0.207333563 | 0.052522006 | 0.257319122 |
| KRT16 | darkmagent | 0.231245044 | 4.36E-07 | 0.208581296 | 5.48E-06 |
| KRT17 | darkmagent | -0.006329974 | 0.891483998 | -0.003165507 | 0.945607296 |
| KRT25 | darkmagent | 0.052266334 | 0.259645821 | 0.042077555 | 0.364264768 |
| KRT27 | darkmagent | 0.098939323 | 0.032548079 | 0.109044062 | 0.018414335 |

| | | | | | |
|---|---|---|---|---|---|
| KRT28 | darkmagent | 0.034147105 | 0.461631945 | 0.035238705 | 0.44742795 |
| KRT5 | darkmagent | 0.061414308 | 0.18521477 | 0.05444016 | 0.240323003 |
| KRT6A | darkmagent | 0.192642635 | 2.77E-05 | 0.183067982 | 6.91E-05 |
| KRT6B | darkmagent | 0.13346266 | 0.003860276 | 0.121267274 | 0.008708957 |
| KRT6C | darkmagent | 0.122948328 | 0.007816064 | 0.094671515 | 0.040856742 |
| KRT71 | darkmagent | 0.03245615 | 0.484122174 | 0.089921394 | 0.052143688 |
| KRT77 | darkmagent | 0.040098805 | 0.387275965 | 0.070816883 | 0.126466961 |
| KRT78 | darkmagent | 0.127174455 | 0.005921796 | 0.133891433 | 0.003746818 |
| KRT9 | darkmagent | 0.163865121 | 0.000376933 | 0.125329428 | 0.006691218 |
| KRTAP3- | darkmagent | 0.089293092 | 0.053815075 | 0.101280986 | 0.028635741 |
| LCE1A | darkmagent | 0.090162235 | 0.051514589 | 0.092486126 | 0.045763415 |
| LCE2B | darkmagent | 0.027965703 | 0.546613643 | 0.043424472 | 0.349099398 |
| LCE3D | darkmagent | 0.077770897 | 0.093213941 | 0.07652224 | 0.098605862 |
| LCE3E | darkmagent | 0.102622568 | 0.026581652 | 0.092689033 | 0.045287919 |
| LIPK | darkmagent | 0.146047379 | 0.001552766 | 0.139956526 | 0.002434925 |
| LY6D | darkmagent | 0.032096366 | 0.488982687 | 0.052897921 | 0.253924412 |
| LYPD3 | darkmagent | 0.0682701 | 0.140725528 | 0.037979435 | 0.412878607 |
| MANSC4 | darkmagent | -0.020274409 | 0.662108146 | -0.026097222 | 0.573741242 |
| MT4 | darkmagent | 0.212652386 | 3.55E-06 | 0.177801468 | 0.000112074 |
| NCCRP1 | darkmagent | -0.098336951 | 0.03362535 | -0.072968513 | 0.115317224 |
| PGLYRP: | darkmagent | 0.112274968 | 0.015204969 | 0.107847731 | 0.019744602 |
| PGLYRP4 | darkmagent | 0.030420742 | 0.511960685 | 0.036242687 | 0.434585587 |
| PI3 | darkmagent | 0.076735464 | 0.097667941 | 0.080197539 | 0.083411057 |
| PKP1 | darkmagent | 0.085053072 | 0.066296239 | 0.071489162 | 0.122896783 |
| PLA2G4E | darkmagent | 0.066016454 | 0.154342728 | 0.068186072 | 0.141216147 |
| PRSS27 | darkmagent | 0.007718442 | 0.867879625 | 0.039314607 | 0.396634759 |
| PSORS1C | darkmagent | 0.108762419 | 0.018720265 | 0.093731433 | 0.042910096 |
| RAET1L | darkmagent | 0.23333111 | 3.41E-07 | 0.227990166 | 6.38E-07 |
| RHCG | darkmagent | 0.019344425 | 0.676711134 | 0.00923131 | 0.842294103 |
| RNASE7 | darkmagent | 0.170778353 | 0.000208956 | 0.183197782 | 6.83E-05 |
| RPTN | darkmagent | 0.183734644 | 6.50E-05 | 0.149269659 | 0.001215393 |
| S100A12 | darkmagent | 0.021140304 | 0.648625557 | 0.025652174 | 0.580295222 |
| S100A2 | darkmagent | 0.085546903 | 0.064730172 | 0.120149433 | 0.009351928 |
| S100A3 | darkmagent | 0.133882261 | 0.003749213 | 0.141067363 | 0.002245993 |
| S100A7 | darkmagent | 0.11033141 | 0.017070854 | 0.113707268 | 0.013946894 |
| S100A7A | darkmagent | 0.011523959 | 0.803843724 | 0.018645752 | 0.687762629 |
| S100A8 | darkmagent | 0.117229846 | 0.011234579 | 0.120733715 | 0.009010773 |
| S100A9 | darkmagent | 0.09251618 | 0.045692724 | 0.099749358 | 0.031145986 |
| S100P | darkmagent | 0.242016808 | 1.19E-07 | 0.276275598 | 1.26E-09 |
| SBSN | darkmagent | 0.096931346 | 0.036257886 | 0.093775927 | 0.042810987 |
| SDR9C7 | darkmagent | 0.05324408 | 0.250826011 | 0.006453617 | 0.88937782 |
| SERPINB | darkmagent | 0.144079003 | 0.001799132 | 0.111799996 | 0.015643616 |
| SERPINB | darkmagent | 0.09913355 | 0.03220707 | 0.055532443 | 0.231004796 |
| SERPINB | darkmagent | 0.089944902 | 0.052082003 | 0.074408118 | 0.108297972 |
| SERPINB | darkmagent | 0.115337839 | 0.012626961 | 0.104285381 | 0.02421286 |

| | | | | | |
|---|---|---|---|---|---|
| SLC6A14 | darkmagent | 0.294363233 | 8.67E-11 | 0.285875341 | 3.11E-10 |
| SLURP1 | darkmagent | 0.054057299 | 0.243650816 | 0.071902511 | 0.120740937 |
| SPINK13 | darkmagent | 0.188745546 | 4.05E-05 | 0.192290423 | 2.87E-05 |
| SPINK5 | darkmagent | 0.201144411 | 1.19E-05 | 0.183692344 | 6.52E-05 |
| SPINK6 | darkmagent | 0.18012136 | 9.07E-05 | 0.130682 | 0.004674726 |
| SPINK7 | darkmagent | 0.142123186 | 0.002078925 | 0.112308938 | 0.015174015 |
| SPINK9 | darkmagent | 0.123002794 | 0.007788554 | 0.092779922 | 0.045076278 |
| SPRR1A | darkmagent | 0.202046832 | 1.08E-05 | 0.180125945 | 9.07E-05 |
| SPRR1B | darkmagent | 0.213624496 | 3.19E-06 | 0.188435405 | 4.17E-05 |
| SPRR2A | darkmagent | 0.15745556 | 0.000638174 | 0.132639914 | 0.004086735 |
| SPRR2B | darkmagent | 0.119804585 | 0.009558628 | 0.069038879 | 0.13629725 |
| SPRR2D | darkmagent | 0.224591248 | 9.43E-07 | 0.195503855 | 2.09E-05 |
| SPRR2E | darkmagent | 0.157180198 | 0.000652489 | 0.125582949 | 0.006580439 |
| SPRR2F | darkmagent | 0.119026314 | 0.010040109 | 0.078194057 | 0.091441106 |
| SPRR2G | darkmagent | 0.084582827 | 0.067816299 | 0.072188218 | 0.119268136 |
| SPRR3 | darkmagent | 0.193957724 | 2.44E-05 | 0.179254094 | 9.82E-05 |
| SPTSSB | darkmagent | 0.224830777 | 9.17E-07 | 0.206837381 | 6.59E-06 |
| TCHH | darkmagent | 0.050198 | 0.279002281 | 0.060466865 | 0.192100003 |
| TCHHL1 | darkmagent | 0.099909894 | 0.030874336 | 0.067289881 | 0.146530648 |
| TGM6 | darkmagent | 0.08445357 | 0.068239088 | 0.079372332 | 0.086646739 |
| TMPRSS | darkmagent | 0.119875699 | 0.009515673 | 0.130733012 | 0.004658485 |
| TNS4 | darkmagent | 0.266167226 | 5.15E-09 | 0.273797418 | 1.78E-09 |
| TRIM29 | darkmagent | -0.077532494 | 0.094224779 | -0.083816899 | 0.070353233 |
| VSIG8 | darkmagent | 0.144307784 | 0.001768762 | 0.125593277 | 0.006575962 |
| XKRX | darkmagent | 0.201485066 | 1.15E-05 | 0.17962359 | 9.50E-05 |
| ZNF750 | darkmagent | 0.263933221 | 6.98E-09 | 0.236452975 | 2.35E-07 |
| ABCB10 | darkolivegr | 0.174351252 | 0.000152657 | 0.143553738 | 0.001870673 |
| ABL2 | darkolivegr | 0.06003605 | 0.195292125 | 0.032512355 | 0.483365235 |
| ACBD3 | darkolivegr | 0.097486845 | 0.035197285 | 0.070131786 | 0.130187492 |
| ADIPOR1 | darkolivegr | 0.287577504 | 2.42E-10 | 0.281919346 | 5.57E-10 |
| ALDH9A | darkolivegr | 0.346863916 | 1.20E-14 | 0.31396615 | 3.84E-12 |
| ANGEL2 | darkolivegr | 0.077167233 | 0.095790469 | 0.04746821 | 0.306012581 |
| ARHGEF | darkolivegr | 0.161305964 | 0.000466215 | 0.138290012 | 0.002745661 |
| ARHGEF | darkolivegr | -0.006104076 | 0.895333986 | -0.04562863 | 0.32515829 |
| ARID4B | darkolivegr | 0.063892249 | 0.168070076 | 0.042661518 | 0.357640008 |
| ATF6 | darkolivegr | 0.046891656 | 0.311931314 | 0.013654038 | 0.768536923 |
| ATP1B1 | darkolivegr | 0.05520603 | 0.233762198 | 0.036612573 | 0.429908246 |
| BLZF1 | darkolivegr | 0.020869953 | 0.652823062 | -0.001674982 | 0.971202867 |
| BROX | darkolivegr | 0.154526936 | 0.000806506 | 0.15108946 | 0.001056092 |
| C1orf198 | darkolivegr | 0.115485518 | 0.012513045 | 0.100980868 | 0.029113459 |
| C1orf27 | darkolivegr | 0.149355043 | 0.001207449 | 0.114439983 | 0.013339446 |
| CAMSAP | darkolivegr | -0.038893253 | 0.401718993 | -0.071292243 | 0.12393431 |
| CD46 | darkolivegr | 0.162605492 | 0.000418673 | 0.139180456 | 0.002575404 |
| CDC42BI | darkolivegr | 0.08742375 | 0.059053355 | 0.055374814 | 0.23233349 |
| CDC42SE | darkolivegr | 0.086423651 | 0.062024788 | 0.061490908 | 0.18466616 |

| | | | | | |
|---|---|---|---|---|---|
| CDC73 | darkolivegr | 0.142625044 | 0.002003568 | 0.117012736 | 0.011387136 |
| CEP170 | darkolivegr | 0.058421386 | 0.207600856 | 0.016710993 | 0.71870883 |
| CEP350 | darkolivegr | 0.085705521 | 0.064233659 | 0.056992763 | 0.218950838 |
| CNST | darkolivegr | 0.019759342 | 0.670180546 | -0.013669858 | 0.768276381 |
| COPA | darkolivegr | 0.160867129 | 0.000483371 | 0.137081452 | 0.002993144 |
| DCAF6 | darkolivegr | 0.117212903 | 0.011246419 | 0.083230647 | 0.072347036 |
| DCAF8 | darkolivegr | -0.052488265 | 0.25762534 | -0.083581337 | 0.071148896 |
| DDX59 | darkolivegr | 0.123132776 | 0.007723251 | 0.090554053 | 0.050504663 |
| DENND1 | darkolivegr | 0.251911637 | 3.42E-08 | 0.239682333 | 1.59E-07 |
| DESI2 | darkolivegr | 0.136383214 | 0.003145206 | 0.115864937 | 0.012224518 |
| DIEXF | darkolivegr | -0.031734143 | 0.493902493 | -0.061095215 | 0.187513056 |
| DISC1 | darkolivegr | 0.102971554 | 0.02606859 | 0.114771339 | 0.01307248 |
| DISP1 | darkolivegr | 0.128722317 | 0.005338653 | 0.102755367 | 0.026385401 |
| EDEM3 | darkolivegr | 0.137963917 | 0.002810533 | 0.109763927 | 0.017652137 |
| EGLN1 | darkolivegr | 0.084287897 | 0.068784151 | 0.072805233 | 0.116135364 |
| ENAH | darkolivegr | 0.161916543 | 0.000443285 | 0.126496666 | 0.006194695 |
| ESRRG | darkolivegr | 0.041144016 | 0.375012915 | 0.05123795 | 0.26915093 |
| ETV6 | darkolivegr | 0.142719277 | 0.0019897 | 0.151650034 | 0.001011046 |
| EXOC8 | darkolivegr | -0.004810808 | 0.917419791 | -0.013810414 | 0.765962712 |
| FAM20B | darkolivegr | -0.018986492 | 0.682364374 | -0.056118637 | 0.226110873 |
| FBXO28 | darkolivegr | 0.120085517 | 0.009389936 | 0.093133046 | 0.044261875 |
| FMO5 | darkolivegr | 0.317706284 | 2.06E-12 | 0.281506381 | 5.91E-10 |
| GLUL | darkolivegr | 0.14626388 | 0.00152765 | 0.13023612 | 0.004818871 |
| GOLPH3L | darkolivegr | 0.086140849 | 0.062887055 | 0.073789242 | 0.111273049 |
| GON4L | darkolivegr | -0.075023369 | 0.105402868 | -0.118198392 | 0.010575826 |
| GORAB | darkolivegr | 0.163110954 | 0.000401432 | 0.13302432 | 0.003979473 |
| GPATCH | darkolivegr | 0.123056929 | 0.007761297 | 0.1055044 | 0.022594265 |
| INTS3 | darkolivegr | -0.031461596 | 0.49762164 | -0.08084088 | 0.080956749 |
| IRF2BP2 | darkolivegr | 0.065636644 | 0.15673276 | 0.058294824 | 0.208588836 |
| IVNS1AB | darkolivegr | 0.209942353 | 4.74E-06 | 0.172448736 | 0.000180572 |
| KCTD3 | darkolivegr | 0.102014052 | 0.027497073 | 0.07681792 | 0.097307148 |
| KDM5B | darkolivegr | 0.16448501 | 0.000357847 | 0.145742446 | 0.001588785 |
| KIFAP3 | darkolivegr | 0.172397789 | 0.000181381 | 0.139545625 | 0.002508408 |
| KLHL12 | darkolivegr | 0.265230192 | 5.85E-09 | 0.241043693 | 1.34E-07 |
| LGALS8 | darkolivegr | 0.170176234 | 0.000220175 | 0.178256103 | 0.000107555 |
| LPGAT1 | darkolivegr | 0.195463673 | 2.10E-05 | 0.194130984 | 2.40E-05 |
| MARK1 | darkolivegr | 0.115787305 | 0.012283069 | 0.092345561 | 0.04609527 |
| MDM4 | darkolivegr | 0.047205334 | 0.308701914 | 0.03171755 | 0.494128496 |
| MIA3 | darkolivegr | 0.169738275 | 0.000228686 | 0.159406143 | 0.000544811 |
| MPZL1 | darkolivegr | 0.300271162 | 3.47E-11 | 0.273546345 | 1.85E-09 |
| MR1 | darkolivegr | 0.086844483 | 0.060759743 | 0.090061168 | 0.051777811 |
| NAV1 | darkolivegr | 0.087989735 | 0.057424585 | 0.088206486 | 0.056810766 |
| NBPF9 | darkolivegr | 0.154814381 | 0.000788327 | 0.110814174 | 0.0165896 |
| NCSTN | darkolivegr | 0.239682798 | 1.59E-07 | 0.211155341 | 4.17E-06 |
| NUCKS1 | darkolivegr | -0.002595592 | 0.955388817 | -0.029929174 | 0.518806567 |

| | | | | | |
|---|---|---|---|---|---|
| OTUD7B | darkolivegr | 0.114869541 | 0.012994271 | 0.09065438 | 0.050248741 |
| PCNXL2 | darkolivegr | 0.028094394 | 0.544768806 | 0.002537143 | 0.956392396 |
| PDE4DIP | darkolivegr | 0.091915784 | 0.047122473 | 0.0751173 | 0.104966307 |
| PFKFB2 | darkolivegr | 0.291678282 | 1.30E-10 | 0.291401885 | 1.36E-10 |
| PGBD2 | darkolivegr | 0.156854861 | 0.000669786 | 0.12578483 | 0.006493405 |
| PIP5K1A | darkolivegr | 0.028992202 | 0.531984774 | -0.013372388 | 0.773179735 |
| POGK | darkolivegr | 0.041535473 | 0.370482367 | 0.003840429 | 0.934034219 |
| POGZ | darkolivegr | -0.054421842 | 0.240481488 | -0.095302428 | 0.039525739 |
| PPP1R15l | darkolivegr | 0.012938598 | 0.780346271 | -0.014401898 | 0.756249441 |
| PPP2R5A | darkolivegr | 0.13531948 | 0.003390392 | 0.123444466 | 0.007568646 |
| RAB29 | darkolivegr | -0.106805943 | 0.020970389 | -0.128229531 | 0.005518438 |
| RAB3GA | darkolivegr | 0.099924288 | 0.030850078 | 0.069955812 | 0.131156687 |
| RABGAP | darkolivegr | 0.232071363 | 3.96E-07 | 0.211234015 | 4.13E-06 |
| RASAL2 | darkolivegr | 0.115022096 | 0.012873593 | 0.078820249 | 0.088867342 |
| RBBP5 | darkolivegr | 0.063797679 | 0.168701805 | 0.038366852 | 0.40812525 |
| RC3H1 | darkolivegr | 0.000377861 | 0.993502296 | -0.002061528 | 0.964561123 |
| RCOR3 | darkolivegr | 0.030317127 | 0.513399765 | -0.002535983 | 0.956412297 |
| RIT1 | darkolivegr | 0.080726426 | 0.081389058 | 0.070563116 | 0.127835344 |
| RNF115 | darkolivegr | -0.026419952 | 0.569010556 | -0.058975922 | 0.203311861 |
| RNF2 | darkolivegr | 0.013549389 | 0.770261035 | -0.02165607 | 0.640648569 |
| RPS6KC1 | darkolivegr | -0.000461275 | 0.992067949 | -0.029582247 | 0.523666315 |
| SCYL3 | darkolivegr | 0.194581576 | 2.29E-05 | 0.147077754 | 0.001436531 |
| SDCCAG | darkolivegr | 0.126733976 | 0.006097888 | 0.09280997 | 0.045006491 |
| SDE2 | darkolivegr | 0.072139684 | 0.119517331 | 0.04665339 | 0.314399098 |
| SHC1 | darkolivegr | -0.010872925 | 0.814718601 | -0.032401141 | 0.484863622 |
| SIPA1L2 | darkolivegr | 0.34654115 | 1.27E-14 | 0.314268052 | 3.65E-12 |
| SLC30A1 | darkolivegr | 0.242820174 | 1.08E-07 | 0.211059639 | 4.21E-06 |
| SMG7 | darkolivegr | 0.097976411 | 0.034284562 | 0.07420182 | 0.109282639 |
| SPRTN | darkolivegr | 0.152280083 | 0.000962537 | 0.143152914 | 0.001927008 |
| SRGAP2 | darkolivegr | 0.014953399 | 0.747227183 | -0.02500844 | 0.58983654 |
| SUCO | darkolivegr | 0.100503194 | 0.029887905 | 0.058551421 | 0.206589291 |
| SWT1 | darkolivegr | 0.050527911 | 0.27585093 | 0.016172541 | 0.727406334 |
| TADA1 | darkolivegr | 0.073830583 | 0.111072332 | 0.029187427 | 0.529225118 |
| TAF5L | darkolivegr | 0.161926742 | 0.000442911 | 0.145072871 | 0.001670582 |
| TOR1AIP | darkolivegr | 0.107327726 | 0.020348425 | 0.070803056 | 0.126541228 |
| TOR1AIP | darkolivegr | 0.090178169 | 0.051473193 | 0.088807779 | 0.055136398 |
| TP53BP2 | darkolivegr | -0.042851104 | 0.355505609 | -0.073333067 | 0.113506868 |
| TRAF5 | darkolivegr | 0.159760207 | 0.000529289 | 0.123017609 | 0.007781086 |
| TROVE2 | darkolivegr | 0.079948706 | 0.084376305 | 0.058809467 | 0.204592481 |
| TUFT1 | darkolivegr | 0.116631613 | 0.011659378 | 0.095291204 | 0.039549093 |
| UAP1 | darkolivegr | 0.280717914 | 6.63E-10 | 0.24267377 | 1.10E-07 |
| UBAP2L | darkolivegr | 0.073365596 | 0.113346422 | 0.057029419 | 0.218654173 |
| UHMK1 | darkolivegr | 0.164598271 | 0.000354459 | 0.145330823 | 0.001638623 |
| VAMP4 | darkolivegr | 0.068707634 | 0.138191938 | 0.040692838 | 0.380276781 |
| WDR26 | darkolivegr | 0.062487693 | 0.177636124 | 0.05355682 | 0.248049441 |

| | | | | | |
|---|---|---|---|---|---|
| XPR1 | darkolivegr | 0.105959793 | 0.022014112 | 0.079369986 | 0.086656078 |
| YOD1 | darkolivegr | 0.140305539 | 0.002374058 | 0.122036975 | 0.008289428 |
| YY1AP1 | darkolivegr | 0.120709895 | 0.009024461 | 0.094463931 | 0.041302866 |
| ZBTB37 | darkolivegr | 0.033714934 | 0.467323995 | 0.014237767 | 0.758941006 |
| ZBTB41 | darkolivegr | 0.150085341 | 0.001141435 | 0.120623557 | 0.009074232 |
| ZC3H11A | darkolivegr | 0.116428625 | 0.011806712 | 0.072957365 | 0.115372938 |
| ZNF124 | darkolivegr | 0.082176043 | 0.076049648 | 0.068430651 | 0.139791735 |
| ZNF281 | darkolivegr | 0.009551889 | 0.836892796 | -0.018499132 | 0.690090413 |
| ZNF669 | darkolivegr | 0.094347466 | 0.041554961 | 0.091305592 | 0.048613793 |
| ZNF678 | darkolivegr | 0.041657866 | 0.369072829 | 0.013912532 | 0.764283077 |
| ALDH3B1 | darkorange | 0.20208551 | 1.08E-05 | 0.200097032 | 1.32E-05 |
| AP1M2 | darkorange | 0.190547607 | 3.40E-05 | 0.212471378 | 3.62E-06 |
| ARHGAP | darkorange | 0.288846581 | 2.00E-10 | 0.283153781 | 4.65E-10 |
| ARHGEF | darkorange | 0.269108459 | 3.44E-09 | 0.265613807 | 5.56E-09 |
| BCR | darkorange | 0.123849224 | 0.007372007 | 0.14738991 | 0.001402928 |
| BSPRY | darkorange | 0.398917228 | 2.89E-19 | 0.41232134 | 1.36E-20 |
| C1orf106 | darkorange | -0.105652789 | 0.022403784 | -0.094239551 | 0.041789708 |
| C1orf210 | darkorange | 0.12621488 | 0.006311435 | 0.131127781 | 0.004534513 |
| C6orf132 | darkorange | -0.117792776 | 0.010847418 | -0.095891836 | 0.038315625 |
| CAMSAP | darkorange | 0.256529262 | 1.87E-08 | 0.264892777 | 6.13E-09 |
| CAPN1 | darkorange | 0.116779528 | 0.011553044 | 0.140262126 | 0.002381553 |
| CBLC | darkorange | 0.066546486 | 0.151053662 | 0.089596642 | 0.053002097 |
| CCDC64B | darkorange | 0.229754295 | 5.19E-07 | 0.241415446 | 1.28E-07 |
| CD24 | darkorange | 0.150428567 | 0.001111573 | 0.163680892 | 0.000382786 |
| CD82 | darkorange | 0.093390712 | 0.043675468 | 0.125676224 | 0.006540098 |
| CD9 | darkorange | 0.010784699 | 0.816195132 | -0.00116281 | 0.980006154 |
| CDC42BP | darkorange | 0.188412304 | 4.18E-05 | 0.206931074 | 6.52E-06 |
| CDCP1 | darkorange | 0.25638894 | 1.91E-08 | 0.265190436 | 5.89E-09 |
| CDH1 | darkorange | 0.241900931 | 1.21E-07 | 0.267313498 | 4.40E-09 |
| CELSR1 | darkorange | 0.098206379 | 0.03386283 | 0.101108273 | 0.028909831 |
| CELSR2 | darkorange | 0.005373889 | 0.907794934 | -0.004610613 | 0.920844751 |
| CGN | darkorange | -0.045953051 | 0.321726586 | -0.058360713 | 0.208074062 |
| CHMP4C | darkorange | 0.101966774 | 0.027569317 | 0.079589358 | 0.085786134 |
| CLDN3 | darkorange | 0.037761715 | 0.415564175 | 0.049942518 | 0.281459375 |
| CLDN4 | darkorange | 0.080950951 | 0.080542749 | 0.096914314 | 0.03629083 |
| CLDN7 | darkorange | 0.167481298 | 0.000277646 | 0.183953126 | 6.37E-05 |
| CNDP2 | darkorange | 0.193255816 | 2.61E-05 | 0.215278602 | 2.67E-06 |
| CNKSR1 | darkorange | 0.101865163 | 0.027725137 | 0.111833412 | 0.015612396 |
| CORO2A | darkorange | 0.182265409 | 7.45E-05 | 0.205381621 | 7.67E-06 |
| CRB3 | darkorange | 0.259508867 | 1.26E-08 | 0.285903781 | 3.10E-10 |
| DDR1 | darkorange | 0.016672582 | 0.719328085 | 0.019377263 | 0.676193386 |
| EFNA1 | darkorange | 0.043895322 | 0.343893611 | 0.025332809 | 0.585019793 |
| ELF3 | darkorange | 0.103427582 | 0.025411035 | 0.111983344 | 0.015472992 |
| ELMO3 | darkorange | 0.087211978 | 0.059672532 | 0.11931687 | 0.009857893 |
| EPCAM | darkorange | 0.134497158 | 0.003591682 | 0.145733262 | 0.001589882 |

| | | | | | |
|---|---|---|---|---|---|
| EPHA1 | darkorange | 0.148619951 | 0.00127743 | 0.155913722 | 0.000722244 |
| EPN3 | darkorange | 0.265208099 | 5.87E-09 | 0.271478775 | 2.47E-09 |
| EPS8L1 | darkorange | -0.044162562 | 0.340961065 | -0.014783908 | 0.749996365 |
| EPS8L2 | darkorange | 0.095586495 | 0.038938544 | 0.101669522 | 0.028027287 |
| ERBB3 | darkorange | 0.065226987 | 0.159341857 | 0.062450829 | 0.177892526 |
| ESRP1 | darkorange | 0.044541323 | 0.336832166 | 0.052981987 | 0.253169518 |
| ESRP2 | darkorange | 0.182562769 | 7.25E-05 | 0.215647773 | 2.56E-06 |
| EVPL | darkorange | 0.144481065 | 0.001746074 | 0.163887012 | 0.000376243 |
| F11R | darkorange | 0.222217302 | 1.23E-06 | 0.229218148 | 5.53E-07 |
| F2RL1 | darkorange | 0.168700366 | 0.000250101 | 0.168917263 | 0.000245477 |
| FAM129E | darkorange | 0.250448859 | 4.13E-08 | 0.274743047 | 1.56E-09 |
| FAM83E | darkorange | 0.093042347 | 0.044469863 | 0.125918607 | 0.006436301 |
| FAM83H | darkorange | 0.14183256 | 0.002123741 | 0.159478201 | 0.000541618 |
| FUT2 | darkorange | 0.26301699 | 7.90E-09 | 0.281858998 | 5.62E-10 |
| FXYD3 | darkorange | 0.192394823 | 2.84E-05 | 0.218918075 | 1.79E-06 |
| GGT6 | darkorange | 0.063988982 | 0.167425725 | 0.075846818 | 0.101624262 |
| GPR39 | darkorange | 0.104950305 | 0.023317982 | 0.122912237 | 0.007834341 |
| GPR56 | darkorange | 0.075489464 | 0.103250654 | 0.09395938 | 0.042404386 |
| GRHL2 | darkorange | 0.262915712 | 8.01E-09 | 0.257970857 | 1.55E-08 |
| GYLTL1I | darkorange | 0.277778619 | 1.01E-09 | 0.238948077 | 1.73E-07 |
| ICA1 | darkorange | 0.218678327 | 1.83E-06 | 0.206549344 | 6.79E-06 |
| ILDR1 | darkorange | 0.184430886 | 6.09E-05 | 0.208388895 | 5.59E-06 |
| IRF6 | darkorange | 0.2218743 | 1.28E-06 | 0.209212619 | 5.13E-06 |
| ITGA3 | darkorange | -0.152274709 | 0.000962941 | -0.137323566 | 0.002941997 |
| JUP | darkorange | 0.045111664 | 0.330675593 | 0.06560583 | 0.156927883 |
| KDF1 | darkorange | 0.170429089 | 0.000215397 | 0.190708198 | 3.35E-05 |
| KIAA152 | darkorange | 0.039172606 | 0.398343855 | 0.06234766 | 0.178611561 |
| KLF5 | darkorange | 0.13392671 | 0.003737619 | 0.14312817 | 0.001930536 |
| KRT18 | darkorange | 0.198841505 | 1.50E-05 | 0.232758446 | 3.65E-07 |
| KRT19 | darkorange | 0.03915488 | 0.398557519 | 0.060802931 | 0.189636601 |
| KRT8 | darkorange | 0.162194545 | 0.000433196 | 0.196092265 | 1.97E-05 |
| KRT80 | darkorange | 0.013383555 | 0.772995488 | 0.015817827 | 0.733155235 |
| KRTCAP | darkorange | 0.085653365 | 0.064396573 | 0.106209546 | 0.021701455 |
| LAD1 | darkorange | 0.282370244 | 5.21E-10 | 0.296397951 | 6.34E-11 |
| LAMB3 | darkorange | -0.012482319 | 0.787904465 | 0.006554954 | 0.887652186 |
| LAMC2 | darkorange | 0.205536809 | 7.55E-06 | 0.208339274 | 5.62E-06 |
| LCN2 | darkorange | 0.0947999 | 0.040582868 | 0.123053077 | 0.007763233 |
| LLGL2 | darkorange | 0.195395359 | 2.12E-05 | 0.210028491 | 4.70E-06 |
| LRG1 | darkorange | 0.130951896 | 0.004589375 | 0.152627394 | 0.000936724 |
| LSR | darkorange | 0.057058326 | 0.21842042 | 0.086585992 | 0.061534232 |
| LZTS3 | darkorange | -0.046868977 | 0.31216565 | -0.057899039 | 0.211700369 |
| MAL2 | darkorange | -0.087292653 | 0.059436025 | -0.095808015 | 0.038485781 |
| MAP7 | darkorange | 0.091901574 | 0.047156762 | 0.098843656 | 0.032717175 |
| MAPK13 | darkorange | 0.199310208 | 1.43E-05 | 0.210277698 | 4.58E-06 |
| MARVEL | darkorange | 0.293399478 | 1.00E-10 | 0.314589103 | 3.46E-12 |

| | | | | | |
|---|---|---|---|---|---|
| MARVEL | darkorange | 0.261550056 | 9.63E-09 | 0.284107381 | 4.04E-10 |
| MISP | darkorange | 0.034422234 | 0.458028464 | 0.063976892 | 0.167506159 |
| MPZL3 | darkorange | 0.165878374 | 0.000318186 | 0.170380061 | 0.000216315 |
| MUC1 | darkorange | 0.039379415 | 0.395856201 | 0.05384245 | 0.245532353 |
| MUC20 | darkorange | -0.14830241 | 0.001308799 | -0.125559991 | 0.006590403 |
| MYH14 | darkorange | -0.000343094 | 0.994100127 | 0.013368839 | 0.773238293 |
| MYO5B | darkorange | 0.1327417 | 0.004058082 | 0.129829394 | 0.004953842 |
| OVOL2 | darkorange | 0.195392973 | 2.12E-05 | 0.210927392 | 4.27E-06 |
| PDZK1IP | darkorange | 0.074387027 | 0.108398316 | 0.091955438 | 0.047026901 |
| PERP | darkorange | 0.274365661 | 1.65E-09 | 0.299239828 | 4.08E-11 |
| PKP3 | darkorange | 0.196041235 | 1.98E-05 | 0.213412732 | 3.27E-06 |
| PPL | darkorange | -0.007619463 | 0.869558672 | 0.004156009 | 0.928627487 |
| PPP1R13l | darkorange | 0.262079063 | 8.97E-09 | 0.27427206 | 1.67E-09 |
| PROM2 | darkorange | 0.155613362 | 0.000739771 | 0.151898301 | 0.000991668 |
| PRR15 | darkorange | 0.2583464 | 1.48E-08 | 0.247004069 | 6.39E-08 |
| PRR15L | darkorange | 0.202119274 | 1.07E-05 | 0.219186568 | 1.73E-06 |
| PRRG2 | darkorange | 0.247174156 | 6.26E-08 | 0.255820115 | 2.06E-08 |
| PRSS22 | darkorange | 0.016767827 | 0.71779289 | 0.017746468 | 0.70208551 |
| PRSS8 | darkorange | 0.170054514 | 0.00022251 | 0.185032362 | 5.75E-05 |
| PSD4 | darkorange | 0.09563701 | 0.038834902 | 0.109302979 | 0.018136948 |
| PTPN3 | darkorange | 0.346980335 | 1.17E-14 | 0.331120375 | 2.06E-13 |
| PTPRF | darkorange | 0.115103539 | 0.012809573 | 0.101268866 | 0.0286549 |
| PVRL4 | darkorange | 0.222479615 | 1.20E-06 | 0.220054474 | 1.57E-06 |
| RAB25 | darkorange | 0.213956155 | 3.08E-06 | 0.227240286 | 6.96E-07 |
| RAB3D | darkorange | 0.184722137 | 5.92E-05 | 0.188217545 | 4.26E-05 |
| RAP1GAl | darkorange | 0.255126186 | 2.25E-08 | 0.258084688 | 1.53E-08 |
| RBBP8NI | darkorange | 0.152579868 | 0.000940218 | 0.157514628 | 0.000635142 |
| RIPK4 | darkorange | 0.076038161 | 0.100761816 | 0.079916458 | 0.084502055 |
| SCNN1A | darkorange | 0.089784646 | 0.052503728 | 0.119275989 | 0.009883351 |
| SEL1L3 | darkorange | 0.314952319 | 3.26E-12 | 0.327698582 | 3.75E-13 |
| SEMA4B | darkorange | 0.242913866 | 1.07E-07 | 0.243249278 | 1.02E-07 |
| SGPP2 | darkorange | 0.107587427 | 0.020044886 | 0.107099882 | 0.020618014 |
| SH2D3A | darkorange | 0.138158405 | 0.002771676 | 0.144390326 | 0.001757921 |
| SLC34A2 | darkorange | -0.009200275 | 0.842817404 | -0.003016047 | 0.948171766 |
| SLC37A1 | darkorange | 0.32550264 | 5.49E-13 | 0.338119034 | 5.93E-14 |
| SLC52A3 | darkorange | 0.085815165 | 0.063892279 | 0.095552326 | 0.039008781 |
| SLPI | darkorange | 0.093063532 | 0.044421208 | 0.128047419 | 0.005586244 |
| SMPDL3l | darkorange | 0.142350459 | 0.002044483 | 0.152112257 | 0.000975244 |
| SPACA4 | darkorange | 0.128756479 | 0.005326388 | 0.161667611 | 0.000452504 |
| SPINT1 | darkorange | 0.237320144 | 2.11E-07 | 0.256994111 | 1.76E-08 |
| SPINT2 | darkorange | 0.065799865 | 0.155702254 | 0.077905774 | 0.092645912 |
| ST14 | darkorange | 0.176221002 | 0.00012921 | 0.194703483 | 2.27E-05 |
| STXBP2 | darkorange | 0.294194305 | 8.90E-11 | 0.328032598 | 3.54E-13 |
| SYNE4 | darkorange | 0.159520247 | 0.000539763 | 0.15130224 | 0.00103878 |
| SYNGR2 | darkorange | 0.128295313 | 0.005494127 | 0.158456317 | 0.000588564 |

| | | | | | |
|---|---|---|---|---|---|
| SYTL1 | darkorange | 0.400566945 | 2.00E-19 | 0.423723411 | 9.01E-22 |
| TACSTD2 | darkorange | 0.123512632 | 0.007535205 | 0.137955667 | 0.002812192 |
| TAGLN2 | darkorange | 0.223691906 | 1.04E-06 | 0.24735351 | 6.11E-08 |
| TJP3 | darkorange | 0.393918791 | 8.74E-19 | 0.408871097 | 3.02E-20 |
| TMC4 | darkorange | 0.164921443 | 0.000344954 | 0.173750263 | 0.000161004 |
| TMEM10 | darkorange | 0.222958667 | 1.14E-06 | 0.226397403 | 7.66E-07 |
| TMEM12 | darkorange | 0.069828993 | 0.131858604 | 0.095592284 | 0.038926655 |
| TMEM13 | darkorange | 0.063529333 | 0.170504044 | 0.06999869 | 0.130920018 |
| TMEM18 | darkorange | 0.234376511 | 3.01E-07 | 0.233929808 | 3.17E-07 |
| TMEM30 | darkorange | -0.017038769 | 0.71343196 | 0.002428764 | 0.958253435 |
| TNK1 | darkorange | 0.314833419 | 3.33E-12 | 0.317336698 | 2.19E-12 |
| TSPAN15 | darkorange | 0.125335829 | 0.0066884 | 0.139453839 | 0.002525097 |
| TSTD1 | darkorange | 0.216197138 | 2.41E-06 | 0.235299155 | 2.69E-07 |
| USP43 | darkorange | 0.290186149 | 1.63E-10 | 0.26003285 | 1.18E-08 |
| VAMP8 | darkorange | 0.075087987 | 0.105102391 | 0.122481674 | 0.008055344 |
| VTCN1 | darkorange | -0.026909165 | 0.56187511 | -0.01868947 | 0.687069131 |
| WFDC2 | darkorange | 0.03026031 | 0.514189777 | 0.067412044 | 0.145797361 |
| WWC1 | darkorange | 0.093738814 | 0.042893642 | 0.103786404 | 0.024903747 |
| YIPF2 | darkorange | -0.002537244 | 0.956390645 | 0.002884902 | 0.950422434 |
| ZBTB42 | darkorange | 0.044009099 | 0.342643125 | 0.038604397 | 0.405226874 |
| ZBTB7B | darkorange | 0.264543325 | 6.43E-09 | 0.256985831 | 1.77E-08 |
| ZFP28 | darkorange2 | -0.176099404 | 0.000130626 | -0.204891777 | 8.07E-06 |
| ZNF112 | darkorange2 | -0.070872533 | 0.126168408 | -0.088816333 | 0.055112877 |
| ZNF134 | darkorange2 | -0.09879524 | 0.032803037 | -0.122566894 | 0.008011165 |
| ZNF155 | darkorange2 | 0.040430497 | 0.383358203 | 0.00214089 | 0.963197791 |
| ZNF17 | darkorange2 | -0.223168469 | 1.11E-06 | -0.235573852 | 2.61E-07 |
| ZNF180 | darkorange2 | -0.090017308 | 0.051892389 | -0.120678658 | 0.00904244 |
| ZNF211 | darkorange2 | -0.035027367 | 0.450158406 | -0.044449056 | 0.337835016 |
| ZNF222 | darkorange2 | -0.045262354 | 0.32906116 | -0.065340692 | 0.158614411 |
| ZNF223 | darkorange2 | -0.131717292 | 0.004354927 | -0.153204214 | 0.000895262 |
| ZNF225 | darkorange2 | 0.007314287 | 0.874739226 | -0.015578129 | 0.737048544 |
| ZNF226 | darkorange2 | 0.032512462 | 0.483363787 | 0.025432558 | 0.583542223 |
| ZNF227 | darkorange2 | -0.041332498 | 0.372827266 | -0.063876027 | 0.168178313 |
| ZNF230 | darkorange2 | -0.011729756 | 0.800413849 | -0.032091675 | 0.489046233 |
| ZNF234 | darkorange2 | -0.027424244 | 0.554409294 | -0.041859257 | 0.366760756 |
| ZNF235 | darkorange2 | -0.103677482 | 0.025056804 | -0.121528018 | 0.008564766 |
| ZNF256 | darkorange2 | -0.181363759 | 8.10E-05 | -0.16981049 | 0.000227262 |
| ZNF283 | darkorange2 | 0.002049876 | 0.964761309 | -0.01233213 | 0.790396731 |
| ZNF284 | darkorange2 | 0.020174944 | 0.663663993 | -0.001398923 | 0.975947444 |
| ZNF304 | darkorange2 | -0.143559222 | 0.001869913 | -0.154155903 | 0.000830544 |
| ZNF329 | darkorange2 | 0.043149617 | 0.35216117 | 0.025750638 | 0.57884219 |
| ZNF347 | darkorange2 | 0.039481194 | 0.394635376 | 0.012646692 | 0.785179315 |
| ZNF350 | darkorange2 | -0.066642542 | 0.150463333 | -0.07027318 | 0.129412775 |
| ZNF415 | darkorange2 | 0.155192876 | 0.000764969 | 0.133320278 | 0.003898635 |
| ZNF416 | darkorange2 | -0.065883597 | 0.155175595 | -0.08146266 | 0.078640554 |

| | | | | | |
|---|---|---|---|---|---|
| ZNF419 | darkorange2 | -0.049364969 | 0.287067753 | -0.063346494 | 0.171740213 |
| ZNF45 | darkorange2 | -0.022628738 | 0.625717657 | -0.060035542 | 0.19529591 |
| ZNF470 | darkorange2 | 0.084021264 | 0.069668851 | 0.053965242 | 0.244455764 |
| ZNF471 | darkorange2 | -0.191611658 | 3.07E-05 | -0.221398279 | 1.35E-06 |
| ZNF528 | darkorange2 | 0.094357333 | 0.041533553 | 0.06081649 | 0.189537696 |
| ZNF547 | darkorange2 | 0.120190471 | 0.009327597 | 0.108604331 | 0.018893919 |
| ZNF548 | darkorange2 | -0.104526442 | 0.02388506 | -0.124659152 | 0.006992192 |
| ZNF549 | darkorange2 | -0.003670141 | 0.936952983 | -0.012023208 | 0.79552971 |
| ZNF550 | darkorange2 | -0.119541906 | 0.00971879 | -0.154811389 | 0.000788514 |
| ZNF583 | darkorange2 | -0.020284824 | 0.661945304 | -0.042563888 | 0.35874228 |
| ZNF606 | darkorange2 | -0.140676683 | 0.002310857 | -0.150087134 | 0.001141277 |
| ZNF610 | darkorange2 | 0.005161359 | 0.911426246 | -0.008457989 | 0.85535349 |
| ZNF613 | darkorange2 | 0.040168239 | 0.386453834 | 0.026165059 | 0.572745327 |
| ZNF614 | darkorange2 | -0.053180604 | 0.251392191 | -0.09623424 | 0.037627162 |
| ZNF615 | darkorange2 | 0.00175968 | 0.969747381 | -0.029126086 | 0.530091443 |
| ZNF616 | darkorange2 | 0.040476056 | 0.382821987 | 0.030440718 | 0.511683476 |
| ZNF649 | darkorange2 | -0.04550182 | 0.326506113 | -0.054663717 | 0.238394683 |
| ZNF665 | darkorange2 | -0.009139385 | 0.84384429 | -0.036277238 | 0.43414744 |
| ZNF766 | darkorange2 | 0.058353688 | 0.208128905 | 0.057977588 | 0.211080198 |
| ZNF773 | darkorange2 | -0.100767079 | 0.029457918 | -0.091114433 | 0.049089039 |
| ZNF776 | darkorange2 | 0.02462565 | 0.595544123 | 0.00676951 | 0.884000362 |
| ZNF814 | darkorange2 | 0.040576874 | 0.381637002 | 0.037270187 | 0.42166484 |
| ZNF836 | darkorange2 | 0.068702356 | 0.138222296 | 0.056397308 | 0.223810401 |
| ZNF880 | darkorange2 | 0.10022021 | 0.030354979 | 0.10498392 | 0.023273511 |
| ZSCAN18 | darkorange2 | -0.18657813 | 4.97E-05 | -0.199252387 | 1.44E-05 |
| AKAP7 | darkred | 0.138413482 | 0.002721456 | 0.129279178 | 0.005141852 |
| ALDH3A | darkred | 0.328560111 | 3.23E-13 | 0.315938151 | 2.77E-12 |
| ALDH6A | darkred | 0.184596144 | 6.00E-05 | 0.183957382 | 6.36E-05 |
| ALG2 | darkred | 0.215344177 | 2.65E-06 | 0.21528353 | 2.67E-06 |
| ANKRA2 | darkred | 0.093268978 | 0.043951696 | 0.080393826 | 0.082655948 |
| ANXA5 | darkred | 0.156938184 | 0.000665316 | 0.140664619 | 0.002312887 |
| AP1G1 | darkred | 0.268934615 | 3.52E-09 | 0.277707168 | 1.02E-09 |
| ARFGAP | darkred | 0.374041105 | 5.91E-17 | 0.348046076 | 9.59E-15 |
| ARFIP1 | darkred | 0.213719955 | 3.16E-06 | 0.197988382 | 1.63E-05 |
| ARHGEF | darkred | 0.127553388 | 0.005773978 | 0.115574655 | 0.012444726 |
| ARL1 | darkred | 0.193165446 | 2.64E-05 | 0.184942638 | 5.80E-05 |
| ARL6IP5 | darkred | 0.341529506 | 3.20E-14 | 0.338067676 | 5.99E-14 |
| ARRDC4 | darkred | 0.187900701 | 4.39E-05 | 0.182082842 | 7.58E-05 |
| ATG4A | darkred | 0.178868559 | 0.000101738 | 0.214532835 | 2.89E-06 |
| ATP8B1 | darkred | 0.215145573 | 2.71E-06 | 0.226746807 | 7.36E-07 |
| BANK1 | darkred | 0.024006285 | 0.604831958 | 0.033182767 | 0.474386059 |
| BHLHE40 | darkred | 0.154172021 | 0.000829486 | 0.134033625 | 0.003709865 |
| C10orf32 | darkred | 0.193842658 | 2.47E-05 | 0.15581816 | 0.000727778 |
| C15orf57 | darkred | 0.181028587 | 8.35E-05 | 0.167316834 | 0.000281571 |
| C20orf27 | darkred | -0.241255063 | 1.31E-07 | -0.248001642 | 5.63E-08 |

| | | | | | |
|---|---|---|---|---|---|
| C9orf156 | darkred | 0.20708448 | 6.42E-06 | 0.21201011 | 3.80E-06 |
| CADPS2 | darkred | 0.018957882 | 0.682817017 | 0.030624925 | 0.509130974 |
| CASC4 | darkred | 0.07526594 | 0.104278402 | 0.057322084 | 0.216295881 |
| CASP7 | darkred | 0.155534237 | 0.000744453 | 0.173864702 | 0.000159383 |
| CAT | darkred | 0.23232981 | 3.84E-07 | 0.227213416 | 6.98E-07 |
| CCNDBP | darkred | 0.024455528 | 0.598088774 | 0.048377554 | 0.296829302 |
| CCPG1 | darkred | 0.135585266 | 0.003327555 | 0.144011704 | 0.001808156 |
| CDC42SE | darkred | 0.218215185 | 1.93E-06 | 0.210182392 | 4.62E-06 |
| CDK7 | darkred | 0.254325075 | 2.50E-08 | 0.226964493 | 7.18E-07 |
| CDYL2 | darkred | 0.230174218 | 4.94E-07 | 0.221588022 | 1.32E-06 |
| CHP1 | darkred | 0.432712327 | 9.86E-23 | 0.431216658 | 1.43E-22 |
| CLCN3 | darkred | 0.268899457 | 3.54E-09 | 0.244356556 | 8.91E-08 |
| CMPK1 | darkred | 0.156852967 | 0.000669888 | 0.148042019 | 0.00133505 |
| CMTM6 | darkred | 0.03881875 | 0.402622015 | 0.019057824 | 0.681236317 |
| COG3 | darkred | 0.164346066 | 0.000362045 | 0.149649921 | 0.001180382 |
| COG6 | darkred | 0.105635186 | 0.022426308 | 0.081577664 | 0.0782181 |
| COX18 | darkred | 0.343664899 | 2.16E-14 | 0.360928468 | 8.16E-16 |
| CPD | darkred | 0.226958116 | 7.19E-07 | 0.209486072 | 4.98E-06 |
| CPNE1 | darkred | -0.220455578 | 1.50E-06 | -0.21946574 | 1.68E-06 |
| CRYZ | darkred | 0.108806249 | 0.018672365 | 0.071093089 | 0.12499053 |
| CTAGE5 | darkred | 0.123847501 | 0.007372834 | 0.107061811 | 0.020663361 |
| CYP4V2 | darkred | 0.159489176 | 0.000541134 | 0.170111137 | 0.000221421 |
| DDAH1 | darkred | 0.349668997 | 7.07E-15 | 0.356391313 | 1.97E-15 |
| DIMT1 | darkred | 0.251824742 | 3.46E-08 | 0.221271724 | 1.37E-06 |
| DNAJB9 | darkred | 0.085367954 | 0.065294115 | 0.078269886 | 0.091126294 |
| DNAJC3 | darkred | 0.20178419 | 1.11E-05 | 0.20071575 | 1.24E-05 |
| DRAM2 | darkred | 0.076783839 | 0.097456142 | 0.055088774 | 0.234758375 |
| DYNLT3 | darkred | 0.005477342 | 0.90602802 | -0.000510711 | 0.991217881 |
| EHMT2 | darkred | -0.110761075 | 0.016641945 | -0.110499935 | 0.016901487 |
| ELL2 | darkred | 0.140346146 | 0.002367067 | 0.151197563 | 0.001047263 |
| ELOVL5 | darkred | 0.224773567 | 9.23E-07 | 0.213515934 | 3.23E-06 |
| ENPP4 | darkred | 0.215394168 | 2.63E-06 | 0.202994155 | 9.82E-06 |
| ERBB2IP | darkred | 0.17645388 | 0.000126539 | 0.152758178 | 0.000927171 |
| ERLEC1 | darkred | 0.149187335 | 0.001223097 | 0.137275457 | 0.002952097 |
| ERO1L | darkred | 0.274529297 | 1.61E-09 | 0.283661354 | 4.31E-10 |
| ETFDH | darkred | 0.287013079 | 2.63E-10 | 0.292887635 | 1.09E-10 |
| FAM107E | darkred | 0.142000332 | 0.002097763 | 0.150241857 | 0.001127727 |
| FAM114A | darkred | 0.095978757 | 0.038139852 | 0.075212231 | 0.104526551 |
| FAM120A | darkred | 0.376888722 | 3.29E-17 | 0.378010593 | 2.61E-17 |
| FBXO4 | darkred | 0.156052777 | 0.000714261 | 0.140821588 | 0.002286602 |
| FPGT | darkred | 0.140409986 | 0.002356115 | 0.115630287 | 0.012402254 |
| GABARA | darkred | 0.050555658 | 0.275587 | 0.07212251 | 0.119605606 |
| GALNT3 | darkred | 0.0568282 | 0.220286235 | 0.044719786 | 0.334897895 |
| GALNT7 | darkred | 0.312005804 | 5.30E-12 | 0.313233575 | 4.33E-12 |
| GFPT1 | darkred | 0.1487691 | 0.001262936 | 0.168342605 | 0.000257908 |

| | | | | | |
|---|---|---|---|---|---|
| GRAMD3 | darkred | 0.099222543 | 0.032051846 | 0.074590289 | 0.107434292 |
| HERPUD | darkred | 0.174385646 | 0.000152192 | 0.189357871 | 3.81E-05 |
| HFE | darkred | 0.217216286 | 2.16E-06 | 0.23694865 | 2.21E-07 |
| HIATL1 | darkred | 0.206382502 | 6.91E-06 | 0.198164109 | 1.61E-05 |
| HIF1A | darkred | 0.178839378 | 0.000102008 | 0.16740472 | 0.000279467 |
| HNF4G | darkred | 0.244598075 | 8.64E-08 | 0.226493211 | 7.58E-07 |
| HSD17B4 | darkred | 0.291290395 | 1.38E-10 | 0.263718946 | 7.19E-09 |
| HSDL2 | darkred | 0.357717787 | 1.52E-15 | 0.334909916 | 1.06E-13 |
| IDH1 | darkred | 0.374306566 | 5.59E-17 | 0.383234469 | 8.72E-18 |
| ILVBL | darkred | -0.257747491 | 1.60E-08 | -0.250869838 | 3.91E-08 |
| ISOC1 | darkred | 0.141195346 | 0.002225108 | 0.120875391 | 0.008929737 |
| IST1 | darkred | 0.159354588 | 0.000547106 | 0.170199657 | 0.000219728 |
| ISYNA1 | darkred | -0.163864748 | 0.000376945 | -0.152383492 | 0.000954783 |
| ITM2B | darkred | -0.159202222 | 0.000553942 | -0.164012943 | 0.000372298 |
| KDELR2 | darkred | 0.030693316 | 0.508184984 | 0.062476738 | 0.177712289 |
| KIAA014 | darkred | 0.165640425 | 0.000324655 | 0.151626985 | 0.001012862 |
| KIAA124 | darkred | 0.305171628 | 1.60E-11 | 0.33380305 | 1.28E-13 |
| KLHL2 | darkred | 0.2139748 | 3.07E-06 | 0.209241994 | 5.11E-06 |
| LACC1 | darkred | 0.132270254 | 0.004192342 | 0.118823861 | 0.010168834 |
| LAMTOR | darkred | 0.099279052 | 0.031953613 | 0.092607781 | 0.045477825 |
| LIN7C | darkred | 0.123213925 | 0.00768273 | 0.124575274 | 0.007030697 |
| LIPA | darkred | 0.085023355 | 0.066391464 | 0.056556933 | 0.222500202 |
| LMAN1 | darkred | 0.037951696 | 0.413220193 | 0.01651481 | 0.721873592 |
| LMBRD1 | darkred | -0.079208854 | 0.08729958 | -0.091512833 | 0.048102923 |
| LNX1 | darkred | 0.246056795 | 7.20E-08 | 0.240241554 | 1.48E-07 |
| LPCAT2 | darkred | 0.293865643 | 9.35E-11 | 0.263679086 | 7.23E-09 |
| MAP1LC | darkred | 0.201613269 | 1.13E-05 | 0.210773776 | 4.34E-06 |
| MAPK6 | darkred | 0.278636456 | 8.95E-10 | 0.259319202 | 1.30E-08 |
| MCCC2 | darkred | 0.326610228 | 4.53E-13 | 0.318506732 | 1.80E-12 |
| MCEE | darkred | 0.161282076 | 0.000467134 | 0.13956594 | 0.002504728 |
| MEGF9 | darkred | 0.190634958 | 3.37E-05 | 0.171498372 | 0.000196244 |
| METAP1 | darkred | 0.29874571 | 4.41E-11 | 0.255938681 | 2.03E-08 |
| MFSD6 | darkred | 0.287519121 | 2.44E-10 | 0.289439132 | 1.83E-10 |
| MKRN1 | darkred | 0.224090564 | 9.98E-07 | 0.22699184 | 7.16E-07 |
| MMAA | darkred | 0.126191434 | 0.006321236 | 0.134443977 | 0.003605066 |
| MOCS2 | darkred | 0.177548098 | 0.000114669 | 0.157281535 | 0.000647187 |
| MPZL2 | darkred | 0.281615887 | 5.82E-10 | 0.272530069 | 2.13E-09 |
| MRRF | darkred | 0.26085447 | 1.06E-08 | 0.252390573 | 3.21E-08 |
| MYLPF | darkred | -0.261686262 | 9.45E-09 | -0.244931322 | 8.29E-08 |
| MYO5C | darkred | 0.337137637 | 7.08E-14 | 0.328411228 | 3.32E-13 |
| NEURL2 | darkred | -0.287108575 | 2.59E-10 | -0.281310122 | 6.08E-10 |
| NIPSNAP | darkred | 0.281294257 | 6.10E-10 | 0.284219049 | 3.97E-10 |
| NUDT12 | darkred | 0.016257286 | 0.726035119 | 0.031872301 | 0.492022885 |
| NUDT9 | darkred | 0.132201508 | 0.004212252 | 0.113239797 | 0.014347045 |
| OMA1 | darkred | 0.157452049 | 0.000638355 | 0.141598777 | 0.00216043 |

| | | | | | |
|---|---|---|---|---|---|
| PACS2 | darkred | -0.071229465 | 0.124266505 | -0.061342967 | 0.185726792 |
| PAFAH2 | darkred | 0.297833966 | 5.08E-11 | 0.306491707 | 1.30E-11 |
| PCED1A | darkred | -0.219738511 | 1.63E-06 | -0.239728688 | 1.58E-07 |
| PEX7 | darkred | 0.3128207 | 4.63E-12 | 0.306200764 | 1.36E-11 |
| PGGT1B | darkred | 0.136843131 | 0.003044277 | 0.115563945 | 0.012452917 |
| PIBF1 | darkred | 0.029511665 | 0.524657863 | 0.000483607 | 0.99168393 |
| PIGB | darkred | 0.215641358 | 2.56E-06 | 0.183761252 | 6.48E-05 |
| PIGV | darkred | 0.176838694 | 0.000122239 | 0.165087819 | 0.000340154 |
| PKN1 | darkred | -0.289731144 | 1.75E-10 | -0.293657082 | 9.66E-11 |
| PNPLA8 | darkred | 0.009063625 | 0.845122332 | -0.003739885 | 0.935757453 |
| POC1B | darkred | 0.310613935 | 6.65E-12 | 0.296394693 | 6.34E-11 |
| PPIP5K1 | darkred | 0.360539704 | 8.80E-16 | 0.330174518 | 2.44E-13 |
| PPM1H | darkred | 0.256132197 | 1.98E-08 | 0.23190151 | 4.04E-07 |
| PRKAG2 | darkred | 0.096025015 | 0.038046588 | 0.064774642 | 0.162260736 |
| PRRC1 | darkred | 0.183252814 | 6.80E-05 | 0.168206737 | 0.000260932 |
| PRRG4 | darkred | 0.139966853 | 0.002433104 | 0.162124456 | 0.000435719 |
| PYROXD | darkred | 0.20873883 | 5.39E-06 | 0.186225589 | 5.14E-05 |
| RAB11A | darkred | 0.240470681 | 1.44E-07 | 0.247576995 | 5.94E-08 |
| RASEF | darkred | 0.218920218 | 1.79E-06 | 0.216625849 | 2.30E-06 |
| RASSF6 | darkred | 0.409106398 | 2.86E-20 | 0.384309287 | 6.94E-18 |
| RB1 | darkred | 0.163556872 | 0.000386773 | 0.156208831 | 0.000705399 |
| REEP5 | darkred | 0.167677541 | 0.000273028 | 0.186097601 | 5.21E-05 |
| REPS2 | darkred | 0.238581564 | 1.81E-07 | 0.22629172 | 7.76E-07 |
| RMDN3 | darkred | 0.291640066 | 1.31E-10 | 0.293373829 | 1.01E-10 |
| RNF6 | darkred | 0.126503614 | 0.006191841 | 0.119437051 | 0.009783386 |
| SAR1B | darkred | 0.247491616 | 6.01E-08 | 0.266665383 | 4.81E-09 |
| SCP2 | darkred | 0.208965767 | 5.26E-06 | 0.193002877 | 2.68E-05 |
| SERINC5 | darkred | 0.357792629 | 1.50E-15 | 0.349273502 | 7.62E-15 |
| SGCB | darkred | 0.289799542 | 1.73E-10 | 0.26178377 | 9.33E-09 |
| SH3GLB1 | darkred | 0.076377621 | 0.099246074 | 0.056117223 | 0.226122594 |
| SIAE | darkred | 0.279566172 | 7.83E-10 | 0.258605649 | 1.43E-08 |
| SLC11A2 | darkred | 0.303027208 | 2.25E-11 | 0.271003657 | 2.64E-09 |
| SLC18B1 | darkred | 0.040337156 | 0.384458246 | 0.023419737 | 0.61368689 |
| SLC26A2 | darkred | 0.29332687 | 1.02E-10 | 0.259202892 | 1.32E-08 |
| SLC30A5 | darkred | 0.150016104 | 0.001147548 | 0.139485043 | 0.002519412 |
| SLC35A3 | darkred | 0.143887154 | 0.001824966 | 0.140824273 | 0.002286154 |
| SLC41A2 | darkred | 0.078669503 | 0.089481555 | 0.062550766 | 0.177198058 |
| SLC44A1 | darkred | 0.24531538 | 7.90E-08 | 0.216888851 | 2.24E-06 |
| SLFN5 | darkred | 0.11293502 | 0.014613335 | 0.111696427 | 0.015740725 |
| SMIM14 | darkred | 0.286676174 | 2.76E-10 | 0.290969005 | 1.45E-10 |
| SMIM15 | darkred | 0.141242694 | 0.002217426 | 0.127238704 | 0.005896496 |
| SNAP23 | darkred | 0.115805242 | 0.012269519 | 0.097723769 | 0.034753027 |
| SOAT1 | darkred | 0.282848275 | 4.86E-10 | 0.272801715 | 2.05E-09 |
| SPCS3 | darkred | 0.075106282 | 0.105017439 | 0.071666077 | 0.12197043 |
| SPPL2A | darkred | 0.213428174 | 3.26E-06 | 0.209169053 | 5.15E-06 |

| | | | | | |
|---|---|---|---|---|---|
| SPRYD7 | darkred | 0.154748648 | 0.000792451 | 0.145500266 | 0.001617936 |
| STAM2 | darkred | 0.065016747 | 0.160693525 | 0.043058645 | 0.353178273 |
| STEAP2 | darkred | 0.163931705 | 0.000374839 | 0.148802952 | 0.001259668 |
| STS | darkred | 0.182459094 | 7.32E-05 | 0.207613757 | 6.07E-06 |
| STX12 | darkred | 0.120051076 | 0.009410473 | 0.104079438 | 0.024495977 |
| SUCLG2 | darkred | 0.222987104 | 1.13E-06 | 0.220001626 | 1.58E-06 |
| SVIP | darkred | 0.228256685 | 6.18E-07 | 0.216628244 | 2.30E-06 |
| SYAP1 | darkred | 0.31832077 | 1.86E-12 | 0.332475294 | 1.63E-13 |
| TADA2B | darkred | 0.207287414 | 6.28E-06 | 0.207375681 | 6.22E-06 |
| TANK | darkred | 0.100901904 | 0.029240282 | 0.078440277 | 0.090422061 |
| TC2N | darkred | 0.08087278 | 0.080836588 | 0.088857223 | 0.055000559 |
| TCEA2 | darkred | -0.309975322 | 7.38E-12 | -0.308290252 | 9.70E-12 |
| TERF2IP | darkred | 0.086418259 | 0.062041139 | 0.091870687 | 0.047231363 |
| TLR3 | darkred | 0.126673403 | 0.006122469 | 0.143184162 | 0.001922561 |
| TMBIM6 | darkred | 0.185107242 | 5.71E-05 | 0.168673468 | 0.000250681 |
| TMED10 | darkred | 0.212527836 | 3.60E-06 | 0.200384424 | 1.28E-05 |
| TMED7 | darkred | 0.041509129 | 0.370786194 | 0.026281545 | 0.571037094 |
| TMEM11 | darkred | 0.152599283 | 0.000938789 | 0.124212476 | 0.007199436 |
| TMEM14 | darkred | 0.35589894 | 2.16E-15 | 0.350405715 | 6.16E-15 |
| TMEM16 | darkred | 0.213996315 | 3.07E-06 | 0.195815384 | 2.03E-05 |
| TMEM16 | darkred | 0.025072786 | 0.588879582 | 0.009564876 | 0.836674139 |
| TMEM17 | darkred | 0.215952259 | 2.48E-06 | 0.211117189 | 4.18E-06 |
| TMEM2 | darkred | 0.228587277 | 5.95E-07 | 0.208213855 | 5.70E-06 |
| TMEM39 | darkred | -0.151546229 | 0.001019251 | -0.172031772 | 0.000187297 |
| TMEM41 | darkred | 0.279224111 | 8.23E-10 | 0.274380697 | 1.64E-09 |
| TMEM50 | darkred | 0.100641263 | 0.029662263 | 0.103985298 | 0.024626343 |
| TMEM59 | darkred | 0.001217066 | 0.979073448 | 0.010227072 | 0.825542235 |
| TMEM87 | darkred | 0.243985044 | 9.33E-08 | 0.204563772 | 8.35E-06 |
| TMEM87 | darkred | 0.136354374 | 0.003151636 | 0.139111842 | 0.002588173 |
| TMEM9B | darkred | 0.269767014 | 3.14E-09 | 0.284033992 | 4.08E-10 |
| TMOD3 | darkred | 0.240866512 | 1.37E-07 | 0.227813255 | 6.51E-07 |
| TMX4 | darkred | 0.177757762 | 0.000112517 | 0.15419942 | 0.000827691 |
| TOB1 | darkred | 0.091109377 | 0.049101663 | 0.086489145 | 0.061826496 |
| TRIM2 | darkred | 0.329663553 | 2.66E-13 | 0.336595303 | 7.80E-14 |
| TSPAN13 | darkred | 0.276881522 | 1.15E-09 | 0.297587692 | 5.28E-11 |
| TVP23B | darkred | 0.216989149 | 2.21E-06 | 0.199903225 | 1.35E-05 |
| TWF1 | darkred | 0.203283305 | 9.53E-06 | 0.17802957 | 0.000109785 |
| UBE2J1 | darkred | 0.077921512 | 0.092579814 | 0.087480519 | 0.058888283 |
| UBXN4 | darkred | 0.052826001 | 0.254571474 | 0.040543039 | 0.382034445 |
| UCKL1 | darkred | -0.157257485 | 0.000648442 | -0.149833656 | 0.001163801 |
| UFL1 | darkred | 0.048505341 | 0.29555368 | 0.034058782 | 0.462792087 |
| UGCG | darkred | 0.075602991 | 0.102731749 | 0.047239796 | 0.308348472 |
| UGDH | darkred | 0.284959409 | 3.56E-10 | 0.273435886 | 1.88E-09 |
| USO1 | darkred | 0.057083972 | 0.218213191 | 0.029291486 | 0.527757147 |
| ZSWIM1 | darkred | -0.114245115 | 0.01349868 | -0.084341974 | 0.068605847 |

| | | | | | |
|---|---|---|---|---|---|
| DPY19L3 | darkslatebl | -0.090277783 | 0.051215022 | -0.124905041 | 0.006880401 |
| EID2B | darkslatebl | -0.196967102 | 1.81E-05 | -0.196512044 | 1.89E-05 |
| HKR1 | darkslatebl | -0.079677966 | 0.085436748 | -0.082460942 | 0.075034522 |
| ZFP14 | darkslatebl | -0.106772435 | 0.021010888 | -0.119961957 | 0.009463801 |
| ZFP30 | darkslatebl | -0.00995461 | 0.830118383 | -0.021887972 | 0.637075282 |
| ZFP82 | darkslatebl | -0.366767846 | 2.57E-16 | -0.362134143 | 6.44E-16 |
| ZNF181 | darkslatebl | -0.293056254 | 1.06E-10 | -0.317486601 | 2.14E-12 |
| ZNF260 | darkslatebl | -0.079521168 | 0.086055791 | -0.085646389 | 0.064418388 |
| ZNF30 | darkslatebl | 0.079834824 | 0.08482106 | 0.043522705 | 0.348009219 |
| ZNF302 | darkslatebl | -0.443436201 | 6.45E-24 | -0.464830003 | 2.08E-26 |
| ZNF311 | darkslatebl | -0.498228427 | 1.14E-30 | -0.505037341 | 1.35E-31 |
| ZNF334 | darkslatebl | -0.464034262 | 2.59E-26 | -0.467171973 | 1.08E-26 |
| ZNF345 | darkslatebl | -0.214528582 | 2.89E-06 | -0.216924825 | 2.23E-06 |
| ZNF382 | darkslatebl | -0.192887531 | 2.71E-05 | -0.207963637 | 5.85E-06 |
| ZNF383 | darkslatebl | -0.176849232 | 0.000122123 | -0.195789499 | 2.03E-05 |
| ZNF404 | darkslatebl | -0.289062656 | 1.94E-10 | -0.286505772 | 2.83E-10 |
| ZNF420 | darkslatebl | -0.166543814 | 0.000300728 | -0.189141893 | 3.89E-05 |
| ZNF461 | darkslatebl | -0.158555715 | 0.000583837 | -0.17852749 | 0.00010494 |
| ZNF507 | darkslatebl | -0.279712888 | 7.67E-10 | -0.315496782 | 2.98E-12 |
| ZNF527 | darkslatebl | -0.137164744 | 0.002975458 | -0.157860141 | 0.000617669 |
| ZNF529 | darkslatebl | -0.188282502 | 4.23E-05 | -0.200357329 | 1.29E-05 |
| ZNF546 | darkslatebl | -0.134104182 | 0.003691651 | -0.152331902 | 0.000958644 |
| ZNF565 | darkslatebl | -0.121736191 | 0.008451177 | -0.144363835 | 0.001761394 |
| ZNF566 | darkslatebl | -0.20822502 | 5.69E-06 | -0.228725332 | 5.86E-07 |
| ZNF567 | darkslatebl | -0.096857734 | 0.036400448 | -0.12165786 | 0.008493759 |
| ZNF568 | darkslatebl | -0.346330622 | 1.32E-14 | -0.345537183 | 1.53E-14 |
| ZNF569 | darkslatebl | -0.072696075 | 0.11668485 | -0.092619829 | 0.045449624 |
| ZNF570 | darkslatebl | -0.031511331 | 0.496941859 | -0.048792667 | 0.292698878 |
| ZNF571 | darkslatebl | -0.100131025 | 0.030503469 | -0.12083372 | 0.008953504 |
| ZNF585A | darkslatebl | -0.167461776 | 0.000278109 | -0.18937445 | 3.81E-05 |
| ZNF585B | darkslatebl | -0.172173887 | 0.000184979 | -0.181933548 | 7.68E-05 |
| ZNF607 | darkslatebl | -0.162058975 | 0.000438089 | -0.169059747 | 0.000242483 |
| ZNF667 | darkslatebl | -0.409202983 | 2.80E-20 | -0.430649285 | 1.65E-22 |
| ZNF780A | darkslatebl | -0.099888214 | 0.030910902 | -0.111098182 | 0.016312064 |
| ZNF780B | darkslatebl | -0.119731988 | 0.009602656 | -0.123570179 | 0.007507076 |
| ZNF790 | darkslatebl | -0.014253567 | 0.758681785 | -0.010957888 | 0.81329732 |
| ZNF793 | darkslatebl | -0.181729944 | 7.83E-05 | -0.19602515 | 1.99E-05 |
| ZNF829 | darkslatebl | -0.40373153 | 9.80E-20 | -0.402736889 | 1.23E-19 |
| ZNF850 | darkslatebl | -0.18681192 | 4.86E-05 | -0.187648974 | 4.49E-05 |
| ZNF879 | darkslatebl | -0.229242712 | 5.51E-07 | -0.260053766 | 1.18E-08 |
| ABCB4 | darkturquoi | -0.019245565 | 0.67827074 | -0.023014305 | 0.619840662 |
| ABHD12I | darkturquoi | 0.11173837 | 0.015701335 | 0.079675427 | 0.085446742 |
| ADAM23 | darkturquoi | 0.090161003 | 0.051517792 | 0.0357036 | 0.44145473 |
| ADAMTS | darkturquoi | 0.212164209 | 3.74E-06 | 0.152715148 | 0.000930304 |
| ADAMTS | darkturquoi | 0.121575166 | 0.008538921 | 0.07247663 | 0.117795661 |

| | | | | | |
|---|---|---|---|---|---|
| ADAMTS | darkturquoi | 0.035554943 | 0.443359783 | 0.021452485 | 0.6437924 |
| AMBN | darkturquoi | 0.061251235 | 0.186386706 | 0.056675293 | 0.221532254 |
| AMOT | darkturquoi | 0.155601975 | 0.000740443 | 0.131120672 | 0.004536719 |
| ANKRD3 | darkturquoi | 0.014070006 | 0.76169511 | -0.020394937 | 0.660224757 |
| APCDD1 | darkturquoi | -0.008824098 | 0.849165712 | -0.020611761 | 0.656842034 |
| ASCL5 | darkturquoi | 0.100156914 | 0.030460302 | 0.082289396 | 0.07564443 |
| AXIN2 | darkturquoi | 0.059581955 | 0.198698526 | 0.028455863 | 0.539603543 |
| BAMBI | darkturquoi | 0.171936644 | 0.000188864 | 0.136351858 | 0.003152197 |
| BEST3 | darkturquoi | 0.079327835 | 0.086824043 | 0.066888398 | 0.148960381 |
| BMP4 | darkturquoi | 0.052623346 | 0.256400891 | 0.038477758 | 0.406770513 |
| BSX | darkturquoi | -0.007703276 | 0.868136861 | -0.023257674 | 0.616143506 |
| C10orf12 | darkturquoi | 0.016915712 | 0.715411471 | 0.015510886 | 0.738141952 |
| C3orf36 | darkturquoi | 0.153518922 | 0.000873362 | 0.142169297 | 0.002071895 |
| CA6 | darkturquoi | 0.165240882 | 0.000335793 | 0.130100816 | 0.004863399 |
| CACNA1 | darkturquoi | 0.071749522 | 0.121535393 | 0.021385548 | 0.64482746 |
| CACNA1 | darkturquoi | 0.092774072 | 0.045089874 | 0.070124157 | 0.130229397 |
| CALML3 | darkturquoi | 0.133040843 | 0.003974921 | 0.092236671 | 0.046353733 |
| CD36 | darkturquoi | 0.003582069 | 0.938462883 | -0.020835697 | 0.653355711 |
| CDX2 | darkturquoi | 0.154842044 | 0.000786598 | 0.120744675 | 0.00900448 |
| CEMIP | darkturquoi | 0.151833868 | 0.000996664 | 0.142173138 | 0.00207131 |
| CHRNA3 | darkturquoi | 0.065441699 | 0.157970307 | 0.043728167 | 0.345736018 |
| CORIN | darkturquoi | -0.013997932 | 0.762879267 | -0.015804594 | 0.733369992 |
| CPNE9 | darkturquoi | -0.011343036 | 0.806862166 | -0.017689169 | 0.703001763 |
| CRYBA4 | darkturquoi | 0.125701819 | 0.006529067 | 0.100219815 | 0.030355635 |
| CRYBB1 | darkturquoi | 0.088561084 | 0.05581832 | 0.060191688 | 0.194134481 |
| CST1 | darkturquoi | 0.153677435 | 0.00086252 | 0.127234114 | 0.0058983 |
| CST2 | darkturquoi | 0.085712896 | 0.06421065 | 0.079949948 | 0.084371467 |
| CST4 | darkturquoi | 0.052564313 | 0.256935501 | 0.023635916 | 0.61041667 |
| CST5 | darkturquoi | -0.029306392 | 0.52754703 | -0.06395783 | 0.167633034 |
| CTD-253 | darkturquoi | -0.008451965 | 0.855455388 | -0.013624339 | 0.769026102 |
| CXCL14 | darkturquoi | 0.269635325 | 3.19E-09 | 0.25651559 | 1.88E-08 |
| DBH | darkturquoi | 0.10831449 | 0.01921595 | 0.078336713 | 0.090849571 |
| DCHS2 | darkturquoi | 0.04966609 | 0.284134356 | 0.020900971 | 0.652340911 |
| DEFA5 | darkturquoi | 0.054651415 | 0.238500511 | 0.014448664 | 0.755483071 |
| DEFA6 | darkturquoi | 0.049156049 | 0.289114903 | 0.015483151 | 0.738593093 |
| DEFB133 | darkturquoi | -0.072059066 | 0.119932155 | -0.080218585 | 0.083329829 |
| DEPDC7 | darkturquoi | -0.003667354 | 0.937000761 | -0.025033766 | 0.589459795 |
| DKK1 | darkturquoi | 0.174664744 | 0.000148466 | 0.162884554 | 0.000409071 |
| DKK4 | darkturquoi | 0.163069483 | 0.000402821 | 0.139266625 | 0.00255945 |
| DLX3 | darkturquoi | -0.049258074 | 0.288113966 | -0.090899707 | 0.049627505 |
| DPEP1 | darkturquoi | 0.055619872 | 0.230270164 | 0.049140505 | 0.289267603 |
| DRAXIN | darkturquoi | 0.024479306 | 0.597732815 | -0.000294127 | 0.994942169 |
| DYNC1I1 | darkturquoi | 0.251469303 | 3.62E-08 | 0.20401937 | 8.83E-06 |
| EDAR | darkturquoi | 0.088996771 | 0.054618675 | 0.088921278 | 0.054824992 |
| EDN3 | darkturquoi | 0.032897264 | 0.478198732 | 0.027454598 | 0.55397084 |

| | | | | | |
|---|---|---|---|---|---|
| EPHA7 | darkturquoi | 0.21695399 | 2.22E-06 | 0.184964689 | 5.79E-05 |
| ERVMER | darkturquoi | 0.018640295 | 0.687849218 | -0.021621246 | 0.641185886 |
| EVX1 | darkturquoi | -0.041515993 | 0.370707016 | -0.029032225 | 0.531418427 |
| FAM159E | darkturquoi | 0.063735362 | 0.169119057 | 0.05267088 | 0.255970975 |
| FAM163E | darkturquoi | 0.058055884 | 0.210463331 | 0.039152343 | 0.398588101 |
| FAM178E | darkturquoi | -0.091439058 | 0.048284268 | -0.092422431 | 0.045913541 |
| FAM84A | darkturquoi | 0.223778796 | 1.03E-06 | 0.187353104 | 4.62E-05 |
| FGF19 | darkturquoi | 0.149359921 | 0.001206997 | 0.11964131 | 0.009657903 |
| FGF20 | darkturquoi | 0.168093016 | 0.000263488 | 0.149313897 | 0.001211271 |
| FGF3 | darkturquoi | 0.047200679 | 0.308749684 | 0.031012385 | 0.503783811 |
| FGF4 | darkturquoi | 0.150055389 | 0.001144076 | 0.104370011 | 0.024097337 |
| FGF8 | darkturquoi | 0.028976522 | 0.532206731 | 0.009298662 | 0.841158697 |
| FOXL1 | darkturquoi | 0.241336974 | 1.29E-07 | 0.235883687 | 2.51E-07 |
| FRMPD1 | darkturquoi | -0.070335445 | 0.129072752 | -0.087358295 | 0.059244161 |
| FSTL4 | darkturquoi | -0.129678916 | 0.005004635 | -0.135621978 | 0.00331896 |
| FZD10 | darkturquoi | 0.240209928 | 1.49E-07 | 0.204796184 | 8.15E-06 |
| GAD1 | darkturquoi | 0.317656113 | 2.08E-12 | 0.291650181 | 1.31E-10 |
| GDNF | darkturquoi | -0.044645287 | 0.33570448 | -0.056613725 | 0.22203538 |
| GLB1L3 | darkturquoi | -0.063017387 | 0.17398213 | -0.051274207 | 0.268811817 |
| GNAT3 | darkturquoi | -0.012838279 | 0.78200629 | -0.024886952 | 0.591645253 |
| GOLGA7 | darkturquoi | 0.035046566 | 0.449909967 | 0.029404861 | 0.526160095 |
| GPCPD1 | darkturquoi | 0.169883632 | 0.000225828 | 0.157030443 | 0.000660399 |
| GPR155 | darkturquoi | -0.096407119 | 0.037283562 | -0.096872821 | 0.036371192 |
| GPR83 | darkturquoi | 0.043919918 | 0.343623036 | 0.021521482 | 0.642726209 |
| GPX2 | darkturquoi | 0.185752241 | 5.38E-05 | 0.181632365 | 7.90E-05 |
| GREM2 | darkturquoi | 0.057604514 | 0.214037427 | 0.015132626 | 0.744302477 |
| GRM3 | darkturquoi | -0.091862126 | 0.047252057 | -0.051944369 | 0.262596427 |
| GRM8 | darkturquoi | 0.067861808 | 0.143121751 | 0.04683156 | 0.312552542 |
| GSDMA | darkturquoi | 0.021456091 | 0.643736663 | 0.003056287 | 0.947481277 |
| HAO2 | darkturquoi | 0.082667366 | 0.074305925 | 0.069874855 | 0.131604432 |
| HPS4 | darkturquoi | 0.161998202 | 0.000440299 | 0.118967671 | 0.010077246 |
| HSPA12A | darkturquoi | -0.049533056 | 0.285427817 | -0.101188199 | 0.028782712 |
| HUNK | darkturquoi | -0.101915674 | 0.027647584 | -0.144725187 | 0.001714562 |
| IGFBPL1 | darkturquoi | 0.008100962 | 0.861396333 | -0.007058612 | 0.879083623 |
| IGFL4 | darkturquoi | -0.01023513 | 0.825406979 | -0.023839748 | 0.607340311 |
| IL10 | darkturquoi | -0.110893065 | 0.016512096 | -0.119849849 | 0.009531267 |
| IL17RD | darkturquoi | 0.00037766 | 0.993505747 | -0.035572425 | 0.443135503 |
| IL23A | darkturquoi | -0.10055793 | 0.029798277 | -0.125001768 | 0.006836865 |
| IL31RA | darkturquoi | 0.027261743 | 0.556759434 | -0.001475605 | 0.97462943 |
| KCNA2 | darkturquoi | -0.06644895 | 0.151654879 | -0.088161934 | 0.056936487 |
| KCNJ9 | darkturquoi | 0.040392393 | 0.383807039 | 0.000250448 | 0.995693259 |
| KIF26B | darkturquoi | 0.254246887 | 2.53E-08 | 0.240480354 | 1.44E-07 |
| KIZ | darkturquoi | 0.099031227 | 0.032386339 | 0.062951201 | 0.174435618 |
| KLK4 | darkturquoi | 0.069298019 | 0.134828976 | 0.044872824 | 0.333244875 |
| KRT31 | darkturquoi | 0.120122048 | 0.009368196 | 0.08362891 | 0.070987619 |

| | | | | | |
|---|---|---|---|---|---|
| KRT74 | darkturquoi | 0.0712935 | 0.123927667 | 0.09452479 | 0.041171648 |
| LAMP5 | darkturquoi | 0.056256845 | 0.224967849 | 0.030610383 | 0.509332231 |
| LEF1 | darkturquoi | 0.210120259 | 4.65E-06 | 0.198490909 | 1.55E-05 |
| LRP4 | darkturquoi | 0.270723362 | 2.75E-09 | 0.243862234 | 9.47E-08 |
| LRRC15 | darkturquoi | 0.022426789 | 0.628805379 | 0.037116234 | 0.42358636 |
| LY6G6C | darkturquoi | 0.080276457 | 0.083106794 | 0.05062299 | 0.27494724 |
| LY6G6D | darkturquoi | 0.015047208 | 0.745695916 | -0.008573567 | 0.853399082 |
| MSX2 | darkturquoi | 0.192853737 | 2.72E-05 | 0.168634805 | 0.000251515 |
| MUCL1 | darkturquoi | 0.14951904 | 0.001192326 | 0.152891054 | 0.000917557 |
| MYH7B | darkturquoi | 0.05689029 | 0.2197817 | 0.026480284 | 0.568128254 |
| NAT16 | darkturquoi | -0.0208009 | 0.653896944 | -0.016531638 | 0.721601948 |
| NKD1 | darkturquoi | 0.060887743 | 0.189018602 | 0.038287821 | 0.409092257 |
| NKD2 | darkturquoi | -0.0952663 | 0.039600955 | -0.097304418 | 0.035542641 |
| NODAL | darkturquoi | -0.016916745 | 0.715394849 | -0.066754452 | 0.149777787 |
| NOTUM | darkturquoi | 0.014582088 | 0.753297913 | -0.012456643 | 0.788330378 |
| NOX3 | darkturquoi | -0.006338418 | 0.891340126 | -0.031928784 | 0.491255558 |
| NRSN1 | darkturquoi | 0.040046202 | 0.387899509 | 0.025150613 | 0.587723085 |
| NTNG2 | darkturquoi | -0.092057745 | 0.046781072 | -0.06253562 | 0.177303179 |
| OSBPL6 | darkturquoi | -0.027569422 | 0.552313782 | -0.039043139 | 0.399905956 |
| PHLDA1 | darkturquoi | 0.263080393 | 7.84E-09 | 0.255956218 | 2.02E-08 |
| PLA2G4D | darkturquoi | 0.098343866 | 0.033612813 | 0.056112659 | 0.226160407 |
| PMCH | darkturquoi | 0.053662818 | 0.247113248 | 0.0278729 | 0.547945914 |
| PROX1 | darkturquoi | 0.052405066 | 0.258381515 | 0.030006488 | 0.517726733 |
| PRR9 | darkturquoi | 0.107465859 | 0.020186481 | 0.109253995 | 0.018189145 |
| PTPRE | darkturquoi | -0.003478944 | 0.940231124 | -0.009458146 | 0.83847145 |
| RASGRF | darkturquoi | 0.119962854 | 0.009463263 | 0.08116438 | 0.079744883 |
| RASL11B | darkturquoi | -0.107131299 | 0.020580657 | -0.145281341 | 0.00164471 |
| RNF43 | darkturquoi | 0.10831145 | 0.019219353 | 0.078873023 | 0.088653115 |
| RP11-520 | darkturquoi | -0.049635802 | 0.28442849 | -0.046691978 | 0.313998564 |
| RUNX2 | darkturquoi | -0.02394602 | 0.605739131 | -0.038739856 | 0.403579586 |
| SCML1 | darkturquoi | -0.02166251 | 0.640549231 | -0.053978772 | 0.244337342 |
| SEZ6L | darkturquoi | 0.033253342 | 0.473446148 | 0.015974808 | 0.730609157 |
| SHISA6 | darkturquoi | 0.073938746 | 0.110548526 | 0.069333299 | 0.134630028 |
| SLC30A1 | darkturquoi | 0.114676825 | 0.013148144 | 0.106781201 | 0.021000287 |
| SLC35D3 | darkturquoi | -0.01547617 | 0.738706663 | -0.018841875 | 0.684653542 |
| SLC6A2 | darkturquoi | 0.186103533 | 5.20E-05 | 0.134125871 | 0.003686069 |
| SLC8A3 | darkturquoi | -0.066118268 | 0.153706753 | -0.078182325 | 0.091489891 |
| SLCO2A1 | darkturquoi | 0.029738162 | 0.521479389 | 0.020954377 | 0.651511086 |
| SLCO5A1 | darkturquoi | 0.115702044 | 0.012347661 | 0.106484028 | 0.021362292 |
| SMPX | darkturquoi | 0.049306082 | 0.287643778 | 0.044558185 | 0.336649109 |
| SOX14 | darkturquoi | 0.190391893 | 3.45E-05 | 0.152113677 | 0.000975135 |
| SP5 | darkturquoi | 0.188832238 | 4.01E-05 | 0.151457218 | 0.001026336 |
| SP6 | darkturquoi | -0.006120024 | 0.895062088 | -0.027785446 | 0.549202872 |
| SP7 | darkturquoi | 0.062354081 | 0.17856675 | 0.055755201 | 0.229136309 |
| SSTR2 | darkturquoi | -0.072292272 | 0.118735243 | -0.084193599 | 0.069095977 |

| | | | | | |
|---|---|---|---|---|---|
| STRIP2 | darkturquoi | 0.157186179 | 0.000652175 | 0.132703527 | 0.004068806 |
| SYT6 | darkturquoi | -0.065101312 | 0.160148809 | -0.09177701 | 0.047458226 |
| TDGF1 | darkturquoi | 0.186448973 | 5.03E-05 | 0.168213619 | 0.000260778 |
| TMEM16 | darkturquoi | -0.145274794 | 0.001645517 | -0.147188382 | 0.001424538 |
| TMEM89 | darkturquoi | 0.073015342 | 0.115083412 | 0.066684131 | 0.150208289 |
| TNFRSF1 | darkturquoi | 0.008622686 | 0.852568739 | -0.018700334 | 0.686896836 |
| TPH1 | darkturquoi | 0.073825544 | 0.111096782 | 0.060331082 | 0.193101931 |
| TPST2 | darkturquoi | 0.177405893 | 0.00011615 | 0.169032298 | 0.000243057 |
| TRAF3IP | darkturquoi | 0.314633362 | 3.44E-12 | 0.273409029 | 1.88E-09 |
| TSPAN11 | darkturquoi | 0.186625215 | 4.95E-05 | 0.165851433 | 0.000318912 |
| TTC9 | darkturquoi | 0.366490891 | 2.72E-16 | 0.353113473 | 3.69E-15 |
| VSNL1 | darkturquoi | 0.088046484 | 0.057263348 | 0.076542747 | 0.098515345 |
| VWA2 | darkturquoi | 0.135154033 | 0.003430048 | 0.119914473 | 0.009492325 |
| WIF1 | darkturquoi | 0.127120335 | 0.005943183 | 0.111409694 | 0.016012335 |
| WNT16 | darkturquoi | -0.068897846 | 0.137101488 | -0.099557385 | 0.031473516 |
| WNT6 | darkturquoi | -0.173424094 | 0.000165712 | -0.184749165 | 5.91E-05 |
| XXbac-BI | darkturquoi | 0.037006823 | 0.424955043 | 0.01412612 | 0.760773563 |
| HIST1H1 | floralwhite | -0.055913432 | 0.227815601 | -0.053064265 | 0.2524322 |
| HIST1H1 | floralwhite | 0.000965851 | 0.983392215 | 0.004511858 | 0.92253479 |
| HIST1H1 | floralwhite | -0.084597012 | 0.067770031 | -0.087105226 | 0.05998668 |
| HIST1H1 | floralwhite | 0.056014925 | 0.226971321 | 0.036081837 | 0.436628701 |
| HIST1H1 | floralwhite | -0.118796517 | 0.010186332 | -0.122555861 | 0.008016873 |
| HIST1H2 | floralwhite | -0.046593716 | 0.315019153 | -0.039474289 | 0.394718118 |
| HIST1H2 | floralwhite | 0.057267259 | 0.21673627 | 0.058762305 | 0.204956381 |
| HIST1H2 | floralwhite | 0.053303605 | 0.250295873 | 0.043531471 | 0.347912037 |
| HIST1H2 | floralwhite | 0.121361711 | 0.008656485 | 0.130692188 | 0.004671478 |
| HIST1H2 | floralwhite | 0.088220167 | 0.056772208 | 0.096714616 | 0.036678985 |
| HIST1H2 | floralwhite | -0.021206641 | 0.647597286 | -0.021348469 | 0.645401108 |
| HIST1H2 | floralwhite | -0.038496814 | 0.406538014 | -0.04938848 | 0.286837986 |
| HIST1H2 | floralwhite | 0.008419736 | 0.856000542 | 0.029181285 | 0.529311832 |
| HIST1H2 | floralwhite | 0.02378054 | 0.608233201 | 0.026785003 | 0.563681994 |
| HIST1H2 | floralwhite | 0.075512945 | 0.103143158 | 0.09020872 | 0.051393899 |
| HIST1H2 | floralwhite | 0.049156969 | 0.289105861 | 0.062377603 | 0.178402654 |
| HIST1H2 | floralwhite | 0.047109226 | 0.309689024 | 0.066039271 | 0.154200029 |
| HIST1H2 | floralwhite | 0.099042111 | 0.032367231 | 0.097575752 | 0.035030013 |
| HIST1H2 | floralwhite | -0.017624391 | 0.704038123 | -0.038407162 | 0.407632551 |
| HIST1H2 | floralwhite | 0.048528139 | 0.295326492 | 0.041170923 | 0.374700418 |
| HIST1H2 | floralwhite | -0.019202636 | 0.678948421 | 0.01447658 | 0.755025705 |
| HIST1H2 | floralwhite | -0.095399388 | 0.039324476 | -0.086867636 | 0.060690767 |
| HIST1H2 | floralwhite | -0.038230171 | 0.409798508 | -0.041196524 | 0.374403242 |
| HIST1H2 | floralwhite | -0.079050065 | 0.087937479 | -0.068634069 | 0.138615457 |
| HIST1H2 | floralwhite | -0.027178679 | 0.557962594 | -0.026106851 | 0.573599835 |
| HIST1H2 | floralwhite | -0.016567594 | 0.721021629 | -0.029916985 | 0.518976915 |
| HIST1H2 | floralwhite | -0.064791177 | 0.162153333 | -0.054391246 | 0.240746372 |
| HIST1H2 | floralwhite | -0.099496117 | 0.031578665 | -0.098255755 | 0.033772859 |

| | | | | | |
|---|---|---|---|---|---|
| HIST1H3 | floralwhite | 0.041908172 | 0.366200539 | 0.05599426 | 0.227143039 |
| HIST1H3 | floralwhite | -0.056431806 | 0.223526776 | -0.063102062 | 0.173403246 |
| HIST1H3 | floralwhite | -0.082249795 | 0.075785798 | -0.06210237 | 0.180329763 |
| HIST1H3 | floralwhite | 0.099279708 | 0.031952473 | 0.107172982 | 0.020531184 |
| HIST1H3 | floralwhite | -0.108923463 | 0.018544795 | -0.093689994 | 0.043002573 |
| HIST1H3 | floralwhite | -0.106132493 | 0.021797502 | -0.10073425 | 0.02951112 |
| HIST1H3 | floralwhite | 0.081306111 | 0.079218592 | 0.084194517 | 0.069092938 |
| HIST1H3 | floralwhite | -0.094420386 | 0.04139697 | -0.09114701 | 0.049007776 |
| HIST1H3 | floralwhite | 0.01708735 | 0.712651014 | 0.020177215 | 0.663628467 |
| HIST1H4 | floralwhite | 0.011195669 | 0.809322894 | 0.029535113 | 0.524328358 |
| HIST1H4 | floralwhite | -0.010821107 | 0.815585745 | -0.012906111 | 0.780883734 |
| HIST1H4 | floralwhite | -0.088457921 | 0.056105556 | -0.091379061 | 0.048432168 |
| HIST1H4 | floralwhite | 0.004338902 | 0.925495526 | 0.019138327 | 0.679964078 |
| HIST1H4 | floralwhite | 0.03930042 | 0.396805311 | 0.034891584 | 0.451917654 |
| HIST1H4 | floralwhite | -0.0578703 | 0.211927599 | -0.050154373 | 0.279420829 |
| HIST1H4 | floralwhite | -0.073265228 | 0.113842054 | -0.065268772 | 0.159074239 |
| HIST1H4 | floralwhite | -0.072402497 | 0.118172779 | -0.05490829 | 0.236297579 |
| HIST1H4 | floralwhite | -0.063563718 | 0.170272309 | -0.054193847 | 0.242460266 |
| HIST1H4 | floralwhite | -0.076501441 | 0.098697734 | -0.07058057 | 0.127740864 |
| HIST2H2 | floralwhite | -0.041388878 | 0.372175014 | -0.039872102 | 0.389967597 |
| HIST2H2 | floralwhite | 0.065030307 | 0.160606082 | 0.082188543 | 0.076004876 |
| HIST2H2 | floralwhite | 0.188143945 | 4.29E-05 | 0.171500191 | 0.000196213 |
| HIST2H2 | floralwhite | 0.054018612 | 0.243988867 | 0.031334436 | 0.499361924 |
| HIST2H3 | floralwhite | -0.052533409 | 0.257215687 | -0.057823507 | 0.212297958 |
| HIST3H2 | floralwhite | -0.098567664 | 0.033209209 | -0.08768205 | 0.058305349 |
| OR2B6 | floralwhite | -0.131833374 | 0.004320331 | -0.123084032 | 0.007747682 |
| AAGAB | green | 0.264300048 | 6.64E-09 | 0.268790592 | 3.59E-09 |
| AARS2 | green | -0.156195744 | 0.000706138 | -0.18050069 | 8.76E-05 |
| ABHD3 | green | 0.091156678 | 0.048983679 | 0.107536104 | 0.020104558 |
| ABTB1 | green | -0.218515008 | 1.87E-06 | -0.22254808 | 1.19E-06 |
| AC00438 | green | 0.142670348 | 0.00199689 | 0.164202136 | 0.000366442 |
| ACAD9 | green | -0.076100614 | 0.100481583 | -0.068475036 | 0.139534424 |
| ACAT2 | green | 0.116475202 | 0.011772761 | 0.100427525 | 0.030012191 |
| ACOXL | green | -0.23310883 | 3.50E-07 | -0.24654667 | 6.77E-08 |
| ACP1 | green | 0.004548545 | 0.921906904 | 0.004732287 | 0.918762948 |
| ACTL6A | green | -0.144453599 | 0.001749652 | -0.163897089 | 0.000375926 |
| ADA | green | -0.272515423 | 2.14E-09 | -0.27181067 | 2.36E-09 |
| ADD2 | green | -0.321183624 | 1.15E-12 | -0.313456956 | 4.17E-12 |
| AGFG2 | green | -0.182102487 | 7.56E-05 | -0.150174968 | 0.001133567 |
| AK2 | green | -0.168400042 | 0.000256639 | -0.151358929 | 0.001034212 |
| ALG8 | green | -0.020788961 | 0.654082699 | -0.054617424 | 0.238793092 |
| ALYREF | green | 0.019878993 | 0.668301879 | 0.020123866 | 0.664463538 |
| ANAPC5 | green | 0.144947041 | 0.001686378 | 0.135789886 | 0.003279902 |
| ANKLE1 | green | -0.310087992 | 7.25E-12 | -0.315928381 | 2.77E-12 |
| ANKRD2 | green | 0.0790855 | 0.087794802 | 0.081784291 | 0.077463709 |

| | | | | | |
|---|---|---|---|---|---|
| ANKRD9 | green | 0.111349818 | 0.016069569 | 0.098742282 | 0.032897174 |
| ANLN | green | 0.061319817 | 0.185893169 | 0.049912759 | 0.281746525 |
| AP5B1 | green | 0.008103894 | 0.861346665 | 0.022947466 | 0.62085774 |
| APH1A | green | 0.002685778 | 0.953840462 | -0.009856729 | 0.831763753 |
| APLP1 | green | -0.192418574 | 2.84E-05 | -0.180645882 | 8.65E-05 |
| APOBEC | green | -0.027063623 | 0.559631239 | -0.023647069 | 0.610248157 |
| ARHGAP | green | 0.07819731 | 0.091427584 | 0.072010067 | 0.120184834 |
| ARHGAP | green | -0.030893773 | 0.505417574 | -0.052430026 | 0.2581545 |
| ARHGAP | green | -0.050243744 | 0.278563877 | -0.062362776 | 0.17850608 |
| ARHGEF | green | -0.073559111 | 0.112395606 | -0.074399732 | 0.108337861 |
| ARHGEF | green | 0.094509066 | 0.041205518 | 0.088863033 | 0.054984614 |
| ARID3B | green | 0.149588002 | 0.001186019 | 0.125808024 | 0.006483472 |
| ARL4C | green | -0.173544117 | 0.000163965 | -0.166841854 | 0.000293202 |
| ARL6IP1 | green | 0.021276195 | 0.646519873 | 0.019955481 | 0.667102018 |
| ARNTL2 | green | -0.157760023 | 0.000622685 | -0.138629368 | 0.002679601 |
| ASF1B | green | 0.15678597 | 0.000673503 | 0.172237014 | 0.000183958 |
| ASNA1 | green | 0.070429434 | 0.128560798 | 0.089650931 | 0.052857784 |
| ASNS | green | -0.011989675 | 0.796087427 | -0.022942693 | 0.620930392 |
| ASPM | green | 0.027741791 | 0.549830838 | 0.010458234 | 0.821664358 |
| ATAD2 | green | -0.079715103 | 0.085290655 | -0.113822249 | 0.013849989 |
| ATAD5 | green | -0.017763431 | 0.701814349 | -0.034371029 | 0.458697923 |
| ATAT1 | green | 0.077513945 | 0.09430379 | 0.06742392 | 0.145726223 |
| ATF5 | green | -0.139751484 | 0.002471345 | -0.11347093 | 0.014147953 |
| ATG4D | green | 0.041720857 | 0.368348689 | 0.061945276 | 0.181436586 |
| ATP1B3 | green | 0.073484231 | 0.112762776 | 0.081055985 | 0.080149292 |
| ATP5C1 | green | 0.080407392 | 0.082603962 | 0.085371573 | 0.065282668 |
| ATPAF1 | green | 0.033767531 | 0.466629195 | 0.023706984 | 0.609343276 |
| AUNIP | green | -0.026723616 | 0.564576368 | -0.028590496 | 0.537685959 |
| AURKA | green | -0.051148217 | 0.269991455 | -0.054077556 | 0.243473937 |
| AURKB | green | 0.131699184 | 0.004360346 | 0.146211555 | 0.001533686 |
| BAK1 | green | -0.176374036 | 0.000127449 | -0.160639791 | 0.000492488 |
| BARD1 | green | 0.066341506 | 0.152319275 | 0.038949242 | 0.401041167 |
| BCL2L12 | green | 0.024321894 | 0.600091075 | 0.031976444 | 0.490608575 |
| BCL6 | green | -0.206409963 | 6.89E-06 | -0.209963236 | 4.73E-06 |
| BEND3 | green | -0.038979318 | 0.400677342 | -0.05893665 | 0.203613479 |
| BIRC5 | green | 0.052379456 | 0.258614593 | 0.058758111 | 0.204988764 |
| BLM | green | 0.016980515 | 0.714368804 | 0.001537077 | 0.973572908 |
| BNIP3 | green | 0.273657016 | 1.82E-09 | 0.268521154 | 3.73E-09 |
| BOLA3 | green | 0.064717712 | 0.162630924 | 0.080438773 | 0.082483816 |
| BORA | green | -0.000524128 | 0.990987172 | -0.008516473 | 0.854364425 |
| BRCA1 | green | 0.21785369 | 2.01E-06 | 0.206280913 | 6.98E-06 |
| BRCA2 | green | -0.008854382 | 0.848654273 | -0.010525879 | 0.820530383 |
| BRI3BP | green | 0.29961114 | 3.85E-11 | 0.297983372 | 4.96E-11 |
| BRIP1 | green | 0.105300228 | 0.022858643 | 0.082582971 | 0.074603108 |
| BUB1 | green | -0.010702704 | 0.817567946 | -0.015480346 | 0.738638728 |

| | | | | | |
|---|---|---|---|---|---|
| BUB1B | green | 0.068266932 | 0.140744002 | 0.062079299 | 0.180492001 |
| BUB3 | green | 0.238421899 | 1.85E-07 | 0.233731191 | 3.25E-07 |
| BYSL | green | -0.094043861 | 0.042218241 | -0.097465379 | 0.035237775 |
| BZW2 | green | -0.05212474 | 0.26094061 | -0.058717422 | 0.205303128 |
| C10orf35 | green | 0.128123604 | 0.005557787 | 0.123738067 | 0.007425549 |
| C11orf84 | green | -0.017403746 | 0.707572249 | -0.025846225 | 0.577433238 |
| C12orf73 | green | -0.037174167 | 0.422862694 | -0.035155405 | 0.448503059 |
| C16orf59 | green | 0.125055009 | 0.006813008 | 0.136017627 | 0.003227594 |
| C16orf89 | green | -0.062375899 | 0.178414534 | -0.029570957 | 0.52382486 |
| C17orf53 | green | -0.008934791 | 0.847296629 | 0.000665728 | 0.988552382 |
| C17orf96 | green | -0.163962543 | 0.000373872 | -0.173316975 | 0.000167286 |
| C18orf54 | green | -0.101119393 | 0.028892116 | -0.135415423 | 0.003367586 |
| C19orf40 | green | -0.096101617 | 0.037892568 | -0.105829782 | 0.022178408 |
| C19orf52 | green | -0.062231539 | 0.179423447 | -0.03955976 | 0.39369453 |
| C19orf57 | green | -0.16057717 | 0.000495028 | -0.183998329 | 6.34E-05 |
| C1orf112 | green | -0.005983052 | 0.897397617 | -0.031461717 | 0.497619984 |
| C1orf115 | green | 0.19036319 | 3.46E-05 | 0.154783462 | 0.000790264 |
| C20orf24 | green | -0.149331569 | 0.001209628 | -0.15031436 | 0.001121429 |
| C2orf68 | green | -0.245896916 | 7.35E-08 | -0.249697124 | 4.54E-08 |
| C4orf46 | green | 0.068257609 | 0.140798373 | 0.040067347 | 0.38764878 |
| C5orf34 | green | -0.194876545 | 2.23E-05 | -0.196556326 | 1.89E-05 |
| C9orf40 | green | 0.089198812 | 0.054069681 | 0.082574944 | 0.074631422 |
| CABLES2 | green | -0.155140253 | 0.000768177 | -0.166519093 | 0.00030136 |
| CADM4 | green | 0.115396954 | 0.012581252 | 0.089972296 | 0.052010197 |
| CAMK2N | green | 0.20544346 | 7.62E-06 | 0.170275157 | 0.000218294 |
| CARS | green | 0.068307414 | 0.140508077 | 0.073121968 | 0.114552436 |
| CASC5 | green | 0.182611904 | 7.21E-05 | 0.174501492 | 0.000150635 |
| CBS | green | -0.190214049 | 3.51E-05 | -0.202949654 | 9.87E-06 |
| CBX2 | green | 0.029066427 | 0.530934688 | 0.017024345 | 0.713663887 |
| CBX5 | green | -0.027800799 | 0.548982099 | -0.053005683 | 0.252957021 |
| CCDC12( | green | -0.021262718 | 0.64672858 | 0.003090424 | 0.946895523 |
| CCDC142 | green | -0.13088092 | 0.004611683 | -0.164893114 | 0.000345778 |
| CCDC15( | green | -0.192087811 | 2.93E-05 | -0.19804751 | 1.62E-05 |
| CCDC18 | green | -0.050468368 | 0.276417903 | -0.070974913 | 0.12562058 |
| CCDC34 | green | 0.067534114 | 0.145067428 | 0.072143556 | 0.119497434 |
| CCDC77 | green | -0.181290801 | 8.15E-05 | -0.20340528 | 9.41E-06 |
| CCNA2 | green | 0.120075864 | 0.009395688 | 0.114750619 | 0.013089035 |
| CCNB1 | green | 0.125539043 | 0.006599506 | 0.134558985 | 0.00357618 |
| CCNB2 | green | 0.108346945 | 0.019179654 | 0.12140067 | 0.008634921 |
| CCNE1 | green | -0.190680556 | 3.36E-05 | -0.166837926 | 0.0002933 |
| CCNE2 | green | 0.078559389 | 0.089932367 | 0.061265588 | 0.186283338 |
| CCNF | green | 0.0724217 | 0.118075001 | 0.084960614 | 0.066592877 |
| CCT3 | green | 0.084636434 | 0.067641585 | 0.055493578 | 0.231331902 |
| CCT6A | green | 0.056653376 | 0.22171126 | 0.046543831 | 0.315538116 |
| CD3EAP | green | -0.058471649 | 0.20720943 | -0.078346745 | 0.09080809 |

| | | | | | |
|---|---|---|---|---|---|
| CD59 | green | -0.011946628 | 0.796803524 | 0.013374904 | 0.773138218 |
| CD81 | green | -0.107228941 | 0.020464931 | -0.114118557 | 0.01360299 |
| CDC123 | green | 0.060745854 | 0.190053336 | 0.041336532 | 0.372780569 |
| CDC20 | green | 0.059434148 | 0.199816589 | 0.072380631 | 0.118284194 |
| CDC25A | green | 0.039592429 | 0.393303711 | 0.032075991 | 0.489258718 |
| CDC25C | green | -0.001424949 | 0.975500108 | 0.002331841 | 0.959917961 |
| CDC45 | green | 0.127381728 | 0.005840525 | 0.12843663 | 0.005442228 |
| CDC6 | green | 0.222126382 | 1.25E-06 | 0.211445127 | 4.04E-06 |
| CDC7 | green | -0.021333061 | 0.645639553 | -0.046719658 | 0.313711458 |
| CDCA2 | green | 0.15235554 | 0.000956873 | 0.146571196 | 0.001492638 |
| CDCA3 | green | 0.065633618 | 0.156751915 | 0.071626262 | 0.122178435 |
| CDCA4 | green | 0.032145634 | 0.488315552 | 0.025330363 | 0.585056042 |
| CDCA5 | green | 0.015899412 | 0.73183165 | 0.017131739 | 0.711937718 |
| CDCA7 | green | 0.294894065 | 7.99E-11 | 0.26870969 | 3.63E-09 |
| CDCA8 | green | 0.046094621 | 0.320236477 | 0.051650248 | 0.265311953 |
| CDH24 | green | 0.14851906 | 0.001287321 | 0.124053045 | 0.007274727 |
| CDK1 | green | 0.12939416 | 0.005102039 | 0.120197462 | 0.009323457 |
| CDK16 | green | 0.004965921 | 0.91476714 | 0.034215633 | 0.460732933 |
| CDK2 | green | 0.001062664 | 0.981727788 | -0.039725752 | 0.391711206 |
| CDK5RA | green | -0.165493016 | 0.000328723 | -0.178635231 | 0.000103918 |
| CDKN2A | green | -0.365279225 | 3.46E-16 | -0.352999634 | 3.77E-15 |
| CDKN3 | green | 0.078075291 | 0.091935929 | 0.076737087 | 0.097660829 |
| CDR2L | green | -0.138094715 | 0.002784347 | -0.132438094 | 0.00414409 |
| CDT1 | green | 0.263618289 | 7.29E-09 | 0.285302252 | 3.39E-10 |
| CELSR3 | green | 0.020094238 | 0.664927486 | -0.001565439 | 0.973085454 |
| CENPA | green | -0.057374034 | 0.215879188 | -0.053699857 | 0.246786696 |
| CENPE | green | 0.00994015 | 0.830361408 | 0.001611425 | 0.972295137 |
| CENPF | green | 0.072416503 | 0.118101458 | 0.068089608 | 0.141780995 |
| CENPH | green | 0.134532277 | 0.003582869 | 0.128369088 | 0.005466978 |
| CENPI | green | -0.015847401 | 0.73267535 | -0.016144248 | 0.727864338 |
| CENPK | green | 0.137625384 | 0.002879352 | 0.126033308 | 0.006387699 |
| CENPL | green | 0.013924883 | 0.76408001 | 0.002915571 | 0.949896061 |
| CENPN | green | 0.162415371 | 0.000425335 | 0.173810386 | 0.00016015 |
| CENPO | green | -0.031798246 | 0.49302991 | -0.031381588 | 0.498716236 |
| CENPP | green | -0.059629201 | 0.198342104 | -0.062206837 | 0.179596507 |
| CENPU | green | 0.174194142 | 0.000154799 | 0.165944431 | 0.000316411 |
| CENPW | green | -0.063466783 | 0.170926192 | -0.052752141 | 0.255237177 |
| CEP55 | green | 0.121138671 | 0.008780868 | 0.11741113 | 0.011108584 |
| CEP72 | green | -0.152177925 | 0.000970253 | -0.157134249 | 0.000654907 |
| CEP85 | green | 0.098082705 | 0.034089079 | 0.065542976 | 0.157326466 |
| CEP89 | green | -0.122771107 | 0.007906178 | -0.125709697 | 0.006525675 |
| CERS1 | green | -0.00051338 | 0.99117199 | -0.023756095 | 0.608602022 |
| CH25H | green | -0.009861329 | 0.831686415 | -0.026693253 | 0.565018993 |
| CHAC2 | green | 0.059576659 | 0.198738509 | 0.0429863 | 0.353988451 |
| CHAF1A | green | 0.302332336 | 2.51E-11 | 0.306421938 | 1.31E-11 |

| | | | | | |
|---|---|---|---|---|---|
| CHAF1B | green | 0.188054633 | 4.32E-05 | 0.183591641 | 6.59E-05 |
| CHEK1 | green | 0.074290193 | 0.108859974 | 0.049713099 | 0.283678246 |
| CHEK2 | green | 0.00399613 | 0.93136624 | -0.016129556 | 0.728102206 |
| CHRAC1 | green | -0.171319147 | 0.000199339 | -0.211890505 | 3.85E-06 |
| CHRNA5 | green | 0.107424672 | 0.02023465 | 0.074181246 | 0.109381227 |
| CIAO1 | green | -0.236950867 | 2.21E-07 | -0.237287368 | 2.12E-07 |
| CILP2 | green | -0.216324742 | 2.38E-06 | -0.230791222 | 4.60E-07 |
| CIRBP | green | 0.145476403 | 0.001620835 | 0.13092528 | 0.004597729 |
| CIT | green | 0.033514218 | 0.469980736 | 0.027056515 | 0.559734408 |
| CKAP2 | green | 0.059791147 | 0.197123927 | 0.035143811 | 0.448652804 |
| CKAP2L | green | 0.015928071 | 0.731366877 | 0.002654228 | 0.954382106 |
| CKAP5 | green | 0.05512423 | 0.234456837 | 0.022124724 | 0.633435925 |
| CKS1B | green | -0.069328632 | 0.134656331 | -0.078057877 | 0.092008663 |
| CKS2 | green | 0.138330434 | 0.002737715 | 0.147733642 | 0.001366763 |
| CLCN2 | green | -0.164728157 | 0.00035061 | -0.171299412 | 0.000199682 |
| CLIC1 | green | 0.012085712 | 0.794490438 | 0.03274998 | 0.48017212 |
| CLPB | green | 0.16471119 | 0.00035111 | 0.183541968 | 6.62E-05 |
| CLPTM1l | green | -0.058688792 | 0.205524537 | -0.04348478 | 0.348429843 |
| CLSPN | green | 0.08617207 | 0.062791379 | 0.067278547 | 0.146598822 |
| CLU | green | -0.034718759 | 0.454162431 | -0.03855958 | 0.405772761 |
| CMC2 | green | 0.186163146 | 5.17E-05 | 0.208682608 | 5.42E-06 |
| CNOT10 | green | 0.0931019 | 0.044333205 | 0.062923726 | 0.174624129 |
| COLGAL | green | -0.033989612 | 0.463701782 | -0.050247206 | 0.278530712 |
| COPS3 | green | 0.235157888 | 2.74E-07 | 0.221443007 | 1.35E-06 |
| COPS7B | green | 0.113105177 | 0.014464135 | 0.098149731 | 0.033966303 |
| COQ2 | green | 0.186763587 | 4.89E-05 | 0.175155141 | 0.000142125 |
| COQ3 | green | 0.000700531 | 0.987953968 | 0.007811042 | 0.866309323 |
| CPSF3 | green | 0.023174168 | 0.617411011 | 0.027532337 | 0.552848706 |
| CPSF4 | green | 0.015004211 | 0.746397644 | 0.010545152 | 0.820207374 |
| CRY2 | green | -0.053756946 | 0.246283964 | -0.072665854 | 0.116837335 |
| CSE1L | green | -0.103075049 | 0.025918086 | -0.129015525 | 0.005234201 |
| CSNK2B- | green | -0.054286427 | 0.241655392 | -0.080117127 | 0.083722002 |
| CSPG5 | green | 0.048796115 | 0.29266473 | 0.03474974 | 0.453759575 |
| CSTF2 | green | 0.086052241 | 0.063159245 | 0.09858898 | 0.033170983 |
| CTPS1 | green | -0.136133699 | 0.003201226 | -0.167591131 | 0.000275052 |
| CUL1 | green | -0.108343752 | 0.019183223 | -0.113899447 | 0.013785261 |
| CWF19L1 | green | -0.025781933 | 0.578380728 | -0.040456703 | 0.383049714 |
| CX3CR1 | green | -0.130968729 | 0.004584099 | -0.136334843 | 0.003155996 |
| CYB5D2 | green | 0.19831497 | 1.58E-05 | 0.187447735 | 4.58E-05 |
| CYP27A1 | green | -0.086074757 | 0.063089987 | -0.080426696 | 0.082530038 |
| DARS2 | green | 0.145178186 | 0.001657465 | 0.114204067 | 0.013532435 |
| DAXX | green | -0.03416903 | 0.461344204 | -0.02457863 | 0.596246943 |
| DAZAP1 | green | 0.253112933 | 2.93E-08 | 0.252610313 | 3.12E-08 |
| DBF4 | green | -0.018710587 | 0.686734236 | -0.023300449 | 0.61549469 |
| DBF4B | green | -0.036800378 | 0.427544586 | -0.037939436 | 0.413371224 |

| | | | | | |
|---|---|---|---|---|---|
| DBN1 | green | -0.058595379 | 0.206248139 | -0.051384532 | 0.267781748 |
| DCAF15 | green | -0.133189106 | 0.003934278 | -0.136495197 | 0.003120356 |
| DCLRE1I | green | 0.067013714 | 0.148198714 | 0.036245101 | 0.434554965 |
| DCUN1D | green | -0.050450381 | 0.276589328 | -0.058065051 | 0.21039119 |
| DDIAS | green | -0.02772846 | 0.550022669 | -0.044877985 | 0.333189221 |
| DDX11 | green | 0.118180861 | 0.010587439 | 0.109231808 | 0.01821283 |
| DDX27 | green | -0.312060192 | 5.25E-12 | -0.318649276 | 1.76E-12 |
| DEPDC1 | green | -0.003176152 | 0.945424669 | -0.012483456 | 0.787885602 |
| DEPDC1I | green | 0.003907116 | 0.932891429 | -0.014113229 | 0.760985242 |
| DFFA | green | -0.047833151 | 0.302304806 | -0.05309059 | 0.252196611 |
| DGAT2 | green | -0.174390826 | 0.000152122 | -0.197236787 | 1.76E-05 |
| DHCR24 | green | 0.037825571 | 0.414775444 | 0.033548818 | 0.469522171 |
| DHCR7 | green | 0.177811913 | 0.000111968 | 0.165471368 | 0.000329325 |
| DHFR | green | 0.17993518 | 9.23E-05 | 0.157064364 | 0.0006586 |
| DHRS12 | green | -0.002486514 | 0.957261739 | -0.017148964 | 0.711660982 |
| DHX37 | green | 0.046254405 | 0.318560074 | 0.052429702 | 0.258157451 |
| DIAPH3 | green | 0.082377641 | 0.075330185 | 0.086364512 | 0.062204295 |
| DLGAP5 | green | 0.017794046 | 0.701325031 | 0.012835045 | 0.782059829 |
| DNA2 | green | 0.068657677 | 0.138479438 | 0.046840934 | 0.312455584 |
| DNAJB11 | green | -0.178281176 | 0.000107311 | -0.173959621 | 0.000158049 |
| DNAJC19 | green | -0.250615418 | 4.04E-08 | -0.239738192 | 1.58E-07 |
| DNAJC9 | green | 0.079147072 | 0.087547331 | 0.049789735 | 0.282935733 |
| DNASE2 | green | 0.119920512 | 0.009488693 | 0.121427459 | 0.008620121 |
| DNMT1 | green | 0.106664299 | 0.021142052 | 0.098923882 | 0.032575322 |
| DNMT3A | green | -0.157364133 | 0.000642895 | -0.152831055 | 0.000921886 |
| DNMT3B | green | 0.036130583 | 0.436008948 | 0.015644379 | 0.735971803 |
| DONSON | green | 0.108290734 | 0.019242555 | 0.083241567 | 0.072309481 |
| DPF1 | green | -0.068715172 | 0.138148602 | -0.070270642 | 0.129426653 |
| DPH2 | green | -0.116688869 | 0.011618116 | -0.117609817 | 0.010971933 |
| DRG1 | green | 0.02379742 | 0.607978582 | 0.015083284 | 0.745107304 |
| DROSHA | green | -0.222508072 | 1.19E-06 | -0.258897676 | 1.37E-08 |
| DSCC1 | green | -0.009567019 | 0.836638062 | -0.040340905 | 0.384414024 |
| DSN1 | green | -0.066755978 | 0.149768454 | -0.080034212 | 0.084043607 |
| DTD1 | green | -0.023239407 | 0.616420678 | -0.020712576 | 0.65527158 |
| DTL | green | 0.224068348 | 1.00E-06 | 0.212246421 | 3.71E-06 |
| DTYMK | green | 0.20059447 | 1.26E-05 | 0.205677487 | 7.44E-06 |
| DUSP9 | green | -0.25549046 | 2.15E-08 | -0.26135468 | 9.88E-09 |
| E2F1 | green | -0.056714776 | 0.221210028 | -0.049265729 | 0.28803896 |
| E2F2 | green | 0.106868936 | 0.020894436 | 0.10192811 | 0.027628519 |
| E2F3 | green | -0.09362479 | 0.043148427 | -0.106049977 | 0.021900766 |
| E2F7 | green | 0.269022733 | 3.48E-09 | 0.259074183 | 1.34E-08 |
| E2F8 | green | 0.073799714 | 0.111222182 | 0.076185428 | 0.100102001 |
| EBP | green | 0.070837957 | 0.126353838 | 0.081998715 | 0.076687107 |
| ECT2 | green | 0.007789977 | 0.866666504 | -0.005374732 | 0.90778054 |
| EIF1AD | green | -0.05989498 | 0.196345762 | -0.054612978 | 0.238831375 |

| | | | | | |
|---|---|---|---|---|---|
| EIF2AK1 | green | 0.13869784 | 0.002666449 | 0.140040073 | 0.002420227 |
| EIF2S2 | green | -0.094220381 | 0.041831523 | -0.097849806 | 0.034518644 |
| ELAVL1 | green | 0.221846965 | 1.29E-06 | 0.215582387 | 2.58E-06 |
| ELOVL6 | green | 0.132080318 | 0.004247559 | 0.126027489 | 0.006390157 |
| EMC1 | green | -0.005202529 | 0.910722658 | -0.026389503 | 0.569456097 |
| EME1 | green | 0.01798444 | 0.698284811 | 0.01453458 | 0.754075751 |
| ENTPD2 | green | 0.305094477 | 1.62E-11 | 0.298741051 | 4.41E-11 |
| EPHX2 | green | -0.042499236 | 0.359473387 | -0.054801696 | 0.237209971 |
| EPT1 | green | 0.008761033 | 0.850230957 | -0.008294621 | 0.858117544 |
| ERAL1 | green | 0.059629893 | 0.198336889 | 0.051808437 | 0.263849052 |
| ERCC6L | green | -0.008768933 | 0.850097494 | -0.00627587 | 0.892405863 |
| ERH | green | 0.142222692 | 0.002063781 | 0.142596318 | 0.002007813 |
| ESCO2 | green | 0.223652535 | 1.05E-06 | 0.200231753 | 1.30E-05 |
| ESM1 | green | 0.087461194 | 0.058944432 | 0.073389474 | 0.11322876 |
| ESPL1 | green | -0.041421294 | 0.371800313 | -0.06349697 | 0.170722361 |
| EVA1C | green | -0.251405674 | 3.65E-08 | -0.231222809 | 4.37E-07 |
| EWSR1 | green | 0.093963219 | 0.042395912 | 0.078377421 | 0.090681337 |
| EXO1 | green | 0.127528617 | 0.005783538 | 0.121350524 | 0.008662687 |
| EXOSC1( | green | -0.096804565 | 0.036503716 | -0.114130783 | 0.013592883 |
| EXOSC2 | green | 0.007557877 | 0.870603698 | -0.01948606 | 0.674479111 |
| EXOSC3 | green | -0.075976336 | 0.101039845 | -0.082043087 | 0.076527193 |
| EZH2 | green | 0.046521357 | 0.315772098 | 0.03399446 | 0.46363799 |
| FABP5 | green | 0.050374871 | 0.277309763 | 0.029685632 | 0.522215669 |
| FADS1 | green | 0.124521284 | 0.007055582 | 0.090108969 | 0.051653176 |
| FADS2 | green | 0.36763789 | 2.16E-16 | 0.351311585 | 5.19E-15 |
| FAF1 | green | -0.010681573 | 0.817921841 | -0.004821291 | 0.91724049 |
| FAM111E | green | 0.185698065 | 5.41E-05 | 0.158198113 | 0.000601009 |
| FAM132E | green | -0.050720751 | 0.274020158 | -0.023976053 | 0.605286971 |
| FAM136A | green | 0.086809337 | 0.060864571 | 0.099754781 | 0.031136777 |
| FAM32A | green | -0.151439066 | 0.001027786 | -0.136192682 | 0.003187902 |
| FAM63A | green | 0.030809517 | 0.506579804 | 0.005976027 | 0.897517414 |
| FAM64A | green | 0.066618399 | 0.150611545 | 0.055470385 | 0.231527256 |
| FAM72A | green | -0.065011971 | 0.16072433 | -0.075667602 | 0.102437353 |
| FAM72B | green | -0.113791671 | 0.013875702 | -0.132909053 | 0.004011366 |
| FAM72C | green | -0.037972863 | 0.412959513 | -0.012137502 | 0.793629587 |
| FAM72D | green | -0.075024272 | 0.105398663 | -0.073547172 | 0.112454086 |
| FAM83D | green | 0.004440327 | 0.92375916 | 0.004871314 | 0.916384941 |
| FANCA | green | 0.182697254 | 7.16E-05 | 0.175026879 | 0.000143758 |
| FANCB | green | -0.057917919 | 0.211551185 | -0.062379893 | 0.178386682 |
| FANCC | green | 0.219813478 | 1.62E-06 | 0.198769939 | 1.51E-05 |
| FANCD2 | green | 0.070853404 | 0.126270969 | 0.055731479 | 0.22933478 |
| FANCE | green | -0.116869728 | 0.011488623 | -0.140717373 | 0.002304023 |
| FANCG | green | 0.091821766 | 0.047349724 | 0.079314252 | 0.086878229 |
| FANCI | green | 0.180983014 | 8.39E-05 | 0.166553864 | 0.000300471 |
| FARSA | green | -0.00027305 | 0.99530459 | 0.00596256 | 0.897747103 |

| | | | | | |
|---|---|---|---|---|---|
| FARSB | green | 0.08141424 | 0.078818972 | 0.059553538 | 0.19891313 |
| FASN | green | 0.15344598 | 0.000878394 | 0.134159075 | 0.003677537 |
| FASTKD | green | -0.045255223 | 0.329137439 | -0.058549568 | 0.206603677 |
| FBN2 | green | 0.004786833 | 0.917829883 | 0.008158266 | 0.860425875 |
| FBXL19 | green | -0.045089749 | 0.330910807 | -0.054682525 | 0.238232949 |
| FBXO31 | green | 0.079545045 | 0.08596129 | 0.086799629 | 0.060893552 |
| FBXO41 | green | -0.13568278 | 0.003304768 | -0.134373684 | 0.003622825 |
| FBXO43 | green | -0.001129956 | 0.980570944 | -0.017771129 | 0.701691305 |
| FBXO45 | green | -0.073463002 | 0.112867044 | -0.080185669 | 0.083456897 |
| FBXO5 | green | -0.060997985 | 0.188217514 | -0.077527442 | 0.094246293 |
| FEN1 | green | 0.170974974 | 0.00020541 | 0.17509532 | 0.000142885 |
| FIGNL1 | green | -0.078296187 | 0.091017305 | -0.104838741 | 0.023466099 |
| FKBP4 | green | -0.098633549 | 0.033091181 | -0.097348944 | 0.035458083 |
| FOXK2 | green | -0.005599537 | 0.903941598 | -0.038999954 | 0.400427818 |
| FOXM1 | green | 0.098780977 | 0.032828367 | 0.095128189 | 0.039889604 |
| FOXRED | green | 0.162273249 | 0.000430379 | 0.141701768 | 0.002144196 |
| FTSJ1 | green | 0.065912959 | 0.154991238 | 0.074787633 | 0.106504822 |
| FUS | green | -0.082564102 | 0.074669683 | -0.095937601 | 0.038222995 |
| GADD45 | green | 0.08624239 | 0.062576324 | 0.069555288 | 0.133383387 |
| GADD45 | green | 0.090244449 | 0.051301295 | 0.08842912 | 0.056185966 |
| GARS | green | 0.02539201 | 0.584142652 | 0.032459218 | 0.484080831 |
| GAS2L3 | green | -0.012879758 | 0.781319809 | -0.02412519 | 0.603043875 |
| GATAD2 | green | 0.027358925 | 0.555353377 | 0.022244392 | 0.631599722 |
| GATB | green | 0.003191171 | 0.945167003 | -0.005484966 | 0.905897819 |
| GATC | green | -0.038518904 | 0.406268584 | -0.03764531 | 0.417004236 |
| GCDH | green | -0.096265069 | 0.037565694 | -0.091360039 | 0.048479138 |
| GEMIN2 | green | -0.05485422 | 0.236760083 | -0.079382033 | 0.08660812 |
| GEN1 | green | -0.046442169 | 0.316597449 | -0.065393568 | 0.158276981 |
| GGH | green | 0.0742515 | 0.109044874 | 0.058701009 | 0.205430042 |
| GID8 | green | -0.195028062 | 2.19E-05 | -0.194836923 | 2.24E-05 |
| GINS1 | green | 0.038936713 | 0.401192793 | 0.037189119 | 0.422676035 |
| GINS2 | green | 0.262411174 | 8.58E-09 | 0.26689001 | 4.67E-09 |
| GINS3 | green | 0.289783829 | 1.74E-10 | 0.269009158 | 3.48E-09 |
| GINS4 | green | -0.015490776 | 0.738469064 | -0.040023464 | 0.388169229 |
| GLA | green | 0.109706333 | 0.017712087 | 0.102716236 | 0.026443099 |
| GLDC | green | -0.219415857 | 1.69E-06 | -0.225237155 | 8.76E-07 |
| GMEB2 | green | -0.128488921 | 0.005423136 | -0.123344026 | 0.007618161 |
| GMNN | green | -0.002971396 | 0.948938015 | -0.000345134 | 0.994065051 |
| GMPS | green | -0.075221996 | 0.1044814 | -0.109860841 | 0.017551659 |
| GNPDA1 | green | 0.138784077 | 0.002649969 | 0.120005408 | 0.009437767 |
| GPC2 | green | -0.092680504 | 0.045307822 | -0.114588365 | 0.01321931 |
| GPER1 | green | 0.017792933 | 0.70134283 | -0.003113643 | 0.94649713 |
| GPR19 | green | -0.324246961 | 6.81E-13 | -0.311732561 | 5.54E-12 |
| GPRIN1 | green | 0.090213011 | 0.051382769 | 0.075508589 | 0.103163094 |
| GPSM2 | green | 0.013825716 | 0.765710953 | -0.00088995 | 0.984697158 |

| | | | | | |
|---|---|---|---|---|---|
| GRK6 | green | 0.002922983 | 0.949768848 | 0.010593307 | 0.819400429 |
| GSG2 | green | 0.140440979 | 0.002350814 | 0.137568502 | 0.002891065 |
| GTF2IRD | green | -0.055179373 | 0.233988404 | -0.073984231 | 0.110328836 |
| GTF3C2 | green | -0.029718273 | 0.5217581 | -0.046215795 | 0.318964632 |
| GTPBP2 | green | -0.025034617 | 0.589447145 | -0.029884946 | 0.519424821 |
| GTPBP3 | green | -0.131200293 | 0.004512069 | -0.149092093 | 0.001232066 |
| GTPBP4 | green | -0.047272766 | 0.308010578 | -0.085220047 | 0.065763274 |
| GTSE1 | green | 0.151830852 | 0.000996898 | 0.14491968 | 0.00168983 |
| H2AFV | green | 0.019490652 | 0.674406794 | 0.025345371 | 0.584833615 |
| H2AFX | green | 0.217917829 | 2.00E-06 | 0.226599684 | 7.49E-07 |
| H2AFY | green | 0.177866687 | 0.000111415 | 0.168982002 | 0.000244112 |
| H2AFZ | green | 0.252580522 | 3.14E-08 | 0.258236378 | 1.50E-08 |
| HAPLN1 | green | 0.104700734 | 0.02365045 | 0.108413038 | 0.019105925 |
| HAUS5 | green | -0.132533711 | 0.004116827 | -0.163092113 | 0.000402063 |
| HAUS8 | green | -0.049112947 | 0.289538457 | -0.047332438 | 0.307399648 |
| HDAC1 | green | 0.043870815 | 0.344163333 | 0.028204271 | 0.543196118 |
| HDAC8 | green | 0.134979445 | 0.003472352 | 0.116211862 | 0.011965859 |
| HDGF | green | -0.042961821 | 0.354262845 | -0.053849221 | 0.245472901 |
| HELLS | green | 0.157172848 | 0.000652876 | 0.134458714 | 0.003601353 |
| HENMT1 | green | -0.45529767 | 2.82E-25 | -0.435281585 | 5.18E-23 |
| HINFP | green | -0.003339873 | 0.942616194 | -0.030026836 | 0.51744272 |
| HJURP | green | 0.025984347 | 0.575400184 | 0.016410697 | 0.723555053 |
| HMCES | green | 0.02498232 | 0.5902252 | 0.02860786 | 0.537438885 |
| HMGA1 | green | -0.111063129 | 0.016346097 | -0.093085105 | 0.044371711 |
| HMGB2 | green | -0.021595847 | 0.641577887 | -0.038565392 | 0.405701944 |
| HMGB3 | green | 0.118208549 | 0.010569104 | 0.12637901 | 0.006243201 |
| HMMR | green | 0.046573401 | 0.315230427 | 0.030775608 | 0.507047943 |
| HN1 | green | 0.177394757 | 0.000116267 | 0.19289633 | 2.71E-05 |
| HNRNPA | green | 0.137088734 | 0.002991594 | 0.143350861 | 0.001898996 |
| HPDL | green | -0.389750051 | 2.17E-18 | -0.387444148 | 3.56E-18 |
| HPRT1 | green | 0.097251245 | 0.035643843 | 0.0946264 | 0.040953353 |
| HSF2BP | green | -0.226327403 | 7.73E-07 | -0.251382711 | 3.66E-08 |
| HSPA14 | green | 0.026558547 | 0.566984691 | 0.004478939 | 0.923098236 |
| HSPA9 | green | 0.066428448 | 0.151781485 | 0.058612441 | 0.206115838 |
| HSPD1 | green | -0.025433017 | 0.583535439 | -0.040484863 | 0.382718382 |
| HTT | green | 0.112307995 | 0.015174873 | 0.11599011 | 0.012130628 |
| IFRD2 | green | 0.090798129 | 0.049883944 | 0.086136722 | 0.06289971 |
| IGFBP4 | green | -0.056643325 | 0.221793388 | -0.050143833 | 0.279522009 |
| ILF2 | green | 0.113796168 | 0.013871918 | 0.088290681 | 0.056573808 |
| ILF3 | green | -0.034741482 | 0.453866935 | -0.068155162 | 0.141396956 |
| ILKAP | green | -0.077386327 | 0.094848851 | -0.065930144 | 0.154883408 |
| IMPA2 | green | 0.059086686 | 0.202462933 | 0.070049301 | 0.130641092 |
| INA | green | -0.118035863 | 0.01068392 | -0.114975578 | 0.012910286 |
| INCENP | green | 0.05036548 | 0.277399452 | 0.039525819 | 0.394100808 |
| INTS8 | green | 0.02084815 | 0.653162056 | 0.005434184 | 0.906765081 |

| | | | | | |
|---|---|---|---|---|---|
| IQGAP3 | green | 0.094544872 | 0.041128429 | 0.082996329 | 0.073156729 |
| IRAK1 | green | -0.048908108 | 0.291557085 | -0.042454139 | 0.359983908 |
| KCNC4 | green | -0.149983469 | 0.00115044 | -0.158605589 | 0.000581479 |
| KCNK6 | green | 0.153954604 | 0.000843861 | 0.150647172 | 0.001092931 |
| KCTD1 | green | -0.140858893 | 0.002280395 | -0.15650498 | 0.000688861 |
| KDM1A | green | 0.067605604 | 0.144641245 | 0.05646177 | 0.223280637 |
| KIAA010 | green | 0.297038218 | 5.74E-11 | 0.305626662 | 1.49E-11 |
| KIAA152 | green | -0.091466724 | 0.048216197 | -0.107346357 | 0.020326517 |
| KIAA168 | green | -0.194469744 | 2.32E-05 | -0.195555087 | 2.08E-05 |
| KIF11 | green | 0.130255887 | 0.004812397 | 0.120457274 | 0.009170778 |
| KIF14 | green | 0.084480818 | 0.068149784 | 0.072833093 | 0.115995445 |
| KIF15 | green | 0.050068426 | 0.28024663 | 0.031546332 | 0.496463759 |
| KIF18A | green | -0.014308851 | 0.757774947 | -0.010441297 | 0.821948336 |
| KIF18B | green | 0.068294481 | 0.140583415 | 0.071140494 | 0.124738485 |
| KIF1A | green | -0.230444368 | 4.79E-07 | -0.253560855 | 2.76E-08 |
| KIF20A | green | 0.070940148 | 0.125806397 | 0.075967249 | 0.101080759 |
| KIF20B | green | 0.048292778 | 0.29767759 | 0.023398887 | 0.6140027 |
| KIF22 | green | -0.013625754 | 0.769002796 | 0.000490466 | 0.991565992 |
| KIF23 | green | 0.008022111 | 0.862732015 | -0.008251845 | 0.858841552 |
| KIF2C | green | -0.028217326 | 0.5430094 | -0.023967464 | 0.605416266 |
| KIF4A | green | 0.049297673 | 0.287726099 | 0.044896951 | 0.332984753 |
| KIF4B | green | 0.072884731 | 0.115736462 | 0.075026339 | 0.105389045 |
| KIFC1 | green | 0.028624088 | 0.537208034 | 0.038004183 | 0.412573979 |
| KLC3 | green | -0.099533886 | 0.03151381 | -0.111441406 | 0.015982095 |
| KLHL14 | green | -0.079380548 | 0.086614031 | -0.064878012 | 0.161590192 |
| KLRG2 | green | -0.256399505 | 1.91E-08 | -0.262094232 | 8.95E-09 |
| KNOP1 | green | -0.02088007 | 0.652665776 | -0.025769057 | 0.578570571 |
| KNSTRN | green | 0.058400925 | 0.207760351 | 0.05138205 | 0.267804893 |
| KNTC1 | green | 0.009320536 | 0.840790014 | -0.015627603 | 0.736244405 |
| KPNA2 | green | 0.106686377 | 0.021115215 | 0.103054058 | 0.02594855 |
| KRI1 | green | -0.13431656 | 0.003637316 | -0.146698278 | 0.001478376 |
| KXD1 | green | 0.121569853 | 0.00854183 | 0.120938767 | 0.008893697 |
| L2HGDH | green | 0.066544517 | 0.151065777 | 0.044168078 | 0.340900698 |
| LAPTM4 | green | -0.030013125 | 0.517634086 | -0.034445586 | 0.457723342 |
| LDHA | green | 0.306551089 | 1.28E-11 | 0.312982769 | 4.51E-12 |
| LEMD2 | green | -0.248757012 | 5.12E-08 | -0.24287073 | 1.07E-07 |
| LETM1 | green | 0.151321189 | 0.001037251 | 0.165601772 | 0.000325717 |
| LIG1 | green | 0.114219069 | 0.01352009 | 0.089723002 | 0.052666704 |
| LIMK1 | green | -0.070617157 | 0.127542983 | -0.064252208 | 0.165681722 |
| LIPG | green | 0.070213442 | 0.129739655 | 0.051495136 | 0.266751793 |
| LMNB1 | green | 0.165289127 | 0.00033443 | 0.156808617 | 0.000672279 |
| LMNB2 | green | 0.217901859 | 2.00E-06 | 0.210066813 | 4.68E-06 |
| LPCAT1 | green | 0.037049968 | 0.424415018 | 0.029893419 | 0.51930635 |
| LRP8 | green | -0.167819634 | 0.00026973 | -0.177117815 | 0.000119206 |
| LRR1 | green | 0.054703406 | 0.238053479 | 0.051489185 | 0.266807139 |

| | | | | | |
|---|---|---|---|---|---|
| LRRC1 | green | 0.020648907 | 0.656263208 | 0.032689977 | 0.480977328 |
| LRRC14E | green | -0.174059342 | 0.000156659 | -0.188061109 | 4.32E-05 |
| LRRC20 | green | 0.286543354 | 2.82E-10 | 0.278887085 | 8.64E-10 |
| LRRC29 | green | 0.06830279 | 0.14053501 | 0.096362031 | 0.037372917 |
| LRRC8D | green | -0.126845172 | 0.006052997 | -0.130668717 | 0.004678963 |
| LSG1 | green | -0.248068576 | 5.59E-08 | -0.238277271 | 1.88E-07 |
| LSM2 | green | 0.033545456 | 0.469566716 | 0.049884282 | 0.282021501 |
| LSM4 | green | 0.085206874 | 0.065805193 | 0.099291441 | 0.031932111 |
| LSM8 | green | -0.084960084 | 0.066594582 | -0.08750739 | 0.058810279 |
| LSS | green | 0.081111407 | 0.079942311 | 0.064963087 | 0.161039891 |
| LYNX1 | green | -0.09618135 | 0.037732817 | -0.076205349 | 0.100013012 |
| LYRM9 | green | 0.023254793 | 0.61618722 | 0.003501847 | 0.939838397 |
| MAD2L1 | green | 0.057554229 | 0.214438291 | 0.059897715 | 0.196325297 |
| MAD2L2 | green | 0.010335567 | 0.823721624 | -0.00696236 | 0.880720067 |
| MAGEF1 | green | -0.236130193 | 2.44E-07 | -0.218569717 | 1.86E-06 |
| MAGOH | green | -0.119307879 | 0.009863486 | -0.124120967 | 0.007242566 |
| MAP4K2 | green | 0.012003246 | 0.795861697 | 0.001335081 | 0.977044809 |
| MAP6D1 | green | -0.171299733 | 0.000199677 | -0.162950804 | 0.000406822 |
| MAP7D2 | green | -0.212607168 | 3.56E-06 | -0.222322222 | 1.22E-06 |
| MARS | green | 0.046829582 | 0.312572997 | 0.042120337 | 0.36377685 |
| MAST1 | green | -0.16559483 | 0.000325908 | -0.17890958 | 0.000101359 |
| MASTL | green | -0.025571578 | 0.581485852 | -0.047327063 | 0.307454644 |
| MCM10 | green | 0.021434615 | 0.644068659 | 0.005954326 | 0.897887534 |
| MCM2 | green | 0.008740598 | 0.850576177 | 0.005254164 | 0.909840345 |
| MCM3 | green | -0.055545672 | 0.23089353 | -0.063313399 | 0.171964684 |
| MCM4 | green | 0.071909274 | 0.12070591 | 0.057766308 | 0.212751306 |
| MCM5 | green | 0.100028758 | 0.030674509 | 0.10554882 | 0.022537098 |
| MCM6 | green | 0.044987202 | 0.332012876 | 0.028217814 | 0.543002428 |
| MCM7 | green | -0.007835669 | 0.865891798 | -0.014500249 | 0.754638007 |
| MCM8 | green | -0.165185622 | 0.000337362 | -0.180809228 | 8.52E-05 |
| MCUR1 | green | -0.195372327 | 2.12E-05 | -0.206972994 | 6.49E-06 |
| MDC1 | green | -0.096420965 | 0.037256159 | -0.110893065 | 0.016512095 |
| MED26 | green | -0.1597997 | 0.000527583 | -0.174649819 | 0.000148663 |
| MED8 | green | -0.082953003 | 0.073307251 | -0.06072779 | 0.190185365 |
| MELK | green | 0.114447309 | 0.013333492 | 0.115675777 | 0.01236762 |
| MEST | green | -0.126473693 | 0.006204139 | -0.124220734 | 0.007195555 |
| METTL1' | green | -0.065322662 | 0.158729594 | -0.079743168 | 0.085180386 |
| METTL2I | green | 0.146980688 | 0.00144713 | 0.148699915 | 0.001269641 |
| MEX3A | green | 0.077580888 | 0.09401888 | 0.04545009 | 0.327056969 |
| MEX3D | green | 0.318593927 | 1.78E-12 | 0.326642964 | 4.51E-13 |
| MGLL | green | 0.057356772 | 0.216017586 | 0.026219515 | 0.571946446 |
| MGME1 | green | -0.124319503 | 0.007149285 | -0.130552184 | 0.004716286 |
| MICB | green | 0.05455966 | 0.239290877 | 0.057716362 | 0.213147737 |
| MIS18A | green | -0.02430031 | 0.600414759 | -0.034255882 | 0.46020536 |
| MKI67 | green | 0.150405171 | 0.001113586 | 0.145112526 | 0.001665632 |

| | | | | | |
|---|---|---|---|---|---|
| MMACH( | green | 0.080256088 | 0.083185242 | 0.081318344 | 0.0791733 |
| MMS22L | green | -0.069454787 | 0.133946671 | -0.081917625 | 0.07698005 |
| MND1 | green | 0.195493438 | 2.10E-05 | 0.194843151 | 2.24E-05 |
| MORC2 | green | -0.022118658 | 0.633529055 | -0.06842721 | 0.139811702 |
| MRGBP | green | -0.232090521 | 3.95E-07 | -0.238018432 | 1.94E-07 |
| MRPL11 | green | -0.127668481 | 0.005729742 | -0.128847005 | 0.005294007 |
| MRPL15 | green | -0.083485632 | 0.071474255 | -0.103492412 | 0.025318727 |
| MRPL3 | green | -0.098888274 | 0.032638217 | -0.108011839 | 0.019557308 |
| MRPL37 | green | 0.066595819 | 0.150750259 | 0.07944481 | 0.086358561 |
| MRPL47 | green | -0.144029905 | 0.001805711 | -0.129863861 | 0.004942274 |
| MRPL48 | green | 0.076416247 | 0.099074758 | 0.041500709 | 0.37088333 |
| MRPS16 | green | 0.073290723 | 0.113715993 | 0.069279136 | 0.134935549 |
| MRPS7 | green | 0.033902869 | 0.464843989 | 0.0383825 | 0.407933948 |
| MRTO4 | green | -0.034932966 | 0.451381089 | -0.031732324 | 0.493927272 |
| MSH2 | green | -0.055571011 | 0.230680517 | -0.075764526 | 0.101996976 |
| MSI1 | green | -0.041169793 | 0.374713536 | -0.049788061 | 0.282951945 |
| MTA2 | green | 0.255175548 | 2.24E-08 | 0.234727922 | 2.89E-07 |
| MTBP | green | -0.193358796 | 2.59E-05 | -0.240575501 | 1.42E-07 |
| MTCH2 | green | 0.243732392 | 9.63E-08 | 0.25359141 | 2.75E-08 |
| MTFR2 | green | -0.033139469 | 0.47496319 | -0.02279229 | 0.62322179 |
| MTHFD1 | green | 0.137891875 | 0.002825051 | 0.113403179 | 0.014206059 |
| MTHFD1 | green | -0.107598226 | 0.02003235 | -0.098298604 | 0.033694946 |
| MTHFD2 | green | 0.013820074 | 0.765803779 | -0.001784083 | 0.969328039 |
| MTIF2 | green | -0.084638899 | 0.067633557 | -0.084370783 | 0.068511013 |
| MYBL1 | green | -0.060921742 | 0.188771272 | -0.087940985 | 0.057563393 |
| MYBL2 | green | 0.078756904 | 0.089125025 | 0.094070431 | 0.04215984 |
| MYCL | green | -0.219938618 | 1.59E-06 | -0.222841367 | 1.15E-06 |
| MYO19 | green | 0.218590458 | 1.85E-06 | 0.195698315 | 2.05E-05 |
| MYO9B | green | -0.098932758 | 0.032559659 | -0.083209693 | 0.072419145 |
| NAA40 | green | 0.041948587 | 0.365738083 | 0.024392629 | 0.599030839 |
| NANP | green | 0.033465811 | 0.470622697 | 0.022847143 | 0.622385679 |
| NAP1L4 | green | 0.202042607 | 1.08E-05 | 0.197866087 | 1.65E-05 |
| NASP | green | -0.068881213 | 0.137196577 | -0.086690691 | 0.061219556 |
| NAT10 | green | 0.018603731 | 0.688429471 | -0.014685069 | 0.751612687 |
| NCAPD2 | green | -0.02260849 | 0.626026949 | -0.040762796 | 0.379457636 |
| NCAPD3 | green | 0.181349009 | 8.11E-05 | 0.156319209 | 0.000699193 |
| NCAPG | green | 0.104708399 | 0.023640179 | 0.097546165 | 0.035085602 |
| NCAPG2 | green | 0.027936307 | 0.547035478 | 0.008493099 | 0.854759685 |
| NCAPH | green | 0.050175696 | 0.279216206 | 0.046888699 | 0.31196186 |
| NCBP2 | green | -0.223104937 | 1.12E-06 | -0.232254893 | 3.87E-07 |
| NDC1 | green | -0.01220893 | 0.792442713 | -0.039374914 | 0.395910245 |
| NDC80 | green | -0.119737467 | 0.009599327 | -0.123124537 | 0.007727376 |
| NDOR1 | green | 0.064012383 | 0.16727013 | 0.060396075 | 0.192621881 |
| NDRG2 | green | -0.032759338 | 0.480046603 | -0.045073726 | 0.33108285 |
| NDUFAF | green | -0.047709726 | 0.303555439 | -0.042650836 | 0.357760507 |

| | | | | | |
|---|---|---|---|---|---|
| NDUFB5 | green | -0.173156719 | 0.000169667 | -0.16455415 | 0.000355775 |
| NEIL3 | green | -0.005454326 | 0.906421075 | -0.016715149 | 0.718641827 |
| NEK2 | green | 0.181365383 | 8.09E-05 | 0.182629001 | 7.20E-05 |
| NELFCD | green | -0.085947517 | 0.063482189 | -0.095537422 | 0.039039453 |
| NETO2 | green | 0.021268246 | 0.646642971 | 0.037960442 | 0.413112465 |
| NFIC | green | 0.235710623 | 2.57E-07 | 0.247791523 | 5.79E-08 |
| NHSL1 | green | 0.198697439 | 1.52E-05 | 0.195880351 | 2.02E-05 |
| NIF3L1 | green | -0.151881698 | 0.000992953 | -0.159264832 | 0.000551123 |
| NIPSNAP | green | 0.021194503 | 0.647785375 | 0.006189748 | 0.893873569 |
| NKIRAS2 | green | 0.181306639 | 8.14E-05 | 0.194750744 | 2.26E-05 |
| NMU | green | -0.101219539 | 0.028732999 | -0.114220519 | 0.013518897 |
| NOMO1 | green | -0.04914152 | 0.28925763 | -0.049491053 | 0.285837021 |
| NOP2 | green | 0.026952755 | 0.561241414 | 0.023824538 | 0.607569633 |
| NOP56 | green | -0.147185594 | 0.00142484 | -0.17229587 | 0.000183011 |
| NPDC1 | green | 0.304305404 | 1.84E-11 | 0.31079226 | 6.46E-12 |
| NPLOC4 | green | -0.132833763 | 0.004032322 | -0.138732259 | 0.00265986 |
| NR1D1 | green | 0.009466098 | 0.838337522 | 0.009665062 | 0.834987797 |
| NR2C2AI | green | 0.008499796 | 0.854646434 | 0.001981308 | 0.96593931 |
| NR6A1 | green | -0.21241961 | 3.64E-06 | -0.233351418 | 3.40E-07 |
| NRD1 | green | -0.069807065 | 0.131980264 | -0.074463682 | 0.108033959 |
| NSUN2 | green | -0.099812169 | 0.031039458 | -0.115852273 | 0.012234052 |
| NSUN4 | green | -0.008987003 | 0.846415331 | -0.011911505 | 0.797387936 |
| NT5DC2 | green | -0.049661207 | 0.284181768 | -0.052694688 | 0.255755836 |
| NUDT15 | green | 0.0568424 | 0.220170774 | 0.045809138 | 0.323245968 |
| NUDT19 | green | -0.024468433 | 0.597895562 | -0.016674966 | 0.719289651 |
| NUDT3 | green | -0.115676295 | 0.012367226 | -0.096766725 | 0.036577362 |
| NUDT5 | green | 0.162753733 | 0.000413547 | 0.153468032 | 0.00087687 |
| NUF2 | green | 0.016572916 | 0.720935749 | 0.000983633 | 0.983086483 |
| NUP107 | green | -0.130538302 | 0.00472075 | -0.15880186 | 0.000572283 |
| NUP155 | green | -0.117243596 | 0.011224978 | -0.132873468 | 0.004021258 |
| NUP210 | green | 0.188418087 | 4.17E-05 | 0.164618481 | 0.000353857 |
| NUP43 | green | 0.098395433 | 0.033519447 | 0.098545401 | 0.033249172 |
| NUP62 | green | 0.044851318 | 0.333476858 | 0.033368964 | 0.471908535 |
| NUP85 | green | -0.068631667 | 0.138629306 | -0.084566477 | 0.067869657 |
| NUP88 | green | 0.206671013 | 6.70E-06 | 0.195698267 | 2.05E-05 |
| NUP93 | green | 0.170013969 | 0.000223293 | 0.176451073 | 0.000126571 |
| NUSAP1 | green | 0.166814176 | 0.000293894 | 0.153645269 | 0.00086471 |
| NXPH4 | green | -0.140496885 | 0.00234128 | -0.136606576 | 0.003095816 |
| NXT1 | green | -0.12715698 | 0.005928694 | -0.113347483 | 0.014253983 |
| ODC1 | green | -0.112615726 | 0.014896958 | -0.118324655 | 0.010492522 |
| OIP5 | green | 0.135391799 | 0.003373189 | 0.135482147 | 0.003351808 |
| ORC1 | green | 0.106935827 | 0.020814044 | 0.102644645 | 0.02654894 |
| ORC6 | green | 0.102520105 | 0.02673393 | 0.110441747 | 0.016959799 |
| OTX1 | green | 0.180674601 | 8.63E-05 | 0.190196255 | 3.52E-05 |
| OXNAD1 | green | -0.136391151 | 0.003143439 | -0.12435064 | 0.007134753 |

| | | | | | |
|---|---|---|---|---|---|
| PA2G4 | green | -0.042742048 | 0.356732399 | -0.062510405 | 0.177478289 |
| PAICS | green | 0.175401114 | 0.000139041 | 0.148146492 | 0.00132446 |
| PARL | green | -0.319146109 | 1.62E-12 | -0.295652561 | 7.11E-11 |
| PARP2 | green | -0.030359722 | 0.512807921 | -0.048941586 | 0.291226528 |
| PARPBP | green | -0.000538581 | 0.990738656 | -0.01362451 | 0.769023287 |
| PASK | green | 0.165948443 | 0.000316304 | 0.150936881 | 0.001068669 |
| PBK | green | 0.226703655 | 7.40E-07 | 0.217553665 | 2.08E-06 |
| PBXIP1 | green | -0.014192351 | 0.759686299 | -0.033814748 | 0.466005934 |
| PCCB | green | 0.148818189 | 0.001258199 | 0.169861667 | 0.000226257 |
| PCK2 | green | 0.216121052 | 2.43E-06 | 0.214361305 | 2.95E-06 |
| PCNA | green | 0.144550808 | 0.001737017 | 0.145898174 | 0.001570296 |
| PCNT | green | -0.04361041 | 0.347037709 | -0.070457998 | 0.128405523 |
| PCNXL3 | green | -0.004965337 | 0.914777133 | 0.005893705 | 0.898921557 |
| PDCD2L | green | -0.053322681 | 0.250126148 | -0.05377653 | 0.246111673 |
| PDCD5 | green | -0.186461327 | 5.03E-05 | -0.197851914 | 1.66E-05 |
| PDE7A | green | 0.095041512 | 0.040071664 | 0.073664321 | 0.111881304 |
| PDHA1 | green | -0.153808677 | 0.000853638 | -0.145141215 | 0.001662059 |
| PDK3 | green | 0.257612295 | 1.63E-08 | 0.265610455 | 5.56E-09 |
| PDLIM4 | green | -0.198673771 | 1.52E-05 | -0.167406 | 0.000279437 |
| PDRG1 | green | -0.182183122 | 7.51E-05 | -0.178986505 | 0.000100652 |
| PDSS1 | green | 0.056828209 | 0.220286158 | 0.02411695 | 0.603167717 |
| PGAM5 | green | 0.188612049 | 4.10E-05 | 0.198265561 | 1.59E-05 |
| PHF5A | green | 0.140030922 | 0.002421833 | 0.123628595 | 0.007478619 |
| PHGDH | green | 0.024743294 | 0.593787326 | -0.001859694 | 0.968028827 |
| PIF1 | green | -0.022138205 | 0.63322896 | -0.01959868 | 0.67270638 |
| PIGX | green | -0.136719003 | 0.003071223 | -0.140716769 | 0.002304124 |
| PKMYT1 | green | 0.1536058 | 0.000867405 | 0.168118836 | 0.000262906 |
| PKN3 | green | 0.217986826 | 1.98E-06 | 0.229979261 | 5.06E-07 |
| PLA2G3 | green | 0.286677736 | 2.76E-10 | 0.260388054 | 1.12E-08 |
| PLCD1 | green | 0.007551432 | 0.870713073 | -0.014567365 | 0.753538952 |
| PLCXD1 | green | 0.011496832 | 0.804296113 | 0.008704124 | 0.85119245 |
| PLK1 | green | 0.090312597 | 0.051125051 | 0.101948855 | 0.027596741 |
| PLK4 | green | 0.15610952 | 0.000711027 | 0.158967242 | 0.000564639 |
| PNP | green | 0.230582373 | 4.71E-07 | 0.212978243 | 3.42E-06 |
| PNPLA7 | green | 0.037193658 | 0.422619374 | 0.032207878 | 0.487473415 |
| POC1A | green | 0.132549535 | 0.004112331 | 0.154563853 | 0.00080415 |
| POLA1 | green | 0.012090921 | 0.79440384 | -0.025436461 | 0.583484443 |
| POLA2 | green | 0.089957088 | 0.05205005 | 0.099804723 | 0.03105207 |
| POLD1 | green | 0.03426598 | 0.460073058 | 0.023437325 | 0.61342053 |
| POLD3 | green | 0.054617938 | 0.238788662 | 0.022318971 | 0.630456525 |
| POLE | green | 0.13026909 | 0.004808077 | 0.108255024 | 0.019282609 |
| POLE2 | green | 0.095094418 | 0.039960454 | 0.081278006 | 0.079322731 |
| POLQ | green | -0.085189049 | 0.065861947 | -0.093650965 | 0.043089826 |
| POLR2D | green | 0.026385512 | 0.569514503 | 0.009260104 | 0.841808653 |
| POMT2 | green | 0.012953229 | 0.780104255 | -0.009454269 | 0.838536758 |

| | | | | | |
|---|---|---|---|---|---|
| POP1 | green | -0.004948623 | 0.915062913 | -0.033242747 | 0.473587193 |
| POP4 | green | -0.217868429 | 2.01E-06 | -0.189556423 | 3.74E-05 |
| PPIA | green | 0.132095015 | 0.004243263 | 0.145144719 | 0.001661623 |
| PPIF | green | 0.048517199 | 0.295435496 | 0.054441093 | 0.240314932 |
| PPIH | green | 0.03344447 | 0.470905874 | 0.039564521 | 0.393637563 |
| PPM1G | green | 0.117608797 | 0.010972631 | 0.130586076 | 0.004705403 |
| PRC1 | green | 0.10914645 | 0.018304204 | 0.091181049 | 0.048922984 |
| PRDX2 | green | 0.216571479 | 2.31E-06 | 0.227793341 | 6.52E-07 |
| PRELID1 | green | 0.041660001 | 0.369048273 | 0.06657449 | 0.150881378 |
| PRICKLE | green | -0.010685332 | 0.817858877 | 0.008877394 | 0.848265693 |
| PRIM1 | green | 0.032753456 | 0.480125492 | 0.007755587 | 0.867249667 |
| PRIM2 | green | -0.143625041 | 0.001860811 | -0.145383662 | 0.001632146 |
| PRPF4 | green | 0.140553406 | 0.002331677 | 0.14044562 | 0.002350021 |
| PRPSAP1 | green | 0.010131066 | 0.827154043 | -0.019426119 | 0.675423377 |
| PRR11 | green | -0.030627572 | 0.509094332 | -0.020131284 | 0.6643474 |
| PRR19 | green | 0.036557912 | 0.43059761 | 0.049753578 | 0.283285886 |
| PSAT1 | green | -0.011489062 | 0.804425712 | -0.023484527 | 0.612705977 |
| PSMA5 | green | 0.018395228 | 0.691741778 | 0.054910535 | 0.236278396 |
| PSMA7 | green | -0.049773534 | 0.283092599 | -0.014441595 | 0.755598899 |
| PSMB2 | green | -0.009563626 | 0.836695193 | 0.035470967 | 0.444438017 |
| PSMC2 | green | -0.001581525 | 0.972809001 | 0.019264648 | 0.677969591 |
| PSMC3IP | green | 0.139592862 | 0.002499859 | 0.117321721 | 0.011170567 |
| PSMD11 | green | 0.055572588 | 0.230667267 | 0.054700201 | 0.238081023 |
| PSMD14 | green | 0.07538437 | 0.103732866 | 0.081514296 | 0.078450647 |
| PSMD2 | green | -0.117818269 | 0.010830169 | -0.088709137 | 0.055408233 |
| PSRC1 | green | -0.001096765 | 0.981141531 | -0.011971967 | 0.796381989 |
| PTBP1 | green | 0.297415745 | 5.42E-11 | 0.293871033 | 9.35E-11 |
| PTDSS1 | green | -0.021802374 | 0.638393263 | -0.061407601 | 0.185262862 |
| PTMA | green | 0.086784259 | 0.060939462 | 0.079441547 | 0.08637152 |
| PTTG1 | green | 0.09395698 | 0.042409684 | 0.108827909 | 0.018648734 |
| PUS7 | green | 0.01948341 | 0.674520858 | -0.021644512 | 0.640826882 |
| PVR | green | 0.166426389 | 0.000303742 | 0.143789378 | 0.001838263 |
| PVRL1 | green | 0.259910249 | 1.20E-08 | 0.250765888 | 3.96E-08 |
| PXMP2 | green | 0.105771348 | 0.022252597 | 0.0901796 | 0.051469478 |
| R3HDM1 | green | 0.084469302 | 0.068187516 | 0.067961775 | 0.14253219 |
| RAB15 | green | 0.157127251 | 0.000655276 | 0.114398454 | 0.013373242 |
| RAB8A | green | -0.041531208 | 0.370531544 | -0.026756693 | 0.564094361 |
| RAC3 | green | 0.043083457 | 0.352900686 | 0.041714603 | 0.368420543 |
| RACGAP | green | 0.053040015 | 0.252649353 | 0.037813192 | 0.414928281 |
| RAD18 | green | -0.058179476 | 0.209492237 | -0.079066579 | 0.087870963 |
| RAD23A | green | -0.077627979 | 0.093818871 | -0.080147656 | 0.083603842 |
| RAD51 | green | 0.140769343 | 0.00229532 | 0.148917953 | 0.001248622 |
| RAD51AI | green | -0.045712677 | 0.324266978 | -0.058408786 | 0.207699066 |
| RAD54B | green | -0.102721784 | 0.026434912 | -0.12337554 | 0.007602595 |
| RAD54L | green | -0.010621619 | 0.818926088 | -0.015328807 | 0.741105314 |

| | | | | | |
|---|---|---|---|---|---|
| RAE1 | green | -0.253350645 | 2.84E-08 | -0.272625309 | 2.10E-09 |
| RANBP1 | green | -0.000249454 | 0.99571036 | -0.007897661 | 0.864840934 |
| RARA | green | 0.169684335 | 0.000229755 | 0.155014728 | 0.000775882 |
| RASD1 | green | 0.150423383 | 0.001112019 | 0.156986796 | 0.000662721 |
| RBL1 | green | -0.016687939 | 0.719080477 | -0.031108399 | 0.502463345 |
| RBM15 | green | -0.042936835 | 0.354543074 | -0.056306186 | 0.224560787 |
| RBM28 | green | -0.157177874 | 0.000652612 | -0.175837482 | 0.000133724 |
| RBPMS | green | -0.025686288 | 0.579791612 | -0.007970619 | 0.863604472 |
| RCC1 | green | 0.078456939 | 0.090353431 | 0.081098777 | 0.079989441 |
| RCC2 | green | 0.141201915 | 0.002224041 | 0.137059288 | 0.002997866 |
| RCCD1 | green | 0.136608372 | 0.003095421 | 0.112327799 | 0.015156852 |
| RCE1 | green | -0.034900479 | 0.451802289 | -0.054687969 | 0.238186145 |
| RCOR2 | green | -0.015414846 | 0.739704533 | -0.031324176 | 0.499502483 |
| RDM1 | green | -0.034244592 | 0.460353313 | -0.013958308 | 0.763530511 |
| RECQL4 | green | 0.099060471 | 0.032335018 | 0.101630265 | 0.028088255 |
| REEP4 | green | 0.144452063 | 0.001749852 | 0.15677732 | 0.000673971 |
| RELT | green | 0.015927726 | 0.731372475 | 0.008764101 | 0.850179122 |
| RFC2 | green | 0.152017565 | 0.000982481 | 0.157988613 | 0.000611286 |
| RFC3 | green | 0.093699968 | 0.042980299 | 0.06911488 | 0.135865359 |
| RFC4 | green | -0.180041141 | 9.14E-05 | -0.187056831 | 4.75E-05 |
| RFC5 | green | 0.003845454 | 0.933948092 | -0.023361304 | 0.614572145 |
| RFT1 | green | -0.005325256 | 0.908625708 | -0.012109977 | 0.794087075 |
| RFWD3 | green | 0.242527709 | 1.12E-07 | 0.232184319 | 3.90E-07 |
| RHNO1 | green | 0.069967259 | 0.131093472 | 0.052347224 | 0.258908134 |
| RMI2 | green | -0.035719683 | 0.441248905 | -0.027266055 | 0.556697005 |
| RNASEH | green | -0.103742591 | 0.024965217 | -0.104192811 | 0.024339767 |
| RNASEH | green | 0.05310274 | 0.25208792 | 0.056311734 | 0.224515048 |
| RNF34 | green | 0.17277925 | 0.000175402 | 0.164285416 | 0.000363892 |
| RNF8 | green | 0.008579334 | 0.853301574 | 0.021338609 | 0.645553689 |
| RNFT2 | green | 0.115447163 | 0.012542543 | 0.115187815 | 0.012743623 |
| RNPS1 | green | -0.03365487 | 0.468118157 | -0.027510677 | 0.55316125 |
| RP11-103 | green | 0.075566184 | 0.102899757 | 0.050537783 | 0.275757008 |
| RPA3 | green | 0.053105388 | 0.252064238 | 0.074120724 | 0.109671641 |
| RPAIN | green | 0.157630688 | 0.000629222 | 0.148216999 | 0.001317357 |
| RPIA | green | 0.083015748 | 0.073089346 | 0.06161513 | 0.183779027 |
| RPL39L | green | -0.230505763 | 4.76E-07 | -0.20549934 | 7.58E-06 |
| RPP38 | green | 0.037496475 | 0.418849754 | 0.033231064 | 0.47374274 |
| RPP40 | green | -0.089798548 | 0.05246703 | -0.102194258 | 0.027223196 |
| RQCD1 | green | 0.204857665 | 8.10E-06 | 0.203904795 | 8.94E-06 |
| RRM1 | green | 0.202857338 | 9.96E-06 | 0.184907457 | 5.82E-05 |
| RRM2 | green | 0.224878062 | 9.12E-07 | 0.221343996 | 1.36E-06 |
| RRP36 | green | 0.016785409 | 0.717509615 | 0.026059592 | 0.574294047 |
| RRS1 | green | -0.139122682 | 0.002586152 | -0.170644935 | 0.000211395 |
| RTKN2 | green | 0.040968428 | 0.377056134 | 0.024709062 | 0.594298269 |
| RUVBL1 | green | -0.059701315 | 0.197798978 | -0.061973274 | 0.181238955 |

| | | | | | |
|---|---|---|---|---|---|
| SAPCD2 | green | 0.330000987 | 2.51E-13 | 0.340237153 | 4.04E-14 |
| SASS6 | green | -0.063068332 | 0.17363367 | -0.086515207 | 0.061747732 |
| SCD | green | 0.364021529 | 4.44E-16 | 0.358735115 | 1.25E-15 |
| SDCCAG | green | 0.190814928 | 3.31E-05 | 0.175059219 | 0.000143345 |
| SEC61A2 | green | -0.101126589 | 0.028880658 | -0.127032745 | 0.005977943 |
| SEMA4D | green | -0.016472007 | 0.722564724 | -0.017822211 | 0.700874995 |
| SENP1 | green | -0.05484601 | 0.236830364 | -0.097111556 | 0.03591088 |
| SEPHS1 | green | 0.042676881 | 0.357466747 | 0.017986483 | 0.698252216 |
| SEPT3 | green | -0.072045291 | 0.120003151 | -0.082525507 | 0.074806009 |
| SERPINB | green | -0.086819787 | 0.060833388 | -0.07776476 | 0.093239854 |
| SETD8 | green | 0.065216002 | 0.159412269 | 0.054616362 | 0.238802238 |
| SFMBT1 | green | -0.193905022 | 2.45E-05 | -0.192939701 | 2.70E-05 |
| SFXN1 | green | 0.187197503 | 4.69E-05 | 0.180684508 | 8.62E-05 |
| SFXN3 | green | 0.001043457 | 0.982057989 | 0.018189384 | 0.695017631 |
| SFXN5 | green | -0.084970131 | 0.066562293 | -0.086379796 | 0.062157861 |
| SGOL1 | green | 0.005206661 | 0.910652043 | -0.004550695 | 0.921870106 |
| SGOL2 | green | -0.007491792 | 0.87172531 | -0.022068366 | 0.634301458 |
| SHCBP1 | green | 0.205931514 | 7.24E-06 | 0.211741589 | 3.91E-06 |
| SHMT2 | green | 0.097899824 | 0.034426004 | 0.086822777 | 0.060824466 |
| SIGMAR | green | 0.160804579 | 0.000485864 | 0.148029359 | 0.001336338 |
| SKA1 | green | 0.106496044 | 0.02134755 | 0.108458473 | 0.019055383 |
| SKA3 | green | 0.141303108 | 0.002207661 | 0.13757623 | 0.002889471 |
| SKP2 | green | 0.083624236 | 0.07100345 | 0.066521333 | 0.151208533 |
| SLBP | green | 0.096921848 | 0.036276254 | 0.083481212 | 0.071489311 |
| SLC16A1 | green | -0.192236006 | 2.89E-05 | -0.191799016 | 3.01E-05 |
| SLC16A1 | green | -0.218252822 | 1.92E-06 | -0.225710261 | 8.29E-07 |
| SLC20A1 | green | 0.199681442 | 1.38E-05 | 0.17330675 | 0.000167437 |
| SLC25A1 | green | -0.119333267 | 0.009847698 | -0.143709544 | 0.001849185 |
| SLC25A3 | green | 0.083763094 | 0.070534328 | 0.095578745 | 0.038954465 |
| SLC27A1 | green | 0.046502682 | 0.315966607 | 0.058976723 | 0.203305715 |
| SLC29A2 | green | -0.057217874 | 0.217133507 | -0.059019385 | 0.202978444 |
| SLC35A2 | green | -0.053897594 | 0.245048459 | -0.02698015 | 0.560843347 |
| SLC44A2 | green | -0.120802993 | 0.008971066 | -0.131768546 | 0.004339621 |
| SLC5A6 | green | 0.012960649 | 0.779981527 | -0.013663273 | 0.768384819 |
| SLMO1 | green | -0.308659669 | 9.13E-12 | -0.32810086 | 3.50E-13 |
| SLX4 | green | 0.239345465 | 1.65E-07 | 0.241305987 | 1.30E-07 |
| SMARCD | green | 0.020210757 | 0.663103635 | 0.00020843 | 0.996415809 |
| SMC1B | green | -0.252148537 | 3.32E-08 | -0.254460143 | 2.46E-08 |
| SMC2 | green | 0.19923434 | 1.44E-05 | 0.167530181 | 0.000276489 |
| SMC4 | green | -0.036901353 | 0.426276861 | -0.073647694 | 0.111962461 |
| SMG5 | green | -0.082874617 | 0.073580215 | -0.075446943 | 0.103445544 |
| SMPD4 | green | -0.093663289 | 0.04306226 | -0.120950596 | 0.008886985 |
| SNRPA1 | green | 0.093132167 | 0.044263888 | 0.104126595 | 0.024430898 |
| SNRPB | green | 0.027539711 | 0.55274232 | 0.030942622 | 0.504744387 |
| SNRPC | green | -0.088233341 | 0.056735095 | -0.074339793 | 0.108623311 |

| | | | | | |
|---|---|---|---|---|---|
| SNRPD1 | green | 0.028360944 | 0.54095753 | 0.024633942 | 0.595420223 |
| SNRPD3 | green | 0.088425827 | 0.056195165 | 0.085832975 | 0.06383697 |
| SOD3 | green | 0.026043498 | 0.57453055 | 0.04851664 | 0.295441074 |
| SOX12 | green | -0.205553122 | 7.53E-06 | -0.193868891 | 2.46E-05 |
| SPAG5 | green | 0.065668619 | 0.156530481 | 0.060987046 | 0.188296889 |
| SPC24 | green | 0.044423967 | 0.338108031 | 0.063966848 | 0.167572999 |
| SPC25 | green | 0.012557208 | 0.786662546 | 0.010742523 | 0.816901204 |
| SPDL1 | green | -0.066013617 | 0.154360473 | -0.098170779 | 0.033927825 |
| SPRYD3 | green | 0.073169444 | 0.114316632 | 0.077472306 | 0.094481358 |
| SRD5A1 | green | -0.102079829 | 0.027396831 | -0.128924445 | 0.005266448 |
| SRRT | green | 0.023635666 | 0.61042045 | 0.021713439 | 0.639763813 |
| STIL | green | 0.08347124 | 0.071523286 | 0.079026429 | 0.088032751 |
| STIP1 | green | 0.015725238 | 0.734658318 | 0.017302233 | 0.709200338 |
| STMN1 | green | 0.021379078 | 0.644927531 | 0.003329545 | 0.94279335 |
| SUMO3 | green | -0.201860896 | 1.10E-05 | -0.212144625 | 3.75E-06 |
| SUV39H1 | green | -0.059946627 | 0.195959536 | -0.029360438 | 0.526785563 |
| SUV39H2 | green | 0.013360052 | 0.77338327 | -0.007063645 | 0.878998068 |
| SYCE2 | green | -0.08809226 | 0.057133562 | -0.089513869 | 0.053222761 |
| SYNGR3 | green | -0.067856704 | 0.143151906 | -0.067166974 | 0.147271237 |
| SYT13 | green | -0.379813454 | 1.79E-17 | -0.382050304 | 1.12E-17 |
| TACC3 | green | 0.037367542 | 0.42045236 | 0.050238048 | 0.278618439 |
| TAF11 | green | -0.122712738 | 0.00793606 | -0.119200626 | 0.009930437 |
| TAF5 | green | -0.008007112 | 0.862986145 | -0.02730312 | 0.55616057 |
| TARS | green | -0.055073932 | 0.234884686 | -0.061058567 | 0.187778354 |
| TATDN2 | green | 0.043243283 | 0.351115865 | 0.040312965 | 0.384743655 |
| TBC1D31 | green | -0.127309313 | 0.005868804 | -0.148673406 | 0.001272218 |
| TCF19 | green | 0.083687424 | 0.070789659 | 0.090316703 | 0.051114449 |
| TCF3 | green | 0.304175232 | 1.87E-11 | 0.312930409 | 4.55E-12 |
| TCOF1 | green | -0.053862861 | 0.245353169 | -0.072079525 | 0.119826775 |
| TDP1 | green | 0.190469373 | 3.43E-05 | 0.180082122 | 9.11E-05 |
| TDRKH | green | -0.029087246 | 0.530640348 | -0.059040194 | 0.202818952 |
| TEAD2 | green | -0.067522988 | 0.145133843 | -0.087180986 | 0.059763592 |
| TEAD4 | green | -0.136569942 | 0.003103868 | -0.127011203 | 0.00598652 |
| TERT | green | 0.086968786 | 0.060390183 | 0.066529111 | 0.151160626 |
| TFAP4 | green | -0.215595219 | 2.58E-06 | -0.209330124 | 5.06E-06 |
| TFG | green | -0.089895273 | 0.052212302 | -0.078818305 | 0.088875242 |
| TFRC | green | 0.08115402 | 0.079783461 | 0.073733858 | 0.111542399 |
| THOC3 | green | -0.043504685 | 0.348209037 | -0.049597518 | 0.284800577 |
| THOC5 | green | -0.096879471 | 0.036358303 | -0.107018582 | 0.020714958 |
| TICRR | green | 0.026341458 | 0.570159438 | 0.014493221 | 0.754753117 |
| TIGD3 | green | 0.145958105 | 0.001563233 | 0.136705446 | 0.003074179 |
| TIMELES | green | 0.0791651 | 0.087474981 | 0.046753012 | 0.313365738 |
| TIMM23 | green | 0.204297972 | 8.58E-06 | 0.213221004 | 3.34E-06 |
| TIMM8A | green | -0.144993215 | 0.001680565 | -0.145249115 | 0.001648685 |
| TIPIN | green | 0.236709395 | 2.27E-07 | 0.210058556 | 4.69E-06 |

| | | | | | |
|---|---|---|---|---|---|
| TK1 | green | 0.140852047 | 0.002281533 | 0.160556117 | 0.000495884 |
| TMEM10 | green | 0.081422346 | 0.078789081 | 0.094804475 | 0.040573139 |
| TMEM10 | green | 0.17389878 | 0.000158903 | 0.154856292 | 0.000785709 |
| TMEM17 | green | -0.12224768 | 0.00817777 | -0.10136076 | 0.028509897 |
| TMEM18 | green | 0.263799072 | 7.11E-09 | 0.255007866 | 2.29E-08 |
| TMEM18 | green | 0.026322382 | 0.570438809 | 0.03764689 | 0.416984667 |
| TMEM19 | green | -0.109744679 | 0.017672153 | -0.144269658 | 0.001773791 |
| TMEM20 | green | -0.026872089 | 0.562414363 | -0.03830317 | 0.408904342 |
| TMEM20 | green | 0.163098112 | 0.000401862 | 0.144983735 | 0.001681757 |
| TMEM38 | green | -0.134217757 | 0.003662503 | -0.128447472 | 0.005438265 |
| TMEM41 | green | -0.341155089 | 3.42E-14 | -0.333912533 | 1.26E-13 |
| TMEM97 | green | 0.12818805 | 0.005533817 | 0.089265546 | 0.053889359 |
| TMPO | green | 0.029384887 | 0.526441284 | 0.016209072 | 0.726815136 |
| TMSB15 | green | -0.075632795 | 0.102595865 | -0.110664636 | 0.016737385 |
| TNFSF12 | green | 0.112181338 | 0.015290574 | 0.12511593 | 0.0067858 |
| TNPO2 | green | -0.020602275 | 0.656989884 | -0.044209592 | 0.340446632 |
| TOM1L2 | green | 0.00873358 | 0.850694748 | 0.02326986 | 0.61595864 |
| TOMM34 | green | -0.033061182 | 0.476007696 | -0.03937031 | 0.395965529 |
| TOMM40 | green | 0.001994493 | 0.965712784 | 0.010099738 | 0.82768015 |
| TOMM5 | green | -0.027281253 | 0.556477012 | -0.030451056 | 0.511540045 |
| TONSL | green | 0.054346125 | 0.241137377 | 0.036672239 | 0.429156483 |
| TOP2A | green | 0.132527905 | 0.004118478 | 0.12060765 | 0.009083429 |
| TOP3A | green | 0.311888807 | 5.40E-12 | 0.302946478 | 2.28E-11 |
| TOPBP1 | green | -0.155535791 | 0.00074436 | -0.182216512 | 7.48E-05 |
| TPCN1 | green | -0.170231 | 0.000219131 | -0.172501792 | 0.000179732 |
| TPGS2 | green | 0.125431458 | 0.006646435 | 0.114744649 | 0.013093808 |
| TPMT | green | 0.089290767 | 0.053821343 | 0.088159074 | 0.056944566 |
| TPRG1L | green | 0.026043788 | 0.574526293 | 0.045946296 | 0.321797802 |
| TPX2 | green | -0.015484306 | 0.738574315 | -0.016066428 | 0.729124546 |
| TRA2B | green | -0.120945505 | 0.008889873 | -0.145212338 | 0.001653232 |
| TRAIP | green | -0.0724552 | 0.11790458 | -0.066417089 | 0.151851669 |
| TRIM59 | green | 0.033878249 | 0.465168459 | 0.025995756 | 0.575232399 |
| TRIP13 | green | -0.07901028 | 0.088097892 | -0.081578946 | 0.0782134 |
| TROAP | green | 0.022032219 | 0.63485687 | 0.026900426 | 0.562002188 |
| TRPC4AI | green | 0.039668819 | 0.392390773 | 0.040068588 | 0.387634073 |
| TTC31 | green | 0.02889657 | 0.533339248 | 0.009552141 | 0.836888545 |
| TTF2 | green | 0.037454728 | 0.419368264 | 0.020911018 | 0.65218476 |
| TTI1 | green | -0.158628603 | 0.000580393 | -0.187596003 | 4.52E-05 |
| TTK | green | -0.103830633 | 0.024841828 | -0.105641295 | 0.022418488 |
| TTL | green | -0.029184802 | 0.529262174 | -0.055481194 | 0.231436199 |
| TTLL4 | green | 0.001939696 | 0.966654235 | -0.021260727 | 0.646759416 |
| TTYH3 | green | 0.109683893 | 0.017735493 | 0.131602587 | 0.004389358 |
| TUBA1C | green | 0.089522404 | 0.053199973 | 0.090843608 | 0.049768992 |
| TUBB | green | -0.059945193 | 0.195970252 | -0.052768568 | 0.255089007 |
| TUBB3 | green | 0.013559286 | 0.77009792 | 0.00292163 | 0.949792068 |

| | | | | | |
|---|---|---|---|---|---|
| TYMS | green | 0.116030268 | 0.012100642 | 0.106053555 | 0.02189628 |
| U2AF2 | green | 0.056086167 | 0.226380011 | 0.033707999 | 0.467415663 |
| UBA1 | green | 0.093570113 | 0.043271055 | 0.117113316 | 0.011316235 |
| UBE2C | green | 0.018881398 | 0.684027639 | 0.021866619 | 0.637403947 |
| UBE2O | green | 0.021430318 | 0.6441351 | 0.00881035 | 0.849397911 |
| UBE2S | green | -0.002068988 | 0.964432977 | -0.000895455 | 0.98460251 |
| UBE2T | green | 0.243747287 | 9.61E-08 | 0.243495726 | 9.91E-08 |
| UBL4A | green | 0.058041634 | 0.210575506 | 0.046491912 | 0.316078831 |
| UCHL1 | green | -0.41323228 | 1.10E-20 | -0.403796961 | 9.65E-20 |
| UCK2 | green | 0.126630982 | 0.006139735 | 0.094951536 | 0.040261397 |
| UFD1L | green | 0.138523071 | 0.002700136 | 0.158995884 | 0.000563325 |
| UHRF1 | green | 0.302194798 | 2.57E-11 | 0.296549448 | 6.19E-11 |
| ULBP1 | green | 0.138264843 | 0.002750619 | 0.121395108 | 0.008637997 |
| UMPS | green | -0.125158407 | 0.006766888 | -0.138547006 | 0.0026955 |
| UNC119 | green | 0.021583007 | 0.641776104 | 0.015242571 | 0.74251016 |
| UNG | green | 0.039515874 | 0.39421991 | 0.004472297 | 0.923211924 |
| USP39 | green | -0.02446666 | 0.597922113 | -0.037639985 | 0.417070184 |
| VDAC1 | green | 0.226402433 | 7.66E-07 | 0.223091576 | 1.12E-06 |
| VDAC3 | green | 0.122317505 | 0.008141065 | 0.095142594 | 0.039859415 |
| VRK1 | green | -0.105429943 | 0.022690369 | -0.11076119 | 0.016641832 |
| VSIG2 | green | -0.18596644 | 5.27E-05 | -0.168151429 | 0.000262172 |
| WBP1L | green | 0.132033905 | 0.004261151 | 0.108426259 | 0.019091206 |
| WDHD1 | green | 0.040884394 | 0.378036413 | 0.015909499 | 0.731668048 |
| WDR53 | green | -0.312417072 | 4.95E-12 | -0.319054105 | 1.64E-12 |
| WDR62 | green | 0.072005183 | 0.120210045 | 0.076669041 | 0.097959353 |
| WDR70 | green | -0.275603654 | 1.38E-09 | -0.288496987 | 2.11E-10 |
| WDR76 | green | 0.240210383 | 1.49E-07 | 0.216431352 | 2.35E-06 |
| WFS1 | green | 0.103402784 | 0.02544642 | 0.077703127 | 0.093500402 |
| WHSC1 | green | 0.161036456 | 0.000476683 | 0.142829723 | 0.001973558 |
| WRAP73 | green | 0.236669028 | 2.29E-07 | 0.235248764 | 2.71E-07 |
| WRNIP1 | green | -0.07007105 | 0.130521374 | -0.071811619 | 0.121212441 |
| XPO5 | green | -0.142475899 | 0.002025698 | -0.159878207 | 0.000524208 |
| XRCC2 | green | 0.006122143 | 0.895025964 | -0.003199936 | 0.945016644 |
| XRCC3 | green | 0.023337605 | 0.614931337 | 0.027612551 | 0.551691999 |
| XXYLT1 | green | -0.105999109 | 0.021964636 | -0.09976015 | 0.031127661 |
| XYLB | green | 0.147959997 | 0.001343418 | 0.13590545 | 0.003253263 |
| YARS | green | 0.09290548 | 0.044785274 | 0.108845885 | 0.018629142 |
| YARS2 | green | 0.205715943 | 7.41E-06 | 0.193252779 | 2.61E-05 |
| YBX1 | green | -0.088961728 | 0.054714364 | -0.098063672 | 0.034124013 |
| YBX2 | green | -0.227569741 | 6.70E-07 | -0.223109798 | 1.12E-06 |
| YKT6 | green | -0.028786619 | 0.534898675 | -0.002303813 | 0.960399341 |
| YRDC | green | -0.008057995 | 0.862124121 | -0.010561289 | 0.819936933 |
| YTHDF1 | green | -0.138262868 | 0.002751008 | -0.130395337 | 0.004766945 |
| ZBTB24 | green | -0.19244125 | 2.83E-05 | -0.208427567 | 5.57E-06 |
| ZBTB7A | green | 0.263771519 | 7.14E-09 | 0.287925139 | 2.29E-10 |

| | | | | | |
|---|---|---|---|---|---|
| ZC3HC1 | green | -0.053503714 | 0.248519403 | -0.069745288 | 0.132323481 |
| ZCCHC1' | green | -0.009146311 | 0.843727465 | 0.016055499 | 0.729301577 |
| ZER1 | green | 0.081451957 | 0.078679967 | 0.096134632 | 0.037826351 |
| ZFP69B | green | 0.009725412 | 0.833972354 | -0.020900975 | 0.652340844 |
| ZGRF1 | green | -0.037574464 | 0.417882113 | -0.051741439 | 0.264467961 |
| ZNF367 | green | 0.155932511 | 0.000721161 | 0.133615704 | 0.003819427 |
| ZNF48 | green | -0.016538613 | 0.721489351 | -0.02000184 | 0.666375188 |
| ZNF492 | green | -0.288562581 | 2.09E-10 | -0.276496872 | 1.22E-09 |
| ZNF530 | green | -0.078743068 | 0.089181393 | -0.087977554 | 0.057459241 |
| ZNF620 | green | -0.087050174 | 0.06014922 | -0.105612444 | 0.022455435 |
| ZNF629 | green | -0.160444693 | 0.00050044 | -0.165315719 | 0.00033368 |
| ZNF695 | green | -0.033052963 | 0.476117429 | -0.042716403 | 0.357021271 |
| ZNF726 | green | -0.183490319 | 6.65E-05 | -0.170342161 | 0.000217028 |
| ZNF862 | green | 0.040240978 | 0.385593723 | 0.024539586 | 0.596830834 |
| ZPR1 | green | -0.133975077 | 0.00372504 | -0.1401099 | 0.002408004 |
| ZSCAN1( | green | 0.084604527 | 0.067745529 | 0.076666503 | 0.097970502 |
| ZWILCH | green | 0.13573987 | 0.003291493 | 0.120951821 | 0.008886291 |
| ZWINT | green | 0.209636851 | 4.90E-06 | 0.203851116 | 8.99E-06 |
| ZYG11A | green | -0.182662522 | 7.18E-05 | -0.19457012 | 2.30E-05 |
| ABCC5 | greenyellov | -0.225314818 | 8.68E-07 | -0.264591791 | 6.38E-09 |
| ACIN1 | greenyellov | 0.049479769 | 0.285947023 | 0.013573985 | 0.769855706 |
| ACTB | greenyellov | 0.219619523 | 1.65E-06 | 0.257708488 | 1.61E-08 |
| ADAT2 | greenyellov | -0.038207406 | 0.410077603 | -0.075592697 | 0.102778715 |
| AGAP10 | greenyellov | 0.052805116 | 0.254759582 | 0.03438059 | 0.458572886 |
| AGAP4 | greenyellov | -0.08626862 | 0.062496264 | -0.119393418 | 0.009810378 |
| AGAP6 | greenyellov | 0.055764516 | 0.229058411 | 0.045742373 | 0.32395243 |
| AHI1 | greenyellov | 0.016843014 | 0.716581794 | -0.002168862 | 0.962717295 |
| AHSA2 | greenyellov | -0.131695408 | 0.004361477 | -0.151490913 | 0.001023649 |
| ALG13 | greenyellov | 0.053858016 | 0.245395688 | 0.059757492 | 0.197376638 |
| ANKRD1 | greenyellov | -0.053132203 | 0.251824506 | -0.083182425 | 0.072513069 |
| ANKRD2 | greenyellov | -0.036236417 | 0.43466512 | -0.062184152 | 0.179755544 |
| ANKRD4 | greenyellov | -0.054964233 | 0.235819726 | -0.057222477 | 0.217096458 |
| ARGLU1 | greenyellov | -0.155713335 | 0.000733894 | -0.18786597 | 4.40E-05 |
| ATG101 | greenyellov | -0.027883581 | 0.547792494 | -0.018670741 | 0.687366204 |
| ATG14 | greenyellov | -0.211193106 | 4.15E-06 | -0.240973165 | 1.35E-07 |
| ATG16L2 | greenyellov | 0.027051912 | 0.559801211 | 0.011295112 | 0.807662204 |
| ATG9A | greenyellov | 0.062114736 | 0.180242852 | 0.069730445 | 0.132406042 |
| ATP6V0I | greenyellov | 0.242945779 | 1.06E-07 | 0.299008442 | 4.23E-11 |
| ATXN3 | greenyellov | -0.196168054 | 1.96E-05 | -0.213080742 | 3.39E-06 |
| BRWD1 | greenyellov | -0.11952398 | 0.009729807 | -0.141628793 | 0.002155687 |
| C12orf29 | greenyellov | 0.004667589 | 0.919869847 | -0.027576849 | 0.552206681 |
| C16orf52 | greenyellov | -0.202356164 | 1.05E-05 | -0.22416023 | 9.90E-07 |
| C1orf52 | greenyellov | -0.226033716 | 7.99E-07 | -0.244620367 | 8.62E-08 |
| C2CD5 | greenyellov | -0.09424707 | 0.041773315 | -0.12664746 | 0.006133023 |
| C5orf28 | greenyellov | -0.097893151 | 0.034438351 | -0.10504177 | 0.023197152 |

| | | | | | |
|---|---|---|---|---|---|
| C5orf45 | greenyellow | -0.035477936 | 0.444348473 | -0.057136497 | 0.217789216 |
| C5orf63 | greenyellow | 0.114417104 | 0.013358056 | 0.118008417 | 0.01070227 |
| C7orf55-L | greenyellow | -0.022743141 | 0.623971371 | -0.052659051 | 0.256077916 |
| C8orf44 | greenyellow | -0.244779534 | 8.45E-08 | -0.267794962 | 4.12E-09 |
| CAPN3 | greenyellow | -0.027694128 | 0.550516869 | -0.041295287 | 0.373258141 |
| CAPRIN2 | greenyellow | -0.14257254 | 0.002011333 | -0.161698879 | 0.000451336 |
| CARD8 | greenyellow | -0.107883131 | 0.019704068 | -0.143554377 | 0.001870585 |
| CATSPEF | greenyellow | -0.038225074 | 0.409860989 | -0.052346481 | 0.258914899 |
| CBWD7 | greenyellow | -0.024183495 | 0.602167953 | -0.040425756 | 0.383414038 |
| CCAR1 | greenyellow | 0.100959645 | 0.029147498 | 0.068741139 | 0.137999377 |
| CCDC14 | greenyellow | -0.005484467 | 0.905906341 | -0.044122654 | 0.341397969 |
| CCDC66 | greenyellow | -0.039725591 | 0.391713125 | -0.061904404 | 0.181725376 |
| CCDC84 | greenyellow | -0.035140176 | 0.448699768 | -0.045541304 | 0.326086057 |
| CCNL1 | greenyellow | -0.244703546 | 8.53E-08 | -0.27630513 | 1.25E-09 |
| CCNL2 | greenyellow | -0.01777772 | 0.701585951 | -0.033034291 | 0.476366759 |
| CCNT2 | greenyellow | -0.093473181 | 0.043489169 | -0.12179987 | 0.008416701 |
| CDC16 | greenyellow | -0.00846195 | 0.855286501 | -0.022797568 | 0.623141318 |
| CEBPZOS | greenyellow | 0.057798331 | 0.212497408 | 0.055811113 | 0.228669004 |
| CENPJ | greenyellow | 0.031416957 | 0.4982322 | -0.005365134 | 0.907944488 |
| CEP162 | greenyellow | 0.082605638 | 0.074523195 | 0.063403919 | 0.171351247 |
| CEP290 | greenyellow | -0.086770015 | 0.060982034 | -0.137905706 | 0.002822259 |
| CEP57L1 | greenyellow | -0.093471302 | 0.043493406 | -0.102164756 | 0.027267872 |
| CEP95 | greenyellow | -0.147404118 | 0.001401416 | -0.175685538 | 0.000135554 |
| CFAP44 | greenyellow | 0.011261552 | 0.808222556 | -0.012712496 | 0.784089078 |
| CLK1 | greenyellow | -0.133334475 | 0.003894794 | -0.147389247 | 0.001402999 |
| CLK4 | greenyellow | -0.174929722 | 0.000145007 | -0.186774522 | 4.88E-05 |
| CSAD | greenyellow | -0.037804173 | 0.415039649 | -0.057961158 | 0.211209807 |
| CXorf23 | greenyellow | 0.022108253 | 0.633688835 | -0.009381088 | 0.839769625 |
| DBNL | greenyellow | 0.046640921 | 0.314528599 | 0.09197511 | 0.046979547 |
| DDX17 | greenyellow | -0.004679095 | 0.919672974 | -0.042521907 | 0.359216901 |
| DDX20 | greenyellow | -0.098686837 | 0.03299598 | -0.132617508 | 0.004093067 |
| DDX26B | greenyellow | -0.144576465 | 0.001733697 | -0.168277595 | 0.000259351 |
| DDX39B | greenyellow | -0.063358347 | 0.171659878 | -0.088647492 | 0.055578675 |
| DDX47 | greenyellow | -0.122282323 | 0.008159541 | -0.140982807 | 0.002259889 |
| DFNB59 | greenyellow | -0.108999227 | 0.018462742 | -0.120789561 | 0.008978752 |
| DMTF1 | greenyellow | -0.071109861 | 0.124901309 | -0.096793802 | 0.03652465 |
| DMXL2 | greenyellow | 0.051253179 | 0.269008453 | 0.010151393 | 0.82681272 |
| DPY19L2 | greenyellow | -0.11488156 | 0.012984727 | -0.124517947 | 0.007057122 |
| EGFL8 | greenyellow | -0.152323383 | 0.000959283 | -0.179940621 | 9.23E-05 |
| EHD1 | greenyellow | 0.000259187 | 0.995542987 | 0.034584592 | 0.455909403 |
| ENOSF1 | greenyellow | 0.102754669 | 0.02638643 | 0.083022164 | 0.073067095 |
| EP400NL | greenyellow | 0.103772239 | 0.024923608 | 0.093192633 | 0.04412568 |
| ESF1 | greenyellow | -0.161596287 | 0.000455178 | -0.186970557 | 4.79E-05 |
| ETHE1 | greenyellow | 0.309729632 | 7.68E-12 | 0.313990065 | 3.82E-12 |
| ETNK1 | greenyellow | -0.014740806 | 0.75070108 | -0.043645473 | 0.346649798 |

| | | | | | |
|---|---|---|---|---|---|
| EXOG | greenyellov | 0.009804553 | 0.83264114 | -0.009624635 | 0.835668185 |
| FAM111/ | greenyellov | 0.117193367 | 0.011260084 | 0.110893167 | 0.016511996 |
| FAM126E | greenyellov | -0.05760569 | 0.214028063 | -0.053024692 | 0.252786637 |
| FAM133E | greenyellov | -0.006978122 | 0.880452046 | -0.040402117 | 0.383692462 |
| FAM76B | greenyellov | -0.072087032 | 0.11978813 | -0.104230474 | 0.024288064 |
| FAM92A | greenyellov | -0.025428492 | 0.583602428 | -0.053371052 | 0.249696128 |
| FANCL | greenyellov | -0.084030983 | 0.069636443 | -0.12231093 | 0.008144515 |
| FASTKD: | greenyellov | -0.192079434 | 2.93E-05 | -0.208988937 | 5.25E-06 |
| FBXW7 | greenyellov | 0.02395725 | 0.605570038 | 0.000802622 | 0.986198628 |
| FNBP4 | greenyellov | -0.060479251 | 0.1920088 | -0.106241887 | 0.021661252 |
| GCFC2 | greenyellov | -0.113977846 | 0.013719798 | -0.134249967 | 0.003654275 |
| GIN1 | greenyellov | -0.043966339 | 0.343112742 | -0.06013466 | 0.194558079 |
| GLMN | greenyellov | -0.061448281 | 0.184971305 | -0.092149518 | 0.046561475 |
| GNRH1 | greenyellov | 0.027023757 | 0.560209978 | 0.032942626 | 0.477591848 |
| GOLGA6 | greenyellov | -0.001241502 | 0.978653388 | -0.019376655 | 0.67620298 |
| GOLGA8 | greenyellov | -0.061982845 | 0.181171434 | -0.088831299 | 0.055071744 |
| GOLGA8 | greenyellov | -0.036205516 | 0.435057249 | -0.057832547 | 0.212226368 |
| GOPC | greenyellov | -0.171350046 | 0.000198802 | -0.200548535 | 1.26E-05 |
| GPR75-A | greenyellov | -0.165223664 | 0.000336281 | -0.172518766 | 0.000179464 |
| GTDC1 | greenyellov | -0.091428598 | 0.048310026 | -0.116202959 | 0.011972436 |
| HAUS3 | greenyellov | -0.062817031 | 0.17535761 | -0.085346963 | 0.065360529 |
| HNRNPA | greenyellov | -0.180765665 | 8.55E-05 | -0.209690104 | 4.87E-06 |
| HNRNPH | greenyellov | -0.021273245 | 0.646565555 | -0.069082399 | 0.136049809 |
| HSPBAP1 | greenyellov | -0.267587308 | 4.24E-09 | -0.29275493 | 1.11E-10 |
| IFT80 | greenyellov | -0.118331459 | 0.01048805 | -0.141247132 | 0.002216708 |
| IQCB1 | greenyellov | -0.061751941 | 0.182805634 | -0.100537316 | 0.029832004 |
| KATNBL | greenyellov | 0.069171554 | 0.135543991 | 0.045372802 | 0.327881108 |
| KIAA090 | greenyellov | -0.088008319 | 0.057371741 | -0.119819657 | 0.00954951 |
| KIAA110 | greenyellov | -0.154758662 | 0.000791821 | -0.195258775 | 2.14E-05 |
| KIN | greenyellov | -0.09880776 | 0.032780816 | -0.114570487 | 0.013233734 |
| KLHL31 | greenyellov | -0.095771273 | 0.038560567 | -0.099096918 | 0.032271152 |
| L3HYPDI | greenyellov | -0.08587612 | 0.063703142 | -0.088563161 | 0.05581255 |
| L3MBTL | greenyellov | -0.303112409 | 2.22E-11 | -0.312944342 | 4.54E-12 |
| LCORL | greenyellov | 0.087818558 | 0.057913215 | 0.079433035 | 0.086405328 |
| LIPT1 | greenyellov | -0.215535367 | 2.59E-06 | -0.22849376 | 6.02E-07 |
| LRRC39 | greenyellov | -0.153348497 | 0.00088516 | -0.159552581 | 0.000538341 |
| LUC7L3 | greenyellov | -0.122691146 | 0.007947139 | -0.148298385 | 0.001309201 |
| 6-Mar | greenyellov | -0.112787949 | 0.014743381 | -0.132217812 | 0.004207523 |
| MARK3 | greenyellov | -0.077450359 | 0.094575056 | -0.0828499 | 0.07366646 |
| METAP1I | greenyellov | -0.099337871 | 0.031851637 | -0.133492627 | 0.003852247 |
| MIPOL1 | greenyellov | -0.05337235 | 0.2496846 | -0.084601826 | 0.067754335 |
| MITD1 | greenyellov | -0.090905771 | 0.04961223 | -0.097605617 | 0.034973973 |
| MSANTD | greenyellov | -0.126674939 | 0.006121844 | -0.162016476 | 0.000439633 |
| MSH5 | greenyellov | -0.039903726 | 0.389591441 | -0.049344349 | 0.287269367 |
| MSS51 | greenyellov | -0.04545078 | 0.327049616 | -0.054459434 | 0.240156324 |

| | | | | | |
|---|---|---|---|---|---|
| MTERF1 | greenyellov | -0.220374349 | 1.52E-06 | -0.232138363 | 3.92E-07 |
| MTERF2 | greenyellov | 0.100528056 | 0.029847166 | 0.076470083 | 0.098836375 |
| MTF2 | greenyellov | -0.15668084 | 0.000679212 | -0.177875647 | 0.000111324 |
| MTRF1 | greenyellov | 0.067578972 | 0.144799897 | 0.055378466 | 0.232302646 |
| MYSM1 | greenyellov | -0.044560166 | 0.3366276 | -0.072764947 | 0.116337921 |
| N4BP2L2 | greenyellov | -0.045599542 | 0.32546714 | -0.063692428 | 0.16940698 |
| NAA16 | greenyellov | -0.00499857 | 0.914208921 | -0.031411232 | 0.498310524 |
| NAPB | greenyellov | -0.243812766 | 9.53E-08 | -0.259016532 | 1.35E-08 |
| NEK3 | greenyellov | -0.127239654 | 0.005896123 | -0.151805063 | 0.000998905 |
| NFATC2I | greenyellov | 0.019852972 | 0.668710263 | 0.006905909 | 0.881680064 |
| NIPSNAP | greenyellov | -0.094722469 | 0.04074786 | -0.107789842 | 0.019811041 |
| NKTR | greenyellov | -0.027091213 | 0.559230892 | -0.055840922 | 0.22842015 |
| NPHP3 | greenyellov | -0.161394232 | 0.000462833 | -0.184005762 | 6.34E-05 |
| NR2C1 | greenyellov | -0.021822689 | 0.638080355 | -0.058204549 | 0.209295625 |
| NSUN6 | greenyellov | -0.0528517 | 0.254340129 | -0.094864916 | 0.040444768 |
| NUPL2 | greenyellov | 0.000459799 | 0.99209332 | -0.02322892 | 0.616579817 |
| OFD1 | greenyellov | -0.066776487 | 0.149643082 | -0.09098086 | 0.049423422 |
| OGT | greenyellov | -0.236062594 | 2.46E-07 | -0.268680328 | 3.65E-09 |
| ORAI1 | greenyellov | 0.136564083 | 0.003105157 | 0.161302684 | 0.000466341 |
| ORC3 | greenyellov | -0.191339719 | 3.15E-05 | -0.197386017 | 1.74E-05 |
| ORMDL1 | greenyellov | -0.060597321 | 0.191140977 | -0.06969035 | 0.132629275 |
| OSGEPL1 | greenyellov | -0.11002329 | 0.017384355 | -0.136302041 | 0.003163333 |
| PAN3 | greenyellov | -0.035762886 | 0.440696272 | -0.082126933 | 0.076225754 |
| PAXBP1 | greenyellov | -0.023421843 | 0.613654992 | -0.059612379 | 0.198468956 |
| PCF11 | greenyellov | -0.10058202 | 0.029758902 | -0.135010918 | 0.003464691 |
| PCMTD1 | greenyellov | -0.089098205 | 0.054342477 | -0.125046906 | 0.006816634 |
| PCMTD2 | greenyellov | -0.236861928 | 2.23E-07 | -0.291554155 | 1.33E-10 |
| PHYKPL | greenyellov | -0.155560927 | 0.00074287 | -0.144513554 | 0.001741849 |
| PIAS2 | greenyellov | -0.056199139 | 0.225444597 | -0.069112353 | 0.135879702 |
| PIGL | greenyellov | 0.016410543 | 0.723557536 | 0.026328249 | 0.570352873 |
| PMS1 | greenyellov | -0.046437519 | 0.316645954 | -0.086709191 | 0.061164093 |
| PNISR | greenyellov | -0.145391431 | 0.001631196 | -0.168010282 | 0.000265363 |
| POC5 | greenyellov | -0.049090146 | 0.289762696 | -0.057513334 | 0.214764688 |
| POLG2 | greenyellov | -0.103769466 | 0.024927497 | -0.116004652 | 0.012119761 |
| POLI | greenyellov | 0.091942254 | 0.047058658 | 0.071299606 | 0.123895392 |
| PPWD1 | greenyellov | -0.008070429 | 0.861913501 | -0.046115216 | 0.320020078 |
| PRPF38B | greenyellov | -0.068610011 | 0.138754177 | -0.099051853 | 0.032350135 |
| PRPF39 | greenyellov | -0.054939943 | 0.236027122 | -0.088074241 | 0.057184622 |
| PTBP2 | greenyellov | -0.342507931 | 2.67E-14 | -0.375166686 | 4.69E-17 |
| PUS10 | greenyellov | -0.072024591 | 0.120109892 | -0.086483831 | 0.061842563 |
| RAD52 | greenyellov | -0.156847589 | 0.000670178 | -0.190698099 | 3.35E-05 |
| RANBP1' | greenyellov | 0.13660992 | 0.003095082 | 0.118864607 | 0.010142809 |
| RASA2 | greenyellov | -0.213656151 | 3.18E-06 | -0.242865686 | 1.07E-07 |
| RBBP6 | greenyellov | -0.088417276 | 0.05621906 | -0.113306181 | 0.014289613 |
| RBM25 | greenyellov | 0.011447144 | 0.80512492 | -0.025771987 | 0.578527362 |

| | | | | | |
|---|---|---|---|---|---|
| RBM39 | greenyellov | -0.189097471 | 3.91E-05 | -0.226487141 | 7.59E-07 |
| RBM5 | greenyellov | 0.083487139 | 0.071469121 | 0.05336745 | 0.249728132 |
| RBM6 | greenyellov | -0.100572815 | 0.029773942 | -0.127246196 | 0.005893552 |
| RIMKLB | greenyellov | -0.169920103 | 0.000225116 | -0.200289648 | 1.29E-05 |
| RNPC3 | greenyellov | -0.28620509 | 2.96E-10 | -0.29535844 | 7.44E-11 |
| RP3-509I | greenyellov | -0.146083033 | 0.001548604 | -0.137309941 | 0.002944854 |
| RSRC2 | greenyellov | -0.022053672 | 0.634527209 | -0.065218099 | 0.159398826 |
| RSRP1 | greenyellov | 0.00036656 | 0.993696612 | -0.001040236 | 0.982113365 |
| SCLT1 | greenyellov | -0.038843466 | 0.402322309 | -0.047625557 | 0.304410271 |
| SENP6 | greenyellov | -0.134633001 | 0.0035577 | -0.171991086 | 0.000187966 |
| SENP7 | greenyellov | -0.105587009 | 0.022488051 | -0.128582139 | 0.005389251 |
| SEPSECS | greenyellov | 0.110774792 | 0.016628409 | 0.089779224 | 0.052518046 |
| SETD4 | greenyellov | -0.070326518 | 0.129121459 | -0.07459096 | 0.107431118 |
| SLC25A2 | greenyellov | -0.230058953 | 5.01E-07 | -0.251613386 | 3.55E-08 |
| SLC25A3 | greenyellov | -0.029680847 | 0.522282772 | -0.067273527 | 0.146629023 |
| SLC35A1 | greenyellov | -0.032623051 | 0.481876309 | -0.030010261 | 0.517674068 |
| SLX4IP | greenyellov | 0.0320303 | 0.489878051 | 0.023396739 | 0.614035237 |
| SMIM8 | greenyellov | -0.049849178 | 0.282360708 | -0.050281281 | 0.278204476 |
| SNX15 | greenyellov | -0.166953548 | 0.000290428 | -0.201451785 | 1.15E-05 |
| SREK1 | greenyellov | 0.033907667 | 0.464780767 | 0.008119203 | 0.861087386 |
| SRRM5 | greenyellov | 0.003646099 | 0.937365138 | -0.00951508 | 0.837512591 |
| SRSF10 | greenyellov | 0.011192839 | 0.809370183 | -0.021636393 | 0.640952152 |
| SRSF11 | greenyellov | -0.092049744 | 0.04680026 | -0.135113939 | 0.003439722 |
| SRSF5 | greenyellov | -0.152816174 | 0.000922963 | -0.180014037 | 9.16E-05 |
| SRSF6 | greenyellov | -0.100029203 | 0.030673762 | -0.145696261 | 0.001594307 |
| STX16 | greenyellov | -0.206158016 | 7.07E-06 | -0.234249516 | 3.06E-07 |
| SUPT20H | greenyellov | 0.059976731 | 0.195734666 | 0.043048616 | 0.353290517 |
| SUPT7L | greenyellov | -0.195298917 | 2.14E-05 | -0.218183925 | 1.94E-06 |
| TARBP1 | greenyellov | 0.035747228 | 0.44089651 | 0.019589358 | 0.672853049 |
| TBC1D32 | greenyellov | -0.154171714 | 0.000829506 | -0.167141089 | 0.000285824 |
| TBRG1 | greenyellov | -0.0313586 | 0.499030981 | -0.047977612 | 0.30084535 |
| TCERG1 | greenyellov | -0.054991617 | 0.235586074 | -0.088822126 | 0.055096952 |
| TCTE3 | greenyellov | -0.184908415 | 5.82E-05 | -0.183725288 | 6.50E-05 |
| THAP9 | greenyellov | 0.005065481 | 0.913065026 | -0.028308051 | 0.541712758 |
| THOC1 | greenyellov | -0.090154355 | 0.051535072 | -0.124598777 | 0.007019889 |
| THOC2 | greenyellov | 0.039338496 | 0.396347669 | 0.006617778 | 0.886582657 |
| THUMPD | greenyellov | -0.057017907 | 0.218747308 | -0.087819571 | 0.057910313 |
| TIA1 | greenyellov | -0.070654719 | 0.127340076 | -0.101524443 | 0.028253171 |
| TIAM2 | greenyellov | -0.116443798 | 0.011795643 | -0.123799306 | 0.007396009 |
| TIGD1 | greenyellov | -0.12287657 | 0.00785244 | -0.135249604 | 0.00340709 |
| TMEM26 | greenyellov | 0.091112006 | 0.049095098 | 0.066078127 | 0.153957251 |
| TRA2A | greenyellov | 0.105399857 | 0.022729302 | 0.087892566 | 0.057701537 |
| TRIM45 | greenyellov | 0.020210415 | 0.663108984 | -0.029366431 | 0.526701158 |
| TRIM52 | greenyellov | -0.083521355 | 0.071352668 | -0.101239421 | 0.028701498 |
| TRMT10I | greenyellov | 0.079768829 | 0.085079661 | 0.06046355 | 0.192124419 |

| | | | | | |
|---|---|---|---|---|---|
| TRMT13 | greenyellow | -0.088405328 | 0.056252463 | -0.109837333 | 0.017575985 |
| TSEN2 | greenyellow | -0.115079257 | 0.012828631 | -0.134308327 | 0.003639409 |
| TTC14 | greenyellow | -0.347376617 | 1.09E-14 | -0.35850153 | 1.31E-15 |
| TTLL3 | greenyellow | -0.058926052 | 0.203694926 | -0.072072138 | 0.119864816 |
| TUBE1 | greenyellow | -0.091116498 | 0.049083885 | -0.125207781 | 0.006744963 |
| TXK | greenyellow | 0.073475245 | 0.1128069 | 0.089588002 | 0.053025095 |
| TYW5 | greenyellow | -0.08996126 | 0.052039115 | -0.1006287 | 0.029682734 |
| U2SURP | greenyellow | -0.139164622 | 0.002578346 | -0.174436089 | 0.000151512 |
| UBN2 | greenyellow | -0.020897871 | 0.652389088 | -0.037549327 | 0.418193859 |
| UPF3A | greenyellow | -0.036597342 | 0.430100274 | -0.065487388 | 0.157679607 |
| USP45 | greenyellow | -0.11367668 | 0.013972774 | -0.106874091 | 0.020888231 |
| USPL1 | greenyellow | -0.003555441 | 0.938919438 | -0.04214628 | 0.363481172 |
| VCPKMT | greenyellow | -0.070077458 | 0.130486117 | -0.095067497 | 0.04001701 |
| VPS18 | greenyellow | 0.255994112 | 2.01E-08 | 0.273222283 | 1.94E-09 |
| WDR27 | greenyellow | -0.142617284 | 0.002004714 | -0.178985607 | 0.00010066 |
| WDR75 | greenyellow | -0.012995711 | 0.779401646 | -0.037730841 | 0.415945834 |
| WSB1 | greenyellow | -0.081635406 | 0.078006686 | -0.111422483 | 0.016000134 |
| YAF2 | greenyellow | -0.077910738 | 0.092625057 | -0.088067967 | 0.057202409 |
| ZBED5 | greenyellow | -0.133695439 | 0.0037983 | -0.156420084 | 0.000693565 |
| ZBED8 | greenyellow | -0.095463475 | 0.039191926 | -0.116454742 | 0.011787664 |
| ZBTB25 | greenyellow | 0.020983764 | 0.651054663 | 0.023828577 | 0.607508728 |
| ZCCHC8 | greenyellow | -0.068692038 | 0.138281642 | -0.092573479 | 0.045558199 |
| ZDHHC1' | greenyellow | -0.172786685 | 0.000175287 | -0.198381088 | 1.57E-05 |
| ZFC3H1 | greenyellow | -0.145695674 | 0.001594377 | -0.173553112 | 0.000163835 |
| ZFP62 | greenyellow | 0.010380828 | 0.822962402 | -0.023191441 | 0.61714874 |
| ZMAT1 | greenyellow | -0.099588494 | 0.031420241 | -0.122345856 | 0.008126204 |
| ZMYM5 | greenyellow | 0.023282277 | 0.615770282 | 0.003673445 | 0.936896338 |
| ZMYM6 | greenyellow | -0.143443822 | 0.00188597 | -0.141192798 | 0.002225522 |
| ZNF10 | greenyellow | -0.050926818 | 0.272072973 | -0.083434174 | 0.071649691 |
| ZNF100 | greenyellow | -0.040451888 | 0.383106377 | -0.054039744 | 0.243804171 |
| ZNF138 | greenyellow | 0.039390051 | 0.395728519 | 0.021102631 | 0.649209798 |
| ZNF140 | greenyellow | 0.020134158 | 0.66430241 | -0.016990603 | 0.714206533 |
| ZNF141 | greenyellow | -0.036369308 | 0.432981129 | -0.052500512 | 0.25751416 |
| ZNF18 | greenyellow | 0.188557655 | 4.12E-05 | 0.168395019 | 0.00025675 |
| ZNF195 | greenyellow | -0.021246859 | 0.646974198 | -0.034576212 | 0.456018633 |
| ZNF202 | greenyellow | -0.081643004 | 0.077978902 | -0.131968399 | 0.004280402 |
| ZNF224 | greenyellow | 0.037689865 | 0.416452696 | 0.030069904 | 0.516841871 |
| ZNF251 | greenyellow | -0.208199191 | 5.71E-06 | -0.244923175 | 8.30E-08 |
| ZNF254 | greenyellow | 0.036808452 | 0.427443133 | 0.023434653 | 0.613460994 |
| ZNF26 | greenyellow | -0.090955387 | 0.049487405 | -0.112972044 | 0.014580757 |
| ZNF266 | greenyellow | 0.040238588 | 0.38562197 | 0.029968549 | 0.518256475 |
| ZNF268 | greenyellow | 0.045028232 | 0.331571646 | 0.033439168 | 0.470976251 |
| ZNF273 | greenyellow | -0.170454184 | 0.000214928 | -0.173259916 | 0.00016813 |
| ZNF326 | greenyellow | -0.097570565 | 0.035039752 | -0.129372194 | 0.005109624 |
| ZNF331 | greenyellow | -0.186945504 | 4.80E-05 | -0.202841962 | 9.98E-06 |

| | | | | | |
|---|---|---|---|---|---|
| ZNF354A | greenyellov | -0.090708198 | 0.050111904 | -0.123661644 | 0.007462562 |
| ZNF354B | greenyellov | -0.115255766 | 0.012690665 | -0.153607514 | 0.000867287 |
| ZNF429 | greenyellov | 0.022037653 | 0.634773352 | 0.035294435 | 0.446709501 |
| ZNF43 | greenyellov | -0.056384454 | 0.223916145 | -0.059561563 | 0.198852505 |
| ZNF430 | greenyellov | -0.121469035 | 0.008597196 | -0.134140315 | 0.003682355 |
| ZNF431 | greenyellov | -0.064542235 | 0.163775953 | -0.077297741 | 0.09522868 |
| ZNF432 | greenyellov | 0.163460559 | 0.000389897 | 0.148147775 | 0.00132433 |
| ZNF433 | greenyellov | 0.059353852 | 0.200425892 | 0.052126573 | 0.260923821 |
| ZNF439 | greenyellov | -0.18113574 | 8.27E-05 | -0.202146171 | 1.07E-05 |
| ZNF44 | greenyellov | -0.133192389 | 0.003933382 | -0.146998821 | 0.001445144 |
| ZNF443 | greenyellov | 0.118355482 | 0.010472272 | 0.095339713 | 0.039448242 |
| ZNF449 | greenyellov | -0.096735335 | 0.03663855 | -0.122510449 | 0.008040403 |
| ZNF493 | greenyellov | -0.048684089 | 0.293775501 | -0.064435118 | 0.164477892 |
| ZNF514 | greenyellov | -0.194257364 | 2.37E-05 | -0.225861818 | 8.15E-07 |
| ZNF577 | greenyellov | -0.087550694 | 0.058684755 | -0.109213195 | 0.018232721 |
| ZNF596 | greenyellov | 0.08584432 | 0.063801758 | 0.066488117 | 0.151413236 |
| ZNF600 | greenyellov | 0.011016189 | 0.812322385 | -0.012908875 | 0.780838011 |
| ZNF605 | greenyellov | -0.055398864 | 0.232130418 | -0.083678032 | 0.070821401 |
| ZNF638 | greenyellov | -0.108536571 | 0.018968781 | -0.149386012 | 0.00120458 |
| ZNF66 | greenyellov | -0.046088143 | 0.320304566 | -0.041332265 | 0.372829963 |
| ZNF675 | greenyellov | -0.021793393 | 0.638531608 | -0.028771605 | 0.535111798 |
| ZNF680 | greenyellov | -0.004457939 | 0.923457682 | -0.0192052 | 0.678907937 |
| ZNF681 | greenyellov | 0.048708582 | 0.293532409 | 0.036338024 | 0.433377229 |
| ZNF682 | greenyellov | 0.035562603 | 0.44326151 | 0.025672661 | 0.579992754 |
| ZNF684 | greenyellov | -0.269739648 | 3.15E-09 | -0.284991634 | 3.55E-10 |
| ZNF700 | greenyellov | 0.021195952 | 0.647762926 | -0.006456471 | 0.889329209 |
| ZNF708 | greenyellov | -0.041114452 | 0.375356462 | -0.04956838 | 0.285083984 |
| ZNF709 | greenyellov | -0.114362211 | 0.013402797 | -0.101417528 | 0.028420634 |
| ZNF721 | greenyellov | 0.076479041 | 0.098796752 | 0.052557109 | 0.257000795 |
| ZNF736 | greenyellov | -0.187678698 | 4.48E-05 | -0.190449558 | 3.43E-05 |
| ZNF738 | greenyellov | -0.112128301 | 0.015339253 | -0.113066781 | 0.014497684 |
| ZNF75A | greenyellov | -0.249907779 | 4.42E-08 | -0.265601967 | 5.56E-09 |
| ZNF763 | greenyellov | -0.082058792 | 0.076470657 | -0.082660706 | 0.074329341 |
| ZNF782 | greenyellov | 0.016429097 | 0.72325779 | 0.000272781 | 0.995309222 |
| ZNF789 | greenyellov | -0.006988038 | 0.880283452 | -0.00822472 | 0.859300737 |
| ZNF799 | greenyellov | 0.094588034 | 0.041035663 | 0.077481507 | 0.094442098 |
| ZNF823 | greenyellov | -0.061595386 | 0.183919821 | -0.085715381 | 0.064202898 |
| ZNF83 | greenyellov | -0.014831284 | 0.749221999 | -0.029560708 | 0.523968804 |
| ZNF84 | greenyellov | -0.001280403 | 0.977984694 | -0.035951662 | 0.438286205 |
| ZNF85 | greenyellov | -0.083022896 | 0.073064558 | -0.078022819 | 0.092155231 |
| ZNF92 | greenyellov | -0.021560709 | 0.642120375 | -0.034727014 | 0.454055069 |
| ZNF93 | greenyellov | 0.072210141 | 0.119155706 | 0.066230459 | 0.153008267 |
| ZRANB2 | greenyellov | -0.165460054 | 0.00032964 | -0.19880876 | 1.50E-05 |
| ZSCAN3( | greenyellov | -0.091722049 | 0.047591753 | -0.145038402 | 0.001674895 |
| ZSCAN9 | greenyellov | -0.013415003 | 0.772476722 | -0.035607705 | 0.442683102 |

| | | | | | |
|---|---|---|---|---|---|
| A1BG | grey | -0.170981411 | 0.000205295 | -0.156410058 | 0.000694123 |
| A4GALT | grey | 0.142895954 | 0.001963936 | 0.143243426 | 0.001914153 |
| AADAC | grey | -0.039931585 | 0.389260263 | -0.04177904 | 0.367680601 |
| AADACL | grey | 0.075654708 | 0.102496047 | 0.058231529 | 0.209084213 |
| AADAT | grey | 0.255602848 | 2.12E-08 | 0.210002463 | 4.71E-06 |
| AANAT | grey | -0.220469228 | 1.50E-06 | -0.218214618 | 1.93E-06 |
| AARD | grey | -0.313127931 | 4.41E-12 | -0.333923989 | 1.26E-13 |
| AATF | grey | -0.00706873 | 0.878911617 | -0.017344065 | 0.708529258 |
| AATK | grey | -0.156466608 | 0.000690984 | -0.177739169 | 0.000112706 |
| ABCA2 | grey | 0.051167165 | 0.269813825 | 0.057387961 | 0.215767575 |
| ABCA3 | grey | -0.25156715 | 3.57E-08 | -0.268200265 | 3.90E-09 |
| ABCA4 | grey | -0.090787371 | 0.049911169 | -0.095040558 | 0.040073671 |
| ABCB5 | grey | -0.153592493 | 0.000868315 | -0.149118827 | 0.001229542 |
| ABCB6 | grey | 0.000652922 | 0.988772585 | 0.000850439 | 0.985376475 |
| ABCB9 | grey | 0.09193637 | 0.047072837 | 0.100953444 | 0.029157452 |
| ABCC12 | grey | -0.054937204 | 0.23605052 | -0.051579556 | 0.265967492 |
| ABCC2 | grey | 0.002823746 | 0.951472094 | -0.004965494 | 0.914774453 |
| ABCC3 | grey | 0.246807681 | 6.55E-08 | 0.257651415 | 1.62E-08 |
| ABCC8 | grey | -0.101424935 | 0.028409004 | -0.091954109 | 0.047030101 |
| ABCD4 | grey | -0.122839473 | 0.007871306 | -0.144692543 | 0.001718745 |
| ABCG1 | grey | 0.150559706 | 0.001100355 | 0.175701784 | 0.000135357 |
| ABCG4 | grey | -0.237003562 | 2.20E-07 | -0.257462451 | 1.66E-08 |
| ABHD1 | grey | -0.218797023 | 1.81E-06 | -0.226195088 | 7.85E-07 |
| ABHD10 | grey | 0.146433896 | 0.001508188 | 0.139089273 | 0.002592386 |
| ABHD14 | grey | -0.040659447 | 0.380668148 | -0.061103481 | 0.187453256 |
| ABHD15 | grey | 0.16390105 | 0.000375802 | 0.164705235 | 0.000351286 |
| ABHD4 | grey | 0.029403866 | 0.526174102 | -0.001895042 | 0.967421471 |
| ABHD8 | grey | -0.046159835 | 0.319551585 | -0.029645698 | 0.522775761 |
| ABLIM1 | grey | -0.086981807 | 0.060351577 | -0.070488217 | 0.128241405 |
| ABO | grey | -0.207857098 | 5.92E-06 | -0.248183919 | 5.51E-08 |
| ABR | grey | 0.230369855 | 4.83E-07 | 0.224625659 | 9.39E-07 |
| AC00231 | grey | 0.021318031 | 0.645872181 | 0.02303283 | 0.619558905 |
| AC00298 | grey | -0.005423529 | 0.906947056 | -0.017740154 | 0.702186453 |
| AC00300 | grey | 0.059812556 | 0.196963297 | 0.046485566 | 0.316144957 |
| AC00300 | grey | -0.161147324 | 0.000472351 | -0.151316355 | 0.001037641 |
| AC00407 | grey | -0.057834524 | 0.212210718 | -0.049143113 | 0.289241975 |
| AC00407 | grey | -0.196665929 | 1.86E-05 | -0.189859619 | 3.64E-05 |
| AC00415 | grey | -0.116105273 | 0.012044809 | -0.126337995 | 0.00626019 |
| AC00475 | grey | -0.102142257 | 0.027301985 | -0.121019059 | 0.008848225 |
| AC00481 | grey | -0.075257702 | 0.104316434 | -0.084143698 | 0.069261461 |
| AC00482 | grey | -0.035782717 | 0.44044274 | -0.042207275 | 0.362786591 |
| AC00638 | grey | -0.107925584 | 0.019655555 | -0.104859413 | 0.023438594 |
| AC00648 | grey | -0.000372839 | 0.993588647 | -0.008099644 | 0.861418648 |
| AC00653 | grey | 0.032205266 | 0.487508733 | 0.022695384 | 0.624700093 |
| AC00716 | grey | -0.109485319 | 0.017943796 | -0.114519845 | 0.013274668 |

| | | | | | |
|---|---|---|---|---|---|
| AC00732 | grey | 0.09581343 | 0.03847477 | 0.091668855 | 0.047721287 |
| AC00868 | grey | -0.07358679 | 0.112260124 | -0.075978964 | 0.101028014 |
| AC00875 | grey | 0.116604248 | 0.011679145 | 0.113454137 | 0.014162336 |
| AC00881 | grey | -0.102995019 | 0.0260344 | -0.106376353 | 0.021494788 |
| AC00891 | grey | 0.026025035 | 0.574801929 | 0.036318474 | 0.433624861 |
| AC00902 | grey | -0.123951843 | 0.007322885 | -0.124112716 | 0.007246466 |
| AC00906 | grey | -0.050058594 | 0.280341201 | -0.01400975 | 0.76268507 |
| AC00913 | grey | 0.090334267 | 0.051069113 | 0.080861273 | 0.080879916 |
| AC00949 | grey | 0.028362981 | 0.540928462 | 0.047792549 | 0.302715833 |
| AC00997 | grey | -0.005084507 | 0.912739795 | 0.022540548 | 0.627065251 |
| AC01018 | grey | -0.031501041 | 0.497082465 | -0.016626685 | 0.720068281 |
| AC01064 | grey | -0.08295012 | 0.073317275 | -0.09244331 | 0.045864285 |
| AC01151 | grey | 0.057420697 | 0.21550539 | 0.074325947 | 0.108689335 |
| AC01153 | grey | -0.08750578 | 0.058814949 | -0.092315461 | 0.046166596 |
| AC01200 | grey | -0.057605409 | 0.214030303 | -0.054801657 | 0.237210305 |
| AC01614 | grey | -0.023018731 | 0.619773333 | -0.025801887 | 0.578086577 |
| AC01708 | grey | -0.050854542 | 0.272754856 | -0.052020371 | 0.261897844 |
| AC01851 | grey | -0.023634547 | 0.610437356 | -0.003240561 | 0.94431971 |
| AC02221 | grey | -0.140722417 | 0.002303177 | -0.143606716 | 0.001863341 |
| AC02243 | grey | -0.209467692 | 4.99E-06 | -0.207602284 | 6.08E-06 |
| AC02436 | grey | 0.02593966 | 0.576057581 | 0.031598889 | 0.495746298 |
| AC02459 | grey | -0.008271808 | 0.85850365 | -0.006963704 | 0.880697205 |
| AC02526 | grey | 0.014658765 | 0.752043023 | 0.007883094 | 0.865087833 |
| AC03745 | grey | -0.058999665 | 0.203129674 | -0.060897308 | 0.188948993 |
| AC05573 | grey | -0.034833882 | 0.452666433 | -0.037026064 | 0.424714168 |
| AC05586 | grey | 0.18249597 | 7.29E-05 | 0.20859794 | 5.47E-06 |
| AC06482 | grey | -0.017852527 | 0.700390702 | -0.008487137 | 0.854860521 |
| AC06853 | grey | -0.034160807 | 0.461452108 | -0.064097316 | 0.166706304 |
| AC07307 | grey | -0.072224814 | 0.119080505 | -0.074305685 | 0.108786011 |
| AC07310 | grey | 0.066012482 | 0.154367576 | 0.036152484 | 0.43573067 |
| AC07333 | grey | -0.038390208 | 0.407839729 | -0.03948898 | 0.394542066 |
| AC07361 | grey | 0.092175335 | 0.046499856 | 0.08872521 | 0.055363861 |
| AC07935 | grey | 0.119571686 | 0.009700513 | 0.125994795 | 0.006403981 |
| AC07990 | grey | -0.05157754 | 0.265986202 | -0.059882808 | 0.19643687 |
| AC08421 | grey | -0.152095473 | 0.000976523 | -0.159069055 | 0.000559981 |
| AC08765 | grey | -0.071677641 | 0.121910072 | -0.074666151 | 0.107076232 |
| AC08765 | grey | -0.000404243 | 0.993048636 | -0.003885392 | 0.933263692 |
| AC09229 | grey | 0.057510103 | 0.214790494 | 0.054265701 | 0.241835412 |
| AC10405 | grey | 0.109842843 | 0.017570281 | 0.067079391 | 0.147800717 |
| AC10438 | grey | 0.042417848 | 0.360395071 | 0.049919598 | 0.281680518 |
| AC10438 | grey | -0.042892082 | 0.355045324 | -0.04405528 | 0.34213639 |
| AC10453 | grey | -0.048418832 | 0.296416846 | -0.062906462 | 0.174742654 |
| AC10458 | grey | -0.168063657 | 0.000264152 | -0.177372147 | 0.000116504 |
| AC10498 | grey | -0.161622943 | 0.000454177 | -0.164080894 | 0.000370185 |
| AC10892 | grey | -0.114477028 | 0.013309363 | -0.119940846 | 0.009476473 |

| | | | | | |
|---|---|---|---|---|---|
| AC10982‹ | grey | -0.296523303 | 6.22E-11 | -0.317185289 | 2.25E-12 |
| AC11061: | grey | -0.0303882 | 0.51241242 | -0.034826805 | 0.452758324 |
| AC13896‹ | grey | -0.089142011 | 0.054223558 | -0.100073051 | 0.030600329 |
| AC23458: | grey | 0.031299157 | 0.499845312 | 0.046070276 | 0.320492408 |
| AC23458: | grey | -0.025909886 | 0.576495779 | -0.021543039 | 0.642393243 |
| AC24137' | grey | 0.00278168 | 0.952194152 | 0.004706154 | 0.919210037 |
| AC24158: | grey | -0.008719392 | 0.850934458 | -0.007475773 | 0.871997218 |
| AC24394: | grey | -0.101779144 | 0.027857639 | -0.096950152 | 0.036221542 |
| AC24423( | grey | -0.029286237 | 0.527831138 | -0.043504908 | 0.348206562 |
| AC25357: | grey | -0.058197072 | 0.209354243 | -0.047188374 | 0.308875959 |
| ACAA1 | grey | 0.203665958 | 9.16E-06 | 0.213481109 | 3.24E-06 |
| ACAN | grey | 0.174041084 | 0.000156913 | 0.188007785 | 4.34E-05 |
| ACAP3 | grey | 0.065486574 | 0.157684782 | 0.092042453 | 0.046817749 |
| ACBD4 | grey | 0.010202108 | 0.825961277 | -0.001155408 | 0.980133397 |
| ACE | grey | 0.04106083 | 0.375980052 | 0.021959031 | 0.635982044 |
| ACE2 | grey | 0.038622064 | 0.405011798 | 0.066559099 | 0.150976043 |
| ACHE | grey | -0.254405612 | 2.48E-08 | -0.231675789 | 4.15E-07 |
| ACKR3 | grey | -0.29058347 | 1.54E-10 | -0.278936696 | 8.58E-10 |
| ACOT1 | grey | 0.0909131 | 0.049593776 | 0.088858948 | 0.054995824 |
| ACOT11 | grey | 0.041002717 | 0.376656599 | 0.021379969 | 0.644913755 |
| ACOT2 | grey | 0.102193751 | 0.027223963 | 0.086794402 | 0.060909162 |
| ACOT4 | grey | 0.11429427 | 0.013458357 | 0.144820656 | 0.001702381 |
| ACOT9 | grey | 0.045375006 | 0.327857581 | 0.046860167 | 0.312256716 |
| ACOX3 | grey | 0.32401541 | 7.08E-13 | 0.300568254 | 3.31E-11 |
| ACP6 | grey | 0.222910653 | 1.14E-06 | 0.208820779 | 5.34E-06 |
| ACPP | grey | -0.079901366 | 0.08456096 | -0.087662798 | 0.058360828 |
| ACR | grey | -0.30273324 | 2.36E-11 | -0.312747646 | 4.69E-12 |
| ACRBP | grey | 0.003482982 | 0.940161883 | -0.000317917 | 0.994533069 |
| ACRC | grey | -0.200579901 | 1.26E-05 | -0.224370841 | 9.67E-07 |
| ACSF2 | grey | -0.199500112 | 1.40E-05 | -0.177237766 | 0.000117924 |
| ACSM3 | grey | -0.018100609 | 0.696432176 | -0.018290211 | 0.693412338 |
| ACSM4 | grey | -0.075648689 | 0.10252346 | -0.072373168 | 0.118322239 |
| ACSS2 | grey | -0.029418881 | 0.525962775 | -0.055911399 | 0.227832532 |
| ACTA1 | grey | 0.038387258 | 0.407875788 | 0.057920707 | 0.211529161 |
| ACTBL2 | grey | -0.007100842 | 0.878365807 | -0.007296624 | 0.875039237 |
| ACTG1 | grey | 0.206168112 | 7.06E-06 | 0.205420179 | 7.64E-06 |
| ACTL8 | grey | -0.270637526 | 2.78E-09 | -0.274377099 | 1.64E-09 |
| ACTN2 | grey | -0.186628464 | 4.95E-05 | -0.185628883 | 5.44E-05 |
| ACTN4 | grey | -0.067986314 | 0.142387756 | -0.067336904 | 0.146248058 |
| ACTR1A | grey | 0.290953857 | 1.46E-10 | 0.313343091 | 4.25E-12 |
| ACTR5 | grey | -0.025766717 | 0.578605082 | -0.036299273 | 0.433868149 |
| ACTRT2 | grey | -0.155684782 | 0.000735568 | -0.150599878 | 0.00109694 |
| ACTRT3 | grey | -0.313832095 | 3.92E-12 | -0.311555841 | 5.70E-12 |
| ACYP1 | grey | 0.022169128 | 0.632754325 | 0.018537941 | 0.689473974 |
| AD00067 | grey | 0.016063125 | 0.729178049 | -0.021709403 | 0.639826042 |

| | | | | | |
|---|---|---|---|---|---|
| ADAD2 | grey | -0.039050152 | 0.399821249 | -0.040909242 | 0.377746386 |
| ADAL | grey | -0.120948778 | 0.008888017 | -0.104112619 | 0.02445017 |
| ADAM15 | grey | 0.278088313 | 9.69E-10 | 0.28259819 | 5.04E-10 |
| ADAM21 | grey | -0.212811473 | 3.49E-06 | -0.232539167 | 3.74E-07 |
| ADAMTS | grey | -0.001187274 | 0.979585588 | -0.02360738 | 0.610847894 |
| ADAMTS | grey | -0.168550315 | 0.000253348 | -0.170305704 | 0.000217716 |
| ADAMTS | grey | -0.179931998 | 9.23E-05 | -0.212821175 | 3.48E-06 |
| ADAMTS | grey | -0.31396064 | 3.84E-12 | -0.326383187 | 4.71E-13 |
| ADAMTS | grey | -0.085651281 | 0.064403091 | -0.105710105 | 0.022330584 |
| ADAMTS | grey | -0.140039157 | 0.002420387 | -0.14102873 | 0.002252332 |
| ADAMTS | grey | -0.053379476 | 0.249621295 | -0.056552797 | 0.222534086 |
| ADAMTS | grey | -0.125074132 | 0.006804457 | -0.128408801 | 0.005452413 |
| ADARB2 | grey | -0.249476684 | 4.67E-08 | -0.245385419 | 7.83E-08 |
| ADCK2 | grey | 0.05650233 | 0.22294777 | 0.050742892 | 0.273810484 |
| ADCK3 | grey | -0.123991621 | 0.007303922 | -0.133978893 | 0.003724049 |
| ADCK4 | grey | -0.024553555 | 0.596621908 | -0.019281662 | 0.677701123 |
| ADCY1 | grey | -0.017454236 | 0.706762991 | 0.011321582 | 0.807220294 |
| ADCY8 | grey | -0.190057089 | 3.57E-05 | -0.193628699 | 2.52E-05 |
| ADI1 | grey | 0.139229787 | 0.002566259 | 0.141694292 | 0.002145371 |
| ADIG | grey | -0.119743325 | 0.009595769 | -0.107659543 | 0.019961298 |
| ADIPOQ | grey | -0.086584973 | 0.061537301 | -0.089486799 | 0.053295095 |
| ADIRF | grey | -0.014531265 | 0.754130033 | 0.026260435 | 0.57134649 |
| ADM | grey | 0.357566743 | 1.57E-15 | 0.371196943 | 1.05E-16 |
| ADM5 | grey | -0.079695658 | 0.085367123 | -0.102493354 | 0.02677381 |
| ADORA1 | grey | -0.137174618 | 0.002973368 | -0.126026163 | 0.006390717 |
| ADORA2 | grey | 0.167223901 | 0.000283813 | 0.178495646 | 0.000105243 |
| ADRA1B | grey | -0.297086747 | 5.70E-11 | -0.300386819 | 3.41E-11 |
| ADRA2B | grey | -0.262150183 | 8.88E-09 | -0.274471069 | 1.62E-09 |
| ADRB1 | grey | -0.016333866 | 0.724796747 | 0.029986537 | 0.518005273 |
| ADSSL1 | grey | -0.143425039 | 0.001888595 | -0.142898258 | 0.001963602 |
| AEN | grey | 0.365594888 | 3.25E-16 | 0.360748294 | 8.45E-16 |
| AF13121 | grey | -0.115449908 | 0.01254043 | -0.117144267 | 0.011294496 |
| AF19677 | grey | -0.014071581 | 0.761669238 | -0.02270093 | 0.624615452 |
| AFF2 | grey | 0.137003894 | 0.003009698 | 0.106478221 | 0.021369419 |
| AFM | grey | -0.064107052 | 0.166641761 | -0.056740339 | 0.221001589 |
| AFMID | grey | 0.117596056 | 0.010981349 | 0.10372134 | 0.024995079 |
| AGA | grey | 0.237294074 | 2.12E-07 | 0.228357285 | 6.11E-07 |
| AGMO | grey | -0.136023237 | 0.003226315 | -0.139234222 | 0.002565439 |
| AGPAT3 | grey | 0.155958363 | 0.000719672 | 0.170584773 | 0.000212503 |
| AGPAT9 | grey | -0.127593416 | 0.005758559 | -0.10544525 | 0.022670583 |
| AGT | grey | -0.079854645 | 0.084743517 | -0.079852313 | 0.084752638 |
| AGTR2 | grey | -0.020093066 | 0.664945842 | -0.032809256 | 0.47937738 |
| AHDC1 | grey | -0.229092538 | 5.61E-07 | -0.222325153 | 1.22E-06 |
| AHNAK2 | grey | -0.168529252 | 0.000253807 | -0.176021145 | 0.000131544 |
| AHRR | grey | -0.110773666 | 0.01662952 | -0.099901331 | 0.030888774 |

| | | | | | |
|---|---|---|---|---|---|
| AIF1L | grey | -0.250153673 | 4.28E-08 | -0.295395468 | 7.40E-11 |
| AIFM2 | grey | 0.176217587 | 0.000129249 | 0.212519125 | 3.60E-06 |
| AIG1 | grey | -0.124670975 | 0.00698678 | -0.118389464 | 0.01044999 |
| AIM1 | grey | 0.102643748 | 0.026550267 | 0.071460049 | 0.123049746 |
| AIM1L | grey | -0.008952703 | 0.846994262 | -0.007664301 | 0.868797981 |
| AIRE | grey | -0.249965427 | 4.39E-08 | -0.235568467 | 2.61E-07 |
| AJ239318 | grey | -0.076715548 | 0.097755243 | -0.083873348 | 0.07016365 |
| AJAP1 | grey | -0.242812713 | 1.08E-07 | -0.26288838 | 8.04E-09 |
| AK5 | grey | -0.058792271 | 0.204725106 | -0.079918574 | 0.084493801 |
| AKAP17/ | grey | 0.05426039 | 0.241881559 | 0.037503089 | 0.418767639 |
| AKAP3 | grey | -0.103534921 | 0.025258357 | -0.117917333 | 0.010763367 |
| AKAP6 | grey | -0.130393064 | 0.004767683 | -0.110579124 | 0.016822411 |
| AKAP8 | grey | -0.153964721 | 0.000843187 | -0.183135069 | 6.87E-05 |
| AKAP8L | grey | -0.275270913 | 1.45E-09 | -0.285025621 | 3.53E-10 |
| AKIP1 | grey | 0.26072286 | 1.08E-08 | 0.261407488 | 9.81E-09 |
| AKR1B1 | grey | -0.379028946 | 2.11E-17 | -0.377435922 | 2.93E-17 |
| AKR1B1( | grey | 0.083799585 | 0.070411465 | 0.083082014 | 0.07285979 |
| AKR1B1: | grey | 0.031485663 | 0.497292625 | 0.055438538 | 0.231795696 |
| AKR1C1 | grey | 0.10498748 | 0.023268807 | 0.093627752 | 0.043141793 |
| AKR1C2 | grey | 0.105942583 | 0.0220358 | 0.109075411 | 0.018380554 |
| AKR1E2 | grey | -0.166030046 | 0.000314125 | -0.150106237 | 0.001139596 |
| AKR7A2 | grey | 0.138402442 | 0.002723613 | 0.13845024 | 0.002714288 |
| AKR7A3 | grey | 0.00552006 | 0.905298546 | 0.010474875 | 0.821385366 |
| AKT2 | grey | -0.207169862 | 6.36E-06 | -0.220958874 | 1.42E-06 |
| AL00872: | grey | -0.064011604 | 0.167275305 | -0.063261949 | 0.172314076 |
| AL02154( | grey | -0.029962023 | 0.518347624 | -0.033403533 | 0.471449342 |
| AL13574: | grey | -0.12515113 | 0.006770124 | -0.147844745 | 0.001355258 |
| AL15883( | grey | 0.027283746 | 0.556440933 | 0.022675658 | 0.625001199 |
| AL16242( | grey | 0.064325167 | 0.165200747 | 0.066245078 | 0.152917427 |
| AL35885: | grey | 0.096694665 | 0.036717958 | 0.115841563 | 0.012242121 |
| AL58974: | grey | -0.182193479 | 7.50E-05 | -0.190256477 | 3.50E-05 |
| AL59180( | grey | 0.008638085 | 0.852308463 | 0.023877174 | 0.606776197 |
| AL64572{ | grey | -0.008734204 | 0.850684199 | -0.015881297 | 0.732125464 |
| AL64592: | grey | -0.055163759 | 0.234120974 | -0.058311988 | 0.208454653 |
| ALAD | grey | 0.309942596 | 7.42E-12 | 0.291982272 | 1.25E-10 |
| ALAS2 | grey | 0.002608912 | 0.955160131 | -0.006463971 | 0.889201482 |
| ALCAM | grey | 0.12052807 | 0.009129562 | 0.102746186 | 0.026398929 |
| ALDH1L | grey | 0.059287561 | 0.200929938 | 0.04428362 | 0.339637908 |
| ALDH1L: | grey | -0.111980293 | 0.015475818 | -0.117287286 | 0.011194521 |
| ALDH2 | grey | 0.12848768 | 0.005423588 | 0.140021095 | 0.002423558 |
| ALDH4A | grey | 0.115709446 | 0.012342041 | 0.101643787 | 0.028067241 |
| ALDH7A | grey | 0.124759527 | 0.006946363 | 0.121611584 | 0.008519006 |
| ALDH8A | grey | -0.074107472 | 0.109735315 | -0.072547768 | 0.117434665 |
| ALG1L2 | grey | -0.254888824 | 2.32E-08 | -0.282672252 | 4.99E-10 |
| ALK | grey | -0.252609208 | 3.12E-08 | -0.249870511 | 4.44E-08 |

| | | | | | |
|---|---|---|---|---|---|
| ALKBH3 | grey | -0.490320824 | 1.28E-29 | -0.48731455 | 3.17E-29 |
| ALKBH6 | grey | -0.093456938 | 0.043525809 | -0.092273212 | 0.046266865 |
| ALLC | grey | -0.056568024 | 0.222409375 | -0.055577156 | 0.230628882 |
| ALOX12 | grey | -0.138887486 | 0.002630329 | -0.160818665 | 0.000485302 |
| ALOXE3 | grey | -0.186914977 | 4.82E-05 | -0.160736839 | 0.000488577 |
| ALPI | grey | 0.044634884 | 0.335817211 | 0.05027948 | 0.278221717 |
| ALPK2 | grey | -0.124157981 | 0.007225092 | -0.116254209 | 0.01193462 |
| ALPK3 | grey | -0.067723696 | 0.143939345 | -0.058155044 | 0.209683944 |
| ALPL | grey | 0.031665901 | 0.494832326 | 0.016113525 | 0.728361768 |
| ALPPL2 | grey | 0.127920213 | 0.005634051 | 0.147052637 | 0.001439267 |
| ALS2CL | grey | -0.057086195 | 0.218195234 | -0.062901311 | 0.174778033 |
| ALS2CR1 | grey | -0.321699278 | 1.05E-12 | -0.317002766 | 2.32E-12 |
| ALX1 | grey | -0.234489583 | 2.97E-07 | -0.223562518 | 1.06E-06 |
| ALX3 | grey | -0.174027626 | 0.0001571 | -0.171448028 | 0.000197109 |
| ALX4 | grey | -0.061544303 | 0.18428445 | -0.084575031 | 0.067841735 |
| AMACR | grey | 0.186747849 | 4.89E-05 | 0.147348765 | 0.001407316 |
| AMBP | grey | 0.198279123 | 1.59E-05 | 0.208453173 | 5.56E-06 |
| AMDHD1 | grey | -0.135679288 | 0.003305581 | -0.142096507 | 0.002083003 |
| AMELX | grey | -0.088970172 | 0.054691294 | -0.09560814 | 0.038894105 |
| AMELY | grey | -0.000806164 | 0.986137721 | 0.007802824 | 0.86644866 |
| AMER2 | grey | -0.14736261 | 0.001405838 | -0.145323185 | 0.001639561 |
| AMER3 | grey | -0.074762501 | 0.106622835 | -0.076144831 | 0.10028355 |
| AMH | grey | 0.417015937 | 4.50E-21 | 0.425788452 | 5.45E-22 |
| AMIGO3 | grey | -0.021797353 | 0.638470598 | -0.01354555 | 0.770324294 |
| AMN | grey | -0.14276499 | 0.001983004 | -0.140958723 | 0.002263861 |
| AMPD2 | grey | -0.344166825 | 1.97E-14 | -0.351938289 | 4.61E-15 |
| AMTN | grey | -0.068024368 | 0.142163991 | -0.066218185 | 0.153084568 |
| AMY1A | grey | -0.061516488 | 0.184483222 | -0.049435608 | 0.286377799 |
| AMY1B | grey | -0.077018627 | 0.096433378 | -0.068300367 | 0.140549121 |
| AMY2A | grey | -0.045735565 | 0.324024525 | -0.033994522 | 0.463637175 |
| AMY2B | grey | -0.189401848 | 3.80E-05 | -0.204720636 | 8.21E-06 |
| ANAPC7 | grey | -0.010076319 | 0.828073484 | -0.022358956 | 0.629843975 |
| ANGPT2 | grey | -0.023214343 | 0.616801067 | -0.022262977 | 0.631314765 |
| ANGPT4 | grey | -0.087048921 | 0.060152924 | -0.109616622 | 0.017805823 |
| ANGPTL4 | grey | 0.287976877 | 2.28E-10 | 0.294995781 | 7.87E-11 |
| ANHX | grey | -0.07576711 | 0.101985255 | -0.075728907 | 0.102158638 |
| ANK1 | grey | 0.111577523 | 0.015852864 | 0.133349904 | 0.003890625 |
| ANKH | grey | -0.139022407 | 0.002604903 | -0.108808547 | 0.018669858 |
| ANKRD1 | grey | 0.043469772 | 0.34859639 | 0.013142705 | 0.776971919 |
| ANKRD1 | grey | -0.027195399 | 0.557720309 | -0.053175807 | 0.251435017 |
| ANKRD1 | grey | 0.008905849 | 0.847785245 | -0.014873297 | 0.748535504 |
| ANKRD1 | grey | -0.074070863 | 0.109911355 | -0.083067638 | 0.072909541 |
| ANKRD2 | grey | -0.184639756 | 5.97E-05 | -0.18771674 | 4.46E-05 |
| ANKRD2 | grey | 0.016838234 | 0.716658772 | 0.014588799 | 0.753188059 |
| ANKRD2 | grey | -0.080182599 | 0.083468757 | -0.081467272 | 0.078623577 |

| | | | | | |
|---|---|---|---|---|---|
| ANKRD2 | grey | -0.065324346 | 0.158718831 | -0.0656078 | 0.156915406 |
| ANKRD3 | grey | -0.281099932 | 6.27E-10 | -0.28347524 | 4.43E-10 |
| ANKRD3 | grey | -0.092085407 | 0.04671479 | -0.12342133 | 0.007580026 |
| ANKRD6 | grey | -0.169660387 | 0.000230232 | -0.182292947 | 7.43E-05 |
| ANKRD7 | grey | -0.422586242 | 1.19E-21 | -0.412101815 | 1.43E-20 |
| ANKS6 | grey | 0.029266698 | 0.528106642 | 0.005039838 | 0.913503394 |
| ANO10 | grey | 0.268208364 | 3.89E-09 | 0.282678396 | 4.98E-10 |
| ANO2 | grey | -0.343591816 | 2.19E-14 | -0.353949951 | 3.14E-15 |
| ANO3 | grey | -0.058952342 | 0.203492923 | -0.051725137 | 0.264618704 |
| ANO7 | grey | 0.003504237 | 0.939797422 | -0.004775438 | 0.918024786 |
| ANO8 | grey | 0.230292973 | 4.88E-07 | 0.203675299 | 9.15E-06 |
| ANP32D | grey | 0.041441674 | 0.371564863 | 0.049091499 | 0.289749382 |
| ANPEP | grey | 0.045842201 | 0.322896488 | 0.095139446 | 0.039866009 |
| ANTXRL | grey | -0.1784943 | 0.000105256 | -0.177189754 | 0.000118436 |
| ANXA1 | grey | 0.302738243 | 2.35E-11 | 0.325697915 | 5.31E-13 |
| ANXA11 | grey | -0.068276364 | 0.140689006 | -0.039276802 | 0.397089346 |
| ANXA2 | grey | 0.347725042 | 1.02E-14 | 0.386195251 | 4.65E-18 |
| ANXA2R | grey | 0.115307047 | 0.012650829 | 0.136125399 | 0.003203105 |
| ANXA3 | grey | 0.052548286 | 0.257080777 | 0.065398898 | 0.158242997 |
| ANXA4 | grey | 0.003942824 | 0.93227958 | -0.012090713 | 0.794407298 |
| ANXA9 | grey | -0.121913949 | 0.00835525 | -0.123362705 | 0.007608931 |
| AOC1 | grey | -0.170624388 | 0.000211773 | -0.163387136 | 0.000392293 |
| AOC2 | grey | 0.135142216 | 0.003432897 | 0.131826366 | 0.004322412 |
| AP00027: | grey | -0.193408109 | 2.57E-05 | -0.198220189 | 1.60E-05 |
| AP00029: | grey | -0.14013406 | 0.002403788 | -0.174977908 | 0.000144387 |
| AP00030 | grey | 0.042922221 | 0.354707035 | 0.027234132 | 0.557159236 |
| AP00032 | grey | -0.029481725 | 0.525078755 | -0.04209869 | 0.364023669 |
| AP00035( | grey | 0.024301993 | 0.600389516 | 0.006250409 | 0.892839735 |
| AP000721 | grey | 0.042431334 | 0.360242251 | 0.070472575 | 0.128326337 |
| AP00341 | grey | -0.078771528 | 0.089065484 | -0.07605011 | 0.100708149 |
| AP00628: | grey | 0.165385306 | 0.000331727 | 0.163190886 | 0.000398767 |
| AP1B1 | grey | 0.28031879 | 7.02E-10 | 0.30297034 | 2.27E-11 |
| AP1G2 | grey | 0.13860783 | 0.002683751 | 0.133306817 | 0.003902279 |
| AP1M1 | grey | -0.129769082 | 0.004974144 | -0.11486721 | 0.012996123 |
| AP2A2 | grey | 0.265296458 | 5.80E-09 | 0.271972507 | 2.31E-09 |
| AP3B2 | grey | -0.347008393 | 1.16E-14 | -0.363950524 | 4.50E-16 |
| AP3S2 | grey | 0.211692842 | 3.93E-06 | 0.221354322 | 1.36E-06 |
| AP4B1 | grey | 0.148694337 | 0.001270183 | 0.153694783 | 0.000861341 |
| AP5S1 | grey | -0.114413121 | 0.013361297 | -0.087867794 | 0.057772319 |
| APBA2 | grey | 0.138792677 | 0.00264833 | 0.113859378 | 0.013818825 |
| APBB3 | grey | 0.022394389 | 0.629301368 | 0.000120143 | 0.997934003 |
| APC2 | grey | 0.016812536 | 0.717072641 | 0.018410252 | 0.691502912 |
| APCDD1 | grey | -0.203561021 | 9.26E-06 | -0.198302763 | 1.58E-05 |
| APCS | grey | -0.068191516 | 0.141184321 | -0.070651762 | 0.12735604 |
| APELA | grey | -0.037137491 | 0.423320745 | -0.037766542 | 0.415504523 |

| | | | | | |
|---|---|---|---|---|---|
| APOA1 | grey | -0.287860885 | 2.32E-10 | -0.281443162 | 5.97E-10 |
| APOA4 | grey | 0.034721848 | 0.454122257 | 0.03427048 | 0.460014109 |
| APOBEC: | grey | -0.126348168 | 0.006255972 | -0.113823968 | 0.013848546 |
| APOL4 | grey | 0.080344898 | 0.082843651 | 0.069965036 | 0.131105744 |
| APOM | grey | -0.12077706 | 0.008985911 | -0.112609007 | 0.014902978 |
| APTX | grey | 0.237969415 | 1.95E-07 | 0.234550932 | 2.95E-07 |
| AQP11 | grey | -0.145673138 | 0.001597078 | -0.159699626 | 0.000531916 |
| AQP12A | grey | 0.01965898 | 0.671757952 | 0.033880764 | 0.465135313 |
| AQP12B | grey | 0.045552973 | 0.325961984 | 0.052090325 | 0.261255987 |
| AQP2 | grey | 0.187374474 | 4.61E-05 | 0.18864777 | 4.08E-05 |
| AQP4 | grey | -0.014883469 | 0.748369314 | -0.039473247 | 0.394730613 |
| AQP6 | grey | 0.074887401 | 0.106037347 | 0.089564562 | 0.05308753 |
| ARAF | grey | -0.081397006 | 0.078882555 | -0.067433201 | 0.145670649 |
| ARAP1 | grey | -0.165341859 | 0.000332945 | -0.170913032 | 0.000206521 |
| ARAP3 | grey | -0.18718401 | 4.70E-05 | -0.192650257 | 2.77E-05 |
| ARC | grey | -0.163716262 | 0.000381656 | -0.16923191 | 0.00023891 |
| AREG | grey | 0.078786067 | 0.089006319 | 0.060262947 | 0.193606135 |
| ARF3 | grey | 0.022473323 | 0.628093316 | 0.02096372 | 0.65136597 |
| ARFGAP: | grey | -0.013817916 | 0.765839291 | -0.024509074 | 0.597287309 |
| ARG2 | grey | 0.101645011 | 0.028065341 | 0.079643342 | 0.085573135 |
| ARHGAP | grey | 0.166899056 | 0.000291778 | 0.182614519 | 7.21E-05 |
| ARHGAP | grey | -0.197401944 | 1.73E-05 | -0.186111475 | 5.20E-05 |
| ARHGAP | grey | -0.14686487 | 0.00145987 | -0.144952532 | 0.001685685 |
| ARHGAP | grey | -0.135518777 | 0.003343175 | -0.122995936 | 0.007792013 |
| ARHGDI( | grey | 0.014085345 | 0.761443164 | 0.011450626 | 0.805066839 |
| ARHGEF | grey | -0.055526259 | 0.231056823 | -0.02554175 | 0.581926786 |
| ARHGEF | grey | -0.041566907 | 0.370120038 | -0.041143305 | 0.375021173 |
| ARHGEF | grey | 0.003170375 | 0.945523778 | -0.0016698 | 0.971291924 |
| ARHGEF | grey | -0.15762342 | 0.000629591 | -0.192100318 | 2.93E-05 |
| ARID3A | grey | 0.030458634 | 0.511434926 | 0.015913694 | 0.731600029 |
| ARID3C | grey | -0.093225325 | 0.044051107 | -0.096060293 | 0.037975591 |
| ARIH2OS | grey | 0.077293713 | 0.095245978 | 0.074050081 | 0.110011391 |
| ARL14 | grey | -0.034204039 | 0.460884964 | -0.04039926 | 0.383726126 |
| ARL14EP | grey | -0.205354048 | 7.69E-06 | -0.200185285 | 1.31E-05 |
| ARL2-SN | grey | -0.070175943 | 0.129945166 | -0.091817522 | 0.047360003 |
| ARL4A | grey | 0.148306671 | 0.001308374 | 0.158126358 | 0.000604511 |
| ARL4D | grey | 0.278601788 | 9.00E-10 | 0.292755519 | 1.11E-10 |
| ARL8A | grey | 0.126835386 | 0.006056936 | 0.121044559 | 0.008833826 |
| ARL9 | grey | 0.079269056 | 0.087058706 | 0.105133339 | 0.023076729 |
| ARMC12 | grey | -0.115275142 | 0.0126756 | -0.119298176 | 0.009869527 |
| ARMC7 | grey | 0.057993732 | 0.210952893 | 0.06936635 | 0.134443855 |
| ARMCX2 | grey | -0.291097954 | 1.42E-10 | -0.322826526 | 8.68E-13 |
| ARMS2 | grey | 0.004687872 | 0.919522808 | 0.004400948 | 0.924433287 |
| ARPC1A | grey | 0.03812016 | 0.411148233 | 0.060315603 | 0.193216393 |
| ARSE | grey | 0.030414918 | 0.512041514 | 0.040663916 | 0.38061575 |

| | | | | | |
|---|---|---|---|---|---|
| ARSF | grey | 0.150757407 | 0.00108364 | 0.129363708 | 0.005112556 |
| ARSG | grey | -0.266304417 | 5.06E-09 | -0.2845497 | 3.79E-10 |
| ARSH | grey | 0.260290664 | 1.14E-08 | 0.23594818 | 2.49E-07 |
| ARSJ | grey | 0.110065866 | 0.017340738 | 0.086795328 | 0.060906398 |
| ART5 | grey | -0.323901643 | 7.22E-13 | -0.33871003 | 5.33E-14 |
| ARX | grey | 0.041019189 | 0.376464756 | 0.045476856 | 0.326771868 |
| AS3MT | grey | 0.146640871 | 0.001484803 | 0.106051585 | 0.02189875 |
| ASAP3 | grey | -0.30873088 | 9.03E-12 | -0.337632517 | 6.48E-14 |
| ASB10 | grey | -0.153480504 | 0.000876009 | -0.137107485 | 0.002987606 |
| ASB13 | grey | 0.074988286 | 0.105566291 | 0.034222467 | 0.460643332 |
| ASB16 | grey | 0.033876906 | 0.465186168 | 0.040598999 | 0.381377257 |
| ASB4 | grey | -0.019012368 | 0.681955091 | 0.017521779 | 0.705680908 |
| ASB6 | grey | -0.039031988 | 0.400040664 | -0.044588074 | 0.336324763 |
| ASB9 | grey | -0.075114078 | 0.104981258 | -0.090195859 | 0.051427266 |
| ASCL1 | grey | 0.048437452 | 0.296230918 | 0.025283553 | 0.585750042 |
| ASCL2 | grey | 0.190646641 | 3.37E-05 | 0.169740756 | 0.000228637 |
| ASCL3 | grey | -0.037975637 | 0.412925365 | -0.043824544 | 0.344672969 |
| ASCL4 | grey | 0.006484289 | 0.888855468 | -0.007608473 | 0.869745134 |
| ASGR1 | grey | -0.192953886 | 2.69E-05 | -0.178601567 | 0.000104236 |
| ASIC1 | grey | 0.191893498 | 2.99E-05 | 0.142244965 | 0.002060405 |
| ASIC2 | grey | 0.007861154 | 0.865459752 | 0.003142103 | 0.946008831 |
| ASIC3 | grey | -0.009375108 | 0.839870373 | -0.007251179 | 0.875811209 |
| ASL | grey | 0.088374416 | 0.056338957 | 0.104678743 | 0.023679942 |
| ASMTL | grey | 0.00527304 | 0.909517814 | 0.008949111 | 0.847054896 |
| ASPG | grey | -0.194169676 | 2.39E-05 | -0.201633244 | 1.13E-05 |
| ASPHD1 | grey | -0.131362815 | 0.004462127 | -0.135333239 | 0.003387113 |
| ASPHD2 | grey | 0.020679793 | 0.655782092 | 0.034404347 | 0.45826226 |
| ASPRV1 | grey | -0.175838342 | 0.000133714 | -0.17014038 | 0.00022086 |
| ASS1 | grey | -0.326608072 | 4.53E-13 | -0.296539372 | 6.20E-11 |
| ASTL | grey | -0.203501241 | 9.32E-06 | -0.234697071 | 2.90E-07 |
| ASTN1 | grey | -0.196149894 | 1.96E-05 | -0.207203062 | 6.34E-06 |
| ASXL3 | grey | -0.218702857 | 1.83E-06 | -0.221793234 | 1.29E-06 |
| ATAD3C | grey | -0.104573104 | 0.023822054 | -0.098524574 | 0.033286595 |
| ATCAY | grey | 0.042289954 | 0.361846406 | 0.048526894 | 0.295338899 |
| ATF3 | grey | -0.157615499 | 0.000629994 | -0.191042842 | 3.24E-05 |
| ATF4 | grey | 0.087590097 | 0.05857073 | 0.065272975 | 0.159047341 |
| ATG10 | grey | 0.198831777 | 1.50E-05 | 0.20679088 | 6.62E-06 |
| ATG13 | grey | -0.106813407 | 0.020961377 | -0.097385335 | 0.0353891 |
| ATG7 | grey | 0.169474707 | 0.000233956 | 0.193083557 | 2.66E-05 |
| ATHL1 | grey | -0.059873373 | 0.196507512 | -0.052989475 | 0.253102354 |
| ATOH1 | grey | 0.078365829 | 0.090729217 | 0.065105757 | 0.160120215 |
| ATOH7 | grey | -0.142008128 | 0.002096563 | -0.142940643 | 0.001957467 |
| ATP10A | grey | -0.217414698 | 2.11E-06 | -0.213628431 | 3.19E-06 |
| ATP10B | grey | -0.028181646 | 0.543519756 | -0.002538765 | 0.956364535 |
| ATP12A | grey | -0.011473436 | 0.804686344 | -0.001379444 | 0.976282253 |

| | | | | | |
|---|---|---|---|---|---|
| ATP13A4 | grey | -0.003775035 | 0.935154977 | -0.010672812 | 0.818068572 |
| ATP13A5 | grey | -0.022441002 | 0.628587854 | -0.012623598 | 0.785562035 |
| ATP1A1 | grey | 0.093581365 | 0.043245795 | 0.089282158 | 0.053844552 |
| ATP1A2 | grey | -0.056343731 | 0.224251389 | -0.070247536 | 0.129553018 |
| ATP1A3 | grey | -0.117165902 | 0.011279321 | -0.098388347 | 0.033532263 |
| ATP1A4 | grey | 0.045336132 | 0.328272589 | 0.030387336 | 0.512424425 |
| ATP2B2 | grey | -0.180974969 | 8.39E-05 | -0.197289419 | 1.75E-05 |
| ATP4A | grey | -0.036361119 | 0.433084793 | -0.050482409 | 0.276284131 |
| ATP5EP2 | grey | 0.127024028 | 0.005981412 | 0.165313681 | 0.000333738 |
| ATP5J2-P | grey | -0.027057038 | 0.559726812 | -0.022345809 | 0.630045358 |
| ATP5SL | grey | 0.049301533 | 0.287688309 | 0.048905202 | 0.291585799 |
| ATP6V0A | grey | 0.175696771 | 0.000135418 | 0.186939551 | 4.81E-05 |
| ATP6V0A | grey | 0.166899642 | 0.000291764 | 0.185391289 | 5.56E-05 |
| ATP6V0E | grey | 0.113113484 | 0.014456885 | 0.157661271 | 0.00062767 |
| ATP6V0E | grey | 0.251982588 | 3.39E-08 | 0.233057308 | 3.52E-07 |
| ATP6V1E | grey | -0.185781244 | 5.36E-05 | -0.173907743 | 0.000158777 |
| ATP6V1C | grey | 0.230798383 | 4.60E-07 | 0.210664155 | 4.39E-06 |
| ATP6V1E | grey | 0.023176288 | 0.617378817 | 0.034799543 | 0.453112373 |
| ATP6V1E | grey | -0.079530129 | 0.086020318 | -0.08231756 | 0.075544021 |
| ATP6V1C | grey | -0.076391299 | 0.099185379 | -0.091717277 | 0.047603362 |
| ATP6V1C | grey | 0.100128639 | 0.030507451 | 0.104777163 | 0.023548202 |
| ATP7B | grey | 0.06295424 | 0.174414776 | 0.054793107 | 0.237283596 |
| ATP8B3 | grey | 0.098929421 | 0.032565546 | 0.097491457 | 0.035188591 |
| ATXN7L | grey | -0.228795624 | 5.81E-07 | -0.241519452 | 1.27E-07 |
| AUTS2 | grey | -0.02790349 | 0.547506588 | -0.038936828 | 0.401191399 |
| AVEN | grey | 0.107904824 | 0.019679266 | 0.114663735 | 0.013158654 |
| AVP | grey | -0.063383307 | 0.171490787 | -0.081801989 | 0.077399366 |
| AVPR1B | grey | 0.135871676 | 0.003261028 | 0.154401975 | 0.000814529 |
| AWAT2 | grey | -0.034564786 | 0.456167605 | -0.025055997 | 0.589129195 |
| AZGP1 | grey | -0.088447347 | 0.056135066 | -0.074095735 | 0.109791729 |
| AZIN2 | grey | -0.276452869 | 1.22E-09 | -0.267842331 | 4.09E-09 |
| B3GALT1 | grey | -0.0608265 | 0.189464708 | -0.054445298 | 0.24027856 |
| B3GALT4 | grey | -0.11427353 | 0.013475357 | -0.059161905 | 0.201887901 |
| B3GAT1 | grey | -0.080447722 | 0.082449578 | -0.078862577 | 0.088695489 |
| B3GNT3 | grey | -0.064361517 | 0.16496151 | -0.033381923 | 0.471736355 |
| B3GNT6 | grey | 0.002979734 | 0.948794917 | 0.039353063 | 0.39617266 |
| B3GNT7 | grey | -0.182666654 | 7.18E-05 | -0.163564927 | 0.000386513 |
| B3GNT8 | grey | -0.18469822 | 5.94E-05 | -0.190493669 | 3.42E-05 |
| B4GALN | grey | 0.031012046 | 0.503788472 | 0.03348807 | 0.470327445 |
| B4GALN | grey | -0.124106514 | 0.007249398 | -0.141481006 | 0.002179131 |
| B4GALT4 | grey | 0.17387544 | 0.000159231 | 0.190633302 | 3.37E-05 |
| BAALC | grey | -0.189343171 | 3.82E-05 | -0.182051119 | 7.60E-05 |
| BAAT | grey | -0.02408341 | 0.603671886 | -0.030441485 | 0.511672832 |
| BAG1 | grey | 0.121310556 | 0.008684873 | 0.155660497 | 0.000736994 |
| BAG3 | grey | 0.154144328 | 0.000831304 | 0.151567193 | 0.001017589 |

| | | | | | |
|---|---|---|---|---|---|
| BAHCC1 | grey | 0.141593438 | 0.002161274 | 0.135052755 | 0.003454531 |
| BAI1 | grey | -0.070747504 | 0.126839939 | -0.088159822 | 0.056942452 |
| BAI2 | grey | -0.149296464 | 0.001212894 | -0.158420397 | 0.000590281 |
| BAIAP2 | grey | -0.204120876 | 8.74E-06 | -0.208470185 | 5.55E-06 |
| BAIAP2L | grey | 0.1365978 | 0.003097743 | 0.149767149 | 0.001169778 |
| BARHL1 | grey | 0.003953437 | 0.932097723 | -0.002621511 | 0.954943813 |
| BARHL2 | grey | -0.124782241 | 0.006936029 | -0.1216153 | 0.008516976 |
| BARX2 | grey | -0.216871579 | 2.24E-06 | -0.212636526 | 3.55E-06 |
| BASP1 | grey | 0.101972506 | 0.027560549 | 0.083969608 | 0.069841324 |
| BATF3 | grey | -0.094049914 | 0.042204932 | -0.079493754 | 0.086164394 |
| BBC3 | grey | 0.362471177 | 6.03E-16 | 0.38442833 | 6.77E-18 |
| BCAM | grey | -0.325726082 | 5.28E-13 | -0.333448226 | 1.37E-13 |
| BCAR3 | grey | -0.00099221 | 0.982939028 | -0.020527642 | 0.658153562 |
| BCAT1 | grey | -0.019104764 | 0.680494384 | -0.017632244 | 0.703912459 |
| BCL11A | grey | -0.218933701 | 1.78E-06 | -0.218803957 | 1.81E-06 |
| BCL2L1 | grey | -0.074221857 | 0.109186696 | -0.054173304 | 0.242639118 |
| BCL2L10 | grey | -0.01787369 | 0.700052699 | -0.018420445 | 0.691340867 |
| BCL2L15 | grey | 0.19662856 | 1.87E-05 | 0.215139388 | 2.71E-06 |
| BCO1 | grey | 0.000159225 | 0.997261943 | 0.031164112 | 0.501697977 |
| BDKRB1 | grey | -0.180596427 | 8.69E-05 | -0.174926682 | 0.000145046 |
| BDNF | grey | 0.103143438 | 0.025819045 | 0.116366605 | 0.011852055 |
| BEAN1 | grey | -0.032986993 | 0.476998681 | -0.008034945 | 0.862514588 |
| BEND4 | grey | -0.079879192 | 0.08464756 | -0.081325442 | 0.07914703 |
| BEND5 | grey | -0.206595058 | 6.76E-06 | -0.208103795 | 5.76E-06 |
| BET1L | grey | 0.215881208 | 2.50E-06 | 0.229741895 | 5.20E-07 |
| BEX1 | grey | -0.223410775 | 1.08E-06 | -0.252313453 | 3.25E-08 |
| BEX2 | grey | -0.514081791 | 7.33E-33 | -0.508290446 | 4.78E-32 |
| BEX4 | grey | -0.486371561 | 4.20E-29 | -0.481241787 | 1.92E-28 |
| BEX5 | grey | 0.292349077 | 1.18E-10 | 0.269521313 | 3.25E-09 |
| BFSP1 | grey | 0.00295677 | 0.949189015 | 0.012508034 | 0.787477965 |
| BHLHA1: | grey | 0.239137165 | 1.69E-07 | 0.239425712 | 1.64E-07 |
| BHLHA9 | grey | -0.140098054 | 0.002410074 | -0.101115502 | 0.028898313 |
| BHLHE2: | grey | 0.051664865 | 0.265176545 | 0.083363034 | 0.071892811 |
| BHLHE2: | grey | -0.077772454 | 0.093207367 | -0.024034759 | 0.604403561 |
| BHMT | grey | -0.193349212 | 2.59E-05 | -0.188370741 | 4.19E-05 |
| BICC1 | grey | -0.1996052 | 1.39E-05 | -0.210633375 | 4.41E-06 |
| BID | grey | 0.029332985 | 0.527172281 | 0.057989188 | 0.210988723 |
| BIN1 | grey | -0.292905224 | 1.08E-10 | -0.2827915 | 4.90E-10 |
| BIRC7 | grey | -0.174951803 | 0.000144722 | -0.170394744 | 0.00021604 |
| BIVM-ER | grey | 0.020552319 | 0.657768712 | 0.008059433 | 0.862099753 |
| BLACE | grey | 0.022974305 | 0.62044925 | 0.064300194 | 0.165365262 |
| BLMH | grey | -0.061087936 | 0.187565722 | -0.061116155 | 0.187361592 |
| BLNK | grey | 0.198051673 | 1.62E-05 | 0.179321723 | 9.76E-05 |
| BLOC1S3 | grey | -0.045739558 | 0.323982244 | -0.045423049 | 0.327345162 |
| BLOC1S4 | grey | 0.049409097 | 0.286636605 | 0.069481329 | 0.133797729 |

| | | | | | |
|---|---|---|---|---|---|
| BLOC1S5 | grey | -0.180838731 | 8.50E-05 | -0.194105631 | 2.40E-05 |
| BLVRA | grey | -0.044007245 | 0.342663483 | -0.046712367 | 0.31378707 |
| BMF | grey | -0.130196635 | 0.004831827 | -0.132497727 | 0.004127068 |
| BMP1 | grey | -0.052392322 | 0.258497484 | -0.073739401 | 0.11151542 |
| BMP3 | grey | -0.181425229 | 8.05E-05 | -0.191348668 | 3.15E-05 |
| BMP5 | grey | 0.134631609 | 0.003558047 | 0.119025937 | 0.010040347 |
| BMP7 | grey | -0.319736424 | 1.47E-12 | -0.346542817 | 1.27E-14 |
| BMP8B | grey | -0.097863691 | 0.034492906 | -0.119698411 | 0.00962308 |
| BMX | grey | 0.084070959 | 0.069503259 | 0.073437289 | 0.112993431 |
| BNC1 | grey | -0.167824177 | 0.000269625 | -0.169998816 | 0.000223586 |
| BNIPL | grey | 0.016463507 | 0.722701993 | -0.001192221 | 0.97950055 |
| BOLA1 | grey | 0.048475614 | 0.295850108 | 0.042119776 | 0.363783244 |
| BPIFA1 | grey | 0.041100743 | 0.375515831 | 0.066785501 | 0.149588008 |
| BPIFA2 | grey | -0.133992034 | 0.003720639 | -0.1008105 | 0.029387677 |
| BPIFB1 | grey | 0.024406615 | 0.598821314 | 0.065095966 | 0.160183204 |
| BPIFB2 | grey | 0.270697673 | 2.76E-09 | 0.271470677 | 2.47E-09 |
| BPIFB3 | grey | -0.048807566 | 0.292551342 | -0.046366971 | 0.317382505 |
| BPIFB4 | grey | -0.012692464 | 0.784420918 | -0.004147953 | 0.928765465 |
| BPIFB6 | grey | 0.147277775 | 0.001414915 | 0.139818048 | 0.002459468 |
| BRD9 | grey | -0.188631015 | 4.09E-05 | -0.201862058 | 1.10E-05 |
| BRDT | grey | -0.093075691 | 0.044393304 | -0.059563921 | 0.198834696 |
| BRINP1 | grey | -0.303923365 | 1.95E-11 | -0.310105164 | 7.22E-12 |
| BRINP2 | grey | -0.001445012 | 0.97515526 | -0.012547168 | 0.786829012 |
| BRINP3 | grey | 0.019801842 | 0.669513014 | 0.002028455 | 0.965129312 |
| BRSK1 | grey | -0.146450192 | 0.001506334 | -0.162341508 | 0.000427949 |
| BRSK2 | grey | -0.054424975 | 0.240454378 | -0.07865438 | 0.089543362 |
| BSCL2 | grey | 0.076438443 | 0.09897642 | 0.047384276 | 0.306869575 |
| BSN | grey | -0.191946547 | 2.97E-05 | -0.218686208 | 1.83E-06 |
| BSPH1 | grey | -0.060439096 | 0.192304594 | -0.05244774 | 0.257993469 |
| BTBD11 | grey | 0.099991602 | 0.030736854 | 0.116243903 | 0.011942216 |
| BTBD16 | grey | 0.082839816 | 0.073701668 | 0.105191519 | 0.023000496 |
| BTBD17 | grey | -0.209409235 | 5.02E-06 | -0.193414697 | 2.57E-05 |
| BTNL8 | grey | -0.09325326 | 0.043987468 | -0.077896807 | 0.092683588 |
| BTNL9 | grey | -0.144575233 | 0.001733856 | -0.171697942 | 0.000192851 |
| BZRAP1 | grey | -0.161358808 | 0.000464188 | -0.198259718 | 1.59E-05 |
| C10orf11 | grey | 0.093881435 | 0.042576741 | 0.098733008 | 0.032913684 |
| C10orf32- | grey | 0.022627649 | 0.625734294 | 0.010801602 | 0.815912189 |
| C10orf55 | grey | -0.074569244 | 0.107533787 | -0.061643315 | 0.183578183 |
| C10orf71 | grey | 0.010037044 | 0.828733246 | 0.025445364 | 0.583352665 |
| C10orf82 | grey | -0.10805638 | 0.019506741 | -0.136289758 | 0.003166083 |
| C10orf90 | grey | -0.037783733 | 0.415292117 | -0.041525244 | 0.370600316 |
| C10orf99 | grey | 0.10282657 | 0.026280693 | 0.154174428 | 0.000829328 |
| C11orf45 | grey | -0.256654552 | 1.84E-08 | -0.260075878 | 1.17E-08 |
| C11orf71 | grey | 0.147503125 | 0.001390921 | 0.140259341 | 0.002382035 |
| C11orf80 | grey | 0.070882268 | 0.126116234 | 0.05311845 | 0.251947443 |

| | | | | | |
|---|---|---|---|---|---|
| C11orf85 | grey | -0.208513375 | 5.52E-06 | -0.219167213 | 1.74E-06 |
| C11orf86 | grey | 0.052704876 | 0.255663805 | 0.028092536 | 0.544795417 |
| C11orf94 | grey | 0.131268086 | 0.004491175 | 0.126642614 | 0.006134996 |
| C12orf43 | grey | 0.026779923 | 0.563755976 | 0.031083922 | 0.502799806 |
| C12orf54 | grey | -0.054869219 | 0.236631718 | -0.070352669 | 0.128978816 |
| C12orf56 | grey | -0.039222422 | 0.397743781 | -0.030310061 | 0.513497984 |
| C12orf65 | grey | 0.072418025 | 0.118093708 | 0.086941612 | 0.060470815 |
| C12orf75 | grey | 0.127632335 | 0.005743602 | 0.144757597 | 0.001710418 |
| C14orf11[illegible] | grey | -0.034922411 | 0.451517909 | -0.026811618 | 0.563294433 |
| C14orf16[illegible] | grey | -0.229486937 | 5.36E-07 | -0.22800952 | 6.36E-07 |
| C14orf17[illegible] | grey | -0.089133516 | 0.0542466 | -0.083131695 | 0.072688071 |
| C14orf18[illegible] | grey | -0.073819199 | 0.111127574 | -0.074161521 | 0.10947581 |
| C15orf27 | grey | -0.166021108 | 0.000314363 | -0.171730962 | 0.000192295 |
| C15orf38- | grey | 0.0802097 | 0.083364113 | 0.029179833 | 0.529332333 |
| C15orf40 | grey | 0.067654009 | 0.144353224 | 0.072293365 | 0.118729657 |
| C15orf59 | grey | -0.090889464 | 0.049653314 | -0.104722018 | 0.023621938 |
| C15orf61 | grey | 0.059348934 | 0.200463258 | 0.086956746 | 0.060425896 |
| C16orf62 | grey | 0.071207226 | 0.124384352 | 0.064866716 | 0.161663366 |
| C16orf74 | grey | -0.13562961 | 0.003317175 | -0.117556543 | 0.011008427 |
| C16orf90 | grey | -0.131040415 | 0.00456169 | -0.123365246 | 0.007607676 |
| C17orf10[illegible] | grey | -0.165250712 | 0.000335515 | -0.171377442 | 0.000198327 |
| C17orf49 | grey | 0.078813957 | 0.088892909 | 0.05477252 | 0.237460132 |
| C17orf58 | grey | -0.097938227 | 0.03435502 | -0.100618766 | 0.029698929 |
| C17orf74 | grey | 0.036329268 | 0.433488128 | 0.036066199 | 0.436827628 |
| C18orf42 | grey | -0.068756491 | 0.137911218 | -0.072086908 | 0.119788769 |
| C19orf12 | grey | -0.277542182 | 1.05E-09 | -0.259011033 | 1.35E-08 |
| C19orf33 | grey | -0.202858361 | 9.96E-06 | -0.181217492 | 8.21E-05 |
| C19orf45 | grey | 0.195102109 | 2.18E-05 | 0.172754154 | 0.000175789 |
| C19orf47 | grey | -0.059648552 | 0.198196254 | -0.054578049 | 0.23913233 |
| C19orf68 | grey | -0.175911647 | 0.00013284 | -0.186581013 | 4.97E-05 |
| C19orf81 | grey | -0.122929319 | 0.007825686 | -0.125863631 | 0.006459713 |
| C19orf83 | grey | -0.02659858 | 0.566400165 | -0.014349951 | 0.757101001 |
| C19orf84 | grey | -0.21478849 | 2.81E-06 | -0.209835278 | 4.80E-06 |
| C1orf116 | grey | 0.192845892 | 2.72E-05 | 0.211380344 | 4.07E-06 |
| C1orf127 | grey | 0.05711145 | 0.21799132 | 0.024544993 | 0.596749959 |
| C1orf145 | grey | -0.185365184 | 5.58E-05 | -0.211500524 | 4.02E-06 |
| C1orf216 | grey | -0.118051229 | 0.010673658 | -0.124291226 | 0.007162504 |
| C1orf226 | grey | 0.082989263 | 0.073181262 | 0.071556545 | 0.122543314 |
| C1orf234 | grey | 0.003311601 | 0.943101128 | 0.019184743 | 0.679230948 |
| C1orf56 | grey | 0.063385843 | 0.171473617 | 0.060072861 | 0.19501787 |
| C1orf61 | grey | -0.120494491 | 0.00914909 | -0.116052537 | 0.012084041 |
| C1orf94 | grey | -0.076129381 | 0.100352709 | -0.07766154 | 0.093676536 |
| C1orf95 | grey | -0.140691881 | 0.002308302 | -0.138102868 | 0.002782722 |
| C1QL2 | grey | -0.196078304 | 1.98E-05 | -0.196986535 | 1.81E-05 |
| C1QL4 | grey | -0.072820779 | 0.116057273 | -0.067991254 | 0.142358689 |

| | | | | | |
|---|---|---|---|---|---|
| C1QTNF3 | grey | -0.176802044 | 0.000122642 | -0.175615016 | 0.000136411 |
| C1QTNF6 | grey | 0.118557855 | 0.010340194 | 0.12660776 | 0.006149206 |
| C1QTNF9 | grey | -0.145946724 | 0.001564572 | -0.13685842 | 0.003040973 |
| C1QTNF9 | grey | -0.024584623 | 0.596157351 | -0.051059659 | 0.270822736 |
| C1RL | grey | 0.011314805 | 0.807333428 | -0.007671636 | 0.868673546 |
| C20orf141 | grey | -0.160342654 | 0.000504646 | -0.162966476 | 0.000406292 |
| C20orf196 | grey | 0.115055339 | 0.012847428 | 0.113345979 | 0.014255279 |
| C20orf202 | grey | -0.092608715 | 0.045475638 | -0.097686094 | 0.034823351 |
| C21orf140 | grey | -0.009743035 | 0.833675877 | -0.001616522 | 0.972207532 |
| C21orf33 | grey | -0.059732098 | 0.197567468 | -0.049291033 | 0.287791113 |
| C22orf29 | grey | 0.018678263 | 0.687246882 | -0.019332567 | 0.676898132 |
| C22orf39 | grey | 0.139995234 | 0.002428105 | 0.147519556 | 0.001389186 |
| C22orf46 | grey | 0.190007392 | 3.58E-05 | 0.174478863 | 0.000150938 |
| C2CD2 | grey | -0.070312564 | 0.129197624 | -0.091684258 | 0.047683748 |
| C2CD4A | grey | 0.131915925 | 0.004295879 | 0.123194765 | 0.00769228 |
| C2CD4B | grey | -0.115032275 | 0.012865576 | -0.095765613 | 0.038572099 |
| C2CD4D | grey | 0.035621481 | 0.442506518 | 0.033321142 | 0.472544169 |
| C2orf43 | grey | -0.465696044 | 1.63E-26 | -0.446053296 | 3.27E-24 |
| C2orf54 | grey | -0.092709782 | 0.04523953 | -0.079792109 | 0.084988366 |
| C2orf66 | grey | 0.081916858 | 0.076982826 | 0.060255627 | 0.193660359 |
| C2orf72 | grey | -0.038097083 | 0.411431693 | -0.070823942 | 0.126429061 |
| C2orf80 | grey | -0.036373215 | 0.432931681 | -0.041405306 | 0.371985094 |
| C2orf82 | grey | -0.00956424 | 0.836684846 | -0.020099217 | 0.664849512 |
| C2orf91 | grey | 0.085019397 | 0.066404155 | 0.10591065 | 0.02207609 |
| C3orf14 | grey | -0.337854689 | 6.22E-14 | -0.314788874 | 3.35E-12 |
| C3orf52 | grey | 0.100698963 | 0.029568399 | 0.089540605 | 0.053151403 |
| C3orf56 | grey | -0.108706593 | 0.018781428 | -0.103846291 | 0.02481994 |
| C3orf67 | grey | -0.341448029 | 3.24E-14 | -0.346302901 | 1.33E-14 |
| C4A | grey | -0.169747387 | 0.000228506 | -0.169480682 | 0.000233835 |
| C4B | grey | -0.119732273 | 0.009602483 | -0.114059834 | 0.013651631 |
| C4BPB | grey | 0.165851278 | 0.000318916 | 0.179339522 | 9.75E-05 |
| C4orf19 | grey | 0.071713899 | 0.121720965 | 0.103859382 | 0.024801651 |
| C4orf36 | grey | -0.108211189 | 0.019331874 | -0.10200596 | 0.027509427 |
| C5 | grey | 0.030058006 | 0.517007828 | 0.011193249 | 0.809363328 |
| C5orf38 | grey | -0.121403901 | 0.008633135 | -0.134926315 | 0.003485319 |
| C5orf46 | grey | 0.069576631 | 0.133263999 | 0.11079484 | 0.016608643 |
| C6orf141 | grey | 0.135032251 | 0.003459507 | 0.112826347 | 0.01470933 |
| C6orf195 | grey | -0.232040033 | 3.97E-07 | -0.233566256 | 3.31E-07 |
| C6orf222 | grey | -0.118339505 | 0.010482763 | -0.1385948 | 0.002686264 |
| C6orf25 | grey | -0.148402284 | 0.001298857 | -0.16558785 | 0.0003261 |
| C6orf58 | grey | -0.063428236 | 0.171186731 | -0.068901553 | 0.137080299 |
| C7orf43 | grey | 0.19197234 | 2.96E-05 | 0.19474243 | 2.26E-05 |
| C8orf22 | grey | 0.006120172 | 0.895059566 | 0.00362878 | 0.937662046 |
| C8orf4 | grey | 0.11195902 | 0.015495534 | 0.092490633 | 0.045752808 |
| C8orf58 | grey | 0.12317969 | 0.007699802 | 0.099298417 | 0.031920008 |

| | | | | | |
|---|---|---|---|---|---|
| C8orf86 | grey | -0.02143495 | 0.644063485 | -0.025457036 | 0.583179907 |
| C8orf89 | grey | -0.216702223 | 2.28E-06 | -0.239776884 | 1.57E-07 |
| C9 | grey | -0.128390105 | 0.005459266 | -0.127511832 | 0.005790025 |
| C9orf129 | grey | -0.037239594 | 0.422046276 | -0.029850979 | 0.519899895 |
| C9orf172 | grey | 0.288345954 | 2.15E-10 | 0.284598508 | 3.76E-10 |
| C9orf173 | grey | -0.048797887 | 0.292647176 | -0.074447121 | 0.108112594 |
| C9orf66 | grey | -0.147419317 | 0.0013998 | -0.136413033 | 0.003138571 |
| C9orf78 | grey | 0.052114651 | 0.261033035 | 0.063488542 | 0.170779252 |
| C9orf91 | grey | 0.257321147 | 1.69E-08 | 0.226345877 | 7.71E-07 |
| C9orf92 | grey | -0.197923665 | 1.64E-05 | -0.190000926 | 3.59E-05 |
| CA10 | grey | 0.140432609 | 0.002352244 | 0.16630069 | 0.000307 |
| CA12 | grey | 0.179716497 | 9.42E-05 | 0.16654961 | 0.00030058 |
| CA14 | grey | -0.026658371 | 0.565527695 | -0.022582484 | 0.626424285 |
| CA2 | grey | 0.168002403 | 0.000265542 | 0.147898052 | 0.00134977 |
| CA4 | grey | -0.152560375 | 0.000941655 | -0.16194296 | 0.000442317 |
| CA9 | grey | 0.088232832 | 0.056736529 | 0.103077472 | 0.025914571 |
| CABIN1 | grey | 0.210446445 | 4.50E-06 | 0.204227691 | 8.65E-06 |
| CABLES1 | grey | -0.027561991 | 0.552420957 | -0.049074979 | 0.289911914 |
| CABP4 | grey | 0.026678571 | 0.56523307 | 0.020492496 | 0.658701858 |
| CABP7 | grey | -0.024350917 | 0.599655958 | -0.047439221 | 0.306308386 |
| CACNA1 | grey | -0.336546016 | 7.87E-14 | -0.339278277 | 4.81E-14 |
| CACNA1 | grey | -0.244072635 | 9.23E-08 | -0.259703907 | 1.23E-08 |
| CACNA1 | grey | 0.038901531 | 0.401618731 | 0.033954533 | 0.464163507 |
| CACNA1 | grey | -0.248430085 | 5.34E-08 | -0.228340091 | 6.12E-07 |
| CACNA1 | grey | -0.049764606 | 0.283179062 | -0.069493927 | 0.133727083 |
| CACNA2 | grey | -0.30969367 | 7.72E-12 | -0.308313715 | 9.66E-12 |
| CACNB1 | grey | -0.039675792 | 0.392307503 | -0.061069724 | 0.187697552 |
| CACNB3 | grey | 0.097508836 | 0.035155847 | 0.065636416 | 0.156734202 |
| CACNG1 | grey | -0.162493467 | 0.000422587 | -0.151527679 | 0.001020724 |
| CACNG3 | grey | -0.054926871 | 0.236138791 | -0.055375466 | 0.23232798 |
| CACNG4 | grey | -0.207105651 | 6.40E-06 | -0.209553495 | 4.94E-06 |
| CACNG5 | grey | 0.070873522 | 0.126163108 | 0.05976582 | 0.197314082 |
| CACNG7 | grey | -0.064479382 | 0.164187559 | -0.057230796 | 0.217029511 |
| CACNG8 | grey | -0.223824744 | 1.03E-06 | -0.205915545 | 7.25E-06 |
| CADM1 | grey | 0.195704426 | 2.05E-05 | 0.162509417 | 0.000422027 |
| CADM2 | grey | -0.091756829 | 0.047507219 | -0.093807088 | 0.042741691 |
| CADPS | grey | -0.081064415 | 0.080117784 | -0.064498902 | 0.164059645 |
| CAGE1 | grey | -0.104395626 | 0.024062467 | -0.084650908 | 0.067594473 |
| CALB1 | grey | -0.043283301 | 0.350669865 | -0.05147911 | 0.266900855 |
| CALB2 | grey | -0.041990642 | 0.365257246 | -0.045520218 | 0.326310344 |
| CALCA | grey | -0.112236577 | 0.015240019 | -0.120698447 | 0.009031047 |
| CALCB | grey | -0.186972558 | 4.79E-05 | -0.186197233 | 5.16E-05 |
| CALHM3 | grey | -0.146474994 | 0.001503518 | -0.174622399 | 0.000149025 |
| CALML6 | grey | 0.158884071 | 0.000568471 | 0.147861631 | 0.001353518 |
| CALY | grey | -0.05638725 | 0.223893135 | -0.053414496 | 0.249310348 |

| | | | | | |
|---|---|---|---|---|---|
| CAMK1D | grey | -0.16713347 | 0.000286009 | -0.167299998 | 0.000281976 |
| CAMK1G | grey | -0.094686972 | 0.040823688 | -0.105850886 | 0.022151667 |
| CAMK2B | grey | -0.174289728 | 0.000153492 | -0.192404479 | 2.84E-05 |
| CAMK2N | grey | -0.020398117 | 0.660175095 | -0.020082769 | 0.665107117 |
| CAMK4 | grey | -0.072293499 | 0.11872897 | -0.066585884 | 0.150811324 |
| CAMKK1 | grey | -0.082323164 | 0.075524054 | -0.107036056 | 0.020694088 |
| CAMKV | grey | -0.104478154 | 0.023950414 | -0.115320411 | 0.012640465 |
| CAMP | grey | 0.122353282 | 0.008122315 | 0.14483343 | 0.001700757 |
| CANT1 | grey | 0.305494182 | 1.52E-11 | 0.286721698 | 2.75E-10 |
| CAPN10 | grey | 0.067493885 | 0.145307674 | 0.055365387 | 0.232413121 |
| CAPN12 | grey | -0.056615278 | 0.222022685 | -0.082667991 | 0.074303728 |
| CAPN13 | grey | 0.157869043 | 0.000617224 | 0.171270428 | 0.000200188 |
| CAPN14 | grey | 0.006144929 | 0.894637534 | -0.000690376 | 0.988128584 |
| CAPN5 | grey | -0.283281029 | 4.56E-10 | -0.275494296 | 1.40E-09 |
| CAPN8 | grey | 0.235758607 | 2.55E-07 | 0.25856141 | 1.43E-08 |
| CAPNS2 | grey | 0.120909868 | 0.008910115 | 0.145357314 | 0.001635373 |
| CAPZA3 | grey | -0.017669395 | 0.703318061 | -0.024223232 | 0.601571309 |
| CARD10 | grey | 0.222506618 | 1.19E-06 | 0.237180423 | 2.15E-07 |
| CARD14 | grey | -0.006076314 | 0.895807295 | -0.03055958 | 0.510035681 |
| CARD6 | grey | -0.06331836 | 0.171931017 | -0.05077479 | 0.273508612 |
| CARNS1 | grey | -0.240492023 | 1.44E-07 | -0.232918152 | 3.58E-07 |
| CARTPT | grey | -0.083141021 | 0.072655874 | -0.073890692 | 0.110780997 |
| CASC10 | grey | -0.230080345 | 5.00E-07 | -0.250241399 | 4.24E-08 |
| CASKIN1 | grey | 0.048525698 | 0.295350814 | 0.03333122 | 0.472410174 |
| CASP14 | grey | 0.098047119 | 0.034154419 | 0.089041667 | 0.054496282 |
| CASP9 | grey | -0.10279717 | 0.026323884 | -0.115473691 | 0.012522134 |
| CASQ1 | grey | -0.023110575 | 0.618377016 | -0.021799879 | 0.638431683 |
| CASR | grey | -0.131928523 | 0.004292159 | -0.128711451 | 0.00534256 |
| CASZ1 | grey | 0.163955108 | 0.000374105 | 0.144347914 | 0.001763484 |
| CATSPEI | grey | -0.004307416 | 0.92603464 | 0.007660633 | 0.868860197 |
| CATSPEI | grey | 0.07908109 | 0.087812549 | 0.098161666 | 0.03394448 |
| CATSPEI | grey | -0.078891788 | 0.088577043 | -0.070838804 | 0.126349292 |
| CBLN2 | grey | -0.10534678 | 0.022798128 | -0.120012015 | 0.009433814 |
| CBLN3 | grey | -0.260403214 | 1.12E-08 | -0.276556001 | 1.21E-09 |
| CBR1 | grey | -0.221231326 | 1.38E-06 | -0.172568758 | 0.000178678 |
| CBR3 | grey | -0.008994878 | 0.846282416 | 0.027546561 | 0.552643501 |
| CBX4 | grey | 0.206931833 | 6.52E-06 | 0.200428079 | 1.28E-05 |
| CBY3 | grey | -0.209764483 | 4.83E-06 | -0.205145552 | 7.86E-06 |
| CC2D1B | grey | 0.040041193 | 0.387958913 | 0.051887722 | 0.263117931 |
| CCBL1 | grey | 0.144249532 | 0.00177645 | 0.132116344 | 0.004237035 |
| CCDC102 | grey | -0.156799148 | 0.00067279 | -0.160504975 | 0.00049797 |
| CCDC109 | grey | -0.017254365 | 0.709968502 | 0.011559737 | 0.803247166 |
| CCDC110 | grey | -0.18331392 | 6.76E-05 | -0.183162169 | 6.85E-05 |
| CCDC116 | grey | -0.012629014 | 0.785472279 | -0.023055091 | 0.619220391 |
| CCDC127 | grey | -0.201256548 | 1.17E-05 | -0.218271348 | 1.92E-06 |

| | | | | | |
|---|---|---|---|---|---|
| CCDC14( | grey | -0.090778296 | 0.049934145 | -0.096231113 | 0.037633401 |
| CCDC154 | grey | -0.107949482 | 0.019628292 | -0.103237842 | 0.025682866 |
| CCDC15: | grey | -0.082796204 | 0.073854105 | -0.0922745 | 0.046263803 |
| CCDC163 | grey | -0.012954146 | 0.780089091 | -0.034928663 | 0.451436869 |
| CCDC16( | grey | -0.178900626 | 0.000101442 | -0.189905329 | 3.62E-05 |
| CCDC169 | grey | -0.093003411 | 0.044559401 | -0.09035412 | 0.051017913 |
| CCDC174 | grey | 0.034124443 | 0.461929468 | 0.014245779 | 0.758809559 |
| CCDC178 | grey | -0.064775069 | 0.162257962 | -0.071691374 | 0.121838421 |
| CCDC183 | grey | 0.135569661 | 0.003331215 | 0.10862749 | 0.018868393 |
| CCDC18: | grey | -0.187174919 | 4.70E-05 | -0.193910774 | 2.45E-05 |
| CCDC42 | grey | -0.159949629 | 0.000521155 | -0.150626859 | 0.001094651 |
| CCDC53 | grey | 0.067565639 | 0.144879378 | 0.062308618 | 0.178884229 |
| CCDC8 | grey | -0.380059235 | 1.70E-17 | -0.387292451 | 3.68E-18 |
| CCDC92 | grey | 0.094639568 | 0.040925135 | 0.096321817 | 0.037452767 |
| CCK | grey | -0.135835733 | 0.00326931 | -0.135857609 | 0.003264267 |
| CCKBR | grey | -0.065236785 | 0.159279074 | -0.066565536 | 0.150936447 |
| CCL11 | grey | -0.003069484 | 0.947254827 | 0.00097219 | 0.983283217 |
| CCL22 | grey | 0.246630607 | 6.70E-08 | 0.280058529 | 7.29E-10 |
| CCL26 | grey | 0.004050816 | 0.930429373 | 0.014836491 | 0.749136894 |
| CCL27 | grey | 0.017001444 | 0.714032175 | 0.019236436 | 0.678414828 |
| CCL28 | grey | -0.082951429 | 0.073312724 | -0.065908685 | 0.155018064 |
| CCNB3 | grey | 0.031582636 | 0.495968116 | 0.019384847 | 0.676073837 |
| CCND1 | grey | 0.265368463 | 5.74E-09 | 0.25476719 | 2.36E-08 |
| CCND3 | grey | -0.255042475 | 2.28E-08 | -0.223901418 | 1.02E-06 |
| CCNI2 | grey | -0.222680887 | 1.17E-06 | -0.217250799 | 2.15E-06 |
| CCNJL | grey | -0.053833287 | 0.245612816 | -0.050405932 | 0.277013259 |
| CCR10 | grey | -0.020016529 | 0.666144965 | -0.012970492 | 0.779818713 |
| CCR9 | grey | 0.094974484 | 0.040212933 | 0.109743508 | 0.017673371 |
| CD177 | grey | -0.01899278 | 0.682264911 | -0.00367855 | 0.936808822 |
| CD1A | grey | 0.05622731 | 0.225211767 | 0.082159296 | 0.076109667 |
| CD207 | grey | 0.067135174 | 0.147463315 | 0.072365975 | 0.118358914 |
| CD22 | grey | -0.389824996 | 2.13E-18 | -0.383984224 | 7.44E-18 |
| CD276 | grey | 0.244320141 | 8.95E-08 | 0.252623418 | 3.12E-08 |
| CD2BP2 | grey | -0.028022271 | 0.545802327 | -0.012510866 | 0.787431002 |
| CD44 | grey | 0.248537835 | 5.26E-08 | 0.261320229 | 9.93E-09 |
| CD55 | grey | -0.041122842 | 0.37525895 | -0.022747926 | 0.623898378 |
| CD58 | grey | 0.177484721 | 0.000115327 | 0.152797308 | 0.00092433 |
| CD5L | grey | 0.010001537 | 0.829329796 | 0.006436134 | 0.889675588 |
| CD83 | grey | -0.202465785 | 1.04E-05 | -0.231051029 | 4.46E-07 |
| CD99 | grey | 0.159478732 | 0.000541595 | 0.181341241 | 8.11E-05 |
| CDA | grey | -0.00904859 | 0.845376015 | -0.011438282 | 0.80527276 |
| CDAN1 | grey | 0.080607076 | 0.08184185 | 0.052796872 | 0.254833867 |
| CDC42EI | grey | 0.040534049 | 0.382140082 | 0.051390432 | 0.267726732 |
| CDC42EI | grey | -0.242683208 | 1.10E-07 | -0.251965264 | 3.40E-08 |
| CDH10 | grey | 0.036524642 | 0.43101752 | 0.016209498 | 0.726808239 |

| | | | | | |
|---|---|---|---|---|---|
| CDH12 | grey | -0.073696914 | 0.111722356 | -0.070985739 | 0.125562759 |
| CDH15 | grey | -0.014400987 | 0.756264368 | 0.02504143 | 0.58934581 |
| CDH16 | grey | -0.096192181 | 0.03771116 | -0.06823114 | 0.140952842 |
| CDH17 | grey | -0.014358719 | 0.756957239 | -0.018918211 | 0.683444852 |
| CDH18 | grey | -0.143936095 | 0.001818344 | -0.139436073 | 0.002528339 |
| CDH19 | grey | -0.223318233 | 1.09E-06 | -0.229220749 | 5.53E-07 |
| CDH2 | grey | -0.049061408 | 0.29004548 | -0.060985199 | 0.188310298 |
| CDH20 | grey | -0.141771393 | 0.002133285 | -0.139065705 | 0.002596791 |
| CDH22 | grey | -0.107151136 | 0.020557101 | -0.127261416 | 0.005887576 |
| CDH26 | grey | 0.117376693 | 0.011132422 | 0.122121502 | 0.008244473 |
| CDH3 | grey | -0.064928343 | 0.16126446 | -0.054793304 | 0.237281903 |
| CDH4 | grey | -0.06577519 | 0.155857708 | -0.09556472 | 0.038983292 |
| CDH6 | grey | -0.287029647 | 2.62E-10 | -0.290925957 | 1.46E-10 |
| CDHR1 | grey | 0.13565008 | 0.003312394 | 0.140665293 | 0.002312774 |
| CDHR5 | grey | 0.062246482 | 0.179318816 | 0.047718351 | 0.303467941 |
| CDIPT | grey | -0.223792024 | 1.03E-06 | -0.209378607 | 5.04E-06 |
| CDK11A | grey | 0.023869007 | 0.606899285 | -0.007227364 | 0.876215798 |
| CDK11B | grey | 0.050813961 | 0.273138219 | 0.044664974 | 0.335491218 |
| CDK18 | grey | 0.056389982 | 0.223870661 | 0.041687319 | 0.368734125 |
| CDK3 | grey | -0.134174542 | 0.003673569 | -0.167214825 | 0.000284032 |
| CDK5R2 | grey | -0.110491367 | 0.016910062 | -0.12646774 | 0.006206589 |
| CDKN1C | grey | -0.22624143 | 7.80E-07 | -0.245672267 | 7.56E-08 |
| CDKN2B | grey | -0.21311892 | 3.37E-06 | -0.216027845 | 2.46E-06 |
| CDKN2C | grey | -0.131850353 | 0.004315291 | -0.116176798 | 0.011991781 |
| CDRT15 | grey | 0.066421568 | 0.151823993 | 0.041153957 | 0.374897442 |
| CDX1 | grey | -0.21811674 | 1.95E-06 | -0.186192844 | 5.16E-05 |
| CDX4 | grey | 0.066422564 | 0.15181784 | 0.0650651 | 0.160381894 |
| CDY2A | grey | -0.058957775 | 0.203451191 | -0.064006956 | 0.167306208 |
| CEACAM | grey | -0.221509356 | 1.34E-06 | -0.241915433 | 1.21E-07 |
| CEACAM | grey | 0.070971547 | 0.125638563 | 0.080954178 | 0.080530636 |
| CEACAM | grey | 0.1604926 | 0.000498476 | 0.186600267 | 4.96E-05 |
| CEACAM | grey | 0.085574516 | 0.06464351 | 0.1145548 | 0.013246402 |
| CEACAM | grey | 0.032857459 | 0.478731626 | 0.073408197 | 0.113136566 |
| CEBPA | grey | -0.20294951 | 9.87E-06 | -0.221357879 | 1.36E-06 |
| CEBPB | grey | -0.194363519 | 2.34E-05 | -0.192491437 | 2.82E-05 |
| CEBPD | grey | -0.077696873 | 0.093526873 | -0.081458858 | 0.078654552 |
| CECR6 | grey | 0.093155176 | 0.044211254 | 0.069658891 | 0.132804634 |
| CELA3A | grey | 0.217049616 | 2.20E-06 | 0.219114847 | 1.75E-06 |
| CELA3B | grey | 0.203358339 | 9.46E-06 | 0.202691356 | 1.01E-05 |
| CELF4 | grey | -0.355120724 | 2.51E-15 | -0.376444865 | 3.60E-17 |
| CELF5 | grey | -0.162124654 | 0.000435712 | -0.16297126 | 0.00040613 |
| CEND1 | grey | -0.136868913 | 0.003038707 | -0.123474235 | 0.007554025 |
| CENPV | grey | -0.215847547 | 2.51E-06 | -0.225751689 | 8.26E-07 |
| CEP250 | grey | 0.249672628 | 4.56E-08 | 0.230614266 | 4.70E-07 |
| CER1 | grey | -0.129945873 | 0.004914845 | -0.134959788 | 0.003477144 |

| | | | | | |
|---|---|---|---|---|---|
| CERS4 | grey | 0.024108271 | 0.603298159 | 0.011209481 | 0.809092191 |
| CERS5 | grey | -0.213860843 | 3.11E-06 | -0.231525001 | 4.22E-07 |
| CETP | grey | -0.078628304 | 0.089650012 | -0.051276422 | 0.268791107 |
| CFC1 | grey | -0.061056778 | 0.187791305 | -0.045080897 | 0.331005845 |
| CFC1B | grey | -0.053194052 | 0.251272167 | -0.051202779 | 0.269480159 |
| CFH | grey | -0.013179375 | 0.776366117 | -0.01318273 | 0.776310689 |
| CFHR1 | grey | -0.012151289 | 0.793400461 | -0.009169789 | 0.843331508 |
| CFHR2 | grey | 0.113157985 | 0.014418104 | 0.128162525 | 0.0055433 |
| CFHR3 | grey | -0.064597732 | 0.163413171 | -0.068455599 | 0.13964706 |
| CFHR5 | grey | 0.041070831 | 0.375863706 | 0.048672745 | 0.293888143 |
| CFTR | grey | -0.070515914 | 0.128091133 | -0.076725775 | 0.097710404 |
| CGA | grey | 0.071753023 | 0.121517169 | 0.067989943 | 0.142366402 |
| CGB | grey | -0.001150675 | 0.980214757 | -0.003181875 | 0.945326495 |
| CGB1 | grey | -0.106851266 | 0.020915716 | -0.109039186 | 0.018419594 |
| CGB2 | grey | -0.134736517 | 0.003532001 | -0.128623992 | 0.005374099 |
| CGB5 | grey | 0.011265342 | 0.808159267 | 0.031368317 | 0.498897925 |
| CGB8 | grey | -0.004510768 | 0.922553443 | 0.020851633 | 0.653107894 |
| CGREF1 | grey | -0.120774607 | 0.008987316 | -0.155432448 | 0.000750516 |
| CH17-140 | grey | 0.012029278 | 0.795428774 | 0.005400235 | 0.907344916 |
| CH17-270 | grey | -0.043183867 | 0.351778714 | -0.042332713 | 0.361360783 |
| CH17-335 | grey | -0.056264434 | 0.224905206 | -0.066797204 | 0.149516524 |
| CH507-42 | grey | -0.087816488 | 0.057919146 | -0.128821491 | 0.005303116 |
| CH507-9I | grey | -0.044432803 | 0.338011867 | -0.037041792 | 0.424517324 |
| CH507-9I | grey | 0.074044128 | 0.110040062 | 0.072873345 | 0.115793529 |
| CH507-9I | grey | -0.033238752 | 0.473640371 | -0.037993798 | 0.412701801 |
| CH507-9I | grey | -0.011774092 | 0.799675432 | -0.035161599 | 0.448423054 |
| CH507-9I | grey | -0.014305864 | 0.757823939 | -0.021163294 | 0.648269115 |
| CHAC1 | grey | 0.003044367 | 0.947685801 | 0.000985572 | 0.983053151 |
| CHAD | grey | -0.163053476 | 0.000403359 | -0.144122408 | 0.001793333 |
| CHAT | grey | 0.03596912 | 0.438063702 | 0.044529812 | 0.336957176 |
| CHCHD5 | grey | -0.326282987 | 4.80E-13 | -0.309093008 | 8.52E-12 |
| CHD3 | grey | 0.124398863 | 0.007112299 | 0.115253286 | 0.012692594 |
| CHD5 | grey | -0.169158423 | 0.000240429 | -0.207183223 | 6.35E-06 |
| CHFR | grey | 0.081826382 | 0.077310759 | 0.08797315 | 0.057471776 |
| CHGB | grey | -0.152170976 | 0.00097078 | -0.161462433 | 0.000460236 |
| CHI3L1 | grey | -0.035907137 | 0.438853969 | -0.016330206 | 0.724855915 |
| CHI3L2 | grey | 0.036972195 | 0.425388767 | 0.040211274 | 0.385944819 |
| CHKB-CI | grey | 0.196542541 | 1.89E-05 | 0.167045804 | 0.000288154 |
| CHL1 | grey | -0.337821667 | 6.26E-14 | -0.344824073 | 1.74E-14 |
| CHMP6 | grey | 0.018779929 | 0.68563499 | 0.018877366 | 0.684091484 |
| CHODL | grey | -0.29969559 | 3.80E-11 | -0.29464483 | 8.30E-11 |
| CHP2 | grey | -0.101700776 | 0.027978828 | -0.115269972 | 0.012679619 |
| CHRFAM | grey | -0.071410715 | 0.12330929 | -0.072666516 | 0.116833996 |
| CHRM1 | grey | -0.055280941 | 0.233127328 | -0.047900082 | 0.301628035 |
| CHRM2 | grey | -0.007900777 | 0.864788107 | -0.000392223 | 0.993255322 |

| | | | | | |
|---|---|---|---|---|---|
| CHRM3 | grey | 0.052186779 | 0.260372754 | 0.002118473 | 0.963582874 |
| CHRNA1 | grey | 0.063581419 | 0.170153109 | 0.070397267 | 0.128735833 |
| CHRNA1 | grey | 0.127721644 | 0.005709413 | 0.125864897 | 0.006459173 |
| CHRNA2 | grey | 0.019541254 | 0.673610089 | -0.015878688 | 0.73216778 |
| CHRNA4 | grey | -0.17157488 | 0.000194936 | -0.172931308 | 0.00017307 |
| CHRNA6 | grey | -0.145637297 | 0.001601382 | -0.15019548 | 0.001131773 |
| CHRNA7 | grey | -0.145923118 | 0.001567353 | -0.160881138 | 0.000482815 |
| CHRNA9 | grey | 0.17842853 | 0.000105886 | 0.169122266 | 0.00024118 |
| CHRNB1 | grey | -0.137339037 | 0.002938756 | -0.153042031 | 0.000906745 |
| CHRNB2 | grey | -0.029657747 | 0.522606731 | -0.040760821 | 0.379480746 |
| CHRNB4 | grey | -0.156988064 | 0.000662654 | -0.173605505 | 0.000163078 |
| CHRND | grey | -0.08667697 | 0.06126072 | -0.080955731 | 0.080524808 |
| CHRNE | grey | -0.177945663 | 0.000110621 | -0.182507554 | 7.28E-05 |
| CHST1 | grey | 0.056347549 | 0.224219937 | 0.08434907 | 0.068582477 |
| CHST10 | grey | -0.088427831 | 0.056189567 | -0.105876119 | 0.02211973 |
| CHST13 | grey | -0.24564966 | 7.58E-08 | -0.239421934 | 1.64E-07 |
| CHST14 | grey | 0.075600759 | 0.102741932 | 0.050896153 | 0.272362134 |
| CHST15 | grey | -0.25300362 | 2.97E-08 | -0.265265661 | 5.83E-09 |
| CHST2 | grey | 0.226008517 | 8.02E-07 | 0.219420346 | 1.69E-06 |
| CHST3 | grey | 0.218268534 | 1.92E-06 | 0.219649998 | 1.65E-06 |
| CHST7 | grey | 0.014413139 | 0.756065214 | 0.034814807 | 0.452914117 |
| CHST8 | grey | -0.156827463 | 0.000671262 | -0.166376133 | 0.000305041 |
| CHURC1 | grey | -0.087857374 | 0.057802112 | -0.117623449 | 0.010962612 |
| CIART | grey | 0.007882951 | 0.865090269 | -0.004285608 | 0.926408042 |
| CIB2 | grey | -0.04493708 | 0.332552398 | -0.089762957 | 0.052561022 |
| CIDEA | grey | -0.115286288 | 0.012666941 | -0.120842479 | 0.008948504 |
| CITED1 | grey | 0.060672081 | 0.190592974 | 0.059728952 | 0.197591121 |
| CKAP4 | grey | 0.157613519 | 0.000630094 | 0.160430967 | 0.000501003 |
| CKB | grey | -0.151805398 | 0.000998879 | -0.116297892 | 0.01190247 |
| CKM | grey | -0.034559686 | 0.456234108 | -0.010486916 | 0.821183492 |
| CKMT1A | grey | 0.110532195 | 0.016869234 | 0.111053709 | 0.016355254 |
| CKMT1B | grey | 0.066987385 | 0.148358492 | 0.078700133 | 0.089356479 |
| CKMT2 | grey | -0.198065363 | 1.62E-05 | -0.196789441 | 1.84E-05 |
| CLC | grey | -0.063339292 | 0.171789046 | -0.097623988 | 0.034939542 |
| CLCA4 | grey | 0.186541368 | 4.99E-05 | 0.166705966 | 0.000296611 |
| CLCF1 | grey | -0.101986349 | 0.027539384 | -0.13046877 | 0.004743167 |
| CLCN4 | grey | -0.336199199 | 8.38E-14 | -0.323721873 | 7.45E-13 |
| CLCNKA | grey | -0.213798327 | 3.13E-06 | -0.200503045 | 1.27E-05 |
| CLCNKB | grey | -0.323921162 | 7.20E-13 | -0.329961584 | 2.53E-13 |
| CLDN1 | grey | -0.250377681 | 4.16E-08 | -0.263482288 | 7.42E-09 |
| CLDN10 | grey | -0.15108521 | 0.00105644 | -0.151028227 | 0.001061123 |
| CLDN14 | grey | 0.021615572 | 0.641273448 | 0.04733499 | 0.307373537 |
| CLDN16 | grey | -0.240108214 | 1.51E-07 | -0.253799401 | 2.68E-08 |
| CLDN17 | grey | 0.048147185 | 0.299138186 | 0.019863093 | 0.668551411 |
| CLDN19 | grey | -0.210284396 | 4.57E-06 | -0.21602244 | 2.46E-06 |

| | | | | | |
|---|---|---|---|---|---|
| CLDN2 | grey | -0.040898882 | 0.377867291 | -0.043851375 | 0.344377389 |
| CLDN23 | grey | 0.108303374 | 0.019228396 | 0.105700656 | 0.022342637 |
| CLDN24 | grey | -0.15522905 | 0.00076277 | -0.144740203 | 0.001712641 |
| CLDN8 | grey | 0.082116358 | 0.076263719 | 0.06034333 | 0.193011402 |
| CLDN9 | grey | -0.28460406 | 3.76E-10 | -0.285072867 | 3.50E-10 |
| CLEC18A | grey | -0.090741558 | 0.050027241 | -0.080965868 | 0.080486775 |
| CLEC18B | grey | -0.160296787 | 0.000506547 | -0.171727216 | 0.000192358 |
| CLEC18C | grey | -0.107441122 | 0.020215399 | -0.107328115 | 0.020347968 |
| CLEC2A | grey | 0.016610992 | 0.720321414 | 0.01380626 | 0.766031073 |
| CLEC2L | grey | -0.130464951 | 0.004744401 | -0.137277839 | 0.002951596 |
| CLEC3A | grey | -0.062931165 | 0.174573075 | -0.064812398 | 0.162015576 |
| CLEC4F | grey | 0.043805988 | 0.344877488 | 0.045672116 | 0.324696927 |
| CLIC3 | grey | 0.121646993 | 0.008499682 | 0.159406803 | 0.000544782 |
| CLIC5 | grey | 0.00573158 | 0.901687757 | -0.011668179 | 0.801439716 |
| CLK2 | grey | 0.04022017 | 0.385839647 | 0.012699263 | 0.784308297 |
| CLK3 | grey | 0.127255027 | 0.005890084 | 0.131752248 | 0.004344483 |
| CLNK | grey | 0.02023313 | 0.662753665 | 0.040729247 | 0.379850328 |
| CLPS | grey | 0.062874249 | 0.174963977 | 0.056824478 | 0.220316507 |
| CLPSL1 | grey | 0.124787345 | 0.006933709 | 0.119707529 | 0.00961753 |
| CLPSL2 | grey | 0.160295339 | 0.000506607 | 0.150341977 | 0.001119038 |
| CLPTM1 | grey | 0.033154903 | 0.474757421 | 0.036805041 | 0.427485985 |
| CLRN3 | grey | 0.093437402 | 0.043569913 | 0.100066093 | 0.030611972 |
| CLSTN1 | grey | 0.007718458 | 0.867879345 | -0.006994881 | 0.880167091 |
| CLSTN3 | grey | -0.147907233 | 0.001348827 | -0.160270244 | 0.00050765 |
| CLTCL1 | grey | -0.123702353 | 0.007442825 | -0.146917216 | 0.001454099 |
| CLUL1 | grey | 0.051410887 | 0.267536079 | 0.036874671 | 0.42661163 |
| CLYBL | grey | 0.02689862 | 0.562028463 | -0.002366438 | 0.959323792 |
| CMBL | grey | 0.152403441 | 0.000953294 | 0.164341963 | 0.00036217 |
| CMC4 | grey | 0.043314339 | 0.350324205 | 0.032890355 | 0.478291209 |
| CMTM2 | grey | 0.051787553 | 0.264041867 | 0.074879384 | 0.106074851 |
| CMTM7 | grey | 0.071239284 | 0.124214502 | 0.043608037 | 0.347063972 |
| CMTM8 | grey | 0.161039899 | 0.000476548 | 0.133255168 | 0.00391629 |
| CNGA1 | grey | 0.153774202 | 0.000855963 | 0.122275018 | 0.008163382 |
| CNGA2 | grey | -0.072452528 | 0.117918168 | -0.07240595 | 0.118155191 |
| CNGB1 | grey | -0.118758022 | 0.010211011 | -0.122604267 | 0.007991859 |
| CNKSR2 | grey | -0.303727362 | 2.01E-11 | -0.281323204 | 6.07E-10 |
| CNN2 | grey | 0.199174866 | 1.45E-05 | 0.220867298 | 1.44E-06 |
| CNNM2 | grey | 0.20196167 | 1.09E-05 | 0.177647828 | 0.000113641 |
| CNPY1 | grey | 0.042129783 | 0.363669167 | -0.022032907 | 0.634846295 |
| CNR1 | grey | -0.246608733 | 6.72E-08 | -0.236474257 | 2.34E-07 |
| CNTD2 | grey | -0.084938946 | 0.066662554 | -0.065809117 | 0.15564399 |
| CNTFR | grey | -0.363594891 | 4.83E-16 | -0.378763844 | 2.23E-17 |
| CNTN6 | grey | -0.048559434 | 0.295014813 | -0.03993743 | 0.389190798 |
| CNTNAP | grey | -0.077022331 | 0.096417312 | -0.085358589 | 0.065323739 |
| CNTNAP | grey | -0.083659683 | 0.070883451 | -0.08629883 | 0.062404159 |

| | | | | | |
|---|---|---|---|---|---|
| CNTROB | grey | 0.074399149 | 0.108340636 | 0.060572847 | 0.191320622 |
| COCH | grey | 0.028979532 | 0.532164125 | 0.053928046 | 0.244781531 |
| COG1 | grey | 0.131974918 | 0.004278482 | 0.123327941 | 0.007626118 |
| COL11A1 | grey | -0.082181551 | 0.076029917 | -0.081851124 | 0.07722097 |
| COL11A2 | grey | -0.256646232 | 1.85E-08 | -0.264530425 | 6.44E-09 |
| COL13A1 | grey | -0.031840618 | 0.492453587 | -0.024395347 | 0.598990111 |
| COL17A1 | grey | 0.279466668 | 7.95E-10 | 0.276485753 | 1.22E-09 |
| COL18A1 | grey | -0.116028244 | 0.012102151 | -0.138952093 | 0.002618125 |
| COL19A1 | grey | -0.062761624 | 0.175739416 | -0.054691011 | 0.238159998 |
| COL22A1 | grey | -0.348565142 | 8.70E-15 | -0.334984833 | 1.04E-13 |
| COL23A1 | grey | -0.252318943 | 3.24E-08 | -0.243122964 | 1.04E-07 |
| COL25A1 | grey | -0.235045648 | 2.78E-07 | -0.228945292 | 5.71E-07 |
| COL26A1 | grey | -0.153895915 | 0.000847781 | -0.170250812 | 0.000218755 |
| COL27A1 | grey | -0.070651148 | 0.127359356 | -0.107388338 | 0.020277227 |
| COL2A1 | grey | 0.077808891 | 0.093053649 | 0.082029429 | 0.076576384 |
| COL4A3 | grey | -0.216849509 | 2.25E-06 | -0.237513315 | 2.06E-07 |
| COL4A4 | grey | -0.272134653 | 2.25E-09 | -0.288828647 | 2.00E-10 |
| COL7A1 | grey | -0.205157764 | 7.85E-06 | -0.18402295 | 6.33E-05 |
| COL8A2 | grey | -0.383497204 | 8.25E-18 | -0.37622354 | 3.77E-17 |
| COL9A1 | grey | -0.297853999 | 5.06E-11 | -0.294974978 | 7.89E-11 |
| COL9A2 | grey | -0.179167647 | 9.90E-05 | -0.177184737 | 0.000118489 |
| COL9A3 | grey | -0.227035733 | 7.12E-07 | -0.231040361 | 4.47E-07 |
| COLEC1( | grey | -0.154725944 | 0.000793879 | -0.156811186 | 0.00067214 |
| COLEC1l | grey | -0.048585853 | 0.294751871 | -0.039527282 | 0.394083295 |
| COLGAL | grey | 0.210514218 | 4.46E-06 | 0.151267745 | 0.001041568 |
| COMMD: | grey | 0.021931133 | 0.636411153 | -0.03841828 | 0.407496715 |
| COMP | grey | -0.164932066 | 0.000344646 | -0.139797845 | 0.002463067 |
| COQ5 | grey | 0.181936243 | 7.68E-05 | 0.191256048 | 3.18E-05 |
| COQ6 | grey | 0.184162866 | 6.24E-05 | 0.194555386 | 2.30E-05 |
| CORO6 | grey | -0.200708079 | 1.24E-05 | -0.212144569 | 3.75E-06 |
| CORO7-P | grey | 0.013501827 | 0.771044991 | 0.012962239 | 0.779955227 |
| CORT | grey | 0.007892542 | 0.864927689 | 0.011147314 | 0.810130745 |
| COX6A2 | grey | -0.255219793 | 2.23E-08 | -0.252696176 | 3.09E-08 |
| COX6B2 | grey | -0.13765109 | 0.002874073 | -0.143783164 | 0.001839111 |
| COX7B2 | grey | -0.137793258 | 0.002845036 | -0.136423349 | 0.003136279 |
| COX8C | grey | -0.152767818 | 0.00092647 | -0.150811035 | 0.001079147 |
| CP | grey | -0.104317501 | 0.024168959 | -0.105920577 | 0.022063558 |
| CPA1 | grey | -0.0575246 | 0.214674735 | -0.04074265 | 0.379693415 |
| CPA2 | grey | -0.072996457 | 0.11517766 | -0.06399204 | 0.16740539 |
| CPA4 | grey | 0.047562609 | 0.30505062 | 0.076654129 | 0.098024872 |
| CPA5 | grey | -0.044825827 | 0.333751951 | -0.029718028 | 0.521761531 |
| CPA6 | grey | 0.054274398 | 0.241759863 | 0.040946352 | 0.377313502 |
| CPAMD8 | grey | -0.267828909 | 4.10E-09 | -0.255904836 | 2.04E-08 |
| CPB2 | grey | -0.005780041 | 0.900860788 | -0.02177155 | 0.638868147 |
| CPLX1 | grey | 0.020337683 | 0.66111915 | 0.036954451 | 0.425611108 |

| | | | | | |
|---|---|---|---|---|---|
| CPLX2 | grey | 0.027119495 | 0.558820628 | 0.00876731 | 0.850124906 |
| CPLX3 | grey | 0.05562802 | 0.23020178 | 0.062497165 | 0.177570284 |
| CPN1 | grey | 0.114073974 | 0.013639905 | 0.101975764 | 0.027555566 |
| CPNE4 | grey | 0.019464481 | 0.674818997 | 0.022250246 | 0.631509967 |
| CPNE7 | grey | 0.204864362 | 8.09E-06 | 0.198754337 | 1.51E-05 |
| CPO | grey | -0.08243266 | 0.075134797 | -0.086957672 | 0.060423149 |
| CPPED1 | grey | -0.005998449 | 0.897135031 | 0.014148335 | 0.760408814 |
| CPS1 | grey | -0.093863918 | 0.042615557 | -0.090059785 | 0.051781419 |
| CPSF4L | grey | -0.035623036 | 0.442486587 | -0.042709151 | 0.357102989 |
| CPT1A | grey | -0.018259228 | 0.693905487 | -0.021500021 | 0.643057756 |
| CPT1B | grey | 0.169039544 | 0.000242905 | 0.153005364 | 0.00090936 |
| CPT1C | grey | -0.189616985 | 3.72E-05 | -0.198199934 | 1.60E-05 |
| CPXM2 | grey | -0.384536936 | 6.62E-18 | -0.390106365 | 2.00E-18 |
| CR2 | grey | -0.208340845 | 5.62E-06 | -0.205569408 | 7.52E-06 |
| CRABP1 | grey | -0.103875152 | 0.024779637 | -0.124568038 | 0.007034028 |
| CRABP2 | grey | -0.284069095 | 4.06E-10 | -0.300252826 | 3.48E-11 |
| CRACR2. | grey | -0.059347035 | 0.200477685 | -0.049527719 | 0.28547979 |
| CRAT | grey | 0.325652916 | 5.35E-13 | 0.329379206 | 2.80E-13 |
| CRB2 | grey | -0.309299921 | 8.23E-12 | -0.319255962 | 1.59E-12 |
| CREG2 | grey | 0.053837997 | 0.245571449 | 0.033668445 | 0.467938605 |
| CRELD1 | grey | -0.026748463 | 0.564214278 | -0.0364235 | 0.432295495 |
| CRH | grey | -0.086220185 | 0.062644168 | -0.041256725 | 0.373704986 |
| CRHR1 | grey | -0.22823782 | 6.20E-07 | -0.223658769 | 1.05E-06 |
| CRIP3 | grey | -0.252494075 | 3.17E-08 | -0.266546701 | 4.89E-09 |
| CRISP1 | grey | -0.032225612 | 0.487233626 | -0.041825322 | 0.367149712 |
| CRNN | grey | -0.063881677 | 0.16814061 | -0.056589201 | 0.22223602 |
| CRP | grey | -0.040185973 | 0.386244032 | -0.041398858 | 0.372059631 |
| CRTAC1 | grey | -0.367901546 | 2.05E-16 | -0.359600879 | 1.06E-15 |
| CRTC1 | grey | -0.120757458 | 0.008997147 | -0.123581986 | 0.007501316 |
| CRTC2 | grey | -0.032645942 | 0.481568725 | -0.0525642 | 0.256936523 |
| CRYAB | grey | -0.228595218 | 5.94E-07 | -0.225971571 | 8.05E-07 |
| CRYBA2 | grey | 0.101655651 | 0.028048816 | 0.077631999 | 0.093801814 |
| CRYBB2 | grey | 0.070820118 | 0.126449591 | 0.085423143 | 0.065119763 |
| CRYGB | grey | -0.067541266 | 0.145024752 | -0.063075308 | 0.173585994 |
| CRYGC | grey | -0.067065696 | 0.147883635 | -0.054827365 | 0.236990033 |
| CRYGN | grey | -0.285340061 | 3.37E-10 | -0.265253048 | 5.84E-09 |
| CRYGS | grey | 0.048719438 | 0.293424708 | 0.050484714 | 0.276262178 |
| CRYM | grey | 0.006671542 | 0.885667514 | 0.010243642 | 0.825264109 |
| CSAG1 | grey | -0.132463065 | 0.004136955 | -0.134344784 | 0.00363015 |
| CSF2 | grey | 0.062286308 | 0.179040175 | 0.095532608 | 0.039049362 |
| CSF3 | grey | 0.221772192 | 1.30E-06 | 0.198211341 | 1.60E-05 |
| CSH1 | grey | -0.037316069 | 0.421093163 | -0.100711249 | 0.029548445 |
| CSH2 | grey | -0.066929668 | 0.148709211 | -0.113007327 | 0.014549769 |
| CSHL1 | grey | -0.064107503 | 0.166638772 | -0.063482339 | 0.170821134 |
| CSN2 | grey | 0.096681303 | 0.036744077 | 0.089408558 | 0.053504615 |

| | | | | | |
|---|---|---|---|---|---|
| CSN3 | grey | -0.06317401 | 0.172912495 | -0.057842585 | 0.212146902 |
| CSRP2 | grey | 0.069189441 | 0.135442679 | 0.033319241 | 0.472569449 |
| CSRP2BP | grey | -0.130116853 | 0.004858102 | -0.148819435 | 0.001258079 |
| CSRP3 | grey | -0.069920774 | 0.131350324 | -0.065420716 | 0.158103951 |
| CST6 | grey | -0.100108482 | 0.030541102 | -0.071037331 | 0.125287495 |
| CST8 | grey | -0.050451704 | 0.276576714 | -0.035407603 | 0.445252579 |
| CST9 | grey | -0.080613341 | 0.081818031 | -0.07560327 | 0.102730476 |
| CT45A1 | grey | -0.120706109 | 0.009026639 | -0.123554114 | 0.007514919 |
| CT45A10 | grey | -0.057060395 | 0.2184037 | -0.076190518 | 0.100079261 |
| CT45A3 | grey | -0.088200886 | 0.056826558 | -0.086735412 | 0.061085554 |
| CT45A5 | grey | -0.094097665 | 0.042100051 | -0.093838259 | 0.042672468 |
| CT45A7 | grey | 0.04479839 | 0.334048212 | 0.052280603 | 0.259515591 |
| CT47A6 | grey | -0.041532377 | 0.370518069 | -0.041563051 | 0.370164481 |
| CT55 | grey | -0.106949502 | 0.020797642 | -0.113329999 | 0.014269056 |
| CT83 | grey | 0.014541725 | 0.753958752 | 0.010963768 | 0.813198973 |
| CTAG1A | grey | -0.1147867 | 0.013060219 | -0.136689625 | 0.003077632 |
| CTAG1B | grey | -0.079271193 | 0.087050166 | -0.082785865 | 0.073890278 |
| CTAG2 | grey | -0.091467615 | 0.048214007 | -0.131344535 | 0.004467719 |
| CTAGE1 | grey | 0.09649944 | 0.037101165 | 0.114793941 | 0.013054443 |
| CTAGE4 | grey | 0.11867921 | 0.010261703 | 0.144114782 | 0.001794351 |
| CTAGE8 | grey | -0.056345431 | 0.224237382 | -0.10172737 | 0.027937652 |
| CTB-50L | grey | 0.091000204 | 0.049374878 | 0.060045543 | 0.195221375 |
| CTB-54O | grey | 0.192841926 | 2.72E-05 | 0.18004016 | 9.14E-05 |
| CTB-60B | grey | -0.087066703 | 0.06010038 | -0.078490415 | 0.090215673 |
| CTB-96E | grey | 0.046634662 | 0.314593612 | 0.049616159 | 0.284619357 |
| CTC-260 | grey | -0.112635928 | 0.014878871 | -0.108868336 | 0.018604698 |
| CTC-273 | grey | 0.038191949 | 0.410267153 | 0.034894767 | 0.451876368 |
| CTC-326 | grey | 0.081505925 | 0.078481409 | 0.066755368 | 0.149772187 |
| CTC-360 | grey | -0.132592033 | 0.004100277 | -0.149351114 | 0.001207814 |
| CTC-398 | grey | -0.03022163 | 0.514727971 | -0.052729393 | 0.255442441 |
| CTC-429 | grey | 0.00206852 | 0.964441011 | 0.010993717 | 0.812698143 |
| CTC-435 | grey | -0.01336356 | 0.773325389 | 0.008684316 | 0.851527163 |
| CTC-490 | grey | 0.055419796 | 0.231953776 | 0.06132387 | 0.185864036 |
| CTC-512 | grey | -0.004451477 | 0.923568312 | -0.008432004 | 0.855793018 |
| CTC1 | grey | 0.273805162 | 1.78E-09 | 0.244690522 | 8.54E-08 |
| CTCFL | grey | -0.275436708 | 1.41E-09 | -0.283753797 | 4.26E-10 |
| CTD-200 | grey | 0.012595252 | 0.786031859 | 0.02420734 | 0.601809897 |
| CTD-200 | grey | -0.016292942 | 0.725458433 | -0.013241962 | 0.775332475 |
| CTD-210 | grey | -0.118228249 | 0.010556075 | -0.139793846 | 0.00246378 |
| CTD-213 | grey | 0.044246015 | 0.340048577 | 0.063222225 | 0.172584208 |
| CTD-213 | grey | -0.013105133 | 0.777592746 | -0.038195561 | 0.410222861 |
| CTD-214 | grey | 0.010789999 | 0.816106412 | -0.005301901 | 0.909024723 |
| CTD-219 | grey | 0.09865817 | 0.033047165 | 0.11464118 | 0.01317678 |
| CTD-219 | grey | 0.038921453 | 0.401377514 | 0.032231467 | 0.487154469 |
| CTD-220 | grey | -0.038667687 | 0.404456717 | -0.032445572 | 0.484264693 |

| | | | | | |
|---|---|---|---|---|---|
| CTD-220 | grey | -0.042198985 | 0.362880949 | -0.060291132 | 0.19339745 |
| CTD-227 | grey | -0.045947304 | 0.321787174 | -0.057366328 | 0.215940962 |
| CTD-234 | grey | -0.024934744 | 0.590933431 | -0.048465094 | 0.295955046 |
| CTD-236 | grey | 0.050488969 | 0.276221645 | 0.025203063 | 0.586944269 |
| CTD-236 | grey | -0.065556906 | 0.157238062 | -0.075505364 | 0.103177857 |
| CTD-241 | grey | 0.002288687 | 0.96065914 | -0.021251275 | 0.646905801 |
| CTD-251 | grey | -0.072471731 | 0.117820555 | -0.074612474 | 0.107329482 |
| CTD-254 | grey | 0.114396848 | 0.01337455 | 0.145473644 | 0.001621171 |
| CTD-256 | grey | -0.013777241 | 0.766508586 | 0.00677805 | 0.883855057 |
| CTD-257 | grey | -0.052129112 | 0.260900562 | -0.063331583 | 0.171841322 |
| CTD-258 | grey | -0.039319872 | 0.396571477 | -0.037942264 | 0.413336371 |
| CTD-258 | grey | 0.09246849 | 0.045804941 | 0.100563075 | 0.029789864 |
| CTD-261 | grey | -0.064282092 | 0.165484584 | -0.073871928 | 0.110871878 |
| CTD-310 | grey | 0.012939915 | 0.780324487 | -0.001503659 | 0.974147263 |
| CTD-314 | grey | 0.025263427 | 0.586048554 | 0.02300526 | 0.619978257 |
| CTD-321 | grey | 0.019485792 | 0.674483334 | 0.024792996 | 0.59304582 |
| CTD-321 | grey | 0.032282515 | 0.486464636 | 0.052351608 | 0.258868192 |
| CTDP1 | grey | 0.109694729 | 0.017724188 | 0.124174651 | 0.007217236 |
| CTDSPL | grey | 0.254191705 | 2.55E-08 | 0.238335019 | 1.87E-07 |
| CTH | grey | 0.219400302 | 1.69E-06 | 0.183813522 | 6.45E-05 |
| CTHRC1 | grey | -0.167641271 | 0.000273876 | -0.162574733 | 0.000419744 |
| CTNND2 | grey | 0.046624805 | 0.314696017 | 0.008393271 | 0.856448261 |
| CTNS | grey | 0.041416741 | 0.371852928 | 0.03276602 | 0.479956997 |
| CTRB1 | grey | 0.023063465 | 0.619093062 | 0.024305713 | 0.600333727 |
| CTRB2 | grey | -0.010535622 | 0.820367096 | -0.016182702 | 0.727241883 |
| CTSA | grey | -0.095145932 | 0.039852421 | -0.080461489 | 0.082396934 |
| CTSE | grey | 0.031978835 | 0.490576131 | 0.079230978 | 0.087211 |
| CTSH | grey | 0.18927786 | 3.84E-05 | 0.165010689 | 0.000342372 |
| CTSV | grey | 0.344754897 | 1.77E-14 | 0.353058945 | 3.72E-15 |
| CTTN | grey | 0.12204814 | 0.008283478 | 0.131079583 | 0.004549488 |
| CTTNBP | grey | -0.220114084 | 1.56E-06 | -0.23959955 | 1.60E-07 |
| CTXN3 | grey | 0.084485576 | 0.068134199 | 0.076330631 | 0.099454801 |
| CUL9 | grey | 0.003063331 | 0.947360393 | -0.007392189 | 0.873416273 |
| CUX2 | grey | -0.065836765 | 0.155469991 | -0.057573046 | 0.214288218 |
| CUZD1 | grey | -0.336210035 | 8.36E-14 | -0.352122766 | 4.45E-15 |
| CWC25 | grey | -0.068379078 | 0.14009117 | -0.088843971 | 0.055036939 |
| CX3CL1 | grey | 0.117302952 | 0.011183618 | 0.105156099 | 0.02304688 |
| CXCL6 | grey | -0.083619094 | 0.071020869 | -0.06722525 | 0.146919734 |
| CXCL8 | grey | 0.17185665 | 0.000190192 | 0.171821521 | 0.000190777 |
| CXCR1 | grey | 0.080204802 | 0.083383017 | 0.097956776 | 0.034320778 |
| CXCR4 | grey | 0.053914585 | 0.244899501 | 0.05578605 | 0.228878397 |
| CXorf38 | grey | -0.098907548 | 0.032604161 | -0.087661037 | 0.058365905 |
| CXorf49 | grey | -0.087907783 | 0.057658091 | -0.086465516 | 0.061897974 |
| CXorf49E | grey | -0.044697644 | 0.335137492 | -0.058748395 | 0.205063797 |
| CXorf67 | grey | -0.196559323 | 1.88E-05 | -0.200284675 | 1.30E-05 |

| | | | | | |
|---|---|---|---|---|---|
| CXXC5 | grey | -0.239166922 | 1.69E-07 | -0.2350727 | 2.77E-07 |
| CYB561D | grey | -0.162750245 | 0.000413667 | -0.16250612 | 0.000422143 |
| CYB5A | grey | 0.213052049 | 3.40E-06 | 0.196390887 | 1.92E-05 |
| CYB5B | grey | 0.214285507 | 2.97E-06 | 0.232771522 | 3.64E-07 |
| CYB5R2 | grey | 0.080687784 | 0.081535436 | 0.105205814 | 0.022981799 |
| CYB5R3 | grey | 0.03991427 | 0.389466077 | 0.047790209 | 0.302739538 |
| CYFIP2 | grey | -0.263898996 | 7.01E-09 | -0.261431747 | 9.78E-09 |
| CYP17A1 | grey | -0.040788527 | 0.379156621 | -0.041971006 | 0.365481704 |
| CYP1A1 | grey | 0.008653301 | 0.852051296 | 0.02109983 | 0.649253252 |
| CYP1A2 | grey | -0.199936446 | 1.34E-05 | -0.199542867 | 1.40E-05 |
| CYP21A2 | grey | -0.040960218 | 0.377151838 | -0.056052415 | 0.226660019 |
| CYP24A1 | grey | 0.129467106 | 0.005076926 | 0.103042671 | 0.025965089 |
| CYP26A1 | grey | 0.0195883 | 0.672869691 | 0.014204282 | 0.759490477 |
| CYP26B1 | grey | -0.081320793 | 0.079164233 | -0.098289746 | 0.03371104 |
| CYP26C1 | grey | -0.296050617 | 6.69E-11 | -0.297872363 | 5.05E-11 |
| CYP27B1 | grey | 0.130306399 | 0.004795888 | 0.145424126 | 0.001627202 |
| CYP27C1 | grey | -0.142792616 | 0.001978968 | -0.128405378 | 0.005453667 |
| CYP2A6 | grey | -0.111677237 | 0.015758776 | -0.095900928 | 0.038297207 |
| CYP2B6 | grey | 0.082295297 | 0.075623384 | 0.068226655 | 0.140979027 |
| CYP2C18 | grey | -0.011323764 | 0.80718386 | -0.012741317 | 0.783611712 |
| CYP2C9 | grey | -0.034039577 | 0.46304457 | -0.038228827 | 0.409814991 |
| CYP2D6 | grey | 0.126798945 | 0.006071624 | 0.139204156 | 0.002571007 |
| CYP2E1 | grey | 0.046257681 | 0.318525766 | 0.029556821 | 0.52402339 |
| CYP2R1 | grey | -0.152021419 | 0.000982186 | -0.180852641 | 8.49E-05 |
| CYP2S1 | grey | -0.312347431 | 5.01E-12 | -0.301557735 | 2.84E-11 |
| CYP2W1 | grey | -0.115566476 | 0.012450981 | -0.104363944 | 0.024105603 |
| CYP3A5 | grey | 0.002293075 | 0.96058377 | -0.006914587 | 0.881532469 |
| CYP3A7 | grey | -0.020894017 | 0.652448985 | -0.021712841 | 0.639773044 |
| CYP4A11 | grey | 0.002252377 | 0.961282779 | 0.002430178 | 0.958229152 |
| CYP4F11 | grey | 0.063093813 | 0.173459576 | 0.055759087 | 0.229103804 |
| CYP4F12 | grey | -0.09416298 | 0.041956948 | -0.088935603 | 0.054785794 |
| CYP4F3 | grey | 0.016325573 | 0.724930824 | 0.015897303 | 0.731865858 |
| CYP4F8 | grey | -0.066695529 | 0.150138445 | -0.090393678 | 0.050916019 |
| CYP4Z1 | grey | 0.035540294 | 0.443547765 | 0.028717468 | 0.53588062 |
| CYSLTR2 | grey | -0.074130214 | 0.109626067 | -0.058402859 | 0.207745268 |
| CYSRT1 | grey | 0.231306371 | 4.33E-07 | 0.248765207 | 5.11E-08 |
| CYTH2 | grey | 0.209570377 | 4.93E-06 | 0.190522273 | 3.41E-05 |
| D4S234E | grey | -0.113558413 | 0.014073234 | -0.133980221 | 0.003723705 |
| DAB1 | grey | -0.179255935 | 9.82E-05 | -0.19175396 | 3.03E-05 |
| DAB2IP | grey | 0.014331843 | 0.757397909 | 0.014193342 | 0.759670036 |
| DACH2 | grey | -0.146235421 | 0.00153093 | -0.14414525 | 0.001790289 |
| DAP | grey | 0.187732651 | 4.46E-05 | 0.207684402 | 6.03E-06 |
| DAPK1 | grey | 0.102619532 | 0.026586153 | 0.088238649 | 0.05672015 |
| DAPK2 | grey | 0.240184958 | 1.49E-07 | 0.240065375 | 1.51E-07 |
| DAPL1 | grey | 0.008676173 | 0.85166476 | -0.010432867 | 0.822089698 |

| | | | | | |
|---|---|---|---|---|---|
| DAZ1 | grey | 0.000949501 | 0.983673301 | -0.016883512 | 0.71592975 |
| DAZ2 | grey | -0.043200072 | 0.351597855 | -0.044901885 | 0.332931574 |
| DBP | grey | -0.177839073 | 0.000111693 | -0.181781952 | 7.79E-05 |
| DBX1 | grey | -0.100087092 | 0.030576846 | -0.093245156 | 0.044005922 |
| DBX2 | grey | -0.062084696 | 0.180454041 | -0.043307862 | 0.350396321 |
| DCAF12I | grey | -0.432065769 | 1.16E-22 | -0.433396646 | 8.31E-23 |
| DCAF4 | grey | -0.24012946 | 1.50E-07 | -0.271330698 | 2.52E-09 |
| DCAF4L2 | grey | -0.119689102 | 0.00962875 | -0.115294698 | 0.012660412 |
| DCAF8L1 | grey | -0.113118017 | 0.014452931 | -0.114369926 | 0.013396501 |
| DCANP1 | grey | 0.012915995 | 0.780720209 | -0.01742286 | 0.707265846 |
| DCBLD1 | grey | 0.200558963 | 1.26E-05 | 0.214370475 | 2.94E-06 |
| DCC | grey | -0.131186642 | 0.004516286 | -0.134860152 | 0.003501528 |
| DCD | grey | 0.094989191 | 0.040181901 | 0.055290487 | 0.233046515 |
| DCLK3 | grey | -0.014892918 | 0.748214966 | -0.032809841 | 0.479369536 |
| DCPS | grey | 0.220714386 | 1.46E-06 | 0.22280103 | 1.16E-06 |
| DCT | grey | -0.077844138 | 0.092905143 | -0.091699432 | 0.047646792 |
| DCTD | grey | 0.227874394 | 6.46E-07 | 0.208512352 | 5.52E-06 |
| DCUN1D | grey | -0.24478053 | 8.45E-08 | -0.267281928 | 4.42E-09 |
| DCX | grey | 0.031495245 | 0.497161674 | 0.025302627 | 0.585467205 |
| DDA1 | grey | -0.125895155 | 0.006446279 | -0.1042389 | 0.02427651 |
| DDB2 | grey | 0.360444773 | 8.97E-16 | 0.376649771 | 3.45E-17 |
| DDC | grey | -0.046559765 | 0.315372296 | -0.056350968 | 0.224191782 |
| DDIT3 | grey | -0.089564025 | 0.053088961 | -0.08337792 | 0.071841884 |
| DDIT4 | grey | 0.279239712 | 8.21E-10 | 0.287301598 | 2.52E-10 |
| DDIT4L | grey | 0.242244854 | 1.16E-07 | 0.22209119 | 1.25E-06 |
| DDN | grey | 0.087282813 | 0.059464831 | 0.06045294 | 0.192202582 |
| DDTL | grey | -0.080700711 | 0.081486443 | -0.091183908 | 0.048915868 |
| DDX19B | grey | 0.186486587 | 5.02E-05 | 0.206814043 | 6.60E-06 |
| DDX25 | grey | -0.306291757 | 1.34E-11 | -0.300939491 | 3.13E-11 |
| DDX43 | grey | -0.076554192 | 0.098464858 | -0.094306054 | 0.041644911 |
| 1-Dec | grey | -0.031937729 | 0.491134086 | -0.038100096 | 0.411394679 |
| DEDD2 | grey | -0.025880745 | 0.576924824 | 0.002204589 | 0.962103612 |
| DEF8 | grey | 0.097847854 | 0.034522265 | 0.087657531 | 0.058376014 |
| DEFA3 | grey | -0.023141721 | 0.617903814 | 0.037240192 | 0.422038817 |
| DEFA4 | grey | -0.024084184 | 0.603660249 | 0.043748866 | 0.345507528 |
| DEFB1 | grey | 0.234853633 | 2.84E-07 | 0.213910623 | 3.10E-06 |
| DEFB103 | grey | -0.029861876 | 0.519747461 | -0.030505778 | 0.510781195 |
| DEFB104 | grey | 0.026564744 | 0.566894197 | 0.019878592 | 0.668308184 |
| DEFB104 | grey | 0.128053963 | 0.005583794 | 0.115378942 | 0.012595163 |
| DEFB105 | grey | -0.004647635 | 0.920211261 | -0.015748584 | 0.734279219 |
| DEFB105 | grey | 0.030575178 | 0.509819638 | 0.0396833 | 0.392217862 |
| DEFB106 | grey | -0.027523679 | 0.552973627 | -0.042004839 | 0.365095008 |
| DEFB107 | grey | -0.035857037 | 0.439493318 | -0.042391264 | 0.360696447 |
| DEFB107 | grey | 0.034377973 | 0.458607103 | -0.00704579 | 0.879301587 |
| DEFB126 | grey | -0.11191328 | 0.015538001 | -0.129920559 | 0.004923296 |

| | | | | | |
|---|---|---|---|---|---|
| DEFB132 | grey | 0.030103853 | 0.516368487 | 0.019735054 | 0.670562157 |
| DEFB136 | grey | 0.026753727 | 0.564137575 | 0.031372894 | 0.498835258 |
| DEFB4A | grey | 0.280716784 | 6.63E-10 | 0.284993218 | 3.55E-10 |
| DEFB4B | grey | 0.142516794 | 0.002019608 | 0.145215694 | 0.001652817 |
| DEGS2 | grey | 0.209938033 | 4.75E-06 | 0.21871075 | 1.83E-06 |
| DENND1 | grey | 0.000324738 | 0.994415784 | 0.012365936 | 0.789835553 |
| DENND4 | grey | -0.022122296 | 0.633473192 | -0.051549517 | 0.266246381 |
| DESI1 | grey | 0.165082403 | 0.000340309 | 0.164824084 | 0.000347792 |
| DGCR6 | grey | 0.003923363 | 0.93261303 | -0.004298825 | 0.926181735 |
| DGCR8 | grey | 0.030391875 | 0.5123614 | 0.014191811 | 0.759695154 |
| DGKA | grey | 0.210037218 | 4.70E-06 | 0.211219704 | 4.14E-06 |
| DGKG | grey | -0.222359747 | 1.21E-06 | -0.237581364 | 2.05E-07 |
| DGKK | grey | -0.045531697 | 0.326188232 | -0.03878707 | 0.403006364 |
| DGKQ | grey | 0.208624113 | 5.46E-06 | 0.231821168 | 4.07E-07 |
| DHDDS | grey | 0.113166189 | 0.014410964 | 0.130290317 | 0.004801139 |
| DHDH | grey | 0.009237953 | 0.842182106 | 0.00734418 | 0.874231547 |
| DHODH | grey | 0.26118014 | 1.01E-08 | 0.26638341 | 5.00E-09 |
| DHRS11 | grey | 0.093898046 | 0.04253996 | 0.105857443 | 0.022143363 |
| DHRS2 | grey | 0.066559514 | 0.150973492 | 0.037699961 | 0.416327773 |
| DHRS7 | grey | 0.254361194 | 2.49E-08 | 0.250396858 | 4.15E-08 |
| DHRS9 | grey | -0.025123241 | 0.588129708 | 0.010549892 | 0.820127942 |
| DHRSX | grey | 0.318051831 | 1.95E-12 | 0.328126227 | 3.48E-13 |
| DIRAS2 | grey | 0.039852452 | 0.39020143 | 0.038334338 | 0.408522918 |
| DIRAS3 | grey | 0.081324727 | 0.079149676 | 0.046345922 | 0.317602487 |
| DIRC3 | grey | -0.279542496 | 7.86E-10 | -0.278519745 | 9.11E-10 |
| DISP2 | grey | -0.027071606 | 0.559515381 | -0.033806559 | 0.466113989 |
| DKFZp43 | grey | 0.01271112 | 0.784111875 | 0.006576365 | 0.887287656 |
| DKK2 | grey | -0.193938693 | 2.44E-05 | -0.171721041 | 0.000192461 |
| DKKL1 | grey | -0.004451581 | 0.923566525 | -0.007650579 | 0.869030767 |
| DLG3 | grey | 0.184445884 | 6.08E-05 | 0.21594783 | 2.48E-06 |
| DLGAP1 | grey | -0.030248288 | 0.514357022 | -0.054474493 | 0.240026148 |
| DLGAP2 | grey | -0.098199318 | 0.033875713 | -0.025368318 | 0.584493606 |
| DLGAP3 | grey | -0.335737228 | 9.10E-14 | -0.34968496 | 7.05E-15 |
| DLGAP4 | grey | -0.048626936 | 0.294343281 | -0.050046497 | 0.280457598 |
| DLK1 | grey | -0.209264817 | 5.10E-06 | -0.197841827 | 1.66E-05 |
| DLK2 | grey | -0.195708015 | 2.05E-05 | -0.195946667 | 2.00E-05 |
| DLL3 | grey | -0.133570247 | 0.003831519 | -0.143269433 | 0.001910474 |
| DLX1 | grey | 0.093615533 | 0.043169167 | 0.104543461 | 0.023862063 |
| DLX2 | grey | 0.088115675 | 0.057067269 | 0.102688506 | 0.026484052 |
| DLX4 | grey | -0.122086789 | 0.008262909 | -0.146325615 | 0.001520556 |
| DLX5 | grey | 0.351005109 | 5.50E-15 | 0.337801089 | 6.28E-14 |
| DLX6 | grey | 0.290207401 | 1.63E-10 | 0.269412963 | 3.29E-09 |
| DMBX1 | grey | -0.0757074 | 0.102256348 | -0.071666068 | 0.121970479 |
| DMC1 | grey | 0.040347349 | 0.384338027 | 0.010089795 | 0.827847145 |
| DMGDH | grey | -0.363356912 | 5.06E-16 | -0.356363205 | 1.98E-15 |

| | | | | | |
|---|---|---|---|---|---|
| DMKN | grey | -0.163133858 | 0.000400667 | -0.16839951 | 0.000256651 |
| DMRT1 | grey | -0.181290098 | 8.15E-05 | -0.180525482 | 8.74E-05 |
| DMRT2 | grey | -0.036569433 | 0.430452262 | -0.035560846 | 0.443284048 |
| DMRT3 | grey | -0.078067681 | 0.091967706 | -0.085594027 | 0.064582335 |
| DMRTA1 | grey | 0.078136453 | 0.091680837 | 0.077786927 | 0.093146285 |
| DMRTA2 | grey | 0.259696117 | 1.23E-08 | 0.270905709 | 2.68E-09 |
| DMRTB1 | grey | -0.109089077 | 0.018365844 | -0.100145471 | 0.030479376 |
| DMRTC1 | grey | -0.127952821 | 0.005621761 | -0.133062277 | 0.003969022 |
| DMRTC1 | grey | -0.109346183 | 0.018091018 | -0.112976023 | 0.014577259 |
| DMTN | grey | 0.198337858 | 1.58E-05 | 0.169883605 | 0.000225828 |
| DMWD | grey | -0.260702375 | 1.08E-08 | -0.261064365 | 1.03E-08 |
| DNAH1 | grey | 0.087961879 | 0.057503867 | 0.085305404 | 0.065492187 |
| DNAH10 | grey | 0.009214347 | 0.842580111 | -0.01284769 | 0.781850534 |
| DNAJB1 | grey | 0.040978854 | 0.376934623 | 0.038276668 | 0.409228836 |
| DNAJB12 | grey | 0.115258393 | 0.012688622 | 0.116684875 | 0.01162099 |
| DNAJC1 | grey | 0.130193201 | 0.004832955 | 0.113142536 | 0.014431557 |
| DNAJC12 | grey | -0.162438551 | 0.000424517 | -0.186380197 | 5.07E-05 |
| DNAJC15 | grey | 0.241027079 | 1.34E-07 | 0.224565584 | 9.46E-07 |
| DNAJC25 | grey | -0.002183793 | 0.962460833 | -0.025845582 | 0.57744271 |
| DNAJC30 | grey | 0.017698326 | 0.702855301 | 0.045568375 | 0.325798275 |
| DNASE1 | grey | 0.007405682 | 0.873187176 | 0.022535295 | 0.627145559 |
| DNER | grey | -0.19517214 | 2.16E-05 | -0.210790033 | 4.33E-06 |
| DNLZ | grey | -0.088117162 | 0.057063062 | -0.072121992 | 0.119608271 |
| DNM1 | grey | -0.34798401 | 9.70E-15 | -0.340272985 | 4.02E-14 |
| DNMBP | grey | 0.261441993 | 9.77E-09 | 0.248651111 | 5.19E-08 |
| DOC2B | grey | -0.163934284 | 0.000374758 | -0.164047686 | 0.000371216 |
| DOCK3 | grey | -0.35135674 | 5.14E-15 | -0.362736168 | 5.72E-16 |
| DOCK6 | grey | -0.098846569 | 0.032712015 | -0.097731804 | 0.034738045 |
| DOK4 | grey | -0.032442743 | 0.484302817 | -0.033333588 | 0.472378694 |
| DOK5 | grey | -0.432210605 | 1.12E-22 | -0.43296611 | 9.26E-23 |
| DOK7 | grey | -0.126095856 | 0.006361335 | -0.126160182 | 0.006334323 |
| DPCR1 | grey | -0.045125171 | 0.330530672 | -0.035369849 | 0.445738318 |
| DPEP3 | grey | -0.109589343 | 0.017834411 | -0.113903437 | 0.013781923 |
| DPP4 | grey | -0.07808539 | 0.091893771 | -0.03958117 | 0.393438383 |
| DPPA2 | grey | -0.074285985 | 0.10888007 | -0.087412637 | 0.059085716 |
| DPPA3 | grey | 0.028781805 | 0.534967002 | 0.040224022 | 0.385794116 |
| DPPA4 | grey | -0.013443855 | 0.772000868 | -0.004820239 | 0.917258485 |
| DPPA5 | grey | -0.112821729 | 0.014713422 | -0.120857049 | 0.008940192 |
| DPRX | grey | -0.02258401 | 0.626400965 | -0.029341532 | 0.527051867 |
| DPYS | grey | -0.116098346 | 0.012049956 | -0.105760067 | 0.022266945 |
| DPYSL5 | grey | -0.163010566 | 0.000404803 | -0.164553958 | 0.000355781 |
| DQX1 | grey | -0.014961933 | 0.747087839 | -0.012763557 | 0.783243396 |
| DRD1 | grey | -0.153621867 | 0.000866307 | -0.156726107 | 0.000676748 |
| DRD2 | grey | -0.216163355 | 2.42E-06 | -0.241909452 | 1.21E-07 |
| DRD5 | grey | -0.153134388 | 0.000900189 | -0.168565136 | 0.000253026 |

| | | | | | |
|---|---|---|---|---|---|
| DRGX | grey | -0.121088961 | 0.008808806 | -0.122904263 | 0.007838384 |
| DSC1 | grey | 0.054075886 | 0.243488521 | 0.074947971 | 0.105754333 |
| DSC3 | grey | -0.153339839 | 0.000885763 | -0.155287911 | 0.000759205 |
| DSCR4 | grey | -0.174943593 | 0.000144828 | -0.157144841 | 0.000654349 |
| DSCR8 | grey | -0.201293569 | 1.17E-05 | -0.188096556 | 4.30E-05 |
| DTNBP1 | grey | -0.122567316 | 0.008010947 | -0.112001802 | 0.015455906 |
| DTX1 | grey | -0.289808125 | 1.73E-10 | -0.257401878 | 1.67E-08 |
| DTX3 | grey | -0.14717387 | 0.001426106 | -0.153607361 | 0.000867298 |
| DTX4 | grey | -0.120899049 | 0.008916269 | -0.126437045 | 0.006219232 |
| DUPD1 | grey | -0.000702977 | 0.987911915 | 0.010724424 | 0.817204243 |
| DUSP10 | grey | 0.174987608 | 0.000144262 | 0.191844559 | 3.00E-05 |
| DUSP13 | grey | -0.041495409 | 0.370944484 | -0.035130847 | 0.448820289 |
| DUSP15 | grey | -0.338335335 | 5.71E-14 | -0.337061323 | 7.18E-14 |
| DUSP2 | grey | -0.184107022 | 6.28E-05 | -0.19199882 | 2.95E-05 |
| DUSP22 | grey | -0.081679045 | 0.077847219 | -0.093897092 | 0.042542073 |
| DUSP27 | grey | -0.03121852 | 0.500951129 | -0.043805146 | 0.344886767 |
| DUSP28 | grey | 0.010989826 | 0.812763207 | 0.020049339 | 0.665630816 |
| DUSP4 | grey | 0.347447222 | 1.07E-14 | 0.332356581 | 1.66E-13 |
| DUSP6 | grey | 0.322299709 | 9.50E-13 | 0.311171777 | 6.07E-12 |
| DUSP8 | grey | 0.176634218 | 0.000124506 | 0.16598989 | 0.000315196 |
| DYNAP | grey | 0.026131029 | 0.573244823 | 0.001033338 | 0.982231956 |
| DYNLL2 | grey | -0.030879176 | 0.505618828 | -0.01761082 | 0.704255317 |
| DYRK1B | grey | -0.130583373 | 0.004706271 | -0.119371857 | 0.009823741 |
| E2F4 | grey | 0.112015284 | 0.015443437 | 0.130410961 | 0.004761877 |
| ECE1 | grey | 0.118814427 | 0.010174868 | 0.11948084 | 0.009756364 |
| ECHDC3 | grey | -0.125705178 | 0.00652762 | -0.117281456 | 0.011198581 |
| EDA | grey | -0.084494297 | 0.06810564 | -0.120132015 | 0.009362272 |
| EDARAD | grey | -0.125097694 | 0.006793934 | -0.094799581 | 0.040583547 |
| EDC3 | grey | 0.049428635 | 0.286445853 | 0.033197625 | 0.474188102 |
| EDDM3A | grey | -0.132576089 | 0.004104795 | -0.107095153 | 0.020623642 |
| EDN2 | grey | 0.058905502 | 0.203852925 | 0.049450195 | 0.286235459 |
| EEF1A2 | grey | -0.199676537 | 1.38E-05 | -0.197464485 | 1.72E-05 |
| EEF1E1-I | grey | -0.063519288 | 0.170571787 | -0.083607905 | 0.071058791 |
| EFNA2 | grey | -0.084992709 | 0.066489783 | -0.085226985 | 0.065741204 |
| EFNA5 | grey | 0.074656877 | 0.107119956 | 0.07221222 | 0.119145049 |
| EFNB1 | grey | -0.291452102 | 1.35E-10 | -0.3091342 | 8.46E-12 |
| EFNB2 | grey | -0.32818298 | 3.45E-13 | -0.316911739 | 2.35E-12 |
| EFNB3 | grey | -0.2466001 | 6.72E-08 | -0.253868442 | 2.65E-08 |
| EFR3B | grey | 0.062057975 | 0.180642051 | 0.047217559 | 0.308576506 |
| EGF | grey | 0.010245281 | 0.8252366 | 0.009188415 | 0.84301739 |
| EGFL6 | grey | -0.251666029 | 3.53E-08 | -0.247773301 | 5.80E-08 |
| EGFLAM | grey | -0.230371028 | 4.83E-07 | -0.234936211 | 2.81E-07 |
| EGR3 | grey | 0.095774293 | 0.038554416 | 0.068332076 | 0.140364501 |
| EGR4 | grey | -0.039168354 | 0.398395106 | -0.048677505 | 0.293840873 |
| EHD3 | grey | -0.242337574 | 1.14E-07 | -0.241327834 | 1.30E-07 |

| | | | | | |
|---|---|---|---|---|---|
| EHD4 | grey | 0.109165765 | 0.018283492 | 0.120766824 | 0.008991776 |
| EIF1AY | grey | 0.056068168 | 0.226529297 | 0.050838182 | 0.272909354 |
| EIF2D | grey | 0.251461994 | 3.62E-08 | 0.232834276 | 3.61E-07 |
| EIF3CL | grey | -0.0098537 | 0.831814681 | -0.02096836 | 0.651293892 |
| EIF5A2 | grey | -0.239180892 | 1.69E-07 | -0.285662687 | 3.21E-10 |
| EIF5AL1 | grey | 0.25593026 | 2.03E-08 | 0.259397815 | 1.28E-08 |
| ELAC2 | grey | 0.358283825 | 1.37E-15 | 0.354420907 | 2.87E-15 |
| ELANE | grey | -0.137425728 | 0.002920655 | -0.100589822 | 0.029746161 |
| ELAVL2 | grey | 0.125468706 | 0.006630154 | 0.086028456 | 0.063232473 |
| ELAVL3 | grey | -0.115981739 | 0.012136886 | -0.112287752 | 0.015193314 |
| ELAVL4 | grey | -0.04246792 | 0.359827846 | -0.039177087 | 0.398289856 |
| ELF4 | grey | -0.247092322 | 6.32E-08 | -0.250576216 | 4.06E-08 |
| ELF5 | grey | 0.299659586 | 3.82E-11 | 0.303707938 | 2.02E-11 |
| ELFN2 | grey | -0.163499485 | 0.000388631 | -0.163815984 | 0.000378486 |
| ELMOD3 | grey | -0.023159287 | 0.617636996 | -0.025678116 | 0.579912233 |
| ELOVL2 | grey | -0.31502413 | 3.22E-12 | -0.307438152 | 1.11E-11 |
| ELOVL3 | grey | -0.232139177 | 3.92E-07 | -0.246247865 | 7.03E-08 |
| ELOVL4 | grey | -0.31137158 | 5.88E-12 | -0.318631594 | 1.77E-12 |
| ELSPBP1 | grey | -0.104744411 | 0.023591972 | -0.103507074 | 0.02529789 |
| EMC4 | grey | 0.03391687 | 0.464659524 | 0.033598038 | 0.46887026 |
| EMC7 | grey | 0.211023864 | 4.23E-06 | 0.216339746 | 2.37E-06 |
| EME2 | grey | 0.16502332 | 0.000342008 | 0.16086535 | 0.000483442 |
| EMID1 | grey | -0.120573869 | 0.009102986 | -0.157240804 | 0.000649313 |
| EMILIN2 | grey | -0.185970509 | 5.27E-05 | -0.155683913 | 0.000735619 |
| EML2 | grey | 0.153545742 | 0.000871519 | 0.158594269 | 0.000582013 |
| EML3 | grey | -0.004049958 | 0.930444066 | -0.009554591 | 0.836847295 |
| EMP2 | grey | 0.146583376 | 0.001491265 | 0.156501512 | 0.000689053 |
| EMX1 | grey | -0.044411574 | 0.338242943 | -0.042999342 | 0.353842307 |
| EMX2 | grey | 0.000840038 | 0.985555306 | -0.056298273 | 0.22462603 |
| EN1 | grey | -0.081190611 | 0.079647265 | -0.092482446 | 0.045772078 |
| EN2 | grey | 0.221253376 | 1.38E-06 | 0.217602736 | 2.07E-06 |
| ENC1 | grey | 0.189895942 | 3.62E-05 | 0.175261821 | 0.00014078 |
| ENDOV | grey | -0.128953783 | 0.005256042 | -0.120113752 | 0.009373129 |
| ENGASE | grey | 0.013727117 | 0.767333604 | -0.014007443 | 0.762722971 |
| ENHO | grey | -0.284694779 | 3.71E-10 | -0.308344242 | 9.61E-12 |
| ENO2 | grey | 0.13541624 | 0.003367392 | 0.131559586 | 0.004402329 |
| ENPP3 | grey | 0.200537005 | 1.26E-05 | 0.190639352 | 3.37E-05 |
| ENPP7 | grey | -0.048566557 | 0.2949439 | -0.030865353 | 0.505809443 |
| ENTHD1 | grey | -0.083653833 | 0.070903244 | -0.056718184 | 0.221182236 |
| ENTPD8 | grey | 0.207395596 | 6.21E-06 | 0.20683444 | 6.59E-06 |
| EPB41L1 | grey | -0.264288077 | 6.65E-09 | -0.253044181 | 2.95E-08 |
| EPHA10 | grey | -0.156273577 | 0.000701752 | -0.139541794 | 0.002509103 |
| EPHA2 | grey | -0.061422259 | 0.185157765 | -0.066527953 | 0.151167761 |
| EPHA8 | grey | -0.045027526 | 0.331579234 | -0.034217983 | 0.460702125 |
| EPHB1 | grey | -0.273040066 | 1.99E-09 | -0.280414732 | 6.93E-10 |

| | | | | | |
|---|---|---|---|---|---|
| EPHB3 | grey | 0.204220845 | 8.65E-06 | 0.172761536 | 0.000175675 |
| EPHB4 | grey | 0.035487665 | 0.444223504 | 0.024595723 | 0.595991415 |
| EPHX3 | grey | -0.180779121 | 8.54E-05 | -0.177814438 | 0.000111942 |
| EPHX4 | grey | -0.245121372 | 8.10E-08 | -0.261126695 | 1.02E-08 |
| EPOR | grey | -0.055707126 | 0.229538654 | -0.08111229 | 0.079939019 |
| EPPK1 | grey | -0.182506072 | 7.28E-05 | -0.179470667 | 9.63E-05 |
| EPS8L3 | grey | 0.163995244 | 0.00037285 | 0.153095967 | 0.000902911 |
| EPYC | grey | -0.073639937 | 0.112000335 | -0.05068909 | 0.274320169 |
| ERAP2 | grey | -0.099251305 | 0.032001815 | -0.062795547 | 0.175505581 |
| ERAS | grey | -0.155075175 | 0.000772163 | -0.148449086 | 0.001294222 |
| ERBB2 | grey | -0.112178271 | 0.015293386 | -0.115458695 | 0.012533667 |
| EREG | grey | 0.053144074 | 0.251718431 | 0.020613928 | 0.656808261 |
| ERICH4 | grey | -0.119006321 | 0.010052756 | -0.123472503 | 0.007554875 |
| ERN2 | grey | 0.14625387 | 0.001528803 | 0.148377732 | 0.001301295 |
| ERO1LB | grey | -0.218632955 | 1.84E-06 | -0.185817705 | 5.34E-05 |
| ERVV-2 | grey | -0.05968056 | 0.197955183 | -0.059552799 | 0.198918711 |
| ESPNL | grey | -0.261684297 | 9.46E-09 | -0.277247714 | 1.09E-09 |
| ESRRB | grey | -0.036407061 | 0.432503414 | -0.038841745 | 0.402343176 |
| ESX1 | grey | -0.126115791 | 0.006352953 | -0.13014121 | 0.004850067 |
| ETNK2 | grey | -0.239885919 | 1.55E-07 | -0.25095501 | 3.87E-08 |
| ETNPPL | grey | 0.053622388 | 0.247470036 | 0.039799869 | 0.390827591 |
| ETS2 | grey | 0.167385841 | 0.000279918 | 0.127860367 | 0.005656669 |
| EVA1A | grey | -0.001711075 | 0.970582621 | 0.003752293 | 0.935544766 |
| EVI5L | grey | 0.126637878 | 0.006136925 | 0.149039364 | 0.001237058 |
| EVL | grey | -0.037248051 | 0.421940807 | -0.059922033 | 0.196143389 |
| EVPLL | grey | -0.179208209 | 9.86E-05 | -0.179404127 | 9.69E-05 |
| EXOC3 | grey | -0.197848617 | 1.66E-05 | -0.170738731 | 0.000209678 |
| EXOC3L4 | grey | -0.204608809 | 8.31E-06 | -0.187390671 | 4.60E-05 |
| EXOSC6 | grey | 0.024163445 | 0.6024691 | 0.050244636 | 0.278555332 |
| EXTL3 | grey | 0.275260521 | 1.45E-09 | 0.267340749 | 4.39E-09 |
| EYA1 | grey | 0.098035896 | 0.034175047 | 0.107824998 | 0.019770669 |
| EYA4 | grey | -0.342570573 | 2.64E-14 | -0.347535239 | 1.05E-14 |
| F2RL2 | grey | 0.053670222 | 0.247047951 | 0.056249137 | 0.225031494 |
| F2RL3 | grey | -0.038901869 | 0.401614636 | 0.002689347 | 0.953779197 |
| F3 | grey | 0.06542101 | 0.158102075 | 0.077558069 | 0.094115921 |
| F5 | grey | 0.112684051 | 0.014835863 | 0.112839478 | 0.014697702 |
| F8A1 | grey | 0.013052251 | 0.778466808 | 0.035663672 | 0.441965945 |
| F8A2 | grey | 0.020059033 | 0.665478932 | 0.028676881 | 0.536457369 |
| F8A3 | grey | -0.062135624 | 0.180096113 | -0.073617444 | 0.112110226 |
| FA2H | grey | 0.311623685 | 5.64E-12 | 0.340814205 | 3.64E-14 |
| FABP1 | grey | -0.029335598 | 0.527135475 | -0.020751818 | 0.654660686 |
| FABP2 | grey | 0.053775946 | 0.246116813 | 0.063803215 | 0.168664782 |
| FABP3 | grey | -0.347910172 | 9.83E-15 | -0.352340907 | 4.27E-15 |
| FABP7 | grey | -0.03870509 | 0.40400198 | -0.023666869 | 0.609949054 |
| FADS3 | grey | -0.262702091 | 8.25E-09 | -0.266574134 | 4.87E-09 |

| | | | | | |
|---|---|---|---|---|---|
| FADS6 | grey | -0.047637868 | 0.304285139 | -0.05509444 | 0.234710169 |
| FAH | grey | -0.069590336 | 0.13318738 | -0.067300032 | 0.146469607 |
| FAHD1 | grey | 0.146050606 | 0.001552389 | 0.160236264 | 0.000509066 |
| FAHD2A | grey | -0.094516659 | 0.041189159 | -0.121666346 | 0.008489137 |
| FAHD2B | grey | -0.165507159 | 0.000328331 | -0.195519561 | 2.09E-05 |
| FAIM2 | grey | 0.191150814 | 3.21E-05 | 0.204824402 | 8.13E-06 |
| FAM107A | grey | -0.299310561 | 4.03E-11 | -0.283420903 | 4.47E-10 |
| FAM109A | grey | 0.069866959 | 0.131648166 | 0.084753964 | 0.067259826 |
| FAM109B | grey | -0.006823637 | 0.88307947 | 0.016756698 | 0.717972213 |
| FAM118A | grey | -0.078963273 | 0.088287727 | -0.084161433 | 0.069202611 |
| FAM124A | grey | -0.184320122 | 6.15E-05 | -0.198310739 | 1.58E-05 |
| FAM127C | grey | -0.096721448 | 0.036665648 | -0.09486946 | 0.04043513 |
| FAM131C | grey | -0.286510853 | 2.83E-10 | -0.285571498 | 3.26E-10 |
| FAM132A | grey | -0.176552651 | 0.000125422 | -0.166418788 | 0.000303938 |
| FAM133A | grey | -0.255924018 | 2.03E-08 | -0.257647546 | 1.62E-08 |
| FAM134A | grey | -0.047309806 | 0.307631262 | -0.05083445 | 0.272944611 |
| FAM134B | grey | -0.099314257 | 0.031892544 | -0.107773043 | 0.019830358 |
| FAM134C | grey | 0.29944241 | 3.95E-11 | 0.318656794 | 1.76E-12 |
| FAM135B | grey | -0.15863447 | 0.000580117 | -0.13319602 | 0.003932392 |
| FAM153A | grey | -0.10442584 | 0.024021392 | -0.12673324 | 0.006098187 |
| FAM153C | grey | -0.155015629 | 0.000775826 | -0.161599347 | 0.000455063 |
| FAM155A | grey | 0.041273659 | 0.373508716 | 0.019476242 | 0.674633751 |
| FAM155B | grey | -0.00398864 | 0.93149458 | -0.041282129 | 0.373410574 |
| FAM156B | grey | -0.091903383 | 0.047152396 | -0.086837614 | 0.060780219 |
| FAM160B | grey | 0.295481156 | 7.30E-11 | 0.284037417 | 4.08E-10 |
| FAM166A | grey | 0.175343775 | 0.000139754 | 0.162738868 | 0.000414058 |
| FAM167A | grey | -0.100282984 | 0.030250832 | -0.12364811 | 0.007469134 |
| FAM170B | grey | 0.095380903 | 0.039362779 | 0.068789642 | 0.137720987 |
| FAM171A | grey | -0.168448461 | 0.000255575 | -0.193246642 | 2.62E-05 |
| FAM171A | grey | 0.033441611 | 0.470943828 | 0.016017981 | 0.729909462 |
| FAM177B | grey | -0.02154518 | 0.642360174 | -0.010793392 | 0.816049621 |
| FAM183B | grey | 0.041512618 | 0.370745945 | 0.021720357 | 0.639657162 |
| FAM189A | grey | 0.131237544 | 0.004500577 | 0.107105084 | 0.020611825 |
| FAM192A | grey | 0.240492851 | 1.44E-07 | 0.246594538 | 6.73E-08 |
| FAM19A | grey | -0.105489326 | 0.022613692 | -0.10055652 | 0.029800581 |
| FAM19A | grey | -0.229496341 | 5.35E-07 | -0.2300236 | 5.03E-07 |
| FAM19A | grey | -0.142251568 | 0.002059405 | -0.139750772 | 0.002471472 |
| FAM209A | grey | -0.136988993 | 0.003012888 | -0.153722823 | 0.000859439 |
| FAM209B | grey | -0.099088381 | 0.032286101 | -0.09250689 | 0.045714565 |
| FAM20A | grey | -0.27667722 | 1.19E-09 | -0.252854498 | 3.03E-08 |
| FAM20C | grey | -0.267276171 | 4.42E-09 | -0.243863734 | 9.47E-08 |
| FAM210B | grey | 0.225198267 | 8.80E-07 | 0.223132045 | 1.11E-06 |
| FAM213A | grey | -0.075722195 | 0.102189122 | -0.08963931 | 0.052888646 |
| FAM213B | grey | -0.053909954 | 0.244940098 | -0.050273626 | 0.278277742 |
| FAM214B | grey | 0.03534154 | 0.446102744 | 0.064755736 | 0.162383601 |

| | | | | | |
|---|---|---|---|---|---|
| FAM215A | grey | -0.137117551 | 0.002985467 | -0.146652248 | 0.001483527 |
| FAM218A | grey | -0.523132826 | 3.64E-34 | -0.526899774 | 1.01E-34 |
| FAM228A | grey | -0.096156636 | 0.037782272 | -0.091068066 | 0.049204897 |
| FAM231A | grey | 0.000910741 | 0.984339699 | -0.003849678 | 0.933875712 |
| FAM231C | grey | 0.004359371 | 0.925145082 | 0.038294423 | 0.409011426 |
| FAM231D | grey | 0.085969137 | 0.063415407 | 0.074522819 | 0.10775353 |
| FAM24B | grey | -0.440280048 | 1.45E-23 | -0.457646702 | 1.49E-25 |
| FAM25C | grey | 0.03186584 | 0.4921107 | 0.002199635 | 0.962188704 |
| FAM3B | grey | -0.138088664 | 0.002785553 | -0.134810505 | 0.003513736 |
| FAM3D | grey | 0.211684659 | 3.94E-06 | 0.23784211 | 1.98E-07 |
| FAM43B | grey | -0.166184831 | 0.000310032 | -0.185091079 | 5.72E-05 |
| FAM46A | grey | -0.176269092 | 0.000128654 | -0.171710308 | 0.000192642 |
| FAM46B | grey | -0.066274038 | 0.152737597 | -0.054994761 | 0.235559254 |
| FAM47E- | grey | -0.056789173 | 0.22060378 | -0.073839111 | 0.111030962 |
| FAM50B | grey | -0.394909459 | 7.03E-19 | -0.377862224 | 2.69E-17 |
| FAM53A | grey | -0.080264257 | 0.083153773 | -0.106090997 | 0.021849379 |
| FAM53B | grey | -0.049327423 | 0.287434936 | -0.047972786 | 0.300894033 |
| FAM57A | grey | 0.370893893 | 1.12E-16 | 0.381117714 | 1.36E-17 |
| FAM57B | grey | 0.012998899 | 0.779348928 | 0.01958019 | 0.672997299 |
| FAM69B | grey | -0.12402316 | 0.007288919 | -0.168156538 | 0.000262057 |
| FAM69C | grey | -0.048398502 | 0.296619943 | 0.015919647 | 0.731503489 |
| FAM71E2 | grey | -0.129910948 | 0.004926508 | -0.156629885 | 0.000681995 |
| FAM71F1 | grey | 0.059109558 | 0.202287957 | 0.048889631 | 0.291739636 |
| FAM71F2 | grey | -0.031558479 | 0.496297886 | -0.02605185 | 0.574407815 |
| FAM83A | grey | -0.052950243 | 0.253454392 | -0.041041125 | 0.376209372 |
| FAM83F | grey | -0.07037503 | 0.128856942 | -0.05367016 | 0.247048491 |
| FAM86C1 | grey | 0.152432328 | 0.000951142 | 0.150192823 | 0.001132006 |
| FAM90A | grey | -0.340820909 | 3.64E-14 | -0.350399747 | 6.16E-15 |
| FAM90A1 | grey | -0.078954115 | 0.088324748 | -0.08160424 | 0.078120741 |
| FAM98C | grey | 0.115643276 | 0.012392356 | 0.127628811 | 0.005744955 |
| FAM9C | grey | 0.014241709 | 0.758876328 | 0.015094168 | 0.744929746 |
| FAT2 | grey | 0.027192146 | 0.557767447 | 0.053066155 | 0.252415278 |
| FATE1 | grey | -0.057909714 | 0.211616011 | -0.061670033 | 0.18338794 |
| FAXC | grey | -0.062161507 | 0.17991441 | -0.043972688 | 0.343042994 |
| FBLIM1 | grey | 0.049877888 | 0.282083264 | 0.045316537 | 0.328481911 |
| FBLL1 | grey | -0.124253207 | 0.007180313 | -0.120966168 | 0.008878156 |
| FBLN2 | grey | -0.30932749 | 8.20E-12 | -0.292971015 | 1.07E-10 |
| FBLN7 | grey | 0.09690024 | 0.036318071 | 0.1078084 | 0.019789721 |
| FBN3 | grey | -0.268816087 | 3.58E-09 | -0.283482105 | 4.43E-10 |
| FBP2 | grey | -0.009079165 | 0.844860137 | 0.004538498 | 0.922078857 |
| FBRS | grey | -0.081893781 | 0.07706636 | -0.085311632 | 0.065472443 |
| FBXL16 | grey | -0.181654916 | 7.88E-05 | -0.167518183 | 0.000276772 |
| FBXO10 | grey | 0.012596148 | 0.78601701 | -0.002321002 | 0.960104124 |
| FBXO2 | grey | -0.259949551 | 1.19E-08 | -0.255582615 | 2.12E-08 |
| FBXO24 | grey | 0.0432953 | 0.350536212 | 0.027897882 | 0.547587122 |

| | | | | | |
|---|---|---|---|---|---|
| FBXO27 | grey | -0.31616495 | 2.67E-12 | -0.315327393 | 3.06E-12 |
| FBXO46 | grey | -0.009020763 | 0.845845572 | -0.025557957 | 0.581687176 |
| FCGR3B | grey | 0.047041738 | 0.310383424 | 0.074449157 | 0.108102927 |
| FCGRT | grey | -0.062166028 | 0.179882686 | -0.06286449 | 0.175031068 |
| FCHSD1 | grey | -0.146771143 | 0.001470255 | -0.171547668 | 0.0001954 |
| FCN2 | grey | -0.086961528 | 0.060411711 | -0.092846874 | 0.044920908 |
| FCN3 | grey | 0.090645131 | 0.050272289 | 0.121126096 | 0.008787927 |
| FCRL2 | grey | 0.153791693 | 0.000854783 | 0.185237944 | 5.64E-05 |
| FCRLB | grey | -0.22278654 | 1.16E-06 | -0.22681967 | 7.30E-07 |
| FDCSP | grey | -0.084688547 | 0.067472093 | -0.068639851 | 0.138582133 |
| FDXACB | grey | 0.204226504 | 8.65E-06 | 0.204608405 | 8.31E-06 |
| FEM1A | grey | 0.109822242 | 0.017591617 | 0.144739223 | 0.001712766 |
| FER1L5 | grey | -0.24753855 | 5.97E-08 | -0.238211917 | 1.90E-07 |
| FERD3L | grey | -0.04272667 | 0.356905606 | -0.044792343 | 0.334113519 |
| FERMT1 | grey | 0.07126697 | 0.124067963 | 0.073436592 | 0.112996861 |
| FETUB | grey | 0.082135744 | 0.076194135 | 0.094603828 | 0.04100176 |
| FEV | grey | 0.018954085 | 0.68287711 | 0.000773562 | 0.986698279 |
| FEZF1 | grey | 0.138965209 | 0.002615654 | 0.147117331 | 0.00143223 |
| FEZF2 | grey | -0.001915489 | 0.967070151 | -0.00584796 | 0.899701946 |
| FFAR1 | grey | -0.289123206 | 1.92E-10 | -0.295454372 | 7.33E-11 |
| FGD1 | grey | -0.13039009 | 0.004768648 | -0.150309102 | 0.001121885 |
| FGF11 | grey | -0.095740798 | 0.038622692 | -0.134747797 | 0.003529211 |
| FGF12 | grey | -0.369932257 | 1.36E-16 | -0.364089144 | 4.38E-16 |
| FGF16 | grey | -0.142909715 | 0.001961942 | -0.144370911 | 0.001760466 |
| FGF17 | grey | -0.069811908 | 0.131953386 | -0.103735149 | 0.02497567 |
| FGF18 | grey | 0.034405596 | 0.458245933 | 0.013607466 | 0.76930406 |
| FGF21 | grey | -0.077267179 | 0.09536 | -0.058158441 | 0.209657281 |
| FGF22 | grey | 0.102095445 | 0.027373079 | 0.134467406 | 0.003599164 |
| FGF23 | grey | 0.026157147 | 0.572861436 | 0.013005514 | 0.77923954 |
| FGF5 | grey | 0.035904426 | 0.438888547 | 0.050755463 | 0.273691487 |
| FGF9 | grey | -0.073478476 | 0.112791035 | -0.087632867 | 0.05844717 |
| FGFBP2 | grey | 0.098626773 | 0.033103302 | 0.096726286 | 0.036656205 |
| FGFBP3 | grey | -0.036041283 | 0.43714468 | -0.044993405 | 0.331946142 |
| FGFR1 | grey | 0.103021827 | 0.025995387 | 0.072160687 | 0.119409442 |
| FGFR2 | grey | -0.075354469 | 0.103870384 | -0.088757023 | 0.05527613 |
| FGFR3 | grey | -0.133050454 | 0.003972275 | -0.12910681 | 0.005202059 |
| FGFR4 | grey | -0.066684247 | 0.150207579 | -0.079246496 | 0.08714891 |
| FGFRL1 | grey | 0.012256783 | 0.791647848 | 0.025210497 | 0.586833931 |
| FGL1 | grey | 0.224781073 | 9.23E-07 | 0.200965505 | 1.21E-05 |
| FHDC1 | grey | 0.040355086 | 0.384246783 | 0.028841117 | 0.534125446 |
| FHL2 | grey | 0.052476043 | 0.25773633 | 0.082043116 | 0.076527088 |
| FHOD1 | grey | 0.235429046 | 2.65E-07 | 0.246771963 | 6.58E-08 |
| FIBCD1 | grey | 0.116822381 | 0.011522399 | 0.117699272 | 0.010910896 |
| FICD | grey | 0.245179173 | 8.04E-08 | 0.252022685 | 3.37E-08 |
| FIGLA | grey | 0.012902615 | 0.780941593 | 0.034337737 | 0.459133483 |

| | | | | | |
|---|---|---|---|---|---|
| FJX1 | grey | -0.325725084 | 5.28E-13 | -0.3318807 | 1.81E-13 |
| FKBP10 | grey | -0.092531794 | 0.045656031 | -0.122145683 | 0.008231652 |
| FKBP11 | grey | 0.150564453 | 0.001099951 | 0.149609775 | 0.001184034 |
| FKBP1B | grey | -0.276044563 | 1.30E-09 | -0.24732454 | 6.14E-08 |
| FKBP1C | grey | -0.176476443 | 0.000126283 | -0.170613621 | 0.000211971 |
| FKBP5 | grey | -0.05746576 | 0.215144849 | -0.078241904 | 0.091242362 |
| FKRP | grey | -0.088637015 | 0.055607688 | -0.103186443 | 0.025756933 |
| FKSG48 | grey | -0.128046233 | 0.005586688 | -0.10900272 | 0.018458967 |
| FKSG51 | grey | -0.123505108 | 0.007538889 | -0.123604742 | 0.007490227 |
| FKSG52 | grey | -0.107543699 | 0.020095718 | -0.117667619 | 0.01093246 |
| FKSG54 | grey | -0.079839034 | 0.084804585 | -0.082082289 | 0.076386135 |
| FKSG56 | grey | -0.04008945 | 0.387386803 | -0.006484536 | 0.888851254 |
| FKSG59 | grey | -0.052027239 | 0.261834775 | -0.063622181 | 0.16987885 |
| FKSG63 | grey | -0.005850201 | 0.899663703 | -0.000393186 | 0.99323876 |
| FKSG66 | grey | -0.10754278 | 0.020096787 | -0.129128164 | 0.005194566 |
| FLG | grey | -0.139109743 | 0.002588564 | -0.144350073 | 0.0017632 |
| FLRT1 | grey | -0.188515071 | 4.14E-05 | -0.192479112 | 2.82E-05 |
| FLRT3 | grey | -0.163959483 | 0.000373968 | -0.178285838 | 0.000107265 |
| FLYWCH | grey | 0.099216273 | 0.032062762 | 0.109128348 | 0.018323632 |
| FMN2 | grey | -0.042556452 | 0.358826314 | -0.039620003 | 0.392974032 |
| FMO3 | grey | 0.002678162 | 0.953971204 | 0.031335485 | 0.499347561 |
| FMR1NB | grey | -0.130663697 | 0.004680565 | -0.1416925 | 0.002145652 |
| FN3K | grey | 0.275787916 | 1.35E-09 | 0.279375864 | 8.05E-10 |
| FNDC4 | grey | -0.251776783 | 3.48E-08 | -0.249655887 | 4.57E-08 |
| FNDC8 | grey | 0.005378129 | 0.907722513 | -0.002654881 | 0.954370893 |
| FNTB | grey | 0.125924104 | 0.006433964 | 0.127286517 | 0.005877731 |
| FO08279( | grey | -0.0751617 | 0.104760448 | -0.069154097 | 0.135642918 |
| FO53875' | grey | -0.063166025 | 0.172966905 | -0.064623223 | 0.16324674 |
| FOLR1 | grey | -0.124635628 | 0.007002972 | -0.092856731 | 0.04489807 |
| FOLR3 | grey | -0.256136957 | 1.97E-08 | -0.260066786 | 1.17E-08 |
| FOXA3 | grey | -0.049564372 | 0.285122978 | -0.041416108 | 0.37186024 |
| FOXC1 | grey | 0.013230858 | 0.77551583 | 0.042455381 | 0.359969844 |
| FOXC2 | grey | 0.167307241 | 0.000281802 | 0.145478625 | 0.001620565 |
| FOXD1 | grey | -0.051931908 | 0.262711087 | -0.024416017 | 0.598680473 |
| FOXD2 | grey | 0.024792826 | 0.593048363 | 0.017355056 | 0.708352981 |
| FOXD3 | grey | -0.168352911 | 0.00025768 | -0.174875159 | 0.000145713 |
| FOXD4 | grey | 0.183299009 | 6.77E-05 | 0.197894062 | 1.65E-05 |
| FOXD4L1 | grey | 0.149513008 | 0.001192879 | 0.134900263 | 0.003491693 |
| FOXD4L4 | grey | 0.055050445 | 0.235084662 | 0.063744986 | 0.169054568 |
| FOXD4L( | grey | 0.105495346 | 0.022605931 | 0.114843249 | 0.01301517 |
| FOXE1 | grey | -0.132867074 | 0.004023038 | -0.117816428 | 0.010831414 |
| FOXF2 | grey | 0.135721687 | 0.003295716 | 0.152085336 | 0.000977296 |
| FOXG1 | grey | 0.010797215 | 0.815985618 | 0.038899809 | 0.401639585 |
| FOXH1 | grey | -0.030522204 | 0.510553531 | -0.039376176 | 0.39589509 |
| FOXI1 | grey | 0.048750326 | 0.2931184 | 0.074430457 | 0.108191768 |

| | | | | | |
|---|---|---|---|---|---|
| FOXI3 | grey | -0.232943414 | 3.57E-07 | -0.24703128 | 6.37E-08 |
| FOXN1 | grey | -0.164567009 | 0.000355391 | -0.179812683 | 9.33E-05 |
| FOXO6 | grey | -0.352920087 | 3.82E-15 | -0.357601188 | 1.56E-15 |
| FOXQ1 | grey | 0.088645351 | 0.055584603 | 0.07577452 | 0.101951653 |
| FOXR2 | grey | -0.083839676 | 0.070276687 | -0.080341417 | 0.08285702 |
| FREM3 | grey | -0.184821873 | 5.87E-05 | -0.190605386 | 3.38E-05 |
| FRG2 | grey | 0.12308711 | 0.007746138 | 0.104148721 | 0.024400414 |
| FRG2B | grey | 0.050516797 | 0.275956699 | 0.045699415 | 0.324407517 |
| FRMD1 | grey | -0.171977688 | 0.000188187 | -0.173411491 | 0.000165897 |
| FRMD4A | grey | -0.09794216 | 0.034347757 | -0.119215311 | 0.009921246 |
| FRMD5 | grey | -0.076908362 | 0.096912633 | -0.081582402 | 0.078200736 |
| FRMPD4 | grey | -0.022845061 | 0.622417406 | -0.006769039 | 0.884008374 |
| FRRS1L | grey | -0.170488848 | 0.000214282 | -0.151298213 | 0.001039105 |
| FRS3 | grey | -0.101583184 | 0.028161524 | -0.121877238 | 0.008374981 |
| FRZB | grey | -0.088218299 | 0.056777471 | -0.060322644 | 0.193164322 |
| FSCN1 | grey | -0.149496797 | 0.001194367 | -0.143787313 | 0.001838545 |
| FSCN2 | grey | 0.064914984 | 0.161350872 | 0.082152464 | 0.076134162 |
| FSCN3 | grey | 0.00300523 | 0.948357384 | 0.00299095 | 0.948602442 |
| FSD1 | grey | -0.270208848 | 2.95E-09 | -0.28269865 | 4.97E-10 |
| FSTL5 | grey | -0.061528853 | 0.184394841 | -0.046363826 | 0.317415373 |
| FTCD | grey | -0.151694747 | 0.00100753 | -0.166340084 | 0.000305975 |
| FTCDNL | grey | -0.151059013 | 0.001058591 | -0.151635236 | 0.001012212 |
| FTHL17 | grey | -0.093949945 | 0.042425216 | -0.090231732 | 0.051334239 |
| FTMT | grey | 0.014650138 | 0.75218419 | 0.020524629 | 0.658200556 |
| FURIN | grey | 0.086238894 | 0.062587003 | 0.092339645 | 0.046109283 |
| FUT1 | grey | -0.139403456 | 0.0025343 | -0.152831786 | 0.000921834 |
| FUT3 | grey | 0.279231604 | 8.22E-10 | 0.320254007 | 1.34E-12 |
| FUT4 | grey | -0.065762531 | 0.155937511 | -0.062704744 | 0.176132006 |
| FUT5 | grey | -0.084440282 | 0.068282676 | -0.069054224 | 0.136209965 |
| FUT6 | grey | 0.255968275 | 2.02E-08 | 0.288441319 | 2.12E-10 |
| FXYD2 | grey | -0.109538927 | 0.017887352 | -0.113857864 | 0.013820094 |
| FXYD4 | grey | -0.009937837 | 0.830400287 | -0.0105542 | 0.820055739 |
| FXYD7 | grey | -0.333966068 | 1.25E-13 | -0.329755014 | 2.62E-13 |
| FZD2 | grey | 0.021929639 | 0.636434141 | 0.031846771 | 0.492369929 |
| FZD8 | grey | 0.199722144 | 1.37E-05 | 0.206547508 | 6.79E-06 |
| FZD9 | grey | -0.047375713 | 0.306957096 | -0.049921558 | 0.281661603 |
| G0S2 | grey | 0.159272068 | 0.000550799 | 0.1722766 | 0.00018332 |
| G6PD | grey | 0.093423294 | 0.043601786 | 0.094096546 | 0.042102506 |
| GAA | grey | 0.052891833 | 0.253979142 | 0.026417372 | 0.569048302 |
| GABBR2 | grey | -0.063827517 | 0.168502299 | -0.034148181 | 0.461617825 |
| GABRA2 | grey | -0.169274576 | 0.000238032 | -0.178923702 | 0.000101229 |
| GABRA3 | grey | -0.239611717 | 1.60E-07 | -0.241766784 | 1.23E-07 |
| GABRA5 | grey | -0.094022392 | 0.042265481 | -0.109642634 | 0.017778599 |
| GABRD | grey | 0.126186799 | 0.006323176 | 0.144649642 | 0.001724257 |
| GABRE | grey | -0.375633458 | 4.26E-17 | -0.39605423 | 5.46E-19 |

| | | | | | |
|---|---|---|---|---|---|
| GABRG1 | grey | -0.098537508 | 0.033263351 | -0.113833287 | 0.013840718 |
| GABRG2 | grey | -0.189041765 | 3.93E-05 | -0.189151076 | 3.89E-05 |
| GABRG3 | grey | -0.091714322 | 0.04761055 | -0.114402766 | 0.013369729 |
| GABRQ | grey | -0.312531162 | 4.86E-12 | -0.327294919 | 4.03E-13 |
| GABRR1 | grey | -0.141161356 | 0.002230637 | -0.14976938 | 0.001169577 |
| GAD2 | grey | -0.001243488 | 0.978619253 | 0.001391822 | 0.976069494 |
| GADD45A | grey | 0.144025309 | 0.001806328 | 0.113787293 | 0.013879387 |
| GADL1 | grey | -0.089084969 | 0.054378451 | -0.079258701 | 0.087100099 |
| GAGE1 | grey | -0.101855453 | 0.027740067 | -0.102798569 | 0.026321827 |
| GAGE12I | grey | -0.081423277 | 0.078785648 | -0.085458681 | 0.065007693 |
| GAGE13 | grey | -0.089988666 | 0.051967325 | -0.070272883 | 0.129414401 |
| GAGE2A | grey | -0.113641271 | 0.014002785 | -0.115211009 | 0.012725524 |
| GAK | grey | 0.216426923 | 2.35E-06 | 0.251764309 | 3.48E-08 |
| GAL | grey | -0.196308944 | 1.93E-05 | -0.213226995 | 3.33E-06 |
| GAL3ST1 | grey | -0.069924853 | 0.131327768 | -0.071391969 | 0.123408025 |
| GAL3ST2 | grey | -0.026207298 | 0.572125627 | -0.021450207 | 0.643827619 |
| GAL3ST3 | grey | -0.390296384 | 1.92E-18 | -0.397136966 | 4.30E-19 |
| GALNT1: | grey | -0.257110978 | 1.74E-08 | -0.246315951 | 6.97E-08 |
| GALNT1 | grey | -0.123777232 | 0.007406645 | -0.123662646 | 0.007462076 |
| GALNT1( | grey | 0.199570296 | 1.39E-05 | 0.179705302 | 9.43E-05 |
| GALNT5 | grey | 0.118423537 | 0.01042769 | 0.139258714 | 0.002560911 |
| GALNT8 | grey | -0.228666104 | 5.90E-07 | -0.234976379 | 2.80E-07 |
| GALNT9 | grey | -0.161848377 | 0.000445792 | -0.165440393 | 0.000330187 |
| GALP | grey | -0.059109315 | 0.202289814 | -0.047992478 | 0.300695436 |
| GALR1 | grey | -0.192591904 | 2.79E-05 | -0.185317008 | 5.60E-05 |
| GALR3 | grey | 0.062355487 | 0.178556935 | 0.074797564 | 0.106458213 |
| GALT | grey | -0.150301947 | 0.001122505 | -0.148820763 | 0.001257951 |
| GAMT | grey | 0.152269115 | 0.000963362 | 0.165600916 | 0.000325741 |
| GAP43 | grey | -0.066927411 | 0.148722938 | -0.067813443 | 0.143407655 |
| GAREML | grey | -0.177984941 | 0.000110229 | -0.189069839 | 3.92E-05 |
| GAS2 | grey | -0.023979432 | 0.605236098 | -0.056199094 | 0.225444966 |
| GAS2L1 | grey | 0.20542644 | 7.63E-06 | 0.229597572 | 5.29E-07 |
| GAST | grey | 0.026087976 | 0.573877045 | 0.017020937 | 0.713718686 |
| GATA2 | grey | -0.065629552 | 0.156777655 | -0.096713481 | 0.036681201 |
| GATA3 | grey | -0.040872104 | 0.378179909 | -0.008978226 | 0.846563463 |
| GATA5 | grey | -0.212327977 | 3.67E-06 | -0.198840786 | 1.50E-05 |
| GATM | grey | 0.022510366 | 0.627526721 | 0.000566123 | 0.990265066 |
| GATSL3 | grey | -0.129641458 | 0.005017352 | -0.144980349 | 0.001682183 |
| GBGT1 | grey | -0.215875631 | 2.50E-06 | -0.248493313 | 5.29E-08 |
| GBP6 | grey | -0.099464836 | 0.031632465 | -0.123806847 | 0.007392379 |
| GBX1 | grey | -0.141014352 | 0.002254695 | -0.14456616 | 0.00173503 |
| GCG | grey | 0.063331257 | 0.171843536 | 0.072268955 | 0.118854492 |
| GCGR | grey | -0.223012395 | 1.13E-06 | -0.239447092 | 1.63E-07 |
| GCK | grey | -0.361873425 | 6.78E-16 | -0.345374308 | 1.58E-14 |
| GCM1 | grey | -0.0974117 | 0.035339196 | -0.086435591 | 0.061988598 |

| | | | | | |
|---|---|---|---|---|---|
| GCNT4 | grey | -0.168044924 | 0.000264576 | -0.169255869 | 0.000238417 |
| GDA | grey | 0.178089698 | 0.000109189 | 0.180897879 | 8.45E-05 |
| GDF11 | grey | -0.130879997 | 0.004611973 | -0.144359695 | 0.001761937 |
| GDF3 | grey | 0.078849906 | 0.088746906 | 0.071093502 | 0.124988333 |
| GDF5OS | grey | 0.043006079 | 0.353766834 | 0.03172386 | 0.494042545 |
| GDF6 | grey | -0.109278642 | 0.018162865 | -0.115295448 | 0.012659829 |
| GDF9 | grey | 0.029412848 | 0.52604768 | 0.024726384 | 0.594039699 |
| GDPD2 | grey | -0.215916952 | 2.49E-06 | -0.216417041 | 2.35E-06 |
| GDPD3 | grey | -0.067275507 | 0.146617111 | -0.061529649 | 0.184389154 |
| GDPD5 | grey | -0.331466357 | 1.94E-13 | -0.318874192 | 1.69E-12 |
| GDPGP1 | grey | 0.25016212 | 4.28E-08 | 0.274986022 | 1.51E-09 |
| GFAP | grey | -0.064661351 | 0.162998032 | -0.055563138 | 0.230746692 |
| GFOD1 | grey | -0.425707746 | 5.56E-22 | -0.415715464 | 6.12E-21 |
| GFRA2 | grey | -0.221002061 | 1.42E-06 | -0.244084641 | 9.21E-08 |
| GFRA3 | grey | 0.027343271 | 0.555579743 | 0.012473055 | 0.788058126 |
| GFRA4 | grey | -0.070688192 | 0.127159472 | -0.076674406 | 0.09793579 |
| GFY | grey | 0.081420717 | 0.078795088 | 0.059449517 | 0.199700124 |
| GGA2 | grey | 0.124370624 | 0.00712544 | 0.133990161 | 0.003721125 |
| GGA3 | grey | -0.187653943 | 4.49E-05 | -0.212751031 | 3.51E-06 |
| GGACT | grey | 0.307853045 | 1.04E-11 | 0.29047485 | 1.57E-10 |
| GGN | grey | -0.116110705 | 0.012040775 | -0.115022937 | 0.012872931 |
| GGT1 | grey | 0.272059519 | 2.28E-09 | 0.253322413 | 2.85E-08 |
| GGT2 | grey | -0.050665715 | 0.274541809 | -0.061729782 | 0.182963034 |
| GGT7 | grey | -0.189693735 | 3.69E-05 | -0.239669138 | 1.59E-07 |
| GGTLC3 | grey | 0.098483533 | 0.033360445 | 0.098448299 | 0.033423958 |
| GH1 | grey | -0.173661086 | 0.000162279 | -0.165672191 | 0.000323784 |
| GH2 | grey | -0.071564476 | 0.122501764 | -0.118767823 | 0.010204722 |
| GHRH | grey | -0.076831497 | 0.097247843 | -0.078885282 | 0.088603412 |
| GHRHR | grey | -0.04543427 | 0.327225551 | -0.043977451 | 0.342990666 |
| GIGYF1 | grey | -0.178099106 | 0.000109096 | -0.203805805 | 9.03E-06 |
| GIMAP1- | grey | -0.019290632 | 0.677559604 | -0.021471456 | 0.643499176 |
| GIPC2 | grey | -0.050406456 | 0.277008254 | -0.073094558 | 0.114688749 |
| GIT1 | grey | -0.052491455 | 0.257596384 | -0.059360435 | 0.200375885 |
| GJA3 | grey | -0.104222825 | 0.024298557 | -0.099097845 | 0.032269529 |
| GJB1 | grey | 0.163966924 | 0.000373735 | 0.176685197 | 0.000123937 |
| GJB3 | grey | -0.141285944 | 0.002210431 | -0.126794148 | 0.006073559 |
| GJB4 | grey | 0.045635509 | 0.325085278 | 0.077693397 | 0.093541587 |
| GJB5 | grey | 0.063029842 | 0.173896894 | 0.076688148 | 0.097875454 |
| GJD4 | grey | -0.069228316 | 0.135222706 | -0.072224452 | 0.119082357 |
| GLB1 | grey | 0.237741623 | 2.01E-07 | 0.225290093 | 8.70E-07 |
| GLDN | grey | -0.167044293 | 0.000288191 | -0.182124263 | 7.55E-05 |
| GLOD5 | grey | -0.172806091 | 0.000174988 | -0.161279016 | 0.000467252 |
| GLP1R | grey | -0.128389918 | 0.005459334 | -0.14194562 | 0.002106203 |
| GLRX | grey | -0.026265972 | 0.57126533 | -0.006501365 | 0.88856467 |
| GLS2 | grey | -0.012896437 | 0.781043812 | -0.042149293 | 0.363446839 |

| | | | | | |
|---|---|---|---|---|---|
| GLTP | grey | 0.312393724 | 4.97E-12 | 0.317686966 | 2.07E-12 |
| GLTPD2 | grey | 0.064205001 | 0.165993481 | 0.06576833 | 0.15590095 |
| GLYATL | grey | 0.267327078 | 4.39E-09 | 0.236736726 | 2.27E-07 |
| GML | grey | -0.089487265 | 0.053293848 | -0.063937885 | 0.167765859 |
| GNAS | grey | 0.086365385 | 0.062201639 | 0.085045262 | 0.066321254 |
| GNAT2 | grey | 0.052534928 | 0.257201908 | 0.040041172 | 0.38795916 |
| GNB3 | grey | -0.152229605 | 0.000966342 | -0.167254323 | 0.000283077 |
| GNG3 | grey | -0.060301174 | 0.193323138 | -0.075484764 | 0.103272181 |
| GNG4 | grey | -0.079686771 | 0.085402093 | -0.083958504 | 0.069878442 |
| GNG8 | grey | -0.168125276 | 0.000262761 | -0.139484826 | 0.002519452 |
| GNGT1 | grey | -0.162853847 | 0.000410118 | -0.159417763 | 0.000544295 |
| GNRH2 | grey | 0.12044198 | 0.009179703 | 0.122157422 | 0.008225435 |
| GOLGA6 | grey | -0.023249027 | 0.616274695 | -0.018708528 | 0.686766886 |
| GOLGA6 | grey | -0.041111978 | 0.375385218 | -0.038083787 | 0.41159506 |
| GOLGA6 | grey | -0.029875639 | 0.519554969 | -0.03757058 | 0.417930273 |
| GOLGA6 | grey | -0.026358787 | 0.569905705 | -0.01133496 | 0.806996978 |
| GOLGA6 | grey | -0.088524838 | 0.055919101 | -0.082917478 | 0.073430857 |
| GOLGA8 | grey | 0.056132126 | 0.225999134 | 0.022676907 | 0.624982132 |
| GOLGA8 | grey | -0.118187131 | 0.010583284 | -0.116605124 | 0.011678512 |
| GOT2 | grey | 0.354400203 | 2.88E-15 | 0.393533508 | 9.50E-19 |
| GP5 | grey | -0.14349029 | 0.001879489 | -0.160324162 | 0.000505411 |
| GP9 | grey | 0.018796228 | 0.685376703 | 0.038518191 | 0.406277285 |
| GPA33 | grey | 0.081599075 | 0.078139654 | 0.099431732 | 0.031689485 |
| GPAT2 | grey | -0.028289071 | 0.541983899 | -0.044026614 | 0.342450887 |
| GPBAR1 | grey | -0.224099386 | 9.97E-07 | -0.211213175 | 4.14E-06 |
| GPC3 | grey | -0.131699139 | 0.004360359 | -0.149796983 | 0.001167093 |
| GPC4 | grey | 0.267098956 | 4.53E-09 | 0.267659226 | 4.20E-09 |
| GPD1 | grey | -0.045414201 | 0.327439493 | -0.040249972 | 0.385487453 |
| GPHA2 | grey | -0.146049782 | 0.001552485 | -0.155780025 | 0.000729997 |
| GPHB5 | grey | -0.026221573 | 0.571916265 | -0.031337985 | 0.499313307 |
| GPN2 | grey | 0.081703752 | 0.07775705 | 0.107867845 | 0.019721563 |
| GPR110 | grey | -0.070615602 | 0.12755139 | -0.050460808 | 0.27648994 |
| GPR111 | grey | -0.05249943 | 0.257523983 | -0.049289895 | 0.287802258 |
| GPR115 | grey | -0.067388082 | 0.14594097 | -0.064845505 | 0.161800836 |
| GPR132 | grey | 0.071966698 | 0.120408824 | 0.105791793 | 0.022226616 |
| GPR139 | grey | -0.085007056 | 0.06644374 | -0.081771172 | 0.077511427 |
| GPR142 | grey | -0.232262457 | 3.87E-07 | -0.233461008 | 3.36E-07 |
| GPR143 | grey | -0.180570457 | 8.71E-05 | -0.19815371 | 1.61E-05 |
| GPR15 | grey | 0.04610941 | 0.320081074 | 0.092582668 | 0.045536658 |
| GPR151 | grey | -0.087232292 | 0.059612906 | -0.032105049 | 0.488865073 |
| GPR156 | grey | -0.326194383 | 4.87E-13 | -0.327109763 | 4.16E-13 |
| GPR157 | grey | 0.244447004 | 8.81E-08 | 0.239917632 | 1.54E-07 |
| GPR158 | grey | -0.324216192 | 6.84E-13 | -0.325042658 | 5.94E-13 |
| GPR160 | grey | -0.033657099 | 0.468088673 | -0.025685527 | 0.57980284 |
| GPR27 | grey | -0.283765967 | 4.25E-10 | -0.307671317 | 1.07E-11 |

| | | | | | |
|---|---|---|---|---|---|
| GPR31 | grey | -0.173877214 | 0.000159206 | -0.151740651 | 0.001003933 |
| GPR35 | grey | 0.184642494 | 5.97E-05 | 0.192608201 | 2.78E-05 |
| GPR37 | grey | 0.15399395 | 0.000841243 | 0.139247229 | 0.002563033 |
| GPR37L1 | grey | -0.0303371 | 0.513122209 | -0.042587808 | 0.358472015 |
| GPR45 | grey | -0.043749112 | 0.34550481 | -0.042304763 | 0.361678168 |
| GPR50 | grey | -0.028861751 | 0.533832831 | -0.024492106 | 0.597541224 |
| GPR55 | grey | 0.038676174 | 0.404353504 | 0.058725954 | 0.205237184 |
| GPR6 | grey | -0.000672822 | 0.988430406 | -0.007425972 | 0.872842675 |
| GPR62 | grey | -0.227657125 | 6.63E-07 | -0.209594033 | 4.92E-06 |
| GPR63 | grey | -0.058299735 | 0.208550436 | -0.049277689 | 0.287921792 |
| GPR64 | grey | -0.142756105 | 0.001984304 | -0.181018751 | 8.36E-05 |
| GPR89A | grey | 0.044940075 | 0.332520142 | 0.04080952 | 0.378911146 |
| GPR89B | grey | -0.016660836 | 0.719517489 | -0.020419231 | 0.659845398 |
| GPR98 | grey | 0.153897536 | 0.000847672 | 0.149574865 | 0.001187218 |
| GPRC5A | grey | 0.123996143 | 0.007301769 | 0.115641547 | 0.012393673 |
| GPRC5B | grey | -0.340901182 | 3.58E-14 | -0.358298854 | 1.36E-15 |
| GPRC5C | grey | -0.140146931 | 0.002401545 | -0.121203153 | 0.008744746 |
| GPRIN2 | grey | -0.396078313 | 5.43E-19 | -0.411008424 | 1.84E-20 |
| GPS2 | grey | 0.000905596 | 0.98442815 | -0.00622557 | 0.893263036 |
| GPSM1 | grey | -0.171606601 | 0.000194397 | -0.185991793 | 5.26E-05 |
| GPX3 | grey | -0.191018111 | 3.25E-05 | -0.177198886 | 0.000118338 |
| GPX7 | grey | -0.075651352 | 0.102511333 | -0.07535405 | 0.103872316 |
| GRAMD1 | grey | -0.017755487 | 0.701941336 | -0.027504942 | 0.553244021 |
| GRAMD1 | grey | -0.199380362 | 1.42E-05 | -0.181579222 | 7.94E-05 |
| GRAMD4 | grey | 0.2102399 | 4.60E-06 | 0.211497745 | 4.02E-06 |
| GRAPL | grey | -0.06564106 | 0.156704814 | -0.079201115 | 0.087330583 |
| GRB14 | grey | 0.125565214 | 0.006588135 | 0.109059129 | 0.018398092 |
| GRB7 | grey | -0.199384057 | 1.42E-05 | -0.208499916 | 5.53E-06 |
| GRHL1 | grey | 0.113807726 | 0.013862196 | 0.130526636 | 0.004724504 |
| GRIA1 | grey | -0.087423207 | 0.059054935 | -0.096300445 | 0.037495263 |
| GRID2 | grey | -0.207277018 | 6.29E-06 | -0.201562122 | 1.14E-05 |
| GRID2IP | grey | -0.210832446 | 4.31E-06 | -0.214492604 | 2.91E-06 |
| GRIK2 | grey | 0.002142641 | 0.963167718 | 0.020860092 | 0.652976365 |
| GRIK3 | grey | -0.045342794 | 0.328201448 | -0.047675019 | 0.303907734 |
| GRIK5 | grey | -0.451730525 | 7.31E-25 | -0.445337587 | 3.94E-24 |
| GRIN2A | grey | -0.177046954 | 0.000119969 | -0.182947331 | 6.99E-05 |
| GRIN2C | grey | -0.068688679 | 0.138300968 | -0.063215415 | 0.172630543 |
| GRIN2D | grey | 0.214217667 | 2.99E-06 | 0.225908751 | 8.11E-07 |
| GRIN3A | grey | -0.071515357 | 0.122759277 | -0.054885545 | 0.236492058 |
| GRIPAP1 | grey | -0.133343549 | 0.003892342 | -0.125172566 | 0.006760594 |
| GRK1 | grey | -0.226985516 | 7.16E-07 | -0.233468055 | 3.35E-07 |
| GRK4 | grey | -0.026869505 | 0.562451968 | -0.037376956 | 0.420335226 |
| GRM1 | grey | -0.036355449 | 0.433156582 | -0.043111174 | 0.352590756 |
| GRM2 | grey | 0.1168401 | 0.011509748 | 0.136458336 | 0.003128516 |
| GRM4 | grey | -0.12051151 | 0.009139188 | -0.121262505 | 0.008711614 |

| | | | | | |
|---|---|---|---|---|---|
| GRM6 | grey | -0.063097184 | 0.173436551 | -0.06702705 | 0.148117831 |
| GRP | grey | 0.032187334 | 0.487751275 | 0.06616345 | 0.153425165 |
| GRPR | grey | 0.152968169 | 0.00091202 | 0.138223639 | 0.002758753 |
| GRTP1 | grey | 0.24732907 | 6.13E-08 | 0.202983254 | 9.83E-06 |
| GSC | grey | 0.054919893 | 0.236198415 | 0.04249537 | 0.35951713 |
| GSDMB | grey | -0.16468626 | 0.000351847 | -0.15393777 | 0.000844983 |
| GSDMC | grey | -0.048976684 | 0.290880246 | -0.042884254 | 0.355133222 |
| GSG1L | grey | -0.11894459 | 0.010091896 | -0.117924428 | 0.010758597 |
| GSR | grey | 0.431647113 | 1.29E-22 | 0.442815367 | 7.57E-24 |
| GSTA2 | grey | 6.22E-05 | 0.998931041 | -0.00556576 | 0.904518259 |
| GSTM1 | grey | -0.025617418 | 0.580808528 | -0.025868639 | 0.577103103 |
| GSTM3 | grey | -0.149454204 | 0.001198284 | -0.126678197 | 0.00612052 |
| GSTM4 | grey | -0.316014918 | 2.73E-12 | -0.289353783 | 1.85E-10 |
| GSTO2 | grey | 0.110250249 | 0.017152949 | 0.092270769 | 0.046272667 |
| GSTT2B | grey | -0.017194395 | 0.710931298 | -0.035618726 | 0.442541819 |
| GSX1 | grey | -0.026324053 | 0.570414329 | -0.024150714 | 0.602660365 |
| GSX2 | grey | 0.069356609 | 0.134498703 | 0.050855448 | 0.272746296 |
| GTF2A1L | grey | -0.13377662 | 0.003776899 | -0.123847217 | 0.007372971 |
| GTF2H2 | grey | 0.026272415 | 0.571170891 | 0.030220069 | 0.514749688 |
| GTSF1 | grey | -0.092278722 | 0.046253777 | -0.088144269 | 0.0569864 |
| GTSF1L | grey | -0.148883076 | 0.001251963 | -0.126948484 | 0.006011555 |
| GUCA1B | grey | -0.15243866 | 0.000950671 | -0.172209724 | 0.000184399 |
| GUCA1C | grey | -0.097991008 | 0.034257662 | -0.103111626 | 0.025865074 |
| GUCA2A | grey | -0.04262135 | 0.358093256 | -0.036972968 | 0.425379079 |
| GUCA2B | grey | 0.003607269 | 0.938030825 | -0.012638427 | 0.785316279 |
| GUCD1 | grey | 0.041921346 | 0.366049757 | 0.066524194 | 0.151190911 |
| GUCY2D | grey | -0.265394545 | 5.72E-09 | -0.246991559 | 6.40E-08 |
| GUSB | grey | 0.023185676 | 0.61723627 | 0.017221729 | 0.710492404 |
| GYG2 | grey | 0.047790128 | 0.302740357 | 0.04642338 | 0.316793484 |
| GYS2 | grey | -0.024683886 | 0.594674177 | -0.037621227 | 0.417302535 |
| H1F0 | grey | 0.139172428 | 0.002576895 | 0.0969393 | 0.03624251 |
| H2AFB1 | grey | 0.021433798 | 0.644081287 | 0.00132077 | 0.977290805 |
| H2AFB2 | grey | -0.058002247 | 0.210885775 | -0.060034795 | 0.195301483 |
| H2AFB3 | grey | 0.03252023 | 0.483259227 | 0.021642744 | 0.640854155 |
| H2AFJ | grey | -0.122217681 | 0.008193585 | -0.087857587 | 0.057801505 |
| H2AFY2 | grey | 0.033053189 | 0.47611441 | 0.012867704 | 0.781519277 |
| H2BFM | grey | -0.21618388 | 2.42E-06 | -0.217166314 | 2.17E-06 |
| H2BFS | grey | -0.009720737 | 0.834051002 | -0.003990931 | 0.931455329 |
| H2BFWT | grey | -0.2231022 | 1.12E-06 | -0.233314497 | 3.41E-07 |
| H3F3C | grey | -0.002444473 | 0.957983675 | 0.012097969 | 0.794286681 |
| HADHA | grey | 0.121612721 | 0.008518385 | 0.129093665 | 0.005206676 |
| HAL | grey | -0.068790957 | 0.137713445 | -0.094990109 | 0.040179963 |
| HAND1 | grey | 0.002086562 | 0.964131061 | 0.005023954 | 0.913774947 |
| HAO1 | grey | 0.064499321 | 0.164056902 | 0.060441357 | 0.192287933 |
| HAP1 | grey | 0.00543264 | 0.906791446 | -0.043500701 | 0.348253228 |

| | | | | | |
|---|---|---|---|---|---|
| HAPLN3 | grey | 0.049452487 | 0.286213095 | 0.060431704 | 0.192359083 |
| HAPLN4 | grey | -0.121606135 | 0.008521983 | -0.13870637 | 0.002664815 |
| HARS | grey | 0.189401834 | 3.80E-05 | 0.185590122 | 5.46E-05 |
| HAS2 | grey | -0.026621687 | 0.566062905 | -0.042187185 | 0.363015274 |
| HAS3 | grey | 0.329291235 | 2.84E-13 | 0.306306119 | 1.33E-11 |
| HAVCR1 | grey | 0.018183402 | 0.695112914 | 0.012976362 | 0.779721638 |
| HBA1 | grey | 0.056767607 | 0.220779396 | 0.060260825 | 0.193621855 |
| HBA2 | grey | 0.051127571 | 0.270185102 | 0.02968457 | 0.522230563 |
| HBB | grey | 0.045015353 | 0.331710098 | 0.015937184 | 0.731219123 |
| HBD | grey | -0.112973119 | 0.014579812 | -0.109527896 | 0.017898954 |
| HBE1 | grey | -0.168083712 | 0.000263698 | -0.181346207 | 8.11E-05 |
| HBM | grey | -0.079916481 | 0.084501968 | -0.088601917 | 0.055704966 |
| HBQ1 | grey | 0.035504355 | 0.444009149 | 0.038380457 | 0.407958913 |
| HBZ | grey | -0.093659058 | 0.043071722 | -0.10058417 | 0.029755391 |
| HCAR1 | grey | -0.26975383 | 3.14E-09 | -0.273402079 | 1.89E-09 |
| HCAR2 | grey | -0.004925254 | 0.915462523 | -0.000868089 | 0.985073018 |
| HCAR3 | grey | 0.065228983 | 0.159329064 | 0.07541137 | 0.103608806 |
| HCG27 | grey | -0.023904076 | 0.606370867 | 0.006477156 | 0.888976936 |
| HCN1 | grey | 0.022586925 | 0.626356421 | 0.008022241 | 0.862729825 |
| HCN2 | grey | 0.028112452 | 0.544510182 | 0.03436687 | 0.458752322 |
| HCN3 | grey | 0.165179303 | 0.000337541 | 0.138767712 | 0.002653089 |
| HCRTR2 | grey | -0.00029438 | 0.994937811 | -0.001702792 | 0.970724965 |
| HDAC11 | grey | -0.159648042 | 0.000534161 | -0.161871587 | 0.000444937 |
| HDAC6 | grey | -0.124599677 | 0.007019475 | -0.14043383 | 0.002352036 |
| HDAC7 | grey | -0.32266643 | 8.92E-13 | -0.328794581 | 3.10E-13 |
| HDC | grey | 0.013090386 | 0.777836469 | -0.010190747 | 0.826151984 |
| HDDC3 | grey | 0.116031721 | 0.012099558 | 0.093527381 | 0.043367094 |
| HDGFL1 | grey | 0.085160865 | 0.065951771 | 0.088555307 | 0.055834374 |
| HDHD1 | grey | 0.044236957 | 0.340147544 | 0.073061879 | 0.114851432 |
| HDLBP | grey | 0.275558058 | 1.39E-09 | 0.281554207 | 5.87E-10 |
| HEBP1 | grey | -0.232605848 | 3.71E-07 | -0.23987343 | 1.55E-07 |
| HECTD3 | grey | 0.118749489 | 0.010216488 | 0.139912784 | 0.002442654 |
| HECW1 | grey | -0.089664107 | 0.052822807 | -0.070592527 | 0.127676169 |
| HELT | grey | -0.111032985 | 0.016375413 | -0.11377066 | 0.013893395 |
| HEMK1 | grey | 0.120324262 | 0.009248662 | 0.111020773 | 0.016387303 |
| HES1 | grey | 0.073040993 | 0.114955499 | 0.097571448 | 0.035038094 |
| HES2 | grey | 0.210964216 | 4.25E-06 | 0.233605038 | 3.30E-07 |
| HES7 | grey | -0.077774468 | 0.093198865 | -0.053789297 | 0.245999396 |
| HEXA | grey | 0.148087309 | 0.001330449 | 0.145411526 | 0.00162874 |
| HEXIM1 | grey | 0.199083822 | 1.46E-05 | 0.20584337 | 7.31E-06 |
| HEXIM2 | grey | 0.195644439 | 2.06E-05 | 0.185172257 | 5.68E-05 |
| HEY1 | grey | 0.181723313 | 7.83E-05 | 0.182102028 | 7.56E-05 |
| HGFAC | grey | 0.055672711 | 0.22982698 | 0.058876295 | 0.20407764 |
| HHIP | grey | 0.113858849 | 0.013819268 | 0.128334653 | 0.005479635 |
| HIRIP3 | grey | -0.031256696 | 0.500427446 | -0.03386244 | 0.465376877 |

| | | | | | |
|---|---|---|---|---|---|
| HIST1H3] | grey | 0.016050744 | 0.729378616 | 0.014772745 | 0.750178854 |
| HIST2H2. | grey | 0.09002498 | 0.051872332 | 0.08199516 | 0.076699928 |
| HIST2H3. | grey | 0.013989066 | 0.763024969 | 0.00416776 | 0.928426226 |
| HIST2H3( | grey | -0.058289793 | 0.208628178 | -0.063204609 | 0.172704095 |
| HIST2H3] | grey | -0.014925371 | 0.747684891 | -0.015808134 | 0.733312545 |
| HIST2H4. | grey | -0.068722518 | 0.138106371 | -0.067896648 | 0.142916071 |
| HIST2H4] | grey | -0.061088253 | 0.187563429 | -0.088819834 | 0.055103254 |
| HIST3H2. | grey | 0.04299474 | 0.353893871 | 0.057289612 | 0.216556638 |
| HK1 | grey | 0.170444271 | 0.000215113 | 0.169809781 | 0.000227276 |
| HKDC1 | grey | 0.079067393 | 0.087867686 | 0.093328152 | 0.043817241 |
| HLCS | grey | 0.258425112 | 1.46E-08 | 0.260898064 | 1.05E-08 |
| HMGCLI | grey | -0.204309483 | 8.57E-06 | -0.199877834 | 1.35E-05 |
| HMGCS2 | grey | 0.033744081 | 0.466938902 | 0.032475645 | 0.483859552 |
| HMGN5 | grey | 0.225857213 | 8.16E-07 | 0.20628344 | 6.98E-06 |
| HMHB1 | grey | -0.043991604 | 0.342835214 | -0.036547453 | 0.430729589 |
| HMP19 | grey | 0.001276498 | 0.978051808 | -0.028531619 | 0.538524129 |
| HMSD | grey | -0.030346288 | 0.512994548 | -0.026775457 | 0.563821031 |
| HMX1 | grey | -0.055648226 | 0.230032273 | -0.062529818 | 0.17734346 |
| HMX2 | grey | 0.100567407 | 0.029782782 | 0.130281726 | 0.004803946 |
| HMX3 | grey | 0.085671485 | 0.064339936 | 0.097856069 | 0.034507033 |
| HNF1B | grey | 0.060369452 | 0.192818416 | 0.054583122 | 0.239088606 |
| HNF4A | grey | 0.012655429 | 0.785034541 | 0.017279253 | 0.709569074 |
| HNRNPC | grey | -0.016307669 | 0.725220301 | -0.029851485 | 0.519892811 |
| HNRNPC | grey | -0.013587751 | 0.76962887 | -0.010247057 | 0.825206792 |
| HOGA1 | grey | -0.117631213 | 0.010957307 | -0.114936004 | 0.012941574 |
| HOMER3 | grey | -0.018421232 | 0.691328359 | -0.018757047 | 0.685997661 |
| HOMEZ | grey | 0.085247949 | 0.065674558 | 0.089095647 | 0.05434943 |
| HORMAI | grey | -0.130139578 | 0.004850605 | -0.129173378 | 0.005178732 |
| HOXA1 | grey | -0.041546894 | 0.370350697 | -0.052526143 | 0.25728159 |
| HOXA10- | grey | 0.164658735 | 0.000352662 | 0.141910476 | 0.00211164 |
| HOXA2 | grey | -0.156260528 | 0.000702486 | -0.174840432 | 0.000146164 |
| HOXA3 | grey | -0.140030826 | 0.00242185 | -0.150849889 | 0.001075902 |
| HOXA5 | grey | -0.102040618 | 0.02745655 | -0.112229523 | 0.015246467 |
| HOXA6 | grey | -0.021666291 | 0.6404909 | -0.043960517 | 0.343176723 |
| HOXA7 | grey | 0.006668458 | 0.885720002 | -0.003495552 | 0.939946337 |
| HOXA9 | grey | 0.123926726 | 0.007334881 | 0.10521536 | 0.022969322 |
| HOXB13 | grey | 0.334758622 | 1.08E-13 | 0.323280741 | 8.03E-13 |
| HOXB7 | grey | -0.088262005 | 0.056654421 | -0.059303767 | 0.200806632 |
| HOXB8 | grey | -0.058098249 | 0.210130087 | -0.036476212 | 0.43162918 |
| HOXB9 | grey | -0.088330121 | 0.056463088 | -0.06664001 | 0.150478874 |
| HOXC10 | grey | 0.189236158 | 3.86E-05 | 0.171003923 | 0.000204893 |
| HOXC11 | grey | 0.236956938 | 2.21E-07 | 0.224138518 | 9.93E-07 |
| HOXC12 | grey | 0.049196991 | 0.288712951 | 0.025401549 | 0.584001365 |
| HOXC13 | grey | 0.077469217 | 0.094494539 | 0.058871029 | 0.204118173 |
| HOXC4 | grey | -0.106929495 | 0.020821642 | -0.112769336 | 0.014759911 |

| | | | | | |
|---|---|---|---|---|---|
| HOXC6 | grey | 0.122611892 | 0.007987925 | 0.098035294 | 0.034176155 |
| HOXC8 | grey | 0.079420808 | 0.08645391 | 0.059386537 | 0.200177707 |
| HOXC9 | grey | 0.163497695 | 0.00038869 | 0.147708919 | 0.001369336 |
| HOXD12 | grey | 0.066752033 | 0.149792579 | 0.043190254 | 0.351707416 |
| HOXD13 | grey | 0.039441995 | 0.395105289 | 0.011334669 | 0.807001833 |
| HP | grey | 0.198769292 | 1.51E-05 | 0.188762188 | 4.04E-05 |
| HPD | grey | -0.208580372 | 5.48E-06 | -0.206002374 | 7.19E-06 |
| HPGD | grey | -0.026324878 | 0.570402249 | -0.035491651 | 0.444172296 |
| HPN | grey | -0.101124303 | 0.028884298 | -0.111250786 | 0.016164624 |
| HPR | grey | 0.031708164 | 0.494256362 | 0.046417521 | 0.316854633 |
| HPS1 | grey | 0.228359721 | 6.11E-07 | 0.256596805 | 1.86E-08 |
| HPS6 | grey | 0.191819684 | 3.01E-05 | 0.197940423 | 1.64E-05 |
| HPSE | grey | -0.27889518 | 8.63E-10 | -0.262908694 | 8.02E-09 |
| HR | grey | -0.113284413 | 0.014308424 | -0.121242735 | 0.008722638 |
| HRASLS | grey | -0.269459481 | 3.27E-09 | -0.290463567 | 1.57E-10 |
| HRASLS5 | grey | -0.040352706 | 0.384274847 | -0.022840263 | 0.622490527 |
| HRH1 | grey | -0.191496543 | 3.10E-05 | -0.166468769 | 0.000302651 |
| HRH3 | grey | 0.024317141 | 0.600162351 | 0.004941396 | 0.915186497 |
| HRK | grey | -0.151410532 | 0.00103007 | -0.157860409 | 0.000617655 |
| HS1BP3 | grey | 0.133977057 | 0.003724526 | 0.130195914 | 0.004832064 |
| HS3ST1 | grey | 0.228955467 | 5.70E-07 | 0.224215582 | 9.84E-07 |
| HS3ST3A | grey | 0.121421167 | 0.008623595 | 0.102179395 | 0.027245695 |
| HS3ST3B | grey | 0.133550428 | 0.003836802 | 0.120889639 | 0.008921623 |
| HS3ST4 | grey | -0.100318854 | 0.030191459 | -0.102385005 | 0.026935859 |
| HS3ST5 | grey | -0.038120851 | 0.411139738 | -0.06159244 | 0.183940833 |
| HSBP1L1 | grey | 0.093772064 | 0.042819584 | 0.116496517 | 0.011757252 |
| HSD11B2 | grey | 0.220521088 | 1.49E-06 | 0.231683653 | 4.14E-07 |
| HSD17B1 | grey | 0.069401433 | 0.134246451 | 0.087541124 | 0.058712476 |
| HSD17B1 | grey | -0.326636572 | 4.51E-13 | -0.324284645 | 6.76E-13 |
| HSD17B2 | grey | 0.216713109 | 2.28E-06 | 0.22613149 | 7.90E-07 |
| HSD3B2 | grey | -0.054420685 | 0.240491506 | -0.049367891 | 0.287039194 |
| HSDL1 | grey | 0.233348646 | 3.40E-07 | 0.222334662 | 1.22E-06 |
| HSF4 | grey | 0.225310575 | 8.68E-07 | 0.212348976 | 3.67E-06 |
| HSFX1 | grey | 0.011195942 | 0.809318339 | -0.014819444 | 0.74941549 |
| HSFX2 | grey | -0.102153144 | 0.027285474 | -0.08493793 | 0.066665824 |
| HSFY1 | grey | -0.014779678 | 0.750065514 | -0.012535824 | 0.78701712 |
| HSFY2 | grey | -0.087353646 | 0.059257734 | -0.091220325 | 0.048825301 |
| HSPA1A | grey | -0.274752399 | 1.56E-09 | -0.281532121 | 5.89E-10 |
| HSPA1B | grey | -0.017484651 | 0.706275641 | -0.006457784 | 0.889306846 |
| HSPA2 | grey | -0.093872654 | 0.042596195 | -0.108591926 | 0.018907606 |
| HSPA6 | grey | 0.054159257 | 0.242761466 | 0.046154137 | 0.319611389 |
| HSPB9 | grey | -0.131850919 | 0.004315123 | -0.102614629 | 0.026593423 |
| HTATIP2 | grey | 0.032684297 | 0.48105359 | 0.05921039 | 0.201517879 |
| HTN1 | grey | -0.037070262 | 0.424161139 | -0.023217202 | 0.616757667 |
| HTN3 | grey | 0.009538904 | 0.837111433 | 0.027840475 | 0.548411785 |

| | | | | | |
|---|---|---|---|---|---|
| HTR1D | grey | 0.129914151 | 0.004925438 | 0.108149199 | 0.019401732 |
| HTR2C | grey | -0.18143217 | 8.05E-05 | -0.171882585 | 0.00018976 |
| HTR3A | grey | -0.170945588 | 0.000205937 | -0.171837531 | 0.00019051 |
| HTR3B | grey | -0.11879219 | 0.010189103 | -0.114548978 | 0.013251106 |
| HTR5A | grey | -0.00668243 | 0.885482208 | -0.033413245 | 0.47132038 |
| HTR6 | grey | -0.14678481 | 0.001468737 | -0.128722664 | 0.005338529 |
| HTR7 | grey | 0.088089052 | 0.057142651 | 0.074251361 | 0.109045536 |
| HUS1B | grey | -0.037500697 | 0.418797342 | -0.043425863 | 0.349083939 |
| HYAL3 | grey | 0.094010839 | 0.04229092 | 0.095865637 | 0.038368741 |
| HYAL4 | grey | 0.109186976 | 0.018260771 | 0.10671764 | 0.021077263 |
| HYI | grey | -0.023023543 | 0.619700147 | -0.035867936 | 0.439354186 |
| HYKK | grey | 0.10691203 | 0.020842612 | 0.106060503 | 0.021887569 |
| HYPK | grey | 0.111755531 | 0.015685243 | 0.08474762 | 0.067280385 |
| HYPM | grey | -0.040369281 | 0.384079429 | -0.005171301 | 0.911256328 |
| IAH1 | grey | 0.111818291 | 0.015626517 | 0.13071993 | 0.004662645 |
| IBA57 | grey | 0.004136789 | 0.928956686 | 0.008577614 | 0.853330664 |
| IBSP | grey | 0.039485616 | 0.39458238 | 0.039820129 | 0.390586261 |
| ICAM5 | grey | -0.052261921 | 0.259686111 | -0.061028476 | 0.187996388 |
| ICOSLG | grey | -0.16237004 | 0.000426938 | -0.189574286 | 3.74E-05 |
| ID1 | grey | 0.124589217 | 0.007024283 | 0.144108167 | 0.001795234 |
| ID2 | grey | 0.169992189 | 0.000223715 | 0.157215647 | 0.00065063 |
| ID3 | grey | 0.134367616 | 0.003624362 | 0.141259202 | 0.002214754 |
| ID4 | grey | -0.11098452 | 0.016422644 | -0.103644993 | 0.025102615 |
| IDO2 | grey | -0.090230544 | 0.051337317 | -0.088711379 | 0.055402041 |
| IER2 | grey | -0.057520363 | 0.214708566 | -0.071865039 | 0.120935148 |
| IER3 | grey | 0.220852523 | 1.44E-06 | 0.212111584 | 3.76E-06 |
| IER5 | grey | 0.242205737 | 1.16E-07 | 0.2322113 | 3.89E-07 |
| IER5L | grey | 0.118532996 | 0.010356338 | 0.114226953 | 0.013513606 |
| IFFO2 | grey | 0.010564962 | 0.819875385 | 0.009254292 | 0.841906638 |
| IFITM10 | grey | 0.027815817 | 0.548766195 | 0.0514892 | 0.266806994 |
| IFITM5 | grey | 0.02572638 | 0.579200015 | 0.024583898 | 0.596168192 |
| IFNA1 | grey | -0.045837037 | 0.322951053 | -0.044718781 | 0.334908763 |
| IFNE | grey | -0.145368621 | 0.001633988 | -0.148053229 | 0.00133391 |
| IFNGR2 | grey | 0.11316879 | 0.014408701 | 0.11547148 | 0.012523834 |
| IFNLR1 | grey | -0.007731592 | 0.867656591 | -0.032781282 | 0.479752354 |
| IGDCC3 | grey | -0.257589621 | 1.63E-08 | -0.284610779 | 3.75E-10 |
| IGF1 | grey | -0.081583511 | 0.078196671 | -0.080286934 | 0.083066471 |
| IGF2BP1 | grey | -0.204521753 | 8.39E-06 | -0.204221691 | 8.65E-06 |
| IGFALS | grey | -0.181273583 | 8.16E-05 | -0.174996439 | 0.000144148 |
| IGFBP1 | grey | -0.162119869 | 0.000435885 | -0.180420863 | 8.83E-05 |
| IGFBP2 | grey | -0.192446596 | 2.83E-05 | -0.179930475 | 9.23E-05 |
| IGFBP3 | grey | 0.024927625 | 0.591039435 | 0.032566399 | 0.482638004 |
| IGFL1 | grey | 0.032669981 | 0.481245833 | 0.029065234 | 0.530951564 |
| IGFL2 | grey | 0.010737463 | 0.816985928 | 0.031979288 | 0.490569988 |
| IGFL3 | grey | -0.003440596 | 0.940888742 | 0.036040791 | 0.437150943 |

| | | | | | |
|---|---|---|---|---|---|
| IGHMBP2 | grey | 0.028856036 | 0.533913879 | 0.002837663 | 0.951233225 |
| IGLON5 | grey | -0.226694509 | 7.41E-07 | -0.232329627 | 3.84E-07 |
| IGSF1 | grey | -0.145322444 | 0.001639652 | -0.150779996 | 0.001081745 |
| IGSF10 | grey | -0.180018311 | 9.16E-05 | -0.200115785 | 1.32E-05 |
| IGSF11 | grey | -0.068990155 | 0.136574685 | -0.048370958 | 0.29689524 |
| IGSF22 | grey | -0.157707594 | 0.000625327 | -0.159786313 | 0.000528161 |
| IGSF23 | grey | -0.173105396 | 0.000170436 | -0.157812344 | 0.000620059 |
| IKBKB | grey | -0.207986484 | 5.84E-06 | -0.238112645 | 1.92E-07 |
| IL10RB | grey | -0.108544893 | 0.018959572 | -0.065754674 | 0.155987054 |
| IL11 | grey | -0.057378073 | 0.21584681 | -0.059848231 | 0.196695838 |
| IL12RB2 | grey | -0.203097778 | 9.72E-06 | -0.188864751 | 4.00E-05 |
| IL13RA2 | grey | -0.140294824 | 0.002375906 | -0.136942326 | 0.003022898 |
| IL17A | grey | 0.067726427 | 0.14392314 | 0.109221322 | 0.018224033 |
| IL17B | grey | -0.024495689 | 0.597487605 | 0.013221665 | 0.775667645 |
| IL17C | grey | 0.148221195 | 0.001316935 | 0.134242761 | 0.003656114 |
| IL17RA | grey | 0.11407861 | 0.013636062 | 0.095228786 | 0.039679181 |
| IL17RB | grey | 0.236496225 | 2.33E-07 | 0.20134373 | 1.16E-05 |
| IL17RC | grey | 0.149989021 | 0.001149948 | 0.145037084 | 0.00167506 |
| IL17RE | grey | 0.149141313 | 0.001227423 | 0.162123033 | 0.000435771 |
| IL17REL | grey | -0.083205323 | 0.072434192 | -0.07983163 | 0.084833561 |
| IL19 | grey | 0.28547636 | 3.30E-10 | 0.285840174 | 3.13E-10 |
| IL1A | grey | 0.130580147 | 0.004707306 | 0.158285064 | 0.000596791 |
| IL1RL2 | grey | -0.257087211 | 1.74E-08 | -0.225413108 | 8.58E-07 |
| IL20 | grey | 0.042610241 | 0.358218667 | 0.032326136 | 0.485875578 |
| IL20RB | grey | -0.093866852 | 0.042609054 | -0.071502499 | 0.122826761 |
| IL22RA1 | grey | -0.174507735 | 0.000150551 | -0.177767764 | 0.000112416 |
| IL22RA2 | grey | -0.051079216 | 0.270639013 | -0.031879922 | 0.491919317 |
| IL27RA | grey | -0.124081497 | 0.00726124 | -0.120643277 | 0.009062843 |
| IL36A | grey | 0.063879953 | 0.168152115 | 0.043094018 | 0.35278257 |
| IL36B | grey | -0.054141277 | 0.242918131 | -0.04507137 | 0.331108151 |
| IL6 | grey | -0.115682195 | 0.012362741 | -0.128599834 | 0.00538284 |
| IL6R | grey | -0.086200205 | 0.062705261 | -0.087272576 | 0.05949481 |
| ILDR2 | grey | 0.044358615 | 0.338819864 | 0.012496594 | 0.787667694 |
| IMPG2 | grey | -0.202149073 | 1.07E-05 | -0.190281707 | 3.49E-05 |
| INCA1 | grey | -0.216514672 | 2.33E-06 | -0.231630051 | 4.17E-07 |
| INF2 | grey | -0.06364771 | 0.169707251 | -0.043289556 | 0.350600191 |
| ING1 | grey | 0.017159474 | 0.711492157 | 0.016007294 | 0.730082643 |
| ING4 | grey | -0.218889196 | 1.79E-06 | -0.227680422 | 6.61E-07 |
| ING5 | grey | -0.140696334 | 0.002307554 | -0.169627526 | 0.000230887 |
| INHBB | grey | -0.036696673 | 0.428848848 | -0.066925872 | 0.148732301 |
| INHBE | grey | -0.091367781 | 0.048460017 | -0.096467149 | 0.037164877 |
| INO80B-\ | grey | 0.116095048 | 0.012052408 | 0.092976057 | 0.044622395 |
| INO80E | grey | 0.16724274 | 0.000283357 | 0.166880455 | 0.000292241 |
| INPP1 | grey | -0.150516508 | 0.001104039 | -0.129399436 | 0.005100219 |
| INPP5J | grey | 0.016108977 | 0.728435421 | -0.004228726 | 0.927382102 |

| | | | | | |
|---|---|---|---|---|---|
| INPP5K | grey | 0.166397325 | 0.000304492 | 0.207059909 | 6.43E-06 |
| INPPL1 | grey | -0.070782908 | 0.126649501 | -0.07931631 | 0.086870017 |
| INS | grey | -0.08100335 | 0.080346268 | -0.084999639 | 0.06646754 |
| INS-IGF2 | grey | -0.120847239 | 0.008945787 | -0.122077898 | 0.008267636 |
| INSC | grey | 0.047517059 | 0.305514542 | 0.054717174 | 0.237935196 |
| INSL4 | grey | 0.086010155 | 0.063288864 | 0.102619212 | 0.026586627 |
| INSM1 | grey | 0.029836609 | 0.520100941 | 0.018526634 | 0.689653553 |
| INSM2 | grey | 0.01906057 | 0.681192906 | 0.02878899 | 0.53486503 |
| INSRR | grey | -0.162765646 | 0.000413137 | -0.161882091 | 0.00044455 |
| INTS5 | grey | 0.170290602 | 0.000218001 | 0.164379198 | 0.00036104 |
| IP6K2 | grey | 0.097989103 | 0.03426117 | 0.107257229 | 0.020431509 |
| IP6K3 | grey | -0.18460383 | 5.99E-05 | -0.183586586 | 6.59E-05 |
| IQCA1 | grey | -0.46987294 | 5.05E-27 | -0.483569184 | 9.66E-29 |
| IQCF5 | grey | 0.000391279 | 0.993271563 | 0.018842472 | 0.684644094 |
| IQSEC2 | grey | 0.076438624 | 0.098975618 | 0.08338373 | 0.071822014 |
| IRAK2 | grey | -0.19705675 | 1.79E-05 | -0.222935539 | 1.14E-06 |
| IRGQ | grey | 0.045797991 | 0.323363852 | 0.054246348 | 0.242003599 |
| IRX2 | grey | -0.120019471 | 0.009429355 | -0.125703884 | 0.006528178 |
| IRX4 | grey | -0.096807638 | 0.03649774 | -0.101870798 | 0.027716477 |
| IRX5 | grey | 0.17832936 | 0.000106843 | 0.188524598 | 4.13E-05 |
| IRX6 | grey | -0.101322014 | 0.028570959 | -0.080065678 | 0.083921445 |
| ISL1 | grey | 0.018331596 | 0.692753824 | 0.017878772 | 0.699971539 |
| ISL2 | grey | 0.094768056 | 0.040650655 | 0.107157721 | 0.020549286 |
| ISLR2 | grey | 0.030702593 | 0.508056739 | 0.020407883 | 0.660022584 |
| ISM1 | grey | -0.12774983 | 0.00569866 | -0.100687213 | 0.029587492 |
| ISM2 | grey | -0.283047006 | 4.72E-10 | -0.304879709 | 1.68E-11 |
| ISY1-RAI | grey | -0.055598239 | 0.230451785 | -0.082986277 | 0.07319163 |
| ITFG2 | grey | -0.205219429 | 7.80E-06 | -0.217108144 | 2.18E-06 |
| ITGA10 | grey | 0.079968315 | 0.084299915 | 0.039645418 | 0.392670305 |
| ITGA2B | grey | -0.014506909 | 0.754528921 | -0.039813095 | 0.390670035 |
| ITGAD | grey | -0.116972522 | 0.011415594 | -0.109938611 | 0.01747139 |
| ITGB3 | grey | -0.099216503 | 0.03206236 | -0.097103716 | 0.035925917 |
| ITGB4 | grey | 0.064189406 | 0.166096573 | 0.079317322 | 0.086865977 |
| ITGB5 | grey | -0.060009679 | 0.195488773 | -0.067156735 | 0.147333059 |
| ITGB6 | grey | 0.081431914 | 0.078753809 | 0.080592302 | 0.081898042 |
| ITIH5 | grey | -0.10337595 | 0.025484758 | -0.085859874 | 0.063753505 |
| ITLN1 | grey | -0.083261976 | 0.072239336 | -0.065906238 | 0.155033424 |
| ITLN2 | grey | 0.097785295 | 0.034638443 | 0.080869417 | 0.08084925 |
| ITPKA | grey | 0.000372281 | 0.993598243 | -0.014421134 | 0.755934181 |
| ITPKC | grey | -0.097142289 | 0.035851984 | -0.107471293 | 0.020180133 |
| ITPRIPL1 | grey | -0.169741134 | 0.000228629 | -0.156097832 | 0.000711692 |
| IVD | grey | 0.229390895 | 5.42E-07 | 0.22296893 | 1.13E-06 |
| IYD | grey | 0.112762041 | 0.014766395 | 0.144970394 | 0.001683436 |
| IZUMO2 | grey | -0.09772611 | 0.03474866 | -0.091882834 | 0.047202012 |
| JADE1 | grey | -0.110432804 | 0.016968776 | -0.106923649 | 0.020828659 |

| | | | | | |
|---|---|---|---|---|---|
| JAG2 | grey | -0.08569426 | 0.064268806 | -0.070633083 | 0.127456924 |
| JAKMIP2 | grey | -0.219784998 | 1.62E-06 | -0.217286228 | 2.14E-06 |
| JAKMIP3 | grey | -0.050487384 | 0.276236746 | -0.065256089 | 0.159155433 |
| JDP2 | grey | -0.133283147 | 0.003908694 | -0.155144877 | 0.000767895 |
| JMJD6 | grey | 0.100270869 | 0.030270908 | 0.107869157 | 0.01972006 |
| JPH1 | grey | -0.132743707 | 0.004057519 | -0.166119552 | 0.000311752 |
| JPH3 | grey | -0.248798631 | 5.09E-08 | -0.253885079 | 2.65E-08 |
| JUN | grey | -0.091764541 | 0.047488492 | -0.125378506 | 0.006669643 |
| JUNB | grey | -0.056680182 | 0.221492339 | -0.063323137 | 0.17189861 |
| KAAG1 | grey | -0.068645259 | 0.13855097 | -0.053274534 | 0.25055469 |
| KAL1 | grey | -0.008655813 | 0.852008852 | -0.020569917 | 0.657494315 |
| KANK4 | grey | 0.074207909 | 0.109253477 | 0.06382749 | 0.168502476 |
| KARS | grey | 0.212384196 | 3.65E-06 | 0.241501386 | 1.27E-07 |
| KAT8 | grey | -0.212879448 | 3.46E-06 | -0.193310327 | 2.60E-05 |
| KAZALD | grey | 0.230641925 | 4.68E-07 | 0.231776767 | 4.10E-07 |
| KAZN | grey | -0.190013492 | 3.58E-05 | -0.188429233 | 4.17E-05 |
| KBTBD1 | grey | -0.100790972 | 0.029419248 | -0.117203528 | 0.011252975 |
| KBTBD1 | grey | -0.114585993 | 0.013221223 | -0.108415212 | 0.019103504 |
| KCNC1 | grey | 0.036521518 | 0.431056962 | 0.008342366 | 0.857309556 |
| KCNC2 | grey | -0.121996836 | 0.008310852 | -0.107083929 | 0.020637005 |
| KCND1 | grey | -0.32097503 | 1.19E-12 | -0.312991704 | 4.51E-12 |
| KCNE2 | grey | -0.286290126 | 2.93E-10 | -0.282332534 | 5.24E-10 |
| KCNE3 | grey | -0.127361284 | 0.005848496 | -0.081473402 | 0.078601017 |
| KCNE5 | grey | -0.108413163 | 0.019105786 | -0.089731304 | 0.052644731 |
| KCNF1 | grey | -0.189116051 | 3.90E-05 | -0.195690539 | 2.06E-05 |
| KCNG1 | grey | -0.295853502 | 6.90E-11 | -0.275606728 | 1.38E-09 |
| KCNG2 | grey | -0.105435408 | 0.022683302 | -0.124909205 | 0.006878522 |
| KCNIP1 | grey | -0.276284121 | 1.25E-09 | -0.284702579 | 3.70E-10 |
| KCNIP2 | grey | -0.165607709 | 0.000325554 | -0.183315938 | 6.76E-05 |
| KCNIP3 | grey | -0.34357148 | 2.20E-14 | -0.365357749 | 3.41E-16 |
| KCNJ1 | grey | -0.042209566 | 0.362760521 | -0.045888048 | 0.322412294 |
| KCNJ11 | grey | 0.084986796 | 0.066508766 | 0.085711529 | 0.064214913 |
| KCNJ12 | grey | -0.324872538 | 6.11E-13 | -0.332947875 | 1.50E-13 |
| KCNJ14 | grey | -0.09116228 | 0.048969723 | -0.092859899 | 0.044890734 |
| KCNJ15 | grey | 0.120306058 | 0.009259367 | 0.127963023 | 0.005617921 |
| KCNJ16 | grey | -0.206970833 | 6.49E-06 | -0.197340641 | 1.74E-05 |
| KCNJ18 | grey | 0.069534436 | 0.133500106 | 0.081948569 | 0.076868154 |
| KCNJ2 | grey | -0.030328795 | 0.51323761 | -0.03489038 | 0.451933272 |
| KCNJ3 | grey | -0.045946896 | 0.321791469 | -0.058031652 | 0.210654107 |
| KCNJ4 | grey | -0.272553487 | 2.13E-09 | -0.280254932 | 7.09E-10 |
| KCNJ5 | grey | -0.074296848 | 0.108828194 | -0.089728619 | 0.052651837 |
| KCNK1 | grey | 0.282571882 | 5.06E-10 | 0.285049544 | 3.52E-10 |
| KCNK12 | grey | -0.08758929 | 0.058573062 | -0.033055468 | 0.476083984 |
| KCNK15 | grey | -0.140758229 | 0.002297179 | -0.165966528 | 0.00031582 |
| KCNK16 | grey | 0.045068412 | 0.331139917 | 0.045525969 | 0.326249159 |

| | | | | | |
|---|---|---|---|---|---|
| KCNK17 | grey | -0.069372711 | 0.134408047 | -0.086653413 | 0.061331443 |
| KCNK18 | grey | -0.066518348 | 0.151226921 | -0.054890047 | 0.236453554 |
| KCNK2 | grey | -0.238732475 | 1.78E-07 | -0.268305378 | 3.84E-09 |
| KCNK5 | grey | 0.008334186 | 0.857447976 | -0.004419864 | 0.924109453 |
| KCNK7 | grey | -0.136071985 | 0.003215221 | -0.133548666 | 0.003837271 |
| KCNK9 | grey | -0.322768004 | 8.77E-13 | -0.321806302 | 1.03E-12 |
| KCNMB4 | grey | -0.161977429 | 0.000441057 | -0.166978262 | 0.000289817 |
| KCNN1 | grey | 0.004085499 | 0.929835241 | -0.028363602 | 0.540919598 |
| KCNN4 | grey | 0.225950693 | 8.07E-07 | 0.230041784 | 5.02E-07 |
| KCNQ1 | grey | -0.010257693 | 0.825028297 | -0.036014817 | 0.437481611 |
| KCNQ2 | grey | -0.10373172 | 0.024980489 | -0.114150183 | 0.013576858 |
| KCNQ3 | grey | -0.200232906 | 1.30E-05 | -0.20982448 | 4.80E-06 |
| KCNQ4 | grey | -0.153798505 | 0.000854323 | -0.178137443 | 0.000108717 |
| KCNS1 | grey | -0.246565063 | 6.75E-08 | -0.247440415 | 6.05E-08 |
| KCNS3 | grey | -0.086823112 | 0.060823468 | -0.120605622 | 0.009084601 |
| KCNT1 | grey | -0.332666283 | 1.57E-13 | -0.337507354 | 6.63E-14 |
| KCNU1 | grey | -0.063438263 | 0.171118934 | -0.05350843 | 0.248477649 |
| KCNV1 | grey | -0.122822649 | 0.007879874 | -0.12559163 | 0.006576675 |
| KCP | grey | -0.227757205 | 6.55E-07 | -0.205166389 | 7.84E-06 |
| KCTD11 | grey | 0.122171181 | 0.008218153 | 0.109120269 | 0.018332309 |
| KCTD13 | grey | 0.155454696 | 0.000749187 | 0.169930108 | 0.000224921 |
| KCTD14 | grey | 0.234191014 | 3.08E-07 | 0.243915238 | 9.41E-08 |
| KCTD16 | grey | -0.118617295 | 0.010301682 | -0.116636765 | 0.01165566 |
| KCTD2 | grey | -0.21874304 | 1.82E-06 | -0.229731422 | 5.21E-07 |
| KCTD21 | grey | -0.009099041 | 0.844524822 | -0.036478729 | 0.431597375 |
| KDELR3 | grey | 0.187287352 | 4.65E-05 | 0.184982083 | 5.78E-05 |
| KDM5D | grey | -0.059885487 | 0.196416813 | -0.058561917 | 0.206507801 |
| KDM8 | grey | -0.139331088 | 0.002547573 | -0.138364363 | 0.002731062 |
| KEAP1 | grey | -0.017341518 | 0.708570108 | -0.008942616 | 0.847164545 |
| KEL | grey | -0.180500132 | 8.77E-05 | -0.185333955 | 5.59E-05 |
| KERA | grey | -0.04543306 | 0.327238446 | -0.073157627 | 0.114375289 |
| KHDC1 | grey | -0.4303232 | 1.79E-22 | -0.413561256 | 1.02E-20 |
| KHDRBS | grey | -0.12822568 | 0.005519864 | -0.133264964 | 0.003913629 |
| KHDRBS | grey | -0.032463136 | 0.484028051 | -0.021786459 | 0.638638433 |
| KIAA004 | grey | 0.054755579 | 0.23760548 | 0.053781055 | 0.246071875 |
| KIAA031 | grey | 0.174169785 | 0.000155134 | 0.171361866 | 0.000198597 |
| KIAA089 | grey | 0.239002382 | 1.72E-07 | 0.232256547 | 3.87E-07 |
| KIAA093 | grey | 0.084685127 | 0.067483206 | 0.094547116 | 0.0411236 |
| KIAA104 | grey | -0.023627563 | 0.610542875 | -0.033448181 | 0.470856632 |
| KIAA116 | grey | -0.040015634 | 0.388262137 | -0.02517624 | 0.587342494 |
| KIAA154 | grey | -0.153908382 | 0.000846947 | -0.161607361 | 0.000454762 |
| KIAA202 | grey | 0.092678301 | 0.045312963 | 0.084481287 | 0.068148245 |
| KIF12 | grey | 0.047392293 | 0.306787644 | 0.05109146 | 0.270524029 |
| KIF1C | grey | 0.10632877 | 0.021553566 | 0.127067936 | 0.005963956 |
| KIF25 | grey | -0.17659436 | 0.000124953 | -0.171906885 | 0.000189357 |

| | | | | | |
|---|---|---|---|---|---|
| KIF5A | grey | -0.33835697 | 5.68E-14 | -0.360859063 | 8.27E-16 |
| KIF5C | grey | -0.115175804 | 0.012753003 | -0.126097797 | 0.006360518 |
| KIF7 | grey | -0.331660156 | 1.88E-13 | -0.365027308 | 3.64E-16 |
| KIFC3 | grey | -0.125297117 | 0.006705455 | -0.126080021 | 0.006368 |
| KIR2DL1 | grey | -0.107499133 | 0.020147639 | -0.097934114 | 0.034362616 |
| KIR2DL3 | grey | -0.104469654 | 0.023961935 | -0.092970963 | 0.044634135 |
| KIR3DL1 | grey | -0.092765714 | 0.045109308 | -0.074329446 | 0.108672646 |
| KIR3DL2 | grey | -0.036239107 | 0.434631007 | -0.012328221 | 0.790461612 |
| KIRREL2 | grey | -0.161968599 | 0.000441379 | -0.160811679 | 0.000485581 |
| KIRREL3 | grey | 0.058192505 | 0.209390051 | 0.047982455 | 0.300796508 |
| KISS1 | grey | 0.159259535 | 0.000551361 | 0.163069637 | 0.000402816 |
| KISS1R | grey | 0.27304895 | 1.98E-09 | 0.286015866 | 3.05E-10 |
| KLC2 | grey | 0.145827696 | 0.001578639 | 0.13384946 | 0.003757789 |
| KLC4 | grey | -0.225656566 | 8.35E-07 | -0.239829486 | 1.56E-07 |
| KLF1 | grey | -0.120424153 | 0.009190117 | -0.104346087 | 0.024129947 |
| KLF14 | grey | -0.061206938 | 0.186705985 | -0.066382926 | 0.152062887 |
| KLF15 | grey | -0.218742737 | 1.82E-06 | -0.251679944 | 3.52E-08 |
| KLHDC7. | grey | 0.184847032 | 5.86E-05 | 0.207199317 | 6.34E-06 |
| KLHDC8. | grey | 0.011103484 | 0.810863155 | -0.011637143 | 0.801956899 |
| KLHDC8 | grey | -0.298083661 | 4.88E-11 | -0.284854513 | 3.62E-10 |
| KLHL1 | grey | -0.163007376 | 0.00040491 | -0.164638534 | 0.000353261 |
| KLHL17 | grey | 0.169222617 | 0.000239102 | 0.159334748 | 0.000547992 |
| KLHL22 | grey | 0.187095127 | 4.74E-05 | 0.187978391 | 4.35E-05 |
| KLHL25 | grey | 0.251744396 | 3.49E-08 | 0.224975252 | 9.02E-07 |
| KLHL29 | grey | -0.235117513 | 2.75E-07 | -0.271615466 | 2.42E-09 |
| KLHL30 | grey | -0.219563591 | 1.66E-06 | -0.210494266 | 4.47E-06 |
| KLHL34 | grey | -0.077180869 | 0.095731645 | -0.055687186 | 0.229705673 |
| KLHL35 | grey | -0.109069236 | 0.018387203 | -0.092888342 | 0.044824901 |
| KLHL40 | grey | -0.12686943 | 0.006043243 | -0.126233025 | 0.006303859 |
| KLK1 | grey | -0.176648658 | 0.000124345 | -0.19494934 | 2.21E-05 |
| KLK10 | grey | -0.040597408 | 0.381395938 | -0.015722454 | 0.73470352 |
| KLK12 | grey | 0.22534424 | 8.65E-07 | 0.206147684 | 7.08E-06 |
| KLK13 | grey | -0.067347135 | 0.146186626 | -0.044174269 | 0.340832957 |
| KLK14 | grey | 0.12692925 | 0.006019251 | 0.127920442 | 0.005633964 |
| KLK15 | grey | 0.02285127 | 0.622322795 | 0.00782145 | 0.866132854 |
| KLK2 | grey | 0.052050043 | 0.261625457 | 0.033778857 | 0.466479651 |
| KLK3 | grey | -0.015228581 | 0.742738139 | -0.044611156 | 0.336074433 |
| KLK5 | grey | -0.25601724 | 2.01E-08 | -0.250665947 | 4.01E-08 |
| KLK6 | grey | -0.364218656 | 4.27E-16 | -0.36460704 | 3.95E-16 |
| KLK7 | grey | -0.343559747 | 2.20E-14 | -0.347104685 | 1.14E-14 |
| KLK8 | grey | -0.342506021 | 2.67E-14 | -0.357188309 | 1.69E-15 |
| KLK9 | grey | -0.015416709 | 0.739674224 | -0.01620408 | 0.726895921 |
| KLKB1 | grey | 0.126514927 | 0.006187197 | 0.116795141 | 0.01154187 |
| KLRF1 | grey | -0.21268131 | 3.54E-06 | -0.207129677 | 6.39E-06 |
| KMO | grey | 0.114133987 | 0.013590235 | 0.087295676 | 0.059427177 |

| | | | | | |
|---|---|---|---|---|---|
| KRBA1 | grey | -0.135898564 | 0.003254845 | -0.170512683 | 0.000213838 |
| KREMEN | grey | -0.122656004 | 0.007965201 | -0.122408764 | 0.008093314 |
| KRT10 | grey | 0.077899173 | 0.092673647 | 0.065137616 | 0.159915389 |
| KRT15 | grey | 0.068116233 | 0.141624919 | 0.068010442 | 0.142245849 |
| KRT20 | grey | 0.04003742 | 0.388003667 | 0.010026686 | 0.828907254 |
| KRT23 | grey | 0.235413055 | 2.66E-07 | 0.223504213 | 1.07E-06 |
| KRT24 | grey | 0.079627986 | 0.08563368 | 0.068408063 | 0.139922822 |
| KRT26 | grey | -0.04140349 | 0.37200608 | 0.05427375 | 0.241765493 |
| KRT3 | grey | -0.008990533 | 0.84635575 | -0.002654027 | 0.954385556 |
| KRT4 | grey | 0.00511063 | 0.912293282 | 0.016665935 | 0.719435266 |
| KRT40 | grey | 0.137408506 | 0.002924243 | 0.141056304 | 0.002247806 |
| KRT72 | grey | -0.086815266 | 0.060846876 | -0.006799287 | 0.883493735 |
| KRT73 | grey | -0.049576852 | 0.28500156 | 0.041489122 | 0.371017038 |
| KRT75 | grey | 0.056248504 | 0.225036718 | 0.069686628 | 0.132650014 |
| KRT76 | grey | -0.08346283 | 0.071551952 | -0.089149016 | 0.05420456 |
| KRT79 | grey | 0.098265938 | 0.03375433 | 0.107057514 | 0.020668485 |
| KRT81 | grey | -0.079226599 | 0.087228525 | -0.044228151 | 0.340243767 |
| KRT86 | grey | -0.067178613 | 0.147200984 | -0.007687687 | 0.868401271 |
| KRTAP1( | grey | -0.063430808 | 0.171169339 | -0.060046551 | 0.195213864 |
| KRTAP2- | grey | -0.193769682 | 2.48E-05 | -0.184498782 | 6.05E-05 |
| KRTAP2( | grey | -0.007504579 | 0.871508257 | 0.005738899 | 0.901562856 |
| KRTAP2 | grey | 0.06181267 | 0.182374782 | 0.056185865 | 0.225554361 |
| KRTAP2 | grey | 0.001971222 | 0.96611259 | 0.005076626 | 0.912874512 |
| KRTAP4- | grey | -0.055646101 | 0.230050092 | -0.033320301 | 0.472555347 |
| KRTAP5- | grey | 0.16727381 | 0.000282607 | 0.157567726 | 0.000632427 |
| KRTAP5- | grey | 0.014183251 | 0.759835652 | -0.004171497 | 0.928362221 |
| KRTAP5- | grey | -0.195882097 | 2.02E-05 | -0.190376022 | 3.46E-05 |
| KRTAP5- | grey | 0.061460469 | 0.184884021 | 0.04701932 | 0.310614314 |
| KRTDAP | grey | -0.169969708 | 0.000224151 | -0.144594697 | 0.00173134 |
| KSR2 | grey | -0.130497034 | 0.004734043 | -0.158140409 | 0.000603824 |
| L1TD1 | grey | -0.005279273 | 0.90941132 | -0.003591988 | 0.938292817 |
| L34079.2 | grey | -0.07279623 | 0.11618061 | -0.07607751 | 0.100585179 |
| L3MBTL | grey | 0.289167955 | 1.91E-10 | 0.300449766 | 3.38E-11 |
| LA16c-30 | grey | -0.075866137 | 0.101536919 | -0.101227105 | 0.028721007 |
| LAMA1 | grey | -0.031578447 | 0.496025298 | -0.030184576 | 0.515243805 |
| LAMA3 | grey | -0.056413676 | 0.223675799 | -0.037102877 | 0.42375331 |
| LAMA5 | grey | -0.135067584 | 0.003450936 | -0.150031605 | 0.001146177 |
| LAMB2 | grey | -0.11357473 | 0.014059336 | -0.121912474 | 0.008356042 |
| LAMC3 | grey | -0.280460819 | 6.88E-10 | -0.258002948 | 1.54E-08 |
| LAMP1 | grey | 0.002502122 | 0.956993735 | 0.006224202 | 0.893286351 |
| LANCL2 | grey | -0.193913461 | 2.45E-05 | -0.212335153 | 3.67E-06 |
| LARP6 | grey | -0.127704695 | 0.005715887 | -0.129990123 | 0.004900102 |
| LBP | grey | -0.125405528 | 0.006657791 | -0.12928567 | 0.005139597 |
| LBX1 | grey | 0.01114818 | 0.810116276 | -0.012634728 | 0.785377582 |
| LBX2 | grey | 0.182827112 | 7.07E-05 | 0.184823439 | 5.87E-05 |

| | | | | | |
|---|---|---|---|---|---|
| LCE1C | grey | 0.072772697 | 0.116298933 | 0.088497717 | 0.055994608 |
| LCE1D | grey | 0.03644878 | 0.431975862 | 0.02316907 | 0.617488424 |
| LCE1E | grey | 0.094332196 | 0.041588109 | 0.083676162 | 0.070827723 |
| LCE1F | grey | 0.009303816 | 0.841071815 | 0.036750381 | 0.42817309 |
| LCE2A | grey | -0.006020411 | 0.896760518 | 0.001221706 | 0.978993689 |
| LCE2D | grey | 0.035146032 | 0.448624114 | 0.033776582 | 0.466509681 |
| LCE4A | grey | 0.011450606 | 0.805067165 | 0.004177611 | 0.928257499 |
| LCE5A | grey | -0.199334013 | 1.43E-05 | -0.204582325 | 8.33E-06 |
| LCMT2 | grey | -0.071113518 | 0.124881867 | -0.059966717 | 0.195809446 |
| LCN1 | grey | -0.170690028 | 0.000210568 | -0.167307568 | 0.000281794 |
| LCN12 | grey | -0.123222983 | 0.007678219 | -0.119682298 | 0.009632895 |
| LCN15 | grey | 0.161207891 | 0.000469999 | 0.109522209 | 0.017904937 |
| LCN8 | grey | 0.049347191 | 0.287241572 | 0.04402557 | 0.342462339 |
| LCN9 | grey | 0.033352746 | 0.472124038 | 0.0300304 | 0.517392988 |
| LCNL1 | grey | -0.192537303 | 2.80E-05 | -0.202100353 | 1.08E-05 |
| LDHC | grey | -0.130026948 | 0.004887864 | -0.125178814 | 0.006757818 |
| LDHD | grey | 0.297391612 | 5.44E-11 | 0.31739081 | 2.17E-12 |
| LDLRAP | grey | 0.095217509 | 0.039702723 | 0.103683243 | 0.025048689 |
| LDOC1 | grey | -0.296162543 | 6.58E-11 | -0.298352681 | 4.68E-11 |
| LEAP2 | grey | 0.158043503 | 0.000608578 | 0.140393135 | 0.002359001 |
| LECT1 | grey | -0.169255787 | 0.000238419 | -0.174027522 | 0.000157102 |
| LEFTY1 | grey | 0.227257558 | 6.94E-07 | 0.239030229 | 1.72E-07 |
| LEMD1 | grey | 0.003890273 | 0.93318004 | 0.017909842 | 0.699475424 |
| LETM2 | grey | 0.07516143 | 0.104761698 | 0.060808262 | 0.189597711 |
| LETMD1 | grey | -0.062765779 | 0.175710759 | -0.088273329 | 0.056622575 |
| LFNG | grey | 0.249902175 | 4.42E-08 | 0.251682604 | 3.52E-08 |
| LGALS12 | grey | 0.07632706 | 0.099470679 | 0.111885872 | 0.015563495 |
| LGALS14 | grey | -0.10767098 | 0.01994807 | -0.122853384 | 0.007864226 |
| LGALS3 | grey | 0.035529063 | 0.443691912 | 0.07347156 | 0.112824998 |
| LGALS4 | grey | -0.073624096 | 0.112077719 | -0.05067583 | 0.274445882 |
| LGALS7 | grey | -0.041389925 | 0.372162907 | -0.028610205 | 0.537405522 |
| LGALS7E | grey | -0.031342636 | 0.499249607 | -0.035385401 | 0.445538195 |
| LGALS9E | grey | 0.02899623 | 0.531927762 | 0.054173026 | 0.242641541 |
| LGALS9C | grey | -0.058918956 | 0.203749475 | -0.022405407 | 0.629132684 |
| LGI1 | grey | -0.065531996 | 0.157396172 | -0.066654133 | 0.150392222 |
| LGR5 | grey | -0.00095971 | 0.983497786 | 0.010306118 | 0.824215699 |
| LGR6 | grey | -0.179312568 | 9.77E-05 | -0.174419087 | 0.000151741 |
| LHFPL1 | grey | 0.027236854 | 0.557119814 | 0.008741984 | 0.850552756 |
| LHFPL3 | grey | -0.15683023 | 0.000671113 | -0.148881933 | 0.001252072 |
| LHFPL4 | grey | -0.043418822 | 0.349162164 | -0.057671524 | 0.213504079 |
| LHX1 | grey | -0.195967346 | 2.00E-05 | -0.195995543 | 1.99E-05 |
| LHX2 | grey | 0.084808392 | 0.067083633 | 0.079871511 | 0.084677576 |
| LHX3 | grey | -0.06224059 | 0.179360066 | -0.071254354 | 0.124134723 |
| LHX5 | grey | -0.020710312 | 0.655306828 | -0.020635589 | 0.656470718 |
| LHX8 | grey | -0.095167502 | 0.039807259 | -0.099646055 | 0.03132187 |

| | | | | | |
|---|---|---|---|---|---|
| LHX9 | grey | 0.054062855 | 0.243602297 | 0.058344169 | 0.208203236 |
| LIF | grey | 0.044011673 | 0.342614874 | 0.044877003 | 0.333199817 |
| LIM2 | grey | -0.119021034 | 0.010043447 | -0.119364515 | 0.009828295 |
| LIME1 | grey | -0.00988128 | 0.831350982 | 0.001589119 | 0.972678479 |
| LIMK2 | grey | -0.049762251 | 0.283201871 | -0.041337454 | 0.372769897 |
| LIMS3 | grey | 0.006486029 | 0.888825826 | 0.034263921 | 0.460100035 |
| LIMS3L | grey | -0.020345756 | 0.660993001 | -0.026841778 | 0.562855419 |
| LIN28A | grey | 0.01933901 | 0.676796524 | 0.000553163 | 0.990487906 |
| LIN37 | grey | -0.218094124 | 1.96E-06 | -0.2396496 | 1.59E-07 |
| LINC0011 | grey | -0.00156688 | 0.973060693 | 0.002960516 | 0.949124719 |
| LINGO1 | grey | -0.153347883 | 0.000885202 | -0.159241655 | 0.000552165 |
| LINGO3 | grey | -0.032369637 | 0.485288532 | -0.023211705 | 0.61684111 |
| LINGO4 | grey | 0.176062219 | 0.000131061 | 0.175213714 | 0.000141385 |
| LIPC | grey | -0.204996165 | 7.98E-06 | -0.179773766 | 9.37E-05 |
| LIPE | grey | -0.104626308 | 0.023750389 | -0.108130953 | 0.019422335 |
| LIPF | grey | -0.010267793 | 0.824858793 | -0.012094952 | 0.794336825 |
| LIPH | grey | -0.084667081 | 0.067541865 | -0.062717051 | 0.176047008 |
| LIPM | grey | 0.323850654 | 7.29E-13 | 0.314945462 | 3.26E-12 |
| LIX1 | grey | -0.29967415 | 3.81E-11 | -0.300493073 | 3.35E-11 |
| LLfos-48I | grey | 0.048364997 | 0.296954847 | 0.058103336 | 0.210090106 |
| LMAN1L | grey | 0.131670588 | 0.004368917 | 0.132620398 | 0.00409225 |
| LMAN2L | grey | -0.159295388 | 0.000549753 | -0.195470244 | 2.10E-05 |
| LMBR1L | grey | -0.001534714 | 0.973613514 | -0.006841179 | 0.88278107 |
| LMNA | grey | 0.011185466 | 0.809493334 | 0.028245844 | 0.542601655 |
| LMNTD2 | grey | 0.279284389 | 8.16E-10 | 0.276262924 | 1.26E-09 |
| LMO1 | grey | -0.161476925 | 0.000459686 | -0.158694146 | 0.000577313 |
| LMO2 | grey | -0.017940967 | 0.698978575 | -0.034741048 | 0.453872574 |
| LMO3 | grey | -0.286082999 | 3.02E-10 | -0.272287453 | 2.21E-09 |
| LMO4 | grey | -0.044238557 | 0.340130061 | -0.025953596 | 0.575852517 |
| LMO7DN | grey | -0.142318337 | 0.002049319 | -0.141209522 | 0.002222805 |
| LMTK3 | grey | 0.128084156 | 0.005572506 | 0.116486247 | 0.011764722 |
| LMX1B | grey | -0.078249045 | 0.091212732 | -0.086929591 | 0.060506512 |
| LOH12CI | grey | 0.044581317 | 0.336398075 | 0.061052185 | 0.187824576 |
| LOX | grey | 0.039962125 | 0.388897405 | 0.055746667 | 0.229207695 |
| LOXL1 | grey | -0.163626842 | 0.000384519 | -0.154707207 | 0.00079506 |
| LOXL3 | grey | -0.284559843 | 3.78E-10 | -0.29266518 | 1.12E-10 |
| LPCAT4 | grey | -0.116639747 | 0.011653509 | -0.124806896 | 0.006924828 |
| LPHN1 | grey | -0.235571855 | 2.61E-07 | -0.242886973 | 1.07E-07 |
| LPHN3 | grey | -0.091072698 | 0.049193313 | -0.106482598 | 0.021364047 |
| LPIN3 | grey | -0.029126729 | 0.530082361 | -0.05688423 | 0.219830907 |
| LPL | grey | -0.173508497 | 0.000164482 | -0.168429786 | 0.000255985 |
| LPO | grey | -0.008403632 | 0.856272965 | -0.02983419 | 0.52013479 |
| LPPR2 | grey | 0.179236222 | 9.84E-05 | 0.180290768 | 8.94E-05 |
| LPPR4 | grey | -0.048025511 | 0.300362475 | -0.015645341 | 0.735956168 |
| LPPR5 | grey | -0.081350029 | 0.079056083 | -0.081819538 | 0.077335614 |

| | | | | | |
|---|---|---|---|---|---|
| LRAT | grey | 0.09092002 | 0.049576355 | 0.098898846 | 0.032619534 |
| LRCOL1 | grey | -0.157657163 | 0.000627879 | -0.172503028 | 0.000179713 |
| LRFN1 | grey | -0.308841717 | 8.87E-12 | -0.325979821 | 5.05E-13 |
| LRFN2 | grey | -0.042331618 | 0.361373214 | -0.011954111 | 0.796679031 |
| LRIT2 | grey | 0.079934158 | 0.084433018 | 0.077824516 | 0.092987792 |
| LRP2 | grey | -0.14401064 | 0.001808299 | -0.140259205 | 0.002382058 |
| LRPAP1 | grey | 0.047227841 | 0.308471051 | 0.039788202 | 0.390966607 |
| LRRC16E | grey | -0.050490346 | 0.276208531 | -0.069867567 | 0.131644795 |
| LRRC24 | grey | 0.014044046 | 0.762121562 | 0.001612359 | 0.972279084 |
| LRRC28 | grey | 0.012740151 | 0.783631026 | 0.012459931 | 0.788275841 |
| LRRC31 | grey | 0.111326002 | 0.016092384 | 0.126867239 | 0.006044124 |
| LRRC37A | grey | -0.136631712 | 0.003090302 | -0.134024913 | 0.003712119 |
| LRRC38 | grey | -0.1756747 | 0.000135685 | -0.180691776 | 8.61E-05 |
| LRRC4 | grey | 0.101403193 | 0.028443152 | 0.100642245 | 0.029660663 |
| LRRC41 | grey | -0.048880649 | 0.291828405 | -0.056933729 | 0.219429213 |
| LRRC4C | grey | -0.173555757 | 0.000163796 | -0.178138591 | 0.000108706 |
| LRRC52 | grey | -0.059859641 | 0.196610358 | -0.082955178 | 0.073299688 |
| LRRC53 | grey | 0.046102623 | 0.320152389 | 0.027950098 | 0.546837554 |
| LRRC55 | grey | -0.04394148 | 0.343385957 | -0.07162982 | 0.122159836 |
| LRRC61 | grey | 0.147976122 | 0.001341769 | 0.149163056 | 0.001225378 |
| LRRC66 | grey | 0.216029241 | 2.46E-06 | 0.193091854 | 2.66E-05 |
| LRRIQ4 | grey | -0.097913892 | 0.034399985 | -0.102572121 | 0.026656532 |
| LRRK1 | grey | -0.196065976 | 1.98E-05 | -0.209256007 | 5.10E-06 |
| LRRN1 | grey | -0.338547704 | 5.49E-14 | -0.363259097 | 5.16E-16 |
| LRRN2 | grey | -0.302432704 | 2.47E-11 | -0.323128761 | 8.24E-13 |
| LRRN4 | grey | -0.21847177 | 1.88E-06 | -0.189691551 | 3.69E-05 |
| LRRTM1 | grey | -0.137422315 | 0.002921366 | -0.150590712 | 0.001097718 |
| LRTM1 | grey | -0.006185904 | 0.893939091 | 0.018824836 | 0.684923449 |
| LRTM2 | grey | 0.157515566 | 0.000635093 | 0.119062963 | 0.010016961 |
| LTB4R | grey | 0.056651533 | 0.221726321 | 0.065214209 | 0.159423762 |
| LTBP3 | grey | -0.245001443 | 8.22E-08 | -0.255001712 | 2.29E-08 |
| LTBR | grey | 0.11343424 | 0.014179394 | 0.103398004 | 0.025453245 |
| LTK | grey | 0.062960335 | 0.174372982 | 0.080871179 | 0.080842615 |
| LUC7L2 | grey | 0.026299401 | 0.570775451 | -0.015728493 | 0.734605451 |
| LURAP1L | grey | 0.144153025 | 0.001789254 | 0.15719036 | 0.000651956 |
| LUZP2 | grey | 0.062613035 | 0.176766375 | 0.025436466 | 0.583484369 |
| LXN | grey | -0.034114712 | 0.462057248 | -0.035167795 | 0.448343046 |
| LY6H | grey | -0.209823703 | 4.80E-06 | -0.223133824 | 1.11E-06 |
| LY6K | grey | -0.193253923 | 2.61E-05 | -0.180407444 | 8.84E-05 |
| LY75-CD | grey | -0.042796461 | 0.356119973 | -0.054222755 | 0.242208743 |
| LYG1 | grey | 0.091409586 | 0.048356873 | 0.108469996 | 0.019042584 |
| LYPD1 | grey | -0.172272287 | 0.00018339 | -0.180702471 | 8.60E-05 |
| LYPD2 | grey | -0.198068888 | 1.62E-05 | -0.164601245 | 0.00035437 |
| LYPD5 | grey | 0.035390655 | 0.44547059 | 0.061491334 | 0.184663112 |
| LYPD6B | grey | -0.272637005 | 2.10E-09 | -0.252443209 | 3.19E-08 |

| | | | | | |
|---|---|---|---|---|---|
| LYPD8 | grey | -0.207341008 | 6.25E-06 | -0.208068733 | 5.79E-06 |
| LYRM1 | grey | 0.067089308 | 0.147740687 | 0.062924423 | 0.174619344 |
| LYZL2 | grey | -0.07080482 | 0.12653175 | -0.029599313 | 0.523426718 |
| LZTR1 | grey | 0.058807289 | 0.204609276 | 0.028112161 | 0.544514344 |
| LZTS1 | grey | -0.011847992 | 0.798445004 | -0.018741073 | 0.686250869 |
| M1AP | grey | 0.021734336 | 0.63944167 | 0.031118214 | 0.502328466 |
| MACROI | grey | -0.323490883 | 7.75E-13 | -0.323666474 | 7.52E-13 |
| MADCAI | grey | -0.019406756 | 0.675728518 | 0.005063633 | 0.913096631 |
| MADD | grey | 0.180660902 | 8.64E-05 | 0.178653173 | 0.000103749 |
| MAEL | grey | 0.056509357 | 0.222890131 | 0.035235283 | 0.447472082 |
| MAFA | grey | -0.125267818 | 0.00671839 | -0.135391792 | 0.00337319 |
| MAFF | grey | 0.084818379 | 0.067051347 | 0.056588975 | 0.222237871 |
| MAG | grey | -0.348126764 | 9.44E-15 | -0.356618193 | 1.88E-15 |
| MAGEA1 | grey | -0.131660105 | 0.004372062 | -0.130948751 | 0.004590361 |
| MAGEA1 | grey | -0.24263846 | 1.10E-07 | -0.241971155 | 1.20E-07 |
| MAGEA1 | grey | -0.214375428 | 2.94E-06 | -0.210133172 | 4.65E-06 |
| MAGEA1 | grey | -0.098884791 | 0.032644375 | -0.103584672 | 0.025187862 |
| MAGEA3 | grey | -0.069916504 | 0.131373939 | -0.073909102 | 0.110691893 |
| MAGEA4 | grey | -0.253543268 | 2.77E-08 | -0.257443782 | 1.66E-08 |
| MAGEA6 | grey | -0.085367556 | 0.065295375 | -0.08986323 | 0.052296573 |
| MAGEA8 | grey | -0.039061313 | 0.399686458 | -0.045570404 | 0.325776705 |
| MAGEA9 | grey | -0.165708677 | 0.000322787 | -0.151970435 | 0.000986102 |
| MAGEA9 | grey | -0.118816626 | 0.010173461 | -0.120586327 | 0.009095769 |
| MAGEB1 | grey | -0.118976506 | 0.010071643 | -0.123906228 | 0.007344684 |
| MAGEB1 | grey | 0.034838879 | 0.452601559 | 0.038856527 | 0.402163982 |
| MAGEB1 | grey | 0.013395759 | 0.772794159 | -0.008521614 | 0.854277491 |
| MAGEB1 | grey | -0.123598072 | 0.007493476 | -0.098842064 | 0.032719995 |
| MAGEB2 | grey | -0.064985779 | 0.160893349 | -0.063326669 | 0.17187465 |
| MAGEB4 | grey | 0.052720359 | 0.255523996 | 0.047728212 | 0.30336791 |
| MAGEB6 | grey | -0.100903983 | 0.029236937 | -0.092667226 | 0.045338821 |
| MAGEC1 | grey | -0.085034609 | 0.066355389 | -0.083314326 | 0.072059661 |
| MAGEC2 | grey | -0.151940741 | 0.00098839 | -0.133651702 | 0.003809875 |
| MAGEC3 | grey | -0.023067242 | 0.619035643 | -0.021160658 | 0.648309973 |
| MAGED1 | grey | -0.120856433 | 0.008940543 | -0.104647992 | 0.023721234 |
| MAGED2 | grey | -0.280446906 | 6.89E-10 | -0.295543496 | 7.23E-11 |
| MAGED4 | grey | -0.160788757 | 0.000486497 | -0.185615392 | 5.45E-05 |
| MAGED4 | grey | -0.119713091 | 0.009614146 | -0.113903673 | 0.013781725 |
| MALSU1 | grey | 0.222891358 | 1.14E-06 | 0.259713877 | 1.23E-08 |
| MAMDC | grey | 0.292666131 | 1.12E-10 | 0.275444809 | 1.41E-09 |
| MAMSTF | grey | -0.275179065 | 1.47E-09 | -0.286647061 | 2.78E-10 |
| MAN2B1 | grey | 0.066555299 | 0.150999428 | 0.080774435 | 0.081207495 |
| MAN2B2 | grey | 0.213545461 | 3.22E-06 | 0.216997852 | 2.21E-06 |
| MAN2C1 | grey | -0.022586299 | 0.626365988 | -0.047418 | 0.306525044 |
| MANBAI | grey | -0.150579185 | 0.001098698 | -0.14322243 | 0.001917128 |
| MANEAI | grey | 0.083394121 | 0.071786489 | 0.064389996 | 0.16477425 |

| | | | | | |
|---|---|---|---|---|---|
| MAOA | grey | 0.007363764 | 0.873898959 | -0.007380265 | 0.873618751 |
| MAP1LC: | grey | -0.051272821 | 0.268824776 | -0.059714883 | 0.197696912 |
| MAP1LC: | grey | -0.163798528 | 0.000379039 | -0.162332936 | 0.000428254 |
| MAP2K7 | grey | 0.029253435 | 0.528293699 | -0.00830267 | 0.857981321 |
| MAP3K1( | grey | -0.093107524 | 0.04432032 | -0.118388618 | 0.010450544 |
| MAP3K1 | grey | -0.00822138 | 0.859357279 | -8.70E-05 | 0.998503822 |
| MAP3K1: | grey | -0.264678862 | 6.31E-09 | -0.275939693 | 1.32E-09 |
| MAP3K6 | grey | 0.081454775 | 0.078669586 | 0.087867 | 0.057774588 |
| MAP3K8 | grey | -0.170821894 | 0.000208166 | -0.175395312 | 0.000139113 |
| MAPK4 | grey | -0.188819798 | 4.02E-05 | -0.20867315 | 5.43E-06 |
| MAPK8IF | grey | 0.003195724 | 0.94508889 | -0.015864632 | 0.732395807 |
| MAPK8IF | grey | 0.031670689 | 0.494767056 | 0.043028053 | 0.353520724 |
| MAPKAF | grey | 0.096747621 | 0.036614591 | 0.110551066 | 0.016850392 |
| MAPT | grey | -0.057704489 | 0.213242059 | -0.080553224 | 0.082046819 |
| MARC2 | grey | 0.241084709 | 1.34E-07 | 0.220673491 | 1.47E-06 |
| 11-Mar | grey | -0.213079592 | 3.39E-06 | -0.219077891 | 1.75E-06 |
| 3-Mar | grey | 0.134852323 | 0.003503451 | 0.164421447 | 0.000359762 |
| MAST3 | grey | -0.049066079 | 0.289999501 | -0.041648655 | 0.369178786 |
| MAT1A | grey | 0.007771981 | 0.866971647 | 0.002372293 | 0.959223226 |
| MATN1 | grey | 0.08467716 | 0.067509098 | 0.063773082 | 0.168866406 |
| MATN3 | grey | -0.092264447 | 0.046287688 | -0.118256547 | 0.010537385 |
| MATN4 | grey | -0.160581765 | 0.000494841 | -0.157429983 | 0.000639491 |
| MAVS | grey | -0.139887451 | 0.002447139 | -0.137009181 | 0.003008567 |
| MBD6 | grey | 0.210367682 | 4.53E-06 | 0.197167817 | 1.77E-05 |
| MC4R | grey | -0.153745857 | 0.000857879 | -0.154107341 | 0.000833739 |
| MC5R | grey | 0.01759062 | 0.704578631 | -0.017461851 | 0.70664096 |
| MCCD1 | grey | -0.150370845 | 0.001116545 | -0.138700659 | 0.002665909 |
| MCOLN3 | grey | -0.374121753 | 5.81E-17 | -0.38150487 | 1.26E-17 |
| MCTP2 | grey | 0.025916979 | 0.576391374 | 0.019428954 | 0.675378712 |
| MDFI | grey | -0.083210777 | 0.072415414 | -0.089098631 | 0.05434132 |
| MDP1 | grey | -0.055435515 | 0.231821186 | -0.07438859 | 0.108390877 |
| ME1 | grey | -0.233437862 | 3.36E-07 | -0.232710851 | 3.67E-07 |
| ME3 | grey | -0.15309097 | 0.000903266 | -0.161510843 | 0.000458401 |
| MECR | grey | 0.035031847 | 0.450100424 | 0.022706086 | 0.624536764 |
| MED10 | grey | -0.193241969 | 2.62E-05 | -0.173503927 | 0.000164548 |
| MED12L | grey | -0.054406564 | 0.240613736 | -0.083786479 | 0.070455572 |
| MED18 | grey | 0.242319537 | 1.15E-07 | 0.267725258 | 4.16E-09 |
| MED29 | grey | -0.116149197 | 0.01201222 | -0.107512269 | 0.020132323 |
| MED9 | grey | 0.157570471 | 0.000632287 | 0.151965804 | 0.000986459 |
| MEF2B | grey | -0.057278243 | 0.216647986 | -0.040217694 | 0.385868927 |
| MEF2BN | grey | -0.133791846 | 0.003772897 | -0.145432735 | 0.001626152 |
| MEGF10 | grey | -0.121742686 | 0.008447655 | -0.120444935 | 0.009177978 |
| MEGF11 | grey | -0.008612479 | 0.852741285 | 0.012621088 | 0.785603627 |
| MEGF6 | grey | -0.19413084 | 2.40E-05 | -0.191376529 | 3.14E-05 |
| MEGF8 | grey | -0.076149722 | 0.100261662 | -0.096355246 | 0.037386381 |

| | | | | | |
|---|---|---|---|---|---|
| MEI4 | grey | -0.16706401 | 0.000287708 | -0.163164897 | 0.000399632 |
| MEPCE | grey | -0.186907328 | 4.82E-05 | -0.193269693 | 2.61E-05 |
| MESDC1 | grey | 0.088868912 | 0.054968486 | 0.090680407 | 0.050182526 |
| MESP2 | grey | 0.103260768 | 0.025649889 | 0.144949028 | 0.001686127 |
| MET | grey | -0.064015876 | 0.167246915 | -0.066832118 | 0.149303412 |
| METRN | grey | 0.02277812 | 0.623437863 | 0.044030984 | 0.342402926 |
| METRNL | grey | -0.354699168 | 2.72E-15 | -0.34170411 | 3.10E-14 |
| METTL1( | grey | 0.068687792 | 0.138306073 | 0.065454049 | 0.157891688 |
| METTL2 | grey | -0.133384382 | 0.003881322 | -0.114778797 | 0.013066526 |
| METTL7I | grey | -0.113132159 | 0.0144406 | -0.113283323 | 0.014309366 |
| MFAP2 | grey | -0.160434795 | 0.000500846 | -0.178640693 | 0.000103867 |
| MFGE8 | grey | -0.090659069 | 0.050236806 | -0.096740896 | 0.036627704 |
| MFI2 | grey | -0.199367725 | 1.42E-05 | -0.185753535 | 5.38E-05 |
| MFRP | grey | -0.031880588 | 0.491910274 | -0.018599201 | 0.688501375 |
| MFSD2A | grey | -0.040733931 | 0.379795487 | -0.060872776 | 0.189127548 |
| MFSD4 | grey | 0.138919021 | 0.002624366 | 0.180239787 | 8.98E-05 |
| MFSD6L | grey | -0.211655065 | 3.95E-06 | -0.22039044 | 1.52E-06 |
| MFSD7 | grey | -0.084113605 | 0.069361411 | -0.06531701 | 0.158765712 |
| MGAT1 | grey | -0.064765111 | 0.162322665 | -0.038154937 | 0.410721264 |
| MGAT3 | grey | -0.262107584 | 8.93E-09 | -0.252582657 | 3.14E-08 |
| MGAT5B | grey | -0.005260144 | 0.909738151 | -0.061556564 | 0.184196883 |
| MGST1 | grey | 0.081862905 | 0.077178245 | 0.096994234 | 0.03613647 |
| MGST3 | grey | 0.206587193 | 6.76E-06 | 0.201356386 | 1.16E-05 |
| MIA | grey | 0.096731567 | 0.036645902 | 0.119804107 | 0.009558917 |
| MIA-RAE | grey | 0.048241485 | 0.298191621 | 0.052799499 | 0.254810196 |
| MICA | grey | 0.0215447 | 0.642367592 | 0.021627157 | 0.641094673 |
| MICALL1 | grey | 0.094553397 | 0.041110091 | 0.110168114 | 0.017236381 |
| MIDN | grey | 0.174487448 | 0.000150823 | 0.18152496 | 7.98E-05 |
| MIEF2 | grey | 0.0265374 | 0.56729358 | 0.047931285 | 0.301312869 |
| MIF4GD | grey | 0.109241252 | 0.018202745 | 0.097858732 | 0.034502096 |
| MINOS1- | grey | -0.007594891 | 0.869975594 | -0.025775659 | 0.578473234 |
| MIOX | grey | -0.00352908 | 0.939371424 | 0.004112725 | 0.929368871 |
| MIXL1 | grey | -0.202552499 | 1.03E-05 | -0.192873632 | 2.71E-05 |
| MKNK1 | grey | -0.081130115 | 0.079872543 | -0.084454274 | 0.06823678 |
| MKNK2 | grey | 0.440523278 | 1.37E-23 | 0.460849188 | 6.23E-26 |
| MKRN2C | grey | 0.233806745 | 3.22E-07 | 0.282738217 | 4.94E-10 |
| MKRN3 | grey | -0.268784825 | 3.59E-09 | -0.264939454 | 6.09E-09 |
| MLC1 | grey | -0.137907156 | 0.002821966 | -0.141350762 | 0.002199985 |
| MLEC | grey | 0.250660139 | 4.02E-08 | 0.237196877 | 2.14E-07 |
| MLF1 | grey | -0.433549739 | 8.00E-23 | -0.438698443 | 2.18E-23 |
| MLH1 | grey | -0.44292025 | 7.37E-24 | -0.44238807 | 8.46E-24 |
| MLLT1 | grey | 0.051179519 | 0.269698051 | 0.037424435 | 0.419744758 |
| MLLT3 | grey | 0.093129409 | 0.044270201 | 0.091661836 | 0.047738402 |
| MLLT6 | grey | -0.038515546 | 0.406309535 | -0.072124777 | 0.119593953 |
| MLNR | grey | 0.035514124 | 0.443883708 | 0.02820207 | 0.543227593 |

| | | | | | |
|---|---|---|---|---|---|
| MLXIP | grey | -0.000988004 | 0.983011346 | -0.026977189 | 0.56088636 |
| MLXIPL | grey | -0.17038092 | 0.000216299 | -0.17288725 | 0.000173743 |
| MMEL1 | grey | 0.01932788 | 0.676972055 | 0.029209545 | 0.528912931 |
| MMP10 | grey | 0.059909212 | 0.196239276 | 0.056517707 | 0.222821664 |
| MMP13 | grey | -0.063601465 | 0.170018191 | -0.030978803 | 0.504246086 |
| MMP17 | grey | 0.032784028 | 0.479715536 | 0.035812935 | 0.440056574 |
| MMP20 | grey | -0.126387775 | 0.006239576 | -0.150768592 | 0.001082701 |
| MMP23B | grey | -0.086255487 | 0.062536339 | -0.091750265 | 0.047523164 |
| MMP27 | grey | -0.120552108 | 0.009115605 | -0.122084329 | 0.008264216 |
| MMP3 | grey | -0.018904474 | 0.683662304 | 0.002918741 | 0.949841665 |
| MMP7 | grey | -0.105281453 | 0.022883088 | -0.110709783 | 0.016692646 |
| MMP8 | grey | -0.084030353 | 0.069638543 | -0.090632702 | 0.050303946 |
| MNX1 | grey | 0.477481417 | 5.73E-28 | 0.483637223 | 9.47E-29 |
| MOCOS | grey | 0.170730626 | 0.000209825 | 0.177684585 | 0.000113264 |
| MOCS1 | grey | -0.028509223 | 0.538843125 | -0.072093295 | 0.11975589 |
| MOGAT1 | grey | 0.046802091 | 0.312857462 | 0.04602656 | 0.320952304 |
| MOGAT2 | grey | -0.024881211 | 0.591730795 | 0.004092278 | 0.92971912 |
| MON1B | grey | 0.387757304 | 3.33E-18 | 0.41428321 | 8.58E-21 |
| MORC4 | grey | 0.057140512 | 0.21775683 | 0.027306303 | 0.556114506 |
| MOS | grey | -0.064479173 | 0.164188929 | -0.06064468 | 0.19079369 |
| MOV10L | grey | -0.052118245 | 0.261000111 | -0.066958495 | 0.148533966 |
| MPC1 | grey | -0.020366238 | 0.66067302 | 0.020419712 | 0.659837883 |
| MPI | grey | 0.27476625 | 1.56E-09 | 0.26573754 | 5.46E-09 |
| MPO | grey | -0.108747033 | 0.018737104 | -0.095657239 | 0.038793463 |
| MPP2 | grey | -0.166880336 | 0.000292244 | -0.203119271 | 9.69E-06 |
| MPP3 | grey | -0.325125552 | 5.85E-13 | -0.33603416 | 8.63E-14 |
| MPPED1 | grey | -0.163065064 | 0.00040297 | -0.162651837 | 0.000417064 |
| MPV17 | grey | -0.000160039 | 0.997247934 | 0.019845498 | 0.668827596 |
| MPV17L | grey | -0.403186349 | 1.11E-19 | -0.390627449 | 1.79E-18 |
| MPZ | grey | -0.270453861 | 2.85E-09 | -0.259780295 | 1.22E-08 |
| MRFAP1l | grey | -0.076492361 | 0.098737862 | -0.082614572 | 0.074491718 |
| MRGPRC | grey | -0.028319259 | 0.541552689 | -0.029804257 | 0.520553726 |
| MRI1 | grey | 0.021954305 | 0.636054721 | 0.005052448 | 0.913287821 |
| MRM1 | grey | 0.067876348 | 0.143035886 | 0.067021782 | 0.148149776 |
| MRO | grey | -0.023069809 | 0.618996613 | -0.040973295 | 0.376999408 |
| MROH2A | grey | -0.024634873 | 0.595406312 | -0.040824875 | 0.378731653 |
| MROH6 | grey | -0.134065527 | 0.003701619 | -0.105863412 | 0.022135808 |
| MROH7-' | grey | -0.154489855 | 0.000808879 | -0.182824831 | 7.07E-05 |
| MROH8 | grey | -0.184378444 | 6.12E-05 | -0.186642737 | 4.94E-05 |
| MRPL38 | grey | -0.05917422 | 0.201793869 | -0.086348578 | 0.062252732 |
| MS4A15 | grey | -0.279326899 | 8.11E-10 | -0.286471994 | 2.85E-10 |
| MS4A6E | grey | -0.066896987 | 0.148908081 | -0.046128829 | 0.319877101 |
| MSGN1 | grey | 0.128215637 | 0.005523585 | 0.126975943 | 0.006000583 |
| MSH4 | grey | 0.114446419 | 0.013334216 | 0.097294349 | 0.035561785 |
| MSLN | grey | -0.183447207 | 6.67E-05 | -0.154383805 | 0.000815701 |

| | | | | | |
|---|---|---|---|---|---|
| MSLNL | grey | -0.188851932 | 4.00E-05 | -0.184563311 | 6.01E-05 |
| MSRA | grey | 0.22123881 | 1.38E-06 | 0.219601927 | 1.66E-06 |
| MST1 | grey | -0.024122833 | 0.603079293 | -0.046790251 | 0.312980034 |
| MST1R | grey | 0.32779818 | 3.69E-13 | 0.342916957 | 2.48E-14 |
| MSTN | grey | 0.023202052 | 0.616987644 | 0.016452604 | 0.722878088 |
| MT1A | grey | -0.009465079 | 0.83835468 | -0.028709154 | 0.535998731 |
| MT1B | grey | 0.089635371 | 0.052899112 | 0.117213231 | 0.011246189 |
| MT1E | grey | -0.116428979 | 0.011806454 | -0.098097062 | 0.034062749 |
| MT1G | grey | 0.061261091 | 0.186315716 | 0.082979369 | 0.07321562 |
| MT1H | grey | -0.07851403 | 0.090118594 | -0.076805858 | 0.09735986 |
| MT1HL1 | grey | 0.019523748 | 0.673885678 | 0.04477635 | 0.33428631 |
| MT1M | grey | -0.065927597 | 0.154899387 | -0.066511404 | 0.151269698 |
| MT3 | grey | 0.134011427 | 0.003715612 | 0.123033842 | 0.007772911 |
| MTA1 | grey | 0.009785192 | 0.832966768 | -0.004474925 | 0.923166947 |
| MTCL1 | grey | -0.066589808 | 0.150787199 | -0.115835231 | 0.012246893 |
| MTFR1L | grey | 0.045774108 | 0.323616505 | 0.043703402 | 0.346009507 |
| MTHFR | grey | -0.14527109 | 0.001645973 | -0.157694886 | 0.000625969 |
| MTRNR2 | grey | -0.04670379 | 0.313876025 | -0.039820752 | 0.390578842 |
| MTRNR2 | grey | -0.033651367 | 0.468164492 | -0.028858619 | 0.533877243 |
| MTRNR2 | grey | 0.033869307 | 0.465286343 | 0.03907647 | 0.399503452 |
| MTRNR2 | grey | -0.042237261 | 0.362445436 | -0.030402935 | 0.512207842 |
| MTRNR2 | grey | 0.041737055 | 0.368162624 | 0.032812665 | 0.479331699 |
| MTRNR2 | grey | 0.052720538 | 0.255522382 | 0.061459556 | 0.18489056 |
| MTRNR2 | grey | 0.060614533 | 0.191014703 | 0.039420284 | 0.395365705 |
| MTRNR2 | grey | -0.067692672 | 0.144123487 | -0.081658264 | 0.077923126 |
| MTSS1 | grey | -0.074447293 | 0.108111781 | -0.106251859 | 0.021648869 |
| MTSS1L | grey | 0.135352552 | 0.003382515 | 0.126971518 | 0.006002349 |
| MUC12 | grey | -0.082635269 | 0.074418836 | -0.063634533 | 0.169795804 |
| MUC15 | grey | -0.113075755 | 0.014489837 | -0.127616339 | 0.005749745 |
| MUC16 | grey | -0.027734399 | 0.549937205 | 0.002757265 | 0.952613268 |
| MUC21 | grey | -0.026757995 | 0.564075399 | 0.003161822 | 0.945670523 |
| MUC3A | grey | -0.133003099 | 0.003985328 | -0.11970406 | 0.009619641 |
| MUC6 | grey | 0.204056979 | 8.80E-06 | 0.216792541 | 2.26E-06 |
| MUC7 | grey | 0.107749527 | 0.019857426 | 0.092436152 | 0.045881167 |
| MUS81 | grey | 0.043531622 | 0.347910366 | 0.020173057 | 0.663693526 |
| MXD4 | grey | 0.127796222 | 0.005681003 | 0.153130272 | 0.000900481 |
| MXRA5 | grey | -0.108187833 | 0.019358169 | -0.092280094 | 0.046250518 |
| MYADM | grey | 0.119104766 | 0.009990617 | 0.091639549 | 0.047792775 |
| MYBPC1 | grey | -0.048738812 | 0.293232552 | -0.043155192 | 0.352098896 |
| MYBPC2 | grey | 0.012512116 | 0.787410259 | 0.016348986 | 0.724552342 |
| MYBPC3 | grey | 0.014962184 | 0.747083738 | 0.040954239 | 0.377221548 |
| MYBPH | grey | -0.109707049 | 0.01771134 | -0.09975181 | 0.031141821 |
| MYBPHL | grey | -0.149410777 | 0.00120229 | -0.162827254 | 0.000411026 |
| MYCN | grey | 0.166203811 | 0.000309533 | 0.158494722 | 0.000586733 |
| MYEOV | grey | 0.208076395 | 5.78E-06 | 0.214669006 | 2.85E-06 |

| | | | | | |
|---|---|---|---|---|---|
| MYF6 | grey | -0.022014286 | 0.635132481 | 0.004039391 | 0.930625099 |
| MYH15 | grey | -0.026753172 | 0.564145665 | -0.056535267 | 0.222677716 |
| MYH3 | grey | -0.192193874 | 2.90E-05 | -0.204101774 | 8.76E-06 |
| MYH6 | grey | -0.121918491 | 0.008352811 | -0.11055076 | 0.016850697 |
| MYH7 | grey | -0.064270606 | 0.165560334 | -0.061288439 | 0.186118856 |
| MYH8 | grey | -0.090903558 | 0.049617803 | -0.083984328 | 0.069792139 |
| MYL1 | grey | -0.051667729 | 0.265150021 | -0.052969636 | 0.253280331 |
| MYL10 | grey | 0.019169348 | 0.679474074 | 0.032036779 | 0.489790193 |
| MYL12A | grey | 0.067141223 | 0.14742676 | 0.08143344 | 0.078748184 |
| MYL12B | grey | -0.03593042 | 0.438557023 | -0.013780528 | 0.76645449 |
| MYL2 | grey | 0.057015589 | 0.218766067 | 0.058405573 | 0.207724111 |
| MYL4 | grey | 0.002738181 | 0.952940864 | -0.00785737 | 0.865523898 |
| MYLK2 | grey | -0.166546637 | 0.000300656 | -0.173169181 | 0.000169481 |
| MYLK4 | grey | -0.208514067 | 5.52E-06 | -0.200642812 | 1.25E-05 |
| MYO15A | grey | -0.175864376 | 0.000133403 | -0.173738635 | 0.00016117 |
| MYO18B | grey | -0.003777076 | 0.935119997 | -0.002837734 | 0.951232004 |
| MYO1A | grey | 0.039670257 | 0.392373607 | 0.043267165 | 0.350849656 |
| MYO1C | grey | 0.228957565 | 5.70E-07 | 0.233084413 | 3.51E-07 |
| MYO3A | grey | -0.219581037 | 1.66E-06 | -0.182279962 | 7.44E-05 |
| MYO3B | grey | 0.10024164 | 0.03031939 | 0.079407014 | 0.086508744 |
| MYO7B | grey | -0.178571906 | 0.000104517 | -0.192091979 | 2.93E-05 |
| MYOC | grey | -0.185776218 | 5.37E-05 | -0.17284073 | 0.000174455 |
| MYOD1 | grey | -0.205803638 | 7.34E-06 | -0.210807048 | 4.33E-06 |
| MYOG | grey | -0.068115376 | 0.141629942 | -0.065294764 | 0.158907942 |
| MYOM3 | grey | -0.185316862 | 5.60E-05 | -0.181715714 | 7.84E-05 |
| MYOT | grey | -0.170963878 | 0.000205609 | -0.184337509 | 6.14E-05 |
| MYOZ1 | grey | -0.310326705 | 6.97E-12 | -0.297510104 | 5.34E-11 |
| MYPOP | grey | 0.066876676 | 0.149031777 | 0.069697039 | 0.132592015 |
| MYRF | grey | -0.152394319 | 0.000953975 | -0.147547888 | 0.001386199 |
| MYRFL | grey | 0.00152634 | 0.973757438 | -0.002240729 | 0.961482843 |
| MYT1 | grey | -0.308497109 | 9.38E-12 | -0.335128281 | 1.01E-13 |
| MYT1L | grey | -0.07713171 | 0.095943839 | -0.05667452 | 0.221538561 |
| NAB2 | grey | -0.25970465 | 1.23E-08 | -0.289299897 | 1.87E-10 |
| NACA2 | grey | 0.09843192 | 0.033453518 | 0.108772226 | 0.018709538 |
| NAGA | grey | 0.277329765 | 1.08E-09 | 0.266088027 | 5.21E-09 |
| NAGS | grey | 0.062188067 | 0.179728092 | 0.045721472 | 0.3241738 |
| NAIF1 | grey | 0.116473416 | 0.011774061 | 0.122600974 | 0.007993559 |
| NALCN | grey | 0.071055734 | 0.125189423 | 0.049136817 | 0.289303835 |
| NANOS1 | grey | -0.069612319 | 0.133064557 | -0.065715107 | 0.156236737 |
| NANOS3 | grey | 0.053970974 | 0.244405586 | 0.069242456 | 0.135142762 |
| NAP1L6 | grey | -0.286396707 | 2.88E-10 | -0.288256059 | 2.18E-10 |
| NAPSA | grey | -0.102346473 | 0.026993692 | -0.105913195 | 0.022072877 |
| NAT1 | grey | 0.230225581 | 4.91E-07 | 0.243684456 | 9.68E-08 |
| NAT6 | grey | -0.006494109 | 0.888688225 | -0.025472289 | 0.582954186 |
| NBPF4 | grey | -0.214591806 | 2.87E-06 | -0.227138224 | 7.04E-07 |

| | | | | | |
|---|---|---|---|---|---|
| NBPF6 | grey | -0.232552998 | 3.74E-07 | -0.246897436 | 6.48E-08 |
| NCALD | grey | 0.129609419 | 0.005028251 | 0.112738851 | 0.014787021 |
| NCAM2 | grey | -0.054178547 | 0.242593464 | -0.061449892 | 0.184959768 |
| NCAN | grey | -0.062899223 | 0.174792373 | -0.060710176 | 0.190314177 |
| NCDN | grey | 0.044544058 | 0.336802468 | 0.048523673 | 0.295370991 |
| NCEH1 | grey | -0.029218895 | 0.528780975 | -0.04186492 | 0.366695867 |
| NCKIPSD | grey | 0.18611302 | 5.20E-05 | 0.182503427 | 7.29E-05 |
| NCR2 | grey | -0.021003188 | 0.650753058 | -0.006354607 | 0.891064325 |
| NDRG3 | grey | -0.087569828 | 0.058629361 | -0.114200219 | 0.013535603 |
| NDST4 | grey | 0.089742213 | 0.05261587 | 0.060691355 | 0.190451875 |
| NDUFA1( | grey | 0.050548856 | 0.275651686 | 0.046721962 | 0.313687572 |
| NDUFA4 | grey | 0.025283696 | 0.585747924 | 0.036768878 | 0.42794051 |
| NEBL | grey | -0.136107393 | 0.003207185 | -0.142162746 | 0.002072892 |
| NECAB2 | grey | -0.104021095 | 0.0245767 | -0.107925125 | 0.01965608 |
| NEDD4L | grey | 0.11968057 | 0.009633949 | 0.127017775 | 0.005983902 |
| NEDD8-M | grey | 0.180081705 | 9.11E-05 | 0.154708897 | 0.000794954 |
| NEFH | grey | -0.318438269 | 1.82E-12 | -0.33616201 | 8.44E-14 |
| NEFL | grey | -0.165462598 | 0.000329569 | -0.151487466 | 0.001023923 |
| NEK8 | grey | 0.09245404 | 0.04583899 | 0.094740404 | 0.040709593 |
| NELL1 | grey | -0.218331873 | 1.91E-06 | -0.223893035 | 1.02E-06 |
| NELL2 | grey | -0.098929892 | 0.032564716 | -0.09291769 | 0.044757059 |
| NETO1 | grey | -0.063907956 | 0.167965324 | -0.066046393 | 0.154155508 |
| NEU4 | grey | -0.118409115 | 0.010437124 | -0.113515767 | 0.014109614 |
| NEUROD | grey | 0.000742307 | 0.987235671 | -0.021926005 | 0.636490052 |
| NEUROD | grey | -0.029049276 | 0.531177241 | -0.038256112 | 0.409480626 |
| NEUROG | grey | 0.054573785 | 0.239169093 | 0.054633478 | 0.238654872 |
| NEUROG | grey | 0.144179085 | 0.001785788 | 0.13613346 | 0.00320128 |
| NEUROG | grey | 0.05349313 | 0.248613147 | 0.051499471 | 0.266711475 |
| NF2 | grey | 0.123908122 | 0.007343778 | 0.127055812 | 0.005968771 |
| NFATC1 | grey | -0.137806871 | 0.00284227 | -0.11182694 | 0.015618439 |
| NFE2 | grey | -0.10090353 | 0.029237665 | -0.116728701 | 0.011589486 |
| NFE2L3 | grey | 0.017451034 | 0.706814294 | -0.003494272 | 0.939968289 |
| NFIB | grey | 0.066379299 | 0.152085327 | 0.074645877 | 0.10717183 |
| NFIX | grey | -0.06167617 | 0.183344261 | -0.071991528 | 0.120280546 |
| NFKBIA | grey | -0.158122992 | 0.000604676 | -0.153601077 | 0.000867728 |
| NFKBID | grey | -0.211479835 | 4.02E-06 | -0.228176801 | 6.24E-07 |
| NFKBIZ | grey | 0.101834238 | 0.027772712 | 0.077947026 | 0.092472735 |
| NFS1 | grey | -0.017624023 | 0.704044012 | -0.027222669 | 0.557325252 |
| NGEF | grey | 0.106780597 | 0.021001017 | 0.098879532 | 0.032653675 |
| NGFRAP | grey | -0.291359367 | 1.37E-10 | -0.277718642 | 1.02E-09 |
| NHLH1 | grey | -0.016341058 | 0.72468049 | -0.025560321 | 0.581652241 |
| NHLH2 | grey | -0.052179611 | 0.260438324 | -0.038652675 | 0.404639316 |
| NHLRC1 | grey | -0.417205887 | 4.30E-21 | -0.397591996 | 3.88E-19 |
| NIM1K | grey | 0.034897259 | 0.451844057 | 0.015175036 | 0.743610953 |
| NINJ2 | grey | -0.068564147 | 0.139018924 | -0.036429522 | 0.432219344 |

| | | | | | |
|---|---|---|---|---|---|
| NIPA1 | grey | 0.398697444 | 3.04E-19 | 0.386800525 | 4.08E-18 |
| NIPAL4 | grey | -0.151089255 | 0.001056109 | -0.14027026 | 0.002380147 |
| NIT1 | grey | 0.15126528 | 0.001041768 | 0.137271207 | 0.00295299 |
| NKAIN1 | grey | -0.047783657 | 0.302805899 | -0.074338923 | 0.108627458 |
| NKAIN2 | grey | -0.113331726 | 0.014267567 | -0.115806445 | 0.012268611 |
| NKAIN3 | grey | -0.049151389 | 0.289160668 | -0.052268846 | 0.259622891 |
| NKAIN4 | grey | -0.289320864 | 1.86E-10 | -0.280036453 | 7.32E-10 |
| NKPD1 | grey | -0.013937679 | 0.763869635 | -0.04673387 | 0.313564121 |
| NKX1-1 | grey | -0.042909267 | 0.354852413 | -0.03431703 | 0.459404504 |
| NKX1-2 | grey | 0.1065759 | 0.021249803 | 0.081897838 | 0.077051671 |
| NKX2-1 | grey | 0.004569612 | 0.921546376 | 0.029541291 | 0.524241564 |
| NKX2-2 | grey | 0.260141972 | 1.16E-08 | 0.240366905 | 1.46E-07 |
| NKX2-3 | grey | 0.018707749 | 0.68677924 | 0.017314795 | 0.708998786 |
| NKX2-4 | grey | -0.100642112 | 0.02966088 | -0.102224314 | 0.027177746 |
| NKX2-5 | grey | -0.08699409 | 0.060315182 | -0.101565088 | 0.028189731 |
| NKX2-8 | grey | -0.09306603 | 0.044415475 | -0.07582322 | 0.101731031 |
| NKX3-1 | grey | 0.149537914 | 0.001190597 | 0.132716274 | 0.004065222 |
| NKX3-2 | grey | -0.049236603 | 0.288324418 | -0.041209615 | 0.374251332 |
| NKX6-1 | grey | -0.096603058 | 0.036897353 | -0.081152356 | 0.079789662 |
| NKX6-2 | grey | -0.154341046 | 0.000818467 | -0.162748172 | 0.000413738 |
| NKX6-3 | grey | 0.122134544 | 0.008237556 | 0.102817445 | 0.026294092 |
| NLE1 | grey | 0.163171404 | 0.000399415 | 0.153320686 | 0.000887099 |
| NLGN4X | grey | -0.070959824 | 0.125701205 | -0.048968569 | 0.290960281 |
| NLGN4Y | grey | -0.017451489 | 0.706807011 | 0.000517321 | 0.991104216 |
| NLRP11 | grey | -0.040087135 | 0.387414243 | -0.050059641 | 0.280331132 |
| NLRP2 | grey | 0.002846074 | 0.951088852 | -0.014727227 | 0.750923136 |
| NLRP4 | grey | -0.065654884 | 0.156617345 | -0.084553081 | 0.067913402 |
| NLRP7 | grey | -0.19329436 | 2.60E-05 | -0.185755463 | 5.38E-05 |
| NLRX1 | grey | 0.265267547 | 5.82E-09 | 0.280981046 | 6.38E-10 |
| NMB | grey | 0.062210109 | 0.179573577 | 0.071812235 | 0.121209239 |
| NME2 | grey | 0.075401264 | 0.103655229 | 0.064025844 | 0.167180671 |
| NME6 | grey | -0.064786669 | 0.162182611 | -0.065957492 | 0.154711934 |
| NMNAT2 | grey | 0.016959076 | 0.714713686 | 0.021159224 | 0.648332208 |
| NMNAT3 | grey | -0.307971437 | 1.02E-11 | -0.3218653 | 1.02E-12 |
| NMRK1 | grey | 0.318952627 | 1.67E-12 | 0.310185779 | 7.13E-12 |
| NMUR2 | grey | 0.035687965 | 0.441654876 | 0.067868835 | 0.143080249 |
| NNMT | grey | -0.029881512 | 0.519472835 | -0.019131385 | 0.680073752 |
| NOG | grey | 0.233441241 | 3.36E-07 | 0.220424744 | 1.51E-06 |
| NOL3 | grey | 0.30674058 | 1.24E-11 | 0.295121716 | 7.72E-11 |
| NOL4 | grey | -0.049824645 | 0.282597937 | -0.06326192 | 0.172314276 |
| NOL4L | grey | -0.392657456 | 1.15E-18 | -0.397117861 | 4.31E-19 |
| NOS1 | grey | -0.211807387 | 3.89E-06 | -0.23303116 | 3.53E-07 |
| NOS2 | grey | -0.017831061 | 0.70073361 | 0.001847551 | 0.968237476 |
| NOV | grey | -0.085279545 | 0.065574215 | -0.075198904 | 0.104588198 |
| NOX4 | grey | -0.156497995 | 0.000689247 | -0.131324736 | 0.004473783 |

| | | | | | |
|---|---|---|---|---|---|
| NOXO1 | grey | 0.023836234 | 0.607393285 | 0.063344984 | 0.171750449 |
| NPAS1 | grey | 0.025784521 | 0.578342579 | 0.03971582 | 0.39182971 |
| NPB | grey | -0.009876402 | 0.831432998 | -0.031322491 | 0.499525569 |
| NPBWR1 | grey | 0.003276317 | 0.943706354 | -0.007263532 | 0.875601359 |
| NPC1L1 | grey | -0.043777492 | 0.345191695 | -0.033099855 | 0.475491565 |
| NPFFR1 | grey | -0.243526117 | 9.88E-08 | -0.255198269 | 2.23E-08 |
| NPIPA1 | grey | -0.106546464 | 0.021285788 | -0.116603263 | 0.011679857 |
| NPIPA2 | grey | 0.025970801 | 0.575599419 | 0.033711238 | 0.467372854 |
| NPIPA3 | grey | -0.010290979 | 0.824469728 | -0.003608216 | 0.938014594 |
| NPIPA5 | grey | -0.03776278 | 0.41555101 | -0.040785399 | 0.379193213 |
| NPIPA7 | grey | -0.081826345 | 0.077310893 | -0.087025016 | 0.060223618 |
| NPIPA8 | grey | -0.018066512 | 0.696975761 | 0.000500894 | 0.991386683 |
| NPIPB11 | grey | 0.020626666 | 0.656609755 | 0.035200271 | 0.447923818 |
| NPIPB15 | grey | -0.036838512 | 0.427065558 | -0.039838499 | 0.390367521 |
| NPIPB6 | grey | 0.040724262 | 0.379908707 | 0.021172225 | 0.648130665 |
| NPIPB7 | grey | 0.012830683 | 0.782132026 | 0.000131226 | 0.99774342 |
| NPIPB9 | grey | -0.120157087 | 0.009347385 | -0.112539304 | 0.014965555 |
| NPM2 | grey | 0.120940146 | 0.008892915 | 0.115360529 | 0.012609399 |
| NPNT | grey | -0.01958688 | 0.672892036 | -0.033584521 | 0.469049244 |
| NPPA | grey | -0.085277278 | 0.065581411 | -0.092730262 | 0.045191812 |
| NPPB | grey | 0.007391375 | 0.873430097 | -0.010585576 | 0.819529963 |
| NPPC | grey | 0.039473204 | 0.394731124 | 0.030563699 | 0.509978618 |
| NPS | grey | 0.074724653 | 0.106800755 | 0.069542516 | 0.133454867 |
| NPSR1 | grey | 0.02381906 | 0.607652228 | 0.036927917 | 0.425943722 |
| NPTX1 | grey | 0.001465457 | 0.974803849 | 0.007798905 | 0.866515112 |
| NPTX2 | grey | -0.247759356 | 5.81E-08 | -0.236165884 | 2.43E-07 |
| NPTXR | grey | -0.40169018 | 1.55E-19 | -0.397472509 | 3.99E-19 |
| NPVF | grey | -0.097917603 | 0.034393125 | -0.095552181 | 0.03900908 |
| NPW | grey | -0.207121069 | 6.39E-06 | -0.185139791 | 5.70E-05 |
| NPY | grey | -0.259721924 | 1.23E-08 | -0.264732104 | 6.26E-09 |
| NQO2 | grey | 0.051230381 | 0.269221755 | 0.08480418 | 0.067097254 |
| NR0B1 | grey | -0.151758357 | 0.001002548 | -0.147052145 | 0.00143932 |
| NR0B2 | grey | -0.059745898 | 0.197463745 | -0.050510275 | 0.276018776 |
| NR1H4 | grey | -0.18973439 | 3.68E-05 | -0.190821826 | 3.31E-05 |
| NR2E1 | grey | 0.030967103 | 0.504407206 | 0.020040019 | 0.665776842 |
| NR5A2 | grey | 0.018876068 | 0.684112041 | 0.012485791 | 0.787846874 |
| NRAP | grey | -0.058758914 | 0.204982562 | -0.062353346 | 0.17857188 |
| NRARP | grey | -0.079857036 | 0.084734166 | -0.086647136 | 0.0613503 |
| NRBP1 | grey | -0.066759192 | 0.149748807 | -0.061708262 | 0.183115987 |
| NRBP2 | grey | -0.16689426 | 0.000291897 | -0.205441068 | 7.62E-06 |
| NRCAM | grey | 0.010709685 | 0.81745105 | -0.003974795 | 0.931731777 |
| NRG1 | grey | -0.14544068 | 0.001625183 | -0.138682304 | 0.002669428 |
| NRG2 | grey | -0.131398075 | 0.004451358 | -0.153310581 | 0.000887804 |
| NRG3 | grey | -0.263955632 | 6.96E-09 | -0.266137679 | 5.17E-09 |
| NRGN | grey | 0.031872552 | 0.492019472 | 0.036537898 | 0.430850177 |

| | | | | | |
|---|---|---|---|---|---|
| NRIP3 | grey | -0.387576648 | 3.46E-18 | -0.365546823 | 3.28E-16 |
| NRL | grey | -0.099789633 | 0.031077644 | -0.070431731 | 0.128548306 |
| NRN1 | grey | 0.218151655 | 1.94E-06 | 0.236176261 | 2.43E-07 |
| NRSN2 | grey | -0.139217926 | 0.002568455 | -0.141954327 | 0.002104858 |
| NRXN1 | grey | -0.101887202 | 0.027691277 | -0.108844121 | 0.018631064 |
| NRXN3 | grey | -0.174199046 | 0.000154732 | -0.16627795 | 0.000307593 |
| NSFL1C | grey | -0.092762559 | 0.045116644 | -0.081230113 | 0.079500444 |
| NSMF | grey | -0.093145704 | 0.044232914 | -0.112194708 | 0.015278324 |
| NT5C1B- | grey | 0.043687114 | 0.346189467 | 0.041158927 | 0.374839717 |
| NT5C3B | grey | 0.102443578 | 0.026848151 | 0.092325812 | 0.046142058 |
| NT5M | grey | -0.053281325 | 0.250494209 | -0.06862439 | 0.138671257 |
| NTF3 | grey | -0.30969616 | 7.72E-12 | -0.316478695 | 2.53E-12 |
| NTF4 | grey | -0.269037489 | 3.47E-09 | -0.267634763 | 4.21E-09 |
| NTN3 | grey | -0.209423614 | 5.01E-06 | -0.224122577 | 9.94E-07 |
| NTN5 | grey | -0.16193994 | 0.000442427 | -0.164704157 | 0.000351318 |
| NTNG1 | grey | -0.129175085 | 0.005178136 | -0.126093825 | 0.006362189 |
| NTRK1 | grey | -0.214851056 | 2.79E-06 | -0.197076577 | 1.79E-05 |
| NTRK2 | grey | 0.071273872 | 0.124031454 | 0.071152176 | 0.124676432 |
| NTS | grey | 0.024239576 | 0.601325995 | 0.03748384 | 0.419006645 |
| NTSR1 | grey | -0.148584713 | 0.001280877 | -0.11939888 | 0.009806996 |
| NUBP1 | grey | 0.194902525 | 2.22E-05 | 0.199860519 | 1.35E-05 |
| NUDCD3 | grey | -0.034700814 | 0.454395877 | -0.028573553 | 0.537927083 |
| NUDT16 | grey | -0.22005065 | 1.57E-06 | -0.171290617 | 0.000199835 |
| NUDT17 | grey | -0.039035426 | 0.399999129 | -0.065596548 | 0.156986694 |
| NUDT6 | grey | 0.074300864 | 0.108809024 | 0.052625857 | 0.256378166 |
| NUPR1L | grey | -0.371322686 | 1.03E-16 | -0.33870975 | 5.33E-14 |
| NUTM2B | grey | -0.052441307 | 0.258051941 | -0.05477207 | 0.23746399 |
| NUTM2D | grey | -0.034087901 | 0.462409425 | -0.044370471 | 0.33869065 |
| NUTM2E | grey | 0.085250801 | 0.065665496 | 0.082399644 | 0.075251997 |
| NUTM2F | grey | 0.065226013 | 0.159348097 | 0.053208699 | 0.251141486 |
| NWD2 | grey | -0.038229383 | 0.40980817 | -0.01939745 | 0.675875186 |
| NXF1 | grey | 0.168294451 | 0.000258976 | 0.133767795 | 0.00377922 |
| NXF2B | grey | -0.065871732 | 0.155250146 | -0.046521441 | 0.315771223 |
| NXN | grey | -0.065028653 | 0.160616747 | -0.072210826 | 0.119152194 |
| NXPE2 | grey | 0.245719153 | 7.51E-08 | 0.202101328 | 1.08E-05 |
| NXPH1 | grey | 0.006104359 | 0.895329147 | 0.008236678 | 0.859098295 |
| NYAP2 | grey | 0.019034698 | 0.681601962 | 0.041822347 | 0.367183817 |
| NYNRIN | grey | -0.31143295 | 5.82E-12 | -0.310951056 | 6.29E-12 |
| NYX | grey | -0.067828251 | 0.143320075 | -0.071467206 | 0.123012128 |
| OAZ2 | grey | 0.036427889 | 0.432239986 | 0.044719058 | 0.33490577 |
| OAZ3 | grey | 0.372445781 | 8.18E-17 | 0.382229529 | 1.08E-17 |
| OBFC1 | grey | 0.193238617 | 2.62E-05 | 0.210211234 | 4.61E-06 |
| OBP2A | grey | 0.06332453 | 0.171889164 | 0.039805982 | 0.390754759 |
| OBP2B | grey | 0.246846114 | 6.52E-08 | 0.195530884 | 2.09E-05 |
| OBSCN | grey | -0.254170918 | 2.55E-08 | -0.286490782 | 2.84E-10 |

| | | | | | |
|---|---|---|---|---|---|
| OBSL1 | grey | -0.154436711 | 0.000812291 | -0.164446649 | 0.000359002 |
| OC90 | grey | -0.033781822 | 0.466440506 | -0.045212804 | 0.329591458 |
| OCA2 | grey | -0.204667453 | 8.26E-06 | -0.204579165 | 8.34E-06 |
| ODAM | grey | -0.050720529 | 0.274022264 | -0.050926021 | 0.272080485 |
| ODF3L2 | grey | 0.168456335 | 0.000255402 | 0.175317151 | 0.000140087 |
| OGDHL | grey | -0.122153317 | 0.008227609 | -0.127684992 | 0.005723421 |
| OLFM2 | grey | -0.25272272 | 3.08E-08 | -0.256397685 | 1.91E-08 |
| OLFM3 | grey | 0.030266442 | 0.514104488 | 0.000709611 | 0.987797852 |
| OLIG1 | grey | 0.027328395 | 0.555794908 | 0.046779364 | 0.313092762 |
| OLIG3 | grey | -0.03636155 | 0.433079343 | -0.02160957 | 0.641366084 |
| OMP | grey | -0.122425413 | 0.00808463 | -0.123543912 | 0.007519904 |
| ONECUT | grey | 0.157461658 | 0.00063786 | 0.172481218 | 0.000180057 |
| ONECUT | grey | -0.074514104 | 0.107794822 | -0.076882989 | 0.097023186 |
| ONECUT | grey | 0.281115909 | 6.26E-10 | 0.296852857 | 5.91E-11 |
| OOEP | grey | -0.043011544 | 0.353705618 | -0.063179092 | 0.172877868 |
| OPA3 | grey | -0.068924537 | 0.136949002 | -0.044651834 | 0.335633547 |
| OPN4 | grey | 0.034773645 | 0.453448856 | 0.061377722 | 0.18547722 |
| OPN5 | grey | -0.137196988 | 0.002968637 | -0.138464925 | 0.002711429 |
| OPRK1 | grey | 0.06846494 | 0.13959292 | 0.040894127 | 0.377922791 |
| OPRL1 | grey | -0.189190048 | 3.88E-05 | -0.194867041 | 2.23E-05 |
| OPTC | grey | 0.029501985 | 0.524793933 | 0.008735195 | 0.850667467 |
| OR10A3 | grey | -0.032044816 | 0.489681238 | -0.036933137 | 0.425878279 |
| OR10G4 | grey | 0.033991194 | 0.463680968 | 0.016996704 | 0.71410841 |
| OR10G9 | grey | -0.052391039 | 0.258509156 | -0.053460834 | 0.248899334 |
| OR10H1 | grey | 0.029754168 | 0.521255149 | 0.032182041 | 0.487822879 |
| OR10Q1 | grey | 0.044871726 | 0.333256717 | 0.041626061 | 0.369438785 |
| OR10R2 | grey | -0.042980483 | 0.354053642 | -0.04294675 | 0.354431855 |
| OR13A1 | grey | -0.159785204 | 0.000528209 | -0.183885454 | 6.41E-05 |
| OR13C2 | grey | -0.015516205 | 0.738055444 | -0.037767078 | 0.415497893 |
| OR14A2 | grey | 0.018667811 | 0.68741268 | 0.041358182 | 0.372530041 |
| OR14K1 | grey | -0.081352363 | 0.079047454 | -0.11965579 | 0.009649061 |
| OR1E1 | grey | -0.026024609 | 0.574808188 | -0.052184218 | 0.260396182 |
| OR1F1 | grey | 0.137229363 | 0.002961802 | 0.150945206 | 0.001067979 |
| OR1N1 | grey | -0.057792766 | 0.212541515 | -0.038987352 | 0.400580183 |
| OR1Q1 | grey | -0.039879669 | 0.389877573 | -0.033350892 | 0.472148678 |
| OR2A1 | grey | 0.026819232 | 0.563183585 | -0.004275708 | 0.926577567 |
| OR2L13 | grey | -0.189944974 | 3.61E-05 | -0.177315027 | 0.000117106 |
| OR2T10 | grey | -0.117799916 | 0.010842585 | -0.096061496 | 0.037973172 |
| OR2T29 | grey | -0.09163509 | 0.047803661 | -0.097553533 | 0.035071751 |
| OR2T33 | grey | -0.089179003 | 0.054123302 | -0.107897934 | 0.01968714 |
| OR2T8 | grey | -0.080785078 | 0.08116729 | -0.100614254 | 0.029706288 |
| OR2V1 | grey | -0.171227181 | 0.000200944 | -0.182402096 | 7.36E-05 |
| OR2W3 | grey | -0.214211692 | 3.00E-06 | -0.241225548 | 1.31E-07 |
| OR3A2 | grey | -0.041676843 | 0.36885457 | -0.037584753 | 0.417754543 |
| OR3A3 | grey | 0.030615506 | 0.509261326 | 0.022470889 | 0.628130542 |

| | | | | | |
|---|---|---|---|---|---|
| OR4C46 | grey | 0.071899477 | 0.120756653 | 0.078866892 | 0.088677983 |
| OR4C6 | grey | -0.044951652 | 0.332395483 | -0.022768238 | 0.623588571 |
| OR4F16 | grey | 0.00571117 | 0.902036086 | -0.004349628 | 0.925311885 |
| OR4F3 | grey | -0.043633459 | 0.346782678 | -0.044785917 | 0.334182944 |
| OR4L1 | grey | -0.069436464 | 0.134049566 | -0.060931385 | 0.188701173 |
| OR4M2 | grey | -0.074048606 | 0.110018493 | -0.076764215 | 0.097542018 |
| OR51A2 | grey | 0.006313759 | 0.891760256 | -0.003715746 | 0.936171211 |
| OR52A5 | grey | -0.02714045 | 0.558516763 | -0.034283861 | 0.459838831 |
| OR56A3 | grey | -0.215291471 | 2.66E-06 | -0.208060455 | 5.79E-06 |
| OR5AN1 | grey | -0.076342121 | 0.09940373 | -0.077105796 | 0.096055846 |
| OR5AU1 | grey | 0.148770881 | 0.001262764 | 0.128776219 | 0.005319312 |
| OR5F1 | grey | -0.054682946 | 0.238229332 | -0.061224125 | 0.186582061 |
| OR5M11 | grey | -0.169993682 | 0.000223686 | -0.167705936 | 0.000272366 |
| OR5T2 | grey | -0.071402097 | 0.123354678 | -0.064928753 | 0.161261806 |
| OR7A5 | grey | 0.099577065 | 0.031439804 | 0.07087748 | 0.126141894 |
| OR7C1 | grey | 0.096177692 | 0.037740133 | 0.066693254 | 0.150152384 |
| OR7D2 | grey | -0.010322881 | 0.823934457 | -0.007044334 | 0.879326341 |
| OR7E24 | grey | -0.036305252 | 0.433792382 | -0.044556593 | 0.336666389 |
| OR9G4 | grey | 0.036985783 | 0.425218546 | 0.032275196 | 0.486563498 |
| ORAI2 | grey | -0.114341766 | 0.013419496 | -0.107029356 | 0.020702088 |
| ORAOV1 | grey | -0.085123362 | 0.066071452 | -0.105135722 | 0.023073602 |
| ORM1 | grey | 0.168928057 | 0.000245249 | 0.159175749 | 0.000555138 |
| ORM2 | grey | 0.246271262 | 7.01E-08 | 0.225367638 | 8.63E-07 |
| ORMDL3 | grey | 0.013766315 | 0.766688396 | 0.000355011 | 0.993895206 |
| OSBP2 | grey | -0.007197386 | 0.876725132 | -0.029953198 | 0.518470906 |
| OSBPL2 | grey | -0.229631163 | 5.27E-07 | -0.228605851 | 5.94E-07 |
| OSBPL7 | grey | -0.061470632 | 0.184811256 | -0.046932495 | 0.31150961 |
| OSGEP | grey | 0.119248213 | 0.009900682 | 0.123842674 | 0.007375153 |
| OSGIN1 | grey | 0.270067266 | 3.01E-09 | 0.287510893 | 2.44E-10 |
| OSTF1 | grey | 0.39111818 | 1.61E-18 | 0.398868659 | 2.92E-19 |
| OSTN | grey | -0.15972272 | 0.000530913 | -0.171055472 | 0.000203975 |
| OTOA | grey | 0.027307993 | 0.556090055 | 0.057932638 | 0.211434935 |
| OTOF | grey | -0.131688963 | 0.004363408 | -0.13278854 | 0.004044957 |
| OTOG | grey | -0.077162029 | 0.095812922 | -0.047520643 | 0.305478017 |
| OTOP1 | grey | -0.03393571 | 0.464411363 | -0.040825157 | 0.378728353 |
| OTOR | grey | -0.024361833 | 0.599492334 | -0.016524776 | 0.721712714 |
| OTOS | grey | 0.063377967 | 0.171526952 | 0.085159472 | 0.065956213 |
| OTP | grey | -0.052731689 | 0.255421722 | -0.033007976 | 0.476718285 |
| OTUD5 | grey | -0.219510524 | 1.67E-06 | -0.201590347 | 1.13E-05 |
| OTX2 | grey | -0.024500848 | 0.59741041 | -0.025434046 | 0.583520194 |
| OVCH2 | grey | -0.090261436 | 0.051257315 | -0.091221736 | 0.048821796 |
| OVOL3 | grey | -0.103946455 | 0.024680309 | -0.10784923 | 0.019742884 |
| OXA1L | grey | 0.027008042 | 0.560438184 | 0.039357991 | 0.396113468 |
| OXCT2 | grey | -0.318575783 | 1.78E-12 | -0.329396881 | 2.79E-13 |
| OXGR1 | grey | -0.137734151 | 0.002857076 | -0.165345874 | 0.000332832 |

| | | | | | |
|---|---|---|---|---|---|
| OXT | grey | -0.203068088 | 9.75E-06 | -0.196143518 | 1.96E-05 |
| P2RX5-T. | grey | -0.042534847 | 0.359070562 | -0.046555964 | 0.315411839 |
| P2RY2 | grey | -0.011295676 | 0.807652784 | 0.005455995 | 0.906392575 |
| P3H1 | grey | -0.018925697 | 0.683326366 | -0.010654009 | 0.818383504 |
| P3H4 | grey | 0.161820186 | 0.000446832 | 0.135019512 | 0.003462601 |
| P4HA2 | grey | 0.180385865 | 8.86E-05 | 0.185156525 | 5.69E-05 |
| PAAF1 | grey | 0.128768203 | 0.005322184 | 0.107547287 | 0.020091542 |
| PABPC1I | grey | -0.155654037 | 0.000737374 | -0.167477354 | 0.000277739 |
| PABPC1I | grey | -0.15158164 | 0.001016445 | -0.144706793 | 0.001716918 |
| PABPC1I | grey | -0.131128681 | 0.004534234 | -0.120429862 | 0.009186781 |
| PABPC3 | grey | 0.10093608 | 0.029185335 | 0.093896833 | 0.042542647 |
| PABPC4 | grey | -0.148937144 | 0.001246788 | -0.17466275 | 0.000148492 |
| PABPN1I | grey | -0.071646517 | 0.122072581 | -0.050437688 | 0.276710338 |
| PACS1 | grey | -0.025202511 | 0.586952465 | -0.017453591 | 0.706773314 |
| PACSIN2 | grey | 0.20512862 | 7.87E-06 | 0.209764013 | 4.83E-06 |
| PACSIN3 | grey | 0.155364232 | 0.000754606 | 0.155705173 | 0.000734372 |
| PADI1 | grey | 0.078211505 | 0.091368592 | 0.072443974 | 0.117961667 |
| PADI2 | grey | 0.130979751 | 0.004580647 | 0.12717024 | 0.005923459 |
| PADI3 | grey | -0.036144464 | 0.435832559 | -0.031553391 | 0.49636736 |
| PAEP | grey | -0.172081263 | 0.000186487 | -0.180258644 | 8.96E-05 |
| PAF1 | grey | -0.045851693 | 0.322796205 | -0.046514776 | 0.315840631 |
| PAGE1 | grey | 0.003825119 | 0.934296594 | 0.007960993 | 0.863767593 |
| PAGE2 | grey | -0.189445332 | 3.78E-05 | -0.17977482 | 9.37E-05 |
| PAGE2B | grey | -0.196111395 | 1.97E-05 | -0.201835174 | 1.11E-05 |
| PAGE3 | grey | -0.075357301 | 0.103857357 | -0.075939242 | 0.101206948 |
| PAGE5 | grey | -0.146054868 | 0.001551891 | -0.147627914 | 0.001377795 |
| PAGR1 | grey | 0.009305929 | 0.841036205 | -0.007641508 | 0.869184654 |
| PAK1 | grey | 0.218426217 | 1.89E-06 | 0.184165832 | 6.24E-05 |
| PAK4 | grey | 0.028778167 | 0.535018646 | 0.036126264 | 0.436063843 |
| PALD1 | grey | -0.171223735 | 0.000201005 | -0.192707989 | 2.76E-05 |
| PALM3 | grey | -0.093543346 | 0.043331192 | -0.098053588 | 0.034142533 |
| PAN2 | grey | -0.102987606 | 0.026045196 | -0.130951776 | 0.004589412 |
| PANK4 | grey | -0.05413934 | 0.242935017 | -0.069218437 | 0.135278583 |
| PANX2 | grey | -0.078043391 | 0.092069203 | -0.061707461 | 0.183121684 |
| PAOX | grey | 0.04745645 | 0.306132553 | 0.078720489 | 0.089273434 |
| PAPL | grey | -0.037403474 | 0.420005376 | -0.033625426 | 0.468507723 |
| PAPPA2 | grey | -0.100092992 | 0.030566984 | -0.109431693 | 0.018000415 |
| PAPSS2 | grey | -0.001393688 | 0.97603743 | 0.014517476 | 0.754355851 |
| PAQR5 | grey | -0.140612462 | 0.002321682 | -0.168045492 | 0.000264563 |
| PAQR6 | grey | 0.2838166 | 4.22E-10 | 0.270084945 | 3.00E-09 |
| PAQR7 | grey | -0.074057144 | 0.109977385 | -0.08308341 | 0.072854959 |
| PAQR8 | grey | -0.193298888 | 2.60E-05 | -0.215937574 | 2.48E-06 |
| PARN | grey | 0.019726257 | 0.670700395 | 0.051829392 | 0.263655684 |
| PARP3 | grey | -0.019542437 | 0.673591463 | 0.001909309 | 0.967176334 |
| PARP6 | grey | -0.172353271 | 0.000182091 | -0.165362478 | 0.000332366 |

| | | | | | |
|---|---|---|---|---|---|
| PARVB | grey | -0.01543566 | 0.739365806 | -0.004615161 | 0.92076691 |
| PASD1 | grey | -0.113069337 | 0.014495449 | -0.102344372 | 0.026996848 |
| PATZ1 | grey | 0.107044153 | 0.020684424 | 0.084154842 | 0.069224475 |
| PAX2 | grey | -0.108423181 | 0.019094632 | -0.117163222 | 0.0112812 |
| PAX3 | grey | -0.107014515 | 0.020719818 | -0.11641984 | 0.011813125 |
| PAX4 | grey | 0.090935168 | 0.049538239 | 0.053910635 | 0.244934124 |
| PAX5 | grey | 0.07522803 | 0.104453505 | 0.077814073 | 0.093031803 |
| PAX6 | grey | 0.258640862 | 1.42E-08 | 0.264248281 | 6.69E-09 |
| PAX7 | grey | 0.012709675 | 0.78413581 | -0.006718442 | 0.884869334 |
| PAX9 | grey | 0.255955508 | 2.02E-08 | 0.255908096 | 2.03E-08 |
| PBX4 | grey | -0.129085134 | 0.005209675 | -0.124863306 | 0.006899262 |
| PC | grey | 0.115620889 | 0.01240942 | 0.120829148 | 0.008956115 |
| PCCA | grey | 0.182025746 | 7.62E-05 | 0.169834647 | 0.000226787 |
| PCDH1 | grey | -0.038239638 | 0.409682486 | -0.013663134 | 0.768387118 |
| PCDH10 | grey | -0.292141183 | 1.22E-10 | -0.286775725 | 2.72E-10 |
| PCDH11X | grey | -0.001554014 | 0.973281805 | -0.022134086 | 0.633292182 |
| PCDH11Y | grey | -0.081424084 | 0.078782673 | -0.092124259 | 0.046621829 |
| PCDH15 | grey | -0.045888401 | 0.322408558 | -0.039176325 | 0.398299046 |
| PCDH19 | grey | 0.118129045 | 0.010621828 | 0.123850861 | 0.007371221 |
| PCDH8 | grey | -0.034748695 | 0.453773159 | -0.048739964 | 0.293221128 |
| PCDHA1 | grey | 0.053442706 | 0.249060071 | 0.039139581 | 0.398741982 |
| PCDHA1 | grey | -0.169418478 | 0.000235094 | -0.182079992 | 7.58E-05 |
| PCDHA1: | grey | -0.130758397 | 0.004650422 | -0.144670255 | 0.001721607 |
| PCDHA1: | grey | -0.066335012 | 0.152359505 | -0.042202566 | 0.362840189 |
| PCDHA2 | grey | -0.024619382 | 0.595637802 | -0.027533067 | 0.552838165 |
| PCDHA3 | grey | -0.071672743 | 0.121935632 | -0.061009456 | 0.188134302 |
| PCDHA4 | grey | 0.113667482 | 0.013980565 | 0.111110463 | 0.016300156 |
| PCDHA6 | grey | -0.024521888 | 0.59709559 | -0.007049295 | 0.879241993 |
| PCDHA7 | grey | -0.077699648 | 0.093515127 | -0.059268238 | 0.201077037 |
| PCDHB1: | grey | 0.03666777 | 0.429212772 | 0.033752357 | 0.466829583 |
| PCDHB1( | grey | -0.067633438 | 0.144475574 | -0.077986463 | 0.092307418 |
| PCDHB2 | grey | -0.011232319 | 0.808710746 | -0.019479524 | 0.67458205 |
| PCDHB3 | grey | -0.105883151 | 0.022110837 | -0.129746549 | 0.004981748 |
| PCDHB6 | grey | -0.307055889 | 1.18E-11 | -0.328281861 | 3.39E-13 |
| PCDHB8 | grey | 0.057947636 | 0.211316525 | 0.033557455 | 0.469407736 |
| PCDHGA | grey | -0.144658645 | 0.001723099 | -0.180455296 | 8.80E-05 |
| PCDHGA | grey | 0.091575372 | 0.047949644 | 0.05665915 | 0.221664097 |
| PCDHGB | grey | -0.009744228 | 0.833655809 | -0.019561522 | 0.673291081 |
| PCDHGB | grey | -0.137503811 | 0.002904439 | -0.143292497 | 0.001907216 |
| PCDHGB | grey | -0.077307559 | 0.095186521 | -0.080171003 | 0.083513566 |
| PCGF2 | grey | 0.082644059 | 0.074387902 | 0.081752794 | 0.077578319 |
| PCK1 | grey | -0.181247484 | 8.18E-05 | -0.164182327 | 0.000367051 |
| PCOLCE2 | grey | -0.280349327 | 6.99E-10 | -0.291819899 | 1.28E-10 |
| PCP4 | grey | -0.034818701 | 0.452863547 | -0.054358077 | 0.241033764 |
| PCP4L1 | grey | 0.135792409 | 0.003279319 | 0.110290051 | 0.017112647 |

| | | | | | |
|---|---|---|---|---|---|
| PCSK1 | grey | -0.077039075 | 0.096344711 | -0.069826224 | 0.131873959 |
| PCSK1N | grey | -0.364373515 | 4.14E-16 | -0.365655042 | 3.21E-16 |
| PCSK5 | grey | -0.14852037 | 0.001287192 | -0.17085566 | 0.000207555 |
| PCSK6 | grey | 0.012887897 | 0.781185127 | -0.021708732 | 0.639836393 |
| PCSK9 | grey | 0.363140309 | 5.28E-16 | 0.362170632 | 6.39E-16 |
| PCTP | grey | 0.13254406 | 0.004113886 | 0.128490421 | 0.005422589 |
| PDCL2 | grey | -0.226442296 | 7.63E-07 | -0.241051415 | 1.34E-07 |
| PDE11A | grey | -0.136687371 | 0.003078124 | -0.1435874 | 0.001866012 |
| PDE3B | grey | -0.118001511 | 0.010706891 | -0.121710395 | 0.00846518 |
| PDE4A | grey | -0.195622555 | 2.07E-05 | -0.167966669 | 0.000266356 |
| PDE4C | grey | -0.121855937 | 0.008386449 | -0.101427045 | 0.028405693 |
| PDE6A | grey | 0.009081422 | 0.844822072 | 0.022567029 | 0.626660463 |
| PDE6B | grey | -0.505618394 | 1.12E-31 | -0.501335857 | 4.33E-31 |
| PDE6D | grey | 0.075598705 | 0.102751303 | 0.056106221 | 0.22621376 |
| PDE6G | grey | -0.281999164 | 5.50E-10 | -0.271023523 | 2.63E-09 |
| PDE9A | grey | -0.11702461 | 0.011378745 | -0.134999478 | 0.003467474 |
| PDGFA | grey | -0.049695203 | 0.28385183 | -0.059810004 | 0.196982444 |
| PDIA2 | grey | 0.03935041 | 0.396204539 | 0.039183018 | 0.398218396 |
| PDIA5 | grey | 0.191075669 | 3.23E-05 | 0.156384404 | 0.000695551 |
| PDILT | grey | -0.048920557 | 0.291434141 | -0.043314749 | 0.350319633 |
| PDX1 | grey | 0.128608298 | 0.005379776 | 0.129872996 | 0.004939211 |
| PDYN | grey | -0.142150732 | 0.002074723 | -0.142472807 | 0.002026159 |
| PDZD7 | grey | -0.021990725 | 0.635494674 | -0.041741057 | 0.368116662 |
| PDZK1 | grey | 0.033928905 | 0.464500983 | 0.006972754 | 0.880543315 |
| PEA15 | grey | 0.055227217 | 0.233582513 | 0.033700559 | 0.467513991 |
| PEBP4 | grey | 0.009766457 | 0.833281887 | 0.009764225 | 0.833319432 |
| PECR | grey | -0.01350016 | 0.771072473 | -0.024056964 | 0.604069571 |
| PEPD | grey | -0.199220174 | 1.44E-05 | -0.212459149 | 3.62E-06 |
| PF4 | grey | -0.006179361 | 0.894050616 | -0.004055455 | 0.930349907 |
| PFKFB3 | grey | 0.041063275 | 0.375951608 | 0.032673534 | 0.48119812 |
| PFKP | grey | 0.168336661 | 0.000258039 | 0.181727619 | 7.83E-05 |
| PFN3 | grey | -0.143368768 | 0.00189648 | -0.151761021 | 0.00100234 |
| PGA4 | grey | -0.118810421 | 0.010177432 | -0.121250546 | 0.008718281 |
| PGA5 | grey | -0.270744721 | 2.74E-09 | -0.275221735 | 1.46E-09 |
| PGAP3 | grey | -0.06550818 | 0.157547445 | -0.061666257 | 0.183414816 |
| PGBD5 | grey | -0.172484857 | 0.00018 | -0.182083176 | 7.58E-05 |
| PGF | grey | -0.208748705 | 5.38E-06 | -0.194632973 | 2.28E-05 |
| PGK2 | grey | 0.073564345 | 0.112369976 | 0.058616068 | 0.20608772 |
| PGPEP1 | grey | 0.002910211 | 0.949988061 | 0.035754511 | 0.440803376 |
| PGPEP1L | grey | -0.085188231 | 0.065864554 | -0.098959729 | 0.032512108 |
| PHACTR | grey | -0.137082833 | 0.00299285 | -0.168537773 | 0.000253622 |
| PHACTR | grey | -0.275093262 | 1.49E-09 | -0.269596607 | 3.21E-09 |
| PHC2 | grey | -0.078132511 | 0.091697262 | -0.06066173 | 0.190668778 |
| PHEX | grey | 0.035193183 | 0.448015292 | 0.017377861 | 0.707987274 |
| PHF11 | grey | 0.113360656 | 0.014242635 | 0.129994952 | 0.004898496 |

| | | | | | |
|---|---|---|---|---|---|
| PHGR1 | grey | -0.022542076 | 0.627041878 | -0.012191885 | 0.792725899 |
| PHKA2 | grey | -0.09359709 | 0.043210514 | -0.120946761 | 0.008889161 |
| PHLDA3 | grey | 0.255318989 | 2.20E-08 | 0.258984291 | 1.36E-08 |
| PHLDB3 | grey | 0.190189857 | 3.52E-05 | 0.135145396 | 0.00343213 |
| PHLPP1 | grey | 0.234081348 | 3.12E-07 | 0.202876055 | 9.94E-06 |
| PHOSPH( | grey | -0.26344079 | 7.46E-09 | -0.254528676 | 2.44E-08 |
| PHOX2A | grey | -0.166541915 | 0.000300776 | -0.181386434 | 8.08E-05 |
| PHOX2B | grey | -0.052402551 | 0.258404403 | -0.062123996 | 0.180177793 |
| PHYH | grey | -0.016335831 | 0.724764988 | -0.022220916 | 0.631959763 |
| PHYHD1 | grey | 0.192768134 | 2.74E-05 | 0.208472675 | 5.54E-06 |
| PI4K2A | grey | 0.100542874 | 0.029822908 | 0.122204709 | 0.008200432 |
| PIEZO1 | grey | -0.147821708 | 0.001357637 | -0.128712754 | 0.005342092 |
| PIGBOS1 | grey | 0.100860809 | 0.029306472 | 0.117781111 | 0.01085532 |
| PIGH | grey | 0.214229935 | 2.99E-06 | 0.194914433 | 2.22E-05 |
| PIGZ | grey | 0.013745977 | 0.76702315 | 0.014761421 | 0.750363988 |
| PIK3C2G | grey | 0.019483232 | 0.674523663 | 0.005448331 | 0.906523462 |
| PILRB | grey | -0.103800259 | 0.024884337 | -0.115219463 | 0.012718934 |
| PIM1 | grey | -0.210395813 | 4.52E-06 | -0.219656038 | 1.65E-06 |
| PIM3 | grey | 0.127983111 | 0.005610366 | 0.12353578 | 0.007523879 |
| PIP | grey | 0.160684652 | 0.000490677 | 0.19438887 | 2.34E-05 |
| PIP4K2B | grey | -0.028201102 | 0.543241441 | -0.071248054 | 0.124168067 |
| PIP5KL1 | grey | -0.232775303 | 3.64E-07 | -0.256043726 | 2.00E-08 |
| PIPOX | grey | -0.299080316 | 4.18E-11 | -0.28548511 | 3.30E-10 |
| PIR | grey | 0.23478265 | 2.87E-07 | 0.214711362 | 2.84E-06 |
| PISD | grey | -0.195686281 | 2.06E-05 | -0.217054717 | 2.20E-06 |
| PITPNC1 | grey | 0.2628613 | 8.07E-09 | 0.25874098 | 1.40E-08 |
| PITPNM2 | grey | -0.136488934 | 0.003121741 | -0.132719911 | 0.0040642 |
| PITRM1 | grey | 0.184013812 | 6.33E-05 | 0.189428408 | 3.79E-05 |
| PITX2 | grey | -0.066456632 | 0.151607461 | -0.06069692 | 0.190411155 |
| PITX3 | grey | -0.07006281 | 0.130566722 | -0.062711114 | 0.176088013 |
| PIWIL1 | grey | 0.015996548 | 0.730256797 | 0.015519863 | 0.737995953 |
| PIWIL4 | grey | -0.269449972 | 3.28E-09 | -0.269796197 | 3.12E-09 |
| PKD1L1 | grey | 0.144986112 | 0.001681458 | 0.122147585 | 0.008230645 |
| PKDCC | grey | 0.226770912 | 7.34E-07 | 0.230084935 | 5.00E-07 |
| PKDREJ | grey | 0.030317548 | 0.513393926 | 0.010213655 | 0.825767432 |
| PKIA | grey | -0.374826673 | 5.03E-17 | -0.385059464 | 5.92E-18 |
| PKIB | grey | -0.173696609 | 0.00016177 | -0.164950634 | 0.000344107 |
| PKNOX1 | grey | -0.184945166 | 5.80E-05 | -0.19619595 | 1.95E-05 |
| PLA1A | grey | -0.055714542 | 0.22947655 | -0.05450426 | 0.239768985 |
| PLA2G15 | grey | 0.041300154 | 0.373201766 | 0.051108044 | 0.270368334 |
| PLA2G16 | grey | -0.176706099 | 0.000123705 | -0.136812337 | 0.003050942 |
| PLA2G2A | grey | -0.106963814 | 0.020780487 | -0.111434222 | 0.015988941 |
| PLA2G2C | grey | 0.224450418 | 9.58E-07 | 0.208467748 | 5.55E-06 |
| PLA2G4A | grey | 0.283141669 | 4.66E-10 | 0.262157029 | 8.87E-09 |
| PLA2G4F | grey | 0.064868694 | 0.161650551 | 0.033748466 | 0.466880976 |

| | | | | | |
|---|---|---|---|---|---|
| PLA2G6 | grey | 0.092556723 | 0.045597503 | 0.081741687 | 0.077618767 |
| PLAC1 | grey | -0.193492393 | 2.55E-05 | -0.205506496 | 7.57E-06 |
| PLAC8L1 | grey | 0.001205737 | 0.979268199 | -0.003143615 | 0.945982888 |
| PLAU | grey | 0.044316336 | 0.339280886 | 0.08377703 | 0.070487386 |
| PLAUR | grey | 0.212561758 | 3.58E-06 | 0.223416552 | 1.08E-06 |
| PLB1 | grey | -0.019923885 | 0.667597561 | -0.021909702 | 0.63674088 |
| PLBD1 | grey | -0.231990487 | 3.99E-07 | -0.212672314 | 3.54E-06 |
| PLCD3 | grey | -0.212393238 | 3.65E-06 | -0.208711722 | 5.41E-06 |
| PLCD4 | grey | -0.12440835 | 0.007107889 | -0.125612672 | 0.00656756 |
| PLCG2 | grey | -0.137344185 | 0.002937679 | -0.144911352 | 0.001690883 |
| PLCH2 | grey | -0.106063196 | 0.021884195 | -0.118946352 | 0.010090777 |
| PLCXD2 | grey | 0.017173958 | 0.71125952 | 0.01662613 | 0.720077224 |
| PLD3 | grey | -0.170820732 | 0.000208187 | -0.183769013 | 6.48E-05 |
| PLD5 | grey | 0.151405542 | 0.00103047 | 0.093873887 | 0.042593464 |
| PLD6 | grey | 0.105327683 | 0.022822936 | 0.087312455 | 0.059378092 |
| PLEK2 | grey | 0.049244604 | 0.288245984 | 0.04476988 | 0.334356233 |
| PLEKHA‹ | grey | -0.253079348 | 2.94E-08 | -0.231738953 | 4.11E-07 |
| PLEKHA( | grey | 0.095499732 | 0.039117104 | 0.107243243 | 0.020448027 |
| PLEKHD | grey | 0.109203201 | 0.018243409 | 0.09583185 | 0.038437332 |
| PLEKHG: | grey | -0.145396354 | 0.001630594 | -0.144189494 | 0.001784406 |
| PLEKHG: | grey | -0.113813665 | 0.013857204 | -0.137005161 | 0.003009427 |
| PLEKHG‹ | grey | -0.045019849 | 0.331661764 | -0.07356646 | 0.112359624 |
| PLEKHG‹ | grey | -0.14031801 | 0.002371909 | -0.141114935 | 0.002238209 |
| PLEKHG: | grey | 0.073708766 | 0.111664596 | 0.052903037 | 0.253878424 |
| PLEKHH: | grey | 0.27376086 | 1.79E-09 | 0.269822733 | 3.11E-09 |
| PLEKHM | grey | 0.322682428 | 8.90E-13 | 0.33460306 | 1.11E-13 |
| PLEKHN | grey | -0.068976543 | 0.136652269 | -0.050801201 | 0.273258834 |
| PLGLB1 | grey | -0.077825437 | 0.092983913 | -0.077488204 | 0.094413531 |
| PLGLB2 | grey | -0.035233553 | 0.447494403 | -0.056536239 | 0.222669748 |
| PLIN1 | grey | 0.01432451 | 0.757518148 | -0.010421761 | 0.822275929 |
| PLIN3 | grey | 0.397931183 | 3.60E-19 | 0.399499357 | 2.54E-19 |
| PLIN5 | grey | 0.05946127 | 0.199611085 | 0.036079057 | 0.436664057 |
| PLK3 | grey | -0.091991614 | 0.046939851 | -0.07109624 | 0.124973767 |
| PLP2 | grey | -0.028914995 | 0.533078151 | 0.005297398 | 0.909101642 |
| PLTP | grey | -0.310456658 | 6.82E-12 | -0.292605831 | 1.13E-10 |
| PLXNA2 | grey | 0.122283159 | 0.008159102 | 0.13299298 | 0.003988122 |
| PLXNA4 | grey | -0.206338086 | 6.94E-06 | -0.21029363 | 4.57E-06 |
| PLXNB3 | grey | 0.045584514 | 0.325626775 | 0.024488856 | 0.597589874 |
| PMAIP1 | grey | 0.050175015 | 0.279222735 | 0.010872996 | 0.814717427 |
| PMEL | grey | -0.073337214 | 0.113486404 | -0.083811581 | 0.070371116 |
| PMFBP1 | grey | 0.010290116 | 0.824484203 | 0.008216835 | 0.859434222 |
| PNCK | grey | -0.137524879 | 0.002900077 | -0.131639941 | 0.004378119 |
| PNMA2 | grey | -0.215916051 | 2.49E-06 | -0.216042144 | 2.45E-06 |
| PNMA3 | grey | -0.493292179 | 5.21E-30 | -0.504443736 | 1.63E-31 |
| PNMA6A | grey | -0.353389633 | 3.50E-15 | -0.354462387 | 2.85E-15 |

| | | | | | |
|---|---|---|---|---|---|
| PNMAL1 | grey | -0.345086352 | 1.66E-14 | -0.360054098 | 9.68E-16 |
| PNMT | grey | -0.178254884 | 0.000107567 | -0.196623886 | 1.87E-05 |
| PNOC | grey | -0.336395175 | 8.09E-14 | -0.318503916 | 1.80E-12 |
| PNPLA3 | grey | 0.120806771 | 0.008968905 | 0.089114303 | 0.054298751 |
| PNPLA4 | grey | 0.027748663 | 0.549731963 | 0.053598969 | 0.247676874 |
| PNPLA5 | grey | -0.223822824 | 1.03E-06 | -0.227756974 | 6.55E-07 |
| PODNL1 | grey | -0.07253625 | 0.117493056 | -0.040057111 | 0.387770145 |
| POF1B | grey | -0.020603704 | 0.656967614 | -0.016756361 | 0.717977642 |
| POLM | grey | -0.097896779 | 0.034431637 | -0.114838977 | 0.013018568 |
| POLN | grey | 0.095615586 | 0.038878828 | 0.090579894 | 0.050438641 |
| POLR2F | grey | -0.180077464 | 9.11E-05 | -0.184385604 | 6.11E-05 |
| POLR2J2 | grey | -0.03235529 | 0.485482101 | -0.048021236 | 0.300405557 |
| POLR2J3 | grey | -0.109985651 | 0.017422995 | -0.111582874 | 0.015847802 |
| POM121L | grey | 0.0373527 | 0.420637077 | 0.031956922 | 0.490873532 |
| POM121L | grey | -0.13062532 | 0.004692831 | -0.118498415 | 0.010378834 |
| POM121L | grey | 0.048356363 | 0.297041187 | 0.052651314 | 0.256147871 |
| POMC | grey | 0.064176121 | 0.166184427 | 0.054228766 | 0.242156464 |
| POMGNT | grey | 0.068000173 | 0.142306229 | 0.055348117 | 0.232559056 |
| POMT1 | grey | 0.124718556 | 0.006965037 | 0.123006464 | 0.007786703 |
| POMZP3 | grey | 0.061700777 | 0.18316921 | 0.078764321 | 0.089094824 |
| PON1 | grey | -0.023480912 | 0.612760687 | -0.012528841 | 0.787132912 |
| POPDC3 | grey | -0.081724959 | 0.077679722 | -0.10085171 | 0.029321145 |
| POTEB2 | grey | 0.024660063 | 0.595029987 | 0.03455172 | 0.456337999 |
| POU3F2 | grey | -0.017520613 | 0.705699582 | -0.023873515 | 0.606831341 |
| POU3F3 | grey | -0.025987111 | 0.575359533 | -0.032718054 | 0.480600461 |
| POU3F4 | grey | -0.073605763 | 0.112167324 | -0.072254069 | 0.118930674 |
| POU4F2 | grey | -0.075363509 | 0.103828795 | -0.069393688 | 0.13429001 |
| POU4F3 | grey | -0.065187904 | 0.159592475 | -0.079192633 | 0.087364573 |
| POU5F1 | grey | -0.023179785 | 0.617325706 | -0.022409482 | 0.629070288 |
| POU5F1E | grey | 0.100025417 | 0.03068011 | 0.057056755 | 0.218433121 |
| PPAN-P2 | grey | 0.163461997 | 0.00038985 | 0.128915111 | 0.005269763 |
| PPAPDC1 | grey | -0.187468158 | 4.57E-05 | -0.178379241 | 0.000106361 |
| PPARG | grey | 0.107936614 | 0.019642968 | 0.066620423 | 0.150599112 |
| PPARGC | grey | -0.312547691 | 4.85E-12 | -0.333397988 | 1.38E-13 |
| PPARGC | grey | -0.195123435 | 2.17E-05 | -0.185897241 | 5.30E-05 |
| PPBP | grey | 0.054875456 | 0.236578356 | 0.050411559 | 0.27695957 |
| PPFIA2 | grey | -0.125208952 | 0.006744444 | -0.127701052 | 0.005717279 |
| PPFIBP2 | grey | 0.031716967 | 0.494136444 | 0.04193796 | 0.365859655 |
| PPIAL4B | grey | 0.011596998 | 0.802625998 | 0.008307205 | 0.857904559 |
| PPIAL4C | grey | 0.140975261 | 0.002261133 | 0.123607153 | 0.007489053 |
| PPIAL4E | grey | -0.065058429 | 0.160424857 | -0.046106721 | 0.320109329 |
| PPIAL4F | grey | -0.025343975 | 0.584854309 | -0.019515199 | 0.674020267 |
| PPIC | grey | 0.059197611 | 0.201615353 | 0.100177947 | 0.030425268 |
| PPIL3 | grey | 0.115554792 | 0.012459921 | 0.087138851 | 0.059887581 |
| PPM1J | grey | 0.130922654 | 0.004598554 | 0.118618582 | 0.01030085 |

| | | | | | |
|---|---|---|---|---|---|
| PPM1N | grey | -0.069680626 | 0.132683463 | -0.081549482 | 0.078321453 |
| PPME1 | grey | 0.335131536 | 1.01E-13 | 0.340617424 | 3.77E-14 |
| PPP1R10 | grey | 0.222547779 | 1.19E-06 | 0.186543931 | 4.99E-05 |
| PPP1R11 | grey | -0.112431437 | 0.015062849 | -0.107837661 | 0.019756146 |
| PPP1R14l | grey | -0.01681962 | 0.71695855 | -0.02204345 | 0.634684283 |
| PPP1R15A | grey | -0.036223608 | 0.434827642 | -0.051188107 | 0.269617587 |
| PPP1R17 | grey | -0.029117646 | 0.5302107 | -0.051054117 | 0.270874814 |
| PPP1R1A | grey | -0.050619319 | 0.27498209 | -0.059101354 | 0.202350708 |
| PPP1R1B | grey | 0.199302514 | 1.43E-05 | 0.201523713 | 1.14E-05 |
| PPP1R1C | grey | -0.088980645 | 0.054662692 | -0.060327372 | 0.193129362 |
| PPP1R26 | grey | 0.082349477 | 0.075430363 | 0.066443828 | 0.151686505 |
| PPP1R37 | grey | 0.125731919 | 0.006516116 | 0.125238751 | 0.006731244 |
| PPP1R3E | grey | -0.115851896 | 0.012234336 | -0.118374431 | 0.010459842 |
| PPP1R3F | grey | -0.229907535 | 5.10E-07 | -0.208174968 | 5.72E-06 |
| PPP1R3G | grey | -0.104101294 | 0.024465796 | -0.102740213 | 0.026407733 |
| PPP1R9B | grey | 0.118297147 | 0.010510621 | 0.108665178 | 0.018826915 |
| PPP2R2B | grey | -0.298302399 | 4.72E-11 | -0.30517263 | 1.60E-11 |
| PPP2R2C | grey | -0.005267327 | 0.909615422 | -0.023644026 | 0.610294136 |
| PPP2R2D | grey | -0.011164174 | 0.809849041 | 0.009562653 | 0.836711566 |
| PPP2R5B | grey | 0.16312187 | 0.000401067 | 0.162449677 | 0.000424126 |
| PPP4R4 | grey | -0.21233027 | 3.67E-06 | -0.213323207 | 3.30E-06 |
| PPP6R2 | grey | 0.218463981 | 1.88E-06 | 0.220256451 | 1.54E-06 |
| PPT2-EGl | grey | -0.137975425 | 0.00280822 | -0.161266349 | 0.00046774 |
| PPY | grey | -0.13328693 | 0.003907668 | -0.13325103 | 0.003917415 |
| PQLC2L | grey | -0.225774776 | 8.23E-07 | -0.200596129 | 1.26E-05 |
| PRAC1 | grey | -0.027757574 | 0.549603764 | -0.032891266 | 0.478279012 |
| PRAC2 | grey | 0.246693564 | 6.65E-08 | 0.246168568 | 7.10E-08 |
| PRAMEF | grey | -0.052551813 | 0.257048804 | -0.064653672 | 0.163048097 |
| PRAP1 | grey | -0.19549282 | 2.10E-05 | -0.197937594 | 1.64E-05 |
| PRCD | grey | 0.00412958 | 0.929080164 | -0.009647707 | 0.835279869 |
| PRDM12 | grey | -0.026092492 | 0.573810724 | -0.014500674 | 0.754631039 |
| PRDM13 | grey | 0.033506422 | 0.470084088 | 0.047085171 | 0.309936409 |
| PRDM15 | grey | -0.206526871 | 6.80E-06 | -0.209971015 | 4.73E-06 |
| PRDM16 | grey | -0.041953854 | 0.365677844 | -0.070368215 | 0.128894076 |
| PRDM6 | grey | -0.236626433 | 2.30E-07 | -0.229816871 | 5.16E-07 |
| PRG2 | grey | -0.178378851 | 0.000106365 | -0.185824196 | 5.34E-05 |
| PRG4 | grey | -0.051870207 | 0.263279331 | -0.0553563 | 0.2324899 |
| PRH1-PR | grey | -0.053042048 | 0.252631148 | -0.102734574 | 0.026416047 |
| PRICKLE | grey | -0.170048966 | 0.000222617 | -0.184395679 | 6.11E-05 |
| PRKAB1 | grey | 0.169305694 | 0.000237394 | 0.176834397 | 0.000122286 |
| PRKACA | grey | -0.071922325 | 0.120638342 | -0.076117522 | 0.100405821 |
| PRKAR1l | grey | -0.2215146 | 1.34E-06 | -0.214263132 | 2.98E-06 |
| PRKCG | grey | -0.203250586 | 9.56E-06 | -0.20736602 | 6.23E-06 |
| PRKCQ | grey | -0.156185441 | 0.000706721 | -0.183672014 | 6.54E-05 |
| PRKG2 | grey | -0.017606199 | 0.704329269 | -0.034298203 | 0.459651004 |

| | | | | | |
|---|---|---|---|---|---|
| PRKX | grey | 0.07120132 | 0.124415661 | 0.077204358 | 0.095630388 |
| PRL | grey | -0.106696413 | 0.021103025 | -0.113152156 | 0.014423178 |
| PRLH | grey | 0.202312876 | 1.05E-05 | 0.221628986 | 1.32E-06 |
| PRM3 | grey | -0.10310915 | 0.02586866 | -0.095319314 | 0.039490626 |
| PRMT2 | grey | -0.104304193 | 0.024187139 | -0.085550905 | 0.064717609 |
| PROCA1 | grey | -0.123833129 | 0.007379738 | -0.116814029 | 0.011528366 |
| PRODH | grey | 0.152758452 | 0.000927151 | 0.144510905 | 0.001742194 |
| PROK2 | grey | -0.109234513 | 0.018209941 | -0.127899692 | 0.005641797 |
| PROKR1 | grey | -0.023692888 | 0.609556108 | -0.025875364 | 0.577004051 |
| PROKR2 | grey | -0.195178506 | 2.16E-05 | -0.195607094 | 2.07E-05 |
| PROL1 | grey | 0.024649237 | 0.59519171 | 0.013167595 | 0.7765607 |
| PROM1 | grey | 0.051373226 | 0.267887179 | 0.035744441 | 0.440932161 |
| PRORY | grey | -0.06705503 | 0.147948245 | -0.075275263 | 0.104235373 |
| PROZ | grey | 0.013377325 | 0.773098284 | 0.04080229 | 0.378995676 |
| PRPH | grey | 0.022890578 | 0.621723948 | 0.032872343 | 0.478532326 |
| PRPS1L1 | grey | -0.017672602 | 0.703266752 | -0.029034857 | 0.531381195 |
| PRR14 | grey | 0.066070213 | 0.154006675 | 0.050959683 | 0.2717633 |
| PRR23D1 | grey | -0.066980168 | 0.148402313 | -0.079023891 | 0.088042987 |
| PRR23D2 | grey | -0.061100791 | 0.187472717 | -0.060193902 | 0.19411805 |
| PRR27 | grey | 0.042044917 | 0.364637263 | 0.038987932 | 0.400573174 |
| PRR35 | grey | -0.003433868 | 0.941004122 | -0.043821067 | 0.344711285 |
| PRR5L | grey | 0.145930173 | 0.001566521 | 0.161648762 | 0.000453209 |
| PRRT3 | grey | -0.005053744 | 0.913265665 | -0.008554294 | 0.853724926 |
| PRRT4 | grey | -0.206234034 | 7.02E-06 | -0.206726015 | 6.66E-06 |
| PRSS1 | grey | -0.236449172 | 2.35E-07 | -0.224846593 | 9.16E-07 |
| PRSS16 | grey | 0.09667351 | 0.03675932 | 0.111126682 | 0.01628444 |
| PRSS21 | grey | -0.29295605 | 1.07E-10 | -0.30285999 | 2.31E-11 |
| PRSS3 | grey | -0.131318028 | 0.004475839 | -0.125206082 | 0.006745716 |
| PRSS33 | grey | -0.002664545 | 0.954204988 | -0.005353726 | 0.908139355 |
| PRSS36 | grey | 0.001246392 | 0.978569327 | 0.017072766 | 0.712885424 |
| PRSS45 | grey | -0.127531064 | 0.005782593 | -0.148794115 | 0.00126052 |
| PRSS50 | grey | -0.294064398 | 9.07E-11 | -0.314605183 | 3.45E-12 |
| PRSS53 | grey | 0.06343011 | 0.171174059 | 0.047720292 | 0.303448245 |
| PRSS56 | grey | 0.090920499 | 0.049575149 | 0.108779881 | 0.018701169 |
| PRSS57 | grey | -0.192518146 | 2.81E-05 | -0.184870879 | 5.84E-05 |
| PRTN3 | grey | -0.04705015 | 0.310296814 | -0.027145195 | 0.558447961 |
| PRY | grey | 0.078070746 | 0.091954908 | 0.06391107 | 0.167944565 |
| PSAP | grey | 0.044246518 | 0.340043082 | 0.041380101 | 0.372276508 |
| PSAPL1 | grey | -0.125424072 | 0.006649668 | -0.108003946 | 0.01956628 |
| PSCA | grey | 0.123503043 | 0.007539901 | 0.166578633 | 0.000299839 |
| PSD2 | grey | -0.144557797 | 0.001736112 | -0.149485296 | 0.001195423 |
| PSEN2 | grey | 0.048145649 | 0.299153621 | 0.039112871 | 0.39906414 |
| PSG4 | grey | -0.065986289 | 0.154531531 | -0.087379837 | 0.059181308 |
| PSMF1 | grey | -0.125787946 | 0.00649207 | -0.115036418 | 0.012862315 |
| PSORS1C | grey | -0.059520053 | 0.199166215 | -0.065848393 | 0.155396857 |

| | | | | | |
|---|---|---|---|---|---|
| PSPN | grey | 0.174010117 | 0.000157344 | 0.194565196 | 2.30E-05 |
| PSTK | grey | -0.254556516 | 2.43E-08 | -0.283047293 | 4.72E-10 |
| PSTPIP2 | grey | 0.117741148 | 0.010882427 | 0.100842569 | 0.029335891 |
| PTCHD2 | grey | 0.124497267 | 0.007066677 | 0.095356362 | 0.03941368 |
| PTCHD4 | grey | 0.139288134 | 0.002555481 | 0.129783801 | 0.004969183 |
| PTF1A | grey | 0.095828531 | 0.038444074 | 0.078344761 | 0.090816291 |
| PTGER1 | grey | -0.179342253 | 9.74E-05 | -0.188881926 | 3.99E-05 |
| PTGES | grey | -0.270859839 | 2.69E-09 | -0.24811297 | 5.55E-08 |
| PTGES3L | grey | -0.071276862 | 0.124015635 | -0.089489087 | 0.053288976 |
| PTGR1 | grey | 0.094462238 | 0.041306522 | 0.090089398 | 0.051704175 |
| PTGS1 | grey | -0.340879091 | 3.60E-14 | -0.329994234 | 2.51E-13 |
| PTGS2 | grey | 0.237335217 | 2.11E-07 | 0.207311112 | 6.27E-06 |
| PTH | grey | -0.029990731 | 0.517946711 | -0.01703803 | 0.713443839 |
| PTH2 | grey | 0.060443041 | 0.192275523 | 0.053030071 | 0.252738441 |
| PTH2R | grey | -0.053034955 | 0.252694685 | -0.053633912 | 0.247368306 |
| PTHLH | grey | 0.079409706 | 0.08649804 | 0.057490434 | 0.214947618 |
| PTK6 | grey | 0.140048132 | 0.002418813 | 0.151298531 | 0.001039079 |
| PTK7 | grey | -0.00435032 | 0.925300037 | -0.025892074 | 0.576758004 |
| PTP4A3 | grey | -0.348944979 | 8.10E-15 | -0.343307239 | 2.31E-14 |
| PTPLA | grey | -0.253342845 | 2.84E-08 | -0.219447478 | 1.68E-06 |
| PTPN18 | grey | 0.189859372 | 3.64E-05 | 0.219913926 | 1.60E-06 |
| PTPN20A | grey | -0.293614317 | 9.72E-11 | -0.301966291 | 2.66E-11 |
| PTPN5 | grey | -0.162718054 | 0.000414775 | -0.167853081 | 0.000268959 |
| PTPRH | grey | 0.108756818 | 0.018726394 | 0.129265441 | 0.005146627 |
| PTPRM | grey | -0.268480199 | 3.75E-09 | -0.264027678 | 6.89E-09 |
| PTPRN | grey | -0.004709238 | 0.919157277 | 0.018403191 | 0.691615172 |
| PTPRS | grey | 0.004120422 | 0.929237033 | 0.011405587 | 0.805818274 |
| PTPRU | grey | -0.201661974 | 1.13E-05 | -0.193324818 | 2.60E-05 |
| PTPRZ1 | grey | 0.018123071 | 0.696074164 | 0.024003433 | 0.604874878 |
| PTRH1 | grey | -0.149963493 | 0.001152213 | -0.173821979 | 0.000159986 |
| PTTG1IP | grey | -0.047111979 | 0.309660715 | -0.055585305 | 0.230560415 |
| PTX3 | grey | -0.391260143 | 1.56E-18 | -0.406444941 | 5.28E-20 |
| PVALB | grey | -0.232678133 | 3.68E-07 | -0.232152278 | 3.92E-07 |
| PWP2 | grey | -0.079352853 | 0.086724321 | -0.085694106 | 0.064269284 |
| PXDC1 | grey | -0.446418995 | 2.97E-24 | -0.446541785 | 2.88E-24 |
| PXDNL | grey | -0.121755875 | 0.008440507 | -0.115645819 | 0.012390419 |
| PXMP4 | grey | -0.05235592 | 0.258828914 | -0.004424731 | 0.924026149 |
| PXN | grey | -0.160581063 | 0.000494869 | -0.157237053 | 0.00064951 |
| PYCR2 | grey | 0.158184028 | 0.000601695 | 0.144857094 | 0.001697753 |
| PYDC1 | grey | -0.1816331 | 7.90E-05 | -0.184137786 | 6.26E-05 |
| PYGB | grey | 0.211634564 | 3.96E-06 | 0.223194183 | 1.11E-06 |
| PYGL | grey | 0.189681166 | 3.70E-05 | 0.159511889 | 0.000540132 |
| PYROXD | grey | 0.00918791 | 0.843025916 | 0.010741482 | 0.816918629 |
| QDPR | grey | 0.092058929 | 0.046778233 | 0.049165836 | 0.289018785 |
| QPCT | grey | -0.123776835 | 0.007406836 | -0.131757247 | 0.004342991 |

| | | | | | |
|---|---|---|---|---|---|
| QPRT | grey | -0.231323579 | 4.32E-07 | -0.193132311 | 2.64E-05 |
| QRFP | grey | -0.131753031 | 0.004344249 | -0.134869283 | 0.003499287 |
| QSOX1 | grey | 0.113874517 | 0.013806135 | 0.126570024 | 0.006164624 |
| QSOX2 | grey | 0.023829925 | 0.607488412 | -0.024040148 | 0.604322484 |
| RAB11FI | grey | 0.270813426 | 2.71E-09 | 0.249231774 | 4.82E-08 |
| RAB34 | grey | -0.017648376 | 0.703654334 | -0.015797976 | 0.733477404 |
| RAB38 | grey | -0.026261234 | 0.571334776 | -0.020561619 | 0.657623697 |
| RAB3A | grey | 0.023545295 | 0.611786577 | 0.024373686 | 0.599314698 |
| RAB3B | grey | 0.094241445 | 0.041785578 | 0.093529316 | 0.043362742 |
| RAB40B | grey | -0.028216803 | 0.543016877 | -0.053245698 | 0.250811591 |
| RAB4B-E | grey | 0.075334015 | 0.103964544 | 0.086515054 | 0.061748196 |
| RAB6B | grey | -0.24707976 | 6.33E-08 | -0.267209004 | 4.47E-09 |
| RAB7B | grey | -0.054108797 | 0.243201328 | -0.021887301 | 0.637085604 |
| RABGEF | grey | -0.055384603 | 0.232250823 | -0.064824155 | 0.16193929 |
| RAC1 | grey | 0.079260994 | 0.087090932 | 0.117351824 | 0.011149664 |
| RAD51A | grey | 0.077323665 | 0.0951174 | 0.088352374 | 0.056400699 |
| RAD51D | grey | 0.303285658 | 2.16E-11 | 0.27593687 | 1.32E-09 |
| RAD51L3 | grey | -0.028861378 | 0.533838129 | -0.041212123 | 0.374222236 |
| RAET1G | grey | 0.043259306 | 0.350937245 | 0.060483728 | 0.191975838 |
| RAG2 | grey | -0.076102599 | 0.100472687 | -0.083278393 | 0.07218295 |
| RAI2 | grey | -0.218695258 | 1.83E-06 | -0.19767216 | 1.69E-05 |
| RALGDS | grey | 0.195346333 | 2.13E-05 | 0.177235478 | 0.000117949 |
| RALYL | grey | -0.157633168 | 0.000629096 | -0.169086029 | 0.000241934 |
| RAP1GA | grey | 0.062410484 | 0.17817345 | 0.087291679 | 0.059438874 |
| RAPGEF | grey | -0.171748705 | 0.000191996 | -0.18436062 | 6.13E-05 |
| RAPGEF | grey | 0.239022405 | 1.72E-07 | 0.241239851 | 1.31E-07 |
| RAPSN | grey | -0.125443162 | 0.006641316 | -0.123137814 | 0.00772073 |
| RARG | grey | -0.223144302 | 1.11E-06 | -0.23186538 | 4.05E-07 |
| RARRES | grey | -0.24608148 | 7.18E-08 | -0.233290722 | 3.42E-07 |
| RASAL1 | grey | -0.188233714 | 4.25E-05 | -0.162019029 | 0.00043954 |
| RASGEF | grey | 0.008364717 | 0.856931364 | -0.010854504 | 0.815026846 |
| RASGEF | grey | -0.127895002 | 0.005643569 | -0.142500565 | 0.002022023 |
| RASL10A | grey | -0.004870665 | 0.916396033 | 0.008900842 | 0.847869789 |
| RASL10B | grey | -0.260480674 | 1.11E-08 | -0.26462352 | 6.36E-09 |
| RASSF1 | grey | 0.072514301 | 0.117604386 | 0.081877251 | 0.077126242 |
| RASSF10 | grey | -0.252943311 | 2.99E-08 | -0.279635505 | 7.75E-10 |
| RAX | grey | -0.143760926 | 0.001842149 | -0.132178449 | 0.004218949 |
| RAX2 | grey | -0.098891256 | 0.032632946 | -0.083453209 | 0.071584754 |
| RBAK-RI | grey | -0.004968924 | 0.914715803 | -0.029928454 | 0.518816632 |
| RBFA | grey | 0.117743618 | 0.01088075 | 0.123284966 | 0.007647412 |
| RBM11 | grey | -0.395943904 | 5.59E-19 | -0.405754393 | 6.18E-20 |
| RBM19 | grey | -0.039441452 | 0.395111805 | -0.051059576 | 0.270823521 |
| RBM3 | grey | 0.203501709 | 9.32E-06 | 0.203366721 | 9.45E-06 |
| RBM4B | grey | 0.108632185 | 0.018863221 | 0.093432667 | 0.043580608 |
| RBP1 | grey | -0.1448602 | 0.001697359 | -0.123983049 | 0.007308005 |

| | | | | | |
|---|---|---|---|---|---|
| RBP2 | grey | -0.115566372 | 0.01245106 | -0.092689587 | 0.045286626 |
| RBP4 | grey | -0.100249323 | 0.030306639 | -0.081408211 | 0.078841212 |
| RCAN1 | grey | 0.142490746 | 0.002023485 | 0.15605531 | 0.000714116 |
| RCVRN | grey | -0.17161707 | 0.000194219 | -0.169417048 | 0.000235123 |
| RD3 | grey | -0.082594944 | 0.074560888 | -0.085996884 | 0.063329784 |
| RDH10 | grey | 0.173008903 | 0.000171892 | 0.167787601 | 0.00027047 |
| RDH11 | grey | 0.209546108 | 4.95E-06 | 0.193828557 | 2.47E-05 |
| RDH13 | grey | -0.057390882 | 0.215744169 | -0.053826901 | 0.245668914 |
| RDH16 | grey | -0.284527498 | 3.80E-10 | -0.277368486 | 1.07E-09 |
| RDH5 | grey | -0.002702449 | 0.953554266 | -0.011825337 | 0.798822151 |
| REC8 | grey | -0.023533399 | 0.611966524 | -0.015029775 | 0.745980404 |
| RECQL5 | grey | -0.208222297 | 5.69E-06 | -0.22160188 | 1.32E-06 |
| REEP2 | grey | -0.211880114 | 3.86E-06 | -0.2220522 | 1.26E-06 |
| REG1A | grey | -0.113970334 | 0.013726059 | -0.11493941 | 0.012938879 |
| REG1B | grey | -0.007850037 | 0.865648203 | -0.003176831 | 0.945413026 |
| REG3A | grey | 0.035192478 | 0.448024399 | 0.022608239 | 0.626030773 |
| REG3G | grey | -0.11454185 | 0.013256868 | -0.11866414 | 0.010271421 |
| REG4 | grey | -0.010811324 | 0.815749478 | -1.50E-05 | 0.999742692 |
| RELA | grey | 0.100358641 | 0.030125718 | 0.096260548 | 0.037574701 |
| RELL2 | grey | 0.001476443 | 0.974615027 | -0.012504518 | 0.787536268 |
| RELN | grey | -0.230178436 | 4.94E-07 | -0.248804483 | 5.09E-08 |
| REN | grey | -0.006245714 | 0.892919752 | 0.037116037 | 0.423588829 |
| REP15 | grey | -0.247886926 | 5.72E-08 | -0.261058259 | 1.03E-08 |
| REPIN1 | grey | 0.225977145 | 8.04E-07 | 0.229123165 | 5.59E-07 |
| RET | grey | 0.019503938 | 0.67419758 | -0.011490927 | 0.804394603 |
| REXO1 | grey | 0.179395321 | 9.70E-05 | 0.179696318 | 9.43E-05 |
| RFPL2 | grey | 0.011060229 | 0.811586132 | -0.003426747 | 0.941126247 |
| RFPL3 | grey | -0.038707359 | 0.403974411 | -0.040109601 | 0.387148058 |
| RFPL3S | grey | -0.091806741 | 0.047386126 | -0.107827869 | 0.019767376 |
| RFX4 | grey | -0.075147507 | 0.104826219 | -0.065980341 | 0.154568778 |
| RFX8 | grey | -0.081247439 | 0.079436114 | -0.065414113 | 0.158146018 |
| RGL2 | grey | 0.141927487 | 0.002109006 | 0.117800221 | 0.010842378 |
| RGL3 | grey | 0.038774019 | 0.403164763 | 0.011997538 | 0.795956636 |
| RGPD2 | grey | -0.007854158 | 0.865578352 | -0.028781813 | 0.534966886 |
| RGPD3 | grey | 0.041728352 | 0.368262589 | -0.002763304 | 0.952509598 |
| RGPD4 | grey | -0.049312626 | 0.287579734 | -0.054477178 | 0.240002945 |
| RGR | grey | 0.274051375 | 1.72E-09 | 0.285227231 | 3.43E-10 |
| RGS10 | grey | 0.154698184 | 0.00079563 | 0.136966493 | 0.003017711 |
| RGS11 | grey | -0.065377397 | 0.158380121 | -0.054502493 | 0.239784239 |
| RGS14 | grey | -0.045715401 | 0.324238119 | -0.040325849 | 0.38459163 |
| RGS16 | grey | -0.049554357 | 0.285220446 | -0.032876806 | 0.478472586 |
| RGS20 | grey | 0.002271637 | 0.96095198 | -0.011830493 | 0.798736324 |
| RGS3 | grey | 0.254589607 | 2.42E-08 | 0.26537534 | 5.74E-09 |
| RGS7 | grey | 0.06490093 | 0.161441809 | 0.05556028 | 0.230770711 |
| RGS8 | grey | -0.104695103 | 0.023657999 | -0.093602563 | 0.043198241 |

| | | | | | |
|---|---|---|---|---|---|
| RGS9BP | grey | -0.15463253 | 0.000799783 | -0.153038443 | 0.000907001 |
| RHBDF1 | grey | 0.207590682 | 6.08E-06 | 0.222777936 | 1.16E-06 |
| RHBDF2 | grey | -0.153302166 | 0.000888392 | -0.138998638 | 0.002609366 |
| RHBDL2 | grey | 0.112876759 | 0.014664731 | 0.112222323 | 0.01525305 |
| RHBDL3 | grey | -0.056146271 | 0.225882001 | -0.102656005 | 0.026532121 |
| RHBG | grey | -0.094225407 | 0.041820557 | -0.099151908 | 0.032174997 |
| RHCE | grey | -0.166222198 | 0.000309051 | -0.148911365 | 0.001249253 |
| RHO | grey | -0.147109122 | 0.001433121 | -0.148857642 | 0.001254404 |
| RHOD | grey | 0.129464473 | 0.005077831 | 0.128873693 | 0.005284495 |
| RHOF | grey | -0.164972745 | 0.000343467 | -0.158478906 | 0.000587487 |
| RHOXF1 | grey | -0.157581312 | 0.000631734 | -0.15739827 | 0.000641128 |
| RHOXF2 | grey | -0.097180891 | 0.035778124 | -0.098068831 | 0.034114541 |
| RHOXF2I | grey | -0.068261073 | 0.140778173 | -0.069179316 | 0.135500018 |
| RIC8A | grey | 0.128924698 | 0.005266359 | 0.125278339 | 0.006713743 |
| RIMBP2 | grey | 0.043928909 | 0.343524164 | 0.04854982 | 0.295110537 |
| RIMBP3 | grey | 0.025357632 | 0.58465193 | -0.003980818 | 0.931628591 |
| RIMBP3E | grey | 0.055641724 | 0.23008681 | 0.043010526 | 0.353717013 |
| RIMBP3C | grey | 0.037288923 | 0.421431343 | 0.061265095 | 0.186286883 |
| RIMKLA | grey | 0.324450899 | 6.57E-13 | 0.292049876 | 1.23E-10 |
| RIMS1 | grey | -0.185135223 | 5.70E-05 | -0.200290061 | 1.29E-05 |
| RIMS2 | grey | 0.07560722 | 0.102712457 | 0.037263459 | 0.421748709 |
| RIMS4 | grey | -0.195418629 | 2.11E-05 | -0.227755781 | 6.55E-07 |
| RINL | grey | -0.196148785 | 1.96E-05 | -0.191365166 | 3.14E-05 |
| RIPK2 | grey | 0.141750411 | 0.002136568 | 0.132998811 | 0.003986511 |
| RIPK3 | grey | 0.00034474 | 0.994071822 | 0.005794683 | 0.900610948 |
| RIPPLY1 | grey | -0.004487677 | 0.922948679 | -0.004867264 | 0.916454212 |
| RIPPLY2 | grey | -0.138031179 | 0.002797039 | -0.122815649 | 0.007883442 |
| RIPPLY3 | grey | 0.204403149 | 8.49E-06 | 0.226407455 | 7.66E-07 |
| RLBP1 | grey | 0.005672881 | 0.902689601 | -0.011843048 | 0.798527309 |
| RLN1 | grey | -0.220914152 | 1.43E-06 | -0.193237255 | 2.62E-05 |
| RLN2 | grey | -0.194728259 | 2.26E-05 | -0.183393568 | 6.71E-05 |
| RMND5B | grey | 0.054848724 | 0.236807127 | 0.053943887 | 0.244642758 |
| RNASE1 | grey | -0.144862662 | 0.001697047 | -0.10801949 | 0.019548614 |
| RNASE1( | grey | -0.012397197 | 0.789316723 | -0.047591397 | 0.304757662 |
| RNASE1: | grey | -0.161067816 | 0.000475454 | -0.154403404 | 0.000814437 |
| RNASEK | grey | 0.216237521 | 2.40E-06 | 0.220384707 | 1.52E-06 |
| RNASEK | grey | 0.196956936 | 1.81E-05 | 0.218329775 | 1.91E-06 |
| RND1 | grey | 0.060368394 | 0.192826228 | 0.036453188 | 0.431920145 |
| RND2 | grey | -0.158104484 | 0.000605582 | -0.167962952 | 0.000266441 |
| RNF10 | grey | 0.101347853 | 0.028530224 | 0.12156236 | 0.008545934 |
| RNF103-( | grey | -0.051683067 | 0.265007989 | -0.035517792 | 0.443836612 |
| RNF128 | grey | 0.067646058 | 0.144400508 | 0.047976665 | 0.300854903 |
| RNF130 | grey | -0.066137172 | 0.153588894 | -0.08381877 | 0.070346944 |
| RNF144B | grey | -0.011344198 | 0.80684278 | -0.024212627 | 0.601730524 |
| RNF151 | grey | -0.070171122 | 0.129971605 | -0.058829276 | 0.204439773 |

| | | | | | |
|---|---|---|---|---|---|
| RNF157 | grey | -0.181444453 | 8.04E-05 | -0.197798142 | 1.67E-05 |
| RNF165 | grey | -0.316709783 | 2.43E-12 | -0.326886725 | 4.32E-13 |
| RNF17 | grey | -0.066891505 | 0.148941457 | -0.074973124 | 0.105636981 |
| RNF175 | grey | 0.151556219 | 0.001018459 | 0.146073585 | 0.001549706 |
| RNF182 | grey | -0.28965422 | 1.77E-10 | -0.312493368 | 4.89E-12 |
| RNF186 | grey | -0.041369971 | 0.372393669 | -0.042381234 | 0.360810191 |
| RNF207 | grey | -0.148198592 | 0.001319208 | -0.15880602 | 0.000572089 |
| RNF212 | grey | -0.389789021 | 2.15E-18 | -0.402007498 | 1.45E-19 |
| RNF215 | grey | -0.063576835 | 0.170183973 | -0.088759549 | 0.055269168 |
| RNF223 | grey | 0.305257129 | 1.58E-11 | 0.30284408 | 2.32E-11 |
| RNF224 | grey | 0.015836933 | 0.732845197 | 0.013691343 | 0.767922587 |
| RNF225 | grey | 0.037278131 | 0.421565821 | 0.031622995 | 0.495417414 |
| RNF24 | grey | 0.037165082 | 0.422976131 | 0.047724134 | 0.303409268 |
| RNF39 | grey | 0.146093088 | 0.001547432 | 0.177137059 | 0.000118999 |
| RNLS | grey | -0.46813991 | 8.24E-27 | -0.447955741 | 1.98E-24 |
| ROBO3 | grey | -0.369931284 | 1.36E-16 | -0.375929106 | 4.01E-17 |
| ROM1 | grey | -0.174133186 | 0.000155638 | -0.184949771 | 5.80E-05 |
| ROPN1 | grey | 0.027925681 | 0.547187999 | 0.022257126 | 0.63140447 |
| ROR2 | grey | 0.003454981 | 0.940642063 | -0.009633288 | 0.835522544 |
| RORC | grey | 0.123122682 | 0.007728304 | 0.106596014 | 0.021225243 |
| ROS1 | grey | 0.017633943 | 0.703885268 | 0.019285298 | 0.67764375 |
| RP1-130F | grey | -0.153913457 | 0.000846607 | -0.17608295 | 0.000130818 |
| RP1-138E | grey | -0.023196403 | 0.617073394 | -0.032311901 | 0.486067759 |
| RP1-309K | grey | 0.021346728 | 0.64542805 | 0.014640115 | 0.752348195 |
| RP1-317E | grey | 0.107506235 | 0.020139357 | 0.092311403 | 0.046176217 |
| RP1-4G17 | grey | -0.005019887 | 0.913844477 | 0.004545826 | 0.921953439 |
| RP11-101 | grey | 0.017452657 | 0.706788285 | 0.036229852 | 0.434748413 |
| RP11-102 | grey | -0.066116885 | 0.153715378 | -0.066829053 | 0.149322115 |
| RP11-106 | grey | -0.056298997 | 0.224620059 | -0.054074672 | 0.243499115 |
| RP11-108 | grey | 0.07758084 | 0.094019083 | 0.113487724 | 0.014133583 |
| RP11-108 | grey | 0.032839084 | 0.478977734 | 0.029988785 | 0.517973885 |
| RP11-109 | grey | 0.062871924 | 0.174979963 | 0.042393228 | 0.360674177 |
| RP11-121 | grey | -0.015147369 | 0.744062062 | -0.021429258 | 0.644151487 |
| RP11-122 | grey | -0.077181237 | 0.09573006 | -0.069703163 | 0.13255791 |
| RP11-123 | grey | 0.158523713 | 0.000585355 | 0.131044737 | 0.004560342 |
| RP11-125 | grey | 0.060436901 | 0.192320775 | 0.056364068 | 0.224083917 |
| RP11-145 | grey | 0.043248196 | 0.351061092 | 0.034486844 | 0.457184536 |
| RP11-152 | grey | -0.008330838 | 0.857504634 | -0.014183495 | 0.759831643 |
| RP11-155 | grey | 0.06941419 | 0.134174725 | 0.065256179 | 0.159154856 |
| RP11-156 | grey | -0.039833499 | 0.390427047 | -0.041439905 | 0.371585288 |
| RP11-159 | grey | 0.043013297 | 0.353685985 | 0.042512972 | 0.359317975 |
| RP11-162 | grey | 0.041380061 | 0.372276965 | 0.007928195 | 0.864323407 |
| RP11-166 | grey | -0.103057435 | 0.025943647 | -0.102381953 | 0.026940435 |
| RP11-176 | grey | -0.203008862 | 9.81E-06 | -0.18257821 | 7.24E-05 |
| RP11-178 | grey | 0.099070472 | 0.032317481 | 0.060989419 | 0.188279672 |

| | | | | | |
|---|---|---|---|---|---|
| RP11-178 | grey | 0.130942736 | 0.004592248 | 0.112793988 | 0.014738021 |
| RP11-192 | grey | 0.030065022 | 0.516909955 | 0.014562405 | 0.753620153 |
| RP11-195 | grey | -0.056132858 | 0.225993071 | -0.059552777 | 0.19891888 |
| RP11-195 | grey | -0.074895871 | 0.105997733 | -0.075612541 | 0.102688192 |
| RP11-196 | grey | 0.11258549 | 0.014924066 | 0.100231456 | 0.030336298 |
| RP11-201 | grey | -0.013180096 | 0.776354212 | 0.010042659 | 0.828638909 |
| RP11-20I | grey | 0.043357998 | 0.349838337 | 0.039699875 | 0.392019997 |
| RP11-20I | grey | 0.016235749 | 0.726383515 | -0.002721453 | 0.953228015 |
| RP11-212 | grey | -0.119507773 | 0.009739776 | -0.110572423 | 0.01682909 |
| RP11-214 | grey | -0.01698017 | 0.714374356 | -0.019217149 | 0.678719292 |
| RP11-215 | grey | -0.062997955 | 0.174115187 | -0.061167048 | 0.186993853 |
| RP11-216 | grey | 0.13215253 | 0.004226489 | 0.108146622 | 0.019404641 |
| RP11-229 | grey | 0.062571645 | 0.177053224 | 0.050747587 | 0.273766041 |
| RP11-234 | grey | -0.028741952 | 0.535532834 | -0.039023827 | 0.400139277 |
| RP11-240 | grey | -0.096919548 | 0.036280704 | -0.117033464 | 0.011372492 |
| RP11-249 | grey | -0.027528796 | 0.552899799 | 0.01519933 | 0.743214914 |
| RP11-257 | grey | -0.071088652 | 0.125014144 | -0.028445809 | 0.539746879 |
| RP11-26J | grey | 0.111178805 | 0.016234023 | 0.082199096 | 0.075967094 |
| RP11-277 | grey | -0.026982508 | 0.560809087 | -0.014373933 | 0.756707833 |
| RP11-295 | grey | -0.025662394 | 0.580144332 | -0.026665907 | 0.565417772 |
| RP11-298 | grey | 0.035602196 | 0.442753728 | 0.04032213 | 0.384635507 |
| RP11-302 | grey | -0.107813667 | 0.019783674 | -0.121012591 | 0.00885188 |
| RP11-302 | grey | -0.040877175 | 0.378120689 | -0.042898048 | 0.354978345 |
| RP11-315 | grey | -0.026347867 | 0.570065583 | -0.019536783 | 0.673680473 |
| RP11-318 | grey | -0.036743089 | 0.428264809 | -0.029280233 | 0.527915787 |
| RP11-322 | grey | 0.08578523 | 0.063985333 | 0.076109138 | 0.100443382 |
| RP11-330 | grey | -0.072741227 | 0.116457316 | -0.064185713 | 0.166120995 |
| RP11-343 | grey | -0.035717722 | 0.441273993 | -0.037081242 | 0.424023817 |
| RP11-343 | grey | 0.098522193 | 0.033290875 | 0.066995015 | 0.148312173 |
| RP11-345 | grey | -0.075005222 | 0.105487375 | -0.087101467 | 0.059997767 |
| RP11-347 | grey | -0.187247933 | 4.67E-05 | -0.155872845 | 0.000724607 |
| RP11-350 | grey | 0.01759275 | 0.704544538 | 0.014355334 | 0.757012743 |
| RP11-35N | grey | 0.07599576 | 0.100952428 | 0.059038853 | 0.202829228 |
| RP11-38C | grey | 0.02100841 | 0.650671974 | 0.026285727 | 0.570975808 |
| RP11-392 | grey | 0.012638079 | 0.785322055 | -0.000801809 | 0.986212597 |
| RP11-403 | grey | 0.031146782 | 0.501935981 | 0.037533663 | 0.41838818 |
| RP11-404 | grey | -0.081310314 | 0.079203029 | -0.071899328 | 0.120757421 |
| RP11-407 | grey | 0.022549653 | 0.626926056 | 0.00993615 | 0.830428642 |
| RP11-411 | grey | 0.026209407 | 0.57209469 | 0.023041017 | 0.619434394 |
| RP11-426 | grey | 0.110219878 | 0.017183759 | 0.086641644 | 0.061366802 |
| RP11-432 | grey | 0.000865343 | 0.985120223 | -0.026327011 | 0.57037101 |
| RP11-438 | grey | -0.024070983 | 0.60385875 | -0.007702973 | 0.868141999 |
| RP11-447 | grey | -0.002903968 | 0.950095197 | -0.007410557 | 0.873104392 |
| RP11-45M | grey | -0.008917694 | 0.847585265 | -0.02068302 | 0.655731834 |
| RP11-463 | grey | 0.069122193 | 0.135823858 | 0.045377017 | 0.327836119 |

| | | | | | |
|---|---|---|---|---|---|
| RP11-468 | grey | 0.00967083 | 0.834890739 | 0.017511259 | 0.705849391 |
| RP11-468 | grey | -0.024320583 | 0.600110738 | -0.021032373 | 0.650299973 |
| RP11-468 | grey | 0.069578476 | 0.133253684 | 0.055817127 | 0.228618788 |
| RP11-475 | grey | 0.117016605 | 0.011384401 | 0.106989446 | 0.020749797 |
| RP11-47I | grey | 0.060044402 | 0.195229873 | 0.064093121 | 0.166734115 |
| RP11-512 | grey | -0.101725257 | 0.027940922 | -0.092095272 | 0.046691171 |
| RP11-514 | grey | -0.066452701 | 0.151631726 | -0.083055949 | 0.072950013 |
| RP11-514 | grey | 0.0164577 | 0.722795777 | 0.014764038 | 0.750321211 |
| RP11-51L | grey | 0.027394933 | 0.554832844 | 0.0310545 | 0.503204388 |
| RP11-529 | grey | 0.072382316 | 0.118275605 | 0.055521253 | 0.231098941 |
| RP11-540 | grey | 0.176908848 | 0.00012147 | 0.141981251 | 0.002100703 |
| RP11-542 | grey | 0.098603197 | 0.033145509 | 0.084836805 | 0.066991808 |
| RP11-548 | grey | -0.009151063 | 0.843647323 | -0.061697295 | 0.183193976 |
| RP11-54C | grey | -0.088243161 | 0.056707448 | -0.072163868 | 0.11939311 |
| RP11-552 | grey | 0.136564749 | 0.003105011 | 0.140274846 | 0.002379355 |
| RP11-565 | grey | 0.001890808 | 0.967494214 | -0.031456442 | 0.497692123 |
| RP11-574 | grey | 0.017650968 | 0.70361286 | -0.013262595 | 0.774991797 |
| RP11-598 | grey | 0.058718675 | 0.205293445 | 0.046044998 | 0.320758274 |
| RP11-5A1 | grey | 0.100291466 | 0.030236783 | 0.08888541 | 0.054923244 |
| RP11-603 | grey | 0.059773355 | 0.197257495 | 0.063710491 | 0.169285804 |
| RP11-613 | grey | 0.005980593 | 0.897439548 | -0.003594691 | 0.938246486 |
| RP11-618 | grey | 0.000117825 | 0.997973854 | -0.027706095 | 0.550344589 |
| RP11-637 | grey | 0.00755292 | 0.870687809 | -0.016776656 | 0.717650633 |
| RP11-644 | grey | -0.132759809 | 0.004053003 | -0.136348104 | 0.003153035 |
| RP11-683 | grey | -0.016184687 | 0.727209765 | -0.05264546 | 0.256200816 |
| RP11-724 | grey | -0.013442525 | 0.772022809 | 0.001007212 | 0.982681121 |
| RP11-73N | grey | 0.061785933 | 0.182564377 | 0.08981137 | 0.052433203 |
| RP11-745 | grey | 0.029041075 | 0.531293241 | 0.015678921 | 0.735410604 |
| RP11-762 | grey | 0.062221781 | 0.179491792 | 0.054747179 | 0.237677568 |
| RP11-77K | grey | 0.119295115 | 0.009871433 | 0.094916497 | 0.040335488 |
| RP11-793 | grey | 0.164736187 | 0.000350373 | 0.170121295 | 0.000221226 |
| RP11-795 | grey | -0.093432366 | 0.043581288 | -0.124253577 | 0.007180139 |
| RP11-831 | grey | 0.008937182 | 0.847256281 | 0.007040814 | 0.879386168 |
| RP11-834 | grey | 0.046977468 | 0.311045662 | 0.032117777 | 0.488692703 |
| RP11-864 | grey | 0.097538244 | 0.035100499 | 0.102533979 | 0.026713267 |
| RP11-872 | grey | -0.167894569 | 0.000268005 | -0.178294726 | 0.000107179 |
| RP11-87C | grey | 0.065696098 | 0.156356797 | 0.04703466 | 0.31045631 |
| RP11-886 | grey | 0.012685849 | 0.784530511 | -0.003306763 | 0.943184117 |
| RP11-934 | grey | 0.01921822 | 0.678702379 | 0.012883341 | 0.781260516 |
| RP11-944 | grey | -0.042586773 | 0.358483702 | -0.041930882 | 0.365940638 |
| RP11-96C | grey | 0.038885689 | 0.401810623 | 0.042092852 | 0.364090258 |
| RP11-977 | grey | 0.124394822 | 0.007114178 | 0.102574562 | 0.026652905 |
| RP13-512 | grey | 0.034339326 | 0.459112686 | 0.027379037 | 0.5550626 |
| RP13-672 | grey | 0.046908109 | 0.311761378 | 0.024147249 | 0.602712411 |
| RP3-468K | grey | -0.040739201 | 0.379733796 | -0.035106923 | 0.449129448 |

| | | | | | |
|---|---|---|---|---|---|
| RP4-539M | grey | 0.010834954 | 0.815354004 | -0.010023506 | 0.828960679 |
| RP4-559A | grey | -0.146123055 | 0.001543944 | -0.139668037 | 0.002486308 |
| RP4-576H | grey | -0.04829153 | 0.297690085 | -0.061541554 | 0.184304089 |
| RP4-583P | grey | -0.067415024 | 0.145779509 | -0.049811931 | 0.282720925 |
| RP4-583P | grey | -0.068982926 | 0.136615883 | -0.08920927 | 0.054041389 |
| RP4-613E | grey | 0.053988548 | 0.244251799 | 0.039984805 | 0.388628064 |
| RP4-734P | grey | -0.121293838 | 0.008694169 | -0.128930346 | 0.005264354 |
| RP4-777C | grey | 0.122870357 | 0.007855597 | 0.095417138 | 0.039287727 |
| RP5-850E | grey | 0.025301303 | 0.585486834 | 0.000473628 | 0.991855521 |
| RP5-874C | grey | 0.060413242 | 0.192495229 | 0.024130253 | 0.602967797 |
| RP5-972E | grey | -0.054120286 | 0.243101129 | -0.064644803 | 0.163105939 |
| RPGRIP1 | grey | -0.070439031 | 0.128508609 | -0.077462501 | 0.094523206 |
| RPH3AL | grey | 0.148668171 | 0.001272728 | 0.169338479 | 0.000236723 |
| RPL10L | grey | 0.106044101 | 0.021908136 | 0.091155051 | 0.048987735 |
| RPL17-C1 | grey | -0.013907067 | 0.764372939 | -0.011068288 | 0.811451422 |
| RPRML | grey | -0.018367608 | 0.692181009 | -0.009487412 | 0.837978542 |
| RPS10-NU | grey | -0.101717214 | 0.027953371 | -0.080618884 | 0.081796962 |
| RPS4Y1 | grey | -0.005564326 | 0.90454274 | -0.010420037 | 0.822304842 |
| RPS4Y2 | grey | -0.015375037 | 0.740352552 | -0.01514832 | 0.744046556 |
| RPS6KL1 | grey | 0.020650027 | 0.656245769 | 0.016973167 | 0.714487003 |
| RRAGA | grey | -0.253044121 | 2.95E-08 | -0.244803551 | 8.42E-08 |
| RRAGD | grey | -0.089760083 | 0.052568618 | -0.064673414 | 0.16291941 |
| RRBP1 | grey | 0.159673194 | 0.000533065 | 0.163590619 | 0.000385684 |
| RRNAD1 | grey | 0.153052125 | 0.000906027 | 0.13361068 | 0.003820761 |
| RRP8 | grey | 0.102777332 | 0.026353062 | 0.108672907 | 0.018818419 |
| RS1 | grey | 0.01454111 | 0.753968822 | 0.009741011 | 0.833709922 |
| RSG1 | grey | 0.029989446 | 0.517964652 | 0.047958496 | 0.301038208 |
| RSPO2 | grey | 0.047220195 | 0.308549469 | 0.03033398 | 0.513165558 |
| RTBDN | grey | -0.104943376 | 0.023327157 | -0.100613567 | 0.029707409 |
| RTEL1-T | grey | -0.084077425 | 0.069481737 | -0.090426977 | 0.050830378 |
| RTL1 | grey | 0.002920096 | 0.949818395 | 0.038949679 | 0.401035882 |
| RTN2 | grey | -0.191076764 | 3.23E-05 | -0.198455893 | 1.56E-05 |
| RTN4R | grey | -0.091326705 | 0.048561541 | -0.08887918 | 0.054940324 |
| RTN4RL1 | grey | 0.09143108 | 0.048303913 | 0.068462115 | 0.139609293 |
| RTN4RL2 | grey | 0.11825131 | 0.010540842 | 0.105106701 | 0.023111705 |
| RTP1 | grey | -0.052998465 | 0.253021739 | -0.037473364 | 0.419136757 |
| RTP3 | grey | -0.100783699 | 0.029431015 | -0.093034522 | 0.044487845 |
| RUNDC3 | grey | -0.201092894 | 1.19E-05 | -0.222374317 | 1.21E-06 |
| RUNX1 | grey | 0.230200182 | 4.93E-07 | 0.223016923 | 1.13E-06 |
| RUNX3 | grey | -0.155941839 | 0.000720623 | -0.119075619 | 0.010008979 |
| RUSC1 | grey | 0.212650831 | 3.55E-06 | 0.205718097 | 7.40E-06 |
| RWDD2E | grey | -0.000155134 | 0.997332286 | 0.001084046 | 0.981360189 |
| RXFP1 | grey | -0.017609238 | 0.704280629 | -0.044289452 | 0.339574238 |
| RXFP3 | grey | -0.063678003 | 0.169503794 | -0.059464781 | 0.199584492 |
| RXFP4 | grey | 0.147051541 | 0.001439386 | 0.138249464 | 0.002753652 |

| | | | | | |
|---|---|---|---|---|---|
| RXRA | grey | -0.091500301 | 0.048133687 | -0.089537566 | 0.05315951 |
| RXRB | grey | -0.228235104 | 6.20E-07 | -0.235369535 | 2.67E-07 |
| RXRG | grey | 0.01787991 | 0.699953361 | -0.002461205 | 0.957696339 |
| RYR1 | grey | -0.371568134 | 9.78E-17 | -0.362218938 | 6.33E-16 |
| S100A10 | grey | 0.12989649 | 0.004931344 | 0.158289596 | 0.000596572 |
| S100A14 | grey | -0.075037466 | 0.105337261 | -0.038172449 | 0.410506368 |
| S100A16 | grey | -0.037456015 | 0.419352277 | 0.004698066 | 0.919348411 |
| S100A4 | grey | 0.001081421 | 0.981405322 | 0.047836481 | 0.30227111 |
| S100A5 | grey | -0.271144955 | 2.59E-09 | -0.257803245 | 1.59E-08 |
| S100A6 | grey | -0.001945495 | 0.966554612 | 0.041550268 | 0.370311809 |
| S1PR2 | grey | -0.10446665 | 0.023966007 | -0.095304458 | 0.039521517 |
| S1PR5 | grey | -0.091910255 | 0.047135811 | -0.089391239 | 0.053551088 |
| SAA1 | grey | 0.082096914 | 0.076333566 | 0.105354362 | 0.022788286 |
| SAA2 | grey | 0.122946065 | 0.007817209 | 0.150055229 | 0.00114409 |
| SAA2-SA | grey | 0.094496008 | 0.041233663 | 0.106163684 | 0.021758577 |
| SAA4 | grey | 0.067923562 | 0.142757332 | 0.097117967 | 0.035898586 |
| SAFB | grey | 0.075259908 | 0.104306247 | 0.057622971 | 0.213890427 |
| SAFB2 | grey | 0.095021936 | 0.040112879 | 0.068920443 | 0.136972383 |
| SAGE1 | grey | -0.031207106 | 0.501107758 | -0.05430765 | 0.24147114 |
| SALL3 | grey | -0.046141244 | 0.319746732 | -0.01379873 | 0.766154972 |
| SALL4 | grey | -0.193757773 | 2.49E-05 | -0.192151705 | 2.91E-05 |
| SAMD11 | grey | -0.242613834 | 1.11E-07 | -0.23604805 | 2.46E-07 |
| SAMD13 | grey | -0.035433478 | 0.444919839 | -0.071599678 | 0.122317467 |
| SAMD14 | grey | -0.095308993 | 0.039512085 | -0.098045756 | 0.034156924 |
| SAMD4B | grey | -0.192951672 | 2.69E-05 | -0.201336001 | 1.16E-05 |
| SAMM50 | grey | 0.338493764 | 5.55E-14 | 0.320426673 | 1.30E-12 |
| SAP30BP | grey | -0.183371264 | 6.72E-05 | -0.175646641 | 0.000136026 |
| SAPCD1 | grey | 0.020512623 | 0.658387849 | 0.023055948 | 0.619207357 |
| SARDH | grey | -0.085113883 | 0.066101727 | -0.095631192 | 0.038846826 |
| SATB2 | grey | 0.279686185 | 7.70E-10 | 0.274578336 | 1.60E-09 |
| SAYSD1 | grey | -0.109315898 | 0.018123203 | -0.119046893 | 0.010027105 |
| SBK2 | grey | -0.164977301 | 0.000343336 | -0.161705377 | 0.000451094 |
| SBNO2 | grey | 0.182800854 | 7.09E-05 | 0.187875903 | 4.40E-05 |
| SBSPON | grey | -0.013161603 | 0.776659689 | -0.046701059 | 0.313904356 |
| SCAMP2 | grey | 0.253200177 | 2.89E-08 | 0.270703804 | 2.75E-09 |
| SCAMP5 | grey | -0.232248053 | 3.87E-07 | -0.254935351 | 2.31E-08 |
| SCARB1 | grey | 0.102971681 | 0.026068403 | 0.083081993 | 0.07285986 |
| SCD5 | grey | 0.150773989 | 0.001082249 | 0.149766896 | 0.001169801 |
| SCEL | grey | -0.094797491 | 0.040587995 | -0.076650166 | 0.098042288 |
| SCG2 | grey | 0.002682824 | 0.953891178 | -0.012672608 | 0.7847499 |
| SCG3 | grey | -0.110687451 | 0.016714763 | -0.10408845 | 0.024483528 |
| SCG5 | grey | -0.296472639 | 6.27E-11 | -0.304504372 | 1.78E-11 |
| SCGB1C1 | grey | -0.002420654 | 0.958392711 | -0.016275145 | 0.725746249 |
| SCGB1C2 | grey | -0.021096473 | 0.649305322 | -0.029254827 | 0.528274065 |
| SCGB1D1 | grey | 0.125544835 | 0.006596988 | 0.151942842 | 0.000988228 |

| | | | | | |
|---|---|---|---|---|---|
| SCGB3A2 | grey | -0.069806542 | 0.131983166 | -0.062134294 | 0.180105458 |
| SCGN | grey | 0.034207189 | 0.460843663 | 0.001672754 | 0.971241168 |
| SCIN | grey | -0.152977237 | 0.000911371 | -0.147331367 | 0.001409175 |
| SCMH1 | grey | 0.118355632 | 0.010472174 | 0.094608537 | 0.040991658 |
| SCN4A | grey | -0.184364587 | 6.13E-05 | -0.184761899 | 5.90E-05 |
| SCN5A | grey | -0.312424131 | 4.95E-12 | -0.314470954 | 3.53E-12 |
| SCN8A | grey | -0.332303615 | 1.68E-13 | -0.33244238 | 1.63E-13 |
| SCN9A | grey | -0.108776265 | 0.018705121 | -0.103909651 | 0.024731535 |
| SCNN1D | grey | -0.174258781 | 0.000153914 | -0.184096387 | 6.28E-05 |
| SCPEP1 | grey | -0.060993746 | 0.188248273 | -0.066086755 | 0.153903388 |
| SCRT1 | grey | -0.093822089 | 0.042708365 | -0.109082364 | 0.018373068 |
| SCRT2 | grey | -0.018303454 | 0.693201598 | -0.025584293 | 0.581297941 |
| SCTR | grey | -0.002857022 | 0.950900946 | 0.004775782 | 0.918018903 |
| SCUBE2 | grey | -0.006414372 | 0.890046241 | -0.004862974 | 0.916527581 |
| SCUBE3 | grey | -0.033598921 | 0.468858573 | -0.060759396 | 0.189954401 |
| SCX | grey | -0.172288939 | 0.000183122 | -0.18052091 | 8.75E-05 |
| SDC4 | grey | 0.01228128 | 0.791241023 | 0.024824421 | 0.592577217 |
| SDCBP2 | grey | 0.194398891 | 2.34E-05 | 0.232653278 | 3.69E-07 |
| SDF2 | grey | -0.029297662 | 0.527670073 | -0.031558442 | 0.496298399 |
| SDK1 | grey | -0.166424236 | 0.000303798 | -0.204539475 | 8.37E-06 |
| SDK2 | grey | -0.256429095 | 1.90E-08 | -0.264344407 | 6.60E-09 |
| SDR16C5 | grey | -0.23852578 | 1.83E-07 | -0.251309768 | 3.69E-08 |
| SDR42E1 | grey | -0.3113735 | 5.88E-12 | -0.280438078 | 6.90E-10 |
| SEC11C | grey | 0.210145898 | 4.64E-06 | 0.211971164 | 3.82E-06 |
| SEC14L2 | grey | 0.067550938 | 0.144967049 | 0.062128134 | 0.180148719 |
| SEC14L4 | grey | -0.209331476 | 5.06E-06 | -0.218030583 | 1.97E-06 |
| SEC14L6 | grey | -0.20224325 | 1.06E-05 | -0.223518785 | 1.07E-06 |
| SEC31B | grey | 0.115473962 | 0.012521926 | 0.113051431 | 0.014511116 |
| SELV | grey | -0.206378342 | 6.91E-06 | -0.204597687 | 8.32E-06 |
| SEMA3A | grey | 0.088321514 | 0.056487236 | 0.126542359 | 0.006175949 |
| SEMA3B | grey | -0.141843376 | 0.002122057 | -0.157269703 | 0.000647804 |
| SEMA3F | grey | -0.014243407 | 0.758848476 | -0.006511226 | 0.888396757 |
| SEMA4F | grey | 0.042404161 | 0.36055022 | 0.004955969 | 0.914937307 |
| SEMA4G | grey | 0.010033298 | 0.828796178 | -0.001902207 | 0.967298366 |
| SEMA5B | grey | 0.018710184 | 0.686740624 | -0.002223973 | 0.961770658 |
| SEMA6A | grey | -0.076742007 | 0.097639273 | -0.107438677 | 0.02021826 |
| SEMA6C | grey | -0.265541615 | 5.61E-09 | -0.270838634 | 2.70E-09 |
| SEMA6D | grey | -0.302598603 | 2.41E-11 | -0.319460949 | 1.54E-12 |
| SEMA7A | grey | 0.050630385 | 0.274877036 | 0.02147338 | 0.643469444 |
| SEMG1 | grey | -0.029724219 | 0.521674769 | -0.011497203 | 0.804289926 |
| SEMG2 | grey | 0.025714165 | 0.579380228 | 0.031790227 | 0.49313903 |
| SENP3-EI | grey | 0.177510924 | 0.000115054 | 0.157164969 | 0.00065329 |
| SEPN1 | grey | 0.362321829 | 6.21E-16 | 0.333589568 | 1.33E-13 |
| SEPT14 | grey | -0.048436061 | 0.296244807 | -0.052438227 | 0.258079946 |
| SEPT5 | grey | -0.160207602 | 0.000510263 | -0.169736321 | 0.000228725 |

| | | | | | |
|---|---|---|---|---|---|
| SEPT6 | grey | -0.084575704 | 0.067839538 | -0.062176061 | 0.179812293 |
| SERF1A | grey | -0.005733988 | 0.901646662 | -0.003548403 | 0.939040109 |
| SERHL2 | grey | 0.024661596 | 0.595007096 | -0.002565187 | 0.955910868 |
| SERINC4 | grey | 0.109260898 | 0.018181781 | 0.108029049 | 0.019537756 |
| SERPINA | grey | 0.137289627 | 0.002949119 | 0.153054817 | 0.000905835 |
| SERPINA | grey | -0.074456603 | 0.108067569 | -0.066846326 | 0.149216756 |
| SERPINA | grey | 0.219062387 | 1.76E-06 | 0.207535846 | 6.12E-06 |
| SERPINA | grey | 0.152918918 | 0.000915553 | 0.14672618 | 0.001475261 |
| SERPINA | grey | -0.091755473 | 0.047510512 | -0.044529309 | 0.336962646 |
| SERPINB | grey | 0.228634255 | 5.92E-07 | 0.256672292 | 1.84E-08 |
| SERPINB | grey | 0.033034106 | 0.476369235 | 0.03693607 | 0.425841507 |
| SERPINB | grey | 0.138435073 | 0.002717244 | 0.120780012 | 0.00898422 |
| SERPINB | grey | 0.024023882 | 0.60456719 | 0.007292677 | 0.875106277 |
| SERPINB | grey | -0.001587948 | 0.972698606 | -0.012884234 | 0.781245735 |
| SERPINB | grey | 0.033441442 | 0.470946067 | 0.041444853 | 0.371528143 |
| SERPINF | grey | -0.015476319 | 0.738704235 | -0.022010873 | 0.635184942 |
| SERPINI1 | grey | -0.188456218 | 4.16E-05 | -0.220841096 | 1.44E-06 |
| SERTAD | grey | -0.111132166 | 0.016279129 | -0.106706495 | 0.021090786 |
| SESN2 | grey | 0.247182385 | 6.25E-08 | 0.249583302 | 4.61E-08 |
| SETD6 | grey | 0.064163894 | 0.166265319 | 0.068917242 | 0.136990665 |
| SEZ6 | grey | -0.021575109 | 0.641898035 | -0.033875519 | 0.465204448 |
| SEZ6L2 | grey | -0.105228638 | 0.022951975 | -0.096921054 | 0.036277791 |
| SF3B4 | grey | 0.083618066 | 0.071024352 | 0.073261122 | 0.113862365 |
| SFRP2 | grey | 0.002274187 | 0.960908175 | 0.035729781 | 0.441119704 |
| SFRP5 | grey | -0.248083284 | 5.58E-08 | -0.265341884 | 5.77E-09 |
| SFSWAP | grey | -0.084669827 | 0.067532936 | -0.108816641 | 0.018661024 |
| SFTA2 | grey | 0.073995454 | 0.110274679 | 0.101366122 | 0.028501454 |
| SFTA3 | grey | -0.001518541 | 0.973891473 | 0.02096396 | 0.651362243 |
| SFTPA1 | grey | -0.053706149 | 0.246731253 | -0.061803594 | 0.182439122 |
| SFTPA2 | grey | 0.051336559 | 0.268229322 | 0.046584793 | 0.315111943 |
| SFTPB | grey | 0.016354263 | 0.72446703 | 0.033437339 | 0.471000525 |
| SFTPC | grey | -0.047529954 | 0.305383158 | -0.04540444 | 0.327543581 |
| SFTPD | grey | -0.094725937 | 0.040740457 | -0.118427247 | 0.010425265 |
| SFXN2 | grey | 0.140517278 | 0.002337811 | 0.09447635 | 0.041276063 |
| SGCD | grey | 0.059595758 | 0.198594353 | 0.041422145 | 0.371790483 |
| SGCG | grey | -0.257415273 | 1.67E-08 | -0.272544636 | 2.13E-09 |
| SGK1 | grey | -0.072339275 | 0.118495139 | -0.092695105 | 0.045273754 |
| SGK2 | grey | 0.053661365 | 0.247126068 | 0.060607262 | 0.191068042 |
| SGK223 | grey | 0.174561919 | 0.000149828 | 0.179188654 | 9.88E-05 |
| SGSH | grey | 0.15538416 | 0.000753409 | 0.171450739 | 0.000197062 |
| SGSM1 | grey | -0.344769954 | 1.76E-14 | -0.346541269 | 1.27E-14 |
| SGSM2 | grey | 0.214517523 | 2.90E-06 | 0.195225448 | 2.15E-05 |
| SH2B1 | grey | -0.03048363 | 0.511088269 | -0.04299451 | 0.353896454 |
| SH3BP2 | grey | -0.146823347 | 0.001464462 | -0.130461024 | 0.00474567 |
| SH3D21 | grey | -0.016941007 | 0.715004409 | -0.030531007 | 0.510431529 |

| | | | | | |
|---|---|---|---|---|---|
| SH3GL2 | grey | -0.097546222 | 0.035085495 | -0.094556646 | 0.041103104 |
| SH3KBP1 | grey | -0.171403902 | 0.000197869 | -0.178598338 | 0.000104267 |
| SH3TC1 | grey | -0.031430254 | 0.498050288 | -0.021612498 | 0.641320884 |
| SH3TC2 | grey | -0.060649386 | 0.190759211 | -0.06834571 | 0.140285171 |
| SHANK1 | grey | -0.168003983 | 0.000265506 | -0.1757981 | 0.000134196 |
| SHBG | grey | -0.07583573 | 0.101674418 | -0.053521323 | 0.248363505 |
| SHC3 | grey | -0.000311425 | 0.994644711 | -0.013168101 | 0.776552345 |
| SHC4 | grey | -0.03694651 | 0.425710632 | -0.035678137 | 0.441780701 |
| SHD | grey | -0.016343471 | 0.724641481 | -0.018479735 | 0.690398586 |
| SHH | grey | 0.175061853 | 0.000143311 | 0.195054238 | 2.19E-05 |
| SHISA2 | grey | -0.020637612 | 0.656439189 | -0.021030022 | 0.650336474 |
| SHISA3 | grey | 0.018673301 | 0.687325594 | 0.010683876 | 0.817883259 |
| SHISA4 | grey | -0.128830918 | 0.005299748 | -0.151674155 | 0.001009148 |
| SHISA9 | grey | -0.105028628 | 0.023214479 | -0.078216218 | 0.091349013 |
| SHOX2 | grey | -0.053818519 | 0.245742548 | -0.034839947 | 0.452587695 |
| SHPK | grey | 0.26138787 | 9.84E-09 | 0.259299088 | 1.30E-08 |
| SHROOM | grey | -0.045770727 | 0.323652289 | -0.07392012 | 0.110638587 |
| SHROOM | grey | -0.012279713 | 0.791267041 | -0.03401556 | 0.463360403 |
| SIAH2 | grey | -0.009099111 | 0.844523644 | -0.008937478 | 0.847251282 |
| SIGLEC1 | grey | 0.099558551 | 0.031471517 | 0.121955892 | 0.008332757 |
| SIK1 | grey | -0.027157132 | 0.558274914 | -0.079180962 | 0.087411358 |
| SIM1 | grey | -0.059138376 | 0.202067646 | -0.033410867 | 0.471351946 |
| SIM2 | grey | 0.067666373 | 0.14427973 | 0.085481047 | 0.064937244 |
| SIRT2 | grey | -0.041781893 | 0.367647869 | -0.040286492 | 0.385056126 |
| SIRT3 | grey | 0.16152229 | 0.000457968 | 0.146435258 | 0.001508033 |
| SIRT4 | grey | -0.148526155 | 0.001286623 | -0.147093571 | 0.001434811 |
| SIX1 | grey | 0.023298616 | 0.615522481 | 0.039870412 | 0.389987701 |
| SIX2 | grey | 0.002083171 | 0.964189321 | 0.036015317 | 0.437475244 |
| SIX3 | grey | -0.142060178 | 0.002088567 | -0.126220619 | 0.006309038 |
| SKAP1 | grey | -0.165767156 | 0.000321194 | -0.126187209 | 0.006323004 |
| SKIDA1 | grey | -0.125072968 | 0.006804977 | -0.153478614 | 0.00087614 |
| SKOR1 | grey | 0.021723614 | 0.639606958 | -0.013131567 | 0.777155939 |
| SLAMF9 | grey | 0.036686044 | 0.428982663 | 0.062223922 | 0.1794768 |
| SLC10A2 | grey | -0.052666496 | 0.256010604 | -0.04537622 | 0.32784463 |
| SLC10A4 | grey | -0.139645367 | 0.002490388 | -0.129744918 | 0.004982299 |
| SLC12A1 | grey | 0.00419522 | 0.927955919 | -0.012984857 | 0.779581143 |
| SLC12A3 | grey | -0.048549071 | 0.295118001 | -0.06247304 | 0.177738002 |
| SLC12A5 | grey | -0.181976577 | 7.65E-05 | -0.194617896 | 2.29E-05 |
| SLC12A9 | grey | 0.052423683 | 0.258212176 | 0.053839992 | 0.24555393 |
| SLC13A2 | grey | 0.061187063 | 0.186849377 | 0.059433295 | 0.199823058 |
| SLC13A3 | grey | -0.08022164 | 0.083318044 | -0.073270727 | 0.113814856 |
| SLC13A5 | grey | -0.315593108 | 2.93E-12 | -0.315850366 | 2.81E-12 |
| SLC14A1 | grey | -0.003980452 | 0.931634851 | 0.018250586 | 0.694043057 |
| SLC14A2 | grey | -0.123610816 | 0.00748727 | -0.124890744 | 0.006886857 |
| SLC15A1 | grey | -0.083397924 | 0.071773491 | -0.093342472 | 0.043784755 |

| | | | | | |
|---|---|---|---|---|---|
| SLC16A1 | grey | 0.158131819 | 0.000604244 | 0.145448457 | 0.001624236 |
| SLC16A1 | grey | 0.054117819 | 0.243122643 | 0.035958597 | 0.438197811 |
| SLC16A5 | grey | -0.254549884 | 2.43E-08 | -0.230262072 | 4.89E-07 |
| SLC16A6 | grey | -0.1948536 | 2.23E-05 | -0.188979382 | 3.96E-05 |
| SLC16A8 | grey | 0.093392744 | 0.043670871 | 0.07749212 | 0.094396828 |
| SLC16A9 | grey | -0.138339737 | 0.00273589 | -0.110639962 | 0.01676188 |
| SLC17A3 | grey | -0.106195669 | 0.021718726 | -0.104696236 | 0.023656481 |
| SLC17A4 | grey | -0.089809186 | 0.052438965 | -0.087292704 | 0.059435875 |
| SLC17A7 | grey | -0.104973816 | 0.02328687 | -0.106617679 | 0.021198818 |
| SLC17A9 | grey | -0.223794252 | 1.03E-06 | -0.223852194 | 1.03E-06 |
| SLC18A1 | grey | -0.022411522 | 0.629039062 | -0.037343479 | 0.420751853 |
| SLC18A2 | grey | 0.005253991 | 0.909843296 | -0.005864958 | 0.899411948 |
| SLC18A3 | grey | -0.057816114 | 0.212356509 | -0.048599336 | 0.294617727 |
| SLC19A3 | grey | -0.196540234 | 1.89E-05 | -0.20901503 | 5.23E-06 |
| SLC1A1 | grey | 0.205756558 | 7.37E-06 | 0.213341473 | 3.29E-06 |
| SLC1A3 | grey | -0.115183922 | 0.012746662 | -0.109210324 | 0.018235791 |
| SLC1A4 | grey | 0.144024226 | 0.001806474 | 0.148552726 | 0.001284013 |
| SLC1A5 | grey | -0.043224689 | 0.351323214 | -0.004604769 | 0.920944746 |
| SLC1A6 | grey | -0.107842093 | 0.019751064 | -0.113365697 | 0.014238295 |
| SLC20A2 | grey | -0.048882861 | 0.291806547 | -0.077441219 | 0.094614097 |
| SLC22A1 | grey | -0.026160354 | 0.572814372 | -0.010820918 | 0.815588894 |
| SLC22A1 | grey | 0.026184112 | 0.572465754 | 0.026989882 | 0.56070196 |
| SLC22A1 | grey | -0.20867831 | 5.42E-06 | -0.197028943 | 1.80E-05 |
| SLC22A1 | grey | 0.259674595 | 1.24E-08 | 0.285796884 | 3.15E-10 |
| SLC22A3 | grey | -0.114833124 | 0.013023226 | -0.086914275 | 0.060552018 |
| SLC22A8 | grey | -0.057733752 | 0.213009652 | -0.069813768 | 0.131943065 |
| SLC23A3 | grey | 0.001123529 | 0.980681431 | 0.009726659 | 0.833951378 |
| SLC24A2 | grey | -0.132117475 | 0.004236705 | -0.139845016 | 0.002454671 |
| SLC25A1 | grey | -0.136108325 | 0.003206973 | -0.137182878 | 0.00297162 |
| SLC25A2 | grey | 0.144577407 | 0.001733575 | 0.177388405 | 0.000116333 |
| SLC25A2 | grey | -0.000125637 | 0.99783952 | -0.015550977 | 0.737489989 |
| SLC25A2 | grey | -0.043325214 | 0.350203133 | -0.073729506 | 0.111563588 |
| SLC25A2 | grey | -0.025212932 | 0.586797791 | -0.020642992 | 0.656355376 |
| SLC25A2 | grey | -0.068393964 | 0.140004691 | -0.061263987 | 0.186294861 |
| SLC25A3 | grey | -0.096263872 | 0.037568078 | -0.1061803 | 0.021737868 |
| SLC25A3 | grey | 0.15280915 | 0.000923472 | 0.122408894 | 0.008093246 |
| SLC25A3 | grey | 0.06208526 | 0.180450072 | 0.078689561 | 0.089399632 |
| SLC25A4 | grey | -0.111703574 | 0.015734006 | -0.109344055 | 0.018093279 |
| SLC25A4 | grey | 0.261734388 | 9.39E-09 | 0.262832434 | 8.10E-09 |
| SLC25A4 | grey | -0.006047371 | 0.896300793 | 0.011888718 | 0.79776715 |
| SLC25A4 | grey | 0.162427236 | 0.000424916 | 0.150221521 | 0.0011295 |
| SLC26A1 | grey | 0.047756819 | 0.303077854 | 0.054588486 | 0.239042379 |
| SLC26A1 | grey | -0.101635099 | 0.028080741 | -0.130859695 | 0.004618372 |
| SLC26A3 | grey | -0.025840306 | 0.577520447 | -0.022646931 | 0.625439804 |
| SLC26A4 | grey | 0.053050388 | 0.252556452 | 0.039403872 | 0.395562641 |

| | | | | | |
|---|---|---|---|---|---|
| SLC26A7 | grey | -0.102580532 | 0.026644035 | -0.112763948 | 0.014764699 |
| SLC26A9 | grey | 0.034520124 | 0.456750172 | 0.068957521 | 0.136760749 |
| SLC27A3 | grey | -0.0896152 | 0.052952728 | -0.082054791 | 0.076485056 |
| SLC28A2 | grey | 0.065556142 | 0.157242912 | 0.058480401 | 0.207141327 |
| SLC29A3 | grey | -0.248150583 | 5.53E-08 | -0.239731515 | 1.58E-07 |
| SLC29A4 | grey | -0.028875001 | 0.533644982 | -0.015004944 | 0.746385675 |
| SLC2A10 | grey | -0.195381985 | 2.12E-05 | -0.189856039 | 3.64E-05 |
| SLC2A11 | grey | -0.112832237 | 0.014704113 | -0.13776681 | 0.002850418 |
| SLC2A14 | grey | -0.047776327 | 0.302880164 | -0.025798067 | 0.578142896 |
| SLC2A9 | grey | 0.037929891 | 0.413488818 | 0.041938025 | 0.365858911 |
| SLC30A2 | grey | -0.094718791 | 0.040755712 | -0.07272241 | 0.116552095 |
| SLC30A3 | grey | -0.12970885 | 0.004994494 | -0.121771255 | 0.008432177 |
| SLC30A8 | grey | -0.125220996 | 0.006739106 | -0.115523405 | 0.012483966 |
| SLC34A3 | grey | -0.055729017 | 0.229355378 | -0.050724363 | 0.273985943 |
| SLC35C1 | grey | 0.199965626 | 1.34E-05 | 0.213206189 | 3.34E-06 |
| SLC35D2 | grey | 0.204677143 | 8.25E-06 | 0.184249215 | 6.19E-05 |
| SLC35E3 | grey | 0.182827367 | 7.07E-05 | 0.187405221 | 4.60E-05 |
| SLC35E4 | grey | -0.207861123 | 5.91E-06 | -0.185550085 | 5.48E-05 |
| SLC35F2 | grey | -0.028823718 | 0.534372242 | -0.025586747 | 0.581261677 |
| SLC35F6 | grey | 0.058416767 | 0.207636851 | 0.105675073 | 0.0223753 |
| SLC35G5 | grey | 0.081556736 | 0.078294842 | 0.077578214 | 0.094030246 |
| SLC35G6 | grey | -0.131286091 | 0.004485641 | -0.148637533 | 0.001275714 |
| SLC38A1 | grey | 0.291583086 | 1.32E-10 | 0.270090648 | 3.00E-09 |
| SLC38A1 | grey | 0.044302416 | 0.339432756 | 0.036524118 | 0.431024128 |
| SLC38A5 | grey | -0.073213352 | 0.11409889 | -0.069943316 | 0.131225722 |
| SLC38A8 | grey | -0.02297749 | 0.620400772 | -0.015616243 | 0.736429016 |
| SLC39A1 | grey | -0.222582256 | 1.18E-06 | -0.216279694 | 2.39E-06 |
| SLC39A2 | grey | -0.078045519 | 0.092060305 | -0.08677494 | 0.060967312 |
| SLC39A5 | grey | -0.177995513 | 0.000110123 | -0.174243905 | 0.000154118 |
| SLC39A8 | grey | 0.042119669 | 0.363784465 | 0.033211614 | 0.474001751 |
| SLC3A1 | grey | -0.026490842 | 0.567973917 | -0.023755726 | 0.608607579 |
| SLC41A3 | grey | -0.058924056 | 0.203710265 | -0.077985121 | 0.09231304 |
| SLC43A2 | grey | 0.182101906 | 7.56E-05 | 0.205678674 | 7.44E-06 |
| SLC43A3 | grey | -0.387286733 | 3.68E-18 | -0.385062662 | 5.92E-18 |
| SLC44A5 | grey | -0.187049181 | 4.76E-05 | -0.195987716 | 2.00E-05 |
| SLC45A2 | grey | 0.003400732 | 0.941572405 | -0.003844748 | 0.933960206 |
| SLC45A3 | grey | 0.019882579 | 0.668245618 | 0.039817475 | 0.390617871 |
| SLC4A11 | grey | 0.040786614 | 0.379178996 | 0.066535411 | 0.151121838 |
| SLC4A3 | grey | -0.264838077 | 6.17E-09 | -0.286226428 | 2.95E-10 |
| SLC5A2 | grey | -0.117604477 | 0.010975587 | -0.111373463 | 0.016046946 |
| SLC5A5 | grey | -0.033057524 | 0.476056526 | -0.024074795 | 0.603801421 |
| SLC5A7 | grey | 0.014315232 | 0.757670301 | 0.011488087 | 0.804441969 |
| SLC5A8 | grey | -0.132637709 | 0.004087358 | -0.106498204 | 0.0213449 |
| SLC6A11 | grey | -0.409881011 | 2.39E-20 | -0.413828376 | 9.54E-21 |
| SLC6A13 | grey | -0.254771626 | 2.36E-08 | -0.2659282 | 5.32E-09 |

| | | | | | |
|---|---|---|---|---|---|
| SLC6A15 | grey | -0.327016344 | 4.22E-13 | -0.336540397 | 7.88E-14 |
| SLC6A18 | grey | -0.016567578 | 0.72102188 | -0.011113282 | 0.810699411 |
| SLC6A20 | grey | 0.159340099 | 0.000547753 | 0.168523067 | 0.000253942 |
| SLC6A3 | grey | -0.192572077 | 2.79E-05 | -0.185032578 | 5.75E-05 |
| SLC6A4 | grey | -0.039461585 | 0.394870407 | -0.052354963 | 0.258837628 |
| SLC6A8 | grey | -0.042778253 | 0.356324832 | -0.020702389 | 0.655430191 |
| SLC6A9 | grey | -0.209937112 | 4.75E-06 | -0.174419691 | 0.000151733 |
| SLC7A10 | grey | -0.310786958 | 6.47E-12 | -0.316487762 | 2.53E-12 |
| SLC7A11 | grey | 0.251316828 | 3.69E-08 | 0.221235269 | 1.38E-06 |
| SLC7A14 | grey | -0.066183575 | 0.153299868 | -0.066121359 | 0.153687479 |
| SLC7A2 | grey | -0.141127839 | 0.002236102 | -0.150223929 | 0.00112929 |
| SLC7A4 | grey | -0.039739366 | 0.391548811 | -0.092950308 | 0.044681761 |
| SLC7A6 | grey | -0.015854572 | 0.732559002 | -0.021560669 | 0.642120985 |
| SLC7A8 | grey | -0.287873685 | 2.31E-10 | -0.304868314 | 1.68E-11 |
| SLC7A9 | grey | -0.266427612 | 4.97E-09 | -0.271676404 | 2.40E-09 |
| SLC8B1 | grey | -0.024385282 | 0.599140924 | -0.019788921 | 0.669715927 |
| SLC9A2 | grey | 0.160751327 | 0.000487996 | 0.155847642 | 0.000726067 |
| SLC9A3 | grey | -0.061964655 | 0.181299781 | -0.073762858 | 0.111401301 |
| SLC9A4 | grey | 0.110459667 | 0.016941822 | 0.10036868 | 0.03010915 |
| SLC9A5 | grey | 0.040096705 | 0.387300844 | 0.039962011 | 0.38889875 |
| SLC9A8 | grey | -0.0090687 | 0.845036707 | -0.012050413 | 0.795077317 |
| SLCO1B3 | grey | 0.09370037 | 0.042979402 | 0.101202573 | 0.028759902 |
| SLCO1C1 | grey | 0.016252881 | 0.726106365 | 0.085483563 | 0.064929324 |
| SLCO4C1 | grey | -0.176407015 | 0.000127072 | -0.174018102 | 0.000157233 |
| SLFN11 | grey | -0.155691601 | 0.000735168 | -0.155175202 | 0.000766045 |
| SLFN13 | grey | -0.327453514 | 3.92E-13 | -0.323042828 | 8.37E-13 |
| SLIT1 | grey | -0.124157699 | 0.007225225 | -0.117966198 | 0.010730552 |
| SLITRK2 | grey | -0.204603384 | 8.32E-06 | -0.213097081 | 3.38E-06 |
| SLITRK4 | grey | -0.198762433 | 1.51E-05 | -0.223837256 | 1.03E-06 |
| SLITRK6 | grey | 0.215357845 | 2.64E-06 | 0.191784263 | 3.02E-05 |
| SLN | grey | -0.091440851 | 0.048279854 | -0.083230824 | 0.072346428 |
| SLX1A | grey | -0.077389023 | 0.094837308 | -0.06419756 | 0.166042666 |
| SLX1A-S | grey | -0.13614821 | 0.003197944 | -0.162271067 | 0.000430457 |
| SMAD6 | grey | -0.2244012 | 9.63E-07 | -0.209950677 | 4.74E-06 |
| SMAGP | grey | -0.007070167 | 0.878887201 | -0.004725379 | 0.918881128 |
| SMARCD | grey | -0.290322954 | 1.60E-10 | -0.303299587 | 2.15E-11 |
| SMCO2 | grey | -0.159531333 | 0.000539275 | -0.157024676 | 0.000660706 |
| SMIM11 | grey | -0.087706035 | 0.05823629 | -0.089598915 | 0.052996049 |
| SMIM19 | grey | 0.077035318 | 0.096360998 | 0.04088374 | 0.37804404 |
| SMIM2 | grey | -0.13648325 | 0.003122998 | -0.134798474 | 0.003516701 |
| SMIM23 | grey | 0.058991162 | 0.203194908 | 0.061122618 | 0.18731486 |
| SMIM24 | grey | -0.070986636 | 0.125557968 | -0.06875568 | 0.137915872 |
| SMIM3 | grey | -0.121130255 | 0.008785592 | -0.113480493 | 0.014139769 |
| SMN2 | grey | -0.009574919 | 0.836505062 | -0.001003895 | 0.982738146 |
| SMO | grey | -0.152208629 | 0.000967928 | -0.167210657 | 0.000284133 |

| | | | | | |
|---|---|---|---|---|---|
| SMPD1 | grey | 0.129269134 | 0.005145343 | 0.147874759 | 0.001352166 |
| SMR3A | grey | 0.096454654 | 0.037189553 | 0.096881477 | 0.036354416 |
| SMR3B | grey | -0.008581902 | 0.85325816 | -0.013808681 | 0.76599124 |
| SMTNL2 | grey | -0.097176869 | 0.035785814 | -0.127667899 | 0.005729965 |
| SMYD1 | grey | -0.021271457 | 0.64659324 | -0.019714296 | 0.670888358 |
| SMYD3 | grey | 0.088137261 | 0.05700621 | 0.077513953 | 0.094303758 |
| SNAI1 | grey | -0.2467356 | 6.61E-08 | -0.239868461 | 1.55E-07 |
| SNAP29 | grey | 0.091507448 | 0.048116141 | 0.072932086 | 0.115499351 |
| SNAP91 | grey | -0.097219796 | 0.035703814 | -0.101893434 | 0.027681707 |
| SNAPC5 | grey | -0.108809794 | 0.018668496 | -0.087251284 | 0.059557204 |
| SNCB | grey | -0.091463041 | 0.048225253 | -0.082414337 | 0.075199823 |
| SNCG | grey | -0.299215674 | 4.10E-11 | -0.298013403 | 4.94E-11 |
| SND1 | grey | 0.173815326 | 0.00016008 | 0.152965936 | 0.00091218 |
| SNRPN | grey | -0.012660821 | 0.784945197 | -0.012815641 | 0.782381032 |
| SNTA1 | grey | -0.229362817 | 5.44E-07 | -0.21831028 | 1.91E-06 |
| SNTB1 | grey | 0.069130141 | 0.135778764 | 0.025508446 | 0.582419282 |
| SNTG1 | grey | -0.082048337 | 0.076508289 | -0.083632266 | 0.070976253 |
| SNX11 | grey | 0.126250413 | 0.006296606 | 0.145568699 | 0.00160965 |
| SNX32 | grey | -0.405371905 | 6.75E-20 | -0.415660661 | 6.20E-21 |
| SOCS3 | grey | -0.252421496 | 3.20E-08 | -0.257316225 | 1.69E-08 |
| SOHLH1 | grey | -0.094967875 | 0.040226885 | -0.08166528 | 0.07789749 |
| SOHLH2 | grey | -0.102254565 | 0.027132067 | -0.09098649 | 0.049409287 |
| SORCS2 | grey | -0.295726828 | 7.03E-11 | -0.299923494 | 3.67E-11 |
| SORCS3 | grey | -0.2069714 | 6.49E-06 | -0.207402736 | 6.21E-06 |
| SORD | grey | 0.276214631 | 1.27E-09 | 0.259105549 | 1.33E-08 |
| SOST | grey | -0.099773233 | 0.031105457 | -0.10244267 | 0.02684951 |
| SOSTDC1 | grey | -0.202023329 | 1.08E-05 | -0.19547445 | 2.10E-05 |
| SOX1 | grey | -0.10278417 | 0.026343001 | -0.102153866 | 0.027284378 |
| SOX11 | grey | -0.303835457 | 1.98E-11 | -0.307930824 | 1.03E-11 |
| SOX13 | grey | 0.090789739 | 0.049905176 | 0.074340518 | 0.108619854 |
| SOX2 | grey | 0.002290648 | 0.960625458 | 0.021278264 | 0.646487838 |
| SOX21 | grey | 0.066381889 | 0.152069302 | 0.085779511 | 0.064003125 |
| SOX8 | grey | -0.114548347 | 0.013251616 | -0.10858664 | 0.018913439 |
| SOX9 | grey | 0.181720243 | 7.83E-05 | 0.16547532 | 0.000329215 |
| SP8 | grey | -0.012467645 | 0.788147868 | -0.005861833 | 0.899465263 |
| SP9 | grey | 0.067749001 | 0.143789278 | 0.016818333 | 0.716979266 |
| SPACA5 | grey | 0.014262944 | 0.758527935 | 0.012814212 | 0.782404683 |
| SPAG11A | grey | 0.094416993 | 0.041404312 | 0.075408971 | 0.103619827 |
| SPAG11B | grey | 0.08256697 | 0.074659562 | 0.062852974 | 0.175110261 |
| SPANXA | grey | -0.042637663 | 0.357909136 | -0.043798149 | 0.344963907 |
| SPANXA | grey | -0.071516263 | 0.122754524 | -0.076101265 | 0.100478663 |
| SPANXB | grey | -0.040847961 | 0.378461896 | -0.014456903 | 0.755348078 |
| SPANXN | grey | 0.080879816 | 0.080810104 | 0.040057497 | 0.387765565 |
| SPANXN | grey | 0.089896693 | 0.052208569 | 0.051166444 | 0.269820579 |
| SPANXN | grey | -0.005528001 | 0.905162967 | 0.013821554 | 0.765779436 |

| | | | | | |
|---|---|---|---|---|---|
| SPANXN | grey | -0.008575381 | 0.853368415 | 0.001002531 | 0.982761593 |
| SPATA20 | grey | 0.131665415 | 0.004370469 | 0.12942549 | 0.00509124 |
| SPATA22 | grey | -0.03999037 | 0.38856199 | -0.05495195 | 0.235924586 |
| SPATA25 | grey | -0.337989077 | 6.07E-14 | -0.358870949 | 1.22E-15 |
| SPATA31 | grey | -0.080362563 | 0.082775842 | -0.067898558 | 0.142904802 |
| SPATA31 | grey | -0.043403224 | 0.34933548 | -0.044664053 | 0.335501191 |
| SPATA31 | grey | -0.076293495 | 0.099620009 | -0.081519075 | 0.078433089 |
| SPATA31 | grey | -0.017496947 | 0.706078653 | -0.020114632 | 0.664608127 |
| SPATA45 | grey | 0.08813089 | 0.057024226 | 0.079199229 | 0.08733814 |
| SPATA8 | grey | -0.051592989 | 0.265842841 | -0.050093961 | 0.280001114 |
| SPATC1L | grey | -0.081593529 | 0.078159969 | -0.068994342 | 0.136550827 |
| SPATS2L | grey | -0.144256106 | 0.001775581 | -0.114329894 | 0.0134292 |
| SPDYC | grey | 0.131783519 | 0.004335159 | 0.153115801 | 0.000901505 |
| SPDYE3 | grey | -0.136863004 | 0.003039983 | -0.143989637 | 0.001811124 |
| SPDYE4 | grey | 0.000837219 | 0.98560378 | 0.022769318 | 0.623572088 |
| SPECC1L | grey | 0.057248054 | 0.216890684 | 0.044608174 | 0.336106771 |
| SPEM1 | grey | 0.019575202 | 0.673075792 | 0.017721166 | 0.702490054 |
| SPERT | grey | -0.192247083 | 2.88E-05 | -0.167435063 | 0.000278744 |
| SPESP1 | grey | -0.134127481 | 0.003685654 | -0.130384342 | 0.004770515 |
| SPHK2 | grey | 0.062297974 | 0.178958618 | 0.053021709 | 0.252813376 |
| SPIN2B | grey | -0.065416608 | 0.158130121 | -0.048388699 | 0.2967179 |
| SPINK1 | grey | -0.014595182 | 0.753083578 | 0.007087577 | 0.878591261 |
| SPINK2 | grey | -0.041818179 | 0.367231617 | -0.049880359 | 0.282059396 |
| SPINK8 | grey | 0.034271119 | 0.460005731 | 0.061583162 | 0.184007027 |
| SPNS2 | grey | 0.040569814 | 0.38171991 | 0.053078081 | 0.25230854 |
| SPOCD1 | grey | -0.088815363 | 0.055115544 | -0.064059618 | 0.166956386 |
| SPOCK3 | grey | -0.203127915 | 9.69E-06 | -0.213452164 | 3.25E-06 |
| SPON1 | grey | -0.374802285 | 5.05E-17 | -0.379680404 | 1.84E-17 |
| SPP2 | grey | -0.086544624 | 0.061658931 | -0.087740053 | 0.058138463 |
| SPR | grey | 0.022179853 | 0.632589744 | 0.036317463 | 0.433637669 |
| SPRED3 | grey | 0.017253698 | 0.709979204 | 0.003458694 | 0.940578396 |
| SPRY4 | grey | 0.180677296 | 8.62E-05 | 0.160331715 | 0.000505098 |
| SPRYD4 | grey | -0.161667869 | 0.000452494 | -0.170922551 | 0.00020635 |
| SPSB4 | grey | -0.209483702 | 4.98E-06 | -0.220055322 | 1.57E-06 |
| SPTBN5 | grey | 0.088067353 | 0.057204149 | 0.091964787 | 0.04700439 |
| SPX | grey | 0.04644513 | 0.316566555 | 0.02169619 | 0.640029784 |
| SQSTM1 | grey | -0.015418007 | 0.739653096 | -0.0188184 | 0.685025405 |
| SRC | grey | -0.020011858 | 0.666218159 | -0.025082917 | 0.588728971 |
| SRMS | grey | 0.261458107 | 9.75E-09 | 0.270681811 | 2.76E-09 |
| SRPK3 | grey | 0.162763948 | 0.000413196 | 0.176294966 | 0.000128356 |
| SRR | grey | 0.274225692 | 1.68E-09 | 0.25125394 | 3.72E-08 |
| SRRM3 | grey | -0.090418206 | 0.050852925 | -0.077591613 | 0.093973297 |
| SRRM4 | grey | -0.155685067 | 0.000735551 | -0.160423128 | 0.000501326 |
| SRXN1 | grey | 0.12610199 | 0.006358754 | 0.088933808 | 0.054790705 |
| SRY | grey | -0.066005256 | 0.154412793 | -0.068104936 | 0.141691125 |

| | | | | | |
|---|---|---|---|---|---|
| SSBP3 | grey | -0.288312873 | 2.17E-10 | -0.292851214 | 1.09E-10 |
| SSC4D | grey | -0.294202709 | 8.88E-11 | -0.304795541 | 1.70E-11 |
| SSH3 | grey | 0.164710697 | 0.000351125 | 0.168111584 | 0.000263069 |
| SSPO | grey | -0.064128031 | 0.166502753 | -0.065896615 | 0.155093837 |
| SST | grey | -0.314149055 | 3.72E-12 | -0.312362885 | 5.00E-12 |
| SSTR4 | grey | -0.073944161 | 0.110522353 | -0.081807708 | 0.077378587 |
| SSTR5 | grey | 0.06999481 | 0.130941421 | 0.086844652 | 0.06075924 |
| SSX2 | grey | -0.069639531 | 0.132912637 | -0.074957197 | 0.105711274 |
| SSX2B | grey | -0.081167502 | 0.07973326 | -0.079420547 | 0.086454946 |
| SSX3 | grey | -0.082730021 | 0.074085919 | -0.077995592 | 0.092269187 |
| SSX5 | grey | -0.042724917 | 0.356925356 | -0.045222212 | 0.329490729 |
| ST20-MT | grey | -0.023587719 | 0.611145092 | -0.040304751 | 0.384840591 |
| ST3GAL1 | grey | -0.16663831 | 0.000298322 | -0.169287761 | 0.000237762 |
| ST3GAL4 | grey | -0.063263572 | 0.172303051 | -0.073341631 | 0.113464612 |
| ST6GAL1 | grey | -0.117010162 | 0.011388956 | -0.105543811 | 0.022543538 |
| ST6GALN | grey | -0.069158949 | 0.135615416 | -0.038256272 | 0.409478672 |
| ST6GALN | grey | -0.227419783 | 6.81E-07 | -0.228396838 | 6.08E-07 |
| ST7 | grey | 0.010627953 | 0.818819973 | -0.007558315 | 0.870596251 |
| ST8SIA2 | grey | -0.179567993 | 9.55E-05 | -0.195753329 | 2.04E-05 |
| ST8SIA5 | grey | -0.194723218 | 2.26E-05 | -0.203405124 | 9.41E-06 |
| ST8SIA6 | grey | 0.010944378 | 0.813523271 | 0.011767416 | 0.799786609 |
| STAC | grey | -0.283145501 | 4.65E-10 | -0.276214061 | 1.27E-09 |
| STAC2 | grey | -0.033645652 | 0.468240099 | -0.034087627 | 0.462413022 |
| STAG3L5 | grey | -0.065971613 | 0.154623449 | -0.084164564 | 0.069192226 |
| STARD3 | grey | -0.043769348 | 0.345281531 | -0.055420233 | 0.231950085 |
| STARD6 | grey | 0.040117056 | 0.387059758 | 0.013504504 | 0.771000861 |
| STAT6 | grey | 0.055392095 | 0.232187564 | 0.05348287 | 0.248704037 |
| STATH | grey | -0.052132176 | 0.260872503 | -0.045384124 | 0.327760291 |
| STC1 | grey | 0.24799159 | 5.64E-08 | 0.222034305 | 1.26E-06 |
| STEAP1E | grey | -0.167647098 | 0.00027374 | -0.176864943 | 0.00012195 |
| STEAP3 | grey | 0.216074428 | 2.44E-06 | 0.210574328 | 4.43E-06 |
| STK11IP | grey | 0.041804557 | 0.367387842 | 0.036986217 | 0.425213102 |
| STK16 | grey | 0.197873306 | 1.65E-05 | 0.204678224 | 8.25E-06 |
| STK25 | grey | 0.102157736 | 0.027278512 | 0.094859465 | 0.040456332 |
| STK32A | grey | 0.012691797 | 0.784431981 | 0.032022222 | 0.489987587 |
| STK32B | grey | 0.063136513 | 0.173168131 | 0.046799624 | 0.312882997 |
| STK32C | grey | -0.155174815 | 0.000766068 | -0.160738996 | 0.000488491 |
| STK39 | grey | 0.104004726 | 0.024599391 | 0.117193074 | 0.01126029 |
| STKLD1 | grey | 0.016104591 | 0.72850644 | 0.017364971 | 0.708193974 |
| STMN2 | grey | -0.011905313 | 0.797490974 | 0.015901981 | 0.731789978 |
| STMN4 | grey | -0.030891081 | 0.505454689 | -0.051475916 | 0.266930572 |
| STOML1 | grey | 0.149017627 | 0.001239121 | 0.177260999 | 0.000117678 |
| STON2 | grey | -0.085975987 | 0.063394261 | -0.080525551 | 0.08215231 |
| STRA6 | grey | 0.005578061 | 0.904308234 | 0.003655349 | 0.93720656 |
| STRA8 | grey | 0.020244654 | 0.662573429 | 0.015094563 | 0.744923295 |

| | | | | | |
|---|---|---|---|---|---|
| STRC | grey | 0.061743248 | 0.182867374 | 0.052209525 | 0.26016477 |
| STX16-N | grey | -0.069842888 | 0.131781554 | -0.088947788 | 0.054752469 |
| STX1A | grey | -0.199527971 | 1.40E-05 | -0.200535459 | 1.26E-05 |
| STYK1 | grey | 0.134977703 | 0.003472776 | 0.173217758 | 0.000168757 |
| SULT1A2 | grey | -0.099471038 | 0.031621792 | -0.041170023 | 0.374710873 |
| SULT1A3 | grey | -0.067987422 | 0.142381233 | -0.071424398 | 0.123237261 |
| SULT1A4 | grey | -0.050883681 | 0.272479799 | -0.025858809 | 0.577247875 |
| SULT1C2 | grey | -0.226349709 | 7.71E-07 | -0.197209941 | 1.77E-05 |
| SULT1E1 | grey | -0.066030357 | 0.154255765 | -0.048959876 | 0.291046047 |
| SULT2B1 | grey | 0.292124858 | 1.22E-10 | 0.301064239 | 3.07E-11 |
| SULT4A1 | grey | -0.159617293 | 0.000535504 | -0.170997969 | 0.000204999 |
| SUMF1 | grey | 0.079843035 | 0.084788931 | 0.05528535 | 0.233090001 |
| SUPT3H | grey | -0.190789706 | 3.32E-05 | -0.218173939 | 1.94E-06 |
| SUPT5H | grey | -0.131015335 | 0.004569518 | -0.115947252 | 0.012162703 |
| SUSD2 | grey | 0.055657197 | 0.229957041 | 0.051724023 | 0.264629 |
| SUSD3 | grey | -0.410537699 | 2.06E-20 | -0.39531534 | 6.43E-19 |
| SUSD4 | grey | -0.170778754 | 0.000208949 | -0.183019707 | 6.95E-05 |
| SV2A | grey | -0.279747791 | 7.63E-10 | -0.288601024 | 2.07E-10 |
| SV2B | grey | -0.208349904 | 5.62E-06 | -0.20742907 | 6.19E-06 |
| SV2C | grey | -0.211513103 | 4.01E-06 | -0.208299445 | 5.65E-06 |
| SVOP | grey | 0.023418885 | 0.61369979 | 0.007873991 | 0.865242136 |
| SYCE1 | grey | -0.017094036 | 0.71254356 | -0.031980515 | 0.490553331 |
| SYCE3 | grey | 0.153594817 | 0.000868156 | 0.160196 | 0.000510748 |
| SYN1 | grey | -0.286672033 | 2.77E-10 | -0.272334178 | 2.19E-09 |
| SYN2 | grey | -0.040470243 | 0.382890375 | -0.071224409 | 0.124293289 |
| SYNDIG1 | grey | -0.325654102 | 5.35E-13 | -0.340022587 | 4.20E-14 |
| SYNDIG1 | grey | -0.09010485 | 0.051663908 | -0.07657628 | 0.098367478 |
| SYNGR1 | grey | -0.165464209 | 0.000329524 | -0.187381085 | 4.61E-05 |
| SYNGR4 | grey | -0.001304204 | 0.97757557 | 0.007873299 | 0.865253881 |
| SYNJ2BP | grey | 0.04376535 | 0.345325639 | 0.031122121 | 0.502274791 |
| SYP | grey | -0.080878229 | 0.08081608 | -0.077673549 | 0.093625647 |
| SYS1-DB | grey | -0.14159237 | 0.002161443 | -0.15695462 | 0.000664438 |
| SYT12 | grey | -0.135321866 | 0.003389823 | -0.118840805 | 0.010158005 |
| SYT14 | grey | -0.207914585 | 5.88E-06 | -0.243797782 | 9.55E-08 |
| SYT17 | grey | -0.055226181 | 0.233591293 | -0.04728811 | 0.307853402 |
| SYT2 | grey | 0.038015715 | 0.412432086 | 0.037516588 | 0.418600075 |
| SYT3 | grey | -0.168039948 | 0.000264689 | -0.161576065 | 0.000455939 |
| SYT4 | grey | 0.002116147 | 0.963622835 | 0.001273641 | 0.97810093 |
| SYT7 | grey | -0.035815584 | 0.440022725 | -0.027795725 | 0.54905506 |
| SYVN1 | grey | 0.082851364 | 0.07366135 | 0.106411284 | 0.021451727 |
| T | grey | 0.013883195 | 0.764765496 | 0.042236264 | 0.362456771 |
| TAAR6 | grey | -0.092011764 | 0.046891425 | -0.08181501 | 0.077352059 |
| TAC1 | grey | 0.04476261 | 0.334434801 | 0.022350882 | 0.629967645 |
| TAC3 | grey | 0.148745336 | 0.001265235 | 0.123141436 | 0.007718917 |
| TADA2A | grey | 0.097428756 | 0.035306943 | 0.070920704 | 0.125910415 |

| | | | | | |
|---|---|---|---|---|---|
| TAF15 | grey | 0.264819085 | 6.19E-09 | 0.241322705 | 1.30E-07 |
| TAF1C | grey | 0.089571287 | 0.053069611 | 0.095805186 | 0.038491535 |
| TAF6L | grey | 0.067813565 | 0.143406934 | 0.029834113 | 0.520135874 |
| TAF7L | grey | -0.15007765 | 0.001142113 | -0.158438179 | 0.00058943 |
| TAGLN3 | grey | -0.044156785 | 0.341024287 | -0.052662558 | 0.256046202 |
| TAL2 | grey | -0.045491472 | 0.326616255 | -0.057970506 | 0.211136059 |
| TAMM41 | grey | 0.089676977 | 0.052788662 | 0.056385146 | 0.223910447 |
| TAS1R1 | grey | 0.020680198 | 0.655775783 | 0.026151835 | 0.572939411 |
| TAS1R3 | grey | -0.08119543 | 0.079629341 | -0.057192791 | 0.217335462 |
| TBATA | grey | 0.053184072 | 0.251361236 | 0.065063555 | 0.160391845 |
| TBC1D10 | grey | -0.15355183 | 0.000871101 | -0.17668449 | 0.000123945 |
| TBC1D10 | grey | 0.034783873 | 0.453315944 | 0.064478263 | 0.164194894 |
| TBC1D13 | grey | -0.042229368 | 0.362535219 | -0.036136836 | 0.435929488 |
| TBC1D16 | grey | 0.113975209 | 0.013721995 | 0.090048843 | 0.051809988 |
| TBC1D2 | grey | 0.228571541 | 5.96E-07 | 0.251930831 | 3.41E-08 |
| TBC1D20 | grey | -0.018678064 | 0.687250039 | -0.02027255 | 0.662137202 |
| TBC1D22 | grey | 0.125533328 | 0.006601992 | 0.139157571 | 0.002579657 |
| TBC1D24 | grey | 0.073981498 | 0.110342027 | 0.082881709 | 0.073555487 |
| TBC1D25 | grey | -0.167702092 | 0.000272456 | -0.171012469 | 0.00020474 |
| TBC1D26 | grey | -0.071229854 | 0.124264443 | -0.101184119 | 0.02878919 |
| TBC1D28 | grey | -0.025238218 | 0.586422544 | -0.014373568 | 0.75671381 |
| TBC1D3 | grey | -0.06393749 | 0.16776849 | -0.079951365 | 0.084365945 |
| TBC1D3E | grey | -0.070467407 | 0.128354407 | -0.070797925 | 0.126568792 |
| TBC1D3C | grey | -0.054649309 | 0.238518629 | -0.048346617 | 0.297138674 |
| TBC1D3I | grey | -0.108627486 | 0.018868396 | -0.107504073 | 0.020141878 |
| TBC1D3E | grey | -0.06804017 | 0.142071155 | -0.09314772 | 0.044228303 |
| TBC1D3F | grey | -0.03980585 | 0.390756332 | -0.043006879 | 0.353757871 |
| TBC1D3C | grey | -0.054990881 | 0.235592347 | -0.054745678 | 0.237690452 |
| TBC1D3I | grey | -0.060648936 | 0.190762503 | -0.0587468 | 0.205076118 |
| TBC1D3I | grey | -0.057123758 | 0.217891984 | -0.058902471 | 0.20387624 |
| TBC1D3K | grey | -0.064311471 | 0.165290959 | -0.066858558 | 0.149142181 |
| TBC1D3L | grey | -0.079435741 | 0.086394579 | -0.104940366 | 0.023331144 |
| TBCC | grey | -0.062280022 | 0.179084134 | -0.042752172 | 0.356618405 |
| TBKBP1 | grey | -0.292132843 | 1.22E-10 | -0.294110943 | 9.01E-11 |
| TBR1 | grey | -0.05266349 | 0.25603778 | -0.018212361 | 0.694651691 |
| TBX1 | grey | -0.091336223 | 0.048538001 | -0.117329068 | 0.011165462 |
| TBX10 | grey | 0.091469036 | 0.048210512 | 0.107887465 | 0.019699112 |
| TBX15 | grey | 0.013663664 | 0.768378377 | -0.007155743 | 0.877432752 |
| TBX18 | grey | 0.231102832 | 4.43E-07 | 0.215797731 | 2.52E-06 |
| TBX19 | grey | -0.137322499 | 0.002942221 | -0.132210049 | 0.004209774 |
| TBX20 | grey | -0.012557067 | 0.78666489 | -0.047906864 | 0.301559514 |
| TBX22 | grey | -0.031346075 | 0.499202502 | -0.026960974 | 0.561121971 |
| TBX4 | grey | -0.122179185 | 0.008213919 | -0.120519239 | 0.009134694 |
| TBX5 | grey | -0.081305894 | 0.079219396 | -0.031214026 | 0.50101279 |
| TBX6 | grey | 0.036449378 | 0.4319683 | 0.048401329 | 0.296591697 |

| | | | | | |
|---|---|---|---|---|---|
| TCAP | grey | -0.162823127 | 0.000411167 | -0.18312084 | 6.88E-05 |
| TCEA3 | grey | -0.002001178 | 0.965597922 | -0.003975321 | 0.931722769 |
| TCEB3CI | grey | -0.022805048 | 0.623027278 | -0.032395289 | 0.484942537 |
| TCERG1I | grey | -0.137225236 | 0.002962673 | -0.140921366 | 0.002270035 |
| TCF24 | grey | 0.020882645 | 0.652625753 | 0.011755473 | 0.799985506 |
| TCF7L1 | grey | -0.077665372 | 0.093660296 | -0.082858913 | 0.073635001 |
| TCL1B | grey | 0.019936571 | 0.667398579 | -0.015372303 | 0.740397068 |
| TCN1 | grey | 0.258100525 | 1.52E-08 | 0.273875832 | 1.76E-09 |
| TCN2 | grey | -0.036836123 | 0.427095561 | -0.059629192 | 0.198342175 |
| TCP10L2 | grey | -0.183629038 | 6.56E-05 | -0.180435673 | 8.82E-05 |
| TCP11 | grey | -0.06706459 | 0.147890335 | -0.068215829 | 0.141042253 |
| TCP11X2 | grey | -0.074812919 | 0.106386186 | -0.051131042 | 0.270152543 |
| TCTA | grey | 0.154587276 | 0.000802658 | 0.196430657 | 1.91E-05 |
| TDO2 | grey | -0.106721801 | 0.021072216 | -0.073722141 | 0.111599448 |
| TDRD12 | grey | -0.186931969 | 4.81E-05 | -0.197305624 | 1.75E-05 |
| TDRD5 | grey | -0.235616883 | 2.59E-07 | -0.242382534 | 1.14E-07 |
| TDRD9 | grey | -0.045432636 | 0.32724297 | -0.041342871 | 0.372707204 |
| TEC | grey | 0.123574336 | 0.007505048 | 0.134406661 | 0.003614484 |
| TECPR1 | grey | 0.195323219 | 2.13E-05 | 0.221257912 | 1.38E-06 |
| TECTA | grey | -0.262382051 | 8.61E-09 | -0.283369342 | 4.50E-10 |
| TECTB | grey | 0.034371938 | 0.458686037 | 0.032802571 | 0.479466969 |
| TEKT5 | grey | 0.11900957 | 0.0100507 | 0.120504683 | 0.009143159 |
| TEN1 | grey | -0.016209699 | 0.72680499 | -0.026672201 | 0.56532598 |
| TEN1-CD | grey | -0.162097426 | 0.000436696 | -0.188627891 | 4.09E-05 |
| TENM1 | grey | -0.15089902 | 0.001071811 | -0.157742385 | 0.000623573 |
| TENM2 | grey | -0.081535092 | 0.07837427 | -0.078614057 | 0.089708326 |
| TENM3 | grey | -0.122210676 | 0.008197282 | -0.130766395 | 0.004647884 |
| TENM4 | grey | -0.279211321 | 8.24E-10 | -0.289671242 | 1.77E-10 |
| TEPP | grey | -0.34272526 | 2.57E-14 | -0.343700413 | 2.15E-14 |
| TESC | grey | 0.227404001 | 6.82E-07 | 0.224577294 | 9.44E-07 |
| TESK1 | grey | 0.143534528 | 0.001873339 | 0.149765444 | 0.001169931 |
| TESK2 | grey | -0.186862952 | 4.84E-05 | -0.209237238 | 5.11E-06 |
| TEX13B | grey | -0.070210113 | 0.129757889 | -0.069298004 | 0.134829061 |
| TEX19 | grey | -0.027109843 | 0.558960631 | -0.0236601 | 0.610051302 |
| TEX22 | grey | 0.032586577 | 0.48236663 | 0.049122045 | 0.289449019 |
| TEX29 | grey | -0.026661042 | 0.565488736 | -0.02096552 | 0.651338003 |
| TEX35 | grey | -0.053406498 | 0.249381343 | -0.04966599 | 0.284135331 |
| TEX36 | grey | 0.127429277 | 0.005822023 | 0.140005821 | 0.002426243 |
| TEX43 | grey | -0.03663416 | 0.429636173 | -0.000869393 | 0.985050594 |
| TFAP2C | grey | -0.023672545 | 0.609863329 | -0.02402973 | 0.604479208 |
| TFAP2E | grey | -0.158076927 | 0.000606934 | -0.1675182 | 0.000276772 |
| TFB1M | grey | 0.245608464 | 7.62E-08 | 0.239523051 | 1.62E-07 |
| TFCP2L1 | grey | 0.085642186 | 0.064431535 | 0.079494451 | 0.086161632 |
| TFF2 | grey | 0.082115255 | 0.076267682 | 0.098299207 | 0.033693852 |
| TFPI2 | grey | 0.13970395 | 0.002479858 | 0.122237363 | 0.008183206 |

| | | | | | |
|---|---|---|---|---|---|
| TG | grey | 0.047074055 | 0.310050774 | 0.051033017 | 0.271073168 |
| TGFA | grey | 0.20598767 | 7.20E-06 | 0.204242553 | 8.63E-06 |
| TGFBR3L | grey | 0.031210248 | 0.501064635 | 0.041069118 | 0.375883632 |
| TGIF1 | grey | 0.150663188 | 0.001091577 | 0.109669745 | 0.017750264 |
| TGIF2 | grey | -0.358313309 | 1.36E-15 | -0.350970528 | 5.53E-15 |
| TGIF2-C2 | grey | -0.193476134 | 2.56E-05 | -0.192526484 | 2.81E-05 |
| TGIF2LY | grey | -0.030621633 | 0.509176526 | -0.034247982 | 0.460308885 |
| TGM1 | grey | -0.229453609 | 5.38E-07 | -0.229508803 | 5.34E-07 |
| TGM5 | grey | 0.063025681 | 0.173925364 | 0.061749994 | 0.182819465 |
| TGM7 | grey | 0.013929342 | 0.764006687 | 0.027248228 | 0.556955116 |
| TH | grey | -0.182122484 | 7.55E-05 | -0.198073472 | 1.62E-05 |
| THADA | grey | -0.291846966 | 1.27E-10 | -0.297608583 | 5.26E-11 |
| THBS3 | grey | -0.273397308 | 1.89E-09 | -0.289883168 | 1.71E-10 |
| THEG | grey | -0.036621233 | 0.429799093 | -0.031458001 | 0.497670805 |
| THEM4 | grey | -0.084199992 | 0.069074801 | -0.110376636 | 0.017025258 |
| THEM5 | grey | -0.056378096 | 0.223968458 | -0.062660045 | 0.176440987 |
| THNSL2 | grey | -0.219755544 | 1.63E-06 | -0.217181475 | 2.16E-06 |
| THRSP | grey | -0.002178008 | 0.962560188 | 0.002562173 | 0.955962607 |
| THSD7B | grey | -0.199011508 | 1.47E-05 | -0.190798477 | 3.32E-05 |
| TICAM1 | grey | 0.256398355 | 1.91E-08 | 0.255554439 | 2.13E-08 |
| TIGD6 | grey | 0.175877356 | 0.000133248 | 0.153775607 | 0.000855868 |
| TIMP4 | grey | -0.156907329 | 0.000666968 | -0.158606122 | 0.000581453 |
| TINAG | grey | -0.047277739 | 0.307959628 | -0.047282832 | 0.307907462 |
| TINAGL1 | grey | 0.132285771 | 0.00418786 | 0.131036652 | 0.004562864 |
| TJAP1 | grey | -0.254666989 | 2.39E-08 | -0.277926684 | 9.92E-10 |
| TKTL1 | grey | -0.179674073 | 9.45E-05 | -0.184269522 | 6.18E-05 |
| TLCD2 | grey | -0.072894968 | 0.115685171 | -0.050285487 | 0.278164224 |
| TLDC1 | grey | 0.069999034 | 0.13091812 | 0.086916072 | 0.060546676 |
| TLDC2 | grey | -0.223790892 | 1.03E-06 | -0.222584207 | 1.18E-06 |
| TLE1 | grey | 0.185600235 | 5.46E-05 | 0.159152274 | 0.0005562 |
| TLE3 | grey | 0.232912024 | 3.58E-07 | 0.229895543 | 5.11E-07 |
| TLE6 | grey | 0.438958264 | 2.04E-23 | 0.4313351 | 1.39E-22 |
| TLL2 | grey | -0.054184309 | 0.242543295 | -0.060083438 | 0.194939119 |
| TLN2 | grey | 0.015629779 | 0.736209044 | -0.004998514 | 0.914209885 |
| TLR5 | grey | 0.157913732 | 0.000614999 | 0.13109881 | 0.004543509 |
| TLX1 | grey | -0.011555664 | 0.803315079 | -0.006519779 | 0.888251117 |
| TLX1NB | grey | -0.113630037 | 0.014012318 | -0.10320978 | 0.025723281 |
| TLX2 | grey | -0.046705691 | 0.313856311 | -0.062238365 | 0.179375643 |
| TLX3 | grey | 0.044065847 | 0.342020516 | 0.039159491 | 0.398501938 |
| TM4SF1 | grey | 0.028746826 | 0.53546362 | 0.031908898 | 0.491525637 |
| TM4SF19 | grey | -0.043740888 | 0.345595584 | -0.032781324 | 0.479751787 |
| TM4SF20 | grey | -0.095929944 | 0.038238478 | -0.092254437 | 0.04631148 |
| TM4SF4 | grey | -0.020174866 | 0.663665218 | -0.021229384 | 0.64724491 |
| TM6SF2 | grey | -0.170486613 | 0.000214323 | -0.182985511 | 6.97E-05 |
| TMBIM1 | grey | -0.159344502 | 0.000547556 | -0.146178242 | 0.00153754 |

| | | | | | |
|---|---|---|---|---|---|
| TMC7 | grey | -0.118559604 | 0.010339059 | -0.134856246 | 0.003502487 |
| TMCC2 | grey | -0.342450116 | 2.70E-14 | -0.349578229 | 7.19E-15 |
| TMCO1 | grey | -0.200245474 | 1.30E-05 | -0.208816734 | 5.35E-06 |
| TMCO4 | grey | 0.091279552 | 0.048678305 | 0.11126778 | 0.016148278 |
| TMCO6 | grey | -0.089916472 | 0.052156612 | -0.121245055 | 0.008721344 |
| TMEM10 | grey | -0.169629781 | 0.000230842 | -0.164963109 | 0.000343746 |
| TMEM10 | grey | 0.103507366 | 0.025297475 | 0.104481919 | 0.023945313 |
| TMEM10 | grey | -0.377734711 | 2.76E-17 | -0.373096564 | 7.17E-17 |
| TMEM11 | grey | 0.099378128 | 0.031782004 | 0.113295273 | 0.014299036 |
| TMEM11 | grey | 0.118904529 | 0.010117368 | 0.112199315 | 0.015274105 |
| TMEM12 | grey | -0.209968706 | 4.73E-06 | -0.218689333 | 1.83E-06 |
| TMEM12 | grey | 0.090042344 | 0.05182696 | 0.10272274 | 0.026433502 |
| TMEM13 | grey | -0.27985513 | 7.51E-10 | -0.263056113 | 7.86E-09 |
| TMEM13 | grey | -0.184967617 | 5.79E-05 | -0.166490652 | 0.000302089 |
| TMEM13 | grey | -0.129232176 | 0.005158207 | -0.114299151 | 0.013454359 |
| TMEM13 | grey | 0.101673439 | 0.02802121 | 0.111493132 | 0.015932878 |
| TMEM14 | grey | 0.066164753 | 0.153417054 | 0.050113009 | 0.279818056 |
| TMEM14 | grey | 0.028162465 | 0.543794222 | 0.031051092 | 0.503251259 |
| TMEM15 | grey | -0.187231963 | 4.67E-05 | -0.190881807 | 3.29E-05 |
| TMEM15 | grey | 0.200957046 | 1.21E-05 | 0.175314958 | 0.000140114 |
| TMEM15 | grey | -0.082261325 | 0.075744615 | -0.07683651 | 0.097225951 |
| TMEM15 | grey | -0.095707187 | 0.038691306 | -0.102044192 | 0.027451101 |
| TMEM15 | grey | -0.081930595 | 0.076933135 | -0.120534292 | 0.009125947 |
| TMEM15 | grey | 0.17931934 | 9.76E-05 | 0.165869177 | 0.000318434 |
| TMEM16 | grey | -0.169983731 | 0.000223879 | -0.182435348 | 7.33E-05 |
| TMEM17 | grey | -0.373978479 | 5.98E-17 | -0.340450084 | 3.89E-14 |
| TMEM17 | grey | -0.121161154 | 0.008768258 | -0.119074095 | 0.01000994 |
| TMEM18 | grey | -0.075574167 | 0.102863301 | -0.080432666 | 0.082507188 |
| TMEM19 | grey | 0.065615416 | 0.15686716 | 0.050735548 | 0.273880021 |
| TMEM19 | grey | -0.082134247 | 0.076199508 | -0.120649583 | 0.009059203 |
| TMEM20 | grey | -0.04982094 | 0.282633777 | -0.042763241 | 0.356493789 |
| TMEM20 | grey | -0.083854908 | 0.070225533 | -0.08248639 | 0.074944389 |
| TMEM21 | grey | 0.221418727 | 1.35E-06 | 0.245446988 | 7.77E-08 |
| TMEM21 | grey | -0.209551837 | 4.94E-06 | -0.21481131 | 2.81E-06 |
| TMEM21 | grey | 0.015092345 | 0.744959484 | 0.002384392 | 0.959015441 |
| TMEM22 | grey | -0.288163899 | 2.21E-10 | -0.270661838 | 2.77E-09 |
| TMEM22 | grey | -0.194265533 | 2.37E-05 | -0.167255832 | 0.000283041 |
| TMEM22 | grey | 0.247728128 | 5.83E-08 | 0.244204116 | 9.08E-08 |
| TMEM23 | grey | 0.005683414 | 0.902509811 | 0.013919694 | 0.764165326 |
| TMEM23 | grey | 0.003091746 | 0.946872838 | 0.013322367 | 0.774005151 |
| TMEM25 | grey | -0.049215748 | 0.288528929 | -0.065081729 | 0.160274824 |
| TMEM25 | grey | -0.037208843 | 0.422429874 | -0.020815044 | 0.65367693 |
| TMEM25 | grey | 0.100312781 | 0.030201504 | 0.065299897 | 0.158875115 |
| TMEM26 | grey | -0.007792218 | 0.866628498 | -0.033451689 | 0.470810076 |
| TMEM26 | grey | 0.028695085 | 0.536198639 | 0.054102561 | 0.24325573 |

| | | | | | |
|---|---|---|---|---|---|
| TMEM26 | grey | -0.033080975 | 0.475743499 | -0.032893383 | 0.47825068 |
| TMEM27 | grey | 0.068101496 | 0.14171129 | 0.050937197 | 0.27197515 |
| TMEM37 | grey | 0.142720478 | 0.001989524 | 0.15815357 | 0.000603181 |
| TMEM42 | grey | -0.119033868 | 0.010035333 | -0.112722267 | 0.014801787 |
| TMEM44 | grey | -0.138606121 | 0.00268408 | -0.138687507 | 0.00266843 |
| TMEM51 | grey | -0.026866707 | 0.562492664 | -0.015200697 | 0.743192631 |
| TMEM59 | grey | -0.186513831 | 5.00E-05 | -0.172013338 | 0.0001876 |
| TMEM61 | grey | -0.002340766 | 0.959764683 | -0.011990839 | 0.79606806 |
| TMEM63 | grey | -0.295937857 | 6.81E-11 | -0.303162538 | 2.20E-11 |
| TMEM72 | grey | 0.136763317 | 0.003061578 | 0.127793929 | 0.005681874 |
| TMEM74 | grey | -0.029814335 | 0.520412653 | -0.045976715 | 0.321477196 |
| TMEM74 | grey | -0.120688085 | 0.009037011 | -0.132101459 | 0.004241381 |
| TMEM79 | grey | 0.14448536 | 0.001745515 | 0.116909204 | 0.011460529 |
| TMEM80 | grey | 0.08127947 | 0.079317302 | 0.064566367 | 0.163618128 |
| TMEM81 | grey | 0.068331646 | 0.140367 | 0.047316032 | 0.307567531 |
| TMEM82 | grey | -0.068433217 | 0.139776851 | -0.061033101 | 0.187962861 |
| TMEM88 | grey | -0.194552078 | 2.30E-05 | -0.192292078 | 2.87E-05 |
| TMEM8C | grey | -0.06489891 | 0.161454885 | -0.080952872 | 0.080535537 |
| TMEM92 | grey | 0.013376718 | 0.773108299 | 0.036171203 | 0.435492891 |
| TMEM98 | grey | -0.001089094 | 0.981273419 | -0.037854987 | 0.414412408 |
| TMEM99 | grey | 0.020973788 | 0.651209594 | 0.025754332 | 0.578787712 |
| TMIE | grey | -0.271129392 | 2.59E-09 | -0.289960389 | 1.69E-10 |
| TMIGD1 | grey | -0.063130063 | 0.173212132 | -0.058424886 | 0.207573581 |
| TMOD4 | grey | -0.059646851 | 0.198209076 | -0.062982868 | 0.17421854 |
| TMPRSS | grey | 0.030923885 | 0.505002553 | 0.022671757 | 0.625060759 |
| TMPRSS | grey | -0.08442974 | 0.06831727 | -0.077946313 | 0.092475727 |
| TMPRSS( | grey | -0.257491062 | 1.65E-08 | -0.251336862 | 3.68E-08 |
| TMSB4X | grey | 0.191927269 | 2.98E-05 | 0.211667843 | 3.94E-06 |
| TMSB4Y | grey | 0.100674084 | 0.029608839 | 0.095133434 | 0.039878609 |
| TMUB2 | grey | 0.146587569 | 0.001490793 | 0.150054779 | 0.00114413 |
| TMX2-C1 | grey | -0.099427918 | 0.031696061 | -0.107636031 | 0.019988517 |
| TNF | grey | -0.106558559 | 0.021270996 | -0.104607847 | 0.023775234 |
| TNFAIP2 | grey | -0.104786495 | 0.023535744 | -0.075932534 | 0.10123719 |
| TNFAIP3 | grey | -0.043593048 | 0.347229884 | -0.052827357 | 0.254559259 |
| TNFAIP6 | grey | -0.15321976 | 0.000894169 | -0.140713878 | 0.002304609 |
| TNFRSF1 | grey | 0.176643677 | 0.0001244 | 0.187194853 | 4.69E-05 |
| TNFRSF1 | grey | 0.049674119 | 0.284056415 | 0.032405911 | 0.484799305 |
| TNFRSF1 | grey | -0.109722292 | 0.017695458 | -0.094749492 | 0.040690214 |
| TNFRSF1 | grey | -0.032753605 | 0.480123494 | -0.052985519 | 0.253137842 |
| TNFRSF1 | grey | -0.00187589 | 0.96775053 | 0.003559406 | 0.93885146 |
| TNFRSF1 | grey | -0.18794 | 4.37E-05 | -0.178146659 | 0.000108627 |
| TNFRSF1 | grey | 0.163880086 | 0.000376462 | 0.197664212 | 1.69E-05 |
| TNFRSF1 | grey | -0.00651407 | 0.888348327 | -0.001601887 | 0.972459051 |
| TNFRSF2 | grey | 0.113777094 | 0.013887975 | 0.114981836 | 0.012905344 |
| TNFRSF2 | grey | -0.032012381 | 0.490121045 | -0.034927232 | 0.451455415 |

| | | | | | |
|---|---|---|---|---|---|
| TNFSF11 | grey | 0.077648595 | 0.093731416 | 0.107154938 | 0.020552588 |
| TNFSF12· | grey | -0.017396521 | 0.707688079 | -0.007086421 | 0.878610913 |
| TNFSF15 | grey | 0.172649264 | 0.000177418 | 0.174104077 | 0.00015604 |
| TNFSF18 | grey | 0.070199171 | 0.129817836 | 0.05436028 | 0.241014665 |
| TNFSF4 | grey | -0.064147635 | 0.166372933 | -0.066578048 | 0.150859501 |
| TNFSF9 | grey | 0.14393168 | 0.00181894 | 0.167944296 | 0.000266867 |
| TNIK | grey | -0.18818109 | 4.27E-05 | -0.186577843 | 4.97E-05 |
| TNIP1 | grey | 0.136901751 | 0.003031627 | 0.165287117 | 0.000334486 |
| TNK2 | grey | -0.327933466 | 3.60E-13 | -0.329267389 | 2.86E-13 |
| TNKS1BI | grey | 0.08841626 | 0.056221899 | 0.088721314 | 0.055374613 |
| TNMD | grey | -0.173417852 | 0.000165803 | -0.168269969 | 0.00025952 |
| TNN | grey | -0.100634278 | 0.029673642 | -0.108690643 | 0.018798935 |
| TNNC1 | grey | 0.061925497 | 0.181576297 | 0.073604344 | 0.112174267 |
| TNNI1 | grey | 0.053630438 | 0.247398974 | 0.040922104 | 0.37759632 |
| TNNI3K | grey | -0.018787739 | 0.685511217 | -0.036149271 | 0.43577148 |
| TNNT2 | grey | 0.132863118 | 0.00402414 | 0.115094332 | 0.012816797 |
| TNNT3 | grey | -0.24515132 | 8.07E-08 | -0.243976541 | 9.34E-08 |
| TOMM40 | grey | -0.109896744 | 0.017514563 | -0.108959184 | 0.018506069 |
| TOR4A | grey | -0.079665856 | 0.085484429 | -0.061019313 | 0.188062819 |
| TOX | grey | -0.139775276 | 0.002467094 | -0.126064946 | 0.006374351 |
| TOX2 | grey | -0.279265812 | 8.18E-10 | -0.260926138 | 1.05E-08 |
| TOX3 | grey | 0.264268139 | 6.67E-09 | 0.259898595 | 1.20E-08 |
| TP53 | grey | 0.261705041 | 9.43E-09 | 0.24914235 | 4.87E-08 |
| TP53I3 | grey | 0.242843553 | 1.07E-07 | 0.252070596 | 3.35E-08 |
| TP53TG3 | grey | -0.0906901 | 0.050157885 | -0.099912965 | 0.030869159 |
| TP53TG3 | grey | 0.051137188 | 0.270094885 | 0.056690734 | 0.221406202 |
| TP53TG3 | grey | -0.304178631 | 1.87E-11 | -0.306802437 | 1.23E-11 |
| TP63 | grey | 0.14280813 | 0.001976704 | 0.137120123 | 0.002984921 |
| TPBG | grey | 0.136152583 | 0.003196955 | 0.162104798 | 0.000436429 |
| TPBGL | grey | -0.20666653 | 6.71E-06 | -0.199153442 | 1.45E-05 |
| TPD52L1 | grey | -0.065967674 | 0.154648127 | -0.055382866 | 0.232265488 |
| TPD52L2 | grey | -0.261502041 | 9.69E-09 | -0.249137842 | 4.88E-08 |
| TPK1 | grey | -0.074573398 | 0.107514144 | -0.070609018 | 0.12758698 |
| TPO | grey | -0.066113279 | 0.15373787 | -0.074437565 | 0.108157992 |
| TPRG1 | grey | 0.042290029 | 0.361845564 | 0.032390969 | 0.485000789 |
| TPTE | grey | -0.122049361 | 0.008282827 | -0.118847409 | 0.010153787 |
| TPTE2 | grey | -0.029493151 | 0.524918116 | 0.032873921 | 0.478511201 |
| TRABD2 | grey | -0.005237933 | 0.91011767 | -0.016594909 | 0.720580891 |
| TRABD2l | grey | -0.102377005 | 0.026947857 | -0.132296071 | 0.004184887 |
| TRAPPC | grey | 0.054710746 | 0.237990419 | 0.010526127 | 0.82052623 |
| TRAPPC | grey | 0.112523062 | 0.01498017 | 0.111001067 | 0.016406506 |
| TREM1 | grey | 0.028464466 | 0.539480902 | 0.05627324 | 0.224832532 |
| TREML2 | grey | -0.160142398 | 0.000512996 | -0.174959372 | 0.000144625 |
| TREX1 | grey | 0.103153276 | 0.025804825 | 0.063463834 | 0.170946112 |
| TREX2 | grey | -0.033277585 | 0.473123519 | -0.039380684 | 0.395840974 |

| | | | | | |
|---|---|---|---|---|---|
| TRHDE | grey | -0.246411406 | 6.89E-08 | -0.243425842 | 1.00E-07 |
| TRIB1 | grey | -0.150229022 | 0.001128846 | -0.178279315 | 0.000107329 |
| TRIB2 | grey | -0.209750458 | 4.84E-06 | -0.223861433 | 1.02E-06 |
| TRIL | grey | -0.192377106 | 2.85E-05 | -0.191249455 | 3.18E-05 |
| TRIM11 | grey | 0.159541709 | 0.000538819 | 0.170153879 | 0.000220602 |
| TRIM16 | grey | 0.231069834 | 4.45E-07 | 0.239648548 | 1.59E-07 |
| TRIM16L | grey | 0.162208681 | 0.000432689 | 0.147555816 | 0.001385364 |
| TRIM17 | grey | -0.161028035 | 0.000477014 | -0.162986168 | 0.000405626 |
| TRIM25 | grey | 0.002750254 | 0.952733618 | -0.008847887 | 0.84876395 |
| TRIM26 | grey | 0.162499662 | 0.000422369 | 0.185273515 | 5.63E-05 |
| TRIM27 | grey | -0.040938379 | 0.377406488 | -0.009458374 | 0.838467617 |
| TRIM36 | grey | 0.204518734 | 8.39E-06 | 0.230002583 | 5.04E-07 |
| TRIM39 | grey | -0.280227774 | 7.12E-10 | -0.296192091 | 6.55E-11 |
| TRIM39-I | grey | 0.091976387 | 0.046976475 | 0.100860596 | 0.029306815 |
| TRIM46 | grey | -0.186184522 | 5.16E-05 | -0.193534268 | 2.54E-05 |
| TRIM47 | grey | -0.152390779 | 0.000954239 | -0.146418675 | 0.001509921 |
| TRIM49D | grey | -0.083775301 | 0.070493207 | -0.085152592 | 0.065978157 |
| TRIM50 | grey | -0.210551157 | 4.45E-06 | -0.201356857 | 1.16E-05 |
| TRIM54 | grey | -0.067876009 | 0.143037886 | -0.033705581 | 0.467447615 |
| TRIM58 | grey | -0.192561473 | 2.80E-05 | -0.228603722 | 5.94E-07 |
| TRIM6-T | grey | -0.075175986 | 0.104694282 | -0.0770969 | 0.09609432 |
| TRIM62 | grey | -0.067232238 | 0.146877627 | -0.047398299 | 0.306726283 |
| TRIM64 | grey | 0.011132159 | 0.810383965 | 0.047818156 | 0.302456561 |
| TRIM65 | grey | 0.110803892 | 0.016599725 | 0.091953979 | 0.047030413 |
| TRIM7 | grey | -0.034287273 | 0.459794137 | -0.004485666 | 0.922983091 |
| TRIM8 | grey | -0.065972625 | 0.15461711 | -0.081132474 | 0.079863749 |
| TRIP10 | grey | 0.150813209 | 0.001078965 | 0.139041788 | 0.002601269 |
| TRIP6 | grey | -0.152950752 | 0.000913268 | -0.148372992 | 0.001301766 |
| TRMT44 | grey | -0.066673738 | 0.15027199 | -0.101503184 | 0.028286402 |
| TRNP1 | grey | -0.246471495 | 6.83E-08 | -0.203848009 | 8.99E-06 |
| TRPC3 | grey | -0.139764941 | 0.002468939 | -0.130179439 | 0.004837479 |
| TRPC6 | grey | 0.07900638 | 0.088113629 | 0.06147816 | 0.184757376 |
| TRPC7 | grey | -0.112914606 | 0.014631325 | -0.092404133 | 0.045956745 |
| TRPM1 | grey | -0.095825034 | 0.038451181 | -0.099503781 | 0.031565495 |
| TRPM6 | grey | 0.104813411 | 0.023499842 | 0.110431206 | 0.016970381 |
| TRPM8 | grey | 0.086856123 | 0.060725056 | 0.100889571 | 0.029260133 |
| TRPV3 | grey | -0.177426832 | 0.000115931 | -0.193255469 | 2.61E-05 |
| TRPV4 | grey | 0.137662737 | 0.002871685 | 0.14722749 | 0.001420321 |
| TRPV6 | grey | 0.016067576 | 0.729105949 | -0.006665878 | 0.885763912 |
| TSC22D4 | grey | -0.118414569 | 0.010433555 | -0.105032313 | 0.02320962 |
| TSHR | grey | -0.185922834 | 5.29E-05 | -0.174661349 | 0.00014851 |
| TSKS | grey | -0.098008102 | 0.034226181 | -0.087298928 | 0.059417661 |
| TSPAN10 | grey | -0.072586709 | 0.117237424 | -0.099170872 | 0.032141895 |
| TSPAN12 | grey | -0.154575898 | 0.000803382 | -0.172579393 | 0.000178511 |
| TSPAN14 | grey | 0.16119324 | 0.000470567 | 0.171205695 | 0.000201321 |

| | | | | | |
|---|---|---|---|---|---|
| TSPEAR | grey | -0.175613465 | 0.00013643 | -0.203515038 | 9.31E-06 |
| TSPY2 | grey | 0.047109171 | 0.309689586 | 0.042366958 | 0.360972132 |
| TSPY8 | grey | -0.043008401 | 0.353740821 | -0.044187299 | 0.34069042 |
| TSR2 | grey | -0.247138301 | 6.28E-08 | -0.237738724 | 2.01E-07 |
| TTBK1 | grey | -0.195749437 | 2.04E-05 | -0.183081447 | 6.91E-05 |
| TTC22 | grey | -0.20140316 | 1.16E-05 | -0.176276521 | 0.000128568 |
| TTC23 | grey | 0.10004894 | 0.030640689 | 0.088664613 | 0.055531294 |
| TTC36 | grey | -0.067536729 | 0.145051826 | -0.070491158 | 0.128225443 |
| TTC38 | grey | 0.458704725 | 1.12E-25 | 0.436451705 | 3.85E-23 |
| TTC39A | grey | 0.05230503 | 0.259292751 | 0.079839228 | 0.084803825 |
| TTC7A | grey | 0.070626098 | 0.127494663 | 0.11536854 | 0.012603204 |
| TTC9B | grey | 0.101493312 | 0.028301844 | 0.089620147 | 0.052939575 |
| TTLL11 | grey | 0.065226525 | 0.159344819 | 0.078462082 | 0.090332257 |
| TTLL2 | grey | -0.148498832 | 0.001289313 | -0.156960056 | 0.000664147 |
| TTPA | grey | 0.01968183 | 0.671398689 | 0.030889522 | 0.505476186 |
| TTYH1 | grey | -0.192407698 | 2.84E-05 | -0.187529727 | 4.54E-05 |
| TUBA1A | grey | -0.028785241 | 0.534918235 | -0.025732738 | 0.57910622 |
| TUBA3C | grey | -0.006698329 | 0.885211616 | -0.006393616 | 0.890399807 |
| TUBA3E | grey | 0.045207538 | 0.329647846 | 0.026604615 | 0.566312074 |
| TUBB2A | grey | -0.201566701 | 1.14E-05 | -0.186789969 | 4.87E-05 |
| TUBB2B | grey | -0.238983929 | 1.73E-07 | -0.252458495 | 3.19E-08 |
| TUBB8 | grey | -0.016225883 | 0.726543135 | -0.03178476 | 0.493213417 |
| TUBGCP( | grey | 0.16507069 | 0.000340645 | 0.162381716 | 0.000426524 |
| TULP1 | grey | -0.259601174 | 1.25E-08 | -0.264811568 | 6.20E-09 |
| TUSC3 | grey | -0.02427658 | 0.600770722 | -0.052349638 | 0.258886139 |
| TXNDC1 | grey | 0.223275243 | 1.10E-06 | 0.262136858 | 8.90E-09 |
| TXNL4B | grey | 0.190683213 | 3.36E-05 | 0.180080838 | 9.11E-05 |
| TXNRD3 | grey | -0.166414104 | 0.000304059 | -0.175266483 | 0.000140721 |
| TYRO3 | grey | -0.09487923 | 0.040414418 | -0.113936229 | 0.013754514 |
| TYRP1 | grey | -0.084291272 | 0.06877301 | -0.068137197 | 0.141502121 |
| TYW1B | grey | 0.047305614 | 0.307674178 | 0.043363286 | 0.349779519 |
| UAP1L1 | grey | 0.279976411 | 7.38E-10 | 0.300545158 | 3.33E-11 |
| UBAC1 | grey | 0.004857273 | 0.916625082 | 0.020787272 | 0.654108963 |
| UBAC2 | grey | 0.060015588 | 0.195444702 | 0.065767662 | 0.155905159 |
| UBASH3] | grey | 0.059697454 | 0.19782803 | 0.084508161 | 0.068060264 |
| UBB | grey | 0.16768793 | 0.000272786 | 0.175257814 | 0.00014083 |
| UBBP4 | grey | 0.204622358 | 8.30E-06 | 0.227114569 | 7.06E-07 |
| UBC | grey | 0.083551899 | 0.071248844 | 0.095360419 | 0.039405261 |
| UBE2D2 | grey | 0.202297852 | 1.05E-05 | 0.219973296 | 1.59E-06 |
| UBE2D4 | grey | 0.00850875 | 0.854495012 | 0.008606575 | 0.852841082 |
| UBE2E2 | grey | -0.403595351 | 1.01E-19 | -0.394158184 | 8.29E-19 |
| UBE2I | grey | 0.051151495 | 0.269960719 | 0.063208702 | 0.172676232 |
| UBE2L3 | grey | 0.205200402 | 7.81E-06 | 0.205897584 | 7.27E-06 |
| UBE2Q2I | grey | -0.036405068 | 0.43252863 | -0.039003066 | 0.400390205 |
| UBE2U | grey | -0.175833414 | 0.000133773 | -0.183874604 | 6.41E-05 |

| | | | | | |
|---|---|---|---|---|---|
| UBIAD1 | grey | 0.092186523 | 0.046473173 | 0.043351346 | 0.349912334 |
| UBOX5 | grey | -0.15051369 | 0.00110428 | -0.153551295 | 0.000871138 |
| UBQLNL | grey | -0.021810066 | 0.638274781 | -0.027039114 | 0.559987008 |
| UCMA | grey | -0.069974058 | 0.131055936 | -0.078638692 | 0.089607512 |
| UCN2 | grey | -0.160185562 | 0.000511185 | -0.142617841 | 0.002004632 |
| UCP1 | grey | -0.109699481 | 0.017719232 | -0.110100671 | 0.017305153 |
| UCP3 | grey | -0.051366482 | 0.267950088 | -0.06298128 | 0.174229419 |
| UGT1A1( | grey | -0.063828901 | 0.168493049 | -0.065935239 | 0.154851454 |
| UGT1A6 | grey | -0.019947348 | 0.667229554 | -0.020748713 | 0.654709022 |
| UGT1A8 | grey | -0.047129659 | 0.309478986 | -0.048962827 | 0.291016929 |
| UGT1A9 | grey | -0.101706276 | 0.027970309 | -0.09964617 | 0.031321673 |
| UGT2A2 | grey | -0.049226501 | 0.288423468 | -0.046584484 | 0.315115155 |
| UGT2A3 | grey | -0.099792206 | 0.031073282 | -0.092089109 | 0.046705926 |
| UGT2B11 | grey | -0.134158048 | 0.003677801 | -0.120227155 | 0.009305894 |
| UGT2B15 | grey | -0.078495599 | 0.090194354 | -0.069017059 | 0.136421436 |
| UGT2B17 | grey | -0.133074559 | 0.003965645 | -0.121124941 | 0.008788576 |
| UGT2B4 | grey | 0.041218985 | 0.374142629 | 0.030489256 | 0.511010252 |
| UGT3A1 | grey | -0.134569788 | 0.003573477 | -0.168819437 | 0.000247553 |
| UGT3A2 | grey | -0.088763931 | 0.055257095 | -0.080715349 | 0.081430997 |
| UGT8 | grey | 0.113290394 | 0.014303253 | 0.114309368 | 0.013445993 |
| ULK3 | grey | 0.251245695 | 3.72E-08 | 0.24064329 | 1.41E-07 |
| UNC13A | grey | -0.030543712 | 0.5102555 | -0.037737559 | 0.415862766 |
| UNC45B | grey | 0.006254671 | 0.892767115 | 0.014344704 | 0.75718703 |
| UNC5A | grey | -0.112449308 | 0.015046691 | -0.096086512 | 0.037922898 |
| UNC5C | grey | 0.05753045 | 0.214628035 | 0.060361838 | 0.192874654 |
| UNC5CL | grey | -0.084339508 | 0.068613968 | -0.09747023 | 0.03522862 |
| UNC80 | grey | -0.222645695 | 1.18E-06 | -0.215251806 | 2.68E-06 |
| UNC93A | grey | 0.197696048 | 1.68E-05 | 0.188856153 | 4.00E-05 |
| UNCX | grey | -0.021708564 | 0.639838979 | -0.027982603 | 0.546371188 |
| UNK | grey | -0.117798717 | 0.010843396 | -0.144963075 | 0.001684357 |
| UPK1A | grey | -0.118331532 | 0.010488002 | -0.127251273 | 0.005891558 |
| UPK2 | grey | -0.169677425 | 0.000229893 | -0.177795726 | 0.000112132 |
| UPK3A | grey | 0.05587165 | 0.228163812 | 0.027034126 | 0.560059412 |
| UPK3B | grey | -0.269172187 | 3.41E-09 | -0.266342815 | 5.03E-09 |
| UPK3BL | grey | -0.092813523 | 0.044998246 | -0.090398274 | 0.050904192 |
| UPP1 | grey | 0.040477593 | 0.382803907 | 0.066969468 | 0.148467298 |
| UQCC1 | grey | -0.256237571 | 1.95E-08 | -0.254264868 | 2.52E-08 |
| UQCRH | grey | 0.244700366 | 8.53E-08 | 0.263579118 | 7.32E-09 |
| UQCRHL | grey | 0.186781111 | 4.88E-05 | 0.202379966 | 1.05E-05 |
| URAD | grey | -0.09794595 | 0.034340759 | -0.106391309 | 0.021476341 |
| URGCP | grey | -0.007533078 | 0.871024558 | -0.011234719 | 0.808670664 |
| URGCP-N | grey | 0.002802122 | 0.951843268 | 0.005320835 | 0.90870124 |
| USB1 | grey | 0.306975255 | 1.20E-11 | 0.342605125 | 2.62E-14 |
| USH2A | grey | 0.050316355 | 0.277868941 | 0.058697169 | 0.205459734 |
| USP11 | grey | -0.356492095 | 1.93E-15 | -0.352196412 | 4.39E-15 |

| | | | | | |
|---|---|---|---|---|---|
| USP17L1: | grey | 0.024173097 | 0.602324131 | 0.022439501 | 0.62861082 |
| USP17L3 | grey | -0.006936608 | 0.88115798 | -0.026477777 | 0.5681649 |
| USP17L4 | grey | -0.022582529 | 0.626423593 | -0.033006699 | 0.47673534 |
| USP17L8 | grey | -0.072217592 | 0.119117514 | -0.074222369 | 0.109184244 |
| USP20 | grey | 0.067974451 | 0.142457568 | 0.077410914 | 0.094743642 |
| USP35 | grey | -0.0929927 | 0.044584058 | -0.121108529 | 0.008797799 |
| USP44 | grey | -0.287454797 | 2.46E-10 | -0.312323329 | 5.03E-12 |
| USP6 | grey | -0.143720838 | 0.001847637 | -0.147682684 | 0.00137207 |
| UTF1 | grey | -0.102235367 | 0.027161048 | -0.103281766 | 0.025619715 |
| UTS2 | grey | -0.042080731 | 0.364228524 | -0.034227003 | 0.46058387 |
| UTS2R | grey | -0.060190669 | 0.194142044 | -0.072204709 | 0.119183556 |
| VAMP1 | grey | -0.096798801 | 0.036514926 | -0.086703843 | 0.061180124 |
| VANGL2 | grey | 0.007940306 | 0.86411816 | -0.017040922 | 0.713397344 |
| VASN | grey | -0.385380172 | 5.53E-18 | -0.364156143 | 4.32E-16 |
| VASP | grey | -0.014182361 | 0.759850261 | -0.002513216 | 0.956803238 |
| VAT1 | grey | 0.010898011 | 0.814298901 | 0.007989471 | 0.863285033 |
| VAV2 | grey | 0.170698609 | 0.000210411 | 0.157141838 | 0.000654507 |
| VAX1 | grey | 0.129545615 | 0.005050022 | 0.101788581 | 0.027843076 |
| VCY | grey | -0.043795329 | 0.344994995 | -0.040738018 | 0.379747639 |
| VCY1B | grey | -0.011006903 | 0.812477649 | 0.007306634 | 0.874869215 |
| VDR | grey | 0.188804806 | 4.02E-05 | 0.21506963 | 2.73E-06 |
| VEGFA | grey | 0.023820089 | 0.607636706 | 0.008499474 | 0.854651875 |
| VGLL1 | grey | -0.242022474 | 1.19E-07 | -0.242417548 | 1.13E-07 |
| VGLL2 | grey | -0.082013788 | 0.076632752 | -0.090692315 | 0.050152256 |
| VIL1 | grey | 0.071422213 | 0.123248762 | 0.054334846 | 0.241235191 |
| VILL | grey | -0.129815018 | 0.004958675 | -0.109116497 | 0.018336362 |
| VIMP | grey | 0.223156986 | 1.11E-06 | 0.21764158 | 2.06E-06 |
| VIPR1 | grey | 0.28547998 | 3.30E-10 | 0.257693464 | 1.61E-08 |
| VLDLR | grey | 0.197060385 | 1.79E-05 | 0.173325106 | 0.000167166 |
| VNN1 | grey | 0.035818758 | 0.439982176 | 0.022894082 | 0.621670577 |
| VOPP1 | grey | -0.001332887 | 0.97708252 | -0.000367048 | 0.993688233 |
| VPREB1 | grey | 0.041962208 | 0.365582308 | 0.050322726 | 0.277808027 |
| VPS26B | grey | 0.365120054 | 3.57E-16 | 0.365077428 | 3.60E-16 |
| VPS33B | grey | 0.019260588 | 0.67803365 | 0.015997576 | 0.730240126 |
| VPS37D | grey | 0.050859528 | 0.272707779 | 0.048248619 | 0.298120094 |
| VPS39 | grey | 0.179170589 | 9.90E-05 | 0.171518613 | 0.000195897 |
| VPS53 | grey | 0.288105459 | 2.23E-10 | 0.309971656 | 7.38E-12 |
| VSIG1 | grey | -0.017163672 | 0.711424718 | -0.000432167 | 0.992568474 |
| VSIG10 | grey | 0.119167444 | 0.009951232 | 0.083281083 | 0.072173712 |
| VSTM1 | grey | -0.08862515 | 0.055640558 | -0.097996642 | 0.034247282 |
| VSTM2A | grey | -0.02865266 | 0.536801693 | -0.042890105 | 0.355067525 |
| VSTM2B | grey | -0.164741535 | 0.000350216 | -0.178706348 | 0.000103249 |
| VSTM2L | grey | -0.223713811 | 1.04E-06 | -0.215205943 | 2.69E-06 |
| VSTM5 | grey | -0.222401659 | 1.21E-06 | -0.229801813 | 5.16E-07 |
| VSX1 | grey | -0.122826632 | 0.007877845 | -0.122490713 | 0.008050648 |

| | | | | | |
|---|---|---|---|---|---|
| VSX2 | grey | -0.047328882 | 0.30743603 | -0.023028757 | 0.619620844 |
| VWA5A | grey | 0.036440278 | 0.432083348 | 0.028623454 | 0.537217044 |
| VWCE | grey | -0.239211834 | 1.68E-07 | -0.268259891 | 3.86E-09 |
| WBP1 | grey | 0.122974596 | 0.007802786 | 0.096067056 | 0.037961993 |
| WDR25 | grey | -0.028727172 | 0.53574276 | 0.022722067 | 0.624292902 |
| WDR59 | grey | 0.170435338 | 0.00021528 | 0.173195689 | 0.000169085 |
| WDR72 | grey | -0.072103539 | 0.119703179 | -0.086341813 | 0.062273305 |
| WDR86 | grey | -0.137620078 | 0.002880443 | -0.151541023 | 0.001019664 |
| WDR91 | grey | -0.000758497 | 0.986957296 | -0.013178639 | 0.776378263 |
| WFDC10l | grey | 0.037256172 | 0.421839558 | 0.052646583 | 0.256190666 |
| WFIKKN | grey | 0.082092204 | 0.076350493 | 0.067691452 | 0.144130735 |
| WIPI1 | grey | 0.162777461 | 0.000412732 | 0.163558695 | 0.000386714 |
| WIPI2 | grey | -0.203155079 | 9.66E-06 | -0.180228326 | 8.99E-05 |
| WISP3 | grey | -0.239959958 | 1.53E-07 | -0.2257873 | 8.22E-07 |
| WNK2 | grey | 0.202743938 | 1.01E-05 | 0.194488626 | 2.31E-05 |
| WNK4 | grey | 0.019626282 | 0.672272179 | 0.02459404 | 0.596016568 |
| WNT1 | grey | -0.036834854 | 0.427111494 | -0.015827079 | 0.733005104 |
| WNT10A | grey | -0.293372385 | 1.01E-10 | -0.299025755 | 4.22E-11 |
| WNT10B | grey | -0.031501083 | 0.497081894 | -0.028923784 | 0.532953631 |
| WNT11 | grey | -0.198985798 | 1.48E-05 | -0.199835989 | 1.36E-05 |
| WNT3 | grey | -0.157840546 | 0.000618648 | -0.150604474 | 0.001096549 |
| WNT3A | grey | 0.046686778 | 0.314052524 | 0.060731809 | 0.190155982 |
| WNT5B | grey | -0.150564754 | 0.001099925 | -0.185846122 | 5.33E-05 |
| WNT7A | grey | -0.322260218 | 9.56E-13 | -0.312562307 | 4.84E-12 |
| WNT7B | grey | -0.044848522 | 0.333507016 | -0.03285498 | 0.478764831 |
| WNT8B | grey | -0.100752702 | 0.029481207 | -0.100851011 | 0.029322271 |
| WSCD1 | grey | -0.109309439 | 0.018130074 | -0.133848987 | 0.003757913 |
| WTIP | grey | -0.369662945 | 1.44E-16 | -0.362720593 | 5.74E-16 |
| WWOX | grey | -0.035217435 | 0.447702329 | -0.036834637 | 0.427114221 |
| XAGE1A | grey | -0.091087795 | 0.049155572 | -0.09685089 | 0.036413728 |
| XAGE1B | grey | -0.103229256 | 0.025695225 | -0.111881665 | 0.015567412 |
| XAGE2B | grey | -0.285824806 | 3.14E-10 | -0.310269281 | 7.03E-12 |
| XAGE3 | grey | -0.138455578 | 0.002713248 | -0.136567252 | 0.00310446 |
| XAGE5 | grey | -0.098393401 | 0.033523121 | -0.096780632 | 0.036550281 |
| XCL1 | grey | -0.215574751 | 2.58E-06 | -0.211972848 | 3.82E-06 |
| XDH | grey | 0.12573861 | 0.006513239 | 0.12246654 | 0.008063213 |
| XIRP1 | grey | 0.067810401 | 0.143425655 | 0.052193851 | 0.26030808 |
| XK | grey | -0.195595619 | 2.07E-05 | -0.181427378 | 8.05E-05 |
| XKR4 | grey | -0.011356901 | 0.806630748 | -0.008111775 | 0.861213198 |
| XKR7 | grey | -0.176333906 | 0.000127908 | -0.177813622 | 0.00011195 |
| XKR8 | grey | 0.027994892 | 0.54619493 | 0.038578525 | 0.405541942 |
| XKR9 | grey | -0.170764241 | 0.000209213 | -0.177752799 | 0.000112568 |
| XRCC6BI | grey | 0.229455543 | 5.38E-07 | 0.198253701 | 1.59E-05 |
| XXbac-BI | grey | 0.011438987 | 0.805261005 | -0.021514131 | 0.642839773 |
| XXbac-BI | grey | 0.18565282 | 5.43E-05 | 0.196885085 | 1.82E-05 |

| | | | | | |
|---|---|---|---|---|---|
| XXbac-BI | grey | -0.127454314 | 0.005812302 | -0.142792328 | 0.00197901 |
| XXcos-LU | grey | 0.09978561 | 0.031084465 | 0.099433829 | 0.03168587 |
| XYLT2 | grey | 0.075111609 | 0.104992718 | 0.052979495 | 0.253191879 |
| YBX3 | grey | -0.191062473 | 3.24E-05 | -0.169761989 | 0.000228217 |
| YIPF7 | grey | -0.028971509 | 0.532277703 | -0.036341838 | 0.433328917 |
| YJEFN3 | grey | -0.041457607 | 0.371380846 | -0.042950216 | 0.354392977 |
| YPEL1 | grey | -0.048995673 | 0.290693009 | -0.068713637 | 0.138157423 |
| YWHAH | grey | 0.127947697 | 0.005623691 | 0.15186098 | 0.000994559 |
| ZAR1 | grey | -0.13710047 | 0.002989097 | -0.136584286 | 0.003100713 |
| ZBED1 | grey | 0.342006482 | 2.93E-14 | 0.331517014 | 1.92E-13 |
| ZBED6CI | grey | -0.034393668 | 0.458401871 | -0.022177547 | 0.632625125 |
| ZBTB12 | grey | -0.131892671 | 0.004302754 | -0.154723648 | 0.000794024 |
| ZBTB22 | grey | -0.240689498 | 1.40E-07 | -0.255256067 | 2.22E-08 |
| ZBTB3 | grey | 0.017220805 | 0.710507237 | 0.025638663 | 0.580494737 |
| ZBTB7C | grey | -0.033490209 | 0.470299076 | -0.013473668 | 0.771509259 |
| ZBTB8B | grey | -0.135603629 | 0.003323253 | -0.150437798 | 0.00111078 |
| ZC3H10 | grey | -0.118392328 | 0.010448114 | -0.132672722 | 0.00407748 |
| ZC3H12A | grey | 0.234123681 | 3.10E-07 | 0.226405072 | 7.66E-07 |
| ZC4H2 | grey | -0.172072485 | 0.00018663 | -0.192764103 | 2.74E-05 |
| ZDHHC1! | grey | -0.277255592 | 1.09E-09 | -0.279182487 | 8.28E-10 |
| ZDHHC9 | grey | 0.198072548 | 1.62E-05 | 0.163593186 | 0.000385602 |
| ZFAND2 | grey | 0.00886118 | 0.848539485 | 0.021810733 | 0.638264499 |
| ZFHX4 | grey | -0.173861124 | 0.000159433 | -0.191003127 | 3.25E-05 |
| ZFP36 | grey | -0.002195862 | 0.962253517 | -0.017064085 | 0.71302496 |
| ZFP91-CN | grey | -0.024305535 | 0.600336396 | -0.047216397 | 0.308588422 |
| ZFP92 | grey | -0.388238998 | 3.00E-18 | -0.410006966 | 2.33E-20 |
| ZFR2 | grey | -0.126043003 | 0.006383606 | -0.119984553 | 0.009450255 |
| ZFY | grey | -0.058785995 | 0.204773532 | -0.066692781 | 0.150155283 |
| ZGPAT | grey | -0.263023107 | 7.90E-09 | -0.271136581 | 2.59E-09 |
| ZIC1 | grey | 0.075438934 | 0.103482284 | 0.075039223 | 0.105329082 |
| ZIC2 | grey | 0.044744945 | 0.334625777 | 0.067561585 | 0.144903551 |
| ZIC3 | grey | -0.046222936 | 0.318889788 | -0.040594804 | 0.381426504 |
| ZIC4 | grey | 0.019215848 | 0.67873983 | 0.036532119 | 0.430923134 |
| ZIC5 | grey | -0.002115658 | 0.963631244 | 0.009176172 | 0.843223855 |
| ZIK1 | grey | -0.398309841 | 3.31E-19 | -0.405184893 | 7.04E-20 |
| ZKSCAN | grey | -0.089737393 | 0.052628619 | -0.10060193 | 0.029726394 |
| ZMIZ2 | grey | 0.068414519 | 0.139885348 | 0.075048576 | 0.105285573 |
| ZMYND8 | grey | 0.095063236 | 0.040025968 | 0.078348921 | 0.090799095 |
| ZNF114 | grey | -0.078502073 | 0.090167737 | -0.094322929 | 0.041608238 |
| ZNF133 | grey | -0.179701543 | 9.43E-05 | -0.192111325 | 2.92E-05 |
| ZNF135 | grey | -0.467369885 | 1.02E-26 | -0.475427644 | 1.04E-27 |
| ZNF185 | grey | -0.19953469 | 1.40E-05 | -0.206738342 | 6.66E-06 |
| ZNF212 | grey | -0.024612748 | 0.595736943 | -0.063219959 | 0.172599622 |
| ZNF215 | grey | -0.02053711 | 0.658005895 | -0.049818072 | 0.282661522 |
| ZNF219 | grey | -0.089737749 | 0.052627678 | -0.105894577 | 0.022096393 |

| | | | | | |
|---|---|---|---|---|---|
| ZNF232 | grey | 0.140483363 | 0.002343583 | 0.114204916 | 0.013531736 |
| ZNF253 | grey | -0.050368407 | 0.277371499 | -0.060352634 | 0.192942648 |
| ZNF263 | grey | 0.047535588 | 0.305325767 | 0.046681349 | 0.314108854 |
| ZNF276 | grey | 0.173110238 | 0.000170364 | 0.168991986 | 0.000243902 |
| ZNF280A | grey | 0.132933402 | 0.00400461 | 0.1059953 | 0.021969425 |
| ZNF282 | grey | 0.289983212 | 1.69E-10 | 0.255916386 | 2.03E-08 |
| ZNF285 | grey | -0.410667782 | 2.00E-20 | -0.447300342 | 2.36E-24 |
| ZNF300 | grey | -0.501066712 | 4.71E-31 | -0.515407918 | 4.75E-33 |
| ZNF321P | grey | 0.045935489 | 0.321911752 | 0.016828957 | 0.716808175 |
| ZNF324 | grey | -0.020673957 | 0.655872987 | -0.034866724 | 0.452240169 |
| ZNF324B | grey | 0.061879518 | 0.181901379 | 0.044036836 | 0.342338718 |
| ZNF335 | grey | -0.031399676 | 0.498468669 | -0.034780469 | 0.453360182 |
| ZNF346 | grey | -0.110097801 | 0.017308085 | -0.111646014 | 0.015788185 |
| ZNF362 | grey | -0.157492496 | 0.000636276 | -0.171519957 | 0.000195874 |
| ZNF365 | grey | -0.10090498 | 0.029235334 | -0.107576888 | 0.020057126 |
| ZNF385A | grey | -0.015411693 | 0.739755858 | -0.018238936 | 0.694228532 |
| ZNF385B | grey | 0.28793972 | 2.29E-10 | 0.248662523 | 5.18E-08 |
| ZNF385C | grey | -0.184246361 | 6.19E-05 | -0.199933354 | 1.34E-05 |
| ZNF394 | grey | -0.217671193 | 2.05E-06 | -0.203314427 | 9.50E-06 |
| ZNF418 | grey | -0.281229191 | 6.15E-10 | -0.281060368 | 6.31E-10 |
| ZNF423 | grey | -0.12657776 | 0.00616146 | -0.117989203 | 0.010715133 |
| ZNF467 | grey | -0.099824341 | 0.03101885 | -0.084173071 | 0.069164014 |
| ZNF486 | grey | -0.140253249 | 0.002383088 | -0.142019696 | 0.002094784 |
| ZNF488 | grey | -0.142800287 | 0.001977848 | -0.152843531 | 0.000920985 |
| ZNF503 | grey | -0.165473907 | 0.000329254 | -0.166420034 | 0.000303906 |
| ZNF513 | grey | 0.07936621 | 0.086671117 | 0.056096413 | 0.226295065 |
| ZNF526 | grey | -0.071708545 | 0.121748873 | -0.054769765 | 0.237483764 |
| ZNF536 | grey | -0.178518158 | 0.000105029 | -0.184248225 | 6.19E-05 |
| ZNF541 | grey | -0.061610527 | 0.183811844 | -0.063092156 | 0.17347089 |
| ZNF544 | grey | -0.318151448 | 1.91E-12 | -0.309661439 | 7.77E-12 |
| ZNF552 | grey | 0.159790667 | 0.000527973 | 0.177297156 | 0.000117295 |
| ZNF554 | grey | 0.038831114 | 0.402472069 | 0.018465638 | 0.69062257 |
| ZNF556 | grey | 0.051396957 | 0.267665908 | 0.044764914 | 0.334409907 |
| ZNF563 | grey | -0.076184809 | 0.10010477 | -0.076566535 | 0.098410431 |
| ZNF574 | grey | -0.036409006 | 0.432478806 | -0.031970703 | 0.490686493 |
| ZNF576 | grey | -0.063425656 | 0.171204186 | -0.059118685 | 0.202218163 |
| ZNF586 | grey | 0.203295367 | 9.52E-06 | 0.220045682 | 1.58E-06 |
| ZNF589 | grey | 0.037077469 | 0.424071006 | 0.028646962 | 0.53688272 |
| ZNF625-Z | grey | -0.086363829 | 0.062206369 | -0.091789323 | 0.047428354 |
| ZNF626 | grey | -0.21042668 | 4.50E-06 | -0.2189838 | 1.77E-06 |
| ZNF648 | grey | -0.096593704 | 0.036915713 | -0.096066858 | 0.037962391 |
| ZNF679 | grey | -0.176719185 | 0.000123559 | -0.175301536 | 0.000140282 |
| ZNF703 | grey | -0.022835527 | 0.6225627 | -0.009958658 | 0.830050351 |
| ZNF71 | grey | -0.027941903 | 0.546955163 | -0.031931475 | 0.491219011 |
| ZNF747 | grey | -0.050947681 | 0.271876357 | -0.034851432 | 0.452438627 |

| | | | | | |
|---|---|---|---|---|---|
| ZNF76 | grey | -0.013349936 | 0.773550192 | 0.000314457 | 0.994592566 |
| ZNF764 | grey | -0.026119556 | 0.573413264 | -0.024067414 | 0.603912406 |
| ZNF768 | grey | 0.001546705 | 0.97340743 | -0.016324419 | 0.724949483 |
| ZNF77 | grey | 0.253704055 | 2.71E-08 | 0.244371257 | 8.89E-08 |
| ZNF771 | grey | -0.14139764 | 0.002192458 | -0.133755065 | 0.00378257 |
| ZNF783 | grey | -0.197440889 | 1.73E-05 | -0.222060373 | 1.26E-06 |
| ZNF785 | grey | -0.085077034 | 0.066219537 | -0.085036723 | 0.066348613 |
| ZNF788 | grey | 0.04314184 | 0.352248046 | 0.027036951 | 0.560018399 |
| ZNF804A | grey | -0.065135606 | 0.159928305 | -0.051350785 | 0.268096541 |
| ZNF853 | grey | -0.256942057 | 1.78E-08 | -0.246588662 | 6.73E-08 |
| ZNF90 | grey | -0.18377339 | 6.47E-05 | -0.158035228 | 0.000608986 |
| ZNRF1 | grey | 0.289084229 | 1.93E-10 | 0.335338779 | 9.77E-14 |
| ZP2 | grey | -0.117773696 | 0.010860344 | -0.102025197 | 0.027480066 |
| ZPLD1 | grey | -0.127784863 | 0.005685321 | -0.122426915 | 0.008083847 |
| ZSCAN1 | grey | -0.158323061 | 0.000594957 | -0.15288319 | 0.000918123 |
| ZSCAN1( | grey | -0.028649224 | 0.536850555 | -0.034180271 | 0.461196725 |
| ZSCAN2 | grey | 0.056645657 | 0.221774337 | 0.041587909 | 0.36987808 |
| ZSCAN31 | grey | -0.036062994 | 0.436868406 | -0.047812423 | 0.302514594 |
| ZSCAN5I | grey | 0.036324156 | 0.433552877 | 0.051582637 | 0.265938901 |
| ZSWIM3 | grey | -0.248122895 | 5.55E-08 | -0.235289715 | 2.70E-07 |
| ZSWIM4 | grey | -0.152683706 | 0.0009326 | -0.160112597 | 0.000514249 |
| ZSWIM5 | grey | 0.156549133 | 0.000686427 | 0.099282688 | 0.0319473 |
| ACAP1 | grey60 | -0.007127749 | 0.877908491 | 0.055163288 | 0.234124972 |
| AGAP2 | grey60 | 0.002185145 | 0.962437595 | 0.046406234 | 0.316972441 |
| AMIGO2 | grey60 | 0.103318907 | 0.025566423 | 0.080325287 | 0.082918985 |
| AMPD1 | grey60 | -0.073697586 | 0.111719076 | -0.038820739 | 0.402597894 |
| ANKRD2 | grey60 | 0.361252762 | 7.65E-16 | 0.362829964 | 5.62E-16 |
| APOBEC: | grey60 | -0.224969749 | 9.03E-07 | -0.205540265 | 7.54E-06 |
| APOBEC: | grey60 | -0.161544104 | 0.000457143 | -0.108206707 | 0.019336918 |
| APOBEC: | grey60 | -0.257731217 | 1.60E-08 | -0.209555405 | 4.94E-06 |
| APOBEC: | grey60 | 0.026178381 | 0.572549836 | 0.081969298 | 0.076793273 |
| ARHGAP | grey60 | -0.007753512 | 0.867284843 | 0.021873351 | 0.637300321 |
| ARHGAP | grey60 | -0.001116354 | 0.980804769 | 0.064554794 | 0.163693807 |
| ARHGDII | grey60 | -0.128607328 | 0.005380127 | -0.06906402 | 0.136154266 |
| ARHGEF | grey60 | 0.067005028 | 0.148251405 | 0.07372831 | 0.111569409 |
| ARRDC5 | grey60 | 0.116286371 | 0.011910943 | 0.160596746 | 0.000494233 |
| ATP2A3 | grey60 | 0.123555543 | 0.007514221 | 0.174935004 | 0.000144939 |
| BATF | grey60 | 0.088996335 | 0.054619865 | 0.144083306 | 0.001798556 |
| BCL11B | grey60 | -0.040190784 | 0.386187125 | -0.000405569 | 0.993025837 |
| BFSP2 | grey60 | -0.071981435 | 0.120332674 | -0.053451727 | 0.248980076 |
| BIN2 | grey60 | -0.061187089 | 0.186849186 | -0.007415709 | 0.873016921 |
| BLK | grey60 | -0.086591156 | 0.06151868 | -0.057613736 | 0.213963974 |
| C11orf21 | grey60 | -0.143676441 | 0.001853732 | -0.115930317 | 0.012175397 |
| C16orf54 | grey60 | -0.026222925 | 0.571896438 | 0.035955392 | 0.438238666 |
| CCL17 | grey60 | 0.105576576 | 0.022501441 | 0.166896716 | 0.000291836 |

| | | | | | |
|---|---|---|---|---|---|
| CCL19 | grey60 | -0.047529349 | 0.305389316 | -0.007118015 | 0.878073925 |
| CCL5 | grey60 | -0.044429901 | 0.338043444 | 0.024597365 | 0.595966873 |
| CCR2 | grey60 | -0.063367408 | 0.171598478 | -0.025086863 | 0.588670315 |
| CCR5 | grey60 | -0.031994584 | 0.490362457 | 0.019669912 | 0.67158605 |
| CCR7 | grey60 | 0.04076545 | 0.379426588 | 0.091077958 | 0.049180162 |
| CD101 | grey60 | 0.105218307 | 0.02296547 | 0.150254066 | 0.001126665 |
| CD19 | grey60 | -0.049999467 | 0.280910407 | -0.006446607 | 0.889497211 |
| CD2 | grey60 | 0.030971688 | 0.504344056 | 0.099231365 | 0.032036493 |
| CD244 | grey60 | -0.024597525 | 0.595964482 | 0.020983787 | 0.651054305 |
| CD247 | grey60 | -0.001332168 | 0.977094886 | 0.065932263 | 0.154870118 |
| CD27 | grey60 | 0.079231166 | 0.087210244 | 0.141778489 | 0.002132175 |
| CD28 | grey60 | -0.021201627 | 0.647674985 | 0.017225654 | 0.710429383 |
| CD37 | grey60 | -0.035108574 | 0.449108118 | 0.028213922 | 0.54305809 |
| CD38 | grey60 | -0.063217394 | 0.172617075 | -0.003435628 | 0.94097394 |
| CD3D | grey60 | 0.075302307 | 0.104110638 | 0.149540899 | 0.001190324 |
| CD3E | grey60 | 0.045411655 | 0.327466637 | 0.110331751 | 0.01707051 |
| CD3G | grey60 | 0.025894109 | 0.576728043 | 0.085367984 | 0.065294021 |
| CD40LG | grey60 | -0.034617417 | 0.455481644 | 0.011035583 | 0.811998144 |
| CD48 | grey60 | -0.052930226 | 0.253634139 | 0.006474631 | 0.88901993 |
| CD5 | grey60 | 0.065656179 | 0.156609154 | 0.100549952 | 0.029811326 |
| CD52 | grey60 | 0.017253043 | 0.70998972 | 0.085627745 | 0.064476724 |
| CD6 | grey60 | -0.033073312 | 0.475845781 | 0.031748257 | 0.493710308 |
| CD7 | grey60 | 0.001633361 | 0.971918151 | 0.069512073 | 0.133625374 |
| CD70 | grey60 | -0.049424291 | 0.286488259 | -0.013524985 | 0.77066326 |
| CD79A | grey60 | 0.017326337 | 0.708813627 | 0.076690605 | 0.097864671 |
| CD79B | grey60 | -0.165172453 | 0.000337736 | -0.126273086 | 0.00628716 |
| CD8A | grey60 | 0.01903755 | 0.681556869 | 0.066859778 | 0.149134747 |
| CD8B | grey60 | -0.009441351 | 0.838754349 | 0.002389572 | 0.958926485 |
| CD96 | grey60 | 0.017731067 | 0.702331745 | 0.081580633 | 0.078207221 |
| CLEC2D | grey60 | -0.126643028 | 0.006134828 | -0.112474548 | 0.015023897 |
| CNR2 | grey60 | 0.004044207 | 0.930542591 | 0.033661032 | 0.468036651 |
| CORO1A | grey60 | -0.001607031 | 0.972370644 | 0.071931252 | 0.120592139 |
| CPNE5 | grey60 | -0.162001185 | 0.00044019 | -0.129954994 | 0.004911803 |
| CRTAM | grey60 | -0.037304571 | 0.421236385 | 0.000627939 | 0.989202147 |
| CST7 | grey60 | -0.006112102 | 0.895197148 | 0.052283379 | 0.259490262 |
| CTLA4 | grey60 | 0.094439889 | 0.041354802 | 0.136320893 | 0.003159114 |
| CTSW | grey60 | -0.051865506 | 0.263322653 | 0.002425667 | 0.958306623 |
| CXCL13 | grey60 | 0.08367195 | 0.070841965 | 0.133395672 | 0.00387828 |
| CXCL9 | grey60 | 0.122255686 | 0.008173554 | 0.162389716 | 0.000426241 |
| CXCR3 | grey60 | 0.048428096 | 0.296324333 | 0.115177556 | 0.012751635 |
| CXCR5 | grey60 | -0.018112074 | 0.696249436 | 0.00781142 | 0.866302909 |
| CXCR6 | grey60 | 0.01596682 | 0.730738654 | 0.072494992 | 0.117702396 |
| CXorf65 | grey60 | -0.030136293 | 0.515916364 | 0.01305094 | 0.778488483 |
| CYTIP | grey60 | -0.038111344 | 0.411256509 | 0.007457976 | 0.872299334 |
| DEF6 | grey60 | 0.161229366 | 0.000469168 | 0.185030703 | 5.76E-05 |

| | | | | | |
|---|---|---|---|---|---|
| DENND1 | grey60 | 0.083819758 | 0.070343621 | 0.091084883 | 0.049162851 |
| DERL3 | grey60 | 0.072210232 | 0.119155241 | 0.100574207 | 0.029771668 |
| DPP9 | grey60 | 0.264178729 | 6.75E-09 | 0.280705948 | 6.64E-10 |
| EAF2 | grey60 | 0.197756368 | 1.67E-05 | 0.215954105 | 2.48E-06 |
| EOMES | grey60 | -0.03624497 | 0.434556623 | 0.000942122 | 0.98380017 |
| FAIM3 | grey60 | 0.040910447 | 0.377732332 | 0.083098747 | 0.072801918 |
| FAM159A | grey60 | -0.141869118 | 0.002118055 | -0.110577374 | 0.016824155 |
| FAM78A | grey60 | -0.075476447 | 0.103310283 | -0.027885356 | 0.547767007 |
| FASLG | grey60 | 0.081512282 | 0.078458047 | 0.113648152 | 0.013996948 |
| FBXO6 | grey60 | 0.185300776 | 5.61E-05 | 0.220806541 | 1.45E-06 |
| FCER2 | grey60 | -0.002640088 | 0.954624862 | 0.023599274 | 0.610970426 |
| FCRL1 | grey60 | -0.005259938 | 0.909741675 | 0.034353585 | 0.45892612 |
| FCRL3 | grey60 | 0.038132036 | 0.411002403 | 0.081457905 | 0.078658061 |
| FCRL4 | grey60 | -0.038891529 | 0.401739882 | 0.016794988 | 0.717355305 |
| FCRL5 | grey60 | -0.012760631 | 0.78329185 | 0.029396791 | 0.526273687 |
| FCRL6 | grey60 | 0.036029393 | 0.437296032 | 0.049782613 | 0.283004687 |
| FCRLA | grey60 | 0.006780396 | 0.883815146 | 0.055340365 | 0.232624585 |
| FMNL1 | grey60 | -0.079067294 | 0.087868084 | -0.030797828 | 0.506741162 |
| FOXP3 | grey60 | 0.07505501 | 0.10525565 | 0.134127747 | 0.003685586 |
| GBP5 | grey60 | 0.04713214 | 0.309453486 | 0.083631942 | 0.07097735 |
| GFI1 | grey60 | 0.019954842 | 0.66711204 | 0.072188789 | 0.119265206 |
| GMFG | grey60 | -0.107632543 | 0.019992557 | -0.05366158 | 0.247124169 |
| GNGT2 | grey60 | -0.145973497 | 0.001561424 | -0.099437955 | 0.03167876 |
| GNLY | grey60 | -0.052203308 | 0.260221611 | -0.008079449 | 0.861760702 |
| GPR114 | grey60 | 0.126622644 | 0.006143134 | 0.15745128 | 0.000638394 |
| GPR171 | grey60 | -0.021914113 | 0.636673014 | 0.015794883 | 0.733527622 |
| GPR174 | grey60 | -0.019191142 | 0.679129906 | 0.023720942 | 0.609132565 |
| GPR25 | grey60 | 0.063265746 | 0.172288276 | 0.111045233 | 0.016363496 |
| GPR68 | grey60 | 0.220921538 | 1.43E-06 | 0.267035684 | 4.57E-09 |
| GPSM3 | grey60 | -0.08598933 | 0.063353086 | -0.030894829 | 0.505403016 |
| GRAP2 | grey60 | -0.047881793 | 0.301812863 | 0.016045507 | 0.729463466 |
| GZMA | grey60 | 0.016479383 | 0.722445596 | 0.067956351 | 0.142564131 |
| GZMB | grey60 | 0.052677748 | 0.255908899 | 0.101808784 | 0.027811921 |
| GZMH | grey60 | -0.014337104 | 0.757311633 | 0.034078554 | 0.462532234 |
| GZMK | grey60 | -0.041083286 | 0.375718825 | -0.00728608 | 0.875218336 |
| GZMM | grey60 | -0.057833099 | 0.212222002 | -0.001231073 | 0.978832664 |
| HCST | grey60 | -0.073813692 | 0.111154306 | -0.023656837 | 0.610100596 |
| HLA-DOB | grey60 | -0.063683236 | 0.169468669 | -0.054548527 | 0.239386906 |
| ICAM3 | grey60 | 0.016592638 | 0.720617532 | 0.064214699 | 0.165929401 |
| ICOS | grey60 | 0.054217763 | 0.242252159 | 0.107201296 | 0.020497639 |
| IFNG | grey60 | 0.083580965 | 0.071150159 | 0.124058698 | 0.007272046 |
| IGJ | grey60 | 0.001021849 | 0.98242947 | 0.069270615 | 0.134983667 |
| IGLL1 | grey60 | 0.008488594 | 0.854835876 | 0.027628242 | 0.551465876 |
| IGLL5 | grey60 | 0.072854111 | 0.115889978 | 0.132897422 | 0.004014597 |
| IKZF3 | grey60 | 0.03369628 | 0.467570567 | 0.052225037 | 0.260023003 |

| | | | | | |
|---|---|---|---|---|---|
| IL12RB1 | grey60 | 0.013721596 | 0.767424491 | 0.07114542 | 0.124712316 |
| IL16 | grey60 | -0.093087448 | 0.044366338 | -0.039098803 | 0.399233891 |
| IL18BP | grey60 | -0.027798277 | 0.549018368 | 0.007978873 | 0.863464605 |
| IL18RAP | grey60 | -0.003902926 | 0.932963227 | 0.038008725 | 0.41251809 |
| IL21R | grey60 | 0.023328315 | 0.615072162 | 0.069964561 | 0.131108366 |
| IL2RB | grey60 | 0.039436698 | 0.395168815 | 0.094252154 | 0.041762233 |
| IL2RG | grey60 | -0.015040568 | 0.745804259 | 0.053438731 | 0.249095323 |
| IL9R | grey60 | 0.06703034 | 0.14809788 | 0.12452442 | 0.007054134 |
| IRF4 | grey60 | -0.015827264 | 0.7330021 | 0.024301583 | 0.600395668 |
| ISG20 | grey60 | 0.071520912 | 0.122730132 | 0.112469097 | 0.015028818 |
| ITGAE | grey60 | 0.199520322 | 1.40E-05 | 0.221992779 | 1.27E-06 |
| ITGAL | grey60 | 0.020394812 | 0.660226712 | 0.074164637 | 0.109460863 |
| ITK | grey60 | -0.015949674 | 0.731016612 | 0.023572698 | 0.611372188 |
| JAKMIP1 | grey60 | 0.101670755 | 0.028025373 | 0.129334414 | 0.005122692 |
| KIF21B | grey60 | 0.040181556 | 0.386296277 | 0.050167144 | 0.279298257 |
| KIR2DL4 | grey60 | -0.016352309 | 0.724498626 | 0.031771083 | 0.493399569 |
| KLRB1 | grey60 | -0.045342063 | 0.328209256 | 0.019358174 | 0.676494342 |
| KLRC1 | grey60 | -0.108209252 | 0.019334054 | -0.060838276 | 0.189378867 |
| KLRC2 | grey60 | -0.004921649 | 0.915524168 | 0.035116838 | 0.449001311 |
| KLRD1 | grey60 | -0.040457745 | 0.383037452 | 0.001931045 | 0.966802875 |
| KLRF2 | grey60 | -0.036358378 | 0.433119496 | -0.040143087 | 0.386751518 |
| LAG3 | grey60 | -0.041968833 | 0.365506549 | 0.010289938 | 0.824487193 |
| LAIR2 | grey60 | 0.031477635 | 0.497402365 | 0.061871136 | 0.181960691 |
| LAT | grey60 | -0.105154412 | 0.023049091 | -0.086347183 | 0.062256973 |
| LAX1 | grey60 | 0.023248378 | 0.616284556 | 0.060186518 | 0.194172858 |
| LCK | grey60 | 0.050743566 | 0.273804106 | 0.101349403 | 0.028527783 |
| LPXN | grey60 | -0.014104348 | 0.761131088 | 0.026411404 | 0.569135617 |
| LTA | grey60 | 0.036441067 | 0.43207337 | 0.089443107 | 0.053412012 |
| LY9 | grey60 | 0.012088371 | 0.794446228 | 0.057255669 | 0.216829449 |
| MAP4K1 | grey60 | -0.254263264 | 2.52E-08 | -0.206752594 | 6.65E-06 |
| MATK | grey60 | -0.136571795 | 0.00310346 | -0.096845201 | 0.036424768 |
| MEI1 | grey60 | -0.036197094 | 0.435164143 | 0.013728053 | 0.767318196 |
| MICAL1 | grey60 | -0.112942113 | 0.014607089 | -0.088592776 | 0.055730325 |
| MLKL | grey60 | 0.146198292 | 0.001535219 | 0.164108219 | 0.000369338 |
| MS4A1 | grey60 | -0.017411961 | 0.707440562 | 0.027460851 | 0.553880541 |
| MYO1G | grey60 | -0.006172721 | 0.894163804 | 0.050759452 | 0.273653739 |
| MYO7A | grey60 | -0.150822627 | 0.001078177 | -0.121289582 | 0.008696537 |
| MZB1 | grey60 | 0.040637433 | 0.380926296 | 0.097811366 | 0.034589986 |
| NCR1 | grey60 | -0.066801317 | 0.149491408 | -0.030599597 | 0.50948154 |
| NCR3 | grey60 | 0.00498608 | 0.914422461 | 0.077415873 | 0.094722434 |
| NKG7 | grey60 | -0.044671609 | 0.335419357 | 0.022544641 | 0.62700268 |
| NLRC3 | grey60 | 0.002142037 | 0.96317809 | 0.050931938 | 0.272024715 |
| NLRP6 | grey60 | 0.067526504 | 0.145112853 | 0.097730667 | 0.034740164 |
| NUGGC | grey60 | 0.028714361 | 0.535924759 | 0.063956097 | 0.167644573 |
| P2RX1 | grey60 | -0.117493835 | 0.01105152 | -0.085247046 | 0.065677428 |

| | | | | | |
|---|---|---|---|---|---|
| P2RX5 | grey60 | -0.109704948 | 0.017713531 | -0.105747854 | 0.022282487 |
| P2RY10 | grey60 | 0.021804856 | 0.638355017 | 0.067080604 | 0.147793368 |
| P2RY8 | grey60 | -0.027299356 | 0.556215033 | 0.012973904 | 0.779762291 |
| PARP15 | grey60 | -0.093740524 | 0.04288983 | -0.053972926 | 0.2443885 |
| PCED1B | grey60 | 0.201546289 | 1.14E-05 | 0.208544631 | 5.50E-06 |
| PDCD1 | grey60 | 0.081852348 | 0.077216529 | 0.125998081 | 0.006402591 |
| PIM2 | grey60 | 0.006254986 | 0.892761735 | 0.064603865 | 0.163373119 |
| PLA2G2I | grey60 | 0.016304481 | 0.725271841 | 0.051681897 | 0.265018826 |
| POU2AF1 | grey60 | 0.002066024 | 0.964483895 | 0.045543052 | 0.32606747 |
| PRF1 | grey60 | -0.012750471 | 0.7834601 | 0.038441054 | 0.407218568 |
| PSTPIP1 | grey60 | -0.028051794 | 0.54537914 | 0.0258356 | 0.57758977 |
| PTPN7 | grey60 | 0.004984962 | 0.91444159 | 0.073005729 | 0.115131379 |
| PYHIN1 | grey60 | 0.010263862 | 0.824924763 | 0.059359569 | 0.200382468 |
| RAB33A | grey60 | -0.175695503 | 0.000135433 | -0.138759573 | 0.002654642 |
| RAB37 | grey60 | -0.015864884 | 0.732391711 | 0.026171932 | 0.572644466 |
| RAC2 | grey60 | -0.104426329 | 0.024020727 | -0.039039881 | 0.399945315 |
| RASAL3 | grey60 | 0.001087746 | 0.981296583 | 0.067987102 | 0.142383119 |
| RHOH | grey60 | 0.037931103 | 0.413473894 | 0.08617257 | 0.062789847 |
| RLTPR | grey60 | -0.101131079 | 0.02887351 | -0.060669639 | 0.190610855 |
| RP11-812 | grey60 | 0.00816603 | 0.860294414 | 0.062467332 | 0.177777703 |
| RTP5 | grey60 | 0.002457301 | 0.95776339 | 0.02783981 | 0.548421343 |
| S1PR4 | grey60 | 0.045775837 | 0.32359821 | 0.121082236 | 0.008812591 |
| SASH3 | grey60 | 0.00290741 | 0.950036133 | 0.059777836 | 0.197223853 |
| SCML4 | grey60 | -0.103703428 | 0.025020272 | -0.05831076 | 0.208464253 |
| SELL | grey60 | -0.004851344 | 0.916726473 | 0.032961536 | 0.477338986 |
| SELPLG | grey60 | 0.002075669 | 0.9643182 | 0.078530918 | 0.090049221 |
| SEPT1 | grey60 | -0.012381529 | 0.789576746 | 0.040426273 | 0.383407943 |
| SH2D1A | grey60 | 0.000339392 | 0.99416379 | 0.049571451 | 0.285054108 |
| SH2D1B | grey60 | -0.040789066 | 0.379150325 | -0.011823013 | 0.798860848 |
| SH2D2A | grey60 | -0.229641558 | 5.26E-07 | -0.190831078 | 3.31E-05 |
| SIRPG | grey60 | 0.072011773 | 0.120176031 | 0.133239893 | 0.003920443 |
| SIT1 | grey60 | 0.074746433 | 0.106698343 | 0.13864671 | 0.002676265 |
| SLA2 | grey60 | -0.000743186 | 0.98722056 | 0.048079405 | 0.299819788 |
| SLAMF1 | grey60 | 0.014880184 | 0.748422986 | 0.065985845 | 0.154534312 |
| SLAMF6 | grey60 | -0.027748518 | 0.54973405 | 0.029738893 | 0.521469148 |
| SLAMF7 | grey60 | -0.004106972 | 0.929467422 | 0.045118721 | 0.330599876 |
| SNAI3 | grey60 | -0.06190881 | 0.181694229 | -0.028813122 | 0.534522584 |
| SNX20 | grey60 | 0.00036943 | 0.993647273 | 0.057814976 | 0.212365528 |
| SOCS1 | grey60 | -0.121111145 | 0.008796328 | -0.075625463 | 0.102629281 |
| SP140 | grey60 | -0.07363567 | 0.112021177 | -0.025162905 | 0.587540515 |
| SPN | grey60 | -0.013863977 | 0.765081578 | 0.035223706 | 0.447621428 |
| SPOCK2 | grey60 | -0.235442807 | 2.65E-07 | -0.198379216 | 1.57E-05 |
| STAT4 | grey60 | -0.056208435 | 0.225367749 | -0.004923292 | 0.915496059 |
| TBC1D10 | grey60 | -0.120151974 | 0.009350419 | -0.053830426 | 0.245637951 |
| TBX21 | grey60 | -0.008776726 | 0.849965846 | 0.050954275 | 0.271814241 |

| | | | | | |
|---|---|---|---|---|---|
| TCL1A | grey60 | -0.06171271 | 0.183084367 | -0.035217349 | 0.447703438 |
| TESPA1 | grey60 | -0.091982027 | 0.046962908 | -0.042347851 | 0.361188945 |
| THEMIS | grey60 | 0.024134825 | 0.602899084 | 0.06026904 | 0.193561006 |
| TIGIT | grey60 | 0.068761324 | 0.137883471 | 0.132792663 | 0.004043804 |
| TMC8 | grey60 | -0.068109577 | 0.141663926 | -0.009496761 | 0.837821094 |
| TMEM15 | grey60 | -0.05911513 | 0.202245348 | -0.015945589 | 0.731082841 |
| TMIGD2 | grey60 | -0.057157148 | 0.217622681 | -0.005005893 | 0.914083724 |
| TNFRSF1 | grey60 | -0.00056866 | 0.990221433 | 0.031637145 | 0.495224427 |
| TNFRSF1 | grey60 | 0.014082886 | 0.761483564 | 0.066643659 | 0.150456479 |
| TNFRSF8 | grey60 | -0.131704642 | 0.004358712 | -0.113980621 | 0.013717486 |
| TNFRSF9 | grey60 | -0.009528841 | 0.837280868 | 0.03923495 | 0.397592954 |
| TRAF1 | grey60 | -0.004608151 | 0.920886864 | 0.027716528 | 0.55019441 |
| TRAF3IP: | grey60 | 0.005746854 | 0.901427108 | 0.057349275 | 0.216077711 |
| TRAT1 | grey60 | -0.010587181 | 0.819503069 | 0.026753353 | 0.564143022 |
| TSPAN32 | grey60 | -0.260823566 | 1.06E-08 | -0.231459404 | 4.25E-07 |
| TTC24 | grey60 | 0.047951489 | 0.30110892 | 0.097315278 | 0.035522001 |
| UBASH3. | grey60 | 0.006571985 | 0.887362234 | 0.064511496 | 0.163977159 |
| VPREB3 | grey60 | -0.015647268 | 0.735924855 | 0.031529102 | 0.496699085 |
| WAS | grey60 | 0.004760731 | 0.918276365 | 0.05608567 | 0.226384137 |
| XCL2 | grey60 | -0.138337961 | 0.002736238 | -0.084922517 | 0.066715424 |
| ZAP70 | grey60 | 0.04795578 | 0.301065616 | 0.0956213 | 0.038867109 |
| ZBED2 | grey60 | -0.11715133 | 0.01128954 | -0.096391644 | 0.037314209 |
| ZBP1 | grey60 | -0.160638167 | 0.000492554 | -0.119139332 | 0.009968879 |
| ZBTB32 | grey60 | -0.076340143 | 0.099412523 | -0.058252369 | 0.208921014 |
| ZC3H12D | grey60 | -0.044735205 | 0.334731108 | -0.001718515 | 0.970454774 |
| ZNF683 | grey60 | 0.036528395 | 0.430970133 | 0.099299951 | 0.031917348 |
| ZNF831 | grey60 | 0.004008621 | 0.931152238 | 0.041873349 | 0.3665993 |
| ABCG8 | ivory | -0.1894252 | 3.79E-05 | -0.191065839 | 3.24E-05 |
| ADRA1A | ivory | 2.95E-05 | 0.999492268 | -0.009671332 | 0.834882285 |
| BMPER | ivory | -0.139335886 | 0.002546691 | -0.164805263 | 0.000348343 |
| C10orf10 | ivory | -0.063511554 | 0.170623951 | -0.061203675 | 0.186729525 |
| C11orf87 | ivory | -0.093899608 | 0.042536505 | -0.109471124 | 0.017958768 |
| CCDC144 | ivory | -0.107593893 | 0.020037379 | -0.122927317 | 0.0078267 |
| CDH7 | ivory | -0.094142856 | 0.042000995 | -0.085605803 | 0.064545435 |
| CDH8 | ivory | -0.112233597 | 0.015242742 | -0.110023526 | 0.017384114 |
| DHRS7C | ivory | 0.011131671 | 0.810392124 | 0.003457772 | 0.940594193 |
| FAM196E | ivory | -0.039944434 | 0.389107567 | -0.040901641 | 0.377835088 |
| FRG2C | ivory | -0.067457855 | 0.145523101 | -0.075313312 | 0.104059916 |
| GAGE12J | ivory | 0.004711925 | 0.919111304 | -0.010170647 | 0.826489432 |
| GBX2 | ivory | 0.080053119 | 0.083970185 | 0.051030748 | 0.271094496 |
| HBG1 | ivory | 0.036535074 | 0.43088583 | 0.043169709 | 0.351936778 |
| KDM4E | ivory | -0.095919596 | 0.038259415 | -0.093279789 | 0.043927105 |
| KHDC1L | ivory | -0.122969997 | 0.007805109 | -0.10130262 | 0.028601566 |
| KRTAP1( | ivory | -0.057135471 | 0.217797487 | -0.067093798 | 0.14771352 |
| KRTAP1: | ivory | -0.076743102 | 0.097634477 | -0.083535139 | 0.071305797 |

| | | | | | |
|---|---|---|---|---|---|
| LEUTX | ivory | -0.043293752 | 0.350553454 | -0.048054751 | 0.300067957 |
| MBD3L2 | ivory | 0.040153623 | 0.386626803 | 0.031598383 | 0.495753212 |
| MBD3L3 | ivory | 0.009160521 | 0.843487808 | -0.009229607 | 0.842322825 |
| MBD3L5 | ivory | -0.027028633 | 0.560139176 | -0.027795768 | 0.549054436 |
| MC3R | ivory | 0.025621471 | 0.580748654 | 0.020413605 | 0.659933235 |
| MYF5 | ivory | 0.054852246 | 0.236776984 | 0.041562337 | 0.370172698 |
| NDST3 | ivory | -0.218985037 | 1.77E-06 | -0.21979799 | 1.62E-06 |
| PAX1 | ivory | 0.00716145 | 0.877335774 | -0.005844921 | 0.899753782 |
| PMP2 | ivory | -0.019345436 | 0.676695187 | -0.034589267 | 0.455848469 |
| PRAMEF | ivory | -0.091912279 | 0.047130929 | -0.089138048 | 0.054234305 |
| PRAMEF | ivory | -0.028342539 | 0.541220265 | -0.034033155 | 0.46312901 |
| PRAMEF | ivory | -0.054426213 | 0.240443668 | -0.04524734 | 0.329221787 |
| PRAMEF | ivory | -0.047854306 | 0.302090787 | -0.051767796 | 0.264224365 |
| PRAMEF | ivory | -0.070766366 | 0.126738453 | -0.06939966 | 0.134256421 |
| PRAMEF | ivory | -0.061919049 | 0.18162186 | -0.06087732 | 0.18909447 |
| PRAMEF | ivory | -0.113791368 | 0.013875958 | -0.110986947 | 0.016420277 |
| PRAMEF | ivory | -0.02122117 | 0.647372158 | -0.022303501 | 0.630693598 |
| PRAMEF | ivory | 0.041502393 | 0.370863906 | 0.03483841 | 0.452607653 |
| PRAMEF | ivory | -0.028699562 | 0.536135029 | -0.030311529 | 0.513477581 |
| PRAMEF | ivory | -0.052506359 | 0.257461094 | -0.049367572 | 0.287042311 |
| PRAMEF | ivory | -0.037737252 | 0.415866558 | -0.02801819 | 0.545860845 |
| PRAMEF | ivory | -0.048999029 | 0.290659932 | -0.047117577 | 0.309603171 |
| RFPL4B | ivory | -0.00969007 | 0.834566989 | -0.022579097 | 0.626476049 |
| SLITRK1 | ivory | -0.051615741 | 0.265631805 | -0.050287094 | 0.278148848 |
| ST8SIA3 | ivory | -0.117236112 | 0.011230203 | -0.117925622 | 0.010757794 |
| TMEM23 | ivory | -0.090035483 | 0.051844883 | -0.080417289 | 0.082566056 |
| TRIM43 | ivory | -0.096341293 | 0.037414077 | -0.085925634 | 0.063549843 |
| TRIM48 | ivory | -0.013860208 | 0.765143576 | 0.010057071 | 0.828396804 |
| TRIM49 | ivory | 0.011172534 | 0.809709381 | 0.035816959 | 0.440005159 |
| TRIM49B | ivory | -0.014142856 | 0.760498774 | -0.011537133 | 0.803624047 |
| TRIM49C | ivory | 0.015983962 | 0.730460784 | 0.026314418 | 0.570555458 |
| TRIM51 | ivory | -0.011369477 | 0.806420857 | 0.002260585 | 0.961141801 |
| TRIM64B | ivory | 0.024792172 | 0.593058113 | 0.02966461 | 0.522510477 |
| USP17L1 | ivory | -0.070949376 | 0.125757056 | -0.074893711 | 0.106007835 |
| WI2-3308 | ivory | -0.079429182 | 0.086420632 | -0.082533881 | 0.074776414 |
| ZDHHC2 | ivory | -0.091356431 | 0.048488052 | -0.093725136 | 0.042924137 |
| ZNF705A | ivory | -0.048388339 | 0.296721503 | -0.032473103 | 0.483893791 |
| ZNF804B | ivory | -0.002833391 | 0.951306546 | -0.012443139 | 0.788554424 |
| ACSM5 | lightcyan | -0.227335497 | 6.88E-07 | -0.220289202 | 1.53E-06 |
| ADAM19 | lightcyan | -0.038874166 | 0.401950223 | -0.023168059 | 0.617503778 |
| ADAMDI | lightcyan | -0.073524373 | 0.112565825 | -0.043020434 | 0.353606043 |
| AF16513 | lightcyan | -0.047542973 | 0.305250547 | -0.040754092 | 0.379559491 |
| AIM2 | lightcyan | -0.08472566 | 0.0673516 | -0.056389908 | 0.223871269 |
| ANKRD4 | lightcyan | -0.130820356 | 0.004630795 | -0.108454693 | 0.019059585 |
| AOAH | lightcyan | -0.136072161 | 0.003215181 | -0.10093126 | 0.029193078 |

| | | | | | |
|---|---|---|---|---|---|
| APBB1IP | lightcyan | -0.087109153 | 0.059975098 | -0.035050866 | 0.449854344 |
| APOL3 | lightcyan | -0.172535328 | 0.000179203 | -0.132045704 | 0.004257692 |
| AQP9 | lightcyan | -0.038143317 | 0.410863894 | -0.025361026 | 0.584601633 |
| ARHGAP | lightcyan | -0.209100826 | 5.19E-06 | -0.180995284 | 8.38E-05 |
| ARHGAP | lightcyan | -0.007992404 | 0.863235344 | 0.048891931 | 0.291716907 |
| ARL11 | lightcyan | -0.160951205 | 0.00048004 | -0.139041045 | 0.002601409 |
| BCL2L14 | lightcyan | 0.01567532 | 0.735469103 | 0.060827912 | 0.189454415 |
| BIRC3 | lightcyan | -0.050395893 | 0.277109063 | -0.039697789 | 0.392044896 |
| BTK | lightcyan | -0.114537839 | 0.01326011 | -0.07672439 | 0.097716475 |
| BTLA | lightcyan | -0.06465953 | 0.163009907 | -0.032913239 | 0.477984961 |
| C3AR1 | lightcyan | -0.108564296 | 0.018938119 | -0.079757342 | 0.085124739 |
| C5orf56 | lightcyan | -0.210951762 | 4.26E-06 | -0.176307537 | 0.000128211 |
| CARD17 | lightcyan | -0.057972179 | 0.211122859 | -0.043498352 | 0.34827928 |
| CASP10 | lightcyan | -0.337351099 | 6.81E-14 | -0.306265391 | 1.34E-11 |
| CASP4 | lightcyan | -0.025489097 | 0.582705496 | 0.003280259 | 0.943638731 |
| CASP5 | lightcyan | 0.012191221 | 0.792736927 | 0.012301215 | 0.790909999 |
| CASS4 | lightcyan | -0.181705679 | 7.84E-05 | -0.1611426 | 0.000472535 |
| CCL13 | lightcyan | 0.069095223 | 0.135976964 | 0.092366842 | 0.0460449 |
| CCR1 | lightcyan | -0.146417977 | 0.00151 | -0.111644019 | 0.015790065 |
| CCR4 | lightcyan | 0.073387872 | 0.113236652 | 0.110669438 | 0.016732622 |
| CCR8 | lightcyan | 0.022314671 | 0.630522424 | 0.038091404 | 0.411501462 |
| CCRL2 | lightcyan | -0.074919369 | 0.1058879 | -0.035978225 | 0.437947692 |
| CD163 | lightcyan | -0.177328896 | 0.000116959 | -0.156507906 | 0.0006887 |
| CD163L1 | lightcyan | -0.180240924 | 8.98E-05 | -0.153282343 | 0.000889779 |
| CD180 | lightcyan | 0.006403882 | 0.890224932 | 0.040704126 | 0.380144545 |
| CD1D | lightcyan | -0.014249197 | 0.75875347 | 0.022072507 | 0.634237852 |
| CD200R1 | lightcyan | -0.130077286 | 0.00487118 | -0.089440386 | 0.053419301 |
| CD209 | lightcyan | -0.030553794 | 0.510115822 | -0.005934422 | 0.898227017 |
| CD226 | lightcyan | -0.043334626 | 0.350098377 | -0.016732758 | 0.718358005 |
| CD274 | lightcyan | -0.092001639 | 0.046915752 | -0.060520881 | 0.191702484 |
| CD4 | lightcyan | -0.055746096 | 0.229212473 | -0.009596534 | 0.836141197 |
| CD53 | lightcyan | -0.014794254 | 0.749827229 | 0.038372338 | 0.408058174 |
| CD69 | lightcyan | -0.056460501 | 0.223291056 | -0.033894392 | 0.464955693 |
| CD80 | lightcyan | -0.066041939 | 0.154183348 | -0.053485759 | 0.248678445 |
| CD84 | lightcyan | -0.062424562 | 0.178075387 | -0.022219148 | 0.631986878 |
| CD86 | lightcyan | -0.066316721 | 0.152472849 | -0.025619104 | 0.58078362 |
| CECR1 | lightcyan | -0.011416303 | 0.805639475 | 0.017004554 | 0.713982161 |
| CLEC12A | lightcyan | -0.066132603 | 0.153617373 | -0.059114025 | 0.2022538 |
| CLEC1B | lightcyan | -0.114849685 | 0.013010051 | -0.108110725 | 0.019445199 |
| CLEC4A | lightcyan | -0.103900667 | 0.024744054 | -0.071912574 | 0.120688821 |
| CLEC4D | lightcyan | -0.054323059 | 0.241337437 | -0.041519199 | 0.370670037 |
| CLEC5A | lightcyan | -0.00869262 | 0.851386835 | 0.01319388 | 0.776126526 |
| CLEC6A | lightcyan | -0.015651999 | 0.735847992 | 0.002063616 | 0.964525263 |
| CLEC7A | lightcyan | 0.001497124 | 0.974259582 | 0.028541218 | 0.538387425 |
| CLECL1 | lightcyan | -0.055389591 | 0.232208703 | -0.01437978 | 0.756611983 |

| | | | | | |
|---|---|---|---|---|---|
| CMKLR1 | lightcyan | -0.162289475 | 0.0004298 | -0.134552088 | 0.003577906 |
| CR1 | lightcyan | -0.035618407 | 0.44254591 | -0.019365504 | 0.67637877 |
| CSF2RA | lightcyan | -0.067925277 | 0.142747223 | -0.03712312 | 0.423500311 |
| CSF2RB | lightcyan | -0.047207312 | 0.308681627 | -0.0142799 | 0.758249789 |
| CTSC | lightcyan | 0.041143949 | 0.375013697 | 0.064275764 | 0.165526317 |
| CTSL | lightcyan | -0.283895742 | 4.17E-10 | -0.267781494 | 4.13E-09 |
| CTSS | lightcyan | 0.079852826 | 0.084750629 | 0.110395653 | 0.017006116 |
| CXCL10 | lightcyan | -0.048543562 | 0.295172858 | -0.012456711 | 0.78832925 |
| CXCL11 | lightcyan | -0.077837893 | 0.092931441 | -0.046002488 | 0.321205722 |
| CXorf21 | lightcyan | -0.154326467 | 0.000819412 | -0.118258021 | 0.010536413 |
| CYBB | lightcyan | -0.060428993 | 0.192379072 | -0.030632375 | 0.509027869 |
| CYLD | lightcyan | 0.045170293 | 0.330046856 | 0.071009455 | 0.125436169 |
| CYSLTR1 | lightcyan | -0.049724557 | 0.283567146 | -0.022472769 | 0.628101794 |
| DAPP1 | lightcyan | -0.006371587 | 0.890775056 | 0.010903975 | 0.81419912 |
| DENND3 | lightcyan | -0.27442885 | 1.63E-09 | -0.259835602 | 1.21E-08 |
| DNAJC5I | lightcyan | -0.018335357 | 0.692694 | 0.001370283 | 0.976439717 |
| DOCK10 | lightcyan | -0.106758604 | 0.021027625 | -0.08319936 | 0.072454724 |
| DOCK2 | lightcyan | -0.047471812 | 0.305975831 | -0.008089214 | 0.861595304 |
| DOCK8 | lightcyan | -0.074588358 | 0.107443415 | -0.050750864 | 0.273735019 |
| DRAM1 | lightcyan | 0.039950873 | 0.389031065 | 0.073291391 | 0.113712695 |
| EMB | lightcyan | -0.129773728 | 0.004972577 | -0.123689339 | 0.00744913 |
| EMR2 | lightcyan | -0.0666749 | 0.150264868 | -0.056641193 | 0.221810817 |
| EMR3 | lightcyan | 0.015873257 | 0.732255879 | 0.047122149 | 0.309556173 |
| EVI2A | lightcyan | -0.093858514 | 0.042627538 | -0.045931008 | 0.321959004 |
| EVI2B | lightcyan | -0.076221081 | 0.099942782 | -0.025822183 | 0.577787468 |
| FAM105A | lightcyan | -0.209450214 | 5.00E-06 | -0.159444189 | 0.000543123 |
| FCAR | lightcyan | -0.035151158 | 0.448557904 | -0.02860486 | 0.537481567 |
| FCGR2A | lightcyan | -0.040616473 | 0.381172193 | -0.005403271 | 0.907293058 |
| FCGR3A | lightcyan | -0.054540656 | 0.239454813 | -0.016649453 | 0.719701052 |
| FGD2 | lightcyan | -0.179803306 | 9.34E-05 | -0.143828947 | 0.001832871 |
| FGL2 | lightcyan | -0.088914452 | 0.054843679 | -0.077284716 | 0.095284626 |
| FGR | lightcyan | -0.103855924 | 0.024806481 | -0.072485301 | 0.117751615 |
| FLT3 | lightcyan | -0.162164455 | 0.000434277 | -0.14845743 | 0.001293398 |
| FLVCR2 | lightcyan | -0.07249903 | 0.117681899 | -0.077162084 | 0.095812684 |
| FPR2 | lightcyan | -0.011514991 | 0.803993276 | -0.00247639 | 0.957435593 |
| FPR3 | lightcyan | -0.101121624 | 0.028888563 | -0.068167567 | 0.141324373 |
| FYB | lightcyan | -0.07294521 | 0.115433707 | -0.031944836 | 0.491037598 |
| GAB3 | lightcyan | -0.171215592 | 0.000201148 | -0.132401575 | 0.004154546 |
| GAPT | lightcyan | -0.137586905 | 0.002887271 | -0.117461657 | 0.011073691 |
| GBP1 | lightcyan | -0.181237644 | 8.19E-05 | -0.155934526 | 0.000721045 |
| GBP2 | lightcyan | -0.123742054 | 0.007423622 | -0.084576967 | 0.067835418 |
| GBP4 | lightcyan | -0.103422834 | 0.025417807 | -0.057655618 | 0.213630591 |
| GNB4 | lightcyan | -0.249160962 | 4.86E-08 | -0.209103708 | 5.19E-06 |
| GPNMB | lightcyan | -0.272888415 | 2.03E-09 | -0.252588941 | 3.13E-08 |
| GPR183 | lightcyan | -0.142465721 | 0.002027216 | -0.102619658 | 0.026585967 |

| | | | | | |
|---|---|---|---|---|---|
| GPR34 | lightcyan | -0.165271085 | 0.000334939 | -0.147378843 | 0.001404107 |
| GPR65 | lightcyan | -0.1037144 | 0.025004837 | -0.058568571 | 0.206456147 |
| GPRIN3 | lightcyan | -0.027675493 | 0.550785204 | -0.012553995 | 0.78671583 |
| HCLS1 | lightcyan | -0.184492192 | 6.05E-05 | -0.144789579 | 0.001706337 |
| HRH2 | lightcyan | -0.214368466 | 2.95E-06 | -0.187152794 | 4.71E-05 |
| HSD11B1 | lightcyan | -0.03527177 | 0.447001604 | -0.017680121 | 0.703146485 |
| HTRA4 | lightcyan | -0.036230409 | 0.434741352 | -0.022388686 | 0.629388687 |
| ICAM1 | lightcyan | -0.081296833 | 0.079252956 | -0.07123978 | 0.124211874 |
| IKZF1 | lightcyan | -0.040936473 | 0.37742871 | 0.01125566 | 0.80832095 |
| IL10RA | lightcyan | -0.048572712 | 0.294882638 | 0.003774168 | 0.935169843 |
| IL12B | lightcyan | -0.051312078 | 0.268457916 | -0.0229002 | 0.62157741 |
| IL15 | lightcyan | -0.232250702 | 3.87E-07 | -0.196162221 | 1.96E-05 |
| IL15RA | lightcyan | -0.204870715 | 8.09E-06 | -0.173050419 | 0.000171264 |
| IL1B | lightcyan | 0.054634921 | 0.238642451 | 0.066491803 | 0.151390507 |
| IL21 | lightcyan | 0.122895297 | 0.007842933 | 0.153507641 | 0.000874139 |
| IL24 | lightcyan | -0.056229414 | 0.225194387 | -0.026358628 | 0.569908026 |
| IL2RA | lightcyan | 0.031999876 | 0.490290657 | 0.061298149 | 0.186048994 |
| IL4R | lightcyan | 0.031813075 | 0.492828174 | 0.057157842 | 0.217617086 |
| IL7R | lightcyan | -0.02061653 | 0.656767709 | 0.010991191 | 0.812740382 |
| INPP5D | lightcyan | -0.071799527 | 0.121275273 | -0.021404667 | 0.644531748 |
| IRF8 | lightcyan | -0.114566803 | 0.013236708 | -0.095052487 | 0.040048573 |
| IRG1 | lightcyan | -0.038878121 | 0.401902307 | -0.028063089 | 0.545217288 |
| ITGB7 | lightcyan | 0.127773176 | 0.005689768 | 0.152021859 | 0.000982152 |
| KBTBD8 | lightcyan | -0.050259128 | 0.278416541 | -0.032689449 | 0.480984424 |
| KCNA3 | lightcyan | -0.048395394 | 0.296650994 | -0.011159504 | 0.809927066 |
| KCNJ10 | lightcyan | -0.027863865 | 0.548075711 | -0.039773601 | 0.39114062 |
| KIAA051 | lightcyan | 0.065898643 | 0.155081102 | 0.099846482 | 0.030981395 |
| KLRC3 | lightcyan | -0.049634411 | 0.284442004 | -0.004157244 | 0.928606343 |
| KLRC4 | lightcyan | -0.02140032 | 0.644598967 | 0.007602068 | 0.869853822 |
| KLRC4-K | lightcyan | -0.066522878 | 0.151199017 | -0.034735414 | 0.45394583 |
| KLRG1 | lightcyan | -0.088222969 | 0.056764312 | -0.092105827 | 0.046665912 |
| KLRK1 | lightcyan | -0.029519037 | 0.524554261 | -0.005395839 | 0.907420006 |
| KYNU | lightcyan | 0.092944913 | 0.044694209 | 0.103693282 | 0.025034553 |
| LACTB | lightcyan | 0.106860387 | 0.020904729 | 0.120241163 | 0.009297619 |
| LCP1 | lightcyan | 0.004848118 | 0.916781658 | 0.039676307 | 0.392301359 |
| LCP2 | lightcyan | -0.029079184 | 0.530754319 | 0.018886218 | 0.683951323 |
| LILRB5 | lightcyan | -0.01402772 | 0.762389785 | -0.018512797 | 0.689873339 |
| LITAF | lightcyan | -0.153784367 | 0.000855277 | -0.118908849 | 0.010114619 |
| LY96 | lightcyan | -0.210125568 | 4.65E-06 | -0.162381962 | 0.000426516 |
| LYN | lightcyan | 0.102385064 | 0.02693577 | 0.113248752 | 0.014339285 |
| LYSMD2 | lightcyan | -0.155341198 | 0.000755991 | -0.141312227 | 0.00220619 |
| LYZ | lightcyan | 0.059370685 | 0.200298049 | 0.095763614 | 0.038576173 |
| MAFB | lightcyan | -0.135929384 | 0.003247771 | -0.110612071 | 0.016789606 |
| 1-Mar | lightcyan | -0.163970086 | 0.000373636 | -0.147583748 | 0.001382427 |
| MCOLN2 | lightcyan | -0.211277934 | 4.11E-06 | -0.188546514 | 4.12E-05 |

| | | | | | |
|---|---|---|---|---|---|
| MEFV | lightcyan | -0.023991757 | 0.605050599 | -0.00770462 | 0.868114059 |
| MMP12 | lightcyan | 0.077039843 | 0.096341381 | 0.099243606 | 0.032015201 |
| MNDA | lightcyan | -0.071254727 | 0.124132748 | -0.039302038 | 0.396785857 |
| MPEG1 | lightcyan | -0.119615659 | 0.009673582 | -0.078511067 | 0.090130769 |
| MPP1 | lightcyan | -0.138604945 | 0.002684307 | -0.159510581 | 0.000540189 |
| MRC1 | lightcyan | -0.123596487 | 0.007494248 | -0.114326128 | 0.013432279 |
| MS4A14 | lightcyan | -0.104896137 | 0.023389797 | -0.091386382 | 0.048414102 |
| MS4A4A | lightcyan | -0.129879003 | 0.004937199 | -0.094347578 | 0.041554719 |
| MS4A6A | lightcyan | -0.098632631 | 0.033092822 | -0.060081014 | 0.194957161 |
| MS4A7 | lightcyan | -0.141297721 | 0.00220853 | -0.11487788 | 0.012987649 |
| MSR1 | lightcyan | -0.136666881 | 0.003082602 | -0.116742096 | 0.011579872 |
| NCF2 | lightcyan | 0.118936042 | 0.010097326 | 0.144854682 | 0.001698059 |
| NCKAP1l | lightcyan | -0.027632539 | 0.551403953 | 0.018287941 | 0.693448468 |
| NECAP2 | lightcyan | -0.015609598 | 0.736537022 | -0.006345142 | 0.891225584 |
| NLRC4 | lightcyan | -0.199689365 | 1.38E-05 | -0.181014737 | 8.36E-05 |
| NLRP3 | lightcyan | -0.148549152 | 0.001284364 | -0.124844381 | 0.00690783 |
| NOD2 | lightcyan | 0.146257477 | 0.001528387 | 0.188256321 | 4.24E-05 |
| NPL | lightcyan | -0.056074701 | 0.226475105 | -0.030445628 | 0.511615348 |
| NRROS | lightcyan | -0.257609086 | 1.63E-08 | -0.247230979 | 6.21E-08 |
| NUB1 | lightcyan | -0.118914984 | 0.010110715 | -0.101781351 | 0.027854233 |
| OLR1 | lightcyan | -0.10362218 | 0.025134826 | -0.081155724 | 0.079777114 |
| P2RY12 | lightcyan | -0.152002024 | 0.000983674 | -0.144052242 | 0.001802715 |
| P2RY13 | lightcyan | -0.036209682 | 0.435004374 | -0.00770084 | 0.868178176 |
| PAG1 | lightcyan | -0.005572774 | 0.904398496 | 0.018709159 | 0.686756885 |
| PATL2 | lightcyan | -0.185111673 | 5.71E-05 | -0.133654894 | 0.00380903 |
| PDCD1L( | lightcyan | -0.150719614 | 0.001086817 | -0.118821022 | 0.010170649 |
| PDE4B | lightcyan | -0.048987422 | 0.290774357 | -0.032989501 | 0.476965158 |
| PIK3AP1 | lightcyan | -0.116876489 | 0.011483807 | -0.100131964 | 0.030501904 |
| PIK3CD | lightcyan | -0.311026891 | 6.22E-12 | -0.266340493 | 5.03E-09 |
| PIK3R5 | lightcyan | -0.119026196 | 0.010040183 | -0.081437747 | 0.078732314 |
| PLA2G7 | lightcyan | -0.107500943 | 0.020145528 | -0.102315874 | 0.027039693 |
| PLEK | lightcyan | -0.056967363 | 0.219156577 | -0.023078621 | 0.618862662 |
| PLXNC1 | lightcyan | -0.187633393 | 4.50E-05 | -0.175155183 | 0.000142124 |
| PREX1 | lightcyan | -0.238524835 | 1.83E-07 | -0.221710344 | 1.31E-06 |
| PRKCB | lightcyan | -0.165669896 | 0.000323847 | -0.141631752 | 0.00215522 |
| PRR33 | lightcyan | -0.137594691 | 0.002885667 | -0.100620007 | 0.029696906 |
| PTGER4 | lightcyan | -0.17078691 | 0.000208801 | -0.162004679 | 0.000440063 |
| PTPN22 | lightcyan | 0.092144176 | 0.046574234 | 0.110927629 | 0.016478239 |
| PTPRC | lightcyan | -0.023496257 | 0.612528456 | 0.017363713 | 0.708214142 |
| RAB27A | lightcyan | 0.009785219 | 0.8329663 | 0.0265738 | 0.566761944 |
| RASGRP | lightcyan | -0.125798938 | 0.006487361 | -0.128978431 | 0.005247313 |
| RASGRP: | lightcyan | -0.121710407 | 0.008465173 | -0.105242507 | 0.022933868 |
| RASSF5 | lightcyan | 0.026128079 | 0.573288127 | 0.040100945 | 0.387250607 |
| RCSD1 | lightcyan | -0.055173375 | 0.23403932 | -0.025643665 | 0.580420878 |
| RGS1 | lightcyan | -0.076385676 | 0.099210327 | -0.051590993 | 0.265861357 |

| | | | | | |
|---|---|---|---|---|---|
| RGS18 | lightcyan | -0.111867336 | 0.015580758 | -0.08772382 | 0.058185128 |
| SAMD3 | lightcyan | -0.177573848 | 0.000114402 | -0.15050138 | 0.001105332 |
| SAMSN1 | lightcyan | -0.060877948 | 0.189089898 | -0.027408129 | 0.554642141 |
| SCIMP | lightcyan | -0.034065231 | 0.462707317 | -0.003838333 | 0.934070137 |
| SERPINB | lightcyan | -0.178923128 | 0.000101234 | -0.143049206 | 0.001941835 |
| SIRPB1 | lightcyan | -0.044201523 | 0.340534857 | -0.015262084 | 0.742192212 |
| SIRPB2 | lightcyan | -0.068217915 | 0.141030068 | -0.056069449 | 0.226518675 |
| SIRPD | lightcyan | -0.060951059 | 0.188558197 | -0.046968121 | 0.311142041 |
| SLA | lightcyan | -0.070648767 | 0.127372215 | -0.017856063 | 0.700334224 |
| SLC2A3 | lightcyan | -0.065422389 | 0.158093292 | -0.045877721 | 0.322521316 |
| SLC31A2 | lightcyan | 0.037461701 | 0.41928163 | 0.032724052 | 0.480519975 |
| SLC46A3 | lightcyan | -0.110081844 | 0.017324394 | -0.113936536 | 0.013754258 |
| SLCO2B1 | lightcyan | -0.139881743 | 0.002448151 | -0.114784923 | 0.013061637 |
| SLFN12L | lightcyan | -0.141649678 | 0.002152393 | -0.109408409 | 0.018025046 |
| SMAP2 | lightcyan | 0.22216046 | 1.24E-06 | 0.235015006 | 2.79E-07 |
| SNX10 | lightcyan | -0.149202978 | 0.001221629 | -0.143607288 | 0.001863262 |
| SOD2 | lightcyan | 0.034198556 | 0.460956871 | 0.040884981 | 0.37802955 |
| SRGN | lightcyan | -0.143283998 | 0.001908416 | -0.101244286 | 0.028693794 |
| ST8SIA4 | lightcyan | -0.176361731 | 0.00012759 | -0.144401229 | 0.001756494 |
| STAMBP | lightcyan | 0.032430637 | 0.484465973 | 0.023462537 | 0.613038822 |
| STAP1 | lightcyan | -0.056658795 | 0.221666996 | -0.030815355 | 0.50649924 |
| STK10 | lightcyan | -0.017828947 | 0.700767373 | 0.029656721 | 0.522621133 |
| STK17B | lightcyan | -0.061459344 | 0.184892074 | -0.047044256 | 0.31035749 |
| STX11 | lightcyan | -0.113959512 | 0.013735082 | -0.084595467 | 0.067775068 |
| SUCNR1 | lightcyan | -0.062347932 | 0.178609666 | -0.039417865 | 0.39539473 |
| TFEC | lightcyan | -0.096038248 | 0.038019944 | -0.064652532 | 0.163055537 |
| TLR1 | lightcyan | -0.086398272 | 0.062101769 | -0.079263337 | 0.087081565 |
| TLR10 | lightcyan | -0.051543659 | 0.266300794 | -0.016870972 | 0.716131639 |
| TLR2 | lightcyan | 0.108270197 | 0.019265582 | 0.101567665 | 0.028185712 |
| TLR6 | lightcyan | -0.02646773 | 0.568311793 | -0.015851196 | 0.732613782 |
| TLR7 | lightcyan | -0.114834895 | 0.013021816 | -0.081361765 | 0.079012704 |
| TLR8 | lightcyan | -0.050935809 | 0.271988232 | -0.027799463 | 0.549001305 |
| TM6SF1 | lightcyan | -0.176265315 | 0.000128698 | -0.157238385 | 0.00064944 |
| TMEM10 | lightcyan | -0.115866858 | 0.012223071 | -0.086657696 | 0.061318582 |
| TMEM14 | lightcyan | -0.203484666 | 9.34E-06 | -0.175269466 | 0.000140684 |
| TMEM71 | lightcyan | -0.078211203 | 0.091369846 | -0.061993363 | 0.181097256 |
| TNFAIP8 | lightcyan | -0.06376222 | 0.168939137 | -0.043678706 | 0.346282381 |
| TNFSF13 | lightcyan | -0.08877485 | 0.055227019 | -0.054465746 | 0.240101755 |
| TNFSF8 | lightcyan | 0.026232976 | 0.571749045 | 0.010187265 | 0.826210451 |
| TNIP3 | lightcyan | 0.079410449 | 0.086495085 | 0.113138736 | 0.014434867 |
| VCAM1 | lightcyan | -0.13556246 | 0.003332905 | -0.110959293 | 0.016447276 |
| VNN2 | lightcyan | -0.033331213 | 0.472410264 | -0.011185104 | 0.809499395 |
| VSIG4 | lightcyan | -0.119679266 | 0.009634743 | -0.102080792 | 0.027395366 |
| WDFY4 | lightcyan | 0.029986579 | 0.518004691 | 0.059815284 | 0.196942839 |
| WIPF1 | lightcyan | -0.179187497 | 9.88E-05 | -0.13991815 | 0.002441704 |

| | | | | | |
|---|---|---|---|---|---|
| XCR1 | lightcyan | -0.098575935 | 0.033194371 | -0.063757139 | 0.168973159 |
| ZNF80 | lightcyan | -0.043101765 | 0.352695938 | -0.026184138 | 0.572465367 |
| A1CF | lightcyan1 | -0.081057958 | 0.080141918 | -0.084906876 | 0.066765788 |
| AFP | lightcyan1 | -0.125037602 | 0.0068208 | -0.123471705 | 0.007555267 |
| AGXT | lightcyan1 | -0.011833318 | 0.798689283 | -0.013809897 | 0.765971223 |
| AHSG | lightcyan1 | -0.081025038 | 0.080265059 | -0.077238017 | 0.095485441 |
| ALB | lightcyan1 | -0.061472938 | 0.184794754 | -0.061099481 | 0.18748219 |
| APOA2 | lightcyan1 | -0.053085358 | 0.252243415 | -0.047629928 | 0.30436584 |
| APOA5 | lightcyan1 | -0.01168149 | 0.801217924 | -0.011180872 | 0.809570093 |
| APOB | lightcyan1 | -0.062713789 | 0.176069538 | -0.064213956 | 0.16593431 |
| APOC3 | lightcyan1 | -0.031669029 | 0.494789686 | -0.026114141 | 0.573492778 |
| APOH | lightcyan1 | 0.006700048 | 0.885182366 | -0.011319999 | 0.807246722 |
| ATP6V0I | lightcyan1 | 0.027492362 | 0.553425594 | 0.045581535 | 0.325658424 |
| C8A | lightcyan1 | 0.021800317 | 0.638424946 | 0.023517601 | 0.61220551 |
| CPN2 | lightcyan1 | -0.036862587 | 0.426763296 | -0.037225889 | 0.422217216 |
| CREB3L3 | lightcyan1 | -0.048952976 | 0.291114127 | -0.037179265 | 0.422799043 |
| CYP3A4 | lightcyan1 | -0.027597858 | 0.551903792 | -0.020898558 | 0.652378406 |
| DCSTAM | lightcyan1 | 0.046734018 | 0.313562579 | 0.045989269 | 0.321344941 |
| DNASE2I | lightcyan1 | -0.076110963 | 0.100435205 | -0.062549532 | 0.177206626 |
| F11 | lightcyan1 | 0.002912176 | 0.949954333 | -0.003410057 | 0.941412471 |
| F2 | lightcyan1 | 0.001276173 | 0.978057402 | -0.005648801 | 0.903100621 |
| FAM169I | lightcyan1 | -0.065284607 | 0.158972909 | -0.059912788 | 0.196212529 |
| FAM9B | lightcyan1 | -0.129722847 | 0.004989758 | -0.13535743 | 0.003381354 |
| FGA | lightcyan1 | 0.021446903 | 0.643878685 | 0.026453935 | 0.568513501 |
| FGB | lightcyan1 | 0.054605726 | 0.238893839 | 0.051091609 | 0.270522622 |
| FGG | lightcyan1 | -0.058261422 | 0.208850151 | -0.058483327 | 0.207118558 |
| GCKR | lightcyan1 | -0.152126855 | 0.000974132 | -0.15342642 | 0.000879747 |
| GP1BA | lightcyan1 | 0.01314069 | 0.777005201 | 0.03408844 | 0.46240234 |
| GPR128 | lightcyan1 | -0.025568921 | 0.581525118 | -0.029484272 | 0.525042951 |
| GPR150 | lightcyan1 | -0.116672522 | 0.011629883 | -0.084042885 | 0.06959677 |
| HFE2 | lightcyan1 | -0.10011677 | 0.030527262 | -0.097820672 | 0.034572704 |
| HRG | lightcyan1 | -0.131752654 | 0.004344361 | -0.123496095 | 0.007543306 |
| ISX | lightcyan1 | -0.05477459 | 0.23744238 | -0.048198782 | 0.298620015 |
| ITIH1 | lightcyan1 | -0.03146392 | 0.497589868 | -0.024620619 | 0.595619311 |
| ITIH3 | lightcyan1 | -0.07310757 | 0.114624023 | -0.064961877 | 0.161047712 |
| KNG1 | lightcyan1 | -0.134240332 | 0.003656734 | -0.147232009 | 0.001419834 |
| LECT2 | lightcyan1 | -0.138978006 | 0.002613246 | -0.1256598 | 0.006547185 |
| MTTP | lightcyan1 | -0.0319718 | 0.4906716 | -0.036293522 | 0.433941025 |
| NAT8 | lightcyan1 | -0.078607228 | 0.089736286 | -0.057067351 | 0.218347484 |
| OCSTAM | lightcyan1 | 0.047477897 | 0.305913768 | 0.048973593 | 0.290910735 |
| PAH | lightcyan1 | -0.033044586 | 0.476229275 | -0.043092119 | 0.352803811 |
| PAQR9 | lightcyan1 | -0.035394854 | 0.445416572 | -0.04937018 | 0.287016821 |
| PGC | lightcyan1 | -0.071435442 | 0.123179152 | -0.090524949 | 0.050579107 |
| PLA2G12 | lightcyan1 | -0.026730334 | 0.564478448 | -0.044366121 | 0.33873806 |
| PSMA8 | lightcyan1 | -0.101159116 | 0.028828912 | -0.095457981 | 0.039203274 |

| | | | | | |
|---|---|---|---|---|---|
| RBM46 | lightcyan1 | -0.028862718 | 0.533819128 | -0.02104832 | 0.650052473 |
| SERPINA | lightcyan1 | -0.112400098 | 0.015091221 | -0.100106087 | 0.030545102 |
| SERPINA | lightcyan1 | -0.07650152 | 0.098697383 | -0.073935743 | 0.110563044 |
| SERPINC | lightcyan1 | 0.029649668 | 0.522720065 | 0.018718676 | 0.686605967 |
| SERPIND | lightcyan1 | -0.02646646 | 0.568330356 | -0.0468686 | 0.312169549 |
| SIGLECL | lightcyan1 | -0.066059457 | 0.154073872 | -0.063263948 | 0.172300494 |
| SLC2A2 | lightcyan1 | -0.09290106 | 0.044795491 | -0.088208465 | 0.056805188 |
| SLC6A19 | lightcyan1 | -0.053466398 | 0.248850015 | -0.056549288 | 0.222562828 |
| SMLR1 | lightcyan1 | -0.094623545 | 0.040959472 | -0.087760135 | 0.058080772 |
| SULT2A1 | lightcyan1 | -0.016631154 | 0.719996185 | 0.001028277 | 0.982318956 |
| TF | lightcyan1 | -0.024920036 | 0.591152457 | -0.016919521 | 0.715350165 |
| TFR2 | lightcyan1 | 0.047818354 | 0.302454557 | 0.056624982 | 0.221943326 |
| TM4SF5 | lightcyan1 | -0.071391025 | 0.123413 | -0.077781769 | 0.093168053 |
| TTR | lightcyan1 | -0.065256579 | 0.159152293 | -0.064546865 | 0.163745668 |
| UGT1A1 | lightcyan1 | -0.058718418 | 0.205295435 | -0.056056519 | 0.226625954 |
| UGT2B1( | lightcyan1 | 0.008191297 | 0.859866598 | 0.024728763 | 0.594004195 |
| VCX | lightcyan1 | -0.164354741 | 0.000361782 | -0.165317272 | 0.000333637 |
| VCX3A | lightcyan1 | -0.145494687 | 0.001618614 | -0.149339852 | 0.001208859 |
| VCX3B | lightcyan1 | -0.134324207 | 0.003635373 | -0.131655403 | 0.004373474 |
| VTN | lightcyan1 | -0.170390538 | 0.000216119 | -0.163138653 | 0.000400507 |
| AAMP | lightgreen | -0.08630636 | 0.062381218 | -0.084719927 | 0.067370202 |
| AAR2 | lightgreen | -0.166235542 | 0.000308701 | -0.172685455 | 0.000176855 |
| AARS | lightgreen | 0.062883888 | 0.174897727 | 0.07534429 | 0.103917237 |
| ABCF1 | lightgreen | -0.181801244 | 7.78E-05 | -0.186351967 | 5.08E-05 |
| ABCF2 | lightgreen | 0.074702245 | 0.106906206 | 0.073313329 | 0.113604315 |
| ABT1 | lightgreen | -0.15646813 | 0.000690899 | -0.14786554 | 0.001353115 |
| ADSL | lightgreen | 0.087024073 | 0.06022641 | 0.076245036 | 0.099835914 |
| AGBL5 | lightgreen | -0.03853203 | 0.406108541 | -0.046338193 | 0.317683292 |
| AGPAT1 | lightgreen | -0.114939768 | 0.012938595 | -0.124968786 | 0.006851683 |
| AHSA1 | lightgreen | 0.01979007 | 0.669697886 | 0.039216165 | 0.397819115 |
| AIMP2 | lightgreen | 0.064662249 | 0.162992183 | 0.064954952 | 0.161092449 |
| ALAS1 | lightgreen | 0.23933229 | 1.66E-07 | 0.248740738 | 5.13E-08 |
| ALG1 | lightgreen | 0.206671034 | 6.70E-06 | 0.214506019 | 2.90E-06 |
| ANKRD2 | lightgreen | -0.012772457 | 0.783096012 | -0.011879847 | 0.797914791 |
| ANP32B | lightgreen | 0.034687917 | 0.454563682 | 0.022584329 | 0.626396087 |
| APEX1 | lightgreen | 0.083067864 | 0.072908759 | 0.057691434 | 0.213345792 |
| APMAP | lightgreen | -0.017443146 | 0.706940705 | -0.043813724 | 0.34479221 |
| ARPC2 | lightgreen | -0.069800739 | 0.132015378 | -0.042707085 | 0.357126268 |
| ATP5A1 | lightgreen | 0.120585457 | 0.009096273 | 0.122445145 | 0.008074348 |
| ATP5B | lightgreen | 0.026072844 | 0.574099341 | 0.028992692 | 0.531977844 |
| ATP5F1 | lightgreen | 0.074636858 | 0.107214379 | 0.067899958 | 0.142896541 |
| ATP5G3 | lightgreen | 0.011797035 | 0.799293377 | 0.024140341 | 0.602816202 |
| ATP6V0C | lightgreen | 0.033857112 | 0.465447132 | 0.016501739 | 0.722084631 |
| C10orf2 | lightgreen | -0.004723583 | 0.918911852 | -0.035853095 | 0.439543648 |
| C16orf91 | lightgreen | 0.026518052 | 0.567576252 | 0.055988188 | 0.227193519 |

| | | | | | |
|---|---|---|---|---|---|
| C1QBP | lightgreen | 0.211641443 | 3.96E-06 | 0.210168636 | 4.63E-06 |
| C2orf47 | lightgreen | -0.130905143 | 0.004604058 | -0.138170303 | 0.002769315 |
| C6orf106 | lightgreen | -0.20726738 | 6.30E-06 | -0.175942063 | 0.000132479 |
| C6orf47 | lightgreen | -0.097854758 | 0.034509462 | -0.102333234 | 0.027013586 |
| C8orf33 | lightgreen | 0.039540239 | 0.393928173 | 0.016510835 | 0.721937766 |
| CALM3 | lightgreen | 0.078624193 | 0.089666836 | 0.086770503 | 0.060980574 |
| CCDC22 | lightgreen | 0.067919243 | 0.1427828 | 0.077154324 | 0.095846177 |
| CCDC51 | lightgreen | 0.04023339 | 0.385683394 | 0.027345119 | 0.555553021 |
| CCDC86 | lightgreen | -0.078038212 | 0.092090854 | -0.091648135 | 0.047771821 |
| CCT2 | lightgreen | 0.051141115 | 0.270058055 | 0.025755881 | 0.578764863 |
| CCT5 | lightgreen | 0.009929643 | 0.83053801 | 0.001046454 | 0.98200647 |
| CCT7 | lightgreen | 0.067240984 | 0.14682494 | 0.081628906 | 0.078030464 |
| CDK4 | lightgreen | -0.025168638 | 0.587455378 | -0.045131736 | 0.330460255 |
| CECR5 | lightgreen | 0.238506398 | 1.83E-07 | 0.218301364 | 1.91E-06 |
| CHCHD2 | lightgreen | 0.113500658 | 0.014122523 | 0.134428934 | 0.00360886 |
| CHCHD3 | lightgreen | -0.078710523 | 0.089314085 | -0.09319338 | 0.044123975 |
| CIAPIN1 | lightgreen | 0.249910092 | 4.42E-08 | 0.273075405 | 1.98E-09 |
| CLP1 | lightgreen | -0.10897128 | 0.018492972 | -0.107245394 | 0.020445486 |
| CNP | lightgreen | 0.180453491 | 8.80E-05 | 0.195069427 | 2.19E-05 |
| CPT2 | lightgreen | 0.228866426 | 5.76E-07 | 0.230153165 | 4.96E-07 |
| CSTF1 | lightgreen | -0.196516822 | 1.89E-05 | -0.194176069 | 2.39E-05 |
| CTD-310: | lightgreen | -0.002059352 | 0.964598518 | 0.008175791 | 0.860129136 |
| CXorf40A | lightgreen | 0.103790864 | 0.024897498 | 0.099158687 | 0.03216316 |
| CXorf40E | lightgreen | 0.002419989 | 0.958404124 | 0.006189783 | 0.89387298 |
| DCAF11 | lightgreen | 0.064435251 | 0.164477018 | 0.072926859 | 0.115525508 |
| DCTN2 | lightgreen | -0.01125995 | 0.808249302 | -0.027056023 | 0.559741548 |
| DCTN3 | lightgreen | -0.002102692 | 0.963853981 | 0.038359912 | 0.408210109 |
| DCTPP1 | lightgreen | 0.073583713 | 0.112275176 | 0.069897898 | 0.131476869 |
| DDOST | lightgreen | 0.091056315 | 0.049234298 | 0.074480183 | 0.107955657 |
| DDX23 | lightgreen | 0.066802285 | 0.149485496 | 0.059767912 | 0.19729837 |
| DHRS13 | lightgreen | 0.239600196 | 1.60E-07 | 0.227290869 | 6.91E-07 |
| DHX16 | lightgreen | -0.061954804 | 0.181369316 | -0.065692628 | 0.156378722 |
| DKC1 | lightgreen | -0.065570878 | 0.157149432 | -0.094763579 | 0.040660191 |
| DOLK | lightgreen | 0.247198352 | 6.24E-08 | 0.263241882 | 7.67E-09 |
| DOLPP1 | lightgreen | 0.39598339 | 5.55E-19 | 0.409871709 | 2.40E-20 |
| EEF2KM' | lightgreen | 0.264684137 | 6.31E-09 | 0.265576185 | 5.58E-09 |
| EIF2B1 | lightgreen | -0.010871409 | 0.814743983 | 0.001028243 | 0.982319548 |
| EIF2B2 | lightgreen | 0.016933299 | 0.715128444 | 0.031270875 | 0.500233013 |
| EIF2B3 | lightgreen | -0.079033565 | 0.088003978 | -0.068054793 | 0.14198528 |
| EIF4A3 | lightgreen | -0.074132588 | 0.109614665 | -0.08509494 | 0.06616227 |
| EIF4G1 | lightgreen | -0.073466339 | 0.112850649 | -0.067473512 | 0.145429459 |
| EMC6 | lightgreen | 0.10383805 | 0.024831458 | 0.090626076 | 0.050320831 |
| EMC8 | lightgreen | 0.22755865 | 6.70E-07 | 0.26889454 | 3.54E-09 |
| ESYT1 | lightgreen | -0.036489996 | 0.431455033 | -0.050130457 | 0.279650452 |
| ETFA | lightgreen | 0.249929448 | 4.41E-08 | 0.26049247 | 1.11E-08 |

| | | | | | |
|---|---|---|---|---|---|
| FADD | lightgreen | 0.053873853 | 0.245256706 | 0.041635753 | 0.369327248 |
| FARS2 | lightgreen | -0.013403133 | 0.772672521 | 0.002790773 | 0.95203808 |
| FDPS | lightgreen | 0.120028344 | 0.00942405 | 0.090852001 | 0.049747804 |
| FN3KRP | lightgreen | -0.014896407 | 0.748157972 | -0.043875554 | 0.344111171 |
| FTH1 | lightgreen | 0.10823958 | 0.019299953 | 0.103641335 | 0.025107778 |
| FTSJ3 | lightgreen | -0.078325596 | 0.09089556 | -0.073130255 | 0.114511248 |
| FXR2 | lightgreen | 0.169064528 | 0.000242383 | 0.14299898 | 0.001949053 |
| GANAB | lightgreen | 0.060877468 | 0.189093393 | 0.020758606 | 0.654555049 |
| GET4 | lightgreen | -0.130838693 | 0.004625001 | -0.138355089 | 0.002732879 |
| GOSR2 | lightgreen | -0.098297238 | 0.033697428 | -0.103451404 | 0.025377082 |
| GPATCH | lightgreen | 0.143057702 | 0.001940616 | 0.128698949 | 0.005347059 |
| GPKOW | lightgreen | -0.089373034 | 0.053599972 | -0.065449081 | 0.157923308 |
| GPN1 | lightgreen | -0.154523826 | 0.000806705 | -0.17350268 | 0.000164566 |
| GRHPR | lightgreen | 0.211632622 | 3.96E-06 | 0.235577138 | 2.61E-07 |
| HCCS | lightgreen | -0.022387528 | 0.629406409 | 0.001649235 | 0.971645347 |
| HDAC3 | lightgreen | -0.001006366 | 0.982695656 | -0.010276287 | 0.824716262 |
| HINT1 | lightgreen | 0.254900852 | 2.32E-08 | 0.260521675 | 1.10E-08 |
| HIRA | lightgreen | 0.018048193 | 0.697267875 | 0.007260991 | 0.87564453 |
| HMGN2 | lightgreen | 0.004921151 | 0.915532681 | -0.006070449 | 0.895907304 |
| HNRNPM | lightgreen | 0.07422441 | 0.109174474 | 0.062016884 | 0.180931442 |
| HSP90AE | lightgreen | -0.163571346 | 0.000386306 | -0.164267418 | 0.000364442 |
| HSPA5 | lightgreen | 0.076363162 | 0.099310262 | 0.065259295 | 0.159134909 |
| HSPA8 | lightgreen | 0.049980033 | 0.281097659 | 0.044681461 | 0.335312679 |
| HTRA2 | lightgreen | -0.104168559 | 0.02437311 | -0.114227463 | 0.013513187 |
| IMMT | lightgreen | -0.029805917 | 0.520530494 | -0.042379125 | 0.360834117 |
| KTI12 | lightgreen | -0.13890261 | 0.002627467 | -0.138660973 | 0.002673523 |
| LDHB | lightgreen | -0.075489834 | 0.103248961 | -0.097934286 | 0.034362298 |
| LGMN | lightgreen | -0.109939743 | 0.017470224 | -0.095530726 | 0.039053237 |
| LRRC42 | lightgreen | -0.081656409 | 0.077929904 | -0.098992829 | 0.032453832 |
| LRRC47 | lightgreen | -0.066024122 | 0.154294761 | -0.078053261 | 0.09202795 |
| MAD2L1 | lightgreen | 0.042411479 | 0.360467266 | 0.046052242 | 0.320682074 |
| MAP7D1 | lightgreen | -0.194285872 | 2.36E-05 | -0.173045365 | 0.00017134 |
| MDH1 | lightgreen | 0.032635657 | 0.481706918 | 0.02775516 | 0.549638492 |
| MDH2 | lightgreen | 0.111172458 | 0.016240155 | 0.131093227 | 0.004545244 |
| MED22 | lightgreen | 0.043044549 | 0.353336047 | 0.033795668 | 0.466257731 |
| MED24 | lightgreen | 0.110677845 | 0.016724285 | 0.083014908 | 0.073092261 |
| MOCS3 | lightgreen | -0.227432003 | 6.80E-07 | -0.217906784 | 2.00E-06 |
| MRPL4 | lightgreen | -0.028562223 | 0.538088364 | -0.016286832 | 0.725557248 |
| MRPL44 | lightgreen | 0.094382362 | 0.041479291 | 0.077447804 | 0.094585967 |
| MRPL45 | lightgreen | 0.05850245 | 0.206969828 | 0.033203594 | 0.474108579 |
| MRPL49 | lightgreen | 0.062106069 | 0.180303765 | 0.049298307 | 0.287719897 |
| MRPS18A | lightgreen | -0.080877623 | 0.080818359 | -0.089574386 | 0.053061355 |
| MRPS18B | lightgreen | -0.064919084 | 0.161324344 | -0.05266108 | 0.256059572 |
| MRPS24 | lightgreen | 0.06769201 | 0.144127418 | 0.05702621 | 0.218680133 |
| MT-ATP6 | lightgreen | 0.012529121 | 0.787128268 | 0.021505152 | 0.642978491 |

| | | | | | |
|---|---|---|---|---|---|
| MT-ATP8 | lightgreen | 0.010227676 | 0.825532084 | 0.00160331 | 0.972434598 |
| MT-CO1 | lightgreen | -0.011541211 | 0.803556063 | -0.015247961 | 0.742422327 |
| MT-CO2 | lightgreen | -0.029650181 | 0.522712868 | -0.007687958 | 0.868396675 |
| MT-CO3 | lightgreen | 0.006295408 | 0.892072949 | 0.011922514 | 0.797204754 |
| MT-CYB | lightgreen | -0.020623012 | 0.656666689 | -0.016783606 | 0.71753867 |
| MT-ND1 | lightgreen | 0.004227399 | 0.927404838 | 0.009239215 | 0.84216083 |
| MT-ND2 | lightgreen | -0.064483884 | 0.164158054 | -0.066204192 | 0.153171587 |
| MT-ND3 | lightgreen | -0.068813053 | 0.137586768 | -0.071162308 | 0.124622637 |
| MT-ND4 | lightgreen | 0.014179867 | 0.759891205 | 0.018348676 | 0.692482121 |
| MT-ND4L | lightgreen | 0.041398047 | 0.372069001 | 0.046236689 | 0.318745664 |
| MT-ND5 | lightgreen | -0.081950595 | 0.076860832 | -0.082044551 | 0.07652192 |
| MT-ND6 | lightgreen | -0.125279152 | 0.006713383 | -0.131350161 | 0.004465997 |
| MTCH1 | lightgreen | -0.216162947 | 2.42E-06 | -0.20376482 | 9.07E-06 |
| MTHFS | lightgreen | 0.036730359 | 0.428424939 | 0.006729795 | 0.884676146 |
| NADK | lightgreen | 0.072404648 | 0.118161822 | 0.063479585 | 0.170839728 |
| NCL | lightgreen | -0.082579984 | 0.074613643 | -0.121472761 | 0.008595144 |
| NHP2 | lightgreen | -0.05599235 | 0.227158916 | -0.072482333 | 0.117766687 |
| NOB1 | lightgreen | 0.156474728 | 0.000690534 | 0.160641981 | 0.0004924 |
| NOC2L | lightgreen | -0.065379748 | 0.15836512 | -0.065008416 | 0.160747264 |
| NOLC1 | lightgreen | 0.084358282 | 0.068552149 | 0.056943571 | 0.21934941 |
| NOP14 | lightgreen | 0.164319198 | 0.000362862 | 0.134801807 | 0.003515879 |
| NTMT1 | lightgreen | 0.150157691 | 0.00113508 | 0.155554227 | 0.000743267 |
| NUP37 | lightgreen | 0.082551538 | 0.07471404 | 0.062219526 | 0.179507595 |
| OGFOD2 | lightgreen | 0.040610759 | 0.381239244 | 0.040082506 | 0.387469101 |
| OS9 | lightgreen | 0.022064617 | 0.634359053 | 0.013694337 | 0.767873294 |
| PARS2 | lightgreen | -0.160971777 | 0.000479228 | -0.16344921 | 0.000390266 |
| PDCD11 | lightgreen | 0.009102729 | 0.844462607 | -0.023530956 | 0.612003467 |
| PDF | lightgreen | 0.102802157 | 0.026316553 | 0.110790635 | 0.016612787 |
| PDIA6 | lightgreen | -0.014721677 | 0.751013906 | -0.034944313 | 0.451234023 |
| PDXP | lightgreen | 0.116396672 | 0.011830054 | 0.106714494 | 0.02108108 |
| PES1 | lightgreen | -0.023054763 | 0.619225369 | -0.020364518 | 0.660699883 |
| PEX10 | lightgreen | -0.001119305 | 0.980754038 | -0.018360694 | 0.692290968 |
| PGAM1 | lightgreen | 0.212932366 | 3.44E-06 | 0.192546135 | 2.80E-05 |
| PGK1 | lightgreen | 0.121883124 | 0.008371815 | 0.123984398 | 0.007307362 |
| PGM1 | lightgreen | 0.154036067 | 0.000838448 | 0.146732745 | 0.001474529 |
| PHB | lightgreen | 0.03017439 | 0.515385657 | 0.031975075 | 0.490627162 |
| PHF23 | lightgreen | 0.232917588 | 3.58E-07 | 0.232985874 | 3.55E-07 |
| PIGS | lightgreen | 0.149681215 | 0.001177543 | 0.136563333 | 0.003105322 |
| PIGT | lightgreen | -0.041391182 | 0.372148373 | -0.039590513 | 0.393326632 |
| PNO1 | lightgreen | -0.078400944 | 0.090584238 | -0.084038962 | 0.069609843 |
| POLR3A | lightgreen | 0.01936906 | 0.676322711 | -0.00882299 | 0.849184427 |
| PPIL1 | lightgreen | -0.038111403 | 0.411255778 | -0.042099558 | 0.364013774 |
| PPP2R5D | lightgreen | 0.040969225 | 0.377046844 | 0.038795946 | 0.40289866 |
| PPP5C | lightgreen | -0.015396172 | 0.740008487 | -0.020190831 | 0.663415396 |
| PRDX4 | lightgreen | 0.178680566 | 0.000103491 | 0.173611989 | 0.000162985 |

| | | | | | |
|---|---|---|---|---|---|
| PRKAG1 | lightgreen | 0.117983746 | 0.010718788 | 0.100730884 | 0.029516581 |
| PSMB5 | lightgreen | 0.173394228 | 0.00016615 | 0.187212144 | 4.68E-05 |
| PSMD1 | lightgreen | -0.014592937 | 0.75312033 | -0.02619337 | 0.572329925 |
| PSMD3 | lightgreen | -0.00585627 | 0.899560169 | -0.004847667 | 0.916789361 |
| PSMD7 | lightgreen | 0.21607517 | 2.44E-06 | 0.21707826 | 2.19E-06 |
| PSMD9 | lightgreen | 0.044291496 | 0.339551933 | 0.047037971 | 0.31042221 |
| RABEPK | lightgreen | 0.173558262 | 0.00016376 | 0.17503144 | 0.0001437 |
| RAN | lightgreen | 0.054259921 | 0.24188564 | 0.03640942 | 0.43247357 |
| RBM15B | lightgreen | 0.131791034 | 0.00433292 | 0.114947922 | 0.012932144 |
| RNF121 | lightgreen | -0.086690218 | 0.061220976 | -0.083836579 | 0.070287088 |
| RNF26 | lightgreen | -0.086958482 | 0.060420746 | -0.104021701 | 0.024575861 |
| RP11-345 | lightgreen | -0.113354526 | 0.014247914 | -0.126299793 | 0.00627605 |
| RPN2 | lightgreen | 0.040155217 | 0.386607941 | 0.044438461 | 0.337950295 |
| RPUSD4 | lightgreen | 0.120947118 | 0.008888959 | 0.092483724 | 0.04576907 |
| RRP12 | lightgreen | 0.154423006 | 0.000813173 | 0.158044134 | 0.000608547 |
| RTCB | lightgreen | 0.065047522 | 0.160495132 | 0.060939867 | 0.188639525 |
| SAE1 | lightgreen | 0.100740674 | 0.029500703 | 0.088008667 | 0.057370753 |
| SAP30 | lightgreen | 0.178889739 | 0.000101542 | 0.1957578 | 2.04E-05 |
| SARS | lightgreen | -0.013556697 | 0.770140593 | -0.008383413 | 0.856615033 |
| SDHA | lightgreen | 0.077595485 | 0.093956846 | 0.084098493 | 0.069411649 |
| SDHB | lightgreen | 0.016143933 | 0.727869426 | 0.023624296 | 0.610592256 |
| SENP3 | lightgreen | -0.030416833 | 0.512014928 | -0.055054101 | 0.235053525 |
| SF3A3 | lightgreen | -0.182092201 | 7.57E-05 | -0.198326091 | 1.58E-05 |
| SF3B2 | lightgreen | -0.023070537 | 0.618985556 | -0.048092118 | 0.299691864 |
| SLC25A3 | lightgreen | 0.055831378 | 0.228499803 | 0.04247928 | 0.359699241 |
| SLC25A4 | lightgreen | 0.259659187 | 1.24E-08 | 0.264415515 | 6.54E-09 |
| SLC25A5 | lightgreen | 0.059865932 | 0.196563232 | 0.055795863 | 0.228796394 |
| SLC29A1 | lightgreen | -0.079250762 | 0.087131846 | -0.090044312 | 0.05182182 |
| SLC35B2 | lightgreen | 0.055915243 | 0.227800513 | 0.037279692 | 0.421546368 |
| SLC39A7 | lightgreen | 0.100748074 | 0.029488708 | 0.113211663 | 0.014371446 |
| SLC4A2 | lightgreen | -0.094989711 | 0.040180803 | -0.118360114 | 0.010469233 |
| SMARCA | lightgreen | -0.067799637 | 0.143489349 | -0.082020496 | 0.076608574 |
| SMARCB | lightgreen | 0.071336069 | 0.123702812 | 0.06368686 | 0.169444343 |
| SMARCD | lightgreen | 0.026282429 | 0.571024147 | 0.030418351 | 0.511993866 |
| SMIM12 | lightgreen | -0.116071952 | 0.012069585 | -0.101227846 | 0.028719834 |
| SNRNP4( | lightgreen | 0.028169165 | 0.543698346 | 0.019961214 | 0.667012117 |
| SPATA2 | lightgreen | -0.242115493 | 1.18E-07 | -0.256329805 | 1.92E-08 |
| SPSB3 | lightgreen | -0.064483283 | 0.164161987 | -0.077294137 | 0.095244155 |
| SRP68 | lightgreen | 0.10926852 | 0.018173654 | 0.088992028 | 0.054631617 |
| SRSF9 | lightgreen | 0.112668626 | 0.014849636 | 0.108963908 | 0.018500954 |
| SSRP1 | lightgreen | 0.039439545 | 0.395134673 | 0.012316007 | 0.79066439 |
| STARD7 | lightgreen | -0.199676476 | 1.38E-05 | -0.205792338 | 7.35E-06 |
| STK19 | lightgreen | -0.088347054 | 0.056415609 | -0.086888413 | 0.060628923 |
| STOML2 | lightgreen | 0.095987176 | 0.038122863 | 0.104281048 | 0.024218788 |
| SURF4 | lightgreen | 0.235115873 | 2.75E-07 | 0.2501003 | 4.31E-08 |

| | | | | | |
|---|---|---|---|---|---|
| TBL2 | lightgreen | 0.093384489 | 0.043689554 | 0.097398925 | 0.03536337 |
| THAP11 | lightgreen | 0.109158852 | 0.018290903 | 0.141815316 | 0.002126427 |
| TMED1 | lightgreen | -0.077475455 | 0.094467919 | -0.095766783 | 0.038569714 |
| TMEM18 | lightgreen | -0.054148328 | 0.24285669 | -0.049368601 | 0.287032254 |
| TMX2 | lightgreen | -0.059399366 | 0.200080358 | -0.060350672 | 0.192957143 |
| TOE1 | lightgreen | 0.039568674 | 0.393587868 | 0.017656985 | 0.703516589 |
| TRAFD1 | lightgreen | -0.092193458 | 0.04645664 | -0.081667721 | 0.077888574 |
| TUBA1B | lightgreen | 0.075816039 | 0.10176354 | 0.05397899 | 0.244335434 |
| UBE2Z | lightgreen | -0.04428012 | 0.339676107 | -0.032288989 | 0.486377178 |
| UBTF | lightgreen | 0.130681576 | 0.004674861 | 0.117170943 | 0.011275789 |
| UQCRFS | lightgreen | -0.170416422 | 0.000215634 | -0.153260307 | 0.000891322 |
| USP5 | lightgreen | 0.021732506 | 0.639469875 | 0.034727949 | 0.454042905 |
| VCP | lightgreen | 0.192015024 | 2.95E-05 | 0.213307052 | 3.31E-06 |
| WDR5 | lightgreen | 0.00985603 | 0.831775513 | -0.007659387 | 0.868881336 |
| WDR61 | lightgreen | 0.194750218 | 2.26E-05 | 0.173389291 | 0.000166222 |
| XRCC6 | lightgreen | 0.149944958 | 0.001153861 | 0.133559013 | 0.003834512 |
| ZDHHC4 | lightgreen | 0.022537225 | 0.627116039 | 0.038814494 | 0.402673637 |
| ZFPL1 | lightgreen | 0.013807647 | 0.766008237 | 2.44E-05 | 0.999579686 |
| ZMAT2 | lightgreen | -0.126349553 | 0.006255398 | -0.124641675 | 0.007000199 |
| ZNF622 | lightgreen | -0.124222215 | 0.00719486 | -0.110747264 | 0.016655584 |
| APOBEC | lightsteelbl | -0.14632242 | 0.001520923 | -0.146155626 | 0.001540161 |
| BST2 | lightsteelbl | -0.235600835 | 2.60E-07 | -0.209661845 | 4.89E-06 |
| C19orf66 | lightsteelbl | -0.280410869 | 6.93E-10 | -0.256616348 | 1.85E-08 |
| CMPK2 | lightsteelbl | -0.237247993 | 2.13E-07 | -0.212204471 | 3.72E-06 |
| DDX58 | lightsteelbl | -0.185576707 | 5.47E-05 | -0.174062644 | 0.000156614 |
| DDX60 | lightsteelbl | -0.149432623 | 0.001200273 | -0.143103939 | 0.001933997 |
| DDX60L | lightsteelbl | -0.079380074 | 0.086615919 | -0.07494828 | 0.105752889 |
| DTX3L | lightsteelbl | -0.198198697 | 1.60E-05 | -0.185205785 | 5.66E-05 |
| EIF2AK2 | lightsteelbl | -0.230493771 | 4.76E-07 | -0.241295144 | 1.30E-07 |
| EPSTI1 | lightsteelbl | -0.219256359 | 1.72E-06 | -0.195146824 | 2.17E-05 |
| FBXO39 | lightsteelbl | -0.090096215 | 0.051686407 | -0.084612006 | 0.067721151 |
| HELZ2 | lightsteelbl | -0.184887005 | 5.83E-05 | -0.179400207 | 9.69E-05 |
| HERC5 | lightsteelbl | -0.442456087 | 8.31E-24 | -0.417869882 | 3.68E-21 |
| HERC6 | lightsteelbl | -0.184329039 | 6.15E-05 | -0.167457993 | 0.000278199 |
| HSH2D | lightsteelbl | -0.068460852 | 0.139616616 | -0.029740943 | 0.521440424 |
| IFI16 | lightsteelbl | -0.291528823 | 1.33E-10 | -0.27341592 | 1.88E-09 |
| IFI27 | lightsteelbl | -0.174257698 | 0.000153929 | -0.143930027 | 0.001819163 |
| IFI44 | lightsteelbl | -0.252516993 | 3.16E-08 | -0.241888978 | 1.21E-07 |
| IFI44L | lightsteelbl | -0.29671221 | 6.04E-11 | -0.276956645 | 1.14E-09 |
| IFI6 | lightsteelbl | -0.164694791 | 0.000351595 | -0.155300499 | 0.000758445 |
| IFIH1 | lightsteelbl | -0.177966269 | 0.000110415 | -0.171249639 | 0.000200551 |
| IFIT1 | lightsteelbl | -0.264192045 | 6.74E-09 | -0.250428055 | 4.14E-08 |
| IFIT2 | lightsteelbl | -0.282977242 | 4.77E-10 | -0.266155637 | 5.16E-09 |
| IFIT3 | lightsteelbl | -0.269729701 | 3.15E-09 | -0.239981398 | 1.53E-07 |
| IFIT5 | lightsteelbl | -0.199042687 | 1.47E-05 | -0.192835243 | 2.72E-05 |

| | | | | | |
|---|---|---|---|---|---|
| IFNB1 | lightsteelblı | -0.19612845 | 1.97E-05 | -0.190584194 | 3.39E-05 |
| IRF7 | lightsteelblı | -0.04437577 | 0.33863291 | -0.018219654 | 0.694535558 |
| IRF9 | lightsteelblı | -0.217206611 | 2.16E-06 | -0.218441423 | 1.88E-06 |
| ISG15 | lightsteelblı | -0.072696912 | 0.116680629 | -0.037752034 | 0.415683827 |
| LAMP3 | lightsteelblı | -0.232646538 | 3.70E-07 | -0.197206667 | 1.77E-05 |
| LAP3 | lightsteelblı | -0.060548005 | 0.191503103 | -0.054337398 | 0.241213052 |
| LY6E | lightsteelblı | -0.251636235 | 3.54E-08 | -0.234439922 | 2.99E-07 |
| MX1 | lightsteelblı | -0.227940301 | 6.41E-07 | -0.208398882 | 5.59E-06 |
| MX2 | lightsteelblı | -0.483554332 | 9.70E-29 | -0.470719289 | 3.98E-27 |
| NMI | lightsteelblı | -0.139118799 | 0.002586876 | -0.12421108 | 0.007200093 |
| OAS1 | lightsteelblı | -0.216581268 | 2.31E-06 | -0.186962055 | 4.80E-05 |
| OAS2 | lightsteelblı | -0.122947888 | 0.007816286 | -0.085537286 | 0.064760379 |
| OAS3 | lightsteelblı | -0.107222816 | 0.020472173 | -0.085615004 | 0.064516614 |
| OASL | lightsteelblı | -0.145861537 | 0.001574628 | -0.107954142 | 0.01962298 |
| OPTN | lightsteelblı | -0.333246709 | 1.42E-13 | -0.312623823 | 4.79E-12 |
| PARP12 | lightsteelblı | -0.234414789 | 3.00E-07 | -0.197967437 | 1.64E-05 |
| PARP14 | lightsteelblı | -0.213480811 | 3.24E-06 | -0.191258498 | 3.18E-05 |
| PARP9 | lightsteelblı | -0.1450947 | 0.001667855 | -0.134852491 | 0.00350341 |
| PLSCR1 | lightsteelblı | -0.264472497 | 6.49E-09 | -0.252217634 | 3.29E-08 |
| RNF213 | lightsteelblı | -0.208214801 | 5.70E-06 | -0.197574859 | 1.70E-05 |
| RSAD2 | lightsteelblı | -0.264100912 | 6.82E-09 | -0.237505819 | 2.07E-07 |
| RTP4 | lightsteelblı | -0.369260418 | 1.56E-16 | -0.32156989 | 1.07E-12 |
| RUFY4 | lightsteelblı | -0.165843898 | 0.000319116 | -0.150561692 | 0.001100186 |
| SAMD9 | lightsteelblı | -0.225318944 | 8.67E-07 | -0.214568732 | 2.88E-06 |
| SAMD9L | lightsteelblı | -0.193738065 | 2.49E-05 | -0.16817086 | 0.000261736 |
| SMTNL1 | lightsteelblı | -0.249792223 | 4.49E-08 | -0.232644116 | 3.70E-07 |
| SP100 | lightsteelblı | -0.25135004 | 3.67E-08 | -0.233694384 | 3.26E-07 |
| SP110 | lightsteelblı | -0.25654204 | 1.87E-08 | -0.229794737 | 5.17E-07 |
| SP140L | lightsteelblı | -0.278349526 | 9.33E-10 | -0.247171744 | 6.26E-08 |
| STAT1 | lightsteelblı | -0.192543333 | 2.80E-05 | -0.176300602 | 0.000128291 |
| STAT2 | lightsteelblı | -0.22938263 | 5.42E-07 | -0.222156896 | 1.24E-06 |
| THEMIS2 | lightsteelblı | -0.262256212 | 8.76E-09 | -0.250446386 | 4.13E-08 |
| TNFSF10 | lightsteelblı | -0.349971041 | 6.68E-15 | -0.326618921 | 4.53E-13 |
| TRIM14 | lightsteelblı | -0.009708278 | 0.834260619 | 0.011703539 | 0.800850583 |
| TRIM21 | lightsteelblı | 0.010428813 | 0.822157667 | 0.030642725 | 0.508884664 |
| TRIM38 | lightsteelblı | -0.131135641 | 0.004532076 | -0.122083153 | 0.008264841 |
| TRIM69 | lightsteelblı | -0.151501418 | 0.001022812 | -0.132570814 | 0.004106291 |
| UBE2L6 | lightsteelblı | -0.308743896 | 9.01E-12 | -0.261901608 | 9.18E-09 |
| USP18 | lightsteelblı | -0.011024082 | 0.812190426 | -0.016401938 | 0.723696577 |
| XAF1 | lightsteelblı | -0.249517546 | 4.65E-08 | -0.23849473 | 1.83E-07 |
| ZNFX1 | lightsteelblı | -0.209249462 | 5.11E-06 | -0.17948802 | 9.62E-05 |
| AASDHP | lightyellow | -0.025036504 | 0.58941907 | -0.053056674 | 0.252500162 |
| ABCA7 | lightyellow | 0.291667969 | 1.31E-10 | 0.304072689 | 1.91E-11 |
| ABCD3 | lightyellow | 0.248590415 | 5.23E-08 | 0.230809789 | 4.59E-07 |
| ABCE1 | lightyellow | -0.014379355 | 0.756618947 | -0.034908599 | 0.451697 |

| | | | | | |
|---|---|---|---|---|---|
| ABHD13 | lightyellow | -0.074725149 | 0.106798424 | -0.088331805 | 0.056458366 |
| ABHD14A | lightyellow | 0.2139065 | 3.10E-06 | 0.212125061 | 3.75E-06 |
| ABHD5 | lightyellow | 0.359410147 | 1.10E-15 | 0.371553826 | 9.81E-17 |
| ABI1 | lightyellow | 0.054007476 | 0.244086241 | 0.02975843 | 0.521195439 |
| ACADM | lightyellow | -0.040948757 | 0.377285457 | -0.065840698 | 0.155445256 |
| ACAT1 | lightyellow | 0.099071133 | 0.032316323 | 0.090497825 | 0.050648569 |
| ACBD5 | lightyellow | 0.255008004 | 2.29E-08 | 0.227406655 | 6.82E-07 |
| ACN9 | lightyellow | 0.126559802 | 0.006168806 | 0.131403177 | 0.004449802 |
| ACSL3 | lightyellow | 0.212114031 | 3.76E-06 | 0.194779889 | 2.25E-05 |
| ACTR2 | lightyellow | 0.008786875 | 0.849794423 | -0.005712173 | 0.902018976 |
| ACTR3 | lightyellow | -0.056449409 | 0.223382156 | -0.059176924 | 0.201773227 |
| ACTR6 | lightyellow | 0.091590487 | 0.047912661 | 0.080477845 | 0.08233442 |
| ADAM10 | lightyellow | 0.080068659 | 0.083909878 | 0.062565663 | 0.177094715 |
| ADH5 | lightyellow | 0.23669514 | 2.28E-07 | 0.236892072 | 2.23E-07 |
| ADIPOR2 | lightyellow | -0.10295335 | 0.02609514 | -0.140454565 | 0.002348494 |
| ADK | lightyellow | 0.253480006 | 2.79E-08 | 0.233086418 | 3.51E-07 |
| ADO | lightyellow | -0.083547362 | 0.071264257 | -0.093483717 | 0.043465418 |
| ADPGK | lightyellow | 0.162078435 | 0.000437383 | 0.137649245 | 0.002874452 |
| AEBP2 | lightyellow | -0.123800836 | 0.007395272 | -0.141372196 | 0.002196541 |
| AFG3L2 | lightyellow | -0.144523091 | 0.001740611 | -0.167726242 | 0.000271893 |
| AFTPH | lightyellow | 0.015000109 | 0.746464592 | 0.002117511 | 0.963599406 |
| AGGF1 | lightyellow | 0.109581743 | 0.017842383 | 0.091016061 | 0.049335116 |
| AGL | lightyellow | 0.0598837 | 0.19643019 | 0.022536447 | 0.627127936 |
| AGPS | lightyellow | 0.103917122 | 0.024721129 | 0.082143697 | 0.076165603 |
| AIDA | lightyellow | -0.009814179 | 0.832479255 | -0.033303744 | 0.472775532 |
| AK4 | lightyellow | 0.038290351 | 0.409061284 | 0.028295967 | 0.541885379 |
| AKIRIN1 | lightyellow | -0.127331075 | 0.005860293 | -0.147625237 | 0.001378075 |
| AKIRIN2 | lightyellow | -0.187247041 | 4.67E-05 | -0.192496062 | 2.81E-05 |
| AKTIP | lightyellow | -0.014425991 | 0.755854596 | -0.011238153 | 0.808613309 |
| ALDH5A | lightyellow | -0.110981236 | 0.016425849 | -0.127225291 | 0.00590177 |
| ALG6 | lightyellow | 0.04708403 | 0.309948147 | 0.029100734 | 0.530449701 |
| ALKBH1 | lightyellow | -0.143838228 | 0.001831608 | -0.157292312 | 0.000646625 |
| AMD1 | lightyellow | 0.101439738 | 0.028385776 | 0.071783452 | 0.121358846 |
| AMMECI | lightyellow | 0.103965872 | 0.02465332 | 0.105354165 | 0.022788541 |
| AMZ2 | lightyellow | -0.038362154 | 0.408182695 | -0.059648539 | 0.198196354 |
| ANKEF1 | lightyellow | 0.037113544 | 0.423619977 | 0.018148952 | 0.69566175 |
| ANKRD1 | lightyellow | 0.1216992 | 0.008471263 | 0.137531063 | 0.002898798 |
| ANKRD3 | lightyellow | 0.114038492 | 0.013669346 | 0.092047691 | 0.046805184 |
| ANXA7 | lightyellow | 0.063344911 | 0.171750947 | 0.057542011 | 0.214535767 |
| AP1AR | lightyellow | 0.081925754 | 0.076950643 | 0.049822126 | 0.282622303 |
| AP1S2 | lightyellow | 0.085243789 | 0.065687778 | 0.055299472 | 0.232970472 |
| AP1S3 | lightyellow | 0.223337353 | 1.09E-06 | 0.226023329 | 8.00E-07 |
| AP3B1 | lightyellow | 0.206921264 | 6.53E-06 | 0.168823364 | 0.000247469 |
| AP3M1 | lightyellow | 0.111499916 | 0.015926432 | 0.079981148 | 0.084249953 |
| AP5M1 | lightyellow | 0.046663526 | 0.314293857 | 0.043852029 | 0.344370188 |

| | | | | | |
|---|---|---|---|---|---|
| API5 | lightyellow | 0.069726167 | 0.132429847 | 0.037857939 | 0.414375987 |
| APIP | lightyellow | 0.116636623 | 0.011655763 | 0.085639553 | 0.064439774 |
| ARCN1 | lightyellow | 0.123252605 | 0.007663482 | 0.103412168 | 0.025433024 |
| ARF4 | lightyellow | 0.147399381 | 0.00140192 | 0.150325767 | 0.001120441 |
| ARF6 | lightyellow | 0.107895865 | 0.019689507 | 0.095674274 | 0.038758595 |
| ARHGAP | lightyellow | 0.175279533 | 0.000140557 | 0.152927978 | 0.000914902 |
| ARL5A | lightyellow | -0.110621003 | 0.016780723 | -0.124105147 | 0.007250045 |
| ARL5B | lightyellow | -0.004730363 | 0.918795864 | -0.03356482 | 0.469310167 |
| ARMC10 | lightyellow | 0.08822268 | 0.056765128 | 0.08901671 | 0.054564289 |
| ARMCX5 | lightyellow | 0.021493488 | 0.643158705 | 0.003366448 | 0.942160396 |
| ARMCX6 | lightyellow | -0.043289634 | 0.350599325 | -0.077499315 | 0.094366151 |
| ARSK | lightyellow | 0.058513165 | 0.206886519 | 0.041182248 | 0.374568943 |
| ASAH2B | lightyellow | 0.148569717 | 0.001282346 | 0.145894167 | 0.001570769 |
| ASCC1 | lightyellow | -0.000614152 | 0.98943922 | -0.024470216 | 0.597868878 |
| ASNSD1 | lightyellow | -0.10276929 | 0.026364898 | -0.118910909 | 0.010113308 |
| ASPH | lightyellow | 0.096376602 | 0.03734402 | 0.062823913 | 0.175310232 |
| ASUN | lightyellow | -0.000159405 | 0.997258843 | -0.028897433 | 0.533327017 |
| ATAD1 | lightyellow | 0.039473733 | 0.39472479 | 0.012230804 | 0.792079351 |
| ATF1 | lightyellow | -0.106781161 | 0.021000336 | -0.141735311 | 0.002138933 |
| ATG3 | lightyellow | 0.083779675 | 0.070478479 | 0.090459703 | 0.050746331 |
| ATG5 | lightyellow | -0.031490811 | 0.497222273 | -0.050750953 | 0.273734176 |
| ATMIN | lightyellow | 0.15925364 | 0.000551626 | 0.155869056 | 0.000724826 |
| ATP2C1 | lightyellow | 0.159122511 | 0.000557549 | 0.125276482 | 0.006714563 |
| ATP6AP2 | lightyellow | -0.138488222 | 0.002706899 | -0.142523487 | 0.002018613 |
| ATP6V1A | lightyellow | 0.078366701 | 0.090725614 | 0.076078904 | 0.100578927 |
| ATP6V1D | lightyellow | 0.136962082 | 0.003018657 | 0.1528765 | 0.000918605 |
| AUH | lightyellow | 0.038541863 | 0.405988681 | 0.012041068 | 0.795232705 |
| AVL9 | lightyellow | 0.110108823 | 0.017296828 | 0.094236573 | 0.041796199 |
| AZI2 | lightyellow | 0.032502715 | 0.483495005 | 0.008676249 | 0.851663482 |
| B3GALN' | lightyellow | -0.006033188 | 0.896542644 | -0.003574656 | 0.938589974 |
| B3GNT2 | lightyellow | -0.078519833 | 0.090094753 | -0.069462327 | 0.133904349 |
| BAZ1A | lightyellow | 0.23909916 | 1.70E-07 | 0.23169934 | 4.13E-07 |
| BBS7 | lightyellow | 0.020078096 | 0.665180318 | -0.010350149 | 0.823477006 |
| BCAS2 | lightyellow | -0.047338924 | 0.307333292 | -0.067775361 | 0.14363308 |
| BCKDHB | lightyellow | 0.040432874 | 0.383330216 | 0.032811215 | 0.47935113 |
| BCL10 | lightyellow | 0.092454499 | 0.045837906 | 0.095342234 | 0.039443006 |
| BCL2L13 | lightyellow | 0.130410399 | 0.004762059 | 0.10639738 | 0.021468858 |
| BCL2L2-I | lightyellow | 0.100012036 | 0.030702554 | 0.085419594 | 0.065130963 |
| BCL3 | lightyellow | 0.128729108 | 0.005336213 | 0.136254702 | 0.003173947 |
| BCLAF1 | lightyellow | 0.036361865 | 0.433075354 | 6.03E-05 | 0.998963392 |
| BFAR | lightyellow | 0.069870512 | 0.131628485 | 0.079186382 | 0.087389629 |
| BIRC2 | lightyellow | -0.012698862 | 0.784314941 | -0.049320619 | 0.287501508 |
| BLOC1S5 | lightyellow | -0.209772332 | 4.83E-06 | -0.238255677 | 1.89E-07 |
| BMI1 | lightyellow | -0.090863135 | 0.049719705 | -0.12302356 | 0.007778088 |
| BMS1 | lightyellow | 0.038181309 | 0.410397671 | 0.003333176 | 0.942731075 |

| | | | | | |
|---|---|---|---|---|---|
| BOD1 | lightyellow | -0.171668035 | 0.000193356 | -0.200589099 | 1.26E-05 |
| BPGM | lightyellow | 0.061875169 | 0.181932152 | 0.060717553 | 0.190260221 |
| BRCC3 | lightyellow | 0.168672255 | 0.000250707 | 0.161472168 | 0.000459866 |
| BRD7 | lightyellow | 0.118168463 | 0.010595658 | 0.098073011 | 0.034106869 |
| BRMS1L | lightyellow | -0.235397143 | 2.66E-07 | -0.258191184 | 1.51E-08 |
| BSDC1 | lightyellow | 0.028382166 | 0.540654661 | 0.001012466 | 0.982590789 |
| BTBD1 | lightyellow | 0.046951187 | 0.311316726 | 0.029376686 | 0.526556753 |
| BTBD10 | lightyellow | 0.077032675 | 0.096372455 | 0.064252429 | 0.165680261 |
| BTG3 | lightyellow | 0.023726212 | 0.609053005 | 0.006286846 | 0.892218825 |
| BUD13 | lightyellow | 0.041001523 | 0.376670508 | 0.008075019 | 0.861835744 |
| BZW1 | lightyellow | -0.058790626 | 0.204737802 | -0.080443186 | 0.082466931 |
| C10orf76 | lightyellow | 0.279440442 | 7.98E-10 | 0.261846464 | 9.25E-09 |
| C10orf88 | lightyellow | -0.030753749 | 0.507349847 | -0.073442535 | 0.112967635 |
| C11orf57 | lightyellow | -0.062774249 | 0.175652362 | -0.088656159 | 0.055554686 |
| C11orf58 | lightyellow | -0.005013152 | 0.913959611 | -0.032396086 | 0.484931785 |
| C12orf4 | lightyellow | -0.034244915 | 0.46034909 | -0.037348171 | 0.420693446 |
| C12orf5 | lightyellow | 0.144298395 | 0.001769999 | 0.146789631 | 0.001468201 |
| C12orf66 | lightyellow | -0.077526407 | 0.094250702 | -0.082487682 | 0.074939814 |
| C16orf72 | lightyellow | 0.044105562 | 0.341585204 | 0.046159264 | 0.319557579 |
| C16orf87 | lightyellow | 0.10596325 | 0.022009758 | 0.111256418 | 0.016159205 |
| C17orf75 | lightyellow | -0.017903754 | 0.699572619 | -0.033764106 | 0.466674419 |
| C18orf32 | lightyellow | 0.078455134 | 0.090360866 | 0.083444357 | 0.071614946 |
| C1D | lightyellow | -0.088213489 | 0.056791025 | -0.095298617 | 0.039533669 |
| C1GALT1 | lightyellow | -0.026177452 | 0.572563472 | -0.012766813 | 0.78318948 |
| C1GALT1 | lightyellow | 0.103260337 | 0.025650507 | 0.097924533 | 0.034380316 |
| C1orf174 | lightyellow | 0.098229172 | 0.033821271 | 0.079195913 | 0.087351429 |
| C21orf2 | lightyellow | -0.020816036 | 0.653661492 | -0.017257594 | 0.709916677 |
| C2orf49 | lightyellow | -0.155244764 | 0.000761817 | -0.188028045 | 4.33E-05 |
| C2orf69 | lightyellow | -0.041456313 | 0.371395787 | -0.056425054 | 0.223582268 |
| C3orf33 | lightyellow | -0.146800218 | 0.001467026 | -0.175067872 | 0.000143234 |
| C3orf38 | lightyellow | 0.025754403 | 0.578786658 | 0.022153401 | 0.632995696 |
| C4orf3 | lightyellow | 0.091680087 | 0.047693911 | 0.107157583 | 0.020549449 |
| C5orf15 | lightyellow | 0.081466726 | 0.078625587 | 0.07397112 | 0.110392124 |
| C5orf22 | lightyellow | -0.039022649 | 0.400153512 | -0.0521028 | 0.261141631 |
| C5orf51 | lightyellow | -0.114504435 | 0.013287146 | -0.129140591 | 0.00519021 |
| C6orf211 | lightyellow | 0.138508598 | 0.002702943 | 0.106782908 | 0.020998223 |
| C7orf49 | lightyellow | -0.077921701 | 0.092579018 | -0.095394639 | 0.039334314 |
| C9orf41 | lightyellow | 0.1373724 | 0.002931778 | 0.120400989 | 0.009203664 |
| C9orf85 | lightyellow | 0.034573122 | 0.456058919 | -0.000835263 | 0.985637411 |
| CAB39 | lightyellow | -0.124647568 | 0.006997499 | -0.124910564 | 0.006877909 |
| CACUL1 | lightyellow | -0.043417113 | 0.349181151 | -0.053467931 | 0.248836424 |
| CALU | lightyellow | -0.100438879 | 0.029993514 | -0.11809343 | 0.010645522 |
| CANX | lightyellow | 0.032290455 | 0.486357384 | -0.000200268 | 0.996556158 |
| CAP1 | lightyellow | -0.057975412 | 0.211097359 | -0.044857643 | 0.333408618 |
| CAPZA1 | lightyellow | 0.120970172 | 0.008875887 | 0.105678017 | 0.02237154 |

| | | | | | |
|---|---|---|---|---|---|
| CAPZA2 | lightyellow | -0.047282278 | 0.307913136 | -0.058682589 | 0.205572533 |
| CASC3 | lightyellow | 0.09793403 | 0.03436277 | 0.064072222 | 0.166872741 |
| CASP3 | lightyellow | 0.258594954 | 1.43E-08 | 0.258904393 | 1.37E-08 |
| CASP8 | lightyellow | -0.033963665 | 0.464043284 | -0.028776093 | 0.535048085 |
| CAST | lightyellow | 0.019611092 | 0.672511119 | 0.033925195 | 0.464549852 |
| CBFB | lightyellow | 0.313780495 | 3.96E-12 | 0.307433187 | 1.11E-11 |
| CBWD2 | lightyellow | -0.116544149 | 0.011722662 | -0.148863641 | 0.001253828 |
| CBWD5 | lightyellow | -0.038310633 | 0.408812998 | -0.052241402 | 0.259873488 |
| CBX1 | lightyellow | -0.036694867 | 0.42887159 | -0.066648811 | 0.150424865 |
| CCBL2 | lightyellow | 0.171486876 | 0.000196441 | 0.151178566 | 0.00104881 |
| CCDC115 | lightyellow | -0.140010981 | 0.002425336 | -0.157038015 | 0.000659997 |
| CCDC126 | lightyellow | 0.04576878 | 0.323672896 | 0.024650151 | 0.595178063 |
| CCDC15 | lightyellow | 0.007322577 | 0.87459843 | -0.021637062 | 0.640941834 |
| CCDC47 | lightyellow | 0.027972476 | 0.546516464 | 0.014102663 | 0.761158752 |
| CCNC | lightyellow | 0.063096098 | 0.173443969 | 0.066701234 | 0.150103497 |
| CCNG1 | lightyellow | 0.110830391 | 0.016573643 | 0.084469548 | 0.06818671 |
| CCNJ | lightyellow | 0.169665357 | 0.000230133 | 0.158357513 | 0.000593298 |
| CCNY | lightyellow | -0.143069611 | 0.001938909 | -0.159014638 | 0.000562466 |
| CCNYL1 | lightyellow | -0.099428597 | 0.03169489 | -0.097046109 | 0.036036574 |
| CCT4 | lightyellow | -0.068807384 | 0.137619265 | -0.083204663 | 0.072436464 |
| CCZ1 | lightyellow | -0.066551572 | 0.151022359 | -0.064093534 | 0.16673138 |
| CCZ1B | lightyellow | -0.055661063 | 0.229924623 | -0.061825828 | 0.18228153 |
| CD164 | lightyellow | -0.01166016 | 0.801573337 | -0.010178413 | 0.826359054 |
| CD2AP | lightyellow | 0.006126852 | 0.894945694 | -0.002236621 | 0.961553402 |
| CDADC1 | lightyellow | 0.067250042 | 0.146770391 | 0.041188478 | 0.374496627 |
| CDC27 | lightyellow | 0.002686014 | 0.953836406 | -0.021381583 | 0.644888788 |
| CDC42EP | lightyellow | -0.113161249 | 0.014415263 | -0.087737563 | 0.058145618 |
| CDC5L | lightyellow | -0.070700376 | 0.127093783 | -0.109856454 | 0.017556196 |
| CDCA7L | lightyellow | -0.138290128 | 0.002745638 | -0.16540022 | 0.000331309 |
| CDK2AP | lightyellow | 0.13030983 | 0.004794769 | 0.13246884 | 0.004135306 |
| CDK8 | lightyellow | -0.00617394 | 0.894143021 | -0.028228559 | 0.542848781 |
| CDK9 | lightyellow | -0.003984786 | 0.931560598 | 0.01182124 | 0.798890367 |
| CDKN1B | lightyellow | -0.224468827 | 9.56E-07 | -0.230721779 | 4.64E-07 |
| CDKN2A | lightyellow | -0.082127368 | 0.076224194 | -0.116446708 | 0.01179352 |
| CDR2 | lightyellow | -0.008020874 | 0.862752973 | -0.014923812 | 0.747710343 |
| CDV3 | lightyellow | -0.150570879 | 0.001099404 | -0.174325494 | 0.000153006 |
| CDYL | lightyellow | -0.214984521 | 2.75E-06 | -0.238455291 | 1.84E-07 |
| CEBPZ | lightyellow | -0.176088937 | 0.000130748 | -0.201940416 | 1.09E-05 |
| CELF1 | lightyellow | -0.031758331 | 0.493573147 | -0.061720648 | 0.183027944 |
| CEP104 | lightyellow | 0.09871402 | 0.032947507 | 0.090757747 | 0.049986198 |
| CEP57 | lightyellow | -0.042887964 | 0.355091569 | -0.08163545 | 0.078006528 |
| CEP63 | lightyellow | -0.11169473 | 0.01574232 | -0.142031025 | 0.002093042 |
| CEP70 | lightyellow | -0.064141651 | 0.166412547 | -0.065547502 | 0.157297735 |
| CEP76 | lightyellow | -0.086053211 | 0.063156259 | -0.099068576 | 0.032320805 |
| CEP78 | lightyellow | 0.14849129 | 0.001290056 | 0.146107569 | 0.001545746 |

| | | | | | |
|---|---|---|---|---|---|
| CERS6 | lightyellow | 0.03293672 | 0.477670842 | 0.004675651 | 0.919731908 |
| CFAP20 | lightyellow | 0.083669943 | 0.070848752 | 0.099476104 | 0.031613075 |
| CHIC2 | lightyellow | 0.017482451 | 0.706310888 | 0.012322821 | 0.790551274 |
| CHML | lightyellow | -0.000244427 | 0.995796793 | -0.027697768 | 0.550464459 |
| CHMP2B | lightyellow | 0.010932648 | 0.813719475 | -0.00660406 | 0.886816173 |
| CHMP3 | lightyellow | 0.070449725 | 0.128450477 | 0.051335868 | 0.26823577 |
| CHMP5 | lightyellow | -0.06341007 | 0.171309622 | -0.062940201 | 0.174511071 |
| CHST12 | lightyellow | -0.16324936 | 0.000396828 | -0.136518312 | 0.003115248 |
| CHUK | lightyellow | 0.128647366 | 0.005365654 | 0.09875032 | 0.032882872 |
| CLCC1 | lightyellow | 0.146067563 | 0.001550409 | 0.121370446 | 0.008651646 |
| CLEC11A | lightyellow | -0.111429195 | 0.015993733 | -0.085887883 | 0.063666696 |
| CLINT1 | lightyellow | -0.044362441 | 0.338778158 | -0.04421492 | 0.340388384 |
| CLN5 | lightyellow | -0.008340815 | 0.857335798 | -0.024478545 | 0.597744201 |
| CLNS1A | lightyellow | -0.060441626 | 0.192285948 | -0.096227318 | 0.037640975 |
| CLPX | lightyellow | 0.064576851 | 0.163549598 | 0.038694985 | 0.404124812 |
| CMAS | lightyellow | -0.025264109 | 0.586038425 | -0.030961589 | 0.504483142 |
| CNBP | lightyellow | -0.291748067 | 1.29E-10 | -0.317002179 | 2.32E-12 |
| CNEP1R1 | lightyellow | 0.213622423 | 3.19E-06 | 0.209175242 | 5.15E-06 |
| CNIH1 | lightyellow | 0.056275351 | 0.224815114 | 0.038018845 | 0.41239358 |
| CNOT11 | lightyellow | 0.25445911 | 2.46E-08 | 0.245740701 | 7.49E-08 |
| CNOT6 | lightyellow | 0.129966719 | 0.004907895 | 0.104120016 | 0.024439968 |
| CNOT8 | lightyellow | 0.067167943 | 0.147265387 | 0.06025786 | 0.193643815 |
| COA1 | lightyellow | -0.05239098 | 0.258509693 | -0.071832648 | 0.121103223 |
| COA7 | lightyellow | -0.160557814 | 0.000495815 | -0.177905367 | 0.000111025 |
| COG8 | lightyellow | 0.140808647 | 0.002288759 | 0.149332684 | 0.001209524 |
| COIL | lightyellow | -0.014942765 | 0.747400836 | -0.044759802 | 0.33446516 |
| COMMD | lightyellow | 0.117005915 | 0.011391959 | 0.107163516 | 0.020542411 |
| COPB1 | lightyellow | 0.118172134 | 0.010593224 | 0.095544185 | 0.039025531 |
| COPB2 | lightyellow | 0.060684114 | 0.190504872 | 0.058404983 | 0.207728713 |
| COPG2 | lightyellow | 0.076100198 | 0.100483448 | 0.031814414 | 0.492809958 |
| COPS4 | lightyellow | 0.086512613 | 0.061755569 | 0.065368842 | 0.158434705 |
| COPS8 | lightyellow | -0.077360662 | 0.094958768 | -0.103917729 | 0.024720284 |
| COQ10B | lightyellow | 0.035843351 | 0.439668073 | 0.043675251 | 0.34632057 |
| COX10 | lightyellow | 0.282047336 | 5.46E-10 | 0.274044131 | 1.72E-09 |
| COX11 | lightyellow | 0.071326698 | 0.123752282 | 0.05419868 | 0.242418196 |
| COX15 | lightyellow | 0.285003811 | 3.54E-10 | 0.283234001 | 4.59E-10 |
| CPOX | lightyellow | 0.072541778 | 0.117465027 | 0.056279487 | 0.224780989 |
| CPSF2 | lightyellow | 0.135976408 | 0.003237004 | 0.130303623 | 0.004796794 |
| CRCP | lightyellow | -0.039972076 | 0.388779213 | -0.045653493 | 0.324894458 |
| CREM | lightyellow | -0.171412138 | 0.000197727 | -0.180503594 | 8.76E-05 |
| CRNKL1 | lightyellow | -0.098362744 | 0.033578607 | -0.100597506 | 0.029733616 |
| CRY1 | lightyellow | -0.034637791 | 0.455216259 | -0.067412556 | 0.145794295 |
| CSNK2A | lightyellow | -0.151163224 | 0.00105006 | -0.179178754 | 9.89E-05 |
| CSNK2A | lightyellow | 0.072836859 | 0.115976545 | 0.085013188 | 0.066424067 |
| CST3 | lightyellow | 0.035668394 | 0.441905478 | 0.057914121 | 0.211581189 |

| | | | | | |
|---|---|---|---|---|---|
| CSTF2T | lightyellow | 0.043262744 | 0.35089893 | 0.017365667 | 0.708182809 |
| CTC-534/ | lightyellow | 0.065893254 | 0.155114945 | 0.062451757 | 0.177886067 |
| CTNNAL | lightyellow | 0.100446543 | 0.029980914 | 0.080905683 | 0.080712804 |
| CTPS2 | lightyellow | -0.004229251 | 0.927373122 | -0.025327469 | 0.585098943 |
| CTR9 | lightyellow | 0.140324844 | 0.002370732 | 0.08562122 | 0.064497152 |
| CUL2 | lightyellow | -0.000966011 | 0.983389451 | -0.022804566 | 0.623034627 |
| CUL4A | lightyellow | 0.048093793 | 0.299675014 | 0.03141178 | 0.498303026 |
| CUL5 | lightyellow | -0.016144162 | 0.727865732 | -0.046437155 | 0.316649754 |
| CWC22 | lightyellow | -0.117328123 | 0.011166119 | -0.151745256 | 0.001003572 |
| CWC27 | lightyellow | -0.020814229 | 0.653689609 | -0.03643757 | 0.432117583 |
| CXADR | lightyellow | 0.234016099 | 3.14E-07 | 0.23453812 | 2.95E-07 |
| CXorf56 | lightyellow | -0.215702537 | 2.55E-06 | -0.227475668 | 6.77E-07 |
| CYB5R4 | lightyellow | 0.213763003 | 3.15E-06 | 0.216747689 | 2.27E-06 |
| D2HGDH | lightyellow | 0.181337194 | 8.12E-05 | 0.170464492 | 0.000214735 |
| DBT | lightyellow | 0.06215727 | 0.179944144 | 0.044534897 | 0.336901951 |
| DCAF12 | lightyellow | 0.12977854 | 0.004970955 | 0.117451546 | 0.011080666 |
| DCK | lightyellow | 0.05420687 | 0.24234693 | 0.029730166 | 0.521591426 |
| DCLRE1/ | lightyellow | 0.166851298 | 0.000292967 | 0.135041642 | 0.003457227 |
| DDX1 | lightyellow | -0.008762583 | 0.850204773 | -0.04680835 | 0.312792681 |
| DDX10 | lightyellow | -0.051377255 | 0.267849605 | -0.091574847 | 0.047950929 |
| DDX18 | lightyellow | -0.132696174 | 0.004070875 | -0.159254529 | 0.000551586 |
| DDX19A | lightyellow | 0.035753234 | 0.440819704 | 0.050411698 | 0.276958245 |
| DDX21 | lightyellow | 0.068973059 | 0.136672135 | 0.030988636 | 0.504110711 |
| DDX3X | lightyellow | 0.054417472 | 0.240519315 | 0.02014134 | 0.664189964 |
| DDX46 | lightyellow | 0.005423426 | 0.90694881 | -0.036509716 | 0.431205976 |
| DDX50 | lightyellow | -0.022068101 | 0.634305528 | -0.053052478 | 0.252537735 |
| DDX52 | lightyellow | -0.020279602 | 0.662026948 | -0.048234644 | 0.298260225 |
| DEK | lightyellow | -0.15139537 | 0.001031285 | -0.179244281 | 9.83E-05 |
| DERA | lightyellow | 0.089058788 | 0.054449669 | 0.089142818 | 0.054221368 |
| DFFB | lightyellow | 0.23516041 | 2.74E-07 | 0.20230748 | 1.05E-05 |
| DHTKD1 | lightyellow | 0.168092507 | 0.0002635 | 0.149080715 | 0.001233142 |
| DHX29 | lightyellow | 0.143312294 | 0.001904424 | 0.124296834 | 0.007159881 |
| DHX32 | lightyellow | 0.260003266 | 1.18E-08 | 0.238697731 | 1.79E-07 |
| DIS3 | lightyellow | 0.039008589 | 0.400323445 | 0.014603605 | 0.752945705 |
| DLAT | lightyellow | 0.11197231 | 0.015483214 | 0.109724749 | 0.017692898 |
| DLD | lightyellow | 0.067400659 | 0.145865578 | 0.058981511 | 0.203268964 |
| DNAAF2 | lightyellow | 0.038026597 | 0.412298215 | 0.021939525 | 0.636282065 |
| DNAJA1 | lightyellow | -0.107263311 | 0.020424329 | -0.100195133 | 0.030396668 |
| DNAJA2 | lightyellow | 0.126118279 | 0.006351907 | 0.131100004 | 0.004543138 |
| DNAJC21 | lightyellow | -0.132506384 | 0.004124602 | -0.155242307 | 0.000761966 |
| DNAJC28 | lightyellow | 0.008694123 | 0.851361442 | -0.000832157 | 0.985690802 |
| DNM1L | lightyellow | -0.044937921 | 0.332543332 | -0.071056729 | 0.125184119 |
| DPH3 | lightyellow | -0.166968905 | 0.000290048 | -0.175516074 | 0.000137622 |
| DPM1 | lightyellow | -0.157451158 | 0.000638401 | -0.182278607 | 7.44E-05 |
| DPY19L1 | lightyellow | 0.177264489 | 0.000117641 | 0.170966257 | 0.000205566 |

| | | | | | |
|---|---|---|---|---|---|
| DR1 | lightyellow | -0.138068956 | 0.002789486 | -0.16116786 | 0.000471552 |
| DRD4 | lightyellow | -0.100034097 | 0.030665559 | -0.099482912 | 0.031601366 |
| DSC2 | lightyellow | 0.241077783 | 1.34E-07 | 0.225939451 | 8.08E-07 |
| DSG2 | lightyellow | 0.191086386 | 3.23E-05 | 0.181413756 | 8.06E-05 |
| DTD2 | lightyellow | 0.07633554 | 0.099432983 | 0.070171487 | 0.129969605 |
| DUS4L | lightyellow | -0.071517766 | 0.122746638 | -0.076650844 | 0.098039307 |
| DUSP23 | lightyellow | 0.146258384 | 0.001528283 | 0.166583839 | 0.000299707 |
| DYNC1L | lightyellow | 0.171394027 | 0.00019804 | 0.164100637 | 0.000369573 |
| E4F1 | lightyellow | 0.098196195 | 0.033881412 | 0.123069523 | 0.007754968 |
| EAF1 | lightyellow | 0.148057287 | 0.001333497 | 0.134482329 | 0.00359541 |
| ECD | lightyellow | 0.072092601 | 0.119759462 | 0.053607692 | 0.247599819 |
| ECHDC1 | lightyellow | -0.054404061 | 0.240635404 | -0.048597905 | 0.294631961 |
| EFTUD1 | lightyellow | 0.266529389 | 4.90E-09 | 0.236106079 | 2.45E-07 |
| EI24 | lightyellow | 0.271055703 | 2.62E-09 | 0.259191629 | 1.32E-08 |
| EIF2AK3 | lightyellow | 0.019303012 | 0.6773643 | -2.50E-05 | 0.99957053 |
| EIF2S1 | lightyellow | 0.079778751 | 0.085040742 | 0.058199422 | 0.209335821 |
| EIF2S3 | lightyellow | 0.007001548 | 0.880053736 | -0.035299744 | 0.446641086 |
| EIF3J | lightyellow | 0.084818708 | 0.067050283 | 0.063859148 | 0.16829099 |
| EIF4E | lightyellow | 0.177014985 | 0.000120315 | 0.152858252 | 0.000919922 |
| EIF4EBP | lightyellow | 0.000467868 | 0.991954572 | -0.034369318 | 0.458720308 |
| EIF4G2 | lightyellow | 0.094844144 | 0.040488846 | 0.069870794 | 0.131626922 |
| EIF5 | lightyellow | -0.101204517 | 0.028756818 | -0.124231413 | 0.007190539 |
| ELAC1 | lightyellow | 0.138539608 | 0.002696932 | 0.133216403 | 0.003926836 |
| ENOPH1 | lightyellow | 0.302298621 | 2.52E-11 | 0.296931439 | 5.84E-11 |
| EPB41L4 | lightyellow | 0.181540573 | 7.97E-05 | 0.185104665 | 5.72E-05 |
| ERCC8 | lightyellow | 0.030929939 | 0.504919138 | 0.006318295 | 0.891682977 |
| ERGIC2 | lightyellow | 0.039381746 | 0.39582822 | 0.027232627 | 0.557181032 |
| ERI1 | lightyellow | 0.255662708 | 2.10E-08 | 0.235252871 | 2.71E-07 |
| ERI2 | lightyellow | 0.144358179 | 0.001762136 | 0.131538598 | 0.004408673 |
| ERLIN1 | lightyellow | 0.216936397 | 2.22E-06 | 0.187894985 | 4.39E-05 |
| ETF1 | lightyellow | 0.005780973 | 0.900844881 | -0.013685967 | 0.768011103 |
| EVA1B | lightyellow | -0.206850392 | 6.58E-06 | -0.176373825 | 0.000127451 |
| EXD2 | lightyellow | 0.052602076 | 0.256593427 | 0.024553597 | 0.596621277 |
| EXD3 | lightyellow | 0.20032244 | 1.29E-05 | 0.190743873 | 3.34E-05 |
| EXOC5 | lightyellow | 0.069652431 | 0.132840661 | 0.055700178 | 0.229596838 |
| EXOC6 | lightyellow | 0.214466723 | 2.91E-06 | 0.205856957 | 7.30E-06 |
| EXT2 | lightyellow | -0.150696563 | 0.001088759 | -0.187860217 | 4.40E-05 |
| FAF2 | lightyellow | 0.037160342 | 0.423035314 | 0.008929359 | 0.847388329 |
| FAM117E | lightyellow | -0.00550218 | 0.905603872 | -0.028544518 | 0.538340444 |
| FAM118E | lightyellow | 0.113127732 | 0.014444459 | 0.111348885 | 0.016070462 |
| FAM120A | lightyellow | 0.151611421 | 0.001014091 | 0.143238627 | 0.001914832 |
| FAM127A | lightyellow | -0.195205962 | 2.16E-05 | -0.185335765 | 5.59E-05 |
| FAM149E | lightyellow | 0.031812636 | 0.492834144 | 0.000377307 | 0.993511814 |
| FAM173E | lightyellow | 0.045977468 | 0.321469259 | 0.036035534 | 0.437217863 |
| FAM175E | lightyellow | -0.034977321 | 0.450806365 | -0.067582762 | 0.144777316 |

| | | | | | |
|---|---|---|---|---|---|
| FAM177A | lightyellow | 0.143902273 | 0.001822918 | 0.143384454 | 0.001894279 |
| FAM185A | lightyellow | 0.125982888 | 0.006409023 | 0.104276309 | 0.024225271 |
| FAM199X | lightyellow | 0.048106317 | 0.299549033 | 0.023173693 | 0.617418215 |
| FAM200A | lightyellow | -0.046498896 | 0.316006059 | -0.062509314 | 0.177485867 |
| FAM200B | lightyellow | 0.100964588 | 0.029139568 | 0.118862801 | 0.010143962 |
| FAM204A | lightyellow | 0.122285092 | 0.008158085 | 0.084525997 | 0.068001918 |
| FAM210A | lightyellow | -0.146756369 | 0.001471898 | -0.174823731 | 0.000146381 |
| FAM21A | lightyellow | -0.09378929 | 0.042781258 | -0.131994127 | 0.004272832 |
| FAM21C | lightyellow | -0.160537251 | 0.000496653 | -0.202055558 | 1.08E-05 |
| FAM220A | lightyellow | -0.045364148 | 0.327973462 | -0.054398242 | 0.240685787 |
| FAM35A | lightyellow | 0.120360825 | 0.009227194 | 0.085723054 | 0.064178968 |
| FAM3C | lightyellow | 0.108176718 | 0.019370693 | 0.09701789 | 0.036090885 |
| FAM45A | lightyellow | 0.085106238 | 0.066126156 | 0.046092261 | 0.320261281 |
| FAM49B | lightyellow | -0.124056312 | 0.007273177 | -0.150246883 | 0.00112729 |
| FAM96A | lightyellow | 0.084838257 | 0.066987117 | 0.077674218 | 0.093622811 |
| FAM98A | lightyellow | 0.16955289 | 0.000232381 | 0.173287007 | 0.000167729 |
| FAM98B | lightyellow | 0.088866404 | 0.054975366 | 0.054278064 | 0.241728021 |
| FAR1 | lightyellow | -0.046747959 | 0.313418088 | -0.052054358 | 0.261585862 |
| FASTKD1 | lightyellow | -0.005566297 | 0.90450909 | -0.01069768 | 0.817652088 |
| FBXO22 | lightyellow | 0.266048322 | 5.24E-09 | 0.271657481 | 2.41E-09 |
| FBXO33 | lightyellow | -0.13212332 | 0.004235 | -0.123252977 | 0.007663297 |
| FBXO34 | lightyellow | 0.033456655 | 0.470744188 | 0.038791114 | 0.402957293 |
| FDX1 | lightyellow | 0.047301531 | 0.30771598 | 0.056112301 | 0.226163375 |
| FECH | lightyellow | 0.214622147 | 2.87E-06 | 0.20032391 | 1.29E-05 |
| FGFR1OP | lightyellow | -0.10300479 | 0.026020175 | -0.123597855 | 0.007493582 |
| FIG4 | lightyellow | 0.055440724 | 0.231777265 | 0.031223099 | 0.500888294 |
| FITM1 | lightyellow | -0.029019314 | 0.531601097 | 0.013641336 | 0.768746118 |
| FNBP1L | lightyellow | 0.188112586 | 4.30E-05 | 0.157323449 | 0.000645005 |
| FREM2 | lightyellow | 0.15448841 | 0.000808971 | 0.110865589 | 0.016539053 |
| FZD3 | lightyellow | 0.150486203 | 0.00110663 | 0.124214321 | 0.007198569 |
| G3BP1 | lightyellow | 0.054608848 | 0.238866951 | 0.023660495 | 0.610045345 |
| G3BP2 | lightyellow | 0.106250172 | 0.021650963 | 0.084293949 | 0.068764178 |
| GABPA | lightyellow | -0.121200316 | 0.008746332 | -0.156148315 | 0.000708824 |
| GABPB1 | lightyellow | -0.138924615 | 0.002623309 | -0.144359656 | 0.001761942 |
| GALNT1 | lightyellow | 0.235760709 | 2.55E-07 | 0.233012478 | 3.54E-07 |
| GART | lightyellow | -0.055208802 | 0.233738682 | -0.081701273 | 0.077766094 |
| GBAS | lightyellow | 0.107851264 | 0.019740553 | 0.103410917 | 0.02543481 |
| GBE1 | lightyellow | 0.313675907 | 4.03E-12 | 0.281381074 | 6.02E-10 |
| GCA | lightyellow | 0.04157166 | 0.370065273 | 0.039058933 | 0.399715196 |
| GCH1 | lightyellow | 0.152528379 | 0.000944017 | 0.158948374 | 0.000565506 |
| GCLM | lightyellow | 0.036292929 | 0.433948546 | 0.039482391 | 0.39462103 |
| GDE1 | lightyellow | 0.187221227 | 4.68E-05 | 0.199995728 | 1.33E-05 |
| GDI2 | lightyellow | 0.097744767 | 0.034713885 | 0.074085886 | 0.10983909 |
| GDPD1 | lightyellow | 0.027938195 | 0.547008381 | -0.000352336 | 0.993941214 |
| GFM2 | lightyellow | 0.204830008 | 8.12E-06 | 0.189295017 | 3.84E-05 |

| | | | | | |
|---|---|---|---|---|---|
| GGNBP2 | lightyellow | 0.051351042 | 0.268094149 | 0.020339346 | 0.661093155 |
| GHITM | lightyellow | 0.128051634 | 0.005584666 | 0.132466305 | 0.00413603 |
| GINM1 | lightyellow | -0.150491437 | 0.001106182 | -0.161526825 | 0.000457796 |
| GLUD1 | lightyellow | 0.029835518 | 0.520116207 | 0.006448239 | 0.889469421 |
| GMCL1 | lightyellow | 0.020480926 | 0.658882392 | -0.006380389 | 0.890625123 |
| GMFB | lightyellow | 0.028072667 | 0.545080041 | -0.000983363 | 0.983091141 |
| GNG10 | lightyellow | -0.062981721 | 0.174226398 | -0.090245083 | 0.051299652 |
| GNPNAT | lightyellow | 0.306431672 | 1.31E-11 | 0.310432019 | 6.85E-12 |
| GOLGA4 | lightyellow | 0.026314352 | 0.570556433 | -0.003358694 | 0.942293394 |
| GOLGA5 | lightyellow | 0.016982444 | 0.714337773 | -0.009749371 | 0.833569292 |
| GOLGA7 | lightyellow | -0.191566332 | 3.08E-05 | -0.223158665 | 1.11E-06 |
| GOLPH3 | lightyellow | 0.015058894 | 0.745505222 | 0.004033093 | 0.930732991 |
| GOLT1B | lightyellow | -0.08261018 | 0.074507189 | -0.096576754 | 0.036949003 |
| GPALPP1 | lightyellow | -0.093711354 | 0.042954884 | -0.11770076 | 0.010909883 |
| GPAM | lightyellow | 0.064503024 | 0.164032644 | 0.011936671 | 0.796969187 |
| GPD2 | lightyellow | 0.053252368 | 0.250752148 | 0.042587872 | 0.358471297 |
| GPN3 | lightyellow | -0.086052096 | 0.06315969 | -0.09341019 | 0.043631408 |
| GPR180 | lightyellow | 0.090283049 | 0.051201404 | 0.079093026 | 0.087764525 |
| GRPEL2 | lightyellow | -0.005941171 | 0.898111902 | -0.026014824 | 0.574952034 |
| GRSF1 | lightyellow | 0.112710042 | 0.01481268 | 0.085784476 | 0.063987678 |
| GSKIP | lightyellow | 0.220338533 | 1.52E-06 | 0.21809703 | 1.96E-06 |
| GSPT1 | lightyellow | 0.169680718 | 0.000229827 | 0.149654729 | 0.001179945 |
| GSTA4 | lightyellow | 0.159859186 | 0.000525024 | 0.130079025 | 0.004870605 |
| GTF2A1 | lightyellow | -0.01695094 | 0.714844591 | -0.040894831 | 0.377914577 |
| GTF2E1 | lightyellow | -0.025615865 | 0.580831474 | -0.040216389 | 0.385884351 |
| GTF2H1 | lightyellow | -0.012520386 | 0.787273125 | -0.016376539 | 0.72410701 |
| GTF2H3 | lightyellow | 0.043530523 | 0.347922547 | 0.017488826 | 0.706208756 |
| GTPBP10 | lightyellow | -0.064658907 | 0.163013965 | -0.088417125 | 0.056219482 |
| GTPBP8 | lightyellow | -0.128047113 | 0.005586358 | -0.137558962 | 0.002893034 |
| GUF1 | lightyellow | 0.19272261 | 2.75E-05 | 0.17872163 | 0.000103106 |
| GYG1 | lightyellow | -0.019960018 | 0.667030873 | -0.008324727 | 0.857608047 |
| HACL1 | lightyellow | 0.102657455 | 0.026529974 | 0.122773941 | 0.007904729 |
| HADHB | lightyellow | 0.145387536 | 0.001631672 | 0.151441183 | 0.001027617 |
| HAT1 | lightyellow | -0.004955634 | 0.914943032 | -0.024796523 | 0.59299322 |
| HAUS2 | lightyellow | 0.166663032 | 0.000297696 | 0.131917351 | 0.004295458 |
| HAUS4 | lightyellow | 0.158814542 | 0.000571693 | 0.115591822 | 0.012431606 |
| HAUS6 | lightyellow | -0.090164551 | 0.05150857 | -0.125718111 | 0.006522054 |
| HBS1L | lightyellow | -0.024608943 | 0.595793812 | -0.045252613 | 0.329165363 |
| HDAC2 | lightyellow | -0.049841273 | 0.282437132 | -0.070094312 | 0.130393422 |
| HDGFRP | lightyellow | -0.193077085 | 2.66E-05 | -0.204125626 | 8.74E-06 |
| HEATR3 | lightyellow | 0.314821618 | 3.33E-12 | 0.323002395 | 8.42E-13 |
| HELQ | lightyellow | -0.048116794 | 0.299443676 | -0.069472601 | 0.133846691 |
| HEXB | lightyellow | 0.265145348 | 5.92E-09 | 0.2577958 | 1.59E-08 |
| HEXDC | lightyellow | 0.062939666 | 0.174514742 | 0.062365997 | 0.178483607 |
| HIAT1 | lightyellow | -0.036880326 | 0.426540669 | -0.072294209 | 0.118725342 |

| | | | | | |
|---|---|---|---|---|---|
| HIBADH | lightyellow | -0.101729322 | 0.027934633 | -0.078299369 | 0.091004124 |
| HIBCH | lightyellow | 0.069438074 | 0.134040517 | 0.071799399 | 0.12127594 |
| HIGD1A | lightyellow | 0.012473631 | 0.78804857 | 0.025724022 | 0.579234792 |
| HINT3 | lightyellow | 0.159125393 | 0.000557419 | 0.142572539 | 0.002011333 |
| HLX | lightyellow | -0.00709056 | 0.878540571 | 0.041981211 | 0.365365044 |
| HMGCR | lightyellow | 0.225513322 | 8.48E-07 | 0.217312918 | 2.13E-06 |
| HMGCS1 | lightyellow | 0.068760994 | 0.137885366 | 0.028758054 | 0.535304187 |
| HNRNPA | lightyellow | -0.044661285 | 0.33553117 | -0.067577293 | 0.144809909 |
| HNRNPD | lightyellow | 0.043921477 | 0.343605889 | -0.007634927 | 0.869296299 |
| HNRNPH | lightyellow | -0.068486606 | 0.139467411 | -0.083506392 | 0.071403574 |
| HNRNPH | lightyellow | -0.151352654 | 0.001034717 | -0.180737585 | 8.58E-05 |
| HNRNPL | lightyellow | -0.023441332 | 0.613359862 | -0.048224467 | 0.298362291 |
| HNRNPR | lightyellow | -0.00834042 | 0.857342478 | -0.043835925 | 0.344547571 |
| HNRNPU | lightyellow | 0.068522156 | 0.139261658 | 0.035544257 | 0.443496905 |
| HOMER1 | lightyellow | 0.014247916 | 0.758774492 | -0.021938947 | 0.636290949 |
| HOOK1 | lightyellow | 0.065991193 | 0.154500826 | 0.039178894 | 0.398268082 |
| HS2ST1 | lightyellow | 0.033945671 | 0.464280185 | 0.006435277 | 0.889690174 |
| HSD17B1 | lightyellow | 0.120497226 | 0.009147498 | 0.104327737 | 0.024154983 |
| HSF2 | lightyellow | -0.089470087 | 0.053339789 | -0.113547476 | 0.014082556 |
| HSP90AA | lightyellow | 0.095394781 | 0.039334019 | 0.099025043 | 0.032397201 |
| HSPA13 | lightyellow | -0.078246665 | 0.091222605 | -0.106099516 | 0.02183872 |
| HSPA4L | lightyellow | 0.174553062 | 0.000149946 | 0.180904128 | 8.45E-05 |
| HSPH1 | lightyellow | -0.019461111 | 0.674872087 | -0.030796409 | 0.506760742 |
| HTATSF1 | lightyellow | -0.096277045 | 0.037541837 | -0.124799556 | 0.006928161 |
| HUS1 | lightyellow | 0.019693228 | 0.671219513 | 0.031034882 | 0.50347425 |
| IDE | lightyellow | 0.331970418 | 1.78E-13 | 0.312015971 | 5.29E-12 |
| IDH3G | lightyellow | 0.085395615 | 0.06520668 | 0.12991766 | 0.004924265 |
| IDI1 | lightyellow | 0.161933025 | 0.000442681 | 0.12198302 | 0.008318238 |
| IDUA | lightyellow | 0.08503688 | 0.066348109 | 0.090249624 | 0.051287893 |
| IFRD1 | lightyellow | -0.120776986 | 0.008985953 | -0.122825969 | 0.007878183 |
| IFT20 | lightyellow | -0.032389984 | 0.485014072 | -0.041419538 | 0.371820609 |
| IFT52 | lightyellow | -0.22842295 | 6.06E-07 | -0.234707648 | 2.89E-07 |
| IFT81 | lightyellow | -0.042461423 | 0.359901417 | -0.062758732 | 0.175759358 |
| IKBIP | lightyellow | -0.138123266 | 0.00277866 | -0.127038934 | 0.005975481 |
| IL13RA1 | lightyellow | -0.059105638 | 0.202317937 | -0.081388637 | 0.078913447 |
| IMMP2L | lightyellow | -0.124866251 | 0.00689793 | -0.160990151 | 0.000478504 |
| IMPACT | lightyellow | -0.086620377 | 0.061430741 | -0.107261204 | 0.020426817 |
| ING2 | lightyellow | 0.047087281 | 0.309914703 | 0.043587035 | 0.347296464 |
| INSIG1 | lightyellow | 0.284739001 | 3.68E-10 | 0.257695201 | 1.61E-08 |
| INSIG2 | lightyellow | -0.054751129 | 0.237643661 | -0.064247103 | 0.165715412 |
| INTS12 | lightyellow | 0.111618456 | 0.01581418 | 0.095695436 | 0.038715319 |
| INTS4 | lightyellow | 0.048604331 | 0.294568047 | 0.010199628 | 0.826002894 |
| IPMK | lightyellow | 0.13942984 | 0.002529477 | 0.126857998 | 0.006047838 |
| IPO11 | lightyellow | 0.091776129 | 0.047460364 | 0.077033591 | 0.096368483 |
| IPO5 | lightyellow | 0.061452425 | 0.184941623 | 0.01588985 | 0.731986734 |

| | | | | | |
|---|---|---|---|---|---|
| IPO7 | lightyellow | 0.158149295 | 0.00060339 | 0.115117591 | 0.012798556 |
| IPO8 | lightyellow | 0.013808996 | 0.765986052 | -0.016113619 | 0.728360246 |
| IREB2 | lightyellow | 0.074850253 | 0.106211219 | 0.059093583 | 0.202410157 |
| ITFG1 | lightyellow | 0.064486544 | 0.164140615 | 0.05941593 | 0.19995471 |
| IWS1 | lightyellow | -0.048625133 | 0.294361201 | -0.078856788 | 0.088718975 |
| JKAMP | lightyellow | 0.168054203 | 0.000264366 | 0.15946364 | 0.000542262 |
| KANSL2 | lightyellow | -0.127533057 | 0.005781824 | -0.15553741 | 0.000744264 |
| KATNA1 | lightyellow | -0.151078208 | 0.001057015 | -0.153482292 | 0.000875886 |
| KCMF1 | lightyellow | -0.033462067 | 0.47067238 | -0.031784661 | 0.49321477 |
| KDELC1 | lightyellow | -0.08927056 | 0.053875833 | -0.114314122 | 0.013442102 |
| KDSR | lightyellow | -0.102058374 | 0.027429493 | -0.117722079 | 0.010895383 |
| KHDRBS | lightyellow | -0.051737489 | 0.264504478 | -0.074750078 | 0.106681209 |
| KIAA002 | lightyellow | 0.025394079 | 0.584112003 | 0.006586259 | 0.887119216 |
| KIAA114 | lightyellow | 0.033031291 | 0.476406833 | 0.003285382 | 0.943550856 |
| KIAA119 | lightyellow | 0.052492426 | 0.257587562 | 0.033285759 | 0.473014774 |
| KIAA127 | lightyellow | 0.152720579 | 0.000929908 | 0.132218378 | 0.004207358 |
| KIAA158 | lightyellow | -0.066540806 | 0.151088624 | -0.086555132 | 0.061627235 |
| KIAA159 | lightyellow | 0.231263843 | 4.35E-07 | 0.220010594 | 1.58E-06 |
| KIAA171 | lightyellow | 0.197943241 | 1.64E-05 | 0.18567023 | 5.42E-05 |
| KIF5B | lightyellow | 0.148608069 | 0.001278592 | 0.120932774 | 0.0088971 |
| KLHL23 | lightyellow | -0.133490658 | 0.003852774 | -0.150021094 | 0.001147107 |
| KLHL7 | lightyellow | 0.015889944 | 0.731985208 | 0.000233337 | 0.995987511 |
| KLHL8 | lightyellow | 0.128848586 | 0.005293444 | 0.088366454 | 0.056361254 |
| KPNA3 | lightyellow | 0.091159997 | 0.04897541 | 0.069747741 | 0.132309837 |
| KPNB1 | lightyellow | 0.153906268 | 0.000847088 | 0.117897704 | 0.010776574 |
| KRAS | lightyellow | 0.011624339 | 0.80217029 | 0.00203176 | 0.965072527 |
| KRCC1 | lightyellow | -0.104935619 | 0.023337433 | -0.13108876 | 0.004546633 |
| KRR1 | lightyellow | -0.094495188 | 0.041235429 | -0.126991361 | 0.00599443 |
| KTN1 | lightyellow | -0.048570894 | 0.294900734 | -0.07151893 | 0.122740534 |
| LAMP2 | lightyellow | 0.14443326 | 0.001752306 | 0.122631901 | 0.007977611 |
| LARP1B | lightyellow | 0.094314392 | 0.041626789 | 0.086162861 | 0.062819585 |
| LARS | lightyellow | 0.128212335 | 0.005524808 | 0.077487529 | 0.094416408 |
| LBR | lightyellow | -0.044078454 | 0.341882289 | -0.076636112 | 0.098104076 |
| LCAT | lightyellow | -0.014759475 | 0.750395808 | -0.010069943 | 0.828180585 |
| LEO1 | lightyellow | 0.278197106 | 9.54E-10 | 0.274225889 | 1.68E-09 |
| LGALSL | lightyellow | 0.085037785 | 0.066345211 | 0.063475747 | 0.170865646 |
| LGR4 | lightyellow | 0.085311025 | 0.065474364 | 0.084380766 | 0.068478175 |
| LHB | lightyellow | 0.074850386 | 0.106210596 | 0.089642744 | 0.052879524 |
| LHPP | lightyellow | 0.054617172 | 0.23879526 | 0.051230783 | 0.269217997 |
| LIN52 | lightyellow | -0.076693036 | 0.097854002 | -0.11362215 | 0.014019015 |
| LIN54 | lightyellow | 0.025954869 | 0.575833791 | 0.027525982 | 0.552940391 |
| LMBR1 | lightyellow | 0.037411813 | 0.419901682 | -0.009155437 | 0.843573548 |
| LMF1 | lightyellow | 0.206782555 | 6.62E-06 | 0.235746221 | 2.55E-07 |
| LRIF1 | lightyellow | -0.2289497 | 5.70E-07 | -0.234499637 | 2.97E-07 |
| LRPPRC | lightyellow | -0.0615761 | 0.184057422 | -0.085018146 | 0.066408166 |

| | | | | | |
|---|---|---|---|---|---|
| LRRC40 | lightyellow | 0.005387631 | 0.907560208 | -0.022577449 | 0.62650122 |
| LRRC57 | lightyellow | 0.200026474 | 1.33E-05 | 0.180945933 | 8.41E-05 |
| LRRC75E | lightyellow | 0.190696954 | 3.35E-05 | 0.161731939 | 0.000450104 |
| LRRC8B | lightyellow | 0.12846351 | 0.005432406 | 0.099044952 | 0.032362244 |
| LRRCC1 | lightyellow | 0.007860172 | 0.865476393 | -0.026127043 | 0.573303336 |
| LRSAM1 | lightyellow | 0.096842805 | 0.036429419 | 0.085341123 | 0.065379019 |
| LTA4H | lightyellow | -0.121984919 | 0.008317222 | -0.155698483 | 0.000734764 |
| LYRM2 | lightyellow | -0.00520653 | 0.910654286 | -0.008049044 | 0.862275738 |
| LZIC | lightyellow | -0.007358705 | 0.873984877 | -0.036520535 | 0.431069363 |
| MAGT1 | lightyellow | -0.02743517 | 0.554251453 | -0.035947138 | 0.438343878 |
| MANBA | lightyellow | 0.041150654 | 0.374935813 | 0.023659493 | 0.610060472 |
| MAP2K1 | lightyellow | 0.117905818 | 0.010771114 | 0.097014543 | 0.036097332 |
| MAPK1IF | lightyellow | 0.060559916 | 0.191415594 | 0.057119373 | 0.217927376 |
| MAPK9 | lightyellow | 0.131566003 | 0.004400391 | 0.115660051 | 0.012379584 |
| MAPRE1 | lightyellow | -0.167696292 | 0.000272591 | -0.190347395 | 3.47E-05 |
| 5-Mar | lightyellow | 0.199729071 | 1.37E-05 | 0.180200444 | 9.01E-05 |
| 7-Mar | lightyellow | -0.101960545 | 0.027578847 | -0.127351034 | 0.005852496 |
| MARCKS | lightyellow | -0.066099722 | 0.153822455 | -0.071602838 | 0.122300934 |
| MAT2B | lightyellow | -0.027688537 | 0.550597369 | -0.031970543 | 0.490688662 |
| MAX | lightyellow | -0.189922695 | 3.61E-05 | -0.184204015 | 6.22E-05 |
| MBD4 | lightyellow | -0.102845023 | 0.026253614 | -0.13196996 | 0.004279942 |
| MBIP | lightyellow | -0.047600463 | 0.304665435 | -0.054641953 | 0.23858193 |
| MC1R | lightyellow | -0.000345852 | 0.994052713 | 3.15E-05 | 0.999458012 |
| MCFD2 | lightyellow | -0.12858871 | 0.005386869 | -0.154616267 | 0.000800815 |
| MCL1 | lightyellow | -0.052670152 | 0.255977549 | -0.065396481 | 0.158258407 |
| MCOLN1 | lightyellow | 0.059634257 | 0.198303988 | 0.076805387 | 0.097361918 |
| MCU | lightyellow | 0.027958102 | 0.54672269 | 0.043447822 | 0.348840067 |
| ME2 | lightyellow | 0.185452816 | 5.53E-05 | 0.171967002 | 0.000188363 |
| MED17 | lightyellow | 0.01008199 | 0.827978236 | -0.018713939 | 0.686681083 |
| MED21 | lightyellow | -0.031767545 | 0.493447725 | -0.056984966 | 0.219013976 |
| MESDC2 | lightyellow | 0.311817456 | 5.46E-12 | 0.283529884 | 4.40E-10 |
| METAP2 | lightyellow | 0.067555452 | 0.144940125 | 0.033695132 | 0.467585742 |
| METTL1( | lightyellow | -0.089159156 | 0.054177071 | -0.106145889 | 0.021780777 |
| METTL1⁴ | lightyellow | -0.016841224 | 0.716610623 | -0.028384237 | 0.540625108 |
| METTL2] | lightyellow | -0.023733789 | 0.608938647 | -0.033754516 | 0.466801067 |
| METTL2/ | lightyellow | 0.097416226 | 0.035330634 | 0.094954671 | 0.040254773 |
| METTL6 | lightyellow | -0.127864381 | 0.005655149 | -0.149369398 | 0.001206118 |
| METTL8 | lightyellow | -0.100728586 | 0.029520307 | -0.115069846 | 0.012836024 |
| METTL9 | lightyellow | 0.019638678 | 0.672077214 | 0.007731795 | 0.867653159 |
| MFAP1 | lightyellow | 0.123274263 | 0.007652724 | 0.104451039 | 0.02398718 |
| MFHAS1 | lightyellow | 0.104321445 | 0.024163573 | 0.067922671 | 0.142762584 |
| MFSD1 | lightyellow | -0.048136321 | 0.299247365 | -0.060154072 | 0.194413815 |
| MFSD11 | lightyellow | 0.016938709 | 0.715041393 | 0.005343552 | 0.908313165 |
| MIB2 | lightyellow | -0.072323671 | 0.118574807 | -0.072791291 | 0.116205434 |
| MICALL2 | lightyellow | 0.120486328 | 0.009153843 | 0.155371104 | 0.000754193 |

| | | | | | |
|---|---|---|---|---|---|
| MICU1 | lightyellow | 0.03507437 | 0.449550329 | 0.037971205 | 0.412979927 |
| MINA | lightyellow | -0.125486771 | 0.00662227 | -0.127461394 | 0.005809556 |
| MINPP1 | lightyellow | 0.126814117 | 0.006065505 | 0.093576637 | 0.043256406 |
| MIOS | lightyellow | 0.127882672 | 0.00564823 | 0.097222009 | 0.035699593 |
| MIS12 | lightyellow | -0.092951995 | 0.044677871 | -0.121871326 | 0.008378162 |
| MKRN2 | lightyellow | -0.063403436 | 0.171354518 | -0.080113346 | 0.083736649 |
| MLLT11 | lightyellow | -0.141660502 | 0.002150687 | -0.144541531 | 0.00173822 |
| MMADH( | lightyellow | -0.022907106 | 0.621472226 | -0.044662583 | 0.335517116 |
| MMD | lightyellow | -0.093104278 | 0.044327757 | -0.129611916 | 0.005027401 |
| MOB1A | lightyellow | -0.048441221 | 0.296193296 | -0.05157276 | 0.266030566 |
| MPHOSP | lightyellow | 0.188398053 | 4.18E-05 | 0.188333119 | 4.21E-05 |
| MPHOSP | lightyellow | 0.047948236 | 0.301141747 | 0.046666734 | 0.314260556 |
| MPP5 | lightyellow | 0.092382107 | 0.046008798 | 0.067710196 | 0.144019451 |
| MRFAP1 | lightyellow | 0.121317943 | 0.008680769 | 0.112321038 | 0.015163003 |
| MRPL19 | lightyellow | 0.114879965 | 0.012985994 | 0.094573061 | 0.041067822 |
| MRPL30 | lightyellow | 0.102342729 | 0.026999316 | 0.093203227 | 0.044101503 |
| MRPL42 | lightyellow | 0.075912941 | 0.101325562 | 0.056571702 | 0.222379259 |
| MRPS23 | lightyellow | -0.050743005 | 0.273809418 | -0.055692448 | 0.229661591 |
| MRPS27 | lightyellow | 0.302015385 | 2.64E-11 | 0.274978574 | 1.51E-09 |
| MRPS30 | lightyellow | -0.033011326 | 0.476673521 | -0.046745074 | 0.313447991 |
| MRPS35 | lightyellow | 0.052937358 | 0.253570084 | 0.041268106 | 0.373573073 |
| MRS2 | lightyellow | -0.051517417 | 0.266544634 | -0.056136134 | 0.225965943 |
| MSH3 | lightyellow | 0.059043882 | 0.202790695 | 0.026404691 | 0.569233841 |
| MSH6 | lightyellow | -0.212209883 | 3.72E-06 | -0.240549992 | 1.43E-07 |
| MSL1 | lightyellow | 0.060739095 | 0.190102729 | 0.013384112 | 0.772986313 |
| MSL3 | lightyellow | 0.028039446 | 0.545556131 | 0.037969324 | 0.413003099 |
| MSMO1 | lightyellow | 0.234228983 | 3.06E-07 | 0.194809469 | 2.24E-05 |
| MTAP | lightyellow | -0.111890762 | 0.015558944 | -0.124577378 | 0.007029729 |
| MTFMT | lightyellow | 0.038368796 | 0.408101477 | 0.028346506 | 0.541163633 |
| MTHFD2 | lightyellow | 0.069687348 | 0.132646005 | 0.080934001 | 0.080606389 |
| MTMR2 | lightyellow | 0.042455343 | 0.359970276 | 0.021960082 | 0.635965878 |
| MTO1 | lightyellow | -0.009555654 | 0.836829412 | -0.019083586 | 0.680829094 |
| MTPN | lightyellow | -0.074518861 | 0.107772282 | -0.09664593 | 0.036813304 |
| MTRF1L | lightyellow | -0.141882507 | 0.002115977 | -0.14718767 | 0.001424615 |
| MTX2 | lightyellow | -0.003346593 | 0.942500945 | -0.01361622 | 0.769159846 |
| MUT | lightyellow | 0.066834179 | 0.149290842 | 0.038559695 | 0.405771358 |
| MYO1B | lightyellow | 0.004173334 | 0.928330756 | -0.007989933 | 0.863277211 |
| MZF1 | lightyellow | 0.107309191 | 0.020370241 | 0.105796119 | 0.022221122 |
| NAA15 | lightyellow | 0.01828613 | 0.693477279 | -0.006038202 | 0.896457141 |
| NAA30 | lightyellow | -0.131481226 | 0.004426055 | -0.139043481 | 0.002600952 |
| NAA50 | lightyellow | -0.085875086 | 0.063706347 | -0.104154456 | 0.024392517 |
| NADK2 | lightyellow | -0.15955507 | 0.000538231 | -0.183318348 | 6.76E-05 |
| NADSYN | lightyellow | 0.28839637 | 2.14E-10 | 0.309075474 | 8.54E-12 |
| NAE1 | lightyellow | 0.156069216 | 0.000713323 | 0.147293631 | 0.001413215 |
| NAF1 | lightyellow | -0.035234378 | 0.447483757 | -0.051207941 | 0.269431823 |

| | | | | | |
|---|---|---|---|---|---|
| NAMPT | lightyellow | -0.057412682 | 0.215569561 | -0.071687771 | 0.121857217 |
| NARS | lightyellow | 0.122662375 | 0.007961924 | 0.11628175 | 0.011914342 |
| NARS2 | lightyellow | -0.030161595 | 0.515563866 | -0.072047028 | 0.119994198 |
| NCK1 | lightyellow | -0.054727393 | 0.23784743 | -0.066790904 | 0.149555001 |
| NCOA5 | lightyellow | -0.088533504 | 0.055894993 | -0.120740424 | 0.00900692 |
| NDFIP2 | lightyellow | 0.088888199 | 0.0549156 | 0.076376717 | 0.099250086 |
| NDST2 | lightyellow | -0.065018684 | 0.160681035 | -0.096887054 | 0.036343608 |
| NECAP1 | lightyellow | 0.082075042 | 0.076412195 | 0.073947386 | 0.110506767 |
| NEDD1 | lightyellow | -0.018515853 | 0.689824797 | -0.056648918 | 0.221747685 |
| NET1 | lightyellow | 0.22472393 | 9.29E-07 | 0.192858152 | 2.72E-05 |
| NFIL3 | lightyellow | -0.181679574 | 7.86E-05 | -0.200875908 | 1.22E-05 |
| NGLY1 | lightyellow | 0.198950448 | 1.48E-05 | 0.187968456 | 4.36E-05 |
| NGRN | lightyellow | -0.020494164 | 0.658675825 | -0.052603087 | 0.256584268 |
| NINJ1 | lightyellow | 0.068262296 | 0.140771039 | 0.091733312 | 0.047564364 |
| NIP7 | lightyellow | 0.032570663 | 0.482580641 | 0.030475039 | 0.511207399 |
| NKIRAS1 | lightyellow | -0.090105922 | 0.051661113 | -0.126614031 | 0.006146647 |
| NKRF | lightyellow | -0.052537865 | 0.257175271 | -0.0855781 | 0.06463227 |
| NLK | lightyellow | 0.017726653 | 0.702402314 | -0.023438456 | 0.613403409 |
| NLN | lightyellow | 0.211799998 | 3.89E-06 | 0.185344189 | 5.59E-05 |
| NMD3 | lightyellow | 0.007223133 | 0.876287675 | -0.027992373 | 0.546231055 |
| NMNAT1 | lightyellow | 0.205127778 | 7.87E-06 | 0.193028813 | 2.67E-05 |
| NNT | lightyellow | 0.051704812 | 0.264806729 | 0.047987105 | 0.300749617 |
| NOC3L | lightyellow | -0.03558509 | 0.442973071 | -0.074068371 | 0.109923347 |
| NOL11 | lightyellow | -0.098708209 | 0.032957864 | -0.132245416 | 0.004199526 |
| NPEPPS | lightyellow | 0.027374278 | 0.555131406 | -0.003450543 | 0.940718167 |
| NPIPB3 | lightyellow | 0.062772021 | 0.175667725 | 0.071762138 | 0.121469728 |
| NPIPB5 | lightyellow | 0.076903842 | 0.096932322 | 0.103075344 | 0.025917657 |
| NR1H2 | lightyellow | 0.071398898 | 0.123371525 | 0.084562107 | 0.067883926 |
| NRAS | lightyellow | -0.122785079 | 0.00789904 | -0.122718465 | 0.007933123 |
| NRBF2 | lightyellow | -0.096098006 | 0.037899817 | -0.115440047 | 0.012548023 |
| NSRP1 | lightyellow | -0.007072834 | 0.878841856 | -0.048633699 | 0.294276059 |
| NT5DC1 | lightyellow | 0.178335988 | 0.000106779 | 0.144527079 | 0.001740094 |
| NUDT21 | lightyellow | 0.08961449 | 0.052954617 | 0.068841419 | 0.137424279 |
| NUDT4 | lightyellow | 0.070518783 | 0.128075575 | 0.042286991 | 0.361880078 |
| NUFIP1 | lightyellow | -0.0391746 | 0.398319835 | -0.067070574 | 0.147854099 |
| NUP50 | lightyellow | 0.0862993 | 0.062402727 | 0.050837724 | 0.272913689 |
| NUP54 | lightyellow | -0.042394224 | 0.360662881 | -0.071594155 | 0.122346367 |
| NUPL1 | lightyellow | 0.103328109 | 0.025553233 | 0.065040701 | 0.160539085 |
| NUS1 | lightyellow | -0.048870838 | 0.291925387 | -0.073038758 | 0.114966643 |
| NXT2 | lightyellow | 0.055147292 | 0.234260846 | 0.026380383 | 0.569589573 |
| OGFOD1 | lightyellow | 0.301763325 | 2.75E-11 | 0.288343694 | 2.16E-10 |
| OLA1 | lightyellow | -0.013860837 | 0.765133218 | -0.049298179 | 0.287721145 |
| ORC5 | lightyellow | 0.086082626 | 0.063065795 | 0.074640604 | 0.107196705 |
| OSBP | lightyellow | 0.149500044 | 0.001194069 | 0.129921876 | 0.004922856 |
| OSBPL3 | lightyellow | -0.096078341 | 0.037939313 | -0.087691355 | 0.058278548 |

| | | | | | |
|---|---|---|---|---|---|
| OXCT1 | lightyellow | 0.097953052 | 0.03432765 | 0.085017597 | 0.066409928 |
| OXSR1 | lightyellow | 0.044661355 | 0.335530413 | 0.004295252 | 0.926242919 |
| P2RX4 | lightyellow | 0.227400548 | 6.83E-07 | 0.228352966 | 6.11E-07 |
| P4HA1 | lightyellow | 0.128758823 | 0.005325547 | 0.108553018 | 0.018950586 |
| PACRGL | lightyellow | -0.01636098 | 0.724358465 | -0.02645455 | 0.568504513 |
| PAFAH1I | lightyellow | 0.10474843 | 0.023586597 | 0.08385379 | 0.070229285 |
| PAIP1 | lightyellow | -0.07316449 | 0.114341218 | -0.096359874 | 0.037377197 |
| PAIP2 | lightyellow | -0.008727496 | 0.850797542 | -0.027531579 | 0.552859634 |
| PANK1 | lightyellow | 0.208954406 | 5.27E-06 | 0.171323171 | 0.000199269 |
| PANK3 | lightyellow | -0.002442006 | 0.95802604 | -0.032520434 | 0.483256474 |
| PANX1 | lightyellow | -0.114519185 | 0.013275202 | -0.134112388 | 0.003689538 |
| PAPD5 | lightyellow | 0.156321342 | 0.000699073 | 0.144738391 | 0.001712872 |
| PAPOLA | lightyellow | -0.021979984 | 0.635659834 | -0.060065063 | 0.195075946 |
| PARP16 | lightyellow | 0.116629581 | 0.011660845 | 0.098796097 | 0.032801515 |
| PBRM1 | lightyellow | 0.082982308 | 0.073205413 | 0.0584144 | 0.207655301 |
| PCGF6 | lightyellow | 0.098225089 | 0.033828713 | 0.065198542 | 0.15952423 |
| PCID2 | lightyellow | -0.135701071 | 0.003300509 | -0.15891301 | 0.000567135 |
| PCMT1 | lightyellow | -0.072165668 | 0.119383866 | -0.055872109 | 0.228159988 |
| PDCD10 | lightyellow | -0.182727118 | 7.14E-05 | -0.186875323 | 4.84E-05 |
| PDCD7 | lightyellow | -0.036191797 | 0.435231392 | -0.04545916 | 0.32696034 |
| PDE12 | lightyellow | 0.162109726 | 0.000436251 | 0.1672881 | 0.000282263 |
| PDHX | lightyellow | 0.263597916 | 7.31E-09 | 0.258729552 | 1.40E-08 |
| PDIK1L | lightyellow | 0.097029742 | 0.036068067 | 0.077230876 | 0.095516178 |
| PDK1 | lightyellow | 0.11034829 | 0.017053824 | 0.099393755 | 0.031755009 |
| PDS5A | lightyellow | 0.20824405 | 5.68E-06 | 0.180761597 | 8.56E-05 |
| PDSS2 | lightyellow | 0.174284864 | 0.000153559 | 0.16465341 | 0.00035282 |
| PDXDC1 | lightyellow | 0.159561821 | 0.000537935 | 0.173311901 | 0.000167361 |
| PDZD8 | lightyellow | 0.302610804 | 2.40E-11 | 0.279662742 | 7.72E-10 |
| PEX13 | lightyellow | 0.115297847 | 0.012657967 | 0.114011315 | 0.013691935 |
| PEX3 | lightyellow | -0.125434671 | 0.00664503 | -0.135151004 | 0.003430778 |
| PFN2 | lightyellow | -0.136584013 | 0.003100773 | -0.126827721 | 0.006060023 |
| PGM2 | lightyellow | 0.158730184 | 0.000575625 | 0.113127456 | 0.0144447 |
| PGM3 | lightyellow | 0.199346343 | 1.42E-05 | 0.189459141 | 3.78E-05 |
| PGRMC1 | lightyellow | -0.082146238 | 0.07615649 | -0.092058401 | 0.046779501 |
| PGRMC2 | lightyellow | 0.114496727 | 0.013293391 | 0.11734627 | 0.011153518 |
| PHAX | lightyellow | 0.050772327 | 0.27353191 | 0.033491976 | 0.470275645 |
| PHF10 | lightyellow | -0.077295907 | 0.095236556 | -0.112499848 | 0.015001079 |
| PHF13 | lightyellow | -0.031053809 | 0.503213889 | -0.073000649 | 0.115156735 |
| PHF14 | lightyellow | 0.013429369 | 0.772239776 | 0.000542291 | 0.99067486 |
| PHF20 | lightyellow | -0.124493899 | 0.007068234 | -0.151339239 | 0.001035796 |
| PHOSPH( | lightyellow | -0.104965637 | 0.023297689 | -0.10236876 | 0.026960228 |
| PHTF2 | lightyellow | -0.067319986 | 0.146349678 | -0.078178875 | 0.091504242 |
| PI4K2B | lightyellow | 0.228352697 | 6.11E-07 | 0.211983046 | 3.81E-06 |
| PIGA | lightyellow | 0.043862404 | 0.344255937 | 0.041441936 | 0.371561835 |
| PIGF | lightyellow | 0.085417922 | 0.065136241 | 0.071325255 | 0.123759906 |

| | | | | | |
|---|---|---|---|---|---|
| PIGK | lightyellow | -0.114129733 | 0.01359375 | -0.151506691 | 0.001022392 |
| PIGN | lightyellow | 0.227874615 | 6.46E-07 | 0.189722166 | 3.68E-05 |
| PIGW | lightyellow | 0.024994077 | 0.590050244 | 0.016617997 | 0.720208419 |
| PIK3CB | lightyellow | -0.023926097 | 0.606039156 | -0.024373167 | 0.599322473 |
| PITPNB | lightyellow | 0.010338067 | 0.823679688 | -0.006679114 | 0.885538646 |
| PLA2G12 | lightyellow | 0.066718155 | 0.149999877 | 0.074843261 | 0.106243967 |
| PLEKHA | lightyellow | 0.069535948 | 0.133491638 | 0.077930661 | 0.092541405 |
| PLOD2 | lightyellow | 0.078541556 | 0.090005544 | 0.079314716 | 0.086876375 |
| PLRG1 | lightyellow | 0.083829533 | 0.070310765 | 0.050890994 | 0.272410799 |
| PLS1 | lightyellow | 0.242702141 | 1.09E-07 | 0.242424515 | 1.13E-07 |
| PLS3 | lightyellow | 0.076075392 | 0.100594682 | 0.074257759 | 0.109014945 |
| PMM2 | lightyellow | 0.095635458 | 0.038838082 | 0.085728993 | 0.064160453 |
| PMPCB | lightyellow | 0.131425322 | 0.004443052 | 0.117717346 | 0.010898601 |
| PNN | lightyellow | -0.020423615 | 0.659776941 | -0.060354863 | 0.192926175 |
| PNPLA2 | lightyellow | -0.048199346 | 0.298614358 | -0.020813263 | 0.653704622 |
| PNPLA6 | lightyellow | 0.098410824 | 0.033491622 | 0.122833164 | 0.007874518 |
| PNPT1 | lightyellow | -0.118988546 | 0.010064012 | -0.135996994 | 0.003232301 |
| PNRC2 | lightyellow | 0.009990939 | 0.829507875 | -0.011510393 | 0.804069955 |
| POLE3 | lightyellow | 0.175948629 | 0.000132401 | 0.156981877 | 0.000662983 |
| POLR2C | lightyellow | 0.066185869 | 0.153285592 | 0.072376124 | 0.118307168 |
| POLR3F | lightyellow | -0.233360157 | 3.40E-07 | -0.237061141 | 2.18E-07 |
| POLR3G | lightyellow | 0.06021775 | 0.193941125 | 0.049914615 | 0.281728617 |
| PON2 | lightyellow | 0.130962711 | 0.004585984 | 0.105048915 | 0.023187736 |
| POT1 | lightyellow | -0.175228656 | 0.000141197 | -0.208851308 | 5.33E-06 |
| PPA1 | lightyellow | 0.125608531 | 0.006569353 | 0.114191631 | 0.013542676 |
| PPAPDC2 | lightyellow | 0.069671327 | 0.132735294 | 0.059342429 | 0.200512682 |
| PPAT | lightyellow | -0.011064293 | 0.811518204 | -0.03967438 | 0.392324372 |
| PPID | lightyellow | 0.155390507 | 0.000753028 | 0.132396772 | 0.004155923 |
| PPIG | lightyellow | -0.071739674 | 0.121586673 | -0.105540984 | 0.022547173 |
| PPIL4 | lightyellow | -0.097222779 | 0.035698122 | -0.115598753 | 0.012426313 |
| PPM1B | lightyellow | -0.025910409 | 0.576488075 | -0.038372465 | 0.408056626 |
| PPP1CB | lightyellow | -0.071887983 | 0.120816203 | -0.085118924 | 0.066085625 |
| PPP1R2 | lightyellow | -0.053798938 | 0.245914635 | -0.064335088 | 0.165135427 |
| PPP2R1B | lightyellow | 0.318527469 | 1.80E-12 | 0.303633489 | 2.04E-11 |
| PPP2R5C | lightyellow | -0.016251107 | 0.726135061 | -0.030691062 | 0.508216146 |
| PPP2R5E | lightyellow | 0.039905936 | 0.389565166 | 0.024955671 | 0.59062186 |
| PPP3R1 | lightyellow | -0.062842233 | 0.175184155 | -0.085670047 | 0.064344427 |
| PPTC7 | lightyellow | -0.100010714 | 0.030704771 | -0.105050133 | 0.023186132 |
| PRCP | lightyellow | -0.096149639 | 0.037796285 | -0.133113531 | 0.003954948 |
| PRDX3 | lightyellow | 0.238389943 | 1.86E-07 | 0.223708469 | 1.04E-06 |
| PRKAR2 | lightyellow | 0.102161961 | 0.027272107 | 0.09409145 | 0.042113687 |
| PRMT3 | lightyellow | 0.022713434 | 0.624424621 | -0.011717478 | 0.800618367 |
| PRMT6 | lightyellow | -0.182069325 | 7.59E-05 | -0.196072752 | 1.98E-05 |
| PRPF18 | lightyellow | -0.059246596 | 0.201241878 | -0.088322802 | 0.056483622 |

| | | | | | |
|---|---|---|---|---|---|
| PRPF38A | lightyellow | -0.108997255 | 0.018464873 | -0.137211482 | 0.002965575 |
| PRPF40A | lightyellow | -0.050893849 | 0.272383872 | -0.07907381 | 0.08784185 |
| PRPS2 | lightyellow | 0.104744409 | 0.023591974 | 0.094622882 | 0.040960894 |
| PRPSAP2 | lightyellow | 0.131100436 | 0.004543004 | 0.117110791 | 0.01131801 |
| PSEN1 | lightyellow | 0.043523494 | 0.348000466 | 0.036321853 | 0.433582053 |
| PSMA6 | lightyellow | -0.050499102 | 0.276125152 | -0.064776974 | 0.162245582 |
| PSMD10 | lightyellow | -0.02292569 | 0.621189242 | -0.017673773 | 0.703248036 |
| PSMD12 | lightyellow | 0.004827999 | 0.91712575 | -0.0048806 | 0.916226142 |
| PSME4 | lightyellow | -0.080893531 | 0.080758503 | -0.068056786 | 0.14197358 |
| PSMG4 | lightyellow | -0.018254416 | 0.69398209 | 0.021210207 | 0.647542023 |
| PSPC1 | lightyellow | 0.148116452 | 0.001327497 | 0.11601429 | 0.012112564 |
| PSPH | lightyellow | 0.169295052 | 0.000237612 | 0.16427649 | 0.000364164 |
| PTBP3 | lightyellow | 0.197267479 | 1.76E-05 | 0.181370222 | 8.09E-05 |
| PTCD2 | lightyellow | 0.049847305 | 0.282378817 | 0.017035043 | 0.713491863 |
| PTER | lightyellow | 0.110872498 | 0.016532271 | 0.096464269 | 0.037170563 |
| PTGES3 | lightyellow | 0.008733607 | 0.850694287 | -0.009437422 | 0.838820532 |
| PTP4A1 | lightyellow | 0.052581736 | 0.256777638 | 0.047803868 | 0.30260121 |
| PTP4A2 | lightyellow | -0.163611831 | 0.000385002 | -0.166397776 | 0.000304481 |
| PTPLB | lightyellow | 0.05338174 | 0.249601179 | 0.018615872 | 0.688236782 |
| PTPN11 | lightyellow | -0.006489143 | 0.888772801 | -0.036904498 | 0.426237415 |
| PWP1 | lightyellow | -0.02530702 | 0.585402075 | -0.048331375 | 0.297291181 |
| PXYLP1 | lightyellow | -0.069655225 | 0.132825078 | -0.105575364 | 0.022502997 |
| PYCARD | lightyellow | 0.010261048 | 0.824971994 | 0.075414344 | 0.103595149 |
| QRSL1 | lightyellow | 0.145488343 | 0.001619384 | 0.14258685 | 0.002009214 |
| R3HCC1L | lightyellow | 0.036112493 | 0.436238883 | 0.017223124 | 0.710470006 |
| RAB10 | lightyellow | 0.015839484 | 0.732803816 | 0.001427518 | 0.975455943 |
| RAB12 | lightyellow | -0.322121545 | 9.79E-13 | -0.344012007 | 2.03E-14 |
| RAB18 | lightyellow | 0.026350156 | 0.57003207 | 0.000246754 | 0.995756775 |
| RAB1A | lightyellow | 0.038890456 | 0.401752872 | 0.038934873 | 0.401215066 |
| RAB21 | lightyellow | -0.091362688 | 0.048472595 | -0.104944494 | 0.023325676 |
| RAB27B | lightyellow | 0.193109082 | 2.65E-05 | 0.165333674 | 0.000333175 |
| RAB28 | lightyellow | -0.033441245 | 0.470948679 | -0.053347788 | 0.249902884 |
| RAB32 | lightyellow | -0.238675513 | 1.79E-07 | -0.219456891 | 1.68E-06 |
| RAB3IP | lightyellow | -0.006702828 | 0.885135049 | -0.031005704 | 0.503875767 |
| RAB5B | lightyellow | -0.141570663 | 0.002164881 | -0.176820496 | 0.000122439 |
| RAB6A | lightyellow | -0.0232186 | 0.616736457 | -0.057068099 | 0.218341433 |
| RAB7A | lightyellow | -0.134895809 | 0.003492784 | -0.13855 | 0.00269492 |
| RAB9A | lightyellow | 0.000646307 | 0.988886321 | -0.000616867 | 0.989392531 |
| RABGGT | lightyellow | -0.091624067 | 0.04783058 | -0.130290736 | 0.004801002 |
| RABL3 | lightyellow | 0.040766536 | 0.379413878 | 0.015945702 | 0.731081013 |
| RAD1 | lightyellow | -0.056786195 | 0.220628026 | -0.073115838 | 0.114582909 |
| RAD51C | lightyellow | -0.014155166 | 0.760296679 | -0.020624266 | 0.656647158 |
| RALA | lightyellow | -0.103698566 | 0.025027114 | -0.109949651 | 0.017460022 |
| RALB | lightyellow | -0.092187875 | 0.046469948 | -0.119910354 | 0.009494803 |
| RANBP9 | lightyellow | -0.052585713 | 0.256741608 | -0.060821438 | 0.189501616 |

| | | | | | |
|---|---|---|---|---|---|
| RAP1A | lightyellow | -0.133606337 | 0.003821916 | -0.143095969 | 0.001935136 |
| RAP1GD! | lightyellow | 0.251880512 | 3.43E-08 | 0.232644233 | 3.70E-07 |
| RAP2A | lightyellow | -0.08287763 | 0.073569708 | -0.09907852 | 0.032303377 |
| RARS2 | lightyellow | -0.004004437 | 0.931223918 | -0.006259484 | 0.892685083 |
| RBBP8 | lightyellow | 0.031385691 | 0.49866007 | -0.004828368 | 0.917119433 |
| RBBP9 | lightyellow | 0.135775269 | 0.003283286 | 0.113965897 | 0.013729758 |
| RBM22 | lightyellow | -0.072169594 | 0.119363713 | -0.088992711 | 0.054629754 |
| RBM27 | lightyellow | -0.149624182 | 0.001182722 | -0.164483379 | 0.000357896 |
| RBM4 | lightyellow | -0.035190841 | 0.448045517 | -0.069825759 | 0.131876539 |
| RBM45 | lightyellow | 0.010862227 | 0.81489762 | -0.004612464 | 0.920813064 |
| RBM7 | lightyellow | 0.107686207 | 0.01993047 | 0.061747228 | 0.182839101 |
| RBMX | lightyellow | -0.05134391 | 0.268160706 | -0.094245495 | 0.041776748 |
| RCL1 | lightyellow | -0.123227994 | 0.007675724 | -0.14279581 | 0.001978502 |
| RCN2 | lightyellow | 0.039523487 | 0.394128735 | -0.003876627 | 0.933413881 |
| RDX | lightyellow | 0.077261132 | 0.095386001 | 0.059202435 | 0.20157855 |
| REEP3 | lightyellow | 0.12148088 | 0.008590675 | 0.103528507 | 0.025267458 |
| REXO2 | lightyellow | 0.186333 | 5.09E-05 | 0.143506335 | 0.001877256 |
| RFK | lightyellow | 0.173208915 | 0.000168888 | 0.149372733 | 0.001205809 |
| RFX5 | lightyellow | 0.021142556 | 0.648590638 | 0.014026755 | 0.76240564 |
| RFXAP | lightyellow | 0.032077675 | 0.489235908 | 0.010007535 | 0.829229021 |
| RGMB | lightyellow | 0.095283046 | 0.039566076 | 0.070990584 | 0.125536893 |
| RHEB | lightyellow | -0.053334025 | 0.250025251 | -0.095079771 | 0.039991215 |
| RHOA | lightyellow | 0.137332149 | 0.002940199 | 0.141245432 | 0.002216983 |
| RHOT1 | lightyellow | 0.084647893 | 0.067604284 | 0.058069156 | 0.210358895 |
| RIOK1 | lightyellow | -0.173718503 | 0.000161457 | -0.192905882 | 2.70E-05 |
| RIOK3 | lightyellow | -0.078874953 | 0.08864529 | -0.100317776 | 0.030193241 |
| RMI1 | lightyellow | 0.216685726 | 2.29E-06 | 0.197020201 | 1.80E-05 |
| RNF138 | lightyellow | -0.052575864 | 0.256830832 | -0.062526034 | 0.177369734 |
| RNF145 | lightyellow | 0.206789376 | 6.62E-06 | 0.191535688 | 3.09E-05 |
| RNF149 | lightyellow | 0.011222486 | 0.808874963 | -0.013170202 | 0.776517641 |
| RNF185 | lightyellow | -0.009569701 | 0.836592909 | -0.03269261 | 0.480941976 |
| RNF219 | lightyellow | -0.059155224 | 0.201938928 | -0.082442873 | 0.075098577 |
| RNFT1 | lightyellow | 0.170912793 | 0.000206526 | 0.157383217 | 0.000641907 |
| RNGTT | lightyellow | -0.041313501 | 0.373047198 | -0.067052307 | 0.147964744 |
| ROGDI | lightyellow | 0.122762008 | 0.007910829 | 0.166377617 | 0.000305002 |
| RP11-347 | lightyellow | 0.053440167 | 0.249082594 | 0.07797394 | 0.09235989 |
| RP11-571 | lightyellow | 0.079705695 | 0.085327645 | 0.05771463 | 0.213161497 |
| RPAP3 | lightyellow | -0.105905125 | 0.022083068 | -0.125799594 | 0.006487081 |
| RPE | lightyellow | -0.118212447 | 0.010566524 | -0.135896586 | 0.0032553 |
| RPL7L1 | lightyellow | -0.030100739 | 0.516411907 | -0.052237878 | 0.259905682 |
| RPRD1A | lightyellow | 0.039094737 | 0.399282952 | -0.010251349 | 0.825134769 |
| RPS6KB1 | lightyellow | -0.049740787 | 0.283409835 | -0.08032955 | 0.082902605 |
| RRAGC | lightyellow | -0.169535344 | 0.000232733 | -0.16867417 | 0.000250666 |
| RRAS | lightyellow | -0.28652744 | 2.83E-10 | -0.257221813 | 1.71E-08 |
| RRAS2 | lightyellow | -0.107861451 | 0.019728883 | -0.124546482 | 0.007043958 |

| | | | | | |
|---|---|---|---|---|---|
| RRM2B | lightyellow | 0.089059908 | 0.054446619 | 0.065445269 | 0.157947579 |
| RRN3 | lightyellow | 0.019241828 | 0.678329721 | 0.007164459 | 0.877284636 |
| RRP1B | lightyellow | -0.070487472 | 0.128245451 | -0.109702179 | 0.017716418 |
| RSU1 | lightyellow | 0.09863215 | 0.033093683 | 0.081935289 | 0.07691616 |
| RTCA | lightyellow | 0.055981151 | 0.227252026 | 0.059922114 | 0.196142777 |
| RTF1 | lightyellow | 0.204078598 | 8.78E-06 | 0.177171455 | 0.000118631 |
| RTN3 | lightyellow | 0.001246123 | 0.978573968 | -0.01327453 | 0.774794755 |
| RWDD2A | lightyellow | -0.016529167 | 0.721641829 | -0.036841676 | 0.427025829 |
| RWDD3 | lightyellow | -0.109288058 | 0.018152834 | -0.136246911 | 0.003175697 |
| SAR1A | lightyellow | 0.039762599 | 0.39127177 | 0.024564998 | 0.596450787 |
| SAV1 | lightyellow | -0.049852002 | 0.282333408 | -0.065758239 | 0.155964575 |
| SBDS | lightyellow | -0.176341612 | 0.00012782 | -0.189057878 | 3.93E-05 |
| SC5D | lightyellow | -0.019037471 | 0.681558119 | -0.066318306 | 0.152463027 |
| SCARB2 | lightyellow | 0.035671125 | 0.441870503 | 0.009264358 | 0.841736941 |
| SCFD1 | lightyellow | 0.043494193 | 0.348325419 | 0.037819943 | 0.414844923 |
| SCO1 | lightyellow | 0.200825666 | 1.23E-05 | 0.197593468 | 1.70E-05 |
| SCYL2 | lightyellow | 0.102917896 | 0.026146915 | 0.090556969 | 0.050497209 |
| SDCBP | lightyellow | -0.031697602 | 0.494400269 | -0.051753542 | 0.26435608 |
| SEC22B | lightyellow | 0.006668606 | 0.885717491 | -0.017897552 | 0.699671657 |
| SEC22C | lightyellow | 0.113202013 | 0.014379825 | 0.096066733 | 0.037962643 |
| SEC23IP | lightyellow | 0.107353377 | 0.020318268 | 0.078056802 | 0.092013154 |
| SEC62 | lightyellow | -0.212754174 | 3.51E-06 | -0.214843193 | 2.80E-06 |
| SEC63 | lightyellow | -0.055558485 | 0.230785801 | -0.0821344 | 0.076198958 |
| SEH1L | lightyellow | -0.023289236 | 0.615664744 | -0.062082537 | 0.180469225 |
| SELO | lightyellow | 0.216271127 | 2.39E-06 | 0.218745754 | 1.82E-06 |
| SELT | lightyellow | -0.010976144 | 0.812992005 | -0.019951708 | 0.66716118 |
| SEPT7 | lightyellow | -0.097539997 | 0.035097202 | -0.119995608 | 0.009443634 |
| SERBP1 | lightyellow | 0.018311927 | 0.693066772 | -0.011982652 | 0.796204239 |
| SERF1B | lightyellow | -0.104099354 | 0.024468473 | -0.122589274 | 0.007999599 |
| SERP1 | lightyellow | -0.022652439 | 0.625355709 | -0.009627501 | 0.835619952 |
| SET | lightyellow | 0.071633483 | 0.122140688 | 0.042127696 | 0.363692958 |
| SETD3 | lightyellow | -0.07781108 | 0.093044422 | -0.075271141 | 0.104254395 |
| SETD9 | lightyellow | 0.183847022 | 6.43E-05 | 0.189035911 | 3.93E-05 |
| SH3GLB1 | lightyellow | 0.175719859 | 0.000135138 | 0.175456242 | 0.000138359 |
| SHQ1 | lightyellow | 0.148181425 | 0.001320936 | 0.129763425 | 0.004976052 |
| SIX4 | lightyellow | 0.101320578 | 0.028573224 | 0.095579864 | 0.038952166 |
| SKAP2 | lightyellow | -0.020873988 | 0.652760319 | -0.023231025 | 0.616547871 |
| SKIV2L2 | lightyellow | 0.03863575 | 0.404845242 | 0.002930248 | 0.949644168 |
| SLC17A5 | lightyellow | 0.138001663 | 0.002802953 | 0.126875752 | 0.006040704 |
| SLC25A1 | lightyellow | 0.050238841 | 0.278610841 | 0.038384735 | 0.407906627 |
| SLC25A1 | lightyellow | 0.163681814 | 0.000382756 | 0.13370728 | 0.003795171 |
| SLC25A2 | lightyellow | -0.019567847 | 0.673191538 | -0.042764636 | 0.356478092 |
| SLC25A4 | lightyellow | 0.087616212 | 0.058495259 | 0.061520703 | 0.184453088 |
| SLC30A9 | lightyellow | 0.102990809 | 0.026040531 | 0.086599495 | 0.061493572 |
| SLC33A1 | lightyellow | -0.014244539 | 0.758829898 | -0.034816816 | 0.452888035 |

| | | | | | |
|---|---|---|---|---|---|
| SLC35A5 | lightyellow | 0.022245934 | 0.631576086 | 0.004295022 | 0.926246847 |
| SLC35B3 | lightyellow | -0.025732421 | 0.579110893 | -0.031341906 | 0.499259603 |
| SLC35G1 | lightyellow | 0.2506708 | 4.01E-08 | 0.190089333 | 3.56E-05 |
| SLC39A9 | lightyellow | 0.270537469 | 2.82E-09 | 0.25842853 | 1.46E-08 |
| SLC4A1A | lightyellow | -0.158089734 | 0.000606306 | -0.17711361 | 0.000119251 |
| SLC9A3R | lightyellow | 0.161439499 | 0.000461108 | 0.210841209 | 4.31E-06 |
| SLMO2 | lightyellow | -0.054016236 | 0.244009646 | -0.070535971 | 0.127982395 |
| SMAP1 | lightyellow | -0.141553772 | 0.002167559 | -0.148327976 | 0.001306248 |
| SMARCA | lightyellow | -0.082076833 | 0.076405755 | -0.113688361 | 0.013962886 |
| SMARCA | lightyellow | 0.030273549 | 0.514005639 | 0.008871534 | 0.848364633 |
| SMARCE | lightyellow | 0.13507782 | 0.003448457 | 0.104231262 | 0.024286984 |
| SMEK1 | lightyellow | -0.016607952 | 0.720370456 | -0.043552039 | 0.347684093 |
| SMEK2 | lightyellow | -0.053513085 | 0.24843643 | -0.07266343 | 0.116849575 |
| SMIM1 | lightyellow | -0.024222362 | 0.601584381 | -0.042135658 | 0.363602216 |
| SMNDC1 | lightyellow | -0.048282849 | 0.297777045 | -0.077006579 | 0.09648565 |
| SMU1 | lightyellow | -0.015300881 | 0.741560148 | -0.02116105 | 0.648303894 |
| SNAPC3 | lightyellow | 0.152816885 | 0.000922912 | 0.142562397 | 0.002012836 |
| SNRNP48 | lightyellow | 0.002817994 | 0.951570834 | -0.009850557 | 0.831867528 |
| SNX14 | lightyellow | 0.162133404 | 0.000435396 | 0.153253432 | 0.000891804 |
| SNX2 | lightyellow | -0.133191426 | 0.003933645 | -0.150438268 | 0.00111074 |
| SNX24 | lightyellow | -0.103985007 | 0.024626748 | -0.106449788 | 0.021404348 |
| SNX25 | lightyellow | 0.100145132 | 0.030479942 | 0.052007852 | 0.262012822 |
| SNX3 | lightyellow | -0.008478595 | 0.855004979 | -0.0054914 | 0.905787949 |
| SNX4 | lightyellow | -0.053174667 | 0.251445197 | -0.066489124 | 0.151407023 |
| SNX5 | lightyellow | -0.098588559 | 0.033171738 | -0.120435947 | 0.009183226 |
| SNX6 | lightyellow | -0.04127887 | 0.373448338 | -0.06435041 | 0.165034584 |
| SNX7 | lightyellow | 0.074611158 | 0.107335696 | 0.066387536 | 0.152034373 |
| SOCS4 | lightyellow | 0.022404696 | 0.62914357 | 0.005261044 | 0.909722774 |
| SORT1 | lightyellow | 0.137831811 | 0.002837208 | 0.138530808 | 0.002698636 |
| SOX4 | lightyellow | 0.171725182 | 0.000192392 | 0.162084607 | 0.00043716 |
| SPATA5L | lightyellow | 0.258660622 | 1.42E-08 | 0.24062931 | 1.41E-07 |
| SPATS2 | lightyellow | 0.319374263 | 1.56E-12 | 0.295501171 | 7.28E-11 |
| SPG21 | lightyellow | 0.024854131 | 0.592134345 | 0.015126749 | 0.744398326 |
| SPIN4 | lightyellow | 0.079989742 | 0.084216506 | 0.075663173 | 0.102457511 |
| SPOP | lightyellow | -0.039403288 | 0.395569645 | -0.058375656 | 0.207957443 |
| SPPL3 | lightyellow | -0.113816834 | 0.01385454 | -0.12463009 | 0.007005512 |
| SPTLC2 | lightyellow | 0.004535008 | 0.922138581 | -0.014358935 | 0.756953711 |
| SPTSSA | lightyellow | 0.062690578 | 0.176229889 | 0.058492759 | 0.207045191 |
| SPTY2D1 | lightyellow | 0.0294455 | 0.525588242 | 0.008186712 | 0.859944213 |
| SRBD1 | lightyellow | 0.070834835 | 0.126370591 | 0.054641817 | 0.238583098 |
| SRFBP1 | lightyellow | 0.061342603 | 0.185729411 | 0.043060439 | 0.353158202 |
| SRI | lightyellow | -0.013534158 | 0.770512054 | -0.011479705 | 0.804581769 |
| SRP54 | lightyellow | 0.098174187 | 0.033921598 | 0.080271767 | 0.083124853 |
| SRP72 | lightyellow | 0.09418473 | 0.041909386 | 0.072334417 | 0.118519935 |
| SRPK1 | lightyellow | 0.017444703 | 0.706915762 | -0.018573778 | 0.688904949 |

| | | | | | |
|---|---|---|---|---|---|
| SRPK2 | lightyellow | 0.162398611 | 0.000425927 | 0.135316235 | 0.003391166 |
| SRRM1 | lightyellow | 0.041030158 | 0.376337038 | 0.011923999 | 0.797180035 |
| SRSF4 | lightyellow | 0.020354662 | 0.660853851 | 0.003753555 | 0.935523149 |
| SSR1 | lightyellow | 0.094976619 | 0.040208427 | 0.088457877 | 0.056105678 |
| SSR3 | lightyellow | 0.00847851 | 0.855006409 | -0.007027167 | 0.879618173 |
| ST7L | lightyellow | -0.116818009 | 0.011525522 | -0.122928643 | 0.007826028 |
| STAM | lightyellow | -0.025306501 | 0.585409774 | -0.049119589 | 0.289473165 |
| STARD3N | lightyellow | -0.121953877 | 0.008333836 | -0.133693018 | 0.00379894 |
| STARD4 | lightyellow | -0.004640533 | 0.92033277 | -0.002236718 | 0.961551745 |
| STAU1 | lightyellow | -0.10797934 | 0.019594275 | -0.1140935 | 0.013623726 |
| STK17A | lightyellow | 0.010238621 | 0.825348387 | 0.033304833 | 0.472761053 |
| STK38L | lightyellow | -0.083840521 | 0.070273849 | -0.100927429 | 0.029199235 |
| STRADB | lightyellow | -0.007488169 | 0.871786806 | -0.023844878 | 0.607262967 |
| STRAP | lightyellow | -0.004920176 | 0.915549342 | -0.021374993 | 0.644990727 |
| STRBP | lightyellow | 0.221313316 | 1.37E-06 | 0.210368764 | 4.53E-06 |
| STT3B | lightyellow | 0.080714278 | 0.081435053 | 0.047239302 | 0.308353533 |
| STX3 | lightyellow | 0.056945561 | 0.21933328 | 0.051790981 | 0.264010209 |
| STXBP3 | lightyellow | -0.136025563 | 0.003225785 | -0.165111007 | 0.00033949 |
| STYX | lightyellow | 0.040682553 | 0.380397301 | 0.031643426 | 0.495138766 |
| SUCLA2 | lightyellow | 0.122389116 | 0.008103574 | 0.100832599 | 0.029351982 |
| SUDS3 | lightyellow | 0.080229927 | 0.083286079 | 0.054424198 | 0.240461106 |
| SUGP2 | lightyellow | 0.010004458 | 0.829280723 | -0.01523086 | 0.742701004 |
| SUGT1 | lightyellow | 0.057388131 | 0.215766214 | 0.044597913 | 0.336218045 |
| SUPT4H1 | lightyellow | -0.079701148 | 0.085345529 | -0.078071077 | 0.091953528 |
| SUZ12 | lightyellow | 0.080295685 | 0.0830328 | 0.040956348 | 0.37719695 |
| SYNCRIP | lightyellow | 0.026697652 | 0.564954847 | -0.001443574 | 0.975179969 |
| SYPL1 | lightyellow | 0.023505804 | 0.612383991 | 0.008892228 | 0.848015227 |
| TAF13 | lightyellow | 0.119647945 | 0.009653851 | 0.127719879 | 0.005710087 |
| TAF1A | lightyellow | -0.117476487 | 0.011063468 | -0.135895693 | 0.003255505 |
| TAF1D | lightyellow | -0.094000538 | 0.042313612 | -0.129756175 | 0.004978498 |
| TAF3 | lightyellow | -0.081709385 | 0.077736502 | -0.082803619 | 0.073828167 |
| TAF7 | lightyellow | 0.066105995 | 0.153783313 | 0.033859036 | 0.465421761 |
| TANGO2 | lightyellow | 0.023405379 | 0.613904353 | 0.050267612 | 0.278335316 |
| TAX1BP1 | lightyellow | -0.082968062 | 0.073254904 | -0.073148148 | 0.11442236 |
| TBC1D17 | lightyellow | -0.079632246 | 0.085616882 | -0.075890507 | 0.101426827 |
| TBK1 | lightyellow | 0.00860825 | 0.852812763 | -0.019786706 | 0.669750726 |
| TBP | lightyellow | -0.180052248 | 9.13E-05 | -0.19426317 | 2.37E-05 |
| TBPL1 | lightyellow | -0.145689111 | 0.001595164 | -0.158109602 | 0.000605331 |
| TCEAL8 | lightyellow | -0.114357328 | 0.013406784 | -0.11987293 | 0.009517342 |
| TCEB3 | lightyellow | 0.006336566 | 0.891371688 | -0.011695646 | 0.800982074 |
| TCIRG1 | lightyellow | 0.111217888 | 0.01619631 | 0.128929328 | 0.005264715 |
| TCP1 | lightyellow | 0.011226952 | 0.80880038 | 0.02207563 | 0.634189873 |
| TCTN3 | lightyellow | 0.284217927 | 3.98E-10 | 0.254089164 | 2.58E-08 |
| TDG | lightyellow | 0.171412399 | 0.000197723 | 0.163132901 | 0.000400699 |
| TEFM | lightyellow | 0.017077615 | 0.712807484 | -0.001482617 | 0.974508919 |

| | | | | | |
|---|---|---|---|---|---|
| TERF1 | lightyellow | -0.133878484 | 0.0037502 | -0.183744942 | 6.49E-05 |
| TERF2 | lightyellow | 0.144727849 | 0.001714221 | 0.133349059 | 0.003890853 |
| TES | lightyellow | -0.010645009 | 0.818534266 | 0.007971529 | 0.863589058 |
| TEX10 | lightyellow | -0.051964879 | 0.262407781 | -0.086560888 | 0.061609879 |
| TFAM | lightyellow | -0.071030864 | 0.125321976 | -0.106882762 | 0.020877797 |
| TFCP2 | lightyellow | -0.064526279 | 0.163880373 | -0.062637614 | 0.176596192 |
| TGDS | lightyellow | -0.046464771 | 0.316361732 | -0.072790909 | 0.116207354 |
| THAP1 | lightyellow | -0.073002619 | 0.115146901 | -0.113744213 | 0.013915693 |
| THAP10 | lightyellow | -0.056403246 | 0.223761561 | -0.069696119 | 0.132597139 |
| THAP5 | lightyellow | -0.017271348 | 0.709695931 | -0.019169539 | 0.679471071 |
| THNSL1 | lightyellow | -0.13150175 | 0.00441983 | -0.156634528 | 0.000681741 |
| THUMPD | lightyellow | 0.170371098 | 0.000216484 | 0.168428995 | 0.000256002 |
| TIAL1 | lightyellow | 0.025995209 | 0.575240441 | -0.002682769 | 0.953892122 |
| TIFA | lightyellow | 0.251762573 | 3.49E-08 | 0.220275987 | 1.54E-06 |
| TIGD2 | lightyellow | 0.203518625 | 9.30E-06 | 0.197919981 | 1.65E-05 |
| TIMM10I | lightyellow | 0.04154754 | 0.370343245 | 0.035699698 | 0.441504669 |
| TIMM21 | lightyellow | 0.020921717 | 0.652018504 | 0.00845592 | 0.85538848 |
| TLK1 | lightyellow | -0.03660306 | 0.430028172 | -0.069539704 | 0.133470609 |
| TLK2 | lightyellow | -0.122298 | 0.008151304 | -0.140587065 | 0.002325976 |
| TM2D1 | lightyellow | -0.159495567 | 0.000540851 | -0.190034974 | 3.57E-05 |
| TM2D3 | lightyellow | 0.138441263 | 0.002716037 | 0.122388004 | 0.008104155 |
| TM7SF3 | lightyellow | 0.206030937 | 7.17E-06 | 0.178003701 | 0.000110042 |
| TM9SF2 | lightyellow | 0.010975269 | 0.813006634 | -0.0060078 | 0.896975564 |
| TM9SF3 | lightyellow | 0.149342948 | 0.001208571 | 0.11724697 | 0.011222624 |
| TMED2 | lightyellow | 0.172964382 | 0.000172567 | 0.160364724 | 0.000503733 |
| TMED5 | lightyellow | 0.079572577 | 0.08585243 | 0.063114226 | 0.173320204 |
| TMED7-T | lightyellow | 0.086757324 | 0.061019983 | 0.047429747 | 0.3064051 |
| TMEM12 | lightyellow | 0.069031342 | 0.136340137 | 0.02574452 | 0.578932419 |
| TMEM12 | lightyellow | 0.019023423 | 0.681780263 | 0.007504308 | 0.871512853 |
| TMEM12 | lightyellow | 0.014650635 | 0.75217606 | -0.010535643 | 0.820366741 |
| TMEM13 | lightyellow | 0.092340582 | 0.046107064 | 0.076639181 | 0.098090581 |
| TMEM14 | lightyellow | -0.036183424 | 0.435337699 | -0.056734814 | 0.22104663 |
| TMEM16 | lightyellow | 0.079133579 | 0.087601516 | 0.058664761 | 0.20571051 |
| TMEM17 | lightyellow | 0.207134552 | 6.38E-06 | 0.238785271 | 1.77E-07 |
| TMEM18 | lightyellow | 0.006085314 | 0.89565385 | -0.016619544 | 0.720183454 |
| TMEM18 | lightyellow | 0.022046953 | 0.634630443 | 0.006457024 | 0.889319785 |
| TMEM19 | lightyellow | -0.096680653 | 0.03674535 | -0.093241074 | 0.044015219 |
| TMEM21 | lightyellow | 0.190339952 | 3.47E-05 | 0.149395617 | 0.001203691 |
| TMEM24 | lightyellow | -0.073029934 | 0.115010636 | -0.060798485 | 0.189669037 |
| TMEM25 | lightyellow | 0.060902382 | 0.188912075 | 0.075443532 | 0.10346119 |
| TMEM26 | lightyellow | -0.056886736 | 0.219810557 | -0.067158581 | 0.147321912 |
| TMEM33 | lightyellow | 0.153094724 | 0.000903 | 0.148855771 | 0.001254584 |
| TMEM38 | lightyellow | -0.101242113 | 0.028697236 | -0.109080845 | 0.018374703 |
| TMEM5 | lightyellow | 0.007886807 | 0.865024904 | -0.031621209 | 0.495441778 |
| TMEM50 | lightyellow | -0.038947115 | 0.401066913 | -0.050836081 | 0.272929205 |

| | | | | | |
|---|---|---|---|---|---|
| TMEM57 | lightyellow | 0.086406455 | 0.062076941 | 0.07401076 | 0.110200858 |
| TMEM69 | lightyellow | -0.065600807 | 0.15695971 | -0.083109942 | 0.072763218 |
| TMLHE | lightyellow | 0.25322303 | 2.89E-08 | 0.238690368 | 1.79E-07 |
| TMTC3 | lightyellow | 0.123930452 | 0.0073331 | 0.096922811 | 0.036274392 |
| TMTC4 | lightyellow | 0.049008366 | 0.290567897 | -0.00668486 | 0.885440838 |
| TMX1 | lightyellow | 0.090301224 | 0.051154429 | 0.062081121 | 0.180479188 |
| TNPO1 | lightyellow | 0.09990515 | 0.030882334 | 0.073834247 | 0.111054556 |
| TOMM70 | lightyellow | -0.185235208 | 5.65E-05 | -0.204499992 | 8.40E-06 |
| TOP1 | lightyellow | 0.099168142 | 0.032146658 | 0.073411331 | 0.113121143 |
| TOP2B | lightyellow | 0.00116374 | 0.979990157 | -0.021320699 | 0.645830884 |
| TPM4 | lightyellow | -0.182590039 | 7.23E-05 | -0.188667189 | 4.08E-05 |
| TRAM1 | lightyellow | -0.123803531 | 0.007393975 | -0.143425407 | 0.001888544 |
| TRAPPC1 | lightyellow | 0.120550708 | 0.009116417 | 0.111905973 | 0.015544793 |
| TRAPPC1 | lightyellow | 0.168164785 | 0.000261872 | 0.130388803 | 0.004769066 |
| TRAPPC2 | lightyellow | 0.107801518 | 0.019797625 | 0.116277224 | 0.011917672 |
| TRAPPC6 | lightyellow | 0.018627835 | 0.688046935 | -0.004587106 | 0.921247003 |
| TRIM24 | lightyellow | 0.129467942 | 0.005076639 | 0.091133664 | 0.049041055 |
| TRIP4 | lightyellow | 0.171803755 | 0.000191074 | 0.155213118 | 0.000763738 |
| TRMT10A | lightyellow | 0.266478242 | 4.94E-09 | 0.225161621 | 8.83E-07 |
| TRMT1L | lightyellow | -6.32E-05 | 0.998914014 | -0.013830544 | 0.765631531 |
| TRMT2B | lightyellow | 0.135533768 | 0.003339647 | 0.140046152 | 0.00241916 |
| TRMT6 | lightyellow | -0.103023018 | 0.025993656 | -0.124670213 | 0.006987129 |
| TRUB1 | lightyellow | 0.033036583 | 0.476336155 | 0.002966374 | 0.949024181 |
| TSG101 | lightyellow | 0.007826199 | 0.866052335 | -0.001853468 | 0.968135796 |
| TSN | lightyellow | -0.107723445 | 0.019887485 | -0.13856555 | 0.002691913 |
| TSPAN31 | lightyellow | -0.216045463 | 2.45E-06 | -0.215891814 | 2.49E-06 |
| TSPAN6 | lightyellow | -0.070007085 | 0.130873719 | -0.074949041 | 0.105749336 |
| TTC19 | lightyellow | 0.359057601 | 1.17E-15 | 0.342418507 | 2.72E-14 |
| TTC27 | lightyellow | -0.131581828 | 0.004395616 | -0.145201086 | 0.001654626 |
| TTC32 | lightyellow | -0.169841259 | 0.000226657 | -0.174177171 | 0.000155032 |
| TTF1 | lightyellow | -0.064428353 | 0.164522301 | -0.089548887 | 0.053129315 |
| TUBD1 | lightyellow | -0.040837232 | 0.378587245 | -0.060760287 | 0.189947892 |
| TUBGCP4 | lightyellow | 0.240202412 | 1.49E-07 | 0.208025597 | 5.81E-06 |
| TVP23C-( | lightyellow | -0.002294943 | 0.960551684 | -0.015926126 | 0.731398419 |
| TWISTNI | lightyellow | -0.037200836 | 0.422529792 | -0.038994374 | 0.400495285 |
| TXLNG | lightyellow | 0.00716142 | 0.877336279 | -0.03162695 | 0.495363475 |
| TXNDC1( | lightyellow | -0.027849402 | 0.548283503 | -0.056735258 | 0.221043005 |
| TXNRD1 | lightyellow | 0.048465407 | 0.295951923 | 0.056780185 | 0.220676963 |
| TYW1 | lightyellow | -0.057498457 | 0.214883521 | -0.082604804 | 0.074526134 |
| TYW3 | lightyellow | 0.029053563 | 0.531116605 | 0.001486708 | 0.9744386 |
| UBA5 | lightyellow | 0.047622164 | 0.304444766 | 0.034849264 | 0.452466768 |
| UBAP1 | lightyellow | 0.042942871 | 0.354475358 | 0.041191029 | 0.374467009 |
| UBE2A | lightyellow | 0.092957668 | 0.044664784 | 0.093727552 | 0.04291875 |
| UBE2D1 | lightyellow | 0.005167293 | 0.911324827 | -0.011488365 | 0.804437331 |
| UBE2D3 | lightyellow | 0.032013162 | 0.490110455 | 0.011205744 | 0.809154604 |

| | | | | | |
|---|---|---|---|---|---|
| UBE2E1 | lightyellow | -0.063506002 | 0.170661413 | -0.090835885 | 0.049788497 |
| UBE2E3 | lightyellow | 0.073603041 | 0.112180635 | 0.075488589 | 0.10325466 |
| UBE2G1 | lightyellow | 0.127638972 | 0.005741055 | 0.104386192 | 0.024075305 |
| UBE2H | lightyellow | 0.02742712 | 0.554367751 | 0.024499965 | 0.597423625 |
| UBE2K | lightyellow | 0.22267111 | 1.17E-06 | 0.219711411 | 1.64E-06 |
| UBE2N | lightyellow | 0.140744271 | 0.002299515 | 0.117077863 | 0.011341182 |
| UBE2Q2 | lightyellow | 0.060479274 | 0.192008629 | 0.034263586 | 0.460104429 |
| UBE2R2 | lightyellow | -0.003798238 | 0.934757301 | 0.015645307 | 0.735956719 |
| UBP1 | lightyellow | -0.056372746 | 0.224012488 | -0.081220785 | 0.079535093 |
| UBR7 | lightyellow | 0.149844122 | 0.001162863 | 0.148879545 | 0.001252301 |
| UBTD2 | lightyellow | -0.050302781 | 0.277998765 | -0.066332106 | 0.152377506 |
| UBXN2A | lightyellow | -0.033429048 | 0.471110575 | -0.045576486 | 0.325712069 |
| UFSP2 | lightyellow | 0.047349864 | 0.307221385 | 0.022917308 | 0.621316879 |
| UNC50 | lightyellow | -0.110498753 | 0.016902669 | -0.129195274 | 0.00517108 |
| UPF2 | lightyellow | -0.069207429 | 0.135340864 | -0.107130309 | 0.020581833 |
| UPRT | lightyellow | 0.073213887 | 0.11409624 | 0.072365975 | 0.118358912 |
| USP1 | lightyellow | -0.074117072 | 0.109689185 | -0.108620769 | 0.018875797 |
| USP14 | lightyellow | -0.027981096 | 0.546392814 | -0.044711926 | 0.334982937 |
| USP16 | lightyellow | -0.051417091 | 0.267478267 | -0.062139608 | 0.180068138 |
| USP28 | lightyellow | -0.041110543 | 0.375401892 | -0.075874729 | 0.101498095 |
| USP38 | lightyellow | -0.015943814 | 0.731111617 | -0.023810804 | 0.607776735 |
| USP48 | lightyellow | 0.015471265 | 0.738786466 | -0.015820447 | 0.733112723 |
| USP7 | lightyellow | 0.203235711 | 9.58E-06 | 0.198411691 | 1.57E-05 |
| UTP15 | lightyellow | 0.150897609 | 0.001071929 | 0.122426111 | 0.008084266 |
| UTP18 | lightyellow | -0.000669457 | 0.988488265 | -0.032843863 | 0.478913723 |
| UTP3 | lightyellow | -0.041467133 | 0.37127085 | -0.054873532 | 0.236594821 |
| UTP6 | lightyellow | 0.033221313 | 0.473872587 | 0.008871458 | 0.848365919 |
| UXS1 | lightyellow | -0.024613106 | 0.595731585 | -0.049983627 | 0.281063023 |
| VAMP7 | lightyellow | 0.01533758 | 0.740962452 | 0.008563943 | 0.853561788 |
| VAPB | lightyellow | -0.086260686 | 0.062520472 | -0.104479872 | 0.023948086 |
| VASH2 | lightyellow | -0.014511813 | 0.7544486 | -0.034385467 | 0.45850911 |
| VDAC2 | lightyellow | -0.005259525 | 0.909748729 | -0.018473712 | 0.690494272 |
| VEZT | lightyellow | 0.077981925 | 0.092326428 | 0.06356043 | 0.17029446 |
| VIPAS39 | lightyellow | -0.014725942 | 0.750944153 | -0.028951423 | 0.532562137 |
| VKORC1 | lightyellow | -0.074519537 | 0.107769079 | -0.089104849 | 0.054324427 |
| VMA21 | lightyellow | -0.063953164 | 0.167664096 | -0.074315277 | 0.108740234 |
| VPS26A | lightyellow | 0.029382933 | 0.526468791 | 0.001709383 | 0.970611705 |
| VPS35 | lightyellow | 0.153312196 | 0.000887691 | 0.145437813 | 0.001625533 |
| VPS4B | lightyellow | 0.040938948 | 0.377399846 | 0.033837345 | 0.465707817 |
| VPS9D1 | lightyellow | 0.127519111 | 0.005787211 | 0.161143325 | 0.000472506 |
| VRK2 | lightyellow | -0.029432378 | 0.525772859 | -0.060735934 | 0.190125836 |
| VTA1 | lightyellow | -0.067806678 | 0.143447681 | -0.072532006 | 0.117514575 |
| VTI1A | lightyellow | 0.068617137 | 0.138713079 | 0.056155268 | 0.225807521 |
| VTI1B | lightyellow | -0.01590409 | 0.731755774 | -0.014595571 | 0.753077217 |
| VWA1 | lightyellow | -0.042375751 | 0.360872381 | -0.029334742 | 0.527147535 |

| | | | | | |
|---|---|---|---|---|---|
| VWA9 | lightyellow | 0.184591351 | 6.00E-05 | 0.175514385 | 0.000137642 |
| WAC | lightyellow | -0.015813826 | 0.733220173 | -0.056894301 | 0.219749138 |
| WAPAL | lightyellow | 0.076229513 | 0.099905155 | 0.043404148 | 0.34932522 |
| WARS2 | lightyellow | 0.104514849 | 0.023900736 | 0.05730676 | 0.216418912 |
| WBP4 | lightyellow | 0.04256142 | 0.358770169 | 0.012347466 | 0.79014214 |
| WBP5 | lightyellow | -0.275802792 | 1.34E-09 | -0.286145881 | 2.99E-10 |
| WDR12 | lightyellow | -0.013023731 | 0.778938319 | -0.04337246 | 0.349677491 |
| WDR13 | lightyellow | -0.077902015 | 0.092661704 | -0.03891393 | 0.401468591 |
| WDR24 | lightyellow | 0.126181296 | 0.006325479 | 0.161562608 | 0.000456446 |
| WDR3 | lightyellow | -0.096670014 | 0.036766158 | -0.127139673 | 0.005935533 |
| WDR41 | lightyellow | 0.131681295 | 0.004365706 | 0.140448678 | 0.002349499 |
| WDR43 | lightyellow | -0.205612609 | 7.49E-06 | -0.245037597 | 8.18E-08 |
| WDSUB1 | lightyellow | -0.062146403 | 0.180020424 | -0.080260471 | 0.083168355 |
| WDYHV1 | lightyellow | -0.057081692 | 0.21823161 | -0.096607483 | 0.036888671 |
| WEE1 | lightyellow | 0.197792399 | 1.67E-05 | 0.153966498 | 0.000843069 |
| WSB2 | lightyellow | 0.356953102 | 1.77E-15 | 0.356233653 | 2.03E-15 |
| WTAP | lightyellow | -0.13356432 | 0.003833098 | -0.154135643 | 0.000831875 |
| XAB2 | lightyellow | 0.094645216 | 0.040913036 | 0.110167086 | 0.017237427 |
| XPNPEP1 | lightyellow | 0.270378735 | 2.88E-09 | 0.261202852 | 1.01E-08 |
| XPOT | lightyellow | 0.012336967 | 0.790316422 | -0.024454726 | 0.598100778 |
| XRCC4 | lightyellow | 0.028230394 | 0.542822547 | 0.007561486 | 0.870542446 |
| XRCC5 | lightyellow | 0.028422655 | 0.540077047 | 1.67E-05 | 0.999712676 |
| YEATS4 | lightyellow | 0.114769148 | 0.01307423 | 0.082827953 | 0.073743108 |
| YES1 | lightyellow | -0.008379796 | 0.856676232 | -0.033162171 | 0.474660546 |
| YIPF4 | lightyellow | -0.271756674 | 2.38E-09 | -0.281687622 | 5.76E-10 |
| YIPF5 | lightyellow | -0.05738314 | 0.215806204 | -0.066177992 | 0.153334621 |
| YIPF6 | lightyellow | -0.020327825 | 0.661273183 | -0.035395431 | 0.445409153 |
| YME1L1 | lightyellow | 0.03837172 | 0.408065733 | 0.001545785 | 0.973423246 |
| YPEL3 | lightyellow | -0.260022115 | 1.18E-08 | -0.233909056 | 3.18E-07 |
| YTHDF2 | lightyellow | 0.089969792 | 0.052016757 | 0.086404803 | 0.062081951 |
| YWHAB | lightyellow | -0.130902577 | 0.004604866 | -0.147884119 | 0.001351203 |
| ZADH2 | lightyellow | 0.224794387 | 9.21E-07 | 0.221108399 | 1.40E-06 |
| ZBTB2 | lightyellow | -0.154378895 | 0.000816019 | -0.17149523 | 0.000196298 |
| ZC3H15 | lightyellow | -0.076495545 | 0.098723788 | -0.096492415 | 0.037115018 |
| ZDHHC2( | lightyellow | 0.184830215 | 5.86E-05 | 0.163081165 | 0.00040243 |
| ZDHHC6 | lightyellow | 0.20328552 | 9.53E-06 | 0.169703489 | 0.000229375 |
| ZFP91 | lightyellow | 0.065420677 | 0.158104199 | 0.034328253 | 0.459257603 |
| ZFPM1 | lightyellow | 0.083743809 | 0.070599327 | 0.09245328 | 0.045840781 |
| ZMPSTE2 | lightyellow | -0.117069705 | 0.011346929 | -0.13856101 | 0.00269279 |
| ZMYM6N | lightyellow | -0.21910433 | 1.75E-06 | -0.237984435 | 1.95E-07 |
| ZMYND1 | lightyellow | -0.029483663 | 0.525051511 | -0.07464022 | 0.107198519 |
| ZNF101 | lightyellow | 0.103844103 | 0.024822996 | 0.122027966 | 0.008294233 |
| ZNF14 | lightyellow | -0.094449238 | 0.0413346 | -0.103691274 | 0.02503738 |
| ZNF174 | lightyellow | 0.093864352 | 0.042614595 | 0.072357451 | 0.118402393 |
| ZNF184 | lightyellow | -0.015811935 | 0.733250857 | -0.034636954 | 0.455227158 |

| | | | | | |
|---|---|---|---|---|---|
| ZNF277 | lightyellow | -0.025414498 | 0.58380962 | -0.036709616 | 0.428685944 |
| ZNF280B | lightyellow | 0.079480948 | 0.086215163 | 0.033258695 | 0.473374906 |
| ZNF322 | lightyellow | -0.134167929 | 0.003675265 | -0.166737869 | 0.000295808 |
| ZNF330 | lightyellow | -0.031179046 | 0.501492924 | -0.042761991 | 0.35650786 |
| ZNF341 | lightyellow | 0.071636969 | 0.122122473 | 0.07983579 | 0.084817281 |
| ZNF35 | lightyellow | 0.077451873 | 0.094568588 | 0.074326826 | 0.108685142 |
| ZNF480 | lightyellow | 0.137380379 | 0.002930112 | 0.144087979 | 0.001797931 |
| ZNF57 | lightyellow | 0.298558008 | 4.54E-11 | 0.287633931 | 2.40E-10 |
| ZNF664 | lightyellow | 0.088428499 | 0.0561877 | 0.063030699 | 0.173891027 |
| ZNF670 | lightyellow | -0.086152823 | 0.062850347 | -0.095369149 | 0.03938715 |
| ZNF830 | lightyellow | -0.011201163 | 0.809231132 | -0.038035591 | 0.41218759 |
| ZNF837 | lightyellow | 0.122529265 | 0.008030646 | 0.115626282 | 0.012405308 |
| ZNHIT3 | lightyellow | 0.085529345 | 0.064785328 | 0.068112159 | 0.141648791 |
| ZNHIT6 | lightyellow | -0.157407585 | 0.000640647 | -0.199886448 | 1.35E-05 |
| ZNRF2 | lightyellow | 0.027385011 | 0.554976258 | -0.000862893 | 0.98516236 |
| ZRANB3 | lightyellow | -0.082072015 | 0.076423082 | -0.125387324 | 0.006665773 |
| ZUFSP | lightyellow | 0.044375386 | 0.338637092 | 0.031354147 | 0.499091951 |
| ZW10 | lightyellow | 0.074824795 | 0.106330505 | 0.03609126 | 0.43650886 |
| ABCC10 | magenta | 0.07215869 | 0.119419697 | 0.068458763 | 0.139628724 |
| ACACA | magenta | 0.234168136 | 3.08E-07 | 0.221786593 | 1.30E-06 |
| ACLY | magenta | 0.407424613 | 4.22E-20 | 0.404647617 | 7.96E-20 |
| ACO2 | magenta | 0.152699508 | 0.000931445 | 0.1496798 | 0.001177671 |
| ACTR1B | magenta | 0.05300596 | 0.252954534 | 0.053518822 | 0.248385643 |
| ACVR1B | magenta | -0.161986401 | 0.000440729 | -0.188787338 | 4.03E-05 |
| ADCY7 | magenta | 0.064977881 | 0.16094434 | 0.065793649 | 0.155741402 |
| ADD1 | magenta | 0.011148351 | 0.810113416 | 0.006202489 | 0.893656421 |
| AGAP1 | magenta | -0.092600478 | 0.045494927 | -0.111072737 | 0.016336763 |
| AKAP1 | magenta | 0.230256916 | 4.90E-07 | 0.21179754 | 3.89E-06 |
| AKT1 | magenta | 0.183796997 | 6.46E-05 | 0.192352592 | 2.85E-05 |
| AMBRA1 | magenta | -0.008940986 | 0.847192055 | -0.027777145 | 0.549322253 |
| AMFR | magenta | 0.102386912 | 0.026933 | 0.098659118 | 0.033045472 |
| ANKFY1 | magenta | 0.132606063 | 0.004096305 | 0.114655465 | 0.013165297 |
| ANKRD1 | magenta | 0.138180016 | 0.002767389 | 0.147342694 | 0.001407964 |
| ANKRD5 | magenta | 0.051954274 | 0.262505308 | 0.033188079 | 0.474315282 |
| AP2A1 | magenta | 0.043578629 | 0.34738955 | 0.053843967 | 0.245519029 |
| AP2B1 | magenta | 0.290487103 | 1.56E-10 | 0.290998453 | 1.45E-10 |
| APP | magenta | 0.143559268 | 0.001869907 | 0.124674313 | 0.006985253 |
| ARHGAP | magenta | -0.033453358 | 0.470787937 | -0.044752044 | 0.334549021 |
| ARHGEF | magenta | 0.160636932 | 0.000492604 | 0.129121096 | 0.005197045 |
| ARID1A | magenta | -0.038132241 | 0.410999886 | -0.039462594 | 0.394858304 |
| ARL6IP4 | magenta | -0.021845069 | 0.637735731 | -0.0156555 | 0.735791093 |
| ATF6B | magenta | -0.091834605 | 0.047318636 | -0.120207358 | 0.009317601 |
| ATG2A | magenta | -0.067433306 | 0.14567002 | -0.060302738 | 0.193311565 |
| ATN1 | magenta | 0.002916331 | 0.94988301 | -0.005945641 | 0.898035661 |
| ATP11A | magenta | 0.107954516 | 0.019622553 | 0.10000243 | 0.030718675 |

| | | | | | |
|---|---|---|---|---|---|
| ATP2A2 | magenta | 0.121027598 | 0.008843401 | 0.118967614 | 0.010077282 |
| ATP9A | magenta | -0.00130601 | 0.977544513 | -0.010720271 | 0.817273785 |
| ATXN10 | magenta | 0.185861501 | 5.32E-05 | 0.144410976 | 0.001755218 |
| ATXN1L | magenta | -0.048982877 | 0.290819177 | -0.042371186 | 0.36092417 |
| ATXN2 | magenta | 0.007134909 | 0.877786803 | 0.008824472 | 0.84915939 |
| ATXN2L | magenta | 0.06250679 | 0.177503403 | 0.061253632 | 0.186369439 |
| ATXN7L: | magenta | 0.044243609 | 0.340074859 | 0.02871969 | 0.535849047 |
| BANP | magenta | 0.066511521 | 0.151268981 | 0.080130034 | 0.083672032 |
| BAP1 | magenta | 0.028162923 | 0.543787671 | 0.042881891 | 0.355159763 |
| BAZ2A | magenta | -0.085499591 | 0.064878881 | -0.109418422 | 0.018014451 |
| BBS2 | magenta | 0.175434433 | 0.000138628 | 0.135004819 | 0.003466174 |
| BCKDHA | magenta | -0.010313799 | 0.824086823 | -0.034631039 | 0.455304192 |
| BCL7C | magenta | -0.022424706 | 0.628837261 | -0.010632207 | 0.818748722 |
| BCL9L | magenta | -0.027773617 | 0.549372988 | -0.040883832 | 0.378042967 |
| BECN1 | magenta | 0.146825427 | 0.001464232 | 0.140194285 | 0.002393308 |
| BRD2 | magenta | 0.034133688 | 0.461808083 | 0.009778219 | 0.833084035 |
| BRD3 | magenta | 0.058478808 | 0.20715372 | 0.039848606 | 0.390247209 |
| BRF2 | magenta | 0.030240488 | 0.514465541 | 0.013495544 | 0.77114858 |
| BRPF1 | magenta | -0.030587529 | 0.509648614 | -0.05797766 | 0.211079631 |
| BRPF3 | magenta | -0.125657967 | 0.006547977 | -0.160722459 | 0.000489155 |
| BTN2A1 | magenta | -0.122420532 | 0.008087175 | -0.128716023 | 0.005340916 |
| C15orf39 | magenta | -0.179822268 | 9.33E-05 | -0.15418735 | 0.000828481 |
| C16orf58 | magenta | -0.005193874 | 0.910870567 | -0.024424531 | 0.598552947 |
| C16orf70 | magenta | 0.09598499 | 0.038127275 | 0.103728667 | 0.024984779 |
| C9orf64 | magenta | 0.154792962 | 0.000789669 | 0.157056143 | 0.000659035 |
| CAD | magenta | -0.130392739 | 0.004767788 | -0.133674109 | 0.003803941 |
| CAMK1 | magenta | -0.087262671 | 0.059523827 | -0.124030345 | 0.007285505 |
| CAMTA2 | magenta | 0.165606575 | 0.000325585 | 0.159608268 | 0.000535899 |
| CARM1 | magenta | 0.071225661 | 0.124286654 | 0.06768276 | 0.144182359 |
| CCDC71 | magenta | 0.052977535 | 0.253209458 | 0.048828056 | 0.292348541 |
| CCDC85( | magenta | -0.143237672 | 0.001914968 | -0.157791293 | 0.000621114 |
| CDC42BI | magenta | -0.055790157 | 0.228844073 | -0.070444778 | 0.12847737 |
| CENPBD | magenta | -0.064227768 | 0.16584307 | -0.077713409 | 0.093456893 |
| CEP170B | magenta | 0.062503669 | 0.177525088 | 0.074548312 | 0.107632821 |
| CES2 | magenta | 0.202720835 | 1.01E-05 | 0.22080476 | 1.45E-06 |
| CHD8 | magenta | -0.019946197 | 0.667247612 | -0.038353356 | 0.408290285 |
| CHKB | magenta | 0.011929169 | 0.79709402 | 0.00602955 | 0.89660467 |
| CHTF8 | magenta | 0.150765121 | 0.001082993 | 0.140178635 | 0.002396027 |
| CIC | magenta | -0.166634249 | 0.000298425 | -0.181609554 | 7.91E-05 |
| CIZ1 | magenta | -0.000556375 | 0.990432687 | -0.013875515 | 0.764891813 |
| CKLF-CM | magenta | 0.034731914 | 0.453991346 | 0.013969581 | 0.763345225 |
| CLCN7 | magenta | 0.056003999 | 0.227062097 | 0.071070029 | 0.125113281 |
| CLDND2 | magenta | 0.021897472 | 0.636929085 | 0.024703397 | 0.594382856 |
| CMIP | magenta | 0.113190099 | 0.014390174 | 0.107407118 | 0.02025521 |
| CMTM1 | magenta | 0.018126159 | 0.696024959 | 0.000963635 | 0.9834303 |

| | | | | | |
|---|---|---|---|---|---|
| CNNM3 | magenta | 0.053575644 | 0.247883004 | 0.038387245 | 0.40787594 |
| CNOT3 | magenta | 0.033751382 | 0.466842453 | 0.032632868 | 0.481744387 |
| CREBBP | magenta | -0.053214172 | 0.251092669 | -0.05579732 | 0.228784222 |
| CRKL | magenta | -0.096678547 | 0.036749469 | -0.129342175 | 0.005120005 |
| CSNK1D | magenta | -0.073875498 | 0.110854583 | -0.08723419 | 0.059607338 |
| CSNK1E | magenta | 0.186774073 | 4.88E-05 | 0.169267883 | 0.00023817 |
| CTBP1 | magenta | 0.102322927 | 0.027029084 | 0.109697344 | 0.01772146 |
| CTD-219 | magenta | -0.027480065 | 0.553603115 | -0.027774388 | 0.5493619 |
| CTDSP2 | magenta | -0.315463969 | 3.00E-12 | -0.33128126 | 2.01E-13 |
| CTNNA1 | magenta | 0.237489074 | 2.07E-07 | 0.246118423 | 7.14E-08 |
| CUL7 | magenta | -0.17019292 | 0.000219856 | -0.174354762 | 0.000152609 |
| CYB561A | magenta | 0.075180617 | 0.104672837 | 0.082400166 | 0.075250143 |
| CYB5D1 | magenta | 0.101875594 | 0.027709107 | 0.086119222 | 0.062953399 |
| CYFIP1 | magenta | 0.286888015 | 2.68E-10 | 0.290944591 | 1.46E-10 |
| CYP51A1 | magenta | 0.132345944 | 0.00417052 | 0.09748044 | 0.035209362 |
| DAG1 | magenta | 0.092774129 | 0.045089743 | 0.074342885 | 0.108608572 |
| DALRD3 | magenta | 0.080364069 | 0.082770064 | 0.079678621 | 0.085434168 |
| DCTN5 | magenta | 0.113018894 | 0.014539623 | 0.130835417 | 0.004626036 |
| DDB1 | magenta | -0.05772941 | 0.213044118 | -0.068719503 | 0.138123706 |
| DDX24 | magenta | -0.059377438 | 0.200246776 | -0.059518759 | 0.199176002 |
| DGCR2 | magenta | 0.172440826 | 0.000180697 | 0.150110582 | 0.001139214 |
| DHX30 | magenta | 0.093778733 | 0.042804744 | 0.089216863 | 0.054020857 |
| DHX38 | magenta | 0.1212616 | 0.008712119 | 0.134423437 | 0.003610247 |
| DHX8 | magenta | 0.233650516 | 3.28E-07 | 0.236352952 | 2.37E-07 |
| DIP2A | magenta | 0.104378456 | 0.024085836 | 0.095182121 | 0.039776675 |
| DLG5 | magenta | 0.128104938 | 0.005564747 | 0.092092995 | 0.046696623 |
| DLST | magenta | 0.074330261 | 0.108668762 | 0.032512344 | 0.483365377 |
| DNAAF5 | magenta | 0.13660517 | 0.003096124 | 0.139964411 | 0.002433535 |
| DNAJA3 | magenta | 0.091396207 | 0.048389863 | 0.096109842 | 0.037876063 |
| DNASE1l | magenta | 0.052448487 | 0.257986686 | 0.027658235 | 0.551033767 |
| DNM2 | magenta | -0.020083335 | 0.665098247 | -0.012165724 | 0.793160585 |
| DPF2 | magenta | -0.136752963 | 0.003063829 | -0.156991469 | 0.000662472 |
| DPH1 | magenta | 0.102370138 | 0.026958159 | 0.080712036 | 0.081443543 |
| DRG2 | magenta | 0.193213231 | 2.62E-05 | 0.191373686 | 3.14E-05 |
| DYNC1H | magenta | 0.07375175 | 0.111455329 | 0.079440345 | 0.086376292 |
| EARS2 | magenta | 0.144410717 | 0.001755252 | 0.157186299 | 0.000652169 |
| EDC4 | magenta | 0.162808915 | 0.000411653 | 0.162233967 | 0.000431783 |
| EEF1A1 | magenta | 0.06788396 | 0.142990951 | 0.035211228 | 0.447782423 |
| EEF1G | magenta | -0.044298469 | 0.339475827 | -0.062826433 | 0.175292883 |
| EFTUD2 | magenta | 0.101540837 | 0.028227568 | 0.114436244 | 0.013342486 |
| EGLN2 | magenta | -0.160240374 | 0.000508894 | -0.140819252 | 0.002286991 |
| EIF3C | magenta | -0.026381584 | 0.569571991 | -0.040214934 | 0.385901547 |
| EIF3L | magenta | 0.077084882 | 0.096146316 | 0.043500455 | 0.348255958 |
| EIF4B | magenta | 0.021264595 | 0.646699506 | -0.036015585 | 0.437471829 |
| EIF4ENIF | magenta | -0.121964158 | 0.008328331 | -0.15157492 | 0.001016977 |

| | | | | | |
|---|---|---|---|---|---|
| EIF4H | magenta | -0.016325027 | 0.724939649 | -0.03967891 | 0.392270274 |
| ELL | magenta | -0.106925265 | 0.020826719 | -0.11180686 | 0.015637198 |
| ELMSAN | magenta | -0.112750775 | 0.014776412 | -0.124207478 | 0.007201786 |
| EP300 | magenta | 0.095152943 | 0.039837738 | 0.063235318 | 0.172495136 |
| EP400 | magenta | 0.020326867 | 0.661288156 | -0.003391244 | 0.941735123 |
| ERF | magenta | -0.159630439 | 0.00053493 | -0.182526429 | 7.27E-05 |
| EXOC7 | magenta | -0.073363682 | 0.113355859 | -0.086890774 | 0.0606219 |
| FAM168E | magenta | -0.152471541 | 0.000948227 | -0.171008068 | 0.000204819 |
| FAM219E | magenta | 0.155075383 | 0.00077215 | 0.127957448 | 0.005620019 |
| FBXL12 | magenta | -0.118985249 | 0.010066101 | -0.13363299 | 0.003814837 |
| FBXL14 | magenta | 0.011350719 | 0.806733926 | -0.005266931 | 0.909622197 |
| FBXL18 | magenta | -0.015608693 | 0.736551736 | 0.012292531 | 0.791054197 |
| FBXO18 | magenta | -0.078322979 | 0.090906388 | -0.108873196 | 0.01859941 |
| FBXW8 | magenta | -0.124296864 | 0.007159867 | -0.146756432 | 0.001471891 |
| FITM2 | magenta | -0.038266003 | 0.409359462 | -0.051833121 | 0.263621286 |
| FLII | magenta | 0.169502815 | 0.000233388 | 0.185464127 | 5.53E-05 |
| FO53875 | magenta | -0.13242833 | 0.004146884 | -0.147274208 | 0.001415298 |
| FOXK1 | magenta | -0.185998352 | 5.25E-05 | -0.188089453 | 4.31E-05 |
| FOXP4 | magenta | -0.21785771 | 2.01E-06 | -0.208436661 | 5.57E-06 |
| FRMD8 | magenta | 0.085047745 | 0.066313301 | 0.096533622 | 0.037033825 |
| FTO | magenta | 0.143389592 | 0.001893559 | 0.144945044 | 0.001686629 |
| GATSL2 | magenta | -0.062666592 | 0.176395705 | -0.070196479 | 0.12983259 |
| GBA2 | magenta | 0.091381799 | 0.048425411 | 0.081796978 | 0.077417581 |
| GBF1 | magenta | 0.269309348 | 3.34E-09 | 0.270278499 | 2.92E-09 |
| GCC1 | magenta | 0.065935483 | 0.154849922 | 0.064725198 | 0.162582205 |
| GCN1L1 | magenta | -0.044432962 | 0.338010129 | -0.045600773 | 0.325454066 |
| GLG1 | magenta | 0.101112478 | 0.028903131 | 0.114017788 | 0.013686552 |
| GLTSCR1 | magenta | 0.086794923 | 0.060907606 | 0.088798789 | 0.055161125 |
| GLYR1 | magenta | 0.068531501 | 0.13920761 | 0.054341578 | 0.241176807 |
| GMPR2 | magenta | -0.021835614 | 0.637881321 | -0.027697445 | 0.550469111 |
| GNA11 | magenta | 0.206247505 | 7.01E-06 | 0.218027974 | 1.97E-06 |
| GNA12 | magenta | -0.026659934 | 0.565504893 | -0.043684133 | 0.346222408 |
| GNL3L | magenta | 0.021934442 | 0.636360248 | 0.023936859 | 0.605877085 |
| GORASP | magenta | 0.073715178 | 0.111633361 | 0.074341024 | 0.108617442 |
| GSE1 | magenta | 0.127764484 | 0.005693077 | 0.105494417 | 0.022607129 |
| GTF3C1 | magenta | 0.033006505 | 0.476737934 | 0.045794486 | 0.323400917 |
| HARBI1 | magenta | 0.15842673 | 0.000589978 | 0.180610111 | 8.68E-05 |
| HAUS7 | magenta | -0.047711686 | 0.303535558 | -0.037986987 | 0.412785627 |
| HCFC1 | magenta | 0.043210064 | 0.351486366 | 0.041299559 | 0.373208658 |
| HECTD4 | magenta | 0.051189599 | 0.269603607 | 0.029598108 | 0.523443631 |
| HERC2 | magenta | 0.034477107 | 0.457311658 | 0.013742854 | 0.767074554 |
| HGS | magenta | -0.012053505 | 0.795025914 | -0.037213828 | 0.422367675 |
| HIC2 | magenta | -0.219178845 | 1.73E-06 | -0.242025304 | 1.19E-07 |
| HNRNPU | magenta | 0.027818896 | 0.548721938 | -0.007188098 | 0.876882954 |
| HUWE1 | magenta | -0.089019139 | 0.054557668 | -0.086686219 | 0.061232972 |

| | | | | | |
|---|---|---|---|---|---|
| IGF2R | magenta | 0.019746588 | 0.670380932 | 0.01318214 | 0.776320444 |
| IKBKG | magenta | -0.103817704 | 0.024859915 | -0.13044221 | 0.004751755 |
| ILK | magenta | -0.03540809 | 0.445246316 | -0.07133873 | 0.123688766 |
| INO80 | magenta | 0.081903254 | 0.077032063 | 0.06147391 | 0.184787796 |
| IP6K1 | magenta | -0.050120429 | 0.279746775 | -0.052407818 | 0.25835648 |
| IPPK | magenta | 0.060704063 | 0.19035889 | 0.045652729 | 0.324902557 |
| IRF2 | magenta | 0.046609209 | 0.314858096 | 0.062744883 | 0.175854897 |
| IRF2BPL | magenta | -0.310680429 | 6.58E-12 | -0.314251927 | 3.66E-12 |
| KAT5 | magenta | -0.030384202 | 0.512467932 | -0.037048511 | 0.424433238 |
| KBTBD4 | magenta | 0.06116322 | 0.18702149 | 0.040068892 | 0.387630464 |
| KCTD5 | magenta | 0.093534595 | 0.04335087 | 0.100886745 | 0.029264684 |
| KDM5C | magenta | 0.166109865 | 0.000312008 | 0.151408291 | 0.001030249 |
| KHNYN | magenta | -0.107720443 | 0.019890947 | -0.133846194 | 0.003758644 |
| KIAA019 | magenta | 0.133809851 | 0.00376817 | 0.118868739 | 0.010140174 |
| KLF13 | magenta | -0.121924197 | 0.008349749 | -0.132198415 | 0.00421315 |
| KLHDC2 | magenta | -0.023280467 | 0.615797737 | -0.046043216 | 0.320777031 |
| KLHL26 | magenta | -0.053576387 | 0.247876434 | -0.048200209 | 0.298605697 |
| KLHL36 | magenta | 0.070872235 | 0.126170003 | 0.096269515 | 0.037556835 |
| KMT2B | magenta | -0.093874823 | 0.04259139 | -0.112871321 | 0.014669536 |
| KMT2D | magenta | -0.036021852 | 0.437392042 | -0.054130302 | 0.243013801 |
| LARS2 | magenta | 0.187439921 | 4.58E-05 | 0.169223887 | 0.000239076 |
| LDB1 | magenta | -0.032385609 | 0.485073081 | -0.064077496 | 0.166837748 |
| LDOC1L | magenta | 0.03220188 | 0.487554534 | 0.006568317 | 0.887424674 |
| LIG3 | magenta | 0.150988908 | 0.001064365 | 0.115649247 | 0.012387808 |
| LLGL1 | magenta | -0.040443465 | 0.383205531 | -0.046147168 | 0.319684538 |
| LPCAT3 | magenta | 0.055316542 | 0.232826041 | 0.024388133 | 0.599098198 |
| LRCH4 | magenta | -0.081478673 | 0.078581622 | -0.079424926 | 0.086437543 |
| LRFN3 | magenta | 0.107477623 | 0.020172741 | 0.092480707 | 0.045776171 |
| LRP3 | magenta | -0.040637179 | 0.380929276 | -0.028859331 | 0.53386715 |
| LRP5 | magenta | 0.155764113 | 0.000730925 | 0.136442535 | 0.00313202 |
| LRRC59 | magenta | 0.307322397 | 1.13E-11 | 0.319682772 | 1.48E-12 |
| M6PR | magenta | -0.025957615 | 0.575793397 | -0.047094033 | 0.309845258 |
| MAEA | magenta | 0.077997886 | 0.092259578 | 0.082955784 | 0.073297579 |
| MAFG | magenta | -0.118034287 | 0.010684973 | -0.139324679 | 0.002548752 |
| MAN2A2 | magenta | 0.046430571 | 0.316718443 | 0.001915494 | 0.967070057 |
| MAPK7 | magenta | 0.142296727 | 0.002052579 | 0.12326171 | 0.007658958 |
| MAZ | magenta | 0.090091368 | 0.05169904 | 0.08104405 | 0.080193924 |
| MBD1 | magenta | -0.035263542 | 0.447107676 | -0.041778584 | 0.367685836 |
| MBTPS1 | magenta | 0.17564274 | 0.000136073 | 0.164870728 | 0.00034643 |
| MCM3AP | magenta | 0.04728003 | 0.307936164 | 0.014945841 | 0.747350601 |
| MED12 | magenta | -0.094806834 | 0.040568121 | -0.103785683 | 0.024904759 |
| MGAT2 | magenta | 0.062210401 | 0.179571529 | 0.030556334 | 0.510080642 |
| MGAT5 | magenta | -0.042690086 | 0.357317864 | -0.053433702 | 0.249139933 |
| MINK1 | magenta | 0.132701269 | 0.004069442 | 0.115251952 | 0.012693633 |
| MLX | magenta | 0.372958842 | 7.37E-17 | 0.374522526 | 5.35E-17 |

| | | | | | |
|---|---|---|---|---|---|
| MLYCD | magenta | 0.044422229 | 0.338126951 | 0.08601249 | 0.063281667 |
| MMGT1 | magenta | -0.064496325 | 0.164076531 | -0.081634437 | 0.078010231 |
| MNT | magenta | 0.012654938 | 0.78504267 | 0.012189357 | 0.792767896 |
| MPPE1 | magenta | 0.123444543 | 0.007568608 | 0.120643861 | 0.009062506 |
| MUL1 | magenta | -0.021239488 | 0.647088379 | -0.029693452 | 0.522106024 |
| MYD88 | magenta | 0.186817495 | 4.86E-05 | 0.196417888 | 1.91E-05 |
| MYO18A | magenta | -0.09370544 | 0.042968083 | -0.122292939 | 0.008153962 |
| MYO1D | magenta | 0.185843827 | 5.33E-05 | 0.180699487 | 8.61E-05 |
| N4BP1 | magenta | 0.066470914 | 0.151519336 | 0.082365361 | 0.075373852 |
| NAPA | magenta | 0.144613395 | 0.001728927 | 0.159146352 | 0.000556468 |
| NCK2 | magenta | -0.031484 | 0.497315366 | -0.038073099 | 0.411726425 |
| NDST1 | magenta | -0.071903164 | 0.120737552 | -0.08357403 | 0.071173695 |
| NELFA | magenta | 0.0475382 | 0.305299167 | 0.038708911 | 0.403955546 |
| NEURL4 | magenta | 0.098487379 | 0.03335352 | 0.083607065 | 0.071061636 |
| NFE2L1 | magenta | -0.031037003 | 0.503445069 | -0.049899465 | 0.281874876 |
| NFYC | magenta | -0.082980848 | 0.073210482 | -0.093848608 | 0.042649508 |
| NISCH | magenta | 0.127922453 | 0.005633206 | 0.117499856 | 0.011047376 |
| NMT1 | magenta | 0.087319072 | 0.059358742 | 0.07655982 | 0.098440036 |
| NOA1 | magenta | 0.181361467 | 8.10E-05 | 0.168438622 | 0.000255791 |
| NOMO2 | magenta | -0.045539091 | 0.326109592 | -0.080931848 | 0.080614477 |
| NOMO3 | magenta | -0.090544958 | 0.050527916 | -0.109814563 | 0.017599576 |
| NOP9 | magenta | -0.075154769 | 0.104792563 | -0.073578455 | 0.112300909 |
| NOTCH1 | magenta | 0.041326804 | 0.372893179 | 0.032562459 | 0.482691 |
| NSF | magenta | 0.362351942 | 6.17E-16 | 0.364429395 | 4.09E-16 |
| NUMA1 | magenta | 0.034174086 | 0.461277867 | 0.010211672 | 0.825800722 |
| OGDH | magenta | 0.117804052 | 0.010839785 | 0.122345533 | 0.008126373 |
| OGG1 | magenta | -0.076167299 | 0.10018304 | -0.110132306 | 0.017272865 |
| OPN3 | magenta | -0.084600126 | 0.067759878 | -0.112979892 | 0.014573859 |
| PAFAH1I | magenta | 0.04805344 | 0.300081163 | 0.035004362 | 0.450456202 |
| PCBP1 | magenta | 0.055197231 | 0.233836845 | 0.038564581 | 0.405711821 |
| PCIF1 | magenta | -0.190076422 | 3.56E-05 | -0.194951089 | 2.21E-05 |
| PELO | magenta | -0.055507417 | 0.23121539 | -0.048011223 | 0.300506461 |
| PI4KA | magenta | 0.058250336 | 0.208936929 | 0.044175619 | 0.340818189 |
| PINK1 | magenta | -0.015638539 | 0.7360667 | -0.016682539 | 0.719167544 |
| PJA1 | magenta | -0.161701171 | 0.00045125 | -0.162357631 | 0.000427377 |
| PLD2 | magenta | 0.163638465 | 0.000384146 | 0.167785953 | 0.000270508 |
| PLEKHM | magenta | -0.12637827 | 0.006243507 | -0.132746259 | 0.004056803 |
| PLXNA3 | magenta | -0.098446163 | 0.033427812 | -0.095105953 | 0.039936242 |
| PNPO | magenta | 0.195955837 | 2.00E-05 | 0.180600635 | 8.68E-05 |
| POLDIP3 | magenta | 0.133808358 | 0.003768562 | 0.112825946 | 0.014709686 |
| POLG | magenta | 0.128263441 | 0.005505894 | 0.098012756 | 0.034217614 |
| POLR1A | magenta | -0.029348158 | 0.526958524 | -0.036836677 | 0.427088607 |
| POLR2A | magenta | 0.093157717 | 0.044205443 | 0.098281406 | 0.033726198 |
| POLR3E | magenta | 0.016018054 | 0.729908268 | 0.024310944 | 0.600255284 |
| POM121 | magenta | 0.003400419 | 0.941577773 | -0.040308531 | 0.384795974 |

| | | | | | |
|---|---|---|---|---|---|
| POM121C | magenta | 0.080844229 | 0.080944126 | 0.053887454 | 0.24513739 |
| PPIL2 | magenta | 0.188114858 | 4.30E-05 | 0.172757866 | 0.000175732 |
| PPP6R1 | magenta | 0.080710576 | 0.081449073 | 0.086121097 | 0.062947645 |
| PRDM4 | magenta | -0.011563888 | 0.803177964 | -0.04069305 | 0.380274306 |
| PRMT5 | magenta | -0.012417717 | 0.788976209 | -0.034665258 | 0.454858612 |
| PRPF8 | magenta | 0.154523797 | 0.000806706 | 0.150507882 | 0.001104776 |
| PRPS1 | magenta | -0.082867475 | 0.073605129 | -0.097804558 | 0.034602633 |
| PRR12 | magenta | 0.069630939 | 0.132960586 | 0.063351828 | 0.171704059 |
| PRRC2B | magenta | 0.022585276 | 0.62638162 | -0.001321343 | 0.97728096 |
| PSKH1 | magenta | 0.019380398 | 0.676143966 | 0.035577226 | 0.443073928 |
| PSME3 | magenta | 0.277413166 | 1.07E-09 | 0.263017038 | 7.90E-09 |
| PTGFRN | magenta | 0.204815553 | 8.13E-06 | 0.18446651 | 6.07E-05 |
| PTPN23 | magenta | 0.101172863 | 0.028807066 | 0.10539854 | 0.022731007 |
| PTPN9 | magenta | 0.175224998 | 0.000141243 | 0.151755063 | 0.001002806 |
| QRICH1 | magenta | 0.055685941 | 0.229716111 | 0.026223434 | 0.571888977 |
| RAB19 | magenta | 0.261173271 | 1.01E-08 | 0.260395553 | 1.12E-08 |
| RAB35 | magenta | 0.108867817 | 0.018605262 | 0.114645863 | 0.013173015 |
| RAB43 | magenta | -0.100446989 | 0.02998018 | -0.102447901 | 0.026841688 |
| RAB5C | magenta | 0.07299276 | 0.115196114 | 0.084947208 | 0.06663598 |
| RAI1 | magenta | 0.109232913 | 0.01821165 | 0.103175391 | 0.025772883 |
| RANBP10 | magenta | 0.171087987 | 0.000203398 | 0.175248408 | 0.000140948 |
| RAPGEF1 | magenta | 0.030866817 | 0.505789263 | 0.026184802 | 0.572455629 |
| RAVER1 | magenta | 0.054495296 | 0.239846406 | 0.042535765 | 0.359060189 |
| RBM14 | magenta | 0.095840285 | 0.038420198 | 0.091235208 | 0.048788327 |
| RBM23 | magenta | -0.036796679 | 0.427591068 | -0.03477982 | 0.453368608 |
| RECQL | magenta | -0.060419272 | 0.19245075 | -0.092914697 | 0.044763975 |
| RETSAT | magenta | 0.360539225 | 8.80E-16 | 0.351533693 | 4.97E-15 |
| RFFL | magenta | 0.22825594 | 6.18E-07 | 0.216439631 | 2.35E-06 |
| RFX1 | magenta | -0.041488044 | 0.371029476 | -0.040918079 | 0.377643279 |
| RGP1 | magenta | -0.095973008 | 0.038151457 | -0.094491219 | 0.041243987 |
| RNF123 | magenta | 0.132795769 | 0.004042935 | 0.117636286 | 0.010953841 |
| RNF19B | magenta | -0.03231929 | 0.485967995 | -0.02029584 | 0.661773098 |
| RNF216 | magenta | -0.139546241 | 0.002508297 | -0.124326134 | 0.007146187 |
| RNF31 | magenta | -0.095237327 | 0.039661361 | -0.10790963 | 0.019673774 |
| RNF40 | magenta | -0.00658724 | 0.887102521 | -0.018307985 | 0.69312949 |
| RNF44 | magenta | -0.157871814 | 0.000617086 | -0.166154207 | 0.000310838 |
| RPAP1 | magenta | 0.24185188 | 1.22E-07 | 0.227536529 | 6.72E-07 |
| RPTOR | magenta | -0.078651405 | 0.089555525 | -0.083432111 | 0.071656734 |
| SAP130 | magenta | -0.002033056 | 0.965050261 | -0.014226952 | 0.759118463 |
| SBF1 | magenta | 0.064355424 | 0.16500159 | 0.057359084 | 0.21599904 |
| SCAP | magenta | 0.070077545 | 0.130485639 | 0.079663237 | 0.085494743 |
| SCRN1 | magenta | -0.331128938 | 2.06E-13 | -0.330801954 | 2.18E-13 |
| SEC14L1 | magenta | 0.180933053 | 8.42E-05 | 0.17136083 | 0.000198615 |
| SEC24C | magenta | 0.079024464 | 0.088040676 | 0.059905887 | 0.196264149 |
| SEMA4C | magenta | -0.138357885 | 0.002732331 | -0.169323426 | 0.000237031 |

| | | | | | |
|---|---|---|---|---|---|
| SEPT8 | magenta | 0.08039909 | 0.082635773 | 0.070129406 | 0.130200566 |
| SEPT9 | magenta | 0.13765746 | 0.002872767 | 0.157127179 | 0.00065528 |
| SETD1A | magenta | -0.097791357 | 0.034627171 | -0.09366468 | 0.043059148 |
| SETD1B | magenta | 0.050974971 | 0.271619324 | 0.047162554 | 0.309141043 |
| SF1 | magenta | 0.014951305 | 0.747261381 | -0.019068113 | 0.681073659 |
| SF3A1 | magenta | -0.042389381 | 0.3607178 | -0.071018308 | 0.125388937 |
| SIX5 | magenta | 0.002445736 | 0.957961987 | -0.010508961 | 0.820813953 |
| SLC25A2 | magenta | -0.034089376 | 0.462390046 | -0.066050138 | 0.154132104 |
| SLC35B1 | magenta | 0.223808612 | 1.03E-06 | 0.235139177 | 2.75E-07 |
| SLC6A6 | magenta | 0.255779493 | 2.07E-08 | 0.268575898 | 3.70E-09 |
| SMARCC | magenta | -0.032410892 | 0.484732146 | -0.063168524 | 0.172949878 |
| SMG6 | magenta | 0.038713125 | 0.403904334 | 0.021768517 | 0.638914874 |
| SMYD4 | magenta | 0.090793924 | 0.049894586 | 0.071837642 | 0.121077301 |
| SNRNP2( | magenta | -0.111893824 | 0.015556094 | -0.11960188 | 0.009682014 |
| SNX12 | magenta | -0.129001243 | 0.005239246 | -0.170199051 | 0.000219739 |
| SOCS7 | magenta | -0.125048299 | 0.00681601 | -0.154628731 | 0.000800024 |
| SOGA1 | magenta | -0.241497067 | 1.27E-07 | -0.262152478 | 8.88E-09 |
| SP2 | magenta | 0.062304716 | 0.178911494 | 0.049170331 | 0.288974646 |
| SPEN | magenta | -0.020273899 | 0.662116114 | -0.043388075 | 0.349503869 |
| SPNS1 | magenta | -0.109593266 | 0.017830297 | -0.119313572 | 0.009859944 |
| SPTAN1 | magenta | 0.002073337 | 0.964358257 | 0.006134969 | 0.894807321 |
| SRRD | magenta | 0.070903048 | 0.126004928 | 0.036340887 | 0.433340972 |
| SRRM2 | magenta | 0.056406765 | 0.223732626 | 0.033761978 | 0.466702519 |
| STRIP1 | magenta | -0.133617511 | 0.003818947 | -0.151848174 | 0.000995553 |
| STRN4 | magenta | 0.174660984 | 0.000148515 | 0.173091166 | 0.00017065 |
| SUN2 | magenta | 0.076658536 | 0.098005504 | 0.05755878 | 0.214401983 |
| SUPT16H | magenta | -0.073445743 | 0.112951866 | -0.109230006 | 0.018214755 |
| SUSD6 | magenta | 0.14587303 | 0.001573268 | 0.160098742 | 0.000514833 |
| SZRD1 | magenta | 0.010472231 | 0.82142968 | 0.012399337 | 0.789281211 |
| TAF6 | magenta | 0.046670343 | 0.314223087 | 0.029060262 | 0.531021873 |
| TAOK2 | magenta | 0.174639416 | 0.0001488 | 0.179985053 | 9.19E-05 |
| TBC1D9E | magenta | 0.155993718 | 0.000717641 | 0.144155532 | 0.00178892 |
| TFIP11 | magenta | -0.009079199 | 0.844859572 | -0.023090969 | 0.618674972 |
| THTPA | magenta | -0.02112076 | 0.648928619 | -0.041874548 | 0.366585567 |
| TIAF1 | magenta | -0.22679252 | 7.32E-07 | -0.233910498 | 3.18E-07 |
| TINF2 | magenta | -0.021747801 | 0.639234133 | -0.030719683 | 0.507820531 |
| TM9SF1 | magenta | 0.076643194 | 0.098072939 | 0.056759116 | 0.220848565 |
| TMEM10 | magenta | 0.061674665 | 0.183354977 | 0.045626924 | 0.325176398 |
| TMEM12 | magenta | -0.064400873 | 0.164702779 | -0.042544283 | 0.358963881 |
| TMEM55 | magenta | 0.006428685 | 0.88980245 | -0.016975701 | 0.714446239 |
| TNFSF13 | magenta | 0.238700431 | 1.79E-07 | 0.213496755 | 3.24E-06 |
| TNRC18 | magenta | 0.034029493 | 0.463177168 | 0.046248987 | 0.318616824 |
| TNS3 | magenta | 0.071716515 | 0.121707332 | 0.076412229 | 0.099092567 |
| TOM1L1 | magenta | 0.30071402 | 3.24E-11 | 0.281473542 | 5.94E-10 |
| TOX4 | magenta | -0.034172432 | 0.461299566 | -0.038750842 | 0.403446166 |

| | | | | | |
|---|---|---|---|---|---|
| TP53I11 | magenta | -0.041148799 | 0.374957353 | -0.021243716 | 0.647022895 |
| TRAF3 | magenta | -0.139689759 | 0.002482405 | -0.141411777 | 0.002190193 |
| TRAF7 | magenta | 0.142054575 | 0.002089427 | 0.158509844 | 0.000586014 |
| TRAM2 | magenta | -0.307882107 | 1.04E-11 | -0.306260524 | 1.34E-11 |
| TRAP1 | magenta | 0.18875416 | 4.04E-05 | 0.182512476 | 7.28E-05 |
| TSSK6 | magenta | 0.102651604 | 0.026538636 | 0.114961417 | 0.012921474 |
| TXNDC5 | magenta | 0.111625882 | 0.015807172 | 0.083119332 | 0.072730772 |
| TYK2 | magenta | 0.138381871 | 0.002727635 | 0.140118271 | 0.002406542 |
| UBE3B | magenta | 0.078406974 | 0.090559361 | 0.05589078 | 0.228004337 |
| UBN1 | magenta | 0.048437556 | 0.296229881 | 0.058305085 | 0.208508614 |
| ULK1 | magenta | -0.074796003 | 0.106465539 | -0.09136381 | 0.048469825 |
| UNC119B | magenta | -0.103275094 | 0.0256293 | -0.123932607 | 0.007332071 |
| UQCRC2 | magenta | 0.116672299 | 0.011630043 | 0.115267089 | 0.01268186 |
| URB1 | magenta | 0.042893695 | 0.355027219 | 0.010192195 | 0.826127674 |
| USP19 | magenta | -0.014305712 | 0.757826438 | -0.028184097 | 0.54348469 |
| USP22 | magenta | 0.040656883 | 0.380698211 | 0.017342207 | 0.70855906 |
| VKORC1 | magenta | 0.15620318 | 0.000705718 | 0.146642216 | 0.001484652 |
| VPRBP | magenta | 0.06442333 | 0.164555276 | 0.039678738 | 0.392272333 |
| VPS37C | magenta | 0.166758954 | 0.000295278 | 0.149289731 | 0.001213521 |
| VPS51 | magenta | -0.039874189 | 0.389942768 | -0.04238055 | 0.360817952 |
| VPS52 | magenta | -0.043194536 | 0.351659633 | -0.067462799 | 0.145493524 |
| WASF2 | magenta | -0.125501394 | 0.006615895 | -0.121704746 | 0.008468248 |
| WASH1 | magenta | -0.116277833 | 0.011917224 | -0.126496216 | 0.00619488 |
| WDR1 | magenta | 0.207319478 | 6.26E-06 | 0.224981806 | 9.02E-07 |
| WDR6 | magenta | 0.144078419 | 0.00179921 | 0.112755137 | 0.014772532 |
| WDR81 | magenta | 0.021357338 | 0.645263872 | 0.018237513 | 0.69425118 |
| WDTC1 | magenta | -0.076670995 | 0.097950773 | -0.07091818 | 0.125923928 |
| WWP2 | magenta | 0.223708225 | 1.04E-06 | 0.245655856 | 7.57E-08 |
| XPC | magenta | 0.044712265 | 0.334979266 | 0.019288193 | 0.677598088 |
| XPO6 | magenta | 0.094840129 | 0.040497372 | 0.117033 | 0.01137282 |
| ZBTB39 | magenta | -0.175140795 | 0.000142307 | -0.209798406 | 4.82E-06 |
| ZC3H4 | magenta | 0.112001768 | 0.015455937 | 0.094784373 | 0.040615909 |
| ZC3H7B | magenta | 0.051346324 | 0.268138172 | 0.043887801 | 0.343976377 |
| ZCCHC14 | magenta | 0.01340954 | 0.772566844 | -0.002248484 | 0.961349648 |
| ZDHHC8 | magenta | -0.045811469 | 0.323221323 | -0.057705151 | 0.213236791 |
| ZFAND3 | magenta | -0.068149391 | 0.141430733 | -0.05855987 | 0.206523692 |
| ZFP64 | magenta | -0.074510488 | 0.107811957 | -0.09417016 | 0.041941242 |
| ZNF319 | magenta | 0.098955306 | 0.032519902 | 0.113078635 | 0.01448732 |
| ZNF384 | magenta | -0.057465777 | 0.21514471 | -0.089128383 | 0.054260531 |
| ZNF496 | magenta | 0.013172421 | 0.776480987 | -0.017353927 | 0.708371093 |
| ZNF500 | magenta | 0.049001295 | 0.290637591 | 0.062610331 | 0.1767851 |
| ZNF592 | magenta | 0.070042387 | 0.130679173 | 0.058353362 | 0.20813145 |
| ZNF646 | magenta | -0.050779243 | 0.273466492 | -0.055250311 | 0.233386772 |
| ZNF668 | magenta | 0.152886882 | 0.000917857 | 0.147639183 | 0.001376615 |
| ZNF710 | magenta | 0.233045772 | 3.53E-07 | 0.217959526 | 1.99E-06 |

| | | | | | |
|---|---|---|---|---|---|
| ZNF74 | magenta | 0.095892808 | 0.038313656 | 0.076608276 | 0.098226549 |
| ZNF740 | magenta | -0.16188631 | 0.000444395 | -0.188177004 | 4.27E-05 |
| ZNF777 | magenta | 0.028253293 | 0.54249518 | 0.011142295 | 0.810214601 |
| ZSWIM8 | magenta | 0.178772449 | 0.000102631 | 0.151899615 | 0.000991566 |
| ZZEF1 | magenta | 0.13733513 | 0.002939575 | 0.142005392 | 0.002096984 |
| ADAM12 | mediumpur | -0.034742674 | 0.453851428 | -0.001572143 | 0.972970235 |
| ADAMTS | mediumpur | -0.197896809 | 1.65E-05 | -0.173914546 | 0.000158681 |
| ADAMTS | mediumpur | -0.083125268 | 0.072710266 | -0.037775909 | 0.415388781 |
| AEBP1 | mediumpur | -0.269181854 | 3.40E-09 | -0.225287083 | 8.71E-07 |
| ANGPTL | mediumpur | -0.223351059 | 1.09E-06 | -0.204783381 | 8.16E-06 |
| ANTXR1 | mediumpur | -0.127932723 | 0.005629333 | -0.118390114 | 0.010449564 |
| BGN | mediumpur | -0.039120495 | 0.398972171 | 0.002326271 | 0.960013631 |
| C3orf80 | mediumpur | -0.078977224 | 0.08823135 | -0.053531563 | 0.248272872 |
| CD248 | mediumpur | -0.171703218 | 0.000192762 | -0.141388211 | 0.00219397 |
| CDH11 | mediumpur | -0.078600206 | 0.089765045 | -0.054234558 | 0.242106096 |
| CHST11 | mediumpur | -0.16497067 | 0.000343527 | -0.162146999 | 0.000434906 |
| COL10A1 | mediumpur | 0.016177058 | 0.727333234 | 0.049354171 | 0.287173325 |
| COL15A1 | mediumpur | 0.001301483 | 0.977622329 | 0.018848262 | 0.684552391 |
| COL1A1 | mediumpur | -0.017419504 | 0.707319637 | 0.026449434 | 0.568579316 |
| COL1A2 | mediumpur | 0.03556745 | 0.443199329 | 0.061287775 | 0.186123631 |
| COL3A1 | mediumpur | -0.08870638 | 0.055415846 | -0.051070497 | 0.270720907 |
| COL4A1 | mediumpur | -0.041941114 | 0.365823564 | -0.039841637 | 0.39033016 |
| COL4A2 | mediumpur | -0.145143328 | 0.001661796 | -0.138664817 | 0.002672785 |
| COL5A1 | mediumpur | -0.146722958 | 0.001475621 | -0.116373632 | 0.01184691 |
| COL5A2 | mediumpur | 0.022988427 | 0.620234356 | 0.047911135 | 0.301516371 |
| COL6A1 | mediumpur | -0.211417437 | 4.05E-06 | -0.178656934 | 0.000103714 |
| COL6A2 | mediumpur | -0.203139213 | 9.67E-06 | -0.164457733 | 0.000358668 |
| COL6A3 | mediumpur | -0.184908896 | 5.82E-05 | -0.153928205 | 0.000845622 |
| COL8A1 | mediumpur | 0.023637597 | 0.610391267 | 0.054755355 | 0.237607399 |
| DIO2 | mediumpur | 0.097373633 | 0.03541127 | 0.103306225 | 0.02558461 |
| DKK3 | mediumpur | -0.16282717 | 0.000411029 | -0.168619552 | 0.000251845 |
| ECM1 | mediumpur | -0.081377019 | 0.078956347 | -0.057586996 | 0.214177019 |
| ETV1 | mediumpur | 0.026169512 | 0.572679976 | 0.015960767 | 0.73083678 |
| F2R | mediumpur | -0.011560571 | 0.803233262 | -0.002053369 | 0.964701292 |
| FAP | mediumpur | -0.171089918 | 0.000203363 | -0.146164512 | 0.001539131 |
| FBN1 | mediumpur | -0.132122975 | 0.004235101 | -0.113023907 | 0.014535228 |
| FN1 | mediumpur | -0.091841797 | 0.04730123 | -0.067343192 | 0.146210299 |
| FNDC1 | mediumpur | -0.002638456 | 0.95465289 | 0.035372626 | 0.445702586 |
| GJA1 | mediumpur | 0.003762982 | 0.935361564 | 0.006491045 | 0.888740418 |
| INHBA | mediumpur | 0.025381104 | 0.584304194 | 0.041933811 | 0.365907115 |
| ISLR | mediumpur | -0.156092161 | 0.000712015 | -0.113510233 | 0.014114341 |
| LAMB1 | mediumpur | 0.133245173 | 0.003919007 | 0.119064551 | 0.010015959 |
| LOXL2 | mediumpur | 0.14674734 | 0.001472903 | 0.163552931 | 0.000386901 |
| LUM | mediumpur | -0.095093652 | 0.039962061 | -0.057545949 | 0.214504347 |
| MMP11 | mediumpur | 0.0019096 | 0.967171332 | 0.045835112 | 0.322971396 |

| | | | | | |
|---|---|---|---|---|---|
| MMP14 | mediumpur | 0.025178786 | 0.587304699 | 0.024462133 | 0.597989882 |
| MMP2 | mediumpur | 0.031589082 | 0.495880143 | 0.03306006 | 0.476022675 |
| MOXD1 | mediumpur | 0.109372154 | 0.018063458 | 0.090319452 | 0.051107351 |
| MXRA8 | mediumpur | -0.29070765 | 1.51E-10 | -0.248156624 | 5.52E-08 |
| NID1 | mediumpur | -0.030867361 | 0.50578176 | -0.020984075 | 0.651049835 |
| NID2 | mediumpur | -0.107728632 | 0.019881504 | -0.096524439 | 0.037051906 |
| NTM | mediumpur | -0.007662449 | 0.868829395 | 0.01957537 | 0.673073153 |
| OLFML2l | mediumpur | 0.02744503 | 0.554109037 | 0.064290982 | 0.165425979 |
| P4HA3 | mediumpur | 0.004892229 | 0.91602726 | 0.014654834 | 0.752107352 |
| PAMR1 | mediumpur | -0.171074868 | 0.00020363 | -0.13541517 | 0.003367646 |
| PCOLCE | mediumpur | -0.202350703 | 1.05E-05 | -0.173639868 | 0.000162584 |
| PDGFB | mediumpur | -0.113036486 | 0.014524204 | -0.132269184 | 0.004192651 |
| PDGFRB | mediumpur | -0.101038807 | 0.029020704 | -0.065858605 | 0.155332649 |
| PMEPA1 | mediumpur | 0.036633029 | 0.429650423 | 0.028203476 | 0.543207486 |
| PRDM1 | mediumpur | -0.007502451 | 0.871544372 | 0.003154055 | 0.945803781 |
| PXDN | mediumpur | -0.339744845 | 4.42E-14 | -0.341285578 | 3.34E-14 |
| SPARC | mediumpur | -0.098868523 | 0.032673149 | -0.066982921 | 0.148385593 |
| SPOCK1 | mediumpur | 0.007498592 | 0.87160988 | -0.01106647 | 0.811481816 |
| SPON2 | mediumpur | -0.027248025 | 0.556958056 | 0.006933417 | 0.88121225 |
| SRPX2 | mediumpur | -0.100774471 | 0.02944595 | -0.058534437 | 0.206721207 |
| SULF1 | mediumpur | 0.078671936 | 0.089471617 | 0.101580389 | 0.028165879 |
| SYDE1 | mediumpur | -0.207463665 | 6.17E-06 | -0.196410426 | 1.91E-05 |
| TGFBI | mediumpur | 0.085697084 | 0.064259988 | 0.092937109 | 0.04471222 |
| THBS1 | mediumpur | -0.08503964 | 0.066339267 | -0.065022557 | 0.160656055 |
| THBS2 | mediumpur | -0.113382565 | 0.014223779 | -0.073922 | 0.110629493 |
| THY1 | mediumpur | -0.178824774 | 0.000102144 | -0.155690384 | 0.000735239 |
| TNC | mediumpur | 0.175843429 | 0.000133653 | 0.176266877 | 0.00012868 |
| VCAN | mediumpur | 0.005966387 | 0.897681827 | -0.020624572 | 0.656642386 |
| WISP1 | mediumpur | -0.121336757 | 0.008670323 | -0.110900464 | 0.016504843 |
| WNT2 | mediumpur | -0.064786634 | 0.162182835 | -0.029950269 | 0.518511817 |
| WNT5A | mediumpur | -0.035175026 | 0.448249689 | -0.036875016 | 0.426607307 |
| ZNF469 | mediumpur | -0.079490277 | 0.086178177 | -0.049496269 | 0.285786192 |
| ABI3 | midnightblu | -0.06112643 | 0.187287305 | -0.006120779 | 0.89504923 |
| ACP5 | midnightblu | -0.142030061 | 0.002093191 | -0.079422578 | 0.086446876 |
| ADAM8 | midnightblu | -0.04837571 | 0.296847738 | -0.003720216 | 0.936094598 |
| ADAP2 | midnightblu | -0.027167139 | 0.558129854 | -0.00175316 | 0.969859413 |
| ADORA3 | midnightblu | -0.134180591 | 0.003672018 | -0.112436308 | 0.015058444 |
| AIF1 | midnightblu | -0.102871124 | 0.026215354 | -0.062447183 | 0.177917895 |
| ALOX15I | midnightblu | -0.20982888 | 4.80E-06 | -0.204537116 | 8.37E-06 |
| ALOX5 | midnightblu | -0.205649712 | 7.46E-06 | -0.189750831 | 3.67E-05 |
| ALOX5A | midnightblu | 0.000582611 | 0.989981561 | 0.044976776 | 0.332125061 |
| AMICA1 | midnightblu | -0.002286837 | 0.960690909 | 0.049211148 | 0.288574049 |
| APOBR | midnightblu | 0.075592893 | 0.102777821 | 0.12770678 | 0.00571509 |
| APOC1 | midnightblu | -0.072018451 | 0.120141571 | -0.030231945 | 0.514584426 |
| APOC2 | midnightblu | -0.074564087 | 0.10755818 | -0.058840501 | 0.204353281 |

| | | | | | |
|---|---|---|---|---|---|
| APOC4-A | midnightblı | -0.066838168 | 0.149266509 | -0.059527112 | 0.199112848 |
| APOE | midnightblı | -0.236353291 | 2.37E-07 | -0.186626655 | 4.95E-05 |
| ARHGAP | midnightblı | -0.178629064 | 0.000103976 | -0.156997661 | 0.000662142 |
| ARRB2 | midnightblı | -0.028859399 | 0.53386618 | -0.013562001 | 0.770053186 |
| ARRDC2 | midnightblı | -0.141125656 | 0.002236458 | -0.120989534 | 0.008864922 |
| AURKC | midnightblı | -0.11963147 | 0.009663915 | -0.110109688 | 0.017295944 |
| BCL2A1 | midnightblı | 0.054451285 | 0.240226788 | 0.100539142 | 0.029829015 |
| C15orf48 | midnightblı | -0.005366935 | 0.907913726 | 0.02948675 | 0.525008105 |
| C19orf35 | midnightblı | -0.055194061 | 0.233863742 | -0.024263477 | 0.60096731 |
| C19orf38 | midnightblı | -0.172302622 | 0.000182902 | -0.127116969 | 0.005944515 |
| C1orf162 | midnightblı | -0.157101915 | 0.000656613 | -0.111721715 | 0.015716966 |
| C1orf54 | midnightblı | -0.125616695 | 0.006565818 | -0.11261892 | 0.014894097 |
| C1QA | midnightblı | -0.116749094 | 0.011574852 | -0.070610817 | 0.127577255 |
| C1QB | midnightblı | -0.062883263 | 0.174902023 | -0.015408338 | 0.739810469 |
| C1QC | midnightblı | -0.066006814 | 0.154403041 | -0.020154003 | 0.663991752 |
| C2 | midnightblı | -0.206212756 | 7.03E-06 | -0.184741117 | 5.91E-05 |
| C3 | midnightblı | -0.005081036 | 0.912799139 | 0.005230391 | 0.910246553 |
| C5AR1 | midnightblı | -0.021872423 | 0.637314615 | 0.006446859 | 0.889492913 |
| CACNA2 | midnightblı | -0.165479425 | 0.000329101 | -0.14621378 | 0.001533428 |
| CAPG | midnightblı | -0.152258214 | 0.000964184 | -0.125514421 | 0.00661022 |
| CARD11 | midnightblı | -0.327170806 | 4.11E-13 | -0.306387566 | 1.32E-11 |
| CARD16 | midnightblı | -0.075044044 | 0.105306656 | -0.027992804 | 0.54622487 |
| CARD9 | midnightblı | -0.161177653 | 0.000471172 | -0.132688283 | 0.004073096 |
| CASP1 | midnightblı | -0.017737961 | 0.70222151 | 0.015097015 | 0.744883306 |
| CCDC88I | midnightblı | 0.078573995 | 0.089872461 | 0.104840251 | 0.02346409 |
| CCL1 | midnightblı | -0.001339122 | 0.976975353 | 0.037514106 | 0.41863088 |
| CCL18 | midnightblı | -0.004359415 | 0.92514433 | 0.026082557 | 0.57395665 |
| CCL2 | midnightblı | -0.112751809 | 0.014775492 | -0.109709397 | 0.017708893 |
| CCL25 | midnightblı | -0.163223926 | 0.00039767 | -0.125186309 | 0.00675449 |
| CCL3 | midnightblı | -0.023454788 | 0.613156124 | 0.018016922 | 0.697766616 |
| CCL3L3 | midnightblı | -0.027100921 | 0.559090051 | 0.013024296 | 0.778928972 |
| CCL4 | midnightblı | -0.018590907 | 0.68863303 | 0.038077401 | 0.411673543 |
| CCL4L1 | midnightblı | -0.017516119 | 0.705771557 | 0.026304681 | 0.57069811 |
| CCL7 | midnightblı | -0.045691762 | 0.324488633 | -0.032074399 | 0.489280297 |
| CCL8 | midnightblı | -0.09202082 | 0.046869673 | -0.078088305 | 0.091881604 |
| CD14 | midnightblı | -0.085210885 | 0.065792426 | -0.036584169 | 0.430266391 |
| CD1B | midnightblı | 0.012493685 | 0.787715949 | 0.031370571 | 0.498867063 |
| CD1C | midnightblı | -0.021019893 | 0.650493705 | 0.01388496 | 0.764736474 |
| CD1E | midnightblı | -0.052295394 | 0.25938064 | -0.027359276 | 0.555348298 |
| CD300A | midnightblı | -0.053782391 | 0.246060123 | -0.00735685 | 0.874016369 |
| CD300C | midnightblı | -0.067624083 | 0.144531239 | -0.036811186 | 0.427408784 |
| CD300E | midnightblı | -0.139179008 | 0.002575673 | -0.15783783 | 0.000618783 |
| CD300LB | midnightblı | -0.121357027 | 0.008659081 | -0.10293524 | 0.026121575 |
| CD300LF | midnightblı | -0.011471804 | 0.804713564 | 0.028156117 | 0.543885071 |
| CD33 | midnightblı | -0.11238399 | 0.015105822 | -0.087724381 | 0.058183513 |

| | | | | | |
|---|---|---|---|---|---|
| CD40 | midnightblu | -0.314133299 | 3.73E-12 | -0.305854435 | 1.43E-11 |
| CD68 | midnightblu | -0.001289873 | 0.977821912 | 0.001613395 | 0.972261282 |
| CD72 | midnightblu | -0.100617344 | 0.029701247 | -0.074078464 | 0.109874787 |
| CD74 | midnightblu | 0.037318802 | 0.42105912 | 0.071511028 | 0.122781995 |
| CEACAM | midnightblu | 0.059289705 | 0.200913622 | 0.093608825 | 0.043184203 |
| CEACAM | midnightblu | -0.040883089 | 0.378051639 | 0.003689607 | 0.936619293 |
| CFD | midnightblu | -0.015271362 | 0.742041035 | 0.020874164 | 0.652757585 |
| CHIT1 | midnightblu | -0.027885385 | 0.547766591 | -0.012869713 | 0.781486043 |
| CIITA | midnightblu | -0.07281744 | 0.116074043 | -0.041836235 | 0.367024596 |
| CLEC10A | midnightblu | -0.032510944 | 0.483384229 | -0.004783139 | 0.917893067 |
| CLEC4E | midnightblu | -0.093441238 | 0.04356125 | -0.072812602 | 0.116098345 |
| CLLU1OS | midnightblu | 0.108471633 | 0.019040766 | 0.129154806 | 0.005185231 |
| COTL1 | midnightblu | 0.028749979 | 0.535418845 | 0.04623847 | 0.318727 |
| CSF1 | midnightblu | -0.12233218 | 0.008133369 | -0.097688819 | 0.034818259 |
| CSF1R | midnightblu | -0.088318332 | 0.056496165 | -0.052718555 | 0.255540287 |
| CSF3R | midnightblu | -0.057575496 | 0.214268689 | -0.026216101 | 0.571996513 |
| CSTA | midnightblu | -0.04112148 | 0.37527478 | -0.027150706 | 0.558368061 |
| CTC-479( | midnightblu | -0.012412166 | 0.789068326 | 0.031609561 | 0.495600685 |
| CTSB | midnightblu | 0.174368657 | 0.000152421 | 0.193393357 | 2.58E-05 |
| CTSD | midnightblu | -0.037529836 | 0.418435676 | -0.000862713 | 0.98516544 |
| CTSZ | midnightblu | 0.084174516 | 0.069159223 | 0.115426243 | 0.012558658 |
| CXCL16 | midnightblu | -0.092556683 | 0.045597597 | -0.062742922 | 0.175868424 |
| CYTH4 | midnightblu | -0.066557166 | 0.15098794 | -0.014754017 | 0.750485052 |
| DOK2 | midnightblu | -0.024983349 | 0.590209884 | 0.032449906 | 0.484206301 |
| DOK3 | midnightblu | 0.089881581 | 0.052248297 | 0.11957462 | 0.009698715 |
| DPEP2 | midnightblu | -0.089272154 | 0.053871533 | -0.04264389 | 0.357838875 |
| EBI3 | midnightblu | -0.080775177 | 0.081204691 | -0.055315964 | 0.232830934 |
| EFHD2 | midnightblu | 0.009145418 | 0.843742532 | 0.056034647 | 0.226807516 |
| EMR1 | midnightblu | -0.163086972 | 0.000402235 | -0.168198129 | 0.000261124 |
| FAM26F | midnightblu | -0.073800499 | 0.111218368 | -0.018118419 | 0.696148315 |
| FBP1 | midnightblu | 0.235270403 | 2.70E-07 | 0.27129419 | 2.54E-09 |
| FCER1A | midnightblu | -0.119322977 | 0.009854094 | -0.108484601 | 0.019026372 |
| FCER1G | midnightblu | -0.037119788 | 0.423541942 | 0.007504399 | 0.871511312 |
| FCGR1A | midnightblu | -0.041813211 | 0.367288589 | -0.012901779 | 0.780955422 |
| FCGR1B | midnightblu | -0.06317027 | 0.17293798 | -0.037357798 | 0.42057363 |
| FCGR2B | midnightblu | -0.0874835 | 0.058879625 | -0.044643413 | 0.335724789 |
| FCN1 | midnightblu | -0.07037245 | 0.128870999 | -0.044681787 | 0.335309143 |
| FERMT3 | midnightblu | -0.031901953 | 0.491619979 | 0.025955581 | 0.575823322 |
| FFAR2 | midnightblu | 0.095119998 | 0.039906778 | 0.108092764 | 0.01946552 |
| FGD3 | midnightblu | 0.072072331 | 0.119863824 | 0.102862435 | 0.026228085 |
| FLT3LG | midnightblu | -0.223906538 | 1.02E-06 | -0.204409773 | 8.48E-06 |
| FOLR2 | midnightblu | -0.190599236 | 3.38E-05 | -0.17801506 | 0.000109929 |
| FPR1 | midnightblu | 0.007751502 | 0.867318928 | 0.026709967 | 0.564775309 |
| FTL | midnightblu | -0.074281189 | 0.108902977 | -0.045019737 | 0.331662967 |
| FUT7 | midnightblu | -0.010998831 | 0.81261262 | 0.037372347 | 0.420392575 |

| | | | | | |
|---|---|---|---|---|---|
| FXYD5 | midnightblu | -0.292326756 | 1.18E-10 | -0.234354067 | 3.02E-07 |
| GAL3ST4 | midnightblu | -0.137822409 | 0.002839116 | -0.121790912 | 0.008421543 |
| GALM | midnightblu | -0.095756263 | 0.038591155 | -0.063573584 | 0.170205863 |
| GM2A | midnightblu | 0.125745287 | 0.006510371 | 0.150415076 | 0.001112734 |
| GNA15 | midnightblu | 0.149508116 | 0.001193328 | 0.181147034 | 8.26E-05 |
| GPR84 | midnightblu | 0.039086935 | 0.39937712 | 0.067428096 | 0.145701215 |
| HAMP | midnightblu | -0.041735834 | 0.368176644 | -0.022604393 | 0.626089531 |
| HAVCR2 | midnightblu | -0.049199667 | 0.288686688 | -0.001516982 | 0.973918267 |
| HCK | midnightblu | -0.114774132 | 0.01307025 | -0.083616618 | 0.07102926 |
| HK3 | midnightblu | -0.017108667 | 0.712308427 | 0.012801529 | 0.782614669 |
| HLA-DM | midnightblu | 0.068199756 | 0.141136162 | 0.107801494 | 0.019797653 |
| HLA-DM | midnightblu | 0.191922536 | 2.98E-05 | 0.197495634 | 1.72E-05 |
| HLA-DO | midnightblu | -0.20316568 | 9.65E-06 | -0.175278902 | 0.000140565 |
| HLA-DP | midnightblu | -0.029537983 | 0.524288035 | -0.000810348 | 0.986065788 |
| HLA-DP | midnightblu | -0.050843359 | 0.272860459 | -0.0084019 | 0.856302267 |
| HLA-DQ | midnightblu | -0.031566854 | 0.49618354 | 0.012597565 | 0.785993526 |
| HLA-DQ | midnightblu | -0.092721797 | 0.04521153 | -0.044427734 | 0.338067032 |
| HLA-DQ | midnightblu | 0.023927801 | 0.606013494 | 0.049328438 | 0.287425006 |
| HLA-DQ | midnightblu | 0.019398937 | 0.675851746 | 0.060108821 | 0.194750225 |
| HLA-DR | midnightblu | -0.002144899 | 0.963128934 | 0.026230819 | 0.571780681 |
| HLA-DR | midnightblu | -0.020206379 | 0.663172125 | 0.014300041 | 0.757919443 |
| HLA-DR | midnightblu | -0.015028676 | 0.74599833 | 0.014297264 | 0.757964986 |
| HMHA1 | midnightblu | 0.142606249 | 0.002006345 | 0.192511101 | 2.81E-05 |
| HMOX1 | midnightblu | -0.011096486 | 0.81098012 | 0.019292681 | 0.677527279 |
| HS3ST2 | midnightblu | -0.083590654 | 0.071117287 | -0.052479126 | 0.257708334 |
| IFI30 | midnightblu | -0.0733161 | 0.113590632 | -0.052536306 | 0.257189408 |
| IGFLR1 | midnightblu | 0.012392926 | 0.789387602 | 0.065791487 | 0.155755022 |
| IGSF6 | midnightblu | 0.014020606 | 0.762506686 | 0.05979295 | 0.197110399 |
| IL18 | midnightblu | 0.118631235 | 0.010292669 | 0.120676262 | 0.00904382 |
| IL27 | midnightblu | 0.107407491 | 0.020254774 | 0.114436652 | 0.013342155 |
| IL32 | midnightblu | -0.048410663 | 0.296498446 | 0.007702571 | 0.868148817 |
| IL4I1 | midnightblu | -0.126996445 | 0.005992403 | -0.092340166 | 0.046108049 |
| INSL3 | midnightblu | -0.173496518 | 0.000164656 | -0.160049261 | 0.000516923 |
| IRF1 | midnightblu | -0.082840923 | 0.073697804 | -0.046004496 | 0.321184578 |
| IRF5 | midnightblu | -0.179920142 | 9.24E-05 | -0.154423615 | 0.000813134 |
| ITGAM | midnightblu | -0.084790447 | 0.067141682 | -0.047059033 | 0.310205376 |
| ITGAX | midnightblu | -0.065728136 | 0.156154486 | -0.023688768 | 0.609618327 |
| ITGB2 | midnightblu | -0.034987971 | 0.450668436 | 0.012620803 | 0.785608351 |
| JAK3 | midnightblu | -0.236431003 | 2.35E-07 | -0.19109751 | 3.23E-05 |
| KCNAB2 | midnightblu | -0.133969836 | 0.003726401 | -0.108662067 | 0.018830336 |
| KCNK13 | midnightblu | 0.005927146 | 0.898351129 | -0.013641089 | 0.76875019 |
| KLHDC7 | midnightblu | -0.050410573 | 0.276968971 | -0.011247685 | 0.808454121 |
| LAIR1 | midnightblu | -0.081284614 | 0.079298236 | -0.042223232 | 0.362605021 |
| LAPTM5 | midnightblu | -0.042248239 | 0.362320581 | 0.02295321 | 0.620770304 |
| LAT2 | midnightblu | -0.028241611 | 0.542662166 | 0.014700796 | 0.751355428 |

| | | | | | |
|---|---|---|---|---|---|
| LGALS2 | midnightblu | -0.078375885 | 0.090687682 | -0.0446044 | 0.336147686 |
| LILRA1 | midnightblu | -0.064601108 | 0.163391124 | -0.060977113 | 0.188368986 |
| LILRA2 | midnightblu | -0.041944384 | 0.365786157 | -0.028754385 | 0.535356284 |
| LILRA4 | midnightblu | -0.057025685 | 0.218684379 | -0.036916105 | 0.426091834 |
| LILRA5 | midnightblu | -0.032323205 | 0.485915139 | -0.003705936 | 0.93633938 |
| LILRA6 | midnightblu | -0.051285448 | 0.268706739 | -0.025337448 | 0.584951033 |
| LILRB1 | midnightblu | -0.064065992 | 0.166914077 | -0.035835277 | 0.439771174 |
| LILRB2 | midnightblu | -0.048067095 | 0.299943683 | -0.022507515 | 0.62757032 |
| LILRB3 | midnightblu | -0.031368006 | 0.498902182 | -0.000878238 | 0.984898522 |
| LILRB4 | midnightblu | -0.022737978 | 0.624050139 | 0.016911917 | 0.71547254 |
| LIMD2 | midnightblu | -0.084536798 | 0.067966608 | -0.053024331 | 0.25278988 |
| LPAR5 | midnightblu | -0.126814376 | 0.0060654 | -0.082074387 | 0.076414552 |
| LRRC25 | midnightblu | -0.089797991 | 0.052468502 | -0.046322467 | 0.317847734 |
| LSP1 | midnightblu | -0.054858324 | 0.236724953 | 0.005343207 | 0.908319057 |
| LST1 | midnightblu | -0.049191162 | 0.288770151 | 7.11E-05 | 0.998776536 |
| LTB | midnightblu | -0.133323766 | 0.003897691 | -0.074747389 | 0.106693846 |
| LY86 | midnightblu | -0.150151084 | 0.001135659 | -0.114692648 | 0.01313545 |
| MARCO | midnightblu | -0.110628737 | 0.016773034 | -0.096997023 | 0.036131091 |
| MCEMP1 | midnightblu | 0.026587657 | 0.566559627 | 0.057557183 | 0.214414727 |
| MILR1 | midnightblu | -0.017701984 | 0.7027968 | 0.018851389 | 0.684502868 |
| MMP25 | midnightblu | -0.019354704 | 0.676549059 | 0.011530682 | 0.803731618 |
| MMP9 | midnightblu | 0.087793233 | 0.057985798 | 0.141360794 | 0.002198372 |
| MYO1F | midnightblu | 0.009733178 | 0.833841706 | 0.064152973 | 0.166337593 |
| NAGK | midnightblu | -0.061880478 | 0.181894592 | -0.058968603 | 0.203368048 |
| NCF1 | midnightblu | -0.019203997 | 0.678926938 | 0.01951241 | 0.674064182 |
| NCF4 | midnightblu | -0.01167938 | 0.801253077 | 0.047758464 | 0.303061185 |
| NEURL3 | midnightblu | -0.049446191 | 0.286274523 | -0.031558276 | 0.496300668 |
| NFAM1 | midnightblu | 0.075099968 | 0.105046754 | 0.102240956 | 0.027152609 |
| NFKBIE | midnightblu | -0.096917814 | 0.036284057 | -0.075280764 | 0.104209989 |
| NR1H3 | midnightblu | -0.032618441 | 0.481938266 | -0.005652546 | 0.903036688 |
| OSCAR | midnightblu | -0.069487925 | 0.133760737 | -0.027931402 | 0.547105872 |
| OSM | midnightblu | 0.018280172 | 0.693572102 | 0.038964314 | 0.400858817 |
| P2RY6 | midnightblu | -0.113120019 | 0.014451184 | -0.069819048 | 0.131913768 |
| PARVG | midnightblu | 0.281871527 | 5.61E-10 | 0.298757211 | 4.40E-11 |
| PIK3R6 | midnightblu | -0.136809875 | 0.003051475 | -0.098048957 | 0.034151041 |
| PILRA | midnightblu | -0.21548316 | 2.61E-06 | -0.18388221 | 6.41E-05 |
| PKD2L1 | midnightblu | -0.070676191 | 0.1272242 | -0.047670671 | 0.303951891 |
| PLCB2 | midnightblu | -0.102835853 | 0.026267067 | -0.065202512 | 0.159498769 |
| PLD4 | midnightblu | -0.152329263 | 0.000958842 | -0.133666855 | 0.003805861 |
| PLEKHO | midnightblu | -0.063778697 | 0.168828822 | -0.026165847 | 0.572733757 |
| PLEKHO: | midnightblu | -0.189540629 | 3.75E-05 | -0.161650795 | 0.000453133 |
| POU2F2 | midnightblu | -0.13284565 | 0.004029007 | -0.095766737 | 0.038569809 |
| PRAM1 | midnightblu | -0.048621178 | 0.294400519 | -0.031673439 | 0.494729574 |
| PSMB10 | midnightblu | 0.107388708 | 0.020276792 | 0.169923973 | 0.00022504 |
| PSME1 | midnightblu | -0.069876942 | 0.131592874 | -0.042530807 | 0.359116249 |

| | | | | | |
|---|---|---|---|---|---|
| PSME2 | midnightblu | 0.05056896 | 0.275460529 | 0.090445545 | 0.050782676 |
| PTAFR | midnightblu | -0.028199704 | 0.543261441 | 0.017221162 | 0.710501502 |
| PTCRA | midnightblu | -0.046977446 | 0.311045884 | -0.033342491 | 0.472260347 |
| PTK2B | midnightblu | 0.114108982 | 0.013610911 | 0.160361573 | 0.000503863 |
| RAB42 | midnightblu | -0.230911759 | 4.53E-07 | -0.222989392 | 1.13E-06 |
| RARRES: | midnightblu | -0.084220836 | 0.069005792 | -0.019005912 | 0.682057198 |
| RASGRP | midnightblu | -0.148017628 | 0.001337533 | -0.135591944 | 0.003325991 |
| RASSF4 | midnightblu | 0.027475445 | 0.553669813 | 0.020428326 | 0.659703387 |
| RELB | midnightblu | -0.194977668 | 2.21E-05 | -0.183089602 | 6.90E-05 |
| RENBP | midnightblu | -0.166355745 | 0.000305569 | -0.148205885 | 0.001318474 |
| RETN | midnightblu | -0.116679396 | 0.011624933 | -0.106946075 | 0.020801751 |
| RGS19 | midnightblu | -0.164747165 | 0.00035005 | -0.131734395 | 0.004349814 |
| RIN3 | midnightblu | -0.138659444 | 0.002673817 | -0.097603872 | 0.034977247 |
| RNASE2 | midnightblu | -0.016479814 | 0.722438649 | 0.01390172 | 0.764460858 |
| RNASE6 | midnightblu | 0.050177313 | 0.279200693 | 0.085693742 | 0.06427042 |
| RNF166 | midnightblu | 0.071823869 | 0.121148809 | 0.096555374 | 0.036991027 |
| S100B | midnightblu | -0.064974211 | 0.16096804 | -0.044479763 | 0.337501051 |
| SAMHD1 | midnightblu | 0.048621668 | 0.29439565 | 0.08327826 | 0.072183408 |
| SDS | midnightblu | -0.080643954 | 0.081701724 | -0.050098536 | 0.279957133 |
| SIGLEC1 | midnightblu | -0.176992493 | 0.000120559 | -0.156868357 | 0.00066906 |
| SIGLEC1 | midnightblu | -0.060815831 | 0.189542506 | -0.036915018 | 0.426105465 |
| SIGLEC1 | midnightblu | 0.022149712 | 0.633052322 | 0.042764722 | 0.356477125 |
| SIGLEC1 | midnightblu | -0.068933985 | 0.13689506 | -0.05116448 | 0.269838985 |
| SIGLEC5 | midnightblu | -0.051264938 | 0.268898479 | -0.024455566 | 0.598088195 |
| SIGLEC7 | midnightblu | -0.065918138 | 0.154958737 | -0.051108522 | 0.270363854 |
| SIGLEC8 | midnightblu | -0.078885473 | 0.088602641 | -0.051407179 | 0.267570632 |
| SIGLEC9 | midnightblu | -0.066169753 | 0.153385915 | -0.037697761 | 0.416354995 |
| SIRPA | midnightblu | -0.149755164 | 0.001170858 | -0.174048569 | 0.000156809 |
| SLAMF8 | midnightblu | 0.014043797 | 0.76212566 | 0.066133987 | 0.153608746 |
| SLC11A1 | midnightblu | -0.073308316 | 0.113629072 | -0.045587521 | 0.325594831 |
| SLC15A3 | midnightblu | -0.208117079 | 5.76E-06 | -0.154497897 | 0.000808364 |
| SLC2A5 | midnightblu | -0.133821795 | 0.003765037 | -0.088401283 | 0.056263773 |
| SLC37A2 | midnightblu | -0.01714904 | 0.71165976 | 0.025239356 | 0.586405655 |
| SLC7A7 | midnightblu | -0.162023193 | 0.000439389 | -0.122844013 | 0.007868995 |
| SMCO4 | midnightblu | -0.132427031 | 0.004147255 | -0.108249907 | 0.019288354 |
| SOWAHI | midnightblu | 0.060756811 | 0.189973287 | 0.079185262 | 0.087394121 |
| SPATC1 | midnightblu | 0.021573082 | 0.641929332 | 0.038555033 | 0.405828169 |
| SPHK1 | midnightblu | -0.122702367 | 0.00794138 | -0.097400402 | 0.035360574 |
| SPI1 | midnightblu | -0.015966869 | 0.730737862 | 0.035820832 | 0.439955681 |
| SPIB | midnightblu | -0.083363336 | 0.071891781 | -0.059475132 | 0.199506116 |
| SQRDL | midnightblu | 0.324879895 | 6.11E-13 | 0.364108164 | 4.36E-16 |
| STAB1 | midnightblu | -0.117632236 | 0.010956608 | -0.076493704 | 0.098731925 |
| STAC3 | midnightblu | 0.12944523 | 0.005084446 | 0.139067763 | 0.002596406 |
| STAT5A | midnightblu | -0.172321525 | 0.000182599 | -0.148652394 | 0.001274265 |
| TBXAS1 | midnightblu | -0.06261836 | 0.176729493 | -0.038788994 | 0.402983019 |

| | | | | | |
|---|---|---|---|---|---|
| TFEB | midnightblu | -0.141836153 | 0.002123181 | -0.096133763 | 0.037828094 |
| TIFAB | midnightblu | -0.01458373 | 0.753271034 | 0.007161479 | 0.877335272 |
| TIMD4 | midnightblu | -0.139368129 | 0.002540772 | -0.119163317 | 0.009953821 |
| TMEM15 | midnightblu | -0.019612985 | 0.672481337 | 0.009165741 | 0.843399779 |
| TMEM17 | midnightblu | -0.382028051 | 1.12E-17 | -0.332781538 | 1.54E-13 |
| TMEM86 | midnightblu | -0.238242186 | 1.89E-07 | -0.210741632 | 4.36E-06 |
| TNFAIP8 | midnightblu | -0.062685711 | 0.176263521 | -0.010628413 | 0.818812279 |
| TNFRSF1 | midnightblu | 0.04610493 | 0.320128144 | 0.080396065 | 0.082647367 |
| TNFRSF1 | midnightblu | -0.15854038 | 0.000584564 | -0.113985972 | 0.013713028 |
| TNFRSF4 | midnightblu | -0.015074807 | 0.745245601 | 0.025749261 | 0.578862505 |
| TNFSF14 | midnightblu | 0.16172042 | 0.000450533 | 0.185975235 | 5.27E-05 |
| TREM2 | midnightblu | -0.045504972 | 0.326472565 | -0.010977938 | 0.812962004 |
| TREML1 | midnightblu | -0.101275727 | 0.028644052 | -0.087923308 | 0.057613795 |
| TRPV2 | midnightblu | -0.039693988 | 0.392090262 | 0.015798393 | 0.733470643 |
| TYMP | midnightblu | 0.036969108 | 0.425427444 | 0.091961869 | 0.047011415 |
| TYROBP | midnightblu | -0.064218331 | 0.165905406 | -0.006488337 | 0.888786533 |
| UBD | midnightblu | -0.053391065 | 0.249518359 | -0.024962371 | 0.590522118 |
| UNC13D | midnightblu | -0.220712198 | 1.46E-06 | -0.203437395 | 9.38E-06 |
| VAMP5 | midnightblu | -0.121233999 | 0.008727513 | -0.066700338 | 0.150108985 |
| VAV1 | midnightblu | -0.118189591 | 0.010581655 | -0.062629529 | 0.176652158 |
| VENTX | midnightblu | -0.147480255 | 0.001393339 | -0.139243235 | 0.002563772 |
| VMO1 | midnightblu | 0.037124647 | 0.423481226 | 0.066535944 | 0.151118554 |
| ZFP57 | midnightblu | -0.070749197 | 0.126830828 | -0.054691146 | 0.23815884 |
| ZMYND1 | midnightblu | -0.198287692 | 1.59E-05 | -0.17692285 | 0.000121317 |
| ACTR10 | orange | -0.105480714 | 0.022624798 | -0.129136307 | 0.005191711 |
| AIMP1 | orange | -0.005887693 | 0.899024103 | -0.014133557 | 0.760651452 |
| AK3 | orange | -0.051872498 | 0.263258214 | -0.08575775 | 0.064070855 |
| ALG5 | orange | 0.200316175 | 1.29E-05 | 0.181733096 | 7.82E-05 |
| ANAPC1( | orange | -0.029717102 | 0.521774514 | -0.03831048 | 0.408814873 |
| ANAPC1: | orange | 0.014830439 | 0.74923581 | 0.010062414 | 0.828307058 |
| ANAPC1( | orange | 0.128806231 | 0.00530857 | 0.130981648 | 0.004580053 |
| AP3S1 | orange | 0.017147624 | 0.711682516 | 0.006957791 | 0.880797758 |
| ARL14EP | orange | -0.063733088 | 0.169134301 | -0.080767568 | 0.081233443 |
| ASF1A | orange | 0.001365493 | 0.976522059 | -0.006786154 | 0.88371717 |
| ATG12 | orange | 0.091481851 | 0.04817901 | 0.08266281 | 0.074321943 |
| ATG4C | orange | -0.102649855 | 0.026541225 | -0.127803679 | 0.005678169 |
| ATP6V1C | orange | 0.118383121 | 0.010454146 | 0.098835549 | 0.032731539 |
| BCDIN3D | orange | 0.090687069 | 0.05016559 | 0.062850484 | 0.175127388 |
| BDH2 | orange | 0.033409411 | 0.471371278 | 0.01670724 | 0.718769326 |
| BET1 | orange | 0.060186136 | 0.194175693 | 0.047050595 | 0.310292225 |
| BTF3 | orange | 0.05954687 | 0.198963508 | 0.02539761 | 0.584059711 |
| C2orf76 | orange | -0.008117675 | 0.861113269 | -0.0261195 | 0.573414087 |
| CAAP1 | orange | 0.023217965 | 0.616746089 | 0.006277063 | 0.892385526 |
| CALM2 | orange | -0.1070637 | 0.020661109 | -0.105924383 | 0.022058755 |
| CAMLG | orange | -0.126987422 | 0.005996002 | -0.161049157 | 0.000476185 |

| | | | | | |
|---|---|---|---|---|---|
| CBR4 | orange | 0.161407278 | 0.000462335 | 0.152384619 | 0.000954699 |
| CCDC112 | orange | -0.038535531 | 0.406065864 | -0.055024315 | 0.235307282 |
| CCDC90I | orange | 0.01809887 | 0.696459891 | -0.000193051 | 0.996680259 |
| CCNH | orange | 0.188547189 | 4.12E-05 | 0.173658184 | 0.000162321 |
| CDC37L1 | orange | -0.026001598 | 0.575146497 | -0.035057903 | 0.449763301 |
| CDC42 | orange | 0.005150439 | 0.911612878 | -0.012791905 | 0.78277401 |
| CETN3 | orange | 0.087516718 | 0.05878322 | 0.059044008 | 0.202789726 |
| CGRRF1 | orange | -0.144534578 | 0.001739121 | -0.158816637 | 0.000571596 |
| CIR1 | orange | -0.203725127 | 9.11E-06 | -0.218798202 | 1.81E-06 |
| COMMD | orange | 0.083539698 | 0.071290302 | 0.056342669 | 0.22426013 |
| COPS2 | orange | 0.079917195 | 0.08449918 | 0.053506142 | 0.248497907 |
| COQ7 | orange | -0.104878136 | 0.023413704 | -0.101440751 | 0.028384186 |
| CRYZL1 | orange | -0.001505989 | 0.974107215 | -0.014267728 | 0.758449464 |
| DARS | orange | -0.013204435 | 0.775952202 | -0.050988597 | 0.271491053 |
| DERL2 | orange | 0.124356321 | 0.007132105 | 0.115001439 | 0.012889876 |
| DNAJC25 | orange | 0.096660185 | 0.036785393 | 0.066331015 | 0.152384265 |
| DPH5 | orange | -0.035455617 | 0.444635269 | -0.055566074 | 0.230722016 |
| DUSP11 | orange | -0.194543554 | 2.30E-05 | -0.211309622 | 4.10E-06 |
| DUT | orange | 0.087213506 | 0.059668045 | 0.071666703 | 0.121967162 |
| EAPP | orange | -0.107018176 | 0.020715443 | -0.108531621 | 0.018974259 |
| EIF1AX | orange | 0.082831651 | 0.07373019 | 0.053602806 | 0.247642979 |
| EIF1B | orange | -0.278475296 | 9.16E-10 | -0.301168325 | 3.02E-11 |
| EIF2A | orange | -0.074327337 | 0.108682707 | -0.10466213 | 0.023702242 |
| EIF3E | orange | -0.036351073 | 0.433211976 | -0.097909067 | 0.034408906 |
| ELMOD2 | orange | 0.127796374 | 0.005680945 | 0.114892243 | 0.01297625 |
| ELP4 | orange | 0.136846355 | 0.00304358 | 0.119599254 | 0.009683621 |
| ERP44 | orange | 0.119852604 | 0.009529604 | 0.111421099 | 0.016001454 |
| ESD | orange | -0.046308564 | 0.317993159 | -0.059839334 | 0.19676252 |
| FOPNL | orange | -0.009885961 | 0.831272287 | -0.028025332 | 0.545758449 |
| FRA10AC | orange | 0.109896469 | 0.017514846 | 0.073750433 | 0.111461739 |
| FXN | orange | 0.224594068 | 9.42E-07 | 0.218274489 | 1.92E-06 |
| GKAP1 | orange | -0.083831748 | 0.070303322 | -0.112491875 | 0.015008267 |
| GPBP1 | orange | -0.073867948 | 0.110891162 | -0.100825434 | 0.029363551 |
| GTF2B | orange | 0.016826243 | 0.716851875 | 0.006458917 | 0.889287553 |
| IER3IP1 | orange | 0.086980306 | 0.060356029 | 0.076745545 | 0.097623773 |
| ISCA1 | orange | -0.067216987 | 0.146969535 | -0.074748063 | 0.106690679 |
| LARP7 | orange | 0.033329551 | 0.472432365 | 0.004077921 | 0.929965051 |
| LIAS | orange | -0.041911833 | 0.366158631 | -0.054017395 | 0.243999505 |
| LYRM5 | orange | 0.069822477 | 0.131894744 | 0.060255651 | 0.19366018 |
| MBD2 | orange | 0.08622052 | 0.062643142 | 0.074499937 | 0.107861971 |
| MEAF6 | orange | -0.050558788 | 0.275557236 | -0.072511641 | 0.117617887 |
| MED31 | orange | 0.19350791 | 2.55E-05 | 0.194568058 | 2.30E-05 |
| MED7 | orange | -0.027480341 | 0.553599125 | -0.030591712 | 0.509590706 |
| MFF | orange | -0.154504477 | 0.000807942 | -0.175165077 | 0.000141999 |
| MICU2 | orange | 0.055195222 | 0.233853895 | 0.022168565 | 0.632762965 |

| | | | | | |
|---|---|---|---|---|---|
| MOB4 | orange | -0.081824047 | 0.077319239 | -0.082281262 | 0.075673448 |
| MORF4L | orange | -0.046553185 | 0.315440765 | -0.074727854 | 0.106785701 |
| MORF4L: | orange | -0.082316594 | 0.075547464 | -0.103785133 | 0.024905529 |
| MRPL50 | orange | 0.176710076 | 0.00012366 | 0.162004625 | 0.000440065 |
| MRPS36 | orange | 0.241307861 | 1.30E-07 | 0.246716284 | 6.63E-08 |
| MSANTD | orange | 0.225705447 | 8.30E-07 | 0.222342629 | 1.22E-06 |
| NAP1L1 | orange | -0.107346724 | 0.020326086 | -0.140704628 | 0.002306161 |
| NDFIP1 | orange | 0.137105134 | 0.002988105 | 0.11654771 | 0.011720079 |
| NDNL2 | orange | -0.063163051 | 0.172987175 | -0.091217075 | 0.048833379 |
| NDUFA5 | orange | -0.025881525 | 0.576913334 | -0.036326121 | 0.433527986 |
| NDUFAF | orange | 0.332577538 | 1.60E-13 | 0.324953096 | 6.03E-13 |
| NDUFS4 | orange | 0.065078277 | 0.160297044 | 0.062971384 | 0.174297239 |
| NFYB | orange | -0.09060942 | 0.050363295 | -0.107729105 | 0.019880959 |
| NKAP | orange | -0.1495033 | 0.00119377 | -0.159611026 | 0.000535778 |
| NOL8 | orange | -0.009633238 | 0.835523393 | -0.050993494 | 0.271444964 |
| NPM1 | orange | -0.03282123 | 0.479216925 | -0.075965415 | 0.101089017 |
| NSA2 | orange | 0.127881017 | 0.005648855 | 0.111951438 | 0.015502566 |
| NT5C3A | orange | 0.014906499 | 0.747993116 | 0.005060015 | 0.913158473 |
| NUDCD2 | orange | -0.00627017 | 0.892502989 | -0.012118874 | 0.793939189 |
| OCIAD1 | orange | 0.031650186 | 0.49504659 | 0.015901064 | 0.731804851 |
| OSTC | orange | 0.170817469 | 0.000208246 | 0.166985497 | 0.000289638 |
| PCNP | orange | -0.185098206 | 5.72E-05 | -0.206204311 | 7.04E-06 |
| PFDN1 | orange | 0.082775603 | 0.0739262 | 0.069880091 | 0.131575439 |
| PIGP | orange | -0.012847097 | 0.781860349 | -0.025316578 | 0.58526038 |
| POLR3GI | orange | 0.131455078 | 0.004433998 | 0.118316859 | 0.010497649 |
| PPHLN1 | orange | -0.08151411 | 0.078451332 | -0.095341812 | 0.039443883 |
| PPP1CC | orange | -0.021993666 | 0.635449462 | -0.049018863 | 0.290464466 |
| PPP2CA | orange | 0.045354657 | 0.328074777 | 0.029898325 | 0.519237756 |
| PPP2R3C | orange | 0.00263086 | 0.954783297 | -0.009017543 | 0.845899908 |
| PPP4R2 | orange | 0.065946247 | 0.154782425 | 0.03407476 | 0.462582096 |
| PRIMPOI | orange | 0.004368214 | 0.924993683 | -0.029328105 | 0.527241037 |
| PSIP1 | orange | -0.144901871 | 0.001692081 | -0.171634974 | 0.000193915 |
| PUS3 | orange | 0.166152495 | 0.000310883 | 0.14953119 | 0.001191213 |
| RAB5A | orange | 0.040456626 | 0.383050614 | 0.024096893 | 0.603469185 |
| RAD17 | orange | 0.053158557 | 0.251589047 | 0.024952225 | 0.590673154 |
| RFESD | orange | -0.061356183 | 0.185631865 | -0.056844569 | 0.22015314 |
| RIOK2 | orange | 0.009513988 | 0.837530985 | -0.010395448 | 0.822717203 |
| RNF14 | orange | 0.175668471 | 0.000135761 | 0.166562099 | 0.000300261 |
| RPS27L | orange | 0.358986247 | 1.19E-15 | 0.365179815 | 3.53E-16 |
| RSL1D1 | orange | 0.008320589 | 0.857678072 | -0.022512626 | 0.62749216 |
| RSL24D1 | orange | 0.016294734 | 0.725429455 | -0.014735649 | 0.750785415 |
| RWDD4 | orange | 0.045442367 | 0.327139255 | 0.033675039 | 0.4678514 |
| SCOC | orange | 0.042659859 | 0.357658718 | 0.03340342 | 0.471450845 |
| SDHD | orange | -0.122972844 | 0.007803671 | -0.137768219 | 0.002850131 |
| SEC11A | orange | 0.283408338 | 4.48E-10 | 0.260620692 | 1.09E-08 |

| | | | | | |
|---|---|---|---|---|---|
| 15-Sep | orange | -0.026633512 | 0.565890364 | -0.052037392 | 0.261741565 |
| SFR1 | orange | 0.017974093 | 0.698449912 | 0.005256083 | 0.909807548 |
| SIAH1 | orange | -0.099429037 | 0.031694132 | -0.103759426 | 0.024941583 |
| SKP1 | orange | -0.043858766 | 0.344296002 | -0.055795459 | 0.228799771 |
| SLU7 | orange | -0.077610759 | 0.093891972 | -0.103203991 | 0.025731624 |
| SMIM20 | orange | 0.145767133 | 0.001585841 | 0.135760789 | 0.00328664 |
| SNAPC1 | orange | -0.005678726 | 0.902589829 | -0.019041231 | 0.681498665 |
| SNW1 | orange | -0.019518205 | 0.673972945 | -0.05281881 | 0.25463623 |
| SPCS2 | orange | 0.074357499 | 0.108538926 | 0.046807804 | 0.312798339 |
| SREK1IP | orange | 0.072808828 | 0.116117302 | 0.069737161 | 0.132368679 |
| SRP14 | orange | 0.185524233 | 5.49E-05 | 0.175601151 | 0.00013658 |
| ST13 | orange | 0.013816865 | 0.765856582 | -0.02074504 | 0.654766187 |
| SYF2 | orange | -0.058445229 | 0.207415107 | -0.07779212 | 0.093124377 |
| TAF12 | orange | 0.099901151 | 0.030889078 | 0.089505047 | 0.053246327 |
| TAF9 | orange | 0.262123191 | 8.91E-09 | 0.260423799 | 1.12E-08 |
| TCAIM | orange | 0.218603977 | 1.85E-06 | 0.203176477 | 9.64E-06 |
| TMA16 | orange | -0.003103831 | 0.946665477 | -0.019757739 | 0.670205728 |
| TMBIM4 | orange | 0.04854664 | 0.295142209 | 0.036968274 | 0.42543789 |
| TMEM23 | orange | -0.134955364 | 0.003478223 | -0.146773704 | 0.00146997 |
| TRMT5 | orange | -0.086350735 | 0.062246172 | -0.11654352 | 0.011723117 |
| TRNT1 | orange | -0.0335542 | 0.46945086 | -0.058557628 | 0.206541096 |
| TXNL1 | orange | 0.161508513 | 0.000458489 | 0.151679059 | 0.001008762 |
| UBA3 | orange | 0.169914954 | 0.000225216 | 0.134937857 | 0.003482498 |
| UBE2B | orange | -0.140610646 | 0.002321989 | -0.157298433 | 0.000646307 |
| UBLCP1 | orange | 0.007673108 | 0.868648583 | -0.01769131 | 0.702967517 |
| UFM1 | orange | 0.114367139 | 0.013398776 | 0.098204177 | 0.033866846 |
| UGP2 | orange | -0.044154528 | 0.341048986 | -0.055558577 | 0.230785026 |
| UIMC1 | orange | -0.047826561 | 0.302371494 | -0.082247004 | 0.075795768 |
| VAPA | orange | -0.071029714 | 0.125328107 | -0.089578794 | 0.053049614 |
| WDR89 | orange | 0.030594166 | 0.509556727 | 0.011059493 | 0.811598438 |
| XPA | orange | 0.022910144 | 0.621425965 | 0.014011075 | 0.76266329 |
| ZCCHC1( | orange | 0.134175367 | 0.003673357 | 0.105411345 | 0.022714429 |
| ZCCHC7 | orange | -0.046621913 | 0.314726072 | -0.071047951 | 0.125230892 |
| ZCCHC9 | orange | -0.09762032 | 0.034946414 | -0.118120552 | 0.010627474 |
| ZCRB1 | orange | 0.031058384 | 0.503150966 | 0.017254277 | 0.709969915 |
| ZFAND6 | orange | 0.019832201 | 0.669036335 | 0.005980995 | 0.897432692 |
| ZNF22 | orange | -0.145184041 | 0.001656739 | -0.176113812 | 0.000130457 |
| BPIFC | orangered4 | -0.035526317 | 0.443727163 | -0.040540471 | 0.382064621 |
| CLCA2 | orangered4 | 0.132169831 | 0.004221455 | 0.119647293 | 0.009654249 |
| FAM26D | orangered4 | -0.061064214 | 0.187737451 | -0.066721481 | 0.149979515 |
| GPRC5D | orangered4 | 0.084290267 | 0.068776328 | 0.082380012 | 0.075321759 |
| IL1F10 | orangered4 | -0.079795128 | 0.084976535 | -0.063966229 | 0.167577123 |
| IL36G | orangered4 | -0.025113824 | 0.588269627 | -0.036605601 | 0.429996145 |
| KRT33A | orangered4 | 0.040119268 | 0.387033566 | 0.015849471 | 0.732641761 |
| KRT33B | orangered4 | 0.016271053 | 0.725812439 | 0.030476865 | 0.511182069 |

| | | | | | |
|---|---|---|---|---|---|
| KRT34 | orangered4 | -0.043606693 | 0.347078842 | -0.02816328 | 0.543782562 |
| KRT36 | orangered4 | 0.046394225 | 0.317097834 | 0.044801624 | 0.334013284 |
| KRT37 | orangered4 | -0.072362479 | 0.118376742 | -0.028402307 | 0.540367296 |
| KRT38 | orangered4 | 0.059464194 | 0.199588941 | 0.051308307 | 0.268493141 |
| KRT39 | orangered4 | -0.045103052 | 0.330768012 | -0.01216471 | 0.793177432 |
| KRT82 | orangered4 | 0.003317643 | 0.942997497 | 0.041099628 | 0.375528789 |
| KRT83 | orangered4 | 0.043403572 | 0.349331616 | 0.022874131 | 0.621974488 |
| KRT84 | orangered4 | 0.007177559 | 0.877062039 | 0.01949605 | 0.674321788 |
| KRT85 | orangered4 | 0.014231154 | 0.759049518 | 0.01408822 | 0.761395949 |
| KRTAP1- | orangered4 | -0.076084779 | 0.100552578 | -0.065718165 | 0.156217427 |
| KRTAP1- | orangered4 | 0.048133124 | 0.299279504 | 0.053669115 | 0.247057709 |
| KRTAP1- | orangered4 | -0.036103194 | 0.436357109 | -0.013885445 | 0.7647285 |
| KRTAP1( | orangered4 | -0.097938109 | 0.034355237 | -0.09363038 | 0.043135907 |
| KRTAP1( | orangered4 | -0.069674029 | 0.132720227 | -0.067033751 | 0.148077201 |
| KRTAP1( | orangered4 | -0.053650387 | 0.247222915 | -0.05608229 | 0.226412163 |
| KRTAP1( | orangered4 | -0.091018206 | 0.049329738 | -0.087842769 | 0.057843897 |
| KRTAP1( | orangered4 | -0.089956371 | 0.052051929 | -0.097414079 | 0.035334695 |
| KRTAP1l | orangered4 | 0.019753672 | 0.670269627 | 0.018549631 | 0.689288348 |
| KRTAP1: | orangered4 | -0.0093599 | 0.840126635 | -0.011873261 | 0.798024401 |
| KRTAP1: | orangered4 | 0.006318632 | 0.891677237 | 0.006395936 | 0.890360279 |
| KRTAP1( | orangered4 | -0.03777715 | 0.415373447 | -0.029411282 | 0.526069725 |
| KRTAP1' | orangered4 | -0.013289654 | 0.774545095 | -0.035829202 | 0.439848765 |
| KRTAP1! | orangered4 | -0.006116727 | 0.895118297 | -0.010498417 | 0.820990699 |
| KRTAP1! | orangered4 | -0.028908056 | 0.533176475 | -0.022750584 | 0.623857832 |
| KRTAP1! | orangered4 | -0.021609254 | 0.641370949 | -0.008548824 | 0.853817395 |
| KRTAP1! | orangered4 | -0.028718376 | 0.535867709 | -0.027856915 | 0.548175563 |
| KRTAP2- | orangered4 | -0.050937512 | 0.271972183 | -0.049042105 | 0.290235525 |
| KRTAP2- | orangered4 | -0.084983067 | 0.06652074 | -0.06459448 | 0.163434409 |
| KRTAP2- | orangered4 | -0.029217679 | 0.528798131 | -0.020493227 | 0.658690444 |
| KRTAP2( | orangered4 | -0.001457505 | 0.974940521 | -0.00142943 | 0.975423084 |
| KRTAP2( | orangered4 | -0.054900085 | 0.236367725 | -0.058010367 | 0.210821782 |
| KRTAP2! | orangered4 | -0.002934795 | 0.949566128 | -0.001426042 | 0.975481317 |
| KRTAP3- | orangered4 | -0.002075781 | 0.964316274 | 0.012120577 | 0.793910888 |
| KRTAP3- | orangered4 | 0.103627716 | 0.025127006 | 0.111026274 | 0.016381946 |
| KRTAP4- | orangered4 | -0.084263621 | 0.068864316 | -0.096884733 | 0.036348106 |
| KRTAP4- | orangered4 | -0.056087136 | 0.226371975 | -0.047906747 | 0.301560701 |
| KRTAP4- | orangered4 | -0.056176677 | 0.225630369 | -0.044437642 | 0.337959205 |
| KRTAP4- | orangered4 | -0.035804299 | 0.44016691 | -0.036734248 | 0.428376008 |
| KRTAP4- | orangered4 | -0.038344247 | 0.408401708 | -0.037827528 | 0.414751289 |
| KRTAP4- | orangered4 | -0.033341499 | 0.472273527 | -0.030003337 | 0.517770717 |
| KRTAP4- | orangered4 | -0.050955979 | 0.271798187 | -0.046371805 | 0.317332005 |
| KRTAP4- | orangered4 | -0.033964047 | 0.464038249 | -0.026430834 | 0.56885136 |
| KRTAP4- | orangered4 | 0.025386132 | 0.584229714 | 0.037627838 | 0.41722063 |
| KRTAP4- | orangered4 | -0.026111049 | 0.57353819 | -0.005305518 | 0.908962926 |
| KRTAP4- | orangered4 | 0.065324369 | 0.158718685 | 0.086417589 | 0.06204317 |

| | | | | | |
|---|---|---|---|---|---|
| KRTAP5- | orangered4 | 0.09059269 | 0.050405977 | 0.086725113 | 0.061116393 |
| KRTAP5- | orangered4 | -0.039047476 | 0.399853568 | -0.034751236 | 0.453740119 |
| KRTAP5- | orangered4 | -0.062456686 | 0.177851766 | -0.067402218 | 0.145856239 |
| KRTAP6- | orangered4 | 0.009947678 | 0.830234876 | 0.012722966 | 0.783915657 |
| KRTAP7- | orangered4 | -0.011569547 | 0.803083619 | -0.007599926 | 0.869890151 |
| KRTAP8- | orangered4 | -0.003053737 | 0.947525021 | -0.004169241 | 0.928400853 |
| KRTAP9- | orangered4 | -0.071487708 | 0.122904418 | -0.068762486 | 0.137876804 |
| KRTAP9- | orangered4 | -0.030403913 | 0.512194267 | -0.029566898 | 0.523881861 |
| KRTAP9- | orangered4 | -0.030059486 | 0.516987181 | -0.010886184 | 0.81449677 |
| KRTAP9- | orangered4 | 0.014490477 | 0.754798074 | 0.029263725 | 0.52814857 |
| KRTAP9- | orangered4 | -0.024041866 | 0.604296654 | -0.025794443 | 0.578196305 |
| KRTAP9- | orangered4 | -0.033273392 | 0.47317931 | -0.017210655 | 0.7106702 |
| KRTAP9- | orangered4 | -0.030378849 | 0.512542277 | -0.025421224 | 0.583710024 |
| LOR | orangered4 | -0.040388368 | 0.383854464 | -0.03259583 | 0.48224222 |
| NEU2 | orangered4 | -0.017509199 | 0.7058824 | -0.003633062 | 0.937588627 |
| R3HDML | orangered4 | -0.061431059 | 0.185094695 | -0.065865206 | 0.155291156 |
| SLC39A1 | orangered4 | -0.080211504 | 0.083357151 | -0.070266254 | 0.129450641 |
| SPRR4 | orangered4 | 0.023997412 | 0.604965488 | 0.039401679 | 0.395588949 |
| WFDC11 | orangered4 | -0.041646137 | 0.369207757 | -0.02456452 | 0.596457929 |
| ABCB8 | paleturquoi | 0.173498149 | 0.000164632 | 0.197633189 | 1.69E-05 |
| ABHD17/ | paleturquoi | 0.269037298 | 3.47E-09 | 0.289373112 | 1.85E-10 |
| ACADS | paleturquoi | 0.289538631 | 1.80E-10 | 0.319081099 | 1.64E-12 |
| ACD | paleturquoi | 0.144326604 | 0.001766285 | 0.165455901 | 0.000329755 |
| ACSF3 | paleturquoi | 0.236511488 | 2.33E-07 | 0.24237881 | 1.14E-07 |
| ADAT3 | paleturquoi | 0.243925487 | 9.40E-08 | 0.263295428 | 7.61E-09 |
| ADRBK1 | paleturquoi | 0.121305631 | 0.008687611 | 0.136951416 | 0.003020946 |
| AKR1A1 | paleturquoi | 0.304436316 | 1.80E-11 | 0.332613794 | 1.59E-13 |
| ALKBH7 | paleturquoi | 0.219695537 | 1.64E-06 | 0.230979332 | 4.50E-07 |
| ANAPC2 | paleturquoi | 0.203932675 | 8.91E-06 | 0.214817088 | 2.81E-06 |
| AP3D1 | paleturquoi | 0.377713586 | 2.77E-17 | 0.387722369 | 3.35E-18 |
| APBA3 | paleturquoi | 0.234506921 | 2.96E-07 | 0.254783904 | 2.36E-08 |
| APRT | paleturquoi | 0.228804733 | 5.80E-07 | 0.26302494 | 7.89E-09 |
| ATP5D | paleturquoi | 0.257281892 | 1.70E-08 | 0.279312895 | 8.12E-10 |
| ATPAF2 | paleturquoi | 0.415069483 | 7.13E-21 | 0.433459179 | 8.18E-23 |
| BSG | paleturquoi | 0.308460523 | 9.43E-12 | 0.306391128 | 1.32E-11 |
| BTBD2 | paleturquoi | 0.274340375 | 1.65E-09 | 0.285311982 | 3.38E-10 |
| C17orf59 | paleturquoi | 0.247428173 | 6.06E-08 | 0.280893173 | 6.46E-10 |
| C19orf24 | paleturquoi | 0.321157387 | 1.15E-12 | 0.358918838 | 1.21E-15 |
| C19orf25 | paleturquoi | 0.28869182 | 2.05E-10 | 0.32518251 | 5.80E-13 |
| C19orf70 | paleturquoi | 0.208554174 | 5.50E-06 | 0.234137665 | 3.10E-07 |
| C9orf114 | paleturquoi | 0.24242439 | 1.13E-07 | 0.237854953 | 1.98E-07 |
| C9orf69 | paleturquoi | 0.171863863 | 0.000190072 | 0.195877427 | 2.02E-05 |
| CACTIN | paleturquoi | 0.262850413 | 8.08E-09 | 0.26851496 | 3.73E-09 |
| CCDC94 | paleturquoi | 0.222773499 | 1.16E-06 | 0.239288208 | 1.66E-07 |
| CD320 | paleturquoi | 0.151643634 | 0.00101155 | 0.146265713 | 0.001527439 |

| | | | | | |
|---|---|---|---|---|---|
| CDC34 | paleturquoi | 0.128652718 | 0.005363721 | 0.148370006 | 0.001302063 |
| CHMP1A | paleturquoi | 0.307982532 | 1.02E-11 | 0.341798616 | 3.04E-14 |
| CLPP | paleturquoi | 0.324108139 | 6.97E-13 | 0.350450709 | 6.10E-15 |
| COG4 | paleturquoi | 0.243448677 | 9.97E-08 | 0.276271009 | 1.26E-09 |
| COX4I1 | paleturquoi | 0.228712163 | 5.86E-07 | 0.271045233 | 2.63E-09 |
| CRELD2 | paleturquoi | 0.392994852 | 1.07E-18 | 0.40860806 | 3.21E-20 |
| CSK | paleturquoi | 0.262883843 | 8.05E-09 | 0.292907868 | 1.08E-10 |
| CSNK1G: | paleturquoi | 0.295769048 | 6.99E-11 | 0.297626819 | 5.24E-11 |
| CTDNEP | paleturquoi | 0.227712892 | 6.59E-07 | 0.236008065 | 2.48E-07 |
| CTU2 | paleturquoi | 0.281209362 | 6.17E-10 | 0.321232928 | 1.14E-12 |
| DAK | paleturquoi | 0.369236144 | 1.57E-16 | 0.369084936 | 1.62E-16 |
| DAPK3 | paleturquoi | 0.177459201 | 0.000115593 | 0.200438618 | 1.28E-05 |
| DDX28 | paleturquoi | 0.061665717 | 0.183418664 | 0.08581649 | 0.063888162 |
| DOHH | paleturquoi | 0.316989846 | 2.32E-12 | 0.342700485 | 2.58E-14 |
| DOK1 | paleturquoi | 0.264590433 | 6.39E-09 | 0.280386648 | 6.96E-10 |
| DOT1L | paleturquoi | 0.366280521 | 2.83E-16 | 0.367768477 | 2.11E-16 |
| DUS2 | paleturquoi | 0.263001477 | 7.92E-09 | 0.285460432 | 3.31E-10 |
| DUS3L | paleturquoi | 0.290349805 | 1.60E-10 | 0.289997197 | 1.68E-10 |
| ELP5 | paleturquoi | 0.104590242 | 0.023798948 | 0.105690667 | 0.022355386 |
| FAM96B | paleturquoi | 0.203643429 | 9.18E-06 | 0.259095474 | 1.34E-08 |
| FASTK | paleturquoi | 0.167463203 | 0.000278075 | 0.191037655 | 3.24E-05 |
| FDXR | paleturquoi | 0.31014463 | 7.18E-12 | 0.325085792 | 5.89E-13 |
| FUK | paleturquoi | 0.344257174 | 1.94E-14 | 0.376918323 | 3.27E-17 |
| FZR1 | paleturquoi | 0.31665279 | 2.46E-12 | 0.338954003 | 5.10E-14 |
| GCHFR | paleturquoi | 0.372082249 | 8.81E-17 | 0.375795385 | 4.12E-17 |
| GMPPA | paleturquoi | 0.218381697 | 1.90E-06 | 0.238820625 | 1.76E-07 |
| GMPPB | paleturquoi | 0.335433817 | 9.61E-14 | 0.368769799 | 1.72E-16 |
| GPX4 | paleturquoi | 0.320810121 | 1.22E-12 | 0.35274185 | 3.96E-15 |
| GTF2F1 | paleturquoi | 0.249586265 | 4.61E-08 | 0.26055071 | 1.10E-08 |
| HMG20B | paleturquoi | 0.443420267 | 6.48E-24 | 0.456208325 | 2.20E-25 |
| HMGCL | paleturquoi | 0.299265265 | 4.06E-11 | 0.328319393 | 3.37E-13 |
| KLF16 | paleturquoi | 0.204370227 | 8.52E-06 | 0.217614037 | 2.06E-06 |
| KLHDC4 | paleturquoi | 0.162897801 | 0.000408621 | 0.186585434 | 4.97E-05 |
| LONP1 | paleturquoi | 0.308105017 | 9.99E-12 | 0.331780948 | 1.84E-13 |
| LSM7 | paleturquoi | 0.362606925 | 5.87E-16 | 0.385942482 | 4.91E-18 |
| MAP2K2 | paleturquoi | 0.291016687 | 1.44E-10 | 0.295572422 | 7.20E-11 |
| MBD3 | paleturquoi | 0.257040305 | 1.75E-08 | 0.270124929 | 2.98E-09 |
| MCAT | paleturquoi | 0.205830745 | 7.32E-06 | 0.231809168 | 4.08E-07 |
| MED16 | paleturquoi | 0.251876171 | 3.43E-08 | 0.269162247 | 3.41E-09 |
| MFSD12 | paleturquoi | 0.274827748 | 1.54E-09 | 0.297806866 | 5.10E-11 |
| MIER2 | paleturquoi | 0.290183513 | 1.64E-10 | 0.292322836 | 1.18E-10 |
| MOB3A | paleturquoi | 0.329630489 | 2.68E-13 | 0.352298992 | 4.30E-15 |
| MPND | paleturquoi | 0.362997904 | 5.43E-16 | 0.384629617 | 6.49E-18 |
| MRPL54 | paleturquoi | 0.273155862 | 1.95E-09 | 0.298763478 | 4.39E-11 |
| MVD | paleturquoi | 0.343097538 | 2.40E-14 | 0.364021002 | 4.44E-16 |

| | | | | | |
|---|---|---|---|---|---|
| MYDGF | paleturquoi | 0.367723548 | 2.12E-16 | 0.379901733 | 1.76E-17 |
| NCLN | paleturquoi | 0.388767419 | 2.68E-18 | 0.411631099 | 1.60E-20 |
| NDUFA1 | paleturquoi | 0.274610186 | 1.59E-09 | 0.299032731 | 4.21E-11 |
| NDUFS7 | paleturquoi | 0.276121128 | 1.28E-09 | 0.314246752 | 3.66E-12 |
| NELFB | paleturquoi | 0.117698839 | 0.010911191 | 0.135453522 | 0.003358569 |
| NHP2L1 | paleturquoi | 0.207777936 | 5.97E-06 | 0.21673384 | 2.27E-06 |
| NUDT18 | paleturquoi | 0.426095665 | 5.06E-22 | 0.457759245 | 1.45E-25 |
| NUTF2 | paleturquoi | 0.276404536 | 1.23E-09 | 0.318323757 | 1.86E-12 |
| OAZ1 | paleturquoi | 0.264302801 | 6.64E-09 | 0.309621307 | 7.82E-12 |
| PARD6A | paleturquoi | 0.338372067 | 5.67E-14 | 0.360070245 | 9.65E-16 |
| PDDC1 | paleturquoi | 0.221818105 | 1.29E-06 | 0.235898417 | 2.51E-07 |
| PEX11G | paleturquoi | 0.338714175 | 5.33E-14 | 0.358182651 | 1.39E-15 |
| PIAS4 | paleturquoi | 0.35423778 | 2.97E-15 | 0.360404898 | 9.04E-16 |
| PLEKHJ1 | paleturquoi | 0.305926743 | 1.42E-11 | 0.324675764 | 6.33E-13 |
| POLR2E | paleturquoi | 0.29300019 | 1.07E-10 | 0.315934571 | 2.77E-12 |
| POLR3H | paleturquoi | 0.194104409 | 2.40E-05 | 0.209557822 | 4.94E-06 |
| POLRMT | paleturquoi | 0.196947542 | 1.81E-05 | 0.231320084 | 4.32E-07 |
| PPP2R4 | paleturquoi | 0.38025335 | 1.63E-17 | 0.391291165 | 1.55E-18 |
| PQLC1 | paleturquoi | 0.148131546 | 0.00132597 | 0.163580835 | 0.000386 |
| PRMT7 | paleturquoi | 0.2806434 | 6.70E-10 | 0.320363183 | 1.32E-12 |
| R3HCC1 | paleturquoi | 0.186442639 | 5.04E-05 | 0.196086148 | 1.98E-05 |
| R3HDM4 | paleturquoi | 0.369362136 | 1.53E-16 | 0.397715062 | 3.78E-19 |
| RAB11B | paleturquoi | 0.164986065 | 0.000343082 | 0.185180279 | 5.68E-05 |
| RANBP3 | paleturquoi | 0.318625946 | 1.77E-12 | 0.320423667 | 1.31E-12 |
| RNF126 | paleturquoi | 0.292692105 | 1.12E-10 | 0.312525527 | 4.87E-12 |
| RPS15 | paleturquoi | 0.292885634 | 1.09E-10 | 0.312261678 | 5.08E-12 |
| RRP7A | paleturquoi | 0.156211016 | 0.000705276 | 0.174969568 | 0.000144494 |
| SDF2L1 | paleturquoi | 0.2593878 | 1.29E-08 | 0.291645311 | 1.31E-10 |
| SF3A2 | paleturquoi | 0.206409423 | 6.89E-06 | 0.207095457 | 6.41E-06 |
| SGSM3 | paleturquoi | 0.3570253 | 1.74E-15 | 0.384088485 | 7.28E-18 |
| SGTA | paleturquoi | 0.374155136 | 5.77E-17 | 0.38502295 | 5.97E-18 |
| SH3GL1 | paleturquoi | 0.382624428 | 9.92E-18 | 0.417072325 | 4.44E-21 |
| SIL1 | paleturquoi | 0.219424985 | 1.69E-06 | 0.235883374 | 2.51E-07 |
| SIRT6 | paleturquoi | 0.390611801 | 1.80E-18 | 0.417568971 | 3.95E-21 |
| SLC2A8 | paleturquoi | 0.153049253 | 0.000906231 | 0.150096145 | 0.001140484 |
| SLC39A3 | paleturquoi | 0.326258929 | 4.82E-13 | 0.349920089 | 6.74E-15 |
| SPATA2L | paleturquoi | 0.319722573 | 1.47E-12 | 0.344179021 | 1.97E-14 |
| SSR4 | paleturquoi | 0.243877218 | 9.45E-08 | 0.27391457 | 1.76E-09 |
| STK11 | paleturquoi | 0.270100805 | 2.99E-09 | 0.273049578 | 1.98E-09 |
| TBCD | paleturquoi | 0.191419045 | 3.13E-05 | 0.20556268 | 7.53E-06 |
| TCF25 | paleturquoi | 0.246145291 | 7.12E-08 | 0.26240993 | 8.58E-09 |
| TEX264 | paleturquoi | 0.318411867 | 1.83E-12 | 0.351272102 | 5.23E-15 |
| THOP1 | paleturquoi | 0.414877726 | 7.46E-21 | 0.412367349 | 1.34E-20 |
| TIMM13 | paleturquoi | 0.294751644 | 8.17E-11 | 0.318504465 | 1.80E-12 |
| TMED3 | paleturquoi | 0.391473412 | 1.49E-18 | 0.387693995 | 3.37E-18 |

| | | | | | |
|---|---|---|---|---|---|
| TMEM20 | paleturquoi | 0.186512756 | 5.00E-05 | 0.228671665 | 5.89E-07 |
| TMEM25 | paleturquoi | 0.197711894 | 1.68E-05 | 0.211820495 | 3.88E-06 |
| TMUB1 | paleturquoi | 0.273608208 | 1.83E-09 | 0.28075517 | 6.59E-10 |
| TP53I13 | paleturquoi | 0.224330609 | 9.71E-07 | 0.226133844 | 7.90E-07 |
| TPGS1 | paleturquoi | 0.116168697 | 0.011997776 | 0.12408929 | 0.007257549 |
| TRAPPC2 | paleturquoi | 0.218034032 | 1.97E-06 | 0.263301864 | 7.60E-09 |
| TRAPPC4 | paleturquoi | 0.236643017 | 2.29E-07 | 0.25304491 | 2.95E-08 |
| TUBG1 | paleturquoi | 0.418244621 | 3.36E-21 | 0.440884295 | 1.25E-23 |
| TUBGCP | paleturquoi | 0.118192084 | 0.010580003 | 0.13158642 | 0.004394231 |
| TXN2 | paleturquoi | 0.282443055 | 5.16E-10 | 0.287485852 | 2.45E-10 |
| UBXN6 | paleturquoi | 0.259077193 | 1.34E-08 | 0.285743522 | 3.17E-10 |
| UQCR11 | paleturquoi | 0.325538496 | 5.45E-13 | 0.358661534 | 1.27E-15 |
| VAC14 | paleturquoi | 0.147670701 | 0.001373321 | 0.1951836 | 2.16E-05 |
| VPS4A | paleturquoi | 0.158556864 | 0.000583783 | 0.195382313 | 2.12E-05 |
| WDR18 | paleturquoi | 0.28621725 | 2.96E-10 | 0.30644193 | 1.31E-11 |
| ZNF358 | paleturquoi | 0.105977755 | 0.021991496 | 0.114359857 | 0.013404719 |
| ZNF414 | paleturquoi | 0.173976441 | 0.000157814 | 0.185890613 | 5.31E-05 |
| ZNF524 | paleturquoi | 0.224015534 | 1.01E-06 | 0.24362309 | 9.76E-08 |
| ZSWIM7 | paleturquoi | 0.380784507 | 1.46E-17 | 0.399561534 | 2.50E-19 |
| AADACL | pink | -0.037192577 | 0.422632869 | -0.051952323 | 0.262523253 |
| ABCG5 | pink | -0.243071689 | 1.04E-07 | -0.235400697 | 2.66E-07 |
| AC00236 | pink | 0.000651739 | 0.988792918 | 0.012783393 | 0.782914932 |
| AC00495 | pink | -0.049280688 | 0.287892421 | -0.063382981 | 0.171492992 |
| AC00577 | pink | -0.048984991 | 0.290798324 | -0.029843115 | 0.52000992 |
| AC00862 | pink | -0.047034015 | 0.310462953 | -0.031742979 | 0.493782172 |
| AC01920 | pink | -0.045462175 | 0.326928224 | -0.035878771 | 0.439215897 |
| AC08776 | pink | -0.021964784 | 0.635893556 | -0.020431247 | 0.659657783 |
| AC09332 | pink | -0.001222867 | 0.978973737 | 0.011318747 | 0.80726762 |
| ACCSL | pink | -0.001717477 | 0.970472599 | 0.006042812 | 0.896378536 |
| ACER1 | pink | -0.023263796 | 0.616050621 | -0.030262069 | 0.51416531 |
| ACSBG2 | pink | -0.046594215 | 0.315013966 | -0.047042922 | 0.310371231 |
| ACTL6B | pink | -0.005836906 | 0.899890528 | 0.001180128 | 0.979708442 |
| ACTL7A | pink | -0.011614941 | 0.802326931 | -0.00480614 | 0.91749963 |
| ACTL7B | pink | -0.0187071 | 0.686789525 | -0.009928977 | 0.830549206 |
| ACTL9 | pink | -0.044460914 | 0.337706025 | -0.039428938 | 0.3952619 |
| ACTN3 | pink | -0.110860162 | 0.016544381 | -0.108474684 | 0.019037379 |
| ADAM30 | pink | -0.052013444 | 0.261961456 | -0.044824917 | 0.333761774 |
| AGRP | pink | 0.103600195 | 0.0251659 | 0.108114227 | 0.019441239 |
| AIPL1 | pink | -0.040176348 | 0.386357894 | -0.038593835 | 0.40535548 |
| AKR1D1 | pink | -0.038055946 | 0.411937274 | -0.02862361 | 0.537214829 |
| AL02257 | pink | -0.064876138 | 0.161602327 | -0.053324958 | 0.250105889 |
| AL35482 | pink | -0.025264605 | 0.58603108 | -0.027552686 | 0.552555162 |
| AL35628 | pink | -0.020105885 | 0.664745099 | -0.013499577 | 0.771082091 |
| AL35714 | pink | -0.010044979 | 0.828599941 | 0.017128535 | 0.711989196 |
| ANGPTL | pink | -0.102896322 | 0.026178465 | -0.065612465 | 0.156885855 |

| | | | | | |
|---|---|---|---|---|---|
| AP00288⁴ | pink | -0.124437707 | 0.007094259 | -0.143326326 | 0.001902448 |
| APOC4 | pink | 0.061158033 | 0.187058955 | 0.051565514 | 0.26609784 |
| APOF | pink | 0.169777731 | 0.000227907 | 0.174201575 | 0.000154697 |
| APOL5 | pink | -0.061266883 | 0.186274009 | -0.06211313 | 0.180254141 |
| ARGFX | pink | -0.031946338 | 0.49101721 | -0.017534767 | 0.705472892 |
| ARL5C | pink | -0.053616293 | 0.247523863 | -0.051172783 | 0.269761166 |
| ARR3 | pink | -0.051098836 | 0.270454772 | -0.053390922 | 0.24951963 |
| ASB11 | pink | -0.080409986 | 0.082594026 | -0.069835307 | 0.131823588 |
| ASGR2 | pink | -0.061008499 | 0.188141239 | -0.050228523 | 0.278709702 |
| ASIC4 | pink | -0.201326136 | 1.17E-05 | -0.192088556 | 2.93E-05 |
| AWAT1 | pink | -0.00564015 | 0.903248287 | 0.024668954 | 0.594897191 |
| BANF2 | pink | -0.12642497 | 0.006224213 | -0.126464677 | 0.00620785 |
| BEST2 | pink | -0.099817466 | 0.031030488 | -0.090210217 | 0.051390015 |
| BHMG1 | pink | -0.056509309 | 0.222890526 | -0.055889219 | 0.228017344 |
| BIRC8 | pink | 0.004520476 | 0.922387302 | 0.007975485 | 0.86352202 |
| BPIFA3 | pink | -0.041308951 | 0.373099883 | -0.036516983 | 0.431114217 |
| BTN1A1 | pink | -0.117537936 | 0.011021198 | -0.095342264 | 0.039442945 |
| BTNL2 | pink | -0.265981072 | 5.28E-09 | -0.264103435 | 6.82E-09 |
| C10orf12( | pink | 0.007356953 | 0.87401462 | 0.000846482 | 0.985444509 |
| C10orf62 | pink | -0.097702995 | 0.034791789 | -0.091222801 | 0.048819149 |
| C11orf40 | pink | -0.024870271 | 0.591893807 | -0.012504223 | 0.787541174 |
| C14orf17' | pink | -0.009549239 | 0.836937414 | -0.005057184 | 0.913206869 |
| C15orf43 | pink | -0.015300692 | 0.741563228 | -0.01500162 | 0.746439933 |
| C15orf53 | pink | 0.011920227 | 0.797242805 | 0.027351662 | 0.555458402 |
| C16orf78 | pink | -0.009616955 | 0.835797455 | -0.009119649 | 0.844177186 |
| C16orf96 | pink | -0.187076212 | 4.74E-05 | -0.182470017 | 7.31E-05 |
| C17orf10! | pink | 0.00195254 | 0.966433563 | 0.014512332 | 0.754440108 |
| C17orf64 | pink | -0.133699836 | 0.003797138 | -0.125652648 | 0.006550274 |
| C17orf98 | pink | -0.001690776 | 0.970931457 | 0.041076393 | 0.375798999 |
| C17orf99 | pink | -0.113390511 | 0.014216946 | -0.098901685 | 0.032614517 |
| C19orf80 | pink | -0.166642792 | 0.000298209 | -0.158885758 | 0.000568393 |
| C1orf177 | pink | -0.111913674 | 0.015537633 | -0.110472292 | 0.016929167 |
| C20orf17: | pink | -0.065330697 | 0.158678255 | -0.063735117 | 0.169120702 |
| C20orf62 | pink | -0.020679907 | 0.65578031 | -0.019502983 | 0.674212622 |
| C2orf57 | pink | -0.077447727 | 0.094586295 | -0.076801743 | 0.09737785 |
| C2orf61 | pink | -0.09386883 | 0.042604669 | -0.082807803 | 0.073813537 |
| C2orf78 | pink | 0.000260813 | 0.995515032 | 0.028090239 | 0.544828321 |
| C2orf83 | pink | -0.065382741 | 0.158346032 | -0.05113898 | 0.270078084 |
| C3orf20 | pink | -0.03313856 | 0.474975322 | -0.032129942 | 0.488527976 |
| C3orf22 | pink | -0.179442964 | 9.66E-05 | -0.167277065 | 0.000282528 |
| C3orf84 | pink | -0.069235765 | 0.135180587 | -0.068047764 | 0.142026553 |
| C4orf50 | pink | -0.038950267 | 0.401028762 | -0.032705381 | 0.480770544 |
| C5orf60 | pink | 0.006465413 | 0.889176922 | 0.017282145 | 0.709522655 |
| C8orf74 | pink | 0.038378331 | 0.407984907 | 0.029240578 | 0.528475054 |
| CA5A | pink | 0.034290553 | 0.459751189 | 0.013487331 | 0.771283978 |

| | | | | | |
|---|---|---|---|---|---|
| CA7 | pink | -0.097378095 | 0.035402816 | -0.090074299 | 0.051743549 |
| CABP5 | pink | -0.033077137 | 0.47579473 | -0.026456936 | 0.568469614 |
| CABS1 | pink | -0.056802101 | 0.220498558 | -0.045245753 | 0.329238767 |
| CACNG2 | pink | -0.008202784 | 0.85967211 | -0.000809416 | 0.986081806 |
| CALR3 | pink | -0.045606118 | 0.325397304 | -0.037429042 | 0.419687485 |
| CATSPEI | pink | -0.119086816 | 0.010001921 | -0.096879922 | 0.036357429 |
| CATSPEI | pink | -0.032209219 | 0.487455275 | -0.021369334 | 0.645078271 |
| CCDC10 | pink | -0.090696467 | 0.050141706 | -0.083957998 | 0.069880133 |
| CCDC18 | pink | -0.060065364 | 0.195073703 | -0.040387885 | 0.383860154 |
| CCDC27 | pink | -0.206112017 | 7.11E-06 | -0.216019699 | 2.46E-06 |
| CCDC63 | pink | -0.025859673 | 0.577235156 | -0.010422306 | 0.822266777 |
| CCDC83 | pink | -0.036236559 | 0.434663328 | -0.010741939 | 0.816910973 |
| CCKAR | pink | -0.079581833 | 0.085815856 | -0.085354056 | 0.06533808 |
| CCT8L2 | pink | -0.031239252 | 0.500666696 | -0.02849487 | 0.539047607 |
| CD300LD | pink | 0.036791556 | 0.427655447 | 0.035154114 | 0.448519724 |
| CDCP2 | pink | 0.004186177 | 0.928110798 | 0.014056924 | 0.761909998 |
| CEACAM | pink | 0.015365129 | 0.740513869 | 0.050603517 | 0.275132161 |
| CEACAM | pink | -0.011951676 | 0.796719536 | -0.005230992 | 0.910236274 |
| CEBPE | pink | -0.071782251 | 0.121365095 | -0.053522662 | 0.248351654 |
| CELA1 | pink | -0.077126741 | 0.095965308 | -0.076073199 | 0.100604518 |
| CELA2A | pink | -0.091409163 | 0.048357917 | -0.093865356 | 0.04261237 |
| CES5A | pink | 0.083215054 | 0.07240069 | 0.061895775 | 0.181786388 |
| CIB3 | pink | -0.074310048 | 0.108765186 | -0.043167647 | 0.351959804 |
| CIB4 | pink | -0.184186733 | 6.23E-05 | -0.189715761 | 3.69E-05 |
| CIDEC | pink | -0.0803584 | 0.08279182 | -0.075915196 | 0.101315392 |
| CLCN1 | pink | -0.212556306 | 3.58E-06 | -0.203285756 | 9.53E-06 |
| CLEC17A | pink | -0.064866571 | 0.161664302 | -0.039402872 | 0.395574633 |
| CLRN2 | pink | -0.055447244 | 0.231722291 | -0.054790353 | 0.237307209 |
| CLVS1 | pink | -0.185509978 | 5.50E-05 | -0.185127926 | 5.70E-05 |
| CNBD2 | pink | -0.107624954 | 0.020001351 | -0.101475273 | 0.028330081 |
| CNTN2 | pink | -0.07700427 | 0.096495671 | -0.075338237 | 0.103945101 |
| CNTNAP | pink | -0.174142225 | 0.000155513 | -0.177673243 | 0.00011338 |
| CPLX4 | pink | -0.035378704 | 0.44562436 | -0.028656725 | 0.536743904 |
| CRLF2 | pink | -0.073160492 | 0.114361068 | -0.070487281 | 0.128246487 |
| CRX | pink | -0.030644675 | 0.508857696 | -0.025136979 | 0.587925604 |
| CRYGA | pink | 0.000513546 | 0.991169122 | 0.003881842 | 0.933324518 |
| CST11 | pink | -0.094929924 | 0.040307082 | -0.077183636 | 0.095719711 |
| CST9L | pink | -0.031411321 | 0.498309317 | -0.02512148 | 0.588155873 |
| CSTL1 | pink | -0.098659612 | 0.033044588 | -0.060549808 | 0.191489849 |
| CT47B1 | pink | -0.091162115 | 0.048970134 | -0.085871105 | 0.063718685 |
| CTD-308 | pink | -0.056897128 | 0.219726185 | -0.001252755 | 0.978459955 |
| CTD-313 | pink | -0.077018203 | 0.096435218 | -0.071546144 | 0.122597824 |
| CTRC | pink | -0.059865109 | 0.1965694 | -0.059171853 | 0.201811938 |
| CU10478 | pink | -0.046047059 | 0.3207366 | -0.035617886 | 0.442552597 |
| CYP11B1 | pink | 0.018159253 | 0.695497628 | 0.020738452 | 0.654868736 |

| | | | | | |
|---|---|---|---|---|---|
| CYP11B2 | pink | -0.035958315 | 0.438201415 | -0.02845669 | 0.539591759 |
| CYP2A13 | pink | -0.102335547 | 0.02701011 | -0.096776342 | 0.036558633 |
| CYP2F1 | pink | -0.087229518 | 0.059621044 | -0.064926266 | 0.161277895 |
| CYP46A1 | pink | -0.288521412 | 2.10E-10 | -0.296892311 | 5.87E-11 |
| CYP4F2 | pink | -0.118718211 | 0.010236589 | -0.114583955 | 0.013222867 |
| DAND5 | pink | -0.078915858 | 0.088479543 | -0.072839174 | 0.115964926 |
| DAO | pink | -0.0448983 | 0.332970217 | -0.04175924 | 0.367907877 |
| DCST1 | pink | 0.189065321 | 3.92E-05 | 0.182354663 | 7.39E-05 |
| DCST2 | pink | 0.265032893 | 6.01E-09 | 0.261454497 | 9.75E-09 |
| DDX4 | pink | -0.1110048 | 0.016402866 | -0.105865075 | 0.022133703 |
| DEFB116 | pink | 0.046082803 | 0.320360703 | 0.043392193 | 0.349458092 |
| DEFB118 | pink | -0.061379367 | 0.185465414 | -0.056748408 | 0.220935823 |
| DEFB119 | pink | -0.022997328 | 0.620098926 | -0.022361545 | 0.629804315 |
| DEFB121 | pink | -0.057483737 | 0.21500114 | -0.050022811 | 0.28068558 |
| DEFB123 | pink | -0.044846663 | 0.333527084 | -0.03477486 | 0.453433064 |
| DGAT2L( | pink | 0.015700908 | 0.735053459 | 0.017649484 | 0.703636601 |
| DMRTC2 | pink | -0.022063012 | 0.634383719 | -0.002138454 | 0.96323965 |
| DNAH17 | pink | -0.249974521 | 4.38E-08 | -0.256484881 | 1.89E-08 |
| DNAJC5( | pink | -0.127132515 | 0.005938364 | -0.126643749 | 0.006134534 |
| DNMT3L | pink | -0.077341318 | 0.095041682 | -0.069139505 | 0.135725654 |
| DSCAM | pink | -0.104519918 | 0.023893881 | -0.074864546 | 0.106144292 |
| DUSP21 | pink | -0.030420405 | 0.511965359 | -0.014859956 | 0.748753468 |
| DUX4 | pink | 0.094746891 | 0.040695759 | 0.075292564 | 0.104155561 |
| EFCAB8 | pink | -0.097169683 | 0.035799555 | -0.092819031 | 0.044985464 |
| EFCAB9 | pink | -0.045897973 | 0.322307527 | -0.055117864 | 0.234510953 |
| ERICH6 | pink | -0.104377558 | 0.024087059 | -0.078806345 | 0.08892385 |
| ETV3L | pink | -0.03138496 | 0.49867008 | -0.042466655 | 0.359842174 |
| FAM129( | pink | -0.12043945 | 0.00918118 | -0.112232997 | 0.01524329 |
| FAM151A | pink | -0.046879967 | 0.31205208 | -0.050508311 | 0.276037479 |
| FAM187E | pink | -0.125229048 | 0.006735539 | -0.123916003 | 0.007340007 |
| FAM205A | pink | -0.040086947 | 0.387416466 | -0.032939994 | 0.477627047 |
| FAM221E | pink | 0.101425809 | 0.028407632 | 0.124380103 | 0.007121027 |
| FAM47A | pink | -0.05513908 | 0.234330622 | -0.052762119 | 0.255147168 |
| FAM47B | pink | 0.051057396 | 0.270844001 | 0.05238945 | 0.258523623 |
| FAM71C | pink | -0.074305934 | 0.108784821 | -0.058268214 | 0.208796997 |
| FBXW12 | pink | -0.077001194 | 0.096509024 | -0.07142399 | 0.123239414 |
| FCAMR | pink | 0.019419568 | 0.675526606 | 0.017860011 | 0.700271158 |
| FFAR3 | pink | 0.012103748 | 0.794190608 | 0.0103938 | 0.822744835 |
| FGF6 | pink | 0.010093616 | 0.827782964 | 0.02736659 | 0.555242548 |
| FNDC7 | pink | -0.107696423 | 0.019918669 | -0.113011084 | 0.014546473 |
| FP325317 | pink | -0.02029729 | 0.661750434 | -0.014574788 | 0.75341743 |
| FSCB | pink | 0.013618967 | 0.769114586 | 0.013104133 | 0.777609276 |
| FSD2 | pink | -0.073804017 | 0.111201285 | -0.076577314 | 0.098362919 |
| G6PC | pink | -0.023597357 | 0.61099939 | -0.026407965 | 0.569185929 |
| GABRB1 | pink | -0.142259549 | 0.002058197 | -0.147064409 | 0.001437984 |

| | | | | | |
|---|---|---|---|---|---|
| GAGE10 | pink | -0.138469811 | 0.002710479 | -0.117698863 | 0.010911175 |
| GATA1 | pink | -0.121438472 | 0.008614043 | -0.120908673 | 0.008910795 |
| GDF2 | pink | -0.004548472 | 0.921908164 | 0.013458183 | 0.771764591 |
| GGTLC1 | pink | 0.060605618 | 0.191080102 | 0.06036417 | 0.192857431 |
| GGTLC2 | pink | 0.043987935 | 0.342875508 | 0.02621173 | 0.572060619 |
| GIP | pink | -0.049923687 | 0.281641055 | -0.048166323 | 0.298945924 |
| GJA10 | pink | -0.039970091 | 0.388802785 | -0.029250801 | 0.528330845 |
| GJA8 | pink | -0.165987556 | 0.000315258 | -0.161570925 | 0.000456132 |
| GJD2 | pink | -0.006605205 | 0.886796674 | -0.027644052 | 0.551238076 |
| GLT6D1 | pink | 0.003350208 | 0.942438932 | -0.003938061 | 0.932361176 |
| GNAT1 | pink | -0.117951639 | 0.010740319 | -0.122940696 | 0.007819926 |
| GOLGA6 | pink | -0.029792031 | 0.520724889 | -0.031140037 | 0.502028636 |
| GOLGA6 | pink | -0.065042516 | 0.16052739 | -0.073243621 | 0.113948975 |
| GOLGA6 | pink | -0.030963143 | 0.504461733 | -0.017124295 | 0.712057308 |
| GOLGA8 | pink | -0.051713868 | 0.264722937 | -0.075672703 | 0.102414137 |
| GOT1L1 | pink | -0.042744689 | 0.356702667 | -0.034787843 | 0.453264371 |
| GPR101 | pink | -0.032452912 | 0.484165801 | -0.020758183 | 0.654561627 |
| GPR119 | pink | -0.023828035 | 0.607516897 | -0.032241262 | 0.487022057 |
| GPR144 | pink | -0.089226338 | 0.053995242 | -0.070837294 | 0.126357397 |
| GPR148 | pink | -0.056644824 | 0.221781139 | -0.053320125 | 0.250148884 |
| GPR152 | pink | -0.04754067 | 0.305274007 | -0.033888384 | 0.465034875 |
| GPR32 | pink | -0.000573288 | 0.990141856 | 0.013078542 | 0.778032226 |
| GPR33 | pink | -0.067976375 | 0.142446239 | -0.038415306 | 0.407533043 |
| GPR42 | pink | -0.028630126 | 0.537122159 | -0.006690161 | 0.885350626 |
| GPX5 | pink | -0.014170626 | 0.760042885 | -0.016053243 | 0.729338131 |
| GRK7 | pink | -0.086014978 | 0.063274 | -0.082408889 | 0.075219164 |
| GRXCR1 | pink | -0.006088782 | 0.895594726 | 0.000445626 | 0.99233704 |
| GSG1 | pink | -0.037039665 | 0.424543933 | -0.029850603 | 0.51990515 |
| H1FNT | pink | -0.111511038 | 0.01591587 | -0.104968027 | 0.023294527 |
| HEATR4 | pink | -0.228543511 | 5.98E-07 | -0.22802889 | 6.35E-07 |
| HIGD2B | pink | 0.042662176 | 0.357632586 | 0.04181433 | 0.367275755 |
| HIPK4 | pink | -0.139465731 | 0.002522929 | -0.140038569 | 0.002420491 |
| HSD3B1 | pink | -0.0374386 | 0.419568684 | -0.042536517 | 0.359051685 |
| HTR1A | pink | -0.052224134 | 0.260031256 | -0.053187378 | 0.251331724 |
| HTR3D | pink | 0.042657588 | 0.357684339 | 0.024258153 | 0.601047198 |
| HZGJ | pink | -0.085997036 | 0.063329313 | -0.078253733 | 0.091193281 |
| IDI2 | pink | -0.041871568 | 0.366619702 | -0.042015946 | 0.364968113 |
| IFNL1 | pink | -0.171428561 | 0.000197444 | -0.163287868 | 0.000395556 |
| IFNL2 | pink | -0.096406995 | 0.037283808 | -0.082744661 | 0.07403459 |
| IFNL3 | pink | -0.135413195 | 0.003368114 | -0.1307132 | 0.004664786 |
| IL1RAPL | pink | -0.031697529 | 0.494401269 | -0.036039338 | 0.437169441 |
| IL22 | pink | -0.035259157 | 0.447164207 | -0.017155427 | 0.71155716 |
| IL31 | pink | -0.0357901 | 0.44034837 | -0.031942387 | 0.491070848 |
| INHBC | pink | -0.156401903 | 0.000694576 | -0.166654289 | 0.000297918 |
| IQCF1 | pink | 0.045840219 | 0.322917436 | 0.04110366 | 0.375481918 |

| | | | | | |
|---|---|---|---|---|---|
| IQCF2 | pink | -0.026441643 | 0.568693264 | -0.017460969 | 0.706655088 |
| IQCF6 | pink | 0.143494375 | 0.001878921 | 0.131506043 | 0.004418528 |
| IRGC | pink | -0.043722166 | 0.345802276 | -0.0323458 | 0.48561017 |
| ITIH6 | pink | -0.143879861 | 0.001825955 | -0.137344176 | 0.002937681 |
| IZUMO1I | pink | -0.05024225 | 0.27857819 | -0.035472598 | 0.444417053 |
| KCNG4 | pink | -0.013067581 | 0.778213397 | -0.002336901 | 0.959831064 |
| KCNH4 | pink | -0.044266343 | 0.339826541 | -0.02746953 | 0.553755218 |
| KCNH5 | pink | 0.019004333 | 0.682082175 | 0.009598205 | 0.836113061 |
| KCNJ6 | pink | -0.069049753 | 0.136235393 | -0.06778137 | 0.143597492 |
| KCNK10 | pink | -0.040173342 | 0.386393465 | -0.031639834 | 0.495187745 |
| KCTD19 | pink | -0.115371235 | 0.01260112 | -0.115170181 | 0.012757397 |
| KHDC3L | pink | -0.169919451 | 0.000225128 | -0.155651184 | 0.000737542 |
| KIF2B | pink | 0.006969204 | 0.880603693 | 0.007283417 | 0.875263579 |
| KIR3DL3 | pink | -0.038186863 | 0.410329541 | -0.021382236 | 0.644878693 |
| KIR3DX1 | pink | -0.043011935 | 0.35370124 | -0.028112807 | 0.544505098 |
| KLHL10 | pink | -0.175976146 | 0.000132075 | -0.185489783 | 5.51E-05 |
| KRT32 | pink | 0.041889522 | 0.36641407 | 0.02110401 | 0.649188412 |
| KRT35 | pink | 0.032809646 | 0.47937215 | 0.005103489 | 0.912415336 |
| KRTAP1- | pink | -0.016247785 | 0.726188804 | -0.021283999 | 0.646399032 |
| KRTAP1( | pink | -0.044160756 | 0.340980824 | -0.049609071 | 0.284688256 |
| KRTAP1( | pink | -0.067682524 | 0.144183759 | -0.066448463 | 0.15165789 |
| KRTAP1( | pink | -0.055912408 | 0.227824127 | -0.054613589 | 0.238826119 |
| KRTAP1( | pink | -0.047586032 | 0.304812246 | -0.05042395 | 0.276841356 |
| KRTAP1( | pink | -0.048339666 | 0.297208212 | -0.047603927 | 0.30463021 |
| KRTAP12 | pink | -0.058048058 | 0.210524928 | -0.044519825 | 0.337065661 |
| KRTAP12 | pink | -0.025170834 | 0.587422778 | -0.023449489 | 0.613236353 |
| KRTAP12 | pink | -0.086222475 | 0.062637167 | -0.07643116 | 0.099008676 |
| KRTAP19 | pink | -0.060271071 | 0.193545964 | -0.064621168 | 0.163260148 |
| KRTAP23 | pink | -0.009405081 | 0.839365365 | -0.005170001 | 0.911278542 |
| KRTAP26 | pink | -0.00788618 | 0.865035534 | -0.00959154 | 0.836225262 |
| KRTAP27 | pink | -0.003988402 | 0.931498656 | 0.004605636 | 0.920929913 |
| KRTAP5- | pink | -0.118687614 | 0.010256286 | -0.120658341 | 0.009054151 |
| KRTAP5- | pink | -0.05849759 | 0.20700762 | -0.046236592 | 0.318746682 |
| KRTAP5- | pink | -0.015121449 | 0.74448476 | -0.01458059 | 0.753322435 |
| KRTAP6- | pink | -0.020952872 | 0.651534478 | -0.014406851 | 0.756168258 |
| KRTAP6- | pink | -0.008785191 | 0.849822858 | -0.005278205 | 0.909429567 |
| KRTAP9- | pink | -0.016409941 | 0.723567266 | -0.0159772 | 0.730570388 |
| LACTBL1 | pink | 0.003975807 | 0.931714438 | 0.008109114 | 0.861258256 |
| LALBA | pink | -0.041203923 | 0.374317376 | -0.031312251 | 0.499665873 |
| LCE3A | pink | -0.049080877 | 0.289853884 | -0.055808783 | 0.228688471 |
| LCE3C | pink | 0.040118514 | 0.387042496 | 0.034962047 | 0.451004226 |
| LCT | pink | -0.02094065 | 0.651724343 | -0.025314547 | 0.585290489 |
| LELP1 | pink | -0.017903387 | 0.699578485 | -0.00403514 | 0.93069792 |
| LEP | pink | -0.068708415 | 0.138187448 | -0.070446024 | 0.128470592 |
| LGALS13 | pink | 0.031966342 | 0.49074568 | 0.039370498 | 0.395963269 |

| | | | | | |
|---|---|---|---|---|---|
| LHFPL5 | pink | 0.031363592 | 0.498962622 | 0.03150809 | 0.496986137 |
| LMOD2 | pink | 0.008648527 | 0.852131979 | 0.015994957 | 0.730282579 |
| LMOD3 | pink | 0.109224424 | 0.018220719 | 0.120832682 | 0.008954097 |
| LOXHD1 | pink | -0.174794484 | 0.000146763 | -0.176065175 | 0.000131027 |
| LRRC10 | pink | 0.040428154 | 0.383385801 | 0.052220474 | 0.260064696 |
| LRRC30 | pink | -0.032363111 | 0.485376567 | -0.026819163 | 0.563184592 |
| LRRC3C | pink | -0.076469317 | 0.098839764 | -0.071338547 | 0.123689736 |
| LY6G6E | pink | -0.045407514 | 0.327510793 | -0.06584866 | 0.155395179 |
| LY6G6F | pink | -0.012228674 | 0.792114735 | -0.01864633 | 0.687753466 |
| LYPD4 | pink | -0.007246081 | 0.875897816 | 0.01449847 | 0.75466715 |
| LYZL4 | pink | 0.07148292 | 0.122929568 | 0.117526922 | 0.011028764 |
| LYZL6 | pink | -0.076433356 | 0.098998949 | -0.057818038 | 0.212341274 |
| MAGEB1 | pink | -0.0694755 | 0.133830429 | -0.045640142 | 0.325036117 |
| MAGEB3 | pink | -0.046751051 | 0.31338605 | -0.044587721 | 0.336328601 |
| MCHR2 | pink | -0.086537416 | 0.06168068 | -0.088076931 | 0.057176996 |
| MDS2 | pink | -0.099615856 | 0.031373447 | -0.075388862 | 0.103712217 |
| MEP1A | pink | 0.046674557 | 0.314179346 | 0.013473823 | 0.771506694 |
| METTL1 | pink | -0.019720371 | 0.670792892 | -0.03646283 | 0.431798285 |
| MMD2 | pink | -0.051881099 | 0.263178954 | -0.05339484 | 0.249484844 |
| MOG | pink | -0.040822409 | 0.378760473 | -0.035641155 | 0.442254392 |
| MOGAT3 | pink | -0.056772468 | 0.220739803 | -0.052397219 | 0.25845292 |
| MPL | pink | -0.187631531 | 4.50E-05 | -0.20171282 | 1.12E-05 |
| MPP4 | pink | -0.076617619 | 0.09818543 | -0.025589217 | 0.581225176 |
| MRGPRD | pink | -0.074232117 | 0.109137592 | -0.052182836 | 0.260408821 |
| MRGPRX | pink | -0.045197415 | 0.329756268 | -0.027824173 | 0.548646084 |
| MRGPRX | pink | -0.062652804 | 0.176491077 | -0.067413718 | 0.14578733 |
| MRGPRX | pink | -0.01373019 | 0.767283009 | -0.000573402 | 0.9901399 |
| MROH7 | pink | -0.22145942 | 1.34E-06 | -0.240296008 | 1.47E-07 |
| MS4A10 | pink | -0.0629036 | 0.174762308 | -0.045337674 | 0.328256118 |
| MS4A18 | pink | -0.095762372 | 0.038578704 | -0.080897851 | 0.080742253 |
| MUC17 | pink | -0.039062334 | 0.399674126 | -0.029621979 | 0.523108577 |
| MYL7 | pink | -0.160330443 | 0.000505151 | -0.141521417 | 0.002172697 |
| MYO16 | pink | -0.155690092 | 0.000735256 | -0.14795733 | 0.001343691 |
| MYPN | pink | 0.006458231 | 0.889299234 | 0.024246295 | 0.601225146 |
| NANOG | pink | -0.05531576 | 0.232832661 | -0.050183305 | 0.279143207 |
| NANOGN | pink | -0.04850955 | 0.295511735 | -0.023214076 | 0.616805116 |
| NANOS2 | pink | -0.071536376 | 0.122649032 | -0.065247558 | 0.159210064 |
| NKX2-6 | pink | -0.027236893 | 0.557119244 | -0.014695331 | 0.751444822 |
| NLRP10 | pink | -0.010935499 | 0.813671787 | -0.002953526 | 0.949244673 |
| NLRP12 | pink | -0.029008871 | 0.531748855 | -0.016627362 | 0.720057354 |
| NLRP13 | pink | -0.028728853 | 0.53571889 | 0.003424083 | 0.94117193 |
| NLRP5 | pink | 0.01567518 | 0.735471368 | 0.015865975 | 0.732374011 |
| NLRP8 | pink | -0.021983602 | 0.635604201 | -0.024022242 | 0.604591864 |
| NLRP9 | pink | -0.000342699 | 0.994106918 | -0.017001961 | 0.714023859 |
| NMRK2 | pink | -0.140734345 | 0.002301177 | -0.13581598 | 0.00327387 |

| | | | | | |
|---|---|---|---|---|---|
| NOBOX | pink | -0.073513159 | 0.112620817 | -0.086614752 | 0.06144766 |
| NPHS1 | pink | -0.136538395 | 0.003110817 | -0.130389274 | 0.004768913 |
| NPY4R | pink | -0.07770188 | 0.093505681 | -0.081223574 | 0.079524735 |
| NR1I2 | pink | -0.313617194 | 4.07E-12 | -0.315657183 | 2.90E-12 |
| NR1I3 | pink | -0.114994342 | 0.012895474 | -0.123911591 | 0.007342118 |
| NT5DC4 | pink | -0.017666134 | 0.703370226 | -0.038341361 | 0.408437003 |
| NUTM1 | pink | -0.107153702 | 0.020554055 | -0.108488527 | 0.019022016 |
| OCM | pink | -0.039288718 | 0.396946025 | -0.035577465 | 0.443070866 |
| OCM2 | pink | -0.050941255 | 0.271936911 | -0.04828668 | 0.297738663 |
| ODF1 | pink | -0.035882843 | 0.439163937 | -0.047649769 | 0.304164209 |
| ODF4 | pink | -0.004200906 | 0.927858546 | 0.009800376 | 0.832711388 |
| OLAH | pink | -0.168133094 | 0.000262585 | -0.16874916 | 0.000249054 |
| OPN1LW | pink | -0.134023494 | 0.003712487 | -0.12849332 | 0.005421533 |
| OPN1MW | pink | -0.00638818 | 0.890492402 | -0.014321349 | 0.757569997 |
| OPRD1 | pink | -0.102425157 | 0.026875709 | -0.096507173 | 0.037085923 |
| OR10A2 | pink | -0.049787061 | 0.282961621 | -0.045422289 | 0.327353254 |
| OR10A4 | pink | -0.076107834 | 0.100449225 | -0.069698826 | 0.132582061 |
| OR10G2 | pink | -0.065395023 | 0.158267706 | -0.050197009 | 0.279011779 |
| OR10G3 | pink | -0.024005158 | 0.604848928 | -0.007325192 | 0.874554014 |
| OR10H2 | pink | -0.064261527 | 0.165620229 | -0.052322888 | 0.259129917 |
| OR10H3 | pink | -0.020606962 | 0.656916827 | -0.019991856 | 0.666531694 |
| OR10H5 | pink | -0.103649088 | 0.025096837 | -0.090656934 | 0.050242241 |
| OR10J3 | pink | -0.01722635 | 0.710418209 | -0.013622622 | 0.769054389 |
| OR10J5 | pink | 0.012894221 | 0.781080478 | 0.007628233 | 0.86940988 |
| OR10K1 | pink | -0.021803435 | 0.638376913 | -0.020627337 | 0.656599303 |
| OR10K2 | pink | -0.005585961 | 0.904173368 | 0.003695627 | 0.936516095 |
| OR10P1 | pink | -0.04497595 | 0.332133943 | -0.039291065 | 0.396917804 |
| OR10S1 | pink | -0.017198075 | 0.71087221 | -0.016038788 | 0.729572313 |
| OR10V1 | pink | -0.056048902 | 0.226689177 | -0.04755822 | 0.305095302 |
| OR10W1 | pink | -0.083946997 | 0.069916926 | -0.071790866 | 0.121320296 |
| OR11A1 | pink | -0.044655434 | 0.335594547 | -0.035998773 | 0.437685928 |
| OR11G2 | pink | -0.023922401 | 0.60609482 | -0.027695668 | 0.550494697 |
| OR11L1 | pink | -0.027088314 | 0.559272952 | -0.021126887 | 0.648833608 |
| OR12D3 | pink | 0.007597661 | 0.869928591 | 0.002761485 | 0.952540826 |
| OR13C8 | pink | -0.002528813 | 0.956535426 | 0.009224984 | 0.84240076 |
| OR13F1 | pink | -0.048453691 | 0.296068828 | -0.054272943 | 0.2417725 |
| OR13J1 | pink | 0.018805722 | 0.685226278 | 0.004817648 | 0.917302793 |
| OR14I1 | pink | -0.035537156 | 0.443588037 | -0.028213008 | 0.543071159 |
| OR14J1 | pink | -0.024258654 | 0.601039685 | -0.020296852 | 0.661757285 |
| OR1A1 | pink | -0.040386147 | 0.383880634 | -0.024565653 | 0.596440981 |
| OR1A2 | pink | -0.015354268 | 0.740690697 | -0.020147239 | 0.66409763 |
| OR1E2 | pink | -0.016243447 | 0.726258975 | -0.016488859 | 0.722292595 |
| OR1G1 | pink | -0.001475047 | 0.974639015 | -0.006163996 | 0.894312514 |
| OR1I1 | pink | -0.035130215 | 0.448828446 | -0.022589037 | 0.626324159 |
| OR1K1 | pink | -0.013331552 | 0.773853559 | -0.000640447 | 0.988987079 |

| | | | | | |
|---|---|---|---|---|---|
| OR1M1 | pink | -0.03581901 | 0.43997896 | -0.032711037 | 0.480694633 |
| OR2AT4 | pink | -0.069978308 | 0.131032478 | -0.053416867 | 0.249289312 |
| OR2B11 | pink | -0.040859872 | 0.378322757 | -0.039381834 | 0.395827166 |
| OR2F2 | pink | -0.044726651 | 0.334823626 | -0.043859944 | 0.344283029 |
| OR2G2 | pink | 0.002603803 | 0.955247848 | 0.005665871 | 0.902809256 |
| OR2G3 | pink | 0.048143002 | 0.29918022 | 0.044609524 | 0.336092122 |
| OR2G6 | pink | -0.08600921 | 0.063291777 | -0.082088345 | 0.076364364 |
| OR2H2 | pink | -0.173638494 | 0.000162603 | -0.176686232 | 0.000123926 |
| OR2L2 | pink | -0.15830075 | 0.000596033 | -0.160724376 | 0.000489078 |
| OR2M4 | pink | -0.041606053 | 0.369669129 | -0.035615259 | 0.442586257 |
| OR2M7 | pink | -0.014266817 | 0.758464415 | -0.013955143 | 0.763582542 |
| OR2T2 | pink | -0.038611301 | 0.405142819 | -0.038042868 | 0.412098092 |
| OR2T27 | pink | -0.050595453 | 0.27520876 | -0.045687913 | 0.324529436 |
| OR2T3 | pink | -0.010120776 | 0.827326842 | -0.008253181 | 0.858818947 |
| OR2T34 | pink | 0.02131147 | 0.645973739 | 0.018828326 | 0.684868166 |
| OR2T35 | pink | -0.039130509 | 0.398851387 | -0.039935161 | 0.389217761 |
| OR2T6 | pink | -0.022884847 | 0.62181125 | -0.01996501 | 0.666952592 |
| OR2T7 | pink | 0.013626799 | 0.768985574 | 0.01548535 | 0.738557332 |
| OR2Y1 | pink | -0.054587851 | 0.23904785 | -0.045542921 | 0.326068869 |
| OR2Z1 | pink | -0.020885931 | 0.652574676 | -0.020023908 | 0.666029311 |
| OR3A1 | pink | -0.033665956 | 0.467971518 | -0.03285205 | 0.47880407 |
| OR4B1 | pink | 0.023685678 | 0.609664994 | 0.012145438 | 0.793497702 |
| OR4C11 | pink | -0.043082781 | 0.352908241 | -0.023630205 | 0.610502957 |
| OR4C12 | pink | -0.028803389 | 0.534660682 | -0.013946567 | 0.76372352 |
| OR4C15 | pink | -0.053164358 | 0.251537245 | -0.04697453 | 0.311075955 |
| OR4C5 | pink | -0.008991599 | 0.846337763 | 0.004138725 | 0.928923534 |
| OR4D1 | pink | -0.033327534 | 0.472459179 | -0.01664873 | 0.719712718 |
| OR4D10 | pink | -0.030265844 | 0.514112813 | -0.028197449 | 0.543293691 |
| OR4D11 | pink | -0.043923551 | 0.343583088 | -0.049108248 | 0.28958466 |
| OR4D5 | pink | 0.008697048 | 0.85131201 | 0.023126568 | 0.618134015 |
| OR4D9 | pink | -0.025381403 | 0.584299765 | -0.026658232 | 0.565529718 |
| OR4K13 | pink | -0.057845153 | 0.212126577 | -0.054296403 | 0.241568769 |
| OR4K14 | pink | 0.022302889 | 0.630702971 | 0.015140917 | 0.74416727 |
| OR4K2 | pink | -0.010549912 | 0.820127593 | -0.003762312 | 0.935373049 |
| OR4M1 | pink | -0.000264768 | 0.99544701 | -0.003845166 | 0.933953028 |
| OR4N2 | pink | -0.040504563 | 0.382486698 | -0.037484627 | 0.418996872 |
| OR4Q3 | pink | 0.023249887 | 0.61626165 | 0.028388163 | 0.540569091 |
| OR51A7 | pink | 0.043490924 | 0.348361679 | 0.066001128 | 0.154438631 |
| OR51D1 | pink | -0.071661427 | 0.121994708 | -0.062978711 | 0.17424702 |
| OR51F2 | pink | -0.033655446 | 0.468110539 | -0.034230681 | 0.460535653 |
| OR51L1 | pink | 0.047345842 | 0.307262527 | 0.04760049 | 0.304665165 |
| OR51S1 | pink | -0.03272223 | 0.480544422 | -0.039332171 | 0.396423668 |
| OR51V1 | pink | 0.001238069 | 0.978712407 | -0.00260999 | 0.955141617 |
| OR52B2 | pink | -0.019846276 | 0.668815379 | -0.007850422 | 0.865641684 |
| OR52I2 | pink | -0.004400476 | 0.924441357 | -0.003651293 | 0.937276096 |

| | | | | | |
|---|---|---|---|---|---|
| OR52K1 | pink | -0.084360225 | 0.068545754 | -0.070462353 | 0.128381859 |
| OR52M1 | pink | 0.028165263 | 0.543754176 | 0.026465728 | 0.568341063 |
| OR52W1 | pink | -0.061587134 | 0.183978685 | -0.062036183 | 0.180795481 |
| OR56B4 | pink | -0.090702566 | 0.05012621 | -0.082528779 | 0.074794444 |
| OR5A1 | pink | -0.030052894 | 0.517079143 | -0.028603538 | 0.537500373 |
| OR5AP2 | pink | -0.044378 | 0.338608611 | -0.041910312 | 0.366176047 |
| OR5B12 | pink | -0.026836526 | 0.562931864 | -0.025384215 | 0.584258106 |
| OR5C1 | pink | 0.005285527 | 0.909304463 | 0.01455126 | 0.753802628 |
| OR5J2 | pink | -0.027140453 | 0.558516712 | -0.021879931 | 0.637199045 |
| OR5M9 | pink | -0.016586574 | 0.720715371 | -0.01473717 | 0.750760541 |
| OR6B2 | pink | -0.055032183 | 0.235240232 | -0.050834998 | 0.272939439 |
| OR6C6 | pink | -0.061532089 | 0.184371712 | -0.081187084 | 0.079660383 |
| OR6C75 | pink | -0.000898286 | 0.984553843 | -0.002859003 | 0.95086695 |
| OR6K2 | pink | -0.072477218 | 0.117792675 | -0.074589213 | 0.107439376 |
| OR6K6 | pink | 0.053483314 | 0.248700104 | 0.032322552 | 0.485923968 |
| OR6M1 | pink | -0.013781635 | 0.766436281 | -0.019578435 | 0.673024921 |
| OR6N2 | pink | -0.00411763 | 0.929284861 | -0.002259971 | 0.961152351 |
| OR6S1 | pink | -0.013324573 | 0.77396874 | -0.011529885 | 0.80374492 |
| OR6T1 | pink | 0.019581229 | 0.672980949 | 0.01761568 | 0.704177528 |
| OR6X1 | pink | -0.105996059 | 0.021968471 | -0.0969499 | 0.036222029 |
| OR6Y1 | pink | -0.037479092 | 0.419065608 | -0.037097057 | 0.423826077 |
| OR7A10 | pink | -0.070403176 | 0.128703663 | -0.061188756 | 0.186837156 |
| OR7C2 | pink | -0.076877404 | 0.097047531 | -0.065948296 | 0.154769581 |
| OR7D4 | pink | -0.075145452 | 0.104835745 | -0.068572823 | 0.138968817 |
| OR7G1 | pink | -0.051260963 | 0.268935655 | -0.049987382 | 0.281026837 |
| OR7G2 | pink | -0.066258538 | 0.15283383 | -0.057038076 | 0.218584147 |
| OR7G3 | pink | -0.073493739 | 0.112716101 | -0.068633761 | 0.138617236 |
| OR8K1 | pink | 0.015718402 | 0.734769323 | 0.024533368 | 0.596923853 |
| OR8K5 | pink | -0.041825163 | 0.367151537 | -0.034697013 | 0.454445332 |
| OR8S1 | pink | -0.067399132 | 0.14587473 | -0.072263265 | 0.118883608 |
| OR9A4 | pink | -0.085944799 | 0.063490591 | -0.080638843 | 0.081721134 |
| OR9Q1 | pink | -0.050874939 | 0.272562299 | -0.039097951 | 0.399244174 |
| OTUD6A | pink | -0.02675992 | 0.56404735 | -0.005592889 | 0.904055088 |
| P2RX3 | pink | -0.067667929 | 0.144270482 | -0.077958275 | 0.092425555 |
| P2RX6 | pink | -0.126346449 | 0.006256685 | -0.116887299 | 0.01147611 |
| PADI4 | pink | -0.043192837 | 0.351678589 | -0.023555477 | 0.611632594 |
| PADI6 | pink | -0.011769427 | 0.799753109 | -0.002244071 | 0.961425447 |
| PANX3 | pink | -0.072075583 | 0.119847075 | -0.064095065 | 0.166721225 |
| PDZD3 | pink | -0.167433696 | 0.000278777 | -0.156633717 | 0.000681785 |
| PEX5L | pink | -0.041668114 | 0.368954954 | -0.044384458 | 0.338538264 |
| PGAM4 | pink | 0.158808035 | 0.000571996 | 0.134184042 | 0.003671134 |
| PGLYRP1 | pink | -0.046068405 | 0.320512079 | -0.032023696 | 0.48996759 |
| PGLYRP2 | pink | -0.104694973 | 0.023658174 | -0.08727055 | 0.059500744 |
| PIWIL3 | pink | -0.05552714 | 0.231049407 | -0.04910837 | 0.289583461 |
| PKLR | pink | -0.025117438 | 0.588215924 | -0.019211864 | 0.678802728 |

| | | | | | |
|---|---|---|---|---|---|
| PLA2G2F | pink | 0.00493981 | 0.915213613 | -0.004695174 | 0.919397889 |
| PNLIPRP | pink | -0.051543479 | 0.266302471 | -0.047584031 | 0.304832598 |
| PP2672 | pink | -0.080999986 | 0.080358869 | -0.065814972 | 0.155607132 |
| PPEF2 | pink | 0.021652092 | 0.640709942 | 0.02773283 | 0.549959789 |
| PPIAL4G | pink | -0.01391828 | 0.764188577 | -0.020207351 | 0.663156923 |
| PPP3R2 | pink | -0.059793475 | 0.197106454 | -0.044730133 | 0.334785964 |
| PRAMEF | pink | -0.016322682 | 0.72497756 | -0.014358775 | 0.756956321 |
| PRAMEF | pink | 0.004162225 | 0.928521031 | 0.006180827 | 0.894025639 |
| PRAMEF | pink | -0.063515923 | 0.170594478 | -0.059873894 | 0.196503611 |
| PRAMEF | pink | -0.054850294 | 0.236793687 | -0.052269617 | 0.259615857 |
| PRDM14 | pink | -0.014407048 | 0.756165034 | -0.015516866 | 0.738044694 |
| PRKACG | pink | -0.023426318 | 0.613587214 | -0.013961219 | 0.763482659 |
| PRKAG3 | pink | -0.043956939 | 0.343216036 | -0.041171902 | 0.374689059 |
| PRM1 | pink | -0.017882943 | 0.699904921 | -0.000116042 | 0.998004512 |
| PRNT | pink | -0.023744614 | 0.608775274 | -0.02061372 | 0.656811502 |
| PRODH2 | pink | -0.057886913 | 0.211796226 | -0.049373892 | 0.28698054 |
| PROP1 | pink | -0.043640193 | 0.346708186 | -0.02295571 | 0.620732253 |
| PRR21 | pink | -0.034750713 | 0.453746924 | -0.017215318 | 0.710595337 |
| PRR23A | pink | 0.013316376 | 0.774104024 | 0.021514875 | 0.642828275 |
| PRR23B | pink | -0.067228898 | 0.146897751 | -0.054052498 | 0.243692752 |
| PRR23C | pink | -0.065436296 | 0.15800471 | -0.051405887 | 0.267582675 |
| PRR30 | pink | -0.016915562 | 0.715413883 | -0.000214142 | 0.996317588 |
| PRSS38 | pink | -0.071720186 | 0.1216882 | -0.071744515 | 0.121561465 |
| PRSS54 | pink | -0.044065637 | 0.342022809 | -0.041290393 | 0.373314835 |
| PRSS55 | pink | 0.032003602 | 0.490240123 | 0.030120861 | 0.516131413 |
| PSG8 | pink | -0.036273523 | 0.434194537 | -0.035211706 | 0.447776255 |
| PSMB11 | pink | -0.029701562 | 0.521992338 | -0.030057275 | 0.517018024 |
| PTCHD3 | pink | -0.095737441 | 0.03862954 | -0.081820217 | 0.077333149 |
| PTX4 | pink | 0.063016911 | 0.173985389 | 0.054543325 | 0.239431783 |
| QRFPR | pink | -0.071165401 | 0.124606219 | -0.070369016 | 0.128889712 |
| RBPJL | pink | -0.196630163 | 1.87E-05 | -0.187258164 | 4.66E-05 |
| RD3L | pink | -0.106581227 | 0.021243296 | -0.108824227 | 0.01865275 |
| RESP18 | pink | -0.030461174 | 0.511399702 | -0.020335101 | 0.66115949 |
| RETNLB | pink | -0.082961678 | 0.073277091 | -0.080435335 | 0.082496974 |
| RFPL4A | pink | -0.068678347 | 0.138360426 | -0.0836629 | 0.070872571 |
| RFPL4AL | pink | -0.074547127 | 0.107638431 | -0.084959788 | 0.066595533 |
| RHAG | pink | -0.051476776 | 0.266922567 | -0.055581462 | 0.230592705 |
| RNF222 | pink | -0.015146995 | 0.744068155 | 0.007123555 | 0.877979761 |
| RP1-27O | pink | -0.110408874 | 0.01699282 | -0.104775024 | 0.023551058 |
| RP11-407 | pink | -0.061073162 | 0.187672661 | -0.043395911 | 0.349416764 |
| RP11-446 | pink | -0.058075948 | 0.210305463 | -0.063934346 | 0.167789436 |
| RP11-477 | pink | -0.012257231 | 0.79164041 | -0.009132692 | 0.843957178 |
| RP11-761 | pink | 0.049636848 | 0.284418333 | 0.067675869 | 0.144223295 |
| RP13-279 | pink | -0.028424161 | 0.540055566 | -0.025450972 | 0.583269651 |
| RP1L1 | pink | 0.01502236 | 0.746101411 | 0.001108112 | 0.980946462 |

| | | | | | |
|---|---|---|---|---|---|
| RP4-533D | pink | 0.001083315 | 0.981372759 | 0.019360684 | 0.676454763 |
| RPH3A | pink | -0.097700161 | 0.034797079 | -0.094424376 | 0.041388341 |
| RPL3L | pink | -0.049393451 | 0.286789425 | -0.048050007 | 0.300115733 |
| RSPH6A | pink | -0.001479197 | 0.974567686 | 0.022194824 | 0.632360026 |
| RTP2 | pink | -0.103265552 | 0.025643012 | -0.095170189 | 0.039801635 |
| S100A7L | pink | 0.012760466 | 0.783294583 | 0.017757631 | 0.701907051 |
| SAG | pink | -0.101269635 | 0.028653685 | -0.092934201 | 0.044718932 |
| SATL1 | pink | -0.073128805 | 0.114518455 | -0.079648006 | 0.085554753 |
| SBK3 | pink | -0.083687551 | 0.07078923 | -0.083468787 | 0.071531645 |
| SCN10A | pink | -0.021465871 | 0.643585491 | -0.016298349 | 0.725371003 |
| SEPT12 | pink | 0.092301226 | 0.04620036 | 0.098516604 | 0.033300926 |
| SERPINE | pink | -0.106450523 | 0.021403444 | -0.102228014 | 0.027172156 |
| SHISA7 | pink | -0.094832363 | 0.040513864 | -0.090431864 | 0.050817819 |
| SLC13A4 | pink | -0.116834265 | 0.011513912 | -0.11384997 | 0.013826715 |
| SLC17A2 | pink | -0.060090961 | 0.194883118 | -0.054211339 | 0.242308051 |
| SLC22A1 | pink | -0.067199693 | 0.147073806 | -0.061597612 | 0.183903941 |
| SLC22A1 | pink | -0.099419745 | 0.031710155 | -0.10248262 | 0.026789825 |
| SLC22A1 | pink | -0.114646752 | 0.0131723 | -0.113152462 | 0.014422912 |
| SLC22A1 | pink | -0.018586738 | 0.688699201 | -0.021200515 | 0.647692214 |
| SLC22A6 | pink | -0.012552185 | 0.786745839 | -0.003186143 | 0.945253264 |
| SLC22A9 | pink | -0.072325551 | 0.118565207 | -0.074446452 | 0.108115776 |
| SLC24A4 | pink | -0.048600985 | 0.294601327 | -0.056456185 | 0.223326502 |
| SLC26A8 | pink | -0.016170678 | 0.7274365 | -0.017601119 | 0.704410578 |
| SLC28A1 | pink | -0.037676162 | 0.41662227 | -0.022682535 | 0.624896226 |
| SLC2A7 | pink | -0.053687705 | 0.246893799 | -0.050842075 | 0.272872586 |
| SLC35G3 | pink | -0.023414724 | 0.613762815 | 0.009539635 | 0.837099117 |
| SLC36A2 | pink | -0.109269402 | 0.018172714 | -0.110982619 | 0.016424499 |
| SLC36A3 | pink | -0.075216478 | 0.104506914 | -0.068366086 | 0.140166683 |
| SLC4A1 | pink | -0.028039626 | 0.545553538 | -0.019477225 | 0.674618269 |
| SLC4A9 | pink | -0.063397784 | 0.171392774 | -0.05921098 | 0.201513375 |
| SLC5A11 | pink | -0.088617674 | 0.055661277 | -0.083018709 | 0.073079076 |
| SLFN14 | pink | -0.087181918 | 0.059760854 | -0.070690066 | 0.127149367 |
| SMCO1 | pink | -0.083000938 | 0.073140731 | -0.074524268 | 0.107746668 |
| SMCP | pink | -0.054681511 | 0.238241664 | -0.06779723 | 0.143503593 |
| SMIM21 | pink | -0.027451593 | 0.554014244 | -0.019900988 | 0.667956764 |
| SOAT2 | pink | -0.199322687 | 1.43E-05 | -0.185655066 | 5.43E-05 |
| SPACA3 | pink | -0.090651434 | 0.050256241 | -0.079076465 | 0.087831162 |
| SPATA21 | pink | 0.03953205 | 0.394026204 | 0.05852769 | 0.206773632 |
| SPATA3 | pink | -0.012582774 | 0.786238705 | -0.010815549 | 0.815678755 |
| SPIC | pink | -0.044283936 | 0.339634455 | -0.034560791 | 0.456219698 |
| SPPL2C | pink | -0.018209545 | 0.69469654 | -0.031070309 | 0.502986972 |
| SPTA1 | pink | -0.068234955 | 0.140930571 | -0.069037243 | 0.136306557 |
| SSMEM1 | pink | -0.071170013 | 0.124581738 | -0.076538999 | 0.098531882 |
| SSX1 | pink | -0.117742001 | 0.010881848 | -0.11046733 | 0.01693414 |
| SUN5 | pink | -0.042908908 | 0.35485644 | -0.040433007 | 0.383328657 |

| | | | | | |
|---|---|---|---|---|---|
| SYCN | pink | -0.094993773 | 0.040172235 | -0.085245768 | 0.065681489 |
| SYNPO2I | pink | -0.106142985 | 0.021784402 | -0.086282139 | 0.062455033 |
| TAAR5 | pink | -0.020955105 | 0.651499791 | -0.009161148 | 0.843477234 |
| TAF1L | pink | -0.019514101 | 0.67403756 | -0.013322627 | 0.774000853 |
| TARM1 | pink | -0.026199392 | 0.572241589 | -0.012944391 | 0.780250436 |
| TAS1R2 | pink | -0.059562142 | 0.198848136 | -0.058229143 | 0.209102903 |
| TAS2R38 | pink | -0.219726601 | 1.63E-06 | -0.217875825 | 2.00E-06 |
| TBC1D21 | pink | -0.06476533 | 0.162321243 | -0.051217424 | 0.269343032 |
| TCP10 | pink | -0.031295266 | 0.499898644 | -0.02322835 | 0.616588473 |
| TEX13A | pink | -0.012687323 | 0.784506096 | -0.013722828 | 0.7674042 |
| TEX37 | pink | -0.005508336 | 0.905498749 | 0.002544088 | 0.956273141 |
| TFDP3 | pink | -0.012840649 | 0.781967074 | -0.012321612 | 0.790571338 |
| TGM4 | pink | 0.032818614 | 0.479251973 | 0.026285789 | 0.570974912 |
| THEGL | pink | -0.140242654 | 0.002384922 | -0.133379261 | 0.003882702 |
| TISP43 | pink | -0.027295229 | 0.556274757 | -0.011486096 | 0.804475179 |
| TKTL2 | pink | -0.144233621 | 0.001778555 | -0.137605008 | 0.002883543 |
| TMC2 | pink | -0.173782038 | 0.000160553 | -0.170562969 | 0.000212906 |
| TMCO2 | pink | -0.034378308 | 0.458602724 | -0.032963951 | 0.477306696 |
| TMEM20 | pink | -0.019642 | 0.672024958 | -0.000725518 | 0.987524343 |
| TMEM22 | pink | -0.046866462 | 0.312191648 | -0.040640664 | 0.380888398 |
| TMEM23 | pink | -0.065017361 | 0.160689567 | -0.069466374 | 0.133881634 |
| TMEM95 | pink | 0.00426594 | 0.926744835 | 0.013744853 | 0.76704165 |
| TMPRSS | pink | -0.125548237 | 0.00659551 | -0.134438238 | 0.003606513 |
| TPRX1 | pink | -0.072834618 | 0.115987793 | -0.070313727 | 0.129191273 |
| TRIM40 | pink | 0.011122782 | 0.810540658 | 0.011717596 | 0.800616408 |
| TRIM42 | pink | -0.052595431 | 0.256653601 | -0.032515484 | 0.483323115 |
| TRIM60 | pink | -0.023341122 | 0.614878023 | -0.020744729 | 0.65477103 |
| TRIM67 | pink | -0.042752242 | 0.356617619 | -0.036533639 | 0.430903943 |
| TRIML1 | pink | 0.004786758 | 0.917831165 | 0.011042727 | 0.811878709 |
| TRIML2 | pink | -0.057828436 | 0.212258922 | -0.053436943 | 0.249111183 |
| TRPV5 | pink | -0.100204794 | 0.030380602 | -0.095725064 | 0.038654799 |
| TSGA10I | pink | -0.001752877 | 0.969864278 | 0.034663192 | 0.454885504 |
| TSGA13 | pink | -0.007535455 | 0.870984216 | 0.004919559 | 0.915559904 |
| TSPAN16 | pink | -0.035882638 | 0.439166553 | -0.031374321 | 0.498815727 |
| TUBAL3 | pink | 0.160116193 | 0.000514098 | 0.141965037 | 0.002103204 |
| TULP2 | pink | -0.174498937 | 0.000150669 | -0.173033015 | 0.000171527 |
| TUSC5 | pink | -0.087679303 | 0.058313261 | -0.079676249 | 0.085443506 |
| TXNDC2 | pink | -0.079792968 | 0.084984999 | -0.083373717 | 0.071856259 |
| UBL4B | pink | -0.132354132 | 0.004168165 | -0.140436682 | 0.002351548 |
| UGT1A3 | pink | -0.031481642 | 0.497347592 | -0.038162273 | 0.410631239 |
| UGT1A4 | pink | -0.057013885 | 0.218779855 | -0.06392336 | 0.167862638 |
| UGT1A5 | pink | -0.083766958 | 0.070521309 | -0.087210462 | 0.059676983 |
| UPB1 | pink | -0.08029799 | 0.083023934 | -0.060353881 | 0.192933436 |
| VHLL | pink | -0.062496226 | 0.17757681 | -0.051683947 | 0.264999845 |
| VRTN | pink | -0.071000097 | 0.12548611 | -0.072933859 | 0.115490485 |

| | | | | | |
|---|---|---|---|---|---|
| WFDC10. | pink | 0.013238312 | 0.775392736 | 0.00055521 | 0.990452716 |
| WFDC12 | pink | -0.062489109 | 0.177626275 | -0.068768761 | 0.137840784 |
| WFDC5 | pink | -0.054111844 | 0.243174752 | -0.061361773 | 0.18559172 |
| WFDC9 | pink | -0.09273979 | 0.045169626 | -0.092185849 | 0.04647478 |
| WNT8A | pink | -0.068222065 | 0.141005833 | -0.065303181 | 0.158854116 |
| XKR3 | pink | -0.053488283 | 0.248656079 | -0.044589361 | 0.336310802 |
| XXbac-B: | pink | -0.034247148 | 0.460319825 | -0.033984998 | 0.463762495 |
| XXbac-BI | pink | -0.035845275 | 0.439643503 | -0.039035465 | 0.399998666 |
| ZAN | pink | -0.044388292 | 0.338496492 | -0.035197298 | 0.447962188 |
| ZCCHC1: | pink | -0.032267479 | 0.486667769 | -0.020926466 | 0.651944712 |
| ZG16 | pink | -0.074017411 | 0.110168794 | -0.054471481 | 0.240052181 |
| ZIM2 | pink | -0.167558212 | 0.000275827 | -0.168083151 | 0.000263711 |
| ZNF645 | pink | -0.039467971 | 0.394793853 | -0.019733732 | 0.670582931 |
| ZNF728 | pink | -0.199865904 | 1.35E-05 | -0.199606087 | 1.39E-05 |
| ZNF729 | pink | -0.165177263 | 0.000337599 | -0.155342812 | 0.000755894 |
| ZNRF4 | pink | 0.020256541 | 0.662387523 | 0.035386822 | 0.445519914 |
| ZP1 | pink | -0.165970459 | 0.000315715 | -0.167525822 | 0.000276592 |
| ZPBP2 | pink | -0.064678229 | 0.162888031 | -0.060615364 | 0.19100861 |
| ZSCAN5( | pink | -0.019959477 | 0.667039346 | -0.003993285 | 0.931414986 |
| ANKRD4 | plum1 | -0.194262136 | 2.37E-05 | -0.232267999 | 3.87E-07 |
| ARFGEF1 | plum1 | 0.004744842 | 0.918548181 | -0.032482071 | 0.483772998 |
| ARMC1 | plum1 | -0.128386833 | 0.005460466 | -0.170874786 | 0.00020721 |
| ATP6V1C | plum1 | -0.156703059 | 0.000678001 | -0.187312182 | 4.64E-05 |
| ATP6V1F | plum1 | 0.030640605 | 0.508914005 | 0.012582821 | 0.786237935 |
| AZIN1 | plum1 | 0.098698733 | 0.032974759 | 0.070599483 | 0.127638544 |
| C8orf76 | plum1 | -0.062417308 | 0.178125913 | -0.07520225 | 0.104572719 |
| CHD7 | plum1 | -0.123832897 | 0.00737985 | -0.147068491 | 0.001437539 |
| COPS5 | plum1 | 0.017182687 | 0.711119329 | -0.012775118 | 0.783051952 |
| CPNE3 | plum1 | -0.022428435 | 0.628780175 | -0.059861811 | 0.196594104 |
| DCAF13 | plum1 | -0.100488735 | 0.02991162 | -0.140138986 | 0.002402929 |
| DERL1 | plum1 | -0.240452922 | 1.44E-07 | -0.246075195 | 7.18E-08 |
| DPY19L4 | plum1 | -0.149014584 | 0.00123941 | -0.191995226 | 2.96E-05 |
| E2F5 | plum1 | -0.084119674 | 0.069341245 | -0.133745404 | 0.003785115 |
| EBAG9 | plum1 | -0.067154021 | 0.147349449 | -0.090104536 | 0.051664724 |
| EIF3H | plum1 | -0.048130652 | 0.299304346 | -0.089225804 | 0.053996686 |
| EMC2 | plum1 | 0.002800136 | 0.951877361 | -0.032448386 | 0.484226787 |
| ENY2 | plum1 | -0.055375732 | 0.232325738 | -0.078477322 | 0.09026953 |
| EXT1 | plum1 | -0.243223071 | 1.03E-07 | -0.244761059 | 8.47E-08 |
| FAM84B | plum1 | -0.092733217 | 0.045184929 | -0.131422796 | 0.004443822 |
| FAM91A | plum1 | -0.123716648 | 0.007435906 | -0.147776142 | 0.001362352 |
| FNTA | plum1 | -0.12870807 | 0.005343776 | -0.168185625 | 0.000261405 |
| IMPA1 | plum1 | -0.028919116 | 0.533019771 | -0.069513949 | 0.133614857 |
| IMPAD1 | plum1 | -0.062124379 | 0.180175102 | -0.088285948 | 0.056587106 |
| KIAA142 | plum1 | -0.197729272 | 1.68E-05 | -0.228068808 | 6.32E-07 |
| LACTB2 | plum1 | -0.082422086 | 0.075172319 | -0.111268302 | 0.016147775 |

| | | | | | |
|---|---|---|---|---|---|
| LYPLA1 | plum1 | 0.078519228 | 0.090097238 | 0.048677826 | 0.293837691 |
| MED30 | plum1 | -0.224335755 | 9.71E-07 | -0.26355326 | 7.35E-09 |
| MRPL13 | plum1 | 0.030335677 | 0.513141982 | 0.01437428 | 0.75670214 |
| MRPS28 | plum1 | -0.03604329 | 0.437119142 | -0.064298042 | 0.165379441 |
| MTDH | plum1 | 0.055644785 | 0.230061127 | 0.029305708 | 0.527556662 |
| MTERF3 | plum1 | -0.070450899 | 0.128444101 | -0.108455773 | 0.019058384 |
| MTFR1 | plum1 | 0.101417711 | 0.028420346 | 0.072777957 | 0.116272479 |
| NBN | plum1 | -0.01541023 | 0.739779673 | -0.04340995 | 0.349260735 |
| NCOA2 | plum1 | -0.077721452 | 0.093422872 | -0.115798672 | 0.012274481 |
| NDUFAF | plum1 | -0.12246562 | 0.008063691 | -0.144466154 | 0.001748016 |
| NSMCE2 | plum1 | -0.199157364 | 1.45E-05 | -0.234577339 | 2.94E-07 |
| NUDCD1 | plum1 | -0.12616589 | 0.006331931 | -0.139618484 | 0.002495233 |
| OSGIN2 | plum1 | -0.080114485 | 0.083732235 | -0.112571192 | 0.014936899 |
| OTUD6B | plum1 | -0.122809231 | 0.007886714 | -0.159174806 | 0.00055518 |
| PABPC1 | plum1 | 0.054443957 | 0.240290157 | 0.024794142 | 0.593028727 |
| PEX2 | plum1 | 0.085436276 | 0.06507833 | 0.042645701 | 0.357818439 |
| PLEKHF2 | plum1 | -0.015844939 | 0.732715294 | -0.036797181 | 0.427584756 |
| POLR2K | plum1 | -0.109631525 | 0.017790221 | -0.123561014 | 0.007511549 |
| PTK2 | plum1 | -0.03192419 | 0.491317942 | -0.065164838 | 0.15974053 |
| RAB2A | plum1 | -0.087647033 | 0.058406291 | -0.0962173 | 0.037660975 |
| RAD21 | plum1 | -0.100499619 | 0.029893767 | -0.140100025 | 0.002409729 |
| RB1CC1 | plum1 | -0.252994866 | 2.97E-08 | -0.29027909 | 1.61E-10 |
| RMDN1 | plum1 | 0.034399696 | 0.45832306 | 0.006103773 | 0.895339145 |
| RNF139 | plum1 | -0.164695304 | 0.00035158 | -0.192737059 | 2.75E-05 |
| SLC25A3 | plum1 | -0.127215588 | 0.005905588 | -0.165399385 | 0.000331333 |
| SQLE | plum1 | -0.001806885 | 0.968936224 | -0.050716981 | 0.27405587 |
| STAU2 | plum1 | -0.029390669 | 0.526359871 | -0.077536126 | 0.094209313 |
| STK3 | plum1 | -0.150504583 | 0.001105058 | -0.192302791 | 2.87E-05 |
| TAF2 | plum1 | -0.065651007 | 0.15664187 | -0.112933909 | 0.014614314 |
| TATDN1 | plum1 | -0.212861623 | 3.47E-06 | -0.26236204 | 8.63E-09 |
| TCEA1 | plum1 | -0.045524944 | 0.326260065 | -0.087619539 | 0.058485649 |
| TCEB1 | plum1 | -0.076866697 | 0.09709422 | -0.081433389 | 0.078748373 |
| TMEM65 | plum1 | -0.15295873 | 0.000912696 | -0.173185386 | 0.000169239 |
| TMEM68 | plum1 | 0.042960178 | 0.354281265 | 0.002295805 | 0.960536884 |
| TMEM70 | plum1 | -0.022741643 | 0.623994219 | -0.046523273 | 0.315752149 |
| TPD52 | plum1 | 0.09394996 | 0.042425185 | 0.085738348 | 0.064131294 |
| TRMT12 | plum1 | -0.155620701 | 0.000739338 | -0.161689535 | 0.000451685 |
| UBE2V2 | plum1 | -0.017407933 | 0.707505129 | -0.050485742 | 0.27625238 |
| UBE2W | plum1 | -0.137029644 | 0.003004193 | -0.178656886 | 0.000103714 |
| UBR5 | plum1 | -0.114495798 | 0.013294144 | -0.148009124 | 0.0013384 |
| UTP23 | plum1 | -0.190272253 | 3.49E-05 | -0.226627491 | 7.46E-07 |
| YWHAZ | plum1 | -0.074671088 | 0.107052964 | -0.093857151 | 0.042630559 |
| ZBTB10 | plum1 | -0.152207317 | 0.000968027 | -0.194938789 | 2.21E-05 |
| ZFAND1 | plum1 | -0.096123136 | 0.037849398 | -0.153154341 | 0.000898779 |
| ZNF16 | plum1 | -0.14478507 | 0.001706912 | -0.184352981 | 6.13E-05 |

| | | | | | |
|---|---|---|---|---|---|
| ZNF250 | plum1 | -0.19301541 | 2.68E-05 | -0.232797017 | 3.63E-07 |
| ZNF572 | plum1 | -0.362799913 | 5.65E-16 | -0.378718944 | 2.25E-17 |
| ZNF623 | plum1 | -0.183817791 | 6.45E-05 | -0.212406908 | 3.64E-06 |
| ZNF7 | plum1 | -0.145087029 | 0.001668813 | -0.181293207 | 8.15E-05 |
| ZNF706 | plum1 | -0.060388128 | 0.192680528 | -0.071967679 | 0.120403756 |
| APOL1 | plum2 | -0.138956888 | 0.002617222 | -0.090649348 | 0.050261551 |
| APOL2 | plum2 | -0.077806794 | 0.093062491 | -0.046409687 | 0.316936399 |
| APOL6 | plum2 | -0.110596806 | 0.016804799 | -0.078990162 | 0.088179095 |
| B2M | plum2 | -0.026687535 | 0.565102365 | -0.013622174 | 0.769061766 |
| BATF2 | plum2 | -0.051652609 | 0.265290078 | -0.009213638 | 0.842592075 |
| BTN2A2 | plum2 | 0.023013519 | 0.619852621 | 0.035391466 | 0.44546016 |
| BTN3A1 | plum2 | -0.048927371 | 0.291366862 | -0.045794044 | 0.323405597 |
| BTN3A2 | plum2 | -0.095285945 | 0.039560041 | -0.086818906 | 0.060836014 |
| BTN3A3 | plum2 | -0.140043339 | 0.002419654 | -0.121397964 | 0.008636418 |
| CFB | plum2 | -0.256765654 | 1.82E-08 | -0.245000884 | 8.22E-08 |
| DHX58 | plum2 | 0.012783085 | 0.782920046 | 0.045421252 | 0.327364317 |
| ETV7 | plum2 | -0.07807341 | 0.091943783 | -0.019872236 | 0.668407925 |
| GBP3 | plum2 | 0.092121866 | 0.046627551 | 0.104113984 | 0.024448287 |
| HLA-A | plum2 | -0.067141464 | 0.147425308 | -0.035101267 | 0.449202556 |
| HLA-B | plum2 | -0.081332123 | 0.079122308 | -0.049260688 | 0.288088348 |
| HLA-C | plum2 | -0.028647581 | 0.53687392 | 0.002213084 | 0.961957687 |
| HLA-E | plum2 | -0.141176072 | 0.002228242 | -0.096122527 | 0.037850619 |
| HLA-F | plum2 | -0.243517391 | 9.89E-08 | -0.203909659 | 8.93E-06 |
| HLA-G | plum2 | -0.037631124 | 0.417179926 | -0.000233677 | 0.995981651 |
| IDO1 | plum2 | 0.031400599 | 0.498456033 | 0.047757682 | 0.303069109 |
| IFI35 | plum2 | -0.147984965 | 0.001340865 | -0.104541752 | 0.023864372 |
| IFITM1 | plum2 | -0.132623551 | 0.004091359 | -0.115545431 | 0.012467088 |
| IFITM2 | plum2 | -0.110495825 | 0.0169056 | -0.074500877 | 0.107857515 |
| IFITM3 | plum2 | -0.276440605 | 1.23E-09 | -0.234453884 | 2.98E-07 |
| LGALS3I | plum2 | -0.159418909 | 0.000544244 | -0.151086042 | 0.001056372 |
| LGALS9 | plum2 | 0.006853741 | 0.882567372 | 0.056050137 | 0.226678919 |
| MVP | plum2 | -0.114651425 | 0.013168544 | -0.063895823 | 0.168046236 |
| NLRC5 | plum2 | -0.074418107 | 0.108250471 | -0.034173591 | 0.461284368 |
| PML | plum2 | -0.128370797 | 0.00546635 | -0.097235764 | 0.035673355 |
| PSMB8 | plum2 | -0.06582646 | 0.155534829 | -0.034897232 | 0.451844399 |
| PSMB9 | plum2 | -0.07306147 | 0.114853468 | -0.021694263 | 0.640059496 |
| SECTM1 | plum2 | -0.017682835 | 0.703103068 | 0.027974603 | 0.546485949 |
| TAP1 | plum2 | -0.052703462 | 0.255676579 | -0.0347294 | 0.454024034 |
| TAP2 | plum2 | -0.20487791 | 8.08E-06 | -0.179916952 | 9.25E-05 |
| TAPBP | plum2 | -0.021284811 | 0.646386459 | 0.028384828 | 0.540616668 |
| TAPBPL | plum2 | -0.200609793 | 1.25E-05 | -0.156839006 | 0.00067064 |
| TRIM22 | plum2 | -0.099401607 | 0.031741452 | -0.082053429 | 0.076489959 |
| UBA7 | plum2 | -0.182481292 | 7.30E-05 | -0.133605159 | 0.003822229 |
| WARS | plum2 | -0.052824775 | 0.254582514 | -0.005450168 | 0.906492083 |
| ABHD12 | purple | 0.123878269 | 0.007358074 | 0.149056047 | 0.001235476 |

| | | | | | |
|---|---|---|---|---|---|
| ACADVL | purple | 0.244902249 | 8.32E-08 | 0.245440088 | 7.78E-08 |
| ADAP1 | purple | 0.189055129 | 3.93E-05 | 0.217677238 | 2.05E-06 |
| ADPRHL | purple | -0.101875994 | 0.027708492 | -0.080022083 | 0.084090735 |
| AMOTL2 | purple | -0.270131854 | 2.98E-09 | -0.301213954 | 2.99E-11 |
| ANKRD3 | purple | 0.10017615 | 0.03042826 | 0.114892448 | 0.012976088 |
| AP4M1 | purple | 0.18170418 | 7.85E-05 | 0.173799462 | 0.000160305 |
| APOA1B | purple | 0.041069304 | 0.375881463 | 0.037940667 | 0.413356048 |
| ARL16 | purple | -0.103450657 | 0.025378146 | -0.088614021 | 0.055671403 |
| ARPC5L | purple | 0.160792221 | 0.000486358 | 0.192464725 | 2.82E-05 |
| ATAD3B | purple | -0.064627234 | 0.163220563 | -0.075694803 | 0.10231361 |
| ATOX1 | purple | 0.041939655 | 0.365840262 | 0.081030362 | 0.080245134 |
| ATP5E | purple | -0.037636749 | 0.417110262 | -0.012485048 | 0.787859202 |
| ATP5G1 | purple | 0.153077872 | 0.000904196 | 0.161697171 | 0.0004514 |
| ATP5H | purple | 0.080860656 | 0.080882239 | 0.088908015 | 0.054861307 |
| ATP5I | purple | -0.003061244 | 0.947396203 | 0.018212586 | 0.694648114 |
| ATP5J2 | purple | 0.130680818 | 0.004675103 | 0.155202245 | 0.000764399 |
| ATP5L | purple | 0.050934537 | 0.272000222 | 0.061063762 | 0.187740729 |
| ATP5O | purple | -0.034906752 | 0.45172095 | -0.031018966 | 0.503693248 |
| ATPIF1 | purple | 0.303485203 | 2.09E-11 | 0.333509156 | 1.35E-13 |
| AURKAI | purple | 0.064811119 | 0.162023875 | 0.089105051 | 0.054323877 |
| BABAM1 | purple | -0.112021086 | 0.015438073 | -0.095753005 | 0.038597797 |
| BAD | purple | 0.088720453 | 0.05537699 | 0.103406771 | 0.025440728 |
| BAX | purple | 0.250347233 | 4.18E-08 | 0.256916865 | 1.78E-08 |
| BCAT2 | purple | 0.045514586 | 0.326370257 | 0.081178042 | 0.079694027 |
| BCS1L | purple | -0.037450177 | 0.419424817 | -0.035771077 | 0.44059154 |
| BNIP1 | purple | 0.188894819 | 3.99E-05 | 0.202219578 | 1.06E-05 |
| BRAT1 | purple | 0.160169317 | 0.000511866 | 0.180095995 | 9.10E-05 |
| BRF1 | purple | 0.137318574 | 0.002943044 | 0.136428543 | 0.003135126 |
| BRICD5 | purple | 0.138498388 | 0.002704925 | 0.146237874 | 0.001530647 |
| BRMS1 | purple | 0.01420973 | 0.759401076 | 0.04812464 | 0.299364782 |
| BTBD6 | purple | 0.191457502 | 3.11E-05 | 0.208843364 | 5.33E-06 |
| C11orf31 | purple | 0.035575362 | 0.443097839 | 0.049488829 | 0.285858705 |
| C12orf45 | purple | 0.25301063 | 2.97E-08 | 0.257287752 | 1.70E-08 |
| C14orf2 | purple | 0.101443158 | 0.028380411 | 0.135372891 | 0.003377679 |
| C16orf13 | purple | 0.149891082 | 0.001158663 | 0.183106914 | 6.89E-05 |
| C16orf95 | purple | 0.200836341 | 1.22E-05 | 0.197236556 | 1.76E-05 |
| C17orf89 | purple | 0.08989757 | 0.052206264 | 0.107306549 | 0.020373352 |
| C19orf73 | purple | 0.274450446 | 1.63E-09 | 0.289161038 | 1.91E-10 |
| C1orf122 | purple | -0.02858247 | 0.537800176 | 0.004617554 | 0.920725968 |
| C1orf35 | purple | 0.057313528 | 0.216364572 | 0.061555268 | 0.18420614 |
| C1orf50 | purple | 0.117769574 | 0.010863139 | 0.138514222 | 0.002701852 |
| C6orf136 | purple | -0.06137482 | 0.18549805 | -0.045410096 | 0.327483261 |
| C7orf26 | purple | 0.025729124 | 0.57915953 | 0.044495071 | 0.337334637 |
| C7orf50 | purple | 0.06867575 | 0.138375378 | 0.098162225 | 0.033943458 |
| C9orf142 | purple | 0.209511758 | 4.97E-06 | 0.221363857 | 1.36E-06 |

| | | | | | |
|---|---|---|---|---|---|
| CARHSP | purple | 0.08812212 | 0.057049034 | 0.115647823 | 0.012388893 |
| CARS2 | purple | 0.291689932 | 1.30E-10 | 0.293284018 | 1.02E-10 |
| CC2D1A | purple | 0.056168608 | 0.225697124 | 0.044736095 | 0.334721484 |
| CCDC137 | purple | 0.042934277 | 0.354571768 | 0.033911625 | 0.464728615 |
| CCDC167 | purple | 0.089432009 | 0.053441744 | 0.111021535 | 0.016386561 |
| CCHCR1 | purple | -0.038915816 | 0.401445757 | -0.018656204 | 0.687596812 |
| CDK5 | purple | 0.248207957 | 5.49E-08 | 0.25440992 | 2.47E-08 |
| CDKN2A | purple | 0.054350697 | 0.241097739 | 0.043156895 | 0.352079877 |
| CDKN2D | purple | -0.025414719 | 0.583806351 | -0.007720634 | 0.867842446 |
| CENPT | purple | 0.085403745 | 0.065181001 | 0.09204958 | 0.046800653 |
| CHCHD1 | purple | 0.261930971 | 9.15E-09 | 0.275392018 | 1.42E-09 |
| CHCHD6 | purple | -0.052267503 | 0.259635151 | -0.063026217 | 0.173921696 |
| CHMP2A | purple | 0.198680646 | 1.52E-05 | 0.239204215 | 1.68E-07 |
| CINP | purple | 0.141655945 | 0.002151405 | 0.16963325 | 0.000230772 |
| CISD3 | purple | 0.04923258 | 0.288363856 | 0.065572925 | 0.157136453 |
| CITED4 | purple | 0.386386525 | 4.46E-18 | 0.383937183 | 7.51E-18 |
| CLN3 | purple | 0.198849619 | 1.50E-05 | 0.203960687 | 8.89E-06 |
| CLN6 | purple | 0.298866782 | 4.32E-11 | 0.302717069 | 2.36E-11 |
| CLTA | purple | 0.115492639 | 0.012507574 | 0.13883837 | 0.00263964 |
| COA3 | purple | 0.217533442 | 2.08E-06 | 0.244613499 | 8.63E-08 |
| COA4 | purple | 0.104891285 | 0.023396238 | 0.112675398 | 0.014843589 |
| COASY | purple | 0.323332802 | 7.96E-13 | 0.354358716 | 2.91E-15 |
| COMMD | purple | 0.019668496 | 0.671608318 | 0.048224912 | 0.298357833 |
| COMMD4 | purple | 0.222821043 | 1.15E-06 | 0.23178755 | 4.09E-07 |
| COMMD7 | purple | -0.059718665 | 0.197668469 | -0.067315716 | 0.14637534 |
| COMMD9 | purple | 0.210677625 | 4.39E-06 | 0.223207034 | 1.10E-06 |
| COPRS | purple | 0.160919338 | 0.0004813 | 0.189321561 | 3.83E-05 |
| COX14 | purple | 0.030150392 | 0.51571992 | 0.062302905 | 0.17892415 |
| COX5B | purple | 0.026308695 | 0.5706393 | 0.062306293 | 0.178900472 |
| COX6A1 | purple | 0.178599098 | 0.00010426 | 0.206541199 | 6.79E-06 |
| COX6B1 | purple | 0.003691679 | 0.936583766 | 0.033536183 | 0.469689592 |
| COX7A2 | purple | 0.156196765 | 0.000706081 | 0.193842334 | 2.47E-05 |
| COX7B | purple | 0.014900261 | 0.748095008 | 0.054719368 | 0.237916353 |
| COX7C | purple | 0.138365738 | 0.002730793 | 0.146460851 | 0.001505123 |
| CRACR2I | purple | 0.335777782 | 9.04E-14 | 0.337712143 | 6.39E-14 |
| CRIP1 | purple | 0.161281383 | 0.000467161 | 0.192575348 | 2.79E-05 |
| CSNK2B | purple | -0.106470112 | 0.021379376 | -0.095021532 | 0.040113729 |
| CTB-55O | purple | 0.208506011 | 5.52E-06 | 0.214737569 | 2.83E-06 |
| DDX51 | purple | -0.036440792 | 0.432076847 | -0.055860498 | 0.22825682 |
| DDX56 | purple | 0.063337882 | 0.171798607 | 0.054680886 | 0.238247039 |
| DNTTIP1 | purple | -0.131670343 | 0.00436899 | -0.112401403 | 0.015090038 |
| DPH7 | purple | 0.031580123 | 0.49600241 | 0.034244196 | 0.460358506 |
| DPM2 | purple | 0.216076146 | 2.44E-06 | 0.237495828 | 2.07E-07 |
| DXO | purple | -0.100968493 | 0.029133304 | -0.093287223 | 0.043910202 |
| ECHS1 | purple | 0.24527431 | 7.94E-08 | 0.273268077 | 1.92E-09 |

| | | | | | |
|---|---|---|---|---|---|
| ECI1 | purple | 0.261035138 | 1.03E-08 | 0.288163601 | 2.21E-10 |
| EDF1 | purple | 0.135452304 | 0.003358857 | 0.152501828 | 0.000945982 |
| EIF3I | purple | 0.136547005 | 0.003108919 | 0.154775104 | 0.000790789 |
| EIF4E2 | purple | 0.209297148 | 5.08E-06 | 0.204199303 | 8.67E-06 |
| EIF5A | purple | 0.237738036 | 2.01E-07 | 0.245261917 | 7.95E-08 |
| ELP6 | purple | 0.2051562 | 7.85E-06 | 0.208610553 | 5.46E-06 |
| ENDOG | purple | 0.272176228 | 2.24E-09 | 0.295049255 | 7.80E-11 |
| ENO3 | purple | 0.09268985 | 0.045286013 | 0.087215338 | 0.059662665 |
| ENTHD2 | purple | -0.035864891 | 0.439393056 | -0.041070119 | 0.375871984 |
| EPC2 | purple | -0.234287006 | 3.04E-07 | -0.257942294 | 1.56E-08 |
| EXOSC5 | purple | -0.076163323 | 0.100200822 | -0.084181397 | 0.069136413 |
| EXOSC7 | purple | 0.060141563 | 0.19450677 | 0.078170739 | 0.09153809 |
| FAAH | purple | 0.294964874 | 7.91E-11 | 0.311501907 | 5.75E-12 |
| FAM162A | purple | 0.163758265 | 0.000380318 | 0.169758899 | 0.000228278 |
| FAM173A | purple | 0.171188195 | 0.000201629 | 0.203912582 | 8.93E-06 |
| FAM195A | purple | 0.204464194 | 8.44E-06 | 0.245776659 | 7.46E-08 |
| FAM50A | purple | 0.136985516 | 0.003013633 | 0.140907588 | 0.002272316 |
| FAM73B | purple | 0.021496043 | 0.643119226 | 0.018475197 | 0.690470691 |
| FDX1L | purple | -0.050211443 | 0.278873391 | -0.042418702 | 0.360385394 |
| FKBPL | purple | -0.103867914 | 0.024789739 | -0.092590946 | 0.045517258 |
| FUOM | purple | -0.182109271 | 7.56E-05 | -0.160059535 | 0.000516488 |
| GADD45G | purple | 0.012134693 | 0.793676262 | 0.02591886 | 0.576363683 |
| GCAT | purple | 0.166082338 | 0.000312737 | 0.192657076 | 2.77E-05 |
| GEMIN7 | purple | -0.036150031 | 0.435761828 | -0.030022029 | 0.517509808 |
| GGA1 | purple | 0.199717228 | 1.37E-05 | 0.198926731 | 1.49E-05 |
| GPATCH | purple | -0.101874125 | 0.027711364 | -0.092283083 | 0.046243421 |
| GSTZ1 | purple | 0.255310763 | 2.20E-08 | 0.268984904 | 3.50E-09 |
| GTF3A | purple | 0.05451469 | 0.239678917 | 0.070259857 | 0.129485621 |
| HDGFRP | purple | 0.131187351 | 0.004516067 | 0.130439557 | 0.004752613 |
| HDHD3 | purple | 0.367513681 | 2.22E-16 | 0.376751814 | 3.38E-17 |
| HEBP2 | purple | 0.240060105 | 1.51E-07 | 0.265376532 | 5.74E-09 |
| HES4 | purple | 0.194688583 | 2.27E-05 | 0.230501499 | 4.76E-07 |
| HGSNAT | purple | -0.084860423 | 0.066915557 | -0.135421584 | 0.003366126 |
| HIGD2A | purple | 0.065486299 | 0.157686527 | 0.07909475 | 0.087757589 |
| HINT2 | purple | 0.135324446 | 0.003389208 | 0.152973087 | 0.000911668 |
| HSD17B1 | purple | 0.055718208 | 0.229445861 | 0.084527453 | 0.067997157 |
| ICT1 | purple | 0.166184779 | 0.000310033 | 0.16642043 | 0.000303896 |
| IGF1R | purple | -0.17963717 | 9.49E-05 | -0.215164365 | 2.70E-06 |
| IMP3 | purple | 0.209153031 | 5.16E-06 | 0.22527823 | 8.72E-07 |
| IPO4 | purple | -0.094982743 | 0.040195505 | -0.090582279 | 0.050432552 |
| JMJD4 | purple | 0.052035299 | 0.261760781 | 0.053059223 | 0.252477336 |
| JSRP1 | purple | 0.335551158 | 9.41E-14 | 0.358449394 | 1.32E-15 |
| KAT2A | purple | 0.068227445 | 0.140974414 | 0.041261633 | 0.373648099 |
| KIRREL | purple | -0.388677245 | 2.73E-18 | -0.388808857 | 2.65E-18 |
| LAGE3 | purple | 0.046484332 | 0.316157814 | 0.056574711 | 0.222354625 |

| | | | | | |
|---|---|---|---|---|---|
| LAMTOR | purple | 0.280885279 | 6.47E-10 | 0.285184052 | 3.45E-10 |
| LAS1L | purple | -0.049820579 | 0.282637263 | -0.048491133 | 0.295695329 |
| 9-Mar | purple | 0.180876589 | 8.47E-05 | 0.209186377 | 5.14E-06 |
| MBD5 | purple | -0.155374458 | 0.000753991 | -0.181898763 | 7.71E-05 |
| MCRS1 | purple | 0.058261554 | 0.208849119 | 0.057283595 | 0.216604982 |
| MEA1 | purple | -0.022281391 | 0.631032475 | 0.000184579 | 0.996825955 |
| MED11 | purple | 0.334166517 | 1.20E-13 | 0.354783951 | 2.68E-15 |
| MED19 | purple | 0.081299318 | 0.079243753 | 0.075290969 | 0.104162919 |
| MED27 | purple | 0.081971729 | 0.076784493 | 0.109639857 | 0.017781503 |
| MEF2A | purple | -0.091686496 | 0.047678295 | -0.117503812 | 0.011044654 |
| MEF2BN | purple | -0.054495648 | 0.239843359 | -0.035355639 | 0.445921224 |
| MESP1 | purple | 0.220112276 | 1.56E-06 | 0.252439728 | 3.19E-08 |
| METTL1 | purple | -0.088834389 | 0.055063257 | -0.094422091 | 0.041393284 |
| MMAB | purple | 0.038438486 | 0.407249924 | 0.044489772 | 0.337392235 |
| MPDU1 | purple | 0.359695099 | 1.04E-15 | 0.385111031 | 5.86E-18 |
| MPV17L2 | purple | 0.060895351 | 0.188963233 | 0.086970389 | 0.060385428 |
| MRPL12 | purple | -0.014387117 | 0.756491718 | -0.011555983 | 0.803309764 |
| MRPL14 | purple | 0.059290615 | 0.200906698 | 0.083549224 | 0.071257931 |
| MRPL16 | purple | 0.193106184 | 2.65E-05 | 0.193090407 | 2.66E-05 |
| MRPL20 | purple | 0.055710596 | 0.229509596 | 0.083131069 | 0.072690233 |
| MRPL21 | purple | -0.063536205 | 0.170457714 | -0.046437302 | 0.316648212 |
| MRPL22 | purple | 0.148073537 | 0.001331847 | 0.166908755 | 0.000291537 |
| MRPL23 | purple | 0.257826237 | 1.58E-08 | 0.276328612 | 1.25E-09 |
| MRPL24 | purple | 0.200953219 | 1.21E-05 | 0.180704461 | 8.60E-05 |
| MRPL27 | purple | 0.119589163 | 0.009689802 | 0.146363449 | 0.001516224 |
| MRPL34 | purple | 0.010233992 | 0.825426082 | 0.017098068 | 0.712478762 |
| MRPL36 | purple | -0.04988255 | 0.282038235 | -0.027687114 | 0.550617863 |
| MRPL41 | purple | 0.250168448 | 4.28E-08 | 0.284777421 | 3.66E-10 |
| MRPL52 | purple | 0.079856083 | 0.084737893 | 0.093029645 | 0.044499055 |
| MRPL53 | purple | 0.060446202 | 0.192252224 | 0.050974098 | 0.271627544 |
| MRPL57 | purple | 0.298429047 | 4.63E-11 | 0.31988631 | 1.43E-12 |
| MRPS15 | purple | 0.13301926 | 0.003980869 | 0.145917728 | 0.001567988 |
| MRPS25 | purple | 0.021832262 | 0.637932936 | 0.019215084 | 0.678751893 |
| MRPS5 | purple | -0.152488645 | 0.000946958 | -0.151451824 | 0.001026767 |
| MSN | purple | -0.215003688 | 2.75E-06 | -0.197797039 | 1.67E-05 |
| MTG2 | purple | -0.110039437 | 0.017367803 | -0.121434031 | 0.008616493 |
| MTMR14 | purple | 0.016304819 | 0.725266384 | 0.023513784 | 0.61226325 |
| MUTYH | purple | 0.004211996 | 0.927668623 | 0.002134787 | 0.963302643 |
| MVK | purple | 0.167422315 | 0.000279048 | 0.171740294 | 0.000192138 |
| MYEOV2 | purple | 0.111501139 | 0.015925271 | 0.135439515 | 0.003361881 |
| MYL6B | purple | 0.004220993 | 0.927514534 | -0.00628952 | 0.892173267 |
| NAA10 | purple | 0.05489046 | 0.236450022 | 0.075884762 | 0.10145277 |
| NAA38 | purple | 0.194461075 | 2.32E-05 | 0.221786129 | 1.30E-06 |
| NABP2 | purple | 0.052565477 | 0.256924955 | 0.067331513 | 0.146280433 |
| NANS | purple | 0.385601117 | 5.28E-18 | 0.418245017 | 3.36E-21 |

| | | | | | |
|---|---|---|---|---|---|
| NAT9 | purple | 0.011804451 | 0.799169896 | -0.009023987 | 0.845791174 |
| NDUFA1 | purple | 0.011286857 | 0.807800028 | 0.030668441 | 0.508528954 |
| NDUFA1: | purple | 0.156567022 | 0.000685443 | 0.180784771 | 8.54E-05 |
| NDUFA1: | purple | 0.087075263 | 0.060075098 | 0.116373549 | 0.011846971 |
| NDUFA2 | purple | 0.18150488 | 7.99E-05 | 0.205104314 | 7.89E-06 |
| NDUFA3 | purple | 0.13366532 | 0.003806268 | 0.168212339 | 0.000260806 |
| NDUFA6 | purple | 0.335608043 | 9.31E-14 | 0.359807757 | 1.02E-15 |
| NDUFA8 | purple | 0.186763276 | 4.89E-05 | 0.206142475 | 7.08E-06 |
| NDUFA9 | purple | 0.004719207 | 0.918986717 | 0.033728808 | 0.467140665 |
| NDUFB1 | purple | 0.019443955 | 0.675142355 | 0.055205825 | 0.23376393 |
| NDUFB2 | purple | 0.073714725 | 0.111635569 | 0.094364212 | 0.041518633 |
| NDUFB4 | purple | 0.186789058 | 4.88E-05 | 0.203789524 | 9.05E-06 |
| NDUFB7 | purple | -0.049513544 | 0.285617865 | -0.030095756 | 0.516481371 |
| NDUFB9 | purple | -0.131146518 | 0.004528704 | -0.137533171 | 0.002898362 |
| NDUFC1 | purple | 0.272887762 | 2.03E-09 | 0.292068942 | 1.23E-10 |
| NDUFS5 | purple | 0.194087862 | 2.41E-05 | 0.218506047 | 1.87E-06 |
| NDUFS6 | purple | -0.032521044 | 0.483248266 | 0.002510577 | 0.956848543 |
| NDUFS8 | purple | 0.130865826 | 0.004616439 | 0.15920294 | 0.000553909 |
| NDUFV3 | purple | 0.157567513 | 0.000632438 | 0.183452562 | 6.67E-05 |
| NELFE | purple | -0.125801809 | 0.006486132 | -0.118903735 | 0.010117874 |
| NME1 | purple | 0.166312236 | 0.000306699 | 0.169334767 | 0.000236799 |
| NOL12 | purple | 0.06864944 | 0.138526884 | 0.062140942 | 0.180058769 |
| NOP10 | purple | 0.323324511 | 7.97E-13 | 0.342575857 | 2.64E-14 |
| NOP16 | purple | 0.037651103 | 0.416932499 | 0.038489358 | 0.406628972 |
| NPRL2 | purple | 0.164253367 | 0.000364871 | 0.170530877 | 0.000213501 |
| NSUN5 | purple | -0.013068687 | 0.778195117 | 0.008754376 | 0.850343403 |
| NTN4 | purple | -0.208078476 | 5.78E-06 | -0.212619812 | 3.56E-06 |
| NUDC | purple | 0.100866777 | 0.029296852 | 0.121732965 | 0.008452928 |
| NUDT1 | purple | 0.02712511 | 0.558739194 | 0.062407585 | 0.178193652 |
| OSMR | purple | -0.096989849 | 0.036144923 | -0.082549169 | 0.074722407 |
| PAM16 | purple | -0.006495449 | 0.888665408 | -0.025042312 | 0.589332703 |
| PANO1 | purple | 0.196183944 | 1.96E-05 | 0.229794487 | 5.17E-07 |
| PARK7 | purple | 0.164530965 | 0.000356469 | 0.160652516 | 0.000491974 |
| PDAP1 | purple | 0.027256082 | 0.556841388 | 0.031107331 | 0.502478027 |
| PDZD11 | purple | 0.090836035 | 0.049788119 | 0.087012425 | 0.060260884 |
| PELP1 | purple | 0.075639772 | 0.102564077 | 0.075166701 | 0.104737281 |
| PFDN6 | purple | -0.064368218 | 0.164917434 | -0.057385801 | 0.215784884 |
| PHPT1 | purple | 0.270493582 | 2.84E-09 | 0.289763671 | 1.74E-10 |
| PICK1 | purple | 0.118603069 | 0.010310888 | 0.131929712 | 0.004291808 |
| PIDD1 | purple | 0.295568642 | 7.21E-11 | 0.295902163 | 6.84E-11 |
| PIGU | purple | -0.068933702 | 0.136896676 | -0.073685935 | 0.111775878 |
| PIN4 | purple | -0.029044708 | 0.531241852 | -0.014287821 | 0.758119868 |
| PMF1 | purple | 0.112426006 | 0.015067763 | 0.10341037 | 0.025435591 |
| PMPCA | purple | 0.188074719 | 4.31E-05 | 0.204090998 | 8.77E-06 |
| PMVK | purple | 0.256571274 | 1.86E-08 | 0.248644825 | 5.19E-08 |

| | | | | | |
|---|---|---|---|---|---|
| POLE4 | purple | -0.017032435 | 0.713533801 | -0.006829421 | 0.882981088 |
| POLR1C | purple | -0.126105758 | 0.00635717 | -0.113783235 | 0.013882803 |
| POLR2J | purple | 0.058684242 | 0.205559745 | 0.077442949 | 0.094606708 |
| POP5 | purple | 0.133903494 | 0.003743671 | 0.158348716 | 0.000593721 |
| POP7 | purple | 0.095391755 | 0.03934029 | 0.098997239 | 0.032446074 |
| PPAN | purple | -0.074932788 | 0.105825219 | -0.081230979 | 0.079497226 |
| PPCDC | purple | 0.247852005 | 5.74E-08 | 0.246737764 | 6.61E-08 |
| PPFIA3 | purple | 0.307499714 | 1.10E-11 | 0.296164712 | 6.57E-11 |
| PPIE | purple | -0.109508893 | 0.017918956 | -0.09687788 | 0.036361386 |
| PPP1R14l | purple | 0.057170764 | 0.217512931 | 0.064606581 | 0.163355382 |
| PPP1R35 | purple | 0.082630714 | 0.074434872 | 0.092073855 | 0.04674246 |
| PPP1R7 | purple | 0.309706396 | 7.71E-12 | 0.343734645 | 2.13E-14 |
| PRADC1 | purple | 0.155646887 | 0.000737795 | 0.20401207 | 8.84E-06 |
| PRKRIP1 | purple | -0.009914332 | 0.830795367 | -0.03283549 | 0.479025878 |
| PSMB1 | purple | 0.009280702 | 0.841461438 | 0.050617717 | 0.274997309 |
| PSMB3 | purple | 0.105451958 | 0.022661916 | 0.135850034 | 0.003266013 |
| PSMB6 | purple | 0.176958481 | 0.000120928 | 0.213701673 | 3.17E-06 |
| PSMB7 | purple | 0.120180595 | 0.009333447 | 0.144386985 | 0.001758359 |
| PSMC4 | purple | -0.04984315 | 0.282418982 | -0.024869647 | 0.591903107 |
| PSMC5 | purple | 0.067547356 | 0.144988415 | 0.088672387 | 0.05550979 |
| PSMD13 | purple | 0.111907877 | 0.015543023 | 0.125671763 | 0.006542022 |
| PSMG3 | purple | 0.203907353 | 8.94E-06 | 0.235679191 | 2.57E-07 |
| PTPMT1 | purple | 0.214236749 | 2.99E-06 | 0.212545779 | 3.59E-06 |
| PTRH2 | purple | 0.040784891 | 0.379199155 | 0.035845252 | 0.439643799 |
| QPCTL | purple | 0.124196633 | 0.007206886 | 0.131611499 | 0.004386674 |
| RAB26 | purple | 0.176793685 | 0.000122734 | 0.202237306 | 1.06E-05 |
| RABEP2 | purple | 0.185994 | 5.26E-05 | 0.200477532 | 1.27E-05 |
| RABL6 | purple | 0.275788654 | 1.35E-09 | 0.284631619 | 3.74E-10 |
| RAD9A | purple | -0.110221308 | 0.017182307 | -0.101997693 | 0.027522052 |
| RASSF7 | purple | 0.186877287 | 4.83E-05 | 0.211433971 | 4.04E-06 |
| RBMS2 | purple | -0.16754272 | 0.000276193 | -0.18673474 | 4.90E-05 |
| RBX1 | purple | 0.185031787 | 5.75E-05 | 0.196680876 | 1.86E-05 |
| REXO4 | purple | 0.027776449 | 0.549332266 | 0.026926634 | 0.561621108 |
| RHBDL1 | purple | 0.194178587 | 2.39E-05 | 0.208582477 | 5.48E-06 |
| RHOT2 | purple | 0.162408351 | 0.000425583 | 0.177909466 | 0.000110984 |
| RNF181 | purple | 0.009080242 | 0.844841975 | 0.056381004 | 0.22394453 |
| RNMTL1 | purple | 0.226658582 | 7.44E-07 | 0.239766049 | 1.57E-07 |
| ROMO1 | purple | 0.041524847 | 0.370604896 | 0.078751217 | 0.089148189 |
| RP1-37E1 | purple | 0.004984309 | 0.914452751 | 0.006296795 | 0.892049311 |
| RP9 | purple | -0.061403247 | 0.185294089 | -0.068588564 | 0.138877932 |
| RPL26L1 | purple | 0.13076281 | 0.004649022 | 0.149714209 | 0.001174556 |
| RPP21 | purple | -0.011897181 | 0.797626297 | -0.00996054 | 0.830018714 |
| RPP25 | purple | 0.001796168 | 0.969120391 | 0.011434781 | 0.805331179 |
| RPS19BP | purple | 0.145826985 | 0.001578723 | 0.158170156 | 0.000602371 |
| RPUSD2 | purple | 0.18769748 | 4.47E-05 | 0.198862907 | 1.50E-05 |

| | | | | | |
|---|---|---|---|---|---|
| RPUSD3 | purple | 0.106193488 | 0.021721442 | 0.099957744 | 0.030793759 |
| RRP1 | purple | -0.090941417 | 0.049522524 | -0.094522472 | 0.04117664 |
| SAP18 | purple | 0.211512109 | 4.01E-06 | 0.215848749 | 2.51E-06 |
| SDHAF1 | purple | -0.041074945 | 0.37581584 | -0.033250197 | 0.473488015 |
| SDHAF2 | purple | 0.035156475 | 0.448489237 | 0.072789265 | 0.11621562 |
| SEC61B | purple | 0.335279239 | 9.88E-14 | 0.350056185 | 6.57E-15 |
| SERF2 | purple | 0.241101804 | 1.33E-07 | 0.271719759 | 2.39E-09 |
| SHFM1 | purple | 0.09982182 | 0.031023118 | 0.122397133 | 0.008099386 |
| SLIRP | purple | 0.065032227 | 0.160593705 | 0.083053372 | 0.072958938 |
| SMAD3 | purple | -0.277547795 | 1.05E-09 | -0.298602023 | 4.51E-11 |
| SMG9 | purple | 0.239013588 | 1.72E-07 | 0.236676724 | 2.28E-07 |
| SMIM4 | purple | 0.127523155 | 0.005785649 | 0.161402757 | 0.000462508 |
| SMUG1 | purple | -0.034493281 | 0.457100504 | -0.042214006 | 0.362709995 |
| SNF8 | purple | 0.048680155 | 0.293814562 | 0.073401101 | 0.113171502 |
| SNRPD2 | purple | -0.040195225 | 0.386134605 | -0.029907947 | 0.519103257 |
| SNRPF | purple | 0.101401334 | 0.028446073 | 0.116487779 | 0.011763608 |
| SOD1 | purple | -0.00167695 | 0.971169049 | 0.025264301 | 0.586035591 |
| SRA1 | purple | 0.270531056 | 2.82E-09 | 0.294541831 | 8.44E-11 |
| SS18L2 | purple | 0.12376331 | 0.00741336 | 0.124211231 | 0.007200021 |
| SSNA1 | purple | 0.231205398 | 4.38E-07 | 0.255081355 | 2.27E-08 |
| SSR2 | purple | 0.274599208 | 1.59E-09 | 0.248927202 | 5.01E-08 |
| SSSCA1 | purple | -0.029579083 | 0.523710738 | 0.002641557 | 0.95459965 |
| SSU72 | purple | 0.062463743 | 0.177802672 | 0.08390378 | 0.070061615 |
| STX10 | purple | 0.047014254 | 0.310666497 | 0.069068288 | 0.136130004 |
| STYXL1 | purple | 0.017679968 | 0.703148938 | 0.049909969 | 0.281773459 |
| SURF1 | purple | 0.210315759 | 4.56E-06 | 0.234811144 | 2.86E-07 |
| SURF2 | purple | 0.104143049 | 0.024408225 | 0.116558793 | 0.011712045 |
| SURF6 | purple | 0.109097744 | 0.018356521 | 0.083084763 | 0.072850281 |
| TACO1 | purple | -0.016116986 | 0.728305731 | 0.002664095 | 0.954212714 |
| TAF10 | purple | 0.176732106 | 0.000123416 | 0.222807107 | 1.15E-06 |
| TCEB2 | purple | 0.073873078 | 0.110866306 | 0.116367741 | 0.011851223 |
| TFPT | purple | 0.115160476 | 0.012764984 | 0.143920162 | 0.001820497 |
| TIMM17I | purple | -0.036596806 | 0.430107032 | 0.006849234 | 0.882644043 |
| TIMM22 | purple | 0.113029332 | 0.014530473 | 0.124628707 | 0.007006146 |
| TIMM44 | purple | 0.112153863 | 0.015315774 | 0.110359555 | 0.017042466 |
| TIMM50 | purple | -0.094397235 | 0.041447075 | -0.083521347 | 0.071352695 |
| TIMM8B | purple | 0.174974124 | 0.000144435 | 0.204867708 | 8.09E-06 |
| TMEM13 | purple | 0.230501766 | 4.76E-07 | 0.241658415 | 1.24E-07 |
| TMEM22 | purple | -0.087907912 | 0.057657723 | -0.085220062 | 0.065763225 |
| TMEM25 | purple | 0.227616515 | 6.66E-07 | 0.243321325 | 1.01E-07 |
| TMEM52 | purple | 0.105557948 | 0.022525366 | 0.079543065 | 0.085969124 |
| TRAPPC: | purple | 0.004141582 | 0.9288746 | 0.023400935 | 0.613971668 |
| TRMT2A | purple | 0.111596445 | 0.015834972 | 0.110240396 | 0.01716294 |
| TRNAU1 | purple | 0.128344902 | 0.005475865 | 0.159188593 | 0.000554557 |
| TSEN54 | purple | 0.003412074 | 0.941377883 | 9.59E-05 | 0.99835075 |

| | | | | | |
|---|---|---|---|---|---|
| TSR3 | purple | -0.01120842 | 0.809109906 | 0.021138048 | 0.648660538 |
| TUBB4B | purple | 0.241320907 | 1.30E-07 | 0.267591819 | 4.24E-09 |
| TUSC2 | purple | 0.011738105 | 0.800274783 | 0.03895008 | 0.401031024 |
| TUT1 | purple | -0.016643399 | 0.719798689 | -0.020793299 | 0.654015202 |
| TXN | purple | 0.289147082 | 1.91E-10 | 0.300658332 | 3.27E-11 |
| TXNDC1' | purple | 0.328777558 | 3.11E-13 | 0.36218845 | 6.37E-16 |
| UBE2F | purple | 0.020034719 | 0.665859892 | 0.040420748 | 0.383473012 |
| UBE2J2 | purple | -0.10551073 | 0.02258611 | -0.096527557 | 0.037045767 |
| UBL5 | purple | 0.03386219 | 0.465380174 | 0.0537229 | 0.246583688 |
| UBL7 | purple | 0.144430563 | 0.001752659 | 0.15557215 | 0.000742206 |
| UCHL3 | purple | 0.166213192 | 0.000309287 | 0.183074691 | 6.91E-05 |
| UCK1 | purple | 0.217065054 | 2.19E-06 | 0.233458816 | 3.36E-07 |
| UQCC2 | purple | -0.010117983 | 0.827373744 | 0.007906169 | 0.864696723 |
| UQCC3 | purple | 0.073427994 | 0.113039149 | 0.077651341 | 0.093719772 |
| UQCRQ | purple | 0.297764113 | 5.13E-11 | 0.32915527 | 2.91E-13 |
| URM1 | purple | 0.186884687 | 4.83E-05 | 0.209096956 | 5.19E-06 |
| UROS | purple | 0.08791479 | 0.057638096 | 0.105510833 | 0.022585977 |
| VARS2 | purple | 0.164678395 | 0.00035208 | 0.172002352 | 0.000187781 |
| VPS25 | purple | 0.236096208 | 2.45E-07 | 0.260300856 | 1.14E-08 |
| WBSCR2 | purple | 0.013407921 | 0.772593537 | 0.022018399 | 0.635069266 |
| WDR34 | purple | 0.35522646 | 2.46E-15 | 0.381220164 | 1.33E-17 |
| WDR46 | purple | -0.119180198 | 0.009943235 | -0.106316091 | 0.021569252 |
| WDR74 | purple | -0.176747338 | 0.000123247 | -0.181184131 | 8.23E-05 |
| WIBG | purple | 0.127598557 | 0.005756581 | 0.122536922 | 0.008026679 |
| WRAP53 | purple | 0.186469554 | 5.03E-05 | 0.200810064 | 1.23E-05 |
| ZBTB38 | purple | -0.147359831 | 0.001406134 | -0.168100599 | 0.000263317 |
| ZBTB8OS | purple | 0.091015876 | 0.04933558 | 0.116736087 | 0.011584184 |
| ZFYVE19 | purple | 0.338859734 | 5.19E-14 | 0.335628651 | 9.28E-14 |
| ZMYND1 | purple | 0.12743294 | 0.0058206 | 0.137729372 | 0.002858051 |
| ZNF296 | purple | 0.006304825 | 0.891912486 | 0.031327504 | 0.499456883 |
| ZNF316 | purple | -0.023427443 | 0.613570184 | -0.017965829 | 0.698581773 |
| ZNF593 | purple | 0.092597293 | 0.045502387 | 0.095618638 | 0.038872569 |
| ZNHIT1 | purple | 0.088210454 | 0.056799581 | 0.113066268 | 0.014498133 |
| A4GNT | red | -0.089120547 | 0.054281799 | -0.077147066 | 0.095877514 |
| AADACL | red | -0.07144156 | 0.123146969 | -0.05853356 | 0.206728026 |
| ABCA12 | red | 0.109661796 | 0.017758568 | 0.103275461 | 0.025628773 |
| ABCA13 | red | -0.104928632 | 0.023346693 | -0.112702806 | 0.014819131 |
| ABRA | red | -0.131338057 | 0.004469702 | -0.132342204 | 0.004171596 |
| AC00504: | red | -0.092314662 | 0.04616849 | -0.090032654 | 0.051852275 |
| AC00547' | red | -0.057342033 | 0.216135806 | -0.044104927 | 0.341592158 |
| AC00548( | red | -0.066342606 | 0.152312462 | -0.066705457 | 0.150077634 |
| AC00611( | red | -0.120270791 | 0.009280138 | -0.112749802 | 0.014777277 |
| AC00704( | red | -0.081305501 | 0.079220852 | -0.062836145 | 0.175226045 |
| AC00755' | red | -0.063944345 | 0.16772283 | -0.046543497 | 0.315541594 |
| AC00790( | red | -0.083241792 | 0.072308707 | -0.075870896 | 0.101515412 |

| | | | | | |
|---|---|---|---|---|---|
| AC00807 | red | -0.068629179 | 0.138643643 | -0.056764942 | 0.220801102 |
| AC00864 | red | -0.013094179 | 0.777773777 | -0.018700648 | 0.686891842 |
| AC00949 | red | -0.025120781 | 0.588166263 | -0.025744184 | 0.578937382 |
| AC01028 | red | -0.06480055 | 0.162092479 | -0.04899282 | 0.290721134 |
| AC01064 | red | -0.167041056 | 0.000288271 | -0.171299888 | 0.000199674 |
| AC01138 | red | -0.02917915 | 0.529341969 | -0.012310035 | 0.790763549 |
| AC01236 | red | -0.100289325 | 0.030240329 | -0.079962033 | 0.084324381 |
| AC01328 | red | -0.074118514 | 0.109682259 | -0.061581051 | 0.184022085 |
| AC01654 | red | -0.081015381 | 0.080301211 | -0.064035006 | 0.167119806 |
| AC01657 | red | -0.054617191 | 0.238795098 | -0.037429634 | 0.419680124 |
| AC01673 | red | -0.01200543 | 0.795825378 | -0.000982926 | 0.983098654 |
| AC01702 | red | -0.103184978 | 0.025759046 | -0.108637725 | 0.018857121 |
| AC01886 | red | 0.022966074 | 0.620574504 | 0.03444485 | 0.457732962 |
| AC01890 | red | -0.048364109 | 0.29696373 | -0.032611126 | 0.482036594 |
| AC02281 | red | -0.080092173 | 0.083818691 | -0.067576841 | 0.144812599 |
| AC02644 | red | -0.080771579 | 0.081218287 | -0.057833281 | 0.212220558 |
| AC06395 | red | -0.003374191 | 0.942027592 | -0.011078332 | 0.811283533 |
| AC06906 | red | -0.090206685 | 0.051399178 | -0.082875983 | 0.073575453 |
| AC06906 | red | -0.080731123 | 0.081371282 | -0.070979564 | 0.125595737 |
| AC06936 | red | -0.187405575 | 4.60E-05 | -0.179703866 | 9.43E-05 |
| AC06949 | red | -0.114704407 | 0.013126023 | -0.098643669 | 0.033073082 |
| AC07313 | red | -0.020063772 | 0.665404691 | -0.014844182 | 0.749011228 |
| AC08739 | red | -0.027163314 | 0.558185301 | -0.020698499 | 0.65549077 |
| AC09215 | red | -0.133708586 | 0.003794827 | -0.118807651 | 0.010179204 |
| AC09283 | red | -0.261842692 | 9.26E-09 | -0.276361461 | 1.24E-09 |
| AC09288 | red | -0.076691907 | 0.097858957 | -0.066560389 | 0.150968106 |
| AC10082 | red | -0.027270255 | 0.556636215 | -0.029102837 | 0.530419975 |
| AC10465 | red | -0.076956008 | 0.096705312 | -0.052519376 | 0.257342975 |
| AC10500 | red | -0.069036898 | 0.136308519 | -0.093210036 | 0.044085969 |
| AC10708 | red | -0.063827461 | 0.168502671 | -0.048988421 | 0.290764504 |
| AC11060 | red | -0.04384381 | 0.344460713 | -0.030239473 | 0.514479668 |
| AC12431 | red | -0.103974625 | 0.024641162 | -0.095271392 | 0.039590346 |
| AC23165 | red | -0.0779538 | 0.092444322 | -0.051063124 | 0.27079018 |
| AC24298 | red | -0.108771508 | 0.018710322 | -0.104977084 | 0.023282549 |
| ACAD11 | red | -0.087548441 | 0.058691278 | -0.10424746 | 0.024264777 |
| ACKR2 | red | -0.046244218 | 0.318666784 | -0.020500199 | 0.658581672 |
| ACMSD | red | -0.106735086 | 0.021056111 | -0.103885811 | 0.024764767 |
| ACPT | red | -0.067431072 | 0.145683398 | -0.070082301 | 0.130459477 |
| ACRV1 | red | -0.120034113 | 0.009420603 | -0.123638228 | 0.007473935 |
| ACSM6 | red | -0.101420941 | 0.028415274 | -0.096196254 | 0.037703019 |
| ACTR3C | red | -0.011900505 | 0.797570991 | -0.021176595 | 0.648062931 |
| ADAM20 | red | -0.095217195 | 0.03970338 | -0.094758545 | 0.040670919 |
| ADAM32 | red | -0.138025477 | 0.00279818 | -0.141658479 | 0.002151006 |
| ADCY10 | red | 0.036907579 | 0.426198767 | 0.03648022 | 0.431578537 |
| ADH1A | red | -0.067583925 | 0.144770384 | -0.059034432 | 0.202863101 |

| | | | | | |
|---|---|---|---|---|---|
| ADH4 | red | 0.175532967 | 0.000137414 | 0.180899245 | 8.45E-05 |
| ADORA2 | red | -0.012885466 | 0.781225342 | 0.005923531 | 0.898412779 |
| AGAP5 | red | 0.068730886 | 0.138058286 | 0.069311068 | 0.134755363 |
| AGBL3 | red | -0.136216806 | 0.003182467 | -0.137873342 | 0.002828797 |
| AGBL4 | red | -0.291292077 | 1.38E-10 | -0.284735363 | 3.68E-10 |
| AL02380( | red | -0.207403458 | 6.21E-06 | -0.19865805 | 1.53E-05 |
| AL04979 | red | -0.058468266 | 0.207235758 | -0.044958732 | 0.332319265 |
| AL04987 | red | -0.060672413 | 0.190590538 | -0.063353638 | 0.17169179 |
| AL10965 | red | 0.0465842 | 0.315118113 | 0.024890045 | 0.591599188 |
| AL13716 | red | -0.07597696 | 0.101037034 | -0.05695507 | 0.219256194 |
| AL13870( | red | 0.031347412 | 0.499184194 | 0.016273353 | 0.725775243 |
| AL13875 | red | 0.005291371 | 0.90920463 | -0.002237374 | 0.961540471 |
| AL13933 | red | -0.062459103 | 0.177834953 | -0.061359034 | 0.185611386 |
| AL35613 | red | -0.005475668 | 0.906056596 | -0.011071184 | 0.811403008 |
| AL35658 | red | -0.026071896 | 0.574113262 | -0.015803893 | 0.733381378 |
| AL36520 | red | -0.100972531 | 0.029126826 | -0.093298661 | 0.043884206 |
| AL44518 | red | -0.034191015 | 0.461055789 | -0.024978738 | 0.590278507 |
| AL59622( | red | -0.011786192 | 0.799473931 | 0.002080163 | 0.964241001 |
| AMZ1 | red | -0.175093765 | 0.000142904 | -0.174040291 | 0.000156924 |
| ANGPTL | red | -0.09387483 | 0.042591375 | -0.081787187 | 0.077453175 |
| ANGPTL | red | -0.093572698 | 0.043265251 | -0.087556978 | 0.058666556 |
| ANKAR | red | -0.164302704 | 0.000363365 | -0.16090185 | 0.000481993 |
| ANKHD1 | red | 0.004386795 | 0.924675578 | 0.015377382 | 0.740314369 |
| ANKHD1 | red | 0.251140464 | 3.78E-08 | 0.24912482 | 4.88E-08 |
| ANKRD2 | red | -0.062585853 | 0.176954719 | -0.06168193 | 0.183303277 |
| ANKRD3 | red | -0.249573055 | 4.61E-08 | -0.243514625 | 9.89E-08 |
| ANKRD3 | red | -0.109234642 | 0.018209803 | -0.107667469 | 0.01995213 |
| ANKRD3 | red | -0.078040365 | 0.092081853 | -0.082333056 | 0.075488823 |
| ANKRD3 | red | -0.082729914 | 0.074086296 | -0.074359905 | 0.108527462 |
| ANKRD5 | red | -0.076652193 | 0.098033378 | -0.077114 | 0.096020374 |
| ANKRD6 | red | -0.088865139 | 0.054978836 | -0.076310018 | 0.099546478 |
| ANKRD6 | red | -0.127467594 | 0.005807152 | -0.124711518 | 0.006968249 |
| ANXA10 | red | -0.042845889 | 0.355564219 | -0.030450692 | 0.511545095 |
| AP00086( | red | -0.13599817 | 0.003232033 | -0.141821966 | 0.002125391 |
| APLF | red | -0.214853141 | 2.79E-06 | -0.239721756 | 1.58E-07 |
| APOBEC | red | -0.07742279 | 0.094692862 | -0.079350389 | 0.086734139 |
| ARG1 | red | -0.06252085 | 0.177405732 | -0.060500033 | 0.191855842 |
| ARHGAP | red | -0.128598962 | 0.005383156 | -0.123362637 | 0.007608965 |
| ARHGAP | red | 0.066529869 | 0.151155963 | 0.032075456 | 0.489265976 |
| ARHGDI | red | 0.129611261 | 0.005027624 | 0.130230823 | 0.004820607 |
| ARL13A | red | -0.065379039 | 0.158369643 | -0.059477939 | 0.199484862 |
| ARL17A | red | -0.037699371 | 0.416335074 | -0.038322235 | 0.408671005 |
| ARL17B | red | -0.055943391 | 0.22756615 | -0.08002363 | 0.084084724 |
| ASB14 | red | -0.02892988 | 0.532867271 | -0.024510192 | 0.597270577 |
| ASPDH | red | -0.13615602 | 0.003196178 | -0.124329364 | 0.007144679 |

| | | | | | |
|---|---|---|---|---|---|
| ASZ1 | red | -0.084841314 | 0.066977244 | -0.08515087 | 0.065983651 |
| ATP2A1 | red | -0.017759748 | 0.701873214 | -0.016029238 | 0.729727058 |
| ATP5L2 | red | -0.030116536 | 0.516191702 | -0.007570204 | 0.870394501 |
| ATRIP | red | -0.021558732 | 0.642150904 | -0.045724123 | 0.324145715 |
| B3GALT2 | red | -0.045960427 | 0.321648836 | -0.031104014 | 0.50252362 |
| B3GAT2 | red | -0.161632758 | 0.000453809 | -0.138861102 | 0.002635327 |
| BBS1 | red | -0.138241905 | 0.002755145 | -0.157546302 | 0.000633521 |
| BCL7B | red | 0.00913989 | 0.843835766 | 0.024109705 | 0.60327661 |
| BLID | red | -0.089253043 | 0.053923108 | -0.082538348 | 0.074760629 |
| BOD1L2 | red | -0.038251631 | 0.409535533 | -0.034657916 | 0.454954197 |
| BOLL | red | -0.078369214 | 0.090715236 | -0.05987884 | 0.196466575 |
| BTBD18 | red | -0.095132425 | 0.039880723 | -0.07873667 | 0.089207464 |
| BTBD8 | red | -0.098657637 | 0.033048117 | -0.108251478 | 0.01928659 |
| C10orf11 | red | -0.083046809 | 0.072981672 | -0.074077081 | 0.109881441 |
| C10orf13 | red | -0.050773909 | 0.273516944 | -0.051724669 | 0.264623028 |
| C11orf42 | red | -0.015314002 | 0.74134644 | -0.018840659 | 0.68467281 |
| C11orf65 | red | -0.03762982 | 0.417196087 | -0.032155462 | 0.488182534 |
| C11orf68 | red | -0.080036505 | 0.084034701 | -0.074141383 | 0.109572443 |
| C12orf40 | red | -0.062802966 | 0.175454473 | -0.055376922 | 0.232315687 |
| C12orf71 | red | -0.067032138 | 0.14808698 | -0.05704035 | 0.218565762 |
| C12orf79 | red | -0.000340916 | 0.994137582 | 0.00854438 | 0.853892543 |
| C14orf18 | red | -0.064282472 | 0.165482079 | -0.055906769 | 0.227871104 |
| C14orf39 | red | -0.14359916 | 0.001864385 | -0.147207826 | 0.00142244 |
| C17orf78 | red | -0.07359641 | 0.112213064 | -0.062710269 | 0.176093847 |
| C18orf63 | red | -0.112623644 | 0.014889867 | -0.102169336 | 0.027260932 |
| C19orf67 | red | -0.023517857 | 0.612201629 | -0.02148278 | 0.643324163 |
| C1orf100 | red | 0.045954821 | 0.321707929 | 0.055544668 | 0.230901977 |
| C1orf105 | red | -0.035462356 | 0.444548658 | -0.034492796 | 0.457106826 |
| C1orf111 | red | -0.048436254 | 0.296242882 | -0.053155215 | 0.251618902 |
| C1orf146 | red | -0.102656616 | 0.026531216 | -0.10530537 | 0.022851951 |
| C1orf195 | red | -0.107581471 | 0.020051803 | -0.097784197 | 0.034640485 |
| C1QTNF | red | -0.085076338 | 0.066221766 | -0.078913613 | 0.088488631 |
| C20orf14 | red | -0.045516233 | 0.326352737 | -0.032939071 | 0.477639396 |
| C3orf30 | red | -0.149771857 | 0.001169354 | -0.132037025 | 0.004260236 |
| C3orf49 | red | -0.088082561 | 0.05716104 | -0.080866195 | 0.080861382 |
| C4orf45 | red | -0.126206663 | 0.006314868 | -0.113700933 | 0.013952251 |
| C4orf51 | red | -0.141696169 | 0.002145076 | -0.131466731 | 0.004430457 |
| C6orf10 | red | -0.07173441 | 0.121614091 | -0.07339622 | 0.113195536 |
| C6orf163 | red | 0.021724555 | 0.63959245 | 0.023118396 | 0.618258183 |
| C7orf25 | red | -0.148776376 | 0.001262233 | -0.145501578 | 0.001617777 |
| C8orf44-S | red | -0.107847533 | 0.019744829 | -0.087686909 | 0.058291352 |
| C9orf131 | red | -0.080128349 | 0.083678555 | -0.060120845 | 0.194660794 |
| C9orf153 | red | -0.095782609 | 0.038537481 | -0.082845887 | 0.073680471 |
| C9orf47 | red | -0.123839699 | 0.007376582 | -0.128986705 | 0.005244385 |
| C9orf84 | red | -0.048165054 | 0.298958668 | -0.037081838 | 0.424016359 |

| | | | | | |
|---|---|---|---|---|---|
| CA1 | red | -0.103593127 | 0.025175898 | -0.094684006 | 0.040830029 |
| CACNB4 | red | -0.075371045 | 0.103794133 | -0.087345048 | 0.059282839 |
| CALCR | red | -0.041992497 | 0.365236049 | -0.032007776 | 0.490183501 |
| CAPZB | red | 0.109970533 | 0.017438535 | 0.130637995 | 0.004688777 |
| CARD18 | red | -0.14160659 | 0.002159195 | -0.135585954 | 0.003327394 |
| CAV3 | red | -0.121372601 | 0.008650453 | -0.11221549 | 0.0152593 |
| CBWD3 | red | -0.089074829 | 0.054406025 | -0.090190706 | 0.051440641 |
| CC2D2B | red | -0.110240607 | 0.017162725 | -0.105283512 | 0.022880405 |
| CCDC144 | red | -0.069641252 | 0.13290303 | -0.063771717 | 0.168875547 |
| CCDC158 | red | -0.286907715 | 2.67E-10 | -0.287446262 | 2.46E-10 |
| CCDC175 | red | -0.062367304 | 0.178474488 | -0.060980019 | 0.188347897 |
| CCDC179 | red | -0.037970727 | 0.412985817 | -0.030340265 | 0.513078223 |
| CCDC38 | red | -0.181088412 | 8.30E-05 | -0.181416888 | 8.06E-05 |
| CCDC54 | red | -0.1456575 | 0.001598955 | -0.133603301 | 0.003822723 |
| CCDC62 | red | -0.01966982 | 0.671587511 | 0.010380339 | 0.822970616 |
| CCDC7 | red | 0.141873754 | 0.002117335 | 0.138067953 | 0.002789686 |
| CCDC70 | red | -0.083764003 | 0.070531263 | -0.089989102 | 0.051966185 |
| CCDC73 | red | -0.149110917 | 0.001230289 | -0.138833789 | 0.00264051 |
| CCDC9 | red | 0.047933699 | 0.3012885 | 0.040439495 | 0.383252267 |
| CCR6 | red | -0.050226478 | 0.278729298 | -0.032778434 | 0.479790533 |
| CCSER1 | red | -0.056257455 | 0.224962814 | -0.063898975 | 0.168025213 |
| CD160 | red | -0.096952059 | 0.036217857 | -0.084633664 | 0.067650601 |
| CD200R1 | red | -0.062253767 | 0.179267823 | -0.042139869 | 0.363554219 |
| CDC37 | red | -0.003978883 | 0.931661746 | 0.007490615 | 0.871745278 |
| CDH9 | red | -0.055697666 | 0.229617876 | -0.043333499 | 0.350110927 |
| CDKL3 | red | -0.127417292 | 0.005826681 | -0.119169501 | 0.009949941 |
| CDKL4 | red | -0.031908059 | 0.491537034 | -0.042249139 | 0.362310348 |
| CDR1 | red | -0.055340248 | 0.232625576 | -0.038474525 | 0.406809971 |
| CDRT15L | red | -0.069810264 | 0.131962505 | -0.06678731 | 0.149576958 |
| CDRT4 | red | -0.01861157 | 0.688305045 | -0.018856441 | 0.68442285 |
| CEACAM | red | 0.014472742 | 0.755088592 | 0.011654475 | 0.801668068 |
| CECR2 | red | -0.244837431 | 8.39E-08 | -0.256331477 | 1.92E-08 |
| CELA2B | red | -0.185282317 | 5.62E-05 | -0.19371468 | 2.50E-05 |
| CELF6 | red | -0.05249929 | 0.257525254 | -0.048132371 | 0.299287067 |
| CFHR4 | red | -0.104738602 | 0.023599743 | -0.088204327 | 0.056816854 |
| CHRM5 | red | -0.076965088 | 0.096665842 | -0.075849355 | 0.10161279 |
| CHRNG | red | -0.077426963 | 0.09467502 | -0.065604867 | 0.156933983 |
| CLCA1 | red | -0.063312386 | 0.17197156 | -0.048221596 | 0.298391091 |
| CLDN18 | red | -0.024342965 | 0.599775157 | -0.018866274 | 0.684267134 |
| CLDN20 | red | -0.08169124 | 0.0778027 | -0.070677104 | 0.127219275 |
| CNPPD1 | red | 0.132359892 | 0.00416651 | 0.135401224 | 0.003370952 |
| COL24A1 | red | -0.152368835 | 0.000955879 | -0.135480312 | 0.003352241 |
| COL6A6 | red | -0.282240752 | 5.31E-10 | -0.281919331 | 5.57E-10 |
| COPS7A | red | 0.108637223 | 0.018857674 | 0.124421097 | 0.007101968 |
| CPNE6 | red | -0.139815129 | 0.002459987 | -0.128517366 | 0.005412776 |

| | | | | | |
|---|---|---|---|---|---|
| CREB3 | red | 0.174019198 | 0.000157217 | 0.206317438 | 6.96E-06 |
| CRYBA1 | red | -0.087205822 | 0.05969061 | -0.095012978 | 0.04013175 |
| CSMD2 | red | 0.018416481 | 0.691403886 | 0.012143156 | 0.793535625 |
| CSMD3 | red | -0.094422759 | 0.041391837 | -0.09571158 | 0.038682332 |
| CSNK1A | red | 0.012470423 | 0.78810179 | 0.004188329 | 0.928073945 |
| CTC-454I | red | -0.100304535 | 0.030215148 | -0.095685471 | 0.038735692 |
| CTC-487I | red | -0.053079807 | 0.252293085 | -0.066308702 | 0.152522561 |
| CTD-211( | red | -0.043892753 | 0.34392188 | -0.030061539 | 0.516958548 |
| CTD-231: | red | -0.140775518 | 0.002294288 | -0.150146915 | 0.001136024 |
| CTD-237( | red | -0.084057739 | 0.069547279 | -0.079693597 | 0.085375233 |
| CTD-252: | red | -0.016644574 | 0.719779736 | -0.02785436 | 0.548212262 |
| CTD-322: | red | -0.07102577 | 0.125349137 | -0.059706506 | 0.197759927 |
| CTDSP1 | red | -0.027391659 | 0.554880154 | -0.036230481 | 0.43474044 |
| CTRL | red | -0.10178426 | 0.027849743 | -0.08235098 | 0.075425016 |
| CYP19A1 | red | -0.108597249 | 0.018901731 | -0.108163167 | 0.019385972 |
| CYP2A7 | red | -0.110358622 | 0.017043407 | -0.081701109 | 0.077766692 |
| CYP2C19 | red | -0.032623255 | 0.481873575 | -0.037136973 | 0.423327213 |
| CYP4A22 | red | -0.089043065 | 0.054492474 | -0.079209343 | 0.087297622 |
| CYP7B1 | red | -0.285087027 | 3.50E-10 | -0.292474155 | 1.16E-10 |
| CYP8B1 | red | -0.13341541 | 0.003872967 | -0.108631928 | 0.018863504 |
| DCAF4L1 | red | -0.007323197 | 0.874587898 | 0.000738789 | 0.98729615 |
| DCDC2C | red | -0.157906363 | 0.000615365 | -0.140490066 | 0.002342441 |
| DCLRE1( | red | -0.064402864 | 0.164689694 | -0.066507222 | 0.151295469 |
| DDI1 | red | -0.088059696 | 0.057225864 | -0.082047932 | 0.076509746 |
| DEFB108 | red | -0.070828843 | 0.126402749 | -0.07475089 | 0.106677393 |
| DEFB113 | red | 0.017906904 | 0.699522335 | 0.034521399 | 0.456733526 |
| DEFB134 | red | 0.017860873 | 0.700257389 | 0.014093742 | 0.761305257 |
| DEFB135 | red | -0.074483728 | 0.107938837 | -0.070000058 | 0.130912471 |
| DGKH | red | -0.030258108 | 0.514220419 | -0.041064416 | 0.375938328 |
| DIRC1 | red | -0.069390215 | 0.134309545 | -0.06051629 | 0.191736249 |
| DKFZp31 | red | -0.044628424 | 0.335887233 | -0.051766938 | 0.264232291 |
| DLEU1 | red | 0.096750557 | 0.036608866 | 0.079726147 | 0.085247251 |
| DLEU7 | red | -0.117700295 | 0.0109102 | -0.103163726 | 0.025789727 |
| DMP1 | red | -0.056983443 | 0.219026312 | -0.049992765 | 0.280974967 |
| DNAH14 | red | -0.000443313 | 0.992376806 | -0.013199274 | 0.776037432 |
| DNAJB7 | red | -0.123304922 | 0.007637518 | -0.111592658 | 0.015838551 |
| DNHD1 | red | -0.186409867 | 5.05E-05 | -0.167033739 | 0.000288451 |
| DNM3 | red | -0.178777654 | 0.000102582 | -0.173188191 | 0.000169197 |
| DRD3 | red | -0.061049844 | 0.187841539 | -0.054722842 | 0.237886517 |
| DSPP | red | -0.056651559 | 0.221726108 | -0.03402524 | 0.463233096 |
| EBLN2 | red | -0.034309054 | 0.459508919 | -0.05328232 | 0.250485348 |
| EDDM3B | red | -0.139095807 | 0.002591165 | -0.121550035 | 0.008552688 |
| EFCAB13 | red | -0.084566931 | 0.067868177 | -0.094263307 | 0.041737935 |
| EFCAB5 | red | -0.020304885 | 0.661631712 | -0.007804863 | 0.866414087 |
| EMD | red | -0.029092945 | 0.530559782 | -0.026781138 | 0.563738287 |

| | | | | | |
|---|---|---|---|---|---|
| EML6 | red | 0.000609928 | 0.989511844 | -0.010146259 | 0.826898923 |
| ENAM | red | -0.142561723 | 0.002012936 | -0.147035643 | 0.00144112 |
| EPB42 | red | -0.060182599 | 0.19420195 | -0.04831686 | 0.297436453 |
| EPGN | red | -0.024598801 | 0.595945398 | -0.040366776 | 0.384108953 |
| EPPIN-W | red | 0.050571031 | 0.275440839 | 0.060060192 | 0.19511223 |
| EQTN | red | -0.188312639 | 4.22E-05 | -0.187806386 | 4.43E-05 |
| ERC2 | red | -0.126853024 | 0.006049838 | -0.147782198 | 0.001361724 |
| ERICH6B | red | -0.008963592 | 0.846810473 | -0.012408603 | 0.789127447 |
| ERVFRD- | red | -0.163986883 | 0.000373111 | -0.153778747 | 0.000855656 |
| ERVV-1 | red | 0.02628083 | 0.571047568 | 0.030094592 | 0.516497603 |
| ERVW-1 | red | -0.004687194 | 0.919534411 | 0.006201985 | 0.893665007 |
| ESR2 | red | -0.08991369 | 0.052163916 | -0.063680453 | 0.169487345 |
| ESYT3 | red | -0.266099371 | 5.20E-09 | -0.287784121 | 2.34E-10 |
| EYS | red | -0.109658938 | 0.017761554 | -0.096676041 | 0.036754369 |
| FABP12 | red | 0.019418883 | 0.675537397 | 0.0047367 | 0.918687459 |
| FAM106A | red | -0.09141549 | 0.048342321 | -0.077529537 | 0.09423737 |
| FAM127E | red | -0.020320044 | 0.661394785 | -0.013237184 | 0.775411371 |
| FAM184E | red | -0.178370806 | 0.000106442 | -0.175755281 | 0.000134711 |
| FAM186E | red | -0.103884652 | 0.024766383 | -0.094505716 | 0.041212737 |
| FAM196A | red | 0.010170695 | 0.826488637 | 0.014784656 | 0.749984134 |
| FAM217A | red | 0.015778023 | 0.733801286 | 0.035370404 | 0.445731181 |
| FAM24A | red | -0.012001747 | 0.795886632 | 0.001270739 | 0.978150819 |
| FAM46D | red | -0.10653949 | 0.021294321 | -0.097943842 | 0.03434465 |
| FANCD2( | red | 0.027698519 | 0.550453656 | 0.024757533 | 0.593574854 |
| FAT3 | red | -0.203067313 | 9.75E-06 | -0.211461684 | 4.03E-06 |
| FBXO40 | red | -0.136384129 | 0.003145003 | -0.126317231 | 0.006268806 |
| FKSG61 | red | -0.062932113 | 0.17456657 | -0.09501797 | 0.040121233 |
| FKSG68 | red | -0.076005406 | 0.10090904 | -0.061590247 | 0.183956476 |
| FKSG70 | red | -0.106139949 | 0.021788191 | -0.110517412 | 0.016884008 |
| FLG2 | red | -0.064283421 | 0.165475824 | -0.060081597 | 0.194952822 |
| FNDC9 | red | -0.099124918 | 0.032222161 | -0.073407431 | 0.113140338 |
| FOXD4L5 | red | 0.013370996 | 0.773202691 | 0.016435904 | 0.723147824 |
| FOXR1 | red | -0.077650169 | 0.093724744 | -0.081745578 | 0.077604598 |
| FP325331 | red | -0.039393985 | 0.395681303 | -0.036780253 | 0.427797509 |
| FPGT-TN | red | -0.081265399 | 0.079369479 | -0.093106602 | 0.044322432 |
| FSBP | red | -0.057797216 | 0.212506245 | -0.062372443 | 0.178438638 |
| FSIP2 | red | -0.080893951 | 0.080756922 | -0.080825938 | 0.08101308 |
| FUT9 | red | -0.125250834 | 0.006725897 | -0.11865569 | 0.010276873 |
| GABRB2 | red | -0.152847696 | 0.000920684 | -0.166444185 | 0.000303283 |
| GABRR2 | red | -0.171556834 | 0.000195244 | -0.185591834 | 5.46E-05 |
| GALNTL | red | -0.084611621 | 0.067722408 | -0.078826339 | 0.088842599 |
| GAPDHS | red | -0.103194452 | 0.025745379 | -0.104067544 | 0.024512415 |
| GBP7 | red | -0.082391104 | 0.075282336 | -0.054346516 | 0.241133985 |
| GCM2 | red | -0.110749568 | 0.016653308 | -0.103138259 | 0.025826534 |
| GCNT7 | red | -0.100446448 | 0.02998107 | -0.089191759 | 0.054088767 |

| | | | | | |
|---|---|---|---|---|---|
| GCSAM | red | -0.058287531 | 0.20864587 | -0.033605068 | 0.468777192 |
| GDI1 | red | 0.000904598 | 0.984445318 | -0.001694468 | 0.970868011 |
| GDPD4 | red | -0.112844943 | 0.014692864 | -0.115787317 | 0.012283061 |
| GHRL | red | -0.099880295 | 0.030924269 | -0.098459461 | 0.033403827 |
| GHSR | red | -0.06079175 | 0.18971818 | -0.068914987 | 0.137003549 |
| GIF | red | -0.077500509 | 0.09436106 | -0.071237256 | 0.124225243 |
| GJA9 | red | -0.018254568 | 0.693979668 | -0.004695213 | 0.919397222 |
| GJE1 | red | -0.097530519 | 0.035115031 | -0.091103466 | 0.049116423 |
| GLIPR1L | red | -0.134474655 | 0.00359734 | -0.129866396 | 0.004941424 |
| GNB2 | red | 0.164274005 | 0.00036424 | 0.206886827 | 6.55E-06 |
| GNRHR | red | -0.071550779 | 0.122573526 | -0.067306678 | 0.146429657 |
| GOLGA8 | red | -0.080721343 | 0.081408303 | -0.077179145 | 0.095739083 |
| GPC5 | red | -0.14906536 | 0.001234595 | -0.1486253 | 0.001276908 |
| GPR113 | red | -0.195530661 | 2.09E-05 | -0.189981185 | 3.59E-05 |
| GPR18 | red | -0.036418902 | 0.432353636 | -0.006646884 | 0.886087207 |
| GPR21 | red | -0.073265571 | 0.113840359 | -0.060509156 | 0.191788721 |
| GPR22 | red | -0.140629793 | 0.002318756 | -0.132408707 | 0.004152502 |
| GPR52 | red | -0.069863239 | 0.131668772 | -0.067585916 | 0.144758518 |
| GPR61 | red | -0.220445889 | 1.51E-06 | -0.203099717 | 9.71E-06 |
| GPR82 | red | -0.121092155 | 0.008807008 | -0.096844819 | 0.03642551 |
| GREB1L | red | 0.029199412 | 0.529055948 | 0.018540181 | 0.689438405 |
| GRIA4 | red | -0.078269036 | 0.091129815 | -0.066592088 | 0.150773191 |
| GRIK1 | red | -0.161645323 | 0.000453338 | -0.156762528 | 0.000674772 |
| GRIN2B | red | -0.163683101 | 0.000382715 | -0.183157746 | 6.86E-05 |
| GRM5 | red | -0.031260039 | 0.500381594 | -0.021726201 | 0.639567066 |
| GS1-114I | red | -0.028759655 | 0.535281455 | -0.030272137 | 0.514025286 |
| GSK3A | red | 0.019714049 | 0.670892246 | 0.013876063 | 0.764882799 |
| GUCY2C | red | -0.133599842 | 0.003823642 | -0.114491928 | 0.013297281 |
| GYPA | red | -0.112038906 | 0.015421609 | -0.108350094 | 0.019176136 |
| GYPB | red | -0.101296937 | 0.02861054 | -0.098494327 | 0.033341009 |
| GYPE | red | -0.205611976 | 7.49E-06 | -0.205920083 | 7.25E-06 |
| H1FX | red | -0.017626392 | 0.7040061 | -0.027976797 | 0.546454482 |
| HCRTR1 | red | -0.099304592 | 0.0319093 | -0.099297088 | 0.031922313 |
| HEPHL1 | red | -0.193543217 | 2.54E-05 | -0.16818871 | 0.000261335 |
| HIGD1C | red | -0.071303664 | 0.12387395 | -0.059866699 | 0.196557491 |
| HIST1H1 | red | -0.087447444 | 0.058984411 | -0.080621836 | 0.081785744 |
| HIST1H2 | red | -0.070685016 | 0.127176602 | -0.060052861 | 0.195166841 |
| HIST3H3 | red | 0.014216425 | 0.759291213 | 0.030442399 | 0.51166016 |
| HIST4H4 | red | -0.170142588 | 0.000220818 | -0.142905398 | 0.001962567 |
| HMGB4 | red | -0.071462412 | 0.123037328 | -0.053808098 | 0.245834121 |
| HRH4 | red | -0.140613188 | 0.00232156 | -0.13301597 | 0.003981776 |
| HRNR | red | -0.173291622 | 0.000167661 | -0.172801973 | 0.000175051 |
| HTR1E | red | -0.242487583 | 1.12E-07 | -0.252840095 | 3.03E-08 |
| HTR1F | red | -0.103873382 | 0.024782106 | -0.093125326 | 0.044279547 |
| IAPP | red | -0.094127255 | 0.042035169 | -0.078518767 | 0.090099133 |

| | | | | | |
|---|---|---|---|---|---|
| IFIT1B | red | -0.126397992 | 0.006235352 | -0.114465304 | 0.013318878 |
| IFNA8 | red | -0.082985586 | 0.073194029 | -0.068129559 | 0.141546855 |
| IFNK | red | -0.082424934 | 0.075162211 | -0.068576006 | 0.138950435 |
| IFNW1 | red | -0.062248052 | 0.179307823 | -0.061450373 | 0.184956324 |
| IL1RAPL: | red | -0.072238987 | 0.1190079 | -0.075613213 | 0.102685127 |
| IL1RL1 | red | -0.040145814 | 0.386719246 | -0.037856067 | 0.414399086 |
| IL5 | red | -0.100263132 | 0.030283735 | -0.09947891 | 0.031608248 |
| IMPG1 | red | -0.057323471 | 0.21628475 | -0.054352952 | 0.241078192 |
| INSL6 | red | -0.051488988 | 0.266808968 | -0.052833845 | 0.254500843 |
| IRF2BP1 | red | -0.074358362 | 0.108534815 | -0.089284985 | 0.053836928 |
| IRGM | red | -0.135194201 | 0.003420382 | -0.131905966 | 0.004298822 |
| ISPD | red | -0.243367695 | 1.01E-07 | -0.237710065 | 2.02E-07 |
| JMJD7 | red | 0.116786324 | 0.011548179 | 0.108911499 | 0.018557781 |
| KCNA7 | red | -0.158239773 | 0.000598985 | -0.153842083 | 0.000851391 |
| KCNG3 | red | -0.117005489 | 0.01139226 | -0.139493737 | 0.00251783 |
| KCNH8 | red | -0.254747653 | 2.37E-08 | -0.272361159 | 2.18E-09 |
| KCNJ13 | red | -0.126004766 | 0.006399762 | -0.129341161 | 0.005120356 |
| KCNMB3 | red | -0.273346052 | 1.90E-09 | -0.273034174 | 1.99E-09 |
| KCNS2 | red | -0.176507752 | 0.000125928 | -0.171364274 | 0.000198555 |
| KCNV2 | red | -0.13507091 | 0.003450131 | -0.129553697 | 0.00504726 |
| KCTD4 | red | -0.078916292 | 0.088477783 | -0.067120964 | 0.147549205 |
| KDELR1 | red | 0.139522078 | 0.00251268 | 0.13669859 | 0.003075675 |
| KIAA040 | red | -0.125298812 | 0.006704708 | -0.11279308 | 0.014738827 |
| KIAA173 | red | -0.153393655 | 0.000882019 | -0.143228862 | 0.001916216 |
| KIAA191 | red | -0.075358325 | 0.103852645 | -0.060646227 | 0.190782354 |
| KLC1 | red | -0.116983848 | 0.011407572 | -0.105137443 | 0.023071343 |
| KRTAP2( | red | -0.066504956 | 0.151309435 | -0.057447606 | 0.215290046 |
| LA16c-43 | red | -0.013824952 | 0.765723532 | 0.007021884 | 0.879707992 |
| LCTL | red | -0.186477069 | 5.02E-05 | -0.18489532 | 5.83E-05 |
| LDHAL6 | red | -0.129078216 | 0.005212108 | -0.119647312 | 0.009654237 |
| LDHAL6l | red | -0.088152928 | 0.056961929 | -0.083113565 | 0.072750698 |
| LENEP | red | -0.013676325 | 0.768169877 | -0.016169455 | 0.727456292 |
| LGSN | red | -0.125882931 | 0.006451486 | -0.089107183 | 0.054318087 |
| LHCGR | red | -0.155549053 | 0.000743574 | -0.147123442 | 0.001431567 |
| LHX4 | red | -0.031529694 | 0.496691 | -0.037633069 | 0.417155835 |
| LIPI | red | -0.199452332 | 1.41E-05 | -0.193268763 | 2.61E-05 |
| LIPN | red | -0.050954084 | 0.271816035 | -0.050155392 | 0.279411046 |
| LRIT3 | red | 0.010082643 | 0.827967266 | 0.003640735 | 0.937457091 |
| LRP1B | red | -0.111441771 | 0.015981747 | -0.106277598 | 0.021616934 |
| LRRC19 | red | -0.129746284 | 0.004981838 | -0.122380227 | 0.008108219 |
| LRRC37A | red | -0.058513841 | 0.206881264 | -0.082209423 | 0.075930139 |
| LRRC37A | red | -0.067674431 | 0.144231844 | -0.085220204 | 0.065762772 |
| LRRC69 | red | -0.043744965 | 0.345550584 | -0.037805943 | 0.415017791 |
| LRRC7 | red | -0.095859527 | 0.038381137 | -0.099718046 | 0.031199209 |
| LRRC75A | red | -0.332510767 | 1.62E-13 | -0.333305202 | 1.40E-13 |

| | | | | | |
|---|---|---|---|---|---|
| LRRC9 | red | -0.2003494 | 1.29E-05 | -0.184845013 | 5.86E-05 |
| LRRD1 | red | -0.100399679 | 0.030058041 | -0.091622547 | 0.047834292 |
| LRRN3 | red | -0.038561707 | 0.405746837 | -0.011211482 | 0.809058774 |
| LRRTM2 | red | -0.129540312 | 0.005051835 | -0.123622908 | 0.007481385 |
| LRRTM4 | red | -0.106692508 | 0.021107768 | -0.105267545 | 0.022901211 |
| LSMEM1 | red | -0.056279395 | 0.224781747 | -0.068689592 | 0.138295717 |
| LYG2 | red | -0.119271798 | 0.009885965 | -0.107674593 | 0.019943893 |
| LZTS2 | red | -0.003865637 | 0.93360222 | -0.0062536 | 0.892785364 |
| MAB21L | red | -0.170370475 | 0.000216495 | -0.164640377 | 0.000353207 |
| MAB21L | red | -0.085433614 | 0.065086727 | -0.073158484 | 0.114371033 |
| MAB21L | red | -0.005436971 | 0.906717469 | -0.008064496 | 0.862013987 |
| MALRD1 | red | -0.209954847 | 4.74E-06 | -0.209236929 | 5.11E-06 |
| MASP2 | red | -0.135783142 | 0.003281463 | -0.145173958 | 0.00165799 |
| MATR3 | red | -0.104202321 | 0.024326704 | -0.114490894 | 0.013298119 |
| MCF2L2 | red | -0.208982495 | 5.25E-06 | -0.194090949 | 2.41E-05 |
| MCMDC | red | -0.216012736 | 2.46E-06 | -0.224034757 | 1.00E-06 |
| MCTP1 | red | -0.029371717 | 0.526626716 | -0.038360736 | 0.408200032 |
| MDGA2 | red | -0.116680318 | 0.01162427 | -0.111365098 | 0.016054946 |
| MEMO1 | red | -0.137565151 | 0.002891756 | -0.15240027 | 0.000953531 |
| MEP1B | red | -0.017356908 | 0.708323285 | -0.020223794 | 0.662899698 |
| METTL2 | red | -0.166214295 | 0.000309258 | -0.160943967 | 0.000480326 |
| MGAM | red | -0.12246638 | 0.008063296 | -0.1166711 | 0.011630907 |
| MIA2 | red | 0.024443292 | 0.598271976 | 0.011002904 | 0.812544517 |
| MLANA | red | -0.062106774 | 0.18029881 | -0.0618028 | 0.182444751 |
| MLF2 | red | 0.056035529 | 0.226800191 | 0.064986273 | 0.160890163 |
| MLN | red | -0.134726547 | 0.003534469 | -0.134207466 | 0.003665135 |
| MMP21 | red | -0.102860929 | 0.026230293 | -0.081928102 | 0.07694215 |
| MOSPD3 | red | 0.072021785 | 0.120124371 | 0.092906845 | 0.044782119 |
| MPG | red | 0.318739407 | 1.73E-12 | 0.343764187 | 2.12E-14 |
| MRAP | red | -0.106636832 | 0.02117548 | -0.095495587 | 0.039125652 |
| MRGPRX | red | -0.115637581 | 0.012396695 | -0.107681059 | 0.019936419 |
| MRPL10 | red | 0.027870609 | 0.54797883 | 0.015530034 | 0.737830549 |
| MS4A12 | red | -0.078319925 | 0.090919027 | -0.060897179 | 0.188949933 |
| MS4A13 | red | -0.115598776 | 0.012426295 | -0.106409201 | 0.021454292 |
| MS4A4E | red | -0.132990967 | 0.003988678 | -0.130600293 | 0.004700845 |
| MS4A5 | red | -0.017656461 | 0.703524972 | -0.011821613 | 0.798884149 |
| MSANTD | red | -0.137466831 | 0.002912108 | -0.128842353 | 0.005295667 |
| MTMR7 | red | -0.151303823 | 0.001038652 | -0.140935694 | 0.002267666 |
| MTNR1A | red | -0.105364073 | 0.022775684 | -0.10318303 | 0.025761858 |
| MTRNR2 | red | 0.007578981 | 0.870245557 | 0.026999513 | 0.560562057 |
| MTRNR2 | red | -0.080400219 | 0.082631446 | -0.07194136 | 0.120539841 |
| MUSK | red | -0.05581092 | 0.228670622 | -0.047283678 | 0.307898796 |
| MYH1 | red | -0.081422076 | 0.078790076 | -0.067485013 | 0.1453607 |
| MYH13 | red | -0.073026592 | 0.1150273 | -0.068374366 | 0.140118555 |
| MYH2 | red | -0.118026096 | 0.010690447 | -0.114507517 | 0.013284649 |

| | | | | | |
|---|---|---|---|---|---|
| MYH4 | red | -0.089594734 | 0.053007175 | -0.077005409 | 0.09649073 |
| MYO1H | red | 0.027151435 | 0.558357496 | 0.012813068 | 0.782423636 |
| NAA11 | red | -0.171368128 | 0.000198488 | -0.162775201 | 0.000412809 |
| NABP1 | red | -0.134955007 | 0.00347831 | -0.158853444 | 0.000569888 |
| NAIP | red | -0.087058449 | 0.060124763 | -0.07533415 | 0.103963922 |
| NBEAL1 | red | -0.060735723 | 0.190127372 | -0.057794352 | 0.212528944 |
| NCR3LG | red | -0.113764806 | 0.013898328 | -0.125107806 | 0.006789423 |
| NEB | red | -0.083444836 | 0.071613314 | -0.068406667 | 0.139930927 |
| NME1-NI | red | 0.071973817 | 0.120372038 | 0.043015635 | 0.35365979 |
| NOTCH2 | red | -0.087548781 | 0.058690295 | -0.084083454 | 0.069461674 |
| NPAP1 | red | -0.235195188 | 2.73E-07 | -0.237967971 | 1.95E-07 |
| NPBWR2 | red | -0.134308015 | 0.003639488 | -0.129713573 | 0.004992895 |
| NPFFR2 | red | -0.170063134 | 0.000222344 | -0.157956828 | 0.00061286 |
| NPHP3-A | red | -0.074381567 | 0.108424308 | -0.086366038 | 0.062199656 |
| NPIPB4 | red | -0.067642033 | 0.144424443 | -0.052320355 | 0.259153005 |
| NPIPB8 | red | -0.047696946 | 0.303685134 | -0.032723094 | 0.480532821 |
| NT5C1B | red | -0.071192095 | 0.124464577 | -0.072947577 | 0.115421871 |
| NUCB1 | red | 0.03944487 | 0.395070809 | 0.043736106 | 0.345648373 |
| NUDT13 | red | 0.160208254 | 0.000510236 | 0.148056693 | 0.001333557 |
| NUP210L | red | -0.059220203 | 0.201443046 | -0.049260397 | 0.288091204 |
| NUTM2A | red | -0.05011383 | 0.279810169 | -0.039286395 | 0.396973961 |
| NUTM2G | red | 0.000508905 | 0.991248931 | -0.005311691 | 0.908857463 |
| NXF2 | red | -0.157871639 | 0.000617095 | -0.126263458 | 0.00629117 |
| NXNL1 | red | -0.174955196 | 0.000144679 | -0.141486867 | 0.002178197 |
| NXPE1 | red | -0.052535037 | 0.257200915 | -0.041605668 | 0.369673554 |
| NXPE4 | red | 0.11248067 | 0.015018373 | 0.104544046 | 0.023861273 |
| OCLM | red | 0.008581088 | 0.853271932 | 0.00676919 | 0.88400581 |
| ODF3 | red | -0.108320543 | 0.019209176 | -0.098432402 | 0.033452646 |
| OFCC1 | red | -0.095699498 | 0.038707018 | -0.077785401 | 0.093152724 |
| OIT3 | red | -0.059197465 | 0.201616465 | -0.053730573 | 0.246516118 |
| OR10A5 | red | -0.044968679 | 0.332212191 | -0.046501576 | 0.315978133 |
| OR10AD | red | -0.090539142 | 0.05054279 | -0.09478975 | 0.040604465 |
| OR13C4 | red | -0.06716055 | 0.147310025 | -0.049213435 | 0.288551618 |
| OR13C9 | red | -0.068300255 | 0.140549779 | -0.049082969 | 0.289833299 |
| OR13D1 | red | -0.031518904 | 0.496838392 | -0.024554821 | 0.596602972 |
| OR13H1 | red | -0.077492749 | 0.094394147 | -0.061721477 | 0.183022055 |
| OR1L4 | red | -0.051270967 | 0.268842103 | -0.037108574 | 0.423682099 |
| OR1L6 | red | -0.074526083 | 0.10773807 | -0.064885037 | 0.161544698 |
| OR2A2 | red | -0.060276405 | 0.193506471 | -0.040952518 | 0.377241607 |
| OR2A42 | red | -0.169974489 | 0.000224058 | -0.174439111 | 0.000151471 |
| OR2A5 | red | -0.021621589 | 0.641180584 | -0.033926345 | 0.464534702 |
| OR2AE1 | red | -0.116023845 | 0.012105433 | -0.106857912 | 0.020907711 |
| OR2AG2 | red | -0.058627189 | 0.206001523 | -0.069935718 | 0.131267709 |
| OR2B2 | red | -0.096655698 | 0.036794175 | -0.083928791 | 0.069977851 |
| OR2D2 | red | -0.07635436 | 0.099349353 | -0.079636 | 0.085602077 |

| | | | | | |
|---|---|---|---|---|---|
| OR2D3 | red | -0.093120914 | 0.044289648 | -0.093239159 | 0.044019581 |
| OR2J2 | red | -0.15374094 | 0.000858212 | -0.150225858 | 0.001129122 |
| OR2M3 | red | -0.041156746 | 0.374865054 | -0.045723302 | 0.324154413 |
| OR2M5 | red | 0.004735709 | 0.918704411 | -0.002135595 | 0.96328875 |
| OR2T12 | red | -0.050647946 | 0.274710374 | -0.04554509 | 0.326045804 |
| OR4S2 | red | -0.067571308 | 0.144845582 | -0.067045133 | 0.148008214 |
| OR51B4 | red | -0.150800063 | 0.001080065 | -0.159636082 | 0.000534683 |
| OR51B6 | red | -0.068625564 | 0.138664486 | -0.055828653 | 0.228522555 |
| OR51I1 | red | -0.098734607 | 0.032910837 | -0.096650764 | 0.036803836 |
| OR51I2 | red | -0.032514873 | 0.483331336 | -0.033960158 | 0.464089443 |
| OR51J1 | red | -0.075858642 | 0.1015708 | -0.069464344 | 0.133893029 |
| OR51M1 | red | -0.079886369 | 0.084619525 | -0.072693808 | 0.116696284 |
| OR51Q1 | red | -0.115100502 | 0.012811956 | -0.113540052 | 0.014088887 |
| OR52B6 | red | -0.065756197 | 0.155977448 | -0.055318451 | 0.232809894 |
| OR52D1 | red | -0.101441523 | 0.028382975 | -0.099012911 | 0.032418518 |
| OR52E4 | red | -0.044884173 | 0.333122504 | -0.04288646 | 0.35510845 |
| OR52E5 | red | -0.055259912 | 0.233305424 | -0.039086698 | 0.399379976 |
| OR52E6 | red | -0.089878042 | 0.052257605 | -0.074911745 | 0.105923525 |
| OR52E8 | red | -0.016829971 | 0.71679184 | -0.02510247 | 0.588438353 |
| OR52H1 | red | -0.09005546 | 0.051792709 | -0.078344509 | 0.090817333 |
| OR52I1 | red | 0.020995042 | 0.650879542 | 0.019001141 | 0.682132665 |
| OR52N1 | red | -0.067053967 | 0.147954681 | -0.064864203 | 0.161679651 |
| OR52N2 | red | -0.065685384 | 0.156424501 | -0.071093784 | 0.124986832 |
| OR52N5 | red | -0.072958605 | 0.115366741 | -0.065139776 | 0.159901504 |
| OR56A1 | red | -0.132780927 | 0.004047088 | -0.132189286 | 0.004215801 |
| OR56B1 | red | -0.054565911 | 0.239236979 | -0.04259444 | 0.358397103 |
| OR5B21 | red | -0.062821045 | 0.175329977 | -0.059985061 | 0.195672481 |
| OR5D16 | red | 0.029089479 | 0.530608786 | 0.049768934 | 0.283137144 |
| OR5P3 | red | -0.062578505 | 0.177005658 | -0.043856876 | 0.344316817 |
| OR6A2 | red | -0.068440944 | 0.139732034 | -0.060755841 | 0.189980368 |
| OR6C70 | red | 0.003897616 | 0.933054218 | 0.016253134 | 0.72610227 |
| OR6C76 | red | -0.063547589 | 0.170380979 | -0.039472429 | 0.394740415 |
| OVCH1 | red | -0.080369403 | 0.082749598 | -0.071028451 | 0.12533484 |
| P2RY4 | red | -0.076072253 | 0.100608762 | -0.043711259 | 0.345922721 |
| PAK6 | red | 0.022405291 | 0.629134449 | -0.00936096 | 0.840108785 |
| PALM2 | red | -0.100448776 | 0.029977243 | -0.093416891 | 0.043616256 |
| PAPOLB | red | -0.07416826 | 0.10944349 | -0.066155434 | 0.153475095 |
| PATE1 | red | -0.073511772 | 0.11262762 | -0.062618203 | 0.17673058 |
| PATE2 | red | -0.118467194 | 0.01039918 | -0.109292619 | 0.018147976 |
| PATE3 | red | -0.090939702 | 0.049526836 | -0.081113632 | 0.079934011 |
| PATE4 | red | -0.085821391 | 0.063872938 | -0.078691614 | 0.089391254 |
| PBOV1 | red | -0.053187209 | 0.251333234 | -0.037300609 | 0.421285737 |
| PCDH9 | red | -0.077614504 | 0.093876071 | -0.066219502 | 0.153076377 |
| PCDHA1( | red | -0.148280685 | 0.001310971 | -0.144106741 | 0.001795424 |
| PCDHA5 | red | -0.07982012 | 0.084878625 | -0.063849748 | 0.168353767 |

| | | | | | |
|---|---|---|---|---|---|
| PCDHA8 | red | -0.069080145 | 0.136062616 | -0.060374821 | 0.192778772 |
| PCDHA9 | red | -0.076651605 | 0.098035963 | -0.056891055 | 0.219775488 |
| PCDHGA | red | -0.120229488 | 0.009304515 | -0.106298302 | 0.021591276 |
| PCDHGA | red | -0.06862486 | 0.138668542 | -0.059989108 | 0.19564227 |
| PCDHGC | red | -0.071092743 | 0.124992371 | -0.080016684 | 0.084111723 |
| PDC | red | -0.074653404 | 0.10713633 | -0.057275397 | 0.21667086 |
| PDE6C | red | -0.032568093 | 0.482615211 | -0.054246091 | 0.242005836 |
| PDZD9 | red | -0.141682231 | 0.002147267 | -0.125009889 | 0.006833222 |
| PEF1 | red | -0.105571088 | 0.022508487 | -0.083236906 | 0.07232551 |
| PFKFB1 | red | 0.192913608 | 2.70E-05 | 0.192399305 | 2.84E-05 |
| PGBD3 | red | -0.048156168 | 0.299047935 | -0.043759854 | 0.345386277 |
| PHKG1 | red | 0.009905798 | 0.830938815 | 0.012056726 | 0.794972362 |
| PKD2L2 | red | -0.117292303 | 0.011191029 | -0.100423621 | 0.030018616 |
| PKHD1 | red | -0.111221658 | 0.016192676 | -0.120880034 | 0.008927092 |
| PLET1 | red | -0.107921667 | 0.019660027 | -0.09465581 | 0.040890352 |
| PLSCR2 | red | -0.218582608 | 1.85E-06 | -0.189267257 | 3.85E-05 |
| POTEE | red | -0.087699444 | 0.058255259 | -0.088849936 | 0.055020561 |
| POTEF | red | -0.064375572 | 0.164869077 | -0.059608465 | 0.198498482 |
| POTEI | red | -0.042853781 | 0.35547553 | -0.034316187 | 0.45941554 |
| POU1F1 | red | -0.053032056 | 0.252720661 | -0.049561217 | 0.28515368 |
| POU5F2 | red | -0.065453012 | 0.157898289 | -0.047823173 | 0.302405785 |
| POU6F2 | red | -0.174738347 | 0.000147497 | -0.181673233 | 7.87E-05 |
| PP2D1 | red | -0.103648844 | 0.025097182 | -0.091631408 | 0.047812652 |
| PRB1 | red | -0.017412526 | 0.707431498 | -0.000494943 | 0.991489012 |
| PRB2 | red | -0.005428844 | 0.906856281 | -0.005813818 | 0.900284444 |
| PRB3 | red | -0.106716144 | 0.021079079 | -0.093717626 | 0.04294089 |
| PRB4 | red | -0.052466813 | 0.257820166 | -0.022509388 | 0.627541682 |
| PRDM7 | red | -0.078490494 | 0.090215348 | -0.079131097 | 0.087611483 |
| PREB | red | -0.023557704 | 0.611598924 | -0.022653317 | 0.625342296 |
| PRH1 | red | -0.159236337 | 0.000552404 | -0.160967837 | 0.000479383 |
| PRH2 | red | -0.042129398 | 0.363673561 | -0.03141117 | 0.498311374 |
| PROX2 | red | -0.069357105 | 0.13449591 | -0.059341466 | 0.20052 |
| PRR26 | red | -0.050144494 | 0.279515668 | -0.060566272 | 0.19136891 |
| PRR34 | red | -0.102964969 | 0.026078191 | -0.096862322 | 0.03639155 |
| PRR4 | red | -0.082055424 | 0.076482777 | -0.064823105 | 0.161946104 |
| PRR5-AR | red | -0.071260035 | 0.124104654 | -0.10191215 | 0.027652988 |
| PRSS37 | red | -0.067154462 | 0.147346787 | -0.062689323 | 0.176238561 |
| PRSS42 | red | -0.192456725 | 2.83E-05 | -0.202128019 | 1.07E-05 |
| PRSS46 | red | -0.196803747 | 1.84E-05 | -0.209718573 | 4.86E-06 |
| PRSS48 | red | -0.081769901 | 0.077516054 | -0.072946293 | 0.115428296 |
| PSG1 | red | -0.050411995 | 0.276955403 | -0.047220017 | 0.308551294 |
| PSG11 | red | -0.051621108 | 0.265582041 | -0.039044219 | 0.399892906 |
| PSG2 | red | -0.053744767 | 0.246391155 | -0.02849037 | 0.539111732 |
| PSG3 | red | -0.076475898 | 0.098810654 | -0.077674527 | 0.093621502 |
| PSG6 | red | -0.079539694 | 0.085982461 | -0.075662367 | 0.102461182 |

| | | | | | |
|---|---|---|---|---|---|
| PSG7 | red | -0.066644453 | 0.150451608 | -0.057057148 | 0.218429942 |
| PTMS | red | -0.02507893 | 0.588788246 | -0.029065947 | 0.530941476 |
| PTTG2 | red | 0.00924397 | 0.842080654 | 0.02961855 | 0.523156699 |
| PVRIG | red | -0.123790429 | 0.007400284 | -0.120692205 | 0.009034639 |
| PXT1 | red | -0.10552972 | 0.022561663 | -0.100941385 | 0.029176814 |
| RAB1B | red | 0.259014139 | 1.35E-08 | 0.272253553 | 2.22E-09 |
| RAB39A | red | -0.174617352 | 0.000149092 | -0.161897701 | 0.000443976 |
| RAB40A | red | -0.324579591 | 6.43E-13 | -0.330888301 | 2.15E-13 |
| RAB40AI | red | -0.137694248 | 0.00286523 | -0.131462262 | 0.004431814 |
| RAD21L1 | red | -0.152741923 | 0.000928353 | -0.138706149 | 0.002664857 |
| RANBP3l | red | -0.171098307 | 0.000203215 | -0.175838903 | 0.000133707 |
| RAPH1 | red | 0.142176472 | 0.002070803 | 0.134737769 | 0.003531692 |
| RBM14-R | red | -0.090069687 | 0.05175558 | -0.11538919 | 0.012587246 |
| RBM44 | red | -0.086477794 | 0.061860826 | -0.099699365 | 0.031230998 |
| RBMXL3 | red | -0.08046926 | 0.082367226 | -0.080866583 | 0.080859919 |
| REC114 | red | -0.077481735 | 0.094441124 | -0.077808385 | 0.093055784 |
| RFPL1 | red | -0.143021476 | 0.001945817 | -0.144423668 | 0.001753559 |
| RFX6 | red | 0.074910947 | 0.105927257 | 0.040724678 | 0.379903833 |
| RGL4 | red | -0.033017131 | 0.476595973 | -0.009040389 | 0.84551439 |
| RGPD1 | red | -0.053024792 | 0.252785745 | -0.065304221 | 0.158847464 |
| RGPD5 | red | -0.035959445 | 0.438187002 | -0.042878104 | 0.355202295 |
| RGPD6 | red | -0.073450721 | 0.112927395 | -0.070789399 | 0.126614612 |
| RGPD8 | red | -0.282208261 | 5.34E-10 | -0.314396476 | 3.57E-12 |
| RGS17 | red | 0.00698112 | 0.880401069 | 0.000177135 | 0.996953961 |
| RHD | red | -0.177260072 | 0.000117687 | -0.167288006 | 0.000282265 |
| RNASE3 | red | -0.117063755 | 0.011351123 | -0.090496568 | 0.050651789 |
| RNF113B | red | -0.073857395 | 0.110942309 | -0.066972123 | 0.148451167 |
| RNF133 | red | -0.032931607 | 0.477739229 | -0.033596596 | 0.468889349 |
| RNF148 | red | -0.057610792 | 0.213987421 | -0.039793107 | 0.390908153 |
| RNF167 | red | 0.119239325 | 0.009906234 | 0.116719024 | 0.011596436 |
| RNF212B | red | -0.094102403 | 0.042089655 | -0.088537432 | 0.055884065 |
| RNH1 | red | 0.14406106 | 0.001801534 | 0.15615844 | 0.00070825 |
| RP11-11N | red | -0.115145863 | 0.012776415 | -0.121411695 | 0.008628827 |
| RP11-12J | red | -0.13119823 | 0.004512706 | -0.13647189 | 0.003125513 |
| RP11-219 | red | -0.065979015 | 0.154577087 | -0.064384741 | 0.164808792 |
| RP11-231 | red | 0.010159804 | 0.826671494 | 0.016541032 | 0.721450308 |
| RP11-248 | red | -0.107918102 | 0.019664098 | -0.101742358 | 0.027914469 |
| RP11-248 | red | -0.116371414 | 0.011848534 | -0.116977839 | 0.011411827 |
| RP11-286 | red | -0.092961176 | 0.044656697 | -0.085752413 | 0.064087477 |
| RP11-287 | red | -0.152473329 | 0.000948094 | -0.165847709 | 0.000319013 |
| RP11-290 | red | -0.096026261 | 0.038044079 | -0.101812091 | 0.027806824 |
| RP11-295 | red | -0.09579336 | 0.038515595 | -0.111331273 | 0.016087332 |
| RP11-314 | red | -0.051046393 | 0.270947413 | -0.035644706 | 0.442208897 |
| RP11-321 | red | -0.01459901 | 0.753020914 | -0.016952916 | 0.714812805 |
| RP11-33C | red | 0.004591269 | 0.921175763 | 0.010206894 | 0.825880933 |

| | | | | | |
|---|---|---|---|---|---|
| RP11-371 | red | -0.054590888 | 0.239021681 | -0.048193586 | 0.298672171 |
| RP11-400 | red | -0.029845534 | 0.519976073 | -0.034617594 | 0.455479342 |
| RP11-434 | red | -0.115186393 | 0.012744733 | -0.126649087 | 0.006132361 |
| RP11-437 | red | -0.06770317 | 0.144061158 | -0.060913109 | 0.188834049 |
| RP11-463 | red | -0.026233414 | 0.571742624 | -0.022459268 | 0.628308338 |
| RP11-468 | red | 0.120880164 | 0.008927018 | 0.120745458 | 0.009004031 |
| RP11-49K | red | -0.060535933 | 0.19159182 | -0.065341588 | 0.158608687 |
| RP11-507 | red | -0.094503692 | 0.041217098 | -0.090303705 | 0.051148019 |
| RP11-508 | red | -0.04415211 | 0.34107545 | -0.053656177 | 0.247171825 |
| RP11-574 | red | -0.104613001 | 0.023768295 | -0.11601192 | 0.012114334 |
| RP11-58C | red | -0.047399972 | 0.306709188 | -0.04759263 | 0.304745116 |
| RP11-599 | red | -0.002548207 | 0.956202419 | 0.001904262 | 0.967263044 |
| RP11-613 | red | -0.07740336 | 0.094775958 | -0.077367278 | 0.094930422 |
| RP11-65E | red | -0.033036538 | 0.476336751 | -0.030366776 | 0.51270994 |
| RP11-690 | red | -0.12164424 | 0.008501183 | -0.113025813 | 0.014533557 |
| RP11-691 | red | -0.078152542 | 0.091613829 | -0.076122392 | 0.100384007 |
| RP11-697 | red | -0.035631993 | 0.442371799 | -0.043482396 | 0.348456298 |
| RP11-849 | red | -0.107478585 | 0.020171618 | -0.122551635 | 0.00801906 |
| RP11-894 | red | -0.147119682 | 0.001431975 | -0.134554546 | 0.003577291 |
| RP3-461F | red | -0.102525724 | 0.02672556 | -0.086377203 | 0.062165737 |
| RP3-468K | red | -0.085092835 | 0.066168999 | -0.071692571 | 0.121832174 |
| RP5-1021 | red | -0.046158577 | 0.319564786 | -0.049626161 | 0.284522158 |
| RP5-1042 | red | -0.002691443 | 0.953743204 | 7.27E-05 | 0.998750412 |
| RP5-1052 | red | -0.069192711 | 0.135424166 | -0.05365919 | 0.247145246 |
| RP5-864K | red | -0.061506355 | 0.184555671 | -0.039021255 | 0.400170355 |
| RPA4 | red | -0.146962521 | 0.001449121 | -0.159961063 | 0.000520667 |
| RPE65 | red | -0.139463925 | 0.002523258 | -0.131752308 | 0.004344465 |
| RPEL1 | red | -0.054764982 | 0.237524795 | -0.038203 | 0.410131631 |
| RRH | red | -0.018279395 | 0.693584474 | -0.031877505 | 0.491952163 |
| S100G | red | 0.122524446 | 0.008033144 | 0.106081234 | 0.021861599 |
| S100Z | red | -0.208193332 | 5.71E-06 | -0.199516695 | 1.40E-05 |
| SARNP | red | -0.084323727 | 0.068665968 | -0.095494514 | 0.039127864 |
| SCAF1 | red | 0.00425643 | 0.926907681 | 0.019054233 | 0.68129309 |
| SCGB2B2 | red | -0.211723443 | 3.92E-06 | -0.207841333 | 5.93E-06 |
| SCHIP1 | red | -0.225472647 | 8.52E-07 | -0.239941743 | 1.54E-07 |
| SCLY | red | 0.035728235 | 0.441139475 | 0.032215733 | 0.487367192 |
| SCN11A | red | -0.303627129 | 2.05E-11 | -0.311335087 | 5.91E-12 |
| SCN2A | red | -0.134957961 | 0.00347759 | -0.107640633 | 0.019983186 |
| SCN3A | red | -0.006833863 | 0.882905518 | -0.002514403 | 0.956782854 |
| SDF4 | red | 0.17625664 | 0.000128798 | 0.177474806 | 0.00011543 |
| SEBOX | red | -0.057532484 | 0.214611798 | -0.046780635 | 0.313079599 |
| SEC16B | red | 0.11193843 | 0.015514637 | 0.100777703 | 0.029440718 |
| SH2D4B | red | -0.083459789 | 0.071562319 | -0.060228658 | 0.193860238 |
| SIX6 | red | -0.108658538 | 0.018834217 | -0.101695831 | 0.02798649 |
| SLC10A1 | red | -0.149092903 | 0.00123199 | -0.128976549 | 0.005247979 |

| | | | | | |
|---|---|---|---|---|---|
| SLC10A3 | red | -0.080457164 | 0.082413467 | -0.10146523 | 0.028345812 |
| SLC10A5 | red | 0.005272956 | 0.909519244 | -0.029550215 | 0.524116185 |
| SLC15A5 | red | -0.098395822 | 0.033518742 | -0.087183558 | 0.059756032 |
| SLC17A1 | red | -0.098308831 | 0.033676374 | -0.095163627 | 0.039815369 |
| SLC1A2 | red | 0.072788669 | 0.116218615 | 0.053318535 | 0.250163026 |
| SLC22A1 | red | -0.252017553 | 3.37E-08 | -0.267158169 | 4.50E-09 |
| SLC24A5 | red | -0.113794514 | 0.01387331 | -0.11518875 | 0.012742892 |
| SLC25A2 | red | 0.039630713 | 0.392846026 | 0.045881893 | 0.32247727 |
| SLC25A5 | red | -0.106868988 | 0.020894372 | -0.120448059 | 0.009176154 |
| SLC26A5 | red | 0.076846297 | 0.097183229 | 0.094865478 | 0.040443576 |
| SLC34A1 | red | -0.089958702 | 0.052045819 | -0.095777316 | 0.038548259 |
| SLC35A4 | red | 0.296727778 | 6.03E-11 | 0.317088963 | 2.29E-12 |
| SLC35F4 | red | -0.161968577 | 0.00044138 | -0.160444955 | 0.000500429 |
| SLC38A4 | red | -0.111592279 | 0.015838909 | -0.135114345 | 0.003439623 |
| SLC38A6 | red | -0.085245342 | 0.065682842 | -0.074140116 | 0.109578524 |
| SLC4A5 | red | -0.116912641 | 0.011458086 | -0.113990842 | 0.013708973 |
| SLC5A12 | red | -0.07082348 | 0.12643154 | -0.074560387 | 0.107575683 |
| SLC5A4 | red | -0.103831879 | 0.024840086 | -0.104511458 | 0.023905323 |
| SLC5A9 | red | 0.001304292 | 0.977574048 | -0.004984608 | 0.914447639 |
| SLC6A7 | red | -0.093457811 | 0.043523841 | -0.072435225 | 0.118006173 |
| SLC9C1 | red | -0.088077889 | 0.057174281 | -0.077493785 | 0.094389727 |
| SLCO1A2 | red | -0.130309054 | 0.004795022 | -0.138795153 | 0.002647859 |
| SLCO1B1 | red | -0.000621132 | 0.989319201 | 0.000686585 | 0.988193761 |
| SLCO6A1 | red | -0.146760768 | 0.001471409 | -0.136248945 | 0.00317524 |
| SLFNL1 | red | -0.158326349 | 0.000594798 | -0.164546067 | 0.000356017 |
| SLX1B | red | -0.064290201 | 0.165431125 | -0.073863447 | 0.110912972 |
| SMCO3 | red | -0.211645088 | 3.95E-06 | -0.192791019 | 2.73E-05 |
| SMIM18 | red | -0.041195283 | 0.374417638 | -0.03001584 | 0.517596189 |
| SNAPC2 | red | 0.182393941 | 7.36E-05 | 0.197735992 | 1.68E-05 |
| SNURF | red | -0.144736325 | 0.001713137 | -0.139123502 | 0.002585999 |
| SNX17 | red | -0.020504277 | 0.658518045 | -0.005369008 | 0.907878317 |
| SOGA3 | red | -0.140963872 | 0.002263011 | -0.146803752 | 0.001466634 |
| SPACA1 | red | -0.067397322 | 0.14588558 | -0.052009556 | 0.261997172 |
| SPATA12 | red | 0.026303935 | 0.57070903 | 0.039611471 | 0.393076018 |
| SPATA32 | red | 0.007929353 | 0.864303783 | 0.008935993 | 0.847276337 |
| SPDYA | red | -0.064278728 | 0.16550677 | -0.058592803 | 0.206268121 |
| SPDYE1 | red | -0.085852458 | 0.063776507 | -0.070061665 | 0.130573028 |
| SPDYE2 | red | -0.059657824 | 0.198126399 | -0.060855017 | 0.18925689 |
| SPDYE2B | red | -0.023645467 | 0.610272358 | -0.018174123 | 0.695260721 |
| SPDYE5 | red | -0.088770738 | 0.055238343 | -0.077392894 | 0.094820742 |
| SPDYE6 | red | -0.051441713 | 0.26724893 | -0.046546196 | 0.315513498 |
| SPECC1L | red | -0.036717806 | 0.42858288 | -0.07358721 | 0.112258065 |
| SPIN2A | red | -0.186269493 | 5.12E-05 | -0.166999553 | 0.000289292 |
| SPINK14 | red | 0.119278068 | 0.009882055 | 0.12819686 | 0.005530547 |
| SPINT3 | red | -0.062232298 | 0.179418129 | -0.045621108 | 0.325238142 |

| | | | | | |
|---|---|---|---|---|---|
| SPINT4 | red | -0.094138246 | 0.042011091 | -0.081329373 | 0.079132481 |
| STH | red | -0.033419558 | 0.471236559 | -0.030456548 | 0.511463868 |
| STK31 | red | -0.195146463 | 2.17E-05 | -0.180345685 | 8.89E-05 |
| STPG2 | red | -0.14006168 | 0.002416439 | -0.137030581 | 0.003003992 |
| STUB1 | red | 0.069209772 | 0.135327606 | 0.091469192 | 0.048210127 |
| SULT1C3 | red | -0.066300355 | 0.152574323 | -0.045548352 | 0.326011114 |
| SUMO4 | red | -0.097851258 | 0.034515952 | -0.082530122 | 0.074789699 |
| SYCP2 | red | -0.218956222 | 1.78E-06 | -0.238560143 | 1.82E-07 |
| SYCP2L | red | -0.2825427 | 5.08E-10 | -0.275827607 | 1.34E-09 |
| SYCP3 | red | -0.074846736 | 0.106227689 | -0.070169455 | 0.129980749 |
| SYN3 | red | -0.176867867 | 0.000121918 | -0.173998093 | 0.000157512 |
| SYNPR | red | -0.09325816 | 0.043976314 | -0.074752249 | 0.106671005 |
| SYT16 | red | -0.147655613 | 0.001374897 | -0.154173383 | 0.000829397 |
| TAAR8 | red | -0.107489822 | 0.020158502 | -0.097545437 | 0.035086972 |
| TAAR9 | red | -0.133623655 | 0.003817315 | -0.122373794 | 0.008111583 |
| TAB1 | red | 0.180346027 | 8.89E-05 | 0.158276224 | 0.000597219 |
| TAC4 | red | -0.05098025 | 0.271569623 | -0.032109777 | 0.488801036 |
| TACR3 | red | -0.114597265 | 0.013212135 | -0.159282484 | 0.000550331 |
| TAS2R10 | red | -0.082989831 | 0.073179287 | -0.087727203 | 0.058175399 |
| TAS2R13 | red | -0.074040757 | 0.110056296 | -0.05996594 | 0.195815251 |
| TAS2R14 | red | -0.184779206 | 5.89E-05 | -0.17317865 | 0.000169339 |
| TAS2R19 | red | -0.180119561 | 9.08E-05 | -0.173731194 | 0.000161276 |
| TAS2R20 | red | -0.141174439 | 0.002228507 | -0.141384316 | 0.002194595 |
| TAS2R3 | red | -0.090571512 | 0.050460049 | -0.07822068 | 0.091330477 |
| TAS2R30 | red | -0.092923247 | 0.044744224 | -0.081559178 | 0.078285884 |
| TAS2R31 | red | -0.136721916 | 0.003070588 | -0.135021122 | 0.00346221 |
| TAS2R4 | red | -0.152962399 | 0.000912433 | -0.150817618 | 0.001078596 |
| TAS2R40 | red | -0.042635313 | 0.357935659 | -0.025052493 | 0.589181294 |
| TAS2R41 | red | -0.079349286 | 0.086738531 | -0.076335732 | 0.099432127 |
| TAS2R42 | red | -0.045240704 | 0.329292795 | -0.021887447 | 0.637083358 |
| TAS2R43 | red | -0.100470662 | 0.029941285 | -0.093332182 | 0.043808096 |
| TAS2R46 | red | -0.104195542 | 0.024336016 | -0.090585814 | 0.050423526 |
| TAS2R50 | red | -0.096689467 | 0.036728116 | -0.081532909 | 0.078382282 |
| TAS2R60 | red | -0.076554265 | 0.098464535 | -0.07295535 | 0.11538301 |
| TAS2R8 | red | -0.028461889 | 0.539517643 | -0.01619051 | 0.727115521 |
| TAS2R9 | red | -0.037440954 | 0.419539425 | -0.029354843 | 0.52686437 |
| TCEB3B | red | -0.131124233 | 0.004535614 | -0.121651478 | 0.008497238 |
| TCEB3C | red | -0.040961078 | 0.377141812 | -0.044872381 | 0.33324966 |
| TCEB3CL | red | -0.048568637 | 0.294923197 | -0.033909274 | 0.464759588 |
| TDRD6 | red | -0.144080626 | 0.001798915 | -0.146631947 | 0.001485804 |
| TEDDM1 | red | -0.05953008 | 0.199090404 | -0.058628105 | 0.205994428 |
| TEX12 | red | -0.106425509 | 0.021434212 | -0.105743583 | 0.022287924 |
| TEX38 | red | -0.130659123 | 0.004682026 | -0.113131057 | 0.01444156 |
| TICAM2 | red | -0.10434486 | 0.02413162 | -0.120548781 | 0.009117535 |
| TM4SF19 | red | -0.06961277 | 0.133062036 | -0.071421804 | 0.123250917 |

| | | | | | |
|---|---|---|---|---|---|
| TMEFF2 | red | -0.196830837 | 1.83E-05 | -0.182666773 | 7.18E-05 |
| TMEM11 | red | -0.060984584 | 0.188314759 | -0.059281229 | 0.200978134 |
| TMEM14 | red | -0.088708756 | 0.055409284 | -0.073940092 | 0.11054202 |
| TMEM17 | red | 0.090609825 | 0.050362262 | 0.095468751 | 0.039181031 |
| TMEM21 | red | 0.295302637 | 7.51E-11 | 0.315084512 | 3.19E-12 |
| TMEM24 | red | -0.122139693 | 0.008234827 | -0.10071292 | 0.029545732 |
| TMEM25 | red | -0.193965764 | 2.44E-05 | -0.19522746 | 2.15E-05 |
| TMEM30 | red | -0.050433818 | 0.276747243 | -0.05717033 | 0.217516427 |
| TMEM52 | red | -0.144964553 | 0.001684171 | -0.136127421 | 0.003202647 |
| TMPRSS | red | -0.064945737 | 0.161152007 | -0.082522004 | 0.074818395 |
| TMPRSS | red | 0.007655558 | 0.868946292 | 0.018739706 | 0.686272553 |
| TMPRSS | red | -0.075028534 | 0.105378827 | -0.077986481 | 0.092307345 |
| TMPRSS | red | -0.116670694 | 0.0116312 | -0.109600174 | 0.017823055 |
| TMPRSS | red | -0.071950917 | 0.120490409 | -0.066166384 | 0.153406896 |
| TNP2 | red | -0.065309143 | 0.158815999 | -0.053663895 | 0.247103746 |
| TOLLIP | red | 0.149433043 | 0.001200234 | 0.178560337 | 0.000104627 |
| TOMM20 | red | -0.079979022 | 0.084258228 | -0.064095338 | 0.166719414 |
| TOP3B | red | -0.083808455 | 0.070381629 | -0.106566967 | 0.021260718 |
| TP53TG5 | red | -0.166595958 | 0.000299398 | -0.162496368 | 0.000422485 |
| TREML4 | red | -0.046660301 | 0.314327342 | -0.035867309 | 0.439362191 |
| TRHR | red | -0.077031269 | 0.096378552 | -0.067048016 | 0.147990744 |
| TRIM34 | red | -0.081123633 | 0.079896711 | -0.099595972 | 0.031407447 |
| TRIM61 | red | -0.224483911 | 9.54E-07 | -0.220291185 | 1.53E-06 |
| TRIM71 | red | -0.172552583 | 0.000178932 | -0.171715277 | 0.000192559 |
| TRIM77 | red | -0.061226617 | 0.186564099 | -0.049248937 | 0.288203505 |
| TRPA1 | red | -0.02032531 | 0.661312493 | -0.026875184 | 0.562369338 |
| TRPC5 | red | -0.095406902 | 0.039308916 | -0.088250767 | 0.05668604 |
| TRPV1 | red | -0.074102458 | 0.109759412 | -0.072068581 | 0.119883138 |
| TSEN34 | red | 0.078414684 | 0.090527557 | 0.067925612 | 0.14274525 |
| TSNAX-I | red | -0.031275519 | 0.500169335 | 0.00080119 | 0.986223233 |
| TSPO2 | red | -0.177795425 | 0.000112135 | -0.156915915 | 0.000666508 |
| TSPYL6 | red | -0.089029217 | 0.0545302 | -0.080511723 | 0.08220506 |
| TSSK1B | red | 0.037581921 | 0.417789658 | 0.030488086 | 0.51102647 |
| TSSK3 | red | -0.111484943 | 0.01594066 | -0.108219884 | 0.019322094 |
| TSSK4 | red | -0.034476583 | 0.457318503 | -0.039797032 | 0.390861387 |
| TTN | red | -0.116121037 | 0.012033104 | -0.109980862 | 0.017427916 |
| TUBA8 | red | -0.184267886 | 6.18E-05 | -0.194778524 | 2.25E-05 |
| TUBB1 | red | -0.061836216 | 0.182207931 | -0.044613841 | 0.336045318 |
| TVP23C | red | -0.003360436 | 0.94226352 | -0.006956879 | 0.880813271 |
| TXNDC8 | red | -0.049896513 | 0.281903381 | -0.031014478 | 0.503755008 |
| TYR | red | 0.01268073 | 0.784615328 | 0.002918574 | 0.949844529 |
| U2AF1 | red | -0.158147163 | 0.000603494 | -0.170045825 | 0.000222678 |
| U51561.1 | red | -0.083042126 | 0.072997898 | -0.076290111 | 0.099635073 |
| UBAP1L | red | -0.087579239 | 0.058602132 | -0.084200518 | 0.069073059 |
| UBQLN3 | red | -0.079109966 | 0.0876964 | -0.071219202 | 0.124320879 |

| | | | | | |
|---|---|---|---|---|---|
| UNC13C | red | -0.127175173 | 0.005921512 | -0.122983236 | 0.007798423 |
| UNC45A | red | 0.055822679 | 0.228572422 | 0.061373363 | 0.185508509 |
| UPP2 | red | -0.063896562 | 0.168041307 | -0.01693594 | 0.715085949 |
| USP17L1 | red | -0.082504978 | 0.074878607 | -0.077316873 | 0.095146545 |
| USP17L2 | red | -0.063991138 | 0.16741139 | -0.055075331 | 0.234872781 |
| USP17L7 | red | -0.021326937 | 0.645734323 | -0.03301595 | 0.476611756 |
| USP50 | red | 0.000358765 | 0.993830656 | 0.003674147 | 0.936884303 |
| UTS2B | red | -0.184306532 | 6.16E-05 | -0.174063988 | 0.000156595 |
| UTY | red | -0.051220617 | 0.269313142 | -0.053834494 | 0.245602215 |
| VN1R1 | red | -0.265086289 | 5.97E-09 | -0.26667522 | 4.80E-09 |
| VWC2L | red | -0.122471436 | 0.008060666 | -0.122339722 | 0.008129417 |
| WBP2 | red | -0.048503722 | 0.295569827 | -0.035743986 | 0.440937979 |
| WBP2NL | red | -0.099503928 | 0.031565241 | -0.121310643 | 0.008684825 |
| WDPCP | red | -0.078981426 | 0.088214376 | -0.082514917 | 0.074843453 |
| WDR64 | red | -0.027470968 | 0.553734448 | -0.027683448 | 0.55067064 |
| WDR87 | red | -0.130269497 | 0.004807944 | -0.130993621 | 0.004576306 |
| WEE2 | red | -0.084616271 | 0.067707253 | -0.077610757 | 0.09389198 |
| WFDC13 | red | -0.079224524 | 0.087236831 | -0.071915755 | 0.12067235 |
| WNK3 | red | -0.349521665 | 7.27E-15 | -0.352643402 | 4.03E-15 |
| XKR6 | red | -0.296746837 | 6.01E-11 | -0.306875784 | 1.22E-11 |
| XRCC1 | red | -0.011162484 | 0.809877292 | -0.02273364 | 0.624116315 |
| XRRA1 | red | 0.053410363 | 0.249347031 | 0.060823633 | 0.18948561 |
| YIPF3 | red | 0.098051969 | 0.034145508 | 0.122745588 | 0.00791923 |
| ZACN | red | -0.092234316 | 0.046359335 | -0.083565888 | 0.071201336 |
| ZBTB20 | red | -0.043985446 | 0.34290285 | -0.04694423 | 0.311388503 |
| ZC2HC1E | red | -0.009476533 | 0.838161754 | -0.003738524 | 0.935780776 |
| ZCCHC1( | red | -0.128654337 | 0.005363137 | -0.1097677 | 0.017648215 |
| ZNF154 | red | -0.185734529 | 5.39E-05 | -0.171846177 | 0.000190366 |
| ZNF177 | red | -0.034298469 | 0.459647519 | -0.048063107 | 0.299983826 |
| ZNF233 | red | -0.113546006 | 0.01408381 | -0.114137912 | 0.013586992 |
| ZNF257 | red | -0.359788425 | 1.02E-15 | -0.349352268 | 7.50E-15 |
| ZNF410 | red | -0.090889318 | 0.049653682 | -0.110756422 | 0.01664654 |
| ZNF483 | red | -0.202963507 | 9.85E-06 | -0.211513431 | 4.01E-06 |
| ZNF506 | red | -0.270643081 | 2.78E-09 | -0.279640728 | 7.75E-10 |
| ZNF559-Z | red | -0.175146121 | 0.000142239 | -0.178343253 | 0.000106708 |
| ZNF564 | red | -0.074975717 | 0.10562489 | -0.080938626 | 0.08058902 |
| ZNF573 | red | -0.077460291 | 0.094532645 | -0.08332432 | 0.072025401 |
| ZNF578 | red | -0.310029956 | 7.31E-12 | -0.291753829 | 1.29E-10 |
| ZNF587B | red | 0.162847867 | 0.000410322 | 0.173219092 | 0.000168737 |
| ZNF716 | red | -0.167270364 | 0.00028269 | -0.16527456 | 0.000334841 |
| ZNF732 | red | -0.23024503 | 4.90E-07 | -0.237694883 | 2.02E-07 |
| ZNF81 | red | -0.074928864 | 0.105843546 | -0.07348766 | 0.112745939 |
| ZNF852 | red | 0.005932118 | 0.898266324 | -0.001986908 | 0.965843089 |
| ZNF878 | red | -0.05232517 | 0.259109116 | -0.048407276 | 0.296532281 |
| ZNF891 | red | -0.116271278 | 0.011922049 | -0.130958413 | 0.004587332 |

| | | | | | |
|---|---|---|---|---|---|
| ZNF98 | red | -0.338898385 | 5.15E-14 | -0.33106518 | 2.08E-13 |
| ZNF99 | red | -0.231343619 | 4.31E-07 | -0.232157501 | 3.92E-07 |
| ZPBP | red | -0.205913152 | 7.26E-06 | -0.195690358 | 2.06E-05 |
| ZRSR1 | red | -0.003094947 | 0.946817921 | -0.006610738 | 0.886702494 |
| ZSCAN2: | red | -0.231948018 | 4.01E-07 | -0.259887247 | 1.20E-08 |
| ZSCAN5I | red | -0.056687812 | 0.221430048 | -0.040987871 | 0.376829554 |
| ABHD14I | royalblue | 0.194326138 | 2.35E-05 | 0.195715309 | 2.05E-05 |
| ACY3 | royalblue | 0.211541659 | 4.00E-06 | 0.244197072 | 9.09E-08 |
| AES | royalblue | 0.244104677 | 9.19E-08 | 0.264899251 | 6.12E-09 |
| ALG10 | royalblue | -0.083658597 | 0.070887126 | -0.097940397 | 0.034351011 |
| ANKLE2 | royalblue | -0.170168054 | 0.000220331 | -0.189610214 | 3.72E-05 |
| ANKRD2 | royalblue | -0.257452844 | 1.66E-08 | -0.28042945 | 6.91E-10 |
| AP3M2 | royalblue | -0.143280983 | 0.001908842 | -0.180623917 | 8.67E-05 |
| ARHGAP | royalblue | -0.346750824 | 1.22E-14 | -0.365853024 | 3.09E-16 |
| ARL6IP6 | royalblue | -0.163484967 | 0.000389103 | -0.199129062 | 1.46E-05 |
| ARMC8 | royalblue | -0.259314363 | 1.30E-08 | -0.276398477 | 1.23E-09 |
| ARSA | royalblue | 0.326075498 | 4.97E-13 | 0.326566763 | 4.57E-13 |
| ASTE1 | royalblue | -0.296814745 | 5.95E-11 | -0.294469664 | 8.53E-11 |
| ATL2 | royalblue | -0.214261179 | 2.98E-06 | -0.23210011 | 3.94E-07 |
| ATP13A3 | royalblue | -0.133496977 | 0.003851082 | -0.146402104 | 0.00151181 |
| ATP6V0A | royalblue | -0.154074819 | 0.000835884 | -0.159447274 | 0.000542987 |
| BOK | royalblue | 0.129695184 | 0.004999121 | 0.156297862 | 0.000700389 |
| BRAP | royalblue | -0.077521767 | 0.094270465 | -0.091088847 | 0.049152943 |
| C17orf10 | royalblue | -0.122110071 | 0.00825054 | -0.132606927 | 0.00409606 |
| C2orf44 | royalblue | -0.13266175 | 0.004080573 | -0.145121423 | 0.001664523 |
| C3orf17 | royalblue | -0.19542455 | 2.11E-05 | -0.224107566 | 9.96E-07 |
| C5orf30 | royalblue | -0.132537561 | 0.004115732 | -0.137997837 | 0.002803721 |
| C6orf62 | royalblue | -0.20377559 | 9.06E-06 | -0.212344716 | 3.67E-06 |
| CBFA2T2 | royalblue | -0.151632419 | 0.001012434 | -0.173122336 | 0.000170182 |
| CCDC6 | royalblue | -0.071921752 | 0.120641304 | -0.061343036 | 0.185726298 |
| CD47 | royalblue | -0.338699909 | 5.34E-14 | -0.331884763 | 1.80E-13 |
| CDKAL1 | royalblue | -0.227470548 | 6.77E-07 | -0.23889232 | 1.75E-07 |
| CEBPG | royalblue | -0.211855388 | 3.87E-06 | -0.242717837 | 1.09E-07 |
| CHN1 | royalblue | -0.2767568 | 1.17E-09 | -0.270756452 | 2.73E-09 |
| CMTR1 | royalblue | -0.277284509 | 1.09E-09 | -0.285531218 | 3.28E-10 |
| COMMD: | royalblue | -0.140818127 | 0.002287178 | -0.167771638 | 0.00027084 |
| DCUN1D | royalblue | -0.229648589 | 5.26E-07 | -0.245386369 | 7.83E-08 |
| DDX55 | royalblue | -0.047471376 | 0.305980286 | -0.070312174 | 0.129199755 |
| DEGS1 | royalblue | -0.253451127 | 2.80E-08 | -0.264550937 | 6.42E-09 |
| DEPDC4 | royalblue | -0.009844124 | 0.831975701 | -0.026888133 | 0.562180975 |
| DHX36 | royalblue | -0.216886502 | 2.24E-06 | -0.245657951 | 7.57E-08 |
| DHX57 | royalblue | -0.150211569 | 0.001130368 | -0.178236512 | 0.000107746 |
| DIRC2 | royalblue | -0.06058273 | 0.191248062 | -0.071925738 | 0.120620672 |
| DLG1 | royalblue | -0.092548558 | 0.045616667 | -0.115782943 | 0.012286367 |
| DNAJC6 | royalblue | -0.276823747 | 1.16E-09 | -0.287586333 | 2.41E-10 |

| | | | | | |
|---|---|---|---|---|---|
| DVL3 | royalblue | -0.157692564 | 0.000626087 | -0.154564965 | 0.000804079 |
| DYRK2 | royalblue | -0.245115202 | 8.10E-08 | -0.256111485 | 1.98E-08 |
| E2F6 | royalblue | -0.189219849 | 3.87E-05 | -0.219485962 | 1.68E-06 |
| EED | royalblue | 0.014640307 | 0.75234506 | 0.008421918 | 0.855963622 |
| EHHADH | royalblue | -0.285737927 | 3.18E-10 | -0.298720054 | 4.42E-11 |
| ELOVL7 | royalblue | -0.236937065 | 2.21E-07 | -0.250931712 | 3.88E-08 |
| ERBB4 | royalblue | -0.167653886 | 0.000273581 | -0.145858624 | 0.001574973 |
| ESCO1 | royalblue | -0.036112881 | 0.436233957 | -0.06992039 | 0.131352447 |
| EXO5 | royalblue | -0.27374129 | 1.80E-09 | -0.296854661 | 5.91E-11 |
| FAM131A | royalblue | -0.294529945 | 8.45E-11 | -0.297356142 | 5.47E-11 |
| FAM216A | royalblue | -0.286605368 | 2.79E-10 | -0.312632091 | 4.78E-12 |
| FAM60A | royalblue | -0.151553501 | 0.001018674 | -0.187299558 | 4.64E-05 |
| FANCM | royalblue | -0.079218914 | 0.087259291 | -0.090851852 | 0.049748179 |
| FAR2 | royalblue | -0.141181895 | 0.002227294 | -0.159688372 | 0.000532405 |
| FASTKD2 | royalblue | -0.130076155 | 0.004871554 | -0.148766966 | 0.001263143 |
| FBXL8 | royalblue | 0.379482792 | 1.92E-17 | 0.409897194 | 2.39E-20 |
| FBXW4 | royalblue | 0.265887733 | 5.35E-09 | 0.279006907 | 8.49E-10 |
| FIGN | royalblue | -0.176820745 | 0.000122436 | -0.186668193 | 4.93E-05 |
| FMR1 | royalblue | -0.192069009 | 2.93E-05 | -0.219995289 | 1.58E-06 |
| FUT8 | royalblue | -0.266661192 | 4.81E-09 | -0.269243689 | 3.37E-09 |
| FXR1 | royalblue | -0.283442247 | 4.46E-10 | -0.292537809 | 1.15E-10 |
| FYTTD1 | royalblue | -0.267831915 | 4.10E-09 | -0.283265905 | 4.57E-10 |
| G2E3 | royalblue | -0.132754469 | 0.0040545 | -0.150015757 | 0.001147579 |
| GFM1 | royalblue | -0.147923515 | 0.001347156 | -0.162506551 | 0.000422128 |
| GHDC | royalblue | 0.207092082 | 6.41E-06 | 0.206123958 | 7.10E-06 |
| GPATCH | royalblue | -0.290687773 | 1.52E-10 | -0.319829679 | 1.44E-12 |
| GPBP1L1 | royalblue | -0.14139348 | 0.002193125 | -0.164010943 | 0.00037236 |
| GPR108 | royalblue | 0.244966372 | 8.25E-08 | 0.259485484 | 1.27E-08 |
| GPR137C | royalblue | -0.104295731 | 0.024198706 | -0.118036474 | 0.010683512 |
| GSK3B | royalblue | 0.023594394 | 0.611044191 | 0.003383086 | 0.941875046 |
| GXYLT1 | royalblue | -0.054394218 | 0.24072063 | -0.087197709 | 0.059714443 |
| HLTF | royalblue | -0.088867259 | 0.05497302 | -0.111958592 | 0.01549593 |
| HPS3 | royalblue | -0.139763865 | 0.002469132 | -0.15398862 | 0.000841597 |
| HYLS1 | royalblue | -0.101934361 | 0.02761894 | -0.11479397 | 0.01305442 |
| ICE1 | royalblue | -0.250446408 | 4.13E-08 | -0.276049059 | 1.30E-09 |
| ITPK1 | royalblue | 0.114233182 | 0.013508486 | 0.152630792 | 0.000936475 |
| IZUMO4 | royalblue | 0.202756894 | 1.01E-05 | 0.21337645 | 3.28E-06 |
| JADE3 | royalblue | -0.164410049 | 0.000360106 | -0.182965746 | 6.98E-05 |
| KIAA022 | royalblue | -0.243086023 | 1.04E-07 | -0.237664534 | 2.03E-07 |
| KLHL24 | royalblue | -0.301509956 | 2.86E-11 | -0.301652239 | 2.80E-11 |
| KPNA1 | royalblue | -0.153630102 | 0.000865745 | -0.175127736 | 0.000142472 |
| KPNA4 | royalblue | -0.185169038 | 5.68E-05 | -0.208753815 | 5.38E-06 |
| LCLAT1 | royalblue | -0.093335019 | 0.043801659 | -0.106346963 | 0.021531077 |
| LENG9 | royalblue | 0.264414189 | 6.54E-09 | 0.274198692 | 1.69E-09 |
| LGALS1 | royalblue | 0.159048358 | 0.000560925 | 0.184062584 | 6.30E-05 |

| | | | | | |
|---|---|---|---|---|---|
| LIN28B | royalblue | -0.133056893 | 0.003970503 | -0.148179265 | 0.001321154 |
| LMF2 | royalblue | 0.257659614 | 1.62E-08 | 0.26873263 | 3.62E-09 |
| LPHN2 | royalblue | -0.306028108 | 1.40E-11 | -0.312222458 | 5.11E-12 |
| LRRC58 | royalblue | -0.032705502 | 0.480768923 | -0.057149312 | 0.217685863 |
| LSM14A | royalblue | -0.175411381 | 0.000138914 | -0.210894128 | 4.29E-06 |
| LYPD6 | royalblue | -0.076477097 | 0.098805351 | -0.070383121 | 0.128812866 |
| MAP3K1: | royalblue | -0.356113714 | 2.08E-15 | -0.350593986 | 5.94E-15 |
| MAPKAP | royalblue | -0.086920446 | 0.060533678 | -0.108945505 | 0.018520891 |
| 2-Mar | royalblue | 0.199064526 | 1.47E-05 | 0.245835789 | 7.40E-08 |
| MB21D1 | royalblue | -0.205882115 | 7.28E-06 | -0.175074812 | 0.000143146 |
| MB21D2 | royalblue | -0.409474707 | 2.63E-20 | -0.434966131 | 5.61E-23 |
| MBOAT2 | royalblue | -0.293413019 | 1.00E-10 | -0.308364832 | 9.58E-12 |
| MBTD1 | royalblue | -0.109679239 | 0.017740351 | -0.1456709 | 0.001597347 |
| MCCC1 | royalblue | -0.175345369 | 0.000139734 | -0.193630969 | 2.52E-05 |
| MED20 | royalblue | -0.082288656 | 0.075647069 | -0.095328858 | 0.039470791 |
| METTL4 | royalblue | -0.165748724 | 0.000321695 | -0.198991646 | 1.48E-05 |
| MFN1 | royalblue | -0.185066257 | 5.74E-05 | -0.193587782 | 2.53E-05 |
| MGAT4A | royalblue | -0.249110111 | 4.89E-08 | -0.24636592 | 6.93E-08 |
| MIS18BP | royalblue | -0.196036445 | 1.99E-05 | -0.228794481 | 5.81E-07 |
| MLK4 | royalblue | -0.068150917 | 0.141421799 | -0.091226744 | 0.048809352 |
| MPP6 | royalblue | -0.179413433 | 9.68E-05 | -0.196692525 | 1.86E-05 |
| MRE11A | royalblue | -0.223655673 | 1.05E-06 | -0.262731428 | 8.21E-09 |
| MTA3 | royalblue | 0.087786789 | 0.058004281 | 0.048349176 | 0.297113081 |
| MTPAP | royalblue | -0.088899224 | 0.054885387 | -0.125999574 | 0.006401959 |
| MURC | royalblue | -0.144101331 | 0.001796147 | -0.178237318 | 0.000107738 |
| MYNN | royalblue | -0.276460387 | 1.22E-09 | -0.299294226 | 4.05E-11 |
| NAA25 | royalblue | -0.11754352 | 0.011017364 | -0.150285919 | 0.001123896 |
| NDUFAF | royalblue | -0.214622785 | 2.87E-06 | -0.229298973 | 5.48E-07 |
| NINL | royalblue | -0.180636962 | 8.66E-05 | -0.202850492 | 9.97E-06 |
| NREP | royalblue | -0.188476832 | 4.15E-05 | -0.204583264 | 8.33E-06 |
| NUP35 | royalblue | -0.122416742 | 0.008089152 | -0.139233218 | 0.002565625 |
| OPA1 | royalblue | -0.164701081 | 0.000351409 | -0.164535744 | 0.000356326 |
| ORC2 | royalblue | -0.018676123 | 0.68728083 | -0.03434489 | 0.459039876 |
| ORC4 | royalblue | -0.164997295 | 0.000342758 | -0.185297748 | 5.61E-05 |
| OSBPL11 | royalblue | -0.218987216 | 1.77E-06 | -0.238978466 | 1.73E-07 |
| OSER1 | royalblue | -0.319001876 | 1.66E-12 | -0.339526161 | 4.60E-14 |
| PAK2 | royalblue | -0.116086147 | 0.012059025 | -0.135174066 | 0.003425224 |
| PANK2 | royalblue | -0.207676776 | 6.03E-06 | -0.205071446 | 7.92E-06 |
| PAPD7 | royalblue | -0.269411132 | 3.30E-09 | -0.293320955 | 1.02E-10 |
| PARD6B | royalblue | -0.225896582 | 8.12E-07 | -0.223151741 | 1.11E-06 |
| PBX2 | royalblue | -0.342591906 | 2.63E-14 | -0.373751054 | 6.27E-17 |
| PCYT1A | royalblue | -0.22022601 | 1.54E-06 | -0.219458609 | 1.68E-06 |
| PEMT | royalblue | 0.201527672 | 1.14E-05 | 0.201072684 | 1.20E-05 |
| PGBD1 | royalblue | -0.169112428 | 0.000241384 | -0.196382872 | 1.92E-05 |
| PHF6 | royalblue | 0.021889948 | 0.637044873 | -0.011198082 | 0.809282599 |

| | | | | | |
|---|---|---|---|---|---|
| PIK3CA | royalblue | -0.329732055 | 2.63E-13 | -0.343509265 | 2.22E-14 |
| PLAG1 | royalblue | -0.416615191 | 4.95E-21 | -0.406708122 | 4.97E-20 |
| PMM1 | royalblue | 0.331789456 | 1.83E-13 | 0.327474352 | 3.90E-13 |
| PPT1 | royalblue | -0.273409829 | 1.88E-09 | -0.302797105 | 2.33E-11 |
| PRELID2 | royalblue | -0.024469504 | 0.597879532 | -0.011510805 | 0.80406308 |
| PRKCI | royalblue | -0.315384241 | 3.04E-12 | -0.3326379 | 1.58E-13 |
| PRKRA | royalblue | 0.000754449 | 0.987026893 | -0.027962694 | 0.546656814 |
| PRTFDC1 | royalblue | -0.29541485 | 7.38E-11 | -0.320351076 | 1.32E-12 |
| PTCD3 | royalblue | 0.016345897 | 0.724602263 | -0.018212467 | 0.694649996 |
| PTPN2 | royalblue | -0.237969907 | 1.95E-07 | -0.264844066 | 6.17E-09 |
| PURB | royalblue | -0.156656341 | 0.000680548 | -0.164976966 | 0.000343345 |
| PVRL3 | royalblue | -0.177177886 | 0.000118562 | -0.172353886 | 0.000182081 |
| QTRTD1 | royalblue | -0.077647149 | 0.093737549 | -0.120915279 | 0.008907039 |
| RAB22A | royalblue | -0.135073716 | 0.003449451 | -0.152581669 | 0.000940085 |
| RAB39B | royalblue | -0.312964935 | 4.53E-12 | -0.320332955 | 1.33E-12 |
| RALGAP | royalblue | -0.208534363 | 5.51E-06 | -0.232653969 | 3.69E-07 |
| REEP6 | royalblue | 0.213054005 | 3.40E-06 | 0.221302256 | 1.37E-06 |
| REST | royalblue | -0.090298343 | 0.051161872 | -0.110951234 | 0.016455152 |
| RIN1 | royalblue | 0.082756146 | 0.073994342 | 0.108903332 | 0.018566651 |
| RNF114 | royalblue | -0.194807215 | 2.24E-05 | -0.197687787 | 1.68E-05 |
| RNF168 | royalblue | -0.093438485 | 0.043567467 | -0.08556781 | 0.064664548 |
| RSRC1 | royalblue | -0.202865111 | 9.95E-06 | -0.190743242 | 3.34E-05 |
| SCML2 | royalblue | -0.293978637 | 9.19E-11 | -0.30004683 | 3.60E-11 |
| SEC22A | royalblue | -0.166934968 | 0.000290887 | -0.171852514 | 0.00019026 |
| SENP2 | royalblue | -0.355549172 | 2.31E-15 | -0.372913035 | 7.44E-17 |
| SENP5 | royalblue | -0.304670853 | 1.73E-11 | -0.298765456 | 4.39E-11 |
| SIKE1 | royalblue | -0.136026678 | 0.003225531 | -0.153677893 | 0.000862489 |
| SIRT5 | royalblue | -0.373441662 | 6.68E-17 | -0.391670488 | 1.43E-18 |
| SLC16A1 | royalblue | -0.219664516 | 1.64E-06 | -0.246489745 | 6.82E-08 |
| SLC25A4 | royalblue | -0.173935257 | 0.00015839 | -0.185724849 | 5.39E-05 |
| SLC25A5 | royalblue | -0.081049011 | 0.080175369 | -0.073966542 | 0.110414233 |
| SLC30A6 | royalblue | -0.204267159 | 8.61E-06 | -0.222491529 | 1.20E-06 |
| SLC38A1 | royalblue | -0.218608164 | 1.85E-06 | -0.226333139 | 7.72E-07 |
| SLC9A7 | royalblue | -0.182909322 | 7.02E-05 | -0.168660403 | 0.000250962 |
| SMC6 | royalblue | -0.066026119 | 0.15428227 | -0.114183485 | 0.013549388 |
| SMIM13 | royalblue | -0.25885066 | 1.38E-08 | -0.292231843 | 1.20E-10 |
| SNIP1 | royalblue | -0.172579029 | 0.000178517 | -0.182483712 | 7.30E-05 |
| SPAST | royalblue | -0.050930089 | 0.272042142 | -0.071778643 | 0.12138386 |
| SRSF12 | royalblue | -0.281898535 | 5.58E-10 | -0.290015682 | 1.68E-10 |
| SSX2IP | royalblue | -0.049976775 | 0.281129064 | -0.086803462 | 0.060882108 |
| STK38 | royalblue | -0.183185731 | 6.84E-05 | -0.185790009 | 5.36E-05 |
| SYCE1L | royalblue | 0.238273369 | 1.88E-07 | 0.228069875 | 6.32E-07 |
| TAF1B | royalblue | -0.223758897 | 1.04E-06 | -0.251495332 | 3.61E-08 |
| TAF4B | royalblue | -0.285936876 | 3.08E-10 | -0.332971616 | 1.49E-13 |
| TBC1D22 | royalblue | -0.097488262 | 0.035194615 | -0.109381117 | 0.018053955 |

| | | | | | |
|---|---|---|---|---|---|
| TBCCD1 | royalblue | -0.08214886 | 0.076147086 | -0.084531658 | 0.067983411 |
| TBL1XR1 | royalblue | -0.286829774 | 2.70E-10 | -0.293295454 | 1.02E-10 |
| TCFL5 | royalblue | -0.275023706 | 1.50E-09 | -0.288097427 | 2.24E-10 |
| TFDP2 | royalblue | -0.17644454 | 0.000126645 | -0.187169913 | 4.70E-05 |
| TLE2 | royalblue | 0.189397393 | 3.80E-05 | 0.176975912 | 0.000120739 |
| TMEM13 | royalblue | -0.36792295 | 2.04E-16 | -0.402705045 | 1.24E-19 |
| TMEM19 | royalblue | -0.124306328 | 0.007155441 | -0.137764924 | 0.002850802 |
| TMEM23 | royalblue | -0.224353684 | 9.69E-07 | -0.255511063 | 2.14E-08 |
| TMSB15I | royalblue | -0.118603912 | 0.010310342 | -0.142331645 | 0.002047315 |
| TOM1 | royalblue | 0.295191304 | 7.64E-11 | 0.303613876 | 2.05E-11 |
| TRADD | royalblue | 0.30876316 | 8.98E-12 | 0.352764457 | 3.94E-15 |
| TRAM1L | royalblue | -0.297985304 | 4.96E-11 | -0.325093998 | 5.89E-13 |
| TTC9C | royalblue | -0.159101975 | 0.000558482 | -0.167400536 | 0.000279567 |
| TTPAL | royalblue | -0.12401936 | 0.007290725 | -0.15145202 | 0.001026751 |
| UBA2 | royalblue | -0.186287066 | 5.11E-05 | -0.228330814 | 6.13E-07 |
| UBE2G2 | royalblue | -0.214843291 | 2.80E-06 | -0.239224314 | 1.68E-07 |
| UBE2V1 | royalblue | -0.179195234 | 9.88E-05 | -0.203722315 | 9.11E-06 |
| UBXN7 | royalblue | -0.238102139 | 1.92E-07 | -0.252743363 | 3.07E-08 |
| URI1 | royalblue | -0.313039146 | 4.47E-12 | -0.317947549 | 1.98E-12 |
| WASF1 | royalblue | -0.254241492 | 2.53E-08 | -0.251974963 | 3.39E-08 |
| WDR33 | royalblue | -0.107452471 | 0.020202128 | -0.125347809 | 0.00668313 |
| WDR88 | royalblue | -0.218488038 | 1.87E-06 | -0.231078345 | 4.45E-07 |
| XPO1 | royalblue | -0.143459284 | 0.001883811 | -0.172524744 | 0.00017937 |
| YEATS2 | royalblue | -0.167674494 | 0.000273099 | -0.186381131 | 5.07E-05 |
| YY1 | royalblue | -0.22049219 | 1.50E-06 | -0.235274726 | 2.70E-07 |
| ZBED9 | royalblue | -0.36304733 | 5.38E-16 | -0.387252293 | 3.71E-18 |
| ZBTB33 | royalblue | -0.100442926 | 0.02998686 | -0.118608476 | 0.010307388 |
| ZC3H8 | royalblue | -0.21839179 | 1.89E-06 | -0.246813254 | 6.55E-08 |
| ZCCHC3 | royalblue | -0.200900941 | 1.22E-05 | -0.215273534 | 2.67E-06 |
| ZDHHC1 | royalblue | -0.09732036 | 0.035512345 | -0.101487855 | 0.028310383 |
| ZDHHC2 | royalblue | -0.030299507 | 0.513644702 | -0.045063223 | 0.331195651 |
| ZFP69 | royalblue | -0.256659964 | 1.84E-08 | -0.272777184 | 2.06E-09 |
| ZKSCAN | royalblue | -0.198092846 | 1.62E-05 | -0.221628038 | 1.32E-06 |
| ZKSCAN | royalblue | -0.045175509 | 0.329990962 | -0.06689847 | 0.148899053 |
| ZNF146 | royalblue | -0.159245773 | 0.00055198 | -0.193663929 | 2.51E-05 |
| ZNF239 | royalblue | -0.201406589 | 1.16E-05 | -0.246175096 | 7.09E-08 |
| ZNF343 | royalblue | -0.289309782 | 1.87E-10 | -0.317267256 | 2.22E-12 |
| ZNF485 | royalblue | -0.035270236 | 0.447021382 | -0.076527509 | 0.098582595 |
| ZNF512 | royalblue | -0.464413164 | 2.33E-26 | -0.453381065 | 4.71E-25 |
| ZNF639 | royalblue | -0.253920971 | 2.64E-08 | -0.264898593 | 6.12E-09 |
| ZNF714 | royalblue | 0.033474682 | 0.470505016 | 0.021913273 | 0.636685929 |
| AACS | saddlebrow | 0.259911342 | 1.20E-08 | 0.269697933 | 3.17E-09 |
| AC11449 | saddlebrow | 0.274260245 | 1.67E-09 | 0.290830837 | 1.48E-10 |
| ACAA2 | saddlebrow | -0.175827032 | 0.00013385 | -0.185178633 | 5.68E-05 |
| ACSL5 | saddlebrow | 0.160997189 | 0.000478226 | 0.153830756 | 0.000852152 |

| | | | | | |
|---|---|---|---|---|---|
| ADAM28 | saddlebrow | 0.304967498 | 1.65E-11 | 0.274123174 | 1.70E-09 |
| ANO1 | saddlebrow | 0.215327531 | 2.65E-06 | 0.209824901 | 4.80E-06 |
| AQP5 | saddlebrow | 0.235857446 | 2.52E-07 | 0.25405617 | 2.59E-08 |
| ARF1 | saddlebrow | 0.325971025 | 5.06E-13 | 0.344018017 | 2.02E-14 |
| ARFIP2 | saddlebrow | 0.396471659 | 4.98E-19 | 0.403090425 | 1.13E-19 |
| ARSD | saddlebrow | 0.401973289 | 1.46E-19 | 0.398324355 | 3.30E-19 |
| ATP2C2 | saddlebrow | 0.432139296 | 1.14E-22 | 0.42750532 | 3.58E-22 |
| B4GALN | saddlebrow | 0.356187279 | 2.05E-15 | 0.363633983 | 4.79E-16 |
| B4GALT1 | saddlebrow | 0.328113736 | 3.49E-13 | 0.330816741 | 2.18E-13 |
| BACE2 | saddlebrow | 0.124062452 | 0.007270266 | 0.128733186 | 0.005334748 |
| BCAS4 | saddlebrow | -0.277615707 | 1.04E-09 | -0.259293298 | 1.30E-08 |
| C1orf64 | saddlebrow | 0.234410237 | 3.00E-07 | 0.262866142 | 8.07E-09 |
| C4BPA | saddlebrow | 0.259022696 | 1.35E-08 | 0.259462022 | 1.27E-08 |
| C5AR2 | saddlebrow | 0.221414152 | 1.35E-06 | 0.231468135 | 4.25E-07 |
| C9orf152 | saddlebrow | 0.440444665 | 1.39E-23 | 0.448236743 | 1.84E-24 |
| CA8 | saddlebrow | 0.294436036 | 8.57E-11 | 0.261532678 | 9.65E-09 |
| CAPN6 | saddlebrow | 0.251907888 | 3.42E-08 | 0.271531984 | 2.45E-09 |
| CCDC129 | saddlebrow | 0.122249234 | 0.008176952 | 0.103060502 | 0.025939194 |
| CEACAM | saddlebrow | 0.370823306 | 1.14E-16 | 0.3681441 | 1.95E-16 |
| CES3 | saddlebrow | 0.391543034 | 1.47E-18 | 0.378662751 | 2.27E-17 |
| CHDH | saddlebrow | 0.340300437 | 4.00E-14 | 0.322928622 | 8.53E-13 |
| CISH | saddlebrow | 0.308361531 | 9.58E-12 | 0.308648996 | 9.15E-12 |
| CLDN6 | saddlebrow | -0.340823476 | 3.63E-14 | -0.351269981 | 5.23E-15 |
| CPVL | saddlebrow | -0.463998889 | 2.61E-26 | -0.462398068 | 4.07E-26 |
| CREB3L1 | saddlebrow | 0.314355311 | 3.60E-12 | 0.322822715 | 8.69E-13 |
| CREB3L4 | saddlebrow | 0.403202657 | 1.10E-19 | 0.40655075 | 5.15E-20 |
| CRYL1 | saddlebrow | 0.33892321 | 5.13E-14 | 0.345780642 | 1.46E-14 |
| CTNNBIP | saddlebrow | 0.379700538 | 1.83E-17 | 0.374303799 | 5.60E-17 |
| CYB561 | saddlebrow | 0.317875732 | 2.00E-12 | 0.33090437 | 2.14E-13 |
| CYB5R1 | saddlebrow | 0.221398699 | 1.35E-06 | 0.215966774 | 2.47E-06 |
| CYP2J2 | saddlebrow | 0.150574485 | 0.001099098 | 0.144664661 | 0.001722326 |
| CYP4X1 | saddlebrow | 0.274689701 | 1.57E-09 | 0.255437786 | 2.16E-08 |
| DACT2 | saddlebrow | 0.162898876 | 0.000408584 | 0.140676721 | 0.002310851 |
| DEPTOR | saddlebrow | 0.247282923 | 6.17E-08 | 0.247703923 | 5.85E-08 |
| DNAJC22 | saddlebrow | 0.294723496 | 8.20E-11 | 0.305359346 | 1.55E-11 |
| DOCK5 | saddlebrow | 0.243430942 | 9.99E-08 | 0.234875517 | 2.84E-07 |
| ENTPD3 | saddlebrow | 0.327626112 | 3.80E-13 | 0.312995549 | 4.50E-12 |
| EPHB2 | saddlebrow | -0.366775623 | 2.57E-16 | -0.369263657 | 1.56E-16 |
| EPHX1 | saddlebrow | 0.391496071 | 1.48E-18 | 0.39510617 | 6.73E-19 |
| ERGIC1 | saddlebrow | 0.350142688 | 6.47E-15 | 0.363355198 | 5.06E-16 |
| ERMN | saddlebrow | 0.306454992 | 1.30E-11 | 0.285121814 | 3.48E-10 |
| ESR1 | saddlebrow | 0.238444675 | 1.84E-07 | 0.243046805 | 1.05E-07 |
| FAM174B | saddlebrow | 0.252234478 | 3.28E-08 | 0.256573915 | 1.86E-08 |
| FAM189A | saddlebrow | 0.15632632 | 0.000698795 | 0.142987163 | 0.001950755 |
| FAM46C | saddlebrow | 0.337126917 | 7.09E-14 | 0.347576361 | 1.05E-14 |

| | | | | | |
|---|---|---|---|---|---|
| FBLN1 | saddlebrow | 0.098892732 | 0.032630337 | 0.099580448 | 0.031434013 |
| FCHO1 | saddlebrow | -0.267195891 | 4.47E-09 | -0.249740558 | 4.52E-08 |
| FOLH1 | saddlebrow | 0.227075894 | 7.09E-07 | 0.23206576 | 3.96E-07 |
| FOXA2 | saddlebrow | 0.40749345 | 4.15E-20 | 0.39971027 | 2.42E-19 |
| GALNT1( | saddlebrow | 0.449178856 | 1.44E-24 | 0.454884763 | 3.15E-25 |
| GALNT1: | saddlebrow | 0.273492743 | 1.86E-09 | 0.286849896 | 2.69E-10 |
| GCNT1 | saddlebrow | 0.281134468 | 6.24E-10 | 0.26107249 | 1.03E-08 |
| GCNT2 | saddlebrow | 0.311986947 | 5.31E-12 | 0.320668822 | 1.25E-12 |
| GCNT3 | saddlebrow | 0.255995632 | 2.01E-08 | 0.288198964 | 2.20E-10 |
| GDF5 | saddlebrow | 0.13983266 | 0.002456867 | 0.145156449 | 0.001660165 |
| GLIS2 | saddlebrow | -0.41762054 | 3.90E-21 | -0.401078442 | 1.78E-19 |
| GLYATL: | saddlebrow | 0.391073559 | 1.63E-18 | 0.39063328 | 1.79E-18 |
| GNB5 | saddlebrow | 0.154816919 | 0.000788168 | 0.14699332 | 0.001445746 |
| GREB1 | saddlebrow | 0.135533262 | 0.003339766 | 0.102080736 | 0.027395452 |
| GRIN1 | saddlebrow | 0.398355186 | 3.28E-19 | 0.400235336 | 2.15E-19 |
| HADH | saddlebrow | 0.315606416 | 2.93E-12 | 0.321489603 | 1.09E-12 |
| HGD | saddlebrow | 0.371607925 | 9.70E-17 | 0.382012694 | 1.13E-17 |
| HID1 | saddlebrow | 0.241691258 | 1.24E-07 | 0.237842486 | 1.98E-07 |
| HIF3A | saddlebrow | -0.48603522 | 4.65E-29 | -0.495652482 | 2.53E-30 |
| HMGA2 | saddlebrow | -0.191565009 | 3.08E-05 | -0.200107537 | 1.32E-05 |
| HOXA4 | saddlebrow | -0.265992796 | 5.28E-09 | -0.276439683 | 1.23E-09 |
| HPX | saddlebrow | 0.191408889 | 3.13E-05 | 0.197622142 | 1.69E-05 |
| IGF2BP2 | saddlebrow | -0.358592134 | 1.29E-15 | -0.363572796 | 4.85E-16 |
| IHH | saddlebrow | 0.274532222 | 1.61E-09 | 0.254758171 | 2.36E-08 |
| IL20RA | saddlebrow | 0.332893805 | 1.51E-13 | 0.336169441 | 8.42E-14 |
| IQCJ-SCI | saddlebrow | -0.316276767 | 2.62E-12 | -0.329255621 | 2.86E-13 |
| ITM2C | saddlebrow | -0.305056783 | 1.63E-11 | -0.322933137 | 8.52E-13 |
| KDM4B | saddlebrow | 0.494969765 | 3.12E-30 | 0.486475221 | 4.08E-29 |
| KIAA132 | saddlebrow | 0.408633857 | 3.19E-20 | 0.418540071 | 3.13E-21 |
| L1CAM | saddlebrow | -0.388612247 | 2.77E-18 | -0.39961452 | 2.47E-19 |
| LRP5L | saddlebrow | 0.214537887 | 2.89E-06 | 0.202453865 | 1.04E-05 |
| LRRC26 | saddlebrow | 0.406275492 | 5.49E-20 | 0.412867823 | 1.20E-20 |
| LRRC3 | saddlebrow | -0.277764186 | 1.02E-09 | -0.300922408 | 3.14E-11 |
| MAL | saddlebrow | -0.178594054 | 0.000104307 | -0.167813952 | 0.000269861 |
| MANSC1 | saddlebrow | 0.372993482 | 7.32E-17 | 0.364404213 | 4.11E-16 |
| MAP2K6 | saddlebrow | 0.303471412 | 2.10E-11 | 0.305749589 | 1.46E-11 |
| MCF2L | saddlebrow | 0.300224065 | 3.50E-11 | 0.283714375 | 4.28E-10 |
| MLPH | saddlebrow | 0.344496912 | 1.85E-14 | 0.361280214 | 7.61E-16 |
| MMP26 | saddlebrow | 0.12381571 | 0.007388114 | 0.08085778 | 0.080893071 |
| MUC5AC | saddlebrow | 0.139492393 | 0.002518075 | 0.185680969 | 5.41E-05 |
| MUC5B | saddlebrow | 0.329329543 | 2.82E-13 | 0.343887238 | 2.07E-14 |
| NAAA | saddlebrow | 0.187808844 | 4.42E-05 | 0.193659964 | 2.51E-05 |
| NCMAP | saddlebrow | 0.265571818 | 5.59E-09 | 0.261357016 | 9.88E-09 |
| PGR | saddlebrow | 0.196433855 | 1.91E-05 | 0.167818894 | 0.000269747 |
| PIGR | saddlebrow | 0.333450929 | 1.37E-13 | 0.354221654 | 2.98E-15 |

| | | | | | |
|---|---|---|---|---|---|
| PIK3R3 | saddlebrow | 0.432015549 | 1.17E-22 | 0.448035926 | 1.94E-24 |
| PLLP | saddlebrow | 0.209251026 | 5.11E-06 | 0.223922293 | 1.02E-06 |
| POC1B-G | saddlebrow | 0.273975838 | 1.74E-09 | 0.265709006 | 5.48E-09 |
| PORCN | saddlebrow | 0.145366354 | 0.001634265 | 0.133071506 | 0.003966484 |
| PPAP2C | saddlebrow | 0.42591544 | 5.29E-22 | 0.441592199 | 1.04E-23 |
| RBBP7 | saddlebrow | 0.301034038 | 3.08E-11 | 0.294492634 | 8.50E-11 |
| RNF183 | saddlebrow | 0.294942461 | 7.93E-11 | 0.30719618 | 1.16E-11 |
| RSPO4 | saddlebrow | -0.42985789 | 2.01E-22 | -0.442901768 | 7.41E-24 |
| SCGB2A | saddlebrow | 0.342956846 | 2.46E-14 | 0.352102952 | 4.47E-15 |
| SELENBI | saddlebrow | 0.19560985 | 2.07E-05 | 0.196434827 | 1.91E-05 |
| SERINC2 | saddlebrow | 0.339160166 | 4.92E-14 | 0.363139773 | 5.28E-16 |
| SH2D4A | saddlebrow | 0.349685112 | 7.05E-15 | 0.339461801 | 4.65E-14 |
| SLC25A3 | saddlebrow | 0.288000363 | 2.27E-10 | 0.276856834 | 1.16E-09 |
| SLC43A1 | saddlebrow | 0.263228859 | 7.68E-09 | 0.246701304 | 6.64E-08 |
| SLC44A3 | saddlebrow | 0.22018598 | 1.55E-06 | 0.204150357 | 8.72E-06 |
| SLC47A1 | saddlebrow | 0.303896673 | 1.96E-11 | 0.272920605 | 2.02E-09 |
| SLC5A1 | saddlebrow | 0.345884402 | 1.43E-14 | 0.335574561 | 9.37E-14 |
| SLC6A12 | saddlebrow | -0.36362976 | 4.80E-16 | -0.359542842 | 1.07E-15 |
| SLCO3A1 | saddlebrow | -0.387467875 | 3.54E-18 | -0.38191891 | 1.15E-17 |
| SPDEF | saddlebrow | 0.449874432 | 1.20E-24 | 0.468793336 | 6.85E-27 |
| SPINK4 | saddlebrow | 0.23838565 | 1.86E-07 | 0.249825587 | 4.47E-08 |
| SRD5A3 | saddlebrow | 0.375853422 | 4.07E-17 | 0.387617232 | 3.43E-18 |
| ST6GALN | saddlebrow | 0.20652677 | 6.80E-06 | 0.244156109 | 9.13E-08 |
| STEAP1 | saddlebrow | 0.30808642 | 1.00E-11 | 0.292908493 | 1.08E-10 |
| STX19 | saddlebrow | 0.247925251 | 5.69E-08 | 0.25099783 | 3.85E-08 |
| STXBP6 | saddlebrow | 0.281736161 | 5.72E-10 | 0.287557766 | 2.42E-10 |
| TBC1D30 | saddlebrow | 0.329523209 | 2.73E-13 | 0.317389869 | 2.17E-12 |
| TFF3 | saddlebrow | 0.431515006 | 1.33E-22 | 0.456099907 | 2.27E-25 |
| TMC5 | saddlebrow | 0.297351969 | 5.47E-11 | 0.311110094 | 6.13E-12 |
| TMEM21 | saddlebrow | 0.384172964 | 7.15E-18 | 0.370117463 | 1.31E-16 |
| TMEM21 | saddlebrow | 0.297151214 | 5.64E-11 | 0.30551839 | 1.51E-11 |
| TMEM62 | saddlebrow | 0.429436272 | 2.23E-22 | 0.441726883 | 1.00E-23 |
| TMEM63 | saddlebrow | 0.305520093 | 1.51E-11 | 0.309462927 | 8.02E-12 |
| TMPRSS | saddlebrow | 0.2706234 | 2.78E-09 | 0.252262511 | 3.27E-08 |
| TNNT1 | saddlebrow | -0.261629933 | 9.52E-09 | -0.25568264 | 2.10E-08 |
| TRIM3 | saddlebrow | 0.304483562 | 1.79E-11 | 0.313682869 | 4.02E-12 |
| TRPM4 | saddlebrow | 0.406550625 | 5.15E-20 | 0.41788074 | 3.67E-21 |
| TSKU | saddlebrow | 0.287967941 | 2.28E-10 | 0.30003793 | 3.60E-11 |
| TSPAN8 | saddlebrow | 0.233709872 | 3.26E-07 | 0.235092261 | 2.76E-07 |
| TUBA3D | saddlebrow | 0.01245174 | 0.788411717 | -0.008044814 | 0.862347395 |
| TUBB4A | saddlebrow | -0.278966769 | 8.54E-10 | -0.29294519 | 1.08E-10 |
| VIM | saddlebrow | 0.136185763 | 0.003189463 | 0.148578713 | 0.001281465 |
| VMAC | saddlebrow | 0.421975914 | 1.38E-21 | 0.435211539 | 5.27E-23 |
| ZG16B | saddlebrow | 0.282773627 | 4.91E-10 | 0.28844541 | 2.12E-10 |
| ZNF516 | saddlebrow | 0.04347965 | 0.348486766 | 0.021343446 | 0.645478837 |

| | | | | | |
|---|---|---|---|---|---|
| AAAS | salmon | 0.053195088 | 0.251262923 | 0.027670833 | 0.550852313 |
| AAK1 | salmon | -0.241019516 | 1.35E-07 | -0.238038219 | 1.94E-07 |
| ABCA1 | salmon | -0.146429662 | 0.00150867 | -0.132618052 | 0.004092913 |
| AC00300 | salmon | -0.069136802 | 0.135740984 | -0.057043751 | 0.218538253 |
| AC07365 | salmon | 0.11079309 | 0.016610368 | 0.095463266 | 0.039192357 |
| AC09015 | salmon | -0.189926669 | 3.61E-05 | -0.202190906 | 1.07E-05 |
| AC09118 | salmon | -0.075731214 | 0.102148159 | -0.072163345 | 0.119395792 |
| AGO1 | salmon | -0.210325707 | 4.55E-06 | -0.208625699 | 5.46E-06 |
| AGO2 | salmon | -0.166415311 | 0.000304028 | -0.191622836 | 3.06E-05 |
| AGO3 | salmon | -0.183288135 | 6.77E-05 | -0.200665436 | 1.25E-05 |
| AK1 | salmon | 0.04055388 | 0.381907068 | 0.070250426 | 0.129537209 |
| AKAP13 | salmon | -0.004414976 | 0.92419314 | -0.014799129 | 0.749747538 |
| ALKBH4 | salmon | -0.026183572 | 0.572473673 | -0.008530885 | 0.854120728 |
| ANKRD6 | salmon | -0.243239556 | 1.02E-07 | -0.252770692 | 3.06E-08 |
| ARF5 | salmon | 0.17700539 | 0.000120419 | 0.192659101 | 2.77E-05 |
| ARFGEF | salmon | -0.076083011 | 0.100560505 | -0.097837971 | 0.034540595 |
| ARHGAP | salmon | 0.148047623 | 0.00133448 | 0.123754319 | 0.007417699 |
| ARPC4 | salmon | 0.132856172 | 0.004026075 | 0.161018337 | 0.000477395 |
| ASXL1 | salmon | -0.356824527 | 1.81E-15 | -0.390580527 | 1.81E-18 |
| ASXL2 | salmon | -0.096324828 | 0.037446783 | -0.118059106 | 0.010668402 |
| ATP5G2 | salmon | 0.195408081 | 2.11E-05 | 0.191947876 | 2.97E-05 |
| ATP6V1F | salmon | 0.059368106 | 0.200317633 | 0.083071258 | 0.07289701 |
| ATP9B | salmon | 0.107754213 | 0.019852029 | 0.101073228 | 0.028965721 |
| ATRAID | salmon | -0.033036144 | 0.476342012 | -0.022265799 | 0.631271502 |
| B3GALN | salmon | 0.134944792 | 0.003480804 | 0.102260306 | 0.027123405 |
| B3GALT | salmon | -0.110424572 | 0.016977044 | -0.115508884 | 0.012495104 |
| BANF1 | salmon | -0.044424685 | 0.338100215 | -0.054734441 | 0.237786915 |
| BIRC6 | salmon | -0.07197815 | 0.120349647 | -0.084541826 | 0.067950175 |
| BMP2K | salmon | 0.050024575 | 0.280668597 | 0.052406374 | 0.258369615 |
| BOD1L1 | salmon | -0.092403131 | 0.045959112 | -0.110528015 | 0.01687341 |
| C10orf12 | salmon | 0.029876356 | 0.519544942 | 0.023967061 | 0.605422323 |
| C12orf10 | salmon | -0.024828905 | 0.592510365 | -0.024741007 | 0.593821456 |
| C14orf1 | salmon | 0.16080583 | 0.000485814 | 0.135380886 | 0.003375779 |
| C19orf53 | salmon | -0.01594378 | 0.731112172 | -0.003005221 | 0.948357548 |
| C1QL3 | salmon | -0.016573321 | 0.720929206 | -0.040226136 | 0.385769125 |
| C2orf16 | salmon | -0.304676826 | 1.73E-11 | -0.301699925 | 2.77E-11 |
| C5orf42 | salmon | -0.193327475 | 2.59E-05 | -0.209603562 | 4.92E-06 |
| C7orf55 | salmon | 0.055329899 | 0.232713079 | 0.066438427 | 0.151719854 |
| CBL | salmon | -0.121055963 | 0.008827394 | -0.135390259 | 0.003373554 |
| CCDC107 | salmon | 0.138725751 | 0.002661105 | 0.139178655 | 0.002575739 |
| CCRN4L | salmon | 0.237442978 | 2.08E-07 | 0.225763908 | 8.24E-07 |
| CD63 | salmon | 0.087237939 | 0.059596338 | 0.099506833 | 0.031560252 |
| CDK5RA | salmon | 0.080676276 | 0.08157907 | 0.070008158 | 0.130867804 |
| CDK6 | salmon | -0.267630847 | 4.21E-09 | -0.262065567 | 8.98E-09 |
| CDKL2 | salmon | -0.258752476 | 1.40E-08 | -0.250612264 | 4.04E-08 |

| | | | | | |
|---|---|---|---|---|---|
| CDKL5 | salmon | -0.217069892 | 2.19E-06 | -0.24349926 | 9.91E-08 |
| CDPF1 | salmon | 0.217113885 | 2.18E-06 | 0.209105038 | 5.18E-06 |
| CH17-14( | salmon | -0.040477149 | 0.382809129 | -0.068846779 | 0.137393593 |
| CLASP1 | salmon | -0.087297638 | 0.059421436 | -0.104560041 | 0.023839678 |
| CNPY3 | salmon | -0.041267833 | 0.373576244 | -0.037721577 | 0.416060398 |
| COBLL1 | salmon | 0.007636256 | 0.869273752 | 0.010871038 | 0.814750184 |
| COPS6 | salmon | 0.018773969 | 0.685729452 | 0.04067205 | 0.380520406 |
| CRYBG3 | salmon | -0.169829004 | 0.000226898 | -0.153438502 | 0.000878911 |
| CTD-258: | salmon | -0.015565455 | 0.737254588 | -0.054593537 | 0.238998854 |
| CYB5RL | salmon | 0.014592734 | 0.753123642 | -0.014101341 | 0.761180464 |
| DGCR14 | salmon | 0.066649762 | 0.150419033 | 0.055065263 | 0.234958478 |
| DGCR6L | salmon | 0.080173167 | 0.083505201 | 0.090830298 | 0.049802612 |
| DHRS4L2 | salmon | 0.076390369 | 0.099189507 | 0.095208685 | 0.039721153 |
| DNAJC17 | salmon | 0.231500025 | 4.23E-07 | 0.23710218 | 2.17E-07 |
| DOCK4 | salmon | -0.235012961 | 2.79E-07 | -0.225815108 | 8.20E-07 |
| DRAP1 | salmon | -0.068152451 | 0.141412821 | -0.062106045 | 0.180303932 |
| DSE | salmon | -0.212625459 | 3.56E-06 | -0.211711082 | 3.93E-06 |
| DYNLRB | salmon | -0.009033275 | 0.845634442 | 0.02297154 | 0.620491321 |
| ECH1 | salmon | 0.062574731 | 0.177031828 | 0.078420819 | 0.090502259 |
| EPB41L4. | salmon | -0.05486866 | 0.236636501 | -0.065496221 | 0.157623451 |
| EPG5 | salmon | -0.050859519 | 0.272707859 | -0.065420864 | 0.158103007 |
| EPHA6 | salmon | -0.223825218 | 1.03E-06 | -0.220440513 | 1.51E-06 |
| ERCC1 | salmon | 0.045787912 | 0.323470457 | 0.041021927 | 0.37643287 |
| ERP29 | salmon | 0.171087556 | 0.000203405 | 0.152052642 | 0.000979794 |
| FAM160A | salmon | 0.045863954 | 0.322666697 | 0.068276522 | 0.140688085 |
| FAM169A | salmon | 0.017570329 | 0.704903465 | -0.004445552 | 0.923669727 |
| FAM83B | salmon | -0.017405233 | 0.707548414 | -0.019981111 | 0.666700139 |
| FAM89B | salmon | 0.000101314 | 0.998257788 | 0.016754983 | 0.717999847 |
| FAU | salmon | -0.011716788 | 0.800629861 | -0.005158877 | 0.91146867 |
| FRK | salmon | -0.0066164 | 0.886606108 | 0.008518088 | 0.854337113 |
| GAFA3 | salmon | 0.115818721 | 0.012259345 | 0.091826118 | 0.047339185 |
| GAN | salmon | 0.026682583 | 0.565174572 | 0.040623917 | 0.381084853 |
| GIT2 | salmon | -0.106240449 | 0.021663038 | -0.116078738 | 0.012064536 |
| GLIS3 | salmon | 0.124147179 | 0.007230188 | 0.133086241 | 0.003962435 |
| GNPTG | salmon | 0.094353759 | 0.041541307 | 0.097704505 | 0.034788969 |
| GOLGA3 | salmon | 0.144305696 | 0.001769037 | 0.161892413 | 0.000444171 |
| GPATCH | salmon | -0.141605183 | 0.002159417 | -0.164247939 | 0.000365037 |
| GPLD1 | salmon | -0.243441377 | 9.98E-08 | -0.270382167 | 2.88E-09 |
| GPR75 | salmon | -0.186381199 | 5.07E-05 | -0.171307287 | 0.000199545 |
| GSTK1 | salmon | 0.279722324 | 7.66E-10 | 0.293213455 | 1.03E-10 |
| HECW2 | salmon | -0.142238724 | 0.00206135 | -0.135627007 | 0.003317784 |
| HELB | salmon | -0.102442655 | 0.026849532 | -0.09747381 | 0.035221867 |
| HERC1 | salmon | -0.065502107 | 0.157586041 | -0.070444666 | 0.128477975 |
| HHLA3 | salmon | 0.118632568 | 0.010291807 | 0.121374129 | 0.008649607 |
| HIPK2 | salmon | -0.103876073 | 0.024778352 | -0.11562649 | 0.012405149 |

| | | | | | |
|---|---|---|---|---|---|
| HIVEP1 | salmon | -0.083398943 | 0.071770009 | -0.087129229 | 0.059915924 |
| HIVEP2 | salmon | -0.212668393 | 3.54E-06 | -0.209152556 | 5.16E-06 |
| HMCN1 | salmon | -0.017203457 | 0.710785783 | -0.027661549 | 0.550986033 |
| IFI27L1 | salmon | 0.130605408 | 0.004699206 | 0.125106413 | 0.006790044 |
| IFI27L2 | salmon | -0.274398611 | 1.64E-09 | -0.268928574 | 3.52E-09 |
| IFNAR2 | salmon | -0.255254061 | 2.22E-08 | -0.240917048 | 1.36E-07 |
| IGF2BP3 | salmon | -0.153536007 | 0.000872188 | -0.171519726 | 0.000195878 |
| INPP4A | salmon | -0.2469617 | 6.42E-08 | -0.265138973 | 5.93E-09 |
| ITGA4 | salmon | 0.094671591 | 0.040856581 | 0.108061012 | 0.019501489 |
| JAGN1 | salmon | 0.007193466 | 0.876791743 | 0.02311676 | 0.618283036 |
| JRK | salmon | -0.143578926 | 0.001867184 | -0.171185225 | 0.000201681 |
| KALRN | salmon | -0.286007689 | 3.05E-10 | -0.300215696 | 3.50E-11 |
| KAT6A | salmon | -0.122106023 | 0.008252689 | -0.152226034 | 0.000966612 |
| KDM5A | salmon | -0.115023594 | 0.012872413 | -0.132535005 | 0.004116459 |
| KIAA035 | salmon | -0.14194332 | 0.002106558 | -0.165798771 | 0.000320336 |
| KIAA201 | salmon | 0.052421119 | 0.258235497 | 0.033674417 | 0.467859627 |
| KIF3B | salmon | 0.066168399 | 0.153394344 | 0.058383425 | 0.207896835 |
| KLHL11 | salmon | 0.072514545 | 0.117603148 | 0.058056437 | 0.210458975 |
| KMT2A | salmon | -0.076791868 | 0.097421025 | -0.106224027 | 0.021683447 |
| KRBA2 | salmon | 0.018527905 | 0.689633368 | 0.030943218 | 0.504736183 |
| LAMTOR | salmon | 0.025850079 | 0.577376467 | 0.054606291 | 0.238888971 |
| LARP4B | salmon | 0.115449771 | 0.012540536 | 0.100702373 | 0.02956286 |
| LENG1 | salmon | -0.044163614 | 0.340949547 | -0.032349265 | 0.485563398 |
| LONRF3 | salmon | 0.078279891 | 0.091084819 | 0.061745575 | 0.182850846 |
| LTB4R2 | salmon | 0.003961701 | 0.931956131 | -0.002805683 | 0.95178214 |
| LY6G5B | salmon | -0.166232825 | 0.000308772 | -0.176634827 | 0.000124499 |
| MACC1 | salmon | -0.193772778 | 2.48E-05 | -0.173210851 | 0.000168859 |
| MACF1 | salmon | -0.107527583 | 0.020114481 | -0.125378553 | 0.006669623 |
| MAP3K9 | salmon | 0.138993745 | 0.002610286 | 0.133200787 | 0.003931092 |
| MICAL3 | salmon | -0.113197269 | 0.014383944 | -0.152971417 | 0.000911787 |
| MICALCl | salmon | 0.154259857 | 0.000823743 | 0.145473766 | 0.001621156 |
| MID2 | salmon | -0.132175211 | 0.004219891 | -0.146101587 | 0.001546442 |
| MIEN1 | salmon | 0.029250517 | 0.528334853 | 0.034141857 | 0.461700833 |
| MKL2 | salmon | -0.066848981 | 0.149200571 | -0.074060847 | 0.109959559 |
| MRPL40 | salmon | 0.201008028 | 1.20E-05 | 0.207720843 | 6.00E-06 |
| MVB12A | salmon | -0.08514515 | 0.0660019 | -0.058858929 | 0.204211337 |
| MYCBP2 | salmon | -0.124476306 | 0.007076373 | -0.142106617 | 0.002081457 |
| MYL6 | salmon | -0.047226177 | 0.308488122 | -0.022730241 | 0.624168173 |
| MYO5A | salmon | -0.243746602 | 9.61E-08 | -0.219700113 | 1.64E-06 |
| MZT2A | salmon | -0.020004666 | 0.666330897 | -0.038508814 | 0.406391639 |
| MZT2B | salmon | 0.083029177 | 0.073042779 | 0.075459345 | 0.103388668 |
| NBPF26 | salmon | -0.132129897 | 0.004233082 | -0.144513473 | 0.00174186 |
| NEDD8 | salmon | -0.018943141 | 0.683050289 | -0.002779452 | 0.952232405 |
| NFAT5 | salmon | 0.005304216 | 0.908985174 | 0.002908624 | 0.950015283 |
| NFKBIL1 | salmon | -0.11144525 | 0.015978433 | -0.125981656 | 0.006409545 |

| | | | | | |
|---|---|---|---|---|---|
| NHSL2 | salmon | -0.058404064 | 0.207735873 | -0.066425498 | 0.151799713 |
| NOD1 | salmon | -0.057369608 | 0.215914667 | -0.090606581 | 0.050370534 |
| NOS1AP | salmon | 0.051506794 | 0.266643388 | 0.024970418 | 0.590402337 |
| NOXA1 | salmon | 0.159773808 | 0.000528701 | 0.154620901 | 0.000800521 |
| NPC2 | salmon | 0.012658897 | 0.784977078 | 0.032899255 | 0.478172087 |
| NT5C | salmon | 0.046449387 | 0.316522156 | 0.038667664 | 0.404456995 |
| OTULIN | salmon | -0.086086271 | 0.063054596 | -0.069076405 | 0.136083869 |
| PCDHAC | salmon | -0.049450889 | 0.286228688 | -0.025783392 | 0.578359225 |
| PCDHGA | salmon | -0.193824056 | 2.47E-05 | -0.194695591 | 2.27E-05 |
| PCDHGB | salmon | -0.111917661 | 0.015533928 | -0.103729006 | 0.024984303 |
| PCDHGC | salmon | 0.094254841 | 0.041756378 | 0.092294575 | 0.046216141 |
| PCLO | salmon | -0.003266364 | 0.943877096 | -0.001363857 | 0.97655017 |
| PCNX | salmon | -0.102421393 | 0.026881343 | -0.128431722 | 0.005444023 |
| PCSK7 | salmon | 0.02112339 | 0.648887834 | 0.023206633 | 0.616918104 |
| PDIA3 | salmon | 0.176696609 | 0.00012381 | 0.174593214 | 0.000149412 |
| PDZD2 | salmon | -0.170075845 | 0.000222099 | -0.20548216 | 7.59E-06 |
| PEAK1 | salmon | -0.051480488 | 0.266888039 | -0.061806375 | 0.182419407 |
| PEBP1 | salmon | 0.182075682 | 7.58E-05 | 0.189216961 | 3.87E-05 |
| PER2 | salmon | 0.067411647 | 0.145799735 | 0.062898748 | 0.17479564 |
| PFDN2 | salmon | 0.161934195 | 0.000442638 | 0.158327414 | 0.000594747 |
| PGLS | salmon | 0.045200098 | 0.329727528 | 0.061870426 | 0.181965715 |
| PHC3 | salmon | -0.212521058 | 3.60E-06 | -0.21209199 | 3.77E-06 |
| PHLPP2 | salmon | -0.097181348 | 0.035777249 | -0.096355978 | 0.037384927 |
| PLEKHM | salmon | -0.154557489 | 0.000804555 | -0.156879967 | 0.000668436 |
| PMF1-BG | salmon | -0.079948004 | 0.08437904 | -0.09449018 | 0.041246229 |
| POMK | salmon | 0.091957309 | 0.047022394 | 0.077335223 | 0.095067821 |
| PPARA | salmon | -0.016541496 | 0.72144283 | -0.031943974 | 0.491049299 |
| PPIB | salmon | 0.264230667 | 6.71E-09 | 0.260370812 | 1.13E-08 |
| PRDX5 | salmon | 0.010708221 | 0.817475574 | 0.029596176 | 0.523470754 |
| PRKCE | salmon | -0.279589722 | 7.81E-10 | -0.280873677 | 6.48E-10 |
| PRR13 | salmon | 0.053610278 | 0.24757698 | 0.064696139 | 0.162771369 |
| PRRC2C | salmon | 0.053999328 | 0.244157496 | 0.022312919 | 0.630549269 |
| PSMB4 | salmon | 0.070687637 | 0.127162465 | 0.088613703 | 0.055672285 |
| PSMD4 | salmon | 0.041477348 | 0.371152929 | 0.054525752 | 0.23958343 |
| PTPN14 | salmon | -0.158495297 | 0.000586706 | -0.168223996 | 0.000260546 |
| PTRHD1 | salmon | 0.016789323 | 0.717446567 | 0.012386286 | 0.789497795 |
| RAB13 | salmon | 0.0870102 | 0.060267469 | 0.079301305 | 0.086929895 |
| RAB24 | salmon | -0.031579914 | 0.496005274 | -0.041353568 | 0.372583426 |
| RAD54L2 | salmon | 0.004158224 | 0.928589557 | -0.012429259 | 0.7887847 |
| RALGAP | salmon | 0.19749888 | 1.72E-05 | 0.188370021 | 4.19E-05 |
| RBM33 | salmon | -0.131873559 | 0.004308412 | -0.154467195 | 0.000810332 |
| REL | salmon | 0.005705569 | 0.902131683 | -0.001759237 | 0.969755 |
| RELL1 | salmon | 0.090170972 | 0.051491887 | 0.09824484 | 0.033792731 |
| RHOC | salmon | 0.270302633 | 2.91E-09 | 0.28401333 | 4.10E-10 |
| RING1 | salmon | -0.147854851 | 0.001354216 | -0.152138945 | 0.000973213 |

| | | | | | |
|---|---|---|---|---|---|
| RNF152 | salmon | -0.128073968 | 0.005576313 | -0.127897928 | 0.005642464 |
| RNF169 | salmon | -0.02197134 | 0.635792738 | -0.048066125 | 0.299953451 |
| RNF25 | salmon | -0.064558468 | 0.163669776 | -0.045931605 | 0.321952707 |
| RP11-101 | salmon | 0.099411624 | 0.031724165 | 0.091879957 | 0.047208962 |
| RP11-449 | salmon | -0.069498011 | 0.133704184 | -0.077633455 | 0.093795635 |
| RP11-849 | salmon | -0.014017524 | 0.762557321 | -0.028250508 | 0.542534986 |
| RPP25L | salmon | 0.099770635 | 0.031109866 | 0.121051726 | 0.008829784 |
| RPS16 | salmon | -0.069659987 | 0.132798521 | -0.068222235 | 0.141004841 |
| RTEL1 | salmon | -0.077795124 | 0.093111707 | -0.107966375 | 0.01960904 |
| SAMD12 | salmon | -0.199524639 | 1.40E-05 | -0.221415728 | 1.35E-06 |
| SAT2 | salmon | 0.261843951 | 9.25E-09 | 0.253553601 | 2.77E-08 |
| SCNM1 | salmon | -0.065197441 | 0.159531292 | -0.054728202 | 0.237840481 |
| SCRN2 | salmon | 0.138406993 | 0.002722724 | 0.134987305 | 0.003470437 |
| SEPW1 | salmon | 0.132605544 | 0.004096452 | 0.184855945 | 5.85E-05 |
| SH3BGRI | salmon | 0.1712068 | 0.000201302 | 0.218711496 | 1.83E-06 |
| SHPRH | salmon | -0.102734615 | 0.026415986 | -0.121058154 | 0.008826159 |
| SHROOM | salmon | -0.194675726 | 2.27E-05 | -0.221882819 | 1.28E-06 |
| SIK2 | salmon | 0.023246969 | 0.616305928 | -0.001832386 | 0.96849805 |
| SIK3 | salmon | -0.074465777 | 0.108024018 | -0.093162504 | 0.044194501 |
| SLC35E2 | salmon | 0.045345525 | 0.328172278 | 0.04847716 | 0.295834684 |
| SLIT2 | salmon | -0.164804602 | 0.000348363 | -0.148550139 | 0.001284267 |
| SOX6 | salmon | 0.035485601 | 0.44425001 | 0.050943919 | 0.271911803 |
| SPAG7 | salmon | 0.0414244 | 0.371764419 | 0.043630937 | 0.346810574 |
| SPIRE1 | salmon | -0.259693761 | 1.23E-08 | -0.259454965 | 1.27E-08 |
| SRGAP1 | salmon | -0.128503908 | 0.005417675 | -0.126572494 | 0.006163613 |
| SRGAP2I | salmon | -0.108888874 | 0.018582361 | -0.156406925 | 0.000694297 |
| SSBP4 | salmon | -0.10014757 | 0.030475876 | -0.078134518 | 0.091688902 |
| STX8 | salmon | 0.202137685 | 1.07E-05 | 0.20634609 | 6.93E-06 |
| SWSAP1 | salmon | -0.14496279 | 0.001684393 | -0.1134552 | 0.014161425 |
| SYDE2 | salmon | -0.268767315 | 3.60E-09 | -0.284324561 | 3.91E-10 |
| SYNE2 | salmon | -0.024699153 | 0.594446218 | -0.040997411 | 0.376718401 |
| SYNGAP | salmon | -0.27306398 | 1.98E-09 | -0.294472532 | 8.53E-11 |
| SYNJ2 | salmon | 0.003496603 | 0.939928317 | 0.006926931 | 0.881322542 |
| SZT2 | salmon | -0.220273369 | 1.54E-06 | -0.231005561 | 4.49E-07 |
| TADA3 | salmon | 0.162643253 | 0.000417362 | 0.164521713 | 0.000356746 |
| TANC1 | salmon | 0.005678965 | 0.902585749 | -0.01070627 | 0.817508245 |
| TANC2 | salmon | -0.368607961 | 1.78E-16 | -0.392924148 | 1.09E-18 |
| TBC1D9 | salmon | -0.029758159 | 0.521199241 | -0.023124495 | 0.618165504 |
| TCP11L1 | salmon | -0.015739773 | 0.73442229 | -0.037332813 | 0.420884645 |
| TCP11L2 | salmon | -0.079590497 | 0.085781633 | -0.07713202 | 0.095942498 |
| TEP1 | salmon | 0.242687132 | 1.10E-07 | 0.245244549 | 7.97E-08 |
| TET1 | salmon | -0.053895246 | 0.245069049 | -0.075002731 | 0.10549898 |
| THAP3 | salmon | 0.025843624 | 0.577471558 | 0.024302431 | 0.600382953 |
| THAP7 | salmon | 0.094366145 | 0.041514443 | 0.081145481 | 0.079815273 |
| TMEM14 | salmon | 0.200049912 | 1.33E-05 | 0.210124315 | 4.65E-06 |

| | | | | | |
|---|---|---|---|---|---|
| TMEM25 | salmon | 0.180305625 | 8.92E-05 | 0.166399241 | 0.000304443 |
| TMPPE | salmon | 0.004343805 | 0.925411592 | 0.02642312 | 0.5689642 |
| TNRC6B | salmon | -0.04032743 | 0.38457298 | -0.045538298 | 0.326118031 |
| TRAPPC1 | salmon | 0.184711118 | 5.93E-05 | 0.20955255 | 4.94E-06 |
| TRAPPC6 | salmon | 0.097821213 | 0.034571699 | 0.10526375 | 0.022906157 |
| TRIO | salmon | -0.215570678 | 2.58E-06 | -0.214728487 | 2.83E-06 |
| TRMT112 | salmon | -0.050306944 | 0.277958948 | -0.046572371 | 0.315241146 |
| TTC28 | salmon | 0.025015211 | 0.589735806 | 0.022718628 | 0.624345367 |
| TULP4 | salmon | 0.091167353 | 0.048957086 | 0.077638349 | 0.093774871 |
| UBXN1 | salmon | -0.080483029 | 0.082314616 | -0.086026529 | 0.063238407 |
| USE1 | salmon | -0.192104569 | 2.92E-05 | -0.192881712 | 2.71E-05 |
| USP24 | salmon | -0.096179066 | 0.037737386 | -0.123342365 | 0.007618983 |
| USP31 | salmon | -0.221934081 | 1.27E-06 | -0.232306392 | 3.85E-07 |
| USP34 | salmon | -0.045042267 | 0.331420798 | -0.077354444 | 0.094985415 |
| USP37 | salmon | 0.036491514 | 0.431435866 | 0.015343794 | 0.74086126 |
| VPS13B | salmon | -0.099000226 | 0.032440821 | -0.128362183 | 0.005469514 |
| WDR7 | salmon | 0.097853502 | 0.034511792 | 0.095679731 | 0.038747433 |
| ZBED6 | salmon | 0.067277229 | 0.146606754 | 0.04245869 | 0.359932371 |
| ZC3HAV | salmon | -0.021737827 | 0.639387855 | -0.021936432 | 0.63632964 |
| ZCCHC1 | salmon | -0.12627126 | 0.006287921 | -0.154987801 | 0.000777544 |
| ZFHX3 | salmon | 0.184858249 | 5.85E-05 | 0.186536415 | 4.99E-05 |
| ZFYVE26 | salmon | -0.005365825 | 0.907932688 | -0.01430405 | 0.757853682 |
| ZHX3 | salmon | -0.13780104 | 0.002843455 | -0.155400665 | 0.000752419 |
| ZMAT5 | salmon | 0.090468015 | 0.050725 | 0.06927274 | 0.134971667 |
| ZNF221 | salmon | 0.066713725 | 0.150027002 | 0.056521017 | 0.22279452 |
| ZNF236 | salmon | 0.041748513 | 0.368031034 | 0.020344374 | 0.661014592 |
| ZNF264 | salmon | 0.04691248 | 0.311716235 | 0.052941866 | 0.253529605 |
| ZNF275 | salmon | -0.142321038 | 0.002048912 | -0.148520839 | 0.001287146 |
| ZNF407 | salmon | -0.091051037 | 0.049247507 | -0.106979175 | 0.02076209 |
| ZNF417 | salmon | -0.059940017 | 0.196008935 | -0.069866197 | 0.131652386 |
| ZNF460 | salmon | -0.086730426 | 0.06110048 | -0.081906621 | 0.077019872 |
| ZNF462 | salmon | -0.111411741 | 0.016010381 | -0.147381581 | 0.001403815 |
| ZNF490 | salmon | -0.007007921 | 0.879945393 | -0.000604629 | 0.989602957 |
| ZNF543 | salmon | -0.017483129 | 0.70630003 | -0.030286243 | 0.513829124 |
| ZNF551 | salmon | -0.081808252 | 0.077376611 | -0.096274228 | 0.037547447 |
| ZNF557 | salmon | 0.116457413 | 0.011785717 | 0.118534568 | 0.010355316 |
| ZNF587 | salmon | 0.014484857 | 0.754890132 | 0.020345067 | 0.661003773 |
| ZNF611 | salmon | -0.030531772 | 0.510420927 | -0.046047335 | 0.32073369 |
| ZNF625 | salmon | 0.003605032 | 0.93806918 | 0.00028736 | 0.995058527 |
| ZNF699 | salmon | -0.005851046 | 0.89964929 | -0.032121096 | 0.488647754 |
| ZNF70 | salmon | -0.092418555 | 0.045922691 | -0.089793059 | 0.052481519 |
| ZNF724P | salmon | 0.012296074 | 0.790995368 | 0.011198104 | 0.809282227 |
| ZNF727 | salmon | -0.36920879 | 1.58E-16 | -0.372829779 | 7.57E-17 |
| ZNF749 | salmon | 0.070839182 | 0.126347266 | 0.059996739 | 0.195585323 |
| ZNF765 | salmon | 0.003848246 | 0.933900256 | 0.000583915 | 0.989959129 |

| | | | | | |
|---|---|---|---|---|---|
| ZNF778 | salmon | 0.009021917 | 0.845826099 | 0.031100164 | 0.502576534 |
| ZNF8 | salmon | -0.072992427 | 0.115197777 | -0.075578947 | 0.102841476 |
| ZNF805 | salmon | -0.050542846 | 0.275708848 | -0.056736885 | 0.221029741 |
| ZNF808 | salmon | -0.012663276 | 0.784904507 | 0.00166419 | 0.971388341 |
| ZNF813 | salmon | 0.051980186 | 0.262267049 | 0.039080071 | 0.39945998 |
| ZNF827 | salmon | 0.008428287 | 0.855855892 | -0.02524971 | 0.586252042 |
| ZNF845 | salmon | 0.013589967 | 0.769592365 | 0.006988213 | 0.88028046 |
| ZNRD1 | salmon | -0.065437918 | 0.157994381 | -0.048107517 | 0.299536965 |
| ZNRF3 | salmon | 0.056416461 | 0.223652903 | 0.024743347 | 0.59378653 |
| ZSCAN2( | salmon | -0.150496826 | 0.001105721 | -0.180160447 | 9.04E-05 |
| ACSL6 | sienna3 | -0.130780073 | 0.004643547 | -0.11225489 | 0.015223291 |
| ADTRP | sienna3 | -0.190031709 | 3.58E-05 | -0.177995778 | 0.000110121 |
| AGTR1 | sienna3 | -0.190385869 | 3.46E-05 | -0.193510265 | 2.55E-05 |
| AICDA | sienna3 | -0.097557837 | 0.035063664 | -0.096424645 | 0.037248879 |
| ALOX12I | sienna3 | -0.176253225 | 0.000128837 | -0.174869361 | 0.000145788 |
| ANKRD1 | sienna3 | -0.12856927 | 0.005393917 | -0.112882636 | 0.014659539 |
| APOBEC | sienna3 | -0.041161613 | 0.374808532 | -0.055913808 | 0.227812463 |
| ARPP21 | sienna3 | -0.104980767 | 0.023277679 | -0.10000717 | 0.030710718 |
| ASMT | sienna3 | -0.05184886 | 0.263476122 | -0.040419651 | 0.383485928 |
| ATP8A2 | sienna3 | -0.318521892 | 1.80E-12 | -0.321145698 | 1.16E-12 |
| AVIL | sienna3 | -0.176746962 | 0.000123251 | -0.188206987 | 4.26E-05 |
| B4GALN' | sienna3 | 0.059671549 | 0.19802303 | 0.055381467 | 0.232277299 |
| BSND | sienna3 | -0.174964888 | 0.000144554 | -0.172021216 | 0.000187471 |
| C11orf53 | sienna3 | -0.034196847 | 0.460979293 | -0.037019321 | 0.424798574 |
| C1orf141 | sienna3 | -0.098662074 | 0.033040191 | -0.098002565 | 0.034236376 |
| C1orf167 | sienna3 | -0.087922873 | 0.057615036 | -0.093352333 | 0.043762396 |
| C5orf58 | sienna3 | -0.127333148 | 0.005859482 | -0.116302625 | 0.011898992 |
| CALHM1 | sienna3 | -0.053564897 | 0.247978012 | -0.070414163 | 0.128643872 |
| CCDC177 | sienna3 | -0.122044353 | 0.008285495 | -0.107175874 | 0.020527756 |
| CERS3 | sienna3 | -0.033524772 | 0.469840829 | -0.050142673 | 0.279533143 |
| CHRNB3 | sienna3 | -0.059646052 | 0.198215095 | -0.067100467 | 0.147673167 |
| CNDP1 | sienna3 | -0.100263433 | 0.030283235 | -0.108698067 | 0.018790784 |
| CNGA3 | sienna3 | -0.083423872 | 0.071684858 | -0.08241312 | 0.075204143 |
| CNIH3 | sienna3 | 0.012398306 | 0.789298327 | 0.002835387 | 0.951272287 |
| CPB1 | sienna3 | -0.073751213 | 0.111457943 | -0.071940708 | 0.120543213 |
| CRHR2 | sienna3 | -0.175195976 | 0.000141609 | -0.176236763 | 0.000129028 |
| CSMD1 | sienna3 | -0.135660601 | 0.003309938 | -0.136649104 | 0.003086492 |
| CTD-254: | sienna3 | 0.023188702 | 0.617190317 | 0.026681176 | 0.565195092 |
| DGKI | sienna3 | -0.091136775 | 0.049033295 | -0.078780165 | 0.089030332 |
| DLG2 | sienna3 | 0.015901963 | 0.731790268 | -0.004543716 | 0.921989558 |
| DNTT | sienna3 | 0.018737866 | 0.686301713 | -0.001184713 | 0.97962962 |
| FAM150A | sienna3 | -0.07523131 | 0.104438346 | -0.068672852 | 0.138392059 |
| FKSG62 | sienna3 | -0.139042684 | 0.002601101 | -0.149215826 | 0.001220425 |
| FOXE3 | sienna3 | -0.096617207 | 0.036869596 | -0.088014087 | 0.057355348 |
| GFI1B | sienna3 | -0.028126684 | 0.544306396 | -0.023022143 | 0.619721436 |

| | | | | | |
|---|---|---|---|---|---|
| GJC3 | sienna3 | -0.265659679 | 5.52E-09 | -0.260710627 | 1.08E-08 |
| GNG13 | sienna3 | -0.06748505 | 0.145360476 | -0.083008275 | 0.07311527 |
| GPR12 | sienna3 | -0.165288124 | 0.000334458 | -0.155385823 | 0.000753309 |
| GPR26 | sienna3 | -0.145631139 | 0.001602123 | -0.144742922 | 0.001712293 |
| HEPACA | sienna3 | 0.016057244 | 0.729273311 | -0.003030434 | 0.947924883 |
| HHIPL2 | sienna3 | -0.016166316 | 0.727507101 | -0.009186348 | 0.843052244 |
| HOXC5 | sienna3 | -0.136309179 | 0.003161735 | -0.149396229 | 0.001203635 |
| HPCA | sienna3 | -0.250571028 | 4.06E-08 | -0.252148123 | 3.32E-08 |
| HTR3C | sienna3 | -0.04537216 | 0.327887949 | -0.061266347 | 0.186277873 |
| HTR3E | sienna3 | -0.060094816 | 0.19485443 | -0.062159252 | 0.179930235 |
| IGSF21 | sienna3 | -0.161953923 | 0.000441916 | -0.151835605 | 0.000996529 |
| IL23R | sienna3 | -0.072474088 | 0.117808578 | -0.056580308 | 0.222308807 |
| IL25 | sienna3 | -0.09260975 | 0.045473217 | -0.094957156 | 0.040249524 |
| IL37 | sienna3 | -0.00620344 | 0.893640211 | -0.000660058 | 0.988649873 |
| IRX1 | sienna3 | -0.141375738 | 0.002195972 | -0.139720722 | 0.002476851 |
| KCNB2 | sienna3 | -0.127358413 | 0.005849616 | -0.135084559 | 0.003446826 |
| KCNH1 | sienna3 | -0.114224249 | 0.01351583 | -0.118323803 | 0.010493083 |
| KCNQ5 | sienna3 | -0.257287979 | 1.70E-08 | -0.24551624 | 7.71E-08 |
| KRT2 | sienna3 | 0.034048179 | 0.462931468 | 0.042497673 | 0.359491071 |
| LANCL3 | sienna3 | -0.033999943 | 0.463565854 | -0.03963192 | 0.392831592 |
| LIPJ | sienna3 | -0.135563887 | 0.00333257 | -0.121242544 | 0.008722744 |
| LRMP | sienna3 | -0.10829381 | 0.019239109 | -0.090060609 | 0.051779269 |
| MAGEA2 | sienna3 | 0.003229363 | 0.944511823 | -0.015109289 | 0.7446831 |
| MAGEA2 | sienna3 | -0.048695724 | 0.293660011 | -0.053868105 | 0.245307146 |
| MEIOB | sienna3 | -0.170316888 | 0.000217505 | -0.168604406 | 0.000252173 |
| MORC1 | sienna3 | -0.061224694 | 0.186577955 | -0.05114377 | 0.270033157 |
| NTSR2 | sienna3 | -0.158784012 | 0.000573113 | -0.160075749 | 0.000515803 |
| NXF5 | sienna3 | -0.055992213 | 0.227160054 | -0.058710409 | 0.205357352 |
| OLIG2 | sienna3 | -0.046695501 | 0.313962014 | -0.051765193 | 0.264248412 |
| PIK3CG | sienna3 | -0.099413424 | 0.031721058 | -0.076166534 | 0.100186462 |
| PIRT | sienna3 | -0.04641407 | 0.31689065 | -0.058860397 | 0.204200032 |
| PNMA5 | sienna3 | -0.142519548 | 0.002019198 | -0.148898062 | 0.001250526 |
| PNPLA1 | sienna3 | -0.092293538 | 0.046218601 | -0.081226389 | 0.079514274 |
| POU2F3 | sienna3 | -0.230091667 | 4.99E-07 | -0.242693826 | 1.10E-07 |
| POU3F1 | sienna3 | 0.035390605 | 0.445471236 | 0.012347865 | 0.790135517 |
| POU4F1 | sienna3 | 0.103800224 | 0.024884386 | 0.094236001 | 0.041797449 |
| PPEF1 | sienna3 | -0.004402016 | 0.92441499 | -0.023697275 | 0.609489872 |
| PSG5 | sienna3 | -0.103671074 | 0.025065835 | -0.108887161 | 0.018584223 |
| PSG9 | sienna3 | -0.044469845 | 0.337608891 | -0.044068304 | 0.341993568 |
| RBFOX1 | sienna3 | -0.152185538 | 0.000969676 | -0.16278875 | 0.000412344 |
| RGS13 | sienna3 | -0.081671593 | 0.077874432 | -0.07266533 | 0.11683998 |
| RGS21 | sienna3 | -0.040986763 | 0.376842463 | -0.03693012 | 0.425916097 |
| RLN3 | sienna3 | -0.121800638 | 0.008416286 | -0.120572166 | 0.009103973 |
| SH2D6 | sienna3 | -0.021279836 | 0.646463486 | -0.022767137 | 0.623605359 |
| SH2D7 | sienna3 | -0.07532206 | 0.104019606 | -0.088325243 | 0.056476773 |

| | | | | | |
|---|---|---|---|---|---|
| SHCBP1I | sienna3 | -0.026381385 | 0.569574901 | -0.016407559 | 0.723605755 |
| SLC22A7 | sienna3 | -0.092249911 | 0.046322242 | -0.090959622 | 0.049476763 |
| SLC4A10 | sienna3 | -0.063280917 | 0.172185208 | -0.02750033 | 0.553310585 |
| SLC6A17 | sienna3 | -0.07883325 | 0.088814528 | -0.073872554 | 0.110868846 |
| SPANXC | sienna3 | -0.02056435 | 0.65758111 | 0.007267937 | 0.875526525 |
| SPANXD | sienna3 | -0.033429125 | 0.471109549 | -0.005112787 | 0.912256404 |
| SPTB | sienna3 | -0.166400188 | 0.000304418 | -0.175278728 | 0.000140568 |
| SSX4B | sienna3 | -0.096430364 | 0.037237566 | -0.103108948 | 0.025868953 |
| ST18 | sienna3 | -0.071066963 | 0.125129608 | -0.064833161 | 0.161880874 |
| TDRD1 | sienna3 | 2.22E-05 | 0.999618096 | 0.002065681 | 0.964489775 |
| TEX11 | sienna3 | -0.107367794 | 0.020301335 | -0.100571862 | 0.029775499 |
| TFAP2B | sienna3 | 0.139323788 | 0.002548916 | 0.123022524 | 0.00777861 |
| TMEM17 | sienna3 | -0.041950195 | 0.365719688 | -0.044390813 | 0.338469034 |
| TMEM24 | sienna3 | -0.035578679 | 0.443055294 | -0.019277352 | 0.67776912 |
| TRPM5 | sienna3 | -0.17881876 | 0.0001022 | -0.178898022 | 0.000101466 |
| USP41 | sienna3 | 0.013351732 | 0.773520553 | -0.015446882 | 0.739183194 |
| VCX2 | sienna3 | -0.076317694 | 0.099512332 | -0.074930166 | 0.105837463 |
| ZFP42 | sienna3 | -0.188825575 | 4.01E-05 | -0.191619136 | 3.07E-05 |
| ZMAT4 | sienna3 | -0.176482078 | 0.000126219 | -0.188522056 | 4.13E-05 |
| ZNF560 | sienna3 | -0.097963327 | 0.034308691 | -0.103383606 | 0.025473815 |
| ZP4 | sienna3 | -0.01569481 | 0.735152495 | -0.022396468 | 0.629269538 |
| AAMDC | skyblue | -0.085557141 | 0.06469803 | -0.078126165 | 0.091723709 |
| AC01568 | skyblue | 0.027078765 | 0.559411493 | 0.055809441 | 0.228682974 |
| ADPRM | skyblue | 0.144583558 | 0.00173278 | 0.126534026 | 0.006179364 |
| ALKBH2 | skyblue | -0.022108342 | 0.633687466 | -0.053440563 | 0.249079079 |
| C11orf1 | skyblue | 0.154390972 | 0.000815239 | 0.141416353 | 0.00218946 |
| C18orf8 | skyblue | -0.022834052 | 0.622585175 | -0.019797035 | 0.669588501 |
| C6orf48 | skyblue | -0.191250981 | 3.18E-05 | -0.221782943 | 1.30E-06 |
| CCNB1IP | skyblue | -0.037845196 | 0.414533216 | -0.064897427 | 0.161464482 |
| CLEC16A | skyblue | -0.038998396 | 0.400446662 | -0.024023777 | 0.604568765 |
| COMMD | skyblue | -0.056012968 | 0.226987573 | -0.058692931 | 0.205492523 |
| CUEDC2 | skyblue | 0.168919575 | 0.000245428 | 0.159770061 | 0.000528863 |
| DEXI | skyblue | -0.136380784 | 0.003145748 | -0.146276214 | 0.00152623 |
| DNAJC5 | skyblue | -0.030906873 | 0.505237004 | -0.003100205 | 0.946727705 |
| DYRK4 | skyblue | 0.037669643 | 0.416702966 | 0.035283341 | 0.446852466 |
| EEF1B2 | skyblue | 0.097534557 | 0.035107434 | 0.067248452 | 0.146779965 |
| EEF1D | skyblue | -0.160519493 | 0.000497377 | -0.203376833 | 9.44E-06 |
| EIF1 | skyblue | 0.093983994 | 0.04235008 | 0.091305635 | 0.048613688 |
| EIF3D | skyblue | 0.040076184 | 0.387544028 | 0.020597675 | 0.657061578 |
| EIF3F | skyblue | 0.104474676 | 0.023955127 | 0.109892002 | 0.017519458 |
| GLTSCR | skyblue | 0.080675568 | 0.081581757 | 0.054965694 | 0.235807259 |
| GNB2L1 | skyblue | 0.103047833 | 0.025957591 | 0.095849463 | 0.038401563 |
| HNRNPA | skyblue | 0.10740764 | 0.020254598 | 0.060387798 | 0.192682967 |
| HSCB | skyblue | 0.087872481 | 0.05775892 | 0.080187042 | 0.083451597 |
| IGBP1 | skyblue | -0.021173777 | 0.648106617 | -0.032488831 | 0.483681957 |

| | | | | | |
|---|---|---|---|---|---|
| IMPDH2 | skyblue | 0.039318905 | 0.396583098 | 0.004483813 | 0.923014809 |
| LYRM4 | skyblue | -0.184883714 | 5.84E-05 | -0.194435955 | 2.33E-05 |
| MALL | skyblue | 0.175542156 | 0.000137301 | 0.212246638 | 3.71E-06 |
| METTL1[illegible] | skyblue | -0.01975164 | 0.670301552 | -0.034270535 | 0.460013387 |
| MTIF3 | skyblue | 0.178300738 | 0.000107121 | 0.178033205 | 0.000109748 |
| NACA | skyblue | 0.051783267 | 0.264081447 | 0.013858543 | 0.76517096 |
| NDUFAF[illegible] | skyblue | 0.090699129 | 0.050134942 | 0.074721008 | 0.106817905 |
| NPAS2 | skyblue | 0.006168926 | 0.894228492 | -0.008289671 | 0.858201322 |
| NPC1 | skyblue | -0.017612458 | 0.704229095 | -0.048377212 | 0.296832722 |
| NUDT2 | skyblue | 0.174987234 | 0.000144267 | 0.172365153 | 0.000181901 |
| PFDN5 | skyblue | -0.059236234 | 0.201320845 | -0.057891802 | 0.211757569 |
| PHB2 | skyblue | -0.019422972 | 0.675472967 | -0.033905537 | 0.464808827 |
| PLBD2 | skyblue | 0.106410526 | 0.021452661 | 0.098607341 | 0.033138087 |
| PLXNA1 | skyblue | -0.072305854 | 0.118665823 | -0.075070515 | 0.10518357 |
| POLL | skyblue | 0.095560927 | 0.038991092 | 0.072423591 | 0.118065378 |
| POLR1D | skyblue | 0.228878231 | 5.75E-07 | 0.206513942 | 6.81E-06 |
| POLR1E | skyblue | -0.084746199 | 0.067284992 | -0.102229954 | 0.027169224 |
| PPARD | skyblue | 0.134060812 | 0.003702837 | 0.131656527 | 0.004373136 |
| PQBP1 | skyblue | -0.184466853 | 6.07E-05 | -0.173960124 | 0.000158042 |
| PRAF2 | skyblue | -0.255136804 | 2.25E-08 | -0.236960735 | 2.21E-07 |
| PRR3 | skyblue | -0.212419891 | 3.64E-06 | -0.231644369 | 4.16E-07 |
| RCN1 | skyblue | 0.032754866 | 0.480106577 | -0.013961433 | 0.763479153 |
| RNF113A | skyblue | -0.024715337 | 0.594204604 | -0.006768765 | 0.884013035 |
| RPL10 | skyblue | 0.034548868 | 0.456375196 | 0.017630484 | 0.703940616 |
| RPL10A | skyblue | 0.038363523 | 0.408165961 | 0.010681395 | 0.817924825 |
| RPL11 | skyblue | 0.0158729 | 0.732261679 | -0.009255882 | 0.841879828 |
| RPL12 | skyblue | 0.121346803 | 0.00866475 | 0.10563679 | 0.022424254 |
| RPL13 | skyblue | 0.173987504 | 0.000157659 | 0.186600346 | 4.96E-05 |
| RPL13A | skyblue | 0.014815522 | 0.749479592 | -0.004674572 | 0.91975036 |
| RPL14 | skyblue | 0.046127895 | 0.31988691 | 0.016360437 | 0.724367247 |
| RPL15 | skyblue | 0.094431637 | 0.04137264 | 0.061346872 | 0.185698738 |
| RPL17 | skyblue | -0.078099606 | 0.091834448 | -0.112314677 | 0.015168791 |
| RPL18 | skyblue | 0.0916295 | 0.04781731 | 0.090433282 | 0.050814178 |
| RPL18A | skyblue | -0.101922373 | 0.027637313 | -0.097118735 | 0.035897114 |
| RPL19 | skyblue | 0.06909771 | 0.135962842 | 0.055083632 | 0.234802135 |
| RPL21 | skyblue | -0.080761331 | 0.081257021 | -0.098206847 | 0.033861975 |
| RPL22 | skyblue | -0.114502709 | 0.013288544 | -0.158187634 | 0.00060152 |
| RPL22L1 | skyblue | 0.266330344 | 5.04E-09 | 0.251984302 | 3.39E-08 |
| RPL23 | skyblue | 0.048527163 | 0.295336216 | 0.027764767 | 0.549500295 |
| RPL23A | skyblue | 0.045821465 | 0.323115646 | 0.020566642 | 0.657545375 |
| RPL24 | skyblue | -0.030501499 | 0.510840513 | -0.050567239 | 0.275476894 |
| RPL26 | skyblue | 0.078929947 | 0.088422511 | 0.044628546 | 0.335885912 |
| RPL27 | skyblue | 0.145046068 | 0.001673935 | 0.143134529 | 0.001929629 |
| RPL27A | skyblue | 0.036957708 | 0.425570291 | 0.029655701 | 0.522635429 |
| RPL28 | skyblue | 0.08510878 | 0.066118032 | 0.096161419 | 0.037772697 |

| | | | | | |
|---|---|---|---|---|---|
| RPL29 | skyblue | 0.19469341 | 2.27E-05 | 0.187566852 | 4.53E-05 |
| RPL3 | skyblue | 0.158645611 | 0.000579592 | 0.130609425 | 0.00469792 |
| RPL30 | skyblue | -0.105596951 | 0.022475297 | -0.136495062 | 0.003120385 |
| RPL31 | skyblue | -0.032402809 | 0.484841124 | -0.054583174 | 0.239088158 |
| RPL32 | skyblue | 0.023570265 | 0.611408975 | 0.012717769 | 0.784001741 |
| RPL34 | skyblue | 0.082112718 | 0.07627679 | 0.077657763 | 0.093692547 |
| RPL35 | skyblue | 0.054109796 | 0.243192619 | 0.049454434 | 0.286194103 |
| RPL35A | skyblue | -0.181228529 | 8.20E-05 | -0.193654555 | 2.51E-05 |
| RPL36 | skyblue | 0.183819696 | 6.45E-05 | 0.197008219 | 1.80E-05 |
| RPL36A | skyblue | -0.089903062 | 0.052191833 | -0.100376332 | 0.030096528 |
| RPL36A-l | skyblue | -0.140022291 | 0.002423348 | -0.119758083 | 0.009586809 |
| RPL36AL | skyblue | 0.093037545 | 0.044480896 | 0.104813983 | 0.02349908 |
| RPL37 | skyblue | -0.073221588 | 0.114058084 | -0.102042072 | 0.027454333 |
| RPL37A | skyblue | -0.030779143 | 0.506999129 | -0.035581077 | 0.443024541 |
| RPL38 | skyblue | -0.049523611 | 0.285519796 | -0.058021041 | 0.210737685 |
| RPL39 | skyblue | -0.108521585 | 0.018985372 | -0.136581342 | 0.00310136 |
| RPL4 | skyblue | 0.088613984 | 0.055671505 | 0.05630449 | 0.224574763 |
| RPL41 | skyblue | -0.059795118 | 0.197094131 | -0.089598624 | 0.052996823 |
| RPL5 | skyblue | -0.080778807 | 0.081190976 | -0.124770115 | 0.006941544 |
| RPL6 | skyblue | 0.011347163 | 0.806793278 | -0.026635056 | 0.565867832 |
| RPL7 | skyblue | -0.099823901 | 0.031019595 | -0.152917484 | 0.000915656 |
| RPL7A | skyblue | 0.211771212 | 3.90E-06 | 0.194513 | 2.31E-05 |
| RPL9 | skyblue | -0.01640788 | 0.723600564 | -0.036239163 | 0.434630296 |
| RPLP0 | skyblue | 0.176170384 | 0.000129797 | 0.15833839 | 0.000594218 |
| RPLP1 | skyblue | 0.124079052 | 0.007262398 | 0.120979216 | 0.008870764 |
| RPLP2 | skyblue | 0.055879271 | 0.228100271 | 0.068627353 | 0.138654175 |
| RPS10 | skyblue | -0.109478906 | 0.017950558 | -0.120227597 | 0.009305633 |
| RPS11 | skyblue | 0.057484329 | 0.214996411 | 0.059933797 | 0.196055428 |
| RPS12 | skyblue | -0.080443671 | 0.082465076 | -0.089690333 | 0.052753246 |
| RPS13 | skyblue | 0.061231301 | 0.186530332 | 0.052360038 | 0.258791408 |
| RPS14 | skyblue | 0.086180646 | 0.062765118 | 0.087130574 | 0.059911961 |
| RPS15A | skyblue | -0.070198143 | 0.129823467 | -0.084694015 | 0.06745433 |
| RPS17 | skyblue | 0.021611474 | 0.641336684 | -0.007922791 | 0.864415007 |
| RPS18 | skyblue | -0.030924598 | 0.50499273 | -0.041318501 | 0.37298931 |
| RPS19 | skyblue | -0.007529724 | 0.871081475 | -0.010380599 | 0.822966246 |
| RPS2 | skyblue | 0.22440973 | 9.62E-07 | 0.23139958 | 4.28E-07 |
| RPS20 | skyblue | -0.119576021 | 0.009697855 | -0.143853825 | 0.001829489 |
| RPS23 | skyblue | -0.034853529 | 0.452411412 | -0.075402879 | 0.103647807 |
| RPS24 | skyblue | -0.022169398 | 0.632750178 | -0.056729788 | 0.221087605 |
| RPS25 | skyblue | -0.032307734 | 0.486124031 | -0.062760739 | 0.175745518 |
| RPS26 | skyblue | 0.028800103 | 0.534707311 | 0.031603274 | 0.495686476 |
| RPS27 | skyblue | -0.032602959 | 0.482146379 | -0.056600179 | 0.222146188 |
| RPS27A | skyblue | -0.173415239 | 0.000165842 | -0.17111268 | 0.00020296 |
| RPS28 | skyblue | 0.18785345 | 4.41E-05 | 0.208550217 | 5.50E-06 |
| RPS29 | skyblue | -0.016879575 | 0.715993139 | -0.016113973 | 0.728354521 |

| | | | | | |
|---|---|---|---|---|---|
| RPS3 | skyblue | 0.016114428 | 0.728347156 | -0.019133591 | 0.680038901 |
| RPS3A | skyblue | -0.028519982 | 0.538689859 | -0.054927889 | 0.236130095 |
| RPS4X | skyblue | 0.14644697 | 0.001506701 | 0.121890533 | 0.008367831 |
| RPS5 | skyblue | 0.070905239 | 0.1259932 | 0.056727365 | 0.221107365 |
| RPS6 | skyblue | 0.067370795 | 0.146044645 | 0.048874244 | 0.291891717 |
| RPS7 | skyblue | -0.009083468 | 0.844787546 | -0.045687068 | 0.324538392 |
| RPS8 | skyblue | -0.016092752 | 0.728698171 | -0.035633086 | 0.442357783 |
| RPS9 | skyblue | 0.093418625 | 0.043612336 | 0.08132723 | 0.079140411 |
| RPSA | skyblue | 0.107059461 | 0.020666164 | 0.112887382 | 0.014655348 |
| RSAD1 | skyblue | 0.212642554 | 3.55E-06 | 0.172217648 | 0.000184271 |
| SLC12A7 | skyblue | 0.001104283 | 0.981012298 | 0.008013903 | 0.862871075 |
| SLC9A1 | skyblue | 0.258682254 | 1.41E-08 | 0.284384443 | 3.88E-10 |
| THG1L | skyblue | 0.037529098 | 0.418444822 | 0.020451104 | 0.659347809 |
| THYN1 | skyblue | 0.117598912 | 0.010979394 | 0.101087916 | 0.028942284 |
| TMEM18 | skyblue | 0.08633735 | 0.062286881 | 0.070635793 | 0.127442284 |
| TMEM18 | skyblue | 0.137487889 | 0.002907739 | 0.135901156 | 0.00325425 |
| TOMM7 | skyblue | -0.008786357 | 0.849803169 | 0.00244585 | 0.957960036 |
| TPT1 | skyblue | 0.14373496 | 0.001845702 | 0.127670313 | 0.005729041 |
| UBA52 | skyblue | -0.023845331 | 0.607256137 | -0.001469974 | 0.97472622 |
| UXT | skyblue | 0.022673888 | 0.625028224 | 0.043287198 | 0.350626462 |
| WDR45 | skyblue | -0.252499587 | 3.17E-08 | -0.229810794 | 5.16E-07 |
| YBEY | skyblue | 0.201739411 | 1.12E-05 | 0.185923398 | 5.29E-05 |
| ZNF32 | skyblue | 0.032782582 | 0.479734913 | 0.018716191 | 0.686645372 |
| ZNF581 | skyblue | 0.011674531 | 0.801333874 | 0.003172288 | 0.945490955 |
| ZNF691 | skyblue | -0.157634272 | 0.00062904 | -0.176802684 | 0.000122635 |
| ABCF3 | skyblue3 | -0.20952209 | 4.96E-06 | -0.195618592 | 2.07E-05 |
| ADCK5 | skyblue3 | 0.136930958 | 0.003025342 | 0.123832763 | 0.007379914 |
| ALG3 | skyblue3 | -0.120444421 | 0.009178278 | -0.091044712 | 0.049263339 |
| AP2M1 | skyblue3 | -0.211148035 | 4.17E-06 | -0.166890377 | 0.000291994 |
| ARHGAP | skyblue3 | 0.013967483 | 0.763379703 | 0.006131396 | 0.894868229 |
| ATAD3A | skyblue3 | 0.155754735 | 0.000731473 | 0.172886796 | 0.00017375 |
| BOP1 | skyblue3 | 0.042912751 | 0.354813301 | 0.045159485 | 0.330162707 |
| C8orf82 | skyblue3 | 0.015282041 | 0.741867063 | 0.022146171 | 0.633106669 |
| CA5B | skyblue3 | 0.116946067 | 0.011434349 | 0.109567257 | 0.017857586 |
| CLUH | skyblue3 | 0.131887071 | 0.004304412 | 0.132285409 | 0.004187965 |
| COMMD | skyblue3 | 0.104479904 | 0.023948043 | 0.117563444 | 0.011003694 |
| CPSF1 | skyblue3 | 0.059847049 | 0.196704701 | 0.047325574 | 0.307469877 |
| CYC1 | skyblue3 | 0.079557369 | 0.08591255 | 0.097366782 | 0.035424255 |
| CYHR1 | skyblue3 | -0.049984875 | 0.281050998 | -0.04684973 | 0.312364627 |
| DGAT1 | skyblue3 | 0.08238811 | 0.075292974 | 0.101382425 | 0.028475802 |
| DNPEP | skyblue3 | -0.033811472 | 0.466049165 | 0.003879712 | 0.933361017 |
| ECE2 | skyblue3 | -0.057592395 | 0.214133986 | -0.036588584 | 0.430210714 |
| EIF2B5 | skyblue3 | -0.0687332 | 0.138044989 | -0.047739245 | 0.303256017 |
| EIF3B | skyblue3 | -0.0457509 | 0.323862149 | -0.029936691 | 0.518701525 |
| ELOVL1 | skyblue3 | 0.131027325 | 0.004565774 | 0.149232409 | 0.001218873 |

| | | | | | |
|---|---|---|---|---|---|
| EXOSC4 | skyblue3 | 0.125678653 | 0.00653905 | 0.142508912 | 0.00202078 |
| FAM229A | skyblue3 | 0.037113122 | 0.423625262 | 0.031938481 | 0.491123875 |
| FBRSL1 | skyblue3 | -0.052595145 | 0.256656186 | -0.064025134 | 0.167185392 |
| FBXL6 | skyblue3 | 0.168875078 | 0.00024637 | 0.166612201 | 0.000298985 |
| GLI4 | skyblue3 | -0.01906544 | 0.681115928 | -0.039436387 | 0.395172549 |
| GPAA1 | skyblue3 | 0.048752979 | 0.293092101 | 0.046080462 | 0.320385312 |
| GPR137 | skyblue3 | 0.063399179 | 0.171383329 | 0.080099003 | 0.083792219 |
| GPT | skyblue3 | -0.003091417 | 0.946878482 | 0.013267237 | 0.77491517 |
| GRINA | skyblue3 | 0.11863251 | 0.010291845 | 0.127892315 | 0.005644584 |
| GRWD1 | skyblue3 | 0.035964946 | 0.438116903 | 0.027623235 | 0.551538033 |
| HGH1 | skyblue3 | 0.162644585 | 0.000417316 | 0.166438597 | 0.000303427 |
| HSF1 | skyblue3 | -0.058485475 | 0.20710185 | -0.047116419 | 0.309615069 |
| IDH3B | skyblue3 | -0.212296713 | 3.69E-06 | -0.199207396 | 1.44E-05 |
| IMP4 | skyblue3 | 0.057541561 | 0.214539356 | 0.073699246 | 0.111710985 |
| INTS1 | skyblue3 | 0.035321678 | 0.446358522 | 0.058327546 | 0.208333071 |
| IPO13 | skyblue3 | 0.020531419 | 0.658094661 | 0.029591739 | 0.523533042 |
| KIFC2 | skyblue3 | 0.12016444 | 0.009343024 | 0.102283782 | 0.027088013 |
| LRFN4 | skyblue3 | -0.04376586 | 0.34532001 | -0.031303395 | 0.499787238 |
| LRRC14 | skyblue3 | 0.003190786 | 0.94517362 | -0.015405666 | 0.739853951 |
| MAF1 | skyblue3 | -0.136987966 | 0.003013108 | -0.124722839 | 0.006963083 |
| MAP3K1 | skyblue3 | -0.003233244 | 0.94444523 | 0.030086098 | 0.516616039 |
| MEN1 | skyblue3 | 0.056301878 | 0.224596306 | 0.028687984 | 0.53629956 |
| MFSD3 | skyblue3 | 0.158405789 | 0.00059098 | 0.163782874 | 0.000379536 |
| MROH1 | skyblue3 | 0.094485112 | 0.04125716 | 0.099472213 | 0.03161977 |
| MYBBP1 | skyblue3 | 0.064578414 | 0.163539385 | 0.048997036 | 0.290679573 |
| NAPRT | skyblue3 | 0.048666949 | 0.293945703 | 0.076865733 | 0.097098425 |
| NAT8L | skyblue3 | -0.203961889 | 8.89E-06 | -0.209117277 | 5.18E-06 |
| NDUFV1 | skyblue3 | -0.043764255 | 0.345337719 | -0.025590016 | 0.581213365 |
| OPLAH | skyblue3 | 0.204781078 | 8.16E-06 | 0.168939149 | 0.000245015 |
| OTUB1 | skyblue3 | 0.017079145 | 0.712782895 | 0.039398897 | 0.395622337 |
| POLD2 | skyblue3 | 0.015024181 | 0.7460717 | 0.036082954 | 0.43661449 |
| POLR2H | skyblue3 | -0.031892036 | 0.491754708 | -0.008743666 | 0.850524353 |
| PPP1R16A | skyblue3 | 0.025644342 | 0.580410874 | 0.044246571 | 0.340042497 |
| PRPF19 | skyblue3 | -0.007834236 | 0.865916091 | -0.033745865 | 0.466915329 |
| PUF60 | skyblue3 | -0.105103117 | 0.023116414 | -0.102379641 | 0.026943903 |
| PYCRL | skyblue3 | 0.12833155 | 0.005480777 | 0.142178499 | 0.002070494 |
| RANGAP | skyblue3 | 0.074412557 | 0.108276859 | 0.088588372 | 0.055742548 |
| RBM38 | skyblue3 | -0.271218176 | 2.56E-09 | -0.264697983 | 6.29E-09 |
| RHPN1 | skyblue3 | 0.170985577 | 0.000205221 | 0.170636206 | 0.000211555 |
| RNF220 | skyblue3 | -0.044353338 | 0.338877386 | -0.047173534 | 0.309028298 |
| SCRIB | skyblue3 | -0.002860578 | 0.950839906 | -0.013271335 | 0.774847513 |
| SCYL1 | skyblue3 | 0.002775664 | 0.952297422 | 0.01804159 | 0.697373166 |
| SHARPIN | skyblue3 | 0.043135979 | 0.35231353 | 0.060392615 | 0.192647415 |
| SKIV2L | skyblue3 | -0.14767029 | 0.001373364 | -0.131451845 | 0.004434981 |
| SLC19A1 | skyblue3 | 0.172681801 | 0.000176911 | 0.174260261 | 0.000153894 |

| | | | | | |
|---|---|---|---|---|---|
| SLC39A4 | skyblue3 | 0.072358195 | 0.118398597 | 0.085300487 | 0.065507778 |
| SLC45A4 | skyblue3 | -0.210083314 | 4.67E-06 | -0.208697685 | 5.41E-06 |
| SLC52A2 | skyblue3 | 0.14353032 | 0.001873923 | 0.154598807 | 0.000801925 |
| SMYD5 | skyblue3 | -0.111518126 | 0.015909143 | -0.117498112 | 0.011048576 |
| TBRG4 | skyblue3 | -0.102269377 | 0.027109726 | -0.078626191 | 0.089658659 |
| THEM6 | skyblue3 | 0.177426306 | 0.000115936 | 0.173455584 | 0.000165252 |
| TIGD5 | skyblue3 | -0.106498818 | 0.021344149 | -0.104898985 | 0.023386015 |
| TMEM24 | skyblue3 | -0.010328178 | 0.823845585 | -0.026274857 | 0.571135108 |
| TRAPPC9 | skyblue3 | 0.045865938 | 0.322645735 | 0.035270278 | 0.447020844 |
| TSNARE | skyblue3 | -0.22789143 | 6.45E-07 | -0.253630266 | 2.74E-08 |
| TSTA3 | skyblue3 | 0.173362096 | 0.000166621 | 0.196579999 | 1.88E-05 |
| TTLL12 | skyblue3 | 0.188502934 | 4.14E-05 | 0.187109105 | 4.73E-05 |
| VARS | skyblue3 | 0.111290282 | 0.016126655 | 0.128900627 | 0.005274911 |
| VPS28 | skyblue3 | 0.093660277 | 0.043068995 | 0.099261961 | 0.031983296 |
| WDR4 | skyblue3 | 0.042985586 | 0.353996446 | 0.046431956 | 0.316703992 |
| ZC3H3 | skyblue3 | -0.018448804 | 0.690890096 | -0.02087177 | 0.652794813 |
| ZFP41 | skyblue3 | -0.152350788 | 0.000957229 | -0.155207687 | 0.000764068 |
| ZNF517 | skyblue3 | -0.112183335 | 0.015288743 | -0.148727489 | 0.001266965 |
| ZNF696 | skyblue3 | -0.132856381 | 0.004026016 | -0.167547895 | 0.000276071 |
| ZNF707 | skyblue3 | -0.205373123 | 7.68E-06 | -0.2190804 | 1.75E-06 |
| AKT1S1 | steelblue | 0.136622973 | 0.003092218 | 0.152768832 | 0.000926396 |
| ALDH16A | steelblue | 0.09015741 | 0.051527131 | 0.088006289 | 0.057377511 |
| ANAPC1 | steelblue | 0.18166986 | 7.87E-05 | 0.214510724 | 2.90E-06 |
| ANAPC1 | steelblue | 0.131060331 | 0.004555482 | 0.137034586 | 0.003003137 |
| AP2S1 | steelblue | 0.225424642 | 8.57E-07 | 0.27440933 | 1.64E-09 |
| ARMC6 | steelblue | 0.026985248 | 0.560769273 | 0.03468125 | 0.454650455 |
| ASCC2 | steelblue | 0.13688646 | 0.003034922 | 0.159096938 | 0.000558711 |
| ASPSCR1 | steelblue | 0.021990243 | 0.635502094 | 0.017476681 | 0.706403335 |
| AUP1 | steelblue | 0.090664966 | 0.0502218 | 0.11348861 | 0.014132825 |
| B3GAT3 | steelblue | 0.138969586 | 0.00261483 | 0.141704106 | 0.002143829 |
| B4GALT | steelblue | 0.085550877 | 0.064717694 | 0.087640245 | 0.058425876 |
| BCKDK | steelblue | 0.12136901 | 0.008652442 | 0.145139823 | 0.001662232 |
| C14orf80 | steelblue | 0.123714152 | 0.007437114 | 0.141954124 | 0.002104889 |
| C17orf62 | steelblue | 0.176880578 | 0.000121779 | 0.198827356 | 1.50E-05 |
| C17orf70 | steelblue | 0.10411313 | 0.024449465 | 0.093803039 | 0.04275069 |
| C19orf48 | steelblue | 0.091342965 | 0.048521331 | 0.088580741 | 0.055763729 |
| C19orf60 | steelblue | 0.051560746 | 0.266142104 | 0.057025376 | 0.218686875 |
| C6orf226 | steelblue | 0.197939087 | 1.64E-05 | 0.241988238 | 1.19E-07 |
| CCDC106 | steelblue | 0.139633353 | 0.002492552 | 0.135063448 | 0.003451938 |
| CENPM | steelblue | 0.323122267 | 8.25E-13 | 0.33424492 | 1.19E-13 |
| CEP131 | steelblue | 0.044836515 | 0.333636583 | 0.032386544 | 0.485060473 |
| CFL1 | steelblue | 0.150686073 | 0.001089644 | 0.171252555 | 0.0002005 |
| CHCHD1 | steelblue | 0.013756454 | 0.766850697 | 0.041350485 | 0.372619101 |
| CHTF18 | steelblue | -0.042245034 | 0.362357031 | -0.026077459 | 0.574031541 |
| COMTD1 | steelblue | 0.067814051 | 0.143404058 | 0.101463149 | 0.028349072 |

| | | | | | |
|---|---|---|---|---|---|
| COX8A | steelblue | 0.157162585 | 0.000653415 | 0.184447507 | 6.08E-05 |
| CTU1 | steelblue | -0.090842605 | 0.049771526 | -0.075581856 | 0.102828194 |
| DCXR | steelblue | 0.134171757 | 0.003674283 | 0.143980828 | 0.00181231 |
| DDX39A | steelblue | 0.011284354 | 0.807841811 | 0.018881524 | 0.684025643 |
| DHX34 | steelblue | -0.021392238 | 0.644723968 | -0.027616319 | 0.5516377 |
| DNPH1 | steelblue | 0.061869784 | 0.181970258 | 0.066152554 | 0.153493039 |
| DUS1L | steelblue | 0.142986429 | 0.00195086 | 0.158534017 | 0.000584866 |
| EIF4EBP1 | steelblue | 0.164690213 | 0.00035173 | 0.179413063 | 9.68E-05 |
| EMC9 | steelblue | 0.187649003 | 4.49E-05 | 0.215704946 | 2.55E-06 |
| ERI3 | steelblue | -0.127558336 | 0.00577207 | -0.114690891 | 0.013136859 |
| FAM195I | steelblue | -0.005659563 | 0.902916923 | -0.02196918 | 0.635825955 |
| FIBP | steelblue | 0.132430384 | 0.004146296 | 0.150676745 | 0.001090431 |
| FLYWCH | steelblue | 0.079955477 | 0.084349921 | 0.097192125 | 0.035756652 |
| FOXRED | steelblue | 0.099680051 | 0.031263895 | 0.104561321 | 0.023837951 |
| GALK1 | steelblue | 0.062987557 | 0.174186409 | 0.080575105 | 0.081963485 |
| GFER | steelblue | 0.245159011 | 8.06E-08 | 0.275351194 | 1.43E-09 |
| GPS1 | steelblue | -0.01400162 | 0.762818665 | -0.001045872 | 0.982016474 |
| HAGH | steelblue | 0.210605212 | 4.42E-06 | 0.241376658 | 1.29E-07 |
| HMBS | steelblue | 0.249180377 | 4.85E-08 | 0.250671526 | 4.01E-08 |
| HMOX2 | steelblue | 0.326928619 | 4.29E-13 | 0.374683437 | 5.18E-17 |
| HRAS | steelblue | 0.190632061 | 3.37E-05 | 0.225831011 | 8.18E-07 |
| HSPBP1 | steelblue | 0.044857022 | 0.333415322 | 0.065304686 | 0.158844493 |
| IRF3 | steelblue | -0.06781137 | 0.143419922 | -0.061839108 | 0.182187444 |
| ISOC2 | steelblue | 0.018365473 | 0.692214956 | 0.05006073 | 0.280320661 |
| ITPA | steelblue | -0.016371469 | 0.724188939 | 2.15E-06 | 0.99996297 |
| KPTN | steelblue | 0.064547467 | 0.163741727 | 0.054775502 | 0.237434554 |
| LAMTOR | steelblue | 0.240146338 | 1.50E-07 | 0.271823768 | 2.35E-09 |
| LRRC45 | steelblue | 0.220324965 | 1.53E-06 | 0.219275591 | 1.72E-06 |
| LSM10 | steelblue | 0.121836253 | 0.008397059 | 0.159212728 | 0.000553468 |
| MLST8 | steelblue | 0.165069254 | 0.000340687 | 0.189688822 | 3.70E-05 |
| MRPL17 | steelblue | 0.204387823 | 8.50E-06 | 0.222807247 | 1.15E-06 |
| MRPL28 | steelblue | 0.072623103 | 0.117053315 | 0.109539511 | 0.017886738 |
| MRPS11 | steelblue | 0.213596133 | 3.20E-06 | 0.219600324 | 1.66E-06 |
| MRPS12 | steelblue | -0.002170515 | 0.962688914 | 0.014041953 | 0.762155953 |
| MRPS26 | steelblue | 0.103045118 | 0.025961534 | 0.114280287 | 0.013469817 |
| MRPS34 | steelblue | 0.2287668 | 5.83E-07 | 0.268366154 | 3.81E-09 |
| MXD3 | steelblue | 0.133079152 | 0.003964383 | 0.140651012 | 0.002315179 |
| NARF | steelblue | 0.207550223 | 6.11E-06 | 0.217378622 | 2.12E-06 |
| NARFL | steelblue | 0.135786663 | 0.003280648 | 0.157582329 | 0.000631682 |
| NAT14 | steelblue | -0.076527222 | 0.098583866 | -0.065137564 | 0.159915719 |
| NCAPH2 | steelblue | 0.133425875 | 0.003870153 | 0.138331818 | 0.002737444 |
| NDUFB1( | steelblue | 0.154730583 | 0.000793587 | 0.197564229 | 1.70E-05 |
| NDUFS3 | steelblue | 0.080099758 | 0.08378929 | 0.101891095 | 0.027685298 |
| NMRAL1 | steelblue | 0.270096038 | 3.00E-09 | 0.304801163 | 1.70E-11 |
| NOC4L | steelblue | 0.098193281 | 0.033886731 | 0.124961876 | 0.00685479 |

| | | | | | |
|---|---|---|---|---|---|
| NOSIP | steelblue | -0.040409354 | 0.383607207 | -0.025290729 | 0.585643623 |
| NTHL1 | steelblue | 0.098424379 | 0.033467135 | 0.126537725 | 0.006177848 |
| NUBP2 | steelblue | 0.233642549 | 3.28E-07 | 0.264562123 | 6.41E-09 |
| NUDT16I | steelblue | 0.064445038 | 0.164412793 | 0.106061545 | 0.021886264 |
| OXLD1 | steelblue | 0.050809295 | 0.273182324 | 0.060553781 | 0.191460658 |
| PAFAH1I | steelblue | 0.230317763 | 4.86E-07 | 0.244163174 | 9.12E-08 |
| PARD3B | steelblue | -0.225474288 | 8.52E-07 | -0.256771391 | 1.82E-08 |
| PCGF1 | steelblue | 0.225621639 | 8.38E-07 | 0.240989187 | 1.35E-07 |
| PCYT2 | steelblue | 0.036790666 | 0.427666623 | 0.050886107 | 0.272456915 |
| PGP | steelblue | 0.187041671 | 4.76E-05 | 0.227174429 | 7.01E-07 |
| PIH1D1 | steelblue | 0.144873085 | 0.001695725 | 0.14416825 | 0.001787228 |
| PNKP | steelblue | 0.16361839 | 0.000384791 | 0.168477325 | 0.000254942 |
| POLR2G | steelblue | 0.16878638 | 0.000248258 | 0.167285701 | 0.00028232 |
| POLR2I | steelblue | 0.059016898 | 0.202997514 | 0.091751105 | 0.047521123 |
| POLR2L | steelblue | 0.116551794 | 0.011717118 | 0.164052201 | 0.000371075 |
| POLR3K | steelblue | 0.221194833 | 1.38E-06 | 0.239300839 | 1.66E-07 |
| PPP1CA | steelblue | 0.161406228 | 0.000462375 | 0.180013382 | 9.16E-05 |
| PPP4C | steelblue | 0.15978439 | 0.000528244 | 0.203956566 | 8.89E-06 |
| PQLC2 | steelblue | 0.141280404 | 0.002211326 | 0.157952091 | 0.000613094 |
| PRPF31 | steelblue | 0.020741843 | 0.654815959 | 0.041258617 | 0.373683061 |
| PSMC3 | steelblue | 0.054693083 | 0.238142193 | 0.078018139 | 0.092174809 |
| PSMD8 | steelblue | 0.017618814 | 0.70412738 | 0.038962238 | 0.400883929 |
| PTGES2 | steelblue | 0.110879204 | 0.01652569 | 0.136909346 | 0.003029991 |
| PTOV1 | steelblue | 0.088094336 | 0.057127682 | 0.105815333 | 0.022196734 |
| RAB40C | steelblue | 0.259953875 | 1.19E-08 | 0.274943208 | 1.52E-09 |
| RFNG | steelblue | -0.021402313 | 0.644568158 | -0.038036321 | 0.412178607 |
| RHBDD3 | steelblue | 0.321766094 | 1.04E-12 | 0.342243807 | 2.80E-14 |
| RPS6KB2 | steelblue | 0.122107912 | 0.008251686 | 0.13606543 | 0.003216711 |
| RPUSD1 | steelblue | 0.07598977 | 0.100979377 | 0.098440189 | 0.033438592 |
| RUVBL2 | steelblue | 0.127168636 | 0.005924092 | 0.153731248 | 0.000858868 |
| SAC3D1 | steelblue | 0.094429143 | 0.041378031 | 0.104431664 | 0.024013481 |
| SAMD1 | steelblue | 0.13272415 | 0.004063009 | 0.120774208 | 0.008987545 |
| SCAND1 | steelblue | 0.080547492 | 0.082068659 | 0.109065355 | 0.018391384 |
| SEC13 | steelblue | 0.176320299 | 0.000128065 | 0.207533662 | 6.12E-06 |
| SF3B5 | steelblue | 0.138459405 | 0.002712503 | 0.179717261 | 9.42E-05 |
| SIRT7 | steelblue | 0.063097187 | 0.173436532 | 0.072217327 | 0.119118872 |
| SIVA1 | steelblue | 0.030155262 | 0.515652084 | 0.063008878 | 0.174040386 |
| SLC25A1 | steelblue | 0.222198383 | 1.24E-06 | 0.229522873 | 5.34E-07 |
| SLC25A3 | steelblue | 0.216324924 | 2.38E-06 | 0.234348887 | 3.02E-07 |
| SLC27A4 | steelblue | 0.301702739 | 2.77E-11 | 0.320296565 | 1.33E-12 |
| SLC37A4 | steelblue | 0.202801336 | 1.00E-05 | 0.172658214 | 0.000177279 |
| SNRNP25 | steelblue | 0.288678607 | 2.05E-10 | 0.318240201 | 1.89E-12 |
| SNRNP7( | steelblue | 0.057788326 | 0.212576713 | 0.043704844 | 0.345993578 |
| SNRPA | steelblue | 0.117951179 | 0.010740628 | 0.114068464 | 0.013644473 |
| SRM | steelblue | 0.068288038 | 0.140620959 | 0.085252047 | 0.065661534 |

| | | | | | |
|---|---|---|---|---|---|
| STRA13 | steelblue | 0.22022109 | 1.54E-06 | 0.232666979 | 3.69E-07 |
| SUV420H | steelblue | -0.039938863 | 0.389173771 | -0.065886188 | 0.155159322 |
| TBL3 | steelblue | 0.049778363 | 0.283045833 | 0.079049751 | 0.087938745 |
| TELO2 | steelblue | 0.221369324 | 1.36E-06 | 0.221524081 | 1.33E-06 |
| THOC6 | steelblue | 0.088954257 | 0.054734783 | 0.089753854 | 0.052585085 |
| TIMM10 | steelblue | 0.109359148 | 0.018077256 | 0.122631656 | 0.007977737 |
| TMEM11 | steelblue | 0.241481135 | 1.27E-07 | 0.260217141 | 1.15E-08 |
| TMEM14 | steelblue | -0.030842526 | 0.506124315 | -0.018410909 | 0.691492462 |
| TMEM16 | steelblue | 0.097864641 | 0.034491146 | 0.128513422 | 0.005414211 |
| TRMU | steelblue | 0.149726357 | 0.001173458 | 0.1550781 | 0.000771983 |
| TUFM | steelblue | 0.108483395 | 0.01902771 | 0.135649496 | 0.00331253 |
| UBE2M | steelblue | 0.074918542 | 0.105891764 | 0.099418831 | 0.031711732 |
| UQCR10 | steelblue | 0.195484524 | 2.10E-05 | 0.234096828 | 3.11E-07 |
| WBSCR1 | steelblue | 0.214539456 | 2.89E-06 | 0.227164617 | 7.02E-07 |
| YDJC | steelblue | 0.037704593 | 0.416270474 | 0.041149103 | 0.374953823 |
| YIF1A | steelblue | 0.101179449 | 0.028796605 | 0.132617416 | 0.004093093 |
| YIF1B | steelblue | 0.176846662 | 0.000122151 | 0.17929096 | 9.79E-05 |
| ZNF428 | steelblue | 0.113570918 | 0.014062581 | 0.113218372 | 0.014365625 |
| ZNF444 | steelblue | 0.013356654 | 0.773439329 | 0.016866871 | 0.716197665 |
| ZNF598 | steelblue | 0.033154325 | 0.474765128 | 0.038894454 | 0.401704443 |
| ZNF784 | steelblue | -0.058973504 | 0.203330427 | -0.064686901 | 0.162831534 |
| ZNF787 | steelblue | -0.064874523 | 0.161612792 | -0.057432497 | 0.21541094 |
| ZNHIT2 | steelblue | 0.093206492 | 0.044094054 | 0.11178795 | 0.015654883 |
| AARSD1 | tan | 0.027090726 | 0.559237949 | 0.002664021 | 0.954213985 |
| ACOT13 | tan | 0.14818882 | 0.001320191 | 0.154713933 | 0.000794636 |
| AL15880 | tan | 0.008483119 | 0.854928467 | -0.023784289 | 0.608176653 |
| ALG12 | tan | 0.215981136 | 2.47E-06 | 0.219222027 | 1.73E-06 |
| ANP32A | tan | 0.125625287 | 0.006562101 | 0.110661355 | 0.016740641 |
| APITD1 | tan | 0.031390606 | 0.498592795 | 0.027167405 | 0.558125994 |
| APITD1-( | tan | -0.019968365 | 0.666899986 | -0.025852645 | 0.577338669 |
| ARPC3 | tan | 0.135127528 | 0.00343644 | 0.145004328 | 0.001679169 |
| ATP5J | tan | -0.021110604 | 0.649086139 | -0.018483905 | 0.690332319 |
| BCAR1 | tan | 0.182786985 | 7.10E-05 | 0.216042182 | 2.45E-06 |
| BCCIP | tan | 0.074111173 | 0.109717528 | 0.052228008 | 0.259995856 |
| BLOC1S2 | tan | 0.139465775 | 0.002522921 | 0.129948836 | 0.004913856 |
| BRE | tan | -0.172845372 | 0.000174384 | -0.17635493 | 0.000127667 |
| BRIX1 | tan | -0.147854253 | 0.001354278 | -0.167774891 | 0.000270764 |
| BRK1 | tan | 0.048689019 | 0.293726563 | 0.059416174 | 0.199952864 |
| BTF3L4 | tan | -0.108161636 | 0.019387699 | -0.128091281 | 0.005569845 |
| BUD31 | tan | -0.038654784 | 0.404613659 | -0.040960578 | 0.377147641 |
| C11orf73 | tan | -0.082243204 | 0.075809346 | -0.086357171 | 0.062226606 |
| C11orf98 | tan | 0.009267799 | 0.841678931 | -0.016496258 | 0.722173123 |
| C14orf16( | tan | 0.165918019 | 0.00031712 | 0.155919123 | 0.000721932 |
| C18orf21 | tan | -0.059033883 | 0.202867308 | -0.064798527 | 0.162105609 |
| C4orf27 | tan | 0.085904403 | 0.063615538 | 0.063610296 | 0.169958778 |

| | | | | | |
|---|---|---|---|---|---|
| C6orf203 | tan | -0.016826308 | 0.716850836 | -0.028676323 | 0.536465301 |
| C7orf73 | tan | 0.02304441 | 0.619382794 | 0.021624049 | 0.641142633 |
| C8orf59 | tan | -0.0867232 | 0.061122121 | -0.116642531 | 0.0116515 |
| CAMTA1 | tan | -0.235270178 | 2.70E-07 | -0.237075731 | 2.18E-07 |
| CBWD1 | tan | -0.080870469 | 0.080845291 | -0.074513976 | 0.107795431 |
| CBX3 | tan | -0.063938268 | 0.167763305 | -0.086637671 | 0.061378743 |
| CCDC23 | tan | -0.251643215 | 3.54E-08 | -0.27021285 | 2.95E-09 |
| CCDC43 | tan | 0.196667189 | 1.86E-05 | 0.185080159 | 5.73E-05 |
| CCDC58 | tan | -0.060993688 | 0.188248688 | -0.084137214 | 0.069282987 |
| CCDC59 | tan | -0.121315524 | 0.008682113 | -0.143278944 | 0.00190913 |
| CCT8 | tan | 0.043417919 | 0.349172195 | 0.020939591 | 0.651740794 |
| CDC26 | tan | 0.037205245 | 0.422474773 | 0.018544015 | 0.68937752 |
| CENPQ | tan | -0.141057574 | 0.002247597 | -0.162797398 | 0.000412048 |
| CFDP1 | tan | 0.073401125 | 0.113171384 | 0.064296421 | 0.16539013 |
| CHCHD4 | tan | 0.052614321 | 0.256482572 | 0.054834819 | 0.23692619 |
| CHCHD7 | tan | -0.278149959 | 9.60E-10 | -0.261786233 | 9.33E-09 |
| CISD1 | tan | 0.027152359 | 0.558344104 | 0.031226867 | 0.500836603 |
| CISD2 | tan | 0.093414272 | 0.043622178 | 0.082293281 | 0.075630574 |
| CKLF | tan | 0.098819734 | 0.032759575 | 0.105344683 | 0.022800851 |
| CMC1 | tan | 0.110841951 | 0.016562275 | 0.105787371 | 0.022232233 |
| CMSS1 | tan | -0.199978134 | 1.34E-05 | -0.217929744 | 1.99E-06 |
| COA5 | tan | -0.221366357 | 1.36E-06 | -0.227210807 | 6.98E-07 |
| COA6 | tan | 0.160251392 | 0.000508435 | 0.159380395 | 0.000545956 |
| COMMD1 | tan | 0.028553188 | 0.538216991 | 0.013692221 | 0.767908132 |
| COX16 | tan | 0.138810317 | 0.002644972 | 0.137899019 | 0.002823609 |
| COX17 | tan | -0.022471035 | 0.628128315 | -0.008548106 | 0.853829541 |
| COX6C | tan | -0.069912232 | 0.131397565 | -0.060951434 | 0.188555479 |
| COX7A2l | tan | -0.090968646 | 0.04945409 | -0.093289002 | 0.043906158 |
| CRIPAK | tan | 0.170049993 | 0.000222597 | 0.194951141 | 2.21E-05 |
| CRIPT | tan | -0.172815707 | 0.00017484 | -0.172693261 | 0.000176733 |
| CRLS1 | tan | -0.078568913 | 0.089893303 | -0.062017101 | 0.180929918 |
| CSTF3 | tan | 0.084057393 | 0.069548431 | 0.052282307 | 0.259500041 |
| CTNNBL | tan | -0.285660887 | 3.21E-10 | -0.275741212 | 1.35E-09 |
| CUTC | tan | 0.042827342 | 0.355772684 | 0.024147267 | 0.602712142 |
| CWC15 | tan | -0.046324222 | 0.317829379 | -0.054808449 | 0.237152095 |
| CYCS | tan | -0.062789079 | 0.175550149 | -0.064154173 | 0.16632965 |
| DAD1 | tan | 0.13614964 | 0.00319762 | 0.136162607 | 0.00319469 |
| DDT | tan | -0.003614445 | 0.937907811 | -0.018605893 | 0.688395155 |
| DECR1 | tan | 0.208913481 | 5.29E-06 | 0.202847268 | 9.97E-06 |
| DENR | tan | -0.01657526 | 0.720897924 | -0.041491633 | 0.370988063 |
| DGUOK | tan | -0.008931388 | 0.847354081 | 0.018492934 | 0.690188875 |
| DNAJC2 | tan | -0.08631 | 0.062370133 | -0.108345108 | 0.019181707 |
| DNAJC7 | tan | 0.178539604 | 0.000104824 | 0.168457305 | 0.000255381 |
| DNAJC8 | tan | 0.013871839 | 0.764952275 | 0.001173582 | 0.979820976 |
| DNTTIP2 | tan | -0.089879911 | 0.05225269 | -0.116957666 | 0.011426122 |

| | | | | | |
|---|---|---|---|---|---|
| DPY30 | tan | -0.023124127 | 0.618171098 | -0.023901139 | 0.606415104 |
| EBNA1Bl | tan | -0.073871225 | 0.110875283 | -0.075525727 | 0.103084679 |
| EBPL | tan | 0.163346788 | 0.000393616 | 0.16591403 | 0.000317227 |
| EEF1E1 | tan | -0.099010334 | 0.032423049 | -0.099909678 | 0.0308747 |
| EEF2 | tan | 0.324547077 | 6.47E-13 | 0.313899133 | 3.88E-12 |
| EID2 | tan | -0.063871397 | 0.168209214 | -0.074736179 | 0.106746547 |
| EIF3M | tan | -0.125678242 | 0.006539228 | -0.149214074 | 0.001220589 |
| EIF5B | tan | -0.0865003 | 0.061792772 | -0.09843951 | 0.033439816 |
| EMC3 | tan | 0.081684914 | 0.077825793 | 0.113012624 | 0.014545122 |
| EXOSC8 | tan | 0.103475951 | 0.025342138 | 0.07612733 | 0.100361894 |
| EXOSC9 | tan | 0.030282124 | 0.513886391 | 0.007007795 | 0.879947536 |
| FAM103/ | tan | 0.087406547 | 0.059103454 | 0.071314944 | 0.123814357 |
| FKBP1A | tan | -0.221738275 | 1.30E-06 | -0.218342351 | 1.90E-06 |
| FKBP3 | tan | 0.059907894 | 0.196249138 | 0.039045207 | 0.399880972 |
| FRG1 | tan | -0.002273531 | 0.960919437 | -0.008543447 | 0.853908321 |
| FTSJ2 | tan | -0.034141118 | 0.461710533 | -0.034637932 | 0.455214414 |
| FUCA2 | tan | 0.012205545 | 0.792498961 | 0.017136818 | 0.71185611 |
| FUNDC1 | tan | -0.094187503 | 0.041903325 | -0.098851378 | 0.032703497 |
| FUNDC2 | tan | -0.221069544 | 1.40E-06 | -0.235160423 | 2.74E-07 |
| GABARA | tan | 0.028891056 | 0.533417406 | 0.004317898 | 0.925855164 |
| GAR1 | tan | 0.061597977 | 0.183901335 | 0.070179048 | 0.12992814 |
| GCSH | tan | -0.032888259 | 0.478319261 | -0.0493946 | 0.2867782 |
| GEMIN6 | tan | -0.145543469 | 0.0016127 | -0.133362878 | 0.003887122 |
| GGCT | tan | 0.230463294 | 4.78E-07 | 0.222741242 | 1.16E-06 |
| GLO1 | tan | -0.102901536 | 0.026170836 | -0.130433838 | 0.004754465 |
| GLRX3 | tan | 0.038202398 | 0.410139014 | 0.026676973 | 0.565256374 |
| GLRX5 | tan | -0.091327743 | 0.048558974 | -0.080378496 | 0.08271472 |
| GNG5 | tan | 0.012283727 | 0.791200393 | 0.010687679 | 0.817819575 |
| GNL2 | tan | -0.060933007 | 0.188689377 | -0.079681413 | 0.085423179 |
| GNL3 | tan | 0.038097155 | 0.41143081 | 0.010394265 | 0.822737044 |
| GPANK1 | tan | -0.16790912 | 0.000267672 | -0.178098256 | 0.000109104 |
| GRPEL1 | tan | 0.152781869 | 0.00092545 | 0.161332613 | 0.000465192 |
| GTF2A2 | tan | 0.173130183 | 0.000170065 | 0.178777987 | 0.000102579 |
| GTF2F2 | tan | 0.095256064 | 0.039622287 | 0.076815671 | 0.097316977 |
| GTF2H4 | tan | -0.154612674 | 0.000801043 | -0.186746381 | 4.90E-05 |
| GTF2H5 | tan | 0.13791827 | 0.002819724 | 0.150469282 | 0.001108079 |
| GTF3C6 | tan | 0.230818425 | 4.58E-07 | 0.238116614 | 1.92E-07 |
| H3F3A | tan | -0.028844585 | 0.534076267 | -0.055926285 | 0.227708558 |
| H3F3B | tan | -0.082105821 | 0.076301566 | -0.099663069 | 0.031292843 |
| HARS2 | tan | 0.057962792 | 0.211196919 | 0.036251134 | 0.434478445 |
| HAUS1 | tan | 0.097318917 | 0.035515087 | 0.077277444 | 0.095315876 |
| HDDC2 | tan | 0.02816586 | 0.543745633 | 0.039145691 | 0.398668301 |
| HESX1 | tan | -0.048057856 | 0.300036695 | -0.069359804 | 0.134480709 |
| HMGB1 | tan | -0.036235402 | 0.434678002 | -0.064226217 | 0.165853313 |
| HMGN1 | tan | 0.017607252 | 0.704312417 | -0.003861382 | 0.933675132 |

| | | | | | |
|---|---|---|---|---|---|
| HMGN3 | tan | -0.027996899 | 0.54616614 | -0.037837623 | 0.414626683 |
| HNRNPA | tan | 0.067508885 | 0.145218063 | 0.044845253 | 0.333542288 |
| HNRNPC | tan | 0.0141975 | 0.75960179 | -0.025023105 | 0.589618377 |
| HNRNPD | tan | 0.038326747 | 0.408615805 | 0.013785764 | 0.766368329 |
| HNRNPL | tan | 0.006069835 | 0.895917764 | -0.035370394 | 0.445731309 |
| HNRNPU | tan | -0.139128622 | 0.002585045 | -0.164148602 | 0.00036809 |
| HRSP12 | tan | 0.079794449 | 0.084979194 | 0.055574113 | 0.230654453 |
| HSP90B1 | tan | -0.016951078 | 0.714842374 | -0.023951606 | 0.605655014 |
| HSPA4 | tan | 0.124857685 | 0.006901806 | 0.118572846 | 0.010330469 |
| HSPB11 | tan | -0.09856635 | 0.033211567 | -0.103609092 | 0.025153321 |
| HSPE1 | tan | 0.010642184 | 0.818581587 | 0.015238531 | 0.742575993 |
| HSPE1-M | tan | -0.05652722 | 0.222743677 | -0.088851873 | 0.055015244 |
| IMMP1L | tan | 0.079173388 | 0.087441733 | 0.0652322 | 0.159308449 |
| INO80C | tan | -0.104676976 | 0.023682313 | -0.106836476 | 0.020933544 |
| ISCA2 | tan | 0.029114683 | 0.530252559 | 0.03375881 | 0.46674435 |
| ITGB1BP | tan | -0.221064176 | 1.41E-06 | -0.224260909 | 9.79E-07 |
| ITGB3BP | tan | 0.067207768 | 0.14702511 | 0.059216605 | 0.201470477 |
| LINC004 | tan | -0.058014501 | 0.210789205 | -0.046128202 | 0.319883686 |
| LLPH | tan | -0.052017415 | 0.261924993 | -0.053221703 | 0.251025501 |
| LSM12 | tan | 0.172078157 | 0.000186538 | 0.143495498 | 0.001878764 |
| LSM3 | tan | 0.04623918 | 0.318719563 | 0.051188762 | 0.269611445 |
| LSM5 | tan | -0.059309368 | 0.200764026 | -0.049110263 | 0.289564849 |
| LSM6 | tan | -0.11643957 | 0.011798726 | -0.140432605 | 0.002352245 |
| LTV1 | tan | -0.089792933 | 0.052481849 | -0.116027543 | 0.012102674 |
| LYAR | tan | -0.086976061 | 0.060368612 | -0.106507438 | 0.021333579 |
| MAGOHI | tan | -0.014980581 | 0.746783376 | -0.015668843 | 0.735574327 |
| MCTS1 | tan | 0.056662563 | 0.221636214 | 0.069432707 | 0.13407067 |
| MED15 | tan | 0.087577967 | 0.058605811 | 0.104364565 | 0.024104757 |
| MED4 | tan | 0.001897748 | 0.96737497 | -0.020192064 | 0.663396098 |
| MED6 | tan | -0.155652052 | 0.000737491 | -0.156674746 | 0.000679544 |
| METTL2 | tan | 0.02317707 | 0.617366931 | 0.0260016 | 0.575146465 |
| METTL5 | tan | 0.095003816 | 0.040151062 | 0.086902462 | 0.060587135 |
| MGRN1 | tan | 0.158223763 | 0.000599762 | 0.191017228 | 3.25E-05 |
| MKKS | tan | -0.062216177 | 0.179531054 | -0.070644511 | 0.127395195 |
| MKL1 | tan | 0.1002494 | 0.030306512 | 0.126421745 | 0.006225543 |
| MNAT1 | tan | 0.093721607 | 0.042932009 | 0.089916631 | 0.052156193 |
| MPHOSP | tan | -0.067039431 | 0.14804277 | -0.085881608 | 0.063686137 |
| MPLKIP | tan | -0.037992593 | 0.412716624 | -0.039068237 | 0.399602844 |
| MRPL1 | tan | 0.058382845 | 0.207901358 | 0.034734955 | 0.453951804 |
| MRPL18 | tan | 0.122802206 | 0.007890298 | 0.135231992 | 0.00341131 |
| MRPL32 | tan | -0.070090217 | 0.130415939 | -0.070545493 | 0.127930797 |
| MRPL33 | tan | -0.156607475 | 0.000683222 | -0.170549075 | 0.000213163 |
| MRPL35 | tan | 0.046270072 | 0.318396012 | 0.050485743 | 0.276252373 |
| MRPL39 | tan | 0.124251489 | 0.007181119 | 0.113006326 | 0.014550647 |
| MRPL46 | tan | -0.052600849 | 0.256604537 | -0.054497075 | 0.23983104 |

| | | | | | |
|---|---|---|---|---|---|
| MRPL51 | tan | -0.02728198 | 0.556466496 | -0.027057977 | 0.559713182 |
| MRPS10 | tan | 0.03143636 | 0.497966761 | 0.012270401 | 0.791421684 |
| MRPS17 | tan | -0.048120386 | 0.299407553 | -0.055398887 | 0.232130226 |
| MRPS18( | tan | -0.06755337 | 0.144952545 | -0.068150677 | 0.141423202 |
| MRPS22 | tan | -0.174554844 | 0.000149922 | -0.18334387 | 6.74E-05 |
| MRPS31 | tan | -0.100854408 | 0.029316793 | -0.124389666 | 0.007116577 |
| MRPS33 | tan | -0.054727806 | 0.237843887 | -0.043561979 | 0.34757397 |
| MRPS6 | tan | -0.160982082 | 0.000478821 | -0.161498841 | 0.000458855 |
| MRPS9 | tan | -0.030255661 | 0.514254451 | -0.04380702 | 0.344866108 |
| MZT1 | tan | 0.091315191 | 0.048590033 | 0.074686498 | 0.106980359 |
| NAA20 | tan | -0.03909249 | 0.399310071 | -0.041530134 | 0.370543932 |
| NCOR2 | tan | -0.004352968 | 0.925254705 | 0.003750108 | 0.935582217 |
| NDUFA4 | tan | 0.022005699 | 0.635264484 | 0.050638053 | 0.274804253 |
| NDUFAB | tan | 0.060855262 | 0.189255106 | 0.091464151 | 0.048222524 |
| NDUFAF | tan | -0.12183059 | 0.008400113 | -0.115883715 | 0.012210392 |
| NDUFB3 | tan | 0.051516683 | 0.266551455 | 0.062890958 | 0.174849151 |
| NDUFB6 | tan | 0.081459193 | 0.078653319 | 0.085077596 | 0.066217741 |
| NDUFC2 | tan | 0.0080973 | 0.861458355 | 0.007928242 | 0.864322614 |
| NFU1 | tan | 0.101539216 | 0.028230098 | 0.129420822 | 0.005092847 |
| NGDN | tan | -0.278958037 | 8.55E-10 | -0.294078139 | 9.06E-11 |
| NIFK | tan | -0.101038981 | 0.029020426 | -0.129393206 | 0.005102369 |
| NIT2 | tan | -0.11883647 | 0.010160774 | -0.112762111 | 0.014766332 |
| NOL10 | tan | -0.072134406 | 0.119544454 | -0.081975905 | 0.076769417 |
| NOL7 | tan | -0.134040792 | 0.003708011 | -0.138860302 | 0.002635479 |
| NOP58 | tan | -0.062480362 | 0.177687087 | -0.096051044 | 0.037994194 |
| NOX1 | tan | 0.079249866 | 0.087135429 | 0.084944805 | 0.066643708 |
| NSMCE4 | tan | 0.072326514 | 0.118560286 | 0.040103964 | 0.387214842 |
| OARD1 | tan | -0.113716095 | 0.013939434 | -0.125485153 | 0.006622976 |
| PBDC1 | tan | 0.084457058 | 0.068227651 | 0.084183971 | 0.069127881 |
| PCBP2 | tan | -0.186236997 | 5.14E-05 | -0.221101135 | 1.40E-06 |
| PDCD2 | tan | 0.009185502 | 0.843066521 | 0.007132712 | 0.877824142 |
| PDCL3 | tan | 0.052568365 | 0.25689878 | 0.025367629 | 0.584503809 |
| PDHB | tan | 0.142809065 | 0.001976568 | 0.155879158 | 0.000724241 |
| PET117 | tan | -0.063682557 | 0.169473224 | -0.063629879 | 0.16982709 |
| PFDN4 | tan | -0.283957014 | 4.13E-10 | -0.300420956 | 3.39E-11 |
| PINX1 | tan | 0.225334637 | 8.66E-07 | 0.202829858 | 9.99E-06 |
| PIP5K1C | tan | 0.196595239 | 1.88E-05 | 0.209887766 | 4.77E-06 |
| PITHD1 | tan | -0.05197865 | 0.262281168 | -0.070056584 | 0.130600993 |
| PLEC | tan | -0.099649841 | 0.031315408 | -0.079663984 | 0.085491802 |
| PLGRKT | tan | 0.097407781 | 0.035346611 | 0.102465161 | 0.026815895 |
| PLXNB2 | tan | 0.239272448 | 1.67E-07 | 0.256244933 | 1.95E-08 |
| POLB | tan | -0.093335759 | 0.043799982 | -0.113691527 | 0.013960207 |
| POMP | tan | 0.099143558 | 0.032189582 | 0.114764094 | 0.013078266 |
| PPA2 | tan | 0.311009446 | 6.24E-12 | 0.300164334 | 3.53E-11 |
| PPCS | tan | 0.115542568 | 0.012469281 | 0.119964631 | 0.009462198 |

| | | | | | |
|---|---|---|---|---|---|
| PPT2 | tan | -0.32748483 | 3.89E-13 | -0.342947154 | 2.46E-14 |
| PRDX1 | tan | 0.195002571 | 2.20E-05 | 0.206320592 | 6.95E-06 |
| PRDX6 | tan | -0.092418768 | 0.045922187 | -0.092570552 | 0.045565063 |
| PSMA1 | tan | 0.008686858 | 0.851484208 | 0.010688393 | 0.817807612 |
| PSMA2 | tan | -0.089348722 | 0.053665314 | -0.075619916 | 0.102654564 |
| PSMA3 | tan | 0.109695996 | 0.017722865 | 0.130279578 | 0.004804648 |
| PSMA4 | tan | 0.099442132 | 0.031671562 | 0.109706045 | 0.017712387 |
| PSMC6 | tan | -0.017872556 | 0.700070804 | -0.027040088 | 0.559972865 |
| PSMD6 | tan | 0.130186811 | 0.004835055 | 0.13702211 | 0.003005802 |
| PSMG1 | tan | 0.160320849 | 0.000505549 | 0.150393192 | 0.001114618 |
| PSMG2 | tan | -0.000850926 | 0.985368113 | -0.013589774 | 0.769595544 |
| PTS | tan | 0.191310969 | 3.16E-05 | 0.174697074 | 0.000148039 |
| PYURF | tan | -0.086774525 | 0.060968552 | -0.081978526 | 0.076759954 |
| RARS | tan | -0.010681088 | 0.817929953 | -0.0108779 | 0.814635363 |
| RBM17 | tan | -0.191586652 | 3.08E-05 | -0.218254929 | 1.92E-06 |
| RBM8A | tan | 0.03251599 | 0.483316302 | 0.01624979 | 0.726156366 |
| RBMX2 | tan | -0.044932177 | 0.332605196 | -0.054916595 | 0.236226603 |
| RDH14 | tan | -0.135142711 | 0.003432777 | -0.141745794 | 0.002137291 |
| RMND1 | tan | 0.05093265 | 0.272018006 | 0.004112276 | 0.929376561 |
| RNASEH | tan | -0.088871503 | 0.054961377 | -0.110145606 | 0.017259306 |
| RNF5 | tan | -0.152868673 | 0.00091917 | -0.138138832 | 0.002775565 |
| RNF7 | tan | -0.071927751 | 0.120610256 | -0.049431407 | 0.286418794 |
| RPA2 | tan | -0.018086108 | 0.69666334 | -0.041555812 | 0.370247904 |
| RPF1 | tan | -0.099049997 | 0.032353391 | -0.118053097 | 0.010672412 |
| RPF2 | tan | -0.062047083 | 0.180718725 | -0.084112235 | 0.069365967 |
| RPP30 | tan | -0.055672613 | 0.229827801 | -0.069686645 | 0.132649921 |
| RPSAP58 | tan | -0.087045215 | 0.06016388 | -0.108532274 | 0.018973537 |
| RTFDC1 | tan | -0.344752679 | 1.77E-14 | -0.354982047 | 2.58E-15 |
| RWDD1 | tan | -0.008338582 | 0.857373582 | 0.006227105 | 0.893236888 |
| SAAL1 | tan | 0.170059173 | 0.00022242 | 0.155233203 | 0.000762518 |
| SEC61G | tan | 0.131703531 | 0.004359045 | 0.152166556 | 0.000971115 |
| SF3B6 | tan | -0.101338642 | 0.02854474 | -0.10739223 | 0.020272662 |
| SFPQ | tan | -0.009205353 | 0.842731764 | -0.026930224 | 0.561568921 |
| SFT2D1 | tan | 0.053721333 | 0.246597496 | 0.065016944 | 0.160692256 |
| SKA2 | tan | 0.013909749 | 0.764328846 | 0.005693933 | 0.902330277 |
| SMIM7 | tan | -0.0647948 | 0.162129806 | -0.064896818 | 0.161468423 |
| SMN1 | tan | 0.023136113 | 0.617989007 | -0.004491317 | 0.922886378 |
| SMS | tan | 0.131008897 | 0.00457153 | 0.139179191 | 0.002575639 |
| SNRNP27 | tan | -0.088031278 | 0.057306516 | -0.11960249 | 0.00968164 |
| SNRPB2 | tan | -0.20580145 | 7.34E-06 | -0.210024854 | 4.70E-06 |
| SNRPE | tan | 0.030158556 | 0.515606197 | 0.014154629 | 0.760305485 |
| SNRPG | tan | 0.065976739 | 0.154591341 | 0.065784608 | 0.155798363 |
| SNUPN | tan | 0.137201677 | 0.002967646 | 0.128352887 | 0.00547293 |
| SPCS1 | tan | 0.198168179 | 1.60E-05 | 0.197630329 | 1.69E-05 |
| SRP19 | tan | 0.110136103 | 0.017268993 | 0.104904854 | 0.023378227 |

| | | | | | |
|---|---|---|---|---|---|
| SRSF1 | tan | 0.051197982 | 0.269525089 | 0.020155438 | 0.66396929 |
| SRSF2 | tan | -0.023089326 | 0.618699941 | -0.051368894 | 0.267927591 |
| SRSF3 | tan | -0.049031739 | 0.290337613 | -0.077689113 | 0.093559726 |
| SRSF7 | tan | 0.006376874 | 0.890684994 | -0.014483894 | 0.754905903 |
| SSB | tan | -0.115190621 | 0.012741432 | -0.141392166 | 0.002193336 |
| SSBP1 | tan | 0.099306814 | 0.031905446 | 0.098175966 | 0.033918348 |
| SUB1 | tan | -0.038752823 | 0.40342211 | -0.052677912 | 0.255907411 |
| SUCLG1 | tan | -0.028022593 | 0.545797722 | -0.017168393 | 0.711348901 |
| SUMO1 | tan | -0.135717193 | 0.00329676 | -0.149627367 | 0.001182432 |
| SUMO2 | tan | -0.114342191 | 0.013419148 | -0.144847107 | 0.00169902 |
| SUPV3L1 | tan | -0.045356931 | 0.328050509 | -0.061412185 | 0.185229991 |
| TARDBP | tan | -0.081920205 | 0.076970717 | -0.107932668 | 0.01964747 |
| TBCA | tan | 0.042825082 | 0.355798097 | 0.024345558 | 0.599736281 |
| TDP2 | tan | -0.002598076 | 0.955346169 | -0.009649698 | 0.835246356 |
| TECPR2 | tan | -0.027571897 | 0.55227809 | -0.026410243 | 0.569152603 |
| TEX30 | tan | -0.042821067 | 0.355843235 | -0.069891507 | 0.131512239 |
| THOC7 | tan | 0.148011043 | 0.001338204 | 0.132701522 | 0.00406937 |
| TIMM9 | tan | -0.026305374 | 0.570687944 | -0.04592239 | 0.322049902 |
| TIMMDC | tan | -0.145409161 | 0.001629029 | -0.138206034 | 0.002762236 |
| TMA7 | tan | 0.065106751 | 0.16011382 | 0.05075294 | 0.273715371 |
| TMEM12 | tan | 0.142905399 | 0.001962567 | 0.145461641 | 0.001622631 |
| TMEM14 | tan | -0.1263721 | 0.00624606 | -0.126153215 | 0.006337244 |
| TMEM14 | tan | -0.13183909 | 0.004318634 | -0.138959327 | 0.002616762 |
| TMEM19 | tan | -0.096437051 | 0.037224343 | -0.093124472 | 0.044281502 |
| TMEM24 | tan | -0.043364408 | 0.349767033 | -0.055321861 | 0.232781053 |
| TMEM60 | tan | 0.005599938 | 0.903934753 | 0.020712008 | 0.655280415 |
| TOMM22 | tan | 0.055905517 | 0.227881538 | 0.04892499 | 0.291390367 |
| TP53RK | tan | -0.13143519 | 0.004440048 | -0.129504161 | 0.005064212 |
| TPRKB | tan | -0.140509905 | 0.002339065 | -0.145906608 | 0.0015693 |
| TRIAP1 | tan | 0.114676545 | 0.013148368 | 0.128109403 | 0.005563081 |
| TRIT1 | tan | -0.238088238 | 1.93E-07 | -0.275712914 | 1.36E-09 |
| TRMT10( | tan | -0.149131119 | 0.001228384 | -0.166903608 | 0.000291665 |
| TSC2 | tan | 0.11066931 | 0.016732748 | 0.11793017 | 0.010754738 |
| TSFM | tan | 0.004021836 | 0.930925839 | 0.002555672 | 0.956074244 |
| TTC1 | tan | -0.036640539 | 0.429555792 | -0.032847936 | 0.478859167 |
| TTC4 | tan | -0.196063701 | 1.98E-05 | -0.23448664 | 2.97E-07 |
| TXNDC1 | tan | 0.05386142 | 0.245365815 | 0.034865653 | 0.452254064 |
| TXNDC9 | tan | -0.129161601 | 0.005182853 | -0.141924646 | 0.002109446 |
| UPF3B | tan | -0.088155738 | 0.056953991 | -0.115647466 | 0.012389165 |
| UQCRB | tan | -0.042733916 | 0.35682399 | -0.069671775 | 0.132732793 |
| UTP11L | tan | -0.050456671 | 0.276529368 | -0.076725859 | 0.097710038 |
| UTP14A | tan | -0.109143467 | 0.018307404 | -0.147963311 | 0.001343079 |
| VBP1 | tan | 0.014133019 | 0.760660289 | -0.002038677 | 0.964953699 |
| VPS29 | tan | 0.00869398 | 0.851363847 | 0.011097962 | 0.810955443 |
| VPS33A | tan | -0.059114768 | 0.202248113 | -0.058028355 | 0.210680069 |

| | | | | | |
|---|---|---|---|---|---|
| YAE1D1 | tan | -0.060698765 | 0.190397654 | -0.063618035 | 0.169906728 |
| ZNF213 | tan | 0.221018593 | 1.41E-06 | 0.248756097 | 5.12E-08 |
| AASS | turquoise | -0.077409138 | 0.094751239 | -0.092011422 | 0.046892246 |
| ABCA10 | turquoise | -0.007469105 | 0.872110415 | -0.015590673 | 0.736844631 |
| ABCA6 | turquoise | -0.157187098 | 0.000652127 | -0.153592918 | 0.000868286 |
| ABCA8 | turquoise | -0.231832392 | 4.07E-07 | -0.232545599 | 3.74E-07 |
| ABCA9 | turquoise | -0.13678844 | 0.003056123 | -0.139153643 | 0.002580387 |
| ABCC1 | turquoise | 0.170539224 | 0.000213346 | 0.14551828 | 0.001615751 |
| ABCC9 | turquoise | -0.193237619 | 2.62E-05 | -0.183396656 | 6.71E-05 |
| ABCD2 | turquoise | -0.229627562 | 5.27E-07 | -0.211037358 | 4.22E-06 |
| ABHD11 | turquoise | 0.091348996 | 0.048506424 | 0.11209656 | 0.015368451 |
| ABHD17( | turquoise | 0.339253917 | 4.83E-14 | 0.364128978 | 4.35E-16 |
| ABI3BP | turquoise | -0.198045481 | 1.62E-05 | -0.193882396 | 2.46E-05 |
| ABLIM3 | turquoise | -0.250780185 | 3.95E-08 | -0.249550804 | 4.63E-08 |
| ABRACL | turquoise | 0.219438594 | 1.69E-06 | 0.233041158 | 3.53E-07 |
| ACACB | turquoise | -0.349721407 | 7.00E-15 | -0.366742152 | 2.58E-16 |
| ACKR4 | turquoise | -0.163136979 | 0.000400563 | -0.137746594 | 0.002854538 |
| ACOX2 | turquoise | -0.093944719 | 0.042436759 | -0.094201334 | 0.041873108 |
| ACSS3 | turquoise | -0.245613902 | 7.61E-08 | -0.252378407 | 3.22E-08 |
| ACTA2 | turquoise | -0.055308511 | 0.232893987 | -0.022672652 | 0.625047092 |
| ACTC1 | turquoise | -0.107263481 | 0.020424129 | -0.106614992 | 0.021202095 |
| ACTG2 | turquoise | -0.080590186 | 0.081906091 | -0.048186402 | 0.298744291 |
| ACTN1 | turquoise | -0.066882358 | 0.148997165 | -0.065755428 | 0.1559823 |
| ACTR3B | turquoise | 0.073784167 | 0.111297714 | 0.030717198 | 0.50785487 |
| ACY1 | turquoise | 0.113443661 | 0.014171315 | 0.108697274 | 0.018791654 |
| ADAM11 | turquoise | -0.204339257 | 8.55E-06 | -0.220987514 | 1.42E-06 |
| ADAM22 | turquoise | -0.053811259 | 0.245806344 | -0.062673543 | 0.176347641 |
| ADAM33 | turquoise | -0.397770101 | 3.73E-19 | -0.400983282 | 1.82E-19 |
| ADAMTS | turquoise | -0.229148717 | 5.57E-07 | -0.233469524 | 3.35E-07 |
| ADAMTS | turquoise | -0.156077202 | 0.000712867 | -0.135630315 | 0.003317011 |
| ADAMTS | turquoise | -0.188835033 | 4.01E-05 | -0.202300756 | 1.05E-05 |
| ADAMTS | turquoise | -0.082696766 | 0.074202626 | -0.075776776 | 0.101941427 |
| ADAMTS | turquoise | -0.094970684 | 0.040220955 | -0.092709362 | 0.045240508 |
| ADAMTS | turquoise | -0.339926733 | 4.28E-14 | -0.333155258 | 1.44E-13 |
| ADAMTS | turquoise | -0.160830933 | 0.000484812 | -0.151816766 | 0.000997994 |
| ADARB1 | turquoise | -0.303641534 | 2.04E-11 | -0.324459497 | 6.56E-13 |
| ADCY2 | turquoise | -0.228612595 | 5.93E-07 | -0.218709215 | 1.83E-06 |
| ADCY3 | turquoise | -0.284948896 | 3.57E-10 | -0.303669701 | 2.03E-11 |
| ADCY5 | turquoise | -0.270760448 | 2.73E-09 | -0.274458837 | 1.63E-09 |
| ADCY9 | turquoise | -0.436639288 | 3.67E-23 | -0.403530638 | 1.03E-19 |
| ADCYAP | turquoise | -0.165750932 | 0.000321635 | -0.168720785 | 0.000249663 |
| ADH1B | turquoise | -0.205264048 | 7.76E-06 | -0.208855811 | 5.32E-06 |
| ADHFE1 | turquoise | -0.370839754 | 1.13E-16 | -0.376594063 | 3.49E-17 |
| ADM2 | turquoise | 0.1502529 | 0.001126766 | 0.152445303 | 0.000950176 |
| ADRA1D | turquoise | -0.299442175 | 3.95E-11 | -0.306799181 | 1.23E-11 |

| | | | | | |
|---|---|---|---|---|---|
| ADRA2A | turquoise | 0.057266501 | 0.216742361 | 0.078329836 | 0.090878018 |
| ADRA2C | turquoise | -0.25754989 | 1.64E-08 | -0.258132627 | 1.52E-08 |
| AFAP1L1 | turquoise | -0.22859436 | 5.95E-07 | -0.228571011 | 5.96E-07 |
| AFAP1L2 | turquoise | -0.280423645 | 6.92E-10 | -0.275787724 | 1.35E-09 |
| AFF3 | turquoise | -0.035486931 | 0.444232923 | -0.049276462 | 0.287933814 |
| AGMAT | turquoise | 0.191180363 | 3.20E-05 | 0.203666857 | 9.16E-06 |
| AGRN | turquoise | -0.031812593 | 0.492834733 | -0.021656517 | 0.64064167 |
| AHCY | turquoise | 0.060281483 | 0.193468869 | 0.048202285 | 0.29858486 |
| AHNAK | turquoise | -0.159618511 | 0.000535451 | -0.173046456 | 0.000171324 |
| AIFM1 | turquoise | 0.054054337 | 0.243676692 | 0.075709118 | 0.102248541 |
| AKAP2 | turquoise | -0.177010406 | 0.000120364 | -0.184094575 | 6.28E-05 |
| AKNAD1 | turquoise | -0.205836222 | 7.31E-06 | -0.196674412 | 1.86E-05 |
| AKT3 | turquoise | -0.259479626 | 1.27E-08 | -0.245703275 | 7.53E-08 |
| ALDH18A | turquoise | 0.405812539 | 6.10E-20 | 0.39927928 | 2.67E-19 |
| ALDH1A | turquoise | -0.089295827 | 0.053807705 | -0.106111447 | 0.021823799 |
| ALDH1A | turquoise | -0.354406577 | 2.88E-15 | -0.347440357 | 1.07E-14 |
| ALDH1A | turquoise | 0.010454613 | 0.821725058 | 0.00933896 | 0.840479511 |
| ALDH1B | turquoise | -0.109495697 | 0.017932858 | -0.115839919 | 0.01224336 |
| ALDOA | turquoise | 0.163706443 | 0.000381969 | 0.207936645 | 5.87E-06 |
| AMIGO1 | turquoise | -0.175796865 | 0.000134211 | -0.183565684 | 6.60E-05 |
| AMPH | turquoise | -0.109924319 | 0.017486117 | -0.126936116 | 0.006016502 |
| ANG | turquoise | 0.178454568 | 0.000105636 | 0.174332464 | 0.000152912 |
| ANGPT1 | turquoise | -0.163473061 | 0.00038949 | -0.152856582 | 0.000920042 |
| ANGPTL | turquoise | -0.11470732 | 0.013123689 | -0.115295729 | 0.012659611 |
| ANK2 | turquoise | -0.155966847 | 0.000719184 | -0.191167294 | 3.20E-05 |
| ANKDD1 | turquoise | -0.327388324 | 3.96E-13 | -0.330872456 | 2.16E-13 |
| ANKRD2 | turquoise | -0.252633994 | 3.11E-08 | -0.269319415 | 3.34E-09 |
| ANKRD3 | turquoise | -0.41143633 | 1.67E-20 | -0.421514063 | 1.54E-21 |
| ANKRD3 | turquoise | 0.049025487 | 0.290399206 | 0.021145779 | 0.648540653 |
| ANKZF1 | turquoise | 0.14913804 | 0.001227732 | 0.144721185 | 0.001715074 |
| ANO4 | turquoise | -0.206393376 | 6.90E-06 | -0.1960454 | 1.98E-05 |
| ANO5 | turquoise | -0.440805777 | 1.27E-23 | -0.452131314 | 6.57E-25 |
| ANO9 | turquoise | 0.058132628 | 0.209859948 | 0.054086272 | 0.243397863 |
| ANTXR2 | turquoise | -0.058482176 | 0.207127516 | -0.055725032 | 0.229388736 |
| ANXA6 | turquoise | -0.182624387 | 7.21E-05 | -0.13810525 | 0.002782247 |
| AOC3 | turquoise | -0.15950061 | 0.000540629 | -0.152387936 | 0.000954451 |
| AOX1 | turquoise | -0.251790735 | 3.47E-08 | -0.267747183 | 4.15E-09 |
| AP1S1 | turquoise | 0.270179132 | 2.96E-09 | 0.289336415 | 1.86E-10 |
| AP4S1 | turquoise | -0.220871736 | 1.44E-06 | -0.20785563 | 5.92E-06 |
| APBA1 | turquoise | -0.180648151 | 8.65E-05 | -0.176130493 | 0.000130262 |
| APBB1 | turquoise | -0.394790601 | 7.21E-19 | -0.400578556 | 1.99E-19 |
| APEH | turquoise | 0.148394577 | 0.001299622 | 0.173724229 | 0.000161376 |
| APEX2 | turquoise | 0.217662485 | 2.05E-06 | 0.239445922 | 1.63E-07 |
| APOD | turquoise | -0.168822556 | 0.000247486 | -0.160692647 | 0.000490355 |
| APOLD1 | turquoise | -0.207924668 | 5.87E-06 | -0.225935233 | 8.08E-07 |

| | | | | | |
|---|---|---|---|---|---|
| AQP8 | turquoise | -0.204826854 | 8.12E-06 | -0.203676814 | 9.15E-06 |
| AR | turquoise | -0.048694038 | 0.293676744 | -0.061969533 | 0.181265356 |
| ARHGAP | turquoise | -0.093097347 | 0.044343641 | -0.07540186 | 0.103652492 |
| ARHGAP | turquoise | -0.290187517 | 1.63E-10 | -0.288722683 | 2.04E-10 |
| ARHGAP | turquoise | -0.444616853 | 4.75E-24 | -0.447323728 | 2.34E-24 |
| ARHGAP | turquoise | -0.14133108 | 0.002203152 | -0.142953431 | 0.00195562 |
| ARHGAP | turquoise | -0.270916584 | 2.67E-09 | -0.269949491 | 3.06E-09 |
| ARHGEF | turquoise | 0.021098199 | 0.649278551 | 0.009737345 | 0.833771603 |
| ARHGEF | turquoise | -0.325437027 | 5.55E-13 | -0.316281247 | 2.62E-12 |
| ARHGEF | turquoise | 0.138679815 | 0.002669906 | 0.123313994 | 0.007633023 |
| ARHGEF | turquoise | -0.265550299 | 5.60E-09 | -0.27528632 | 1.45E-09 |
| ARHGEF | turquoise | -0.068664471 | 0.138440314 | -0.094403562 | 0.041433377 |
| ARHGEF | turquoise | -0.110345611 | 0.017056526 | -0.137344428 | 0.002937628 |
| ARHGEF | turquoise | 0.075432976 | 0.103509622 | 0.073863635 | 0.110912062 |
| ARL10 | turquoise | -0.407020284 | 4.63E-20 | -0.417677066 | 3.85E-21 |
| ARMC9 | turquoise | -0.051542371 | 0.266312759 | -0.057994358 | 0.210947964 |
| ARMCX1 | turquoise | -0.493717896 | 4.57E-30 | -0.487470936 | 3.03E-29 |
| ARMCX4 | turquoise | -0.383337234 | 8.53E-18 | -0.393282265 | 1.00E-18 |
| ARRB1 | turquoise | -0.328520769 | 3.25E-13 | -0.285113379 | 3.48E-10 |
| ARSI | turquoise | -0.287211797 | 2.55E-10 | -0.266730223 | 4.77E-09 |
| ART4 | turquoise | -0.155533543 | 0.000744494 | -0.138182194 | 0.002766957 |
| ARTN | turquoise | 0.243822404 | 9.52E-08 | 0.232071227 | 3.96E-07 |
| ARVCF | turquoise | 0.190212526 | 3.51E-05 | 0.168756135 | 0.000248905 |
| ASB1 | turquoise | -0.203436651 | 9.38E-06 | -0.216363002 | 2.37E-06 |
| ASB2 | turquoise | -0.160741887 | 0.000488374 | -0.125584113 | 0.006579935 |
| ASB5 | turquoise | -0.142692673 | 0.001993606 | -0.141683175 | 0.002147118 |
| ASPA | turquoise | -0.231987206 | 4.00E-07 | -0.237104555 | 2.17E-07 |
| ASPN | turquoise | -0.077700867 | 0.093509967 | -0.0472126 | 0.308627379 |
| ATIC | turquoise | 0.127298993 | 0.005872844 | 0.113763031 | 0.013899824 |
| ATL1 | turquoise | -0.041138756 | 0.375074021 | -0.048818424 | 0.292443861 |
| ATOH8 | turquoise | -0.25221506 | 3.29E-08 | -0.262927316 | 8.00E-09 |
| ATP13A1 | turquoise | 0.127336483 | 0.005858179 | 0.146427413 | 0.001508926 |
| ATP13A2 | turquoise | 0.278814676 | 8.73E-10 | 0.279339934 | 8.09E-10 |
| ATP1B2 | turquoise | -0.347154859 | 1.13E-14 | -0.334164961 | 1.20E-13 |
| ATP6AP1 | turquoise | 0.11726582 | 0.011209476 | 0.130956099 | 0.004588057 |
| ATP6V0E | turquoise | 0.094080644 | 0.04213741 | 0.121753724 | 0.008441672 |
| ATP6V1C | turquoise | -0.279644137 | 7.74E-10 | -0.294092294 | 9.04E-11 |
| ATP8B2 | turquoise | -0.326663169 | 4.49E-13 | -0.322209259 | 9.64E-13 |
| ATP8B4 | turquoise | -0.179541762 | 9.57E-05 | -0.174359166 | 0.00015255 |
| ATRNL1 | turquoise | -0.245633399 | 7.59E-08 | -0.257930796 | 1.56E-08 |
| AVPR1A | turquoise | -0.227888337 | 6.45E-07 | -0.21513546 | 2.71E-06 |
| AVPR2 | turquoise | -0.318182701 | 1.90E-12 | -0.321160274 | 1.15E-12 |
| AXIN1 | turquoise | 0.201618347 | 1.13E-05 | 0.204882034 | 8.08E-06 |
| AXL | turquoise | -0.300966671 | 3.11E-11 | -0.280077464 | 7.27E-10 |
| B3GALTI | turquoise | 0.004056466 | 0.93033258 | -0.013954925 | 0.763586122 |

| | | | | | |
|---|---|---|---|---|---|
| B3GNT4 | turquoise | 0.187539641 | 4.54E-05 | 0.200190075 | 1.31E-05 |
| B4GAT1 | turquoise | -0.141634154 | 0.002154841 | -0.168146876 | 0.000262275 |
| BACH2 | turquoise | -0.349392068 | 7.45E-15 | -0.348110132 | 9.47E-15 |
| BAG2 | turquoise | -0.001363352 | 0.976558866 | -0.003158811 | 0.945722175 |
| BAI3 | turquoise | -0.263705557 | 7.20E-09 | -0.287299665 | 2.52E-10 |
| BAIAP2L | turquoise | 0.254149964 | 2.56E-08 | 0.268806841 | 3.58E-09 |
| BCAN | turquoise | -0.150469767 | 0.001108038 | -0.163930365 | 0.000374881 |
| BCHE | turquoise | -0.251274622 | 3.71E-08 | -0.26018959 | 1.15E-08 |
| BCL2 | turquoise | -0.035399166 | 0.445361107 | -0.101578463 | 0.028168881 |
| BDH1 | turquoise | 0.08261261 | 0.074498629 | 0.137791108 | 0.002845474 |
| BEGAIN | turquoise | -0.153898136 | 0.000847632 | -0.133350831 | 0.003890374 |
| BEND6 | turquoise | -0.350803657 | 5.71E-15 | -0.344829748 | 1.74E-14 |
| BEST1 | turquoise | -0.234152418 | 3.09E-07 | -0.233651979 | 3.28E-07 |
| BHLHE41 | turquoise | -0.258620065 | 1.42E-08 | -0.240631944 | 1.41E-07 |
| BHMT2 | turquoise | -0.396388755 | 5.07E-19 | -0.38824291 | 3.00E-18 |
| BIK | turquoise | 0.059446514 | 0.199722876 | 0.081009486 | 0.080323284 |
| BMP6 | turquoise | -0.162900575 | 0.000408526 | -0.178952511 | 0.000100964 |
| BNC2 | turquoise | -0.261654413 | 9.49E-09 | -0.260105703 | 1.17E-08 |
| BOC | turquoise | -0.224148903 | 9.92E-07 | -0.213976465 | 3.07E-06 |
| BPI | turquoise | -0.19444462 | 2.32E-05 | -0.182263955 | 7.45E-05 |
| BST1 | turquoise | -0.296456826 | 6.28E-11 | -0.281794698 | 5.67E-10 |
| BTBD19 | turquoise | -0.34541144 | 1.56E-14 | -0.331189613 | 2.04E-13 |
| BTG1 | turquoise | 0.062759703 | 0.175752666 | 0.045334046 | 0.32829487 |
| BVES | turquoise | -0.186811212 | 4.87E-05 | -0.199919143 | 1.34E-05 |
| C11orf95 | turquoise | -0.323230688 | 8.10E-13 | -0.341714609 | 3.09E-14 |
| C11orf96 | turquoise | -0.112312607 | 0.015170674 | -0.094925556 | 0.04031632 |
| C14orf28 | turquoise | -0.042605784 | 0.358269002 | -0.046441408 | 0.316605378 |
| C15orf52 | turquoise | -0.269922551 | 3.07E-09 | -0.269901732 | 3.08E-09 |
| C16orf45 | turquoise | -0.310335101 | 6.96E-12 | -0.29039682 | 1.58E-10 |
| C16orf86 | turquoise | -0.084572863 | 0.067848813 | -0.067703187 | 0.144061057 |
| C17orf51 | turquoise | -0.354263402 | 2.96E-15 | -0.368371693 | 1.87E-16 |
| C17orf61- | turquoise | 0.026773094 | 0.563855442 | 0.016570633 | 0.720972584 |
| C19orf54 | turquoise | 0.186980274 | 4.79E-05 | 0.201027012 | 1.20E-05 |
| C1orf159 | turquoise | 0.139565596 | 0.002504791 | 0.134007751 | 0.003716564 |
| C1orf21 | turquoise | -0.066932302 | 0.148693193 | -0.08212168 | 0.076244612 |
| C1orf53 | turquoise | 0.076917759 | 0.096871719 | 0.096720213 | 0.036668058 |
| C1QTNF1 | turquoise | -0.243802946 | 9.54E-08 | -0.203360341 | 9.46E-06 |
| C1QTNF2 | turquoise | -0.200444243 | 1.27E-05 | -0.194475735 | 2.32E-05 |
| C1QTNF4 | turquoise | -0.225416414 | 8.58E-07 | -0.22177863 | 1.30E-06 |
| C1QTNF7 | turquoise | -0.244581416 | 8.66E-08 | -0.236742925 | 2.27E-07 |
| C1R | turquoise | -0.305428519 | 1.54E-11 | -0.26925276 | 3.37E-09 |
| C1S | turquoise | -0.310505636 | 6.77E-12 | -0.266649281 | 4.82E-09 |
| C2CD4C | turquoise | -0.163476988 | 0.000389362 | -0.166208542 | 0.000309409 |
| C2orf15 | turquoise | -0.034641829 | 0.45516367 | -0.030597914 | 0.509504829 |
| C2orf40 | turquoise | -0.331478661 | 1.94E-13 | -0.334435702 | 1.15E-13 |

| | | | | | |
|---|---|---|---|---|---|
| C2orf74 | turquoise | -0.400565383 | 2.00E-19 | -0.377802305 | 2.72E-17 |
| C3orf18 | turquoise | -0.155013157 | 0.000775979 | -0.180191843 | 9.02E-05 |
| C3orf70 | turquoise | -0.225288959 | 8.70E-07 | -0.2188753 | 1.79E-06 |
| C4orf32 | turquoise | -0.009992899 | 0.829474942 | -0.013253421 | 0.775143264 |
| C7 | turquoise | -0.208659023 | 5.44E-06 | -0.208726503 | 5.40E-06 |
| C7orf31 | turquoise | -0.430414438 | 1.75E-22 | -0.432727362 | 9.83E-23 |
| C8orf46 | turquoise | -0.301610249 | 2.81E-11 | -0.305663287 | 1.48E-11 |
| C8orf88 | turquoise | -0.383599131 | 8.07E-18 | -0.38803271 | 3.14E-18 |
| C9orf3 | turquoise | -0.274276049 | 1.67E-09 | -0.263279508 | 7.63E-09 |
| CA11 | turquoise | -0.174325778 | 0.000153002 | -0.195014147 | 2.20E-05 |
| CA3 | turquoise | -0.269451137 | 3.28E-09 | -0.294043569 | 9.10E-11 |
| CAB39L | turquoise | -0.092068568 | 0.046755131 | -0.100985443 | 0.029106125 |
| CABP1 | turquoise | -0.210928948 | 4.27E-06 | -0.217391781 | 2.11E-06 |
| CACNA1 | turquoise | -0.151892117 | 0.000992146 | -0.146005739 | 0.00155764 |
| CACNA1 | turquoise | -0.196005645 | 1.99E-05 | -0.204485147 | 8.42E-06 |
| CACNA1 | turquoise | -0.173529573 | 0.000164176 | -0.161361026 | 0.000464103 |
| CACNA2 | turquoise | -0.283624375 | 4.34E-10 | -0.280382366 | 6.96E-10 |
| CACNB2 | turquoise | -0.254839882 | 2.34E-08 | -0.2604593 | 1.11E-08 |
| CADM3 | turquoise | -0.192856569 | 2.72E-05 | -0.203710326 | 9.12E-06 |
| CALCOC | turquoise | -0.18104031 | 8.34E-05 | -0.200131851 | 1.32E-05 |
| CALD1 | turquoise | -0.132522837 | 0.004119919 | -0.122098499 | 0.008256685 |
| CALHM2 | turquoise | -0.266210536 | 5.12E-09 | -0.262180778 | 8.85E-09 |
| CALR | turquoise | 0.018608429 | 0.688354911 | 0.02860485 | 0.537481709 |
| CAMK2A | turquoise | -0.229643792 | 5.26E-07 | -0.237474833 | 2.07E-07 |
| CAMK2C | turquoise | -0.095150596 | 0.039842653 | -0.12468191 | 0.006981778 |
| CAMKM' | turquoise | 0.131961652 | 0.004282389 | 0.124599976 | 0.007019338 |
| CAND2 | turquoise | -0.275545639 | 1.39E-09 | -0.292489299 | 1.15E-10 |
| CAPN15 | turquoise | 0.149549926 | 0.001189497 | 0.158249127 | 0.000598531 |
| CASKIN2 | turquoise | -0.136096495 | 0.003209656 | -0.151711447 | 0.00100622 |
| CASP6 | turquoise | 0.299812319 | 3.73E-11 | 0.324947117 | 6.04E-13 |
| CASQ2 | turquoise | -0.160406126 | 0.000502025 | -0.164743586 | 0.000350155 |
| CAV1 | turquoise | -0.189962623 | 3.60E-05 | -0.190175552 | 3.53E-05 |
| CAV2 | turquoise | -0.238421538 | 1.85E-07 | -0.229047045 | 5.64E-07 |
| CBLN1 | turquoise | -0.135747561 | 0.003289708 | -0.137778099 | 0.00284812 |
| CBLN4 | turquoise | -0.230049657 | 5.02E-07 | -0.224268009 | 9.78E-07 |
| CBX6 | turquoise | -0.303181685 | 2.20E-11 | -0.314261132 | 3.66E-12 |
| CBX7 | turquoise | -0.225332659 | 8.66E-07 | -0.226477249 | 7.59E-07 |
| CBX8 | turquoise | 0.182358556 | 7.39E-05 | 0.166857599 | 0.00029281 |
| CCBE1 | turquoise | -0.240973268 | 1.35E-07 | -0.261361352 | 9.87E-09 |
| CCDC134 | turquoise | 0.164017444 | 0.000372157 | 0.171586674 | 0.000194736 |
| CCDC136 | turquoise | -0.276982964 | 1.14E-09 | -0.283330784 | 4.53E-10 |
| CCDC141 | turquoise | -0.11849957 | 0.010378081 | -0.103755046 | 0.024947729 |
| CCDC149 | turquoise | -0.010056672 | 0.828403505 | -0.017570488 | 0.704900925 |
| CCDC152 | turquoise | -0.262628626 | 8.33E-09 | -0.271741127 | 2.38E-09 |
| CCDC181 | turquoise | -0.38705645 | 3.87E-18 | -0.387972756 | 3.18E-18 |

| | | | | | |
|---|---|---|---|---|---|
| CCDC184 | turquoise | -0.259296798 | 1.30E-08 | -0.238511277 | 1.83E-07 |
| CCDC3 | turquoise | -0.291660721 | 1.31E-10 | -0.270093617 | 3.00E-09 |
| CCDC36 | turquoise | -0.27079252 | 2.72E-09 | -0.265794271 | 5.42E-09 |
| CCDC64 | turquoise | 0.105952318 | 0.022023529 | 0.109241447 | 0.018202537 |
| CCDC80 | turquoise | -0.230704946 | 4.65E-07 | -0.205788555 | 7.35E-06 |
| CCDC85A | turquoise | -0.016953874 | 0.71479738 | -0.057345667 | 0.216106652 |
| CCIN | turquoise | -0.296234864 | 6.50E-11 | -0.293799336 | 9.45E-11 |
| CCL14 | turquoise | -0.210364607 | 4.53E-06 | -0.217187833 | 2.16E-06 |
| CCL16 | turquoise | -0.165868434 | 0.000318454 | -0.164070621 | 0.000370503 |
| CCL21 | turquoise | -0.145560788 | 0.001610606 | -0.110287879 | 0.017114844 |
| CCL23 | turquoise | -0.200908143 | 1.22E-05 | -0.1874426 | 4.58E-05 |
| CCND2 | turquoise | -0.181960514 | 7.66E-05 | -0.144068168 | 0.001800582 |
| CD200 | turquoise | -0.106655582 | 0.021152656 | -0.08048114 | 0.08232183 |
| CD300LG | turquoise | -0.183793443 | 6.46E-05 | -0.193320455 | 2.60E-05 |
| CD302 | turquoise | -0.233913402 | 3.18E-07 | -0.227546048 | 6.71E-07 |
| CD99L2 | turquoise | -0.252074237 | 3.35E-08 | -0.26108942 | 1.02E-08 |
| CDC42EP | turquoise | -0.184658208 | 5.96E-05 | -0.209235101 | 5.11E-06 |
| CDC42EP | turquoise | -0.210181109 | 4.62E-06 | -0.170393718 | 0.000216059 |
| CDH13 | turquoise | -0.072215808 | 0.119126654 | -0.07235749 | 0.118402192 |
| CDH23 | turquoise | -0.245327206 | 7.89E-08 | -0.235998663 | 2.48E-07 |
| CDIP1 | turquoise | -0.26203592 | 9.02E-09 | -0.247048805 | 6.35E-08 |
| CDO1 | turquoise | -0.292001795 | 1.24E-10 | -0.295992378 | 6.75E-11 |
| CDON | turquoise | -0.282944481 | 4.79E-10 | -0.319139932 | 1.62E-12 |
| CELF2 | turquoise | -0.03149868 | 0.497114723 | -0.024246513 | 0.601221884 |
| CEP112 | turquoise | -0.22190812 | 1.28E-06 | -0.225653958 | 8.35E-07 |
| CES1 | turquoise | -0.102607146 | 0.026604523 | -0.123951945 | 0.007322836 |
| CFL2 | turquoise | -0.2237717 | 1.03E-06 | -0.235452614 | 2.65E-07 |
| CGNL1 | turquoise | 0.113983999 | 0.013714672 | 0.092383315 | 0.046005941 |
| CHKA | turquoise | 0.087825311 | 0.057893874 | 0.07357422 | 0.112321636 |
| CHPF2 | turquoise | 0.131606414 | 0.004388205 | 0.146799451 | 0.001467111 |
| CHPT1 | turquoise | -0.203416998 | 9.40E-06 | -0.211241881 | 4.13E-06 |
| CHRD | turquoise | -0.234358537 | 3.02E-07 | -0.219405799 | 1.69E-06 |
| CHRDL1 | turquoise | -0.261703664 | 9.43E-09 | -0.269215761 | 3.39E-09 |
| CHRDL2 | turquoise | -0.140232189 | 0.002386734 | -0.130422669 | 0.004758082 |
| CHSY3 | turquoise | -0.190361687 | 3.46E-05 | -0.177918731 | 0.000110891 |
| CIDEB | turquoise | -0.17886995 | 0.000101725 | -0.197352356 | 1.74E-05 |
| CILP | turquoise | -0.234453234 | 2.98E-07 | -0.224543752 | 9.48E-07 |
| CLDN11 | turquoise | -0.236728457 | 2.27E-07 | -0.239578924 | 1.61E-07 |
| CLEC4G | turquoise | -0.118431467 | 0.010422506 | -0.105800959 | 0.022214976 |
| CLEC4M | turquoise | -0.215158205 | 2.70E-06 | -0.223600877 | 1.06E-06 |
| CLIP2 | turquoise | -0.191997178 | 2.96E-05 | -0.193396751 | 2.58E-05 |
| CLIP3 | turquoise | -0.363081217 | 5.34E-16 | -0.344632225 | 1.81E-14 |
| CLMP | turquoise | -0.246616791 | 6.71E-08 | -0.231305125 | 4.33E-07 |
| CLSTN2 | turquoise | -0.290988756 | 1.45E-10 | -0.276879683 | 1.15E-09 |
| CLVS2 | turquoise | -0.042606448 | 0.358261498 | -0.05077725 | 0.273485338 |

| | | | | | |
|---|---|---|---|---|---|
| CMA1 | turquoise | -0.170030054 | 0.000222982 | -0.169865405 | 0.000226184 |
| CMTM3 | turquoise | -0.125231896 | 0.006734278 | -0.103992611 | 0.024616195 |
| CMTM5 | turquoise | -0.222570806 | 1.19E-06 | -0.242491195 | 1.12E-07 |
| CMYA5 | turquoise | -0.200882814 | 1.22E-05 | -0.21342149 | 3.26E-06 |
| CNN1 | turquoise | -0.156220642 | 0.000704733 | -0.126156338 | 0.006335934 |
| CNNM1 | turquoise | -0.301826727 | 2.72E-11 | -0.295166278 | 7.66E-11 |
| CNPY4 | turquoise | -0.026024961 | 0.574803018 | -0.050510377 | 0.276017814 |
| CNRIP1 | turquoise | -0.250701644 | 3.99E-08 | -0.232319288 | 3.84E-07 |
| CNTN1 | turquoise | 0.023888566 | 0.606604547 | 0.010764824 | 0.816527845 |
| CNTN4 | turquoise | -0.199564366 | 1.39E-05 | -0.18672671 | 4.90E-05 |
| CNTNAP | turquoise | -0.077644232 | 0.093749919 | -0.073981795 | 0.110340592 |
| COL12A1 | turquoise | 0.044880456 | 0.333162575 | 0.053324608 | 0.250109007 |
| COL14A1 | turquoise | -0.117842905 | 0.010813522 | -0.11397796 | 0.013719703 |
| COL16A1 | turquoise | -0.323572306 | 7.64E-13 | -0.310425813 | 6.86E-12 |
| COL4A6 | turquoise | -0.205612688 | 7.49E-06 | -0.222062658 | 1.26E-06 |
| COLEC12 | turquoise | -0.250987879 | 3.85E-08 | -0.261512768 | 9.68E-09 |
| COPG1 | turquoise | 0.165215505 | 0.000336513 | 0.187302141 | 4.64E-05 |
| COPZ1 | turquoise | 0.239148884 | 1.69E-07 | 0.244535956 | 8.71E-08 |
| COPZ2 | turquoise | -0.326744198 | 4.43E-13 | -0.286971145 | 2.65E-10 |
| COQ9 | turquoise | 0.315059587 | 3.20E-12 | 0.347938389 | 9.78E-15 |
| CORO2B | turquoise | -0.319615453 | 1.50E-12 | -0.30688362 | 1.22E-11 |
| COX4I2 | turquoise | -0.289181375 | 1.90E-10 | -0.275088548 | 1.49E-09 |
| COX5A | turquoise | 0.224236786 | 9.82E-07 | 0.237289402 | 2.12E-07 |
| COX7A1 | turquoise | -0.200129202 | 1.32E-05 | -0.170773803 | 0.000209039 |
| CPA3 | turquoise | -0.151120143 | 0.001053579 | -0.109506411 | 0.01792157 |
| CPE | turquoise | -0.285309382 | 3.38E-10 | -0.276987623 | 1.13E-09 |
| CPEB1 | turquoise | -0.346869185 | 1.19E-14 | -0.353227192 | 3.61E-15 |
| CPED1 | turquoise | -0.1963842 | 1.92E-05 | -0.192322084 | 2.86E-05 |
| CPQ | turquoise | -0.161580157 | 0.000455785 | -0.181693885 | 7.85E-05 |
| CPXM1 | turquoise | -0.335794887 | 9.01E-14 | -0.294823117 | 8.08E-11 |
| CPZ | turquoise | -0.193173597 | 2.63E-05 | -0.179668576 | 9.46E-05 |
| CREBRF | turquoise | -0.277829043 | 1.01E-09 | -0.286372309 | 2.89E-10 |
| CRHBP | turquoise | -0.269222829 | 3.38E-09 | -0.279386653 | 8.04E-10 |
| CRIM1 | turquoise | -0.300590245 | 3.30E-11 | -0.300816324 | 3.19E-11 |
| CRISPLD | turquoise | -0.131093566 | 0.004545139 | -0.112901516 | 0.014642871 |
| CRMP1 | turquoise | -0.281588189 | 5.84E-10 | -0.283937897 | 4.14E-10 |
| CRTAP | turquoise | -0.076931539 | 0.09681174 | -0.095726141 | 0.0386526 |
| CSDC2 | turquoise | -0.255418772 | 2.17E-08 | -0.244225993 | 9.05E-08 |
| CSGALN | turquoise | -0.02398081 | 0.605215357 | -0.045182423 | 0.329916872 |
| CSRNP1 | turquoise | -0.040072494 | 0.387587765 | -0.036100159 | 0.436395697 |
| CSRP1 | turquoise | -0.05147964 | 0.266895929 | -0.049045276 | 0.290204302 |
| CSTB | turquoise | 0.2082281 | 5.69E-06 | 0.239555623 | 1.61E-07 |
| CTB-133( | turquoise | -0.124184631 | 0.007212536 | -0.128220394 | 0.005521822 |
| CTD-220 | turquoise | -0.140945148 | 0.002266103 | -0.128958031 | 0.005254536 |
| CTF1 | turquoise | 0.034168059 | 0.461356949 | 0.05693208 | 0.219442592 |

| | | | | | |
|---|---|---|---|---|---|
| CTGF | turquoise | -0.048868332 | 0.291950165 | -0.036095088 | 0.436460171 |
| CTIF | turquoise | -0.024515868 | 0.597185657 | -0.033404569 | 0.471435577 |
| CTNNA3 | turquoise | -0.235775184 | 2.55E-07 | -0.247861499 | 5.73E-08 |
| CTSF | turquoise | -0.335230027 | 9.96E-14 | -0.36279558 | 5.65E-16 |
| CTSG | turquoise | -0.220333362 | 1.53E-06 | -0.206380642 | 6.91E-06 |
| CTSK | turquoise | -0.133316996 | 0.003899523 | -0.097774698 | 0.034658154 |
| CTSO | turquoise | -0.128236683 | 0.00551579 | -0.116241837 | 0.011943739 |
| CUX1 | turquoise | -0.181298851 | 8.14E-05 | -0.212620315 | 3.56E-06 |
| CXCL12 | turquoise | -0.283414771 | 4.47E-10 | -0.268290287 | 3.85E-09 |
| CXorf57 | turquoise | -0.194122083 | 2.40E-05 | -0.19692358 | 1.82E-05 |
| CYBRD1 | turquoise | -0.31912363 | 1.63E-12 | -0.322732284 | 8.82E-13 |
| CYP1B1 | turquoise | -0.272875981 | 2.03E-09 | -0.248326674 | 5.41E-08 |
| CYP2U1 | turquoise | -0.151368717 | 0.001033425 | -0.139441738 | 0.002527304 |
| CYR61 | turquoise | -0.089546966 | 0.053134438 | -0.096994013 | 0.036136894 |
| CYS1 | turquoise | -0.226367157 | 7.69E-07 | -0.239334321 | 1.65E-07 |
| CYTL1 | turquoise | -0.305437027 | 1.53E-11 | -0.3154569 | 3.00E-12 |
| DAAM2 | turquoise | -0.08211296 | 0.076275922 | -0.125430467 | 0.006646869 |
| DAB2 | turquoise | -0.189374693 | 3.81E-05 | -0.17740273 | 0.000116183 |
| DACT1 | turquoise | -0.111463262 | 0.015961283 | -0.101297005 | 0.028610431 |
| DACT3 | turquoise | -0.24233084 | 1.15E-07 | -0.231460909 | 4.25E-07 |
| DAGLB | turquoise | 0.045229779 | 0.329409722 | 0.082960966 | 0.073279566 |
| DBI | turquoise | 0.224617277 | 9.40E-07 | 0.229208539 | 5.54E-07 |
| DBNDD1 | turquoise | 0.319450928 | 1.54E-12 | 0.343305018 | 2.31E-14 |
| DBNDD2 | turquoise | -0.194114718 | 2.40E-05 | -0.20752016 | 6.13E-06 |
| DCAF12I | turquoise | -0.292651494 | 1.13E-10 | -0.293186844 | 1.04E-10 |
| DCHS1 | turquoise | -0.186318758 | 5.10E-05 | -0.19023613 | 3.51E-05 |
| DCLK1 | turquoise | -0.139786047 | 0.002465171 | -0.155510287 | 0.000745875 |
| DCLK2 | turquoise | -0.23482969 | 2.85E-07 | -0.23985149 | 1.55E-07 |
| DCN | turquoise | -0.214806519 | 2.81E-06 | -0.193030854 | 2.67E-05 |
| DCP1B | turquoise | -0.139233889 | 0.0025655 | -0.15848154 | 0.000587361 |
| DDR2 | turquoise | -0.262010928 | 9.05E-09 | -0.245912007 | 7.33E-08 |
| DDX54 | turquoise | 0.115974833 | 0.012142052 | 0.125121019 | 0.006783532 |
| DEFB124 | turquoise | -0.286563638 | 2.81E-10 | -0.281952681 | 5.54E-10 |
| DENND2. | turquoise | -0.261662015 | 9.48E-09 | -0.252836485 | 3.03E-08 |
| DENND2. | turquoise | 0.009668879 | 0.834923572 | 0.046912643 | 0.31171455 |
| DES | turquoise | -0.190646844 | 3.37E-05 | -0.163222079 | 0.000397731 |
| DET1 | turquoise | -0.093912335 | 0.042508344 | -0.108438156 | 0.019077969 |
| DFNA5 | turquoise | -0.342510033 | 2.67E-14 | -0.345292838 | 1.60E-14 |
| DGKB | turquoise | -0.18474095 | 5.91E-05 | -0.18984871 | 3.64E-05 |
| DIO3 | turquoise | -0.164784774 | 0.000348944 | -0.168642858 | 0.000251341 |
| DIP2C | turquoise | -0.206501892 | 6.82E-06 | -0.244923152 | 8.30E-08 |
| DIRAS1 | turquoise | -0.179401821 | 9.69E-05 | -0.190289712 | 3.49E-05 |
| DIXDC1 | turquoise | -0.180909351 | 8.44E-05 | -0.184771039 | 5.90E-05 |
| DLC1 | turquoise | -0.016633243 | 0.719962489 | -0.030438281 | 0.511717295 |
| DLG4 | turquoise | -0.103541396 | 0.025249172 | -0.131403903 | 0.00444958 |

| | | | | | |
|---|---|---|---|---|---|
| DLL1 | turquoise | -0.056657615 | 0.221676633 | -0.080377175 | 0.082719787 |
| DMD | turquoise | -0.183698935 | 6.52E-05 | -0.188018754 | 4.34E-05 |
| DMPK | turquoise | -0.114820247 | 0.013033478 | -0.130914866 | 0.004601001 |
| DNAJB5 | turquoise | -0.248766233 | 5.11E-08 | -0.252589489 | 3.13E-08 |
| DNAJC18 | turquoise | -0.308673472 | 9.11E-12 | -0.340746905 | 3.69E-14 |
| DNASE1l | turquoise | -0.082208593 | 0.075933105 | -0.08187667 | 0.077128349 |
| DOK6 | turquoise | -0.139556491 | 0.00250644 | -0.130343171 | 0.004783902 |
| DPP3 | turquoise | 0.196905136 | 1.82E-05 | 0.199935264 | 1.34E-05 |
| DPP6 | turquoise | -0.193343594 | 2.59E-05 | -0.196632032 | 1.87E-05 |
| DPT | turquoise | -0.210380594 | 4.53E-06 | -0.205585073 | 7.51E-06 |
| DPYD | turquoise | -0.158542034 | 0.000584486 | -0.142488683 | 0.002023792 |
| DPYSL2 | turquoise | -0.124127887 | 0.007239296 | -0.131049353 | 0.004558903 |
| DPYSL3 | turquoise | -0.10012027 | 0.030521419 | -0.105927999 | 0.022054192 |
| DPYSL4 | turquoise | -0.157624154 | 0.000629554 | -0.158089927 | 0.000606296 |
| DRP2 | turquoise | -0.25293219 | 3.00E-08 | -0.249730084 | 4.52E-08 |
| DSCAML | turquoise | -0.339539635 | 4.59E-14 | -0.319720154 | 1.47E-12 |
| DSEL | turquoise | -0.291759589 | 1.29E-10 | -0.309774575 | 7.62E-12 |
| DSP | turquoise | 0.132601858 | 0.004097495 | 0.1186223 | 0.010298445 |
| DSTN | turquoise | -0.079064925 | 0.087877622 | -0.081257217 | 0.079399833 |
| DTNA | turquoise | -0.378813696 | 2.20E-17 | -0.391512043 | 1.48E-18 |
| DTNB | turquoise | 0.274793406 | 1.55E-09 | 0.293655347 | 9.66E-11 |
| DUSP1 | turquoise | -0.116861569 | 0.011494436 | -0.120273699 | 0.009278424 |
| DUSP3 | turquoise | -0.067620198 | 0.14455436 | -0.045475026 | 0.326791356 |
| DZIP1 | turquoise | -0.353621576 | 3.35E-15 | -0.382052968 | 1.12E-17 |
| EBF1 | turquoise | -0.170416681 | 0.000215629 | -0.168808401 | 0.000247788 |
| EBF2 | turquoise | -0.013043683 | 0.778608458 | -0.025533422 | 0.582049912 |
| EBF3 | turquoise | -0.190067577 | 3.56E-05 | -0.206037013 | 7.16E-06 |
| EBF4 | turquoise | -0.42406539 | 8.30E-22 | -0.417563885 | 3.95E-21 |
| ECM2 | turquoise | -0.206806864 | 6.61E-06 | -0.189855787 | 3.64E-05 |
| EDA2R | turquoise | 0.014912615 | 0.747893229 | 0.00285929 | 0.950862016 |
| EDEM2 | turquoise | 0.084622052 | 0.067688419 | 0.074202896 | 0.109277485 |
| EDIL3 | turquoise | -0.122369752 | 0.008113697 | -0.108113587 | 0.019441962 |
| EDNRA | turquoise | -0.217894536 | 2.00E-06 | -0.201378794 | 1.16E-05 |
| EDNRB | turquoise | -0.144062308 | 0.001801366 | -0.156496454 | 0.000689332 |
| EFCC1 | turquoise | -0.161820933 | 0.000446805 | -0.157691008 | 0.000626165 |
| EFEMP1 | turquoise | -0.329846823 | 2.58E-13 | -0.32099011 | 1.19E-12 |
| EFEMP2 | turquoise | -0.314448458 | 3.54E-12 | -0.282489293 | 5.12E-10 |
| EFHD1 | turquoise | -0.207851179 | 5.92E-06 | -0.206218219 | 7.03E-06 |
| EFNA3 | turquoise | 0.106118428 | 0.021815073 | 0.095531068 | 0.039052533 |
| EFNA4 | turquoise | 0.228314193 | 6.14E-07 | 0.209990704 | 4.72E-06 |
| EFS | turquoise | -0.318220996 | 1.89E-12 | -0.33056508 | 2.28E-13 |
| EGR1 | turquoise | -0.028637796 | 0.537013068 | -0.055363446 | 0.232429523 |
| EGR2 | turquoise | -0.156916067 | 0.0006665 | -0.161449433 | 0.00046073 |
| EHBP1L1 | turquoise | -0.128222924 | 0.005520885 | -0.118421493 | 0.010429027 |
| EHD2 | turquoise | -0.193716452 | 2.50E-05 | -0.164468136 | 0.000358354 |

| | | | | | |
|---|---|---|---|---|---|
| EID3 | turquoise | -0.358729056 | 1.25E-15 | -0.355303931 | 2.43E-15 |
| ELK3 | turquoise | -0.174586531 | 0.000149501 | -0.171336824 | 0.000199031 |
| ELN | turquoise | -0.108957952 | 0.018507404 | -0.08713709 | 0.059892766 |
| EMILIN1 | turquoise | -0.165068241 | 0.000340716 | -0.137495588 | 0.002906143 |
| EMILIN3 | turquoise | -0.168394399 | 0.000256764 | -0.15431266 | 0.000820308 |
| EML1 | turquoise | -0.373547801 | 6.53E-17 | -0.374609691 | 5.26E-17 |
| EMP3 | turquoise | -0.18303206 | 6.94E-05 | -0.137282067 | 0.002950707 |
| ENO1 | turquoise | 0.13461988 | 0.00356097 | 0.140743688 | 0.002299612 |
| ENOX1 | turquoise | -0.220273863 | 1.54E-06 | -0.223523863 | 1.06E-06 |
| ENPEP | turquoise | -0.224402549 | 9.63E-07 | -0.227759359 | 6.55E-07 |
| ENPP1 | turquoise | -0.077707326 | 0.09348263 | -0.105162389 | 0.023038637 |
| ENTPD6 | turquoise | 0.225511308 | 8.49E-07 | 0.218145188 | 1.95E-06 |
| EPB41L3 | turquoise | -0.285669327 | 3.21E-10 | -0.273889308 | 1.76E-09 |
| EPDR1 | turquoise | -0.104342142 | 0.024135327 | -0.121963508 | 0.008328679 |
| EPHA3 | turquoise | 0.029209732 | 0.528910285 | 0.014622924 | 0.752629512 |
| EPHA5 | turquoise | -0.157488611 | 0.000636476 | -0.157952582 | 0.00061307 |
| EPHB6 | turquoise | -0.3014741 | 2.87E-11 | -0.312667773 | 4.75E-12 |
| EPM2A | turquoise | -0.334008601 | 1.24E-13 | -0.352041072 | 4.52E-15 |
| EPO | turquoise | -0.007239322 | 0.876012636 | -0.035099334 | 0.449227554 |
| ESPN | turquoise | 0.086906734 | 0.060574434 | 0.111100914 | 0.016309414 |
| ESRRA | turquoise | 0.136218289 | 0.003182134 | 0.13791074 | 0.002821243 |
| EVC2 | turquoise | -0.361315615 | 7.56E-16 | -0.37011229 | 1.31E-16 |
| EXTL1 | turquoise | -0.260652655 | 1.09E-08 | -0.26949292 | 3.26E-09 |
| EZH1 | turquoise | -0.126493041 | 0.006196185 | -0.151408912 | 0.0010302 |
| EZR | turquoise | 0.129976133 | 0.004904759 | 0.159935252 | 0.000521768 |
| F10 | turquoise | -0.317710118 | 2.06E-12 | -0.318267842 | 1.88E-12 |
| F12 | turquoise | 0.173093181 | 0.00017062 | 0.173456943 | 0.000165232 |
| F13A1 | turquoise | -0.163412501 | 0.000391464 | -0.163212455 | 0.000398051 |
| F8 | turquoise | -0.208591378 | 5.47E-06 | -0.230489456 | 4.77E-07 |
| FAAH2 | turquoise | -0.028903505 | 0.533240972 | -0.035000417 | 0.450507268 |
| FAM101E | turquoise | -0.005797402 | 0.900564555 | -0.01555168 | 0.737478558 |
| FAM110A | turquoise | 0.300787618 | 3.20E-11 | 0.311683296 | 5.59E-12 |
| FAM110E | turquoise | -0.504280767 | 1.71E-31 | -0.509431725 | 3.31E-32 |
| FAM114A | turquoise | 0.008088577 | 0.8616061 | 0.031006431 | 0.503865756 |
| FAM117A | turquoise | -0.231985657 | 4.00E-07 | -0.257416909 | 1.67E-08 |
| FAM129A | turquoise | 0.050756837 | 0.273678484 | 0.062834784 | 0.175235409 |
| FAM131E | turquoise | -0.244594762 | 8.65E-08 | -0.234982444 | 2.80E-07 |
| FAM13C | turquoise | -0.049609008 | 0.284688868 | -0.038928109 | 0.401296929 |
| FAM162E | turquoise | -0.32538116 | 5.60E-13 | -0.304609567 | 1.75E-11 |
| FAM180A | turquoise | -0.141701783 | 0.002144194 | -0.124173836 | 0.00721762 |
| FAM180E | turquoise | -0.190274277 | 3.49E-05 | -0.197329256 | 1.75E-05 |
| FAM198A | turquoise | -0.153009973 | 0.000909031 | -0.150930376 | 0.001069208 |
| FAM198E | turquoise | 0.021521785 | 0.642721533 | 0.014443455 | 0.75556842 |
| FAM19A: | turquoise | -0.277414774 | 1.07E-09 | -0.278716614 | 8.85E-10 |
| FAM212E | turquoise | 0.003566813 | 0.938724451 | -0.002256165 | 0.961217713 |

| | | | | | |
|---|---|---|---|---|---|
| FAM219A | turquoise | -0.249908397 | 4.42E-08 | -0.244474443 | 8.78E-08 |
| FAM228E | turquoise | -0.162903633 | 0.000408422 | -0.168119959 | 0.00026288 |
| FAM26E | turquoise | -0.145972471 | 0.001561544 | -0.120110235 | 0.009375221 |
| FAM43A | turquoise | -0.215386762 | 2.64E-06 | -0.205792717 | 7.35E-06 |
| FAM49A | turquoise | -0.258621902 | 1.42E-08 | -0.241222378 | 1.31E-07 |
| FAM65A | turquoise | -0.040206133 | 0.386005606 | -0.021850438 | 0.637653064 |
| FAM65B | turquoise | -0.220829527 | 1.44E-06 | -0.204917723 | 8.05E-06 |
| FAM65C | turquoise | -0.298223092 | 4.78E-11 | -0.293568211 | 9.79E-11 |
| FAM69A | turquoise | -0.33491932 | 1.05E-13 | -0.326535092 | 4.59E-13 |
| FAM71E1 | turquoise | -0.116519573 | 0.011740497 | -0.10263703 | 0.026560219 |
| FAM78B | turquoise | 0.06596096 | 0.154690201 | 0.044421891 | 0.338130631 |
| FAM83G | turquoise | 0.191076395 | 3.23E-05 | 0.18221551 | 7.48E-05 |
| FAM89A | turquoise | -0.353430672 | 3.47E-15 | -0.346209724 | 1.35E-14 |
| FAT4 | turquoise | -0.100801805 | 0.029401731 | -0.100967912 | 0.029134236 |
| FAXDC2 | turquoise | -0.33837737 | 5.66E-14 | -0.337367033 | 6.79E-14 |
| FBLN5 | turquoise | -0.289632269 | 1.78E-10 | -0.261441509 | 9.77E-09 |
| FBXL22 | turquoise | -0.165483929 | 0.000328976 | -0.162705302 | 0.000415215 |
| FBXL7 | turquoise | -0.189579476 | 3.73E-05 | -0.18682589 | 4.86E-05 |
| FBXO17 | turquoise | -0.449613069 | 1.28E-24 | -0.453000986 | 5.21E-25 |
| FBXO32 | turquoise | -0.181119865 | 8.28E-05 | -0.178035133 | 0.000109729 |
| FERMT2 | turquoise | -0.159818001 | 0.000526795 | -0.170426134 | 0.000215452 |
| FES | turquoise | -0.293828279 | 9.41E-11 | -0.266917586 | 4.65E-09 |
| FEZ1 | turquoise | -0.224764453 | 9.24E-07 | -0.194779917 | 2.25E-05 |
| FGF1 | turquoise | -0.169051314 | 0.000242659 | -0.149108713 | 0.001230497 |
| FGF10 | turquoise | -0.212857702 | 3.47E-06 | -0.224379854 | 9.66E-07 |
| FGF13 | turquoise | -0.092057703 | 0.046781172 | -0.142525215 | 0.002018356 |
| FGF2 | turquoise | -0.22985678 | 5.13E-07 | -0.217491592 | 2.09E-06 |
| FGF7 | turquoise | -0.182003705 | 7.63E-05 | -0.183796978 | 6.46E-05 |
| FHL1 | turquoise | -0.300584327 | 3.31E-11 | -0.289905538 | 1.71E-10 |
| FHL3 | turquoise | -0.241406785 | 1.28E-07 | -0.233334124 | 3.41E-07 |
| FHL5 | turquoise | -0.124034168 | 0.007283689 | -0.13204123 | 0.004259003 |
| FIBIN | turquoise | -0.034066537 | 0.462690164 | -0.024765227 | 0.593460053 |
| FIGF | turquoise | -0.076421413 | 0.099051861 | -0.092240574 | 0.046344448 |
| FILIP1 | turquoise | -0.190047443 | 3.57E-05 | -0.183788527 | 6.47E-05 |
| FILIP1L | turquoise | -0.184179504 | 6.23E-05 | -0.169661898 | 0.000230201 |
| FKBP7 | turquoise | -0.203843561 | 9.00E-06 | -0.210274135 | 4.58E-06 |
| FLAD1 | turquoise | 0.221746613 | 1.30E-06 | 0.232915965 | 3.58E-07 |
| FLNA | turquoise | -0.192491238 | 2.82E-05 | -0.17570354 | 0.000135336 |
| FLNC | turquoise | -0.265661176 | 5.52E-09 | -0.258472809 | 1.45E-08 |
| FLRT2 | turquoise | -0.187610938 | 4.51E-05 | -0.189588069 | 3.73E-05 |
| FMNL3 | turquoise | -0.295252969 | 7.56E-11 | -0.295996492 | 6.75E-11 |
| FMO1 | turquoise | -0.06114541 | 0.187150139 | -0.058217132 | 0.209197004 |
| FMOD | turquoise | -0.049954706 | 0.281341824 | -0.034534112 | 0.456567667 |
| FNBP1 | turquoise | -0.358596347 | 1.28E-15 | -0.339600193 | 4.54E-14 |
| FNDC5 | turquoise | -0.300918662 | 3.14E-11 | -0.30185806 | 2.71E-11 |

| | | | | | |
|---|---|---|---|---|---|
| FOS | turquoise | -0.002787741 | 0.952090117 | -0.029111995 | 0.530290543 |
| FOSB | turquoise | -0.084209616 | 0.069042931 | -0.11701554 | 0.011385154 |
| FOSL2 | turquoise | -0.244891234 | 8.33E-08 | -0.252802801 | 3.05E-08 |
| FOXI2 | turquoise | -0.091777013 | 0.047458218 | -0.088465699 | 0.056083857 |
| FOXJ2 | turquoise | -0.071029711 | 0.125328122 | -0.080557019 | 0.082032362 |
| FOXL2 | turquoise | -0.218802681 | 1.81E-06 | -0.206171144 | 7.06E-06 |
| FOXL2NI | turquoise | -0.205824959 | 7.32E-06 | -0.200036625 | 1.33E-05 |
| FOXN3 | turquoise | -0.104242662 | 0.024271354 | -0.110541142 | 0.016860298 |
| FOXO1 | turquoise | -0.069201309 | 0.135375497 | -0.078702501 | 0.089346816 |
| FOXO4 | turquoise | -0.266850718 | 4.69E-09 | -0.302957297 | 2.27E-11 |
| FOXP1 | turquoise | 0.007990251 | 0.86327182 | 0.037600728 | 0.417556538 |
| FOXP2 | turquoise | -0.141293202 | 0.002209259 | -0.145255246 | 0.001647928 |
| FRAT2 | turquoise | 0.098836556 | 0.032729754 | 0.103466478 | 0.025355618 |
| FREM1 | turquoise | -0.220224587 | 1.54E-06 | -0.228168334 | 6.25E-07 |
| FRMD3 | turquoise | -0.222563356 | 1.19E-06 | -0.214921088 | 2.77E-06 |
| FRMD6 | turquoise | -0.073804887 | 0.111197057 | -0.089467299 | 0.053347248 |
| FRMD7 | turquoise | -0.149856685 | 0.001161738 | -0.154143293 | 0.000831372 |
| FRY | turquoise | -0.213513724 | 3.23E-06 | -0.205608957 | 7.49E-06 |
| FSTL1 | turquoise | -0.131212671 | 0.004508247 | -0.133129997 | 0.003950436 |
| FSTL3 | turquoise | -0.214373051 | 2.94E-06 | -0.187002995 | 4.78E-05 |
| FXYD1 | turquoise | -0.332121539 | 1.73E-13 | -0.338050303 | 6.01E-14 |
| FXYD6 | turquoise | -0.412541943 | 1.29E-20 | -0.41637513 | 5.24E-21 |
| FYCO1 | turquoise | -0.03289092 | 0.478283648 | -0.046711815 | 0.313792793 |
| FYN | turquoise | -0.244309952 | 8.96E-08 | -0.262476619 | 8.50E-09 |
| FZD4 | turquoise | -0.204536523 | 8.37E-06 | -0.199231513 | 1.44E-05 |
| FZD7 | turquoise | -0.23493326 | 2.82E-07 | -0.254086138 | 2.58E-08 |
| GAB2 | turquoise | -0.214860641 | 2.79E-06 | -0.24100634 | 1.35E-07 |
| GABARA | turquoise | -0.192194589 | 2.90E-05 | -0.182859357 | 7.05E-05 |
| GABBR1 | turquoise | -0.316501246 | 2.52E-12 | -0.32617193 | 4.89E-13 |
| GABRB3 | turquoise | -0.162185869 | 0.000433508 | -0.18337282 | 6.72E-05 |
| GALE | turquoise | 0.198215586 | 1.60E-05 | 0.223746853 | 1.04E-06 |
| GALNT1: | turquoise | -0.125841005 | 0.006469371 | -0.118496766 | 0.010379907 |
| GALNT1 | turquoise | -0.204338672 | 8.55E-06 | -0.210047354 | 4.69E-06 |
| GALNT6 | turquoise | 0.115855669 | 0.012231495 | 0.135259166 | 0.0034048 |
| GAPDH | turquoise | 0.280127553 | 7.22E-10 | 0.290242886 | 1.62E-10 |
| GARNL3 | turquoise | -0.258660508 | 1.42E-08 | -0.289208689 | 1.89E-10 |
| GAS1 | turquoise | -0.266416016 | 4.98E-09 | -0.248740324 | 5.13E-08 |
| GAS6 | turquoise | -0.342591918 | 2.63E-14 | -0.338063776 | 5.99E-14 |
| GAS7 | turquoise | -0.238905927 | 1.74E-07 | -0.240343383 | 1.46E-07 |
| GATA6 | turquoise | -0.203218283 | 9.60E-06 | -0.200664186 | 1.25E-05 |
| GCOM1 | turquoise | -0.087144419 | 0.059871183 | -0.109067923 | 0.018388618 |
| GCSAML | turquoise | -0.209433175 | 5.01E-06 | -0.194121195 | 2.40E-05 |
| GDAP1L1 | turquoise | -0.213425381 | 3.26E-06 | -0.215808329 | 2.52E-06 |
| GDF10 | turquoise | -0.161850241 | 0.000445723 | -0.16054628 | 0.000496285 |
| GDF15 | turquoise | 0.335900161 | 8.84E-14 | 0.355511028 | 2.33E-15 |

| | | | | | |
|---|---|---|---|---|---|
| GDF7 | turquoise | -0.17298079 | 0.000172318 | -0.168152951 | 0.000262138 |
| GEM | turquoise | -0.079204033 | 0.087318891 | -0.070472938 | 0.128324365 |
| GFPT2 | turquoise | -0.191892625 | 2.99E-05 | -0.1941538 | 2.39E-05 |
| GFRA1 | turquoise | -0.288709925 | 2.04E-10 | -0.292928812 | 1.08E-10 |
| GGT5 | turquoise | -0.099576998 | 0.031439919 | -0.042401603 | 0.360579217 |
| GHR | turquoise | -0.269739838 | 3.15E-09 | -0.270073523 | 3.01E-09 |
| GIPC1 | turquoise | 0.038150435 | 0.410776523 | 0.063004806 | 0.174068266 |
| GJC1 | turquoise | -0.177823867 | 0.000111847 | -0.177428972 | 0.000115908 |
| GJC2 | turquoise | -0.207555132 | 6.11E-06 | -0.194443889 | 2.32E-05 |
| GLI1 | turquoise | -0.041978028 | 0.365401422 | -0.0345413 | 0.456473897 |
| GLI2 | turquoise | -0.294615474 | 8.34E-11 | -0.275748157 | 1.35E-09 |
| GLI3 | turquoise | -0.359496264 | 1.08E-15 | -0.36559218 | 3.25E-16 |
| GLIPR1 | turquoise | -0.235301271 | 2.69E-07 | -0.209152267 | 5.16E-06 |
| GLIPR1L | turquoise | -0.138197675 | 0.00276389 | -0.144634219 | 0.001726243 |
| GLIPR2 | turquoise | -0.278813742 | 8.73E-10 | -0.245654377 | 7.57E-08 |
| GLIS1 | turquoise | -0.295355674 | 7.44E-11 | -0.280379967 | 6.96E-10 |
| GLP2R | turquoise | -0.099597984 | 0.031404004 | -0.113756748 | 0.01390512 |
| GLRA4 | turquoise | -0.150845607 | 0.001076259 | -0.157177668 | 0.000652622 |
| GLT1D1 | turquoise | -0.256588817 | 1.86E-08 | -0.25767091 | 1.61E-08 |
| GLT8D2 | turquoise | -0.229674024 | 5.24E-07 | -0.225544361 | 8.45E-07 |
| GMDS | turquoise | 0.248351892 | 5.39E-08 | 0.290753676 | 1.50E-10 |
| GMIP | turquoise | 0.283057465 | 4.71E-10 | 0.310383352 | 6.91E-12 |
| GNA14 | turquoise | 0.016798797 | 0.71729394 | 0.036111482 | 0.436251734 |
| GNAL | turquoise | -0.270958264 | 2.66E-09 | -0.278600099 | 9.00E-10 |
| GNAO1 | turquoise | -0.181889071 | 7.71E-05 | -0.175034357 | 0.000143663 |
| GNAZ | turquoise | -0.28391331 | 4.16E-10 | -0.307196204 | 1.16E-11 |
| GNG11 | turquoise | -0.063527739 | 0.170514793 | -0.058368706 | 0.208011682 |
| GNG2 | turquoise | -0.131386702 | 0.004454829 | -0.104234065 | 0.02428314 |
| GNG7 | turquoise | -0.205737666 | 7.39E-06 | -0.214500979 | 2.90E-06 |
| GOLT1A | turquoise | 0.23470158 | 2.89E-07 | 0.238547315 | 1.82E-07 |
| GOT1 | turquoise | 0.288288823 | 2.17E-10 | 0.286881853 | 2.68E-10 |
| GPI | turquoise | 0.133957407 | 0.003729631 | 0.151406102 | 0.001030425 |
| GPIHBP1 | turquoise | -0.243056727 | 1.05E-07 | -0.253876569 | 2.65E-08 |
| GPM6A | turquoise | -0.270737211 | 2.74E-09 | -0.279583502 | 7.81E-10 |
| GPR1 | turquoise | -0.179075774 | 9.98E-05 | -0.186154605 | 5.18E-05 |
| GPR124 | turquoise | -0.165976525 | 0.000315553 | -0.146097112 | 0.001546963 |
| GPR133 | turquoise | -0.29401345 | 9.15E-11 | -0.295234204 | 7.59E-11 |
| GPR137B | turquoise | -0.21914687 | 1.74E-06 | -0.189681448 | 3.70E-05 |
| GPR146 | turquoise | -0.092621008 | 0.045446866 | -0.087829624 | 0.057881525 |
| GPR153 | turquoise | -0.290132271 | 1.65E-10 | -0.298885182 | 4.31E-11 |
| GPR161 | turquoise | -0.207581342 | 6.09E-06 | -0.226704397 | 7.40E-07 |
| GPR17 | turquoise | -0.264026141 | 6.89E-09 | -0.257632972 | 1.62E-08 |
| GPR173 | turquoise | -0.458573286 | 1.16E-25 | -0.471071609 | 3.60E-27 |
| GPR176 | turquoise | -0.204283487 | 8.60E-06 | -0.185860459 | 5.32E-05 |
| GPR182 | turquoise | -0.189204282 | 3.87E-05 | -0.198296621 | 1.58E-05 |

| | | | | | |
|---|---|---|---|---|---|
| GPR20 | turquoise | -0.302152139 | 2.58E-11 | -0.295949332 | 6.79E-11 |
| GPR78 | turquoise | -0.245013871 | 8.21E-08 | -0.246064152 | 7.19E-08 |
| GPRASP1 | turquoise | -0.259952348 | 1.19E-08 | -0.263079973 | 7.84E-09 |
| GPRASP2 | turquoise | -0.245993908 | 7.26E-08 | -0.263290353 | 7.62E-09 |
| GPT2 | turquoise | 0.332668076 | 1.57E-13 | 0.355790898 | 2.21E-15 |
| GRASP | turquoise | -0.40014015 | 2.20E-19 | -0.409882898 | 2.39E-20 |
| GREM1 | turquoise | -0.1326455 | 0.004085158 | -0.125203487 | 0.006746867 |
| GRIA3 | turquoise | -0.251911058 | 3.42E-08 | -0.234364709 | 3.01E-07 |
| GRID1 | turquoise | -0.232584422 | 3.72E-07 | -0.213938034 | 3.09E-06 |
| GRK5 | turquoise | -0.15620208 | 0.00070578 | -0.13987864 | 0.002448701 |
| GRM7 | turquoise | -0.191668581 | 3.05E-05 | -0.191514531 | 3.10E-05 |
| GS1-259F | turquoise | -0.078655371 | 0.089539311 | -0.087265503 | 0.059515531 |
| GSN | turquoise | -0.038043866 | 0.412085814 | -0.013327677 | 0.773917518 |
| GSPT2 | turquoise | -0.452804815 | 5.49E-25 | -0.44551901 | 3.76E-24 |
| GSS | turquoise | 0.290143216 | 1.65E-10 | 0.295502503 | 7.28E-11 |
| GSTM2 | turquoise | -0.434802105 | 5.84E-23 | -0.433368275 | 8.37E-23 |
| GSTM5 | turquoise | -0.263938742 | 6.98E-09 | -0.261041647 | 1.03E-08 |
| GTF2IRD | turquoise | -0.140354843 | 0.002365572 | -0.16171157 | 0.000450863 |
| GUCY1A | turquoise | -0.256738831 | 1.82E-08 | -0.251017728 | 3.84E-08 |
| GUCY1B | turquoise | -0.074755483 | 0.10665581 | -0.076578014 | 0.098359836 |
| GYS1 | turquoise | 0.254370199 | 2.49E-08 | 0.257028367 | 1.76E-08 |
| H6PD | turquoise | -0.053152459 | 0.251643522 | -0.061351346 | 0.185666602 |
| HAAO | turquoise | -0.307235946 | 1.15E-11 | -0.295976011 | 6.77E-11 |
| HABP4 | turquoise | -0.060920208 | 0.188782432 | -0.093005068 | 0.044555587 |
| HAND2 | turquoise | -0.278300746 | 9.40E-10 | -0.25939946 | 1.28E-08 |
| HAPLN2 | turquoise | -0.1964349 | 1.91E-05 | -0.205681976 | 7.43E-06 |
| HAS1 | turquoise | -0.167341556 | 0.000280978 | -0.165649432 | 0.000324408 |
| HBEGF | turquoise | -0.05091477 | 0.272186555 | -0.057040059 | 0.218568112 |
| HDAC5 | turquoise | -0.242462145 | 1.13E-07 | -0.239444928 | 1.63E-07 |
| HEPH | turquoise | -0.151047156 | 0.001059565 | -0.146551209 | 0.001494892 |
| HES6 | turquoise | 0.343737808 | 2.13E-14 | 0.337208961 | 6.99E-14 |
| HGF | turquoise | -0.149017969 | 0.001239088 | -0.168522164 | 0.000253962 |
| HHIPL1 | turquoise | -0.326669854 | 4.49E-13 | -0.318046794 | 1.95E-12 |
| HIC1 | turquoise | -0.164348538 | 0.00036197 | -0.137414791 | 0.002922933 |
| HILPDA | turquoise | 0.285099738 | 3.49E-10 | 0.288951515 | 1.97E-10 |
| HIP1R | turquoise | 0.233236633 | 3.45E-07 | 0.23594356 | 2.49E-07 |
| HK2 | turquoise | 0.248662797 | 5.18E-08 | 0.255925502 | 2.03E-08 |
| HLF | turquoise | -0.213626808 | 3.19E-06 | -0.231370751 | 4.30E-07 |
| HMCN2 | turquoise | -0.226726085 | 7.38E-07 | -0.226610671 | 7.48E-07 |
| HN1L | turquoise | 0.30819774 | 9.84E-12 | 0.320523365 | 1.28E-12 |
| HNMT | turquoise | -0.081220886 | 0.079534718 | -0.077917386 | 0.092597136 |
| HOOK2 | turquoise | 0.033633744 | 0.468397647 | 0.008228179 | 0.85924218 |
| HOPX | turquoise | -0.137485098 | 0.002908317 | -0.100786277 | 0.029426844 |
| HOXA10 | turquoise | 0.164672054 | 0.000352267 | 0.14639661 | 0.001512436 |
| HOXA11 | turquoise | 0.116552778 | 0.011716405 | 0.121350083 | 0.008662931 |

| | | | | | |
|---|---|---|---|---|---|
| HOXA13 | turquoise | -0.110302335 | 0.017100224 | -0.111959051 | 0.015495505 |
| HPGDS | turquoise | -0.208470752 | 5.55E-06 | -0.193886386 | 2.46E-05 |
| HPSE2 | turquoise | -0.232701153 | 3.67E-07 | -0.224552528 | 9.47E-07 |
| HRC | turquoise | -0.136586993 | 0.003100118 | -0.137918876 | 0.002819602 |
| HRCT1 | turquoise | -0.131776491 | 0.004337253 | -0.109123053 | 0.018329319 |
| HS6ST1 | turquoise | -0.061061575 | 0.187756566 | -0.068073764 | 0.141873939 |
| HS6ST3 | turquoise | -0.027362039 | 0.555308355 | -0.041499559 | 0.370896598 |
| HSD17B1 | turquoise | -0.197293667 | 1.75E-05 | -0.225457071 | 8.54E-07 |
| HSD17B1 | turquoise | -0.193407213 | 2.57E-05 | -0.199493384 | 1.40E-05 |
| HSD17B6 | turquoise | -0.223044527 | 1.12E-06 | -0.221427095 | 1.35E-06 |
| HSPA12E | turquoise | -0.276872614 | 1.15E-09 | -0.257304507 | 1.69E-08 |
| HSPB2 | turquoise | -0.349096557 | 7.87E-15 | -0.330627134 | 2.25E-13 |
| HSPB2-C | turquoise | -0.176191593 | 0.000129551 | -0.168079762 | 0.000263788 |
| HSPB3 | turquoise | -0.18752616 | 4.55E-05 | -0.191041934 | 3.24E-05 |
| HSPB6 | turquoise | -0.339125277 | 4.95E-14 | -0.32902787 | 2.98E-13 |
| HSPB7 | turquoise | -0.213681591 | 3.17E-06 | -0.200084698 | 1.32E-05 |
| HSPB8 | turquoise | -0.159065537 | 0.000560141 | -0.144557793 | 0.001736113 |
| HTR1B | turquoise | -0.126697734 | 0.006112584 | -0.090116909 | 0.051632498 |
| HTR2A | turquoise | -0.128017385 | 0.005597498 | -0.127917195 | 0.005635189 |
| HTR2B | turquoise | -0.1738868 | 0.000159071 | -0.160322726 | 0.000505471 |
| HTRA1 | turquoise | 0.028263851 | 0.54234428 | 0.025432197 | 0.583547569 |
| HTRA3 | turquoise | -0.115081561 | 0.012826821 | -0.057817165 | 0.212348187 |
| IDH2 | turquoise | 0.306435181 | 1.31E-11 | 0.336073069 | 8.57E-14 |
| IGDCC4 | turquoise | -0.27460454 | 1.59E-09 | -0.270750236 | 2.74E-09 |
| IGF2 | turquoise | -0.327315149 | 4.01E-13 | -0.311417355 | 5.83E-12 |
| IGFBP5 | turquoise | -0.099209618 | 0.032074351 | -0.084184703 | 0.069125455 |
| IGFBP6 | turquoise | -0.367833827 | 2.08E-16 | -0.354930186 | 2.61E-15 |
| IGFN1 | turquoise | -0.1401458 | 0.002401742 | -0.135250476 | 0.003406881 |
| IGIP | turquoise | -0.064601585 | 0.163388006 | -0.060059779 | 0.195115302 |
| IGSF8 | turquoise | 0.160537062 | 0.00049666 | 0.145146941 | 0.001661347 |
| IGSF9 | turquoise | 0.047599989 | 0.304670255 | 0.015359884 | 0.740599255 |
| IGSF9B | turquoise | -0.26740052 | 4.35E-09 | -0.283526543 | 4.40E-10 |
| IKBKE | turquoise | 0.141873354 | 0.002117397 | 0.153713573 | 0.000860066 |
| IL11RA | turquoise | -0.282176445 | 5.36E-10 | -0.287387681 | 2.49E-10 |
| IL17D | turquoise | -0.260364694 | 1.13E-08 | -0.271337192 | 2.52E-09 |
| INMT | turquoise | -0.109283733 | 0.018157441 | -0.092657315 | 0.045361973 |
| INMT-FA | turquoise | -0.097017854 | 0.036090955 | -0.095597979 | 0.03891496 |
| INPP5A | turquoise | -0.188850874 | 4.01E-05 | -0.21284377 | 3.47E-06 |
| IQSEC1 | turquoise | -0.07658078 | 0.098347645 | -0.088659636 | 0.055545064 |
| IQSEC3 | turquoise | -0.429676378 | 2.10E-22 | -0.430539586 | 1.69E-22 |
| IRS1 | turquoise | -0.177799706 | 0.000112091 | -0.193728431 | 2.49E-05 |
| IRS2 | turquoise | -0.369838948 | 1.39E-16 | -0.393655171 | 9.25E-19 |
| IRS4 | turquoise | -0.140961577 | 0.00226339 | -0.144933054 | 0.001688142 |
| ISCU | turquoise | -0.197091018 | 1.79E-05 | -0.173779831 | 0.000160584 |
| ITGA11 | turquoise | -0.02295212 | 0.620786887 | 0.007349984 | 0.874132974 |

| | | | | | |
|---|---|---|---|---|---|
| ITGA7 | turquoise | -0.340022191 | 4.20E-14 | -0.34479667 | 1.75E-14 |
| ITGA8 | turquoise | -0.166248675 | 0.000308358 | -0.172448419 | 0.000180577 |
| ITGA9 | turquoise | -0.057786341 | 0.212592449 | -0.06303781 | 0.173842379 |
| ITGB1BP | turquoise | -0.156040765 | 0.000714947 | -0.155703207 | 0.000734487 |
| ITGBL1 | turquoise | 0.041853532 | 0.366826352 | 0.059488145 | 0.19940761 |
| ITM2A | turquoise | -0.2396046 | 1.60E-07 | -0.221967615 | 1.27E-06 |
| ITPKB | turquoise | 0.134967708 | 0.003475212 | 0.10369422 | 0.025033232 |
| ITPR1 | turquoise | -0.060340502 | 0.193032299 | -0.077824525 | 0.092987757 |
| ITPRIP | turquoise | -0.01972447 | 0.670728477 | -0.034102407 | 0.462218859 |
| JADE2 | turquoise | -0.164651103 | 0.000352888 | -0.169071476 | 0.000242238 |
| JAM2 | turquoise | -0.138040963 | 0.002795081 | -0.141637003 | 0.002154392 |
| JAM3 | turquoise | -0.232240737 | 3.88E-07 | -0.218793474 | 1.81E-06 |
| JAZF1 | turquoise | -0.144836155 | 0.001700411 | -0.135987054 | 0.003234571 |
| JPH2 | turquoise | -0.25781167 | 1.58E-08 | -0.253445733 | 2.80E-08 |
| JPH4 | turquoise | -0.250876298 | 3.91E-08 | -0.252002237 | 3.38E-08 |
| KANK2 | turquoise | -0.155770424 | 0.000730557 | -0.15627832 | 0.000701486 |
| KATNAL | turquoise | -0.187837912 | 4.41E-05 | -0.200211762 | 1.31E-05 |
| KBTBD1 | turquoise | -0.187586317 | 4.52E-05 | -0.187701746 | 4.47E-05 |
| KCNA1 | turquoise | -0.200577788 | 1.26E-05 | -0.197207304 | 1.77E-05 |
| KCNA4 | turquoise | -0.137141474 | 0.002980389 | -0.14912174 | 0.001229268 |
| KCNA5 | turquoise | -0.218164556 | 1.94E-06 | -0.214721749 | 2.83E-06 |
| KCNAB1 | turquoise | -0.186020049 | 5.24E-05 | -0.17655895 | 0.000125351 |
| KCNAB3 | turquoise | -0.164537925 | 0.00035626 | -0.167023133 | 0.000288711 |
| KCNB1 | turquoise | -0.179805633 | 9.34E-05 | -0.202065009 | 1.08E-05 |
| KCND2 | turquoise | -0.252283001 | 3.26E-08 | -0.253393003 | 2.82E-08 |
| KCND3 | turquoise | -0.043031075 | 0.353486885 | -0.042581734 | 0.358540627 |
| KCNE4 | turquoise | -0.086535875 | 0.06168533 | -0.074925875 | 0.105857506 |
| KCNH2 | turquoise | -0.406114811 | 5.69E-20 | -0.408732199 | 3.12E-20 |
| KCNJ8 | turquoise | -0.187897141 | 4.39E-05 | -0.173663548 | 0.000162244 |
| KCNK3 | turquoise | -0.218608819 | 1.85E-06 | -0.204883692 | 8.08E-06 |
| KCNMA1 | turquoise | -0.079923973 | 0.084472739 | -0.077933744 | 0.092528465 |
| KCNMB1 | turquoise | -0.146759905 | 0.001471505 | -0.123474809 | 0.007553744 |
| KCNN2 | turquoise | 0.120090881 | 0.009386741 | 0.08890513 | 0.054869209 |
| KCTD10 | turquoise | -0.137506561 | 0.002903869 | -0.149087609 | 0.00123249 |
| KCTD12 | turquoise | -0.354097778 | 3.05E-15 | -0.355441276 | 2.36E-15 |
| KCTD15 | turquoise | -0.200115249 | 1.32E-05 | -0.233005785 | 3.54E-07 |
| KCTD17 | turquoise | -0.27311981 | 1.96E-09 | -0.275145383 | 1.47E-09 |
| KCTD7 | turquoise | -0.195106597 | 2.18E-05 | -0.206453773 | 6.86E-06 |
| KCTD8 | turquoise | -0.222685818 | 1.17E-06 | -0.224316345 | 9.73E-07 |
| KIAA121 | turquoise | -0.254283142 | 2.51E-08 | -0.243688878 | 9.68E-08 |
| KIAA146 | turquoise | -0.180902129 | 8.45E-05 | -0.172826865 | 0.000174668 |
| KIAA161 | turquoise | 0.010850643 | 0.815091458 | 0.001980417 | 0.965954613 |
| KIAA164 | turquoise | -0.156442883 | 0.000692299 | -0.160905814 | 0.000481836 |
| KIAA175 | turquoise | -0.14939175 | 0.001204049 | -0.118547177 | 0.010347126 |
| KIF26A | turquoise | -0.190566511 | 3.40E-05 | -0.197766217 | 1.67E-05 |

| | | | | | |
|---|---|---|---|---|---|
| KIT | turquoise | -0.107684471 | 0.019932475 | -0.128600761 | 0.005382504 |
| KLF17 | turquoise | -0.157806643 | 0.000620345 | -0.154543097 | 0.000805474 |
| KLF2 | turquoise | -0.093507998 | 0.043410719 | -0.059659877 | 0.198110939 |
| KLF4 | turquoise | 0.050580467 | 0.275351154 | 0.038236138 | 0.409725384 |
| KLF7 | turquoise | -0.200999249 | 1.20E-05 | -0.211067314 | 4.21E-06 |
| KLF9 | turquoise | -0.162326262 | 0.000428491 | -0.162379121 | 0.000426616 |
| KLHDC1 | turquoise | -0.193833754 | 2.47E-05 | -0.20922991 | 5.12E-06 |
| KLHL13 | turquoise | -0.109472762 | 0.01795704 | -0.104674728 | 0.023685329 |
| KLHL21 | turquoise | -0.088680249 | 0.05548805 | -0.088016473 | 0.057348568 |
| KLHL33 | turquoise | -0.177852147 | 0.000111561 | -0.181359917 | 8.10E-05 |
| KLHL38 | turquoise | -0.19319239 | 2.63E-05 | -0.190435393 | 3.44E-05 |
| KLHL4 | turquoise | -0.211416985 | 4.05E-06 | -0.220915346 | 1.43E-06 |
| KRT222 | turquoise | -0.085295087 | 0.065524901 | -0.069675726 | 0.132710773 |
| KRT7 | turquoise | -0.158178703 | 0.000601955 | -0.142613816 | 0.002005226 |
| KSR1 | turquoise | -0.269862579 | 3.10E-09 | -0.282084889 | 5.43E-10 |
| KY | turquoise | -0.179423101 | 9.67E-05 | -0.181015086 | 8.36E-05 |
| LAMA2 | turquoise | -0.301194438 | 3.00E-11 | -0.292962961 | 1.07E-10 |
| LAMA4 | turquoise | -0.09029646 | 0.051166737 | -0.095313804 | 0.039502081 |
| LAMTOR | turquoise | 0.053417268 | 0.249285748 | 0.06551584 | 0.157498778 |
| LATS2 | turquoise | -0.227816166 | 6.51E-07 | -0.230265823 | 4.89E-07 |
| LAYN | turquoise | -0.384066789 | 7.31E-18 | -0.363793664 | 4.64E-16 |
| LBH | turquoise | -0.209677791 | 4.88E-06 | -0.181918743 | 7.69E-05 |
| LCN10 | turquoise | -0.204237123 | 8.64E-06 | -0.219543879 | 1.67E-06 |
| LCN6 | turquoise | -0.175952173 | 0.000132359 | -0.188532686 | 4.13E-05 |
| LDB2 | turquoise | -0.160951107 | 0.000480044 | -0.157467896 | 0.00063754 |
| LDB3 | turquoise | -0.050220804 | 0.278783671 | -0.058532922 | 0.206732978 |
| LDLRAD | turquoise | -0.183131222 | 6.87E-05 | -0.182971739 | 6.98E-05 |
| LDLRAD | turquoise | -0.041025642 | 0.376389619 | -0.047051472 | 0.310283204 |
| LEFTY2 | turquoise | -0.065077819 | 0.160299994 | -0.064140393 | 0.166420882 |
| LEPR | turquoise | -0.274271766 | 1.67E-09 | -0.27774571 | 1.02E-09 |
| LGI2 | turquoise | -0.3103685 | 6.92E-12 | -0.333070428 | 1.46E-13 |
| LGI3 | turquoise | -0.182957284 | 6.99E-05 | -0.184848726 | 5.85E-05 |
| LGI4 | turquoise | -0.210210629 | 4.61E-06 | -0.203049367 | 9.76E-06 |
| LHFP | turquoise | -0.162260823 | 0.000430822 | -0.147925361 | 0.001346966 |
| LIFR | turquoise | -0.2725701 | 2.12E-09 | -0.294375797 | 8.65E-11 |
| LIMS2 | turquoise | -0.231584645 | 4.19E-07 | -0.211830679 | 3.88E-06 |
| LIN7A | turquoise | -0.012233328 | 0.792037428 | -0.024199149 | 0.601932886 |
| LINGO2 | turquoise | -0.240573273 | 1.42E-07 | -0.254195274 | 2.54E-08 |
| LIX1L | turquoise | 0.00032493 | 0.994412476 | -0.007095474 | 0.878457046 |
| LMCD1 | turquoise | -0.298577661 | 4.52E-11 | -0.273244931 | 1.93E-09 |
| LMOD1 | turquoise | -0.177158278 | 0.000118772 | -0.15555439 | 0.000743258 |
| LNP1 | turquoise | -0.401881848 | 1.49E-19 | -0.404201911 | 8.80E-20 |
| LONRF2 | turquoise | -0.220671967 | 1.47E-06 | -0.220656651 | 1.47E-06 |
| LOXL4 | turquoise | -0.247065339 | 6.34E-08 | -0.2332859 | 3.43E-07 |
| LPAR1 | turquoise | 0.142078526 | 0.002085755 | 0.142898076 | 0.001963628 |

| | | | | | |
|---|---|---|---|---|---|
| LPAR2 | turquoise | 0.141193548 | 0.0022254 | 0.169828392 | 0.00022691 |
| LPAR4 | turquoise | -0.194708068 | 2.27E-05 | -0.195442814 | 2.11E-05 |
| LPAR6 | turquoise | -0.02240018 | 0.629212699 | -0.040390286 | 0.383831866 |
| LPP | turquoise | -0.10702944 | 0.020701988 | -0.100190486 | 0.0304044 |
| LRCH2 | turquoise | -0.4168534 | 4.68E-21 | -0.418299286 | 3.32E-21 |
| LRFN5 | turquoise | -0.260591933 | 1.09E-08 | -0.273363272 | 1.90E-09 |
| LRP1 | turquoise | -0.197327125 | 1.75E-05 | -0.192310692 | 2.87E-05 |
| LRRC2 | turquoise | 0.015811106 | 0.733264308 | -0.017745771 | 0.702096654 |
| LRRC32 | turquoise | -0.194555353 | 2.30E-05 | -0.165109534 | 0.000339532 |
| LRRC4B | turquoise | -0.240145616 | 1.50E-07 | -0.227505201 | 6.75E-07 |
| LRRC70 | turquoise | -0.171252056 | 0.000200509 | -0.180539946 | 8.73E-05 |
| LRRC8C | turquoise | -0.150240494 | 0.001127846 | -0.139820396 | 0.00245905 |
| LRRK2 | turquoise | -0.259095635 | 1.34E-08 | -0.258916829 | 1.37E-08 |
| LRRN4Cl | turquoise | -0.232332841 | 3.84E-07 | -0.224324286 | 9.72E-07 |
| LSAMP | turquoise | -0.254201328 | 2.54E-08 | -0.265946632 | 5.31E-09 |
| LSMEM2 | turquoise | -0.121785756 | 0.008424331 | -0.121154803 | 0.008771818 |
| LTBP2 | turquoise | -0.18216977 | 7.52E-05 | -0.168775813 | 0.000248484 |
| LTBP4 | turquoise | -0.220081776 | 1.57E-06 | -0.211679964 | 3.94E-06 |
| LTC4S | turquoise | -0.1385795 | 0.002689217 | -0.140890418 | 0.002275162 |
| LURAP1 | turquoise | -0.20987874 | 4.78E-06 | -0.203481099 | 9.34E-06 |
| LYPLA2 | turquoise | 0.05209924 | 0.261174268 | 0.090650127 | 0.050259568 |
| LYPLAL1 | turquoise | -0.219361788 | 1.70E-06 | -0.195942541 | 2.00E-05 |
| LYSMD4 | turquoise | -0.133740355 | 0.003786445 | -0.165854992 | 0.000318816 |
| LYVE1 | turquoise | -0.207576599 | 6.09E-06 | -0.20207057 | 1.08E-05 |
| MAF | turquoise | -0.147499499 | 0.001391304 | -0.147796607 | 0.001360232 |
| MAGEE1 | turquoise | -0.315393261 | 3.03E-12 | -0.328208117 | 3.43E-13 |
| MAGEE2 | turquoise | -0.236799408 | 2.25E-07 | -0.234130743 | 3.10E-07 |
| MAGEH1 | turquoise | -0.531453229 | 2.12E-35 | -0.508242035 | 4.85E-32 |
| MAGEL2 | turquoise | -0.292617292 | 1.13E-10 | -0.300507368 | 3.35E-11 |
| MAGI2 | turquoise | -0.400119074 | 2.21E-19 | -0.388528026 | 2.82E-18 |
| MAMDC2 | turquoise | -0.267622874 | 4.22E-09 | -0.273174546 | 1.95E-09 |
| MAMLD1 | turquoise | -0.259193287 | 1.32E-08 | -0.266364892 | 5.01E-09 |
| MAN1A1 | turquoise | -0.152840646 | 0.000921193 | -0.155017841 | 0.00077569 |
| MAN1C1 | turquoise | -0.028698733 | 0.536146801 | -0.056356293 | 0.224147934 |
| MANF | turquoise | 0.142573711 | 0.00201116 | 0.141003271 | 0.002256518 |
| MAOB | turquoise | -0.112525342 | 0.014978118 | -0.103418097 | 0.025424564 |
| MAP10 | turquoise | -0.355337497 | 2.41E-15 | -0.346991743 | 1.17E-14 |
| MAP1B | turquoise | 0.022734311 | 0.624106078 | 0.021985561 | 0.635574076 |
| MAP2K3 | turquoise | 0.300795235 | 3.20E-11 | 0.334996217 | 1.04E-13 |
| MAP3K1: | turquoise | -0.302986692 | 2.26E-11 | -0.322885078 | 8.59E-13 |
| MAP3K3 | turquoise | -0.247199106 | 6.24E-08 | -0.263367174 | 7.54E-09 |
| MAP3K7( | turquoise | -0.158669019 | 0.000578492 | -0.132991364 | 0.003988568 |
| MAP7D3 | turquoise | -0.37700895 | 3.21E-17 | -0.395225704 | 6.55E-19 |
| MAPK10 | turquoise | -0.286365058 | 2.89E-10 | -0.290113692 | 1.65E-10 |
| MAPK3 | turquoise | -0.222451137 | 1.20E-06 | -0.182898397 | 7.02E-05 |

| | | | | | |
|---|---|---|---|---|---|
| MAPKAP | turquoise | 0.06645879 | 0.151594141 | 0.081101913 | 0.079977737 |
| MAPKBP | turquoise | 0.084844414 | 0.066967233 | 0.061956659 | 0.181356217 |
| MAPRE2 | turquoise | 0.058929349 | 0.203669581 | 0.042682315 | 0.357405483 |
| MARCKS | turquoise | 0.253490386 | 2.79E-08 | 0.248043732 | 5.60E-08 |
| MARVEL | turquoise | -0.190781274 | 3.33E-05 | -0.18574024 | 5.38E-05 |
| MAS1 | turquoise | -0.174545685 | 0.000150044 | -0.178454263 | 0.000105639 |
| MAS1L | turquoise | -0.154376923 | 0.000816146 | -0.146810405 | 0.001465897 |
| MASP1 | turquoise | -0.174208127 | 0.000154607 | -0.170573007 | 0.000212721 |
| MAST4 | turquoise | 0.121215304 | 0.008737954 | 0.116278724 | 0.011916568 |
| MATN2 | turquoise | -0.202201779 | 1.07E-05 | -0.193474302 | 2.56E-05 |
| MBOAT7 | turquoise | 0.188601159 | 4.10E-05 | 0.183948235 | 6.37E-05 |
| MCHR1 | turquoise | -0.10183929 | 0.027764934 | -0.09014848 | 0.051550346 |
| MDFIC | turquoise | -0.158379053 | 0.000592263 | -0.14014921 | 0.002401148 |
| MDGA1 | turquoise | -0.331447703 | 1.95E-13 | -0.309051177 | 8.57E-12 |
| MECP2 | turquoise | -0.293185151 | 1.04E-10 | -0.299605628 | 3.85E-11 |
| MEDAG | turquoise | -0.182281233 | 7.44E-05 | -0.163719767 | 0.000381544 |
| MEF2C | turquoise | -0.223460017 | 1.07E-06 | -0.210148469 | 4.64E-06 |
| MEF2D | turquoise | -0.041918549 | 0.366081769 | -0.05144208 | 0.267245512 |
| MEIS2 | turquoise | -0.068653748 | 0.138502068 | -0.06314429 | 0.173115086 |
| MEIS3 | turquoise | -0.185113141 | 5.71E-05 | -0.168104446 | 0.00026323 |
| MEOX1 | turquoise | -0.255208056 | 2.23E-08 | -0.254906551 | 2.32E-08 |
| MEOX2 | turquoise | -0.188156279 | 4.28E-05 | -0.175748451 | 0.000134793 |
| METTL7A | turquoise | 0.010254636 | 0.825079599 | 0.000319429 | 0.994507063 |
| MEX3B | turquoise | 0.043360435 | 0.349811228 | 0.029290004 | 0.527778033 |
| MFAP4 | turquoise | -0.30780274 | 1.05E-11 | -0.281465765 | 5.95E-10 |
| MFAP5 | turquoise | -0.213095909 | 3.38E-06 | -0.213230456 | 3.33E-06 |
| MGARP | turquoise | -0.143827881 | 0.001833016 | -0.14731698 | 0.001410714 |
| MGAT4B | turquoise | 0.044300363 | 0.339455165 | 0.051334455 | 0.268248962 |
| MGP | turquoise | -0.083173093 | 0.072545235 | -0.04456227 | 0.33660476 |
| MICAL2 | turquoise | -0.013970934 | 0.763322974 | 0.009910817 | 0.830854458 |
| MICU3 | turquoise | -0.189433346 | 3.79E-05 | -0.207484824 | 6.15E-06 |
| MITF | turquoise | -0.083677235 | 0.070824095 | -0.098346698 | 0.03360768 |
| MME | turquoise | -0.181354463 | 8.10E-05 | -0.172689125 | 0.000176797 |
| MMP15 | turquoise | 0.193027765 | 2.67E-05 | 0.226642021 | 7.45E-07 |
| MMP16 | turquoise | -0.140786174 | 0.002292508 | -0.136505668 | 0.003118041 |
| MMP19 | turquoise | -0.248616166 | 5.21E-08 | -0.231847126 | 4.06E-07 |
| MMP24 | turquoise | -0.23139509 | 4.28E-07 | -0.236980135 | 2.20E-07 |
| MMP28 | turquoise | -0.192452883 | 2.83E-05 | -0.169392443 | 0.000235623 |
| MN1 | turquoise | -0.182288914 | 7.43E-05 | -0.188328715 | 4.21E-05 |
| MOB3C | turquoise | -0.38900698 | 2.54E-18 | -0.370760212 | 1.15E-16 |
| MOGS | turquoise | 0.14695854 | 0.001449558 | 0.153799105 | 0.000854283 |
| MOV10 | turquoise | -0.024294558 | 0.600501034 | -0.000705749 | 0.987864251 |
| MPRIP | turquoise | -0.08992548 | 0.052132961 | -0.100214616 | 0.030364274 |
| MRAS | turquoise | -0.32453087 | 6.48E-13 | -0.329178289 | 2.90E-13 |
| MRC2 | turquoise | -0.197393372 | 1.73E-05 | -0.177191117 | 0.000118421 |

| | | | | | |
|---|---|---|---|---|---|
| MREG | turquoise | 0.157202783 | 0.000651304 | 0.14667982 | 0.001480439 |
| MRGPRE | turquoise | -0.154608868 | 0.000801285 | -0.153099047 | 0.000902693 |
| MRGPRF | turquoise | -0.268665013 | 3.65E-09 | -0.248800158 | 5.09E-08 |
| MRVI1 | turquoise | -0.171731104 | 0.000192292 | -0.152344353 | 0.000957711 |
| MS4A2 | turquoise | -0.181417342 | 8.06E-05 | -0.165165872 | 0.000337924 |
| MSC | turquoise | -0.018614003 | 0.688266434 | 0.024093043 | 0.603527068 |
| MSRB3 | turquoise | -0.141899232 | 0.002113382 | -0.141424094 | 0.002188221 |
| MSTO1 | turquoise | 0.145574393 | 0.001608962 | 0.129238005 | 0.005156176 |
| MT1F | turquoise | 0.099652934 | 0.031310132 | 0.110379741 | 0.017022131 |
| MTFP1 | turquoise | 0.180691762 | 8.61E-05 | 0.190869941 | 3.30E-05 |
| MTL5 | turquoise | -0.114579031 | 0.01322684 | -0.157936714 | 0.000613857 |
| MTMR11 | turquoise | -0.101097284 | 0.028927346 | -0.121538991 | 0.008558745 |
| MTUS2 | turquoise | -0.170349655 | 0.000216887 | -0.179713276 | 9.42E-05 |
| MTX1 | turquoise | 0.199691399 | 1.38E-05 | 0.187970871 | 4.36E-05 |
| MUM1 | turquoise | 0.107329755 | 0.020346039 | 0.095955226 | 0.03818737 |
| MUM1L1 | turquoise | -0.263465575 | 7.44E-09 | -0.257625701 | 1.62E-08 |
| MUSTN1 | turquoise | -0.067814852 | 0.143399324 | -0.077311435 | 0.095169884 |
| MVB12B | turquoise | -0.143330029 | 0.001901926 | -0.163534721 | 0.00038749 |
| MXRA7 | turquoise | -0.143222698 | 0.00191709 | -0.155594267 | 0.000740898 |
| MYADM | turquoise | -0.106466135 | 0.02138426 | -0.086270216 | 0.062491394 |
| MYH11 | turquoise | -0.113248719 | 0.014339315 | -0.09241556 | 0.045929761 |
| MYL3 | turquoise | -0.15925001 | 0.000551789 | -0.164719015 | 0.000350879 |
| MYL9 | turquoise | -0.219871219 | 1.61E-06 | -0.195181169 | 2.16E-05 |
| MYLK | turquoise | -0.199601568 | 1.39E-05 | -0.188763275 | 4.04E-05 |
| MYO10 | turquoise | 0.126185242 | 0.006323827 | 0.115421936 | 0.012561979 |
| MYOCD | turquoise | -0.186120245 | 5.19E-05 | -0.187987021 | 4.35E-05 |
| MYOM1 | turquoise | -0.296875873 | 5.89E-11 | -0.298637092 | 4.48E-11 |
| MYOM2 | turquoise | -0.006443164 | 0.889555843 | 0.003762264 | 0.935373865 |
| MYOZ2 | turquoise | -0.142269621 | 0.002056674 | -0.145624053 | 0.001602975 |
| MYOZ3 | turquoise | -0.294695285 | 8.24E-11 | -0.293607765 | 9.73E-11 |
| MYZAP | turquoise | -0.127059875 | 0.005967157 | -0.135926996 | 0.003248319 |
| N4BP2L1 | turquoise | -0.344749674 | 1.77E-14 | -0.348842965 | 8.26E-15 |
| NAALAD | turquoise | -0.311959624 | 5.34E-12 | -0.3182068 | 1.90E-12 |
| NAALAD | turquoise | -0.318948897 | 1.67E-12 | -0.306643888 | 1.26E-11 |
| NACAD | turquoise | -0.29945238 | 3.95E-11 | -0.301880801 | 2.70E-11 |
| NACC1 | turquoise | 0.030509986 | 0.510722871 | 0.038091838 | 0.41149614 |
| NACC2 | turquoise | -0.031126796 | 0.502210556 | -0.006634969 | 0.886290014 |
| NAP1L2 | turquoise | -0.264495527 | 6.47E-09 | -0.270775067 | 2.73E-09 |
| NAP1L3 | turquoise | -0.304376853 | 1.82E-11 | -0.309122318 | 8.47E-12 |
| NAP1L5 | turquoise | -0.076492631 | 0.098736666 | -0.09358177 | 0.043244886 |
| NAT2 | turquoise | -0.149804498 | 0.001166418 | -0.151017306 | 0.001062022 |
| NAV3 | turquoise | -0.200927929 | 1.21E-05 | -0.222271253 | 1.23E-06 |
| NBEA | turquoise | -0.187141592 | 4.71E-05 | -0.194536263 | 2.30E-05 |
| NBEAL2 | turquoise | 0.25229667 | 3.25E-08 | 0.255602067 | 2.12E-08 |
| NBL1 | turquoise | -0.251306886 | 3.70E-08 | -0.216201285 | 2.41E-06 |

| | | | | | |
|---|---|---|---|---|---|
| NCAM1 | turquoise | -0.185939659 | 5.28E-05 | -0.208221245 | 5.69E-06 |
| NCS1 | turquoise | -0.239935961 | 1.54E-07 | -0.230010292 | 5.04E-07 |
| NDN | turquoise | -0.238360494 | 1.86E-07 | -0.20807753 | 5.78E-06 |
| NDNF | turquoise | -0.216333815 | 2.38E-06 | -0.213949395 | 3.08E-06 |
| NDP | turquoise | -0.035781494 | 0.440458377 | -0.023325073 | 0.615121323 |
| NDRG4 | turquoise | -0.298688456 | 4.45E-11 | -0.293929094 | 9.26E-11 |
| NECAB1 | turquoise | -0.356767238 | 1.83E-15 | -0.362813227 | 5.63E-16 |
| NECAB3 | turquoise | -0.103615213 | 0.02514467 | -0.103001716 | 0.02602465 |
| NEDD4 | turquoise | -0.059485737 | 0.199425833 | -0.072110944 | 0.119665084 |
| NEFM | turquoise | -0.177274672 | 0.000117533 | -0.192045029 | 2.94E-05 |
| NEGR1 | turquoise | -0.144883942 | 0.00169435 | -0.149157845 | 0.001225868 |
| NEK6 | turquoise | 0.359363923 | 1.11E-15 | 0.373142114 | 7.10E-17 |
| NES | turquoise | -0.217946668 | 1.99E-06 | -0.218887103 | 1.79E-06 |
| NEU1 | turquoise | 0.131183726 | 0.004517188 | 0.13534579 | 0.003384124 |
| NEURL1 | turquoise | -0.266366799 | 5.01E-09 | -0.276688738 | 1.18E-09 |
| NEURL1I | turquoise | -0.100705897 | 0.029557135 | -0.101100078 | 0.028922892 |
| NEXN | turquoise | -0.223601697 | 1.06E-06 | -0.218493204 | 1.87E-06 |
| NFASC | turquoise | -0.136905631 | 0.003030791 | -0.133411998 | 0.003873885 |
| NFATC2 | turquoise | -0.223056889 | 1.12E-06 | -0.203136173 | 9.68E-06 |
| NFATC4 | turquoise | -0.191410118 | 3.13E-05 | -0.213100126 | 3.38E-06 |
| NGF | turquoise | -0.20296785 | 9.85E-06 | -0.199312836 | 1.43E-05 |
| NGFR | turquoise | -0.1669189 | 0.000291286 | -0.162050609 | 0.000438392 |
| NIPAL2 | turquoise | -0.162341037 | 0.000427966 | -0.158082604 | 0.000606656 |
| NKAPL | turquoise | -0.220547105 | 1.49E-06 | -0.227567525 | 6.70E-07 |
| NLGN1 | turquoise | -0.344078287 | 2.00E-14 | -0.339377693 | 4.73E-14 |
| NLGN2 | turquoise | -0.151133758 | 0.001052466 | -0.189637223 | 3.71E-05 |
| NLGN3 | turquoise | -0.195095448 | 2.18E-05 | -0.191400337 | 3.13E-05 |
| NLRP1 | turquoise | -0.371249208 | 1.04E-16 | -0.35050736 | 6.04E-15 |
| NMT2 | turquoise | -0.391879874 | 1.36E-18 | -0.406098716 | 5.71E-20 |
| NMUR1 | turquoise | -0.221563675 | 1.33E-06 | -0.20496105 | 8.01E-06 |
| NNAT | turquoise | -0.266796145 | 4.73E-09 | -0.245869774 | 7.37E-08 |
| NOSTRIN | turquoise | -0.101410549 | 0.028431594 | -0.08005934 | 0.083946038 |
| NOVA1 | turquoise | -0.384101653 | 7.26E-18 | -0.386579345 | 4.28E-18 |
| NPAS4 | turquoise | -0.161785845 | 0.000448103 | -0.163295683 | 0.000395298 |
| NPR1 | turquoise | -0.424750318 | 7.02E-22 | -0.417779194 | 3.76E-21 |
| NPR2 | turquoise | -0.288350462 | 2.15E-10 | -0.297478507 | 5.37E-11 |
| NPR3 | turquoise | -0.06677027 | 0.149681083 | -0.060702675 | 0.190369046 |
| NPY1R | turquoise | -0.206280709 | 6.98E-06 | -0.201710471 | 1.12E-05 |
| NPY5R | turquoise | -0.15096538 | 0.001066309 | -0.148685068 | 0.001271084 |
| NR2F1 | turquoise | -0.180005891 | 9.17E-05 | -0.170643586 | 0.00021142 |
| NR2F2 | turquoise | -0.123375018 | 0.007602852 | -0.106733307 | 0.021058267 |
| NR2F6 | turquoise | -0.115394521 | 0.01258313 | -0.094592476 | 0.041026126 |
| NR3C1 | turquoise | -0.436480718 | 3.83E-23 | -0.416295532 | 5.34E-21 |
| NR3C2 | turquoise | -0.147941107 | 0.001345352 | -0.141004839 | 0.00225626 |
| NR4A1 | turquoise | 0.019573372 | 0.673104597 | -0.019684603 | 0.671355085 |

| | | | | | |
|---|---|---|---|---|---|
| NR4A2 | turquoise | 0.141559402 | 0.002166666 | 0.135317875 | 0.003390775 |
| NR4A3 | turquoise | -0.063427916 | 0.171188896 | -0.09062335 | 0.050327779 |
| NRIP2 | turquoise | -0.142248719 | 0.002059836 | -0.132109166 | 0.00423913 |
| NRP2 | turquoise | -0.010111652 | 0.827480054 | -0.020368841 | 0.660632354 |
| NRXN2 | turquoise | -0.332035137 | 1.76E-13 | -0.314336733 | 3.61E-12 |
| NT5DC3 | turquoise | -0.056129378 | 0.2260219 | -0.084904344 | 0.066773945 |
| NT5E | turquoise | 0.25886608 | 1.38E-08 | 0.25316095 | 2.91E-08 |
| NTAN1 | turquoise | -0.003616087 | 0.937879657 | 0.001041746 | 0.982087407 |
| NTRK3 | turquoise | -0.26221048 | 8.81E-09 | -0.255242338 | 2.22E-08 |
| NUAK1 | turquoise | -0.288351474 | 2.15E-10 | -0.281087681 | 6.28E-10 |
| NUAK2 | turquoise | -0.113443068 | 0.014171823 | -0.122321932 | 0.008138743 |
| NUDT10 | turquoise | -0.352352588 | 4.26E-15 | -0.343345887 | 2.29E-14 |
| NUDT11 | turquoise | -0.340048619 | 4.18E-14 | -0.349943412 | 6.72E-15 |
| NUDT7 | turquoise | 0.031688575 | 0.494523276 | 0.023163249 | 0.617576827 |
| NUMBL | turquoise | -0.180587243 | 8.70E-05 | -0.195157035 | 2.17E-05 |
| NXPE3 | turquoise | -0.336646098 | 7.73E-14 | -0.339541524 | 4.59E-14 |
| NXPH3 | turquoise | -0.208171895 | 5.72E-06 | -0.205344357 | 7.70E-06 |
| NYAP1 | turquoise | -0.399459331 | 2.56E-19 | -0.413042692 | 1.15E-20 |
| OAF | turquoise | -0.286879736 | 2.68E-10 | -0.312298189 | 5.05E-12 |
| OCIAD2 | turquoise | 0.274601053 | 1.59E-09 | 0.304002746 | 1.93E-11 |
| OGN | turquoise | -0.229205039 | 5.54E-07 | -0.230702536 | 4.65E-07 |
| OLFM1 | turquoise | -0.202573793 | 1.03E-05 | -0.204594783 | 8.32E-06 |
| OLFML1 | turquoise | -0.255953351 | 2.02E-08 | -0.230948095 | 4.52E-07 |
| OLFML2A | turquoise | -0.129228355 | 0.005159539 | -0.124957058 | 0.006856958 |
| OLFML3 | turquoise | -0.113318359 | 0.014279099 | -0.105727377 | 0.022308566 |
| OMD | turquoise | -0.209179796 | 5.14E-06 | -0.209192545 | 5.14E-06 |
| OPCML | turquoise | -0.097628 | 0.034932027 | -0.106031315 | 0.02192418 |
| OPN1SW | turquoise | -0.150782 | 0.001081577 | -0.159502371 | 0.000540551 |
| OR5K2 | turquoise | -0.152538791 | 0.000943248 | -0.172495705 | 0.000179828 |
| ORMDL2 | turquoise | 0.17135813 | 0.000198662 | 0.193007318 | 2.68E-05 |
| OSBPL1A | turquoise | -0.224161248 | 9.90E-07 | -0.254476069 | 2.45E-08 |
| OSBPL5 | turquoise | -0.040871819 | 0.378183237 | -0.039650348 | 0.392611407 |
| OSR1 | turquoise | 0.034817351 | 0.452881085 | 0.007729013 | 0.867700339 |
| OSR2 | turquoise | -0.294165229 | 8.94E-11 | -0.283112529 | 4.68E-10 |
| OTC | turquoise | -0.201364696 | 1.16E-05 | -0.204741214 | 8.20E-06 |
| OTOP2 | turquoise | -0.09223256 | 0.046363513 | -0.090365336 | 0.050989005 |
| OTUD7A | turquoise | -0.22786116 | 6.47E-07 | -0.259170754 | 1.32E-08 |
| OVOL1 | turquoise | -0.000931086 | 0.983989912 | -0.01464024 | 0.752346156 |
| OXER1 | turquoise | -0.320238324 | 1.35E-12 | -0.316512671 | 2.52E-12 |
| OXSM | turquoise | 0.296029944 | 6.71E-11 | 0.302155524 | 2.58E-11 |
| P2RY1 | turquoise | -0.109584453 | 0.017839539 | -0.121545144 | 0.00855537 |
| P2RY14 | turquoise | -0.100826712 | 0.029361488 | -0.085230451 | 0.065730181 |
| P3H2 | turquoise | -0.296896128 | 5.87E-11 | -0.308464179 | 9.43E-12 |
| P3H3 | turquoise | -0.179811874 | 9.34E-05 | -0.173219534 | 0.00016873 |
| PABPC5 | turquoise | -0.252190498 | 3.30E-08 | -0.254908231 | 2.32E-08 |

| | | | | | |
|---|---|---|---|---|---|
| PACSIN1 | turquoise | 0.181233171 | 8.19E-05 | 0.160960263 | 0.000479682 |
| PAGE4 | turquoise | -0.235585204 | 2.60E-07 | -0.235279011 | 2.70E-07 |
| PAK3 | turquoise | -0.257509795 | 1.65E-08 | -0.259667341 | 1.24E-08 |
| PALLD | turquoise | 0.111091381 | 0.016318662 | 0.108756735 | 0.018726484 |
| PALM | turquoise | -0.342188656 | 2.83E-14 | -0.318552194 | 1.79E-12 |
| PALM2-A | turquoise | -0.157272078 | 0.00064768 | -0.164519943 | 0.000356799 |
| PAPLN | turquoise | -0.424357793 | 7.73E-22 | -0.427816536 | 3.32E-22 |
| PAPPA | turquoise | -0.177431593 | 0.000115881 | -0.173703995 | 0.000161664 |
| PAQR4 | turquoise | 0.402554693 | 1.28E-19 | 0.410409052 | 2.12E-20 |
| PARK2 | turquoise | -0.083555553 | 0.071236432 | -0.125227495 | 0.006736227 |
| PARM1 | turquoise | -0.043706692 | 0.345973166 | -0.066021264 | 0.154312638 |
| PARVA | turquoise | -0.028716848 | 0.535889426 | -0.024762589 | 0.593499409 |
| PAX8 | turquoise | -0.029964383 | 0.518314671 | -0.031652369 | 0.495016824 |
| PBX3 | turquoise | -0.266365335 | 5.01E-09 | -0.261696566 | 9.44E-09 |
| PCBD1 | turquoise | 0.192698041 | 2.76E-05 | 0.191540467 | 3.09E-05 |
| PCBP3 | turquoise | -0.311234968 | 6.01E-12 | -0.307463568 | 1.11E-11 |
| PCDH18 | turquoise | -0.101589152 | 0.028152228 | -0.086750038 | 0.061041779 |
| PCDH20 | turquoise | -0.049656596 | 0.284226539 | -0.074958503 | 0.105705185 |
| PCDHB10 | turquoise | -0.129387999 | 0.005104165 | -0.139052335 | 0.002599294 |
| PCDHB11 | turquoise | -0.00245453 | 0.957810972 | -0.021255147 | 0.646845839 |
| PCDHB12 | turquoise | -0.162361774 | 0.00042723 | -0.173792017 | 0.000160411 |
| PCDHB14 | turquoise | -0.003553391 | 0.938954586 | -0.013376991 | 0.773103796 |
| PCDHB15 | turquoise | -0.30930796 | 8.22E-12 | -0.307178055 | 1.16E-11 |
| PCDHB4 | turquoise | -0.235132997 | 2.75E-07 | -0.235982823 | 2.48E-07 |
| PCDHB5 | turquoise | -0.289343322 | 1.86E-10 | -0.289976872 | 1.69E-10 |
| PCDHB7 | turquoise | -0.192674685 | 2.77E-05 | -0.188838263 | 4.01E-05 |
| PCDHB9 | turquoise | -0.009291696 | 0.841276109 | -0.004280971 | 0.926487448 |
| PCDHGA | turquoise | -0.172604988 | 0.00017811 | -0.163194991 | 0.000398631 |
| PCDHGA | turquoise | -0.241908813 | 1.21E-07 | -0.246293082 | 6.99E-08 |
| PCDHGA | turquoise | -0.2424853 | 1.12E-07 | -0.242792199 | 1.08E-07 |
| PCDHGA | turquoise | -0.210529746 | 4.46E-06 | -0.203049473 | 9.76E-06 |
| PCDHGA | turquoise | -0.236403248 | 2.36E-07 | -0.233732805 | 3.25E-07 |
| PCDHGA | turquoise | -0.193641167 | 2.52E-05 | -0.200988837 | 1.21E-05 |
| PCDHGA | turquoise | -0.121631789 | 0.008507975 | -0.161367407 | 0.000463859 |
| PCDHGB | turquoise | -0.132537603 | 0.004115721 | -0.117270818 | 0.011205992 |
| PCDHGB | turquoise | -0.080333923 | 0.082885805 | -0.077073761 | 0.096194452 |
| PDCD6 | turquoise | -0.029877771 | 0.519525153 | -0.004940671 | 0.915198899 |
| PDE10A | turquoise | -0.068593752 | 0.138847986 | -0.075127942 | 0.104916937 |
| PDE1A | turquoise | -0.198094395 | 1.62E-05 | -0.208061366 | 5.79E-06 |
| PDE1B | turquoise | -0.226260437 | 7.79E-07 | -0.215750035 | 2.53E-06 |
| PDE1C | turquoise | -0.194114462 | 2.40E-05 | -0.19919068 | 1.45E-05 |
| PDE2A | turquoise | -0.228226758 | 6.20E-07 | -0.236071554 | 2.46E-07 |
| PDE5A | turquoise | -0.152858923 | 0.000919873 | -0.135217507 | 0.003414784 |
| PDE7B | turquoise | -0.259317262 | 1.30E-08 | -0.260156451 | 1.16E-08 |
| PDE8B | turquoise | -0.162186929 | 0.000433469 | -0.164163794 | 0.000367622 |

| | | | | | |
|---|---|---|---|---|---|
| PDGFD | turquoise | -0.085392498 | 0.065216528 | -0.091076479 | 0.049183858 |
| PDGFRA | turquoise | -0.107913206 | 0.01966969 | -0.103154875 | 0.025802514 |
| PDGFRL | turquoise | -0.103633658 | 0.025118615 | -0.107520067 | 0.020123236 |
| PDIA4 | turquoise | 0.159481556 | 0.00054147 | 0.159937827 | 0.000521658 |
| PDK4 | turquoise | -0.111159568 | 0.016252614 | -0.098322723 | 0.033651158 |
| PDLIM2 | turquoise | -0.047124132 | 0.309535787 | -0.037268199 | 0.421689624 |
| PDLIM3 | turquoise | -0.175384758 | 0.000139244 | -0.15616546 | 0.000707852 |
| PDLIM7 | turquoise | -0.249607812 | 4.59E-08 | -0.231086131 | 4.44E-07 |
| PDPN | turquoise | -0.289833017 | 1.72E-10 | -0.281584698 | 5.84E-10 |
| PDZD4 | turquoise | -0.244722686 | 8.51E-08 | -0.244138684 | 9.15E-08 |
| PDZRN3 | turquoise | -0.033576245 | 0.469158834 | -0.039981534 | 0.3886669 |
| PDZRN4 | turquoise | -0.206328004 | 6.95E-06 | -0.193982486 | 2.43E-05 |
| PEAR1 | turquoise | -0.147309766 | 0.001411486 | -0.144836074 | 0.001700421 |
| PEG10 | turquoise | -0.068753926 | 0.137925947 | -0.069290158 | 0.134873334 |
| PEG3 | turquoise | -0.299258746 | 4.07E-11 | -0.305055073 | 1.63E-11 |
| PELI3 | turquoise | -0.084301666 | 0.068738714 | -0.102042255 | 0.027454054 |
| PENK | turquoise | -0.189769017 | 3.67E-05 | -0.195572637 | 2.08E-05 |
| PER1 | turquoise | -0.162988516 | 0.000405547 | -0.177153011 | 0.000118828 |
| PER3 | turquoise | -0.335953625 | 8.76E-14 | -0.347968814 | 9.73E-15 |
| PFKFB4 | turquoise | 0.254763028 | 2.36E-08 | 0.23920775 | 1.68E-07 |
| PGAP2 | turquoise | 0.338797959 | 5.25E-14 | 0.329452415 | 2.76E-13 |
| PGD | turquoise | 0.242318238 | 1.15E-07 | 0.237071075 | 2.18E-07 |
| PGM5 | turquoise | -0.144898565 | 0.001692499 | -0.146570253 | 0.001492744 |
| PHF1 | turquoise | -0.209500664 | 4.97E-06 | -0.216746681 | 2.27E-06 |
| PHF21B | turquoise | -0.171771388 | 0.000191616 | -0.197363409 | 1.74E-05 |
| PHLDA2 | turquoise | 0.278153143 | 9.60E-10 | 0.30934264 | 8.18E-12 |
| PHLDB2 | turquoise | -0.06214765 | 0.180011675 | -0.093695831 | 0.042989536 |
| PHYHIP | turquoise | -0.271549112 | 2.45E-09 | -0.263717301 | 7.19E-09 |
| PI15 | turquoise | -0.164048498 | 0.000371191 | -0.172174792 | 0.000184964 |
| PI16 | turquoise | -0.178085043 | 0.000109235 | -0.180746744 | 8.57E-05 |
| PIANP | turquoise | -0.195897491 | 2.01E-05 | -0.201262431 | 1.17E-05 |
| PID1 | turquoise | -0.245119222 | 8.10E-08 | -0.250853901 | 3.92E-08 |
| PIEZO2 | turquoise | -0.175866794 | 0.000133374 | -0.16040897 | 0.000501908 |
| PIK3IP1 | turquoise | -0.17639014 | 0.000127265 | -0.174363425 | 0.000152492 |
| PINLYP | turquoise | -0.24811617 | 5.55E-08 | -0.234553654 | 2.95E-07 |
| PITX1 | turquoise | 0.138222022 | 0.002759073 | 0.163912406 | 0.000375445 |
| PIWIL2 | turquoise | -0.1423888 | 0.002038725 | -0.152240447 | 0.000965524 |
| pk | turquoise | -0.024141428 | 0.602799877 | -0.055759997 | 0.229096201 |
| PKD1 | turquoise | -0.152956303 | 0.00091287 | -0.149657455 | 0.001179698 |
| PKD2 | turquoise | -0.144544655 | 0.001737815 | -0.174716283 | 0.000147787 |
| PKIG | turquoise | -0.116857605 | 0.011497262 | -0.099691966 | 0.031243597 |
| PKM | turquoise | 0.20597423 | 7.21E-06 | 0.235098849 | 2.76E-07 |
| PKNOX2 | turquoise | -0.235848894 | 2.52E-07 | -0.229948133 | 5.08E-07 |
| PLA2R1 | turquoise | -0.033799678 | 0.466204807 | -0.056985367 | 0.219010729 |
| PLAC9 | turquoise | -0.226850814 | 7.27E-07 | -0.216128517 | 2.43E-06 |

| | | | | | |
|---|---|---|---|---|---|
| PLAT | turquoise | -0.19134141 | 3.15E-05 | -0.164508605 | 0.000357139 |
| PLCL1 | turquoise | -0.26412867 | 6.80E-09 | -0.261424499 | 9.79E-09 |
| PLEKHG( | turquoise | -0.077442207 | 0.094609877 | -0.084970457 | 0.066561246 |
| PLEKHH. | turquoise | -0.181235735 | 8.19E-05 | -0.193350463 | 2.59E-05 |
| PLIN4 | turquoise | -0.106731305 | 0.021060693 | -0.126550682 | 0.00617254 |
| PLK5 | turquoise | -0.17290591 | 0.000173458 | -0.183944498 | 6.37E-05 |
| PLN | turquoise | -0.152594941 | 0.000939109 | -0.134071329 | 0.003700122 |
| PLOD1 | turquoise | 0.225515954 | 8.48E-07 | 0.208342353 | 5.62E-06 |
| PLP1 | turquoise | -0.142397466 | 0.002037425 | -0.151456691 | 0.001026378 |
| PLSCR4 | turquoise | -0.311149095 | 6.09E-12 | -0.297450588 | 5.39E-11 |
| PLXDC1 | turquoise | -0.075267552 | 0.104270958 | -0.058825926 | 0.204465597 |
| PLXDC2 | turquoise | -0.207921029 | 5.88E-06 | -0.188162849 | 4.28E-05 |
| PMP22 | turquoise | -0.111647388 | 0.015786889 | -0.095007153 | 0.040144027 |
| PNKD | turquoise | -0.054035659 | 0.24383987 | -0.05278421 | 0.254947989 |
| PNMAL2 | turquoise | -0.444485892 | 4.91E-24 | -0.447525969 | 2.22E-24 |
| PODN | turquoise | -0.267994314 | 4.01E-09 | -0.252587736 | 3.13E-08 |
| PODXL2 | turquoise | 0.13704637 | 0.003000621 | 0.112378482 | 0.015110817 |
| POLDIP2 | turquoise | 0.106941452 | 0.020807296 | 0.117656103 | 0.010940314 |
| POMGNT | turquoise | -0.215174429 | 2.70E-06 | -0.210531529 | 4.45E-06 |
| POPDC2 | turquoise | -0.162909647 | 0.000408218 | -0.16096144 | 0.000479636 |
| POR | turquoise | 0.120624326 | 0.009073788 | 0.136942758 | 0.003022806 |
| POSTN | turquoise | -0.105987179 | 0.021979639 | -0.069904293 | 0.131441482 |
| POU6F1 | turquoise | -0.20254443 | 1.03E-05 | -0.217591934 | 2.07E-06 |
| PPAPDC: | turquoise | -0.250579558 | 4.06E-08 | -0.245046145 | 8.17E-08 |
| PPFIA4 | turquoise | 0.14413002 | 0.001792318 | 0.121632259 | 0.008507718 |
| PPM1F | turquoise | -0.170819734 | 0.000208205 | -0.178908322 | 0.000101371 |
| PPM1K | turquoise | -0.234779779 | 2.87E-07 | -0.205645033 | 7.46E-06 |
| PPP1R12l | turquoise | -0.161402538 | 0.000462516 | -0.157643322 | 0.00062858 |
| PPP1R12( | turquoise | -0.250123695 | 4.30E-08 | -0.264727536 | 6.27E-09 |
| PPP1R14 | turquoise | -0.161436416 | 0.000461225 | -0.167648792 | 0.0002737 |
| PPP1R18 | turquoise | -0.137330383 | 0.002940569 | -0.112348779 | 0.015137781 |
| PPP1R3C | turquoise | -0.226451487 | 7.62E-07 | -0.225268223 | 8.73E-07 |
| PRAME | turquoise | 0.13544805 | 0.003359862 | 0.113623165 | 0.014018153 |
| PRDM11 | turquoise | -0.360821591 | 8.33E-16 | -0.363681539 | 4.75E-16 |
| PRDM5 | turquoise | -0.323059045 | 8.34E-13 | -0.336611376 | 7.78E-14 |
| PRDM8 | turquoise | -0.154001662 | 0.00084073 | -0.152739491 | 0.00092853 |
| PRELP | turquoise | -0.170687337 | 0.000210617 | -0.151193297 | 0.001047611 |
| PRICKLE | turquoise | -0.116871627 | 0.01148727 | -0.157784759 | 0.000621442 |
| PRICKLE | turquoise | -0.190094219 | 3.55E-05 | -0.224943348 | 9.06E-07 |
| PRIMA1 | turquoise | -0.36156818 | 7.20E-16 | -0.364846924 | 3.77E-16 |
| PRKAR2l | turquoise | -0.299974495 | 3.64E-11 | -0.298883123 | 4.31E-11 |
| PRKCA | turquoise | -0.016719636 | 0.718569508 | -0.048585449 | 0.29475589 |
| PRKCD | turquoise | 0.057623313 | 0.213887709 | 0.074596375 | 0.107405529 |
| PRKCDB | turquoise | -0.280856378 | 6.50E-10 | -0.244569541 | 8.67E-08 |
| PRKCZ | turquoise | 0.187393849 | 4.60E-05 | 0.188342711 | 4.20E-05 |

| | | | | | |
|---|---|---|---|---|---|
| PRKD1 | turquoise | -0.301343831 | 2.93E-11 | -0.315004869 | 3.23E-12 |
| PRKG1 | turquoise | -0.18624341 | 5.13E-05 | -0.189805056 | 3.65E-05 |
| PRLHR | turquoise | -0.128497652 | 0.005419954 | -0.132751038 | 0.004055462 |
| PRLR | turquoise | -0.010921135 | 0.813912052 | -0.0138705 | 0.764974284 |
| PRND | turquoise | -0.097023836 | 0.036079436 | -0.091852824 | 0.047274553 |
| PRNP | turquoise | -0.436006384 | 4.31E-23 | -0.444305867 | 5.15E-24 |
| PROB1 | turquoise | -0.208110053 | 5.76E-06 | -0.192684071 | 2.76E-05 |
| PROCR | turquoise | -0.222940467 | 1.14E-06 | -0.199947453 | 1.34E-05 |
| PROS1 | turquoise | -0.295804796 | 6.95E-11 | -0.294057671 | 9.08E-11 |
| PROSER2 | turquoise | 0.191895404 | 2.98E-05 | 0.195641829 | 2.07E-05 |
| PRPH2 | turquoise | -0.161826341 | 0.000446605 | -0.175997734 | 0.00013182 |
| PRR16 | turquoise | 0.02566672 | 0.580080465 | 0.031655593 | 0.494972865 |
| PRR36 | turquoise | 0.265469078 | 5.67E-09 | 0.257910359 | 1.56E-08 |
| PRR7 | turquoise | 0.100570688 | 0.029777418 | 0.101041196 | 0.029016886 |
| PRRG3 | turquoise | -0.343987695 | 2.04E-14 | -0.346950805 | 1.18E-14 |
| PRRT1 | turquoise | -0.072417952 | 0.118094082 | -0.072871159 | 0.115804483 |
| PRRT2 | turquoise | -0.146871769 | 0.001459108 | -0.143647487 | 0.001857717 |
| PRRX1 | turquoise | 0.126680801 | 0.006119462 | 0.137295709 | 0.002947842 |
| PRSS23 | turquoise | -0.05461196 | 0.238840145 | -0.061364927 | 0.185569075 |
| PRSS35 | turquoise | -0.184459041 | 6.07E-05 | -0.172427795 | 0.000180904 |
| PRUNE2 | turquoise | -0.232765713 | 3.64E-07 | -0.22759963 | 6.67E-07 |
| PRX | turquoise | -0.222447452 | 1.20E-06 | -0.241080912 | 1.34E-07 |
| PSD | turquoise | -0.294246478 | 8.83E-11 | -0.28894534 | 1.97E-10 |
| PTCH1 | turquoise | 0.049165233 | 0.289024702 | 0.028617023 | 0.537308537 |
| PTCH2 | turquoise | 0.020531034 | 0.658100668 | 0.024544181 | 0.596762114 |
| PTCHD1 | turquoise | -0.112966337 | 0.014585774 | -0.119716893 | 0.009611833 |
| PTGDR | turquoise | -0.197909393 | 1.65E-05 | -0.162137612 | 0.000435244 |
| PTGDR2 | turquoise | -0.1218044 | 0.008414253 | -0.129995197 | 0.004898415 |
| PTGDS | turquoise | -0.190969311 | 3.27E-05 | -0.1666824 | 0.000297206 |
| PTGER2 | turquoise | -0.074210613 | 0.109240528 | -0.061105811 | 0.187436401 |
| PTGER3 | turquoise | -0.173756048 | 0.000160922 | -0.17800645 | 0.000110015 |
| PTGES3L | turquoise | -0.11373882 | 0.013920243 | -0.101395712 | 0.028454909 |
| PTGFR | turquoise | -0.184536888 | 6.03E-05 | -0.1859397 | 5.28E-05 |
| PTGIS | turquoise | -0.257545874 | 1.64E-08 | -0.245541734 | 7.68E-08 |
| PTH1R | turquoise | -0.259267505 | 1.31E-08 | -0.258850729 | 1.38E-08 |
| PTN | turquoise | -0.146476391 | 0.001503359 | -0.152656202 | 0.000934612 |
| PTPN21 | turquoise | -0.171062513 | 0.00020385 | -0.192329237 | 2.86E-05 |
| PTPN6 | turquoise | 0.139422553 | 0.002530808 | 0.174901405 | 0.000145373 |
| PTPRD | turquoise | -0.022846315 | 0.622398292 | -0.012579438 | 0.786294017 |
| PTPRN2 | turquoise | -0.196630247 | 1.87E-05 | -0.188678028 | 4.07E-05 |
| PTPRO | turquoise | -0.20813595 | 5.74E-06 | -0.197160598 | 1.78E-05 |
| PTPRR | turquoise | 0.005195506 | 0.910842671 | 0.012222434 | 0.792218379 |
| PTRF | turquoise | -0.191089757 | 3.23E-05 | -0.165399405 | 0.000331332 |
| PURG | turquoise | -0.275746771 | 1.35E-09 | -0.285601355 | 3.24E-10 |
| PYCR1 | turquoise | 0.296484622 | 6.26E-11 | 0.282974416 | 4.77E-10 |

| | | | | | |
|---|---|---|---|---|---|
| PYGM | turquoise | -0.261621759 | 9.54E-09 | -0.256405782 | 1.91E-08 |
| RAB11FI | turquoise | -0.040534514 | 0.382134621 | -0.032554036 | 0.482804311 |
| RAB11FI | turquoise | -0.267969093 | 4.02E-09 | -0.269905125 | 3.08E-09 |
| RAB17 | turquoise | 0.111192945 | 0.01622037 | 0.128889437 | 0.005278891 |
| RAB20 | turquoise | 0.209105675 | 5.18E-06 | 0.239220285 | 1.68E-07 |
| RAB23 | turquoise | -0.110938283 | 0.016467815 | -0.127017701 | 0.005983932 |
| RAB31 | turquoise | -0.295845696 | 6.90E-11 | -0.258140869 | 1.52E-08 |
| RAB3C | turquoise | -0.051829976 | 0.263650294 | -0.058821844 | 0.204497054 |
| RAB3IL1 | turquoise | -0.351723362 | 4.80E-15 | -0.343504432 | 2.23E-14 |
| RAB41 | turquoise | -0.170164503 | 0.000220399 | -0.168854043 | 0.000246817 |
| RAB6C | turquoise | -0.198048777 | 1.62E-05 | -0.204244038 | 8.63E-06 |
| RAB9B | turquoise | -0.258605805 | 1.43E-08 | -0.264987322 | 6.05E-09 |
| RADIL | turquoise | -0.241753739 | 1.23E-07 | -0.257960423 | 1.55E-08 |
| RAMP1 | turquoise | -0.179717385 | 9.42E-05 | -0.158577575 | 0.000582802 |
| RAPGEF4 | turquoise | -0.14498108 | 0.001682091 | -0.165187299 | 0.000337314 |
| RARRES: | turquoise | -0.313419079 | 4.20E-12 | -0.275166066 | 1.47E-09 |
| RASA3 | turquoise | -0.247861359 | 5.73E-08 | -0.249848863 | 4.45E-08 |
| RASA4 | turquoise | -0.261847168 | 9.25E-09 | -0.259657893 | 1.24E-08 |
| RASA4B | turquoise | -0.153495408 | 0.000874981 | -0.167340195 | 0.000281011 |
| RASD2 | turquoise | -0.081948451 | 0.076868582 | -0.070063265 | 0.130564221 |
| RASGRF: | turquoise | -0.054763989 | 0.237533313 | -0.067107191 | 0.147632496 |
| RASL11A | turquoise | -0.083893694 | 0.07009542 | -0.07648141 | 0.098786279 |
| RASL12 | turquoise | -0.246776769 | 6.58E-08 | -0.22925021 | 5.51E-07 |
| RASSF2 | turquoise | -0.170787327 | 0.000208793 | -0.184879696 | 5.84E-05 |
| RASSF8 | turquoise | -0.274706852 | 1.57E-09 | -0.277709582 | 1.02E-09 |
| RBFOX2 | turquoise | -0.089363656 | 0.053625168 | -0.102508089 | 0.026751837 |
| RBFOX3 | turquoise | -0.246982618 | 6.41E-08 | -0.243056228 | 1.05E-07 |
| RBM20 | turquoise | -0.118933515 | 0.010098932 | -0.113602612 | 0.014035616 |
| RBM24 | turquoise | -0.046480227 | 0.316200608 | -0.055720954 | 0.229422873 |
| RBM47 | turquoise | 0.255225017 | 2.22E-08 | 0.281881544 | 5.60E-10 |
| RBMS3 | turquoise | -0.212813238 | 3.49E-06 | -0.207819997 | 5.94E-06 |
| RBP7 | turquoise | -0.21200961 | 3.80E-06 | -0.238478503 | 1.84E-07 |
| RBPMS2 | turquoise | -0.014635545 | 0.752422984 | -0.013375238 | 0.773132716 |
| RCAN2 | turquoise | -0.214372252 | 2.94E-06 | -0.222645863 | 1.18E-06 |
| RCBTB2 | turquoise | -0.28909902 | 1.93E-10 | -0.293019773 | 1.06E-10 |
| RECK | turquoise | -0.294713659 | 8.22E-11 | -0.293269277 | 1.02E-10 |
| REEP1 | turquoise | -0.128110706 | 0.005562596 | -0.135600055 | 0.00332409 |
| REM1 | turquoise | -0.286812921 | 2.71E-10 | -0.271228005 | 2.56E-09 |
| RER1 | turquoise | 0.097830162 | 0.034555087 | 0.104609652 | 0.023772804 |
| RERG | turquoise | -0.284118704 | 4.03E-10 | -0.279929333 | 7.43E-10 |
| RERGL | turquoise | -0.206602605 | 6.75E-06 | -0.220181194 | 1.55E-06 |
| RFTN2 | turquoise | -0.257111574 | 1.74E-08 | -0.240815186 | 1.38E-07 |
| RGAG4 | turquoise | -0.26467439 | 6.31E-09 | -0.266371194 | 5.01E-09 |
| RGL1 | turquoise | 0.063094665 | 0.173453756 | 0.035096492 | 0.449264285 |
| RGMA | turquoise | -0.252377624 | 3.22E-08 | -0.250842508 | 3.92E-08 |

| | | | | | |
|---|---|---|---|---|---|
| RGN | turquoise | -0.383702851 | 7.90E-18 | -0.389065129 | 2.51E-18 |
| RGS2 | turquoise | -0.149499075 | 0.001194158 | -0.148177685 | 0.001321313 |
| RGS4 | turquoise | -0.210243558 | 4.59E-06 | -0.177855909 | 0.000111523 |
| RGS9 | turquoise | -0.339089219 | 4.98E-14 | -0.364923755 | 3.71E-16 |
| RHOB | turquoise | 0.026304113 | 0.570706427 | 0.003042928 | 0.947710497 |
| RHOBTB | turquoise | 0.039115944 | 0.399027068 | 0.01727863 | 0.70957907 |
| RHOV | turquoise | 0.211072892 | 4.20E-06 | 0.214608045 | 2.87E-06 |
| RHPN2 | turquoise | -0.010603441 | 0.819230642 | -0.019273452 | 0.677830658 |
| RIC3 | turquoise | -0.376004803 | 3.95E-17 | -0.38232649 | 1.06E-17 |
| RILPL1 | turquoise | -0.176682259 | 0.00012397 | -0.182331339 | 7.40E-05 |
| RIMS3 | turquoise | -0.275937688 | 1.32E-09 | -0.301598657 | 2.82E-11 |
| RITA1 | turquoise | 0.111116232 | 0.016294564 | 0.124611382 | 0.007014098 |
| RNASE4 | turquoise | 0.001616492 | 0.97220805 | -0.011713686 | 0.800681528 |
| RNF112 | turquoise | -0.293004349 | 1.07E-10 | -0.292250156 | 1.20E-10 |
| RNF144A | turquoise | -0.268127177 | 3.94E-09 | -0.288191188 | 2.21E-10 |
| RORB | turquoise | -0.202766589 | 1.01E-05 | -0.194190101 | 2.38E-05 |
| RP11-136 | turquoise | -0.182333721 | 7.40E-05 | -0.202620758 | 1.02E-05 |
| RP11-20J | turquoise | -0.240072792 | 1.51E-07 | -0.244139215 | 9.15E-08 |
| RP11-216 | turquoise | -0.075470479 | 0.103337634 | -0.089790614 | 0.052487972 |
| RP11-244 | turquoise | -0.150120574 | 0.001138336 | -0.158815974 | 0.000571627 |
| RP11-903 | turquoise | -0.062426007 | 0.178065322 | -0.090188462 | 0.051446467 |
| RP6-24A2 | turquoise | -0.144958274 | 0.001684962 | -0.151865231 | 0.000994229 |
| RPN1 | turquoise | -0.028207797 | 0.543145683 | 0.000924966 | 0.984095136 |
| RPRM | turquoise | -0.232427686 | 3.79E-07 | -0.227795081 | 6.52E-07 |
| RPS6KA1 | turquoise | 0.377502423 | 2.89E-17 | 0.395184316 | 6.61E-19 |
| RPS6KA2 | turquoise | -0.127105135 | 0.005949202 | -0.123332682 | 0.007623772 |
| RPS6KA6 | turquoise | -0.454773805 | 3.24E-25 | -0.458775499 | 1.10E-25 |
| RSPO1 | turquoise | -0.257975954 | 1.55E-08 | -0.258438405 | 1.46E-08 |
| RSPO3 | turquoise | -0.340717269 | 3.71E-14 | -0.345273534 | 1.61E-14 |
| RTKN | turquoise | 0.125295969 | 0.006705962 | 0.128805636 | 0.005308783 |
| RTN4IP1 | turquoise | 0.236478491 | 2.34E-07 | 0.256741596 | 1.82E-08 |
| RUNDC3 | turquoise | -0.112470707 | 0.015027364 | -0.107625208 | 0.020001057 |
| RUNX1T | turquoise | -0.218660475 | 1.84E-06 | -0.213924755 | 3.09E-06 |
| RUSC2 | turquoise | -0.234621076 | 2.92E-07 | -0.231506122 | 4.23E-07 |
| RYR2 | turquoise | -0.124459348 | 0.007084226 | -0.097442727 | 0.035280542 |
| RYR3 | turquoise | -0.177403026 | 0.00011618 | -0.192417014 | 2.84E-05 |
| S100A11 | turquoise | 0.282345446 | 5.23E-10 | 0.307088289 | 1.18E-11 |
| S1PR3 | turquoise | -0.140006497 | 0.002426124 | -0.141848979 | 0.002121186 |
| SAMD10 | turquoise | 0.110750431 | 0.016652456 | 0.122353575 | 0.008122162 |
| SAMD4A | turquoise | -0.355137554 | 2.50E-15 | -0.352540652 | 4.11E-15 |
| SAMD5 | turquoise | -0.279342103 | 8.09E-10 | -0.263080865 | 7.83E-09 |
| SAP30L | turquoise | -0.199871856 | 1.35E-05 | -0.215325177 | 2.65E-06 |
| SATB1 | turquoise | -0.104875719 | 0.023416916 | -0.095879081 | 0.038341477 |
| SBK1 | turquoise | 0.18638311 | 5.07E-05 | 0.196836736 | 1.83E-05 |
| SCARA3 | turquoise | -0.078904519 | 0.088525463 | -0.089436776 | 0.053428971 |

| | | | | | |
|---|---|---|---|---|---|
| SCARA5 | turquoise | -0.144208875 | 0.001781834 | -0.153733106 | 0.000858742 |
| SCARF2 | turquoise | -0.234683008 | 2.90E-07 | -0.213978507 | 3.07E-06 |
| SCN1B | turquoise | -0.208733969 | 5.39E-06 | -0.20400438 | 8.85E-06 |
| SCN2B | turquoise | -0.305739021 | 1.46E-11 | -0.315968468 | 2.75E-12 |
| SCN3B | turquoise | -0.274512942 | 1.61E-09 | -0.277609636 | 1.04E-09 |
| SCN4B | turquoise | -0.097061308 | 0.03600735 | -0.117877702 | 0.010790047 |
| SCN7A | turquoise | -0.145677089 | 0.001596604 | -0.144246172 | 0.001776894 |
| SCRG1 | turquoise | -0.136162871 | 0.00319463 | -0.124967162 | 0.006852413 |
| SDC1 | turquoise | 0.321941139 | 1.01E-12 | 0.321915581 | 1.01E-12 |
| SDPR | turquoise | -0.286311392 | 2.92E-10 | -0.275493265 | 1.40E-09 |
| SEC61A1 | turquoise | 0.253086167 | 2.94E-08 | 0.261034346 | 1.03E-08 |
| SEL1L2 | turquoise | -0.173057699 | 0.000171154 | -0.179959121 | 9.21E-05 |
| SELE | turquoise | -0.08680591 | 0.060874802 | -0.090570013 | 0.050463879 |
| SELM | turquoise | -0.071795751 | 0.121294901 | -0.061015122 | 0.188093208 |
| SEMA4A | turquoise | 0.101898993 | 0.027673175 | 0.122514873 | 0.008038108 |
| SEMA5A | turquoise | -0.075301793 | 0.104113006 | -0.063877446 | 0.168168843 |
| SEPHS2 | turquoise | 0.133710896 | 0.003794217 | 0.153948985 | 0.000844235 |
| SEPT4 | turquoise | 0.026623395 | 0.566037981 | 0.043518089 | 0.348060396 |
| SERP2 | turquoise | -0.381788913 | 1.18E-17 | -0.388964714 | 2.57E-18 |
| SERPINF | turquoise | -0.144036583 | 0.001804815 | -0.122109769 | 0.0082507 |
| SERPING | turquoise | -0.328407672 | 3.32E-13 | -0.308787716 | 8.95E-12 |
| SERTAD4 | turquoise | 0.040052622 | 0.387823369 | 0.002513959 | 0.956790471 |
| SERTM1 | turquoise | -0.035635222 | 0.442330418 | -0.061645084 | 0.183565579 |
| SETBP1 | turquoise | -0.245280219 | 7.94E-08 | -0.257849476 | 1.58E-08 |
| SETD7 | turquoise | -0.076748458 | 0.097611013 | -0.092987766 | 0.04459542 |
| SFN | turquoise | 0.388241285 | 3.00E-18 | 0.398431461 | 3.22E-19 |
| SFRP1 | turquoise | -0.288038312 | 2.26E-10 | -0.29699671 | 5.78E-11 |
| SFRP4 | turquoise | -0.139877013 | 0.00244899 | -0.110430744 | 0.016970845 |
| SGCA | turquoise | -0.116173545 | 0.011994188 | -0.097174571 | 0.035790207 |
| SGCE | turquoise | -0.265774696 | 5.43E-09 | -0.272417642 | 2.17E-09 |
| SGIP1 | turquoise | -0.179874013 | 9.28E-05 | -0.176586043 | 0.000125046 |
| SH3BGRL | turquoise | 0.007791675 | 0.866637699 | -0.015189505 | 0.743375071 |
| SH3BP5 | turquoise | -0.198177097 | 1.60E-05 | -0.227561019 | 6.70E-07 |
| SH3PXD2 | turquoise | -0.179651702 | 9.47E-05 | -0.177595199 | 0.000114182 |
| SHC2 | turquoise | -0.14326157 | 0.001911585 | -0.138896595 | 0.002628605 |
| SHF | turquoise | -0.186822441 | 4.86E-05 | -0.201077045 | 1.20E-05 |
| SHMT1 | turquoise | 0.245930565 | 7.31E-08 | 0.235104455 | 2.76E-07 |
| SIDT2 | turquoise | -0.163487934 | 0.000389006 | -0.163862764 | 0.000377008 |
| SIGLEC6 | turquoise | -0.191778743 | 3.02E-05 | -0.175022056 | 0.00014382 |
| SLC10A6 | turquoise | -0.014671204 | 0.75183951 | -0.023703638 | 0.609393804 |
| SLC12A4 | turquoise | -0.041060209 | 0.375987278 | -0.023598897 | 0.610976115 |
| SLC12A8 | turquoise | 0.099695875 | 0.031236941 | 0.097858318 | 0.034502864 |
| SLC16A2 | turquoise | -0.203789113 | 9.05E-06 | -0.199883552 | 1.35E-05 |
| SLC16A3 | turquoise | 0.060023565 | 0.19538521 | 0.099945501 | 0.030814359 |
| SLC16A7 | turquoise | -0.270154511 | 2.97E-09 | -0.260058893 | 1.18E-08 |

| | | | | | |
|---|---|---|---|---|---|
| SLC1A7 | turquoise | -0.198091629 | 1.62E-05 | -0.206266195 | 6.99E-06 |
| SLC22A1 | turquoise | -0.342028004 | 2.92E-14 | -0.36350802 | 4.91E-16 |
| SLC22A3 | turquoise | -0.258769133 | 1.40E-08 | -0.266954658 | 4.62E-09 |
| SLC23A2 | turquoise | -0.304672141 | 1.73E-11 | -0.318594638 | 1.78E-12 |
| SLC24A3 | turquoise | -0.234607825 | 2.93E-07 | -0.239629854 | 1.60E-07 |
| SLC25A2 | turquoise | 0.212971116 | 3.43E-06 | 0.22876583 | 5.83E-07 |
| SLC25A4 | turquoise | -0.202092693 | 1.08E-05 | -0.215218182 | 2.69E-06 |
| SLC26A1 | turquoise | -0.074834561 | 0.106284734 | -0.100600484 | 0.029728755 |
| SLC26A6 | turquoise | 0.237185094 | 2.15E-07 | 0.221197545 | 1.38E-06 |
| SLC2A4 | turquoise | -0.339650153 | 4.50E-14 | -0.347943357 | 9.77E-15 |
| SLC2A6 | turquoise | 0.080246841 | 0.083220872 | 0.083480786 | 0.071490762 |
| SLC35F1 | turquoise | -0.195953472 | 2.00E-05 | -0.214188097 | 3.00E-06 |
| SLC35G2 | turquoise | -0.208331309 | 5.63E-06 | -0.207078818 | 6.42E-06 |
| SLC38A7 | turquoise | 0.306472342 | 1.30E-11 | 0.325236865 | 5.74E-13 |
| SLC39A1 | turquoise | 0.255598274 | 2.12E-08 | 0.259524456 | 1.26E-08 |
| SLC45A1 | turquoise | -0.241609068 | 1.25E-07 | -0.24598863 | 7.26E-08 |
| SLC50A1 | turquoise | 0.166582046 | 0.000299753 | 0.177585888 | 0.000114278 |
| SLC51B | turquoise | -0.094242929 | 0.041782341 | -0.079493201 | 0.086166586 |
| SLC6A1 | turquoise | 0.019182698 | 0.679263248 | 0.028987165 | 0.532056073 |
| SLC7A3 | turquoise | -0.16568698 | 0.000323379 | -0.14863062 | 0.001276388 |
| SLC7A5 | turquoise | 0.359066884 | 1.17E-15 | 0.345318494 | 1.59E-14 |
| SLC8A1 | turquoise | -0.39600899 | 5.51E-19 | -0.414176077 | 8.80E-21 |
| SLC8A2 | turquoise | -0.16792288 | 0.000267357 | -0.169117563 | 0.000241277 |
| SLC9B2 | turquoise | -0.044768095 | 0.334375523 | -0.074384878 | 0.108408547 |
| SLIT3 | turquoise | -0.198613381 | 1.53E-05 | -0.22207493 | 1.25E-06 |
| SLITRK3 | turquoise | -0.188691677 | 4.07E-05 | -0.196874535 | 1.83E-05 |
| SMAD9 | turquoise | 0.091296726 | 0.048635752 | 0.062710273 | 0.176093821 |
| SMARCA | turquoise | 0.12852484 | 0.005410057 | 0.126190966 | 0.006321432 |
| SMIM10 | turquoise | -0.406579216 | 5.12E-20 | -0.418369123 | 3.26E-21 |
| SMOC2 | turquoise | -0.108933809 | 0.018533572 | -0.121573215 | 0.008539989 |
| SMPDL3 | turquoise | -0.115614564 | 0.012414245 | -0.10309519 | 0.025888884 |
| SMTN | turquoise | -0.132922683 | 0.004007583 | -0.132369142 | 0.004163852 |
| SNAI2 | turquoise | -0.046376406 | 0.31728394 | -0.061507703 | 0.184546032 |
| SNAP25 | turquoise | -0.37304561 | 7.24E-17 | -0.379896073 | 1.76E-17 |
| SNCA | turquoise | -0.352495316 | 4.15E-15 | -0.357680476 | 1.53E-15 |
| SNCAIP | turquoise | -0.130600369 | 0.004700821 | -0.139753369 | 0.002471008 |
| SNED1 | turquoise | -0.238734301 | 1.78E-07 | -0.246752866 | 6.60E-08 |
| SNPH | turquoise | -0.128598641 | 0.005383272 | -0.146537638 | 0.001496425 |
| SNTG2 | turquoise | -0.21307863 | 3.39E-06 | -0.190432693 | 3.44E-05 |
| SNX21 | turquoise | -0.105877928 | 0.022117442 | -0.122359307 | 0.008119161 |
| SNX33 | turquoise | 0.032663434 | 0.481333757 | 0.045868797 | 0.322615544 |
| SOBP | turquoise | -0.153733035 | 0.000858747 | -0.165130262 | 0.00033894 |
| SOCS2 | turquoise | -0.170726192 | 0.000209906 | -0.172559732 | 0.00017882 |
| SORBS1 | turquoise | -0.184368328 | 6.12E-05 | -0.202594757 | 1.02E-05 |
| SORBS3 | turquoise | -0.081092758 | 0.080011912 | -0.070093871 | 0.130395844 |

| | | | | | |
|---|---|---|---|---|---|
| SORCS1 | turquoise | -0.16693965 | 0.000290771 | -0.168520485 | 0.000253998 |
| SOX15 | turquoise | -0.227206242 | 6.98E-07 | -0.225232029 | 8.76E-07 |
| SOX7 | turquoise | -0.074160626 | 0.109480103 | -0.091711306 | 0.04761789 |
| SPAG4 | turquoise | 0.212809457 | 3.49E-06 | 0.235546284 | 2.62E-07 |
| SPARCL1 | turquoise | -0.187444014 | 4.58E-05 | -0.183379975 | 6.72E-05 |
| SPEG | turquoise | -0.397900566 | 3.63E-19 | -0.42572816 | 5.53E-22 |
| SPP1 | turquoise | 0.072834499 | 0.115988389 | 0.064084177 | 0.166793429 |
| SPSB1 | turquoise | -0.218171138 | 1.94E-06 | -0.192984188 | 2.68E-05 |
| SPTBN2 | turquoise | 0.043698392 | 0.346064854 | 0.043136652 | 0.352306003 |
| SPTBN4 | turquoise | -0.258086353 | 1.53E-08 | -0.264632787 | 6.35E-09 |
| SREBF1 | turquoise | 0.44301622 | 7.19E-24 | 0.450896686 | 9.13E-25 |
| SRF | turquoise | -0.188703849 | 4.06E-05 | -0.187241529 | 4.67E-05 |
| SRL | turquoise | -0.232911468 | 3.58E-07 | -0.211273713 | 4.11E-06 |
| SRPRB | turquoise | 0.240168498 | 1.49E-07 | 0.258722231 | 1.40E-08 |
| SRPX | turquoise | -0.339672765 | 4.48E-14 | -0.327692975 | 3.76E-13 |
| SSBP2 | turquoise | -0.150815276 | 0.001078792 | -0.127414003 | 0.00582796 |
| SSC5D | turquoise | -0.187697281 | 4.47E-05 | -0.15239594 | 0.000953854 |
| SSPN | turquoise | -0.359432572 | 1.09E-15 | -0.342762225 | 2.55E-14 |
| SSTR1 | turquoise | -0.327406362 | 3.95E-13 | -0.335255876 | 9.92E-14 |
| SSTR3 | turquoise | -0.202087305 | 1.08E-05 | -0.195511732 | 2.09E-05 |
| ST3GAL2 | turquoise | -0.161958484 | 0.000441749 | -0.16323456 | 0.000397318 |
| ST3GAL3 | turquoise | -0.23807144 | 1.93E-07 | -0.251254991 | 3.72E-08 |
| ST3GAL5 | turquoise | -0.158070676 | 0.000607241 | -0.15333086 | 0.000886389 |
| ST5 | turquoise | -0.13519326 | 0.003420608 | -0.153440776 | 0.000878754 |
| ST6GAL2 | turquoise | -0.135010166 | 0.003464874 | -0.1488444 | 0.001255677 |
| ST6GALN | turquoise | -0.263808514 | 7.10E-09 | -0.28215776 | 5.38E-10 |
| ST6GALN | turquoise | -0.256863911 | 1.79E-08 | -0.236794036 | 2.25E-07 |
| ST8SIA1 | turquoise | -0.278138963 | 9.62E-10 | -0.258814418 | 1.39E-08 |
| STAB2 | turquoise | -0.152696525 | 0.000931663 | -0.164417454 | 0.000359883 |
| STARD13 | turquoise | -0.045977518 | 0.321468739 | -0.056803239 | 0.220489291 |
| STARD5 | turquoise | -0.014979669 | 0.746798273 | -0.026172634 | 0.572634164 |
| STARD9 | turquoise | -0.21314066 | 3.37E-06 | -0.227513676 | 6.74E-07 |
| STAT5B | turquoise | -0.09023726 | 0.051319916 | -0.102805442 | 0.026311725 |
| STC2 | turquoise | 0.123438821 | 0.007571421 | 0.107991593 | 0.01958033 |
| STEAP4 | turquoise | -0.051325978 | 0.268328106 | -0.055675727 | 0.229801699 |
| STMN3 | turquoise | -0.212095554 | 3.77E-06 | -0.220141819 | 1.56E-06 |
| STON1 | turquoise | -0.375230337 | 4.63E-17 | -0.380471074 | 1.56E-17 |
| STON1-G | turquoise | -0.178775033 | 0.000102607 | -0.174503421 | 0.000150609 |
| STX1B | turquoise | -0.293133607 | 1.05E-10 | -0.29296203 | 1.07E-10 |
| STX2 | turquoise | -0.103891502 | 0.02475683 | -0.120140568 | 0.009357192 |
| STXBP1 | turquoise | -0.252355329 | 3.23E-08 | -0.293215716 | 1.03E-10 |
| SULF2 | turquoise | -0.097158794 | 0.035820387 | -0.092901586 | 0.044794275 |
| SUSD5 | turquoise | -0.249207723 | 4.83E-08 | -0.2517496 | 3.49E-08 |
| SVEP1 | turquoise | -0.134964672 | 0.003475953 | -0.116963332 | 0.011422106 |
| SVIL | turquoise | -0.179136634 | 9.93E-05 | -0.19564275 | 2.06E-05 |

| | | | | | |
|---|---|---|---|---|---|
| SYK | turquoise | 0.222516968 | 1.19E-06 | 0.230665866 | 4.67E-07 |
| SYNC | turquoise | -0.179167102 | 9.90E-05 | -0.174245962 | 0.000154089 |
| SYNE1 | turquoise | -0.269298091 | 3.35E-09 | -0.256824126 | 1.80E-08 |
| SYNE3 | turquoise | -0.202842336 | 9.97E-06 | -0.19388681 | 2.46E-05 |
| SYNM | turquoise | -0.196661241 | 1.87E-05 | -0.208848176 | 5.33E-06 |
| SYNPO | turquoise | -0.241282618 | 1.30E-07 | -0.189807493 | 3.65E-05 |
| SYNPO2 | turquoise | -0.177967932 | 0.000110399 | -0.162596237 | 0.000418995 |
| SYPL2 | turquoise | -0.099715396 | 0.031203716 | -0.098865333 | 0.032678795 |
| SYT1 | turquoise | -0.124213768 | 0.007198829 | -0.148463839 | 0.001292765 |
| SYT15 | turquoise | -0.245446931 | 7.77E-08 | -0.24847078 | 5.31E-08 |
| SYT9 | turquoise | -0.256002771 | 2.01E-08 | -0.26239073 | 8.60E-09 |
| SYTL4 | turquoise | -0.102683205 | 0.026491887 | -0.119340685 | 0.009843089 |
| SYTL5 | turquoise | -0.123549817 | 0.007517018 | -0.13181817 | 0.004324848 |
| TACC1 | turquoise | -0.143799155 | 0.001836929 | -0.159949499 | 0.00052116 |
| TACR2 | turquoise | -0.145921741 | 0.001567515 | -0.150207004 | 0.001130767 |
| TAGLN | turquoise | -0.14092844 | 0.002268865 | -0.106801945 | 0.020975218 |
| TAL1 | turquoise | -0.21495426 | 2.76E-06 | -0.217146191 | 2.17E-06 |
| TALDO1 | turquoise | 0.277854613 | 1.00E-09 | 0.299352556 | 4.01E-11 |
| TARBP2 | turquoise | -0.117530702 | 0.011026167 | -0.116659775 | 0.011639067 |
| TBC1D1 | turquoise | -0.0931958 | 0.04411845 | -0.101031271 | 0.029032754 |
| TBC1D2E | turquoise | -0.169697898 | 0.000229486 | -0.173446928 | 0.000165378 |
| TBC1D7 | turquoise | 0.124492399 | 0.007068928 | 0.143221498 | 0.00191726 |
| TBL1X | turquoise | -0.142644469 | 0.002000702 | -0.162008675 | 0.000439917 |
| TBX2 | turquoise | -0.159719038 | 0.000531073 | -0.129866507 | 0.004941386 |
| TBX3 | turquoise | -0.177633714 | 0.000113786 | -0.182338833 | 7.40E-05 |
| TCAF2 | turquoise | 0.02282505 | 0.622722376 | 0.020001737 | 0.66637681 |
| TCEAL1 | turquoise | -0.104279042 | 0.024221531 | -0.128590329 | 0.005386283 |
| TCEAL2 | turquoise | -0.219308199 | 1.71E-06 | -0.237538929 | 2.06E-07 |
| TCEAL3 | turquoise | -0.135733113 | 0.003293061 | -0.131129698 | 0.004533919 |
| TCEAL4 | turquoise | -0.154840093 | 0.00078672 | -0.16902354 | 0.00024324 |
| TCEAL5 | turquoise | -0.429214628 | 2.35E-22 | -0.43928163 | 1.88E-23 |
| TCEAL6 | turquoise | -0.186316436 | 5.10E-05 | -0.195254603 | 2.15E-05 |
| TCEAL7 | turquoise | -0.257604236 | 1.63E-08 | -0.247468552 | 6.03E-08 |
| TCF21 | turquoise | -0.159139433 | 0.000556782 | -0.160514275 | 0.00049759 |
| TCF23 | turquoise | -0.227222808 | 6.97E-07 | -0.228107338 | 6.29E-07 |
| TCF4 | turquoise | -0.191959944 | 2.97E-05 | -0.179203774 | 9.87E-05 |
| TCF7L2 | turquoise | -0.197870467 | 1.65E-05 | -0.193816407 | 2.47E-05 |
| TDRD10 | turquoise | -0.202046567 | 1.08E-05 | -0.217322758 | 2.13E-06 |
| TEAD3 | turquoise | -0.210524393 | 4.46E-06 | -0.220744206 | 1.46E-06 |
| TEF | turquoise | -0.16625242 | 0.00030826 | -0.177003936 | 0.000120435 |
| TEX15 | turquoise | -0.223083855 | 1.12E-06 | -0.227760842 | 6.55E-07 |
| TFAP2A | turquoise | 0.248818881 | 5.08E-08 | 0.229167017 | 5.56E-07 |
| TFPI | turquoise | -0.071566072 | 0.122493403 | -0.06847474 | 0.139536142 |
| TGFB1I1 | turquoise | -0.155016467 | 0.000775775 | -0.125627106 | 0.006561314 |
| TGFB3 | turquoise | -0.03992019 | 0.389395707 | -0.047111251 | 0.309668199 |

| | | | | | |
|---|---|---|---|---|---|
| TGFBR3 | turquoise | -0.27998077 | 7.38E-10 | -0.288344691 | 2.16E-10 |
| TGM2 | turquoise | -0.204213419 | 8.66E-06 | -0.176100789 | 0.000130609 |
| THBS4 | turquoise | -0.103258126 | 0.025653686 | -0.088121407 | 0.057051051 |
| THPO | turquoise | -0.220004634 | 1.58E-06 | -0.213004305 | 3.42E-06 |
| THRA | turquoise | -0.179149136 | 9.92E-05 | -0.195311844 | 2.13E-05 |
| THRB | turquoise | -0.388006706 | 3.15E-18 | -0.393438202 | 9.71E-19 |
| TIAM1 | turquoise | -0.307855942 | 1.04E-11 | -0.311913456 | 5.38E-12 |
| TIMP2 | turquoise | -0.334886311 | 1.06E-13 | -0.311397887 | 5.85E-12 |
| TIMP3 | turquoise | -0.229426164 | 5.40E-07 | -0.227981168 | 6.38E-07 |
| TK2 | turquoise | 0.134177989 | 0.003672685 | 0.138636833 | 0.002678165 |
| TKT | turquoise | 0.206757275 | 6.64E-06 | 0.224672147 | 9.34E-07 |
| TLCD1 | turquoise | 0.15313463 | 0.000900172 | 0.175597535 | 0.000136624 |
| TLE4 | turquoise | -0.319174355 | 1.61E-12 | -0.290303233 | 1.61E-10 |
| TLL1 | turquoise | -0.193929224 | 2.45E-05 | -0.189573152 | 3.74E-05 |
| TLN1 | turquoise | -0.100858829 | 0.029309664 | -0.090588297 | 0.050417188 |
| TM7SF2 | turquoise | 0.166386286 | 0.000304778 | 0.173827679 | 0.000159906 |
| TMC6 | turquoise | -0.07685941 | 0.097126006 | -0.044972144 | 0.332174897 |
| TMEM11 | turquoise | -0.196909046 | 1.82E-05 | -0.153697479 | 0.000861158 |
| TMEM13 | turquoise | 0.323469545 | 7.78E-13 | 0.318595198 | 1.78E-12 |
| TMEM13 | turquoise | -0.309567761 | 7.88E-12 | -0.323653339 | 7.54E-13 |
| TMEM15 | turquoise | -0.004957683 | 0.914907998 | -0.005858246 | 0.899526463 |
| TMEM17 | turquoise | 0.110032643 | 0.017374765 | 0.093059595 | 0.044430246 |
| TMEM19 | turquoise | -0.165559239 | 0.00032689 | -0.169906542 | 0.00022538 |
| TMEM20 | turquoise | -0.226850779 | 7.27E-07 | -0.2117005 | 3.93E-06 |
| TMEM20 | turquoise | -0.192376827 | 2.85E-05 | -0.187009066 | 4.77E-05 |
| TMEM21 | turquoise | -0.125843189 | 0.006468438 | -0.125622122 | 0.00656347 |
| TMEM22 | turquoise | 0.103799359 | 0.024885597 | 0.102527232 | 0.026723313 |
| TMEM23 | turquoise | 0.237806248 | 1.99E-07 | 0.264425178 | 6.53E-09 |
| TMEM24 | turquoise | -0.237398283 | 2.09E-07 | -0.225951943 | 8.07E-07 |
| TMEM24 | turquoise | -0.409609004 | 2.55E-20 | -0.430144371 | 1.87E-22 |
| TMEM25 | turquoise | -0.195692533 | 2.05E-05 | -0.182912896 | 7.01E-05 |
| TMEM35 | turquoise | -0.055810636 | 0.228672993 | -0.070088744 | 0.130424038 |
| TMEM43 | turquoise | -0.173616401 | 0.000162921 | -0.199048816 | 1.47E-05 |
| TMEM47 | turquoise | -0.111718047 | 0.01572041 | -0.086204202 | 0.062693037 |
| TMEM55 | turquoise | -0.242257366 | 1.16E-07 | -0.237406322 | 2.09E-07 |
| TMEM63 | turquoise | 0.282242332 | 5.31E-10 | 0.293021131 | 1.06E-10 |
| TMEM88 | turquoise | -0.065789393 | 0.155768215 | -0.058515969 | 0.206864727 |
| TMEM8A | turquoise | 0.23654206 | 2.32E-07 | 0.269696438 | 3.17E-09 |
| TMEM8B | turquoise | -0.096246604 | 0.037602499 | -0.105319471 | 0.02283361 |
| TMOD1 | turquoise | -0.160239293 | 0.000508939 | -0.1368827 | 0.003035733 |
| TMOD2 | turquoise | -0.230866121 | 4.56E-07 | -0.234474691 | 2.97E-07 |
| TMPRSS4 | turquoise | 0.095493955 | 0.039129016 | 0.117715006 | 0.010900192 |
| TNFAIP8 | turquoise | -0.160293954 | 0.000506665 | -0.162176146 | 0.000433857 |
| TNRC6C | turquoise | -0.212438577 | 3.63E-06 | -0.21366101 | 3.18E-06 |
| TNS1 | turquoise | -0.278698858 | 8.87E-10 | -0.268474966 | 3.75E-09 |

| | | | | | |
|---|---|---|---|---|---|
| TNS2 | turquoise | -0.131169512 | 0.004521584 | -0.117480964 | 0.011060384 |
| TNXB | turquoise | -0.227311271 | 6.90E-07 | -0.218847592 | 1.80E-06 |
| TPI1 | turquoise | 0.233238823 | 3.45E-07 | 0.260279717 | 1.14E-08 |
| TPM1 | turquoise | -0.181225232 | 8.20E-05 | -0.174393972 | 0.000152079 |
| TPM2 | turquoise | -0.1490144 | 0.001239428 | -0.141655031 | 0.002151549 |
| TPPP2 | turquoise | -0.167877792 | 0.000268391 | -0.17315903 | 0.000169633 |
| TPRN | turquoise | 0.10984731 | 0.017565658 | 0.120303935 | 0.009260617 |
| TPSAB1 | turquoise | -0.119288814 | 0.009875358 | -0.06949276 | 0.133733623 |
| TPSB2 | turquoise | -0.135480823 | 0.00335212 | -0.105675731 | 0.022374459 |
| TPSD1 | turquoise | -0.090090462 | 0.0517014 | -0.072596717 | 0.117186772 |
| TPSG1 | turquoise | -0.187844385 | 4.41E-05 | -0.2009288 | 1.21E-05 |
| TRAF4 | turquoise | 0.165688851 | 0.000323328 | 0.168481139 | 0.000254858 |
| TRANK1 | turquoise | -0.213750247 | 3.15E-06 | -0.194747906 | 2.26E-05 |
| TRDN | turquoise | -0.162496243 | 0.000422489 | -0.165381791 | 0.000331825 |
| TRERF1 | turquoise | -0.287252243 | 2.54E-10 | -0.284885997 | 3.60E-10 |
| TRH | turquoise | -0.217116853 | 2.18E-06 | -0.239915147 | 1.54E-07 |
| TRIM4 | turquoise | -0.220465688 | 1.50E-06 | -0.219408324 | 1.69E-06 |
| TRIM56 | turquoise | -0.213497075 | 3.24E-06 | -0.20139572 | 1.16E-05 |
| TRIM63 | turquoise | -0.146333179 | 0.001519689 | -0.151737331 | 0.001004192 |
| TRIM9 | turquoise | -0.081901122 | 0.07703978 | -0.090066978 | 0.05176265 |
| TRIOBP | turquoise | -0.058370843 | 0.207995004 | -0.058768546 | 0.204908197 |
| TRO | turquoise | -0.569831687 | 1.47E-41 | -0.580678301 | 1.89E-43 |
| TRPC1 | turquoise | -0.368370807 | 1.87E-16 | -0.377548224 | 2.87E-17 |
| TRPC4 | turquoise | -0.170377072 | 0.000216372 | -0.160533293 | 0.000496814 |
| TRPM2 | turquoise | 0.251429637 | 3.64E-08 | 0.258138077 | 1.52E-08 |
| TRPM3 | turquoise | -0.145248137 | 0.001648806 | -0.157206667 | 0.000651101 |
| TRUB2 | turquoise | 0.243296244 | 1.02E-07 | 0.24863189 | 5.20E-08 |
| TSACC | turquoise | 0.036458467 | 0.431853419 | 0.036018536 | 0.437434259 |
| TSC22D3 | turquoise | -0.355107814 | 2.52E-15 | -0.329787094 | 2.61E-13 |
| TSHB | turquoise | -0.186405013 | 5.06E-05 | -0.183308849 | 6.76E-05 |
| TSHZ2 | turquoise | -0.197716444 | 1.68E-05 | -0.180917015 | 8.44E-05 |
| TSHZ3 | turquoise | -0.259726637 | 1.23E-08 | -0.25068232 | 4.00E-08 |
| TSLP | turquoise | -0.132906774 | 0.004011999 | -0.143565496 | 0.001869044 |
| TSPAN17 | turquoise | 0.040220942 | 0.385830522 | 0.065656082 | 0.156609769 |
| TSPAN18 | turquoise | -0.307430204 | 1.11E-11 | -0.302211112 | 2.56E-11 |
| TSPAN2 | turquoise | -0.332679534 | 1.57E-13 | -0.323540052 | 7.68E-13 |
| TSPAN33 | turquoise | -0.034882105 | 0.452040613 | -0.045545614 | 0.326040227 |
| TSPAN4 | turquoise | -0.287628872 | 2.40E-10 | -0.247096172 | 6.32E-08 |
| TSPAN5 | turquoise | 0.060396765 | 0.192616789 | 0.030372102 | 0.512635975 |
| TSPAN7 | turquoise | -0.23719697 | 2.14E-07 | -0.246620217 | 6.71E-08 |
| TSPAN9 | turquoise | -0.034337031 | 0.459142721 | -0.01581505 | 0.733200301 |
| TSPYL2 | turquoise | -0.190535883 | 3.41E-05 | -0.208642923 | 5.45E-06 |
| TSPYL5 | turquoise | -0.440522614 | 1.37E-23 | -0.445195104 | 4.09E-24 |
| TSSC1 | turquoise | 0.199938887 | 1.34E-05 | 0.191312024 | 3.16E-05 |
| TTYH2 | turquoise | -0.263062712 | 7.85E-09 | -0.279660459 | 7.73E-10 |

| | | | | | |
|---|---|---|---|---|---|
| TUB | turquoise | -0.390937153 | 1.67E-18 | -0.393704125 | 9.16E-19 |
| TUBB6 | turquoise | -0.326254173 | 4.82E-13 | -0.305933518 | 1.42E-11 |
| TUBG2 | turquoise | -0.03723851 | 0.42205979 | -0.045976162 | 0.321483022 |
| TUSC1 | turquoise | -0.240295469 | 1.47E-07 | -0.233571926 | 3.31E-07 |
| TVP23A | turquoise | -0.153096169 | 0.000902897 | -0.157454857 | 0.00063821 |
| TWIST1 | turquoise | -0.085861676 | 0.063747917 | -0.075138001 | 0.104870286 |
| TWIST2 | turquoise | -0.318325028 | 1.86E-12 | -0.313516331 | 4.13E-12 |
| TXLNB | turquoise | -0.280138778 | 7.21E-10 | -0.282490376 | 5.12E-10 |
| TXNDC1: | turquoise | 0.00730498 | 0.874897306 | -0.011499383 | 0.804253579 |
| TXNIP | turquoise | 0.015004771 | 0.746388502 | 0.005380185 | 0.907687384 |
| TXNL4A | turquoise | 0.142698289 | 0.001992781 | 0.168624692 | 0.000251734 |
| TYSND1 | turquoise | 0.158255768 | 0.000598209 | 0.147046834 | 0.001439899 |
| UBE2QL1 | turquoise | -0.046167438 | 0.319471793 | -0.073813126 | 0.111157056 |
| UCP2 | turquoise | 0.177900065 | 0.000111079 | 0.207952643 | 5.86E-06 |
| UNC93B1 | turquoise | 0.002945648 | 0.949379874 | 0.031123793 | 0.502251803 |
| USF1 | turquoise | 0.167518468 | 0.000276766 | 0.18949843 | 3.76E-05 |
| USP27X | turquoise | -0.333758089 | 1.29E-13 | -0.353304369 | 3.55E-15 |
| USP51 | turquoise | -0.399744616 | 2.40E-19 | -0.418213379 | 3.39E-21 |
| UST | turquoise | -0.340526795 | 3.84E-14 | -0.352301251 | 4.30E-15 |
| VAMP2 | turquoise | 0.059393924 | 0.200121649 | 0.060574902 | 0.191305537 |
| VASH1 | turquoise | -0.242318653 | 1.15E-07 | -0.240852533 | 1.37E-07 |
| VAT1L | turquoise | -0.147685009 | 0.001371828 | -0.128652365 | 0.005363849 |
| VAV3 | turquoise | 0.201197654 | 1.18E-05 | 0.200220643 | 1.30E-05 |
| VGLL3 | turquoise | -0.206346937 | 6.93E-06 | -0.200079165 | 1.32E-05 |
| VIP | turquoise | -0.212766916 | 3.50E-06 | -0.211204075 | 4.15E-06 |
| VIPR2 | turquoise | -0.172393059 | 0.000181456 | -0.15641464 | 0.000693868 |
| VPS37B | turquoise | 0.306711947 | 1.25E-11 | 0.328573846 | 3.22E-13 |
| VSTM4 | turquoise | -0.192779329 | 2.74E-05 | -0.178895103 | 0.000101493 |
| VWC2 | turquoise | -0.217337139 | 2.13E-06 | -0.219456929 | 1.68E-06 |
| WBSCR1 | turquoise | -0.287782669 | 2.34E-10 | -0.29769534 | 5.19E-11 |
| WDR17 | turquoise | -0.297305517 | 5.51E-11 | -0.317178363 | 2.25E-12 |
| WDR55 | turquoise | 0.34034537 | 3.96E-14 | 0.328338374 | 3.36E-13 |
| WIPF3 | turquoise | -0.330354865 | 2.36E-13 | -0.328534917 | 3.24E-13 |
| WISP2 | turquoise | -0.175259967 | 0.000140803 | -0.199915234 | 1.35E-05 |
| WNT2B | turquoise | -0.210472981 | 4.48E-06 | -0.214937293 | 2.77E-06 |
| WNT4 | turquoise | -0.278480225 | 9.16E-10 | -0.270630692 | 2.78E-09 |
| WNT9B | turquoise | -0.188791041 | 4.03E-05 | -0.193306708 | 2.60E-05 |
| WSCD2 | turquoise | -0.185451866 | 5.53E-05 | -0.1815622 | 7.95E-05 |
| WT1 | turquoise | -0.411805748 | 1.53E-20 | -0.407290523 | 4.35E-20 |
| WWC3 | turquoise | -0.211987178 | 3.81E-06 | -0.225214186 | 8.78E-07 |
| WWTR1 | turquoise | -0.242293996 | 1.15E-07 | -0.247553078 | 5.96E-08 |
| XKR5 | turquoise | -0.040593489 | 0.381441941 | -0.065100665 | 0.160152969 |
| XPNPEP2 | turquoise | -0.129265198 | 0.005146712 | -0.1277902 | 0.005683292 |
| XYLT1 | turquoise | -0.230111312 | 4.98E-07 | -0.220415184 | 1.51E-06 |
| YIPF1 | turquoise | 0.208537535 | 5.51E-06 | 0.234123635 | 3.10E-07 |

| | | | | | |
|---|---|---|---|---|---|
| YPEL4 | turquoise | -0.328863124 | 3.06E-13 | -0.333306324 | 1.40E-13 |
| YWHAE | turquoise | 0.29736981 | 5.46E-11 | 0.282908256 | 4.82E-10 |
| ZBTB16 | turquoise | -0.203934596 | 8.91E-06 | -0.210410489 | 4.51E-06 |
| ZBTB4 | turquoise | 0.002149777 | 0.963045131 | -0.017796631 | 0.701283731 |
| ZBTB46 | turquoise | -0.446199199 | 3.14E-24 | -0.43790445 | 2.67E-23 |
| ZBTB47 | turquoise | -0.171075073 | 0.000203627 | -0.180169194 | 9.04E-05 |
| ZC3H12B | turquoise | -0.334672993 | 1.10E-13 | -0.34192993 | 2.97E-14 |
| ZCCHC1[illegible] | turquoise | -0.072874255 | 0.115788967 | -0.113893063 | 0.013790603 |
| ZCCHC1[illegible] | turquoise | -0.192500302 | 2.81E-05 | -0.203709571 | 9.12E-06 |
| ZCCHC2[illegible] | turquoise | -0.26110249 | 1.02E-08 | -0.247058982 | 6.35E-08 |
| ZCCHC5 | turquoise | -0.122642082 | 0.007972366 | -0.122813665 | 0.007884453 |
| ZDBF2 | turquoise | -0.375621438 | 4.27E-17 | -0.384681253 | 6.42E-18 |
| ZDHHC1[illegible] | turquoise | -0.264509208 | 6.46E-09 | -0.270296966 | 2.91E-09 |
| ZDHHC1[illegible] | turquoise | -0.345872453 | 1.44E-14 | -0.339510496 | 4.61E-14 |
| ZDHHC1[illegible] | turquoise | 0.248752842 | 5.12E-08 | 0.250675538 | 4.01E-08 |
| ZEB1 | turquoise | -0.172586172 | 0.000178405 | -0.167837886 | 0.000269309 |
| ZEB2 | turquoise | -0.241575003 | 1.26E-07 | -0.219222523 | 1.73E-06 |
| ZFPM2 | turquoise | -0.327993892 | 3.57E-13 | -0.324605373 | 6.40E-13 |
| ZFYVE21 | turquoise | 0.003656676 | 0.937183803 | 0.011578719 | 0.802930704 |
| ZNF165 | turquoise | -0.102111703 | 0.027348371 | -0.111570169 | 0.015859822 |
| ZNF208 | turquoise | -0.305449058 | 1.53E-11 | -0.309938663 | 7.42E-12 |
| ZNF229 | turquoise | -0.459421998 | 9.20E-26 | -0.472850466 | 2.17E-27 |
| ZNF25 | turquoise | -0.095370465 | 0.039384421 | -0.126278333 | 0.006284977 |
| ZNF34 | turquoise | -0.262397128 | 8.59E-09 | -0.280145315 | 7.20E-10 |
| ZNF385D | turquoise | -0.189335632 | 3.82E-05 | -0.188162224 | 4.28E-05 |
| ZNF438 | turquoise | -0.140495049 | 0.002341593 | -0.143456124 | 0.001884252 |
| ZNF454 | turquoise | -0.262280363 | 8.73E-09 | -0.286912624 | 2.67E-10 |
| ZNF501 | turquoise | -0.37446755 | 5.41E-17 | -0.371364741 | 1.02E-16 |
| ZNF502 | turquoise | -0.390004295 | 2.05E-18 | -0.397670416 | 3.82E-19 |
| ZNF521 | turquoise | -0.132485311 | 0.004130607 | -0.13610571 | 0.003207566 |
| ZNF540 | turquoise | -0.125284782 | 0.006710898 | -0.129723265 | 0.004989617 |
| ZNF559 | turquoise | -0.234919385 | 2.82E-07 | -0.258338408 | 1.48E-08 |
| ZNF575 | turquoise | -0.05846855 | 0.207233546 | -0.06086261 | 0.189201578 |
| ZNF582 | turquoise | -0.32175044 | 1.04E-12 | -0.312005485 | 5.30E-12 |
| ZNF662 | turquoise | -0.496643722 | 1.86E-30 | -0.497686726 | 1.35E-30 |
| ZNF672 | turquoise | 0.301543813 | 2.84E-11 | 0.313898789 | 3.88E-12 |
| ZNF676 | turquoise | -0.281610117 | 5.82E-10 | -0.285978004 | 3.07E-10 |
| ZNF677 | turquoise | -0.377459934 | 2.92E-17 | -0.397385853 | 4.07E-19 |
| ZNF781 | turquoise | -0.163391198 | 0.00039216 | -0.206238439 | 7.01E-06 |
| ZNF835 | turquoise | -0.398231999 | 3.37E-19 | -0.394615762 | 7.50E-19 |
| ZNF843 | turquoise | -0.304446095 | 1.80E-11 | -0.29917533 | 4.12E-11 |
| ZYX | turquoise | -0.100291797 | 0.030236234 | -0.079468794 | 0.086263367 |
| A3GALT[illegible] | violet | -0.116008688 | 0.012116747 | -0.110084534 | 0.017321644 |
| ADAD1 | violet | -0.037980655 | 0.412863584 | -0.032329057 | 0.485836154 |
| ADAM7 | violet | -0.100738035 | 0.029504983 | -0.075063373 | 0.105216769 |

| | | | | | |
|---|---|---|---|---|---|
| AGBL1 | violet | -0.062848846 | 0.17513866 | -0.039400655 | 0.395601245 |
| ANKRD3 | violet | -0.081145809 | 0.07981405 | -0.08824271 | 0.056708716 |
| ASB17 | violet | -0.042486145 | 0.359621528 | -0.022060155 | 0.634427611 |
| ASB18 | violet | -0.043659153 | 0.346498519 | -0.044648552 | 0.335669111 |
| ATP11AU | violet | -0.066642321 | 0.150464688 | -0.041955196 | 0.365662491 |
| ATXN3L | violet | -0.073280719 | 0.113765448 | -0.05722328 | 0.217089997 |
| BAGE5 | violet | -0.131382919 | 0.004455984 | -0.124457624 | 0.007085025 |
| BRS3 | violet | -0.083831119 | 0.070305437 | -0.097916055 | 0.034395987 |
| C1orf185 | violet | -0.064014385 | 0.16725682 | -0.038208262 | 0.410067102 |
| C7orf33 | violet | -0.110650103 | 0.016751809 | -0.10160694 | 0.028124534 |
| CABP2 | violet | -0.136539966 | 0.00311047 | -0.099133782 | 0.032206664 |
| CCDC168 | violet | -0.026186922 | 0.572424527 | -0.014580002 | 0.753332071 |
| CCDC79 | violet | -0.045282819 | 0.328842295 | -0.033007164 | 0.476729129 |
| CNBD1 | violet | -0.075779357 | 0.101929726 | -0.059051869 | 0.202729501 |
| CRB1 | violet | -0.103491832 | 0.025319551 | -0.110806915 | 0.016596748 |
| CRYAA | violet | -0.0271038 | 0.559048291 | -0.034198209 | 0.460961426 |
| CYLC2 | violet | -0.043387367 | 0.349511739 | -0.025869577 | 0.577089283 |
| CYP7A1 | violet | -0.06167491 | 0.183353234 | -0.065925031 | 0.154915486 |
| DAOA | violet | -0.037083778 | 0.423992102 | -0.021730333 | 0.639503379 |
| DDX3Y | violet | -0.070875354 | 0.126153286 | -0.058443043 | 0.207432136 |
| DDX53 | violet | 0.009957945 | 0.830062336 | 0.032781197 | 0.479753483 |
| DEFB110 | violet | -0.063782523 | 0.168803214 | -0.051687575 | 0.264966256 |
| DEFB128 | violet | -0.044732384 | 0.334761616 | -0.01677645 | 0.717653954 |
| DNAH8 | violet | -0.100896501 | 0.029248977 | -0.096490002 | 0.037119777 |
| DNAJB8 | violet | -0.113151841 | 0.014423453 | -0.087083925 | 0.060049527 |
| EFCAB3 | violet | -0.011086149 | 0.811152888 | -0.007672216 | 0.868663709 |
| G6PC2 | violet | -0.076574557 | 0.098375069 | -0.063538752 | 0.170440541 |
| GABRR3 | violet | -0.075469882 | 0.103340367 | -0.064610949 | 0.163326861 |
| GAGE2E | violet | -0.080259824 | 0.083170848 | -0.061126415 | 0.187287415 |
| GFRAL | violet | -0.053637047 | 0.247340633 | -0.04342232 | 0.349123298 |
| GPR112 | violet | -0.123123375 | 0.007727957 | -0.123188402 | 0.007695454 |
| GPR141 | violet | 0.027992425 | 0.546230316 | 0.040061832 | 0.387714168 |
| GPR149 | violet | -0.064540196 | 0.163789298 | -0.060156057 | 0.194399064 |
| HEMGN | violet | -0.060806946 | 0.189607306 | -0.044977275 | 0.332119691 |
| HIST1H4 | violet | 0.010011262 | 0.829166403 | 0.014315927 | 0.757658903 |
| HNRNPC | violet | 0.013891373 | 0.76463101 | 0.017715331 | 0.702583358 |
| HORMAI | violet | -0.040159732 | 0.386554503 | -0.035553591 | 0.443377135 |
| HSF5 | violet | -0.054579495 | 0.239119864 | -0.025743986 | 0.578940299 |
| HTR4 | violet | -0.095615646 | 0.038878706 | -0.092657442 | 0.045361675 |
| IFNA2 | violet | -0.128416513 | 0.005449589 | -0.116086012 | 0.012059125 |
| IFNA21 | violet | -0.030683743 | 0.508317339 | -0.015301012 | 0.741558027 |
| IL17F | violet | 0.027851765 | 0.548249561 | 0.055066928 | 0.234944311 |
| IL26 | violet | -0.01124525 | 0.808494784 | 0.017537262 | 0.705432938 |
| IL3 | violet | -0.051206428 | 0.269445988 | -0.045544714 | 0.326049802 |
| IQCF3 | violet | -0.02915451 | 0.529689919 | -0.024684936 | 0.5946585 |

| | | | | | |
|---|---|---|---|---|---|
| KRTAP21 | violet | -0.061754574 | 0.18278694 | -0.043150101 | 0.352155754 |
| KRTAP24 | violet | -0.056016288 | 0.226959998 | -0.035358922 | 0.445878968 |
| MBL2 | violet | -0.041561779 | 0.370179138 | -0.028349324 | 0.541123408 |
| MGAT4D | violet | -0.086925156 | 0.060519685 | -0.073609585 | 0.112148638 |
| MLIP | violet | -0.135478673 | 0.003352628 | -0.123109815 | 0.007734751 |
| MROH2B | violet | -0.081783774 | 0.077465587 | -0.067370157 | 0.146048473 |
| NLRP14 | violet | -0.201148645 | 1.19E-05 | -0.215361723 | 2.64E-06 |
| NME8 | violet | -0.049148228 | 0.289191724 | -0.035356311 | 0.445912573 |
| NOTO | violet | -0.024134001 | 0.602911465 | -0.016464176 | 0.722691182 |
| NPHS2 | violet | -0.127674806 | 0.00572732 | -0.116755628 | 0.011570167 |
| OOSP2 | violet | -0.013919484 | 0.764168781 | -0.01108056 | 0.811246299 |
| OPRM1 | violet | -0.024734508 | 0.593918449 | -0.015400292 | 0.739941427 |
| OR11H4 | violet | -0.185004327 | 5.77E-05 | -0.179190768 | 9.88E-05 |
| OR11H6 | violet | -0.085011784 | 0.06642857 | -0.074682507 | 0.106999161 |
| OR13G1 | violet | -0.10972898 | 0.017688493 | -0.096390499 | 0.037316479 |
| OR1L8 | violet | -0.053615851 | 0.247527762 | -0.044394194 | 0.338432213 |
| OR1N2 | violet | -0.054619033 | 0.238779234 | -0.040856999 | 0.378356311 |
| OR2A12 | violet | -0.072067627 | 0.119888052 | -0.052708201 | 0.255633778 |
| OR2B3 | violet | -0.075681874 | 0.102372411 | -0.050570888 | 0.275442202 |
| OR4K15 | violet | -0.030752087 | 0.507372798 | -0.026936018 | 0.561484686 |
| OR4K17 | violet | -0.094260156 | 0.041744798 | -0.083211039 | 0.072414509 |
| OR56A5 | violet | -0.114011322 | 0.013691929 | -0.106536167 | 0.021298389 |
| OR5K3 | violet | -0.068897661 | 0.137102546 | -0.054776204 | 0.237428537 |
| OR5L2 | violet | -0.050964347 | 0.271719373 | -0.04215989 | 0.363326116 |
| OR5M8 | violet | -0.058689869 | 0.205516205 | -0.045104918 | 0.330747983 |
| OR5T1 | violet | -0.063068438 | 0.173632946 | -0.050024387 | 0.280670415 |
| OR6B1 | violet | -0.060378114 | 0.192754455 | -0.045953071 | 0.321726375 |
| OR7A17 | violet | -0.065511491 | 0.157526409 | -0.059761844 | 0.197343945 |
| OR8B12 | violet | -0.03707679 | 0.424079497 | -0.015261821 | 0.742196481 |
| OR8B3 | violet | -0.045034042 | 0.331509197 | -0.025391062 | 0.584156692 |
| OR8D1 | violet | -0.035518405 | 0.443828742 | -0.011794464 | 0.799336186 |
| OR8D4 | violet | 0.028440854 | 0.539817529 | 0.046932712 | 0.311507369 |
| OR8J3 | violet | -0.049148315 | 0.289190865 | -0.019777175 | 0.669900419 |
| OR9A2 | violet | -0.065827452 | 0.155528586 | -0.056892098 | 0.21976702 |
| OTOGL | violet | -0.186729928 | 4.90E-05 | -0.188126759 | 4.29E-05 |
| PLA2G2E | violet | -0.02996404 | 0.518319461 | -0.013621039 | 0.769080453 |
| PLCZ1 | violet | 0.01508275 | 0.745116016 | 0.029110102 | 0.530317302 |
| PNLIPRP | violet | -0.057443686 | 0.215321401 | -0.039219146 | 0.397783227 |
| POTEC | violet | -0.06920952 | 0.135329027 | -0.064292368 | 0.165416845 |
| POTEG | violet | -0.017604031 | 0.704363976 | -0.007504285 | 0.871513254 |
| POTEH | violet | 0.020451062 | 0.659348462 | 0.071372107 | 0.123512704 |
| POTEJ | violet | -0.056142783 | 0.225910884 | -0.050926887 | 0.272072319 |
| POTEM | violet | 0.002582101 | 0.955620447 | 0.042582089 | 0.358536622 |
| PRDM9 | violet | -0.119006667 | 0.010052537 | -0.114842683 | 0.01301562 |
| RBMXL2 | violet | -0.145509216 | 0.00161685 | -0.138093263 | 0.002784636 |

| | | | | | |
|---|---|---|---|---|---|
| RDH8 | violet | -0.107371817 | 0.020296611 | -0.099519442 | 0.031538599 |
| RGSL1 | violet | -0.054822713 | 0.237029877 | -0.046318682 | 0.317887319 |
| RIT2 | violet | -0.050494175 | 0.276172069 | -0.030206777 | 0.51493471 |
| RNASE11 | violet | -0.051487785 | 0.266820154 | -0.051475091 | 0.26693825 |
| RP11-310 | violet | -0.038340318 | 0.408449769 | -0.029739861 | 0.521455586 |
| RP11-506 | violet | -0.014528639 | 0.754173038 | -0.006639314 | 0.886216057 |
| RXFP2 | violet | -0.088269559 | 0.056633176 | -0.080534038 | 0.082119944 |
| SAMD7 | violet | -0.16313975 | 0.00040047 | -0.146848463 | 0.001461683 |
| SERPINB | violet | -0.073788721 | 0.111275581 | -0.067539393 | 0.145035926 |
| SLC13A1 | violet | -0.07585767 | 0.101575192 | -0.069354752 | 0.134509158 |
| SLC22A2 | violet | -0.033874299 | 0.465220526 | -0.027785875 | 0.549196698 |
| SLC22A2 | violet | -0.062299102 | 0.178950736 | -0.044253834 | 0.339963156 |
| SLC25A3 | violet | -0.029791423 | 0.520733394 | -0.01283608 | 0.782042692 |
| SPACA7 | violet | -0.019543143 | 0.673580361 | -0.012586343 | 0.786179543 |
| SPAM1 | violet | 0.02462775 | 0.595512742 | 0.032461854 | 0.484045319 |
| SPATA16 | violet | -0.115493546 | 0.012506878 | -0.101958043 | 0.027582676 |
| SPATA31 | violet | -0.072213382 | 0.119139092 | -0.055113378 | 0.2345491 |
| SPATA9 | violet | -0.09558475 | 0.038942129 | -0.099841353 | 0.030990068 |
| SPO11 | violet | -0.076386395 | 0.099207137 | -0.057718893 | 0.213127634 |
| SPZ1 | violet | -0.053876218 | 0.245235958 | -0.061186729 | 0.186851785 |
| SULT6B1 | violet | -0.089663818 | 0.052823574 | -0.081528301 | 0.078399205 |
| TAS2R39 | violet | -0.062203622 | 0.179619041 | -0.046123437 | 0.319933723 |
| TAS2R7 | violet | -0.050599514 | 0.275170183 | -0.036522237 | 0.43104788 |
| TBC1D29 | violet | -0.051847562 | 0.263488096 | -0.038639076 | 0.404804766 |
| TBPL2 | violet | -0.096634988 | 0.03683474 | -0.077546844 | 0.094163687 |
| TDRD15 | violet | 0.023965351 | 0.605448071 | 0.030876233 | 0.505659411 |
| TEX14 | violet | -0.106479544 | 0.021367796 | -0.09261469 | 0.045461652 |
| TGIF2LX | violet | -0.093590739 | 0.043224761 | -0.078077981 | 0.091924699 |
| TNR | violet | -0.032037767 | 0.489776801 | -0.023761831 | 0.60851546 |
| TOPAZ1 | violet | -0.037612348 | 0.417412545 | -0.026466946 | 0.568323244 |
| TPH2 | violet | -0.057352966 | 0.216048103 | -0.052119835 | 0.26098554 |
| TRIM64C | violet | -0.065715018 | 0.156237297 | -0.050094226 | 0.279998563 |
| UGT1A7 | violet | -0.125384373 | 0.006667068 | -0.11676598 | 0.011562748 |
| UMOD | violet | 0.013062301 | 0.778300676 | 0.020135637 | 0.664279248 |
| USP26 | violet | 0.033715874 | 0.467311579 | 0.044052432 | 0.342167625 |
| USP9Y | violet | -0.01464348 | 0.752293134 | 0.005069585 | 0.91299487 |
| WFDC8 | violet | 0.015775567 | 0.733841147 | 0.008887804 | 0.848089915 |
| ZNF157 | violet | -0.326567594 | 4.57E-13 | -0.31988778 | 1.43E-12 |
| ZSWIM2 | violet | -0.116173852 | 0.011993961 | -0.103464289 | 0.025358734 |
| AC01065 | white | -0.045732409 | 0.32405795 | -0.053708907 | 0.24670695 |
| AC10407 | white | -0.144924337 | 0.001689242 | -0.134415646 | 0.003612214 |
| AC11271 | white | -0.07876147 | 0.089106432 | -0.070313901 | 0.129190326 |
| AC11875 | white | -7.71E-05 | 0.998673805 | 0.001601258 | 0.972469859 |
| ACOT12 | white | -0.013301428 | 0.774350749 | -0.024604601 | 0.595858705 |
| ACOT6 | white | -0.097955256 | 0.034323582 | -0.058716358 | 0.205311354 |

| | | | | | |
|---|---|---|---|---|---|
| ACSM2A | white | -0.067388642 | 0.145937611 | -0.060773428 | 0.189851922 |
| ACSM2B | white | -0.080710254 | 0.081450294 | -0.073784516 | 0.111296015 |
| ACTRT1 | white | -0.07051033 | 0.128121419 | -0.069664604 | 0.132772773 |
| AGXT2 | white | -0.077188767 | 0.095697588 | -0.098670284 | 0.033025527 |
| AHSP | white | -0.000102285 | 0.998241091 | -0.009483418 | 0.838045799 |
| AL13333 | white | -0.094875474 | 0.04042238 | -0.096273198 | 0.0375495 |
| AL35482 | white | -0.091635982 | 0.047801483 | -0.076824985 | 0.097276285 |
| ART1 | white | -0.063262517 | 0.17231022 | -0.056978286 | 0.219068079 |
| ASB15 | white | -0.045875748 | 0.322542142 | -0.032651702 | 0.481491353 |
| BEND2 | white | -0.021235572 | 0.647149051 | -0.013026901 | 0.778885902 |
| C12orf50 | white | -0.024370761 | 0.599358523 | -0.022482687 | 0.627950059 |
| C16orf92 | white | -0.033906153 | 0.464800713 | -0.027163787 | 0.558178444 |
| C17orf47 | white | -0.090481686 | 0.050689938 | -0.098028685 | 0.034188308 |
| C4orf26 | white | -0.144537036 | 0.001738802 | -0.144729705 | 0.001713984 |
| C5orf47 | white | -0.118218119 | 0.010562773 | -0.103271097 | 0.025635042 |
| C9orf57 | white | -0.053181085 | 0.251387896 | -0.042348523 | 0.361181323 |
| CETN1 | white | -0.020383847 | 0.66039795 | -0.010974459 | 0.813020178 |
| CGB7 | white | -0.097617289 | 0.034952094 | -0.115854297 | 0.012232528 |
| CLLU1 | white | -0.030809314 | 0.50658261 | -0.030039827 | 0.517261444 |
| COL20A1 | white | -0.185923697 | 5.29E-05 | -0.195606486 | 2.07E-05 |
| COL6A5 | white | -0.143908864 | 0.001822026 | -0.14814401 | 0.00132471 |
| CTAGE1 | white | 0.030439349 | 0.511702474 | 0.032173274 | 0.487941495 |
| CTD-307 | white | 0.056651338 | 0.221727919 | 0.036051611 | 0.437013242 |
| CXorf66 | white | -0.043779968 | 0.345164387 | -0.038045501 | 0.412065708 |
| CYP3A43 | white | -0.020883159 | 0.652617759 | -0.036850841 | 0.42691075 |
| DAZL | white | -0.023327754 | 0.615080677 | -0.007308915 | 0.874830481 |
| DEFB125 | white | -0.004939357 | 0.915221358 | -0.004448532 | 0.923618709 |
| DUXA | white | -0.054191433 | 0.242481274 | -0.040414616 | 0.383545228 |
| EIF4E1B | white | -0.080445717 | 0.082457249 | -0.074624376 | 0.10727329 |
| FAM186 | white | -0.036093332 | 0.436482502 | -0.031741732 | 0.493799153 |
| FAM25E | white | -0.023005339 | 0.619977048 | -0.022256723 | 0.631410646 |
| FAM47C | white | -0.00047281 | 0.991869594 | 0.006462101 | 0.889233324 |
| FAM71D | white | 0.046076937 | 0.320422374 | 0.053819423 | 0.245734612 |
| FAM9A | white | -0.085492375 | 0.064901586 | -0.081688676 | 0.07781206 |
| FBXO47 | white | -0.155499514 | 0.000746516 | -0.141895462 | 0.002113967 |
| FLJ20019 | white | -0.082740586 | 0.074048875 | -0.081717151 | 0.077708186 |
| GABRA1 | white | -0.109550856 | 0.017874813 | -0.104232567 | 0.024285195 |
| GABRA6 | white | -0.067745907 | 0.143807619 | -0.060555659 | 0.191446866 |
| GK2 | white | -0.043797352 | 0.344972692 | -0.032143388 | 0.488345959 |
| GKN1 | white | -0.057104486 | 0.218047533 | -0.05221766 | 0.260090414 |
| GLYAT | white | -0.084431072 | 0.068312898 | -0.077275277 | 0.09532519 |
| GOLGA6 | white | -0.065397022 | 0.158254957 | -0.067867808 | 0.143086315 |
| GOLGA8 | white | -0.081729885 | 0.077661769 | -0.07908679 | 0.087789612 |
| GRIFIN | white | -0.133718282 | 0.003792267 | -0.108971732 | 0.018492482 |
| GSC2 | white | -0.090091492 | 0.051698716 | -0.081956532 | 0.076839384 |

| | | | | | |
|---|---|---|---|---|---|
| GUCA1A | white | -0.057819856 | 0.212326873 | -0.044374897 | 0.33864242 |
| HBG2 | white | -0.052341252 | 0.258962545 | -0.09796123 | 0.034312559 |
| HCRT | white | -0.065901197 | 0.155065071 | -0.041114712 | 0.37535344 |
| HEPACA | white | -0.049450929 | 0.286228295 | -0.041162653 | 0.374796452 |
| HES3 | white | 0.006268771 | 0.892526832 | 0.008139984 | 0.860735456 |
| HNRNPC | white | 0.043685098 | 0.346211744 | 0.01052543 | 0.820537917 |
| HSPC047 | white | -0.190367302 | 3.46E-05 | -0.195750521 | 2.04E-05 |
| IFNA13 | white | -0.10011724 | 0.030526477 | -0.098501886 | 0.033327404 |
| IFNA6 | white | -0.135845603 | 0.003267034 | -0.14096904 | 0.002262159 |
| IL13 | white | 0.090905774 | 0.049612224 | 0.067269656 | 0.146652321 |
| IZUMO1 | white | -0.156066328 | 0.000713487 | -0.167481204 | 0.000277648 |
| IZUMO3 | white | -0.02399071 | 0.605066353 | -0.014357321 | 0.756980165 |
| KCNK4 | white | -0.094460446 | 0.041310391 | -0.087692005 | 0.058276677 |
| KIAA102 | white | 0.041975346 | 0.365432085 | 0.018951002 | 0.682925892 |
| KRTAP5- | white | -0.092631967 | 0.045421227 | -0.087973687 | 0.057470248 |
| LCE2C | white | -0.007258359 | 0.875689231 | -0.008263886 | 0.858637744 |
| LYZL1 | white | -0.038788729 | 0.402986238 | -0.038418947 | 0.407488562 |
| MAGEB5 | white | 0.008217782 | 0.859418186 | 0.020160065 | 0.663896873 |
| MIP | white | -0.044067345 | 0.342004089 | -0.02810638 | 0.544597138 |
| MS4A3 | white | -0.017880466 | 0.699944483 | 0.007615884 | 0.869619388 |
| MTRNR2 | white | -0.02233112 | 0.630270382 | -0.013595198 | 0.769506173 |
| MUC22 | white | -0.09354244 | 0.043333228 | -0.088697866 | 0.055439362 |
| NANOGP | white | 0.019402136 | 0.67580133 | -0.010071349 | 0.828156972 |
| NEUROD | white | -0.129705142 | 0.004995749 | -0.129837525 | 0.004951111 |
| NRN1L | white | -0.050035203 | 0.280566286 | -0.040801253 | 0.379007802 |
| OPALIN | white | -0.004989071 | 0.914371333 | -0.004803838 | 0.917539003 |
| OR10G7 | white | -0.049843497 | 0.282415634 | -0.056626412 | 0.221931641 |
| OR11H1 | white | -0.083614263 | 0.071037242 | -0.083042384 | 0.072997005 |
| OR11H12 | white | 0.010291777 | 0.824456338 | 0.026799946 | 0.563464387 |
| OR1D2 | white | -0.05363881 | 0.247325072 | -0.05585532 | 0.228300012 |
| OR1D5 | white | -0.004984845 | 0.914443579 | -0.000730345 | 0.987441345 |
| OR2H1 | white | -0.049176087 | 0.288918125 | -0.041852446 | 0.366838801 |
| OR2K2 | white | 0.044723021 | 0.334862892 | 0.021833817 | 0.637908996 |
| OR2T4 | white | -0.111201979 | 0.016211652 | -0.104843173 | 0.023460201 |
| OR4D2 | white | -0.013118472 | 0.777372321 | -0.01303659 | 0.778725722 |
| OR4F4 | white | -0.003875847 | 0.933427257 | -0.018172706 | 0.695283301 |
| OR4K5 | white | -0.070236222 | 0.12961493 | -0.043684216 | 0.346221486 |
| OR51H1 | white | -0.060346859 | 0.19298532 | -0.052353498 | 0.258850979 |
| OR52A1 | white | -0.030436643 | 0.511740018 | -0.032142969 | 0.48835163 |
| OR5AS1 | white | -0.036203209 | 0.435086523 | -0.031374633 | 0.498811449 |
| OR5D18 | white | -0.085385881 | 0.065237438 | -0.088286344 | 0.056585993 |
| OR5H2 | white | -0.067950644 | 0.142597745 | -0.065576104 | 0.157116291 |
| OR5T3 | white | 0.004316374 | 0.925881249 | 0.015823863 | 0.73305728 |
| OR5V1 | white | -0.049640086 | 0.284386879 | -0.050261529 | 0.278393552 |
| OR6C2 | white | -0.070402429 | 0.128707731 | -0.061591015 | 0.183950996 |

| | | | | | |
|---|---|---|---|---|---|
| OR6C68 | white | -0.055627952 | 0.230202357 | -0.060363254 | 0.192864192 |
| OR6J1 | white | -0.049903886 | 0.281832189 | -0.044878255 | 0.333186315 |
| OR6N1 | white | -0.032004166 | 0.490232465 | -0.028248766 | 0.542559892 |
| OR6V1 | white | -0.086269214 | 0.06249445 | -0.083769227 | 0.070513666 |
| OR8H1 | white | -0.053475848 | 0.248766259 | -0.043594854 | 0.347209893 |
| OR8H2 | white | -0.011028561 | 0.812115535 | -0.009495803 | 0.837837213 |
| OTOP3 | white | 0.01911571 | 0.680321417 | 0.023585931 | 0.611172116 |
| PDE6H | white | -0.076863479 | 0.097108257 | -0.06880718 | 0.137620433 |
| PGA3 | white | -0.091450032 | 0.048257258 | -0.093178274 | 0.044158468 |
| PKD1L3 | white | -0.033292833 | 0.472920667 | -0.027727502 | 0.550036462 |
| PNLIP | white | 0.051870964 | 0.263272352 | 0.060239048 | 0.193783218 |
| PPP1R27 | white | -0.101713545 | 0.027959051 | -0.097272535 | 0.035603293 |
| PPP5D1 | white | -0.16293199 | 0.000407459 | -0.151632058 | 0.001012462 |
| PRG3 | white | -0.049558212 | 0.285182922 | -0.046886118 | 0.311988525 |
| PRR25 | white | -0.071586911 | 0.122384279 | -0.048359815 | 0.297006665 |
| PRSS58 | white | -0.073084113 | 0.114740726 | -0.074273239 | 0.108940958 |
| RP1-66C1 | white | -0.107512244 | 0.020132352 | -0.098680273 | 0.033007693 |
| RP11-322 | white | -0.036951118 | 0.425652874 | -0.035737072 | 0.441026425 |
| RP11-385 | white | 0.078721401 | 0.089269712 | 0.09746215 | 0.035243868 |
| RP11-545 | white | 0.039305507 | 0.396744148 | 0.032684031 | 0.481057163 |
| RP11-546 | white | -0.017422507 | 0.707271512 | -0.019958439 | 0.667055633 |
| RP5-907C | white | 0.034192849 | 0.461031731 | 0.004069478 | 0.930109687 |
| SHOX | white | -0.032607867 | 0.482080401 | -0.035427123 | 0.445001549 |
| SKOR2 | white | -0.112629124 | 0.01488496 | -0.107468065 | 0.020183904 |
| SLC22A2 | white | -0.072581993 | 0.117261297 | -0.074769111 | 0.106591784 |
| SLC5A10 | white | -0.064901382 | 0.161438887 | -0.058106094 | 0.210068425 |
| SLCO1B7 | white | 0.013216727 | 0.775749188 | 0.005135075 | 0.911875468 |
| SMIM9 | white | -0.032950516 | 0.477486339 | -0.020967523 | 0.651306894 |
| SPATA19 | white | -0.136258472 | 0.003173101 | -0.108606002 | 0.018892077 |
| SPATA31 | white | -0.057526424 | 0.214660172 | -0.051635208 | 0.265451328 |
| SPHKAP | white | -0.117342635 | 0.011156042 | -0.117705829 | 0.010906434 |
| SSX7 | white | -0.0107855 | 0.816181715 | -0.000964039 | 0.983423368 |
| SUN3 | white | -0.150063009 | 0.001143404 | -0.15366489 | 0.000863374 |
| SYCP1 | white | -0.129992572 | 0.004899288 | -0.128602575 | 0.005381847 |
| TAT | white | -0.088237716 | 0.056722778 | -0.088609058 | 0.055685162 |
| TBL1Y | white | 0.026596267 | 0.566433931 | 0.018150914 | 0.695630484 |
| TEX101 | white | 0.031862435 | 0.492156988 | 0.010214607 | 0.825751464 |
| TEX28 | white | -0.011132619 | 0.810376287 | 0.005741204 | 0.901523529 |
| TEX40 | white | -0.07453824 | 0.107680501 | -0.075480917 | 0.103289807 |
| TFAP2D | white | -0.026943807 | 0.561371474 | -0.012595259 | 0.786031744 |
| TLR9 | white | -0.183017027 | 6.95E-05 | -0.18155823 | 7.95E-05 |
| TPD52L3 | white | -0.083810422 | 0.070375013 | -0.064008124 | 0.167298439 |
| TTLL8 | white | -0.094204899 | 0.041865322 | -0.08393896 | 0.069943814 |
| USP29 | white | -0.039798145 | 0.390848132 | -0.040009562 | 0.388334182 |
| ZAR1L | white | -0.135271216 | 0.003401917 | -0.122567434 | 0.008010886 |

| | | | | | |
|---|---|---|---|---|---|
| ZIM3 | white | -0.047218841 | 0.308563355 | -0.043069074 | 0.353061585 |
| ZNF534 | white | -0.061753548 | 0.182794227 | -0.066050257 | 0.154131357 |
| ZNF735 | white | -0.025665427 | 0.580099549 | -0.022808317 | 0.622977446 |
| ADSS | yellowgreen | 0.092710006 | 0.045239008 | 0.06302179 | 0.173951998 |
| AHCTF1 | yellowgreen | 0.001389888 | 0.976102742 | -0.037287483 | 0.421449277 |
| ANP32E | yellowgreen | 0.179134414 | 9.93E-05 | 0.150076097 | 0.00114225 |
| ARPC5 | yellowgreen | 0.069033886 | 0.13632566 | 0.070833939 | 0.126375401 |
| ARV1 | yellowgreen | 0.1517458 | 0.00100353 | 0.148490211 | 0.001290162 |
| B4GALT3 | yellowgreen | 0.191146247 | 3.21E-05 | 0.165160697 | 0.000338071 |
| BPNT1 | yellowgreen | 0.246557167 | 6.76E-08 | 0.251902433 | 3.42E-08 |
| C1orf131 | yellowgreen | 0.153288141 | 0.000889373 | 0.141490281 | 0.002177653 |
| C1orf43 | yellowgreen | 0.264136781 | 6.79E-09 | 0.24662682 | 6.70E-08 |
| C1orf74 | yellowgreen | 0.151521325 | 0.001021229 | 0.116571653 | 0.011702729 |
| CACYBP | yellowgreen | 0.212683038 | 3.54E-06 | 0.182751527 | 7.12E-05 |
| CCSAP | yellowgreen | 0.101931143 | 0.027623871 | 0.092701181 | 0.045259582 |
| CHD1L | yellowgreen | 0.168727335 | 0.000249522 | 0.12371671 | 0.007435876 |
| CHTOP | yellowgreen | 0.015922264 | 0.731461043 | -0.013007813 | 0.779201518 |
| CNIH4 | yellowgreen | 0.164461924 | 0.000358541 | 0.175560209 | 0.00013708 |
| COG2 | yellowgreen | 0.165156647 | 0.000338187 | 0.142968359 | 0.001953465 |
| COX20 | yellowgreen | 0.039742693 | 0.391509127 | 0.017630623 | 0.703938403 |
| CREG1 | yellowgreen | -0.070210804 | 0.129754102 | -0.109276618 | 0.018165022 |
| DAP3 | yellowgreen | 0.117809644 | 0.010836002 | 0.102836864 | 0.026265584 |
| DEDD | yellowgreen | 0.19752045 | 1.71E-05 | 0.165974494 | 0.000315607 |
| DUSP12 | yellowgreen | 0.004898868 | 0.915913723 | -0.022885696 | 0.621798319 |
| ENSA | yellowgreen | 0.092832134 | 0.044955076 | 0.083177217 | 0.072531017 |
| EPRS | yellowgreen | 0.166905662 | 0.000291614 | 0.137927114 | 0.002817941 |
| FAM189B | yellowgreen | -0.043173169 | 0.351898139 | -0.074290913 | 0.108856533 |
| FH | yellowgreen | 0.310592992 | 6.67E-12 | 0.319770241 | 1.46E-12 |
| FLVCR1 | yellowgreen | 0.219727059 | 1.63E-06 | 0.18417569 | 6.24E-05 |
| GALNT2 | yellowgreen | 0.082668854 | 0.074300693 | 0.076991268 | 0.096552114 |
| GBA | yellowgreen | 0.091852067 | 0.047276383 | 0.081548653 | 0.078324497 |
| GGPS1 | yellowgreen | 0.212717387 | 3.52E-06 | 0.198172326 | 1.60E-05 |
| GLMP | yellowgreen | -0.105970407 | 0.022000746 | -0.135602399 | 0.003323541 |
| GLRX2 | yellowgreen | 0.25134472 | 3.68E-08 | 0.251827191 | 3.46E-08 |
| GNPAT | yellowgreen | 0.273652387 | 1.82E-09 | 0.270087397 | 3.00E-09 |
| HAX1 | yellowgreen | 0.142903447 | 0.00196285 | 0.132327371 | 0.004175865 |
| HSD17B7 | yellowgreen | 0.244484747 | 8.77E-08 | 0.219902095 | 1.60E-06 |
| IARS2 | yellowgreen | 0.222281575 | 1.23E-06 | 0.196455334 | 1.90E-05 |
| INTS7 | yellowgreen | 0.259777295 | 1.22E-08 | 0.248282064 | 5.44E-08 |
| ISG20L2 | yellowgreen | 0.072970451 | 0.115307538 | 0.040466204 | 0.382937906 |
| JTB | yellowgreen | 0.174170166 | 0.000155128 | 0.180830817 | 8.50E-05 |
| LHX4-AS | yellowgreen | 0.112313583 | 0.015169786 | 0.102118068 | 0.027338701 |
| LIN9 | yellowgreen | -0.042702267 | 0.357180572 | -0.066677984 | 0.150245965 |
| MARC1 | yellowgreen | 0.140600791 | 0.002323654 | 0.127771419 | 0.005690437 |
| METTL1: | yellowgreen | 0.149825557 | 0.001164527 | 0.142260899 | 0.002057993 |

| | | | | | |
|---|---|---|---|---|---|
| METTL1{ | yellowgreer | -0.011912024 | 0.797379296 | -0.046413623 | 0.31689531 |
| MPC2 | yellowgreer | 0.239285663 | 1.66E-07 | 0.231922205 | 4.03E-07 |
| MRPL9 | yellowgreer | 0.006032477 | 0.896554763 | -0.027001749 | 0.560529584 |
| MRPS14 | yellowgreer | 0.130839858 | 0.004624633 | 0.12224234 | 0.008180583 |
| NDUFS2 | yellowgreer | 0.24602987 | 7.22E-08 | 0.235871615 | 2.52E-07 |
| NSL1 | yellowgreer | 0.092393806 | 0.045981143 | 0.057385461 | 0.215787608 |
| NTPCR | yellowgreer | 0.261843143 | 9.26E-09 | 0.251963533 | 3.40E-08 |
| NUP133 | yellowgreer | 0.087207272 | 0.059686351 | 0.056119719 | 0.226101913 |
| NVL | yellowgreer | 0.127234613 | 0.005898104 | 0.09760065 | 0.034983289 |
| PAK1IP1 | yellowgreer | 0.129888487 | 0.004934023 | 0.102497193 | 0.026768083 |
| PARP1 | yellowgreer | 0.154130462 | 0.000832216 | 0.132228866 | 0.004204319 |
| PEX11B | yellowgreer | 0.185100346 | 5.72E-05 | 0.178948371 | 0.000101002 |
| PEX19 | yellowgreer | -0.011924661 | 0.797169025 | -0.035298392 | 0.446658501 |
| PIGC | yellowgreer | 0.065278043 | 0.159014908 | 0.033351149 | 0.472145267 |
| PIGM | yellowgreer | 0.13782569 | 0.00283845 | 0.108929417 | 0.018538335 |
| POLR3C | yellowgreer | -0.0933013 | 0.043878211 | -0.133091501 | 0.003960991 |
| PRCC | yellowgreer | -0.000402305 | 0.993081966 | -0.017170603 | 0.711313399 |
| PRPF3 | yellowgreer | -0.043822898 | 0.344691113 | -0.066427906 | 0.151784834 |
| PYGO2 | yellowgreer | 0.116416809 | 0.011815339 | 0.094853465 | 0.040469062 |
| RAB4A | yellowgreer | 0.179034185 | 0.000100216 | 0.182282846 | 7.44E-05 |
| RABIF | yellowgreer | 0.33885938 | 5.19E-14 | 0.343115192 | 2.39E-14 |
| RBM34 | yellowgreer | 0.153058881 | 0.000905546 | 0.146984262 | 0.001446738 |
| RFWD2 | yellowgreer | 0.014748723 | 0.750571626 | -0.006107222 | 0.895280339 |
| RNPEP | yellowgreer | 0.359021692 | 1.18E-15 | 0.350683175 | 5.84E-15 |
| RRP15 | yellowgreer | 0.028674791 | 0.536487067 | 0.001093181 | 0.981203156 |
| SCAMP3 | yellowgreer | 0.153568365 | 0.000869967 | 0.144096797 | 0.001796753 |
| SCCPDH | yellowgreer | 0.108774601 | 0.018706941 | 0.085891909 | 0.063654225 |
| SDHC | yellowgreer | 0.099108476 | 0.032250921 | 0.071040309 | 0.125271621 |
| SH3BP5L | yellowgreer | 0.176553552 | 0.000125412 | 0.17635991 | 0.00012761 |
| SLC25A4 | yellowgreer | 0.102696706 | 0.026471937 | 0.08642414 | 0.062023306 |
| SMYD2 | yellowgreer | 0.31101048 | 6.23E-12 | 0.309609041 | 7.83E-12 |
| SNAP47 | yellowgreer | 0.023219263 | 0.616726381 | 0.006194752 | 0.893788294 |
| SNAPIN | yellowgreer | 0.113793387 | 0.013874258 | 0.094040252 | 0.042226179 |
| SRP9 | yellowgreer | 0.264056284 | 6.87E-09 | 0.254723504 | 2.37E-08 |
| STX6 | yellowgreer | 0.367919018 | 2.04E-16 | 0.355886285 | 2.17E-15 |
| TARS2 | yellowgreer | 0.026360639 | 0.569878584 | 0.013717218 | 0.767496567 |
| TATDN3 | yellowgreer | 0.272247602 | 2.22E-09 | 0.24261361 | 1.11E-07 |
| TBCE | yellowgreer | 0.168725772 | 0.000249556 | 0.137927929 | 0.002817777 |
| TFB2M | yellowgreer | 0.083549195 | 0.071258031 | 0.055461018 | 0.231606184 |
| TIMM17/ | yellowgreer | 0.162583249 | 0.000419448 | 0.153639429 | 0.000865109 |
| TIPRL | yellowgreer | 0.111332005 | 0.01608663 | 0.094332548 | 0.041587346 |
| TMEM18 | yellowgreer | 0.242175644 | 1.17E-07 | 0.224689768 | 9.32E-07 |
| TOMM20 | yellowgreer | 0.136516354 | 0.00311568 | 0.098531001 | 0.033275043 |
| TOR3A | yellowgreer | 0.36419493 | 4.29E-16 | 0.348654394 | 8.55E-15 |
| TPM3 | yellowgreer | 0.164431841 | 0.000359448 | 0.139090552 | 0.002592147 |

| | | | | | |
|---|---|---|---|---|---|
| TSEN15 | yellowgreen | 0.154051249 | 0.000837443 | 0.119480062 | 0.009756843 |
| TSNAX | yellowgreen | 0.030402549 | 0.512213201 | 0.019285209 | 0.677645163 |
| TTC13 | yellowgreen | 0.044115635 | 0.341474853 | 0.018310287 | 0.693092866 |
| UBE2Q1 | yellowgreen | 0.07918156 | 0.08740896 | 0.050512857 | 0.275994201 |
| UBQLN4 | yellowgreen | 0.006154414 | 0.894475855 | -0.041920703 | 0.366057115 |
| UCHL5 | yellowgreen | 0.161848973 | 0.00044577 | 0.133520428 | 0.003844811 |
| USP21 | yellowgreen | 0.03650867 | 0.431219178 | 0.006724177 | 0.884771738 |
| VPS45 | yellowgreen | 0.138675818 | 0.002670673 | 0.123697469 | 0.007445191 |
| VPS72 | yellowgreen | 0.008773841 | 0.850014594 | -0.0081688 | 0.860247506 |

| GS.cg07773116 | p.GS.cg07773116 | GS.cg14720763 | p.GS.cg14720763 | GS.cg20186445 |
|---|---|---|---|---|
| -0.142396252 | 0.002037607 | -0.102310337 | 0.027048024 | -0.11707949 |
| -0.177886497 | 0.000111215 | -0.159695777 | 0.000532083 | -0.165125343 |
| -0.154170768 | 0.000829568 | -0.1386361 | 0.002678306 | -0.13624427 |
| -0.11270933 | 0.014813315 | -0.090285513 | 0.051195034 | -0.09443133 |
| -0.279499896 | 7.91E-10 | -0.254780881 | 2.36E-08 | -0.272211901 |
| -0.168457867 | 0.000255368 | -0.147512803 | 0.001389899 | -0.141167627 |
| -0.121134957 | 0.008782952 | -0.123806768 | 0.007392416 | -0.135632345 |
| -0.166039139 | 0.000313883 | -0.149096604 | 0.00123164 | -0.143565238 |
| -0.125214543 | 0.006741965 | -0.113450734 | 0.014165252 | -0.109812214 |
| -0.284190813 | 3.99E-10 | -0.251957571 | 3.40E-08 | -0.27062187 |
| -0.111210163 | 0.016203758 | -0.097647557 | 0.034895409 | -0.100481142 |
| -0.173377336 | 0.000166397 | -0.172485189 | 0.000179994 | -0.178933502 |
| -0.237323952 | 2.11E-07 | -0.237025878 | 2.19E-07 | -0.23350034 |
| -0.140329252 | 0.002369973 | -0.112664092 | 0.014853687 | -0.06113615 |
| -0.141786773 | 0.002130881 | -0.137262947 | 0.002954728 | -0.139227756 |
| -0.156554938 | 0.000686107 | -0.134719866 | 0.003536124 | -0.136065153 |
| -0.112131794 | 0.015336043 | -0.115702837 | 0.012347059 | -0.12272438 |
| -0.099225156 | 0.032047297 | -0.082603259 | 0.07453158 | -0.083019002 |
| -0.087250901 | 0.059558328 | -0.109019397 | 0.018440951 | -0.111965388 |
| -0.076182966 | 0.100113004 | -0.050778644 | 0.273472154 | -0.063114589 |
| -0.223095766 | 1.12E-06 | -0.214121248 | 3.03E-06 | -0.208289158 |
| -0.13949092 | 0.002518343 | -0.156681373 | 0.000679183 | -0.11768828 |
| -0.070940314 | 0.125805513 | -0.038689539 | 0.404191008 | -0.03362143 |
| -0.063608775 | 0.169969014 | -0.07230792 | 0.118655268 | -0.057413478 |
| -0.034827444 | 0.452750018 | -0.011406291 | 0.80580653 | 0.002400389 |
| -0.20649249 | 6.83E-06 | -0.186370649 | 5.07E-05 | -0.190243653 |
| -0.095874423 | 0.038350921 | -0.074791417 | 0.106487061 | -0.093211265 |
| -0.232910075 | 3.58E-07 | -0.219323702 | 1.71E-06 | -0.214821192 |
| -0.116286759 | 0.011910657 | -0.122522 | 0.008034412 | -0.137312551 |
| -0.081341523 | 0.079087538 | -0.079264968 | 0.087075048 | -0.082888137 |
| -0.142537039 | 0.002016599 | -0.125811622 | 0.006481932 | -0.115126635 |
| -0.263093964 | 7.82E-09 | -0.211093135 | 4.19E-06 | -0.203066273 |
| -0.19525714 | 2.15E-05 | -0.172247441 | 0.00018379 | -0.201976798 |
| -0.137637774 | 0.002876807 | -0.113411936 | 0.014198536 | -0.104420879 |
| -0.039523154 | 0.39413272 | -0.022980416 | 0.620356246 | -0.016585991 |
| -0.050885997 | 0.272457949 | -0.043450528 | 0.348810021 | -0.039859006 |
| -0.13155409 | 0.004403989 | -0.118025499 | 0.010690846 | -0.129297747 |
| -0.043603358 | 0.347115763 | -0.032285619 | 0.486422697 | -0.036026459 |
| -0.066179637 | 0.153324379 | -0.046535467 | 0.315625182 | -0.033920021 |
| -0.159834791 | 0.000526072 | -0.123398154 | 0.007591441 | -0.127024935 |
| -0.081561009 | 0.078279167 | -0.078547256 | 0.08998215 | -0.111931839 |
| -0.213834836 | 3.12E-06 | -0.19526564 | 2.14E-05 | -0.194681915 |
| -0.202391105 | 1.04E-05 | -0.213411861 | 3.27E-06 | -0.218748125 |
| -0.120108349 | 0.009376343 | -0.086992839 | 0.060318888 | -0.118895866 |

| | | | | |
|---|---|---|---|---|
| 0.137477602 | 0.002909873 | 0.184170201 | 6.24E-05 | 0.190460198 |
| 0.024760613 | 0.593528902 | 0.093475245 | 0.043484515 | 0.123251312 |
| -0.036961871 | 0.425518122 | -0.005222424 | 0.910382694 | -0.022989642 |
| -0.029923288 | 0.518888831 | 0.011032618 | 0.812047707 | 0.025948154 |
| -0.037249114 | 0.421927556 | -0.009901875 | 0.831004765 | 0.010369741 |
| 0.145682653 | 0.001595937 | 0.214914558 | 2.78E-06 | 0.218034309 |
| -0.029545087 | 0.524188228 | -0.023829886 | 0.607488987 | -0.04584114 |
| 0.069813737 | 0.131943236 | 0.106103261 | 0.021834036 | 0.14202734 |
| 0.106541927 | 0.021291339 | 0.154887674 | 0.000783753 | 0.108781036 |
| 0.194345709 | 2.35E-05 | 0.241469594 | 1.27E-07 | 0.258111248 |
| 0.043145207 | 0.352210432 | 0.108958736 | 0.018506555 | 0.133895869 |
| 0.037376961 | 0.420335167 | 0.10558382 | 0.022492142 | 0.098752894 |
| -0.160745243 | 0.00048824 | -0.140606903 | 0.002322621 | -0.137140509 |
| 0.21811058 | 1.95E-06 | 0.266616172 | 4.84E-09 | 0.274019435 |
| -0.024620746 | 0.59561741 | 0.003576084 | 0.938565492 | -0.030330678 |
| 0.286259621 | 2.94E-10 | 0.326759236 | 4.42E-13 | 0.366601435 |
| 0.092286778 | 0.046234648 | 0.152174621 | 0.000970504 | 0.126764582 |
| -0.227848798 | 6.48E-07 | -0.188775155 | 4.03E-05 | -0.18553156 |
| -0.082786891 | 0.073886688 | -0.041478297 | 0.37114198 | -0.036999546 |
| -0.089490491 | 0.053285224 | -0.100813425 | 0.02938295 | -0.105170595 |
| -0.119105024 | 0.009990454 | -0.089826415 | 0.052393537 | -0.114217543 |
| 0.194478604 | 2.32E-05 | 0.216028584 | 2.46E-06 | 0.213550733 |
| 0.0405351 | 0.38212773 | 0.068567757 | 0.138998074 | 0.040353916 |
| 0.021465236 | 0.64359531 | -0.015622504 | 0.736327272 | -0.008837681 |
| -0.233972749 | 3.16E-07 | -0.207162053 | 6.37E-06 | -0.228160607 |
| -0.215635485 | 2.57E-06 | -0.168336382 | 0.000258046 | -0.169214423 |
| 0.182299041 | 7.43E-05 | 0.184712695 | 5.93E-05 | 0.199506727 |
| -0.042300686 | 0.36172448 | -0.02632677 | 0.570374539 | -0.004477617 |
| -0.211583617 | 3.98E-06 | -0.144067053 | 0.001800731 | -0.124915212 |
| -0.082863077 | 0.073620472 | -0.010658382 | 0.818310255 | -0.000752137 |
| -0.229317683 | 5.47E-07 | -0.198850964 | 1.50E-05 | -0.179268989 |
| 0.046799875 | 0.312880401 | 0.087283051 | 0.059464133 | 0.109642103 |
| -0.173258546 | 0.000168151 | -0.130843118 | 0.004623604 | -0.124188492 |
| -0.033797862 | 0.466228769 | -0.027532923 | 0.552840251 | -0.017196401 |
| -0.093609048 | 0.043183702 | -0.032005374 | 0.490216087 | -0.05851467 |
| 0.044407357 | 0.33828886 | 0.097604922 | 0.034975278 | 0.0830836 |
| 0.075501322 | 0.103196355 | 0.103125514 | 0.025844971 | 0.110710201 |
| 0.012514541 | 0.787370048 | -0.002158915 | 0.96288816 | 0.040590137 |
| -0.09289453 | 0.04481059 | -0.065254332 | 0.159166683 | -0.044408847 |
| 0.038816923 | 0.402644173 | 0.065195329 | 0.159544844 | 0.087616272 |
| -0.038700593 | 0.404056644 | 0.01732737 | 0.70879705 | 0.008113771 |
| -0.052949139 | 0.253464298 | -0.007528888 | 0.871095672 | -0.004268688 |
| -0.003089125 | 0.946917809 | 0.045685042 | 0.324559875 | 0.033268577 |
| -0.171299434 | 0.000199682 | -0.098333157 | 0.03363223 | -0.098507241 |
| -0.05438578 | 0.240793713 | -0.026139823 | 0.57311572 | 0.001008637 |

| | | | | |
|---|---|---|---|---|
| -0.029586582 | 0.523605445 | 0.008118494 | 0.861099397 | 0.008490656 |
| 0.065508742 | 0.157543873 | 0.098628998 | 0.03309932 | 0.11789366 |
| -0.277910952 | 9.94E-10 | -0.237309019 | 2.12E-07 | -0.220145397 |
| -0.062071627 | 0.180545975 | -0.04990286 | 0.281842093 | -0.064930682 |
| 0.11502075 | 0.012874654 | 0.161621031 | 0.000454248 | 0.14910896 |
| -0.07984071 | 0.084798026 | -0.004700721 | 0.919302978 | -0.019953859 |
| 0.048776784 | 0.292856199 | 0.059639064 | 0.198267757 | 0.050064891 |
| -0.174047147 | 0.000156829 | -0.115825001 | 0.012254608 | -0.156792995 |
| -0.158014535 | 0.000610006 | -0.099809231 | 0.031044435 | -0.08393193 |
| -0.177247121 | 0.000117825 | -0.18494091 | 5.80E-05 | -0.164620393 |
| 0.003233234 | 0.944445415 | 0.020915335 | 0.652117683 | 0.034388694 |
| -0.05186458 | 0.263331194 | -0.046260778 | 0.318493328 | -0.064326157 |
| -0.191568567 | 3.08E-05 | -0.144495304 | 0.001744221 | -0.098439744 |
| -0.096299352 | 0.037497437 | -0.055682606 | 0.229744051 | -0.024556207 |
| -0.075579118 | 0.102840696 | -0.080066635 | 0.083917728 | -0.097132861 |
| 0.034818752 | 0.452862883 | 0.063202853 | 0.172716049 | 0.093067516 |
| 0.042032851 | 0.364775035 | 0.070188652 | 0.129875487 | 0.066689413 |
| -0.155529729 | 0.00074472 | -0.11901529 | 0.01004708 | -0.130982578 |
| -0.018389546 | 0.691832136 | 0.043264071 | 0.350884143 | 0.074830123 |
| -0.25474168 | 2.37E-08 | -0.262920627 | 8.01E-09 | -0.226729082 |
| 0.019717044 | 0.670845173 | 0.035071515 | 0.449587251 | 0.008026745 |
| -0.03652943 | 0.430957067 | -0.004822515 | 0.917219552 | 0.023447221 |
| 0.128542722 | 0.005403556 | 0.068049709 | 0.142015133 | 0.073174665 |
| -0.164772295 | 0.000349311 | -0.0880278 | 0.057316392 | -0.077589781 |
| 0.077108789 | 0.096042904 | 0.133354182 | 0.00388947 | 0.157009163 |
| -0.093858123 | 0.042628405 | -0.093057097 | 0.044435984 | -0.087546369 |
| 0.078330649 | 0.090874656 | 0.126443731 | 0.006216476 | 0.142240868 |
| 0.018932192 | 0.683223568 | 0.076441215 | 0.098964143 | 0.090209592 |
| 0.040550986 | 0.381941073 | 0.067148072 | 0.147385383 | 0.056093088 |
| 0.061566154 | 0.184128415 | 0.003238995 | 0.944346575 | 0.03756672 |
| 0.130089836 | 0.004867028 | 0.144866324 | 0.001696582 | 0.144447757 |
| -0.20010894 | 1.32E-05 | -0.188542413 | 4.13E-05 | -0.196999907 |
| 0.093897627 | 0.042540889 | 0.037691157 | 0.416436704 | 0.018137369 |
| 0.007613944 | 0.869652308 | -0.015511354 | 0.738134351 | -0.014736353 |
| 0.002110468 | 0.963720386 | 0.03454374 | 0.456442071 | 0.044339148 |
| 0.058850792 | 0.204274003 | 0.055974745 | 0.227305292 | 0.082652545 |
| -0.085198964 | 0.065830374 | -0.029566465 | 0.523887947 | -0.021243595 |
| -0.187904343 | 4.38E-05 | -0.233605473 | 3.30E-07 | -0.28181892 |
| -0.036994893 | 0.425104436 | -0.072865388 | 0.115833422 | -0.100435332 |
| 0.200748862 | 1.24E-05 | 0.153659451 | 0.000863744 | 0.195030848 |
| -0.249032316 | 4.94E-08 | -0.190092472 | 3.55E-05 | -0.185238112 |
| 0.045596414 | 0.325500357 | 0.050853426 | 0.272765385 | 0.059414003 |
| -0.082924339 | 0.07340697 | -0.045362937 | 0.327986389 | -0.041869551 |
| 0.183294563 | 6.77E-05 | 0.190951684 | 3.27E-05 | 0.217312692 |
| -0.097680083 | 0.034834582 | -0.075029739 | 0.10537322 | -0.074968703 |

| | | | | |
|---|---|---|---|---|
| -0.078399975 | 0.090588234 | -0.080820232 | 0.081034603 | -0.101187241 |
| 0.012380014 | 0.789601896 | 0.081732824 | 0.07765106 | 0.088834406 |
| 0.029311159 | 0.527479841 | 0.028838777 | 0.534158639 | 0.018721684 |
| -0.001485021 | 0.974467592 | -0.000644008 | 0.988925851 | 0.019640017 |
| 0.2544216 | 2.47E-08 | 0.238378851 | 1.86E-07 | 0.27353578 |
| -0.136372336 | 0.00314763 | -0.158308489 | 0.00059566 | -0.154889069 |
| 0.047112386 | 0.309656532 | 0.020631809 | 0.656529608 | 0.017966216 |
| -0.188787373 | 4.03E-05 | -0.140312403 | 0.002372875 | -0.169219867 |
| -0.095913271 | 0.038272217 | -0.113878846 | 0.013802508 | -0.111085739 |
| -0.010890764 | 0.814420139 | 0.03927922 | 0.397060261 | 0.035229694 |
| -0.073423407 | 0.113061718 | -0.024451011 | 0.598156405 | -0.037221159 |
| -0.003504007 | 0.939801362 | -0.029944293 | 0.518595317 | -0.022107721 |
| 0.202524717 | 1.03E-05 | 0.209509815 | 4.97E-06 | 0.211544668 |
| -0.034001883 | 0.463540318 | 0.024628825 | 0.595496691 | 0.029829447 |
| 0.06588307 | 0.155178909 | 0.077352554 | 0.094993512 | 0.127904102 |
| -0.13912084 | 0.002586495 | -0.103297616 | 0.025596961 | -0.062448974 |
| -0.015388627 | 0.740131316 | 0.022985774 | 0.620274722 | -0.008285526 |
| 0.160136242 | 0.000513254 | 0.176910275 | 0.000121454 | 0.223889826 |
| -0.096388001 | 0.037321429 | -0.070056296 | 0.130602583 | -0.078052679 |
| -0.067650521 | 0.144373963 | -0.069953357 | 0.131170247 | -0.061779307 |
| -0.012179555 | 0.792930756 | 0.018941859 | 0.683070568 | 0.019365789 |
| 0.123118321 | 0.007730489 | 0.130611919 | 0.004697121 | 0.116085951 |
| -0.107374655 | 0.020293281 | -0.090023066 | 0.051877333 | -0.12327667 |
| -0.092914317 | 0.044764854 | -0.114329133 | 0.013429822 | -0.107733089 |
| 0.103149289 | 0.025810587 | 0.114537782 | 0.013260157 | 0.140024511 |
| -0.112111192 | 0.015354984 | -0.063692508 | 0.169406442 | -0.068498081 |
| -0.044796727 | 0.334066174 | -0.052772469 | 0.255053838 | -0.087115928 |
| 0.114126308 | 0.013596581 | 0.158900123 | 0.00056773 | 0.148168945 |
| 0.097199626 | 0.035742324 | 0.070055333 | 0.130607882 | 0.079221032 |
| 0.063083047 | 0.173533114 | 0.088191827 | 0.056852106 | 0.0783206 |
| 0.209859537 | 4.79E-06 | 0.170729277 | 0.00020985 | 0.162046525 |
| -0.124031673 | 0.007284874 | -0.114146912 | 0.013579559 | -0.114913374 |
| 0.098626028 | 0.033104636 | 0.148989447 | 0.0012418 | 0.135540785 |
| -0.001888189 | 0.967539215 | 0.058113278 | 0.210011967 | 0.056229343 |
| -0.053451578 | 0.248981393 | -0.004771933 | 0.918084752 | 0.031747328 |
| -0.063341691 | 0.171772779 | -0.015066508 | 0.745380999 | -0.003532232 |
| -0.033662681 | 0.468014841 | -0.0312188 | 0.50094728 | -0.038904727 |
| -0.202012109 | 1.09E-05 | -0.147190973 | 0.001424259 | -0.149366181 |
| 0.090366346 | 0.050986402 | 0.134820992 | 0.003511154 | 0.141181724 |
| 0.157816609 | 0.000619845 | 0.196328141 | 1.93E-05 | 0.192783674 |
| -0.107785202 | 0.019816375 | -0.077109729 | 0.096038836 | -0.065153455 |
| 0.070889509 | 0.126077443 | 0.021234738 | 0.647161971 | 0.043871415 |
| 0.047902484 | 0.301603769 | 0.118786697 | 0.010192623 | 0.118164732 |
| -0.0312255 | 0.500855357 | 0.042131605 | 0.363648407 | 0.049247203 |
| -0.043079218 | 0.352948096 | 0.007012966 | 0.87985961 | 0.031199504 |

| | | | | |
|---|---|---|---|---|
| -0.251987602 | 3.39E-08 | -0.222631422 | 1.18E-06 | -0.217877414 |
| -0.162922843 | 0.00040777 | -0.190109417 | 3.55E-05 | -0.200472809 |
| 0.139224404 | 0.002567256 | 0.144735118 | 0.001713291 | 0.114131126 |
| -0.101022101 | 0.029047423 | -0.046474126 | 0.316264201 | -0.042715521 |
| -0.123566073 | 0.00750908 | -0.109816793 | 0.017597265 | -0.098418631 |
| -0.143229783 | 0.001916086 | -0.071143989 | 0.124719919 | -0.074481764 |
| 0.013249235 | 0.775212389 | 0.093909494 | 0.042514628 | 0.101239973 |
| -0.038848077 | 0.402266415 | 0.021999044 | 0.635366784 | 0.016103616 |
| -0.047469332 | 0.306001128 | -0.047465216 | 0.306043114 | -0.033623559 |
| -0.230364966 | 4.84E-07 | -0.204817298 | 8.13E-06 | -0.229007941 |
| 0.133830913 | 0.003762647 | 0.111768484 | 0.015673107 | 0.078250539 |
| -0.219635918 | 1.65E-06 | -0.174812517 | 0.000146527 | -0.207602486 |
| 0.077715401 | 0.093448466 | 0.107155814 | 0.020551549 | 0.129877582 |
| -0.203788577 | 9.05E-06 | -0.20224953 | 1.06E-05 | -0.197624339 |
| -0.038077151 | 0.411676622 | 0.023320571 | 0.615189579 | 0.034711201 |
| -0.063683441 | 0.169467292 | 0.007427706 | 0.872813242 | 0.020350625 |
| -0.044200195 | 0.340549385 | 0.014021009 | 0.762500064 | 0.00845248 |
| 0.22975312 | 5.19E-07 | 0.232151611 | 3.92E-07 | 0.223021221 |
| 0.161884478 | 0.000444462 | 0.165083397 | 0.000340281 | 0.191862946 |
| -0.001594624 | 0.972583871 | 0.043449222 | 0.348824518 | 0.054670454 |
| -0.075671792 | 0.102418284 | -0.054928662 | 0.236123486 | -0.050175867 |
| -0.059118078 | 0.202222802 | -0.021435271 | 0.644058513 | -0.007651622 |
| -0.012844423 | 0.781904598 | 0.02921301 | 0.528864025 | 0.051459038 |
| -0.074659622 | 0.107107014 | -0.026714504 | 0.564709174 | -0.02637956 |
| -0.009820963 | 0.832365173 | 0.039476489 | 0.394691759 | 0.053881812 |
| 0.098229185 | 0.033821248 | 0.170932361 | 0.000206174 | 0.162162735 |
| 0.057218197 | 0.217130906 | 0.068552036 | 0.139088901 | 0.043409314 |
| -0.036031461 | 0.4372697 | 0.015137575 | 0.744221772 | 0.010709251 |
| 0.160292215 | 0.000506737 | 0.18708525 | 4.74E-05 | 0.178133698 |
| 0.170263867 | 0.000218508 | 0.215015355 | 2.75E-06 | 0.23170578 |
| 0.057747261 | 0.212902425 | 0.059384518 | 0.200193031 | 0.067706662 |
| 0.035149112 | 0.448584331 | 0.102523652 | 0.026728645 | 0.082476175 |
| -0.107259136 | 0.020429257 | -0.113087957 | 0.014479173 | -0.119084045 |
| -0.186800425 | 4.87E-05 | -0.176735362 | 0.00012338 | -0.18936393 |
| -0.074275599 | 0.108929683 | -0.096556932 | 0.036987964 | -0.112419944 |
| -0.160327682 | 0.000505265 | -0.157300699 | 0.000646189 | -0.132337858 |
| 0.043335368 | 0.350090126 | 0.045955612 | 0.321699588 | 0.051603074 |
| -0.153967271 | 0.000843017 | -0.108418195 | 0.019100183 | -0.135787023 |
| 0.178131041 | 0.00010878 | 0.238697662 | 1.79E-07 | 0.281288468 |
| -0.158576681 | 0.000582845 | -0.158400751 | 0.000591222 | -0.128657596 |
| -0.108064133 | 0.019497951 | -0.066849583 | 0.149196897 | -0.050816449 |
| -0.060186188 | 0.194175306 | -0.034060077 | 0.462775071 | -0.065371231 |
| -0.095006491 | 0.040145421 | -0.040301623 | 0.384877515 | -0.027874972 |
| -0.147568266 | 0.001384055 | -0.120554287 | 0.009114341 | -0.129573297 |
| -0.030374824 | 0.51259817 | 0.016418912 | 0.723422338 | 0.020890971 |

| | | | | |
|---|---|---|---|---|
| -0.085246371 | 0.065679572 | -0.056490678 | 0.223043355 | -0.067423141 |
| 0.029805774 | 0.520532484 | 0.010021652 | 0.828991834 | 0.042593159 |
| -0.085702946 | 0.064241693 | -0.067122526 | 0.147539765 | -0.095290769 |
| 0.048277682 | 0.297828814 | 0.024644807 | 0.59525789 | 0.051930887 |
| -0.089703117 | 0.052719366 | -0.025861102 | 0.577214103 | -0.023038606 |
| -0.275212249 | 1.46E-09 | -0.216577876 | 2.31E-06 | -0.216497322 |
| 0.105563346 | 0.022518431 | 0.134048778 | 0.003705946 | 0.131022666 |
| -0.02746735 | 0.553786701 | -0.010368484 | 0.823169448 | -0.020353542 |
| -0.232842179 | 3.61E-07 | -0.168580823 | 0.000252685 | -0.152736872 |
| 0.161586964 | 0.000455528 | 0.158114704 | 0.000605082 | 0.179301114 |
| -0.012208482 | 0.79245016 | 0.009404402 | 0.83937681 | 0.018049996 |
| -0.019942095 | 0.66731194 | 0.02026179 | 0.662305454 | 0.05314275 |
| 0.073196318 | 0.114183325 | 0.122121157 | 0.008244656 | 0.143899079 |
| -0.074109661 | 0.109724796 | -0.082453232 | 0.075061849 | -0.065966276 |
| 0.140861681 | 0.002279932 | 0.141075882 | 0.002244597 | 0.167335941 |
| -0.097578165 | 0.035025481 | -0.030150609 | 0.515716894 | -0.007600683 |
| 0.012161139 | 0.793236775 | 0.071483465 | 0.122926703 | 0.075148263 |
| 0.103593977 | 0.025174695 | 0.128426731 | 0.005445849 | 0.128281161 |
| 0.126533926 | 0.006179405 | 0.186191328 | 5.16E-05 | 0.179371786 |
| 0.071391836 | 0.123408728 | 0.072625435 | 0.117041526 | 0.110240173 |
| -0.051151233 | 0.269963177 | -0.009117533 | 0.844212883 | 0.025335247 |
| -0.168726543 | 0.000249539 | -0.122877093 | 0.007852175 | -0.118027014 |
| -0.205135093 | 7.87E-06 | -0.172726547 | 0.000176217 | -0.185033784 |
| -0.044619155 | 0.335987703 | -0.001012766 | 0.982585636 | -0.032038785 |
| -0.121814546 | 0.008408773 | -0.078286909 | 0.091055738 | -0.099240017 |
| 0.165389476 | 0.00033161 | 0.210458648 | 4.49E-06 | 0.208077075 |
| 0.044926229 | 0.332669269 | 0.01731511 | 0.708993728 | 0.022732536 |
| -0.11383619 | 0.013838281 | -0.098415408 | 0.03348334 | -0.08707668 |
| 0.035863645 | 0.439408958 | 0.070640938 | 0.127414495 | 0.070392631 |
| 0.064673112 | 0.162921379 | 0.094741595 | 0.040707053 | 0.095344187 |
| 0.114603364 | 0.01320722 | 0.169275126 | 0.000238021 | 0.181169978 |
| -0.077675465 | 0.093617528 | -0.007685536 | 0.86843777 | -0.017984758 |
| 0.096225799 | 0.037644007 | 0.114180039 | 0.013552229 | 0.167310428 |
| -0.246899178 | 6.48E-08 | -0.222641905 | 1.18E-06 | -0.236319816 |
| 0.132873433 | 0.004021268 | 0.174506377 | 0.000150569 | 0.185043001 |
| 0.157710579 | 0.000625177 | 0.186382436 | 5.07E-05 | 0.193038663 |
| 0.03376381 | 0.466678328 | 0.03467558 | 0.454724249 | 0.01952252 |
| 0.158298113 | 0.000596161 | 0.216697998 | 2.28E-06 | 0.187957168 |
| 0.196363957 | 1.92E-05 | 0.239105829 | 1.70E-07 | 0.269388353 |
| -0.037232962 | 0.422128983 | 0.003680157 | 0.936781287 | 0.014764108 |
| -0.177328183 | 0.000116967 | -0.174771522 | 0.000147063 | -0.190385314 |
| 0.001390346 | 0.976094859 | 0.062229354 | 0.179438746 | 0.088421404 |
| -0.12744383 | 0.005816371 | -0.081955 | 0.076844915 | -0.077362944 |
| 0.011408388 | 0.805771525 | 0.038480383 | 0.40673848 | 0.079484588 |
| -0.100082152 | 0.030585107 | -0.027229622 | 0.557224554 | -0.037778223 |

| | | | | |
|---|---|---|---|---|
| -0.085920571 | 0.063565505 | -0.052030933 | 0.261800861 | -0.105090457 |
| 0.090992442 | 0.049394351 | 0.126857324 | 0.006048109 | 0.161050976 |
| 0.168437029 | 0.000255826 | 0.188405694 | 4.18E-05 | 0.20527853 |
| 0.096862994 | 0.036390246 | 0.151650828 | 0.001010983 | 0.096506901 |
| -0.024482851 | 0.597679748 | 0.029300255 | 0.527633529 | 0.040652551 |
| 0.068379318 | 0.14008978 | 0.104308626 | 0.024181082 | 0.122801093 |
| -0.080319634 | 0.082940711 | -0.011456499 | 0.804968864 | -0.011349299 |
| 0.165721253 | 0.000322444 | 0.263198655 | 7.71E-09 | 0.278996563 |
| 0.036874248 | 0.426616942 | 0.057456647 | 0.215217722 | 0.03998922 |
| 0.105954633 | 0.022020613 | 0.093333782 | 0.043804466 | 0.095637073 |
| -0.051591211 | 0.265859334 | -0.007716604 | 0.86791079 | -0.006125403 |
| -0.016481273 | 0.722415082 | 0.016057413 | 0.729270586 | 0.011159634 |
| 0.091163554 | 0.04896655 | 0.019086637 | 0.680780874 | -0.013588603 |
| -0.166807976 | 0.000294049 | -0.113367727 | 0.014236547 | -0.11634566 |
| 0.005456716 | 0.906380264 | -0.047970726 | 0.300914811 | -0.022632662 |
| 0.253353782 | 2.84E-08 | 0.230929401 | 4.53E-07 | 0.214850442 |
| -0.160074331 | 0.000515863 | -0.124478171 | 0.00707551 | -0.101988309 |
| -0.053831265 | 0.245630578 | 0.003657482 | 0.937169996 | -0.001158329 |
| -0.058570934 | 0.206437807 | 1.09E-05 | 0.999811964 | -0.034038749 |
| -0.053102238 | 0.252092417 | -0.003178148 | 0.945390423 | 0.010971818 |
| -0.058688549 | 0.205526419 | -0.013878553 | 0.764841846 | -0.017645175 |
| 0.021508856 | 0.64292126 | 0.046804091 | 0.312836764 | 0.061552785 |
| 0.122235458 | 0.00818421 | 0.156310353 | 0.000699689 | 0.162898686 |
| -0.071653541 | 0.122035892 | -0.0576559 | 0.213628351 | -0.026753341 |
| -0.051904516 | 0.262963252 | 0.009855098 | 0.83179118 | 0.00713776 |
| 0.031122446 | 0.50227032 | 0.033814322 | 0.466011555 | 0.037983614 |
| -0.058292981 | 0.208603249 | -0.093424433 | 0.043599211 | -0.119010203 |
| -0.099107911 | 0.032251911 | -0.06486562 | 0.161670464 | -0.070043842 |
| 0.09963117 | 0.031347283 | 0.13241249 | 0.004151419 | 0.140387543 |
| 0.206417745 | 6.88E-06 | 0.233792282 | 3.23E-07 | 0.228396553 |
| -0.028243083 | 0.542641134 | -0.014468776 | 0.755153552 | -0.013731388 |
| 0.022706574 | 0.624529314 | -0.004899988 | 0.915894583 | 0.001288554 |
| -0.043593498 | 0.347224907 | -0.078702768 | 0.089345724 | -0.074708389 |
| -0.134421585 | 0.003610714 | -0.103440189 | 0.025393061 | -0.112441665 |
| 0.06767415 | 0.144233511 | 0.114307219 | 0.013447752 | 0.106091176 |
| 0.096811569 | 0.036490099 | 0.145522382 | 0.001615254 | 0.160256118 |
| 0.040575521 | 0.381652898 | 0.091898007 | 0.047165372 | 0.08297029 |
| -0.205636473 | 7.47E-06 | -0.216250685 | 2.40E-06 | -0.235640487 |
| 0.079209614 | 0.087296538 | 0.13007343 | 0.004872456 | 0.126729896 |
| 0.146982187 | 0.001446965 | 0.194367464 | 2.34E-05 | 0.2080382 |
| -0.010910421 | 0.814091294 | 0.059937929 | 0.196024542 | 0.069315244 |
| 0.13720719 | 0.002966482 | 0.130622562 | 0.004693714 | 0.108145693 |
| -0.108871201 | 0.018601581 | -0.058806889 | 0.204612359 | -0.064020747 |
| 0.030273693 | 0.514003637 | 0.037508613 | 0.418699067 | 0.084195937 |
| -0.260236168 | 1.15E-08 | -0.240590051 | 1.42E-07 | -0.272638898 |

| | | | | |
|---|---|---|---|---|
| -0.104060303 | 0.024522427 | -0.065365393 | 0.158456711 | -0.028556322 |
| -0.004684177 | 0.919586022 | 0.056419891 | 0.223624707 | 0.063617549 |
| -0.11002385 | 0.017383781 | -0.13666173 | 0.003083729 | -0.13817939 |
| -0.0877094 | 0.058226608 | -0.032802554 | 0.479467199 | -0.033111527 |
| -0.046773405 | 0.313154476 | 0.047715214 | 0.303499762 | 0.043172862 |
| -0.172975595 | 0.000172397 | -0.137208579 | 0.002966188 | -0.1026629 |
| -0.10262936 | 0.026571585 | -0.051697327 | 0.264875989 | -0.071203173 |
| -0.084961896 | 0.066588758 | -0.027366011 | 0.555250915 | -0.019254361 |
| -0.016486419 | 0.72233199 | 0.049716881 | 0.283641571 | 0.031379682 |
| -0.071521534 | 0.122726869 | -0.045897014 | 0.322317652 | -0.042580432 |
| 0.076417885 | 0.099067499 | 0.050482619 | 0.276282129 | 0.060891633 |
| -0.050573897 | 0.275413604 | -0.009634942 | 0.835494713 | -0.008259967 |
| -0.054843079 | 0.236855464 | -0.00019301 | 0.996680973 | 0.000991444 |
| 0.15498017 | 0.000778016 | 0.188419269 | 4.17E-05 | 0.253998032 |
| -0.002538495 | 0.956369168 | 0.009617957 | 0.835780588 | -0.001602432 |
| -0.016264765 | 0.725914145 | -0.01526141 | 0.74220319 | -0.039128482 |
| 0.050500824 | 0.276108759 | 0.088665635 | 0.055528468 | 0.091195958 |
| -0.130957024 | 0.004587767 | -0.106432345 | 0.0214258 | -0.052036946 |
| -0.041463879 | 0.371308425 | -0.000617165 | 0.989387412 | 0.037691247 |
| 0.067973451 | 0.142463454 | 0.063963531 | 0.16759508 | 0.077025927 |
| -0.015195868 | 0.743271332 | -0.003234329 | 0.944426624 | 0.045756418 |
| 0.06008343 | 0.194939181 | 0.088411471 | 0.056235288 | 0.080763719 |
| 0.038465102 | 0.40692497 | 0.070396346 | 0.128740845 | 0.089298085 |
| -0.194152721 | 2.39E-05 | -0.146208727 | 0.001534012 | -0.132866446 |
| -0.221814782 | 1.29E-06 | -0.159869292 | 0.00052459 | -0.178743516 |
| 0.338945423 | 5.11E-14 | 0.377573438 | 2.85E-17 | 0.372412735 |
| -0.212490242 | 3.61E-06 | -0.177999184 | 0.000110087 | -0.170115223 |
| -0.329764123 | 2.62E-13 | -0.304976283 | 1.65E-11 | -0.282883709 |
| -0.083790787 | 0.070441072 | -0.033959197 | 0.464102094 | -0.039840818 |
| -0.213226485 | 3.33E-06 | -0.169815838 | 0.000227157 | -0.199867372 |
| 0.16646385 | 0.000302777 | 0.244666568 | 8.57E-08 | 0.265659545 |
| -0.065668722 | 0.156529831 | -0.000164695 | 0.997167876 | -0.016223777 |
| -0.080100289 | 0.083787233 | -0.028460212 | 0.539541545 | -0.022613206 |
| 0.102766396 | 0.026369159 | 0.103741241 | 0.024967113 | 0.089074833 |
| -0.185172571 | 5.68E-05 | -0.14752533 | 0.001388577 | -0.127553258 |
| -0.117569688 | 0.010999412 | -0.064471848 | 0.164236948 | -0.057120784 |
| 0.033785125 | 0.4663969 | 0.100312265 | 0.030202357 | 0.095129677 |
| -0.064316712 | 0.165256433 | -0.056175377 | 0.225641122 | -0.073537638 |
| 0.163823598 | 0.000378245 | 0.194474448 | 2.32E-05 | 0.209125546 |
| -0.133313632 | 0.003900433 | -0.108373131 | 0.019150413 | -0.1143694 |
| 0.215728728 | 2.54E-06 | 0.286748367 | 2.73E-10 | 0.266305485 |
| 0.185260219 | 5.63E-05 | 0.161223891 | 0.00046938 | 0.18147593 |
| -0.042574883 | 0.358618027 | -0.012525609 | 0.787186507 | -0.034145395 |
| 0.017337086 | 0.708641206 | 0.018870578 | 0.684198968 | -0.010941826 |
| 0.124399951 | 0.007111793 | 0.178837466 | 0.000102026 | 0.177897754 |

| | | | | |
|---|---|---|---|---|
| -0.242459274 | 1.13E-07 | -0.211223623 | 4.14E-06 | -0.237655043 |
| 0.238801427 | 1.77E-07 | 0.285615423 | 3.23E-10 | 0.303590883 |
| -0.013122208 | 0.777310579 | 0.022306019 | 0.630654998 | -0.015452484 |
| 0.175556707 | 0.000137123 | 0.192536214 | 2.80E-05 | 0.195108797 |
| 0.047698587 | 0.303668484 | 0.078893764 | 0.088569036 | 0.080242848 |
| 0.107358859 | 0.020311827 | 0.128430807 | 0.005444358 | 0.167888364 |
| 0.248282037 | 5.44E-08 | 0.278232649 | 9.49E-10 | 0.274196165 |
| -0.016204467 | 0.726889659 | 0.00738035 | 0.873617316 | 0.04058132 |
| -0.014063879 | 0.761795761 | 0.018781132 | 0.685615928 | 0.053509683 |
| -0.134137516 | 0.003683074 | -0.066132065 | 0.153620724 | -0.056851267 |
| -0.102045904 | 0.027448492 | -0.058688813 | 0.205524379 | -0.064693606 |
| -0.007418943 | 0.872962015 | 0.010231301 | 0.825471239 | 0.002508144 |
| 0.13878314 | 0.002650147 | 0.120141779 | 0.009356472 | 0.091923392 |
| -0.154516638 | 0.000807164 | -0.121186199 | 0.00875423 | -0.087510337 |
| 0.036062165 | 0.436878947 | 0.086452004 | 0.061938881 | 0.107344234 |
| -0.004720576 | 0.918963307 | -0.031759991 | 0.493550552 | 0.029364704 |
| -0.007761352 | 0.867151887 | -0.009585769 | 0.836322402 | 0.023032302 |
| -0.0303042 | 0.513579456 | 0.016306101 | 0.725245658 | 0.007039379 |
| -0.031864264 | 0.49213213 | -0.003857103 | 0.933748463 | -0.018032958 |
| -0.000512134 | 0.991193401 | 0.04879568 | 0.292669033 | 0.082962271 |
| 0.071732726 | 0.121622865 | 0.122499291 | 0.008046194 | 0.125656341 |
| -0.224453734 | 9.58E-07 | -0.176078128 | 0.000130875 | -0.184157018 |
| 0.092095064 | 0.04669167 | 0.070568776 | 0.127804701 | 0.075173515 |
| -0.102692668 | 0.026477902 | -0.085508896 | 0.064849614 | -0.090687406 |
| 0.040782056 | 0.379232313 | -0.003051135 | 0.947569678 | -0.021411118 |
| 0.044123237 | 0.341391583 | 0.080659912 | 0.081641148 | 0.065366947 |
| 0.008533739 | 0.85407246 | 0.007865238 | 0.86539052 | -0.005209217 |
| -0.059275722 | 0.201020058 | -0.000506589 | 0.991288751 | 0.005881559 |
| 0.091201042 | 0.048873239 | 0.123970919 | 0.007313786 | 0.113540488 |
| -0.233645083 | 3.28E-07 | -0.195568152 | 2.08E-05 | -0.196858959 |
| -0.044831253 | 0.333693385 | 0.001500887 | 0.974194898 | -0.005862252 |
| -0.050885385 | 0.272463721 | 0.018562394 | 0.689085683 | 0.023131357 |
| -0.02706969 | 0.559543196 | -0.009497568 | 0.837807502 | -0.044364323 |
| -0.136060232 | 0.003217892 | -0.162264022 | 0.000430708 | -0.207636414 |
| 0.113240204 | 0.014346692 | 0.130409844 | 0.004762239 | 0.156959843 |
| 0.09299503 | 0.044578692 | 0.098788765 | 0.032814534 | 0.064852695 |
| -0.00752748 | 0.871119571 | -0.039680525 | 0.392250996 | -0.04269107 |
| 0.027096171 | 0.559158959 | -0.005502036 | 0.905606319 | -0.017277557 |
| 0.023681998 | 0.609720564 | 0.066661449 | 0.150347347 | 0.066141765 |
| -0.065638267 | 0.156722486 | -0.04544689 | 0.327091066 | -0.051031357 |
| -0.077441585 | 0.094612534 | -0.117152964 | 0.011288394 | -0.084441989 |
| 0.145674315 | 0.001596937 | 0.133082133 | 0.003963564 | 0.173287836 |
| -0.177357704 | 0.000116656 | -0.124917304 | 0.006874868 | -0.133216048 |
| -0.089675314 | 0.052793073 | -0.058035949 | 0.210620264 | -0.078980319 |
| 0.007928944 | 0.864310714 | 0.025016863 | 0.589711234 | -0.005356761 |

| | | | | |
|---|---|---|---|---|
| 0.001784815 | 0.969315461 | 0.013968876 | 0.763356806 | 0.009022965 |
| -0.173307658 | 0.000167424 | -0.106197253 | 0.021716754 | -0.128388879 |
| 0.101406867 | 0.028437378 | 0.153654372 | 0.00086409 | 0.174958304 |
| -0.188338039 | 4.21E-05 | -0.170154439 | 0.000220591 | -0.20218572 |
| -0.167859414 | 0.000268813 | -0.114207414 | 0.01352968 | -0.085022503 |
| 0.00864013 | 0.852273898 | 0.046670298 | 0.314223555 | 0.047817667 |
| 0.08080042 | 0.08110936 | 0.073993076 | 0.110286153 | 0.068973597 |
| 0.183916907 | 6.39E-05 | 0.20937569 | 5.04E-06 | 0.206417027 |
| 0.097892513 | 0.034439531 | 0.132507591 | 0.004124258 | 0.139213989 |
| -0.032072791 | 0.489302084 | -0.009863623 | 0.831647839 | 0.014236499 |
| 0.09124638 | 0.048760591 | 0.127022838 | 0.005981886 | 0.173987972 |
| 0.097439204 | 0.035287198 | 0.14120504 | 0.002223533 | 0.143251825 |
| -0.013014854 | 0.779085103 | 0.040909607 | 0.377742127 | 0.039397607 |
| -0.161548329 | 0.000456984 | -0.12387941 | 0.007357527 | -0.111879043 |
| -0.005691795 | 0.90236677 | 0.034748548 | 0.453775061 | 0.037816463 |
| -0.03156368 | 0.496226878 | -0.019411421 | 0.675654991 | -0.009115073 |
| 0.116143139 | 0.012016709 | 0.188688305 | 4.07E-05 | 0.211271143 |
| 0.085035051 | 0.066353973 | 0.162859764 | 0.000409916 | 0.167835491 |
| 0.13554333 | 0.003337399 | 0.104878327 | 0.02341345 | 0.118928409 |
| 0.168468116 | 0.000255144 | 0.1247608 | 0.006945783 | 0.147305951 |
| 0.146587348 | 0.001490818 | 0.115044719 | 0.012855782 | 0.137191138 |
| -0.009504783 | 0.837685992 | -0.013430488 | 0.772221325 | -0.022053011 |
| -0.194510505 | 2.31E-05 | -0.135291765 | 0.003397006 | -0.098179826 |
| 0.196135456 | 1.97E-05 | 0.183288998 | 6.77E-05 | 0.179604277 |
| -0.270333374 | 2.90E-09 | -0.279773353 | 7.60E-10 | -0.293386332 |
| 0.09562767 | 0.038854048 | 0.126809933 | 0.006067191 | 0.147257431 |
| 0.03687727 | 0.426579019 | 0.101576173 | 0.028172449 | 0.06853281 |
| -0.006050532 | 0.896246899 | 0.038309341 | 0.408828815 | 0.059435524 |
| 0.056032353 | 0.226826567 | 0.08798849 | 0.057428126 | 0.076735716 |
| 0.005499343 | 0.905652306 | 0.043823724 | 0.344682008 | 0.033339418 |
| 0.022175394 | 0.632658164 | 0.032625933 | 0.481837584 | 0.012990763 |
| 0.229934717 | 5.09E-07 | 0.200051157 | 1.33E-05 | 0.192698946 |
| 0.05328412 | 0.250469322 | 0.053854936 | 0.245422724 | 0.024136335 |
| -0.036833429 | 0.427129393 | -0.01336428 | 0.773313511 | -0.004107705 |
| -0.169961013 | 0.00022432 | -0.140255355 | 0.002382724 | -0.156978318 |
| -0.12009068 | 0.00938686 | -0.107209862 | 0.020487499 | -0.068883397 |
| -0.013138319 | 0.777044377 | -0.048184778 | 0.298760599 | -0.027142229 |
| -0.009899412 | 0.831046174 | 0.02543209 | 0.583549155 | 0.01125669 |
| -0.071536263 | 0.122649623 | -0.039299555 | 0.396815711 | -0.029531386 |
| 0.046927061 | 0.311565703 | 0.09744893 | 0.035268826 | 0.09171199 |
| 0.103011231 | 0.026010801 | 0.164430273 | 0.000359495 | 0.156368289 |
| -0.178240868 | 0.000107703 | -0.129784248 | 0.004969032 | -0.153104767 |
| -0.058196495 | 0.209358764 | -0.026113631 | 0.573500267 | -0.027324052 |
| 0.348984761 | 8.04E-15 | 0.372028491 | 8.91E-17 | 0.401653875 |
| -0.085656286 | 0.064387439 | -0.048162218 | 0.298987153 | -0.050091695 |

| | | | | |
|---|---|---|---|---|
| 0.036374705 | 0.432912824 | 0.091212923 | 0.048843697 | 0.086827539 |
| 0.060559513 | 0.191418549 | 0.05463603 | 0.23863291 | 0.015444875 |
| 0.018374508 | 0.692071264 | -0.013682124 | 0.768074395 | -0.0297827 |
| 0.197618288 | 1.70E-05 | 0.218423883 | 1.89E-06 | 0.187226801 |
| -0.114151327 | 0.013575913 | -0.170046154 | 0.000222671 | -0.206440881 |
| -0.058363142 | 0.208055103 | -0.104717094 | 0.023628531 | -0.086587377 |
| -0.072701597 | 0.116657003 | -0.052299109 | 0.259346753 | -0.075001042 |
| 0.267575518 | 4.25E-09 | 0.24823402 | 5.47E-08 | 0.26422311 |
| -0.094241324 | 0.041785842 | -0.110382119 | 0.017019736 | -0.089496421 |
| -0.118185991 | 0.01058404 | -0.100077992 | 0.030592064 | -0.123330222 |
| 0.070548018 | 0.127917119 | 0.124655973 | 0.006993648 | 0.106352478 |
| 0.067943663 | 0.142638867 | 0.066492011 | 0.151389225 | 0.055383801 |
| 0.136659694 | 0.003084174 | 0.17850815 | 0.000105124 | 0.183999649 |
| 0.042253537 | 0.362260336 | 0.074049801 | 0.110012738 | 0.0435057 |
| -0.119549585 | 0.009714074 | -0.084633728 | 0.067650394 | -0.070231407 |
| -0.111543388 | 0.015885186 | -0.059811657 | 0.196970044 | -0.058869205 |
| 0.068307202 | 0.140509313 | 0.117826241 | 0.010824779 | 0.151246996 |
| 0.098859661 | 0.032688832 | 0.11195011 | 0.015503798 | 0.098985698 |
| -0.175738561 | 0.000134913 | -0.142395025 | 0.002037791 | -0.166859751 |
| 0.136094138 | 0.003210191 | 0.172855821 | 0.000174224 | 0.195740505 |
| -0.028045749 | 0.545465778 | -0.077621188 | 0.093847692 | -0.066938735 |
| 0.111435994 | 0.015987253 | 0.095286515 | 0.039558854 | 0.095754542 |
| 0.043381069 | 0.349581758 | 0.077802151 | 0.093082071 | 0.080502352 |
| -0.096725991 | 0.036656781 | -0.09689487 | 0.036328469 | -0.097871129 |
| 0.106389768 | 0.021478242 | 0.153090628 | 0.00090329 | 0.169348599 |
| -0.324833294 | 6.16E-13 | -0.302833812 | 2.32E-11 | -0.324030427 |
| -0.26265803 | 8.29E-09 | -0.229290952 | 5.48E-07 | -0.244672219 |
| -0.033093364 | 0.475578173 | 0.007511122 | 0.871397203 | 0.01234879 |
| -0.161998794 | 0.000440277 | -0.115651314 | 0.012386235 | -0.093090836 |
| -0.027739805 | 0.54985942 | -0.002807902 | 0.951744047 | -0.000784254 |
| 0.024280135 | 0.600717391 | 0.07070095 | 0.127090686 | 0.09672497 |
| 0.115206636 | 0.012728935 | 0.154912651 | 0.0007822 | 0.147444521 |
| 0.09357012 | 0.043271037 | 0.083052748 | 0.072961099 | 0.089154937 |
| 0.054861859 | 0.2366947 | 0.007841193 | 0.865798133 | 0.023933943 |
| -0.230957838 | 4.51E-07 | -0.17984456 | 9.31E-05 | -0.19631908 |
| 0.085279608 | 0.065574014 | 0.116983724 | 0.01140766 | 0.128532254 |
| -0.039197722 | 0.398041242 | 0.003129795 | 0.946220009 | -0.033553038 |
| 0.159427197 | 0.000543876 | 0.21524915 | 2.68E-06 | 0.23998295 |
| 0.086780844 | 0.060949665 | 0.153913962 | 0.000846573 | 0.151614517 |
| 0.139099474 | 0.002590481 | 0.149514656 | 0.001192728 | 0.142425939 |
| -0.073966034 | 0.110416684 | -0.072098722 | 0.119727965 | -0.09460981 |
| 0.041306399 | 0.373129444 | -0.003005325 | 0.948355768 | 0.019588871 |
| -0.203586068 | 9.24E-06 | -0.137907783 | 0.00282184 | -0.164295433 |
| 0.052034775 | 0.261765587 | 0.033303996 | 0.472772184 | 0.028367109 |
| 0.041396679 | 0.372084818 | 0.082468767 | 0.075006796 | 0.090872774 |

| | | | | |
|---|---|---|---|---|
| 0.170876212 | 0.000207184 | 0.22785363 | 6.48E-07 | 0.225956348 |
| 0.036781331 | 0.427783953 | 0.04973684 | 0.283448082 | 0.085117606 |
| 0.058568383 | 0.206457607 | 0.072301359 | 0.118688795 | 0.075834904 |
| -0.185028346 | 5.76E-05 | -0.194684761 | 2.27E-05 | -0.189779892 |
| -0.001269384 | 0.978174105 | 0.035372265 | 0.445707227 | 0.074781486 |
| 0.064584182 | 0.163501693 | 0.080988256 | 0.080402828 | 0.054603006 |
| 0.097694416 | 0.034807807 | 0.118423801 | 0.010427517 | 0.084906022 |
| -0.04311179 | 0.352583869 | 0.01998726 | 0.666603738 | 0.041831679 |
| -0.291597994 | 1.32E-10 | -0.268789464 | 3.59E-09 | -0.275559 |
| -0.200795455 | 1.23E-05 | -0.190146534 | 3.54E-05 | -0.216243778 |
| 0.054329405 | 0.24128238 | 0.051478327 | 0.266908143 | 0.034950995 |
| 0.012310571 | 0.790754657 | 0.05417841 | 0.242594655 | 0.041746977 |
| -0.172027374 | 0.00018737 | -0.124603038 | 0.007017931 | -0.137032447 |
| -0.03622375 | 0.434825839 | -0.021244385 | 0.647012527 | -0.003762502 |
| 0.029487741 | 0.524994179 | 0.056229397 | 0.225194531 | 0.067879127 |
| 0.02265102 | 0.62537737 | 0.085364761 | 0.065304213 | 0.091076272 |
| 0.145127774 | 0.001663732 | 0.188987122 | 3.95E-05 | 0.209235078 |
| 0.072101833 | 0.119711954 | 0.107631869 | 0.019993338 | 0.14113366 |
| -0.04299416 | 0.353900366 | -0.038409415 | 0.407605024 | -0.041569101 |
| -0.136755906 | 0.003063189 | -0.098591633 | 0.033166228 | -0.115595893 |
| 0.039249512 | 0.397417685 | 0.098498177 | 0.03333408 | 0.123290608 |
| 0.254645897 | 2.40E-08 | 0.21849742 | 1.87E-06 | 0.228805978 |
| 0.042534509 | 0.359074381 | 0.037556188 | 0.418108747 | 0.033498031 |
| -0.129269998 | 0.005145043 | -0.119718818 | 0.009610662 | -0.114523155 |
| -0.104050847 | 0.024535507 | -0.061720517 | 0.183028873 | -0.0771438 |
| 0.096111687 | 0.037872361 | 0.112913806 | 0.014632031 | 0.101146474 |
| -0.056000229 | 0.22709343 | -0.032824301 | 0.479175783 | -0.043805485 |
| -0.195309771 | 2.13E-05 | -0.150421788 | 0.001112156 | -0.159841925 |
| 0.073276588 | 0.113785871 | 0.087514609 | 0.058789339 | 0.078155068 |
| -0.062052418 | 0.180681163 | -0.02514356 | 0.587827848 | -0.060325568 |
| -0.066832392 | 0.14930174 | -0.005871611 | 0.899298446 | 0.014368891 |
| 0.230133605 | 4.97E-07 | 0.243473593 | 9.94E-08 | 0.230520131 |
| 0.130710064 | 0.004665785 | 0.066001473 | 0.154436468 | 0.074804994 |
| -0.057237829 | 0.216972932 | 0.010072739 | 0.828133618 | 0.013750676 |
| 0.203155932 | 9.66E-06 | 0.236813459 | 2.25E-07 | 0.265315922 |
| -0.095444464 | 0.039231207 | -0.050470754 | 0.276395168 | -0.051799912 |
| -0.053910173 | 0.244938177 | 0.001979821 | 0.965964852 | 0.010981813 |
| -0.028631357 | 0.537104638 | 0.028891618 | 0.533409427 | 0.027086654 |
| -0.206459136 | 6.85E-06 | -0.130325376 | 0.004789699 | -0.121042915 |
| -0.061057259 | 0.187787822 | -0.009004602 | 0.846118298 | 0.00258166 |
| -0.165417462 | 0.000330827 | -0.139978703 | 0.002431016 | -0.143024623 |
| -0.039770733 | 0.391174799 | 0.001163855 | 0.979988191 | -2.02E-05 |
| -0.011229401 | 0.808759483 | 0.033176926 | 0.474463891 | 0.019556162 |
| -0.243271557 | 1.02E-07 | -0.205285358 | 7.75E-06 | -0.187727399 |
| -0.143238757 | 0.001914814 | -0.127346629 | 0.005854216 | -0.110344834 |

| | | | | |
|---|---|---|---|---|
| 0.079927317 | 0.084459696 | 0.045887778 | 0.322415135 | 0.010729037 |
| -0.251183356 | 3.75E-08 | -0.216934146 | 2.22E-06 | -0.256115459 |
| -0.031945708 | 0.491025764 | -0.000546463 | 0.990603117 | -0.041097823 |
| -0.100761149 | 0.029467521 | -0.120722595 | 0.009017161 | -0.118409025 |
| -0.072354254 | 0.118418699 | -0.085541195 | 0.064748099 | -0.084745511 |
| 0.015048098 | 0.74568138 | 0.072973402 | 0.115292796 | 0.071652243 |
| -0.041678733 | 0.36883284 | -0.001826223 | 0.968603948 | 0.047212551 |
| -0.150551643 | 0.001101042 | -0.106219664 | 0.021688871 | -0.138927123 |
| -0.207309281 | 6.27E-06 | -0.156062126 | 0.000713727 | -0.156660838 |
| -0.002034147 | 0.965031525 | 0.064947589 | 0.161140033 | 0.102621548 |
| 0.15041277 | 0.001112932 | 0.122991742 | 0.007794129 | 0.132343325 |
| 0.140206347 | 0.002391214 | 0.167633493 | 0.000274058 | 0.155664326 |
| -0.047656159 | 0.304099294 | -0.019691578 | 0.67124545 | -0.011090829 |
| -0.026892082 | 0.562123541 | 0.014015754 | 0.76258641 | -0.006364023 |
| 0.234383377 | 3.01E-07 | 0.259835522 | 1.21E-08 | 0.29513831 |
| -0.153841926 | 0.000851401 | -0.163102579 | 0.000401712 | -0.198315471 |
| -0.08187342 | 0.077140126 | -0.103105661 | 0.025873712 | -0.138424795 |
| 0.211860366 | 3.86E-06 | 0.269693352 | 3.17E-09 | 0.293005831 |
| -0.31340744 | 4.21E-12 | -0.289042197 | 1.94E-10 | -0.267904905 |
| -0.112877409 | 0.014664156 | -0.154165416 | 0.000829919 | -0.160410672 |
| -0.079441419 | 0.086372025 | -0.059978968 | 0.195717966 | -0.107426208 |
| -0.126800587 | 0.006070961 | -0.147520719 | 0.001389063 | -0.154632227 |
| -0.019604306 | 0.67261786 | 0.000795709 | 0.986317473 | 0.005790907 |
| -0.108000908 | 0.019569734 | -0.090962016 | 0.049470747 | -0.08050656 |
| 0.193215198 | 2.62E-05 | 0.225788176 | 8.22E-07 | 0.2218734 |
| -0.038497767 | 0.406526388 | -0.033650808 | 0.468171883 | -0.043898347 |
| 0.372222065 | 8.56E-17 | 0.414762267 | 7.66E-21 | 0.419883752 |
| 0.257123833 | 1.73E-08 | 0.291452128 | 1.35E-10 | 0.271140343 |
| 0.028298309 | 0.541851919 | 0.092813378 | 0.044998584 | 0.108419732 |
| 0.119987583 | 0.00944844 | 0.207445419 | 6.18E-06 | 0.182637691 |
| 0.057613402 | 0.213966628 | 0.064246737 | 0.165717827 | 0.070192063 |
| 0.17272483 | 0.000176243 | 0.184952302 | 5.80E-05 | 0.194837479 |
| -0.068909473 | 0.137035043 | 0.017489686 | 0.706194983 | 0.028208385 |
| -0.048235657 | 0.298250063 | 0.007358588 | 0.873986863 | 0.032163017 |
| -0.110989053 | 0.016418222 | -0.056324367 | 0.224410919 | -0.057536116 |
| 0.069828719 | 0.131860119 | 0.075737317 | 0.102120447 | 0.090886605 |
| 0.127713247 | 0.005712619 | 0.153967369 | 0.000843011 | 0.128282023 |
| 0.077579254 | 0.094025828 | 0.056156795 | 0.225794887 | 0.053903254 |
| 0.069976769 | 0.13104097 | 0.083129913 | 0.072694227 | 0.119512719 |
| 0.2204986 | 1.50E-06 | 0.207598707 | 6.08E-06 | 0.247676493 |
| 0.024512915 | 0.597229834 | 0.061493389 | 0.184648407 | 0.1068792 |
| 0.105387585 | 0.0227452 | 0.155323349 | 0.000757066 | 0.142881911 |
| 0.058875764 | 0.204081727 | 0.062574435 | 0.177033879 | 0.046497238 |
| -0.044328781 | 0.339145137 | -0.016259864 | 0.725993409 | -0.01292851 |
| -0.224233688 | 9.82E-07 | -0.219029821 | 1.76E-06 | -0.21866905 |

| | | | | |
|---|---|---|---|---|
| 0.168192244 | 0.000261256 | 0.166880795 | 0.000292232 | 0.150252448 |
| -0.2030362 | 9.78E-06 | -0.171499936 | 0.000196217 | -0.146964812 |
| 0.270246214 | 2.93E-09 | 0.320413389 | 1.31E-12 | 0.352892847 |
| 0.014041451 | 0.762164196 | 0.070013494 | 0.130838383 | 0.099992349 |
| -0.006981239 | 0.880399044 | 0.076592643 | 0.098295386 | 0.100035577 |
| 0.101769882 | 0.027871938 | 0.166222003 | 0.000309056 | 0.189619676 |
| 0.134734395 | 0.003532526 | 0.126344701 | 0.006257409 | 0.13034437 |
| 0.036203607 | 0.435081477 | 0.074137477 | 0.109591191 | 0.063232434 |
| -0.055409544 | 0.232040279 | -0.047131287 | 0.309462261 | -0.029517578 |
| -0.085415949 | 0.065142467 | -0.117683537 | 0.010921611 | -0.151084722 |
| -0.01949831 | 0.674286203 | -0.022326431 | 0.630342222 | -0.035011268 |
| -0.026245183 | 0.57157007 | 0.005614769 | 0.903681559 | 0.039217977 |
| 0.029558634 | 0.523997925 | 0.049032713 | 0.290328025 | 0.066581425 |
| 0.080222278 | 0.083315581 | 0.105520581 | 0.022573425 | 0.068676684 |
| -0.102920757 | 0.026142733 | -0.070533996 | 0.127993099 | -0.064153725 |
| 0.092488345 | 0.045758192 | 0.14018985 | 0.002394078 | 0.173793222 |
| 0.002495136 | 0.957113686 | 0.032791458 | 0.47961593 | -0.006491942 |
| 0.107553252 | 0.020084604 | 0.146629259 | 0.001486106 | 0.170184814 |
| 0.027614962 | 0.551657255 | 0.060856739 | 0.189244341 | 0.052818893 |
| -0.124340246 | 0.007139601 | -0.044750969 | 0.334560648 | -0.051982793 |
| -0.217753258 | 2.03E-06 | -0.202776902 | 1.00E-05 | -0.188666288 |
| 0.03874239 | 0.403548805 | 0.090208355 | 0.051394845 | 0.134836767 |
| 0.025896886 | 0.576687154 | 0.074094589 | 0.109797239 | 0.086366944 |
| 0.283377539 | 4.50E-10 | 0.281296946 | 6.09E-10 | 0.295176879 |
| 0.205974926 | 7.21E-06 | 0.180493688 | 8.77E-05 | 0.148704546 |
| -0.080130986 | 0.083668344 | -0.078249587 | 0.091210481 | -0.133536648 |
| -0.158425453 | 0.000590039 | -0.151530539 | 0.001020497 | -0.140701952 |
| 0.10164755 | 0.028061397 | 0.071017429 | 0.125393625 | 0.082505268 |
| -0.025884615 | 0.576867828 | 0.043157959 | 0.352067987 | 0.036978346 |
| 0.022999836 | 0.620060777 | 0.052600368 | 0.256608888 | 0.024190798 |
| 0.007253853 | 0.87576579 | 0.056012162 | 0.226994271 | 0.065128427 |
| 0.154054406 | 0.000837234 | 0.13913294 | 0.002584241 | 0.1722317 |
| 0.03646811 | 0.431731552 | 0.094633646 | 0.040937822 | 0.079535431 |
| -0.03736707 | 0.420458237 | -0.009403618 | 0.839390026 | -0.01914321 |
| 0.012039353 | 0.795261228 | 0.064068567 | 0.166896994 | 0.089167104 |
| -0.075805788 | 0.101809959 | -0.007045151 | 0.879312453 | -0.017249971 |
| 0.064549666 | 0.163727346 | 0.072917166 | 0.115574018 | 0.080152522 |
| 0.020804451 | 0.653841708 | 0.044801997 | 0.334009246 | 0.045561656 |
| 0.214075876 | 3.04E-06 | 0.276024138 | 1.30E-09 | 0.3014319 |
| 0.19789946 | 1.65E-05 | 0.21840757 | 1.89E-06 | 0.24406331 |
| -0.054912526 | 0.23626137 | -0.009437708 | 0.83881572 | -0.023676409 |
| -0.134624014 | 0.00355994 | -0.091594865 | 0.047901952 | -0.057489272 |
| 0.013676436 | 0.768168054 | 0.015530872 | 0.737816911 | 0.01162939 |
| -0.011397401 | 0.805954872 | 0.028786528 | 0.534899974 | 0.069327925 |
| 0.135862478 | 0.003263146 | 0.18635112 | 5.08E-05 | 0.227849294 |

| | | | | |
|---|---|---|---|---|
| 0.077460652 | 0.094531103 | 0.063589627 | 0.170097855 | 0.008077648 |
| 0.033705092 | 0.467454079 | 0.080724351 | 0.081396913 | 0.079144979 |
| 0.007725395 | 0.867761696 | 0.028969885 | 0.532300705 | 0.036629593 |
| 0.242128013 | 1.17E-07 | 0.267705804 | 4.17E-09 | 0.304585117 |
| 0.159357491 | 0.000546977 | 0.159087534 | 0.000559139 | 0.193777474 |
| -0.165035785 | 0.000341649 | -0.149038432 | 0.001237146 | -0.120683286 |
| 0.007547015 | 0.870788026 | -0.014702047 | 0.751334978 | -0.003675868 |
| -0.121498861 | 0.008580783 | -0.048671255 | 0.293902934 | -0.064840342 |
| 0.093058657 | 0.0444324 | 0.12843873 | 0.00544146 | 0.110239426 |
| 0.261177343 | 1.01E-08 | 0.286714463 | 2.75E-10 | 0.269061761 |
| -0.071366452 | 0.123542521 | -0.06151981 | 0.184459476 | -0.041787586 |
| -0.008669 | 0.851785994 | -0.018875404 | 0.684122551 | -0.016687131 |
| -0.130029933 | 0.004886874 | -0.069841382 | 0.131789901 | -0.083444515 |
| 0.205925674 | 7.25E-06 | 0.247698905 | 5.85E-08 | 0.268109402 |
| 0.032188112 | 0.487740759 | 0.093915214 | 0.042501974 | 0.136340141 |
| 0.129335381 | 0.005122357 | 0.185030615 | 5.76E-05 | 0.176164255 |
| -0.002520985 | 0.956669826 | 0.007497906 | 0.871621522 | 0.002194035 |
| -0.034914021 | 0.451626688 | 0.029291863 | 0.527751828 | 0.038059022 |
| 0.010884141 | 0.814530957 | 0.065101115 | 0.160150076 | 0.078722484 |
| -0.408658754 | 3.18E-20 | -0.360595996 | 8.70E-16 | -0.359008344 |
| 0.113273482 | 0.014317877 | 0.133332561 | 0.003895312 | 0.129808893 |
| -0.137957334 | 0.002811857 | -0.089886546 | 0.052235243 | -0.090247004 |
| -0.228253378 | 6.19E-07 | -0.179613438 | 9.51E-05 | -0.207190585 |
| 0.176062439 | 0.000131059 | 0.232189426 | 3.90E-07 | 0.24155034 |
| -0.084977638 | 0.066538176 | -0.063722844 | 0.16920297 | -0.058351486 |
| 0.066946943 | 0.148604175 | 0.035242504 | 0.447378956 | 0.034437975 |
| -0.054968187 | 0.23578598 | -0.013956739 | 0.763556298 | -0.010097927 |
| -0.015054417 | 0.74557828 | 0.045016538 | 0.331697357 | 0.057791859 |
| -0.206387713 | 6.90E-06 | -0.254186605 | 2.55E-08 | -0.237009015 |
| 0.030943388 | 0.50473384 | 0.082802241 | 0.073832988 | 0.091604845 |
| 0.046715351 | 0.313756125 | 0.05756723 | 0.214334595 | 0.039455672 |
| 0.018737366 | 0.686309642 | 0.04565855 | 0.324840806 | 0.076341001 |
| -0.175205116 | 0.000141493 | -0.156725999 | 0.000676754 | -0.153066692 |
| 0.047291761 | 0.307816018 | 0.059958248 | 0.195872709 | 0.052721961 |
| -0.116748559 | 0.011575236 | -0.108978511 | 0.018485146 | -0.130999687 |
| -0.09949658 | 0.031577868 | -0.050820323 | 0.273078088 | -0.053654182 |
| -0.050037109 | 0.280547947 | -0.005675592 | 0.902643319 | -0.028672751 |
| -0.042327815 | 0.361416391 | 0.017728737 | 0.702368997 | -0.006948603 |
| -0.034733918 | 0.453965287 | 0.005692279 | 0.902358509 | 0.000115061 |
| 0.02218825 | 0.632460887 | -0.011048365 | 0.811784454 | 0.017953078 |
| 0.274589039 | 1.60E-09 | 0.339070324 | 5.00E-14 | 0.350528052 |
| -0.158736721 | 0.00057532 | -0.090047715 | 0.051812932 | -0.074003834 |
| 0.188155164 | 4.28E-05 | 0.20091224 | 1.22E-05 | 0.200391135 |
| -0.172356841 | 0.000182034 | -0.175980009 | 0.00013203 | -0.180801412 |
| 0.021189511 | 0.647862738 | 0.067677896 | 0.144211257 | 0.044440025 |

| | | | | |
|---|---|---|---|---|
| -0.108713245 | 0.018774131 | -0.10514201 | 0.023065353 | -0.114399191 |
| -0.228496222 | 6.01E-07 | -0.216705117 | 2.28E-06 | -0.188920163 |
| 0.112725963 | 0.014798495 | 0.168813978 | 0.000247669 | 0.167744037 |
| -0.204953155 | 8.02E-06 | -0.190155228 | 3.53E-05 | -0.193448763 |
| -0.163251988 | 0.000396741 | -0.146477895 | 0.001503189 | -0.185577439 |
| 0.158490118 | 0.000586953 | 0.14020178 | 0.002392007 | 0.148225588 |
| 0.114754641 | 0.01308582 | 0.14198575 | 0.00210001 | 0.1650896 |
| -0.107343497 | 0.020329879 | -0.068218132 | 0.1410288 | -0.110110821 |
| -0.021296115 | 0.646211441 | -0.053398126 | 0.249455664 | -0.057731063 |
| -0.002849599 | 0.951028359 | 0.018310141 | 0.693095191 | 0.014914465 |
| 0.169483559 | 0.000233777 | 0.131103033 | 0.004542197 | 0.151435896 |
| 0.015795332 | 0.733520324 | 0.020959502 | 0.651431483 | 0.022313194 |
| 0.258099288 | 1.52E-08 | 0.256529287 | 1.87E-08 | 0.28281633 |
| -0.146323467 | 0.001520803 | -0.163123241 | 0.000401021 | -0.17689878 |
| 0.154344748 | 0.000818228 | 0.205889105 | 7.27E-06 | 0.206143158 |
| 0.022769197 | 0.623573942 | 0.091340421 | 0.04852762 | 0.091703909 |
| 0.054602015 | 0.238925808 | 0.073704264 | 0.111686536 | 0.09729122 |
| 0.012311357 | 0.790741603 | 0.083119712 | 0.072729458 | 0.070855813 |
| -0.023882015 | 0.606703248 | 0.007481852 | 0.871894025 | 0.009970943 |
| -0.075422865 | 0.103556027 | -0.049157626 | 0.289099405 | -0.059257629 |
| 0.00079397 | 0.98634738 | -0.011226248 | 0.808812132 | -0.036167114 |
| 0.120493316 | 0.009149774 | 0.160285533 | 0.000507014 | 0.152015035 |
| 0.184439668 | 6.08E-05 | 0.24959148 | 4.60E-08 | 0.270414506 |
| -0.055144783 | 0.234282162 | -0.000161353 | 0.99722535 | 0.003389334 |
| -0.023319659 | 0.615203394 | -0.008306148 | 0.857922459 | -0.037349008 |
| -0.136665884 | 0.00308282 | -0.069843577 | 0.131777734 | -0.05237023 |
| 0.074901458 | 0.105971612 | 0.153275092 | 0.000890286 | 0.157382327 |
| -0.031633142 | 0.495279018 | 0.003413023 | 0.941361617 | 0.003034903 |
| -0.03178716 | 0.493180758 | 0.016650914 | 0.719677502 | 0.027667873 |
| -0.040280337 | 0.385128804 | 0.001950984 | 0.966460294 | -0.006509205 |
| -0.241088593 | 1.33E-07 | -0.214190048 | 3.00E-06 | -0.218490023 |
| 0.209054 | 5.21E-06 | 0.287167165 | 2.57E-10 | 0.283598088 |
| 0.054804293 | 0.237187709 | 0.07585894 | 0.101569451 | 0.106772683 |
| 0.014618184 | 0.752707095 | 0.044751199 | 0.334558162 | 0.037919968 |
| -0.124275804 | 0.007169724 | -0.136644165 | 0.003087573 | -0.16446258 |
| 0.094222578 | 0.04182673 | 0.094829515 | 0.040519914 | 0.107634084 |
| -0.181093251 | 8.30E-05 | -0.14313359 | 0.001929763 | -0.122500517 |
| -0.102836267 | 0.02626646 | -0.062628029 | 0.17666254 | -0.080287798 |
| -0.094705631 | 0.040783814 | -0.049052869 | 0.290129541 | -0.061474068 |
| 0.275027358 | 1.50E-09 | 0.26335082 | 7.55E-09 | 0.256964854 |
| 0.084725352 | 0.0673526 | 0.14483647 | 0.001700371 | 0.162027165 |
| 0.003455213 | 0.940638089 | 0.035639261 | 0.442278663 | 0.021568299 |
| 0.158280894 | 0.000596993 | 0.182461958 | 7.31E-05 | 0.194087137 |
| 0.268495644 | 3.74E-09 | 0.304549509 | 1.77E-11 | 0.329021851 |
| 0.100307355 | 0.030210481 | 0.096188658 | 0.037718202 | 0.150311146 |

| | | | | |
|---|---|---|---|---|
| -0.1286076 | 0.005380029 | -0.11610711 | 0.012043445 | -0.13284121 |
| 0.129635 | 0.005019547 | 0.122699526 | 0.007942837 | 0.120394683 |
| 0.096442231 | 0.037214104 | 0.132813202 | 0.004038062 | 0.116542969 |
| 0.143327099 | 0.001902339 | 0.084500065 | 0.068086759 | 0.106880745 |
| 0.118492698 | 0.010382556 | 0.156232831 | 0.000704045 | 0.149954332 |
| -0.120935926 | 0.008895311 | -0.09759953 | 0.03498539 | -0.093904889 |
| -0.151866784 | 0.000994109 | -0.137250651 | 0.002957316 | -0.146694616 |
| 0.219249113 | 1.72E-06 | 0.263459194 | 7.44E-09 | 0.289136365 |
| 0.047472219 | 0.305971685 | 0.114206311 | 0.013530588 | 0.163573886 |
| -0.024882513 | 0.5917114 | -0.021012412 | 0.650609841 | -0.040005554 |
| -0.081597419 | 0.07814572 | -0.007695817 | 0.868263379 | -0.008812505 |
| 0.027902314 | 0.547523483 | 0.047733032 | 0.303319027 | 0.045977369 |
| -0.157989915 | 0.000611222 | -0.118203742 | 0.010572284 | -0.104623197 |
| 0.002715228 | 0.953334892 | 0.082999782 | 0.073144744 | 0.078898279 |
| -0.048464603 | 0.295959945 | 0.003925064 | 0.932583877 | 0.012811935 |
| 0.094785142 | 0.040614273 | 0.157552345 | 0.000633212 | 0.15554522 |
| -0.0160411 | 0.729534853 | 0.004611332 | 0.920832437 | 0.027029943 |
| -0.15389831 | 0.00084762 | -0.110984901 | 0.016422273 | -0.110008651 |
| 0.113227705 | 0.014357529 | 0.150690141 | 0.001089301 | 0.144468636 |
| 0.167199213 | 0.000284411 | 0.244292037 | 8.98E-08 | 0.265402344 |
| -0.019508201 | 0.674130462 | -0.023700781 | 0.609436929 | -0.02901177 |
| 0.168765676 | 0.0002487 | 0.1896517 | 3.71E-05 | 0.200458797 |
| -0.144311465 | 0.001768278 | -0.080939251 | 0.080586674 | -0.040996869 |
| -0.086223595 | 0.062633746 | -0.054373325 | 0.240901619 | -0.034919431 |
| -0.116792182 | 0.011543988 | -0.048719401 | 0.293425075 | -0.021168627 |
| 0.089378278 | 0.053585887 | 0.117223333 | 0.011239129 | 0.133923873 |
| 0.211712283 | 3.93E-06 | 0.254916396 | 2.32E-08 | 0.293183442 |
| 0.009130334 | 0.84399695 | 0.075653134 | 0.102503215 | 0.078149692 |
| -0.010145982 | 0.826903572 | -0.01941028 | 0.675672974 | -0.024908927 |
| -0.131940182 | 0.004288718 | -0.094190523 | 0.041896726 | -0.109268454 |
| -0.004927506 | 0.915424005 | -0.032475484 | 0.483861719 | -0.01986623 |
| 0.011811146 | 0.799058427 | 0.087748146 | 0.058115209 | 0.10140992 |
| -0.067062072 | 0.147905589 | -0.040088046 | 0.387403451 | -0.056329388 |
| -0.078585892 | 0.089823695 | -0.041538315 | 0.370449598 | -0.031031203 |
| 0.13978347 | 0.002465631 | 0.145631361 | 0.001602096 | 0.159758884 |
| 0.138287563 | 0.002746143 | 0.177831914 | 0.000111765 | 0.18034389 |
| -0.174279084 | 0.000153637 | -0.183272878 | 6.78E-05 | -0.210298606 |
| 0.016772972 | 0.717709996 | 0.068861425 | 0.13730977 | 0.055922121 |
| -0.150584481 | 0.001098248 | -0.079885671 | 0.084622248 | -0.088992849 |
| -0.053680746 | 0.246955152 | 0.003782387 | 0.935028971 | 0.021472264 |
| -0.21357057 | 3.21E-06 | -0.14317708 | 0.001923568 | -0.12281662 |
| 0.020071393 | 0.665285309 | 0.02133211 | 0.645654274 | 0.036185485 |
| -0.173906393 | 0.000158796 | -0.215378524 | 2.64E-06 | -0.218627568 |
| -0.23666999 | 2.29E-07 | -0.18293755 | 7.00E-05 | -0.209051567 |
| -0.178275032 | 0.00010737 | -0.171785349 | 0.000191382 | -0.162164035 |

| | | | | |
|---|---|---|---|---|
| -0.174189818 | 0.000154858 | -0.171293394 | 0.000199787 | -0.166412508 |
| 0.043620022 | 0.346931334 | 0.038117795 | 0.411177269 | 0.056561778 |
| 0.077788826 | 0.093138275 | 0.136553533 | 0.00310748 | 0.135945843 |
| 0.109313595 | 0.018125653 | 0.148575375 | 0.001281792 | 0.136855766 |
| -0.008261095 | 0.858684979 | 0.022614423 | 0.625936308 | 0.037357871 |
| -0.179087096 | 9.97E-05 | -0.124788404 | 0.006933228 | -0.139447645 |
| 0.144130673 | 0.001792231 | 0.177379783 | 0.000116424 | 0.196972698 |
| -0.11169846 | 0.015738813 | -0.109043733 | 0.01841469 | -0.083151197 |
| 0.167125571 | 0.000286202 | 0.200711531 | 1.24E-05 | 0.197451167 |
| 0.237436045 | 2.08E-07 | 0.202174128 | 1.07E-05 | 0.206663152 |
| -0.158830268 | 0.000570963 | -0.153993505 | 0.000841272 | -0.160559868 |
| -0.263117575 | 7.80E-09 | -0.188758079 | 4.04E-05 | -0.17826982 |
| -0.195242129 | 2.15E-05 | -0.185695509 | 5.41E-05 | -0.178610556 |
| 0.049923491 | 0.28164295 | 0.088657956 | 0.055549714 | 0.113524334 |
| -0.188391293 | 4.19E-05 | -0.159397323 | 0.000545203 | -0.128856393 |
| -0.083758204 | 0.070550803 | -0.014562913 | 0.753611841 | -0.029365884 |
| 0.00307731 | 0.947120541 | 0.029706969 | 0.521916532 | 0.009936631 |
| 0.073064188 | 0.114839932 | 0.071277785 | 0.124010757 | 0.079686085 |
| -0.136273201 | 0.003169795 | -0.088100247 | 0.057110942 | -0.085998416 |
| 0.094763638 | 0.040660067 | 0.095741265 | 0.038621739 | 0.098404859 |
| 0.144472494 | 0.00174719 | 0.144092465 | 0.001797332 | 0.158082315 |
| -0.091566598 | 0.047971125 | -0.035722813 | 0.441208846 | -0.031213585 |
| 0.036544557 | 0.430766141 | 0.023069395 | 0.619002917 | 0.03803818 |
| 0.069027893 | 0.136359766 | 0.066419381 | 0.151837502 | 0.068995077 |
| 0.021844986 | 0.637737009 | 0.080567174 | 0.081993683 | 0.104013995 |
| -0.106068348 | 0.02187774 | -0.050940044 | 0.271948324 | -0.049241303 |
| -0.042551628 | 0.358880844 | 0.026353337 | 0.569985491 | 0.043095148 |
| 0.021634876 | 0.640975565 | 0.055141931 | 0.234306392 | 0.034436395 |
| -0.185726462 | 5.39E-05 | -0.121956103 | 0.008332644 | -0.121713921 |
| 0.208596543 | 5.47E-06 | 0.188505076 | 4.14E-05 | 0.228996738 |
| 0.078805795 | 0.088926089 | 0.103032703 | 0.025979574 | 0.112123285 |
| -0.174872364 | 0.000145749 | -0.161405814 | 0.000462391 | -0.197572913 |
| -0.254974739 | 2.30E-08 | -0.213926163 | 3.09E-06 | -0.241862622 |
| -0.082814811 | 0.073789037 | -0.055237879 | 0.233492127 | -0.10009719 |
| -0.110434799 | 0.016966774 | -0.043807069 | 0.344865568 | -0.040662126 |
| 0.040616836 | 0.381167935 | 0.091719143 | 0.047598823 | 0.096073907 |
| -0.228628489 | 5.92E-07 | -0.197210735 | 1.77E-05 | -0.193401611 |
| -0.100838868 | 0.029341864 | -0.054857795 | 0.236729485 | -0.066388226 |
| -0.24409661 | 9.20E-08 | -0.208095846 | 5.77E-06 | -0.222819349 |
| 0.042777386 | 0.356334588 | 0.069780702 | 0.132126644 | 0.043801194 |
| -0.208575863 | 5.48E-06 | -0.156260931 | 0.000702463 | -0.174612621 |
| -0.038276511 | 0.40923076 | 0.014463274 | 0.755243703 | 0.051972615 |
| -0.027288094 | 0.556378003 | -0.078435214 | 0.090442926 | -0.107126465 |
| 0.152021559 | 0.000982175 | 0.115440504 | 0.012547671 | 0.124202197 |
| -0.01161017 | 0.802406441 | 0.000155833 | 0.997320274 | 0.001464376 |

| | | | | |
|---|---|---|---|---|
| 0.020197793 | 0.663306453 | 0.029911978 | 0.519046898 | 0.051898703 |
| -0.007339548 | 0.874310206 | -0.015552149 | 0.737470926 | -0.045664092 |
| -0.223518897 | 1.07E-06 | -0.150204386 | 0.001130995 | -0.150047819 |
| -0.31652253 | 2.51E-12 | -0.285325398 | 3.38E-10 | -0.266107164 |
| -0.197971571 | 1.64E-05 | -0.161482946 | 0.000459457 | -0.19045079 |
| 0.112484781 | 0.015014664 | 0.110951893 | 0.016454507 | 0.124411665 |
| 0.049111385 | 0.289553813 | 0.081989578 | 0.076720067 | 0.098990261 |
| -0.15351758 | 0.000873455 | -0.09856847 | 0.033207762 | -0.096046933 |
| -0.005309727 | 0.908891011 | -0.065144661 | 0.159870119 | -0.071076232 |
| -0.012648065 | 0.785156562 | 0.085291963 | 0.065534814 | 0.0691584 |
| -0.019844547 | 0.668842523 | 0.043751495 | 0.345478519 | 0.057023001 |
| 0.011155501 | 0.80999396 | -0.058932966 | 0.203641783 | -0.056676966 |
| 0.066926793 | 0.1487267 | 0.07842211 | 0.090496935 | 0.071042366 |
| -0.198923264 | 1.49E-05 | -0.139920403 | 0.002441306 | -0.119473361 |
| 0.180718665 | 8.59E-05 | 0.128289859 | 0.005496139 | 0.126370361 |
| -0.027275641 | 0.556558242 | 0.013100401 | 0.777670951 | 0.035496391 |
| 0.100552857 | 0.029806574 | 0.055551365 | 0.230845666 | 0.034755253 |
| -0.053778831 | 0.246091436 | -0.012982148 | 0.779625948 | -0.028995808 |
| -0.083603388 | 0.071074104 | -0.106057694 | 0.021891091 | -0.131274303 |
| -0.046238109 | 0.318730787 | 0.014940129 | 0.74744387 | 0.007323965 |
| -0.141376449 | 0.002195858 | -0.057860294 | 0.212006757 | -0.055120824 |
| -0.070210673 | 0.129754823 | -0.024035098 | 0.604398462 | -0.051814216 |
| -0.053249418 | 0.250778436 | -0.020037813 | 0.665811421 | -0.041377395 |
| -0.146406181 | 0.001511345 | -0.151354064 | 0.001034603 | -0.184746426 |
| -0.156583946 | 0.000684513 | -0.114656964 | 0.013164092 | -0.09637634 |
| -0.085917283 | 0.063575678 | -0.000205886 | 0.996459549 | -0.005979117 |
| 0.019534173 | 0.673721551 | 0.078235794 | 0.091267721 | 0.115781925 |
| -0.109778628 | 0.017636864 | -0.032830675 | 0.479090376 | -0.044941914 |
| -0.114263375 | 0.013483689 | -0.059484678 | 0.19943385 | -0.054404334 |
| -0.055745311 | 0.229219038 | -0.036334928 | 0.433416435 | -0.039154183 |
| 0.061075167 | 0.187658146 | 0.022825497 | 0.622715575 | 0.02022996 |
| -0.172711186 | 0.000176455 | -0.174602133 | 0.000149294 | -0.161924849 |
| 0.119523765 | 0.009729939 | 0.16622615 | 0.000308947 | 0.160308156 |
| -0.071871162 | 0.120903396 | -0.03997556 | 0.388737834 | -0.039869761 |
| -0.011296896 | 0.807632418 | 0.037945305 | 0.413298919 | 0.016997474 |
| -0.116052102 | 0.012084365 | -0.074535801 | 0.107692049 | -0.064414722 |
| -0.204618083 | 8.30E-06 | -0.204481808 | 8.42E-06 | -0.239452981 |
| -0.069579046 | 0.133250494 | 0.002663982 | 0.954214656 | 0.010865986 |
| -0.154096258 | 0.000834469 | -0.099501351 | 0.03156967 | -0.120688213 |
| -0.151107929 | 0.001054579 | -0.072447654 | 0.117942951 | -0.118521861 |
| -0.023663022 | 0.610007171 | 0.0535764 | 0.247876319 | 0.061178338 |
| -0.233101164 | 3.50E-07 | -0.216954495 | 2.22E-06 | -0.229468631 |
| 0.080663196 | 0.081628689 | 0.144544784 | 0.001737798 | 0.143272838 |
| 0.103844083 | 0.024823024 | 0.088753001 | 0.055287215 | 0.086764535 |
| -0.1345473 | 0.003579105 | -0.056677602 | 0.221513398 | -0.070224391 |

| | | | | |
|---|---|---|---|---|
| -0.062682617 | 0.176284908 | -0.000650525 | 0.9888138 | 0.005471791 |
| -0.146822112 | 0.001464599 | -0.110290235 | 0.017112461 | -0.146360396 |
| -0.055012403 | 0.23540882 | -0.012945718 | 0.78022849 | -0.0234261 |
| -0.149821015 | 0.001164935 | -0.070617867 | 0.127539147 | -0.075970268 |
| -0.049471292 | 0.28602968 | 0.025492369 | 0.582657087 | 0.026330926 |
| -0.023715739 | 0.609211112 | 0.054265354 | 0.241838426 | 0.020659974 |
| 0.099734189 | 0.03117176 | 0.166106124 | 0.000312107 | 0.194219461 |
| -0.045373926 | 0.327869107 | -0.017285559 | 0.709467874 | -0.034341993 |
| -0.081347262 | 0.079066315 | -0.103247295 | 0.025669263 | -0.128169297 |
| -0.170159193 | 0.0002205 | -0.195698216 | 2.05E-05 | -0.230521061 |
| -0.025925949 | 0.57625935 | 0.043622139 | 0.346907909 | 0.048743903 |
| -0.141561036 | 0.002166407 | -0.063159724 | 0.173009856 | -0.060899542 |
| -0.337374261 | 6.79E-14 | -0.273232167 | 1.93E-09 | -0.276639039 |
| -0.123795468 | 0.007397857 | -0.058523065 | 0.20680957 | -0.044792592 |
| -0.144995788 | 0.001680242 | -0.080426252 | 0.082531738 | -0.08218967 |
| -0.015359746 | 0.740601507 | 0.04159593 | 0.369785697 | 0.063239022 |
| -0.119169445 | 0.009949977 | -0.047166668 | 0.309098803 | -0.028910746 |
| 0.007283805 | 0.875256982 | 0.059115684 | 0.202241113 | 0.082248724 |
| -0.03858721 | 0.405436165 | 0.005173975 | 0.911210633 | 0.03235223 |
| -0.132406403 | 0.004153163 | -0.083214048 | 0.072404155 | -0.070662967 |
| -0.247415313 | 6.07E-08 | -0.200863963 | 1.22E-05 | -0.181037363 |
| -0.029612151 | 0.52324651 | 0.010252195 | 0.825120563 | 0.025641915 |
| -0.181892907 | 7.71E-05 | -0.134176448 | 0.00367308 | -0.138469836 |
| -0.150127259 | 0.001137749 | -0.202059163 | 1.08E-05 | -0.205925027 |
| -0.085293156 | 0.065531027 | -0.031926642 | 0.491284645 | -0.018194998 |
| -0.056541147 | 0.222629529 | -0.057950179 | 0.211296448 | -0.074127297 |
| 0.016685584 | 0.719118438 | 0.093485293 | 0.043461866 | 0.098628721 |
| -0.01849658 | 0.69013095 | 0.036190592 | 0.435246692 | 0.017874496 |
| 0.138124539 | 0.002778407 | 0.082559263 | 0.074686766 | 0.030616893 |
| 0.080592896 | 0.081895779 | 0.1208431 | 0.008948149 | 0.140675212 |
| 0.047527412 | 0.305409052 | 0.106113395 | 0.021821364 | 0.129605165 |
| 0.303689937 | 2.03E-11 | 0.246998262 | 6.40E-08 | 0.267857557 |
| -0.010216092 | 0.825726539 | 0.053678103 | 0.246978451 | 0.057805348 |
| -0.172435716 | 0.000180778 | -0.151491429 | 0.001023607 | -0.129586572 |
| -0.132177551 | 0.00421921 | -0.10004233 | 0.030651762 | -0.107280105 |
| -0.035174824 | 0.448252294 | 0.008843489 | 0.848838223 | 0.01699391 |
| -0.01742145 | 0.707288455 | 0.058171754 | 0.20955281 | 0.044820567 |
| -0.097823547 | 0.034567366 | -0.034293007 | 0.45971905 | -0.046036819 |
| 0.129710346 | 0.004993987 | 0.177912748 | 0.000110951 | 0.191887862 |
| -0.128924731 | 0.005266347 | -0.103631482 | 0.025121687 | -0.107071352 |
| -0.182679056 | 7.17E-05 | -0.122378409 | 0.008109169 | -0.102433961 |
| 0.024126782 | 0.603019953 | 0.00300408 | 0.94837713 | 0.002097778 |
| 0.003027234 | 0.947979799 | 0.060093488 | 0.194864312 | 0.052951734 |
| -0.219049177 | 1.76E-06 | -0.151899123 | 0.000991604 | -0.127840359 |
| -0.229862046 | 5.13E-07 | -0.192069757 | 2.93E-05 | -0.217146857 |

| | | | | |
|---|---|---|---|---|
| -0.188659235 | 4.08E-05 | -0.125655265 | 0.006549143 | -0.148255812 |
| -0.105716076 | 0.022322971 | -0.063514742 | 0.170602448 | -0.060527423 |
| -0.060153743 | 0.194416258 | -0.037938142 | 0.413387165 | -0.031858979 |
| -0.079712449 | 0.085301089 | -0.009605922 | 0.835983166 | -0.005491087 |
| 0.033821689 | 0.465914357 | 0.048852573 | 0.292105991 | 0.054161509 |
| -0.10088142 | 0.029273258 | -0.053119315 | 0.251939708 | -0.065413197 |
| -0.089848283 | 0.052335922 | -0.019646754 | 0.671950209 | 0.010850324 |
| -0.092130792 | 0.046606212 | -0.0297387 | 0.52147185 | -0.025582496 |
| 0.0412337 | 0.373971952 | 0.099313395 | 0.031894038 | 0.090946296 |
| -0.06786104 | 0.143126288 | -0.043336202 | 0.350080838 | -0.06425235 |
| -0.368206523 | 1.93E-16 | -0.313563265 | 4.10E-12 | -0.309710839 |
| 0.104544943 | 0.023860061 | 0.063876648 | 0.168174171 | 0.077839467 |
| -0.212980795 | 3.42E-06 | -0.147541843 | 0.001386836 | -0.179382262 |
| -0.125017131 | 0.006829974 | -0.055076209 | 0.234865303 | -0.039439099 |
| -0.022039533 | 0.634744463 | 0.0199777 | 0.666753619 | 0.029372207 |
| -0.333073037 | 1.46E-13 | -0.31167836 | 5.59E-12 | -0.291922281 |
| -0.133330795 | 0.003895789 | -0.065020332 | 0.160670402 | -0.07271115 |
| -0.037817668 | 0.414873011 | 0.023898171 | 0.60645983 | 0.008922328 |
| 0.02796819 | 0.546577952 | 0.077217827 | 0.095572367 | 0.076170472 |
| 0.033787286 | 0.466368366 | -0.049627761 | 0.284506611 | -0.038764858 |
| -0.082439818 | 0.075109411 | -0.033145639 | 0.474880924 | -0.031576972 |
| -0.511079079 | 1.95E-32 | -0.512328061 | 1.30E-32 | -0.530482921 |
| -0.282002242 | 5.50E-10 | -0.254761914 | 2.36E-08 | -0.25588923 |
| -0.193737592 | 2.49E-05 | -0.154155317 | 0.000830582 | -0.154158035 |
| -0.214277419 | 2.97E-06 | -0.138585025 | 0.00268815 | -0.130246209 |
| -0.026115226 | 0.573476851 | 0.055131082 | 0.234398593 | 0.055175552 |
| 0.183119528 | 6.88E-05 | 0.133615162 | 0.003819571 | 0.078954281 |
| -0.06481435 | 0.162002906 | 0.030797815 | 0.506741334 | 0.066839175 |
| -0.178048462 | 0.000109597 | -0.111478182 | 0.015947089 | -0.098345335 |
| -0.150849122 | 0.001075966 | -0.094816894 | 0.040546734 | -0.081613111 |
| 0.128005992 | 0.005601773 | 0.057222089 | 0.217099579 | 0.067508828 |
| -0.116748838 | 0.011575036 | -0.075806754 | 0.101805586 | -0.078521469 |
| 0.073859543 | 0.110931894 | 0.00851559 | 0.854379352 | -0.056505933 |
| -0.029585552 | 0.523619904 | 0.011554931 | 0.803327292 | 0.022339931 |
| -0.061784517 | 0.182574425 | 0.005257167 | 0.909789022 | 0.034060719 |
| -0.067180996 | 0.147186601 | -0.004000071 | 0.931298731 | -0.003823215 |
| -0.101674088 | 0.028020203 | -0.040657159 | 0.380694977 | -0.008773029 |
| -0.125134537 | 0.00677751 | -0.102641225 | 0.026554006 | -0.132549155 |
| 0.081041771 | 0.080202449 | 0.120637214 | 0.009066343 | 0.137855426 |
| 0.177231209 | 0.000117994 | 0.15898124 | 0.000563997 | 0.18519923 |
| -0.097969656 | 0.034297019 | -0.035788028 | 0.440374861 | -0.022569508 |
| -0.026636296 | 0.56584974 | 0.02940783 | 0.526118313 | 0.014339885 |
| 0.135473803 | 0.003353777 | 0.120232072 | 0.009302989 | 0.111719409 |
| -0.261530449 | 9.65E-09 | -0.218703393 | 1.83E-06 | -0.188738183 |
| -0.124340889 | 0.007139301 | -0.112331474 | 0.015153509 | -0.065825349 |

| | | | | |
|---|---|---|---|---|
| -0.068929177 | 0.136922506 | -0.036577402 | 0.43035174 | -0.054601504 |
| -0.241341589 | 1.29E-07 | -0.166309181 | 0.000306779 | -0.165490017 |
| 0.034230049 | 0.460543936 | 0.123208012 | 0.007685676 | 0.160417621 |
| -0.132319491 | 0.004178135 | -0.092980473 | 0.04461222 | -0.108192948 |
| -0.218097679 | 1.96E-06 | -0.153690166 | 0.000861655 | -0.168908426 |
| -0.230268659 | 4.89E-07 | -0.170580238 | 0.000212587 | -0.162570721 |
| -0.265826221 | 5.40E-09 | -0.241758261 | 1.23E-07 | -0.235264629 |
| -0.115602462 | 0.012423481 | -0.066416084 | 0.151857878 | -0.113016308 |
| 0.106886686 | 0.020873076 | 0.162278357 | 0.000430197 | 0.143694963 |
| 0.116255573 | 0.011933615 | 0.060733997 | 0.190139992 | 0.10542958 |
| -0.075026951 | 0.105386194 | -0.016680578 | 0.719199164 | -0.015139939 |
| 0.131781002 | 0.004335908 | 0.104003981 | 0.024600423 | 0.119357078 |
| -0.097879011 | 0.034464527 | -0.052116969 | 0.261011798 | -0.076278601 |
| -0.152171961 | 0.000970705 | -0.096577081 | 0.03694836 | -0.088357452 |
| -0.028313995 | 0.541627872 | 0.012477423 | 0.787985674 | 0.00642853 |
| -0.033466012 | 0.470620031 | -0.002703302 | 0.953539619 | 0.000186399 |
| 0.06630864 | 0.152522944 | 0.016002374 | 0.730162378 | 0.021561713 |
| -0.0507166 | 0.274059479 | 0.003024745 | 0.948022517 | 0.016536541 |
| 0.193980205 | 2.43E-05 | 0.176121069 | 0.000130372 | 0.170583751 |
| -0.092287283 | 0.04623345 | -0.048897132 | 0.291665523 | -0.088531873 |
| 0.101796073 | 0.02783152 | 0.172860206 | 0.000174157 | 0.169592919 |
| -0.152223037 | 0.000966839 | -0.113725441 | 0.013931539 | -0.141035869 |
| 0.188883805 | 3.99E-05 | 0.208754159 | 5.38E-06 | 0.180152459 |
| -0.111193635 | 0.016219704 | -0.045823764 | 0.323091342 | -0.040463096 |
| 0.197808882 | 1.66E-05 | 0.148845702 | 0.001255551 | 0.181932454 |
| -0.173507141 | 0.000164501 | -0.122045453 | 0.008284909 | -0.111883203 |
| -0.000324691 | 0.99441658 | 0.061340716 | 0.18574297 | 0.040596169 |
| -0.108650578 | 0.018842973 | -0.057118238 | 0.217936533 | -0.067268357 |
| 0.028300922 | 0.541814597 | 0.077743556 | 0.093329427 | 0.106909743 |
| -0.079949683 | 0.084372497 | -0.058068541 | 0.210363726 | -0.049984074 |
| -0.078957346 | 0.088311684 | -0.018037815 | 0.697433377 | -0.020701391 |
| 0.065548287 | 0.157292754 | 0.020118091 | 0.664553964 | 0.025116229 |
| -0.06611347 | 0.153736681 | -0.006086883 | 0.895627102 | -0.042280574 |
| -0.11991302 | 0.009493199 | -0.082113564 | 0.076273753 | -0.068530117 |
| -0.044988411 | 0.331999868 | 0.004661944 | 0.91996643 | 0.012069837 |
| 0.016260282 | 0.725986658 | 0.066623816 | 0.150578282 | 0.07770497 |
| -0.328245208 | 3.41E-13 | -0.270379272 | 2.88E-09 | -0.285621931 |
| -0.109714203 | 0.017703885 | -0.056496791 | 0.222993205 | -0.092219606 |
| -0.137954429 | 0.002812441 | -0.126446999 | 0.00621513 | -0.144223276 |
| -0.159959687 | 0.000520726 | -0.104888981 | 0.023399299 | -0.102922751 |
| -0.115146751 | 0.01277572 | -0.158074437 | 0.000607057 | -0.12677065 |
| -0.21638424 | 2.36E-06 | -0.186016852 | 5.24E-05 | -0.200982352 |
| -0.164872127 | 0.000346389 | -0.140077628 | 0.002413646 | -0.137363775 |
| 0.044522557 | 0.337035983 | 0.011748526 | 0.800101209 | 0.002501162 |
| -0.12674047 | 0.006095259 | -0.07549733 | 0.103214635 | -0.042943973 |

| | | | | |
|---|---|---|---|---|
| -0.179105949 | 9.96E-05 | -0.127716684 | 0.005711307 | -0.129445367 |
| 0.009141687 | 0.843805463 | 0.056397368 | 0.223809908 | 0.054565201 |
| 0.149342944 | 0.001208572 | 0.226972957 | 7.17E-07 | 0.242471919 |
| -0.128828836 | 0.005300492 | -0.106296572 | 0.021593419 | -0.081135766 |
| 0.162137614 | 0.000435244 | 0.126736217 | 0.006096981 | 0.14228385 |
| 0.041643849 | 0.369234079 | 0.073437907 | 0.112990393 | 0.091954959 |
| -0.151351384 | 0.001034819 | -0.111423434 | 0.015999227 | -0.084606678 |
| -0.19053388 | 3.41E-05 | -0.136421797 | 0.003136624 | -0.122215354 |
| -0.068817181 | 0.137563117 | -0.064960285 | 0.161057991 | -0.076052164 |
| -0.234763536 | 2.87E-07 | -0.184816793 | 5.87E-05 | -0.191343753 |
| -0.16734731 | 0.00028084 | -0.124088224 | 0.007258054 | -0.115919183 |
| 0.20747551 | 6.16E-06 | 0.166162854 | 0.00031061 | 0.158433954 |
| -0.217132274 | 2.18E-06 | -0.169459434 | 0.000234264 | -0.183633956 |
| 0.038763357 | 0.4032942 | -0.004727904 | 0.918837933 | 0.010561748 |
| -0.163681132 | 0.000382778 | -0.08253536 | 0.074771188 | -0.093196672 |
| -0.047269318 | 0.308045904 | -0.003497381 | 0.93991498 | 0.018988822 |
| -0.095201973 | 0.039735175 | -0.052804545 | 0.254764731 | -0.046540393 |
| -0.168875423 | 0.000246363 | -0.112728064 | 0.014796624 | -0.106622806 |
| 0.022876222 | 0.621942624 | 0.062761281 | 0.175741782 | 0.087542944 |
| -0.142831283 | 0.001973331 | -0.087206469 | 0.059688709 | -0.098011769 |
| -0.074517298 | 0.107779688 | -0.021699566 | 0.639977717 | -0.021809081 |
| -0.191976591 | 2.96E-05 | -0.130734625 | 0.004657972 | -0.145534015 |
| -0.243739827 | 9.62E-08 | -0.183393648 | 6.71E-05 | -0.178375665 |
| -0.246083238 | 7.18E-08 | -0.195439735 | 2.11E-05 | -0.179973045 |
| -0.057901281 | 0.211682649 | -0.008616419 | 0.852674684 | -0.011797273 |
| -0.084700117 | 0.067434511 | -0.016622614 | 0.720133946 | -0.044817345 |
| 0.039687341 | 0.39216962 | 0.097063514 | 0.036003109 | 0.101167214 |
| -0.072013116 | 0.1201691 | -0.034740906 | 0.453874423 | -0.070849135 |
| -0.138815536 | 0.00264398 | -0.073304099 | 0.113649904 | -0.050888964 |
| -0.046211558 | 0.319009046 | 0.018970495 | 0.682617457 | 0.015957896 |
| -0.05704072 | 0.218562768 | 0.011055207 | 0.811670091 | 0.026125479 |
| -0.153850381 | 0.000850833 | -0.104156222 | 0.024390087 | -0.090664612 |
| -0.014764336 | 0.750316342 | 0.044969596 | 0.33220232 | 0.049067918 |
| -0.148405815 | 0.001298507 | -0.093842036 | 0.042664086 | -0.10464087 |
| 0.110104719 | 0.017301019 | 0.104636705 | 0.023736406 | 0.134226002 |
| -0.22794787 | 6.41E-07 | -0.168979159 | 0.000244172 | -0.191418409 |
| -0.065896016 | 0.155097599 | -0.114847525 | 0.013011769 | -0.139218046 |
| -0.070490947 | 0.128226589 | -0.029821757 | 0.520308786 | -0.04181058 |
| 0.157955181 | 0.000612941 | 0.140924769 | 0.002269472 | 0.20481691 |
| -0.147694132 | 0.001370876 | -0.105048645 | 0.023188092 | -0.058638098 |
| -0.012254366 | 0.791687991 | 0.054684681 | 0.238214411 | 0.030222768 |
| 0.425365308 | 6.05E-22 | 0.392799327 | 1.12E-18 | 0.382240743 |
| -0.050306618 | 0.277962068 | 0.025934124 | 0.576139042 | 0.053754422 |
| -0.170563814 | 0.000212891 | -0.099607675 | 0.031387432 | -0.084587921 |
| -0.336458023 | 8.00E-14 | -0.312120479 | 5.20E-12 | -0.331684541 |

| | | | | |
|---|---|---|---|---|
| 0.071495819 | 0.122861827 | 0.106194884 | 0.021719703 | 0.088727596 |
| -0.225670674 | 8.33E-07 | -0.161951083 | 0.000442019 | -0.148935249 |
| -0.197591812 | 1.70E-05 | -0.149593082 | 0.001185556 | -0.14500939 |
| 0.05980279 | 0.197036564 | 0.09276058 | 0.045121247 | 0.090901492 |
| -0.355358868 | 2.40E-15 | -0.362230665 | 6.32E-16 | -0.358876314 |
| -0.072192177 | 0.119247824 | -0.016904268 | 0.715595648 | -0.004466825 |
| 0.192689215 | 2.76E-05 | 0.146020711 | 0.001555886 | 0.165144794 |
| -0.002770178 | 0.952391601 | 0.052820898 | 0.254617428 | 0.041859406 |
| 0.193052385 | 2.67E-05 | 0.189981114 | 3.59E-05 | 0.171605099 |
| 0.005452723 | 0.906448458 | 0.03072823 | 0.507702417 | 0.054769002 |
| -0.127213434 | 0.005906435 | -0.080395156 | 0.082650848 | -0.075385042 |
| -0.139686753 | 0.002482945 | -0.101914437 | 0.027649481 | -0.110794423 |
| -0.043884558 | 0.344012061 | 0.015516722 | 0.738047031 | 0.039264816 |
| -0.082179464 | 0.076037393 | -0.049875068 | 0.28211051 | -0.029370286 |
| 0.005925516 | 0.898378923 | 0.061131296 | 0.187252129 | 0.056961194 |
| -0.063368471 | 0.171591276 | -0.022897134 | 0.621624099 | -0.025957621 |
| 0.126037198 | 0.006386057 | 0.190280894 | 3.49E-05 | 0.183721535 |
| -0.021659585 | 0.640594357 | 0.024766309 | 0.593443909 | 0.081104955 |
| 0.318635469 | 1.76E-12 | 0.293602694 | 9.74E-11 | 0.309893659 |
| 0.279486888 | 7.92E-10 | 0.22550561 | 8.49E-07 | 0.239682925 |
| 0.276048906 | 1.30E-09 | 0.258734186 | 1.40E-08 | 0.255251756 |
| -0.064558757 | 0.163667889 | -0.026166789 | 0.572719941 | -0.02904244 |
| 0.122982554 | 0.007798767 | 0.150274589 | 0.00112488 | 0.130502109 |
| 0.083650169 | 0.070915644 | 0.121329276 | 0.008674475 | 0.110349869 |
| -0.339948953 | 4.26E-14 | -0.306328095 | 1.33E-11 | -0.33274509 |
| 0.078917417 | 0.088473228 | 0.066055517 | 0.154098484 | 0.079340087 |
| 0.130562065 | 0.004713111 | 0.060768917 | 0.189884863 | 0.079021094 |
| -0.153893873 | 0.000847917 | -0.127959188 | 0.005619364 | -0.095146294 |
| -0.286599805 | 2.80E-10 | -0.225255634 | 8.74E-07 | -0.219784282 |
| -0.233463016 | 3.35E-07 | -0.169280539 | 0.00023791 | -0.184500178 |
| 0.062445288 | 0.177931086 | 0.09384369 | 0.042660417 | 0.106281394 |
| -0.082463008 | 0.075027204 | -0.035309069 | 0.446520944 | -0.057217903 |
| -0.155670048 | 0.000736433 | -0.101796047 | 0.02783156 | -0.111778561 |
| 0.116020502 | 0.012107928 | 0.103363013 | 0.025503259 | 0.111995232 |
| -0.006936433 | 0.88116095 | 0.062742066 | 0.175874333 | 0.098457158 |
| 0.018222962 | 0.694482877 | 0.083677603 | 0.070822852 | 0.058670851 |
| -0.094985376 | 0.040189948 | -0.024612648 | 0.595738431 | -0.022661083 |
| -0.134740176 | 0.003531096 | -0.060685249 | 0.190496571 | -0.076040147 |
| -0.098015629 | 0.034212327 | -0.049413391 | 0.286594674 | -0.024310954 |
| -0.073556956 | 0.112406162 | -0.049556174 | 0.285202762 | -0.001357748 |
| -0.163658037 | 0.000383518 | -0.134563009 | 0.003575173 | -0.120040706 |
| -0.111611877 | 0.015820393 | -0.074654347 | 0.107131886 | -0.105320405 |
| -0.150455536 | 0.001109258 | -0.11498835 | 0.012900202 | -0.121059599 |
| -0.064401657 | 0.164697629 | 0.003591472 | 0.938301675 | 0.021259424 |
| -0.217185991 | 2.16E-06 | -0.157420238 | 0.000639994 | -0.156867833 |

| | | | | |
|---|---|---|---|---|
| -0.167452154 | 0.000278338 | -0.140056491 | 0.002417348 | -0.147615978 |
| 0.013532156 | 0.770545061 | -0.025694089 | 0.579676482 | -0.015170116 |
| -0.222016302 | 1.26E-06 | -0.168828923 | 0.000247351 | -0.169322936 |
| 0.114383895 | 0.013385108 | 0.12039101 | 0.009209505 | 0.129049012 |
| 0.346365112 | 1.31E-14 | 0.335935511 | 8.78E-14 | 0.310678719 |
| 0.205600731 | 7.50E-06 | 0.175572963 | 0.000136924 | 0.157374176 |
| 0.078826589 | 0.088841583 | 0.114491475 | 0.013297648 | 0.119873774 |
| -0.016799606 | 0.717280919 | 0.01557725 | 0.73706283 | 0.032410119 |
| -0.025306966 | 0.585402873 | 0.028428539 | 0.53999313 | 0.054375778 |
| -0.070143302 | 0.130124262 | -0.062373772 | 0.178429371 | -0.03102903 |
| -0.071729082 | 0.121641843 | -0.032786339 | 0.479684553 | -0.011772755 |
| -0.139431327 | 0.002529205 | -0.090108818 | 0.051653571 | -0.072775464 |
| -0.161746331 | 0.000449569 | -0.107281432 | 0.020402952 | -0.113052364 |
| -0.06670362 | 0.150088881 | 0.00254314 | 0.956289409 | 0.03113282 |
| -0.09158256 | 0.047932053 | -0.049607253 | 0.28470593 | -0.012189032 |
| -0.057089218 | 0.218170823 | -0.004083587 | 0.929867986 | 0.01401357 |
| 0.058705642 | 0.205394208 | 0.086023642 | 0.063247301 | 0.107301017 |
| -0.076942318 | 0.096764847 | -0.015218826 | 0.742897126 | 0.012905003 |
| -0.019523134 | 0.67389534 | 0.046719261 | 0.313715575 | 0.055687765 |
| -0.155904109 | 0.000722799 | -0.177039804 | 0.000120046 | -0.159229822 |
| -0.044544149 | 0.336801487 | 0.003237591 | 0.944370673 | 0.032107908 |
| 0.037536577 | 0.418352024 | 0.080222158 | 0.083316046 | 0.07893966 |
| 0.126547753 | 0.006173739 | 0.094209344 | 0.041855616 | 0.078188861 |
| 0.137052581 | 0.002999296 | 0.078331983 | 0.090869138 | 0.079758267 |
| 0.11805636 | 0.010670234 | 0.062512833 | 0.177461422 | 0.06911531 |
| -0.024393139 | 0.599023198 | 0.053437817 | 0.249103433 | 0.057706129 |
| -0.067869816 | 0.143074457 | 0.010509231 | 0.820809439 | 0.031714091 |
| 0.093775168 | 0.042812675 | 0.121832284 | 0.008399199 | 0.145492972 |
| 0.399508235 | 2.53E-19 | 0.379452824 | 1.93E-17 | 0.382201063 |
| -0.181139122 | 8.27E-05 | -0.156080854 | 0.000712659 | -0.123348579 |
| -0.052417286 | 0.25827036 | 0.006544919 | 0.887823048 | 0.010950609 |
| -0.059256841 | 0.201163833 | -0.012116756 | 0.793974389 | -0.029734901 |
| -0.329781488 | 2.61E-13 | -0.2901269 | 1.65E-10 | -0.279002983 |
| -0.091301424 | 0.048624116 | -0.026893395 | 0.562104448 | -0.032710507 |
| 0.011067398 | 0.811466299 | 0.074999198 | 0.105515439 | 0.050308883 |
| 0.087611885 | 0.058507759 | 0.03221568 | 0.487367908 | 0.026158981 |
| -0.043404378 | 0.349322665 | 0.029798763 | 0.520630642 | 0.047380849 |
| 0.044405259 | 0.338311705 | 0.015889942 | 0.731985235 | 0.02627819 |
| 0.037447854 | 0.419453681 | 0.029541308 | 0.524241322 | 0.033329945 |
| 0.106621789 | 0.021193808 | 0.091322714 | 0.048571416 | 0.081263971 |
| -0.126244285 | 0.006299161 | -0.053783293 | 0.24605219 | -0.021998656 |
| -0.099810608 | 0.031042102 | -0.075293903 | 0.104149388 | -0.105806152 |
| -0.084301622 | 0.068738861 | -0.025505538 | 0.582462289 | -0.019206847 |
| 0.100198933 | 0.030390347 | 0.141125192 | 0.002236534 | 0.143127671 |
| -0.05284227 | 0.254424999 | -0.009128771 | 0.844023312 | -0.001560968 |

| | | | | |
|---|---|---|---|---|
| -0.065129718 | 0.159966145 | 0.015376915 | 0.74032198 | 0.001903394 |
| 0.089662677 | 0.052826601 | 0.043570528 | 0.347479263 | 0.05094392 |
| -0.202672208 | 1.02E-05 | -0.157053315 | 0.000659185 | -0.185338064 |
| -0.182028051 | 7.61E-05 | -0.185925807 | 5.29E-05 | -0.21138511 |
| -0.214495228 | 2.91E-06 | -0.154166816 | 0.000829827 | -0.158054884 |
| 0.000596649 | 0.989740179 | 0.040176063 | 0.386361263 | 0.044732067 |
| -0.08242735 | 0.07515364 | -0.007703301 | 0.868136426 | -0.02156695 |
| -0.22675715 | 7.35E-07 | -0.177417457 | 0.000116029 | -0.183651628 |
| 0.212448596 | 3.63E-06 | 0.188289412 | 4.23E-05 | 0.222426271 |
| 0.132914744 | 0.004009786 | 0.10669631 | 0.02110315 | 0.143759709 |
| -0.151390117 | 0.001031707 | -0.112609603 | 0.014902444 | -0.112265054 |
| -0.099689554 | 0.031247706 | -0.029846044 | 0.519968929 | -0.041078132 |
| -0.031390123 | 0.498599416 | -0.02306027 | 0.619141635 | -0.009468351 |
| 0.277772668 | 1.01E-09 | 0.263591392 | 7.31E-09 | 0.286330082 |
| 0.232841425 | 3.61E-07 | 0.194018579 | 2.42E-05 | 0.203306979 |
| -0.027547772 | 0.552626035 | 0.037458251 | 0.419324493 | 0.03297864 |
| -0.157987964 | 0.000611318 | -0.13017744 | 0.004838137 | -0.100922628 |
| -0.225023444 | 8.97E-07 | -0.18863735 | 4.09E-05 | -0.177668177 |
| 0.005694522 | 0.902320225 | 0.092306738 | 0.046187283 | 0.092843395 |
| 0.083693126 | 0.070770391 | 0.12292049 | 0.007830158 | 0.118109159 |
| 0.211325787 | 4.09E-06 | 0.201919824 | 1.10E-05 | 0.204826267 |
| 0.05688517 | 0.219823274 | 0.004943203 | 0.915155593 | 0.016665481 |
| -0.136705497 | 0.003074168 | -0.077767118 | 0.093229896 | -0.089171163 |
| -0.008934326 | 0.847304485 | -0.042408928 | 0.360496184 | -0.063549907 |
| 0.097676578 | 0.034841132 | 0.176532803 | 0.000125646 | 0.176594715 |
| -0.053288443 | 0.250430833 | -0.001569482 | 0.973015966 | -0.011570259 |
| -0.043274 | 0.350773494 | -0.066422431 | 0.151818661 | -0.082984858 |
| -0.063244602 | 0.172431998 | -0.007798638 | 0.866519636 | 0.001015455 |
| -0.100075794 | 0.03059574 | -0.052694969 | 0.255753291 | -0.065516538 |
| -0.133816184 | 0.003766508 | -0.111228119 | 0.01618645 | -0.124945876 |
| -0.253201704 | 2.89E-08 | -0.199539125 | 1.40E-05 | -0.220719836 |
| -0.090290241 | 0.051182811 | -0.026822935 | 0.563129681 | -0.011886843 |
| 0.081392324 | 0.078899836 | 0.11922475 | 0.009915343 | 0.150459324 |
| 0.154160339 | 0.000830253 | 0.125724103 | 0.006519476 | 0.138879645 |
| -0.023938234 | 0.605856377 | -0.041043655 | 0.37617993 | -0.044116358 |
| -0.070633878 | 0.127452629 | -0.13271057 | 0.004066826 | -0.168756185 |
| -0.039193031 | 0.398097754 | 0.00715973 | 0.877364996 | 0.008154014 |
| -0.269377353 | 3.31E-09 | -0.235851673 | 2.52E-07 | -0.239724785 |
| -0.244605655 | 8.63E-08 | -0.196607352 | 1.88E-05 | -0.180965075 |
| -0.11853685 | 0.010353834 | -0.070258931 | 0.129490688 | -0.042438108 |
| -0.011066495 | 0.8114814 | -0.045588726 | 0.325582025 | -0.078128611 |
| -0.109844402 | 0.017568667 | -0.036850322 | 0.426917278 | -0.040174944 |
| -0.09188158 | 0.04720504 | -0.035672761 | 0.441849542 | -0.066050606 |
| -0.028225616 | 0.542890859 | -0.069185443 | 0.135465323 | -0.070240733 |
| -0.125285772 | 0.006710461 | -0.073801124 | 0.11121533 | -0.032912057 |

| | | | | |
|---|---|---|---|---|
| 0.137186049 | 0.002970949 | 0.12609523 | 0.006361598 | 0.149426053 |
| -0.097923824 | 0.034381627 | -0.067652254 | 0.144363662 | -0.070689694 |
| 0.142181575 | 0.002070026 | 0.195529764 | 2.09E-05 | 0.209247435 |
| 0.00629651 | 0.892054157 | 0.07714153 | 0.095901422 | 0.11810176 |
| -0.069682904 | 0.132670765 | -0.045238485 | 0.329316541 | -0.038685588 |
| -0.132246743 | 0.004199142 | -0.05977741 | 0.197227051 | -0.070080494 |
| -0.144923748 | 0.001689317 | -0.06146097 | 0.184880431 | -0.049824411 |
| -0.544011415 | 2.50E-37 | -0.535378343 | 5.40E-36 | -0.525516453 |
| 0.025442074 | 0.583401353 | -0.018632149 | 0.687978477 | -0.024109729 |
| -0.178890387 | 0.000101536 | -0.122609786 | 0.007989012 | -0.137742146 |
| 0.128296758 | 0.005493594 | 0.194938292 | 2.21E-05 | 0.164591299 |
| 0.101949322 | 0.027596025 | 0.076158024 | 0.100224521 | 0.077749096 |
| -0.146286093 | 0.001525094 | -0.091510934 | 0.048107585 | -0.091480633 |
| -0.042339275 | 0.361286287 | 0.03146264 | 0.497607367 | 0.019664378 |
| -0.086122013 | 0.062944835 | -0.008217413 | 0.859424433 | 0.001665798 |
| 0.03679423 | 0.427621843 | 0.002686587 | 0.953826564 | 0.015945111 |
| -0.13877014 | 0.002652626 | -0.103365613 | 0.025499541 | -0.09757855 |
| 0.082415183 | 0.075196819 | 0.147916989 | 0.001347825 | 0.150097718 |
| 0.059515384 | 0.199201527 | 0.083668723 | 0.070852876 | 0.11418767 |
| 0.022748562 | 0.623888676 | 0.047642037 | 0.304242777 | 0.060976285 |
| -0.001162379 | 0.980013552 | 0.040556411 | 0.381877335 | 0.053235382 |
| 0.193541818 | 2.54E-05 | 0.159405995 | 0.000544818 | 0.134026814 |
| -0.108402148 | 0.019118056 | -0.017892366 | 0.699754456 | -0.025545735 |
| -0.094995547 | 0.040168494 | -0.044443093 | 0.337899887 | -0.03269295 |
| 0.222815415 | 1.15E-06 | 0.254082908 | 2.58E-08 | 0.275030913 |
| -0.141451504 | 0.002183839 | -0.076055509 | 0.100683911 | -0.052588629 |
| 0.028218204 | 0.542996853 | 0.08226914 | 0.075716713 | 0.088203444 |
| -0.016703562 | 0.718828613 | 0.051310177 | 0.268475673 | 0.043853563 |
| -0.148354489 | 0.001303606 | -0.124795677 | 0.006929923 | -0.122233621 |
| -0.031688259 | 0.494527594 | 0.022528986 | 0.627242003 | 0.024973492 |
| -0.124296882 | 0.007159858 | -0.129495614 | 0.005067142 | -0.147134436 |
| -0.083067222 | 0.072910981 | -0.091237296 | 0.048783144 | -0.086634775 |
| -0.07092602 | 0.125881972 | -0.000310272 | 0.994664531 | -0.026654135 |
| -0.190362708 | 3.46E-05 | -0.147887262 | 0.001350879 | -0.155822969 |
| 0.156710229 | 0.000677611 | 0.19253709 | 2.80E-05 | 0.157818673 |
| -0.144173068 | 0.001786588 | -0.074114821 | 0.109700001 | -0.068601204 |
| -0.065448667 | 0.157925947 | 0.00734091 | 0.874287081 | 0.034510395 |
| -0.185519755 | 5.50E-05 | -0.174690738 | 0.000148123 | -0.169578063 |
| -0.078062575 | 0.091989035 | -0.013215868 | 0.775763372 | 0.003899566 |
| -0.120247291 | 0.009294001 | -0.063198577 | 0.172745164 | -0.052876743 |
| -0.181032051 | 8.35E-05 | -0.123752822 | 0.007418422 | -0.14167866 |
| -0.0953213 | 0.039486498 | -0.018560659 | 0.689113226 | -0.010021059 |
| -0.309794737 | 7.60E-12 | -0.292747579 | 1.11E-10 | -0.330433158 |
| -0.116028639 | 0.012101856 | -0.042800509 | 0.356074428 | -0.034265262 |
| -0.118102366 | 0.010639573 | -0.074300613 | 0.108810222 | -0.050717069 |

| | | | | |
|---|---|---|---|---|
| 0.043898916 | 0.343854064 | 0.002351389 | 0.959582238 | -0.029968969 |
| -0.143913297 | 0.001821426 | -0.064794836 | 0.162129577 | -0.067532561 |
| -0.12461021 | 0.007014636 | -0.070461529 | 0.128386338 | -0.061705516 |
| 0.119118212 | 0.009982156 | 0.162422719 | 0.000425076 | 0.204397099 |
| -0.188065563 | 4.32E-05 | -0.162036364 | 0.00043891 | -0.169417448 |
| -0.047614778 | 0.304519856 | 0.014816551 | 0.749462772 | 0.007623961 |
| -0.02695364 | 0.561228564 | -0.100039837 | 0.030655939 | -0.075448054 |
| 0.01216964 | 0.793095519 | -0.064340737 | 0.165098243 | -0.064040385 |
| -0.188995247 | 3.95E-05 | -0.137829958 | 0.002837584 | -0.145286314 |
| -0.211989748 | 3.81E-06 | -0.166534045 | 0.000300978 | -0.173091195 |
| -0.059396473 | 0.200102309 | -0.012942344 | 0.780284306 | -0.012878127 |
| -0.112453889 | 0.015042553 | -0.042761587 | 0.356512412 | -0.054811446 |
| 0.053164211 | 0.251538556 | 0.098063309 | 0.034124679 | 0.115398394 |
| -0.102112903 | 0.027346547 | -0.13826547 | 0.002750495 | -0.147604198 |
| 0.204029054 | 8.83E-06 | 0.175315855 | 0.000140103 | 0.177355078 |
| -0.054732107 | 0.237806952 | 0.011277912 | 0.80794938 | 0.016190443 |
| -0.247533039 | 5.98E-08 | -0.170828343 | 0.000208049 | -0.193995168 |
| -0.046335864 | 0.317707643 | 0.000426448 | 0.992666809 | -0.002065601 |
| 0.075190158 | 0.10462867 | 0.139107055 | 0.002589066 | 0.150918984 |
| -0.034808335 | 0.452998179 | 0.023793671 | 0.608035133 | 0.007662965 |
| -0.160079312 | 0.000515653 | -0.099224737 | 0.032048027 | -0.105271127 |
| -0.045358192 | 0.328037045 | 0.018935911 | 0.683164698 | 0.025170698 |
| -0.038216766 | 0.409962843 | 0.015097923 | 0.744868495 | 0.039414676 |
| -0.084067141 | 0.069515971 | -0.16650072 | 0.000301831 | -0.165078712 |
| -0.162392919 | 0.000426128 | -0.147745508 | 0.00136553 | -0.135627582 |
| -0.088263887 | 0.056649128 | -0.030368449 | 0.512686701 | -0.050945606 |
| -0.066312574 | 0.152498557 | 0.011435663 | 0.805316468 | 0.011297857 |
| 0.292951212 | 1.08E-10 | 0.265618095 | 5.55E-09 | 0.275410336 |
| 0.032748107 | 0.480197239 | 0.081407764 | 0.078842862 | 0.056461825 |
| -0.122912974 | 0.007833967 | -0.095673075 | 0.038761049 | -0.105367062 |
| -0.14744735 | 0.001396824 | -0.101888309 | 0.027689576 | -0.113093269 |
| 0.047214561 | 0.308607256 | 0.094263376 | 0.041737785 | 0.112765345 |
| -0.145234772 | 0.001650457 | -0.078547759 | 0.089980086 | -0.071558747 |
| -0.231805119 | 4.08E-07 | -0.201038307 | 1.20E-05 | -0.229725633 |
| -0.019951438 | 0.667165412 | 0.007800147 | 0.866494051 | 0.008546342 |
| 0.067352867 | 0.146152223 | 0.102766376 | 0.026369188 | 0.102533044 |
| 0.027795013 | 0.549065301 | -0.022967189 | 0.620557544 | -0.06180637 |
| -0.008175393 | 0.860135878 | -0.043723302 | 0.345789732 | -0.020085931 |
| 0.047948326 | 0.301140846 | 0.141610746 | 0.002158538 | 0.157367196 |
| 0.05134376 | 0.268162108 | 0.007185953 | 0.876919392 | 0.014836168 |
| 0.023116326 | 0.618289623 | 0.087539572 | 0.058716972 | 0.100022135 |
| -0.182838648 | 7.06E-05 | -0.222204046 | 1.24E-06 | -0.221948279 |
| 0.155625657 | 0.000739045 | 0.121893045 | 0.00836648 | 0.107989887 |
| -0.158148866 | 0.000603411 | -0.137949665 | 0.0028134 | -0.110203087 |
| -0.006577312 | 0.887271541 | 0.077082407 | 0.096157027 | 0.101816694 |

| | | | | |
|---|---|---|---|---|
| -0.034379919 | 0.45858166 | 0.032660173 | 0.481377562 | 0.048301943 |
| -0.066497326 | 0.151356461 | -0.01406673 | 0.761748933 | -0.018393465 |
| 0.070121347 | 0.130244835 | 0.142357059 | 0.002043491 | 0.146819355 |
| -0.12062769 | 0.009071844 | -0.077237957 | 0.0954857 | -0.086230238 |
| -0.083316773 | 0.072051269 | -0.041788107 | 0.367576566 | -0.045140183 |
| 0.133928373 | 0.003737186 | 0.114567668 | 0.01323601 | 0.119218188 |
| -0.04711561 | 0.309623395 | 0.015089849 | 0.745000191 | 0.049379018 |
| -0.122254651 | 0.008174099 | -0.06710223 | 0.1476625 | -0.050916741 |
| 0.054821275 | 0.237042198 | 0.11409854 | 0.013619553 | 0.132966724 |
| 0.061956143 | 0.181359861 | 0.103837942 | 0.024831608 | 0.139781556 |
| -0.009019372 | 0.845869043 | 0.03571551 | 0.441302299 | 0.060234143 |
| 0.212000185 | 3.81E-06 | 0.22531777 | 8.68E-07 | 0.270857831 |
| -0.143994553 | 0.001810462 | -0.054477086 | 0.240003743 | -0.056836246 |
| 0.062871637 | 0.174981934 | 0.087370902 | 0.05920737 | 0.0936231 |
| 0.049530138 | 0.285456235 | 0.07852018 | 0.090093326 | 0.084208749 |
| 0.163739614 | 0.000380911 | 0.234625018 | 2.92E-07 | 0.23004671 |
| 0.071904724 | 0.120729475 | 0.125476462 | 0.006626768 | 0.131987618 |
| 0.028342007 | 0.541227856 | 0.066018503 | 0.154329906 | 0.043065527 |
| 0.037678443 | 0.416594043 | 0.073152497 | 0.114400759 | 0.068437973 |
| -0.127543969 | 0.005777612 | -0.062620877 | 0.176712061 | -0.082104388 |
| -0.091241723 | 0.048772152 | -0.042010321 | 0.365032374 | -0.057111159 |
| -0.284166726 | 4.01E-10 | -0.270945776 | 2.66E-09 | -0.2609747 |
| -0.097972034 | 0.034292633 | -0.051853595 | 0.263432467 | -0.042031688 |
| 0.079697737 | 0.085358946 | 0.129103663 | 0.005203164 | 0.112423644 |
| -0.064845523 | 0.16180072 | -0.042382961 | 0.360790604 | -0.016362968 |
| -0.191018399 | 3.25E-05 | -0.162960399 | 0.000406497 | -0.210368489 |
| -0.228480429 | 6.02E-07 | -0.162375597 | 0.000426741 | -0.167648034 |
| -0.160440831 | 0.000500598 | -0.101923283 | 0.027635918 | -0.12920769 |
| 0.023119948 | 0.618234599 | 0.07884068 | 0.088784358 | 0.068569754 |
| -0.029864935 | 0.519704674 | -0.072389529 | 0.118238846 | -0.049981893 |
| -0.038543513 | 0.405968562 | 0.035522465 | 0.443776622 | 0.008012549 |
| -0.111759252 | 0.015681756 | -0.049823907 | 0.282605077 | -0.025669475 |
| 0.160092121 | 0.000515112 | 0.194152277 | 2.39E-05 | 0.185829393 |
| 0.234939453 | 2.81E-07 | 0.291031202 | 1.44E-10 | 0.299155582 |
| -0.154732796 | 0.000793448 | -0.104899291 | 0.02338561 | -0.151532597 |
| -0.109500834 | 0.017927444 | -0.079995805 | 0.084192915 | -0.091584168 |
| -0.088615261 | 0.055667966 | -0.009357717 | 0.840163427 | -0.003915157 |
| -0.005533855 | 0.905063009 | 0.061501015 | 0.184593862 | 0.060741915 |
| 0.034555845 | 0.456284206 | 0.093916289 | 0.042499597 | 0.076832597 |
| 0.092164022 | 0.046526849 | 0.145097062 | 0.00166756 | 0.140404806 |
| -0.107430739 | 0.020227548 | -0.040286029 | 0.385061599 | -0.031605638 |
| 0.120716974 | 0.009020391 | 0.158119364 | 0.000604853 | 0.146831397 |
| -0.032667563 | 0.481278306 | -0.015292827 | 0.741691345 | 0.002851358 |
| -0.239774522 | 1.57E-07 | -0.173403311 | 0.000166016 | -0.150963133 |
| -0.009545016 | 0.837008517 | -0.002247017 | 0.961374838 | -0.038457245 |

| | | | | |
|---|---|---|---|---|
| 0.134631518 | 0.00355807 | 0.095413762 | 0.039294714 | 0.052120551 |
| -0.192833319 | 2.72E-05 | -0.138115569 | 0.002780192 | -0.182550844 |
| -0.007907596 | 0.864672543 | 0.050355823 | 0.277491705 | 0.055168764 |
| 0.081013188 | 0.08030942 | 0.0814249 | 0.078779663 | 0.062849293 |
| 0.076953194 | 0.096717546 | 0.056874258 | 0.219911895 | 0.009923672 |
| 0.027904363 | 0.547494054 | -0.027982015 | 0.546379625 | -0.041718667 |
| -0.120372677 | 0.009220245 | -0.099524226 | 0.031530386 | -0.085759429 |
| 0.17967987 | 9.45E-05 | 0.175148166 | 0.000142213 | 0.182717336 |
| 0.111675341 | 0.01576056 | 0.070720403 | 0.126985863 | 0.069710908 |
| 0.230674531 | 4.66E-07 | 0.163108053 | 0.000401529 | 0.155526345 |
| 0.032345945 | 0.485608208 | 0.073872624 | 0.110868506 | 0.058524594 |
| -0.104641719 | 0.023729665 | -0.092785885 | 0.04506242 | -0.113672929 |
| -0.13403873 | 0.003708544 | -0.101527309 | 0.028248693 | -0.061270277 |
| -0.167582878 | 0.000275247 | -0.137717046 | 0.002860569 | -0.135291505 |
| -0.155230565 | 0.000762678 | -0.091873245 | 0.047225181 | -0.097627857 |
| -0.116319717 | 0.011886437 | -0.051552433 | 0.266219306 | -0.069364876 |
| 0.005566858 | 0.904499504 | 0.054847086 | 0.236821152 | 0.05553001 |
| -0.432472391 | 1.05E-22 | -0.396188067 | 5.30E-19 | -0.426593258 |
| 0.024775041 | 0.593313644 | 0.058757571 | 0.204992934 | 0.054784866 |
| 0.07904416 | 0.087961271 | 0.115857453 | 0.012230151 | 0.136652977 |
| -0.142113316 | 0.002080433 | -0.1109308 | 0.016475136 | -0.107212853 |
| -0.165972793 | 0.000315652 | -0.123498909 | 0.007541927 | -0.109408214 |
| -0.232814827 | 3.62E-07 | -0.148140398 | 0.001325075 | -0.157673205 |
| -0.122455642 | 0.008068883 | -0.03603817 | 0.437184312 | -0.026376289 |
| 0.079400811 | 0.086533412 | 0.106586179 | 0.021237249 | 0.092952835 |
| -0.038730046 | 0.403698753 | 0.029863955 | 0.519718376 | 0.031724943 |
| -0.199228623 | 1.44E-05 | -0.146300687 | 0.001523417 | -0.131713973 |
| -0.348746663 | 8.41E-15 | -0.317134277 | 2.27E-12 | -0.310581887 |
| 0.050475975 | 0.276345418 | 0.122281912 | 0.008159757 | 0.120553574 |
| -0.114245661 | 0.013498232 | -0.066641568 | 0.15046931 | -0.085258006 |
| -0.017069794 | 0.712933187 | -0.060447339 | 0.192243847 | -0.077351979 |
| -0.104143478 | 0.024407634 | -0.042791293 | 0.356178105 | -0.006052867 |
| -0.158298112 | 0.000596161 | -0.090680182 | 0.050183098 | -0.097741021 |
| -0.032034753 | 0.489817666 | 0.018977759 | 0.682502529 | -0.001740881 |
| -0.059135922 | 0.202086404 | -0.016644376 | 0.719782938 | -0.048452682 |
| 0.012323094 | 0.790546738 | -0.016186997 | 0.72717237 | -0.029395196 |
| 0.139918596 | 0.002441625 | 0.066799849 | 0.149500371 | 0.085643822 |
| 0.01377611 | 0.766527191 | 0.031693565 | 0.494455283 | 0.085940917 |
| 0.055997109 | 0.227119364 | 0.114206282 | 0.013530612 | 0.105347572 |
| 0.037545627 | 0.418239757 | 0.101753244 | 0.027897641 | 0.109796935 |
| 0.077240323 | 0.095475517 | 0.139571657 | 0.002503694 | 0.150068214 |
| -0.19598084 | 2.00E-05 | -0.151620689 | 0.001013359 | -0.160356359 |
| -0.080816805 | 0.081047531 | -0.037128317 | 0.423435365 | -0.04835404 |
| -0.147830024 | 0.001356778 | -0.111483425 | 0.015942103 | -0.106445011 |
| 0.000905602 | 0.984428058 | 0.050234074 | 0.278656516 | 0.041705923 |

| | | | | |
|---|---|---|---|---|
| -0.219135288 | 1.74E-06 | -0.215979991 | 2.47E-06 | -0.22562616 |
| -0.111596758 | 0.015834676 | -0.03592823 | 0.438584944 | -0.041623392 |
| -0.06112539 | 0.187294824 | -0.006898956 | 0.881798319 | -0.006979947 |
| -0.12272868 | 0.007927888 | -0.107063024 | 0.020661915 | -0.117254155 |
| -0.03185879 | 0.492206533 | 0.032901521 | 0.478141761 | 0.033990977 |
| 0.258035991 | 1.54E-08 | 0.214603634 | 2.87E-06 | 0.204116977 |
| 0.075555831 | 0.102947055 | 0.119121604 | 0.009980022 | 0.136420895 |
| -0.140925336 | 0.002269378 | -0.088894107 | 0.054899406 | -0.108292294 |
| -0.083270666 | 0.072209483 | -0.022619403 | 0.625860246 | -0.036392882 |
| -0.07426984 | 0.108957202 | -0.016755297 | 0.717994791 | -0.05895992 |
| -0.051469811 | 0.26698738 | -0.01267449 | 0.784718702 | -0.005588475 |
| -0.09517217 | 0.039797492 | -0.02332527 | 0.615118331 | -0.012241267 |
| 0.020626975 | 0.656604947 | 0.067593116 | 0.144715626 | 0.099534236 |
| -0.085309333 | 0.06547973 | -0.060772056 | 0.189861944 | -0.061522123 |
| 0.11187485 | 0.015573758 | 0.155876222 | 0.000724411 | 0.17569703 |
| -0.148139906 | 0.001325125 | -0.095520172 | 0.039074976 | -0.106662173 |
| 0.173864942 | 0.000159379 | 0.124107901 | 0.007248743 | 0.160598188 |
| -0.147032358 | 0.001441479 | -0.089975078 | 0.052002908 | -0.110004395 |
| -0.161489718 | 0.000459201 | -0.127961272 | 0.00561858 | -0.124258456 |
| 0.002114263 | 0.963655204 | 0.052847708 | 0.254376055 | 0.031446724 |
| -0.19153806 | 3.09E-05 | -0.142995483 | 0.001949556 | -0.143850874 |
| 0.004674252 | 0.919755845 | 0.024674018 | 0.59482155 | 0.001902827 |
| -0.040274651 | 0.385195952 | 0.006117833 | 0.895099441 | -0.033515135 |
| -0.103562187 | 0.025219701 | -0.043090474 | 0.352822198 | -0.044974253 |
| 0.113484265 | 0.014136542 | 0.146416189 | 0.001510204 | 0.148125173 |
| 0.156572437 | 0.000685145 | 0.136977434 | 0.003015365 | 0.126534803 |
| -0.284284784 | 3.94E-10 | -0.231444495 | 4.26E-07 | -0.201193979 |
| 0.093202143 | 0.044103977 | 0.07241 | 0.11813457 | 0.068324019 |
| -0.067034149 | 0.148074791 | -0.030905264 | 0.505259183 | -0.017814092 |
| -0.191280821 | 3.17E-05 | -0.146184153 | 0.001536855 | -0.180144226 |
| -0.380561902 | 1.53E-17 | -0.344998674 | 1.69E-14 | -0.341297827 |
| -0.016099997 | 0.728580842 | 0.029450998 | 0.525510901 | 0.023231785 |
| -0.214319269 | 2.96E-06 | -0.161970129 | 0.000441323 | -0.15572626 |
| -0.045683583 | 0.324575338 | 0.014602633 | 0.75296161 | 0.017507524 |
| -0.004985777 | 0.91442765 | 0.041288124 | 0.373341123 | 0.062429585 |
| 0.047444814 | 0.306251297 | 0.113982215 | 0.013716158 | 0.136625193 |
| -0.235178098 | 2.73E-07 | -0.187835502 | 4.41E-05 | -0.204380846 |
| -0.3970387 | 4.39E-19 | -0.363943716 | 4.51E-16 | -0.362908078 |
| -0.092163539 | 0.046528 | -0.061633772 | 0.18364617 | -0.070308653 |
| 0.090068765 | 0.051757986 | 0.135816031 | 0.003273858 | 0.151104214 |
| -0.135985442 | 0.00323494 | -0.123607148 | 0.007489056 | -0.080921483 |
| 0.109446594 | 0.017984667 | 0.148705154 | 0.001269132 | 0.127983932 |
| 0.288579023 | 2.08E-10 | 0.319173424 | 1.61E-12 | 0.335612729 |
| -0.019780238 | 0.669852311 | 0.040106446 | 0.387185438 | 0.05381886 |
| 0.120816161 | 0.008963536 | 0.180806025 | 8.52E-05 | 0.180903945 |

| | | | | |
|---|---|---|---|---|
| -0.171842883 | 0.000190421 | -0.11909267 | 0.009998233 | -0.102221138 |
| 0.122740776 | 0.007921693 | 0.191975161 | 2.96E-05 | 0.202263909 |
| -0.034419637 | 0.458062406 | 0.030774904 | 0.507057673 | 0.050705913 |
| 0.019050008 | 0.681359881 | 0.029488277 | 0.524986641 | 0.03911409 |
| 0.005702375 | 0.902186194 | 0.024315404 | 0.600188395 | 0.018720621 |
| -0.07133205 | 0.123724028 | -0.015050674 | 0.745639355 | -0.03493583 |
| -0.11757859 | 0.010993311 | -0.039650096 | 0.392614422 | -0.034366719 |
| -0.272262244 | 2.21E-09 | -0.223090766 | 1.12E-06 | -0.200345181 |
| -0.25643981 | 1.90E-08 | -0.198584846 | 1.54E-05 | -0.189654726 |
| 0.094536726 | 0.041145956 | 0.14058223 | 0.002326794 | 0.137552422 |
| -0.198312459 | 1.58E-05 | -0.160200763 | 0.000510549 | -0.16329117 |
| 0.179565202 | 9.55E-05 | 0.125579642 | 0.006581874 | 0.148157882 |
| -0.099900255 | 0.030890589 | -0.051195611 | 0.26954729 | -0.022143645 |
| -0.033836283 | 0.465721831 | 0.038673558 | 0.404385317 | 0.005810812 |
| 0.024646352 | 0.595234807 | 0.051076392 | 0.270665535 | 0.012839858 |
| 0.023698011 | 0.609478756 | 0.060355519 | 0.192921334 | 0.056610594 |
| -0.04885979 | 0.292034622 | -0.008220526 | 0.859371741 | -0.040583924 |
| -0.331809918 | 1.83E-13 | -0.288919019 | 1.98E-10 | -0.279066863 |
| -0.034850173 | 0.452454962 | 0.029633752 | 0.522943364 | 0.04868224 |
| -0.121677204 | 0.008483226 | -0.074995083 | 0.105534615 | -0.091845486 |
| -0.222830512 | 1.15E-06 | -0.191207963 | 3.19E-05 | -0.224066362 |
| -0.013720877 | 0.767436326 | 0.008273545 | 0.858474258 | 0.028893713 |
| -0.095824133 | 0.038453011 | -0.129570881 | 0.005041391 | -0.132765859 |
| -0.097747539 | 0.03470872 | -0.089043948 | 0.05449007 | -0.087197572 |
| -0.045111207 | 0.330680492 | 0.018437579 | 0.691068517 | 0.017567506 |
| -0.111658012 | 0.015776878 | -0.081269193 | 0.079355409 | -0.059721235 |
| -0.352541972 | 4.11E-15 | -0.325854073 | 5.16E-13 | -0.321353317 |
| -0.076453163 | 0.098911245 | -0.017390933 | 0.707777679 | -0.021674727 |
| 0.060539446 | 0.191565999 | 0.096863186 | 0.036389874 | 0.099657852 |
| -0.186848321 | 4.85E-05 | -0.130332803 | 0.004787279 | -0.13722779 |
| -0.328422053 | 3.31E-13 | -0.279799984 | 7.57E-10 | -0.29011605 |
| -0.145728455 | 0.001590456 | -0.108503003 | 0.019005962 | -0.125107686 |
| -0.147023533 | 0.001442442 | -0.109301312 | 0.018138722 | -0.081633609 |
| -0.170212527 | 0.000219483 | -0.103336099 | 0.025541787 | -0.118208801 |
| -0.050536291 | 0.275771199 | -0.003191548 | 0.945160539 | -0.026096946 |
| -0.203387099 | 9.43E-06 | -0.189548035 | 3.75E-05 | -0.193660715 |
| -0.011925718 | 0.797151434 | 0.00326728 | 0.943861376 | -0.002815649 |
| -0.014513492 | 0.754421108 | 0.049513894 | 0.285614451 | 0.048328408 |
| -0.015780978 | 0.733753325 | -0.054353228 | 0.241075797 | -0.010057392 |
| -0.096366985 | 0.037363092 | -0.037664182 | 0.416770569 | -0.06176347 |
| -0.165607219 | 0.000325567 | -0.125612543 | 0.006567616 | -0.114168857 |
| -0.067630878 | 0.144490809 | 0.006505671 | 0.888491352 | 0.023686051 |
| -0.17701263 | 0.00012034 | -0.121997804 | 0.008310335 | -0.108842219 |
| -0.082204208 | 0.075948797 | -0.040411068 | 0.383587024 | -0.023270424 |
| -0.01931616 | 0.677156899 | 0.055319394 | 0.232801919 | 0.069777323 |

| | | | | |
|---|---|---|---|---|
| 0.093833576 | 0.042682862 | 0.142043753 | 0.002091087 | 0.155298122 |
| -0.072952 | 0.11539976 | -0.071447759 | 0.123114366 | -0.070248603 |
| -0.063458874 | 0.170979623 | 0.007891017 | 0.864953536 | 0.015879142 |
| -0.005429448 | 0.906845969 | 0.030462646 | 0.511379277 | 0.03314936 |
| 0.16714634 | 0.000285696 | 0.136659868 | 0.003084136 | 0.136526952 |
| -0.00630308 | 0.891942212 | 0.060505271 | 0.191817298 | 0.0442813 |
| -0.128112001 | 0.005562112 | -0.073176265 | 0.114282784 | -0.086728572 |
| 0.159049956 | 0.000560852 | 0.207546645 | 6.11E-06 | 0.205344051 |
| -0.026729208 | 0.564494867 | -0.018568984 | 0.688981049 | 0.009920457 |
| -0.126082405 | 0.006366996 | -0.059738391 | 0.197520162 | -0.064731544 |
| -0.292661989 | 1.12E-10 | -0.250549434 | 4.07E-08 | -0.22354281 |
| -0.268883536 | 3.55E-09 | -0.20603688 | 7.16E-06 | -0.206513002 |
| -0.237593952 | 2.04E-07 | -0.156177151 | 0.00070719 | -0.126564492 |
| -0.177043289 | 0.000120009 | -0.114520112 | 0.013274452 | -0.097626205 |
| -0.127955766 | 0.005620652 | -0.112304974 | 0.015177624 | -0.111030683 |
| 0.108366303 | 0.019158034 | 0.112203968 | 0.015269845 | 0.110538159 |
| 0.007298233 | 0.875011908 | 0.062760636 | 0.175746226 | 0.069192357 |
| -0.102082648 | 0.027392542 | -0.074612513 | 0.107329297 | -0.064135943 |
| -0.202889393 | 9.93E-06 | -0.135080583 | 0.003447788 | -0.144374357 |
| 0.061537102 | 0.184335894 | 0.094020743 | 0.042269111 | 0.09298959 |
| 0.045554942 | 0.32594106 | 0.102478345 | 0.026796207 | 0.088837606 |
| 0.076980584 | 0.096598513 | 0.133287841 | 0.003907421 | 0.11860319 |
| 0.202690127 | 1.01E-05 | 0.169425929 | 0.000234943 | 0.201254425 |
| 0.040765849 | 0.379421911 | 0.01228507 | 0.791178078 | 0.032501644 |
| -0.009808309 | 0.832577965 | 0.042767012 | 0.356451341 | 0.043630596 |
| -0.165742381 | 0.000321868 | -0.116171759 | 0.01199551 | -0.091054309 |
| 0.033006918 | 0.476732422 | 0.110274744 | 0.017128136 | 0.125112196 |
| 0.187012483 | 4.77E-05 | 0.202821918 | 1.00E-05 | 0.206812736 |
| 0.154038158 | 0.00083831 | 0.203704201 | 9.13E-06 | 0.208073079 |
| -0.066136558 | 0.153592718 | 0.003086389 | 0.946964749 | -0.020898134 |
| 0.020697384 | 0.655508133 | -0.00500096 | 0.914168065 | -0.018929165 |
| 0.13181115 | 0.004326935 | 0.218179042 | 1.94E-06 | 0.252263573 |
| -0.080985008 | 0.080415 | -0.037088936 | 0.423927611 | -0.048635491 |
| -0.109929202 | 0.017481085 | -0.043933163 | 0.343477387 | -0.062793534 |
| -0.027283122 | 0.556449974 | 0.023734079 | 0.608934265 | 0.019801772 |
| -0.092290879 | 0.046224912 | -0.050468501 | 0.276416635 | -0.070728767 |
| -0.13439971 | 0.003616241 | -0.088880503 | 0.054936697 | -0.115712061 |
| -0.193424284 | 2.57E-05 | -0.145576139 | 0.001608751 | -0.126409648 |
| -0.14785272 | 0.001354436 | -0.080985032 | 0.08041491 | -0.066465107 |
| -0.077852703 | 0.092869085 | -0.018377286 | 0.69202709 | 0.000853422 |
| -0.222245236 | 1.23E-06 | -0.197411322 | 1.73E-05 | -0.199986646 |
| 0.037775952 | 0.41538825 | 0.090302772 | 0.051150428 | 0.120856088 |
| -0.052922448 | 0.253704004 | -0.009550347 | 0.836918762 | -0.02029707 |
| -0.126703069 | 0.006110419 | -0.070364816 | 0.1289126 | -0.091075707 |
| -0.324004134 | 7.10E-13 | -0.28870153 | 2.04E-10 | -0.29785632 |

| | | | | |
|---|---|---|---|---|
| -0.068812774 | 0.137588369 | -0.03093575 | 0.504839066 | -0.078531292 |
| -0.113704578 | 0.013949169 | -0.091966802 | 0.04699954 | -0.096284889 |
| 0.019786121 | 0.669759903 | 0.048506244 | 0.29554468 | 0.103783771 |
| 0.017226814 | 0.710410773 | 0.070927721 | 0.125872874 | 0.054273892 |
| -0.005234877 | 0.910169888 | -0.001291091 | 0.977800962 | 0.013175507 |
| -0.231447206 | 4.26E-07 | -0.171384749 | 0.000198201 | -0.1601155 |
| -0.097276307 | 0.035596112 | -0.079912684 | 0.084516782 | -0.046725577 |
| -0.13421087 | 0.003664264 | -0.065193296 | 0.159557882 | -0.055716802 |
| -0.19503535 | 2.19E-05 | -0.160091777 | 0.000515127 | -0.156376651 |
| -0.014820837 | 0.749392726 | 0.061492404 | 0.184655453 | 0.065032832 |
| -0.174711632 | 0.000147848 | -0.132779585 | 0.004047464 | -0.116254874 |
| 0.011763969 | 0.799844014 | 0.079794018 | 0.084980884 | 0.083197898 |
| 0.096891203 | 0.036335572 | 0.150031648 | 0.001146173 | 0.138264939 |
| -0.001607009 | 0.972371029 | 0.051997966 | 0.262103644 | 0.041096172 |
| -0.016153175 | 0.727719821 | 0.039875955 | 0.389921747 | 0.003069237 |
| -0.124231777 | 0.007190369 | -0.076342082 | 0.099403903 | -0.057876516 |
| 0.171759769 | 0.000191811 | 0.129997168 | 0.004897759 | 0.149791713 |
| 0.130805546 | 0.00463548 | 0.09873786 | 0.032905045 | 0.069124188 |
| -0.130851946 | 0.004620817 | -0.073219468 | 0.114068588 | -0.090426739 |
| -0.128728463 | 0.005336445 | -0.04989047 | 0.281961734 | -0.044090297 |
| -0.02943395 | 0.525750734 | 0.014850435 | 0.74890905 | 0.051456255 |
| -0.166530952 | 0.000301057 | -0.097897655 | 0.034430017 | -0.082688199 |
| 0.000852942 | 0.985333437 | 0.017303834 | 0.70917464 | 0.036102336 |
| -0.096990023 | 0.036144588 | -0.029725045 | 0.521663196 | -0.033739508 |
| -0.047506286 | 0.305624327 | 0.000195512 | 0.99663794 | -0.000538408 |
| 0.210302625 | 4.56E-06 | 0.178333854 | 0.000106799 | 0.188619412 |
| -0.132792991 | 0.004043712 | -0.081431231 | 0.078756326 | -0.099158031 |
| -0.158786716 | 0.000572987 | -0.087896634 | 0.05768992 | -0.101181833 |
| -0.131224117 | 0.004504716 | -0.101491124 | 0.028305267 | -0.106624299 |
| 0.093341129 | 0.0437878 | 0.142377696 | 0.002040391 | 0.13401792 |
| -0.123655905 | 0.007465348 | -0.061896617 | 0.181780439 | -0.048872632 |
| 0.023487604 | 0.612659405 | 0.027092975 | 0.559205323 | 0.021280618 |
| -0.276398035 | 1.23E-09 | -0.247433639 | 6.05E-08 | -0.253948219 |
| 0.251761566 | 3.49E-08 | 0.242830594 | 1.08E-07 | 0.246085339 |
| -0.114728644 | 0.013106612 | -0.053810089 | 0.24581663 | -0.054097828 |
| -0.173813346 | 0.000160109 | -0.100383465 | 0.030084764 | -0.112112074 |
| -0.171642245 | 0.000193792 | -0.164691495 | 0.000351692 | -0.150254668 |
| 0.102666682 | 0.026516322 | 0.138837173 | 0.002639868 | 0.121710358 |
| -0.156114466 | 0.000710746 | -0.102970866 | 0.026069592 | -0.094866192 |
| -0.019768483 | 0.670036962 | 0.056206813 | 0.225381159 | 0.059898168 |
| -0.063267582 | 0.172275799 | -0.044279374 | 0.339684259 | -0.068758115 |
| 0.22548022 | 8.52E-07 | 0.232751874 | 3.65E-07 | 0.248004485 |
| -0.086798999 | 0.060895433 | -0.147038008 | 0.001440862 | -0.187462858 |
| 0.260911624 | 1.05E-08 | 0.240655459 | 1.41E-07 | 0.270192494 |
| 0.198053298 | 1.62E-05 | 0.160197144 | 0.0005107 | 0.171495671 |

| | | | | |
|---|---|---|---|---|
| 0.144490312 | 0.001744871 | 0.117704278 | 0.01090749 | 0.10904425 |
| -0.078782581 | 0.089020501 | -0.045500147 | 0.32652392 | -0.044659065 |
| -0.268260851 | 3.86E-09 | -0.221309053 | 1.37E-06 | -0.191785532 |
| 0.076503095 | 0.098690423 | 0.131445678 | 0.004436856 | 0.131327698 |
| -0.228900236 | 5.74E-07 | -0.183344932 | 6.74E-05 | -0.183927186 |
| 0.089203363 | 0.054057368 | 0.032691045 | 0.480962989 | 0.031168881 |
| 0.09994616 | 0.03081325 | 0.069780299 | 0.132128883 | 0.098399715 |
| 0.145820868 | 0.001579449 | 0.092351155 | 0.046082026 | 0.119259688 |
| -0.041993686 | 0.365222455 | -0.028259785 | 0.542402387 | -0.069048866 |
| -0.173303476 | 0.000167486 | -0.133791999 | 0.003772857 | -0.176429579 |
| -0.165852228 | 0.000318891 | -0.105461534 | 0.02264955 | -0.096344382 |
| -0.183216263 | 6.82E-05 | -0.109355231 | 0.018081413 | -0.103304663 |
| 0.066651246 | 0.150409932 | 0.159950414 | 0.000521121 | 0.15903882 |
| -0.097893408 | 0.034437875 | -0.031297286 | 0.499870965 | -0.049524258 |
| -0.252666766 | 3.10E-08 | -0.197965894 | 1.64E-05 | -0.24631013 |
| -0.122739034 | 0.007922585 | -0.095831851 | 0.03843733 | -0.061888082 |
| 0.220796949 | 1.45E-06 | 0.284661679 | 3.72E-10 | 0.273047493 |
| -0.197228096 | 1.76E-05 | -0.153810004 | 0.000853549 | -0.136204256 |
| 0.022356455 | 0.629882278 | 0.037936739 | 0.41340444 | 0.048295791 |
| -0.147759042 | 0.001364125 | -0.126863227 | 0.006045736 | -0.100441439 |
| -0.024836582 | 0.59239592 | 0.001952556 | 0.966433291 | 0.032519331 |
| -0.133508557 | 0.003847984 | -0.103763278 | 0.024936177 | -0.097665002 |
| 0.073505341 | 0.11265917 | 0.114831962 | 0.013024151 | 0.108168798 |
| -0.132185278 | 0.004216965 | -0.086669607 | 0.06128282 | -0.106969277 |
| -0.048571163 | 0.294898058 | 0.030101973 | 0.516394703 | 0.005288838 |
| -0.055862746 | 0.228238067 | 0.010215705 | 0.82573302 | 0.038229872 |
| -0.063576215 | 0.170188148 | -0.028896448 | 0.533340983 | -0.037779698 |
| 0.267595112 | 4.23E-09 | 0.242757456 | 1.09E-07 | 0.23386323 |
| -0.024798009 | 0.592971061 | 0.01507186 | 0.745293667 | 0.016266924 |
| -0.267160732 | 4.50E-09 | -0.243813747 | 9.53E-08 | -0.241602285 |
| 0.162916877 | 0.000407972 | 0.174886392 | 0.000145568 | 0.187627708 |
| 0.05249474 | 0.25756656 | 0.037934899 | 0.413427111 | 0.027682353 |
| 0.082533579 | 0.074777482 | 0.119134999 | 0.009971602 | 0.104275725 |
| -0.079244008 | 0.087158863 | -0.044770235 | 0.3343524 | -0.051762158 |
| -0.092458832 | 0.045827696 | -0.043886819 | 0.343987176 | -0.052359588 |
| -0.086605024 | 0.061476931 | -0.010020868 | 0.829005012 | -0.051610142 |
| -0.110400726 | 0.017001013 | -0.039470959 | 0.394758037 | -0.05102747 |
| -0.136891882 | 0.003033753 | -0.058014741 | 0.210787316 | -0.078006952 |
| -0.142517921 | 0.00201944 | -0.089333188 | 0.053707096 | -0.132328942 |
| 0.208653515 | 5.44E-06 | 0.254591194 | 2.42E-08 | 0.256079063 |
| -0.105088403 | 0.023135756 | -0.03583932 | 0.439719546 | -0.070817261 |
| -0.036084137 | 0.436599448 | 0.042595541 | 0.358384669 | 0.00361813 |
| 0.052599832 | 0.256613748 | 0.123828436 | 0.007381994 | 0.111785206 |
| -0.024747232 | 0.593728556 | 0.009362987 | 0.840074623 | 0.010843625 |
| -0.014305934 | 0.757822787 | 0.004742157 | 0.91859411 | 0.01930281 |

| | | | | |
|---|---|---|---|---|
| -0.032318306 | 0.485981291 | 0.001395347 | 0.976008899 | -0.027867663 |
| -0.137674478 | 0.002869278 | -0.109734038 | 0.017683227 | -0.14428199 |
| 0.158914801 | 0.000567053 | 0.096711868 | 0.036684351 | 0.130883731 |
| -0.032481821 | 0.483776366 | 0.03595055 | 0.43830038 | 0.040533087 |
| -0.036830386 | 0.427167614 | 0.009630972 | 0.835561519 | -0.025281223 |
| -0.139715147 | 0.002477851 | -0.085458145 | 0.065009384 | -0.072085028 |
| -0.072367062 | 0.118353372 | -0.023639704 | 0.610359426 | -0.030218021 |
| -0.127475294 | 0.005804167 | -0.079519043 | 0.086064207 | -0.057211636 |
| -0.076152046 | 0.100251266 | -0.014809235 | 0.749582355 | 0.000647452 |
| -0.045521554 | 0.326296123 | -0.025503981 | 0.582485325 | -0.049683241 |
| 0.168835076 | 0.00024722 | 0.215719445 | 2.54E-06 | 0.247224328 |
| 0.021292046 | 0.646274443 | 0.011081376 | 0.811232651 | 0.006518123 |
| 0.138976715 | 0.002613488 | 0.103216929 | 0.02571298 | 0.094289547 |
| -0.002510941 | 0.956842291 | 0.05691919 | 0.219547148 | 0.07714436 |
| -0.019245032 | 0.678279164 | 0.05375889 | 0.246266863 | 0.051116374 |
| -0.089709371 | 0.052702799 | 0.00324101 | 0.944312018 | -0.013925352 |
| 0.042293512 | 0.361805992 | 0.071542886 | 0.122614901 | 0.087874162 |
| 0.016920513 | 0.715334202 | 0.041597232 | 0.369770706 | 0.085186842 |
| 0.068735644 | 0.138030945 | 0.101433837 | 0.028395034 | 0.118173262 |
| -0.254662307 | 2.39E-08 | -0.19284027 | 2.72E-05 | -0.195387319 |
| 0.279528486 | 7.87E-10 | 0.233410935 | 3.38E-07 | 0.254710459 |
| -0.097677469 | 0.034839467 | -0.041781009 | 0.367658004 | -0.03567036 |
| 0.132201626 | 0.004212218 | 0.112092575 | 0.015372119 | 0.130415056 |
| 0.237228191 | 2.14E-07 | 0.18197413 | 7.65E-05 | 0.204843247 |
| -0.251512093 | 3.60E-08 | -0.209834602 | 4.80E-06 | -0.203229088 |
| -0.127680843 | 0.005725009 | -0.065189193 | 0.159584206 | -0.058641009 |
| -0.127377235 | 0.005842276 | -0.05527144 | 0.23320778 | -0.060293698 |
| 0.039738006 | 0.391565032 | 0.115288506 | 0.012665219 | 0.123850001 |
| 0.081362878 | 0.07900859 | 0.03695651 | 0.425585301 | 0.047900789 |
| -0.145488159 | 0.001619406 | -0.099986272 | 0.030745806 | -0.085104171 |
| 0.100298968 | 0.030224363 | 0.056057508 | 0.226617749 | 0.083790394 |
| -0.047521924 | 0.305464966 | 0.022489681 | 0.627843082 | 0.006832107 |
| -0.127490675 | 0.00579821 | -0.105047027 | 0.023190224 | -0.078976509 |
| 0.051917149 | 0.26284693 | 0.013321526 | 0.774019026 | 0.003494296 |
| 0.276371593 | 1.24E-09 | 0.240431819 | 1.45E-07 | 0.231085109 |
| 0.292871661 | 1.09E-10 | 0.238042195 | 1.94E-07 | 0.233148079 |
| -0.037459672 | 0.419306842 | 0.006047054 | 0.896306202 | -0.017593156 |
| -0.071475935 | 0.12296626 | -0.053321361 | 0.250137893 | -0.060052088 |
| -0.082489095 | 0.074934813 | -0.054964854 | 0.235814429 | -0.03150196 |
| 0.0490713 | 0.28994812 | 0.052499267 | 0.25752546 | 0.059822854 |
| -0.083442064 | 0.071622771 | -0.043815113 | 0.344776899 | -0.073530124 |
| -0.058948303 | 0.203523948 | -0.006067699 | 0.895954177 | 0.017652034 |
| -0.001945973 | 0.966546396 | 0.029913323 | 0.519028099 | 0.002326361 |
| -0.124745188 | 0.006952893 | -0.064727457 | 0.162567508 | -0.052648627 |
| -0.24799151 | 5.64E-08 | -0.19140402 | 3.13E-05 | -0.200142178 |

| | | | | |
|---|---|---|---|---|
| 0.103818861 | 0.024858296 | 0.041467136 | 0.371270824 | 0.051205021 |
| 0.018711398 | 0.68672138 | 0.054269925 | 0.241798721 | 0.037034287 |
| -0.354077429 | 3.07E-15 | -0.328522162 | 3.25E-13 | -0.354578341 |
| -0.12985518 | 0.004945185 | -0.074427462 | 0.108205999 | -0.048116385 |
| -0.133421939 | 0.003871212 | -0.07997948 | 0.084256443 | -0.106280219 |
| -0.103641838 | 0.025107068 | -0.036030799 | 0.437278139 | -0.023322975 |
| 0.283951286 | 4.13E-10 | 0.287857225 | 2.32E-10 | 0.280272866 |
| -0.286543036 | 2.82E-10 | -0.24262482 | 1.10E-07 | -0.229362075 |
| -0.271966408 | 2.31E-09 | -0.257163691 | 1.72E-08 | -0.247325048 |
| 0.010542399 | 0.820253508 | 0.089594421 | 0.05300801 | 0.078496447 |
| -0.234628539 | 2.92E-07 | -0.184990296 | 5.78E-05 | -0.181656546 |
| -0.025644194 | 0.580413061 | 0.043403375 | 0.349333802 | 0.038021225 |
| -0.155474768 | 0.00074799 | -0.111470385 | 0.015954505 | -0.077125691 |
| 0.034829824 | 0.452719121 | 0.019379475 | 0.676158524 | -0.020269999 |
| -0.257348897 | 1.68E-08 | -0.237335109 | 2.11E-07 | -0.243717047 |
| -0.061331166 | 0.185811597 | -0.009613863 | 0.835849502 | -0.007996444 |
| -0.129911546 | 0.004926309 | -0.083811623 | 0.070370974 | -0.089407907 |
| -0.197222744 | 1.76E-05 | -0.128163052 | 0.005543103 | -0.138356832 |
| -0.011308001 | 0.807447017 | -0.008906003 | 0.847782648 | -0.007350375 |
| 0.068203722 | 0.141112984 | 0.051176443 | 0.269726874 | 0.082411334 |
| -0.045568858 | 0.32579314 | -0.019073285 | 0.680991916 | -0.010572201 |
| -0.085745919 | 0.064107705 | -0.004568131 | 0.921571726 | -0.000730128 |
| -0.091859332 | 0.047258813 | -0.067065198 | 0.147886655 | -0.049209877 |
| -0.033838708 | 0.465689846 | -0.013792381 | 0.766259438 | -0.00400035 |
| 0.035864526 | 0.43939772 | 0.104622713 | 0.023755225 | 0.098230364 |
| -0.0972432 | 0.035659177 | -0.059856914 | 0.196630783 | -0.030228894 |
| 0.030270474 | 0.514048406 | 0.092428876 | 0.045898332 | 0.086264881 |
| 0.0186539 | 0.687633361 | 0.064791763 | 0.162149527 | 0.046458146 |
| -0.152803068 | 0.000923913 | -0.092166768 | 0.046520295 | -0.086099658 |
| 0.12975986 | 0.004977255 | 0.160909375 | 0.000481695 | 0.149384934 |
| 0.033608321 | 0.468734123 | 0.085298349 | 0.065514555 | 0.097522627 |
| 0.00131909 | 0.977319675 | 0.021638039 | 0.640926749 | 0.004001577 |
| -0.126325803 | 0.006265248 | -0.071802249 | 0.121261127 | -0.064443136 |
| -0.231357949 | 4.30E-07 | -0.153652929 | 0.000864188 | -0.159062612 |
| 0.014059064 | 0.761874852 | 0.004289903 | 0.926334512 | -0.032512862 |
| -0.089574803 | 0.053060243 | -0.03477602 | 0.453417993 | -0.042346696 |
| -0.393081595 | 1.05E-18 | -0.37418629 | 5.73E-17 | -0.399628675 |
| 0.113402071 | 0.014207011 | 0.157774025 | 0.000621981 | 0.140445437 |
| -0.063460011 | 0.170971944 | -0.017238223 | 0.710227609 | -0.022293751 |
| 0.041619629 | 0.369512822 | 0.017253977 | 0.709974723 | 0.013191042 |
| 0.168919001 | 0.00024544 | 0.111024661 | 0.016383517 | 0.124199161 |
| 0.270465262 | 2.85E-09 | 0.229744109 | 5.20E-07 | 0.206316517 |
| 0.311924669 | 5.37E-12 | 0.272867973 | 2.03E-09 | 0.265577343 |
| -0.120566054 | 0.009107516 | -0.098691758 | 0.032987201 | -0.079548709 |
| -0.050469829 | 0.276403975 | -0.017926888 | 0.699203305 | 0.0037426 |

| | | | | |
|---|---|---|---|---|
| 0.014940047 | 0.74744521 | 0.028598042 | 0.53757858 | 0.058264113 |
| -0.191587157 | 3.08E-05 | -0.129991323 | 0.004899703 | -0.137581944 |
| -0.154362471 | 0.00081708 | -0.11086486 | 0.016539768 | -0.091777368 |
| -0.181755086 | 7.81E-05 | -0.152930848 | 0.000914696 | -0.139470703 |
| -0.229758904 | 5.19E-07 | -0.164745136 | 0.00035011 | -0.174005234 |
| -0.151307053 | 0.001038391 | -0.112524638 | 0.014978751 | -0.114435082 |
| 0.30485334 | 1.68E-11 | 0.376891284 | 3.29E-17 | 0.391990334 |
| -0.11856606 | 0.01033487 | -0.058307758 | 0.208487713 | -0.091130316 |
| -0.100157703 | 0.030458986 | -0.040626498 | 0.381054575 | -0.036442244 |
| 0.213017135 | 3.41E-06 | 0.189985546 | 3.59E-05 | 0.21170215 |
| -0.052362934 | 0.258765034 | 0.009917205 | 0.830747077 | 0.034057575 |
| -0.253497211 | 2.79E-08 | -0.157935923 | 0.000613896 | -0.169103874 |
| -0.067274679 | 0.146622092 | -0.000402911 | 0.99307154 | -0.004690547 |
| 0.106012629 | 0.021947645 | 0.082091332 | 0.076353628 | 0.071249891 |
| -0.029416363 | 0.525998212 | 0.043885694 | 0.343999558 | 0.037107416 |
| -0.150267304 | 0.001125513 | -0.082473066 | 0.074991571 | -0.101301582 |
| 0.025290089 | 0.585653118 | 0.054894449 | 0.236415909 | 0.053475893 |
| -0.361122199 | 7.85E-16 | -0.327441827 | 3.92E-13 | -0.327439931 |
| 0.005897854 | 0.898850766 | 0.073184859 | 0.114240152 | 0.10317968 |
| 0.13530818 | 0.003393087 | 0.173247841 | 0.000168309 | 0.214194506 |
| 0.161799846 | 0.000447585 | 0.212526813 | 3.60E-06 | 0.202559476 |
| -0.089978835 | 0.051993069 | -0.043908762 | 0.343745749 | -0.071047555 |
| -0.189491438 | 3.77E-05 | -0.178834812 | 0.000102051 | -0.18023742 |
| 0.074345008 | 0.108598453 | 0.136175575 | 0.003191762 | 0.153166307 |
| -0.170166987 | 0.000220351 | -0.134130281 | 0.003684935 | -0.144711532 |
| 0.089704497 | 0.05271571 | 0.165674491 | 0.000323721 | 0.181608819 |
| -0.239525766 | 1.62E-07 | -0.283028281 | 4.73E-10 | -0.313133263 |
| -0.040032211 | 0.388065459 | 0.006218142 | 0.893389643 | 0.029859726 |
| 0.077233703 | 0.09550401 | 0.043754964 | 0.345440239 | 0.025404254 |
| -0.246818529 | 6.54E-08 | -0.217262966 | 2.15E-06 | -0.250688477 |
| -0.276578246 | 1.20E-09 | -0.234070104 | 3.12E-07 | -0.258423645 |
| -0.306819285 | 1.23E-11 | -0.251990142 | 3.38E-08 | -0.224869684 |
| 0.04873226 | 0.29329753 | 0.1232608 | 0.00765941 | 0.109037912 |
| -0.150824495 | 0.001078021 | -0.071670432 | 0.121947698 | -0.074998796 |
| -0.10265367 | 0.026535578 | -0.061833419 | 0.182227742 | -0.07144012 |
| 0.140101955 | 0.002409392 | 0.068508666 | 0.139339706 | 0.060727003 |
| -0.30406161 | 1.91E-11 | -0.265694123 | 5.49E-09 | -0.249687671 |
| -0.044588826 | 0.336316607 | 0.023654281 | 0.610139205 | 0.024513723 |
| -0.096220762 | 0.037654061 | -0.039088314 | 0.399360474 | -0.040659894 |
| -0.115807269 | 0.012267989 | -0.054237712 | 0.242078678 | -0.048209745 |
| 0.057944414 | 0.211341957 | 0.106425705 | 0.021433972 | 0.112489523 |
| -0.165340737 | 0.000332977 | -0.110366984 | 0.01703498 | -0.109234151 |
| -0.231311402 | 4.33E-07 | -0.19708227 | 1.79E-05 | -0.210572608 |
| 0.148175071 | 0.001321576 | 0.17227341 | 0.000183372 | 0.195497488 |
| 0.070567616 | 0.127810978 | 0.094748198 | 0.040692973 | 0.06615329 |

| | | | | |
|---|---|---|---|---|
| 0.002054584 | 0.964680417 | 0.034726615 | 0.454060251 | 0.051312274 |
| 0.197389383 | 1.73E-05 | 0.169420571 | 0.000235052 | 0.175403108 |
| -0.336200084 | 8.38E-14 | -0.298765229 | 4.39E-11 | -0.292938822 |
| 0.002850675 | 0.951009882 | -0.031736333 | 0.493872681 | -0.002321099 |
| -0.059045256 | 0.202780164 | -0.013093024 | 0.777792878 | -0.004818526 |
| -0.11300308 | 0.014553496 | -0.044483912 | 0.337455941 | -0.022456643 |
| -0.170436935 | 0.00021525 | -0.104069743 | 0.024509376 | -0.115266236 |
| -0.015607308 | 0.736574242 | 0.042770731 | 0.356409483 | 0.05074086 |
| -0.045146032 | 0.330306942 | -0.053384421 | 0.249577366 | -0.081694852 |
| -0.197190789 | 1.77E-05 | -0.129406714 | 0.005097709 | -0.112601869 |
| -0.098595962 | 0.033158471 | -0.047445595 | 0.306243324 | -0.061675805 |
| -0.010546771 | 0.82018024 | 0.033425825 | 0.471153364 | 0.046273677 |
| -0.130914028 | 0.004601264 | -0.04958546 | 0.284917827 | -0.083919678 |
| -0.183212937 | 6.82E-05 | -0.164251059 | 0.000364942 | -0.158012749 |
| -0.060256588 | 0.19365324 | 0.00281629 | 0.95160008 | -0.009551336 |
| -0.164563119 | 0.000355507 | -0.084930913 | 0.066688402 | -0.081333258 |
| -0.122044101 | 0.00828563 | -0.042143807 | 0.363509347 | -0.044638983 |
| -0.065359981 | 0.158491252 | -0.119053799 | 0.010022745 | -0.09951496 |
| 0.075400447 | 0.103658982 | 0.022533288 | 0.627176238 | 0.004767678 |
| -0.236041639 | 2.47E-07 | -0.15714533 | 0.000654323 | -0.155119454 |
| -0.220606329 | 1.48E-06 | -0.157337242 | 0.000644289 | -0.168604385 |
| 0.048695375 | 0.293663468 | 0.014494221 | 0.754736743 | 0.022726055 |
| -0.05576435 | 0.229059798 | 0.013038101 | 0.77870074 | 0.019898004 |
| -0.138711893 | 0.002663757 | -0.068737366 | 0.138021051 | -0.081052212 |
| -0.163432445 | 0.000390813 | -0.176941864 | 0.000121109 | -0.228373958 |
| -0.311794683 | 5.48E-12 | -0.277800041 | 1.01E-09 | -0.292673472 |
| -0.191178411 | 3.20E-05 | -0.119696631 | 0.009624164 | -0.119210486 |
| -0.140346336 | 0.002367035 | -0.094098366 | 0.042098511 | -0.072294353 |
| -0.100260963 | 0.030287331 | -0.021057036 | 0.649917207 | -0.028296891 |
| 0.190394941 | 3.45E-05 | 0.134056652 | 0.003703912 | 0.098911494 |
| -0.002865388 | 0.950757349 | -0.030455886 | 0.511473048 | 0.011802484 |
| -0.093167064 | 0.044184079 | -0.015419689 | 0.739625712 | 0.001283477 |
| -0.042538749 | 0.359026444 | -0.006842857 | 0.882752519 | -0.009450807 |
| 0.033994843 | 0.463632946 | 0.074279612 | 0.108910512 | 0.084349659 |
| -0.308892719 | 8.80E-12 | -0.266789382 | 4.73E-09 | -0.27291764 |
| -0.03004148 | 0.517238382 | 0.046243574 | 0.318673532 | 0.058098569 |
| -0.099281024 | 0.031950189 | -0.040930195 | 0.377501934 | -0.00468324 |
| -0.104449742 | 0.023988939 | -0.062122115 | 0.180191007 | -0.053937354 |
| -0.080633542 | 0.081741267 | -0.043134317 | 0.352332094 | -0.049575509 |
| 0.145595928 | 0.001606363 | 0.09623534 | 0.037624967 | 0.077778463 |
| -0.03400302 | 0.463525364 | 0.031525127 | 0.496753379 | 0.012715455 |
| 0.174233052 | 0.000154266 | 0.123257854 | 0.007660874 | 0.105502632 |
| -0.05635638 | 0.224147218 | -0.086325791 | 0.062322054 | -0.1030641 |
| -0.038828097 | 0.402508655 | 0.009437871 | 0.838812977 | -0.014110292 |
| 0.136712087 | 0.003072731 | 0.110568172 | 0.016833329 | 0.098276617 |

| | | | | |
|---|---|---|---|---|
| -0.158165238 | 0.000602611 | -0.112717879 | 0.014805696 | -0.119141711 |
| -0.302597374 | 2.41E-11 | -0.227512788 | 6.74E-07 | -0.189921584 |
| 0.159064004 | 0.000560211 | 0.151813315 | 0.000998262 | 0.190381476 |
| -0.2850127 | 3.54E-10 | -0.223485045 | 1.07E-06 | -0.242623305 |
| -0.04763131 | 0.304351798 | 0.003538585 | 0.939208446 | 0.003585973 |
| -0.012586471 | 0.786177427 | -0.044367415 | 0.338723953 | -0.050415104 |
| -0.154260975 | 0.00082367 | -0.153598339 | 0.000867915 | -0.109958841 |
| -0.09068756 | 0.050164342 | -0.011570163 | 0.803073341 | -0.014546894 |
| -0.140011424 | 0.002425258 | -0.111325175 | 0.016093177 | -0.100672428 |
| -0.060721422 | 0.190231927 | 0.007672681 | 0.868655828 | 0.041759703 |
| -0.08599799 | 0.063326373 | -0.006333783 | 0.891419102 | -0.016155143 |
| -0.201486884 | 1.15E-05 | -0.130663643 | 0.004680583 | -0.1534946 |
| -0.148476089 | 0.001291555 | -0.086619956 | 0.061432007 | -0.120831415 |
| -0.103406913 | 0.025440525 | -0.134568599 | 0.003573774 | -0.125118105 |
| 0.007748763 | 0.867365382 | 0.083189939 | 0.072487176 | 0.104881293 |
| -0.083457699 | 0.071569444 | -0.020381376 | 0.660436547 | 0.01000078 |
| 0.20600875 | 7.18E-06 | 0.158819706 | 0.000571453 | 0.173333042 |
| -0.066601949 | 0.150712591 | -0.033660201 | 0.468047635 | -0.012582755 |
| -0.003423956 | 0.941174109 | 0.0642077 | 0.165975645 | 0.074564067 |
| -0.26466291 | 6.32E-09 | -0.232533578 | 3.75E-07 | -0.270433784 |
| -0.164283112 | 0.000363962 | -0.104710725 | 0.023637062 | -0.110747121 |
| -0.251751515 | 3.49E-08 | -0.209262712 | 5.10E-06 | -0.205525929 |
| -0.023698514 | 0.609471169 | 0.035849164 | 0.439593847 | 0.042730105 |
| -0.099222289 | 0.032052288 | -0.08215014 | 0.076142493 | -0.117766683 |
| 0.030002867 | 0.517777275 | -0.027194681 | 0.557730717 | -0.031791284 |
| -0.017325071 | 0.708833943 | 0.058516786 | 0.206858373 | 0.077804952 |
| -0.019101071 | 0.680552742 | 0.036948361 | 0.42568743 | 0.039271877 |
| -0.081709318 | 0.077736748 | -0.056979966 | 0.219054474 | -0.076478086 |
| -0.171976379 | 0.000188208 | -0.129588424 | 0.005035406 | -0.152013473 |
| -0.013070654 | 0.778162613 | 0.061170737 | 0.186967218 | 0.084193469 |
| -0.194023197 | 2.42E-05 | -0.1716252 | 0.000194081 | -0.209018542 |
| -0.096540156 | 0.037020965 | -0.052953734 | 0.253423055 | -0.068793059 |
| -0.153425901 | 0.000879783 | -0.14965893 | 0.001179564 | -0.131422878 |
| -0.022319368 | 0.630450438 | -0.032332165 | 0.485794196 | -0.022048517 |
| -0.21488517 | 2.78E-06 | -0.209784088 | 4.82E-06 | -0.236318819 |
| 0.096780097 | 0.036551322 | 0.120276148 | 0.00927698 | 0.135928868 |
| -0.086955798 | 0.060428709 | -0.039698991 | 0.392030542 | -0.020965232 |
| -0.028635485 | 0.537045936 | -0.079518371 | 0.086066868 | -0.107725506 |
| 0.003340323 | 0.942608475 | -0.018665817 | 0.6874443 | -0.016489397 |
| -0.187161652 | 4.71E-05 | -0.139762782 | 0.002469325 | -0.151828938 |
| -0.116695206 | 0.011613557 | -0.03644844 | 0.431980163 | -0.027720436 |
| -0.078638359 | 0.089608875 | -0.034309135 | 0.459507863 | -0.035651653 |
| -0.131036271 | 0.004562983 | -0.166243998 | 0.00030848 | -0.185833057 |
| -0.163772902 | 0.000379852 | -0.105013803 | 0.02323404 | -0.136123796 |
| -0.206999259 | 6.48E-06 | -0.158606315 | 0.000581444 | -0.185917899 |

| | | | | |
|---|---|---|---|---|
| -0.405422666 | 6.67E-20 | -0.376172408 | 3.81E-17 | -0.396653423 |
| -0.142214396 | 0.00206504 | -0.102416801 | 0.026888217 | -0.110598095 |
| 0.02892275 | 0.53296828 | 0.067215976 | 0.146975629 | 0.113098686 |
| 0.071103452 | 0.124935399 | 0.126644941 | 0.006134049 | 0.147066937 |
| 0.002364689 | 0.959353817 | 0.064423376 | 0.164554973 | 0.051640927 |
| -0.090675059 | 0.050196127 | -0.02348539 | 0.612692905 | -0.036914526 |
| -0.099342763 | 0.031843169 | -0.002030433 | 0.965095326 | 0.014029722 |
| 0.016412393 | 0.723527645 | 0.098878734 | 0.032655086 | 0.115380834 |
| -0.020455224 | 0.659283498 | -0.08064295 | 0.081705538 | -0.079720498 |
| 0.147514059 | 0.001389766 | 0.07784703 | 0.092892969 | 0.097278545 |
| -0.065596344 | 0.156987987 | -0.02614331 | 0.57306454 | 0.003033825 |
| -0.229486168 | 5.36E-07 | -0.202491761 | 1.03E-05 | -0.175775734 |
| 0.152179867 | 0.000970106 | 0.127215645 | 0.005905565 | 0.123225287 |
| -0.365885107 | 3.07E-16 | -0.340221307 | 4.06E-14 | -0.340288044 |
| -0.034546706 | 0.456403393 | -0.014692298 | 0.751494433 | -0.0280005 |
| -0.144915642 | 0.00169034 | -0.088617837 | 0.055660824 | -0.081822404 |
| -0.10324955 | 0.025666019 | -0.064295413 | 0.16539677 | -0.088732234 |
| -0.328043131 | 3.53E-13 | -0.27120235 | 2.57E-09 | -0.295457153 |
| -0.24297353 | 1.06E-07 | -0.196067016 | 1.98E-05 | -0.205277691 |
| 0.045013604 | 0.331728904 | 0.05196187 | 0.262435451 | 0.051510365 |
| -0.18406062 | 6.30E-05 | -0.137038109 | 0.003002385 | -0.120772832 |
| -0.101063488 | 0.02898127 | -0.049503426 | 0.28571644 | -0.062899869 |
| -0.323926657 | 7.19E-13 | -0.257821811 | 1.58E-08 | -0.2750756 |
| -0.076118218 | 0.100402704 | -0.06006218 | 0.195097418 | -0.045448188 |
| 0.019231955 | 0.678485565 | 0.080675164 | 0.081583288 | 0.055721108 |
| -0.248638316 | 5.20E-08 | -0.220118579 | 1.56E-06 | -0.197504882 |
| -0.034563427 | 0.456185336 | 0.028377985 | 0.540714323 | 0.017014175 |
| -0.14608472 | 0.001548407 | -0.080057371 | 0.083953681 | -0.093297012 |
| -0.010890623 | 0.814422497 | -0.007232743 | 0.876124419 | -0.025241538 |
| -0.090877175 | 0.049684292 | -0.035432054 | 0.444938152 | -0.059081789 |
| 0.102287506 | 0.027082401 | 0.151732992 | 0.001004532 | 0.164449609 |
| -0.279124067 | 8.35E-10 | -0.208301718 | 5.65E-06 | -0.206669285 |
| -0.403482462 | 1.04E-19 | -0.358837321 | 1.23E-15 | -0.333087101 |
| -0.101194454 | 0.028772784 | -0.046746555 | 0.313432642 | -0.040129647 |
| -0.109561038 | 0.017864116 | -0.045108435 | 0.330710247 | -0.035021276 |
| 0.154719113 | 0.00079431 | 0.11280326 | 0.014729795 | 0.096289547 |
| -0.199808968 | 1.36E-05 | -0.17208812 | 0.000186375 | -0.182025089 |
| 0.190319566 | 3.48E-05 | 0.15200602 | 0.000983367 | 0.188035817 |
| -0.334075491 | 1.22E-13 | -0.308394216 | 9.53E-12 | -0.311276512 |
| 0.296651646 | 6.10E-11 | 0.247794187 | 5.78E-08 | 0.252582163 |
| -0.157868252 | 0.000617264 | -0.090906397 | 0.049610655 | -0.083367107 |
| -0.147051937 | 0.001439343 | -0.081268252 | 0.079358898 | -0.090019602 |
| -0.05988897 | 0.196390747 | -0.015973277 | 0.730633986 | -0.019325389 |
| -0.113416933 | 0.014194246 | -0.084222783 | 0.068999347 | -0.045628892 |
| -0.14695626 | 0.001449808 | -0.098004376 | 0.034233041 | -0.096705365 |

| | | | | |
|---|---|---|---|---|
| -0.017813253 | 0.701018126 | 0.021224539 | 0.647319973 | 0.017927907 |
| -0.059687305 | 0.197904412 | -0.009809095 | 0.832564757 | -0.005356565 |
| 0.009385495 | 0.839695368 | -0.032380628 | 0.485140268 | -0.004961536 |
| -0.137273467 | 0.002952515 | -0.091229441 | 0.048802652 | -0.084095469 |
| -0.276184281 | 1.27E-09 | -0.236836588 | 2.24E-07 | -0.26394038 |
| -0.071409177 | 0.123317392 | -0.119536302 | 0.009722233 | -0.140005726 |
| -0.131595053 | 0.004391628 | -0.102449317 | 0.02683957 | -0.121794623 |
| 0.137528795 | 0.002899267 | 0.090627552 | 0.050317069 | 0.045690158 |
| 0.08897091 | 0.054689279 | 0.150354834 | 0.001117927 | 0.151493116 |
| -0.292285108 | 1.19E-10 | -0.262708294 | 8.24E-09 | -0.26178664 |
| 0.076593826 | 0.098290177 | 0.059240919 | 0.201285136 | 0.062417074 |
| -0.161387171 | 0.000463103 | -0.106653185 | 0.021155573 | -0.095694017 |
| -0.046058743 | 0.320613694 | -0.008557004 | 0.853679093 | -0.012896579 |
| -0.221284779 | 1.37E-06 | -0.159930997 | 0.000521949 | -0.145488536 |
| 0.200850142 | 1.22E-05 | 0.27342868 | 1.88E-09 | 0.292169488 |
| -0.029944468 | 0.518592866 | -0.026192006 | 0.572349944 | -0.052910211 |
| 0.034014702 | 0.463371697 | 0.088190272 | 0.056856495 | 0.098584587 |
| -0.058935024 | 0.203625969 | 0.009499981 | 0.837766866 | 0.022293299 |
| -0.335958019 | 8.75E-14 | -0.292644214 | 1.13E-10 | -0.28485044 |
| -0.056445512 | 0.223414168 | 0.027434055 | 0.554267563 | 0.060041096 |
| -0.254870471 | 2.33E-08 | -0.191509908 | 3.10E-05 | -0.17494735 |
| -0.186561448 | 4.98E-05 | -0.125895243 | 0.006446242 | -0.134035788 |
| -0.035142696 | 0.448667204 | 0.050982281 | 0.271550508 | 0.040624228 |
| -0.1468299 | 0.001463737 | -0.090804575 | 0.049867639 | -0.085819935 |
| -0.161772539 | 0.000448596 | -0.079488479 | 0.086185305 | -0.08018163 |
| -0.105592764 | 0.022480667 | -0.133708531 | 0.003794841 | -0.130214425 |
| -0.214556633 | 2.89E-06 | -0.166974613 | 0.000289907 | -0.177277352 |
| 0.002498445 | 0.957056866 | 0.079689333 | 0.08539201 | 0.082828206 |
| -0.114954444 | 0.012926987 | -0.049740966 | 0.283408098 | -0.065706506 |
| -0.170961094 | 0.000205659 | -0.11254125 | 0.014963805 | -0.121320329 |
| -0.190240074 | 3.50E-05 | -0.138347985 | 0.002734272 | -0.128411277 |
| 0.019981965 | 0.666686754 | 0.062543477 | 0.177248641 | 0.079495014 |
| -0.059885844 | 0.19641414 | -0.025623101 | 0.580724583 | -0.011698862 |
| 0.265159329 | 5.91E-09 | 0.226452365 | 7.62E-07 | 0.202607812 |
| 0.06577697 | 0.155846494 | 0.094869405 | 0.040435249 | 0.090511917 |
| -0.128277095 | 0.00550085 | -0.094719666 | 0.040753844 | -0.07407193 |
| 0.209098734 | 5.19E-06 | 0.182844823 | 7.06E-05 | 0.166617065 |
| -0.066377857 | 0.152094247 | 0.006263418 | 0.892618056 | -0.0056181 |
| 0.119195306 | 0.009933768 | 0.168763335 | 0.000248751 | 0.158915035 |
| -0.094166636 | 0.04194895 | -0.029575231 | 0.523764833 | -0.011360747 |
| -0.024384351 | 0.599154868 | 0.052460086 | 0.257881281 | 0.039561106 |
| 0.007259019 | 0.875678026 | 0.080393507 | 0.08265717 | 0.101639743 |
| -0.118364911 | 0.010466086 | -0.037804153 | 0.415039899 | -0.045796929 |
| -0.039452487 | 0.394979485 | -0.009005249 | 0.846107387 | 0.041449751 |
| -0.006047693 | 0.896295304 | 0.015591795 | 0.736826386 | 0.004728224 |

| | | | | |
|---|---|---|---|---|
| -0.271452609 | 2.48E-09 | -0.221925662 | 1.28E-06 | -0.259270262 |
| -0.130185153 | 0.004835601 | -0.06299144 | 0.174159811 | -0.066791646 |
| -0.116112516 | 0.01203943 | -0.0583935 | 0.207818252 | -0.046243913 |
| -0.054450949 | 0.240229686 | 0.007435161 | 0.872686673 | 0.006143662 |
| 0.144331169 | 0.001765685 | 0.18063308 | 8.66E-05 | 0.182772062 |
| -0.208645942 | 5.44E-06 | -0.147389413 | 0.001402981 | -0.171648343 |
| -0.119686207 | 0.009630513 | -0.079494604 | 0.086161026 | -0.085377124 |
| 0.024000074 | 0.604925426 | 0.012465879 | 0.788177166 | -0.019883325 |
| -0.110150555 | 0.017254263 | -0.098791929 | 0.032808915 | -0.083279828 |
| 0.125984896 | 0.006408172 | 0.174144879 | 0.000155476 | 0.175823252 |
| 0.058229275 | 0.209101863 | 0.126432515 | 0.0062211 | 0.182675021 |
| 0.079988773 | 0.084220275 | 0.143738696 | 0.00184519 | 0.156409952 |
| -0.126628856 | 0.006140602 | -0.044831768 | 0.333687821 | -0.029547559 |
| -0.051371968 | 0.267898917 | 0.041571783 | 0.370063854 | 0.0713983 |
| -0.034673912 | 0.454745962 | 0.038138501 | 0.410923023 | 0.03629717 |
| -0.135756681 | 0.003287593 | -0.073639996 | 0.112000049 | -0.076792349 |
| -0.073812831 | 0.111158487 | -0.02344171 | 0.613354132 | -0.039856594 |
| -0.087662299 | 0.058362266 | -0.076607779 | 0.098228739 | -0.075738904 |
| -0.008598034 | 0.85298545 | 0.046046063 | 0.320747079 | 0.02479483 |
| 0.033508177 | 0.470060816 | 0.076686314 | 0.097883506 | 0.030570711 |
| -0.006908095 | 0.881642882 | 0.027476941 | 0.553648218 | 0.017612725 |
| -0.167883884 | 0.000268251 | -0.136803613 | 0.003052832 | -0.13110208 |
| -0.047423665 | 0.306467201 | -0.092956972 | 0.04466639 | -0.092611472 |
| -0.107653712 | 0.019968045 | -0.046446052 | 0.316556942 | -0.069037247 |
| -0.155942476 | 0.000720587 | -0.089339984 | 0.053688813 | -0.107119336 |
| 0.218993782 | 1.77E-06 | 0.200453413 | 1.27E-05 | 0.214436021 |
| 0.068712963 | 0.138161298 | 0.065574582 | 0.157125943 | 0.052233643 |
| -0.067417904 | 0.145762253 | -0.082308196 | 0.075577393 | -0.11183917 |
| 0.042637714 | 0.357908562 | 0.083729213 | 0.070648558 | 0.106506568 |
| 0.167266108 | 0.000282793 | 0.139827136 | 0.00245785 | 0.146799981 |
| -0.348609535 | 8.63E-15 | -0.346220745 | 1.35E-14 | -0.352594821 |
| 0.091225716 | 0.048811906 | 0.072467757 | 0.117840746 | 0.058562539 |
| -0.136871065 | 0.003038243 | -0.058064078 | 0.210398843 | -0.048846943 |
| -0.217190859 | 2.16E-06 | -0.195387714 | 2.12E-05 | -0.185447773 |
| 0.050614829 | 0.275024726 | 0.094276204 | 0.041709852 | 0.101295058 |
| 0.079237324 | 0.087185605 | 0.163021589 | 0.000404431 | 0.143155912 |
| 0.043680115 | 0.346266806 | 0.07588024 | 0.101473199 | 0.045494377 |
| -0.177740189 | 0.000112696 | -0.147957393 | 0.001343684 | -0.170653767 |
| -0.001299377 | 0.977658528 | 0.053605008 | 0.247623525 | 0.039756664 |
| 0.276208778 | 1.27E-09 | 0.257541526 | 1.64E-08 | 0.280089647 |
| 0.198290891 | 1.58E-05 | 0.162743779 | 0.000413889 | 0.183306316 |
| -0.210152003 | 4.64E-06 | -0.187983973 | 4.35E-05 | -0.23235465 |
| 0.040654454 | 0.380726684 | 0.063514366 | 0.170604982 | 0.064900105 |
| -0.022493199 | 0.627789273 | 0.055588615 | 0.230532615 | 0.072195614 |
| -0.162928101 | 0.000407591 | -0.126959434 | 0.006007177 | -0.124040112 |

| | | | | |
|---|---|---|---|---|
| 0.173735064 | 0.000161221 | 0.109497749 | 0.017930694 | 0.144874885 |
| 0.065254849 | 0.159163371 | 0.056307225 | 0.224552214 | 0.073623218 |
| -0.21774799 | 2.03E-06 | -0.166189734 | 0.000309903 | -0.193135941 |
| 0.076499116 | 0.098708007 | 0.027878299 | 0.547868363 | -0.006088298 |
| -0.203937065 | 8.91E-06 | -0.143853094 | 0.001829588 | -0.142571377 |
| 0.019170786 | 0.679451377 | 0.082202591 | 0.075954584 | 0.078733557 |
| -0.221209513 | 1.38E-06 | -0.175162407 | 0.000142033 | -0.148496384 |
| -0.007519396 | 0.871256773 | 0.043005513 | 0.353773174 | 0.022078362 |
| -0.199633207 | 1.38E-05 | -0.157847224 | 0.000618314 | -0.184335083 |
| -0.246612943 | 6.71E-08 | -0.193119111 | 2.65E-05 | -0.238694479 |
| 0.10234377 | 0.026997752 | 0.148754545 | 0.001264344 | 0.151248725 |
| -0.313408503 | 4.21E-12 | -0.275705781 | 1.36E-09 | -0.261098088 |
| -0.258702036 | 1.41E-08 | -0.233109353 | 3.50E-07 | -0.256225377 |
| -0.169756823 | 0.000228319 | -0.123958978 | 0.00731948 | -0.11908068 |
| 0.037321925 | 0.421020232 | 0.081646365 | 0.077966614 | 0.10516303 |
| -0.172936244 | 0.000172995 | -0.132404602 | 0.004153679 | -0.145635712 |
| -0.037289467 | 0.421424564 | -0.013213617 | 0.775800553 | -0.010621597 |
| -0.111552183 | 0.015876852 | -0.037975094 | 0.412932046 | -0.036265028 |
| -0.092138426 | 0.04658797 | -0.058379462 | 0.207927756 | -0.048927815 |
| 0.043147575 | 0.352183976 | 0.097784044 | 0.03464077 | 0.099046398 |
| -0.102279646 | 0.027094245 | -0.038354099 | 0.408281207 | -0.056195576 |
| -0.008292007 | 0.858161775 | 0.038850694 | 0.402234691 | 0.060519794 |
| -0.090269593 | 0.05123621 | -0.028657928 | 0.536726795 | -0.023143821 |
| -0.156539215 | 0.000686973 | -0.070475565 | 0.128310097 | -0.077379271 |
| -0.05258541 | 0.256744358 | 0.024753245 | 0.593638833 | 0.022889446 |
| -0.056718922 | 0.221176215 | -0.009129341 | 0.844013704 | -0.043932117 |
| -0.041821613 | 0.367192234 | 0.0325722 | 0.482559973 | 0.053082842 |
| -0.063657478 | 0.169641621 | 0.005562298 | 0.904577358 | -0.000793049 |
| -0.223517127 | 1.07E-06 | -0.174454462 | 0.000151265 | -0.168293353 |
| -0.191053247 | 3.24E-05 | -0.132337392 | 0.00417298 | -0.140837751 |
| -0.123655633 | 0.00746548 | -0.108180908 | 0.019365971 | -0.115318307 |
| -0.094687196 | 0.040823208 | -0.024915635 | 0.591217999 | -0.011017359 |
| -0.153175924 | 0.000897256 | -0.103897131 | 0.024748983 | -0.100187832 |
| -0.172232368 | 0.000184033 | -0.137811443 | 0.002841341 | -0.160358289 |
| -0.107162697 | 0.020543382 | -0.042063516 | 0.364424962 | -0.051351112 |
| -0.203039434 | 9.77E-06 | -0.156390892 | 0.000695189 | -0.1347395 |
| 0.057862691 | 0.21198779 | 0.044312351 | 0.339324361 | 0.022411543 |
| -0.097648766 | 0.034893147 | -0.072562506 | 0.117359985 | -0.058380015 |
| -0.260922118 | 1.05E-08 | -0.211409738 | 4.05E-06 | -0.205170675 |
| 0.046862836 | 0.312229125 | 0.114976977 | 0.012909181 | 0.108921077 |
| -0.23612306 | 2.44E-07 | -0.209154432 | 5.16E-06 | -0.214690177 |
| -0.171020544 | 0.000204597 | -0.115756439 | 0.012306418 | -0.11117853 |
| -0.240270302 | 1.48E-07 | -0.212926374 | 3.44E-06 | -0.228733736 |
| -0.142976779 | 0.001952251 | -0.121277589 | 0.008703212 | -0.092210345 |
| -0.004363783 | 0.925069548 | 0.056502702 | 0.222944716 | 0.043133005 |

| | | | | |
|---|---|---|---|---|
| -0.19057954 | 3.39E-05 | -0.141741469 | 0.002137968 | -0.171226056 |
| -0.189095683 | 3.91E-05 | -0.12353633 | 0.00752361 | -0.122009336 |
| -0.045582896 | 0.325643968 | 0.028478735 | 0.539277532 | 0.010856226 |
| -0.032578881 | 0.482470124 | -0.009214107 | 0.842584161 | -0.001772977 |
| -0.058642107 | 0.205885944 | -0.00799782 | 0.863143581 | -0.013337986 |
| -0.242618539 | 1.11E-07 | -0.161188048 | 0.000470768 | -0.183435941 |
| -0.124307583 | 0.007154854 | -0.08915388 | 0.054191374 | -0.096506754 |
| -0.089594972 | 0.053006543 | -0.017802434 | 0.701190993 | -0.002612144 |
| -0.155386221 | 0.000753285 | -0.148842887 | 0.001255822 | -0.135224822 |
| -0.222013719 | 1.26E-06 | -0.175153241 | 0.000142149 | -0.199145497 |
| -0.074256633 | 0.109020329 | 0.006308511 | 0.891849687 | 0.02953913 |
| -0.183535119 | 6.62E-05 | -0.161552598 | 0.000456823 | -0.179524783 |
| -0.250918835 | 3.88E-08 | -0.211012486 | 4.23E-06 | -0.220106966 |
| -0.018769464 | 0.685800837 | 0.023585774 | 0.611174498 | 0.064082436 |
| -0.088506088 | 0.055971293 | -0.044952543 | 0.332385892 | -0.02972729 |
| -0.106601554 | 0.021218484 | -0.091074637 | 0.049188465 | -0.061855051 |
| 0.109554329 | 0.017871164 | 0.153467082 | 0.000876935 | 0.161546625 |
| 0.039243124 | 0.397494559 | 0.05561495 | 0.230311476 | 0.053095931 |
| -0.085069417 | 0.066243911 | -0.044255831 | 0.339941344 | -0.028414653 |
| 0.375962473 | 3.98E-17 | 0.37310977 | 7.15E-17 | 0.366465849 |
| 0.224677277 | 9.34E-07 | 0.217253093 | 2.15E-06 | 0.237914049 |
| 0.191096677 | 3.23E-05 | 0.224335129 | 9.71E-07 | 0.227201197 |
| -0.21939414 | 1.69E-06 | -0.183506935 | 6.64E-05 | -0.214353551 |
| -0.207755888 | 5.98E-06 | -0.168019822 | 0.000265146 | -0.174134931 |
| -0.239908394 | 1.54E-07 | -0.193412162 | 2.57E-05 | -0.191370621 |
| -0.017916421 | 0.699370395 | 0.032995513 | 0.476884814 | 0.027219947 |
| -0.075041489 | 0.105318541 | -0.02705155 | 0.559806473 | -0.022462647 |
| 0.01946636 | 0.674789395 | 0.079505253 | 0.086118826 | 0.099688474 |
| -0.067693295 | 0.14411979 | -0.105939409 | 0.022039802 | -0.115327038 |
| 0.054921365 | 0.236185835 | 0.060443897 | 0.192269214 | 0.061872214 |
| -0.170641652 | 0.000211455 | -0.116873953 | 0.011485612 | -0.141335582 |
| -0.065550404 | 0.15727932 | 0.017021377 | 0.713711613 | -0.010261333 |
| 0.092960121 | 0.04465913 | 0.168503798 | 0.000254363 | 0.139640578 |
| -0.021454124 | 0.643767067 | -0.042608157 | 0.358242203 | -0.048708586 |
| -0.2558499 | 2.05E-08 | -0.186254629 | 5.13E-05 | -0.171682679 |
| -0.054729742 | 0.237827259 | -0.024927724 | 0.591037954 | -0.013865705 |
| -0.087448661 | 0.058980872 | -0.041076301 | 0.375800068 | -0.032369329 |
| 0.196318883 | 1.93E-05 | 0.258551026 | 1.44E-08 | 0.255367849 |
| -0.048304591 | 0.297559292 | -0.061984514 | 0.181159665 | -0.054460427 |
| 0.057108317 | 0.218016606 | 0.054858503 | 0.236723423 | 0.041130183 |
| -0.032909311 | 0.478037519 | 0.015678977 | 0.735409697 | -0.007003943 |
| -0.042168026 | 0.363233439 | 0.019185518 | 0.679218722 | 0.02481978 |
| -0.091784539 | 0.04743996 | -0.064990758 | 0.160861209 | -0.067680628 |
| -0.267791482 | 4.12E-09 | -0.210330295 | 4.55E-06 | -0.220422058 |
| -0.152120894 | 0.000974586 | -0.117619794 | 0.010965111 | -0.130657124 |

| | | | | |
|---|---|---|---|---|
| -0.150354091 | 0.001117991 | -0.145723788 | 0.001591014 | -0.164984717 |
| -0.102554657 | 0.026682496 | -0.02005421 | 0.665554502 | -0.027387384 |
| -0.061894141 | 0.181797947 | 0.006750339 | 0.884326553 | 0.014923475 |
| -0.05514013 | 0.234321698 | -0.044491099 | 0.337377814 | -0.08644342 |
| 0.103680297 | 0.025052839 | 0.177251776 | 0.000117776 | 0.171733846 |
| -0.09357681 | 0.043256019 | -0.065424417 | 0.158080367 | -0.058882133 |
| -0.210668533 | 4.39E-06 | -0.185303479 | 5.61E-05 | -0.185789075 |
| -0.196342326 | 1.93E-05 | -0.143507293 | 0.001877123 | -0.176396646 |
| 0.007670195 | 0.868697994 | 0.086795067 | 0.060907175 | 0.110522204 |
| -0.223788872 | 1.03E-06 | -0.189022986 | 3.94E-05 | -0.216782285 |
| -0.13897441 | 0.002613922 | -0.075770322 | 0.101970688 | -0.062661471 |
| -0.182685983 | 7.16E-05 | -0.135947386 | 0.003243645 | -0.128248991 |
| -0.100264907 | 0.030280791 | -0.049613966 | 0.284640674 | -0.019865196 |
| -0.123065734 | 0.007756871 | -0.05829363 | 0.208598178 | -0.084695917 |
| -0.069669748 | 0.132744091 | -0.113709274 | 0.013945199 | -0.110606401 |
| -0.217568045 | 2.07E-06 | -0.156094037 | 0.000711908 | -0.143113703 |
| -0.11991801 | 0.009490198 | -0.031284023 | 0.500052751 | 0.003721926 |
| -0.021423956 | 0.64423347 | 0.033006331 | 0.476740268 | 0.034090297 |
| -0.00682281 | 0.883093543 | -0.007300783 | 0.874968601 | 0.010960292 |
| 0.000219966 | 0.996217423 | 0.057422342 | 0.215492219 | 0.063563909 |
| 0.05092037 | 0.272133761 | 0.125757502 | 0.006505126 | 0.160800456 |
| 0.051052613 | 0.270888956 | 0.116449605 | 0.011791408 | 0.13968888 |
| -0.00925955 | 0.841818002 | 0.018204633 | 0.694774761 | -0.054402803 |
| -0.245356516 | 7.86E-08 | -0.181927127 | 7.69E-05 | -0.175800381 |
| 0.173429991 | 0.000165626 | 0.112190034 | 0.015282606 | 0.118146349 |
| 0.090762967 | 0.049972972 | 0.167833327 | 0.000269414 | 0.199524201 |
| -0.156672229 | 0.000679681 | -0.085223241 | 0.065753112 | -0.074902258 |
| -0.186529092 | 5.00E-05 | -0.169837745 | 0.000226726 | -0.190146311 |
| 0.045635075 | 0.325089891 | 0.018773631 | 0.685734795 | 0.029214308 |
| 0.031121072 | 0.502289192 | 0.08491849 | 0.066728389 | 0.083147581 |
| -0.165559289 | 0.000326888 | -0.223216773 | 1.10E-06 | -0.227853544 |
| -0.16966934 | 0.000230053 | -0.110386795 | 0.017015029 | -0.113680062 |
| -0.006142739 | 0.894674864 | 0.058948852 | 0.203519728 | 0.05166412 |
| -0.377291613 | 3.02E-17 | -0.352839394 | 3.88E-15 | -0.384970151 |
| -0.179617179 | 9.50E-05 | -0.134573188 | 0.003572627 | -0.147606314 |
| -0.069116046 | 0.135858745 | -0.028952171 | 0.532551542 | -0.004831025 |
| -0.333943941 | 1.25E-13 | -0.311741863 | 5.53E-12 | -0.326500833 |
| -0.068404474 | 0.139943658 | -0.003010074 | 0.948274266 | 0.016368797 |
| 0.131023433 | 0.004566989 | 0.103068053 | 0.025928235 | 0.096639375 |
| -0.143607974 | 0.001863168 | -0.075063192 | 0.105217607 | -0.094327617 |
| 0.06030862 | 0.193268045 | 0.012987237 | 0.779541779 | 0.004720047 |
| -0.099333614 | 0.031859008 | -0.035595389 | 0.442841003 | -0.023686644 |
| 0.155232891 | 0.000762537 | 0.106233158 | 0.021672096 | 0.115629451 |
| -0.016004125 | 0.730134 | 0.01579968 | 0.73344976 | -0.010824814 |
| -0.027778904 | 0.549296957 | 0.023958814 | 0.605546485 | 0.043919806 |

| | | | | |
|---|---|---|---|---|
| -0.370251775 | 1.28E-16 | -0.31736547 | 2.18E-12 | -0.302707451 |
| 0.105808406 | 0.022205524 | 0.146671567 | 0.001481363 | 0.168519303 |
| 0.181958079 | 7.66E-05 | 0.142037509 | 0.002092046 | 0.152923969 |
| 0.02864525 | 0.536907071 | 0.083504218 | 0.071410973 | 0.07722428 |
| 0.125740236 | 0.006512541 | 0.081627216 | 0.078036647 | 0.086186935 |
| 0.19876268 | 1.51E-05 | 0.183996802 | 6.34E-05 | 0.185554527 |
| -0.236098561 | 2.45E-07 | -0.196735337 | 1.85E-05 | -0.206989823 |
| 0.009887151 | 0.831252295 | 0.064691793 | 0.162799668 | 0.063860971 |
| -0.198294977 | 1.58E-05 | -0.120006409 | 0.009437168 | -0.121522054 |
| -0.299608568 | 3.85E-11 | -0.234800724 | 2.86E-07 | -0.227535589 |
| 0.143029162 | 0.001944712 | 0.118450451 | 0.010410106 | 0.135752531 |
| 0.113269322 | 0.014321477 | 0.075887097 | 0.101442226 | 0.040107459 |
| 0.094426612 | 0.041383504 | 0.147085771 | 0.001435659 | 0.181126305 |
| -0.189782828 | 3.66E-05 | -0.133613459 | 0.003820023 | -0.132571619 |
| -0.204567575 | 8.35E-06 | -0.172176998 | 0.000184929 | -0.184059412 |
| 0.286266476 | 2.94E-10 | 0.281190958 | 6.19E-10 | 0.295393552 |
| 0.046256536 | 0.318537752 | 0.117842697 | 0.010813662 | 0.109654228 |
| -0.299033644 | 4.21E-11 | -0.289093095 | 1.93E-10 | -0.27532137 |
| 0.108693413 | 0.018795894 | 0.142032197 | 0.002092862 | 0.169581456 |
| -0.006764025 | 0.884093691 | 0.053365134 | 0.24974871 | 0.066209974 |
| 0.170096173 | 0.000221708 | 0.156386004 | 0.000695462 | 0.148918925 |
| -0.052326016 | 0.259101401 | 0.015114806 | 0.744593107 | 0.042811578 |
| -0.167451448 | 0.000278354 | -0.121673917 | 0.008485015 | -0.105460577 |
| -0.12407541 | 0.007264123 | -0.150649536 | 0.001092731 | -0.129218632 |
| -0.110264792 | 0.017138214 | -0.059228196 | 0.201382108 | -0.061243293 |
| 0.253381217 | 2.83E-08 | 0.258099816 | 1.52E-08 | 0.260881738 |
| -0.054806887 | 0.237165484 | 0.007196931 | 0.87673286 | 0.052090173 |
| -0.070836333 | 0.126362554 | -0.001441177 | 0.975221165 | -0.016584493 |
| -0.126681234 | 0.006119286 | -0.163331077 | 0.000394132 | -0.167913222 |
| -0.443108884 | 7.02E-24 | -0.401820242 | 1.51E-19 | -0.441977503 |
| 0.329731952 | 2.63E-13 | 0.315408391 | 3.02E-12 | 0.318317556 |
| 0.155860743 | 0.000725307 | 0.115038963 | 0.012860312 | 0.090792875 |
| -0.087774332 | 0.058040019 | -0.039925813 | 0.389328867 | -0.013108681 |
| -0.180910503 | 8.44E-05 | -0.145457471 | 0.001623138 | -0.142233008 |
| 0.012629881 | 0.78545791 | 0.09419711 | 0.041882333 | 0.084462259 |
| -0.065280862 | 0.158996868 | 0.003895462 | 0.933091134 | 0.008552861 |
| -0.049057789 | 0.290081102 | -0.001627297 | 0.972022365 | 0.015975646 |
| 0.034700251 | 0.454403198 | 0.113257321 | 0.014331864 | 0.160301704 |
| -0.125108708 | 0.006789021 | -0.061860271 | 0.182037593 | -0.076610858 |
| -0.006790988 | 0.883634926 | -0.013756487 | 0.76685015 | -0.004398568 |
| -0.092877423 | 0.044850165 | -0.039890628 | 0.389747213 | -0.019323449 |
| -0.20267934 | 1.01E-05 | -0.177341134 | 0.00011683 | -0.183674714 |
| -0.005837704 | 0.899876921 | 0.049655446 | 0.284237702 | 0.047514314 |
| -0.071977617 | 0.120352402 | -0.016274448 | 0.725757533 | -0.051712481 |
| 0.027474387 | 0.553685087 | 0.084793741 | 0.067131023 | 0.096521703 |

| | | | | |
|---|---|---|---|---|
| -0.08170443 | 0.077754576 | -0.000317676 | 0.994537207 | -0.004370268 |
| -0.060657298 | 0.190701244 | 0.003972589 | 0.931769582 | -0.000832608 |
| -0.174186683 | 0.000154901 | -0.185698819 | 5.40E-05 | -0.191503381 |
| -0.157876632 | 0.000616846 | -0.127139761 | 0.005935498 | -0.145398466 |
| -0.13255711 | 0.00411018 | -0.14743024 | 0.00139864 | -0.179614025 |
| 0.04223041 | 0.362523365 | 0.113559207 | 0.014072557 | 0.12177734 |
| 0.160194462 | 0.000510812 | 0.208304981 | 5.64E-06 | 0.25248256 |
| 0.259394643 | 1.28E-08 | 0.210341956 | 4.55E-06 | 0.23942556 |
| -0.09655504 | 0.036991684 | -0.038123618 | 0.411105759 | -0.017841446 |
| -0.318499279 | 1.81E-12 | -0.271395136 | 2.50E-09 | -0.287201569 |
| 0.341960886 | 2.95E-14 | 0.310193891 | 7.12E-12 | 0.297241763 |
| -0.286127248 | 3.00E-10 | -0.235255423 | 2.71E-07 | -0.230226721 |
| 0.010091843 | 0.827812752 | -0.002530863 | 0.956500224 | -0.01402578 |
| 0.141803889 | 0.002128209 | 0.142849387 | 0.001970697 | 0.125592735 |
| -0.130822001 | 0.004630275 | -0.058711488 | 0.205349006 | -0.05374185 |
| -0.237015824 | 2.19E-07 | -0.197223346 | 1.76E-05 | -0.197921649 |
| -0.124543006 | 0.00704556 | -0.088108888 | 0.057086478 | -0.101972127 |
| -0.059894532 | 0.196349115 | 0.028352159 | 0.541082935 | 0.05132309 |
| -0.158239944 | 0.000598977 | -0.073961636 | 0.110437925 | -0.088627023 |
| -0.075580414 | 0.102834778 | -0.027802223 | 0.548961627 | -0.037934881 |
| 0.016761868 | 0.717888905 | 0.030039605 | 0.517264536 | 0.016223105 |
| -0.093997682 | 0.042319907 | -0.031048443 | 0.503287704 | -0.057360174 |
| 0.156225875 | 0.000704438 | 0.14397635 | 0.001812913 | 0.147991272 |
| -0.304972478 | 1.65E-11 | -0.247737076 | 5.83E-08 | -0.24076404 |
| 0.221366675 | 1.36E-06 | 0.207510638 | 6.14E-06 | 0.201381139 |
| -0.096467853 | 0.037163486 | -0.057963246 | 0.211193333 | -0.050561468 |
| 0.002393805 | 0.9588538 | 0.058733543 | 0.205178536 | 0.070591777 |
| 0.03198698 | 0.490465618 | 0.003093696 | 0.946839378 | 0.010426061 |
| -0.016069799 | 0.729069937 | -0.066680243 | 0.150232116 | -0.065478571 |
| 0.025288237 | 0.585680576 | 0.029428991 | 0.525820512 | 0.038650589 |
| -0.026012238 | 0.574990059 | 0.039209773 | 0.397896101 | 0.049380144 |
| -0.063629028 | 0.169832813 | -0.002144395 | 0.963137591 | 0.026572794 |
| -0.116780446 | 0.011552387 | -0.042388648 | 0.360726116 | -0.03594149 |
| 0.092192698 | 0.04645845 | 0.179767362 | 9.37E-05 | 0.174982002 |
| -0.190887927 | 3.29E-05 | -0.148413518 | 0.001297743 | -0.167376843 |
| -0.031679767 | 0.494643324 | 0.0278429 | 0.548376935 | 0.036942159 |
| -0.00818006 | 0.860056846 | -0.044147414 | 0.341126857 | -0.010438672 |
| 0.121430543 | 0.008618418 | 0.181337372 | 8.12E-05 | 0.185295426 |
| -0.000753638 | 0.987040848 | -0.081010732 | 0.080318621 | -0.104726875 |
| -0.187911442 | 4.38E-05 | -0.103134252 | 0.025832329 | -0.098997225 |
| 0.101294871 | 0.028613802 | 0.154782896 | 0.0007903 | 0.151347497 |
| -0.165641743 | 0.000324618 | -0.102631159 | 0.026568918 | -0.092103648 |
| -0.063611857 | 0.169948278 | -0.020012712 | 0.666204785 | 0.01860795 |
| 0.23037535 | 4.83E-07 | 0.197234101 | 1.76E-05 | 0.202865302 |
| -0.065241979 | 0.159245799 | -0.08689584 | 0.060606828 | -0.085044079 |

| | | | | |
|---|---|---|---|---|
| -0.11481345 | 0.013038892 | -0.183157318 | 6.86E-05 | -0.206214732 |
| -0.165350652 | 0.000332698 | -0.102432776 | 0.026864307 | -0.115358677 |
| -0.095958766 | 0.038180219 | -0.103347421 | 0.025525574 | -0.077869769 |
| -0.038967292 | 0.400822799 | 0.013062613 | 0.778295521 | -0.012475134 |
| -0.163607842 | 0.00038513 | -0.09730529 | 0.035540983 | -0.111674913 |
| 0.092861845 | 0.044886228 | 0.152709356 | 0.000930726 | 0.137504795 |
| -0.179105997 | 9.96E-05 | -0.127502222 | 0.005793742 | -0.118134186 |
| -0.209794508 | 4.82E-06 | -0.155438986 | 0.000750125 | -0.175781818 |
| -0.059778053 | 0.197222218 | -0.030587072 | 0.509654944 | -0.004386251 |
| -0.039717776 | 0.391806362 | 0.034247773 | 0.460311626 | 0.046624471 |
| -0.227798234 | 6.52E-07 | -0.178161801 | 0.000108478 | -0.170450819 |
| 0.002450003 | 0.957888719 | 0.046957574 | 0.311250831 | 0.072232671 |
| 0.041520023 | 0.370660538 | 0.09667248 | 0.036761335 | 0.107087386 |
| 0.055640079 | 0.230100608 | 0.08470666 | 0.067413264 | 0.071759756 |
| -0.078706178 | 0.089331809 | -0.043686262 | 0.346198874 | -0.025860153 |
| -0.366895301 | 2.51E-16 | -0.352562289 | 4.09E-15 | -0.372836646 |
| 0.120877204 | 0.008928704 | 0.145701512 | 0.001593678 | 0.157033938 |
| -0.188671561 | 4.07E-05 | -0.124430642 | 0.007097537 | -0.182123594 |
| 0.042541053 | 0.359000394 | 0.008368749 | 0.856863138 | 0.042788065 |
| -0.156246444 | 0.000703279 | -0.112261636 | 0.015217133 | -0.107318711 |
| -0.018826439 | 0.684898066 | -0.067262469 | 0.146695576 | -0.077280805 |
| -0.133578833 | 0.003829232 | -0.09500059 | 0.040157862 | -0.101233376 |
| -0.00882203 | 0.849200642 | 0.033039494 | 0.476297281 | 0.017246384 |
| 0.046499472 | 0.316000057 | 0.098185285 | 0.033901329 | 0.104140844 |
| -0.042727647 | 0.356894601 | 0.00461684 | 0.920738187 | -0.001988921 |
| 0.01369599 | 0.767846065 | 0.006251723 | 0.892817344 | -0.01471127 |
| -0.000697803 | 0.98800087 | 0.082619498 | 0.074474364 | 0.082501941 |
| -0.118513194 | 0.010369214 | -0.056636197 | 0.221851647 | -0.042686822 |
| -0.05008156 | 0.280120329 | 0.009314335 | 0.840894528 | 0.025802327 |
| -0.23245639 | 3.78E-07 | -0.193964513 | 2.44E-05 | -0.181408898 |
| 0.090619682 | 0.050337129 | 0.156611673 | 0.000682992 | 0.188522347 |
| -0.075492047 | 0.103238826 | -0.032151287 | 0.488239038 | -0.065279667 |
| -0.154787262 | 0.000790026 | -0.08248137 | 0.074962163 | -0.099458961 |
| 0.00362671 | 0.937697529 | 0.031460398 | 0.49763802 | 0.02407744 |
| -0.115960106 | 0.012153075 | -0.079865597 | 0.084700694 | -0.066332289 |
| 0.064058762 | 0.166962064 | 0.121160373 | 0.008768696 | 0.129141588 |
| -0.029605299 | 0.523342682 | 0.048761366 | 0.293008972 | 0.065718064 |
| -0.023889368 | 0.606592458 | 0.036220004 | 0.434873369 | 0.04271453 |
| -0.082628017 | 0.074444367 | -0.025089395 | 0.588632685 | -0.051289426 |
| -0.199673893 | 1.38E-05 | -0.16212382 | 0.000435742 | -0.175363228 |
| -0.04110452 | 0.375471915 | 0.018437611 | 0.691068006 | 0.010871517 |
| -0.058361146 | 0.208070683 | -0.020799971 | 0.653911398 | -0.046085782 |
| -0.084811722 | 0.067072868 | -0.049633519 | 0.284450669 | -0.0475475 |
| -0.189755217 | 3.67E-05 | -0.115947003 | 0.012162889 | -0.083722417 |
| -0.044738478 | 0.334695712 | 0.007462439 | 0.872223581 | 0.000895668 |

| | | | | |
|---|---|---|---|---|
| 0.006018869 | 0.896786815 | -0.018891961 | 0.683860393 | -0.022451757 |
| 0.138460931 | 0.002712206 | 0.170840021 | 0.000207838 | 0.158768202 |
| -0.040411893 | 0.383577299 | 0.016365843 | 0.724279876 | 0.022252643 |
| -0.317088022 | 2.29E-12 | -0.281830121 | 5.64E-10 | -0.284134752 |
| -0.042388425 | 0.36072864 | 0.009607206 | 0.835961555 | 0.027618691 |
| 0.066771405 | 0.149674144 | 0.115364015 | 0.012606703 | 0.11648411 |
| -0.080435927 | 0.082494706 | -0.011140125 | 0.810250867 | -0.018511196 |
| -0.180663209 | 8.64E-05 | -0.133935794 | 0.003735254 | -0.138631264 |
| -0.172171327 | 0.000185021 | -0.097616949 | 0.034952731 | -0.09057843 |
| -0.270305303 | 2.91E-09 | -0.229434745 | 5.39E-07 | -0.241245233 |
| -0.236720604 | 2.27E-07 | -0.219337999 | 1.70E-06 | -0.207612297 |
| -0.172641156 | 0.000177545 | -0.101129167 | 0.028876553 | -0.102425265 |
| 0.160146028 | 0.000512843 | 0.201050217 | 1.20E-05 | 0.233286178 |
| -0.193378586 | 2.58E-05 | -0.133348412 | 0.003891028 | -0.123784226 |
| 0.008873551 | 0.848330578 | 0.009831287 | 0.832191566 | 0.033351008 |
| -0.096500382 | 0.037099308 | -0.032581785 | 0.482431067 | -0.024905673 |
| -0.174310758 | 0.000153206 | -0.111340552 | 0.016078442 | -0.113938052 |
| -0.188140703 | 4.29E-05 | -0.123182339 | 0.00769848 | -0.116622526 |
| 0.106735347 | 0.021055795 | 0.113993555 | 0.013706713 | 0.123620788 |
| -0.031973152 | 0.490653248 | 0.010377359 | 0.823020583 | -0.004472889 |
| -0.179950104 | 9.22E-05 | -0.12488425 | 0.006889791 | -0.124144349 |
| -0.194652827 | 2.28E-05 | -0.147872279 | 0.001352421 | -0.127426892 |
| -0.063578378 | 0.170173584 | -0.034215251 | 0.460737938 | -0.020936211 |
| -0.178067095 | 0.000109412 | -0.127427455 | 0.005822731 | -0.137765488 |
| 0.180020202 | 9.16E-05 | 0.192817105 | 2.73E-05 | 0.207694299 |
| -0.084439297 | 0.068285906 | -0.017060872 | 0.713076618 | -0.006336919 |
| -0.191721527 | 3.04E-05 | -0.134997389 | 0.003467982 | -0.121573436 |
| -0.271420826 | 2.49E-09 | -0.219377945 | 1.70E-06 | -0.250452078 |
| -0.149245128 | 0.001217684 | -0.071100911 | 0.124948915 | -0.039599754 |
| -0.169733023 | 0.00022879 | -0.116216345 | 0.011962549 | -0.113746935 |
| -0.050039808 | 0.280521971 | 0.027545993 | 0.552651702 | -0.003737297 |
| 0.021183522 | 0.647955573 | 0.066955859 | 0.148549985 | 0.043196433 |
| 0.032787023 | 0.479675382 | 0.082480536 | 0.074965113 | 0.064317851 |
| 0.081769759 | 0.077516569 | 0.141828163 | 0.002124426 | 0.163867444 |
| -0.019767959 | 0.67004519 | 0.05212002 | 0.260983846 | 0.042905425 |
| 0.330637287 | 2.25E-13 | 0.270834986 | 2.70E-09 | 0.293947358 |
| 0.065348721 | 0.158563141 | 0.088078505 | 0.057172535 | 0.108517199 |
| -0.104169459 | 0.024371871 | -0.059423125 | 0.199900152 | -0.068152306 |
| 0.248446733 | 5.32E-08 | 0.210927905 | 4.27E-06 | 0.230581014 |
| 0.088372604 | 0.05634403 | 0.045797032 | 0.323373995 | 0.029844937 |
| -0.080087469 | 0.083836926 | -0.029069452 | 0.530891917 | -0.038516375 |
| -0.032403343 | 0.484833925 | 0.014192945 | 0.759676554 | 0.026659839 |
| -0.06393195 | 0.167805397 | -0.00661866 | 0.88656764 | 0.030119621 |
| -0.075711152 | 0.102239296 | -0.020069611 | 0.665313226 | 0.00305423 |
| -0.4199277 | 2.25E-21 | -0.402230091 | 1.38E-19 | -0.416563856 |

| | | | | |
|---|---|---|---|---|
| -0.076193849 | 0.100064376 | -0.04193074 | 0.365942258 | -0.051144295 |
| -0.042625375 | 0.358047822 | 0.041568905 | 0.370097021 | 0.057184205 |
| -0.017123986 | 0.712062285 | 0.035010847 | 0.450372238 | 0.086312381 |
| -0.145093501 | 0.001668005 | -0.099605084 | 0.031391862 | -0.105178294 |
| -0.027255849 | 0.55684477 | 0.042398212 | 0.36061766 | 0.049056173 |
| -0.162812854 | 0.000411519 | -0.12970208 | 0.004996786 | -0.134560484 |
| -0.012668772 | 0.784813451 | 0.026313955 | 0.570562243 | 0.028701109 |
| -0.108595585 | 0.018903567 | -0.066892244 | 0.148936958 | -0.049237872 |
| -0.455857717 | 2.42E-25 | -0.407319634 | 4.32E-20 | -0.360357756 |
| -0.315584605 | 2.94E-12 | -0.252320241 | 3.24E-08 | -0.241473045 |
| -0.014574796 | 0.753417295 | 0.058811603 | 0.20457601 | 0.065822281 |
| -0.199823853 | 1.36E-05 | -0.143944357 | 0.001817228 | -0.144543146 |
| -0.088857993 | 0.054998444 | -0.018257693 | 0.693929915 | 0.022154828 |
| -0.19203474 | 2.94E-05 | -0.12210453 | 0.008253482 | -0.097322069 |
| 0.022707873 | 0.624509492 | 0.081429981 | 0.078760936 | 0.09744543 |
| -0.070614188 | 0.127559033 | -0.033868952 | 0.465291021 | -0.038405681 |
| -0.010263277 | 0.824934583 | 0.001478608 | 0.974577818 | 0.019397715 |
| -0.223888828 | 1.02E-06 | -0.166640215 | 0.000298274 | -0.163160986 |
| 0.042450017 | 0.36003059 | 0.080831963 | 0.080990362 | 0.098638919 |
| -0.13457472 | 0.003572244 | -0.060469709 | 0.192079055 | -0.086533533 |
| -0.204006828 | 8.85E-06 | -0.15278063 | 0.00092554 | -0.155045102 |
| -0.190863734 | 3.30E-05 | -0.129330447 | 0.005124066 | -0.144994869 |
| -0.058535513 | 0.20671285 | -0.019043968 | 0.68145539 | 0.003592094 |
| -0.082766316 | 0.073958719 | -0.038433176 | 0.407314768 | -0.068841665 |
| -0.245760441 | 7.47E-08 | -0.203096636 | 9.72E-06 | -0.193927088 |
| -0.181422116 | 8.05E-05 | -0.142807669 | 0.001976772 | -0.137628072 |
| -0.070109971 | 0.130307339 | -0.011593987 | 0.802676194 | 0.011876046 |
| -0.133343222 | 0.00389243 | -0.073697472 | 0.111719632 | -0.02925922 |
| -0.177398495 | 0.000116227 | -0.13288363 | 0.004018431 | -0.095308073 |
| 0.106713386 | 0.021082424 | 0.123969241 | 0.007314586 | 0.14015072 |
| 0.136445966 | 0.003131259 | 0.095634333 | 0.038840388 | 0.128074679 |
| -0.313627925 | 4.06E-12 | -0.269370444 | 3.31E-09 | -0.287597852 |
| -0.112313127 | 0.015170201 | -0.090534034 | 0.050555858 | -0.086194219 |
| -0.05704862 | 0.218498888 | -0.015612635 | 0.736487665 | -0.008011816 |
| -0.137729055 | 0.002858116 | -0.066400887 | 0.151951814 | -0.037344109 |
| -0.39314371 | 1.04E-18 | -0.325041899 | 5.94E-13 | -0.314990598 |
| -0.124655943 | 0.006993662 | -0.084162396 | 0.069199417 | -0.10397949 |
| -0.205871187 | 7.29E-06 | -0.182963185 | 6.98E-05 | -0.213369415 |
| -0.05644869 | 0.22338806 | -0.016849098 | 0.716483828 | -0.008531437 |
| -0.069651364 | 0.132846616 | 0.008104713 | 0.861332799 | 0.019881272 |
| -0.110305818 | 0.017096703 | -0.043593667 | 0.347223035 | -0.011091981 |
| -0.191223825 | 3.19E-05 | -0.137609509 | 0.002882617 | -0.115062947 |
| -0.054685376 | 0.238208441 | 0.016878475 | 0.716010845 | 0.041224219 |
| -0.092064284 | 0.046765396 | -0.028300816 | 0.541816109 | -0.015796385 |
| -0.07233613 | 0.118511193 | -0.021257729 | 0.646805839 | 0.018961782 |

| | | | | |
|---|---|---|---|---|
| -0.262580839 | 8.38E-09 | -0.223427231 | 1.08E-06 | -0.204481378 |
| -0.144597616 | 0.001730963 | -0.111523458 | 0.015904084 | -0.113500171 |
| -0.124573322 | 0.007031595 | -0.050415934 | 0.276917827 | -0.042973173 |
| -0.051155148 | 0.269926472 | -0.001197152 | 0.979415777 | 0.005911158 |
| -0.416038604 | 5.67E-21 | -0.358610986 | 1.28E-15 | -0.377055093 |
| -0.205105443 | 7.89E-06 | -0.136720631 | 0.003070868 | -0.129211891 |
| -0.152318299 | 0.000959665 | -0.0861919 | 0.062730674 | -0.047877507 |
| 0.019572679 | 0.673115494 | -0.023253606 | 0.616205229 | -0.01021377 |
| -0.102191752 | 0.027226989 | -0.03596067 | 0.438171396 | -0.0404422 |
| -0.037232275 | 0.422137555 | 0.01581205 | 0.733248986 | 0.03249889 |
| -0.097052755 | 0.036023793 | -0.022286153 | 0.630959472 | 0.001303465 |
| -0.264134876 | 6.79E-09 | -0.200958929 | 1.21E-05 | -0.212373377 |
| -0.134777382 | 0.003521902 | -0.075995685 | 0.100952765 | -0.064343807 |
| -0.105255739 | 0.022916604 | -0.041988738 | 0.365279004 | -0.012834422 |
| -0.205041009 | 7.95E-06 | -0.140231519 | 0.00238685 | -0.130684948 |
| -0.07678054 | 0.097470575 | -0.007506806 | 0.871470462 | -0.000145046 |
| 0.085963088 | 0.063434088 | 0.150376265 | 0.001116077 | 0.174694452 |
| -0.024399664 | 0.598925443 | 0.0566847 | 0.221455448 | 0.069894559 |
| -0.014670743 | 0.751847052 | 0.05287937 | 0.254091199 | 0.064109637 |
| -0.149796875 | 0.001167103 | -0.110714 | 0.016688473 | -0.093370509 |
| -0.301406353 | 2.91E-11 | -0.228010131 | 6.36E-07 | -0.243344113 |
| 0.038704081 | 0.404014244 | 0.056630294 | 0.2218999 | 0.056357288 |
| -0.060665687 | 0.190639795 | 0.026084216 | 0.573932274 | 0.059654066 |
| -0.097225822 | 0.035692317 | -0.06180076 | 0.182459216 | -0.055059316 |
| 0.024290423 | 0.600563059 | 0.084522196 | 0.068014349 | 0.119494578 |
| 0.102902632 | 0.026169233 | 0.152935391 | 0.00091437 | 0.174452641 |
| -0.127408313 | 0.005830174 | -0.181167049 | 8.24E-05 | -0.158727033 |
| 0.085964273 | 0.063430429 | 0.063291458 | 0.172113622 | 0.087969454 |
| -0.144771005 | 0.001708706 | -0.101461162 | 0.028352185 | -0.08108112 |
| -0.175770465 | 0.000134528 | -0.151697912 | 0.001007282 | -0.137026223 |
| 0.13654301 | 0.003109799 | 0.149824785 | 0.001164596 | 0.163106307 |
| -0.003347662 | 0.942482608 | 0.080901127 | 0.080729934 | 0.110242926 |
| -0.114457955 | 0.013324845 | -0.052145752 | 0.260748192 | -0.021408562 |
| -0.200217363 | 1.30E-05 | -0.145958331 | 0.001563206 | -0.141255037 |
| 0.007120716 | 0.878028012 | -0.030258587 | 0.514213752 | -0.037265268 |
| -0.066017475 | 0.154336338 | -0.034279394 | 0.459897341 | -0.023846219 |
| -0.097360069 | 0.035436982 | -0.027658926 | 0.551023804 | -0.013269054 |
| -0.271911679 | 2.33E-09 | -0.207587736 | 6.09E-06 | -0.217649191 |
| 0.132967424 | 0.003995187 | 0.183903267 | 6.40E-05 | 0.182517577 |
| 0.09035925 | 0.051004689 | 0.073768565 | 0.111373549 | 0.077130801 |
| -0.005078658 | 0.91283978 | 0.058435335 | 0.207492173 | 0.065771012 |
| -0.152127925 | 0.000974051 | -0.102663415 | 0.026521154 | -0.115471871 |
| -0.121733246 | 0.008452775 | -0.078355349 | 0.090772526 | -0.057614724 |
| -0.472484361 | 2.41E-27 | -0.437041258 | 3.32E-23 | -0.389786658 |
| -0.184583008 | 6.00E-05 | -0.138920309 | 0.002624122 | -0.148508602 |

| | | | | |
|---|---|---|---|---|
| -0.125597099 | 0.006574305 | -0.084038277 | 0.069612126 | -0.044955596 |
| -0.01432936 | 0.757438621 | 0.036930244 | 0.425914542 | 0.026273954 |
| -0.02111352 | 0.649040904 | 0.04404125 | 0.342290292 | 0.072721987 |
| -0.293734161 | 9.54E-11 | -0.242110761 | 1.18E-07 | -0.240806365 |
| -0.026665333 | 0.565426136 | 0.031764182 | 0.493493502 | 0.040034728 |
| -0.447603762 | 2.18E-24 | -0.430790729 | 1.59E-22 | -0.430690566 |
| -0.29825242 | 4.76E-11 | -0.247448161 | 6.04E-08 | -0.284850186 |
| -0.112863656 | 0.014676312 | -0.066730296 | 0.149925564 | -0.046585009 |
| -0.205229279 | 7.79E-06 | -0.178547218 | 0.000104752 | -0.181421569 |
| -0.057747606 | 0.212899684 | -0.011348722 | 0.80676727 | -0.001814239 |
| -0.003294357 | 0.943396918 | 0.008565878 | 0.853529068 | 0.014024409 |
| 0.037126765 | 0.423454765 | 0.064875744 | 0.161604881 | 0.066991389 |
| -0.333852624 | 1.27E-13 | -0.317817001 | 2.02E-12 | -0.33850412 |
| -0.11174716 | 0.015693091 | -0.099843032 | 0.030987228 | -0.09197715 |
| -0.158451084 | 0.000588814 | -0.113103361 | 0.01446572 | -0.098385704 |
| -0.029033453 | 0.531401056 | 0.031625765 | 0.495379633 | 0.028086527 |
| 0.182493871 | 7.29E-05 | 0.231025173 | 4.47E-07 | 0.256309586 |
| -0.014476044 | 0.755034496 | 0.035484544 | 0.444263589 | 0.025094747 |
| 0.023686743 | 0.609648909 | 0.057087316 | 0.218186182 | 0.070686216 |
| -0.110046744 | 0.017360316 | -0.054166268 | 0.242700398 | -0.055195478 |
| -0.195542677 | 2.09E-05 | -0.144107282 | 0.001795352 | -0.142227837 |
| 0.000125538 | 0.997841226 | -0.038714752 | 0.403884572 | -0.032820771 |
| -0.092610516 | 0.045471423 | -0.031415196 | 0.498256296 | -0.034663874 |
| 0.020077666 | 0.665187054 | -0.016903956 | 0.715600671 | -0.046460762 |
| -0.447053441 | 2.51E-24 | -0.395886371 | 5.67E-19 | -0.412055523 |
| -0.049110359 | 0.289563908 | -0.02630483 | 0.570695924 | -0.024599707 |
| -0.356678568 | 1.86E-15 | -0.299404286 | 3.98E-11 | -0.30197782 |
| 0.066012794 | 0.154365626 | 0.122442728 | 0.008075607 | 0.150284856 |
| -0.006719874 | 0.884844964 | 0.059915799 | 0.196190007 | 0.03071721 |
| 0.000209148 | 0.996403462 | 0.022722897 | 0.624280225 | 0.04563386 |
| -0.249165754 | 4.86E-08 | -0.219737131 | 1.63E-06 | -0.225909486 |
| -0.153792828 | 0.000854706 | -0.107336638 | 0.020337943 | -0.114680391 |
| -0.263267257 | 7.64E-09 | -0.194509601 | 2.31E-05 | -0.211359002 |
| -0.217962857 | 1.99E-06 | -0.142772913 | 0.001981846 | -0.144154348 |
| 0.025441733 | 0.583406404 | 0.073260826 | 0.113863833 | 0.05606495 |
| -0.059870337 | 0.196530244 | -0.040071244 | 0.387602588 | -0.062822582 |
| -0.076412943 | 0.099089401 | -0.050285211 | 0.278166863 | -0.083220314 |
| -0.071572794 | 0.122458194 | -0.070254934 | 0.12951255 | -0.101415122 |
| -0.156840969 | 0.000670534 | -0.182977884 | 6.97E-05 | -0.219421552 |
| 0.133585644 | 0.003827419 | 0.168232144 | 0.000260364 | 0.192988331 |
| -0.013311112 | 0.774190899 | -0.07440631 | 0.108306571 | -0.120773879 |
| 0.119012496 | 0.010048848 | 0.116834871 | 0.01151348 | 0.103308588 |
| 0.034853013 | 0.4524181 | 0.026073416 | 0.574090934 | 0.052658184 |
| -0.026037975 | 0.574611721 | -0.028933052 | 0.53282233 | -0.059605792 |
| -0.098257723 | 0.033769277 | -0.09523143 | 0.039673665 | -0.103234286 |

| | | | | |
|---|---|---|---|---|
| -0.075265058 | 0.104282473 | -0.02893007 | 0.532864584 | -0.052581335 |
| 0.195341972 | 2.13E-05 | 0.210318108 | 4.56E-06 | 0.165993141 |
| 0.088946098 | 0.054757089 | 0.088274199 | 0.05662013 | 0.081401984 |
| -0.096830671 | 0.036452981 | -0.064564218 | 0.163632177 | -0.035285147 |
| -0.030665301 | 0.508572383 | -0.042859708 | 0.355408936 | -0.047425521 |
| 0.087720642 | 0.058194268 | 0.079260006 | 0.087094882 | 0.121452829 |
| 0.014698051 | 0.751400331 | 0.014293858 | 0.758020855 | 0.036317644 |
| -0.112129974 | 0.015337715 | -0.097883293 | 0.034456597 | -0.098947026 |
| 0.104463524 | 0.023970245 | 0.045820084 | 0.323130239 | 0.044146519 |
| 0.053338631 | 0.249984294 | 0.019379593 | 0.676156663 | -0.005420132 |
| 0.058714028 | 0.205329372 | 0.06844658 | 0.13969935 | 0.06469908 |
| -0.022790865 | 0.623243528 | -0.024908612 | 0.591322598 | -0.1028533 |
| -0.113225067 | 0.014359816 | -0.135833425 | 0.003269843 | -0.144805992 |
| -0.090612262 | 0.050356045 | -0.080976478 | 0.080446982 | -0.042861255 |
| 0.053698838 | 0.246795675 | 0.072373034 | 0.118322919 | 0.102256103 |
| -0.034362097 | 0.458814756 | -0.031333045 | 0.499380982 | -0.009294934 |
| 0.032351523 | 0.485532933 | 0.038287365 | 0.409097845 | 0.02555419 |
| 0.070837508 | 0.126356246 | 0.106041691 | 0.021911159 | 0.063812303 |
| 0.144357343 | 0.001762246 | 0.156154291 | 0.000708485 | 0.139446601 |
| 0.113514426 | 0.01411076 | 0.116502276 | 0.011753065 | 0.104411977 |
| 0.102308858 | 0.02705025 | 0.102693325 | 0.026476931 | 0.080957524 |
| 0.005623504 | 0.903532452 | 0.004368381 | 0.924990818 | -0.011083492 |
| -0.197124024 | 1.78E-05 | -0.160275734 | 0.000507422 | -0.156065025 |
| -0.096882369 | 0.036352687 | -0.072126084 | 0.119587231 | -0.073219638 |
| 0.317628427 | 2.09E-12 | 0.352672232 | 4.01E-15 | 0.307558129 |
| 0.059771691 | 0.197269993 | 0.073809077 | 0.111176711 | 0.058578261 |
| 0.119584289 | 0.009692788 | 0.121655905 | 0.008494825 | 0.119065521 |
| 0.116038278 | 0.012094668 | 0.111354528 | 0.016065061 | 0.122409023 |
| 0.090552182 | 0.050509444 | 0.106598995 | 0.021221605 | 0.111305955 |
| 0.084553679 | 0.067911451 | 0.077947102 | 0.092472415 | 0.076547374 |
| 0.014511036 | 0.754461324 | 0.056347897 | 0.224217077 | 0.025387366 |
| 0.081393411 | 0.078895826 | 0.087640702 | 0.058424558 | 0.090809004 |
| 0.059502599 | 0.199298236 | 0.052509953 | 0.257428483 | 0.014689829 |
| 0.191021287 | 3.25E-05 | 0.20576184 | 7.37E-06 | 0.170709414 |
| -0.109255659 | 0.018187369 | -0.143820863 | 0.001833971 | -0.187090779 |
| 0.067782553 | 0.14359049 | 0.093721863 | 0.042931437 | 0.064069385 |
| 0.125505797 | 0.006613977 | 0.155442249 | 0.00074993 | 0.156559736 |
| 0.090137486 | 0.051578943 | 0.095357886 | 0.039410516 | 0.04427039 |
| 0.17650955 | 0.000125908 | 0.177899564 | 0.000111084 | 0.128125955 |
| 0.0697986 | 0.132027253 | 0.097292609 | 0.035565094 | 0.086805219 |
| 0.134881193 | 0.003496366 | 0.10627249 | 0.021623269 | 0.108780105 |
| -0.098566482 | 0.03321133 | -0.09745202 | 0.035262992 | -0.123727233 |
| 0.049521341 | 0.285541906 | 0.063133312 | 0.173189967 | 0.05348908 |
| -0.074674124 | 0.107038655 | -0.017021274 | 0.713713277 | -0.003879372 |
| 0.084664574 | 0.067550018 | 0.135720216 | 0.003296057 | 0.140681432 |

| | | | | |
|---|---|---|---|---|
| 0.024390894 | 0.599056836 | 0.054101505 | 0.243264937 | 0.024117044 |
| 0.22076886 | 1.45E-06 | 0.246395767 | 6.90E-08 | 0.253833567 |
| 0.08855093 | 0.055846537 | 0.074124914 | 0.109651517 | 0.088893285 |
| 0.105092587 | 0.023130255 | 0.109319769 | 0.018119086 | 0.099008006 |
| -0.185004917 | 5.77E-05 | -0.145371608 | 0.001633622 | -0.169905517 |
| 0.176047724 | 0.000131232 | 0.205350271 | 7.69E-06 | 0.221948934 |
| 0.278884589 | 8.64E-10 | 0.295457733 | 7.33E-11 | 0.31015856 |
| 0.057053187 | 0.218461968 | 0.094069276 | 0.042162377 | 0.099254006 |
| -0.083315613 | 0.072055246 | -0.067226388 | 0.146912875 | -0.067394306 |
| -0.248691619 | 5.16E-08 | -0.237000689 | 2.20E-07 | -0.223073148 |
| 0.217912566 | 2.00E-06 | 0.222717992 | 1.17E-06 | 0.22363536 |
| -0.115113165 | 0.012802025 | -0.152875093 | 0.000918707 | -0.152427622 |
| 0.086212785 | 0.06266679 | 0.076904721 | 0.096928491 | 0.06791047 |
| 0.063574577 | 0.170199176 | 0.048899729 | 0.291639865 | -0.002038771 |
| 0.023093009 | 0.618643974 | 0.028020339 | 0.545830032 | 0.022230833 |
| -0.149573966 | 0.0011873 | -0.140273489 | 0.002379589 | -0.185448663 |
| 0.063418034 | 0.171255738 | 0.056162841 | 0.225744845 | 0.052607686 |
| -0.017502842 | 0.705984232 | -0.031164977 | 0.501686103 | -0.030995278 |
| 0.159881362 | 0.000524073 | 0.164794872 | 0.000348648 | 0.182548836 |
| 0.104741283 | 0.023596156 | 0.11086425 | 0.016540367 | 0.110483578 |
| 0.322803364 | 8.71E-13 | 0.245674838 | 7.55E-08 | 0.270802647 |
| -0.031266368 | 0.500294815 | -0.032626148 | 0.481834694 | -0.054965916 |
| 0.234599863 | 2.93E-07 | 0.265850045 | 5.38E-09 | 0.216615391 |
| -0.052672571 | 0.255955687 | -0.023535747 | 0.611930999 | -0.07091619 |
| 0.115582774 | 0.01243852 | 0.147476366 | 0.00139375 | 0.112178137 |
| 0.235903877 | 2.51E-07 | 0.255320673 | 2.20E-08 | 0.226714795 |
| -0.139660482 | 0.002487667 | -0.126728223 | 0.006100219 | -0.108585125 |
| -0.238271911 | 1.88E-07 | -0.1973673 | 1.74E-05 | -0.226049512 |
| 0.214046125 | 3.05E-06 | 0.259190342 | 1.32E-08 | 0.24386842 |
| 0.068667046 | 0.138425485 | 0.074582225 | 0.107472409 | 0.104735578 |
| -0.047477976 | 0.305912966 | -0.011194336 | 0.809345175 | -0.017560497 |
| -0.001515848 | 0.973937761 | 0.019991361 | 0.666539453 | 0.00908569 |
| 0.12142464 | 0.008621677 | 0.140574295 | 0.002328137 | 0.14494128 |
| 0.012241507 | 0.791901562 | 0.029331936 | 0.52718707 | 0.023394534 |
| 0.111506138 | 0.015920523 | 0.151155791 | 0.001050667 | 0.158708793 |
| 0.051264397 | 0.268903539 | 0.045378749 | 0.327817643 | 0.061292008 |
| 0.215531771 | 2.59E-06 | 0.240903492 | 1.37E-07 | 0.245950497 |
| -0.006611174 | 0.886695072 | 0.007842044 | 0.865783719 | 0.020365403 |
| -0.142840491 | 0.001971991 | -0.129203932 | 0.005168057 | -0.116324454 |
| 0.015042895 | 0.745766296 | -0.013122931 | 0.777298643 | -0.025977213 |
| -0.102418166 | 0.026886173 | -0.165314259 | 0.000333721 | -0.159444484 |
| -0.024422076 | 0.59858971 | -0.037953443 | 0.413198667 | -0.030780578 |
| -0.017863372 | 0.70021748 | 0.012730427 | 0.783792081 | -0.028567618 |
| 0.053202188 | 0.251199575 | 0.066329612 | 0.152392957 | 0.095579329 |
| 0.079805574 | 0.084935597 | 0.094083645 | 0.04213082 | 0.086675431 |

| | | | | |
|---|---|---|---|---|
| -0.011900838 | 0.797565449 | 0.010988033 | 0.812793188 | -0.004195647 |
| 0.079289656 | 0.08697641 | 0.111325493 | 0.016092871 | 0.071442949 |
| 0.141370932 | 0.002196744 | 0.136773046 | 0.003059465 | 0.145263973 |
| 0.1917163 | 3.04E-05 | 0.189337823 | 3.82E-05 | 0.146048531 |
| -0.004619229 | 0.920697316 | 0.021257307 | 0.646812375 | 0.006030681 |
| 0.087132647 | 0.059905855 | 0.081044805 | 0.080191101 | 0.104989884 |
| 0.195075759 | 2.18E-05 | 0.185825524 | 5.34E-05 | 0.15412361 |
| 0.105806842 | 0.022207508 | 0.088463623 | 0.056089647 | 0.085795574 |
| 0.016631321 | 0.719993491 | -0.019073356 | 0.680990787 | -0.003536071 |
| 0.08277402 | 0.073931741 | 0.07115133 | 0.124680929 | 0.044105931 |
| -0.076716786 | 0.097749816 | -0.039014506 | 0.40025193 | -0.053553123 |
| 0.165044144 | 0.000341408 | 0.219356907 | 1.70E-06 | 0.228703909 |
| 0.011984741 | 0.7961695 | 0.030532347 | 0.51041296 | 0.014005539 |
| -0.014235492 | 0.758978329 | -0.004032326 | 0.930746131 | -0.037886508 |
| -0.102137485 | 0.027309226 | -0.069723788 | 0.13244309 | -0.060565822 |
| -0.118810406 | 0.010177441 | -0.101626944 | 0.028093418 | -0.131852654 |
| -0.003097865 | 0.946767847 | 0.001938378 | 0.966676887 | 0.01947637 |
| -0.306732533 | 1.25E-11 | -0.34484281 | 1.74E-14 | -0.387336773 |
| 0.041656659 | 0.369086712 | 0.060070812 | 0.195033129 | 0.04972433 |
| 0.00908604 | 0.844744152 | 0.034764699 | 0.453565121 | 0.037642815 |
| 0.161889115 | 0.000444292 | 0.186951777 | 4.80E-05 | 0.196374205 |
| -0.089524495 | 0.05319439 | -0.069830263 | 0.131851556 | -0.081289036 |
| 1.11E-05 | 0.999809848 | -0.002685505 | 0.953845148 | -0.011429744 |
| 0.031382888 | 0.498698443 | 0.03627841 | 0.434132589 | 0.038603063 |
| -0.102746522 | 0.026398434 | -0.115237126 | 0.012705173 | -0.140573161 |
| -0.085332041 | 0.065407775 | -0.097633084 | 0.034922504 | -0.117351524 |
| 0.126994502 | 0.005993177 | 0.120601145 | 0.009087192 | 0.115623265 |
| 0.165861104 | 0.000318651 | 0.179220715 | 9.85E-05 | 0.186968781 |
| 0.094913395 | 0.040342053 | 0.076336655 | 0.099428026 | 0.06981742 |
| 0.119830837 | 0.009542751 | 0.125428063 | 0.006647921 | 0.145284696 |
| -0.043307601 | 0.350399228 | -0.035443266 | 0.444794008 | -0.039615161 |
| -0.040332094 | 0.38451795 | -0.028842727 | 0.534102619 | -0.005797268 |
| -0.040424993 | 0.383423017 | -0.051316741 | 0.268414372 | -0.063243585 |
| -0.037455456 | 0.419359223 | -0.071030449 | 0.125324187 | -0.071153495 |
| -0.102842454 | 0.026257383 | -0.086638432 | 0.061376456 | -0.079949938 |
| 0.134111615 | 0.003689737 | 0.184261369 | 6.19E-05 | 0.151808681 |
| -0.096669638 | 0.036766894 | -0.101945095 | 0.027602499 | -0.093635462 |
| -0.176816999 | 0.000122477 | -0.153888937 | 0.000848248 | -0.188952122 |
| 0.135869084 | 0.003261625 | 0.145722838 | 0.001591127 | 0.103116279 |
| 0.031389949 | 0.498601786 | 0.040165118 | 0.386490773 | 0.04310732 |
| -0.047286732 | 0.307867523 | -0.015994386 | 0.73029183 | -0.022577997 |
| -0.023620085 | 0.610655892 | -0.009404432 | 0.839376303 | -0.020297752 |
| 0.104519402 | 0.023894579 | 0.103536209 | 0.02525653 | 0.114545736 |
| 0.258452045 | 1.45E-08 | 0.258833824 | 1.38E-08 | 0.278935159 |
| 0.133695595 | 0.003798259 | 0.158356651 | 0.000593339 | 0.164434985 |

| | | | | |
|---|---|---|---|---|
| 0.121190219 | 0.008751981 | 0.117897882 | 0.010776455 | 0.11561621 |
| 0.141875803 | 0.002117017 | 0.130317945 | 0.004792122 | 0.132032162 |
| 0.085886362 | 0.063671408 | 0.059516028 | 0.199196657 | 0.041069808 |
| 0.122566277 | 0.008011484 | 0.126410001 | 0.006230391 | 0.158442511 |
| 0.170458432 | 0.000214848 | 0.199949992 | 1.34E-05 | 0.256985439 |
| 0.076888767 | 0.096998001 | 0.090352387 | 0.051022381 | 0.113600611 |
| 0.256912498 | 1.78E-08 | 0.260212786 | 1.15E-08 | 0.299773947 |
| -0.12845491 | 0.005435547 | -0.117232369 | 0.011232817 | -0.169533606 |
| -0.026862184 | 0.56255847 | -0.017069629 | 0.712935849 | -0.029558502 |
| 0.109023995 | 0.018435987 | 0.094543266 | 0.041131884 | 0.076458782 |
| 0.120424587 | 0.009189863 | 0.152382954 | 0.000954823 | 0.128163707 |
| 0.112506168 | 0.014995384 | 0.098371553 | 0.033562657 | 0.093952743 |
| 0.026996319 | 0.560608453 | 0.061695237 | 0.183208616 | 0.025598697 |
| -0.03719958 | 0.422545462 | -0.020105605 | 0.664749475 | -0.029733253 |
| 0.107163939 | 0.020541909 | 0.104766002 | 0.023563109 | 0.109184137 |
| 0.138928748 | 0.002622529 | 0.134344967 | 0.003630103 | 0.133120613 |
| 0.097204438 | 0.035733133 | 0.097768247 | 0.03467016 | 0.105759518 |
| -0.018693636 | 0.687003061 | -0.040050731 | 0.387845792 | -0.05199671 |
| -0.107403276 | 0.020259713 | -0.101860085 | 0.027732945 | -0.117849509 |
| 0.090633988 | 0.050300671 | 0.099996619 | 0.030728429 | 0.106511177 |
| -0.067753614 | 0.143761932 | -0.121128161 | 0.008786768 | -0.132789503 |
| 0.059041531 | 0.202808703 | 0.04237288 | 0.360904949 | 0.051187492 |
| -0.067114672 | 0.147587251 | -0.049998413 | 0.280920554 | -0.044304244 |
| 0.10137505 | 0.028487403 | 0.088042745 | 0.057273961 | 0.083531565 |
| 0.093870937 | 0.0426 | 0.092756081 | 0.045131713 | 0.09094944 |
| 0.081296076 | 0.079255761 | 0.081919596 | 0.076972918 | 0.090624241 |
| -0.07636458 | 0.099303968 | -0.055138446 | 0.234336013 | -0.087261194 |
| -0.033363828 | 0.471976773 | -0.047433757 | 0.306364162 | -0.029728953 |
| 0.283277457 | 4.56E-10 | 0.243780492 | 9.57E-08 | 0.25972031 |
| 0.083698706 | 0.070751541 | 0.080877873 | 0.080817418 | 0.052459474 |
| -0.014752034 | 0.750517485 | -0.03093686 | 0.504823776 | -0.054849872 |
| 0.041354906 | 0.372567941 | 0.029649072 | 0.522728424 | 0.007830505 |
| 0.080542497 | 0.082087698 | 0.077367871 | 0.094927883 | 0.082297471 |
| 0.063616749 | 0.169915375 | 0.084753421 | 0.067261586 | 0.109654767 |
| 0.049999936 | 0.280905881 | 0.050438069 | 0.276706704 | 0.02626622 |
| 0.175635235 | 0.000136164 | 0.207048516 | 6.44E-06 | 0.195020202 |
| 0.105795004 | 0.022222537 | 0.11567005 | 0.012371976 | 0.115927904 |
| 0.043281317 | 0.350691974 | 0.042053351 | 0.364540988 | 0.026559405 |
| 0.006940511 | 0.881091608 | -0.009411871 | 0.839250969 | -0.0100997 |
| -0.046496577 | 0.316030217 | -0.031547004 | 0.496454574 | -0.050914657 |
| 0.084796452 | 0.067122252 | 0.094770202 | 0.040646084 | 0.084236619 |
| 0.033786267 | 0.466381817 | 0.046261172 | 0.318489198 | 0.017482454 |
| 0.003218012 | 0.944706535 | 0.018831408 | 0.684819351 | 0.007174576 |
| 0.094884932 | 0.040402331 | 0.105964261 | 0.022008484 | 0.089529948 |
| -0.009539306 | 0.837104653 | -0.019969088 | 0.666888644 | -0.034772751 |

| | | | | |
|---|---|---|---|---|
| 0.253197032 | 2.90E-08 | 0.272785991 | 2.06E-09 | 0.290510285 |
| 0.018526272 | 0.689659299 | 0.001701515 | 0.970746901 | -0.009438545 |
| 0.083204234 | 0.07243794 | 0.082062359 | 0.076457821 | 0.072136293 |
| 0.013513126 | 0.770858729 | 0.03407084 | 0.462633604 | 0.041813502 |
| 0.123578945 | 0.0075028 | 0.138041126 | 0.002795049 | 0.129701851 |
| -0.190919792 | 3.28E-05 | -0.201633683 | 1.13E-05 | -0.188220773 |
| 0.250287015 | 4.21E-08 | 0.238199447 | 1.90E-07 | 0.245484575 |
| 0.102232598 | 0.02716523 | 0.115786753 | 0.012283487 | 0.113984483 |
| -0.065309371 | 0.158814542 | -0.077621693 | 0.093845549 | -0.089641319 |
| 0.075619921 | 0.102654544 | 0.074831954 | 0.106296948 | 0.080124318 |
| 0.10209132 | 0.027379352 | 0.104822315 | 0.023487976 | 0.104776119 |
| 0.000208268 | 0.996418582 | -0.00611903 | 0.895079036 | 0.025689215 |
| -0.067459741 | 0.145511818 | -0.081579923 | 0.078209822 | -0.066261538 |
| 0.159883235 | 0.000523992 | 0.167180142 | 0.000284874 | 0.177585942 |
| 0.037146314 | 0.423210528 | 0.043809475 | 0.344839047 | 0.058564302 |
| -0.242335319 | 1.14E-07 | -0.285482975 | 3.30E-10 | -0.268727346 |
| -0.270047613 | 3.02E-09 | -0.270101118 | 2.99E-09 | -0.295596463 |
| 0.189881808 | 3.63E-05 | 0.170345745 | 0.000216961 | 0.165804294 |
| 0.348332195 | 9.09E-15 | 0.344009306 | 2.03E-14 | 0.325421819 |
| 0.074603037 | 0.107374057 | 0.068730003 | 0.138063361 | 0.065142909 |
| 0.054525903 | 0.23958212 | 0.055061462 | 0.234990845 | 0.058493714 |
| 0.294805422 | 8.10E-11 | 0.302262628 | 2.54E-11 | 0.307050875 |
| -0.065816753 | 0.155595921 | -0.028829002 | 0.534297285 | -0.049637907 |
| 0.102689361 | 0.026482789 | 0.100567972 | 0.029781858 | 0.098743688 |
| -0.028134385 | 0.544196142 | 0.025152379 | 0.58769685 | 0.028910032 |
| 0.082626145 | 0.074450956 | 0.088305914 | 0.056531024 | 0.090213632 |
| 0.022424508 | 0.628840283 | -0.012521955 | 0.787247093 | 0.021653845 |
| 0.08781299 | 0.057929167 | 0.084739137 | 0.067307887 | 0.076031933 |
| 0.055032814 | 0.23523486 | 0.037309158 | 0.421179241 | 0.027044888 |
| 0.197294193 | 1.75E-05 | 0.220153257 | 1.56E-06 | 0.250998001 |
| -0.020604163 | 0.65696046 | 0.018804596 | 0.685244111 | 0.019146598 |
| -0.0071226 | 0.877995994 | -0.006518756 | 0.888268529 | -0.010573416 |
| 0.055682272 | 0.22974685 | 0.061696756 | 0.183197807 | 0.057711282 |
| 0.143889346 | 0.001824669 | 0.163544203 | 0.000387183 | 0.157821617 |
| 0.103318755 | 0.025566641 | 0.112667036 | 0.014851057 | 0.10157597 |
| 0.359228149 | 1.14E-15 | 0.384608613 | 6.52E-18 | 0.401957159 |
| 0.044921532 | 0.332719866 | 0.056714952 | 0.221208598 | 0.051369136 |
| 0.061667923 | 0.183402962 | 0.084371613 | 0.068508281 | 0.085687113 |
| 0.098057984 | 0.034134458 | 0.115202955 | 0.012731807 | 0.123431457 |
| 0.041362919 | 0.372475236 | 0.08069778 | 0.081497553 | 0.067994383 |
| -0.044918125 | 0.332756574 | -0.051081115 | 0.270621177 | -0.054087347 |
| -0.016041069 | 0.729535358 | -0.010202378 | 0.825956735 | -0.034558044 |
| -0.01254991 | 0.786783548 | 0.00294708 | 0.949355295 | 0.026894827 |
| 0.25507754 | 2.27E-08 | 0.252483504 | 3.18E-08 | 0.290524134 |
| 0.034043108 | 0.462998132 | 0.037752604 | 0.415676781 | 0.028054596 |

| | | | | |
|---|---|---|---|---|
| 0.085182558 | 0.065882628 | 0.109385227 | 0.0180496 | 0.104743006 |
| 0.083416443 | 0.071710225 | 0.083318416 | 0.072045639 | 0.070163031 |
| 0.056228845 | 0.225199086 | 0.086866692 | 0.060693577 | 0.080831847 |
| -0.094604582 | 0.041000142 | -0.090942899 | 0.049518797 | -0.109586945 |
| 0.021517875 | 0.642781927 | -0.003673409 | 0.936896958 | 0.000427437 |
| 0.066892862 | 0.148933196 | 0.104661617 | 0.023702931 | 0.070940415 |
| -0.088686857 | 0.055469783 | -0.067169607 | 0.147255339 | -0.065767337 |
| 0.021428423 | 0.644164398 | 0.017988381 | 0.698221935 | 0.009929682 |
| 0.092434594 | 0.045884842 | 0.094362005 | 0.04152342 | 0.074239195 |
| -0.113515519 | 0.014109826 | -0.091440604 | 0.048280463 | -0.117208172 |
| 0.128349151 | 0.005474303 | 0.140732298 | 0.00230152 | 0.136423019 |
| 0.122211337 | 0.008196933 | 0.123517004 | 0.007533065 | 0.112414498 |
| 0.125844038 | 0.006468076 | 0.134435429 | 0.003607221 | 0.134781586 |
| 0.075408293 | 0.103622941 | 0.086786856 | 0.060931704 | 0.067994193 |
| 0.029147305 | 0.529791677 | 0.030514299 | 0.510663083 | 0.024413072 |
| -0.250103943 | 4.31E-08 | -0.270031815 | 3.02E-09 | -0.308179384 |
| 0.003234881 | 0.944417149 | -0.017006015 | 0.713958654 | -0.021198615 |
| -0.082176248 | 0.076048914 | -0.083786017 | 0.070457127 | -0.068062238 |
| 0.123091325 | 0.007744023 | 0.098318099 | 0.033659549 | 0.082250976 |
| -0.198203077 | 1.60E-05 | -0.20357726 | 9.25E-06 | -0.222519523 |
| -0.004592258 | 0.921158843 | -0.014789551 | 0.749904114 | -0.00679154 |
| 0.2527925 | 3.05E-08 | 0.238942402 | 1.74E-07 | 0.251436099 |
| -0.20812367 | 5.75E-06 | -0.22108092 | 1.40E-06 | -0.237090618 |
| 0.15684222 | 0.000670467 | 0.150149701 | 0.00113578 | 0.13860505 |
| -0.020997641 | 0.650839186 | -0.028367531 | 0.540863519 | -0.06787604 |
| -0.041437459 | 0.371613544 | -0.058034451 | 0.210632059 | -0.086331235 |
| 0.224105799 | 9.96E-07 | 0.243267709 | 1.02E-07 | 0.219802062 |
| 0.149590299 | 0.001185809 | 0.156571409 | 0.000685202 | 0.171151133 |
| 0.281337509 | 6.06E-10 | 0.241957393 | 1.20E-07 | 0.246726982 |
| 0.100438794 | 0.029993655 | 0.129652775 | 0.005013507 | 0.142582943 |
| 0.092885244 | 0.044832067 | 0.083741976 | 0.070605509 | 0.072853726 |
| 0.197693711 | 1.68E-05 | 0.172846152 | 0.000174372 | 0.184024954 |
| -0.169517078 | 0.000233101 | -0.195226701 | 2.15E-05 | -0.212013824 |
| 0.032069916 | 0.489341049 | 0.024260728 | 0.60100857 | -0.013914735 |
| 0.094286874 | 0.041686628 | 0.094086023 | 0.0421256 | 0.079004272 |
| 0.126960502 | 0.006006751 | 0.130070963 | 0.004873273 | 0.133234529 |
| 0.131410354 | 0.004447613 | 0.152936759 | 0.000914271 | 0.14962549 |
| -0.061661545 | 0.183448363 | -0.028205421 | 0.543179667 | -0.077761524 |
| 0.410343851 | 2.15E-20 | 0.415342655 | 6.69E-21 | 0.439485479 |
| -0.073024217 | 0.115039143 | -0.062674481 | 0.176341156 | -0.0466746 |
| 0.135957465 | 0.003241338 | 0.156907773 | 0.000666944 | 0.130223276 |
| 0.166326635 | 0.000306325 | 0.169637447 | 0.000230689 | 0.183536875 |
| -0.084879639 | 0.066853568 | -0.023836494 | 0.607389364 | -0.021709386 |
| 0.176972142 | 0.00012078 | 0.15799818 | 0.000610813 | 0.162606471 |
| -0.04643188 | 0.316704786 | -0.065714432 | 0.156240998 | -0.020916092 |

| | | | | |
|---|---|---|---|---|
| -0.239859725 | 1.55E-07 | -0.214832861 | 2.80E-06 | -0.180694454 |
| -0.135308354 | 0.003393046 | -0.130111882 | 0.004859743 | -0.153325508 |
| 0.019807317 | 0.669427046 | 0.008770131 | 0.850077266 | 0.029158709 |
| 0.046238465 | 0.318727058 | 0.080496019 | 0.082265004 | 0.085326496 |
| -0.149185759 | 0.001223245 | -0.153406899 | 0.0008811 | -0.116985772 |
| 0.242818234 | 1.08E-07 | 0.273529904 | 1.85E-09 | 0.239676721 |
| -0.145112443 | 0.001665642 | -0.16195439 | 0.000441898 | -0.135133911 |
| -0.02939994 | 0.526229366 | -0.028963079 | 0.532397075 | -0.045205251 |
| 0.040640752 | 0.38088737 | 0.051162345 | 0.269859002 | 0.028403756 |
| 0.055723059 | 0.229405248 | 0.053764979 | 0.246213285 | 0.069041665 |
| 0.066599747 | 0.150726119 | 0.080593884 | 0.081892021 | 0.080355689 |
| 0.151528768 | 0.001020637 | 0.147511884 | 0.001389996 | 0.139068398 |
| 0.02360295 | 0.610914857 | 0.008529111 | 0.854150717 | -0.002680837 |
| -0.032338563 | 0.485707835 | -0.042836527 | 0.355669433 | -0.056485218 |
| 0.043455686 | 0.348752755 | 0.036553684 | 0.430650957 | 0.046579077 |
| -0.076503795 | 0.09868733 | -0.057959759 | 0.211220848 | -0.078575788 |
| 0.069053437 | 0.136214439 | 0.071559729 | 0.122526628 | 0.056643279 |
| -0.018598286 | 0.688515892 | 0.001081683 | 0.981400826 | -0.02139212 |
| 0.048934797 | 0.291293547 | 0.053938105 | 0.244693404 | 0.037809646 |
| -0.001970384 | 0.966126996 | 0.013682828 | 0.768062791 | -0.006543156 |
| -0.032889442 | 0.478303432 | -0.019289651 | 0.677575081 | -0.010046392 |
| 0.09984994 | 0.030975548 | 0.078372201 | 0.090702895 | 0.081019783 |
| 0.111752847 | 0.015687759 | 0.103020149 | 0.025997827 | 0.098067794 |
| -0.229542233 | 5.32E-07 | -0.194945015 | 2.21E-05 | -0.190683598 |
| 0.063397942 | 0.171391705 | 0.053471956 | 0.248800747 | 0.025510728 |
| 0.11332373 | 0.014274464 | 0.084590909 | 0.067789931 | 0.103393557 |
| 0.114325263 | 0.013432987 | 0.097533246 | 0.035109899 | 0.121899506 |
| 0.004815406 | 0.917341144 | 0.024574381 | 0.596310482 | 0.02885696 |
| -0.063242859 | 0.17244385 | -0.074428498 | 0.108201076 | -0.072298141 |
| 0.13588792 | 0.003257291 | 0.128780872 | 0.005317645 | 0.140807017 |
| 0.122613482 | 0.007987105 | 0.124034545 | 0.00728351 | 0.090266197 |
| 0.050533133 | 0.275801248 | 0.018468635 | 0.690574945 | 0.010588284 |
| 0.316616685 | 2.47E-12 | 0.31763235 | 2.09E-12 | 0.333815146 |
| -0.092330863 | 0.046130088 | -0.086439143 | 0.061977836 | -0.101427876 |
| -0.059264644 | 0.201104409 | -0.045990516 | 0.32133181 | -0.071393344 |
| 0.051789375 | 0.264025042 | 0.045453618 | 0.327019381 | 0.04738813 |
| 0.026478096 | 0.568160238 | 0.057575821 | 0.214266095 | 0.073494219 |
| -0.086325453 | 0.062323084 | -0.097931664 | 0.034367141 | -0.065867461 |
| 0.081979698 | 0.076755723 | 0.12141685 | 0.008625979 | 0.079916844 |
| -0.043011704 | 0.353703824 | -0.003405397 | 0.941492397 | -0.007135024 |
| 0.096988951 | 0.036146656 | 0.092376966 | 0.046020953 | 0.091988869 |
| 0.102384702 | 0.026936314 | 0.072975167 | 0.115283977 | 0.091113389 |
| 0.081120249 | 0.079909332 | 0.093265899 | 0.043958701 | 0.060003288 |
| -0.019318361 | 0.677122192 | 0.012780832 | 0.782957335 | -0.027683149 |
| -0.124166427 | 0.007221111 | -0.102214573 | 0.027192469 | -0.091324822 |

| | | | | |
|---|---|---|---|---|
| -0.099109033 | 0.032249947 | -0.096143326 | 0.03780893 | -0.09956906 |
| -0.024289704 | 0.600573846 | -0.046357598 | 0.317480458 | -0.033778147 |
| -0.025524556 | 0.58218102 | -0.025376421 | 0.584373555 | -0.007800775 |
| 0.157502467 | 0.000635765 | 0.175108557 | 0.000142716 | 0.142078498 |
| 0.259449102 | 1.27E-08 | 0.259035922 | 1.35E-08 | 0.259527162 |
| 0.040128783 | 0.386920877 | 0.05435021 | 0.241101959 | 0.044878308 |
| 0.052730476 | 0.255432673 | 0.077781195 | 0.093170473 | 0.095997676 |
| 0.000840396 | 0.985549152 | -0.010306944 | 0.824201849 | -0.015667495 |
| -0.186059857 | 5.22E-05 | -0.177150651 | 0.000118854 | -0.183173453 |
| -0.051387052 | 0.267758249 | -0.001124496 | 0.980664801 | -0.004360073 |
| 0.22317426 | 1.11E-06 | 0.212217012 | 3.72E-06 | 0.21265279 |
| 0.000615461 | 0.989416703 | 0.025314653 | 0.585288921 | 0.000316731 |
| 0.075185181 | 0.104651707 | 0.085929237 | 0.0635387 | 0.109259586 |
| 0.208454167 | 5.55E-06 | 0.214992 | 2.75E-06 | 0.257759206 |
| 0.049493933 | 0.285808954 | 0.063889723 | 0.168086925 | 0.079809584 |
| -0.011340719 | 0.806900851 | -0.023461828 | 0.613049551 | -0.072465994 |
| -0.128008983 | 0.00560065 | -0.131297685 | 0.004482081 | -0.155954806 |
| -0.05397225 | 0.244394421 | -0.034602716 | 0.455673189 | -0.058748519 |
| -0.02913564 | 0.529956465 | -0.057915095 | 0.21157349 | -0.027786467 |
| -0.104308395 | 0.024181398 | -0.084612051 | 0.067721006 | -0.107831247 |
| -0.044676057 | 0.335371193 | -0.016713814 | 0.718663352 | -0.014091478 |
| -0.020287091 | 0.661909863 | -0.064682215 | 0.162862061 | -0.083250106 |
| 0.005115266 | 0.912214038 | -0.017359981 | 0.708274001 | -0.026913255 |
| -0.032480353 | 0.483796135 | 0.00275636 | 0.952628803 | 0.022649791 |
| 0.030854559 | 0.505958328 | 0.021541249 | 0.642420897 | 0.053977804 |
| -0.047660366 | 0.304056556 | 0.004627585 | 0.920554318 | 0.011611097 |
| 0.284135081 | 4.02E-10 | 0.293150595 | 1.04E-10 | 0.271362311 |
| 0.142819829 | 0.001974999 | 0.158234789 | 0.000599227 | 0.11675076 |
| -0.048129711 | 0.299313806 | -0.052691755 | 0.255782328 | -0.060145324 |
| 0.167689363 | 0.000272752 | 0.161059822 | 0.000475767 | 0.15829875 |
| 0.12019622 | 0.009324193 | 0.135709977 | 0.003298438 | 0.115301395 |
| 0.242141365 | 1.17E-07 | 0.263958632 | 6.96E-09 | 0.248848956 |
| 0.093263291 | 0.043964636 | 0.136556997 | 0.003106717 | 0.140067357 |
| 0.075966871 | 0.101082463 | 0.107487746 | 0.020160925 | 0.087194648 |
| 0.00804589 | 0.862329176 | 0.025403628 | 0.583970575 | 0.01418629 |
| 0.010084488 | 0.827936285 | 0.01494726 | 0.747327433 | -0.009079965 |
| 0.028503419 | 0.538925802 | 0.053309535 | 0.2502431 | 0.059326568 |
| -0.201252056 | 1.17E-05 | -0.171362477 | 0.000198586 | -0.19604077 |
| 0.004066637 | 0.930158353 | -0.018298243 | 0.693284511 | -0.04226361 |
| 0.282550188 | 5.08E-10 | 0.23724915 | 2.13E-07 | 0.218859422 |
| 0.125197371 | 0.00674958 | 0.137964669 | 0.002810382 | 0.12877574 |
| -0.105582075 | 0.022494382 | -0.110516741 | 0.016884678 | -0.138677793 |
| 0.26600435 | 5.27E-09 | 0.284917194 | 3.59E-10 | 0.34513561 |
| -0.003083329 | 0.947017268 | 0.028136975 | 0.544159065 | 0.012696637 |
| -0.132945972 | 0.004001126 | -0.10906515 | 0.018391605 | -0.147039758 |

| | | | | |
|---|---|---|---|---|
| -0.024295099 | 0.600492927 | -0.020319479 | 0.661403625 | -0.036089169 |
| -0.022613065 | 0.62595705 | -0.023043446 | 0.619397452 | -0.077723433 |
| 0.212599228 | 3.57E-06 | 0.211654711 | 3.95E-06 | 0.208235659 |
| 0.125662027 | 0.006546224 | 0.14569254 | 0.001594753 | 0.171406072 |
| 0.121960262 | 0.008330417 | 0.097503968 | 0.035165017 | 0.08779349 |
| 0.357657038 | 1.54E-15 | 0.341926926 | 2.97E-14 | 0.383777671 |
| -0.022531972 | 0.627196361 | -0.045448852 | 0.32707016 | -0.109302706 |
| 0.086874974 | 0.060668919 | 0.073023548 | 0.115042483 | 0.098049797 |
| 0.037086366 | 0.423959743 | 0.036689332 | 0.428941262 | 0.048083812 |
| 0.075112452 | 0.104988805 | 0.10341555 | 0.025428199 | 0.09507757 |
| 0.129945862 | 0.004914848 | 0.130742781 | 0.00465538 | 0.135065358 |
| 0.117653678 | 0.010941968 | 0.105124727 | 0.023088031 | 0.10187897 |
| 0.047063776 | 0.310156559 | 0.031896388 | 0.491695589 | 0.027294952 |
| 0.11584107 | 0.012242492 | 0.141642365 | 0.002153546 | 0.126067205 |
| 0.066428124 | 0.15178349 | 0.072845122 | 0.115935077 | 0.06135007 |
| -0.080855967 | 0.0808999 | -0.076197126 | 0.100049737 | -0.094889104 |
| 0.091905987 | 0.047146112 | 0.092867937 | 0.044872122 | 0.084550826 |
| 0.054512604 | 0.239696932 | 0.063728994 | 0.169161743 | 0.036655015 |
| 0.013670483 | 0.768266093 | 0.036689537 | 0.428938687 | 0.019338943 |
| -0.038955251 | 0.400968468 | -0.013942553 | 0.763789499 | -0.033026248 |
| 0.057711163 | 0.213189034 | 0.073713994 | 0.111639129 | 0.069503295 |
| 0.086569414 | 0.061584179 | 0.08362898 | 0.07098738 | 0.067943632 |
| -0.013075356 | 0.778084885 | -0.007574223 | 0.870326305 | 0.002057143 |
| 0.09210044 | 0.046678803 | 0.0996411 | 0.031330327 | 0.112263235 |
| 0.122088123 | 0.008262199 | 0.154516797 | 0.000807154 | 0.118850174 |
| 0.058797809 | 0.204682389 | 0.086454012 | 0.0619328 | 0.111794073 |
| 0.016861267 | 0.716287882 | 0.060597146 | 0.191142257 | 0.067456266 |
| 0.138280335 | 0.002747566 | 0.149321792 | 0.001210537 | 0.156551762 |
| -0.122447865 | 0.008072931 | -0.088521704 | 0.055927821 | -0.085270663 |
| -0.016095147 | 0.728659393 | -0.04262469 | 0.358055555 | -0.04236283 |
| 0.264021927 | 6.90E-09 | 0.249014857 | 4.95E-08 | 0.274614613 |
| -0.103011076 | 0.026011026 | -0.093355008 | 0.043756333 | -0.095370334 |
| 0.051558494 | 0.266163022 | 0.046935798 | 0.311475524 | 0.033902169 |
| 0.112560368 | 0.01494662 | 0.120808944 | 0.008967662 | 0.11318697 |
| -0.004661018 | 0.919982268 | 0.024564246 | 0.596462026 | 0.045224167 |
| 0.043626074 | 0.346864374 | 0.057554129 | 0.214439085 | 0.033091705 |
| 0.058673836 | 0.205640265 | 0.072417884 | 0.118094426 | 0.086338922 |
| 0.068519312 | 0.139278109 | 0.075567443 | 0.102894007 | 0.097641678 |
| 0.032787398 | 0.47967035 | 0.056776078 | 0.220710404 | 0.080128065 |
| 0.031566643 | 0.496186429 | 0.027834167 | 0.54850244 | 0.034624828 |
| 0.230953296 | 4.51E-07 | 0.239936621 | 1.54E-07 | 0.247967515 |
| -0.036817383 | 0.427330928 | 0.014010595 | 0.762671182 | 0.021581592 |
| -0.083192065 | 0.072479854 | -0.067924725 | 0.142750477 | -0.063827933 |
| -0.048222196 | 0.298385078 | -0.051684777 | 0.26499216 | -0.053040596 |
| 0.071027528 | 0.125339765 | 0.061234419 | 0.186507863 | 0.038852964 |

| | | | | |
|---|---|---|---|---|
| -0.002034098 | 0.965032368 | -0.015129161 | 0.744358978 | -0.043825145 |
| -0.193274979 | 2.61E-05 | -0.141071526 | 0.00224531 | -0.145320454 |
| -0.066713314 | 0.150029517 | -0.013910745 | 0.764312466 | -0.031116732 |
| 0.184883182 | 5.84E-05 | 0.140540028 | 0.002333947 | 0.108371148 |
| 0.064498387 | 0.164063017 | 0.068516903 | 0.139292047 | 0.067680317 |
| 0.37443529 | 5.45E-17 | 0.360884658 | 8.23E-16 | 0.359656809 |
| 0.027897889 | 0.547587014 | 0.050010541 | 0.280803737 | 0.028898538 |
| 0.127278537 | 0.005880859 | 0.132587427 | 0.004101582 | 0.12368935 |
| -0.101803204 | 0.027820524 | -0.071840789 | 0.121060963 | -0.088141086 |
| -0.05051402 | 0.275983129 | -0.035809997 | 0.440094113 | -0.07015873 |
| 0.019073259 | 0.680992323 | 0.071529992 | 0.122682506 | 0.072190996 |
| 0.136269297 | 0.003170671 | 0.122638241 | 0.007974344 | 0.149745185 |
| -0.056130126 | 0.2260157 | -0.026975917 | 0.560904842 | -0.041539862 |
| 0.033156145 | 0.474740875 | 0.052209774 | 0.2601625 | 0.049362465 |
| -0.019198812 | 0.679008801 | -0.01847772 | 0.690430592 | -0.040573271 |
| 0.200176087 | 1.31E-05 | 0.184740493 | 5.91E-05 | 0.207954322 |
| -0.009282354 | 0.841433578 | -0.004944499 | 0.915133434 | -0.012189082 |
| 0.072760573 | 0.116359933 | 0.07427401 | 0.108937276 | 0.074324922 |
| 0.199256533 | 1.44E-05 | 0.23058833 | 4.71E-07 | 0.217566045 |
| 0.078186391 | 0.09147298 | 0.065648933 | 0.156654995 | 0.059357591 |
| 0.001031005 | 0.982272057 | 0.033993946 | 0.463644748 | -0.007620662 |
| -0.01205901 | 0.794934371 | -0.00106269 | 0.981727336 | -0.007397348 |
| 0.013083324 | 0.777953192 | 0.012997239 | 0.77937637 | 0.012744563 |
| -0.036051533 | 0.437014239 | -0.003079004 | 0.94709147 | -0.009413846 |
| -0.136885836 | 0.003035056 | -0.114578032 | 0.013227645 | -0.104324465 |
| 0.19698172 | 1.81E-05 | 0.201250029 | 1.17E-05 | 0.211113162 |
| -0.183528391 | 6.62E-05 | -0.21641377 | 2.36E-06 | -0.261688708 |
| 0.265990222 | 5.28E-09 | 0.26736392 | 4.37E-09 | 0.248622487 |
| -0.123021116 | 0.007779319 | -0.129783623 | 0.004969242 | -0.156284256 |
| 0.274962995 | 1.51E-09 | 0.294785587 | 8.13E-11 | 0.342360806 |
| 0.167203649 | 0.000284303 | 0.167934156 | 0.000267099 | 0.186028728 |
| 0.091447776 | 0.04826281 | 0.089366018 | 0.053618822 | 0.090762667 |
| 0.069551152 | 0.133406533 | 0.082909897 | 0.073457255 | 0.079287183 |
| 0.101746008 | 0.027908826 | 0.10025602 | 0.03029553 | 0.102444309 |
| 0.072156055 | 0.119433227 | 0.079108035 | 0.087704164 | 0.067745423 |
| -0.125999187 | 0.006402123 | -0.101882165 | 0.027699013 | -0.129851641 |
| 0.132005324 | 0.004269541 | 0.131272275 | 0.004489887 | 0.160026008 |
| 0.127821835 | 0.005671275 | 0.159667708 | 0.000533304 | 0.155075011 |
| -0.140031109 | 0.0024218 | -0.165647218 | 0.000324468 | -0.169192142 |
| 0.063357573 | 0.171665118 | 0.10415521 | 0.02439148 | 0.092781205 |
| -0.319965147 | 1.41E-12 | -0.32143915 | 1.10E-12 | -0.336105333 |
| 0.084330122 | 0.068644894 | 0.080659835 | 0.081641441 | 0.126744313 |
| -0.010514013 | 0.820729268 | 0.005081156 | 0.912797085 | 0.0391587 |
| 0.214931726 | 2.77E-06 | 0.223304017 | 1.09E-06 | 0.242333267 |
| -0.068124313 | 0.141577581 | -0.077109755 | 0.096038726 | -0.110742078 |

| | | | | |
|---|---|---|---|---|
| -0.109120208 | 0.018332375 | -0.146258981 | 0.001528214 | -0.158028435 |
| -0.082430326 | 0.075143081 | -0.058695962 | 0.205469077 | -0.083850279 |
| -0.07892782 | 0.088431118 | -0.100793249 | 0.029415565 | -0.119317023 |
| 0.08403147 | 0.069634817 | 0.074736263 | 0.106746152 | 0.032821946 |
| -0.005342871 | 0.908324797 | -0.025382605 | 0.584281956 | -0.015753385 |
| 0.069160789 | 0.135604989 | 0.068326551 | 0.140396654 | 0.068873238 |
| 0.245598228 | 7.63E-08 | 0.249993864 | 4.37E-08 | 0.265918097 |
| 0.040655806 | 0.380710833 | 0.052975954 | 0.253223648 | 0.078310169 |
| 0.175967809 | 0.000132174 | 0.206933781 | 6.52E-06 | 0.228735051 |
| -0.008484897 | 0.854898404 | -0.017491924 | 0.706159125 | -0.012274351 |
| 0.078672741 | 0.089468329 | 0.087002558 | 0.060290098 | 0.076898882 |
| 0.061400928 | 0.185310718 | 0.116193132 | 0.011979699 | 0.12990617 |
| -0.12130014 | 0.008690664 | -0.053732359 | 0.246500397 | -0.07023207 |
| -0.005486144 | 0.905877705 | -0.022061697 | 0.634403914 | -0.029869398 |
| 0.102792709 | 0.026330443 | 0.106008203 | 0.021953206 | 0.102719514 |
| 0.089375047 | 0.053594564 | 0.085181669 | 0.06588546 | 0.077557095 |
| 0.208004153 | 5.83E-06 | 0.216006268 | 2.46E-06 | 0.215925903 |
| 0.030819973 | 0.506435506 | 0.029768471 | 0.521054809 | 0.016105103 |
| -0.34522374 | 1.62E-14 | -0.32288461 | 8.59E-13 | -0.324810167 |
| -0.022994345 | 0.620144308 | -0.032929988 | 0.477760884 | -0.044263601 |
| -0.025536825 | 0.581999594 | -0.011499783 | 0.804246912 | 0.019321488 |
| -0.027111933 | 0.558930317 | -0.034760768 | 0.45361621 | -0.060302334 |
| 0.094528428 | 0.041163815 | 0.085093943 | 0.066165457 | 0.080254677 |
| -0.042717813 | 0.357005387 | -0.02778491 | 0.549210571 | -0.05876534 |
| 0.079834279 | 0.084823194 | 0.112122964 | 0.015344159 | 0.106649131 |
| 0.105092867 | 0.023129887 | 0.108306877 | 0.019224473 | 0.114469291 |
| -0.052607513 | 0.256544198 | -0.010262068 | 0.824954874 | -0.02272303 |
| 0.02952486 | 0.524472429 | 0.00647765 | 0.888968525 | 0.037686228 |
| -0.015242416 | 0.742512687 | 0.019645212 | 0.671974455 | 0.021505479 |
| -0.04461467 | 0.336036335 | -0.032114168 | 0.488741576 | -0.04281708 |
| 0.020910404 | 0.652194306 | 0.026637506 | 0.565832075 | -0.030620362 |
| 0.091687036 | 0.047676981 | 0.107033586 | 0.020697037 | 0.084099661 |
| 0.215436028 | 2.62E-06 | 0.20689028 | 6.55E-06 | 0.170202469 |
| 0.080662618 | 0.081630882 | 0.071255727 | 0.124127454 | 0.06083716 |
| -0.019940049 | 0.667344032 | -0.026207893 | 0.572116895 | -0.016194809 |
| 0.090209356 | 0.051392248 | 0.102306047 | 0.027054482 | 0.104974948 |
| 0.001523415 | 0.973807705 | 0.012786802 | 0.782858495 | 0.018552689 |
| 0.109528005 | 0.017898839 | 0.102032886 | 0.027468339 | 0.109897496 |
| 0.045982518 | 0.321416057 | 0.052419514 | 0.258250097 | 0.074465963 |
| 0.133424519 | 0.003870518 | 0.133193756 | 0.003933009 | 0.140283653 |
| -0.053942114 | 0.244658283 | -0.027736427 | 0.549908019 | -0.038112111 |
| -0.242233114 | 1.16E-07 | -0.20698375 | 6.49E-06 | -0.19646516 |
| -0.015910445 | 0.731652715 | -0.018046773 | 0.697290516 | -0.009942377 |
| 0.307074016 | 1.18E-11 | 0.315717273 | 2.87E-12 | 0.342775086 |
| 0.130601485 | 0.004700463 | 0.13263549 | 0.004087985 | 0.15398455 |

| | | | | |
|---|---|---|---|---|
| 0.020595226 | 0.657099755 | 0.026320225 | 0.570470401 | 0.013403935 |
| 0.11716777 | 0.011278012 | 0.118676644 | 0.010263357 | 0.112354435 |
| 0.056737779 | 0.221022457 | 0.098922237 | 0.032578225 | 0.087001662 |
| -0.001424927 | 0.975500471 | 0.017776798 | 0.701600691 | 0.014140113 |
| 0.007652461 | 0.868998842 | -0.014483243 | 0.754916563 | -0.039917089 |
| 0.05488448 | 0.236501166 | 0.054535067 | 0.239503034 | 0.031678763 |
| -0.040249734 | 0.385490263 | -0.025929455 | 0.576207751 | -0.056018843 |
| 0.101931937 | 0.027622654 | 0.108236948 | 0.019302911 | 0.110450094 |
| 0.057364294 | 0.215957272 | 0.058414083 | 0.207657775 | 0.03838009 |
| 0.037807016 | 0.415004536 | 0.052734039 | 0.255400513 | 0.091067509 |
| -0.193118431 | 2.65E-05 | -0.183481777 | 6.65E-05 | -0.15965926 |
| -0.046167486 | 0.319471289 | -0.079950184 | 0.084370546 | -0.06266045 |
| 0.026653248 | 0.565602416 | 0.05128345 | 0.268725408 | 0.055112265 |
| -0.050483115 | 0.276277402 | -0.051575113 | 0.266008725 | -0.058234668 |
| -0.043622659 | 0.34690216 | -0.008906486 | 0.847774485 | -0.01318597 |
| 0.096827307 | 0.036459516 | 0.061661339 | 0.183449831 | 0.061512285 |
| 0.056978103 | 0.219069561 | 0.052707588 | 0.255639316 | 0.105362309 |
| 0.191129751 | 3.22E-05 | 0.189666837 | 3.70E-05 | 0.187209042 |
| 0.062564202 | 0.177104846 | 0.045306575 | 0.328588362 | 0.081104202 |
| -0.028366287 | 0.540881269 | -0.022382437 | 0.629484373 | -0.018305774 |
| 0.102827274 | 0.026279659 | 0.11158352 | 0.015847191 | 0.098208283 |
| 0.1328905 | 0.004016521 | 0.141087134 | 0.002242754 | 0.11494209 |
| 0.113655589 | 0.013990643 | 0.072700203 | 0.116664034 | 0.095626878 |
| 0.02355692 | 0.61161078 | 0.037309952 | 0.421169358 | 0.052213432 |
| 0.150769933 | 0.001082589 | 0.164895102 | 0.00034572 | 0.153991004 |
| 0.078320825 | 0.090915303 | 0.112220515 | 0.015254704 | 0.114614289 |
| -0.114763231 | 0.013078956 | -0.100154513 | 0.030464302 | -0.128333546 |
| -0.095168101 | 0.039806006 | -0.081021897 | 0.080276814 | -0.086877738 |
| 0.219903446 | 1.60E-06 | 0.25902074 | 1.35E-08 | 0.246533723 |
| -0.252095582 | 3.34E-08 | -0.286439943 | 2.86E-10 | -0.274990508 |
| 0.024323269 | 0.600070458 | 0.04182572 | 0.367145146 | 0.03237472 |
| -0.246179869 | 7.09E-08 | -0.203942458 | 8.90E-06 | -0.208355752 |
| 0.144367067 | 0.00176097 | 0.146628024 | 0.001486244 | 0.150907488 |
| 0.086669368 | 0.061283536 | 0.028033347 | 0.545643542 | 0.023944157 |
| 0.133905533 | 0.003743139 | 0.130954902 | 0.004588432 | 0.13266324 |
| 0.261106543 | 1.02E-08 | 0.264944741 | 6.09E-09 | 0.271990891 |
| -0.205982461 | 7.20E-06 | -0.192819933 | 2.73E-05 | -0.217874108 |
| 0.216801153 | 2.26E-06 | 0.241859228 | 1.21E-07 | 0.265404756 |
| 0.215365914 | 2.64E-06 | 0.235085752 | 2.76E-07 | 0.264524101 |
| 0.07526666 | 0.104275078 | 0.055603101 | 0.230410953 | 0.015097988 |
| -0.078084116 | 0.091899087 | -0.095849066 | 0.038402369 | -0.106559794 |
| 0.044350417 | 0.338909221 | 0.070107114 | 0.130323043 | 0.028697175 |
| -0.003484547 | 0.940135051 | 0.002334578 | 0.959870962 | 0.009562864 |
| 0.105199193 | 0.022990458 | 0.103296903 | 0.025597984 | 0.073342278 |
| -0.061019033 | 0.188064846 | -0.090261615 | 0.051256851 | -0.081646201 |

| | | | | |
|---|---|---|---|---|
| 0.099994812 | 0.030731462 | 0.128702511 | 0.005345776 | 0.134364482 |
| 0.017811359 | 0.701048384 | 0.083851234 | 0.070237869 | 0.099553451 |
| 0.179526762 | 9.58E-05 | 0.206952461 | 6.51E-06 | 0.203957299 |
| 0.144517239 | 0.001741371 | 0.189193514 | 3.88E-05 | 0.16796427 |
| 0.057828204 | 0.212260762 | 0.025741037 | 0.578983796 | 0.047536886 |
| 0.089657375 | 0.052840673 | 0.113007385 | 0.014549719 | 0.102966632 |
| -0.246490321 | 6.82E-08 | -0.226494274 | 7.58E-07 | -0.253640623 |
| 0.030446247 | 0.511606761 | -0.003941735 | 0.932298228 | 0.003504328 |
| -0.067776712 | 0.143625081 | -0.036947767 | 0.425694872 | -0.039869125 |
| -0.075086898 | 0.10510745 | -0.047495922 | 0.305729975 | -0.059520438 |
| -0.05307678 | 0.252320183 | -0.039313636 | 0.39664643 | -0.048581644 |
| -0.027602025 | 0.551843724 | -0.015683554 | 0.735335334 | -0.006006638 |
| -0.127495521 | 0.005796335 | -0.099691678 | 0.031244088 | -0.107777075 |
| 0.278008622 | 9.80E-10 | 0.283313155 | 4.54E-10 | 0.298257602 |
| 0.239344145 | 1.65E-07 | 0.282330242 | 5.24E-10 | 0.278955507 |
| 0.088176384 | 0.056895686 | 0.073926295 | 0.110608725 | 0.063054554 |
| -0.039601547 | 0.393194676 | -0.053609043 | 0.247587884 | -0.086971837 |
| -0.003979684 | 0.931648015 | -0.023766205 | 0.608449461 | 0.010577634 |
| -0.050214558 | 0.278843539 | -0.118097295 | 0.010642948 | -0.15937604 |
| -0.186220264 | 5.15E-05 | -0.159679953 | 0.000532771 | -0.182158754 |
| -0.151689996 | 0.001007903 | -0.149680041 | 0.001177649 | -0.154954155 |
| -0.047123807 | 0.30953913 | -0.024547399 | 0.596713981 | 0.009504384 |
| -0.019013138 | 0.681942904 | -0.003275066 | 0.943727823 | -0.014548861 |
| -0.049790342 | 0.282929862 | -0.031431099 | 0.49803873 | -0.065532379 |
| -0.023422995 | 0.613637533 | -0.019729948 | 0.670642383 | -0.053891724 |
| -0.022233614 | 0.631765016 | -0.016659298 | 0.71954229 | -0.010086922 |
| -0.006123246 | 0.895007176 | -0.060490888 | 0.191923133 | -0.056651029 |
| 0.037978926 | 0.412884868 | 0.026361577 | 0.569864853 | 0.013272683 |
| -0.105346432 | 0.02279858 | -0.093136637 | 0.044253658 | -0.0972623 |
| 0.095811466 | 0.038478762 | 0.122641214 | 0.007972814 | 0.125549576 |
| -0.080232056 | 0.083277868 | -0.080682623 | 0.081555002 | -0.080927659 |
| -0.001365005 | 0.976530448 | -0.004199783 | 0.927877768 | -0.007251038 |
| -0.170789869 | 0.000208747 | -0.122023362 | 0.008296688 | -0.12638216 |
| -0.021257803 | 0.646804705 | 0.011428119 | 0.805442315 | -0.018973665 |
| 0.040539783 | 0.382072702 | 0.021238956 | 0.64709663 | 0.029782923 |
| -0.011202849 | 0.809202968 | 0.022858368 | 0.622214638 | -0.011339245 |
| -0.164058714 | 0.000370873 | -0.184395141 | 6.11E-05 | -0.172727312 |
| -0.058088779 | 0.210204551 | -0.068899298 | 0.13709319 | -0.089005724 |
| 0.183870992 | 6.42E-05 | 0.168501921 | 0.000254404 | 0.238036138 |
| -0.110395341 | 0.017006431 | -0.123741485 | 0.007423898 | -0.08508876 |
| 0.033839491 | 0.465679517 | 0.089081938 | 0.054386692 | 0.113007089 |
| -0.072695935 | 0.116685557 | -0.091716855 | 0.047604387 | -0.107639595 |
| -0.044698292 | 0.335130481 | -0.066850679 | 0.149190213 | -0.108946234 |
| 0.038582232 | 0.405496793 | 0.048732582 | 0.293294332 | 0.068294389 |
| 0.175086938 | 0.000142991 | 0.180716378 | 8.59E-05 | 0.223892618 |

| | | | | |
|---|---|---|---|---|
| 0.229840458 | 5.14E-07 | 0.2659579 | 5.30E-09 | 0.222257857 |
| 0.161511668 | 0.000458369 | 0.174694134 | 0.000148078 | 0.193116013 |
| -0.016585954 | 0.720725365 | -0.02351915 | 0.612182076 | -0.039379238 |
| -0.120772942 | 0.00898827 | -0.143705966 | 0.001849676 | -0.1571938 |
| -0.113177747 | 0.014400911 | -0.111021128 | 0.016386958 | -0.108759507 |
| -0.110640306 | 0.016761538 | -0.144994934 | 0.001680349 | -0.147928219 |
| -0.022551252 | 0.626901613 | -0.032520179 | 0.483259907 | -0.024212249 |
| -0.028129728 | 0.544262816 | -0.027484813 | 0.553534569 | -0.029469315 |
| -0.074303177 | 0.108797982 | -0.084222883 | 0.068999018 | -0.133694658 |
| -0.116888893 | 0.011474976 | -0.151027677 | 0.001061168 | -0.160314299 |
| 0.308233078 | 9.79E-12 | 0.315322349 | 3.07E-12 | 0.331265343 |
| -0.018066203 | 0.696980682 | -0.037780402 | 0.415333263 | -0.02078576 |
| -0.107266804 | 0.020420208 | -0.102490564 | 0.026777973 | -0.128916996 |
| -0.064178801 | 0.166166703 | -0.066446454 | 0.151670287 | -0.076515805 |
| -0.123004559 | 0.007787664 | -0.10089917 | 0.029244682 | -0.098724693 |
| 0.302791363 | 2.34E-11 | 0.324182579 | 6.88E-13 | 0.323436242 |
| -0.012902336 | 0.780946207 | -0.014354248 | 0.75703055 | -0.006625522 |
| 0.077910435 | 0.09262633 | 0.077047646 | 0.096307563 | 0.089963896 |
| -0.023206663 | 0.616917636 | -0.029431224 | 0.525789084 | -0.04244358 |
| 0.08347266 | 0.071518448 | 0.044578362 | 0.336430136 | 0.033084949 |
| 0.032286086 | 0.486416392 | 0.059578001 | 0.198728378 | 0.047452716 |
| 0.060252992 | 0.193679885 | 0.060744779 | 0.190061195 | 0.078621484 |
| -0.041839768 | 0.366984103 | 0.008053201 | 0.862205323 | 0.021890901 |
| -0.20244002 | 1.04E-05 | -0.178997424 | 0.000100552 | -0.207775434 |
| -0.079940006 | 0.084410218 | -0.042763218 | 0.356494053 | -0.054726091 |
| -0.004436918 | 0.923817528 | 0.052787075 | 0.25492216 | 0.067455599 |
| 0.129365579 | 0.00511191 | 0.142823868 | 0.001974411 | 0.117954258 |
| 0.005163809 | 0.91138437 | 0.010058504 | 0.828372745 | -0.009802105 |
| -0.091189681 | 0.048901502 | -0.131581529 | 0.004395706 | -0.154193536 |
| 0.142128761 | 0.002078074 | 0.155695197 | 0.000734957 | 0.142172445 |
| 0.150168654 | 0.00113412 | 0.146506607 | 0.001499934 | 0.150189713 |
| 0.054419376 | 0.240502829 | 0.04599208 | 0.321315339 | 0.064654863 |
| 0.026736838 | 0.564383673 | 0.045178523 | 0.329958664 | 0.018279842 |
| 0.059261639 | 0.201127291 | 0.057322411 | 0.216293255 | 0.053547741 |
| -0.011657674 | 0.801614762 | 0.014519873 | 0.754316604 | -0.039626626 |
| 0.068206344 | 0.141097667 | 0.064621946 | 0.163255073 | 0.07042844 |
| 0.035636848 | 0.442309588 | 0.00644921 | 0.889452871 | -0.018926487 |
| -0.020383392 | 0.660405058 | 0.045359722 | 0.328020705 | 0.052944056 |
| -0.044151556 | 0.341081522 | -0.016876597 | 0.716041084 | -0.006465248 |
| -0.029354035 | 0.526875749 | -0.028192846 | 0.543359542 | -0.027278917 |
| 0.114427063 | 0.013349953 | 0.095787869 | 0.038526772 | 0.08698573 |
| 0.132446082 | 0.004141807 | 0.126939614 | 0.006015103 | 0.11854889 |
| 0.035215028 | 0.447733393 | 0.048600007 | 0.294611055 | 0.035869951 |
| 0.078415316 | 0.09052495 | 0.080605989 | 0.081845984 | 0.110891574 |
| -0.001118801 | 0.980762713 | 0.030527717 | 0.510477129 | 0.024650306 |

| | | | | |
|---|---|---|---|---|
| 0.107973577 | 0.019600837 | 0.129693766 | 0.004999602 | 0.15184734 |
| 0.121906689 | 0.008359148 | 0.131797126 | 0.004331107 | 0.116740853 |
| 0.124118615 | 0.007243677 | 0.07870271 | 0.089345962 | 0.031440205 |
| 0.027996156 | 0.5461768 | 0.026242656 | 0.571607125 | 0.014146498 |
| -0.041148784 | 0.374957533 | -0.035060972 | 0.449723604 | -0.052007573 |
| -0.108561409 | 0.018941309 | -0.092672985 | 0.045325375 | -0.094073161 |
| 0.239660357 | 1.59E-07 | 0.236990169 | 2.20E-07 | 0.249845968 |
| -0.14098956 | 0.002258776 | -0.131431124 | 0.004441285 | -0.125322599 |
| 0.091607695 | 0.047870582 | 0.095346151 | 0.039434875 | 0.07595106 |
| 0.149816041 | 0.001165381 | 0.136900143 | 0.003031973 | 0.132552393 |
| -0.026460934 | 0.568411151 | -0.045848879 | 0.322825931 | -0.025550041 |
| -0.192736043 | 2.75E-05 | -0.16657832 | 0.000299847 | -0.208185882 |
| -0.117529593 | 0.011026929 | -0.100995878 | 0.029089406 | -0.122348403 |
| 0.155984083 | 0.000718194 | 0.160462853 | 0.000499695 | 0.163634625 |
| -0.095554703 | 0.039003892 | -0.121356971 | 0.008659112 | -0.163090621 |
| -0.03448167 | 0.457252081 | 0.008204688 | 0.859639876 | 0.044759094 |
| -0.032380816 | 0.485137723 | -0.047768758 | 0.302956858 | -0.060731714 |
| 0.044660895 | 0.335535395 | 0.071558366 | 0.122533774 | 0.057185405 |
| -0.024069238 | 0.603884978 | 0.019196381 | 0.67904719 | 0.007956399 |
| -0.022041662 | 0.634711756 | -0.018886359 | 0.683949102 | -0.050742102 |
| 0.091767113 | 0.047482248 | 0.083234296 | 0.072334485 | 0.097796573 |
| -0.081600453 | 0.078134606 | -0.045306356 | 0.328590703 | -0.067779253 |
| -0.036225496 | 0.434803681 | -8.92E-05 | 0.998465892 | 0.011195384 |
| -0.073661671 | 0.111894236 | -0.042054003 | 0.364533543 | -0.024748065 |
| 0.146962129 | 0.001449164 | 0.138675495 | 0.002670735 | 0.136955793 |
| -0.025412743 | 0.583835602 | -0.057539049 | 0.214559407 | -0.035221958 |
| -0.076776868 | 0.097486641 | -0.092290907 | 0.046224847 | -0.100101798 |
| -0.152349412 | 0.000957332 | -0.145300321 | 0.001642373 | -0.171000576 |
| 0.045104271 | 0.330754932 | 0.014154963 | 0.760300005 | 0.016785236 |
| 0.127038656 | 0.005975592 | 0.115420479 | 0.012563102 | 0.119711073 |
| 0.067102023 | 0.147663753 | 0.116486354 | 0.011764645 | 0.135891827 |
| -0.015359475 | 0.740605916 | -0.01010624 | 0.82757095 | -0.012362372 |
| 0.180493398 | 8.77E-05 | 0.203080509 | 9.73E-06 | 0.190156899 |
| -0.017069165 | 0.712943301 | 0.013748267 | 0.766985448 | -0.007145106 |
| 0.207873886 | 5.91E-06 | 0.18854657 | 4.12E-05 | 0.210194337 |
| -0.023510538 | 0.61231236 | -0.000315173 | 0.994580259 | 0.029255899 |
| -0.151606589 | 0.001014472 | -0.1452878 | 0.001643914 | -0.180113447 |
| -0.057661119 | 0.213586832 | -0.023138097 | 0.617958867 | -0.010995786 |
| 0.040818498 | 0.378806191 | 0.042705783 | 0.357140945 | 0.020358589 |
| 0.162804079 | 0.000411819 | 0.138702028 | 0.002665647 | 0.090234106 |
| 0.119872387 | 0.00951767 | 0.150225811 | 0.001129126 | 0.187872137 |
| 0.075784067 | 0.101908374 | 0.051613989 | 0.265648044 | 0.054778459 |
| -0.091763512 | 0.047490991 | -0.102168585 | 0.027262071 | -0.151893806 |
| 0.083160277 | 0.07258943 | 0.089190771 | 0.054091442 | 0.039346209 |
| 0.126876497 | 0.006040404 | 0.113535163 | 0.014093058 | 0.134738495 |

| | | | | |
|---|---|---|---|---|
| 0.055431755 | 0.231852898 | 0.087395869 | 0.059134569 | 0.065591041 |
| -0.16973316 | 0.000228787 | -0.105332642 | 0.022816491 | -0.118317873 |
| 0.08221907 | 0.075895627 | 0.094703799 | 0.040787727 | 0.08276393 |
| 0.107325549 | 0.020350986 | 0.125388773 | 0.006665138 | 0.080666684 |
| -0.191197698 | 3.19E-05 | -0.158687128 | 0.000577642 | -0.191642215 |
| -0.088502434 | 0.05598147 | -0.116062355 | 0.012076729 | -0.087414633 |
| -0.028741327 | 0.535541713 | -0.062956194 | 0.174401376 | -0.08064438 |
| 0.042635078 | 0.357938307 | 0.072451881 | 0.117921455 | 0.101430744 |
| 0.033215813 | 0.473945832 | 0.039479855 | 0.394651418 | 0.029811967 |
| 0.071690457 | 0.121843201 | 0.079091291 | 0.087771506 | 0.083340532 |
| -0.02558257 | 0.581323402 | -0.052609363 | 0.256527455 | -0.079076918 |
| -0.174632107 | 0.000148897 | -0.196156542 | 1.96E-05 | -0.179314038 |
| 0.093756773 | 0.042853628 | 0.063370694 | 0.171576218 | 0.086342267 |
| 0.096229521 | 0.037636579 | 0.108798735 | 0.01868057 | 0.100840917 |
| 0.025290604 | 0.585645475 | 0.057911685 | 0.211600431 | 0.08761265 |
| 0.201785208 | 1.11E-05 | 0.212806011 | 3.49E-06 | 0.178912375 |
| 0.079744043 | 0.085176949 | 0.08511578 | 0.066095667 | 0.101254711 |
| 0.064269677 | 0.165566461 | 0.037142696 | 0.423255718 | 0.046572361 |
| 0.057272349 | 0.216695354 | 0.037483708 | 0.419008289 | 0.092736223 |
| 0.084162518 | 0.06919901 | 0.08324369 | 0.072302181 | 0.083370385 |
| 0.071775126 | 0.121402154 | 0.065526121 | 0.157433478 | 0.066596302 |
| 0.032822786 | 0.479196072 | 0.05585582 | 0.228295844 | 0.052273639 |
| -0.242207669 | 1.16E-07 | -0.218651089 | 1.84E-06 | -0.244598318 |
| 0.143955735 | 0.001815692 | 0.149565731 | 0.001188052 | 0.150734185 |
| -0.006087934 | 0.895609185 | 0.011517699 | 0.803948118 | 0.020695967 |
| 0.10249927 | 0.026764986 | 0.110106144 | 0.017299564 | 0.095432875 |
| 0.143794668 | 0.001837541 | 0.147410312 | 0.001400757 | 0.15216869 |
| 0.09157009 | 0.047962574 | 0.114500462 | 0.013290365 | 0.112054974 |
| -0.051505973 | 0.26665102 | -0.003650955 | 0.937281879 | 0.010140543 |
| 0.203727359 | 9.10E-06 | 0.200078887 | 1.32E-05 | 0.205722757 |
| 0.059431529 | 0.199836442 | 0.068715393 | 0.138147332 | 0.069886391 |
| -0.022559739 | 0.626771898 | -0.01337362 | 0.773159411 | -0.044829722 |
| 0.117453489 | 0.011079326 | 0.113881208 | 0.01380053 | 0.114324628 |
| 0.093473687 | 0.043488029 | 0.064289468 | 0.165435956 | 0.05106623 |
| 0.069482535 | 0.133790964 | 0.073107344 | 0.114625144 | 0.074588208 |
| -0.091722377 | 0.047590956 | -0.083781822 | 0.070471249 | -0.080808068 |
| -0.025300419 | 0.585499953 | -0.005300541 | 0.909047947 | -0.026265344 |
| 0.168777884 | 0.000248439 | 0.190768328 | 3.33E-05 | 0.196987508 |
| 0.197868975 | 1.65E-05 | 0.173057282 | 0.000171161 | 0.168559626 |
| 0.092785062 | 0.045064335 | 0.109192996 | 0.018254328 | 0.108827284 |
| 0.244766626 | 8.46E-08 | 0.258252472 | 1.49E-08 | 0.263909848 |
| 0.067561045 | 0.144906772 | 0.070839876 | 0.126343541 | 0.00451969 |
| -0.082447158 | 0.075083381 | -0.021970079 | 0.635812135 | -0.01894427 |
| -0.228318954 | 6.14E-07 | -0.203193242 | 9.62E-06 | -0.232907752 |
| 0.149838637 | 0.001163354 | 0.219205259 | 1.73E-06 | 0.246717217 |

| | | | | |
|---|---|---|---|---|
| -0.011789848 | 0.799413056 | 0.011419292 | 0.805589598 | -0.009869793 |
| -0.060023752 | 0.195383816 | -0.022909855 | 0.621430376 | -0.018830911 |
| 0.089758405 | 0.052573054 | 0.102121659 | 0.027333248 | 0.089372563 |
| -0.132868145 | 0.00402274 | -0.136311344 | 0.00316125 | -0.129548762 |
| 0.089265083 | 0.053890609 | 0.087978694 | 0.057455998 | 0.082680649 |
| -0.01943535 | 0.675277921 | -0.03645477 | 0.431900152 | -0.015901565 |
| -0.037121084 | 0.423525747 | -0.07227372 | 0.118830115 | -0.047932291 |
| 0.044912962 | 0.332812205 | 0.044054182 | 0.342148431 | 0.039365666 |
| -0.095788971 | 0.038524529 | -0.09721056 | 0.035721444 | -0.10261758 |
| -0.036027075 | 0.437325543 | -0.064955361 | 0.161089807 | -0.057692269 |
| -0.166941073 | 0.000290736 | -0.191491798 | 3.10E-05 | -0.161937125 |
| -0.035745428 | 0.440919543 | -0.028415433 | 0.540180061 | -0.003464508 |
| 0.201738483 | 1.12E-05 | 0.19273839 | 2.75E-05 | 0.171953959 |
| -0.072132644 | 0.11955351 | -0.073364216 | 0.113353227 | -0.083524781 |
| 0.042026894 | 0.364843067 | 0.073858004 | 0.110939355 | 0.047516373 |
| 0.203938759 | 8.91E-06 | 0.161059476 | 0.000475781 | 0.16697778 |
| -0.037824144 | 0.414793066 | -0.03422946 | 0.460551657 | -0.030103751 |
| 0.008350605 | 0.857170148 | 0.031741559 | 0.493801515 | 0.008673987 |
| 0.314640183 | 3.43E-12 | 0.349536511 | 7.25E-15 | 0.369738876 |
| 0.002111664 | 0.96369985 | -0.045216862 | 0.329548008 | -0.027547032 |
| -0.046102026 | 0.320158665 | -0.04748657 | 0.305825322 | -0.042613419 |
| -0.262006958 | 9.05E-09 | -0.231735323 | 4.12E-07 | -0.242293579 |
| 0.005559734 | 0.904621138 | 0.043146328 | 0.352197909 | 0.050052729 |
| 0.207435505 | 6.19E-06 | 0.192843606 | 2.72E-05 | 0.215215322 |
| 0.044790291 | 0.334135687 | 0.041189019 | 0.37449034 | 0.024082703 |
| 0.245046249 | 8.17E-08 | 0.237253764 | 2.13E-07 | 0.2547493 |
| 0.156093627 | 0.000711931 | 0.135064221 | 0.003451751 | 0.108443508 |
| 0.194435196 | 2.33E-05 | 0.224567607 | 9.45E-07 | 0.224763445 |
| -0.026044912 | 0.574509772 | 0.009041576 | 0.845494369 | 0.004641745 |
| -0.064861857 | 0.161694851 | -0.047928573 | 0.301340258 | -0.032281574 |
| 0.020048107 | 0.665650114 | 0.037601254 | 0.41755002 | 0.070518853 |
| -0.033776305 | 0.466513336 | -0.02825315 | 0.542497222 | -0.044844242 |
| -0.161507626 | 0.000458522 | -0.168934571 | 0.000245111 | -0.168877338 |
| 0.014967079 | 0.747003815 | 0.033805752 | 0.466124645 | 0.019910985 |
| -0.078124286 | 0.091731539 | -0.090624592 | 0.050324613 | -0.104496115 |
| 0.1039454 | 0.024681776 | 0.087074016 | 0.060078781 | 0.086154306 |
| 0.123246393 | 0.007666571 | 0.106281192 | 0.021612478 | 0.108350679 |
| 0.062977728 | 0.174253761 | 0.085651409 | 0.06440269 | 0.129900187 |
| 0.035019734 | 0.450257202 | 0.036278794 | 0.434127716 | 0.029459903 |
| 0.097870108 | 0.034481017 | 0.100601733 | 0.029726716 | 0.144165159 |
| 0.236158596 | 2.43E-07 | 0.251805435 | 3.47E-08 | 0.25827855 |
| 0.046485821 | 0.3161423 | 0.067860841 | 0.143127465 | 0.06033138 |
| -0.016699664 | 0.718891452 | 0.012392799 | 0.789389708 | -0.004374054 |
| -0.088369186 | 0.0563536 | -0.110129368 | 0.017275861 | -0.138365216 |
| 0.135995522 | 0.003232637 | 0.117617506 | 0.010966675 | 0.143712852 |

| | | | | |
|---|---|---|---|---|
| 0.253151617 | 2.91E-08 | 0.295612035 | 7.16E-11 | 0.329605794 |
| 0.156672249 | 0.00067968 | 0.179518325 | 9.59E-05 | 0.182706805 |
| -0.042218591 | 0.362657827 | -0.003987713 | 0.931510461 | 0.010663799 |
| 0.210080008 | 4.67E-06 | 0.234201666 | 3.07E-07 | 0.187781725 |
| -0.150410668 | 0.001113113 | -0.152588135 | 0.000939609 | -0.175343962 |
| 0.249101557 | 4.90E-08 | 0.236067802 | 2.46E-07 | 0.227356293 |
| 0.208456313 | 5.55E-06 | 0.240238527 | 1.48E-07 | 0.214107621 |
| 0.068272057 | 0.140714117 | 0.077486378 | 0.094421317 | 0.091285438 |
| 0.027665152 | 0.55093413 | 0.036206104 | 0.435049784 | 0.039006362 |
| -0.181592158 | 7.93E-05 | -0.222881873 | 1.14E-06 | -0.236180704 |
| 0.047105109 | 0.309731355 | 0.060895832 | 0.188959737 | 0.079621464 |
| -0.019998702 | 0.666424375 | -0.065908729 | 0.155017787 | -0.073052113 |
| -0.135128247 | 0.003436267 | -0.140184206 | 0.002395059 | -0.106772223 |
| -0.031237362 | 0.500692621 | -0.036782609 | 0.427767895 | -0.055258648 |
| 0.205719199 | 7.40E-06 | 0.24985426 | 4.45E-08 | 0.267605081 |
| 0.091458453 | 0.048236539 | 0.112153752 | 0.015315876 | 0.096230915 |
| -0.011581132 | 0.802890485 | 0.032195921 | 0.487635132 | 0.007977387 |
| 0.063022804 | 0.173945056 | 0.084350919 | 0.068576389 | 0.067103619 |
| -0.150989553 | 0.001064312 | -0.197658322 | 1.69E-05 | -0.221836659 |
| 0.082607284 | 0.074517395 | 0.084526825 | 0.067999211 | 0.086719965 |
| 0.003715101 | 0.936182276 | 0.04037075 | 0.384062116 | 0.080350051 |
| -0.082136448 | 0.076191609 | -0.051529837 | 0.266429209 | -0.086176486 |
| 0.128557157 | 0.005398313 | 0.13605327 | 0.003219476 | 0.14824416 |
| -0.040831594 | 0.378653125 | 0.012501664 | 0.7875836 | -0.024625275 |
| 0.014283185 | 0.758195912 | 0.026391234 | 0.569430753 | 0.019615507 |
| 0.004061329 | 0.930249276 | 0.018960256 | 0.682779459 | 0.016725224 |
| -0.061729275 | 0.182966638 | -0.064320485 | 0.165231585 | -0.057511647 |
| 0.057176149 | 0.217469529 | 0.048015828 | 0.30046005 | 0.083533115 |
| -0.022370018 | 0.62967455 | 0.013808347 | 0.765996728 | -0.00604818 |
| 0.071560174 | 0.122524297 | 0.081078732 | 0.08006429 | 0.062517808 |
| 0.410930462 | 1.88E-20 | 0.404218813 | 8.77E-20 | 0.362402143 |
| -0.079405418 | 0.086515089 | -0.089725737 | 0.052659464 | -0.086847397 |
| -0.101590969 | 0.028149399 | -0.071794341 | 0.121302231 | -0.096261138 |
| -0.12499285 | 0.006840869 | -0.15527266 | 0.000760127 | -0.18365078 |
| -0.02902152 | 0.531569875 | -0.055902476 | 0.227906869 | -0.047559411 |
| 0.244536903 | 8.71E-08 | 0.218802054 | 1.81E-06 | 0.208489228 |
| -0.052146031 | 0.260745638 | -0.045143863 | 0.330330201 | -0.070273205 |
| 0.078527023 | 0.090065216 | 0.051883333 | 0.263158375 | 0.088365436 |
| 0.170972945 | 0.000205447 | 0.196727956 | 1.85E-05 | 0.200805432 |
| 0.128474244 | 0.005428489 | 0.133319012 | 0.003898977 | 0.129302526 |
| 0.205475191 | 7.59E-06 | 0.214994918 | 2.75E-06 | 0.187296887 |
| 0.050227454 | 0.278719938 | 0.04511563 | 0.330633043 | 0.052991896 |
| 0.061885833 | 0.181856707 | 0.066102798 | 0.153803256 | 0.048660943 |
| 0.234835371 | 2.85E-07 | 0.26413092 | 6.80E-09 | 0.279317884 |
| 0.045236441 | 0.329338417 | 0.080609444 | 0.081832847 | 0.090759664 |

| | | | | |
|---|---|---|---|---|
| 0.127628559 | 0.005745052 | 0.105228737 | 0.022951846 | 0.067622216 |
| -0.063719679 | 0.16922419 | -0.062213027 | 0.17955313 | -0.071512638 |
| 0.087692398 | 0.058275546 | 0.087356266 | 0.059250085 | 0.116099059 |
| 0.14715984 | 0.001427624 | 0.159645365 | 0.000534278 | 0.156301399 |
| -0.023967743 | 0.605412055 | -0.02897236 | 0.532265663 | -0.034139492 |
| 0.079592827 | 0.085772432 | 0.100056096 | 0.030628707 | 0.10898706 |
| 0.114187043 | 0.013546456 | 0.117645714 | 0.010947404 | 0.122988445 |
| 0.062463676 | 0.177803134 | 0.107588507 | 0.020043632 | 0.136397056 |
| 0.049358671 | 0.287129323 | 0.045357882 | 0.328040353 | 0.035255735 |
| -0.25429929 | 2.51E-08 | -0.249588809 | 4.60E-08 | -0.242988516 |
| -0.144773318 | 0.001708411 | -0.175788024 | 0.000134317 | -0.18701259 |
| 0.206148078 | 7.08E-06 | 0.207304806 | 6.27E-06 | 0.201928575 |
| -0.063624951 | 0.169860223 | -0.042224071 | 0.362595478 | -0.043404665 |
| 0.020338317 | 0.661109231 | -0.003173264 | 0.94547422 | 0.060773049 |
| 0.06578336 | 0.155806227 | 0.114758053 | 0.013083093 | 0.129568741 |
| 0.008485369 | 0.854890423 | 0.058166799 | 0.20959169 | 0.091751396 |
| 0.126173032 | 0.006328939 | 0.163655547 | 0.000383598 | 0.196363333 |
| 0.155190109 | 0.000765137 | 0.222925442 | 1.14E-06 | 0.193746162 |
| -0.237297202 | 2.12E-07 | -0.249223778 | 4.82E-08 | -0.273328144 |
| 0.178367455 | 0.000106474 | 0.149974212 | 0.001151261 | 0.167861206 |
| 0.029376961 | 0.526552873 | 0.035188157 | 0.448080163 | 0.069754913 |
| 0.039457785 | 0.394915964 | 0.048089545 | 0.299717751 | 0.063996798 |
| 0.256748936 | 1.82E-08 | 0.251358269 | 3.67E-08 | 0.24972817 |
| 0.12532507 | 0.006693136 | 0.150807892 | 0.001079409 | 0.149411322 |
| -0.016044241 | 0.729483964 | -0.03733743 | 0.42082717 | -0.046868166 |
| 0.115320009 | 0.012640776 | 0.125110966 | 0.006788014 | 0.101998555 |
| -0.081929154 | 0.076938347 | -0.090297121 | 0.051165031 | -0.078992489 |
| -0.118742917 | 0.010220709 | -0.109206526 | 0.018239852 | -0.140125571 |
| 0.237867958 | 1.98E-07 | 0.257143049 | 1.73E-08 | 0.298677363 |
| -0.017996769 | 0.698088105 | 0.010806306 | 0.815833458 | 0.026736959 |
| 0.100079869 | 0.030588924 | 0.11574812 | 0.012312718 | 0.118606075 |
| 0.078804723 | 0.088930446 | 0.077844641 | 0.092903025 | 0.066848408 |
| 0.062872605 | 0.174975281 | 0.082119475 | 0.07625253 | 0.070815432 |
| 0.074930555 | 0.105835648 | 0.069349466 | 0.134538934 | 0.055808557 |
| 0.124954761 | 0.006857992 | 0.167086408 | 0.000287159 | 0.172661233 |
| 0.128684087 | 0.00535241 | 0.174713866 | 0.000147819 | 0.209319325 |
| 0.111666352 | 0.015769023 | 0.089049336 | 0.054475398 | 0.066444478 |
| 0.009552947 | 0.83687499 | 0.007014283 | 0.879837226 | 0.024930027 |
| 0.096640222 | 0.036824485 | 0.142788739 | 0.001979534 | 0.144890656 |
| 0.243339905 | 1.01E-07 | 0.248378118 | 5.37E-08 | 0.242336624 |
| 0.055713356 | 0.229486485 | 0.077500239 | 0.094362209 | 0.015555125 |
| 0.146504237 | 0.001500203 | 0.119950389 | 0.009470743 | 0.137698355 |
| 0.176270992 | 0.000128632 | 0.19119695 | 3.19E-05 | 0.191236404 |
| 0.094226906 | 0.041817286 | 0.073824311 | 0.111102765 | 0.104054186 |
| 0.166981689 | 0.000289732 | 0.195815904 | 2.03E-05 | 0.17926021 |

| | | | | |
|---|---|---|---|---|
| 0.134955104 | 0.003478287 | 0.157778125 | 0.000621775 | 0.207433291 |
| 0.332851459 | 1.52E-13 | 0.34521441 | 1.62E-14 | 0.318187835 |
| 0.055036845 | 0.235200514 | 0.045875536 | 0.322544387 | 0.02045389 |
| -0.201595002 | 1.13E-05 | -0.180453418 | 8.80E-05 | -0.198448066 |
| -0.055403704 | 0.232089565 | -0.053793993 | 0.245958109 | -0.020697126 |
| -0.01498838 | 0.746656067 | 0.013368365 | 0.773246109 | -0.022184605 |
| 0.002686127 | 0.953834472 | 0.029256421 | 0.528251573 | 0.010138756 |
| -0.097056868 | 0.036015885 | -0.065301777 | 0.158863095 | -0.086226718 |
| 0.301319435 | 2.95E-11 | 0.331141907 | 2.06E-13 | 0.353380332 |
| 0.056182039 | 0.225586005 | 0.056461242 | 0.223284973 | 0.040149954 |
| -0.063701663 | 0.169345017 | -0.063386192 | 0.171471248 | -0.062599642 |
| 0.082156927 | 0.076118157 | 0.083457508 | 0.071570095 | 0.077746054 |
| -0.035221141 | 0.447654516 | -0.004394605 | 0.924541863 | -0.0172932 |
| -0.058316322 | 0.208420777 | -0.092503267 | 0.045723085 | -0.083356378 |
| 0.09789611 | 0.034432875 | 0.093296258 | 0.043889668 | 0.101718515 |
| 0.072784902 | 0.116237553 | 0.083686068 | 0.070794239 | 0.077090848 |
| 0.02603791 | 0.574612674 | 0.01743991 | 0.70699257 | 0.013961668 |
| 0.006519291 | 0.888259424 | 0.032554208 | 0.48280199 | 0.034229315 |
| -0.103146281 | 0.025814935 | -0.088553622 | 0.055839055 | -0.087204042 |
| 0.05966873 | 0.198044261 | 0.049659919 | 0.284194266 | -0.000982871 |
| -0.041516461 | 0.370701615 | -0.071711616 | 0.121732867 | -0.097657808 |
| 0.021998765 | 0.635371082 | 0.02210303 | 0.633769034 | 0.001859158 |
| 0.033201685 | 0.474134012 | 0.03031552 | 0.513422111 | 0.04147521 |
| -0.136519002 | 0.003115096 | -0.14241933 | 0.00203415 | -0.153382562 |
| -0.140685725 | 0.002309337 | -0.179703513 | 9.43E-05 | -0.190209161 |
| 0.029801613 | 0.520590745 | -0.000843886 | 0.985489142 | -0.019768403 |
| -0.039226566 | 0.39769389 | -0.010855444 | 0.815011119 | -0.021343377 |
| -0.054422378 | 0.240476855 | -0.014839149 | 0.749093468 | -0.056670899 |
| -0.089566993 | 0.053081051 | -0.052599084 | 0.256620515 | -0.080818623 |
| -0.235946581 | 2.49E-07 | -0.243220758 | 1.03E-07 | -0.268057934 |
| 0.084489757 | 0.068120506 | 0.091128161 | 0.04905478 | 0.140975347 |
| 0.076991405 | 0.096551519 | 0.073411186 | 0.113121857 | 0.095111209 |
| 0.113186176 | 0.014393583 | 0.141673779 | 0.002148597 | 0.172837866 |
| 0.07152507 | 0.122708324 | 0.065981171 | 0.154563582 | 0.063528418 |
| -0.057995501 | 0.210938953 | -0.066991649 | 0.148332607 | -0.067791163 |
| -0.108426642 | 0.019090779 | -0.097529052 | 0.03511779 | -0.123920153 |
| 0.107266693 | 0.020420339 | 0.131109011 | 0.00454034 | 0.131888992 |
| 0.108078664 | 0.019481486 | 0.115268267 | 0.012680944 | 0.071102662 |
| 0.098642851 | 0.033074545 | 0.093212681 | 0.044079935 | 0.10028748 |
| -0.22232188 | 1.22E-06 | -0.191516693 | 3.10E-05 | -0.205732806 |
| 0.094636218 | 0.040932313 | 0.096572667 | 0.036957032 | 0.047694143 |
| 0.156988439 | 0.000662634 | 0.161855186 | 0.000445541 | 0.164390237 |
| 0.008879682 | 0.848227057 | 0.026949261 | 0.561292198 | 0.011555426 |
| 0.064555882 | 0.163686691 | 0.054591895 | 0.239013004 | 0.089369425 |
| 0.026656631 | 0.565553072 | 0.044130869 | 0.341308009 | 0.033851836 |

| | | | | |
|---|---|---|---|---|
| 0.07681777 | 0.097307802 | 0.113545392 | 0.014084333 | 0.107918368 |
| 0.047496869 | 0.305720324 | 0.057145457 | 0.217716942 | 0.089133614 |
| -0.187354696 | 4.62E-05 | -0.178501109 | 0.000105191 | -0.186374096 |
| -0.072887671 | 0.115721727 | -0.065249148 | 0.159199879 | -0.043746333 |
| -0.090239047 | 0.051315287 | -0.071018518 | 0.125387818 | -0.056660279 |
| -0.06576695 | 0.155909649 | -0.085784482 | 0.06398766 | -0.122364004 |
| 0.17123823 | 0.000200751 | 0.165600045 | 0.000325765 | 0.172764704 |
| 0.117451584 | 0.01108064 | 0.118798403 | 0.010185124 | 0.119058686 |
| 0.085436118 | 0.065078828 | 0.086284491 | 0.062447861 | 0.08728873 |
| 0.140317916 | 0.002371925 | 0.171290452 | 0.000199838 | 0.145546269 |
| -0.018170723 | 0.695314885 | -0.002316609 | 0.960179562 | 0.053240491 |
| 0.094658705 | 0.040884154 | 0.137162975 | 0.002975832 | 0.11550009 |
| -0.13279624 | 0.004042803 | -0.162115165 | 0.000436055 | -0.197701532 |
| 0.107390253 | 0.02027498 | 0.114128123 | 0.013595081 | 0.155606566 |
| 0.045776546 | 0.323590712 | 0.056244558 | 0.225069298 | 0.068782204 |
| 0.126755778 | 0.006089063 | 0.123099152 | 0.007740096 | 0.120165602 |
| -0.038996017 | 0.400475417 | -0.024820718 | 0.592632427 | -0.060599074 |
| 0.098457382 | 0.033407575 | 0.097853481 | 0.034511832 | 0.093405199 |
| -0.076851696 | 0.097159665 | -0.082129247 | 0.076217449 | -0.088845341 |
| -0.132022852 | 0.004264394 | -0.134311 | 0.003638729 | -0.147929451 |
| -0.000529532 | 0.990894245 | 0.018727819 | 0.686461004 | 0.013234182 |
| 0.143141079 | 0.001928695 | 0.197035343 | 1.80E-05 | 0.168442962 |
| 0.047581688 | 0.304856445 | 0.030536418 | 0.510356554 | 0.014437398 |
| 0.004144866 | 0.928818345 | -0.005202016 | 0.910731429 | -0.015781256 |
| 0.125095565 | 0.006794885 | 0.152272739 | 0.000963089 | 0.136643429 |
| -0.010521106 | 0.82061039 | -0.005354605 | 0.908124339 | -0.042392664 |
| -0.107643912 | 0.01997939 | -0.099783315 | 0.031088356 | -0.119252465 |
| 5.28E-05 | 0.999091759 | 0.002915803 | 0.949892088 | -0.010512741 |
| -0.087058517 | 0.060124563 | -0.061610098 | 0.183814904 | -0.066359349 |
| 0.086808723 | 0.060866404 | 0.124865185 | 0.006898412 | 0.121130071 |
| -0.013868977 | 0.764999332 | -0.027143165 | 0.558477396 | -0.065471413 |
| 0.153459573 | 0.000877454 | 0.144121305 | 0.001793481 | 0.137371 |
| 0.011958432 | 0.796607143 | -6.82E-05 | 0.998827874 | -0.006856148 |
| 0.0043398 | 0.925480147 | -0.003004459 | 0.948370623 | -0.01024952 |
| 0.020787345 | 0.654107827 | 0.023197066 | 0.617063337 | 0.03411376 |
| 0.082074601 | 0.07641378 | 0.102479475 | 0.026794521 | 0.097616099 |
| 0.095644875 | 0.038818785 | 0.103421542 | 0.025419649 | 0.098191926 |
| -0.065325335 | 0.158712515 | -0.084015519 | 0.069688017 | -0.090679366 |
| -0.012161732 | 0.79322692 | -0.036195526 | 0.435184049 | -0.004967858 |
| 0.111001596 | 0.016405989 | 0.110487621 | 0.016913812 | 0.098516816 |
| 0.237812759 | 1.99E-07 | 0.281677242 | 5.77E-10 | 0.273135186 |
| 0.013958958 | 0.763519824 | 0.021627909 | 0.641083056 | 0.037677901 |
| 0.361160641 | 7.79E-16 | 0.352211593 | 4.37E-15 | 0.390883365 |
| 0.365931267 | 3.04E-16 | 0.372260023 | 8.50E-17 | 0.404700149 |
| 0.134408188 | 0.003614098 | 0.127092299 | 0.005954289 | 0.104678902 |

| | | | | |
|---|---|---|---|---|
| 0.120123885 | 0.009367104 | 0.125416844 | 0.006652833 | 0.169427735 |
| 0.049002594 | 0.290624787 | 0.04415822 | 0.341008581 | 0.06702259 |
| 0.250485673 | 4.11E-08 | 0.277978153 | 9.84E-10 | 0.290060514 |
| 0.191119634 | 3.22E-05 | 0.208895062 | 5.30E-06 | 0.218808603 |
| -0.037974211 | 0.412942918 | -0.047482075 | 0.305871161 | -0.061024708 |
| 0.200459454 | 1.27E-05 | 0.165492135 | 0.000328748 | 0.18735686 |
| 0.050445684 | 0.276634106 | 0.041911359 | 0.366164064 | 0.070541283 |
| -0.285594963 | 3.24E-10 | -0.268260343 | 3.86E-09 | -0.30451085 |
| 0.081663496 | 0.077904008 | 0.073069552 | 0.114813217 | 0.083104342 |
| 0.048642632 | 0.294187283 | 0.044125308 | 0.341368906 | 0.045197956 |
| 0.074943901 | 0.105773333 | 0.056246284 | 0.225055045 | 0.053309552 |
| 0.020630947 | 0.656543039 | 0.025074014 | 0.588861322 | 0.047202572 |
| -0.035949019 | 0.438319896 | -0.042686574 | 0.357357459 | -0.023990483 |
| 0.091807771 | 0.047383631 | 0.061142652 | 0.187170067 | 0.076969837 |
| 0.18306728 | 6.91E-05 | 0.199570928 | 1.39E-05 | 0.195966946 |
| 0.099669966 | 0.031281084 | 0.104653786 | 0.023713449 | 0.086521488 |
| -0.002799308 | 0.951891567 | -0.015223353 | 0.742823348 | 0.006731125 |
| -0.175234176 | 0.000141127 | -0.173574389 | 0.000163527 | -0.14458212 |
| -0.085928556 | 0.063540806 | -0.086719354 | 0.061133642 | -0.120964246 |
| 0.114057541 | 0.013653533 | 0.147872823 | 0.001352365 | 0.145176365 |
| -0.000562206 | 0.990332424 | 0.024485214 | 0.597644378 | 0.026193857 |
| -0.08592527 | 0.063550969 | -0.114824099 | 0.013030411 | -0.118318072 |
| 0.109829038 | 0.017584576 | 0.085772258 | 0.064025694 | 0.105264897 |
| -0.0605658 | 0.19137237 | -0.063583766 | 0.170137309 | -0.073371953 |
| -0.097456366 | 0.035254785 | -0.106178153 | 0.021740542 | -0.082835248 |
| 0.264040591 | 6.88E-09 | 0.25448293 | 2.45E-08 | 0.285745024 |
| -0.14329546 | 0.001906798 | -0.114876156 | 0.012989018 | -0.102852274 |
| 0.152854164 | 0.000920217 | 0.179157793 | 9.91E-05 | 0.199890587 |
| 0.098462786 | 0.033397831 | 0.088981116 | 0.054661406 | 0.097937194 |
| 0.20818758 | 5.71E-06 | 0.187945709 | 4.37E-05 | 0.241124386 |
| -0.144399482 | 0.001756722 | -0.121476332 | 0.008593178 | -0.112257605 |
| 0.193297208 | 2.60E-05 | 0.201583784 | 1.13E-05 | 0.208870942 |
| 0.169094334 | 0.000241761 | 0.197085151 | 1.79E-05 | 0.250600461 |
| -0.073078036 | 0.114770976 | -0.044890022 | 0.333059449 | -0.012715989 |
| 0.187720627 | 4.46E-05 | 0.219108977 | 1.75E-06 | 0.209173382 |
| 0.03152338 | 0.496777253 | 0.029489805 | 0.524965158 | 0.043177451 |
| 0.153188154 | 0.000896393 | 0.161553424 | 0.000456792 | 0.163841999 |
| 0.087767075 | 0.058060849 | 0.104882152 | 0.023408369 | 0.042659221 |
| 0.193813903 | 2.47E-05 | 0.188817739 | 4.02E-05 | 0.189659324 |
| 0.109411103 | 0.018022195 | 0.09943232 | 0.031688473 | 0.060548932 |
| 0.030744001 | 0.507484507 | 0.032225544 | 0.487234537 | 0.049013989 |
| 0.275408295 | 1.42E-09 | 0.288069389 | 2.25E-10 | 0.264437445 |
| -0.15736567 | 0.000642815 | -0.133710227 | 0.003794393 | -0.126955826 |
| 0.126085183 | 0.006365827 | 0.135285029 | 0.003398615 | 0.12268346 |
| 0.054032265 | 0.243869533 | 0.02110855 | 0.649118 | 0.044386577 |

| | | | | |
|---|---|---|---|---|
| -0.004566372 | 0.921601824 | 0.009959068 | 0.830043456 | 0.012070754 |
| -0.162791574 | 0.000412247 | -0.160217551 | 0.000509847 | -0.19241891 |
| 0.01501572 | 0.746209789 | -0.011593037 | 0.802692036 | -0.003356761 |
| 0.111380941 | 0.016039797 | 0.137289081 | 0.002949234 | 0.133185897 |
| 0.030248952 | 0.514347789 | 0.05149115 | 0.266788859 | -0.001528163 |
| -0.074783594 | 0.106523778 | -0.099137328 | 0.032200468 | -0.145149577 |
| 0.059359179 | 0.200385427 | 0.056316354 | 0.224476964 | 0.108177363 |
| 0.087401189 | 0.059119066 | 0.088267025 | 0.056640301 | 0.03581593 |
| -0.133937892 | 0.003734708 | -0.174648066 | 0.000148686 | -0.177972879 |
| -0.019906887 | 0.667864215 | 0.008272948 | 0.858484366 | -0.015745778 |
| 0.017425576 | 0.707222313 | 0.005919044 | 0.89848932 | 0.00334131 |
| -0.02981743 | 0.520369338 | -0.045289157 | 0.328774538 | -0.036556645 |
| 0.043373647 | 0.349664286 | 0.057728258 | 0.213053266 | 0.044117288 |
| 0.050781911 | 0.273441256 | 0.025618736 | 0.580789052 | 0.05055384 |
| 0.250003689 | 4.37E-08 | 0.188870037 | 4.00E-05 | 0.184583543 |
| -0.105336878 | 0.022810988 | -0.082092663 | 0.076348844 | -0.119454877 |
| 0.094588572 | 0.041034507 | 0.04830896 | 0.297515546 | 0.0250654 |
| -0.018410756 | 0.691494896 | -0.016022821 | 0.729831036 | -0.014360612 |
| -0.004380376 | 0.924785464 | -0.010091687 | 0.827815368 | -0.013872199 |
| 0.13858643 | 0.002687879 | 0.152491688 | 0.000946733 | 0.148082039 |
| -0.049796223 | 0.282872933 | -0.106387567 | 0.021480956 | -0.136777171 |
| -0.072296764 | 0.118712278 | -0.078595674 | 0.089783612 | -0.089927845 |
| 0.039624513 | 0.392920123 | 0.038266062 | 0.409358734 | 0.047111481 |
| 0.205489852 | 7.58E-06 | 0.161926096 | 0.000442934 | 0.154609718 |
| 0.277631061 | 1.03E-09 | 0.28798007 | 2.28E-10 | 0.301218012 |
| 0.049815814 | 0.282683363 | 0.044828881 | 0.333718987 | 0.048896584 |
| 0.052316857 | 0.259184901 | 0.052538548 | 0.257169073 | 0.037546039 |
| 0.013021697 | 0.778971954 | -0.022195153 | 0.632354982 | 0.016331558 |
| 0.284456221 | 3.84E-10 | 0.328262086 | 3.40E-13 | 0.330933938 |
| -0.121196031 | 0.008748729 | -0.145656717 | 0.001599049 | -0.162266835 |
| 0.144189636 | 0.001784387 | 0.162390875 | 0.0004262 | 0.193131749 |
| -0.111524469 | 0.015903125 | -0.138191249 | 0.002765163 | -0.138594183 |
| -0.210475095 | 4.48E-06 | -0.222279319 | 1.23E-06 | -0.234049687 |
| 0.249576708 | 4.61E-08 | 0.257488572 | 1.65E-08 | 0.257336256 |
| 0.00907287 | 0.844966343 | 0.020753728 | 0.65463097 | 0.021218739 |
| -0.335119603 | 1.02E-13 | -0.347301614 | 1.10E-14 | -0.348227531 |
| -0.135932704 | 0.00324701 | -0.118867757 | 0.0101408 | -0.126418972 |
| -0.278749095 | 8.81E-10 | -0.257520192 | 1.65E-08 | -0.256645991 |
| 0.050473222 | 0.276371649 | 0.053977041 | 0.244352485 | 0.048845773 |
| 0.106857555 | 0.02090814 | 0.12190947 | 0.008357655 | 0.108322662 |
| 0.260325767 | 1.13E-08 | 0.282093282 | 5.43E-10 | 0.294142642 |
| -0.071645406 | 0.122078386 | -0.083351593 | 0.071931974 | -0.085811533 |
| 0.268218131 | 3.89E-09 | 0.244227181 | 9.05E-08 | 0.255670485 |
| -0.058571967 | 0.20642979 | -0.056884929 | 0.219825229 | -0.08923551 |
| 0.082976934 | 0.07322408 | 0.144734579 | 0.00171336 | 0.143103015 |

| | | | | |
|---|---|---|---|---|
| 6.28E-05 | 0.998920074 | 0.032766188 | 0.479954736 | -0.019602188 |
| -0.21164372 | 3.95E-06 | -0.146100933 | 0.001546518 | -0.129448524 |
| 0.078459875 | 0.090341341 | 0.072764436 | 0.116340494 | 0.064236996 |
| 0.135715305 | 0.003297199 | 0.139492488 | 0.002518057 | 0.130002078 |
| 0.074987278 | 0.105570988 | 0.072735233 | 0.116487499 | 0.058040859 |
| -0.081768558 | 0.077520939 | -0.044180383 | 0.340766072 | -0.057915001 |
| 0.009112535 | 0.844297194 | -0.031848346 | 0.492348519 | -0.035408632 |
| 0.005754144 | 0.901302695 | -0.00387492 | 0.933443144 | -0.048608891 |
| 0.301300627 | 2.95E-11 | 0.299284212 | 4.05E-11 | 0.33318963 |
| 0.151040074 | 0.001060148 | 0.206448042 | 6.86E-06 | 0.246886017 |
| -0.004501101 | 0.922718904 | -0.040982708 | 0.376889716 | -0.07075197 |
| 0.054558342 | 0.239302246 | 0.071712278 | 0.121729413 | 0.051573716 |
| -0.057042734 | 0.218546481 | -0.025281192 | 0.585785065 | -0.017144562 |
| -0.175127751 | 0.000142472 | -0.161345139 | 0.000464711 | -0.179138666 |
| -0.189430777 | 3.79E-05 | -0.156849053 | 0.000670099 | -0.167480228 |
| 0.134870004 | 0.00349911 | 0.127412853 | 0.005828408 | 0.128553063 |
| -0.133298052 | 0.003904653 | -0.143667707 | 0.001854933 | -0.17592177 |
| -0.115641588 | 0.012393642 | -0.084610165 | 0.067727152 | -0.089076584 |
| -0.012215022 | 0.792341515 | 0.014447076 | 0.755509093 | 0.037582195 |
| 0.093705264 | 0.042968477 | 0.093444218 | 0.043554521 | 0.083976934 |
| 0.053361667 | 0.249779519 | 0.0493291 | 0.28741853 | 0.053264581 |
| 0.051658615 | 0.265234432 | 0.054492969 | 0.239866503 | 0.053817486 |
| 0.2269331 | 7.21E-07 | 0.245459655 | 7.76E-08 | 0.238677749 |
| 0.205678722 | 7.44E-06 | 0.257333608 | 1.69E-08 | 0.275419784 |
| -0.333302921 | 1.40E-13 | -0.316531838 | 2.51E-12 | -0.288787372 |
| 0.050556383 | 0.275580105 | 0.020640095 | 0.65640051 | -0.013876755 |
| 0.153363554 | 0.000884111 | 0.132579707 | 0.00410377 | 0.138880475 |
| -0.167553108 | 0.000275948 | -0.198978823 | 1.48E-05 | -0.217602131 |
| -0.027915654 | 0.54733194 | -0.070386671 | 0.128793532 | -0.083559721 |
| -0.207281439 | 6.29E-06 | -0.201950272 | 1.09E-05 | -0.246750782 |
| -0.080927041 | 0.080632533 | -0.103521215 | 0.025277809 | -0.051909219 |
| 0.158054956 | 0.000608014 | 0.166692857 | 0.000296942 | 0.166342233 |
| 0.229328018 | 5.46E-07 | 0.228875836 | 5.75E-07 | 0.219796943 |
| -0.093286891 | 0.043910958 | -0.076024971 | 0.10082108 | -0.108890874 |
| -0.102479053 | 0.026795151 | -0.078510647 | 0.090132495 | -0.086209815 |
| -0.01113002 | 0.810419714 | -0.004647354 | 0.920216067 | -0.000183057 |
| 0.200331917 | 1.29E-05 | 0.228046186 | 6.34E-07 | 0.222102468 |
| 0.09288629 | 0.044829648 | 0.095289722 | 0.039552177 | 0.124648546 |
| -0.152297905 | 0.000961196 | -0.189729933 | 3.68E-05 | -0.202034088 |
| 0.086156119 | 0.062840243 | 0.082734868 | 0.074068921 | 0.073565764 |
| -0.149283397 | 0.001214111 | -0.158667153 | 0.000578579 | -0.199159634 |
| 0.116983555 | 0.01140778 | 0.091513738 | 0.048100703 | 0.08737547 |
| 0.101303359 | 0.028600398 | 0.097997878 | 0.034245007 | 0.095722898 |
| 0.04025685 | 0.385406192 | 0.068132813 | 0.141527796 | 0.073971791 |
| 0.049965324 | 0.281239449 | 0.075373528 | 0.10378271 | 0.06430159 |

| | | | | |
|---|---|---|---|---|
| -0.305253959 | 1.58E-11 | -0.293346537 | 1.01E-10 | -0.284077189 |
| 0.013676846 | 0.768161305 | 0.003920991 | 0.932653668 | 0.015532975 |
| 0.064028947 | 0.167160061 | 0.060937184 | 0.188659024 | 0.062189589 |
| 0.027264183 | 0.556724115 | 0.034976921 | 0.450811549 | 0.036170393 |
| 0.140999607 | 0.002257122 | 0.126862978 | 0.006045836 | 0.135643502 |
| -0.040736493 | 0.379765497 | -0.067016073 | 0.148184399 | -0.070358757 |
| 0.017872525 | 0.7000713 | 0.064531762 | 0.163844489 | 0.052731284 |
| 0.29124685 | 1.39E-10 | 0.280182356 | 7.16E-10 | 0.269089793 |
| 0.264615646 | 6.36E-09 | 0.266404131 | 4.99E-09 | 0.241040961 |
| -0.217398576 | 2.11E-06 | -0.230103668 | 4.99E-07 | -0.223696021 |
| 0.021454244 | 0.643765209 | 0.047687813 | 0.303777836 | 0.055793919 |
| 0.112128555 | 0.015339019 | 0.111358098 | 0.016061643 | 0.116261617 |
| -0.058543376 | 0.20665177 | -0.066821594 | 0.149367626 | -0.078984472 |
| 0.063481886 | 0.170824192 | 0.052581451 | 0.256780221 | 0.036013242 |
| 0.184397833 | 6.11E-05 | 0.215864375 | 2.50E-06 | 0.192485454 |
| 0.028480135 | 0.539257586 | 0.081714558 | 0.077717638 | 0.118239587 |
| 0.235257179 | 2.71E-07 | 0.246753529 | 6.60E-08 | 0.26785945 |
| 0.067744052 | 0.143818618 | 0.041001294 | 0.37667317 | 0.027710038 |
| 0.039801244 | 0.390811206 | 0.052552901 | 0.257038941 | 0.034951142 |
| 0.024770138 | 0.593386795 | 0.008059964 | 0.862090754 | 0.026350916 |
| 0.063223036 | 0.172578685 | 0.038311471 | 0.408802741 | 0.043037655 |
| 0.220247857 | 1.54E-06 | 0.247778684 | 5.79E-08 | 0.23151575 |
| -0.105963823 | 0.022009037 | -0.07799283 | 0.092280751 | -0.097535448 |
| -0.01941011 | 0.675675656 | -0.073055342 | 0.114883993 | -0.1141431 |
| -0.082271938 | 0.075706725 | -0.076242825 | 0.099845773 | -0.01584279 |
| -0.014377865 | 0.756643369 | -0.050878439 | 0.272529268 | -0.045080378 |
| -0.123916692 | 0.007339678 | -0.135726004 | 0.003294713 | -0.114729493 |
| -0.025227895 | 0.586575729 | -0.039692844 | 0.392103926 | -0.05659129 |
| 0.119194666 | 0.009934169 | 0.138119433 | 0.002779423 | 0.13710474 |
| 0.014543113 | 0.753936026 | 0.036392362 | 0.432689376 | 0.01803106 |
| -0.07893406 | 0.088405866 | -0.09748159 | 0.035207194 | -0.081991125 |
| 0.260790431 | 1.07E-08 | 0.291152533 | 1.41E-10 | 0.322035125 |
| -0.142264667 | 0.002057423 | -0.140980452 | 0.002260277 | -0.110163313 |
| -0.121264681 | 0.008710402 | -0.143043256 | 0.001942689 | -0.117681847 |
| 0.046194516 | 0.319187744 | 0.016130925 | 0.728080039 | 0.011700422 |
| 0.16308921 | 0.00040216 | 0.165202429 | 0.000336884 | 0.129188382 |
| -0.129089947 | 0.005207983 | -0.082906745 | 0.073468235 | -0.068810845 |
| 0.276573801 | 1.20E-09 | 0.294302978 | 8.75E-11 | 0.304594276 |
| -0.009955888 | 0.830096897 | -0.048980253 | 0.290845046 | -0.087248754 |
| -0.058440076 | 0.207455244 | -0.089758306 | 0.052573313 | -0.106672251 |
| 0.067717012 | 0.143979003 | 0.074162108 | 0.109472996 | 0.044849405 |
| -0.057707773 | 0.21321596 | -0.060230454 | 0.193846922 | -0.076450685 |
| 0.041874821 | 0.36658244 | 0.047086611 | 0.309921604 | 0.038362155 |
| 0.153742331 | 0.000858118 | 0.155373648 | 0.00075404 | 0.139861042 |
| 0.358996449 | 1.19E-15 | 0.362013191 | 6.59E-16 | 0.334721306 |

| | | | | |
|---|---|---|---|---|
| -0.192854154 | 2.72E-05 | -0.1686355 | 0.0002515 | -0.155448 |
| -0.048665078 | 0.293964285 | -0.069662657 | 0.13278363 | -0.114905508 |
| 0.105922034 | 0.02206172 | 0.091964565 | 0.047004926 | 0.093068726 |
| -0.038592118 | 0.405376389 | -0.009004836 | 0.846114351 | -0.02890196 |
| 0.033067442 | 0.475924126 | 0.061724019 | 0.183003986 | 0.054985477 |
| 0.201821382 | 1.11E-05 | 0.211480507 | 4.02E-06 | 0.19925027 |
| -0.021625671 | 0.641117605 | -0.069621175 | 0.133015102 | -0.098607154 |
| 0.027109072 | 0.558971812 | 0.03098896 | 0.504106244 | -0.001562765 |
| 0.073463835 | 0.112862951 | 0.080200397 | 0.083400022 | 0.047657573 |
| 0.113975109 | 0.013722078 | 0.117492513 | 0.011052431 | 0.06997926 |
| 0.030612478 | 0.509303226 | 0.024467886 | 0.597903749 | 0.044945457 |
| 0.011437475 | 0.805286231 | 0.032924732 | 0.477831193 | 0.014762644 |
| 0.003336605 | 0.942672255 | 0.015746233 | 0.734317393 | 0.016041286 |
| -0.058495526 | 0.207023673 | -0.063073509 | 0.173598286 | -0.066789601 |
| -0.039546136 | 0.393857578 | -0.025781748 | 0.578383449 | -0.025803802 |
| 0.091769049 | 0.047477549 | 0.097639809 | 0.034909913 | 0.100681929 |
| 0.176192793 | 0.000129537 | 0.178299699 | 0.000107131 | 0.13528868 |
| -0.085136899 | 0.066028231 | -0.088835378 | 0.055060539 | -0.104227737 |
| 0.002555142 | 0.956083335 | 0.010177788 | 0.826369553 | 0.017246678 |
| 0.160991532 | 0.000478449 | 0.200353306 | 1.29E-05 | 0.223520158 |
| 0.048298644 | 0.297618838 | 0.037448918 | 0.419440459 | 0.021622874 |
| 0.087330824 | 0.059324394 | 0.118176821 | 0.010590116 | 0.104621744 |
| -0.000723107 | 0.987565791 | 0.00929307 | 0.841252954 | 0.007882796 |
| 0.085050413 | 0.066304754 | 0.095218647 | 0.039700347 | 0.085526569 |
| 0.076161346 | 0.100209664 | 0.0981815 | 0.033908239 | 0.076493165 |
| 0.063338297 | 0.171795789 | 0.071131796 | 0.124784703 | 0.053929382 |
| -0.269746984 | 3.15E-09 | -0.270683495 | 2.76E-09 | -0.291817499 |
| -0.143872057 | 0.001827013 | -0.150428727 | 0.00111156 | -0.150164036 |
| -0.16393569 | 0.000374714 | -0.159831642 | 0.000526208 | -0.182458269 |
| -0.077379609 | 0.09487761 | -0.056014238 | 0.226977022 | -0.050381044 |
| 0.067188883 | 0.147139014 | 0.052772949 | 0.255049507 | 0.067045973 |
| -0.000626173 | 0.989232516 | 0.051505144 | 0.266658731 | 0.103624839 |
| 0.204187974 | 8.68E-06 | 0.228401387 | 6.08E-07 | 0.252239917 |
| 0.093702033 | 0.04297569 | 0.084983968 | 0.066517847 | 0.08275542 |
| -0.008843322 | 0.848841044 | 0.012102693 | 0.79420815 | 0.032977342 |
| -0.042064315 | 0.364415851 | -0.069478555 | 0.133813292 | -0.116285015 |
| 0.010889087 | 0.814448206 | 0.052967075 | 0.253303311 | 0.064683092 |
| -0.017561423 | 0.705046056 | -0.013955822 | 0.763571381 | -0.031115865 |
| 0.164616839 | 0.000353906 | 0.132001452 | 0.004270678 | 0.087233895 |
| -0.147972899 | 0.001342098 | -0.113313095 | 0.014283643 | -0.137207278 |
| 0.141613654 | 0.002158078 | 0.172448822 | 0.00018057 | 0.16593011 |
| 0.09680876 | 0.03649556 | 0.078483327 | 0.090244828 | 0.0633182 |
| 0.028880873 | 0.533561736 | 0.00671055 | 0.885003643 | 0.00562787 |
| 0.216016193 | 2.46E-06 | 0.236611607 | 2.30E-07 | 0.223637446 |
| -0.170274343 | 0.000218309 | -0.151914103 | 0.000990446 | -0.15151227 |

| | | | | |
|---|---|---|---|---|
| 0.263848519 | 7.06E-09 | 0.263807838 | 7.10E-09 | 0.213486322 |
| -0.136873928 | 0.003037625 | -0.097770186 | 0.034666552 | -0.106042031 |
| 0.007983716 | 0.863382553 | -0.01968675 | 0.671321344 | -0.051482772 |
| -0.133114455 | 0.003954694 | -0.143994025 | 0.001810533 | -0.119269999 |
| 0.145184893 | 0.001656633 | 0.153060221 | 0.000905451 | 0.175089035 |
| -0.014907296 | 0.747980098 | -0.063503402 | 0.170678959 | -0.064454587 |
| 0.058679445 | 0.205596861 | 0.072355787 | 0.118410878 | 0.032582791 |
| -0.00806446 | 0.862014598 | -0.017519832 | 0.705712077 | 0.01677879 |
| -0.093435506 | 0.043574194 | -0.098567933 | 0.033208726 | -0.140346609 |
| 0.008613946 | 0.852716483 | 0.040626256 | 0.381057412 | 0.07708654 |
| -0.072585444 | 0.117243823 | -0.06325129 | 0.172386532 | -0.105203644 |
| -0.05184092 | 0.263549346 | -0.072737393 | 0.116476623 | -0.054735973 |
| 0.041076295 | 0.375800141 | 0.050493969 | 0.276174033 | 0.012207687 |
| 0.094754705 | 0.040679102 | 0.088148069 | 0.056975661 | 0.032506492 |
| -0.108485182 | 0.019025727 | -0.098084105 | 0.034086511 | -0.119511848 |
| 0.069582352 | 0.133232013 | 0.083961345 | 0.069868943 | 0.089332887 |
| 0.067992553 | 0.142351052 | 0.06921124 | 0.135319299 | 0.05501426 |
| -0.113990884 | 0.013708938 | -0.09764607 | 0.034898192 | -0.113197043 |
| 0.17779867 | 0.000112102 | 0.15364838 | 0.000864498 | 0.173481404 |
| -0.14148228 | 0.002178928 | -0.137160791 | 0.002976295 | -0.176019426 |
| 0.123332938 | 0.007623645 | 0.085506099 | 0.06485841 | 0.057415428 |
| 0.06273444 | 0.175926963 | 0.025229967 | 0.586544974 | -0.017655537 |
| -0.144641868 | 0.001725258 | -0.108038653 | 0.019526852 | -0.09689678 |
| 0.255320812 | 2.20E-08 | 0.282077984 | 5.44E-10 | 0.302074833 |
| 0.09177344 | 0.047466891 | 0.090622108 | 0.050330945 | 0.088929163 |
| 0.04986291 | 0.282227985 | 0.059494498 | 0.19935953 | 0.031020506 |
| 0.08086996 | 0.080847207 | 0.058993924 | 0.203173713 | 0.064906362 |
| -0.150065016 | 0.001143227 | -0.128010608 | 0.005600041 | -0.114122824 |
| -0.02884582 | 0.534058754 | -0.015676627 | 0.735447859 | -0.000586188 |
| 0.038920927 | 0.401383881 | 0.050570432 | 0.275446537 | 0.044815828 |
| 0.093564798 | 0.043282989 | 0.108738098 | 0.01874689 | 0.105187734 |
| 0.053949845 | 0.24459058 | 0.058428871 | 0.207542533 | 0.040824167 |
| 0.044894342 | 0.33301288 | 0.061170709 | 0.186967418 | 0.060645209 |
| 0.225661763 | 8.34E-07 | 0.227388067 | 6.84E-07 | 0.215394117 |
| -0.110675038 | 0.016727068 | -0.102235339 | 0.027161091 | -0.129033763 |
| 0.074724237 | 0.106802712 | 0.073013954 | 0.115090337 | 0.064416877 |
| 0.051317228 | 0.268409817 | 0.018617236 | 0.688215131 | -0.008964618 |
| 0.119453485 | 0.009773237 | 0.129132291 | 0.005193119 | 0.11541776 |
| -0.081453015 | 0.07867607 | -0.098375182 | 0.033556086 | -0.135199669 |
| 0.087265776 | 0.059514731 | 0.122903118 | 0.007838965 | 0.100089701 |
| 0.197435891 | 1.73E-05 | 0.222492724 | 1.20E-06 | 0.276404542 |
| 0.197496174 | 1.72E-05 | 0.212917475 | 3.45E-06 | 0.220561639 |
| 0.01953959 | 0.673636283 | 0.033913708 | 0.464701178 | 0.0163508 |
| 0.243737101 | 9.62E-08 | 0.242947036 | 1.06E-07 | 0.234884473 |
| 0.031204823 | 0.501139087 | 0.031872638 | 0.492018303 | 0.014672683 |

| | | | | |
|---|---|---|---|---|
| -0.24469144 | 8.54E-08 | -0.234187317 | 3.08E-07 | -0.269551726 |
| 0.238110405 | 1.92E-07 | 0.247136099 | 6.29E-08 | 0.260982762 |
| -0.051817786 | 0.26376277 | -0.087919833 | 0.057623709 | -0.11262255 |
| -0.178270678 | 0.000107413 | -0.220156411 | 1.56E-06 | -0.255174424 |
| 0.137353293 | 0.002935773 | 0.141860352 | 0.002119417 | 0.14960201 |
| 0.080059646 | 0.083944852 | 0.080865892 | 0.080862524 | 0.082517567 |
| 0.069777398 | 0.132144998 | 0.080225171 | 0.083304421 | 0.064530415 |
| -0.070782799 | 0.126650088 | -0.071750348 | 0.121531095 | -0.087931856 |
| 0.157293959 | 0.00064654 | 0.162641018 | 0.000417439 | 0.150981697 |
| 0.081901929 | 0.077036858 | 0.084283823 | 0.068797599 | 0.084343102 |
| -0.330522065 | 2.29E-13 | -0.298756112 | 4.40E-11 | -0.303238443 |
| 0.00153662 | 0.973580761 | 0.040857728 | 0.378347798 | 0.048881625 |
| -0.014072291 | 0.761657574 | 0.009898881 | 0.831055098 | 0.012306179 |
| -0.013069514 | 0.778181446 | -0.008828625 | 0.849089258 | -0.015726784 |
| -0.019991246 | 0.666541262 | 0.039448239 | 0.395030415 | 0.036970507 |
| -0.029621152 | 0.523120181 | 0.00520489 | 0.910682319 | 0.01928887 |
| 0.023186858 | 0.617218318 | 0.066547617 | 0.151046697 | 0.042438828 |
| 0.096533868 | 0.037033342 | 0.118048704 | 0.010675344 | 0.125024361 |
| -0.056751845 | 0.220907811 | -0.057427231 | 0.215453087 | -0.077296599 |
| 0.107853472 | 0.019738023 | 0.117513541 | 0.011037962 | 0.105361219 |
| 0.057906918 | 0.211638098 | 0.082746176 | 0.074029281 | 0.076222144 |
| 0.128628804 | 0.005372359 | 0.12713691 | 0.005936625 | 0.152336275 |
| -0.009735631 | 0.83380043 | 0.005936069 | 0.898198927 | 0.01328453 |
| -0.236690093 | 2.28E-07 | -0.221657493 | 1.31E-06 | -0.23085086 |
| 0.070528927 | 0.128020576 | 0.076013725 | 0.100871631 | 0.061514969 |
| 0.063322149 | 0.171905314 | 0.147332405 | 0.001409064 | 0.152621343 |
| 0.13227949 | 0.004189674 | 0.149741886 | 0.001172056 | 0.173342326 |
| -0.037006578 | 0.424958116 | -0.042567445 | 0.358702081 | -0.043548631 |
| -0.071019633 | 0.125381869 | -0.081695483 | 0.077787216 | -0.111376628 |
| 0.099699686 | 0.031230452 | 0.139877879 | 0.002448836 | 0.148354534 |
| 0.15615979 | 0.000708173 | 0.190305974 | 3.48E-05 | 0.198409169 |
| 0.02951492 | 0.524612114 | 0.075325726 | 0.104002717 | 0.077157085 |
| 0.269482143 | 3.26E-09 | 0.285982733 | 3.06E-10 | 0.319559117 |
| -0.010602883 | 0.819239989 | 0.032790177 | 0.479633102 | 0.035717286 |
| 0.106852706 | 0.020913982 | 0.131585125 | 0.004394621 | 0.129710297 |
| 0.170081447 | 0.000221991 | 0.210311547 | 4.56E-06 | 0.24662261 |
| 0.212881169 | 3.46E-06 | 0.241026731 | 1.35E-07 | 0.250821767 |
| 0.175498858 | 0.000137833 | 0.210844532 | 4.31E-06 | 0.221028691 |
| 0.273108071 | 1.97E-09 | 0.306355312 | 1.32E-11 | 0.29790231 |
| 0.237020712 | 2.19E-07 | 0.262760899 | 8.18E-09 | 0.262149175 |
| 0.061610647 | 0.183810991 | 0.111720204 | 0.015718385 | 0.119164159 |
| 0.142281825 | 0.002054829 | 0.157519155 | 0.00063491 | 0.176061875 |
| 0.006664335 | 0.885790173 | 0.054376658 | 0.240872737 | 0.093367098 |
| 0.186398457 | 5.06E-05 | 0.240516687 | 1.43E-07 | 0.286066968 |
| 0.060728427 | 0.190180713 | 0.087205566 | 0.059691363 | 0.087615129 |

| | | | | |
|---|---|---|---|---|
| 0.157196353 | 0.000651641 | 0.207294417 | 6.28E-06 | 0.224005875 |
| 0.164108861 | 0.000369318 | 0.196110959 | 1.97E-05 | 0.19208996 |
| 0.357972729 | 1.45E-15 | 0.362800098 | 5.65E-16 | 0.39377156 |
| 0.16540782 | 0.000331097 | 0.216374356 | 2.37E-06 | 0.253931011 |
| 0.268700842 | 3.64E-09 | 0.252340051 | 3.24E-08 | 0.275293438 |
| -0.008909008 | 0.847731913 | 0.037790697 | 0.415206085 | 0.065859563 |
| 0.276885762 | 1.15E-09 | 0.306992085 | 1.20E-11 | 0.322242456 |
| 0.289661983 | 1.77E-10 | 0.318942101 | 1.68E-12 | 0.339631767 |
| 0.131951857 | 0.004285276 | 0.17439564 | 0.000152057 | 0.152486997 |
| 0.186004748 | 5.25E-05 | 0.184167611 | 6.24E-05 | 0.217032598 |
| -0.047688356 | 0.303772328 | -0.013398681 | 0.772745965 | 0.014968989 |
| 0.130544422 | 0.004718781 | 0.160242909 | 0.000508789 | 0.149825541 |
| 0.057032619 | 0.218628288 | 0.097261639 | 0.035624041 | 0.101691944 |
| 0.170670707 | 0.000210922 | 0.241047497 | 1.34E-07 | 0.263323864 |
| -0.087658916 | 0.058372021 | -0.039416795 | 0.395407569 | -0.027838254 |
| 0.220662027 | 1.47E-06 | 0.211034058 | 4.22E-06 | 0.216056611 |
| 0.106982772 | 0.020757785 | 0.154088377 | 0.000834989 | 0.173806382 |
| 0.000290243 | 0.995008946 | 0.022714303 | 0.624411366 | 0.058868602 |
| 0.087670048 | 0.058339929 | 0.114332244 | 0.013427278 | 0.115845321 |
| 0.063319503 | 0.171923264 | 0.099189383 | 0.032109609 | 0.09113064 |
| 0.145356755 | 0.001635441 | 0.193465778 | 2.56E-05 | 0.200220805 |
| 0.051163429 | 0.269848838 | 0.096975319 | 0.036172951 | 0.09469805 |
| 0.0730361 | 0.114979889 | 0.105002048 | 0.023249559 | 0.084213755 |
| 0.107464489 | 0.020188082 | 0.119463018 | 0.009767354 | 0.136954081 |
| 0.086628345 | 0.061406777 | 0.109318506 | 0.01812043 | 0.125428667 |
| 0.357135739 | 1.70E-15 | 0.370649658 | 1.18E-16 | 0.414941556 |
| 0.096498069 | 0.037103868 | 0.121618962 | 0.008514977 | 0.153221806 |
| 0.253372949 | 2.83E-08 | 0.290813243 | 1.49E-10 | 0.298695194 |
| 0.015114523 | 0.744597722 | 0.051559618 | 0.266152581 | 0.0380341 |
| 0.145299934 | 0.00164242 | 0.191296618 | 3.16E-05 | 0.196288443 |
| -0.010276968 | 0.824704827 | 0.046076409 | 0.320427925 | 0.053201387 |
| 0.161890456 | 0.000444243 | 0.188345048 | 4.20E-05 | 0.214938109 |
| 0.122331611 | 0.008133668 | 0.137916004 | 0.002820181 | 0.148040158 |
| -0.022844746 | 0.622422212 | 0.00081208 | 0.986036004 | -0.007563387 |
| -0.063048573 | 0.173768759 | -0.035883084 | 0.439160857 | -0.058435695 |
| -0.014301565 | 0.757894451 | 0.003774257 | 0.935168309 | 0.007136808 |
| -0.05386361 | 0.24534659 | -0.04164716 | 0.369195985 | -0.055266776 |
| -0.116129068 | 0.012027145 | -0.089408765 | 0.05350406 | -0.09592804 |
| 0.020421653 | 0.659807572 | 0.042842421 | 0.355603187 | 0.024056167 |
| -0.125840669 | 0.006469515 | -0.084423084 | 0.06833912 | -0.106132944 |
| -0.076601725 | 0.09825539 | -0.054774039 | 0.237447109 | -0.06213454 |
| -0.020464992 | 0.659131055 | 0.00065169 | 0.988793762 | -0.025153087 |
| -0.033370283 | 0.471891007 | -0.004911926 | 0.915690432 | -0.02641118 |
| 0.002956026 | 0.94920178 | 0.01448163 | 0.754942995 | 0.003048633 |
| -0.026947981 | 0.561310808 | 0.003137004 | 0.946096317 | -0.023764638 |

| | | | | |
|---|---|---|---|---|
| -0.045988338 | 0.321354745 | -0.008740301 | 0.850581192 | -0.030360118 |
| -0.016850425 | 0.716462453 | 0.015416908 | 0.739670982 | 0.005154174 |
| -0.052898709 | 0.253917325 | -0.016191453 | 0.72710025 | -0.039426378 |
| -0.101656099 | 0.02804812 | -0.053797416 | 0.245928018 | -0.063945641 |
| 0.013945152 | 0.763746774 | 0.056915951 | 0.219573426 | 0.043525957 |
| -0.05492654 | 0.236141619 | -0.013738148 | 0.767152016 | -0.031327515 |
| -0.068397173 | 0.139986055 | -0.054070422 | 0.243536219 | -0.049372234 |
| -0.044978843 | 0.332102816 | -0.033053828 | 0.476105877 | -0.035893238 |
| -0.053763503 | 0.246226274 | -0.044718229 | 0.334914736 | -0.060916955 |
| -0.048102509 | 0.299587338 | -0.02876932 | 0.53514423 | -0.025467714 |
| -0.148317528 | 0.00130729 | -0.125574981 | 0.006583896 | -0.149150966 |
| 0.006221044 | 0.893340173 | 0.035181635 | 0.448164364 | 0.011059254 |
| -0.131335093 | 0.00447061 | -0.125441097 | 0.006642219 | -0.132008603 |
| -0.095300708 | 0.039529318 | -0.052731889 | 0.255419915 | -0.06955352 |
| -0.022643061 | 0.625498907 | -0.001276068 | 0.978059201 | -0.013186124 |
| -0.152882045 | 0.000918206 | -0.116255845 | 0.011933415 | -0.13865836 |
| -0.011834558 | 0.798668641 | -0.010713552 | 0.817386291 | -0.048839511 |
| -0.024665675 | 0.594946162 | -0.019234604 | 0.678443746 | -0.03564103 |
| 0.014831742 | 0.749214508 | 0.006674918 | 0.885610055 | 0.024151411 |
| -0.119620681 | 0.009670511 | -0.102621626 | 0.026583048 | -0.103374264 |
| -0.039421506 | 0.395351053 | -0.028958481 | 0.532462186 | -0.041527054 |
| -0.051135666 | 0.270109167 | -0.021564159 | 0.642067103 | -0.046244747 |
| -0.221670199 | 1.31E-06 | -0.192816952 | 2.73E-05 | -0.237329977 |
| -0.032978918 | 0.477106607 | -0.00933297 | 0.840580459 | -0.029375378 |
| 0.012158556 | 0.793279694 | 0.061384946 | 0.185425382 | 0.046071267 |
| -0.004343751 | 0.925412509 | 0.026684491 | 0.565146748 | 0.023439859 |
| -0.049198422 | 0.288698905 | -0.003594663 | 0.938246963 | -0.019290641 |
| -0.17426051 | 0.000153891 | -0.172267137 | 0.000183472 | -0.193546521 |
| -0.047504042 | 0.305647196 | -0.015052569 | 0.745608439 | -0.03601226 |
| -0.039922182 | 0.389372022 | -0.008169465 | 0.860236248 | -0.030953374 |
| -0.002999384 | 0.948457711 | 0.006747416 | 0.884376292 | -0.003041497 |
| -0.033219836 | 0.473892257 | -0.002276092 | 0.960875465 | -0.029055131 |
| -0.010857487 | 0.814976923 | 0.01333504 | 0.773796006 | 0.002071726 |
| -0.028100687 | 0.544678665 | -0.010017157 | 0.829067359 | -0.022046497 |
| -0.017470337 | 0.706504983 | -0.025306792 | 0.58540545 | -0.018074332 |
| -0.148407104 | 0.001298379 | -0.128175972 | 0.005538302 | -0.147872635 |
| -0.13344984 | 0.003863716 | -0.106217986 | 0.021690958 | -0.10257624 |
| -0.0780504 | 0.092039905 | -0.060644435 | 0.190795483 | -0.063211429 |
| -0.038381952 | 0.407940642 | -0.008358666 | 0.857033741 | -0.012213644 |
| -0.056124392 | 0.226063199 | -0.014039643 | 0.762193899 | -0.054421447 |
| -0.037217487 | 0.42232203 | -0.01547188 | 0.738776463 | -0.04756955 |
| -0.096155882 | 0.037783783 | -0.051389628 | 0.267734234 | -0.070132404 |
| 0.072309178 | 0.118648841 | 0.082482797 | 0.074957108 | 0.085800943 |
| -0.046888612 | 0.311962757 | -0.029088073 | 0.530628655 | -0.031358516 |
| -0.046480805 | 0.316194575 | -0.022708681 | 0.624497162 | -0.049289483 |

| | | | | |
|---|---|---|---|---|
| -0.061139984 | 0.187189341 | -0.018317466 | 0.69297864 | -0.049073634 |
| -0.080455155 | 0.082421152 | -0.051543819 | 0.266299307 | -0.069594809 |
| -0.071315507 | 0.123811385 | -0.064372515 | 0.164889172 | -0.078454081 |
| 0.001569685 | 0.973012485 | -0.007532441 | 0.871035363 | -0.036935885 |
| 0.032460157 | 0.484068181 | 0.019759092 | 0.67018448 | 0.022887263 |
| -0.070830295 | 0.126394961 | -0.057437076 | 0.215374294 | -0.063628068 |
| -0.051849642 | 0.263468907 | -0.017756208 | 0.701929812 | -0.0429684 |
| -0.129364787 | 0.005112183 | -0.117568471 | 0.011000247 | -0.130919743 |
| -0.086139364 | 0.062891606 | -0.08363376 | 0.070971193 | -0.098795944 |
| -0.160865991 | 0.000483417 | -0.151594675 | 0.001015414 | -0.155830569 |
| -0.14329663 | 0.001906633 | -0.094615869 | 0.040975932 | -0.115248853 |
| -0.141031637 | 0.002251854 | -0.119112382 | 0.009985824 | -0.112672518 |
| -0.0125902 | 0.786115613 | 0.026379638 | 0.569600473 | 0.01033994 |
| -0.055152889 | 0.234213301 | -0.035500264 | 0.444061678 | -0.016523363 |
| -0.042161801 | 0.363304349 | -0.013815076 | 0.765886013 | -0.036964777 |
| -0.014776829 | 0.75011208 | -0.028478047 | 0.539287336 | 0.012066982 |
| 0.04947417 | 0.286001614 | 0.024229296 | 0.601480292 | 0.05942289 |
| 0.004384691 | 0.924711595 | 0.036905826 | 0.426220757 | 0.000415319 |
| -0.022974126 | 0.620451975 | 0.007747132 | 0.867393052 | -0.018363628 |
| -0.031423947 | 0.498136568 | 0.001864794 | 0.967941199 | -0.021503088 |
| -0.074257425 | 0.109016545 | -0.061962513 | 0.181314901 | -0.035235305 |
| -0.048930235 | 0.291338578 | -0.022065409 | 0.634346892 | -0.028479305 |
| -0.093083074 | 0.044376367 | -0.038226934 | 0.409838195 | -0.058842105 |
| -0.067475946 | 0.145414906 | -0.059009086 | 0.203057418 | -0.056804012 |
| 0.022330701 | 0.630276794 | 0.073068804 | 0.114816944 | 0.048423146 |
| -0.045951395 | 0.321744047 | -0.051438186 | 0.267281774 | -0.088055094 |
| -0.027005767 | 0.560471229 | -0.002208991 | 0.962028006 | 0.005260769 |
| -0.019260984 | 0.678027405 | -0.011272452 | 0.808040552 | -0.040336854 |
| -0.225036203 | 8.96E-07 | -0.213070832 | 3.39E-06 | -0.244012943 |
| -0.043585837 | 0.347309728 | -0.030516978 | 0.510625949 | -0.052824143 |
| -0.05788802 | 0.211787472 | -0.036000596 | 0.437662712 | -0.055467129 |
| -0.100781524 | 0.029434534 | -0.093719472 | 0.042936772 | -0.092518357 |
| -0.041638515 | 0.369295466 | -0.023497702 | 0.612506598 | -0.023595874 |
| -0.09444123 | 0.041351904 | -0.068205652 | 0.141101706 | -0.081113481 |
| -0.05156417 | 0.266110317 | -0.015011351 | 0.746281093 | -0.033476956 |
| -0.0681066 | 0.141681372 | -0.026942727 | 0.561387172 | -0.04170999 |
| -0.01191679 | 0.797299998 | -0.000493745 | 0.991509605 | -0.01137583 |
| -0.080284537 | 0.083075693 | -0.088400396 | 0.056266254 | -0.090320803 |
| -0.046596827 | 0.314986814 | -0.023152149 | 0.617745408 | -0.023033925 |
| 0.047115597 | 0.309623526 | 0.077042092 | 0.096331632 | 0.063728357 |
| 0.000589559 | 0.989862087 | 0.019718255 | 0.670826143 | 0.032713124 |
| -0.067997601 | 0.142321359 | -0.047734556 | 0.303303571 | -0.058963701 |
| -0.002445389 | 0.957967946 | 0.013846975 | 0.765361228 | 0.025770084 |
| 0.005712933 | 0.902006004 | 0.033285451 | 0.473018868 | -0.018764124 |
| 0.019112842 | 0.680366746 | 0.014379853 | 0.756610778 | -0.025516264 |

| | | | | |
|---|---|---|---|---|
| 0.013819267 | 0.765817061 | 0.027638829 | 0.551313326 | -0.003953372 |
| -0.031832281 | 0.492566954 | -0.012008796 | 0.795769393 | -0.01164703 |
| -0.120342524 | 0.009237935 | -0.087186076 | 0.059748631 | -0.097670323 |
| -0.049093261 | 0.289732051 | -0.016426438 | 0.723300742 | -0.052085109 |
| -0.017631412 | 0.703925764 | 0.000215497 | 0.996294274 | 0.004108415 |
| -0.078577227 | 0.089859214 | -0.05221417 | 0.260122319 | -0.081352375 |
| -0.085508796 | 0.064849928 | -0.057541412 | 0.21454055 | -0.058405762 |
| 0.046835348 | 0.312513359 | 0.050668294 | 0.274517347 | 0.038162349 |
| 0.037605134 | 0.417501939 | 0.042318059 | 0.361527165 | 0.069184747 |
| -0.025926099 | 0.576257142 | 0.013270852 | 0.774855481 | -0.009947747 |
| 0.011716813 | 0.800629439 | 0.031208829 | 0.501084116 | 0.014245794 |
| -0.023064276 | 0.619080731 | 0.01021492 | 0.825746197 | 0.022469089 |
| -0.047602017 | 0.304649628 | -0.025404085 | 0.583963818 | -0.032828172 |
| -0.035982601 | 0.437891936 | -0.020759535 | 0.654540585 | -0.035442401 |
| -0.015618258 | 0.736396276 | 0.018357947 | 0.692334659 | -0.009550168 |
| -0.028576112 | 0.53789066 | -0.029482136 | 0.525072983 | -0.01513094 |
| -0.03687872 | 0.426560826 | -0.008000843 | 0.863092349 | -0.02553365 |
| -0.017043758 | 0.713351742 | 0.005222927 | 0.91037409 | -0.018284139 |
| -1.10E-05 | 0.999809998 | 0.025865892 | 0.577143554 | 0.001691773 |
| -0.046061928 | 0.320580197 | -0.027222746 | 0.557324135 | -0.036642666 |
| 0.040358687 | 0.384204328 | 0.056462141 | 0.22327759 | 0.045105606 |
| -0.038153939 | 0.410733514 | -0.006370877 | 0.890787161 | -0.035834233 |
| -0.086786348 | 0.06093322 | -0.045991594 | 0.321320454 | -0.062902338 |
| -0.024818787 | 0.592661213 | 0.029686546 | 0.522202849 | 0.002045532 |
| -0.018044803 | 0.69732193 | 0.019219069 | 0.678688983 | -0.001193484 |
| -0.067389183 | 0.14593437 | -0.037866282 | 0.414273063 | -0.058808504 |
| -0.12890427 | 0.005273616 | -0.104874073 | 0.023419104 | -0.123875298 |
| -0.07787831 | 0.092761351 | -0.039017486 | 0.400215912 | -0.062831538 |
| -0.048619784 | 0.29441438 | -0.012111504 | 0.794061697 | -0.008537599 |
| -0.101359952 | 0.028511168 | -0.064197624 | 0.166042245 | -0.040026423 |
| -0.089019156 | 0.05455762 | -0.069390488 | 0.134308011 | -0.06186821 |
| -0.084663459 | 0.067553646 | -0.061879436 | 0.181901959 | -0.069278789 |
| -0.096776963 | 0.036557423 | -0.052180912 | 0.260426418 | -0.070372445 |
| -0.054146955 | 0.242868652 | -0.039382241 | 0.395822278 | -0.02260627 |
| -0.082793981 | 0.073861879 | -0.065083067 | 0.16026621 | -0.092463476 |
| 0.006485237 | 0.888839323 | 0.006552641 | 0.887691575 | 0.038315846 |
| -0.051571892 | 0.266038626 | -0.009509973 | 0.837598597 | -0.034535733 |
| -0.057661357 | 0.21358494 | -0.019934615 | 0.667429256 | -0.032894976 |
| -0.04090076 | 0.377845372 | -0.023992808 | 0.605034782 | -0.013664109 |
| -0.099158805 | 0.032162955 | -0.084187768 | 0.069115297 | -0.112201268 |
| -0.081350235 | 0.079055323 | -0.040196527 | 0.386119199 | -0.070786652 |
| -0.094509644 | 0.041204272 | -0.066385521 | 0.152046838 | -0.095306101 |
| -0.066136209 | 0.153594891 | -0.054949977 | 0.235941434 | -0.067223095 |
| -0.065965525 | 0.154661593 | -0.020182466 | 0.663546284 | -0.047924403 |
| -0.034354728 | 0.458911157 | -0.001006681 | 0.982690236 | -0.013592189 |

| | | | | |
|---|---|---|---|---|
| -0.024015557 | 0.604692452 | 0.000599342 | 0.98969387 | -0.032020532 |
| -0.056891894 | 0.219768681 | -0.053965218 | 0.244455975 | -0.060846121 |
| -0.071659809 | 0.12200316 | -0.056860611 | 0.220022761 | -0.064993871 |
| -0.060138262 | 0.194531302 | -0.038155927 | 0.410709113 | -0.044722743 |
| -0.064631847 | 0.163190457 | -0.02885143 | 0.533979186 | -0.049108431 |
| -0.028382043 | 0.540656409 | 0.000587027 | 0.989905621 | -0.017507894 |
| -0.022711286 | 0.624457404 | 0.007577086 | 0.870277714 | -0.008733755 |
| -0.028373137 | 0.540783503 | -0.014617942 | 0.752711052 | -0.025975822 |
| -2.68E-05 | 0.999538866 | 0.02175976 | 0.639049825 | 0.002026589 |
| -0.034887207 | 0.45197443 | -0.003225356 | 0.944580547 | -0.038781068 |
| -0.050899618 | 0.27232945 | -0.02093177 | 0.651862302 | -0.027472221 |
| -0.051579376 | 0.265969158 | -0.019031342 | 0.681655029 | -0.041910745 |
| -0.03831482 | 0.408761747 | -0.019835782 | 0.668980113 | -0.02643683 |
| -0.009862806 | 0.831661578 | 0.015312049 | 0.741378251 | 0.010539352 |
| -0.053198745 | 0.251230287 | -0.05369456 | 0.246833378 | -0.04576895 |
| -0.072811001 | 0.116106385 | -0.040229442 | 0.385730061 | -0.057320566 |
| -0.044779015 | 0.33425752 | -0.01772634 | 0.702407319 | -0.038166465 |
| -0.050965052 | 0.271712728 | -0.022513401 | 0.627480321 | -0.054967013 |
| -0.025294466 | 0.585588208 | 1.15E-05 | 0.999801763 | -0.007531467 |
| -0.033726775 | 0.467167534 | 0.003473296 | 0.940327992 | -0.013819278 |
| -0.0456059 | 0.325399617 | -0.025999173 | 0.575182154 | -0.047319402 |
| -0.061837892 | 0.182196061 | -0.036597238 | 0.430101586 | -0.060955033 |
| 0.025947218 | 0.575946359 | 0.028225552 | 0.542891778 | 0.052526227 |
| -0.021650333 | 0.64073707 | 0.020541134 | 0.65794314 | -0.005418462 |
| -0.071732706 | 0.121622966 | -0.053771295 | 0.246157719 | -0.08237195 |
| 0.022317919 | 0.630472646 | 0.049187438 | 0.288806699 | 0.043779614 |
| -0.051362113 | 0.267990845 | -0.017056057 | 0.713154015 | -0.048749747 |
| -0.08751218 | 0.058796383 | -0.072203748 | 0.119188481 | -0.089971085 |
| -0.139694755 | 0.002481508 | -0.095063453 | 0.040025511 | -0.118878261 |
| -0.109914214 | 0.017496536 | -0.078137294 | 0.091677333 | -0.095900025 |
| -0.054730061 | 0.237824518 | -0.018327405 | 0.692820501 | -0.039067444 |
| 0.021121831 | 0.648912011 | 0.041712865 | 0.368440517 | 0.042418344 |
| -0.054077687 | 0.243472796 | -0.028531958 | 0.538519297 | -0.043414252 |
| -0.027617354 | 0.551622776 | -0.007170205 | 0.877186994 | -0.028867603 |
| -0.061063793 | 0.187740499 | -0.028605463 | 0.537472985 | -0.04328189 |
| -0.063497127 | 0.170721306 | -0.031596123 | 0.49578405 | -0.052092244 |
| -0.044885561 | 0.333107536 | -0.020730019 | 0.655000012 | -0.043849453 |
| -0.052265831 | 0.25965042 | -0.008261386 | 0.858680065 | -0.033265699 |
| -0.033835775 | 0.465728536 | -0.003582885 | 0.938448898 | -0.025939003 |
| -0.038601806 | 0.405258414 | 0.003816203 | 0.934449393 | -0.025841964 |
| -0.042381378 | 0.360808557 | -0.042444055 | 0.360098127 | -0.057835984 |
| -0.04043621 | 0.383290939 | -0.010244651 | 0.825247181 | -0.014237007 |
| 0.032904561 | 0.47810109 | 0.0699534 | 0.131170009 | 0.03722922 |
| 0.010031509 | 0.828826233 | 0.02109209 | 0.649373314 | -0.002654113 |
| -0.055337458 | 0.232649165 | -0.032092456 | 0.48903565 | -0.063637367 |

| | | | | |
|---|---|---|---|---|
| -0.040770552 | 0.379366885 | -0.008315766 | 0.85775968 | -0.024580948 |
| -0.052190112 | 0.260342274 | -0.027114085 | 0.558899103 | -0.047692442 |
| 0.002470892 | 0.957530007 | 0.015341805 | 0.740893645 | -0.001539445 |
| -0.042285314 | 0.361899135 | -0.009624846 | 0.835664636 | -0.02294118 |
| -0.033093774 | 0.475572705 | -0.006646625 | 0.886091617 | -0.011631816 |
| 0.017613396 | 0.704214082 | 0.044225188 | 0.340276153 | 0.025296715 |
| -0.070058657 | 0.130589581 | -0.043361582 | 0.349798473 | -0.053088398 |
| 0.001401542 | 0.975902424 | 0.025221741 | 0.586667057 | 0.014662496 |
| 0.0309731 | 0.504324623 | 0.074328906 | 0.10867522 | 0.051996918 |
| 0.0566183 | 0.221997966 | 0.10540701 | 0.022720041 | 0.084363473 |
| -0.003454419 | 0.940651691 | 0.018078858 | 0.696778919 | 0.00399754 |
| -0.018957982 | 0.68281544 | -0.045664101 | 0.32478193 | -0.058824001 |
| -0.02889672 | 0.533337122 | -0.001549392 | 0.973361239 | -0.022657384 |
| -0.034962425 | 0.450999338 | -0.002437249 | 0.958107728 | -0.024577589 |
| -0.023804637 | 0.607869726 | 0.005327326 | 0.90859036 | -0.021891753 |
| -0.041885681 | 0.36645806 | -0.058597523 | 0.206231516 | -0.083126729 |
| -0.042936553 | 0.354546231 | -0.018008839 | 0.697895558 | -0.027338122 |
| -0.050440004 | 0.276688255 | -0.04885706 | 0.292061619 | -0.048911495 |
| -0.060737011 | 0.19011796 | -0.035374214 | 0.445682145 | -0.046323894 |
| -0.025424052 | 0.583668151 | 0.007974666 | 0.863535906 | -0.012687868 |
| -0.066016153 | 0.154344609 | -0.044918597 | 0.332751486 | -0.053577723 |
| -0.020590113 | 0.657179459 | -0.025684758 | 0.579814193 | -0.018985164 |
| -0.049613707 | 0.284643191 | -0.041872118 | 0.366613407 | -0.040303229 |
| -0.017324854 | 0.708837413 | 0.013131623 | 0.777155008 | -0.016000814 |
| -0.102125241 | 0.027327809 | -0.059945311 | 0.195969373 | -0.068739877 |
| 0.011340369 | 0.806906694 | 0.052100286 | 0.261164679 | 0.013207504 |
| -0.053741154 | 0.246422964 | -0.035689484 | 0.441635425 | -0.036396007 |
| -0.142405958 | 0.002036152 | -0.130672557 | 0.004677738 | -0.138822283 |
| -0.035350879 | 0.445982496 | -0.004627082 | 0.920562924 | -0.030963382 |
| 0.022954579 | 0.620749465 | 0.060483059 | 0.191980762 | 0.040502145 |
| -0.010209374 | 0.8258393 | 0.001488048 | 0.974415563 | -0.00963484 |
| -0.052253886 | 0.259759473 | -0.02542855 | 0.583601566 | -0.031089248 |
| -0.020921769 | 0.652017694 | 0.017882537 | 0.699911405 | -0.023146616 |
| -0.025247921 | 0.586278576 | 0.003994967 | 0.931386166 | 0.015390401 |
| -0.056527031 | 0.222745224 | -0.055354844 | 0.232502209 | -0.065963361 |
| 0.009329111 | 0.840645495 | 0.020061022 | 0.665447777 | 0.016804666 |
| -0.094136371 | 0.042015196 | -0.074830291 | 0.106304745 | -0.087107453 |
| -0.047872293 | 0.301908894 | -0.01442997 | 0.75578938 | -0.027813396 |
| -0.110868384 | 0.016536309 | -0.114200427 | 0.013535432 | -0.148904304 |
| -0.042744772 | 0.356701727 | -0.016279221 | 0.72568034 | -0.037430108 |
| -0.024818173 | 0.592670375 | -0.009231827 | 0.842285384 | -0.005718026 |
| 0.034775945 | 0.453418965 | 0.071635406 | 0.122130636 | 0.065486416 |
| -0.050557011 | 0.275574136 | -0.023007905 | 0.619938014 | -0.040742074 |
| -0.033230385 | 0.473751783 | -0.0382884 | 0.409085176 | -0.046529612 |
| -0.055473623 | 0.231499971 | -0.053565231 | 0.247975058 | -0.060335259 |

| | | | | |
|---|---|---|---|---|
| -0.069071889 | 0.136109536 | -0.05067352 | 0.27446779 | -0.060880521 |
| 0.017128983 | 0.711981985 | 0.04852391 | 0.295368622 | 0.023237104 |
| 7.16E-05 | 0.998768289 | 0.008921242 | 0.847525376 | -0.013434394 |
| 0.012011789 | 0.795719622 | 0.013906465 | 0.76438284 | 0.030463307 |
| -0.018074896 | 0.696842084 | -0.015002689 | 0.746422487 | 0.013627759 |
| -0.010443571 | 0.821910213 | 0.015289358 | 0.741747855 | -0.037663329 |
| 0.005172785 | 0.911230967 | 0.020942582 | 0.651694327 | 0.017099761 |
| 0.000797927 | 0.986279349 | 0.023312826 | 0.615306997 | -0.015723595 |
| -0.008901365 | 0.847860947 | 0.002060144 | 0.9645849 | -0.012085191 |
| 0.008516518 | 0.854363654 | 0.007274947 | 0.875407456 | 0.023915693 |
| -0.032698129 | 0.480867889 | 0.005124336 | 0.912059019 | -0.022941025 |
| -0.037295368 | 0.421351031 | -0.070106598 | 0.130325878 | -0.06614413 |
| 0.03586338 | 0.439412341 | 0.047942057 | 0.301204121 | 0.045810828 |
| -0.076805857 | 0.097359863 | -0.065048257 | 0.160490395 | -0.069909438 |
| -0.102926941 | 0.026133698 | -0.101635509 | 0.028080104 | -0.118850584 |
| -0.053198745 | 0.251230287 | -0.05369456 | 0.246833378 | -0.04576895 |
| -0.057758574 | 0.212812657 | -0.026160024 | 0.572819215 | -0.04660601 |
| -0.013815868 | 0.765872981 | -0.043275929 | 0.350751999 | -0.067593948 |
| 0.011378452 | 0.80627108 | 0.023905737 | 0.606345838 | 0.020888924 |
| -0.103695281 | 0.025031738 | -0.063377839 | 0.171527821 | -0.08420219 |
| -0.066840916 | 0.149249747 | -0.051542582 | 0.266310802 | -0.075542166 |
| -0.035708168 | 0.441396257 | -0.023259428 | 0.616116892 | -0.031356721 |
| -0.092727215 | 0.045198907 | -0.054751763 | 0.23763822 | -0.074442078 |
| -0.082542519 | 0.074745894 | -0.065045755 | 0.160506516 | -0.066698003 |
| 0.032108045 | 0.488824491 | 0.079207754 | 0.087303985 | 0.038603614 |
| 0.011327309 | 0.807124698 | 0.050127999 | 0.279674057 | 0.038582438 |
| -0.046849838 | 0.312363513 | -0.016578108 | 0.720851968 | -0.039287175 |
| -0.155003166 | 0.000776595 | -0.128464941 | 0.005431884 | -0.132702987 |
| -0.22154456 | 1.33E-06 | -0.180583662 | 8.70E-05 | -0.200141298 |
| -0.279942276 | 7.42E-10 | -0.234550526 | 2.95E-07 | -0.243668211 |
| -0.184689614 | 5.94E-05 | -0.158616818 | 0.000580949 | -0.149401185 |
| -0.128162925 | 0.005543151 | -0.106825422 | 0.020946877 | -0.097755253 |
| -0.1269193 | 0.006023236 | -0.106031196 | 0.021924329 | -0.105174302 |
| 0.059050444 | 0.202740418 | 0.069267626 | 0.135000547 | 0.086256152 |
| -0.126748724 | 0.006091917 | -0.085110598 | 0.066112225 | -0.110151327 |
| -0.293362974 | 1.01E-10 | -0.302931424 | 2.28E-11 | -0.333446724 |
| -0.018890166 | 0.683888813 | -0.028838686 | 0.534159922 | -0.029826743 |
| 0.033072412 | 0.475857795 | 0.062652698 | 0.176491807 | 0.072658935 |
| -0.141287823 | 0.002210128 | -0.124903559 | 0.00688107 | -0.13493273 |
| -0.187059295 | 4.75E-05 | -0.136801143 | 0.003053368 | -0.143815656 |
| -0.147996145 | 0.001339724 | -0.109026504 | 0.018433278 | -0.123069269 |
| -0.14757308 | 0.001383548 | -0.108846658 | 0.0186283 | -0.127048892 |
| -0.235656408 | 2.58E-07 | -0.233844326 | 3.21E-07 | -0.212991141 |
| 0.112013843 | 0.015444769 | 0.173561999 | 0.000163706 | 0.197125764 |
| -0.207106028 | 6.40E-06 | -0.166819459 | 0.000293762 | -0.164462783 |

| | | | | |
|---|---|---|---|---|
| -0.166099593 | 0.00031228 | -0.13619246 | 0.003187953 | -0.141171027 |
| -0.068334587 | 0.140349886 | -0.039765112 | 0.391241805 | -0.031721075 |
| -0.195885338 | 2.02E-05 | -0.204896398 | 8.07E-06 | -0.181427464 |
| 0.011507015 | 0.80412629 | 0.022831717 | 0.622620761 | 0.031556556 |
| -0.180325429 | 8.91E-05 | -0.18003212 | 9.15E-05 | -0.225893072 |
| -0.110698477 | 0.016703841 | -0.059169862 | 0.201827144 | -0.063359938 |
| -0.078005947 | 0.092225832 | -0.083744039 | 0.070598554 | -0.094010813 |
| -0.127251178 | 0.005891595 | -0.145696073 | 0.00159433 | -0.16062576 |
| -0.162665988 | 0.000416574 | -0.157505226 | 0.000635623 | -0.170908017 |
| -0.314053009 | 3.78E-12 | -0.315111995 | 3.18E-12 | -0.328984321 |
| -0.174612043 | 0.000149163 | -0.141432961 | 0.002186803 | -0.16065194 |
| -0.101973131 | 0.027559593 | -0.056621798 | 0.221969366 | -0.050090976 |
| -0.099929933 | 0.030840569 | -0.102531784 | 0.026716535 | -0.159973111 |
| -0.0283685 | 0.540849688 | 0.002168697 | 0.962720128 | 0.017267446 |
| -0.089129178 | 0.054258372 | -0.097254515 | 0.035637612 | -0.129367517 |
| -0.187936431 | 4.37E-05 | -0.199083776 | 1.46E-05 | -0.174736071 |
| -0.13464639 | 0.003554367 | -0.115468576 | 0.012526067 | -0.120122567 |
| -0.229287524 | 5.48E-07 | -0.192825594 | 2.73E-05 | -0.176001355 |
| -0.216630644 | 2.30E-06 | -0.189561526 | 3.74E-05 | -0.199045437 |
| -0.182894983 | 7.03E-05 | -0.154221891 | 0.000826221 | -0.166465813 |
| -0.172860634 | 0.00017415 | -0.152648212 | 0.000935197 | -0.172711581 |
| -0.224076728 | 1.00E-06 | -0.1774891 | 0.000115281 | -0.196248516 |
| -0.084054514 | 0.069558021 | -0.054266493 | 0.241828529 | -0.064546245 |
| -0.013058609 | 0.778361707 | 0.00099434 | 0.982902414 | 0.024737849 |
| -0.232428255 | 3.79E-07 | -0.199590736 | 1.39E-05 | -0.197496702 |
| 0.074361865 | 0.108518127 | 0.085867137 | 0.063730985 | 0.110847423 |
| -0.344940609 | 1.71E-14 | -0.318743379 | 1.73E-12 | -0.328833368 |
| -0.046919062 | 0.311648276 | -0.010605349 | 0.819198666 | -0.026113284 |
| -0.05055443 | 0.275598679 | -0.024077554 | 0.603759933 | -0.0112388 |
| -0.115075854 | 0.012831304 | -0.086683831 | 0.061240135 | -0.096695546 |
| -0.13991305 | 0.002442606 | -0.121829355 | 0.008400779 | -0.111013994 |
| -0.186861982 | 4.84E-05 | -0.154980092 | 0.00077802 | -0.156548575 |
| -0.153456558 | 0.000877662 | -0.183350852 | 6.73E-05 | -0.18467359 |
| -0.074644677 | 0.107177491 | -0.074200853 | 0.109287271 | -0.075716147 |
| -0.096675629 | 0.036755176 | -0.100667218 | 0.029620008 | -0.084184093 |
| -0.160564969 | 0.000495524 | -0.115808186 | 0.012267297 | -0.112361579 |
| -0.23156622 | 4.20E-07 | -0.192577734 | 2.79E-05 | -0.198609362 |
| -0.1865407 | 4.99E-05 | -0.152498858 | 0.000946202 | -0.140870644 |
| -0.296644181 | 6.10E-11 | -0.265665844 | 5.52E-09 | -0.274823787 |
| -0.042298485 | 0.361749489 | 0.01048859 | 0.821155444 | 0.034598936 |
| 0.076376071 | 0.099252954 | 0.102317143 | 0.027037785 | 0.105861795 |
| -0.028196089 | 0.543313147 | 0.007890814 | 0.864956986 | 0.000815362 |
| -0.08658317 | 0.061542731 | -0.070014971 | 0.130830244 | -0.074178429 |
| -0.051337288 | 0.268222513 | -0.024945116 | 0.590778987 | -0.049774902 |
| 0.074485774 | 0.107929133 | 0.085674188 | 0.064331488 | 0.090963962 |

| | | | | |
|---|---|---|---|---|
| -0.064719907 | 0.162616635 | -0.049236257 | 0.288327806 | -0.050506727 |
| -0.188258059 | 4.24E-05 | -0.156394759 | 0.000694974 | -0.161954803 |
| -0.16622391 | 0.000309006 | -0.165160395 | 0.00033808 | -0.120357372 |
| -0.06383798 | 0.168432378 | -0.065965135 | 0.15466404 | -0.045517576 |
| -0.15074482 | 0.001084697 | -0.146945312 | 0.00145101 | -0.146482515 |
| -0.233956657 | 3.16E-07 | -0.212863757 | 3.47E-06 | -0.217985261 |
| -0.174838868 | 0.000146184 | -0.14938077 | 0.001205065 | -0.17566031 |
| -0.022866635 | 0.622088681 | 0.014910515 | 0.747927529 | 0.0280284 |
| -0.202122232 | 1.07E-05 | -0.17122978 | 0.000200899 | -0.176156568 |
| -0.017936399 | 0.699051477 | 0.019256141 | 0.678103836 | 0.028210149 |
| 0.044527544 | 0.336981816 | 0.029526005 | 0.524456343 | 0.045664778 |
| -0.155406644 | 0.000752061 | -0.128904108 | 0.005273673 | -0.156354979 |
| -0.028406479 | 0.540307777 | -0.006282729 | 0.892288992 | -0.03742066 |
| -0.102122638 | 0.027331761 | -0.081394002 | 0.078893643 | -0.102293096 |
| -0.160354107 | 0.000504172 | -0.126169828 | 0.006330281 | -0.143319055 |
| -0.143678231 | 0.001853486 | -0.108257145 | 0.019280228 | -0.132348914 |
| -0.107632097 | 0.019993074 | -0.085719019 | 0.064191551 | -0.109965072 |
| -0.178162998 | 0.000108466 | -0.151062076 | 0.001058339 | -0.166089524 |
| -0.123942623 | 0.007327286 | -0.119689515 | 0.009628498 | -0.101881503 |
| -0.127084534 | 0.005957368 | -0.131949031 | 0.004286109 | -0.113012263 |
| -0.099843405 | 0.030986597 | -0.093966574 | 0.042388507 | -0.052461524 |
| 0.075188759 | 0.104635145 | 0.087213293 | 0.05966867 | 0.089574926 |
| -0.144699204 | 0.001717891 | -0.102194525 | 0.027222792 | -0.109455313 |
| 0.06298285 | 0.174218662 | 0.073181491 | 0.11425686 | 0.08960977 |
| -0.259262367 | 1.31E-08 | -0.231782994 | 4.09E-07 | -0.22670928 |
| -0.082355877 | 0.07540759 | -0.041100419 | 0.375519595 | -0.057975354 |
| 0.076521841 | 0.098607623 | 0.131797058 | 0.004331127 | 0.133976016 |
| -0.201970273 | 1.09E-05 | -0.18933346 | 3.82E-05 | -0.201531571 |
| -0.214362969 | 2.95E-06 | -0.184565645 | 6.01E-05 | -0.198732797 |
| -0.116140814 | 0.012018434 | -0.077845868 | 0.092897861 | -0.064335056 |
| -0.327387985 | 3.96E-13 | -0.304143802 | 1.88E-11 | -0.339299536 |
| -0.117767621 | 0.010864463 | -0.087564172 | 0.058645731 | -0.089026065 |
| -0.127790051 | 0.005683349 | -0.13240148 | 0.004154573 | -0.136235501 |
| -0.183593832 | 6.58E-05 | -0.150736536 | 0.001085394 | -0.132414813 |
| -0.144616996 | 0.001728463 | -0.130206033 | 0.004828741 | -0.104766981 |
| -0.214899078 | 2.78E-06 | -0.183744531 | 6.49E-05 | -0.163177931 |
| -0.21081631 | 4.32E-06 | -0.19627514 | 1.94E-05 | -0.172456658 |
| -0.171895613 | 0.000189544 | -0.174725341 | 0.000147668 | -0.152625641 |
| -0.174927413 | 0.000145037 | -0.172298004 | 0.000182976 | -0.13882112 |
| -0.039364614 | 0.396033929 | -0.090375875 | 0.050961855 | -0.124004402 |
| -0.267470433 | 4.31E-09 | -0.242702043 | 1.09E-07 | -0.261268002 |
| -0.12692525 | 0.006020853 | -0.109631255 | 0.017790503 | -0.140375782 |
| -0.221198731 | 1.38E-06 | -0.1837825 | 6.47E-05 | -0.236831492 |
| -0.19499664 | 2.20E-05 | -0.160690138 | 0.000490456 | -0.18711366 |
| -0.125646191 | 0.006553063 | -0.090841898 | 0.049773312 | -0.099235082 |

| | | | | |
|---|---|---|---|---|
| -0.036645795 | 0.42948957 | 0.000797057 | 0.986294305 | 0.009083156 |
| -0.114470553 | 0.013314618 | -0.14334702 | 0.001899536 | -0.171106068 |
| -0.026951239 | 0.561263451 | -0.029097098 | 0.530501089 | -0.037890533 |
| -0.234429961 | 2.99E-07 | -0.212461243 | 3.62E-06 | -0.192585076 |
| -0.203185834 | 9.63E-06 | -0.154799116 | 0.000789283 | -0.19408689 |
| -0.063618024 | 0.169906803 | -0.023542337 | 0.611831316 | 0.030569715 |
| -0.093433107 | 0.043579613 | -0.07847062 | 0.090297111 | -0.072729002 |
| -0.159765035 | 0.00052908 | -0.135699984 | 0.003300762 | -0.151826818 |
| -0.150828747 | 0.001077666 | -0.113171112 | 0.014406681 | -0.106667286 |
| -0.255518261 | 2.14E-08 | -0.226457592 | 7.61E-07 | -0.22645523 |
| -0.247662824 | 5.88E-08 | -0.234880219 | 2.83E-07 | -0.255087555 |
| -0.194659685 | 2.28E-05 | -0.178974946 | 0.000100758 | -0.196633082 |
| 0.115799743 | 0.012273672 | 0.119081189 | 0.010005468 | 0.12679103 |
| -0.1989312 | 1.49E-05 | -0.173022302 | 0.000171689 | -0.171404998 |
| 0.050449667 | 0.276596129 | 0.05148644 | 0.266832672 | 0.021893535 |
| -0.214076469 | 3.04E-06 | -0.18288941 | 7.03E-05 | -0.190873673 |
| -0.163540523 | 0.000387302 | -0.151619838 | 0.001013426 | -0.160654996 |
| -0.233632676 | 3.29E-07 | -0.192643802 | 2.77E-05 | -0.202689692 |
| 0.006990683 | 0.880238477 | 0.016010811 | 0.730025652 | -0.007513741 |
| -0.110437425 | 0.016964138 | -0.083386639 | 0.07181207 | -0.069876609 |
| -0.304946642 | 1.66E-11 | -0.28926651 | 1.88E-10 | -0.278562913 |
| -0.103163215 | 0.025790465 | -0.06480837 | 0.162041719 | -0.077083877 |
| 0.194218044 | 2.38E-05 | 0.201944012 | 1.09E-05 | 0.235138018 |
| 0.062745677 | 0.175849417 | 0.08809592 | 0.057123195 | 0.10510169 |
| -0.161956028 | 0.000441839 | -0.152183243 | 0.00096985 | -0.164189378 |
| -0.104254881 | 0.02425461 | -0.083789157 | 0.070446558 | -0.076600757 |
| -0.142897387 | 0.001963728 | -0.103249092 | 0.025666678 | -0.089530029 |
| -0.060446425 | 0.192250586 | -0.045517054 | 0.326343996 | -0.043135794 |
| -0.246313852 | 6.97E-08 | -0.220044846 | 1.58E-06 | -0.250604215 |
| 0.121768141 | 0.008433863 | 0.109657798 | 0.017762746 | 0.122108258 |
| -0.15077086 | 0.001082511 | -0.174263358 | 0.000153852 | -0.175569024 |
| -0.056017626 | 0.226948877 | -0.026288141 | 0.57094044 | -0.041258945 |
| 0.080866222 | 0.08086128 | 0.118610191 | 0.010306278 | 0.098298892 |
| -0.136721416 | 0.003070697 | -0.131548944 | 0.004405545 | -0.136342894 |
| 0.124880968 | 0.006891274 | 0.166943561 | 0.000290675 | 0.145410779 |
| -0.129135027 | 0.00519216 | -0.111719122 | 0.015719401 | -0.118674924 |
| -0.138761407 | 0.002654292 | -0.098123281 | 0.034014709 | -0.078169143 |
| -0.159577279 | 0.000537257 | -0.157582011 | 0.000631698 | -0.156775719 |
| 0.057518432 | 0.214723979 | 0.052682495 | 0.255865996 | 0.064227178 |
| -0.177228275 | 0.000118025 | -0.165026329 | 0.000341921 | -0.191336403 |
| -0.133132741 | 0.003949684 | -0.112796233 | 0.014736029 | -0.154209325 |
| -0.169807797 | 0.000227315 | -0.140153775 | 0.002400353 | -0.17118322 |
| -0.256216426 | 1.95E-08 | -0.234426383 | 2.99E-07 | -0.252180031 |
| -0.083743075 | 0.070601803 | -0.067854322 | 0.143165978 | -0.065025994 |
| -0.075439409 | 0.103480104 | -0.026811123 | 0.563301639 | -0.015281079 |

| | | | | |
|---|---|---|---|---|
| -0.277831949 | 1.01E-09 | -0.220389847 | 1.52E-06 | -0.229036075 |
| -0.137806456 | 0.002842354 | -0.11405943 | 0.013651966 | -0.101870985 |
| -0.079392097 | 0.086568073 | -0.054814015 | 0.237104403 | -0.039057536 |
| -0.114651884 | 0.013168175 | -0.086237486 | 0.062591302 | -0.12282255 |
| -0.135859271 | 0.003263885 | -0.100735639 | 0.029508869 | -0.104801536 |
| 0.000126086 | 0.997831799 | 0.015866194 | 0.732370459 | 0.049507104 |
| -0.070392654 | 0.128760947 | -0.084392781 | 0.06843867 | -0.068638404 |
| -0.000994497 | 0.982899718 | 0.01460707 | 0.752888997 | 0.03663424 |
| -0.149656275 | 0.001179805 | -0.085297522 | 0.065517179 | -0.123333762 |
| -0.153366147 | 0.000883931 | -0.120947072 | 0.008888984 | -0.157807712 |
| -0.101875255 | 0.027709627 | -0.084513159 | 0.068043909 | -0.095493681 |
| -0.228183426 | 6.24E-07 | -0.19784014 | 1.66E-05 | -0.2232391 |
| -0.212713787 | 3.52E-06 | -0.177828219 | 0.000111803 | -0.172080785 |
| 0.053980379 | 0.244323274 | 0.028764141 | 0.535217766 | 0.047423574 |
| -0.202480922 | 1.04E-05 | -0.170980893 | 0.000205304 | -0.201895519 |
| -0.222613663 | 1.18E-06 | -0.242287834 | 1.15E-07 | -0.258196426 |
| -0.192818847 | 2.73E-05 | -0.168836561 | 0.000247188 | -0.189481769 |
| -0.123845724 | 0.007373688 | -0.089439373 | 0.053422013 | -0.123594354 |
| -0.058326893 | 0.208338175 | -0.020123104 | 0.664475478 | -0.016308837 |
| -0.143542776 | 0.001872194 | -0.10332088 | 0.025563595 | -0.144379585 |
| -0.10509736 | 0.02312398 | -0.104055596 | 0.024528938 | -0.089881045 |
| -0.101494701 | 0.028299671 | -0.068489873 | 0.139448494 | -0.064529669 |
| -0.142976078 | 0.001952352 | -0.119496373 | 0.009746794 | -0.130427528 |
| -0.182082477 | 7.58E-05 | -0.124778791 | 0.006937598 | -0.130892375 |
| -0.108448851 | 0.019066077 | -0.099169563 | 0.032144177 | -0.08250134 |
| -0.091893353 | 0.047176607 | -0.025209635 | 0.586846726 | -0.028671319 |
| -0.246159026 | 7.11E-08 | -0.223081224 | 1.12E-06 | -0.226747846 |
| 0.08866091 | 0.055541538 | 0.160622593 | 0.000493185 | 0.162421304 |
| -0.146766278 | 0.001470796 | -0.099232288 | 0.032034887 | -0.12393446 |
| -0.130308483 | 0.004795208 | -0.086431031 | 0.062002417 | -0.077746644 |
| -0.160497392 | 0.00049828 | -0.1589955 | 0.000563343 | -0.159382481 |
| -0.033911005 | 0.46473679 | -0.006581311 | 0.887203459 | 0.007486973 |
| -0.189628998 | 3.72E-05 | -0.173899173 | 0.000158897 | -0.164889894 |
| -0.124854849 | 0.006903089 | -0.095811949 | 0.03847778 | -0.071534566 |
| -0.016525547 | 0.72170026 | 0.008690586 | 0.851421214 | 0.015991974 |
| -0.030200177 | 0.515026581 | -0.005149692 | 0.911625634 | 0.003531037 |
| -0.140713271 | 0.002304711 | -0.104211023 | 0.024314754 | -0.104163798 |
| -0.055063522 | 0.234973306 | -0.014940281 | 0.747441392 | -0.034467912 |
| 0.068841384 | 0.137424481 | 0.142262608 | 0.002057734 | 0.153263538 |
| 0.130399755 | 0.004765511 | 0.168592036 | 0.000252442 | 0.188148166 |
| -0.165847097 | 0.000319029 | -0.117300665 | 0.011185209 | -0.131270095 |
| -0.017379518 | 0.70796071 | 0.003144534 | 0.945967133 | -0.003450131 |
| 0.088985654 | 0.054649016 | 0.069085465 | 0.136032389 | 0.097422761 |
| 0.045055645 | 0.331277059 | 0.095225056 | 0.039686967 | 0.09651582 |
| 0.025402582 | 0.583986074 | 0.022931435 | 0.621101773 | 0.022796234 |

| | | | | |
|---|---|---|---|---|
| -0.193402347 | 2.58E-05 | -0.171102614 | 0.000203139 | -0.188192743 |
| -0.125053533 | 0.006813668 | -0.085417866 | 0.065136417 | -0.082813098 |
| -0.029236931 | 0.5285265 | 0.00525632 | 0.909803506 | 0.028675722 |
| -0.054853781 | 0.236763843 | -0.01943744 | 0.675244995 | -0.033225518 |
| 0.042440838 | 0.36013457 | 0.090926606 | 0.049559782 | 0.104889925 |
| 0.126111718 | 0.006354664 | 0.118136476 | 0.01061689 | 0.0714927 |
| -0.100316729 | 0.030194973 | -0.064468893 | 0.164256321 | -0.069592272 |
| -0.084671645 | 0.067527026 | -0.022037666 | 0.634773161 | -0.050168888 |
| -0.022483532 | 0.627937133 | 0.029812506 | 0.520438254 | 0.053305898 |
| 0.003944767 | 0.932246284 | 0.071215559 | 0.124340185 | 0.059674031 |
| -0.214775874 | 2.82E-06 | -0.216699948 | 2.28E-06 | -0.208505378 |
| -0.107635172 | 0.019989512 | -0.109329167 | 0.018109096 | -0.146347208 |
| 0.073319121 | 0.113575713 | 0.143266262 | 0.001910922 | 0.133960438 |
| -0.081255681 | 0.079405529 | -0.014514522 | 0.754404238 | -0.042540415 |
| 0.148453763 | 0.00129376 | 0.149920032 | 0.00115608 | 0.148632928 |
| 0.050857891 | 0.272723234 | 0.116251178 | 0.011936854 | 0.114178427 |
| -0.0579848 | 0.21102332 | -0.026160629 | 0.572810334 | -0.027406948 |
| -0.028617628 | 0.537299923 | 0.012658853 | 0.784977803 | -0.011557068 |
| -0.011911074 | 0.797395111 | 0.030017166 | 0.517577685 | 0.040849691 |
| 0.008288666 | 0.858218327 | 0.060671721 | 0.190595607 | 0.037931385 |
| 0.028933315 | 0.532818608 | 0.082920966 | 0.073418712 | 0.078205016 |
| -0.057720325 | 0.213116263 | 0.000186962 | 0.996784961 | 0.036701651 |
| 0.003752009 | 0.935549641 | 0.053627032 | 0.247429043 | 0.025431492 |
| -0.047394946 | 0.306760543 | -0.00480184 | 0.917573188 | -0.038722521 |
| -0.032085192 | 0.489134064 | 0.001300924 | 0.977631937 | -0.024377243 |
| -0.089998935 | 0.051940447 | -0.057800556 | 0.212479775 | -0.063652731 |
| 0.015782004 | 0.73373666 | 0.061979792 | 0.181192968 | 0.035208954 |
| 0.017412859 | 0.707426163 | 0.06821786 | 0.14103039 | 0.071440181 |
| -0.076714013 | 0.097761976 | -0.078233589 | 0.091276876 | -0.066749839 |
| -0.046039208 | 0.320819195 | -0.047494008 | 0.30574949 | -0.083416957 |
| 0.145306357 | 0.00164163 | 0.208557663 | 5.49E-06 | 0.188031826 |
| -0.074597431 | 0.107400542 | -0.049235328 | 0.288336919 | -0.034135031 |
| -0.046724511 | 0.313661145 | 0.031805841 | 0.49292659 | 0.024242884 |
| -0.10964786 | 0.017773134 | -0.07453152 | 0.107712319 | -0.11329359 |
| -0.028460252 | 0.53954097 | 0.004621409 | 0.920659998 | -0.001383006 |
| -0.08746307 | 0.058938979 | -0.056912854 | 0.219598558 | -0.064967667 |
| -0.064649649 | 0.163074336 | 0.002697249 | 0.953643537 | -0.016038009 |
| 0.027666296 | 0.550917653 | 0.083038757 | 0.073009571 | 0.074286714 |
| -0.096709256 | 0.036689453 | -0.062317981 | 0.17881881 | -0.084187673 |
| 0.294837256 | 8.06E-11 | 0.308843653 | 8.87E-12 | 0.305176868 |
| 0.116564929 | 0.011707599 | 0.165383976 | 0.000331764 | 0.169964161 |
| -0.111676562 | 0.015759412 | -0.063516137 | 0.170593038 | -0.062883781 |
| -0.025823763 | 0.577764188 | 0.020625385 | 0.656629713 | 0.000892664 |
| -0.043106821 | 0.352639419 | -0.008605399 | 0.852860948 | -0.055986445 |
| 0.06372174 | 0.169210368 | 0.122554594 | 0.008017529 | 0.130613798 |

| | | | | |
|---|---|---|---|---|
| 0.124352356 | 0.007133953 | 0.176332647 | 0.000127923 | 0.161219729 |
| -0.049223463 | 0.288453258 | 0.011900132 | 0.797577197 | 0.025082872 |
| -0.179951674 | 9.22E-05 | -0.121945232 | 0.008338469 | -0.165442798 |
| -0.004130554 | 0.929063482 | 0.060341755 | 0.193023038 | 0.06614093 |
| -0.060017241 | 0.19543237 | -0.021374138 | 0.64500395 | -0.023535809 |
| 0.274961617 | 1.51E-09 | 0.300698351 | 3.25E-11 | 0.278529809 |
| 0.080776907 | 0.081198155 | 0.116246606 | 0.011940223 | 0.113199473 |
| -0.066148566 | 0.153517886 | -0.071438027 | 0.123165552 | -0.087454498 |
| 0.106530428 | 0.021305414 | 0.139533165 | 0.002510668 | 0.131973089 |
| 0.09907541 | 0.032308826 | 0.111484194 | 0.015941373 | 0.086334644 |
| 0.032145943 | 0.488311367 | 0.095130721 | 0.039884296 | 0.108722195 |
| 0.09271795 | 0.045220493 | 0.149955775 | 0.001152899 | 0.144952529 |
| -0.02288887 | 0.621749973 | 0.035619394 | 0.442533268 | 0.032737195 |
| -0.054275677 | 0.241748751 | -0.028072586 | 0.545081211 | -0.031773901 |
| 0.040709814 | 0.380077909 | 0.080753514 | 0.081286575 | 0.080008214 |
| -0.200485382 | 1.27E-05 | -0.196082403 | 1.98E-05 | -0.1799923 |
| 0.186537118 | 4.99E-05 | 0.229575515 | 5.30E-07 | 0.222777214 |
| -0.142171573 | 0.002071548 | -0.107749655 | 0.019857278 | -0.105234551 |
| 0.110030693 | 0.017376764 | 0.155292906 | 0.000758903 | 0.165864462 |
| 0.162289657 | 0.000429794 | 0.197703885 | 1.68E-05 | 0.202269113 |
| -0.047934018 | 0.301285278 | -0.015158506 | 0.743880462 | -0.029676771 |
| 0.105575552 | 0.022502756 | 0.093650427 | 0.04309103 | 0.073517281 |
| 8.12E-05 | 0.998603167 | 0.005801136 | 0.900500838 | -0.002845239 |
| -0.017476854 | 0.706400559 | 0.00581017 | 0.900346694 | 0.014830482 |
| -0.116476363 | 0.011771916 | -0.097229946 | 0.03568445 | -0.097807832 |
| 0.057360123 | 0.215990709 | 0.061886469 | 0.181852206 | 0.03237067 |
| -0.082085859 | 0.0763733 | -0.040428521 | 0.383381468 | -0.077617371 |
| 0.025248896 | 0.586264119 | 0.067190707 | 0.147128009 | 0.062516982 |
| -0.06648469 | 0.151434368 | -0.014424535 | 0.755878444 | -0.027535662 |
| -0.060959428 | 0.18849741 | -0.004347018 | 0.925356568 | 0.001078029 |
| 0.103640958 | 0.025108309 | 0.141797546 | 0.002129199 | 0.156933769 |
| 0.111758128 | 0.015682809 | 0.1390538 | 0.002599019 | 0.161489656 |
| 0.147864352 | 0.001353237 | 0.177067091 | 0.000119752 | 0.179652964 |
| 0.119521855 | 0.009731114 | 0.13275615 | 0.004054029 | 0.109796831 |
| 0.081212734 | 0.07956501 | 0.140847501 | 0.002282289 | 0.122872812 |
| -0.084428726 | 0.068320597 | -0.03233478 | 0.485758891 | -0.019109223 |
| 0.081848763 | 0.077229534 | 0.133026085 | 0.003978987 | 0.125632636 |
| 0.078550212 | 0.089970017 | 0.091535065 | 0.048048388 | 0.083937009 |
| -0.037513062 | 0.418643841 | -0.032389039 | 0.48502682 | -0.037625169 |
| 0.016479487 | 0.722443918 | 0.038935192 | 0.401211205 | 0.031818276 |
| -0.087644151 | 0.058414607 | -0.048079361 | 0.299820227 | -0.047542132 |
| -0.013596173 | 0.769490113 | 0.044430158 | 0.338040654 | 0.042156943 |
| 0.143291932 | 0.001907296 | 0.16183794 | 0.000446177 | 0.168179449 |
| 0.0539708 | 0.244407112 | 0.132136599 | 0.004231129 | 0.140225274 |
| -0.092991088 | 0.044587771 | -0.056959264 | 0.219222199 | -0.055159363 |

| | | | | |
|---|---|---|---|---|
| -0.159185549 | 0.000554695 | -0.114231132 | 0.01351017 | -0.131297762 |
| -0.024045613 | 0.604240282 | 0.027169378 | 0.558097407 | 0.01693754 |
| -0.042299009 | 0.36174353 | -0.027290252 | 0.556346782 | -0.045138135 |
| -0.023327983 | 0.615077209 | 0.03092432 | 0.504996562 | 0.039164274 |
| 0.131113812 | 0.004538849 | 0.180118349 | 9.08E-05 | 0.195152184 |
| -0.119509992 | 0.009738411 | -0.099530148 | 0.031520223 | -0.100309618 |
| -0.085807984 | 0.063914592 | -0.049161319 | 0.289063143 | -0.067484898 |
| 0.091606601 | 0.047873258 | 0.131090217 | 0.00454618 | 0.140546494 |
| 0.088916391 | 0.05483837 | 0.15041788 | 0.001112492 | 0.149149386 |
| 0.165942569 | 0.000316461 | 0.193956887 | 2.44E-05 | 0.190588757 |
| -0.102480146 | 0.026793519 | -0.062871632 | 0.174981969 | -0.071967916 |
| -0.020252021 | 0.662458212 | 0.019179111 | 0.679319893 | 0.013541355 |
| -0.073660755 | 0.111898704 | -0.010784988 | 0.816190297 | -0.025595454 |
| 0.042877031 | 0.355214343 | 0.106164207 | 0.021757925 | 0.1031676 |
| -0.03300684 | 0.476733464 | 0.012286274 | 0.791158097 | 0.010869891 |
| -0.013104969 | 0.777595456 | 0.045548604 | 0.32600844 | 0.039764135 |
| 0.101350925 | 0.028525385 | 0.132082251 | 0.004246993 | 0.119090177 |
| -0.043190028 | 0.351709946 | -0.044852147 | 0.333467915 | -0.050322258 |
| 0.076204936 | 0.100014857 | 0.134444181 | 0.003605014 | 0.13479117 |
| -0.021417883 | 0.644327363 | 0.011039768 | 0.811928176 | -0.006014332 |
| -0.121915937 | 0.008354182 | -0.049775218 | 0.283076292 | -0.065249591 |
| -0.03724884 | 0.421930977 | -0.010524558 | 0.820552534 | -0.030342198 |
| 0.293545094 | 9.82E-11 | 0.311118891 | 6.12E-12 | 0.30980051 |
| 0.087017538 | 0.060245749 | 0.113978289 | 0.013719429 | 0.113359737 |
| -0.044701732 | 0.335093251 | -0.004082777 | 0.929881867 | -0.019571774 |
| -0.062063666 | 0.180601994 | -0.004208854 | 0.927722423 | -0.01667978 |
| -0.060694896 | 0.190425968 | -0.007331789 | 0.874441982 | -0.026388976 |
| -0.043102505 | 0.352687669 | 0.021437521 | 0.64402373 | 0.020541838 |
| -0.031560786 | 0.49626639 | 0.020409522 | 0.65999699 | 0.008827045 |
| 0.124891712 | 0.00688642 | 0.173116811 | 0.000170265 | 0.133511601 |
| -0.201788066 | 1.11E-05 | -0.163647853 | 0.000383844 | -0.161064606 |
| -0.043315236 | 0.35031421 | 0.021474964 | 0.64344495 | 0.020638227 |
| 0.030931873 | 0.504892479 | 0.013672635 | 0.76823065 | -0.022501912 |
| -8.00E-05 | 0.998624028 | 0.021713626 | 0.63976093 | 0.029771533 |
| 0.07891077 | 0.088500146 | 0.075714634 | 0.102223473 | 0.059229426 |
| 0.099273819 | 0.031962698 | 0.119856819 | 0.00952706 | 0.136480148 |
| 0.117307853 | 0.011180209 | 0.136534657 | 0.003111641 | 0.118835991 |
| 0.04882572 | 0.292371654 | 0.127098107 | 0.005951987 | 0.112122012 |
| 0.021906548 | 0.63678942 | 0.075399533 | 0.103663181 | 0.078832761 |
| -0.137875445 | 0.002828372 | -0.102107973 | 0.027354037 | -0.101690378 |
| 0.087677479 | 0.058318516 | 0.143975057 | 0.001813087 | 0.155507411 |
| 0.11163504 | 0.015798532 | 0.135560983 | 0.003333252 | 0.12032557 |
| -0.044311093 | 0.339338081 | -0.010416248 | 0.822368377 | -0.013897931 |
| 0.09337924 | 0.043701438 | 0.135003591 | 0.003466473 | 0.131647198 |
| -0.140429045 | 0.002352854 | -0.091260713 | 0.048725022 | -0.094550963 |

| | | | | |
|---|---|---|---|---|
| -0.198943509 | 1.48E-05 | -0.15650518 | 0.00068885 | -0.156101995 |
| -0.099464725 | 0.031632655 | -0.068796656 | 0.137680768 | -0.0642574 |
| -0.126908894 | 0.006027405 | -0.114113832 | 0.013606898 | -0.146794014 |
| -0.135865119 | 0.003262538 | -0.134935381 | 0.003483103 | -0.137932832 |
| -0.175369791 | 0.00013943 | -0.118711564 | 0.010240865 | -0.130263967 |
| 0.076073847 | 0.100601613 | 0.09563205 | 0.038845067 | 0.094456359 |
| -0.032415567 | 0.484669112 | -0.028136783 | 0.544161814 | -0.025150722 |
| -0.059724132 | 0.197627361 | -0.027596533 | 0.551922885 | -0.020854813 |
| -0.061855241 | 0.182073201 | 0.003406031 | 0.941481531 | 0.015375285 |
| 0.152372741 | 0.000955587 | 0.163604305 | 0.000385244 | 0.161517057 |
| 0.062531562 | 0.177331352 | 0.107920148 | 0.019661761 | 0.092382369 |
| 0.144921966 | 0.001689542 | 0.182166622 | 7.52E-05 | 0.171457263 |
| 0.139808713 | 0.00246113 | 0.163622732 | 0.000384651 | 0.182823554 |
| -0.035399446 | 0.445357496 | -0.032535197 | 0.483057784 | -0.030627874 |
| -0.000127371 | 0.997809694 | 0.015877317 | 0.732190024 | 0.012893654 |
| 0.148869899 | 0.001253227 | 0.159916045 | 0.000522588 | 0.200673244 |
| 0.065417007 | 0.158127581 | 0.047731769 | 0.30333183 | 0.05377272 |
| 0.19363157 | 2.52E-05 | 0.212034121 | 3.79E-06 | 0.235898929 |
| -0.014718805 | 0.751060885 | 0.019820009 | 0.669227744 | 0.037657859 |
| 0.102949503 | 0.026100753 | 0.093414675 | 0.043621266 | 0.081363456 |
| 0.040060613 | 0.387728619 | 0.055892862 | 0.227986989 | 0.073642071 |
| 0.044239586 | 0.340118816 | 0.068605399 | 0.138780785 | 0.055130863 |
| 0.108397409 | 0.019123337 | 0.146800124 | 0.001467037 | 0.140672037 |
| 0.115976079 | 0.012141121 | 0.152527879 | 0.000944054 | 0.184149642 |
| 0.090041217 | 0.051829904 | 0.105443659 | 0.022672638 | 0.110650808 |
| 0.005454552 | 0.906417213 | 0.00272671 | 0.95313777 | 0.032784417 |
| 0.061330217 | 0.185818412 | 0.111549508 | 0.015879386 | 0.146883497 |
| 0.079638859 | 0.085590805 | 0.093189246 | 0.044133412 | 0.094046983 |
| 0.152726658 | 0.000929465 | 0.17343571 | 0.000165542 | 0.199724283 |
| 0.225830403 | 8.18E-07 | 0.256904206 | 1.78E-08 | 0.255928224 |
| 0.224901246 | 9.10E-07 | 0.229115033 | 5.60E-07 | 0.252463596 |
| 0.035220862 | 0.447658122 | 0.053618619 | 0.24750332 | 0.104818227 |
| 0.048877251 | 0.29186199 | 0.043779788 | 0.345166374 | 0.087973197 |
| 0.04311014 | 0.352602306 | 0.050503408 | 0.276084158 | 0.06590144 |
| 0.03269218 | 0.480947757 | 0.038652317 | 0.404643671 | 0.009091958 |
| 0.008131433 | 0.860880274 | 0.019502363 | 0.674222386 | 0.024585141 |
| 0.138581314 | 0.002688867 | 0.164980521 | 0.000343243 | 0.189163994 |
| 0.054679501 | 0.238258943 | 0.079776918 | 0.085047931 | 0.067787026 |
| 0.045902561 | 0.322259115 | 0.067296011 | 0.146493785 | 0.065197059 |
| 0.040177655 | 0.386342431 | 0.053433708 | 0.249139887 | 0.078361746 |
| 0.046076409 | 0.320427918 | 0.038238832 | 0.409692367 | 0.061426089 |
| 0.198248709 | 1.59E-05 | 0.200997887 | 1.20E-05 | 0.233722568 |
| -0.041071575 | 0.375855043 | -0.045183126 | 0.329909343 | -0.007603307 |
| 0.056101912 | 0.226249479 | 0.044624326 | 0.335931652 | 0.043109171 |
| 0.102244064 | 0.027147916 | 0.099474367 | 0.031616064 | 0.097721989 |

| | | | | |
|---|---|---|---|---|
| 0.031953995 | 0.490913261 | 0.017355309 | 0.708348919 | 0.043229372 |
| 0.045256211 | 0.329126876 | 0.029215871 | 0.528823651 | 0.074288313 |
| 0.164486473 | 0.000357803 | 0.164753994 | 0.000349849 | 0.19934674 |
| 0.108156803 | 0.019393151 | 0.108705292 | 0.018782855 | 0.141953619 |
| 0.100945261 | 0.029170588 | 0.10712716 | 0.020585576 | 0.117005499 |
| 0.091213461 | 0.048842362 | 0.041927638 | 0.365977752 | 0.02463801 |
| 0.044348953 | 0.338925182 | 0.056142799 | 0.225910755 | 0.061913511 |
| 0.117518384 | 0.011034632 | 0.124823489 | 0.006917299 | 0.138160214 |
| 0.139035489 | 0.00260245 | 0.117361549 | 0.011142919 | 0.142081567 |
| 0.088326109 | 0.056474345 | 0.10942651 | 0.018005895 | 0.105125714 |
| 0.069447763 | 0.133986106 | 0.074393231 | 0.108368793 | 0.08880502 |
| 0.016245624 | 0.726223758 | 0.029267749 | 0.528091816 | 0.03427872 |
| 0.0642601 | 0.165629642 | 0.07137932 | 0.123474685 | 0.076515916 |
| 0.065401643 | 0.158225497 | 0.082430875 | 0.075141131 | 0.095472952 |
| 0.132075349 | 0.004249012 | 0.144866069 | 0.001696615 | 0.153083906 |
| 0.038754468 | 0.403402136 | 0.038859679 | 0.402125788 | 0.037711915 |
| 0.069141363 | 0.135715116 | 0.07322803 | 0.114026176 | 0.080506932 |
| -0.049511261 | 0.285640106 | -0.039767767 | 0.391210154 | -0.023598327 |
| 0.173140744 | 0.000169906 | 0.194884619 | 2.23E-05 | 0.217822591 |
| -0.048255432 | 0.298051788 | -0.065619491 | 0.156841357 | -0.093460723 |
| 0.092509205 | 0.045709121 | 0.121327036 | 0.008675719 | 0.127324416 |
| 0.031915056 | 0.491441995 | 0.057032845 | 0.21862646 | 0.033484567 |
| 0.08839448 | 0.056282804 | 0.081736613 | 0.077637252 | 0.07828908 |
| 0.083759584 | 0.070546154 | 0.074112945 | 0.109709013 | 0.104889535 |
| 0.089931043 | 0.052118361 | 0.065419237 | 0.15811337 | 0.077309113 |
| -0.001688989 | 0.970962166 | 0.016402498 | 0.723687533 | 0.028292196 |
| 0.091959236 | 0.047017754 | 0.107117558 | 0.020596988 | 0.119497391 |
| 0.210460083 | 4.49E-06 | 0.212992663 | 3.42E-06 | 0.252243663 |
| -0.02635431 | 0.569971251 | -0.007913229 | 0.864577061 | 0.02349281 |
| 0.134002311 | 0.003717974 | 0.182985112 | 6.97E-05 | 0.184747377 |
| 0.125892395 | 0.006447454 | 0.170491839 | 0.000214226 | 0.187388636 |
| -0.01658146 | 0.720797875 | 0.020626494 | 0.656612435 | 0.02564721 |
| 0.098377752 | 0.033551435 | 0.076321166 | 0.099496889 | 0.08513568 |
| 0.119065354 | 0.010015453 | 0.10722479 | 0.020469839 | 0.148709772 |
| 0.097073122 | 0.035984648 | 0.108208716 | 0.019334657 | 0.1217782 |
| 0.015585154 | 0.736934347 | 0.016704394 | 0.718815205 | 0.025970542 |
| 0.093530817 | 0.043359367 | 0.112292476 | 0.015189008 | 0.118978567 |
| 0.088774105 | 0.055229069 | 0.085838275 | 0.063820516 | 0.096026119 |
| 0.24936395 | 4.74E-08 | 0.265304079 | 5.80E-09 | 0.264183584 |
| 0.056067483 | 0.22653498 | 0.068690079 | 0.138292915 | 0.101190799 |
| 0.004802965 | 0.917553938 | 0.031848427 | 0.492347418 | 0.045234238 |
| 0.101855231 | 0.027740408 | 0.104120826 | 0.024438851 | 0.133164743 |
| 0.046003005 | 0.321200275 | 0.071027956 | 0.125337481 | 0.103742527 |
| 0.062934678 | 0.174548968 | 0.082645604 | 0.074382465 | 0.089884617 |
| 0.085550645 | 0.064718424 | 0.101609464 | 0.028120606 | 0.119820031 |

| | | | | |
|---|---|---|---|---|
| 0.287308039 | 2.52E-10 | 0.310269113 | 7.03E-12 | 0.300912747 |
| 0.042356862 | 0.361086682 | 0.056166051 | 0.225718284 | 0.062067817 |
| 0.173898774 | 0.000158903 | 0.186657539 | 4.94E-05 | 0.196620504 |
| 0.13168494 | 0.004364613 | 0.166450254 | 0.000303127 | 0.203312101 |
| 0.096609861 | 0.036884004 | 0.16192428 | 0.000443001 | 0.178224479 |
| 0.08405514 | 0.069555936 | 0.127977449 | 0.005612495 | 0.151735226 |
| 0.085272439 | 0.065596771 | 0.126138195 | 0.006343544 | 0.124802008 |
| 0.150207082 | 0.00113076 | 0.179010364 | 0.000100434 | 0.204597448 |
| 0.146601986 | 0.001489171 | 0.174099632 | 0.000156101 | 0.215321728 |
| 0.104472043 | 0.023958696 | 0.135349974 | 0.003383128 | 0.157868942 |
| 0.061374629 | 0.185499423 | 0.07243878 | 0.117988089 | 0.109537419 |
| 0.160635624 | 0.000492657 | 0.197225386 | 1.76E-05 | 0.229910813 |
| 0.100904372 | 0.029236312 | 0.133929796 | 0.003736815 | 0.162179251 |
| 0.054201041 | 0.242397657 | 0.086623673 | 0.061420829 | 0.113895749 |
| 0.052435021 | 0.258109088 | 0.06417794 | 0.1661724 | 0.082448084 |
| 0.147604108 | 0.00138029 | 0.176813972 | 0.000122511 | 0.204416631 |
| 0.194744572 | 2.26E-05 | 0.219708704 | 1.64E-06 | 0.241414116 |
| 0.050971207 | 0.271654764 | 0.029991889 | 0.517930549 | 0.051524887 |
| 0.066657108 | 0.150373968 | 0.080758554 | 0.081267518 | 0.103470146 |
| 0.068372055 | 0.14013199 | 0.090694343 | 0.050147102 | 0.087445106 |
| 0.126456833 | 0.006211079 | 0.128367464 | 0.005467574 | 0.143791109 |
| 0.242951045 | 1.06E-07 | 0.22896359 | 5.70E-07 | 0.274941184 |
| -0.051381677 | 0.267808372 | -0.068683868 | 0.138328653 | -0.074174856 |
| 0.122799359 | 0.00789175 | 0.132543978 | 0.004113909 | 0.152560015 |
| 0.161233636 | 0.000469003 | 0.187636828 | 4.50E-05 | 0.203571269 |
| 0.221439972 | 1.35E-06 | 0.208564302 | 5.49E-06 | 0.273310644 |
| 0.13799964 | 0.002803359 | 0.203516432 | 9.31E-06 | 0.165074542 |
| -0.008643858 | 0.852210894 | 0.048003408 | 0.300585239 | 0.054690541 |
| 0.064108926 | 0.16662934 | 0.11817998 | 0.010588022 | 0.094908079 |
| 0.270411721 | 2.87E-09 | 0.27560266 | 1.38E-09 | 0.28865818 |
| 0.289946797 | 1.69E-10 | 0.35111407 | 5.39E-15 | 0.33428417 |
| -0.003311096 | 0.943109784 | 0.071773112 | 0.121412629 | 0.061766592 |
| 0.105201118 | 0.02298794 | 0.152682742 | 0.00093267 | 0.151487707 |
| -0.043779658 | 0.345167811 | -0.018493224 | 0.690184262 | -0.028946083 |
| -0.002868854 | 0.95069787 | 0.046942099 | 0.311410496 | 0.054643082 |
| -0.007445234 | 0.872515645 | 0.049502486 | 0.285725602 | 0.033010971 |
| 0.039447526 | 0.39503897 | 0.062915819 | 0.174678408 | 0.045850446 |
| -0.021537753 | 0.642474889 | 0.040469737 | 0.382896331 | 0.00947357 |
| 0.114426237 | 0.013350625 | 0.15898398 | 0.000563871 | 0.148841684 |
| 0.076858088 | 0.097131777 | 0.113686092 | 0.013964807 | 0.103037611 |
| 0.080466635 | 0.082377261 | 0.137939634 | 0.002815419 | 0.132835862 |
| -0.122085631 | 0.008263524 | -0.058826314 | 0.204462604 | -0.050937664 |
| 0.135406425 | 0.003369719 | 0.177111991 | 0.000119268 | 0.161799982 |
| 0.03083084 | 0.50628555 | 0.08376341 | 0.070533261 | 0.074870153 |
| 0.056928646 | 0.219470444 | 0.080042227 | 0.084012479 | 0.081339512 |

| | | | | |
|---|---|---|---|---|
| 0.087125584 | 0.059926665 | 0.144836956 | 0.001700309 | 0.132215907 |
| -0.031119099 | 0.502316305 | 0.035227979 | 0.447566309 | 0.048708136 |
| 0.023197773 | 0.617052605 | 0.089131028 | 0.054253353 | 0.083038337 |
| -0.0249974 | 0.590000794 | 0.025624088 | 0.58071001 | 0.019886645 |
| 0.111797463 | 0.015645984 | 0.163217302 | 0.00039789 | 0.156836472 |
| 0.072806469 | 0.116129153 | 0.115755666 | 0.012307003 | 0.105605942 |
| -0.115524756 | 0.01248293 | -0.069304636 | 0.134791644 | -0.085033748 |
| 0.087387738 | 0.05915827 | 0.133352601 | 0.003889896 | 0.106915266 |
| 0.207722161 | 6.00E-06 | 0.269128023 | 3.43E-09 | 0.253736903 |
| 0.081188483 | 0.079655182 | 0.140697754 | 0.002307316 | 0.131354053 |
| -0.06477201 | 0.162277832 | -0.00995899 | 0.830044774 | -0.047657564 |
| 0.071912447 | 0.12068948 | 0.096986269 | 0.036151828 | 0.111650388 |
| 0.073561623 | 0.112383305 | 0.099803429 | 0.031054263 | 0.1101479 |
| 0.075732909 | 0.102140464 | 0.148820347 | 0.001257991 | 0.135393196 |
| 0.034253114 | 0.460241644 | 0.071111331 | 0.124893495 | 0.076040077 |
| 0.082844466 | 0.07368543 | 0.135153012 | 0.003430294 | 0.153969521 |
| 0.034596413 | 0.455755333 | 0.064459493 | 0.164317967 | 0.043256564 |
| 0.140197601 | 0.002392732 | 0.15628768 | 0.00070096 | 0.14493536 |
| -0.032350126 | 0.48555178 | 0.022763768 | 0.623656744 | -0.005825584 |
| -0.081559312 | 0.078285389 | -0.032405393 | 0.484806287 | -0.037645043 |
| 0.059292912 | 0.200889219 | 0.126771684 | 0.006082632 | 0.111249997 |
| 0.282172848 | 5.36E-10 | 0.33909049 | 4.98E-14 | 0.297434594 |
| 0.062277615 | 0.179100971 | 0.109431238 | 0.018000896 | 0.133473866 |
| 0.062410973 | 0.178170046 | 0.096804979 | 0.036502911 | 0.068881917 |
| -0.141921965 | 0.002109861 | -0.080696097 | 0.081503929 | -0.086653709 |
| 0.080583661 | 0.081930918 | 0.151187886 | 0.001048051 | 0.147406962 |
| 0.056464087 | 0.223261609 | 0.114713976 | 0.013118357 | 0.109070232 |
| -0.091157658 | 0.048981238 | -0.034584602 | 0.455909269 | -0.049217761 |
| 0.044404959 | 0.338314975 | 0.092352199 | 0.046079555 | 0.056168468 |
| 0.136303931 | 0.003162909 | 0.183763486 | 6.48E-05 | 0.19791421 |
| 0.049185366 | 0.288827043 | 0.105141008 | 0.023066668 | 0.090913212 |
| 0.063919428 | 0.167888848 | 0.120213145 | 0.009314177 | 0.156291234 |
| 0.086808761 | 0.06086629 | 0.164946965 | 0.000344214 | 0.167463295 |
| 0.183561858 | 6.60E-05 | 0.252750738 | 3.07E-08 | 0.248960299 |
| 0.15409929 | 0.000834269 | 0.195920091 | 2.01E-05 | 0.171716135 |
| 0.186878063 | 4.83E-05 | 0.221096469 | 1.40E-06 | 0.195553522 |
| 0.04240251 | 0.360568937 | 0.103421368 | 0.025419898 | 0.10627962 |
| -0.016783789 | 0.717535712 | 0.028167205 | 0.543726398 | 0.029144863 |
| 0.105045042 | 0.02319284 | 0.165681951 | 0.000323517 | 0.186085485 |
| 0.239750783 | 1.57E-07 | 0.290521572 | 1.55E-10 | 0.284557231 |
| 0.054676415 | 0.238285482 | 0.113214954 | 0.01436859 | 0.079763469 |
| 0.04605369 | 0.320666838 | 0.078196039 | 0.091432869 | 0.094883703 |
| 0.108353211 | 0.019172654 | 0.153840774 | 0.000851479 | 0.135717708 |
| 0.175321034 | 0.000140038 | 0.196966413 | 1.81E-05 | 0.221159218 |
| -0.051300398 | 0.26856703 | -0.016500796 | 0.722099854 | -0.023615169 |

| | | | | |
|---|---|---|---|---|
| 0.033140267 | 0.474952562 | 0.075647471 | 0.102529005 | 0.100696321 |
| -0.031532046 | 0.496658872 | 0.011529735 | 0.803747419 | 0.018630338 |
| 0.066065889 | 0.154033689 | 0.089891894 | 0.052221183 | 0.073126071 |
| 0.275151509 | 1.47E-09 | 0.30851993 | 9.34E-12 | 0.291989205 |
| 0.10313907 | 0.025825361 | 0.160918296 | 0.000481341 | 0.145746934 |
| -0.05073018 | 0.273930856 | 0.007979268 | 0.863457928 | 0.006308166 |
| -0.018697756 | 0.68693772 | 0.027341455 | 0.555606003 | 0.0272571 |
| -0.147676161 | 0.001372751 | -0.09456235 | 0.041090843 | -0.084935661 |
| -0.016162218 | 0.727573435 | 0.023286273 | 0.615709678 | 0.001733527 |
| 0.137226721 | 0.00296236 | 0.180183182 | 9.02E-05 | 0.121797197 |
| -0.111540363 | 0.015888052 | -0.087364726 | 0.059225391 | -0.11714516 |
| 0.03967008 | 0.392375712 | 0.09276734 | 0.045105526 | 0.100266067 |
| 0.193807836 | 2.48E-05 | 0.231703995 | 4.13E-07 | 0.228627589 |
| 0.050245649 | 0.278545632 | 0.117849225 | 0.010809255 | 0.110109783 |
| 0.01242488 | 0.788857354 | 0.065945652 | 0.154786156 | 0.045722839 |
| -0.066165896 | 0.153409935 | 0.011729106 | 0.800424678 | -0.005118209 |
| -0.038857719 | 0.402149533 | 0.019569447 | 0.673166362 | 0.010059965 |
| 0.026982534 | 0.560808715 | 0.067455419 | 0.145537674 | 0.073464287 |
| -0.09789668 | 0.03443182 | -0.041461568 | 0.371335112 | -0.049222127 |
| -0.064170379 | 0.166222417 | 0.003934809 | 0.932416897 | -0.008705071 |
| -0.066114685 | 0.153729099 | -0.000385596 | 0.993369286 | -0.008771964 |
| 0.117024065 | 0.01137913 | 0.195943747 | 2.00E-05 | 0.176298916 |
| 0.085110504 | 0.066112525 | 0.132265056 | 0.004193844 | 0.107318907 |
| 0.016254352 | 0.726082574 | 0.072633209 | 0.117002229 | 0.067219203 |
| -0.054365979 | 0.240965274 | 0.008125575 | 0.860979481 | -0.025332809 |
| 0.254860463 | 2.33E-08 | 0.314469567 | 3.53E-12 | 0.341751321 |
| 0.194002231 | 2.43E-05 | 0.236914064 | 2.22E-07 | 0.241807872 |
| 0.059052552 | 0.202724277 | 0.093766445 | 0.042832091 | 0.082040592 |
| 0.106505342 | 0.021336149 | 0.162086171 | 0.000437103 | 0.149669117 |
| -0.038060258 | 0.411884266 | -0.001888064 | 0.967541358 | -0.001211328 |
| 0.019647828 | 0.671933306 | 0.066822048 | 0.149364855 | 0.078391264 |
| -0.04617886 | 0.319351952 | 0.03191695 | 0.491416265 | 0.034511491 |
| 0.003907016 | 0.932893135 | 0.072448274 | 0.117939796 | 0.048930702 |
| 0.154645567 | 0.000798957 | 0.199575716 | 1.39E-05 | 0.159753504 |
| 0.030488052 | 0.511026948 | 0.113007997 | 0.014549181 | 0.089194246 |
| 0.041376046 | 0.3723234 | 0.0898787 | 0.052255874 | 0.07077259 |
| -0.091995219 | 0.046931185 | -0.04245084 | 0.360021269 | -0.056593805 |
| 0.120935289 | 0.008895672 | 0.160469632 | 0.000499417 | 0.159706741 |
| 0.022246656 | 0.631565017 | 0.082164783 | 0.076089998 | 0.07609162 |
| 0.098720055 | 0.032936753 | 0.122635785 | 0.007975609 | 0.110408306 |
| 0.243044878 | 1.05E-07 | 0.294930264 | 7.95E-11 | 0.275820856 |
| 0.04933936 | 0.287318162 | 0.07630972 | 0.099547803 | 0.069424764 |
| 0.13924176 | 0.002564044 | 0.186932019 | 4.81E-05 | 0.157251966 |
| -0.007654911 | 0.868957274 | 0.064788557 | 0.162170349 | 0.057742822 |
| 0.02187837 | 0.637223074 | 0.073142833 | 0.114448757 | 0.060374423 |

| | | | | |
|---|---|---|---|---|
| 0.067966795 | 0.142502631 | 0.084129186 | 0.069309648 | 0.089141358 |
| 0.09775821 | 0.034688845 | 0.15000223 | 0.001148777 | 0.133738102 |
| 0.076404152 | 0.099128378 | 0.131327451 | 0.004472951 | 0.108692694 |
| -0.060572601 | 0.191322431 | 0.022493242 | 0.627788615 | 0.022823869 |
| 0.085587959 | 0.064601355 | 0.158229614 | 0.000599478 | 0.139100321 |
| 0.025957003 | 0.575802398 | 0.103551468 | 0.025234892 | 0.092033624 |
| 0.02818714 | 0.543441167 | 0.080712607 | 0.081441382 | 0.070775128 |
| -0.043075812 | 0.352986205 | 0.002965594 | 0.949037582 | 0.002094321 |
| 0.029078406 | 0.530765317 | 0.084671322 | 0.067528075 | 0.094872719 |
| -0.054373895 | 0.240896676 | 0.032637148 | 0.481686883 | 0.026705349 |
| 0.218806017 | 1.81E-06 | 0.182146437 | 7.53E-05 | 0.216867489 |
| 0.225401977 | 8.59E-07 | 0.194947133 | 2.21E-05 | 0.19175531 |
| 0.34047233 | 3.87E-14 | 0.322708705 | 8.86E-13 | 0.293121898 |
| 0.295214549 | 7.61E-11 | 0.288808293 | 2.01E-10 | 0.267031259 |
| 0.122333653 | 0.008132598 | 0.11595663 | 0.012155678 | 0.130471861 |
| 0.420974005 | 1.75E-21 | 0.41127644 | 1.73E-20 | 0.416932997 |
| -0.061427912 | 0.185117247 | -0.063378405 | 0.171523986 | -0.111985556 |
| 0.155113861 | 0.000769791 | 0.14755774 | 0.001385162 | 0.121478524 |
| -0.074200173 | 0.109290532 | -0.088439125 | 0.056158022 | -0.12936687 |
| 0.29075898 | 1.50E-10 | 0.265212626 | 5.87E-09 | 0.253533276 |
| 0.159452732 | 0.000542745 | 0.14202475 | 0.002094007 | 0.113137754 |
| 0.09640393 | 0.037289875 | 0.063829799 | 0.168487046 | 0.070505715 |
| 0.240137883 | 1.50E-07 | 0.221046516 | 1.41E-06 | 0.238103154 |
| 0.154763344 | 0.000791527 | 0.166952413 | 0.000290456 | 0.151830137 |
| 0.188998035 | 3.95E-05 | 0.139251448 | 0.002562253 | 0.095039552 |
| 0.051246362 | 0.269072222 | 0.011183979 | 0.809518187 | 0.003889231 |
| 0.222716354 | 1.17E-06 | 0.18619389 | 5.16E-05 | 0.201431009 |
| 0.27510516 | 1.48E-09 | 0.276823288 | 1.16E-09 | 0.281016938 |
| 0.261704031 | 9.43E-09 | 0.248899462 | 5.03E-08 | 0.24713745 |
| 0.072175496 | 0.119333416 | 0.084592984 | 0.067783165 | 0.098550254 |
| 0.012503525 | 0.787552736 | -0.013153451 | 0.776794373 | -0.006994751 |
| -0.014806897 | 0.749620563 | -0.034075137 | 0.462577138 | -0.056957677 |
| 0.104678909 | 0.023679719 | 0.100173812 | 0.030432152 | 0.102761208 |
| 0.068311033 | 0.140486998 | 0.033387133 | 0.47166716 | 0.025478932 |
| 0.13075408 | 0.004651792 | 0.105232789 | 0.022946554 | 0.076183104 |
| 0.22401334 | 1.01E-06 | 0.194693188 | 2.27E-05 | 0.168295408 |
| 0.18005748 | 9.13E-05 | 0.208890233 | 5.30E-06 | 0.193442853 |
| 0.108038472 | 0.019527058 | 0.106715911 | 0.021079361 | 0.092392082 |
| 0.246029407 | 7.22E-08 | 0.240749976 | 1.39E-07 | 0.195650944 |
| 0.282883812 | 4.83E-10 | 0.258656272 | 1.42E-08 | 0.267946418 |
| 0.044992325 | 0.331957765 | 0.015629956 | 0.736206164 | 0.011931653 |
| 0.055764176 | 0.229061253 | 0.049193054 | 0.288751589 | 0.025884387 |
| 0.143769168 | 0.001841022 | 0.129835839 | 0.004951677 | 0.102981259 |
| 0.16281138 | 0.000411569 | 0.137735129 | 0.002856877 | 0.09264303 |
| 0.154854794 | 0.000785802 | 0.128228743 | 0.005518729 | 0.133135727 |

| | | | | |
|---|---|---|---|---|
| 0.135717759 | 0.003296628 | 0.125274865 | 0.006715277 | 0.143990308 |
| 0.266962415 | 4.62E-09 | 0.257302981 | 1.69E-08 | 0.271467875 |
| -0.038748555 | 0.40347393 | -0.017867627 | 0.700149525 | -0.056607728 |
| 0.126881346 | 0.006038457 | 0.113168456 | 0.014408992 | 0.096594963 |
| 0.134867706 | 0.003499674 | 0.083704656 | 0.070731447 | 0.06796494 |
| 0.041731444 | 0.368227063 | 0.020552604 | 0.657764261 | 0.05600399 |
| 0.219709002 | 1.64E-06 | 0.187055948 | 4.75E-05 | 0.203283944 |
| 0.172213172 | 0.000184343 | 0.166283248 | 0.000307455 | 0.142223877 |
| 0.211678104 | 3.94E-06 | 0.210416558 | 4.51E-06 | 0.213720989 |
| 0.183546503 | 6.61E-05 | 0.184615989 | 5.98E-05 | 0.170641047 |
| 0.251809646 | 3.46E-08 | 0.263555666 | 7.35E-09 | 0.261933275 |
| 0.149256071 | 0.001216661 | 0.139129231 | 0.002584932 | 0.109685546 |
| 0.174574994 | 0.000149654 | 0.13774424 | 0.002855018 | 0.142812756 |
| 0.299874803 | 3.69E-11 | 0.291435938 | 1.35E-10 | 0.283279591 |
| 0.249127421 | 4.88E-08 | 0.219571567 | 1.66E-06 | 0.208870667 |
| 0.07170723 | 0.121755731 | 0.072769053 | 0.116317266 | 0.057383361 |
| 0.119404454 | 0.009803545 | 0.12290698 | 0.007837006 | 0.091707136 |
| 0.115429212 | 0.01255637 | 0.084754972 | 0.067256559 | 0.093816779 |
| 0.231512497 | 4.23E-07 | 0.249184026 | 4.85E-08 | 0.262308273 |
| 0.219590744 | 1.66E-06 | 0.216070701 | 2.45E-06 | 0.271278104 |
| 0.167052129 | 0.000287999 | 0.200530371 | 1.26E-05 | 0.222042833 |
| 0.208063888 | 5.79E-06 | 0.202062515 | 1.08E-05 | 0.19545021 |
| 0.182187722 | 7.50E-05 | 0.193957469 | 2.44E-05 | 0.220883834 |
| -0.118803042 | 0.010182154 | -0.14738359 | 0.001403601 | -0.168869813 |
| 0.063158987 | 0.173014876 | 0.054311764 | 0.241435442 | 0.043378027 |
| 0.204694738 | 8.24E-06 | 0.174448734 | 0.000151342 | 0.177719745 |
| 0.099583209 | 0.031429286 | 0.063726306 | 0.169179762 | 0.032526103 |
| 0.111692493 | 0.015744424 | 0.126346738 | 0.006256565 | 0.139959834 |
| 0.276675641 | 1.19E-09 | 0.200773005 | 1.23E-05 | 0.207345324 |
| 0.063442633 | 0.17108939 | 0.045800104 | 0.3233415 | 0.043572234 |
| 0.231765425 | 4.10E-07 | 0.168422407 | 0.000256147 | 0.170625875 |
| 0.098896201 | 0.032624207 | 0.048545732 | 0.29515125 | 0.024058186 |
| 0.142264103 | 0.002057508 | 0.107120777 | 0.020593162 | 0.087009235 |
| 0.315688767 | 2.89E-12 | 0.307068526 | 1.18E-11 | 0.282357813 |
| 0.021163326 | 0.648268615 | 0.011440629 | 0.80523361 | -0.016302279 |
| 0.158276985 | 0.000597182 | 0.176988828 | 0.000120598 | 0.204944739 |
| 0.11779079 | 0.010848763 | 0.119484199 | 0.009754294 | 0.102769061 |
| 0.236428132 | 2.35E-07 | 0.208912129 | 5.29E-06 | 0.19743105 |
| 0.186412359 | 5.05E-05 | 0.173478683 | 0.000164915 | 0.130570757 |
| 0.116216473 | 0.011962454 | 0.04903196 | 0.29033544 | 0.057918533 |
| -0.041894406 | 0.366358141 | -0.039841329 | 0.390333833 | -0.051154671 |
| -0.057377869 | 0.215848447 | -0.072344255 | 0.11846972 | -0.095378331 |
| 0.119317019 | 0.009857799 | 0.13030173 | 0.004797412 | 0.108508471 |
| 0.24252259 | 1.12E-07 | 0.223365623 | 1.08E-06 | 0.211095433 |
| 0.304318423 | 1.83E-11 | 0.291592343 | 1.32E-10 | 0.305438617 |

| | | | | |
|---|---|---|---|---|
| 0.270745153 | 2.74E-09 | 0.25775342 | 1.60E-08 | 0.264798761 |
| 0.089340591 | 0.053687182 | 0.068147474 | 0.141441953 | 0.043852864 |
| 0.143980822 | 0.001812311 | 0.15188933 | 0.000992362 | 0.176846351 |
| 0.085931874 | 0.063530547 | 0.067865083 | 0.143102408 | 0.029802925 |
| -0.111353164 | 0.016066366 | -0.119631879 | 0.009663665 | -0.149831049 |
| 0.037105839 | 0.423716292 | 0.021336406 | 0.645587787 | 0.00541167 |
| 0.133392889 | 0.00387903 | 0.134917378 | 0.003487504 | 0.13465211 |
| 0.217838055 | 2.01E-06 | 0.203465749 | 9.35E-06 | 0.208405873 |
| 0.130464499 | 0.004744547 | 0.142474024 | 0.002025977 | 0.076154252 |
| 0.304552788 | 1.77E-11 | 0.290342833 | 1.60E-10 | 0.29458123 |
| 0.230472974 | 4.77E-07 | 0.204133809 | 8.73E-06 | 0.202265683 |
| -0.00633739 | 0.891357639 | -0.007019098 | 0.879755367 | -0.002836997 |
| 0.285664578 | 3.21E-10 | 0.26745082 | 4.32E-09 | 0.272918749 |
| 0.156292221 | 0.000700706 | 0.154809287 | 0.000788646 | 0.154540471 |
| 0.211256726 | 4.12E-06 | 0.217434719 | 2.10E-06 | 0.255337069 |
| 0.234980106 | 2.80E-07 | 0.224094178 | 9.98E-07 | 0.212563109 |
| 0.231304353 | 4.33E-07 | 0.222430295 | 1.20E-06 | 0.244422409 |
| 0.034205718 | 0.46086295 | 0.01547067 | 0.738796153 | 0.026562572 |
| 0.167175067 | 0.000284997 | 0.153107774 | 0.000902074 | 0.165096607 |
| 0.141280911 | 0.002211244 | 0.128928545 | 0.005264993 | 0.094867596 |
| 0.331610496 | 1.89E-13 | 0.348933029 | 8.12E-15 | 0.356364938 |
| 0.116662658 | 0.011636989 | 0.105604714 | 0.022465343 | 0.110891516 |
| 0.220322844 | 1.53E-06 | 0.208061875 | 5.79E-06 | 0.229982418 |
| 0.214838493 | 2.80E-06 | 0.210901971 | 4.28E-06 | 0.216868662 |
| 0.19886709 | 1.50E-05 | 0.190840772 | 3.31E-05 | 0.186597449 |
| 0.253991753 | 2.61E-08 | 0.26264992 | 8.30E-09 | 0.24124399 |
| 0.16440357 | 0.000360302 | 0.1197344 | 0.00960119 | 0.145266496 |
| 0.079314245 | 0.086878256 | 0.078272723 | 0.09111453 | 0.066162076 |
| 0.136784615 | 0.003056953 | 0.120208573 | 0.009316881 | 0.100050661 |
| 0.319469877 | 1.53E-12 | 0.321032413 | 1.18E-12 | 0.330072138 |
| 0.230040554 | 5.02E-07 | 0.236677391 | 2.28E-07 | 0.245497045 |
| 0.123260238 | 0.007659689 | 0.130131233 | 0.004853357 | 0.112183959 |
| 0.161539014 | 0.000457336 | 0.155224851 | 0.000763025 | 0.140949708 |
| -0.013580336 | 0.76975105 | -0.018010014 | 0.697876807 | -0.015884333 |
| 0.369751833 | 1.41E-16 | 0.350609702 | 5.92E-15 | 0.344960738 |
| 0.079309168 | 0.086898515 | 0.098664364 | 0.033036099 | 0.084472783 |
| 0.149619431 | 0.001183155 | 0.130985986 | 0.004578695 | 0.083196295 |
| 0.145028289 | 0.001676163 | 0.136427659 | 0.003135322 | 0.138903404 |
| 0.16282443 | 0.000411122 | 0.1495013 | 0.001193953 | 0.144117786 |
| 0.252443335 | 3.19E-08 | 0.223373458 | 1.08E-06 | 0.244492225 |
| 0.098433258 | 0.033451102 | 0.070612188 | 0.127569844 | 0.055036765 |
| 0.208098231 | 5.77E-06 | 0.185756547 | 5.38E-05 | 0.182254792 |
| 0.349861864 | 6.82E-15 | 0.31953465 | 1.52E-12 | 0.307724873 |
| 0.167503467 | 0.00027712 | 0.147340567 | 0.001408191 | 0.146203638 |
| 0.171196747 | 0.000201478 | 0.144852061 | 0.001698391 | 0.128858148 |

| | | | | |
|---|---|---|---|---|
| 0.403364188 | 1.06E-19 | 0.399426936 | 2.58E-19 | 0.406683825 |
| 0.139967144 | 0.002433053 | 0.117747413 | 0.010878173 | 0.128452599 |
| 0.292777862 | 1.10E-10 | 0.256545311 | 1.87E-08 | 0.232575254 |
| 0.412691558 | 1.25E-20 | 0.413311377 | 1.08E-20 | 0.405848939 |
| 0.155991803 | 0.000717751 | 0.15008965 | 0.001141056 | 0.154856675 |
| 0.206083471 | 7.13E-06 | 0.220856723 | 1.44E-06 | 0.225829831 |
| 0.110385217 | 0.017016618 | 0.097374156 | 0.035410279 | 0.076696537 |
| 0.061274302 | 0.186220602 | 0.045798209 | 0.323361548 | 0.051838857 |
| 0.265693381 | 5.50E-09 | 0.263091783 | 7.82E-09 | 0.249703062 |
| -0.014505939 | 0.754544806 | -0.007516998 | 0.871297475 | -0.017483614 |
| 0.314608166 | 3.45E-12 | 0.311385084 | 5.86E-12 | 0.317814695 |
| 0.185190808 | 5.67E-05 | 0.180493674 | 8.77E-05 | 0.122510599 |
| 0.245915589 | 7.33E-08 | 0.227893686 | 6.45E-07 | 0.212301204 |
| 0.222157061 | 1.24E-06 | 0.267396916 | 4.35E-09 | 0.284069824 |
| 0.180158746 | 9.04E-05 | 0.108476652 | 0.019035194 | 0.090261793 |
| -0.03395768 | 0.464122069 | -0.02969899 | 0.522028387 | -0.024461293 |
| 0.049658607 | 0.284207005 | 0.034977739 | 0.450800962 | 0.013955009 |
| 0.131628348 | 0.004381604 | 0.110792961 | 0.016610495 | 0.087104055 |
| 0.030179493 | 0.515314594 | 0.000900348 | 0.984518379 | -0.003585338 |
| 0.048895118 | 0.291685423 | 0.045134519 | 0.330430409 | 0.025225911 |
| 0.278045276 | 9.75E-10 | 0.273258408 | 1.93E-09 | 0.262906882 |
| -0.255410432 | 2.17E-08 | -0.206712073 | 6.67E-06 | -0.182786262 |
| -0.116645399 | 0.011649431 | -0.076328386 | 0.099464785 | -0.080752504 |
| -0.13605042 | 0.003220124 | -0.111599206 | 0.015832362 | -0.108065251 |
| -0.032895878 | 0.478217288 | 0.031374375 | 0.498814986 | 0.031292222 |
| -0.262502172 | 8.47E-09 | -0.232388641 | 3.81E-07 | -0.235072509 |
| -0.144630186 | 0.001726762 | -0.091212441 | 0.048844896 | -0.108861321 |
| -0.089082816 | 0.054384307 | -0.050099747 | 0.279945498 | -0.042253114 |
| -0.084877413 | 0.066860748 | -0.037427057 | 0.419712165 | -0.056596349 |
| -0.164881551 | 0.000346114 | -0.142370968 | 0.002041401 | -0.141559296 |
| -0.051125307 | 0.27020634 | -0.010932597 | 0.813720328 | 0.002455733 |
| -0.004184834 | 0.928133791 | 0.041990454 | 0.365259397 | 0.023187783 |
| -0.072191175 | 0.119252966 | -0.030803657 | 0.506660694 | -0.049962391 |
| -0.04495997 | 0.332305932 | -0.01439327 | 0.756390856 | -0.025732309 |
| -0.066947509 | 0.14860073 | -0.013758437 | 0.766818058 | -0.02462225 |
| -0.141426831 | 0.002187783 | -0.084548403 | 0.067928687 | -0.107796875 |
| -0.176079017 | 0.000130864 | -0.170745056 | 0.000209562 | -0.179494158 |
| -0.029078953 | 0.530757586 | 0.014704212 | 0.751299557 | -0.00401232 |
| -0.023091005 | 0.618674435 | 0.006606977 | 0.886766511 | 0.008276749 |
| -0.178923804 | 0.000101228 | -0.151348311 | 0.001035066 | -0.153713927 |
| -0.021088729 | 0.649425454 | 0.039082574 | 0.399429759 | 0.02571425 |
| -0.037237753 | 0.422069233 | 0.02091633 | 0.652102216 | 0.032983805 |
| -0.100855189 | 0.029315534 | -0.0774686 | 0.094497175 | -0.079404135 |
| 0.078500488 | 0.090174253 | 0.112667219 | 0.014850893 | 0.141013039 |
| -0.101883584 | 0.027696833 | -0.069308961 | 0.13476725 | -0.079818408 |

| | | | | |
|---|---|---|---|---|
| -0.076113819 | 0.100422409 | -0.075372713 | 0.103786458 | -0.061452136 |
| -0.076153748 | 0.10024365 | -0.022116229 | 0.633566353 | -0.030528187 |
| -0.010048742 | 0.828536724 | 0.041364752 | 0.372454033 | 0.074890327 |
| -0.251513783 | 3.60E-08 | -0.211544245 | 4.00E-06 | -0.202896707 |
| 0.015493292 | 0.738428139 | 0.062184721 | 0.179751553 | 0.078053829 |
| 0.082886189 | 0.073539866 | 0.096549623 | 0.03700234 | 0.107468513 |
| -0.160688133 | 0.000490536 | -0.128834267 | 0.005298553 | -0.122094097 |
| -0.041103724 | 0.375481165 | -0.028598585 | 0.537570844 | -0.010211719 |
| -0.156524886 | 0.000687763 | -0.096317378 | 0.037461589 | -0.129665027 |
| -0.094585932 | 0.041040175 | -0.04289094 | 0.355058144 | -0.019101907 |
| -0.205413053 | 7.64E-06 | -0.17151328 | 0.000195988 | -0.165043839 |
| -0.058797437 | 0.204685258 | -0.046083821 | 0.320349998 | -0.011757258 |
| -0.024979391 | 0.590268796 | 0.008731271 | 0.850733767 | 0.032961025 |
| -0.140982264 | 0.002259978 | -0.071947511 | 0.120508027 | -0.067792423 |
| -0.091411919 | 0.048351123 | -0.023747683 | 0.608728952 | -0.004255794 |
| -0.038589829 | 0.405404261 | 0.012252815 | 0.791713743 | 0.038396652 |
| -0.109226432 | 0.018218573 | -0.060520569 | 0.191704776 | -0.057804956 |
| -0.05756724 | 0.214334512 | -0.020127457 | 0.664407315 | -0.029295789 |
| 0.005336778 | 0.908428886 | 0.039424278 | 0.395317793 | 0.050197767 |
| -0.103833213 | 0.024838221 | -0.094021149 | 0.042268216 | -0.109418272 |
| -0.028940306 | 0.532719589 | 0.01754976 | 0.705232792 | 0.014672275 |
| 0.02268434 | 0.624868665 | 0.022851962 | 0.622312241 | 0.031309839 |
| -0.015284762 | 0.741822739 | 0.029652658 | 0.522678125 | 0.065487918 |
| 0.043495973 | 0.348305667 | 0.081199271 | 0.079615058 | 0.100767142 |
| -0.229671942 | 5.24E-07 | -0.20665209 | 6.72E-06 | -0.210855046 |
| 0.122551442 | 0.00801916 | 0.162120566 | 0.00043586 | 0.131397915 |
| 0.308015311 | 1.01E-11 | 0.34034423 | 3.97E-14 | 0.32807833 |
| 0.133238964 | 0.003920696 | 0.180844352 | 8.49E-05 | 0.18726378 |
| 0.157467932 | 0.000637538 | 0.210432524 | 4.50E-06 | 0.212138629 |
| 0.046860202 | 0.312256353 | 0.098552854 | 0.033235789 | 0.096214888 |
| 0.101286603 | 0.028626864 | 0.16955368 | 0.000232365 | 0.156411281 |
| 0.225795834 | 8.21E-07 | 0.284373895 | 3.89E-10 | 0.285794576 |
| 0.299523421 | 3.90E-11 | 0.370334855 | 1.26E-16 | 0.381552056 |
| 0.131682031 | 0.004365485 | 0.215308263 | 2.66E-06 | 0.207511351 |
| 0.040196073 | 0.386124568 | 0.121275762 | 0.00870423 | 0.13601346 |
| 0.170970122 | 0.000205497 | 0.203271371 | 9.54E-06 | 0.200604306 |
| 0.298080161 | 4.89E-11 | 0.340329779 | 3.98E-14 | 0.344127569 |
| 0.130728986 | 0.004659765 | 0.192570113 | 2.79E-05 | 0.19407912 |
| 0.193773515 | 2.48E-05 | 0.217320472 | 2.13E-06 | 0.204657365 |
| 0.219230445 | 1.73E-06 | 0.228015607 | 6.36E-07 | 0.235025272 |
| 0.009831628 | 0.83218582 | 0.031309158 | 0.499708263 | 0.029298648 |
| 0.113864799 | 0.01381428 | 0.138127143 | 0.002777889 | 0.168444411 |
| 0.103247449 | 0.025669042 | 0.159143499 | 0.000556597 | 0.181403491 |
| 0.145627044 | 0.001602615 | 0.206217016 | 7.03E-06 | 0.187787934 |
| -0.194721542 | 2.26E-05 | -0.218184032 | 1.94E-06 | -0.24828834 |

| | | | | |
|---|---|---|---|---|
| 0.182347724 | 7.39E-05 | 0.233637473 | 3.29E-07 | 0.199500569 |
| 0.042340136 | 0.361276507 | 0.072549722 | 0.117424759 | 0.039423054 |
| 0.01774574 | 0.70209715 | 0.081204445 | 0.079595821 | 0.07516622 |
| 0.165560459 | 0.000326856 | 0.180055413 | 9.13E-05 | 0.169534805 |
| 0.193639523 | 2.52E-05 | 0.255193351 | 2.23E-08 | 0.234931127 |
| 0.011986862 | 0.796134221 | 0.062787333 | 0.17556218 | 0.042029581 |
| 0.098088967 | 0.034077593 | 0.132798395 | 0.004042201 | 0.138605673 |
| 0.190693315 | 3.35E-05 | 0.226111168 | 7.92E-07 | 0.224316531 |
| 0.228585045 | 5.95E-07 | 0.210091018 | 4.67E-06 | 0.254518969 |
| 0.187751859 | 4.45E-05 | 0.237129424 | 2.16E-07 | 0.239505274 |
| 0.397899135 | 3.63E-19 | 0.412663106 | 1.25E-20 | 0.465555197 |
| 0.195241413 | 2.15E-05 | 0.261102055 | 1.02E-08 | 0.271425285 |
| 0.140783481 | 0.002292958 | 0.191984607 | 2.96E-05 | 0.160193417 |
| -0.015458695 | 0.73899098 | 0.02944879 | 0.525541962 | 0.037763914 |
| 0.057420687 | 0.215505466 | 0.16669795 | 0.000296814 | 0.170898016 |
| 0.017945596 | 0.698904681 | 0.099073881 | 0.032311506 | 0.105080371 |
| 0.291262727 | 1.39E-10 | 0.328049335 | 3.53E-13 | 0.362867693 |
| 0.192292419 | 2.87E-05 | 0.242658017 | 1.10E-07 | 0.225457178 |
| -0.200634033 | 1.25E-05 | -0.218966922 | 1.78E-06 | -0.23360234 |
| 0.05367067 | 0.247043995 | 0.104001363 | 0.024604055 | 0.098303809 |
| 0.077344699 | 0.095027188 | 0.099126081 | 0.032220127 | 0.125973536 |
| 0.155132371 | 0.000768659 | 0.178060652 | 0.000109476 | 0.16284144 |
| 0.333356477 | 1.39E-13 | 0.359320022 | 1.12E-15 | 0.351558193 |
| 0.202616214 | 1.02E-05 | 0.243745178 | 9.61E-08 | 0.258123925 |
| 0.046408621 | 0.316947523 | 0.09013422 | 0.051587441 | 0.088286724 |
| 0.14618218 | 0.001537084 | 0.223367876 | 1.08E-06 | 0.211568776 |
| 0.072934431 | 0.11548762 | 0.086100573 | 0.063010655 | 0.078305833 |
| -0.020184731 | 0.663510844 | 0.034694223 | 0.454481627 | 0.017237504 |
| -0.091153638 | 0.048991255 | -0.123058329 | 0.007760593 | -0.121675319 |
| 0.161164824 | 0.00047167 | 0.189483164 | 3.77E-05 | 0.162376742 |
| 0.187063186 | 4.75E-05 | 0.215942462 | 2.48E-06 | 0.241463412 |
| 0.211906301 | 3.84E-06 | 0.229368267 | 5.43E-07 | 0.213487614 |
| 0.106967107 | 0.020776543 | 0.163624597 | 0.000384591 | 0.173432977 |
| 0.093957926 | 0.042407596 | 0.144248215 | 0.001776624 | 0.154153504 |
| 0.262469531 | 8.51E-09 | 0.282426059 | 5.17E-10 | 0.288316974 |
| 0.280919891 | 6.44E-10 | 0.318368847 | 1.84E-12 | 0.312654032 |
| 0.121603313 | 0.008523525 | 0.16389861 | 0.000375878 | 0.153344779 |
| 0.02871249 | 0.535951332 | 0.103684834 | 0.025046449 | 0.096397696 |
| 0.361267174 | 7.63E-16 | 0.41438326 | 8.38E-21 | 0.392123615 |
| 0.076117014 | 0.100408094 | 0.141063934 | 0.002246554 | 0.154393218 |
| 0.099158106 | 0.032164175 | 0.155562912 | 0.000742753 | 0.138292517 |
| 0.036172802 | 0.435472584 | 0.06358505 | 0.170128663 | 0.060988092 |
| 0.053741712 | 0.246418044 | 0.075246453 | 0.104368384 | 0.053784827 |
| 0.269249815 | 3.37E-09 | 0.340074718 | 4.16E-14 | 0.322475551 |
| 0.176574093 | 0.000125181 | 0.209879717 | 4.78E-06 | 0.161547218 |

| | | | | |
|---|---|---|---|---|
| 0.046292348 | 0.31816283 | 0.096656928 | 0.036791769 | 0.095252942 |
| 0.164675905 | 0.000352153 | 0.192919854 | 2.70E-05 | 0.19920942 |
| 0.195265538 | 2.14E-05 | 0.282016157 | 5.49E-10 | 0.229997597 |
| 0.181313525 | 8.13E-05 | 0.21387664 | 3.11E-06 | 0.202131131 |
| 0.120031259 | 0.009422308 | 0.172143282 | 0.000185476 | 0.180636302 |
| 0.235373797 | 2.67E-07 | 0.28315501 | 4.65E-10 | 0.240831086 |
| 0.236246956 | 2.41E-07 | 0.289770047 | 1.74E-10 | 0.297243 |
| 0.287776458 | 2.35E-10 | 0.324694182 | 6.31E-13 | 0.36184837 |
| 0.390304081 | 1.92E-18 | 0.399831275 | 2.36E-19 | 0.382997138 |
| -0.218576202 | 1.86E-06 | -0.251136547 | 3.78E-08 | -0.271689784 |
| 0.114795639 | 0.013053089 | 0.139031122 | 0.002603269 | 0.144531376 |
| 0.092958921 | 0.044661897 | 0.153978745 | 0.000842253 | 0.160697759 |
| -0.135571353 | 0.003330818 | -0.188319862 | 4.21E-05 | -0.179251487 |
| -0.23540452 | 2.66E-07 | -0.177998262 | 0.000110096 | -0.17047433 |
| 0.028382387 | 0.540651502 | 0.072867197 | 0.115824349 | 0.042988832 |
| 0.139564539 | 0.002504982 | 0.177216732 | 0.000118148 | 0.158379969 |
| 0.33158442 | 1.90E-13 | 0.351309751 | 5.19E-15 | 0.332800391 |
| 0.175244828 | 0.000140993 | 0.216274894 | 2.39E-06 | 0.230344736 |
| 0.084605721 | 0.067741637 | 0.12865524 | 0.005362811 | 0.140473689 |
| 0.033367297 | 0.47193068 | 0.092691145 | 0.045282992 | 0.093807206 |
| 0.069433661 | 0.134065306 | 0.124418318 | 0.007103259 | 0.126893917 |
| 0.034982452 | 0.450739915 | 0.08508222 | 0.066202948 | 0.092320613 |
| -0.02338302 | 0.614243085 | 0.03377903 | 0.466477364 | 0.028582043 |
| -0.116673105 | 0.011629463 | -0.075038589 | 0.105332031 | -0.085692746 |
| 0.220668152 | 1.47E-06 | 0.246963477 | 6.42E-08 | 0.259171314 |
| 0.195846276 | 2.02E-05 | 0.288102966 | 2.23E-10 | 0.303949924 |
| 0.154056462 | 0.000837098 | 0.181693565 | 7.85E-05 | 0.209516143 |
| 0.217132765 | 2.18E-06 | 0.270166675 | 2.97E-09 | 0.296842909 |
| 0.320607858 | 1.27E-12 | 0.318684308 | 1.75E-12 | 0.339560914 |
| 0.146961908 | 0.001449188 | 0.17251611 | 0.000179506 | 0.161849134 |
| 0.152938387 | 0.000914155 | 0.198790168 | 1.51E-05 | 0.194029118 |
| 0.221018426 | 1.41E-06 | 0.278999266 | 8.50E-10 | 0.303651101 |
| 0.300158177 | 3.53E-11 | 0.306727446 | 1.25E-11 | 0.297562692 |
| 0.259018719 | 1.35E-08 | 0.242343798 | 1.14E-07 | 0.22552715 |
| 0.099043483 | 0.032364822 | 0.14876992 | 0.001262857 | 0.135919359 |
| 0.133785691 | 0.003774514 | 0.158514241 | 0.000585805 | 0.188101567 |
| 0.22516343 | 8.83E-07 | 0.245074597 | 8.14E-08 | 0.286614113 |
| 0.23104854 | 4.46E-07 | 0.257068358 | 1.75E-08 | 0.260728565 |
| -0.268776547 | 3.60E-09 | -0.262788827 | 8.15E-09 | -0.270425986 |
| 0.281411907 | 5.99E-10 | 0.323796069 | 7.35E-13 | 0.347311133 |
| -0.269589437 | 3.22E-09 | -0.2685918 | 3.69E-09 | -0.306350267 |
| 0.23131166 | 4.33E-07 | 0.274827327 | 1.54E-09 | 0.286594807 |
| -0.018749401 | 0.686118848 | 0.006277443 | 0.892379067 | 0.021517468 |
| 0.078196889 | 0.091429334 | 0.116672093 | 0.011630192 | 0.129448025 |
| 0.148029843 | 0.001336289 | 0.18320398 | 6.83E-05 | 0.166225309 |

| | | | | |
|---|---|---|---|---|
| -0.085478617 | 0.064944895 | -0.086464831 | 0.061900048 | -0.075761112 |
| 0.262992661 | 7.93E-09 | 0.297631387 | 5.24E-11 | 0.319993558 |
| -0.234470031 | 2.98E-07 | -0.23897476 | 1.73E-07 | -0.241289429 |
| 0.271714735 | 2.39E-09 | 0.309375255 | 8.13E-12 | 0.321812905 |
| 0.094021944 | 0.042266466 | 0.149563304 | 0.001188274 | 0.136590802 |
| -0.06775243 | 0.143768949 | 0.016309444 | 0.725191602 | 0.01263028 |
| 0.150565872 | 0.00109983 | 0.221939485 | 1.27E-06 | 0.215887952 |
| 0.103313147 | 0.025574681 | 0.142485477 | 0.00202427 | 0.17213081 |
| -0.253310217 | 2.85E-08 | -0.280453255 | 6.89E-10 | -0.303370578 |
| -0.012589625 | 0.786125143 | 0.023499805 | 0.612474762 | 0.01564171 |
| 0.26221726 | 8.80E-09 | 0.300621944 | 3.29E-11 | 0.321740029 |
| 0.339062348 | 5.00E-14 | 0.354695474 | 2.73E-15 | 0.360611316 |
| 0.171146297 | 0.000202367 | 0.247166066 | 6.26E-08 | 0.246978242 |
| 0.085076424 | 0.066221491 | 0.079609382 | 0.085707075 | 0.099003912 |
| 0.133927062 | 0.003737527 | 0.195805865 | 2.03E-05 | 0.191313201 |
| 0.220927014 | 1.43E-06 | 0.195103495 | 2.18E-05 | 0.153163613 |
| 0.176832985 | 0.000122301 | 0.232239789 | 3.88E-07 | 0.214025997 |
| 0.224948267 | 9.05E-07 | 0.246930266 | 6.45E-08 | 0.254584568 |
| 0.216542383 | 2.32E-06 | 0.243185923 | 1.03E-07 | 0.21956533 |
| 0.390627829 | 1.79E-18 | 0.417735471 | 3.79E-21 | 0.421645588 |
| 0.072917241 | 0.115573643 | 0.148773633 | 0.001262498 | 0.168007697 |
| 0.192854869 | 2.72E-05 | 0.181103981 | 8.29E-05 | 0.175871081 |
| 0.223658496 | 1.05E-06 | 0.228111766 | 6.29E-07 | 0.25348621 |
| 0.277307508 | 1.08E-09 | 0.298937114 | 4.28E-11 | 0.298716392 |
| 0.090263418 | 0.051252185 | 0.136910178 | 0.003029812 | 0.138686715 |
| 0.277815395 | 1.01E-09 | 0.298641789 | 4.48E-11 | 0.271107834 |
| 0.173251272 | 0.000168259 | 0.218241603 | 1.93E-06 | 0.204353073 |
| 0.300229961 | 3.49E-11 | 0.342832668 | 2.52E-14 | 0.362053721 |
| 0.196175284 | 1.96E-05 | 0.265019786 | 6.02E-09 | 0.28442435 |
| 0.001575632 | 0.972910273 | 0.084169651 | 0.069175356 | 0.083878115 |
| 0.215172704 | 2.70E-06 | 0.244794505 | 8.43E-08 | 0.266782711 |
| 0.243499442 | 9.91E-08 | 0.277429125 | 1.06E-09 | 0.297529111 |
| 0.013660165 | 0.768436009 | 0.065655847 | 0.156611253 | 0.026046876 |
| 0.177646183 | 0.000113657 | 0.230943277 | 4.52E-07 | 0.280359819 |
| 0.111712914 | 0.015725231 | 0.179154253 | 9.91E-05 | 0.15472529 |
| 0.138811215 | 0.002644802 | 0.168010515 | 0.000265357 | 0.154578433 |
| 0.051145308 | 0.270018739 | 0.102009895 | 0.027503417 | 0.088627096 |
| 0.192339305 | 2.86E-05 | 0.230695576 | 4.65E-07 | 0.239593755 |
| 0.088727166 | 0.055358466 | 0.109936747 | 0.01747331 | 0.117902467 |
| 0.251095004 | 3.80E-08 | 0.285311492 | 3.38E-10 | 0.283668896 |
| 0.116616654 | 0.01167018 | 0.143748339 | 0.00184387 | 0.136289444 |
| 0.042264276 | 0.362138245 | 0.125134977 | 0.006777314 | 0.121696861 |
| 0.260743119 | 1.07E-08 | 0.333745014 | 1.30E-13 | 0.28044112 |
| 0.011578201 | 0.802939337 | 0.083226438 | 0.072361516 | 0.079629109 |
| 0.169756547 | 0.000228325 | 0.240928076 | 1.36E-07 | 0.226182181 |

| | | | | |
|---|---|---|---|---|
| 0.143976029 | 0.001812956 | 0.164841333 | 0.000347288 | 0.167870047 |
| 0.021414779 | 0.644375373 | 0.084921361 | 0.066719146 | 0.065340224 |
| 0.165728945 | 0.000322234 | 0.196166648 | 1.96E-05 | 0.174356952 |
| 0.189607836 | 3.72E-05 | 0.231146507 | 4.41E-07 | 0.189838135 |
| 0.061275753 | 0.186210152 | 0.125245386 | 0.006728308 | 0.13127393 |
| 0.192033123 | 2.94E-05 | 0.236885375 | 2.23E-07 | 0.221442603 |
| 0.209444774 | 5.00E-06 | 0.225123128 | 8.87E-07 | 0.230706156 |
| 0.340363053 | 3.95E-14 | 0.312284132 | 5.06E-12 | 0.330007764 |
| 0.170316782 | 0.000217507 | 0.194096206 | 2.41E-05 | 0.210449776 |
| 0.054464463 | 0.240112844 | 0.101605881 | 0.028126183 | 0.094358118 |
| 0.093249146 | 0.043996836 | 0.12135528 | 0.00866005 | 0.087264432 |
| -0.326670018 | 4.49E-13 | -0.342569747 | 2.64E-14 | -0.335631465 |
| 0.049972892 | 0.281166489 | 0.078909644 | 0.088504705 | 0.091827569 |
| 0.145181013 | 0.001657115 | 0.170247557 | 0.000218817 | 0.121649096 |
| 0.177322153 | 0.000117031 | 0.18588468 | 5.31E-05 | 0.171878451 |
| 0.128304139 | 0.005490873 | 0.18415659 | 6.25E-05 | 0.20900777 |
| -0.004624592 | 0.920605534 | 0.042433548 | 0.360217166 | 0.0363342 |
| 0.064434509 | 0.164481892 | 0.111786811 | 0.015655949 | 0.155393479 |
| 0.316668154 | 2.45E-12 | 0.375247532 | 4.61E-17 | 0.361577167 |
| 0.173454211 | 0.000165272 | 0.208622022 | 5.46E-06 | 0.227110392 |
| -0.031188348 | 0.501365227 | 0.012460706 | 0.788262987 | 0.021676774 |
| 0.158735103 | 0.000575395 | 0.215023388 | 2.74E-06 | 0.213736459 |
| 0.185895769 | 5.31E-05 | 0.23523182 | 2.72E-07 | 0.249539081 |
| -0.163903427 | 0.000375727 | -0.164919027 | 0.000345024 | -0.167635603 |
| 0.230902252 | 4.54E-07 | 0.258260807 | 1.49E-08 | 0.278867204 |
| 0.095149373 | 0.039845214 | 0.096055958 | 0.03798431 | 0.098142364 |
| -0.015266892 | 0.742113864 | 0.026954111 | 0.561221718 | -0.000766766 |
| 0.187390554 | 4.60E-05 | 0.22557967 | 8.42E-07 | 0.239356809 |
| 0.116946102 | 0.011434324 | 0.177235056 | 0.000117953 | 0.14261982 |
| 0.249554064 | 4.62E-08 | 0.269540789 | 3.24E-09 | 0.279898007 |
| 0.177591015 | 0.000114225 | 0.241828037 | 1.22E-07 | 0.251486884 |
| 0.092977425 | 0.044619242 | 0.172123904 | 0.000185791 | 0.169300582 |
| 0.107739002 | 0.019869551 | 0.148902254 | 0.001250125 | 0.096992732 |
| 0.273018307 | 1.99E-09 | 0.331139455 | 2.06E-13 | 0.339965635 |
| 0.264458082 | 6.50E-09 | 0.294099602 | 9.03E-11 | 0.289959545 |
| 0.187157862 | 4.71E-05 | 0.231698874 | 4.13E-07 | 0.217614669 |
| 0.16590234 | 0.000317541 | 0.212394207 | 3.65E-06 | 0.201505193 |
| 0.046557696 | 0.315393824 | 0.09623495 | 0.037625746 | 0.098694181 |
| 0.001918922 | 0.967011158 | 0.049531838 | 0.285439677 | 0.051507042 |
| -0.107224498 | 0.020470184 | -0.136226015 | 0.003180395 | -0.161872845 |
| -0.00058736 | 0.989899896 | 0.050859944 | 0.272703847 | 0.049841515 |
| 0.004721931 | 0.918940114 | 0.046053624 | 0.320667535 | 0.066070067 |
| 0.267988978 | 4.01E-09 | 0.309394909 | 8.11E-12 | 0.276697813 |
| -0.003276688 | 0.943699988 | 0.051735904 | 0.264519128 | 0.065654376 |
| -0.088382583 | 0.056316094 | -0.108383459 | 0.019138891 | -0.108491922 |

| | | | | |
|---|---|---|---|---|
| -0.192458764 | 2.83E-05 | -0.118269998 | 0.010528512 | -0.108436327 |
| -0.22352917 | 1.06E-06 | -0.203110611 | 9.70E-06 | -0.212305465 |
| -0.111045819 | 0.016362926 | -0.091109282 | 0.0491019 | -0.098384591 |
| -0.178772479 | 0.00010263 | -0.117866152 | 0.010797834 | -0.122079382 |
| -0.085253649 | 0.065656446 | -0.026648166 | 0.565676552 | -0.018170615 |
| -0.38315068 | 8.87E-18 | -0.36113009 | 7.84E-16 | -0.370510601 |
| -0.330170105 | 2.44E-13 | -0.280009189 | 7.35E-10 | -0.309453415 |
| -0.148690926 | 0.001270514 | -0.11355115 | 0.014079424 | -0.089295355 |
| 0.061180478 | 0.186896895 | 0.075159459 | 0.104770833 | 0.05876244 |
| -0.453270278 | 4.85E-25 | -0.443731954 | 5.97E-24 | -0.466534022 |
| -0.487333767 | 3.15E-29 | -0.481706986 | 1.67E-28 | -0.520116342 |
| -0.46047231 | 6.91E-26 | -0.446939508 | 2.59E-24 | -0.482886838 |
| -0.269648569 | 3.19E-09 | -0.235994204 | 2.48E-07 | -0.230950113 |
| -0.228188273 | 6.23E-07 | -0.220346699 | 1.52E-06 | -0.206335943 |
| -0.235118424 | 2.75E-07 | -0.186261187 | 5.13E-05 | -0.192026349 |
| -0.251606381 | 3.56E-08 | -0.258833669 | 1.38E-08 | -0.304467495 |
| -0.227502982 | 6.75E-07 | -0.191230982 | 3.18E-05 | -0.180437718 |
| -0.205112842 | 7.89E-06 | -0.178111746 | 0.000108971 | -0.173474881 |
| -0.343098829 | 2.40E-14 | -0.291316804 | 1.38E-10 | -0.291819522 |
| -0.19408733 | 2.41E-05 | -0.149153284 | 0.001226297 | -0.14592596 |
| -0.23881224 | 1.76E-07 | -0.209177701 | 5.15E-06 | -0.207525156 |
| -0.189400885 | 3.80E-05 | -0.157466701 | 0.000637601 | -0.156476873 |
| -0.173514446 | 0.000164395 | -0.146768354 | 0.001470565 | -0.139833967 |
| -0.268232428 | 3.88E-09 | -0.230512276 | 4.75E-07 | -0.22523747 |
| -0.147883212 | 0.001351296 | -0.112934063 | 0.014614178 | -0.106630379 |
| -0.361310077 | 7.57E-16 | -0.353453113 | 3.45E-15 | -0.355981886 |
| -0.113743253 | 0.013916503 | -0.08323247 | 0.072340765 | -0.086424061 |
| -0.121880878 | 0.008373023 | -0.068246893 | 0.140860894 | -0.038072851 |
| -0.159950263 | 0.000521128 | -0.113064918 | 0.014499314 | -0.110500735 |
| -0.225375869 | 8.62E-07 | -0.17948808 | 9.62E-05 | -0.183972094 |
| -0.209950395 | 4.74E-06 | -0.175703513 | 0.000135336 | -0.17814577 |
| -0.176797012 | 0.000122698 | -0.168813396 | 0.000247681 | -0.179908651 |
| -0.431100352 | 1.47E-22 | -0.402001232 | 1.45E-19 | -0.432774261 |
| -0.175303555 | 0.000140257 | -0.102998925 | 0.026028713 | -0.111244898 |
| -0.168174559 | 0.000261653 | -0.126040263 | 0.006384763 | -0.125067844 |
| -0.085035965 | 0.066351043 | -0.0422485 | 0.362317608 | -0.027646875 |
| -0.237392295 | 2.09E-07 | -0.190280366 | 3.49E-05 | -0.184563966 |
| -0.385995966 | 4.85E-18 | -0.390934571 | 1.68E-18 | -0.408301326 |
| -0.217688442 | 2.05E-06 | -0.21253537 | 3.59E-06 | -0.198376101 |
| -0.293399416 | 1.00E-10 | -0.261389064 | 9.84E-09 | -0.255798189 |
| -0.093749492 | 0.042869847 | -0.025408278 | 0.583901725 | -0.02254136 |
| 0.06973918 | 0.132357452 | 0.07477497 | 0.10656427 | 0.122821382 |
| 0.006632624 | 0.886329939 | 0.055750048 | 0.229179407 | 0.092600556 |
| 0.135217567 | 0.00341477 | 0.135041657 | 0.003457223 | 0.197868892 |
| 0.037284429 | 0.421487338 | 0.075804685 | 0.101814955 | 0.128418104 |

| | | | | |
|---|---|---|---|---|
| -0.025720872 | 0.579281272 | -0.003943529 | 0.932267485 | 0.036682206 |
| 0.051094415 | 0.270496277 | 0.025976186 | 0.575520212 | 0.065635457 |
| 0.102749922 | 0.026393424 | 0.135745599 | 0.003290163 | 0.151693531 |
| -0.003845695 | 0.93394397 | -0.041175305 | 0.374649543 | 0.017692862 |
| -0.102923073 | 0.026139349 | -0.052442748 | 0.258038846 | -0.006395275 |
| 0.067826177 | 0.143332337 | 0.072543794 | 0.117454808 | 0.113529839 |
| -0.044341087 | 0.339010948 | 0.024303278 | 0.600370252 | 0.053482629 |
| 0.087390471 | 0.059150302 | 0.132454435 | 0.004139419 | 0.159513169 |
| 0.067663609 | 0.144296156 | 0.042026568 | 0.364846793 | 0.088502313 |
| -0.011131938 | 0.810387664 | 0.017307148 | 0.709121468 | 0.060830839 |
| -0.041577534 | 0.369997598 | -0.035851206 | 0.439567775 | 0.010767873 |
| 0.004847452 | 0.916793049 | -0.002730139 | 0.953078915 | 0.031256437 |
| 0.107100062 | 0.0206178 | 0.127074515 | 0.005961344 | 0.163695318 |
| 0.096302476 | 0.037491223 | 0.132908518 | 0.004011515 | 0.170368219 |
| -1.88E-05 | 0.999675873 | 0.009709403 | 0.834241698 | 0.070432627 |
| 0.064792029 | 0.162147804 | 0.069091645 | 0.135997287 | 0.08321844 |
| 0.083561286 | 0.07121696 | 0.074837343 | 0.106271695 | 0.144398862 |
| -0.01229207 | 0.791061841 | 0.013252463 | 0.775159078 | 0.0114625 |
| 0.118424194 | 0.010427261 | 0.131133202 | 0.004532832 | 0.148582791 |
| 0.104246283 | 0.024266391 | 0.124202329 | 0.007204207 | 0.155845944 |
| 0.027455275 | 0.55396106 | 0.027320826 | 0.555904404 | 0.061734796 |
| -0.055072645 | 0.234895637 | -0.044675867 | 0.335373251 | 0.013310964 |
| -0.012610893 | 0.785772607 | -0.032317224 | 0.485995888 | -0.004180141 |
| 0.060172938 | 0.194273678 | 0.103985384 | 0.024626225 | 0.116662507 |
| 0.032270415 | 0.486628103 | 0.079238373 | 0.087181406 | 0.07518903 |
| 0.090801739 | 0.049874812 | 0.111106122 | 0.016304364 | 0.156007937 |
| 0.051609608 | 0.265688678 | 0.058070798 | 0.210345977 | 0.098042242 |
| 0.010792346 | 0.816067122 | 0.007853849 | 0.865583593 | 0.058615417 |
| -0.064922708 | 0.161300904 | -0.058335035 | 0.208274569 | -0.023501896 |
| -0.028928197 | 0.532891118 | -0.011068627 | 0.811445749 | -0.005187971 |
| 0.218279366 | 1.92E-06 | 0.23797346 | 1.95E-07 | 0.277186834 |
| 0.066108586 | 0.153767146 | 0.090189636 | 0.05144342 | 0.108309273 |
| -0.01065937 | 0.81829371 | 0.025261759 | 0.586073295 | 0.047067265 |
| 0.016119709 | 0.728261632 | 0.016186099 | 0.727186904 | 0.071402928 |
| 0.019589068 | 0.672857612 | 0.001947004 | 0.966528672 | 0.067283296 |
| -0.105192446 | 0.022999283 | -0.096239602 | 0.037616465 | -0.064251226 |
| -0.036326663 | 0.433521116 | -0.042453401 | 0.359992269 | 0.016409611 |
| 0.12308713 | 0.007746127 | 0.166844958 | 0.000293125 | 0.182496963 |
| 0.100905549 | 0.029234418 | 0.12763408 | 0.005742932 | 0.163328267 |
| -0.090860918 | 0.049725299 | -0.104423254 | 0.024024905 | -0.063573825 |
| 0.054176494 | 0.24261134 | 0.040323001 | 0.38462523 | 0.06107741 |
| -0.022580433 | 0.62645563 | -0.019444089 | 0.675140234 | 0.014113065 |
| 0.167753474 | 0.000271261 | 0.223052999 | 1.12E-06 | 0.248384318 |
| 0.056113578 | 0.226152797 | 0.087717996 | 0.058201876 | 0.096534047 |
| -0.033497781 | 0.47019867 | -0.026823523 | 0.563121129 | 0.031389657 |

| | | | | |
|---|---|---|---|---|
| 0.125020173 | 0.00682861 | 0.169531483 | 0.000232811 | 0.222791608 |
| -0.052643956 | 0.256214424 | -0.030062082 | 0.516950971 | 0.02812458 |
| -0.008575383 | 0.853368377 | -0.062274786 | 0.179120755 | -0.008575015 |
| 0.028848663 | 0.534018427 | 0.047411533 | 0.306591098 | 0.07346102 |
| 0.024573427 | 0.59632474 | 0.034785906 | 0.453289534 | 0.055601261 |
| -0.065385115 | 0.158330886 | -0.102480465 | 0.026793042 | -0.07235736 |
| 0.131835297 | 0.00431976 | 0.184680259 | 5.95E-05 | 0.226601413 |
| 0.104519901 | 0.023893904 | 0.101856884 | 0.027737866 | 0.150575426 |
| 0.103973124 | 0.024643246 | 0.111788032 | 0.015654807 | 0.17661217 |
| 0.016341074 | 0.724680228 | 0.018993644 | 0.682251239 | 0.056271909 |
| 0.10292878 | 0.02613101 | 0.101907779 | 0.027659694 | 0.153018747 |
| -0.007285274 | 0.875232031 | -0.01901106 | 0.681975769 | 0.032238628 |
| 0.233688462 | 3.27E-07 | 0.210799885 | 4.33E-06 | 0.254936442 |
| -0.080963748 | 0.080494727 | -0.09609448 | 0.037906897 | -0.063908979 |
| -0.159314059 | 0.000548917 | -0.167622268 | 0.000274321 | -0.13208498 |
| 0.161962298 | 0.000441609 | 0.180835944 | 8.50E-05 | 0.22933754 |
| 0.262851405 | 8.08E-09 | 0.274001002 | 1.73E-09 | 0.32977746 |
| -0.08708551 | 0.060044847 | -0.086175446 | 0.06278104 | -0.044144275 |
| -0.090926505 | 0.049560034 | -0.066812882 | 0.1494208 | -0.059222911 |
| 0.012022149 | 0.795547331 | -0.021418941 | 0.644311005 | 0.01205456 |
| 0.046543482 | 0.315541748 | 0.02293051 | 0.621115857 | 0.040228429 |
| 0.089763656 | 0.052559173 | 0.147323504 | 0.001410016 | 0.182682579 |
| -0.074581731 | 0.107474744 | -0.09194022 | 0.04706356 | -0.084089091 |
| 0.020789166 | 0.654079502 | -0.006334665 | 0.891404065 | 0.048007585 |
| 0.196807789 | 1.84E-05 | 0.211345193 | 4.08E-06 | 0.198434432 |
| -0.02381377 | 0.607732002 | 0.011428619 | 0.805433975 | 0.060620695 |
| -0.10054796 | 0.029814584 | -0.115958198 | 0.012154504 | -0.085370993 |
| 0.035439879 | 0.44483756 | 0.035891234 | 0.439056851 | 0.076350318 |
| 0.014317403 | 0.757634699 | 0.015262175 | 0.742190721 | 0.029530184 |
| 0.050155535 | 0.279409677 | 0.045129091 | 0.33048863 | 0.077044712 |
| 0.059837376 | 0.196777194 | 0.11879464 | 0.010187534 | 0.158760338 |
| -0.086464459 | 0.061901175 | -0.094319652 | 0.041615359 | -0.056500174 |
| -0.146238461 | 0.001530579 | -0.136329084 | 0.003157283 | -0.113917198 |
| -0.005091059 | 0.912627812 | -0.021921779 | 0.636555062 | 0.003912563 |
| -0.017003913 | 0.71399247 | -0.025887325 | 0.57682792 | 0.011160173 |
| -0.127909064 | 0.005638258 | -0.119980655 | 0.009452591 | -0.113014491 |
| -0.114147016 | 0.013579472 | -0.070988905 | 0.125545856 | -0.002072161 |
| -0.095098056 | 0.039952816 | -0.103903202 | 0.024740521 | -0.090218519 |
| -0.023935634 | 0.605895531 | -0.025074271 | 0.588857497 | 0.018574944 |
| -0.094627415 | 0.040951178 | -0.086257528 | 0.06253011 | -0.03658541 |
| 0.013231377 | 0.775507261 | -0.011429395 | 0.805421039 | 0.052877824 |
| 0.186585108 | 4.97E-05 | 0.241788441 | 1.22E-07 | 0.253622464 |
| 0.028536899 | 0.538448926 | 0.070070641 | 0.130523623 | 0.105204841 |
| 0.043177629 | 0.351848353 | 0.056829841 | 0.220272888 | 0.064257515 |
| 0.06495554 | 0.16108865 | 0.088394882 | 0.056281679 | 0.129532145 |

| | | | | |
|---|---|---|---|---|
| 0.085018087 | 0.066408355 | 0.036073461 | 0.436735237 | 0.057734058 |
| -0.025519495 | 0.582255857 | 0.004747981 | 0.918494473 | 0.056503113 |
| 0.13235356 | 0.00416833 | 0.175251251 | 0.000140912 | 0.217186102 |
| 0.196093132 | 1.97E-05 | 0.250860603 | 3.91E-08 | 0.269665513 |
| 0.015291619 | 0.741711026 | 0.024483081 | 0.597676302 | 0.041366672 |
| 0.032721675 | 0.480551864 | 0.051306387 | 0.268511085 | 0.084905734 |
| -0.009831391 | 0.832189804 | -0.020526083 | 0.658177881 | 0.016950633 |
| 0.121256235 | 0.008715109 | 0.15997669 | 0.000520002 | 0.197427579 |
| 0.166408506 | 0.000304204 | 0.158635524 | 0.000580067 | 0.159120563 |
| 0.014966709 | 0.74700986 | 0.024819245 | 0.592654394 | 0.066810247 |
| -0.029026852 | 0.531494443 | -0.051307029 | 0.268505088 | -0.019483141 |
| -0.003628772 | 0.937662177 | 0.030549279 | 0.510178376 | 0.072052654 |
| -0.09194836 | 0.047043947 | -0.106619184 | 0.021196984 | -0.093449877 |
| -0.056256126 | 0.22497379 | -0.070684128 | 0.12718139 | 0.015243961 |
| -0.021368293 | 0.64509438 | -0.008534985 | 0.854051397 | 0.017576595 |
| -0.032121316 | 0.488644774 | -0.032689403 | 0.480985032 | 0.010208761 |
| 0.012607772 | 0.78582434 | -0.008074236 | 0.861849014 | 0.045563829 |
| -0.095993088 | 0.038110939 | -0.104434774 | 0.024009257 | -0.089126922 |
| -0.048997544 | 0.290674563 | -0.048195169 | 0.298656281 | -0.015154742 |
| 0.217616133 | 2.06E-06 | 0.230968143 | 4.51E-07 | 0.281001634 |
| 0.058446348 | 0.207406396 | 0.063923546 | 0.167861399 | 0.104824476 |
| 0.006637998 | 0.886238452 | 0.014252675 | 0.75869641 | 0.052297678 |
| 0.017177758 | 0.711198475 | 0.049266024 | 0.288036066 | 0.05834743 |
| 0.086225651 | 0.062627462 | 0.091362585 | 0.048472851 | 0.109543768 |
| -0.017227161 | 0.710405194 | -0.001361275 | 0.976594557 | 0.018870542 |
| 0.036144806 | 0.435828218 | 0.077467621 | 0.094501351 | 0.117813134 |
| -0.213598702 | 3.20E-06 | -0.175702116 | 0.000135353 | -0.107428947 |
| 0.048496897 | 0.295637858 | 0.099025241 | 0.032396853 | 0.112144086 |
| -0.042605647 | 0.358270539 | -0.068641216 | 0.13857427 | -0.043235804 |
| -0.060376117 | 0.192769204 | -0.043051093 | 0.353262798 | -0.013272419 |
| -0.069086282 | 0.136027747 | -0.049362034 | 0.287096449 | -0.02086051 |
| 0.020664068 | 0.656027023 | -0.001856408 | 0.968085285 | 0.046014391 |
| 0.033889151 | 0.465024764 | 0.085584181 | 0.0646132 | 0.076791332 |
| 0.107843254 | 0.019749733 | 0.101242985 | 0.028695855 | 0.117537647 |
| -0.048032555 | 0.300291511 | -0.040924911 | 0.37756358 | 0.005874106 |
| 0.093585184 | 0.043237224 | 0.158061416 | 0.000607697 | 0.185020374 |
| -0.065604181 | 0.15693833 | -0.100102248 | 0.030551516 | -0.053359183 |
| -0.010477816 | 0.821336053 | 0.013868313 | 0.765010256 | 0.040972139 |
| 0.08667351 | 0.061271104 | 0.098933504 | 0.032558343 | 0.113758708 |
| 0.034878025 | 0.452093547 | 0.047898711 | 0.301641889 | 0.055657256 |
| 0.159883421 | 0.000523984 | 0.153781734 | 0.000855455 | 0.195905684 |
| 0.128165446 | 0.005542214 | 0.155826328 | 0.000727304 | 0.190984386 |
| -0.011263911 | 0.808183159 | -0.034570116 | 0.456098121 | -0.005197775 |
| 0.013899331 | 0.764500142 | 0.027124181 | 0.558752674 | 0.059129951 |
| -0.10483308 | 0.023473637 | -0.091767883 | 0.047480379 | -0.071224467 |

| | | | | |
|---|---|---|---|---|
| 0.136681002 | 0.003079515 | 0.153699398 | 0.000861028 | 0.166199299 |
| -0.081343775 | 0.079079208 | -0.092982219 | 0.044608198 | -0.050324865 |
| 0.119381663 | 0.009817661 | 0.177111176 | 0.000119277 | 0.196375286 |
| -0.154374649 | 0.000816293 | -0.147253303 | 0.001417544 | -0.137925675 |
| 0.020057069 | 0.665509707 | 0.040684707 | 0.380372063 | 0.074533603 |
| -0.085535639 | 0.064765552 | -0.058393095 | 0.207821413 | -0.000810086 |
| 0.020470287 | 0.659048417 | 0.051910535 | 0.262907829 | 0.070261953 |
| 0.130870341 | 0.004615016 | 0.172270548 | 0.000183418 | 0.190075741 |
| 0.223104836 | 1.12E-06 | 0.287987622 | 2.27E-10 | 0.313578962 |
| 0.105022064 | 0.023223139 | 0.143483642 | 0.001880415 | 0.197978111 |
| 0.302647761 | 2.39E-11 | 0.343459691 | 2.24E-14 | 0.383882065 |
| 0.103031864 | 0.025980794 | 0.09700914 | 0.036107741 | 0.115379762 |
| 0.091290823 | 0.048650374 | 0.10726974 | 0.020416743 | 0.147018612 |
| 0.046734473 | 0.313557867 | 0.096447097 | 0.037204485 | 0.134985387 |
| -0.104922536 | 0.023354773 | -0.095344421 | 0.039438466 | -0.057032341 |
| -0.202917461 | 9.90E-06 | -0.197046842 | 1.80E-05 | -0.189760686 |
| 0.017402721 | 0.707588688 | -0.011525794 | 0.803813132 | 0.038531778 |
| -0.069304638 | 0.134791631 | -0.06153628 | 0.184341768 | -0.048279486 |
| -0.004067656 | 0.930140885 | -0.013643555 | 0.768709569 | -0.004905504 |
| -0.119636429 | 0.009660884 | -0.087746124 | 0.058121018 | -0.075210646 |
| 0.015190758 | 0.743354638 | 0.038872201 | 0.401974034 | 0.058146195 |
| -0.128773879 | 0.00532015 | -0.102947652 | 0.026103454 | -0.125916517 |
| -0.083151911 | 0.072618293 | -0.050111158 | 0.279835845 | -0.039903118 |
| 0.026743542 | 0.564285982 | 0.058745932 | 0.20508282 | 0.052920692 |
| 0.020217842 | 0.662992794 | 0.050221346 | 0.278778472 | 0.058945986 |
| 0.119338805 | 0.009844256 | 0.118435419 | 0.010419924 | 0.129328125 |
| 0.05484396 | 0.23684792 | 0.07519602 | 0.104601543 | 0.091642042 |
| -0.055732239 | 0.229328416 | -0.034794655 | 0.453175873 | -0.013233944 |
| -0.062492675 | 0.17760149 | -0.03193439 | 0.49117943 | -0.038599761 |
| 0.010880586 | 0.814590426 | 0.023427147 | 0.613574668 | 0.022058652 |
| 0.013092972 | 0.777793734 | 0.009040316 | 0.845515631 | 0.034059441 |
| 0.060220945 | 0.193917429 | 0.0796326 | 0.085615484 | 0.080654862 |
| 0.025154965 | 0.587658448 | 0.048416882 | 0.296436327 | 0.056182201 |
| 0.044150146 | 0.341096955 | 0.049796286 | 0.282872326 | 0.053910193 |
| 0.064113323 | 0.166600198 | 0.085317563 | 0.065453643 | 0.115487841 |
| -0.049390552 | 0.286817746 | -0.022366493 | 0.629728532 | -0.007094146 |
| 0.03735607 | 0.420595137 | 0.052627554 | 0.256362812 | 0.055993427 |
| 0.004026172 | 0.930851556 | 0.002762623 | 0.952521292 | -0.001357791 |
| -0.108505537 | 0.019003153 | -0.09010387 | 0.051666459 | -0.07955088 |
| -0.041150733 | 0.374934887 | -0.037309643 | 0.421173207 | -0.039273149 |
| -0.065866424 | 0.155283501 | -0.062200921 | 0.179637976 | -0.080312567 |
| -0.044554066 | 0.336693818 | -0.015141706 | 0.744154402 | -0.0199006 |
| -0.042838708 | 0.355644919 | -0.044009523 | 0.342638467 | -0.005595133 |
| -0.09100823 | 0.049354749 | -0.061612494 | 0.183797818 | -0.06889748 |
| -0.107166554 | 0.020538807 | -0.086263462 | 0.062512001 | -0.096792682 |

| | | | | |
|---|---|---|---|---|
| 0.005076534 | 0.912876092 | 0.032441814 | 0.484315336 | 0.05442334 |
| -0.086049917 | 0.063166396 | -0.074141496 | 0.1095719 | -0.056095085 |
| -0.078595883 | 0.089782756 | -0.059571628 | 0.198776499 | -0.064002591 |
| 0.073264907 | 0.113843644 | 0.097578329 | 0.035025174 | 0.111278234 |
| -0.117973716 | 0.01072551 | -0.110620691 | 0.016781033 | -0.098619061 |
| -0.090010456 | 0.051910309 | -0.093442988 | 0.043557298 | -0.094808253 |
| 0.059348607 | 0.20046574 | 0.075173969 | 0.104703622 | 0.084392507 |
| -0.112106971 | 0.015358868 | -0.087518948 | 0.058776754 | -0.082601151 |
| -0.022842938 | 0.622449755 | -0.001270368 | 0.978157191 | 0.016822017 |
| -0.021186405 | 0.647910879 | -0.007784966 | 0.866751464 | 0.021276671 |
| -0.050289503 | 0.278125799 | -0.0297395 | 0.521460635 | -0.004123749 |
| -0.123459732 | 0.007561145 | -0.099352097 | 0.031827016 | -0.085570106 |
| -0.016543261 | 0.721414332 | 0.0112 | 0.809250555 | 0.0129515 |
| -0.018673381 | 0.687324313 | 0.018497705 | 0.690113075 | 0.043446748 |
| -0.075060781 | 0.105228819 | -0.058154982 | 0.209684433 | -0.041542849 |
| -0.085349057 | 0.065353903 | -0.062346464 | 0.178619913 | -0.067296146 |
| -0.066975525 | 0.148430508 | -0.073662614 | 0.111889635 | -0.072005261 |
| -0.07689856 | 0.096955329 | -0.026493943 | 0.567928586 | -0.053518623 |
| -0.09510432 | 0.039939668 | -0.072390147 | 0.118235697 | -0.067993615 |
| -0.084694678 | 0.067452176 | -0.047595171 | 0.304719267 | -0.037520565 |
| 0.050004729 | 0.280859721 | 0.056904836 | 0.219663621 | 0.071248464 |
| 0.13502995 | 0.003460065 | 0.150449557 | 0.001109771 | 0.182622647 |
| -0.005435167 | 0.90674828 | 0.058902514 | 0.203875905 | 0.054482928 |
| -0.0746673 | 0.107070818 | -0.046521389 | 0.315771764 | -0.047611273 |
| -0.092038277 | 0.046827768 | -0.089097512 | 0.05434436 | -0.101807648 |
| -0.126188513 | 0.006322459 | -0.132725045 | 0.004062758 | -0.138643268 |
| 0.236248294 | 2.40E-07 | 0.257808646 | 1.58E-08 | 0.270464678 |
| -0.150247821 | 0.001127208 | -0.141013793 | 0.002254787 | -0.1689296 |
| 0.147820076 | 0.001357805 | 0.128912721 | 0.005270613 | 0.113486381 |
| -0.204279461 | 8.60E-06 | -0.221431775 | 1.35E-06 | -0.224271287 |
| 0.152102941 | 0.000975953 | 0.150098753 | 0.001140255 | 0.144990116 |
| -0.033564103 | 0.469319667 | -0.055705888 | 0.229549021 | -0.070128788 |
| 0.133594907 | 0.003824955 | 0.118382896 | 0.010454294 | 0.131846433 |
| -0.238849456 | 1.76E-07 | -0.244733006 | 8.50E-08 | -0.248553223 |
| 0.046341853 | 0.317645022 | 0.006992931 | 0.880200248 | -0.011433709 |
| -0.136096178 | 0.003209728 | -0.127743031 | 0.005701252 | -0.148583804 |
| -0.228245215 | 6.19E-07 | -0.261720183 | 9.41E-09 | -0.275615032 |
| -0.314761387 | 3.37E-12 | -0.329902785 | 2.56E-13 | -0.331129096 |
| -0.08664294 | 0.061362909 | -0.111296916 | 0.016120286 | -0.174434093 |
| -0.102210528 | 0.027198584 | -0.125049828 | 0.006815326 | -0.165799503 |
| -0.076435731 | 0.098988429 | -0.047233564 | 0.308412364 | -0.028044219 |
| 0.058840731 | 0.204351503 | 0.046904246 | 0.311801267 | 0.015859875 |
| 0.133218704 | 0.003926209 | 0.15976301 | 0.000529168 | 0.147526977 |
| -0.291422926 | 1.36E-10 | -0.311407006 | 5.84E-12 | -0.327072297 |
| 0.072834615 | 0.115987806 | 0.088139745 | 0.056999188 | 0.082028804 |

| | | | | |
|---|---|---|---|---|
| 0.103905387 | 0.024737476 | 0.070869957 | 0.126182216 | 0.10833817 |
| 0.077931775 | 0.092536729 | 0.075358895 | 0.10385002 | 0.067345146 |
| 0.028721307 | 0.535826079 | 0.024399859 | 0.598922526 | 0.022816992 |
| 0.001602649 | 0.972445951 | 0.00906485 | 0.845101661 | -0.021025687 |
| -0.172110164 | 0.000186015 | -0.212182798 | 3.73E-06 | -0.198559258 |
| -0.009122818 | 0.84412373 | -0.000995742 | 0.982878318 | -0.035533155 |
| 0.0675681 | 0.144864708 | 0.078668874 | 0.089484126 | 0.084405148 |
| -0.037452194 | 0.419399751 | -0.022147426 | 0.633087399 | -0.02543781 |
| -0.043649347 | 0.346606946 | -0.051044241 | 0.270967644 | -0.059084495 |
| -0.080909452 | 0.080698634 | -0.083762808 | 0.070535291 | -0.07142659 |
| 0.122521543 | 0.008034649 | 0.091416891 | 0.048338869 | 0.090706275 |
| 0.103882678 | 0.024769136 | 0.114254013 | 0.013491373 | 0.168189523 |
| -0.168028205 | 0.000264956 | -0.204490995 | 8.41E-06 | -0.188377683 |
| 0.013664603 | 0.768362921 | 0.019932173 | 0.66746755 | 0.01637222 |
| -0.110162337 | 0.017242262 | -0.151385122 | 0.001032108 | -0.14604257 |
| 0.193990987 | 2.43E-05 | 0.161074121 | 0.000475207 | 0.157253251 |
| 0.108548724 | 0.018955335 | 0.087880177 | 0.057736927 | 0.080651011 |
| 0.031440451 | 0.49791081 | 0.008814055 | 0.849335323 | -0.015059769 |
| 0.015761511 | 0.734069345 | 0.037306893 | 0.421207451 | 0.025104648 |
| -0.071500264 | 0.12283849 | -0.077457538 | 0.094544398 | -0.080557328 |
| -0.050357439 | 0.277476264 | -0.019302526 | 0.677371962 | -0.021351992 |
| 0.023929167 | 0.605992925 | 0.039641881 | 0.392712565 | 0.067319133 |
| -0.049697018 | 0.283834218 | -0.138538743 | 0.002697099 | -0.144958753 |
| 0.101122161 | 0.028887708 | 0.052998353 | 0.253022739 | 0.047348342 |
| 0.077753191 | 0.093288715 | 0.062154582 | 0.179963014 | 0.095973121 |
| 0.12037716 | 0.009217618 | 0.090021782 | 0.051880693 | 0.086460632 |
| 0.024109087 | 0.603285896 | 0.012069309 | 0.794763142 | 0.031580502 |
| 0.004843259 | 0.916864753 | -0.019377755 | 0.676185638 | -0.020555806 |
| -0.003908707 | 0.932864161 | -0.032989237 | 0.476968691 | -0.047839325 |
| 0.156235575 | 0.000703891 | 0.13074156 | 0.004655768 | 0.136361737 |
| -0.091146132 | 0.049009964 | -0.118187971 | 0.010582728 | -0.172604401 |
| 0.043813846 | 0.344790867 | 0.071166014 | 0.124602964 | 0.065824843 |
| 0.062816526 | 0.175361092 | 0.025467258 | 0.583028631 | 0.017555519 |
| -0.202030073 | 1.08E-05 | -0.197763961 | 1.67E-05 | -0.209183767 |
| -0.041163436 | 0.374787353 | -0.044360976 | 0.338794128 | -0.040552837 |
| 0.076337395 | 0.099424735 | 0.054937327 | 0.236049466 | 0.051931743 |
| 0.010259361 | 0.825000309 | 0.013557299 | 0.770130673 | 0.021058344 |
| 0.277560231 | 1.05E-09 | 0.286213116 | 2.96E-10 | 0.273154756 |
| 0.132145543 | 0.004228523 | 0.077685697 | 0.093574189 | 0.070492036 |
| -0.029791936 | 0.520726217 | 0.019980067 | 0.666716513 | 0.001149034 |
| 0.204944981 | 8.03E-06 | 0.233008589 | 3.54E-07 | 0.221727611 |
| -0.039143237 | 0.398697892 | -0.000207869 | 0.996425446 | -0.002906306 |
| 0.342325665 | 2.76E-14 | 0.331344879 | 1.98E-13 | 0.315438532 |
| 0.048633729 | 0.294275762 | 0.080905506 | 0.080713469 | 0.098810491 |
| 0.000325059 | 0.994410252 | -0.00084699 | 0.985435774 | -0.012174986 |

| | | | | |
|---|---|---|---|---|
| 0.058861728 | 0.20418978 | 0.067533138 | 0.145073257 | 0.075033691 |
| 0.257464017 | 1.66E-08 | 0.28210362 | 5.42E-10 | 0.251460556 |
| -0.082459274 | 0.075040435 | -0.085650025 | 0.064407018 | -0.100257181 |
| -0.032415513 | 0.484669842 | -0.023896878 | 0.606479306 | -0.050715003 |
| 0.111957563 | 0.015496885 | 0.096833549 | 0.03644739 | 0.121923323 |
| -0.0289524 | 0.532548298 | -0.051941935 | 0.262618815 | -0.018964075 |
| 0.000501867 | 0.991369956 | -0.042141858 | 0.363531562 | -0.040427409 |
| 0.145382877 | 0.001632242 | 0.115384725 | 0.012590695 | 0.121269867 |
| -0.027156239 | 0.558287851 | -0.032481659 | 0.483778553 | -0.059754893 |
| -0.001547408 | 0.973395346 | -0.001812308 | 0.968843047 | -0.010154171 |
| -0.17943051 | 9.67E-05 | -0.209785543 | 4.82E-06 | -0.183903108 |
| -0.164887626 | 0.000345937 | -0.110743463 | 0.016659339 | -0.108600903 |
| -0.085075768 | 0.06622359 | -0.093242177 | 0.044012707 | -0.099343173 |
| -0.021579514 | 0.641830026 | -0.060434591 | 0.192337804 | -0.065566351 |
| -0.155525132 | 0.000744993 | -0.162105381 | 0.000436408 | -0.177717204 |
| -0.034275486 | 0.459948523 | 0.002556996 | 0.956051508 | -0.013782396 |
| 0.147752749 | 0.001364778 | 0.16678409 | 0.000294647 | 0.168493077 |
| -0.065781138 | 0.155820223 | -0.090258525 | 0.05126485 | -0.151543254 |
| -0.234152391 | 3.09E-07 | -0.272917172 | 2.02E-09 | -0.254845096 |
| -0.006526585 | 0.888135227 | 0.046791785 | 0.312964149 | 0.068723719 |
| -0.177466508 | 0.000115516 | -0.17791426 | 0.000110936 | -0.193410469 |
| 0.067732794 | 0.143885378 | 0.095529845 | 0.039055051 | 0.087358179 |
| -0.144151247 | 0.00178949 | -0.163667943 | 0.0003832 | -0.165947211 |
| 0.066617458 | 0.150617323 | 0.069337678 | 0.134605349 | 0.100930708 |
| 0.165322349 | 0.000333494 | 0.188469334 | 4.15E-05 | 0.181766843 |
| 0.063852388 | 0.168336134 | 0.058458215 | 0.207313995 | 0.076585766 |
| 0.154062432 | 0.000836703 | 0.1937319 | 2.49E-05 | 0.194898487 |
| -0.164814311 | 0.000348078 | -0.184992044 | 5.78E-05 | -0.201872446 |
| 0.00808055 | 0.861742054 | -0.013258629 | 0.775057287 | 0.008716082 |
| -0.053406791 | 0.249378738 | -0.041969358 | 0.365500545 | -0.041729761 |
| -0.000473762 | 0.991853222 | -0.026422346 | 0.568975534 | -0.012858429 |
| -0.10837073 | 0.019153093 | -0.129086001 | 0.00520937 | -0.12794838 |
| -0.212886217 | 3.46E-06 | -0.193086883 | 2.66E-05 | -0.195906038 |
| -0.07803371 | 0.092109676 | -0.034952729 | 0.451124961 | -0.050321018 |
| 0.078119183 | 0.091752808 | 0.068245082 | 0.140871462 | 0.070716555 |
| -0.214465533 | 2.91E-06 | -0.204975517 | 8.00E-06 | -0.201702103 |
| 0.127604634 | 0.005754244 | 0.131403046 | 0.004449841 | 0.129140381 |
| 0.161005228 | 0.00047791 | 0.135816974 | 0.00327364 | 0.13046997 |
| 0.130078507 | 0.004870776 | 0.112299509 | 0.015182601 | 0.113165512 |
| -0.154229089 | 0.000825751 | -0.17838339 | 0.000106321 | -0.184236432 |
| 0.096216867 | 0.037661839 | 0.102266097 | 0.027114672 | 0.088815824 |
| 0.124471488 | 0.007078603 | 0.093246679 | 0.044002455 | 0.081580322 |
| 0.086369479 | 0.062189202 | 0.07468015 | 0.107010264 | 0.067400941 |
| 0.070847498 | 0.12630265 | 0.075010365 | 0.105463418 | 0.067126204 |
| -0.015340986 | 0.740906985 | -0.057855521 | 0.212044519 | -0.063943045 |

| | | | | |
|---|---|---|---|---|
| -0.001964175 | 0.966233664 | -0.003199166 | 0.945029843 | -0.019380936 |
| -0.147159404 | 0.001427671 | -0.134750404 | 0.003528567 | -0.123266036 |
| 0.055410029 | 0.232036185 | 0.075135405 | 0.104882327 | 0.06122339 |
| 0.100803441 | 0.029399086 | 0.06484674 | 0.161792826 | 0.06103173 |
| 0.040467095 | 0.382927415 | 0.026980938 | 0.560831899 | 0.049115605 |
| 0.020129985 | 0.664367732 | -0.012732842 | 0.783752084 | -0.000605164 |
| 0.139180533 | 0.00257539 | 0.127904539 | 0.005639966 | 0.135636004 |
| 0.220931156 | 1.43E-06 | 0.219901039 | 1.60E-06 | 0.222301385 |
| -0.035677137 | 0.44179351 | -0.013692919 | 0.767896628 | -0.029253041 |
| 0.160919883 | 0.000481278 | 0.160621815 | 0.000493216 | 0.160434785 |
| 0.093296671 | 0.043888728 | 0.05716564 | 0.217554225 | 0.068508891 |
| 0.026452802 | 0.568530069 | 0.014952901 | 0.747235309 | 0.024043966 |
| 0.046364109 | 0.317412419 | 0.016032745 | 0.72967023 | 0.01178847 |
| 0.264780432 | 6.22E-09 | 0.303435554 | 2.11E-11 | 0.290771121 |
| 0.076776314 | 0.097489067 | 0.055376998 | 0.232315046 | 0.049157038 |
| 0.110971201 | 0.016435645 | 0.132136282 | 0.004231221 | 0.139823409 |
| 0.133269605 | 0.003912369 | 0.122577442 | 0.008005712 | 0.129047893 |
| 0.05835684 | 0.208104298 | 0.017414344 | 0.707402351 | 0.020503618 |
| -0.005960784 | 0.897777385 | -0.015827776 | 0.732993791 | -0.011307544 |
| -0.13920904 | 0.002570102 | -0.138710465 | 0.002664031 | -0.165662461 |
| -0.296210336 | 6.53E-11 | -0.326765164 | 4.41E-13 | -0.375400915 |
| 0.110503098 | 0.016898322 | 0.086106587 | 0.062992187 | 0.086557615 |
| -0.099395388 | 0.031752189 | -0.112953624 | 0.014596957 | -0.13666406 |
| 0.316793511 | 2.40E-12 | 0.271864524 | 2.34E-09 | 0.27168475 |
| -0.035483614 | 0.444275532 | -0.013802985 | 0.76608496 | 0.018038478 |
| -0.029776482 | 0.520942611 | -0.059339402 | 0.200535689 | -0.060338527 |
| -0.009072777 | 0.844967916 | 0.009905622 | 0.830941776 | 0.022456562 |
| 0.067920404 | 0.142775953 | 0.074494162 | 0.107889351 | 0.068674421 |
| 0.145866274 | 0.001574067 | 0.117471925 | 0.011066612 | 0.130502867 |
| -0.008582097 | 0.853254877 | 0.001789859 | 0.969228792 | -0.00568755 |
| 0.12633879 | 0.00625986 | 0.139004108 | 0.002608338 | 0.142603507 |
| -0.002089069 | 0.964088001 | 0.022763012 | 0.623668265 | 0.00588904 |
| 0.164446196 | 0.000359015 | 0.167134987 | 0.000285972 | 0.17391221 |
| -0.013118517 | 0.777371577 | -0.016690947 | 0.719031981 | -0.031924552 |
| -0.05952162 | 0.199154372 | -0.033257487 | 0.473390981 | -0.043622727 |
| 0.166715233 | 0.000296378 | 0.172571202 | 0.000178639 | 0.183243838 |
| -0.018620015 | 0.688171023 | -0.057917313 | 0.211555973 | -0.056840189 |
| 0.123108822 | 0.007735249 | 0.108504436 | 0.019004373 | 0.126068368 |
| -0.090071359 | 0.051751219 | -0.125290933 | 0.006708183 | -0.147929273 |
| 0.114529759 | 0.013266646 | 0.116964009 | 0.011421626 | 0.094933446 |
| -0.145709096 | 0.001592771 | -0.142125574 | 0.002078561 | -0.137798039 |
| -0.047767065 | 0.302974012 | -0.059202272 | 0.201579799 | -0.02263188 |
| -0.050610726 | 0.275063696 | -0.029879317 | 0.519503538 | 0.004405876 |
| 0.072866929 | 0.115825695 | 0.065350217 | 0.158553587 | 0.065840306 |
| 0.311350886 | 5.90E-12 | 0.316196403 | 2.65E-12 | 0.312411418 |

| | | | | |
|---|---|---|---|---|
| 0.21724778 | 2.15E-06 | 0.207341807 | 6.25E-06 | 0.194133875 |
| 0.061352673 | 0.185657073 | 0.061551222 | 0.184235035 | 0.072482134 |
| -0.010394045 | 0.822740737 | 0.002451702 | 0.957859532 | -0.005538624 |
| -0.190307705 | 3.48E-05 | -0.191269802 | 3.17E-05 | -0.187260563 |
| 0.095513191 | 0.039089359 | 0.100316536 | 0.030195293 | 0.103504102 |
| -0.210742872 | 4.36E-06 | -0.201829638 | 1.11E-05 | -0.232356288 |
| -0.205725656 | 7.40E-06 | -0.229733642 | 5.21E-07 | -0.235471804 |
| 0.049832127 | 0.282525569 | 0.099916848 | 0.030862615 | 0.132325542 |
| 0.056394404 | 0.223834285 | 0.017446505 | 0.706886883 | 0.043750323 |
| -0.000756985 | 0.986983295 | 0.037206301 | 0.422461594 | 0.05474081 |
| 0.019074946 | 0.680965648 | 0.026741623 | 0.564313937 | 0.022315281 |
| 0.032359882 | 0.485420134 | 0.041261043 | 0.373654935 | 0.049676038 |
| -0.034650591 | 0.455049567 | -0.050456671 | 0.276529372 | -0.082092351 |
| 0.172982769 | 0.000172288 | 0.141623409 | 0.002156537 | 0.145139948 |
| -0.127244372 | 0.005894269 | -0.165874275 | 0.000318296 | -0.164706688 |
| 0.099655022 | 0.03130657 | 0.061441192 | 0.185022091 | 0.009836566 |
| 0.1983889 | 1.57E-05 | 0.190057895 | 3.57E-05 | 0.195698562 |
| 0.001117733 | 0.980781064 | -0.007030179 | 0.879566981 | -0.054606847 |
| 0.082106826 | 0.076297955 | 0.090694792 | 0.050145961 | 0.100330671 |
| -0.019985841 | 0.666625994 | -0.00838655 | 0.856561956 | -0.052369158 |
| 0.217689675 | 2.05E-06 | 0.213156653 | 3.36E-06 | 0.199711218 |
| 0.036984476 | 0.42523491 | 0.075431064 | 0.103518396 | 0.091420126 |
| 0.012865554 | 0.781554866 | -0.024652154 | 0.595148137 | -0.036005447 |
| 0.187327061 | 4.63E-05 | 0.214336878 | 2.96E-06 | 0.246897086 |
| 0.104512232 | 0.023904276 | 0.083568646 | 0.071191972 | 0.095180534 |
| 0.167649211 | 0.00027369 | 0.19387714 | 2.46E-05 | 0.198075108 |
| 0.045231954 | 0.32938644 | 0.014733061 | 0.75082773 | 0.009378014 |
| 0.031403719 | 0.498413333 | 0.004927469 | 0.915424637 | 0.026565404 |
| 0.089623797 | 0.052929872 | 0.046120819 | 0.319961226 | 0.008128941 |
| -0.087648657 | 0.058401606 | -0.050532791 | 0.275804505 | -0.057697602 |
| -0.099770078 | 0.031110811 | -0.104350576 | 0.024123825 | -0.103653013 |
| -0.067468847 | 0.145457351 | -0.041751863 | 0.367992571 | -0.058716478 |
| 0.055017775 | 0.235363024 | 0.044423715 | 0.338110778 | 0.048863827 |
| 0.107580208 | 0.020053269 | 0.13247233 | 0.00413431 | 0.101383512 |
| -0.133212901 | 0.00392779 | -0.123623741 | 0.00748098 | -0.139637967 |
| -0.092993305 | 0.044582666 | -0.104630418 | 0.02374486 | -0.100192127 |
| -0.056129976 | 0.226016941 | -0.033442089 | 0.470937477 | -0.034166315 |
| -0.137260987 | 0.00295514 | -0.108695995 | 0.018793059 | -0.136854076 |
| 0.067588222 | 0.144744778 | 0.118232716 | 0.010553123 | 0.186749984 |
| -0.097542249 | 0.035092966 | -0.083659481 | 0.070884136 | -0.088477351 |
| 0.117531812 | 0.011025404 | 0.133241849 | 0.003919911 | 0.135258619 |
| -0.005579711 | 0.904280061 | -0.019359574 | 0.676472261 | -0.037339585 |
| 0.248630703 | 5.20E-08 | 0.250344568 | 4.18E-08 | 0.255905418 |
| -0.011653039 | 0.801691988 | 0.00107723 | 0.981477378 | -0.019291324 |
| -0.059089179 | 0.202443853 | -0.034661307 | 0.454910044 | -0.045826061 |

| | | | | |
|---|---|---|---|---|
| -0.095971522 | 0.038154458 | -0.102728646 | 0.026424789 | -0.065358815 |
| -0.104809608 | 0.023504912 | -0.142579903 | 0.002010242 | -0.142957822 |
| 0.053088683 | 0.252213671 | 0.077506377 | 0.094336044 | 0.068317262 |
| -0.046683731 | 0.314084139 | -0.049528802 | 0.285469247 | -0.045206381 |
| -0.060826484 | 0.189464827 | -0.032149481 | 0.488263486 | -0.019941889 |
| 0.143959086 | 0.00181524 | 0.101653122 | 0.028052742 | 0.122216314 |
| -0.265134719 | 5.93E-09 | -0.261949758 | 9.12E-09 | -0.318605496 |
| -0.00414502 | 0.928815707 | 0.010184658 | 0.826254209 | 0.005506611 |
| 0.012261971 | 0.791561683 | 0.013541623 | 0.770389026 | 0.00413372 |
| -0.045424312 | 0.327331697 | -0.036677455 | 0.429090798 | -0.035472723 |
| -0.15446522 | 0.000810459 | -0.154916589 | 0.000781955 | -0.198925722 |
| 0.11312895 | 0.014443397 | 0.069593138 | 0.133171723 | 0.043236188 |
| 0.191253727 | 3.18E-05 | 0.144136659 | 0.001791433 | 0.193488095 |
| 0.186087119 | 5.21E-05 | 0.182435951 | 7.33E-05 | 0.180667891 |
| -0.080295937 | 0.083031829 | -0.017092857 | 0.7125625 | -0.01077136 |
| 0.097544367 | 0.035088983 | 0.07678326 | 0.097458677 | 0.05671393 |
| 0.062944041 | 0.174484731 | 0.099986589 | 0.030745273 | 0.096618674 |
| 0.021266662 | 0.646667503 | 0.014189299 | 0.759736394 | 0.019942677 |
| 0.046738257 | 0.313518644 | 0.069225812 | 0.135236866 | 0.070524616 |
| -0.156072156 | 0.000713155 | -0.184149919 | 6.25E-05 | -0.181385736 |
| -0.215547266 | 2.59E-06 | -0.230490017 | 4.77E-07 | -0.256313646 |
| 0.039390416 | 0.395724135 | 0.079554891 | 0.085922348 | 0.069691882 |
| 0.150117585 | 0.001138599 | 0.10903506 | 0.018424045 | 0.119268094 |
| 0.13460463 | 0.003564773 | 0.131266499 | 0.004491663 | 0.114771329 |
| -0.19837813 | 1.57E-05 | -0.184114645 | 6.27E-05 | -0.165189033 |
| 0.014667309 | 0.751903246 | 0.036626508 | 0.429732605 | 0.044234866 |
| 0.100713153 | 0.029545353 | 0.11943845 | 0.009782522 | 0.118367117 |
| -0.077046565 | 0.096312247 | -0.083216656 | 0.072395178 | -0.07974782 |
| -0.06980978 | 0.131965195 | -0.086176515 | 0.062777768 | -0.105797167 |
| -0.015728387 | 0.734607174 | 0.009277487 | 0.841515619 | 0.014643736 |
| -0.266958354 | 4.62E-09 | -0.226649678 | 7.45E-07 | -0.219724243 |
| -0.008868647 | 0.848413382 | -0.018645804 | 0.687761805 | -0.009687864 |
| -0.066236604 | 0.152970081 | -0.054544684 | 0.239420053 | -0.073689975 |
| -0.001883725 | 0.967615923 | -0.017705086 | 0.702747196 | -0.020575734 |
| 0.214896998 | 2.78E-06 | 0.22941393 | 5.40E-07 | 0.221974474 |
| 0.210662619 | 4.39E-06 | 0.197244137 | 1.76E-05 | 0.193300355 |
| -0.220111771 | 1.56E-06 | -0.253456449 | 2.80E-08 | -0.283201695 |
| 0.006974155 | 0.880519496 | -0.039426672 | 0.395289078 | -0.052507511 |
| 0.1223399 | 0.008129324 | 0.10769253 | 0.019923165 | 0.111776941 |
| -0.119251055 | 0.009898908 | -0.112758438 | 0.014769598 | -0.101839219 |
| 0.238588945 | 1.81E-07 | 0.244105609 | 9.19E-08 | 0.266945609 |
| 0.10649308 | 0.021351186 | 0.102554425 | 0.02668284 | 0.083366848 |
| 0.106644291 | 0.021166398 | 0.073161349 | 0.11435681 | 0.078860198 |
| 0.00475612 | 0.918355239 | 0.026590311 | 0.566520873 | 0.006263757 |
| -0.027167161 | 0.558129539 | -0.068629309 | 0.138642897 | -0.053241702 |

| | | | | |
|---|---|---|---|---|
| 0.127700486 | 0.005717496 | 0.103405071 | 0.025443155 | 0.141308229 |
| -0.033043639 | 0.476241925 | -0.059027708 | 0.202914636 | -0.088097464 |
| 0.17380998 | 0.000160156 | 0.210419348 | 4.51E-06 | 0.225661892 |
| 0.136847472 | 0.003043339 | 0.129667342 | 0.005008561 | 0.139348273 |
| -0.046457826 | 0.316434146 | -0.015911333 | 0.731638318 | -0.007597789 |
| 0.019683292 | 0.671375704 | 0.017652296 | 0.703591618 | 0.016395262 |
| 0.30211853 | 2.60E-11 | 0.289221701 | 1.89E-10 | 0.287487352 |
| -0.04454032 | 0.336843059 | -0.030387986 | 0.5124154 | -0.061639191 |
| 0.021830027 | 0.637967351 | 0.022844766 | 0.622421897 | 0.014205424 |
| 0.072098044 | 0.119731454 | 0.055871937 | 0.228161419 | 0.05013037 |
| 0.007716986 | 0.867904323 | 0.005907454 | 0.898687009 | -8.36E-05 |
| 0.130510977 | 0.004729548 | 0.119689595 | 0.009628449 | 0.13491632 |
| 0.212958013 | 3.43E-06 | 0.232728732 | 3.66E-07 | 0.232248489 |
| 0.079919921 | 0.084488546 | 0.085552498 | 0.064712605 | 0.081668421 |
| -0.017541751 | 0.705361046 | -0.0320496 | 0.48961639 | -0.049080605 |
| -0.222423052 | 1.21E-06 | -0.215683226 | 2.55E-06 | -0.254824291 |
| 0.065644462 | 0.156683285 | 0.11653678 | 0.011728007 | 0.086544288 |
| 0.137306798 | 0.002945514 | 0.140714018 | 0.002304585 | 0.128630145 |
| -0.090571539 | 0.050459979 | -0.061616951 | 0.183766047 | -0.096278959 |
| -0.001763155 | 0.969687669 | 0.017699279 | 0.702840066 | 0.00021311 |
| -0.073411819 | 0.113118738 | -0.064052945 | 0.167000681 | -0.08089179 |
| 0.058421176 | 0.20760249 | 0.039185494 | 0.398188555 | 0.04443834 |
| 0.055181096 | 0.233973778 | 0.071198099 | 0.124432741 | 0.051706621 |
| 0.041261481 | 0.373649858 | 0.070455 | 0.128421813 | 0.124112588 |
| 0.284396291 | 3.87E-10 | 0.320816568 | 1.22E-12 | 0.380130976 |
| 0.00172472 | 0.970348133 | 0.025024997 | 0.589590231 | -0.002187279 |
| 0.20244336 | 1.04E-05 | 0.197450422 | 1.72E-05 | 0.183746226 |
| -0.013407937 | 0.772593276 | -0.034339049 | 0.459116314 | -0.052160994 |
| 0.104930224 | 0.023344583 | 0.079189793 | 0.087375957 | 0.086419245 |
| -0.129648952 | 0.005014805 | -0.145261064 | 0.00164721 | -0.161951625 |
| -0.018194027 | 0.694943683 | 0.009370188 | 0.839953282 | 0.014178253 |
| 0.065808184 | 0.155649866 | 0.05617548 | 0.225640264 | 0.064437434 |
| -0.031226263 | 0.500844888 | -0.027966678 | 0.546599646 | -0.063756947 |
| -0.101513949 | 0.02826957 | -0.1003624 | 0.030119514 | -0.110567446 |
| 0.002389082 | 0.958934907 | -0.012914162 | 0.780750529 | -0.039429612 |
| -0.046321084 | 0.317862197 | -0.059449939 | 0.199696926 | -0.077224784 |
| 0.043560568 | 0.347589597 | 0.029684863 | 0.522226453 | 0.014275191 |
| 0.196799014 | 1.84E-05 | 0.177555262 | 0.000114595 | 0.191579367 |
| -0.05224833 | 0.259810211 | -0.050808675 | 0.273188182 | -0.052971568 |
| 0.183154641 | 6.86E-05 | 0.219741316 | 1.63E-06 | 0.211076075 |
| 0.051959298 | 0.262459099 | 0.071587944 | 0.122378873 | 0.079091201 |
| -0.094266592 | 0.04173078 | -0.111152245 | 0.016259696 | -0.124683286 |
| 0.097977233 | 0.034283047 | 0.092156503 | 0.046544796 | 0.08903759 |
| 0.162540915 | 0.000420925 | 0.170224989 | 0.000219246 | 0.188611704 |
| 0.055713663 | 0.229483916 | 0.024436047 | 0.598380473 | 0.004789791 |

| | | | | |
|---|---|---|---|---|
| 0.080625471 | 0.08177193 | 0.050176887 | 0.279204775 | 0.080110138 |
| 0.158574919 | 0.000582928 | 0.144444186 | 0.00175088 | 0.169269785 |
| -0.042820084 | 0.355854289 | -0.040945289 | 0.377325904 | -0.046750819 |
| -0.047057976 | 0.310216248 | -0.030391859 | 0.512361625 | 0.009818207 |
| -0.078940238 | 0.088380872 | -0.064279727 | 0.165500183 | -0.060396931 |
| 0.044284772 | 0.339625332 | 0.084329826 | 0.068645868 | 0.080745852 |
| -0.146653592 | 0.001483376 | -0.132650536 | 0.004083736 | -0.138892751 |
| -0.00145899 | 0.974915005 | -0.007687326 | 0.868407395 | -0.011634983 |
| -0.074701112 | 0.106911542 | -0.065822266 | 0.155561224 | -0.064870012 |
| -0.078561037 | 0.089925604 | -0.038871064 | 0.401987807 | -0.063695239 |
| 0.196625477 | 1.87E-05 | 0.179723399 | 9.41E-05 | 0.173211139 |
| -0.12053052 | 0.009128138 | -0.070714333 | 0.127018563 | -0.080169622 |
| -0.051779601 | 0.264115305 | -0.095390158 | 0.039343599 | -0.103505039 |
| -0.029881527 | 0.519472628 | -0.02795546 | 0.546760603 | -0.002617363 |
| 0.118976026 | 0.010071947 | 0.109575171 | 0.017849279 | 0.10537842 |
| 0.116092704 | 0.01205415 | 0.155224643 | 0.000763038 | 0.16690023 |
| 0.099264969 | 0.03197807 | 0.10216338 | 0.027269957 | 0.090141417 |
| -0.07371467 | 0.111635837 | -0.074374223 | 0.108459269 | -0.097167907 |
| 0.075802992 | 0.101822621 | 0.069962476 | 0.131119883 | 0.084359546 |
| 0.096075772 | 0.037944476 | 0.073960601 | 0.110442923 | 0.084444032 |
| 0.046880912 | 0.312042317 | 0.036829271 | 0.427181609 | 0.042584091 |
| 0.003843496 | 0.933981659 | 0.012845621 | 0.781884774 | -0.013907328 |
| 0.043514096 | 0.348104672 | 0.024769965 | 0.593389365 | 0.036678514 |
| -0.022993938 | 0.620150506 | 0.002749009 | 0.952754976 | 0.001321171 |
| -0.021787241 | 0.638626383 | -0.034245966 | 0.460335304 | -0.04203828 |
| -0.072583281 | 0.117254774 | -0.075499567 | 0.103204394 | -0.091751951 |
| -0.087734765 | 0.05815366 | -0.059311166 | 0.200750351 | -0.067189129 |
| -0.066898429 | 0.1488993 | -0.054032951 | 0.243863531 | -0.045356341 |
| 0.045792497 | 0.323421964 | 0.042421112 | 0.360358086 | 0.070813629 |
| -0.164157355 | 0.00036782 | -0.185243755 | 5.64E-05 | -0.200059375 |
| 0.043070326 | 0.353047571 | 0.042961062 | 0.354271353 | 0.037647385 |
| 0.272739185 | 2.07E-09 | 0.249431325 | 4.70E-08 | 0.273856991 |
| 0.271967415 | 2.31E-09 | 0.300674989 | 3.26E-11 | 0.2911568 |
| -0.033908159 | 0.464774279 | -0.033117888 | 0.475250997 | -0.015969196 |
| 0.096822234 | 0.036469371 | 0.095855959 | 0.038388378 | 0.117177197 |
| -0.178592069 | 0.000104326 | -0.212677728 | 3.54E-06 | -0.227890732 |
| -0.093441195 | 0.043561346 | -0.110527749 | 0.016873676 | -0.133601872 |
| 0.035466389 | 0.44449684 | 0.018350084 | 0.69245972 | -0.006786969 |
| -0.056010385 | 0.227009036 | -0.050882963 | 0.272486575 | -0.076949253 |
| 0.125725823 | 0.006518737 | 0.144268976 | 0.001773881 | 0.124888869 |
| -0.124210628 | 0.007200305 | -0.114640761 | 0.013177117 | -0.113385384 |
| -0.020826957 | 0.653491629 | -0.009795039 | 0.832801141 | 0.016693084 |
| -0.288601264 | 2.07E-10 | -0.303607509 | 2.05E-11 | -0.332168012 |
| 0.080325863 | 0.082916771 | 0.079057475 | 0.087907627 | 0.087296347 |
| 0.014072173 | 0.761659519 | 0.040830796 | 0.378662458 | 0.016604161 |

| | | | | |
|---|---|---|---|---|
| 0.042219902 | 0.362642911 | -0.003228891 | 0.944519904 | 0.007882349 |
| 0.132556933 | 0.00411023 | 0.150145963 | 0.001136108 | 0.158241015 |
| -0.059641392 | 0.198250211 | -0.086430962 | 0.062002628 | -0.063855824 |
| -0.022145883 | 0.633111089 | -0.0366823 | 0.429029804 | -0.041674673 |
| -0.011280189 | 0.807911358 | -0.016964835 | 0.71462105 | -0.016764414 |
| -0.083893167 | 0.070097187 | -0.109121907 | 0.01833055 | -0.142252931 |
| -0.06346351 | 0.170948306 | -0.039501187 | 0.39439582 | -0.047793368 |
| 0.147487561 | 0.001392566 | 0.153465715 | 0.00087703 | 0.15207223 |
| 0.006731356 | 0.88464957 | 0.043181256 | 0.351807859 | 0.013405244 |
| 0.215532351 | 2.59E-06 | 0.184325733 | 6.15E-05 | 0.213926078 |
| 0.202313481 | 1.05E-05 | 0.18206002 | 7.59E-05 | 0.183075818 |
| 0.265090239 | 5.97E-09 | 0.269721957 | 3.16E-09 | 0.253540513 |
| 0.058921924 | 0.20372666 | 0.073133867 | 0.1144933 | 0.10425441 |
| -0.125273526 | 0.006715868 | -0.126284261 | 0.006282509 | -0.148044907 |
| -0.014204031 | 0.759494596 | -0.0470764 | 0.310026653 | -0.052688414 |
| 0.045533296 | 0.326171228 | 0.043866848 | 0.34420701 | 0.032717051 |
| 0.077060548 | 0.09625167 | 0.097068711 | 0.035993123 | 0.130267777 |
| -0.006714629 | 0.884934218 | -0.024284237 | 0.60065586 | -0.065145644 |
| 0.104235399 | 0.024281311 | 0.138953897 | 0.002617786 | 0.151622904 |
| -0.40464889 | 7.95E-20 | -0.419844114 | 2.29E-21 | -0.45837966 |
| -0.060309552 | 0.193261151 | -0.041667296 | 0.368964363 | -0.014981562 |
| 0.046383446 | 0.317210403 | 0.013473248 | 0.771516174 | 0.018285433 |
| 0.088045558 | 0.057265976 | 0.051961951 | 0.262434705 | 0.022958779 |
| -0.053334181 | 0.250023867 | -0.088985765 | 0.054648711 | -0.108704393 |
| -0.019452786 | 0.675003229 | -0.012878027 | 0.781348452 | -0.030227845 |
| 0.121661536 | 0.008491757 | 0.105114515 | 0.023101439 | 0.115498757 |
| 0.048985456 | 0.290793742 | 0.042883085 | 0.355146347 | 0.045607298 |
| 0.190908584 | 3.28E-05 | 0.170281831 | 0.000218167 | 0.180039469 |
| 0.179455419 | 9.64E-05 | 0.144959166 | 0.00168485 | 0.136066036 |
| -0.331825611 | 1.82E-13 | -0.364718696 | 3.87E-16 | -0.408140775 |
| 0.149413995 | 0.001201993 | 0.119102916 | 0.009991781 | 0.094653989 |
| -0.252197751 | 3.30E-08 | -0.240743649 | 1.39E-07 | -0.244151083 |
| 0.012054098 | 0.795016053 | 0.04250751 | 0.35937976 | 0.038339849 |
| 0.118097972 | 0.010642498 | 0.094514533 | 0.04119374 | 0.074214267 |
| 0.031832787 | 0.492560078 | -0.003674059 | 0.936885809 | -0.025819308 |
| 0.117979709 | 0.010721493 | 0.122528947 | 0.008030811 | 0.126941757 |
| 0.119310224 | 0.009862027 | 0.129588658 | 0.005035326 | 0.101675032 |
| -0.078350904 | 0.090790895 | -0.07072094 | 0.12698297 | -0.056424254 |
| 0.086274583 | 0.062478076 | 0.113869684 | 0.013810184 | 0.095925229 |
| -0.065712443 | 0.15625356 | -0.05879567 | 0.204698885 | -0.051517624 |
| -0.023020187 | 0.619751186 | -0.050066135 | 0.280268668 | -0.083323737 |
| 0.121679919 | 0.008481749 | 0.117851904 | 0.010807447 | 0.082188069 |
| -0.165913882 | 0.000317231 | -0.162198498 | 0.000433054 | -0.110281567 |
| 0.066910894 | 0.14882343 | 0.061590797 | 0.183952553 | 0.057529402 |
| 0.00444722 | 0.923641165 | 0.019543668 | 0.673572083 | 0.02983813 |

| | | | | |
|---|---|---|---|---|
| 0.101953931 | 0.027588969 | 0.096379382 | 0.037338511 | 0.093581971 |
| 0.047543099 | 0.305249272 | 0.004580806 | 0.921354813 | -0.042130924 |
| -0.118151903 | 0.010606645 | -0.103702316 | 0.025021837 | -0.147817528 |
| 0.123735515 | 0.007426782 | 0.139106299 | 0.002589207 | 0.161953453 |
| -0.200494393 | 1.27E-05 | -0.184836615 | 5.86E-05 | -0.160909772 |
| 0.016951488 | 0.714835769 | 0.038854989 | 0.402182624 | 0.063865203 |
| 0.314322029 | 3.62E-12 | 0.304901049 | 1.67E-11 | 0.307270476 |
| -0.096922728 | 0.036274552 | -0.078204222 | 0.091398855 | -0.087842813 |
| -0.217768733 | 2.03E-06 | -0.210586427 | 4.43E-06 | -0.214587984 |
| 0.105822636 | 0.02218747 | 0.119705676 | 0.009618658 | 0.126616135 |
| 0.071349752 | 0.123630607 | 0.085923615 | 0.063556089 | 0.086135259 |
| 0.022124142 | 0.633444862 | 0.043160924 | 0.352034873 | 0.052061936 |
| -0.008835719 | 0.848969438 | -0.000468561 | 0.991942665 | -0.00856854 |
| 0.087664598 | 0.058355639 | 0.071299423 | 0.123896363 | 0.0712995 |
| -0.239124096 | 1.70E-07 | -0.273569359 | 1.84E-09 | -0.240176446 |
| 0.089505597 | 0.053244856 | 0.076425359 | 0.099034378 | 0.077460674 |
| -0.004199798 | 0.92787752 | 0.035583492 | 0.442993567 | 0.04837692 |
| 0.016887964 | 0.715858092 | -0.008173433 | 0.860169051 | -0.018711247 |
| 0.022267298 | 0.631248509 | 0.012352947 | 0.790051148 | 0.015826513 |
| 0.005614201 | 0.903691249 | -0.015760581 | 0.734084444 | -0.027758644 |
| 0.078228932 | 0.091296209 | 0.061837166 | 0.182201204 | 0.048465849 |
| 0.06454854 | 0.163734707 | 0.077677372 | 0.093609451 | 0.077569041 |
| 0.057026784 | 0.218675485 | 0.038089486 | 0.411525032 | 0.025113803 |
| -0.051114409 | 0.270308597 | -0.063271281 | 0.172250667 | -0.093321854 |
| -0.108230411 | 0.019310258 | -0.079330772 | 0.086812332 | -0.092532034 |
| -0.198258844 | 1.59E-05 | -0.246922179 | 6.46E-08 | -0.256959037 |
| -0.025876907 | 0.576981332 | -0.027902262 | 0.547524221 | -0.015682167 |
| 0.051064433 | 0.270777883 | 0.042201252 | 0.362855138 | 0.066441974 |
| 0.011882712 | 0.797867112 | 0.016349896 | 0.724537622 | 0.014636522 |
| 0.101981177 | 0.02754729 | 0.116595556 | 0.01168543 | 0.110708064 |
| -0.100693172 | 0.029577807 | -0.124321085 | 0.007148546 | -0.145128687 |
| 0.158691048 | 0.000577458 | 0.130891189 | 0.004608449 | 0.128525097 |
| 0.058601928 | 0.206197349 | 0.076982383 | 0.096590701 | 0.070030445 |
| -0.055378943 | 0.232298616 | -0.037738336 | 0.41585316 | -0.034857133 |
| 0.3235517 | 7.67E-13 | 0.310370297 | 6.92E-12 | 0.319005752 |
| -0.22418842 | 9.87E-07 | -0.231080098 | 4.45E-07 | -0.258094073 |
| 0.177257053 | 0.000117719 | 0.155894836 | 0.000723335 | 0.169117547 |
| 0.148135224 | 0.001325598 | 0.120522526 | 0.009132783 | 0.106835695 |
| -0.02671032 | 0.564770171 | -0.060911641 | 0.188844729 | -0.066166183 |
| 0.03322398 | 0.473837066 | 0.048906738 | 0.291570627 | 0.080822605 |
| 0.164469199 | 0.000358323 | 0.159313229 | 0.000548954 | 0.163207147 |
| 0.203128421 | 9.69E-06 | 0.22136697 | 1.36E-06 | 0.219747018 |
| 0.041554931 | 0.370258059 | 0.070837413 | 0.12635676 | 0.022449624 |
| -0.104066402 | 0.024513994 | -0.12877459 | 0.005319896 | -0.166533733 |
| 0.058307724 | 0.208487982 | 0.075504177 | 0.103183288 | 0.051183087 |

| | | | | |
|---|---|---|---|---|
| 0.000554396 | 0.990466717 | 0.025234224 | 0.58648181 | 0.017130935 |
| -0.188659651 | 4.08E-05 | -0.169234805 | 0.00023885 | -0.186873947 |
| 0.269914463 | 3.07E-09 | 0.27434361 | 1.65E-09 | 0.292727208 |
| 0.067591996 | 0.144722298 | 0.04889129 | 0.291723246 | 0.068729744 |
| -0.107447239 | 0.020208245 | -0.122342954 | 0.008127724 | -0.119783559 |
| -0.226513451 | 7.56E-07 | -0.228234339 | 6.20E-07 | -0.23222683 |
| 0.080449072 | 0.082444414 | 0.046550905 | 0.315464492 | 0.028471182 |
| 0.146527312 | 0.001497592 | 0.082525749 | 0.074805154 | 0.083851104 |
| -0.078174111 | 0.091524058 | -0.081440593 | 0.078721828 | -0.094942029 |
| 0.085400341 | 0.065191751 | 0.089985834 | 0.05197474 | 0.089901485 |
| -0.093330465 | 0.043811993 | -0.080179777 | 0.083479662 | -0.099480204 |
| -0.023122357 | 0.618197988 | -0.014041195 | 0.76216841 | -0.004717026 |
| 0.066170979 | 0.153378282 | 0.072613933 | 0.11709968 | 0.05783169 |
| 0.002102529 | 0.963856776 | -0.022685994 | 0.624843415 | 0.002284333 |
| -0.236242998 | 2.41E-07 | -0.255689602 | 2.09E-08 | -0.246807715 |
| -0.112498227 | 0.015002541 | -0.123269616 | 0.007655031 | -0.134662121 |
| 0.017028351 | 0.713599477 | 0.007673227 | 0.868646557 | 0.024007867 |
| -0.164078934 | 0.000370245 | -0.186137956 | 5.19E-05 | -0.164417838 |
| -0.184187143 | 6.23E-05 | -0.227918644 | 6.43E-07 | -0.2042717 |
| 0.091261376 | 0.048723378 | 0.069696372 | 0.13259573 | 0.059162417 |
| -0.192930006 | 2.70E-05 | -0.203651107 | 9.18E-06 | -0.175355478 |
| -0.041571188 | 0.370070714 | -0.030143927 | 0.51580999 | -0.020241054 |
| 0.021427927 | 0.644172061 | 0.031649392 | 0.495057405 | 0.029174006 |
| 0.039928025 | 0.389302577 | 0.027119696 | 0.558817722 | 0.008312924 |
| -0.054562173 | 0.23926921 | -0.0432162 | 0.351417909 | -0.06388923 |
| 0.09569797 | 0.03871014 | 0.082274605 | 0.075697205 | 0.073839973 |
| 0.111566292 | 0.015863492 | 0.118873783 | 0.010136957 | 0.102648852 |
| 0.052850544 | 0.254350531 | 0.059505313 | 0.199277706 | 0.041534504 |
| 0.040793978 | 0.379092872 | 0.014433974 | 0.755723768 | -0.010182389 |
| -0.175299758 | 0.000140304 | -0.157530959 | 0.000634305 | -0.158534925 |
| -0.170986902 | 0.000205197 | -0.17294924 | 0.000172797 | -0.203493306 |
| -0.102407505 | 0.026902138 | -0.091595427 | 0.047900578 | -0.087597222 |
| -0.122271587 | 0.008165186 | -0.151041698 | 0.001060014 | -0.169153929 |
| -0.052167561 | 0.260548572 | -0.045876305 | 0.322536269 | -0.071972314 |
| 0.130156545 | 0.004845014 | 0.122857319 | 0.007862225 | 0.120245652 |
| -0.150316811 | 0.001121217 | -0.142666215 | 0.001997498 | -0.138185187 |
| -0.043705589 | 0.345985353 | -0.052250504 | 0.259790359 | -0.078028871 |
| 0.141024989 | 0.002252947 | 0.125243609 | 0.006729094 | 0.138703269 |
| -0.008970432 | 0.846695016 | 0.002768801 | 0.952415241 | 0.05911112 |
| 0.285271215 | 3.40E-10 | 0.319416229 | 1.55E-12 | 0.328171314 |
| 0.057062909 | 0.218383382 | 0.046900347 | 0.311841537 | 0.06960502 |
| -0.120669597 | 0.009047661 | -0.126156333 | 0.006335936 | -0.123209667 |
| 0.089962002 | 0.052037168 | 0.090864878 | 0.049715309 | 0.064069152 |
| -0.023715305 | 0.609217656 | -0.023609123 | 0.610821551 | -0.029894959 |
| 0.155580171 | 0.000741731 | 0.149582232 | 0.001186545 | 0.157836731 |

| | | | | |
|---|---|---|---|---|
| 0.070535608 | 0.127984363 | 0.073828109 | 0.111084334 | 0.080129364 |
| -0.085877516 | 0.063698816 | -0.059641543 | 0.198249072 | -0.059634377 |
| 0.194611126 | 2.29E-05 | 0.181075291 | 8.31E-05 | 0.196111669 |
| -0.072260549 | 0.118897508 | -0.017786184 | 0.701450672 | -0.026229155 |
| -0.188386696 | 4.19E-05 | -0.216365933 | 2.37E-06 | -0.244909916 |
| -0.09167699 | 0.047701458 | -0.114470419 | 0.013314726 | -0.14442621 |
| -0.057765669 | 0.212756373 | -0.096061399 | 0.037973367 | -0.093415586 |
| -0.101108858 | 0.028908899 | -0.092304394 | 0.046192842 | -0.09525326 |
| 0.118647754 | 0.010281997 | 0.07987764 | 0.084653626 | 0.073837696 |
| -0.114145454 | 0.013580762 | -0.143018308 | 0.001946272 | -0.151955213 |
| 0.061904376 | 0.181725573 | 0.048330509 | 0.297299844 | 0.072468733 |
| 0.064865608 | 0.161670544 | 0.059367505 | 0.200322192 | 0.059537805 |
| 0.056520187 | 0.222801326 | 0.033115388 | 0.475284356 | 0.027746235 |
| 0.000787521 | 0.986458261 | -0.027925804 | 0.547186237 | -0.037790896 |
| -0.073481871 | 0.112774362 | -0.05293645 | 0.253578242 | -0.056854607 |
| -0.082604784 | 0.074526205 | -0.11320181 | 0.014380001 | -0.048342114 |
| 0.218395705 | 1.89E-06 | 0.223183794 | 1.11E-06 | 0.259964085 |
| -0.238081235 | 1.93E-07 | -0.215574416 | 2.58E-06 | -0.208935777 |
| 0.286826419 | 2.70E-10 | 0.262403206 | 8.58E-09 | 0.252037602 |
| 0.0189708 | 0.682612623 | -0.006076907 | 0.895797195 | -0.027569003 |
| 0.145278756 | 0.001645028 | 0.153601247 | 0.000867716 | 0.138317304 |
| -0.105406586 | 0.02272059 | -0.099749503 | 0.03114574 | -0.096099817 |
| 0.042200934 | 0.362858765 | 0.016774954 | 0.717678057 | 0.016128145 |
| -0.0611436 | 0.187163212 | -0.075086007 | 0.105111589 | -0.077930077 |
| -0.070353442 | 0.128974602 | -0.049288692 | 0.287814041 | -0.051831201 |
| 0.124443731 | 0.007091465 | 0.088191899 | 0.056851906 | 0.086864022 |
| -0.225903634 | 8.11E-07 | -0.25724888 | 1.71E-08 | -0.231800056 |
| 0.220550559 | 1.49E-06 | 0.219198048 | 1.73E-06 | 0.21727081 |
| -0.08271634 | 0.074133913 | -0.087254978 | 0.059546376 | -0.086279322 |
| 0.016689426 | 0.719056498 | 0.022977881 | 0.620394823 | 0.021022049 |
| 0.024990304 | 0.590106391 | 0.058568934 | 0.20645333 | 0.038078492 |
| 0.143847928 | 0.00183029 | 0.167744936 | 0.000271459 | 0.18596152 |
| -0.089669514 | 0.052808458 | -0.084072544 | 0.069497982 | -0.074575348 |
| -0.005115499 | 0.912210057 | -0.002807652 | 0.951748341 | 0.006715766 |
| -0.021372004 | 0.64503697 | -0.026693788 | 0.565011188 | -0.026506445 |
| 0.160598951 | 0.000494143 | 0.184277325 | 6.18E-05 | 0.192327874 |
| 0.10590411 | 0.022084349 | 0.107888174 | 0.019698301 | 0.107794027 |
| 0.040373191 | 0.384033342 | 0.050089891 | 0.280040238 | 0.031997838 |
| 0.066015075 | 0.154351351 | 0.057382891 | 0.215808197 | 0.055581556 |
| -0.239751863 | 1.57E-07 | -0.236061889 | 2.46E-07 | -0.239618006 |
| -0.03835433 | 0.408278373 | -0.012414739 | 0.78902563 | -0.015319848 |
| -0.110988803 | 0.016418466 | -0.114632453 | 0.013183799 | -0.12546361 |
| 0.086550272 | 0.061641893 | 0.071776546 | 0.121394768 | 0.069978993 |
| -0.076724944 | 0.09771405 | -0.047444346 | 0.306256074 | -0.038034506 |
| -0.048564043 | 0.294968932 | -0.04705011 | 0.310297222 | -0.050871778 |

| | | | | |
|---|---|---|---|---|
| -0.132472491 | 0.004134264 | -0.146943238 | 0.001451238 | -0.182711672 |
| 0.015739127 | 0.734432777 | 0.013756822 | 0.766844645 | 0.00241823 |
| 0.198719979 | 1.52E-05 | 0.195042896 | 2.19E-05 | 0.181227376 |
| -0.076328955 | 0.099462252 | -0.087574738 | 0.058615153 | -0.089340762 |
| -0.006977252 | 0.880466849 | 0.004919031 | 0.91556893 | 0.02988784 |
| 0.220544344 | 1.49E-06 | 0.230576599 | 4.72E-07 | 0.254551421 |
| 0.220501751 | 1.50E-06 | 0.230873696 | 4.56E-07 | 0.213418336 |
| -0.125760134 | 0.006503996 | -0.139981647 | 0.002430497 | -0.155986247 |
| 0.004145461 | 0.928808155 | 0.011026642 | 0.812147623 | 0.018477532 |
| 0.150680242 | 0.001090136 | 0.144611454 | 0.001729177 | 0.199169086 |
| -0.087178233 | 0.059771688 | -0.103971106 | 0.02464605 | -0.115901016 |
| -0.01196895 | 0.796432171 | -0.049003715 | 0.290613735 | -0.043236525 |
| 0.064342674 | 0.165085495 | 0.034898199 | 0.451831868 | 0.028973769 |
| -0.120529283 | 0.009128857 | -0.127102338 | 0.00595031 | -0.163728978 |
| 0.259622149 | 1.25E-08 | 0.272912135 | 2.02E-09 | 0.295774056 |
| -0.110031304 | 0.017376138 | -0.118042217 | 0.010679675 | -0.124500872 |
| 0.001826525 | 0.968598747 | -0.008755446 | 0.850325329 | 0.016707936 |
| 0.053641315 | 0.247302967 | 0.021680411 | 0.640273114 | 0.005817672 |
| -0.213298465 | 3.31E-06 | -0.232471312 | 3.77E-07 | -0.218605346 |
| -0.059733231 | 0.197558955 | -0.059840348 | 0.19675492 | -0.054664447 |
| -0.082886545 | 0.073538625 | -0.065287079 | 0.158957098 | -0.093382466 |
| -0.030687524 | 0.508265067 | 0.014371 | 0.756755905 | -0.011723354 |
| -0.061938149 | 0.181486919 | -0.091150982 | 0.048997876 | -0.046337836 |
| -0.005079204 | 0.912830443 | 0.052404643 | 0.258385363 | 0.049787258 |
| 0.013207718 | 0.775897981 | -0.00845986 | 0.855321851 | -0.020686679 |
| -0.118264887 | 0.010531882 | -0.122806045 | 0.007888339 | -0.119871414 |
| 0.171678912 | 0.000193172 | 0.164946989 | 0.000344213 | 0.16769687 |
| 0.019739236 | 0.670496441 | 0.013869647 | 0.764988323 | 0.008709477 |
| -0.152071441 | 0.000978357 | -0.1423726 | 0.002041156 | -0.146388749 |
| -0.128172335 | 0.005539653 | -0.100004175 | 0.030715744 | -0.112007763 |
| 0.189263622 | 3.85E-05 | 0.191902539 | 2.98E-05 | 0.183798358 |
| 0.095942988 | 0.038212103 | 0.119683966 | 0.009631879 | 0.100115542 |
| 0.068932761 | 0.136902046 | 0.065846276 | 0.155410173 | 0.040185943 |
| -0.070562749 | 0.127837332 | -0.050758319 | 0.273664466 | -0.077457927 |
| 0.137286003 | 0.00294988 | 0.175370632 | 0.00013942 | 0.216235905 |
| 0.152960628 | 0.00091256 | 0.161863497 | 0.000445234 | 0.178697056 |
| 0.15993611 | 0.000521731 | 0.156499634 | 0.000689157 | 0.168269352 |
| -0.089070355 | 0.054418194 | -0.150122836 | 0.001138138 | -0.144462853 |
| -0.083611208 | 0.071047595 | -0.11538719 | 0.012588791 | -0.140828406 |
| -0.112892157 | 0.014651132 | -0.113482106 | 0.014138388 | -0.116033593 |
| 0.145250852 | 0.00164847 | 0.138445786 | 0.002715156 | 0.142993359 |
| 0.137322387 | 0.002942245 | 0.135542666 | 0.003337555 | 0.116287972 |
| 0.109227442 | 0.018217494 | 0.093479527 | 0.043474862 | 0.110217174 |
| 0.193355268 | 2.59E-05 | 0.188287639 | 4.23E-05 | 0.185592904 |
| -0.103900817 | 0.024743845 | -0.085700377 | 0.06424971 | -0.125734499 |

| | | | | |
|---|---|---|---|---|
| 0.008013614 | 0.862875974 | -0.041347089 | 0.372658394 | -0.050964837 |
| 0.167165935 | 0.000285219 | 0.165142602 | 0.000338587 | 0.180435635 |
| -0.283435071 | 4.46E-10 | -0.302552072 | 2.43E-11 | -0.32570428 |
| -0.046604319 | 0.314908925 | -0.014904041 | 0.748033271 | -0.032175459 |
| -0.000126597 | 0.997823014 | 0.008033185 | 0.862544412 | 0.005332248 |
| 0.142827439 | 0.001973891 | 0.134921842 | 0.003486413 | 0.163989525 |
| 0.233639111 | 3.29E-07 | 0.216173093 | 2.42E-06 | 0.229197336 |
| -0.088179218 | 0.056887686 | -0.05304729 | 0.252584196 | -0.039749405 |
| 0.199269695 | 1.44E-05 | 0.186494808 | 5.01E-05 | 0.159960868 |
| 0.254196384 | 2.54E-08 | 0.22301581 | 1.13E-06 | 0.215555026 |
| 0.164600006 | 0.000354407 | 0.167196667 | 0.000284473 | 0.150175596 |
| -0.043051157 | 0.353262079 | -0.031890814 | 0.491771312 | -0.047045791 |
| 0.024075616 | 0.603789077 | -0.001140893 | 0.980382922 | -0.001283542 |
| 0.02463822 | 0.59535631 | -0.019751914 | 0.670297242 | -0.052248634 |
| -0.129039225 | 0.005225839 | -0.178015374 | 0.000109926 | -0.193078212 |
| 0.046675934 | 0.314165059 | 0.065531064 | 0.157402087 | 0.097669594 |
| -0.053178255 | 0.251413164 | -0.129730007 | 0.004987337 | -0.14784575 |
| 0.287378701 | 2.49E-10 | 0.262909535 | 8.02E-09 | 0.28191272 |
| -0.205931287 | 7.24E-06 | -0.203181975 | 9.63E-06 | -0.197567833 |
| -0.124067705 | 0.007267775 | -0.173074941 | 0.000170894 | -0.192176642 |
| 0.058462725 | 0.207278887 | 0.065465691 | 0.157817604 | 0.056468076 |
| 0.248894774 | 5.03E-08 | 0.211972103 | 3.82E-06 | 0.19570334 |
| 0.130153917 | 0.004845879 | 0.145732305 | 0.001589996 | 0.144174485 |
| 0.009789561 | 0.832893273 | -0.024392925 | 0.5990264 | 7.07E-05 |
| 0.003359905 | 0.942272627 | -0.016448801 | 0.722939512 | -0.023669185 |
| -0.156577186 | 0.000684884 | -0.154005458 | 0.000840478 | -0.141110302 |
| 0.210109877 | 4.66E-06 | 0.16969969 | 0.00022945 | 0.160929724 |
| 0.198345806 | 1.58E-05 | 0.207383701 | 6.22E-06 | 0.21818841 |
| 0.248611641 | 5.21E-08 | 0.27051467 | 2.83E-09 | 0.291904517 |
| -0.047434206 | 0.306359575 | -0.014766628 | 0.750278863 | -0.001048144 |
| 0.031043715 | 0.503352738 | -0.000582714 | 0.989979777 | 0.012024901 |
| 0.135383061 | 0.003375263 | 0.111500011 | 0.015926342 | 0.101686225 |
| 0.144991778 | 0.001680746 | 0.154254478 | 0.000824094 | 0.155227133 |
| 0.242736377 | 1.09E-07 | 0.233588987 | 3.31E-07 | 0.240172514 |
| 0.015035157 | 0.745892566 | 0.043769195 | 0.345283215 | 0.034080649 |
| 0.164843156 | 0.000347235 | 0.135611905 | 0.003321316 | 0.13529264 |
| 0.004134162 | 0.929001691 | 0.009812535 | 0.832506906 | 0.013206166 |
| 0.137413482 | 0.002923206 | 0.108696333 | 0.018792688 | 0.096132075 |
| 0.069040731 | 0.136286714 | 0.046501857 | 0.315975208 | 0.031016539 |
| 0.000459158 | 0.992104343 | 0.061029477 | 0.187989132 | 0.048912495 |
| 0.150115131 | 0.001138815 | 0.130667257 | 0.004679429 | 0.13167061 |
| 0.08775851 | 0.058085439 | 0.115610424 | 0.012417404 | 0.096855293 |
| -0.083367125 | 0.071878815 | -0.073065211 | 0.114834834 | -0.076387163 |
| -0.017095119 | 0.712526157 | 0.013768387 | 0.766654293 | 0.012212972 |
| -0.045947185 | 0.321788425 | -0.021160427 | 0.64831356 | -0.013369174 |

| | | | | |
|---|---|---|---|---|
| -0.01739716 | 0.707677833 | 0.002245485 | 0.961401165 | -0.000468669 |
| -0.162991677 | 0.00040544 | -0.196181113 | 1.96E-05 | -0.228588252 |
| 0.164253332 | 0.000364872 | 0.157034172 | 0.000660201 | 0.141296605 |
| 0.122594969 | 0.007996658 | 0.077745338 | 0.093321893 | 0.042581887 |
| 0.047402146 | 0.306686984 | 0.029325391 | 0.527279285 | 0.028621728 |
| 0.161367182 | 0.000463867 | 0.107415727 | 0.020245125 | 0.120570799 |
| 0.12197787 | 0.008320993 | 0.114179269 | 0.013552863 | 0.115724973 |
| 0.205223528 | 7.80E-06 | 0.193636837 | 2.52E-05 | 0.224039567 |
| 0.152284768 | 0.000962184 | 0.090606366 | 0.050371083 | 0.041136321 |
| 0.072816962 | 0.116076443 | 0.024540557 | 0.596816311 | 0.010782218 |
| 0.043222561 | 0.351346953 | 0.052703284 | 0.255678186 | 0.032942509 |
| -0.151286177 | 0.001040078 | -0.13051787 | 0.004727327 | -0.149380216 |
| 0.153938855 | 0.000844911 | 0.156545083 | 0.00068665 | 0.147156591 |
| -0.020656259 | 0.656148668 | 0.02577842 | 0.578432524 | 0.00109176 |
| -0.012049051 | 0.795099977 | -0.007356937 | 0.874014893 | -0.019820318 |
| 0.084956512 | 0.066606063 | 0.051403483 | 0.267605078 | 0.033969832 |
| -0.026542526 | 0.567218698 | -0.034298108 | 0.459652244 | -0.021763268 |
| 0.099346892 | 0.031836022 | 0.070462707 | 0.128379935 | 0.023148261 |
| 0.028487889 | 0.539147088 | -0.009140435 | 0.843826582 | -0.040973744 |
| 0.064062547 | 0.166936945 | 0.026391127 | 0.56943233 | -0.000299826 |
| 0.085290142 | 0.065540589 | 0.052208206 | 0.26017683 | 0.010312655 |
| 0.123127595 | 0.007725845 | 0.142943333 | 0.001957078 | 0.133453243 |
| 0.066059592 | 0.154073027 | 0.054078694 | 0.243464008 | 0.056526675 |
| 0.102037426 | 0.027461415 | 0.092895024 | 0.044809448 | 0.082103375 |
| -0.058688321 | 0.205528185 | -0.078359701 | 0.090754539 | -0.100156205 |
| 0.012057294 | 0.794962914 | -0.003372333 | 0.942059467 | -0.011821233 |
| 0.277341928 | 1.08E-09 | 0.294737923 | 8.19E-11 | 0.304110863 |
| -0.039641056 | 0.392722423 | -0.050180645 | 0.27916873 | -0.021716323 |
| 0.116311014 | 0.011892828 | 0.087416668 | 0.059073977 | 0.074049545 |
| 0.14767547 | 0.001372823 | 0.095243151 | 0.039649211 | 0.091772655 |
| 0.021754529 | 0.639130438 | 0.02054691 | 0.657853061 | 0.022167472 |
| 0.168604202 | 0.000252178 | 0.190344114 | 3.47E-05 | 0.18442233 |
| 0.235699713 | 2.57E-07 | 0.228208327 | 6.22E-07 | 0.263995814 |
| 0.125936411 | 0.006428735 | 0.116190474 | 0.011981664 | 0.099709478 |
| 0.044797881 | 0.334053707 | 0.068793711 | 0.137697654 | 0.098605096 |
| 0.126532788 | 0.006179871 | 0.142320786 | 0.00204895 | 0.153848941 |
| 0.012972968 | 0.779777762 | -0.003356865 | 0.94232476 | -0.033826998 |
| 0.048693127 | 0.293685785 | 0.029558545 | 0.523999183 | 0.026856089 |
| 0.061303848 | 0.186007999 | 0.060055221 | 0.195149263 | 0.052177613 |
| -0.090283195 | 0.051201027 | -0.044413367 | 0.338223421 | -0.058350246 |
| -0.021457003 | 0.643722562 | -0.046099818 | 0.32018186 | -0.076696157 |
| 0.161756191 | 0.000449203 | 0.143631167 | 0.001859966 | 0.149618117 |
| -0.045668202 | 0.32473843 | -0.039008104 | 0.400329297 | -0.047426267 |
| -0.119502044 | 0.009743302 | -0.096990775 | 0.036143137 | -0.11369137 |
| 0.00703879 | 0.879420582 | 0.004023948 | 0.930889659 | -0.015709663 |

| | | | | |
|---|---|---|---|---|
| -0.227618102 | 6.66E-07 | -0.244359297 | 8.90E-08 | -0.271665604 |
| 0.004241494 | 0.92716346 | 0.001607429 | 0.97236381 | -0.011525482 |
| 0.128737549 | 0.005333181 | 0.127924561 | 0.00563241 | 0.154635969 |
| 0.124924074 | 0.006871815 | 0.171478881 | 0.000196578 | 0.161934211 |
| -0.033469067 | 0.470579501 | 0.00284394 | 0.951125481 | -0.008100901 |
| -0.044075321 | 0.341916635 | -0.007967238 | 0.863661773 | -0.036118408 |
| -0.141010082 | 0.002255398 | -0.125063176 | 0.006809355 | -0.162189271 |
| -0.015214675 | 0.742964795 | 0.00824668 | 0.858928998 | -0.013182125 |
| 0.107811403 | 0.019786273 | 0.103068097 | 0.025928172 | 0.085494156 |
| 0.147932115 | 0.001346274 | 0.132222339 | 0.00420621 | 0.137924707 |
| 0.119769045 | 0.009580159 | 0.117274166 | 0.011203659 | 0.142223194 |
| -0.008895979 | 0.847951896 | -0.028150534 | 0.543964982 | -0.041835581 |
| -0.03724945 | 0.421923367 | -0.053401903 | 0.249422132 | -0.03028935 |
| -0.008206212 | 0.85961407 | -0.019090047 | 0.680726965 | -0.035101064 |
| 0.123501826 | 0.007540497 | 0.084437434 | 0.068292019 | 0.097030475 |
| 0.193317053 | 2.60E-05 | 0.142827431 | 0.001973892 | 0.14984268 |
| 0.06188734 | 0.181846046 | 0.010435932 | 0.82203829 | 0.021092569 |
| 0.168794071 | 0.000248094 | 0.146744296 | 0.001473242 | 0.15034208 |
| 0.048349472 | 0.297110119 | 0.081630914 | 0.078023118 | 0.093935754 |
| -0.150605122 | 0.001096495 | -0.165436971 | 0.000330283 | -0.187796962 |
| 0.018057596 | 0.697117934 | 0.009792844 | 0.832838068 | -0.004472285 |
| -0.026823714 | 0.563118346 | -0.008524613 | 0.854226776 | -0.017582831 |
| 0.212131992 | 3.75E-06 | 0.233449512 | 3.36E-07 | 0.250500903 |
| 0.037704272 | 0.41627444 | 0.041268745 | 0.373565675 | 0.058088671 |
| -0.013200919 | 0.776010258 | -0.035602837 | 0.4427455 | -0.038839189 |
| -0.067530469 | 0.145089184 | -0.093693594 | 0.042994533 | -0.091485478 |
| 0.085728811 | 0.064161019 | 0.047942545 | 0.3011992 | 0.051222849 |
| 0.197998723 | 1.63E-05 | 0.198987825 | 1.48E-05 | 0.178896827 |
| -0.028777374 | 0.535029908 | -0.00464701 | 0.920221948 | 0.000679745 |
| 0.133062696 | 0.003968906 | 0.101125387 | 0.028882572 | 0.120862792 |
| -0.001404301 | 0.975855009 | -0.027959002 | 0.546709786 | -0.047921063 |
| 0.058619766 | 0.206059053 | 0.068985102 | 0.13660348 | 0.069464909 |
| 0.089813138 | 0.052428541 | 0.080803443 | 0.081097948 | 0.056809888 |
| 0.082130706 | 0.076212215 | 0.124982342 | 0.006845589 | 0.157724899 |
| 0.058820184 | 0.204509851 | 0.080300441 | 0.083014505 | 0.078533221 |
| -0.186325225 | 5.09E-05 | -0.197760141 | 1.67E-05 | -0.241824722 |
| 0.026493348 | 0.567937287 | 0.031615261 | 0.49552293 | 0.031864467 |
| -0.084798765 | 0.067114769 | -0.096732058 | 0.036644943 | -0.109294976 |
| 0.208304358 | 5.64E-06 | 0.194622365 | 2.28E-05 | 0.207823289 |
| 0.174688537 | 0.000148152 | 0.207861948 | 5.91E-06 | 0.211624651 |
| 0.236398499 | 2.36E-07 | 0.231797362 | 4.09E-07 | 0.232876413 |
| 0.035156495 | 0.44848898 | 0.020452143 | 0.659331588 | 0.015799411 |
| -0.12521212 | 0.00674304 | -0.14547296 | 0.001621254 | -0.139956008 |
| 0.004232271 | 0.927321402 | 0.026990372 | 0.560694847 | 0.037422833 |
| -0.026747359 | 0.564230359 | -0.041013566 | 0.376530241 | -0.067051202 |

| | | | | |
|---|---|---|---|---|
| 0.357056526 | 1.73E-15 | 0.323466224 | 7.78E-13 | 0.328371173 |
| -0.098072567 | 0.034107684 | -0.065707032 | 0.156287728 | -0.068279656 |
| 0.354702915 | 2.72E-15 | 0.332657251 | 1.57E-13 | 0.388829366 |
| 0.180126771 | 9.07E-05 | 0.186915972 | 4.82E-05 | 0.192735791 |
| -0.126024828 | 0.006391281 | -0.101454767 | 0.028362208 | -0.102541084 |
| -0.034963674 | 0.450983157 | -0.023713808 | 0.60924026 | -0.010991764 |
| -0.119069923 | 0.010012571 | -0.077930913 | 0.092540346 | -0.064719122 |
| 0.01026009 | 0.824988065 | 0.022737267 | 0.624060979 | 0.031479868 |
| -0.058895527 | 0.20392965 | -0.083834028 | 0.07029566 | -0.069622913 |
| -0.101390184 | 0.028463599 | -0.118135659 | 0.010617433 | -0.102644998 |
| 0.095622975 | 0.038863674 | 0.096444191 | 0.037210229 | 0.067481238 |
| -0.172021157 | 0.000187472 | -0.163701233 | 0.000382136 | -0.185029113 |
| 0.194174542 | 2.39E-05 | 0.192972428 | 2.69E-05 | 0.194262501 |
| 0.039817528 | 0.390617235 | 0.02713199 | 0.558639429 | 0.007147539 |
| -0.079717086 | 0.085282859 | -0.097777827 | 0.034652334 | -0.099032537 |
| 0.009129184 | 0.84401636 | 0.011286091 | 0.807812817 | 0.010527884 |
| -0.010836521 | 0.815327773 | 0.005017895 | 0.913878536 | -0.008222328 |
| 0.220006487 | 1.58E-06 | 0.225276614 | 8.72E-07 | 0.221598229 |
| 0.142193498 | 0.002068214 | 0.096375541 | 0.037346124 | 0.098519073 |
| 0.195865292 | 2.02E-05 | 0.160525401 | 0.000497136 | 0.172175392 |
| 0.126331877 | 0.006262727 | 0.105486238 | 0.022617673 | 0.105478562 |
| 0.133131626 | 0.00394999 | 0.134152483 | 0.003679229 | 0.140095792 |
| 0.042668026 | 0.357566601 | 0.069747644 | 0.132310377 | 0.075876963 |
| 0.100475534 | 0.029933286 | 0.099089393 | 0.032284328 | 0.090693158 |
| -0.222653501 | 1.17E-06 | -0.209268457 | 5.10E-06 | -0.189891798 |
| -0.188061194 | 4.32E-05 | -0.201236011 | 1.18E-05 | -0.22800807 |
| 0.169698796 | 0.000229468 | 0.194279058 | 2.36E-05 | 0.211795339 |
| -0.087713126 | 0.058215888 | -0.070683963 | 0.127182282 | -0.125503613 |
| 0.12358521 | 0.007499745 | 0.112562397 | 0.014944798 | 0.105115642 |
| 0.078007559 | 0.092219085 | 0.050544924 | 0.275689079 | 0.032099166 |
| 0.007216713 | 0.876396749 | -0.060452605 | 0.192205049 | -0.059906379 |
| 0.008246722 | 0.858928279 | -0.006621738 | 0.88651524 | -0.038620046 |
| -0.099073919 | 0.032311441 | -0.132172235 | 0.004220756 | -0.130815394 |
| 0.033593522 | 0.46893005 | 0.003965281 | 0.931894788 | 0.019319569 |
| -0.271848587 | 2.35E-09 | -0.303016517 | 2.25E-11 | -0.318216658 |
| 0.235698659 | 2.57E-07 | 0.238818716 | 1.76E-07 | 0.259679102 |
| -0.032665538 | 0.481305501 | -0.024033006 | 0.604429921 | -0.003515923 |
| -0.224453947 | 9.58E-07 | -0.249593502 | 4.60E-08 | -0.257437478 |
| 0.146228347 | 0.001531746 | 0.205323513 | 7.72E-06 | 0.198127898 |
| -0.052964866 | 0.253323138 | -0.039727427 | 0.391691222 | -0.037495981 |
| -0.039941692 | 0.389140149 | -0.054057361 | 0.243650276 | -0.097126243 |
| -0.108819526 | 0.018657877 | -0.07991396 | 0.084511804 | -0.104820434 |
| 0.144881162 | 0.001694702 | 0.114345266 | 0.013416635 | 0.106385575 |
| 0.058904293 | 0.203862223 | 0.026350167 | 0.570031907 | 0.015463093 |
| -0.032654449 | 0.481454442 | -0.076537406 | 0.098538913 | -0.096486576 |

| | | | | |
|---|---|---|---|---|
| 0.039557392 | 0.393722867 | 0.02629261 | 0.570874959 | 0.018583922 |
| 0.090265845 | 0.051245906 | 0.084113585 | 0.069361477 | 0.082843356 |
| 0.046809783 | 0.312777855 | 0.028883852 | 0.533519506 | 0.019973824 |
| -0.193577586 | 2.53E-05 | -0.221408658 | 1.35E-06 | -0.221040114 |
| 0.088205328 | 0.056814033 | 0.065086179 | 0.160246185 | 0.063949126 |
| 0.082947596 | 0.073326053 | 0.0442956 | 0.339507139 | 0.046725536 |
| 0.032316532 | 0.486005236 | 0.009893684 | 0.831142462 | 0.010048252 |
| -0.072091572 | 0.119764761 | -0.055958349 | 0.227441679 | -0.072915577 |
| 0.102396054 | 0.026919296 | 0.09290367 | 0.044789458 | 0.077531509 |
| -0.049403573 | 0.286690552 | -0.07891948 | 0.088464877 | -0.105687692 |
| 0.070560532 | 0.127849339 | 0.043295255 | 0.35053671 | 0.0174752 |
| 0.096684597 | 0.036737638 | 0.106217792 | 0.021691198 | 0.085820007 |
| 0.061758049 | 0.18276227 | 0.025620903 | 0.580757054 | 0.022222982 |
| -0.029356167 | 0.526845717 | -0.010503098 | 0.820912236 | 0.007526894 |
| -0.138906776 | 0.00262668 | -0.162907137 | 0.000408303 | -0.215146476 |
| 0.013768983 | 0.766644497 | -0.011777638 | 0.799616368 | -0.050470282 |
| -0.021970127 | 0.635811394 | 0.017211445 | 0.710657519 | 0.009978224 |
| -0.076375534 | 0.099255337 | -0.087255118 | 0.059545964 | -0.093022019 |
| -0.026970339 | 0.560985896 | -0.052530382 | 0.25724314 | -0.069699752 |
| -0.304819175 | 1.69E-11 | -0.356605786 | 1.89E-15 | -0.394616223 |
| 0.068892268 | 0.137133369 | 0.036210223 | 0.434997501 | 0.037050227 |
| -0.163704473 | 0.000382032 | -0.147447781 | 0.001396779 | -0.129710345 |
| -0.053917515 | 0.244873823 | -0.030767275 | 0.50716302 | -0.008717029 |
| -0.033268101 | 0.473249728 | -0.02698334 | 0.560797003 | -0.047870893 |
| -0.000145872 | 0.997491553 | 0.024088268 | 0.60359886 | 0.044212023 |
| -0.107088082 | 0.02063206 | -0.10522982 | 0.022950431 | -0.124407479 |
| 0.135813162 | 0.003274521 | 0.124542814 | 0.007045648 | 0.086223071 |
| 0.268072272 | 3.97E-09 | 0.283611469 | 4.35E-10 | 0.309928482 |
| -0.018093352 | 0.696547861 | -0.055848659 | 0.228355585 | -0.058844364 |
| 0.123480866 | 0.007550772 | 0.174684971 | 0.000148199 | 0.18920614 |
| -0.040290697 | 0.385006483 | -0.031430523 | 0.49804661 | -0.03820608 |
| -0.141753073 | 0.002136151 | -0.115859836 | 0.012228357 | -0.079264395 |
| -0.083765821 | 0.070525138 | -0.107908264 | 0.019675336 | -0.138672813 |
| 0.051488933 | 0.266809485 | 0.048503861 | 0.295568435 | 0.080428116 |
| -0.166840165 | 0.000293244 | -0.208015269 | 5.82E-06 | -0.224671711 |
| -0.080031533 | 0.084054017 | -0.0608124 | 0.189567524 | -0.089324197 |
| 0.11712796 | 0.011305944 | 0.099783699 | 0.031087705 | 0.10562178 |
| -0.016877071 | 0.716033448 | -0.03787662 | 0.414145536 | -0.049215599 |
| -0.16591601 | 0.000317174 | -0.104694243 | 0.023659152 | -0.099745007 |
| 0.024075036 | 0.603797807 | 0.021076314 | 0.649618064 | 0.03582111 |
| 0.164716439 | 0.000350955 | 0.155103883 | 0.000770402 | 0.1507168 |
| 0.094978236 | 0.040205014 | 0.082633681 | 0.074424426 | 0.070738006 |
| 0.220618996 | 1.48E-06 | 0.185135024 | 5.70E-05 | 0.204346063 |
| -0.105172724 | 0.0230251 | -0.122693562 | 0.007945898 | -0.145538801 |
| 0.221042351 | 1.41E-06 | 0.233568276 | 3.31E-07 | 0.237964642 |

| | | | | |
|---|---|---|---|---|
| 0.19092609 | 3.28E-05 | 0.154866668 | 0.000785062 | 0.142996061 |
| 0.085264185 | 0.065622981 | 0.094373246 | 0.041499047 | 0.087385861 |
| 0.207485669 | 6.15E-06 | 0.187565756 | 4.53E-05 | 0.161208193 |
| -0.092100652 | 0.046678294 | -0.099546187 | 0.031492712 | -0.128017668 |
| 0.269487902 | 3.26E-09 | 0.286105718 | 3.01E-10 | 0.271341941 |
| 0.043886466 | 0.343991065 | -0.004308905 | 0.926009145 | 0.031212583 |
| -0.135879582 | 0.003259209 | -0.116061995 | 0.012076997 | -0.120360642 |
| -0.001117258 | 0.980789242 | -0.031854942 | 0.492258845 | -0.022202074 |
| 0.108558324 | 0.01894472 | 0.135696686 | 0.00330153 | 0.159467164 |
| -0.041657635 | 0.369075479 | -0.112973812 | 0.014579203 | -0.13713235 |
| -0.264959584 | 6.07E-09 | -0.305249394 | 1.58E-11 | -0.338074522 |
| 0.110292735 | 0.017109932 | 0.098076765 | 0.034099979 | 0.120585472 |
| 0.005023848 | 0.913776753 | 0.055146326 | 0.234269052 | 0.027879165 |
| -0.188432677 | 4.17E-05 | -0.118439577 | 0.010417207 | -0.098955172 |
| 0.10001787 | 0.030692766 | 0.116497942 | 0.011756216 | 0.113823291 |
| -0.059111461 | 0.202273407 | -0.045651971 | 0.324910601 | -0.019536782 |
| -0.01419166 | 0.759697631 | 0.017198435 | 0.710866427 | 0.026539535 |
| -0.032267992 | 0.48666083 | -0.049518495 | 0.285569633 | -0.051470227 |
| 0.069493713 | 0.133728281 | 0.014784874 | 0.749980573 | 0.002754154 |
| 0.021124836 | 0.648865407 | 0.007991065 | 0.86325803 | -0.029182036 |
| 0.061771526 | 0.1826666 | 0.040661304 | 0.380646371 | 0.049545761 |
| 0.123477022 | 0.007552658 | 0.12169022 | 0.008476145 | 0.128962825 |
| 0.277419538 | 1.07E-09 | 0.318905364 | 1.69E-12 | 0.326975844 |
| -0.173400488 | 0.000166058 | -0.140715112 | 0.002304402 | -0.158112722 |
| -0.142322744 | 0.002048655 | -0.154760248 | 0.000791721 | -0.162219333 |
| 0.057844715 | 0.212130044 | 0.077362919 | 0.094949098 | 0.128501061 |
| 0.074414092 | 0.10826956 | 0.105050337 | 0.023185863 | 0.083469623 |
| 0.057709996 | 0.213198301 | 0.058185891 | 0.209441922 | 0.035403877 |
| 0.017810567 | 0.701061034 | -0.007949795 | 0.863957354 | -0.01564319 |
| -0.138810757 | 0.002644889 | -0.106846034 | 0.020922021 | -0.12798356 |
| -0.055920437 | 0.227757259 | -0.066539844 | 0.151094544 | -0.078942387 |
| 0.002487413 | 0.957246307 | 0.040586922 | 0.381519034 | 0.031913649 |
| -0.041759211 | 0.367908208 | -0.061559402 | 0.184176618 | -0.08046098 |
| 0.047151293 | 0.309256705 | 0.008950966 | 0.847023597 | 0.016023697 |
| 0.042080591 | 0.364230129 | 0.054501491 | 0.239792894 | 0.040069516 |
| 0.014771757 | 0.75019501 | 0.025257082 | 0.586142669 | 0.009174237 |
| 0.023922995 | 0.606085872 | 0.039811509 | 0.390688925 | 0.042968488 |
| -0.174915248 | 0.000145194 | -0.151797537 | 0.000999491 | -0.157150084 |
| -0.090451555 | 0.050767244 | -0.094991531 | 0.040176964 | -0.106243851 |
| -0.047988746 | 0.300733064 | -0.03047791 | 0.511167588 | -0.034415634 |
| 0.033316815 | 0.472601705 | 0.006374184 | 0.890730812 | -0.002810108 |
| 0.099473372 | 0.031617776 | 0.093281157 | 0.043923995 | 0.120886134 |
| 0.136070339 | 0.003215595 | 0.125436654 | 0.006644162 | 0.098269528 |
| -0.054996307 | 0.235546067 | -0.069926476 | 0.131318795 | -0.064123812 |
| -0.00362378 | 0.937747759 | -0.027145272 | 0.558446843 | 0.011905841 |

| | | | | |
|---|---|---|---|---|
| 0.132195763 | 0.00421392 | 0.131857186 | 0.004313265 | 0.113642618 |
| 0.042573203 | 0.358637009 | 0.050762163 | 0.273628086 | 0.050564028 |
| 0.12336779 | 0.00760642 | 0.113768382 | 0.013895314 | 0.11140087 |
| 0.050854649 | 0.272753839 | 0.009993101 | 0.829471548 | 0.013886019 |
| 0.013091551 | 0.777817217 | 0.022766081 | 0.623621464 | 0.023815003 |
| 0.045427427 | 0.327298483 | 0.004314221 | 0.925918106 | -0.005708909 |
| 0.25960519 | 1.25E-08 | 0.238794631 | 1.77E-07 | 0.24515454 |
| 0.106445559 | 0.021409547 | 0.073227891 | 0.114026865 | 0.055422291 |
| -0.368977977 | 1.65E-16 | -0.401294797 | 1.70E-19 | -0.419736861 |
| 0.119968982 | 0.009459588 | 0.133333742 | 0.003894992 | 0.122436385 |
| 0.132752327 | 0.004055101 | 0.136568821 | 0.003104114 | 0.159400324 |
| 0.303840179 | 1.98E-11 | 0.319377446 | 1.56E-12 | 0.312742832 |
| 0.163233079 | 0.000397367 | 0.152193773 | 0.000969052 | 0.123854723 |
| -0.112367288 | 0.015120974 | -0.096066419 | 0.037963274 | -0.132998743 |
| 0.056697105 | 0.221354203 | -0.002634041 | 0.954728678 | 0.022448872 |
| 0.050472941 | 0.276374325 | 0.01626562 | 0.725900317 | 0.018961504 |
| -0.037565475 | 0.41799358 | -0.042939566 | 0.354512436 | -0.022459391 |
| 0.281547036 | 5.88E-10 | 0.249905942 | 4.42E-08 | 0.232881555 |
| 0.10487548 | 0.023417234 | 0.098531492 | 0.033274161 | 0.114942804 |
| -0.110768641 | 0.016634478 | -0.097168362 | 0.035802082 | -0.110246562 |
| -0.194845827 | 2.23E-05 | -0.147103197 | 0.001433765 | -0.182557113 |
| 0.061762342 | 0.18273179 | 0.091914567 | 0.047125408 | 0.125794384 |
| 0.02021185 | 0.663086544 | 0.039916557 | 0.389438886 | 0.038986721 |
| -0.299417073 | 3.97E-11 | -0.313841073 | 3.92E-12 | -0.315168553 |
| 0.113809493 | 0.013860711 | 0.096065989 | 0.037964139 | 0.075556182 |
| -0.305997336 | 1.40E-11 | -0.273612773 | 1.83E-09 | -0.281948406 |
| 0.225109439 | 8.89E-07 | 0.2570811 | 1.74E-08 | 0.247734394 |
| 0.030413677 | 0.512058741 | 0.059029733 | 0.202899115 | 0.104186277 |
| 0.129009234 | 0.005236422 | 0.146687022 | 0.001479634 | 0.156335516 |
| 0.257510185 | 1.65E-08 | 0.251537261 | 3.59E-08 | 0.253422033 |
| -0.038657515 | 0.404580432 | -0.051762608 | 0.264272301 | -0.077210809 |
| -0.121441488 | 0.008612379 | -0.128858395 | 0.005289946 | -0.147463918 |
| 0.029570101 | 0.523836882 | 0.016820452 | 0.716945139 | 0.013916454 |
| 0.032566189 | 0.482640818 | 0.04309471 | 0.352774834 | 0.0176911 |
| -0.136733921 | 0.003067973 | -0.135693718 | 0.003302221 | -0.118606068 |
| 0.141731722 | 0.002139496 | 0.17018194 | 0.000220066 | 0.158088684 |
| 0.14576328 | 0.0015863 | 0.114981083 | 0.012905939 | 0.098857396 |
| 0.184017038 | 6.33E-05 | 0.186082392 | 5.21E-05 | 0.200217365 |
| -0.055303981 | 0.232932313 | -0.069885291 | 0.131546647 | -0.101760942 |
| -0.187398105 | 4.60E-05 | -0.217762637 | 2.03E-06 | -0.2353392 |
| -0.0064138 | 0.890055988 | -0.012202601 | 0.792547855 | -0.025546438 |
| 0.013960191 | 0.763499561 | 0.006180494 | 0.894031308 | -0.005678393 |
| -0.116051239 | 0.012085008 | -0.107339764 | 0.020334267 | -0.145030792 |
| -0.218070733 | 1.96E-06 | -0.177629616 | 0.000113828 | -0.188789027 |
| 0.304335747 | 1.83E-11 | 0.286398364 | 2.88E-10 | 0.290349193 |

| | | | | |
|---|---|---|---|---|
| -0.042383693 | 0.360782309 | -0.043891992 | 0.343930248 | -0.074735932 |
| 0.010820288 | 0.815599439 | -0.025546217 | 0.581860737 | -0.005160483 |
| 0.05635894 | 0.224126142 | 0.05948502 | 0.19943126 | 0.071654344 |
| -0.08518395 | 0.065878192 | -0.030046433 | 0.517169274 | 0.003324485 |
| -0.094068094 | 0.042164974 | -0.046474987 | 0.316255221 | -0.038368293 |
| 0.147818135 | 0.001358006 | 0.157262479 | 0.000648181 | 0.151257642 |
| -0.065269437 | 0.159069983 | -0.069301636 | 0.134808569 | -0.032816612 |
| -0.27894788 | 8.56E-10 | -0.281944102 | 5.55E-10 | -0.297708659 |
| -0.068334365 | 0.140351178 | -0.070854373 | 0.126265773 | -0.082261868 |
| -0.152956318 | 0.000912869 | -0.099435403 | 0.031683158 | -0.090111261 |
| -0.210555985 | 4.44E-06 | -0.199873844 | 1.35E-05 | -0.185394055 |
| -0.044160055 | 0.340988493 | -0.017755626 | 0.701939104 | -0.03380753 |
| -0.216663532 | 2.29E-06 | -0.196772319 | 1.85E-05 | -0.189610739 |
| 0.014557262 | 0.753704358 | 0.025408962 | 0.5838916 | 0.031694069 |
| -0.12126146 | 0.008712197 | -0.118510722 | 0.010370822 | -0.124108789 |
| 0.052446068 | 0.25800867 | 0.053880896 | 0.245194918 | 0.073841772 |
| 0.106554622 | 0.02127581 | 0.124954812 | 0.006857969 | 0.137871756 |
| 0.208428726 | 5.57E-06 | 0.204488589 | 8.41E-06 | 0.21244428 |
| -0.138628593 | 0.002679751 | -0.179974757 | 9.20E-05 | -0.183409793 |
| -0.259123153 | 1.33E-08 | -0.231953685 | 4.01E-07 | -0.237669825 |
| -0.019820316 | 0.669222923 | 0.026972312 | 0.560957227 | 0.038312738 |
| 0.243268842 | 1.02E-07 | 0.238997478 | 1.72E-07 | 0.248114676 |
| -0.088627142 | 0.055635036 | -0.055366869 | 0.232400604 | -0.041685815 |
| 0.038597926 | 0.405305667 | 0.057899567 | 0.211696196 | 0.043783306 |
| -0.11873221 | 0.010227589 | -0.087756939 | 0.058089951 | -0.104113214 |
| 0.023741076 | 0.608828659 | 0.043116424 | 0.352532068 | 0.05053673 |
| -0.049157826 | 0.289097441 | 0.022381914 | 0.629492376 | 0.019132103 |
| -0.17864119 | 0.000103862 | -0.161211637 | 0.000469854 | -0.141203575 |
| 0.044262804 | 0.339865184 | 0.068335216 | 0.140346227 | 0.063110518 |
| -0.144388762 | 0.001758126 | -0.073266873 | 0.113833917 | -0.078361409 |
| -0.106644817 | 0.021165758 | -0.056371161 | 0.224025534 | -0.019872782 |
| -0.103519789 | 0.025279833 | -0.072885525 | 0.115732484 | -0.042467197 |
| -0.22878553 | 5.81E-07 | -0.165214605 | 0.000336538 | -0.178424027 |
| 0.025241781 | 0.586369672 | -0.000421632 | 0.992749623 | -0.029770357 |
| -0.299946536 | 3.65E-11 | -0.244985974 | 8.23E-08 | -0.227372386 |
| -0.010095477 | 0.827751709 | 0.03024588 | 0.514390528 | 0.022517925 |
| 0.090895525 | 0.04963804 | 0.051980844 | 0.262260999 | 0.060234112 |
| 0.278763674 | 8.79E-10 | 0.280264606 | 7.08E-10 | 0.266634563 |
| -0.283910242 | 4.16E-10 | -0.214486687 | 2.91E-06 | -0.20388756 |
| -0.188680965 | 4.07E-05 | -0.129037744 | 0.005226361 | -0.127034476 |
| -0.030240989 | 0.51445858 | 0.023262291 | 0.616073466 | -0.003195041 |
| -0.263291632 | 7.61E-09 | -0.203059787 | 9.75E-06 | -0.209230524 |
| -0.290529142 | 1.55E-10 | -0.246848174 | 6.52E-08 | -0.230667491 |
| -0.154595361 | 0.000802144 | -0.093779718 | 0.042802552 | -0.10671276 |
| -0.091724332 | 0.047586199 | -0.065064476 | 0.160385911 | -0.100017961 |

| | | | | |
|---|---|---|---|---|
| -0.042879914 | 0.355181968 | -0.028722588 | 0.535807873 | -0.05543555 |
| 0.069473903 | 0.133839389 | 0.101130218 | 0.028874881 | 0.111319169 |
| -0.052128453 | 0.260906598 | -0.016631256 | 0.719994548 | -0.032747341 |
| -0.284217525 | 3.98E-10 | -0.25908708 | 1.34E-08 | -0.263714649 |
| -0.015402019 | 0.739913321 | -0.001106078 | 0.980981425 | -0.040799044 |
| -0.180275132 | 8.95E-05 | -0.161304685 | 0.000466264 | -0.148562528 |
| -0.159300965 | 0.000549503 | -0.102124948 | 0.027328253 | -0.115323349 |
| -0.066608823 | 0.150670361 | -0.042255726 | 0.362235444 | -0.04293992 |
| -0.062006827 | 0.181002328 | -0.029783455 | 0.520844963 | -0.042948037 |
| 0.038178597 | 0.410430946 | 0.086929312 | 0.06050734 | 0.102598981 |
| -0.062065235 | 0.180590954 | -0.021917145 | 0.636626367 | -0.022268036 |
| -0.107820364 | 0.019775987 | -0.050811284 | 0.273163516 | -0.055611208 |
| -0.061510442 | 0.184526448 | -0.046065949 | 0.320537903 | -0.031418829 |
| -0.269043914 | 3.47E-09 | -0.236150646 | 2.43E-07 | -0.25170799 |
| -0.059654963 | 0.198147957 | -0.026077751 | 0.574027252 | -0.020475174 |
| -0.171280043 | 0.00020002 | -0.108508008 | 0.019000414 | -0.101520001 |
| -0.097586973 | 0.035008948 | -0.041750428 | 0.368009042 | -0.028380426 |
| 0.025943923 | 0.575994852 | 0.010520691 | 0.820617347 | 0.050679863 |
| -0.043590611 | 0.347256868 | 0.028290287 | 0.541966528 | 0.025012633 |
| -0.012624899 | 0.78554047 | 0.053917435 | 0.244874524 | 0.07193239 |
| -0.167003936 | 0.000289184 | -0.109751617 | 0.017664936 | -0.1002727 |
| -0.10483722 | 0.023468125 | -0.059815391 | 0.196942034 | -0.082612548 |
| -0.221297761 | 1.37E-06 | -0.171855971 | 0.000190203 | -0.16588357 |
| -0.040220795 | 0.38583226 | -0.010210961 | 0.825812661 | -0.000511373 |
| -0.191711493 | 3.04E-05 | -0.141487335 | 0.002178122 | -0.139766774 |
| -0.218299444 | 1.91E-06 | -0.179386804 | 9.70E-05 | -0.190495985 |
| -0.049019926 | 0.290453994 | -0.042050389 | 0.364574796 | -0.054971181 |
| -0.046204332 | 0.319084807 | -0.009316761 | 0.84085364 | 0.023142099 |
| 0.089966271 | 0.052025983 | 0.082503675 | 0.074883216 | 0.067161227 |
| -0.11603674 | 0.012095815 | -0.024416794 | 0.598668832 | -0.018419414 |
| -0.168637892 | 0.000251449 | -0.110536943 | 0.016864491 | -0.109189663 |
| -0.209822005 | 4.80E-06 | -0.142224113 | 0.002063565 | -0.174212554 |
| -0.103499435 | 0.025308745 | -0.065934971 | 0.154853132 | -0.082969362 |
| -0.168856478 | 0.000246765 | -0.12717254 | 0.005922551 | -0.12522104 |
| -0.191850421 | 3.00E-05 | -0.158315874 | 0.000595303 | -0.118870448 |
| -0.123643634 | 0.007471308 | -0.079778748 | 0.085040755 | -0.079621038 |
| -0.015740598 | 0.734408893 | 0.038077544 | 0.411671781 | 0.052089268 |
| -0.189029742 | 3.94E-05 | -0.128255184 | 0.005508946 | -0.130552998 |
| -0.200500744 | 1.27E-05 | -0.164010678 | 0.000372368 | -0.176250029 |
| 0.065317992 | 0.158759436 | 0.030859431 | 0.505891118 | 0.006089962 |
| 0.069464786 | 0.133890544 | 0.107194538 | 0.02050564 | 0.107599575 |
| 0.081723802 | 0.07768394 | 0.09080031 | 0.049878426 | 0.097387873 |
| -0.179803999 | 9.34E-05 | -0.152616927 | 0.000937492 | -0.161504163 |
| 0.345162575 | 1.64E-14 | 0.340107089 | 4.14E-14 | 0.317957857 |
| -0.092008352 | 0.046899621 | -0.019796789 | 0.669592367 | -0.030017941 |

| | | | | |
|---|---|---|---|---|
| -0.08118854 | 0.079654968 | 0.007664757 | 0.868790252 | 0.018930562 |
| 0.114432524 | 0.013345511 | 0.12950199 | 0.005064956 | 0.118218876 |
| -0.063206309 | 0.172692526 | -0.015370037 | 0.740433948 | -0.042206261 |
| -0.046972549 | 0.311096378 | -0.00334738 | 0.942487443 | -0.018794795 |
| -0.149164126 | 0.001225277 | -0.086375105 | 0.062172108 | -0.078632069 |
| -0.092934575 | 0.044718069 | -0.05463426 | 0.238648139 | -0.035938163 |
| -0.110263976 | 0.01713904 | -0.071815337 | 0.121193126 | -0.101634018 |
| -0.191897536 | 2.98E-05 | -0.165042189 | 0.000341464 | -0.201516065 |
| -0.053250611 | 0.250767803 | 0.004489891 | 0.922910774 | 0.031281896 |
| -0.129831938 | 0.004952987 | -0.079938706 | 0.084415283 | -0.07177359 |
| -0.159469817 | 0.000541989 | -0.119042032 | 0.010030175 | -0.127237174 |
| -0.075766131 | 0.101989699 | -0.033163637 | 0.474640996 | -0.049875309 |
| -0.089466692 | 0.053348873 | -0.057039794 | 0.218570255 | -0.062394048 |
| 0.015305257 | 0.741488873 | 0.051683771 | 0.265001474 | 0.024682217 |
| -0.044766078 | 0.334397325 | -0.01391017 | 0.764321916 | -0.003344404 |
| -0.129721605 | 0.004990178 | -0.102708075 | 0.026455146 | -0.072646717 |
| -0.104068041 | 0.024511728 | -0.064543532 | 0.163767471 | -0.051247946 |
| -0.23940306 | 1.64E-07 | -0.176266781 | 0.000128681 | -0.177099452 |
| -0.189946628 | 3.60E-05 | -0.159047972 | 0.000560943 | -0.172803051 |
| -0.121900318 | 0.008362572 | -0.107295533 | 0.020386329 | -0.088875698 |
| -0.103519875 | 0.025279711 | -0.063871673 | 0.168207374 | -0.070866837 |
| -0.249303698 | 4.77E-08 | -0.191507712 | 3.10E-05 | -0.198951631 |
| -0.08729873 | 0.059418239 | -0.018780604 | 0.685624293 | -0.03369365 |
| -0.301436787 | 2.89E-11 | -0.274562271 | 1.60E-09 | -0.284769319 |
| -0.18074811 | 8.57E-05 | -0.131826675 | 0.00432232 | -0.137091439 |
| -0.095202461 | 0.039734155 | -0.056344922 | 0.224241579 | -0.074509008 |
| -0.01151465 | 0.803998964 | 0.045856004 | 0.32275066 | 0.066630306 |
| -0.137076861 | 0.002994121 | -0.100787982 | 0.029424086 | -0.108678821 |
| -0.216000038 | 2.46E-06 | -0.158061759 | 0.00060768 | -0.17111489 |
| -0.153081221 | 0.000903958 | -0.089625591 | 0.052925101 | -0.097906865 |
| -0.158172321 | 0.000602266 | -0.119338124 | 0.00984468 | -0.105207938 |
| -0.121838558 | 0.008395816 | -0.06461139 | 0.163323977 | -0.085857688 |
| -0.324784885 | 6.21E-13 | -0.295699411 | 7.06E-11 | -0.322719529 |
| 0.012309606 | 0.790770667 | 0.089661842 | 0.052828817 | 0.092354395 |
| -0.228159331 | 6.25E-07 | -0.191076065 | 3.23E-05 | -0.230458128 |
| -0.16778385 | 0.000270557 | -0.151853915 | 0.000995107 | -0.172981861 |
| -0.163159034 | 0.000399827 | -0.1160092 | 0.012116365 | -0.137209629 |
| -0.150141787 | 0.001136474 | -0.113286818 | 0.014306344 | -0.117935622 |
| -0.138264802 | 0.002750627 | -0.098489055 | 0.033350501 | -0.075038056 |
| -0.150052084 | 0.001144368 | -0.112218497 | 0.015256549 | -0.104926085 |
| -0.111632923 | 0.015800529 | -0.048773394 | 0.292889791 | -0.066578755 |
| -0.108213448 | 0.019329333 | -0.084788685 | 0.067147385 | -0.103330882 |
| -0.218202767 | 1.93E-06 | -0.160770167 | 0.000487241 | -0.146592936 |
| -0.051757413 | 0.264320303 | -0.04513967 | 0.330375158 | -0.051614241 |
| -0.12116725 | 0.008764842 | -0.061430411 | 0.185099337 | -0.0546417 |

| | | | | |
|---|---|---|---|---|
| -0.200123187 | 1.32E-05 | -0.208942859 | 5.27E-06 | -0.23134635 |
| 0.091734441 | 0.047561619 | 0.107410417 | 0.020251345 | 0.097518477 |
| -0.223446222 | 1.07E-06 | -0.175529831 | 0.000137453 | -0.168274364 |
| -0.011440598 | 0.805234126 | 0.055798652 | 0.228773094 | 0.070446129 |
| -0.110406056 | 0.016995653 | -0.039731019 | 0.391648371 | -0.042117896 |
| -0.137151572 | 0.002978248 | -0.05605769 | 0.226616239 | -0.049690208 |
| -0.081633975 | 0.078011923 | -0.012002092 | 0.795880905 | -0.008468305 |
| -0.283187046 | 4.62E-10 | -0.239064172 | 1.71E-07 | -0.244634925 |
| -0.227190438 | 7.00E-07 | -0.143624825 | 0.001860841 | -0.141608934 |
| -0.012436081 | 0.788671521 | 0.02987477 | 0.51956712 | 0.015680055 |
| -0.187210186 | 4.68E-05 | -0.118788829 | 0.010191257 | -0.108903647 |
| -0.100570053 | 0.029778456 | -0.038974825 | 0.400731676 | -0.029681465 |
| -0.211980971 | 3.81E-06 | -0.178167983 | 0.000108417 | -0.176702116 |
| -0.098775942 | 0.032837314 | -0.06183719 | 0.182201034 | -0.022547585 |
| -0.135130746 | 0.003435664 | -0.074798784 | 0.106452491 | -0.061635684 |
| -0.037349621 | 0.420675407 | 0.022793978 | 0.623196062 | -0.014066797 |
| -0.089170994 | 0.054144995 | -0.108434268 | 0.019082295 | -0.072753819 |
| -0.262601394 | 8.36E-09 | -0.247393608 | 6.08E-08 | -0.243818549 |
| 0.211008289 | 4.23E-06 | 0.167445495 | 0.000278496 | 0.149476192 |
| -0.202602108 | 1.02E-05 | -0.18254958 | 7.26E-05 | -0.198622574 |
| -0.080658872 | 0.081645096 | -0.063676773 | 0.169512053 | -0.072633405 |
| -0.142002404 | 0.002097444 | -0.121478773 | 0.008591834 | -0.112551587 |
| -0.143293652 | 0.001907053 | -0.075985099 | 0.101000401 | -0.055542136 |
| -0.095993597 | 0.038109911 | -0.037283445 | 0.421499605 | -0.022283623 |
| -0.174521339 | 0.000150369 | -0.107051713 | 0.020675404 | -0.107174186 |
| -0.168819282 | 0.000247556 | -0.099410643 | 0.031725857 | -0.098327635 |
| -0.319031189 | 1.65E-12 | -0.262411242 | 8.58E-09 | -0.258612955 |
| -0.133398209 | 0.003877597 | -0.119789544 | 0.009567735 | -0.160921435 |
| -0.113420347 | 0.014191315 | -0.084190284 | 0.069106961 | -0.061857316 |
| -0.027895442 | 0.547622156 | 0.009458648 | 0.838462994 | 0.021137234 |
| -0.094751954 | 0.040684966 | -0.053428594 | 0.249185253 | -0.052956024 |
| -0.192858705 | 2.72E-05 | -0.147973384 | 0.001342049 | -0.15369091 |
| -0.082340002 | 0.075464089 | -0.049394567 | 0.286778525 | -0.055073254 |
| -0.153202828 | 0.00089536 | -0.101259852 | 0.028669158 | -0.118047492 |
| -0.027144108 | 0.558463716 | 0.039871401 | 0.389975931 | 0.074541354 |
| -0.082055751 | 0.076481599 | -0.028305624 | 0.541747423 | -0.020195986 |
| -0.114439961 | 0.013339465 | -0.088147479 | 0.056977328 | -0.0761812 |
| -0.123545695 | 0.007519032 | -0.074998574 | 0.105518345 | -0.069717983 |
| -0.370463954 | 1.22E-16 | -0.337911212 | 6.16E-14 | -0.363664415 |
| -0.106492232 | 0.021352226 | -0.063176503 | 0.172895505 | -0.073299124 |
| -0.210463525 | 4.49E-06 | -0.193837157 | 2.47E-05 | -0.175489603 |
| 0.108520223 | 0.01898688 | 0.125752339 | 0.006507342 | 0.135265439 |
| -0.268065923 | 3.97E-09 | -0.215754256 | 2.53E-06 | -0.217517598 |
| -0.138279838 | 0.002747664 | -0.10153683 | 0.028233824 | -0.096407318 |
| -0.06163523 | 0.183635781 | -0.019956322 | 0.667088832 | -0.000289505 |

| | | | | |
|---|---|---|---|---|
| -0.240944534 | 1.36E-07 | -0.184052398 | 6.31E-05 | -0.204286214 |
| -0.02255364 | 0.62686511 | 0.061270848 | 0.186245463 | 0.070438964 |
| -0.160229353 | 0.000509354 | -0.123735007 | 0.007427028 | -0.108739065 |
| -0.192544895 | 2.80E-05 | -0.151194137 | 0.001047542 | -0.17402701 |
| -0.304131254 | 1.89E-11 | -0.261721437 | 9.41E-09 | -0.290357496 |
| -0.152865623 | 0.00091939 | -0.15557572 | 0.000741995 | -0.152989916 |
| -0.086639855 | 0.061372179 | -0.034680788 | 0.454656467 | -0.034320261 |
| -0.02177227 | 0.638857052 | 0.010848107 | 0.815133891 | -0.00625926 |
| -0.070881351 | 0.126121148 | -0.031860455 | 0.492183898 | -0.039325904 |
| -0.201858279 | 1.10E-05 | -0.138830156 | 0.002641201 | -0.148118356 |
| -0.187386783 | 4.61E-05 | -0.128960458 | 0.005253676 | -0.110809882 |
| 0.02542238 | 0.583692906 | 0.084396163 | 0.068427554 | 0.104207761 |
| -0.081310494 | 0.079202362 | -0.049652889 | 0.28426253 | -0.082381044 |
| -0.282896353 | 4.83E-10 | -0.254866415 | 2.33E-08 | -0.250773738 |
| -0.105638286 | 0.02242234 | -0.044263686 | 0.339855551 | -0.045955717 |
| -0.056234493 | 0.225152428 | -0.02543482 | 0.583508736 | -0.030736213 |
| -0.022193778 | 0.632376081 | 0.020311645 | 0.661526059 | 0.037699897 |
| -0.091624737 | 0.047828943 | -0.037304838 | 0.421233052 | -0.05071554 |
| -0.1878764 | 4.40E-05 | -0.157081262 | 0.000657705 | -0.186730281 |
| -0.02922286 | 0.528725034 | 0.030486103 | 0.511053972 | 0.030064337 |
| 0.01509406 | 0.744931507 | 0.010722901 | 0.817229739 | 0.00695663 |
| -0.054366201 | 0.240963348 | 0.012734886 | 0.78371823 | 0.006514029 |
| -0.165629124 | 0.000324965 | -0.113259712 | 0.014329794 | -0.103751437 |
| -0.208415519 | 5.58E-06 | -0.166507442 | 0.000301658 | -0.171654147 |
| -0.169537296 | 0.000232694 | -0.11909095 | 0.009999317 | -0.125506702 |
| -0.250592169 | 4.05E-08 | -0.226947839 | 7.19E-07 | -0.225391816 |
| -0.035988737 | 0.437813771 | 0.039334104 | 0.396400442 | 0.052673489 |
| -0.222981735 | 1.13E-06 | -0.222093905 | 1.25E-06 | -0.205247048 |
| 0.030404512 | 0.512185953 | 0.059515519 | 0.199200505 | 0.026898407 |
| -0.19876072 | 1.51E-05 | -0.166489307 | 0.000302123 | -0.159124792 |
| -0.082852159 | 0.073658574 | -0.047136813 | 0.309405469 | -0.025533745 |
| -0.098721732 | 0.032933766 | -0.063610568 | 0.169956951 | -0.058700671 |
| -0.196129881 | 1.97E-05 | -0.183210475 | 6.82E-05 | -0.197747182 |
| -0.080307584 | 0.082987036 | -0.028201279 | 0.543238908 | 6.42E-06 |
| -0.142608265 | 0.002006047 | -0.107458177 | 0.020195458 | -0.101863486 |
| -0.009656049 | 0.83513948 | 0.030571435 | 0.509871478 | 0.040803814 |
| -0.093757386 | 0.042852263 | -0.064134371 | 0.166460761 | -0.068719185 |
| -0.161773163 | 0.000448573 | -0.109394385 | 0.018039897 | -0.08729221 |
| -0.148715807 | 0.001268098 | -0.114201491 | 0.013534556 | -0.122779874 |
| -0.143023628 | 0.001945508 | -0.166485124 | 0.000302231 | -0.141213683 |
| -0.008781458 | 0.849885912 | 0.057649473 | 0.213679481 | 0.086697311 |
| 0.006953851 | 0.88086476 | 0.072785195 | 0.116236078 | 0.099103016 |
| -0.089682401 | 0.052774277 | -0.053067463 | 0.252403571 | -0.013204598 |
| -0.138136138 | 0.0027761 | -0.086657829 | 0.061318182 | -0.092104478 |
| 0.008518941 | 0.854322678 | 0.056586387 | 0.222259047 | 0.06904798 |

| | | | | |
|---|---|---|---|---|
| -0.15628118 | 0.000701325 | -0.092055805 | 0.046785725 | -0.096686571 |
| -0.151967676 | 0.000986315 | -0.110751396 | 0.016651502 | -0.122868605 |
| -0.374987296 | 4.86E-17 | -0.332588983 | 1.59E-13 | -0.349852334 |
| -0.099964932 | 0.03078167 | -0.055701708 | 0.229584029 | -0.069081137 |
| -0.135515944 | 0.003343842 | -0.082226743 | 0.075868186 | -0.095710507 |
| 0.052726098 | 0.255472184 | 0.070218764 | 0.129710507 | 0.085062984 |
| -0.1150886 | 0.012821296 | -0.078080586 | 0.091913824 | -0.092679989 |
| -0.187145508 | 4.71E-05 | -0.144880108 | 0.001694836 | -0.153587683 |
| -0.065914374 | 0.154982355 | -0.021496369 | 0.643114184 | -0.014950143 |
| -0.113320283 | 0.014277439 | -0.049494526 | 0.285803178 | -0.060851991 |
| -0.120729545 | 0.009013167 | -0.074209159 | 0.109247488 | -0.106060049 |
| -0.121815822 | 0.008408083 | -0.027964116 | 0.546636405 | -0.019765272 |
| -0.139920558 | 0.002441278 | -0.100833835 | 0.029349987 | -0.083141537 |
| 0.239236631 | 1.67E-07 | 0.249284784 | 4.79E-08 | 0.269612498 |
| -0.169150198 | 0.0002406 | -0.152276236 | 0.000962826 | -0.155148084 |
| -0.042303793 | 0.361689196 | -0.025656915 | 0.580225224 | -0.021628393 |
| -0.137410361 | 0.002923857 | -0.092616683 | 0.045456988 | -0.088472594 |
| -0.1194365 | 0.009783727 | -0.071219933 | 0.124317007 | -0.075841953 |
| -0.221773643 | 1.30E-06 | -0.159535775 | 0.00053908 | -0.144689149 |
| -0.154052443 | 0.000837364 | -0.125540803 | 0.006598741 | -0.117497296 |
| -0.048333202 | 0.2972729 | -0.004635389 | 0.920420784 | 0.016567639 |
| -0.090035375 | 0.051845166 | -0.061821008 | 0.182315681 | -0.077925607 |
| -0.243069833 | 1.05E-07 | -0.180696354 | 8.61E-05 | -0.186378196 |
| -0.204011252 | 8.84E-06 | -0.148573566 | 0.001281969 | -0.151024858 |
| -0.070649983 | 0.127365649 | -0.036118603 | 0.436161214 | 0.000268335 |
| -0.16368894 | 0.000382528 | -0.123922923 | 0.007336699 | -0.116365183 |
| -0.064185927 | 0.166119575 | 0.00489309 | 0.916012534 | 0.009774657 |
| -0.191753937 | 3.03E-05 | -0.145751187 | 0.001587742 | -0.14662036 |
| -0.127926263 | 0.005631769 | -0.09741215 | 0.035338344 | -0.062117965 |
| -0.086194675 | 0.062722182 | -0.044167879 | 0.340902882 | -0.046597338 |
| -0.002875846 | 0.95057785 | 0.030667276 | 0.508545064 | 0.029408658 |
| -0.050129927 | 0.279655547 | -0.007624061 | 0.869480659 | 0.004816871 |
| -0.100904238 | 0.029236527 | -0.062198855 | 0.179652452 | -0.046294787 |
| 0.106173234 | 0.021746672 | 0.152129843 | 0.000973905 | 0.185676191 |
| -0.091485251 | 0.048170655 | -0.056884057 | 0.219832315 | -0.027489497 |
| -0.170839816 | 0.000207842 | -0.132198595 | 0.004213098 | -0.089357097 |
| 0.005299776 | 0.909061017 | 0.030870128 | 0.505743601 | 0.026995579 |
| -0.291065417 | 1.43E-10 | -0.251659166 | 3.53E-08 | -0.231439252 |
| -0.035555468 | 0.443353055 | 0.02526061 | 0.58609033 | 0.033705239 |
| -0.139251946 | 0.002562161 | -0.099472455 | 0.031619353 | -0.103440846 |
| -0.011474914 | 0.804661686 | 0.024536973 | 0.59686992 | 0.03608549 |
| -0.026116731 | 0.573454753 | 0.019573383 | 0.673104412 | 0.030493828 |
| -0.172440294 | 0.000180705 | -0.149876034 | 0.001160007 | -0.175972271 |
| -0.161667341 | 0.000452514 | -0.096838514 | 0.03643775 | -0.107606604 |
| -0.234291718 | 3.04E-07 | -0.190529751 | 3.41E-05 | -0.185876762 |

| | | | | |
|---|---|---|---|---|
| -0.145616631 | 0.001603869 | -0.12266851 | 0.007958769 | -0.113257102 |
| -0.172178431 | 0.000184905 | -0.135248985 | 0.003407238 | -0.137244456 |
| -0.028072721 | 0.545079273 | 0.013301785 | 0.774344848 | 0.027226321 |
| -0.124063155 | 0.007269932 | -0.070267983 | 0.129441191 | -0.062169196 |
| -0.162578477 | 0.000419614 | -0.109291404 | 0.018149271 | -0.119271203 |
| -0.131334678 | 0.004470737 | -0.074781481 | 0.106533701 | -0.066709011 |
| 0.089354165 | 0.053650678 | 0.117493214 | 0.011051948 | 0.152379774 |
| 0.009538368 | 0.83712046 | 0.048761503 | 0.293007614 | 0.058178457 |
| -0.26992384 | 3.07E-09 | -0.2116128 | 3.97E-06 | -0.204458641 |
| -0.194795083 | 2.25E-05 | -0.139075741 | 0.002594914 | -0.142179779 |
| 0.029316756 | 0.527400971 | 0.09446104 | 0.04130911 | 0.114060243 |
| -0.166623799 | 0.000298691 | -0.121053504 | 0.008828781 | -0.101528952 |
| -0.103671677 | 0.025064985 | -0.047868162 | 0.301950666 | -0.054519134 |
| -0.274190961 | 1.69E-09 | -0.236256978 | 2.40E-07 | -0.206932687 |
| -0.163612077 | 0.000384994 | -0.108514926 | 0.018992748 | -0.100273114 |
| 0.001678099 | 0.971149303 | 0.039392298 | 0.395701552 | 0.063925599 |
| -0.009715489 | 0.834139294 | 0.001719625 | 0.970435686 | 0.010809023 |
| -0.135251587 | 0.003406615 | -0.099971803 | 0.030770119 | -0.072621226 |
| -0.189682175 | 3.70E-05 | -0.124467657 | 0.007080377 | -0.127738629 |
| -0.090542964 | 0.050533017 | -0.044818377 | 0.333832378 | -0.043356322 |
| -0.063665801 | 0.169585723 | -0.011903683 | 0.797518095 | -0.022090181 |
| -0.032606663 | 0.48209658 | 0.003855475 | 0.933776363 | -0.008716839 |
| -0.010352116 | 0.823444005 | 0.042173153 | 0.363175052 | 0.042732814 |
| -0.045838386 | 0.322936799 | -0.003432681 | 0.94102449 | 0.024669674 |
| -0.306197824 | 1.36E-11 | -0.261294977 | 9.96E-09 | -0.291350953 |
| -0.021499263 | 0.643069472 | 0.026380859 | 0.569582595 | 0.016842825 |
| -0.104213366 | 0.024311538 | -0.035607118 | 0.442690616 | -0.042579296 |
| -0.158218859 | 0.0006 | -0.134700647 | 0.003540887 | -0.122115174 |
| -0.001902845 | 0.967287401 | 0.049594409 | 0.284830802 | 0.060169234 |
| -0.234834764 | 2.85E-07 | -0.183755968 | 6.49E-05 | -0.202235587 |
| -0.154360158 | 0.00081723 | -0.13216489 | 0.004222892 | -0.111162309 |
| -0.262190918 | 8.83E-09 | -0.247011709 | 6.38E-08 | -0.254163405 |
| -0.141422812 | 0.002188426 | -0.103083393 | 0.025905984 | -0.108398877 |
| -0.040304555 | 0.384842905 | 0.008259923 | 0.85870483 | 0.010816885 |
| 0.004293278 | 0.926276715 | 0.024069717 | 0.603877781 | -0.009131604 |
| 0.023400372 | 0.613980194 | 0.079257655 | 0.087104282 | 0.090225261 |
| -0.106017616 | 0.02194138 | -0.061842995 | 0.182159915 | -0.074084177 |
| -0.050007747 | 0.280830652 | -0.042139088 | 0.363563118 | -0.021205572 |
| -0.093375887 | 0.043709029 | -0.054125674 | 0.243054152 | -0.019695954 |
| -0.129029699 | 0.005229199 | -0.09493945 | 0.040286939 | -0.082625793 |
| -0.049449088 | 0.28624626 | -0.017225825 | 0.710426646 | -0.025611833 |
| -1.85E-05 | 0.999682577 | 0.028164612 | 0.543763495 | 0.066058934 |
| -0.218405282 | 1.89E-06 | -0.162013902 | 0.000439727 | -0.178586902 |
| -0.214888002 | 2.78E-06 | -0.143071972 | 0.001938571 | -0.113020877 |
| -0.117672512 | 0.010929123 | -0.059702837 | 0.197787527 | -0.037377944 |

| | | | | |
|---|---|---|---|---|
| -0.147773082 | 0.001362669 | -0.167871571 | 0.000268533 | -0.163561883 |
| 0.140684422 | 0.002309556 | 0.128676173 | 0.005355262 | 0.143406051 |
| -0.045811005 | 0.323226227 | -0.026507532 | 0.567729981 | -0.035742488 |
| 0.020440649 | 0.659511019 | 0.062086903 | 0.180438519 | 0.063134188 |
| 0.141946821 | 0.002106017 | 0.19359971 | 2.53E-05 | 0.238214956 |
| -0.195383965 | 2.12E-05 | -0.218041091 | 1.97E-06 | -0.223333693 |
| -0.284056807 | 4.07E-10 | -0.301233956 | 2.99E-11 | -0.319646162 |
| 0.001630998 | 0.971958751 | -0.022909091 | 0.621442003 | -0.025285298 |
| -0.201849523 | 1.10E-05 | -0.200073794 | 1.32E-05 | -0.182786101 |
| 0.1028865 | 0.026192839 | 0.046601984 | 0.314933203 | 0.040772321 |
| -0.202895752 | 9.92E-06 | -0.250386953 | 4.16E-08 | -0.265000357 |
| -0.120801905 | 0.008971688 | -0.11736318 | 0.011141788 | -0.100491067 |
| -0.140696098 | 0.002307594 | -0.138547755 | 0.002695355 | -0.158406289 |
| 0.025922534 | 0.576309606 | 0.011882681 | 0.797867629 | -0.000331638 |
| 0.120163242 | 0.009343734 | 0.090097677 | 0.051682596 | 0.097374411 |
| -0.079625786 | 0.085642358 | -0.049935929 | 0.281522941 | -0.062072775 |
| 0.029274683 | 0.527994046 | 0.016241511 | 0.726290291 | 0.015210143 |
| 0.268540448 | 3.72E-09 | 0.302951578 | 2.28E-11 | 0.259893921 |
| -0.105642873 | 0.022416469 | -0.096698607 | 0.036710255 | -0.102680126 |
| -0.204157213 | 8.71E-06 | -0.163946332 | 0.00037438 | -0.153430791 |
| 0.153206848 | 0.000895077 | 0.128595069 | 0.005384565 | 0.167320193 |
| -0.288817281 | 2.01E-10 | -0.265588588 | 5.57E-09 | -0.256747305 |
| -0.231343452 | 4.31E-07 | -0.232543941 | 3.74E-07 | -0.233013816 |
| 0.09474548 | 0.040698768 | 0.109817372 | 0.017596665 | 0.146704516 |
| -0.031592352 | 0.495835508 | -0.019344186 | 0.676714904 | -0.060239443 |
| 0.167338923 | 0.000281041 | 0.194188056 | 2.38E-05 | 0.163365808 |
| 0.009235032 | 0.842231345 | 0.028377023 | 0.540728052 | 0.032917291 |
| -0.038647188 | 0.404706071 | -0.051020955 | 0.271186598 | -0.045278129 |
| -0.070566929 | 0.127814699 | -0.07765404 | 0.093708329 | -0.071194376 |
| -0.228946834 | 5.71E-07 | -0.221059996 | 1.41E-06 | -0.228256814 |
| 0.211570015 | 3.99E-06 | 0.234687097 | 2.90E-07 | 0.232576685 |
| 0.008629922 | 0.852446449 | 0.032500112 | 0.483530063 | 0.013994685 |
| -0.001762085 | 0.969706054 | -0.025873379 | 0.577033284 | -0.017603536 |
| 0.020833027 | 0.653397228 | 0.033980905 | 0.46381636 | 0.046901703 |
| -0.153722612 | 0.000859453 | -0.142025188 | 0.002093939 | -0.154796581 |
| -0.053411981 | 0.249332673 | -0.03970497 | 0.391959189 | -0.052740788 |
| -0.18869138 | 4.07E-05 | -0.169469691 | 0.000234057 | -0.200990258 |
| -0.120003793 | 0.009438734 | -0.097123749 | 0.035887502 | -0.124236939 |
| -0.107585056 | 0.020047639 | -0.152205035 | 0.0009682 | -0.126760719 |
| -0.070374929 | 0.12885749 | -0.080422468 | 0.082546226 | -0.083731723 |
| -0.014920591 | 0.747762951 | -0.048123973 | 0.29937149 | -0.039610683 |
| -0.093060346 | 0.044428524 | -0.092628354 | 0.045429677 | -0.123652212 |
| -0.025820423 | 0.577813399 | -0.016650637 | 0.719681962 | -0.007152936 |
| 0.018386112 | 0.691886736 | 0.021659766 | 0.64059155 | 0.02547413 |
| -0.110618453 | 0.016783259 | -0.124147299 | 0.007230131 | -0.108783708 |

| | | | | |
|---|---|---|---|---|
| 0.118003287 | 0.010705703 | 0.146272886 | 0.001526613 | 0.092998407 |
| -0.107952503 | 0.019624847 | -0.107586433 | 0.02004604 | -0.085092949 |
| 0.101309201 | 0.028591176 | 0.112417657 | 0.015075318 | 0.112411785 |
| -0.092235624 | 0.046356224 | -0.106516211 | 0.021322828 | -0.105885101 |
| 0.016763054 | 0.717869785 | 0.00254388 | 0.956276707 | 0.028856581 |
| -0.118580201 | 0.010325701 | -0.10189553 | 0.02767849 | -0.124085033 |
| -0.019719181 | 0.670811588 | -0.0484583 | 0.296022841 | -0.057409792 |
| 0.07578541 | 0.101902285 | 0.059556236 | 0.198892749 | 0.071256282 |
| 0.03375057 | 0.466853188 | 0.015754491 | 0.734183311 | 0.035182262 |
| 0.005776148 | 0.900927214 | 0.005139637 | 0.911797493 | -0.008756373 |
| -0.023343936 | 0.614835365 | -0.013976997 | 0.763223328 | -0.033089865 |
| -0.073298973 | 0.113675229 | -0.059442887 | 0.199750358 | -0.084059029 |
| 0.07738971 | 0.09483437 | 0.082796357 | 0.073853568 | 0.059522987 |
| -0.09211153 | 0.046652269 | -0.08562816 | 0.064475425 | -0.079746342 |
| -0.041834544 | 0.367043986 | -0.059990131 | 0.195634634 | -0.051031155 |
| 0.007006337 | 0.879972311 | -0.007324043 | 0.874573541 | -0.032197312 |
| -0.052236951 | 0.259914152 | -0.050217329 | 0.278816976 | -0.059186582 |
| -0.000781576 | 0.98656048 | -0.025122767 | 0.588136749 | -0.014515179 |
| -0.158867129 | 0.000569255 | -0.156597824 | 0.000683751 | -0.15052291 |
| -0.195541822 | 2.09E-05 | -0.195727679 | 2.05E-05 | -0.22931273 |
| 0.022387138 | 0.629412379 | 0.029293847 | 0.527723862 | 0.04007145 |
| 0.016424987 | 0.723324188 | 0.002082784 | 0.964195977 | -0.016329278 |
| 0.005946538 | 0.898020371 | 0.017006092 | 0.71395742 | 0.007091527 |
| -0.062619186 | 0.176723771 | -0.044217869 | 0.340356152 | -0.057395229 |
| -0.008486506 | 0.854871195 | -0.016131661 | 0.728068116 | -0.027796382 |
| 0.191461039 | 3.11E-05 | 0.208640328 | 5.45E-06 | 0.193867257 |
| -0.029801321 | 0.520594828 | -0.02948075 | 0.525092478 | -0.016038124 |
| -0.029875756 | 0.519553339 | 0.002144884 | 0.963129196 | -0.035956583 |
| -0.085682102 | 0.064306769 | -0.090215858 | 0.051375386 | -0.083776443 |
| 0.062013125 | 0.180957936 | 0.037323388 | 0.421002012 | 0.048368925 |
| -0.03588862 | 0.439090208 | -0.043552533 | 0.347678622 | -0.038001698 |
| 0.100022587 | 0.030684856 | 0.140062109 | 0.002416363 | 0.115145021 |
| 0.098530444 | 0.033276044 | 0.104366844 | 0.024101652 | 0.112529249 |
| -0.041253024 | 0.373747887 | -0.055941278 | 0.227583736 | -0.051254423 |
| -0.136794643 | 0.003054777 | -0.147618562 | 0.001378775 | -0.145977918 |
| -0.083836168 | 0.07028847 | -0.077032434 | 0.096373499 | -0.080438859 |
| 0.010716031 | 0.817344792 | -0.00791154 | 0.864605682 | 0.001194504 |
| 0.036371813 | 0.432949422 | 0.049144611 | 0.289227258 | 0.057405317 |
| 0.091239162 | 0.048778509 | 0.0894539 | 0.053383112 | 0.09060771 |
| 0.034632518 | 0.455284931 | 0.053921738 | 0.244836805 | 0.033763292 |
| -0.058837167 | 0.204378966 | -0.048894828 | 0.291688286 | -0.04688217 |
| -0.048838624 | 0.292243967 | -0.040407215 | 0.383632406 | -0.045254898 |
| -0.193298259 | 2.60E-05 | -0.169222555 | 0.000239103 | -0.17073195 |
| -0.156123158 | 0.000710252 | -0.168449167 | 0.000255559 | -0.166726993 |
| -0.062136717 | 0.180088442 | -0.068792195 | 0.137706347 | -0.099756267 |

| | | | | |
|---|---|---|---|---|
| -0.306480023 | 1.30E-11 | -0.316456175 | 2.54E-12 | -0.303903379 |
| -0.048551951 | 0.295089322 | -0.010811884 | 0.815740107 | -0.020918277 |
| -0.103737699 | 0.024972088 | -0.128522288 | 0.005410985 | -0.097749654 |
| 0.077076824 | 0.096181193 | 0.061555077 | 0.184207503 | 0.029966488 |
| -0.015010702 | 0.746291685 | 0.002188946 | 0.962372317 | -0.016907431 |
| -0.006495259 | 0.888668649 | -0.005666409 | 0.902800061 | -0.014568057 |
| -0.030598853 | 0.509491842 | -0.034744343 | 0.453829741 | -0.03186544 |
| -0.05001571 | 0.280753959 | -0.045609583 | 0.3253605 | -0.100805887 |
| -0.070377485 | 0.128843568 | -0.076976835 | 0.096614798 | -0.043035686 |
| -0.043453775 | 0.348773968 | -0.063501923 | 0.170688937 | -0.052977399 |
| 0.195619829 | 2.07E-05 | 0.210059171 | 4.68E-06 | 0.216303814 |
| 0.161819291 | 0.000446865 | 0.165904164 | 0.000317492 | 0.182107739 |
| 0.107300072 | 0.020380981 | 0.091110564 | 0.049098699 | 0.091419787 |
| -0.033414152 | 0.471308333 | -0.002541865 | 0.956311309 | 4.22E-05 |
| -0.003741046 | 0.935737559 | 0.00978722 | 0.832932649 | 0.034256113 |
| 0.094807669 | 0.040566347 | 0.0901856 | 0.051453896 | 0.051858509 |
| -0.154639728 | 0.000799327 | -0.20426017 | 8.62E-06 | -0.263348009 |
| -0.259685456 | 1.24E-08 | -0.289760953 | 1.74E-10 | -0.294662034 |
| 0.075967226 | 0.101080863 | 0.115456485 | 0.012535368 | 0.092726198 |
| 0.04593113 | 0.321957716 | 0.048819539 | 0.292432825 | 0.032542551 |
| 0.09288008 | 0.044844016 | 0.135186111 | 0.003422327 | 0.104556736 |
| 0.100152039 | 0.030468426 | 0.135544542 | 0.003337114 | 0.123255488 |
| 0.070988368 | 0.125548721 | 0.050198969 | 0.278992986 | 0.061679756 |
| 0.272226281 | 2.23E-09 | 0.310591377 | 6.68E-12 | 0.332939347 |
| 0.170248199 | 0.000218805 | 0.209823056 | 4.80E-06 | 0.216208731 |
| -0.061447154 | 0.18497938 | -0.073128185 | 0.114521535 | -0.078177044 |
| -0.274346761 | 1.65E-09 | -0.30042489 | 3.39E-11 | -0.32808346 |
| -0.033546671 | 0.46955061 | -0.018664178 | 0.687470305 | -0.007298965 |
| -0.22194424 | 1.27E-06 | -0.203599677 | 9.23E-06 | -0.205385112 |
| -0.161944018 | 0.000442278 | -0.163223107 | 0.000397697 | -0.197577276 |
| -0.033324032 | 0.472505735 | -0.003629958 | 0.93764184 | -0.027720427 |
| -0.056697921 | 0.221347545 | -0.070182328 | 0.129910156 | -0.065954402 |
| 0.023551956 | 0.611685843 | -0.025695653 | 0.579653403 | -0.003237724 |
| 0.056829765 | 0.220273504 | 0.064276895 | 0.16551886 | 0.034109449 |
| 0.027126266 | 0.558722444 | 0.03594739 | 0.438340666 | -0.011052875 |
| 0.196533 | 1.89E-05 | 0.201906038 | 1.10E-05 | 0.207795655 |
| -0.236659656 | 2.29E-07 | -0.258095503 | 1.53E-08 | -0.282980567 |
| -0.18154651 | 7.96E-05 | -0.139062083 | 0.002597469 | -0.198821095 |
| -0.03204095 | 0.489733657 | -0.050902697 | 0.272300406 | -0.053979302 |
| 0.257311813 | 1.69E-08 | 0.266007844 | 5.26E-09 | 0.308391678 |
| 0.009416272 | 0.839176835 | 0.008652882 | 0.852058383 | -0.023598897 |
| -0.103702436 | 0.025021668 | -0.141195698 | 0.00222505 | -0.167225692 |
| -0.310059261 | 7.28E-12 | -0.308819476 | 8.90E-12 | -0.317591765 |
| 0.037390199 | 0.420170478 | 0.043120982 | 0.352481114 | 0.021340187 |
| -0.028149779 | 0.543975786 | -0.007575972 | 0.870296629 | -0.002427419 |

| | | | | |
|---|---|---|---|---|
| -0.065259687 | 0.159132396 | -0.044263064 | 0.339862352 | -0.043982538 |
| -0.115884181 | 0.012210042 | -0.100623222 | 0.029691663 | -0.116527211 |
| 0.32213831 | 9.76E-13 | 0.299112114 | 4.16E-11 | 0.277212269 |
| -0.245497629 | 7.72E-08 | -0.217753047 | 2.03E-06 | -0.226077437 |
| -0.081246622 | 0.07943915 | -0.055991708 | 0.227164252 | -0.032310185 |
| -0.196945732 | 1.81E-05 | -0.181628145 | 7.90E-05 | -0.167103561 |
| -0.222457375 | 1.20E-06 | -0.214710905 | 2.84E-06 | -0.202560407 |
| -0.353808542 | 3.23E-15 | -0.335460274 | 9.56E-14 | -0.336416934 |
| -0.090928245 | 0.049555656 | -0.105763408 | 0.022262696 | -0.080337375 |
| -0.166881582 | 0.000292213 | -0.148864996 | 0.001253698 | -0.143318408 |
| -0.154107601 | 0.000833721 | -0.121528842 | 0.008564313 | -0.069100139 |
| -0.199012231 | 1.47E-05 | -0.151452705 | 0.001026696 | -0.126989904 |
| -0.235965917 | 2.49E-07 | -0.241664212 | 1.24E-07 | -0.254370481 |
| 0.10209213 | 0.027378121 | 0.064920838 | 0.161312998 | 0.053632242 |
| -0.109634277 | 0.017787341 | -0.098787846 | 0.032816167 | -0.147232406 |
| 0.002246434 | 0.961384863 | -0.02355211 | 0.611683519 | -0.039665928 |
| 0.006383508 | 0.890571981 | -0.010742531 | 0.816901073 | -0.007383669 |
| -0.165969017 | 0.000315753 | -0.17614114 | 0.000130138 | -0.199268997 |
| 0.185233667 | 5.65E-05 | 0.153900965 | 0.000847443 | 0.137754009 |
| -0.117686101 | 0.010919864 | -0.126332771 | 0.006262356 | -0.121677005 |
| -0.101173301 | 0.02880637 | -0.071965758 | 0.120413684 | -0.085449098 |
| 0.006407172 | 0.890168893 | 0.023015426 | 0.619823615 | -0.008393792 |
| 0.353820476 | 3.22E-15 | 0.325865048 | 5.15E-13 | 0.373572898 |
| -0.116672995 | 0.011629542 | -0.11413016 | 0.013593397 | -0.098267741 |
| -0.072379555 | 0.118289676 | -0.099824333 | 0.031018863 | -0.15558368 |
| 0.163305939 | 0.00039496 | 0.171544209 | 0.00019546 | 0.17619012 |
| -0.299300119 | 4.04E-11 | -0.289530856 | 1.80E-10 | -0.320341321 |
| -0.226961785 | 7.18E-07 | -0.25359142 | 2.75E-08 | -0.278845759 |
| 0.003414149 | 0.941342297 | 0.003040142 | 0.947758309 | 0.002588639 |
| -0.166439376 | 0.000303407 | -0.168955578 | 0.000244668 | -0.156069251 |
| 0.331938708 | 1.79E-13 | 0.355126659 | 2.51E-15 | 0.389208484 |
| -0.183729802 | 6.50E-05 | -0.16587062 | 0.000318395 | -0.128434089 |
| -0.010344504 | 0.823571703 | -0.001949681 | 0.966482688 | -0.017276592 |
| 0.058755355 | 0.205010043 | 0.060324508 | 0.19315054 | 0.133515528 |
| -0.06511069 | 0.160088484 | -0.049440387 | 0.286331162 | -0.06474553 |
| 0.123861855 | 0.007365945 | 0.140014017 | 0.002424802 | 0.10854816 |
| 0.139056872 | 0.002598444 | 0.194895814 | 2.22E-05 | 0.236447118 |
| -0.158566537 | 0.000583325 | -0.137313786 | 0.002944048 | -0.144251285 |
| 0.188599688 | 4.10E-05 | 0.19063967 | 3.37E-05 | 0.148746859 |
| -0.076165975 | 0.100188964 | -0.06248259 | 0.1776716 | -0.117556325 |
| -0.025768195 | 0.578583279 | -0.06108699 | 0.187572571 | -0.07542041 |
| -0.099626143 | 0.031355869 | -0.069006643 | 0.136480753 | -0.032089791 |
| -0.198354732 | 1.57E-05 | -0.202791123 | 1.00E-05 | -0.23034799 |
| -0.160832699 | 0.000484742 | -0.162742048 | 0.000413949 | -0.171032056 |
| -0.103272515 | 0.025633006 | -0.093036516 | 0.044483262 | -0.105607899 |

| | | | | |
|---|---|---|---|---|
| -0.252971897 | 2.98E-08 | -0.267543036 | 4.27E-09 | -0.287454743 |
| 0.20688147 | 6.56E-06 | 0.214455282 | 2.92E-06 | 0.184138002 |
| -0.086670536 | 0.061280031 | -0.130894443 | 0.004607425 | -0.138546049 |
| 0.071455518 | 0.123073566 | 0.101818529 | 0.027796904 | 0.10296273 |
| 0.016390464 | 0.723881984 | 0.009715419 | 0.83414048 | -0.006974721 |
| -0.274182651 | 1.69E-09 | -0.244516624 | 8.73E-08 | -0.249473822 |
| -0.088423748 | 0.056200974 | -0.073552502 | 0.112427976 | -0.065660821 |
| -0.239064617 | 1.71E-07 | -0.242391713 | 1.14E-07 | -0.25552042 |
| -0.092359796 | 0.046061572 | -0.084371908 | 0.068507311 | -0.070193273 |
| 0.061522236 | 0.184442131 | 0.049183564 | 0.288844728 | 0.05738241 |
| -0.148308225 | 0.001308218 | -0.13903556 | 0.002602437 | -0.128820492 |
| -0.124020342 | 0.007290258 | -0.093717624 | 0.042940894 | -0.118130571 |
| -0.16254665 | 0.000420725 | -0.137365029 | 0.002933319 | -0.168682644 |
| -0.251623502 | 3.55E-08 | -0.265813164 | 5.41E-09 | -0.288065021 |
| 0.259944752 | 1.19E-08 | 0.288015867 | 2.26E-10 | 0.266510122 |
| -0.393236799 | 1.01E-18 | -0.389490335 | 2.29E-18 | -0.375868824 |
| 0.126082519 | 0.006366948 | 0.126405766 | 0.00623214 | 0.091945341 |
| 0.062652915 | 0.176490306 | 0.047777517 | 0.302868109 | 0.059760309 |
| 0.122466936 | 0.008063007 | 0.122427559 | 0.008083511 | 0.110198033 |
| 0.129018755 | 0.005233061 | 0.13769251 | 0.002865586 | 0.115561777 |
| -0.156189427 | 0.000706496 | -0.135917335 | 0.003250535 | -0.138836069 |
| 0.156848721 | 0.000670117 | 0.139457471 | 0.002524435 | 0.137217332 |
| 0.060348867 | 0.192970484 | 0.030339854 | 0.513083941 | 0.002962229 |
| -0.185153392 | 5.69E-05 | -0.193443185 | 2.57E-05 | -0.218604004 |
| -0.046104585 | 0.320131777 | -0.066327188 | 0.152407981 | -0.078072332 |
| -0.016169533 | 0.727455028 | -0.036141667 | 0.435868092 | -0.025590828 |
| -0.102281917 | 0.027090822 | -0.127141766 | 0.005934705 | -0.150751051 |
| 0.006758472 | 0.884188163 | 0.001300593 | 0.977637634 | 0.028619466 |
| 0.060192259 | 0.194130247 | 0.063166609 | 0.172962931 | 0.07015688 |
| 0.072369045 | 0.118343261 | 0.064650806 | 0.16306679 | 0.096964196 |
| -0.18631807 | 5.10E-05 | -0.175668082 | 0.000135765 | -0.191789399 |
| 0.032287268 | 0.486400435 | 0.023441262 | 0.613360914 | 0.013664319 |
| -0.036691348 | 0.428915889 | -0.047955871 | 0.301064702 | -0.007527772 |
| -0.054552074 | 0.239356308 | -0.059592164 | 0.198621474 | -0.052152109 |
| 0.268509756 | 3.73E-09 | 0.288621353 | 2.07E-10 | 0.308405724 |
| -0.001472083 | 0.974689971 | -0.029332369 | 0.527180963 | 0.012644103 |
| 0.059413246 | 0.199975069 | 0.113527721 | 0.014099408 | 0.116951507 |
| 0.067428167 | 0.145700788 | 0.049137836 | 0.289293826 | 0.053545912 |
| -0.104785678 | 0.023536834 | -0.075825471 | 0.101720842 | -0.088200501 |
| 0.184114434 | 6.27E-05 | 0.179011906 | 0.00010042 | 0.149143029 |
| 0.123165496 | 0.007706889 | 0.087871342 | 0.057762177 | 0.101091431 |
| 0.101252729 | 0.02868043 | 0.116962051 | 0.011423014 | 0.118235683 |
| -0.084903742 | 0.066775883 | -0.064128472 | 0.166499832 | -0.071773708 |
| -0.254077805 | 2.58E-08 | -0.25128169 | 3.71E-08 | -0.276578202 |
| -0.221579848 | 1.33E-06 | -0.246365069 | 6.93E-08 | -0.260424426 |

| | | | | |
|---|---|---|---|---|
| -0.446569754 | 2.85E-24 | -0.465137961 | 1.91E-26 | -0.493673746 |
| -0.088373295 | 0.056342095 | -0.090831638 | 0.049799226 | -0.100335371 |
| -0.032762055 | 0.480010159 | -0.066850082 | 0.149193858 | -0.0375912 |
| -0.161658803 | 0.000452833 | -0.128779147 | 0.005318263 | -0.141620995 |
| -0.155740036 | 0.000732331 | -0.177427686 | 0.000115922 | -0.200683707 |
| 0.116511787 | 0.011746153 | 0.092410212 | 0.045942389 | 0.031282372 |
| -0.11449583 | 0.013294118 | -0.12071905 | 0.009019198 | -0.115895783 |
| -0.090744803 | 0.050019013 | -0.07643533 | 0.098990208 | -0.051475309 |
| -0.052580547 | 0.256788411 | -0.058423853 | 0.207581634 | 0.017675838 |
| 0.157566829 | 0.000632473 | 0.143380503 | 0.001894834 | 0.128936447 |
| -0.05394851 | 0.244602269 | -0.032157098 | 0.488160393 | -0.059816994 |
| -0.356468542 | 1.94E-15 | -0.313836058 | 3.92E-12 | -0.33526492 |
| -0.239101696 | 1.70E-07 | -0.231992009 | 3.99E-07 | -0.243287269 |
| -0.183154979 | 6.86E-05 | -0.199921837 | 1.34E-05 | -0.185930225 |
| -0.073228217 | 0.114025246 | -0.063259021 | 0.172333978 | -0.083225549 |
| 0.123915959 | 0.007340029 | 0.17204016 | 0.00018716 | 0.186536218 |
| 0.229828204 | 5.15E-07 | 0.248357787 | 5.38E-08 | 0.198826013 |
| -0.135836685 | 0.003269091 | -0.121565486 | 0.008544222 | -0.150577072 |
| -0.067547629 | 0.144986786 | -0.093908207 | 0.042517475 | -0.089594697 |
| 0.016079067 | 0.728919821 | -0.004291553 | 0.926306255 | -0.00029333 |
| -0.158626958 | 0.000580471 | -0.143714113 | 0.001848559 | -0.157002717 |
| -0.067665666 | 0.144283928 | -0.077247428 | 0.095444944 | -0.083400047 |
| 0.378161794 | 2.52E-17 | 0.383119476 | 8.93E-18 | 0.413712808 |
| -0.041808542 | 0.367342141 | -0.034582641 | 0.455934837 | -0.013721797 |
| -0.130662461 | 0.00468096 | -0.11779493 | 0.01084596 | -0.151666337 |
| -0.308189183 | 9.86E-12 | -0.333426059 | 1.37E-13 | -0.346859555 |
| -0.05668033 | 0.221491129 | -0.073561065 | 0.112386037 | -0.064637871 |
| -0.052967735 | 0.253297391 | -0.066492073 | 0.151388841 | -0.049139503 |
| -0.062469038 | 0.17776584 | -0.074447964 | 0.108108591 | -0.060654894 |
| -0.014335491 | 0.757338096 | -0.036595448 | 0.430124151 | -0.049672258 |
| -0.207051769 | 6.44E-06 | -0.190652466 | 3.37E-05 | -0.202919809 |
| 0.002350459 | 0.959598207 | -0.016657622 | 0.719569317 | -0.022326294 |
| -0.068991756 | 0.136565564 | -0.037811549 | 0.414948561 | -0.020299116 |
| -0.135008503 | 0.003465278 | -0.144169015 | 0.001787127 | -0.092659707 |
| 0.259172061 | 1.32E-08 | 0.26043726 | 1.12E-08 | 0.289028882 |
| -0.100701542 | 0.02956421 | -0.083073553 | 0.072889065 | -0.082623056 |
| 0.148675895 | 0.001271976 | 0.122595877 | 0.00799619 | 0.120980811 |
| -0.113167002 | 0.014410257 | -0.135182549 | 0.003423183 | -0.139463344 |
| -0.008589937 | 0.853122325 | 0.017785956 | 0.701454319 | 0.044325801 |
| -0.032083626 | 0.489155274 | -0.042987205 | 0.353978309 | -0.032058868 |
| -0.045853497 | 0.322777151 | -0.048899621 | 0.291640933 | 0.005096079 |
| -0.077309842 | 0.095176723 | -0.054461853 | 0.240135405 | -0.08041865 |
| -0.175376118 | 0.000139352 | -0.175048842 | 0.000143477 | -0.175942705 |
| 0.019126637 | 0.680148777 | 0.027602117 | 0.551842392 | 0.013531118 |
| -0.116676208 | 0.011627229 | -0.062808714 | 0.175414882 | -0.085774063 |

| | | | | |
|---|---|---|---|---|
| -0.050665999 | 0.274539113 | -0.038468367 | 0.406885121 | -0.077782032 |
| -0.283882899 | 4.18E-10 | -0.29127987 | 1.39E-10 | -0.291187937 |
| -0.094915392 | 0.040337826 | -0.107451031 | 0.020203812 | -0.09968384 |
| -0.149192825 | 0.001222582 | -0.153669669 | 0.000863049 | -0.175758989 |
| -0.432242611 | 1.11E-22 | -0.42429887 | 7.84E-22 | -0.440755773 |
| 0.001024474 | 0.982384339 | 0.031754876 | 0.493620192 | -0.00413159 |
| 0.244309332 | 8.96E-08 | 0.258361365 | 1.47E-08 | 0.295219055 |
| -0.376299673 | 3.71E-17 | -0.35225781 | 4.34E-15 | -0.36192603 |
| -0.061253997 | 0.186366809 | -0.036575361 | 0.430377486 | -0.054844242 |
| -0.001936129 | 0.966715517 | 0.002205032 | 0.962096009 | -0.003502471 |
| 0.192370048 | 2.85E-05 | 0.19218631 | 2.90E-05 | 0.232998059 |
| 0.030049227 | 0.517130296 | 0.041399782 | 0.372048949 | 0.044463516 |
| 0.143622518 | 0.00186116 | 0.114229727 | 0.013511326 | 0.05990117 |
| -0.154675995 | 0.000797031 | -0.188191483 | 4.27E-05 | -0.192218765 |
| 0.301917602 | 2.68E-11 | 0.308523823 | 9.34E-12 | 0.316975739 |
| -0.034902258 | 0.451779218 | -0.046642194 | 0.314515371 | -0.082080442 |
| 0.370491039 | 1.22E-16 | 0.375528733 | 4.35E-17 | 0.360025555 |
| 0.122906529 | 0.007837235 | 0.159267029 | 0.000551025 | 0.118065778 |
| 0.067891112 | 0.142948734 | 0.068003543 | 0.142286409 | 0.051060862 |
| 0.056630974 | 0.221894344 | 0.072619173 | 0.117073183 | 0.004558647 |
| -0.094613231 | 0.040981589 | -0.087075622 | 0.060074039 | -0.139417953 |
| -0.136992181 | 0.003012205 | -0.151813192 | 0.000998272 | -0.172695937 |
| 0.092421261 | 0.045916304 | 0.143912315 | 0.001821559 | 0.15082116 |
| -0.146277157 | 0.001526122 | -0.16249496 | 0.000422534 | -0.211133245 |
| -0.186524663 | 5.00E-05 | -0.138399474 | 0.002724193 | -0.151963869 |
| 0.008482392 | 0.854940758 | 0.041665846 | 0.368981043 | 0.037872424 |
| -0.046177492 | 0.319366301 | -0.035399483 | 0.44535702 | -0.034457977 |
| 0.018449822 | 0.69087392 | -0.009424641 | 0.839035843 | 0.020884218 |
| 0.080226654 | 0.083298703 | 0.020226635 | 0.662855251 | 0.043743624 |
| -0.084063999 | 0.069526432 | -0.085745924 | 0.064107689 | -0.099941182 |
| 0.153276767 | 0.000890169 | 0.148878816 | 0.001252371 | 0.169178732 |
| 0.322735437 | 8.82E-13 | 0.310047744 | 7.29E-12 | 0.30296837 |
| 0.165173472 | 0.000337707 | 0.176180846 | 0.000129676 | 0.128768434 |
| -0.090133299 | 0.051589836 | -0.103022358 | 0.025994615 | -0.138165162 |
| 0.231415985 | 4.27E-07 | 0.288506418 | 2.10E-10 | 0.267344696 |
| -0.334906051 | 1.06E-13 | -0.367943228 | 2.03E-16 | -0.368325247 |
| 0.216822836 | 2.25E-06 | 0.222435228 | 1.20E-06 | 0.218642132 |
| 0.137551456 | 0.002894584 | 0.163269995 | 0.000396146 | 0.160281551 |
| -0.058574737 | 0.206408289 | -0.064910127 | 0.161382294 | -0.110688051 |
| 0.106991868 | 0.020746899 | 0.083610519 | 0.07104993 | 0.130729188 |
| -0.031316758 | 0.499604117 | -0.018608042 | 0.688361051 | 0.014383192 |
| 0.006248177 | 0.892877773 | 0.040535475 | 0.382123321 | 0.015450603 |
| -0.21199689 | 3.81E-06 | -0.221503157 | 1.34E-06 | -0.211499168 |
| -0.013235513 | 0.775438954 | -0.046660518 | 0.314325082 | -0.069911223 |
| -0.080617587 | 0.081801889 | -0.048900264 | 0.29163458 | -0.024747578 |

| | | | | |
|---|---|---|---|---|
| -0.287207023 | 2.55E-10 | -0.304293707 | 1.84E-11 | -0.310018206 |
| 0.019966531 | 0.666928745 | 0.032946762 | 0.477536532 | 0.032694988 |
| -0.124915885 | 0.006875508 | -0.1251763 | 0.006758935 | -0.135624861 |
| 0.020133846 | 0.664307296 | 0.050564697 | 0.275501054 | 0.075373964 |
| -0.107690177 | 0.019925883 | -0.141113443 | 0.002238453 | -0.140744951 |
| 0.216645287 | 2.30E-06 | 0.219541771 | 1.67E-06 | 0.244414793 |
| -0.143141368 | 0.001928654 | -0.129142652 | 0.005189488 | -0.156282894 |
| 0.062668415 | 0.176383098 | 0.032472106 | 0.483907218 | 0.014628715 |
| 0.089296521 | 0.053805834 | 0.062873878 | 0.174966525 | 0.041385416 |
| 0.165125929 | 0.000339063 | 0.141528223 | 0.002171616 | 0.1805479 |
| -0.004929324 | 0.915392926 | -0.050966775 | 0.271696506 | -0.015807184 |
| 0.098971112 | 0.032492057 | 0.04749391 | 0.305750485 | 0.072752964 |
| -0.044450285 | 0.337821644 | -0.035251762 | 0.447259561 | -0.063230976 |
| -0.153961676 | 0.00084339 | -0.139925964 | 0.002440323 | -0.171343969 |
| -0.185150005 | 5.69E-05 | -0.182660823 | 7.18E-05 | -0.195189681 |
| -0.182283953 | 7.44E-05 | -0.192760648 | 2.74E-05 | -0.176364941 |
| 0.050369126 | 0.277364628 | 0.080267119 | 0.083142749 | 0.091902413 |
| 0.053571931 | 0.247915823 | 0.013502529 | 0.771033425 | 0.018014731 |
| -0.021631685 | 0.641024791 | -0.022552495 | 0.62688261 | -0.032773846 |
| 0.067070656 | 0.147853601 | 0.098860121 | 0.032688018 | 0.108817245 |
| 0.165722351 | 0.000322414 | 0.186041459 | 5.23E-05 | 0.180550584 |
| -0.164288472 | 0.000363799 | -0.16961957 | 0.000231045 | -0.200573235 |
| -0.117857656 | 0.010803565 | -0.156413798 | 0.000693915 | -0.159842974 |
| -0.102988266 | 0.026044235 | -0.122161793 | 0.008223121 | -0.142681244 |
| 0.019860057 | 0.668599058 | -0.016069153 | 0.729080401 | 0.01063628 |
| 0.026938746 | 0.56144504 | 0.012647019 | 0.785173902 | -0.047961877 |
| 0.013023496 | 0.778942208 | 0.040011051 | 0.388316517 | -0.039290893 |
| 7.14E-05 | 0.998773034 | 0.014951121 | 0.747264382 | -0.001019888 |
| -0.17296881 | 0.0001725 | -0.1802247 | 8.99E-05 | -0.17891199 |
| -0.017483297 | 0.706297342 | 0.004818873 | 0.917281849 | 0.041438617 |
| -0.106899138 | 0.020858105 | -0.111167931 | 0.01624453 | -0.113865996 |
| 0.011712365 | 0.800703545 | 0.027480289 | 0.55359988 | 0.061964284 |
| -0.014826133 | 0.749306181 | -0.019478288 | 0.674601515 | -0.037332683 |
| -0.210132014 | 4.65E-06 | -0.200226123 | 1.30E-05 | -0.203386613 |
| -0.070425297 | 0.128583298 | -0.066120051 | 0.153695637 | -0.065811912 |
| 0.13862527 | 0.00268039 | 0.155093826 | 0.000771019 | 0.161668952 |
| 0.277208766 | 1.10E-09 | 0.280811685 | 6.54E-10 | 0.27549993 |
| 0.122784259 | 0.007899459 | 0.0969688 | 0.036185533 | 0.110312462 |
| 0.083075646 | 0.072881824 | 0.106169302 | 0.021751574 | 0.075821106 |
| -0.14116504 | 0.002230037 | -0.15208717 | 0.000977156 | -0.130683124 |
| 0.078479751 | 0.090259539 | 0.085089772 | 0.066178793 | 0.046330113 |
| -0.376412079 | 3.63E-17 | -0.334482171 | 1.14E-13 | -0.292256798 |
| -0.036975472 | 0.425347703 | -0.034075452 | 0.462573 | -0.005633496 |
| 0.15508451 | 0.00077159 | 0.101007362 | 0.029071014 | 0.053174617 |
| 0.051429392 | 0.267363678 | 0.069680973 | 0.132681526 | 0.034642976 |

| | | | | |
|---|---|---|---|---|
| 0.147023835 | 0.00144241 | 0.127973766 | 0.00561388 | 0.154150986 |
| -0.332045249 | 1.75E-13 | -0.292532496 | 1.15E-10 | -0.268263855 |
| 0.239575913 | 1.61E-07 | 0.2495772 | 4.61E-08 | 0.267200532 |
| 0.034765692 | 0.453552216 | 0.076258844 | 0.099774356 | 0.117061059 |
| -0.335228497 | 9.97E-14 | -0.330800256 | 2.18E-13 | -0.339173426 |
| 0.039326078 | 0.396496882 | 0.046612674 | 0.314822078 | 0.032235733 |
| 0.084903218 | 0.066777572 | 0.094472442 | 0.041284494 | 0.152921091 |
| -0.336707887 | 7.65E-14 | -0.305859077 | 1.43E-11 | -0.323609551 |
| -0.163092509 | 0.00040205 | -0.161616874 | 0.000454404 | -0.168552273 |
| 0.047613861 | 0.304529188 | 0.091050373 | 0.049249169 | 0.07301587 |
| -0.005434655 | 0.906757035 | 0.02912902 | 0.53004998 | 0.033082013 |
| 0.022784825 | 0.623335615 | 0.015638913 | 0.736060624 | 0.009321882 |
| -0.028632686 | 0.537085743 | -0.055386793 | 0.232232325 | -0.04872142 |
| -0.016072893 | 0.729019828 | -0.027506535 | 0.553221027 | -0.089211786 |
| 0.05226277 | 0.259678364 | 0.065270475 | 0.159063339 | 0.05432123 |
| 0.167201575 | 0.000284354 | 0.164328931 | 0.000362566 | 0.191856208 |
| -0.048502384 | 0.295583159 | -0.043881419 | 0.344046608 | -0.045565458 |
| -0.003166496 | 0.945590332 | -0.005403116 | 0.907295708 | 0.021416822 |
| -0.183484718 | 6.65E-05 | -0.17299642 | 0.000172081 | -0.200968594 |
| 0.113594509 | 0.014042506 | 0.12898676 | 0.005244366 | 0.182461065 |
| -0.015681148 | 0.73537442 | -0.001585201 | 0.972745817 | 0.00751736 |
| 0.028464881 | 0.539474995 | 0.001314481 | 0.97739891 | -0.013957305 |
| 0.157930267 | 0.000614177 | 0.170960741 | 0.000205665 | 0.094864562 |
| 0.040567371 | 0.381748607 | 0.018269569 | 0.693740877 | 0.014655237 |
| -0.230645269 | 4.68E-07 | -0.202773372 | 1.00E-05 | -0.197850005 |
| -0.093740143 | 0.042890679 | -0.107888214 | 0.019698254 | -0.143248034 |
| 0.015318758 | 0.741268971 | 0.040994137 | 0.376756549 | 0.022924935 |
| -0.211896418 | 3.85E-06 | -0.154831999 | 0.000787225 | -0.17753067 |
| -0.225134539 | 8.86E-07 | -0.274304966 | 1.66E-09 | -0.339312212 |
| -0.216206829 | 2.41E-06 | -0.219144535 | 1.74E-06 | -0.2191307 |
| -0.260415284 | 1.12E-08 | -0.216851149 | 2.24E-06 | -0.209461417 |
| -0.242235093 | 1.16E-07 | -0.223146507 | 1.11E-06 | -0.226374739 |
| -0.044720852 | 0.334886357 | -0.096007151 | 0.038082582 | -0.107390642 |
| 0.029702609 | 0.521977659 | 0.022213559 | 0.632072619 | 0.036919049 |
| -0.164333554 | 0.000362425 | -0.134140546 | 0.003682296 | -0.163230349 |
| 0.063871431 | 0.168208988 | 0.066123337 | 0.153675142 | 0.079869367 |
| 0.166864743 | 0.000292632 | 0.172485799 | 0.000179985 | 0.200080333 |
| -0.079514408 | 0.08608256 | -0.107662747 | 0.019957591 | -0.109977151 |
| 0.139008618 | 0.002607491 | 0.186950425 | 4.80E-05 | 0.180444143 |
| -0.072782147 | 0.116251404 | -0.070989886 | 0.125540618 | -0.066320342 |
| 0.062249879 | 0.179295033 | 0.088235149 | 0.056730006 | 0.071370681 |
| -0.166143065 | 0.000311131 | -0.163855463 | 0.000377238 | -0.163492955 |
| -0.183905084 | 6.40E-05 | -0.163106271 | 0.000401589 | -0.228143302 |
| 0.042202698 | 0.362838681 | 0.060012531 | 0.1954675 | 0.003485187 |
| -0.011928468 | 0.797105681 | -0.019690352 | 0.67126471 | -0.006430061 |

| | | | | |
|---|---|---|---|---|
| -0.005825741 | 0.90008102 | 0.028713529 | 0.53593657 | -0.01740853 |
| 0.02716904 | 0.558102297 | 0.008230644 | 0.859200455 | -0.024792511 |
| 0.136395309 | 0.003142514 | 0.096556249 | 0.036989307 | 0.095295338 |
| -0.092475055 | 0.045789479 | -0.042691335 | 0.357303784 | -0.061578953 |
| -0.045692907 | 0.324476491 | -0.073274278 | 0.113797293 | -0.117452081 |
| 0.014363025 | 0.756886654 | 0.048739623 | 0.293224518 | 0.032757632 |
| -0.163823875 | 0.000378236 | -0.169702945 | 0.000229386 | -0.171902059 |
| -0.055094374 | 0.234710731 | -0.0644184 | 0.164587651 | -0.021852375 |
| 0.145896896 | 0.001570447 | 0.102672989 | 0.026506993 | 0.139868404 |
| 0.021129616 | 0.648791281 | -0.000976779 | 0.983204322 | -0.024394664 |
| 0.067281366 | 0.146581861 | 0.066431555 | 0.151762296 | 0.049928735 |
| 0.164198331 | 0.000366559 | 0.181285977 | 8.15E-05 | 0.194482688 |
| 0.215790181 | 2.52E-06 | 0.214929739 | 2.77E-06 | 0.17632284 |
| 0.181437259 | 8.04E-05 | 0.148944726 | 0.001246064 | 0.13320933 |
| 0.267051392 | 4.56E-09 | 0.24105642 | 1.34E-07 | 0.244025013 |
| -0.146637995 | 0.001485125 | -0.17910295 | 9.96E-05 | -0.201893562 |
| 0.219597973 | 1.66E-06 | 0.210043942 | 4.69E-06 | 0.237794521 |
| 0.025840692 | 0.577514758 | 0.031001571 | 0.503932654 | 0.033670022 |
| -0.08200867 | 0.076651204 | -0.114687488 | 0.013139588 | -0.08661193 |
| -0.108173662 | 0.019374138 | -0.081704318 | 0.077754984 | -0.091561678 |
| 0.064477652 | 0.164198896 | 0.074474037 | 0.107984817 | 0.095246687 |
| 0.047914825 | 0.301479095 | 0.055681385 | 0.229754287 | 0.063534423 |
| 0.108931894 | 0.018535648 | 0.122266625 | 0.008167796 | 0.090796826 |
| -0.247127752 | 6.29E-08 | -0.228374612 | 6.10E-07 | -0.249858309 |
| -0.093115402 | 0.044302272 | -0.082172161 | 0.076063558 | -0.022928517 |
| 0.109649839 | 0.017771065 | 0.099154758 | 0.032170021 | 0.12469076 |
| -0.064036544 | 0.167109593 | -0.075269209 | 0.104263313 | -0.053322274 |
| 0.14060298 | 0.002323284 | 0.147525036 | 0.001388608 | 0.13094092 |
| -0.04082272 | 0.378756844 | -0.028017666 | 0.545868349 | -0.035443156 |
| -0.043052841 | 0.353243226 | -0.034088585 | 0.462400441 | -0.074844497 |
| -0.250266328 | 4.22E-08 | -0.256270773 | 1.94E-08 | -0.285454922 |
| -0.1092847 | 0.01815641 | -0.08213965 | 0.076180122 | -0.067829819 |
| -0.041267561 | 0.373579393 | -0.051745527 | 0.264430165 | -0.099439212 |
| -0.083753093 | 0.070568031 | -0.05145411 | 0.267133513 | -0.089435507 |
| 0.024953289 | 0.59065731 | 0.00396365 | 0.931922735 | -0.059242159 |
| 0.01892516 | 0.683334858 | 0.02775484 | 0.549643096 | 0.032174837 |
| -0.186173772 | 5.17E-05 | -0.176149461 | 0.000130041 | -0.17454227 |
| -0.199740604 | 1.37E-05 | -0.176539282 | 0.000125572 | -0.200711701 |
| 0.01997333 | 0.666822135 | 0.012509634 | 0.787451427 | 0.015327203 |
| -0.163452665 | 0.000390154 | -0.169733698 | 0.000228777 | -0.14141676 |
| 0.168831471 | 0.000247297 | 0.19892996 | 1.49E-05 | 0.187312991 |
| -0.164658492 | 0.000352669 | -0.183664164 | 6.54E-05 | -0.196605413 |
| -0.021003362 | 0.650750344 | 0.005711852 | 0.902024445 | -0.029991696 |
| 0.151806101 | 0.000998824 | 0.141985199 | 0.002100094 | 0.133082319 |
| 0.158152964 | 0.00060321 | 0.147902331 | 0.00134933 | 0.165515856 |

| | | | | |
|---|---|---|---|---|
| 0.141146485 | 0.00223306 | 0.14208814 | 0.002084283 | 0.126188642 |
| -0.084033442 | 0.069628245 | -0.092854705 | 0.044902763 | -0.078249415 |
| -0.143100554 | 0.001934481 | -0.197452731 | 1.72E-05 | -0.162428612 |
| -0.158922767 | 0.000566685 | -0.194794634 | 2.25E-05 | -0.216248577 |
| 0.180457912 | 8.80E-05 | 0.202537906 | 1.03E-05 | 0.1403522 |
| 0.023744936 | 0.60877041 | -0.009561096 | 0.836737777 | 0.032820649 |
| -0.087971383 | 0.057476806 | -0.133449053 | 0.003863927 | -0.126542976 |
| -0.132191839 | 0.004215059 | -0.193148301 | 2.64E-05 | -0.233649809 |
| 0.000591078 | 0.989835961 | 0.046855667 | 0.312303244 | 0.100099188 |
| -0.043389364 | 0.349489536 | -0.065307794 | 0.158824623 | -0.087848162 |
| 0.381575585 | 1.24E-17 | 0.377624802 | 2.82E-17 | 0.372198434 |
| -0.299131293 | 4.15E-11 | -0.333341169 | 1.39E-13 | -0.36485048 |
| -0.043977141 | 0.342994077 | -0.023909722 | 0.606285806 | -0.007060642 |
| -0.104658325 | 0.023707352 | -0.045456845 | 0.326985004 | -0.02302023 |
| -0.21883893 | 1.80E-06 | -0.24412359 | 9.17E-08 | -0.207462937 |
| -0.008647027 | 0.852157345 | -0.051696363 | 0.264884913 | -0.07814253 |
| -0.03028131 | 0.513897718 | -0.019862456 | 0.668561412 | -0.024029859 |
| 0.197705588 | 1.68E-05 | 0.2416396 | 1.25E-07 | 0.211595273 |
| 0.034571128 | 0.456084917 | 0.064957817 | 0.161073939 | 0.005823427 |
| -0.182766183 | 7.11E-05 | -0.16294611 | 0.000406981 | -0.158403689 |
| 0.093266954 | 0.043956302 | 0.082210711 | 0.075925528 | 0.118351696 |
| 0.000433088 | 0.992552623 | -0.017409411 | 0.707481432 | -0.021089235 |
| -0.067039848 | 0.148040241 | -0.078400153 | 0.090587501 | -0.081256206 |
| -0.27235654 | 2.19E-09 | -0.249011822 | 4.96E-08 | -0.234593156 |
| 0.197184608 | 1.77E-05 | 0.223721021 | 1.04E-06 | 0.226059124 |
| -0.31476018 | 3.37E-12 | -0.296891587 | 5.88E-11 | -0.242308788 |
| -0.497348565 | 1.50E-30 | -0.510623762 | 2.25E-32 | -0.5237471 |
| -0.524051988 | 2.67E-34 | -0.501636261 | 3.94E-31 | -0.499586529 |
| 0.210666201 | 4.39E-06 | 0.25761502 | 1.63E-08 | 0.291647309 |
| -0.020613619 | 0.656813072 | -0.005084784 | 0.912735061 | 0.01058703 |
| 0.239161996 | 1.69E-07 | 0.263283332 | 7.62E-09 | 0.267634804 |
| -0.077372686 | 0.094907259 | -0.128790144 | 0.005314325 | -0.156958885 |
| 0.03360119 | 0.468828522 | 0.058740306 | 0.205126281 | 0.061356552 |
| -0.021079843 | 0.649563315 | -0.072898725 | 0.115666351 | -0.084487249 |
| -0.188601759 | 4.10E-05 | -0.163099786 | 0.000401806 | -0.192333615 |
| -0.208458442 | 5.55E-06 | -0.186012665 | 5.25E-05 | -0.203244479 |
| 0.093636655 | 0.043121855 | 0.074172197 | 0.109424611 | 0.037722952 |
| -0.268586427 | 3.69E-09 | -0.259367958 | 1.29E-08 | -0.312789313 |
| -0.12748066 | 0.005802088 | -0.159047262 | 0.000560975 | -0.187841093 |
| -0.030866516 | 0.505793402 | -0.031968719 | 0.490713412 | 0.017597049 |
| 0.067343051 | 0.146211145 | 0.049065384 | 0.290006346 | 0.022282239 |
| -0.097336966 | 0.035480812 | -0.096224627 | 0.037646346 | -0.066524198 |
| 0.187672784 | 4.48E-05 | 0.193275661 | 2.61E-05 | 0.20040739 |
| -0.018236121 | 0.694273353 | -0.038235768 | 0.409729915 | -0.041243301 |
| 0.057315836 | 0.216346041 | 0.045151071 | 0.330252911 | 0.039347878 |

| | | | | |
|---|---|---|---|---|
| -0.199186852 | 1.45E-05 | -0.204136282 | 8.73E-06 | -0.189647061 |
| -0.006778242 | 0.883851779 | -0.016502543 | 0.722071641 | -0.042188666 |
| -0.203366044 | 9.45E-06 | -0.165994758 | 0.000315066 | -0.1443588 |
| -0.119615753 | 0.009673524 | -0.09986204 | 0.030955099 | -0.067725932 |
| -0.234414329 | 3.00E-07 | -0.215722659 | 2.54E-06 | -0.194524934 |
| 0.111421147 | 0.016001408 | 0.091684155 | 0.047683998 | 0.151204703 |
| -0.332323155 | 1.67E-13 | -0.358061131 | 1.43E-15 | -0.328845153 |
| -0.081489255 | 0.078542699 | -0.095737631 | 0.038629151 | -0.10184352 |
| 0.0863563 | 0.062229253 | 0.084475009 | 0.068168813 | 0.090523405 |
| -0.187839161 | 4.41E-05 | -0.166730491 | 0.000295993 | -0.172684269 |
| -0.005362781 | 0.907984687 | 0.00843039 | 0.855820325 | 0.003983792 |
| 0.006082009 | 0.895710202 | 0.028652555 | 0.536803187 | 0.037640397 |
| 0.079698629 | 0.085355436 | 0.072533882 | 0.117505064 | 0.041857022 |
| -0.119369335 | 0.009825305 | -0.118646816 | 0.010282603 | -0.126216452 |
| 0.05200649 | 0.26202534 | 0.052926618 | 0.253666544 | 0.035654112 |
| 0.230261844 | 4.89E-07 | 0.267577291 | 4.25E-09 | 0.275432681 |
| -0.042319275 | 0.361513357 | -0.039099399 | 0.399226697 | -0.056116736 |
| -0.022568717 | 0.626634674 | -0.017151771 | 0.711615898 | -0.004618068 |
| 0.12962748 | 0.005022104 | 0.148333872 | 0.00130566 | 0.141894261 |
| -0.181326405 | 8.12E-05 | -0.179768675 | 9.37E-05 | -0.191991605 |
| -0.039690551 | 0.392131296 | -0.093787751 | 0.042784682 | -0.084410714 |
| -0.335793656 | 9.01E-14 | -0.295131099 | 7.71E-11 | -0.302357442 |
| -0.048768563 | 0.292937659 | -0.029155009 | 0.529682869 | -0.005300601 |
| -0.000759267 | 0.986944062 | -0.00945356 | 0.838548705 | 0.024003388 |
| -0.218317509 | 1.91E-06 | -0.175326613 | 0.000139968 | -0.159419872 |
| -0.060394095 | 0.192636493 | -0.067984137 | 0.142400563 | -0.064589904 |
| 0.066622355 | 0.150587252 | 0.078871472 | 0.088659405 | 0.073891704 |
| -0.20497458 | 8.00E-06 | -0.216995588 | 2.21E-06 | -0.20169445 |
| -0.068372829 | 0.140127491 | -0.065901879 | 0.15506079 | -0.066321977 |
| 0.122849717 | 0.007866092 | 0.055195727 | 0.233849611 | 0.096659144 |
| 0.10289256 | 0.026183969 | 0.102196209 | 0.027220243 | 0.07918241 |
| -0.147905132 | 0.001349043 | -0.212076582 | 3.77E-06 | -0.231727093 |
| 0.008615778 | 0.852685517 | -0.027106007 | 0.559016262 | -0.097492421 |
| -0.159807524 | 0.000527246 | -0.174551173 | 0.000149971 | -0.166966154 |
| -0.231928157 | 4.02E-07 | -0.217006081 | 2.21E-06 | -0.188292448 |
| 0.083536958 | 0.071299615 | 0.085668143 | 0.064350379 | 0.091751135 |
| 0.007191481 | 0.876825466 | 0.00737345 | 0.873734475 | 0.005889507 |
| -0.063057393 | 0.173708449 | -0.04760525 | 0.304616753 | -0.072920837 |
| 0.004859865 | 0.91658075 | -0.015286646 | 0.741792036 | 0.009880575 |
| -0.20407056 | 8.79E-06 | -0.189353124 | 3.82E-05 | -0.131121103 |
| 0.008526396 | 0.854196624 | -0.018550164 | 0.689279883 | -0.032232773 |
| 0.156789222 | 0.000673327 | 0.153426874 | 0.000879716 | 0.116725742 |
| -0.20382604 | 9.01E-06 | -0.252011017 | 3.38E-08 | -0.264926705 |
| 0.078063326 | 0.091985898 | 0.085276649 | 0.065583408 | 0.147875347 |
| 0.069534682 | 0.133498729 | 0.038379504 | 0.407970572 | 0.068884825 |

| | | | | |
|---|---|---|---|---|
| -0.244263753 | 9.01E-08 | -0.23169826 | 4.13E-07 | -0.224476662 |
| 0.010397107 | 0.822689381 | 0.03280275 | 0.47946457 | 0.049911857 |
| 0.116246383 | 0.011940388 | 0.109375772 | 0.018059622 | 0.127462678 |
| 0.040493916 | 0.382611906 | 0.030643043 | 0.50888027 | 0.019195841 |
| -0.099070303 | 0.032317779 | -0.049841703 | 0.282432981 | -0.03770245 |
| -0.029227323 | 0.528662057 | -0.035735906 | 0.441041338 | -0.036449727 |
| 0.068551994 | 0.139089141 | 0.061725022 | 0.182996862 | 0.068240422 |
| 0.144188775 | 0.001784501 | 0.17409857 | 0.000156116 | 0.137360416 |
| -0.030225551 | 0.514673393 | -0.026768653 | 0.563920142 | -0.034175091 |
| -0.217650497 | 2.06E-06 | -0.21026533 | 4.58E-06 | -0.219828312 |
| -0.112620776 | 0.014892435 | -0.098689845 | 0.032990614 | -0.081711816 |
| -0.09699961 | 0.036126105 | -0.072059324 | 0.119930829 | -0.081075896 |
| -0.136925642 | 0.003026485 | -0.165219339 | 0.000336404 | -0.172563489 |
| 0.03521948 | 0.447675948 | 0.080995988 | 0.08037385 | 0.067836106 |
| 0.041458182 | 0.37137421 | 0.073920762 | 0.110635485 | 0.0593684 |
| -0.162331432 | 0.000428307 | -0.129963447 | 0.004908985 | -0.102442096 |
| 0.114214486 | 0.01352386 | 0.077638241 | 0.09377533 | 0.080746319 |
| -0.013491565 | 0.771214183 | 0.045793315 | 0.323413312 | 0.070446181 |
| -0.081312957 | 0.079193244 | -0.129771077 | 0.004973471 | -0.138106144 |
| -0.17165709 | 0.000193541 | -0.15199728 | 0.000984038 | -0.132607817 |
| -0.185710535 | 5.40E-05 | -0.133507677 | 0.00384822 | -0.164949959 |
| 0.049703269 | 0.283773585 | 0.086636131 | 0.061383372 | 0.06733253 |
| -0.098563412 | 0.033216838 | -0.092398643 | 0.045969714 | -0.099366527 |
| 0.025151911 | 0.587703801 | 0.026123335 | 0.573357784 | 0.023878782 |
| -0.063522132 | 0.170552601 | -0.089420752 | 0.053471915 | -0.06334115 |
| -0.265649662 | 5.53E-09 | -0.264382376 | 6.57E-09 | -0.295163159 |
| -0.140481023 | 0.002343982 | -0.159107975 | 0.00055821 | -0.213835674 |
| 0.134875388 | 0.00349779 | 0.137906398 | 0.002822119 | 0.204684476 |
| -0.043322362 | 0.350234889 | -0.060394806 | 0.192631241 | -0.056855666 |
| -0.144485219 | 0.001745533 | -0.16757283 | 0.000275483 | -0.193719011 |
| -0.07582811 | 0.101708897 | -0.135538339 | 0.003338572 | -0.136316521 |
| 0.017502481 | 0.705990013 | -0.0032148 | 0.944761641 | -0.021374105 |
| -0.208906498 | 5.30E-06 | -0.221171072 | 1.39E-06 | -0.220931117 |
| 0.221082722 | 1.40E-06 | 0.239420109 | 1.64E-07 | 0.194405819 |
| 0.077578168 | 0.094030443 | 0.045099186 | 0.330809509 | 0.071200091 |
| -0.216010417 | 2.46E-06 | -0.226788362 | 7.33E-07 | -0.213628039 |
| -0.132193483 | 0.004214582 | -0.103673689 | 0.02506215 | -0.114261441 |
| 0.084643747 | 0.067617776 | 0.088044518 | 0.057268927 | 0.068933869 |
| 0.037587272 | 0.417723325 | 0.000802165 | 0.986206483 | 0.002847723 |
| 0.032757482 | 0.480071488 | 0.02927637 | 0.527970258 | 0.046632173 |
| -0.086630473 | 0.06140038 | -0.089994071 | 0.051953177 | -0.123776667 |
| -0.080088385 | 0.083833375 | -0.072067108 | 0.119890725 | -0.078945769 |
| -0.15846934 | 0.000587943 | -0.138210029 | 0.002761445 | -0.159802781 |
| -0.194306899 | 2.36E-05 | -0.199932243 | 1.34E-05 | -0.208558449 |
| -0.064245078 | 0.16572878 | -0.089233537 | 0.05397579 | -0.093079934 |

| | | | | |
|---|---|---|---|---|
| -0.230849428 | 4.57E-07 | -0.187014551 | 4.77E-05 | -0.181144666 |
| 0.06151194 | 0.184515735 | 0.074745802 | 0.106701307 | 0.110673542 |
| -0.161652522 | 0.000453068 | -0.152067264 | 0.000978676 | -0.14806818 |
| -0.055873884 | 0.228145184 | -0.070858226 | 0.126245108 | -0.030829294 |
| -0.047548626 | 0.305192988 | -0.012059856 | 0.794920319 | -0.010689177 |
| -0.132567395 | 0.004107261 | -0.1514645 | 0.001025755 | -0.165337549 |
| 0.078252945 | 0.091196549 | 0.080534073 | 0.082119811 | 0.11871344 |
| -0.098331768 | 0.03363475 | -0.094823316 | 0.040533086 | -0.100479101 |
| 0.014040562 | 0.762178797 | -0.00964518 | 0.83532239 | -0.014742198 |
| -0.003841021 | 0.93402407 | -0.038334325 | 0.408523082 | -0.055227878 |
| -0.042317834 | 0.361529714 | -0.020323259 | 0.661344542 | 0.005999917 |
| 0.097860479 | 0.034498859 | 0.140280412 | 0.002378393 | 0.145981202 |
| 0.097904717 | 0.034416952 | 0.151050772 | 0.001059268 | 0.192974147 |
| -0.074878507 | 0.106078953 | -0.052563432 | 0.256943488 | -0.081160063 |
| 0.124032023 | 0.007284707 | 0.134011316 | 0.003715641 | 0.132642909 |
| -0.115133379 | 0.012786187 | -0.116828916 | 0.011517731 | -0.119278738 |
| 0.043526701 | 0.347964912 | 0.057047928 | 0.218504481 | 0.016336597 |
| -0.432526995 | 1.03E-22 | -0.456366971 | 2.11E-25 | -0.460182776 |
| -0.054942429 | 0.236005897 | -0.076233925 | 0.09988547 | -0.094292986 |
| 0.058223413 | 0.209147787 | 0.064847214 | 0.161789756 | 0.073567182 |
| -0.029265103 | 0.528129129 | -0.041160246 | 0.374824405 | -0.059764472 |
| -0.049308827 | 0.287616911 | -0.029679291 | 0.522304591 | -0.036605515 |
| 0.021712043 | 0.639785349 | -0.035631108 | 0.44238313 | -0.027554789 |
| 0.090165612 | 0.051505813 | 0.135546671 | 0.003336614 | 0.103090222 |
| -0.286299811 | 2.92E-10 | -0.311808229 | 5.47E-12 | -0.326859035 |
| 0.101709095 | 0.027965943 | 0.112385967 | 0.015104029 | 0.115004182 |
| -0.118259479 | 0.01053545 | -0.121054148 | 0.008828418 | -0.115595383 |
| -0.305372064 | 1.55E-11 | -0.327344471 | 3.99E-13 | -0.336369933 |
| -0.146797006 | 0.001467383 | -0.132697956 | 0.004070374 | -0.183338381 |
| -0.097241874 | 0.035661705 | -0.073223535 | 0.114048437 | -0.126731832 |
| 0.183856595 | 6.42E-05 | 0.194488247 | 2.31E-05 | 0.179857979 |
| 0.09793576 | 0.034359575 | 0.145879075 | 0.001572553 | 0.096633148 |
| -0.169970266 | 0.00022414 | -0.109641648 | 0.01777963 | -0.108957083 |
| 0.006157081 | 0.894430388 | 0.036720672 | 0.428546822 | 0.026718478 |
| -0.133660501 | 0.003807544 | -0.151086403 | 0.001056342 | -0.131453904 |
| 0.107562866 | 0.020073423 | 0.092792847 | 0.045046249 | 0.09832345 |
| 0.12563363 | 0.006558492 | 0.152986555 | 0.000910704 | 0.131519754 |
| -0.266745044 | 4.76E-09 | -0.238615301 | 1.81E-07 | -0.257820592 |
| -0.051793549 | 0.263986496 | -0.081771073 | 0.077511788 | -0.130471878 |
| -0.175746733 | 0.000134814 | -0.165494911 | 0.000328671 | -0.154476489 |
| -0.113936414 | 0.013754359 | -0.082150854 | 0.076139932 | -0.066694142 |
| 0.219932919 | 1.60E-06 | 0.186374428 | 5.07E-05 | 0.186948897 |
| 0.025173941 | 0.587376641 | 0.021642986 | 0.640850428 | 0.011749117 |
| 0.074982734 | 0.105592171 | 0.105783178 | 0.022237561 | 0.104910223 |
| 0.049063274 | 0.290027105 | 0.08310736 | 0.072772142 | 0.117931539 |

| | | | | |
|---|---|---|---|---|
| -0.015045185 | 0.745728925 | -0.022769681 | 0.623566562 | -0.02406126 |
| -0.275108768 | 1.48E-09 | -0.228623484 | 5.93E-07 | -0.219525235 |
| -0.043120099 | 0.352490989 | -0.077105169 | 0.096058557 | -0.1331544 |
| -0.077926069 | 0.092560679 | -0.029666477 | 0.522484294 | -0.039509258 |
| 0.323261308 | 8.06E-13 | 0.289836688 | 1.72E-10 | 0.276763004 |
| -0.063997003 | 0.167372379 | -0.102939631 | 0.026115163 | -0.054408386 |
| -0.15838296 | 0.000592075 | -0.132110554 | 0.004238725 | -0.156383981 |
| 0.04525351 | 0.329155768 | 0.052864467 | 0.254225252 | 0.060715116 |
| 0.179591666 | 9.53E-05 | 0.221286039 | 1.37E-06 | 0.237130435 |
| -0.175457038 | 0.000138349 | -0.178875445 | 0.000101674 | -0.195869168 |
| 0.151183472 | 0.00104841 | 0.135850528 | 0.003265899 | 0.151406639 |
| 0.207617578 | 6.07E-06 | 0.205723756 | 7.40E-06 | 0.184584302 |
| -0.056054862 | 0.22663971 | -0.05592578 | 0.22771276 | -0.044895507 |
| 0.150926717 | 0.001069512 | 0.156542321 | 0.000686802 | 0.161653826 |
| -0.148140639 | 0.001325051 | -0.133522775 | 0.003844184 | -0.162579456 |
| 0.154784555 | 0.000790196 | 0.101612454 | 0.028115954 | 0.09987729 |
| 0.165868253 | 0.000318458 | 0.191947486 | 2.97E-05 | 0.205491171 |
| -0.046286393 | 0.31822516 | -0.036028894 | 0.437302384 | -0.033543639 |
| 0.001988178 | 0.965821275 | 0.027194906 | 0.557727461 | 0.01848674 |
| 0.003964843 | 0.931902289 | -0.04513529 | 0.330422142 | -0.029363775 |
| -0.33610274 | 8.53E-14 | -0.33256402 | 1.60E-13 | -0.35209296 |
| -0.21950674 | 1.67E-06 | -0.25924515 | 1.31E-08 | -0.264776672 |
| 0.035581411 | 0.443020257 | 0.081634133 | 0.078011344 | 0.03739225 |
| -0.166486107 | 0.000302206 | -0.209614307 | 4.91E-06 | -0.255709532 |
| -0.081504271 | 0.07848749 | -0.052286814 | 0.259458916 | -0.06077074 |
| -0.309653597 | 7.78E-12 | -0.3418826 | 3.00E-14 | -0.324825989 |
| -0.075422372 | 0.103558294 | -0.075395972 | 0.103679543 | -0.055422091 |
| 0.023139734 | 0.617933995 | 0.050590053 | 0.275260066 | 0.086404807 |
| -0.130351708 | 0.004781124 | -0.143452537 | 0.001884753 | -0.165027979 |
| -0.059911324 | 0.196223483 | -0.055289408 | 0.233055656 | -0.063448062 |
| -0.205857014 | 7.30E-06 | -0.222990814 | 1.13E-06 | -0.226510415 |
| 0.0366458 | 0.429489506 | 0.071697625 | 0.121805812 | 0.062615081 |
| -0.078226935 | 0.091304502 | -0.072733387 | 0.116496798 | -0.073079725 |
| -0.18626891 | 5.12E-05 | -0.199198982 | 1.45E-05 | -0.221010342 |
| 0.109783632 | 0.017631667 | 0.126171306 | 0.006329662 | 0.206301887 |
| -0.116281343 | 0.011914641 | -0.095818211 | 0.038465048 | -0.09286996 |
| -0.097356302 | 0.035444125 | -0.103937043 | 0.0246934 | -0.059036211 |
| -0.082191387 | 0.075994693 | -0.077963537 | 0.092403492 | -0.088339971 |
| -0.057690067 | 0.213356664 | -0.073734506 | 0.111539244 | -0.046857099 |
| -0.08150004 | 0.07850304 | -0.058211893 | 0.209238059 | -0.044273095 |
| -0.148640022 | 0.001275471 | -0.129001756 | 0.005239064 | -0.127930959 |
| -0.204605489 | 8.31E-06 | -0.19997186 | 1.34E-05 | -0.199058278 |
| -0.100329761 | 0.030173425 | -0.14677949 | 0.001469328 | -0.151180083 |
| 0.146623373 | 0.001486767 | 0.139558016 | 0.002506163 | 0.15939319 |
| -0.046700304 | 0.313912183 | -0.055583979 | 0.230571559 | -0.071661037 |

| | | | | |
|---|---|---|---|---|
| -0.133334157 | 0.00389488 | -0.139828642 | 0.002457582 | -0.17105168 |
| -0.159006331 | 0.000562846 | -0.156943266 | 0.000665044 | -0.105548362 |
| -0.197455448 | 1.72E-05 | -0.218161021 | 1.94E-06 | -0.187059097 |
| 0.005877778 | 0.899193245 | -0.029053562 | 0.531116626 | -0.018930842 |
| -0.060808282 | 0.189597564 | -0.077123143 | 0.095980853 | -0.056855561 |
| -0.053782045 | 0.24606317 | -0.018992794 | 0.682264693 | -0.080420704 |
| -0.088318544 | 0.056495571 | -0.10602727 | 0.021929258 | -0.112125635 |
| 0.161517294 | 0.000458157 | 0.143963481 | 0.001814647 | 0.137487087 |
| 0.276714085 | 1.18E-09 | 0.317324281 | 2.20E-12 | 0.29567137 |
| 0.053916818 | 0.24487993 | 0.045698055 | 0.32442193 | 0.059208062 |
| -0.056292134 | 0.224676658 | -0.073695255 | 0.111730441 | -0.074077283 |
| 0.169363144 | 0.00023622 | 0.168713303 | 0.000249823 | 0.16627202 |
| -0.0027051 | 0.953508746 | 0.019911405 | 0.667793339 | 0.013835405 |
| -0.242691696 | 1.10E-07 | -0.251033283 | 3.83E-08 | -0.284203143 |
| 0.252294017 | 3.25E-08 | 0.249424516 | 4.70E-08 | 0.249184079 |
| 0.082545856 | 0.074734108 | 0.120441869 | 0.009179768 | 0.13929176 |
| -0.036755451 | 0.428109327 | 0.004563127 | 0.92165735 | -0.00434667 |
| 0.233285764 | 3.43E-07 | 0.240414046 | 1.45E-07 | 0.219693592 |
| 0.030142299 | 0.515832674 | 0.001833345 | 0.968481567 | -0.001694116 |
| -0.070268983 | 0.129435724 | -0.009130754 | 0.843989873 | -0.046310048 |
| -0.218585397 | 1.85E-06 | -0.242340557 | 1.14E-07 | -0.255847853 |
| -0.042122138 | 0.363756314 | -0.08886762 | 0.05497203 | -0.08862284 |
| -0.225142837 | 8.85E-07 | -0.254623853 | 2.41E-08 | -0.241031248 |
| 0.01632484 | 0.724942666 | 0.010987153 | 0.812807911 | 0.044749796 |
| 0.090339774 | 0.051054908 | 0.106699026 | 0.021099853 | 0.109781723 |
| -0.116251803 | 0.011936393 | -0.087821823 | 0.057903863 | -0.104270341 |
| -0.058975163 | 0.203317694 | -0.020732755 | 0.654957424 | -0.032100102 |
| -0.172704131 | 0.000176564 | -0.144126136 | 0.001792836 | -0.141799098 |
| 0.175221887 | 0.000141282 | 0.193133611 | 2.64E-05 | 0.177494695 |
| 0.036891418 | 0.426401497 | 0.008964495 | 0.84679523 | -0.016442475 |
| 0.088687675 | 0.055467522 | 0.125854134 | 0.006463765 | 0.084868539 |
| -0.079349015 | 0.086739611 | -0.081214617 | 0.079558014 | -0.092092602 |
| -0.141702034 | 0.002144154 | -0.136408978 | 0.003139473 | -0.117481105 |
| -0.262022651 | 9.04E-09 | -0.251182977 | 3.75E-08 | -0.27004044 |
| -0.120269369 | 0.009280976 | -0.14839004 | 0.001300072 | -0.189973633 |
| 0.086089365 | 0.063045088 | 0.0536166 | 0.247521144 | 0.001451583 |
| 0.241542663 | 1.26E-07 | 0.194999555 | 2.20E-05 | 0.204627416 |
| -0.191003283 | 3.25E-05 | -0.201305197 | 1.17E-05 | -0.227996528 |
| 0.051892953 | 0.263069744 | 0.051626299 | 0.265533908 | 0.053134662 |
| 0.11784201 | 0.010814126 | 0.137520316 | 0.002901021 | 0.13589614 |
| -0.205600425 | 7.50E-06 | -0.184759371 | 5.90E-05 | -0.186560729 |
| 0.020647446 | 0.656285978 | 0.02193377 | 0.636370595 | 0.001837046 |
| -0.134470862 | 0.003598294 | -0.143160456 | 0.001925934 | -0.170730978 |
| -0.004276515 | 0.926563755 | -0.022185684 | 0.632500269 | -0.013343715 |
| -0.218528483 | 1.87E-06 | -0.202810258 | 1.00E-05 | -0.22092196 |

| | | | | |
|---|---|---|---|---|
| -0.088124999 | 0.057040887 | -0.094785035 | 0.0406145 | -0.087194002 |
| -0.124281757 | 0.007166936 | -0.12799208 | 0.005606997 | -0.112901666 |
| -0.127227283 | 0.005900986 | -0.115918736 | 0.012184085 | -0.093939653 |
| -0.019423202 | 0.675469346 | -0.011925878 | 0.79714878 | -0.009839442 |
| -0.167441037 | 0.000278602 | -0.170484644 | 0.00021436 | -0.171127599 |
| -0.108028365 | 0.019538533 | -0.074341262 | 0.108616307 | -0.09883488 |
| -0.05303626 | 0.252682996 | 0.013322054 | 0.774010313 | 0.036062505 |
| -0.144756427 | 0.001710567 | -0.096544449 | 0.037012518 | -0.072124351 |
| 0.14117488 | 0.002228435 | 0.11061539 | 0.016786305 | 0.121695875 |
| -0.195392961 | 2.12E-05 | -0.17936686 | 9.72E-05 | -0.201603386 |
| -0.133674463 | 0.003803847 | -0.141252154 | 0.002215895 | -0.168159225 |
| 0.031243843 | 0.500603729 | 0.054757522 | 0.237588804 | 0.07965646 |
| -0.382390801 | 1.04E-17 | -0.390660682 | 1.78E-18 | -0.404364176 |
| 0.062834173 | 0.175239613 | 0.061448171 | 0.184972093 | 0.092025619 |
| -0.12217435 | 0.008216477 | -0.129863939 | 0.004942247 | -0.143178818 |
| -0.099685665 | 0.03125433 | -0.082532544 | 0.07478114 | -0.058399803 |
| -0.016588997 | 0.720676275 | 0.011058401 | 0.811616696 | 0.028154187 |
| 0.243657279 | 9.72E-08 | 0.247077543 | 6.33E-08 | 0.28120957 |
| -0.010228615 | 0.825516328 | -0.006763793 | 0.884097627 | 0.010145239 |
| 0.05017213 | 0.279250415 | 0.032733577 | 0.480392158 | 0.028818837 |
| -0.02166487 | 0.640512817 | -0.024638601 | 0.595350608 | -0.079128046 |
| -0.011402677 | 0.805866828 | 0.012882537 | 0.781273818 | 0.027130645 |
| 0.204330907 | 8.55E-06 | 0.203851853 | 8.99E-06 | 0.264968318 |
| -0.171557975 | 0.000195225 | -0.204920423 | 8.05E-06 | -0.247761704 |
| -0.20682725 | 6.59E-06 | -0.204609088 | 8.31E-06 | -0.228088853 |
| -0.051734123 | 0.264535603 | -0.053214829 | 0.251086807 | -0.063901991 |
| -0.026064809 | 0.574217397 | 0.001103576 | 0.981024439 | -0.020338904 |
| 0.07671724 | 0.097747827 | 0.128581451 | 0.0053895 | 0.092431573 |
| -0.028495371 | 0.53904047 | -0.0068225 | 0.883098827 | -0.00809828 |
| 0.051236562 | 0.269163914 | 0.070531257 | 0.128007944 | 0.054643556 |
| 0.043415889 | 0.34919475 | 0.060187119 | 0.194168398 | 0.062322484 |
| -0.3747309 | 5.13E-17 | -0.386676556 | 4.19E-18 | -0.404493516 |
| 0.204401937 | 8.49E-06 | 0.208705655 | 5.41E-06 | 0.243265921 |
| -0.049324087 | 0.287467578 | -0.04971167 | 0.283692106 | -0.031984517 |
| 0.238054904 | 1.93E-07 | 0.276125706 | 1.28E-09 | 0.26161321 |
| -0.03508504 | 0.449412344 | -0.02185135 | 0.637639028 | -0.04534063 |
| 0.165870075 | 0.000318409 | 0.172395535 | 0.000181417 | 0.173968377 |
| 0.009278386 | 0.84150047 | -0.009276719 | 0.841528571 | 0.012794319 |
| -0.236516373 | 2.33E-07 | -0.197008373 | 1.80E-05 | -0.20872753 |
| 0.144553273 | 0.001736698 | 0.133275977 | 0.00391064 | 0.162771891 |
| 0.022312732 | 0.630552136 | 0.015576772 | 0.737070597 | 0.010840651 |
| 0.028317744 | 0.541574329 | 0.042649527 | 0.357775274 | 0.080076482 |
| 0.093551377 | 0.043313141 | 0.08417158 | 0.069168959 | 0.029286606 |
| -0.218728876 | 1.82E-06 | -0.223274911 | 1.10E-06 | -0.258044927 |
| -0.02027803 | 0.662051527 | 0.007411766 | 0.873083866 | 0.027556976 |

| | | | | |
|---|---|---|---|---|
| -0.079837829 | 0.084809299 | -0.064975003 | 0.160962928 | -0.075984237 |
[truncated: 481,926 more chars]
