# Supplementary material for: Comprehensive Analysis of GDF10 Methylation Site-Associated Genes as Prognostic Markers for Endometrial Cancer
Source: J Oncol. 2022 Oct 10;2022:7117083. doi: 10.1155/2022/7117083 (PMC9576415; doi:10.1155/2022/7117083)
Supplement: Supplementary Materials — are shown as Supplemental Figures S1-3 and Supplemental Tables S1-7. Supplemental Figure S1: Cox regression independent prognostic analyses. Supplemental Figure S2: Analysis of expression correlations between the risk model and clinicopathological factors. Supplemental Figure S3: Kaplan-Meier survival analysis of patients with different clinicopathologic features between high- and low-risk groups. (a–i) The different OS of UCEC patients with different ages, tumor stages, and grades between high-risk and low-risk groups according to GDF10 methylation. The abscissa shows survival months, and the vertical axis shows overall survival probability. Supplemental Table S1: 48 modules were identified. Supplemental Table S2: The saddlebrown module positively correlated with the methylation sites the most. Supplemental Table S3: 991 genes were upregulated in DEGs. Supplemental Table S4: 1260 genes were downregulated in DEGs. Supplemental Table S5: 44 DEGMRGs were identified. Supplemental Table S6: All UCEC samples in the training set were divided into the high- and low-risk groups. Supplemental Table S7: Risk scores were significantly different between patients classified by age, tumor stage, and grade. [file Supplemental Table S7.pdf › Supplemental Table S7.pdf]

submitter_id.samples	age	Stage	Grade
TCGA-AJ-A3NE-01A	46.4575	Stage IA	G3
TCGA-AX-A1C7-01A	77.2548	Stage IB	G3
TCGA-AX-A3G8-01A	76.4356	Stage IA	G3
TCGA-EO-A2CG-01A	69.2575	Stage IA	G3
TCGA-AW-A1PO-01A	66.3699	Stage IIIA	G3
TCGA-AX-A1C5-01A	47.6247	Stage IIIC	G3
TCGA-AJ-A8CW-01A	64.5315	Stage IA	G2
TCGA-FI-A2EY-01A	63.1288	Stage IIIA	G3
TCGA-A5-A2K3-01A	68.2384	Stage IA	G3
TCGA-BG-A222-01A	49.9178	Stage IA	G3
TCGA-A5-A7WJ-01A	64.9945	Stage IA	G2
TCGA-EY-A548-01A	83.6822	Stage IA	G1
TCGA-B5-A0K8-01A	71.211	Stage IB	G3
TCGA-BG-A18B-01A	53.5616	Stage I	G2
TCGA-AX-A2IN-01A	63.7671	Stage IC	G3
TCGA-EO-A2CH-01A	73.8795	Stage IVB	G3
TCGA-D1-A1NX-01A	66.9616	Stage IIIC	G3
TCGA-AX-A0IS-01A	52.5288	Stage IC	G2
TCGA-AP-A1DQ-01A	76.2055	Stage IIIC	High Grade
TCGA-B5-A11O-01A	62.2466	Stage IA	G1
TCGA-PG-A6IB-01A	68.4082	Stage IA	G3
TCGA-AX-A2HF-01A	67.6411	Stage IB	G3
TCGA-EY-A72D-01A	87.1726	Stage II	G3
TCGA-AP-A1DK-01A	53.4493	Stage IA	G3
TCGA-D1-A103-01A	87.2356	Stage IB	G1
TCGA-B5-A11G-01A	71.2192	Stage IA	G1
TCGA-BG-A0YU-01A	37.3808	Stage IA	G1
TCGA-AJ-A2QO-01A	85.5397	Stage IB	G3
TCGA-BG-A220-01A	69.4411	Stage IA	G3
TCGA-DF-A2KU-01A	NA	Stage IB	G3
TCGA-AJ-A3NC-01A	63.9808	Stage IA	G3
TCGA-EO-A3B0-01A	43.7096	Stage IIIC	G3
TCGA-FI-A3PV-01A	68.2137	Stage IA	High Grade
TCGA-D1-A165-01A	64.4548	Stage IIIA	G1
TCGA-D1-A1O5-01A	61.863	Stage IA	G1
TCGA-D1-A17C-01A	78.2767	Stage IA	G1
TCGA-B5-A11W-01A	61.274	Stage IB	G2
TCGA-EY-A1GS-01A	71.2658	Stage IVB	G3
TCGA-D1-A3DH-01A	71.0548	Stage IA	G3
TCGA-EO-A3L0-01A	76.7205	Stage IA	G3
TCGA-SJ-A6ZJ-01A	61.9096	Stage IB	G2
TCGA-BS-A0V6-01A	55.8055	Stage IB	G1
TCGA-BG-A2AE-01A	57.7233	Stage IB	G3
TCGA-AX-A2HD-01A	69.8603	Stage IIIA	G3

TCGA-AJ-A3TW-01A	78.2438	Stage IB	G3
TCGA-BK-A26L-01A	71.2384	Stage IIIC	G3
TCGA-D1-A2G6-01A	53.5534	Stage IIIC	G3
TCGA-AJ-A3QS-01A	57.9781	Stage IIIA	G3
TCGA-AX-A1CF-01A	69.3151	Stage IVB	G3
TCGA-DI-A2QU-01A	67.0904	Stage IVB	G3
TCGA-AX-A1CP-01A	84.211	Stage IA	G3
TCGA-AX-A1CN-01A	54.411	Stage IIB	G3
TCGA-BG-A3PP-01A	82.8137	Stage II	G3
TCGA-D1-A16J-01A	61.1479	Stage IIB	G2
TCGA-EO-A3KX-01A	80.1644	Stage IA	G3
TCGA-D1-A0ZP-01A	59.126	Stage IVA	G3
TCGA-D1-A0ZR-01A	57.9644	Stage IB	G2
TCGA-D1-A2G0-01A	70.063	Stage IB	G3
TCGA-B5-A3F9-01A	56.2274	Stage IB	G3
TCGA-D1-A102-01A	49.2904	Stage IB	G2
TCGA-D1-A17L-01A	81.9151	Stage IA	G1
TCGA-BG-A0YV-01A	67.3068	Stage IIIA	G3
TCGA-AJ-A8CV-01A	58.4548	Stage IB	G2
TCGA-A5-A2K7-01A	41.0411	Stage II	G3
TCGA-EO-A22U-01A	83.7178	Stage IB	G3
TCGA-D1-A3JQ-01A	61.0877	Stage IIIC	G3
TCGA-D1-A16G-01A	74.1507	Stage IV	High Grade
TCGA-AX-A3G6-01A	67.6192	Stage IA	G2
TCGA-SL-A6JA-01A	77.811	Stage IB	G3
TCGA-AX-A06D-01A	82.2575	Stage IIIA	G2
TCGA-AX-A0IZ-01A	53.0521	Stage IB	G3
TCGA-FI-A2CY-01A	60.5425	Stage IA	G3
TCGA-A5-A1OJ-01A	31.8767	Stage IA	G2
TCGA-D1-A16Y-01A	56.0411	Stage IB	G1
TCGA-EY-A214-01A	66.126	Stage IIIC	G3
TCGA-D1-A16Q-01A	54.4466	Stage II	G2
TCGA-D1-A2G5-01A	50.1973	Stage IB	G3
TCGA-FI-A2EX-01A	58.874	Stage IIIA	G3
TCGA-QS-A5YR-01A	61.2932	Stage IIIB	G2
TCGA-EY-A549-01A	78.274	Stage IA	G3
TCGA-QS-A8F1-01A	85.989	Stage IIIA	G3
TCGA-EO-A1Y5-01A	63.7014	Stage IIIC	G3
TCGA-BK-A6W4-01A	62.3726	Stage IA	G2
TCGA-AJ-A3NF-01A	60.8493	Stage IIIC	G3
TCGA-A5-A0G1-01A	67.0603	Stage IA	G3
TCGA-AJ-A2QK-01A	65.6027	Stage IA	G3
TCGA-B5-A3FB-01A	73.3671	Stage IB	G3
TCGA-AJ-A3EL-01A	47.7863	Stage IA	G3
TCGA-EY-A212-01A	84.0548	Stage IIIC	G3

TCGA-AP-A1E1-01A	74.1699	Stage IA	G2
TCGA-AX-A2H7-01A	87.9452	Stage IC	G3
TCGA-AJ-A23N-01A	70.6082	Stage IIIA	G3
TCGA-QS-A5YQ-01A	55.9589	Stage IIIA	G1
TCGA-AX-A1CI-01A	61.8685	Stage IB	G1
TCGA-AX-A3GB-01A	73.2164	Stage IB	G3
TCGA-AP-A0LS-01A	63.3589	Stage IA	G2
TCGA-B5-A121-01A	57.5836	Stage IIIA	G1
TCGA-BS-A0UL-01A	54.7452	Stage IA	G1
TCGA-PG-A5BC-01A	72.4274	Stage II	G3
TCGA-EY-A1GM-01A	60.3014	Stage IIIA	G3
TCGA-EO-A3AU-01A	72.3452	Stage II	G3
TCGA-FI-A2F9-01A	79.1397	Stage IA	G3
TCGA-AP-A5FX-01A	68.6658	Stage IIIC	G3
TCGA-E6-A2P8-01A	53.2822	Stage IV	G3
TCGA-EY-A1GF-01A	75.2055	Stage IA	G2
TCGA-DI-A1NN-01A	63.0849	Stage IIIC	G3
TCGA-BK-A56F-01A	75.4055	Stage IB	G1
TCGA-DF-A2KZ-01A	87.5973	Stage IB	G3
TCGA-BG-A0W2-01A	57.5288	Stage IA	G1
TCGA-EO-A22T-01A	56.4603	Stage II	G3
TCGA-AX-A3FS-01A	82.4712	Stage IA	G3
TCGA-B5-A0KB-01A	62.1068	Stage IB	G3
TCGA-AX-A3G1-01A	85.0329	Stage IC	G3
TCGA-EY-A1GU-01A	66.5863	Stage IB	G2
TCGA-EY-A1GT-01A	68.9068	Stage IIIA	G1
TCGA-A5-A0VO-01A	64.5123	Stage IB	G2
TCGA-EY-A1GD-01A	51.063	Stage IA	G1
TCGA-E6-A1LZ-01A	76.8603	Stage IA	G3
TCGA-BG-A187-01A	66.0247	Stage IB	G2
TCGA-AJ-A23O-01A	69.6795	Stage IB	G3
TCGA-4E-A92E-01A	54.2959	Stage I	G1
TCGA-D1-A0ZU-01A	34.1315	Stage IB	G2
TCGA-AJ-A3IA-01A	78	Stage IB	G3
TCGA-DF-A2KV-01A	55.2329	Stage IB	G3
TCGA-D1-A0ZS-01A	54.3041	Stage IB	G3
TCGA-PG-A915-01A	60.6	Stage IA	G3
TCGA-EY-A1G8-01A	83.6082	Stage IB	G3
TCGA-A5-A2K5-01A	76.8685	Stage IA	G3
TCGA-AJ-A3NG-01A	83.1534	Stage II	G3
TCGA-B5-A11N-01A	69.7041	Stage IA	G1
TCGA-5B-A90C-01A	69.9123	Stage IA	G2
TCGA-AP-A1E4-01A	54.2849	Stage IB	G2
TCGA-B5-A11J-01A	64.3425	Stage IA	G2
TCGA-B5-A11I-01A	65.3616	Stage IVB	G3

TCGA-BK-A4ZD-01A	42.5644	Stage IA	G3
TCGA-AX-A0J0-01A	47.8521	Stage IB	G2
TCGA-B5-A5OE-01A	68.2329	Stage IIIC	G3
TCGA-EY-A547-01A	75.8521	Stage IA	G3
TCGA-FI-A2F8-01A	64.7918	Stage II	G3
TCGA-B5-A5OD-01A	67.8767	Stage IIIC	G3
TCGA-A5-A0GG-01A	76.189	Stage IA	G1
TCGA-EY-A3L3-01A	82.5699	Stage IA	G3
TCGA-B5-A11S-01A	63.2466	Stage IA	G2
TCGA-AX-A3G9-01A	64.0027	Stage IIIC	G3
TCGA-E6-A8L9-01A	61.1863	Stage IB	G3
TCGA-D1-A17A-01A	59.0685	Stage IIA	G3
TCGA-AJ-A5DW-01A	56.7425	Stage IA	G2
TCGA-D1-A177-01A	70.1425	Stage IB	G2
TCGA-D1-A3DG-01A	81.5644	Stage IVB	G3
TCGA-AP-A3K1-01A	56.4219	Stage IVB	High Grade
TCGA-AJ-A2QL-01A	60.4301	Stage IIIC	G3
TCGA-B5-A1N2-01A	70.1699	Stage IIIC	G3
TCGA-KP-A3W3-01A	72.0822	Stage IB	G3
TCGA-A5-A1OK-01A	63.5041	Stage IIIA	G3
TCGA-D1-A1O8-01A	70.6548	Stage IIIC	G3
TCGA-BG-A0VW-01A	77.7616	Stage IA	G1
TCGA-D1-A167-01A	70.0685	Stage IC	G2
TCGA-FI-A2F4-01A	64.7151	Stage IC	G3
TCGA-AX-A2HA-01A	36.0055	Stage IB	G3
TCGA-A5-A3LO-01A	64.1123	Stage IB	G3
TCGA-BK-A13B-01A	58.8849	Stage IB	G3
TCGA-DI-A1BY-01A	63.3233	Stage IIB	G2
TCGA-K6-A3WQ-01A	60.526	Stage IVB	G3
TCGA-AX-A1CE-01A	60.189	Stage IB	G3
TCGA-EO-A22Y-01A	68.8411	Stage IA	G3
TCGA-AX-A3FT-01A	64.5014	Stage IB	G3
TCGA-D1-A15Z-01A	72.9808	Stage IB	G2
TCGA-BG-A0MH-01A	61.5753	Stage IC	G2
TCGA-D1-A15V-01A	68.1808	Stage IB	G3
TCGA-JU-AAVI-01A	61.0658	Stage IIIC	G3
TCGA-QF-A5YT-01A	57.4822	Stage II	G1
TCGA-D1-A16S-01A	70.3836	Stage IIIC	High Grade
TCGA-BK-A0CA-01A	61.3096	Stage IIIC	G3
TCGA-AX-A3G7-01A	71.7452	Stage IVB	G3
TCGA-SL-A6J9-01A	73.1534	Stage IB	G3
TCGA-AJ-A3I9-01A	52.4356	Stage II	G3
TCGA-B5-A11E-01A	53.3096	Stage IB	G3
TCGA-BG-A221-01A	84.8219	Stage IB	G3
TCGA-A5-A1OG-01A	65.6329	Stage IIIC	G3

TCGA-BG-A0MA-01A	60.3726	Stage IB	G3
TCGA-D1-A1NY-01A	67.6247	Stage IB	G3
TCGA-B5-A11Q-01A	64.4877	Stage IIIA	G1
TCGA-AJ-A3BD-01A	57.5288	Stage IIIC	G3
TCGA-A5-A2K2-01A	77.9616	Stage IA	G3
TCGA-AX-A3G3-01A	58.3781	Stage IIIA	G3
TCGA-B5-A11V-01A	64.7397	Stage IA	G1
TCGA-AJ-A5DV-01A	65.4575	Stage IA	G3
TCGA-D1-A15W-01A	59.0274	Stage IIIC	G1
TCGA-E6-A1LX-01A	40.3589	Stage IB	G3
TCGA-EY-A4KR-01A	56.6274	Stage IIIC	G3
TCGA-D1-A17H-01A	61.3397	Stage IB	G1
TCGA-B5-A1MU-01A	79.526	Stage IVB	G3
TCGA-AX-A06H-01A	61.0137	Stage IIIA	G1
TCGA-AX-A1C4-01A	52.6274	Stage IB	G2
TCGA-EY-A2ON-01A	61.9836	Stage IVB	G3
TCGA-PG-A916-01A	70.1836	Stage I	G2
TCGA-AX-A3G4-01A	60.5452	Stage IIIC	G3
TCGA-DF-A2L0-01A	60.8219	Stage IIIC	G3
TCGA-D1-A17T-01A	67.5397	Stage IA	G2
TCGA-AJ-A3OL-01A	55.1096	Stage IIIA	G3
TCGA-AP-A05O-01A	68.4329	Stage IB	G3
TCGA-A5-A1OF-01A	47.4192	Stage IA	High Grade
TCGA-BG-A0W1-01A	89.726	Stage II	G2
TCGA-BG-A0VZ-01A	58.6685	Stage IIIA	G1
TCGA-AJ-A3EJ-01A	74.8411	Stage IIIC	G3
TCGA-D1-A1O7-01A	60.3753	Stage IA	G1
TCGA-EY-A1GH-01A	70.6904	Stage IA	G2
TCGA-EO-A1Y8-01A	87.5918	Stage IA	G3
TCGA-A5-A3LP-01A	74.3315	Stage IB	G3
TCGA-B5-A11F-01A	55.263	Stage IA	G2
TCGA-D1-A1O0-01A	77.1562	Stage IA	G3
TCGA-EC-A1NJ-01A	73.9781	Stage IA	G2
TCGA-EO-A22X-01A	36.2877	Stage IIIA	G3
TCGA-E6-A1M0-01A	56.5178	Stage IIIC	G3
TCGA-AX-A3FZ-01A	33.7836	Stage IIIB	G3
TCGA-BS-A0VI-01A	58.0767	Stage IIB	G3
TCGA-KP-A3W1-01A	76.4438	Stage IA	G3
TCGA-KP-A3W0-01A	72.2685	Stage IB	G3
TCGA-BS-A0V7-01A	48.7945	Stage IB	G1
TCGA-A5-A0GR-01A	69.4603	Stage IA	G2
TCGA-EO-A3AS-01A	86.1644	Stage IA	G3
TCGA-D1-A1NS-01A	53.8027	Stage IA	G1
TCGA-D1-A176-01A	67.1808	Stage IB	G2
TCGA-5S-A9Q8-01A	51.4658	Stage IIIA	G2

TCGA-EY-A2OP-01A	63.2603	Stage IA	G3
TCGA-EC-A24G-01A	57.1425	Stage II	G3
TCGA-B5-A11Z-01A	61.6575	Stage IA	G1
TCGA-B5-A1MY-01A	62.4795	Stage IIIC	G3
TCGA-EO-A3KU-01A	68.811	Stage II	G3
TCGA-EY-A2OQ-01A	61.1205	Stage IA	G3
TCGA-D1-A16R-01A	39.6301	Stage IA	G1
TCGA-D1-A162-01A	69.9808	Stage IB	G1
TCGA-BG-A3EW-01A	62.2055	Stage III	G3
TCGA-DF-A2KR-01A	84.9507	Stage IIIC	G3
TCGA-QF-A5YS-01A	57.0986	Stage II	G2
TCGA-BK-A139-01A	74.1836	Stage IB	G3
TCGA-BS-A0UA-01A	68.1425	Stage IB	G1
TCGA-AJ-A3BI-01A	67.4603	Stage IA	G3
TCGA-D1-A16E-01A	73.5863	Stage IB	G1
TCGA-EY-A1GX-01A	76.6082	Stage IIIA	G2
TCGA-SJ-A6ZI-01A	64.8027	Stage IB	G1
TCGA-EY-A3QX-01A	64.8301	Stage IVB	G3
TCGA-EY-A5W2-01A	72.5973	Stage IB	G3
TCGA-EO-A3KW-01A	72.5808	Stage IA	G3
TCGA-EY-A1GC-01A	62.0712	Stage IB	G3
TCGA-AP-A1DP-01A	70.1397	Stage IA	G2
TCGA-BG-A2L7-01A	68.7425	Stage IA	G3
TCGA-AP-A0LF-01A	82.2658	Stage IVB	G3
TCGA-D1-A17S-01A	59.7096	Stage IA	G1
TCGA-D1-A16O-01A	44.1479	Stage IB	G1
TCGA-BS-A0T9-01A	39.0493	Stage IVB	G3
TCGA-EO-A1Y7-01A	65.9863	Stage IB	G3
TCGA-D1-A15X-01A	45.2932	Stage IA	High Grade
TCGA-AJ-A3NH-01A	90.0575	Stage IB	G3
TCGA-AJ-A3OK-01A	73.5644	Stage IA	G3
TCGA-EY-A1GI-01A	52.3753	Stage IB	G2
TCGA-D1-A101-01A	60.4877	Stage IB	G2
TCGA-A5-A0GQ-01A	76.926	Stage IB	G1
TCGA-FI-A2EW-01A	71.9644	Stage IIB	G3
TCGA-B5-A5OC-01A	71.5315	Stage IIIB	G3
TCGA-AX-A1CJ-01A	59.3288	Stage IC	G2
TCGA-D1-A16V-01A	78.4521	Stage IIIC	G3
TCGA-DF-A2KN-01A	NA	Stage IC	G3
TCGA-EY-A215-01A	60.4822	Stage IB	G3
TCGA-AX-A2H5-01A	67.8274	Stage IIIC	G3
TCGA-AX-A1CA-01A	70.7315	Stage IB	G3
TCGA-D1-A0ZO-01A	75.2932	Stage IB	G1
TCGA-FI-A2D4-01A	44.2822	Stage IIIC	G3
TCGA-FI-A2D5-01A	56.6192	Stage IVB	G3

TCGA-AX-A1CC-01A	65.1918	Stage IC	G3
TCGA-EO-A3AV-01A	51.2685	Stage IIIC	G3
TCGA-AX-A1C8-01A	77.9507	Stage IIIC	G3
TCGA-A5-A1OH-01A	86.1671	Stage IIIA	G3
TCGA-D1-A16N-01A	51.4712	Stage IIIA	G3
TCGA-B5-A11R-01A	51.7205	Stage II	G3
TCGA-D1-A17F-01A	68.811	Stage IA	G2
TCGA-BS-A0WQ-01A	55.1534	Stage IA	G2
TCGA-B5-A1MW-01A	55.2959	Stage IA	G3
TCGA-H5-A2HR-01A	71.1945	Stage IB	G3
TCGA-D1-A0ZV-01A	58.5589	Stage IB	G2
TCGA-AP-A1E0-01A	40.1452	Stage IIIC	G2
TCGA-BG-A0VV-01A	53.6466	Stage IA	G1
TCGA-FI-A2EU-01A	68.0466	Stage IA	G3
TCGA-PG-A917-01A	74.2575	Stage IA	G2
TCGA-EY-A54A-01A	68.0055	Stage IIIC	G3
TCGA-BS-A0V8-01A	68.4411	Stage IIB	G2
TCGA-AX-A1CK-01A	58.0822	Stage IB	G2
TCGA-AP-A1E3-01A	45.326	Stage IIIC	G3
TCGA-D1-A1NU-01A	74.7178	Stage IA	G3
TCGA-D1-A169-01A	63.6658	Stage IV	G2
TCGA-BG-A2AD-01A	63.189	Stage IIIB	G3
TCGA-BG-A0VX-01A	58.6986	Stage IB	G1
TCGA-BG-A18A-01A	74.2082	Stage IA	G1
TCGA-EY-A210-01A	82.4521	Stage IIIC	G3
TCGA-AX-A2HC-01A	53.3836	Stage IIIC	G3
TCGA-AX-A1CR-01A	70.7178	Stage IIB	G3
TCGA-D1-A17B-01A	69.874	Stage IB	G2
TCGA-AJ-A3BG-01A	65.3726	Stage IA	G3
TCGA-AX-A2H4-01A	66.3945	Stage IIIA	G3
TCGA-DI-A1NO-01A	68.4822	Stage IIIA	G3
TCGA-AJ-A3BK-01A	68.337	Stage IC	G3
TCGA-AJ-A2QM-01A	67.8795	Stage II	G3
TCGA-D1-A17Q-01A	54.8356	Stage IA	G2
TCGA-2E-A9G8-01A	NA	Stage III	G2
TCGA-AX-A2H8-01A	64.2411	Stage IB	G3
TCGA-B5-A3S1-01A	71.3178	Stage IIIC	G3
TCGA-D1-A0ZZ-01A	80.6027	Stage IB	G3
TCGA-AX-A3GI-01A	66.8164	Stage IIIC	G3
TCGA-BK-A13C-01A	47.4438	Stage IB	G2
TCGA-AX-A2HH-01A	60.0658	Stage IA	G3
TCGA-FI-A2D6-01A	74.137	Stage IB	G3
TCGA-AX-A2HG-01A	56.9068	Stage IC	G3
TCGA-EO-A3AZ-01A	80.0932	Stage IB	G3
TCGA-D1-A1NW-01A	72.7562	Stage IVB	G3

TCGA-BG-A18C-01A	72.8822	Stage IA	G2
TCGA-BS-A0U5-01A	76.5616	Stage IA	G1
TCGA-EY-A1GL-01A	51.4	Stage II	G2
TCGA-PG-A7D5-01A	63.0301	Stage IA	G3
